# Functional and Transcriptional Characterization of Human Embryonic Stem Cell-Derived Endothelial Cells for Treatment of Myocardial Infarction

**DOI:** 10.1371/journal.pone.0008443

**Published:** 2009-12-31

**Authors:** Zongjin Li, Kitchener D. Wilson, Bryan Smith, Daniel L. Kraft, Fangjun Jia, Mei Huang, Xiaoyan Xie, Robert C. Robbins, Sanjiv S. Gambhir, Irving L. Weissman, Joseph C. Wu

**Affiliations:** 1 Department of Radiology and Molecular Imaging Program at Stanford (MIPS), Stanford University School of Medicine, Stanford, California, United States of America; 2 Department of Bioengineering, Stanford University School of Medicine, Stanford, California, United States of America; 3 Department of Developmental Biology, Stanford University School of Medicine, Stanford, California, United States of America; 4 Department of Cardiothoracic Surgery, Stanford University School of Medicine, Stanford, California, United States of America; 5 Institute for Stem Cell Biology and Regenerative Medicine, Stanford University School of Medicine, Stanford, California, United States of America; 6 Department of Medicine, Division of Cardiology, Stanford University School of Medicine, Stanford, California, United States of America; 7 Nankai University School of Medicine, Tianjin, China; Istituto Dermopatico dell'Immacolata, Italy

## Abstract

**Background:**

Differentiation of human embryonic stem cells into endothelial cells (hESC-ECs) has the potential to provide an unlimited source of cells for novel transplantation therapies of ischemic diseases by supporting angiogenesis and vasculogenesis. However, the endothelial differentiation efficiency of the conventional embryoid body (EB) method is low while the 2-dimensional method of co-culturing with mouse embryonic fibroblasts (MEFs) require animal product, both of which can limit the future clinical application of hESC-ECs. Moreover, to fully understand the beneficial effects of stem cell therapy, investigators must be able to track the functional biology and physiology of transplanted cells in *living* subjects over time.

**Methodology:**

In this study, we developed an extracellular matrix (ECM) culture system for increasing endothelial differentiation and free from contaminating animal cells. We investigated the transcriptional changes that occur during endothelial differentiation of hESCs using whole genome microarray, and compared to human umbilical vein endothelial cells (HUVECs). We also showed functional vascular formation by hESC-ECs in a mouse dorsal window model. Moreover, our study is the *first* so far to transplant hESC-ECs in a myocardial infarction model and monitor cell fate using molecular imaging methods.

**Conclusion:**

Taken together, we report a more efficient method for derivation of hESC-ECs that express appropriate patterns of endothelial genes, form functional vessels *in vivo*, and improve cardiac function. These studies suggest that hESC-ECs may provide a novel therapy for ischemic heart disease in the future.

## Introduction

Ischemic heart disease is a leading cause of mortality and morbidity worldwide. Current treatments fail to address the underlying scarring and cell loss, which is a major cause of heart failure after infarction [1], [2]. Therapeutic angiogenesis and/or vasculogenesis using cellular transplantation is a promising and novel strategy to increase blood flow in patients with severe ischemic heart disease and peripheral vascular disease. In several studies related to peripheral vascular disease, endothelial progenitor cells (EPCs) derived from patients that are then used for autologous transplantation therapy have been shown to foster the formation of arterial collaterals and promote the regeneration of ischemic tissues [3], [4], [5]. However, difficulties in obtaining sufficient numbers of adult EPCs from patients, as well as impaired function of these cells in patients with co-morbid diseases, may limit autologous stem cell therapies [6], [7]. With their capacity for unlimited self-renewal and pluripotency, human embryonic stem cells (hESCs) may provide an alternate source of therapeutic cells by allowing the derivation of large numbers of endothelial cells for various tissue repair and cell replacement therapies.

Several methods have been used to successfully differentiate hESCs into endothelial cells, including three-dimensional (3D) embryoid body (EB) formation and two-dimensional (2D) culture system [6], [8], [9], [10], [11], [12]. However, the endothelial differentiation efficiency of previous 3D embryoid body method is low and the 2D method by co-culture with mouse cells suffers from contamination with animal material. Thus, new methods should be introduced to increase the differentiation efficiency and ensure animal free derived products. In this study, we developed an extracellular matrix culture system for increasing endothelial differentiation and ensuring animal free hESC-ECs. We designed a staged protocol that involved EB formation (stage 1) and expansion of endothelial lineage by subculturing EBs in collagen (stage 2). We then derive a highly pure endothelial population by CD31/CD144 double sorting using flow cytometry. In order to define at a molecular level the changes occurring at each stage of hESC differentiation to endothelial cell progeny, and to validate that these cells are similar to human umbilical vein endothelial cells (HUVECs), we also perform transcriptional profiling using whole human genome microarrays and real-time PCR arrays. Finally, to fully understand the beneficial effects of stem cell therapy, one must also be able to track the transplanted cells in *living* subjects over time in order to better understand their behavior and function *in vivo*. Therefore, we performed multi-modality imaging in a murine dorsal window model and a murine myocardial ischemia model to assess hESC-EC fate and function.

## Materials and Methods

### Two-step in vitro differentiation of hESC-ECs

**Figure 1:**
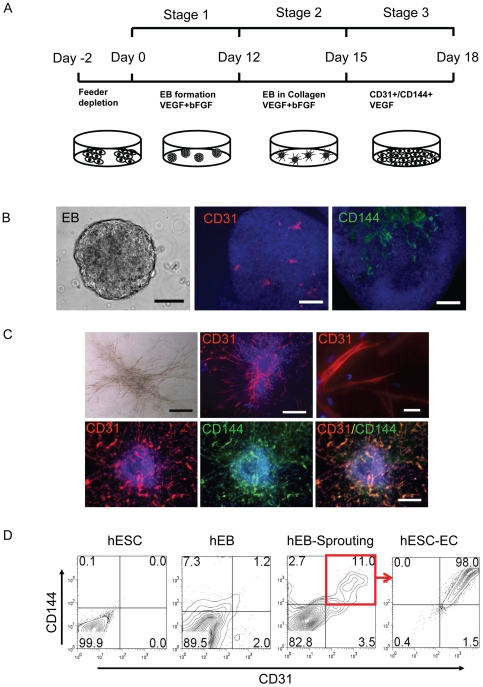
Specification of hESC differentiation into endothelial lineage. (A) An outline of the protocol used for the differentiation of hESCs to the endothelial lineage. Undifferentiated hESCs were grown to 60%–70% confluence on Matrigel and subcultured in low attachment dish with differentiation medium supplement VEGF and bFGF, shown as day 0. At day 15, EBs in collagen were collected and digested and CD31+/CD144+ cells were isolated by FACS and then sub-cultured in EGM-2 medium with 5% Knockout SRTM to expand and induce endothelial maturation. (B) Whole-mount immunochemistry of day-12 EB. Areas of CD31 (red) and CD144 (green) expression within EBs are organized in elongated clusters and channels. Cell nuclei stained with DAPI (blue). Scale bars = 100 µm. (C) Endothelial differentiation of sprouting EBs in collagen. Representative sprouting EBs after 3 days of culturing in collagen matrix (upper panel, left). Whole-mount immunostaining shows CD31 sprouting with channel-like vessel structures and CD31 cell clumps (upper panel). These sproutings are also CD31 and CD144 double positive. Scale bars = 20 µm (top panel, right one) and 100 µm (others). (D) Kinetic expression of CD31 and CD144 during the two-step hESC differentiation procedures. CD31/CD144 expression was triggered and increased from 1–3% to 10–15% after EBs were subcultured in collagen. hESC-ECs were enriched by isolation CD31+/CD144+ cells after FACS (right panel). Experiments were performed in triplicates.

Undifferentiated hESCs (H9 line from Wicell, passages 35 to 45) were grown on Matrigel-coated plates in mTeSR1 medium (Stem Cell Technologies, Vancouver, BC, Canada) as previously described [13]. To induce endothelial differentiation, undifferentiated hESCs were cultured in differentiation medium containing Iscove's modified Dulbecco's medium (IMDM) and 15% Knockout™ Serum Replacement (Knockout™ SR) (Invitrogen, Carlsbad, CA), 1× BIT (BSA, insulin, transferring; Stem Cell Technologies), 0.1 mM nonessential amino acids, 2 mM L-glutamine, 450 µM monothioglycerol (Sigma, St. Louis, MO), 50 U/ml penicillin, and 50 µg/ml streptomycin, supplemented with 20 ng/ml bFGF (R&D Systems Inc., Minneapolis) and 50 ng/ml VEGF (R&D Systems Inc.), in ultra-low attachment plates (Corning Incorporated, Corning, NY) for the formation of suspended embryoid bodies (EBs) as previously described [10], [12]. EB sprouting differentiation in collagen type I was performed as described [14], with minor modifications. Briefly, 12 days-old EBs were harvested, and then suspended into rat tail collagen type I (Becton Dickinson, San Jose, CA) at a final concentration of 1.5 mg/ml collagen. After thoroughly mixing EBs into collagen, 1.5 ml/well of mixture was added into six-well plate. The plates were incubated at 37°C for 30 min, allowing gel polymerization prior to addition of medium. After gel formation, each dish was supplemented with EGM-2 medium (Lonza, Basel, Switzerland) and 5% Knockout™ SR with additional 50 ng/ml VEGF and 20 ng/ml bFGF. The cultures were then incubated for 3 days without media change (**Figure 1A**).

### Flow cytometry sorting (FCS) of hESC-ECs

Single cell suspension from hEB-sprouting was obtained by treatment with 0.25% collagenase I (Invitrogen, Carlsbad, CA) at 37°C for 20–30 min, then with 0.56 units/ml Liberase Blendzyme IV (Roche Diagnostics, Indianapolis) at 37°C for 10–20 minutes. Cells were passed through a 40-µm cell strainer (BD Falcon, San Diego) [15]. Cells were incubated with mouse anti-human CD31 (BD) and rabbit anti-human CD144 (Abcam, Cambridge, MA). The CD31^+^/CD144^+^ cells were isolated using FACScan (Becton Dickinson). To generate hESC-ECs, the isolated CD31^+^/CD144^+^ cells from hEB-sprouting were grown on 4 g/cm^2^ human fibronectin (Calbiochem, San Diego, CA) coated plates in EGM-2 (Lonza) and 5% Knockout SR™ with additional 5 ng/ml VEGF. The medium was changed every 2–3 days.

### Functional and biological characterization of hESC-ECs

Flow cytometry analysis, immunostaining, DiI-ac-LDL uptake assay, and Matrigel assay were used to confirm endothelial cell phenotype within these CD31^+^/CD144^+^ purified hES cells. Antibodies used for flow cytometry analysis were phycoerythrin (PE) conjugated anti-CD31, CD34 (BD Pharmingen) and Allophycocyanin (APC) conjugated anti-KDR, CD133 (R&D Systems), APC conjugated anti-mouse IgG2a, and rabbit anti-human CD144 (Abcam, Cambridge, MA). The stained cells were analyzed using FACS Vantage (Becton-Dickinson, MA). Dead cells stained by propidium-iodide (PI) were excluded from the analysis. Isotype-identical antibodies served as controls (BD Pharmingen). For immunostaining, the cells were fixed with 4% paraformaldehyde in PBS at room temperature for 15 minutes. The fixed cells were permeated by 1% Triton 100 and incubated with 2% BSA for 30 minutes to block nonspecific binding, and stained for 1 hour with the primary antibodies: CD31 (BD Pharmingen), CD144 (Abcam), and vWF (Chemicon International Inc), respectively. The cells were then incubated for 30 minutes with either Alexa 594-conjugated donkey anti-mouse secondary antibody or Alexa 488-conjugated donkey anti-rabbit secondary antibody (Invitrogen), and counter stained with DAPI. Immunostaining of EBs and EB sproutings were performed as previously described with minor modifications [14]. The EBs were fixed in methanol and DMSO (4∶1) at 4°C overnight. For staining, the rehydrated EBs were first blocked by two incubations in PBSBT (2% BSA and 0.2% Tween-20 in PBS), then with PBSBT containing mouse anti-human MoAb CD31 (BD Pharmingen) or rabbit anti-human CD144 (Abcam, Cambridge, MA) overnight at 4°C. The EBs were washed five times in PBSBT each for 1 hour at 4°C for the initial three washes and at room temperature for the final two. The primary antibody was labeled by incubating the EBs with Alexa 594-conjugated donkey anti-mouse IgG or Alexa 488-conjugated donkey anti-rabbit IgG (Invitrogen) in PBSBT overnight at 4°C and nuclear counterstained with DAPI.

For DiI-ac-LDL uptake assay, hESC-ECs were incubated with 10 µg/ml of DiI-Ac-LDL (Molecular Probes, Eugene, OR) at 37°C for 6 hours. After washing with PBS twice, cells were fixed and counterstained with DAPI (4, 6-diamidino-2-phenylindole) as described [11]. The formation of endothelial tubes was assessed by seeding cells in 24-well plates coated with Matrigel (BD Pharmingen) and incubating them at 37° for 12 hours as described [16].

### Microarray hybridization and data acquisition

Total RNA samples were isolated in Trizol (Invitrogen) followed by purification over a Qiagen RNeasy column (Qiagen) from undifferentiated hESCs, day 12 EBs, hESC-ECs (after CD31/CD144 sort), and HUVECs. Four samples from each group (for a total of 16 unique samples) were harvested for RNA isolation. Using Agilent Low RNA Input Fluorescent Linear Amplification Kits, cDNA was reverse transcribed from each of 16 RNA samples representing four biological quadruplicates, as well as the pooled reference control, and cRNA was then transcribed and fluorescently labeled with Cy5/Cy3. cRNA was purified using an RNeasy kit (Qiagen, Valencia, CA, USA). 825 ng of Cy3- and Cy5- labeled and amplified cRNA was hybridized to Agilent 4×44K whole human genome microarrays (G4112F) and processed according to the manufacturer's instructions. The array was scanned using Agilent G2505B DNA microarray scanner. The image files were extracted using Agilent Feature Extraction software version 9.5.1 applying LOWESS background subtraction and dye-normalization. The data were analyzed using GeneSpring GX 7.3.1. (Agilent Technologies, Santa Clara, CA) with multiple testing correction to identify genes which had statistically significant changes in expression between stages, and K-means clustering to identify clusters of genes having unique temporal expression profiles. For hierarchical clustering, we used Pearson correlation for similarity measure and average linkage clustering. Gene Ontology (GO) overrepresentation analysis was performed also using the GeneSpring software. For confirmation of microarray results, RT-PCR assays were performed using the human endothelial cell biology RT^2^ Profiler™ PCR Array (SuperArray Bioscience, Frederick, MD) on an ABI PRISM 7900 HT (Applied Biosystems, Foster City, CA). Data analysis is available at the company website: http://www.superarray.com/pcr/arrayanalysis.php.

### In vivo angiogenesis of hESC-ECs

To track transplanted cells *in vivo*, hESCs were transduced with a self-inactivating lentiviral vector carrying a human ubiquitin promoter driving firefly luciferase and enhanced green fluorescence protein (Fluc-eGFP) double fusion reporter gene as described [12]. For Matrigel plug analysis, 1×10^6^ hESC-ECs suspended in 1 ml Matrigel were subcutaneous injected into SCID Beige mice (n = 5) and plugs were harvested at day 14 [17]. For dorsal skin fold chamber (n = 10 animals), 1×10^6^ hESC-ECs were suspended in 1 ml solution of rat-tail type I collagen (at final concentration 1.5 mg/ml) and Matrigel (1∶1 ratio) at 4°C. The cell suspension was pipetted into 12-well plates and warmed to 37°C for 30 minutes to allow polymerization of collagen and Matrigel and 1 ml EGM-2 medium with 5% Knockout SR™ was added after that. After one day of culture in 5% CO_2_, a skin puncher was applied to create circular disk-shape pieces of the construct (8-mm diameter), and they were implanted into the dorsal skin fold windows in SCID mice. The titanium dorsal skin fold chamber (APJ Trading Co. Inc, Ventura, Ca) surgically mounted onto each mouse was described previously [18]. Briefly, mice were anesthetized with inhaled isoflurane (2%–3%), and two symmetrical titanium frames were implanted to sandwich the extended double layer of the skin. One layer was removed in a 10-mm-diameter circular area. The remaining layer, consisting of epidermis, subcutaneous tissue, and striated skin muscle, was covered with a glass coverslip incorporated in one of the titanium frames. The animals were housed one per cage and had free access to water and food throughout the experiment. A recovery period of 2 days was allowed before the collagen/Matrigel patch was transplanted. Multiple laser-scanning intravital microscopy (IV-100, Olympus, Center Valley, PA) was used to visualize and quantify the morphological changes of Fluc-eGFP positive hESC-ECs. The perfused vessels were highlighted by vein injection of Angiosense 680 (VisEn Medical, Woburn, MA), indicating the formation of functional engineered vessels [19].

### Transplantation of hESC-ECs into ischemic myocardium

Animal protocols were approved by the Stanford University Animal Care and Use Committee guidelines. All surgical procedures were performed on 8–10 week old female SCID Beige mice (Charles River Laboratories, Wilmington, MA) by a single experienced micro-surgeon. Following induction with inhaled isoflurane (2% to 3%), mice were intubated and ventilated and anesthesia was maintained with inhaled isoflurane (1% to 2.5%). A left thoracotomy was performed followed by ligation of the middle of left anterior descending (LAD) artery for 30 minutes followed by reperfusion. Infarction was visually confirmed by blanching of the anterolateral region of the left ventricle along with dyskinesis. After 30 minutes, 1×10^6^ hESC-ECs stably expressing eGFP-Fluc were injected intramyocardially into 3 sites near the peri-infarct zone at 20 µl of total volume (n = 28). Control animals received PBS injection instead (n = 15). For sham operated animals (n = 5), open thoracotomy was performed but without ligation of the LAD artery.

### Bioluminescence imaging of transplanted cell survival

Cardiac bioluminescence imaging was performed using the Xenogen IVIS 200 system by a blinded investigator (XX). After intraperitoneal injection of the reporter probe D-Luciferin (150 mg Luciferin/kg body weight), animals were imaged for 1–10 minutes. The same mice were imaged for up to 8 weeks. Bioluminescence signal was quantified in units of maximum photons per second per cm square per steridian (photons/sec/cm^2^/sr) as described [16].

### Left ventricular functional analysis with echocardiogram and pressure-volume (PV) loop

Animals were scanned on baseline (day −7) and days 2, 14, 28, and 56, post-operatively by a blinded investigator (MH) using the Siemens-Acuson Sequoia C512 system equipped with a multi-frequency (8–14 MHz) 15L8 transducer. Animals were anesthetized with inhaled 2% isoflurane. Analysis of M-mode images was performed. Left ventricular end diastolic diameter (LVEDd) and end-systolic diameter (LVESd) were measured and used to calculate fractional shortening (FS) by the following formula: FS = [LVEDd−LVESd]/LVEDd [16]. At the end of the study (day 56), invasive hemodynamic measurements were performed as described [20]. Briefly, after midline neck incision, a conductance 1.4 conductance catheter (Millar Instruments, Houston, TX, USA) was introduced into the left ventricle through the right carotid artery. After stabilization, the signals were continuously recorded at a sampling rate of 1000/s using pressure-volume conductance system coupled to a PowerLab/4SP analog to digital converter (ADInstruments). Data were analyzed by using a cardiac pressure-volume analysis program (PVAN 3.4; Millar Instruments, Houston, TX, USA) and Chart/Scope Software (AD Instruments, Colorado Springs, CO, USA).

### Positron emission tomography (PET) imaging of cardiac viability

Small animal microPET imaging was carried out at day −7 (baseline) and days 2, 14, 28, and 56, post-operatively. Mice were fasted for 3 hours prior to radioisotope injection and then were injected with 140±17 µCi of F18-fluorodeoxyglucose ([^18^F]-FDG) via the tail vein. At 45 to 60 minutes post-injection, animals were anesthetized with inhaled 2% isoflurane and imaged with a Vista microPET system (GE Health Care, Chalfont St. Giles, United Kingdom). Images were reconstructed by filtered back projection (FBP) and analyzed by image software Amide (http://amide.sourceforge.net/index.html). Three-dimensional regions of interest (ROIs) were drawn encompassing the heart. Counts/pixel/min were converted to counts/ml/min (assuming a tissue density of 1 g/ml) with a calibration constant derived from scanning a cylindrical phantom [21]. For each ROI, counts/ml/min were then converted to counts/gram/min and divided by the injected dose to obtain the image ROI-derived [^18^F]-FDG percentage injected dose per gram of heart (% ID/g). To measure the myocardial infarction size, [^18^F]-FDG PET images were assembled into polar maps [22]. The size of perfusion defects was measured as the myocardium with 50% of maximum activity and expressed as percent total myocardium polar map by GE Healthcare Advantage Workstation (GE Healthcare, UK).

### Histological analysis

Explanted hearts from study and control groups were embedded into OCT compound (Miles Scientific). Frozen sections (5 µm thick) were processed for immunostaining. To track GFP^+^ hESC-ECs in hearts, rabbit anti-GFP antibody (Invitrogen), rat anti-mouse CD31 antibody (BD Pharmingen), anti-α-sarcomeric actin (α-SA) antibody (Sigma), and anti-smooth muscle actin (SMA) antibody (Labvision) were used. Alexa Fluor 488, Alexa Fluor 647, and Alexa Fluor 594-conjugated secondary antibodies were applied appropriately. DAPI was used for nuclear counterstaining. To detect microvascular density (MVD) in the peri-infarct area, a rat anti-mouse CD31 was used. The number of capillary vessels was counted by a blinded investigator (FJ) in ten randomly selected areas using a fluorescence microscope (200x). To measure circumferential fibrosis area, Masson's trichrome staining was performed [23]. Explanted hearts from study and control groups were harvested after euthanasia at 8 weeks after ischemia reperfusion for assessment. Hearts embedded in paraffin were cut into 5-µm slice, five to six sections from apex to base were subjected to staining. Images were taken for each section to calculate the fibrotic and nonfibrotic areas. The measurement of area of fibrosis was determined by Image J software (NIH) performed on 3 separate sections, and the averages were used for statistics analysis.

### Statistical analysis

Statistics were calculated using SPSS 16.0 (SPSS Inc., Chicago, IL, USA). Descriptive statistics included mean and standard error. Two-way repeated measures ANOVA and two-tailed Student's *t*–test were used. Differences were considered significant at *P* values of <0.05.

## Results

### Two step endothelial differentiation of hESCs

Major challenges for hESC-based therapies are the generation of sufficient numbers of differentiated cells and animal product free culture system. Several previous studies have shown that the efficiency of hESC-EC differentiation by the 3D EB model is about 1%–3% [10], [12]. To increase endothelial differentiation efficiency and avoid contamination from mouse embryonic fibroblasts (MEFs), we developed a novel two-step differentiation process with serum free culture system. hESCs were first cultured in Petri dishes with differentiation medium for 12 days to induce spontaneous EB formation (**Figure 1A**). Whole-mount immunostaining confirmed that CD31^+^ and CD144^+^ cells were organized into channel-like structures within day-12 EBs (**Figure 1B**). These data demonstrate that some cells within EBs can spontaneously differentiate into endothelial cells that are then able to form blood vessel-like structures, confirming previous reports from our lab and others [8], [10], [12]. After step 1, EBs were embedded into collagen I and subcultured for an additional 3 days. We observed sprouting outgrowths from these EBs in collagen I that stained positive for CD31/CD144 (**Figure 1C**). CD31/CD144 expression increased swiftly to 10–15% after subculture in collagen as confirmed by FACS analysis, as compared to only 1–2% using the conventional EB culturing technique (**Figure 1D**). After sorting, CD31^+^/CD144^+^ cells were further expanded as a nearly pure population (98±3%) and these cells were used for subsequent experiments.

### Characterization of hESC-ECs

**Figure 2:**
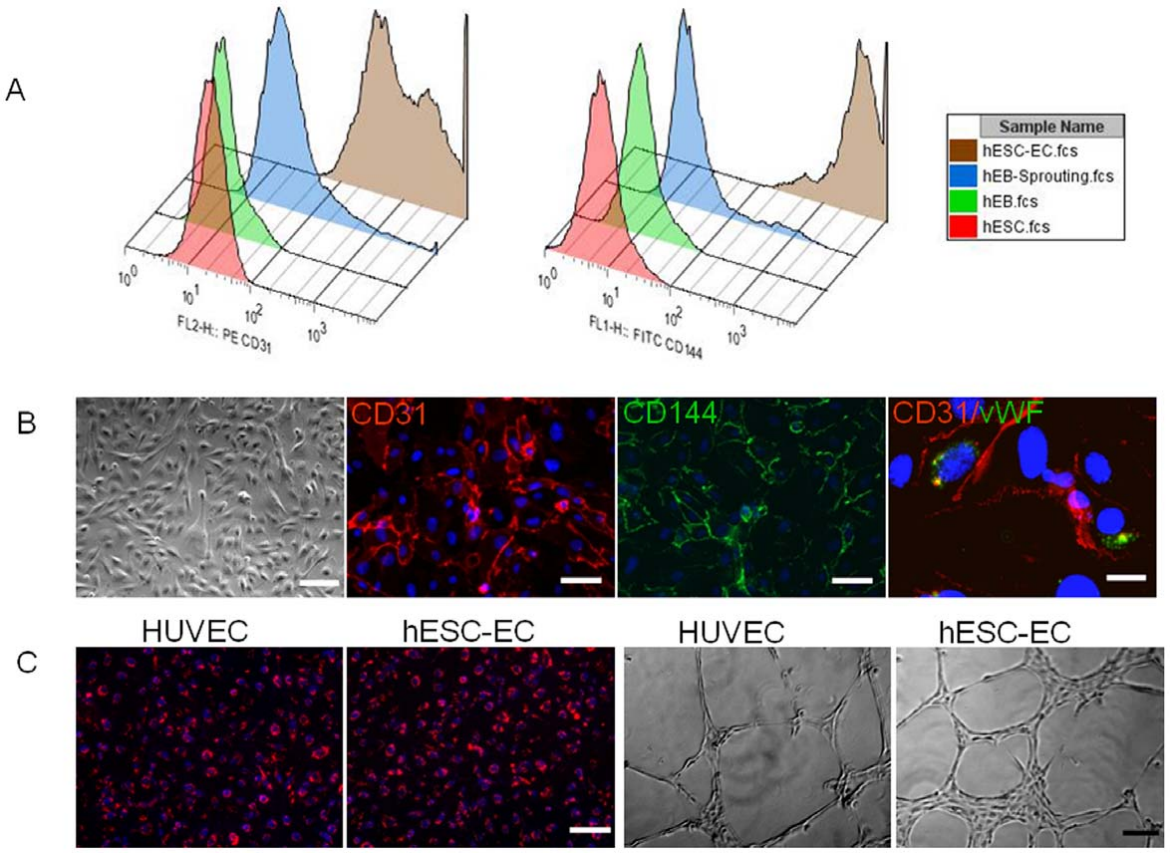
In vitro characterization of hESC-ECs. (A) During hESC differentiation and endothelial isolation, FACS analysis shows CD31 and CD144 increase significantly. (B) Morphogenesis shows the cells possess cobblestone-like morphology. Histology shows CD31 and CD144 are expressed on cell membranes, and vWF in the cytoplasm. Scale bars = 20 µm (left), 10 µm (middle two), 5 µm (right). (C) Similar to HUVECs, hESC-ECs also can uptake ac-DiI-LDL and form tube-like structures on Matrigel. Scale bars = 20 µm.

To determine whether hESC-ECs resemble HUVEC in endothelial markers and angiogenesis potential, we expanded FACS sorted CD31^+^/CD144^+^ cells on fibronectin-coated plates supplemented with endothelial cell medium EGM-2. For FACS analysis, we examined CD31, CD144, CD34, CD133, and Flk-1, which are known to be markers for endothelial differentiation of ES cells [10], [12]. As expected, CD31 and CD144 were upregulated during the two-step differentiation and expressed robustly after sorting (**Figure 2A**). In contrast, the embryonic marker Oct-4 was downregulated during hESC-EC differentiation (data not show). We also observed Flk-1 and CD133 were expressed at high level on undifferentiated hESCs (**Figure S1**), consistent with previous reports [10], [12]. hESC-ECs morphologically resembled HUVECs, which were uniformly flat, adherent, and cobblestone-like in appearance. When CD31^+^/CD144^+^ cells were cultured in endothelial growth medium, the majority of cells were adherent and expressed endothelial markers (CD31, CD144) at the endothelial cell adherent junctions as well as von Willebrand factor (vWF) located within the cytoplasm (**Figure 2B**). The CD31^+^/CD144^+^ cells also uptake DiI-acetylated low-density lipoprotein, and rapidly formed vascular network-like structures when placed on Matrigel (**Figure 2C**). Taken together, these data confirm that these differentiated cells were of endothelial lineage.

### Gene profiles of hESC-EC differentiation

**Figure 3:**
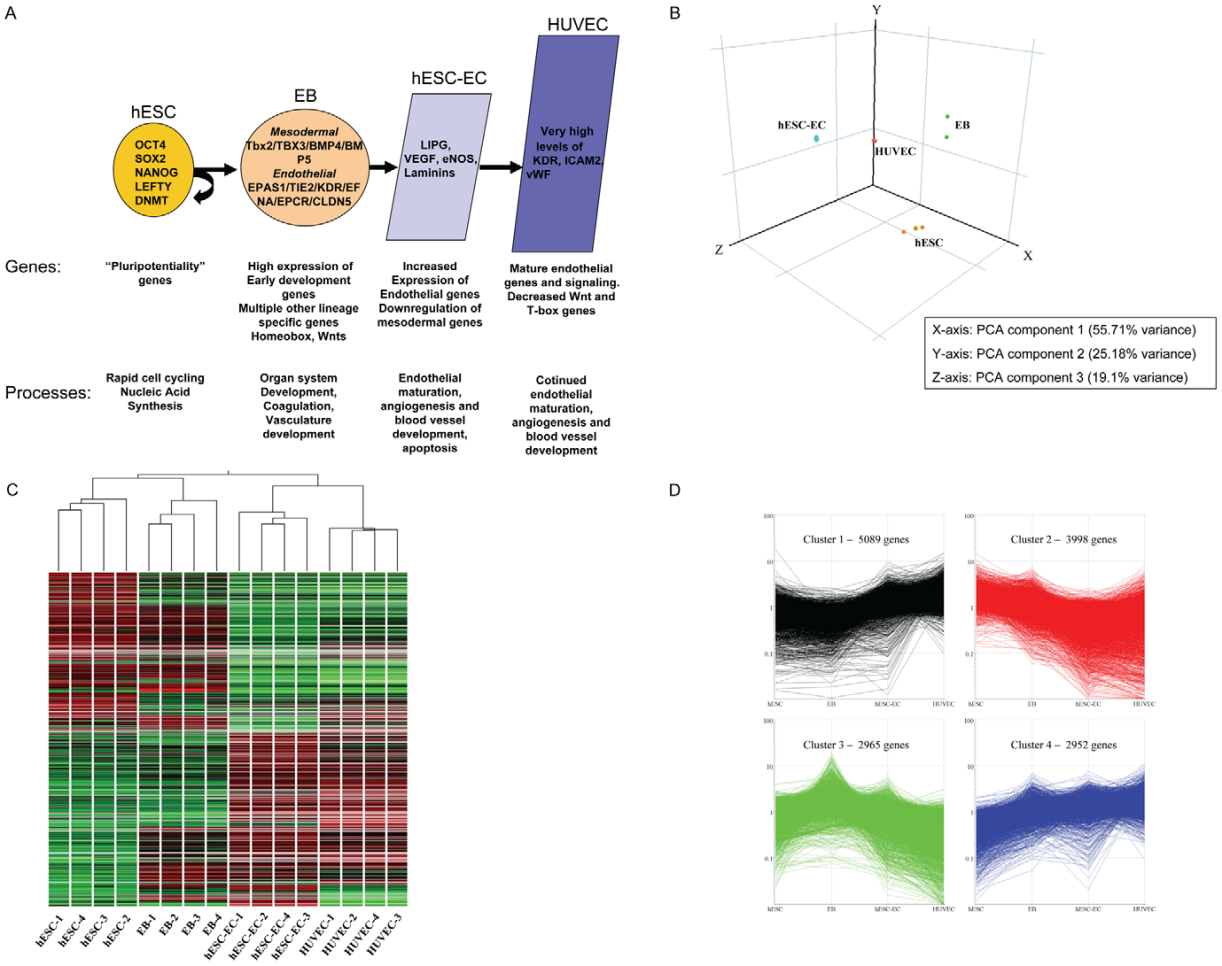
Major themes in gene expression profiles at each stage of differentiation. (A) hESCs express high levels of pluripotency-associated genes including Oct4, Sox2, NANOG, Lefty, and DNMT. At the EB stage, the cells express high levels of mesodermal master regulators such as Tbx2, and BMP4 as well as very enriched levels of endothelial specific master regulators including EPAS1, TIE2, KDR, and EFNA. This population also expresses genes from other cell layers, and many developmental genes from Wnt and homeobox families. hESC-ECs downregulate early mesodermal genes and express more endothelial specific genes, while HUVECs have the highest levels of mature endothelial gene expression with very few other developmental lineages represented. (B) Principal Components Analysis (PCA) shows that replicate experiments of each cell type are very similar while differentiation groups separate significantly along components 1 and 2. (C) Hierarchical Clustering Analysis - Cells from each developmental stage cluster relatively close to each other, with the most distance between hESCs and HUVECs. (D) K-means clustering analysis identifies major trends in gene expression across the time course.

In order to define at a molecular level the changes occurring at each stage of hESC differentiation into endothelial cells, we next performed transcriptional profiling using whole human genome microarrays on (i) undifferentiated hESCs, (ii) day-12 EBs, (iii) hESC-ECs after CD31/CD144 sort, and (iv) HUVECs as positive control (n = 4/group). The resulting data were analyzed using GeneSpring GX 7.3.1 to identify genes which had changed expression significantly between stages. A summary of our major findings is shown in **Figure 3A**. To obtain an overview of the transcriptional landscape, we looked at the data using principal components analysis (PCA), a dimensional reduction technique which identifies “principal components” or major trends in gene expression in the overall data (**Figure 3B**). PCA demonstrates that each of the four replicates from each stage has very similar transcriptional profiles to one another, but distinctly different between stages, as expected. A hierarchical clustering overview of the microarray experiments as a whole (**Figure 3C**) likewise shows that the overall gene expressions among replicates of each stage are very similar, with progressive differences between more distantly separated stages. Of particular interest is the close clustering of hESC-ECs and HUVECs, indicating a high degree of similarity between their respective expression profiles. Using K-means clustering analysis (**Figure 3D**), we can easily identify four major trends in the microarray data. Additional analysis and data can be found in **Supplemental Microarray Analysis S1 and Tables S1, S3, S3**, and **S4**.

### qPCR confirmation of microarray results and analysis of specific gene expression changes on sprouting EBs in collagen

**Figure 4:**
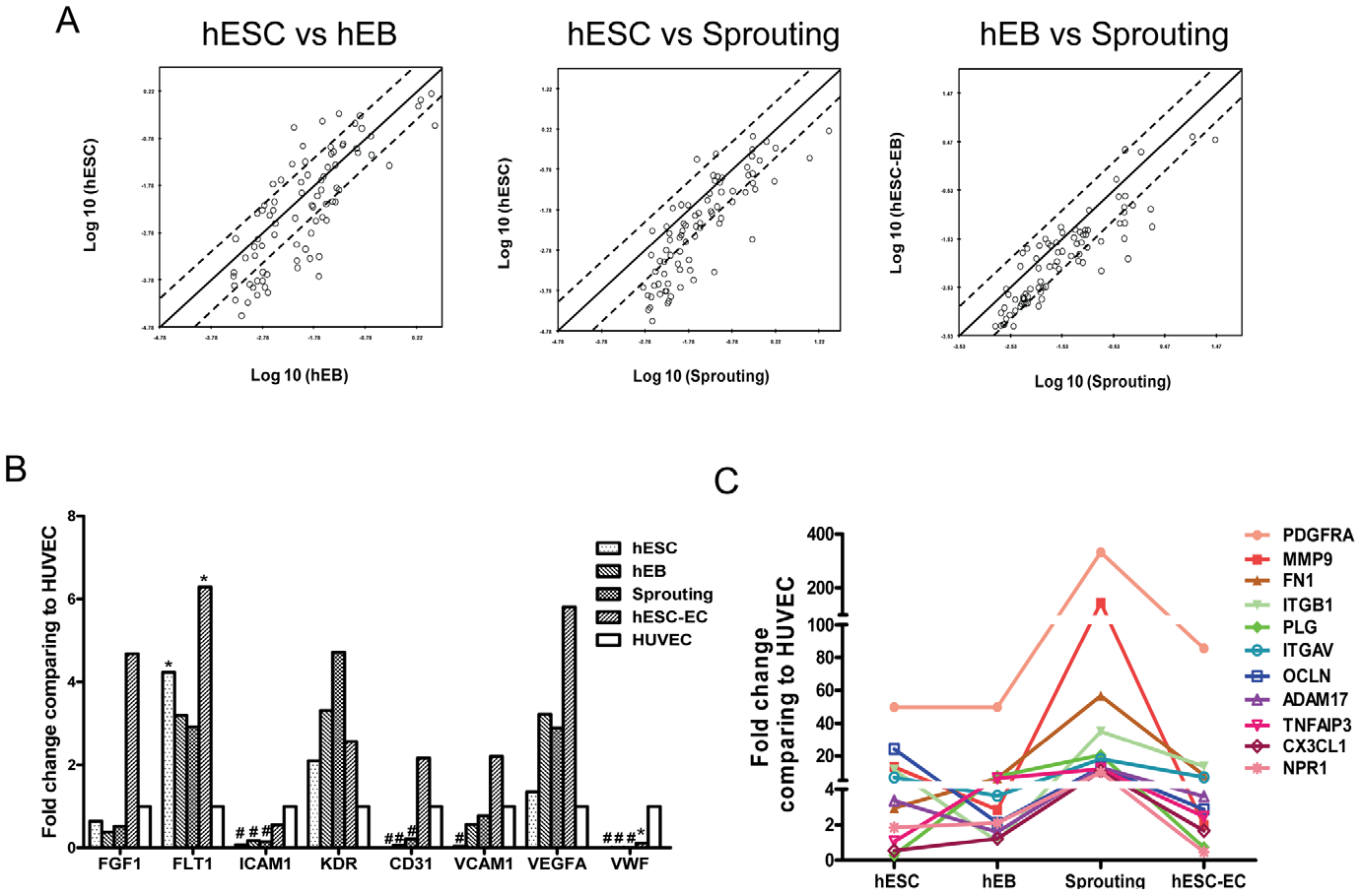
Quantitative PCR confirmation of endothelial gene expression in hESC, EB, EB sprouting, hESC-EC, and HUVEC. (A) Scatter plots of the endothelial related gene-expression were compared between hESC vs EB, hESC vs EB sprouting, and EB vs sprouting. Endothelial related genes expression was analyzed by Human Endothelial Cell Biology RT2 Profiler PCR Array. The array includes 84 genes related to endothelial cell biology. The lines indicate the diagonal and 4-fold changes between the two samples. (B) Kinetic expression of selected endothelial genes among hESC, hEB, hEB sprouting, hESC-EC, and HUVEC. Compared to HUVEC, hESC-ECs express abundant endothelial gene except adult endothelial gene vWF. *P<0.05; #*P*<0.01 compared with HUVECs. (C) After EB embedded into collagen, endothelial differentiation was triggered. Note endothelial activation related gene expression increased swiftly regardless of the level at the beginning.

To investigate changes in endothelial related genes during endothelial cell differentiation in collagen, we performed quantitative real-time PCR analysis using the Human Endothelial Cell Biology PCR array on undifferentiated hESCs, EBs, EB sprouting, hESC-ECs, and HUVEC as a positive control. Analysis of scatter plot demonstrated 28 genes were upregulated when day-12 EBs were embedded into collagen type I for an additional 3 days (**Figure 4A**). Analysis of scatter plots also revealed that 30 genes were upregulated and 8 genes downregulated after undifferentiated hESCs differentiated into EBs, and 33 genes upregulated and 2 genes downregulated after hESCs differentiated into EB sprouting. Quantitative analysis showed after endothelial induction, ICAM1, CD31, VCAM1, and vWF were significantly upregulated, while FGF1, Flt1, KDR and VEGFA were expressed in undifferentiated cells and upregulated with endothelial differentiation of hESCs (**Figure 4B**), which were also consistent with the microarray data. To explore the mechanism of promotion of endothelial differentiation in collagen, quantitative analysis was carried out and showed that endothelial cell activation genes were upregulated swiftly and endothelial differentiation was triggered after hEBs were embedded into collagen (**Figure 4C**).

### In vivo vasculogenic potential of hESC-ECs

**Figure 5:**
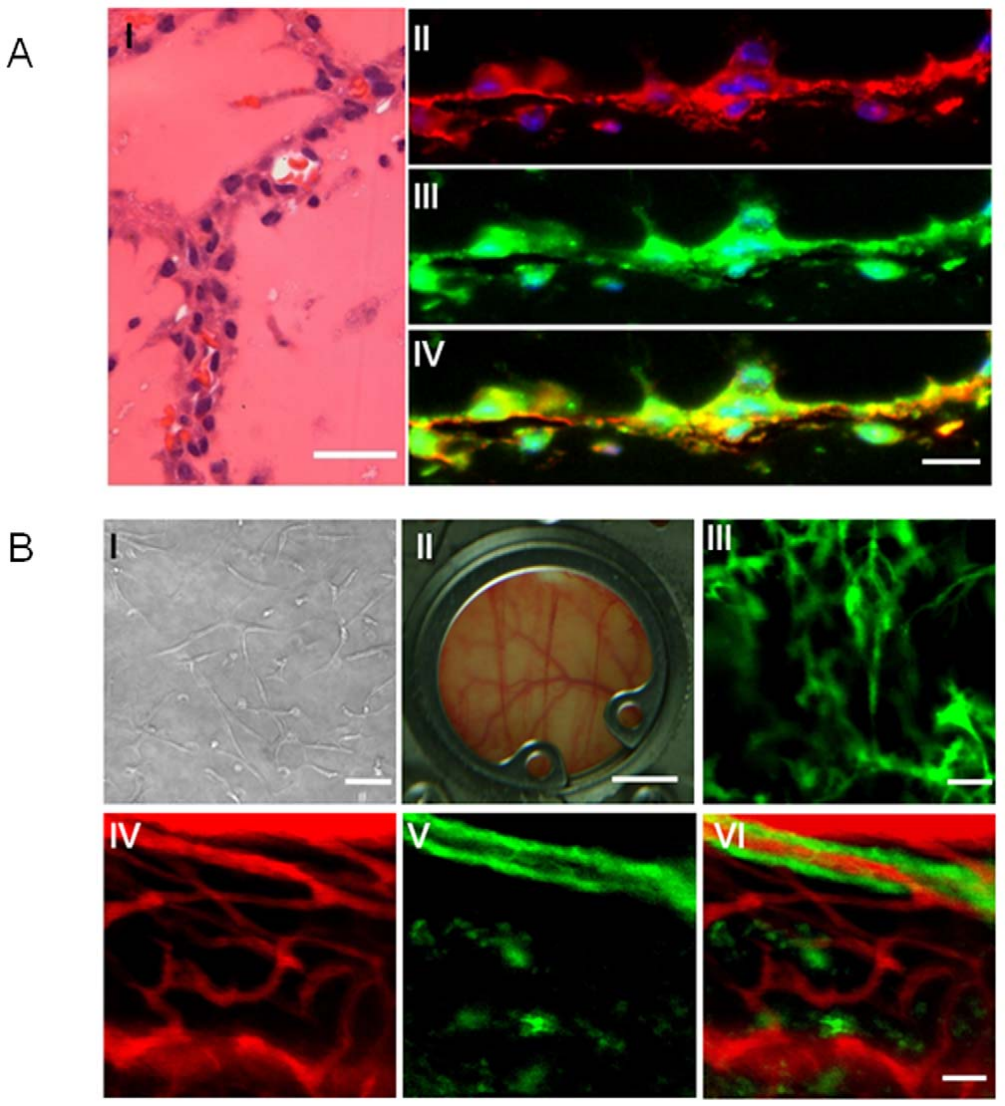
Demonstration of functional vessels in vivo using Matrigel plug and dorsal window chamber. (A) Matrigel plug with hESC-ECs were implanted subcutaneous injection in the dorsal region of 8-week-old SCID mice. (I) HE stain of Matrigel plug. Some of these microvessels have mouse blood cells in their lumen. (II–IV) 14-day Matrigel plugs were stained with anti-human CD31 (red) and anti-GFP (green) antibodies, showing microvessels that are immunoreactive with these human-specific antibodies. Scale bars = 20 µm. (B) Dorsal window chamber model in SCID mice. GFP+ hESC-ECs were cultured in a mix of collagen and Matrigel for 1 day (left, upper panel), and implanted into dorsal windows in SCID mice (middle, upper panel). Images were taken at day 2, 14 and 21 after implantation. After 14 days, Angiosense 680 was injected by tail vein to highlight perfused vessels within the dorsal window. Green, hESC-ECs expressing GFP; red, functional blood vessels with contrast enhanced by Angiosense 680. Scale bars = 20 mm (middle, upper panel), 20 µm (others).

To evaluate whether hESC-ECs are capable of forming functional blood vessels *in vivo*, we used Matrigel plug and tissue-engineered vessel models in immunodeficient SCID mice that allow dynamic and long-term observation of blood vessels derived from implanted cells [8], [17]. For Matrigel plug assay, two weeks after subcutaneous injection, Matrigel plugs were harvested and histology performed. Hematoxylin and eosin (H&E) staining showed microvessels that contained murine blood cells in the vessel lumens. Harvested Matrigel plugs were also stained with anti-human CD31 and anti-GFP antibodies (**Figure 5A**). For tissue-engineered vessel formation assay, hESC-ECs were cultured in collagen and Matrigel for one day and tube-like structures can be formed (**Figure 5B–I**). After the collagen and Matrigel patch was implanted into mouse dorsal windows (**Figure 5B–II**), cord-like networks formation by the GFP-labeled hESC-ECs can be observed by intravital microscope (**Figure 5B–III**). To test whether the engineered vascular networks integrated with the host murine circulatory system, we next injected Angiosense 680 intravenously to enhance the contrast of perfused vessels. Importantly, several of the hESC-EC derived vessels formed conduits that contained blood flow from day 12 to day 60 (**Figure 5B** **, III–VI**). Importantly, these data suggest that transplanted hESC-ECs have angiogenic potential and are able to integrate into the host vasculature *without* co-transplantation of supporting fibroblasts (**Video S1**).

### Assessment of left ventricular function following hESC-EC transplantation

Previous studies have demonstrated that hESC-derived cardiomyocyte transplantation can improve cardiac function after myocardial infarction [24], [25]. To understand the therapeutic potential of hESC-ECs for treatment of ischemia heart disease, we subjected adult SCID mice to LAD ischemia-reperfusion followed by injection with either 1×10^6^ hESC-ECs (n = 28) or PBS (n = 15). For sham operated animals (n = 5), open thoracotomy was performed without LAD ligation and without injection of hESC-ECs or PBS. Echocardiograms were performed at baseline, day 2, day 14, day 28 and day 56 post transplantation (**Figure S2**). At day 2 following infarction, there was a significant decrease in the fractional shortening (FS) in both the hESC-EC treated group and control group, consistent with successful induction of myocardial infarction and subsequent loss of cardiac contractility. By day 14, we observed a significant improvement in the fractional shortening for the hESC-EC treated group compared to control group (27.8±3.0 vs. 23.9±2.0; *P*<0.05). However, by day 56, there was no significant difference between the treatment group and control group (31.4±5.6 vs. 29.8±3.7; *P* = 0.26).

### Assessment of cardiac viability and contractility following hESC-EC therapy

Metabolic activity and deficits in the heart can be detected using the [^18^F]-FDG radiotracer with a microPET system [22]. This technique can be used to accurately detect changes in cardiac viability following infarctions in small animal models [26]. Therefore, we performed [^18^F]-FDG PET scans at baseline, day 2, day 14, day 28, and day 56 post infarction for hESC-EC treated and control animals. We observed a significant reduction in cardiac [^18^F]-FDG uptake for both groups at day 2, confirming successful myocardial infarction. At day 14, there was a significant increase in cardiac viability (indicated by increased [^18^F]-FDG uptake) in the hESC-EC treated group compared to controls (26.3±1.8 vs. 22.4±3.4; *P*<0.05). However, by day 28 to day 56 there was no significant difference between the treatment group and control (**Figure S3A–B**). Analysis of the [^18^F]-FDG PET images using polar-map reconstruction revealed reduced infarction size on the hESC-EC transplantation group compared to the PBS group at day 14, but there was no significant difference at subsequent time points (**Figure S3C–D**). Overall, the trends for changes in functional contractility and metabolic activity were very similar based on the echocardiography and microPET imaging results, respectively. These findings were further corroborated using pressure volume (PV) loops at week 8, which showed a decrease in ejection fraction for both hESC-EC treated and PBS control groups compared to sham non-infarcted group as expected. However, no significant difference was seen between hESC-EC treated and PBS control groups at week 8 (**Figure S4**). This was associated with higher left ventricular end diastolic pressure (EDP) and left ventricular end diastolic volume (EDV) in both groups compared to sham (n = 5). Interestingly, EDV is lower in the hESC-EC group (n = 8) compared to PBS group (n = 6) (*P* = 0.03), indicating that hESC-EC transplantation may have exerted some benefit on cardiac diastolic function. Finally, as highlighted in **Table S5**, there was no significant difference in other functional parameters (e.g., cardiac output and stroke volume) between hESC-EC treated mice and PBS control mice.

### Correlation of hESC-EC survival and cardiac recovery

**Figure 6:**
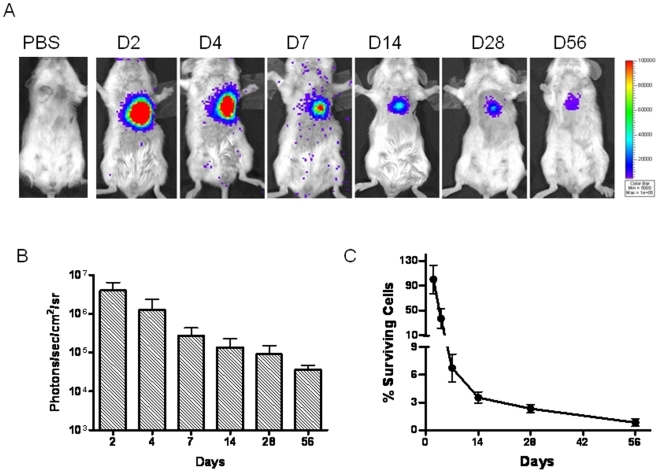
Molecular imaging of hESC-EC fate after transplantation. (A) A representative animal injected with 1×10^6^ hESC-ECs shows significant bioluminescence activity at day 2, which decreases progressively over the following 8 weeks. A representative control animal injected with PBS shows no imaging signals as expected. (B) Detailed quantitative analysis of signals from all animals (n = 28) transplanted with hESC-ECs (signal activity is expressed as photons/sec/cm^2^/sr). (C) Donor cell survival plotted as % signal activity from day 2 to week 8.

In order to better understand the mechanism for lack of long-term efficacy, we next evaluated the survival of hESC-ECs using non-invasive bioluminescence imaging. Injection of hESC-ECs into infarcted myocardium resulted in a robust bioluminescence signal at day 2. However, serial imaging of the same animals out to 8 weeks demonstrated a significant decay in bioluminescence, suggesting acute donor cell death. When normalized to the signal on day 2 after injection, the percent bioluminescence signals were 36.8±15.5 at day 4, 6.7±1.5 at day 7, 3.5±0.6 at day 14, 2.3±0.5 at day 28, and 0.8±0.4 at day 56 (**Figure 6**). Thus, we believe less than 1% of transplanted hESC-ECs survived beyond 8 weeks.

### Histological assessment of infarct size and hESC-EC engraftment

**Figure 7:**
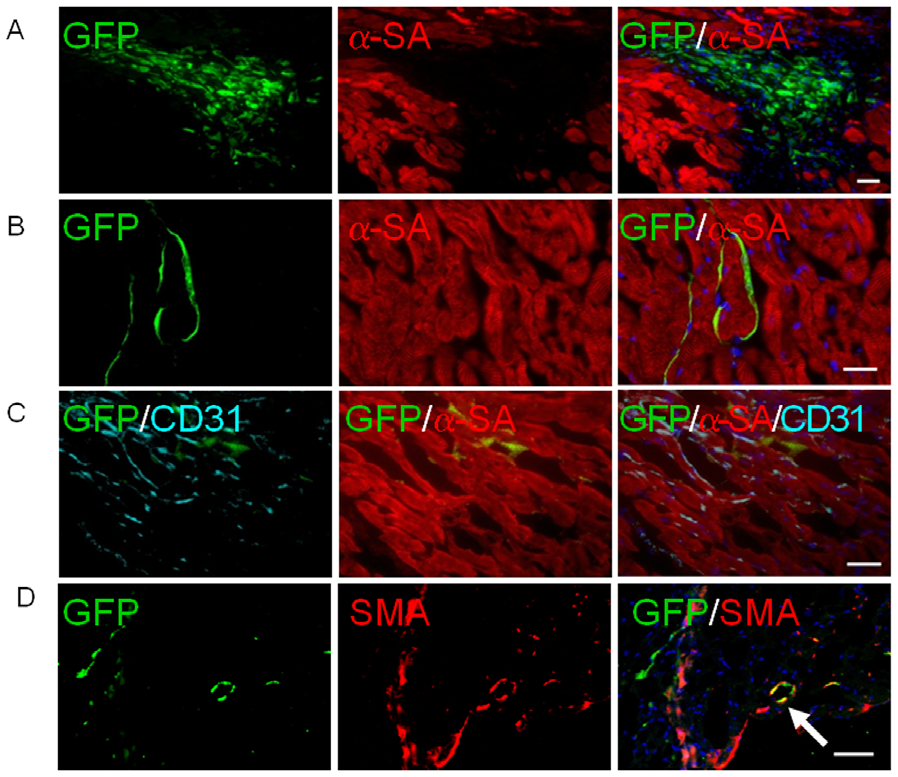
Confirmation of engrafted hESC-ECs by immunofluorescence. (A) Fate of hESC-ECs within the recipient myocardium 4 days after injection showed clump formation. (B, C, D) At day 28, transplanted hESC-ECs differentiate into vasculature and smooth muscle cells (arrow), and integrate with host myocardium as confirmed by GFP, mouse CD31, α-sarcomeric actin (α-SA) and smooth muscle actin (SMA) co-staining. However, this population became significantly rare compared to day 4 due to donor cell death. Scale bar = 20 µm.

Histological analysis of the myocardium was performed by examining thin sections of the gross specimen and via immunofluorescent microscopic examination. Microscopic examination showed the presence of GFP^+^ hESC-ECs within myocardium. At day 4, clumps of hESC-ECs can be found in the interstitial spaces between cardiomyocytes, as seen with α-sarcomeric actin (α-SA) and GFP double staining (**Figure 7A**). At day 28, GFP, α-SA, and mouse-specific CD31 triple staining revealed that some of the hESC-ECs had formed microvessel structures, integrated with host vasculature, and differentiated into smooth muscle but not cardiac cells (**Figure 7B–D**). However, the overall frequency of GFP^+^ cells was significantly decreased at day 28 (**Figure 7B–C**), which is consistent with the decrease in bioluminescence signals measured over this same period of time and likely due to massive cell death. Moreover, immunostaining with mouse CD31 and α-SA at day 4 showed no host vasculature extension into the hESC-EC clumps (**Figure S5**), which suggests no direct nutrient transport to transplanted cells other than diffusion. Finally, examination of the explanted hearts showed a downward trend in infarct size and an upward trend in microvascular density (MVD) in the hESC-EC treated group, though these differences with controls were not statistically significant (**Figure S6**).

## Discussion

Therapeutic angiogenesis/vasculogenesis is promising for ischemia diseases. Due to the limited source of endothelial progenitor cells, hESCs represent a new and exciting avenue of angiogenic therapy. However, traditional spontaneous 3D method by EB differentiation of hESCs into endothelial cells is inefficient and the 2D method by co-culture with animal materials such as MEFs will limit its future clinic usage [6], [8], [9], [10], [11], [12]. Therefore, we sought to develop an extracellular matrix (ECM) culture system for increasing endothelial differentiation of hESCs and ensuring animal product free cells. In the past decade, there has been an increasing consensus that the ECM contains critical signals that regulate endothelial differentiation and blood vessel formation during development and disease progression [27], [28], [29], [30], [31], [32].

Here we describe a two-step differentiation procedure that takes advantage of collagen I to efficiently promote differentiation of hESCs into endothelial lineage cells. Compared to previous 3D EB methods [10], [12], this new protocol is more efficient for deriving hESC-ECs, and may be utilized to generate large numbers of endothelial cells for therapeutic application. Our qRT-PCR data confirmed that angiogenesis from EB sprouting in collagen triggered release of gelatinase, matrix metalloproteinases (MMPs), tissue inhibitors of MMP (TIMP), and upregulated endothelial progenitor markers. We believe changes in endothelial gene expression and signaling lead to events that not only stimulate vascular morphogenesis but also promote endothelial differentiation. Global gene expression profiling of hESCs during directed endothelial differentiation revealed significant enrichment of a number of endothelial genes and appropriate biological processes that correspond to each differentiation stage. Results were confirmed using qPCR analysis. Though hESC-ECs and HUVECs revealed a high degree of similarity between their respective expression profiles, hESC-ECs do not resemble HUVEC completely. Previous reports have shown that hemodynamic forces such as fluid shear stress can affect gene transcription *in vivo* and *in vitro* [33], [34]. This suggests that shear stress on HUVEC may contribute to the discrepancy of expression profiles between HUVEC and hESC-ECs (which have not been exposed to hemodynamic forces during the 3-week differentiation process). Thus, to generate more functional and mature endothelial cells, shear stress conditioning of hESC-ECs may be needed in the future. Importantly, our microarray analysis provides a greater understanding of the patterns of activation of specific genes during endothelial differentiation, and identifies novel gene targets that may be used to enhance endothelial differentiation in the future.

To understand whether hESC-ECs also possess vasculogenic ability *in vivo*, we next evaluated the *in vivo* vascular formation potential of hESC-ECs after transplantation in mouse dorsal window chamber model. Previous data showed that supporting fibroblast cells were needed to stabilize engineered blood vessels [8], [35]. However, there are obvious drawbacks in using mouse support cells in the study of vessel maturation of human cell. Here we introduced collagen type I and Matrigel, without support cells, and showed hESC-ECs can form functional vasculature and are stable up to 2 months post transplantation. At present, we speculate that Matrigel, which is abundant in growth factor and extracellular matrix other than collagen I, may help stabilize the newly formed blood vessels.

An important observation in the present study is that the engraftment of hESC-ECs can improve cardiac function short-term. In recent years, several animal studies have shown that transplantation of EPCs isolated from the peripheral blood or bone marrow can improve cardiac function [36], [37], [38], [39]. In this study, we have shown that transplantation of hESC-ECs can also result in significant improvements in cardiac function at week 2. However, this was not sustained at a significant level beyond 4 weeks. We believe this is likely due to massive cell death within the first few weeks of transplantation, as indicated by our serial bioluminescence imaging studies to assess longitudinal cell fate. These findings indicate that other mechanisms such as activation of paracrine pathways may play an important role on cardiac performance amelioration at week 2 [1], but additional studies will be needed in the future to test this hypothesis. Indeed, one can speculate that the acute donor cell death phenomenon may also explain the lack of long-term functional benefits in several of the clinical trials completed so far [40]. The reasons for acute donor cell death are likely multi-factorial, and may involve immunogenicity due to xenotransplantation of human cells (note SCID mice still have natural killer cells), as well as the lack of regional nutrients and signaling factors for maintenance of cell viability. Following direct intramyocardial injection, the transplanted cells formed clumps as confirmed by histology. This is in contrast to the Matrigel plug and dorsal skin fold chamber data shown in Figure 5, which however is a static and artificial environment loaded with collagen and Matrigel, and does not reflect the stress imposed upon hESC-ECs within a fast-beating ischemic heart. Without adequate host blood supply, nutrient transportation to the hESC-ECs is only by diffusion and is likely inadequate. The survival of transplanted cells could further be exacerbated in the context of myocardial infarction. Altogether, these factors contributed to the loss of bioluminescence imaging signals over time and the lack of long-term benefit in cardiac function, which is also consistent with recent findings based on transplantation of hESC-derived cardiomyocytes [25], [41]. Because of these obstacles, the development of a strategy to alleviate apoptotic cell death may be of primary importance for hESC-ECs therapy. Recent work on hESC-derived cardiomyocyte therapy revealed that Matrigel combined with pro-survival growth factor cocktail resulted in reliable formation of substantial myocardial grafts and significant functional improvement [25]. Thus, tissue engineering techniques, rather than direct stem cell transplantation, may prove to be a more viable approach in the future [42].

In summary, our results show that (***i***) endothelial differentiation efficiency was increased by a two-step differentiation of hESCs and with animal product free procedure by using serum free culture medium, (***ii***) hESC-ECs possess functional vasculogenic ability *in vivo*, and (***iii***) transplantation of hESC-ECs can significantly improve short-term cardiac function at 2 weeks. However, the numbers of surviving hESC-ECs in infarcted tissue decreased significantly after the first few weeks, demonstrating the requirement for close long-term follow-up in all cardiac cell transplantation studies. These data suggest that prolonged heart function recovery may require more permanent graft survival of transplanted cells. Alternative transplantation protocols with larger numbers of hESC-ECs, multiple injection time points, or addition of matrix or prosurvival factors to prevent donor cell death after transplantation could eventually lead to the realization of sustained enhancement of heart function post myocardial infarction.

## Supporting Information

Figure S1 Flow cytometric analysis of endothelial differentiation of hESCs. Single representative sample of triplicates are shown here. HUVECs were used as positive control. Isotype-matched antibodies were used in flow cytometry for background fluorescence.
(0.22 MB TIF)

Figure S2 Echocardiographic evaluation of cardiac contractility. (A) Representative M-mode echocardiographic data of infarcted hearts receiving PBS (n = 15) vs. hESC-ECs (n = 28) at baseline, day 14, and day 56. (B) Comparison of fractional shortening (FS) between the two groups 7 days before (baseline), 2 days, 14 days, 28 days, and 56 days after infarction. *P<0.05 vs. PBS group at day 14.
(0.30 MB TIF)

Figure S3 [18F]-FDG PET imaging of cardiac viability. (A) Representative image in a normal mouse heart and at day 14 and day 56 in infarcted hearts receiving PBS vs. hESC-ECs. (B) Detailed quantitative analysis of signals from all animals transplanted with hESC-ECs and PBS group. *P<0.05 vs. PBS group at day 14. (C, D) Representative polar map of the microPET images obtained from mice treated with PBS vs. hESC-ECs. Measurements are based on 50% thresholds. *P<0.05 vs. PBS group at day 14.
(0.28 MB TIF)

Figure S4 Functional evaluation of transplanted hESC-EC. (A) Representative pressure volume (P–V) loops measured from sham (n = 5), hESC-EC (n = 8), or PBS (n = 6) treated at day 56. Curvilinear end-systolic P–V relations in hESC-ECs treated mice were shifted to the left, indicating enhanced contractility. (B–D) Invasive hemodynamic assessment of ejection fraction (EF), end-systolic pressure (ESP), end-diastolic pressure (EDP), end-systolic volume (ESV), end-diastolic volume (EDV) in the 3 groups of mice at day 56. *P<0.05; #*P*<0.01 compared with sham; &*P*<0.05 compared with PBS group.
(0.12 MB TIF)

Figure S5 Confirmation of engrafted hESC-ECs by immunostaining. Clump formation of injected hESC-ECs was observed and immunostaining with mouse CD31 (red) and α-sarcomeric actin (green) at day 4 demonstrated no extension of host vasculature into injected cell clumps. Scale bar = 20 µm.
(1.29 MB TIF)

Figure S6 Functional evaluation of transplanted hESC-EC. (A–B) Representative Masson's trichrome stain of hearts injected with hESC-ECs versus PBS. There is no significant difference of the area of fibrosis between hESC-EC and PBS groups. *P = 0.14 vs. PBS group. Scale bar = 500 µm. (C–D) Quantitative analysis of capillary density also showed no significantly difference in both groups. Nuclear staining is identified by DAPI (blue). Data are expressed as mean±SEM. *P = 0.21 vs. PBS group. Scale bar = 10 µm.
(0.40 MB TIF)

Table S1 Significant gene lists from microarray data.
(6.51 MB PDF)

Table S2 Over-represented gene ontology (GO) terms in significant gene lists from microarray data.
(0.90 MB PDF)

Table S3 K-means clustering significant gene lists.
(4.69 MB PDF)

Table S4 Over-represented GO terms in the K-means clustering significant gene lists.
(1.25 MB PDF)

Table S5 Values are means Â± SD. MI, myocardial infarction; P–V, pressure-volume; HR, heart rate; ESP, end-systolic pressure; EDP, end-diastolic pressure; ESV, end-systolic volume; EDV, end-diastolic volume; SV, stroke volume; SW, stroke work; CO, cardiac output; dP/dtmax, maximum first derivative of change in pressure rise with respect to time; dP/dtmin, maximum first derivative of change in pressure fall with respect to time; Tau-Glantz, time constant of fall in ventricular pressure by Glantz method; PAMP, preload-adjusted maximal power; Ea, arterial elastance. *P<0.05 versus sham; #*P*<0.01 versus sham; *P*>0.05, PBS versus hESC-EC group.
(0.06 MB TIF)

Supplemental Microarray Analysis S1 (0.15 MB DOC)

Video S1 Blood flow in hESC-ECs engineered vessels.
(0.21 MB MOV)
