# Supplementary figures and images for: Functional and Transcriptional Characterization of Human Embryonic Stem Cell-Derived Endothelial Cells for Treatment of Myocardial Infarction
Source: PLoS One. 2009 Dec 31;4(12):e8443. doi: 10.1371/journal.pone.0008443 (PMC2795856; doi:10.1371/journal.pone.0008443)

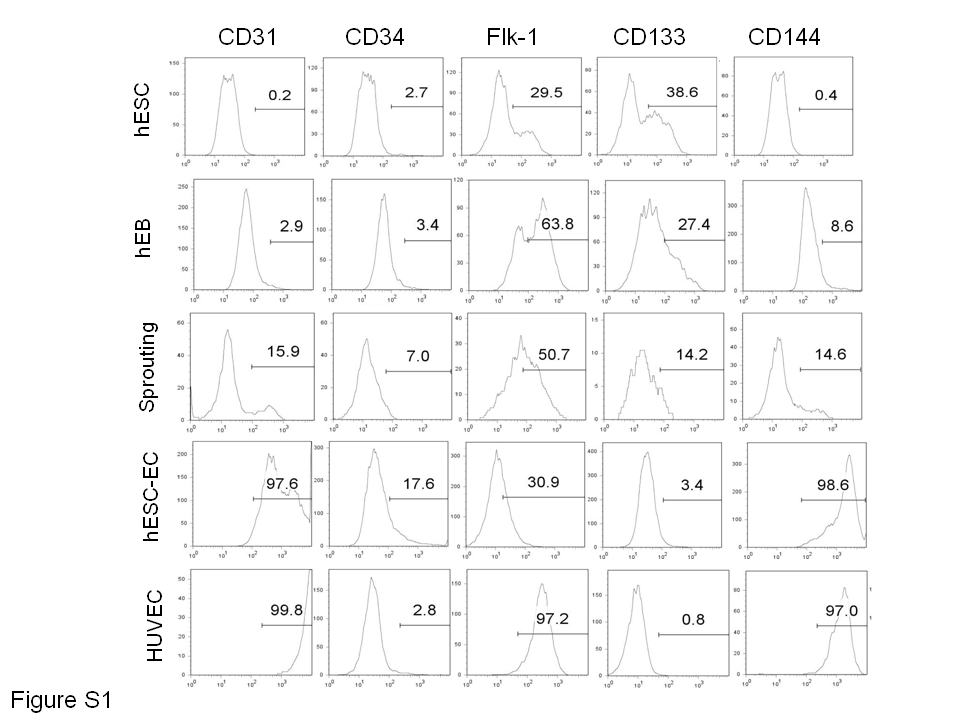

Supplement: Figure S1 — Flow cytometric analysis of endothelial differentiation of hESCs. Single representative sample of triplicates are shown here. HUVECs were used as positive control. Isotype-matched antibodies were used in flow cytometry for background fluorescence. (0.22 MB TIF) [file pone.0008443.s001.tif]

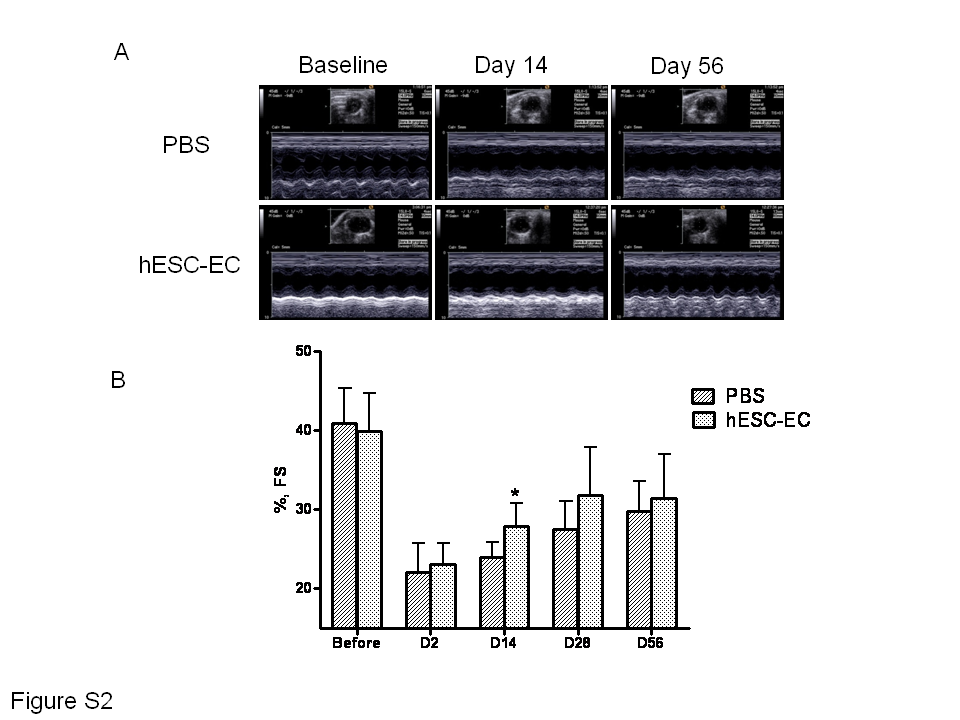

Supplement: Figure S2 — Echocardiographic evaluation of cardiac contractility. (A) Representative M-mode echocardiographic data of infarcted hearts receiving PBS (n = 15) vs. hESC-ECs (n = 28) at baseline, day 14, and day 56. (B) Comparison of fractional shortening (FS) between the two groups 7 days before (baseline), 2 days, 14 days, 28 days, and 56 days after infarction. *P<0.05 vs. PBS group at day 14. (0.30 MB TIF) [file pone.0008443.s002.tif]

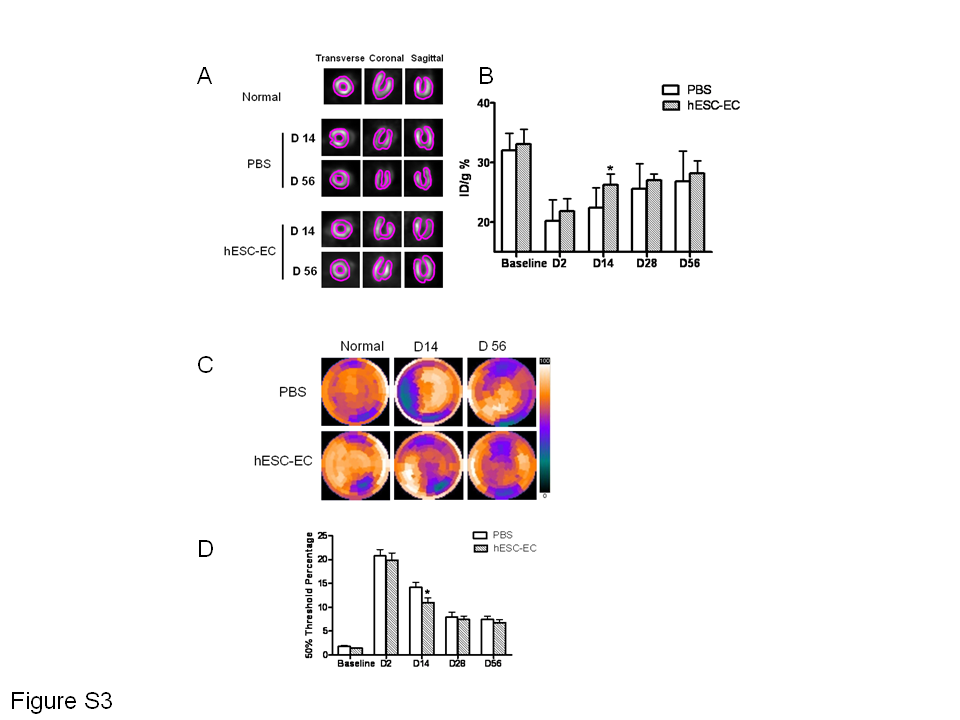

Supplement: Figure S3 — [18F]-FDG PET imaging of cardiac viability. (A) Representative image in a normal mouse heart and at day 14 and day 56 in infarcted hearts receiving PBS vs. hESC-ECs. (B) Detailed quantitative analysis of signals from all animals transplanted with hESC-ECs and PBS group. *P<0.05 vs. PBS group at day 14. (C, D) Representative polar map of the microPET images obtained from mice treated with PBS vs. hESC-ECs. Measurements are based on 50% thresholds. *P<0.05 vs. PBS group at day 14. (0.28 MB TIF) [file pone.0008443.s003.tif]

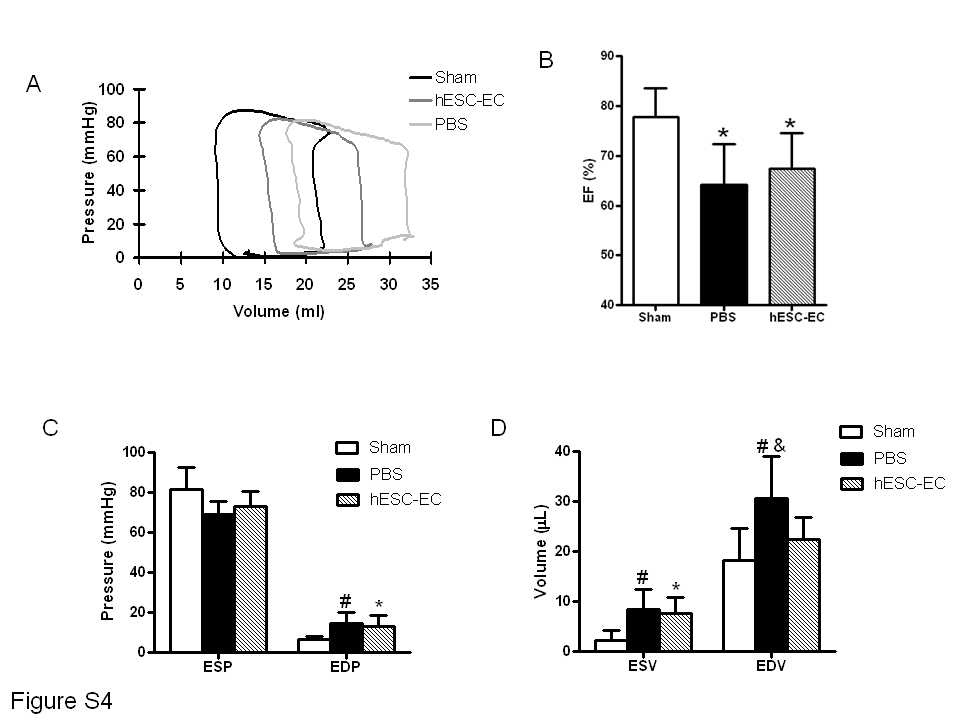

Supplement: Figure S4 — Functional evaluation of transplanted hESC-EC. (A) Representative pressure volume (P–V) loops measured from sham (n = 5), hESC-EC (n = 8), or PBS (n = 6) treated at day 56. Curvilinear end-systolic P–V relations in hESC-ECs treated mice were shifted to the left, indicating enhanced contractility. (B–D) Invasive hemodynamic assessment of ejection fraction (EF), end-systolic pressure (ESP), end-diastolic pressure (EDP), end-systolic volume (ESV), end-diastolic volume (EDV) in the 3 groups of mice at day 56. *P<0.05; #P<0.01 compared with sham; &P<0.05 compared with PBS group. (0.12 MB TIF) [file pone.0008443.s004.tif]

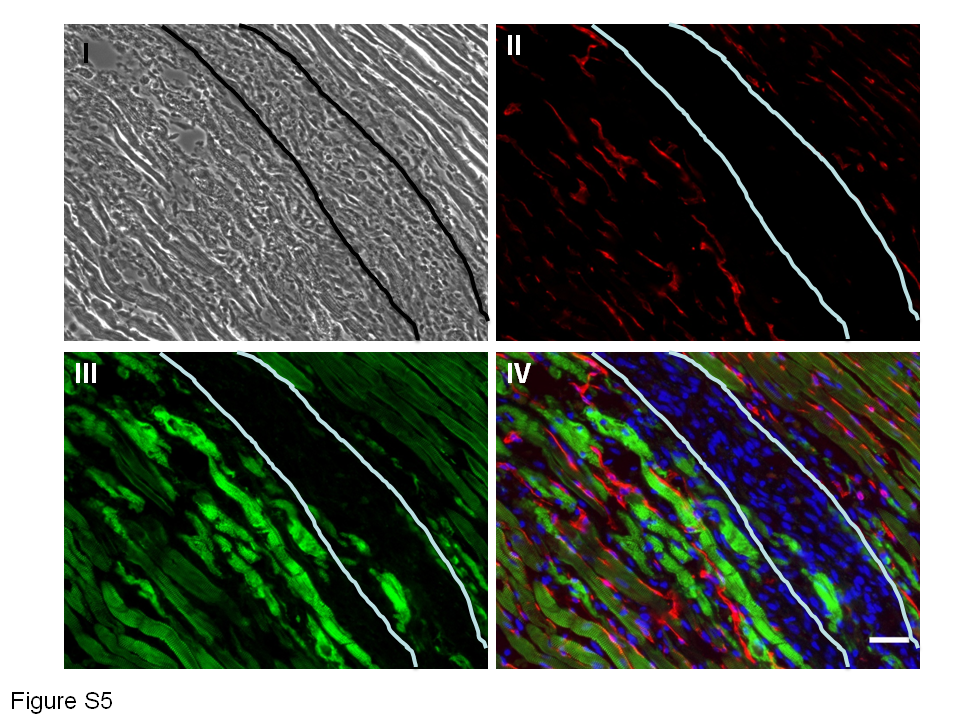

Supplement: Figure S5 — Confirmation of engrafted hESC-ECs by immunostaining. Clump formation of injected hESC-ECs was observed and immunostaining with mouse CD31 (red) and α-sarcomeric actin (green) at day 4 demonstrated no extension of host vasculature into injected cell clumps. Scale bar = 20 µm. (1.29 MB TIF) [file pone.0008443.s005.tif]

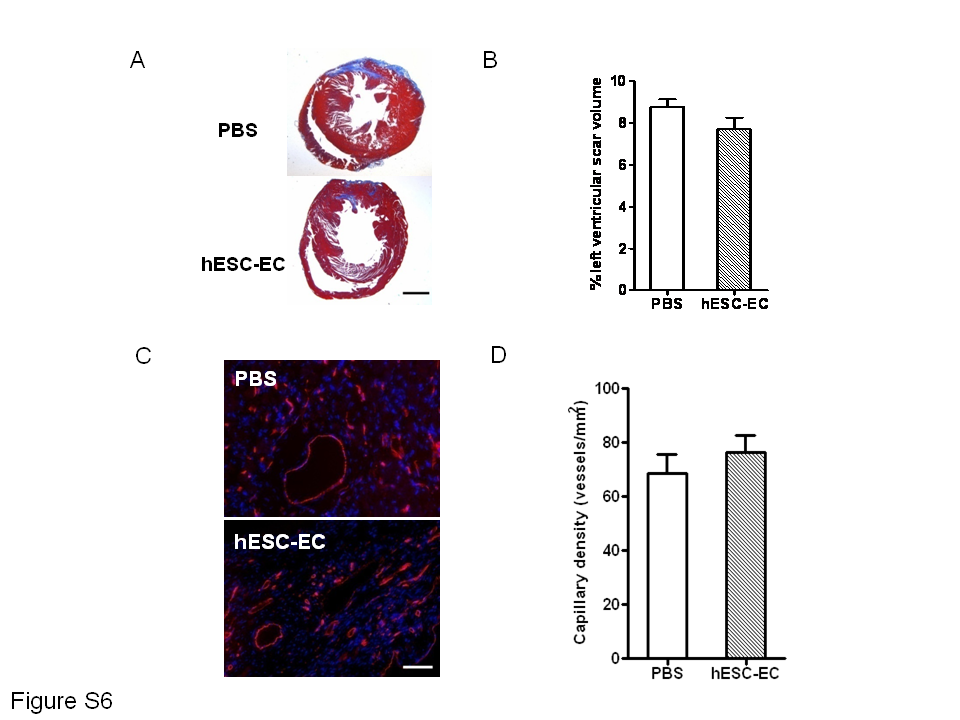

Supplement: Figure S6 — Functional evaluation of transplanted hESC-EC. (A–B) Representative Masson's trichrome stain of hearts injected with hESC-ECs versus PBS. There is no significant difference of the area of fibrosis between hESC-EC and PBS groups. *P = 0.14 vs. PBS group. Scale bar = 500 µm. (C–D) Quantitative analysis of capillary density also showed no significantly difference in both groups. Nuclear staining is identified by DAPI (blue). Data are expressed as mean±SEM. *P = 0.21 vs. PBS group. Scale bar = 10 µm. (0.40 MB TIF) [file pone.0008443.s006.tif]

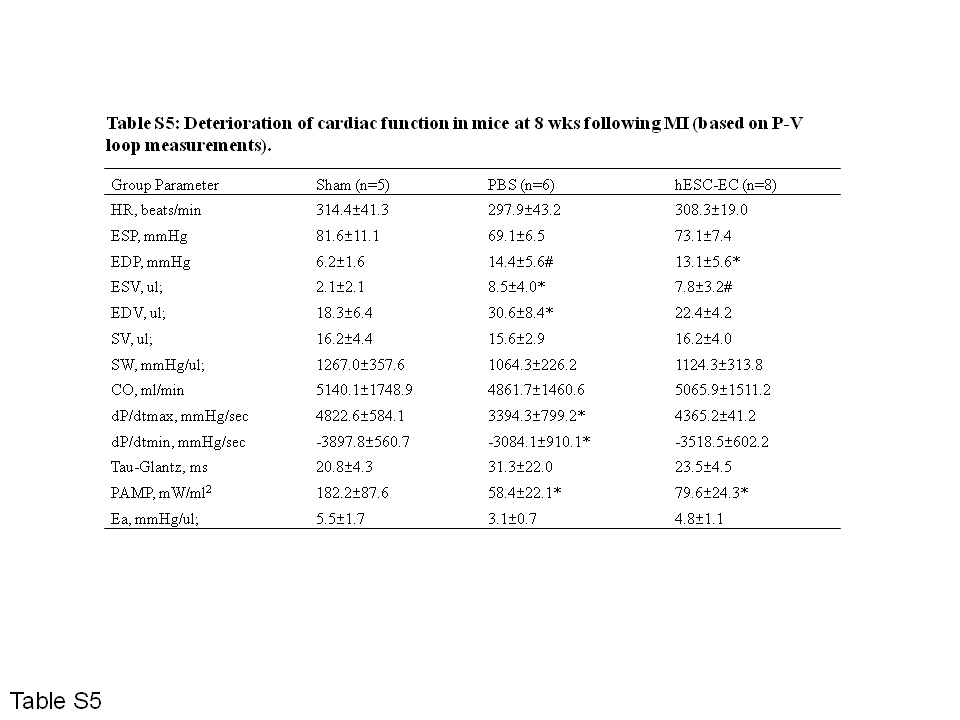

Supplement: Table S5 — Values are means Â± SD. MI, myocardial infarction; P–V, pressure-volume; HR, heart rate; ESP, end-systolic pressure; EDP, end-diastolic pressure; ESV, end-systolic volume; EDV, end-diastolic volume; SV, stroke volume; SW, stroke work; CO, cardiac output; dP/dtmax, maximum first derivative of change in pressure rise with respect to time; dP/dtmin, maximum first derivative of change in pressure fall with respect to time; Tau-Glantz, time constant of fall in ventricular pressure by Glantz method; PAMP, preload-adjusted maximal power; Ea, arterial elastance. *P<0.05 versus sham; #P<0.01 versus sham; P>0.05, PBS versus hESC-EC group. (0.06 MB TIF) [file pone.0008443.s011.tif]
