# Supplementary material for: Functional and Transcriptional Characterization of Human Embryonic Stem Cell-Derived Endothelial Cells for Treatment of Myocardial Infarction
Source: PLoS One. 2009 Dec 31;4(12):e8443. doi: 10.1371/journal.pone.0008443 (PMC2795856; doi:10.1371/journal.pone.0008443)
Supplement: Table S1 — Significant gene lists from microarray data. (6.51 MB PDF) [file pone.0008443.s007.pdf]

Supplemental Table S1A

Genes upregulated in hESC vs. EB

P-Value ≤ 0.015 with multiple testing correction (Benjamini and Hochberg), Fold Change ≥ 2.0

Gene Name	*P*-value	Fold change	Common	Genbank	Description	RefSeq
A_23_P119593	1.39E-04	3.46	NM_024794	NM_024794	Homo sapiens abhydrolase domain containing 9 (ABHD9), mRNA [NM_024794]	NM_024794
A_23_P500614	1.49E-04	18.08	NM_001243	NM_001243	Homo sapiens tumor necrosis factor receptor superfamily, member 8 (TNFRSF8), transcript variant 1, mRNA [NM_001243]	NM_001243
A_32_P151875	1.80E-04	3.19	NM_020781	NM_020781	Homo sapiens zinc finger protein 398 (ZNF398), transcript variant 2, mRNA [NM_020781]	NM_020781
A_23_P125265	1.90E-04	2.21	NM_002266	NM_002266	Homo sapiens karyopherin alpha 2 (RAG cohort 1, importin alpha 1) (KPNA2), mRNA [NM_002266]	NM_002266
A_23_P433785	1.99E-04	3.65	NM_002561	NM_002561	Homo sapiens purinergic receptor P2X, ligand-gated ion channel, 5 (P2RX5), transcript variant 1, mRNA [NM_002561]	NM_002561
A_32_P24068	2.41E-04	8.40	THC2285742			
A_23_P57588	2.95E-04	2.62	NM_016426	NM_016426	Homo sapiens G-2 and S-phase expressed 1 (GTSE1), mRNA [NM_016426]	NM_016426
A_23_P323751	2.95E-04	3.31	NM_030919	NM_030919	Homo sapiens chromosome 20 open reading frame 129 (C20orf129), mRNA [NM_030919]	NM_030919
A_24_P916586	2.98E-04	5.15	BC010091	BC010091	Homo sapiens bicaudal D homolog 1 (Drosophila), mRNA (cDNA clone IMAGE:3050215), with apparent retained intron. [BC010091]	
A_23_P873	2.98E-04	6.29	NM_004848	NM_004848	Homo sapiens chromosome 1 open reading frame 38 (C1orf38), mRNA [NM_004848]	NM_004848
A_32_P148745	2.98E-04	7.25	AK027618	AK027618	Homo sapiens cDNA FLJ14712 fis, clone NT2RP3000825, weakly similar to NEUROGENIC LOCUS NOTCH 3 PROTEIN. [AK027618]	XM_371878
A_24_P218979	3.02E-04	2.18	NM_031299	NM_031299	Homo sapiens cell division cycle associated 3 (CDCA3), mRNA [NM_031299]	NM_031299
A_23_P212397	3.02E-04	2.31	NM_015340	NM_015340	Homo sapiens leucyl-tRNA synthetase 2, mitochondrial (LARS2), nuclear gene encoding mitochondrial protein, mRNA [NM_015340]	NM_015340
A_23_P206830	3.02E-04	2.39	NM_016069	NM_016069	Homo sapiens mitochondria-associated protein involved in granulocyte-macrophage colony-stimulating factor signal transduction (Magmas), nuclear gene encoding mitochondrial protein, mRNA [NM_016069]	NM_016069
A_24_P923102	3.02E-04	2.42	BC016950	BC016950	Homo sapiens kelch-like 23 (Drosophila), mRNA (cDNA clone IMAGE:3854163), complete cds. [BC016950]	
A_23_P92082	3.02E-04	2.43	NM_001064	NM_001064	Homo sapiens transketolase (Wernicke-Korsakoff syndrome) (TKT), mRNA [NM_001064]	NM_001064
A_23_P104651	3.02E-04	2.66	NM_080668	NM_080668	Homo sapiens cell division cycle associated 5 (CDCA5), mRNA [NM_080668]	NM_080668
A_23_P56922	3.02E-04	2.75	NM_002157	NM_002157	Homo sapiens heat shock 10kDa protein 1 (chaperonin 10) (HSPE1), mRNA [NM_002157]	NM_002157
A_23_P345065	3.02E-04	3.52	NM_016510	NM_016510	Homo sapiens selenocysteine lyase (SCLY), mRNA [NM_016510]	NM_016510
A_23_P426511	3.02E-04	4.18	NM_138698	NM_138698	Homo sapiens prematurely terminated mRNA decay factor-like (LOC91431), mRNA [NM_138698]	NM_138698
A_23_P91640	3.02E-04	4.29	NM_020437	NM_020437	Homo sapiens similar to aspartate beta hydroxylase (ASPH) (LOC57168), mRNA [NM_020437]	NM_020437
A_23_P12784	3.02E-04	6.76	NM_012083	NM_012083	Homo sapiens frequently rearranged in advanced T-cell lymphomas 2 (FRAT2), mRNA [NM_012083]	NM_012083
A_32_P114918	3.02E-04	8.93	BM665043	BM665043	UI-E-CQ1-aev-p-07-0-UI.s1 UI-E-CQ1 Homo sapiens cDNA clone UI-E-CQ1-aev-p-07-0-UI 3', mRNA sequence [BM665043]	
A_23_P254816	3.03E-04	2.01	NM_004609	NM_004609	Homo sapiens transcription factor 15 (basic helix-loop-helix) (TCF15), mRNA [NM_004609]	NM_004609
A_23_P212284	3.10E-04	2.67	NM_015426	NM_015426	Homo sapiens WD repeat domain 51A (WDR51A), mRNA [NM_015426]	NM_015426
A_23_P213620	3.70E-04	4.76	NM_004576	NM_004576	Homo sapiens protein phosphatase 2 (formerly 2A), regulatory subunit B (PR 52), beta isoform (PPP2R2B), transcript variant 1, mRNA [NM_004576]	NM_004576
A_23_P85703	3.97E-04	2.72	NM_005686	NM_005686	Homo sapiens SRY (sex determining region Y)-box 13 (SOX13), mRNA [NM_005686]	NM_005686
A_24_P393844	3.97E-04	3.11	NM_001384	NM_001384	Homo sapiens DPH2 homolog (S. cerevisiae) (DPH2), transcript variant 1, mRNA [NM_001384]	NM_001384
A_23_P63980	4.00E-04	2.57	NM_024036	NM_024036	Homo sapiens leucine rich repeat and fibronectin type III domain containing 4 (LRFN4), mRNA [NM_024036]	NM_024036
A_32_P399546	4.00E-04	5.62	AF256215	AF256215	Homo sapiens cycle-like factor CLIF mRNA, complete cds. [AF256215]	
A_24_P372672	4.01E-04	2.24	NM_001931	NM_001931	Homo sapiens dihydrolipoamide S-acetyltransferase (E2 component of pyruvate dehydrogenase complex) (DLAT), mRNA [NM_001931]	NM_001931
A_23_P252371	4.01E-04	2.48	NM_002894	NM_002894	Homo sapiens retinoblastoma binding protein 8 (RBBP8), transcript variant 1, mRNA [NM_002894]	NM_002894
A_23_P41327	4.01E-04	3.32	NM_017816	NM_017816	Homo sapiens hypothetical protein FLJ20425 (LYAR), mRNA [NM_017816]	NM_017816

A_32_P211248	4.01E-04	8.47	AJ276555	AJ276555	Homo sapiens mRNA for hypothetical protein (ORF1), clone 00275. [AJ276555]	
A_32_P211253	4.01E-04	12.99	ENST00000331096			
A_23_P376557	4.21E-04	7.35	NM_022718	NM_022718	Homo sapiens matrix metalloproteinase 25 (MMP25), transcript variant 2, mRNA [NM_022718]	NM_022718
A_24_P85775	4.26E-04	6.25	NM_004848	NM_004848	Homo sapiens chromosome 1 open reading frame 38 (C1orf38), mRNA [NM_004848]	NM_004848
A_23_P422831	4.32E-04	10.57	NM_004816	NM_004816	Homo sapiens chromosome 9 open reading frame 61 (C9orf61), mRNA [NM_004816]	NM_004816
A_23_P325661	4.33E-04	2.01	NM_003435	NM_003435	Homo sapiens zinc finger protein 134 (clone pHZ-15) (ZNF134), mRNA [NM_003435]	NM_003435
A_23_P93269	4.33E-04	3.33	NM_003447	NM_003447	Homo sapiens zinc finger protein 165 (ZNF165), mRNA [NM_003447]	NM_003447
A_23_P5435	4.33E-04	4.46	AY358993	AY358993	Homo sapiens clone DNA129535 MRV222 (UNQ3066) mRNA, complete cds. [AY358993]	
A_23_P134946	4.72E-04	2.84	NM_014665	NM_014665	Homo sapiens leucine rich repeat containing 14 (LRRC14), mRNA [NM_014665]	NM_014665
A_23_P213350	4.72E-04	4.59	A_23_P213350			
A_23_P409093	4.72E-04	4.59	NM_178826	NM_178826	Homo sapiens transmembrane protein 16D (TMEM16D), mRNA [NM_178826]	NM_178826
A_32_P505730	4.72E-04	8.70	BC017967	BC017967	Homo sapiens major histocompatibility complex, class II, DP beta 2 (pseudogene), mRNA (cDNA clone MGC:24119 IMAGE:4663904), complete cds. [BC017967]	
A_23_P88740	4.75E-04	2.46	NM_018455	NM_018455	Homo sapiens uncharacterized bone marrow protein BM039 (BM039), mRNA [NM_018455]	NM_018455
A_23_P107154	4.75E-04	2.66	NM_032582	NM_032582	Homo sapiens ubiquitin specific protease 32 (USP32), mRNA [NM_032582]	NM_032582
A_23_P155969	4.75E-04	2.78	NM_014264	NM_014264	Homo sapiens polo-like kinase 4 (Drosophila) (PLK4), mRNA [NM_014264]	NM_014264
A_23_P93690	4.75E-04	2.85	NM_182776	NM_182776	Homo sapiens MCM7 minichromosome maintenance deficient 7 (S. cerevisiae) (MCM7), transcript variant 2, mRNA [NM_182776]	NM_182776
A_23_P104617	4.75E-04	4.93	NM_152312	NM_152312	Homo sapiens glycosyltransferase-like 1B (GYLTL1B), mRNA [NM_152312]	NM_152312
A_23_P31536	4.86E-04	2.65	NM_003143	NM_003143	Homo sapiens single-stranded DNA binding protein 1 (SSBP1), mRNA [NM_003143]	NM_003143
A_23_P44139	4.86E-04	2.97	NM_000947	NM_000947	Homo sapiens primase, polypeptide 2A, 58kDa (PRIM2A), mRNA [NM_000947]	NM_000947
A_32_P221822	4.86E-04	3.05	AB007976	AB007976	Homo sapiens mRNA, chromosome 1 specific transcript KIAA0507. [AB007976]	
A_32_P459533	4.86E-04	3.21	NM_015122	NM_015122	Homo sapiens FCH domain only 1 (FCHO1), mRNA [NM_015122]	NM_015122
A_23_P209337	4.86E-04	3.37	NM_145280	NM_145280	Homo sapiens similar to hepatocellular carcinoma-associated antigen HCA557b (LOC151194), mRNA [NM_145280]	NM_145280
A_23_P343954	4.86E-04	3.55	NM_006546	NM_006546	Homo sapiens IGF-II mRNA-binding protein 1 (IMP-1), mRNA [NM_006546]	NM_006546
A_23_P90612	4.86E-04	3.82	NM_005915	NM_005915	Homo sapiens MCM6 minichromosome maintenance deficient 6 (MIS5 homolog, S. pombe) (S. cerevisiae) (MCM6), mRNA [NM_005915]	NM_005915
A_32_P55462	4.86E-04	4.18	CR593500	CR593500	full-length cDNA clone CS0DF014YD20 of Fetal brain of Homo sapiens (human). [CR593500]	XM_373788
A_23_P78664	5.03E-04	3.15	NM_138998	NM_138998	Homo sapiens DEAD (Asp-Glu-Ala-Asp) box polypeptide 39 (DDX39), transcript variant 2, mRNA [NM_138998]	NM_138998
A_23_P71727	5.03E-04	3.66	NM_001827	NM_001827	Homo sapiens CDC28 protein kinase regulatory subunit 2 (CKS2), mRNA [NM_001827]	NM_001827
A_23_P7353	5.06E-04	2.96	NM_178043	NM_178043	Homo sapiens La ribonucleoprotein domain family, member 2 (LARP2), transcript variant 2, mRNA [NM_178043]	NM_178043
A_32_P221429	5.15E-04	4.02	THC2374165			
A_24_P124662	5.26E-04	2.15	NM_139078	NM_139078	Homo sapiens mitogen-activated protein kinase-activated protein kinase 5 (MAPKAPK5), transcript variant 2, mRNA [NM_139078]	NM_139078
A_23_P23318	5.26E-04	2.35	NM_006784	NM_006784	Homo sapiens WD repeat domain 3 (WDR3), mRNA [NM_006784]	NM_006784
A_32_P209230	5.26E-04	2.51	NM_133467	NM_133467	Homo sapiens Cbp/p300-interacting transactivator, with Glu/Asp-rich carboxy-terminal domain, 4 (CITED4), mRNA [NM_133467]	NM_133467
A_23_P75071	5.26E-04	2.87	NM_016195	NM_016195	Homo sapiens M-phase phosphoprotein 1 (MPHOSPH1), mRNA [NM_016195]	NM_016195
A_24_P169688	5.26E-04	2.90	NM_005931	NM_005931	Homo sapiens MHC class I polypeptide-related sequence B (MICB), mRNA [NM_005931]	NM_005931
A_23_P70168	5.26E-04	2.92	NM_152295	NM_152295	Homo sapiens threonyl-tRNA synthetase (TARS), mRNA [NM_152295]	NM_152295
A_24_P912871	5.26E-04	2.93	A_24_P912871			
A_23_P92765	5.26E-04	3.65	CR604116	CR604116	full-length cDNA clone CS0DF027YM18 of Fetal brain of Homo sapiens (human). [CR604116]	
A_23_P75786	5.26E-04	4.69	NM_016582	NM_016582	Homo sapiens solute carrier family 15, member 3 (SLC15A3), mRNA [NM_016582]	NM_016582
A_24_P192434	5.26E-04	10.18	NM_017489	NM_017489	Homo sapiens telomeric repeat binding factor (NIMA-interacting) 1 (TERF1), transcript variant 1, mRNA [NM_017489]	NM_017489

A_23_P154065	5.26E-04	12.30	NM_006000	NM_006000	Homo sapiens tubulin, alpha 1 (testis specific) (TUBA1), mRNA [NM_006000]	NM_006000
A_23_P109393	5.27E-04	2.30	NM_001670	NM_001670	Homo sapiens armadillo repeat gene deletes in velocardiofacial syndrome (ARVCF), mRNA [NM_001670]	NM_001670
A_23_P92320	5.27E-04	2.43	NM_017426	NM_017426	Homo sapiens nucleoporin 54kDa (NUP54), mRNA [NM_017426]	NM_017426
A_24_P307466	5.27E-04	2.70	ENST00000332917			
A_23_P206077	5.27E-04	2.88	NM_022767	NM_022767	Homo sapiens interferon stimulated exonuclease gene 20kDa-like 1 (ISG20L1), mRNA [NM_022767]	NM_022767
A_23_P256384	5.27E-04	3.80	NM_021144	NM_021144	Homo sapiens PC4 and SFRS1 interacting protein 1 (PSIP1), transcript variant 1, mRNA [NM_021144]	NM_021144
A_23_P138655	5.27E-04	13.46	NM_057157	NM_057157	Homo sapiens cytochrome P450, family 26, subfamily A, polypeptide 1 (CYP26A1), transcript variant 2, mRNA [NM_057157]	NM_057157
A_23_P154526	5.29E-04	5.13	NM_004490	NM_004490	Homo sapiens growth factor receptor-bound protein 14 (GRB14), mRNA [NM_004490]	NM_004490
A_23_P211748	5.34E-04	2.87	NM_005513	NM_005513	Homo sapiens general transcription factor IIE, polypeptide 1 (alpha subunit, 56kD) (GTF2E1), mRNA [NM_005513]	NM_005513
A_23_P37954	5.34E-04	3.08	NM_001761	NM_001761	Homo sapiens cyclin F (CCNF), mRNA [NM_001761]	NM_001761
A_23_P46351	5.34E-04	3.38	NM_006862	NM_006862	Homo sapiens tudor and KH domain containing (TDRKH), mRNA [NM_006862]	NM_006862
A_32_P160972	5.39E-04	2.63	BC014953	BC014953	Homo sapiens chromosome 6 open reading frame 115, mRNA (cDNA clone MGC:22978 IMAGE:4849571), complete cds. [BC014953]	XM_371848
A_23_P12601	5.39E-04	2.78	NM_018706	NM_018706	Homo sapiens dehydrogenase E1 and transketolase domain containing 1 (DHTKD1), mRNA [NM_018706]	NM_018706
A_23_P216556	5.39E-04	3.21	NM_018424	NM_018424	Homo sapiens erythrocyte membrane protein band 4.1 like 4B (EPB41L4B), transcript variant 1, mRNA [NM_018424]	NM_018424
A_32_P206541	5.39E-04	4.20	AK128714	AK128714	Homo sapiens cDNA FLJ46881 fis, clone UTERU3015647, moderately similar to Embigin precursor. [AK128714]	
A_23_P14986	5.39E-04	10.00	NM_000196	NM_000196	Homo sapiens hydroxysteroid (11-beta) dehydrogenase 2 (HSD11B2), mRNA [NM_000196]	NM_000196
A_32_P143245	5.40E-04	2.39	NM_001012507	NM_001012507	Homo sapiens chromosome 6 open reading frame 173 (C6orf173), mRNA [NM_001012507]	NM_001012507
A_32_P144710	5.54E-04	3.00	NM_176880	NM_176880	Homo sapiens TR4 orphan receptor associated protein TRA16 (TRA16), mRNA [NM_176880]	NM_176880
A_32_P151782	5.54E-04	3.24	BG033002	BG033002	602300107F1 NIH_MGC_87 Homo sapiens cDNA clone IMAGE:4401960 5', mRNA sequence [BG033002]	
A_23_P109636	5.54E-04	4.29	NM_015541	NM_015541	Homo sapiens leucine-rich repeats and immunoglobulin-like domains 1 (LRIG1), mRNA [NM_015541]	NM_015541
A_23_P160881	5.54E-04	11.56	NM_001009568	NM_001009568	Homo sapiens sphingomyelin phosphodiesterase, acid-like 3B (SMPDL3B), transcript variant 2, mRNA [NM_001009568]	NM_001009568
A_23_P153651	5.71E-04	3.41	NM_024333	NM_024333	Homo sapiens fibronectin type III and SPRY domain containing 1 (FSD1), mRNA [NM_024333]	NM_024333
A_23_P353744	5.74E-04	3.27	NM_032239	NM_032239	Homo sapiens La ribonucleoprotein domain family, member 2 (LARP2), transcript variant 3, mRNA [NM_032239]	NM_032239
A_24_P814444	5.76E-04	3.05	THC2407545			
A_24_P804951	5.76E-04	10.00	XM_498560	XM_498560	PREDICTED: Homo sapiens LOC440132 (LOC440132), mRNA [XM_498560]	XM_498560
A_23_P17163	5.78E-04	2.09	S63912	S63912	D10S102=FBRNP [human, fetal brain, mRNA, 3043 nt]. [S63912]	XM_370728
A_23_P408239	5.84E-04	2.20	AL117400	AL117400	Homo sapiens mRNA; cDNA DKFZp434O051 (from clone DKFZp434O051). [AL117400]	
A_23_P90634	5.84E-04	5.13	NM_152523	NM_152523	Homo sapiens hypothetical protein FLJ40432 (FLJ40432), mRNA [NM_152523]	NM_152523
A_23_P58747	5.85E-04	5.88	NM_015111	NM_015111	Homo sapiens Nedd4 binding protein 3 (N4BP3), mRNA [NM_015111]	NM_015111
A_24_P346126	5.87E-04	2.82	BC021174	BC021174	Homo sapiens small EDRK-rich factor 1A (telomeric), mRNA (cDNA clone MGC:32975 IMAGE:4824358), complete cds. [BC021174]	
A_23_P214111	5.88E-04	4.72	NM_022113	NM_022113	Homo sapiens kinesin family member 13A (KIF13A), mRNA [NM_022113]	NM_022113
A_32_P217510	5.90E-04	2.12	NM_032168	NM_032168	Homo sapiens WD repeat domain 75 (WDR75), mRNA [NM_032168]	NM_032168
A_23_P46539	5.91E-04	2.40	NM_032636	NM_032636	Homo sapiens differential display and activated by p53 (DDA3), transcript variant 1, mRNA [NM_032636]	NM_032636
A_23_P22027	5.91E-04	2.40	NM_198336	NM_198336	Homo sapiens insulin induced gene 1 (INSIG1), transcript variant 2, mRNA [NM_198336]	NM_198336
A_24_P187948	5.91E-04	2.44	NM_197966	NM_197966	Homo sapiens BH3 interacting domain death agonist (BID), transcript variant 1, mRNA [NM_197966]	NM_197966
A_23_P206290	5.91E-04	6.90	NM_153837	NM_153837	Homo sapiens G protein-coupled receptor 114 (GPR114), mRNA [NM_153837]	NM_153837
A_23_P69537	5.93E-04	6.17	NM_006681	NM_006681	Homo sapiens neuromedin U (NMU), mRNA [NM_006681]	NM_006681
A_23_P74115	5.96E-04	2.62	NM_003579	NM_003579	Homo sapiens RAD54-like (S. cerevisiae) (RAD54L), mRNA [NM_003579]	NM_003579
A_32_P103837	5.98E-04	2.62	NM_021238	NM_021238	Homo sapiens family with sequence similarity 60, member A (FAM60A), mRNA [NM_021238]	NM_021238
A_32_P195291	6.05E-04	2.72	CR603272	CR603272	full-length cDNA clone CS0DC013YI10 of Neuroblastoma Cot 25-normalized of Homo sapiens (human). [CR603272]	
A_23_P254702	6.05E-04	2.77	NM_003472	NM_003472	Homo sapiens DEK oncogene (DNA binding) (DEK), mRNA [NM_003472]	NM_003472

A_23_P211909	6.05E-04	2.90	NM_002670	NM_002670	Homo sapiens plastin 1 (I isoform) (PLS1), mRNA [NM_002670]	NM_002670
A_23_P150092	6.05E-04	5.08	NM_012247	NM_012247	Homo sapiens selenophosphate synthetase 1 (SEPHS1), mRNA [NM_012247]	NM_012247
A_23_P312840	6.17E-04	5.65	NM_020796	NM_020796	Homo sapiens sema domain, transmembrane domain (TM), and cytoplasmic domain, (semaphorin) 6A (SEMA6A), mRNA [NM_020796]	NM_020796
A_23_P333951	6.18E-04	3.07	BC017415	BC017415	Homo sapiens chromosome 1 open reading frame 67, mRNA (cDNA clone IMAGE:4655328). [BC017415]	
A_23_P131706	6.25E-04	2.19	NM_212552	NM_212552	Homo sapiens bolA-like 3 (E. coli) (BOLA3), mRNA [NM_212552]	NM_212552
A_32_P135985	6.33E-04	9.35	NM_003212	NM_003212	Homo sapiens teratocarcinoma-derived growth factor 1 (TDGF1), mRNA [NM_003212]	NM_003212
A_23_P85250	6.37E-04	5.49	NM_013230	NM_013230	Homo sapiens CD24 antigen (small cell lung carcinoma cluster 4 antigen) (CD24), mRNA [NM_013230]	NM_013230
A_32_P137382	6.61E-04	2.53	BX089701	BX089701	BX089701 BX089701 NCI_CGAP_Lu24 Homo sapiens cDNA clone IMAGp998I115809 ; IMAGE:2341330, mRNA sequence [BX089701]	
A_24_P367397	6.61E-04	2.74	A_24_P367397			
A_23_P166306	6.61E-04	2.91	NM_000071	NM_000071	Homo sapiens cystathionine-beta-synthase (CBS), mRNA [NM_000071]	NM_000071
A_23_P27947	6.61E-04	3.24	NM_032346	NM_032346	Homo sapiens hypothetical protein MGC13096 (MGC13096), mRNA [NM_032346]	NM_032346
A_23_P379614	6.61E-04	3.32	NM_007280	NM_007280	Homo sapiens Opa interacting protein 5 (OIP5), mRNA [NM_007280]	NM_007280
A_23_P21706	6.61E-04	3.91	NM_001905	NM_001905	Homo sapiens CTP synthase (CTPS), mRNA [NM_001905]	NM_001905
A_23_P115492	6.69E-04	11.76	NM_024749	NM_024749	Homo sapiens hypothetical protein FLJ12505 (FLJ12505), mRNA [NM_024749]	NM_024749
A_23_P208595	6.74E-04	2.67	NM_000527	NM_000527	Homo sapiens low density lipoprotein receptor (familial hypercholesterolemia) (LDLR), mRNA [NM_000527]	NM_000527
A_24_P365901	6.76E-04	2.82	NM_178562	NM_178562	Homo sapiens hypothetical protein MGC50844 (MGC50844), mRNA [NM_178562]	NM_178562
A_32_P232647	6.76E-04	2.88	A_32_P232647			
A_23_P66473	6.76E-04	6.06	NM_181671	NM_181671	Homo sapiens phosphatidylinositol transfer protein, cytoplasmic 1 (PITPNC1), transcript variant 2, mRNA [NM_181671]	NM_181671
A_24_P379104	6.76E-04	10.18	NM_006875	NM_006875	Homo sapiens pim-2 oncogene (PIM2), mRNA [NM_006875]	NM_006875
A_23_P218827	6.77E-04	2.62	NM_006596	NM_006596	Homo sapiens polymerase (DNA directed), theta (POLQ), transcript variant 1, mRNA [NM_006596]	NM_006596
A_23_P88439	6.84E-04	2.43	NM_152332	NM_152332	Homo sapiens membrane targeting (tandem) C2 domain containing 1 (MTAC2D1), mRNA [NM_152332]	NM_152332
A_23_P163992	6.85E-04	2.66	NM_005310	NM_005310	Homo sapiens growth factor receptor-bound protein 7 (GRB7), mRNA [NM_005310]	NM_005310
A_23_P119214	6.86E-04	2.17	NM_006351	NM_006351	Homo sapiens translocase of inner mitochondrial membrane 44 homolog (yeast) (TIMM44), mRNA [NM_006351]	NM_006351
A_23_P254612	6.89E-04	2.53	NM_006716	NM_006716	Homo sapiens activator of S phase kinase (ASK), mRNA [NM_006716]	NM_006716
A_32_P231226	6.89E-04	3.94	AK095046	AK095046	Homo sapiens cDNA FLJ37727 fis, clone BRHIP2019972. [AK095046]	
A_23_P160537	6.92E-04	3.38	NM_024037	NM_024037	Homo sapiens chromosome 1 open reading frame 135 (C1orf135), mRNA [NM_024037]	NM_024037
A_24_P568645	6.93E-04	2.77	A_24_P568645			
A_23_P216149	6.93E-04	11.68	NM_017489	NM_017489	Homo sapiens telomeric repeat binding factor (NIMA-interacting) 1 (TERF1), transcript variant 1, mRNA [NM_017489]	NM_017489
A_23_P100056	6.98E-04	7.14	NM_194272	NM_194272	Homo sapiens RNA binding protein with multiple splicing 2 (RBPMS2), mRNA [NM_194272]	NM_194272
A_23_P96325	7.11E-04	3.37	NM_001009954	NM_001009954	Homo sapiens FLJ20105 protein (FLJ20105), transcript variant 2, mRNA [NM_001009954]	NM_001009954
A_23_P13914	7.12E-04	2.99	NM_032656	NM_032656	Homo sapiens DEAH (Asp-Glu-Ala-His) box polypeptide 37 (DHX37), mRNA [NM_032656]	NM_032656
A_23_P323094	7.41E-04	8.40	NM_004426	NM_004426	Homo sapiens polyhomeotic-like 1 (Drosophila) (PHC1), mRNA [NM_004426]	NM_004426
A_23_P37399	7.43E-04	2.17	NM_014216	NM_014216	Homo sapiens inositol 1,3,4-triphosphate 5/6 kinase (ITPK1), mRNA [NM_014216]	NM_014216
A_23_P26557	7.43E-04	3.89	NM_025108	NM_025108	Homo sapiens hypothetical protein FLJ13909 (FLJ13909), mRNA [NM_025108]	NM_025108
A_32_P112970	7.45E-04	3.91	THC2441612			
A_24_P286868	7.51E-04	2.46	CR592483	CR592483	full-length cDNA clone CS0DL004YD15 of B cells (Ramos cell line) Cot 25-normalized of Homo sapiens (human). [CR592483]	
A_32_P506600	7.51E-04	2.51	NM_006325	NM_006325	Homo sapiens RAN, member RAS oncogene family (RAN), mRNA [NM_006325]	NM_006325
A_24_P945283	7.53E-04	2.60	AB033058	AB033058	Homo sapiens mRNA for KIAA1232 protein, partial cds. [AB033058]	
A_23_P108574	7.56E-04	2.99	NM_005813	NM_005813	Homo sapiens protein kinase D3 (PRKD3), mRNA [NM_005813]	NM_005813
A_24_P205137	7.61E-04	2.85	BC011498	BC011498	Homo sapiens histone deacetylase 6, mRNA (cDNA clone IMAGE:4179066), complete cds. [BC011498]	

A_24_P724886	7.61E-04	3.05	THC2334650		BQ437598 AGENCOURT_7826771 NIH_MGC_67 Homo sapiens cDNA clone IMAGE:6153024 5', mRNA sequence [BQ437598]	
A_24_P261734	7.61E-04	3.97	CR594705	CR594705	full-length cDNA clone CS0DC003YJ07 of Neuroblastoma Cot 25-normalized of Homo sapiens (human). [CR594705]	
A_24_P152404	7.66E-04	2.74	BC032118	BC032118	Homo sapiens cDNA clone IMAGE:5016307, partial cds. [BC032118]	
A_23_P57293	7.74E-04	2.38	ENST00000270201		Homo sapiens chromosome 21 C21orf108 mRNA, partial cds. [AF231919]	
A_23_P126426	7.74E-04	4.61	NM_001384	NM_001384	Homo sapiens DPH2 homolog (S. cerevisiae) (DPH2), transcript variant 1, mRNA [NM_001384]	NM_001384
A_32_P199263	7.85E-04	3.27	BC073929	BC073929	Homo sapiens cDNA clone IMAGE:5196961, partial cds. [BC073929]	
A_23_P161152	7.90E-04	2.33	NM_014317	NM_014317	Homo sapiens trans-prenyltransferase (TPRT), mRNA [NM_014317]	NM_014317
A_23_P132277	7.90E-04	2.97	NM_006739	NM_006739	Homo sapiens MCM5 minichromosome maintenance deficient 5, cell division cycle 46 (S. cerevisiae) (MCM5), mRNA [NM_006739]	NM_006739
A_23_P203283	7.90E-04	3.95	AK125902	AK125902	Homo sapiens cDNA FLJ43914 fis, clone TESTI4011161. [AK125902]	
A_32_P154361	7.97E-04	10.46	THC2415390			
A_23_P154447	8.07E-04	2.16	NM_015934	NM_015934	Homo sapiens nucleolar protein NOP5/NOP58 (NOP5/NOP58), mRNA [NM_015934]	NM_015934
A_24_P415208	8.07E-04	2.26	BC060806	BC060806	Homo sapiens cDNA clone MGC:71628 IMAGE:30336414, complete cds. [BC060806]	
A_24_P698136	8.07E-04	3.52	AK125299	AK125299	Homo sapiens cDNA FLJ43309 fis, clone NT2RI2004618, highly similar to Cytosolic acyl coenzyme A thioester hydrolase (EC 3.1.2.2). [AK125299]	XR_000194
A_23_P102071	8.07E-04	4.67	AK027315	AK027315	Homo sapiens cDNA FLJ14409 fis, clone HEMBA1004408, moderately similar to PEPTIDYL-PROLYL CIS-TRANS ISOMERASE 10 (EC 5.2.1.8). [AK027315]	
A_23_P359854	8.07E-04	6.10	AB046773	AB046773	Homo sapiens mRNA for KIAA1553 protein, partial cds. [AB046773]	XM_166320
A_23_P141035	8.07E-04	6.94	NM_005769	NM_005769	Homo sapiens carbohydrate (N-acetylglucosamine 6-O) sulfotransferase 4 (CHST4), mRNA [NM_005769]	NM_005769
A_24_P392475	8.07E-04	12.25	ENST00000321892		Homo sapiens cDNA: FLJ23531 fis, clone LNG06065. [AK027184]	
A_24_P111096	8.07E-04	20.12	NM_004566	NM_004566	Homo sapiens 6-phosphofructo-2-kinase/fructose-2,6-biphosphatase 3 (PFKFB3), mRNA [NM_004566]	NM_004566
A_32_P155645	8.10E-04	3.50	NM_152549	NM_152549	Homo sapiens hypothetical protein MGC39633 (MGC39633), mRNA [NM_152549]	NM_152549
A_23_P28625	8.24E-04	2.36	NM_018256	NM_018256	Homo sapiens WD repeat domain 12 (WDR12), mRNA [NM_018256]	NM_018256
A_23_P18196	8.25E-04	2.37	NM_002916	NM_002916	Homo sapiens replication factor C (activator 1) 4, 37kDa (RFC4), transcript variant 1, mRNA [NM_002916]	NM_002916
A_32_P24165	8.25E-04	3.48	NM_001018115	NM_001018115	Homo sapiens Fanconi anemia, complementation group D2 (FANCD2), transcript variant 2, mRNA [NM_001018115]	NM_001018115
A_24_P232696	8.28E-04	2.66	NM_139071	NM_139071	Homo sapiens SWI/SNF related, matrix associated, actin dependent regulator of chromatin, subfamily d, member 1 (SMARCD1), transcript variant 2, mRNA [NM_139071]	NM_139071
A_23_P204246	8.45E-04	9.43	NM_004426	NM_004426	Homo sapiens polyhomeotic-like 1 (Drosophila) (PHC1), mRNA [NM_004426]	NM_004426
A_23_P118815	8.50E-04	2.03	NM_001012271	NM_001012271	Homo sapiens baculoviral IAP repeat-containing 5 (survivin) (BIRC5), transcript variant 3, mRNA [NM_001012271]	NM_001012271
A_23_P132175	8.53E-04	2.79	NM_023004	NM_023004	Homo sapiens reticulon 4 receptor (RTN4R), mRNA [NM_023004]	NM_023004
A_23_P100220	8.54E-04	3.00	NM_024939	NM_024939	Homo sapiens hypothetical protein FLJ21918 (FLJ21918), mRNA [NM_024939]	NM_024939
A_24_P226949	8.59E-04	3.02	NM_017645	NM_017645	Homo sapiens family with sequence similarity 29, member A (FAM29A), mRNA [NM_017645]	NM_017645
A_24_P222184	8.60E-04	2.44	AK091439	AK091439	Homo sapiens cDNA FLJ34120 fis, clone FCBBF3009541. [AK091439]	
A_23_P379945	8.64E-04	3.18	AB040942	AB040942	Homo sapiens mRNA for KIAA1509 protein, partial cds. [AB040942]	XM_029353
A_23_P117494	8.64E-04	4.59	NM_005956	NM_005956	Homo sapiens methylenetetrahydrofolate dehydrogenase (NADP+ dependent) 1, methenyltetrahydrofolate cyclohydrolase, formyltetrahydrofolate synthetase (MTHFD1), mRNA [NM_005956]	NM_005956
A_24_P712350	8.90E-04	3.27	NM_001821	NM_001821	Homo sapiens choroideremia-like (Rab escort protein 2) (CHML), mRNA [NM_001821]	NM_001821
A_23_P15284	8.97E-04	2.02	NM_024109	NM_024109	Homo sapiens hypothetical protein MGC2654 (MGC2654), mRNA [NM_024109]	NM_024109
A_24_P187750	9.01E-04	2.10	NM_001659	NM_001659	Homo sapiens ADP-ribosylation factor 3 (ARF3), mRNA [NM_001659]	NM_001659
A_24_P134235	9.09E-04	3.08	NM_003685	NM_003685	Homo sapiens KH-type splicing regulatory protein (FUSE binding protein 2) (KHSRP), mRNA [NM_003685]	NM_003685
A_24_P183150	9.09E-04	4.59	NM_002090	NM_002090	Homo sapiens chemokine (C-X-C motif) ligand 3 (CXCL3), mRNA [NM_002090]	NM_002090
A_23_P7636	9.11E-04	2.07	NM_004219	NM_004219	Homo sapiens pituitary tumor-transforming 1 (PTTG1), mRNA [NM_004219]	NM_004219
A_23_P122947	9.11E-04	2.16	NM_015060	NM_015060	Homo sapiens KIAA0241 protein (KIAA0241), mRNA [NM_015060]	NM_015060
A_24_P876864	9.11E-04	2.34	BC061590	BC061590	Homo sapiens cDNA clone MGC:75203 IMAGE:6502529, complete cds. [BC061590]	

A_23_P1043	9.11E-04	2.56	NM_018265	NM_018265	Homo sapiens chromosome 1 open reading frame 106 (C1orf106), mRNA [NM_018265]	NM_018265
A_24_P149266	9.11E-04	3.34	NM_020804	NM_020804	Homo sapiens protein kinase C and casein kinase substrate in neurons 1 (PACSIN1), mRNA [NM_020804]	NM_020804
A_23_P110031	9.11E-04	3.69	NM_016089	NM_016089	Homo sapiens zinc finger protein 589 (ZNF589), mRNA [NM_016089]	NM_016089
A_24_P341731	9.11E-04	5.59	A_24_P341731			
A_23_P54540	9.14E-04	2.36	NM_001013703	NM_001013703	Homo sapiens eukaryotic translation initiation factor 2 alpha kinase 4 (EIF2AK4), mRNA [NM_001013703]	NM_001013703
A_23_P48585	9.14E-04	3.38	NM_005407	NM_005407	Homo sapiens sal-like 2 (Drosophila) (SALL2), mRNA [NM_005407]	NM_005407
A_24_P105102	9.16E-04	2.65	NM_182687	NM_182687	Homo sapiens protein kinase, membrane associated tyrosine/threonine 1 (PKMYT1), transcript variant 2, mRNA [NM_182687]	NM_182687
A_23_P25163	9.23E-04	2.70	NM_172178	NM_172178	Homo sapiens mitochondrial ribosomal protein L42 (MRPL42), nuclear gene encoding mitochondrial protein, transcript variant 3, mRNA [NM_172178]	NM_172178
A_24_P128085	9.27E-04	3.37	BC044642	BC044642	Homo sapiens membrane associated DNA binding protein, mRNA (cDNA clone MGC:52176 IMAGE:5455191), complete cds. [BC044642]	
A_23_P78170	9.30E-04	2.85	NM_014520	NM_014520	Homo sapiens MYB binding protein (P160) 1a (MYBBP1A), mRNA [NM_014520]	NM_014520
A_23_P136817	9.31E-04	2.49	NM_014708	NM_014708	Homo sapiens kinetochore associated 1 (KNTC1), mRNA [NM_014708]	NM_014708
A_23_P213359	9.31E-04	2.83	NM_002587	NM_002587	Homo sapiens protocadherin 1 (cadherin-like 1) (PCDH1), transcript variant 1, mRNA [NM_002587]	NM_002587
A_23_P314151	9.31E-04	2.85	NM_004741	NM_004741	Homo sapiens nucleolar and coiled-body phosphoprotein 1 (NOLC1), mRNA [NM_004741]	NM_004741
A_23_P366468	9.38E-04	2.53	ENST00000246228			
A_23_P14493	9.43E-04	2.30	NM_018139	NM_018139	Homo sapiens chromosome 14 open reading frame 104 (C14orf104), mRNA [NM_018139]	NM_018139
A_23_P152353	9.52E-04	2.26	NM_133451	NM_133451	Homo sapiens KIAA1970 protein (KIAA1970), mRNA [NM_133451]	NM_133451
A_23_P365817	9.53E-04	2.67	NM_138689	NM_138689	Homo sapiens protein phosphatase 1, regulatory (inhibitor) subunit 14B (PPP1R14B), mRNA [NM_138689]	NM_138689
A_23_P64898	9.57E-04	2.67	NM_005810	NM_005810	Homo sapiens killer cell lectin-like receptor subfamily G, member 1 (KLRG1), mRNA [NM_005810]	NM_005810
A_23_P143535	9.62E-04	5.10	NM_033661	NM_033661	Homo sapiens WD repeat domain 4 (WDR4), transcript variant 2, mRNA [NM_033661]	NM_033661
A_32_P65473	9.64E-04	5.99	ENST00000330640		Homo sapiens, clone IMAGE:2899977, mRNA, partial cds. [BC022980]	
A_23_P80098	9.68E-04	2.93	NM_000819	NM_000819	Homo sapiens phosphoribosylglycinamide formyltransferase, phosphoribosylglycinamide synthetase, phosphoribosylaminoimidazole synthetase (GART), transcript variant 1, mRNA [NM_000819]	NM_000819
A_23_P152047	9.70E-04	2.06	NM_138967	NM_138967	Homo sapiens secretory carrier membrane protein 5 (SCAMP5), mRNA [NM_138967]	NM_138967
A_23_P98431	9.72E-04	2.02	NM_000190	NM_000190	Homo sapiens hydroxymethylbilane synthase (HMBS), transcript variant 1, mRNA [NM_000190]	NM_000190
A_23_P116533	9.72E-04	2.03	NM_015055	NM_015055	Homo sapiens SWAP-70 protein (SWAP70), mRNA [NM_015055]	NM_015055
A_23_P256021	9.72E-04	2.15	NM_031206	NM_031206	Homo sapiens LAS1-like (S. cerevisiae) (LAS1L), mRNA [NM_031206]	NM_031206
A_32_P117322	9.72E-04	2.28	BG192275	BG192275	RST11383 Athersys RAGE Library Homo sapiens cDNA, mRNA sequence [BG192275]	
A_23_P129313	9.72E-04	2.31	ENST00000249760		Homo sapiens isovaleryl Coenzyme A dehydrogenase, mRNA (cDNA clone MGC:3496 IMAGE:3627787), complete cds. [BC017202]	
A_24_P680947	9.72E-04	2.51	ENST00000335534		Homo sapiens hypothetical protein LOC146909, mRNA (cDNA clone IMAGE:4587138), partial cds. [BC067365]	
A_23_P397341	9.72E-04	2.74	NM_152341	NM_152341	Homo sapiens progestin and adipoQ receptor family member IV (PAQR4), mRNA [NM_152341]	NM_152341
A_23_P120467	9.72E-04	3.10	NM_199427	NM_199427	Homo sapiens zinc finger protein 64 homolog (mouse) (ZFP64), transcript variant 4, mRNA [NM_199427]	NM_199427
A_23_P72187	9.72E-04	3.27	NM_004229	NM_004229	Homo sapiens cofactor required for Sp1 transcriptional activation, subunit 2, 150kDa (CRSP2), mRNA [NM_004229]	NM_004229
A_23_P46396	9.72E-04	3.34	NM_021190	NM_021190	Homo sapiens polypyrimidine tract binding protein 2 (PTBP2), mRNA [NM_021190]	NM_021190
A_23_P23356	9.72E-04	3.42	NM_016052	NM_016052	Homo sapiens CGI-115 protein (CGI-115), mRNA [NM_016052]	NM_016052
A_24_P272073	9.72E-04	4.08	ENST00000335078			
A_24_P56557	9.72E-04	5.65	NM_080650	NM_080650	Homo sapiens similar to RIKEN cDNA 5730421E18 gene (MGC14798), mRNA [NM_080650]	NM_080650
A_23_P1374	9.72E-04	8.47	NM_006257	NM_006257	Homo sapiens protein kinase C, theta (PRKCQ), mRNA [NM_006257]	NM_006257
A_32_P220696	9.72E-04	11.14	NM_017489	NM_017489	Homo sapiens telomeric repeat binding factor (NIMA-interacting) 1 (TERF1), transcript variant 1, mRNA [NM_017489]	NM_017489
A_23_P87513	9.79E-04	2.26	NM_012463	NM_012463	Homo sapiens ATPase, H+ transporting, lysosomal V0 subunit a isoform 2 (ATP6V0A2), mRNA [NM_012463]	NM_012463

A_23_P217637	9.82E-04	2.85	NM_004085	NM_004085	Homo sapiens translocase of inner mitochondrial membrane 8 homolog A (yeast) (TIMM8A), nuclear gene encoding mitochondrial protein, mRNA [NM_004085]	NM_004085
A_23_P301995	9.85E-04	2.43	NM_173083	NM_173083	Homo sapiens lin-9 homolog (C. elegans) (LIN9), mRNA [NM_173083]	NM_173083
A_24_P217834	9.89E-04	2.03	NM_003530	NM_003530	Homo sapiens histone 1, H3d (HIST1H3D), mRNA [NM_003530]	NM_003530
A_24_P171549	9.89E-04	3.85	NM_031942	NM_031942	Homo sapiens cell division cycle associated 7 (CDCA7), transcript variant 1, mRNA [NM_031942]	NM_031942
A_24_P287826	9.89E-04	5.43	NM_018124	NM_018124	Homo sapiens ring finger and WD repeat domain 3 (RFWD3), mRNA [NM_018124]	NM_018124
A_23_P146347	9.92E-04	2.55	NM_017645	NM_017645	Homo sapiens family with sequence similarity 29, member A (FAM29A), mRNA [NM_017645]	NM_017645
A_23_P415643	9.95E-04	3.06	NM_152652	NM_152652	Homo sapiens zinc finger protein 553 (ZNF553), mRNA [NM_152652]	NM_152652
A_23_P91991	9.98E-04	2.02	NM_138381	NM_138381	Homo sapiens hypothetical protein BC008322 (MGC15763), mRNA [NM_138381]	NM_138381
A_32_P41574	9.98E-04	2.55	CR626729	CR626729	full-length cDNA clone CS0DF009YE11 of Fetal brain of Homo sapiens (human). [CR626729]	
A_23_P252748	9.98E-04	3.82	NM_007152	NM_007152	Homo sapiens zinc finger protein 195 (ZNF195), mRNA [NM_007152]	NM_007152
A_23_P99731	1.01E-03	2.16	A_23_P99731			
A_23_P433063	1.03E-03	2.02	NM_033064	NM_033064	Homo sapiens ataxia, cerebellar, Cayman type (caytaxin) (ATCAY), mRNA [NM_033064]	NM_033064
A_24_P28165	1.03E-03	2.02	NM_015071	NM_015071	Homo sapiens Rho GTPase activating protein 26 (ARHGAP26), mRNA [NM_015071]	NM_015071
A_23_P23303	1.04E-03	2.82	NM_003686	NM_003686	Homo sapiens exonuclease 1 (EXO1), transcript variant 3, mRNA [NM_003686]	NM_003686
A_23_P336513	1.04E-03	4.44	NM_015465	NM_015465	Homo sapiens gem (nuclear organelle) associated protein 5 (GEMIN5), mRNA [NM_015465]	NM_015465
A_24_P150160	1.05E-03	2.35	NM_004265	NM_004265	Homo sapiens fatty acid desaturase 2 (FADS2), mRNA [NM_004265]	NM_004265
A_23_P133146	1.05E-03	2.86	NM_002106	NM_002106	Homo sapiens H2A histone family, member Z (H2AFZ), mRNA [NM_002106]	NM_002106
A_23_P136504	1.05E-03	8.47	NM_030631	NM_030631	Homo sapiens solute carrier family 25 (mitochondrial oxodicarboxylate carrier), member 21 (SLC25A21), mRNA [NM_030631]	NM_030631
A_23_P101332	1.06E-03	2.09	NM_023008	NM_023008	Homo sapiens hypothetical protein FLJ12949 (FLJ12949), transcript variant 1, mRNA [NM_023008]	NM_023008
A_23_P1956	1.06E-03	2.12	ENST00000307366		Homo sapiens, Similar to RIKEN cDNA 0610006I08 gene, clone IMAGE:3855124, mRNA, partial cds. [BC008643]	XM_495878
A_24_P462899	1.06E-03	2.24	NM_001012507	NM_001012507	Homo sapiens chromosome 6 open reading frame 173 (C6orf173), mRNA [NM_001012507]	NM_001012507
A_23_P120103	1.06E-03	2.44	NM_002252	NM_002252	Homo sapiens potassium voltage-gated channel, delayed-rectifier, subfamily S, member 3 (KCNS3), mRNA [NM_002252]	NM_002252
A_23_P133123	1.06E-03	3.29	NM_032117	NM_032117	Homo sapiens GAJ protein (GAJ), mRNA [NM_032117]	NM_032117
A_23_P116123	1.06E-03	3.73	NM_001274	NM_001274	Homo sapiens CHK1 checkpoint homolog (S. pombe) (CHEK1), mRNA [NM_001274]	NM_001274
A_24_P407742	1.07E-03	2.77	A_24_P407742			
A_23_P344853	1.07E-03	3.08	ENST00000296126		Homo sapiens KIAA0007 mRNA, partial cds. [D26488]	XM_087089
A_23_P102320	1.07E-03	3.30	NM_138285	NM_138285	Homo sapiens nucleoporin 35kDa (NUP35), transcript variant 1, mRNA [NM_138285]	NM_138285
A_23_P97265	1.07E-03	4.46	NM_017725	NM_017725	Homo sapiens G patch domain containing 4 (GPATC4), transcript variant 3, mRNA [NM_017725]	NM_017725
A_23_P125705	1.07E-03	6.94	NM_021963	NM_021963	Homo sapiens nucleosome assembly protein 1-like 2 (NAP1L2), mRNA [NM_021963]	NM_021963
A_23_P121106	1.07E-03	7.94	NM_003865	NM_003865	Homo sapiens homeo box (expressed in ES cells) 1 (HESX1), mRNA [NM_003865]	NM_003865
A_32_P55860	1.08E-03	2.01	NM_182620	NM_182620	Homo sapiens family with sequence similarity 33, member A (FAM33A), mRNA [NM_182620]	NM_182620
A_24_P252705	1.08E-03	2.99	NM_004412	NM_004412	Homo sapiens DNA (cytosine-5-)-methyltransferase 2 (DNMT2), transcript variant a, mRNA [NM_004412]	NM_004412
A_24_P137713	1.08E-03	3.65	NM_030899	NM_030899	Homo sapiens zinc finger protein 323 (ZNF323), transcript variant 1, mRNA [NM_030899]	NM_030899
A_24_P683011	1.08E-03	4.02	BM696546	BM696546	UI-E-DW0-agk-i-01-0-UI.r1 UI-E-DW0 Homo sapiens cDNA clone UI-E-DW0-agk-i-01-0-UI 5', mRNA sequence [BM696546]	
A_23_P130141	1.08E-03	4.12	NM_020162	NM_020162	Homo sapiens DEAH (Asp-Glu-Ala-His) box polypeptide 33 (DHX33), mRNA [NM_020162]	NM_020162
A_23_P49972	1.08E-03	4.65	NM_001254	NM_001254	Homo sapiens CDC6 cell division cycle 6 homolog (S. cerevisiae) (CDC6), mRNA [NM_001254]	NM_001254
A_23_P72059	1.08E-03	5.49	ENST00000359236		Homo sapiens cDNA FLJ20674 fis, clone KAIA4450. [AK000681]	
A_32_P171328	1.10E-03	2.16	NM_014501	NM_014501	Homo sapiens ubiquitin-conjugating enzyme E2S (UBE2S), mRNA [NM_014501]	NM_014501
A_24_P8075	1.10E-03	2.44	AY251274	AY251274	Homo sapiens unknown mRNA. [AY251274]	
A_24_P83758	1.10E-03	3.77	ENST00000292728		Homo sapiens mRNA for KIAA1653 protein, partial cds. [AB051440]	XM_496493

A_32_P192970	1.10E-03	5.35	NM_170726	NM_170726	Homo sapiens aldehyde dehydrogenase 4 family, member A1 (ALDH4A1), nuclear gene encoding mitochondrial protein, transcript variant P5CDhS, mRNA [NM_170726]	NM_170726
A_23_P168167	1.11E-03	3.70	NM_032511	NM_032511	Homo sapiens chromosome 6 open reading frame 168 (C6orf168), mRNA [NM_032511]	NM_032511
A_23_P134517	1.12E-03	2.43	NM_033224	NM_033224	Homo sapiens purine-rich element binding protein B (PURB), mRNA [NM_033224]	NM_033224
A_23_P165130	1.12E-03	2.67	NM_032683	NM_032683	Homo sapiens hypothetical protein MGC12972 (FKSG24), mRNA [NM_032683]	NM_032683
A_23_P92093	1.12E-03	2.96	NM_001407	NM_001407	Homo sapiens cadherin, EGF LAG seven-pass G-type receptor 3 (flamingo homolog, Drosophila) (CELSR3), mRNA [NM_001407]	NM_001407
A_32_P6221	1.13E-03	2.01	ENST00000359244		Homo sapiens cDNA FLJ31209 fis, clone KIDNE2003377. [AK055771]	
A_23_P94380	1.13E-03	4.74	NM_001002260	NM_001002260	Homo sapiens chromosome 9 open reading frame 58 (C9orf58), transcript variant 2, mRNA [NM_001002260]	NM_001002260
A_23_P124417	1.13E-03	6.06	NM_004336	NM_004336	Homo sapiens BUB1 budding uninhibited by benzimidazoles 1 homolog (yeast) (BUB1), mRNA [NM_004336]	NM_004336
A_23_P104676	1.14E-03	2.15	NM_005877	NM_005877	Homo sapiens splicing factor 3a, subunit 1, 120kDa (SF3A1), transcript variant 1, mRNA [NM_005877]	NM_005877
A_32_P7783	1.14E-03	2.52	AW377662	AW377662	AW377662 PM0-CT0237-141099-001-e02 CT0237 Homo sapiens cDNA, mRNA sequence [AW377662]	
A_23_P123596	1.14E-03	3.17	NM_000170	NM_000170	Homo sapiens glycine dehydrogenase (decarboxylating; glycine decarboxylase, glycine cleavage system protein P) (GLDC), mRNA [NM_000170]	NM_000170
A_23_P101374	1.14E-03	3.53	NM_030622	NM_030622	Homo sapiens cytochrome P450, family 2, subfamily S, polypeptide 1 (CYP2S1), mRNA [NM_030622]	NM_030622
A_23_P323749	1.14E-03	3.98	NM_025049	NM_025049	Homo sapiens chromosome 15 open reading frame 20 (C15orf20), mRNA [NM_025049]	NM_025049
A_32_P19294	1.14E-03	4.29	NM_144669	NM_144669	Homo sapiens hypothetical protein FLJ31978 (FLJ31978), mRNA [NM_144669]	NM_144669
A_24_P149036	1.14E-03	5.32	NM_001387	NM_001387	Homo sapiens dihydropyrimidinase-like 3 (DPYSL3), mRNA [NM_001387]	NM_001387
A_24_P323598	1.15E-03	2.21	NM_001017420	NM_001017420	Homo sapiens establishment of cohesion 1 homolog 2 (S. cerevisiae) (ESCO2), mRNA [NM_001017420]	NM_001017420
A_23_P314591	1.15E-03	2.62	NM_006166	NM_006166	Homo sapiens nuclear transcription factor Y, beta (NFYB), mRNA [NM_006166]	NM_006166
A_23_P77066	1.15E-03	5.24	NM_022807	NM_022807	Homo sapiens small nuclear ribonucleoprotein polypeptide N (SNRPN), transcript variant 4, mRNA [NM_022807]	NM_022807
A_23_P119964	1.15E-03	5.38	NM_005760	NM_005760	Homo sapiens CCAAT/enhancer binding protein zeta (CEBPZ), mRNA [NM_005760]	NM_005760
A_23_P54736	1.15E-03	5.81	NM_016541	NM_016541	Homo sapiens guanine nucleotide binding protein (G protein), gamma 13 (GNG13), mRNA [NM_016541]	NM_016541
A_23_P130466	1.16E-03	2.38	NM_021089	NM_021089	Homo sapiens zinc finger protein 8 (clone HF.18) (ZNF8), mRNA [NM_021089]	NM_021089
A_23_P67391	1.16E-03	2.44	NM_007059	NM_007059	Homo sapiens kaptin (actin binding protein) (KPTN), mRNA [NM_007059]	NM_007059
A_23_P122197	1.16E-03	2.46	NM_031966	NM_031966	Homo sapiens cyclin B1 (CCNB1), mRNA [NM_031966]	NM_031966
A_23_P215517	1.16E-03	4.44	BC009555	BC009555	Homo sapiens kelch-like 7 (Drosophila), mRNA (cDNA clone IMAGE:3899090), complete cds. [BC009555]	
A_23_P152919	1.17E-03	2.33	NM_002532	NM_002532	Homo sapiens nucleoporin 88kDa (NUP88), mRNA [NM_002532]	NM_002532
A_24_P326739	1.19E-03	4.95	NM_013267	NM_013267	Homo sapiens glutaminase 2 (liver, mitochondrial) (GLS2), nuclear gene encoding mitochondrial protein, transcript variant 1, mRNA [NM_013267]	NM_013267
A_24_P102362	1.20E-03	2.48	ENST00000320159		Homo sapiens zinc finger protein 553, mRNA (cDNA clone IMAGE:3833591), partial cds. [BC007393]	
A_24_P664891	1.20E-03	33.11	AF020589	AF020589	Homo sapiens cytochrome C oxidase subunit VIa homolog mRNA, complete cds. [AF020589]	
A_24_P542375	1.21E-03	2.09	NM_002823	NM_002823	Homo sapiens prothymosin, alpha (gene sequence 28) (PTMA), mRNA [NM_002823]	NM_002823
A_24_P100664	1.21E-03	2.92	NM_170784	NM_170784	Homo sapiens McKusick-Kaufman syndrome (MKKS), transcript variant 2, mRNA [NM_170784]	NM_170784
A_23_P319970	1.21E-03	3.17	BC034271	BC034271	Homo sapiens Fanconi anemia, complementation group C, mRNA (cDNA clone IMAGE:4777682), with apparent retained intron. [BC034271]	
A_23_P364107	1.22E-03	2.58	NM_018353	NM_018353	Homo sapiens chromosome 14 open reading frame 106 (C14orf106), mRNA [NM_018353]	NM_018353
A_23_P154266	1.23E-03	2.05	NM_017952	NM_017952	Homo sapiens FLJ20758 protein (FLJ20758), mRNA [NM_017952]	NM_017952
A_23_P17204	1.23E-03	2.71	NM_022662	NM_022662	Homo sapiens anaphase promoting complex subunit 1 (ANAPC1), mRNA [NM_022662]	NM_022662
A_24_P935881	1.23E-03	2.99	NM_022978	NM_022978	Homo sapiens small EDRK-rich factor 1B (centromeric) (SERF1B), mRNA [NM_022978]	NM_022978
A_23_P146187	1.23E-03	5.95	NM_015169	NM_015169	Homo sapiens RRS1 ribosome biogenesis regulator homolog (S. cerevisiae) (RRS1), mRNA [NM_015169]	NM_015169
A_24_P935203	1.23E-03	56.82	THC2273930		AF285161 polyubiquitin C {Mus musculus;} , partial (41%) [THC2273930]	
A_24_P325035	1.24E-03	2.26	AK092090	AK092090	Homo sapiens cDNA FLJ34771 fis, clone NT2NE2003150. [AK092090]	
A_24_P161494	1.24E-03	2.56	ENST00000327591		PREDICTED: Homo sapiens similar to 40S ribosomal protein S26 (LOC401470), mRNA [XM_376787]	XM_376787
A_23_P10385	1.24E-03	2.99	NM_016448	NM_016448	Homo sapiens denticleless homolog (Drosophila) (DTL), mRNA [NM_016448]	NM_016448

A_23_P214533	1.24E-03	2.99	NM_030899	NM_030899	Homo sapiens zinc finger protein 323 (ZNF323), transcript variant 1, mRNA [NM_030899]	NM_030899
A_23_P99930	1.24E-03	3.44	NM_017858	NM_017858	Homo sapiens timeless-interacting protein (FLJ20516), mRNA [NM_017858]	NM_017858
A_24_P274795	1.24E-03	3.88	NM_018719	NM_018719	Homo sapiens cell division cycle associated 7-like (CDCA7L), mRNA [NM_018719]	NM_018719
A_23_P416468	1.24E-03	5.78	ENST00000268043		Homo sapiens DNA helicase homolog (PIF1) mRNA, partial cds. [AF108138]	
A_23_P312224	1.26E-03	2.45	NM_019022	NM_019022	Homo sapiens thioredoxin domain containing 10 (TXNDC10), mRNA [NM_019022]	NM_019022
A_32_P181722	1.26E-03	2.59	CR606587	CR606587	full-length cDNA clone CS0DI044YA04 of Placenta Cot 25-normalized of Homo sapiens (human). [CR606587]	
A_23_P252362	1.26E-03	2.78	NM_016640	NM_016640	Homo sapiens mitochondrial ribosomal protein S30 (MRPS30), nuclear gene encoding mitochondrial protein, mRNA [NM_016640]	NM_016640
A_24_P258073	1.27E-03	2.81	NM_021830	NM_021830	Homo sapiens progressive external ophthalmoplegia 1 (PEO1), mRNA [NM_021830]	NM_021830
A_23_P57306	1.27E-03	3.13	NM_005441	NM_005441	Homo sapiens chromatin assembly factor 1, subunit B (p60) (CHAF1B), mRNA [NM_005441]	NM_005441
A_23_P88331	1.27E-03	3.65	NM_014750	NM_014750	Homo sapiens discs, large homolog 7 (Drosophila) (DLG7), mRNA [NM_014750]	NM_014750
A_23_P90275	1.27E-03	3.82	NM_022467	NM_022467	Homo sapiens carbohydrate (N-acetylgalactosamine 4-0) sulfotransferase 8 (CHST8), mRNA [NM_022467]	NM_022467
A_23_P392384	1.27E-03	4.12	NM_001002260	NM_001002260	Homo sapiens chromosome 9 open reading frame 58 (C9orf58), transcript variant 2, mRNA [NM_001002260]	NM_001002260
A_32_P7974	1.27E-03	8.40	A_32_P7974			
A_23_P156025	1.27E-03	9.01	ENST00000302057		Homo sapiens homeodomain protein IRXA2 (IRX2) mRNA, complete cds. [AY335940]	
A_23_P94795	1.28E-03	2.92	NM_003213	NM_003213	Homo sapiens TEA domain family member 4 (TEAD4), transcript variant 1, mRNA [NM_003213]	NM_003213
A_24_P29876	1.28E-03	3.40	NM_018361	NM_018361	Homo sapiens 1-acylglycerol-3-phosphate O-acyltransferase 5 (lysophosphatidic acid acyltransferase, epsilon) (AGPAT5), mRNA [NM_018361]	NM_018361
A_24_P205589	1.30E-03	4.05	NM_181864	NM_181864	Homo sapiens brain acyl-CoA hydrolase (BACH), transcript variant hBACHb, mRNA [NM_181864]	NM_181864
A_24_P178523	1.30E-03	4.52	A_24_P178523			
A_23_P422851	1.31E-03	4.35	NM_138375	NM_138375	Homo sapiens Cdk5 and Abl enzyme substrate 1 (CABLES1), mRNA [NM_138375]	NM_138375
A_23_P41942	1.31E-03	16.75	NM_006467	NM_006467	Homo sapiens polymerase (RNA) III (DNA directed) polypeptide G (32kD) (POLR3G), mRNA [NM_006467]	NM_006467
A_23_P8241	1.32E-03	2.18	NM_012177	NM_012177	Homo sapiens F-box protein 5 (FBXO5), mRNA [NM_012177]	NM_012177
A_24_P377499	1.32E-03	2.44	NM_145323	NM_145323	Homo sapiens oxysterol binding protein-like 3 (OSBPL3), transcript variant 5, mRNA [NM_145323]	NM_145323
A_23_P430181	1.32E-03	2.58	NM_024784	NM_024784	Homo sapiens zinc finger and BTB domain containing 3 (ZBTB3), mRNA [NM_024784]	NM_024784
A_23_P325625	1.32E-03	2.72	NM_173690	NM_173690	Homo sapiens chromosome 9 open reading frame 126 (C9orf126), mRNA [NM_173690]	NM_173690
A_24_P41975	1.32E-03	5.32	AK056402	AK056402	Homo sapiens cDNA FLJ31840 fis, clone NT2RP7000109, highly similar to Homo sapiens putative RNA binding protein mRNA. [AK056402]	
A_24_P347624	1.34E-03	4.85	NM_022804	NM_022804	Homo sapiens SNRPN upstream reading frame (SNURF), transcript variant 2, mRNA [NM_022804]	NM_022804
A_23_P416112	1.35E-03	2.31	NM_152617	NM_152617	Homo sapiens ring finger protein 168 (RNF168), mRNA [NM_152617]	NM_152617
A_24_P901084	1.35E-03	2.84	XM_374965	XM_374965	PREDICTED: Homo sapiens similar to BC004636 protein (LOC400010), mRNA [XM_374965]	XM_374965
A_24_P928765	1.35E-03	8.70	THC2341283		A40201 artifact-warning sequence (translated ALU class A) - human {Homo sapiens;} , partial (13%) [THC2341283]	
A_23_P355447	1.35E-03	15.53	NM_174976	NM_174976	Homo sapiens zinc finger, DHHC-type containing 22 (ZDHHC22), mRNA [NM_174976]	NM_174976
A_23_P63789	1.36E-03	2.16	NM_001005414	NM_001005414	Homo sapiens ZW10 interactor (ZWINT), transcript variant 4, mRNA [NM_001005414]	NM_001005414
A_23_P339705	1.36E-03	2.93	NM_173636	NM_173636	Homo sapiens WD repeat domain 62 (WDR62), mRNA [NM_173636]	NM_173636
A_24_P187954	1.37E-03	2.24	NM_213654	NM_213654	Homo sapiens armadillo repeat containing 8 (ARMC8), mRNA [NM_213654]	NM_213654
A_23_P378526	1.37E-03	2.64	NM_016434	NM_016434	Homo sapiens regulator of telomere elongation helicase 1 (RTEL1), transcript variant 1, mRNA [NM_016434]	NM_016434
A_32_P74964	1.37E-03	3.26	AK055101	AK055101	Homo sapiens cDNA FLJ30539 fis, clone BRAWH2001255. [AK055101]	
A_23_P348146	1.37E-03	4.50	NM_144595	NM_144595	Homo sapiens hypothetical protein FLJ30046 (FLJ30046), mRNA [NM_144595]	NM_144595
A_23_P259692	1.38E-03	2.87	NM_058179	NM_058179	Homo sapiens phosphoserine aminotransferase 1 (PSAT1), transcript variant 1, mRNA [NM_058179]	NM_058179
A_23_P128532	1.38E-03	3.36	NM_152318	NM_152318	Homo sapiens hypothetical protein MGC40397 (MGC40397), mRNA [NM_152318]	NM_152318
A_24_P938169	1.38E-03	20.45	AF136171	AF136171	Homo sapiens heparin-binding protein HBp15 mRNA, complete cds. [AF136171]	
A_23_P98261	1.39E-03	2.06	NM_006396	NM_006396	Homo sapiens Sjogren's syndrome/scleroderma autoantigen 1 (SSSCA1), mRNA [NM_006396]	NM_006396
A_23_P364537	1.39E-03	2.10	NM_175066	NM_175066	Homo sapiens DEAD (Asp-Glu-Ala-Asp) box polypeptide 51 (DDX51), mRNA [NM_175066]	NM_175066

A_23_P345830	1.39E-03	2.25	NM_014929	NM_014929	Homo sapiens KIAA0971 (KIAA0971), mRNA [NM_014929]	NM_014929
A_24_P123347	1.40E-03	2.43	NM_002703	NM_002703	Homo sapiens phosphoribosyl pyrophosphate amidotransferase (PPAT), mRNA [NM_002703]	NM_002703
A_23_P213166	1.40E-03	2.61	NM_138698	NM_138698	Homo sapiens prematurely terminated mRNA decay factor-like (LOC91431), mRNA [NM_138698]	NM_138698
A_23_P204541	1.40E-03	5.62	BX648591	BX648591	Homo sapiens mRNA; cDNA DKFZp686G14198 (from clone DKFZp686G14198). [BX648591]	
A_23_P76761	1.41E-03	2.84	NM_003384	NM_003384	Homo sapiens vaccinia related kinase 1 (VRK1), mRNA [NM_003384]	NM_003384
A_24_P58894	1.41E-03	3.23	XM_378054	XM_378054	PREDICTED: Homo sapiens similar to hypothetical protein (LOC402360), mRNA [XM_378054]	XM_378054
A_23_P74914	1.41E-03	3.92	NM_014777	NM_014777	Homo sapiens KIAA0133 (KIAA0133), mRNA [NM_014777]	NM_014777
A_23_P37676	1.41E-03	5.43	NM_007223	NM_007223	Homo sapiens putative G protein coupled receptor (GPR), mRNA [NM_007223]	NM_007223
A_23_P5415	1.42E-03	2.10	NM_021824	NM_021824	Homo sapiens NIF3 NGG1 interacting factor 3-like 1 (S. pombe) (NIF3L1), mRNA [NM_021824]	NM_021824
A_24_P460195	1.42E-03	2.29	A_24_P460195			
A_24_P917866	1.42E-03	2.31	NM_003011	NM_003011	Homo sapiens SET translocation (myeloid leukemia-associated) (SET), mRNA [NM_003011]	NM_003011
A_32_P59262	1.42E-03	2.38	BM986990	BM986990	BM986990 UI-H-CO0-aqe-h-08-0-UI.s1 NCI_CGAP_Sub9 Homo sapiens cDNA clone IMAGE:3104077 3', mRNA sequence [BM986990]	
A_32_P72181	1.42E-03	2.79	BC035184	BC035184	Homo sapiens cDNA clone IMAGE:5266408, partial cds. [BC035184]	
A_23_P257863	1.42E-03	3.03	NM_012073	NM_012073	Homo sapiens chaperonin containing TCP1, subunit 5 (epsilon) (CCT5), mRNA [NM_012073]	NM_012073
A_24_P113144	1.42E-03	3.30	NM_024857	NM_024857	Homo sapiens chromosome 17 open reading frame 41 (C17orf41), mRNA [NM_024857]	NM_024857
A_24_P683013	1.42E-03	3.60	BM696546	BM696546	UI-E-DW0-agk-i-01-0-UI.r1 UI-E-DW0 Homo sapiens cDNA clone UI-E-DW0-agk-i-01-0-UI 5', mRNA sequence [BM696546]	
A_23_P429491	1.42E-03	4.50	NM_145018	NM_145018	Homo sapiens hypothetical protein FLJ25416 (FLJ25416), mRNA [NM_145018]	NM_145018
A_32_P170664	1.45E-03	2.06	AK024898	AK024898	Homo sapiens cDNA: FLJ21245 fis, clone COL01184. [AK024898]	
A_23_P9392	1.45E-03	2.31	NM_016390	NM_016390	Homo sapiens chromosome 9 open reading frame 114 (C9orf114), mRNA [NM_016390]	NM_016390
A_23_P74954	1.45E-03	4.00	NM_018715	NM_018715	Homo sapiens regulator of chromosome condensation 2 (RCC2), mRNA [NM_018715]	NM_018715
A_23_P121234	1.45E-03	4.26	A_23_P121234			
A_23_P18818	1.47E-03	4.48	NM_015455	NM_015455	Homo sapiens CCR4-NOT transcription complex, subunit 6 (CNOT6), mRNA [NM_015455]	NM_015455
A_23_P404778	1.47E-03	7.04	NM_012465	NM_012465	Homo sapiens tolloid-like 2 (TLL2), mRNA [NM_012465]	NM_012465
A_23_P148984	1.48E-03	2.76	NM_018122	NM_018122	Homo sapiens aspartyl-tRNA synthetase 2 (mitochondrial) (DARS2), mRNA [NM_018122]	NM_018122
A_23_P254288	1.49E-03	2.10	A_23_P254288			
A_23_P207999	1.49E-03	11.40	NM_021127	NM_021127	Homo sapiens phorbol-12-myristate-13-acetate-induced protein 1 (PMAIP1), mRNA [NM_021127]	NM_021127
A_23_P210747	1.50E-03	2.13	NM_015939	NM_015939	Homo sapiens CGI-09 protein (CGI-09), mRNA [NM_015939]	NM_015939
A_23_P383835	1.50E-03	2.14	NM_005891	NM_005891	Homo sapiens acetyl-Coenzyme A acetyltransferase 2 (acetoacetyl Coenzyme A thiolase) (ACAT2), mRNA [NM_005891]	NM_005891
A_32_P202588	1.50E-03	2.24	BU620016	BU620016	UI-H-FH1-bfr-e-10-0-UI.s1 NCI_CGAP_FH1 Homo sapiens cDNA clone UI-H-FH1-bfr-e-10-0-UI 3', mRNA sequence [BU620016]	
A_23_P32558	1.50E-03	2.37	NM_017588	NM_017588	Homo sapiens WD repeat domain 5 (WDR5), transcript variant 1, mRNA [NM_017588]	NM_017588
A_24_P850336	1.50E-03	2.59	A_24_P850336			
A_23_P406425	1.50E-03	3.08	NM_173359	NM_173359	Homo sapiens eukaryotic translation initiation factor 4E member 3 (EIF4E3), mRNA [NM_173359]	NM_173359
A_23_P115167	1.50E-03	5.03	NM_015350	NM_015350	Homo sapiens leucine rich repeat containing 8 family, member B (LRRC8B), mRNA [NM_015350]	NM_015350
A_23_P66732	1.50E-03	5.05	NM_031965	NM_031965	Homo sapiens germ cell associated 2 (haspin) (GSG2), mRNA [NM_031965]	NM_031965
A_23_P434900	1.52E-03	2.11	NM_144570	NM_144570	Homo sapiens chromosome 16 open reading frame 34 (C16orf34), mRNA [NM_144570]	NM_144570
A_24_P515319	1.52E-03	2.58	AL832996	AL832996	Homo sapiens mRNA; cDNA DKFZp666M073 (from clone DKFZp666M073). [AL832996]	XM_496956
A_23_P143994	1.52E-03	2.83	NM_001018115	NM_001018115	Homo sapiens Fanconi anemia, complementation group D2 (FANCD2), transcript variant 2, mRNA [NM_001018115]	NM_001018115
A_23_P258321	1.53E-03	2.49	NM_015969	NM_015969	Homo sapiens mitochondrial ribosomal protein S17 (MRPS17), nuclear gene encoding mitochondrial protein, mRNA [NM_015969]	NM_015969
A_23_P132956	1.53E-03	2.89	NM_004181	NM_004181	Homo sapiens ubiquitin carboxyl-terminal esterase L1 (ubiquitin thiolesterase) (UCHL1), mRNA [NM_004181]	NM_004181
A_23_P155989	1.54E-03	2.05	NM_022145	NM_022145	Homo sapiens leucine zipper protein FKSG14 (FKSG14), mRNA [NM_022145]	NM_022145

A_24_P922631	1.54E-03	4.61	BC092511	BC092511	Homo sapiens cDNA clone IMAGE:5271968. [BC092511]	XM_059672
A_23_P90601	1.55E-03	3.77	NM_182915	NM_182915	Homo sapiens STEAP family member 3 (STEAP3), transcript variant 1, mRNA [NM_182915]	NM_182915
A_23_P43800	1.55E-03	4.12	NM_015201	NM_015201	Homo sapiens block of proliferation 1 (BOP1), mRNA [NM_015201]	NM_015201
A_23_P252155	1.57E-03	2.54	NM_018387	NM_018387	Homo sapiens spermatid perinuclear RNA binding protein (STRBP), mRNA [NM_018387]	NM_018387
A_23_P145541	1.60E-03	2.15	NM_014845	NM_014845	Homo sapiens KIAA0274 (KIAA0274), mRNA [NM_014845]	NM_014845
A_23_P383060	1.60E-03	2.30	NM_021095	NM_021095	Homo sapiens solute carrier family 5 (sodium-dependent vitamin transporter), member 6 (SLC5A6), mRNA [NM_021095]	NM_021095
A_23_P70991	1.60E-03	2.61	NM_006303	NM_006303	Homo sapiens JTV1 gene (JTV1), mRNA [NM_006303]	NM_006303
A_24_P127063	1.60E-03	2.72	ENST00000321482			
A_23_P156996	1.60E-03	2.84	AF086442	AF086442	Homo sapiens full length insert cDNA clone ZD81B04. [AF086442]	XM_499391
A_23_P349416	1.61E-03	2.44	NM_001982	NM_001982	Homo sapiens v-erb-b2 erythroblastic leukemia viral oncogene homolog 3 (avian) (ERBB3), transcript variant 1, mRNA [NM_001982]	NM_001982
A_23_P55073	1.61E-03	2.92	NM_015462	NM_015462	Homo sapiens DKFZP586L0724 protein (DKFZP586L0724), mRNA [NM_015462]	NM_015462
A_24_P364970	1.61E-03	3.40	NM_020162	NM_020162	Homo sapiens DEAH (Asp-Glu-Ala-His) box polypeptide 33 (DHX33), mRNA [NM_020162]	NM_020162
A_23_P45699	1.62E-03	2.53	NM_003902	NM_003902	Homo sapiens far upstream element (FUSE) binding protein 1 (FUBP1), mRNA [NM_003902]	NM_003902
A_23_P216655	1.62E-03	2.63	NM_014788	NM_014788	Homo sapiens tripartite motif-containing 14 (TRIM14), transcript variant 1, mRNA [NM_014788]	NM_014788
A_23_P153197	1.62E-03	2.65	NM_170695	NM_170695	Homo sapiens TGFB-induced factor (TALE family homeobox) (TGIF), transcript variant 1, mRNA [NM_170695]	NM_170695
A_24_P392496	1.62E-03	3.07	ENST00000315293			XM_372099
A_23_P22647	1.62E-03	4.69	NM_015686	NM_015686	Homo sapiens transmembrane protein 28 (TMEM28), mRNA [NM_015686]	NM_015686
A_24_P222043	1.62E-03	6.13	NM_002131	NM_002131	Homo sapiens high mobility group AT-hook 1 (HMGA1), transcript variant 2, mRNA [NM_002131]	NM_002131
A_23_P3237	1.63E-03	2.29	NM_025165	NM_025165	Homo sapiens elongation factor RNA polymerase II-like 3 (ELL3), mRNA [NM_025165]	NM_025165
A_32_P169500	1.63E-03	2.42	THC2433217		ALU1_HUMAN (P39188) Alu subfamily J sequence contamination warning entry, partial (14%) [THC2433217]	
A_32_P103633	1.63E-03	3.08	NM_004526	NM_004526	Homo sapiens MCM2 minichromosome maintenance deficient 2, mitotin (S. cerevisiae) (MCM2), mRNA [NM_004526]	NM_004526
A_23_P108948	1.63E-03	3.18	NM_018000	NM_018000	Homo sapiens dilute suppressor (DSU), mRNA [NM_018000]	NM_018000
A_23_P137578	1.63E-03	3.61	NM_015176	NM_015176	Homo sapiens F-box protein 28 (FBXO28), mRNA [NM_015176]	NM_015176
A_23_P12199	1.63E-03	5.10	NM_052943	NM_052943	Homo sapiens family with sequence similarity 46, member B (FAM46B), mRNA [NM_052943]	NM_052943
A_23_P55045	1.64E-03	2.31	NM_021734	NM_021734	Homo sapiens solute carrier family 25 (mitochondrial deoxynucleotide carrier), member 19 (SLC25A19), mRNA [NM_021734]	NM_021734
A_23_P122775	1.65E-03	2.07	NM_032730	NM_032730	Homo sapiens reticulon 4 interacting protein 1 (RTN4IP1), nuclear gene encoding mitochondrial protein, mRNA [NM_032730]	NM_032730
A_23_P152055	1.65E-03	2.51	NM_024580	NM_024580	Homo sapiens elongation factor Tu GTP binding domain containing 1 (EFTUD1), mRNA [NM_024580]	NM_024580
A_23_P165247	1.65E-03	2.56	NM_170711	NM_170711	Homo sapiens DAZ associated protein 1 (DAZAP1), transcript variant 1, mRNA [NM_170711]	NM_170711
A_32_P1614	1.65E-03	3.24	AK023018	AK023018	Homo sapiens cDNA FLJ12956 fis, clone NT2RP2005501. [AK023018]	
A_23_P108042	1.65E-03	4.22	NM_004829	NM_004829	Homo sapiens natural cytotoxicity triggering receptor 1 (NCR1), mRNA [NM_004829]	NM_004829
A_23_P160200	1.65E-03	4.29	NM_023070	NM_023070	Homo sapiens zinc finger protein 643 (ZNF643), mRNA [NM_023070]	NM_023070
A_23_P156562	1.65E-03	6.25	A_23_P156562			
A_23_P130470	1.66E-03	2.36	NM_021089	NM_021089	Homo sapiens zinc finger protein 8 (clone HF.18) (ZNF8), mRNA [NM_021089]	NM_021089
A_24_P234196	1.66E-03	3.34	NM_001034	NM_001034	Homo sapiens ribonucleotide reductase M2 polypeptide (RRM2), mRNA [NM_001034]	NM_001034
A_32_P227400	1.67E-03	2.40	A_32_P227400			
A_23_P1387	1.67E-03	2.58	NM_032900	NM_032900	Homo sapiens Rho GTPase activating protein 19 (ARHGAP19), mRNA [NM_032900]	NM_032900
A_23_P405761	1.67E-03	3.12	NM_012250	NM_012250	Homo sapiens related RAS viral (r-ras) oncogene homolog 2 (RRAS2), mRNA [NM_012250]	NM_012250
A_23_P415443	1.67E-03	3.38	NM_015341	NM_015341	Homo sapiens barren homolog (Drosophila) (BRRN1), mRNA [NM_015341]	NM_015341
A_23_P252740	1.67E-03	5.71	NM_024094	NM_024094	Homo sapiens defective in sister chromatid cohesion homolog 1 (S. cerevisiae) (DCC1), mRNA [NM_024094]	NM_024094
A_23_P110851	1.67E-03	5.88	NM_003219	NM_003219	Homo sapiens telomerase reverse transcriptase (TERT), transcript variant 1, mRNA [NM_003219]	NM_003219

A_23_P9152	1.68E-03	2.16	NM_005772	NM_005772	Homo sapiens RNA terminal phosphate cyclase-like 1 (RCL1), mRNA [NM_005772]	NM_005772
A_23_P416965	1.68E-03	2.67	NM_015398	NM_015398	Homo sapiens DKFZP564J102 protein (DKFZP564J102), transcript variant 1, mRNA [NM_015398]	NM_015398
A_32_P87531	1.68E-03	3.80	BC042869	BC042869	Homo sapiens chromosome 1 open reading frame 67, mRNA (cDNA clone MGC:51214 IMAGE:5270407), complete cds. [BC042869]	XM_290922
A_23_P380998	1.69E-03	2.09	NM_015361	NM_015361	Homo sapiens R3H domain (binds single-stranded nucleic acids) containing (R3HDM), mRNA [NM_015361]	NM_015361
A_23_P122579	1.69E-03	2.10	NM_001350	NM_001350	Homo sapiens death-associated protein 6 (DAXX), mRNA [NM_001350]	NM_001350
A_23_P82941	1.69E-03	2.22	NM_006421	NM_006421	Homo sapiens ADP-ribosylation factor guanine nucleotide-exchange factor 1(brefeldin A-inhibited) (ARFGEF1), mRNA [NM_006421]	NM_006421
A_23_P143748	1.69E-03	2.43	NM_015140	NM_015140	Homo sapiens KIAA0153 protein (KIAA0153), mRNA [NM_015140]	NM_015140
A_23_P402604	1.69E-03	2.54	NM_012393	NM_012393	Homo sapiens phosphoribosylformylglycinamidine synthase (FGAR amidotransferase) (PFAS), mRNA [NM_012393]	NM_012393
A_24_P200000	1.69E-03	3.31	NM_182915	NM_182915	Homo sapiens STEAP family member 3 (STEAP3), transcript variant 1, mRNA [NM_182915]	NM_182915
A_23_P393034	1.70E-03	11.17	NM_005329	NM_005329	Homo sapiens hyaluronan synthase 3 (HAS3), transcript variant 1, mRNA [NM_005329]	NM_005329
A_24_P252497	1.71E-03	2.06	NM_025195	NM_025195	Homo sapiens tribbles homolog 1 (Drosophila) (TRIB1), mRNA [NM_025195]	NM_025195
A_23_P18384	1.71E-03	2.77	NM_213654	NM_213654	Homo sapiens armadillo repeat containing 8 (ARMC8), mRNA [NM_213654]	NM_213654
A_32_P38645	1.71E-03	6.67	NM_182970	NM_182970	Homo sapiens regulating synaptic membrane exocytosis 4 (RIMS4), mRNA [NM_182970]	NM_182970
A_23_P300797	1.73E-03	2.26	NM_173529	NM_173529	Homo sapiens chromosome 18 open reading frame 54 (C18orf54), mRNA [NM_173529]	NM_173529
A_23_P141434	1.73E-03	2.26	NM_000664	NM_000664	Homo sapiens acetyl-Coenzyme A carboxylase alpha (ACACA), transcript variant 6, mRNA [NM_000664]	NM_000664
A_23_P99253	1.73E-03	3.70	NM_004664	NM_004664	Homo sapiens lin-7 homolog A (C. elegans) (LIN7A), mRNA [NM_004664]	NM_004664
A_23_P349147	1.73E-03	8.13	ENST00000305423		Homo sapiens MUC3B mRNA for intestinal mucin, partial cds. [AB038783]	XM_168578
A_23_P136721	1.73E-03	19.84	U88896	U88896	Human endogenous retrovirus H protease/integrase-derived ORF1, ORF2, and putative envelope protein mRNA, complete cds. [U88896]	
A_32_P51894	1.75E-03	2.21	CR624054	CR624054	full-length cDNA clone CS0DC029YL12 of Neuroblastoma Cot 25-normalized of Homo sapiens (human). [CR624054]	
A_24_P359205	1.75E-03	2.85	BC010094	BC010094	Homo sapiens phosphatidylinositol glycan, class Q, mRNA (cDNA clone IMAGE:3357878), partial cds. [BC010094]	
A_23_P320261	1.76E-03	2.07	NM_033317	NM_033317	Homo sapiens dermokine (ZD52F10), mRNA [NM_033317]	NM_033317
A_23_P214907	1.76E-03	2.39	NM_015440	NM_015440	Homo sapiens methylenetetrahydrofolate dehydrogenase (NADP+ dependent) 1-like (MTHFD1L), mRNA [NM_015440]	NM_015440
A_24_P278762	1.76E-03	2.40	NM_016534	NM_016534	Homo sapiens apoptosis-related protein PNAS-1 (FLJ39616), mRNA [NM_016534]	NM_016534
A_23_P154929	1.76E-03	2.65	NM_197966	NM_197966	Homo sapiens BH3 interacting domain death agonist (BID), transcript variant 1, mRNA [NM_197966]	NM_197966
A_24_P91852	1.76E-03	4.13	NM_006520	NM_006520	Homo sapiens t-complex-associated-testis-expressed 1-like (TCTE1L), mRNA [NM_006520]	NM_006520
A_23_P218965	1.77E-03	2.16	NM_020117	NM_020117	Homo sapiens leucyl-tRNA synthetase (LARS), mRNA [NM_020117]	NM_020117
A_23_P69738	1.78E-03	3.28	NM_023940	NM_023940	Homo sapiens RAS-like, family 11, member B (RASL11B), mRNA [NM_023940]	NM_023940
A_24_P90022	1.78E-03	5.46	BC064610	BC064610	Homo sapiens cDNA clone IMAGE:5548247, partial cds. [BC064610]	
A_23_P152181	1.79E-03	2.01	NM_018119	NM_018119	Homo sapiens polymerase (RNA) III (DNA directed) polypeptide E (80kD) (POLR3E), mRNA [NM_018119]	NM_018119
A_23_P108028	1.79E-03	2.13	NM_007145	NM_007145	Homo sapiens zinc finger protein 146 (ZNF146), mRNA [NM_007145]	NM_007145
A_24_P921933	1.79E-03	3.13	CR611166	CR611166	full-length cDNA clone CS0CAP007YF02 of Thymus of Homo sapiens (human). [CR611166]	
A_23_P136787	1.79E-03	5.24	NM_032336	NM_032336	Homo sapiens SLD5 homolog (SLD5), mRNA [NM_032336]	NM_032336
A_23_P41267	1.80E-03	2.08	BC010526	BC010526	Homo sapiens hypothetical LOC401127, mRNA (cDNA clone MGC:18091 IMAGE:4151462), complete cds. [BC010526]	
A_24_P102726	1.80E-03	2.08	NM_000309	NM_000309	Homo sapiens protoporphyrinogen oxidase (PPOX), nuclear gene encoding mitochondrial protein, mRNA [NM_000309]	NM_000309
A_23_P135357	1.80E-03	2.11	NM_018146	NM_018146	Homo sapiens RNA methyltransferase like 1 (RNMTL1), mRNA [NM_018146]	NM_018146
A_23_P20683	1.80E-03	2.51	NM_014878	NM_014878	Homo sapiens KIAA0020 (KIAA0020), mRNA [NM_014878]	NM_014878
A_23_P144726	1.80E-03	3.27	NM_016391	NM_016391	Homo sapiens hypothetical protein HSPC111 (HSPC111), mRNA [NM_016391]	NM_016391
A_23_P114826	1.82E-03	2.01	NM_031280	NM_031280	Homo sapiens mitochondrial ribosomal protein S15 (MRPS15), nuclear gene encoding mitochondrial protein, mRNA [NM_031280]	NM_031280
A_23_P502425	1.82E-03	2.06	NM_020409	NM_020409	Homo sapiens mitochondrial ribosomal protein L47 (MRPL47), nuclear gene encoding mitochondrial protein, transcript variant 1, mRNA [NM_020409]	NM_020409

A_24_P127462	1.82E-03	3.40	A_24_P127462			
A_23_P112159	1.82E-03	3.66	NM_012154	NM_012154	Homo sapiens eukaryotic translation initiation factor 2C, 2 (EIF2C2), mRNA [NM_012154]	NM_012154
A_23_P87482	1.83E-03	2.07	NM_024068	NM_024068	Homo sapiens hypothetical protein MGC2731 (MGC2731), mRNA [NM_024068]	NM_024068
A_23_P109794	1.83E-03	2.12	NM_015199	NM_015199	Homo sapiens ankyrin repeat domain 28 (ANKRD28), mRNA [NM_015199]	NM_015199
A_23_P162476	1.83E-03	2.12	NM_031299	NM_031299	Homo sapiens cell division cycle associated 3 (CDCA3), mRNA [NM_031299]	NM_031299
A_23_P41917	1.83E-03	2.32	NM_004272	NM_004272	Homo sapiens homer homolog 1 (Drosophila) (HOMER1), mRNA [NM_004272]	NM_004272
A_23_P109171	1.83E-03	3.26	NM_001195	NM_001195	Homo sapiens beaded filament structural protein 1, filensin (BFSP1), mRNA [NM_001195]	NM_001195
A_23_P97046	1.83E-03	3.66	NM_000478	NM_000478	Homo sapiens alkaline phosphatase, liver/bone/kidney (ALPL), mRNA [NM_000478]	NM_000478
A_23_P98042	1.83E-03	3.95	A_23_P98042			
A_23_P45799	1.83E-03	6.02	NM_004153	NM_004153	Homo sapiens origin recognition complex, subunit 1-like (yeast) (ORC1L), mRNA [NM_004153]	NM_004153
A_23_P123413	1.83E-03	18.66	NM_014729	NM_014729	Homo sapiens thymus high mobility group box protein TOX (TOX), mRNA [NM_014729]	NM_014729
A_23_P1948	1.84E-03	2.23	NM_024099	NM_024099	Homo sapiens hypothetical protein MGC2477 (MGC2477), mRNA [NM_024099]	NM_024099
A_24_P552987	1.84E-03	2.38	A_24_P552987			
A_23_P110802	1.84E-03	2.44	NM_022909	NM_022909	Homo sapiens centromere protein H (CENPH), mRNA [NM_022909]	NM_022909
A_23_P253524	1.84E-03	2.72	NM_001813	NM_001813	Homo sapiens centromere protein E, 312kDa (CENPE), mRNA [NM_001813]	NM_001813
A_32_P148199	1.84E-03	5.05	BM667833	BM667833	BM667833 UI-E-DW0-agj-g-19-0-UI.s1 UI-E-DW0 Homo sapiens cDNA clone UI-E-DW0-agj-g-19-0-UI 3', mRNA sequence [BM667833]	
A_23_P108294	1.84E-03	5.24	NM_177543	NM_177543	Homo sapiens phosphatidic acid phosphatase type 2C (PPAP2C), transcript variant 3, mRNA [NM_177543]	NM_177543
A_23_P28153	1.84E-03	5.95	AF175767	AF175767	Homo sapiens putative selenocysteine lyase (SCLY) mRNA, complete cds. [AF175767]	
A_32_P232559	1.84E-03	6.21	AY007155	AY007155	Homo sapiens clone CDABP0095 mRNA sequence. [AY007155]	XM_498474
A_23_P42718	1.84E-03	12.64	NM_004289	NM_004289	Homo sapiens nuclear factor (erythroid-derived 2)-like 3 (NFE2L3), mRNA [NM_004289]	NM_004289
A_32_P11499	1.85E-03	2.12	NM_003348	NM_003348	Homo sapiens ubiquitin-conjugating enzyme E2N (UBC13 homolog, yeast) (UBE2N), mRNA [NM_003348]	NM_003348
A_24_P253755	1.85E-03	2.16	NM_004278	NM_004278	Homo sapiens phosphatidylinositol glycan, class L (PIGL), mRNA [NM_004278]	NM_004278
A_24_P208794	1.85E-03	2.17	NM_001640	NM_001640	Homo sapiens N-acylaminoacyl-peptide hydrolase (APEH), mRNA [NM_001640]	NM_001640
A_24_P101402	1.85E-03	2.31	NM_006392	NM_006392	Homo sapiens nucleolar protein 5A (56kDa with KKE/D repeat) (NOL5A), mRNA [NM_006392]	NM_006392
A_24_P385341	1.85E-03	2.33	NM_014388	NM_014388	Homo sapiens chromosome 1 open reading frame 107 (C1orf107), mRNA [NM_014388]	NM_014388
A_24_P736638	1.85E-03	2.65	A_24_P736638			
A_23_P114783	1.85E-03	2.73	NM_001618	NM_001618	Homo sapiens poly (ADP-ribose) polymerase family, member 1 (PARP1), mRNA [NM_001618]	NM_001618
A_23_P74950	1.85E-03	3.70	NM_018715	NM_018715	Homo sapiens regulator of chromosome condensation 2 (RCC2), mRNA [NM_018715]	NM_018715
A_23_P344568	1.85E-03	6.13	NM_145019	NM_145019	Homo sapiens hypothetical protein FLJ30707 (FLJ30707), mRNA [NM_145019]	NM_145019
A_32_P72351	1.85E-03	7.81	AK026140	AK026140	Homo sapiens cDNA: FLJ22487 fis, clone HRC10931. [AK026140]	
A_23_P204745	1.86E-03	3.09	NM_139071	NM_139071	Homo sapiens SWI/SNF related, matrix associated, actin dependent regulator of chromatin, subfamily d, member 1 (SMARCD1), transcript variant 2, mRNA [NM_139071]	NM_139071
A_23_P203947	1.87E-03	2.04	NM_030655	NM_030655	Homo sapiens DEAD/H (Asp-Glu-Ala-Asp/His) box polypeptide 11 (CHL1-like helicase homolog, S. cerevisiae) (DDX11), transcript variant 3, mRNA [NM_030655]	NM_030655
A_23_P150069	1.88E-03	2.16	NM_001009943	NM_001009943	Homo sapiens ankyrin repeat domain 16 (ANKRD16), transcript variant 4, mRNA [NM_001009943]	NM_001009943
A_23_P425750	1.88E-03	2.33	NM_033415	NM_033415	Homo sapiens armadillo repeat containing 6 (ARMC6), mRNA [NM_033415]	NM_033415
A_32_P120084	1.88E-03	2.47	NM_014167	NM_014167	Homo sapiens HSPC128 protein (HSPC128), mRNA [NM_014167]	NM_014167
A_24_P52921	1.88E-03	2.63	NM_005504	NM_005504	Homo sapiens branched chain aminotransferase 1, cytosolic (BCAT1), mRNA [NM_005504]	NM_005504
A_23_P363936	1.88E-03	2.90	NM_014278	NM_014278	Homo sapiens heat shock 70kDa protein 4-like (HSPA4L), mRNA [NM_014278]	NM_014278
A_24_P924185	1.88E-03	3.92	S81524	S81524	RC1=NADH dehydrogenase subunit 3 homolog/ND3 homolog [human, renal cell carcinoma, mRNA Mitochondrial Partial, 65 nt]. [S81524]	
A_23_P74895	1.88E-03	7.09	NM_024674	NM_024674	Homo sapiens lin-28 homolog (C. elegans) (LIN28), mRNA [NM_024674]	NM_024674
A_23_P34325	1.88E-03	24.51	NM_033300	NM_033300	Homo sapiens low density lipoprotein receptor-related protein 8, apolipoprotein e receptor (LRP8), transcript variant 2, mRNA [NM_033300]	NM_033300

A_23_P39574	1.89E-03	2.07	NM_173466	NM_173466	Homo sapiens hypothetical protein DKFZp434P055 (DKFZp434P055), mRNA [NM_173466]	NM_173466
A_23_P5339	1.89E-03	2.16	NM_030577	NM_030577	Homo sapiens hypothetical protein MGC10993 (MGC10993), mRNA [NM_030577]	NM_030577
A_23_P345707	1.89E-03	3.08	NM_152259	NM_152259	Homo sapiens leucine-rich repeat kinase 1 (MGC45866), mRNA [NM_152259]	NM_152259
A_24_P119201	1.89E-03	3.25	NM_015832	NM_015832	Homo sapiens methyl-CpG binding domain protein 2 (MBD2), transcript variant testis-specific, mRNA [NM_015832]	NM_015832
A_23_P97161	1.89E-03	3.98	NM_003594	NM_003594	Homo sapiens transcription termination factor, RNA polymerase II (TTF2), mRNA [NM_003594]	NM_003594
A_24_P569294	1.90E-03	2.11	NM_021107	NM_021107	Homo sapiens mitochondrial ribosomal protein S12 (MRPS12), nuclear gene encoding mitochondrial protein, transcript variant 1, mRNA [NM_021107]	NM_021107
A_24_P20120	1.90E-03	2.20	NM_018084	NM_018084	Homo sapiens KIAA1212 (KIAA1212), mRNA [NM_018084]	NM_018084
A_24_P355493	1.90E-03	2.40	NM_022126	NM_022126	Homo sapiens phospholysine phosphohistidine inorganic pyrophosphate phosphatase (LHPP), mRNA [NM_022126]	NM_022126
A_23_P121945	1.90E-03	2.60	NM_001001502	NM_001001502	Homo sapiens synuclein, beta (SNCB), transcript variant 1, mRNA [NM_001001502]	NM_001001502
A_23_P43726	1.91E-03	2.28	NM_015231	NM_015231	Homo sapiens nucleoporin 160kDa (NUP160), mRNA [NM_015231]	NM_015231
A_23_P45917	1.91E-03	2.29	NM_001826	NM_001826	Homo sapiens CDC28 protein kinase regulatory subunit 1B (CKS1B), mRNA [NM_001826]	NM_001826
A_32_P107797	1.91E-03	2.81	A_32_P107797			
A_23_P111373	1.91E-03	3.01	NM_020662	NM_020662	Homo sapiens MRS2-like, magnesium homeostasis factor (S. cerevisiae) (MRS2L), mRNA [NM_020662]	NM_020662
A_23_P64184	1.91E-03	3.23	A_23_P64184			
A_32_P89415	1.91E-03	3.62	A_32_P89415			
A_24_P13041	1.91E-03	4.10	NM_145307	NM_145307	Homo sapiens pleckstrin homology domain containing, family K member 1 (PLEKHK1), mRNA [NM_145307]	NM_145307
A_23_P14295	1.92E-03	9.26	NM_020181	NM_020181	Homo sapiens chromosome 14 open reading frame 162 (C14orf162), mRNA [NM_020181]	NM_020181
A_23_P87759	1.93E-03	2.25	NM_006331	NM_006331	Homo sapiens C2f protein (C2F), mRNA [NM_006331]	NM_006331
A_24_P38081	1.93E-03	2.36	NM_004117	NM_004117	Homo sapiens FK506 binding protein 5 (FKBP5), mRNA [NM_004117]	NM_004117
A_23_P252201	1.93E-03	3.55	NM_018456	NM_018456	Homo sapiens ELL associated factor 2 (EAF2), mRNA [NM_018456]	NM_018456
A_32_P207789	1.93E-03	7.04	BQ017638	BQ017638	BQ017638 UI-H-DI0-auv-p-03-0-UI.s1 NCI_CGAP_DI0 Homo sapiens cDNA clone IMAGE:5875058 3', mRNA sequence [BQ017638]	
A_32_P3914	1.94E-03	2.11	THC2437430		ALU7_HUMAN (P39194) Alu subfamily SQ sequence contamination warning entry, partial (17%) [THC2437430]	
A_23_P202392	1.94E-03	2.28	NM_024670	NM_024670	Homo sapiens suppressor of variegation 3-9 homolog 2 (Drosophila) (SUV39H2), mRNA [NM_024670]	NM_024670
A_32_P831181	1.94E-03	2.81	NM_080626	NM_080626	Homo sapiens BRI3 binding protein (BRI3BP), mRNA [NM_080626]	NM_080626
A_23_P56865	1.95E-03	2.43	NM_006773	NM_006773	Homo sapiens DEAD (Asp-Glu-Ala-Asp) box polypeptide 18 (DDX18), mRNA [NM_006773]	NM_006773
A_23_P377267	1.95E-03	5.43	AB007940	AB007940	Homo sapiens mRNA for KIAA0471 protein, partial cds. [AB007940]	
A_23_P200222	1.95E-03	13.57	NM_033300	NM_033300	Homo sapiens low density lipoprotein receptor-related protein 8, apolipoprotein e receptor (LRP8), transcript variant 2, mRNA [NM_033300]	NM_033300
A_23_P142389	1.96E-03	2.36	NM_205834	NM_205834	Homo sapiens liver-specific bHLH-Zip transcription factor (LISCH7), transcript variant 2, mRNA [NM_205834]	NM_205834
A_23_P21230	1.97E-03	2.13	NM_015294	NM_015294	Homo sapiens tripartite motif-containing 37 (TRIM37), transcript variant 1, mRNA [NM_015294]	NM_015294
A_24_P354468	1.97E-03	2.89	NM_002106	NM_002106	Homo sapiens H2A histone family, member Z (H2AFZ), mRNA [NM_002106]	NM_002106
A_24_P671842	1.97E-03	3.21	A_24_P671842			
A_23_P163711	1.97E-03	5.08	NM_031478	NM_031478	Homo sapiens family with sequence similarity 57, member B (FAM57B), mRNA [NM_031478]	NM_031478
A_23_P27239	1.97E-03	14.75	NM_002548	NM_002548	Homo sapiens olfactory receptor, family 1, subfamily D, member 2 (OR1D2), mRNA [NM_002548]	NM_002548
A_32_P77502	1.98E-03	2.05	S63912	S63912	D10S102=FBRNP [human, fetal brain, mRNA, 3043 nt]. [S63912]	XM_370728
A_23_P127079	1.98E-03	2.09	NM_015062	NM_015062	Homo sapiens peroxisome proliferative activated receptor, gamma, coactivator-related 1 (PPRC1), mRNA [NM_015062]	NM_015062
A_32_P63086	1.98E-03	2.13	BC041913	BC041913	Homo sapiens, clone IMAGE:5299642, mRNA. [BC041913]	
A_24_P270376	1.98E-03	2.54	NM_012345	NM_012345	Homo sapiens nuclear fragile X mental retardation protein interacting protein 1 (NUFIP1), mRNA [NM_012345]	NM_012345
A_23_P17393	1.98E-03	2.65	NM_001316	NM_001316	Homo sapiens CSE1 chromosome segregation 1-like (yeast) (CSE1L), transcript variant 1, mRNA [NM_001316]	NM_001316
A_23_P202143	1.98E-03	2.71	BC006769	BC006769	Homo sapiens nucleolar and coiled-body phosphoprotein 1, mRNA (cDNA clone MGC:5049 IMAGE:2900024), complete cds. [BC006769]	
A_23_P89755	1.98E-03	2.91	NM_016271	NM_016271	Homo sapiens ring finger protein 138 (RNF138), transcript variant 1, mRNA [NM_016271]	NM_016271

A_24_P68019	1.98E-03	2.99	NM_138347	NM_138347	Homo sapiens zinc finger protein 551 (ZNF551), mRNA [NM_138347]	NM_138347
A_23_P136805	1.98E-03	3.01	NM_014783	NM_014783	Homo sapiens Rho GTPase activating protein 11A (ARHGAP11A), mRNA [NM_014783]	NM_014783
A_24_P504405	1.98E-03	3.85	NM_003095	NM_003095	Homo sapiens small nuclear ribonucleoprotein polypeptide F (SNRPF), mRNA [NM_003095]	NM_003095
A_23_P74034	1.99E-03	2.31	NM_014412	NM_014412	Homo sapiens calcyclin binding protein (CACYBP), transcript variant 1, mRNA [NM_014412]	NM_014412
A_24_P327499	1.99E-03	2.43	NM_014657	NM_014657	Homo sapiens KIAA0406 gene product (KIAA0406), mRNA [NM_014657]	NM_014657
A_23_P117623	1.99E-03	2.60	NM_018319	NM_018319	Homo sapiens tyrosyl-DNA phosphodiesterase 1 (TDP1), transcript variant 1, mRNA [NM_018319]	NM_018319
A_23_P211015	1.99E-03	2.70	NM_017447	NM_017447	Homo sapiens chromosome 21 open reading frame 91 (C21orf91), mRNA [NM_017447]	NM_017447
A_24_P867201	1.99E-03	3.52	CR613944	CR613944	full-length cDNA clone CS0CAP007YK21 of Thymus of Homo sapiens (human). [CR613944]	
A_23_P141315	2.00E-03	2.23	NM_001014445	NM_001014445	Homo sapiens notchless homolog 1 (Drosophila) (NLE1), transcript variant 2, mRNA [NM_001014445]	NM_001014445
A_23_P411335	2.00E-03	2.49	NM_152524	NM_152524	Homo sapiens shugoshin-like 2 (S. pombe) (SGOL2), mRNA [NM_152524]	NM_152524
A_24_P353619	2.01E-03	2.96	NM_000478	NM_000478	Homo sapiens alkaline phosphatase, liver/bone/kidney (ALPL), mRNA [NM_000478]	NM_000478
A_24_P412088	2.01E-03	3.45	NM_182751	NM_182751	Homo sapiens MCM10 minichromosome maintenance deficient 10 (S. cerevisiae) (MCM10), transcript variant 1, mRNA [NM_182751]	NM_182751
A_23_P117068	2.01E-03	3.60	NM_003095	NM_003095	Homo sapiens small nuclear ribonucleoprotein polypeptide F (SNRPF), mRNA [NM_003095]	NM_003095
A_23_P50807	2.02E-03	2.02	NM_021107	NM_021107	Homo sapiens mitochondrial ribosomal protein S12 (MRPS12), nuclear gene encoding mitochondrial protein, transcript variant 1, mRNA [NM_021107]	NM_021107
A_23_P323743	2.02E-03	2.99	NM_025049	NM_025049	Homo sapiens chromosome 15 open reading frame 20 (C15orf20), mRNA [NM_025049]	NM_025049
A_23_P157333	2.02E-03	5.18	NM_005232	NM_005232	Homo sapiens EPH receptor A1 (EPHA1), mRNA [NM_005232]	NM_005232
A_32_P94	2.02E-03	22.99	CR602878	CR602878	full-length cDNA clone CS0DF035YB23 of Fetal brain of Homo sapiens (human). [CR602878]	
A_24_P122524	2.04E-03	2.69	NM_006784	NM_006784	Homo sapiens WD repeat domain 3 (WDR3), mRNA [NM_006784]	NM_006784
A_23_P205101	2.04E-03	3.18	NM_025138	NM_025138	Homo sapiens chromosome 13 open reading frame 23 (C13orf23), transcript variant 1, mRNA [NM_025138]	NM_025138
A_32_P168388	2.04E-03	3.66	AK123765	AK123765	Homo sapiens cDNA FLJ41771 fis, clone IMR322009807. [AK123765]	
A_23_P310317	2.05E-03	2.08	NM_001008656	NM_001008656	Homo sapiens Treacher Collins-Franceschetti syndrome 1 (TCOF1), transcript variant 1, mRNA [NM_001008656]	NM_001008656
A_23_P152753	2.05E-03	2.70	AY358101	AY358101	Homo sapiens clone DNA108695 Wpep3002 (UNQ3002) mRNA, complete cds. [AY358101]	
A_24_P333306	2.05E-03	2.72	AK023737	AK023737	Homo sapiens cDNA FLJ13675 fis, clone PLACE1011875, highly similar to Homo sapiens mRNA for KIAA0580 protein. [AK023737]	
A_23_P168747	2.05E-03	3.42	AK000318	AK000318	Homo sapiens cDNA FLJ20311 fis, clone HEP07319. [AK000318]	
A_23_P52017	2.06E-03	2.11	NM_018136	NM_018136	Homo sapiens asp (abnormal spindle)-like, microcephaly associated (Drosophila) (ASPM), mRNA [NM_018136]	NM_018136
A_23_P54389	2.06E-03	2.19	NM_024611	NM_024611	Homo sapiens NMDA receptor regulated 2 (NARG2), transcript variant 1, mRNA [NM_024611]	NM_024611
A_23_P320250	2.06E-03	2.30	NM_025109	NM_025109	Homo sapiens myosin head domain containing 1 (MYOHD1), mRNA [NM_025109]	NM_025109
A_23_P99837	2.06E-03	2.47	NM_017437	NM_017437	Homo sapiens cleavage and polyadenylation specific factor 2, 100kDa (CPSF2), mRNA [NM_017437]	NM_017437
A_24_P238499	2.06E-03	2.49	NM_001012716	NM_001012716	Homo sapiens chromosome 18 open reading frame 56 (C18orf56), mRNA [NM_001012716]	NM_001012716
A_23_P19210	2.06E-03	2.51	NM_032194	NM_032194	Homo sapiens brix domain containing 1 (BXDC1), mRNA [NM_032194]	NM_032194
A_23_P385861	2.06E-03	2.82	NM_152562	NM_152562	Homo sapiens cell division cycle associated 2 (CDCA2), mRNA [NM_152562]	NM_152562
A_24_P33989	2.06E-03	5.24	NM_022351	NM_022351	Homo sapiens EF-hand calcium binding protein 1 (EFCBP1), mRNA [NM_022351]	NM_022351
A_23_P90273	2.06E-03	5.88	NM_022467	NM_022467	Homo sapiens carbohydrate (N-acetylgalactosamine 4-0) sulfotransferase 8 (CHST8), mRNA [NM_022467]	NM_022467
A_24_P50458	2.06E-03	16.86	NM_017489	NM_017489	Homo sapiens telomeric repeat binding factor (NIMA-interacting) 1 (TERF1), transcript variant 1, mRNA [NM_017489]	NM_017489
A_23_P205393	2.07E-03	2.13	NM_175748	NM_175748	Homo sapiens chromosome 14 open reading frame 130 (C14orf130), transcript variant 2, mRNA [NM_175748]	NM_175748
A_24_P211351	2.07E-03	2.16	A_24_P211351			
A_23_P139486	2.07E-03	2.17	NM_004642	NM_004642	Homo sapiens CDK2-associated protein 1 (CDK2AP1), mRNA [NM_004642]	NM_004642
A_24_P14485	2.07E-03	2.61	NM_017495	NM_017495	Homo sapiens RNA-binding region (RNP1, RRM) containing 1 (RNPC1), transcript variant 1, mRNA [NM_017495]	NM_017495
A_23_P209987	2.07E-03	3.28	NM_019014	NM_019014	Homo sapiens polymerase (RNA) I polypeptide B, 128kDa (POLR1B), mRNA [NM_019014]	NM_019014
A_23_P7873	2.07E-03	3.32	NM_002388	NM_002388	Homo sapiens MCM3 minichromosome maintenance deficient 3 (S. cerevisiae) (MCM3), mRNA [NM_002388]	NM_002388

A_24_P698141	2.07E-03	3.68	AK125299	AK125299	Homo sapiens cDNA FLJ43309 fis, clone NT2RI2004618, highly similar to Cytosolic acyl coenzyme A thioester hydrolase (EC 3.1.2.2). [AK125299]	XR_000194
A_24_P245815	2.07E-03	4.42	AL161993	AL161993	Homo sapiens mRNA; cDNA DKFZp761P039 (from clone DKFZp761P039); partial cds. [AL161993]	
A_23_P32328	2.08E-03	2.04	NM_020408	NM_020408	Homo sapiens chromosome 6 open reading frame 149 (C6orf149), mRNA [NM_020408]	NM_020408
A_23_P166964	2.08E-03	2.09	NM_006286	NM_006286	Homo sapiens transcription factor Dp-2 (E2F dimerization partner 2) (TFDP2), mRNA [NM_006286]	NM_006286
A_23_P121122	2.08E-03	2.30	NM_025146	NM_025146	Homo sapiens Mak3 homolog (S. cerevisiae) (MAK3), mRNA [NM_025146]	NM_025146
A_32_P53486	2.08E-03	2.41	BC022832	BC022832	Homo sapiens bolA-like 2 (E. coli), mRNA (cDNA clone IMAGE:4647797), with apparent retained intron. [BC022832]	
A_23_P202316	2.08E-03	2.80	NM_001007793	NM_001007793	Homo sapiens BUB3 budding uninhibited by benzimidazoles 3 homolog (yeast) (BUB3), transcript variant 2, mRNA [NM_001007793]	NM_001007793
A_24_P379969	2.08E-03	14.47	NM_003106	NM_003106	Homo sapiens SRY (sex determining region Y)-box 2 (SOX2), mRNA [NM_003106]	NM_003106
A_23_P113811	2.09E-03	16.95	A_23_P113811			
A_24_P377225	2.10E-03	2.00	NM_022832	NM_022832	Homo sapiens ubiquitin specific protease 46 (USP46), mRNA [NM_022832]	NM_022832
A_23_P208737	2.10E-03	2.13	NM_004497	NM_004497	Homo sapiens forkhead box A3 (FOXA3), mRNA [NM_004497]	NM_004497
A_32_P108474	2.10E-03	2.38	NM_002940	NM_002940	Homo sapiens ATP-binding cassette, sub-family E (OABP), member 1 (ABCE1), mRNA [NM_002940]	NM_002940
A_23_P111228	2.10E-03	2.89	NM_017421	NM_017421	Homo sapiens coenzyme Q3 homolog, methyltransferase (yeast) (COQ3), mRNA [NM_017421]	NM_017421
A_32_P86578	2.11E-03	2.16	BC032913	BC032913	Homo sapiens hypothetical gene supported by BC032913; BC048425, mRNA (cDNA clone IMAGE:5265535). [BC032913]	XM_374002
A_23_P142407	2.11E-03	2.75	NM_033204	NM_033204	Homo sapiens zinc finger protein 101 (ZNF101), mRNA [NM_033204]	NM_033204
A_24_P261169	2.11E-03	3.21	NM_006378	NM_006378	Homo sapiens sema domain, immunoglobulin domain (Ig), transmembrane domain (TM) and short cytoplasmic domain, (semaphorin) 4D (SEMA4D), mRNA [NM_006378]	NM_006378
A_24_P607880	2.11E-03	5.88	AL832758	AL832758	Homo sapiens mRNA; cDNA DKFZp686C0927 (from clone DKFZp686C0927). [AL832758]	
A_32_P137966	2.12E-03	2.48	NM_152317	NM_152317	Homo sapiens DEP domain containing 4 (DEPDC4), mRNA [NM_152317]	NM_152317
A_23_P397999	2.12E-03	5.38	NM_003468	NM_003468	Homo sapiens frizzled homolog 5 (Drosophila) (FZD5), mRNA [NM_003468]	NM_003468
A_23_P154688	2.12E-03	7.41	NM_032034	NM_032034	Homo sapiens solute carrier family 4, sodium bicarbonate transporter-like, member 11 (SLC4A11), mRNA [NM_032034]	NM_032034
A_23_P115149	2.13E-03	2.16	NM_024102	NM_024102	Homo sapiens WD repeat domain 77 (WDR77), mRNA [NM_024102]	NM_024102
A_24_P176131	2.13E-03	2.97	NM_052950	NM_052950	Homo sapiens WD repeat and FYVE domain containing 2 (WDFY2), mRNA [NM_052950]	NM_052950
A_24_P902509	2.13E-03	4.57	BC004277	BC004277	Homo sapiens cDNA clone MGC:10837 IMAGE:3615489, complete cds. [BC004277]	
A_23_P140821	2.13E-03	7.25	NM_016948	NM_016948	Homo sapiens par-6 partitioning defective 6 homolog alpha (C.elegans) (PARD6A), mRNA [NM_016948]	NM_016948
A_23_P314712	2.13E-03	8.55	NM_012189	NM_012189	Homo sapiens calcium binding tyrosine-(Y)-phosphorylation regulated (fibrousheathin 2) (CABYR), transcript variant 1, mRNA [NM_012189]	NM_012189
A_24_P636834	2.14E-03	2.20	A_24_P636834			
A_23_P122863	2.14E-03	2.30	NM_001001555	NM_001001555	Homo sapiens growth factor receptor-bound protein 10 (GRB10), transcript variant 4, mRNA [NM_001001555]	NM_001001555
A_24_P775659	2.14E-03	2.43	CR593500	CR593500	full-length cDNA clone CS0DF014YD20 of Fetal brain of Homo sapiens (human). [CR593500]	XM_373788
A_23_P112341	2.14E-03	2.47	NM_024945	NM_024945	Homo sapiens chromosome 9 open reading frame 76 (C9orf76), mRNA [NM_024945]	NM_024945
A_23_P134176	2.14E-03	2.74	NM_000636	NM_000636	Homo sapiens superoxide dismutase 2, mitochondrial (SOD2), nuclear gene encoding mitochondrial protein, transcript variant 1, mRNA [NM_000636]	NM_000636
A_23_P67042	2.14E-03	2.82	NM_017947	NM_017947	Homo sapiens molybdenum cofactor sulfurase (MOCOS), mRNA [NM_017947]	NM_017947
A_32_P145010	2.14E-03	2.92	CR594811	CR594811	full-length cDNA clone CS0DL006YD08 of B cells (Ramos cell line) Cot 25-normalized of Homo sapiens (human). [CR594811]	
A_23_P64121	2.15E-03	2.24	NM_152314	NM_152314	Homo sapiens hypothetical protein MGC34830 (MGC34830), mRNA [NM_152314]	NM_152314
A_23_P103628	2.15E-03	2.82	BC011983	BC011983	Homo sapiens protein BAP28, mRNA (cDNA clone IMAGE:3863792), complete cds. [BC011983]	XM_375853
A_32_P34941	2.16E-03	2.46	THC2378378			
A_23_P73577	2.16E-03	4.02	ENST00000297871		Homo sapiens t-complex-associated-testis-expressed 1-like, mRNA (cDNA clone MGC:5007 IMAGE:3448623), complete cds. [BC000968]	
A_23_P259451	2.17E-03	2.18	NM_007080	NM_007080	Homo sapiens LSM6 homolog, U6 small nuclear RNA associated (S. cerevisiae) (LSM6), mRNA [NM_007080]	NM_007080
A_23_P375	2.17E-03	2.32	NM_018101	NM_018101	Homo sapiens cell division cycle associated 8 (CDCA8), mRNA [NM_018101]	NM_018101

A_23_P424	2.18E-03	2.38	NM_018650	NM_018650	Homo sapiens MAP/microtubule affinity-regulating kinase 1 (MARK1), mRNA [NM_018650]	NM_018650
A_24_P170103	2.18E-03	2.46	A_24_P170103			
A_32_P7193	2.18E-03	4.42	A_32_P7193			
A_24_P404458	2.18E-03	6.06	NM_014446	NM_014446	Homo sapiens integrin beta 1 binding protein 3 (ITGB1BP3), transcript variant 1, mRNA [NM_014446]	NM_014446
A_24_P349616	2.20E-03	2.65	BC042297	BC042297	Homo sapiens upstream binding transcription factor, RNA polymerase I, mRNA (cDNA clone MGC:48801 IMAGE:4509695), complete cds. [BC042297]	
A_24_P171983	2.20E-03	3.37	NM_014165	NM_014165	Homo sapiens chromosome 6 open reading frame 66 (C6orf66), mRNA [NM_014165]	NM_014165
A_24_P55148	2.20E-03	7.75	NM_021058	NM_021058	Homo sapiens histone 1, H2bj (HIST1H2BJ), mRNA [NM_021058]	NM_021058
A_23_P91590	2.21E-03	2.18	NM_002882	NM_002882	Homo sapiens RAN binding protein 1 (RANBP1), mRNA [NM_002882]	NM_002882
A_24_P224526	2.21E-03	2.35	NM_022344	NM_022344	Homo sapiens protein kinase Njmu-R1 (NJMU-R1), mRNA [NM_022344]	NM_022344
A_23_P259344	2.21E-03	3.22	NM_031890	NM_031890	Homo sapiens cat eye syndrome chromosome region, candidate 6 (CECR6), mRNA [NM_031890]	NM_031890
A_23_P256047	2.22E-03	3.31	NM_022096	NM_022096	Homo sapiens ankyrin repeat domain 5 (ANKRD5), transcript variant 1, mRNA [NM_022096]	NM_022096
A_23_P133386	2.22E-03	9.71	NM_006909	NM_006909	Homo sapiens Ras protein-specific guanine nucleotide-releasing factor 2 (RASGRF2), mRNA [NM_006909]	NM_006909
A_32_P179771	2.23E-03	2.53	AI088710	AI088710	AI088710 qa12c10.x1 NCI_CGAP_Brn23 Homo sapiens cDNA clone IMAGE:1686546 3' similar to gb:L06132 OUTER MITOCHONDRIAL MEMBRANE PROTEIN PORIN (HUMAN);, mRNA sequence [AI088710]	
A_23_P76034	2.23E-03	2.72	NM_203286	NM_203286	Homo sapiens poliovirus receptor-related 1 (herpesvirus entry mediator C; nectin) (PVRL1), transcript variant 3, mRNA [NM_203286]	NM_203286
A_32_P210202	2.23E-03	2.96	NM_203394	NM_203394	Homo sapiens E2F transcription factor 7 (E2F7), mRNA [NM_203394]	NM_203394
A_23_P32175	2.23E-03	3.04	NM_014368	NM_014368	Homo sapiens LIM homeobox 6 (LHX6), transcript variant 1, mRNA [NM_014368]	NM_014368
A_24_P109417	2.23E-03	5.56	NM_198545	NM_198545	Homo sapiens hypothetical gene supported by AK075558; BC021286 (LOC374946), mRNA [NM_198545]	NM_198545
A_23_P311201	2.24E-03	2.00	ENST00000341154		Homo sapiens TLS-associated protein TASR-1 mRNA, complete cds, alternative transcript. [AF419331]	
A_23_P151497	2.24E-03	2.15	NM_152307	NM_152307	Homo sapiens chromosome 14 open reading frame 172 (C14orf172), mRNA [NM_152307]	NM_152307
A_23_P128084	2.24E-03	2.62	NM_002206	NM_002206	Homo sapiens integrin, alpha 7 (ITGA7), mRNA [NM_002206]	NM_002206
A_23_P37942	2.24E-03	4.90	NM_021195	NM_021195	Homo sapiens claudin 6 (CLDN6), mRNA [NM_021195]	NM_021195
A_24_P91991	2.24E-03	7.09	NM_178557	NM_178557	Homo sapiens hypothetical protein FLJ37478 (FLJ37478), mRNA [NM_178557]	NM_178557
A_23_P139864	2.25E-03	2.67	NM_031289	NM_031289	Homo sapiens germ cell associated 1 (GSG1), transcript variant 1, mRNA [NM_031289]	NM_031289
A_23_P7361	2.25E-03	2.69	NM_024090	NM_024090	Homo sapiens ELOVL family member 6, elongation of long chain fatty acids (FEN1/Elo2, SUR4/Elo3-like, yeast) (ELOVL6), mRNA [NM_024090]	NM_024090
A_23_P14216	2.25E-03	3.16	ENST00000239860		Q9H1T5 (Q9H1T5) OTTHUMP00000042268, complete [THC2436901]	
A_24_P109351	2.25E-03	3.19	NM_001009814	NM_001009814	Homo sapiens KIAA0564 protein (KIAA0564), transcript variant 2, mRNA [NM_001009814]	NM_001009814
A_23_P435407	2.25E-03	12.89	NM_001448	NM_001448	Homo sapiens glypican 4 (GPC4), mRNA [NM_001448]	NM_001448
A_23_P216355	2.26E-03	2.09	NM_013432	NM_013432	Homo sapiens nuclear factor of kappa light polypeptide gene enhancer in B-cells inhibitor-like 2 (NFKBIL2), mRNA [NM_013432]	NM_013432
A_23_P203949	2.26E-03	2.36	NM_004399	NM_004399	Homo sapiens DEAD/H (Asp-Glu-Ala-Asp/His) box polypeptide 11 (CHL1-like helicase homolog, S. cerevisiae) (DDX11), transcript variant 2, mRNA [NM_004399]	NM_004399
A_24_P793228	2.26E-03	2.77	XR_000292	XR_000292	PREDICTED: Homo sapiens similar to ATP-binding cassette sub-family E member 1 (RNase L inhibitor) (Ribonuclease 4 inhibitor) (RNS4I) (LOC442517), mRNA [XR_000292]	XR_000292
A_23_P372308	2.26E-03	4.12	NM_020211	NM_020211	Homo sapiens RGM domain family, member A (RGMA), mRNA [NM_020211]	NM_020211
A_23_P103361	2.26E-03	25.91	NM_005356	NM_005356	Homo sapiens lymphocyte-specific protein tyrosine kinase (LCK), mRNA [NM_005356]	NM_005356
A_23_P69586	2.27E-03	2.32	NM_005245	NM_005245	Homo sapiens FAT tumor suppressor homolog 1 (Drosophila) (FAT), mRNA [NM_005245]	NM_005245
A_24_P306896	2.27E-03	2.48	A_24_P306896			
A_32_P207428	2.27E-03	3.89	BC007307	BC007307	Homo sapiens, Similar to zinc finger protein 268, clone IMAGE:3352268, mRNA, partial cds. [BC007307]	XM_039908
A_23_P88731	2.28E-03	2.26	NM_002875	NM_002875	Homo sapiens RAD51 homolog (RecA homolog, E. coli) (S. cerevisiae) (RAD51), transcript variant 1, mRNA [NM_002875]	NM_002875
A_32_P91156	2.28E-03	2.26	A_32_P91156			
A_24_P38572	2.28E-03	2.36	NM_130793	NM_130793	Homo sapiens nucleolar protein family 6 (RNA-associated) (NOL6), transcript variant beta, mRNA [NM_130793]	NM_130793

A_23_P115636	2.28E-03	2.73	NM_004412	NM_004412	Homo sapiens DNA (cytosine-5-)-methyltransferase 2 (DNMT2), transcript variant a, mRNA [NM_004412]	NM_004412
A_24_P225970	2.28E-03	2.87	NM_001012409	NM_001012409	Homo sapiens shugoshin-like 1 (S. pombe) (SGOL1), transcript variant A1, mRNA [NM_001012409]	NM_001012409
A_23_P161474	2.28E-03	3.92	NM_182751	NM_182751	Homo sapiens MCM10 minichromosome maintenance deficient 10 (S. cerevisiae) (MCM10), transcript variant 1, mRNA [NM_182751]	NM_182751
A_23_P317056	2.28E-03	4.33	ENST00000361681			
A_23_P99515	2.28E-03	8.13	NM_032849	NM_032849	Homo sapiens hypothetical protein FLJ14834 (FLJ14834), mRNA [NM_032849]	NM_032849
A_23_P310350	2.29E-03	2.15	NM_013276	NM_013276	Homo sapiens carbohydrate kinase-like (CARKL), mRNA [NM_013276]	NM_013276
A_23_P18579	2.29E-03	2.19	NM_006607	NM_006607	Homo sapiens pituitary tumor-transforming 2 (PTTG2), mRNA [NM_006607]	NM_006607
A_23_P259127	2.29E-03	5.65	NM_017697	NM_017697	Homo sapiens hypothetical protein FLJ20171 (FLJ20171), mRNA [NM_017697]	NM_017697
A_24_P409881	2.30E-03	2.65	ENST00000217341		PREDICTED: Homo sapiens similar to hypothetical protein (LOC338756), mRNA [XM_291989]	XM_291989
A_23_P429950	2.30E-03	18.48	NM_000216	NM_000216	Homo sapiens Kallmann syndrome 1 sequence (KAL1), mRNA [NM_000216]	NM_000216
A_23_P332326	2.31E-03	2.00	NM_153213	NM_153213	Homo sapiens Rho guanine nucleotide exchange factor (GEF) 19 (ARHGEF19), mRNA [NM_153213]	NM_153213
A_23_P56140	2.31E-03	2.09	NM_001319	NM_001319	Homo sapiens casein kinase 1, gamma 2 (CSNK1G2), mRNA [NM_001319]	NM_001319
A_23_P88904	2.31E-03	2.38	NM_002528	NM_002528	Homo sapiens nth endonuclease III-like 1 (E. coli) (NTHL1), mRNA [NM_002528]	NM_002528
A_32_P19806	2.31E-03	3.80	NM_003777	NM_003777	Homo sapiens dynein, axonemal, heavy polypeptide 11 (DNAH11), mRNA [NM_003777]	NM_003777
A_23_P163227	2.31E-03	7.94	NM_020990	NM_020990	Homo sapiens creatine kinase, mitochondrial 1B (CKMT1B), nuclear gene encoding mitochondrial protein, mRNA [NM_020990]	NM_020990
A_24_P366107	2.32E-03	3.18	ENST00000260916		Homo sapiens, clone IMAGE:4385301, mRNA, partial cds. [BC028188]	XM_166103
A_23_P28772	2.32E-03	3.48	NM_018478	NM_018478	Homo sapiens chromosome 20 open reading frame 35 (C20orf35), mRNA [NM_018478]	NM_018478
A_32_P202134	2.33E-03	2.46	AB051463	AB051463	Homo sapiens mRNA for KIAA1676 protein, partial cds. [AB051463]	
A_24_P186274	2.33E-03	2.84	NM_001007563	NM_001007563	Homo sapiens insulin-like growth factor binding protein-like 1 (IGFBPL1), mRNA [NM_001007563]	NM_001007563
A_23_P60130	2.33E-03	4.52	NM_052886	NM_052886	Homo sapiens mal, T-cell differentiation protein 2 (MAL2), mRNA [NM_052886]	NM_052886
A_32_P142440	2.33E-03	7.04	NM_174936	NM_174936	Homo sapiens proprotein convertase subtilisin/kexin type 9 (PCSK9), mRNA [NM_174936]	NM_174936
A_23_P127915	2.34E-03	3.52	NM_030906	NM_030906	Homo sapiens serine/threonine kinase 33 (STK33), mRNA [NM_030906]	NM_030906
A_24_P66027	2.35E-03	2.11	NM_004900	NM_004900	Homo sapiens apolipoprotein B mRNA editing enzyme, catalytic polypeptide-like 3B (APOBEC3B), mRNA [NM_004900]	NM_004900
A_23_P122254	2.35E-03	2.43	NM_005124	NM_005124	Homo sapiens nucleoporin 153kDa (NUP153), mRNA [NM_005124]	NM_005124
A_32_P122590	2.35E-03	7.69	BC035844	BC035844	Homo sapiens, clone IMAGE:5745916, mRNA. [BC035844]	
A_23_P160618	2.38E-03	2.40	NM_003975	NM_003975	Homo sapiens SH2 domain protein 2A (SH2D2A), mRNA [NM_003975]	NM_003975
A_23_P395460	2.38E-03	4.44	NM_004321	NM_004321	Homo sapiens kinesin family member 1A (KIF1A), mRNA [NM_004321]	NM_004321
A_32_P23795	2.39E-03	2.01	BF960831	BF960831	BF960831 QV2-NN0045-071200-530-e10 NN0045 Homo sapiens cDNA, mRNA sequence [BF960831]	
A_24_P686965	2.39E-03	4.95	AK124869	AK124869	Homo sapiens cDNA FLJ42879 fis, clone BRHIP3001283. [AK124869]	XM_375698
A_23_P134008	2.40E-03	2.35	BC005991	BC005991	Homo sapiens ubiquitin specific protease 45, mRNA (cDNA clone MGC:14793 IMAGE:4047601), complete cds. [BC005991]	
A_24_P176374	2.40E-03	2.64	NM_030928	NM_030928	Homo sapiens DNA replication factor (CDT1), mRNA [NM_030928]	NM_030928
A_32_P72394	2.40E-03	3.95	NM_002735	NM_002735	Homo sapiens protein kinase, cAMP-dependent, regulatory, type I, beta (PRKAR1B), mRNA [NM_002735]	NM_002735
A_23_P301051	2.42E-03	3.34	ENST00000244221		Homo sapiens mRNA for KIAA1155 protein, partial cds. [AB032981]	XM_376062
A_23_P385063	2.42E-03	3.48	NM_058246	NM_058246	Homo sapiens DnaJ (Hsp40) homolog, subfamily B, member 6 (DNAJB6), transcript variant 1, mRNA [NM_058246]	NM_058246
A_23_P48835	2.43E-03	2.34	NM_138555	NM_138555	Homo sapiens kinesin family member 23 (KIF23), transcript variant 1, mRNA [NM_138555]	NM_138555
A_23_P16683	2.43E-03	2.54	NM_017722	NM_017722	Homo sapiens hypothetical protein FLJ20244 (FLJ20244), mRNA [NM_017722]	NM_017722
A_23_P322196	2.43E-03	2.77	NM_138400	NM_138400	Homo sapiens nucleolar protein with MIF4G domain 1 (NOM1), mRNA [NM_138400]	NM_138400
A_24_P206047	2.43E-03	2.78	NM_001151	NM_001151	Homo sapiens solute carrier family 25 (mitochondrial carrier; adenine nucleotide translocator), member 4 (SLC25A4), nuclear gene encoding mitochondrial protein, mRNA [NM_001151]	NM_001151
A_23_P377664	2.44E-03	2.26	AB053306	AB053306	Homo sapiens ALS2 mRNA, complete cds, short form. [AB053306]	

A_24_P235049	2.44E-03	2.48	NM_015440	NM_015440	Homo sapiens methylenetetrahydrofolate dehydrogenase (NADP+ dependent) 1-like (MTHFD1L), mRNA [NM_015440]	NM_015440
A_24_P941336	2.44E-03	2.74	NM_018128	NM_018128	Homo sapiens hypothetical protein FLJ10534 (FLJ10534), mRNA [NM_018128]	NM_018128
A_23_P64129	2.44E-03	3.60	NM_006410	NM_006410	Homo sapiens HIV-1 Tat interactive protein 2, 30kDa (HTATIP2), mRNA [NM_006410]	NM_006410
A_23_P48495	2.44E-03	4.27	NM_199206	NM_199206	Homo sapiens T-cell leukemia/lymphoma 1B (TCL1B), transcript variant 2, mRNA [NM_199206]	NM_199206
A_23_P1145	2.45E-03	2.15	NM_018109	NM_018109	Homo sapiens PAP associated domain containing 1 (PAPD1), mRNA [NM_018109]	NM_018109
A_23_P347508	2.45E-03	4.81	NM_023077	NM_023077	Homo sapiens chromosome 1 open reading frame 163 (C1orf163), mRNA [NM_023077]	NM_023077
A_32_P202092	2.46E-03	2.84	THC2406182			
A_23_P82959	2.46E-03	3.80	NM_003923	NM_003923	Homo sapiens forkhead box H1 (FOXH1), mRNA [NM_003923]	NM_003923
A_23_P22378	2.46E-03	6.71	NM_003108	NM_003108	Homo sapiens SRY (sex determining region Y)-box 11 (SOX11), mRNA [NM_003108]	NM_003108
A_32_P221305	2.47E-03	2.19	BC073935	BC073935	Homo sapiens cDNA clone IMAGE:5219247, partial cds. [BC073935]	XM_498535
A_23_P254733	2.47E-03	3.03	NM_024629	NM_024629	Homo sapiens MLF1 interacting protein (MLF1IP), mRNA [NM_024629]	NM_024629
A_32_P137399	2.47E-03	3.75	ENST00000335142		human full-length cDNA clone CS0DK001YK16 of HeLa cells of Homo sapiens (human). [BX248296]	XM_375081
A_24_P267664	2.48E-03	2.27	NM_153754	NM_153754	Homo sapiens chromosome 21 open reading frame 88 (C21orf88), mRNA [NM_153754]	NM_153754
A_23_P312174	2.48E-03	2.33	NM_015120	NM_015120	Homo sapiens Alstrom syndrome 1 (ALMS1), mRNA [NM_015120]	NM_015120
A_23_P501134	2.49E-03	2.65	NM_002311	NM_002311	Homo sapiens ligase III, DNA, ATP-dependent (LIG3), transcript variant beta, mRNA [NM_002311]	NM_002311
A_23_P28318	2.49E-03	3.48	NM_018607	NM_018607	Homo sapiens hypothetical protein PRO1853 (PRO1853), transcript variant 2, mRNA [NM_018607]	NM_018607
A_24_P929137	2.51E-03	2.35	THC2361427			
A_24_P376556	2.51E-03	2.89	NM_018947	NM_018947	Homo sapiens cytochrome c, somatic (CYCS), nuclear gene encoding mitochondrial protein, mRNA [NM_018947]	NM_018947
A_23_P51085	2.51E-03	3.25	NM_020675	NM_020675	Homo sapiens spindle pole body component 25 homolog (S. cerevisiae) (SPBC25), mRNA [NM_020675]	NM_020675
A_23_P12816	2.51E-03	3.98	NM_018063	NM_018063	Homo sapiens helicase, lymphoid-specific (HELLS), mRNA [NM_018063]	NM_018063
A_23_P211459	2.52E-03	2.06	NM_014323	NM_014323	Homo sapiens zinc finger protein 278 (ZNF278), transcript variant 1, mRNA [NM_014323]	NM_014323
A_23_P148807	2.54E-03	2.16	NM_003503	NM_003503	Homo sapiens CDC7 cell division cycle 7 (S. cerevisiae) (CDC7), mRNA [NM_003503]	NM_003503
A_23_P64343	2.54E-03	2.49	NM_012456	NM_012456	Homo sapiens translocase of inner mitochondrial membrane 10 homolog (yeast) (TIMM10), mRNA [NM_012456]	NM_012456
A_23_P119677	2.54E-03	2.64	NM_001533	NM_001533	Homo sapiens heterogeneous nuclear ribonucleoprotein L (HNRPL), transcript variant 1, mRNA [NM_001533]	NM_001533
A_23_P115861	2.54E-03	2.70	NM_145312	NM_145312	Homo sapiens zinc finger protein 485 (ZNF485), mRNA [NM_145312]	NM_145312
A_24_P252310	2.54E-03	4.26	NM_014690	NM_014690	Homo sapiens KIAA0773 gene product (KIAA0773), mRNA [NM_014690]	NM_014690
A_23_P159851	2.55E-03	5.32	NM_032803	NM_032803	Homo sapiens solute carrier family 7 (cationic amino acid transporter, y+ system), member 3 (SLC7A3), mRNA [NM_032803]	NM_032803
A_32_P203878	2.55E-03	10.45	THC2373072		CB243285 UI-CF-FN0-agc-l-12-0-UI.s1 UI-CF-FN0 Homo sapiens cDNA clone UI-CF-FN0-agc-l-12-0-UI 3', mRNA sequence [CB243285]	
A_23_P100092	2.56E-03	2.10	NM_152455	NM_152455	Homo sapiens zinc finger protein 690 (ZNF690), mRNA [NM_152455]	NM_152455
A_23_P59005	2.56E-03	2.18	NM_000593	NM_000593	Homo sapiens transporter 1, ATP-binding cassette, sub-family B (MDR/TAP) (TAP1), mRNA [NM_000593]	NM_000593
A_23_P138465	2.57E-03	3.11	NM_004741	NM_004741	Homo sapiens nucleolar and coiled-body phosphoprotein 1 (NOLC1), mRNA [NM_004741]	NM_004741
A_24_P924752	2.57E-03	37.88	M14087	M14087	Human HL14 gene encoding beta-galactoside-binding lectin, 3' end, clone 2. [M14087]	
A_24_P757638	2.58E-03	2.03	BI520212	BI520212	603071460F1 NIH_MGC_119 Homo sapiens cDNA clone IMAGE:5163326 5', mRNA sequence [BI520212]	
A_24_P341504	2.58E-03	2.08	NM_017619	NM_017619	Homo sapiens RNA-binding region (RNP1, RRM) containing 3 (RNPC3), mRNA [NM_017619]	NM_017619
A_23_P80902	2.58E-03	2.33	NM_020242	NM_020242	Homo sapiens kinesin family member 15 (KIF15), mRNA [NM_020242]	NM_020242
A_23_P89249	2.58E-03	2.54	NM_001005862	NM_001005862	Homo sapiens v-erb-b2 erythroblastic leukemia viral oncogene homolog 2, neuro/glioblastoma derived oncogene homolog (avian) (ERBB2), transcript variant 2, mRNA [NM_001005862]	NM_001005862
A_23_P44684	2.58E-03	2.96	NM_018098	NM_018098	Homo sapiens epithelial cell transforming sequence 2 oncogene (ECT2), mRNA [NM_018098]	NM_018098
A_23_P253752	2.58E-03	3.70	NM_138419	NM_138419	Homo sapiens family with sequence similarity 54, member A (FAM54A), mRNA [NM_138419]	NM_138419
A_24_P146603	2.58E-03	3.89	NM_004728	NM_004728	Homo sapiens DEAD (Asp-Glu-Ala-Asp) box polypeptide 21 (DDX21), mRNA [NM_004728]	NM_004728

A_32_P49631	2.59E-03	2.22	CR594811	CR594811	full-length cDNA clone CS0DL006YD08 of B cells (Ramos cell line) Cot 25-normalized of Homo sapiens (human). [CR594811]	
A_23_P91346	2.59E-03	2.33	BC008667	BC008667	Homo sapiens cDNA clone MGC:17708 IMAGE:3868595, complete cds. [BC008667]	
A_23_P343261	2.59E-03	2.36	NM_153709	NM_153709	Homo sapiens hypothetical protein MGC40168 (MGC40168), mRNA [NM_153709]	NM_153709
A_23_P301079	2.59E-03	2.82	NM_182620	NM_182620	Homo sapiens family with sequence similarity 33, member A (FAM33A), mRNA [NM_182620]	NM_182620
A_32_P135243	2.59E-03	2.92	NM_015440	NM_015440	Homo sapiens methylenetetrahydrofolate dehydrogenase (NADP+ dependent) 1-like (MTHFD1L), mRNA [NM_015440]	NM_015440
A_23_P208310	2.59E-03	2.93	NM_012099	NM_012099	Homo sapiens CD3E antigen, epsilon polypeptide associated protein (CD3EAP), mRNA [NM_012099]	NM_012099
A_32_P218671	2.59E-03	3.16	BG575983	BG575983	602597328F1 NIH_MGC_87 Homo sapiens cDNA clone IMAGE:4705974 5', mRNA sequence [BG575983]	
A_23_P216396	2.60E-03	2.24	NM_014285	NM_014285	Homo sapiens exosome component 2 (EXOSC2), mRNA [NM_014285]	NM_014285
A_32_P50815	2.60E-03	2.26	AK054852	AK054852	Homo sapiens cDNA FLJ30290 fis, clone BRACE2002884. [AK054852]	
A_24_P652609	2.60E-03	2.43	AK054645	AK054645	Homo sapiens cDNA FLJ30083 fis, clone BGGI12001097, weakly similar to Homo sapiens contactin associated protein (Caspr) mRNA. [AK054645]	XM_372097
A_23_P400449	2.60E-03	2.52	NM_020927	NM_020927	Homo sapiens KIAA1576 protein (KIAA1576), mRNA [NM_020927]	NM_020927
A_23_P140434	2.60E-03	4.07	NM_018728	NM_018728	Homo sapiens myosin VC (MYO5C), mRNA [NM_018728]	NM_018728
A_24_P184799	2.60E-03	5.62	NM_004086	NM_004086	Homo sapiens coagulation factor C homolog, cochlin (Limulus polyphemus) (COCH), mRNA [NM_004086]	NM_004086
A_24_P126181	2.61E-03	2.34	NM_176880	NM_176880	Homo sapiens TR4 orphan receptor associated protein TRA16 (TRA16), mRNA [NM_176880]	NM_176880
A_23_P81121	2.61E-03	2.41	NM_005033	NM_005033	Homo sapiens exosome component 9 (EXOSC9), mRNA [NM_005033]	NM_005033
A_23_P301247	2.61E-03	3.58	NM_003517	NM_003517	Homo sapiens histone 2, H2ac (HIST2H2AC), mRNA [NM_003517]	NM_003517
A_23_P401675	2.62E-03	2.02	NM_144724	NM_144724	Homo sapiens MARVEL domain containing 2 (MARVELD2), mRNA [NM_144724]	NM_144724
A_32_P30693	2.62E-03	2.32	NM_003707	NM_003707	Homo sapiens RuvB-like 1 (E. coli) (RUVBL1), mRNA [NM_003707]	NM_003707
A_23_P387471	2.62E-03	2.64	NM_005931	NM_005931	Homo sapiens MHC class I polypeptide-related sequence B (MICB), mRNA [NM_005931]	NM_005931
A_23_P82181	2.63E-03	2.57	NM_018292	NM_018292	Homo sapiens glutaminyl-tRNA synthase (glutamine-hydrolyzing)-like 1 (QRSL1), mRNA [NM_018292]	NM_018292
A_23_P208158	2.63E-03	2.98	NM_001392	NM_001392	Homo sapiens dystrobrevin, alpha (DTNA), transcript variant 7, mRNA [NM_001392]	NM_001392
A_24_P375609	2.63E-03	3.10	NM_001970	NM_001970	Homo sapiens eukaryotic translation initiation factor 5A (EIF5A), mRNA [NM_001970]	NM_001970
A_23_P22499	2.63E-03	4.67	NM_019067	NM_019067	Homo sapiens guanine nucleotide binding protein-like 3 (nucleolar)-like (GNL3L), mRNA [NM_019067]	NM_019067
A_23_P66787	2.64E-03	2.30	U18197	U18197	Human ATP:citrate lyase mRNA, complete cds. [U18197]	
A_23_P426398	2.64E-03	2.94	NM_138575	NM_138575	Homo sapiens Bcl-XL-binding protein v68 (MGC5352), mRNA [NM_138575]	NM_138575
A_24_P655268	2.64E-03	3.21	CR609905	CR609905	full-length cDNA clone CS0DD006YF13 of Neuroblastoma Cot 50-normalized of Homo sapiens (human). [CR609905]	
A_23_P88909	2.64E-03	3.83	NM_004209	NM_004209	Homo sapiens synaptogyrin 3 (SYNGR3), mRNA [NM_004209]	NM_004209
A_23_P28590	2.65E-03	2.15	NM_024622	NM_024622	Homo sapiens hypothetical protein FLJ21901 (FLJ21901), mRNA [NM_024622]	NM_024622
A_23_P108200	2.65E-03	2.63	NM_002866	NM_002866	Homo sapiens RAB3A, member RAS oncogene family (RAB3A), mRNA [NM_002866]	NM_002866
A_23_P23155	2.65E-03	4.61	NM_018836	NM_018836	Homo sapiens adherens junction associated protein 1 (AJAP1), mRNA [NM_018836]	NM_018836
A_24_P878388	2.66E-03	2.00	XM_373338	XM_373338	PREDICTED: Homo sapiens similar to bA92K2.2 (similar to ubiquitin) (LOC392425), mRNA [XM_373338]	XM_373338
A_23_P131866	2.66E-03	2.19	NM_198433	NM_198433	Homo sapiens serine/threonine kinase 6 (STK6), transcript variant 1, mRNA [NM_198433]	NM_198433
A_23_P51051	2.66E-03	2.85	NM_005081	NM_005081	Homo sapiens zinc finger protein 142 (clone pHZ-49) (ZNF142), mRNA [NM_005081]	NM_005081
A_23_P122674	2.66E-03	3.50	NM_017906	NM_017906	Homo sapiens PAK1 interacting protein 1 (PAK1IP1), mRNA [NM_017906]	NM_017906
A_23_P398073	2.67E-03	3.25	NM_177968	NM_177968	Homo sapiens protein phosphatase 1B (formerly 2C), magnesium-dependent, beta isoform (PPM1B), transcript variant 2, mRNA [NM_177968]	NM_177968
A_23_P251795	2.67E-03	4.13	NM_152742	NM_152742	Homo sapiens glypican 2 (cerebroglycan) (GPC2), mRNA [NM_152742]	NM_152742
A_23_P211436	2.68E-03	2.05	NM_005877	NM_005877	Homo sapiens splicing factor 3a, subunit 1, 120kDa (SF3A1), transcript variant 1, mRNA [NM_005877]	NM_005877
A_23_P205789	2.68E-03	2.50	NM_002041	NM_002041	Homo sapiens GA binding protein transcription factor, beta subunit 2 (GABPB2), transcript variant gamma-1, mRNA [NM_002041]	NM_002041
A_23_P7679	2.68E-03	2.78	NM_153485	NM_153485	Homo sapiens nucleoporin 155kDa (NUP155), transcript variant 1, mRNA [NM_153485]	NM_153485
A_23_P6362	2.69E-03	2.10	NM_198440	NM_198440	Homo sapiens Der1-like domain family, member 3 (DERL3), transcript variant 1, mRNA [NM_198440]	NM_198440

A_24_P728604	2.69E-03	2.13	XM_208930	XM_208930	PREDICTED: Homo sapiens similar to RIKEN cDNA 4930511J11 (LOC283953), mRNA [XM_208930]	XM_208930
A_24_P942112	2.69E-03	2.27	NM_019014	NM_019014	Homo sapiens polymerase (RNA) I polypeptide B, 128kDa (POLR1B), mRNA [NM_019014]	NM_019014
A_24_P263443	2.70E-03	2.31	ENST00000259550			
A_23_P256148	2.70E-03	3.08	NM_024595	NM_024595	Homo sapiens chromosome 1 open reading frame 108 (C1orf108), mRNA [NM_024595]	NM_024595
A_23_P131215	2.70E-03	3.52	NM_006891	NM_006891	Homo sapiens crystallin, gamma D (CRYGD), mRNA [NM_006891]	NM_006891
A_32_P135043	2.70E-03	4.69	THC2376245			
A_23_P211302	2.70E-03	5.56	NM_033661	NM_033661	Homo sapiens WD repeat domain 4 (WDR4), transcript variant 2, mRNA [NM_033661]	NM_033661
A_23_P58280	2.71E-03	2.12	NM_018983	NM_018983	Homo sapiens nucleolar protein family A, member 1 (H/ACA small nucleolar RNPs) (NOLA1), transcript variant 1, mRNA [NM_018983]	NM_018983
A_23_P46924	2.72E-03	2.38	NM_001007793	NM_001007793	Homo sapiens BUB3 budding uninhibited by benzimidazoles 3 homolog (yeast) (BUB3), transcript variant 2, mRNA [NM_001007793]	NM_001007793
A_24_P922808	2.73E-03	4.07	ENST00000346571		Homo sapiens chromosome 1 open reading frame 121, mRNA (cDNA clone IMAGE:4718788), partial cds. [BC020640]	
A_23_P209298	2.74E-03	2.05	BC032822	BC032822	Homo sapiens erythrocyte membrane protein band 4.1 like 5, mRNA (cDNA clone MGC:26029 IMAGE:4827274), complete cds. [BC032822]	
A_24_P71244	2.74E-03	2.26	NM_005026	NM_005026	Homo sapiens phosphoinositide-3-kinase, catalytic, delta polypeptide (PIK3CD), mRNA [NM_005026]	NM_005026
A_23_P141863	2.74E-03	3.55	NM_014480	NM_014480	Homo sapiens zinc finger protein 544 (ZNF544), mRNA [NM_014480]	NM_014480
A_24_P914000	2.74E-03	4.46	BC001437	BC001437	Homo sapiens hypothetical protein BC001437, mRNA (cDNA clone MGC:1013 IMAGE:3138594), complete cds. [BC001437]	
A_23_P253046	2.74E-03	4.90	NM_006759	NM_006759	Homo sapiens UDP-glucose pyrophosphorylase 2 (UGP2), transcript variant 1, mRNA [NM_006759]	NM_006759
A_23_P207445	2.76E-03	2.49	NM_002758	NM_002758	Homo sapiens mitogen-activated protein kinase kinase 6 (MAP2K6), transcript variant 1, mRNA [NM_002758]	NM_002758
A_23_P202773	2.76E-03	2.70	NM_016611	NM_016611	Homo sapiens potassium channel, subfamily K, member 4 (KCNK4), transcript variant 1, mRNA [NM_016611]	NM_016611
A_23_P256142	2.76E-03	2.97	NM_024595	NM_024595	Homo sapiens chromosome 1 open reading frame 108 (C1orf108), mRNA [NM_024595]	NM_024595
A_23_P46903	2.76E-03	3.12	NM_172171	NM_172171	Homo sapiens calcium/calmodulin-dependent protein kinase (CaM kinase) II gamma (CAMK2G), transcript variant 1, mRNA [NM_172171]	NM_172171
A_23_P212825	2.77E-03	3.77	NM_057175	NM_057175	Homo sapiens NMDA receptor regulated 1 (NARG1), mRNA [NM_057175]	NM_057175
A_32_P152696	2.77E-03	4.39	A_32_P152696			
A_32_P318086	2.78E-03	2.03	NM_025207	NM_025207	Homo sapiens FAD-synthetase (PP591), transcript variant 1, mRNA [NM_025207]	NM_025207
A_23_P143285	2.79E-03	2.34	AK000809	AK000809	Homo sapiens cDNA FLJ20802 fis, clone ADSU01223. [AK000809]	
A_32_P59673	2.80E-03	2.17	NM_199136	NM_199136	Homo sapiens hypothetical protein MGC72075 (MGC72075), mRNA [NM_199136]	NM_199136
A_23_P151337	2.81E-03	2.34	NM_005887	NM_005887	Homo sapiens deleted in lymphocytic leukemia, 1 (DLEU1), mRNA [NM_005887]	NM_005887
A_23_P103070	2.82E-03	2.11	NM_003405	NM_003405	Homo sapiens tyrosine 3-monooxygenase/tryptophan 5-monooxygenase activation protein, eta polypeptide (YWHAH), mRNA [NM_003405]	NM_003405
A_32_P36582	2.82E-03	2.28	AK123649	AK123649	Homo sapiens cDNA FLJ41655 fis, clone FEBRA2025477. [AK123649]	
A_32_P79707	2.82E-03	2.54	AI022288	AI022288	AI022288 ow63d08.x1 Soares_senescent_fibroblasts_NbHSF Homo sapiens cDNA clone IMAGE:1651503 3', mRNA sequence [AI022288]	
A_24_P323941	2.82E-03	2.65	NM_001012971	NM_001012971	Homo sapiens chromosome 20 open reading frame 106 (C20orf106), mRNA [NM_001012971]	NM_001012971
A_32_P109572	2.83E-03	2.38	NM_001533	NM_001533	Homo sapiens heterogeneous nuclear ribonucleoprotein L (HNRPL), transcript variant 1, mRNA [NM_001533]	NM_001533
A_24_P35891	2.83E-03	2.62	NM_016423	NM_016423	Homo sapiens zinc finger protein 219 (ZNF219), mRNA [NM_016423]	NM_016423
A_23_P121423	2.83E-03	5.05	NM_001789	NM_001789	Homo sapiens cell division cycle 25A (CDC25A), transcript variant 1, mRNA [NM_001789]	NM_001789
A_23_P137143	2.84E-03	2.21	NM_001363	NM_001363	Homo sapiens dyskeratosis congenita 1, dyskerin (DKC1), mRNA [NM_001363]	NM_001363
A_23_P252855	2.85E-03	2.09	NM_018321	NM_018321	Homo sapiens brix domain containing 2 (BXDC2), mRNA [NM_018321]	NM_018321
A_23_P133216	2.85E-03	4.22	NM_020726	NM_020726	Homo sapiens neurolysin (metallopeptidase M3 family) (NLN), mRNA [NM_020726]	NM_020726
A_23_P53345	2.86E-03	5.49	NM_020183	NM_020183	Homo sapiens aryl hydrocarbon receptor nuclear translocator-like 2 (ARNTL2), mRNA [NM_020183]	NM_020183
A_23_P380526	2.86E-03	8.47	NM_018189	NM_018189	Homo sapiens developmental pluripotency associated 4 (DPPA4), mRNA [NM_018189]	NM_018189
A_24_P258051	2.88E-03	2.54	NM_032844	NM_032844	Homo sapiens microtubule associated serine/threonine kinase-like (MASTL), mRNA [NM_032844]	NM_032844

A_23_P50990	2.89E-03	2.49	NM_024322	NM_024322	Homo sapiens hypothetical protein MGC11266 (MGC11266), mRNA [NM_024322]	NM_024322
A_23_P161615	2.90E-03	2.00	NM_002689	NM_002689	Homo sapiens polymerase (DNA directed), alpha 2 (70kD subunit) (POLA2), mRNA [NM_002689]	NM_002689
A_32_P35512	2.90E-03	2.62	NM_003142	NM_003142	Homo sapiens Sjogren syndrome antigen B (autoantigen La) (SSB), mRNA [NM_003142]	NM_003142
A_23_P72737	2.90E-03	2.74	NM_003641	NM_003641	Homo sapiens interferon induced transmembrane protein 1 (9-27) (IFITM1), mRNA [NM_003641]	NM_003641
A_23_P362759	2.90E-03	2.75	NM_018699	NM_018699	Homo sapiens PR domain containing 5 (PRDM5), mRNA [NM_018699]	NM_018699
A_23_P398770	2.91E-03	2.55	AK055921	AK055921	Homo sapiens cDNA FLJ31359 fis, clone MESAN2000501, weakly similar to Homo sapiens DNA cytosine methyltransferase 3 alpha (DNMT3A) mRNA. [AK055921]	
A_23_P43820	2.91E-03	3.06	NM_032793	NM_032793	Homo sapiens major facilitator superfamily domain containing 2 (MFSD2), mRNA [NM_032793]	NM_032793
A_23_P145006	2.92E-03	19.38	NM_054023	NM_054023	Homo sapiens secretoglobin, family 3A, member 2 (SCGB3A2), mRNA [NM_054023]	NM_054023
A_23_P163458	2.94E-03	2.07	NM_139265	NM_139265	Homo sapiens EH-domain containing 4 (EHD4), mRNA [NM_139265]	NM_139265
A_23_P128304	2.94E-03	2.56	NM_001003398	NM_001003398	Homo sapiens bicaudal D homolog 1 (Drosophila) (BICD1), transcript variant 2, mRNA [NM_001003398]	NM_001003398
A_23_P218918	2.94E-03	7.14	NM_002006	NM_002006	Homo sapiens fibroblast growth factor 2 (basic) (FGF2), mRNA [NM_002006]	NM_002006
A_23_P165408	2.95E-03	3.01	NM_144711	NM_144711	Homo sapiens kelch-like 23 (Drosophila) (KLHL23), mRNA [NM_144711]	NM_144711
A_23_P13946	2.96E-03	2.87	NM_005475	NM_005475	Homo sapiens lymphocyte adaptor protein (LNK), mRNA [NM_005475]	NM_005475
A_23_P487	2.97E-03	2.47	NM_012474	NM_012474	Homo sapiens uridine-cytidine kinase 2 (UCK2), mRNA [NM_012474]	NM_012474
A_23_P166698	2.97E-03	3.69	NM_016305	NM_016305	Homo sapiens synovial sarcoma translocation gene on chromosome 18-like 2 (SS18L2), mRNA [NM_016305]	NM_016305
A_23_P36795	2.97E-03	5.35	NM_005639	NM_005639	Homo sapiens synaptotagmin I (SYT1), mRNA [NM_005639]	NM_005639
A_32_P223140	2.99E-03	2.36	NM_145313	NM_145313	Homo sapiens RasGEF domain family, member 1A (RASGEF1A), mRNA [NM_145313]	NM_145313
A_23_P163099	2.99E-03	2.62	NM_002692	NM_002692	Homo sapiens polymerase (DNA directed), epsilon 2 (p59 subunit) (POLE2), mRNA [NM_002692]	NM_002692
A_23_P139983	2.99E-03	2.71	NM_022459	NM_022459	Homo sapiens exportin 4 (XPO4), mRNA [NM_022459]	NM_022459
A_23_P346086	2.99E-03	3.30	CR589996	CR589996	full-length cDNA clone CS0DF019YO17 of Fetal brain of Homo sapiens (human). [CR589996]	
A_23_P318396	2.99E-03	3.50	NM_198700	NM_198700	Homo sapiens CUG triplet repeat, RNA binding protein 1 (CUGBP1), transcript variant 2, mRNA [NM_198700]	NM_198700
A_23_P35444	2.99E-03	4.33	NM_032727	NM_032727	Homo sapiens internexin neuronal intermediate filament protein, alpha (INA), mRNA [NM_032727]	NM_032727
A_23_P104563	3.01E-03	4.65	NM_001876	NM_001876	Homo sapiens carnitine palmitoyltransferase 1A (liver) (CPT1A), nuclear gene encoding mitochondrial protein, mRNA [NM_001876]	NM_001876
A_24_P633575	3.02E-03	2.02	AF086329	AF086329	Homo sapiens full length insert cDNA clone ZD54C08. [AF086329]	
A_24_P241373	3.02E-03	2.38	BC006177	BC006177	Homo sapiens metastasis associated 1, mRNA (cDNA clone IMAGE:4054392), complete cds. [BC006177]	
A_23_P215525	3.02E-03	2.41	NM_145323	NM_145323	Homo sapiens oxysterol binding protein-like 3 (OSBPL3), transcript variant 5, mRNA [NM_145323]	NM_145323
A_23_P94689	3.02E-03	2.60	NM_030914	NM_030914	Homo sapiens chromosome 9 open reading frame 74 (C9orf74), mRNA [NM_030914]	NM_030914
A_23_P132874	3.03E-03	2.02	NM_032359	NM_032359	Homo sapiens hypothetical protein MGC4308 (MGC4308), mRNA [NM_032359]	NM_032359
A_23_P1431	3.03E-03	2.48	NM_004329	NM_004329	Homo sapiens bone morphogenetic protein receptor, type IA (BMPR1A), mRNA [NM_004329]	NM_004329
A_24_P296254	3.03E-03	3.26	NM_014783	NM_014783	Homo sapiens Rho GTPase activating protein 11A (ARHGAP11A), mRNA [NM_014783]	NM_014783
A_23_P12363	3.03E-03	3.94	NM_005012	NM_005012	Homo sapiens receptor tyrosine kinase-like orphan receptor 1 (ROR1), mRNA [NM_005012]	NM_005012
A_24_P205263	3.03E-03	4.44	NM_016649	NM_016649	Homo sapiens chromosome 20 open reading frame 6 (C20orf6), mRNA [NM_016649]	NM_016649
A_32_P194264	3.03E-03	4.74	NM_001008708	NM_001008708	Homo sapiens similar to RIKEN cDNA 2510006C20 gene (LOC494143), mRNA [NM_001008708]	NM_001008708
A_23_P343719	3.04E-03	2.40	NM_000932	NM_000932	Homo sapiens phospholipase C, beta 3 (phosphatidylinositol-specific) (PLCB3), mRNA [NM_000932]	NM_000932
A_23_P402610	3.04E-03	2.67	NM_012393	NM_012393	Homo sapiens phosphoribosylformylglycinamidine synthase (FGAR amidotransferase) (PFAS), mRNA [NM_012393]	NM_012393
A_23_P335039	3.04E-03	2.91	NM_133474	NM_133474	Homo sapiens KIAA1982 protein (KIAA1982), mRNA [NM_133474]	NM_133474
A_32_P84242	3.04E-03	3.21	CR936791	CR936791	Homo sapiens mRNA; cDNA DKFZp781C2356 (from clone DKFZp781C2356). [CR936791]	XM_032571
A_23_P73982	3.04E-03	3.26	NM_018087	NM_018087	Homo sapiens transmembrane protein 48 (TMEM48), mRNA [NM_018087]	NM_018087
A_24_P50328	3.04E-03	3.79	A_24_P50328			
A_24_P942493	3.04E-03	4.95	NM_015253	NM_015253	Homo sapiens KIAA0523 protein (KIAA0523), mRNA [NM_015253]	NM_015253
A_23_P322756	3.05E-03	2.43	AB051463	AB051463	Homo sapiens mRNA for KIAA1676 protein, partial cds. [AB051463]	

A_24_P107291	3.05E-03	3.26	NM_181699	NM_181699	Homo sapiens protein phosphatase 2 (formerly 2A), regulatory subunit A (PR 65), beta isoform (PPP2R1B), transcript variant 2, mRNA [NM_181699]	NM_181699
A_23_P386942	3.06E-03	2.02	NM_145173	NM_145173	Homo sapiens DIRAS family, GTP-binding RAS-like 1 (DIRAS1), mRNA [NM_145173]	NM_145173
A_23_P156842	3.06E-03	3.94	NM_004280	NM_004280	Homo sapiens eukaryotic translation elongation factor 1 epsilon 1 (EEF1E1), mRNA [NM_004280]	NM_004280
A_23_P341471	3.07E-03	2.02	NM_003348	NM_003348	Homo sapiens ubiquitin-conjugating enzyme E2N (UBC13 homolog, yeast) (UBE2N), mRNA [NM_003348]	NM_003348
A_23_P127186	3.07E-03	2.13	NM_206862	NM_206862	Homo sapiens transforming, acidic coiled-coil containing protein 2 (TACC2), transcript variant 1, mRNA [NM_206862]	NM_206862
A_32_P232381	3.08E-03	2.34	THC2401087			
A_23_P308305	3.08E-03	2.93	ENST00000317571		Homo sapiens cDNA FLJ90394 fis, clone NT2RP2005632. [AK074875]	
A_32_P200934	3.08E-03	4.88	AK021443	AK021443	Homo sapiens cDNA FLJ11381 fis, clone HEMBA1000501. [AK021443]	
A_23_P107963	3.08E-03	5.38	NM_000148	NM_000148	Homo sapiens fucosyltransferase 1 (galactoside 2-alpha-L-fucosyltransferase) (FUT1), mRNA [NM_000148]	NM_000148
A_24_P382001	3.09E-03	2.57	NM_016310	NM_016310	Homo sapiens polymerase (RNA) III (DNA directed) polypeptide K, 12.3 kDa (POLR3K), mRNA [NM_016310]	NM_016310
A_32_P147189	3.10E-03	4.29	BC071732	BC071732	Homo sapiens cDNA clone IMAGE:3862422, partial cds. [BC071732]	
A_23_P308954	3.11E-03	2.33	NM_030639	NM_030639	Homo sapiens basic helix-loop-helix domain containing, class B, 9 (BHLHB9), mRNA [NM_030639]	NM_030639
A_23_P162378	3.11E-03	2.34	NM_016122	NM_016122	Homo sapiens NY-REN-58 antigen (NY-REN-58), mRNA [NM_016122]	NM_016122
A_23_P251771	3.11E-03	3.70	NM_025215	NM_025215	Homo sapiens pseudouridylate synthase 1 (PUS1), transcript variant 1, mRNA [NM_025215]	NM_025215
A_23_P345118	3.12E-03	2.02	NM_002648	NM_002648	Homo sapiens pim-1 oncogene (PIM1), mRNA [NM_002648]	NM_002648
A_32_P101235	3.12E-03	2.06	NM_013282	NM_013282	Homo sapiens ubiquitin-like, containing PHD and RING finger domains, 1 (UHRF1), mRNA [NM_013282]	NM_013282
A_24_P711050	3.12E-03	2.07	A_24_P711050			
A_24_P53519	3.12E-03	3.09	NM_005483	NM_005483	Homo sapiens chromatin assembly factor 1, subunit A (p150) (CHAF1A), mRNA [NM_005483]	NM_005483
A_23_P33539	3.12E-03	9.71	NM_020546	NM_020546	Homo sapiens adenylate cyclase 2 (brain) (ADCY2), mRNA [NM_020546]	NM_020546
A_23_P125001	3.13E-03	2.69	NM_005052	NM_005052	Homo sapiens ras-related C3 botulinum toxin substrate 3 (rho family, small GTP binding protein Rac3) (RAC3), mRNA [NM_005052]	NM_005052
A_24_P284783	3.14E-03	2.04	NM_014337	NM_014337	Homo sapiens peptidylprolyl isomerase (cyclophilin)-like 2 (PPIL2), transcript variant 1, mRNA [NM_014337]	NM_014337
A_24_P283294	3.14E-03	2.64	NM_018141	NM_018141	Homo sapiens mitochondrial ribosomal protein S10 (MRPS10), nuclear gene encoding mitochondrial protein, mRNA [NM_018141]	NM_018141
A_23_P65555	3.14E-03	3.02	NM_021818	NM_021818	Homo sapiens salvador homolog 1 (Drosophila) (SAV1), mRNA [NM_021818]	NM_021818
A_23_P28664	3.14E-03	3.73	NM_018084	NM_018084	Homo sapiens KIAA1212 (KIAA1212), mRNA [NM_018084]	NM_018084
A_23_P217820	3.14E-03	3.94	NM_014762	NM_014762	Homo sapiens 24-dehydrocholesterol reductase (DHCR24), mRNA [NM_014762]	NM_014762
A_23_P21033	3.15E-03	2.06	NM_003875	NM_003875	Homo sapiens guanine monphosphate synthetase (GMPS), mRNA [NM_003875]	NM_003875
A_23_P50477	3.17E-03	2.20	NM_138639	NM_138639	Homo sapiens BCL2-like 12 (proline rich) (BCL2L12), transcript variant 1, mRNA [NM_138639]	NM_138639
A_23_P67151	3.17E-03	2.30	NM_058164	NM_058164	Homo sapiens olfactomedin 2 (OLFM2), mRNA [NM_058164]	NM_058164
A_23_P25313	3.17E-03	2.84	NM_080626	NM_080626	Homo sapiens BRI3 binding protein (BRI3BP), mRNA [NM_080626]	NM_080626
A_24_P131293	3.18E-03	2.11	NM_002699	NM_002699	Homo sapiens POU domain, class 3, transcription factor 1 (POU3F1), mRNA [NM_002699]	NM_002699
A_24_P64329	3.18E-03	2.25	NM_173575	NM_173575	Homo sapiens serine/threonine kinase 32C (STK32C), mRNA [NM_173575]	NM_173575
A_24_P118452	3.18E-03	5.24	NM_012247	NM_012247	Homo sapiens selenophosphate synthetase 1 (SEPHS1), mRNA [NM_012247]	NM_012247
A_23_P398947	3.18E-03	16.00	NM_205848	NM_205848	Homo sapiens synaptotagmin VI (SYT6), mRNA [NM_205848]	NM_205848
A_23_P28953	3.18E-03	18.87	NM_175850	NM_175850	Homo sapiens DNA (cytosine-5-)-methyltransferase 3 beta (DNMT3B), transcript variant 6, mRNA [NM_175850]	NM_175850
A_32_P141418	3.19E-03	7.58	NM_018076	NM_018076	Homo sapiens armadillo repeat containing 4 (ARMC4), mRNA [NM_018076]	NM_018076
A_24_P13533	3.20E-03	2.60	NM_203467	NM_203467	Homo sapiens peptidylprolyl isomerase (cyclophilin)-like 5 (PPIL5), transcript variant 3, mRNA [NM_203467]	NM_203467
A_32_P72341	3.21E-03	2.02	BX537987	BX537987	Homo sapiens mRNA; cDNA DKFZp686N1030 (from clone DKFZp686N1030). [BX537987]	
A_23_P28886	3.21E-03	3.02	NM_002592	NM_002592	Homo sapiens proliferating cell nuclear antigen (PCNA), transcript variant 1, mRNA [NM_002592]	NM_002592
A_23_P91930	3.21E-03	3.42	NM_024638	NM_024638	Homo sapiens queuine tRNA-ribosyltransferase domain containing 1 (QTRTD1), mRNA [NM_024638]	NM_024638
A_24_P282237	3.23E-03	2.20	NM_000947	NM_000947	Homo sapiens primase, polypeptide 2A, 58kDa (PRIM2A), mRNA [NM_000947]	NM_000947
A_32_P204205	3.23E-03	2.87	NM_017420	NM_017420	Homo sapiens sine oculis homeobox homolog 4 (Drosophila) (SIX4), mRNA [NM_017420]	NM_017420

A_32_P182395	3.23E-03	3.79	THC2434943		Q8WUP8 (Q8WUP8) MGC21881 protein (Fragment), partial (36%) [THC2434943]	
A_23_P213592	3.24E-03	3.19	NM_014901	NM_014901	Homo sapiens ring finger protein 44 (RNF44), mRNA [NM_014901]	NM_014901
A_24_P179646	3.24E-03	19.12	M96956	M96956	Human (clone CR-3) teratocarcinoma-derived growth factor 3 (TDGF3) mRNA, complete cds. [M96956]	
A_23_P203075	3.25E-03	2.72	NM_020886	NM_020886	Homo sapiens ubiquitin specific protease 28 (USP28), mRNA [NM_020886]	NM_020886
A_23_P170491	3.26E-03	2.04	NM_005879	NM_005879	Homo sapiens TRAF interacting protein (TRIP), mRNA [NM_005879]	NM_005879
A_23_P424002	3.26E-03	2.16	NM_002697	NM_002697	Homo sapiens POU domain, class 2, transcription factor 1 (POU2F1), mRNA [NM_002697]	NM_002697
A_23_P16225	3.26E-03	2.17	NM_017682	NM_017682	Homo sapiens vitelliform macular dystrophy 2-like 1 (VMD2L1), mRNA [NM_017682]	NM_017682
A_23_P45560	3.26E-03	2.62	NM_000273	NM_000273	Homo sapiens G protein-coupled receptor 143 (GPR143), mRNA [NM_000273]	NM_000273
A_23_P121480	3.26E-03	2.97	NM_001004196	NM_001004196	Homo sapiens CD200 antigen (CD200), transcript variant 2, mRNA [NM_001004196]	NM_001004196
A_23_P121276	3.27E-03	2.22	NM_003157	NM_003157	Homo sapiens NIMA (never in mitosis gene a)-related kinase 4 (NEK4), mRNA [NM_003157]	NM_003157
A_23_P9574	3.27E-03	2.85	NM_018098	NM_018098	Homo sapiens epithelial cell transforming sequence 2 oncogene (ECT2), mRNA [NM_018098]	NM_018098
A_32_P81674	3.27E-03	17.39	AK091593	AK091593	Homo sapiens cDNA FLJ34274 fis, clone FEBRA2003327. [AK091593]	
A_23_P86133	3.28E-03	2.20	NM_002946	NM_002946	Homo sapiens replication protein A2, 32kDa (RPA2), mRNA [NM_002946]	NM_002946
A_23_P121182	3.28E-03	2.54	NM_012260	NM_012260	Homo sapiens 2-hydroxyphytanoyl-CoA lyase (HPCL2), mRNA [NM_012260]	NM_012260
A_23_P170337	3.28E-03	3.03	NM_003748	NM_003748	Homo sapiens aldehyde dehydrogenase 4 family, member A1 (ALDH4A1), nuclear gene encoding mitochondrial protein, transcript variant P5CDhL, mRNA [NM_003748]	NM_003748
A_24_P96897	3.28E-03	5.95	CR594520	CR594520	full-length cDNA clone CS0DF016YO07 of Fetal brain of Homo sapiens (human). [CR594520]	
A_23_P69030	3.28E-03	7.63	NM_001850	NM_001850	Homo sapiens collagen, type VIII, alpha 1 (COL8A1), transcript variant 1, mRNA [NM_001850]	NM_001850
A_32_P153773	3.30E-03	2.59	AB209467	AB209467	Homo sapiens mRNA for calcium channel, voltage-dependent, L type, alpha 1B subunit variant protein. [AB209467]	
A_23_P163117	3.31E-03	2.09	NM_024644	NM_024644	Homo sapiens chromosome 14 open reading frame 169 (C14orf169), mRNA [NM_024644]	NM_024644
A_23_P360605	3.31E-03	2.29	BC040542	BC040542	Homo sapiens KIAA0802, mRNA (cDNA clone MGC:39663 IMAGE:5268201), complete cds. [BC040542]	
A_23_P34800	3.31E-03	2.57	NM_172164	NM_172164	Homo sapiens nuclear autoantigenic sperm protein (histone-binding) (NASP), transcript variant 1, mRNA [NM_172164]	NM_172164
A_23_P26629	3.31E-03	2.97	NM_013258	NM_013258	Homo sapiens PYD and CARD domain containing (PYCARD), transcript variant 1, mRNA [NM_013258]	NM_013258
A_32_P92814	3.32E-03	3.36	BQ929021	BQ929021	AGENCOURT_10034103 NIH_MGC_40 Homo sapiens cDNA clone IMAGE:6482800 5', mRNA sequence [BQ929021]	
A_32_P184933	3.33E-03	2.29	NM_014501	NM_014501	Homo sapiens ubiquitin-conjugating enzyme E2S (UBE2S), mRNA [NM_014501]	NM_014501
A_23_P253412	3.35E-03	2.41	NM_019051	NM_019051	Homo sapiens mitochondrial ribosomal protein L50 (MRPL50), nuclear gene encoding mitochondrial protein, mRNA [NM_019051]	NM_019051
A_23_P407614	3.35E-03	2.56	NM_152901	NM_152901	Homo sapiens pyrin domain containing 1 (PYDC1), mRNA [NM_152901]	NM_152901
A_24_P12660	3.35E-03	3.10	THC2303284		Q6GLA9 (Q6GLA9) MGC69246 protein, partial (21%) [THC2303284]	
A_32_P141374	3.37E-03	2.19	NM_178831	NM_178831	Homo sapiens opposite strand transcription unit to STAG3 (GATS), mRNA [NM_178831]	NM_178831
A_23_P108641	3.38E-03	2.38	NM_032822	NM_032822	Homo sapiens hypothetical protein FLJ14668 (FLJ14668), mRNA [NM_032822]	NM_032822
A_24_P289404	3.38E-03	2.40	NM_001029	NM_001029	Homo sapiens ribosomal protein S26 (RPS26), mRNA [NM_001029]	NM_001029
A_32_P91821	3.38E-03	2.47	BM041657	BM041657	BM041657 603614719T1 NIH_MGC_108 Homo sapiens cDNA clone IMAGE:5556485 3', mRNA sequence [BM041657]	
A_23_P57379	3.38E-03	2.78	NM_003504	NM_003504	Homo sapiens CDC45 cell division cycle 45-like (S. cerevisiae) (CDC45L), mRNA [NM_003504]	NM_003504
A_32_P56874	3.39E-03	2.06	BG502322	BG502322	BG502322 602550205F1 NIH_MGC_61 Homo sapiens cDNA clone IMAGE:4657992 5', mRNA sequence [BG502322]	
A_32_P149432	3.39E-03	2.16	NM_001416	NM_001416	Homo sapiens eukaryotic translation initiation factor 4A, isoform 1 (EIF4A1), mRNA [NM_001416]	NM_001416
A_23_P143817	3.40E-03	2.24	NM_053025	NM_053025	Homo sapiens myosin, light polypeptide kinase (MYLK), transcript variant 1, mRNA [NM_053025]	NM_053025
A_23_P128174	3.40E-03	2.62	NM_175623	NM_175623	Homo sapiens RAB3A interacting protein (rabin3) (RAB3IP), transcript variant alpha 2, mRNA [NM_175623]	NM_175623
A_23_P77079	3.40E-03	2.71	NM_016132	NM_016132	Homo sapiens myelin expression factor 2 (MYEF2), mRNA [NM_016132]	NM_016132
A_24_P904484	3.40E-03	7.94	NM_001001873	NM_001001873	Homo sapiens hypothetical protein LOC283174 (LOC283174), mRNA [NM_001001873]	NM_001001873
A_23_P3963	3.41E-03	2.02	NM_014603	NM_014603	Homo sapiens paraneoplastic antigen (HUMPPA), mRNA [NM_014603]	NM_014603

A_23_P88691	3.41E-03	4.63	NM_000745	NM_000745	Homo sapiens cholinergic receptor, nicotinic, alpha polypeptide 5 (CHRNA5), mRNA [NM_000745]	NM_000745
A_32_P216715	3.41E-03	16.69	BM666601	BM666601	UI-E-CQ1-aew-l-18-0-UI.s1 UI-E-CQ1 Homo sapiens cDNA clone UI-E-CQ1-aew-l-18-0-UI 3', mRNA sequence [BM666601]	
A_23_P76145	3.41E-03	20.70	AF256215	AF256215	Homo sapiens cycle-like factor CLIF mRNA, complete cds. [AF256215]	
A_24_P363100	3.42E-03	2.63	NM_173670	NM_173670	Homo sapiens RGM domain family, member B (RGMB), transcript variant 2, mRNA [NM_173670]	NM_173670
A_23_P340909	3.42E-03	2.96	BC013418	BC013418	Homo sapiens chromosome 13 open reading frame 3, mRNA (cDNA clone MGC:4832 IMAGE:3604003), complete cds. [BC013418]	
A_23_P58321	3.43E-03	2.93	NM_001237	NM_001237	Homo sapiens cyclin A2 (CCNA2), mRNA [NM_001237]	NM_001237
A_23_P106922	3.44E-03	7.81	NM_021615	NM_021615	Homo sapiens carbohydrate (N-acetylglucosamine 6-O) sulfotransferase 6 (CHST6), mRNA [NM_021615]	NM_021615
A_23_P102109	3.44E-03	14.04	NM_025019	NM_025019	Homo sapiens tubulin, alpha 4 (TUBA4), mRNA [NM_025019]	NM_025019
A_23_P213471	3.45E-03	2.40	NM_024830	NM_024830	Homo sapiens hypothetical protein FLJ12443 (FLJ12443), mRNA [NM_024830]	NM_024830
A_24_P75220	3.45E-03	2.87	NM_004742	NM_004742	Homo sapiens membrane associated guanylate kinase, WW and PDZ domain containing 1 (MAGI1), mRNA [NM_004742]	NM_004742
A_32_P18668	3.45E-03	3.52	NM_002867	NM_002867	Homo sapiens RAB3B, member RAS oncogene family (RAB3B), mRNA [NM_002867]	NM_002867
A_32_P147949	3.48E-03	2.16	THC2441537			
A_24_P32560	3.49E-03	2.09	NM_198573	NM_198573	Homo sapiens GAAI470 (UNQ470), mRNA [NM_198573]	NM_198573
A_24_P97129	3.49E-03	2.42	NM_001006946	NM_001006946	Homo sapiens syndecan 1 (SDC1), transcript variant 1, mRNA [NM_001006946]	NM_001006946
A_23_P200015	3.49E-03	2.82	NM_174858	NM_174858	Homo sapiens adenylate kinase 5 (AK5), transcript variant 1, mRNA [NM_174858]	NM_174858
A_23_P374844	3.49E-03	22.57	NM_015973	NM_015973	Homo sapiens galanin (GAL), mRNA [NM_015973]	NM_015973
A_23_P389919	3.51E-03	2.00	NM_014919	NM_014919	Homo sapiens Wolf-Hirschhorn syndrome candidate 1 (WHSC1), transcript variant 4, mRNA [NM_014919]	NM_014919
A_23_P90762	3.51E-03	2.01	NM_013233	NM_013233	Homo sapiens serine threonine kinase 39 (STE20/SPS1 homolog, yeast) (STK39), mRNA [NM_013233]	NM_013233
A_24_P59569	3.51E-03	2.14	BC000922	BC000922	Homo sapiens hypothetical gene supported by BC000922, mRNA (cDNA clone IMAGE:3447073), partial cds. [BC000922]	XM_378793
A_24_P942335	3.51E-03	2.36	BC002881	BC002881	Homo sapiens leucine-rich repeat kinase 1, mRNA (cDNA clone IMAGE:3940845), partial cds. [BC002881]	
A_23_P107903	3.51E-03	2.46	AK126263	AK126263	Homo sapiens cDNA FLJ44275 fis, clone TOVAR2002800. [AK126263]	XM_290867
A_23_P124542	3.51E-03	2.79	M26004	M26004	Human CR2/CD21/C3d/Epstein-Barr virus receptor mRNA, complete cds. [M26004]	
A_24_P158193	3.51E-03	23.87	A_24_P158193			
A_23_P424561	3.52E-03	2.05	NM_133639	NM_133639	Homo sapiens ras homolog gene family, member V (RHOV), mRNA [NM_133639]	NM_133639
A_24_P876862	3.52E-03	2.10	BC061590	BC061590	Homo sapiens cDNA clone MGC:75203 IMAGE:6502529, complete cds. [BC061590]	
A_23_P30799	3.52E-03	3.13	NM_021018	NM_021018	Homo sapiens histone 1, H3f (HIST1H3F), mRNA [NM_021018]	NM_021018
A_24_P472055	3.52E-03	4.48	AK027134	AK027134	Homo sapiens cDNA: FLJ23481 fis, clone KAIA03003. [AK027134]	
A_23_P62081	3.52E-03	7.94	NM_003020	NM_003020	Homo sapiens secretory granule, neuroendocrine protein 1 (7B2 protein) (SGNE1), mRNA [NM_003020]	NM_003020
A_23_P251730	3.53E-03	2.34	NM_001010986	NM_001010986	Homo sapiens ATPase, Class VI, type 11C (ATP11C), transcript variant 2, mRNA [NM_001010986]	NM_001010986
A_23_P166023	3.53E-03	2.47	NM_002623	NM_002623	Homo sapiens prefoldin 4 (PFDN4), mRNA [NM_002623]	NM_002623
A_23_P111961	3.53E-03	2.53	NM_032509	NM_032509	Homo sapiens RNA binding motif protein 13 (RBM13), mRNA [NM_032509]	NM_032509
A_32_P169353	3.54E-03	2.73	A_32_P169353			
A_24_P902052	3.54E-03	3.95	CR611723	CR611723	full-length cDNA clone CS0DK002YP11 of HeLa cells Cot 25-normalized of Homo sapiens (human). [CR611723]	
A_23_P137103	3.56E-03	2.17	NM_001416	NM_001416	Homo sapiens eukaryotic translation initiation factor 4A, isoform 1 (EIF4A1), mRNA [NM_001416]	NM_001416
A_23_P16262	3.56E-03	2.64	NM_004533	NM_004533	Homo sapiens myosin binding protein C, fast type (MYBPC2), mRNA [NM_004533]	NM_004533
A_23_P12113	3.56E-03	3.77	NM_014053	NM_014053	Homo sapiens feline leukemia virus subgroup C cellular receptor (FLVCR), mRNA [NM_014053]	NM_014053
A_23_P102508	3.57E-03	2.45	NM_021095	NM_021095	Homo sapiens solute carrier family 5 (sodium-dependent vitamin transporter), member 6 (SLC5A6), mRNA [NM_021095]	NM_021095
A_24_P59147	3.57E-03	12.42	ENST00000332560			
A_23_P5945	3.58E-03	2.25	NM_021931	NM_021931	Homo sapiens DEAH (Asp-Glu-Ala-His) box polypeptide 35 (DHX35), mRNA [NM_021931]	NM_021931
A_32_P760762	3.58E-03	2.72	A_32_P760762			

A_24_P119577	3.59E-03	2.04	NM_023923	NM_023923	Homo sapiens phosphatase and actin regulator 4 (PHACTR4), mRNA [NM_023923]	NM_023923
A_23_P369328	3.59E-03	2.30	NM_145306	NM_145306	Homo sapiens chromosome 10 open reading frame 35 (C10orf35), mRNA [NM_145306]	NM_145306
A_23_P213441	3.59E-03	3.21	NM_032175	NM_032175	Homo sapiens Src-associated protein SAW (FLJ12787), mRNA [NM_032175]	NM_032175
A_32_P108666	3.59E-03	6.25	AK091731	AK091731	Homo sapiens cDNA FLJ34412 fis, clone HEART2002432. [AK091731]	
A_24_P299911	3.60E-03	2.98	NM_015148	NM_015148	Homo sapiens PAS domain containing serine/threonine kinase (PASK), mRNA [NM_015148]	NM_015148
A_23_P49136	3.61E-03	2.03	NM_139174	NM_139174	Homo sapiens testis nuclear RNA-binding protein-like (LOC161931), mRNA [NM_139174]	NM_139174
A_32_P41553	3.62E-03	2.70	NM_144627	NM_144627	Homo sapiens SSTK-interacting protein (SSTK-IP), mRNA [NM_144627]	NM_144627
A_23_P98022	3.62E-03	3.16	NM_012238	NM_012238	Homo sapiens sirtuin (silent mating type information regulation 2 homolog) 1 (S. cerevisiae) (SIRT1), mRNA [NM_012238]	NM_012238
A_23_P351204	3.62E-03	3.36	NM_172109	NM_172109	Homo sapiens potassium voltage-gated channel, KQT-like subfamily, member 2 (KCNQ2), transcript variant 5, mRNA [NM_172109]	NM_172109
A_23_P112026	3.62E-03	44.64	NM_002164	NM_002164	Homo sapiens indoleamine-pyrrole 2,3 dioxygenase (INDO), mRNA [NM_002164]	NM_002164
A_23_P391228	3.63E-03	6.33	NM_152496	NM_152496	Homo sapiens mannosidase, endo-alpha-like (MANEAL), mRNA [NM_152496]	NM_152496
A_32_P72447	3.66E-03	2.76	NM_014501	NM_014501	Homo sapiens ubiquitin-conjugating enzyme E2S (UBE2S), mRNA [NM_014501]	NM_014501
A_23_P433132	3.66E-03	3.95	NM_173853	NM_173853	Homo sapiens keratinocyte associated protein 3 (KRTCAP3), mRNA [NM_173853]	NM_173853
A_23_P146237	3.68E-03	2.14	NM_015024	NM_015024	Homo sapiens exportin 7 (XPO7), mRNA [NM_015024]	NM_015024
A_24_P94402	3.68E-03	2.50	NM_005378	NM_005378	Homo sapiens v-myc myelocytomatosis viral related oncogene, neuroblastoma derived (avian) (MYCN), mRNA [NM_005378]	NM_005378
A_23_P344281	3.69E-03	2.04	NM_001010879	NM_001010879	Homo sapiens zinc finger protein interacting with K protein 1 (ZIK1), mRNA [NM_001010879]	NM_001010879
A_23_P19142	3.69E-03	2.54	NM_004137	NM_004137	Homo sapiens potassium large conductance calcium-activated channel, subfamily M, beta member 1 (KCNMB1), mRNA [NM_004137]	NM_004137
A_23_P102925	3.69E-03	2.72	NM_005049	NM_005049	Homo sapiens PWP2 periodic tryptophan protein homolog (yeast) (PWP2H), mRNA [NM_005049]	NM_005049
A_24_P226116	3.69E-03	2.84	NM_057175	NM_057175	Homo sapiens NMDA receptor regulated 1 (NARG1), mRNA [NM_057175]	NM_057175
A_24_P219474	3.69E-03	5.03	NM_144677	NM_144677	Homo sapiens mannosyl (alpha-1,6-)-glycoprotein beta-1,6-N-acetyl-glucosaminyltransferase, isoenzyme B (MGAT5B), transcript variant 1, mRNA [NM_144677]	NM_144677
A_32_P168431	3.72E-03	2.43	ENST00000358081		PREDICTED: Homo sapiens similar to 40S ribosomal protein S26 (LOC441486), mRNA [XM_497095]	XM_497095
A_24_P335358	3.72E-03	4.02	NM_025215	NM_025215	Homo sapiens pseudouridylate synthase 1 (PUS1), transcript variant 1, mRNA [NM_025215]	NM_025215
A_24_P944714	3.72E-03	4.90	AL390129	AL390129	Homo sapiens mRNA; cDNA DKFZp761K0912 (from clone DKFZp761K0912). [AL390129]	
A_23_P15647	3.73E-03	2.31	NM_016231	NM_016231	Homo sapiens nemo like kinase (NLK), mRNA [NM_016231]	NM_016231
A_23_P121222	3.73E-03	2.54	NM_020165	NM_020165	Homo sapiens RAD18 homolog (S. cerevisiae) (RAD18), mRNA [NM_020165]	NM_020165
A_23_P141856	3.73E-03	3.45	NM_014480	NM_014480	Homo sapiens zinc finger protein 544 (ZNF544), mRNA [NM_014480]	NM_014480
A_23_P117912	3.74E-03	2.05	CR618466	CR618466	full-length cDNA clone CS0DI031YH01 of Placenta Cot 25-normalized of Homo sapiens (human). [CR618466]	
A_23_P92642	3.75E-03	2.73	NM_024668	NM_024668	Homo sapiens ankyrin repeat and KH domain containing 1 (ANKHD1), transcript variant 3, mRNA [NM_024668]	NM_024668
A_23_P64404	3.76E-03	2.04	NM_021727	NM_021727	Homo sapiens fatty acid desaturase 3 (FADS3), mRNA [NM_021727]	NM_021727
A_32_P177955	3.76E-03	2.41	BC030123	BC030123	Homo sapiens, clone IMAGE:4815474, mRNA. [BC030123]	XM_499157
A_23_P314691	3.77E-03	2.14	NM_152382	NM_152382	Homo sapiens hypothetical protein FLJ37953 (FLJ37953), mRNA [NM_152382]	NM_152382
A_24_P722216	3.77E-03	2.37	NM_018706	NM_018706	Homo sapiens dehydrogenase E1 and transketolase domain containing 1 (DHTKD1), mRNA [NM_018706]	NM_018706
A_23_P370989	3.77E-03	5.10	NM_005914	NM_005914	Homo sapiens MCM4 minichromosome maintenance deficient 4 (S. cerevisiae) (MCM4), transcript variant 1, mRNA [NM_005914]	NM_005914
A_24_P409182	3.78E-03	7.04	ENST00000303979			
A_24_P303594	3.79E-03	3.02	NM_020445	NM_020445	Homo sapiens ARP3 actin-related protein 3 homolog B (yeast) (ACTR3B), mRNA [NM_020445]	NM_020445
A_23_P25097	3.80E-03	2.05	NM_032338	NM_032338	Homo sapiens hypothetical protein MGC14817 (MGC14817), mRNA [NM_032338]	NM_032338
A_23_P129358	3.80E-03	4.22	NM_024860	NM_024860	Homo sapiens hypothetical protein FLJ21148 (FLJ21148), mRNA [NM_024860]	NM_024860
A_24_P413941	3.81E-03	2.30	NM_153689	NM_153689	Homo sapiens hypothetical protein FLJ38973 (FLJ38973), mRNA [NM_153689]	NM_153689
A_32_P51084	3.81E-03	2.31	NM_015135	NM_015135	Homo sapiens nucleoporin 205kDa (NUP205), mRNA [NM_015135]	NM_015135
A_32_P122703	3.81E-03	2.82	NM_173582	NM_173582	Homo sapiens phosphoglucomutase 2-like 1 (PGM2L1), mRNA [NM_173582]	NM_173582

A_32_P159289	3.81E-03	3.30	A_32_P159289			
A_23_P159877	3.81E-03	12.82	NM_171998	NM_171998	Homo sapiens RAB39B, member RAS oncogene family (RAB39B), mRNA [NM_171998]	NM_171998
A_23_P89812	3.82E-03	2.10	NM_018235	NM_018235	Homo sapiens CNDP dipeptidase 2 (metallopeptidase M20 family) (CNDP2), mRNA [NM_018235]	NM_018235
A_24_P366535	3.82E-03	2.30	ENST00000312855			
A_24_P638294	3.82E-03	2.50	NM_001029	NM_001029	Homo sapiens ribosomal protein S26 (RPS26), mRNA [NM_001029]	NM_001029
A_24_P362931	3.82E-03	9.43	NM_003615	NM_003615	Homo sapiens solute carrier family 4, sodium bicarbonate cotransporter, member 7 (SLC4A7), mRNA [NM_003615]	NM_003615
A_24_P160874	3.83E-03	2.07	NM_001948	NM_001948	Homo sapiens dUTP pyrophosphatase (DUT), nuclear gene encoding mitochondrial protein, transcript variant 2, mRNA [NM_001948]	NM_001948
A_23_P259586	3.84E-03	3.14	NM_003318	NM_003318	Homo sapiens TTK protein kinase (TTK), mRNA [NM_003318]	NM_003318
A_23_P25587	3.85E-03	32.26	NM_007015	NM_007015	Homo sapiens leukocyte cell derived chemotaxin 1 (LECT1), transcript variant 1, mRNA [NM_007015]	NM_007015
A_23_P502747	3.86E-03	2.34	NM_170692	NM_170692	Homo sapiens RAS protein activator like 2 (RASAL2), transcript variant 2, mRNA [NM_170692]	NM_170692
A_23_P163481	3.86E-03	3.83	NM_001211	NM_001211	Homo sapiens BUB1 budding uninhibited by benzimidazoles 1 homolog beta (yeast) (BUB1B), mRNA [NM_001211]	NM_001211
A_23_P307430	3.87E-03	2.05	NM_152344	NM_152344	Homo sapiens hypothetical protein FLJ30656 (FLJ30656), mRNA [NM_152344]	NM_152344
A_23_P66158	3.87E-03	2.83	CR625565	CR625565	full-length cDNA clone CS0DI060YI16 of Placenta Cot 25-normalized of Homo sapiens (human). [CR625565]	
A_23_P117540	3.87E-03	3.04	NM_145251	NM_145251	Homo sapiens serine/threonine/tyrosine interacting protein (STYX), mRNA [NM_145251]	NM_145251
A_24_P873688	3.88E-03	2.16	BC039021	BC039021	Homo sapiens cDNA clone IMAGE:6043059, partial cds. [BC039021]	
A_23_P320190	3.88E-03	2.23	NM_173646	NM_173646	Homo sapiens hypothetical protein FLJ39660 (FLJ39660), mRNA [NM_173646]	NM_173646
A_23_P356598	3.88E-03	6.99	NM_018057	NM_018057	Homo sapiens solute carrier family 6, member 15 (SLC6A15), transcript variant 2, mRNA [NM_018057]	NM_018057
A_23_P55917	3.89E-03	2.04	NM_032298	NM_032298	Homo sapiens synaptotagmin III (SYT3), mRNA [NM_032298]	NM_032298
A_23_P357995	3.89E-03	2.36	NM_178547	NM_178547	Homo sapiens zinc finger and BTB domain containing 8 opposite strand (ZBTB8OS), mRNA [NM_178547]	NM_178547
A_32_P16989	3.89E-03	2.54	A_32_P16989			
A_24_P113572	3.89E-03	4.72	NM_138415	NM_138415	Homo sapiens PHD finger protein 21B (PHF21B), mRNA [NM_138415]	NM_138415
A_24_P306585	3.89E-03	5.32	CD359021	CD359021	AGENCOURT_14276711 NIH_MGC_180 Homo sapiens cDNA clone IMAGE:30390221 5', mRNA sequence [CD359021]	
A_23_P10127	3.89E-03	6.21	NM_003012	NM_003012	Homo sapiens secreted frizzled-related protein 1 (SFRP1), mRNA [NM_003012]	NM_003012
A_32_P95729	3.90E-03	2.28	NM_018193	NM_018193	Homo sapiens hypothetical protein FLJ10719 (FLJ10719), mRNA [NM_018193]	NM_018193
A_23_P93258	3.90E-03	2.73	NM_003537	NM_003537	Homo sapiens histone 1, H3b (HIST1H3B), mRNA [NM_003537]	NM_003537
A_24_P625382	3.90E-03	2.83	NM_003651	NM_003651	Homo sapiens cold shock domain protein A (CSDA), mRNA [NM_003651]	NM_003651
A_24_P861099	3.90E-03	3.16	AK094718	AK094718	Homo sapiens cDNA FLJ37399 fis, clone BRAMY2027587. [AK094718]	
A_24_P942604	3.91E-03	2.11	NM_006306	NM_006306	Homo sapiens SMC1 structural maintenance of chromosomes 1-like 1 (yeast) (SMC1L1), mRNA [NM_006306]	NM_006306
A_23_P43079	3.91E-03	2.23	NM_017864	NM_017864	Homo sapiens hypothetical protein FLJ20530 (FLJ20530), mRNA [NM_017864]	NM_017864
A_32_P4943	3.91E-03	2.48	NM_021927	NM_021927	Homo sapiens hypothetical protein FLJ13220 (FLJ13220), mRNA [NM_021927]	NM_021927
A_23_P407115	3.91E-03	2.67	NM_138687	NM_138687	Homo sapiens phosphatidylinositol-4-phosphate 5-kinase, type II, beta (PIP5K2B), transcript variant 2, mRNA [NM_138687]	NM_138687
A_23_P102183	3.91E-03	2.68	L48692	L48692	Homo sapiens (clone p5-23-3) mRNA. [L48692]	
A_24_P765053	3.91E-03	2.86	THC2370447		ALU5_HUMAN (P39192) Alu subfamily SC sequence contamination warning entry, partial (13%) [THC2370447]	
A_32_P135007	3.94E-03	2.19	AL110237	AL110237	Homo sapiens mRNA; cDNA DKFZp566D224 (from clone DKFZp566D224). [AL110237]	
A_32_P3290	3.94E-03	2.22	NM_017645	NM_017645	Homo sapiens family with sequence similarity 29, member A (FAM29A), mRNA [NM_017645]	NM_017645
A_32_P100464	3.94E-03	2.28	THC2340639		Q6MX26 (Q6MX26) PE-PGRS FAMILY PROTEIN, partial (6%) [THC2340639]	
A_32_P188193	3.94E-03	11.01	THC2445517			
A_23_P25638	3.95E-03	2.15	NM_024546	NM_024546	Homo sapiens chromosome 13 open reading frame 7 (C13orf7), mRNA [NM_024546]	NM_024546
A_32_P222857	3.95E-03	2.25	NM_002139	NM_002139	Homo sapiens RNA binding motif protein, X-linked (RBMX), mRNA [NM_002139]	NM_002139
A_23_P206441	3.96E-03	2.56	NM_000135	NM_000135	Homo sapiens Fanconi anemia, complementation group A (FANCA), transcript variant 1, mRNA [NM_000135]	NM_000135
A_23_P124327	3.96E-03	2.62	NM_022455	NM_022455	Homo sapiens nuclear receptor binding SET domain protein 1 (NSD1), transcript variant 2, mRNA [NM_022455]	NM_022455

A_24_P100517	3.96E-03	3.42	NM_178448	NM_178448	Homo sapiens chromosome 9 open reading frame 140 (C9orf140), mRNA [NM_178448]	NM_178448
A_23_P202945	3.96E-03	3.51	NM_014155	NM_014155	Homo sapiens BTB (POZ) domain containing 15 (BTBD15), mRNA [NM_014155]	NM_014155
A_23_P158829	3.97E-03	2.55	NM_004313	NM_004313	Homo sapiens arrestin, beta 2 (ARRB2), transcript variant 1, mRNA [NM_004313]	NM_004313
A_23_P100868	3.98E-03	2.43	AK026518	AK026518	Homo sapiens cDNA: FLJ22865 fis, clone KAT02171. [AK026518]	
A_23_P130316	3.98E-03	2.57	NM_004671	NM_004671	Homo sapiens protein inhibitor of activated STAT, 2 (PIAS2), transcript variant beta, mRNA [NM_004671]	NM_004671
A_32_P155052	3.98E-03	3.08	BC032684	BC032684	Homo sapiens chromosome 18 open reading frame 17, mRNA (cDNA clone IMAGE:5582870), partial cds. [BC032684]	
A_23_P257104	3.99E-03	2.24	NM_002486	NM_002486	Homo sapiens nuclear cap binding protein subunit 1, 80kDa (NCBP1), mRNA [NM_002486]	NM_002486
A_23_P23206	3.99E-03	3.38	NM_006341	NM_006341	Homo sapiens MAD2 mitotic arrest deficient-like 2 (yeast) (MAD2L2), mRNA [NM_006341]	NM_006341
A_23_P251505	4.00E-03	2.01	NM_148956	NM_148956	Homo sapiens NOL1/NOP2/Sun domain family, member 5 (NSUN5), transcript variant 1, mRNA [NM_148956]	NM_148956
A_23_P253932	4.00E-03	2.02	NM_018147	NM_018147	Homo sapiens Fas apoptotic inhibitory molecule (FAIM), mRNA [NM_018147]	NM_018147
A_23_P15285	4.00E-03	2.20	NM_024109	NM_024109	Homo sapiens hypothetical protein MGC2654 (MGC2654), mRNA [NM_024109]	NM_024109
A_23_P138426	4.00E-03	2.44	BC042943	BC042943	Homo sapiens USP6 N-terminal like, mRNA (cDNA clone MGC:41831 IMAGE:5296060), complete cds. [BC042943]	XM_374768
A_24_P42122	4.00E-03	2.48	NM_001533	NM_001533	Homo sapiens heterogeneous nuclear ribonucleoprotein L (HNRPL), transcript variant 1, mRNA [NM_001533]	NM_001533
A_24_P240187	4.00E-03	3.75	NM_020873	NM_020873	Homo sapiens leucine rich repeat neuronal 1 (LRRN1), mRNA [NM_020873]	NM_020873
A_24_P351283	4.00E-03	5.43	NM_018000	NM_018000	Homo sapiens dilute suppressor (DSU), mRNA [NM_018000]	NM_018000
A_24_P345498	4.01E-03	2.06	NM_021141	NM_021141	Homo sapiens X-ray repair complementing defective repair in Chinese hamster cells 5 (double-strand-break rejoining; Ku autoantigen, 80kDa) (XRCC5), mRNA [NM_021141]	NM_021141
A_23_P252335	4.01E-03	2.58	NM_018944	NM_018944	Homo sapiens chromosome 21 open reading frame 45 (C21orf45), mRNA [NM_018944]	NM_018944
A_23_P430839	4.02E-03	2.36	NM_023002	NM_023002	Homo sapiens hyaluronan and proteoglycan link protein 4 (HAPLN4), mRNA [NM_023002]	NM_023002
A_23_P116942	4.02E-03	2.71	NM_002286	NM_002286	Homo sapiens lymphocyte-activation gene 3 (LAG3), mRNA [NM_002286]	NM_002286
A_24_P411186	4.03E-03	2.16	NM_022893	NM_022893	Homo sapiens B-cell CLL/lymphoma 11A (zinc finger protein) (BCL11A), transcript variant 1, mRNA [NM_022893]	NM_022893
A_23_P122600	4.03E-03	2.30	NM_207409	NM_207409	Homo sapiens AAAL3045 (UNQ3045), mRNA [NM_207409]	NM_207409
A_23_P73787	4.03E-03	2.41	NM_153183	NM_153183	Homo sapiens nudix (nucleoside diphosphate linked moiety X)-type motif 10 (NUDT10), mRNA [NM_153183]	NM_153183
A_32_P78491	4.03E-03	7.52	NM_004956	NM_004956	Homo sapiens ets variant gene 1 (ETV1), mRNA [NM_004956]	NM_004956
A_24_P936252	4.03E-03	7.81	THC2269172			
A_23_P104138	4.04E-03	2.54	BC007286	BC007286	Homo sapiens hypothetical protein MGC15634, mRNA (cDNA clone MGC:15634 IMAGE:3344302), complete cds. [BC007286]	
A_24_P344307	4.04E-03	3.29	NM_176863	NM_176863	Homo sapiens proteasome (prosome, macropain) activator subunit 3 (PA28 gamma; Ki) (PSME3), transcript variant 2, mRNA [NM_176863]	NM_176863
A_23_P379071	4.05E-03	2.70	NM_145032	NM_145032	Homo sapiens F-box and leucine-rich repeat protein 13 (FBXL13), mRNA [NM_145032]	NM_145032
A_23_P26037	4.05E-03	8.13	NM_032892	NM_032892	Homo sapiens FERM domain containing 5 (FRMD5), mRNA [NM_032892]	NM_032892
A_24_P467449	4.06E-03	2.05	THC2437930		ALU7_HUMAN (P39194) Alu subfamily SQ sequence contamination warning entry, partial (4%) [THC2437930]	
A_23_P430842	4.06E-03	2.15	NM_023002	NM_023002	Homo sapiens hyaluronan and proteoglycan link protein 4 (HAPLN4), mRNA [NM_023002]	NM_023002
A_24_P56194	4.08E-03	2.19	NM_001310	NM_001310	Homo sapiens cAMP responsive element binding protein-like 2 (CREBL2), mRNA [NM_001310]	NM_001310
A_24_P382253	4.08E-03	2.20	NM_018170	NM_018170	Homo sapiens hypothetical protein FLJ10656 (P15RS), mRNA [NM_018170]	NM_018170
A_23_P5171	4.10E-03	2.05	A_23_P5171			
A_23_P217411	4.10E-03	2.11	NM_006306	NM_006306	Homo sapiens SMC1 structural maintenance of chromosomes 1-like 1 (yeast) (SMC1L1), mRNA [NM_006306]	NM_006306
A_23_P313828	4.10E-03	2.20	NM_181716	NM_181716	Homo sapiens proline rich 6 (PRR6), mRNA [NM_181716]	NM_181716
A_23_P252962	4.10E-03	2.88	NM_001001132	NM_001001132	Homo sapiens intersectin 1 (SH3 domain protein) (ITSN1), transcript variant 2, mRNA [NM_001001132]	NM_001001132
A_23_P12336	4.13E-03	2.24	NM_018137	NM_018137	Homo sapiens HMT1 hnRNP methyltransferase-like 6 (S. cerevisiae) (HRMT1L6), mRNA [NM_018137]	NM_018137
A_24_P76521	4.13E-03	2.48	AK056691	AK056691	Homo sapiens cDNA FLJ32129 fis, clone PEBLM2000213, weakly similar to Mus musculus genes for integrin aM290, hapsin. [AK056691]	
A_32_P176550	4.14E-03	2.55	NM_152405	NM_152405	Homo sapiens junction-mediating and regulatory protein (JMY), mRNA [NM_152405]	NM_152405
A_24_P374154	4.14E-03	2.62	NM_005877	NM_005877	Homo sapiens splicing factor 3a, subunit 1, 120kDa (SF3A1), transcript variant 1, mRNA [NM_005877]	NM_005877

A_24_P638453	4.15E-03	2.40	A_24_P638453			
A_23_P89327	4.16E-03	2.40	NM_020652	NM_020652	Homo sapiens zinc finger protein 286 (ZNF286), mRNA [NM_020652]	NM_020652
A_23_P258612	4.16E-03	4.02	NM_016529	NM_016529	Homo sapiens ATPase, aminophospholipid transporter-like, Class I, type 8A, member 2 (ATP8A2), mRNA [NM_016529]	NM_016529
A_23_P216517	4.17E-03	3.34	NM_032818	NM_032818	Homo sapiens chromosome 9 open reading frame 100 (C9orf100), mRNA [NM_032818]	NM_032818
A_24_P659113	4.17E-03	4.08	NM_152523	NM_152523	Homo sapiens hypothetical protein FLJ40432 (FLJ40432), mRNA [NM_152523]	NM_152523
A_32_P116813	4.21E-03	2.03	NM_020408	NM_020408	Homo sapiens chromosome 6 open reading frame 149 (C6orf149), mRNA [NM_020408]	NM_020408
A_23_P58898	4.21E-03	2.11	NM_012115	NM_012115	Homo sapiens CASP8 associated protein 2 (CASP8AP2), mRNA [NM_012115]	NM_012115
A_23_P255653	4.21E-03	4.98	NM_003844	NM_003844	Homo sapiens tumor necrosis factor receptor superfamily, member 10a (TNFRSF10A), mRNA [NM_003844]	NM_003844
A_23_P154022	4.22E-03	2.08	NM_025203	NM_025203	Homo sapiens hypothetical protein FLJ21945 (FLJ21945), mRNA [NM_025203]	NM_025203
A_23_P317657	4.22E-03	2.62	NM_024005	NM_024005	Homo sapiens DEAD (Asp-Glu-Ala-Asp) box polypeptide 3, X-linked (DDX3X), transcript variant 1, mRNA [NM_024005]	NM_024005
A_32_P70135	4.22E-03	2.65	NM_025138	NM_025138	Homo sapiens chromosome 13 open reading frame 23 (C13orf23), transcript variant 1, mRNA [NM_025138]	NM_025138
A_23_P202496	4.23E-03	2.01	NM_022451	NM_022451	Homo sapiens chromosome 10 open reading frame 117 (C10orf117), mRNA [NM_022451]	NM_022451
A_24_P557019	4.23E-03	2.74	THC2406147		BF476310 naa21a07.x1 NCI_CGAP_Pr28 Homo sapiens cDNA clone IMAGE:3255444 3' similar to contains Alu repetitive element;contains element MIR MIR repetitive element ;, mRNA sequence [BF476310]	
A_32_P167396	4.23E-03	2.77	BC039399	BC039399	Homo sapiens, clone IMAGE:5300185, mRNA. [BC039399]	
A_23_P108463	4.23E-03	3.05	NM_001006657	NM_001006657	Homo sapiens WD repeat domain 35 (WDR35), transcript variant 1, mRNA [NM_001006657]	NM_001006657
A_23_P39704	4.24E-03	2.28	NM_006276	NM_006276	Homo sapiens splicing factor, arginine/serine-rich 7, 35kDa (SFRS7), mRNA [NM_006276]	NM_006276
A_23_P107644	4.24E-03	2.36	NM_006938	NM_006938	Homo sapiens small nuclear ribonucleoprotein D1 polypeptide 16kDa (SNRPD1), mRNA [NM_006938]	NM_006938
A_23_P91328	4.24E-03	2.54	NM_006392	NM_006392	Homo sapiens nucleolar protein 5A (56kDa with KKE/D repeat) (NOL5A), mRNA [NM_006392]	NM_006392
A_23_P37503	4.24E-03	2.60	NM_004998	NM_004998	Homo sapiens myosin IE (MYO1E), mRNA [NM_004998]	NM_004998
A_23_P41674	4.24E-03	2.92	NM_152407	NM_152407	Homo sapiens GrpE-like 2, mitochondrial (E. coli) (GRPEL2), mRNA [NM_152407]	NM_152407
A_23_P68087	4.25E-03	2.83	NM_004044	NM_004044	Homo sapiens 5-aminoimidazole-4-carboxamide ribonucleotide formyltransferase/IMP cyclohydrolase (ATIC), mRNA [NM_004044]	NM_004044
A_23_P52082	4.27E-03	2.10	NM_015434	NM_015434	Homo sapiens chromosome 1 open reading frame 73 (C1orf73), mRNA [NM_015434]	NM_015434
A_23_P334218	4.27E-03	2.11	NM_145647	NM_145647	Homo sapiens WD repeat domain 67 (WDR67), mRNA [NM_145647]	NM_145647
A_32_P38623	4.27E-03	2.24	BC037849	BC037849	Homo sapiens cDNA clone IMAGE:4815736, partial cds. [BC037849]	
A_23_P123544	4.27E-03	2.43	NM_017444	NM_017444	Homo sapiens chromatin accessibility complex 1 (CHRAC1), mRNA [NM_017444]	NM_017444
A_23_P63379	4.27E-03	2.43	NM_012113	NM_012113	Homo sapiens carbonic anhydrase XIV (CA14), mRNA [NM_012113]	NM_012113
A_24_P63608	4.27E-03	2.49	NM_017838	NM_017838	Homo sapiens nucleolar protein family A, member 2 (H/ACA small nucleolar RNPs) (NOLA2), mRNA [NM_017838]	NM_017838
A_23_P319719	4.27E-03	2.56	NM_138575	NM_138575	Homo sapiens Bcl-XL-binding protein v68 (MGC5352), mRNA [NM_138575]	NM_138575
A_23_P9135	4.27E-03	2.88	NM_033655	NM_033655	Homo sapiens contactin associated protein-like 3 (CNTNAP3), mRNA [NM_033655]	NM_033655
A_23_P143190	4.30E-03	2.21	NM_002466	NM_002466	Homo sapiens v-myb myeloblastosis viral oncogene homolog (avian)-like 2 (MYBL2), mRNA [NM_002466]	NM_002466
A_23_P110598	4.30E-03	2.58	NM_139281	NM_139281	Homo sapiens WD repeat domain 36 (WDR36), mRNA [NM_139281]	NM_139281
A_23_P133995	4.30E-03	2.83	NM_016059	NM_016059	Homo sapiens peptidylprolyl isomerase (cyclophilin)-like 1 (PPIL1), mRNA [NM_016059]	NM_016059
A_24_P212314	4.30E-03	2.86	A_24_P212314			
A_23_P55564	4.31E-03	2.54	BC006340	BC006340	Homo sapiens cDNA clone IMAGE:4079754, complete cds. [BC006340]	
A_23_P105028	4.32E-03	2.07	NM_015459	NM_015459	Homo sapiens DKFZP564J0863 protein (DKFZP564J0863), mRNA [NM_015459]	NM_015459
A_24_P179336	4.32E-03	2.47	AY029066	AY029066	Homo sapiens Humanin (HN1) mRNA, complete cds. [AY029066]	
A_23_P15202	4.33E-03	2.03	NM_001361	NM_001361	Homo sapiens dihydroorotate dehydrogenase (DHODH), nuclear gene encoding mitochondrial protein, transcript variant 1, mRNA [NM_001361]	NM_001361
A_32_P353677	4.33E-03	2.60	AF343078	AF343078	Homo sapiens TOB3 mRNA, complete cds. [AF343078]	
A_24_P573978	4.34E-03	4.17	THC2371907		CCHU cytochrome c [validated] - human {Homo sapiens;} , partial (85%) [THC2371907]	
A_24_P166613	4.36E-03	2.38	NM_017549	NM_017549	Homo sapiens ependymin related protein 1 (zebrafish) (EPDR1), mRNA [NM_017549]	NM_017549

A_23_P141484	4.36E-03	2.83	NM_018182	NM_018182	Homo sapiens hypothetical protein FLJ10700 (FLJ10700), mRNA [NM_018182]	NM_018182
A_23_P9192	4.37E-03	2.26	NM_005156	NM_005156	Homo sapiens ROD1 regulator of differentiation 1 (S. pombe) (ROD1), mRNA [NM_005156]	NM_005156
A_24_P385739	4.37E-03	2.42	NM_020390	NM_020390	Homo sapiens eukaryotic translation initiation factor 5A2 (EIF5A2), mRNA [NM_020390]	NM_020390
A_23_P5903	4.40E-03	5.15	NM_016354	NM_016354	Homo sapiens solute carrier organic anion transporter family, member 4A1 (SLCO4A1), mRNA [NM_016354]	NM_016354
A_23_P208812	4.41E-03	2.75	NM_014910	NM_014910	Homo sapiens zinc finger protein 507 (ZNF507), mRNA [NM_014910]	NM_014910
A_32_P71447	4.43E-03	2.23	NM_015261	NM_015261	Homo sapiens KIAA0056 protein (hCAP-D3), mRNA [NM_015261]	NM_015261
A_23_P115482	4.43E-03	2.36	NM_014176	NM_014176	Homo sapiens ubiquitin-conjugating enzyme E2T (putative) (UBE2T), mRNA [NM_014176]	NM_014176
A_24_P19544	4.44E-03	2.13	NM_013233	NM_013233	Homo sapiens serine threonine kinase 39 (STE20/SPS1 homolog, yeast) (STK39), mRNA [NM_013233]	NM_013233
A_24_P926935	4.44E-03	2.42	THC2339234		ALU8_HUMAN (P39195) Alu subfamily SX sequence contamination warning entry, partial (5%) [THC2339234]	
A_24_P16071	4.44E-03	5.13	A_24_P16071			
A_23_P48358	4.45E-03	2.16	NM_000282	NM_000282	Homo sapiens propionyl Coenzyme A carboxylase, alpha polypeptide (PCCA), mRNA [NM_000282]	NM_000282
A_32_P38404	4.45E-03	2.27	BC015133	BC015133	Homo sapiens cDNA clone IMAGE:3934193, partial cds. [BC015133]	
A_23_P82823	4.45E-03	2.99	NM_017884	NM_017884	Homo sapiens PIN2-interacting protein 1 (PINX1), mRNA [NM_017884]	NM_017884
A_23_P373119	4.45E-03	8.40	ENST00000324884		GB|NM_178467.1|NP_848562.1 high-mobility group (nonhistone chromosomal) protein 4-like [Homo sapiens] [NP798948]	
A_23_P5551	4.46E-03	2.18	NM_005381	NM_005381	Homo sapiens nucleolin (NCL), mRNA [NM_005381]	NM_005381
A_23_P68547	4.46E-03	2.57	NM_182802	NM_182802	Homo sapiens MCM8 minichromosome maintenance deficient 8 (S. cerevisiae) (MCM8), transcript variant 2, mRNA [NM_182802]	NM_182802
A_23_P419714	4.46E-03	4.65	NM_152322	NM_152322	Homo sapiens BTB (POZ) domain containing 11 (BTBD11), transcript variant 1, mRNA [NM_152322]	NM_152322
A_23_P146217	4.47E-03	2.06	NM_004874	NM_004874	Homo sapiens BCL2-associated athanogene 4 (BAG4), mRNA [NM_004874]	NM_004874
A_24_P227091	4.47E-03	2.12	NM_004523	NM_004523	Homo sapiens kinesin family member 11 (KIF11), mRNA [NM_004523]	NM_004523
A_24_P340771	4.47E-03	2.13	A_24_P340771			
A_23_P39067	4.47E-03	2.21	NM_003121	NM_003121	Homo sapiens Spi-B transcription factor (Spi-1/PU.1 related) (SPIB), mRNA [NM_003121]	NM_003121
A_32_P154091	4.47E-03	2.99	AK124295	AK124295	Homo sapiens cDNA FLJ42301 fis, clone TOVAR2002514. [AK124295]	XM_374260
A_23_P388798	4.47E-03	7.09	AF467442	AF467442	Homo sapiens Smith-Magenis syndrome chromosome region candidate 5 protein (SMCR5) mRNA, complete cds. [AF467442]	
A_23_P29680	4.47E-03	13.51	NM_024046	NM_024046	Homo sapiens CaM kinase-like vesicle-associated (CAMKV), mRNA [NM_024046]	NM_024046
A_23_P207400	4.48E-03	2.03	NM_007295	NM_007295	Homo sapiens breast cancer 1, early onset (BRCA1), transcript variant BRCA1b, mRNA [NM_007295]	NM_007295
A_24_P55391	4.48E-03	2.20	BC082970	BC082970	Homo sapiens cDNA clone IMAGE:6598034. [BC082970]	
A_24_P401473	4.48E-03	2.77	AK057719	AK057719	Homo sapiens cDNA FLJ33157 fis, clone UTERU2000393. [AK057719]	
A_32_P48086	4.48E-03	3.16	BC015434	BC015434	Homo sapiens, clone IMAGE:4414697, mRNA. [BC015434]	
A_24_P193257	4.49E-03	2.38	THC2437177			
A_24_P237117	4.51E-03	2.02	NM_172318	NM_172318	Homo sapiens potassium voltage-gated channel, subfamily G, member 1 (KCNG1), transcript variant 2, mRNA [NM_172318]	NM_172318
A_23_P1552	4.51E-03	3.60	NM_001814	NM_001814	Homo sapiens cathepsin C (CTSC), transcript variant 1, mRNA [NM_001814]	NM_001814
A_23_P6878	4.54E-03	2.04	NM_004186	NM_004186	Homo sapiens sema domain, immunoglobulin domain (Ig), short basic domain, secreted, (semaphorin) 3F (SEMA3F), mRNA [NM_004186]	NM_004186
A_23_P218835	4.54E-03	2.87	NM_002268	NM_002268	Homo sapiens karyopherin alpha 4 (importin alpha 3) (KPNA4), mRNA [NM_002268]	NM_002268
A_23_P132417	4.55E-03	2.26	NM_018385	NM_018385	Homo sapiens hypothetical protein FLJ11301 (FLJ11301), mRNA [NM_018385]	NM_018385
A_23_P218331	4.56E-03	2.31	NM_001017916	NM_001017916	Homo sapiens cytochrome b-561 (CYB561), transcript variant 2, mRNA [NM_001017916]	NM_001017916
A_24_P58899	4.57E-03	2.39	A_24_P58899			
A_24_P600036	4.58E-03	3.44	A_24_P600036			
A_24_P193011	4.58E-03	4.03	NM_053056	NM_053056	Homo sapiens cyclin D1 (PRAD1: parathyroid adenomatosis 1) (CCND1), mRNA [NM_053056]	NM_053056
A_24_P301846	4.59E-03	2.54	NM_175085	NM_175085	Homo sapiens phosphoribosylglycinamide formyltransferase, phosphoribosylglycinamide synthetase, phosphoribosylaminoimidazole synthetase (GART), transcript variant 2, mRNA [NM_175085]	NM_175085

A_24_P941148	4.59E-03	2.55	NM_017645	NM_017645	Homo sapiens family with sequence similarity 29, member A (FAM29A), mRNA [NM_017645]	NM_017645
A_24_P179013	4.59E-03	2.60	ENST00000333462		GB|AL031963.40|CAD70623.1 dJ40E16.3 (novel gene similar to D. melanogaster CG5327) [Homo sapiens] [NP1083521]	
A_32_P226205	4.59E-03	5.03	NM_033400	NM_033400	Homo sapiens zinc finger homeobox 2 (ZFHX2), mRNA [NM_033400]	NM_033400
A_23_P134366	4.59E-03	5.46	NM_004956	NM_004956	Homo sapiens ets variant gene 1 (ETV1), mRNA [NM_004956]	NM_004956
A_23_P69188	4.60E-03	2.08	NM_206831	NM_206831	Homo sapiens zinc finger, CSL-type containing 2 (ZCSL2), mRNA [NM_206831]	NM_206831
A_23_P216900	4.60E-03	3.58	ENST00000344523		H.sapiens mRNA for hRTR/hGCNF protein. [X99975]	
A_24_P305556	4.61E-03	3.16	NM_182802	NM_182802	Homo sapiens MCM8 minichromosome maintenance deficient 8 (S. cerevisiae) (MCM8), transcript variant 2, mRNA [NM_182802]	NM_182802
A_23_P42096	4.62E-03	2.42	NM_005922	NM_005922	Homo sapiens mitogen-activated protein kinase kinase kinase 4 (MAP3K4), transcript variant 1, mRNA [NM_005922]	NM_005922
A_23_P353717	4.62E-03	2.46	NM_152308	NM_152308	Homo sapiens hypothetical protein MGC24665 (MGC24665), mRNA [NM_152308]	NM_152308
A_32_P65571	4.62E-03	4.57	BX648855	BX648855	Homo sapiens mRNA; cDNA DKFZp686L05231 (from clone DKFZp686L05231). [BX648855]	
A_23_P72330	4.63E-03	2.38	A_23_P72330			
A_23_P25224	4.63E-03	2.70	NM_003651	NM_003651	Homo sapiens cold shock domain protein A (CSDA), mRNA [NM_003651]	NM_003651
A_24_P148653	4.63E-03	2.88	NM_018385	NM_018385	Homo sapiens hypothetical protein FLJ11301 (FLJ11301), mRNA [NM_018385]	NM_018385
A_24_P50139	4.63E-03	10.17	A_24_P50139			
A_32_P25273	4.64E-03	2.40	NM_002156	NM_002156	Homo sapiens heat shock 60kDa protein 1 (chaperonin) (HSPD1), nuclear gene encoding mitochondrial protein, transcript variant 1, mRNA [NM_002156]	NM_002156
A_23_P212844	4.65E-03	2.75	NM_006342	NM_006342	Homo sapiens transforming, acidic coiled-coil containing protein 3 (TACC3), mRNA [NM_006342]	NM_006342
A_23_P406616	4.66E-03	2.01	NM_175884	NM_175884	Homo sapiens hypothetical protein FLJ36031 (FLJ36031), mRNA [NM_175884]	NM_175884
A_23_P17844	4.66E-03	2.29	NM_002854	NM_002854	Homo sapiens parvalbumin (PVALB), mRNA [NM_002854]	NM_002854
A_23_P212159	4.66E-03	3.05	NM_024923	NM_024923	Homo sapiens nucleoporin 210kDa (NUP210), mRNA [NM_024923]	NM_024923
A_23_P88630	4.66E-03	3.16	NM_000057	NM_000057	Homo sapiens Bloom syndrome (BLM), mRNA [NM_000057]	NM_000057
A_23_P111206	4.66E-03	3.82	NM_004117	NM_004117	Homo sapiens FK506 binding protein 5 (FKBP5), mRNA [NM_004117]	NM_004117
A_23_P253446	4.66E-03	4.10	NM_002045	NM_002045	Homo sapiens growth associated protein 43 (GAP43), mRNA [NM_002045]	NM_002045
A_32_P41127	4.67E-03	2.51	NM_002622	NM_002622	Homo sapiens prefoldin 1 (PFDN1), mRNA [NM_002622]	NM_002622
A_32_P168375	4.67E-03	2.84	THC2404028			
A_23_P143348	4.67E-03	3.61	NM_021220	NM_021220	Homo sapiens ovo-like 2 (Drosophila) (OVOL2), mRNA [NM_021220]	NM_021220
A_24_P78153	4.68E-03	2.11	AK027509	AK027509	Homo sapiens cDNA FLJ14603 fis, clone NT2RP1000357. [AK027509]	
A_23_P145197	4.68E-03	2.18	NM_004053	NM_004053	Homo sapiens bystin-like (BYSL), mRNA [NM_004053]	NM_004053
A_24_P614579	4.68E-03	3.23	BC000206	BC000206	Homo sapiens, clone IMAGE:3350658, mRNA. [BC000206]	
A_32_P110505	4.69E-03	2.02	A_32_P110505			
A_23_P425104	4.71E-03	2.11	NM_058243	NM_058243	Homo sapiens bromodomain containing 4 (BRD4), transcript variant long, mRNA [NM_058243]	NM_058243
A_24_P100387	4.71E-03	2.15	NM_203391	NM_203391	Homo sapiens glycerol kinase (GK), transcript variant 1, mRNA [NM_203391]	NM_203391
A_23_P258814	4.71E-03	2.16	ENST00000328644			
A_23_P323685	4.71E-03	4.59	NM_003543	NM_003543	Homo sapiens histone 1, H4h (HIST1H4H), mRNA [NM_003543]	NM_003543
A_24_P184803	4.71E-03	5.08	NM_004086	NM_004086	Homo sapiens coagulation factor C homolog, cochlin (Limulus polyphemus) (COCH), mRNA [NM_004086]	NM_004086
A_23_P324327	4.71E-03	6.33	NM_016235	NM_016235	Homo sapiens G protein-coupled receptor, family C, group 5, member B (GPRC5B), mRNA [NM_016235]	NM_016235
A_23_P253762	4.71E-03	6.45	BC004565	BC004565	Homo sapiens hypothetical protein MGC12935, mRNA (cDNA clone IMAGE:4309284), partial cds. [BC004565]	
A_23_P373799	4.73E-03	2.11	NM_020943	NM_020943	Homo sapiens KIAA1604 protein (KIAA1604), mRNA [NM_020943]	NM_020943
A_32_P114574	4.73E-03	2.26	NM_014412	NM_014412	Homo sapiens calcyclin binding protein (CACYBP), transcript variant 1, mRNA [NM_014412]	NM_014412
A_24_P246710	4.73E-03	2.50	NM_017954	NM_017954	Homo sapiens Ca2+-dependent activator protein for secretion 2 (CADPS2), transcript variant 1, mRNA [NM_017954]	NM_017954
A_23_P127652	4.74E-03	2.75	NM_003455	NM_003455	Homo sapiens zinc finger protein 202 (ZNF202), mRNA [NM_003455]	NM_003455

A_32_P63365	4.74E-03	3.13	BE697496	BE697496	BE697496 RC0-CT0428-310700-031-g12 CT0428 Homo sapiens cDNA, mRNA sequence [BE697496]	
A_32_P156851	4.76E-03	2.36	NM_005822	NM_005822	Homo sapiens Down syndrome critical region gene 1-like 1 (DSCR1L1), mRNA [NM_005822]	NM_005822
A_23_P74688	4.77E-03	2.16	NM_031921	NM_031921	Homo sapiens ATPase family, AAA domain containing 3B (ATAD3B), mRNA [NM_031921]	NM_031921
A_23_P32684	4.77E-03	2.33	AF116619	AF116619	Homo sapiens PRO1051 mRNA, complete cds. [AF116619]	
A_23_P351215	4.77E-03	2.54	NM_005414	NM_005414	Homo sapiens SKI-like (SKIL), mRNA [NM_005414]	NM_005414
A_23_P88303	4.77E-03	4.27	NM_021979	NM_021979	Homo sapiens heat shock 70kDa protein 2 (HSPA2), mRNA [NM_021979]	NM_021979
A_24_P256579	4.78E-03	2.16	AB051533	AB051533	Homo sapiens mRNA for KIAA1746 protein, partial cds. [AB051533]	
A_32_P155035	4.78E-03	3.83	AK096500	AK096500	Homo sapiens cDNA FLJ39181 fis, clone OCBBF2004235. [AK096500]	
A_24_P934546	4.78E-03	7.41	NM_003013	NM_003013	Homo sapiens secreted frizzled-related protein 2 (SFRP2), mRNA [NM_003013]	NM_003013
A_23_P120316	4.79E-03	2.00	NM_006636	NM_006636	Homo sapiens methylenetetrahydrofolate dehydrogenase (NADP+ dependent) 2, methenyltetrahydrofolate cyclohydrolase (MTHFD2), nuclear gene encoding mitochondrial protein, mRNA [NM_006636]	NM_006636
A_23_P76435	4.80E-03	2.07	NM_176818	NM_176818	Homo sapiens hypothetical protein 15E1.2 (15E1.2), mRNA [NM_176818]	NM_176818
A_23_P170352	4.80E-03	2.16	NM_002949	NM_002949	Homo sapiens mitochondrial ribosomal protein L12 (MRPL12), nuclear gene encoding mitochondrial protein, mRNA [NM_002949]	NM_002949
A_23_P259094	4.80E-03	2.54	NM_025231	NM_025231	Homo sapiens zinc finger protein 435 (ZNF435), mRNA [NM_025231]	NM_025231
A_24_P193295	4.82E-03	2.29	NM_198686	NM_198686	Homo sapiens RAB15, member RAS onocogene family (RAB15), mRNA [NM_198686]	NM_198686
A_24_P35935	4.82E-03	4.10	AK055438	AK055438	Homo sapiens cDNA FLJ30876 fis, clone FEBRA2004412. [AK055438]	
A_24_P807883	4.83E-03	2.03	THC2279305			
A_24_P938303	4.84E-03	2.54	U57365	U57365	Human GRP/bombesin receptor mRNA, partial cds. [U57365]	
A_23_P116091	4.84E-03	3.31	NM_130443	NM_130443	Homo sapiens dipeptidylpeptidase 3 (DPP3), transcript variant 2, mRNA [NM_130443]	NM_130443
A_23_P406025	4.84E-03	3.58	NM_015225	NM_015225	Homo sapiens KIAA0367 (KIAA0367), mRNA [NM_015225]	NM_015225
A_23_P415411	4.84E-03	4.07	NM_003545	NM_003545	Homo sapiens histone 1, H4e (HIST1H4E), mRNA [NM_003545]	NM_003545
A_32_P179148	4.85E-03	14.10	CN431194	CN431194	CN431194 328775669 GRN_ES Homo sapiens cDNA 5', mRNA sequence [CN431194]	
A_24_P255654	4.86E-03	2.24	A_24_P255654			
A_23_P132889	4.86E-03	4.18	NM_001014809	NM_001014809	Homo sapiens collapsin response mediator protein 1 (CRMP1), transcript variant 1, mRNA [NM_001014809]	NM_001014809
A_24_P620521	4.87E-03	2.10	A_24_P620521			
A_23_P304489	4.87E-03	2.19	NM_015381	NM_015381	Homo sapiens family with sequence similarity 19 (chemokine (C-C motif)-like), member A5 (FAM19A5), mRNA [NM_015381]	NM_015381
A_24_P840868	4.87E-03	2.58	A_24_P840868			
A_32_P213831	4.87E-03	2.81	NM_020704	NM_020704	Homo sapiens family with sequence similarity 40, member B (FAM40B), mRNA [NM_020704]	NM_020704
A_24_P31235	4.87E-03	3.11	NM_001970	NM_001970	Homo sapiens eukaryotic translation initiation factor 5A (EIF5A), mRNA [NM_001970]	NM_001970
A_23_P121702	4.87E-03	3.58	NM_001014446	NM_001014446	Homo sapiens OCIA domain containing 2 (OCIAD2), transcript variant 1, mRNA [NM_001014446]	NM_001014446
A_23_P131518	4.88E-03	2.23	CR621399	CR621399	full-length cDNA clone CS0DI010YB22 of Placenta Cot 25-normalized of Homo sapiens (human). [CR621399]	
A_23_P395582	4.88E-03	10.94	NM_174900	NM_174900	Homo sapiens zinc finger protein 42 (ZFP42), mRNA [NM_174900]	NM_174900
A_23_P204380	4.91E-03	3.18	NM_024312	NM_024312	Homo sapiens MGC4170 protein (MGC4170), mRNA [NM_024312]	NM_024312
A_24_P73962	4.94E-03	2.98	THC2400647		PSDB_HUMAN 26S proteasome non-ATPase regulatory subunit 11 (26S proteasomeregulatory subunit S9) (26S proteasome regulatory subunit p44.5). {Homo sapiens;} , partial (39%) [THC2400647]	
A_24_P67494	5.00E-03	2.13	XM_496705	XM_496705	PREDICTED: Homo sapiens similar to karyopherin alpha 2 (LOC339991), mRNA [XM_496705]	XM_496705
A_24_P195454	5.00E-03	2.23	A_24_P195454			
A_23_P67952	5.01E-03	4.50	S49953	S49953	N-cym=DNA-binding transcriptional activator homolog {oncogene} [human, Kelly neuroblastoma cell line, mRNA, 778 nt]. [S49953]	
A_32_P154021	5.02E-03	3.10	AK054939	AK054939	Homo sapiens cDNA FLJ30377 fis, clone BRACE2007952. [AK054939]	
A_24_P165965	5.03E-03	2.02	NM_007275	NM_007275	Homo sapiens tumor suppressor candidate 2 (TUSC2), mRNA [NM_007275]	NM_007275
A_23_P216108	5.03E-03	2.95	NM_020478	NM_020478	Homo sapiens ankyrin 1, erythrocytic (ANK1), transcript variant 5, mRNA [NM_020478]	NM_020478

A_23_P31273	5.03E-03	3.23	NM_001635	NM_001635	Homo sapiens amphiphysin (Stiff-Man syndrome with breast cancer 128kDa autoantigen) (AMPH), transcript variant 1, mRNA [NM_001635]	NM_001635
A_23_P49878	5.03E-03	4.24	NM_019013	NM_019013	Homo sapiens family with sequence similarity 64, member A (FAM64A), mRNA [NM_019013]	NM_019013
A_32_P121226	5.03E-03	25.58	A_32_P121226			
A_23_P128991	5.06E-03	3.82	NM_031210	NM_031210	Homo sapiens chromosome 14 open reading frame 156 (C14orf156), mRNA [NM_031210]	NM_031210
A_23_P111517	5.06E-03	5.81	NM_022479	NM_022479	Homo sapiens Williams-Beuren syndrome chromosome region 17 (WBSCR17), mRNA [NM_022479]	NM_022479
A_24_P147263	5.07E-03	2.65	AB033029	AB033029	Homo sapiens mRNA for KIAA1203 protein, partial cds. [AB033029]	
A_23_P157527	5.08E-03	2.07	NM_033402	NM_033402	Homo sapiens KIAA1764 protein (KIAA1764), mRNA [NM_033402]	NM_033402
A_24_P32672	5.08E-03	2.09	NM_016050	NM_016050	Homo sapiens mitochondrial ribosomal protein L11 (MRPL11), nuclear gene encoding mitochondrial protein, transcript variant 1, mRNA [NM_016050]	NM_016050
A_24_P42071	5.08E-03	2.11	ENST00000332925			
A_32_P28365	5.08E-03	2.17	NM_172164	NM_172164	Homo sapiens nuclear autoantigenic sperm protein (histone-binding) (NASP), transcript variant 1, mRNA [NM_172164]	NM_172164
A_23_P123905	5.08E-03	2.18	NM_016042	NM_016042	Homo sapiens exosome component 3 (EXOSC3), transcript variant 1, mRNA [NM_016042]	NM_016042
A_24_P383834	5.08E-03	2.68	A_24_P383834			
A_23_P41255	5.08E-03	4.27	NM_203505	NM_203505	Homo sapiens Ras-GTPase activating protein SH3 domain-binding protein 2 (G3BP2), transcript variant 1, mRNA [NM_203505]	NM_203505
A_23_P52410	5.08E-03	5.56	NM_145307	NM_145307	Homo sapiens pleckstrin homology domain containing, family K member 1 (PLEKHK1), mRNA [NM_145307]	NM_145307
A_32_P139196	5.08E-03	5.56	NM_213723	NM_213723	Homo sapiens chromosome 13 open reading frame 25 (C13orf25), transcript variant 2, mRNA [NM_213723]	NM_213723
A_32_P86533	5.10E-03	5.99	AF038185	AF038185	Homo sapiens clone 23700 mRNA sequence. [AF038185]	
A_24_P267452	5.11E-03	6.10	NM_012099	NM_012099	Homo sapiens CD3E antigen, epsilon polypeptide associated protein (CD3EAP), mRNA [NM_012099]	NM_012099
A_23_P162525	5.11E-03	7.58	NM_014503	NM_014503	Homo sapiens down-regulated in metastasis (DRIM), mRNA [NM_014503]	NM_014503
A_23_P141893	5.12E-03	4.07	NM_178494	NM_178494	Homo sapiens hypothetical protein FLJ40125 (FLJ40125), mRNA [NM_178494]	NM_178494
A_24_P99071	5.13E-03	2.16	NM_002271	NM_002271	Homo sapiens RAN binding protein 5 (RANBP5), mRNA [NM_002271]	NM_002271
A_24_P336853	5.13E-03	2.27	NM_020143	NM_020143	Homo sapiens putatative 28 kDa protein (LOC56902), mRNA [NM_020143]	NM_020143
A_23_P50368	5.13E-03	2.70	NM_206818	NM_206818	Homo sapiens osteoclast-associated receptor (OSCAR), transcript variant 1, mRNA [NM_206818]	NM_206818
A_32_P227027	5.13E-03	3.06	THC2288599			
A_24_P50666	5.13E-03	3.82	A_24_P50666			
A_32_P192430	5.14E-03	2.28	NM_001826	NM_001826	Homo sapiens CDC28 protein kinase regulatory subunit 1B (CKS1B), mRNA [NM_001826]	NM_001826
A_23_P217236	5.14E-03	3.55	NM_005342	NM_005342	Homo sapiens high-mobility group box 3 (HMGB3), mRNA [NM_005342]	NM_005342
A_23_P131935	5.18E-03	2.55	NM_017671	NM_017671	Homo sapiens chromosome 20 open reading frame 42 (C20orf42), mRNA [NM_017671]	NM_017671
A_23_P91414	5.19E-03	2.29	NM_080625	NM_080625	Homo sapiens chromosome 20 open reading frame 160 (C20orf160), mRNA [NM_080625]	NM_080625
A_23_P219105	5.19E-03	2.95	NM_023111	NM_023111	Homo sapiens fibroblast growth factor receptor 1 (fms-related tyrosine kinase 2, Pfeiffer syndrome) (FGFR1), transcript variant 9, mRNA [NM_023111]	NM_023111
A_23_P128663	5.20E-03	2.49	NM_014363	NM_014363	Homo sapiens spastic ataxia of Charlevoix-Saguenay (sacsin) (SACS), mRNA [NM_014363]	NM_014363
A_24_P813147	5.20E-03	3.76	NM_177987	NM_177987	Homo sapiens tubulin, beta 8 (TUBB8), mRNA [NM_177987]	NM_177987
A_23_P71558	5.23E-03	2.07	NM_004260	NM_004260	Homo sapiens RecQ protein-like 4 (RECQL4), mRNA [NM_004260]	NM_004260
A_24_P129483	5.23E-03	2.08	NM_173492	NM_173492	Homo sapiens phosphatidylinositol-4-phosphate 5-kinase-like 1 (PIP5KL1), mRNA [NM_173492]	NM_173492
A_24_P187304	5.23E-03	2.43	ENST00000329599			
A_32_P147790	5.24E-03	2.00	BC071732	BC071732	Homo sapiens cDNA clone IMAGE:3862422, partial cds. [BC071732]	
A_23_P164826	5.24E-03	2.15	NM_006397	NM_006397	Homo sapiens ribonuclease H2, large subunit (RNASEH2A), mRNA [NM_006397]	NM_006397
A_23_P1102	5.24E-03	10.54	NM_001100	NM_001100	Homo sapiens actin, alpha 1, skeletal muscle (ACTA1), mRNA [NM_001100]	NM_001100
A_23_P12874	5.25E-03	2.05	NM_012341	NM_012341	Homo sapiens GTP binding protein 4 (GTPBP4), mRNA [NM_012341]	NM_012341
A_23_P431360	5.25E-03	2.28	NM_016423	NM_016423	Homo sapiens zinc finger protein 219 (ZNF219), mRNA [NM_016423]	NM_016423
A_24_P410117	5.26E-03	2.02	ENST00000331861			

A_23_P216200	5.26E-03	2.19	NM_018361	NM_018361	Homo sapiens 1-acylglycerol-3-phosphate O-acyltransferase 5 (lysophosphatidic acid acyltransferase, epsilon) (AGPAT5), mRNA [NM_018361]	NM_018361
A_23_P312179	5.26E-03	2.81	NM_015120	NM_015120	Homo sapiens Alstrom syndrome 1 (ALMS1), mRNA [NM_015120]	NM_015120
A_23_P99285	5.26E-03	3.83	NM_006143	NM_006143	Homo sapiens G protein-coupled receptor 19 (GPR19), mRNA [NM_006143]	NM_006143
A_23_P156861	5.26E-03	4.05	NM_012419	NM_012419	Homo sapiens regulator of G-protein signalling 17 (RGS17), mRNA [NM_012419]	NM_012419
A_24_P935782	5.26E-03	4.67	NM_001008727	NM_001008727	Homo sapiens zinc finger protein 121 (clone ZHC32) (ZNF121), mRNA [NM_001008727]	NM_001008727
A_23_P86956	5.28E-03	3.05	NM_007037	NM_007037	Homo sapiens a disintegrin-like and metalloprotease (reprolysin type) with thrombospondin type 1 motif, 8 (ADAMTS8), mRNA [NM_007037]	NM_007037
A_23_P142174	5.29E-03	2.06	NM_004497	NM_004497	Homo sapiens forkhead box A3 (FOXA3), mRNA [NM_004497]	NM_004497
A_23_P132226	5.32E-03	2.11	NM_001008566	NM_001008566	Homo sapiens tyrosylprotein sulfotransferase 2 (TPST2), transcript variant 1, mRNA [NM_001008566]	NM_001008566
A_23_P338912	5.32E-03	2.37	NM_007350	NM_007350	Homo sapiens pleckstrin homology-like domain, family A, member 1 (PHLDA1), mRNA [NM_007350]	NM_007350
A_24_P402588	5.32E-03	2.54	NM_138553	NM_138553	Homo sapiens B-cell CLL/lymphoma 11A (zinc finger protein) (BCL11A), transcript variant 5, mRNA [NM_138553]	NM_138553
A_24_P277576	5.32E-03	2.59	NM_004237	NM_004237	Homo sapiens thyroid hormone receptor interactor 13 (TRIP13), mRNA [NM_004237]	NM_004237
A_23_P88184	5.32E-03	3.00	NM_018167	NM_018167	Homo sapiens BTB (POZ) domain containing 7 (BTBD7), transcript variant 2, mRNA [NM_018167]	NM_018167
A_23_P90089	5.32E-03	3.02	NM_013976	NM_013976	Homo sapiens glutaryl-Coenzyme A dehydrogenase (GCDH), nuclear gene encoding mitochondrial protein, transcript variant 2, mRNA [NM_013976]	NM_013976
A_24_P311856	5.32E-03	5.75	NM_001851	NM_001851	Homo sapiens collagen, type IX, alpha 1 (COL9A1), transcript variant 1, mRNA [NM_001851]	NM_001851
A_23_P343927	5.33E-03	2.49	NM_175065	NM_175065	Homo sapiens histone 2, H2ab (HIST2H2AB), mRNA [NM_175065]	NM_175065
A_23_P218079	5.33E-03	2.80	NM_018976	NM_018976	Homo sapiens solute carrier family 38, member 2 (SLC38A2), mRNA [NM_018976]	NM_018976
A_23_P356021	5.33E-03	3.26	NM_152633	NM_152633	Homo sapiens Fanconi anemia, complementation group B (FANCB), transcript variant 2, mRNA [NM_152633]	NM_152633
A_24_P290314	5.33E-03	3.29	ENST00000332498			
A_23_P60271	5.34E-03	2.31	NM_006444	NM_006444	Homo sapiens SMC2 structural maintenance of chromosomes 2-like 1 (yeast) (SMC2L1), mRNA [NM_006444]	NM_006444
A_23_P88362	5.34E-03	2.91	NM_152329	NM_152329	Homo sapiens peptidylprolyl isomerase (cyclophilin)-like 5 (PPIL5), transcript variant 1, mRNA [NM_152329]	NM_152329
A_23_P126457	5.39E-03	2.02	NM_030980	NM_030980	Homo sapiens interferon stimulated exonuclease gene 20kDa-like 2 (ISG20L2), mRNA [NM_030980]	NM_030980
A_32_P30905	5.39E-03	2.11	BC032420	BC032420	Homo sapiens WDFY family member 4, mRNA (cDNA clone MGC:40604 IMAGE:5221804), complete cds. [BC032420]	
A_23_P41380	5.39E-03	2.28	NM_002940	NM_002940	Homo sapiens ATP-binding cassette, sub-family E (OABP), member 1 (ABCE1), mRNA [NM_002940]	NM_002940
A_23_P165343	5.39E-03	2.43	NM_002830	NM_002830	Homo sapiens protein tyrosine phosphatase, non-receptor type 4 (megakaryocyte) (PTPN4), mRNA [NM_002830]	NM_002830
A_23_P71319	5.41E-03	2.11	NM_004462	NM_004462	Homo sapiens farnesyl-diphosphate farnesyltransferase 1 (FDFT1), mRNA [NM_004462]	NM_004462
A_24_P39843	5.42E-03	2.11	NM_001017916	NM_001017916	Homo sapiens cytochrome b-561 (CYB561), transcript variant 2, mRNA [NM_001017916]	NM_001017916
A_23_P415510	5.42E-03	2.16	NM_005558	NM_005558	Homo sapiens ladinin 1 (LAD1), mRNA [NM_005558]	NM_005558
A_24_P213478	5.42E-03	4.46	NM_020796	NM_020796	Homo sapiens sema domain, transmembrane domain (TM), and cytoplasmic domain, (semaphorin) 6A (SEMA6A), mRNA [NM_020796]	NM_020796
A_23_P109072	5.42E-03	6.62	NM_020436	NM_020436	Homo sapiens sal-like 4 (Drosophila) (SALL4), mRNA [NM_020436]	NM_020436
A_23_P145657	5.43E-03	2.62	NM_012447	NM_012447	Homo sapiens stromal antigen 3 (STAG3), mRNA [NM_012447]	NM_012447
A_23_P31135	5.45E-03	2.10	BC000408	BC000408	Homo sapiens acetyl-Coenzyme A acetyltransferase 2 (acetoacetyl Coenzyme A thiolase), mRNA (cDNA clone MGC:8573 IMAGE:2823036), complete cds. [BC000408]	
A_23_P47839	5.46E-03	2.28	NM_020936	NM_020936	Homo sapiens DEAD (Asp-Glu-Ala-Asp) box polypeptide 55 (DDX55), mRNA [NM_020936]	NM_020936
A_24_P394510	5.46E-03	2.33	NM_021066	NM_021066	Homo sapiens histone 1, H2aj (HIST1H2AJ), mRNA [NM_021066]	NM_021066
A_23_P201988	5.47E-03	3.39	NM_032844	NM_032844	Homo sapiens microtubule associated serine/threonine kinase-like (MASTL), mRNA [NM_032844]	NM_032844
A_23_P101671	5.48E-03	4.41	AB058775	AB058775	Homo sapiens mRNA for KIAA1872 protein, partial cds. [AB058775]	
A_24_P265088	5.50E-03	2.65	NM_032512	NM_032512	Homo sapiens PDZ domain containing 4 (PDZK4), mRNA [NM_032512]	NM_032512
A_24_P68008	5.50E-03	4.85	NM_198545	NM_198545	Homo sapiens hypothetical gene supported by AK075558; BC021286 (LOC374946), mRNA [NM_198545]	NM_198545
A_23_P74981	5.51E-03	2.62	NM_033213	NM_033213	Homo sapiens zinc finger protein 670 (ZNF670), mRNA [NM_033213]	NM_033213
A_24_P41570	5.51E-03	2.72	NM_002106	NM_002106	Homo sapiens H2A histone family, member Z (H2AFZ), mRNA [NM_002106]	NM_002106

A_23_P29655	5.52E-03	2.48	NM_020685	NM_020685	Homo sapiens chromosome 3 open reading frame 14 (C3orf14), mRNA [NM_020685]	NM_020685
A_24_P940509	5.52E-03	2.54	AK022793	AK022793	Homo sapiens cDNA FLJ12731 fis, clone NT2RP2000108. [AK022793]	
A_24_P415260	5.52E-03	2.89	ENST00000354185		Homo sapiens cDNA FLJ36123 fis, clone TESTI2022874, weakly similar to ZINC FINGER PROTEIN 135. [AK093442]	
A_24_P940599	5.52E-03	3.01	BC032643	BC032643	Homo sapiens synaptotagmin binding, cytoplasmic RNA interacting protein, mRNA (cDNA clone MGC:45213 IMAGE:5495201), complete cds. [BC032643]	
A_24_P405190	5.53E-03	2.18	NM_201626	NM_201626	Homo sapiens ubiquitin specific protease 33 (USP33), transcript variant 3, mRNA [NM_201626]	NM_201626
A_23_P131202	5.53E-03	3.28	NM_018645	NM_018645	Homo sapiens hairy and enhancer of split 6 (Drosophila) (HES6), mRNA [NM_018645]	NM_018645
A_23_P325690	5.53E-03	5.62	NM_144698	NM_144698	Homo sapiens ankyrin repeat domain 35 (ANKRD35), mRNA [NM_144698]	NM_144698
A_32_P11181	5.54E-03	2.60	THC2401405		P2G4_HUMAN (Q9UQ80) Proliferation-associated protein 2G4 (Cell cycle protein p38-2G4 homolog) (hG4-1), partial (97%) [THC2401405]	
A_24_P270999	5.56E-03	2.77	NM_000334	NM_000334	Homo sapiens sodium channel, voltage-gated, type IV, alpha (SCN4A), mRNA [NM_000334]	NM_000334
A_23_P360777	5.59E-03	2.16	NM_013957	NM_013957	Homo sapiens neuregulin 1 (NRG1), transcript variant HRG-beta2, mRNA [NM_013957]	NM_013957
A_23_P210686	5.59E-03	2.58	NM_033089	NM_033089	Homo sapiens zinc finger, CCHC domain containing 3 (ZCCHC3), mRNA [NM_033089]	NM_033089
A_24_P191047	5.61E-03	2.03	NM_019060	NM_019060	Homo sapiens chromosome 1 open reading frame 42 (C1orf42), mRNA [NM_019060]	NM_019060
A_23_P39616	5.61E-03	2.34	NM_006190	NM_006190	Homo sapiens origin recognition complex, subunit 2-like (yeast) (ORC2L), mRNA [NM_006190]	NM_006190
A_24_P7642	5.64E-03	4.46	NM_001444	NM_001444	Homo sapiens fatty acid binding protein 5 (psoriasis-associated) (FABP5), mRNA [NM_001444]	NM_001444
A_23_P423074	5.64E-03	6.06	CR936791	CR936791	Homo sapiens mRNA; cDNA DKFZp781C2356 (from clone DKFZp781C2356). [CR936791]	XM_032571
A_23_P324461	5.65E-03	2.16	NM_177401	NM_177401	Homo sapiens midnolin (MIDN), mRNA [NM_177401]	NM_177401
A_23_P3775	5.66E-03	2.48	NM_018233	NM_018233	Homo sapiens hypothetical protein FLJ10826 (FLJ10826), mRNA [NM_018233]	NM_018233
A_23_P218108	5.66E-03	2.71	NM_052950	NM_052950	Homo sapiens WD repeat and FYVE domain containing 2 (WDFY2), mRNA [NM_052950]	NM_052950
A_23_P379794	5.67E-03	3.37	NM_178517	NM_178517	Homo sapiens phosphatidylinositol glycan, class W (PIGW), mRNA [NM_178517]	NM_178517
A_24_P254705	5.67E-03	3.95	NM_020394	NM_020394	Homo sapiens zinc finger protein 695 (ZNF695), mRNA [NM_020394]	NM_020394
A_23_P146997	5.68E-03	6.99	NM_018360	NM_018360	Homo sapiens chromosome X open reading frame 15 (CXorf15), mRNA [NM_018360]	NM_018360
A_24_P391230	5.69E-03	2.23	NM_052954	NM_052954	Homo sapiens cysteine/tyrosine-rich 1 (CYYR1), mRNA [NM_052954]	NM_052954
A_23_P28068	5.70E-03	2.04	NM_133644	NM_133644	Homo sapiens GTP binding protein 3 (mitochondrial) (GTPBP3), transcript variant IV, mRNA [NM_133644]	NM_133644
A_24_P346855	5.70E-03	2.05	NM_002417	NM_002417	Homo sapiens antigen identified by monoclonal antibody Ki-67 (MKI67), mRNA [NM_002417]	NM_002417
A_23_P168211	5.70E-03	2.06	NM_030796	NM_030796	Homo sapiens EGFR-coamplified and overexpressed protein (ECOP), mRNA [NM_030796]	NM_030796
A_23_P55948	5.70E-03	2.42	AB033031	AB033031	Homo sapiens mRNA for KIAA1205 protein, partial cds. [AB033031]	
A_32_P64936	5.70E-03	2.46	X05126	X05126	Human fibroblast mRNA fragment with Alu sequence (pRHF11). [X05126]	
A_32_P109296	5.70E-03	2.68	NM_152259	NM_152259	Homo sapiens leucine-rich repeat kinase 1 (MGC45866), mRNA [NM_152259]	NM_152259
A_23_P211997	5.70E-03	2.93	NM_032970	NM_032970	Homo sapiens SEC22 vesicle trafficking protein-like 3 (S. cerevisiae) (SEC22L3), transcript variant 1, mRNA [NM_032970]	NM_032970
A_23_P39088	5.71E-03	2.10	NM_198319	NM_198319	Homo sapiens HMT1 hnRNP methyltransferase-like 2 (S. cerevisiae) (HRMT1L2), transcript variant 2, mRNA [NM_198319]	NM_198319
A_23_P106822	5.71E-03	2.23	NM_014062	NM_014062	Homo sapiens nin one binding protein (NOB1P), mRNA [NM_014062]	NM_014062
A_23_P40049	5.72E-03	2.16	NM_004341	NM_004341	Homo sapiens carbamoyl-phosphate synthetase 2, aspartate transcarbamylase, and dihydroorotase (CAD), mRNA [NM_004341]	NM_004341
A_23_P164258	5.72E-03	2.23	NM_016518	NM_016518	Homo sapiens pipecolic acid oxidase (PIPOX), mRNA [NM_016518]	NM_016518
A_24_P22562	5.72E-03	2.29	BC069097	BC069097	Homo sapiens apoptosis related protein, mRNA (cDNA clone MGC:95372 IMAGE:7216911), complete cds. [BC069097]	XM_498424
A_32_P165363	5.72E-03	3.77	THC2283809			
A_23_P215175	5.74E-03	2.16	NM_005692	NM_005692	Homo sapiens ATP-binding cassette, sub-family F (GCN20), member 2 (ABCF2), nuclear gene encoding mitochondrial protein, transcript variant 2, mRNA [NM_005692]	NM_005692
A_23_P83149	5.75E-03	2.31	NM_016014	NM_016014	Homo sapiens chromosome 9 open reading frame 77 (C9orf77), transcript variant 1, mRNA [NM_016014]	NM_016014
A_23_P59069	5.76E-03	2.39	NM_003527	NM_003527	Homo sapiens histone 1, H2bo (HIST1H2BO), mRNA [NM_003527]	NM_003527
A_24_P214231	5.78E-03	2.82	NM_003035	NM_003035	Homo sapiens TAL1 (SCL) interrupting locus (SIL), mRNA [NM_003035]	NM_003035

A_24_P886197	5.81E-03	2.70	BC036230	BC036230	Homo sapiens chromosome 9 open reading frame 122, mRNA (cDNA clone IMAGE:5288595), partial cds. [BC036230]	
A_32_P106315	5.81E-03	2.70	BX641009	BX641009	Homo sapiens mRNA; cDNA DKFZp686D13227 (from clone DKFZp686D13227) [BX641009]	
A_23_P81369	5.82E-03	2.00	NM_023038	NM_023038	Homo sapiens a disintegrin and metalloproteinase domain 19 (meltrin beta) (ADAM19), transcript variant 1, mRNA [NM_023038]	NM_023038
A_24_P196519	5.83E-03	2.77	NM_002451	NM_002451	Homo sapiens methylthioadenosine phosphorylase (MTAP), mRNA [NM_002451]	NM_002451
A_23_P361419	5.84E-03	2.08	NM_018369	NM_018369	Homo sapiens DEP domain containing 1B (DEPDC1B), mRNA [NM_018369]	NM_018369
A_23_P116387	5.84E-03	2.15	NM_020238	NM_020238	Homo sapiens inner centromere protein antigens 135/155kDa (INCENP), mRNA [NM_020238]	NM_020238
A_23_P129075	5.84E-03	2.60	NM_024908	NM_024908	Homo sapiens WD repeat domain 76 (WDR76), mRNA [NM_024908]	NM_024908
A_24_P419132	5.84E-03	2.81	NM_006733	NM_006733	Homo sapiens FSH primary response (LRPR1 homolog, rat) 1 (FSHPRH1), mRNA [NM_006733]	NM_006733
A_23_P319583	5.85E-03	2.25	NM_014747	NM_014747	Homo sapiens regulating synaptic membrane exocytosis 3 (RIMS3), mRNA [NM_014747]	NM_014747
A_23_P5845	5.86E-03	2.65	NM_000221	NM_000221	Homo sapiens ketohexokinase (fructokinase) (KHK), transcript variant a, mRNA [NM_000221]	NM_000221
A_24_P138713	5.86E-03	2.93	NM_182922	NM_182922	Homo sapiens hypothetical protein FLJ20718 (FLJ20718), transcript variant 2, mRNA [NM_182922]	NM_182922
A_24_P397107	5.87E-03	2.74	NM_001789	NM_001789	Homo sapiens cell division cycle 25A (CDC25A), transcript variant 1, mRNA [NM_001789]	NM_001789
A_23_P152984	5.88E-03	2.29	NM_005782	NM_005782	Homo sapiens THO complex 4 (THOC4), mRNA [NM_005782]	NM_005782
A_23_P91491	5.88E-03	2.71	NM_021254	NM_021254	Homo sapiens chromosome 21 open reading frame 59 (C21orf59), transcript variant 2, mRNA [NM_021254]	NM_021254
A_32_P119197	5.89E-03	2.06	BF686720	BF686720	602143863F1 NIH_MGC_46 Homo sapiens cDNA clone IMAGE:4304846 5', mRNA sequence [BF686720]	
A_23_P1936	5.89E-03	2.28	NM_025080	NM_025080	Homo sapiens asparaginase like 1 (ASRGL1), mRNA [NM_025080]	NM_025080
A_24_P74731	5.89E-03	3.31	NM_021120	NM_021120	Homo sapiens discs, large homolog 3 (neuroendocrine-dlg, Drosophila) (DLG3), mRNA [NM_021120]	NM_021120
A_23_P359540	5.93E-03	3.98	NM_003540	NM_003540	Homo sapiens histone 1, H4f (HIST1H4F), mRNA [NM_003540]	NM_003540
A_24_P20506	5.94E-03	3.53	NM_001014809	NM_001014809	Homo sapiens collapsin response mediator protein 1 (CRMP1), transcript variant 1, mRNA [NM_001014809]	NM_001014809
A_24_P273647	5.94E-03	4.39	AL833749	AL833749	Homo sapiens mRNA; cDNA DKFZp666L166 (from clone DKFZp666L166). [AL833749]	XM_085463
A_24_P587882	5.95E-03	2.04	A_24_P587882			
A_32_P197698	5.95E-03	2.77	AK055939	AK055939	Homo sapiens cDNA FLJ31377 fis, clone NESOP1000087. [AK055939]	
A_23_P215875	5.97E-03	2.26	NM_015420	NM_015420	Homo sapiens WD repeats and SOF1 domain containing (WDSOF1), mRNA [NM_015420]	NM_015420
A_23_P250385	5.97E-03	8.77	NM_005322	NM_005322	Homo sapiens histone 1, H1b (HIST1H1B), mRNA [NM_005322]	NM_005322
A_23_P388168	6.00E-03	3.31	NM_002867	NM_002867	Homo sapiens RAB3B, member RAS oncogene family (RAB3B), mRNA [NM_002867]	NM_002867
A_23_P98399	6.01E-03	2.69	NM_213621	NM_213621	Homo sapiens 5-hydroxytryptamine (serotonin) receptor 3A (HTR3A), transcript variant 1, mRNA [NM_213621]	NM_213621
A_23_P329133	6.01E-03	2.98	NM_002080	NM_002080	Homo sapiens glutamic-oxaloacetic transaminase 2, mitochondrial (aspartate aminotransferase 2) (GOT2), mRNA [NM_002080]	NM_002080
A_23_P7423	6.02E-03	2.14	NM_017755	NM_017755	Homo sapiens NOL1/NOP2/Sun domain family, member 2 (NSUN2), mRNA [NM_017755]	NM_017755
A_23_P150667	6.02E-03	2.79	NM_031217	NM_031217	Homo sapiens kinesin family member 18A (KIF18A), mRNA [NM_031217]	NM_031217
A_23_P144684	6.04E-03	2.02	NM_032290	NM_032290	Homo sapiens ankyrin repeat domain 32 (ANKRD32), mRNA [NM_032290]	NM_032290
A_24_P25040	6.04E-03	3.38	A_24_P25040			
A_32_P57702	6.05E-03	2.34	THC2304000		GCSH_HUMAN (P23434) Glycine cleavage system H protein, mitochondrial precursor, partial (80%) [THC2304000]	
A_24_P24685	6.07E-03	8.47	ENST00000329156			
A_23_P408996	6.10E-03	2.99	AK131269	AK131269	Homo sapiens cDNA FLJ16207 fis, clone CTONG2019822. [AK131269]	XM_371801
A_32_P175557	6.11E-03	4.39	R01145	R01145	ye88e07.s1 Soares fetal liver spleen 1NFLS Homo sapiens cDNA clone IMAGE:124836 3', mRNA sequence [R01145]	
A_23_P333484	6.12E-03	2.21	NM_003536	NM_003536	Homo sapiens histone 1, H3h (HIST1H3H), mRNA [NM_003536]	NM_003536
A_23_P366376	6.12E-03	39.22	NM_003212	NM_003212	Homo sapiens teratocarcinoma-derived growth factor 1 (TDGF1), mRNA [NM_003212]	NM_003212
A_23_P66063	6.16E-03	2.14	NM_020786	NM_020786	Homo sapiens pyruvate dehydrogenase phosphatase isoenzyme 2 (PDP2), mRNA [NM_020786]	NM_020786
A_23_P133332	6.16E-03	2.14	NM_015084	NM_015084	Homo sapiens mitochondrial ribosomal protein S27 (MRPS27), nuclear gene encoding mitochondrial protein, mRNA [NM_015084]	NM_015084

A_23_P57570	6.16E-03	2.60	NM_017436	NM_017436	Homo sapiens alpha 1,4-galactosyltransferase (globotriaosylceramide synthase) (A4GALT), mRNA [NM_017436]	NM_017436
A_23_P100196	6.17E-03	2.36	NM_005153	NM_005153	Homo sapiens ubiquitin specific protease 10 (USP10), mRNA [NM_005153]	NM_005153
A_24_P217365	6.19E-03	2.11	NM_015199	NM_015199	Homo sapiens ankyrin repeat domain 28 (ANKRD28), mRNA [NM_015199]	NM_015199
A_23_P427217	6.19E-03	2.17	NM_004241	NM_004241	Homo sapiens jumonji domain containing 1C (JMJD1C), mRNA [NM_004241]	NM_004241
A_23_P25873	6.19E-03	3.44	NM_007086	NM_007086	Homo sapiens WD repeat and HMG-box DNA binding protein 1 (WDHD1), transcript variant 1, mRNA [NM_007086]	NM_007086
A_23_P151700	6.20E-03	3.86	NM_199421	NM_199421	Homo sapiens suppressor of cytokine signaling 4 (SOCS4), transcript variant 1, mRNA [NM_199421]	NM_199421
A_24_P662972	6.20E-03	4.69	THC2355570			
A_24_P592544	6.22E-03	2.42	THC2399998		Q5XI42 (Q5XI42) Fatty aldehyde dehydrogenase-like, partial (5%) [THC2399998]	
A_23_P80566	6.22E-03	4.18	NM_006506	NM_006506	Homo sapiens RAS p21 protein activator 2 (RASA2), mRNA [NM_006506]	NM_006506
A_24_P64233	6.23E-03	2.86	NM_000692	NM_000692	Homo sapiens aldehyde dehydrogenase 1 family, member B1 (ALDH1B1), nuclear gene encoding mitochondrial protein, mRNA [NM_000692]	NM_000692
A_24_P20292	6.23E-03	3.12	CR626252	CR626252	full-length cDNA clone CS0DD001YO10 of Neuroblastoma Cot 50-normalized of Homo sapiens (human). [CR626252]	
A_23_P110837	6.23E-03	3.39	NM_016358	NM_016358	Homo sapiens iroquois homeobox protein 4 (IRX4), mRNA [NM_016358]	NM_016358
A_32_P81676	6.23E-03	8.93	AL832535	AL832535	Homo sapiens mRNA; cDNA DKFZp547J1816 (from clone DKFZp547J1816). [AL832535]	
A_23_P28733	6.25E-03	2.12	NM_002895	NM_002895	Homo sapiens retinoblastoma-like 1 (p107) (RBL1), transcript variant 1, mRNA [NM_002895]	NM_002895
A_32_P71768	6.25E-03	2.84	BM504117	BM504117	BM504117 ih21d06.x1 Human insulinoma Homo sapiens cDNA 3' similar to SW:HMG1_CRIGR P07156 HIGH MOBILITY GROUP PROTEIN HMG1 ;, mRNA sequence [BM504117]	
A_23_P350689	6.27E-03	6.37	NM_173570	NM_173570	Homo sapiens zinc finger, DHHC-type containing 23 (ZDHHC23), mRNA [NM_173570]	NM_173570
A_23_P85543	6.31E-03	2.09	NM_007212	NM_007212	Homo sapiens ring finger protein 2 (RNF2), mRNA [NM_007212]	NM_007212
A_23_P41629	6.31E-03	2.80	AB095949	AB095949	Homo sapiens mRNA for KIAA2029 protein. [AB095949]	
A_23_P326760	6.33E-03	7.35	NM_015460	NM_015460	Homo sapiens myosin VIIA and Rab interacting protein (MYRIP), mRNA [NM_015460]	NM_015460
A_23_P63153	6.34E-03	2.13	NM_007204	NM_007204	Homo sapiens DEAD (Asp-Glu-Ala-Asp) box polypeptide 20 (DDX20), mRNA [NM_007204]	NM_007204
A_23_P29985	6.34E-03	4.27	AK022953	AK022953	Homo sapiens cDNA FLJ12891 fis, clone NT2RP2004142. [AK022953]	
A_23_P200325	6.36E-03	11.25	NM_014857	NM_014857	Homo sapiens RAB GTPase activating protein 1-like (RABGAP1L), mRNA [NM_014857]	NM_014857
A_24_P230176	6.37E-03	2.72	BC009369	BC009369	Homo sapiens similar to RIKEN cDNA 3110023B02, mRNA (cDNA clone MGC:16597 IMAGE:4110481), complete cds. [BC009369]	XM_375500
A_24_P564462	6.40E-03	2.39	CN430223	CN430223	17000600171867 GRN_PREHEP Homo sapiens cDNA 5', mRNA sequence [CN430223]	
A_23_P132468	6.40E-03	5.81	NM_003615	NM_003615	Homo sapiens solute carrier family 4, sodium bicarbonate cotransporter, member 7 (SLC4A7), mRNA [NM_003615]	NM_003615
A_23_P308731	6.40E-03	6.62	NM_138328	NM_138328	Homo sapiens rhomboid, veinlet-like 4 (Drosophila) (RHBDL4), mRNA [NM_138328]	NM_138328
A_24_P67681	6.40E-03	7.75	ENST00000332148		PREDICTED: Homo sapiens similar to High mobility group protein 4 (HMG-4) (High mobility group protein 2a) (HMG-2a) (LOC441795), mRNA [XM_497547]	XM_497547
A_23_P21966	6.41E-03	2.40	NM_022455	NM_022455	Homo sapiens nuclear receptor binding SET domain protein 1 (NSD1), transcript variant 2, mRNA [NM_022455]	NM_022455
A_23_P161918	6.41E-03	2.75	NM_024098	NM_024098	Homo sapiens hypothetical protein MGC2574 (MGC2574), mRNA [NM_024098]	NM_024098
A_32_P151800	6.41E-03	3.41	NM_207418	NM_207418	Homo sapiens family with sequence similarity 72, member A (FAM72A), mRNA [NM_207418]	NM_207418
A_23_P12965	6.42E-03	2.12	NM_002033	NM_002033	Homo sapiens fucosyltransferase 4 (alpha (1,3) fucosyltransferase, myeloid-specific) (FUT4), mRNA [NM_002033]	NM_002033
A_23_P82474	6.43E-03	3.44	NM_001002926	NM_001002926	Homo sapiens TWIST neighbor (TWISTNB), mRNA [NM_001002926]	NM_001002926
A_24_P182620	6.45E-03	2.39	NM_001408	NM_001408	Homo sapiens cadherin, EGF LAG seven-pass G-type receptor 2 (flamingo homolog, Drosophila) (CELSR2), mRNA [NM_001408]	NM_001408
A_23_P60101	6.47E-03	2.20	NM_030895	NM_030895	Homo sapiens zinc finger protein 696 (ZNF696), mRNA [NM_030895]	NM_030895
A_23_P214798	6.48E-03	2.05	NM_006372	NM_006372	Homo sapiens synaptotagmin binding, cytoplasmic RNA interacting protein (SYNCRIP), mRNA [NM_006372]	NM_006372
A_23_P34527	6.48E-03	2.35	NM_025207	NM_025207	Homo sapiens FAD-synthetase (PP591), transcript variant 1, mRNA [NM_025207]	NM_025207
A_23_P157914	6.48E-03	4.26	NM_153267	NM_153267	Homo sapiens MAM domain containing 2 (MAMDC2), mRNA [NM_153267]	NM_153267
A_24_P106728	6.49E-03	2.29	NM_019042	NM_019042	Homo sapiens hypothetical protein FLJ20485 (FLJ20485), mRNA [NM_019042]	NM_019042

A_24_P239364	6.49E-03	2.36	AB004064	AB004064	Homo sapiens mRNA for tomoregulin, complete cds. [AB004064]	
A_24_P235360	6.49E-03	2.59	NM_017884	NM_017884	Homo sapiens PIN2-interacting protein 1 (PINX1), mRNA [NM_017884]	NM_017884
A_32_P210516	6.49E-03	5.85	AK024566	AK024566	Homo sapiens cDNA: FLJ20913 fis, clone ADSE00630. [AK024566]	
A_32_P80610	6.50E-03	2.11	AK124080	AK124080	Homo sapiens cDNA FLJ42086 fis, clone TESOP1000127. [AK124080]	
A_24_P239140	6.50E-03	3.64	NM_000819	NM_000819	Homo sapiens phosphoribosylglycinamide formyltransferase, phosphoribosylglycinamide synthetase, phosphoribosylaminoimidazole synthetase (GART), transcript variant 1, mRNA [NM_000819]	NM_000819
A_23_P169479	6.50E-03	23.53	CR595826	CR595826	full-length cDNA clone CS0DC021YA11 of Neuroblastoma Cot 25-normalized of Homo sapiens (human). [CR595826]	
A_24_P76313	6.53E-03	2.65	NM_207334	NM_207334	Homo sapiens family with sequence similarity 43, member B (FAM43B), mRNA [NM_207334]	NM_207334
A_23_P63829	6.54E-03	2.07	NM_016299	NM_016299	Homo sapiens heat shock 70kDa protein 14 (HSPA14), mRNA [NM_016299]	NM_016299
A_23_P142154	6.54E-03	2.53	NM_031485	NM_031485	Homo sapiens glutamate-rich WD repeat containing 1 (GRWD1), mRNA [NM_031485]	NM_031485
A_23_P410587	6.54E-03	2.56	NM_024900	NM_024900	Homo sapiens PHD finger protein 17 (PHF17), transcript variant S, mRNA [NM_024900]	NM_024900
A_23_P350005	6.54E-03	3.51	NM_173553	NM_173553	Homo sapiens hypothetical protein FLJ25801 (FLJ25801), mRNA [NM_173553]	NM_173553
A_32_P51781	6.56E-03	2.22	A_32_P51781			
A_23_P434944	6.57E-03	2.99	NM_004516	NM_004516	Homo sapiens interleukin enhancer binding factor 3, 90kDa (ILF3), transcript variant 2, mRNA [NM_004516]	NM_004516
A_23_P119084	6.57E-03	3.06	NM_138347	NM_138347	Homo sapiens zinc finger protein 551 (ZNF551), mRNA [NM_138347]	NM_138347
A_23_P137586	6.58E-03	2.03	NM_006582	NM_006582	Homo sapiens glucocorticoid modulatory element binding protein 1 (GMEB1), transcript variant 1, mRNA [NM_006582]	NM_006582
A_23_P56553	6.58E-03	2.54	NM_024770	NM_024770	Homo sapiens hypothetical protein FLJ13984 (FLJ13984), mRNA [NM_024770]	NM_024770
A_23_P2293	6.59E-03	3.04	NM_033647	NM_033647	Homo sapiens helicase (DNA) B (HELB), mRNA [NM_033647]	NM_033647
A_23_P34366	6.59E-03	4.07	NM_000864	NM_000864	Homo sapiens 5-hydroxytryptamine (serotonin) receptor 1D (HTR1D), mRNA [NM_000864]	NM_000864
A_24_P233995	6.60E-03	2.11	NM_022746	NM_022746	Homo sapiens MOCO sulphurase C-terminal domain containing 1 (MOSC1), mRNA [NM_022746]	NM_022746
A_23_P392383	6.60E-03	2.42	NM_033551	NM_033551	Homo sapiens La ribonucleoprotein domain family, member 1 (LARP1), mRNA [NM_033551]	NM_033551
A_24_P393449	6.60E-03	2.57	NM_004938	NM_004938	Homo sapiens death-associated protein kinase 1 (DAPK1), mRNA [NM_004938]	NM_004938
A_32_P133090	6.60E-03	2.98	XM_379210	XM_379210	PREDICTED: Homo sapiens hypothetical LOC401085 (LOC401085), mRNA [XM_379210]	XM_379210
A_23_P54622	6.61E-03	2.02	NM_007317	NM_007317	Homo sapiens kinesin family member 22 (KIF22), mRNA [NM_007317]	NM_007317
A_23_P84448	6.64E-03	4.05	NM_025019	NM_025019	Homo sapiens tubulin, alpha 4 (TUBA4), mRNA [NM_025019]	NM_025019
A_23_P52278	6.65E-03	2.29	NM_004523	NM_004523	Homo sapiens kinesin family member 11 (KIF11), mRNA [NM_004523]	NM_004523
A_23_P70445	6.65E-03	2.34	NM_003532	NM_003532	Homo sapiens histone 1, H3e (HIST1H3E), mRNA [NM_003532]	NM_003532
A_23_P10121	6.65E-03	5.78	NM_003012	NM_003012	Homo sapiens secreted frizzled-related protein 1 (SFRP1), mRNA [NM_003012]	NM_003012
A_23_P435029	6.66E-03	5.26	BC015544	BC015544	Homo sapiens, histone gene complex 1, clone MGC:9629 IMAGE:3913365, mRNA, complete cds. [BC015544]	
A_24_P332647	6.67E-03	2.45	AK095421	AK095421	Homo sapiens cDNA FLJ38102 fis, clone D3OST2000618, moderately similar to Drosophila melanogaster slingshot mRNA. [AK095421]	
A_23_P52298	6.67E-03	2.85	NM_006993	NM_006993	Homo sapiens nucleophosmin/nucleoplasmin, 3 (NPM3), mRNA [NM_006993]	NM_006993
A_23_P112078	6.68E-03	2.67	NM_004225	NM_004225	Homo sapiens malignant fibrous histiocytoma amplified sequence 1 (MFHAS1), mRNA [NM_004225]	NM_004225
A_23_P133956	6.68E-03	2.79	NM_002263	NM_002263	Homo sapiens kinesin family member C1 (KIFC1), mRNA [NM_002263]	NM_002263
A_24_P134626	6.69E-03	4.03	NM_018360	NM_018360	Homo sapiens chromosome X open reading frame 15 (CXorf15), mRNA [NM_018360]	NM_018360
A_32_P99690	6.71E-03	3.70	BM709498	BM709498	UI-E-CQ1-afy-b-13-0-UI.r1 UI-E-CQ1 Homo sapiens cDNA clone UI-E-CQ1-afy-b-13-0-UI 5', mRNA sequence [BM709498]	
A_23_P112673	6.71E-03	4.35	NM_017975	NM_017975	Homo sapiens Zwilch, kinetochore associated, homolog (Drosophila) (ZWILCH), mRNA [NM_017975]	NM_017975
A_24_P124550	6.71E-03	4.41	NM_053056	NM_053056	Homo sapiens cyclin D1 (PRAD1: parathyroid adenomatosis 1) (CCND1), mRNA [NM_053056]	NM_053056
A_23_P89343	6.75E-03	2.13	NM_152244	NM_152244	Homo sapiens sorting nexin 11 (SNX11), transcript variant 1, mRNA [NM_152244]	NM_152244
A_23_P30377	6.75E-03	2.15	NM_014829	NM_014829	Homo sapiens DEAD (Asp-Glu-Ala-Asp) box polypeptide 46 (DDX46), mRNA [NM_014829]	NM_014829
A_23_P119778	6.75E-03	2.42	NM_020342	NM_020342	Homo sapiens solute carrier family 39 (zinc transporter), member 10 (SLC39A10), mRNA [NM_020342]	NM_020342

A_23_P132378	6.77E-03	2.23	NM_014246	NM_014246	Homo sapiens cadherin, EGF LAG seven-pass G-type receptor 1 (flamingo homolog, Drosophila) (CELSR1), mRNA [NM_014246]	NM_014246
A_24_P53824	6.77E-03	3.09	NM_014636	NM_014636	Homo sapiens Ral GEF with PH domain and SH3 binding motif 1 (RALGPS1), mRNA [NM_014636]	NM_014636
A_24_P323434	6.78E-03	2.63	NM_152562	NM_152562	Homo sapiens cell division cycle associated 2 (CDCA2), mRNA [NM_152562]	NM_152562
A_24_P200427	6.83E-03	3.33	NM_006452	NM_006452	Homo sapiens phosphoribosylaminoimidazole carboxylase, phosphoribosylaminoimidazole succinocarboxamide synthetase (PAICS), mRNA [NM_006452]	NM_006452
A_32_P175183	6.84E-03	2.88	BC071729	BC071729	Homo sapiens BTB (POZ) domain containing 15, mRNA (cDNA clone MGC:88058 IMAGE:5163748), complete cds. [BC071729]	
A_32_P69296	6.85E-03	2.12	AK130071	AK130071	Homo sapiens cDNA FLJ26561 fis, clone LNF03981. [AK130071]	
A_32_P99275	6.86E-03	2.30	NM_016258	NM_016258	Homo sapiens YTH domain family, member 2 (YTHDF2), mRNA [NM_016258]	NM_016258
A_24_P364025	6.86E-03	2.56	NM_003338	NM_003338	Homo sapiens ubiquitin-conjugating enzyme E2D 1 (UBC4/5 homolog, yeast) (UBE2D1), mRNA [NM_003338]	NM_003338
A_23_P110196	6.86E-03	2.58	NM_016323	NM_016323	Homo sapiens hect domain and RLD 5 (HERC5), mRNA [NM_016323]	NM_016323
A_24_P307695	6.88E-03	3.12	NM_033402	NM_033402	Homo sapiens KIAA1764 protein (KIAA1764), mRNA [NM_033402]	NM_033402
A_24_P935652	6.90E-03	2.06	CR606629	CR606629	full-length cDNA clone CS0DJ007YF12 of T cells (Jurkat cell line) Cot 10-normalized of Homo sapiens (human). [CR606629]	
A_32_P24656	6.90E-03	3.73	THC2403568		BC005089 LOC285069 protein {Homo sapiens;} , partial (68%) [THC2403568]	
A_24_P301186	6.91E-03	2.40	NM_001008726	NM_001008726	Homo sapiens chromosome 14 open reading frame 150 (C14orf150), transcript variant 1, mRNA [NM_001008726]	NM_001008726
A_24_P181108	6.91E-03	2.58	NM_018093	NM_018093	Homo sapiens WD repeat domain 74 (WDR74), mRNA [NM_018093]	NM_018093
A_23_P127153	6.91E-03	5.05	U88048	U88048	Human clone KiSS-16 unknown product mRNA, complete cds. [U88048]	
A_24_P8267	6.92E-03	3.14	BC010906	BC010906	Homo sapiens mediator of RNA polymerase II transcription, subunit 9 homolog (yeast), mRNA (cDNA clone IMAGE:4095249). [BC010906]	
A_23_P4294	6.93E-03	2.05	NM_014519	NM_014519	Homo sapiens zinc finger protein 232 (ZNF232), mRNA [NM_014519]	NM_014519
A_32_P9127	6.93E-03	6.67	THC2406727			
A_24_P911571	6.93E-03	9.09	BC011671	BC011671	Homo sapiens guanine nucleotide binding protein (G protein), beta 5, mRNA (cDNA clone IMAGE:4131809), complete cds. [BC011671]	
A_32_P88605	6.93E-03	10.91	A_32_P88605			
A_32_P885123	6.97E-03	2.86	A_32_P885123			
A_23_P402134	7.00E-03	4.42	NM_080764	NM_080764	Homo sapiens suppressor of hairy wing homolog 2 (Drosophila) (SUHW2), mRNA [NM_080764]	NM_080764
A_23_P7528	7.00E-03	6.10	NM_001387	NM_001387	Homo sapiens dihydropyrimidinase-like 3 (DPYSL3), mRNA [NM_001387]	NM_001387
A_24_P223384	7.00E-03	6.29	NM_003513	NM_003513	Homo sapiens histone 1, H2ab (HIST1H2AB), mRNA [NM_003513]	NM_003513
A_24_P67063	7.00E-03	7.35	A_24_P67063			
A_23_P129031	7.03E-03	2.33	NM_004993	NM_004993	Homo sapiens ataxin 3 (ATXN3), transcript variant 1, mRNA [NM_004993]	NM_004993
A_23_P258088	7.03E-03	4.52	NM_020804	NM_020804	Homo sapiens protein kinase C and casein kinase substrate in neurons 1 (PACSIN1), mRNA [NM_020804]	NM_020804
A_23_P133739	7.03E-03	5.13	NM_148959	NM_148959	Homo sapiens HUS1 checkpoint homolog b (S. pombe) (HUS1B), mRNA [NM_148959]	NM_148959
A_24_P526177	7.04E-03	3.07	NM_003211	NM_003211	Homo sapiens thymine-DNA glycosylase (TDG), transcript variant 1, mRNA [NM_003211]	NM_003211
A_23_P155185	7.05E-03	2.62	ENST00000256031		Homo sapiens cDNA FLJ90613 fis, clone PLACE1001949, highly similar to Probable cation-transporting ATPase 3 (EC3.6.3.-). [AK075094]	
A_24_P349756	7.06E-03	2.41	A_24_P349756			
A_24_P707102	7.06E-03	5.10	THC2283468		Q8WC13 (Q8WC13) ATPase 6 (Fragment), partial (7%) [THC2283468]	
A_24_P383680	7.07E-03	2.16	A_24_P383680			
A_23_P102471	7.07E-03	3.09	NM_000251	NM_000251	Homo sapiens mutS homolog 2, colon cancer, nonpolyposis type 1 (E. coli) (MSH2), mRNA [NM_000251]	NM_000251
A_23_P256231	7.08E-03	2.07	NM_032145	NM_032145	Homo sapiens F-box protein 30 (FBXO30), mRNA [NM_032145]	NM_032145
A_24_P160466	7.08E-03	2.99	NM_052899	NM_052899	Homo sapiens G protein-regulated inducer of neurite outgrowth 1 (KIAA1893), mRNA [NM_052899]	NM_052899
A_24_P84608	7.09E-03	2.11	A_24_P84608			
A_23_P335329	7.10E-03	5.29	NM_004485	NM_004485	Homo sapiens guanine nucleotide binding protein (G protein), gamma 4 (GNG4), mRNA [NM_004485]	NM_004485
A_23_P162010	7.11E-03	4.26	NM_176875	NM_176875	Homo sapiens cholecystokinin B receptor (CCKBR), mRNA [NM_176875]	NM_176875

A_24_P527404	7.12E-03	2.50	NM_004329	NM_004329	Homo sapiens bone morphogenetic protein receptor, type IA (BMPR1A), mRNA [NM_004329]	NM_004329
A_23_P109001	7.12E-03	3.08	NM_014657	NM_014657	Homo sapiens KIAA0406 gene product (KIAA0406), mRNA [NM_014657]	NM_014657
A_32_P208599	7.13E-03	2.48	AA393589	AA393589	AA393589 zt72d07.r1 Soares_testis_NHT Homo sapiens cDNA clone IMAGE:727885 5', mRNA sequence [AA393589]	
A_24_P322611	7.13E-03	2.99	NM_080797	NM_080797	Homo sapiens death associated transcription factor 1 (DATF1), transcript variant 3, mRNA [NM_080797]	NM_080797
A_32_P228331	7.14E-03	2.68	A_32_P228331			
A_32_P204722	7.16E-03	2.38	BM723328	BM723328	UI-E-EJ0-aio-o-14-0-UI.r1 UI-E-EJ0 Homo sapiens cDNA clone UI-E-EJ0-aio-o-14-0-UI 5', mRNA sequence [BM723328]	
A_24_P68631	7.16E-03	2.54	NM_175065	NM_175065	Homo sapiens histone 2, H2ab (HIST2H2AB), mRNA [NM_175065]	NM_175065
A_24_P914649	7.21E-03	2.04	BX108121	BX108121	BX108121 Soares_testis_NHT Homo sapiens cDNA clone IMAGp998B051795, mRNA sequence [BX108121]	
A_23_P46871	7.21E-03	2.25	NM_018344	NM_018344	Homo sapiens solute carrier family 29 (nucleoside transporters), member 3 (SLC29A3), mRNA [NM_018344]	NM_018344
A_23_P256455	7.24E-03	2.33	NM_002947	NM_002947	Homo sapiens replication protein A3, 14kDa (RPA3), mRNA [NM_002947]	NM_002947
A_23_P102235	7.24E-03	2.39	NM_003096	NM_003096	Homo sapiens small nuclear ribonucleoprotein polypeptide G (SNRPG), mRNA [NM_003096]	NM_003096
A_23_P153745	7.25E-03	2.38	NM_006332	NM_006332	Homo sapiens interferon, gamma-inducible protein 30 (IFI30), mRNA [NM_006332]	NM_006332
A_23_P106575	7.25E-03	2.90	ENST00000245206		Homo sapiens cDNA FLJ26346 fis, clone HRT04038, highly similar to Homo sapiens titin (TTN), transcript variant N2-B. [AK129856]	
A_24_P450172	7.28E-03	2.80	AK095151	AK095151	Homo sapiens cDNA FLJ37832 fis, clone BRSSN2009630. [AK095151]	
A_23_P252236	7.28E-03	6.21	NM_000892	NM_000892	Homo sapiens kallikrein B, plasma (Fletcher factor) 1 (KLKB1), mRNA [NM_000892]	NM_000892
A_24_P878419	7.28E-03	7.58	A_24_P878419			
A_23_P365705	7.29E-03	4.31	NM_024086	NM_024086	Homo sapiens hypothetical protein MGC3329 (MGC3329), mRNA [NM_024086]	NM_024086
A_32_P130968	7.30E-03	2.43	BC035377	BC035377	Homo sapiens, clone IMAGE:4826240, mRNA. [BC035377]	
A_23_P164718	7.32E-03	2.12	NM_004596	NM_004596	Homo sapiens small nuclear ribonucleoprotein polypeptide A (SNRPA), mRNA [NM_004596]	NM_004596
A_24_P560519	7.33E-03	2.02	AK024346	AK024346	Homo sapiens cDNA FLJ14284 fis, clone PLACE1005898. [AK024346]	
A_23_P88484	7.33E-03	2.14	NM_001948	NM_001948	Homo sapiens dUTP pyrophosphatase (DUT), nuclear gene encoding mitochondrial protein, transcript variant 2, mRNA [NM_001948]	NM_001948
A_23_P20622	7.34E-03	2.34	NM_003671	NM_003671	Homo sapiens CDC14 cell division cycle 14 homolog B (S. cerevisiae) (CDC14B), transcript variant 1, mRNA [NM_003671]	NM_003671
A_24_P258633	7.34E-03	2.35	NM_006086	NM_006086	Homo sapiens tubulin, beta 3 (TUBB3), mRNA [NM_006086]	NM_006086
A_23_P132619	7.34E-03	3.24	NM_000916	NM_000916	Homo sapiens oxytocin receptor (OXTR), mRNA [NM_000916]	NM_000916
A_23_P138461	7.34E-03	3.38	NM_021830	NM_021830	Homo sapiens progressive external ophthalmoplegia 1 (PEO1), mRNA [NM_021830]	NM_021830
A_23_P43197	7.35E-03	7.52	NM_004929	NM_004929	Homo sapiens calbindin 1, 28kDa (CALB1), mRNA [NM_004929]	NM_004929
A_23_P14508	7.38E-03	2.05	ENST00000256367		Homo sapiens tetratricopeptide repeat domain 9, mRNA (cDNA clone IMAGE:5763935), partial cds. [BC047950]	XM_027236
A_23_P363399	7.38E-03	4.93	NM_030674	NM_030674	Homo sapiens solute carrier family 38, member 1 (SLC38A1), mRNA [NM_030674]	NM_030674
A_23_P3186	7.40E-03	2.20	NM_003858	NM_003858	Homo sapiens cyclin K (CCNK), mRNA [NM_003858]	NM_003858
A_24_P418203	7.40E-03	3.46	NM_033655	NM_033655	Homo sapiens contactin associated protein-like 3 (CNTNAP3), mRNA [NM_033655]	NM_033655
A_24_P56887	7.41E-03	2.17	NM_015986	NM_015986	Homo sapiens cytokine receptor-like factor 3 (CRLF3), mRNA [NM_015986]	NM_015986
A_23_P134295	7.41E-03	2.50	NM_198949	NM_198949	Homo sapiens nudix (nucleoside diphosphate linked moiety X)-type motif 1 (NUDT1), transcript variant 2B, mRNA [NM_198949]	NM_198949
A_23_P88880	7.41E-03	2.76	NM_015069	NM_015069	Homo sapiens zinc finger protein 423 (ZNF423), mRNA [NM_015069]	NM_015069
A_23_P355075	7.42E-03	2.19	AK023669	AK023669	Homo sapiens cDNA FLJ13607 fis, clone PLACE1010624. [AK023669]	
A_24_P900730	7.43E-03	2.54	A_24_P900730			
A_23_P31055	7.43E-03	3.77	NM_198887	NM_198887	Homo sapiens nucleoporin 43kDa (NUP43), transcript variant 1, mRNA [NM_198887]	NM_198887
A_24_P20873	7.43E-03	4.35	NM_003495	NM_003495	Homo sapiens histone 1, H4i (HIST1H4I), mRNA [NM_003495]	NM_003495
A_23_P253571	7.46E-03	2.12	NM_032758	NM_032758	Homo sapiens PHD finger protein 5A (PHF5A), mRNA [NM_032758]	NM_032758
A_23_P46118	7.46E-03	4.41	NM_001821	NM_001821	Homo sapiens choroideremia-like (Rab escort protein 2) (CHML), mRNA [NM_001821]	NM_001821
A_24_P307974	7.47E-03	2.55	BC033728	BC033728	Homo sapiens cDNA clone MGC:45470 IMAGE:5166848, complete cds. [BC033728]	

A_23_P256956	7.48E-03	2.17	NM_005733	NM_005733	Homo sapiens kinesin family member 20A (KIF20A), mRNA [NM_005733]	NM_005733
A_23_P102351	7.48E-03	2.41	NM_004854	NM_004854	Homo sapiens carbohydrate sulfotransferase 10 (CHST10), mRNA [NM_004854]	NM_004854
A_23_P414855	7.48E-03	3.86	NM_033224	NM_033224	Homo sapiens purine-rich element binding protein B (PURB), mRNA [NM_033224]	NM_033224
A_23_P210969	7.49E-03	2.07	NM_016436	NM_016436	Homo sapiens PHD finger protein 20 (PHF20), mRNA [NM_016436]	NM_016436
A_24_P96780	7.49E-03	5.41	NM_016343	NM_016343	Homo sapiens centromere protein F, 350/400ka (mitosin) (CENPF), mRNA [NM_016343]	NM_016343
A_23_P160336	7.50E-03	47.39	NM_020997	NM_020997	Homo sapiens left-right determination factor 1 (LEFTY1), mRNA [NM_020997]	NM_020997
A_23_P138881	7.51E-03	9.90	NM_001104	NM_001104	Homo sapiens actinin, alpha 3 (ACTN3), mRNA [NM_001104]	NM_001104
A_32_P174083	7.53E-03	2.62	NM_018947	NM_018947	Homo sapiens cytochrome c, somatic (CYCS), nuclear gene encoding mitochondrial protein, mRNA [NM_018947]	NM_018947
A_32_P22702	7.55E-03	3.16	NM_033222	NM_033222	Homo sapiens PC4 and SFRS1 interacting protein 1 (PSIP1), transcript variant 2, mRNA [NM_033222]	NM_033222
A_23_P19182	7.56E-03	2.99	NM_016606	NM_016606	Homo sapiens chromosome 5 open reading frame 19 (C5orf19), mRNA [NM_016606]	NM_016606
A_23_P63999	7.58E-03	2.21	CR614032	CR614032	full-length cDNA clone CS0DL003YC18 of B cells (Ramos cell line) Cot 25-normalized of Homo sapiens (human). [CR614032]	
A_32_P227673	7.58E-03	2.80	THC2305334			
A_32_P105195	7.59E-03	2.26	NM_014829	NM_014829	Homo sapiens DEAD (Asp-Glu-Ala-Asp) box polypeptide 46 (DDX46), mRNA [NM_014829]	NM_014829
A_32_P10100	7.59E-03	2.43	A_32_P10100			
A_24_P922606	7.63E-03	2.02	NM_015231	NM_015231	Homo sapiens nucleoporin 160kDa (NUP160), mRNA [NM_015231]	NM_015231
A_23_P80062	7.63E-03	2.48	NM_003185	NM_003185	Homo sapiens TAF4 RNA polymerase II, TATA box binding protein (TBP)-associated factor, 135kDa (TAF4), mRNA [NM_003185]	NM_003185
A_24_P257201	7.63E-03	3.80	NM_016503	NM_016503	Homo sapiens mitochondrial ribosomal protein L30 (MRPL30), nuclear gene encoding mitochondrial protein, transcript variant 2, mRNA [NM_016503]	NM_016503
A_23_P92786	7.63E-03	3.98	ENST00000265271		Homo sapiens RNA binding motif protein 27, mRNA (cDNA clone IMAGE:3458303), partial cds. [BC033524]	XM_291128
A_24_P159635	7.64E-03	2.24	NM_030576	NM_030576	Homo sapiens hypothetical protein MGC10986 (MGC10986), mRNA [NM_030576]	NM_030576
A_23_P417994	7.64E-03	7.69	NM_152429	NM_152429	Homo sapiens chromosome 10 open reading frame 13 (C10orf13), mRNA [NM_152429]	NM_152429
A_23_P335813	7.66E-03	2.13	NM_016272	NM_016272	Homo sapiens transducer of ERBB2, 2 (TOB2), mRNA [NM_016272]	NM_016272
A_23_P408195	7.66E-03	10.66	NM_152399	NM_152399	Homo sapiens hypothetical protein FLJ30834 (FLJ30834), mRNA [NM_152399]	NM_152399
A_23_P14863	7.71E-03	2.16	NM_024111	NM_024111	Homo sapiens hypothetical protein MGC4504 (MGC4504), mRNA [NM_024111]	NM_024111
A_23_P118246	7.71E-03	2.17	NM_016095	NM_016095	Homo sapiens DNA replication complex GINS protein PSF2 (Pfs2), mRNA [NM_016095]	NM_016095
A_23_P99985	7.71E-03	2.17	NM_002128	NM_002128	Homo sapiens high-mobility group box 1 (HMGB1), mRNA [NM_002128]	NM_002128
A_23_P423480	7.71E-03	2.88	NM_173555	NM_173555	Homo sapiens trypsin domain containing 1 (TYSND1), mRNA [NM_173555]	NM_173555
A_23_P78311	7.71E-03	3.29	NM_031216	NM_031216	Homo sapiens SEH1-like (S. cerevisiae) (SEH1L), transcript variant 2, mRNA [NM_031216]	NM_031216
A_32_P181891	7.71E-03	11.42	AK097571	AK097571	Homo sapiens cDNA FLJ40252 fis, clone TESTI2024299. [AK097571]	
A_23_P145824	7.72E-03	4.76	AK000075	AK000075	Homo sapiens cDNA FLJ20068 fis, clone COL01755. [AK000075]	XM_371933
A_23_P63681	7.73E-03	2.60	NM_004969	NM_004969	Homo sapiens insulin-degrading enzyme (IDE), mRNA [NM_004969]	NM_004969
A_24_P117029	7.75E-03	2.71	NM_000527	NM_000527	Homo sapiens low density lipoprotein receptor (familial hypercholesterolemia) (LDLR), mRNA [NM_000527]	NM_000527
A_32_P221832	7.76E-03	2.43	CD630738	CD630738	CD630738 56066364H1 FLP Homo sapiens cDNA, mRNA sequence [CD630738]	
A_24_P158946	7.78E-03	9.71	NM_139241	NM_139241	Homo sapiens FYVE, RhoGEF and PH domain containing 4 (FGD4), mRNA [NM_139241]	NM_139241
A_23_P255376	7.80E-03	2.81	NM_017918	NM_017918	Homo sapiens hypothetical protein FLJ20647 (FLJ20647), mRNA [NM_017918]	NM_017918
A_24_P211151	7.81E-03	2.92	NM_020158	NM_020158	Homo sapiens exosome component 5 (EXOSC5), mRNA [NM_020158]	NM_020158
A_32_P207147	7.82E-03	11.07	A_32_P207147			
A_32_P6917	7.84E-03	2.46	NM_025207	NM_025207	Homo sapiens FAD-synthetase (PP591), transcript variant 1, mRNA [NM_025207]	NM_025207
A_32_P221748	7.84E-03	3.07	BX350880	BX350880	BX350880 Homo sapiens NEUROBLASTOMA COT 25-NORMALIZED Homo sapiens cDNA clone CS0DC005YN21 3-PRIME, mRNA sequence [BX350880]	
A_24_P392833	7.84E-03	7.81	ENST00000361761			
A_23_P54834	7.85E-03	2.30	NM_016101	NM_016101	Homo sapiens comparative gene identification transcript 37 (CGI-37), mRNA [NM_016101]	NM_016101
A_24_P163477	7.85E-03	2.43	NM_018292	NM_018292	Homo sapiens glutaminyl-tRNA synthase (glutamine-hydrolyzing)-like 1 (QRSL1), mRNA [NM_018292]	NM_018292

A_24_P772103	7.85E-03	2.99	AK094724	AK094724	Homo sapiens cDNA FLJ37405 fis, clone BRAMY2028269. [AK094724]	
A_32_P164661	7.85E-03	3.40	AI302935	AI302935	AI302935 qn53c07.x1 NCI_CGAP_Kid5 Homo sapiens cDNA clone IMAGE:1901964 3', mRNA sequence [AI302935]	
A_23_P121441	7.85E-03	3.85	NM_014893	NM_014893	Homo sapiens neuroligin 4, Y-linked (NLGN4Y), mRNA [NM_014893]	NM_014893
A_23_P40693	7.89E-03	2.37	NM_001429	NM_001429	Homo sapiens E1A binding protein p300 (EP300), mRNA [NM_001429]	NM_001429
A_23_P53057	7.89E-03	2.52	NM_013250	NM_013250	Homo sapiens zinc finger protein 215 (ZNF215), mRNA [NM_013250]	NM_013250
A_23_P108437	7.90E-03	6.76	AK024850	AK024850	Homo sapiens cDNA: FLJ21197 fis, clone COL00201. [AK024850]	
A_23_P311087	7.92E-03	3.12	NM_012482	NM_012482	Homo sapiens zinc finger protein 281 (ZNF281), mRNA [NM_012482]	NM_012482
A_23_P149200	7.92E-03	3.36	NM_001255	NM_001255	Homo sapiens CDC20 cell division cycle 20 homolog (S. cerevisiae) (CDC20), mRNA [NM_001255]	NM_001255
A_32_P83520	7.93E-03	2.13	A_32_P83520			
A_24_P210244	7.93E-03	2.61	U09088	U09088	Human thymopoietin gamma mRNA, complete cds. [U09088]	
A_24_P367326	7.96E-03	2.04	ENST00000332687			
A_23_P8539	7.97E-03	2.48	NM_139179	NM_139179	Homo sapiens KCCR13L (LOC221955), mRNA [NM_139179]	NM_139179
A_23_P250313	7.97E-03	2.54	AB040957	AB040957	Homo sapiens mRNA for KIAA1524 protein, partial cds. [AB040957]	
A_23_P131825	7.97E-03	9.80	NM_003279	NM_003279	Homo sapiens troponin C2, fast (TNNC2), mRNA [NM_003279]	NM_003279
A_24_P706340	7.98E-03	3.40	L10374	L10374	Human (clone CTG-A4) mRNA sequence. [L10374]	
A_23_P16337	8.04E-03	2.24	NM_005500	NM_005500	Homo sapiens SUMO-1 activating enzyme subunit 1 (SAE1), mRNA [NM_005500]	NM_005500
A_23_P152136	8.04E-03	2.29	NM_022770	NM_022770	Homo sapiens hypothetical protein FLJ13912 (FLJ13912), mRNA [NM_022770]	NM_022770
A_32_P221256	8.06E-03	2.05	NM_203481	NM_203481	Homo sapiens hypothetical LOC403340 (MGC70870), mRNA [NM_203481]	NM_203481
A_23_P134237	8.07E-03	3.66	NM_002889	NM_002889	Homo sapiens retinoic acid receptor responder (tazarotene induced) 2 (RARRES2), mRNA [NM_002889]	NM_002889
A_24_P100351	8.08E-03	2.77	NM_019067	NM_019067	Homo sapiens guanine nucleotide binding protein-like 3 (nucleolar)-like (GNL3L), mRNA [NM_019067]	NM_019067
A_24_P213325	8.09E-03	7.19	A_24_P213325			
A_23_P93282	8.10E-03	2.44	NM_003535	NM_003535	Homo sapiens histone 1, H3j (HIST1H3J), mRNA [NM_003535]	NM_003535
A_23_P365060	8.10E-03	5.92	NM_014611	NM_014611	Homo sapiens MDN1, midasin homolog (yeast) (MDN1), mRNA [NM_014611]	NM_014611
A_24_P307580	8.11E-03	4.63	AF092095	AF092095	Homo sapiens alternatively spliced product of metastasis-suppressor gene CC3 (TC3) mRNA, complete cds. [AF092095]	
A_23_P162970	8.12E-03	2.15	NM_024658	NM_024658	Homo sapiens importin 4 (IPO4), mRNA [NM_024658]	NM_024658
A_23_P309545	8.12E-03	2.78	NM_006879	NM_006879	Homo sapiens Mdm2, transformed 3T3 cell double minute 2, p53 binding protein (mouse) (MDM2), transcript variant MDM2b, mRNA [NM_006879]	NM_006879
A_23_P30805	8.13E-03	3.70	NM_021968	NM_021968	Homo sapiens histone 1, H4j (HIST1H4J), mRNA [NM_021968]	NM_021968
A_24_P93948	8.13E-03	7.63	BC035285	BC035285	Homo sapiens cDNA clone IMAGE:5197468, with apparent retained intron. [BC035285]	
A_23_P70417	8.14E-03	2.25	NM_018141	NM_018141	Homo sapiens mitochondrial ribosomal protein S10 (MRPS10), nuclear gene encoding mitochondrial protein, mRNA [NM_018141]	NM_018141
A_23_P134147	8.14E-03	2.57	NM_014797	NM_014797	Homo sapiens zinc finger and BTB domain containing 24 (ZBTB24), mRNA [NM_014797]	NM_014797
A_23_P129301	8.15E-03	2.86	NM_018097	NM_018097	Homo sapiens chromosome 15 open reading frame 25 (C15orf25), mRNA [NM_018097]	NM_018097
A_24_P165423	8.16E-03	2.88	NM_052960	NM_052960	Homo sapiens retinol binding protein 7, cellular (RBP7), mRNA [NM_052960]	NM_052960
A_32_P113736	8.17E-03	2.02	THC2282618			
A_23_P92132	8.19E-03	2.17	NM_006764	NM_006764	Homo sapiens interferon-related developmental regulator 2 (IFRD2), mRNA [NM_006764]	NM_006764
A_23_P324453	8.19E-03	2.24	NM_004876	NM_004876	Homo sapiens zinc finger protein 254 (ZNF254), mRNA [NM_004876]	NM_004876
A_23_P87049	8.19E-03	4.31	NM_003105	NM_003105	Homo sapiens sortilin-related receptor, L(DLR class) A repeats-containing (SORL1), mRNA [NM_003105]	NM_003105
A_23_P74269	8.22E-03	2.04	NM_003132	NM_003132	Homo sapiens spermidine synthase (SRM), mRNA [NM_003132]	NM_003132
A_23_P120667	8.22E-03	2.36	NM_021219	NM_021219	Homo sapiens junctional adhesion molecule 2 (JAM2), mRNA [NM_021219]	NM_021219
A_23_P35656	8.22E-03	2.48	BC003519	BC003519	Homo sapiens weakly similar to zinc finger protein 195, mRNA (cDNA clone IMAGE:3606289), partial cds. [BC003519]	
A_24_P220897	8.22E-03	3.61	ENST00000302091		Homo sapiens cDNA FLJ25874 fis, clone CBR02446. [AK098740]	XM_114430

A_23_P127033	8.22E-03	8.13	NM_024693	NM_024693	Homo sapiens enoyl Coenzyme A hydratase domain containing 3 (ECHDC3), mRNA [NM_024693]	NM_024693
A_23_P140807	8.26E-03	2.04	NM_002801	NM_002801	Homo sapiens proteasome (prosome, macropain) subunit, beta type, 10 (PSMB10), mRNA [NM_002801]	NM_002801
A_23_P501276	8.28E-03	2.66	NM_001069	NM_001069	Homo sapiens tubulin, beta 2 (TUBB2), mRNA [NM_001069]	NM_001069
A_32_P146815	8.28E-03	3.06	BC062473	BC062473	Homo sapiens cDNA clone IMAGE:30374677, partial cds. [BC062473]	
A_23_P201238	8.28E-03	3.37	NM_030918	NM_030918	Homo sapiens sorting nexin family member 27 (SNX27), mRNA [NM_030918]	NM_030918
A_24_P557534	8.29E-03	3.91	ENST00000361789		Homo sapiens clone 35w unknown mRNA; mitochondrial gene for mitochondrial product. [AF391805]	
A_32_P169142	8.32E-03	2.19	A_32_P169142			
A_24_P210513	8.33E-03	2.34	NM_018167	NM_018167	Homo sapiens BTB (POZ) domain containing 7 (BTBD7), transcript variant 2, mRNA [NM_018167]	NM_018167
A_23_P51966	8.33E-03	2.37	A_23_P51966			
A_23_P10701	8.33E-03	2.96	NM_173075	NM_173075	Homo sapiens amyloid beta (A4) precursor protein-binding, family B, member 2 (Fe65-like) (APBB2), mRNA [NM_173075]	NM_173075
A_23_P41280	8.34E-03	2.18	NM_006452	NM_006452	Homo sapiens phosphoribosylaminoimidazole carboxylase, phosphoribosylaminoimidazole succinocarboxamide synthetase (PAICS), mRNA [NM_006452]	NM_006452
A_23_P211326	8.34E-03	3.97	AK091754	AK091754	Homo sapiens cDNA FLJ34435 fis, clone HLUNG2000955. [AK091754]	
A_24_P297098	8.35E-03	2.02	NM_016436	NM_016436	Homo sapiens PHD finger protein 20 (PHF20), mRNA [NM_016436]	NM_016436
A_23_P53603	8.35E-03	2.09	NM_001681	NM_001681	Homo sapiens ATPase, Ca++ transporting, cardiac muscle, slow twitch 2 (ATP2A2), transcript variant 2, mRNA [NM_001681]	NM_001681
A_24_P177631	8.35E-03	3.85	AL832120	AL832120	Homo sapiens mRNA; cDNA DKFZp686B2110 (from clone DKFZp686B2110). [AL832120]	XM_291344
A_24_P668974	8.35E-03	38.02	CD048206	CD048206	AGENCOURT_13966160 NIH_MGC_172 Homo sapiens cDNA 5', mRNA sequence [CD048206]	
A_32_P143057	8.36E-03	2.27	AI267511	AI267511	AI267511 aq65g08.x1 Stanley Frontal SN pool 2 Homo sapiens cDNA clone IMAGE:2035838 similar to gb:X16560 CYTOCHROME C OXIDASE POLYPEPTIDE VIIC PRECURSOR (HUMAN);, mRNA sequence [AI267511]	
A_24_P392723	8.36E-03	3.19	BC033082	BC033082	Homo sapiens KIAA1922 protein, mRNA (cDNA clone MGC:45647 IMAGE:2960715), complete cds. [BC033082]	XM_057040
A_23_P367043	8.37E-03	2.24	ENST00000324993		Homo sapiens CDC14 cell division cycle 14 homolog C (S. cerevisiae), mRNA (cDNA clone IMAGE:4826219), partial cds. [BC028690]	
A_24_P4678	8.38E-03	2.08	NM_014384	NM_014384	Homo sapiens acyl-Coenzyme A dehydrogenase family, member 8 (ACAD8), mRNA [NM_014384]	NM_014384
A_24_P856273	8.38E-03	3.38	A_24_P856273			
A_23_P46333	8.39E-03	2.27	NM_007358	NM_007358	Homo sapiens metal response element binding transcription factor 2 (MTF2), mRNA [NM_007358]	NM_007358
A_23_P126825	8.39E-03	2.40	NM_003051	NM_003051	Homo sapiens solute carrier family 16 (monocarboxylic acid transporters), member 1 (SLC16A1), mRNA [NM_003051]	NM_003051
A_23_P436281	8.39E-03	4.07	NM_003548	NM_003548	Homo sapiens histone 2, H4 (HIST2H4), mRNA [NM_003548]	NM_003548
A_23_P3885	8.40E-03	2.18	NM_024109	NM_024109	Homo sapiens hypothetical protein MGC2654 (MGC2654), mRNA [NM_024109]	NM_024109
A_23_P25534	8.41E-03	2.14	THC2310223		Q5VW08 (Q5VW08) OTTHUMP00000042245, partial (19%) [THC2310223]	
A_32_P22622	8.42E-03	2.23	NM_003703	NM_003703	Homo sapiens chromosome 4 open reading frame 9 (C4orf9), mRNA [NM_003703]	NM_003703
A_23_P34877	8.45E-03	2.07	NM_022768	NM_022768	Homo sapiens RNA binding motif protein 15 (RBM15), mRNA [NM_022768]	NM_022768
A_23_P309701	8.45E-03	2.76	NM_002828	NM_002828	Homo sapiens protein tyrosine phosphatase, non-receptor type 2 (PTPN2), transcript variant 1, mRNA [NM_002828]	NM_002828
A_24_P571864	8.47E-03	4.55	A_24_P571864			
A_23_P159741	8.48E-03	2.13	NM_017745	NM_017745	Homo sapiens BCL6 co-repressor (BCOR), transcript variant 1, mRNA [NM_017745]	NM_017745
A_23_P66872	8.49E-03	2.06	NM_015721	NM_015721	Homo sapiens gem (nuclear organelle) associated protein 4 (GEMIN4), mRNA [NM_015721]	NM_015721
A_24_P524452	8.49E-03	2.16	CA310244	CA310244	UI-H-FT1-big-b-11-0-UI.s1 NCI_CGAP_FT1 Homo sapiens cDNA clone UI-H-FT1-big-b-11-0-UI 3', mRNA sequence [CA310244]	
A_24_P913828	8.49E-03	2.17	M55405	M55405	Homo sapiens mucin (MUC-3) mRNA, partial cds. [M55405]	
A_24_P750636	8.49E-03	2.37	THC2267012			
A_24_P246863	8.49E-03	2.45	A_24_P246863			
A_23_P343963	8.49E-03	2.71	NM_138435	NM_138435	Homo sapiens family with sequence similarity 83, member F (FAM83F), mRNA [NM_138435]	NM_138435
A_24_P179339	8.50E-03	6.02	AY029066	AY029066	Homo sapiens Humanin (HN1) mRNA, complete cds. [AY029066]	
A_23_P209449	8.52E-03	3.32	NM_003507	NM_003507	Homo sapiens frizzled homolog 7 (Drosophila) (FZD7), mRNA [NM_003507]	NM_003507

A_24_P917836	8.53E-03	2.17	AF034187	AF034187	Homo sapiens clone 2.2H12 Ndr Ser/Thr kinase-like protein mRNA, partial cds. [AF034187]	
A_24_P153643	8.53E-03	6.41	NM_004947	NM_004947	Homo sapiens dedicator of cytokinesis 3 (DOCK3), mRNA [NM_004947]	NM_004947
A_23_P358417	8.55E-03	2.59	NM_004085	NM_004085	Homo sapiens translocase of inner mitochondrial membrane 8 homolog A (yeast) (TIMM8A), nuclear gene encoding mitochondrial protein, mRNA [NM_004085]	NM_004085
A_24_P127701	8.55E-03	2.99	XM_378015	XM_378015	PREDICTED: Homo sapiens similar to chromosome 11 open reading frame2; chromosome 11 open reading frame2 (LOC402330), mRNA [XM_378015]	XM_378015
A_23_P25019	8.55E-03	3.57	NM_000946	NM_000946	Homo sapiens primase, polypeptide 1, 49kDa (PRIM1), mRNA [NM_000946]	NM_000946
A_24_P943922	8.56E-03	2.04	NM_020925	NM_020925	Homo sapiens von Willebrand factor type A and cache domain containing 1 (VWCD1), mRNA [NM_020925]	NM_020925
A_23_P164415	8.56E-03	2.83	AK000760	AK000760	Homo sapiens cDNA FLJ20753 fis, clone HEP02714. [AK000760]	
A_23_P219161	8.56E-03	6.71	NM_006334	NM_006334	Homo sapiens olfactomedin 1 (OLFM1), transcript variant 2, mRNA [NM_006334]	NM_006334
A_23_P7402	8.58E-03	2.24	NM_178140	NM_178140	Homo sapiens PDZ domain containing 3 (PDZK3), transcript variant 1, mRNA [NM_178140]	NM_178140
A_24_P594094	8.60E-03	2.03	A_24_P594094			
A_23_P158148	8.62E-03	2.01	NM_001762	NM_001762	Homo sapiens chaperonin containing TCP1, subunit 6A (zeta 1) (CCT6A), transcript variant 1, mRNA [NM_001762]	NM_001762
A_23_P204033	8.62E-03	2.15	NM_007007	NM_007007	Homo sapiens cleavage and polyadenylation specific factor 6, 68kDa (CPSF6), mRNA [NM_007007]	NM_007007
A_23_P33303	8.62E-03	2.17	NM_005026	NM_005026	Homo sapiens phosphoinositide-3-kinase, catalytic, delta polypeptide (PIK3CD), mRNA [NM_005026]	NM_005026
A_24_P175589	8.62E-03	3.12	BC003519	BC003519	Homo sapiens weakly similar to zinc finger protein 195, mRNA (cDNA clone IMAGE:3606289), partial cds. [BC003519]	
A_23_P7752	8.62E-03	7.63	NM_020796	NM_020796	Homo sapiens sema domain, transmembrane domain (TM), and cytoplasmic domain, (semaphorin) 6A (SEMA6A), mRNA [NM_020796]	NM_020796
A_32_P221569	8.63E-03	2.05	BM989272	BM989272	BM989272 UI-H-DP0-ats-f-05-0-UI.s1 NCI_CGAP_Fs1 Homo sapiens cDNA clone IMAGE:5863684 3', mRNA sequence [BM989272]	
A_32_P5673	8.63E-03	3.18	BG336702	BG336702	602405126F1 NIH_MGC_21 Homo sapiens cDNA clone IMAGE:4542655 5', mRNA sequence [BG336702]	
A_32_P70519	8.63E-03	4.42	BP420721	BP420721	BP420721 Homo sapiens small intestine Homo sapiens cDNA clone HIE04034r 3', mRNA sequence [BP420721]	
A_24_P49597	8.64E-03	2.54	ENST00000299756		PREDICTED: Homo sapiens similar to Chain A, Crystal Structure Of The R463a Mutant Of Human Glutamate Dehydrogenase (LOC390859), mRNA [XM_372695]	XM_372695
A_32_P74847	8.64E-03	38.02	CD048206	CD048206	AGENCOURT_13966160 NIH_MGC_172 Homo sapiens cDNA 5', mRNA sequence [CD048206]	
A_24_P42453	8.66E-03	5.13	BC000845	BC000845	Homo sapiens cDNA clone IMAGE:3457769, partial cds. [BC000845]	
A_23_P35148	8.68E-03	3.31	NM_005645	NM_005645	Homo sapiens TAF13 RNA polymerase II, TATA box binding protein (TBP)-associated factor, 18kDa (TAF13), mRNA [NM_005645]	NM_005645
A_32_P203786	8.69E-03	5.43	BC071630	BC071630	Homo sapiens cDNA clone IMAGE:4476024, partial cds. [BC071630]	
A_23_P325547	8.70E-03	2.02	NM_020175	NM_020175	Homo sapiens dihydrouridine synthase 3-like (S. cerevisiae) (DUS3L), mRNA [NM_020175]	NM_020175
A_24_P945181	8.70E-03	2.22	NM_013286	NM_013286	Homo sapiens RNA binding motif protein 15B (RBM15B), mRNA [NM_013286]	NM_013286
A_23_P254688	8.74E-03	2.21	NM_023943	NM_023943	Homo sapiens hypothetical protein MGC3040 (MGC3040), mRNA [NM_023943]	NM_023943
A_24_P926053	8.75E-03	2.45	AY358690	AY358690	Homo sapiens clone DNA62876 LPPA601 (UNQ601) mRNA, complete cds. [AY358690]	
A_23_P370569	8.75E-03	2.55	NM_152440	NM_152440	Homo sapiens hypothetical protein FLJ32549 (FLJ32549), mRNA [NM_152440]	NM_152440
A_24_P734720	8.75E-03	5.41	NM_152523	NM_152523	Homo sapiens hypothetical protein FLJ40432 (FLJ40432), mRNA [NM_152523]	NM_152523
A_23_P92140	8.76E-03	2.31	NM_002468	NM_002468	Homo sapiens myeloid differentiation primary response gene (88) (MYD88), mRNA [NM_002468]	NM_002468
A_23_P131227	8.76E-03	2.34	NM_017735	NM_017735	Homo sapiens hypothetical protein FLJ20272 (FLJ20272), mRNA [NM_017735]	NM_017735
A_24_P197964	8.76E-03	2.58	NM_014788	NM_014788	Homo sapiens tripartite motif-containing 14 (TRIM14), transcript variant 1, mRNA [NM_014788]	NM_014788
A_24_P287941	8.79E-03	2.01	NM_013290	NM_013290	Homo sapiens TBP-1 interacting protein (TBPIP), transcript variant 1, mRNA [NM_013290]	NM_013290
A_24_P479065	8.79E-03	2.10	THC2306368		Q9UND3 (Q9UND3) Nuclear pore complex interacting protein NPIP, partial (40%) [THC2306368]	
A_23_P256855	8.79E-03	2.99	NM_020750	NM_020750	Homo sapiens exportin 5 (XPO5), mRNA [NM_020750]	NM_020750
A_32_P85433	8.79E-03	4.31	BQ130701	BQ130701	BQ130701 ij86c06.y1 Human insulinoma Homo sapiens cDNA clone IMAGE:5778418 5' similar to SW:CTGF_HUMAN P29279 CONNECTIVE TISSUE GROWTH FACTOR PRECURSOR. [1] ;, mRNA sequence [BQ130701]	
A_24_P41339	8.80E-03	2.39	A_24_P41339			
A_23_P50376	8.80E-03	2.91	NM_018555	NM_018555	Homo sapiens zinc finger protein 331 (ZNF331), mRNA [NM_018555]	NM_018555

A_23_P19192	8.81E-03	2.25	NM_006330	NM_006330	Homo sapiens lysophospholipase I (LYPLA1), mRNA [NM_006330]	NM_006330
A_32_P178537	8.81E-03	4.98	G36631	G36631	SHGC-53577 Human Homo sapiens STS cDNA, sequence tagged site. [G36631]	
A_23_P200901	8.82E-03	2.26	NM_004436	NM_004436	Homo sapiens endosulfine alpha (ENSA), transcript variant 3, mRNA [NM_004436]	NM_004436
A_23_P206474	8.82E-03	2.56	NM_032271	NM_032271	Homo sapiens TNF receptor-associated factor 7 (TRAF7), transcript variant 1, mRNA [NM_032271]	NM_032271
A_23_P142918	8.82E-03	3.29	NM_153689	NM_153689	Homo sapiens hypothetical protein FLJ38973 (FLJ38973), mRNA [NM_153689]	NM_153689
A_32_P24762	8.86E-03	2.48	THC2272599		CA438893 UI-H-DH0-aut-f-13-0-UI.s1 NCI_CGAP_DH0 Homo sapiens cDNA clone UI-H-DH0-aut-f-13-0-UI 3', mRNA sequence [CA438893]	
A_23_P166508	8.87E-03	8.62	BC038245	BC038245	Homo sapiens, clone IMAGE:5241654, mRNA. [BC038245]	XM_086879
A_23_P7941	8.88E-03	2.24	NM_015950	NM_015950	Homo sapiens mitochondrial ribosomal protein L2 (MRPL2), nuclear gene encoding mitochondrial protein, mRNA [NM_015950]	NM_015950
A_24_P367259	8.89E-03	2.32	A_24_P367259			
A_24_P332532	8.92E-03	2.87	A_24_P332532			
A_24_P345131	8.92E-03	4.18	D86984	D86984	Human mRNA for KIAA0231 gene, partial cds. [D86984]	
A_24_P148503	8.92E-03	5.08	AK024850	AK024850	Homo sapiens cDNA: FLJ21197 fis, clone COL00201. [AK024850]	
A_24_P915007	8.93E-03	2.00	NM_052876	NM_052876	Homo sapiens BTB (POZ) domain containing 14B (BTBD14B), mRNA [NM_052876]	NM_052876
A_24_P213794	8.93E-03	3.27	NM_012118	NM_012118	Homo sapiens CCR4 carbon catabolite repression 4-like (S. cerevisiae) (CCRN4L), mRNA [NM_012118]	NM_012118
A_32_P139654	8.95E-03	2.16	NM_198935	NM_198935	Homo sapiens synovial sarcoma translocation gene on chromosome 18-like 1 (SS18L1), transcript variant 1, mRNA [NM_198935]	NM_198935
A_24_P71834	8.97E-03	2.12	NM_016141	NM_016141	Homo sapiens dynein, cytoplasmic, light intermediate polypeptide 1 (DNCLI1), mRNA [NM_016141]	NM_016141
A_24_P15621	8.97E-03	2.41	NM_198857	NM_198857	Homo sapiens similar to sodium- and chloride-dependent creatine transporter (FLJ43855), mRNA [NM_198857]	NM_198857
A_23_P15603	8.98E-03	2.98	NM_024864	NM_024864	Homo sapiens hypothetical protein FLJ22578 (FLJ22578), mRNA [NM_024864]	NM_024864
A_23_P49865	8.98E-03	3.03	NM_015982	NM_015982	Homo sapiens germ cell specific Y-box binding protein (YBX2), mRNA [NM_015982]	NM_015982
A_23_P68786	8.98E-03	3.24	NM_016335	NM_016335	Homo sapiens proline dehydrogenase (oxidase) 1 (PRODH), nuclear gene encoding mitochondrial protein, mRNA [NM_016335]	NM_016335
A_32_P49764	8.98E-03	10.50	THC2394165		DBP_HUMAN (Q10586) D-site-binding protein (Albumin D box-binding protein) (TAXREB302), partial (6%) [THC2394165]	
A_23_P501080	8.99E-03	2.26	NM_007139	NM_007139	Homo sapiens zinc finger protein 92 (HTF12) (ZNF92), mRNA [NM_007139]	NM_007139
A_24_P263036	9.01E-03	3.29	NM_003748	NM_003748	Homo sapiens aldehyde dehydrogenase 4 family, member A1 (ALDH4A1), nuclear gene encoding mitochondrial protein, transcript variant P5CDhL, mRNA [NM_003748]	NM_003748
A_23_P90041	9.01E-03	5.49	NM_033297	NM_033297	Homo sapiens NACHT, leucine rich repeat and PYD containing 12 (NALP12), transcript variant 1, mRNA [NM_033297]	NM_033297
A_32_P187245	9.03E-03	2.46	AA669846	AA669846	AA669846 ag36h02.s1 Human bone marrow stromal cells Homo sapiens cDNA clone IMAGE:1118931 3', mRNA sequence [AA669846]	
A_24_P357169	9.03E-03	2.60	NM_031308	NM_031308	Homo sapiens epiplakin 1 (EPPK1), mRNA [NM_031308]	NM_031308
A_32_P61729	9.06E-03	2.26	NM_201430	NM_201430	Homo sapiens reticulon 3 (RTN3), transcript variant 4, mRNA [NM_201430]	NM_201430
A_24_P336754	9.07E-03	2.10	NM_021960	NM_021960	Homo sapiens myeloid cell leukemia sequence 1 (BCL2-related) (MCL1), transcript variant 1, mRNA [NM_021960]	NM_021960
A_32_P132438	9.08E-03	2.24	NM_139275	NM_139275	Homo sapiens A kinase (PRKA) anchor protein 1 (AKAP1), nuclear gene encoding mitochondrial protein, transcript variant 2, mRNA [NM_139275]	NM_139275
A_23_P100632	9.08E-03	2.32	NM_001002033	NM_001002033	Homo sapiens hematological and neurological expressed 1 (HN1), transcript variant 3, mRNA [NM_001002033]	NM_001002033
A_24_P136653	9.08E-03	14.04	NM_004289	NM_004289	Homo sapiens nuclear factor (erythroid-derived 2)-like 3 (NFE2L3), mRNA [NM_004289]	NM_004289
A_23_P35099	9.09E-03	2.52	NM_016183	NM_016183	Homo sapiens chromosome 1 open reading frame 33 (C1orf33), mRNA [NM_016183]	NM_016183
A_32_P205329	9.10E-03	2.38	AF088007	AF088007	Homo sapiens full length insert cDNA clone YY74A01. [AF088007]	
A_23_P9894	9.11E-03	2.21	NM_005788	NM_005788	Homo sapiens HMT1 hnRNP methyltransferase-like 3 (S. cerevisiae) (HRMT1L3), mRNA [NM_005788]	NM_005788
A_23_P103476	9.12E-03	2.04	NM_013319	NM_013319	Homo sapiens UbiA prenyltransferase domain containing 1 (UBIAD1), mRNA [NM_013319]	NM_013319
A_23_P132863	9.12E-03	2.24	ENST00000306024		Homo sapiens MDS017 (MDS017) mRNA, complete cds. [AF182418]	
A_32_P225625	9.13E-03	2.58	AA971667	AA971667	AA971667 op85c06.s1 Soares_NFL_T_GBC_S1 Homo sapiens cDNA clone IMAGE:1583626 3', mRNA sequence [AA971667]	
A_32_P235727	9.21E-03	2.09	A_32_P235727			

A_24_P679409	9.22E-03	2.03	NM_005839	NM_005839	Homo sapiens serine/arginine repetitive matrix 1 (SRRM1), mRNA [NM_005839]	NM_005839
A_24_P178963	9.23E-03	2.02	ENST00000332844		PREDICTED: Homo sapiens similar to unc-93 homolog B1; unc93 (C.elegans) homolog B; unc-93 related protein; unc93 (C. elegans) homolog B1 (LOC285479), mRNA [XM_211908]	XM_211908
A_23_P17354	9.24E-03	2.82	NM_024034	NM_024034	Homo sapiens ganglioside-induced differentiation-associated protein 1-like 1 (GDAP1L1), mRNA [NM_024034]	NM_024034
A_24_P417984	9.25E-03	2.05	NM_138689	NM_138689	Homo sapiens protein phosphatase 1, regulatory (inhibitor) subunit 14B (PPP1R14B), mRNA [NM_138689]	NM_138689
A_24_P23522	9.25E-03	2.24	NM_182529	NM_182529	Homo sapiens THAP domain containing 5 (THAP5), mRNA [NM_182529]	NM_182529
A_23_P206612	9.25E-03	2.34	AK126447	AK126447	Homo sapiens cDNA FLJ44483 fis, clone UTERU2033375. [AK126447]	
A_24_P919460	9.25E-03	2.72	AB065089	AB065089	Homo sapiens OK/KNS-cl.7 mRNA for ribosomal protein S2, complete cds. [AB065089]	
A_24_P410605	9.26E-03	2.30	BC080541	BC080541	Homo sapiens receptor tyrosine kinase-like orphan receptor 1, mRNA (cDNA clone IMAGE:5477978), complete cds. [BC080541]	
A_32_P167577	9.32E-03	2.24	A_32_P167577			
A_32_P148672	9.34E-03	2.44	NM_006938	NM_006938	Homo sapiens small nuclear ribonucleoprotein D1 polypeptide 16kDa (SNRPD1), mRNA [NM_006938]	NM_006938
A_32_P167631	9.34E-03	2.81	THC2320257			
A_23_P36464	9.34E-03	2.94	NM_018164	NM_018164	Homo sapiens chromosome 12 open reading frame 11 (C12orf11), mRNA [NM_018164]	NM_018164
A_32_P112104	9.34E-03	4.26	NM_003074	NM_003074	Homo sapiens SWI/SNF related, matrix associated, actin dependent regulator of chromatin, subfamily c, member 1 (SMARCC1), mRNA [NM_003074]	NM_003074
A_24_P380629	9.37E-03	2.38	NM_007189	NM_007189	Homo sapiens ATP-binding cassette, sub-family F (GCN20), member 2 (ABCF2), nuclear gene encoding mitochondrial protein, transcript variant 1, mRNA [NM_007189]	NM_007189
A_32_P141238	9.40E-03	4.72	NM_020373	NM_020373	Homo sapiens transmembrane protein 16B (TMEM16B), mRNA [NM_020373]	NM_020373
A_24_P172993	9.43E-03	2.16	NM_022066	NM_022066	Homo sapiens likely ortholog of mouse ubiquitin-conjugating enzyme E2-230K (E2-230K), mRNA [NM_022066]	NM_022066
A_23_P8649	9.45E-03	2.98	NM_152789	NM_152789	Homo sapiens hypothetical protein MGC40405 (MGC40405), mRNA [NM_152789]	NM_152789
A_24_P258235	9.47E-03	2.24	NM_001004739	NM_001004739	Homo sapiens olfactory receptor, family 5, subfamily L, member 2 (OR5L2), mRNA [NM_001004739]	NM_001004739
A_23_P27167	9.49E-03	2.05	NM_002936	NM_002936	Homo sapiens ribonuclease H1 (RNASEH1), mRNA [NM_002936]	NM_002936
A_23_P502488	9.49E-03	2.15	NM_032852	NM_032852	Homo sapiens APG4 autophagy 4 homolog C (S. cerevisiae) (APG4C), transcript variant 1, mRNA [NM_032852]	NM_032852
A_23_P148475	9.51E-03	2.32	NM_012310	NM_012310	Homo sapiens kinesin family member 4A (KIF4A), mRNA [NM_012310]	NM_012310
A_23_P59138	9.51E-03	50.00	NM_002701	NM_002701	Homo sapiens POU domain, class 5, transcription factor 1 (POU5F1), transcript variant 1, mRNA [NM_002701]	NM_002701
A_24_P38944	9.52E-03	2.74	NM_024098	NM_024098	Homo sapiens hypothetical protein MGC2574 (MGC2574), mRNA [NM_024098]	NM_024098
A_23_P405885	9.53E-03	3.82	NM_138815	NM_138815	Homo sapiens developmental pluripotency associated 2 (DPPA2), mRNA [NM_138815]	NM_138815
A_23_P171385	9.56E-03	8.13	NM_032335	NM_032335	Homo sapiens PHD finger protein 6 (PHF6), transcript variant 3, mRNA [NM_032335]	NM_032335
A_23_P209962	9.57E-03	2.61	NM_024624	NM_024624	Homo sapiens SMC6 structural maintenance of chromosomes 6-like 1 (yeast) (SMC6L1), mRNA [NM_024624]	NM_024624
A_23_P215658	9.58E-03	2.01	NM_030900	NM_030900	Homo sapiens transforming growth factor beta regulator 4 (TBRG4), transcript variant 2, mRNA [NM_030900]	NM_030900
A_23_P5611	9.58E-03	2.08	NM_018151	NM_018151	Homo sapiens RAP1 interacting factor homolog (yeast) (RIF1), mRNA [NM_018151]	NM_018151
A_24_P739355	9.58E-03	2.18	THC2442931			
A_23_P127522	9.58E-03	2.54	NM_145014	NM_145014	Homo sapiens hydrolethalus syndrome 1 (HYLS1), mRNA [NM_145014]	NM_145014
A_23_P395426	9.58E-03	2.76	NM_022105	NM_022105	Homo sapiens death associated transcription factor 1 (DATF1), transcript variant 1, mRNA [NM_022105]	NM_022105
A_23_P406385	9.58E-03	5.46	NM_153350	NM_153350	Homo sapiens F-box and leucine-rich repeat protein 16 (FBXL16), mRNA [NM_153350]	NM_153350
A_23_P2262	9.61E-03	2.23	NM_004264	NM_004264	Homo sapiens SRB7 suppressor of RNA polymerase B homolog (yeast) (SURB7), mRNA [NM_004264]	NM_004264
A_23_P369701	9.64E-03	2.05	ENST00000258884		Homo sapiens hypothetical protein from EUROIMAGE 588495, mRNA (cDNA clone IMAGE:30346383), partial cds. [BC059401]	XM_051862
A_23_P393051	9.64E-03	2.56	NM_152365	NM_152365	Homo sapiens chromosome 1 open reading frame 172 (C1orf172), mRNA [NM_152365]	NM_152365
A_24_P860797	9.64E-03	4.10	ENST00000244221		Homo sapiens mRNA for KIAA1155 protein, partial cds. [AB032981]	XM_376062
A_24_P350656	9.64E-03	5.38	NM_001005389	NM_001005389	Homo sapiens neurofascin (NFASC), transcript variant 3, mRNA [NM_001005389]	NM_001005389
A_32_P3476	9.64E-03	6.71	NM_203400	NM_203400	Homo sapiens similar to candidate mediator of the p53-dependent G2 arrest (LOC388394), mRNA [NM_203400]	NM_203400

A_32_P36143	9.65E-03	2.22	CR602569	CR602569	full-length cDNA clone CS0DC001YL20 of Neuroblastoma Cot 25-normalized of Homo sapiens (human). [CR602569]	
A_24_P306788	9.68E-03	2.74	A_24_P306788			
A_23_P117882	9.68E-03	3.24	NM_004378	NM_004378	Homo sapiens cellular retinoic acid binding protein 1 (CRABP1), mRNA [NM_004378]	NM_004378
A_32_P148914	9.70E-03	2.22	BC037255	BC037255	Homo sapiens hypothetical LOC389634, mRNA (cDNA clone IMAGE:4157715). [BC037255]	
A_24_P490704	9.71E-03	3.00	THC2430670		ALU1_HUMAN (P39188) Alu subfamily J sequence contamination warning entry, partial (8%) [THC2430670]	
A_24_P32085	9.73E-03	2.20	NM_024761	NM_024761	Homo sapiens MOB1, Mps One Binder kinase activator-like 2B (yeast) (MOBKL2B), mRNA [NM_024761]	NM_024761
A_24_P489399	9.75E-03	2.19	A_24_P489399			
A_24_P20200	9.75E-03	2.48	NM_017958	NM_017958	Homo sapiens pleckstrin homology domain containing, family B (evectins) member 2 (PLEKHB2), mRNA [NM_017958]	NM_017958
A_23_P209269	9.76E-03	2.69	NM_002706	NM_002706	Homo sapiens protein phosphatase 1B (formerly 2C), magnesium-dependent, beta isoform (PPM1B), transcript variant 1, mRNA [NM_002706]	NM_002706
A_23_P334282	9.77E-03	2.06	NM_017593	NM_017593	Homo sapiens BMP2 inducible kinase (BMP2K), transcript variant 2, mRNA [NM_017593]	NM_017593
A_23_P302709	9.77E-03	3.23	NM_005126	NM_005126	Homo sapiens nuclear receptor subfamily 1, group D, member 2 (NR1D2), mRNA [NM_005126]	NM_005126
A_23_P119344	9.82E-03	2.68	NM_003598	NM_003598	Homo sapiens TEA domain family member 2 (TEAD2), mRNA [NM_003598]	NM_003598
A_23_P153266	9.84E-03	2.30	NM_006114	NM_006114	Homo sapiens translocase of outer mitochondrial membrane 40 homolog (yeast) (TOMM40), mRNA [NM_006114]	NM_006114
A_24_P4171	9.86E-03	3.47	NM_023111	NM_023111	Homo sapiens fibroblast growth factor receptor 1 (fms-related tyrosine kinase 2, Pfeiffer syndrome) (FGFR1), transcript variant 9, mRNA [NM_023111]	NM_023111
A_32_P112380	9.88E-03	2.54	NM_178518	NM_178518	Homo sapiens hypothetical protein FLJ36878 (FLJ36878), mRNA [NM_178518]	NM_178518
A_23_P121447	9.91E-03	3.09	NM_032487	NM_032487	Homo sapiens actin related protein M1 (ARPM1), mRNA [NM_032487]	NM_032487
A_32_P98979	9.91E-03	15.41	THC2281332			
A_24_P200603	9.92E-03	2.32	NM_032151	NM_032151	Homo sapiens 6-pyruvoyl-tetrahydropterin synthase/dimerization cofactor of hepatocyte nuclear factor 1 alpha (TCF1) 2 (PCBD2), mRNA [NM_032151]	NM_032151
A_24_P330625	9.93E-03	2.75	NM_032815	NM_032815	Homo sapiens nuclear factor of activated T-cells, cytoplasmic, calcineurin-dependent 2 interacting protein (NFATC2IP), mRNA [NM_032815]	NM_032815
A_32_P36412	9.94E-03	2.16	A_32_P36412			
A_24_P289170	9.97E-03	2.16	ENST00000282163		full-length cDNA clone CS0DK012YL12 of HeLa cells Cot 25-normalized of Homo sapiens (human). [CR606047]	XM_496003
A_23_P203957	9.98E-03	2.85	NM_031920	NM_031920	Homo sapiens ARG99 protein (ARG99), transcript variant 1, mRNA [NM_031920]	NM_031920
A_23_P39263	9.99E-03	2.60	NM_173480	NM_173480	Homo sapiens hypothetical protein LOC126295 (LOC126295), mRNA [NM_173480]	NM_173480
A_24_P929745	1.00E-02	2.82	AK021842	AK021842	Homo sapiens cDNA FLJ11780 fis, clone HEMBA1005931, weakly similar to ZINC FINGER PROTEIN 83. [AK021842]	
A_23_P380839	1.00E-02	3.02	NM_020193	NM_020193	Homo sapiens chromosome 11 open reading frame 30 (C11orf30), mRNA [NM_020193]	NM_020193
A_23_P62021	1.00E-02	5.15	L12350	L12350	Human thrombospondin 2 (THBS2) mRNA, complete cds. [L12350]	
A_23_P201939	1.01E-02	2.24	NM_005167	NM_005167	Homo sapiens protein phosphatase 1J (PP2C domain containing) (PPM1J), mRNA [NM_005167]	NM_005167
A_24_P335221	1.01E-02	2.33	NM_201430	NM_201430	Homo sapiens reticulon 3 (RTN3), transcript variant 4, mRNA [NM_201430]	NM_201430
A_23_P433504	1.01E-02	2.87	AK091315	AK091315	Homo sapiens cDNA FLJ33996 fis, clone DFNES2008881. [AK091315]	
A_23_P127805	1.01E-02	3.06	THC2438100		ALU2_HUMAN (P39189) Alu subfamily SB sequence contamination warning entry, partial (6%) [THC2438100]	
A_24_P165259	1.01E-02	3.28	NM_013328	NM_013328	Homo sapiens pyrroline-5-carboxylate reductase family, member 2 (PYCR2), mRNA [NM_013328]	NM_013328
A_24_P260443	1.01E-02	5.49	NM_003248	NM_003248	Homo sapiens thrombospondin 4 (THBS4), mRNA [NM_003248]	NM_003248
A_23_P396765	1.01E-02	7.35	NM_173582	NM_173582	Homo sapiens phosphoglucomutase 2-like 1 (PGM2L1), mRNA [NM_173582]	NM_173582
A_24_P139152	1.01E-02	7.63	AL359062	AL359062	Homo sapiens mRNA full length insert cDNA clone EUROIMAGE 1913076. [AL359062]	
A_24_P324488	1.02E-02	2.01	A_24_P324488			
A_23_P164000	1.02E-02	2.04	NM_015670	NM_015670	Homo sapiens SUMO1/sentrin/SMT3 specific protease 3 (SENP3), mRNA [NM_015670]	NM_015670
A_23_P428326	1.02E-02	2.29	AK000755	AK000755	Homo sapiens cDNA FLJ20748 fis, clone HEP05772. [AK000755]	
A_24_P6903	1.02E-02	2.33	NM_001017992	NM_001017992	Homo sapiens similar to RIKEN cDNA 4732495G21 gene (DKFZp686D0972), mRNA [NM_001017992]	NM_001017992
A_23_P426021	1.02E-02	3.25	NM_015187	NM_015187	Homo sapiens KIAA0746 protein (KIAA0746), mRNA [NM_015187]	NM_015187
A_23_P82478	1.03E-02	2.00	NM_019042	NM_019042	Homo sapiens hypothetical protein FLJ20485 (FLJ20485), mRNA [NM_019042]	NM_019042

A_24_P375132	1.03E-02	2.02	A_24_P375132			
A_23_P394567	1.03E-02	2.04	NM_020853	NM_020853	Homo sapiens KIAA1467 protein (KIAA1467), mRNA [NM_020853]	NM_020853
A_23_P360769	1.03E-02	2.33	NM_002372	NM_002372	Homo sapiens mannosidase, alpha, class 2A, member 1 (MAN2A1), mRNA [NM_002372]	NM_002372
A_24_P727884	1.03E-02	2.34	U10991	U10991	Human G2 protein mRNA, partial cds. [U10991]	XM_039515
A_32_P4018	1.03E-02	2.58	AK000776	AK000776	Homo sapiens cDNA FLJ20769 fis, clone COL06674. [AK000776]	
A_23_P20337	1.03E-02	2.83	NM_001386	NM_001386	Homo sapiens dihydropyrimidinase-like 2 (DPYSL2), mRNA [NM_001386]	NM_001386
A_23_P251421	1.03E-02	2.85	NM_031942	NM_031942	Homo sapiens cell division cycle associated 7 (CDCA7), transcript variant 1, mRNA [NM_031942]	NM_031942
A_23_P30813	1.03E-02	3.00	NM_003541	NM_003541	Homo sapiens histone 1, H4k (HIST1H4K), mRNA [NM_003541]	NM_003541
A_24_P161655	1.04E-02	2.01	A_24_P161655			
A_24_P195400	1.04E-02	2.20	A_24_P195400			
A_24_P538590	1.04E-02	2.23	AK092032	AK092032	Homo sapiens cDNA FLJ34713 fis, clone MESAN2004138. [AK092032]	
A_24_P257099	1.04E-02	2.44	NM_018410	NM_018410	Homo sapiens hypothetical protein DKFZp762E1312 (DKFZp762E1312), mRNA [NM_018410]	NM_018410
A_32_P104617	1.04E-02	2.58	AK055960	AK055960	Homo sapiens cDNA FLJ31398 fis, clone NT2NE1000175. [AK055960]	
A_24_P126931	1.04E-02	2.62	A_24_P126931			
A_24_P84698	1.04E-02	2.65	A_24_P84698			
A_23_P107087	1.04E-02	2.86	NM_018128	NM_018128	Homo sapiens hypothetical protein FLJ10534 (FLJ10534), mRNA [NM_018128]	NM_018128
A_23_P17269	1.04E-02	3.23	NM_018084	NM_018084	Homo sapiens KIAA1212 (KIAA1212), mRNA [NM_018084]	NM_018084
A_24_P226755	1.04E-02	16.34	NM_014729	NM_014729	Homo sapiens thymus high mobility group box protein TOX (TOX), mRNA [NM_014729]	NM_014729
A_24_P525749	1.04E-02	19.65	AK091547	AK091547	Homo sapiens cDNA FLJ34228 fis, clone FCBBF3025417. [AK091547]	
A_23_P32938	1.05E-02	2.10	NM_004398	NM_004398	Homo sapiens DEAD (Asp-Glu-Ala-Asp) box polypeptide 10 (DDX10), mRNA [NM_004398]	NM_004398
A_24_P860842	1.05E-02	2.38	BC027988	BC027988	Homo sapiens hypothetical protein LOC253842, mRNA (cDNA clone IMAGE:4993690), partial cds. [BC027988]	
A_32_P191290	1.05E-02	2.39	BM045853	BM045853	603624848F1 NIH_MGC_40 Homo sapiens cDNA clone IMAGE:5451514 5', mRNA sequence [BM045853]	
A_23_P87919	1.05E-02	2.54	NM_032590	NM_032590	Homo sapiens F-box and leucine-rich repeat protein 10 (FBXL10), transcript variant 1, mRNA [NM_032590]	NM_032590
A_23_P52939	1.05E-02	2.58	NM_003627	NM_003627	Homo sapiens solute carrier family 43, member 1 (SLC43A1), mRNA [NM_003627]	NM_003627
A_23_P151426	1.05E-02	2.72	NM_002015	NM_002015	Homo sapiens forkhead box O1A (rhabdomyosarcoma) (FOXO1A), mRNA [NM_002015]	NM_002015
A_24_P7211	1.05E-02	2.72	NM_033551	NM_033551	Homo sapiens La ribonucleoprotein domain family, member 1 (LARP1), mRNA [NM_033551]	NM_033551
A_23_P413923	1.05E-02	2.86	NM_022160	NM_022160	Homo sapiens DMRT-like family A1 (DMRTA1), mRNA [NM_022160]	NM_022160
A_23_P125157	1.05E-02	2.95	NM_032815	NM_032815	Homo sapiens nuclear factor of activated T-cells, cytoplasmic, calcineurin-dependent 2 interacting protein (NFATC2IP), mRNA [NM_032815]	NM_032815
A_32_P114483	1.05E-02	3.97	NM_153344	NM_153344	Homo sapiens chromosome 6 open reading frame 141 (C6orf141), mRNA [NM_153344]	NM_153344
A_24_P332504	1.06E-02	2.13	A_24_P332504			
A_24_P354300	1.06E-02	2.28	NM_015426	NM_015426	Homo sapiens WD repeat domain 51A (WDR51A), mRNA [NM_015426]	NM_015426
A_23_P142518	1.06E-02	2.40	NM_052876	NM_052876	Homo sapiens BTB (POZ) domain containing 14B (BTBD14B), mRNA [NM_052876]	NM_052876
A_24_P718833	1.06E-02	4.15	BC039479	BC039479	Homo sapiens, clone IMAGE:5534210, mRNA. [BC039479]	
A_23_P68669	1.06E-02	5.78	NM_024944	NM_024944	Homo sapiens chondrolectin (CHODL), mRNA [NM_024944]	NM_024944
A_23_P140362	1.06E-02	34.13	NM_018228	NM_018228	Homo sapiens chromosome 14 open reading frame 115 (C14orf115), mRNA [NM_018228]	NM_018228
A_23_P1322	1.07E-02	2.04	NM_031436	NM_031436	Homo sapiens aldo-keto reductase family 1, member C-like 2 (AKR1CL2), mRNA [NM_031436]	NM_031436
A_24_P169258	1.07E-02	2.31	NM_005993	NM_005993	Homo sapiens tubulin-specific chaperone d (TBCD), mRNA [NM_005993]	NM_005993
A_23_P211064	1.07E-02	2.42	NM_058191	NM_058191	Homo sapiens chromosome 21 open reading frame 66 (C21orf66), transcript variant 4, mRNA [NM_058191]	NM_058191
A_23_P100788	1.07E-02	2.48	NM_012448	NM_012448	Homo sapiens signal transducer and activator of transcription 5B (STAT5B), mRNA [NM_012448]	NM_012448
A_24_P250333	1.08E-02	2.04	NM_004596	NM_004596	Homo sapiens small nuclear ribonucleoprotein polypeptide A (SNRPA), mRNA [NM_004596]	NM_004596
A_23_P205959	1.08E-02	2.04	NM_000693	NM_000693	Homo sapiens aldehyde dehydrogenase 1 family, member A3 (ALDH1A3), mRNA [NM_000693]	NM_000693
A_24_P95007	1.08E-02	2.13	NM_032999	NM_032999	Homo sapiens general transcription factor II, i (GTF2I), transcript variant 1, mRNA [NM_032999]	NM_032999

A_23_P47788	1.08E-02	2.35	NM_005371	NM_005371	Homo sapiens methyltransferase like 1 (METTL1), transcript variant 1, mRNA [NM_005371]	NM_005371
A_24_P46093	1.08E-02	2.48	AB209172	AB209172	Homo sapiens mRNA for solute carrier family 6 (neurotransmitter transporter, taurine), member 6 variant protein. [AB209172]	
A_23_P140405	1.08E-02	2.62	NM_005197	NM_005197	Homo sapiens checkpoint suppressor 1 (CHES1), mRNA [NM_005197]	NM_005197
A_23_P205986	1.08E-02	2.78	NM_000875	NM_000875	Homo sapiens insulin-like growth factor 1 receptor (IGF1R), mRNA [NM_000875]	NM_000875
A_23_P36397	1.08E-02	2.93	NM_000785	NM_000785	Homo sapiens cytochrome P450, family 27, subfamily B, polypeptide 1 (CYP27B1), nuclear gene encoding mitochondrial protein, mRNA [NM_000785]	NM_000785
A_32_P225768	1.08E-02	3.28	A_32_P225768			
A_23_P256279	1.09E-02	2.18	NM_145267	NM_145267	Homo sapiens chromosome 6 open reading frame 57 (C6orf57), mRNA [NM_145267]	NM_145267
A_23_P99204	1.09E-02	2.38	NM_016534	NM_016534	Homo sapiens apoptosis-related protein PNAS-1 (FLJ39616), mRNA [NM_016534]	NM_016534
A_23_P420417	1.09E-02	2.45	NM_138463	NM_138463	Homo sapiens hypothetical protein BC014072 (LOC116238), mRNA [NM_138463]	NM_138463
A_32_P197524	1.10E-02	2.02	THC2367807			
A_24_P944040	1.10E-02	2.28	NM_015056	NM_015056	Homo sapiens KIAA0179 (KIAA0179), mRNA [NM_015056]	NM_015056
A_24_P461497	1.10E-02	3.62	ENST00000338852		PREDICTED: Homo sapiens similar to actin 3 - fruit fly (Drosophila melanogaster) (fragments) (LOC441836), mRNA [XM_497605]	XM_497605
A_24_P926450	1.10E-02	38.61	THC2274524			
A_32_P143323	1.11E-02	2.11	CR613267	CR613267	full-length cDNA clone CS0DL011YP14 of B cells (Ramos cell line) Cot 25-normalized of Homo sapiens (human). [CR613267]	
A_23_P114649	1.11E-02	2.36	NM_021933	NM_021933	Homo sapiens invasion inhibitory protein 45 (IIP45), transcript variant 1, mRNA [NM_021933]	NM_021933
A_23_P330727	1.11E-02	2.53	NM_182612	NM_182612	Homo sapiens hypothetical protein FLJ34283 (FLJ34283), mRNA [NM_182612]	NM_182612
A_23_P28878	1.11E-02	2.72	AK022713	AK022713	Homo sapiens cDNA FLJ12651 fis, clone NT2RM4002062, moderately similar to ASPARTYL-TRNA SYNTHETASE (EC 6.1.1.12). [AK022713]	
A_24_P392109	1.11E-02	2.72	NM_018455	NM_018455	Homo sapiens uncharacterized bone marrow protein BM039 (BM039), mRNA [NM_018455]	NM_018455
A_23_P215625	1.11E-02	3.82	NM_021723	NM_021723	Homo sapiens a disintegrin and metalloproteinase domain 22 (ADAM22), transcript variant 1, mRNA [NM_021723]	NM_021723
A_23_P136786	1.11E-02	5.08	NM_032336	NM_032336	Homo sapiens SLD5 homolog (SLD5), mRNA [NM_032336]	NM_032336
A_32_P215113	1.12E-02	2.01	NM_001017928	NM_001017928	Homo sapiens hypothetical LOC131076 (LOC131076), mRNA [NM_001017928]	NM_001017928
A_24_P239731	1.12E-02	2.10	NM_004776	NM_004776	Homo sapiens UDP-Gal:betaGlcNAc beta 1,4- galactosyltransferase, polypeptide 5 (B4GALT5), mRNA [NM_004776]	NM_004776
A_23_P103414	1.12E-02	2.13	NM_016258	NM_016258	Homo sapiens YTH domain family, member 2 (YTHDF2), mRNA [NM_016258]	NM_016258
A_23_P46337	1.12E-02	2.22	NM_014388	NM_014388	Homo sapiens chromosome 1 open reading frame 107 (C1orf107), mRNA [NM_014388]	NM_014388
A_24_P312072	1.12E-02	2.33	NM_020408	NM_020408	Homo sapiens chromosome 6 open reading frame 149 (C6orf149), mRNA [NM_020408]	NM_020408
A_23_P79231	1.12E-02	2.82	NM_134442	NM_134442	Homo sapiens cAMP responsive element binding protein 1 (CREB1), transcript variant B, mRNA [NM_134442]	NM_134442
A_23_P328766	1.12E-02	3.69	NM_145287	NM_145287	Homo sapiens zinc finger protein 519 (ZNF519), mRNA [NM_145287]	NM_145287
A_24_P913760	1.13E-02	2.01	THC2278264			
A_23_P143047	1.13E-02	2.10	NM_080653	NM_080653	Homo sapiens ATPase, H+ transporting, lysosomal 31kDa, V1 subunit E isoform 2 (ATP6V1E2), mRNA [NM_080653]	NM_080653
A_32_P63166	1.13E-02	2.28	W45382	W45382	W45382 zc80e10.s1 Pancreatic Islet Homo sapiens cDNA clone IMAGE:328650 3' similar to gb:D13748 EUKARYOTIC INITIATION FACTOR 4A-I (HUMAN);, mRNA sequence [W45382]	
A_24_P494658	1.13E-02	2.28	A_24_P494658			
A_24_P125839	1.13E-02	2.40	NM_017447	NM_017447	Homo sapiens chromosome 21 open reading frame 91 (C21orf91), mRNA [NM_017447]	NM_017447
A_24_P375691	1.13E-02	2.52	AF289566	AF289566	Homo sapiens clone pp6455 unknown mRNA. [AF289566]	
A_24_P67552	1.13E-02	2.67	A_24_P67552			
A_24_P50908	1.13E-02	3.82	AK074866	AK074866	Homo sapiens cDNA FLJ90385 fis, clone NT2RP2005247, weakly similar to ZINC-FINGER PROTEIN RFP. [AK074866]	
A_23_P51376	1.13E-02	10.74	NM_024522	NM_024522	Homo sapiens hypothetical protein FLJ12650 (FLJ12650), mRNA [NM_024522]	NM_024522
A_23_P204640	1.13E-02	59.88	NM_024865	NM_024865	Homo sapiens Nanog homeobox (NANOG), mRNA [NM_024865]	NM_024865
A_23_P112241	1.14E-02	2.00	NM_012266	NM_012266	Homo sapiens DnaJ (Hsp40) homolog, subfamily B, member 5 (DNAJB5), mRNA [NM_012266]	NM_012266

A_23_P139919	1.14E-02	2.03	NM_018413	NM_018413	Homo sapiens carbohydrate (chondroitin 4) sulfotransferase 11 (CHST11), mRNA [NM_018413]	NM_018413
A_23_P135157	1.14E-02	2.10	NM_004253	NM_004253	Homo sapiens phospholipase A2-activating protein (PLAA), mRNA [NM_004253]	NM_004253
A_24_P153542	1.14E-02	2.18	AK024684	AK024684	Homo sapiens cDNA: FLJ21031 fis, clone CAE07336. [AK024684]	
A_23_P96249	1.14E-02	2.28	NM_020180	NM_020180	Homo sapiens bruno-like 4, RNA binding protein (Drosophila) (BRUNOL4), mRNA [NM_020180]	NM_020180
A_32_P116271	1.14E-02	2.34	NM_205857	NM_205857	Homo sapiens chromosome 4 open reading frame 12 (C4orf12), mRNA [NM_205857]	NM_205857
A_32_P162443	1.14E-02	2.46	A_32_P162443			
A_24_P246361	1.14E-02	2.87	NM_006633	NM_006633	Homo sapiens IQ motif containing GTPase activating protein 2 (IQGAP2), mRNA [NM_006633]	NM_006633
A_24_P273561	1.14E-02	3.45	NM_018169	NM_018169	Homo sapiens hypothetical protein FLJ10652 (FLJ10652), mRNA [NM_018169]	NM_018169
A_24_P827491	1.14E-02	3.76	NM_006191	NM_006191	Homo sapiens proliferation-associated 2G4, 38kDa (PA2G4), mRNA [NM_006191]	NM_006191
A_23_P143926	1.14E-02	4.41	BC014794	BC014794	Homo sapiens unc-51-like kinase 4 (C. elegans), mRNA (cDNA clone MGC:16994 IMAGE:4343362), complete cds. [BC014794]	
A_24_P278853	1.15E-02	2.10	NM_032813	NM_032813	Homo sapiens hypothetical protein FLJ14624 (FLJ14624), mRNA [NM_032813]	NM_032813
A_23_P54626	1.15E-02	2.20	NM_032830	NM_032830	Homo sapiens cirrhosis, autosomal recessive 1A (cirhin) (CIRH1A), mRNA [NM_032830]	NM_032830
A_24_P927537	1.15E-02	2.30	THC2269654			
A_23_P420373	1.15E-02	2.39	NM_175630	NM_175630	Homo sapiens DNA (cytosine-5-)-methyltransferase 3 alpha (DNMT3A), transcript variant 4, mRNA [NM_175630]	NM_175630
A_32_P116840	1.15E-02	2.53	NM_203356	NM_203356	Homo sapiens CTAGE family, member 5 (CTAGE5), transcript variant 4, mRNA [NM_203356]	NM_203356
A_32_P128496	1.15E-02	2.56	T40959	T40959	T40959 ya15a04.s1 Stratagene liver (#937224) Homo sapiens cDNA clone IMAGE:61518 3', mRNA sequence [T40959]	
A_23_P214121	1.15E-02	3.12	NM_001634	NM_001634	Homo sapiens adenosylmethionine decarboxylase 1 (AMD1), mRNA [NM_001634]	NM_001634
A_32_P77343	1.15E-02	5.78	CR749256	CR749256	Homo sapiens mRNA; cDNA DKFZp781P0919 (from clone DKFZp781P0919). [CR749256]	
A_32_P58796	1.16E-02	2.16	NM_145809	NM_145809	Homo sapiens TL132 protein (LOC220594), mRNA [NM_145809]	NM_145809
A_23_P421563	1.16E-02	2.16	NM_014463	NM_014463	Homo sapiens LSM3 homolog, U6 small nuclear RNA associated (S. cerevisiae) (LSM3), mRNA [NM_014463]	NM_014463
A_24_P749042	1.16E-02	2.21	A_24_P749042			
A_23_P26439	1.16E-02	2.50	NM_024043	NM_024043	Homo sapiens hypothetical protein MGC3101 (MGC3101), mRNA [NM_024043]	NM_024043
A_23_P429624	1.16E-02	2.58	NM_178545	NM_178545	Homo sapiens transmembrane protein 52 (TMEM52), mRNA [NM_178545]	NM_178545
A_32_P98162	1.16E-02	2.83	A_32_P98162			
A_23_P116414	1.16E-02	5.68	NM_007069	NM_007069	Homo sapiens HRAS-like suppressor 3 (HRASLS3), mRNA [NM_007069]	NM_007069
A_23_P211727	1.16E-02	7.46	NM_004113	NM_004113	Homo sapiens fibroblast growth factor 12 (FGF12), transcript variant 2, mRNA [NM_004113]	NM_004113
A_23_P19712	1.17E-02	2.02	NM_015895	NM_015895	Homo sapiens geminin, DNA replication inhibitor (GMNN), mRNA [NM_015895]	NM_015895
A_23_P317591	1.17E-02	2.06	NM_006080	NM_006080	Homo sapiens sema domain, immunoglobulin domain (Ig), short basic domain, secreted, (semaphorin) 3A (SEMA3A), mRNA [NM_006080]	NM_006080
A_32_P310335	1.17E-02	2.21	AK056079	AK056079	Homo sapiens cDNA FLJ31517 fis, clone NT2RI2000007. [AK056079]	
A_23_P78302	1.17E-02	2.27	NM_003204	NM_003204	Homo sapiens nuclear factor (erythroid-derived 2)-like 1 (NFE2L1), mRNA [NM_003204]	NM_003204
A_23_P319423	1.17E-02	2.62	NM_003740	NM_003740	Homo sapiens potassium channel, subfamily K, member 5 (KCNK5), mRNA [NM_003740]	NM_003740
A_23_P81392	1.17E-02	2.88	NM_015238	NM_015238	Homo sapiens KIBRA protein (KIBRA), mRNA [NM_015238]	NM_015238
A_24_P144601	1.17E-02	37.59	NM_002701	NM_002701	Homo sapiens POU domain, class 5, transcription factor 1 (POU5F1), transcript variant 1, mRNA [NM_002701]	NM_002701
A_24_P903680	1.18E-02	2.05	XM_496957	XM_496957	PREDICTED: Homo sapiens similar to FLJ10408 protein (LOC441328), mRNA [XM_496957]	XM_496957
A_32_P218249	1.18E-02	2.07	NM_003211	NM_003211	Homo sapiens thymine-DNA glycosylase (TDG), transcript variant 1, mRNA [NM_003211]	NM_003211
A_24_P803801	1.18E-02	2.13	NM_032999	NM_032999	Homo sapiens general transcription factor II, i (GTF2I), transcript variant 1, mRNA [NM_032999]	NM_032999
A_23_P120325	1.18E-02	2.23	NM_002254	NM_002254	Homo sapiens kinesin family member 3C (KIF3C), mRNA [NM_002254]	NM_002254
A_23_P213344	1.18E-02	2.24	NM_005219	NM_005219	Homo sapiens diaphanous homolog 1 (Drosophila) (DIAPH1), mRNA [NM_005219]	NM_005219
A_23_P126291	1.18E-02	2.33	NM_003094	NM_003094	Homo sapiens small nuclear ribonucleoprotein polypeptide E (SNRPE), mRNA [NM_003094]	NM_003094
A_23_P335988	1.18E-02	2.40	NM_014235	NM_014235	Homo sapiens ubiquitin-like 4 (UBL4), mRNA [NM_014235]	NM_014235
A_24_P247978	1.18E-02	2.46	NM_016089	NM_016089	Homo sapiens zinc finger protein 589 (ZNF589), mRNA [NM_016089]	NM_016089

A_32_P145968	1.18E-02	2.70	AA291137	AA291137	AA291137 zs46b08.s1 NCI_CGAP_GCB1 Homo sapiens cDNA clone IMAGE:700503 3', mRNA sequence [AA291137]	
A_23_P315286	1.19E-02	2.01	NM_138774	NM_138774	Homo sapiens chromosome 19 open reading frame 22 (C19orf22), mRNA [NM_138774]	NM_138774
A_23_P37704	1.19E-02	2.14	NM_030928	NM_030928	Homo sapiens DNA replication factor (CDT1), mRNA [NM_030928]	NM_030928
A_24_P214841	1.19E-02	37.17	NM_002701	NM_002701	Homo sapiens POU domain, class 5, transcription factor 1 (POU5F1), transcript variant 1, mRNA [NM_002701]	NM_002701
A_23_P50108	1.20E-02	2.01	NM_006101	NM_006101	Homo sapiens kinetochore associated 2 (KNTC2), mRNA [NM_006101]	NM_006101
A_23_P123193	1.20E-02	2.05	NM_020445	NM_020445	Homo sapiens ARP3 actin-related protein 3 homolog B (yeast) (ACTR3B), mRNA [NM_020445]	NM_020445
A_23_P18931	1.20E-02	2.10	NM_002372	NM_002372	Homo sapiens mannosidase, alpha, class 2A, member 1 (MAN2A1), mRNA [NM_002372]	NM_002372
A_24_P142743	1.20E-02	2.86	NM_004368	NM_004368	Homo sapiens calponin 2 (CNN2), transcript variant 1, mRNA [NM_004368]	NM_004368
A_23_P102769	1.20E-02	2.95	NM_052865	NM_052865	Homo sapiens chromosome 20 open reading frame 72 (C20orf72), mRNA [NM_052865]	NM_052865
A_24_P272515	1.20E-02	3.22	XM_293018	XM_293018	PREDICTED: Homo sapiens similar to Fatty acid-binding protein, epidermal (E-FABP) (Psoriasis-associated fatty acid-binding protein homolog) (PA-FABP) (LOC344332), mRNA [XM_293018]	XM_293018
A_23_P76245	1.20E-02	5.85	NM_014191	NM_014191	Homo sapiens sodium channel, voltage gated, type VIII, alpha (SCN8A), mRNA [NM_014191]	NM_014191
A_24_P253251	1.21E-02	2.60	NM_003045	NM_003045	Homo sapiens solute carrier family 7 (cationic amino acid transporter, y+ system), member 1 (SLC7A1), mRNA [NM_003045]	NM_003045
A_32_P229221	1.21E-02	5.56	AB209318	AB209318	Homo sapiens mRNA for glutamate receptor, ionotropic, delta 2 variant protein. [AB209318]	
A_32_P78101	1.21E-02	7.69	NM_032880	NM_032880	Homo sapiens immunoglobin superfamily, member 21 (IGSF21), mRNA [NM_032880]	NM_032880
A_23_P81212	1.22E-02	2.08	NM_016067	NM_016067	Homo sapiens mitochondrial ribosomal protein S18C (MRPS18C), nuclear gene encoding mitochondrial protein, mRNA [NM_016067]	NM_016067
A_23_P60016	1.22E-02	2.16	AF095289	AF095289	Homo sapiens pituitary tumor transforming gene protein 3 (PTTG3) mRNA, complete cds. [AF095289]	
A_24_P853366	1.22E-02	2.16	A_24_P853366			
A_24_P561223	1.22E-02	2.17	THC2308977		R18C_MOUSE (Q8R2L5) 28S ribosomal protein S18c, mitochondrial precursor (MRP-S18-c) (Mrps18c) (MRP-S18-1), partial (20%) [THC2308977]	
A_24_P143543	1.22E-02	2.48	ENST00000273340			
A_23_P426279	1.22E-02	2.55	BU944300	BU944300	AGENCOURT_10545189 NIH_MGC_107 Homo sapiens cDNA clone IMAGE:6728554 5', mRNA sequence [BU944300]	
A_24_P365349	1.22E-02	2.95	A_24_P365349			
A_24_P15754	1.22E-02	3.00	NM_006114	NM_006114	Homo sapiens translocase of outer mitochondrial membrane 40 homolog (yeast) (TOMM40), mRNA [NM_006114]	NM_006114
A_23_P216448	1.22E-02	3.07	NM_005596	NM_005596	Homo sapiens nuclear factor I/B (NFIB), mRNA [NM_005596]	NM_005596
A_23_P48264	1.22E-02	5.03	NM_181783	NM_181783	Homo sapiens SMILE protein (SMILE), mRNA [NM_181783]	NM_181783
A_23_P4101	1.22E-02	6.33	BC088374	BC088374	Homo sapiens cDNA clone IMAGE:6171379, containing frame-shift errors. [BC088374]	
A_23_P70007	1.23E-02	2.04	NM_012484	NM_012484	Homo sapiens hyaluronan-mediated motility receptor (RHAMM) (HMMR), transcript variant 1, mRNA [NM_012484]	NM_012484
A_23_P334892	1.23E-02	2.14	NM_178518	NM_178518	Homo sapiens hypothetical protein FLJ36878 (FLJ36878), mRNA [NM_178518]	NM_178518
A_24_P313504	1.23E-02	2.18	NM_005030	NM_005030	Homo sapiens polo-like kinase 1 (Drosophila) (PLK1), mRNA [NM_005030]	NM_005030
A_24_P7157	1.24E-02	2.24	NM_020734	NM_020734	Homo sapiens family with sequence similarity 80, member B (FAM80B), mRNA [NM_020734]	NM_020734
A_23_P135294	1.24E-02	2.27	NM_000692	NM_000692	Homo sapiens aldehyde dehydrogenase 1 family, member B1 (ALDH1B1), nuclear gene encoding mitochondrial protein, mRNA [NM_000692]	NM_000692
A_32_P167459	1.24E-02	2.45	AK021744	AK021744	Homo sapiens cDNA FLJ11682 fis, clone HEMBA1004880. [AK021744]	
A_23_P52978	1.24E-02	3.21	NM_014502	NM_014502	Homo sapiens PRP19/PSO4 pre-mRNA processing factor 19 homolog (S. cerevisiae) (PRPF19), mRNA [NM_014502]	NM_014502
A_32_P179740	1.24E-02	4.20	AB033044	AB033044	Homo sapiens mRNA for KIAA1218 protein, partial cds. [AB033044]	
A_32_P142407	1.25E-02	2.08	CB853422	CB853422	UI-CF-FN0-agf-a-15-0-UI.s1 UI-CF-FN0 Homo sapiens cDNA clone UI-CF-FN0-agf-a-15-0-UI 3', mRNA sequence [CB853422]	
A_32_P54018	1.25E-02	2.15	NM_020696	NM_020696	Homo sapiens KIAA1143 (KIAA1143), mRNA [NM_020696]	NM_020696
A_24_P233256	1.25E-02	5.68	NM_023074	NM_023074	Homo sapiens zinc finger protein 649 (ZNF649), mRNA [NM_023074]	NM_023074
A_23_P208208	1.25E-02	6.13	NM_023074	NM_023074	Homo sapiens zinc finger protein 649 (ZNF649), mRNA [NM_023074]	NM_023074
A_23_P10025	1.25E-02	10.08	NM_006159	NM_006159	Homo sapiens NEL-like 2 (chicken) (NELL2), mRNA [NM_006159]	NM_006159

A_23_P17814	1.25E-02	20.92	NM_015715	NM_015715	Homo sapiens phospholipase A2, group III (PLA2G3), mRNA [NM_015715]	NM_015715
A_32_P49699	1.26E-02	2.15	THC2404479		Q9BUA6 (Q9BUA6) Myosin light chain 2, lymphocyte-specific, partial (7%) [THC2404479]	
A_32_P189093	1.26E-02	2.38	AI090167	AI090167	AI090167 qb33g12.x1 Soares_pregnant_uterus_NbHPU Homo sapiens cDNA clone IMAGE:1698118 3', mRNA sequence [AI090167]	
A_32_P202778	1.26E-02	2.65	A_32_P202778			
A_23_P340263	1.26E-02	2.79	NM_173662	NM_173662	Homo sapiens ring finger protein 175 (RNF175), mRNA [NM_173662]	NM_173662
A_23_P344719	1.26E-02	3.47	NM_003898	NM_003898	Homo sapiens synaptojanin 2 (SYNJ2), mRNA [NM_003898]	NM_003898
A_24_P502652	1.26E-02	3.75	A_24_P502652			
A_23_P123478	1.26E-02	5.81	NM_002603	NM_002603	Homo sapiens phosphodiesterase 7A (PDE7A), transcript variant 1, mRNA [NM_002603]	NM_002603
A_23_P209356	1.27E-02	2.02	NM_014946	NM_014946	Homo sapiens spastin (SPAST), transcript variant 1, mRNA [NM_014946]	NM_014946
A_24_P920555	1.27E-02	2.12	BM994983	BM994983	BM994983 UI-H-ED0-awz-n-15-0-UI.s1 NCI_CGAP_ED0 Homo sapiens cDNA clone IMAGE:5825870 3', mRNA sequence [BM994983]	
A_23_P255637	1.27E-02	2.18	A_23_P255637			
A_23_P203392	1.27E-02	2.36	NM_025080	NM_025080	Homo sapiens asparaginase like 1 (ASRGL1), mRNA [NM_025080]	NM_025080
A_24_P361167	1.27E-02	2.38	NM_020920	NM_020920	Homo sapiens chromodomain helicase DNA binding protein 8 (CHD8), mRNA [NM_020920]	NM_020920
A_24_P85123	1.27E-02	2.60	AK024440	AK024440	Homo sapiens mRNA for FLJ00030 protein, partial cds. [AK024440]	
A_32_P71788	1.27E-02	3.18	NM_002014	NM_002014	Homo sapiens FK506 binding protein 4, 59kDa (FKBP4), mRNA [NM_002014]	NM_002014
A_24_P201381	1.27E-02	3.25	NM_152429	NM_152429	Homo sapiens chromosome 10 open reading frame 13 (C10orf13), mRNA [NM_152429]	NM_152429
A_24_P166407	1.28E-02	3.09	NM_003544	NM_003544	Homo sapiens histone 1, H4b (HIST1H4B), mRNA [NM_003544]	NM_003544
A_23_P95718	1.28E-02	3.75	ENST00000269142		H.sapiens TAFII105 mRNA, partial. [Y09321]	XM_290809
A_23_P42563	1.28E-02	4.03	NM_012470	NM_012470	Homo sapiens transportin 3 (TNPO3), mRNA [NM_012470]	NM_012470
A_23_P24044	1.29E-02	2.38	NM_017649	NM_017649	Homo sapiens cyclin M2 (CNNM2), transcript variant 1, mRNA [NM_017649]	NM_017649
A_23_P101480	1.29E-02	2.59	NM_014975	NM_014975	Homo sapiens microtubule associated serine/threonine kinase 1 (MAST1), mRNA [NM_014975]	NM_014975
A_23_P59836	1.29E-02	2.77	NM_018396	NM_018396	Homo sapiens methyltransferase like 2 (METTL2), mRNA [NM_018396]	NM_018396
A_23_P209649	1.29E-02	2.83	NM_014929	NM_014929	Homo sapiens KIAA0971 (KIAA0971), mRNA [NM_014929]	NM_014929
A_23_P200267	1.29E-02	4.03	AB007895	AB007895	Homo sapiens KIAA0435 mRNA, partial cds. [AB007895]	
A_23_P360213	1.29E-02	4.31	ENST00000361227		Homo sapiens mitochondrial mRNA for AD 1, partial cds. [AB017116]	
A_23_P154675	1.30E-02	2.04	NM_198216	NM_198216	Homo sapiens small nuclear ribonucleoprotein polypeptides B and B1 (SNRPB), transcript variant 1, mRNA [NM_198216]	NM_198216
A_23_P259189	1.30E-02	2.04	NM_013943	NM_013943	Homo sapiens chloride intracellular channel 4 (CLIC4), mRNA [NM_013943]	NM_013943
A_23_P16609	1.30E-02	2.24	AL136548	AL136548	Homo sapiens mRNA; cDNA DKFZp761G18121 (from clone DKFZp761G18121). [AL136548]	
A_32_P197942	1.30E-02	2.36	AK125261	AK125261	Homo sapiens cDNA FLJ43271 fis, clone KIDNE2002882, highly similar to Homo sapiens Cadherin. [AK125261]	
A_23_P349310	1.30E-02	2.76	NM_014494	NM_014494	Homo sapiens trinucleotide repeat containing 6A (TNRC6A), transcript variant 1, mRNA [NM_014494]	NM_014494
A_23_P206598	1.30E-02	2.83	THC2314346		Q6P4C2 (Q6P4C2) RBBP6 protein (Fragment), partial (31%) [THC2314346]	
A_24_P221485	1.30E-02	2.87	A_24_P221485			
A_32_P31744	1.30E-02	3.16	AK021664	AK021664	Homo sapiens cDNA FLJ11602 fis, clone HEMBA1003908. [AK021664]	
A_24_P340659	1.30E-02	44.25	AF268613	AF268613	Homo sapiens POU 5 domain protein (POU5FLC1) mRNA, complete cds. [AF268613]	
A_32_P175539	1.31E-02	2.00	NM_002902	NM_002902	Homo sapiens reticulocalbin 2, EF-hand calcium binding domain (RCN2), mRNA [NM_002902]	NM_002902
A_24_P372123	1.31E-02	2.08	NM_018396	NM_018396	Homo sapiens methyltransferase like 2 (METTL2), mRNA [NM_018396]	NM_018396
A_32_P162709	1.31E-02	2.13	CR624517	CR624517	full-length cDNA clone CS0DC002YA18 of Neuroblastoma Cot 25-normalized of Homo sapiens (human). [CR624517]	
A_23_P139198	1.31E-02	2.19	NM_033101	NM_033101	Homo sapiens lectin, galactoside-binding, soluble, 12 (galectin 12) (LGALS12), mRNA [NM_033101]	NM_033101
A_23_P413888	1.31E-02	2.66	NM_001029858	NM_001029858	Homo sapiens solute carrier family 35, member F1 (SLC35F1), mRNA [NM_001029858]	NM_001029858
A_24_P333525	1.31E-02	7.69	NM_014857	NM_014857	Homo sapiens RAB GTPase activating protein 1-like (RABGAP1L), mRNA [NM_014857]	NM_014857
A_32_P213002	1.32E-02	2.10	THC2364440		U84B_HUMAN (Q9UH99) Sad1/unc-84-like protein 2 (Rab5 interacting protein) (Rab5IP), partial (12%) [THC2364440]	

A_23_P137423	1.32E-02	2.22	NM_052868	NM_052868	Homo sapiens immunoglobulin superfamily, member 8 (IGSF8), mRNA [NM_052868]	NM_052868
A_23_P170574	1.32E-02	2.40	ENST00000332281		Homo sapiens, Similar to snail homolog 3 (Drosophila), clone IMAGE:5209145, mRNA, partial cds. [BC041461]	XM_370995
A_23_P70785	1.32E-02	2.42	NM_001624	NM_001624	Homo sapiens absent in melanoma 1 (AIM1), mRNA [NM_001624]	NM_001624
A_32_P218355	1.32E-02	3.55	AK074567	AK074567	Homo sapiens cDNA FLJ90086 fis, clone HEMBA1005145. [AK074567]	XM_371820
A_32_P199049	1.32E-02	6.99	THC2314201		Q7QYY0 (Q7QYY0) GLP_164_21502_20957, partial (9%) [THC2314201]	
A_32_P148476	1.33E-02	2.05	ENST00000304245		PREDICTED: Homo sapiens similar to Ran-specific GTPase-activating protein (Ran binding protein 1) (RanBP1) (LOC389842), mRNA [XM_372200]	XM_372200
A_23_P73992	1.33E-02	2.25	BC029660	BC029660	Homo sapiens ubiquitin specific protease 24, mRNA (cDNA clone MGC:29848 IMAGE:4995223), complete cds. [BC029660]	XM_371254
A_24_P551842	1.33E-02	3.51	ENST00000361789		Human mitochondrial cytochrome b gene, partial cds. [M28016]	
A_24_P48856	1.33E-02	3.70	NM_000071	NM_000071	Homo sapiens cystathionine-beta-synthase (CBS), mRNA [NM_000071]	NM_000071
A_23_P122906	1.33E-02	3.92	NM_015570	NM_015570	Homo sapiens autism susceptibility candidate 2 (AUTS2), mRNA [NM_015570]	NM_015570
A_24_P221335	1.34E-02	2.13	A_24_P221335			
A_24_P516215	1.34E-02	2.16	NM_014062	NM_014062	Homo sapiens nin one binding protein (NOB1P), mRNA [NM_014062]	NM_014062
A_24_P47988	1.34E-02	2.29	NM_025165	NM_025165	Homo sapiens elongation factor RNA polymerase II-like 3 (ELL3), mRNA [NM_025165]	NM_025165
A_32_P198518	1.34E-02	2.55	THC2304443		HSU36501 SP100-B {Homo sapiens;} , partial (65%) [THC2304443]	
A_23_P19219	1.34E-02	2.57	NM_014034	NM_014034	Homo sapiens ASF1 anti-silencing function 1 homolog A (S. cerevisiae) (ASF1A), mRNA [NM_014034]	NM_014034
A_23_P27688	1.34E-02	3.92	BC022233	BC022233	Homo sapiens, clone IMAGE:4401286, mRNA. [BC022233]	XM_373810
A_24_P314477	1.34E-02	6.33	NM_178012	NM_178012	Homo sapiens tubulin, beta polypeptide paralog (RP11-506K6.1), mRNA [NM_178012]	NM_178012
A_32_P170206	1.35E-02	2.09	AA451676	AA451676	AA451676 zx44b03.s1 Soares_total_fetus_Nb2HF8_9w Homo sapiens cDNA clone IMAGE:789293 3', mRNA sequence [AA451676]	
A_24_P854913	1.35E-02	2.10	THC2275676			
A_23_P91783	1.35E-02	2.20	NM_181335	NM_181335	Homo sapiens Rho GTPase activating protein 8 (ARHGAP8), transcript variant 2, mRNA [NM_181335]	NM_181335
A_32_P135790	1.35E-02	2.54	BU729734	BU729734	UI-E-CK1-afj-k-04-0-UI.s1 UI-E-CK1 Homo sapiens cDNA clone UI-E-CK1-afj-k-04-0-UI 3', mRNA sequence [BU729734]	
A_23_P36364	1.35E-02	2.79	NM_033209	NM_033209	Homo sapiens Thy-1 co-transcribed (LOC94105), mRNA [NM_033209]	NM_033209
A_23_P250767	1.35E-02	2.87	NM_019061	NM_019061	Homo sapiens myotubularin related protein 12 (MTMR12), mRNA [NM_019061]	NM_019061
A_23_P406928	1.35E-02	3.42	ENST00000361567		Homo sapiens NADH dehydrogenase subunit 5 (MTND5) mRNA, RNA 5, complete cds; mitochondrial gene for mitochondrial product. [AF339085]	
A_32_P104334	1.35E-02	10.00	AW972815	AW972815	EST384910 MAGE resequences, MAGL Homo sapiens cDNA, mRNA sequence [AW972815]	
A_24_P349590	1.36E-02	2.02	A_24_P349590			
A_32_P117860	1.36E-02	2.04	W05707	W05707	W05707 za87h03.r1 Soares_fetal_lung_NbHL19W Homo sapiens cDNA clone IMAGE:299573 5' similar to SW:TCPD_MOUSE P80315 T-COMPLEX PROTEIN 1, DELTA SUBUNIT ;, mRNA sequence [W05707]	
A_23_P401	1.36E-02	2.08	NM_016343	NM_016343	Homo sapiens centromere protein F, 350/400ka (mitosin) (CENPF), mRNA [NM_016343]	NM_016343
A_24_P119036	1.36E-02	2.14	ENST00000219746		Homo sapiens cDNA: FLJ23353 fis, clone HEP14321, highly similar to HSU80736 Homo sapiens CAGF9 mRNA. [AK027006]	XM_049037
A_23_P47155	1.36E-02	2.15	NM_015368	NM_015368	Homo sapiens pannexin 1 (PANX1), mRNA [NM_015368]	NM_015368
A_23_P68610	1.36E-02	2.29	NM_012112	NM_012112	Homo sapiens TPX2, microtubule-associated, homolog (Xenopus laevis) (TPX2), mRNA [NM_012112]	NM_012112
A_23_P326319	1.36E-02	2.36	NM_033201	NM_033201	Homo sapiens chromosome 16 open reading frame 45 (C16orf45), mRNA [NM_033201]	NM_033201
A_24_P303390	1.36E-02	2.93	NM_025256	NM_025256	Homo sapiens euchromatic histone-lysine N-methyltransferase 2 (EHMT2), transcript variant NG36/G9a-SPI, mRNA [NM_025256]	NM_025256
A_23_P26072	1.36E-02	3.66	AF118084	AF118084	Homo sapiens PRO1914 mRNA, complete cds. [AF118084]	
A_24_P332081	1.36E-02	4.37	NM_194303	NM_194303	Homo sapiens chromosome 10 open reading frame 39 (C10orf39), mRNA [NM_194303]	NM_194303
A_24_P943113	1.38E-02	2.05	NM_173359	NM_173359	Homo sapiens eukaryotic translation initiation factor 4E member 3 (EIF4E3), mRNA [NM_173359]	NM_173359
A_23_P399255	1.38E-02	2.27	NM_152737	NM_152737	Homo sapiens ring finger protein 182 (RNF182), mRNA [NM_152737]	NM_152737
A_23_P39441	1.38E-02	2.40	NM_133473	NM_133473	Homo sapiens zinc finger protein 431 (ZNF431), mRNA [NM_133473]	NM_133473

A_23_P334751	1.38E-02	2.72	NM_152490	NM_152490	Homo sapiens UDP-GalNAc:betaGlcNAc beta 1,3-galactosaminyltransferase, polypeptide 2 (B3GALNT2), mRNA [NM_152490]	NM_152490
A_24_P205242	1.38E-02	3.98	BC034378	BC034378	Homo sapiens ribosomal protein L23, mRNA (cDNA clone MGC:34067 IMAGE:5186030), complete cds. [BC034378]	
A_23_P137484	1.38E-02	26.74	NM_019079	NM_019079	Homo sapiens hypothetical protein FLJ10884 (ECAT11), mRNA [NM_019079]	NM_019079
A_23_P257335	1.39E-02	2.07	NM_006558	NM_006558	Homo sapiens KH domain containing, RNA binding, signal transduction associated 3 (KHDRBS3), mRNA [NM_006558]	NM_006558
A_23_P52793	1.39E-02	2.28	NM_002716	NM_002716	Homo sapiens protein phosphatase 2 (formerly 2A), regulatory subunit A (PR 65), beta isoform (PPP2R1B), transcript variant 1, mRNA [NM_002716]	NM_002716
A_24_P933319	1.39E-02	2.70	AK002107	AK002107	Homo sapiens cDNA FLJ11245 fis, clone PLACE1008629. [AK002107]	
A_23_P151150	1.39E-02	2.89	NM_202002	NM_202002	Homo sapiens forkhead box M1 (FOXM1), transcript variant 1, mRNA [NM_202002]	NM_202002
A_24_P114249	1.39E-02	4.63	NM_004482	NM_004482	Homo sapiens UDP-N-acetyl-alpha-D-galactosamine:polypeptide N-acetylgalactosaminyltransferase 3 (GalNAc-T3) (GALNT3), mRNA [NM_004482]	NM_004482
A_24_P132703	1.39E-02	4.81	ENST00000259676		full-length cDNA clone CS0DB009YI22 of Neuroblastoma Cot 10-normalized of Homo sapiens (human). [CR607939]	
A_23_P167553	1.40E-02	2.13	NM_000791	NM_000791	Homo sapiens dihydrofolate reductase (DHFR), mRNA [NM_000791]	NM_000791
A_23_P106158	1.40E-02	2.14	NM_024884	NM_024884	Homo sapiens L-2-hydroxyglutarate dehydrogenase (L2HGDH), mRNA [NM_024884]	NM_024884
A_23_P111343	1.40E-02	2.69	NM_014739	NM_014739	Homo sapiens BCL2-associated transcription factor 1 (BCLAF1), mRNA [NM_014739]	NM_014739
A_23_P433690	1.40E-02	2.90	NM_018555	NM_018555	Homo sapiens zinc finger protein 331 (ZNF331), mRNA [NM_018555]	NM_018555
A_23_P17593	1.40E-02	2.92	NM_001794	NM_001794	Homo sapiens cadherin 4, type 1, R-cadherin (retinal) (CDH4), mRNA [NM_001794]	NM_001794
A_24_P93901	1.40E-02	3.18	BC063531	BC063531	Homo sapiens SIN3 homolog B, transcription regulator (yeast), mRNA (cDNA clone IMAGE:4417458), complete cds. [BC063531]	
A_24_P343621	1.40E-02	4.00	NM_024693	NM_024693	Homo sapiens enoyl Coenzyme A hydratase domain containing 3 (ECHDC3), mRNA [NM_024693]	NM_024693
A_23_P48663	1.40E-02	5.24	NM_021728	NM_021728	Homo sapiens orthodenticle homolog 2 (Drosophila) (OTX2), transcript variant 1, mRNA [NM_021728]	NM_021728
A_32_P154473	1.40E-02	6.21	ENST00000334436		Homo sapiens mRNA for KIAA0531 protein, partial cds. [AB011103]	
A_23_P329798	1.40E-02	9.26	NM_005454	NM_005454	Homo sapiens cerberus 1 homolog, cysteine knot superfamily (Xenopus laevis) (CER1), mRNA [NM_005454]	NM_005454
A_24_P383640	1.40E-02	45.87	AF268617	AF268617	Homo sapiens POU 5 domain protein (POU5FLC12) mRNA, complete cds. [AF268617]	XR_000266
A_23_P73297	1.41E-02	2.13	NM_004742	NM_004742	Homo sapiens membrane associated guanylate kinase, WW and PDZ domain containing 1 (MAGI1), mRNA [NM_004742]	NM_004742
A_23_P110253	1.41E-02	2.22	NM_000222	NM_000222	Homo sapiens v-kit Hardy-Zuckerman 4 feline sarcoma viral oncogene homolog (KIT), mRNA [NM_000222]	NM_000222
A_24_P332326	1.41E-02	2.27	A_24_P332326			
A_24_P159323	1.41E-02	2.49	NM_022770	NM_022770	Homo sapiens hypothetical protein FLJ13912 (FLJ13912), mRNA [NM_022770]	NM_022770
A_23_P47790	1.41E-02	2.52	NM_005371	NM_005371	Homo sapiens methyltransferase like 1 (METTL1), transcript variant 1, mRNA [NM_005371]	NM_005371
A_24_P891276	1.41E-02	2.61	A_24_P891276			
A_32_P42406	1.41E-02	2.93	BC018676	BC018676	Homo sapiens, clone IMAGE:4337652, mRNA. [BC018676]	
A_23_P413051	1.41E-02	3.13	NM_144603	NM_144603	Homo sapiens NADPH oxidase organizer 1 (NOXO1), transcript variant a, mRNA [NM_144603]	NM_144603
A_24_P263803	1.41E-02	5.85	ENST00000308819		PREDICTED: Homo sapiens similar to Chloride intracellular channel protein 1 (Nuclear chloride ion channel 27) (NCC27) (p64 CLCP) (Chloride channel ABP) (LOC390363), mRNA [XM_495936]	XM_495936
A_32_P70315	1.41E-02	6.02	NM_003256	NM_003256	Homo sapiens tissue inhibitor of metalloproteinase 4 (TIMP4), mRNA [NM_003256]	NM_003256
A_24_P371962	1.42E-02	2.03	NM_001634	NM_001634	Homo sapiens adenosylmethionine decarboxylase 1 (AMD1), mRNA [NM_001634]	NM_001634
A_24_P100742	1.42E-02	2.13	NM_014189	NM_014189	Homo sapiens adducin 1 (alpha) (ADD1), transcript variant 2, mRNA [NM_014189]	NM_014189
A_23_P142697	1.42E-02	2.19	NM_014640	NM_014640	Homo sapiens tubulin tyrosine ligase-like family, member 4 (TTLL4), mRNA [NM_014640]	NM_014640
A_23_P11237	1.42E-02	2.21	NM_004606	NM_004606	Homo sapiens TAF1 RNA polymerase II, TATA box binding protein (TBP)-associated factor, 250kDa (TAF1), transcript variant 1, mRNA [NM_004606]	NM_004606
A_23_P35194	1.42E-02	2.22	NM_001412	NM_001412	Homo sapiens eukaryotic translation initiation factor 1A, X-linked (EIF1AX), mRNA [NM_001412]	NM_001412
A_23_P60899	1.42E-02	2.41	NM_024831	NM_024831	Homo sapiens nuclear receptor coactivator 6 interacting protein (NCOA6IP), mRNA [NM_024831]	NM_024831
A_32_P175979	1.42E-02	2.43	THC2277837			
A_23_P47058	1.42E-02	31.45	NM_022034	NM_022034	Homo sapiens CUB and zona pellucida-like domains 1 (CUZD1), mRNA [NM_022034]	NM_022034

A_32_P132563	1.42E-02	45.05	NM_002701	NM_002701	Homo sapiens POU domain, class 5, transcription factor 1 (POU5F1), transcript variant 1, mRNA [NM_002701]	NM_002701
A_23_P88435	1.43E-02	2.00	NM_005197	NM_005197	Homo sapiens checkpoint suppressor 1 (CHES1), mRNA [NM_005197]	NM_005197
A_23_P33927	1.43E-02	2.02	AF334945	AF334945	Homo sapiens FKSG43 (FKSG43) mRNA, complete cds. [AF334945]	
A_24_P315066	1.43E-02	2.14	NM_031449	NM_031449	Homo sapiens hypothetical protein DKFZp761I2123 (DKFZp761I2123), transcript variant 1, mRNA [NM_031449]	NM_031449
A_23_P357207	1.43E-02	2.87	NM_138409	NM_138409	Homo sapiens chromosome 6 open reading frame 117 (C6orf117), mRNA [NM_138409]	NM_138409
A_24_P255845	1.43E-02	3.79	A_24_P255845			
A_23_P85218	1.43E-02	7.04	NM_005634	NM_005634	Homo sapiens SRY (sex determining region Y)-box 3 (SOX3), mRNA [NM_005634]	NM_005634
A_23_P89727	1.44E-02	2.04	NM_048368	NM_048368	Homo sapiens CTD (carboxy-terminal domain, RNA polymerase II, polypeptide A) phosphatase, subunit 1 (CTDP1), transcript variant FCP1b, mRNA [NM_048368]	NM_048368
A_32_P53670	1.44E-02	2.04	AA442488	AA442488	zv59a10.r1 Soares_testis_NHT Homo sapiens cDNA clone IMAGE:757914 5' similar to contains Alu repetitive element;, mRNA sequence [AA442488]	
A_23_P336479	1.44E-02	2.31	NM_005754	NM_005754	Homo sapiens Ras-GTPase-activating protein SH3-domain-binding protein (G3BP), transcript variant 1, mRNA [NM_005754]	NM_005754
A_23_P120146	1.44E-02	2.46	NM_004257	NM_004257	Homo sapiens transforming growth factor, beta receptor associated protein 1 (TGFBRAP1), mRNA [NM_004257]	NM_004257
A_32_P187458	1.44E-02	3.47	BQ233242	BQ233242	BQ233242 AGENCOURT_7283259 NIH_MGC_70 Homo sapiens cDNA clone IMAGE:6017276 5', mRNA sequence [BQ233242]	
A_24_P163237	1.44E-02	4.22	NM_020225	NM_020225	Homo sapiens storkhead box 2 (STOX2), mRNA [NM_020225]	NM_020225
A_23_P162719	1.45E-02	3.26	NM_030932	NM_030932	Homo sapiens diaphanous homolog 3 (Drosophila) (DIAPH3), mRNA [NM_030932]	NM_030932
A_23_P382654	1.45E-02	3.37	NM_022091	NM_022091	Homo sapiens dJ467N11.1 protein (DJ467N11.1), mRNA [NM_022091]	NM_022091
A_24_P29001	1.46E-02	2.08	NM_014463	NM_014463	Homo sapiens LSM3 homolog, U6 small nuclear RNA associated (S. cerevisiae) (LSM3), mRNA [NM_014463]	NM_014463
A_24_P212851	1.46E-02	2.12	NM_003072	NM_003072	Homo sapiens SWI/SNF related, matrix associated, actin dependent regulator of chromatin, subfamily a, member 4 (SMARCA4), mRNA [NM_003072]	NM_003072
A_24_P388528	1.46E-02	2.58	NM_173216	NM_173216	Homo sapiens ST6 beta-galactosamide alpha-2,6-sialyltranferase 1 (ST6GAL1), transcript variant 1, mRNA [NM_173216]	NM_173216
A_23_P431252	1.46E-02	3.11	NM_032505	NM_032505	Homo sapiens T-cell activation kelch repeat protein (TA-KRP), mRNA [NM_032505]	NM_032505
A_32_P188860	1.46E-02	4.22	AK125591	AK125591	Homo sapiens cDNA FLJ43603 fis, clone SPLEN2005767. [AK125591]	
A_32_P191696	1.47E-02	2.07	T12588	T12588	CHR90108 Chromosome 9 exon II Homo sapiens cDNA clone P94_53 5' and 3', mRNA sequence [T12588]	
A_23_P66540	1.47E-02	2.19	NM_014308	NM_014308	Homo sapiens phosphoinositide-3-kinase, regulatory subunit 5, p101 (PIK3R5), mRNA [NM_014308]	NM_014308
A_23_P211785	1.47E-02	2.33	NM_003420	NM_003420	Homo sapiens zinc finger protein 35 (clone HF.10) (ZNF35), mRNA [NM_003420]	NM_003420
A_24_P332780	1.47E-02	2.60	A_24_P332780			
A_23_P44794	1.47E-02	3.62	NM_138453	NM_138453	Homo sapiens RAB3C, member RAS oncogene family (RAB3C), mRNA [NM_138453]	NM_138453
A_23_P149834	1.48E-02	2.02	NM_007055	NM_007055	Homo sapiens polymerase (RNA) III (DNA directed) polypeptide A, 155kDa (POLR3A), mRNA [NM_007055]	NM_007055
A_32_P198330	1.48E-02	2.09	AL832348	AL832348	Homo sapiens mRNA; cDNA DKFZp451A086 (from clone DKFZp451A086). [AL832348]	
A_23_P61881	1.48E-02	2.11	NM_006321	NM_006321	Homo sapiens ariadne homolog 2 (Drosophila) (ARIH2), mRNA [NM_006321]	NM_006321
A_24_P118247	1.48E-02	2.16	NM_001012762	NM_001012762	Homo sapiens hypothetical protein LOC348180 (LOC348180), transcript variant 2, mRNA [NM_001012762]	NM_001012762
A_24_P25346	1.48E-02	2.22	NM_032830	NM_032830	Homo sapiens cirrhosis, autosomal recessive 1A (cirhin) (CIRH1A), mRNA [NM_032830]	NM_032830
A_23_P99710	1.48E-02	2.27	NM_014749	NM_014749	Homo sapiens KIAA0586 (KIAA0586), mRNA [NM_014749]	NM_014749
A_32_P171348	1.48E-02	2.38	A_32_P171348			
A_24_P196400	1.48E-02	2.65	NM_017934	NM_017934	Homo sapiens pleckstrin homology domain interacting protein (PHIP), mRNA [NM_017934]	NM_017934
A_23_P138706	1.48E-02	2.79	NM_000681	NM_000681	Homo sapiens adrenergic, alpha-2A-, receptor (ADRA2A), mRNA [NM_000681]	NM_000681
A_24_P178093	1.48E-02	3.00	NM_006114	NM_006114	Homo sapiens translocase of outer mitochondrial membrane 40 homolog (yeast) (TOMM40), mRNA [NM_006114]	NM_006114
A_23_P109950	1.48E-02	3.45	NM_001106	NM_001106	Homo sapiens activin A receptor, type IIB (ACVR2B), mRNA [NM_001106]	NM_001106
A_24_P307486	1.49E-02	2.02	ENST00000309556		PREDICTED: Homo sapiens similar to peptidyl-Pro cis trans isomerase (LOC128192), mRNA [XM_060887]	XM_060887
A_23_P169249	1.49E-02	2.04	NM_017585	NM_017585	Homo sapiens solute carrier family 2 (facilitated glucose transporter), member 6 (SLC2A6), mRNA [NM_017585]	NM_017585

A_23_P167005	1.49E-02	2.32	NM_014373	NM_014373	Homo sapiens G protein-coupled receptor 160 (GPR160), mRNA [NM_014373]	NM_014373
A_32_P78285	1.49E-02	2.48	A_32_P78285			
A_24_P660811	1.49E-02	2.54	NM_001013651	NM_001013651	Homo sapiens hypothetical gene supported by AK128318 (LOC389607), mRNA [NM_001013651]	NM_001013651
A_32_P222277	1.49E-02	2.67	BX096810	BX096810	BX096810 BX096810 NCI_CGAP_Co3 Homo sapiens cDNA clone IMAGp998G175770 ; IMAGE:2326312, mRNA sequence [BX096810]	
A_23_P331028	1.49E-02	3.11	AK025280	AK025280	Homo sapiens cDNA: FLJ21627 fis, clone COL08058. [AK025280]	
A_24_P557232	1.49E-02	3.42	CB111670	CB111670	K-EST0153390 L5HLK1 Homo sapiens cDNA clone L5HLK1-3-D02 5', mRNA sequence [CB111670]	
A_23_P380208	1.50E-02	3.64	NM_024621	NM_024621	Homo sapiens ventricular zone expressed PH domain homolog 1 (zebrafish) (VEPH1), mRNA [NM_024621]	NM_024621

Supplemental Table S1B

Genes upregulated in EB vs. hESC

P-Value ≤ 0.015 with multiple testing correction (Benjamini and Hochberg), Fold Change ≥ 2.0

Gene Name	*P* -value	Fold change	Common	Genbank	Description	RefSeq
A_23_P143885	4.15E-06	15.01	NM_019555	NM_019555	Homo sapiens Rho guanine nucleotide exchange factor (GEF) 3 (ARHGEF3), mRNA [NM_019555]	NM_019555
A_24_P190472	1.39E-04	29.89	NM_003064	NM_003064	Homo sapiens secretory leukocyte protease inhibitor (antileukoproteinase) (SLPI), mRNA [NM_003064]	NM_003064
A_23_P128574	1.39E-04	16.54	NM_017993	NM_017993	Homo sapiens hypothetical protein FLJ10094 (FLJ10094), mRNA [NM_017993]	NM_017993
A_23_P19936	1.39E-04	5.532	NM_006854	NM_006854	Homo sapiens KDEL (Lys-Asp-Glu-Leu) endoplasmic reticulum protein retention receptor 2 (KDELR2), mRNA [NM_006854]	NM_006854
A_24_P189533	1.39E-04	5.137	ENST00000278505		Homo sapiens mRNA for KIAA0830 protein, partial cds. [AB020637]	XM_290546
A_23_P76743	1.39E-04	4.094	AX781433	AX781433	Sequence 16 from Patent EP1321519. [AX781433]	
A_23_P115407	1.39E-04	4.006	NM_146421	NM_146421	Homo sapiens glutathione S-transferase M1 (GSTM1), transcript variant 2, mRNA [NM_146421]	NM_146421
A_23_P59616	1.39E-04	3.978	NM_173537	NM_173537	Homo sapiens GTF2I repeat domain containing 2 (GTF2IRD2), mRNA [NM_173537]	NM_173537
A_32_P156171	1.39E-04	3.91	THC2341051			
A_24_P54879	1.39E-04	2.708	NM_005506	NM_005506	Homo sapiens scavenger receptor class B, member 2 (SCARB2), mRNA [NM_005506]	NM_005506
A_23_P19030	1.49E-04	18.73	ENST00000328668		full-length cDNA clone CS0DI041YK16 of Placenta Cot 25-normalized of Homo sapiens (human). [CR621346]	
A_23_P162640	1.80E-04	4.908	NM_031412	NM_031412	Homo sapiens GABA(A) receptor-associated protein like 1 (GABARAPL1), mRNA [NM_031412]	NM_031412
A_23_P133517	1.88E-04	21.32	NM_002310	NM_002310	Homo sapiens leukemia inhibitory factor receptor (LIFR), mRNA [NM_002310]	NM_002310
A_23_P128919	1.99E-04	33.79	NM_002306	NM_002306	Homo sapiens lectin, galactoside-binding, soluble, 3 (galectin 3) (LGALS3), mRNA [NM_002306]	NM_002306
A_24_P330518	2.21E-04	21.61	NM_001218	NM_001218	Homo sapiens carbonic anhydrase XII (CA12), transcript variant 1, mRNA [NM_001218]	NM_001218
A_32_P220700	2.21E-04	12.94	THC2440162			
A_23_P114084	2.35E-04	14.96	NM_000444	NM_000444	Homo sapiens phosphate regulating endopeptidase homolog, X-linked (hypophosphatemia, vitamin D resistant rickets) (PHEX), transcript variant 1843529, mRNA [NM_000444]	NM_000444
A_23_P158318	2.35E-04	3.184	NM_004560	NM_004560	Homo sapiens receptor tyrosine kinase-like orphan receptor 2 (ROR2), mRNA [NM_004560]	NM_004560
A_23_P300220	2.95E-04	8.843	NM_145008	NM_145008	Homo sapiens yippee-like 4 (Drosophila) (YPEL4), mRNA [NM_145008]	NM_145008
A_23_P46639	2.95E-04	6.137	NM_000562	NM_000562	Homo sapiens complement component 8, alpha polypeptide (C8A), mRNA [NM_000562]	NM_000562
A_23_P62920	2.95E-04	4.684	NM_014970	NM_014970	Homo sapiens kinesin-associated protein 3 (KIFAP3), mRNA [NM_014970]	NM_014970
A_23_P114232	2.95E-04	3.27	NM_006406	NM_006406	Homo sapiens peroxiredoxin 4 (PRDX4), mRNA [NM_006406]	NM_006406
A_23_P53567	2.95E-04	2.929	NM_016053	NM_016053	Homo sapiens CGI-116 protein (CGI-116), mRNA [NM_016053]	NM_016053
A_23_P353005	2.95E-04	2.608	NM_152553	NM_152553	Homo sapiens IBR domain containing 1 (IBRDC1), mRNA [NM_152553]	NM_152553
A_23_P151690	2.95E-04	2.284	NM_018477	NM_018477	Homo sapiens actin-related protein 10 homolog (S. cerevisiae) (ACTR10), mRNA [NM_018477]	NM_018477
A_23_P363778	2.98E-04	56.38	NM_001463	NM_001463	Homo sapiens frizzled-related protein (FRZB), mRNA [NM_001463]	NM_001463
A_23_P55544	2.98E-04	3.405	NM_133459	NM_133459	Homo sapiens collagen and calcium binding EGF domains 1 (CCBE1), mRNA [NM_133459]	NM_133459
A_23_P91230	3.02E-04	43.03	NM_003064	NM_003064	Homo sapiens secretory leukocyte protease inhibitor (antileukoproteinase) (SLPI), mRNA [NM_003064]	NM_003064
A_23_P97181	3.02E-04	39.83	NM_022469	NM_022469	Homo sapiens gremlin 2, cysteine knot superfamily, homolog (Xenopus laevis) (GREM2), mRNA [NM_022469]	NM_022469
A_24_P89426	3.02E-04	21.9	NM_019101	NM_019101	Homo sapiens apolipoprotein M (APOM), mRNA [NM_019101]	NM_019101
A_24_P62530	3.02E-04	21.22	NM_021205	NM_021205	Homo sapiens ras homolog gene family, member U (RHOU), mRNA [NM_021205]	NM_021205
A_23_P171117	3.02E-04	15.4	NM_024657	NM_024657	Homo sapiens MORC family CW-type zinc finger 4 (MORC4), mRNA [NM_024657]	NM_024657
A_23_P149613	3.02E-04	10.34	NM_002021	NM_002021	Homo sapiens flavin containing monooxygenase 1 (FMO1), mRNA [NM_002021]	NM_002021
A_23_P204296	3.02E-04	9.013	NM_032918	NM_032918	Homo sapiens RAS-like, estrogen-regulated, growth inhibitor (RERG), mRNA [NM_032918]	NM_032918
A_23_P83277	3.02E-04	8.783	NM_004512	NM_004512	Homo sapiens interleukin 11 receptor, alpha (IL11RA), transcript variant 1, mRNA [NM_004512]	NM_004512
A_32_P167471	3.02E-04	7.319	AK125038	AK125038	Homo sapiens cDNA FLJ43048 fis, clone BRTHA3004502. [AK125038]	
A_23_P27040	3.02E-04	6.635	NM_015544	NM_015544	Homo sapiens DKFZP564K1964 protein (DKFZP564K1964), mRNA [NM_015544]	NM_015544

A_24_P229726	3.02E-04	5.921	A_24_P229726			
A_24_P229728	3.02E-04	4.818	A_24_P229728			
A_23_P36689	3.02E-04	4.378	NM_006992	NM_006992	Homo sapiens B7 gene (B7), transcript variant 2, mRNA [NM_006992]	NM_006992
A_32_P452655	3.02E-04	4.115	NM_009587	NM_009587	Homo sapiens lectin, galactoside-binding, soluble, 9 (galectin 9) (LGALS9), transcript variant long, mRNA [NM_009587]	NM_009587
A_23_P114903	3.02E-04	3.057	NM_002155	NM_002155	Homo sapiens heat shock 70kDa protein 6 (HSP70B') (HSPA6), mRNA [NM_002155]	NM_002155
A_23_P258048	3.02E-04	2.71	AK056630	AK056630	Homo sapiens cDNA FLJ32068 fis, clone OCBBF1000114. [AK056630]	
A_23_P204238	3.02E-04	2.461	NM_018416	NM_018416	Homo sapiens forkhead box J2 (FOXJ2), mRNA [NM_018416]	NM_018416
A_23_P125164	3.02E-04	2.132	NM_002012	NM_002012	Homo sapiens fragile histidine triad gene (FHIT), mRNA [NM_002012]	NM_002012
A_23_P17053	3.10E-04	5.922	NM_019618	NM_019618	Homo sapiens interleukin 1 family, member 9 (IL1F9), mRNA [NM_019618]	NM_019618
A_23_P33356	3.56E-04	22.25	AF261918	AF261918	Homo sapiens disintegrin metalloproteinase with thrombospondin repeats (ADAMTS9) mRNA, complete cds. [AF261918]	
A_24_P343233	3.56E-04	7.186	NM_002124	NM_002124	Homo sapiens major histocompatibility complex, class II, DR beta 1 (HLA-DRB1), mRNA [NM_002124]	NM_002124
A_23_P78405	3.60E-04	4.623	NM_006033	NM_006033	Homo sapiens lipase, endothelial (LIPG), mRNA [NM_006033]	NM_006033
A_23_P204286	3.70E-04	18.63	NM_000900	NM_000900	Homo sapiens matrix Gla protein (MGP), mRNA [NM_000900]	NM_000900
A_23_P31765	3.70E-04	12.46	NM_006823	NM_006823	Homo sapiens protein kinase (cAMP-dependent, catalytic) inhibitor alpha (PKIA), transcript variant 1, mRNA [NM_006823]	NM_006823
A_24_P247931	3.70E-04	2.885	NM_183420	NM_183420	Homo sapiens F-box protein 25 (FBXO25), transcript variant 2, mRNA [NM_183420]	NM_183420
A_23_P169494	3.81E-04	13.16	NM_000607	NM_000607	Homo sapiens orosomucoid 1 (ORM1), mRNA [NM_000607]	NM_000607
A_24_P817209	3.81E-04	3.296	AK125077	AK125077	Homo sapiens cDNA FLJ43087 fis, clone BRTHA3019105. [AK125077]	
A_23_P170273	3.81E-04	2.522	NM_014933	NM_014933	Homo sapiens SEC31-like 1 (S. cerevisiae) (SEC31L1), transcript variant 1, mRNA [NM_014933]	NM_014933
A_32_P229493	3.95E-04	6.631	BC004287	BC004287	Homo sapiens, clone IMAGE:3618365, mRNA. [BC004287]	
A_23_P114172	3.97E-04	12.3	NM_017752	NM_017752	Homo sapiens FLJ20298 protein (FLJ20298), transcript variant 1, mRNA [NM_017752]	NM_017752
A_23_P5983	3.97E-04	8.89	NM_006227	NM_006227	Homo sapiens phospholipid transfer protein (PLTP), transcript variant 1, mRNA [NM_006227]	NM_006227
A_23_P70719	3.97E-04	6.252	NM_000426	NM_000426	Homo sapiens laminin, alpha 2 (merosin, congenital muscular dystrophy) (LAMA2), mRNA [NM_000426]	NM_000426
A_23_P373031	3.97E-04	3.146	NM_000719	NM_000719	Homo sapiens calcium channel, voltage-dependent, L type, alpha 1C subunit (CACNA1C), mRNA [NM_000719]	NM_000719
A_23_P218706	3.97E-04	2.623	NM_024325	NM_024325	Homo sapiens zinc finger protein 343 (ZNF343), mRNA [NM_024325]	NM_024325
A_23_P334123	3.97E-04	2.39	NM_030790	NM_030790	Homo sapiens T-cell immunomodulatory protein (CDA08), mRNA [NM_030790]	NM_030790
A_23_P218463	3.97E-04	2.057	NM_013376	NM_013376	Homo sapiens SERTA domain containing 1 (SERTAD1), mRNA [NM_013376]	NM_013376
A_23_P138885	4.01E-04	11.72	NM_203371	NM_203371	Homo sapiens similar to RIKEN cDNA 1110018M03 (LOC387758), mRNA [NM_203371]	NM_203371
A_23_P75790	4.01E-04	10.57	NM_013279	NM_013279	Homo sapiens chromosome 11 open reading frame 9 (C11orf9), mRNA [NM_013279]	NM_013279
A_23_P34710	4.01E-04	4.939	ENST00000357180		full-length cDNA clone CS0DF032YO23 of Fetal brain of Homo sapiens (human). [CR604521]	
A_32_P186157	4.01E-04	4.817	NM_030626	NM_030626	Homo sapiens leucine rich repeat containing 27 (LRRC27), mRNA [NM_030626]	NM_030626
A_23_P60283	4.19E-04	3.116	NM_000380	NM_000380	Homo sapiens xeroderma pigmentosum, complementation group A (XPA), mRNA [NM_000380]	NM_000380
A_23_P410613	4.26E-04	10.65	NM_152261	NM_152261	Homo sapiens hypothetical protein MGC17943 (MGC17943), mRNA [NM_152261]	NM_152261
A_23_P205228	4.32E-04	12.64	NM_000053	NM_000053	Homo sapiens ATPase, Cu++ transporting, beta polypeptide (Wilson disease) (ATP7B), transcript variant 1, mRNA [NM_000053]	NM_000053
A_23_P45099	4.33E-04	10.49	NM_002125	NM_002125	Homo sapiens major histocompatibility complex, class II, DR beta 5 (HLA-DRB5), mRNA [NM_002125]	NM_002125
A_23_P144677	4.33E-04	8.619	AF178574	AF178574	Homo sapiens MSTP146 (MST146) mRNA, complete cds. [AF178574]	
A_23_P19102	4.33E-04	6.7	AK021777	AK021777	Homo sapiens cDNA FLJ11715 fis, clone HEMBA1005223. [AK021777]	
A_32_P228804	4.33E-04	4.372	BX649112	BX649112	Homo sapiens mRNA; cDNA DKFZp686E02109 (from clone DKFZp686E02109). [BX649112]	
A_23_P94591	4.33E-04	4.272	NM_032928	NM_032928	Homo sapiens hypothetical protein MGC14141 (MGC14141), mRNA [NM_032928]	NM_032928
A_23_P390032	4.33E-04	3.797	NM_153226	NM_153226	Homo sapiens transmembrane protein 20 (TMEM20), mRNA [NM_153226]	NM_153226
A_24_P756494	4.46E-04	9.055	AK057923	AK057923	Homo sapiens cDNA FLJ25194 fis, clone REC04095. [AK057923]	

A_23_P383532	4.46E-04	5.424	AK090988	AK090988	Homo sapiens cDNA FLJ33669 fis, clone BRAMY2028740. [AK090988]	
A_24_P50057	4.46E-04	4.958	A_24_P50057			
A_23_P71867	4.46E-04	4.131	NM_147162	NM_147162	Homo sapiens interleukin 11 receptor, alpha (IL11RA), transcript variant 2, mRNA [NM_147162]	NM_147162
A_23_P145965	4.48E-04	5.454	NM_003596	NM_003596	Homo sapiens tyrosylprotein sulfotransferase 1 (TPST1), mRNA [NM_003596]	NM_003596
A_32_P215856	4.48E-04	4.41	A_32_P215856			
A_23_P67799	4.48E-04	3.36	BC046362	BC046362	Homo sapiens transmembrane protein 37, mRNA (cDNA clone MGC:50757 IMAGE:5221396), complete cds. [BC046362]	
A_23_P73721	4.55E-04	3.5	NM_016656	NM_016656	Homo sapiens Ras-related GTP binding B (RRAGB), transcript variant RAGBl, mRNA [NM_016656]	NM_016656
A_23_P40217	4.64E-04	2.405	NM_018431	NM_018431	Homo sapiens docking protein 5 (DOK5), transcript variant 1, mRNA [NM_018431]	NM_018431
A_23_P21382	4.71E-04	4.285	NM_002292	NM_002292	Homo sapiens laminin, beta 2 (laminin S) (LAMB2), mRNA [NM_002292]	NM_002292
A_23_P106602	4.73E-04	8.727	NM_031476	NM_031476	Homo sapiens cysteine-rich secretory protein LCCL domain containing 2 (CRISPLD2), mRNA [NM_031476]	NM_031476
A_32_P147063	4.73E-04	6.238	AL355688	AL355688	Homo sapiens EST from clone 208499, full insert. [AL355688]	
A_24_P273799	4.75E-04	21.34	AL713659	AL713659	Homo sapiens mRNA; cDNA DKFZp667D1012 (from clone DKFZp667D1012). [AL713659]	
A_24_P340066	4.75E-04	11.23	NM_001421	NM_001421	Homo sapiens E74-like factor 4 (ets domain transcription factor) (ELF4), mRNA [NM_001421]	NM_001421
A_23_P9932	4.75E-04	9.823	NM_145341	NM_145341	Homo sapiens programmed cell death 4 (neoplastic transformation inhibitor) (PDCD4), transcript variant 2, mRNA [NM_145341]	NM_145341
A_23_P145336	4.75E-04	7.219	V00522	V00522	Human mRNA encoding major histocompatibility complex gene HLA-DR beta-I. [V00522]	
A_23_P13364	4.75E-04	5.315	NM_005013	NM_005013	Homo sapiens nucleobindin 2 (NUCB2), mRNA [NM_005013]	NM_005013
A_23_P254917	4.75E-04	4.063	NM_006149	NM_006149	Homo sapiens lectin, galactoside-binding, soluble, 4 (galectin 4) (LGALS4), mRNA [NM_006149]	NM_006149
A_23_P135634	4.75E-04	3.892	AF217963	AF217963	Homo sapiens NRAGE mRNA, complete cds. [AF217963]	
A_23_P201619	4.75E-04	3.681	AL080111	AL080111	Homo sapiens mRNA; cDNA DKFZp586G2222 (from clone DKFZp586G2222). [AL080111]	
A_23_P133133	4.77E-04	4.851	NM_025144	NM_025144	Homo sapiens alpha-kinase 1 (ALPK1), mRNA [NM_025144]	NM_025144
A_24_P370472	4.86E-04	8.855	NM_021983	NM_021983	Homo sapiens major histocompatibility complex, class II, DR beta 4 (HLA-DRB4), mRNA [NM_021983]	NM_021983
A_23_P68155	4.86E-04	5.777	NM_022168	NM_022168	Homo sapiens interferon induced with helicase C domain 1 (IFIH1), mRNA [NM_022168]	NM_022168
A_23_P401084	4.86E-04	5.267	NM_174945	NM_174945	Homo sapiens zinc finger protein 575 (ZNF575), mRNA [NM_174945]	NM_174945
A_23_P32454	4.86E-04	3.559	NM_003235	NM_003235	Homo sapiens thyroglobulin (TG), mRNA [NM_003235]	NM_003235
A_24_P39378	4.86E-04	2.61	NM_004748	NM_004748	Homo sapiens cell cycle progression 1 (CCPG1), transcript variant 1, mRNA [NM_004748]	NM_004748
A_23_P218111	4.98E-04	11.47	NM_001002236	NM_001002236	Homo sapiens serine (or cysteine) proteinase inhibitor, clade A (alpha-1 antiproteinase, antitrypsin), member 1 (SERPINA1), transcript variant 2, mRNA [NM_001002236]	NM_001002236
A_23_P255812	4.98E-04	7.789	NM_016127	NM_016127	Homo sapiens hypothetical protein MGC8721 (MGC8721), mRNA [NM_016127]	NM_016127
A_24_P298179	4.98E-04	7.41	ENST00000354530			
A_24_P397928	4.98E-04	3.22	NM_147780	NM_147780	Homo sapiens cathepsin B (CTSB), transcript variant 2, mRNA [NM_147780]	NM_147780
A_23_P64873	5.00E-04	190.8	NM_001920	NM_001920	Homo sapiens decorin (DCN), transcript variant A1, mRNA [NM_001920]	NM_001920
A_23_P20494	5.01E-04	17.86	NM_006096	NM_006096	Homo sapiens N-myc downstream regulated gene 1 (NDRG1), mRNA [NM_006096]	NM_006096
A_23_P17955	5.08E-04	4.711	NM_012157	NM_012157	Homo sapiens F-box and leucine-rich repeat protein 2 (FBXL2), mRNA [NM_012157]	NM_012157
A_23_P303155	5.08E-04	3.53	NM_032824	NM_032824	Homo sapiens hypothetical protein FLJ14681 (FLJ14681), mRNA [NM_032824]	NM_032824
A_23_P250164	5.09E-04	14.37	NM_000187	NM_000187	Homo sapiens homogentisate 1,2-dioxygenase (homogentisate oxidase) (HGD), mRNA [NM_000187]	NM_000187
A_23_P84018	5.09E-04	7.217	NM_000943	NM_000943	Homo sapiens peptidylprolyl isomerase C (cyclophilin C) (PPIC), mRNA [NM_000943]	NM_000943
A_23_P37914	5.15E-04	7.965	NM_052944	NM_052944	Homo sapiens solute carrier family 5 (sodium/glucose cotransporter), member 11 (SLC5A11), mRNA [NM_052944]	NM_052944
A_23_P353704	5.15E-04	2.894	NM_019593	NM_019593	Homo sapiens hypothetical protein KIAA1434 (KIAA1434), mRNA [NM_019593]	NM_019593
A_23_P130974	5.15E-04	2.336	NM_025249	NM_025249	Homo sapiens KIAA1683 (KIAA1683), mRNA [NM_025249]	NM_025249
A_24_P320699	5.24E-04	23.93	NM_001013398	NM_001013398	Homo sapiens insulin-like growth factor binding protein 3 (IGFBP3), transcript variant 1, mRNA [NM_001013398]	NM_001013398
A_32_P206123	5.26E-04	147.2	NM_000301	NM_000301	Homo sapiens plasminogen (PLG), mRNA [NM_000301]	NM_000301
A_23_P30693	5.26E-04	95.74	NM_000301	NM_000301	Homo sapiens plasminogen (PLG), mRNA [NM_000301]	NM_000301

A_23_P256504	5.26E-04	35.74	NM_001633	NM_001633	Homo sapiens alpha-1-microglobulin/bikunin precursor (AMBP), mRNA [NM_001633]	NM_001633
A_24_P418908	5.26E-04	10.8	AK021798	AK021798	Homo sapiens cDNA FLJ11736 fis, clone HEMBA1005468. [AK021798]	
A_24_P748377	5.26E-04	7.713	AL833456	AL833456	Homo sapiens mRNA; cDNA DKFZp686I18116 (from clone DKFZp686I18116). [AL833456]	
A_32_P159023	5.26E-04	6.432	THC2285720		BX114329 BX114329 Soares_NhHMPu_S1 Homo sapiens cDNA clone IMAGp998G064741 ; IMAGE:1932317, mRNA sequence [BX114329]	
A_23_P141180	5.26E-04	5.427	AK055959	AK055959	Homo sapiens cDNA FLJ31397 fis, clone NT2NE1000163. [AK055959]	
A_23_P65779	5.26E-04	5.33	NM_022369	NM_022369	Homo sapiens stimulated by retinoic acid gene 6 homolog (mouse) (STRA6), mRNA [NM_022369]	NM_022369
A_23_P99741	5.26E-04	4.422	NM_004196	NM_004196	Homo sapiens cyclin-dependent kinase-like 1 (CDC2-related kinase) (CDKL1), mRNA [NM_004196]	NM_004196
A_23_P305140	5.26E-04	4.253	NM_144591	NM_144591	Homo sapiens chromosome 10 open reading frame 32 (C10orf32), mRNA [NM_144591]	NM_144591
A_32_P45297	5.26E-04	3.63	THC2441058		Q81D10 (Q81D10) Phage protein, partial (21%) [THC2441058]	
A_23_P335452	5.26E-04	3.353	NM_153367	NM_153367	Homo sapiens chromosome 10 open reading frame 56 (C10orf56), mRNA [NM_153367]	NM_153367
A_23_P106405	5.26E-04	3.224	NM_002487	NM_002487	Homo sapiens necdin homolog (mouse) (NDN), mRNA [NM_002487]	NM_002487
A_24_P106297	5.26E-04	2.835	NM_014324	NM_014324	Homo sapiens alpha-methylacyl-CoA racemase (AMACR), transcript variant 1, mRNA [NM_014324]	NM_014324
A_23_P250800	5.26E-04	2.559	NM_006100	NM_006100	Homo sapiens ST3 beta-galactoside alpha-2,3-sialyltransferase 6 (ST3GAL6), mRNA [NM_006100]	NM_006100
A_23_P19590	5.26E-04	2.435	NM_003379	NM_003379	Homo sapiens villin 2 (ezrin) (VIL2), mRNA [NM_003379]	NM_003379
A_24_P408981	5.26E-04	2.427	ENST00000276672			
A_32_P214503	5.26E-04	2.165	BC038512	BC038512	Homo sapiens cDNA clone IMAGE:5262734, partial cds. [BC038512]	
A_24_P383660	5.26E-04	2.091	ENST00000312751			
A_23_P162861	5.27E-04	45.84	ENST00000332273		Sequence 1 from Patent WO03046006. [AX772926]	
A_23_P250444	5.27E-04	22.61	NM_000166	NM_000166	Homo sapiens gap junction protein, beta 1, 32kDa (connexin 32, Charcot-Marie-Tooth neuropathy, X-linked) (GJB1), mRNA [NM_000166]	NM_000166
A_24_P746314	5.27E-04	19.22	THC2403712		aspartate aminotranferase {Xylella fastidiosa Temecula1;} , partial (4%) [THC2403712]	
A_23_P99642	5.27E-04	18.37	NM_003982	NM_003982	Homo sapiens solute carrier family 7 (cationic amino acid transporter, y+ system), member 7 (SLC7A7), mRNA [NM_003982]	NM_003982
A_23_P94338	5.27E-04	18.34	NM_006209	NM_006209	Homo sapiens ectonucleotide pyrophosphatase/phosphodiesterase 2 (autotaxin) (ENPP2), mRNA [NM_006209]	NM_006209
A_23_P152262	5.27E-04	13.99	NM_004413	NM_004413	Homo sapiens dipeptidase 1 (renal) (DPEP1), mRNA [NM_004413]	NM_004413
A_23_P31006	5.27E-04	9.276	NM_002125	NM_002125	Homo sapiens major histocompatibility complex, class II, DR beta 5 (HLA-DRB5), mRNA [NM_002125]	NM_002125
A_23_P205900	5.27E-04	5.145	NM_001012338	NM_001012338	Homo sapiens neurotrophic tyrosine kinase, receptor, type 3 (NTRK3), transcript variant 1, mRNA [NM_001012338]	NM_001012338
A_24_P623814	5.27E-04	4.816	AK023526	AK023526	Homo sapiens cDNA FLJ13464 fis, clone PLACE1003478. [AK023526]	
A_23_P1782	5.27E-04	4.579	NM_002231	NM_002231	Homo sapiens CD82 antigen (CD82), transcript variant 1, mRNA [NM_002231]	NM_002231
A_32_P167239	5.27E-04	3.357	NM_152406	NM_152406	Homo sapiens hypothetical protein FLJ36748 (FLJ36748), mRNA [NM_152406]	NM_152406
A_23_P94159	5.27E-04	3.244	NM_183421	NM_183421	Homo sapiens F-box protein 25 (FBXO25), transcript variant 1, mRNA [NM_183421]	NM_183421
A_24_P379750	5.27E-04	2.782	NM_002357	NM_002357	Homo sapiens MAX dimerization protein 1 (MXD1), mRNA [NM_002357]	NM_002357
A_23_P132718	5.27E-04	2.765	NM_004636	NM_004636	Homo sapiens sema domain, immunoglobulin domain (Ig), short basic domain, secreted, (semaphorin) 3B (SEMA3B), transcript variant 1, mRNA [NM_004636]	NM_004636
A_23_P211522	5.27E-04	2.48	NM_145738	NM_145738	Homo sapiens synaptogyrin 1 (SYNGR1), transcript variant 1c, mRNA [NM_145738]	NM_145738
A_23_P375372	5.30E-04	115.6	NM_021871	NM_021871	Homo sapiens fibrinogen alpha chain (FGA), transcript variant alpha, mRNA [NM_021871]	NM_021871
A_24_P335202	5.30E-04	7.246	NM_015424	NM_015424	Homo sapiens chordin-like 2 (CHRDL2), mRNA [NM_015424]	NM_015424
A_32_P94801	5.30E-04	2.471	THC2308876			
A_24_P402222	5.34E-04	5.836	NM_022555	NM_022555	Homo sapiens major histocompatibility complex, class II, DR beta 3 (HLA-DRB3), mRNA [NM_022555]	NM_022555
A_24_P338648	5.34E-04	3.239	NM_001177	NM_001177	Homo sapiens ADP-ribosylation factor-like 1 (ARL1), mRNA [NM_001177]	NM_001177
A_24_P62469	5.34E-04	2.636	AK122589	AK122589	Homo sapiens mRNA for FLJ00414 protein. [AK122589]	XM_371214
A_23_P168587	5.39E-04	3.704	NM_138771	NM_138771	Homo sapiens alpha-1,3(6)-mannosylglycoprotein beta-1,6-N-acetyl-glucosaminyltransferase-like (LOC90693), mRNA [NM_138771]	NM_138771
A_24_P45367	5.39E-04	3.231	NM_020448	NM_020448	Homo sapiens NIPA-like domain containing 3 (NPAL3), mRNA [NM_020448]	NM_020448

A_23_P78099	5.45E-04	17.11	NM_000638	NM_000638	Homo sapiens vitronectin (serum spreading factor, somatomedin B, complement S-protein) (VTN), mRNA [NM_000638]	NM_000638
A_23_P378722	5.45E-04	7.992	NM_002970	NM_002970	Homo sapiens spermidine/spermine N1-acetyltransferase (SAT), mRNA [NM_002970]	NM_002970
A_23_P46238	5.45E-04	5.217	NM_033440	NM_033440	Homo sapiens elastase 2A (ELA2A), mRNA [NM_033440]	NM_033440
A_23_P257144	5.45E-04	2.227	ENST00000252804		Homo sapiens Melanoma associated gene, mRNA (cDNA clone IMAGE:3948774), partial cds. [BC009496]	XM_056455
A_23_P24414	5.49E-04	3.615	NM_016938	NM_016938	Homo sapiens EGF-containing fibulin-like extracellular matrix protein 2 (EFEMP2), mRNA [NM_016938]	NM_016938
A_23_P148088	5.54E-04	229.5	NM_000509	NM_000509	Homo sapiens fibrinogen gamma chain (FGG), transcript variant gamma-A, mRNA [NM_000509]	NM_000509
A_23_P31124	5.54E-04	62.05	NM_030820	NM_030820	Homo sapiens collagen, type XXI, alpha 1 (COL21A1), mRNA [NM_030820]	NM_030820
A_23_P217737	5.54E-04	14.47	L06133	L06133	Human putative Cu++-transporting P-type ATPase mRNA, complete cds. [L06133]	
A_24_P941572	5.54E-04	10.76	AK126071	AK126071	Homo sapiens cDNA FLJ44083 fis, clone TESTI4040939. [AK126071]	
A_23_P166297	5.54E-04	9.699	NM_207630	NM_207630	Homo sapiens ATP-binding cassette, sub-family G (WHITE), member 1 (ABCG1), transcript variant 1, mRNA [NM_207630]	NM_207630
A_23_P9485	5.54E-04	9.099	NM_000608	NM_000608	Homo sapiens orosomucoid 2 (ORM2), mRNA [NM_000608]	NM_000608
A_32_P77252	5.54E-04	5.948	THC2375310			
A_23_P70355	5.54E-04	4.194	NM_004568	NM_004568	Homo sapiens serine (or cysteine) proteinase inhibitor, clade B (ovalbumin), member 6 (SERPINB6), mRNA [NM_004568]	NM_004568
A_23_P81660	5.54E-04	3.826	NM_018368	NM_018368	Homo sapiens LMBR1 domain containing 1 (LMBRD1), mRNA [NM_018368]	NM_018368
A_32_P89352	5.54E-04	3.641	AK125899	AK125899	Homo sapiens cDNA FLJ43911 fis, clone TESTI4010928. [AK125899]	
A_23_P50504	5.54E-04	2.869	NM_000146	NM_000146	Homo sapiens ferritin, light polypeptide (FTL), mRNA [NM_000146]	NM_000146
A_32_P123527	5.54E-04	2.746	THC2366170		ALU5_HUMAN (P39192) Alu subfamily SC sequence contamination warning entry, partial (3%) [THC2366170]	
A_23_P134953	5.54E-04	2.39	NM_001122	NM_001122	Homo sapiens adipose differentiation-related protein (ADFP), mRNA [NM_001122]	NM_001122
A_23_P147423	5.63E-04	16.59	NM_182920	NM_182920	Homo sapiens a disintegrin-like and metalloprotease (reprolysin type) with thrombospondin type 1 motif, 9 (ADAMTS9), mRNA [NM_182920]	NM_182920
A_23_P27994	5.70E-04	3.621	NM_003332	NM_003332	Homo sapiens TYRO protein tyrosine kinase binding protein (TYROBP), transcript variant 1, mRNA [NM_003332]	NM_003332
A_23_P253896	5.71E-04	76.58	NM_198278	NM_198278	Homo sapiens likely ortholog of mouse nephronectin (NPNT), mRNA [NM_198278]	NM_198278
A_23_P19938	5.71E-04	6.373	NM_006854	NM_006854	Homo sapiens KDEL (Lys-Asp-Glu-Leu) endoplasmic reticulum protein retention receptor 2 (KDELR2), mRNA [NM_006854]	NM_006854
A_23_P149975	5.71E-04	5.656	NM_031453	NM_031453	Homo sapiens chromosome 10 open reading frame 45 (C10orf45), mRNA [NM_031453]	NM_031453
A_23_P108835	5.71E-04	3.125	NM_016061	NM_016061	Homo sapiens yippee-like 5 (Drosophila) (YPEL5), mRNA [NM_016061]	NM_016061
A_24_P74371	5.71E-04	2.024	NM_000308	NM_000308	Homo sapiens protective protein for beta-galactosidase (galactosialidosis) (PPGB), mRNA [NM_000308]	NM_000308
A_23_P50276	5.74E-04	18.48	NM_031917	NM_031917	Homo sapiens angiopoietin-like 6 (ANGPTL6), mRNA [NM_031917]	NM_031917
A_23_P132915	5.76E-04	5.029	NM_138389	NM_138389	Homo sapiens hypothetical protein BC001096 (LOC92689), mRNA [NM_138389]	NM_138389
A_23_P122052	5.76E-04	2.213	NM_001008397	NM_001008397	Homo sapiens similar to RIKEN cDNA 2310016C16 (LOC493869), mRNA [NM_001008397]	NM_001008397
A_24_P180243	5.78E-04	12.85	NM_053039	NM_053039	Homo sapiens UDP glucuronosyltransferase 2 family, polypeptide B28 (UGT2B28), mRNA [NM_053039]	NM_053039
A_24_P358406	5.78E-04	2.17	A_24_P358406			
A_24_P282060	5.85E-04	4.443	AK093006	AK093006	Homo sapiens cDNA FLJ35687 fis, clone SPLEN2019349. [AK093006]	
A_23_P254626	5.88E-04	2.972	NM_003919	NM_003919	Homo sapiens sarcoglycan, epsilon (SGCE), mRNA [NM_003919]	NM_003919
A_23_P120710	5.91E-04	3.984	NM_003316	NM_003316	Homo sapiens tetratricopeptide repeat domain 3 (TTC3), transcript variant 1, mRNA [NM_003316]	NM_003316
A_24_P612441	5.91E-04	3.944	CR591566	CR591566	full-length cDNA clone CS0DL004YB03 of B cells (Ramos cell line) Cot 25-normalized of Homo sapiens (human). [CR591566]	
A_23_P151915	5.91E-04	3.684	ENST00000313774		Homo sapiens glucosaminyl (N-acetyl) transferase 3, mucin type, mRNA (cDNA clone MGC:9086 IMAGE:3851937), complete cds. [BC017032]	
A_24_P320796	5.91E-04	3.246	NM_182827	NM_182827	Homo sapiens FK506 binding protein 9-like (FKBP9L), mRNA [NM_182827]	NM_182827
A_23_P394154	5.91E-04	2.252	NM_173457	NM_173457	Homo sapiens phosphodiesterase 8A (PDE8A), transcript variant 5, mRNA [NM_173457]	NM_173457
A_24_P247454	5.91E-04	2.098	A_24_P247454			
A_23_P27107	5.91E-04	2.094	NM_003963	NM_003963	Homo sapiens transmembrane 4 L six family member 5 (TM4SF5), mRNA [NM_003963]	NM_003963

A_24_P206624	5.96E-04	3.701	NM_022974	NM_022974	Homo sapiens fibroblast growth factor receptor 2 (bacteria-expressed kinase, keratinocyte growth factor receptor, craniofacial dysostosis 1, Crouzon syndrome, Pfeiffer syndrome, Jackson-Weiss syndrome) (FGFR2), transcript variant 7, mRNA [NM_022974]	NM_022974
A_23_P130113	5.99E-04	32.82	NM_080912	NM_080912	Homo sapiens asialoglycoprotein receptor 2 (ASGR2), transcript variant H2', mRNA [NM_080912]	NM_080912
A_23_P57227	6.03E-04	2.271	NM_003098	NM_003098	Homo sapiens syntrophin, alpha 1 (dystrophin-associated protein A1, 59kDa, acidic component) (SNTA1), mRNA [NM_003098]	NM_003098
A_23_P52266	6.05E-04	27.27	NM_001548	NM_001548	Homo sapiens interferon-induced protein with tetratricopeptide repeats 1 (IFIT1), transcript variant 2, mRNA [NM_001548]	NM_001548
A_24_P117620	6.05E-04	3.74	NM_018584	NM_018584	Homo sapiens calcium/calmodulin-dependent protein kinase II inhibitor 1 (CAMK2N1), mRNA [NM_018584]	NM_018584
A_24_P347566	6.05E-04	2.61	NM_015059	NM_015059	Homo sapiens talin 2 (TLN2), mRNA [NM_015059]	NM_015059
A_24_P406693	6.09E-04	13.17	NM_000917	NM_000917	Homo sapiens procollagen-proline, 2-oxoglutarate 4-dioxygenase (proline 4-hydroxylase), alpha polypeptide I (P4HA1), transcript variant 1, mRNA [NM_000917]	NM_000917
A_23_P155514	6.11E-04	81.89	NM_001622	NM_001622	Homo sapiens alpha-2-HS-glycoprotein (AHSG), mRNA [NM_001622]	NM_001622
A_24_P323072	6.12E-04	5.14	NM_178332	NM_178332	Homo sapiens gonadotropin-releasing hormone 2 (GNRH2), transcript variant 2, mRNA [NM_178332]	NM_178332
A_24_P251841	6.18E-04	10.57	ENST00000274031		Homo sapiens mRNA for KIAA1717 protein, partial cds. [AB051504]	
A_24_P158314	6.21E-04	6.083	NM_032293	NM_032293	Homo sapiens GTPase activating Rap/RanGAP domain-like 3 (GARNL3), mRNA [NM_032293]	NM_032293
A_32_P141969	6.24E-04	4.772	AK094929	AK094929	Homo sapiens cDNA FLJ37610 fis, clone BRCOC2011398. [AK094929]	
A_23_P154379	6.24E-04	4.424	NM_003960	NM_003960	Homo sapiens N-acetyltransferase 8 (camello like) (NAT8), mRNA [NM_003960]	NM_003960
A_23_P211207	6.29E-04	2.079	NM_015833	NM_015833	Homo sapiens adenosine deaminase, RNA-specific, B1 (RED1 homolog rat) (ADARB1), transcript variant DRABA2b, mRNA [NM_015833]	NM_015833
A_23_P27035	6.33E-04	6.154	NM_015544	NM_015544	Homo sapiens DKFZP564K1964 protein (DKFZP564K1964), mRNA [NM_015544]	NM_015544
A_23_P70290	6.33E-04	2.807	NM_018247	NM_018247	Homo sapiens transmembrane protein 30A (TMEM30A), mRNA [NM_018247]	NM_018247
A_24_P321525	6.40E-04	17.86	NM_032918	NM_032918	Homo sapiens RAS-like, estrogen-regulated, growth inhibitor (RERG), mRNA [NM_032918]	NM_032918
A_32_P126222	6.45E-04	4.375	AW302758	AW302758	AW302758 xr55g08.x1 NCI_CGAP_Ov26 Homo sapiens cDNA clone IMAGE:2764094 3', mRNA sequence [AW302758]	
A_24_P418687	6.45E-04	2.104	A_24_P418687			
A_23_P31810	6.46E-04	14.25	NM_005195	NM_005195	Homo sapiens CCAAT/enhancer binding protein (C/EBP), delta (CEBPD), mRNA [NM_005195]	NM_005195
A_23_P93141	6.61E-04	93.21	NM_153699	NM_153699	Homo sapiens glutathione S-transferase A5 (GSTA5), mRNA [NM_153699]	NM_153699
A_24_P145009	6.61E-04	19.61	NM_001013723	NM_001013723	Homo sapiens hypothetical gene supported by AK094370 (LOC441208), mRNA [NM_001013723]	NM_001013723
A_23_P64785	6.61E-04	15.68	NM_152320	NM_152320	Homo sapiens zinc finger protein 641 (ZNF641), mRNA [NM_152320]	NM_152320
A_32_P95034	6.61E-04	10.14	BC091525	BC091525	Homo sapiens cDNA clone IMAGE:30512240. [BC091525]	
A_23_P31399	6.61E-04	5.597	NM_000305	NM_000305	Homo sapiens paraoxonase 2 (PON2), transcript variant 1, mRNA [NM_000305]	NM_000305
A_23_P374104	6.61E-04	5.322	NM_012098	NM_012098	Homo sapiens angiopoietin-like 2 (ANGPTL2), mRNA [NM_012098]	NM_012098
A_23_P127220	6.61E-04	4.466	NM_021800	NM_021800	Homo sapiens DnaJ (Hsp40) homolog, subfamily C, member 12 (DNAJC12), transcript variant 1, mRNA [NM_021800]	NM_021800
A_24_P767725	6.61E-04	3.46	XM_496156	XM_496156	PREDICTED: Homo sapiens similar to rhophilin-like protein; RhoB effector; rhophilin-2; rhophilin 2 (LOC440368), mRNA [XM_496156]	XM_496156
A_23_P47879	6.61E-04	3.189	NM_003153	NM_003153	Homo sapiens signal transducer and activator of transcription 6, interleukin-4 induced (STAT6), mRNA [NM_003153]	NM_003153
A_23_P53439	6.61E-04	3.076	NM_138432	NM_138432	Homo sapiens serine dehydratase-like (SDSL), mRNA [NM_138432]	NM_138432
A_23_P75369	6.61E-04	2.858	NM_006019	NM_006019	Homo sapiens T-cell, immune regulator 1, ATPase, H+ transporting, lysosomal V0 protein a isoform 3 (TCIRG1), transcript variant 1, mRNA [NM_006019]	NM_006019
A_24_P364591	6.61E-04	2.573	NM_006329	NM_006329	Homo sapiens fibulin 5 (FBLN5), mRNA [NM_006329]	NM_006329
A_32_P497742	6.61E-04	2.543	AK091271	AK091271	Homo sapiens cDNA FLJ33952 fis, clone CTONG2018614. [AK091271]	
A_24_P161827	6.61E-04	2.124	A_24_P161827			
A_23_P159986	6.65E-04	10.19	BC007360	BC007360	Homo sapiens hypothetical protein MGC16121, mRNA (cDNA clone MGC:16121 IMAGE:3627113), complete cds. [BC007360]	
A_23_P252903	6.65E-04	6.525	NM_022464	NM_022464	Homo sapiens endoplasmic reticulum chaperone SIL1, homolog of yeast (SIL1), mRNA [NM_022464]	NM_022464
A_32_P187009	6.65E-04	4.441	AK026295	AK026295	Homo sapiens cDNA: FLJ22642 fis, clone HSI06970. [AK026295]	

A_32_P203749	6.69E-04	39.13	AF086547	AF086547	Homo sapiens full length insert cDNA clone ZE12B03. [AF086547]	
A_23_P300033	6.69E-04	12.79	NM_006206	NM_006206	Homo sapiens platelet-derived growth factor receptor, alpha polypeptide (PDGFRA), mRNA [NM_006206]	NM_006206
A_32_P150748	6.69E-04	6.021	G31710	G31710	sWSS2257 Eric D. Green Homo sapiens STS cDNA, sequence tagged site. [G31710]	
A_23_P365614	6.69E-04	5.355	NM_004557	NM_004557	Homo sapiens Notch homolog 4 (Drosophila) (NOTCH4), mRNA [NM_004557]	NM_004557
A_24_P31929	6.69E-04	2.145	NM_000950	NM_000950	Homo sapiens proline rich Gla (G-carboxyglutamic acid) 1 (PRRG1), mRNA [NM_000950]	NM_000950
A_24_P16230	6.69E-04	2.07	ENST00000331037			
A_23_P217379	6.76E-04	4.747	NM_033641	NM_033641	Homo sapiens collagen, type IV, alpha 6 (COL4A6), transcript variant B, mRNA [NM_033641]	NM_033641
A_24_P816844	6.80E-04	9.559	THC2433066			
A_24_P133288	6.80E-04	6.029	X97675	X97675	H.sapiens mRNA for plakophilin 2a and b. [X97675]	
A_24_P940006	6.80E-04	3.75	NM_001406	NM_001406	Homo sapiens ephrin-B3 (EFNB3), mRNA [NM_001406]	NM_001406
A_23_P373708	6.80E-04	2.238	NM_173624	NM_173624	Homo sapiens hypothetical protein FLJ40504 (FLJ40504), mRNA [NM_173624]	NM_173624
A_23_P27381	6.82E-04	7.593	NM_005786	NM_005786	Homo sapiens serologically defined colon cancer antigen 33 (SDCCAG33), mRNA [NM_005786]	NM_005786
A_23_P209408	6.84E-04	3.957	NM_032977	NM_032977	Homo sapiens caspase 10, apoptosis-related cysteine protease (CASP10), transcript variant D, mRNA [NM_032977]	NM_032977
A_23_P8834	6.85E-04	5.273	NM_001979	NM_001979	Homo sapiens epoxide hydrolase 2, cytoplasmic (EPHX2), mRNA [NM_001979]	NM_001979
A_23_P72643	6.85E-04	5.154	NM_003816	NM_003816	Homo sapiens a disintegrin and metalloproteinase domain 9 (meltrin gamma) (ADAM9), transcript variant 1, mRNA [NM_003816]	NM_003816
A_24_P922948	6.85E-04	2.644	ENST00000216214		Homo sapiens cDNA FLJ43037 fis, clone BRTHA3002933, highly similar to Homo sapiens uroplakin 3 (UPK3). [AK125027]	
A_24_P40721	6.87E-04	6.444	ENST00000217204		Q5TD12 (Q5TD12) OTTHUMP00000030291 (Fragment), partial (46%) [THC2403720]	
A_32_P27535	6.89E-04	2.618	ENST00000290997		Homo sapiens hypothetical protein FLJ11724, mRNA (cDNA clone IMAGE:5299340), partial cds. [BC045676]	
A_23_P70843	6.98E-04	2.165	NM_199186	NM_199186	Homo sapiens 2,3-bisphosphoglycerate mutase (BPGM), transcript variant 2, mRNA [NM_199186]	NM_199186
A_23_P120435	7.04E-04	4.905	NM_080614	NM_080614	Homo sapiens WAP four-disulfide core domain 3 (WFDC3), transcript variant 1, mRNA [NM_080614]	NM_080614
A_23_P20864	7.21E-04	12.61	NM_012098	NM_012098	Homo sapiens angiopoietin-like 2 (ANGPTL2), mRNA [NM_012098]	NM_012098
A_23_P354217	7.30E-04	2.752	NM_153266	NM_153266	Homo sapiens hypothetical protein MGC33486 (MGC33486), mRNA [NM_153266]	NM_153266
A_23_P330611	7.31E-04	4.548	NM_003387	NM_003387	Homo sapiens Wiskott-Aldrich syndrome protein interacting protein (WASPIP), mRNA [NM_003387]	NM_003387
A_23_P65240	7.43E-04	3.7	NM_001845	NM_001845	Homo sapiens collagen, type IV, alpha 1 (COL4A1), mRNA [NM_001845]	NM_001845
A_23_P4223	7.43E-04	3.193	NM_005831	NM_005831	Homo sapiens nuclear domain 10 protein (NDP52), mRNA [NM_005831]	NM_005831
A_23_P371107	7.43E-04	2.958	NM_014392	NM_014392	Homo sapiens DNA segment on chromosome 4 (unique) 234 expressed sequence (D4S234E), mRNA [NM_014392]	NM_014392
A_24_P195164	7.46E-04	2.191	A_24_P195164			
A_23_P134426	7.51E-04	13.96	NM_001005340	NM_001005340	Homo sapiens glycoprotein (transmembrane) nmb (GPNMB), transcript variant 1, mRNA [NM_001005340]	NM_001005340
A_24_P940115	7.51E-04	7.734	NM_182643	NM_182643	Homo sapiens deleted in liver cancer 1 (DLC1), transcript variant 1, mRNA [NM_182643]	NM_182643
A_23_P103877	7.56E-04	4.496	CR622769	CR622769	full-length cDNA clone CS0DI025YD24 of Placenta Cot 25-normalized of Homo sapiens (human). [CR622769]	
A_32_P122136	7.61E-04	4.293	AK057596	AK057596	Homo sapiens cDNA FLJ33034 fis, clone THYMU2000236. [AK057596]	XM_498456
A_24_P247074	7.61E-04	3.111	A_24_P247074			
A_23_P316472	7.61E-04	2.341	NM_144666	NM_144666	Homo sapiens hypothetical protein FLJ32752 (FLJ32752), mRNA [NM_144666]	NM_144666
A_24_P845223	7.65E-04	8.494	M27126	M27126	Human lymphocyte antigen (DRw8) mRNA. [M27126]	
A_24_P942517	7.65E-04	2.833	ENST00000246024		Homo sapiens mRNA for KIAA1162 protein, partial cds. [AB032988]	
A_23_P120973	7.74E-04	3.863	NM_017911	NM_017911	Homo sapiens chromosome 22 open reading frame 8 (C22orf8), mRNA [NM_017911]	NM_017911
A_24_P456723	7.74E-04	2.12	BC039374	BC039374	Homo sapiens, clone IMAGE:5271446, mRNA. [BC039374]	
A_23_P166677	7.76E-04	2.5	NM_022736	NM_022736	Homo sapiens major facilitator superfamily domain containing 1 (MFSD1), mRNA [NM_022736]	NM_022736
A_23_P39971	7.76E-04	2.063	NM_015701	NM_015701	Homo sapiens chromosome 2 open reading frame 30 (C2orf30), mRNA [NM_015701]	NM_015701
A_23_P397208	7.90E-04	2.927	NM_000848	NM_000848	Homo sapiens glutathione S-transferase M2 (muscle) (GSTM2), mRNA [NM_000848]	NM_000848
A_24_P414371	7.90E-04	2.384	NM_000944	NM_000944	Homo sapiens protein phosphatase 3 (formerly 2B), catalytic subunit, alpha isoform (calcineurin A alpha) (PPP3CA), mRNA [NM_000944]	NM_000944

A_23_P139632	7.90E-04	2.175	NM_002076	NM_002076	Homo sapiens glucosamine (N-acetyl)-6-sulfatase (Sanfilippo disease IIID) (GNS), mRNA [NM_002076]	NM_002076
A_23_P203183	8.07E-04	67.16	NM_000040	NM_000040	Homo sapiens apolipoprotein C-III (APOC3), mRNA [NM_000040]	NM_000040
A_24_P396702	8.07E-04	10.51	NM_014880	NM_014880	Homo sapiens CD302 antigen (CD302), mRNA [NM_014880]	NM_014880
A_23_P205177	8.07E-04	5.044	NM_000504	NM_000504	Homo sapiens coagulation factor X (F10), mRNA [NM_000504]	NM_000504
A_23_P95599	8.07E-04	4.541	NM_001012731	NM_001012731	Homo sapiens hypothetical protein LOC283874 (LOC283874), mRNA [NM_001012731]	NM_001012731
A_24_P129232	8.07E-04	3.651	NM_020755	NM_020755	Homo sapiens tumor differentially expressed 2 (TDE2), mRNA [NM_020755]	NM_020755
A_23_P70794	8.07E-04	3.445	NM_016277	NM_016277	Homo sapiens RAB23, member RAS oncogene family (RAB23), transcript variant 1, mRNA [NM_016277]	NM_016277
A_23_P79331	8.07E-04	2.865	NM_022152	NM_022152	Homo sapiens PP1201 protein (PP1201), mRNA [NM_022152]	NM_022152
A_24_P322741	8.07E-04	2.016	NM_000628	NM_000628	Homo sapiens interleukin 10 receptor, beta (IL10RB), mRNA [NM_000628]	NM_000628
A_23_P6335	8.08E-04	5.692	NM_000185	NM_000185	Homo sapiens serine (or cysteine) proteinase inhibitor, clade D (heparin cofactor), member 1 (SERPIND1), mRNA [NM_000185]	NM_000185
A_32_P217051	8.09E-04	13.75	THC2330484			
A_32_P159651	8.10E-04	10.11	NM_003884	NM_003884	Homo sapiens p300/CBP-associated factor (PCAF), mRNA [NM_003884]	NM_003884
A_23_P255884	8.10E-04	3.699	NM_198252	NM_198252	Homo sapiens gelsolin (amyloidosis, Finnish type) (GSN), transcript variant 2, mRNA [NM_198252]	NM_198252
A_23_P335958	8.24E-04	6.951	NM_152529	NM_152529	Homo sapiens G protein-coupled receptor 155 (GPR155), mRNA [NM_152529]	NM_152529
A_32_P107777	8.25E-04	20.98	THC2339241		Q822A8 (Q822A8) Ribonucleoside-diphosphate reductase, beta subunit, partial (5%) [THC2339241]	
A_24_P66592	8.25E-04	2.869	NM_173515	NM_173515	Homo sapiens CNKSR family member 3 (CNKSR3), mRNA [NM_173515]	NM_173515
A_24_P331727	8.27E-04	2.622	NM_181836	NM_181836	Homo sapiens transmembrane emp24 protein transport domain containing 7 (TMED7), mRNA [NM_181836]	NM_181836
A_23_P136355	8.28E-04	10.44	NM_018194	NM_018194	Homo sapiens hedgehog acyltransferase (HHAT), mRNA [NM_018194]	NM_018194
A_24_P944570	8.28E-04	3.67	AF200348	AF200348	Homo sapiens melanoma-associated antigen MG50 mRNA, partial cds. [AF200348]	
A_23_P128855	8.29E-04	15.28	NM_014579	NM_014579	Homo sapiens solute carrier family 39 (zinc transporter), member 2 (SLC39A2), mRNA [NM_014579]	NM_014579
A_24_P88801	8.39E-04	2.021	NM_000272	NM_000272	Homo sapiens nephronophthisis 1 (juvenile) (NPHP1), transcript variant 1, mRNA [NM_000272]	NM_000272
A_23_P344884	8.41E-04	6.343	BC036557	BC036557	Homo sapiens KIAA1394 protein, mRNA (cDNA clone MGC:39385 IMAGE:4310128), complete cds. [BC036557]	XM_208522
A_23_P356965	8.41E-04	3.264	ENST00000327026		Homo sapiens mRNA for FLJ00130 protein. [AK074059]	
A_32_P8604	8.53E-04	4.03	AK093691	AK093691	Homo sapiens cDNA FLJ36372 fis, clone THYMU2008072. [AK093691]	
A_24_P358474	8.53E-04	2.01	A_24_P358474			
A_23_P407695	8.60E-04	40.98	NM_176782	NM_176782	Homo sapiens chromosome 1 open reading frame 179 (C1orf179), mRNA [NM_176782]	NM_176782
A_24_P896373	8.60E-04	4.095	THC2300907			
A_23_P138635	8.64E-04	5.729	NM_004052	NM_004052	Homo sapiens BCL2/adenovirus E1B 19kDa interacting protein 3 (BNIP3), nuclear gene encoding mitochondrial protein, mRNA [NM_004052]	NM_004052
A_23_P211631	8.70E-04	6.001	NM_006486	NM_006486	Homo sapiens fibulin 1 (FBLN1), transcript variant D, mRNA [NM_006486]	NM_006486
A_24_P71700	8.70E-04	3.394	NM_145166	NM_145166	Homo sapiens zinc finger protein 651 (ZNF651), mRNA [NM_145166]	NM_145166
A_24_P564396	8.73E-04	4.045	THC2335868		ALU5_HUMAN (P39192) Alu subfamily SC sequence contamination warning entry, partial (8%) [THC2335868]	
A_23_P23855	8.73E-04	2.273	NM_032305	NM_032305	Homo sapiens polymerase (RNA) III (DNA directed) polypeptide G (32kD) like (POLR3GL), mRNA [NM_032305]	NM_032305
A_23_P22143	8.77E-04	2.795	NM_000283	NM_000283	Homo sapiens phosphodiesterase 6B, cGMP-specific, rod, beta (congenital stationary night blindness 3, autosomal dominant) (PDE6B), mRNA [NM_000283]	NM_000283
A_23_P309599	8.78E-04	3.835	AK026760	AK026760	Homo sapiens cDNA: FLJ23107 fis, clone LNG07738. [AK026760]	
A_32_P56001	8.89E-04	22.6	NM_012072	NM_012072	Homo sapiens complement component 1, q subcomponent, receptor 1 (C1QR1), mRNA [NM_012072]	NM_012072
A_23_P157580	8.90E-04	2.003	NM_005625	NM_005625	Homo sapiens syndecan binding protein (syntenin) (SDCBP), transcript variant 1, mRNA [NM_005625]	NM_005625
A_23_P396666	8.97E-04	2.234	NM_015079	NM_015079	Homo sapiens KIAA1055 protein (KIAA1055), mRNA [NM_015079]	NM_015079
A_23_P37441	9.01E-04	9.997	NM_004048	NM_004048	Homo sapiens beta-2-microglobulin (B2M), mRNA [NM_004048]	NM_004048
A_23_P138899	9.01E-04	2.25	NM_016451	NM_016451	Homo sapiens coatomer protein complex, subunit beta (COPB), mRNA [NM_016451]	NM_016451
A_24_P303647	9.04E-04	4.058	ENST00000297145		Homo sapiens mRNA; cDNA DKFZp762M126 (from clone DKFZp762M126). [AL834437]	
A_32_P52609	9.04E-04	3.39	NM_145693	NM_145693	Homo sapiens lipin 1 (LPIN1), mRNA [NM_145693]	NM_145693

A_24_P300394	9.10E-04	94.12	NM_000846	NM_000846	Homo sapiens glutathione S-transferase A2 (GSTA2), mRNA [NM_000846]	NM_000846
A_23_P215634	9.11E-04	53.52	NM_001013398	NM_001013398	Homo sapiens insulin-like growth factor binding protein 3 (IGFBP3), transcript variant 1, mRNA [NM_001013398]	NM_001013398
A_24_P397386	9.11E-04	18.38	BX648635	BX648635	Homo sapiens mRNA; cDNA DKFZp686C03120 (from clone DKFZp686C03120). [BX648635]	
A_23_P66798	9.11E-04	8.463	NM_002276	NM_002276	Homo sapiens keratin 19 (KRT19), mRNA [NM_002276]	NM_002276
A_32_P227870	9.11E-04	7.805	BC042520	BC042520	Homo sapiens, clone IMAGE:4828750, mRNA. [BC042520]	
A_32_P159535	9.11E-04	5.884	THC2303047		RS24_HUMAN (P62847) 40S ribosomal protein S24, partial (77%) [THC2303047]	
A_23_P50498	9.11E-04	3.06	NM_000146	NM_000146	Homo sapiens ferritin, light polypeptide (FTL), mRNA [NM_000146]	NM_000146
A_23_P385206	9.11E-04	2.75	NM_177424	NM_177424	Homo sapiens syntaxin 12 (STX12), mRNA [NM_177424]	NM_177424
A_23_P91910	9.16E-04	13.39	NM_020353	NM_020353	Homo sapiens phospholipid scramblase 4 (PLSCR4), mRNA [NM_020353]	NM_020353
A_23_P155857	9.16E-04	3.178	NM_198041	NM_198041	Homo sapiens nudix (nucleoside diphosphate linked moiety X)-type motif 6 (NUDT6), transcript variant 2, mRNA [NM_198041]	NM_198041
A_24_P395610	9.28E-04	3.52	NM_201433	NM_201433	Homo sapiens growth arrest-specific 7 (GAS7), transcript variant c, mRNA [NM_201433]	NM_201433
A_24_P372913	9.31E-04	12.55	NM_000545	NM_000545	Homo sapiens transcription factor 1, hepatic; LF-B1, hepatic nuclear factor (HNF1), albumin proximal factor (TCF1), mRNA [NM_000545]	NM_000545
A_24_P104174	9.31E-04	2.333	NM_016281	NM_016281	Homo sapiens TAO kinase 3 (TAOK3), mRNA [NM_016281]	NM_016281
A_24_P105391	9.31E-04	2.277	NM_001017922	NM_001017922	Homo sapiens erythroblast membrane-associated protein (ERMAP), transcript variant 1, mRNA [NM_001017922]	NM_001017922
A_24_P920715	9.39E-04	33.95	A_24_P920715			
A_23_P80570	9.41E-04	12.5	NM_001086	NM_001086	Homo sapiens arylacetamide deacetylase (esterase) (AADAC), mRNA [NM_001086]	NM_001086
A_24_P368023	9.43E-04	3.321	BC039457	BC039457	Homo sapiens cDNA clone IMAGE:5312122, partial cds. [BC039457]	
A_23_P252817	9.54E-04	71.81	NM_001048	NM_001048	Homo sapiens somatostatin (SST), mRNA [NM_001048]	NM_001048
A_24_P344961	9.54E-04	8.46	NM_133265	NM_133265	Homo sapiens angiomotin (AMOT), mRNA [NM_133265]	NM_133265
A_24_P914434	9.62E-04	7.866	NM_000849	NM_000849	Homo sapiens glutathione S-transferase M3 (brain) (GSTM3), mRNA [NM_000849]	NM_000849
A_32_P192480	9.65E-04	11.16	CR627122	CR627122	Homo sapiens mRNA; cDNA DKFZp779M2422 (from clone DKFZp779M2422). [CR627122]	
A_23_P84334	9.66E-04	2.108	NM_018266	NM_018266	Homo sapiens transmembrane protein 39A (TMEM39A), mRNA [NM_018266]	NM_018266
A_23_P12884	9.70E-04	12.26	NM_005308	NM_005308	Homo sapiens G protein-coupled receptor kinase 5 (GRK5), mRNA [NM_005308]	NM_005308
A_24_P520767	9.70E-04	3.284	BC036441	BC036441	Homo sapiens hypothetical protein LOC149351, mRNA (cDNA clone IMAGE:5273076), with apparent retained intron. [BC036441]	XM_378876
A_24_P170763	9.70E-04	2.891	NM_015299	NM_015299	Homo sapiens KIAA0323 (KIAA0323), mRNA [NM_015299]	NM_015299
A_23_P163567	9.72E-04	15.61	NM_018667	NM_018667	Homo sapiens sphingomyelin phosphodiesterase 3, neutral membrane (neutral sphingomyelinase II) (SMPD3), mRNA [NM_018667]	NM_018667
A_23_P308519	9.72E-04	11.4	NM_004252	NM_004252	Homo sapiens solute carrier family 9 (sodium/hydrogen exchanger), isoform 3 regulator 1 (SLC9A3R1), mRNA [NM_004252]	NM_004252
A_24_P566916	9.72E-04	8.543	CR618687	CR618687	full-length cDNA clone CS0CAP008YE23 of Thymus of Homo sapiens (human). [CR618687]	
A_24_P364807	9.72E-04	7.187	BX641069	BX641069	Homo sapiens mRNA; cDNA DKFZp686H22112 (from clone DKFZp686H22112). [BX641069]	
A_23_P106773	9.72E-04	6.364	NM_177528	NM_177528	Homo sapiens sulfotransferase family, cytosolic, 1A, phenol-preferring, member 2 (SULT1A2), transcript variant 2, mRNA [NM_177528]	NM_177528
A_24_P163590	9.72E-04	4.774	NM_152556	NM_152556	Homo sapiens hypothetical protein FLJ31818 (FLJ31818), mRNA [NM_152556]	NM_152556
A_24_P201353	9.72E-04	4.184	NM_144591	NM_144591	Homo sapiens chromosome 10 open reading frame 32 (C10orf32), mRNA [NM_144591]	NM_144591
A_32_P385587	9.72E-04	3.833	NM_000032	NM_000032	Homo sapiens aminolevulinate, delta-, synthase 2 (sideroblastic/hypochromic anemia) (ALAS2), nuclear gene encoding mitochondrial protein, mRNA [NM_000032]	NM_000032
A_23_P412392	9.72E-04	3.334	NM_004892	NM_004892	Homo sapiens SEC22 vesicle trafficking protein-like 1 (S. cerevisiae) (SEC22L1), mRNA [NM_004892]	NM_004892
A_23_P56709	9.72E-04	3.302	NM_005667	NM_005667	Homo sapiens ring finger protein 103 (RNF103), mRNA [NM_005667]	NM_005667
A_24_P180424	9.72E-04	2.953	NM_018247	NM_018247	Homo sapiens transmembrane protein 30A (TMEM30A), mRNA [NM_018247]	NM_018247
A_32_P110016	9.72E-04	2.932	A_32_P110016			
A_23_P4096	9.89E-04	12.39	NM_000717	NM_000717	Homo sapiens carbonic anhydrase IV (CA4), mRNA [NM_000717]	NM_000717
A_24_P242391	9.89E-04	2.577	NM_003953	NM_003953	Homo sapiens myelin protein zero-like 1 (MPZL1), transcript variant 1, mRNA [NM_003953]	NM_003953
A_24_P267293	9.92E-04	4.331	NM_015077	NM_015077	Homo sapiens sterile alpha and TIR motif containing 1 (SARM1), mRNA [NM_015077]	NM_015077

A_23_P32029	9.92E-04	2.98	NM_007001	NM_007001	Homo sapiens solute carrier family 35, member D2 (SLC35D2), mRNA [NM_007001]	NM_007001
A_24_P110558	9.95E-04	6.544	NM_001007189	NM_001007189	Homo sapiens similar to bovine IgA regulatory protein (LOC492311), mRNA [NM_001007189]	NM_001007189
A_23_P254756	9.98E-04	4.228	NM_006016	NM_006016	Homo sapiens CD164 antigen, sialomucin (CD164), mRNA [NM_006016]	NM_006016
A_23_P212500	1.01E-03	177.3	NM_001063	NM_001063	Homo sapiens transferrin (TF), mRNA [NM_001063]	NM_001063
A_24_P379413	1.01E-03	19.7	NM_000565	NM_000565	Homo sapiens interleukin 6 receptor (IL6R), transcript variant 1, mRNA [NM_000565]	NM_000565
A_23_P100660	1.01E-03	9.404	NM_002615	NM_002615	Homo sapiens serine (or cysteine) proteinase inhibitor, clade F (alpha-2 antiplasmin, pigment epithelium derived factor), member 1 (SERPINF1), mRNA [NM_002615]	NM_002615
A_24_P772488	1.01E-03	8.022	THC2311602			
A_23_P92042	1.01E-03	6.805	NM_002222	NM_002222	Homo sapiens inositol 1,4,5-triphosphate receptor, type 1 (ITPR1), mRNA [NM_002222]	NM_002222
A_24_P838743	1.01E-03	4.733	NM_004568	NM_004568	Homo sapiens serine (or cysteine) proteinase inhibitor, clade B (ovalbumin), member 6 (SERPINB6), mRNA [NM_004568]	NM_004568
A_23_P201368	1.01E-03	4.182	NM_004388	NM_004388	Homo sapiens chitobiase, di-N-acetyl- (CTBS), mRNA [NM_004388]	NM_004388
A_23_P356731	1.01E-03	3.632	NM_152509	NM_152509	Homo sapiens hypothetical protein FLJ31568 (FLJ31568), mRNA [NM_152509]	NM_152509
A_32_P41924	1.01E-03	3.622	AF086011	AF086011	Homo sapiens full length insert cDNA clone YW18A11. [AF086011]	
A_23_P92710	1.01E-03	3.231	NM_014899	NM_014899	Homo sapiens Rho-related BTB domain containing 3 (RHOBTB3), mRNA [NM_014899]	NM_014899
A_24_P936758	1.01E-03	2.833	AK074614	AK074614	Homo sapiens cDNA FLJ90133 fis, clone HEMBB1000567. [AK074614]	
A_24_P924484	1.02E-03	5.997	K03200	K03200	Human melanoma-associated antigen p97 (melanotransferrin) mRNA, 3' flank. [K03200]	
A_23_P65401	1.02E-03	3.034	NM_021914	NM_021914	Homo sapiens cofilin 2 (muscle) (CFL2), transcript variant 1, mRNA [NM_021914]	NM_021914
A_32_P120330	1.03E-03	10.02	THC2329478			
A_23_P117286	1.03E-03	3.331	NM_006493	NM_006493	Homo sapiens ceroid-lipofuscinosis, neuronal 5 (CLN5), mRNA [NM_006493]	NM_006493
A_32_P108592	1.03E-03	2.072	THC2312637		moxR3 {Mycobacterium smegmatis str. MC2 155;} , partial (3%) [THC2312637]	
A_24_P402242	1.04E-03	86	NM_000090	NM_000090	Homo sapiens collagen, type III, alpha 1 (Ehlers-Danlos syndrome type IV, autosomal dominant) (COL3A1), mRNA [NM_000090]	NM_000090
A_23_P372834	1.04E-03	36.12	NM_000385	NM_000385	Homo sapiens aquaporin 1 (channel-forming integral protein, 28kDa) (AQP1), transcript variant 2, mRNA [NM_000385]	NM_000385
A_32_P2161	1.04E-03	3.373	AK023040	AK023040	Homo sapiens cDNA FLJ12978 fis, clone NT2RP2006321. [AK023040]	
A_23_P169197	1.04E-03	3.033	NM_032303	NM_032303	Homo sapiens hydroxysteroid dehydrogenase like 2 (HSDL2), mRNA [NM_032303]	NM_032303
A_23_P81880	1.04E-03	2.237	NM_005730	NM_005730	Homo sapiens CTD (carboxy-terminal domain, RNA polymerase II, polypeptide A) small phosphatase 2 (CTDSP2), mRNA [NM_005730]	NM_005730
A_24_P24645	1.04E-03	2.073	XM_497978	XM_497978	PREDICTED: Homo sapiens similar to Keratin, type I cytoskeletal 18 (Cytokeratin 18) (K18) (CK 18) (LOC132391), mRNA [XM_497978]	XM_497978
A_24_P879740	1.04E-03	2.061	CR595522	CR595522	full-length cDNA clone CS0DC001YI12 of Neuroblastoma Cot 25-normalized of Homo sapiens (human). [CR595522]	
A_23_P212508	1.05E-03	81.48	NM_001063	NM_001063	Homo sapiens transferrin (TF), mRNA [NM_001063]	NM_001063
A_23_P120227	1.05E-03	4.995	NM_030915	NM_030915	Homo sapiens likely ortholog of mouse limb-bud and heart gene (LBH), mRNA [NM_030915]	NM_030915
A_24_P322191	1.05E-03	4.582	NM_005831	NM_005831	Homo sapiens nuclear domain 10 protein (NDP52), mRNA [NM_005831]	NM_005831
A_24_P4816	1.05E-03	4.178	NM_031412	NM_031412	Homo sapiens GABA(A) receptor-associated protein like 1 (GABARAPL1), mRNA [NM_031412]	NM_031412
A_24_P95154	1.05E-03	2.015	NM_178234	NM_178234	Homo sapiens tumor suppressor candidate 3 (TUSC3), transcript variant 2, mRNA [NM_178234]	NM_178234
A_32_P11372	1.06E-03	19.34	NM_174950	NM_174950	Homo sapiens hypothetical protein FLJ30435 (FLJ30435), mRNA [NM_174950]	NM_174950
A_23_P110957	1.06E-03	16.84	NM_001452	NM_001452	Homo sapiens forkhead box F2 (FOXF2), mRNA [NM_001452]	NM_001452
A_23_P414654	1.06E-03	15.57	NM_175738	NM_175738	Homo sapiens RAB37, member RAS oncogene family (RAB37), mRNA [NM_175738]	NM_175738
A_24_P161733	1.06E-03	2.205	A_24_P161733			
A_32_P186348	1.07E-03	3.967	THC2388093		STPHRG deoxyribodipyrimidine photolyase {Salmonella typhimurium;} , partial (4%) [THC2388093]	
A_24_P383480	1.08E-03	14.53	NM_000313	NM_000313	Homo sapiens protein S (alpha) (PROS1), mRNA [NM_000313]	NM_000313
A_24_P359942	1.08E-03	9.134	NM_014483	NM_014483	Homo sapiens RNA binding motif, single stranded interacting protein (RBMS3), transcript variant 2, mRNA [NM_014483]	NM_014483
A_32_P15288	1.08E-03	6.416	THC2285720		BX114329 BX114329 Soares_NhHMPu_S1 Homo sapiens cDNA clone IMAGp998G064741 ; IMAGE:1932317, mRNA sequence [BX114329]	

A_32_P3214	1.08E-03	5.471	THC2433340		Q93KJ6 (Q93KJ6) Dissimilatory (Bi-)sulfite reductase beta subunit (Fragment), partial (5%) [THC2433340]	
A_23_P422724	1.08E-03	5.145	NM_000943	NM_000943	Homo sapiens peptidylprolyl isomerase C (cyclophilin C) (PPIC), mRNA [NM_000943]	NM_000943
A_24_P802145	1.08E-03	2.665	THC2339772			
A_24_P329065	1.08E-03	2.653	NM_007048	NM_007048	Homo sapiens butyrophilin, subfamily 3, member A1 (BTN3A1), transcript variant 1, mRNA [NM_007048]	NM_007048
A_32_P131929	1.08E-03	2.47	AK000028	AK000028	Homo sapiens cDNA FLJ20021 fis, clone ADSE01233. [AK000028]	XM_028217
A_23_P431789	1.08E-03	2.461	NM_001660	NM_001660	Homo sapiens ADP-ribosylation factor 4 (ARF4), mRNA [NM_001660]	NM_001660
A_23_P365119	1.08E-03	2.308	NM_152675	NM_152675	Homo sapiens hypothetical protein FLJ23754 (FLJ23754), mRNA [NM_152675]	NM_152675
A_24_P233560	1.10E-03	8.775	A_24_P233560			
A_23_P342275	1.10E-03	8.583	NM_006988	NM_006988	Homo sapiens a disintegrin-like and metalloprotease (reprolysin type) with thrombospondin type 1 motif, 1 (ADAMTS1), mRNA [NM_006988]	NM_006988
A_23_P211039	1.10E-03	7.919	NM_006988	NM_006988	Homo sapiens a disintegrin-like and metalloprotease (reprolysin type) with thrombospondin type 1 motif, 1 (ADAMTS1), mRNA [NM_006988]	NM_006988
A_23_P26890	1.10E-03	6.033	NM_032950	NM_032950	Homo sapiens matrix metalloproteinase 28 (MMP28), transcript variant 2, mRNA [NM_032950]	NM_032950
A_23_P32036	1.10E-03	4.429	NM_017881	NM_017881	Homo sapiens chromosome 9 open reading frame 95 (C9orf95), mRNA [NM_017881]	NM_017881
A_23_P304682	1.10E-03	4.141	ENST00000320378		Homo sapiens cDNA FLJ39084 fis, clone NT2RP7018871. [AK096403]	
A_23_P94030	1.10E-03	3.964	NM_002291	NM_002291	Homo sapiens laminin, beta 1 (LAMB1), mRNA [NM_002291]	NM_002291
A_24_P152649	1.10E-03	3.533	AK092522	AK092522	Homo sapiens cDNA FLJ35203 fis, clone PLACE6018441, moderately similar to Mus musculus peroxisomal long chain acyl-CoA thioesterase Ib (Pte1b) gene. [AK092522]	
A_32_P140475	1.10E-03	3.409	NM_020802	NM_020802	Homo sapiens KIAA1377 protein (KIAA1377), mRNA [NM_020802]	NM_020802
A_23_P76015	1.10E-03	2.751	NM_014786	NM_014786	Homo sapiens Rho guanine nucleotide exchange factor (GEF) 17 (ARHGEF17), mRNA [NM_014786]	NM_014786
A_32_P186678	1.11E-03	2.178	A_32_P186678			
A_32_P112546	1.12E-03	2.611	A_32_P112546			
A_23_P306105	1.12E-03	2.179	NM_020474	NM_020474	Homo sapiens UDP-N-acetyl-alpha-D-galactosamine:polypeptide N-acetylgalactosaminyltransferase 1 (GalNAc-T1) (GALNT1), mRNA [NM_020474]	NM_020474
A_23_P116187	1.13E-03	44.5	NM_031938	NM_031938	Homo sapiens beta-carotene dioxygenase 2 (BCDO2), mRNA [NM_031938]	NM_031938
A_23_P70307	1.13E-03	18.61	NM_022138	NM_022138	Homo sapiens SPARC related modular calcium binding 2 (SMOC2), mRNA [NM_022138]	NM_022138
A_23_P131435	1.13E-03	14.76	NM_014880	NM_014880	Homo sapiens CD302 antigen (CD302), mRNA [NM_014880]	NM_014880
A_23_P170453	1.13E-03	5.508	NM_001900	NM_001900	Homo sapiens cystatin D (CST5), mRNA [NM_001900]	NM_001900
A_32_P181166	1.13E-03	3.484	NM_174896	NM_174896	Homo sapiens chromosome 1 open reading frame 162 (C1orf162), mRNA [NM_174896]	NM_174896
A_23_P157371	1.13E-03	2.585	NM_014888	NM_014888	Homo sapiens family with sequence similarity 3, member C (FAM3C), mRNA [NM_014888]	NM_014888
A_24_P273857	1.14E-03	6.466	NM_012082	NM_012082	Homo sapiens zinc finger protein, multitype 2 (ZFPM2), mRNA [NM_012082]	NM_012082
A_24_P944458	1.14E-03	5.077	NM_016133	NM_016133	Homo sapiens insulin induced gene 2 (INSIG2), mRNA [NM_016133]	NM_016133
A_23_P376211	1.14E-03	4.562	NM_007270	NM_007270	Homo sapiens FK506 binding protein 9, 63 kDa (FKBP9), mRNA [NM_007270]	NM_007270
A_23_P21644	1.14E-03	3.607	NM_016245	NM_016245	Homo sapiens dehydrogenase/reductase (SDR family) member 8 (DHRS8), mRNA [NM_016245]	NM_016245
A_24_P391526	1.14E-03	3.142	NM_001005333	NM_001005333	Homo sapiens melanoma antigen family D, 1 (MAGED1), transcript variant 1, mRNA [NM_001005333]	NM_001005333
A_24_P253318	1.14E-03	2.168	NM_018477	NM_018477	Homo sapiens actin-related protein 10 homolog (S. cerevisiae) (ACTR10), mRNA [NM_018477]	NM_018477
A_23_P102258	1.14E-03	2.065	NM_053050	NM_053050	Homo sapiens mitochondrial ribosomal protein L53 (MRPL53), nuclear gene encoding mitochondrial protein, mRNA [NM_053050]	NM_053050
A_23_P155509	1.15E-03	423.2	NM_001622	NM_001622	Homo sapiens alpha-2-HS-glycoprotein (AHSG), mRNA [NM_001622]	NM_001622
A_23_P83007	1.15E-03	15.56	NM_203403	NM_203403	Homo sapiens chromosome 9 open reading frame 150 (C9orf150), mRNA [NM_203403]	NM_203403
A_23_P391906	1.15E-03	4.743	BC044246	BC044246	Homo sapiens KIAA1913, mRNA (cDNA clone MGC:50847 IMAGE:5760073), complete cds. [BC044246]	
A_23_P217447	1.15E-03	4.195	NM_002294	NM_002294	Homo sapiens lysosomal-associated membrane protein 2 (LAMP2), transcript variant LAMP2A, mRNA [NM_002294]	NM_002294
A_23_P258887	1.15E-03	2.523	NM_012190	NM_012190	Homo sapiens aldehyde dehydrogenase 1 family, member L1 (ALDH1L1), mRNA [NM_012190]	NM_012190
A_23_P154585	1.15E-03	2.439	NM_033421	NM_033421	Homo sapiens chromosome 20 open reading frame 161 (C20orf161), transcript variant 1, mRNA [NM_033421]	NM_033421

A_32_P42989	1.16E-03	27.74	A_32_P42989			
A_23_P253661	1.16E-03	8.091	NM_024902	NM_024902	Homo sapiens hypothetical protein FLJ13236 (FLJ13236), mRNA [NM_024902]	NM_024902
A_23_P434212	1.17E-03	5.81	NM_177529	NM_177529	Homo sapiens sulfotransferase family, cytosolic, 1A, phenol-preferring, member 1 (SULT1A1), transcript variant 2, mRNA [NM_177529]	NM_177529
A_23_P122068	1.17E-03	3.09	NM_181435	NM_181435	Homo sapiens C1q and tumor necrosis factor related protein 3 (C1QTNF3), transcript variant 2, mRNA [NM_181435]	NM_181435
A_24_P255954	1.17E-03	2.141	A_24_P255954			
A_23_P72025	1.18E-03	4.168	NM_000387	NM_000387	Homo sapiens solute carrier family 25 (carnitine/acylcarnitine translocase), member 20 (SLC25A20), nuclear gene encoding mitochondrial protein, mRNA [NM_000387]	NM_000387
A_24_P839239	1.18E-03	3.844	AK002036	AK002036	Homo sapiens cDNA FLJ11174 fis, clone PLACE1007367. [AK002036]	
A_24_P118608	1.18E-03	2.49	NM_032437	NM_032437	Homo sapiens KIAA1799 protein (KIAA1799), mRNA [NM_032437]	NM_032437
A_24_P923483	1.20E-03	60.78	BC020867	BC020867	Homo sapiens solute carrier family 6 (neurotransmitter transporter, GABA), member 13, mRNA (cDNA clone IMAGE:4594185), complete cds. [BC020867]	
A_23_P72117	1.20E-03	15.25	NM_006714	NM_006714	Homo sapiens sphingomyelin phosphodiesterase, acid-like 3A (SMPDL3A), mRNA [NM_006714]	NM_006714
A_24_P200023	1.20E-03	5.518	NM_000877	NM_000877	Homo sapiens interleukin 1 receptor, type I (IL1R1), mRNA [NM_000877]	NM_000877
A_23_P211493	1.20E-03	3.978	NM_153609	NM_153609	Homo sapiens transmembrane protease, serine 6 (TMPRSS6), mRNA [NM_153609]	NM_153609
A_23_P75283	1.21E-03	32.84	NM_006744	NM_006744	Homo sapiens retinol binding protein 4, plasma (RBP4), mRNA [NM_006744]	NM_006744
A_23_P123454	1.21E-03	4.537	NM_024815	NM_024815	Homo sapiens nudix (nucleoside diphosphate linked moiety X)-type motif 18 (NUDT18), mRNA [NM_024815]	NM_024815
A_23_P89145	1.21E-03	2.175	NM_018300	NM_018300	Homo sapiens zinc finger protein 83 (HPF1) (ZNF83), mRNA [NM_018300]	NM_018300
A_24_P264293	1.21E-03	2.158	A_24_P264293			
A_23_P135417	1.22E-03	105.9	NM_145740	NM_145740	Homo sapiens glutathione S-transferase A1 (GSTA1), mRNA [NM_145740]	NM_145740
A_23_P146274	1.22E-03	29.13	NM_007029	NM_007029	Homo sapiens stathmin-like 2 (STMN2), mRNA [NM_007029]	NM_007029
A_32_P228886	1.22E-03	3.305	BX115350	BX115350	BX115350 Soares fetal liver spleen 1NFLS Homo sapiens cDNA clone IMAGp998L11127, mRNA sequence [BX115350]	
A_23_P213085	1.22E-03	2.51	AK075364	AK075364	Homo sapiens cDNA PSEC0051 fis, clone NT2RP2000168. [AK075364]	
A_23_P41487	1.23E-03	11.88	NM_015130	NM_015130	Homo sapiens KIAA0882 protein (KIAA0882), mRNA [NM_015130]	NM_015130
A_23_P110212	1.23E-03	3.09	NM_001995	NM_001995	Homo sapiens acyl-CoA synthetase long-chain family member 1 (ACSL1), mRNA [NM_001995]	NM_001995
A_23_P45361	1.23E-03	2.415	NM_012084	NM_012084	Homo sapiens glutamate dehydrogenase 2 (GLUD2), mRNA [NM_012084]	NM_012084
A_23_P94879	1.24E-03	56.65	NM_000506	NM_000506	Homo sapiens coagulation factor II (thrombin) (F2), mRNA [NM_000506]	NM_000506
A_24_P355568	1.24E-03	12.25	NM_005330	NM_005330	Homo sapiens hemoglobin, epsilon 1 (HBE1), mRNA [NM_005330]	NM_005330
A_23_P109934	1.24E-03	4.576	NM_016173	NM_016173	Homo sapiens HemK methyltransferase family member 1 (HEMK1), mRNA [NM_016173]	NM_016173
A_23_P6413	1.24E-03	4.56	NM_080430	NM_080430	Homo sapiens selenoprotein M (SELM), mRNA [NM_080430]	NM_080430
A_24_P102080	1.24E-03	3.516	AF070629	AF070629	Homo sapiens clone 24484 RAB2 mRNA, partial cds. [AF070629]	
A_24_P192840	1.24E-03	3.199	THC2437510		ALU4_HUMAN (P39191) Alu subfamily SB2 sequence contamination warning entry, partial (27%) [THC2437510]	
A_24_P308506	1.24E-03	2.097	NM_016347	NM_016347	Homo sapiens putative N-acetyltransferase Camello 2 (CML2), mRNA [NM_016347]	NM_016347
A_23_P12928	1.25E-03	47.03	ENST00000340797		Homo sapiens solute carrier family 5 (sodium/glucose cotransporter), member 12, mRNA (cDNA clone IMAGE:5187504), partial cds. [BC041454]	
A_24_P296689	1.25E-03	3.346	AK124426	AK124426	Homo sapiens cDNA FLJ42435 fis, clone BLADE2006849. [AK124426]	
A_23_P56898	1.26E-03	7.661	NM_003937	NM_003937	Homo sapiens kynureninase (L-kynurenine hydrolase) (KYNU), mRNA [NM_003937]	NM_003937
A_32_P429876	1.26E-03	6.27	AK092378	AK092378	Homo sapiens cDNA FLJ35059 fis, clone OCBBF2018827. [AK092378]	
A_23_P343594	1.26E-03	5.287	BC067300	BC067300	Homo sapiens cDNA clone IMAGE:30389677, partial cds. [BC067300]	
A_24_P627415	1.26E-03	3.304	THC2268216		NCB2_HUMAN (P80303) Nucleobindin 2 precursor (DNA-binding protein NEFA), partial (49%) [THC2268216]	
A_24_P358131	1.26E-03	2.14	ENST00000351766			
A_24_P145134	1.26E-03	2.008	BC010943	BC010943	Homo sapiens oncostatin M receptor, mRNA (cDNA clone IMAGE:4043935), complete cds. [BC010943]	
A_23_P58205	1.27E-03	473.6	NM_001134	NM_001134	Homo sapiens alpha-fetoprotein (AFP), mRNA [NM_001134]	NM_001134
A_24_P270424	1.27E-03	23.83	AK124946	AK124946	Homo sapiens cDNA FLJ42956 fis, clone BRSTN2009899. [AK124946]	

A_23_P79289	1.27E-03	16.01	NM_014900	NM_014900	Homo sapiens COBL-like 1 (COBLL1), mRNA [NM_014900]	NM_014900
A_23_P211504	1.27E-03	8.578	NM_016657	NM_016657	Homo sapiens KDEL (Lys-Asp-Glu-Leu) endoplasmic reticulum protein retention receptor 3 (KDELR3), transcript variant 2, mRNA [NM_016657]	NM_016657
A_23_P104224	1.27E-03	5.511	NM_138933	NM_138933	Homo sapiens apobec-1 complementation factor (ACF), transcript variant 3, mRNA [NM_138933]	NM_138933
A_23_P98876	1.27E-03	4.84	NM_173596	NM_173596	Homo sapiens solute carrier family 39 (metal ion transporter), member 5 (SLC39A5), mRNA [NM_173596]	NM_173596
A_23_P25746	1.27E-03	4.778	BC007251	BC007251	Homo sapiens chromosome 14 open reading frame 128, mRNA (cDNA clone MGC:15504 IMAGE:2990071), complete cds. [BC007251]	
A_23_P78265	1.27E-03	4.18	NM_004138	NM_004138	Homo sapiens keratin, hair, acidic, 3A (KRTHA3A), mRNA [NM_004138]	NM_004138
A_23_P102571	1.27E-03	3.758	NM_020062	NM_020062	Homo sapiens SLC2A4 regulator (SLC2A4RG), mRNA [NM_020062]	NM_020062
A_32_P42236	1.27E-03	3.685	XM_370839	XM_370839	PREDICTED: Homo sapiens similar to hypothetical protein (LOC440234), mRNA [XM_370839]	XM_370839
A_24_P902195	1.27E-03	3.574	A_24_P902195			
A_23_P361569	1.27E-03	3.32	NM_025181	NM_025181	Homo sapiens solute carrier family 35, member F5 (SLC35F5), mRNA [NM_025181]	NM_025181
A_24_P257348	1.27E-03	3.315	NM_006407	NM_006407	Homo sapiens ADP-ribosylation-like factor 6 interacting protein 5 (ARL6IP5), mRNA [NM_006407]	NM_006407
A_23_P69941	1.27E-03	2.593	AK002097	AK002097	Homo sapiens cDNA FLJ11235 fis, clone PLACE1008488. [AK002097]	XM_496773
A_24_P371194	1.27E-03	2.567	NM_053050	NM_053050	Homo sapiens mitochondrial ribosomal protein L53 (MRPL53), nuclear gene encoding mitochondrial protein, mRNA [NM_053050]	NM_053050
A_23_P127128	1.27E-03	2.52	NM_022365	NM_022365	Homo sapiens DnaJ (Hsp40) homolog, subfamily C, member 1 (DNAJC1), mRNA [NM_022365]	NM_022365
A_24_P265051	1.28E-03	7.289	AK095606	AK095606	Homo sapiens cDNA FLJ38287 fis, clone FCBBF3008362, moderately similar to PLEXIN 4 PRECURSOR. [AK095606]	XM_039393
A_24_P321766	1.28E-03	3.81	NM_000624	NM_000624	Homo sapiens serine (or cysteine) proteinase inhibitor, clade A (alpha-1 antiproteinase, antitrypsin), member 5 (SERPINA5), mRNA [NM_000624]	NM_000624
A_23_P121926	1.29E-03	37.99	NM_005410	NM_005410	Homo sapiens selenoprotein P, plasma, 1 (SEPP1), mRNA [NM_005410]	NM_005410
A_23_P52362	1.29E-03	4.528	NM_003055	NM_003055	Homo sapiens solute carrier family 18 (vesicular acetylcholine), member 3 (SLC18A3), mRNA [NM_003055]	NM_003055
A_23_P123228	1.30E-03	4.578	NM_000111	NM_000111	Homo sapiens solute carrier family 26, member 3 (SLC26A3), mRNA [NM_000111]	NM_000111
A_32_P180958	1.30E-03	4.562	THC2401540		ALU8_HUMAN (P39195) Alu subfamily SX sequence contamination warning entry, partial (27%) [THC2401540]	
A_24_P166789	1.30E-03	4.48	THC2361388			
A_24_P252078	1.31E-03	3.344	BC067086	BC067086	Homo sapiens cDNA clone MGC:71335 IMAGE:6088873, complete cds. [BC067086]	
A_23_P103433	1.31E-03	2.643	NM_145047	NM_145047	Homo sapiens chromosome 1 open reading frame 102 (C1orf102), transcript variant 1, mRNA [NM_145047]	NM_145047
A_23_P14673	1.31E-03	2.523	NM_020962	NM_020962	Homo sapiens likely ortholog of mouse neighbor of Punc E11 (NOPE), mRNA [NM_020962]	NM_020962
A_24_P944616	1.31E-03	2.323	AK023129	AK023129	Homo sapiens cDNA FLJ13067 fis, clone NT2RP3001712, highly similar to Homo sapiens HP1-BP74 protein mRNA. [AK023129]	
A_23_P301521	1.32E-03	6.633	AK055602	AK055602	Homo sapiens cDNA FLJ31040 fis, clone HSYRA2000224. [AK055602]	
A_24_P328646	1.32E-03	2.016	NM_005778	NM_005778	Homo sapiens RNA binding motif protein 5 (RBM5), mRNA [NM_005778]	NM_005778
A_23_P387184	1.33E-03	3.039	ENST00000343505		Homo sapiens mRNA for KIAA1357 protein, partial cds. [AB037778]	XM_496826
A_23_P23748	1.33E-03	2.055	NM_014969	NM_014969	Homo sapiens WD repeat domain 47 (WDR47), mRNA [NM_014969]	NM_014969
A_23_P114423	1.34E-03	16.82	NM_004683	NM_004683	Homo sapiens regucalcin (senescence marker protein-30) (RGN), transcript variant 1, mRNA [NM_004683]	NM_004683
A_32_P85539	1.34E-03	12.15	THC2364429			
A_23_P162589	1.34E-03	7.227	NM_001017535	NM_001017535	Homo sapiens vitamin D (1,25- dihydroxyvitamin D3) receptor (VDR), transcript variant 2, mRNA [NM_001017535]	NM_001017535
A_24_P277349	1.34E-03	2.426	NM_014933	NM_014933	Homo sapiens SEC31-like 1 (S. cerevisiae) (SEC31L1), transcript variant 1, mRNA [NM_014933]	NM_014933
A_23_P62932	1.35E-03	6.295	NM_001677	NM_001677	Homo sapiens ATPase, Na+/K+ transporting, beta 1 polypeptide (ATP1B1), transcript variant 1, mRNA [NM_001677]	NM_001677
A_23_P204980	1.35E-03	3.601	NM_020121	NM_020121	Homo sapiens UDP-glucose ceramide glucosyltransferase-like 2 (UGCGL2), mRNA [NM_020121]	NM_020121
A_24_P345451	1.35E-03	3.577	NM_024843	NM_024843	Homo sapiens cytochrome b reductase 1 (CYBRD1), mRNA [NM_024843]	NM_024843
A_23_P48936	1.35E-03	2.94	NM_005902	NM_005902	Homo sapiens SMAD, mothers against DPP homolog 3 (Drosophila) (SMAD3), mRNA [NM_005902]	NM_005902
A_23_P212522	1.35E-03	2.372	NM_014616	NM_014616	Homo sapiens ATPase, Class VI, type 11B (ATP11B), mRNA [NM_014616]	NM_014616
A_24_P745352	1.35E-03	2.206	A_24_P745352			

A_23_P131676	1.36E-03	22.49	NM_020311	NM_020311	Homo sapiens chemokine orphan receptor 1 (CMKOR1), mRNA [NM_020311]	NM_020311
A_24_P213684	1.36E-03	3.85	AB062434	AB062434	Homo sapiens mRNA for OK/SW-CL.24, complete cds. [AB062434]	
A_24_P379649	1.36E-03	2.47	NR_002229	NR_002229	Homo sapiens ribosomal protein L23a pseudogene 13 (RPL23AP13) on chromosome 2 [NR_002229]	NR_002229
A_23_P99320	1.36E-03	2.428	NM_000224	NM_000224	Homo sapiens keratin 18 (KRT18), transcript variant 1, mRNA [NM_000224]	NM_000224
A_23_P19894	1.37E-03	48.91	NM_198098	NM_198098	Homo sapiens aquaporin 1 (channel-forming integral protein, 28kDa) (AQP1), transcript variant 1, mRNA [NM_198098]	NM_198098
A_32_P228341	1.37E-03	4.649	AK055783	AK055783	Homo sapiens cDNA FLJ31221 fis, clone KIDNE2004279. [AK055783]	
A_23_P404259	1.37E-03	2.237	NM_001008397	NM_001008397	Homo sapiens similar to RIKEN cDNA 2310016C16 (LOC493869), mRNA [NM_001008397]	NM_001008397
A_24_P471242	1.38E-03	2.1	A_24_P471242			
A_23_P21207	1.39E-03	8.598	NM_003335	NM_003335	Homo sapiens ubiquitin-activating enzyme E1-like (UBE1L), mRNA [NM_003335]	NM_003335
A_24_P211565	1.39E-03	6.832	NM_031910	NM_031910	Homo sapiens C1q and tumor necrosis factor related protein 6 (C1QTNF6), transcript variant 1, mRNA [NM_031910]	NM_031910
A_24_P251053	1.39E-03	2.662	NM_024329	NM_024329	Homo sapiens EF-hand domain family, member D2 (EFHD2), mRNA [NM_024329]	NM_024329
A_23_P12044	1.39E-03	2.647	NM_018022	NM_018022	Homo sapiens transmembrane protein 51 (TMEM51), mRNA [NM_018022]	NM_018022
A_23_P203819	1.40E-03	3.076	NM_005895	NM_005895	Homo sapiens golgi autoantigen, golgin subfamily a, 3 (GOLGA3), mRNA [NM_005895]	NM_005895
A_23_P204879	1.40E-03	2.326	NM_030925	NM_030925	Homo sapiens calcium binding protein 39-like (CAB39L), mRNA [NM_030925]	NM_030925
A_24_P478940	1.41E-03	3.614	A_24_P478940			
A_32_P4626	1.41E-03	2.75	AF143325	AF143325	Homo sapiens clone IMAGE:110436 mRNA sequence. [AF143325]	
A_23_P399265	1.42E-03	14.94	S82024	S82024	SCG10=neuron-specific growth-associated protein/stathmin homolog [human, embryo, mRNA, 696 nt]. [S82024]	
A_23_P54376	1.42E-03	2.973	NM_004809	NM_004809	Homo sapiens stomatin (EPB72)-like 1 (STOML1), mRNA [NM_004809]	NM_004809
A_23_P367899	1.42E-03	2.459	NM_000121	NM_000121	Homo sapiens erythropoietin receptor (EPOR), mRNA [NM_000121]	NM_000121
A_23_P92623	1.42E-03	2.406	NM_000944	NM_000944	Homo sapiens protein phosphatase 3 (formerly 2B), catalytic subunit, alpha isoform (calcineurin A alpha) (PPP3CA), mRNA [NM_000944]	NM_000944
A_23_P200067	1.42E-03	2.138	NM_004442	NM_004442	Homo sapiens EPH receptor B2 (EPHB2), transcript variant 2, mRNA [NM_004442]	NM_004442
A_24_P302249	1.43E-03	66.26	NM_001643	NM_001643	Homo sapiens apolipoprotein A-II (APOA2), mRNA [NM_001643]	NM_001643
A_23_P118254	1.43E-03	21.83	NM_001451	NM_001451	Homo sapiens forkhead box F1 (FOXF1), mRNA [NM_001451]	NM_001451
A_23_P257003	1.43E-03	5.738	NM_006200	NM_006200	Homo sapiens proprotein convertase subtilisin/kexin type 5 (PCSK5), mRNA [NM_006200]	NM_006200
A_23_P166640	1.43E-03	2.958	NM_006407	NM_006407	Homo sapiens ADP-ribosylation-like factor 6 interacting protein 5 (ARL6IP5), mRNA [NM_006407]	NM_006407
A_23_P129704	1.43E-03	2.637	NM_018975	NM_018975	Homo sapiens telomeric repeat binding factor 2, interacting protein (TERF2IP), mRNA [NM_018975]	NM_018975
A_23_P20752	1.44E-03	4.775	NM_178432	NM_178432	Homo sapiens cell cycle related kinase (CCRK), transcript variant 1, mRNA [NM_178432]	NM_178432
A_24_P279489	1.44E-03	3.102	AK123107	AK123107	Homo sapiens cDNA FLJ41112 fis, clone BRACE1000239. [AK123107]	
A_23_P215931	1.44E-03	2.559	NM_015344	NM_015344	Homo sapiens leptin receptor overlapping transcript-like 1 (LEPROTL1), mRNA [NM_015344]	NM_015344
A_24_P277807	1.44E-03	2.371	NM_152828	NM_152828	Homo sapiens sorting nexin 3 (SNX3), transcript variant 3, mRNA [NM_152828]	NM_152828
A_32_P175098	1.44E-03	2.134	CR613736	CR613736	full-length cDNA clone CS0DI072YA21 of Placenta Cot 25-normalized of Homo sapiens (human). [CR613736]	
A_23_P258912	1.44E-03	2.116	NM_003970	NM_003970	Homo sapiens myomesin (M-protein) 2, 165kDa (MYOM2), mRNA [NM_003970]	NM_003970
A_23_P7642	1.45E-03	25.13	NM_003118	NM_003118	Homo sapiens secreted protein, acidic, cysteine-rich (osteonectin) (SPARC), mRNA [NM_003118]	NM_003118
A_23_P154338	1.45E-03	4.959	NM_025202	NM_025202	Homo sapiens EF-hand domain family, member D1 (EFHD1), mRNA [NM_025202]	NM_025202
A_23_P417331	1.46E-03	2.929	NM_004586	NM_004586	Homo sapiens ribosomal protein S6 kinase, 90kDa, polypeptide 3 (RPS6KA3), mRNA [NM_004586]	NM_004586
A_24_P151032	1.46E-03	2.439	NM_002476	NM_002476	Homo sapiens myosin, light polypeptide 4, alkali; atrial, embryonic (MYL4), transcript variant 2, mRNA [NM_002476]	NM_002476
A_23_P113212	1.47E-03	4.866	NM_018004	NM_018004	Homo sapiens transmembrane protein 45A (TMEM45A), mRNA [NM_018004]	NM_018004
A_23_P114929	1.47E-03	4.748	NM_015415	NM_015415	Homo sapiens brain protein 44 (BRP44), mRNA [NM_015415]	NM_015415
A_32_P197720	1.48E-03	4.835	CR738137	CR738137	CR738137 Soares_testis_NHT Homo sapiens cDNA clone IMAGp971D1846 ; IMAGE:731424 5', mRNA sequence [CR738137]	
A_24_P247233	1.48E-03	2.188	XM_498018	XM_498018	PREDICTED: Homo sapiens similar to Keratin, type I cytoskeletal 18 (Cytokeratin 18) (K18) (CK 18) (LOC391827), mRNA [XM_498018]	XM_498018

A_32_P47107	1.48E-03	2.186	CR610759	CR610759	full-length cDNA clone CS0DI026YO05 of Placenta Cot 25-normalized of Homo sapiens (human). [CR610759]	
A_23_P203191	1.49E-03	31.52	NM_000039	NM_000039	Homo sapiens apolipoprotein A-I (APOA1), mRNA [NM_000039]	NM_000039
A_23_P143867	1.49E-03	2.539	NM_007114	NM_007114	Homo sapiens TATA element modulatory factor 1 (TMF1), mRNA [NM_007114]	NM_007114
A_23_P120794	1.50E-03	8.956	NM_004173	NM_004173	Homo sapiens solute carrier family 7 (cationic amino acid transporter, y+ system), member 4 (SLC7A4), mRNA [NM_004173]	NM_004173
A_23_P26847	1.50E-03	5.175	NM_000346	NM_000346	Homo sapiens SRY (sex determining region Y)-box 9 (campomelic dysplasia, autosomal sex-reversal) (SOX9), mRNA [NM_000346]	NM_000346
A_24_P75920	1.50E-03	3.14	ENST00000327804		Homo sapiens hypothetical protein LOC126075, mRNA (cDNA clone IMAGE:5171832). [BC038439]	XM_496289
A_23_P157299	1.50E-03	2.336	NM_001129	NM_001129	Homo sapiens AE binding protein 1 (AEBP1), mRNA [NM_001129]	NM_001129
A_23_P31584	1.50E-03	2.058	NM_022777	NM_022777	Homo sapiens RAB, member RAS oncogene family-like 5 (RABL5), mRNA [NM_022777]	NM_022777
A_24_P355944	1.51E-03	3.062	NM_004093	NM_004093	Homo sapiens ephrin-B2 (EFNB2), mRNA [NM_004093]	NM_004093
A_23_P69383	1.52E-03	4.892	NM_031458	NM_031458	Homo sapiens poly (ADP-ribose) polymerase family, member 9 (PARP9), mRNA [NM_031458]	NM_031458
A_23_P42265	1.53E-03	22.72	NM_019101	NM_019101	Homo sapiens apolipoprotein M (APOM), mRNA [NM_019101]	NM_019101
A_24_P926993	1.53E-03	12.04	A_24_P926993			
A_24_P105191	1.53E-03	7.225	NM_147175	NM_147175	Homo sapiens heparan sulfate 6-O-sulfotransferase 2 (HS6ST2), transcript variant S, mRNA [NM_147175]	NM_147175
A_24_P97785	1.53E-03	3.864	CR611332	CR611332	full-length cDNA clone CS0DF014YA22 of Fetal brain of Homo sapiens (human). [CR611332]	
A_23_P79836	1.53E-03	3.441	NM_006811	NM_006811	Homo sapiens tumor differentially expressed 1 (TDE1), transcript variant 1, mRNA [NM_006811]	NM_006811
A_32_P223859	1.54E-03	8.53	NM_006714	NM_006714	Homo sapiens sphingomyelin phosphodiesterase, acid-like 3A (SMPDL3A), mRNA [NM_006714]	NM_006714
A_23_P146554	1.54E-03	8.458	NM_000954	NM_000954	Homo sapiens prostaglandin D2 synthase 21kDa (brain) (PTGDS), mRNA [NM_000954]	NM_000954
A_23_P170719	1.54E-03	5.828	A_23_P170719			
A_32_P221590	1.54E-03	4.08	A_32_P221590			
A_32_P90709	1.54E-03	3.781	BC045657	BC045657	Homo sapiens, clone IMAGE:5270591, mRNA. [BC045657]	
A_24_P49183	1.54E-03	3.488	NM_017820	NM_017820	Homo sapiens hypothetical protein FLJ20433 (FLJ20433), mRNA [NM_017820]	NM_017820
A_23_P348894	1.54E-03	2.87	NM_006364	NM_006364	Homo sapiens Sec23 homolog A (S. cerevisiae) (SEC23A), mRNA [NM_006364]	NM_006364
A_32_P4814	1.54E-03	2.582	NM_032508	NM_032508	Homo sapiens family with sequence similarity 11, member A (FAM11A), mRNA [NM_032508]	NM_032508
A_23_P95221	1.55E-03	86.31	NM_005577	NM_005577	Homo sapiens lipoprotein, Lp(a) (LPA), mRNA [NM_005577]	NM_005577
A_23_P114814	1.55E-03	11.42	NM_021205	NM_021205	Homo sapiens ras homolog gene family, member U (RHOU), mRNA [NM_021205]	NM_021205
A_24_P344516	1.55E-03	6.431	NM_024924	NM_024924	Homo sapiens hypothetical protein FLJ12985 (FLJ12985), mRNA [NM_024924]	NM_024924
A_23_P137532	1.55E-03	2.609	NM_000302	NM_000302	Homo sapiens procollagen-lysine 1, 2-oxoglutarate 5-dioxygenase 1 (PLOD1), mRNA [NM_000302]	NM_000302
A_23_P71170	1.55E-03	2.24	NM_018646	NM_018646	Homo sapiens transient receptor potential cation channel, subfamily V, member 6 (TRPV6), mRNA [NM_018646]	NM_018646
A_23_P202978	1.57E-03	10.16	NM_033292	NM_033292	Homo sapiens caspase 1, apoptosis-related cysteine protease (interleukin 1, beta, convertase) (CASP1), transcript variant alpha, mRNA [NM_033292]	NM_033292
A_23_P251412	1.57E-03	4.92	NM_006998	NM_006998	Homo sapiens secretagogin, EF-hand calcium binding protein (SCGN), mRNA [NM_006998]	NM_006998
A_24_P103803	1.58E-03	5.534	NM_001497	NM_001497	Homo sapiens UDP-Gal:betaGlcNAc beta 1,4- galactosyltransferase, polypeptide 1 (B4GALT1), mRNA [NM_001497]	NM_001497
A_23_P363034	1.58E-03	3.448	ENST00000307201		Homo sapiens hypothetical protein LOC116236, mRNA (cDNA clone MGC:21749 IMAGE:4537124), complete cds. [BC012476]	
A_23_P67971	1.59E-03	2.594	NM_138801	NM_138801	Homo sapiens galactose mutarotase (aldose 1-epimerase) (GALM), mRNA [NM_138801]	NM_138801
A_23_P1083	1.60E-03	7.18	NM_002060	NM_002060	Homo sapiens gap junction protein, alpha 4, 37kDa (connexin 37) (GJA4), mRNA [NM_002060]	NM_002060
A_23_P145238	1.60E-03	3.061	NM_080593	NM_080593	Homo sapiens histone 1, H2bk (HIST1H2BK), mRNA [NM_080593]	NM_080593
A_23_P126474	1.60E-03	2.741	NM_003145	NM_003145	Homo sapiens signal sequence receptor, beta (translocon-associated protein beta) (SSR2), mRNA [NM_003145]	NM_003145
A_23_P328652	1.60E-03	2.173	NM_153240	NM_153240	Homo sapiens nephronophthisis 3 (adolescent) (NPHP3), mRNA [NM_153240]	NM_153240
A_24_P211064	1.60E-03	2.01	NM_020648	NM_020648	Homo sapiens twisted gastrulation homolog 1 (Drosophila) (TWSG1), mRNA [NM_020648]	NM_020648
A_24_P589028	1.61E-03	15.81	AK026750	AK026750	Homo sapiens cDNA: FLJ23097 fis, clone LNG07418. [AK026750]	
A_24_P842962	1.61E-03	14.91	THC2379232			

A_23_P23996	1.61E-03	11.03	NM_000429	NM_000429	Homo sapiens methionine adenosyltransferase I, alpha (MAT1A), mRNA [NM_000429]	NM_000429
A_23_P170280	1.61E-03	7.401	NM_020453	NM_020453	Homo sapiens ATPase, Class V, type 10D (ATP10D), mRNA [NM_020453]	NM_020453
A_24_P795371	1.62E-03	12.17	AK000872	AK000872	Homo sapiens cDNA FLJ10010 fis, clone HEMBA1000302. [AK000872]	XM_498625
A_23_P139500	1.62E-03	9.429	NM_030762	NM_030762	Homo sapiens basic helix-loop-helix domain containing, class B, 3 (BHLHB3), mRNA [NM_030762]	NM_030762
A_23_P253949	1.62E-03	3.475	AL137321	AL137321	Homo sapiens mRNA; cDNA DKFZp434P231 (from clone DKFZp434P231). [AL137321]	
A_23_P214139	1.62E-03	2.425	NM_002912	NM_002912	Homo sapiens REV3-like, catalytic subunit of DNA polymerase zeta (yeast) (REV3L), mRNA [NM_002912]	NM_002912
A_32_P125568	1.63E-03	6.751	THC2416690		Q9EQG5 (Q9EQG5) Nedd4-binding brain specific protein BEAN (Fragment), partial (5%) [THC2416690]	
A_32_P44453	1.63E-03	2.671	NM_002194	NM_002194	Homo sapiens inositol polyphosphate-1-phosphatase (INPP1), mRNA [NM_002194]	NM_002194
A_32_P101653	1.63E-03	2.43	XM_499110	XM_499110	PREDICTED: Homo sapiens LOC441345 (LOC441345), mRNA [XM_499110]	XM_499110
A_32_P56661	1.65E-03	23.17	AK074614	AK074614	Homo sapiens cDNA FLJ90133 fis, clone HEMBB1000567. [AK074614]	
A_32_P146764	1.65E-03	9.733	THC2382264			
A_23_P58815	1.65E-03	8.519	NM_012176	NM_012176	Homo sapiens F-box protein 4 (FBXO4), transcript variant 1, mRNA [NM_012176]	NM_012176
A_24_P753161	1.65E-03	7.18	NM_001204	NM_001204	Homo sapiens bone morphogenetic protein receptor, type II (serine/threonine kinase) (BMPR2), transcript variant 1, mRNA [NM_001204]	NM_001204
A_32_P101420	1.65E-03	5.513	A_32_P101420			
A_24_P223518	1.65E-03	3.878	NM_007270	NM_007270	Homo sapiens FK506 binding protein 9, 63 kDa (FKBP9), mRNA [NM_007270]	NM_007270
A_23_P168828	1.65E-03	3.366	NM_005655	NM_005655	Homo sapiens Kruppel-like factor 10 (KLF10), mRNA [NM_005655]	NM_005655
A_23_P123732	1.65E-03	2.768	NM_001001551	NM_001001551	Homo sapiens chromosome 9 open reading frame 103 (C9orf103), mRNA [NM_001001551]	NM_001001551
A_23_P156049	1.65E-03	2.231	NM_000521	NM_000521	Homo sapiens hexosaminidase B (beta polypeptide) (HEXB), mRNA [NM_000521]	NM_000521
A_32_P62145	1.66E-03	10.61	BC013423	BC013423	Homo sapiens, clone IMAGE:3892140, mRNA. [BC013423]	
A_23_P39814	1.66E-03	2.573	NM_004882	NM_004882	Homo sapiens CBF1 interacting corepressor (CIR), transcript variant 1, mRNA [NM_004882]	NM_004882
A_23_P52101	1.66E-03	2.421	NM_016243	NM_016243	Homo sapiens cytochrome b5 reductase 1 (CYB5R1), mRNA [NM_016243]	NM_016243
A_32_P125832	1.67E-03	10.53	AK123079	AK123079	Homo sapiens cDNA FLJ41084 fis, clone ADRGL2010974. [AK123079]	
A_24_P230466	1.67E-03	2.062	A_24_P230466			
A_23_P424080	1.68E-03	2.539	NM_032312	NM_032312	Homo sapiens Yip1 domain family, member 4 (YIPF4), mRNA [NM_032312]	NM_032312
A_24_P306704	1.68E-03	2.236	A_24_P306704			
A_24_P416370	1.69E-03	26.96	NM_024015	NM_024015	Homo sapiens homeo box B4 (HOXB4), mRNA [NM_024015]	NM_024015
A_23_P50946	1.69E-03	25.82	NM_005855	NM_005855	Homo sapiens receptor (calcitonin) activity modifying protein 1 (RAMP1), mRNA [NM_005855]	NM_005855
A_23_P73012	1.69E-03	5.092	NM_032823	NM_032823	Homo sapiens chromosome 9 open reading frame 3 (C9orf3), mRNA [NM_032823]	NM_032823
A_32_P31771	1.69E-03	3.565	NM_030650	NM_030650	Homo sapiens KIAA1715 (KIAA1715), mRNA [NM_030650]	NM_030650
A_23_P20002	1.69E-03	3.058	NM_020319	NM_020319	Homo sapiens ankyrin repeat and MYND domain containing 2 (ANKMY2), mRNA [NM_020319]	NM_020319
A_24_P937139	1.69E-03	3.006	U97060	U97060	Homo sapiens Slug mRNA, partial cds. [U97060]	
A_32_P134427	1.69E-03	2.918	BC048201	BC048201	Homo sapiens, clone IMAGE:3660074, mRNA. [BC048201]	
A_24_P50567	1.69E-03	2.62	A_24_P50567			
A_24_P390055	1.69E-03	2.437	AL834278	AL834278	Homo sapiens mRNA; cDNA DKFZp547N1013 (from clone DKFZp547N1013). [AL834278]	
A_23_P4190	1.70E-03	4.282	NM_025149	NM_025149	Homo sapiens hypothetical protein FLJ20920 (FLJ20920), mRNA [NM_025149]	NM_025149
A_23_P21363	1.71E-03	6.553	NM_024060	NM_024060	Homo sapiens AHNAK nucleoprotein (desmoyokin) (AHNAK), transcript variant 2, mRNA [NM_024060]	NM_024060
A_23_P65262	1.72E-03	4.236	NM_033111	NM_033111	Homo sapiens CG016 (LOC88523), mRNA [NM_033111]	NM_033111
A_23_P214300	1.73E-03	43.72	NM_000846	NM_000846	Homo sapiens glutathione S-transferase A2 (GSTA2), mRNA [NM_000846]	NM_000846
A_23_P212061	1.73E-03	6.5	NM_007289	NM_007289	Homo sapiens membrane metallo-endopeptidase (neutral endopeptidase, enkephalinase, CALLA, CD10) (MME), transcript variant 2b, mRNA [NM_007289]	NM_007289
A_23_P208866	1.73E-03	5.149	NM_004877	NM_004877	Homo sapiens glia maturation factor, gamma (GMFG), mRNA [NM_004877]	NM_004877
A_23_P99996	1.73E-03	3.039	NM_019066	NM_019066	Homo sapiens MAGE-like 2 (MAGEL2), mRNA [NM_019066]	NM_019066
A_23_P391264	1.73E-03	2.912	NM_007047	NM_007047	Homo sapiens butyrophilin, subfamily 3, member A2 (BTN3A2), mRNA [NM_007047]	NM_007047

A_24_P109214	1.75E-03	3.417	NM_001645	NM_001645	Homo sapiens apolipoprotein C-I (APOC1), mRNA [NM_001645]	NM_001645
A_23_P122937	1.75E-03	2.559	NM_014800	NM_014800	Homo sapiens engulfment and cell motility 1 (ced-12 homolog, C. elegans) (ELMO1), transcript variant 1, mRNA [NM_014800]	NM_014800
A_24_P355246	1.76E-03	7.544	AK023096	AK023096	Homo sapiens cDNA FLJ13034 fis, clone NT2RP3001232. [AK023096]	
A_23_P36445	1.76E-03	3.266	NM_006815	NM_006815	Homo sapiens coated vesicle membrane protein (RNP24), mRNA [NM_006815]	NM_006815
A_23_P92929	1.77E-03	20.45	NM_016644	NM_016644	Homo sapiens mesenchymal stem cell protein DSC54 (LOC51334), mRNA [NM_016644]	NM_016644
A_23_P6935	1.77E-03	4.386	NM_198793	NM_198793	Homo sapiens CD47 antigen (Rh-related antigen, integrin-associated signal transducer) (CD47), transcript variant 2, mRNA [NM_198793]	NM_198793
A_32_P20454	1.77E-03	3.793	THC2369831		Q99JH4 (Q99JH4) TAFII140 protein (Fragment), partial (4%) [THC2369831]	
A_23_P95790	1.78E-03	5.321	NM_017625	NM_017625	Homo sapiens intelectin 1 (galactofuranose binding) (ITLN1), mRNA [NM_017625]	NM_017625
A_24_P792988	1.78E-03	2.015	A_24_P792988			
A_32_P40288	1.79E-03	4.231	NM_052913	NM_052913	Homo sapiens KIAA1913 (KIAA1913), mRNA [NM_052913]	NM_052913
A_23_P91379	1.79E-03	2.919	NM_018993	NM_018993	Homo sapiens Ras and Rab interactor 2 (RIN2), mRNA [NM_018993]	NM_018993
A_23_P14804	1.79E-03	2.812	NM_005724	NM_005724	Homo sapiens tetraspanin 3 (TSPAN3), transcript variant 1, mRNA [NM_005724]	NM_005724
A_23_P138665	1.79E-03	2.374	NM_005271	NM_005271	Homo sapiens glutamate dehydrogenase 1 (GLUD1), mRNA [NM_005271]	NM_005271
A_23_P253495	1.80E-03	29.66	NM_000847	NM_000847	Homo sapiens glutathione S-transferase A3 (GSTA3), mRNA [NM_000847]	NM_000847
A_32_P144381	1.80E-03	18.63	A_32_P144381			
A_23_P129695	1.80E-03	7.368	NM_138440	NM_138440	Homo sapiens slit-like 2 (Drosophila) (SLITL2), mRNA [NM_138440]	NM_138440
A_24_P944640	1.80E-03	3.401	NM_020909	NM_020909	Homo sapiens erythrocyte membrane protein band 4.1 like 5 (EPB41L5), mRNA [NM_020909]	NM_020909
A_23_P36448	1.80E-03	2.757	NM_006815	NM_006815	Homo sapiens coated vesicle membrane protein (RNP24), mRNA [NM_006815]	NM_006815
A_23_P66454	1.80E-03	2.623	NM_018530	NM_018530	Homo sapiens gasdermin-like (GSDML), mRNA [NM_018530]	NM_018530
A_24_P303080	1.80E-03	2.493	THC2370211			
A_24_P209765	1.80E-03	2.28	NM_007234	NM_007234	Homo sapiens dynactin 3 (p22) (DCTN3), transcript variant 1, mRNA [NM_007234]	NM_007234
A_23_P21976	1.81E-03	6.337	NM_001897	NM_001897	Homo sapiens chondroitin sulfate proteoglycan 4 (melanoma-associated) (CSPG4), mRNA [NM_001897]	NM_001897
A_23_P138435	1.81E-03	4.858	NM_020338	NM_020338	Homo sapiens retinoic acid induced 17 (RAI17), mRNA [NM_020338]	NM_020338
A_32_P178635	1.81E-03	2.778	BC033590	BC033590	Homo sapiens, clone IMAGE:4344826, mRNA. [BC033590]	
A_23_P301855	1.82E-03	5.45	NM_002338	NM_002338	Homo sapiens limbic system-associated membrane protein (LSAMP), mRNA [NM_002338]	NM_002338
A_32_P107617	1.82E-03	5.018	NM_003019	NM_003019	Homo sapiens surfactant, pulmonary-associated protein D (SFTPD), mRNA [NM_003019]	NM_003019
A_23_P129188	1.82E-03	3.278	NM_033429	NM_033429	Homo sapiens calmodulin-like 4 (CALML4), mRNA [NM_033429]	NM_033429
A_32_P105549	1.83E-03	51.33	NM_001630	NM_001630	Homo sapiens annexin A8 (ANXA8), mRNA [NM_001630]	NM_001630
A_23_P150343	1.83E-03	46.6	NM_003063	NM_003063	Homo sapiens sarcolipin (SLN), mRNA [NM_003063]	NM_003063
A_24_P406754	1.83E-03	14.54	NM_032211	NM_032211	Homo sapiens lysyl oxidase-like 4 (LOXL4), mRNA [NM_032211]	NM_032211
A_23_P205531	1.83E-03	11.34	NM_194430	NM_194430	Homo sapiens ribonuclease, RNase A family, 4 (RNASE4), transcript variant 1, mRNA [NM_194430]	NM_194430
A_23_P65918	1.83E-03	7.078	NM_002220	NM_002220	Homo sapiens inositol 1,4,5-trisphosphate 3-kinase A (ITPKA), mRNA [NM_002220]	NM_002220
A_23_P421493	1.83E-03	4.227	NM_020995	NM_020995	Homo sapiens haptoglobin-related protein (HPR), mRNA [NM_020995]	NM_020995
A_23_P70297	1.83E-03	3.378	AB023174	AB023174	Homo sapiens mRNA for KIAA0957 protein, partial cds. [AB023174]	
A_32_P2392	1.83E-03	2.939	NM_181076	NM_181076	Homo sapiens 88-kDa golgi protein (GM88), transcript variant 2, mRNA [NM_181076]	NM_181076
A_23_P363968	1.83E-03	2.828	NM_016546	NM_016546	Homo sapiens complement component 1, r subcomponent-like (C1RL), mRNA [NM_016546]	NM_016546
A_23_P136196	1.83E-03	2.766	NM_018317	NM_018317	Homo sapiens TBC1 domain family, member 19 (TBC1D19), mRNA [NM_018317]	NM_018317
A_32_P150040	1.83E-03	2.528	CR606969	CR606969	full-length cDNA clone CS0DF003YF10 of Fetal brain of Homo sapiens (human). [CR606969]	
A_23_P24535	1.83E-03	2.405	AK023921	AK023921	Homo sapiens cDNA FLJ13859 fis, clone THYRO1001033, weakly similar to TRANSFORMATION-SENSITIVE PROTEIN IEF SSP 3521. [AK023921]	
A_23_P358555	1.83E-03	2.367	NM_032356	NM_032356	Homo sapiens hypothetical protein MGC14151 (MGC14151), mRNA [NM_032356]	NM_032356
A_24_P281443	1.83E-03	2.138	A_24_P281443			

A_23_P38864	1.83E-03	2.136	NM_006423	NM_006423	Homo sapiens Rab acceptor 1 (prenylated) (RABAC1), mRNA [NM_006423]	NM_006423
A_23_P14184	1.83E-03	2.107	NM_018676	NM_018676	Homo sapiens thrombospondin, type I, domain containing 1 (THSD1), transcript variant 1, mRNA [NM_018676]	NM_018676
A_23_P15182	1.84E-03	5.388	NM_012106	NM_012106	Homo sapiens ADP-ribosylation factor-like 2 binding protein (ARL2BP), mRNA [NM_012106]	NM_012106
A_23_P37785	1.84E-03	3.639	BC070103	BC070103	Homo sapiens potassium channel tetramerisation domain containing 19, mRNA (cDNA clone IMAGE:5268205). [BC070103]	XM_085367
A_32_P135336	1.84E-03	3.379	XM_370965	XM_370965	PREDICTED: Homo sapiens similar to hypothetical protein BC011981 (LOC388242), mRNA [XM_370965]	XM_370965
A_23_P155417	1.84E-03	3.033	NM_032750	NM_032750	Homo sapiens hypothetical protein MGC15429 (MGC15429), mRNA [NM_032750]	NM_032750
A_23_P250379	1.84E-03	2.31	NM_015274	NM_015274	Homo sapiens mannosidase, alpha, class 2B, member 2 (MAN2B2), mRNA [NM_015274]	NM_015274
A_24_P186746	1.84E-03	2.107	A_24_P186746			
A_32_P46981	1.85E-03	11.59	BC017654	BC017654	Homo sapiens, clone IMAGE:3887603, mRNA. [BC017654]	
A_23_P103454	1.85E-03	3.426	NM_032264	NM_032264	Homo sapiens hypothetical protein AE2 (AE2), mRNA [NM_032264]	NM_032264
A_24_P139993	1.85E-03	3.169	NM_032977	NM_032977	Homo sapiens caspase 10, apoptosis-related cysteine protease (CASP10), transcript variant D, mRNA [NM_032977]	NM_032977
A_23_P144578	1.85E-03	2.904	NM_138335	NM_138335	Homo sapiens glucosamine-6-phosphate deaminase 2 (GNPDA2), mRNA [NM_138335]	NM_138335
A_23_P502224	1.85E-03	2.527	NM_000398	NM_000398	Homo sapiens cytochrome b5 reductase 3 (CYB5R3), transcript variant M, mRNA [NM_000398]	NM_000398
A_24_P584463	1.85E-03	2.204	A_24_P584463			
A_23_P259054	1.85E-03	2.133	NM_153816	NM_153816	Homo sapiens sorting nexin 14 (SNX14), transcript variant 1, mRNA [NM_153816]	NM_153816
A_23_P346670	1.86E-03	3.399	AK000249	AK000249	Homo sapiens cDNA FLJ20242 fis, clone COLF6369. [AK000249]	
A_23_P18223	1.86E-03	3.076	NM_002215	NM_002215	Homo sapiens inter-alpha (globulin) inhibitor H1 (ITIH1), mRNA [NM_002215]	NM_002215
A_24_P97104	1.87E-03	15.74	NM_001935	NM_001935	Homo sapiens dipeptidylpeptidase 4 (CD26, adenosine deaminase complexing protein 2) (DPP4), mRNA [NM_001935]	NM_001935
A_23_P14774	1.87E-03	6.297	NM_148979	NM_148979	Homo sapiens cathepsin H (CTSH), transcript variant 2, mRNA [NM_148979]	NM_148979
A_23_P136573	1.87E-03	3.379	NM_003896	NM_003896	Homo sapiens ST3 beta-galactoside alpha-2,3-sialyltransferase 5 (ST3GAL5), mRNA [NM_003896]	NM_003896
A_23_P110961	1.87E-03	3.211	NM_016098	NM_016098	Homo sapiens brain protein 44-like (BRP44L), mRNA [NM_016098]	NM_016098
A_23_P94230	1.88E-03	15.76	NM_015364	NM_015364	Homo sapiens lymphocyte antigen 96 (LY96), mRNA [NM_015364]	NM_015364
A_23_P156890	1.88E-03	14.06	NM_003206	NM_003206	Homo sapiens transcription factor 21 (TCF21), transcript variant 2, mRNA [NM_003206]	NM_003206
A_23_P116435	1.88E-03	11.08	NM_016412	NM_016412	Homo sapiens insulin-like growth factor 2 antisense (IGF2AS), mRNA [NM_016412]	NM_016412
A_23_P381714	1.88E-03	4.935	NM_198584	NM_198584	Homo sapiens carbonic anhydrase XIII (CA13), mRNA [NM_198584]	NM_198584
A_23_P166248	1.89E-03	4.425	NM_004414	NM_004414	Homo sapiens Down syndrome critical region gene 1 (DSCR1), transcript variant 1, mRNA [NM_004414]	NM_004414
A_23_P143713	1.89E-03	3.43	NM_021822	NM_021822	Homo sapiens apolipoprotein B mRNA editing enzyme, catalytic polypeptide-like 3G (APOBEC3G), mRNA [NM_021822]	NM_021822
A_23_P338919	1.89E-03	3.276	NM_005876	NM_005876	Homo sapiens aortic preferentially expressed gene 1 (APEG1), mRNA [NM_005876]	NM_005876
A_24_P116233	1.89E-03	2.831	NM_020456	NM_020456	Homo sapiens chromosome 13 open reading frame 1 (C13orf1), mRNA [NM_020456]	NM_020456
A_24_P361457	1.89E-03	2.29	NM_173627	NM_173627	Homo sapiens hypothetical protein FLJ35220 (FLJ35220), mRNA [NM_173627]	NM_173627
A_23_P370588	1.90E-03	22.32	NM_024016	NM_024016	Homo sapiens homeo box B8 (HOXB8), mRNA [NM_024016]	NM_024016
A_23_P311869	1.90E-03	5.525	NM_003896	NM_003896	Homo sapiens ST3 beta-galactoside alpha-2,3-sialyltransferase 5 (ST3GAL5), mRNA [NM_003896]	NM_003896
A_24_P294842	1.90E-03	3.533	NM_000332	NM_000332	Homo sapiens ataxin 1 (ATXN1), mRNA [NM_000332]	NM_000332
A_24_P256063	1.90E-03	2.159	ENST00000332292			
A_23_P58588	1.91E-03	35.98	NM_003062	NM_003062	Homo sapiens slit homolog 3 (Drosophila) (SLIT3), mRNA [NM_003062]	NM_003062
A_23_P31893	1.91E-03	12.39	NM_003033	NM_003033	Homo sapiens ST3 beta-galactoside alpha-2,3-sialyltransferase 1 (ST3GAL1), transcript variant 1, mRNA [NM_003033]	NM_003033
A_23_P104624	1.91E-03	7.204	ENST00000278505		Homo sapiens mRNA for KIAA0830 protein, partial cds. [AB020637]	XM_290546
A_23_P95060	1.91E-03	7.181	NM_004443	NM_004443	Homo sapiens EPH receptor B3 (EPHB3), mRNA [NM_004443]	NM_004443
A_23_P145555	1.91E-03	5.09	NM_138569	NM_138569	Homo sapiens chromosome 6 open reading frame 142 (C6orf142), mRNA [NM_138569]	NM_138569
A_23_P207299	1.91E-03	4.289	NM_016125	NM_016125	Homo sapiens PTD016 protein (LOC51136), mRNA [NM_016125]	NM_016125

A_32_P133916	1.91E-03	3.961	THC2405066		predicted protein {Methanosarcina acetivorans C2A;} , partial (13%) [THC2405066]	
A_23_P156970	1.91E-03	3.382	NM_002402	NM_002402	Homo sapiens mesoderm specific transcript homolog (mouse) (MEST), transcript variant 1, mRNA [NM_002402]	NM_002402
A_24_P205268	1.91E-03	2.291	NM_015299	NM_015299	Homo sapiens KIAA0323 (KIAA0323), mRNA [NM_015299]	NM_015299
A_24_P102389	1.91E-03	2.07	NM_001004127	NM_001004127	Homo sapiens similar to hypothetical protein B230397C21 (LOC440138), mRNA [NM_001004127]	NM_001004127
A_23_P115261	1.93E-03	85.96	NM_000029	NM_000029	Homo sapiens angiotensinogen (serine (or cysteine) proteinase inhibitor, clade A (alpha-1 antiproteinase, antitrypsin), member 8) (AGT), mRNA [NM_000029]	NM_000029
A_24_P108863	1.93E-03	13.51	NM_006746	NM_006746	Homo sapiens sex comb on midleg-like 1 (Drosophila) (SCML1), mRNA [NM_006746]	NM_006746
A_23_P35414	1.93E-03	9.371	NM_005398	NM_005398	Homo sapiens protein phosphatase 1, regulatory (inhibitor) subunit 3C (PPP1R3C), mRNA [NM_005398]	NM_005398
A_23_P57199	1.93E-03	3.44	NM_080920	NM_080920	Homo sapiens gamma-glutamyltransferase-like activity 4 (GGTLA4), transcript variant C, mRNA [NM_080920]	NM_080920
A_23_P23941	1.93E-03	2.857	NM_001007094	NM_001007094	Homo sapiens zinc finger protein 37a (KOX 21) (ZNF37A), transcript variant 1, mRNA [NM_001007094]	NM_001007094
A_23_P39718	1.93E-03	2.3	NM_005102	NM_005102	Homo sapiens fasciculation and elongation protein zeta 2 (zygin II) (FEZ2), mRNA [NM_005102]	NM_005102
A_24_P26069	1.93E-03	2.209	NM_152275	NM_152275	Homo sapiens hypothetical protein FLJ13946 (FLJ13946), mRNA [NM_152275]	NM_152275
A_23_P34546	1.94E-03	3.22	NM_006642	NM_006642	Homo sapiens serologically defined colon cancer antigen 8 (SDCCAG8), mRNA [NM_006642]	NM_006642
A_23_P215024	1.95E-03	6.42	NM_018214	NM_018214	Homo sapiens leucine rich repeat containing 1 (LRRC1), mRNA [NM_018214]	NM_018214
A_24_P330773	1.95E-03	4.08	NM_005831	NM_005831	Homo sapiens nuclear domain 10 protein (NDP52), mRNA [NM_005831]	NM_005831
A_24_P120537	1.95E-03	3.381	NM_152550	NM_152550	Homo sapiens SH3 domain containing ring finger 2 (SH3RF2), mRNA [NM_152550]	NM_152550
A_24_P265856	1.95E-03	3.25	NM_020654	NM_020654	Homo sapiens SUMO1/sentrin specific protease 7 (SENP7), mRNA [NM_020654]	NM_020654
A_23_P351679	1.95E-03	2.829	NM_004614	NM_004614	Homo sapiens thymidine kinase 2, mitochondrial (TK2), mRNA [NM_004614]	NM_004614
A_24_P236935	1.95E-03	2.776	NM_001012964	NM_001012964	Homo sapiens kallikrein 6 (neurosin, zyme) (KLK6), transcript variant B, mRNA [NM_001012964]	NM_001012964
A_23_P56759	1.95E-03	2.062	NM_016618	NM_016618	Homo sapiens hypothetical protein LOC51315 (LOC51315), mRNA [NM_016618]	NM_016618
A_32_P45168	1.96E-03	3.143	NR_002211	NR_002211	Homo sapiens Meis1, myeloid ecotropic viral integration site 1 homolog 4 (mouse) (MEIS4) on chromosome 17 [NR_002211]	NR_002211
A_24_P192727	1.97E-03	4.915	AF333487	AF333487	Homo sapiens FKSG40 (FKSG40) mRNA, complete cds. [AF333487]	XM_498494
A_23_P250564	1.97E-03	3.729	NM_005400	NM_005400	Homo sapiens protein kinase C, epsilon (PRKCE), mRNA [NM_005400]	NM_005400
A_23_P357929	1.97E-03	3.354	NM_015139	NM_015139	Homo sapiens solute carrier family 35 (UDP-glucuronic acid/UDP-N-acetylgalactosamine dual transporter), member D1 (SLC35D1), mRNA [NM_015139]	NM_015139
A_23_P201979	1.97E-03	2.73	NM_183013	NM_183013	Homo sapiens cAMP responsive element modulator (CREM), transcript variant 19, mRNA [NM_183013]	NM_183013
A_23_P118916	1.97E-03	2.657	AK026031	AK026031	Homo sapiens cDNA: FLJ22378 fis, clone HRC07430. [AK026031]	
A_32_P100947	1.98E-03	49.33	AI985214	AI985214	AI985214 wr98h11.x1 NCI_CGAP_Kid11 Homo sapiens cDNA clone IMAGE:2495781 3', mRNA sequence [AI985214]	
A_23_P91552	1.98E-03	13.23	NM_206965	NM_206965	Homo sapiens formiminotransferase cyclodeaminase (FTCD), transcript variant A, mRNA [NM_206965]	NM_206965
A_24_P89887	1.98E-03	4.444	NM_032823	NM_032823	Homo sapiens chromosome 9 open reading frame 3 (C9orf3), mRNA [NM_032823]	NM_032823
A_32_P178842	1.98E-03	3.755	BM906215	BM906215	AGENCOURT_6621270 NIH_MGC_125 Homo sapiens cDNA clone IMAGE:5590136 5', mRNA sequence [BM906215]	
A_24_P300952	1.98E-03	2.744	NM_001642	NM_001642	Homo sapiens amyloid beta (A4) precursor-like protein 2 (APLP2), mRNA [NM_001642]	NM_001642
A_23_P31798	1.98E-03	2.57	NM_000015	NM_000015	Homo sapiens N-acetyltransferase 2 (arylamine N-acetyltransferase) (NAT2), mRNA [NM_000015]	NM_000015
A_24_P288685	1.98E-03	2.334	NM_001560	NM_001560	Homo sapiens interleukin 13 receptor, alpha 1 (IL13RA1), mRNA [NM_001560]	NM_001560
A_24_P630490	1.99E-03	2.507	BC020859	BC020859	Homo sapiens, Similar to deafness, autosomal dominant 5 homolog (human), clone IMAGE:4551670, mRNA. [BC020859]	
A_23_P83751	1.99E-03	2.261	NM_024806	NM_024806	Homo sapiens hypothetical protein FLJ23554 (FLJ23554), transcript variant 1, mRNA [NM_024806]	NM_024806
A_24_P618928	1.99E-03	2.167	CR602022	CR602022	full-length cDNA clone CS0DJ004YB09 of T cells (Jurkat cell line) Cot 10-normalized of Homo sapiens (human). [CR602022]	
A_23_P354074	2.00E-03	17.56	NM_000081	NM_000081	Homo sapiens lysosomal trafficking regulator (LYST), transcript variant 1, mRNA [NM_000081]	NM_000081
A_23_P117298	2.01E-03	8.507	NM_000131	NM_000131	Homo sapiens coagulation factor VII (serum prothrombin conversion accelerator) (F7), transcript variant 1, mRNA [NM_000131]	NM_000131
A_23_P76658	2.01E-03	5.712	NM_052818	NM_052818	Homo sapiens hypothetical gene CG018 (CG018), mRNA [NM_052818]	NM_052818

A_23_P417148	2.01E-03	3.273	NM_030665	NM_030665	Homo sapiens retinoic acid induced 1 (RAI1), mRNA [NM_030665]	NM_030665
A_32_P136967	2.01E-03	3.248	ENST00000328046		Homo sapiens mRNA for KIAA1677 protein, partial cds. [AB051464]	
A_32_P104263	2.01E-03	2.154	THC2432735			
A_24_P42517	2.02E-03	4.152	NM_006854	NM_006854	Homo sapiens KDEL (Lys-Asp-Glu-Leu) endoplasmic reticulum protein retention receptor 2 (KDELR2), mRNA [NM_006854]	NM_006854
A_23_P4649	2.02E-03	3.219	NM_001645	NM_001645	Homo sapiens apolipoprotein C-I (APOC1), mRNA [NM_001645]	NM_001645
A_24_P795594	2.02E-03	2.653	AB096249	AB096249	Homo sapiens LOH11CR1J gene, loss of heterozygosity, 11, chromosomal region 1 gene J product. [AB096249]	
A_24_P171075	2.02E-03	2.25	NM_183013	NM_183013	Homo sapiens cAMP responsive element modulator (CREM), transcript variant 19, mRNA [NM_183013]	NM_183013
A_24_P835500	2.02E-03	2.217	AK026647	AK026647	Homo sapiens cDNA: FLJ22994 fis, clone KAT11918. [AK026647]	
A_23_P15357	2.02E-03	2.103	NM_005567	NM_005567	Homo sapiens lectin, galactoside-binding, soluble, 3 binding protein (LGALS3BP), mRNA [NM_005567]	NM_005567
A_23_P340251	2.02E-03	2.018	NM_002865	NM_002865	Homo sapiens RAB2, member RAS oncogene family (RAB2), mRNA [NM_002865]	NM_002865
A_23_P150379	2.03E-03	6.033	NM_144765	NM_144765	Homo sapiens epithelial V-like antigen 1 (EVA1), transcript variant 2, mRNA [NM_144765]	NM_144765
A_23_P258862	2.04E-03	12.88	NM_145341	NM_145341	Homo sapiens programmed cell death 4 (neoplastic transformation inhibitor) (PDCD4), transcript variant 2, mRNA [NM_145341]	NM_145341
A_24_P892402	2.05E-03	7.891	AK057652	AK057652	Homo sapiens cDNA FLJ33090 fis, clone TRACH2000559. [AK057652]	
A_32_P211558	2.05E-03	2.35	THC2312818		CJ11_MOUSE (Q9D9B4) Protein C10orf11 homolog, partial (95%) [THC2312818]	
A_23_P329890	2.05E-03	2.245	NM_174926	NM_174926	Homo sapiens hypothetical protein MGC17839 (MGC17839), mRNA [NM_174926]	NM_174926
A_24_P941268	2.05E-03	2.063	AK057568	AK057568	Homo sapiens cDNA FLJ33006 fis, clone THYMU1000316. [AK057568]	
A_24_P943792	2.06E-03	7.76	NM_001777	NM_001777	Homo sapiens CD47 antigen (Rh-related antigen, integrin-associated signal transducer) (CD47), transcript variant 1, mRNA [NM_001777]	NM_001777
A_23_P146077	2.06E-03	6.581	NM_018660	NM_018660	Homo sapiens zinc finger protein 395 (ZNF395), mRNA [NM_018660]	NM_018660
A_32_P62963	2.06E-03	6.055	ENST00000323509			
A_24_P272594	2.06E-03	5.27	NM_014994	NM_014994	Homo sapiens mitogen activated protein kinase binding protein 1 (MAPKBP1), mRNA [NM_014994]	NM_014994
A_23_P208389	2.06E-03	2.765	NM_021913	NM_021913	Homo sapiens AXL receptor tyrosine kinase (AXL), transcript variant 1, mRNA [NM_021913]	NM_021913
A_24_P80776	2.06E-03	2.607	AK129879	AK129879	Homo sapiens cDNA FLJ26369 fis, clone HRT06001. [AK129879]	
A_23_P119464	2.06E-03	2.544	NM_033103	NM_033103	Homo sapiens rhophilin, Rho GTPase binding protein 2 (RHPN2), mRNA [NM_033103]	NM_033103
A_32_P190036	2.06E-03	2.059	XM_497783	XM_497783	PREDICTED: Homo sapiens similar to transcript increased in spermiogenesis 78 (LOC441964), mRNA [XM_497783]	XM_497783
A_23_P372234	2.07E-03	14.66	NM_001218	NM_001218	Homo sapiens carbonic anhydrase XII (CA12), transcript variant 1, mRNA [NM_001218]	NM_001218
A_23_P151805	2.07E-03	13.47	NM_006329	NM_006329	Homo sapiens fibulin 5 (FBLN5), mRNA [NM_006329]	NM_006329
A_24_P295010	2.07E-03	10.91	NM_004155	NM_004155	Homo sapiens serine (or cysteine) proteinase inhibitor, clade B (ovalbumin), member 9 (SERPINB9), mRNA [NM_004155]	NM_004155
A_23_P395438	2.07E-03	9.03	NM_053044	NM_053044	Homo sapiens HtrA serine peptidase 3 (HTRA3), mRNA [NM_053044]	NM_053044
A_24_P826046	2.07E-03	6.894	AK024177	AK024177	Homo sapiens cDNA FLJ14115 fis, clone MAMMA1001760. [AK024177]	XM_499165
A_32_P154121	2.07E-03	4.36	THC2340670			
A_24_P148750	2.07E-03	3.67	NM_004844	NM_004844	Homo sapiens SH3-domain binding protein 5 (BTK-associated) (SH3BP5), transcript variant 1, mRNA [NM_004844]	NM_004844
A_24_P224488	2.07E-03	3.308	AK055986	AK055986	Homo sapiens cDNA FLJ31424 fis, clone NT2NE2000392. [AK055986]	
A_23_P70670	2.07E-03	3.159	NM_004233	NM_004233	Homo sapiens CD83 antigen (activated B lymphocytes, immunoglobulin superfamily) (CD83), mRNA [NM_004233]	NM_004233
A_24_P7750	2.07E-03	2.972	A_24_P7750			
A_32_P4608	2.07E-03	2.722	A_32_P4608			
A_23_P102575	2.07E-03	2.539	NM_020062	NM_020062	Homo sapiens SLC2A4 regulator (SLC2A4RG), mRNA [NM_020062]	NM_020062
A_23_P112135	2.07E-03	2.289	NM_014294	NM_014294	Homo sapiens translocation associated membrane protein 1 (TRAM1), mRNA [NM_014294]	NM_014294
A_23_P98910	2.07E-03	2.057	NM_006152	NM_006152	Homo sapiens lymphoid-restricted membrane protein (LRMP), mRNA [NM_006152]	NM_006152
A_23_P252541	2.08E-03	40.96	NM_177403	NM_177403	Homo sapiens RAB7B, member RAS oncogene family (RAB7B), mRNA [NM_177403]	NM_177403
A_23_P2492	2.08E-03	10.99	NM_001734	NM_001734	Homo sapiens complement component 1, s subcomponent (C1S), transcript variant 1, mRNA [NM_001734]	NM_001734

A_23_P10194	2.08E-03	2.928	NM_201575	NM_201575	Homo sapiens seizure related 6 homolog (mouse)-like 2 (SEZ6L2), transcript variant 2, mRNA [NM_201575]	NM_201575
A_23_P99226	2.08E-03	2.696	NM_012240	NM_012240	Homo sapiens sirtuin (silent mating type information regulation 2 homolog) 4 (S. cerevisiae) (SIRT4), mRNA [NM_012240]	NM_012240
A_24_P209772	2.08E-03	2.187	NM_007234	NM_007234	Homo sapiens dynactin 3 (p22) (DCTN3), transcript variant 1, mRNA [NM_007234]	NM_007234
A_23_P26173	2.08E-03	2.161	NM_007364	NM_007364	Homo sapiens transmembrane emp24 domain containing 3 (TMED3), mRNA [NM_007364]	NM_007364
A_24_P281683	2.08E-03	2.124	A_24_P281683			
A_23_P212779	2.09E-03	11.24	NM_015393	NM_015393	Homo sapiens DKFZP564O0823 protein (DKFZP564O0823), mRNA [NM_015393]	NM_015393
A_23_P215944	2.09E-03	6.591	NM_147780	NM_147780	Homo sapiens cathepsin B (CTSB), transcript variant 2, mRNA [NM_147780]	NM_147780
A_32_P190737	2.09E-03	3.483	AK057981	AK057981	Homo sapiens cDNA FLJ25252 fis, clone STM03814. [AK057981]	
A_23_P105002	2.09E-03	2.837	NM_000327	NM_000327	Homo sapiens retinal outer segment membrane protein 1 (ROM1), mRNA [NM_000327]	NM_000327
A_23_P70148	2.09E-03	2.316	NM_182977	NM_182977	Homo sapiens nicotinamide nucleotide transhydrogenase (NNT), mRNA [NM_182977]	NM_182977
A_24_P93855	2.09E-03	2.135	NM_001012426	NM_001012426	Homo sapiens forkhead box P4 (FOXP4), transcript variant 1, mRNA [NM_001012426]	NM_001012426
A_23_P422911	2.09E-03	2.113	NM_153456	NM_153456	Homo sapiens heparan sulfate 6-O-sulfotransferase 3 (HS6ST3), mRNA [NM_153456]	NM_153456
A_32_P80850	2.10E-03	3.034	NM_021110	NM_021110	Homo sapiens collagen, type XIV, alpha 1 (undulin) (COL14A1), mRNA [NM_021110]	NM_021110
A_24_P114671	2.11E-03	3.085	AL117599	AL117599	Homo sapiens mRNA; cDNA DKFZp564I0463 (from clone DKFZp564I0463). [AL117599]	
A_32_P33937	2.11E-03	2.416	ENST00000355190		Homo sapiens mRNA; cDNA DKFZp686L01145 (from clone DKFZp686L01145). [BX538108]	
A_23_P258978	2.11E-03	2.275	NM_002077	NM_002077	Homo sapiens golgi autoantigen, golgin subfamily a, 1 (GOLGA1), mRNA [NM_002077]	NM_002077
A_23_P31873	2.11E-03	2.204	NM_001002233	NM_001002233	Homo sapiens RAB11 family interacting protein 1 (class I) (RAB11FIP1), transcript variant 2, mRNA [NM_001002233]	NM_001002233
A_23_P30069	2.13E-03	3.196	AK023743	AK023743	Homo sapiens cDNA FLJ13681 fis, clone PLACE2000014, weakly similar to HYPOTHETICAL HELICASE C28H8.3 IN CHROMOSOME III. [AK023743]	
A_23_P46928	2.13E-03	2.286	NM_002627	NM_002627	Homo sapiens phosphofructokinase, platelet (PFKP), mRNA [NM_002627]	NM_002627
A_23_P103588	2.14E-03	6.393	NM_005518	NM_005518	Homo sapiens 3-hydroxy-3-methylglutaryl-Coenzyme A synthase 2 (mitochondrial) (HMGCS2), mRNA [NM_005518]	NM_005518
A_23_P12572	2.14E-03	4.122	NM_033339	NM_033339	Homo sapiens caspase 7, apoptosis-related cysteine protease (CASP7), transcript variant gamma, mRNA [NM_033339]	NM_033339
A_23_P19657	2.14E-03	3.433	NM_032832	NM_032832	Homo sapiens low density lipoprotein receptor-related protein 11 (LRP11), mRNA [NM_032832]	NM_032832
A_23_P65823	2.14E-03	2.391	AF180519	AF180519	Homo sapiens GABA-A receptor-associated protein mRNA, complete cds. [AF180519]	
A_23_P15402	2.14E-03	2.034	NM_133491	NM_133491	Homo sapiens spermidine/spermine N1-acetyltransferase 2 (SAT2), mRNA [NM_133491]	NM_133491
A_23_P166421	2.14E-03	2.005	NM_031937	NM_031937	Homo sapiens TBC1 domain family, member 10A (TBC1D10A), mRNA [NM_031937]	NM_031937
A_23_P93641	2.15E-03	106	NM_020299	NM_020299	Homo sapiens aldo-keto reductase family 1, member B10 (aldose reductase) (AKR1B10), mRNA [NM_020299]	NM_020299
A_23_P7342	2.15E-03	18.35	NM_001075	NM_001075	Homo sapiens UDP glucuronosyltransferase 2 family, polypeptide B10 (UGT2B10), mRNA [NM_001075]	NM_001075
A_32_P205431	2.15E-03	8.999	AF085962	AF085962	Homo sapiens full length insert cDNA clone YS02G11. [AF085962]	
A_24_P639671	2.15E-03	3.789	AK095831	AK095831	Homo sapiens cDNA FLJ38512 fis, clone HCHON2000503. [AK095831]	
A_23_P400378	2.15E-03	3.107	NM_170699	NM_170699	Homo sapiens G protein-coupled bile acid receptor 1 (GPBAR1), mRNA [NM_170699]	NM_170699
A_23_P258944	2.15E-03	3.087	NM_012328	NM_012328	Homo sapiens DnaJ (Hsp40) homolog, subfamily B, member 9 (DNAJB9), mRNA [NM_012328]	NM_012328
A_24_P323084	2.15E-03	2.435	NM_178519	NM_178519	Homo sapiens hypothetical protein FLJ39421 (FLJ39421), mRNA [NM_178519]	NM_178519
A_23_P143514	2.15E-03	2.078	BC004343	BC004343	Homo sapiens chromosome 21 open reading frame 122, mRNA (cDNA clone MGC:10960 IMAGE:3633193), complete cds. [BC004343]	
A_24_P387869	2.16E-03	2.119	BC029749	BC029749	Homo sapiens, clone IMAGE:4513287, mRNA, partial cds. [BC029749]	
A_32_P96692	2.17E-03	3.848	THC2340838		Q9BX12 (Q9BX12) GTP binding protein 2 (Fragment), partial (26%) [THC2340838]	
A_23_P27584	2.17E-03	2.554	NM_001020818	NM_001020818	Homo sapiens myeloid-associated differentiation marker (MYADM), transcript variant 1, mRNA [NM_001020818]	NM_001020818
A_32_P161855	2.18E-03	15.59	NM_018689	NM_018689	Homo sapiens KIAA1199 (KIAA1199), mRNA [NM_018689]	NM_018689
A_23_P148047	2.18E-03	7.522	NM_000958	NM_000958	Homo sapiens prostaglandin E receptor 4 (subtype EP4) (PTGER4), mRNA [NM_000958]	NM_000958
A_23_P303803	2.18E-03	3.843	NM_152474	NM_152474	Homo sapiens chromosome 19 open reading frame 18 (C19orf18), mRNA [NM_152474]	NM_152474
A_32_P71675	2.18E-03	3.797	BC035379	BC035379	Homo sapiens, clone IMAGE:5162922, mRNA. [BC035379]	XM_373823

A_24_P910050	2.18E-03	3.077	THC2273889			
A_23_P142533	2.20E-03	210.8	NM_000090	NM_000090	Homo sapiens collagen, type III, alpha 1 (Ehlers-Danlos syndrome type IV, autosomal dominant) (COL3A1), mRNA [NM_000090]	NM_000090
A_24_P643776	2.20E-03	8.685	THC2437143			
A_23_P372874	2.20E-03	3.013	NM_005979	NM_005979	Homo sapiens S100 calcium binding protein A13 (S100A13), transcript variant 2, mRNA [NM_005979]	NM_005979
A_32_P112623	2.21E-03	2.704	NM_175923	NM_175923	Homo sapiens hypothetical protein MGC42630 (MGC42630), mRNA [NM_175923]	NM_175923
A_24_P69691	2.22E-03	5.938	NM_145011	NM_145011	Homo sapiens zinc finger protein 25 (KOX 19) (ZNF25), mRNA [NM_145011]	NM_145011
A_23_P42498	2.22E-03	2.233	NM_152828	NM_152828	Homo sapiens sorting nexin 3 (SNX3), transcript variant 3, mRNA [NM_152828]	NM_152828
A_23_P45365	2.23E-03	4.768	NM_033380	NM_033380	Homo sapiens collagen, type IV, alpha 5 (Alport syndrome) (COL4A5), transcript variant 2, mRNA [NM_033380]	NM_033380
A_23_P125117	2.23E-03	4.404	NM_173546	NM_173546	Homo sapiens hypothetical protein MGC35097 (MGC35097), mRNA [NM_173546]	NM_173546
A_24_P102981	2.23E-03	2.452	NM_006736	NM_006736	Homo sapiens DnaJ (Hsp40) homolog, subfamily B, member 2 (DNAJB2), mRNA [NM_006736]	NM_006736
A_24_P510502	2.24E-03	9.15	N53226	N53226	N53226 yv57e10.s1 Soares fetal liver spleen 1NFLS Homo sapiens cDNA clone IMAGE:246858 3' similar to gb:X07868_rna1 PUTATIVE INSULIN-LIKE GROWTH FACTOR II ASSOCIATED (HUMAN);, mRNA sequence [N53226]	
A_23_P16976	2.24E-03	4.827	NM_001153	NM_001153	Homo sapiens annexin A4 (ANXA4), mRNA [NM_001153]	NM_001153
A_32_P213946	2.24E-03	4.139	A_32_P213946			
A_23_P107412	2.24E-03	3.174	NM_000918	NM_000918	Homo sapiens procollagen-proline, 2-oxoglutarate 4-dioxygenase (proline 4-hydroxylase), beta polypeptide (protein disulfide isomerase-associated 1) (P4HB), mRNA [NM_000918]	NM_000918
A_23_P133470	2.24E-03	3.096	NM_014819	NM_014819	Homo sapiens praja 2, RING-H2 motif containing (PJA2), mRNA [NM_014819]	NM_014819
A_24_P240487	2.25E-03	4.992	NM_000301	NM_000301	Homo sapiens plasminogen (PLG), mRNA [NM_000301]	NM_000301
A_23_P55477	2.25E-03	4.318	NM_000676	NM_000676	Homo sapiens adenosine A2b receptor (ADORA2B), mRNA [NM_000676]	NM_000676
A_32_P122285	2.25E-03	4.22	A_32_P122285			
A_24_P53778	2.25E-03	3.589	NM_080878	NM_080878	Homo sapiens intelectin 2 (ITLN2), mRNA [NM_080878]	NM_080878
A_32_P41021	2.25E-03	2.477	NM_015338	NM_015338	Homo sapiens additional sex combs like 1 (Drosophila) (ASXL1), mRNA [NM_015338]	NM_015338
A_23_P371787	2.25E-03	2.417	NM_014734	NM_014734	Homo sapiens KIAA0247 (KIAA0247), mRNA [NM_014734]	NM_014734
A_23_P209032	2.25E-03	2.193	NM_018443	NM_018443	Homo sapiens zinc finger protein 302 (ZNF302), transcript variant 1, mRNA [NM_018443]	NM_018443
A_23_P83159	2.25E-03	2.169	NM_018847	NM_018847	Homo sapiens kelch-like 9 (Drosophila) (KLHL9), mRNA [NM_018847]	NM_018847
A_24_P365807	2.26E-03	5.107	NM_004429	NM_004429	Homo sapiens ephrin-B1 (EFNB1), mRNA [NM_004429]	NM_004429
A_24_P294124	2.26E-03	3.153	NM_014755	NM_014755	Homo sapiens SERTA domain containing 2 (SERTAD2), mRNA [NM_014755]	NM_014755
A_23_P170857	2.26E-03	2.709	NM_002182	NM_002182	Homo sapiens interleukin 1 receptor accessory protein (IL1RAP), transcript variant 1, mRNA [NM_002182]	NM_002182
A_23_P125423	2.27E-03	4.589	NM_001733	NM_001733	Homo sapiens complement component 1, r subcomponent (C1R), mRNA [NM_001733]	NM_001733
A_24_P940135	2.27E-03	3.931	BC024007	BC024007	Homo sapiens chitobiase, di-N-acetyl-, mRNA (cDNA clone IMAGE:4823479), complete cds. [BC024007]	
A_24_P195785	2.27E-03	3.675	NM_033380	NM_033380	Homo sapiens collagen, type IV, alpha 5 (Alport syndrome) (COL4A5), transcript variant 2, mRNA [NM_033380]	NM_033380
A_23_P251499	2.27E-03	3.405	NM_002593	NM_002593	Homo sapiens procollagen C-endopeptidase enhancer (PCOLCE), mRNA [NM_002593]	NM_002593
A_24_P270144	2.27E-03	2.898	NM_001780	NM_001780	Homo sapiens CD63 antigen (melanoma 1 antigen) (CD63), mRNA [NM_001780]	NM_001780
A_23_P47616	2.27E-03	2.869	NM_004476	NM_004476	Homo sapiens folate hydrolase (prostate-specific membrane antigen) 1 (FOLH1), transcript variant 1, mRNA [NM_004476]	NM_004476
A_23_P109881	2.27E-03	2.078	NM_002218	NM_002218	Homo sapiens inter-alpha (globulin) inhibitor H4 (plasma Kallikrein-sensitive glycoprotein) (ITIH4), mRNA [NM_002218]	NM_002218
A_23_P342910	2.28E-03	3.205	ENST00000328046		Homo sapiens mRNA for KIAA1677 protein, partial cds. [AB051464]	
A_24_P118531	2.28E-03	2.22	BP290435	BP290435	BP290435 BP290435 Sugano cDNA library, lung fibroblast Homo sapiens cDNA clone LNF05072, mRNA sequence [BP290435]	
A_24_P401124	2.28E-03	2.144	A_24_P401124			
A_23_P388150	2.29E-03	16.53	NM_032562	NM_032562	Homo sapiens phospholipase A2, group XIIB (PLA2G12B), mRNA [NM_032562]	NM_032562
A_23_P144911	2.29E-03	13.79	NM_152403	NM_152403	Homo sapiens hypothetical protein FLJ39155 (FLJ39155), transcript variant 1, mRNA [NM_152403]	NM_152403

A_23_P168771	2.29E-03	4.798	NM_020879	NM_020879	Homo sapiens KIAA1505 protein (KIAA1505), mRNA [NM_020879]	NM_020879
A_23_P257417	2.29E-03	2.864	NM_152385	NM_152385	Homo sapiens hypothetical protein FLJ31438 (FLJ31438), mRNA [NM_152385]	NM_152385
A_24_P858698	2.29E-03	2.82	NM_002501	NM_002501	Homo sapiens nuclear factor I/X (CCAAT-binding transcription factor) (NFIX), mRNA [NM_002501]	NM_002501
A_32_P79584	2.29E-03	2.608	NM_001011553	NM_001011553	Homo sapiens septin 7 (SEPT7), transcript variant 2, mRNA [NM_001011553]	NM_001011553
A_23_P258037	2.29E-03	2.602	NM_018433	NM_018433	Homo sapiens jumonji domain containing 1A (JMJD1A), mRNA [NM_018433]	NM_018433
A_24_P622468	2.29E-03	2.467	BU076424	BU076424	BU076424 im51d09.x1 HR85 islet Homo sapiens cDNA clone IMAGE:6038465 3' similar to SW:MIC2_HUMAN P14209 T-CELL SURFACE GLYCOPROTEIN E2 PRECURSOR ;, mRNA sequence [BU076424]	
A_23_P210100	2.31E-03	6.112	NM_019885	NM_019885	Homo sapiens cytochrome P450, family 26, subfamily B, polypeptide 1 (CYP26B1), mRNA [NM_019885]	NM_019885
A_24_P174755	2.31E-03	5.968	NM_003060	NM_003060	Homo sapiens solute carrier family 22 (organic cation transporter), member 5 (SLC22A5), mRNA [NM_003060]	NM_003060
A_23_P126613	2.31E-03	4.971	NM_080429	NM_080429	Homo sapiens aquaporin 10 (AQP10), mRNA [NM_080429]	NM_080429
A_23_P218555	2.32E-03	7.056	NM_005253	NM_005253	Homo sapiens FOS-like antigen 2 (FOSL2), mRNA [NM_005253]	NM_005253
A_24_P212539	2.32E-03	3.221	NM_138801	NM_138801	Homo sapiens galactose mutarotase (aldose 1-epimerase) (GALM), mRNA [NM_138801]	NM_138801
A_23_P52697	2.33E-03	17.49	NM_020404	NM_020404	Homo sapiens CD248 antigen, endosialin (CD248), mRNA [NM_020404]	NM_020404
A_32_P184268	2.33E-03	6.249	THC2432515			
A_23_P142560	2.34E-03	10.11	NM_014795	NM_014795	Homo sapiens zinc finger homeobox 1b (ZFHX1B), mRNA [NM_014795]	NM_014795
A_24_P13790	2.34E-03	2.002	NM_003869	NM_003869	Homo sapiens carboxylesterase 2 (intestine, liver) (CES2), transcript variant 1, mRNA [NM_003869]	NM_003869
A_23_P68006	2.35E-03	10.94	NM_000877	NM_000877	Homo sapiens interleukin 1 receptor, type I (IL1R1), mRNA [NM_000877]	NM_000877
A_23_P211233	2.35E-03	10.46	NM_001849	NM_001849	Homo sapiens collagen, type VI, alpha 2 (COL6A2), transcript variant 2C2, mRNA [NM_001849]	NM_001849
A_32_P115606	2.35E-03	10.33	AK131385	AK131385	Homo sapiens cDNA FLJ16460 fis, clone BRCAN2018240. [AK131385]	
A_23_P126782	2.35E-03	5.784	NM_001993	NM_001993	Homo sapiens coagulation factor III (thromboplastin, tissue factor) (F3), mRNA [NM_001993]	NM_001993
A_23_P14302	2.35E-03	3.213	NM_024633	NM_024633	Homo sapiens chromosome 14 open reading frame 139 (C14orf139), mRNA [NM_024633]	NM_024633
A_24_P262201	2.35E-03	3.079	NM_001017389	NM_001017389	Homo sapiens sulfotransferase family, cytosolic, 1A, phenol-preferring, member 4 (SULT1A4), transcript variant 1, mRNA [NM_001017389]	NM_001017389
A_23_P395075	2.35E-03	2.697	NM_018433	NM_018433	Homo sapiens jumonji domain containing 1A (JMJD1A), mRNA [NM_018433]	NM_018433
A_32_P19608	2.36E-03	2.389	THC2439900			
A_23_P157495	2.38E-03	7.173	NM_005605	NM_005605	Homo sapiens protein phosphatase 3 (formerly 2B), catalytic subunit, gamma isoform (calcineurin A gamma) (PPP3CC), mRNA [NM_005605]	NM_005605
A_24_P354954	2.38E-03	3.017	NM_138771	NM_138771	Homo sapiens alpha-1,3(6)-mannosylglycoprotein beta-1,6-N-acetyl-glucosaminyltransferase-like (LOC90693), mRNA [NM_138771]	NM_138771
A_23_P366254	2.40E-03	3.993	NM_019848	NM_019848	Homo sapiens solute carrier family 10 (sodium/bile acid cotransporter family), member 3 (SLC10A3), mRNA [NM_019848]	NM_019848
A_24_P337397	2.41E-03	2.772	NM_023039	NM_023039	Homo sapiens ankyrin repeat, family A (RFXANK-like), 2 (ANKRA2), mRNA [NM_023039]	NM_023039
A_23_P356466	2.42E-03	5.191	BC004219	BC004219	Homo sapiens cDNA clone IMAGE:3354271, complete cds. [BC004219]	
A_23_P217428	2.43E-03	44.62	NM_001174	NM_001174	Homo sapiens Rho GTPase activating protein 6 (ARHGAP6), transcript variant 2, mRNA [NM_001174]	NM_001174
A_23_P7144	2.44E-03	15.73	NM_001511	NM_001511	Homo sapiens chemokine (C-X-C motif) ligand 1 (melanoma growth stimulating activity, alpha) (CXCL1), mRNA [NM_001511]	NM_001511
A_32_P228618	2.44E-03	8.19	NM_001003793	NM_001003793	Homo sapiens RNA binding motif, single stranded interacting protein (RBMS3), transcript variant 1, mRNA [NM_001003793]	NM_001003793
A_23_P395054	2.45E-03	35.91	BC008813	BC008813	Homo sapiens annexin A8, mRNA (cDNA clone MGC:10405 IMAGE:3958020), complete cds. [BC008813]	
A_23_P98350	2.45E-03	20.67	NM_001165	NM_001165	Homo sapiens baculoviral IAP repeat-containing 3 (BIRC3), transcript variant 1, mRNA [NM_001165]	NM_001165
A_23_P108143	2.45E-03	4.069	NM_000156	NM_000156	Homo sapiens guanidinoacetate N-methyltransferase (GAMT), transcript variant 1, mRNA [NM_000156]	NM_000156
A_23_P253052	2.45E-03	3.584	NM_031462	NM_031462	Homo sapiens CD99 antigen-like 2 (CD99L2), transcript variant 1, mRNA [NM_031462]	NM_031462
A_24_P298894	2.45E-03	2.308	ENST00000332888			
A_24_P73075	2.45E-03	2.174	NM_017868	NM_017868	Homo sapiens tetratricopeptide repeat domain 12 (TTC12), mRNA [NM_017868]	NM_017868
A_32_P156237	2.47E-03	25.59	A_32_P156237			
A_23_P1331	2.47E-03	7.6	NM_005203	NM_005203	Homo sapiens collagen, type XIII, alpha 1 (COL13A1), transcript variant 1, mRNA [NM_005203]	NM_005203

A_23_P12755	2.47E-03	3.909	NM_032211	NM_032211	Homo sapiens lysyl oxidase-like 4 (LOXL4), mRNA [NM_032211]	NM_032211
A_23_P51754	2.47E-03	2.608	NM_001010935	NM_001010935	Homo sapiens RAP1A, member of RAS oncogene family (RAP1A), transcript variant 1, mRNA [NM_001010935]	NM_001010935
A_23_P321511	2.47E-03	2.482	NM_178450	NM_178450	Homo sapiens membrane-associated ring finger (C3HC4) 3 (MARCH3), mRNA [NM_178450]	NM_178450
A_23_P151529	2.47E-03	2.13	NM_020215	NM_020215	Homo sapiens chromosome 14 open reading frame 132 (C14orf132), mRNA [NM_020215]	NM_020215
A_24_P943393	2.49E-03	19.48	NM_001620	NM_001620	Homo sapiens AHNAK nucleoprotein (desmoyokin) (AHNAK), transcript variant 1, mRNA [NM_001620]	NM_001620
A_23_P306730	2.49E-03	3.046	L02932	L02932	Human peroxisome proliferator activated receptor mRNA, complete cds. [L02932]	
A_23_P69339	2.50E-03	3.151	NM_001607	NM_001607	Homo sapiens acetyl-Coenzyme A acyltransferase 1 (peroxisomal 3-oxoacyl-Coenzyme A thiolase) (ACAA1), nuclear gene encoding mitochondrial protein, mRNA [NM_001607]	NM_001607
A_24_P305623	2.52E-03	2.747	NM_006134	NM_006134	Homo sapiens transmembrane protein 50B (TMEM50B), mRNA [NM_006134]	NM_006134
A_23_P80040	2.52E-03	2.546	NM_006404	NM_006404	Homo sapiens protein C receptor, endothelial (EPCR) (PROCR), mRNA [NM_006404]	NM_006404
A_24_P154868	2.54E-03	25.11	NM_005588	NM_005588	Homo sapiens meprin A, alpha (PABA peptide hydrolase) (MEP1A), mRNA [NM_005588]	NM_005588
A_24_P612446	2.54E-03	3.097	CR591566	CR591566	full-length cDNA clone CS0DL004YB03 of B cells (Ramos cell line) Cot 25-normalized of Homo sapiens (human). [CR591566]	
A_23_P319572	2.54E-03	2.783	NM_005122	NM_005122	Homo sapiens nuclear receptor subfamily 1, group I, member 3 (NR1I3), mRNA [NM_005122]	NM_005122
A_23_P40866	2.55E-03	5.153	NM_015642	NM_015642	Homo sapiens zinc finger and BTB domain containing 20 (ZBTB20), mRNA [NM_015642]	NM_015642
A_23_P145	2.55E-03	3.012	NM_000191	NM_000191	Homo sapiens 3-hydroxymethyl-3-methylglutaryl-Coenzyme A lyase (hydroxymethylglutaricaciduria) (HMGCL), mRNA [NM_000191]	NM_000191
A_23_P211244	2.56E-03	2.061	NM_206962	NM_206962	Homo sapiens HMT1 hnRNP methyltransferase-like 1 (S. cerevisiae) (HRMT1L1), transcript variant 1, mRNA [NM_206962]	NM_206962
A_32_P223777	2.57E-03	32.19	THC2315176		IL6B_HUMAN (P40189) Interleukin-6 receptor beta chain precursor (IL-6R-beta) (Interleukin 6 signal transducer) (Membrane glycoprotein 130) (gp130) (Oncostatin M receptor) (CDw130) (CD130 antigen), partial (27%) [THC2315176]	
A_23_P15394	2.57E-03	4.617	NM_001251	NM_001251	Homo sapiens CD68 antigen (CD68), mRNA [NM_001251]	NM_001251
A_23_P114983	2.58E-03	5.266	NM_032588	NM_032588	Homo sapiens tripartite motif-containing 63 (TRIM63), mRNA [NM_032588]	NM_032588
A_23_P347632	2.58E-03	3.24	NM_014751	NM_014751	Homo sapiens metastasis suppressor 1 (MTSS1), mRNA [NM_014751]	NM_014751
A_24_P196592	2.58E-03	2.666	NM_024302	NM_024302	Homo sapiens matrix metalloproteinase 28 (MMP28), transcript variant 1, mRNA [NM_024302]	NM_024302
A_23_P88865	2.58E-03	2.54	NM_144601	NM_144601	Homo sapiens chemokine-like factor super family 3 (CKLFSF3), transcript variant 1, mRNA [NM_144601]	NM_144601
A_24_P610945	2.59E-03	6.881	ENST00000311197			
A_24_P303770	2.59E-03	4.1	NM_147780	NM_147780	Homo sapiens cathepsin B (CTSB), transcript variant 2, mRNA [NM_147780]	NM_147780
A_23_P61674	2.59E-03	2.98	NM_020666	NM_020666	Homo sapiens CDC-like kinase 4 (CLK4), mRNA [NM_020666]	NM_020666
A_23_P48886	2.59E-03	2.445	NM_001110	NM_001110	Homo sapiens a disintegrin and metalloproteinase domain 10 (ADAM10), mRNA [NM_001110]	NM_001110
A_23_P207650	2.59E-03	2.337	NM_000018	NM_000018	Homo sapiens acyl-Coenzyme A dehydrogenase, very long chain (ACADVL), nuclear gene encoding mitochondrial protein, mRNA [NM_000018]	NM_000018
A_24_P312041	2.59E-03	2.306	NM_006718	NM_006718	Homo sapiens pleiomorphic adenoma gene-like 1 (PLAGL1), transcript variant 2, mRNA [NM_006718]	NM_006718
A_24_P228027	2.59E-03	2.092	NM_144611	NM_144611	Homo sapiens hypothetical protein MGC32124 (MGC32124), mRNA [NM_144611]	NM_144611
A_23_P209564	2.60E-03	5.546	NM_024843	NM_024843	Homo sapiens cytochrome b reductase 1 (CYBRD1), mRNA [NM_024843]	NM_024843
A_24_P942321	2.60E-03	2.579	NM_018027	NM_018027	Homo sapiens FERM domain containing 4A (FRMD4A), mRNA [NM_018027]	NM_018027
A_23_P68970	2.60E-03	2.502	NM_014570	NM_014570	Homo sapiens ADP-ribosylation factor GTPase activating protein 3 (ARFGAP3), mRNA [NM_014570]	NM_014570
A_23_P90659	2.60E-03	2.013	NM_014713	NM_014713	Homo sapiens lysosomal-associated protein transmembrane 4 alpha (LAPTM4A), mRNA [NM_014713]	NM_014713
A_23_P43337	2.61E-03	14.65	NM_144966	NM_144966	Homo sapiens FRAS1 related extracellular matrix 1 (FREM1), mRNA [NM_144966]	NM_144966
A_24_P315120	2.61E-03	7.094	NM_001855	NM_001855	Homo sapiens collagen, type XV, alpha 1 (COL15A1), mRNA [NM_001855]	NM_001855
A_23_P416711	2.61E-03	5.354	NM_152996	NM_152996	Homo sapiens ST6 (alpha-N-acetyl-neuraminyl-2,3-beta-galactosyl-1,3)-N-acetylgalactosaminide alpha-2,6-sialyltransferase 3 (ST6GALNAC3), mRNA [NM_152996]	NM_152996
A_23_P410408	2.62E-03	4.13	NM_175883	NM_175883	Homo sapiens olfactory receptor, family 7, subfamily D, member 2 (OR7D2), mRNA [NM_175883]	NM_175883
A_23_P350491	2.62E-03	2.113	NM_198794	NM_198794	Homo sapiens mitogen-activated protein kinase kinase kinase kinase 5 (MAP4K5), transcript variant 2, mRNA [NM_198794]	NM_198794

A_23_P320739	2.63E-03	11.71	NM_002397	NM_002397	Homo sapiens MADS box transcription enhancer factor 2, polypeptide C (myocyte enhancer factor 2C) (MEF2C), mRNA [NM_002397]	NM_002397
A_23_P323943	2.63E-03	9.858	NM_178498	NM_178498	Homo sapiens solute carrier family 5 (sodium/glucose cotransporter), member 12 (SLC5A12), mRNA [NM_178498]	NM_178498
A_24_P48723	2.63E-03	7.783	NM_000961	NM_000961	Homo sapiens prostaglandin I2 (prostacyclin) synthase (PTGIS), mRNA [NM_000961]	NM_000961
A_23_P390172	2.63E-03	6.203	NM_021133	NM_021133	Homo sapiens ribonuclease L (2',5'-oligoisoadenylate synthetase-dependent) (RNASEL), mRNA [NM_021133]	NM_021133
A_24_P929083	2.63E-03	4.761	THC2275899		BC073800 BTB (POZ) domain containing 4 {Homo sapiens;} , partial (3%) [THC2275899]	
A_24_P285768	2.63E-03	2.347	NM_014674	NM_014674	Homo sapiens ER degradation enhancer, mannosidase alpha-like 1 (EDEM1), mRNA [NM_014674]	NM_014674
A_32_P55344	2.63E-03	2.232	A_32_P55344			
A_24_P328969	2.64E-03	4.902	NM_030799	NM_030799	Homo sapiens Yip1 domain family, member 5 (YIPF5), transcript variant 2, mRNA [NM_030799]	NM_030799
A_23_P76901	2.64E-03	4.055	NM_015549	NM_015549	Homo sapiens pleckstrin homology domain containing, family G (with RhoGef domain) member 3 (PLEKHG3), mRNA [NM_015549]	NM_015549
A_24_P330886	2.64E-03	3.754	NM_016125	NM_016125	Homo sapiens PTD016 protein (LOC51136), mRNA [NM_016125]	NM_016125
A_24_P135322	2.64E-03	2.88	NM_003873	NM_003873	Homo sapiens neuropilin 1 (NRP1), transcript variant 1, mRNA [NM_003873]	NM_003873
A_23_P119202	2.64E-03	2.244	NM_001252	NM_001252	Homo sapiens tumor necrosis factor (ligand) superfamily, member 7 (TNFSF7), mRNA [NM_001252]	NM_001252
A_23_P168610	2.64E-03	2.191	NM_014399	NM_014399	Homo sapiens tetraspanin 13 (TSPAN13), mRNA [NM_014399]	NM_014399
A_24_P356601	2.65E-03	3.065	NM_006460	NM_006460	Homo sapiens hexamethylene bis-acetamide inducible 1 (HEXIM1), mRNA [NM_006460]	NM_006460
A_32_P169406	2.66E-03	21.65	CR615016	CR615016	full-length cDNA clone CS0DB006YM19 of Neuroblastoma Cot 10-normalized of Homo sapiens (human). [CR615016]	XM_378360
A_24_P362904	2.66E-03	4.16	NM_004567	NM_004567	Homo sapiens 6-phosphofructo-2-kinase/fructose-2,6-biphosphatase 4 (PFKFB4), mRNA [NM_004567]	NM_004567
A_32_P179317	2.66E-03	3.513	BF761348	BF761348	BF761348 RC2-CS0018-041000-015-g01 CS0018 Homo sapiens cDNA, mRNA sequence [BF761348]	
A_24_P238131	2.66E-03	3.185	ENST00000309558		Homo sapiens mRNA for KIAA0527 protein, partial cds. [AB011099]	XM_171054
A_23_P103282	2.66E-03	3.166	NM_004872	NM_004872	Homo sapiens transmembrane protein 59 (TMEM59), mRNA [NM_004872]	NM_004872
A_23_P168130	2.66E-03	2.42	NM_054111	NM_054111	Homo sapiens inositol hexaphosphate kinase 3 (IHPK3), mRNA [NM_054111]	NM_054111
A_32_P22637	2.66E-03	2.312	THC2280227			
A_23_P79591	2.67E-03	110.9	NM_000384	NM_000384	Homo sapiens apolipoprotein B (including Ag(x) antigen) (APOB), mRNA [NM_000384]	NM_000384
A_24_P90005	2.67E-03	6.68	NM_005203	NM_005203	Homo sapiens collagen, type XIII, alpha 1 (COL13A1), transcript variant 1, mRNA [NM_005203]	NM_005203
A_23_P217151	2.67E-03	2.903	NM_017776	NM_017776	Homo sapiens zinc finger protein 673 (ZNF673), mRNA [NM_017776]	NM_017776
A_23_P88309	2.67E-03	2.277	NM_153811	NM_153811	Homo sapiens solute carrier family 38, member 6 (SLC38A6), mRNA [NM_153811]	NM_153811
A_23_P2884	2.68E-03	6.926	NM_004569	NM_004569	Homo sapiens phosphatidylinositol glycan, class H (PIGH), mRNA [NM_004569]	NM_004569
A_32_P69136	2.68E-03	3.86	XM_498851	XM_498851	PREDICTED: Homo sapiens LOC440765 (LOC440765), mRNA [XM_498851]	XM_498851
A_23_P77228	2.68E-03	3.59	AK090443	AK090443	Homo sapiens mRNA for FLJ00364 protein. [AK090443]	
A_32_P219520	2.68E-03	2.586	NM_014350	NM_014350	Homo sapiens tumor necrosis factor, alpha-induced protein 8 (TNFAIP8), mRNA [NM_014350]	NM_014350
A_24_P129341	2.69E-03	111.4	NM_020299	NM_020299	Homo sapiens aldo-keto reductase family 1, member B10 (aldose reductase) (AKR1B10), mRNA [NM_020299]	NM_020299
A_23_P500861	2.69E-03	8.484	NM_182961	NM_182961	Homo sapiens spectrin repeat containing, nuclear envelope 1 (SYNE1), transcript variant longest, mRNA [NM_182961]	NM_182961
A_24_P64653	2.69E-03	3.002	NM_152637	NM_152637	Homo sapiens hypothetical protein MGC17301 (MGC17301), mRNA [NM_152637]	NM_152637
A_32_P58755	2.70E-03	3.542	BM726522	BM726522	UI-E-EJ0-aii-c-04-0-UI.r1 UI-E-EJ0 Homo sapiens cDNA clone UI-E-EJ0-aii-c-04-0-UI 5', mRNA sequence [BM726522]	
A_32_P163125	2.70E-03	2.926	NM_147156	NM_147156	Homo sapiens transmembrane protein 23 (TMEM23), mRNA [NM_147156]	NM_147156
A_23_P29153	2.70E-03	2.806	NM_014433	NM_014433	Homo sapiens rhabdoid tumor deletion region gene 1 (RTDR1), mRNA [NM_014433]	NM_014433
A_23_P83659	2.70E-03	2.289	NM_003399	NM_003399	Homo sapiens X-prolyl aminopeptidase (aminopeptidase P) 2, membrane-bound (XPNPEP2), mRNA [NM_003399]	NM_003399
A_23_P4082	2.71E-03	2.352	NM_006584	NM_006584	Homo sapiens chaperonin containing TCP1, subunit 6B (zeta 2) (CCT6B), mRNA [NM_006584]	NM_006584
A_24_P294233	2.72E-03	10.62	NM_014905	NM_014905	Homo sapiens glutaminase (GLS), mRNA [NM_014905]	NM_014905
A_24_P281605	2.72E-03	2.373	A_24_P281605			
A_24_P313334	2.72E-03	2.057	AK094846	AK094846	Homo sapiens cDNA FLJ37527 fis, clone BRCAN2011946. [AK094846]	
A_32_P147149	2.73E-03	4.913	AF088062	AF088062	Homo sapiens full length insert cDNA clone ZD74E10. [AF088062]	

A_32_P194372	2.73E-03	4.459	AK129547	AK129547	Homo sapiens cDNA FLJ26036 fis, clone PRS00145. [AK129547]	
A_23_P201628	2.73E-03	2.472	NM_002293	NM_002293	Homo sapiens laminin, gamma 1 (formerly LAMB2) (LAMC1), mRNA [NM_002293]	NM_002293
A_24_P192805	2.74E-03	11.79	NM_001007232	NM_001007232	Homo sapiens inhibitory caspase recruitment domain (CARD) protein (INCA), mRNA [NM_001007232]	NM_001007232
A_24_P347431	2.74E-03	11.25	NM_004496	NM_004496	Homo sapiens forkhead box A1 (FOXA1), mRNA [NM_004496]	NM_004496
A_23_P66432	2.74E-03	3.064	NM_032646	NM_032646	Homo sapiens tweety homolog 2 (Drosophila) (TTYH2), transcript variant 1, mRNA [NM_032646]	NM_032646
A_23_P256172	2.75E-03	2.274	NM_199417	NM_199417	Homo sapiens nuclear protein E3-3 (DKFZP564J0123), transcript variant 5, mRNA [NM_199417]	NM_199417
A_23_P137016	2.76E-03	6.933	NM_002970	NM_002970	Homo sapiens spermidine/spermine N1-acetyltransferase (SAT), mRNA [NM_002970]	NM_002970
A_23_P6909	2.76E-03	6.5	NM_178445	NM_178445	Homo sapiens chemokine (C-C motif) receptor-like 1 (CCRL1), transcript variant 1, mRNA [NM_178445]	NM_178445
A_23_P12514	2.76E-03	3.315	NM_175744	NM_175744	Homo sapiens ras homolog gene family, member C (RHOC), mRNA [NM_175744]	NM_175744
A_32_P92642	2.76E-03	3.013	BE710245	BE710245	BE710245 IL3-HT0619-120700-212-G05 HT0619 Homo sapiens cDNA, mRNA sequence [BE710245]	
A_23_P134755	2.76E-03	2.929	NM_014112	NM_014112	Homo sapiens trichorhinophalangeal syndrome I (TRPS1), mRNA [NM_014112]	NM_014112
A_23_P161399	2.76E-03	2.86	NM_130439	NM_130439	Homo sapiens MAX interactor 1 (MXI1), transcript variant 2, mRNA [NM_130439]	NM_130439
A_23_P215787	2.76E-03	2.233	NM_012257	NM_012257	Homo sapiens HMG-box transcription factor 1 (HBP1), mRNA [NM_012257]	NM_012257
A_32_P71736	2.77E-03	6.531	NM_004569	NM_004569	Homo sapiens phosphatidylinositol glycan, class H (PIGH), mRNA [NM_004569]	NM_004569
A_23_P217510	2.79E-03	2.439	NM_002414	NM_002414	Homo sapiens CD99 antigen (CD99), mRNA [NM_002414]	NM_002414
A_24_P350060	2.79E-03	2.33	ENST00000330461		PREDICTED: Homo sapiens similar to Keratin, type I cytoskeletal 18 (Cytokeratin 18) (K18) (CK 18) (LOC391819), mRNA [XM_498013]	XM_498013
A_23_P2573	2.80E-03	3.816	NM_032256	NM_032256	Homo sapiens hypothetical protein DKFZp434K2435 (DKFZp434K2435), mRNA [NM_032256]	NM_032256
A_23_P217088	2.80E-03	2.78	NM_000476	NM_000476	Homo sapiens adenylate kinase 1 (AK1), mRNA [NM_000476]	NM_000476
A_23_P207520	2.81E-03	8.137	NM_000088	NM_000088	Homo sapiens collagen, type I, alpha 1 (COL1A1), mRNA [NM_000088]	NM_000088
A_23_P139912	2.82E-03	4.107	NM_002178	NM_002178	Homo sapiens insulin-like growth factor binding protein 6 (IGFBP6), mRNA [NM_002178]	NM_002178
A_23_P82503	2.82E-03	3.734	ENST00000362013		Homo sapiens MEF3L1 mRNA for MEF3 like 1, complete cds. [AB049150]	XM_499343
A_23_P117546	2.82E-03	2.575	AK129920	AK129920	Homo sapiens cDNA FLJ26410 fis, clone HRT09622. [AK129920]	
A_23_P146417	2.82E-03	2.05	NM_032012	NM_032012	Homo sapiens chromosome 9 open reading frame 5 (C9orf5), mRNA [NM_032012]	NM_032012
A_23_P71480	2.83E-03	10.28	NM_005218	NM_005218	Homo sapiens defensin, beta 1 (DEFB1), mRNA [NM_005218]	NM_005218
A_23_P102060	2.83E-03	8.563	NM_006751	NM_006751	Homo sapiens sperm specific antigen 2 (SSFA2), mRNA [NM_006751]	NM_006751
A_24_P396231	2.83E-03	3.724	BC040653	BC040653	Homo sapiens cDNA clone IMAGE:4797120, partial cds. [BC040653]	
A_23_P63038	2.83E-03	2.961	NM_022356	NM_022356	Homo sapiens leucine proline-enriched proteoglycan (leprecan) 1 (LEPRE1), mRNA [NM_022356]	NM_022356
A_23_P29555	2.83E-03	2.105	NM_183352	NM_183352	Homo sapiens SEC13-like 1 (S. cerevisiae) (SEC13L1), transcript variant 2, mRNA [NM_183352]	NM_183352
A_24_P179351	2.84E-03	2.787	NM_003295	NM_003295	Homo sapiens tumor protein, translationally-controlled 1 (TPT1), mRNA [NM_003295]	NM_003295
A_24_P120303	2.84E-03	2.167	NM_018266	NM_018266	Homo sapiens transmembrane protein 39A (TMEM39A), mRNA [NM_018266]	NM_018266
A_24_P942636	2.84E-03	2.031	NM_014423	NM_014423	Homo sapiens AF4/FMR2 family, member 4 (AFF4), mRNA [NM_014423]	NM_014423
A_24_P171873	2.85E-03	5.808	NM_012176	NM_012176	Homo sapiens F-box protein 4 (FBXO4), transcript variant 1, mRNA [NM_012176]	NM_012176
A_23_P165333	2.85E-03	4.518	NM_139346	NM_139346	Homo sapiens bridging integrator 1 (BIN1), transcript variant 4, mRNA [NM_139346]	NM_139346
A_24_P230057	2.85E-03	2.118	ENST00000327707			
A_32_P74477	2.86E-03	16.86	THC2403217			
A_23_P212756	2.86E-03	2.848	NM_001004057	NM_001004057	Homo sapiens G protein-coupled receptor kinase 4 (GRK4), transcript variant 3, mRNA [NM_001004057]	NM_001004057
A_23_P168592	2.86E-03	2.014	NM_138771	NM_138771	Homo sapiens alpha-1,3(6)-mannosylglycoprotein beta-1,6-N-acetyl-glucosaminyltransferase-like (LOC90693), mRNA [NM_138771]	NM_138771
A_23_P66891	2.88E-03	2.584	NM_012121	NM_012121	Homo sapiens CDC42 effector protein (Rho GTPase binding) 4 (CDC42EP4), mRNA [NM_012121]	NM_012121
A_24_P290153	2.88E-03	2.584	NM_000495	NM_000495	Homo sapiens collagen, type IV, alpha 5 (Alport syndrome) (COL4A5), transcript variant 1, mRNA [NM_000495]	NM_000495
A_23_P20484	2.90E-03	10.53	NM_201553	NM_201553	Homo sapiens fibrinogen-like 1 (FGL1), transcript variant 4, mRNA [NM_201553]	NM_201553
A_23_P125109	2.90E-03	2.337	A_23_P125109			
A_24_P416177	2.91E-03	3.952	NM_001114	NM_001114	Homo sapiens adenylate cyclase 7 (ADCY7), mRNA [NM_001114]	NM_001114

A_32_P213917	2.91E-03	2.968	BF343856	BF343856	602015535F1 NCI_CGAP_Brn64 Homo sapiens cDNA clone IMAGE:4151048 5', mRNA sequence [BF343856]	
A_23_P156431	2.92E-03	17.73	NM_005907	NM_005907	Homo sapiens mannosidase, alpha, class 1A, member 1 (MAN1A1), mRNA [NM_005907]	NM_005907
A_23_P24004	2.92E-03	16.24	NM_001547	NM_001547	Homo sapiens interferon-induced protein with tetratricopeptide repeats 2 (IFIT2), mRNA [NM_001547]	NM_001547
A_23_P206371	2.92E-03	2.13	NM_003946	NM_003946	Homo sapiens nucleolar protein 3 (apoptosis repressor with CARD domain) (NOL3), mRNA [NM_003946]	NM_003946
A_24_P314688	2.93E-03	2.995	NM_018218	NM_018218	Homo sapiens ubiquitin specific protease 40 (USP40), mRNA [NM_018218]	NM_018218
A_24_P268993	2.93E-03	2.789	NM_052971	NM_052971	Homo sapiens liver-expressed antimicrobial peptide 2 (LEAP-2), mRNA [NM_052971]	NM_052971
A_23_P330578	2.93E-03	2.42	NM_144620	NM_144620	Homo sapiens leucine rich repeat containing 39 (LRRC39), mRNA [NM_144620]	NM_144620
A_23_P330461	2.94E-03	2.611	NM_144686	NM_144686	Homo sapiens transmembrane channel-like 4 (TMC4), mRNA [NM_144686]	NM_144686
A_24_P336728	2.94E-03	2.43	NM_014873	NM_014873	Homo sapiens lysophosphatidylglycerol acyltransferase 1 (LPGAT1), mRNA [NM_014873]	NM_014873
A_23_P24157	2.94E-03	2.215	NM_032709	NM_032709	Homo sapiens chromosome 10 open reading frame 33 (C10orf33), mRNA [NM_032709]	NM_032709
A_24_P405850	2.94E-03	2.094	NM_006320	NM_006320	Homo sapiens progesterone receptor membrane component 2 (PGRMC2), mRNA [NM_006320]	NM_006320
A_23_P66739	2.95E-03	24.64	NM_177550	NM_177550	Homo sapiens solute carrier family 13 (sodium-dependent citrate transporter), member 5 (SLC13A5), mRNA [NM_177550]	NM_177550
A_23_P321501	2.95E-03	4.265	NM_182908	NM_182908	Homo sapiens dehydrogenase/reductase (SDR family) member 2 (DHRS2), transcript variant 1, mRNA [NM_182908]	NM_182908
A_24_P221445	2.95E-03	2.365	NM_181354	NM_181354	Homo sapiens oxidation resistance 1 (OXR1), mRNA [NM_181354]	NM_181354
A_32_P50587	2.96E-03	3.066	THC2448178			
A_23_P8571	2.97E-03	3.025	NM_080744	NM_080744	Homo sapiens scavenger receptor cysteine rich domain containing, group B (4 domains) (SRCRB4D), mRNA [NM_080744]	NM_080744
A_23_P53467	2.99E-03	5.287	NM_201612	NM_201612	Homo sapiens IKK interacting protein (IKIP), transcript variant 2, mRNA [NM_201612]	NM_201612
A_23_P26928	2.99E-03	2.173	ENST00000357776		Homo sapiens cDNA FLJ13094 fis, clone NT2RP3002163. [AK023156]	
A_23_P64539	3.02E-03	26.53	NM_000559	NM_000559	Homo sapiens hemoglobin, gamma A (HBG1), mRNA [NM_000559]	NM_000559
A_23_P421379	3.02E-03	11.66	NM_000612	NM_000612	Homo sapiens insulin-like growth factor 2 (somatomedin A) (IGF2), mRNA [NM_000612]	NM_000612
A_24_P37946	3.02E-03	2.045	BX537394	BX537394	Homo sapiens mRNA; cDNA DKFZp686F23142 (from clone DKFZp686F23142); complete cds. [BX537394]	
A_23_P80048	3.03E-03	5.064	NR_001442	NR_001442	Homo sapiens fer-1-like 4 (C. elegans) (FER1L4) on chromosome 20 [NR_001442]	NR_001442
A_23_P41390	3.03E-03	4.881	NM_018986	NM_018986	Homo sapiens SH3 domain and tetratricopeptide repeats 1 (SH3TC1), mRNA [NM_018986]	NM_018986
A_23_P408271	3.03E-03	4.439	NM_016245	NM_016245	Homo sapiens dehydrogenase/reductase (SDR family) member 8 (DHRS8), mRNA [NM_016245]	NM_016245
A_32_P90047	3.03E-03	2.768	BC062790	BC062790	Homo sapiens hypothetical gene supported by BC038466; BC062790, mRNA (cDNA clone IMAGE:4775221). [BC062790]	XM_379250
A_23_P24345	3.03E-03	2.721	NM_152264	NM_152264	Homo sapiens solute carrier family 39 (zinc transporter), member 13 (SLC39A13), mRNA [NM_152264]	NM_152264
A_23_P25003	3.03E-03	2.573	NM_002956	NM_002956	Homo sapiens restin (Reed-Steinberg cell-expressed intermediate filament-associated protein) (RSN), transcript variant 1, mRNA [NM_002956]	NM_002956
A_24_P384369	3.03E-03	2.029	ENST00000330875			
A_23_P360964	3.04E-03	11.67	NM_145056	NM_145056	Homo sapiens thymus expressed gene 3-like (MGC15476), mRNA [NM_145056]	NM_145056
A_32_P171530	3.04E-03	6.978	BC038371	BC038371	Homo sapiens, clone IMAGE:4829282, mRNA. [BC038371]	
A_23_P122531	3.04E-03	2.588	NM_016947	NM_016947	Homo sapiens chromosome 6 open reading frame 48 (C6orf48), mRNA [NM_016947]	NM_016947
A_23_P96383	3.05E-03	4.506	NM_006307	NM_006307	Homo sapiens sushi-repeat-containing protein, X-linked (SRPX), mRNA [NM_006307]	NM_006307
A_23_P204702	3.05E-03	2.074	NM_003217	NM_003217	Homo sapiens testis enhanced gene transcript (BAX inhibitor 1) (TEGT), mRNA [NM_003217]	NM_003217
A_23_P145463	3.06E-03	6.255	NM_015948	NM_015948	Homo sapiens solute carrier family 35, member B3 (SLC35B3), mRNA [NM_015948]	NM_015948
A_23_P314115	3.06E-03	3.257	NM_005180	NM_005180	Homo sapiens polycomb group ring finger 4 (PCGF4), mRNA [NM_005180]	NM_005180
A_23_P210176	3.06E-03	2.364	NM_000210	NM_000210	Homo sapiens integrin, alpha 6 (ITGA6), mRNA [NM_000210]	NM_000210
A_32_P32254	3.07E-03	7.465	NM_001848	NM_001848	Homo sapiens collagen, type VI, alpha 1 (COL6A1), mRNA [NM_001848]	NM_001848
A_24_P943301	3.07E-03	5.149	ENST00000292357		Homo sapiens cDNA FLJ25943 fis, clone JTH10559. [AK098809]	
A_23_P52806	3.10E-03	13.94	NM_012104	NM_012104	Homo sapiens beta-site APP-cleaving enzyme 1 (BACE1), transcript variant a, mRNA [NM_012104]	NM_012104
A_23_P98402	3.10E-03	3.445	NM_015996	NM_015996	Homo sapiens SID1 transmembrane family, member 2 (SIDT2), mRNA [NM_015996]	NM_015996

A_23_P92184	3.10E-03	2.333	NM_019069	NM_019069	Homo sapiens WD repeat domain 5B (WDR5B), mRNA [NM_019069]	NM_019069
A_24_P240732	3.11E-03	4.995	NM_017813	NM_017813	Homo sapiens myo-inositol monophosphatase A3 (IMPA3), mRNA [NM_017813]	NM_017813
A_23_P93236	3.11E-03	3.914	NM_006355	NM_006355	Homo sapiens tripartite motif-containing 38 (TRIM38), mRNA [NM_006355]	NM_006355
A_24_P410686	3.11E-03	2.913	CR616528	CR616528	full-length cDNA clone CS0DC014YA20 of Neuroblastoma Cot 25-normalized of Homo sapiens (human). [CR616528]	
A_23_P313603	3.11E-03	2.527	AK056855	AK056855	Homo sapiens cDNA FLJ32293 fis, clone PROST2001739. [AK056855]	XM_374902
A_23_P21485	3.12E-03	12.07	NM_017933	NM_017933	Homo sapiens hypothetical protein FLJ20701 (FLJ20701), mRNA [NM_017933]	NM_017933
A_23_P311740	3.12E-03	3.179	NM_015089	NM_015089	Homo sapiens p53-associated parkin-like cytoplasmic protein (PARC), mRNA [NM_015089]	NM_015089
A_23_P29029	3.12E-03	3.076	NM_032910	NM_032910	Homo sapiens chromosome 21 open reading frame 119 (C21orf119), mRNA [NM_032910]	NM_032910
A_23_P377957	3.13E-03	7.828	NM_138444	NM_138444	Homo sapiens potassium channel tetramerisation domain containing 12 (KCTD12), mRNA [NM_138444]	NM_138444
A_32_P95147	3.13E-03	7.446	BC038559	BC038559	Homo sapiens, clone IMAGE:3851018, mRNA. [BC038559]	
A_32_P166921	3.13E-03	4.638	AV692191	AV692191	AV692191 AV692191 GKC Homo sapiens cDNA clone GKCFFD09 5', mRNA sequence [AV692191]	
A_32_P119348	3.13E-03	4.079	AK130049	AK130049	Homo sapiens cDNA FLJ26539 fis, clone KDN09310. [AK130049]	
A_23_P10902	3.14E-03	134.8	NM_001463	NM_001463	Homo sapiens frizzled-related protein (FRZB), mRNA [NM_001463]	NM_001463
A_32_P215938	3.14E-03	3.728	BC048343	BC048343	Homo sapiens G-protein signalling modulator 1 (AGS3-like, C. elegans), mRNA (cDNA clone IMAGE:5242824), complete cds. [BC048343]	
A_23_P144369	3.14E-03	2.456	NM_153757	NM_153757	Homo sapiens nucleosome assembly protein 1-like 5 (NAP1L5), mRNA [NM_153757]	NM_153757
A_23_P500892	3.14E-03	2.312	NM_003320	NM_003320	Homo sapiens tubby homolog (mouse) (TUB), transcript variant 1, mRNA [NM_003320]	NM_003320
A_32_P162494	3.14E-03	2.087	THC2375981		Q5SSW7 (Q5SSW7) CAMP responsive elment binding protein-like 1, partial (5%) [THC2375981]	
A_24_P63290	3.15E-03	2.21	NM_198562	NM_198562	Homo sapiens FLJ43654 protein (FLJ43654), mRNA [NM_198562]	NM_198562
A_23_P43150	3.17E-03	11.61	NM_001017926	NM_001017926	Homo sapiens zinc fingers and homeoboxes 1 (ZHX1), transcript variant 1, mRNA [NM_001017926]	NM_001017926
A_24_P209455	3.17E-03	6.41	NM_018326	NM_018326	Homo sapiens GTPase, IMAP family member 4 (GIMAP4), mRNA [NM_018326]	NM_018326
A_23_P54649	3.17E-03	3.432	NM_153425	NM_153425	Homo sapiens TNFRSF1A-associated via death domain (TRADD), transcript variant 2, mRNA [NM_153425]	NM_153425
A_23_P28906	3.17E-03	2.512	AB046810	AB046810	Homo sapiens mRNA for KIAA1590 protein, partial cds. [AB046810]	
A_24_P403501	3.17E-03	2.189	S80864	S80864	cytochrome c-like polypeptide [human, lung adenocarcinoma A549, mRNA, 1041 nt]. [S80864]	
A_23_P375494	3.18E-03	10.62	NM_004364	NM_004364	Homo sapiens CCAAT/enhancer binding protein (C/EBP), alpha (CEBPA), mRNA [NM_004364]	NM_004364
A_24_P329795	3.19E-03	6.353	NM_007021	NM_007021	Homo sapiens chromosome 10 open reading frame 10 (C10orf10), mRNA [NM_007021]	NM_007021
A_23_P377434	3.21E-03	5.603	AK027341	AK027341	Homo sapiens cDNA FLJ14435 fis, clone HEMBA1007085. [AK027341]	XM_376567
A_24_P6030	3.22E-03	4.943	NM_058182	NM_058182	Homo sapiens chromosome 21 open reading frame 51 (C21orf51), mRNA [NM_058182]	NM_058182
A_23_P6321	3.22E-03	4.235	NM_003277	NM_003277	Homo sapiens claudin 5 (transmembrane protein deleted in velocardiofacial syndrome) (CLDN5), mRNA [NM_003277]	NM_003277
A_23_P135616	3.22E-03	3.865	NM_016930	NM_016930	Homo sapiens syntaxin 18 (STX18), mRNA [NM_016930]	NM_016930
A_23_P63319	3.23E-03	2.668	NM_003101	NM_003101	Homo sapiens sterol O-acyltransferase (acyl-Coenzyme A: cholesterol acyltransferase) 1 (SOAT1), transcript variant 688113, mRNA [NM_003101]	NM_003101
A_23_P309515	3.23E-03	2.49	NM_152387	NM_152387	Homo sapiens potassium channel tetramerisation domain containing 18 (KCTD18), mRNA [NM_152387]	NM_152387
A_32_P204330	3.23E-03	2.123	AK093982	AK093982	Homo sapiens cDNA FLJ36663 fis, clone UTERU2002826. [AK093982]	
A_23_P145644	3.24E-03	84.03	NM_000790	NM_000790	Homo sapiens dopa decarboxylase (aromatic L-amino acid decarboxylase) (DDC), mRNA [NM_000790]	NM_000790
A_23_P159325	3.24E-03	8.076	NM_016109	NM_016109	Homo sapiens angiopoietin-like 4 (ANGPTL4), transcript variant 2, mRNA [NM_016109]	NM_016109
A_23_P218770	3.24E-03	4.045	NM_002872	NM_002872	Homo sapiens ras-related C3 botulinum toxin substrate 2 (rho family, small GTP binding protein Rac2) (RAC2), mRNA [NM_002872]	NM_002872
A_24_P332953	3.24E-03	3.378	A_24_P332953			
A_23_P92073	3.25E-03	2.419	NM_001003935	NM_001003935	Homo sapiens poly (ADP-ribose) polymerase family, member 3 (PARP3), transcript variant 3, mRNA [NM_001003935]	NM_001003935
A_23_P91390	3.26E-03	48.17	NM_000361	NM_000361	Homo sapiens thrombomodulin (THBD), mRNA [NM_000361]	NM_000361
A_23_P42397	3.26E-03	16.89	NM_153362	NM_153362	Homo sapiens protease, serine, 35 (PRSS35), mRNA [NM_153362]	NM_153362
A_23_P170649	3.26E-03	8.815	NM_153225	NM_153225	Homo sapiens RPE-spondin (RPESP), mRNA [NM_153225]	NM_153225

A_32_P109029	3.26E-03	8.142	NM_022036	NM_022036	Homo sapiens G protein-coupled receptor, family C, group 5, member C (GPRC5C), transcript variant 1, mRNA [NM_022036]	NM_022036
A_23_P76983	3.26E-03	5.686	NM_025057	NM_025057	Homo sapiens chromosome 14 open reading frame 45 (C14orf45), mRNA [NM_025057]	NM_025057
A_24_P481783	3.26E-03	2.864	AK023802	AK023802	Homo sapiens cDNA FLJ13740 fis, clone PLACE3000199. [AK023802]	
A_23_P18082	3.26E-03	2.285	NM_032806	NM_032806	Homo sapiens glycosyltransferase (AGO61), mRNA [NM_032806]	NM_032806
A_24_P236235	3.27E-03	15.61	NM_013231	NM_013231	Homo sapiens fibronectin leucine rich transmembrane protein 2 (FLRT2), mRNA [NM_013231]	NM_013231
A_23_P117363	3.27E-03	14.55	NM_001756	NM_001756	Homo sapiens serine (or cysteine) proteinase inhibitor, clade A (alpha-1 antiproteinase, antitrypsin), member 6 (SERPINA6), mRNA [NM_001756]	NM_001756
A_23_P31896	3.27E-03	12.93	NM_003033	NM_003033	Homo sapiens ST3 beta-galactoside alpha-2,3-sialyltransferase 1 (ST3GAL1), transcript variant 1, mRNA [NM_003033]	NM_003033
A_23_P101642	3.27E-03	12.41	NM_002842	NM_002842	Homo sapiens protein tyrosine phosphatase, receptor type, H (PTPRH), mRNA [NM_002842]	NM_002842
A_24_P810290	3.27E-03	3.137	AK098668	AK098668	Homo sapiens cDNA FLJ25802 fis, clone TST07145. [AK098668]	XM_113641
A_23_P389102	3.27E-03	2.931	NM_015194	NM_015194	Homo sapiens myosin ID (MYO1D), mRNA [NM_015194]	NM_015194
A_23_P32135	3.27E-03	2.824	NM_018956	NM_018956	Homo sapiens chromosome 9 open reading frame 9 (C9orf9), mRNA [NM_018956]	NM_018956
A_24_P714618	3.27E-03	2.79	BC011779	BC011779	Homo sapiens cDNA clone IMAGE:3941306, partial cds. [BC011779]	
A_23_P79622	3.28E-03	11.09	NM_016105	NM_016105	Homo sapiens FK506 binding protein 7 (FKBP7), transcript variant 1, mRNA [NM_016105]	NM_016105
A_24_P392991	3.28E-03	7.134	NM_005557	NM_005557	Homo sapiens keratin 16 (focal non-epidermolytic palmoplantar keratoderma) (KRT16), mRNA [NM_005557]	NM_005557
A_24_P303354	3.28E-03	2.876	NM_021064	NM_021064	Homo sapiens histone 1, H2ag (HIST1H2AG), mRNA [NM_021064]	NM_021064
A_23_P37988	3.28E-03	2.806	NM_152727	NM_152727	Homo sapiens copine II (CPNE2), mRNA [NM_152727]	NM_152727
A_23_P129005	3.28E-03	2.75	BC008219	BC008219	Homo sapiens KIAA1305, mRNA (cDNA clone MGC:17689 IMAGE:3865984), complete cds. [BC008219]	XM_370756
A_23_P326987	3.28E-03	2.028	AF318337	AF318337	Homo sapiens pp13759 mRNA, complete cds. [AF318337]	
A_23_P25475	3.30E-03	33.46	NM_003578	NM_003578	Homo sapiens sterol O-acyltransferase 2 (SOAT2), mRNA [NM_003578]	NM_003578
A_23_P217611	3.31E-03	4.649	NM_016607	NM_016607	Homo sapiens armadillo repeat containing, X-linked 3 (ARMCX3), transcript variant 1, mRNA [NM_016607]	NM_016607
A_32_P116556	3.32E-03	10.21	AB058761	AB058761	Homo sapiens mRNA for KIAA1858 protein, partial cds. [AB058761]	XM_040592
A_32_P111587	3.32E-03	3.112	AF086098	AF086098	Homo sapiens full length insert cDNA clone YZ88E12. [AF086098]	
A_23_P106898	3.32E-03	2.47	NM_152288	NM_152288	Homo sapiens hypothetical protein MGC13024 (MGC13024), mRNA [NM_152288]	NM_152288
A_32_P208078	3.32E-03	2.293	BC011614	BC011614	Homo sapiens 5,10-methylenetetrahydrofolate reductase (NADPH), mRNA (cDNA clone IMAGE:3949285), partial cds. [BC011614]	
A_23_P61688	3.33E-03	5.329	NM_006598	NM_006598	Homo sapiens solute carrier family 12 (potassium/chloride transporters), member 7 (SLC12A7), mRNA [NM_006598]	NM_006598
A_32_P157846	3.33E-03	3.712	AK092260	AK092260	Homo sapiens cDNA FLJ34941 fis, clone NT2RP7007480. [AK092260]	
A_24_P82200	3.34E-03	11.84	NM_170676	NM_170676	Homo sapiens Meis1, myeloid ecotropic viral integration site 1 homolog 2 (mouse) (MEIS2), transcript variant d, mRNA [NM_170676]	NM_170676
A_23_P83028	3.34E-03	6.599	NM_021111	NM_021111	Homo sapiens reversion-inducing-cysteine-rich protein with kazal motifs (RECK), mRNA [NM_021111]	NM_021111
A_23_P57323	3.34E-03	6.55	NM_004449	NM_004449	Homo sapiens v-ets erythroblastosis virus E26 oncogene like (avian) (ERG), transcript variant 2, mRNA [NM_004449]	NM_004449
A_32_P227921	3.34E-03	5.85	THC2283605			
A_32_P66222	3.35E-03	2.802	NM_001012421	NM_001012421	Homo sapiens OTTHUMP00000064580 (LOC441430), mRNA [NM_001012421]	NM_001012421
A_24_P942469	3.35E-03	2.698	AK129652	AK129652	Homo sapiens cDNA FLJ26141 fis, clone TST03911. [AK129652]	
A_23_P130626	3.35E-03	2.533	NM_024075	NM_024075	Homo sapiens tRNA splicing endonuclease 34 homolog (SEN34, S. cerevisiae) (TSEN34), mRNA [NM_024075]	NM_024075
A_23_P124619	3.36E-03	31.01	NM_020672	NM_020672	Homo sapiens S100 calcium binding protein A14 (S100A14), mRNA [NM_020672]	NM_020672
A_23_P257638	3.36E-03	2.008	NM_006384	NM_006384	Homo sapiens calcium and integrin binding 1 (calmyrin) (CIB1), mRNA [NM_006384]	NM_006384
A_23_P77000	3.37E-03	2.647	NM_014909	NM_014909	Homo sapiens KIAA1036 (KIAA1036), mRNA [NM_014909]	NM_014909
A_23_P399078	3.38E-03	7.272	NM_000362	NM_000362	Homo sapiens tissue inhibitor of metalloproteinase 3 (Sorsby fundus dystrophy, pseudoinflammatory) (TIMP3), mRNA [NM_000362]	NM_000362
A_24_P634530	3.38E-03	3.112	THC2281463			
A_23_P137984	3.39E-03	8.193	NM_002966	NM_002966	Homo sapiens S100 calcium binding protein A10 (annexin II ligand, calpactin I, light polypeptide (p11)) (S100A10), mRNA [NM_002966]	NM_002966

A_23_P122852	3.39E-03	6.14	NM_003078	NM_003078	Homo sapiens SWI/SNF related, matrix associated, actin dependent regulator of chromatin, subfamily d, member 3 (SMARCD3), transcript variant 2, mRNA [NM_003078]	NM_003078
A_24_P410453	3.40E-03	14.81	NM_033071	NM_033071	Homo sapiens spectrin repeat containing, nuclear envelope 1 (SYNE1), transcript variant longer, mRNA [NM_033071]	NM_033071
A_23_P27013	3.40E-03	6.881	NM_024017	NM_024017	Homo sapiens homeo box B9 (HOXB9), mRNA [NM_024017]	NM_024017
A_23_P360804	3.40E-03	5.976	NM_020939	NM_020939	Homo sapiens copine V (CPNE5), mRNA [NM_020939]	NM_020939
A_23_P388433	3.40E-03	3.858	ENST00000296496		full-length cDNA clone CS0DI016YF21 of Placenta Cot 25-normalized of Homo sapiens (human). [CR597270]	
A_23_P55936	3.40E-03	3.535	NM_004107	NM_004107	Homo sapiens Fc fragment of IgG, receptor, transporter, alpha (FCGRT), mRNA [NM_004107]	NM_004107
A_23_P167051	3.40E-03	2.65	NM_014556	NM_014556	Homo sapiens Ellis van Creveld syndrome (EVC), transcript variant 1, mRNA [NM_014556]	NM_014556
A_24_P414376	3.41E-03	3.258	THC2371729			
A_23_P127027	3.41E-03	2.014	NM_001081	NM_001081	Homo sapiens cubilin (intrinsic factor-cobalamin receptor) (CUBN), mRNA [NM_001081]	NM_001081
A_32_P88965	3.42E-03	11.51	BX537651	BX537651	Homo sapiens mRNA; cDNA DKFZp686C0390 (from clone DKFZp686C0390). [BX537651]	
A_23_P85180	3.42E-03	3.281	NM_003492	NM_003492	Homo sapiens chromosome X open reading frame 12 (CXorf12), mRNA [NM_003492]	NM_003492
A_32_P157208	3.42E-03	2.546	AY343891	AY343891	Homo sapiens chromosome 2 mRNA sequence. [AY343891]	
A_24_P305570	3.43E-03	13.24	NM_018993	NM_018993	Homo sapiens Ras and Rab interactor 2 (RIN2), mRNA [NM_018993]	NM_018993
A_23_P96285	3.43E-03	6.895	NM_022912	NM_022912	Homo sapiens chromosome 2 open reading frame 23 (C2orf23), mRNA [NM_022912]	NM_022912
A_24_P30194	3.43E-03	3.958	NM_012420	NM_012420	Homo sapiens interferon-induced protein with tetratricopeptide repeats 5 (IFIT5), mRNA [NM_012420]	NM_012420
A_23_P44466	3.43E-03	3.247	NM_024781	NM_024781	Homo sapiens chromosome 18 open reading frame 14 (C18orf14), mRNA [NM_024781]	NM_024781
A_23_P350187	3.43E-03	3.089	ENST00000265149		Homo sapiens mRNA for KIAA1546 protein, partial cds. [AB046766]	XM_042301
A_23_P395609	3.45E-03	14.54	NM_147189	NM_147189	Homo sapiens hypothetical protein MGC39325 (MGC39325), mRNA [NM_147189]	NM_147189
A_32_P155247	3.45E-03	3.13	NM_000146	NM_000146	Homo sapiens ferritin, light polypeptide (FTL), mRNA [NM_000146]	NM_000146
A_24_P333901	3.45E-03	3.01	NM_015284	NM_015284	Homo sapiens KIAA0467 (KIAA0467), mRNA [NM_015284]	NM_015284
A_23_P52552	3.45E-03	2.453	NM_004281	NM_004281	Homo sapiens BCL2-associated athanogene 3 (BAG3), mRNA [NM_004281]	NM_004281
A_24_P66001	3.46E-03	2.198	NM_001003684	NM_001003684	Homo sapiens ubiquinol-cytochrome c reductase complex (7.2 kD) (UCRC), transcript variant 2, mRNA [NM_001003684]	NM_001003684
A_23_P406227	3.48E-03	6.474	AY203928	AY203928	Homo sapiens FP17581 mRNA, complete cds. [AY203928]	
A_24_P105761	3.48E-03	2.559	NM_018433	NM_018433	Homo sapiens jumonji domain containing 1A (JMJD1A), mRNA [NM_018433]	NM_018433
A_23_P37127	3.49E-03	12.06	NM_004496	NM_004496	Homo sapiens forkhead box A1 (FOXA1), mRNA [NM_004496]	NM_004496
A_23_P417363	3.49E-03	11.63	AK057267	AK057267	Homo sapiens cDNA FLJ32705 fis, clone TESTI2000600, weakly similar to RESTIN. [AK057267]	
A_32_P161762	3.49E-03	7.968	NM_004348	NM_004348	Homo sapiens runt-related transcription factor 2 (RUNX2), transcript variant 3, mRNA [NM_004348]	NM_004348
A_23_P70660	3.49E-03	6.505	NM_017633	NM_017633	Homo sapiens family with sequence similarity 46, member A (FAM46A), mRNA [NM_017633]	NM_017633
A_32_P75695	3.49E-03	3.649	AK022008	AK022008	Homo sapiens cDNA FLJ11946 fis, clone HEMBB1000709. [AK022008]	
A_23_P336644	3.49E-03	2.409	NM_145034	NM_145034	Homo sapiens torsin A interacting protein 2 (TOR1AIP2), mRNA [NM_145034]	NM_145034
A_24_P391586	3.50E-03	3.926	NM_178507	NM_178507	Homo sapiens NS5ATP13TP2 protein (NS5ATP13TP2), mRNA [NM_178507]	NM_178507
A_32_P196036	3.50E-03	2.668	CR594200	CR594200	full-length cDNA clone CS0DF031YH08 of Fetal brain of Homo sapiens (human). [CR594200]	
A_24_P270728	3.50E-03	2.2	NM_012385	NM_012385	Homo sapiens p8 protein (candidate of metastasis 1) (P8), mRNA [NM_012385]	NM_012385
A_23_P34637	3.51E-03	9.607	NM_000537	NM_000537	Homo sapiens renin (REN), mRNA [NM_000537]	NM_000537
A_23_P58770	3.51E-03	9.606	NM_004821	NM_004821	Homo sapiens heart and neural crest derivatives expressed 1 (HAND1), mRNA [NM_004821]	NM_004821
A_23_P200815	3.51E-03	5.242	BF678528	BF678528	602085985F1 NIH_MGC_83 Homo sapiens cDNA clone IMAGE:4250205 5', mRNA sequence [BF678528]	
A_23_P159255	3.51E-03	4.708	NM_002845	NM_002845	Homo sapiens protein tyrosine phosphatase, receptor type, M (PTPRM), mRNA [NM_002845]	NM_002845
A_23_P9523	3.51E-03	3.702	NM_022128	NM_022128	Homo sapiens ribokinase (RBKS), mRNA [NM_022128]	NM_022128
A_24_P307827	3.51E-03	2.305	AK126887	AK126887	Homo sapiens cDNA FLJ44939 fis, clone BRAMY3018754, weakly similar to Mus musculus junction-mediating and regulatory protein (Jmy-pending). [AK126887]	XM_058720
A_23_P91891	3.51E-03	2.17	NM_004766	NM_004766	Homo sapiens coatomer protein complex, subunit beta 2 (beta prime) (COPB2), mRNA [NM_004766]	NM_004766
A_23_P382065	3.52E-03	10.63	NM_016242	NM_016242	Homo sapiens endomucin (EMCN), mRNA [NM_016242]	NM_016242

A_23_P60488	3.52E-03	3.49	NM_002540	NM_002540	Homo sapiens outer dense fiber of sperm tails 2 (ODF2), transcript variant 1, mRNA [NM_002540]	NM_002540
A_23_P216869	3.52E-03	2.128	NM_197977	NM_197977	Homo sapiens zinc finger protein 189 (ZNF189), mRNA [NM_197977]	NM_197977
A_23_P373521	3.53E-03	10.62	NM_021973	NM_021973	Homo sapiens heart and neural crest derivatives expressed 2 (HAND2), mRNA [NM_021973]	NM_021973
A_23_P141044	3.53E-03	2.264	NM_145271	NM_145271	Homo sapiens zinc finger protein 688 (ZNF688), mRNA [NM_145271]	NM_145271
A_23_P216596	3.54E-03	10.25	AK075235	AK075235	Homo sapiens cDNA FLJ90754 fis, clone PLACE4000354, weakly similar to COMPLEMENT RECEPTOR TYPE 1 PRECURSOR. [AK075235]	
A_23_P219197	3.54E-03	3.885	NM_134427	NM_134427	Homo sapiens regulator of G-protein signalling 3 (RGS3), transcript variant 4, mRNA [NM_134427]	NM_134427
A_23_P140146	3.54E-03	2.684	NM_032036	NM_032036	Homo sapiens family with sequence similarity 14, member A (FAM14A), mRNA [NM_032036]	NM_032036
A_23_P112554	3.55E-03	35.59	NM_001855	NM_001855	Homo sapiens collagen, type XV, alpha 1 (COL15A1), mRNA [NM_001855]	NM_001855
A_23_P96590	3.55E-03	3.286	NM_014710	NM_014710	Homo sapiens G protein-coupled receptor associated sorting protein 1 (GPRASP1), mRNA [NM_014710]	NM_014710
A_24_P379858	3.56E-03	2.931	NM_001607	NM_001607	Homo sapiens acetyl-Coenzyme A acyltransferase 1 (peroxisomal 3-oxoacyl-Coenzyme A thiolase) (ACAA1), nuclear gene encoding mitochondrial protein, mRNA [NM_001607]	NM_001607
A_23_P168388	3.57E-03	6.832	NM_175571	NM_175571	Homo sapiens GTPase, IMAP family member 8 (GIMAP8), mRNA [NM_175571]	NM_175571
A_23_P118042	3.57E-03	3.275	NM_018296	NM_018296	Homo sapiens leucine rich repeat containing 36 (LRRC36), mRNA [NM_018296]	NM_018296
A_23_P156748	3.57E-03	2.229	NM_015245	NM_015245	Homo sapiens ankyrin repeat and sterile alpha motif domain containing 1 (ANKS1), mRNA [NM_015245]	NM_015245
A_23_P341532	3.57E-03	2.037	NM_031915	NM_031915	Homo sapiens SET domain, bifurcated 2 (SETDB2), mRNA [NM_031915]	NM_031915
A_24_P923011	3.58E-03	11.31	NM_000301	NM_000301	Homo sapiens plasminogen (PLG), mRNA [NM_000301]	NM_000301
A_23_P138514	3.59E-03	6.079	NM_012071	NM_012071	Homo sapiens COMM domain containing 3 (COMMD3), mRNA [NM_012071]	NM_012071
A_32_P27020	3.61E-03	4.392	NM_173638	NM_173638	Homo sapiens hypothetical protein MGC8902 (MGC8902), mRNA [NM_173638]	NM_173638
A_23_P41634	3.61E-03	2.741	NM_023039	NM_023039	Homo sapiens ankyrin repeat, family A (RFXANK-like), 2 (ANKRA2), mRNA [NM_023039]	NM_023039
A_23_P62881	3.61E-03	2.498	NM_032291	NM_032291	Homo sapiens SH3-domain GRB2-like (endophilin) interacting protein 1 (SGIP1), mRNA [NM_032291]	NM_032291
A_32_P190181	3.62E-03	3.637	THC2345075			
A_24_P37519	3.62E-03	2.149	NM_020347	NM_020347	Homo sapiens leucine zipper transcription factor-like 1 (LZTFL1), mRNA [NM_020347]	NM_020347
A_23_P158024	3.63E-03	2.155	NM_007234	NM_007234	Homo sapiens dynactin 3 (p22) (DCTN3), transcript variant 1, mRNA [NM_007234]	NM_007234
A_23_P34375	3.65E-03	5.262	NM_003196	NM_003196	Homo sapiens transcription elongation factor A (SII), 3 (TCEA3), mRNA [NM_003196]	NM_003196
A_23_P30666	3.65E-03	2.72	NM_014452	NM_014452	Homo sapiens tumor necrosis factor receptor superfamily, member 21 (TNFRSF21), mRNA [NM_014452]	NM_014452
A_24_P365015	3.67E-03	18.57	NM_006361	NM_006361	Homo sapiens homeo box B13 (HOXB13), mRNA [NM_006361]	NM_006361
A_24_P317762	3.67E-03	2.197	NM_002346	NM_002346	Homo sapiens lymphocyte antigen 6 complex, locus E (LY6E), mRNA [NM_002346]	NM_002346
A_23_P3651	3.68E-03	10.1	NM_005332	NM_005332	Homo sapiens hemoglobin, zeta (HBZ), mRNA [NM_005332]	NM_005332
A_23_P101407	3.68E-03	7.109	NM_000064	NM_000064	Homo sapiens complement component 3 (C3), mRNA [NM_000064]	NM_000064
A_24_P298360	3.68E-03	3.867	NM_021070	NM_021070	Homo sapiens latent transforming growth factor beta binding protein 3 (LTBP3), mRNA [NM_021070]	NM_021070
A_23_P342869	3.68E-03	3.551	NM_020066	NM_020066	Homo sapiens formin 2 (FMN2), mRNA [NM_020066]	NM_020066
A_23_P56356	3.69E-03	4.389	BC042674	BC042674	Homo sapiens phospholipase B1, mRNA (cDNA clone MGC:35447 IMAGE:5191712), complete cds. [BC042674]	
A_23_P129209	3.69E-03	2.649	NM_002168	NM_002168	Homo sapiens isocitrate dehydrogenase 2 (NADP+), mitochondrial (IDH2), mRNA [NM_002168]	NM_002168
A_24_P52697	3.70E-03	21.38	NR_002196	NR_002196	Homo sapiens H19, imprinted maternally expressed untranslated mRNA (H19) on chromosome 11 [NR_002196]	NR_002196
A_23_P169117	3.70E-03	2.096	NM_006570	NM_006570	Homo sapiens Ras-related GTP binding A (RRAGA), mRNA [NM_006570]	NM_006570
A_23_P21134	3.72E-03	4.473	NM_004083	NM_004083	Homo sapiens DNA-damage-inducible transcript 3 (DDIT3), mRNA [NM_004083]	NM_004083
A_23_P14072	3.72E-03	2.593	NM_002273	NM_002273	Homo sapiens keratin 8 (KRT8), mRNA [NM_002273]	NM_002273
A_23_P13137	3.72E-03	2.547	AY358815	AY358815	Homo sapiens clone DNA108923 SFVP2550 (UNQ2550) mRNA, complete cds. [AY358815]	
A_23_P76350	3.72E-03	2.176	NM_002075	NM_002075	Homo sapiens guanine nucleotide binding protein (G protein), beta polypeptide 3 (GNB3), mRNA [NM_002075]	NM_002075
A_24_P235429	3.73E-03	11.55	NM_005502	NM_005502	Homo sapiens ATP-binding cassette, sub-family A (ABC1), member 1 (ABCA1), mRNA [NM_005502]	NM_005502
A_23_P103011	3.73E-03	2.163	NM_004914	NM_004914	Homo sapiens RAB36, member RAS oncogene family (RAB36), mRNA [NM_004914]	NM_004914
A_23_P502930	3.73E-03	2.031	NM_006712	NM_006712	Homo sapiens Fas-activated serine/threonine kinase (FASTK), transcript variant 1, mRNA [NM_006712]	NM_006712

A_24_P107897	3.76E-03	2.28	NM_015914	NM_015914	Homo sapiens thioredoxin domain containing 11 (TXNDC11), mRNA [NM_015914]	NM_015914
A_23_P156402	3.76E-03	2.188	NM_003551	NM_003551	Homo sapiens non-metastatic cells 5, protein expressed in (nucleoside-diphosphate kinase) (NME5), mRNA [NM_003551]	NM_003551
A_23_P58642	3.77E-03	11.03	NM_002653	NM_002653	Homo sapiens paired-like homeodomain transcription factor 1 (PITX1), mRNA [NM_002653]	NM_002653
A_24_P260101	3.77E-03	6.996	NM_007289	NM_007289	Homo sapiens membrane metallo-endopeptidase (neutral endopeptidase, enkephalinase, CALLA, CD10) (MME), transcript variant 2b, mRNA [NM_007289]	NM_007289
A_24_P722068	3.77E-03	2.286	THC2268343			
A_23_P72411	3.78E-03	7.641	NM_178033	NM_178033	Homo sapiens cytochrome P450, family 4, subfamily X, polypeptide 1 (CYP4X1), mRNA [NM_178033]	NM_178033
A_23_P216966	3.78E-03	6.689	NM_000962	NM_000962	Homo sapiens prostaglandin-endoperoxide synthase 1 (prostaglandin G/H synthase and cyclooxygenase) (PTGS1), transcript variant 1, mRNA [NM_000962]	NM_000962
A_32_P166693	3.78E-03	3.923	THC2265769		Q9GEJ2 (Q9GEJ2) NADH dehydrogenase subunit F (Fragment), partial (5%) [THC2265769]	
A_23_P16817	3.78E-03	3.494	NM_004071	NM_004071	Homo sapiens CDC-like kinase 1 (CLK1), transcript variant 1, mRNA [NM_004071]	NM_004071
A_23_P78479	3.78E-03	3.443	NM_015363	NM_015363	Homo sapiens zinc finger, imprinted 2 (ZIM2), mRNA [NM_015363]	NM_015363
A_23_P384355	3.78E-03	2.693	BG547557	BG547557	BG547557 602575410F1 NIH_MGC_77 Homo sapiens cDNA clone IMAGE:4703546 5', mRNA sequence [BG547557]	
A_32_P17635	3.78E-03	2.48	THC2427841		Q9JM93 (Q9JM93) SRp25 nuclear protein (ADP-ribosylation factor-like 6 interacting protein 4), partial (7%) [THC2427841]	
A_32_P106615	3.79E-03	68.98	AW268902	AW268902	AW268902 xv48h10.x1 Soares_NFL_T_GBC_S1 Homo sapiens cDNA clone IMAGE:2816419 3', mRNA sequence [AW268902]	
A_24_P525917	3.79E-03	8.656	AK025758	AK025758	Homo sapiens cDNA: FLJ22105 fis, clone HEP17660. [AK025758]	
A_24_P762886	3.80E-03	3.218	AB007954	AB007954	Homo sapiens mRNA, chromosome 1 specific transcript KIAA0485. [AB007954]	
A_23_P53137	3.81E-03	24.75	NM_000559	NM_000559	Homo sapiens hemoglobin, gamma A (HBG1), mRNA [NM_000559]	NM_000559
A_32_P107372	3.81E-03	4.168	NM_002053	NM_002053	Homo sapiens guanylate binding protein 1, interferon-inducible, 67kDa (GBP1), mRNA [NM_002053]	NM_002053
A_24_P85200	3.81E-03	3.112	NM_194278	NM_194278	Homo sapiens chromosome 14 open reading frame 43 (C14orf43), mRNA [NM_194278]	NM_194278
A_23_P353865	3.81E-03	2.575	AB041269	AB041269	Homo sapiens mRNA for keratin 19, partial cds, isolate:K19-141. [AB041269]	
A_24_P408321	3.81E-03	2.344	NM_144498	NM_144498	Homo sapiens oxysterol binding protein-like 2 (OSBPL2), transcript variant 2, mRNA [NM_144498]	NM_144498
A_23_P207742	3.82E-03	3.487	NM_003250	NM_003250	Homo sapiens thyroid hormone receptor, alpha (erythroblastic leukemia viral (v-erb-a) oncogene homolog, avian) (THRA), transcript variant 2, mRNA [NM_003250]	NM_003250
A_32_P196520	3.82E-03	2.328	AK023159	AK023159	Homo sapiens cDNA FLJ13097 fis, clone NT2RP3002173. [AK023159]	
A_32_P114372	3.82E-03	2.319	AL133627	AL133627	Homo sapiens mRNA; cDNA DKFZp434K0722 (from clone DKFZp434K0722). [AL133627]	
A_32_P167122	3.82E-03	2.285	NM_018254	NM_018254	Homo sapiens REST corepressor 3 (RCOR3), mRNA [NM_018254]	NM_018254
A_23_P64980	3.83E-03	5.792	NM_016615	NM_016615	Homo sapiens solute carrier family 6 (neurotransmitter transporter, GABA), member 13 (SLC6A13), mRNA [NM_016615]	NM_016615
A_24_P195974	3.83E-03	2.141	A_24_P195974			
A_23_P19020	3.85E-03	4.458	NM_005460	NM_005460	Homo sapiens synuclein, alpha interacting protein (synphilin) (SNCAIP), mRNA [NM_005460]	NM_005460
A_32_P154321	3.85E-03	3.894	AW804939	AW804939	AW804939 QV4-UM0094-060400-159-g07 UM0094 Homo sapiens cDNA, mRNA sequence [AW804939]	
A_24_P928830	3.86E-03	8.177	A_24_P928830			
A_23_P37514	3.87E-03	2.253	BC011905	BC011905	Homo sapiens DKFZP434H132 protein, mRNA (cDNA clone IMAGE:4121832), complete cds. [BC011905]	
A_32_P67610	3.87E-03	2.243	CR605444	CR605444	full-length cDNA clone CS0DK008YC22 of HeLa cells Cot 25-normalized of Homo sapiens (human). [CR605444]	
A_32_P129527	3.87E-03	2.007	NM_018341	NM_018341	Homo sapiens chromosome 6 open reading frame 70 (C6orf70), mRNA [NM_018341]	NM_018341
A_32_P111072	3.88E-03	44.03	THC2389705			
A_23_P139476	3.88E-03	2.864	NM_001780	NM_001780	Homo sapiens CD63 antigen (melanoma 1 antigen) (CD63), mRNA [NM_001780]	NM_001780
A_23_P7212	3.90E-03	17.85	NM_000204	NM_000204	Homo sapiens I factor (complement) (IF), mRNA [NM_000204]	NM_000204
A_32_P44808	3.90E-03	2.789	AK124941	AK124941	Homo sapiens cDNA FLJ42951 fis, clone BRSTN2007765. [AK124941]	
A_24_P294832	3.90E-03	2.254	NM_003463	NM_003463	Homo sapiens protein tyrosine phosphatase type IVA, member 1 (PTP4A1), mRNA [NM_003463]	NM_003463
A_23_P136232	3.91E-03	5.841	NM_017813	NM_017813	Homo sapiens myo-inositol monophosphatase A3 (IMPA3), mRNA [NM_017813]	NM_017813
A_23_P133648	3.91E-03	3.646	NM_016255	NM_016255	Homo sapiens family with sequence similarity 8, member A1 (FAM8A1), mRNA [NM_016255]	NM_016255

A_23_P200792	3.93E-03	2.568	NM_024408	NM_024408	Homo sapiens Notch homolog 2 (Drosophila) (NOTCH2), mRNA [NM_024408]	NM_024408
A_32_P57002	3.93E-03	2.145	A_32_P57002			
A_23_P161998	3.94E-03	13.48	NM_000613	NM_000613	Homo sapiens hemopexin (HPX), mRNA [NM_000613]	NM_000613
A_24_P333857	3.94E-03	6.987	NM_032291	NM_032291	Homo sapiens SH3-domain GRB2-like (endophilin) interacting protein 1 (SGIP1), mRNA [NM_032291]	NM_032291
A_24_P295791	3.94E-03	4.245	NM_032564	NM_032564	Homo sapiens diacylglycerol O-acyltransferase homolog 2 (mouse) (DGAT2), mRNA [NM_032564]	NM_032564
A_24_P254437	3.94E-03	3.453	CA312433	CA312433	CA312433 UI-CF-FN0-afk-i-18-0-UI.s1 UI-CF-FN0 Homo sapiens cDNA clone UI-CF-FN0-afk-i-18-0-UI 3', mRNA sequence [CA312433]	
A_24_P398940	3.94E-03	2.533	NM_138423	NM_138423	Homo sapiens cancer susceptibility candidate 4 (CASC4), transcript variant 1, mRNA [NM_138423]	NM_138423
A_32_P58029	3.94E-03	2.501	A_32_P58029			
A_24_P286054	3.94E-03	2.309	AB002303	AB002303	Homo sapiens mRNA for KIAA0305 gene, partial cds. [AB002303]	
A_23_P122815	3.94E-03	2.035	NM_001219	NM_001219	Homo sapiens calumenin (CALU), mRNA [NM_001219]	NM_001219
A_24_P234732	3.96E-03	2.125	NM_006454	NM_006454	Homo sapiens MAX dimerization protein 4 (MXD4), mRNA [NM_006454]	NM_006454
A_32_P96213	3.97E-03	2.604	NM_003295	NM_003295	Homo sapiens tumor protein, translationally-controlled 1 (TPT1), mRNA [NM_003295]	NM_003295
A_23_P210900	3.97E-03	2.073	NM_018677	NM_018677	Homo sapiens acetyl-Coenzyme A synthetase 2 (ADP forming) (ACAS2), transcript variant 1, mRNA [NM_018677]	NM_018677
A_23_P127964	3.97E-03	2.055	NM_199418	NM_199418	Homo sapiens prolylcarboxypeptidase (angiotensinase C) (PRCP), transcript variant 2, mRNA [NM_199418]	NM_199418
A_23_P92222	3.98E-03	15.25	NM_152673	NM_152673	Homo sapiens mucin 20 (MUC20), mRNA [NM_152673]	NM_152673
A_23_P255601	3.99E-03	2.046	NM_032508	NM_032508	Homo sapiens family with sequence similarity 11, member A (FAM11A), mRNA [NM_032508]	NM_032508
A_24_P216294	4.00E-03	4.405	NM_000099	NM_000099	Homo sapiens cystatin C (amyloid angiopathy and cerebral hemorrhage) (CST3), mRNA [NM_000099]	NM_000099
A_23_P115366	4.00E-03	4.244	NM_016308	NM_016308	Homo sapiens cytidylate kinase (CMPK), mRNA [NM_016308]	NM_016308
A_24_P373152	4.00E-03	3.536	NM_021914	NM_021914	Homo sapiens cofilin 2 (muscle) (CFL2), transcript variant 1, mRNA [NM_021914]	NM_021914
A_32_P7015	4.00E-03	3.397	NM_012339	NM_012339	Homo sapiens tetraspanin 15 (TSPAN15), mRNA [NM_012339]	NM_012339
A_24_P541919	4.00E-03	3.181	AF086301	AF086301	Homo sapiens full length insert cDNA clone ZD50F09. [AF086301]	
A_23_P98282	4.00E-03	3.168	NM_006946	NM_006946	Homo sapiens spectrin, beta, non-erythrocytic 2 (SPTBN2), mRNA [NM_006946]	NM_006946
A_23_P59700	4.00E-03	2.648	NM_005868	NM_005868	Homo sapiens BET1 homolog (S. cerevisiae) (BET1), mRNA [NM_005868]	NM_005868
A_32_P32835	4.00E-03	2.534	NM_145274	NM_145274	Homo sapiens hypothetical protein MGC21518 (MGC21518), mRNA [NM_145274]	NM_145274
A_23_P209669	4.00E-03	2.412	NM_201266	NM_201266	Homo sapiens neuropilin 2 (NRP2), transcript variant 1, mRNA [NM_201266]	NM_201266
A_23_P255827	4.00E-03	2.147	NM_021631	NM_021631	Homo sapiens apoptosis inhibitor (FKSG2), mRNA [NM_021631]	NM_021631
A_24_P334640	4.00E-03	2.109	NM_133367	NM_133367	Homo sapiens progestin and adipoQ receptor family member VIII (PAQR8), mRNA [NM_133367]	NM_133367
A_23_P45821	4.01E-03	6.643	THC2281747		Q96KY5 (Q96KY5) MGC27169 protein, complete [THC2281747]	
A_23_P35456	4.01E-03	3.578	NM_014631	NM_014631	Homo sapiens SH3 multiple domains 1 (SH3MD1), mRNA [NM_014631]	NM_014631
A_23_P256735	4.01E-03	3.306	NM_016134	NM_016134	Homo sapiens plasma glutamate carboxypeptidase (PGCP), mRNA [NM_016134]	NM_016134
A_32_P70468	4.01E-03	2.371	AK124072	AK124072	Homo sapiens cDNA FLJ42078 fis, clone SYNOV2020085. [AK124072]	
A_23_P67432	4.01E-03	2.346	NM_152478	NM_152478	Homo sapiens zinc finger protein 583 (ZNF583), mRNA [NM_152478]	NM_152478
A_23_P16866	4.02E-03	5.555	NM_007127	NM_007127	Homo sapiens villin 1 (VIL1), mRNA [NM_007127]	NM_007127
A_23_P363472	4.02E-03	4.517	AB032991	AB032991	Homo sapiens mRNA for KIAA1165 protein, partial cds. [AB032991]	
A_32_P154445	4.02E-03	4.223	CR590573	CR590573	full-length cDNA clone CS0DI042YD07 of Placenta Cot 25-normalized of Homo sapiens (human). [CR590573]	
A_23_P202334	4.02E-03	3.671	NM_023028	NM_023028	Homo sapiens fibroblast growth factor receptor 2 (bacteria-expressed kinase, keratinocyte growth factor receptor, craniofacial dysostosis 1, Crouzon syndrome, Pfeiffer syndrome, Jackson-Weiss syndrome) (FGFR2), transcript variant 10, mRNA [NM_023028]	NM_023028
A_23_P107432	4.02E-03	2.386	NM_207453	NM_207453	Homo sapiens FLJ35934 protein (FLJ35934), mRNA [NM_207453]	NM_207453
A_24_P844100	4.02E-03	2.049	A_24_P844100			
A_24_P391591	4.03E-03	4.628	AK057596	AK057596	Homo sapiens cDNA FLJ33034 fis, clone THYMU2000236. [AK057596]	XM_499585
A_24_P40551	4.03E-03	3.558	BC015794	BC015794	Homo sapiens brain expressed X-linked-like 1, mRNA (cDNA clone IMAGE:3909303), partial cds. [BC015794]	XM_043653

A_23_P384761	4.03E-03	2.815	NM_002052	NM_002052	Homo sapiens GATA binding protein 4 (GATA4), mRNA [NM_002052]	NM_002052
A_32_P196047	4.03E-03	2.516	NM_181787	NM_181787	Homo sapiens dpy-19-like 4 (C. elegans) (DPY19L4), mRNA [NM_181787]	NM_181787
A_24_P116587	4.03E-03	2.319	NM_201575	NM_201575	Homo sapiens seizure related 6 homolog (mouse)-like 2 (SEZ6L2), transcript variant 2, mRNA [NM_201575]	NM_201575
A_23_P310956	4.04E-03	6.956	NM_058175	NM_058175	Homo sapiens collagen, type VI, alpha 2 (COL6A2), transcript variant 2C2a', mRNA [NM_058175]	NM_058175
A_23_P108932	4.04E-03	5.445	NR_002229	NR_002229	Homo sapiens ribosomal protein L23a pseudogene 13 (RPL23AP13) on chromosome 2 [NR_002229]	NR_002229
A_23_P37359	4.04E-03	2.477	NM_014430	NM_014430	Homo sapiens cell death-inducing DFFA-like effector b (CIDEB), mRNA [NM_014430]	NM_014430
A_23_P202327	4.05E-03	22.35	NM_003474	NM_003474	Homo sapiens a disintegrin and metalloproteinase domain 12 (meltrin alpha) (ADAM12), transcript variant 1, mRNA [NM_003474]	NM_003474
A_23_P142455	4.05E-03	2.542	NM_012237	NM_012237	Homo sapiens sirtuin (silent mating type information regulation 2 homolog) 2 (S. cerevisiae) (SIRT2), transcript variant 1, mRNA [NM_012237]	NM_012237
A_23_P63541	4.06E-03	7.465	AK001019	AK001019	Homo sapiens cDNA FLJ10157 fis, clone HEMBA1003461. [AK001019]	XM_371354
A_32_P177040	4.06E-03	3.874	NM_175064	NM_175064	Homo sapiens Williams Beuren syndrome chromosome region 19 (WBSCR19), mRNA [NM_175064]	NM_175064
A_23_P342688	4.06E-03	2.367	NM_032874	NM_032874	Homo sapiens KIAA1984 (KIAA1984), mRNA [NM_032874]	NM_032874
A_24_P463989	4.07E-03	8.848	THC2376306		Q7QRW0 (Q7QRW0) GLP_69_6195_6536, partial (14%) [THC2376306]	
A_23_P153037	4.07E-03	2.947	NM_020787	NM_020787	Homo sapiens zinc finger protein 624 (ZNF624), mRNA [NM_020787]	NM_020787
A_24_P11462	4.08E-03	2.041	NM_052998	NM_052998	Homo sapiens arginine decarboxylase (ADC), mRNA [NM_052998]	NM_052998
A_23_P379649	4.09E-03	3.132	NM_001003940	NM_001003940	Homo sapiens Bcl2 modifying factor (BMF), transcript variant 1, mRNA [NM_001003940]	NM_001003940
A_23_P216340	4.10E-03	7.524	NM_006748	NM_006748	Homo sapiens Src-like-adaptor (SLA), mRNA [NM_006748]	NM_006748
A_23_P61886	4.10E-03	7.246	AK055659	AK055659	Homo sapiens cDNA FLJ31097 fis, clone IMR321000210. [AK055659]	
A_24_P234871	4.13E-03	7.085	A_24_P234871			
A_23_P99693	4.13E-03	4.521	NM_014950	NM_014950	Homo sapiens zinc finger and BTB domain containing 1 (ZBTB1), mRNA [NM_014950]	NM_014950
A_23_P38015	4.13E-03	3.687	NM_022452	NM_022452	Homo sapiens fibrosin 1 (FBS1), mRNA [NM_022452]	NM_022452
A_24_P264031	4.13E-03	2.313	AK126415	AK126415	Homo sapiens cDNA FLJ44451 fis, clone UTERU2023039. [AK126415]	
A_23_P121011	4.13E-03	2.084	NM_033027	NM_033027	Homo sapiens AXIN1 up-regulated 1 (AXUD1), mRNA [NM_033027]	NM_033027
A_23_P81805	4.14E-03	2.358	NM_001025366	NM_001025366	Homo sapiens vascular endothelial growth factor (VEGF), transcript variant 1, mRNA [NM_001025366]	NM_001025366
A_24_P11315	4.15E-03	3.155	NM_020190	NM_020190	Homo sapiens olfactomedin-like 3 (OLFML3), mRNA [NM_020190]	NM_020190
A_23_P357504	4.16E-03	3.247	ENST00000328520		Q765F0 (Q765F0) PreC/core protein, partial (13%) [THC2251776]	
A_23_P120572	4.16E-03	2.964	NM_018993	NM_018993	Homo sapiens Ras and Rab interactor 2 (RIN2), mRNA [NM_018993]	NM_018993
A_24_P262127	4.17E-03	5.806	NM_004165	NM_004165	Homo sapiens Ras-related associated with diabetes (RRAD), mRNA [NM_004165]	NM_004165
A_23_P86171	4.17E-03	4.092	NM_004474	NM_004474	Homo sapiens forkhead box D2 (FOXD2), mRNA [NM_004474]	NM_004474
A_24_P738130	4.17E-03	3.477	THC2432280		Q66KD2 (Q66KD2) Lasp1-prov protein, partial (10%) [THC2432280]	
A_32_P110872	4.17E-03	2.666	BC001077	BC001077	Homo sapiens hypothetical protein BC004360, mRNA (cDNA clone MGC:2727 IMAGE:2822295), complete cds. [BC001077]	XM_373431
A_24_P233915	4.17E-03	2.595	NM_001001653	NM_001001653	Homo sapiens mediator of RNA polymerase II transcription, subunit 8 homolog (yeast) (MED8), transcript variant 4, mRNA [NM_001001653]	NM_001001653
A_23_P86434	4.17E-03	2.297	BC007866	BC007866	Homo sapiens hypothetical protein MGC14425, mRNA (cDNA clone MGC:14425 IMAGE:4302902), complete cds. [BC007866]	
A_32_P73071	4.17E-03	2.155	THC2453866			
A_23_P69267	4.17E-03	2.125	NM_015407	NM_015407	Homo sapiens abhydrolase domain containing 14A (ABHD14A), mRNA [NM_015407]	NM_015407
A_24_P719948	4.17E-03	2.119	AK023629	AK023629	Homo sapiens cDNA FLJ13567 fis, clone PLACE1008331. [AK023629]	
A_24_P118196	4.20E-03	3.739	ENST00000308603		PREDICTED: Homo sapiens similar to CG9996-PA (LOC389129), mRNA [XM_371647]	XM_371647
A_23_P100711	4.21E-03	6.408	NM_000304	NM_000304	Homo sapiens peripheral myelin protein 22 (PMP22), transcript variant 1, mRNA [NM_000304]	NM_000304
A_23_P156425	4.21E-03	2.488	NM_005907	NM_005907	Homo sapiens mannosidase, alpha, class 1A, member 1 (MAN1A1), mRNA [NM_005907]	NM_005907
A_24_P341546	4.21E-03	2.319	A_24_P341546			
A_32_P13823	4.22E-03	4.205	THC2411757			

A_32_P220798	4.23E-03	37.14	NM_001773	NM_001773	Homo sapiens CD34 antigen (CD34), mRNA [NM_001773]	NM_001773
A_23_P328545	4.23E-03	4.599	NM_014211	NM_014211	Homo sapiens gamma-aminobutyric acid (GABA) A receptor, pi (GABRP), mRNA [NM_014211]	NM_014211
A_32_P128974	4.24E-03	3.271	AW971123	AW971123	EST383210 MAGE resequences, MAGL Homo sapiens cDNA, mRNA sequence [AW971123]	
A_23_P413180	4.24E-03	3.119	NM_017440	NM_017440	Homo sapiens Mdm4, transformed 3T3 cell double minute 1, p53 binding protein (mouse) (MDM1), transcript variant 1, mRNA [NM_017440]	NM_017440
A_23_P343330	4.24E-03	2.723	AK056277	AK056277	Homo sapiens cDNA FLJ31715 fis, clone NT2RI2006553. [AK056277]	
A_32_P49035	4.24E-03	2.18	A_32_P49035			
A_23_P99747	4.25E-03	6.46	NM_004196	NM_004196	Homo sapiens cyclin-dependent kinase-like 1 (CDC2-related kinase) (CDKL1), mRNA [NM_004196]	NM_004196
A_23_P57856	4.26E-03	3.476	NM_138931	NM_138931	Homo sapiens B-cell CLL/lymphoma 6 (zinc finger protein 51) (BCL6), transcript variant 2, mRNA [NM_138931]	NM_138931
A_32_P192594	4.26E-03	2.145	CR624390	CR624390	full-length cDNA clone CS0DC018YB19 of Neuroblastoma Cot 25-normalized of Homo sapiens (human). [CR624390]	XM_378399
A_24_P234094	4.26E-03	2.11	NM_003929	NM_003929	Homo sapiens RAB7, member RAS oncogene family-like 1 (RAB7L1), mRNA [NM_003929]	NM_003929
A_23_P150609	4.27E-03	40.72	NM_001007139	NM_001007139	Homo sapiens putative insulin-like growth factor II associated protein (LOC492304), mRNA [NM_001007139]	NM_001007139
A_23_P111888	4.27E-03	27.99	NM_138455	NM_138455	Homo sapiens collagen triple helix repeat containing 1 (CTHRC1), mRNA [NM_138455]	NM_138455
A_23_P202219	4.27E-03	7.632	NM_015916	NM_015916	Homo sapiens family with sequence similarity 26, member B (FAM26B), mRNA [NM_015916]	NM_015916
A_32_P135450	4.27E-03	3.424	A_32_P135450			
A_32_P121234	4.27E-03	2.843	A_32_P121234			
A_32_P192922	4.27E-03	2.363	AL050061	AL050061	Homo sapiens mRNA; cDNA DKFZp566J123 (from clone DKFZp566J123). [AL050061]	
A_32_P125402	4.28E-03	3.714	THC2314822		NOA1_HUMAN (Q9NY12) Nucleolar protein family A member 1 (snoRNP protein GAR1) (H/ACA ribonucleoprotein GAR1), partial (18%) [THC2314822]	
A_23_P36345	4.28E-03	2.342	NM_006645	NM_006645	Homo sapiens START domain containing 10 (STARD10), mRNA [NM_006645]	NM_006645
A_24_P255123	4.28E-03	2.011	A_24_P255123			
A_24_P157926	4.29E-03	21.32	NM_006290	NM_006290	Homo sapiens tumor necrosis factor, alpha-induced protein 3 (TNFAIP3), mRNA [NM_006290]	NM_006290
A_23_P162746	4.29E-03	16.33	NM_015974	NM_015974	Homo sapiens crystallin, lambda 1 (CRYL1), mRNA [NM_015974]	NM_015974
A_24_P643587	4.29E-03	2.926	NM_024095	NM_024095	Homo sapiens ankyrin repeat and SOCS box-containing 8 (ASB8), mRNA [NM_024095]	NM_024095
A_24_P376309	4.29E-03	2.716	BC008564	BC008564	Homo sapiens cDNA clone IMAGE:4178802, partial cds. [BC008564]	
A_23_P52647	4.29E-03	2.187	NM_006795	NM_006795	Homo sapiens EH-domain containing 1 (EHD1), mRNA [NM_006795]	NM_006795
A_32_P59606	4.30E-03	7.797	THC2401724		A30241 ribosomal protein L7a - mouse {Mus musculus;} , partial (11%) [THC2401724]	
A_23_P49627	4.30E-03	3.482	NM_006039	NM_006039	Homo sapiens mannose receptor, C type 2 (MRC2), mRNA [NM_006039]	NM_006039
A_24_P208091	4.30E-03	2.214	D87449	D87449	Human mRNA for KIAA0260 gene, partial cds. [D87449]	
A_32_P35906	4.30E-03	2.127	BQ010172	BQ010172	BQ010172 UI-H-ED0-axz-j-15-0-UI.s1 NCI_CGAP_ED0 Homo sapiens cDNA clone IMAGE:5835758 3', mRNA sequence [BQ010172]	
A_23_P137196	4.31E-03	3.002	NM_001560	NM_001560	Homo sapiens interleukin 13 receptor, alpha 1 (IL13RA1), mRNA [NM_001560]	NM_001560
A_23_P35412	4.32E-03	6.353	NM_001549	NM_001549	Homo sapiens interferon-induced protein with tetratricopeptide repeats 3 (IFIT3), mRNA [NM_001549]	NM_001549
A_24_P544882	4.32E-03	2.974	AK026687	AK026687	Homo sapiens cDNA: FLJ23034 fis, clone LNG02018. [AK026687]	
A_32_P126259	4.33E-03	7.582	THC2443836			
A_23_P86195	4.33E-03	5.23	NM_152369	NM_152369	Homo sapiens hypothetical protein MGC45474 (MGC45474), mRNA [NM_152369]	NM_152369
A_23_P405267	4.33E-03	4.91	AK057922	AK057922	Homo sapiens cDNA FLJ25193 fis, clone JTH00761. [AK057922]	
A_24_P683905	4.33E-03	3.866	AK001829	AK001829	Homo sapiens cDNA FLJ10967 fis, clone PLACE1000798. [AK001829]	
A_23_P165707	4.33E-03	3.609	NM_174898	NM_174898	Homo sapiens hypothetical protein LOC129530 (LOC129530), mRNA [NM_174898]	NM_174898
A_23_P334709	4.33E-03	2.887	NM_007270	NM_007270	Homo sapiens FK506 binding protein 9, 63 kDa (FKBP9), mRNA [NM_007270]	NM_007270
A_23_P27515	4.33E-03	2.863	NM_012268	NM_012268	Homo sapiens phospholipase D family, member 3 (PLD3), mRNA [NM_012268]	NM_012268
A_32_P73217	4.33E-03	2.182	NM_020141	NM_020141	Homo sapiens chromosome 1 open reading frame 119 (C1orf119), mRNA [NM_020141]	NM_020141
A_23_P6674	4.34E-03	2.879	NM_020169	NM_020169	Homo sapiens latexin (LXN), mRNA [NM_020169]	NM_020169
A_24_P160263	4.39E-03	2.75	NM_201222	NM_201222	Homo sapiens melanoma antigen family D, 2 (MAGED2), transcript variant 3, mRNA [NM_201222]	NM_201222
A_24_P88554	4.41E-03	2.251	NM_003846	NM_003846	Homo sapiens peroxisomal biogenesis factor 11B (PEX11B), mRNA [NM_003846]	NM_003846

A_24_P144134	4.43E-03	4.341	A_24_P144134			
A_23_P68486	4.43E-03	2.868	NM_080821	NM_080821	Homo sapiens chromosome 20 open reading frame 108 (C20orf108), mRNA [NM_080821]	NM_080821
A_23_P149562	4.44E-03	9.634	NM_004815	NM_004815	Homo sapiens Rho GTPase activating protein 29 (ARHGAP29), mRNA [NM_004815]	NM_004815
A_24_P188218	4.45E-03	7.135	NM_002476	NM_002476	Homo sapiens myosin, light polypeptide 4, alkali; atrial, embryonic (MYL4), transcript variant 2, mRNA [NM_002476]	NM_002476
A_23_P158725	4.46E-03	31.24	NM_004207	NM_004207	Homo sapiens solute carrier family 16 (monocarboxylic acid transporters), member 3 (SLC16A3), mRNA [NM_004207]	NM_004207
A_23_P164057	4.46E-03	31.1	NM_002404	NM_002404	Homo sapiens microfibrillar-associated protein 4 (MFAP4), mRNA [NM_002404]	NM_002404
A_23_P150807	4.46E-03	3.376	NM_003621	NM_003621	Homo sapiens PTPRF interacting protein, binding protein 2 (liprin beta 2) (PPFIBP2), mRNA [NM_003621]	NM_003621
A_24_P364066	4.46E-03	3.095	BC030112	BC030112	Homo sapiens, clone IMAGE:4799578, mRNA. [BC030112]	
A_23_P218225	4.46E-03	2.083	NM_014298	NM_014298	Homo sapiens quinolinate phosphoribosyltransferase (nicotinate-nucleotide pyrophosphorylase (carboxylating)) (QPRT), mRNA [NM_014298]	NM_014298
A_23_P60166	4.47E-03	6.384	NM_022783	NM_022783	Homo sapiens DEP domain containing 6 (DEPDC6), mRNA [NM_022783]	NM_022783
A_23_P137470	4.47E-03	3.519	NM_020808	NM_020808	Homo sapiens signal-induced proliferation-associated 1 like 2 (SIPA1L2), mRNA [NM_020808]	NM_020808
A_23_P29023	4.48E-03	8.028	NM_032910	NM_032910	Homo sapiens chromosome 21 open reading frame 119 (C21orf119), mRNA [NM_032910]	NM_032910
A_32_P208403	4.48E-03	4.383	NM_053064	NM_053064	Homo sapiens guanine nucleotide binding protein (G protein), gamma 2 (GNG2), mRNA [NM_053064]	NM_053064
A_23_P148609	4.48E-03	3.589	NM_021796	NM_021796	Homo sapiens placenta-specific 1 (PLAC1), mRNA [NM_021796]	NM_021796
A_23_P102160	4.48E-03	2.135	AF435956	AF435956	Homo sapiens unknown mRNA. [AF435956]	
A_23_P337168	4.48E-03	2.096	NM_153013	NM_153013	Homo sapiens hypothetical protein FLJ30596 (FLJ30596), mRNA [NM_153013]	NM_153013
A_23_P358709	4.50E-03	4.336	AB033060	AB033060	Homo sapiens mRNA for KIAA1234 protein, partial cds. [AB033060]	
A_32_P179854	4.50E-03	4.297	CR607153	CR607153	full-length cDNA clone CS0DJ012YN17 of T cells (Jurkat cell line) Cot 10-normalized of Homo sapiens (human). [CR607153]	
A_23_P19592	4.50E-03	2.667	NM_015599	NM_015599	Homo sapiens phosphoglucomutase 3 (PGM3), mRNA [NM_015599]	NM_015599
A_23_P101093	4.51E-03	9.57	NM_016429	NM_016429	Homo sapiens coatomer protein complex, subunit zeta 2 (COPZ2), mRNA [NM_016429]	NM_016429
A_23_P346006	4.52E-03	3.174	NM_020739	NM_020739	Homo sapiens cell cycle progression 1 (CCPG1), transcript variant 2, mRNA [NM_020739]	NM_020739
A_24_P419039	4.52E-03	2.108	NM_020766	NM_020766	Homo sapiens protocadherin 19 (PCDH19), mRNA [NM_020766]	NM_020766
A_23_P18966	4.53E-03	12.03	NM_001017973	NM_001017973	Homo sapiens procollagen-proline, 2-oxoglutarate 4-dioxygenase (proline 4-hydroxylase), alpha polypeptide II (P4HA2), transcript variant 2, mRNA [NM_001017973]	NM_001017973
A_23_P4662	4.54E-03	4.463	NM_005178	NM_005178	Homo sapiens B-cell CLL/lymphoma 3 (BCL3), mRNA [NM_005178]	NM_005178
A_32_P40615	4.54E-03	2.416	BC049371	BC049371	Homo sapiens hypothetical protein FLJ22313, mRNA (cDNA clone IMAGE:4821170), partial cds. [BC049371]	
A_23_P161563	4.54E-03	2.211	NM_022337	NM_022337	Homo sapiens RAB38, member RAS oncogene family (RAB38), mRNA [NM_022337]	NM_022337
A_23_P217109	4.55E-03	3.686	NM_001860	NM_001860	Homo sapiens solute carrier family 31 (copper transporters), member 2 (SLC31A2), mRNA [NM_001860]	NM_001860
A_32_P98752	4.55E-03	3.438	THC2446811			
A_24_P218814	4.55E-03	2.862	NM_002905	NM_002905	Homo sapiens retinol dehydrogenase 5 (11-cis and 9-cis) (RDH5), mRNA [NM_002905]	NM_002905
A_23_P63798	4.55E-03	2.424	NM_001300	NM_001300	Homo sapiens Kruppel-like factor 6 (KLF6), transcript variant 2, mRNA [NM_001300]	NM_001300
A_32_P195387	4.56E-03	3	AK097956	AK097956	Homo sapiens cDNA FLJ40637 fis, clone THYMU2015984. [AK097956]	
A_23_P359430	4.56E-03	2.729	NM_015383	NM_015383	Homo sapiens hypothetical protein DJ328E19.C1.1 (DJ328E19.C1.1), mRNA [NM_015383]	NM_015383
A_23_P52797	4.56E-03	2.236	NM_030770	NM_030770	Homo sapiens transmembrane protease, serine 5 (spinesin) (TMPRSS5), mRNA [NM_030770]	NM_030770
A_23_P43276	4.57E-03	14.24	NM_032777	NM_032777	Homo sapiens G protein-coupled receptor 124 (GPR124), mRNA [NM_032777]	NM_032777
A_23_P84016	4.57E-03	2.666	W31297	W31297	W31297 zb64a03.r1 Soares_fetal_lung_NbHL19W Homo sapiens cDNA clone IMAGE:308332 5' similar to gb:M36341 ADP-RIBOSYLATION FACTOR 4 (HUMAN);, mRNA sequence [W31297]	
A_24_P876522	4.58E-03	3.557	AK022110	AK022110	Homo sapiens cDNA FLJ12048 fis, clone HEMBB1001990. [AK022110]	
A_24_P86389	4.58E-03	2.925	NM_003514	NM_003514	Homo sapiens histone 1, H2am (HIST1H2AM), mRNA [NM_003514]	NM_003514
A_23_P122216	4.59E-03	19	NM_002317	NM_002317	Homo sapiens lysyl oxidase (LOX), mRNA [NM_002317]	NM_002317
A_23_P217866	4.59E-03	7.199	AK094968	AK094968	Homo sapiens cDNA FLJ37649 fis, clone BRHIP2000534, moderately similar to GAMMA-INTERFERON-INDUCIBLE PROTEIN IFI-16. [AK094968]	

A_23_P60627	4.59E-03	5.303	NM_001141	NM_001141	Homo sapiens arachidonate 15-lipoxygenase, second type (ALOX15B), mRNA [NM_001141]	NM_001141
A_24_P912228	4.59E-03	2.958	AK095564	AK095564	Homo sapiens cDNA FLJ38245 fis, clone FCBBF2007186. [AK095564]	
A_24_P780319	4.59E-03	2.142	A_24_P780319			
A_23_P209360	4.60E-03	7.578	ENST00000288548		Homo sapiens mRNA for KIAA1921 protein, partial cds. [AB067508]	
A_23_P3424	4.60E-03	2.744	NM_017793	NM_017793	Homo sapiens ribonuclease P 25kDa subunit (RPP25), mRNA [NM_017793]	NM_017793
A_23_P23924	4.61E-03	4.349	NM_001748	NM_001748	Homo sapiens calpain 2, (m/II) large subunit (CAPN2), mRNA [NM_001748]	NM_001748
A_23_P3274	4.61E-03	3.43	NM_004884	NM_004884	Homo sapiens putative neuronal cell adhesion molecule (PUNC), mRNA [NM_004884]	NM_004884
A_23_P167040	4.61E-03	3.009	NM_006810	NM_006810	Homo sapiens protein disulfide isomerase family A, member 5 (PDIA5), mRNA [NM_006810]	NM_006810
A_23_P166	4.61E-03	2.645	NM_145279	NM_145279	Homo sapiens MOB1, Mps One Binder kinase activator-like 2C (yeast) (MOBKL2C), transcript variant 1, mRNA [NM_145279]	NM_145279
A_23_P33894	4.62E-03	2.762	NM_201222	NM_201222	Homo sapiens melanoma antigen family D, 2 (MAGED2), transcript variant 3, mRNA [NM_201222]	NM_201222
A_23_P381979	4.62E-03	2.6	NM_003605	NM_003605	Homo sapiens O-linked N-acetylglucosamine (GlcNAc) transferase (UDP-N-acetylglucosamine:polypeptide-N-acetylglucosaminyl transferase) (OGT), transcript variant 3, mRNA [NM_003605]	NM_003605
A_23_P256470	4.63E-03	5.978	NM_000905	NM_000905	Homo sapiens neuropeptide Y (NPY), mRNA [NM_000905]	NM_000905
A_23_P53390	4.63E-03	5.681	NM_002837	NM_002837	Homo sapiens protein tyrosine phosphatase, receptor type, B (PTPRB), mRNA [NM_002837]	NM_002837
A_24_P148836	4.63E-03	3.455	NM_173546	NM_173546	Homo sapiens hypothetical protein MGC35097 (MGC35097), mRNA [NM_173546]	NM_173546
A_32_P127248	4.63E-03	3.093	CR936742	CR936742	Homo sapiens mRNA; cDNA DKFZp686G1442 (from clone DKFZp686G1442). [CR936742]	
A_23_P370651	4.64E-03	3.073	NM_014883	NM_014883	Homo sapiens family with sequence similarity 13, member A1 (FAM13A1), transcript variant 1, mRNA [NM_014883]	NM_014883
A_24_P350686	4.65E-03	6.845	NM_052864	NM_052864	Homo sapiens TRAF-interacting protein with a forkhead-associated domain (TIFA), mRNA [NM_052864]	NM_052864
A_24_P203000	4.65E-03	4.523	NM_000878	NM_000878	Homo sapiens interleukin 2 receptor, beta (IL2RB), mRNA [NM_000878]	NM_000878
A_23_P40025	4.65E-03	2.637	NM_014764	NM_014764	Homo sapiens DAZ associated protein 2 (DAZAP2), mRNA [NM_014764]	NM_014764
A_32_P140656	4.66E-03	19.83	CR621148	CR621148	full-length cDNA clone CS0DC029YJ02 of Neuroblastoma Cot 25-normalized of Homo sapiens (human). [CR621148]	
A_23_P167595	4.66E-03	2.382	NM_003337	NM_003337	Homo sapiens ubiquitin-conjugating enzyme E2B (RAD6 homolog) (UBE2B), mRNA [NM_003337]	NM_003337
A_23_P157569	4.66E-03	2.363	NM_144650	NM_144650	Homo sapiens alcohol dehydrogenase, iron containing, 1 (ADHFE1), mRNA [NM_144650]	NM_144650
A_23_P122976	4.66E-03	2.118	NM_002069	NM_002069	Homo sapiens guanine nucleotide binding protein (G protein), alpha inhibiting activity polypeptide 1 (GNAI1), mRNA [NM_002069]	NM_002069
A_23_P61987	4.68E-03	6.083	NM_025268	NM_025268	Homo sapiens hole gene (MGC4659), mRNA [NM_025268]	NM_025268
A_23_P380614	4.68E-03	4.02	NM_006045	NM_006045	Homo sapiens ATPase, Class II, type 9A (ATP9A), mRNA [NM_006045]	NM_006045
A_23_P115417	4.69E-03	10.88	NM_015149	NM_015149	Homo sapiens ral guanine nucleotide dissociation stimulator-like 1 (RGL1), mRNA [NM_015149]	NM_015149
A_24_P238525	4.69E-03	2.673	ENST00000324709			
A_23_P75800	4.69E-03	2.378	NM_013401	NM_013401	Homo sapiens RAB3A interacting protein (rabin3)-like 1 (RAB3IL1), mRNA [NM_013401]	NM_013401
A_23_P376686	4.69E-03	2.349	NM_019602	NM_019602	Homo sapiens butyrophilin-like 2 (MHC class II associated) (BTNL2), mRNA [NM_019602]	NM_019602
A_23_P355244	4.70E-03	10.41	NM_017654	NM_017654	Homo sapiens sterile alpha motif domain containing 9 (SAMD9), mRNA [NM_017654]	NM_017654
A_23_P49708	4.70E-03	3.267	NM_002087	NM_002087	Homo sapiens granulin (GRN), transcript variant 1, mRNA [NM_002087]	NM_002087
A_24_P570049	4.73E-03	9.232	NM_001001930	NM_001001930	Homo sapiens peroxisome proliferative activated receptor, alpha (PPARA), transcript variant 6, mRNA [NM_001001930]	NM_001001930
A_23_P42375	4.73E-03	2.877	NM_006834	NM_006834	Homo sapiens RAB32, member RAS oncogene family (RAB32), mRNA [NM_006834]	NM_006834
A_23_P5300	4.74E-03	2.937	NM_001875	NM_001875	Homo sapiens carbamoyl-phosphate synthetase 1, mitochondrial (CPS1), mRNA [NM_001875]	NM_001875
A_23_P143935	4.75E-03	7.239	NM_025163	NM_025163	Homo sapiens SMP3 mannosyltransferase (SMP3), mRNA [NM_025163]	NM_025163
A_23_P48217	4.77E-03	8.331	NM_030817	NM_030817	Homo sapiens hypothetical protein DKFZp434F0318 (DKFZP434F0318), mRNA [NM_030817]	NM_030817
A_24_P23625	4.77E-03	3.823	ENST00000245519		Homo sapiens heparan sulfate (glucosamine) 3-O-sulfotransferase 3B1, mRNA (cDNA clone MGC:71688 IMAGE:30343352), complete cds. [BC063301]	
A_23_P112162	4.77E-03	2.751	NM_012079	NM_012079	Homo sapiens diacylglycerol O-acyltransferase homolog 1 (mouse) (DGAT1), mRNA [NM_012079]	NM_012079
A_23_P121533	4.78E-03	51.72	NM_012445	NM_012445	Homo sapiens spondin 2, extracellular matrix protein (SPON2), mRNA [NM_012445]	NM_012445

A_23_P62634	4.78E-03	4.518	NM_138617	NM_138617	Homo sapiens Rhesus blood group, CcEe antigens (RHCE), transcript variant 4, mRNA [NM_138617]	NM_138617
A_23_P79545	4.78E-03	2.723	NM_003849	NM_003849	Homo sapiens succinate-CoA ligase, GDP-forming, alpha subunit (SUCLG1), mRNA [NM_003849]	NM_003849
A_24_P304723	4.78E-03	2.423	NM_000942	NM_000942	Homo sapiens peptidylprolyl isomerase B (cyclophilin B) (PPIB), mRNA [NM_000942]	NM_000942
A_23_P131417	4.78E-03	2.396	NM_005336	NM_005336	Homo sapiens high density lipoprotein binding protein (vigilin) (HDLBP), mRNA [NM_005336]	NM_005336
A_32_P35486	4.81E-03	5.778	THC2317058			
A_32_P154601	4.82E-03	3.122	AK125850	AK125850	Homo sapiens cDNA FLJ43862 fis, clone TESTI4007775. [AK125850]	
A_23_P76402	4.82E-03	2.446	NM_024549	NM_024549	Homo sapiens hypothetical protein FLJ21127 (FLJ21127), mRNA [NM_024549]	NM_024549
A_23_P167017	4.82E-03	2.432	NM_022135	NM_022135	Homo sapiens popeye domain containing 2 (POPDC2), mRNA [NM_022135]	NM_022135
A_24_P48069	4.83E-03	7.188	NM_018110	NM_018110	Homo sapiens docking protein 4 (DOK4), mRNA [NM_018110]	NM_018110
A_23_P156061	4.84E-03	3.881	NM_005575	NM_005575	Homo sapiens leucyl/cystinyl aminopeptidase (LNPEP), transcript variant 1, mRNA [NM_005575]	NM_005575
A_24_P269619	4.84E-03	2.154	NM_001359	NM_001359	Homo sapiens 2,4-dienoyl CoA reductase 1, mitochondrial (DECR1), nuclear gene encoding mitochondrial protein, mRNA [NM_001359]	NM_001359
A_23_P88404	4.85E-03	21.54	NM_003239	NM_003239	Homo sapiens transforming growth factor, beta 3 (TGFB3), mRNA [NM_003239]	NM_003239
A_23_P201808	4.86E-03	9.979	NM_003713	NM_003713	Homo sapiens phosphatidic acid phosphatase type 2B (PPAP2B), transcript variant 1, mRNA [NM_003713]	NM_003713
A_32_P84333	4.86E-03	3.791	BG620191	BG620191	BG620191 602618456F1 NIH_MGC_79 Homo sapiens cDNA clone IMAGE:4732285 5', mRNA sequence [BG620191]	
A_23_P88849	4.87E-03	7.535	NM_004165	NM_004165	Homo sapiens Ras-related associated with diabetes (RRAD), mRNA [NM_004165]	NM_004165
A_24_P75019	4.87E-03	5.623	AF274938	AF274938	Homo sapiens PNAS-13 mRNA, complete cds. [AF274938]	XM_496861
A_24_P410797	4.89E-03	6.749	AK125979	AK125979	Homo sapiens cDNA FLJ43991 fis, clone TESTI4019843, highly similar to Rattus norvegicus huntingtin-associated protein interacting protein (duo) (Hapip). [AK125979]	
A_24_P703642	4.90E-03	2.732	THC2406981		Q9LCV5 (Q9LCV5) Atp operon (Fragment), partial (3%) [THC2406981]	
A_24_P282578	4.90E-03	2.23	NM_003145	NM_003145	Homo sapiens signal sequence receptor, beta (translocon-associated protein beta) (SSR2), mRNA [NM_003145]	NM_003145
A_23_P56654	4.93E-03	2.619	NM_032601	NM_032601	Homo sapiens methylmalonyl CoA epimerase (MCEE), mRNA [NM_032601]	NM_032601
A_23_P55011	4.93E-03	2.495	BC014156	BC014156	Homo sapiens cDNA clone IMAGE:4337090, partial cds. [BC014156]	
A_23_P21804	4.93E-03	2.172	A_23_P21804			
A_23_P200030	4.94E-03	3.102	NM_003838	NM_003838	Homo sapiens fucose-1-phosphate guanylyltransferase (FPGT), mRNA [NM_003838]	NM_003838
A_32_P140501	4.94E-03	2.881	A_32_P140501			
A_23_P22134	4.95E-03	133.3	NM_001717	NM_001717	Homo sapiens basonuclin 1 (BNC1), mRNA [NM_001717]	NM_001717
A_24_P40626	4.95E-03	55.23	NM_022469	NM_022469	Homo sapiens gremlin 2, cysteine knot superfamily, homolog (Xenopus laevis) (GREM2), mRNA [NM_022469]	NM_022469
A_23_P126836	4.95E-03	6.314	NM_003326	NM_003326	Homo sapiens tumor necrosis factor (ligand) superfamily, member 4 (tax-transcriptionally activated glycoprotein 1, 34kDa) (TNFSF4), mRNA [NM_003326]	NM_003326
A_32_P213692	4.95E-03	2.33	ENST00000216468		Homo sapiens cDNA: FLJ22042 fis, clone HEP09065. [AK025695]	
A_24_P335901	4.95E-03	2.063	NM_006631	NM_006631	Homo sapiens zinc finger protein 266 (ZNF266), mRNA [NM_006631]	NM_006631
A_23_P397376	5.00E-03	7.671	NM_005360	NM_005360	Homo sapiens v-maf musculoaponeurotic fibrosarcoma oncogene homolog (avian) (MAF), mRNA [NM_005360]	NM_005360
A_23_P32975	5.00E-03	3.145	NM_013379	NM_013379	Homo sapiens dipeptidylpeptidase 7 (DPP7), mRNA [NM_013379]	NM_013379
A_24_P942945	5.00E-03	2.788	NM_198569	NM_198569	Homo sapiens G protein-coupled receptor 126 (GPR126), mRNA [NM_198569]	NM_198569
A_23_P204016	5.00E-03	2.581	NM_000725	NM_000725	Homo sapiens calcium channel, voltage-dependent, beta 3 subunit (CACNB3), mRNA [NM_000725]	NM_000725
A_32_P62137	5.00E-03	2.562	A_32_P62137			
A_23_P218086	5.00E-03	2.525	AB032995	AB032995	Homo sapiens mRNA for KIAA1169 protein, partial cds. [AB032995]	
A_23_P87752	5.00E-03	2.223	NM_014262	NM_014262	Homo sapiens leprecan-like 2 (LEPREL2), mRNA [NM_014262]	NM_014262
A_23_P433016	5.01E-03	3.375	NM_001996	NM_001996	Homo sapiens fibulin 1 (FBLN1), transcript variant C, mRNA [NM_001996]	NM_001996
A_32_P168464	5.01E-03	2.417	AK056476	AK056476	Homo sapiens cDNA FLJ31914 fis, clone NT2RP7004884. [AK056476]	
A_23_P160025	5.02E-03	7.183	NM_005531	NM_005531	Homo sapiens interferon, gamma-inducible protein 16 (IFI16), mRNA [NM_005531]	NM_005531
A_23_P307544	5.02E-03	4.17	AK055196	AK055196	Homo sapiens cDNA FLJ30634 fis, clone CTONG2002453. [AK055196]	XM_498440
A_24_P125469	5.03E-03	3.709	NM_006033	NM_006033	Homo sapiens lipase, endothelial (LIPG), mRNA [NM_006033]	NM_006033

A_23_P141394	5.03E-03	2.717	NM_017983	NM_017983	Homo sapiens WD40 repeat protein Interacting with phosphoInositides of 49kDa (WIPI49), mRNA [NM_017983]	NM_017983
A_23_P81993	5.03E-03	2.303	NM_178508	NM_178508	Homo sapiens chromosome 6 open reading frame 1 (C6orf1), transcript variant 1, mRNA [NM_178508]	NM_178508
A_23_P213745	5.04E-03	49.85	NM_004887	NM_004887	Homo sapiens chemokine (C-X-C motif) ligand 14 (CXCL14), mRNA [NM_004887]	NM_004887
A_23_P12343	5.04E-03	9.463	NM_000849	NM_000849	Homo sapiens glutathione S-transferase M3 (brain) (GSTM3), mRNA [NM_000849]	NM_000849
A_23_P206022	5.05E-03	7.937	NM_001004439	NM_001004439	Homo sapiens integrin, alpha 11 (ITGA11), transcript variant 1, mRNA [NM_001004439]	NM_001004439
A_23_P75523	5.05E-03	2.285	NM_203330	NM_203330	Homo sapiens CD59 antigen p18-20 (antigen identified by monoclonal antibodies 16.3A5, EJ16, EJ30, EL32 and G344) (CD59), transcript variant 1, mRNA [NM_203330]	NM_203330
A_23_P131614	5.06E-03	20.78	NM_004369	NM_004369	Homo sapiens collagen, type VI, alpha 3 (COL6A3), transcript variant 1, mRNA [NM_004369]	NM_004369
A_23_P51711	5.06E-03	3.025	NM_015849	NM_015849	Homo sapiens elastase 2B (ELA2B), mRNA [NM_015849]	NM_015849
A_32_P206899	5.07E-03	16.03	THC2340845			
A_23_P159237	5.07E-03	10.3	AB209066	AB209066	Homo sapiens mRNA for G protein-coupled receptor 20 variant protein. [AB209066]	
A_23_P415021	5.07E-03	5.339	NM_014033	NM_014033	Homo sapiens DKFZP586A0522 protein (DKFZP586A0522), mRNA [NM_014033]	NM_014033
A_23_P253982	5.08E-03	8.029	NM_002141	NM_002141	Homo sapiens homeo box A4 (HOXA4), mRNA [NM_002141]	NM_002141
A_23_P110122	5.08E-03	4.337	NM_004354	NM_004354	Homo sapiens cyclin G2 (CCNG2), mRNA [NM_004354]	NM_004354
A_24_P289954	5.08E-03	4.327	NM_001024455	NM_001024455	Homo sapiens retrotransposon gag domain containing 4 (RGAG4), mRNA [NM_001024455]	NM_001024455
A_23_P148175	5.08E-03	3.328	ENST00000264546		Homo sapiens FERM domain containing 4B, mRNA (cDNA clone IMAGE:4508579), partial cds. [BC028291]	XM_114303
A_24_P356592	5.08E-03	3.158	NM_212472	NM_212472	Homo sapiens protein kinase, cAMP-dependent, regulatory, type I, alpha (tissue specific extinguisher 1) (PRKAR1A), transcript variant 3, mRNA [NM_212472]	NM_212472
A_23_P409623	5.08E-03	2.855	NM_003621	NM_003621	Homo sapiens PTPRF interacting protein, binding protein 2 (liprin beta 2) (PPFIBP2), mRNA [NM_003621]	NM_003621
A_23_P69670	5.08E-03	2.023	NM_018366	NM_018366	Homo sapiens cappuccino homolog (mouse) (CNO), mRNA [NM_018366]	NM_018366
A_32_P193166	5.09E-03	2.684	AA495894	AA495894	AA495894 zw04g12.r1 Soares_NhHMPu_S1 Homo sapiens cDNA clone IMAGE:768358 5' similar to contains Alu repetitive element;, mRNA sequence [AA495894]	
A_24_P394533	5.10E-03	3.257	NM_000434	NM_000434	Homo sapiens sialidase 1 (lysosomal sialidase) (NEU1), mRNA [NM_000434]	NM_000434
A_24_P71973	5.11E-03	7.592	NM_002253	NM_002253	Homo sapiens kinase insert domain receptor (a type III receptor tyrosine kinase) (KDR), mRNA [NM_002253]	NM_002253
A_23_P64611	5.12E-03	3.998	NM_176798	NM_176798	Homo sapiens pyrimidinergic receptor P2Y, G-protein coupled, 6 (P2RY6), transcript variant 2, mRNA [NM_176798]	NM_176798
A_23_P135271	5.12E-03	2.103	ENST00000199168		Homo sapiens beta-1,4-galactosyltransferase I mRNA, 3' UTR. [AY864848]	
A_23_P375476	5.13E-03	5.074	NM_175907	NM_175907	Homo sapiens zinc binding alcohol dehydrogenase, domain containing 2 (ZADH2), mRNA [NM_175907]	NM_175907
A_23_P46618	5.13E-03	4.46	NM_025179	NM_025179	Homo sapiens plexin A2 (PLXNA2), mRNA [NM_025179]	NM_025179
A_23_P102454	5.13E-03	2.719	NM_016133	NM_016133	Homo sapiens insulin induced gene 2 (INSIG2), mRNA [NM_016133]	NM_016133
A_32_P179526	5.15E-03	5.818	BC010934	BC010934	Homo sapiens zinc finger and BTB domain containing 20, mRNA (cDNA clone IMAGE:4291354), partial cds. [BC010934]	
A_23_P99163	5.15E-03	2.996	NM_018370	NM_018370	Homo sapiens hypothetical protein FLJ11259 (FLJ11259), mRNA [NM_018370]	NM_018370
A_23_P360744	5.16E-03	4.786	NM_000448	NM_000448	Homo sapiens recombination activating gene 1 (RAG1), mRNA [NM_000448]	NM_000448
A_23_P83781	5.16E-03	2.713	NM_004762	NM_004762	Homo sapiens pleckstrin homology, Sec7 and coiled-coil domains 1(cytohesin 1) (PSCD1), transcript variant 1, mRNA [NM_004762]	NM_004762
A_23_P253368	5.17E-03	4.975	NM_018951	NM_018951	Homo sapiens homeo box A10 (HOXA10), transcript variant 1, mRNA [NM_018951]	NM_018951
A_23_P359214	5.18E-03	6.627	AK096715	AK096715	Homo sapiens cDNA FLJ39396 fis, clone PLACE6008640. [AK096715]	
A_23_P1461	5.18E-03	3.819	NM_001008211	NM_001008211	Homo sapiens optineurin (OPTN), transcript variant 1, mRNA [NM_001008211]	NM_001008211
A_32_P106056	5.18E-03	3.679	THC2432340		BC007443 FKBP9 protein {Homo sapiens;} , partial (31%) [THC2432340]	
A_23_P30283	5.18E-03	2.607	NM_198507	NM_198507	Homo sapiens HGS_RE408 (UNQ1912), mRNA [NM_198507]	NM_198507
A_23_P217114	5.18E-03	2.438	NM_001003945	NM_001003945	Homo sapiens aminolevulinate, delta-, dehydratase (ALAD), transcript variant 1, mRNA [NM_001003945]	NM_001003945
A_23_P5405	5.18E-03	2.019	NM_172070	NM_172070	Homo sapiens zinc finger protein 650 (ZNF650), mRNA [NM_172070]	NM_172070
A_23_P42065	5.19E-03	2.106	NM_014452	NM_014452	Homo sapiens tumor necrosis factor receptor superfamily, member 21 (TNFRSF21), mRNA [NM_014452]	NM_014452
A_23_P304450	5.21E-03	12.6	NM_005257	NM_005257	Homo sapiens GATA binding protein 6 (GATA6), mRNA [NM_005257]	NM_005257

A_23_P164958	5.21E-03	2.209	NM_032040	NM_032040	Homo sapiens coiled-coil domain containing 8 (CCDC8), mRNA [NM_032040]	NM_032040
A_23_P401238	5.21E-03	2.158	ENST00000319246		Homo sapiens mRNA for KIAA0725 protein, partial cds. [AB018268]	
A_23_P74619	5.22E-03	2.296	NM_003944	NM_003944	Homo sapiens selenium binding protein 1 (SELENBP1), mRNA [NM_003944]	NM_003944
A_32_P68586	5.22E-03	2.024	NM_001177	NM_001177	Homo sapiens ADP-ribosylation factor-like 1 (ARL1), mRNA [NM_001177]	NM_001177
A_32_P18440	5.23E-03	14.13	BX641020	BX641020	Homo sapiens mRNA; cDNA DKFZp686G23148 (from clone DKFZp686G23148). [BX641020]	
A_32_P156746	5.23E-03	3.392	AI888033	AI888033	wm27e09.x1 NCI_CGAP_Ut4 Homo sapiens cDNA clone IMAGE:2437192 3' similar to contains element PTR7 repetitive element ;, mRNA sequence [AI888033]	
A_23_P209232	5.24E-03	52.2	NM_024692	NM_024692	Homo sapiens restin-like 2 (RSNL2), mRNA [NM_024692]	NM_024692
A_32_P122494	5.24E-03	6.615	AI652920	AI652920	AI652920 wb40g09.x1 NCI_CGAP_GC6 Homo sapiens cDNA clone IMAGE:2308192 3' similar to SW:NMA_HUMAN Q13145 PUTATIVE TRANSMEMBRANE PROTEIN NMA PRECURSOR. ;, mRNA sequence [AI652920]	
A_24_P224727	5.25E-03	10.56	NM_004364	NM_004364	Homo sapiens CCAAT/enhancer binding protein (C/EBP), alpha (CEBPA), mRNA [NM_004364]	NM_004364
A_23_P420692	5.25E-03	4.955	NM_015053	NM_015053	Homo sapiens protein tyrosine phosphatase, receptor type, f polypeptide (PTPRF), interacting protein (liprin), alpha 4 (PPFIA4), mRNA [NM_015053]	NM_015053
A_24_P357576	5.25E-03	2.229	NM_019600	NM_019600	Homo sapiens hypothetical protein FLJ10980 (FLJ10980), mRNA [NM_019600]	NM_019600
A_23_P125107	5.25E-03	2.2	NM_005514	NM_005514	Homo sapiens major histocompatibility complex, class I, B (HLA-B), mRNA [NM_005514]	NM_005514
A_23_P77859	5.26E-03	8.463	NM_203411	NM_203411	Homo sapiens similar to RIKEN cDNA 2600017H02 (LOC92162), mRNA [NM_203411]	NM_203411
A_23_P300150	5.26E-03	3.145	NM_172387	NM_172387	Homo sapiens nuclear factor of activated T-cells, cytoplasmic, calcineurin-dependent 1 (NFATC1), transcript variant 3, mRNA [NM_172387]	NM_172387
A_32_P132317	5.29E-03	35.41	A_32_P132317			
A_23_P105923	5.29E-03	12.16	NM_001362	NM_001362	Homo sapiens deiodinase, iodothyronine, type III (DIO3), mRNA [NM_001362]	NM_001362
A_24_P278234	5.29E-03	5.55	AB075864	AB075864	Homo sapiens mRNA for KIAA1984 protein. [AB075864]	
A_23_P428738	5.29E-03	3.207	NM_001145	NM_001145	Homo sapiens angiogenin, ribonuclease, RNase A family, 5 (ANG), mRNA [NM_001145]	NM_001145
A_23_P106682	5.29E-03	2.99	NM_001424	NM_001424	Homo sapiens epithelial membrane protein 2 (EMP2), mRNA [NM_001424]	NM_001424
A_32_P210642	5.29E-03	2.116	NM_201446	NM_201446	Homo sapiens EGF-like-domain, multiple 7 (EGFL7), transcript variant 2, mRNA [NM_201446]	NM_201446
A_23_P8482	5.30E-03	2.47	NM_001011553	NM_001011553	Homo sapiens septin 7 (SEPT7), transcript variant 2, mRNA [NM_001011553]	NM_001011553
A_24_P327886	5.31E-03	6.937	NM_003196	NM_003196	Homo sapiens transcription elongation factor A (SII), 3 (TCEA3), mRNA [NM_003196]	NM_003196
A_24_P380679	5.31E-03	3.922	NM_182597	NM_182597	Homo sapiens hypothetical protein FLJ39575 (FLJ39575), mRNA [NM_182597]	NM_182597
A_32_P13337	5.31E-03	3.683	THC2408277			
A_24_P105913	5.31E-03	2.489	THC2268988		AY151386 NAP1 {Homo sapiens;} , complete [THC2268988]	
A_23_P112481	5.32E-03	11.36	NM_004925	NM_004925	Homo sapiens aquaporin 3 (AQP3), mRNA [NM_004925]	NM_004925
A_23_P316511	5.32E-03	10.7	NM_002146	NM_002146	Homo sapiens homeo box B3 (HOXB3), mRNA [NM_002146]	NM_002146
A_23_P156180	5.32E-03	8.278	NM_003059	NM_003059	Homo sapiens solute carrier family 22 (organic cation transporter), member 4 (SLC22A4), mRNA [NM_003059]	NM_003059
A_24_P364072	5.32E-03	4.046	THC2364724			
A_24_P394569	5.32E-03	2.582	AK056855	AK056855	Homo sapiens cDNA FLJ32293 fis, clone PROST2001739. [AK056855]	XM_374902
A_24_P40306	5.33E-03	10.34	NM_000185	NM_000185	Homo sapiens serine (or cysteine) proteinase inhibitor, clade D (heparin cofactor), member 1 (SERPIND1), mRNA [NM_000185]	NM_000185
A_23_P86021	5.33E-03	2.722	NM_003944	NM_003944	Homo sapiens selenium binding protein 1 (SELENBP1), mRNA [NM_003944]	NM_003944
A_23_P94365	5.33E-03	2.111	NM_174922	NM_174922	Homo sapiens aarF domain containing kinase 5 (ADCK5), mRNA [NM_174922]	NM_174922
A_24_P762613	5.34E-03	38.75	AK021543	AK021543	Homo sapiens cDNA FLJ11481 fis, clone HEMBA1001803. [AK021543]	
A_23_P18806	5.34E-03	2.751	NM_030799	NM_030799	Homo sapiens Yip1 domain family, member 5 (YIPF5), transcript variant 2, mRNA [NM_030799]	NM_030799
A_23_P142322	5.34E-03	2.435	NM_001280	NM_001280	Homo sapiens cold inducible RNA binding protein (CIRBP), mRNA [NM_001280]	NM_001280
A_32_P184488	5.37E-03	3.144	ENST00000292140		Homo sapiens cDNA clone IMAGE:4299555, partial cds. [BC007947]	
A_32_P180435	5.38E-03	5.019	NM_175064	NM_175064	Homo sapiens Williams Beuren syndrome chromosome region 19 (WBSCR19), mRNA [NM_175064]	NM_175064
A_24_P98411	5.38E-03	3.179	NM_005347	NM_005347	Homo sapiens heat shock 70kDa protein 5 (glucose-regulated protein, 78kDa) (HSPA5), mRNA [NM_005347]	NM_005347

A_23_P120153	5.38E-03	2.469	NM_173647	NM_173647	Homo sapiens ring finger protein 149 (RNF149), mRNA [NM_173647]	NM_173647
A_23_P307392	5.38E-03	2.464	NM_012074	NM_012074	Homo sapiens D4, zinc and double PHD fingers, family 3 (DPF3), mRNA [NM_012074]	NM_012074
A_23_P113613	5.38E-03	2.238	NM_022842	NM_022842	Homo sapiens CUB domain containing protein 1 (CDCP1), transcript variant 1, mRNA [NM_022842]	NM_022842
A_24_P931944	5.39E-03	10.64	AK128814	AK128814	Homo sapiens cDNA FLJ46049 fis, clone SYNOV2020463. [AK128814]	
A_24_P106910	5.39E-03	3.645	ENST00000331920		Homo sapiens cDNA FLJ42602 fis, clone BRACE3011271, moderately similar to Patched protein. [AK124593]	
A_32_P13370	5.39E-03	3.16	AK091672	AK091672	Homo sapiens cDNA FLJ34353 fis, clone FEBRA2011665. [AK091672]	
A_24_P177763	5.40E-03	2.04	NM_030911	NM_030911	Homo sapiens cytidine and dCMP deaminase domain containing 1 (CDADC1), mRNA [NM_030911]	NM_030911
A_24_P392925	5.41E-03	4.606	NM_001014985	NM_001014985	Homo sapiens hypothetical LOC388323 (LOC388323), mRNA [NM_001014985]	NM_001014985
A_24_P411899	5.42E-03	3.423	NM_183419	NM_183419	Homo sapiens ring finger protein 19 (RNF19), transcript variant 1, mRNA [NM_183419]	NM_183419
A_23_P328069	5.42E-03	2.293	NM_000195	NM_000195	Homo sapiens Hermansky-Pudlak syndrome 1 (HPS1), transcript variant 1, mRNA [NM_000195]	NM_000195
A_23_P162466	5.43E-03	5.227	NM_004572	NM_004572	Homo sapiens plakophilin 2 (PKP2), transcript variant 2b, mRNA [NM_004572]	NM_004572
A_23_P404965	5.43E-03	2.381	AK095071	AK095071	Homo sapiens cDNA FLJ37752 fis, clone BRHIP2023309. [AK095071]	
A_23_P4474	5.44E-03	2.567	NM_016097	NM_016097	Homo sapiens immediate early response 3 interacting protein 1 (IER3IP1), mRNA [NM_016097]	NM_016097
A_23_P70648	5.45E-03	9.989	NM_000865	NM_000865	Homo sapiens 5-hydroxytryptamine (serotonin) receptor 1E (HTR1E), mRNA [NM_000865]	NM_000865
A_23_P105442	5.46E-03	6.212	NM_181711	NM_181711	Homo sapiens GRP1 (general receptor for phosphoinositides 1)-associated scaffold protein (GRASP), mRNA [NM_181711]	NM_181711
A_24_P226700	5.48E-03	2.167	BX648950	BX648950	Homo sapiens mRNA; cDNA DKFZp686E1648 (from clone DKFZp686E1648). [BX648950]	
A_24_P299685	5.48E-03	2.147	NM_198389	NM_198389	Homo sapiens podoplanin (PDPN), transcript variant 2, mRNA [NM_198389]	NM_198389
A_32_P5542	5.49E-03	4.474	AF131782	AF131782	Homo sapiens clone 24941 mRNA sequence. [AF131782]	
A_32_P209702	5.50E-03	5.6	THC2283850			
A_24_P595460	5.50E-03	4.262	AK097398	AK097398	Homo sapiens cDNA FLJ40079 fis, clone TESTI2001498, highly similar to DNA-BINDING PROTEIN NEFA PRECURSOR. [AK097398]	
A_23_P382154	5.50E-03	2.892	NM_176794	NM_176794	Homo sapiens mitochondrial ribosomal protein L43 (MRPL43), nuclear gene encoding mitochondrial protein, transcript variant 4, mRNA [NM_176794]	NM_176794
A_23_P46812	5.51E-03	4.188	NM_014912	NM_014912	Homo sapiens cytoplasmic polyadenylation element binding protein 3 (CPEB3), mRNA [NM_014912]	NM_014912
A_23_P20427	5.51E-03	3.898	NM_015178	NM_015178	Homo sapiens Rho-related BTB domain containing 2 (RHOBTB2), mRNA [NM_015178]	NM_015178
A_24_P33077	5.53E-03	5.476	NM_002145	NM_002145	Homo sapiens homeo box B2 (HOXB2), mRNA [NM_002145]	NM_002145
A_24_P183128	5.53E-03	5.288	NM_016619	NM_016619	Homo sapiens placenta-specific 8 (PLAC8), mRNA [NM_016619]	NM_016619
A_23_P207280	5.53E-03	3.661	NM_006380	NM_006380	Homo sapiens amyloid beta precursor protein (cytoplasmic tail) binding protein 2 (APPBP2), mRNA [NM_006380]	NM_006380
A_24_P115183	5.53E-03	2.648	NM_001305	NM_001305	Homo sapiens claudin 4 (CLDN4), mRNA [NM_001305]	NM_001305
A_24_P97703	5.54E-03	2.089	NM_000919	NM_000919	Homo sapiens peptidylglycine alpha-amidating monooxygenase (PAM), transcript variant 1, mRNA [NM_000919]	NM_000919
A_24_P365975	5.55E-03	42.24	NM_005202	NM_005202	Homo sapiens collagen, type VIII, alpha 2 (COL8A2), mRNA [NM_005202]	NM_005202
A_23_P71328	5.55E-03	4.289	NM_030583	NM_030583	Homo sapiens matrilin 2 (MATN2), transcript variant 2, mRNA [NM_030583]	NM_030583
A_32_P76853	5.55E-03	2.408	XM_370839	XM_370839	PREDICTED: Homo sapiens similar to hypothetical protein (LOC440234), mRNA [XM_370839]	XM_370839
A_23_P205031	5.56E-03	5.404	NM_001846	NM_001846	Homo sapiens collagen, type IV, alpha 2 (COL4A2), mRNA [NM_001846]	NM_001846
A_23_P257649	5.56E-03	2.868	NM_002899	NM_002899	Homo sapiens retinol binding protein 1, cellular (RBP1), mRNA [NM_002899]	NM_002899
A_24_P282031	5.57E-03	2.127	NM_015914	NM_015914	Homo sapiens thioredoxin domain containing 11 (TXNDC11), mRNA [NM_015914]	NM_015914
A_23_P339818	5.59E-03	8.272	NM_183376	NM_183376	Homo sapiens arrestin domain containing 4 (ARRDC4), mRNA [NM_183376]	NM_183376
A_23_P35617	5.59E-03	7.983	NM_016341	NM_016341	Homo sapiens phospholipase C, epsilon 1 (PLCE1), mRNA [NM_016341]	NM_016341
A_24_P405205	5.59E-03	4.68	NM_001001396	NM_001001396	Homo sapiens ATPase, Ca++ transporting, plasma membrane 4 (ATP2B4), transcript variant 1, mRNA [NM_001001396]	NM_001001396
A_23_P83798	5.59E-03	4.227	NM_006982	NM_006982	Homo sapiens cartilage paired-class homeoprotein 1 (CART1), mRNA [NM_006982]	NM_006982
A_23_P308189	5.59E-03	2.322	NM_032264	NM_032264	Homo sapiens hypothetical protein AE2 (AE2), mRNA [NM_032264]	NM_032264
A_23_P140069	5.60E-03	2.585	NM_012158	NM_012158	Homo sapiens F-box and leucine-rich repeat protein 3 (FBXL3), mRNA [NM_012158]	NM_012158
A_23_P211850	5.60E-03	2.233	NM_020676	NM_020676	Homo sapiens abhydrolase domain containing 6 (ABHD6), mRNA [NM_020676]	NM_020676

A_23_P204751	5.61E-03	4.548	NM_020039	NM_020039	Homo sapiens amiloride-sensitive cation channel 2, neuronal (ACCN2), transcript variant 1, mRNA [NM_020039]	NM_020039
A_24_P788772	5.61E-03	3.268	THC2373524		APE_HUMAN (P02649) Apolipoprotein E precursor (Apo-E), partial (50%) [THC2373524]	
A_23_P55107	5.61E-03	3.048	NM_014683	NM_014683	Homo sapiens unc-51-like kinase 2 (C. elegans) (ULK2), mRNA [NM_014683]	NM_014683
A_23_P216522	5.61E-03	2.464	NM_016446	NM_016446	Homo sapiens chromosome 9 open reading frame 127 (C9orf127), mRNA [NM_016446]	NM_016446
A_23_P49975	5.61E-03	2.318	NM_000421	NM_000421	Homo sapiens keratin 10 (epidermolytic hyperkeratosis; keratosis palmaris et plantaris) (KRT10), mRNA [NM_000421]	NM_000421
A_23_P148959	5.61E-03	2.121	NM_032027	NM_032027	Homo sapiens TM2 domain containing 1 (TM2D1), mRNA [NM_032027]	NM_032027
A_24_P101651	5.61E-03	2.095	NM_001025306	NM_001025306	Homo sapiens CSAG family, member 4 (CSAG4), mRNA [NM_001025306]	NM_001025306
A_32_P62342	5.61E-03	2.082	BC039145	BC039145	Homo sapiens hypothetical protein LOC283464, mRNA (cDNA clone MGC:21651 IMAGE:4508300), complete cds. [BC039145]	XM_290597
A_24_P57631	5.62E-03	9.441	NM_004484	NM_004484	Homo sapiens glypican 3 (GPC3), mRNA [NM_004484]	NM_004484
A_24_P90097	5.63E-03	3.071	NM_016824	NM_016824	Homo sapiens adducin 3 (gamma) (ADD3), transcript variant 1, mRNA [NM_016824]	NM_016824
A_24_P772330	5.63E-03	2.558	AK093903	AK093903	Homo sapiens cDNA FLJ36584 fis, clone TRACH2013450. [AK093903]	
A_23_P415006	5.63E-03	2.513	NM_015470	NM_015470	Homo sapiens RAB11 family interacting protein 5 (class I) (RAB11FIP5), mRNA [NM_015470]	NM_015470
A_32_P184888	5.64E-03	3.741	THC2343253			
A_23_P212968	5.69E-03	95.41	NM_001073	NM_001073	Homo sapiens UDP glucuronosyltransferase 2 family, polypeptide B11 (UGT2B11), mRNA [NM_001073]	NM_001073
A_23_P34142	5.69E-03	4.46	NM_016303	NM_016303	Homo sapiens WW domain binding protein 5 (WBP5), transcript variant 1, mRNA [NM_016303]	NM_016303
A_32_P24832	5.69E-03	2.811	NM_020190	NM_020190	Homo sapiens olfactomedin-like 3 (OLFML3), mRNA [NM_020190]	NM_020190
A_24_P503669	5.70E-03	2.451	AK093628	AK093628	Homo sapiens cDNA FLJ36309 fis, clone THYMU2004986. [AK093628]	
A_23_P67847	5.72E-03	2.143	NM_024572	NM_024572	Homo sapiens UDP-N-acetyl-alpha-D-galactosamine:polypeptide N-acetylgalactosaminyltransferase 14 (GalNAc-T14) (GALNT14), mRNA [NM_024572]	NM_024572
A_23_P31143	5.73E-03	6.433	NM_001003395	NM_001003395	Homo sapiens tumor protein D52-like 1 (TPD52L1), transcript variant 2, mRNA [NM_001003395]	NM_001003395
A_23_P79302	5.73E-03	3.24	NM_177964	NM_177964	Homo sapiens hypothetical protein LOC130576 (LOC130576), mRNA [NM_177964]	NM_177964
A_23_P202275	5.73E-03	2.547	NM_145202	NM_145202	Homo sapiens proline-rich acidic protein 1 (PRAP1), mRNA [NM_145202]	NM_145202
A_23_P212497	5.74E-03	2.986	NM_032169	NM_032169	Homo sapiens putative acyl-CoA dehydrogenase (FLJ12592), mRNA [NM_032169]	NM_032169
A_32_P220161	5.74E-03	2.723	BI497361	BI497361	df135e07.y1 Morton Fetal Cochlea Homo sapiens cDNA clone IMAGE:2537964 5', mRNA sequence [BI497361]	
A_24_P190894	5.74E-03	2.075	NM_001658	NM_001658	Homo sapiens ADP-ribosylation factor 1 (ARF1), transcript variant 4, mRNA [NM_001658]	NM_001658
A_23_P24215	5.75E-03	3.885	AJ404330	AJ404330	Homo sapiens partial mRNA for KIAA0608 transcript, 3' UTR. [AJ404330]	XM_051081
A_23_P32404	5.76E-03	2.242	NM_002201	NM_002201	Homo sapiens interferon stimulated exonuclease gene 20kDa (ISG20), mRNA [NM_002201]	NM_002201
A_32_P78208	5.77E-03	2.488	AK123627	AK123627	Homo sapiens cDNA FLJ41633 fis, clone FCBBF3003435. [AK123627]	
A_24_P407866	5.77E-03	2.322	NM_006455	NM_006455	Homo sapiens synaptonemal complex protein SC65 (SC65), mRNA [NM_006455]	NM_006455
A_23_P331379	5.77E-03	2.158	NM_006154	NM_006154	Homo sapiens neural precursor cell expressed, developmentally down-regulated 4 (NEDD4), transcript variant 1, mRNA [NM_006154]	NM_006154
A_24_P119141	5.78E-03	20.75	NM_000313	NM_000313	Homo sapiens protein S (alpha) (PROS1), mRNA [NM_000313]	NM_000313
A_23_P36753	5.79E-03	2.626	NM_000690	NM_000690	Homo sapiens aldehyde dehydrogenase 2 family (mitochondrial) (ALDH2), nuclear gene encoding mitochondrial protein, mRNA [NM_000690]	NM_000690
A_23_P381954	5.80E-03	2.57	NM_000121	NM_000121	Homo sapiens erythropoietin receptor (EPOR), mRNA [NM_000121]	NM_000121
A_23_P57836	5.81E-03	5.088	A_23_P57836			
A_24_P410363	5.84E-03	2.031	NM_022051	NM_022051	Homo sapiens egl nine homolog 1 (C. elegans) (EGLN1), mRNA [NM_022051]	NM_022051
A_23_P3312	5.86E-03	5.425	NM_005545	NM_005545	Homo sapiens immunoglobulin superfamily containing leucine-rich repeat (ISLR), transcript variant 1, mRNA [NM_005545]	NM_005545
A_23_P167237	5.86E-03	3.172	NM_031911	NM_031911	Homo sapiens C1q and tumor necrosis factor related protein 7 (C1QTNF7), mRNA [NM_031911]	NM_031911
A_23_P74550	5.86E-03	2.154	NM_178454	NM_178454	Homo sapiens hypothetical protein MGC54289 (MGC54289), mRNA [NM_178454]	NM_178454
A_24_P406034	5.86E-03	2.097	NM_006416	NM_006416	Homo sapiens solute carrier family 35 (CMP-sialic acid transporter), member A1 (SLC35A1), mRNA [NM_006416]	NM_006416
A_23_P200477	5.86E-03	2.031	AB043587	AB043587	Homo sapiens gcp60 mRNA for golgi resident protein GCP60, complete cds. [AB043587]	
A_23_P111701	5.87E-03	16.65	NM_004126	NM_004126	Homo sapiens guanine nucleotide binding protein (G protein), gamma 11 (GNG11), mRNA [NM_004126]	NM_004126

A_24_P401830	5.89E-03	10.97	AK057725	AK057725	Homo sapiens cDNA FLJ33163 fis, clone UTERU2000541. [AK057725]	
A_24_P131646	5.89E-03	4.203	NM_000258	NM_000258	Homo sapiens myosin, light polypeptide 3, alkali; ventricular, skeletal, slow (MYL3), mRNA [NM_000258]	NM_000258
A_23_P158470	5.89E-03	2.891	NM_203370	NM_203370	Homo sapiens similar to RIKEN cDNA 6530418L21 (LOC389119), mRNA [NM_203370]	NM_203370
A_24_P67806	5.89E-03	2.487	ENST00000333549		Homo sapiens, clone IMAGE:3909623, mRNA, partial cds. [BC015894]	
A_24_P264909	5.89E-03	2.466	NM_019080	NM_019080	Homo sapiens Nedd4 family interacting protein 2 (NDFIP2), mRNA [NM_019080]	NM_019080
A_23_P347048	5.89E-03	2.335	NM_030791	NM_030791	Homo sapiens sphingosine-1-phosphate phosphatase 1 (SGPP1), mRNA [NM_030791]	NM_030791
A_32_P405942	5.89E-03	2.248	CR620977	CR620977	full-length cDNA clone CS0CAP004YK15 of Thymus of Homo sapiens (human). [CR620977]	
A_32_P87809	5.91E-03	2.583	BC041636	BC041636	Homo sapiens, clone IMAGE:4860560, mRNA. [BC041636]	XM_377935
A_23_P368886	5.93E-03	4.922	NM_175856	NM_175856	Homo sapiens chondroitin sulfate synthase 3 (CSS3), mRNA [NM_175856]	NM_175856
A_23_P91802	5.93E-03	3.347	NM_001953	NM_001953	Homo sapiens endothelial cell growth factor 1 (platelet-derived) (ECGF1), mRNA [NM_001953]	NM_001953
A_23_P11874	5.94E-03	3.171	NM_003953	NM_003953	Homo sapiens myelin protein zero-like 1 (MPZL1), transcript variant 1, mRNA [NM_003953]	NM_003953
A_23_P431591	5.95E-03	4.094	NM_001014279	NM_001014279	Homo sapiens similar to annexin II receptor (LOC389289), mRNA [NM_001014279]	NM_001014279
A_23_P106016	5.99E-03	2.944	NM_002742	NM_002742	Homo sapiens protein kinase D1 (PRKD1), mRNA [NM_002742]	NM_002742
A_24_P15973	5.99E-03	2.379	A_24_P15973			
A_24_P700052	6.03E-03	9.684	AK098638	AK098638	Homo sapiens cDNA FLJ25772 fis, clone TST06461. [AK098638]	
A_23_P132027	6.03E-03	3.215	NM_003116	NM_003116	Homo sapiens sperm associated antigen 4 (SPAG4), mRNA [NM_003116]	NM_003116
A_24_P306726	6.03E-03	2.43	NM_003295	NM_003295	Homo sapiens tumor protein, translationally-controlled 1 (TPT1), mRNA [NM_003295]	NM_003295
A_24_P236956	6.03E-03	2.195	BC007960	BC007960	Homo sapiens RAB3D, member RAS oncogene family, mRNA (cDNA clone IMAGE:4301650), partial cds. [BC007960]	
A_23_P310590	6.05E-03	3.548	NM_178507	NM_178507	Homo sapiens NS5ATP13TP2 protein (NS5ATP13TP2), mRNA [NM_178507]	NM_178507
A_24_P96593	6.05E-03	2.231	NM_005665	NM_005665	Homo sapiens ecotropic viral integration site 5 (EVI5), mRNA [NM_005665]	NM_005665
A_23_P430068	6.06E-03	3.023	NM_006474	NM_006474	Homo sapiens podoplanin (PDPN), transcript variant 1, mRNA [NM_006474]	NM_006474
A_23_P210253	6.06E-03	2.316	NM_152879	NM_152879	Homo sapiens diacylglycerol kinase, delta 130kDa (DGKD), transcript variant 2, mRNA [NM_152879]	NM_152879
A_23_P202737	6.08E-03	2.831	NM_003772	NM_003772	Homo sapiens jerky homolog-like (mouse) (JRKL), mRNA [NM_003772]	NM_003772
A_24_P115199	6.09E-03	2.506	AK022628	AK022628	Homo sapiens cDNA FLJ12566 fis, clone NT2RM4000852. [AK022628]	
A_23_P62115	6.10E-03	5.454	NM_003254	NM_003254	Homo sapiens tissue inhibitor of metalloproteinase 1 (erythroid potentiating activity, collagenase inhibitor) (TIMP1), mRNA [NM_003254]	NM_003254
A_23_P112061	6.10E-03	5.414	BC012452	BC012452	Homo sapiens hypothetical protein FLJ32731, mRNA (cDNA clone MGC:16804 IMAGE:3880903), complete cds. [BC012452]	XM_372038
A_24_P376391	6.10E-03	3.093	NM_015103	NM_015103	Homo sapiens plexin D1 (PLXND1), mRNA [NM_015103]	NM_015103
A_24_P54390	6.12E-03	34.3	NM_170672	NM_170672	Homo sapiens RAS guanyl releasing protein 3 (calcium and DAG-regulated) (RASGRP3), mRNA [NM_170672]	NM_170672
A_23_P422766	6.12E-03	6.579	NM_172193	NM_172193	Homo sapiens kelch domain containing 1 (KLHDC1), mRNA [NM_172193]	NM_172193
A_23_P423309	6.12E-03	3.842	NM_016580	NM_016580	Homo sapiens protocadherin 12 (PCDH12), mRNA [NM_016580]	NM_016580
A_23_P10995	6.12E-03	2.925	NM_014483	NM_014483	Homo sapiens RNA binding motif, single stranded interacting protein (RBMS3), transcript variant 2, mRNA [NM_014483]	NM_014483
A_24_P418408	6.13E-03	4.196	NM_198552	NM_198552	Homo sapiens chromosome 1 open reading frame 153 (C1orf153), mRNA [NM_198552]	NM_198552
A_24_P217848	6.15E-03	2.014	NM_003510	NM_003510	Homo sapiens histone 1, H2ak (HIST1H2AK), mRNA [NM_003510]	NM_003510
A_32_P123743	6.16E-03	8.005	THC2376725			
A_23_P28263	6.18E-03	3.47	NM_021198	NM_021198	Homo sapiens CTD (carboxy-terminal domain, RNA polymerase II, polypeptide A) small phosphatase 1 (CTDSP1), transcript variant 1, mRNA [NM_021198]	NM_021198
A_23_P113005	6.19E-03	5.169	NM_004428	NM_004428	Homo sapiens ephrin-A1 (EFNA1), transcript variant 1, mRNA [NM_004428]	NM_004428
A_32_P226620	6.20E-03	9.04	THC2441546			
A_23_P6771	6.20E-03	5.636	NM_014583	NM_014583	Homo sapiens LIM and cysteine-rich domains 1 (LMCD1), mRNA [NM_014583]	NM_014583
A_32_P141768	6.20E-03	3.593	NM_001012733	NM_001012733	Homo sapiens 1-acylglycerol-3-phosphate O-acyltransferase 4 (lysophosphatidic acid acyltransferase, delta) (AGPAT4), transcript variant 2, mRNA [NM_001012733]	NM_001012733
A_24_P30314	6.20E-03	2.51	NM_020680	NM_020680	Homo sapiens SCY1-like 1 (S. cerevisiae) (SCYL1), mRNA [NM_020680]	NM_020680

A_23_P127879	6.21E-03	2.001	NM_016526	NM_016526	Homo sapiens blocked early in transport 1 homolog (S. cerevisiae) like (BET1L), mRNA [NM_016526]	NM_016526
A_23_P30363	6.23E-03	21.53	NM_004199	NM_004199	Homo sapiens procollagen-proline, 2-oxoglutarate 4-dioxygenase (proline 4-hydroxylase), alpha polypeptide II (P4HA2), transcript variant 1, mRNA [NM_004199]	NM_004199
A_24_P760368	6.23E-03	4.64	THC2434152		Q9HBV3 (Q9HBV3) PP3111, partial (17%) [THC2434152]	
A_23_P257043	6.23E-03	3.435	NM_005261	NM_005261	Homo sapiens GTP binding protein overexpressed in skeletal muscle (GEM), transcript variant 1, mRNA [NM_005261]	NM_005261
A_23_P211598	6.23E-03	2.471	NM_002676	NM_002676	Homo sapiens phosphomannomutase 1 (PMM1), mRNA [NM_002676]	NM_002676
A_23_P371835	6.25E-03	17.4	NM_080473	NM_080473	Homo sapiens GATA binding protein 5 (GATA5), mRNA [NM_080473]	NM_080473
A_24_P277934	6.27E-03	31.45	NM_000089	NM_000089	Homo sapiens collagen, type I, alpha 2 (COL1A2), mRNA [NM_000089]	NM_000089
A_23_P58419	6.27E-03	5.181	NM_002253	NM_002253	Homo sapiens kinase insert domain receptor (a type III receptor tyrosine kinase) (KDR), mRNA [NM_002253]	NM_002253
A_23_P127642	6.27E-03	2.299	NM_015313	NM_015313	Homo sapiens Rho guanine nucleotide exchange factor (GEF) 12 (ARHGEF12), mRNA [NM_015313]	NM_015313
A_23_P428129	6.29E-03	21.69	NM_000076	NM_000076	Homo sapiens cyclin-dependent kinase inhibitor 1C (p57, Kip2) (CDKN1C), mRNA [NM_000076]	NM_000076
A_23_P136012	6.29E-03	2.888	NM_012180	NM_012180	Homo sapiens F-box protein 8 (FBXO8), mRNA [NM_012180]	NM_012180
A_23_P85008	6.31E-03	7.035	NM_000898	NM_000898	Homo sapiens monoamine oxidase B (MAOB), nuclear gene encoding mitochondrial protein, mRNA [NM_000898]	NM_000898
A_23_P83976	6.31E-03	3.672	NM_145036	NM_145036	Homo sapiens hypothetical protein MGC33887 (MGC33887), mRNA [NM_145036]	NM_145036
A_24_P38387	6.31E-03	2.932	NM_006096	NM_006096	Homo sapiens N-myc downstream regulated gene 1 (NDRG1), mRNA [NM_006096]	NM_006096
A_24_P255252	6.31E-03	2.555	ENST00000309246			
A_23_P333138	6.31E-03	2.522	ENST00000261188		O99981 (O99981) NADH dehydrogenase subunit 4 , partial (5%) [THC2339600]	
A_23_P97932	6.31E-03	2.291	NM_012228	NM_012228	Homo sapiens methionine sulfoxide reductase B2 (MSRB2), mRNA [NM_012228]	NM_012228
A_23_P310022	6.32E-03	3.141	NM_019590	NM_019590	Homo sapiens KIAA1217 (KIAA1217), mRNA [NM_019590]	NM_019590
A_23_P11543	6.32E-03	2.884	NM_000147	NM_000147	Homo sapiens fucosidase, alpha-L- 1, tissue (FUCA1), mRNA [NM_000147]	NM_000147
A_24_P403561	6.33E-03	3.7	NM_002334	NM_002334	Homo sapiens low density lipoprotein receptor-related protein 4 (LRP4), mRNA [NM_002334]	NM_002334
A_32_P91042	6.33E-03	2.003	CR610374	CR610374	full-length cDNA clone CS0DI065YP13 of Placenta Cot 25-normalized of Homo sapiens (human). [CR610374]	
A_32_P212764	6.34E-03	5.518	AA554768	AA554768	AA554768 ni37h02.s1 NCI_CGAP_Lu1 Homo sapiens cDNA clone IMAGE:979059 3' similar to gb:D29805 N-ACETYLLACTOSAMINE SYNTHASE (HUMAN);, mRNA sequence [AA554768]	
A_24_P324314	6.34E-03	2.337	NM_001008529	NM_001008529	Homo sapiens matrix-remodelling associated 7 (MXRA7), transcript variant 2, mRNA [NM_001008529]	NM_001008529
A_32_P157945	6.34E-03	2.241	NM_004415	NM_004415	Homo sapiens desmoplakin (DSP), transcript variant 1, mRNA [NM_004415]	NM_004415
A_24_P870509	6.36E-03	17.68	AF086261	AF086261	Homo sapiens full length insert cDNA clone ZD42A11. [AF086261]	
A_23_P417144	6.36E-03	8.538	NM_030665	NM_030665	Homo sapiens retinoic acid induced 1 (RAI1), mRNA [NM_030665]	NM_030665
A_24_P513262	6.36E-03	2.191	AK026485	AK026485	Homo sapiens cDNA: FLJ22832 fis, clone KAIA4195. [AK026485]	
A_23_P420981	6.36E-03	2.056	NM_174891	NM_174891	Homo sapiens chromosome 14 open reading frame 79 (C14orf79), mRNA [NM_174891]	NM_174891
A_23_P30634	6.38E-03	2.029	NM_021813	NM_021813	Homo sapiens BTB and CNC homology 1, basic leucine zipper transcription factor 2 (BACH2), mRNA [NM_021813]	NM_021813
A_23_P23611	6.39E-03	7.663	NM_001008219	NM_001008219	Homo sapiens amylase, alpha 1C; salivary (AMY1C), mRNA [NM_001008219]	NM_001008219
A_32_P101264	6.39E-03	3.394	THC2375782			
A_24_P390833	6.39E-03	2.353	NM_023075	NM_023075	Homo sapiens metallophosphoesterase 1 (MPPE1), transcript variant 1, mRNA [NM_023075]	NM_023075
A_23_P10647	6.41E-03	3.766	NM_018659	NM_018659	Homo sapiens cytokine-like 1 (CYTL1), mRNA [NM_018659]	NM_018659
A_23_P131899	6.41E-03	2.422	NM_080489	NM_080489	Homo sapiens syndecan binding protein (syntenin) 2 (SDCBP2), transcript variant 1, mRNA [NM_080489]	NM_080489
A_23_P501538	6.42E-03	15.57	NM_153631	NM_153631	Homo sapiens homeo box A3 (HOXA3), transcript variant 2, mRNA [NM_153631]	NM_153631
A_24_P56240	6.42E-03	4.176	NM_153634	NM_153634	Homo sapiens copine VIII (CPNE8), mRNA [NM_153634]	NM_153634
A_23_P79398	6.42E-03	2.73	NM_004633	NM_004633	Homo sapiens interleukin 1 receptor, type II (IL1R2), transcript variant 1, mRNA [NM_004633]	NM_004633
A_24_P42136	6.42E-03	2.097	NM_000224	NM_000224	Homo sapiens keratin 18 (KRT18), transcript variant 1, mRNA [NM_000224]	NM_000224
A_23_P216325	6.43E-03	3.935	NM_004315	NM_004315	Homo sapiens N-acylsphingosine amidohydrolase (acid ceramidase) 1 (ASAH1), transcript variant 2, mRNA [NM_004315]	NM_004315
A_23_P351724	6.44E-03	5.153	NM_022648	NM_022648	Homo sapiens tensin 1 (TNS1), mRNA [NM_022648]	NM_022648

A_32_P217261	6.47E-03	2.32	AK023660	AK023660	Homo sapiens cDNA FLJ13598 fis, clone PLACE1009921. [AK023660]	
A_24_P70888	6.47E-03	2.024	AB002313	AB002313	Human mRNA for KIAA0315 gene, partial cds. [AB002313]	XM_371474
A_23_P102391	6.48E-03	161.9	NM_014585	NM_014585	Homo sapiens solute carrier family 40 (iron-regulated transporter), member 1 (SLC40A1), mRNA [NM_014585]	NM_014585
A_23_P41021	6.49E-03	2.155	NM_007184	NM_007184	Homo sapiens nischarin (NISCH), mRNA [NM_007184]	NM_007184
A_24_P22746	6.52E-03	7.044	THC2336861		Q96FJ6 (Q96FJ6) Zinc binding alcohol dehydrogenase, domain containing 2, complete [THC2336861]	
A_23_P200043	6.52E-03	3.023	NM_014704	NM_014704	Homo sapiens glycine-, glutamate-, thienylcyclohexylpiperidine-binding protein (GlyBP), mRNA [NM_014704]	NM_014704
A_24_P191971	6.53E-03	3.131	AK027248	AK027248	Homo sapiens cDNA: FLJ23595 fis, clone LNG15262. [AK027248]	
A_23_P48029	6.54E-03	4.016	NM_016184	NM_016184	Homo sapiens C-type lectin domain family 4, member A (CLEC4A), transcript variant 1, mRNA [NM_016184]	NM_016184
A_23_P218190	6.54E-03	2.502	NM_000070	NM_000070	Homo sapiens calpain 3, (p94) (CAPN3), transcript variant 1, mRNA [NM_000070]	NM_000070
A_32_P104053	6.56E-03	3.065	THC2372182		O13102 (O13102) Activin type IIB receptor precursor, partial (5%) [THC2372182]	
A_23_P134395	6.56E-03	2.681	NM_032988	NM_032988	Homo sapiens transducin (beta)-like 2 (TBL2), transcript variant 2, mRNA [NM_032988]	NM_032988
A_23_P13713	6.57E-03	11.22	NM_006262	NM_006262	Homo sapiens peripherin (PRPH), mRNA [NM_006262]	NM_006262
A_23_P93780	6.57E-03	8.842	NM_001010931	NM_001010931	Homo sapiens hepatocyte growth factor (hepapoietin A; scatter factor) (HGF), transcript variant 2, mRNA [NM_001010931]	NM_001010931
A_32_P169550	6.57E-03	5.652	BX099371	BX099371	BX099371 BX099371 NCI_CGAP_Lu24 Homo sapiens cDNA clone IMAGp998O205798 ; IMAGE:2337259, mRNA sequence [BX099371]	
A_24_P240065	6.57E-03	2.609	NM_016079	NM_016079	Homo sapiens vacuolar protein sorting 24 (yeast) (VPS24), transcript variant 1, mRNA [NM_016079]	NM_016079
A_32_P228348	6.58E-03	2.967	NM_207505	NM_207505	Homo sapiens FLJ45248 protein (FLJ45248), mRNA [NM_207505]	NM_207505
A_23_P59397	6.58E-03	2.732	NM_031924	NM_031924	Homo sapiens radial spokehead-like 2 (RSHL2), mRNA [NM_031924]	NM_031924
A_23_P205188	6.58E-03	2.706	NM_017905	NM_017905	Homo sapiens transmembrane and coiled-coil domains 3 (TMCO3), mRNA [NM_017905]	NM_017905
A_23_P144796	6.58E-03	2.196	NM_003687	NM_003687	Homo sapiens PDZ and LIM domain 4 (PDLIM4), mRNA [NM_003687]	NM_003687
A_23_P319792	6.59E-03	2.347	NM_019001	NM_019001	Homo sapiens 5'-3' exoribonuclease 1 (XRN1), mRNA [NM_019001]	NM_019001
A_24_P257151	6.60E-03	4.499	NM_004071	NM_004071	Homo sapiens CDC-like kinase 1 (CLK1), transcript variant 1, mRNA [NM_004071]	NM_004071
A_23_P146855	6.60E-03	2.113	NM_001585	NM_001585	Homo sapiens chromosome 22 open reading frame 1 (C22orf1), mRNA [NM_001585]	NM_001585
A_23_P11800	6.61E-03	3.116	BC020630	BC020630	Homo sapiens calcium/calmodulin-dependent protein kinase II inhibitor 1, mRNA (cDNA clone MGC:22256 IMAGE:4703846), complete cds. [BC020630]	
A_24_P119745	6.62E-03	5.959	NM_212482	NM_212482	Homo sapiens fibronectin 1 (FN1), transcript variant 1, mRNA [NM_212482]	NM_212482
A_23_P157620	6.62E-03	2.05	NM_022749	NM_022749	Homo sapiens retinoic acid induced 16 (RAI16), mRNA [NM_022749]	NM_022749
A_23_P121646	6.63E-03	2.012	NM_006424	NM_006424	Homo sapiens solute carrier family 34 (sodium phosphate), member 2 (SLC34A2), mRNA [NM_006424]	NM_006424
A_23_P152305	6.65E-03	8.557	NM_001797	NM_001797	Homo sapiens cadherin 11, type 2, OB-cadherin (osteoblast) (CDH11), mRNA [NM_001797]	NM_001797
A_23_P363316	6.66E-03	21.54	NM_002147	NM_002147	Homo sapiens homeo box B5 (HOXB5), mRNA [NM_002147]	NM_002147
A_23_P218774	6.67E-03	6.249	NM_002872	NM_002872	Homo sapiens ras-related C3 botulinum toxin substrate 2 (rho family, small GTP binding protein Rac2) (RAC2), mRNA [NM_002872]	NM_002872
A_32_P20221	6.67E-03	3.985	AK095738	AK095738	Homo sapiens cDNA FLJ38419 fis, clone FEBRA2009846. [AK095738]	
A_23_P41888	6.67E-03	2.132	NM_032921	NM_032921	Homo sapiens hypothetical protein MGC15875 (MGC15875), transcript variant 1, mRNA [NM_032921]	NM_032921
A_24_P271527	6.67E-03	2.098	NM_014876	NM_014876	Homo sapiens KIAA0063 gene product (KIAA0063), mRNA [NM_014876]	NM_014876
A_23_P400580	6.67E-03	2.094	AB040883	AB040883	Homo sapiens mRNA for KIAA1450 protein, partial cds. [AB040883]	
A_24_P236003	6.68E-03	4.648	NM_177925	NM_177925	Homo sapiens H2A histone family, member J (H2AFJ), transcript variant 2, mRNA [NM_177925]	NM_177925
A_23_P64617	6.68E-03	3.811	NM_012193	NM_012193	Homo sapiens frizzled homolog 4 (Drosophila) (FZD4), mRNA [NM_012193]	NM_012193
A_32_P35031	6.68E-03	2.577	A_32_P35031			
A_23_P335695	6.69E-03	2.499	AK023854	AK023854	Homo sapiens cDNA FLJ13792 fis, clone THYRO1000072, weakly similar to MYOSIN LIGHT CHAIN KINASE, SMOOTH MUSCLE AND NON-MUSCLE ISOZYMES (EC 2.7.1.117). [AK023854]	
A_23_P218858	6.70E-03	13.58	NM_015429	NM_015429	Homo sapiens ABI gene family, member 3 (NESH) binding protein (ABI3BP), mRNA [NM_015429]	NM_015429
A_23_P257834	6.72E-03	106	NM_000477	NM_000477	Homo sapiens albumin (ALB), mRNA [NM_000477]	NM_000477

A_24_P383450	6.74E-03	2.921	NM_203434	NM_203434	Homo sapiens immediate early response 5-like (IER5L), mRNA [NM_203434]	NM_203434
A_23_P254512	6.75E-03	14.05	NM_004428	NM_004428	Homo sapiens ephrin-A1 (EFNA1), transcript variant 1, mRNA [NM_004428]	NM_004428
A_24_P148796	6.76E-03	5.913	NM_020998	NM_020998	Homo sapiens macrophage stimulating 1 (hepatocyte growth factor-like) (MST1), mRNA [NM_020998]	NM_020998
A_24_P639679	6.76E-03	3.17	AK095831	AK095831	Homo sapiens cDNA FLJ38512 fis, clone HCHON2000503. [AK095831]	
A_32_P129894	6.77E-03	6.036	ENST00000223618		Homo sapiens mRNA for MEGF9, partial cds. [AB011542]	XM_376905
A_24_P57700	6.79E-03	2.742	NM_015035	NM_015035	Homo sapiens zinc fingers and homeoboxes 3 (ZHX3), mRNA [NM_015035]	NM_015035
A_24_P89971	6.80E-03	2.849	NM_033161	NM_033161	Homo sapiens surfeit 4 (SURF4), mRNA [NM_033161]	NM_033161
A_23_P115573	6.80E-03	2.269	NM_198149	NM_198149	Homo sapiens transmembrane protein 58 (TMEM58), mRNA [NM_198149]	NM_198149
A_23_P92687	6.84E-03	2.259	NM_004394	NM_004394	Homo sapiens death-associated protein (DAP), mRNA [NM_004394]	NM_004394
A_23_P431268	6.86E-03	6.892	NM_014935	NM_014935	Homo sapiens pleckstrin homology domain containing, family A member 6 (PLEKHA6), mRNA [NM_014935]	NM_014935
A_24_P134488	6.86E-03	2.588	NM_052880	NM_052880	Homo sapiens HGFL gene (MGC17330), mRNA [NM_052880]	NM_052880
A_23_P29816	6.87E-03	4.165	NM_015900	NM_015900	Homo sapiens phospholipase A1 member A (PLA1A), mRNA [NM_015900]	NM_015900
A_24_P90774	6.89E-03	2.048	NM_001008224	NM_001008224	Homo sapiens uveal autoantigen with coiled-coil domains and ankyrin repeats (UACA), transcript variant 2, mRNA [NM_001008224]	NM_001008224
A_23_P64173	6.90E-03	41.31	NM_001017534	NM_001017534	Homo sapiens CARD only protein (COPI), transcript variant 1, mRNA [NM_001017534]	NM_001017534
A_23_P97990	6.90E-03	3.897	NM_002775	NM_002775	Homo sapiens protease, serine, 11 (IGF binding) (PRSS11), mRNA [NM_002775]	NM_002775
A_24_P567349	6.90E-03	3.353	THC2279364		ALU8_HUMAN (P39195) Alu subfamily SX sequence contamination warning entry, partial (4%) [THC2279364]	
A_23_P200780	6.91E-03	20.24	NM_003243	NM_003243	Homo sapiens transforming growth factor, beta receptor III (betaglycan, 300kDa) (TGFBR3), mRNA [NM_003243]	NM_003243
A_23_P83818	6.91E-03	16.45	NM_000093	NM_000093	Homo sapiens collagen, type V, alpha 1 (COL5A1), mRNA [NM_000093]	NM_000093
A_23_P212258	6.91E-03	13.87	NM_000893	NM_000893	Homo sapiens kininogen 1 (KNG1), mRNA [NM_000893]	NM_000893
A_32_P30717	6.91E-03	2.753	THC2317093			
A_23_P27285	6.91E-03	2.333	NM_138608	NM_138608	Homo sapiens metallophosphoesterase 1 (MPPE1), transcript variant 2, mRNA [NM_138608]	NM_138608
A_23_P11244	6.91E-03	2.196	NM_145119	NM_145119	Homo sapiens praja 1 (PJA1), mRNA [NM_145119]	NM_145119
A_32_P205913	6.92E-03	2.628	A_32_P205913			
A_23_P377245	6.93E-03	6.682	ENST00000311990		Homo sapiens full length insert cDNA clone YR69B11. [AF085941]	
A_32_P4581	6.93E-03	4.149	AK130118	AK130118	Homo sapiens cDNA FLJ26608 fis, clone LVR00914. [AK130118]	
A_23_P387031	6.93E-03	3.183	NM_173465	NM_173465	Homo sapiens collagen, type XXIII, alpha 1 (COL23A1), mRNA [NM_173465]	NM_173465
A_24_P854758	6.93E-03	2.302	THC2367825		Q9N3X9 (Q9N3X9) Collagen protein 115, partial (5%) [THC2367825]	
A_24_P297182	6.95E-03	7.963	NM_004121	NM_004121	Homo sapiens gamma-glutamyltransferase-like activity 1 (GGTLA1), mRNA [NM_004121]	NM_004121
A_32_P196263	6.96E-03	36.1	NM_182920	NM_182920	Homo sapiens a disintegrin-like and metalloprotease (reprolysin type) with thrombospondin type 1 motif, 9 (ADAMTS9), mRNA [NM_182920]	NM_182920
A_32_P43878	6.96E-03	3.962	THC2379275			
A_32_P184417	6.96E-03	3.546	BE181102	BE181102	CM3-HT0629-260400-161-b05 HT0629 Homo sapiens cDNA, mRNA sequence [BE181102]	
A_23_P117506	6.99E-03	2.173	NM_016029	NM_016029	Homo sapiens dehydrogenase/reductase (SDR family) member 7 (DHRS7), mRNA [NM_016029]	NM_016029
A_24_P604649	6.99E-03	2.003	NM_000291	NM_000291	Homo sapiens phosphoglycerate kinase 1 (PGK1), mRNA [NM_000291]	NM_000291
A_23_P361014	7.00E-03	14.75	NM_020856	NM_020856	Homo sapiens zinc finger protein 537 (ZNF537), mRNA [NM_020856]	NM_020856
A_23_P351270	7.01E-03	3.929	NM_002581	NM_002581	Homo sapiens pregnancy-associated plasma protein A, pappalysin 1 (PAPPA), mRNA [NM_002581]	NM_002581
A_32_P58977	7.02E-03	5.813	AW068592	AW068592	AW068592 cn21d01.x1 Normal Human Trabecular Bone Cells Homo sapiens cDNA clone NHTBC_cn21d01 random, mRNA sequence [AW068592]	
A_23_P58251	7.03E-03	5.134	NM_001014448	NM_001014448	Homo sapiens carboxypeptidase Z (CPZ), transcript variant 3, mRNA [NM_001014448]	NM_001014448
A_23_P47991	7.03E-03	2.362	NM_015335	NM_015335	Homo sapiens thyroid hormone receptor associated protein 2 (THRAP2), mRNA [NM_015335]	NM_015335
A_23_P33723	7.04E-03	2.573	NM_004244	NM_004244	Homo sapiens CD163 antigen (CD163), transcript variant 1, mRNA [NM_004244]	NM_004244
A_24_P943205	7.05E-03	4.923	ENST00000313624		Homo sapiens mRNA; cDNA DKFZp667P0410 (from clone DKFZp667P0410). [AL831953]	
A_23_P422732	7.05E-03	3.56	NM_145172	NM_145172	Homo sapiens WD repeat domain 63 (WDR63), mRNA [NM_145172]	NM_145172

A_23_P210482	7.05E-03	2.765	NM_000022	NM_000022	Homo sapiens adenosine deaminase (ADA), mRNA [NM_000022]	NM_000022
A_23_P425304	7.05E-03	2.491	NM_016169	NM_016169	Homo sapiens suppressor of fused homolog (Drosophila) (SUFU), mRNA [NM_016169]	NM_016169
A_23_P421011	7.06E-03	6.226	NM_030929	NM_030929	Homo sapiens Kazal-type serine protease inhibitor domain 1 (KAZALD1), mRNA [NM_030929]	NM_030929
A_24_P32715	7.06E-03	2.013	ENST00000313481			
A_23_P43283	7.07E-03	4.827	NM_032777	NM_032777	Homo sapiens G protein-coupled receptor 124 (GPR124), mRNA [NM_032777]	NM_032777
A_24_P278393	7.07E-03	2.537	NM_183013	NM_183013	Homo sapiens cAMP responsive element modulator (CREM), transcript variant 19, mRNA [NM_183013]	NM_183013
A_24_P212997	7.08E-03	2.221	A_24_P212997			
A_23_P206454	7.10E-03	2.178	NM_145039	NM_145039	Homo sapiens hypothetical protein MGC16385 (MGC16385), mRNA [NM_145039]	NM_145039
A_24_P392201	7.13E-03	2.87	NM_198517	NM_198517	Homo sapiens TBC1 domain family, member 10C (TBC1D10C), mRNA [NM_198517]	NM_198517
A_23_P390504	7.15E-03	4.841	NM_001453	NM_001453	Homo sapiens forkhead box C1 (FOXC1), mRNA [NM_001453]	NM_001453
A_23_P143964	7.16E-03	5.425	NM_004844	NM_004844	Homo sapiens SH3-domain binding protein 5 (BTK-associated) (SH3BP5), transcript variant 1, mRNA [NM_004844]	NM_004844
A_23_P147950	7.16E-03	3.499	AK057293	AK057293	Homo sapiens cDNA FLJ32731 fis, clone TESTI2001134. [AK057293]	XM_372038
A_32_P40999	7.16E-03	2.657	A_32_P40999			
A_24_P942092	7.16E-03	2.431	AK024362	AK024362	Homo sapiens cDNA FLJ14300 fis, clone PLACE1011891. [AK024362]	
A_23_P215341	7.16E-03	2.354	NM_017946	NM_017946	Homo sapiens FK506 binding protein 14, 22 kDa (FKBP14), mRNA [NM_017946]	NM_017946
A_23_P205355	7.18E-03	2.546	NM_000624	NM_000624	Homo sapiens serine (or cysteine) proteinase inhibitor, clade A (alpha-1 antiproteinase, antitrypsin), member 5 (SERPINA5), mRNA [NM_000624]	NM_000624
A_23_P420326	7.20E-03	2.804	NM_153756	NM_153756	Homo sapiens fibronectin type III domain containing 5 (FNDC5), mRNA [NM_153756]	NM_153756
A_24_P208436	7.21E-03	3.208	NM_001003683	NM_001003683	Homo sapiens phosphodiesterase 1A, calmodulin-dependent (PDE1A), transcript variant 2, mRNA [NM_001003683]	NM_001003683
A_24_P15610	7.24E-03	2.006	XM_372991	XM_372991	PREDICTED: Homo sapiens similar to coated vesicle membrane protein (LOC391540), mRNA [XM_372991]	XM_372991
A_23_P8175	7.27E-03	17.4	NM_006718	NM_006718	Homo sapiens pleiomorphic adenoma gene-like 1 (PLAGL1), transcript variant 2, mRNA [NM_006718]	NM_006718
A_32_P222684	7.27E-03	8.639	ENST00000261364		Homo sapiens PR-domain zinc finger protein 6 isoform A (PRDM6) mRNA, partial cds; alternatively spliced. [AF272898]	
A_23_P39682	7.27E-03	3.711	NM_001079	NM_001079	Homo sapiens zeta-chain (TCR) associated protein kinase 70kDa (ZAP70), transcript variant 1, mRNA [NM_001079]	NM_001079
A_32_P100683	7.27E-03	2.735	AK092338	AK092338	Homo sapiens cDNA FLJ35019 fis, clone OCBBF2014541. [AK092338]	
A_23_P217228	7.27E-03	2.211	NM_016157	NM_016157	Homo sapiens trophinin (TRO), transcript variant 3, mRNA [NM_016157]	NM_016157
A_23_P86012	7.28E-03	16.9	NM_001017402	NM_001017402	Homo sapiens laminin, beta 3 (LAMB3), transcript variant 2, mRNA [NM_001017402]	NM_001017402
A_23_P97700	7.28E-03	11.26	NM_006472	NM_006472	Homo sapiens thioredoxin interacting protein (TXNIP), mRNA [NM_006472]	NM_006472
A_23_P138725	7.29E-03	2.004	NM_031484	NM_031484	Homo sapiens MARVEL domain containing 1 (MARVELD1), mRNA [NM_031484]	NM_031484
A_23_P208991	7.31E-03	3.51	NM_002579	NM_002579	Homo sapiens paralemmin (PALM), mRNA [NM_002579]	NM_002579
A_23_P32955	7.32E-03	4.172	U08023	U08023	Human cellular proto-oncogene (c-mer) mRNA, complete cds. [U08023]	
A_23_P345692	7.34E-03	6.263	NM_138284	NM_138284	Homo sapiens interleukin 17D (IL17D), mRNA [NM_138284]	NM_138284
A_24_P887092	7.34E-03	4.774	THC2339791			
A_23_P154627	7.34E-03	4.571	AF230201	AF230201	Homo sapiens OVC10-2 mRNA, complete cds. [AF230201]	
A_23_P391396	7.34E-03	3.174	NM_001005463	NM_001005463	Homo sapiens early B-cell factor 3 (EBF3), mRNA [NM_001005463]	NM_001005463
A_23_P205046	7.34E-03	2.117	NM_017664	NM_017664	Homo sapiens ankyrin repeat domain 10 (ANKRD10), mRNA [NM_017664]	NM_017664
A_32_P181107	7.36E-03	12.93	A_32_P181107			
A_23_P501831	7.36E-03	7.09	NM_032385	NM_032385	Homo sapiens chromosome 5 open reading frame 4 (C5orf4), transcript variant 2, mRNA [NM_032385]	NM_032385
A_23_P3784	7.36E-03	3.042	ENST00000358530		Homo sapiens mRNA for KIAA1972 protein. [AB075852]	
A_24_P637651	7.37E-03	8.925	THC2314600			
A_23_P105794	7.37E-03	7.955	NM_033255	NM_033255	Homo sapiens epithelial stromal interaction 1 (breast) (EPSTI1), mRNA [NM_033255]	NM_033255
A_23_P155335	7.37E-03	7.142	NM_002662	NM_002662	Homo sapiens phospholipase D1, phophatidylcholine-specific (PLD1), mRNA [NM_002662]	NM_002662
A_23_P135548	7.39E-03	3.656	NM_000110	NM_000110	Homo sapiens dihydropyrimidine dehydrogenase (DPYD), mRNA [NM_000110]	NM_000110

A_32_P192842	7.40E-03	4.107	AI476245	AI476245	AI476245 ti72d11.x1 NCI_CGAP_Kid11 Homo sapiens cDNA clone IMAGE:2137557 3', mRNA sequence [AI476245]	
A_23_P381261	7.43E-03	3.814	NM_139247	NM_139247	Homo sapiens adenylate cyclase 4 (ADCY4), mRNA [NM_139247]	NM_139247
A_23_P30687	7.44E-03	5.477	NM_004155	NM_004155	Homo sapiens serine (or cysteine) proteinase inhibitor, clade B (ovalbumin), member 9 (SERPINB9), mRNA [NM_004155]	NM_004155
A_24_P122337	7.45E-03	4.129	NM_080737	NM_080737	Homo sapiens synaptotagmin-like 4 (granuphilin-a) (SYTL4), mRNA [NM_080737]	NM_080737
A_23_P502174	7.45E-03	2.264	NM_015522	NM_015522	Homo sapiens dynein 2 light intermediate chain (D2LIC), transcript variant 2, mRNA [NM_015522]	NM_015522
A_23_P159125	7.45E-03	2.031	NM_004695	NM_004695	Homo sapiens solute carrier family 16 (monocarboxylic acid transporters), member 5 (SLC16A5), mRNA [NM_004695]	NM_004695
A_23_P79488	7.46E-03	5.914	NM_172311	NM_172311	Homo sapiens stoned B/TFIIA-alpha/beta-like factor (SALF), mRNA [NM_172311]	NM_172311
A_24_P2584	7.46E-03	3.423	BC001892	BC001892	Homo sapiens chromosome 1 open reading frame 63, transcript variant 2, mRNA (cDNA clone MGC:1827 IMAGE:3534629), complete cds. [BC001892]	
A_23_P70998	7.48E-03	2.343	ENST00000297227		full-length cDNA clone CS0DF028YG12 of Fetal brain of Homo sapiens (human). [CR590623]	
A_23_P200737	7.49E-03	18.43	NM_005613	NM_005613	Homo sapiens regulator of G-protein signalling 4 (RGS4), mRNA [NM_005613]	NM_005613
A_32_P131449	7.49E-03	7.489	A_32_P131449			
A_23_P36745	7.49E-03	4.05	NM_000690	NM_000690	Homo sapiens aldehyde dehydrogenase 2 family (mitochondrial) (ALDH2), nuclear gene encoding mitochondrial protein, mRNA [NM_000690]	NM_000690
A_23_P211910	7.49E-03	3.548	NM_182943	NM_182943	Homo sapiens procollagen-lysine, 2-oxoglutarate 5-dioxygenase 2 (PLOD2), transcript variant 1, mRNA [NM_182943]	NM_182943
A_24_P235520	7.49E-03	2.329	NM_175923	NM_175923	Homo sapiens hypothetical protein MGC42630 (MGC42630), mRNA [NM_175923]	NM_175923
A_24_P12865	7.49E-03	2.153	NM_000155	NM_000155	Homo sapiens galactose-1-phosphate uridylyltransferase (GALT), transcript variant 1, mRNA [NM_000155]	NM_000155
A_24_P381604	7.50E-03	3.241	NM_021999	NM_021999	Homo sapiens integral membrane protein 2B (ITM2B), mRNA [NM_021999]	NM_021999
A_23_P255194	7.50E-03	2.154	NM_014827	NM_014827	Homo sapiens zinc finger CCCH-type containing 11A (ZC3H11A), mRNA [NM_014827]	NM_014827
A_24_P67395	7.50E-03	2.027	NM_002273	NM_002273	Homo sapiens keratin 8 (KRT8), mRNA [NM_002273]	NM_002273
A_32_P34750	7.53E-03	2.82	AV702101	AV702101	AV702101 ADB Homo sapiens cDNA clone ADBCGB06 5', mRNA sequence [AV702101]	
A_23_P256542	7.53E-03	2.583	NM_014367	NM_014367	Homo sapiens growth and transformation-dependent protein (E2IG5), mRNA [NM_014367]	NM_014367
A_24_P408047	7.53E-03	2.197	NM_020904	NM_020904	Homo sapiens pleckstrin homology domain containing, family A (phosphoinositide binding specific) member 4 (PLEKHA4), mRNA [NM_020904]	NM_020904
A_24_P602871	7.56E-03	18.45	THC2278542			
A_23_P420442	7.56E-03	6.731	NM_153618	NM_153618	Homo sapiens sema domain, transmembrane domain (TM), and cytoplasmic domain, (semaphorin) 6D (SEMA6D), transcript variant 4, mRNA [NM_153618]	NM_153618
A_32_P49508	7.56E-03	5.867	NM_019036	NM_019036	Homo sapiens 3-hydroxymethyl-3-methylglutaryl-Coenzyme A lyase-like 1 (HMGCLL1), mRNA [NM_019036]	NM_019036
A_32_P137632	7.56E-03	5.414	BC018548	BC018548	Homo sapiens F-box and leucine-rich repeat protein 17, mRNA (cDNA clone IMAGE:4215262), partial cds. [BC018548]	
A_23_P109034	7.56E-03	3.722	NM_002999	NM_002999	Homo sapiens syndecan 4 (amphiglycan, ryudocan) (SDC4), mRNA [NM_002999]	NM_002999
A_23_P13094	7.57E-03	7.82	NM_002425	NM_002425	Homo sapiens matrix metalloproteinase 10 (stromelysin 2) (MMP10), mRNA [NM_002425]	NM_002425
A_32_P151544	7.57E-03	2.45	NM_000224	NM_000224	Homo sapiens keratin 18 (KRT18), transcript variant 1, mRNA [NM_000224]	NM_000224
A_23_P9883	7.57E-03	2.187	NM_004895	NM_004895	Homo sapiens cold autoinflammatory syndrome 1 (CIAS1), transcript variant 1, mRNA [NM_004895]	NM_004895
A_24_P304636	7.58E-03	7.007	NM_004755	NM_004755	Homo sapiens ribosomal protein S6 kinase, 90kDa, polypeptide 5 (RPS6KA5), transcript variant 1, mRNA [NM_004755]	NM_004755
A_23_P343671	7.60E-03	13.31	ENST00000265539		Homo sapiens mRNA; cDNA DKFZp686E0486 (from clone DKFZp686E0486). [BX647822]	
A_32_P23145	7.60E-03	5.884	BM983766	BM983766	BM983766 UI-CF-DU1-aay-k-01-0-UI.s1 UI-CF-DU1 Homo sapiens cDNA clone UI-CF-DU1-aay-k-01-0-UI 3', mRNA sequence [BM983766]	
A_23_P88819	7.60E-03	3.842	NM_017458	NM_017458	Homo sapiens major vault protein (MVP), transcript variant 1, mRNA [NM_017458]	NM_017458
A_23_P82814	7.62E-03	6.546	NM_058229	NM_058229	Homo sapiens F-box protein 32 (FBXO32), transcript variant 1, mRNA [NM_058229]	NM_058229
A_23_P121082	7.63E-03	3.186	NM_000158	NM_000158	Homo sapiens glucan (1,4-alpha-), branching enzyme 1 (glycogen branching enzyme, Andersen disease, glycogen storage disease type IV) (GBE1), mRNA [NM_000158]	NM_000158
A_23_P34144	7.64E-03	40.79	NM_014061	NM_014061	Homo sapiens melanoma antigen family H, 1 (MAGEH1), mRNA [NM_014061]	NM_014061
A_24_P208567	7.64E-03	13.53	NM_003855	NM_003855	Homo sapiens interleukin 18 receptor 1 (IL18R1), mRNA [NM_003855]	NM_003855
A_23_P58835	7.64E-03	2.553	NM_005242	NM_005242	Homo sapiens coagulation factor II (thrombin) receptor-like 1 (F2RL1), mRNA [NM_005242]	NM_005242

A_23_P2920	7.65E-03	4.911	NM_001085	NM_001085	Homo sapiens serine (or cysteine) proteinase inhibitor, clade A (alpha-1 antiproteinase, antitrypsin), member 3 (SERPINA3), mRNA [NM_001085]	NM_001085
A_32_P156006	7.66E-03	2.921	BC018099	BC018099	Homo sapiens, clone IMAGE:4796019, mRNA. [BC018099]	
A_23_P502350	7.66E-03	2.447	NM_000635	NM_000635	Homo sapiens regulatory factor X, 2 (influences HLA class II expression) (RFX2), transcript variant 1, mRNA [NM_000635]	NM_000635
A_32_P210572	7.66E-03	2.408	NM_001024594	NM_001024594	Homo sapiens chromosome 1 open reading frame 53 (C1orf53), mRNA [NM_001024594]	NM_001024594
A_23_P30567	7.68E-03	6.907	NM_001882	NM_001882	Homo sapiens corticotropin releasing hormone binding protein (CRHBP), mRNA [NM_001882]	NM_001882
A_23_P99853	7.68E-03	2.165	NM_019600	NM_019600	Homo sapiens hypothetical protein FLJ10980 (FLJ10980), mRNA [NM_019600]	NM_019600
A_23_P204277	7.69E-03	7.361	NM_177925	NM_177925	Homo sapiens H2A histone family, member J (H2AFJ), transcript variant 2, mRNA [NM_177925]	NM_177925
A_24_P48204	7.70E-03	28.52	NM_003004	NM_003004	Homo sapiens secreted and transmembrane 1 (SECTM1), mRNA [NM_003004]	NM_003004
A_23_P132486	7.70E-03	3.343	NM_017897	NM_017897	Homo sapiens hypothetical protein FLJ20604 (KS), mRNA [NM_017897]	NM_017897
A_23_P377616	7.70E-03	2.158	AK128423	AK128423	Homo sapiens cDNA FLJ46566 fis, clone THYMU3040829, moderately similar to Cold-inducible RNA-binding protein. [AK128423]	
A_23_P30175	7.70E-03	2.045	NM_018695	NM_018695	Homo sapiens erbb2 interacting protein (ERBB2IP), transcript variant 2, mRNA [NM_018695]	NM_018695
A_32_P69166	7.71E-03	3.377	NM_182603	NM_182603	Homo sapiens ankyrin repeat domain 42 (ANKRD42), mRNA [NM_182603]	NM_182603
A_23_P100486	7.71E-03	2.226	NM_206824	NM_206824	Homo sapiens vitamin K epoxide reductase complex, subunit 1 (VKORC1), transcript variant 2, mRNA [NM_206824]	NM_206824
A_23_P80382	7.73E-03	3.057	NM_015366	NM_015366	Homo sapiens proline rich protein 5 (PRR5), transcript variant 2, mRNA [NM_015366]	NM_015366
A_23_P158593	7.75E-03	31.06	NM_000093	NM_000093	Homo sapiens collagen, type V, alpha 1 (COL5A1), mRNA [NM_000093]	NM_000093
A_23_P257871	7.75E-03	5.444	NM_001343	NM_001343	Homo sapiens disabled homolog 2, mitogen-responsive phosphoprotein (Drosophila) (DAB2), mRNA [NM_001343]	NM_001343
A_23_P51986	7.75E-03	5.125	NM_006697	NM_006697	Homo sapiens myotubularin related protein 11 (MTMR11), transcript variant 1, mRNA [NM_006697]	NM_006697
A_23_P103981	7.75E-03	4.375	NM_003516	NM_003516	Homo sapiens histone 2, H2aa (HIST2H2AA), mRNA [NM_003516]	NM_003516
A_23_P207221	7.76E-03	3.496	NM_018242	NM_018242	Homo sapiens hypothetical protein FLJ10847 (FLJ10847), mRNA [NM_018242]	NM_018242
A_23_P352717	7.76E-03	2.159	NM_182541	NM_182541	Homo sapiens transmembrane protein 31 (TMEM31), mRNA [NM_182541]	NM_182541
A_24_P558135	7.77E-03	3.072	BE328864	BE328864	BE328864 hv98a08.x1 NCI_CGAP_Lu24 Homo sapiens cDNA clone IMAGE:3181430 3', mRNA sequence [BE328864]	
A_23_P325411	7.79E-03	2.147	THC2310302		Q9Y474 (Q9Y474) DNA-binding protein, complete [THC2310302]	
A_23_P149153	7.80E-03	2.448	NM_022359	NM_022359	Homo sapiens phosphodiesterase 4D interacting protein (myomegalin) (PDE4DIP), transcript variant 3, mRNA [NM_022359]	NM_022359
A_23_P26024	7.82E-03	5.217	NM_032413	NM_032413	Homo sapiens normal mucosa of esophagus specific 1 (NMES1), transcript variant 2, mRNA [NM_032413]	NM_032413
A_24_P76512	7.82E-03	2.404	AK054626	AK054626	Homo sapiens cDNA FLJ30064 fis, clone ADRGL2000323. [AK054626]	
A_24_P407311	7.83E-03	2.28	NM_014584	NM_014584	Homo sapiens ERO1-like (S. cerevisiae) (ERO1L), mRNA [NM_014584]	NM_014584
A_23_P206760	7.84E-03	4.422	NM_005143	NM_005143	Homo sapiens haptoglobin (HP), mRNA [NM_005143]	NM_005143
A_24_P849801	7.84E-03	2.118	NM_000983	NM_000983	Homo sapiens ribosomal protein L22 (RPL22), mRNA [NM_000983]	NM_000983
A_23_P130333	7.85E-03	297.9	NM_000371	NM_000371	Homo sapiens transthyretin (prealbumin, amyloidosis type I) (TTR), mRNA [NM_000371]	NM_000371
A_32_P213103	7.85E-03	24.2	CA414006	CA414006	CA414006 UI-H-EZ0-ban-f-19-0-UI.s1 NCI_CGAP_Ch1 Homo sapiens cDNA clone UI-H-EZ0-ban-f-19-0-UI 3', mRNA sequence [CA414006]	
A_23_P433855	7.85E-03	16.22	NM_005613	NM_005613	Homo sapiens regulator of G-protein signalling 4 (RGS4), mRNA [NM_005613]	NM_005613
A_23_P53198	7.85E-03	5.026	NM_032564	NM_032564	Homo sapiens diacylglycerol O-acyltransferase homolog 2 (mouse) (DGAT2), mRNA [NM_032564]	NM_032564
A_23_P101272	7.85E-03	2.504	NM_017908	NM_017908	Homo sapiens zinc finger protein 446 (ZNF446), mRNA [NM_017908]	NM_017908
A_32_P193378	7.88E-03	13.01	AK055370	AK055370	Homo sapiens cDNA FLJ30808 fis, clone FEBRA2001383. [AK055370]	
A_23_P145916	7.88E-03	3.568	NM_001129	NM_001129	Homo sapiens AE binding protein 1 (AEBP1), mRNA [NM_001129]	NM_001129
A_24_P508946	7.88E-03	2.876	A_24_P508946			
A_23_P76731	7.89E-03	2.56	NM_014226	NM_014226	Homo sapiens renal tumor antigen (RAGE), mRNA [NM_014226]	NM_014226
A_23_P354591	7.89E-03	2.236	NM_033446	NM_033446	Homo sapiens chromosome 9 open reading frame 28 (C9orf28), transcript variant 1, mRNA [NM_033446]	NM_033446
A_23_P92842	7.89E-03	2.021	NM_016103	NM_016103	Homo sapiens SAR1a gene homolog 2 (S. cerevisiae) (SARA2), mRNA [NM_016103]	NM_016103

A_23_P120902	7.90E-03	75.77	NM_006498	NM_006498	Homo sapiens lectin, galactoside-binding, soluble, 2 (galectin 2) (LGALS2), mRNA [NM_006498]	NM_006498
A_24_P353905	7.90E-03	4.169	NM_032348	NM_032348	Homo sapiens matrix-remodelling associated 8 (MXRA8), mRNA [NM_032348]	NM_032348
A_23_P120566	7.90E-03	2.597	NM_004587	NM_004587	Homo sapiens ribosome binding protein 1 homolog 180kDa (dog) (RRBP1), mRNA [NM_004587]	NM_004587
A_23_P74229	7.90E-03	2.441	NM_032017	NM_032017	Homo sapiens serine/threonine kinase 40 (STK40), mRNA [NM_032017]	NM_032017
A_24_P880043	7.91E-03	4.383	AK055625	AK055625	Homo sapiens cDNA FLJ31063 fis, clone HSYRA2001105. [AK055625]	
A_23_P418083	7.91E-03	2.845	NM_181714	NM_181714	Homo sapiens chromosome 6 open reading frame 152 (C6orf152), mRNA [NM_181714]	NM_181714
A_24_P944383	7.91E-03	2.119	AL050107	AL050107	Homo sapiens mRNA; cDNA DKFZp586I1419 (from clone DKFZp586I1419); partial cds. [AL050107]	
A_23_P37205	7.92E-03	2.876	NM_201535	NM_201535	Homo sapiens NDRG family member 2 (NDRG2), transcript variant 1, mRNA [NM_201535]	NM_201535
A_23_P348121	7.94E-03	7.364	NM_005253	NM_005253	Homo sapiens FOS-like antigen 2 (FOSL2), mRNA [NM_005253]	NM_005253
A_24_P10137	7.95E-03	7.219	NM_014059	NM_014059	Homo sapiens response gene to complement 32 (RGC32), mRNA [NM_014059]	NM_014059
A_32_P100379	7.96E-03	8.029	AA599881	AA599881	AA599881 ag32e07.s1 Human bone marrow stromal cells Homo sapiens cDNA clone IMAGE:1091268 3' similar to gb:M21574 ALPHA PLATELET-DERIVED GROWTH FACTOR RECEPTOR PRECURSOR (HUMAN);, mRNA sequence [AA599881]	
A_23_P384748	7.96E-03	3.614	NM_172069	NM_172069	Homo sapiens pleckstrin homology domain containing, family H (with MyTH4 domain) member 2 (PLEKHH2), mRNA [NM_172069]	NM_172069
A_24_P251411	7.96E-03	3.515	NM_032824	NM_032824	Homo sapiens hypothetical protein FLJ14681 (FLJ14681), mRNA [NM_032824]	NM_032824
A_23_P259442	7.96E-03	3.154	NM_001873	NM_001873	Homo sapiens carboxypeptidase E (CPE), mRNA [NM_001873]	NM_001873
A_24_P124370	7.97E-03	3.781	NM_018222	NM_018222	Homo sapiens parvin, alpha (PARVA), mRNA [NM_018222]	NM_018222
A_23_P10182	7.98E-03	8.525	NM_003500	NM_003500	Homo sapiens acyl-Coenzyme A oxidase 2, branched chain (ACOX2), mRNA [NM_003500]	NM_003500
A_32_P6172	7.99E-03	5.034	BC036622	BC036622	Homo sapiens cDNA clone IMAGE:5286843, partial cds. [BC036622]	
A_23_P361773	8.00E-03	2.359	NM_001760	NM_001760	Homo sapiens cyclin D3 (CCND3), mRNA [NM_001760]	NM_001760
A_32_P205859	8.02E-03	2.256	NM_198490	NM_198490	Homo sapiens RAB43, member RAS oncogene family (RAB43), mRNA [NM_198490]	NM_198490
A_24_P127641	8.03E-03	2.885	AK128882	AK128882	Homo sapiens cDNA FLJ46914 fis, clone SPLEN2027852. [AK128882]	XM_499014
A_23_P42168	8.03E-03	2.051	NM_005586	NM_005586	Homo sapiens MyoD family inhibitor (MDFI), mRNA [NM_005586]	NM_005586
A_23_P133000	8.07E-03	21.4	AK021601	AK021601	Homo sapiens cDNA FLJ11539 fis, clone HEMBA1002748. [AK021601]	XM_496724
A_23_P159382	8.07E-03	2.524	NM_014822	NM_014822	Homo sapiens SEC24 related gene family, member D (S. cerevisiae) (SEC24D), mRNA [NM_014822]	NM_014822
A_23_P73114	8.10E-03	25.46	NM_000313	NM_000313	Homo sapiens protein S (alpha) (PROS1), mRNA [NM_000313]	NM_000313
A_23_P571	8.11E-03	14.71	NM_006516	NM_006516	Homo sapiens solute carrier family 2 (facilitated glucose transporter), member 1 (SLC2A1), mRNA [NM_006516]	NM_006516
A_24_P355649	8.12E-03	10.35	NM_002017	NM_002017	Homo sapiens Friend leukemia virus integration 1 (FLI1), mRNA [NM_002017]	NM_002017
A_24_P96403	8.12E-03	3.73	NM_001001890	NM_001001890	Homo sapiens runt-related transcription factor 1 (acute myeloid leukemia 1; aml1 oncogene) (RUNX1), transcript variant 2, mRNA [NM_001001890]	NM_001001890
A_23_P48198	8.12E-03	2.196	NM_031302	NM_031302	Homo sapiens glycosyltransferase 8 domain containing 2 (GLT8D2), mRNA [NM_031302]	NM_031302
A_23_P338233	8.13E-03	2.812	NM_181708	NM_181708	Homo sapiens hypothetical protein LOC144233 (LOC144233), mRNA [NM_181708]	NM_181708
A_32_P205616	8.18E-03	2.74	A_32_P205616			
A_23_P109682	8.18E-03	2.394	NM_022461	NM_022461	Homo sapiens 5-azacytidine induced 2 (AZI2), mRNA [NM_022461]	NM_022461
A_23_P396804	8.18E-03	2.34	NM_198893	NM_198893	Homo sapiens zinc finger protein 160 (ZNF160), transcript variant 2, mRNA [NM_198893]	NM_198893
A_24_P80532	8.19E-03	3.995	NM_004354	NM_004354	Homo sapiens cyclin G2 (CCNG2), mRNA [NM_004354]	NM_004354
A_23_P344531	8.19E-03	3.565	ENST00000307662		Homo sapiens mRNA for KIAA1029 protein, partial cds. [AB028952]	
A_32_P51237	8.22E-03	35.33	NM_181712	NM_181712	Homo sapiens ankyrin repeat domain 38 (ANKRD38), mRNA [NM_181712]	NM_181712
A_24_P890536	8.22E-03	12.34	CR627148	CR627148	Homo sapiens mRNA; cDNA DKFZp779F2127 (from clone DKFZp779F2127). [CR627148]	
A_23_P204671	8.22E-03	8.248	THC2280612		Q6UWF2 (Q6UWF2) Macrophage antigen h, complete [THC2280612]	
A_32_P129950	8.22E-03	3.298	NM_001012754	NM_001012754	Homo sapiens similar to RIKEN cDNA 8030451K01 (LOC387921), transcript variant 1, mRNA [NM_001012754]	NM_001012754
A_23_P427083	8.22E-03	2.775	NM_173622	NM_173622	Homo sapiens hypothetical protein FLJ36674 (FLJ36674), mRNA [NM_173622]	NM_173622
A_24_P280113	8.31E-03	3.691	NM_001560	NM_001560	Homo sapiens interleukin 13 receptor, alpha 1 (IL13RA1), mRNA [NM_001560]	NM_001560

A_23_P147388	8.31E-03	2.978	NM_015254	NM_015254	Homo sapiens kinesin family member 13B (KIF13B), mRNA [NM_015254]	NM_015254
A_32_P425876	8.31E-03	2.354	AK095904	AK095904	Homo sapiens cDNA FLJ38585 fis, clone HCHON2009191. [AK095904]	
A_24_P304629	8.31E-03	2.002	NM_014909	NM_014909	Homo sapiens KIAA1036 (KIAA1036), mRNA [NM_014909]	NM_014909
A_23_P16806	8.35E-03	2.515	BC004487	BC004487	Homo sapiens hypothetical protein MGC10701, mRNA (cDNA clone MGC:10701 IMAGE:3832541), complete cds. [BC004487]	
A_24_P356830	8.36E-03	2.564	AK129956	AK129956	Homo sapiens cDNA FLJ26446 fis, clone KDN02743. [AK129956]	
A_23_P61529	8.38E-03	2.923	NM_000404	NM_000404	Homo sapiens galactosidase, beta 1 (GLB1), transcript variant 179423, mRNA [NM_000404]	NM_000404
A_32_P2883	8.39E-03	2.322	THC2269190		Q7PWY5 (Q7PWY5) ENSANGP00000012061, partial (5%) [THC2269190]	
A_23_P48339	8.40E-03	3.765	NM_175605	NM_175605	Homo sapiens tetratricopeptide repeat domain 10 (TTC10), transcript variant 1, mRNA [NM_175605]	NM_175605
A_23_P93722	8.42E-03	2.773	AK074077	AK074077	Homo sapiens mRNA for FLJ00148 protein. [AK074077]	
A_32_P75661	8.42E-03	2.387	THC2309312			
A_24_P185854	8.43E-03	5.06	NM_004010	NM_004010	Homo sapiens dystrophin (muscular dystrophy, Duchenne and Becker types) (DMD), transcript variant Dp427p2, mRNA [NM_004010]	NM_004010
A_23_P170667	8.43E-03	2.548	NM_181718	NM_181718	Homo sapiens hypothetical protein LOC253982 (LOC253982), mRNA [NM_181718]	NM_181718
A_32_P517749	8.45E-03	6.8	NM_004586	NM_004586	Homo sapiens ribosomal protein S6 kinase, 90kDa, polypeptide 3 (RPS6KA3), mRNA [NM_004586]	NM_004586
A_24_P287272	8.46E-03	2.401	BC034418	BC034418	Homo sapiens hypothetical protein FLJ20719, mRNA (cDNA clone MGC:34369 IMAGE:3896523), complete cds. [BC034418]	XM_498427
A_23_P157038	8.46E-03	2.224	NM_152755	NM_152755	Homo sapiens hypothetical protein MGC40499 (MGC40499), mRNA [NM_152755]	NM_152755
A_24_P129277	8.47E-03	3.209	NM_006092	NM_006092	Homo sapiens caspase recruitment domain family, member 4 (CARD4), mRNA [NM_006092]	NM_006092
A_23_P40847	8.48E-03	2.325	NM_004267	NM_004267	Homo sapiens carbohydrate (N-acetylglucosamine-6-O) sulfotransferase 2 (CHST2), mRNA [NM_004267]	NM_004267
A_23_P10391	8.49E-03	7.137	NM_000393	NM_000393	Homo sapiens collagen, type V, alpha 2 (COL5A2), mRNA [NM_000393]	NM_000393
A_32_P406142	8.49E-03	3.737	AK025975	AK025975	Homo sapiens cDNA: FLJ22322 fis, clone HRC05532. [AK025975]	
A_23_P426636	8.49E-03	3.017	NM_001620	NM_001620	Homo sapiens AHNAK nucleoprotein (desmoyokin) (AHNAK), transcript variant 1, mRNA [NM_001620]	NM_001620
A_24_P935103	8.49E-03	2.499	NM_001116	NM_001116	Homo sapiens adenylate cyclase 9 (ADCY9), mRNA [NM_001116]	NM_001116
A_23_P122650	8.49E-03	2.38	A_23_P122650			
A_32_P104460	8.49E-03	2.03	NM_019007	NM_019007	Homo sapiens armadillo repeat containing, X-linked 6 (ARMCX6), transcript variant 1, mRNA [NM_019007]	NM_019007
A_32_P116488	8.51E-03	2.742	THC2283842			
A_24_P319736	8.53E-03	5.571	NM_002398	NM_002398	Homo sapiens Meis1, myeloid ecotropic viral integration site 1 homolog (mouse) (MEIS1), mRNA [NM_002398]	NM_002398
A_23_P430120	8.53E-03	2.142	NM_001430	NM_001430	Homo sapiens endothelial PAS domain protein 1 (EPAS1), mRNA [NM_001430]	NM_001430
A_23_P148737	8.55E-03	7.596	NM_004997	NM_004997	Homo sapiens myosin binding protein H (MYBPH), mRNA [NM_004997]	NM_004997
A_23_P257993	8.55E-03	4.679	NM_004944	NM_004944	Homo sapiens deoxyribonuclease I-like 3 (DNASE1L3), mRNA [NM_004944]	NM_004944
A_24_P38143	8.57E-03	2.425	NM_017651	NM_017651	Homo sapiens Abelson helper integration site (AHI1), mRNA [NM_017651]	NM_017651
A_23_P201319	8.58E-03	2.935	NM_032890	NM_032890	Homo sapiens dispatched homolog 1 (Drosophila) (DISP1), mRNA [NM_032890]	NM_032890
A_23_P33196	8.59E-03	14.08	NM_000393	NM_000393	Homo sapiens collagen, type V, alpha 2 (COL5A2), mRNA [NM_000393]	NM_000393
A_24_P940517	8.59E-03	2.409	NM_016297	NM_016297	Homo sapiens prenylcysteine oxidase 1 (PCYOX1), mRNA [NM_016297]	NM_016297
A_23_P19348	8.60E-03	2.997	NM_014780	NM_014780	Homo sapiens cullin 7 (CUL7), mRNA [NM_014780]	NM_014780
A_23_P71067	8.61E-03	3.614	NM_000474	NM_000474	Homo sapiens twist homolog 1 (acrocephalosyndactyly 3; Saethre-Chotzen syndrome) (Drosophila) (TWIST1), mRNA [NM_000474]	NM_000474
A_32_P211353	8.62E-03	7.641	THC2272851		Q7V9T5 (Q7V9T5) Uncharacterized membrane protein, partial (10%) [THC2272851]	
A_24_P384588	8.62E-03	2.462	NM_001012452	NM_001012452	Homo sapiens hypothetical protein FLJ32679 (FLJ32679), mRNA [NM_001012452]	NM_001012452
A_24_P362737	8.62E-03	2.305	NM_032682	NM_032682	Homo sapiens forkhead box P1 (FOXP1), transcript variant 1, mRNA [NM_032682]	NM_032682
A_32_P109242	8.63E-03	2.083	AK055302	AK055302	Homo sapiens cDNA FLJ30740 fis, clone FEBRA2000319. [AK055302]	
A_32_P177300	8.64E-03	14.18	BQ186377	BQ186377	UI-E-EJ1-ajr-a-19-0-UI.r1 UI-E-EJ1 Homo sapiens cDNA clone UI-E-EJ1-ajr-a-19-0-UI 5', mRNA sequence [BQ186377]	
A_32_P117185	8.64E-03	3.121	THC2406981		Q9LCV5 (Q9LCV5) Atp operon (Fragment), partial (3%) [THC2406981]	

A_23_P37484	8.65E-03	2.29	NM_014918	NM_014918	Homo sapiens carbohydrate (chondroitin) synthase 1 (CHSY1), mRNA [NM_014918]	NM_014918
A_23_P426305	8.66E-03	2.651	NM_003734	NM_003734	Homo sapiens amine oxidase, copper containing 3 (vascular adhesion protein 1) (AOC3), mRNA [NM_003734]	NM_003734
A_24_P706752	8.67E-03	2.341	A_24_P706752			
A_32_P170925	8.67E-03	2.014	ENST00000360201		Homo sapiens thioredoxin reductase 3, mRNA (cDNA clone IMAGE:5270485), partial cds. [BC050032]	XM_051264
A_23_P315571	8.68E-03	2.953	NM_015150	NM_015150	Homo sapiens raft-linking protein (RAFTLIN), mRNA [NM_015150]	NM_015150
A_24_P414556	8.68E-03	2.519	NM_012382	NM_012382	Homo sapiens osmosis responsive factor (OSRF), mRNA [NM_012382]	NM_012382
A_23_P115732	8.69E-03	3.113	L13689	L13689	Human prot-oncogene (BMI-1) mRNA, complete cds. [L13689]	
A_23_P78342	8.69E-03	2.548	NM_005570	NM_005570	Homo sapiens lectin, mannose-binding, 1 (LMAN1), mRNA [NM_005570]	NM_005570
A_23_P81219	8.70E-03	4.767	NM_016619	NM_016619	Homo sapiens placenta-specific 8 (PLAC8), mRNA [NM_016619]	NM_016619
A_24_P281264	8.70E-03	2.094	A_24_P281264			
A_32_P123088	8.71E-03	2.787	NM_021649	NM_021649	Homo sapiens toll-like receptor adaptor molecule 2 (TICAM2), mRNA [NM_021649]	NM_021649
A_32_P204048	8.72E-03	6.392	CR601260	CR601260	full-length cDNA clone CS0DM001YA20 of Fetal liver of Homo sapiens (human). [CR601260]	
A_23_P69617	8.74E-03	4.864	NM_003728	NM_003728	Homo sapiens unc-5 homolog C (C. elegans) (UNC5C), mRNA [NM_003728]	NM_003728
A_24_P257022	8.74E-03	2.136	NM_000364	NM_000364	Homo sapiens troponin T2, cardiac (TNNT2), transcript variant 1, mRNA [NM_000364]	NM_000364
A_32_P351277	8.75E-03	2.396	BC004968	BC004968	Homo sapiens, clone IMAGE:3543963, mRNA. [BC004968]	XM_496109
A_24_P47467	8.75E-03	2.102	NM_024092	NM_024092	Homo sapiens hypothetical protein MGC5508 (MGC5508), mRNA [NM_024092]	NM_024092
A_23_P110571	8.75E-03	2.04	ENST00000261569		Human mRNA for KIAA0303 gene, partial cds. [AB002301]	XM_291141
A_23_P106425	8.76E-03	2.255	NM_182616	NM_182616	Homo sapiens hypothetical protein MGC61550 (MGC61550), mRNA [NM_182616]	NM_182616
A_23_P51690	8.77E-03	4.063	NM_020407	NM_020407	Homo sapiens Rhesus blood group, B glycoprotein (RHBG), mRNA [NM_020407]	NM_020407
A_23_P4551	8.78E-03	3.436	NM_015559	NM_015559	Homo sapiens SET binding protein 1 (SETBP1), mRNA [NM_015559]	NM_015559
A_23_P114947	8.78E-03	3.298	NM_002923	NM_002923	Homo sapiens regulator of G-protein signalling 2, 24kDa (RGS2), mRNA [NM_002923]	NM_002923
A_23_P82868	8.79E-03	30.34	NM_000930	NM_000930	Homo sapiens plasminogen activator, tissue (PLAT), transcript variant 1, mRNA [NM_000930]	NM_000930
A_24_P109661	8.79E-03	2.191	A_24_P109661			
A_32_P178499	8.79E-03	2.122	NM_001012984	NM_001012984	Homo sapiens hypothetical gene supported by BC032064; BC041612 (LOC388284), mRNA [NM_001012984]	NM_001012984
A_23_P434352	8.80E-03	3.136	NM_001750	NM_001750	Homo sapiens calpastatin (CAST), transcript variant 1, mRNA [NM_001750]	NM_001750
A_23_P20285	8.80E-03	2.562	NM_021630	NM_021630	Homo sapiens PDZ and LIM domain 2 (mystique) (PDLIM2), transcript variant 2, mRNA [NM_021630]	NM_021630
A_23_P10081	8.80E-03	2.112	NM_182896	NM_182896	Homo sapiens ADP-ribosylation factor-like 2-like 1 (ARL2L1), transcript variant 1, mRNA [NM_182896]	NM_182896
A_24_P12521	8.80E-03	2.104	NM_138811	NM_138811	Homo sapiens chromosome 7 open reading frame 31 (C7orf31), mRNA [NM_138811]	NM_138811
A_24_P933151	8.81E-03	3.703	S69023	S69023	HOX B6=class I homeobox {fragment M2, homeodomain} [human, MCF7 cells, mRNA Partial, 117 nt]. [S69023]	
A_23_P55179	8.81E-03	2.046	NM_001661	NM_001661	Homo sapiens ADP-ribosylation factor 4-like (ARF4L), mRNA [NM_001661]	NM_001661
A_24_P78590	8.82E-03	3.129	NM_172373	NM_172373	Homo sapiens E74-like factor 1 (ets domain transcription factor) (ELF1), mRNA [NM_172373]	NM_172373
A_24_P391987	8.82E-03	2.896	NM_001001701	NM_001001701	Homo sapiens HCV F-transactivated protein 1 (LOC401152), mRNA [NM_001001701]	NM_001001701
A_23_P73801	8.83E-03	2.752	NM_001006640	NM_001006640	Homo sapiens transcription elongation factor A (SII)-like 1 (TCEAL1), transcript variant 3, mRNA [NM_001006640]	NM_001006640
A_24_P21985	8.85E-03	2.277	NM_018416	NM_018416	Homo sapiens forkhead box J2 (FOXJ2), mRNA [NM_018416]	NM_018416
A_23_P206733	8.86E-03	3.85	NM_001266	NM_001266	Homo sapiens carboxylesterase 1 (monocyte/macrophage serine esterase 1) (CES1), transcript variant 3, mRNA [NM_001266]	NM_001266
A_24_P945215	8.86E-03	2.73	BC010351	BC010351	Homo sapiens USP6 N-terminal like, mRNA (cDNA clone IMAGE:4047207), partial cds. [BC010351]	
A_23_P218434	8.88E-03	2.836	NM_015919	NM_015919	Homo sapiens zinc finger protein 226 (ZNF226), mRNA [NM_015919]	NM_015919
A_32_P171043	8.89E-03	10.71	BI090438	BI090438	602853723F1 NIH_MGC_10 Homo sapiens cDNA clone IMAGE:4995269 5', mRNA sequence [BI090438]	
A_23_P55666	8.89E-03	5.85	AK023047	AK023047	Homo sapiens cDNA FLJ12985 fis, clone NT2RP3000050, moderately similar to ZINC FINGER PROTEIN 91. [AK023047]	
A_24_P156113	8.89E-03	3.956	NM_014601	NM_014601	Homo sapiens EH-domain containing 2 (EHD2), mRNA [NM_014601]	NM_014601
A_24_P755069	8.89E-03	3.456	A_24_P755069			

A_23_P31064	8.90E-03	2.118	NM_015529	NM_015529	Homo sapiens monooxygenase, DBH-like 1 (MOXD1), mRNA [NM_015529]	NM_015529
A_24_P481824	8.92E-03	3.175	AF086017	AF086017	Homo sapiens full length insert cDNA clone YW19E12. [AF086017]	
A_23_P98900	8.94E-03	2.072	NM_025140	NM_025140	Homo sapiens limkain beta 2 (FLJ22471), mRNA [NM_025140]	NM_025140
A_32_P106864	8.96E-03	3.694	AA303143	AA303143	AA303143 EST13031 Uterus tumor I Homo sapiens cDNA 5' end similar to hypothetical protein KIAA0222, mRNA sequence [AA303143]	
A_24_P201404	8.96E-03	2.31	NM_014039	NM_014039	Homo sapiens PTD012 protein (PTD012), mRNA [NM_014039]	NM_014039
A_23_P163467	8.98E-03	5.58	NM_207380	NM_207380	Homo sapiens FLJ43339 protein (FLJ43339), mRNA [NM_207380]	NM_207380
A_23_P54846	8.98E-03	5.035	NM_014685	NM_014685	Homo sapiens homocysteine-inducible, endoplasmic reticulum stress-inducible, ubiquitin-like domain member 1 (HERPUD1), transcript variant 1, mRNA [NM_014685]	NM_014685
A_32_P149492	8.98E-03	4.943	AF419616	AF419616	Homo sapiens clone DKFZp564A057 AG02 mRNA, partial cds. [AF419616]	XM_496399
A_23_P152995	8.98E-03	4.784	NM_001045	NM_001045	Homo sapiens solute carrier family 6 (neurotransmitter transporter, serotonin), member 4 (SLC6A4), mRNA [NM_001045]	NM_001045
A_24_P692600	8.98E-03	3.411	BU615415	BU615415	UI-H-FH0-bco-j-01-0-UI.s1 NCI_CGAP_FH0 Homo sapiens cDNA clone UI-H-FH0-bco-j-01-0-UI 3', mRNA sequence [BU615415]	
A_23_P402000	8.98E-03	2.56	AK091585	AK091585	Homo sapiens cDNA FLJ34266 fis, clone FEBRA2002682, moderately similar to ZINC FINGER PROTEIN 91. [AK091585]	XM_030378
A_32_P77098	8.98E-03	2.379	NM_001003682	NM_001003682	Homo sapiens cDNA DKFZp434C184 gene (TTMB), mRNA [NM_001003682]	NM_001003682
A_23_P335848	8.98E-03	2.308	NM_057169	NM_057169	Homo sapiens G protein-coupled receptor kinase interactor 2 (GIT2), transcript variant 1, mRNA [NM_057169]	NM_057169
A_32_P137266	8.98E-03	2.108	NM_032437	NM_032437	Homo sapiens KIAA1799 protein (KIAA1799), mRNA [NM_032437]	NM_032437
A_23_P118722	9.01E-03	3.18	NM_001671	NM_001671	Homo sapiens asialoglycoprotein receptor 1 (ASGR1), mRNA [NM_001671]	NM_001671
A_23_P8196	9.01E-03	3.054	CR591849	CR591849	full-length cDNA clone CS0DC003YO01 of Neuroblastoma Cot 25-normalized of Homo sapiens (human). [CR591849]	
A_24_P355816	9.03E-03	3.488	NM_018370	NM_018370	Homo sapiens hypothetical protein FLJ11259 (FLJ11259), mRNA [NM_018370]	NM_018370
A_23_P123672	9.05E-03	2.545	NM_014290	NM_014290	Homo sapiens tudor domain containing 7 (TDRD7), mRNA [NM_014290]	NM_014290
A_23_P37375	9.06E-03	4.867	NM_004755	NM_004755	Homo sapiens ribosomal protein S6 kinase, 90kDa, polypeptide 5 (RPS6KA5), transcript variant 1, mRNA [NM_004755]	NM_004755
A_24_P919304	9.06E-03	2.472	BC012204	BC012204	Homo sapiens fibronectin type III domain containing 3B, mRNA (cDNA clone IMAGE:3882800), complete cds. [BC012204]	
A_23_P165848	9.08E-03	4.352	NM_007046	NM_007046	Homo sapiens elastin microfibril interfacer 1 (EMILIN1), mRNA [NM_007046]	NM_007046
A_23_P103756	9.08E-03	2.499	NM_002557	NM_002557	Homo sapiens oviductal glycoprotein 1, 120kDa (mucin 9, oviductin) (OVGP1), mRNA [NM_002557]	NM_002557
A_32_P186725	9.08E-03	2.306	THC2433670		G01958 homeobox protein - human (fragment) {Homo sapiens;} , partial (5%) [THC2433670]	
A_23_P53866	9.10E-03	3.581	NM_020751	NM_020751	Homo sapiens component of oligomeric golgi complex 6 (COG6), mRNA [NM_020751]	NM_020751
A_23_P70867	9.11E-03	2.291	NM_016038	NM_016038	Homo sapiens Shwachman-Bodian-Diamond syndrome (SBDS), mRNA [NM_016038]	NM_016038
A_24_P707530	9.13E-03	2.692	THC2336549		S10030 urease 11K chain - Ureaplasma urealyticum {Ureaplasma urealyticum;} , partial (10%) [THC2336549]	
A_24_P316257	9.13E-03	2.366	NM_176677	NM_176677	Homo sapiens hypothetical protein FLJ36208 (FLJ36208), mRNA [NM_176677]	NM_176677
A_23_P24616	9.14E-03	11.23	NM_170601	NM_170601	Homo sapiens cytosolic sialic acid 9-O-acetylesterase homolog (CSE-C), mRNA [NM_170601]	NM_170601
A_24_P928052	9.17E-03	13.31	NM_003873	NM_003873	Homo sapiens neuropilin 1 (NRP1), transcript variant 1, mRNA [NM_003873]	NM_003873
A_32_P69475	9.17E-03	4.845	AK023633	AK023633	Homo sapiens cDNA FLJ13571 fis, clone PLACE1008405. [AK023633]	
A_32_P38323	9.20E-03	4.007	NM_004155	NM_004155	Homo sapiens serine (or cysteine) proteinase inhibitor, clade B (ovalbumin), member 9 (SERPINB9), mRNA [NM_004155]	NM_004155
A_23_P373100	9.21E-03	3.452	BC020879	BC020879	Homo sapiens hypothetical protein MGC24103, mRNA (cDNA clone MGC:24103 IMAGE:4613905), complete cds. [BC020879]	
A_23_P136916	9.22E-03	2.059	NM_017883	NM_017883	Homo sapiens WD repeat domain 13 (WDR13), mRNA [NM_017883]	NM_017883
A_23_P62831	9.23E-03	3.212	NM_018166	NM_018166	Homo sapiens chromosome 1 open reading frame 78 (C1orf78), mRNA [NM_018166]	NM_018166
A_23_P116235	9.23E-03	3.013	NM_001012334	NM_001012334	Homo sapiens midkine (neurite growth-promoting factor 2) (MDK), transcript variant 1, mRNA [NM_001012334]	NM_001012334
A_32_P231493	9.23E-03	2.811	AF339771	AF339771	Homo sapiens clone IMAGE:1257951, mRNA sequence. [AF339771]	
A_23_P44257	9.23E-03	2.39	NM_017845	NM_017845	Homo sapiens COMM domain containing 8 (COMMD8), mRNA [NM_017845]	NM_017845
A_23_P48295	9.23E-03	2.189	NM_030911	NM_030911	Homo sapiens cytidine and dCMP deaminase domain containing 1 (CDADC1), mRNA [NM_030911]	NM_030911

A_23_P115246	9.23E-03	2.159	NM_003665	NM_003665	Homo sapiens ficolin (collagen/fibrinogen domain containing) 3 (Hakata antigen) (FCN3), transcript variant 1, mRNA [NM_003665]	NM_003665
A_24_P156922	9.28E-03	8.062	NM_001007098	NM_001007098	Homo sapiens sterol carrier protein 2 (SCP2), transcript variant 2, mRNA [NM_001007098]	NM_001007098
A_23_P30956	9.30E-03	2.247	NM_015323	NM_015323	Homo sapiens KIAA0776 (KIAA0776), mRNA [NM_015323]	NM_015323
A_23_P254654	9.31E-03	13.27	NM_004669	NM_004669	Homo sapiens chloride intracellular channel 3 (CLIC3), mRNA [NM_004669]	NM_004669
A_23_P304000	9.32E-03	2.365	NM_015026	NM_015026	Homo sapiens KIAA1040 protein (KIAA1040), mRNA [NM_015026]	NM_015026
A_24_P265274	9.34E-03	8.38	NM_000089	NM_000089	Homo sapiens collagen, type I, alpha 2 (COL1A2), mRNA [NM_000089]	NM_000089
A_23_P331928	9.34E-03	2.677	AK095888	AK095888	Homo sapiens cDNA FLJ38569 fis, clone HCHON2006459. [AK095888]	
A_23_P81522	9.35E-03	2.915	NM_012382	NM_012382	Homo sapiens osmosis responsive factor (OSRF), mRNA [NM_012382]	NM_012382
A_24_P489649	9.37E-03	3.224	XM_067448	XM_067448	PREDICTED: Homo sapiens similar to MEST (LOC131572), mRNA [XM_067448]	XM_067448
A_23_P141248	9.37E-03	2.523	NM_021947	NM_021947	Homo sapiens serine racemase (SRR), mRNA [NM_021947]	NM_021947
A_23_P306987	9.39E-03	5.889	NM_031439	NM_031439	Homo sapiens SRY (sex determining region Y)-box 7 (SOX7), mRNA [NM_031439]	NM_031439
A_32_P129265	9.40E-03	4.651	U92981	U92981	Homo sapiens clone DT1P1B6 mRNA, CAG repeat region. [U92981]	
A_23_P329198	9.40E-03	4.022	NM_022837	NM_022837	Homo sapiens hypothetical protein FLJ22833 (FLJ22833), mRNA [NM_022837]	NM_022837
A_23_P334864	9.40E-03	2.038	NM_173822	NM_173822	Homo sapiens hypothetical protein MGC39518 (MGC39518), mRNA [NM_173822]	NM_173822
A_23_P382584	9.41E-03	3.509	NM_001819	NM_001819	Homo sapiens chromogranin B (secretogranin 1) (CHGB), mRNA [NM_001819]	NM_001819
A_23_P101806	9.42E-03	3.4	NM_182983	NM_182983	Homo sapiens hepsin (transmembrane protease, serine 1) (HPN), transcript variant 1, mRNA [NM_182983]	NM_182983
A_23_P53126	9.44E-03	7.241	NM_005574	NM_005574	Homo sapiens LIM domain only 2 (rhombotin-like 1) (LMO2), mRNA [NM_005574]	NM_005574
A_23_P11843	9.46E-03	8.51	NM_201630	NM_201630	Homo sapiens leucine rich repeat neuronal 5 (LRRN5), transcript variant 2, mRNA [NM_201630]	NM_201630
A_32_P192389	9.46E-03	2.588	AW844496	AW844496	AW844496 RC2-CN0051-210200-015-f06 CN0051 Homo sapiens cDNA, mRNA sequence [AW844496]	
A_24_P123190	9.49E-03	12.09	NM_002662	NM_002662	Homo sapiens phospholipase D1, phophatidylcholine-specific (PLD1), mRNA [NM_002662]	NM_002662
A_23_P341938	9.51E-03	3.025	NM_005450	NM_005450	Homo sapiens noggin (NOG), mRNA [NM_005450]	NM_005450
A_24_P787947	9.51E-03	2.906	NM_001005404	NM_001005404	Homo sapiens yippee-like 2 (Drosophila) (YPEL2), mRNA [NM_001005404]	NM_001005404
A_23_P214739	9.51E-03	2.649	NM_012160	NM_012160	Homo sapiens F-box and leucine-rich repeat protein 4 (FBXL4), mRNA [NM_012160]	NM_012160
A_23_P408913	9.51E-03	2.224	NM_152517	NM_152517	Homo sapiens hypothetical protein FLJ30990 (FLJ30990), mRNA [NM_152517]	NM_152517
A_23_P161125	9.51E-03	2.106	NM_020963	NM_020963	Homo sapiens Mov10, Moloney leukemia virus 10, homolog (mouse) (MOV10), mRNA [NM_020963]	NM_020963
A_24_P312119	9.52E-03	2.559	A_24_P312119			
A_24_P95070	9.53E-03	13.65	ENST00000222543		Homo sapiens cDNA FLJ26323 fis, clone HRT00813, highly similar to Tissue factor pathway inhibitor 2 precursor (TFPI-2). [AK129833]	
A_32_P2303	9.53E-03	4.287	A_32_P2303			
A_23_P11685	9.54E-03	2.968	NM_024420	NM_024420	Homo sapiens phospholipase A2, group IVA (cytosolic, calcium-dependent) (PLA2G4A), mRNA [NM_024420]	NM_024420
A_32_P231617	9.56E-03	3.723	NM_014220	NM_014220	Homo sapiens transmembrane 4 L six family member 1 (TM4SF1), mRNA [NM_014220]	NM_014220
A_23_P74852	9.56E-03	2.456	AL035301	AL035301	H.sapiens gene from PAC 106H8. [AL035301]	
A_24_P687594	9.58E-03	3.56	ENST00000281794		Homo sapiens mRNA; cDNA DKFZp762F237 (from clone DKFZp762F237). [CR627381]	
A_24_P639629	9.58E-03	3.073	AK056245	AK056245	Homo sapiens cDNA FLJ31683 fis, clone NT2RI2005353. [AK056245]	
A_23_P258972	9.58E-03	2.536	NM_002077	NM_002077	Homo sapiens golgi autoantigen, golgin subfamily a, 1 (GOLGA1), mRNA [NM_002077]	NM_002077
A_23_P206310	9.58E-03	2.126	NM_014732	NM_014732	Homo sapiens KIAA0513 (KIAA0513), mRNA [NM_014732]	NM_014732
A_23_P33173	9.58E-03	2.08	NM_004487	NM_004487	Homo sapiens golgi autoantigen, golgin subfamily b, macrogolgin (with transmembrane signal), 1 (GOLGB1), mRNA [NM_004487]	NM_004487
A_24_P85539	9.61E-03	4.633	NM_212482	NM_212482	Homo sapiens fibronectin 1 (FN1), transcript variant 1, mRNA [NM_212482]	NM_212482
A_23_P4041	9.62E-03	2.153	NM_021213	NM_021213	Homo sapiens phosphatidylcholine transfer protein (PCTP), mRNA [NM_021213]	NM_021213
A_23_P123608	9.62E-03	2.004	NM_004972	NM_004972	Homo sapiens Janus kinase 2 (a protein tyrosine kinase) (JAK2), mRNA [NM_004972]	NM_004972
A_24_P778649	9.63E-03	2.64	BX640923	BX640923	Homo sapiens mRNA; cDNA DKFZp686B01123 (from clone DKFZp686B01123). [BX640923]	
A_24_P326398	9.64E-03	7.188	NM_173689	NM_173689	Homo sapiens crumbs homolog 2 (Drosophila) (CRB2), mRNA [NM_173689]	NM_173689

A_23_P421032	9.64E-03	6.378	NM_174977	NM_174977	Homo sapiens SEC14-like 4 (S. cerevisiae) (SEC14L4), mRNA [NM_174977]	NM_174977
A_23_P213518	9.64E-03	3.036	NM_173060	NM_173060	Homo sapiens calpastatin (CAST), transcript variant 2, mRNA [NM_173060]	NM_173060
A_23_P205913	9.64E-03	2.463	NM_004727	NM_004727	Homo sapiens solute carrier family 24 (sodium/potassium/calcium exchanger), member 1 (SLC24A1), mRNA [NM_004727]	NM_004727
A_23_P168669	9.64E-03	2.311	NM_021151	NM_021151	Homo sapiens carnitine O-octanoyltransferase (CROT), mRNA [NM_021151]	NM_021151
A_24_P86755	9.65E-03	6.673	NM_018956	NM_018956	Homo sapiens chromosome 9 open reading frame 9 (C9orf9), mRNA [NM_018956]	NM_018956
A_23_P156826	9.65E-03	5.353	NM_032744	NM_032744	Homo sapiens chromosome 6 open reading frame 105 (C6orf105), mRNA [NM_032744]	NM_032744
A_23_P147641	9.65E-03	2.287	NM_003195	NM_003195	Homo sapiens transcription elongation factor A (SII), 2 (TCEA2), transcript variant 1, mRNA [NM_003195]	NM_003195
A_24_P389916	9.70E-03	12.76	NM_005512	NM_005512	Homo sapiens leucine rich repeat containing 32 (LRRC32), mRNA [NM_005512]	NM_005512
A_23_P90510	9.70E-03	3.877	NM_138393	NM_138393	Homo sapiens chromosome 19 open reading frame 32 (C19orf32), mRNA [NM_138393]	NM_138393
A_24_P169634	9.71E-03	2.64	AF019382	AF019382	Homo sapiens mannose-binding protein-A pseudogene (MBL1P1) mRNA sequence. [AF019382]	XR_000276
A_23_P303210	9.74E-03	3.141	NM_153687	NM_153687	Homo sapiens IKK interacting protein (IKIP), transcript variant 1, mRNA [NM_153687]	NM_153687
A_23_P118266	9.75E-03	4.563	NM_006885	NM_006885	Homo sapiens AT-binding transcription factor 1 (ATBF1), mRNA [NM_006885]	NM_006885
A_23_P204736	9.75E-03	2.045	NM_005276	NM_005276	Homo sapiens glycerol-3-phosphate dehydrogenase 1 (soluble) (GPD1), mRNA [NM_005276]	NM_005276
A_23_P381577	9.76E-03	8.557	NM_145011	NM_145011	Homo sapiens zinc finger protein 25 (KOX 19) (ZNF25), mRNA [NM_145011]	NM_145011
A_23_P207911	9.77E-03	7.085	NM_016113	NM_016113	Homo sapiens transient receptor potential cation channel, subfamily V, member 2 (TRPV2), mRNA [NM_016113]	NM_016113
A_23_P13222	9.77E-03	2.273	NM_002901	NM_002901	Homo sapiens reticulocalbin 1, EF-hand calcium binding domain (RCN1), mRNA [NM_002901]	NM_002901
A_23_P254254	9.79E-03	2.007	NM_000199	NM_000199	Homo sapiens N-sulfoglucosamine sulfohydrolase (sulfamidase) (SGSH), mRNA [NM_000199]	NM_000199
A_23_P342709	9.80E-03	2.537	NM_152676	NM_152676	Homo sapiens F-box protein 15 (FBXO15), mRNA [NM_152676]	NM_152676
A_32_P82650	9.83E-03	26.86	BM701175	BM701175	UI-E-EJ0-ahj-e-13-0-UI.r1 UI-E-EJ0 Homo sapiens cDNA clone UI-E-EJ0-ahj-e-13-0-UI 5', mRNA sequence [BM701175]	
A_32_P35452	9.83E-03	4.886	THC2279497		HSCLOCK17 clock {Homo sapiens;} , partial (3%) [THC2279497]	
A_23_P34548	9.83E-03	3.837	NM_006642	NM_006642	Homo sapiens serologically defined colon cancer antigen 8 (SDCCAG8), mRNA [NM_006642]	NM_006642
A_24_P268160	9.84E-03	2.211	NM_178454	NM_178454	Homo sapiens hypothetical protein MGC54289 (MGC54289), mRNA [NM_178454]	NM_178454
A_23_P374695	9.85E-03	12.01	NM_000459	NM_000459	Homo sapiens TEK tyrosine kinase, endothelial (venous malformations, multiple cutaneous and mucosal) (TEK), mRNA [NM_000459]	NM_000459
A_23_P94533	9.85E-03	3.033	NM_001912	NM_001912	Homo sapiens cathepsin L (CTSL), transcript variant 1, mRNA [NM_001912]	NM_001912
A_23_P158096	9.85E-03	2.074	AK021957	AK021957	Homo sapiens cDNA FLJ11895 fis, clone HEMBA1007301, weakly similar to COLLAGEN ALPHA 1(III) CHAIN. [AK021957]	
A_23_P202484	9.86E-03	7.254	NM_032772	NM_032772	Homo sapiens zinc finger protein 503 (ZNF503), mRNA [NM_032772]	NM_032772
A_23_P85682	9.88E-03	2.221	NM_005595	NM_005595	Homo sapiens nuclear factor I/A (NFIA), mRNA [NM_005595]	NM_005595
A_23_P314101	9.90E-03	3.217	NM_019601	NM_019601	Homo sapiens sushi domain containing 2 (SUSD2), mRNA [NM_019601]	NM_019601
A_24_P126325	9.94E-03	4.158	NM_207035	NM_207035	Homo sapiens chromosome 1 open reading frame 63 (C1orf63), transcript variant 1, mRNA [NM_207035]	NM_207035
A_23_P61823	9.94E-03	2.019	NM_130781	NM_130781	Homo sapiens RAB24, member RAS oncogene family (RAB24), mRNA [NM_130781]	NM_130781
A_32_P23838	9.97E-03	3.167	NM_207352	NM_207352	Homo sapiens cytochrome P450, family 4, subfamily V, polypeptide 2 (CYP4V2), mRNA [NM_207352]	NM_207352
A_24_P156490	9.97E-03	2.855	NM_002247	NM_002247	Homo sapiens potassium large conductance calcium-activated channel, subfamily M, alpha member 1 (KCNMA1), transcript variant 2, mRNA [NM_002247]	NM_002247
A_24_P923142	9.98E-03	7.871	BC020784	BC020784	Homo sapiens similar to RIKEN cDNA 1200014N16 gene, mRNA (cDNA clone IMAGE:4773508), complete cds. [BC020784]	
A_24_P128001	9.99E-03	6.653	NM_018660	NM_018660	Homo sapiens zinc finger protein 395 (ZNF395), mRNA [NM_018660]	NM_018660
A_23_P157460	9.99E-03	3.142	NM_018660	NM_018660	Homo sapiens zinc finger protein 395 (ZNF395), mRNA [NM_018660]	NM_018660
A_23_P52121	1.00E-02	6.427	NM_002614	NM_002614	Homo sapiens PDZ domain containing 1 (PDZK1), mRNA [NM_002614]	NM_002614
A_24_P45446	1.00E-02	5.586	NM_052941	NM_052941	Homo sapiens guanylate binding protein 4 (GBP4), mRNA [NM_052941]	NM_052941
A_24_P350838	1.00E-02	4.657	X62009	X62009	Homo sapiens partial mRNA for fibrillin 5. [X62009]	
A_23_P15272	1.00E-02	2.628	BC050733	BC050733	Homo sapiens ATP-binding cassette, sub-family C (CFTR/MRP), member 6, mRNA (cDNA clone IMAGE:6141205), complete cds. [BC050733]	

A_32_P225328	1.01E-02	6.464	A_32_P225328			
A_24_P385134	1.01E-02	5.641	AF389338	AF389338	Homo sapiens acyl-CoA-desaturase mRNA, complete cds. [AF389338]	
A_23_P53557	1.01E-02	2.857	NM_002342	NM_002342	Homo sapiens lymphotoxin beta receptor (TNFR superfamily, member 3) (LTBR), mRNA [NM_002342]	NM_002342
A_23_P124300	1.01E-02	2.612	NM_017429	NM_017429	Homo sapiens beta-carotene 15,15'-monooxygenase 1 (BCMO1), mRNA [NM_017429]	NM_017429
A_32_P387905	1.01E-02	2.377	AK024224	AK024224	Homo sapiens cDNA FLJ14162 fis, clone NT2RM4002504. [AK024224]	
A_23_P147918	1.01E-02	2.223	NM_080388	NM_080388	Homo sapiens S100 calcium binding protein A16 (S100A16), mRNA [NM_080388]	NM_080388
A_23_P394605	1.01E-02	2.087	ENST00000265341		Homo sapiens mRNA; cDNA DKFZp564I062 (from clone DKFZp564I062). [AL080064]	
A_23_P81241	1.01E-02	2.041	NM_030571	NM_030571	Homo sapiens Nedd4 family interacting protein 1 (NDFIP1), mRNA [NM_030571]	NM_030571
A_23_P69573	1.02E-02	17.82	NM_000856	NM_000856	Homo sapiens guanylate cyclase 1, soluble, alpha 3 (GUCY1A3), mRNA [NM_000856]	NM_000856
A_23_P118571	1.02E-02	10.38	NM_025237	NM_025237	Homo sapiens sclerosteosis (SOST), mRNA [NM_025237]	NM_025237
A_23_P200670	1.02E-02	5.624	NM_207014	NM_207014	Homo sapiens WD repeat domain 78 (WDR78), transcript variant 2, mRNA [NM_207014]	NM_207014
A_23_P329261	1.02E-02	3.569	NM_000891	NM_000891	Homo sapiens potassium inwardly-rectifying channel, subfamily J, member 2 (KCNJ2), mRNA [NM_000891]	NM_000891
A_23_P101871	1.02E-02	3.241	NM_024907	NM_024907	Homo sapiens F-box protein 17 (FBXO17), transcript variant 2, mRNA [NM_024907]	NM_024907
A_24_P106166	1.02E-02	3.02	A_24_P106166			
A_23_P46470	1.02E-02	2.583	NM_018948	NM_018948	Homo sapiens mitogen-inducible gene 6 (MIG-6), mRNA [NM_018948]	NM_018948
A_23_P343935	1.02E-02	2.521	NM_022051	NM_022051	Homo sapiens egl nine homolog 1 (C. elegans) (EGLN1), mRNA [NM_022051]	NM_022051
A_23_P39840	1.02E-02	2.475	NM_006634	NM_006634	Homo sapiens vesicle-associated membrane protein 5 (myobrevin) (VAMP5), mRNA [NM_006634]	NM_006634
A_32_P192984	1.02E-02	2.286	AK128396	AK128396	Homo sapiens cDNA FLJ46539 fis, clone THYMU3037836. [AK128396]	
A_23_P348063	1.02E-02	2.215	NM_004711	NM_004711	Homo sapiens synaptogyrin 1 (SYNGR1), transcript variant 1a, mRNA [NM_004711]	NM_004711
A_23_P15864	1.03E-02	20.68	NM_003927	NM_003927	Homo sapiens methyl-CpG binding domain protein 2 (MBD2), transcript variant 1, mRNA [NM_003927]	NM_003927
A_32_P181061	1.03E-02	3.578	BE612504	BE612504	BE612504 601452004F1 NIH_MGC_66 Homo sapiens cDNA clone IMAGE:3855793 5', mRNA sequence [BE612504]	
A_23_P80068	1.03E-02	3.022	NM_006806	NM_006806	Homo sapiens BTG family, member 3 (BTG3), mRNA [NM_006806]	NM_006806
A_24_P79040	1.03E-02	2.955	NM_144691	NM_144691	Homo sapiens calpain 12 (CAPN12), mRNA [NM_144691]	NM_144691
A_23_P214185	1.03E-02	2.78	NM_016021	NM_016021	Homo sapiens ubiquitin-conjugating enzyme E2, J1 (UBC6 homolog, yeast) (UBE2J1), mRNA [NM_016021]	NM_016021
A_23_P138717	1.03E-02	2.717	NM_001005339	NM_001005339	Homo sapiens regulator of G-protein signalling 10 (RGS10), transcript variant 1, mRNA [NM_001005339]	NM_001005339
A_32_P78131	1.03E-02	2.703	THC2305027			
A_23_P53176	1.03E-02	2.443	NM_016725	NM_016725	Homo sapiens folate receptor 1 (adult) (FOLR1), transcript variant 1, mRNA [NM_016725]	NM_016725
A_23_P77381	1.03E-02	2.435	NM_018110	NM_018110	Homo sapiens docking protein 4 (DOK4), mRNA [NM_018110]	NM_018110
A_23_P59349	1.03E-02	2.22	NM_016217	NM_016217	Homo sapiens headcase homolog (Drosophila) (HECA), mRNA [NM_016217]	NM_016217
A_24_P912985	1.03E-02	2.158	A_24_P912985			
A_32_P37089	1.03E-02	2.146	AK097700	AK097700	Homo sapiens cDNA FLJ40381 fis, clone TESTI2035688. [AK097700]	
A_23_P60296	1.03E-02	2.131	NM_012383	NM_012383	Homo sapiens osteoclast stimulating factor 1 (OSTF1), mRNA [NM_012383]	NM_012383
A_23_P19723	1.04E-02	14.76	NM_021073	NM_021073	Homo sapiens bone morphogenetic protein 5 (BMP5), mRNA [NM_021073]	NM_021073
A_32_P74409	1.04E-02	13.43	ENST00000339446		Homo sapiens hypothetical LOC387763, mRNA (cDNA clone IMAGE:6272440), partial cds. [BC052560]	XM_373497
A_23_P81898	1.04E-02	8.422	NM_006398	NM_006398	Homo sapiens ubiquitin D (UBD), mRNA [NM_006398]	NM_006398
A_23_P140170	1.04E-02	2.696	NM_006364	NM_006364	Homo sapiens Sec23 homolog A (S. cerevisiae) (SEC23A), mRNA [NM_006364]	NM_006364
A_23_P80827	1.04E-02	2.35	NM_001011537	NM_001011537	Homo sapiens forty-two-three domain containing 1 (FYTTD1), transcript variant 2, mRNA [NM_001011537]	NM_001011537
A_24_P64362	1.04E-02	2.125	AK000161	AK000161	Homo sapiens cDNA FLJ20154 fis, clone COL08740. [AK000161]	
A_24_P3627	1.04E-02	2.02	A_24_P3627			
A_23_P13772	1.05E-02	4.521	NM_016569	NM_016569	Homo sapiens T-box 3 (ulnar mammary syndrome) (TBX3), transcript variant 2, mRNA [NM_016569]	NM_016569
A_23_P204937	1.05E-02	4.153	NM_014059	NM_014059	Homo sapiens response gene to complement 32 (RGC32), mRNA [NM_014059]	NM_014059
A_23_P18078	1.05E-02	4.02	NM_002888	NM_002888	Homo sapiens retinoic acid receptor responder (tazarotene induced) 1 (RARRES1), transcript variant 2, mRNA [NM_002888]	NM_002888

A_24_P23411	1.05E-02	2.804	NM_016607	NM_016607	Homo sapiens armadillo repeat containing, X-linked 3 (ARMCX3), transcript variant 1, mRNA [NM_016607]	NM_016607
A_23_P108522	1.05E-02	2.762	BC064950	BC064950	Homo sapiens chromosome 2 open reading frame 17, mRNA (cDNA clone IMAGE:6048607), partial cds. [BC064950]	
A_23_P382148	1.05E-02	2.71	NM_004161	NM_004161	Homo sapiens RAB1A, member RAS oncogene family (RAB1A), mRNA [NM_004161]	NM_004161
A_23_P99540	1.05E-02	2.666	NM_004926	NM_004926	Homo sapiens zinc finger protein 36, C3H type-like 1 (ZFP36L1), mRNA [NM_004926]	NM_004926
A_32_P190097	1.05E-02	2.66	AI467970	AI467970	AI467970 tj79c03.x1 Soares_NSF_F8_9W_OT_PA_P_S1 Homo sapiens cDNA clone IMAGE:2147716 3', mRNA sequence [AI467970]	
A_23_P416289	1.05E-02	2.437	NM_033425	NM_033425	Homo sapiens DIX domain containing 1 (DIXDC1), mRNA [NM_033425]	NM_033425
A_24_P118938	1.05E-02	2.353	ENST00000359676		Homo sapiens mRNA; cDNA DKFZp566G1424 (from clone DKFZp566G1424). [AL122043]	
A_23_P98786	1.05E-02	2.314	NM_000260	NM_000260	Homo sapiens myosin VIIA (Usher syndrome 1B (autosomal recessive, severe)) (MYO7A), mRNA [NM_000260]	NM_000260
A_24_P123720	1.05E-02	2.275	A_24_P123720			
A_23_P200928	1.05E-02	2.019	NM_002508	NM_002508	Homo sapiens nidogen 1 (NID1), mRNA [NM_002508]	NM_002508
A_24_P370702	1.06E-02	20.09	NM_018284	NM_018284	Homo sapiens guanylate binding protein 3 (GBP3), mRNA [NM_018284]	NM_018284
A_24_P168398	1.06E-02	2.921	NM_003451	NM_003451	Homo sapiens zinc finger protein 177 (ZNF177), mRNA [NM_003451]	NM_003451
A_23_P502170	1.06E-02	2.403	NM_015522	NM_015522	Homo sapiens dynein 2 light intermediate chain (D2LIC), transcript variant 2, mRNA [NM_015522]	NM_015522
A_32_P25050	1.06E-02	2.315	NM_172037	NM_172037	Homo sapiens retinol dehydrogenase 10 (all-trans) (RDH10), mRNA [NM_172037]	NM_172037
A_23_P132115	1.06E-02	2.263	NM_173354	NM_173354	Homo sapiens SNF1-like kinase (SNF1LK), mRNA [NM_173354]	NM_173354
A_24_P935491	1.07E-02	454.6	NM_000090	NM_000090	Homo sapiens collagen, type III, alpha 1 (Ehlers-Danlos syndrome type IV, autosomal dominant) (COL3A1), mRNA [NM_000090]	NM_000090
A_24_P349039	1.07E-02	2.922	NM_020754	NM_020754	Homo sapiens Cdc42 GTPase-activating protein (CDGAP), mRNA [NM_020754]	NM_020754
A_23_P7791	1.07E-02	2.914	NM_024576	NM_024576	Homo sapiens opioid growth factor receptor-like 1 (OGFRL1), mRNA [NM_024576]	NM_024576
A_32_P94722	1.07E-02	2.679	AK126242	AK126242	Homo sapiens cDNA FLJ44254 fis, clone TKIDN2009641. [AK126242]	
A_32_P60065	1.08E-02	57.61	NM_004101	NM_004101	Homo sapiens coagulation factor II (thrombin) receptor-like 2 (F2RL2), mRNA [NM_004101]	NM_004101
A_23_P39766	1.08E-02	9.458	NM_014905	NM_014905	Homo sapiens glutaminase (GLS), mRNA [NM_014905]	NM_014905
A_23_P393856	1.08E-02	5.447	AK021929	AK021929	Homo sapiens cDNA FLJ11867 fis, clone HEMBA1006976, weakly similar to H.sapiens mRNA for Gal-beta(1-3/1-4)GlcNAc alpha-2.3-sialyltransferase. [AK021929]	
A_23_P109143	1.08E-02	3.488	NM_000311	NM_000311	Homo sapiens prion protein (p27-30) (Creutzfeld-Jakob disease, Gerstmann-Strausler-Scheinker syndrome, fatal familial insomnia) (PRNP), transcript variant 1, mRNA [NM_000311]	NM_000311
A_23_P134925	1.08E-02	3.311	NM_004331	NM_004331	Homo sapiens BCL2/adenovirus E1B 19kDa interacting protein 3-like (BNIP3L), mRNA [NM_004331]	NM_004331
A_32_P108826	1.08E-02	2.299	NM_194314	NM_194314	Homo sapiens zinc finger and BTB domain containing 41 (ZBTB41), mRNA [NM_194314]	NM_194314
A_23_P83414	1.08E-02	2.133	NM_002709	NM_002709	Homo sapiens protein phosphatase 1, catalytic subunit, beta isoform (PPP1CB), transcript variant 1, mRNA [NM_002709]	NM_002709
A_23_P23947	1.08E-02	2.116	NM_005204	NM_005204	Homo sapiens mitogen-activated protein kinase kinase kinase 8 (MAP3K8), mRNA [NM_005204]	NM_005204
A_23_P392575	1.09E-02	9.048	NM_080671	NM_080671	Homo sapiens potassium voltage-gated channel, Isk-related family, member 4 (KCNE4), mRNA [NM_080671]	NM_080671
A_23_P16523	1.09E-02	6.212	NM_004864	NM_004864	Homo sapiens growth differentiation factor 15 (GDF15), mRNA [NM_004864]	NM_004864
A_23_P431638	1.09E-02	4.002	BC029662	BC029662	Homo sapiens chromosome 20 open reading frame 142, mRNA (cDNA clone IMAGE:4933017), with apparent retained intron. [BC029662]	
A_32_P50066	1.09E-02	3.336	CR749274	CR749274	Homo sapiens mRNA; cDNA DKFZp781M044 (from clone DKFZp781M044). [CR749274]	
A_23_P392541	1.09E-02	3.287	NM_015087	NM_015087	Homo sapiens spastic paraplegia 20, spartin (Troyer syndrome) (SPG20), mRNA [NM_015087]	NM_015087
A_24_P402779	1.09E-02	2.945	NM_001003935	NM_001003935	Homo sapiens poly (ADP-ribose) polymerase family, member 3 (PARP3), transcript variant 3, mRNA [NM_001003935]	NM_001003935
A_23_P58912	1.09E-02	2.71	NM_006416	NM_006416	Homo sapiens solute carrier family 35 (CMP-sialic acid transporter), member A1 (SLC35A1), mRNA [NM_006416]	NM_006416
A_23_P433753	1.09E-02	2.668	NM_212472	NM_212472	Homo sapiens protein kinase, cAMP-dependent, regulatory, type I, alpha (tissue specific extinguisher 1) (PRKAR1A), transcript variant 3, mRNA [NM_212472]	NM_212472
A_24_P256583	1.09E-02	2.474	NM_194291	NM_194291	Homo sapiens hypothetical protein BC017881 (LOC157378), mRNA [NM_194291]	NM_194291
A_23_P8561	1.09E-02	2.04	NM_020684	NM_020684	Homo sapiens rhomboid, veinlet-like 7 (Drosophila) (RHBDL7), mRNA [NM_020684]	NM_020684
A_23_P383009	1.10E-02	5.163	NM_000599	NM_000599	Homo sapiens insulin-like growth factor binding protein 5 (IGFBP5), mRNA [NM_000599]	NM_000599

A_24_P166663	1.10E-02	3.818	NM_001259	NM_001259	Homo sapiens cyclin-dependent kinase 6 (CDK6), mRNA [NM_001259]	NM_001259
A_23_P168882	1.10E-02	3.263	NM_033285	NM_033285	Homo sapiens tumor protein p53 inducible nuclear protein 1 (TP53INP1), mRNA [NM_033285]	NM_033285
A_23_P307310	1.10E-02	3.057	NM_013227	NM_013227	Homo sapiens aggrecan 1 (chondroitin sulfate proteoglycan 1, large aggregating proteoglycan, antigen identified by monoclonal antibody A0122) (AGC1), transcript variant 2, mRNA [NM_013227]	NM_013227
A_23_P62959	1.10E-02	2.969	NM_012396	NM_012396	Homo sapiens pleckstrin homology-like domain, family A, member 3 (PHLDA3), mRNA [NM_012396]	NM_012396
A_23_P159012	1.10E-02	2.849	NM_023039	NM_023039	Homo sapiens ankyrin repeat, family A (RFXANK-like), 2 (ANKRA2), mRNA [NM_023039]	NM_023039
A_23_P338981	1.10E-02	2.452	NM_134268	NM_134268	Homo sapiens cytoglobin (CYGB), mRNA [NM_134268]	NM_134268
A_24_P551067	1.11E-02	4.572	CR603951	CR603951	full-length cDNA clone CS0DM011YC22 of Fetal liver of Homo sapiens (human). [CR603951]	XM_499585
A_32_P54442	1.11E-02	3.295	NM_019015	NM_019015	Homo sapiens chondroitin sulfate glucuronyltransferase (CSGlcA-T), mRNA [NM_019015]	NM_019015
A_23_P385105	1.11E-02	2.47	NM_032726	NM_032726	Homo sapiens phospholipase C, delta 4 (PLCD4), mRNA [NM_032726]	NM_032726
A_23_P21457	1.11E-02	2.273	NM_015938	NM_015938	Homo sapiens NMD3 homolog (S. cerevisiae) (NMD3), mRNA [NM_015938]	NM_015938
A_24_P915806	1.12E-02	3.618	NM_001024074	NM_001024074	Homo sapiens histamine N-methyltransferase (HNMT), transcript variant 2, mRNA [NM_001024074]	NM_001024074
A_24_P374834	1.12E-02	2.953	AB188491	AB188491	Homo sapiens mRNA, clone: TH020D07. [AB188491]	XM_166659
A_23_P399797	1.12E-02	2.368	AF071111	AF071111	Homo sapiens DAMS mRNA, complete cds. [AF071111]	
A_24_P521994	1.12E-02	2.279	NM_017644	NM_017644	Homo sapiens DRE1 protein (DRE1), mRNA [NM_017644]	NM_017644
A_23_P106145	1.12E-02	2.222	NM_014584	NM_014584	Homo sapiens ERO1-like (S. cerevisiae) (ERO1L), mRNA [NM_014584]	NM_014584
A_32_P32722	1.12E-02	2.103	CR603845	CR603845	full-length cDNA clone CS0DI026YH22 of Placenta Cot 25-normalized of Homo sapiens (human). [CR603845]	
A_23_P202939	1.12E-02	2.014	NM_001642	NM_001642	Homo sapiens amyloid beta (A4) precursor-like protein 2 (APLP2), mRNA [NM_001642]	NM_001642
A_24_P484894	1.13E-02	4.443	BC045718	BC045718	Homo sapiens, clone IMAGE:4797078, mRNA. [BC045718]	
A_24_P662177	1.13E-02	3.93	THC2448843		O57150 (O57150) H88, partial (32%) [THC2448843]	
A_23_P209527	1.13E-02	3.434	THC2310298		A31642 villin [validated] - human {Homo sapiens;} , partial (23%) [THC2310298]	
A_23_P359630	1.13E-02	3.051	NM_002665	NM_002665	Homo sapiens plasminogen-like B1 (PLGLB1), mRNA [NM_002665]	NM_002665
A_23_P7706	1.13E-02	2.75	NM_198321	NM_198321	Homo sapiens UDP-N-acetyl-alpha-D-galactosamine:polypeptide N-acetylgalactosaminyltransferase 10 (GalNAc-T10) (GALNT10), transcript variant 1, mRNA [NM_198321]	NM_198321
A_24_P497186	1.13E-02	2.593	BC020516	BC020516	Homo sapiens interferon regulatory factor 2 binding protein 2, mRNA (cDNA clone IMAGE:3882977), partial cds. [BC020516]	
A_32_P60585	1.13E-02	2.116	AK002023	AK002023	Homo sapiens cDNA FLJ11161 fis, clone PLACE1007021. [AK002023]	
A_23_P8640	1.14E-02	20.02	NM_001505	NM_001505	Homo sapiens G protein-coupled receptor 30 (GPR30), mRNA [NM_001505]	NM_001505
A_23_P86283	1.14E-02	9.323	NM_006762	NM_006762	Homo sapiens lysosomal associated multispanning membrane protein 5 (LAPTM5), mRNA [NM_006762]	NM_006762
A_24_P333421	1.14E-02	7.047	AB011115	AB011115	Homo sapiens mRNA for KIAA0543 protein, partial cds. [AB011115]	XM_376720
A_23_P335661	1.14E-02	3.383	AB028976	AB028976	Homo sapiens mRNA for KIAA1053 protein, partial cds. [AB028976]	
A_23_P216708	1.14E-02	3.126	NM_018339	NM_018339	Homo sapiens riboflavin kinase (RFK), mRNA [NM_018339]	NM_018339
A_23_P452	1.14E-02	2.754	NM_001014796	NM_001014796	Homo sapiens discoidin domain receptor family, member 2 (DDR2), transcript variant 1, mRNA [NM_001014796]	NM_001014796
A_23_P329271	1.14E-02	2.615	NM_002386	NM_002386	Homo sapiens melanocortin 1 receptor (alpha melanocyte stimulating hormone receptor) (MC1R), mRNA [NM_002386]	NM_002386
A_24_P125273	1.14E-02	2.604	NM_001661	NM_001661	Homo sapiens ADP-ribosylation factor 4-like (ARF4L), mRNA [NM_001661]	NM_001661
A_23_P157809	1.14E-02	2.406	NM_012212	NM_012212	Homo sapiens leukotriene B4 12-hydroxydehydrogenase (LTB4DH), mRNA [NM_012212]	NM_012212
A_23_P322562	1.14E-02	2.331	NM_004210	NM_004210	Homo sapiens neuralized-like (Drosophila) (NEURL), mRNA [NM_004210]	NM_004210
A_24_P178834	1.14E-02	2.167	AK091178	AK091178	Homo sapiens cDNA FLJ33859 fis, clone CTONG2006223, moderately similar to KERATIN, TYPE II CYTOSKELETAL 8. [AK091178]	XM_496418
A_23_P404481	1.15E-02	8.444	NM_001400	NM_001400	Homo sapiens endothelial differentiation, sphingolipid G-protein-coupled receptor, 1 (EDG1), mRNA [NM_001400]	NM_001400
A_32_P92445	1.15E-02	4.84	AK092715	AK092715	Homo sapiens cDNA FLJ35396 fis, clone SKNSH2003483. [AK092715]	
A_24_P218905	1.15E-02	3.819	NM_006675	NM_006675	Homo sapiens tetraspanin 9 (TSPAN9), mRNA [NM_006675]	NM_006675
A_32_P153833	1.15E-02	2.623	AK098597	AK098597	Homo sapiens cDNA FLJ25731 fis, clone TST05584. [AK098597]	

A_23_P167983	1.15E-02	2.575	ENST00000314088		Homo sapiens histone 1, H2ac, mRNA (cDNA clone MGC:1730 IMAGE:2988620), complete cds. [BC017379]	
A_24_P942648	1.15E-02	2.423	AL133642	AL133642	Homo sapiens mRNA; cDNA DKFZp586G1721 (from clone DKFZp586G1721). [AL133642]	
A_23_P51339	1.15E-02	2.293	NM_007034	NM_007034	Homo sapiens DnaJ (Hsp40) homolog, subfamily B, member 4 (DNAJB4), mRNA [NM_007034]	NM_007034
A_23_P168909	1.16E-02	61.94	NM_012082	NM_012082	Homo sapiens zinc finger protein, multitype 2 (ZFPM2), mRNA [NM_012082]	NM_012082
A_24_P551302	1.16E-02	3.178	BC038432	BC038432	Homo sapiens cDNA clone IMAGE:5162874, partial cds. [BC038432]	
A_23_P205499	1.16E-02	3.031	NM_014045	NM_014045	Homo sapiens low density lipoprotein receptor-related protein 10 (LRP10), mRNA [NM_014045]	NM_014045
A_24_P318897	1.16E-02	2.875	NM_033421	NM_033421	Homo sapiens chromosome 20 open reading frame 161 (C20orf161), transcript variant 1, mRNA [NM_033421]	NM_033421
A_23_P124962	1.16E-02	2.667	NM_152832	NM_152832	Homo sapiens Mouse Mammary Turmor Virus Receptor homolog 1 (MTVR1), mRNA [NM_152832]	NM_152832
A_32_P50984	1.16E-02	2.521	THC2378893			
A_32_P48526	1.16E-02	2.165	THC2341087			
A_23_P215790	1.16E-02	2.071	NM_005228	NM_005228	Homo sapiens epidermal growth factor receptor (erythroblastic leukemia viral (v-erb-b) oncogene homolog, avian) (EGFR), transcript variant 1, mRNA [NM_005228]	NM_005228
A_32_P86318	1.16E-02	2.051	NM_198490	NM_198490	Homo sapiens RAB43, member RAS oncogene family (RAB43), mRNA [NM_198490]	NM_198490
A_32_P218766	1.17E-02	2.407	BC027471	BC027471	Homo sapiens LOC440173, mRNA (cDNA clone MGC:34689 IMAGE:4995424), complete cds. [BC027471]	
A_23_P114883	1.18E-02	50.12	NM_002023	NM_002023	Homo sapiens fibromodulin (FMOD), mRNA [NM_002023]	NM_002023
A_23_P38167	1.18E-02	10.13	NM_022036	NM_022036	Homo sapiens G protein-coupled receptor, family C, group 5, member C (GPRC5C), transcript variant 1, mRNA [NM_022036]	NM_022036
A_23_P133359	1.18E-02	3.458	NM_030613	NM_030613	Homo sapiens zinc finger protein 2 homolog (mouse) (ZFP2), mRNA [NM_030613]	NM_030613
A_24_P376139	1.18E-02	2.948	AK057798	AK057798	Homo sapiens cDNA FLJ25069 fis, clone CBL05145. [AK057798]	
A_24_P143171	1.18E-02	2.535	NM_031442	NM_031442	Homo sapiens transmembrane protein 47 (TMEM47), mRNA [NM_031442]	NM_031442
A_23_P361448	1.18E-02	2.481	NM_144665	NM_144665	Homo sapiens sestrin 3 (SESN3), mRNA [NM_144665]	NM_144665
A_23_P201264	1.18E-02	2.37	AF088049	AF088049	Homo sapiens full length insert cDNA clone ZD62D09. [AF088049]	
A_23_P250002	1.18E-02	2.146	NM_020771	NM_020771	Homo sapiens HECT domain and ankyrin repeat containing, E3 ubiquitin protein ligase 1 (HACE1), mRNA [NM_020771]	NM_020771
A_24_P823514	1.19E-02	5.967	AK092875	AK092875	Homo sapiens cDNA FLJ35556 fis, clone SPLEN2004844. [AK092875]	
A_23_P382081	1.19E-02	4.417	NM_144722	NM_144722	Homo sapiens KPL2 protein (FLJ23577), transcript variant 2, mRNA [NM_144722]	NM_144722
A_24_P156993	1.19E-02	3.666	NM_139346	NM_139346	Homo sapiens bridging integrator 1 (BIN1), transcript variant 4, mRNA [NM_139346]	NM_139346
A_23_P368718	1.19E-02	2.251	NM_020456	NM_020456	Homo sapiens chromosome 13 open reading frame 1 (C13orf1), mRNA [NM_020456]	NM_020456
A_32_P196837	1.20E-02	8.032	AA449494	AA449494	AA449494 zx08h11.s1 Soares_total_fetus_Nb2HF8_9w Homo sapiens cDNA clone IMAGE:785925 3' similar to gb:U15981 ADRENAL SPECIFIC 30 KD PROTEIN (HUMAN);, mRNA sequence [AA449494]	
A_23_P110941	1.20E-02	3.347	NM_001512	NM_001512	Homo sapiens glutathione S-transferase A4 (GSTA4), mRNA [NM_001512]	NM_001512
A_24_P313109	1.20E-02	2.79	NM_016617	NM_016617	Homo sapiens ubiquitin-fold modifier 1 (UFM1), mRNA [NM_016617]	NM_016617
A_24_P928510	1.20E-02	2.771	AF075119	AF075119	Homo sapiens full length insert cDNA ZD26E01. [AF075119]	
A_23_P254079	1.20E-02	2.347	NM_003943	NM_003943	Homo sapiens genethonin 1 (GENX-3414), mRNA [NM_003943]	NM_003943
A_32_P101860	1.20E-02	2.116	A_32_P101860			
A_32_P161681	1.20E-02	2.087	NM_001008528	NM_001008528	Homo sapiens matrix-remodelling associated 7 (MXRA7), transcript variant 1, mRNA [NM_001008528]	NM_001008528
A_24_P347418	1.20E-02	2.084	THC2277808		AE001161 B. burgdorferi predicted coding region BB0600 {Borrelia burgdorferi B31;} , partial (4%) [THC2277808]	
A_23_P52207	1.21E-02	14.63	NM_012342	NM_012342	Homo sapiens BMP and activin membrane-bound inhibitor homolog (Xenopus laevis) (BAMBI), mRNA [NM_012342]	NM_012342
A_24_P918044	1.21E-02	11.17	AK023572	AK023572	Homo sapiens cDNA FLJ13510 fis, clone PLACE1005146. [AK023572]	
A_24_P270033	1.21E-02	6.556	ENST00000278949		Homo sapiens cDNA FLJ38080 fis, clone CTONG2016185. [AK095399]	
A_24_P932680	1.21E-02	3.591	AK093521	AK093521	Homo sapiens cDNA FLJ36202 fis, clone TESTI2028296. [AK093521]	
A_23_P207058	1.21E-02	3.539	NM_003955	NM_003955	Homo sapiens suppressor of cytokine signaling 3 (SOCS3), mRNA [NM_003955]	NM_003955
A_23_P91350	1.21E-02	3.36	ENST00000265183		Homo sapiens mRNA for KIAA1434 protein, partial cds. [AB037855]	
A_32_P491904	1.21E-02	2.399	AK091057	AK091057	Homo sapiens cDNA FLJ33738 fis, clone BRAWH2018527. [AK091057]	

A_23_P23234	1.21E-02	2.219	BC000988	BC000988	Homo sapiens hypothetical protein MGC5457, mRNA (cDNA clone MGC:5457 IMAGE:3450898), complete cds. [BC000988]	
A_23_P140907	1.21E-02	2.211	NM_021259	NM_021259	Homo sapiens transmembrane protein 8 (five membrane-spanning domains) (TMEM8), mRNA [NM_021259]	NM_021259
A_32_P319200	1.21E-02	2.171	NM_199127	NM_199127	Homo sapiens gamma-glutamyltransferase-like 4 (GGTL4), transcript variant 1, mRNA [NM_199127]	NM_199127
A_23_P11192	1.21E-02	2.028	NM_006357	NM_006357	Homo sapiens ubiquitin-conjugating enzyme E2E 3 (UBC4/5 homolog, yeast) (UBE2E3), transcript variant 1, mRNA [NM_006357]	NM_006357
A_23_P167096	1.22E-02	11.8	NM_005429	NM_005429	Homo sapiens vascular endothelial growth factor C (VEGFC), mRNA [NM_005429]	NM_005429
A_24_P162319	1.22E-02	7.12	NM_006746	NM_006746	Homo sapiens sex comb on midleg-like 1 (Drosophila) (SCML1), mRNA [NM_006746]	NM_006746
A_24_P367602	1.22E-02	2.842	AK092260	AK092260	Homo sapiens cDNA FLJ34941 fis, clone NT2RP7007480. [AK092260]	
A_23_P145957	1.22E-02	2.611	NM_022445	NM_022445	Homo sapiens thiamin pyrophosphokinase 1 (TPK1), mRNA [NM_022445]	NM_022445
A_23_P73660	1.22E-02	2.479	NM_001011658	NM_001011658	Homo sapiens trafficking protein particle complex 2 (TRAPPC2), transcript variant 1, mRNA [NM_001011658]	NM_001011658
A_23_P76480	1.23E-02	19.94	BF213738	BF213738	BF213738 601847628F1 NIH_MGC_55 Homo sapiens cDNA clone IMAGE:4078519 5', mRNA sequence [BF213738]	
A_23_P88602	1.23E-02	15.85	NM_170677	NM_170677	Homo sapiens Meis1, myeloid ecotropic viral integration site 1 homolog 2 (mouse) (MEIS2), transcript variant a, mRNA [NM_170677]	NM_170677
A_23_P164451	1.23E-02	9.793	NM_005994	NM_005994	Homo sapiens T-box 2 (TBX2), mRNA [NM_005994]	NM_005994
A_32_P32413	1.23E-02	3.643	AK123972	AK123972	Homo sapiens cDNA FLJ41978 fis, clone SKNSH2000482. [AK123972]	
A_24_P270357	1.23E-02	3.3	NM_006493	NM_006493	Homo sapiens ceroid-lipofuscinosis, neuronal 5 (CLN5), mRNA [NM_006493]	NM_006493
A_23_P150207	1.23E-02	2.202	NM_004322	NM_004322	Homo sapiens BCL2-antagonist of cell death (BAD), transcript variant 1, mRNA [NM_004322]	NM_004322
A_23_P53257	1.23E-02	2.143	BX647344	BX647344	Homo sapiens mRNA; cDNA DKFZp779O1812 (from clone DKFZp779O1812). [BX647344]	
A_32_P47874	1.24E-02	6.532	THC2440787			
A_32_P179859	1.24E-02	4.689	BC034428	BC034428	Homo sapiens, clone IMAGE:4821804, mRNA, partial cds. [BC034428]	
A_23_P428139	1.24E-02	3.475	NM_004093	NM_004093	Homo sapiens ephrin-B2 (EFNB2), mRNA [NM_004093]	NM_004093
A_23_P82929	1.24E-02	3.456	NM_002514	NM_002514	Homo sapiens nephroblastoma overexpressed gene (NOV), mRNA [NM_002514]	NM_002514
A_23_P116512	1.24E-02	3.313	NM_024841	NM_024841	Homo sapiens hypothetical protein FLJ14213 (FLJ14213), mRNA [NM_024841]	NM_024841
A_23_P370544	1.24E-02	3.04	AJ549812	AJ549812	Homo sapiens mRNA for hypothetical protein (FL25415 gene). [AJ549812]	XM_371586
A_23_P10591	1.24E-02	2.323	NM_001004431	NM_001004431	Homo sapiens meteorin, glial cell differentiation regulator-like (METRNL), mRNA [NM_001004431]	NM_001004431
A_24_P245838	1.25E-02	7.887	AK125361	AK125361	Homo sapiens cDNA FLJ43371 fis, clone NTONG2005969. [AK125361]	
A_23_P136347	1.25E-02	6.821	NM_004447	NM_004447	Homo sapiens epidermal growth factor receptor pathway substrate 8 (EPS8), mRNA [NM_004447]	NM_004447
A_23_P125643	1.25E-02	4.654	NM_024087	NM_024087	Homo sapiens ankyrin repeat and SOCS box-containing 9 (ASB9), mRNA [NM_024087]	NM_024087
A_23_P45999	1.25E-02	3.145	NM_012168	NM_012168	Homo sapiens F-box protein 2 (FBXO2), mRNA [NM_012168]	NM_012168
A_23_P386320	1.25E-02	2.933	NM_033316	NM_033316	Homo sapiens antigen p97 (melanoma associated) identified by monoclonal antibodies 133.2 and 96.5 (MFI2), transcript variant 2, mRNA [NM_033316]	NM_033316
A_24_P37903	1.26E-02	113.9	THC2371798			
A_23_P500998	1.26E-02	21.76	NM_152739	NM_152739	Homo sapiens homeo box A9 (HOXA9), transcript variant 1, mRNA [NM_152739]	NM_152739
A_24_P77904	1.26E-02	11.47	NM_018951	NM_018951	Homo sapiens homeo box A10 (HOXA10), transcript variant 1, mRNA [NM_018951]	NM_018951
A_24_P511686	1.26E-02	5.792	CR616845	CR616845	full-length cDNA clone CS0DF020YJ04 of Fetal brain of Homo sapiens (human). [CR616845]	XM_378309
A_23_P90311	1.26E-02	5.363	NM_182919	NM_182919	Homo sapiens toll-like receptor adaptor molecule 1 (TICAM1), transcript variant 2, mRNA [NM_182919]	NM_182919
A_23_P363896	1.26E-02	2.818	NM_152350	NM_152350	Homo sapiens hypothetical protein MGC40157 (MGC40157), mRNA [NM_152350]	NM_152350
A_23_P44244	1.26E-02	2.4	NM_003069	NM_003069	Homo sapiens SWI/SNF related, matrix associated, actin dependent regulator of chromatin, subfamily a, member 1 (SMARCA1), transcript variant 1, mRNA [NM_003069]	NM_003069
A_32_P212024	1.26E-02	2.039	THC2365796			
A_23_P211926	1.27E-02	9.251	NM_003392	NM_003392	Homo sapiens wingless-type MMTV integration site family, member 5A (WNT5A), mRNA [NM_003392]	NM_003392
A_23_P100730	1.27E-02	3.271	NM_003726	NM_003726	Homo sapiens src family associated phosphoprotein 1 (SCAP1), mRNA [NM_003726]	NM_003726
A_24_P220947	1.27E-02	2.485	NM_001353	NM_001353	Homo sapiens aldo-keto reductase family 1, member C1 (dihydrodiol dehydrogenase 1; 20-alpha (3-alpha)-hydroxysteroid dehydrogenase) (AKR1C1), mRNA [NM_001353]	NM_001353

A_23_P211247	1.27E-02	2.389	NM_206962	NM_206962	Homo sapiens HMT1 hnRNP methyltransferase-like 1 (S. cerevisiae) (HRMT1L1), transcript variant 1, mRNA [NM_206962]	NM_206962
A_32_P164061	1.27E-02	2.239	A_32_P164061			
A_23_P372974	1.27E-02	2.119	NM_152402	NM_152402	Homo sapiens translocation associated membrane protein 1-like 1 (TRAM1L1), mRNA [NM_152402]	NM_152402
A_24_P325992	1.28E-02	11.27	NM_002310	NM_002310	Homo sapiens leukemia inhibitory factor receptor (LIFR), mRNA [NM_002310]	NM_002310
A_23_P153146	1.28E-02	4.503	NM_004361	NM_004361	Homo sapiens cadherin 7, type 2 (CDH7), transcript variant b, mRNA [NM_004361]	NM_004361
A_23_P79510	1.28E-02	3.58	NM_016079	NM_016079	Homo sapiens vacuolar protein sorting 24 (yeast) (VPS24), transcript variant 1, mRNA [NM_016079]	NM_016079
A_23_P37391	1.28E-02	3	AK093969	AK093969	Homo sapiens cDNA FLJ36650 fis, clone UTERU2000023. [AK093969]	
A_23_P54144	1.29E-02	6.291	NM_001202	NM_001202	Homo sapiens bone morphogenetic protein 4 (BMP4), transcript variant 1, mRNA [NM_001202]	NM_001202
A_32_P12430	1.29E-02	3.268	NM_015087	NM_015087	Homo sapiens spastic paraplegia 20, spartin (Troyer syndrome) (SPG20), mRNA [NM_015087]	NM_015087
A_23_P207493	1.29E-02	2.907	NM_016424	NM_016424	Homo sapiens cisplatin resistance-associated overexpressed protein (CROP), transcript variant 1, mRNA [NM_016424]	NM_016424
A_23_P74088	1.29E-02	2.894	NM_006983	NM_006983	Homo sapiens matrix metalloproteinase 23B (MMP23B), mRNA [NM_006983]	NM_006983
A_24_P251351	1.29E-02	2.872	NM_004161	NM_004161	Homo sapiens RAB1A, member RAS oncogene family (RAB1A), mRNA [NM_004161]	NM_004161
A_24_P186401	1.29E-02	2.251	BC022254	BC022254	Homo sapiens DNA cross-link repair 1C (PSO2 homolog, S. cerevisiae), mRNA (cDNA clone MGC:22216 IMAGE:4272155), complete cds. [BC022254]	
A_32_P7308	1.29E-02	2.233	THC2407640			
A_24_P373562	1.29E-02	2.051	NM_018404	NM_018404	Homo sapiens centaurin, alpha 2 (CENTA2), mRNA [NM_018404]	NM_018404
A_23_P99063	1.30E-02	640.7	NM_002345	NM_002345	Homo sapiens lumican (LUM), mRNA [NM_002345]	NM_002345
A_23_P124084	1.30E-02	7.236	NM_005576	NM_005576	Homo sapiens lysyl oxidase-like 1 (LOXL1), mRNA [NM_005576]	NM_005576
A_24_P236799	1.30E-02	3.632	NM_006868	NM_006868	Homo sapiens RAB31, member RAS oncogene family (RAB31), mRNA [NM_006868]	NM_006868
A_23_P415401	1.30E-02	3.492	NM_001206	NM_001206	Homo sapiens Kruppel-like factor 9 (KLF9), mRNA [NM_001206]	NM_001206
A_24_P79403	1.30E-02	3.017	NM_002619	NM_002619	Homo sapiens platelet factor 4 (chemokine (C-X-C motif) ligand 4) (PF4), mRNA [NM_002619]	NM_002619
A_23_P54447	1.30E-02	2.458	NM_030944	NM_030944	Homo sapiens chromosome 15 open reading frame 5 (C15orf5), mRNA [NM_030944]	NM_030944
A_32_P42253	1.30E-02	2.32	THC2356023			
A_24_P405981	1.30E-02	2.229	CR598481	CR598481	full-length cDNA clone CS0DD001YH15 of Neuroblastoma Cot 50-normalized of Homo sapiens (human). [CR598481]	
A_23_P129801	1.31E-02	3.824	NM_006822	NM_006822	Homo sapiens RAB40B, member RAS oncogene family (RAB40B), mRNA [NM_006822]	NM_006822
A_32_P128023	1.31E-02	3.457	AK125351	AK125351	Homo sapiens cDNA FLJ43361 fis, clone NT2RP7015512. [AK125351]	
A_24_P630640	1.31E-02	2.549	AK126509	AK126509	Homo sapiens cDNA FLJ44545 fis, clone UTERU3005970. [AK126509]	
A_24_P155196	1.31E-02	2.264	THC2315644		BC036504 C9orf96 protein {Homo sapiens;} , partial (28%) [THC2315644]	
A_23_P256473	1.32E-02	15.14	NM_006379	NM_006379	Homo sapiens sema domain, immunoglobulin domain (Ig), short basic domain, secreted, (semaphorin) 3C (SEMA3C), mRNA [NM_006379]	NM_006379
A_24_P896205	1.32E-02	6.514	XM_498676	XM_498676	PREDICTED: Homo sapiens LOC440450 (LOC440450), mRNA [XM_498676]	XM_498676
A_23_P30614	1.32E-02	5.806	NM_002667	NM_002667	Homo sapiens phospholamban (PLN), mRNA [NM_002667]	NM_002667
A_23_P53588	1.32E-02	4.987	NM_030775	NM_030775	Homo sapiens wingless-type MMTV integration site family, member 5B (WNT5B), transcript variant 2, mRNA [NM_030775]	NM_030775
A_24_P895528	1.32E-02	3.6	AK022035	AK022035	Homo sapiens cDNA FLJ11973 fis, clone HEMBB1001221. [AK022035]	
A_23_P35791	1.32E-02	2.912	NM_003942	NM_003942	Homo sapiens ribosomal protein S6 kinase, 90kDa, polypeptide 4 (RPS6KA4), transcript variant 1, mRNA [NM_003942]	NM_003942
A_23_P303810	1.32E-02	2.31	AK098569	AK098569	Homo sapiens cDNA FLJ25703 fis, clone TST04744. [AK098569]	XM_376254
A_32_P235796	1.32E-02	2.126	NM_152618	NM_152618	Homo sapiens hypothetical protein FLJ35630 (FLJ35630), mRNA [NM_152618]	NM_152618
A_24_P775249	1.32E-02	2.121	NM_001008234	NM_001008234	Homo sapiens hypothetical gene supported by BC036588 (LOC400657), mRNA [NM_001008234]	NM_001008234
A_32_P235159	1.32E-02	2.083	CR623684	CR623684	full-length cDNA clone CS0DK004YM05 of HeLa cells Cot 25-normalized of Homo sapiens (human). [CR623684]	XM_087225
A_23_P169039	1.33E-02	30.47	NM_003068	NM_003068	Homo sapiens snail homolog 2 (Drosophila) (SNAI2), mRNA [NM_003068]	NM_003068
A_23_P15876	1.33E-02	13.99	NM_052947	NM_052947	Homo sapiens heart alpha-kinase (HAK), mRNA [NM_052947]	NM_052947
A_24_P68079	1.33E-02	4.43	ENST00000301807		Homo sapiens mRNA for KIAA0342 protein, partial cds. [AB002340]	XM_047357

A_32_P226356	1.33E-02	4.004	THC2317058			
A_24_P204244	1.33E-02	2.833	NR_001562	NR_001562	Homo sapiens annexin A2 pseudogene 1 (ANXA2P1) on chromosome 4 [NR_001562]	NR_001562
A_23_P142294	1.33E-02	2.42	NM_014297	NM_014297	Homo sapiens ethylmalonic encephalopathy 1 (ETHE1), mRNA [NM_014297]	NM_014297
A_23_P213562	1.33E-02	2.212	NM_001992	NM_001992	Homo sapiens coagulation factor II (thrombin) receptor (F2R), mRNA [NM_001992]	NM_001992
A_23_P210158	1.34E-02	4.85	THC2278254		Q9VK63 (Q9VK63) CG5776-PA (LD25466p), partial (3%) [THC2278254]	
A_24_P945059	1.34E-02	3.048	BC022556	BC022556	Homo sapiens myc target 1, mRNA (cDNA clone MGC:26933 IMAGE:4793564), complete cds. [BC022556]	
A_24_P208045	1.34E-02	2.589	NM_025191	NM_025191	Homo sapiens chromosome 1 open reading frame 22 (C1orf22), mRNA [NM_025191]	NM_025191
A_24_P98251	1.34E-02	2.139	NM_017890	NM_017890	Homo sapiens vacuolar protein sorting 13B (yeast) (VPS13B), transcript variant 5, mRNA [NM_017890]	NM_017890
A_32_P140489	1.35E-02	28.15	NM_001001557	NM_001001557	Homo sapiens growth differentiation factor 6 (GDF6), mRNA [NM_001001557]	NM_001001557
A_32_P139708	1.35E-02	20.43	THC2290220			
A_32_P214011	1.35E-02	15.02	THC2277187		Q6PKF4 (Q6PKF4) C20orf77 protein (Fragment), partial (5%) [THC2277187]	
A_23_P14083	1.35E-02	6.696	NM_181847	NM_181847	Homo sapiens adhesion molecule with Ig-like domain 2 (AMIGO2), mRNA [NM_181847]	NM_181847
A_24_P216654	1.35E-02	3.051	NM_003101	NM_003101	Homo sapiens sterol O-acyltransferase (acyl-Coenzyme A: cholesterol acyltransferase) 1 (SOAT1), transcript variant 688113, mRNA [NM_003101]	NM_003101
A_23_P301984	1.35E-02	2.71	NM_001007090	NM_001007090	Homo sapiens hypothetical protein FLJ25402 (FLJ25402), mRNA [NM_001007090]	NM_001007090
A_23_P32444	1.36E-02	10.17	NM_032348	NM_032348	Homo sapiens matrix-remodelling associated 8 (MXRA8), mRNA [NM_032348]	NM_032348
A_24_P845631	1.36E-02	7.1	A_24_P845631			
A_23_P90333	1.36E-02	6.01	XM_292765	XM_292765	PREDICTED: Homo sapiens zinc finger protein 404 (ZNF404), mRNA [XM_292765]	XM_292765
A_24_P243749	1.36E-02	5.914	NM_002612	NM_002612	Homo sapiens pyruvate dehydrogenase kinase, isoenzyme 4 (PDK4), mRNA [NM_002612]	NM_002612
A_23_P306215	1.36E-02	5.769	NM_145175	NM_145175	Homo sapiens family with sequence similarity 84, member A (FAM84A), mRNA [NM_145175]	NM_145175
A_24_P579356	1.36E-02	4.049	NM_001010000	NM_001010000	Homo sapiens Rho GTPase activating protein 28 (ARHGAP28), transcript variant 1, mRNA [NM_001010000]	NM_001010000
A_32_P10311	1.36E-02	3.934	AF305816	AF305816	Homo sapiens PRO0633 mRNA, complete cds. [AF305816]	
A_23_P212688	1.36E-02	3.2	NM_005241	NM_005241	Homo sapiens ecotropic viral integration site 1 (EVI1), mRNA [NM_005241]	NM_005241
A_23_P47077	1.36E-02	2.128	NM_004281	NM_004281	Homo sapiens BCL2-associated athanogene 3 (BAG3), mRNA [NM_004281]	NM_004281
A_24_P184445	1.36E-02	2.046	NM_022791	NM_022791	Homo sapiens matrix metalloproteinase 19 (MMP19), transcript variant rasi-6, mRNA [NM_022791]	NM_022791
A_24_P633543	1.36E-02	2.01	AK127450	AK127450	Homo sapiens cDNA FLJ45542 fis, clone BRTHA2033320. [AK127450]	XM_498467
A_23_P211835	1.38E-02	5.225	D13814	D13814	Homo sapiens mRNA for angiotensin II type 1b receptor, complete cds. [D13814]	
A_24_P118211	1.38E-02	4.868	AF289610	AF289610	Homo sapiens clone pp9372 unknown mRNA. [AF289610]	
A_24_P380061	1.38E-02	4.248	NM_031305	NM_031305	Homo sapiens Rho GTPase activating protein 24 (ARHGAP24), mRNA [NM_031305]	NM_031305
A_24_P217572	1.38E-02	3.538	NM_001957	NM_001957	Homo sapiens endothelin receptor type A (EDNRA), mRNA [NM_001957]	NM_001957
A_23_P258018	1.38E-02	3.211	NM_002477	NM_002477	Homo sapiens myosin, light polypeptide 5, regulatory (MYL5), mRNA [NM_002477]	NM_002477
A_23_P87401	1.38E-02	2.974	NM_030792	NM_030792	Homo sapiens glycerophosphodiester phosphodiesterase domain containing 5 (GDPD5), mRNA [NM_030792]	NM_030792
A_24_P128361	1.39E-02	6.473	AF289562	AF289562	Homo sapiens clone pp6337 unknown mRNA. [AF289562]	
A_24_P10214	1.39E-02	4.696	NM_014178	NM_014178	Homo sapiens syntaxin binding protein 6 (amisyn) (STXBP6), mRNA [NM_014178]	NM_014178
A_24_P8116	1.39E-02	3.441	NM_199511	NM_199511	Homo sapiens steroid sensitive gene 1 (URB), transcript variant 1, mRNA [NM_199511]	NM_199511
A_23_P417383	1.39E-02	2.404	NM_152792	NM_152792	Homo sapiens hypothetical protein FLJ25084 (FLJ25084), mRNA [NM_152792]	NM_152792
A_24_P944390	1.39E-02	2.395	AL050107	AL050107	Homo sapiens mRNA; cDNA DKFZp586I1419 (from clone DKFZp586I1419); partial cds. [AL050107]	
A_23_P2801	1.39E-02	2.281	NM_172373	NM_172373	Homo sapiens E74-like factor 1 (ets domain transcription factor) (ELF1), mRNA [NM_172373]	NM_172373
A_32_P171793	1.39E-02	2.149	THC2317110			
A_24_P263259	1.39E-02	2.109	AK093969	AK093969	Homo sapiens cDNA FLJ36650 fis, clone UTERU2000023. [AK093969]	
A_23_P154919	1.40E-02	5.882	BC005107	BC005107	Homo sapiens chromosome 21 open reading frame 105, mRNA (cDNA clone IMAGE:3840937), partial cds. [BC005107]	
A_23_P336342	1.40E-02	4.005	ENST00000316708		Homo sapiens cDNA FLJ42585 fis, clone BRACE3009237. [AK124576]	
A_24_P280558	1.40E-02	3.818	ENST00000309771		Homo sapiens melanoma-associated antigen mRNA, partial cds. [AF543495]	

A_23_P98455	1.40E-02	3.15	NM_014622	NM_014622	Homo sapiens loss of heterozygosity, 11, chromosomal region 2, gene A (LOH11CR2A), transcript variant 1, mRNA [NM_014622]	NM_014622
A_23_P3911	1.40E-02	2.864	NM_020405	NM_020405	Homo sapiens plexin domain containing 1 (PLXDC1), mRNA [NM_020405]	NM_020405
A_23_P152955	1.40E-02	2.748	NM_017622	NM_017622	Homo sapiens hypothetical protein FLJ20014 (FLJ20014), mRNA [NM_017622]	NM_017622
A_23_P26386	1.40E-02	2.391	NM_016140	NM_016140	Homo sapiens brain specific protein (CGI-38), mRNA [NM_016140]	NM_016140
A_24_P794447	1.41E-02	9.524	AK021552	AK021552	Homo sapiens cDNA FLJ11490 fis, clone HEMBA1001918. [AK021552]	XM_378316
A_23_P107775	1.41E-02	4.466	NM_139172	NM_139172	Homo sapiens MDAC1 (MDAC1), mRNA [NM_139172]	NM_139172
A_23_P211957	1.41E-02	3.153	NM_001024847	NM_001024847	Homo sapiens transforming growth factor, beta receptor II (70/80kDa) (TGFBR2), transcript variant 1, mRNA [NM_001024847]	NM_001024847
A_23_P423864	1.41E-02	2.514	NM_198040	NM_198040	Homo sapiens polyhomeotic-like 2 (Drosophila) (PHC2), transcript variant 1, mRNA [NM_198040]	NM_198040
A_24_P268676	1.41E-02	2.444	NM_003670	NM_003670	Homo sapiens basic helix-loop-helix domain containing, class B, 2 (BHLHB2), mRNA [NM_003670]	NM_003670
A_23_P111360	1.41E-02	2.153	NM_004506	NM_004506	Homo sapiens heat shock transcription factor 2 (HSF2), mRNA [NM_004506]	NM_004506
A_23_P167367	1.42E-02	18.88	NM_153426	NM_153426	Homo sapiens paired-like homeodomain transcription factor 2 (PITX2), transcript variant 2, mRNA [NM_153426]	NM_153426
A_23_P27306	1.42E-02	18.26	NM_030781	NM_030781	Homo sapiens collectin sub-family member 12 (COLEC12), transcript variant II, mRNA [NM_030781]	NM_030781
A_32_P111524	1.42E-02	5.885	BC033539	BC033539	Homo sapiens, clone IMAGE:4819052, mRNA. [BC033539]	
A_32_P77742	1.42E-02	4.487	THC2338229			
A_23_P145074	1.42E-02	4.394	NM_006813	NM_006813	Homo sapiens proline-rich nuclear receptor coactivator 1 (PNRC1), mRNA [NM_006813]	NM_006813
A_32_P220671	1.42E-02	3.709	BE005242	BE005242	BE005242 CM1-BN0116-030400-171-h08 BN0116 Homo sapiens cDNA, mRNA sequence [BE005242]	
A_23_P24784	1.42E-02	3.55	NM_003282	NM_003282	Homo sapiens troponin I, skeletal, fast (TNNI2), mRNA [NM_003282]	NM_003282
A_23_P147826	1.42E-02	3.396	NM_021183	NM_021183	Homo sapiens RAP2C, member of RAS oncogene family (RAP2C), mRNA [NM_021183]	NM_021183
A_23_P2203	1.42E-02	2.695	NM_016281	NM_016281	Homo sapiens TAO kinase 3 (TAOK3), mRNA [NM_016281]	NM_016281
A_23_P102950	1.43E-02	4.598	NM_080860	NM_080860	Homo sapiens testis specific A2 homolog (mouse) (TSGA2), mRNA [NM_080860]	NM_080860
A_24_P515815	1.43E-02	3.14	AI263220	AI263220	AI263220 qz36f10.x1 NCI_CGAP_Kid11 Homo sapiens cDNA clone IMAGE:2029003 3' similar to TR:Q14041 Q14041 COLLAGEN VI ALPHA-1 N-TERMINAL GLOBULAR DOMAIN PRECURSOR ;, mRNA sequence [AI263220]	
A_23_P374339	1.43E-02	2.748	NM_152794	NM_152794	Homo sapiens hypoxia inducible factor 3, alpha subunit (HIF3A), transcript variant 1, mRNA [NM_152794]	NM_152794
A_24_P417189	1.43E-02	2.565	NM_001395	NM_001395	Homo sapiens dual specificity phosphatase 9 (DUSP9), mRNA [NM_001395]	NM_001395
A_23_P97064	1.43E-02	2.503	NM_018438	NM_018438	Homo sapiens F-box protein 6 (FBXO6), mRNA [NM_018438]	NM_018438
A_24_P46334	1.43E-02	2.244	NM_032547	NM_032547	Homo sapiens short coiled-coil protein (SCOC), mRNA [NM_032547]	NM_032547
A_24_P930963	1.43E-02	2.216	CR616309	CR616309	full-length cDNA clone CS0DF015YK23 of Fetal brain of Homo sapiens (human). [CR616309]	
A_24_P75072	1.43E-02	2.077	NM_014311	NM_014311	Homo sapiens single-strand selective monofunctional uracil DNA glycosylase (SMUG1), mRNA [NM_014311]	NM_014311
A_23_P427023	1.44E-02	6.296	NM_130759	NM_130759	Homo sapiens GTPase, IMAP family member 1 (GIMAP1), mRNA [NM_130759]	NM_130759
A_23_P120243	1.44E-02	2.605	NM_024501	NM_024501	Homo sapiens homeo box D1 (HOXD1), mRNA [NM_024501]	NM_024501
A_24_P401392	1.44E-02	2.225	XM_372695	XM_372695	PREDICTED: Homo sapiens similar to Chain A, Crystal Structure Of The R463a Mutant Of Human Glutamate Dehydrogenase (LOC390859), mRNA [XM_372695]	XM_372695
A_24_P52111	1.44E-02	2.085	NM_130781	NM_130781	Homo sapiens RAB24, member RAS oncogene family (RAB24), mRNA [NM_130781]	NM_130781
A_23_P120513	1.45E-02	2.513	NM_080739	NM_080739	Homo sapiens chromosome 20 open reading frame 141 (C20orf141), mRNA [NM_080739]	NM_080739
A_23_P412389	1.45E-02	2.196	NM_033649	NM_033649	Homo sapiens fibroblast growth factor 18 (FGF18), transcript variant 2, mRNA [NM_033649]	NM_033649
A_23_P58082	1.46E-02	25.09	NM_199511	NM_199511	Homo sapiens steroid sensitive gene 1 (URB), transcript variant 1, mRNA [NM_199511]	NM_199511
A_23_P156327	1.46E-02	11.45	NM_000358	NM_000358	Homo sapiens transforming growth factor, beta-induced, 68kDa (TGFBI), mRNA [NM_000358]	NM_000358
A_32_P151557	1.46E-02	4.284	AA779434	AA779434	AA779434 af22b10.s1 Soares_total_fetus_Nb2HF8_9w Homo sapiens cDNA clone IMAGE:1032379 3', mRNA sequence [AA779434]	
A_32_P23125	1.46E-02	3.323	THC2435906		Q8BU53 (Q8BU53) Mus musculus 2 days pregnant adult female oviduct cDNA, RIKEN full-length enriched library, clone:E230038K10 product:procollagen-proline, 2-oxoglutarate 4-dioxygenase (proline 4-hydroxylase), alpha II polypeptide, full insert sequence,...	
A_23_P168836	1.46E-02	2.436	NM_173174	NM_173174	Homo sapiens PTK2B protein tyrosine kinase 2 beta (PTK2B), transcript variant 1, mRNA [NM_173174]	NM_173174

A_23_P210690	1.46E-02	2.266	NM_021158	NM_021158	Homo sapiens tribbles homolog 3 (Drosophila) (TRIB3), mRNA [NM_021158]	NM_021158
A_23_P62901	1.46E-02	2.16	NM_006763	NM_006763	Homo sapiens BTG family, member 2 (BTG2), mRNA [NM_006763]	NM_006763
A_24_P913146	1.47E-02	9.609	NM_139211	NM_139211	Homo sapiens homeodomain-only protein (HOP), transcript variant 2, mRNA [NM_139211]	NM_139211
A_24_P384379	1.47E-02	3.491	A_24_P384379			
A_24_P6517	1.47E-02	3.162	NM_001029884	NM_001029884	Homo sapiens pleckstrin homology domain containing, family G (with RhoGef domain) member 1 (PLEKHG1), mRNA [NM_001029884]	NM_001029884
A_23_P16409	1.47E-02	2.677	NM_144691	NM_144691	Homo sapiens calpain 12 (CAPN12), mRNA [NM_144691]	NM_144691
A_24_P912799	1.48E-02	2.842	AF056434	AF056434	Homo sapiens clone FBD8 Cri-du-chat critical region mRNA. [AF056434]	
A_24_P677525	1.48E-02	2.703	AF336795	AF336795	Homo sapiens NM-4 mRNA, complete cds. [AF336795]	
A_24_P80135	1.48E-02	2.145	NM_014369	NM_014369	Homo sapiens protein tyrosine phosphatase, non-receptor type 18 (brain-derived) (PTPN18), mRNA [NM_014369]	NM_014369
A_23_P10873	1.49E-02	5.308	NM_003263	NM_003263	Homo sapiens toll-like receptor 1 (TLR1), mRNA [NM_003263]	NM_003263
A_32_P213930	1.49E-02	3.112	THC2280739		ALU7_HUMAN (P39194) Alu subfamily SQ sequence contamination warning entry, partial (19%) [THC2280739]	
A_23_P125303	1.49E-02	2.864	NM_001555	NM_001555	Homo sapiens immunoglobulin superfamily, member 1 (IGSF1), transcript variant 1, mRNA [NM_001555]	NM_001555
A_24_P911775	1.49E-02	2.769	AI521071	AI521071	AI521071 to70e08.x1 NCI_CGAP_Gas4 Homo sapiens cDNA clone IMAGE:2183654 3', mRNA sequence [AI521071]	
A_23_P204850	1.49E-02	2.38	NM_000321	NM_000321	Homo sapiens retinoblastoma 1 (including osteosarcoma) (RB1), mRNA [NM_000321]	NM_000321
A_23_P206228	1.50E-02	2.056	NM_017684	NM_017684	Homo sapiens vacuolar protein sorting 13C (yeast) (VPS13C), transcript variant 1A, mRNA [NM_017684]	NM_017684
A_23_P301372	1.50E-02	2.011	NM_153365	NM_153365	Homo sapiens hypothetical protein FLJ90013 (FLJ90013), mRNA [NM_153365]	NM_153365

Supplemental Table S1C

Genes upregulated in EB vs. hESC-EC

P-Value ≤ 0.015 with multiple testing correction (Benjamini and Hochberg), Fold Change ≥ 2.0

Gene Name	*P* -value	Fold change	Common	Genbank	Description	RefSeq
A_23_P67847	9.67E-07	49.34	NM_024572	NM_024572	Homo sapiens UDP-N-acetyl-alpha-D-galactosamine:polypeptide N-acetylgalactosaminyltransferase 14 (GalNAc-T14) (GALNT14), mRNA [NM_024572]	NM_024572
A_32_P122373	9.67E-07	13.69	ENST00000334429		Homo sapiens cDNA FLJ43241 fis, clone HEART1000010, weakly similar to Hepatocyte growth factor-like protein precursor. [AK125231]	
A_23_P154919	9.67E-07	11.96	BC005107	BC005107	Homo sapiens chromosome 21 open reading frame 105, mRNA (cDNA clone IMAGE:3840937), partial cds. [BC005107]	
A_23_P117190	1.07E-06	42.09	NM_013238	NM_013238	Homo sapiens DnaJ (Hsp40) homolog, subfamily C, member 15 (DNAJC15), mRNA [NM_013238]	NM_013238
A_23_P37205	1.34E-06	13.35	NM_201535	NM_201535	Homo sapiens NDRG family member 2 (NDRG2), transcript variant 1, mRNA [NM_201535]	NM_201535
A_32_P108655	2.16E-06	65.93	NM_001005353	NM_001005353	Homo sapiens adenylate kinase 3-like 1 (AK3L1), nuclear gene encoding mitochondrial protein, transcript variant 1, mRNA [NM_001005353]	NM_001005353
A_24_P403561	2.16E-06	19.13	NM_002334	NM_002334	Homo sapiens low density lipoprotein receptor-related protein 4 (LRP4), mRNA [NM_002334]	NM_002334
A_24_P610945	2.16E-06	8.536	ENST00000311197			
A_24_P182461	2.62E-06	33.84	NM_001542	NM_001542	Homo sapiens immunoglobulin superfamily, member 3 (IGSF3), transcript variant 1, mRNA [NM_001542]	NM_001542
A_23_P44724	2.94E-06	14.11	NM_001321	NM_001321	Homo sapiens cysteine and glycine-rich protein 2 (CSRP2), mRNA [NM_001321]	NM_001321
A_24_P334208	2.98E-06	160.7	A_24_P334208			
A_23_P22526	2.98E-06	47.01	NM_014799	NM_014799	Homo sapiens hephaestin (HEPH), transcript variant 2, mRNA [NM_014799]	NM_014799
A_23_P41424	2.98E-06	18.48	NM_022154	NM_022154	Homo sapiens solute carrier family 39 (zinc transporter), member 8 (SLC39A8), mRNA [NM_022154]	NM_022154
A_23_P146077	2.98E-06	10.86	NM_018660	NM_018660	Homo sapiens zinc finger protein 395 (ZNF395), mRNA [NM_018660]	NM_018660
A_24_P886336	2.98E-06	7.053	BC029907	BC029907	Homo sapiens, clone IMAGE:5175565, mRNA. [BC029907]	
A_32_P12183	2.98E-06	5.9	NM_198284	NM_198284	Homo sapiens hypothetical protein LOC349114 (LOC349114), mRNA [NM_198284]	NM_198284
A_23_P104054	3.20E-06	6.184	NM_016227	NM_016227	Homo sapiens chromosome 1 open reading frame 9 (C1orf9), transcript variant 2, mRNA [NM_016227]	NM_016227
A_24_P738130	3.25E-06	5.386	THC2432280		Q66KD2 (Q66KD2) Lasp1-prov protein, partial (10%) [THC2432280]	
A_23_P79562	3.29E-06	211.2	NM_001443	NM_001443	Homo sapiens fatty acid binding protein 1, liver (FABP1), mRNA [NM_001443]	NM_001443
A_23_P363778	3.29E-06	96.08	NM_001463	NM_001463	Homo sapiens frizzled-related protein (FRZB), mRNA [NM_001463]	NM_001463
A_23_P2283	3.29E-06	76.98	NM_001006667	NM_001006667	Homo sapiens tachykinin 3 (neuromedin K, neurokinin beta) (TAC3), mRNA [NM_001006667]	NM_001006667
A_24_P339429	3.29E-06	22.22	AK024229	AK024229	Homo sapiens cDNA FLJ14167 fis, clone NT2RP2001214. [AK024229]	
A_23_P64792	3.29E-06	13.67	NM_014505	NM_014505	Homo sapiens potassium large conductance calcium-activated channel, subfamily M, beta member 4 (KCNMB4), mRNA [NM_014505]	NM_014505
A_23_P359630	3.29E-06	3.899	NM_002665	NM_002665	Homo sapiens plasminogen-like B1 (PLGLB1), mRNA [NM_002665]	NM_002665
A_24_P309415	3.29E-06	3.489	NM_052932	NM_052932	Homo sapiens pro-oncosis receptor inducing membrane injury gene (PORIMIN), mRNA [NM_052932]	NM_052932
A_23_P406341	3.33E-06	32.46	NM_001001936	NM_001001936	Homo sapiens KIAA1914 (KIAA1914), transcript variant 1, mRNA [NM_001001936]	NM_001001936
A_23_P398172	3.33E-06	5.417	NM_020819	NM_020819	Homo sapiens KIAA1411 (KIAA1411), mRNA [NM_020819]	NM_020819
A_23_P304450	3.64E-06	63.02	NM_005257	NM_005257	Homo sapiens GATA binding protein 6 (GATA6), mRNA [NM_005257]	NM_005257
A_23_P98930	3.88E-06	9.406	NM_018169	NM_018169	Homo sapiens hypothetical protein FLJ10652 (FLJ10652), mRNA [NM_018169]	NM_018169
A_23_P158318	4.06E-06	8.584	NM_004560	NM_004560	Homo sapiens receptor tyrosine kinase-like orphan receptor 2 (ROR2), mRNA [NM_004560]	NM_004560
A_23_P217228	4.12E-06	4.548	NM_016157	NM_016157	Homo sapiens trophinin (TRO), transcript variant 3, mRNA [NM_016157]	NM_016157
A_24_P105191	4.45E-06	216	NM_147175	NM_147175	Homo sapiens heparan sulfate 6-O-sulfotransferase 2 (HS6ST2), transcript variant S, mRNA [NM_147175]	NM_147175
A_23_P206359	4.45E-06	179.1	NM_004360	NM_004360	Homo sapiens cadherin 1, type 1, E-cadherin (epithelial) (CDH1), mRNA [NM_004360]	NM_004360
A_24_P206624	4.45E-06	47.3	NM_022974	NM_022974	Homo sapiens fibroblast growth factor receptor 2 (bacteria-expressed kinase, keratinocyte growth factor receptor, craniofacial dysostosis 1, Crouzon syndrome, Pfeiffer syndrome, Jackson-Weiss syndrome) (FGFR2), transcript variant 7, mRNA [NM_022974]	NM_022974

A_23_P395054	4.45E-06	26.7	BC008813	BC008813	Homo sapiens annexin A8, mRNA (cDNA clone MGC:10405 IMAGE:3958020), complete cds. [BC008813]	
A_23_P152305	4.45E-06	24.4	NM_001797	NM_001797	Homo sapiens cadherin 11, type 2, OB-cadherin (osteoblast) (CDH11), mRNA [NM_001797]	NM_001797
A_32_P178966	4.45E-06	11.61	THC2373940			
A_32_P157945	4.45E-06	10.5	NM_004415	NM_004415	Homo sapiens desmoplakin (DSP), transcript variant 1, mRNA [NM_004415]	NM_004415
A_23_P37623	4.45E-06	8.323	NM_181076	NM_181076	Homo sapiens 88-kDa golgi protein (GM88), transcript variant 2, mRNA [NM_181076]	NM_181076
A_23_P393645	4.45E-06	6.76	NM_139025	NM_139025	Homo sapiens a disintegrin-like and metalloprotease (reprolysin type) with thrombospondin type 1 motif, 13 (ADAMTS13), transcript variant 1, mRNA [NM_139025]	NM_139025
A_23_P70307	4.49E-06	186.5	NM_022138	NM_022138	Homo sapiens SPARC related modular calcium binding 2 (SMOC2), mRNA [NM_022138]	NM_022138
A_23_P145437	4.49E-06	8.662	NM_017934	NM_017934	Homo sapiens pleckstrin homology domain interacting protein (PHIP), mRNA [NM_017934]	NM_017934
A_23_P146456	4.70E-06	23.47	NM_001333	NM_001333	Homo sapiens cathepsin L2 (CTSL2), mRNA [NM_001333]	NM_001333
A_23_P15876	4.81E-06	12.65	NM_052947	NM_052947	Homo sapiens heart alpha-kinase (HAK), mRNA [NM_052947]	NM_052947
A_23_P2492	5.03E-06	19.7	NM_001734	NM_001734	Homo sapiens complement component 1, s subcomponent (C1S), transcript variant 1, mRNA [NM_001734]	NM_001734
A_23_P64873	5.22E-06	279.9	NM_001920	NM_001920	Homo sapiens decorin (DCN), transcript variant A1, mRNA [NM_001920]	NM_001920
A_23_P51187	5.22E-06	16.42	NM_002744	NM_002744	Homo sapiens protein kinase C, zeta (PRKCZ), mRNA [NM_002744]	NM_002744
A_23_P211136	5.22E-06	8.908	NM_018963	NM_018963	Homo sapiens bromodomain and WD repeat domain containing 1 (BRWD1), transcript variant 1, mRNA [NM_018963]	NM_018963
A_32_P106117	5.22E-06	4.067	NM_015044	NM_015044	Homo sapiens golgi associated, gamma adaptin ear containing, ARF binding protein 2 (GGA2), transcript variant 1, mRNA [NM_015044]	NM_015044
A_32_P229493	5.33E-06	63.48	BC004287	BC004287	Homo sapiens, clone IMAGE:3618365, mRNA. [BC004287]	
A_32_P105549	5.33E-06	33.17	NM_001630	NM_001630	Homo sapiens annexin A8 (ANXA8), mRNA [NM_001630]	NM_001630
A_24_P167063	5.33E-06	8.946	NM_014803	NM_014803	Homo sapiens zinc finger protein 518 (ZNF518), mRNA [NM_014803]	NM_014803
A_23_P257164	5.33E-06	6.621	NM_000481	NM_000481	Homo sapiens aminomethyltransferase (glycine cleavage system protein T) (AMT), mRNA [NM_000481]	NM_000481
A_23_P20463	5.33E-06	3.34	NM_006265	NM_006265	Homo sapiens RAD21 homolog (S. pombe) (RAD21), mRNA [NM_006265]	NM_006265
A_23_P8513	5.43E-06	55.88	NM_013322	NM_013322	Homo sapiens sorting nexin 10 (SNX10), mRNA [NM_013322]	NM_013322
A_32_P58937	5.53E-06	9.111	AL133577	AL133577	Homo sapiens mRNA; cDNA DKFZp434G0972 (from clone DKFZp434G0972). [AL133577]	
A_23_P87709	5.54E-06	39.42	NM_024829	NM_024829	Homo sapiens hypothetical protein FLJ22662 (FLJ22662), mRNA [NM_024829]	NM_024829
A_23_P163306	5.56E-06	18.66	NM_032866	NM_032866	Homo sapiens cingulin-like 1 (CGNL1), mRNA [NM_032866]	NM_032866
A_23_P5435	5.56E-06	10.11	AY358993	AY358993	Homo sapiens clone DNA129535 MRV222 (UNQ3066) mRNA, complete cds. [AY358993]	
A_23_P115922	5.56E-06	3.199	NM_004096	NM_004096	Homo sapiens eukaryotic translation initiation factor 4E binding protein 2 (EIF4EBP2), mRNA [NM_004096]	NM_004096
A_32_P97169	5.76E-06	224.8	BX640888	BX640888	Homo sapiens mRNA; cDNA DKFZp686H20120 (from clone DKFZp686H20120). [BX640888]	
A_24_P40626	5.76E-06	112.5	NM_022469	NM_022469	Homo sapiens gremlin 2, cysteine knot superfamily, homolog (Xenopus laevis) (GREM2), mRNA [NM_022469]	NM_022469
A_23_P22735	5.76E-06	62.79	NM_032621	NM_032621	Homo sapiens brain expressed X-linked 2 (BEX2), mRNA [NM_032621]	NM_032621
A_23_P126075	5.76E-06	44.88	NM_002245	NM_002245	Homo sapiens potassium channel, subfamily K, member 1 (KCNK1), mRNA [NM_002245]	NM_002245
A_23_P257834	5.76E-06	21.27	NM_000477	NM_000477	Homo sapiens albumin (ALB), mRNA [NM_000477]	NM_000477
A_23_P7882	5.76E-06	18.49	BC022217	BC022217	Homo sapiens chromosome 6 open reading frame 85, mRNA (cDNA clone IMAGE:3846727), complete cds. [BC022217]	
A_23_P19134	5.76E-06	16.41	NM_032119	NM_032119	Homo sapiens monogenic, audiogenic seizure susceptibility 1 homolog (mouse) (MASS1), mRNA [NM_032119]	NM_032119
A_23_P19030	5.76E-06	15.05	ENST00000328668		full-length cDNA clone CS0DI041YK16 of Placenta Cot 25-normalized of Homo sapiens (human). [CR621346]	
A_23_P95029	5.76E-06	10.27	NM_021021	NM_021021	Homo sapiens syntrophin, beta 1 (dystrophin-associated protein A1, 59kDa, basic component 1) (SNTB1), mRNA [NM_021021]	NM_021021
A_23_P13753	5.76E-06	9.767	NM_006163	NM_006163	Homo sapiens nuclear factor (erythroid-derived 2), 45kDa (NFE2), mRNA [NM_006163]	NM_006163
A_24_P389916	5.76E-06	8.902	NM_005512	NM_005512	Homo sapiens leucine rich repeat containing 32 (LRRC32), mRNA [NM_005512]	NM_005512
A_23_P415652	5.76E-06	6.119	NM_024642	NM_024642	Homo sapiens UDP-N-acetyl-alpha-D-galactosamine:polypeptide N-acetylgalactosaminyltransferase 12 (GalNAc-T12) (GALNT12), mRNA [NM_024642]	NM_024642
A_23_P151529	5.76E-06	5.919	NM_020215	NM_020215	Homo sapiens chromosome 14 open reading frame 132 (C14orf132), mRNA [NM_020215]	NM_020215
A_23_P123448	5.76E-06	5.349	AL136588	AL136588	Homo sapiens mRNA; cDNA DKFZp761D112 (from clone DKFZp761D112). [AL136588]	

A_23_P143981	5.76E-06	4.372	NM_001004019	NM_001004019	Homo sapiens fibulin 2 (FBLN2), transcript variant 1, mRNA [NM_001004019]	NM_001004019
A_23_P66719	5.76E-06	3.28	NM_144683	NM_144683	Homo sapiens hypothetical protein MGC23280 (MGC23280), mRNA [NM_144683]	NM_144683
A_23_P404211	5.76E-06	2.939	ENST00000267430		Homo sapiens mRNA for KIAA1596 protein, partial cds. [AB046816]	XM_048128
A_32_P93045	5.93E-06	28.6	AL080082	AL080082	Homo sapiens mRNA; cDNA DKFZp564G1162 (from clone DKFZp564G1162). [AL080082]	
A_23_P106131	5.93E-06	4.181	NM_182926	NM_182926	Homo sapiens kinectin 1 (kinesin receptor) (KTN1), mRNA [NM_182926]	NM_182926
A_32_P69368	6.16E-06	100.3	NM_002166	NM_002166	Homo sapiens inhibitor of DNA binding 2, dominant negative helix-loop-helix protein (ID2), mRNA [NM_002166]	NM_002166
A_23_P150343	6.16E-06	39.89	NM_003063	NM_003063	Homo sapiens sarcolipin (SLN), mRNA [NM_003063]	NM_003063
A_23_P144656	6.16E-06	29.05	NM_006727	NM_006727	Homo sapiens cadherin 10, type 2 (T2-cadherin) (CDH10), mRNA [NM_006727]	NM_006727
A_23_P35617	6.20E-06	29.29	NM_016341	NM_016341	Homo sapiens phospholipase C, epsilon 1 (PLCE1), mRNA [NM_016341]	NM_016341
A_32_P89371	6.20E-06	3.95	NM_005054	NM_005054	Homo sapiens RAN binding protein 2-like 1 (RANBP2L1), transcript variant 1, mRNA [NM_005054]	NM_005054
A_24_P402588	6.20E-06	3.421	NM_138553	NM_138553	Homo sapiens B-cell CLL/lymphoma 11A (zinc finger protein) (BCL11A), transcript variant 5, mRNA [NM_138553]	NM_138553
A_23_P20743	6.39E-06	10.12	NM_032342	NM_032342	Homo sapiens chromosome 9 open reading frame 125 (C9orf125), mRNA [NM_032342]	NM_032342
A_23_P216038	6.39E-06	4.451	NM_032205	NM_032205	Homo sapiens PHD finger protein 20-like 1 (PHF20L1), transcript variant 2, mRNA [NM_032205]	NM_032205
A_23_P134925	6.39E-06	4.132	NM_004331	NM_004331	Homo sapiens BCL2/adenovirus E1B 19kDa interacting protein 3-like (BNIP3L), mRNA [NM_004331]	NM_004331
A_24_P166473	6.39E-06	3.687	AK023682	AK023682	Homo sapiens cDNA FLJ13620 fis, clone PLACE1010947. [AK023682]	
A_24_P148796	6.50E-06	9.733	NM_020998	NM_020998	Homo sapiens macrophage stimulating 1 (hepatocyte growth factor-like) (MST1), mRNA [NM_020998]	NM_020998
A_24_P245838	6.55E-06	72	AK125361	AK125361	Homo sapiens cDNA FLJ43371 fis, clone NTONG2005969. [AK125361]	
A_24_P329487	6.60E-06	84.96	NM_174911	NM_174911	Homo sapiens family with sequence similarity 84, member B (FAM84B), mRNA [NM_174911]	NM_174911
A_24_P418250	6.65E-06	8.797	NM_203306	NM_203306	Homo sapiens hypothetical protein MGC39606 (MGC39606), mRNA [NM_203306]	NM_203306
A_32_P45375	6.65E-06	5.736	AF037219	AF037219	Homo sapiens PIX1 mRNA sequence. [AF037219]	
A_23_P62932	6.76E-06	16.68	NM_001677	NM_001677	Homo sapiens ATPase, Na+/K+ transporting, beta 1 polypeptide (ATP1B1), transcript variant 1, mRNA [NM_001677]	NM_001677
A_32_P27917	6.76E-06	4.655	BC009415	BC009415	Homo sapiens kinesin family member 26A, mRNA (cDNA clone MGC:14884 IMAGE:3502885), complete cds. [BC009415]	XM_050278
A_32_P129265	6.82E-06	5.645	U92981	U92981	Homo sapiens clone DT1P1B6 mRNA, CAG repeat region. [U92981]	
A_24_P115007	6.87E-06	8.189	NM_170740	NM_170740	Homo sapiens aldehyde dehydrogenase 5 family, member A1 (succinate-semialdehyde dehydrogenase) (ALDH5A1), nuclear gene encoding mitochondrial protein, transcript variant 1, mRNA [NM_170740]	NM_170740
A_23_P2181	6.99E-06	12.38	NM_001001336	NM_001001336	Homo sapiens cytochrome b5 reductase 2 (CYB5R2), transcript variant 2, mRNA [NM_001001336]	NM_001001336
A_23_P159255	7.01E-06	2.985	NM_002845	NM_002845	Homo sapiens protein tyrosine phosphatase, receptor type, M (PTPRM), mRNA [NM_002845]	NM_002845
A_24_P456944	7.01E-06	2.773	AK123446	AK123446	Homo sapiens cDNA FLJ41452 fis, clone BRSTN2010363. [AK123446]	
A_23_P72411	7.35E-06	29.96	NM_178033	NM_178033	Homo sapiens cytochrome P450, family 4, subfamily X, polypeptide 1 (CYP4X1), mRNA [NM_178033]	NM_178033
A_24_P371962	7.35E-06	2.824	NM_001634	NM_001634	Homo sapiens adenosylmethionine decarboxylase 1 (AMD1), mRNA [NM_001634]	NM_001634
A_23_P31143	7.36E-06	62.2	NM_001003395	NM_001003395	Homo sapiens tumor protein D52-like 1 (TPD52L1), transcript variant 2, mRNA [NM_001003395]	NM_001003395
A_23_P42265	7.36E-06	43.12	NM_019101	NM_019101	Homo sapiens apolipoprotein M (APOM), mRNA [NM_019101]	NM_019101
A_23_P43337	7.36E-06	24.7	NM_144966	NM_144966	Homo sapiens FRAS1 related extracellular matrix 1 (FREM1), mRNA [NM_144966]	NM_144966
A_23_P217297	7.36E-06	11.75	NM_021998	NM_021998	Homo sapiens zinc finger protein 6 (CMPX1) (ZNF6), mRNA [NM_021998]	NM_021998
A_23_P371107	7.36E-06	11.74	NM_014392	NM_014392	Homo sapiens DNA segment on chromosome 4 (unique) 234 expressed sequence (D4S234E), mRNA [NM_014392]	NM_014392
A_24_P11315	7.36E-06	8.122	NM_020190	NM_020190	Homo sapiens olfactomedin-like 3 (OLFML3), mRNA [NM_020190]	NM_020190
A_23_P118722	7.36E-06	7.992	NM_001671	NM_001671	Homo sapiens asialoglycoprotein receptor 1 (ASGR1), mRNA [NM_001671]	NM_001671
A_23_P420326	7.36E-06	7.42	NM_153756	NM_153756	Homo sapiens fibronectin type III domain containing 5 (FNDC5), mRNA [NM_153756]	NM_153756
A_23_P33173	7.36E-06	6.419	NM_004487	NM_004487	Homo sapiens golgi autoantigen, golgin subfamily b, macrogolgin (with transmembrane signal), 1 (GOLGB1), mRNA [NM_004487]	NM_004487
A_32_P74955	7.36E-06	5.387	NM_152641	NM_152641	Homo sapiens AT rich interactive domain 2 (ARID, RFX-like) (ARID2), mRNA [NM_152641]	NM_152641
A_24_P347566	7.38E-06	5.869	NM_015059	NM_015059	Homo sapiens talin 2 (TLN2), mRNA [NM_015059]	NM_015059

A_24_P940006	7.43E-06	11.39	NM_001406	NM_001406	Homo sapiens ephrin-B3 (EFNB3), mRNA [NM_001406]	NM_001406
A_32_P10133	7.44E-06	15.77	AK022045	AK022045	Homo sapiens cDNA FLJ11983 fis, clone HEMBB1001337. [AK022045]	
A_23_P212779	7.62E-06	31.97	NM_015393	NM_015393	Homo sapiens DKFZP564O0823 protein (DKFZP564O0823), mRNA [NM_015393]	NM_015393
A_23_P56050	7.62E-06	14.12	M19308	M19308	Human slow skeletal muscle troponin T mRNA, clone M1. [M19308]	
A_23_P166566	7.62E-06	8.306	NM_024768	NM_024768	Homo sapiens hypothetical protein FLJ12057 (FLJ12057), mRNA [NM_024768]	NM_024768
A_23_P87036	7.67E-06	38.29	NM_000482	NM_000482	Homo sapiens apolipoprotein A-IV (APOA4), mRNA [NM_000482]	NM_000482
A_32_P223319	7.67E-06	4.765	NM_052911	NM_052911	Homo sapiens establishment of cohesion 1 homolog 1 (S. cerevisiae) (ESCO1), mRNA [NM_052911]	NM_052911
A_23_P94319	7.68E-06	10.43	NM_014867	NM_014867	Homo sapiens KIAA0711 gene product (KIAA0711), mRNA [NM_014867]	NM_014867
A_23_P156708	7.80E-06	6.9	NM_019105	NM_019105	Homo sapiens tenascin XB (TNXB), transcript variant XB, mRNA [NM_019105]	NM_019105
A_23_P68759	7.80E-06	4.97	NM_003225	NM_003225	Homo sapiens trefoil factor 1 (breast cancer, estrogen-inducible sequence expressed in) (TFF1), mRNA [NM_003225]	NM_003225
A_23_P205489	7.80E-06	4.84	NM_182728	NM_182728	Homo sapiens solute carrier family 7 (cationic amino acid transporter, y+ system), member 8 (SLC7A8), transcript variant 2, mRNA [NM_182728]	NM_182728
A_23_P37484	7.80E-06	2.774	NM_014918	NM_014918	Homo sapiens carbohydrate (chondroitin) synthase 1 (CHSY1), mRNA [NM_014918]	NM_014918
A_24_P169773	7.80E-06	2.523	AF338232	AF338232	Homo sapiens CTAGE-4 protein mRNA, complete cds. [AF338232]	XM_496933
A_24_P55295	7.82E-06	5.281	NM_000165	NM_000165	Homo sapiens gap junction protein, alpha 1, 43kDa (connexin 43) (GJA1), mRNA [NM_000165]	NM_000165
A_24_P136711	7.99E-06	7.638	BC030757	BC030757	Homo sapiens, clone IMAGE:4797534, mRNA, partial cds. [BC030757]	
A_23_P382043	7.99E-06	3.813	NM_152729	NM_152729	Homo sapiens 5'-nucleotidase, cytosolic II-like 1 (NT5C2L1), mRNA [NM_152729]	NM_152729
A_23_P99063	8.06E-06	223.8	NM_002345	NM_002345	Homo sapiens lumican (LUM), mRNA [NM_002345]	NM_002345
A_23_P414654	8.06E-06	33.65	NM_175738	NM_175738	Homo sapiens RAB37, member RAS oncogene family (RAB37), mRNA [NM_175738]	NM_175738
A_32_P109242	8.06E-06	16.33	AK055302	AK055302	Homo sapiens cDNA FLJ30740 fis, clone FEBRA2000319. [AK055302]	
A_23_P120902	8.14E-06	114.2	NM_006498	NM_006498	Homo sapiens lectin, galactoside-binding, soluble, 2 (galectin 2) (LGALS2), mRNA [NM_006498]	NM_006498
A_23_P253896	8.14E-06	75.63	NM_198278	NM_198278	Homo sapiens likely ortholog of mouse nephronectin (NPNT), mRNA [NM_198278]	NM_198278
A_23_P11843	8.14E-06	38.01	NM_201630	NM_201630	Homo sapiens leucine rich repeat neuronal 5 (LRRN5), transcript variant 2, mRNA [NM_201630]	NM_201630
A_23_P28015	8.14E-06	19.03	NM_144693	NM_144693	Homo sapiens zinc finger protein 558 (ZNF558), mRNA [NM_144693]	NM_144693
A_32_P127501	8.14E-06	12.24	AV753543	AV753543	AV753543 AV753543 NPD Homo sapiens cDNA clone NPDBEC03 5', mRNA sequence [AV753543]	
A_23_P251043	8.14E-06	9.467	NM_024893	NM_024893	Homo sapiens chromosome 20 open reading frame 39 (C20orf39), mRNA [NM_024893]	NM_024893
A_23_P372234	8.18E-06	6.773	NM_001218	NM_001218	Homo sapiens carbonic anhydrase XII (CA12), transcript variant 1, mRNA [NM_001218]	NM_001218
A_23_P67799	8.40E-06	13.62	BC046362	BC046362	Homo sapiens transmembrane protein 37, mRNA (cDNA clone MGC:50757 IMAGE:5221396), complete cds. [BC046362]	
A_23_P403398	8.40E-06	9.416	NR_002186	NR_002186	Homo sapiens hypothetical protein DKFZp586I1420 (DKFZp586I1420) on chromosome 7 [NR_002186]	NR_002186
A_23_P253221	8.40E-06	5.179	NM_032995	NM_032995	Homo sapiens Rho guanine nucleotide exchange factor (GEF) 4 (ARHGEF4), transcript variant 2, mRNA [NM_032995]	NM_032995
A_32_P42574	8.46E-06	3.551	NM_032800	NM_032800	Homo sapiens hypothetical protein FLJ14525 (FLJ14525), mRNA [NM_032800]	NM_032800
A_24_P29277	8.67E-06	5.74	AK125387	AK125387	Homo sapiens cDNA FLJ43397 fis, clone OCBBF2009788. [AK125387]	
A_23_P52207	8.68E-06	38.35	NM_012342	NM_012342	Homo sapiens BMP and activin membrane-bound inhibitor homolog (Xenopus laevis) (BAMBI), mRNA [NM_012342]	NM_012342
A_23_P201808	8.75E-06	45.81	NM_003713	NM_003713	Homo sapiens phosphatidic acid phosphatase type 2B (PPAP2B), transcript variant 1, mRNA [NM_003713]	NM_003713
A_23_P99996	8.75E-06	5.98	NM_019066	NM_019066	Homo sapiens MAGE-like 2 (MAGEL2), mRNA [NM_019066]	NM_019066
A_23_P83579	8.75E-06	4.786	NM_014862	NM_014862	Homo sapiens aryl-hydrocarbon receptor nuclear translocator 2 (ARNT2), mRNA [NM_014862]	NM_014862
A_23_P209360	8.91E-06	6.764	ENST00000288548		Homo sapiens mRNA for KIAA1921 protein, partial cds. [AB067508]	
A_24_P236235	8.91E-06	5.17	NM_013231	NM_013231	Homo sapiens fibronectin leucine rich transmembrane protein 2 (FLRT2), mRNA [NM_013231]	NM_013231
A_24_P497244	9.03E-06	8.788	AL050210	AL050210	Homo sapiens mRNA; cDNA DKFZp586G1023 (from clone DKFZp586G1023). [AL050210]	
A_24_P20630	9.06E-06	145.1	NM_016269	NM_016269	Homo sapiens lymphoid enhancer-binding factor 1 (LEF1), mRNA [NM_016269]	NM_016269
A_24_P942786	9.10E-06	6.691	AK024870	AK024870	Homo sapiens cDNA: FLJ21217 fis, clone COL00536. [AK024870]	

A_23_P115261	9.12E-06	49.74	NM_000029	NM_000029	Homo sapiens angiotensinogen (serine (or cysteine) proteinase inhibitor, clade A (alpha-1 antiproteinase, antitrypsin), member 8) (AGT), mRNA [NM_000029]	NM_000029
A_23_P57658	9.14E-06	25.55	NM_020386	NM_020386	Homo sapiens HRAS-like suppressor (HRASLS), mRNA [NM_020386]	NM_020386
A_32_P220523	9.14E-06	8.979	THC2439328			
A_23_P28485	9.20E-06	33.49	NM_012198	NM_012198	Homo sapiens grancalcin, EF-hand calcium binding protein (GCA), mRNA [NM_012198]	NM_012198
A_23_P304897	9.20E-06	16.12	NM_000623	NM_000623	Homo sapiens bradykinin receptor B2 (BDKRB2), mRNA [NM_000623]	NM_000623
A_23_P14774	9.20E-06	14.08	NM_148979	NM_148979	Homo sapiens cathepsin H (CTSH), transcript variant 2, mRNA [NM_148979]	NM_148979
A_23_P73540	9.20E-06	4.156	NM_033626	NM_033626	Homo sapiens JM11 protein (JM11), mRNA [NM_033626]	NM_033626
A_23_P31124	9.22E-06	123.6	NM_030820	NM_030820	Homo sapiens collagen, type XXI, alpha 1 (COL21A1), mRNA [NM_030820]	NM_030820
A_23_P571	9.22E-06	15.86	NM_006516	NM_006516	Homo sapiens solute carrier family 2 (facilitated glucose transporter), member 1 (SLC2A1), mRNA [NM_006516]	NM_006516
A_23_P19590	9.22E-06	4.099	NM_003379	NM_003379	Homo sapiens villin 2 (ezrin) (VIL2), mRNA [NM_003379]	NM_003379
A_23_P99582	9.70E-06	5.637	NM_002687	NM_002687	Homo sapiens pinin, desmosome associated protein (PNN), mRNA [NM_002687]	NM_002687
A_24_P555510	9.70E-06	4.74	THC2437881		Q9NBA9 (Q9NBA9) Stretchin-MLCK (Fragment), partial (5%) [THC2437881]	
A_23_P7282	9.70E-06	4.142	ENST00000323570		Homo sapiens cDNA FLJ38038 fis, clone CTONG2013907. [AK095357]	
A_23_P131664	9.70E-06	4.04	NM_006267	NM_006267	Homo sapiens RAN binding protein 2 (RANBP2), mRNA [NM_006267]	NM_006267
A_23_P429689	9.70E-06	3.3	BC016987	BC016987	Homo sapiens KIAA1327 protein, mRNA (cDNA clone MGC:21978 IMAGE:4395670), complete cds. [BC016987]	
A_23_P253661	9.70E-06	2.641	NM_024902	NM_024902	Homo sapiens hypothetical protein FLJ13236 (FLJ13236), mRNA [NM_024902]	NM_024902
A_23_P354827	9.85E-06	5.216	NM_153231	NM_153231	Homo sapiens zinc finger protein 550 (ZNF550), mRNA [NM_153231]	NM_153231
A_32_P231086	9.91E-06	4.426	NM_198181	NM_198181	Homo sapiens hypothetical protein LOC440295 (LOC440295), mRNA [NM_198181]	NM_198181
A_23_P201551	9.91E-06	3.59	NM_006113	NM_006113	Homo sapiens vav 3 oncogene (VAV3), mRNA [NM_006113]	NM_006113
A_23_P427217	9.96E-06	3.498	NM_004241	NM_004241	Homo sapiens jumonji domain containing 1C (JMJD1C), mRNA [NM_004241]	NM_004241
A_32_P123743	1.03E-05	24.64	THC2376725			
A_23_P64785	1.03E-05	10.99	NM_152320	NM_152320	Homo sapiens zinc finger protein 641 (ZNF641), mRNA [NM_152320]	NM_152320
A_32_P214503	1.03E-05	7.452	BC038512	BC038512	Homo sapiens cDNA clone IMAGE:5262734, partial cds. [BC038512]	
A_32_P107617	1.03E-05	6.151	NM_003019	NM_003019	Homo sapiens surfactant, pulmonary-associated protein D (SFTPD), mRNA [NM_003019]	NM_003019
A_32_P16315	1.03E-05	4.654	ENST00000308911		full-length cDNA clone CS0DE002YA12 of Placenta of Homo sapiens (human). [CR603249]	XM_371684
A_32_P196263	1.04E-05	61.45	NM_182920	NM_182920	Homo sapiens a disintegrin-like and metalloprotease (reprolysin type) with thrombospondin type 1 motif, 9 (ADAMTS9), mRNA [NM_182920]	NM_182920
A_23_P344481	1.04E-05	30.58	NM_152709	NM_152709	Homo sapiens storkhead box 1 (STOX1), mRNA [NM_152709]	NM_152709
A_23_P91250	1.04E-05	24.11	NM_199441	NM_199441	Homo sapiens zinc finger protein 334 (ZNF334), transcript variant 2, mRNA [NM_199441]	NM_199441
A_23_P157726	1.04E-05	3.439	NM_017925	NM_017925	Homo sapiens chromosome 9 open reading frame 55 (C9orf55), mRNA [NM_017925]	NM_017925
A_24_P287756	1.04E-05	3.015	NM_007006	NM_007006	Homo sapiens nudix (nucleoside diphosphate linked moiety X)-type motif 21 (NUDT21), mRNA [NM_007006]	NM_007006
A_23_P212968	1.05E-05	94.86	NM_001073	NM_001073	Homo sapiens UDP glucuronosyltransferase 2 family, polypeptide B11 (UGT2B11), mRNA [NM_001073]	NM_001073
A_23_P11806	1.05E-05	50.61	NM_080629	NM_080629	Homo sapiens collagen, type XI, alpha 1 (COL11A1), transcript variant B, mRNA [NM_080629]	NM_080629
A_23_P120504	1.05E-05	2.961	NM_018354	NM_018354	Homo sapiens chromosome 20 open reading frame 46 (C20orf46), mRNA [NM_018354]	NM_018354
A_23_P217098	1.07E-05	7.11	NM_033305	NM_033305	Homo sapiens vacuolar protein sorting 13A (yeast) (VPS13A), transcript variant A, mRNA [NM_033305]	NM_033305
A_24_P132099	1.07E-05	3.324	NM_138730	NM_138730	Homo sapiens high mobility group nucleosomal binding domain 3 (HMGN3), transcript variant 2, mRNA [NM_138730]	NM_138730
A_24_P89426	1.08E-05	31.59	NM_019101	NM_019101	Homo sapiens apolipoprotein M (APOM), mRNA [NM_019101]	NM_019101
A_23_P99405	1.08E-05	6.393	NM_003453	NM_003453	Homo sapiens zinc finger protein 198 (ZNF198), mRNA [NM_003453]	NM_003453
A_24_P706314	1.08E-05	3.844	ENST00000306311		full-length cDNA clone CS0DJ006YK13 of T cells (Jurkat cell line) Cot 10-normalized of Homo sapiens (human). [CR620336]	
A_24_P236251	1.10E-05	70.44	NM_003836	NM_003836	Homo sapiens delta-like 1 homolog (Drosophila) (DLK1), mRNA [NM_003836]	NM_003836
A_23_P114670	1.11E-05	6.92	NM_014448	NM_014448	Homo sapiens Rho guanine exchange factor (GEF) 16 (ARHGEF16), mRNA [NM_014448]	NM_014448
A_24_P87036	1.15E-05	54.57	NM_018043	NM_018043	Homo sapiens transmembrane protein 16A (TMEM16A), mRNA [NM_018043]	NM_018043

A_23_P319133	1.16E-05	2.753	NM_018981	NM_018981	Homo sapiens DnaJ (Hsp40) homolog, subfamily C, member 10 (DNAJC10), mRNA [NM_018981]	NM_018981
A_24_P188218	1.20E-05	13.06	NM_002476	NM_002476	Homo sapiens myosin, light polypeptide 4, alkali; atrial, embryonic (MYL4), transcript variant 2, mRNA [NM_002476]	NM_002476
A_23_P205057	1.20E-05	10.83	NM_014459	NM_014459	Homo sapiens protocadherin 17 (PCDH17), mRNA [NM_014459]	NM_014459
A_23_P52986	1.20E-05	7.958	NM_152718	NM_152718	Homo sapiens hypothetical protein FLJ32009 (FLJ32009), mRNA [NM_152718]	NM_152718
A_23_P25121	1.20E-05	7.163	NM_016594	NM_016594	Homo sapiens FK506 binding protein 11, 19 kDa (FKBP11), mRNA [NM_016594]	NM_016594
A_23_P45345	1.20E-05	2.16	NM_014500	NM_014500	Homo sapiens HIV TAT specific factor 1 (HTATSF1), mRNA [NM_014500]	NM_014500
A_23_P134237	1.24E-05	8.373	NM_002889	NM_002889	Homo sapiens retinoic acid receptor responder (tazarotene induced) 2 (RARRES2), mRNA [NM_002889]	NM_002889
A_23_P212617	1.25E-05	3.535	NM_003234	NM_003234	Homo sapiens transferrin receptor (p90, CD71) (TFRC), mRNA [NM_003234]	NM_003234
A_23_P380951	1.27E-05	7.611	NM_144689	NM_144689	Homo sapiens zinc finger protein 420 (ZNF420), mRNA [NM_144689]	NM_144689
A_23_P90333	1.28E-05	5.779	XM_292765	XM_292765	PREDICTED: Homo sapiens zinc finger protein 404 (ZNF404), mRNA [XM_292765]	XM_292765
A_32_P34404	1.29E-05	3.816	THC2279933			
A_32_P221429	1.30E-05	9.664	THC2374165			
A_23_P114883	1.31E-05	59.06	NM_002023	NM_002023	Homo sapiens fibromodulin (FMOD), mRNA [NM_002023]	NM_002023
A_23_P146274	1.31E-05	42.27	NM_007029	NM_007029	Homo sapiens stathmin-like 2 (STMN2), mRNA [NM_007029]	NM_007029
A_32_P233799	1.31E-05	14.2	AB007953	AB007953	Homo sapiens mRNA, chromosome 1 specific transcript KIAA0484. [AB007953]	
A_23_P352266	1.31E-05	5.175	NM_000633	NM_000633	Homo sapiens B-cell CLL/lymphoma 2 (BCL2), nuclear gene encoding mitochondrial protein, transcript variant alpha, mRNA [NM_000633]	NM_000633
A_23_P85250	1.35E-05	58.38	NM_013230	NM_013230	Homo sapiens CD24 antigen (small cell lung carcinoma cluster 4 antigen) (CD24), mRNA [NM_013230]	NM_013230
A_32_P3998	1.35E-05	3.143	NM_001004301	NM_001004301	Homo sapiens FLJ16542 protein (FLJ16542), mRNA [NM_001004301]	NM_001004301
A_32_P82895	1.35E-05	2.968	AB075837	AB075837	Homo sapiens mRNA for KIAA1957 protein. [AB075837]	XM_065166
A_23_P408285	1.36E-05	27.43	NM_153026	NM_153026	Homo sapiens prickle-like 1 (Drosophila) (PRICKLE1), mRNA [NM_153026]	NM_153026
A_23_P120227	1.36E-05	25.14	NM_030915	NM_030915	Homo sapiens likely ortholog of mouse limb-bud and heart gene (LBH), mRNA [NM_030915]	NM_030915
A_24_P370472	1.36E-05	23.31	NM_021983	NM_021983	Homo sapiens major histocompatibility complex, class II, DR beta 4 (HLA-DRB4), mRNA [NM_021983]	NM_021983
A_23_P130113	1.36E-05	22.63	NM_080912	NM_080912	Homo sapiens asialoglycoprotein receptor 2 (ASGR2), transcript variant H2', mRNA [NM_080912]	NM_080912
A_23_P71328	1.36E-05	20.9	NM_030583	NM_030583	Homo sapiens matrilin 2 (MATN2), transcript variant 2, mRNA [NM_030583]	NM_030583
A_23_P213166	1.36E-05	7.43	NM_138698	NM_138698	Homo sapiens prematurely terminated mRNA decay factor-like (LOC91431), mRNA [NM_138698]	NM_138698
A_24_P289383	1.36E-05	6.428	NM_017780	NM_017780	Homo sapiens chromodomain helicase DNA binding protein 7 (CHD7), mRNA [NM_017780]	NM_017780
A_23_P218637	1.36E-05	5.66	NM_005054	NM_005054	Homo sapiens RAN binding protein 2-like 1 (RANBP2L1), transcript variant 1, mRNA [NM_005054]	NM_005054
A_23_P40217	1.36E-05	5.419	NM_018431	NM_018431	Homo sapiens docking protein 5 (DOK5), transcript variant 1, mRNA [NM_018431]	NM_018431
A_23_P162640	1.36E-05	4.781	NM_031412	NM_031412	Homo sapiens GABA(A) receptor-associated protein like 1 (GABARAPL1), mRNA [NM_031412]	NM_031412
A_24_P392201	1.36E-05	4.272	NM_198517	NM_198517	Homo sapiens TBC1 domain family, member 10C (TBC1D10C), mRNA [NM_198517]	NM_198517
A_32_P179746	1.36E-05	4.189	THC2405620		Q6DN13 (Q6DN13) MCTP1S, partial (5%) [THC2405620]	
A_23_P384056	1.36E-05	3.433	NM_022757	NM_022757	Homo sapiens coiled-coil domain containing 14 (CCDC14), mRNA [NM_022757]	NM_022757
A_24_P932632	1.36E-05	3.02	CR617018	CR617018	full-length cDNA clone CS0DG001YH13 of B cells (Ramos cell line) of Homo sapiens (human). [CR617018]	
A_23_P397376	1.40E-05	24.24	NM_005360	NM_005360	Homo sapiens v-maf musculoaponeurotic fibrosarcoma oncogene homolog (avian) (MAF), mRNA [NM_005360]	NM_005360
A_23_P431268	1.40E-05	16.02	NM_014935	NM_014935	Homo sapiens pleckstrin homology domain containing, family A member 6 (PLEKHA6), mRNA [NM_014935]	NM_014935
A_23_P417942	1.40E-05	4.772	NM_001024948	NM_001024948	Homo sapiens formin binding protein 1-like (FNBP1L), transcript variant 1, mRNA [NM_001024948]	NM_001024948
A_23_P372467	1.40E-05	3.857	NM_152392	NM_152392	Homo sapiens AHA1, activator of heat shock 90kDa protein ATPase homolog 2 (yeast) (AHSA2), mRNA [NM_152392]	NM_152392
A_23_P79482	1.40E-05	3.482	NM_001822	NM_001822	Homo sapiens chimerin (chimaerin) 1 (CHN1), transcript variant 1, mRNA [NM_001822]	NM_001822
A_32_P163036	1.40E-05	2.536	ENST00000344142		Homo sapiens cDNA PSEC0178 fis, clone OVARC1000636, moderately similar to Sterile alpha motif domain containing protein 4. [AK075484]	XM_291016
A_23_P151426	1.42E-05	6.342	NM_002015	NM_002015	Homo sapiens forkhead box O1A (rhabdomyosarcoma) (FOXO1A), mRNA [NM_002015]	NM_002015
A_23_P79289	1.44E-05	18.05	NM_014900	NM_014900	Homo sapiens COBL-like 1 (COBLL1), mRNA [NM_014900]	NM_014900

A_23_P52017	1.44E-05	3.624	NM_018136	NM_018136	Homo sapiens asp (abnormal spindle)-like, microcephaly associated (Drosophila) (ASPM), mRNA [NM_018136]	NM_018136
A_23_P45875	1.44E-05	3.463	NM_003292	NM_003292	Homo sapiens translocated promoter region (to activated MET oncogene) (TPR), mRNA [NM_003292]	NM_003292
A_24_P390928	1.44E-05	2.476	NM_024108	NM_024108	Homo sapiens hypothetical protein MGC2650 (MGC2650), mRNA [NM_024108]	NM_024108
A_23_P128543	1.44E-05	2.252	NM_014166	NM_014166	Homo sapiens mediator of RNA polymerase II transcription, subunit 4 homolog (yeast) (MED4), mRNA [NM_014166]	NM_014166
A_23_P209320	1.44E-05	2.002	NM_022817	NM_022817	Homo sapiens period homolog 2 (Drosophila) (PER2), transcript variant 1, mRNA [NM_022817]	NM_022817
A_32_P33083	1.47E-05	7.107	NM_016378	NM_016378	Homo sapiens variable charge, X-linked 2 (VCX2), mRNA [NM_016378]	NM_016378
A_24_P920447	1.47E-05	3.865	NM_020215	NM_020215	Homo sapiens chromosome 14 open reading frame 132 (C14orf132), mRNA [NM_020215]	NM_020215
A_24_P128361	1.48E-05	34.23	AF289562	AF289562	Homo sapiens clone pp6337 unknown mRNA. [AF289562]	
A_32_P81173	1.48E-05	11.26	AL050376	AL050376	Homo sapiens mRNA; cDNA DKFZp586J101 (from clone DKFZp586J101). [AL050376]	
A_23_P371266	1.50E-05	12.69	NM_015569	NM_015569	Homo sapiens dynamin 3 (DNM3), mRNA [NM_015569]	NM_015569
A_23_P48561	1.50E-05	9.684	NM_005864	NM_005864	Homo sapiens embryonal Fyn-associated substrate (EFS), transcript variant 1, mRNA [NM_005864]	NM_005864
A_23_P377267	1.50E-05	6.236	AB007940	AB007940	Homo sapiens mRNA for KIAA0471 protein, partial cds. [AB007940]	
A_23_P136909	1.50E-05	4.929	NM_030763	NM_030763	Homo sapiens nucleosomal binding protein 1 (NSBP1), mRNA [NM_030763]	NM_030763
A_23_P83134	1.53E-05	38.89	NM_002048	NM_002048	Homo sapiens growth arrest-specific 1 (GAS1), mRNA [NM_002048]	NM_002048
A_23_P1043	1.53E-05	5.614	NM_018265	NM_018265	Homo sapiens chromosome 1 open reading frame 106 (C1orf106), mRNA [NM_018265]	NM_018265
A_32_P61684	1.53E-05	4.973	NM_018440	NM_018440	Homo sapiens phosphoprotein associated with glycosphingolipid microdomains 1 (PAG1), mRNA [NM_018440]	NM_018440
A_23_P9472	1.54E-05	6.801	NM_033305	NM_033305	Homo sapiens vacuolar protein sorting 13A (yeast) (VPS13A), transcript variant A, mRNA [NM_033305]	NM_033305
A_23_P106675	1.54E-05	3.801	NM_002661	NM_002661	Homo sapiens phospholipase C, gamma 2 (phosphatidylinositol-specific) (PLCG2), mRNA [NM_002661]	NM_002661
A_23_P250982	1.54E-05	2.533	NM_016048	NM_016048	Homo sapiens isochorismatase domain containing 1 (ISOC1), mRNA [NM_016048]	NM_016048
A_23_P55518	1.55E-05	3.102	NM_005904	NM_005904	Homo sapiens SMAD, mothers against DPP homolog 7 (Drosophila) (SMAD7), mRNA [NM_005904]	NM_005904
A_23_P137470	1.56E-05	12.05	NM_020808	NM_020808	Homo sapiens signal-induced proliferation-associated 1 like 2 (SIPA1L2), mRNA [NM_020808]	NM_020808
A_24_P481783	1.56E-05	3.719	AK023802	AK023802	Homo sapiens cDNA FLJ13740 fis, clone PLACE3000199. [AK023802]	
A_23_P61886	1.56E-05	2.992	AK055659	AK055659	Homo sapiens cDNA FLJ31097 fis, clone IMR321000210. [AK055659]	
A_23_P49155	1.57E-05	21.16	NM_001793	NM_001793	Homo sapiens cadherin 3, type 1, P-cadherin (placental) (CDH3), mRNA [NM_001793]	NM_001793
A_23_P216257	1.58E-05	32.68	NM_005079	NM_005079	Homo sapiens tumor protein D52 (TPD52), transcript variant 3, mRNA [NM_005079]	NM_005079
A_23_P50946	1.58E-05	28.66	NM_005855	NM_005855	Homo sapiens receptor (calcitonin) activity modifying protein 1 (RAMP1), mRNA [NM_005855]	NM_005855
A_23_P53891	1.58E-05	15.02	NM_001730	NM_001730	Homo sapiens Kruppel-like factor 5 (intestinal) (KLF5), mRNA [NM_001730]	NM_001730
A_23_P500501	1.59E-05	31.55	NM_000142	NM_000142	Homo sapiens fibroblast growth factor receptor 3 (achondroplasia, thanatophoric dwarfism) (FGFR3), transcript variant 1, mRNA [NM_000142]	NM_000142
A_23_P144911	1.59E-05	18.91	NM_152403	NM_152403	Homo sapiens hypothetical protein FLJ39155 (FLJ39155), transcript variant 1, mRNA [NM_152403]	NM_152403
A_23_P25674	1.59E-05	12.24	NM_001823	NM_001823	Homo sapiens creatine kinase, brain (CKB), mRNA [NM_001823]	NM_001823
A_23_P5875	1.59E-05	3.769	NM_000939	NM_000939	Homo sapiens proopiomelanocortin (adrenocorticotropin/ beta-lipotropin/ alpha-melanocyte stimulating hormone/ beta-melanocyte stimulating hormone/ beta-endorphin) (POMC), mRNA [NM_000939]	NM_000939
A_23_P214977	1.59E-05	3.467	NM_007214	NM_007214	Homo sapiens SEC63-like (S. cerevisiae) (SEC63), mRNA [NM_007214]	NM_007214
A_23_P149775	1.59E-05	3.158	NM_018287	NM_018287	Homo sapiens Rho GTPase activating protein 12 (ARHGAP12), mRNA [NM_018287]	NM_018287
A_23_P18276	1.59E-05	2.421	NM_005778	NM_005778	Homo sapiens RNA binding motif protein 5 (RBM5), mRNA [NM_005778]	NM_005778
A_23_P110253	1.60E-05	13.6	NM_000222	NM_000222	Homo sapiens v-kit Hardy-Zuckerman 4 feline sarcoma viral oncogene homolog (KIT), mRNA [NM_000222]	NM_000222
A_23_P211909	1.60E-05	7.099	NM_002670	NM_002670	Homo sapiens plastin 1 (I isoform) (PLS1), mRNA [NM_002670]	NM_002670
A_23_P212002	1.60E-05	5.902	NM_001012651	NM_001012651	Homo sapiens natural killer-tumor recognition sequence (NKTR), transcript variant 2, mRNA [NM_001012651]	NM_001012651
A_23_P143127	1.60E-05	5.369	NM_019063	NM_019063	Homo sapiens echinoderm microtubule associated protein like 4 (EML4), mRNA [NM_019063]	NM_019063
A_23_P108028	1.60E-05	2.803	NM_007145	NM_007145	Homo sapiens zinc finger protein 146 (ZNF146), mRNA [NM_007145]	NM_007145
A_23_P110433	1.60E-05	2.638	NM_015342	NM_015342	Homo sapiens peptidylprolyl isomerase domain and WD repeat containing 1 (PPWD1), mRNA [NM_015342]	NM_015342

A_23_P53646	1.60E-05	2.561	NM_139207	NM_139207	Homo sapiens nucleosome assembly protein 1-like 1 (NAP1L1), transcript variant 1, mRNA [NM_139207]	NM_139207
A_32_P141768	1.62E-05	8.085	NM_001012733	NM_001012733	Homo sapiens 1-acylglycerol-3-phosphate O-acyltransferase 4 (lysophosphatidic acid acyltransferase, delta) (AGPAT4), transcript variant 2, mRNA [NM_001012733]	NM_001012733
A_23_P430068	1.63E-05	180.8	NM_006474	NM_006474	Homo sapiens podoplanin (PDPN), transcript variant 1, mRNA [NM_006474]	NM_006474
A_24_P98371	1.63E-05	5.946	NM_033222	NM_033222	Homo sapiens PC4 and SFRS1 interacting protein 1 (PSIP1), transcript variant 2, mRNA [NM_033222]	NM_033222
A_32_P88349	1.65E-05	4.756	CR620599	CR620599	full-length cDNA clone CS0DF021YI14 of Fetal brain of Homo sapiens (human). [CR620599]	XM_498811
A_23_P213171	1.67E-05	60.88	NM_000253	NM_000253	Homo sapiens microsomal triglyceride transfer protein (large polypeptide, 88kDa) (MTP), mRNA [NM_000253]	NM_000253
A_24_P299685	1.68E-05	51.66	NM_198389	NM_198389	Homo sapiens podoplanin (PDPN), transcript variant 2, mRNA [NM_198389]	NM_198389
A_24_P128001	1.68E-05	10.92	NM_018660	NM_018660	Homo sapiens zinc finger protein 395 (ZNF395), mRNA [NM_018660]	NM_018660
A_24_P89512	1.68E-05	2.983	NM_014739	NM_014739	Homo sapiens BCL2-associated transcription factor 1 (BCLAF1), mRNA [NM_014739]	NM_014739
A_24_P936319	1.70E-05	6.944	BC030115	BC030115	Homo sapiens, clone IMAGE:4801326, mRNA. [BC030115]	
A_23_P81660	1.70E-05	3.212	NM_018368	NM_018368	Homo sapiens LMBR1 domain containing 1 (LMBRD1), mRNA [NM_018368]	NM_018368
A_23_P201996	1.70E-05	2.366	NM_100264	NM_100264	Homo sapiens WW domain containing adaptor with coiled-coil (WAC), transcript variant 2, mRNA [NM_100264]	NM_100264
A_32_P108722	1.71E-05	2.324	AK127572	AK127572	Homo sapiens cDNA FLJ45665 fis, clone CTONG2027959. [AK127572]	
A_32_P32722	1.72E-05	2.031	CR603845	CR603845	full-length cDNA clone CS0DI026YH22 of Placenta Cot 25-normalized of Homo sapiens (human). [CR603845]	
A_23_P166826	1.75E-05	3.178	NM_018403	NM_018403	Homo sapiens decapping enzyme (DCP1A), mRNA [NM_018403]	NM_018403
A_23_P112554	1.77E-05	24.24	NM_001855	NM_001855	Homo sapiens collagen, type XV, alpha 1 (COL15A1), mRNA [NM_001855]	NM_001855
A_23_P202269	1.78E-05	20.58	NM_020987	NM_020987	Homo sapiens ankyrin 3, node of Ranvier (ankyrin G) (ANK3), transcript variant 1, mRNA [NM_020987]	NM_020987
A_23_P250800	1.80E-05	6.122	NM_006100	NM_006100	Homo sapiens ST3 beta-galactoside alpha-2,3-sialyltransferase 6 (ST3GAL6), mRNA [NM_006100]	NM_006100
A_32_P205944	1.80E-05	2.619	NM_005054	NM_005054	Homo sapiens RAN binding protein 2-like 1 (RANBP2L1), transcript variant 1, mRNA [NM_005054]	NM_005054
A_23_P84610	1.80E-05	2.117	NM_015450	NM_015450	Homo sapiens POT1 protection of telomeres 1 homolog (S. pombe) (POT1), mRNA [NM_015450]	NM_015450
A_24_P174503	1.82E-05	2.587	NM_000481	NM_000481	Homo sapiens aminomethyltransferase (glycine cleavage system protein T) (AMT), mRNA [NM_000481]	NM_000481
A_32_P34495	1.83E-05	25.57	AK026418	AK026418	Homo sapiens cDNA: FLJ22765 fis, clone KAIA1180. [AK026418]	
A_24_P789425	1.83E-05	13.01	AK026966	AK026966	Homo sapiens cDNA: FLJ23313 fis, clone HEP11919. [AK026966]	
A_23_P344884	1.83E-05	4.483	BC036557	BC036557	Homo sapiens KIAA1394 protein, mRNA (cDNA clone MGC:39385 IMAGE:4310128), complete cds. [BC036557]	XM_208522
A_23_P201319	1.84E-05	2.943	NM_032890	NM_032890	Homo sapiens dispatched homolog 1 (Drosophila) (DISP1), mRNA [NM_032890]	NM_032890
A_32_P52227	1.85E-05	8.789	THC2314457		Q6DIG3 (Q6DIG3) Hedgehog interacting protein, partial (3%) [THC2314457]	
A_32_P172141	1.86E-05	7.772	NM_016952	NM_016952	Homo sapiens cell adhesion molecule-related/down-regulated by oncogenes (CDON), mRNA [NM_016952]	NM_016952
A_23_P409623	1.86E-05	6.735	NM_003621	NM_003621	Homo sapiens PTPRF interacting protein, binding protein 2 (liprin beta 2) (PPFIBP2), mRNA [NM_003621]	NM_003621
A_24_P141736	1.87E-05	3.154	THC2336852		AMP2_HUMAN (P50579) Methionine aminopeptidase 2 (MetAP 2) (Peptidase M 2) (Initiation factor 2 associated 67 kDa glycoprotein) (p67) (p67eIF2) , partial (89%) [THC2336852]	
A_23_P163697	1.89E-05	13.48	NM_016524	NM_016524	Homo sapiens synaptotagmin XVII (SYT17), mRNA [NM_016524]	NM_016524
A_23_P19829	1.92E-05	2.351	NM_013440	NM_013440	Homo sapiens paired immunoglobin-like type 2 receptor beta (PILRB), transcript variant 1, mRNA [NM_013440]	NM_013440
A_23_P88580	1.93E-05	6.715	NM_006465	NM_006465	Homo sapiens AT rich interactive domain 3B (BRIGHT- like) (ARID3B), mRNA [NM_006465]	NM_006465
A_24_P38316	1.94E-05	30.97	THC2374505			
A_24_P579356	1.95E-05	41.45	NM_001010000	NM_001010000	Homo sapiens Rho GTPase activating protein 28 (ARHGAP28), transcript variant 1, mRNA [NM_001010000]	NM_001010000
A_32_P17145	1.95E-05	14.09	THC2440409			
A_23_P65779	1.95E-05	7.069	NM_022369	NM_022369	Homo sapiens stimulated by retinoic acid gene 6 homolog (mouse) (STRA6), mRNA [NM_022369]	NM_022369
A_23_P300150	1.95E-05	5.245	NM_172387	NM_172387	Homo sapiens nuclear factor of activated T-cells, cytoplasmic, calcineurin-dependent 1 (NFATC1), transcript variant 3, mRNA [NM_172387]	NM_172387
A_23_P354894	1.95E-05	4.642	NM_152603	NM_152603	Homo sapiens zinc finger protein 567 (ZNF567), mRNA [NM_152603]	NM_152603
A_23_P122615	1.95E-05	4.27	NM_032870	NM_032870	Homo sapiens chromosome 6 open reading frame 111 (C6orf111), mRNA [NM_032870]	NM_032870
A_23_P424582	1.95E-05	2.874	NM_138934	NM_138934	Homo sapiens palmitoyl-protein thioesterase 2 (PPT2), transcript variant 3, mRNA [NM_138934]	NM_138934
A_23_P215419	1.98E-05	5.625	NM_004968	NM_004968	Homo sapiens islet cell autoantigen 1, 69kDa (ICA1), transcript variant 2, mRNA [NM_004968]	NM_004968

A_24_P62469	1.98E-05	2.167	AK122589	AK122589	Homo sapiens mRNA for FLJ00414 protein. [AK122589]	XM_371214
A_23_P339119	1.99E-05	3.929	NM_024560	NM_024560	Homo sapiens FLJ21963 protein (FLJ21963), mRNA [NM_024560]	NM_024560
A_23_P27023	2.01E-05	26.71	NM_032932	NM_032932	Homo sapiens RAB11 family interacting protein 4 (class II) (RAB11FIP4), mRNA [NM_032932]	NM_032932
A_32_P169406	2.01E-05	22.28	CR615016	CR615016	full-length cDNA clone CS0DB006YM19 of Neuroblastoma Cot 10-normalized of Homo sapiens (human). [CR615016]	XM_378360
A_24_P190472	2.01E-05	21.3	NM_003064	NM_003064	Homo sapiens secretory leukocyte protease inhibitor (antileukoproteinase) (SLPI), mRNA [NM_003064]	NM_003064
A_32_P206899	2.01E-05	18.83	THC2340845			
A_23_P65518	2.01E-05	14.18	NM_016651	NM_016651	Homo sapiens dapper, antagonist of beta-catenin, homolog 1 (Xenopus laevis) (DACT1), mRNA [NM_016651]	NM_016651
A_23_P310921	2.01E-05	3.411	NM_002589	NM_002589	Homo sapiens BH-protocadherin (brain-heart) (PCDH7), transcript variant a, mRNA [NM_002589]	NM_002589
A_24_P307785	2.01E-05	3.129	AK098491	AK098491	Homo sapiens cDNA FLJ25625 fis, clone STM02974. [AK098491]	
A_23_P164210	2.01E-05	3.056	NM_032258	NM_032258	Homo sapiens TBC1 domain family, member 3 (TBC1D3), mRNA [NM_032258]	NM_032258
A_23_P427122	2.03E-05	8.512	NM_053017	NM_053017	Homo sapiens ADP-ribosyltransferase 5 (ART5), mRNA [NM_053017]	NM_053017
A_24_P100996	2.06E-05	68.42	ENST00000324559		Homo sapiens mRNA; cDNA DKFZp451A148 (from clone DKFZp451A148). [AL833271]	
A_24_P62530	2.06E-05	36.57	NM_021205	NM_021205	Homo sapiens ras homolog gene family, member U (RHOU), mRNA [NM_021205]	NM_021205
A_23_P105923	2.06E-05	24.57	NM_001362	NM_001362	Homo sapiens deiodinase, iodothyronine, type III (DIO3), mRNA [NM_001362]	NM_001362
A_32_P162443	2.06E-05	21.36	A_32_P162443			
A_23_P71537	2.06E-05	3.52	NM_024790	NM_024790	Homo sapiens centrosome spindle pole associated protein (CSPP), mRNA [NM_024790]	NM_024790
A_23_P11331	2.06E-05	3.082	NM_153333	NM_153333	Homo sapiens transcription elongation factor A (SII)-like 8 (TCEAL8), transcript variant 1, mRNA [NM_153333]	NM_153333
A_32_P60223	2.06E-05	2.925	NM_032329	NM_032329	Homo sapiens inhibitor of growth family, member 5 (ING5), mRNA [NM_032329]	NM_032329
A_24_P240137	2.09E-05	5.227	NM_004487	NM_004487	Homo sapiens golgi autoantigen, golgin subfamily b, macrogolgin (with transmembrane signal), 1 (GOLGB1), mRNA [NM_004487]	NM_004487
A_24_P636441	2.11E-05	3.34	AK022223	AK022223	Homo sapiens cDNA FLJ12161 fis, clone MAMMA1000576. [AK022223]	
A_23_P311144	2.11E-05	2.347	NM_144978	NM_144978	Homo sapiens hypothetical protein FLJ32745 (FLJ32745), mRNA [NM_144978]	NM_144978
A_24_P294233	2.12E-05	2.764	NM_014905	NM_014905	Homo sapiens glutaminase (GLS), mRNA [NM_014905]	NM_014905
A_23_P129064	2.14E-05	64.03	NM_001482	NM_001482	Homo sapiens glycine amidinotransferase (L-arginine:glycine amidinotransferase) (GATM), mRNA [NM_001482]	NM_001482
A_32_P108544	2.14E-05	4.978	NM_001024457	NM_001024457	Homo sapiens Ran binding protein 2-like 2 (RANBP2L2), mRNA [NM_001024457]	NM_001024457
A_32_P129660	2.14E-05	4.287	NM_181453	NM_181453	Homo sapiens GRIP and coiled-coil domain containing 2 (GCC2), transcript variant 1, mRNA [NM_181453]	NM_181453
A_23_P5742	2.14E-05	3.69	NM_024584	NM_024584	Homo sapiens hypothetical protein FLJ13646 (FLJ13646), mRNA [NM_024584]	NM_024584
A_24_P944723	2.15E-05	5.036	AK021676	AK021676	Homo sapiens cDNA FLJ11614 fis, clone HEMBA1004015. [AK021676]	
A_23_P166929	2.15E-05	4.252	NM_005025	NM_005025	Homo sapiens serine (or cysteine) proteinase inhibitor, clade I (neuroserpin), member 1 (SERPINI1), mRNA [NM_005025]	NM_005025
A_23_P19592	2.15E-05	2.28	NM_015599	NM_015599	Homo sapiens phosphoglucomutase 3 (PGM3), mRNA [NM_015599]	NM_015599
A_32_P51518	2.17E-05	9.912	AK098220	AK098220	Homo sapiens cDNA FLJ40901 fis, clone UTERU2003704. [AK098220]	
A_32_P206293	2.17E-05	3.978	BC070371	BC070371	Homo sapiens HLA complex group 12, mRNA (cDNA clone IMAGE:4657554). [BC070371]	
A_32_P429876	2.19E-05	86.86	AK092378	AK092378	Homo sapiens cDNA FLJ35059 fis, clone OCBBF2018827. [AK092378]	
A_23_P200780	2.19E-05	30.44	NM_003243	NM_003243	Homo sapiens transforming growth factor, beta receptor III (betaglycan, 300kDa) (TGFBR3), mRNA [NM_003243]	NM_003243
A_23_P369328	2.19E-05	7.088	NM_145306	NM_145306	Homo sapiens chromosome 10 open reading frame 35 (C10orf35), mRNA [NM_145306]	NM_145306
A_32_P115277	2.19E-05	4.897	THC2279466			
A_24_P737553	2.19E-05	4.198	AK023774	AK023774	Homo sapiens cDNA FLJ13712 fis, clone PLACE2000394. [AK023774]	
A_23_P213877	2.19E-05	2.391	NM_023073	NM_023073	Homo sapiens hypothetical protein FLJ13231 (FLJ13231), mRNA [NM_023073]	NM_023073
A_23_P130149	2.26E-05	2.668	NM_001976	NM_001976	Homo sapiens enolase 3 (beta, muscle) (ENO3), transcript variant 1, mRNA [NM_001976]	NM_001976
A_23_P388150	2.27E-05	17.43	NM_032562	NM_032562	Homo sapiens phospholipase A2, group XIIB (PLA2G12B), mRNA [NM_032562]	NM_032562
A_23_P168909	2.30E-05	7.51	NM_012082	NM_012082	Homo sapiens zinc finger protein, multitype 2 (ZFPM2), mRNA [NM_012082]	NM_012082
A_23_P14798	2.32E-05	11.17	NM_002499	NM_002499	Homo sapiens neogenin homolog 1 (chicken) (NEO1), mRNA [NM_002499]	NM_002499
A_23_P40078	2.32E-05	2.434	NM_003400	NM_003400	Homo sapiens exportin 1 (CRM1 homolog, yeast) (XPO1), mRNA [NM_003400]	NM_003400

A_23_P52266	2.36E-05	15.27	NM_001548	NM_001548	Homo sapiens interferon-induced protein with tetratricopeptide repeats 1 (IFIT1), transcript variant 2, mRNA [NM_001548]	NM_001548
A_23_P80594	2.38E-05	10.23	NM_015184	NM_015184	Homo sapiens phospholipase C-like 2 (PLCL2), mRNA [NM_015184]	NM_015184
A_23_P146990	2.41E-05	2.737	NM_007013	NM_007013	Homo sapiens WW domain containing E3 ubiquitin protein ligase 1 (WWP1), mRNA [NM_007013]	NM_007013
A_23_P369343	2.42E-05	35.7	NM_144505	NM_144505	Homo sapiens kallikrein 8 (neuropsin/ovasin) (KLK8), transcript variant 2, mRNA [NM_144505]	NM_144505
A_24_P767725	2.42E-05	8.981	XM_496156	XM_496156	PREDICTED: Homo sapiens similar to rhophilin-like protein; RhoB effector; rhophilin-2; rhophilin 2 (LOC440368), mRNA [XM_496156]	XM_496156
A_23_P397999	2.43E-05	9.77	NM_003468	NM_003468	Homo sapiens frizzled homolog 5 (Drosophila) (FZD5), mRNA [NM_003468]	NM_003468
A_23_P142075	2.43E-05	6.528	NM_001611	NM_001611	Homo sapiens acid phosphatase 5, tartrate resistant (ACP5), mRNA [NM_001611]	NM_001611
A_23_P337790	2.43E-05	4.11	NM_173082	NM_173082	Homo sapiens SNF2 histone linker PHD RING helicase (SHPRH), mRNA [NM_173082]	NM_173082
A_23_P154740	2.43E-05	2.353	NM_018474	NM_018474	Homo sapiens chromosome 20 open reading frame 19 (C20orf19), mRNA [NM_018474]	NM_018474
A_23_P139687	2.49E-05	24.9	NM_152321	NM_152321	Homo sapiens hypothetical protein FLJ32115 (FLJ32115), mRNA [NM_152321]	NM_152321
A_23_P159237	2.49E-05	14.74	AB209066	AB209066	Homo sapiens mRNA for G protein-coupled receptor 20 variant protein. [AB209066]	
A_23_P218928	2.49E-05	4.516	NM_016613	NM_016613	Homo sapiens hypothetical protein DKFZp434L142 (DKFZp434L142), mRNA [NM_016613]	NM_016613
A_23_P419213	2.50E-05	4.409	NM_020817	NM_020817	Homo sapiens KIAA1407 (KIAA1407), mRNA [NM_020817]	NM_020817
A_24_P665185	2.52E-05	12.25	AL832142	AL832142	Homo sapiens mRNA; cDNA DKFZp686A22111 (from clone DKFZp686A22111). [AL832142]	
A_24_P153880	2.52E-05	4.597	NM_198181	NM_198181	Homo sapiens hypothetical protein LOC440295 (LOC440295), mRNA [NM_198181]	NM_198181
A_23_P171143	2.52E-05	2.861	NM_003270	NM_003270	Homo sapiens tetraspanin 6 (TSPAN6), mRNA [NM_003270]	NM_003270
A_23_P401	2.54E-05	3.503	NM_016343	NM_016343	Homo sapiens centromere protein F, 350/400ka (mitosin) (CENPF), mRNA [NM_016343]	NM_016343
A_23_P8571	2.55E-05	10.98	NM_080744	NM_080744	Homo sapiens scavenger receptor cysteine rich domain containing, group B (4 domains) (SRCRB4D), mRNA [NM_080744]	NM_080744
A_32_P137336	2.55E-05	10.51	BC013077	BC013077	Homo sapiens, clone IMAGE:3459334, mRNA. [BC013077]	
A_23_P27381	2.56E-05	3.516	NM_005786	NM_005786	Homo sapiens serologically defined colon cancer antigen 33 (SDCCAG33), mRNA [NM_005786]	NM_005786
A_23_P401106	2.56E-05	2.034	NM_002599	NM_002599	Homo sapiens phosphodiesterase 2A, cGMP-stimulated (PDE2A), mRNA [NM_002599]	NM_002599
A_23_P356494	2.57E-05	6.061	NM_006846	NM_006846	Homo sapiens serine protease inhibitor, Kazal type 5 (SPINK5), mRNA [NM_006846]	NM_006846
A_23_P200298	2.57E-05	5.123	NM_000028	NM_000028	Homo sapiens amylo-1, 6-glucosidase, 4-alpha-glucanotransferase (glycogen debranching enzyme, glycogen storage disease type III) (AGL), transcript variant 4, mRNA [NM_000028]	NM_000028
A_23_P209527	2.58E-05	22.8	THC2310298		A31642 villin [validated] - human {Homo sapiens;} , partial (23%) [THC2310298]	
A_23_P53126	2.60E-05	26.84	NM_005574	NM_005574	Homo sapiens LIM domain only 2 (rhombotin-like 1) (LMO2), mRNA [NM_005574]	NM_005574
A_32_P84242	2.60E-05	20.75	CR936791	CR936791	Homo sapiens mRNA; cDNA DKFZp781C2356 (from clone DKFZp781C2356). [CR936791]	XM_032571
A_23_P412764	2.60E-05	9.946	AB075838	AB075838	Homo sapiens mRNA for KIAA1958 protein. [AB075838]	
A_23_P205713	2.65E-05	9.37	NM_014178	NM_014178	Homo sapiens syntaxin binding protein 6 (amisyn) (STXBP6), mRNA [NM_014178]	NM_014178
A_24_P225308	2.66E-05	4.522	NM_016374	NM_016374	Homo sapiens AT rich interactive domain 4B (RBP1- like) (ARID4B), transcript variant 1, mRNA [NM_016374]	NM_016374
A_24_P391591	2.71E-05	5.358	AK057596	AK057596	Homo sapiens cDNA FLJ33034 fis, clone THYMU2000236. [AK057596]	XM_499585
A_23_P10902	2.72E-05	451.1	NM_001463	NM_001463	Homo sapiens frizzled-related protein (FRZB), mRNA [NM_001463]	NM_001463
A_23_P259741	2.73E-05	8.446	NM_002971	NM_002971	Homo sapiens special AT-rich sequence binding protein 1 (binds to nuclear matrix/scaffold-associating DNA's) (SATB1), mRNA [NM_002971]	NM_002971
A_23_P46315	2.73E-05	6.243	NM_198459	NM_198459	Homo sapiens FLJ37099 protein (FLJ37099), mRNA [NM_198459]	NM_198459
A_32_P31633	2.73E-05	4.681	NM_012433	NM_012433	Homo sapiens splicing factor 3b, subunit 1, 155kDa (SF3B1), transcript variant 1, mRNA [NM_012433]	NM_012433
A_23_P30294	2.74E-05	23.08	NM_001801	NM_001801	Homo sapiens cysteine dioxygenase, type I (CDO1), mRNA [NM_001801]	NM_001801
A_24_P242299	2.74E-05	4.264	NM_005455	NM_005455	Homo sapiens zinc finger protein 265 (ZNF265), transcript variant 2, mRNA [NM_005455]	NM_005455
A_23_P307400	2.74E-05	3.856	NM_138363	NM_138363	Homo sapiens hypothetical protein BC009518 (LOC90799), mRNA [NM_138363]	NM_138363
A_23_P140328	2.74E-05	3.826	NM_004713	NM_004713	Homo sapiens serologically defined colon cancer antigen 1 (SDCCAG1), mRNA [NM_004713]	NM_004713
A_24_P280558	2.75E-05	5.05	ENST00000309771		Homo sapiens melanoma-associated antigen mRNA, partial cds. [AF543495]	

A_23_P31765	2.78E-05	14.77	NM_006823	NM_006823	Homo sapiens protein kinase (cAMP-dependent, catalytic) inhibitor alpha (PKIA), transcript variant 1, mRNA [NM_006823]	NM_006823
A_23_P391443	2.78E-05	7.163	AB032983	AB032983	Homo sapiens mRNA for KIAA1157 protein, partial cds. [AB032983]	XM_350880
A_23_P501080	2.78E-05	4.424	NM_007139	NM_007139	Homo sapiens zinc finger protein 92 (HTF12) (ZNF92), mRNA [NM_007139]	NM_007139
A_23_P58588	2.79E-05	51.52	NM_003062	NM_003062	Homo sapiens slit homolog 3 (Drosophila) (SLIT3), mRNA [NM_003062]	NM_003062
A_24_P923483	2.79E-05	30.18	BC020867	BC020867	Homo sapiens solute carrier family 6 (neurotransmitter transporter, GABA), member 13, mRNA (cDNA clone IMAGE:4594185), complete cds. [BC020867]	
A_24_P630039	2.79E-05	7.702	CR600369	CR600369	full-length cDNA clone CS0DF025YM09 of Fetal brain of Homo sapiens (human). [CR600369]	
A_23_P144165	2.79E-05	4.287	NM_014648	NM_014648	Homo sapiens zinc finger DAZ interacting protein 3 (DZIP3), mRNA [NM_014648]	NM_014648
A_23_P204980	2.79E-05	3.736	NM_020121	NM_020121	Homo sapiens UDP-glucose ceramide glucosyltransferase-like 2 (UGCGL2), mRNA [NM_020121]	NM_020121
A_24_P350890	2.79E-05	3.248	NM_006267	NM_006267	Homo sapiens RAN binding protein 2 (RANBP2), mRNA [NM_006267]	NM_006267
A_23_P97584	2.79E-05	2.462	NM_014597	NM_014597	Homo sapiens estrogen receptor binding protein (ERBP), mRNA [NM_014597]	NM_014597
A_23_P75430	2.79E-05	2.356	NM_020179	NM_020179	Homo sapiens FN5 protein (FN5), mRNA [NM_020179]	NM_020179
A_23_P74467	2.79E-05	2.093	NM_014949	NM_014949	Homo sapiens KIAA0907 (KIAA0907), mRNA [NM_014949]	NM_014949
A_32_P140475	2.80E-05	7.761	NM_020802	NM_020802	Homo sapiens KIAA1377 protein (KIAA1377), mRNA [NM_020802]	NM_020802
A_32_P405942	2.84E-05	34.36	CR620977	CR620977	full-length cDNA clone CS0CAP004YK15 of Thymus of Homo sapiens (human). [CR620977]	
A_23_P150807	2.84E-05	10.95	NM_003621	NM_003621	Homo sapiens PTPRF interacting protein, binding protein 2 (liprin beta 2) (PPFIBP2), mRNA [NM_003621]	NM_003621
A_23_P89431	2.84E-05	10.54	NM_002982	NM_002982	Homo sapiens chemokine (C-C motif) ligand 2 (CCL2), mRNA [NM_002982]	NM_002982
A_23_P82950	2.84E-05	4.272	NM_006197	NM_006197	Homo sapiens pericentriolar material 1 (PCM1), mRNA [NM_006197]	NM_006197
A_23_P341532	2.84E-05	3.685	NM_031915	NM_031915	Homo sapiens SET domain, bifurcated 2 (SETDB2), mRNA [NM_031915]	NM_031915
A_24_P90878	2.84E-05	2.545	NM_017736	NM_017736	Homo sapiens THUMP domain containing 1 (THUMPD1), mRNA [NM_017736]	NM_017736
A_23_P24433	2.86E-05	6.057	NM_003793	NM_003793	Homo sapiens cathepsin F (CTSF), mRNA [NM_003793]	NM_003793
A_23_P8664	2.87E-05	3.871	NM_021145	NM_021145	Homo sapiens cyclin D binding myb-like transcription factor 1 (DMTF1), mRNA [NM_021145]	NM_021145
A_23_P113245	2.87E-05	3.363	D14041	D14041	Homo sapiens mRNA for H-2K binding factor-2, complete cds. [D14041]	
A_23_P161297	2.88E-05	13.86	NM_018245	NM_018245	Homo sapiens oxoglutarate dehydrogenase-like (OGDHL), mRNA [NM_018245]	NM_018245
A_23_P148990	2.88E-05	3.713	NM_031935	NM_031935	Homo sapiens hemicentin 1 (HMCN1), mRNA [NM_031935]	NM_031935
A_23_P424269	2.88E-05	2.555	NM_020207	NM_020207	Homo sapiens chromosome 9 open reading frame 102 (C9orf102), mRNA [NM_020207]	NM_020207
A_23_P209146	2.89E-05	7.413	NM_003430	NM_003430	Homo sapiens zinc finger protein 91 (HPF7, HTF10) (ZNF91), mRNA [NM_003430]	NM_003430
A_23_P116435	2.89E-05	6.28	NM_016412	NM_016412	Homo sapiens insulin-like growth factor 2 antisense (IGF2AS), mRNA [NM_016412]	NM_016412
A_24_P109244	2.89E-05	2.095	NM_015026	NM_015026	Homo sapiens KIAA1040 protein (KIAA1040), mRNA [NM_015026]	NM_015026
A_24_P403501	2.90E-05	3.513	S80864	S80864	cytochrome c-like polypeptide [human, lung adenocarcinoma A549, mRNA, 1041 nt]. [S80864]	
A_23_P107116	2.91E-05	25.91	NM_007148	NM_007148	Homo sapiens zinc finger protein 179 (ZNF179), mRNA [NM_007148]	NM_007148
A_24_P253003	2.91E-05	20.46	NM_004626	NM_004626	Homo sapiens wingless-type MMTV integration site family, member 11 (WNT11), mRNA [NM_004626]	NM_004626
A_23_P218111	2.93E-05	10.82	NM_001002236	NM_001002236	Homo sapiens serine (or cysteine) proteinase inhibitor, clade A (alpha-1 antiproteinase, antitrypsin), member 1 (SERPINA1), transcript variant 2, mRNA [NM_001002236]	NM_001002236
A_24_P85942	2.93E-05	3.505	NM_181453	NM_181453	Homo sapiens GRIP and coiled-coil domain containing 2 (GCC2), transcript variant 1, mRNA [NM_181453]	NM_181453
A_23_P341418	2.93E-05	2.276	AK098818	AK098818	Homo sapiens cDNA FLJ25952 fis, clone SYN00911. [AK098818]	
A_24_P48069	2.95E-05	4.276	NM_018110	NM_018110	Homo sapiens docking protein 4 (DOK4), mRNA [NM_018110]	NM_018110
A_24_P355246	2.98E-05	68.66	AK023096	AK023096	Homo sapiens cDNA FLJ13034 fis, clone NT2RP3001232. [AK023096]	
A_23_P164057	2.98E-05	46.59	NM_002404	NM_002404	Homo sapiens microfibrillar-associated protein 4 (MFAP4), mRNA [NM_002404]	NM_002404
A_23_P132910	2.98E-05	24.51	NM_019027	NM_019027	Homo sapiens RNA-binding protein (FLJ20273), mRNA [NM_019027]	NM_019027
A_32_P229818	2.98E-05	16.61	AK022044	AK022044	Homo sapiens cDNA FLJ11982 fis, clone HEMBB1001335. [AK022044]	
A_23_P101806	2.99E-05	9.11	NM_182983	NM_182983	Homo sapiens hepsin (transmembrane protease, serine 1) (HPN), transcript variant 1, mRNA [NM_182983]	NM_182983
A_23_P430658	3.02E-05	8.818	NM_014571	NM_014571	Homo sapiens hairy/enhancer-of-split related with YRPW motif-like (HEYL), mRNA [NM_014571]	NM_014571

A_23_P200710	3.02E-05	4.501	NM_002646	NM_002646	Homo sapiens phosphoinositide-3-kinase, class 2, beta polypeptide (PIK3C2B), mRNA [NM_002646]	NM_002646
A_23_P313734	3.02E-05	3.954	AB051518	AB051518	Homo sapiens mRNA for KIAA1731 protein, partial cds. [AB051518]	XM_374922
A_32_P20982	3.02E-05	2.414	ENST00000342168		Homo sapiens cDNA FLJ45371 fis, clone BRHIP3017855, highly similar to Homo sapiens nuclear pore complex interacting protein (NPIP). [AK128772]	XM_290670
A_23_P17053	3.03E-05	5.225	NM_019618	NM_019618	Homo sapiens interleukin 1 family, member 9 (IL1F9), mRNA [NM_019618]	NM_019618
A_23_P110661	3.06E-05	2.811	NM_015360	NM_015360	Homo sapiens superkiller viralicidic activity 2-like 2 (S. cerevisiae) (SKIV2L2), mRNA [NM_015360]	NM_015360
A_24_P116535	3.09E-05	5.99	NM_002428	NM_002428	Homo sapiens matrix metalloproteinase 15 (membrane-inserted) (MMP15), mRNA [NM_002428]	NM_002428
A_24_P345781	3.09E-05	5.361	THC2428103		Q9W056 (Q9W056) CG1139-PA (LP06969p), partial (4%) [THC2428103]	
A_24_P937855	3.09E-05	3.201	CR936771	CR936771	Homo sapiens mRNA; cDNA DKFZp686A0668 (from clone DKFZp686A0668). [CR936771]	
A_32_P42224	3.10E-05	63.72	BX097190	BX097190	BX097190 Soares placenta Nb2HP Homo sapiens cDNA clone IMAGp998G19212, mRNA sequence [BX097190]	
A_23_P119964	3.10E-05	3.452	NM_005760	NM_005760	Homo sapiens CCAAT/enhancer binding protein zeta (CEBPZ), mRNA [NM_005760]	NM_005760
A_23_P103511	3.16E-05	19.23	AK125122	AK125122	Homo sapiens cDNA FLJ43132 fis, clone CTONG3005813. [AK125122]	XM_378908
A_23_P257583	3.17E-05	10.58	NM_015689	NM_015689	Homo sapiens KIAA1277 (KIAA1277), mRNA [NM_015689]	NM_015689
A_23_P107432	3.20E-05	9.549	NM_207453	NM_207453	Homo sapiens FLJ35934 protein (FLJ35934), mRNA [NM_207453]	NM_207453
A_32_P65473	3.21E-05	20.63	ENST00000330640		Homo sapiens, clone IMAGE:2899977, mRNA, partial cds. [BC022980]	
A_23_P122906	3.22E-05	5.281	NM_015570	NM_015570	Homo sapiens autism susceptibility candidate 2 (AUTS2), mRNA [NM_015570]	NM_015570
A_23_P213883	3.22E-05	5.049	NM_133433	NM_133433	Homo sapiens Nipped-B homolog (Drosophila) (NIPBL), transcript variant A, mRNA [NM_133433]	NM_133433
A_23_P60130	3.24E-05	57.38	NM_052886	NM_052886	Homo sapiens mal, T-cell differentiation protein 2 (MAL2), mRNA [NM_052886]	NM_052886
A_23_P82503	3.24E-05	6.279	ENST00000362013		Homo sapiens MEF3L1 mRNA for MEF3 like 1, complete cds. [AB049150]	XM_499343
A_23_P30395	3.25E-05	4.382	AL161991	AL161991	Homo sapiens mRNA; cDNA DKFZp761C169 (from clone DKFZp761C169). [AL161991]	
A_23_P148768	3.26E-05	2.255	NM_000130	NM_000130	Homo sapiens coagulation factor V (proaccelerin, labile factor) (F5), mRNA [NM_000130]	NM_000130
A_32_P2161	3.27E-05	8.762	AK023040	AK023040	Homo sapiens cDNA FLJ12978 fis, clone NT2RP2006321. [AK023040]	
A_23_P159053	3.27E-05	2.483	NM_002873	NM_002873	Homo sapiens RAD17 homolog (S. pombe) (RAD17), transcript variant 8, mRNA [NM_002873]	NM_002873
A_23_P14673	3.28E-05	10.21	NM_020962	NM_020962	Homo sapiens likely ortholog of mouse neighbor of Punc E11 (NOPE), mRNA [NM_020962]	NM_020962
A_23_P68155	3.28E-05	7.836	NM_022168	NM_022168	Homo sapiens interferon induced with helicase C domain 1 (IFIH1), mRNA [NM_022168]	NM_022168
A_23_P9574	3.28E-05	2.259	NM_018098	NM_018098	Homo sapiens epithelial cell transforming sequence 2 oncogene (ECT2), mRNA [NM_018098]	NM_018098
A_32_P122494	3.30E-05	9.863	AI652920	AI652920	AI652920 wb40g09.x1 NCI_CGAP_GC6 Homo sapiens cDNA clone IMAGE:2308192 3' similar to SW:NMA_HUMAN Q13145 PUTATIVE TRANSMEMBRANE PROTEIN NMA PRECURSOR. ;, mRNA sequence [AI652920]	
A_24_P652700	3.30E-05	3.37	BX648822	BX648822	Homo sapiens mRNA; cDNA DKFZp686C15165 (from clone DKFZp686C15165). [BX648822]	
A_23_P138507	3.33E-05	2.91	NM_001786	NM_001786	Homo sapiens cell division cycle 2, G1 to S and G2 to M (CDC2), transcript variant 1, mRNA [NM_001786]	NM_001786
A_24_P943062	3.34E-05	2.345	NM_014615	NM_014615	Homo sapiens KIAA0182 protein (KIAA0182), mRNA [NM_014615]	NM_014615
A_23_P131676	3.36E-05	350.5	NM_020311	NM_020311	Homo sapiens chemokine orphan receptor 1 (CMKOR1), mRNA [NM_020311]	NM_020311
A_23_P54144	3.37E-05	14.18	NM_001202	NM_001202	Homo sapiens bone morphogenetic protein 4 (BMP4), transcript variant 1, mRNA [NM_001202]	NM_001202
A_32_P81806	3.37E-05	13.67	THC2406779			
A_23_P129466	3.37E-05	9.228	NM_024997	NM_024997	Homo sapiens activating transcription factor 7 interacting protein 2 (ATF7IP2), mRNA [NM_024997]	NM_024997
A_32_P3742	3.37E-05	8.358	AK127804	AK127804	Homo sapiens cDNA FLJ45905 fis, clone OCBBF3026576. [AK127804]	
A_23_P27040	3.39E-05	5.19	NM_015544	NM_015544	Homo sapiens DKFZP564K1964 protein (DKFZP564K1964), mRNA [NM_015544]	NM_015544
A_23_P50217	3.42E-05	2.234	NM_024833	NM_024833	Homo sapiens zinc finger protein 671 (ZNF671), mRNA [NM_024833]	NM_024833
A_23_P131383	3.43E-05	5.04	NM_018062	NM_018062	Homo sapiens Fanconi anemia, complementation group L (FANCL), mRNA [NM_018062]	NM_018062
A_23_P71053	3.44E-05	4.656	NM_016447	NM_016447	Homo sapiens membrane protein, palmitoylated 6 (MAGUK p55 subfamily member 6) (MPP6), mRNA [NM_016447]	NM_016447
A_24_P845223	3.45E-05	25.65	M27126	M27126	Human lymphocyte antigen (DRw8) mRNA. [M27126]	
A_23_P202520	3.45E-05	3.248	NM_001003408	NM_001003408	Homo sapiens actin binding LIM protein 1 (ABLIM1), transcript variant 3, mRNA [NM_001003408]	NM_001003408
A_32_P104432	3.48E-05	13.48	NM_203306	NM_203306	Homo sapiens hypothetical protein MGC39606 (MGC39606), mRNA [NM_203306]	NM_203306

A_24_P655849	3.63E-05	4.95	BF514799	BF514799	UI-H-BW1-anj-a-01-0-UI.s1 NCI_CGAP_Sub7 Homo sapiens cDNA clone IMAGE:3082272 3', mRNA sequence [BF514799]	
A_23_P36865	3.63E-05	4.896	NM_025114	NM_025114	Homo sapiens centrosome protein cep290 (Cep290), mRNA [NM_025114]	NM_025114
A_23_P210330	3.66E-05	4.975	ENST00000238875		full-length cDNA clone CS0DL009YB17 of B cells (Ramos cell line) Cot 25-normalized of Homo sapiens (human). [CR593568]	
A_32_P182186	3.66E-05	4.95	BC041955	BC041955	Homo sapiens, clone IMAGE:5301910, mRNA. [BC041955]	
A_23_P96936	3.67E-05	2.622	NM_017673	NM_017673	Homo sapiens chromosome 1 open reading frame 26 (C1orf26), mRNA [NM_017673]	NM_017673
A_24_P392661	3.67E-05	2.099	A_24_P392661			
A_32_P190181	3.68E-05	3.631	THC2345075			
A_23_P38167	3.69E-05	31.18	NM_022036	NM_022036	Homo sapiens G protein-coupled receptor, family C, group 5, member C (GPRC5C), transcript variant 1, mRNA [NM_022036]	NM_022036
A_23_P39574	3.69E-05	4.967	NM_173466	NM_173466	Homo sapiens hypothetical protein DKFZp434P055 (DKFZp434P055), mRNA [NM_173466]	NM_173466
A_24_P76300	3.69E-05	4.716	NM_003444	NM_003444	Homo sapiens zinc finger protein 154 (pHZ-92) (ZNF154), mRNA [NM_003444]	NM_003444
A_23_P366559	3.69E-05	3.813	NM_144973	NM_144973	Homo sapiens hypothetical protein MGC24039 (MGC24039), mRNA [NM_144973]	NM_144973
A_24_P227230	3.75E-05	3.898	NM_014333	NM_014333	Homo sapiens immunoglobulin superfamily, member 4 (IGSF4), mRNA [NM_014333]	NM_014333
A_23_P308519	3.76E-05	15.15	NM_004252	NM_004252	Homo sapiens solute carrier family 9 (sodium/hydrogen exchanger), isoform 3 regulator 1 (SLC9A3R1), mRNA [NM_004252]	NM_004252
A_23_P83298	3.76E-05	8.225	NM_016307	NM_016307	Homo sapiens paired related homeobox 2 (PRRX2), mRNA [NM_016307]	NM_016307
A_23_P390734	3.76E-05	2.598	NM_015633	NM_015633	Homo sapiens FGFR1 oncogene partner 2 (FGFR1OP2), mRNA [NM_015633]	NM_015633
A_23_P370666	3.80E-05	11.82	NM_080661	NM_080661	Homo sapiens similar to RIKEN cDNA 0610008P16 gene (MGC15937), mRNA [NM_080661]	NM_080661
A_23_P133517	3.80E-05	8.359	NM_002310	NM_002310	Homo sapiens leukemia inhibitory factor receptor (LIFR), mRNA [NM_002310]	NM_002310
A_23_P7697	3.85E-05	4.047	NM_003100	NM_003100	Homo sapiens sorting nexin 2 (SNX2), mRNA [NM_003100]	NM_003100
A_23_P120345	3.86E-05	3.293	NM_020651	NM_020651	Homo sapiens pellino homolog 1 (Drosophila) (PELI1), mRNA [NM_020651]	NM_020651
A_23_P201998	3.86E-05	2.716	THC2372371			
A_32_P177040	3.86E-05	2.384	NM_175064	NM_175064	Homo sapiens Williams Beuren syndrome chromosome region 19 (WBSCR19), mRNA [NM_175064]	NM_175064
A_23_P11652	3.88E-05	2.655	NM_003368	NM_003368	Homo sapiens ubiquitin specific protease 1 (USP1), transcript variant 1, mRNA [NM_003368]	NM_003368
A_23_P21485	3.89E-05	15.36	NM_017933	NM_017933	Homo sapiens hypothetical protein FLJ20701 (FLJ20701), mRNA [NM_017933]	NM_017933
A_32_P88965	3.89E-05	6.389	BX537651	BX537651	Homo sapiens mRNA; cDNA DKFZp686C0390 (from clone DKFZp686C0390). [BX537651]	
A_23_P160318	3.89E-05	6.206	NM_001856	NM_001856	Homo sapiens collagen, type XVI, alpha 1 (COL16A1), mRNA [NM_001856]	NM_001856
A_24_P133991	3.89E-05	5.254	NM_015208	NM_015208	Homo sapiens ankyrin repeat domain 12 (ANKRD12), mRNA [NM_015208]	NM_015208
A_23_P347070	3.89E-05	5.05	NM_018440	NM_018440	Homo sapiens phosphoprotein associated with glycosphingolipid microdomains 1 (PAG1), mRNA [NM_018440]	NM_018440
A_32_P233304	3.89E-05	2.287	NM_173083	NM_173083	Homo sapiens lin-9 homolog (C. elegans) (LIN9), mRNA [NM_173083]	NM_173083
A_24_P153456	3.91E-05	15.95	NM_024786	NM_024786	Homo sapiens zinc finger, DHHC-type containing 11 (ZDHHC11), mRNA [NM_024786]	NM_024786
A_24_P941148	3.95E-05	3.371	NM_017645	NM_017645	Homo sapiens family with sequence similarity 29, member A (FAM29A), mRNA [NM_017645]	NM_017645
A_23_P93988	3.95E-05	2.036	NM_005435	NM_005435	Homo sapiens Rho guanine nucleotide exchange factor (GEF) 5 (ARHGEF5), transcript variant 1, mRNA [NM_005435]	NM_005435
A_23_P306890	3.97E-05	3.412	NM_007195	NM_007195	Homo sapiens polymerase (DNA directed) iota (POLI), mRNA [NM_007195]	NM_007195
A_24_P80500	3.97E-05	3.085	NM_020139	NM_020139	Homo sapiens dehydrogenase/reductase (SDR family) member 6 (DHRS6), mRNA [NM_020139]	NM_020139
A_23_P149975	4.06E-05	6.408	NM_031453	NM_031453	Homo sapiens chromosome 10 open reading frame 45 (C10orf45), mRNA [NM_031453]	NM_031453
A_24_P411186	4.07E-05	12.25	NM_022893	NM_022893	Homo sapiens B-cell CLL/lymphoma 11A (zinc finger protein) (BCL11A), transcript variant 1, mRNA [NM_022893]	NM_022893
A_23_P20752	4.07E-05	3.023	NM_178432	NM_178432	Homo sapiens cell cycle related kinase (CCRK), transcript variant 1, mRNA [NM_178432]	NM_178432
A_24_P120934	4.11E-05	3.665	NM_006705	NM_006705	Homo sapiens growth arrest and DNA-damage-inducible, gamma (GADD45G), mRNA [NM_006705]	NM_006705
A_24_P97104	4.14E-05	14.15	NM_001935	NM_001935	Homo sapiens dipeptidylpeptidase 4 (CD26, adenosine deaminase complexing protein 2) (DPP4), mRNA [NM_001935]	NM_001935
A_23_P115091	4.14E-05	10.66	NM_020387	NM_020387	Homo sapiens RAB25, member RAS oncogene family (RAB25), mRNA [NM_020387]	NM_020387

A_23_P154526	4.14E-05	7.999	NM_004490	NM_004490	Homo sapiens growth factor receptor-bound protein 14 (GRB14), mRNA [NM_004490]	NM_004490
A_32_P29200	4.14E-05	2.5	AK093416	AK093416	Homo sapiens cDNA FLJ36097 fis, clone TESTI2020956. [AK093416]	
A_23_P65823	4.14E-05	2.041	AF180519	AF180519	Homo sapiens GABA-A receptor-associated protein mRNA, complete cds. [AF180519]	
A_23_P378364	4.15E-05	4.224	NM_032457	NM_032457	Homo sapiens BH-protocadherin (brain-heart) (PCDH7), transcript variant c, mRNA [NM_032457]	NM_032457
A_24_P289366	4.15E-05	3.791	NM_203463	NM_203463	Homo sapiens LAG1 longevity assurance homolog 6 (S. cerevisiae) (LASS6), mRNA [NM_203463]	NM_203463
A_23_P348636	4.15E-05	3.766	NM_001454	NM_001454	Homo sapiens forkhead box J1 (FOXJ1), mRNA [NM_001454]	NM_001454
A_23_P95060	4.16E-05	13.74	NM_004443	NM_004443	Homo sapiens EPH receptor B3 (EPHB3), mRNA [NM_004443]	NM_004443
A_23_P128855	4.17E-05	11.79	NM_014579	NM_014579	Homo sapiens solute carrier family 39 (zinc transporter), member 2 (SLC39A2), mRNA [NM_014579]	NM_014579
A_32_P23872	4.17E-05	2.49	A_32_P23872			
A_23_P115022	4.20E-05	20.28	NM_144626	NM_144626	Homo sapiens hypothetical protein MGC17299 (MGC17299), mRNA [NM_144626]	NM_144626
A_24_P921144	4.20E-05	3.889	BC056662	BC056662	Homo sapiens cDNA clone IMAGE:4045027, partial cds. [BC056662]	
A_23_P137514	4.21E-05	2.512	NM_016389	NM_016389	Homo sapiens influenza virus NS1A binding protein (IVNS1ABP), transcript variant 2, mRNA [NM_016389]	NM_016389
A_24_P241792	4.23E-05	7.679	NM_198893	NM_198893	Homo sapiens zinc finger protein 160 (ZNF160), transcript variant 2, mRNA [NM_198893]	NM_198893
A_24_P91852	4.24E-05	18.48	NM_006520	NM_006520	Homo sapiens t-complex-associated-testis-expressed 1-like (TCTE1L), mRNA [NM_006520]	NM_006520
A_23_P75790	4.28E-05	8.676	NM_013279	NM_013279	Homo sapiens chromosome 11 open reading frame 9 (C11orf9), mRNA [NM_013279]	NM_013279
A_23_P379054	4.28E-05	8.411	NM_148960	NM_148960	Homo sapiens claudin 19 (CLDN19), mRNA [NM_148960]	NM_148960
A_23_P93629	4.32E-05	13.32	NM_015905	NM_015905	Homo sapiens tripartite motif-containing 24 (TRIM24), transcript variant 1, mRNA [NM_015905]	NM_015905
A_24_P778844	4.37E-05	4.012	AK124841	AK124841	Homo sapiens cDNA FLJ42851 fis, clone BRHIP2005719. [AK124841]	
A_24_P7202	4.38E-05	4.704	NM_020738	NM_020738	Homo sapiens kinase D-interacting substance of 220 kDa (KIDINS220), mRNA [NM_020738]	NM_020738
A_32_P95914	4.39E-05	4.186	CR749603	CR749603	Homo sapiens mRNA; cDNA DKFZp686C20164 (from clone DKFZp686C20164). [CR749603]	
A_24_P925565	4.40E-05	8.937	THC2438118			
A_24_P941787	4.40E-05	5.88	NM_003913	NM_003913	Homo sapiens PRP4 pre-mRNA processing factor 4 homolog B (yeast) (PRPF4B), transcript variant 1, mRNA [NM_003913]	NM_003913
A_24_P74070	4.40E-05	3.228	NM_032510	NM_032510	Homo sapiens par-6 partitioning defective 6 homolog gamma (C. elegans) (PARD6G), mRNA [NM_032510]	NM_032510
A_23_P253464	4.40E-05	2.675	NM_139076	NM_139076	Homo sapiens hypothetical protein FLJ13614 (FLJ13614), mRNA [NM_139076]	NM_139076
A_32_P4018	4.41E-05	5.111	AK000776	AK000776	Homo sapiens cDNA FLJ20769 fis, clone COL06674. [AK000776]	
A_23_P363316	4.43E-05	2.022	NM_002147	NM_002147	Homo sapiens homeo box B5 (HOXB5), mRNA [NM_002147]	NM_002147
A_24_P161973	4.46E-05	3.079	NM_015205	NM_015205	Homo sapiens ATPase, Class VI, type 11A (ATP11A), transcript variant 1, mRNA [NM_015205]	NM_015205
A_24_P181998	4.46E-05	2.58	ENST00000255896		F lambda 8=Ig lambda-like gene/beta-glucuronidase exon 11 homolog {5' region} [human, fetal liver, mRNA Partial, 452 nt]. [S82637]	
A_24_P157720	4.46E-05	2.067	BQ188033	BQ188033	BQ188033 UI-E-EJ1-aju-o-13-0-UI.r1 UI-E-EJ1 Homo sapiens cDNA clone UI-E-EJ1-aju-o-13-0-UI 5', mRNA sequence [BQ188033]	
A_24_P886040	4.50E-05	16.81	NM_152624	NM_152624	Homo sapiens DCP2 decapping enzyme homolog (S. cerevisiae) (DCP2), mRNA [NM_152624]	NM_152624
A_32_P113584	4.50E-05	5.484	AB011102	AB011102	Homo sapiens mRNA for KIAA0530 protein, partial cds. [AB011102]	
A_23_P319583	4.51E-05	9.157	NM_014747	NM_014747	Homo sapiens regulating synaptic membrane exocytosis 3 (RIMS3), mRNA [NM_014747]	NM_014747
A_24_P489480	4.51E-05	2.645	BC062438	BC062438	Homo sapiens cDNA clone IMAGE:6494968, containing frame-shift errors. [BC062438]	
A_32_P111072	4.52E-05	30.45	THC2389705			
A_23_P40108	4.52E-05	15.45	NM_001853	NM_001853	Homo sapiens collagen, type IX, alpha 3 (COL9A3), mRNA [NM_001853]	NM_001853
A_23_P102471	4.54E-05	2.818	NM_000251	NM_000251	Homo sapiens mutS homolog 2, colon cancer, nonpolyposis type 1 (E. coli) (MSH2), mRNA [NM_000251]	NM_000251
A_23_P146134	4.54E-05	2.684	NM_024025	NM_024025	Homo sapiens dual specificity phosphatase 26 (putative) (DUSP26), mRNA [NM_024025]	NM_024025
A_23_P159390	4.54E-05	2.049	NM_007027	NM_007027	Homo sapiens topoisomerase (DNA) II binding protein 1 (TOPBP1), mRNA [NM_007027]	NM_007027
A_24_P26073	4.58E-05	2.396	NM_133259	NM_133259	Homo sapiens leucine-rich PPR-motif containing (LRPPRC), mRNA [NM_133259]	NM_133259
A_23_P86822	4.60E-05	2.574	NM_014679	NM_014679	Homo sapiens translokin (PIG8), mRNA [NM_014679]	NM_014679
A_24_P561165	4.63E-05	22.79	A_24_P561165			

A_24_P541919	4.63E-05	4.327	AF086301	AF086301	Homo sapiens full length insert cDNA clone ZD50F09. [AF086301]	
A_23_P324813	4.63E-05	3.65	NM_181844	NM_181844	Homo sapiens B-cell CLL/lymphoma 6, member B (zinc finger protein) (BCL6B), mRNA [NM_181844]	NM_181844
A_23_P8834	4.64E-05	33.17	NM_001979	NM_001979	Homo sapiens epoxide hydrolase 2, cytoplasmic (EPHX2), mRNA [NM_001979]	NM_001979
A_23_P342275	4.64E-05	10.41	NM_006988	NM_006988	Homo sapiens a disintegrin-like and metalloprotease (reprolysin type) with thrombospondin type 1 motif, 1 (ADAMTS1), mRNA [NM_006988]	NM_006988
A_23_P119562	4.64E-05	5.567	NM_001928	NM_001928	Homo sapiens D component of complement (adipsin) (DF), mRNA [NM_001928]	NM_001928
A_23_P125717	4.64E-05	3.695	NM_004538	NM_004538	Homo sapiens nucleosome assembly protein 1-like 3 (NAP1L3), mRNA [NM_004538]	NM_004538
A_23_P70818	4.64E-05	2.709	NM_005631	NM_005631	Homo sapiens smoothened homolog (Drosophila) (SMO), mRNA [NM_005631]	NM_005631
A_32_P45168	4.65E-05	5.145	NR_002211	NR_002211	Homo sapiens Meis1, myeloid ecotropic viral integration site 1 homolog 4 (mouse) (MEIS4) on chromosome 17 [NR_002211]	NR_002211
A_23_P139388	4.66E-05	3.424	NM_016578	NM_016578	Homo sapiens hepatitis B virus x associated protein (HBXAP), mRNA [NM_016578]	NM_016578
A_24_P943802	4.71E-05	2.426	AK128047	AK128047	Homo sapiens cDNA FLJ46167 fis, clone TESTI4003179. [AK128047]	
A_24_P944640	4.72E-05	6.638	NM_020909	NM_020909	Homo sapiens erythrocyte membrane protein band 4.1 like 5 (EPB41L5), mRNA [NM_020909]	NM_020909
A_24_P303420	4.77E-05	6.009	NM_001010871	NM_001010871	Homo sapiens hypothetical protein LOC221442 (LOC221442), mRNA [NM_001010871]	NM_001010871
A_24_P91916	4.77E-05	2.955	NM_018698	NM_018698	Homo sapiens nuclear transport factor 2-like export factor 2 (NXT2), mRNA [NM_018698]	NM_018698
A_24_P48403	4.78E-05	3.378	NM_005433	NM_005433	Homo sapiens v-yes-1 Yamaguchi sarcoma viral oncogene homolog 1 (YES1), mRNA [NM_005433]	NM_005433
A_23_P5441	4.79E-05	2.631	NM_005689	NM_005689	Homo sapiens ATP-binding cassette, sub-family B (MDR/TAP), member 6 (ABCB6), nuclear gene encoding mitochondrial protein, mRNA [NM_005689]	NM_005689
A_24_P220454	4.84E-05	2.076	NM_181552	NM_181552	Homo sapiens cut-like 1, CCAAT displacement protein (Drosophila) (CUTL1), transcript variant 1, mRNA [NM_181552]	NM_181552
A_32_P123514	4.86E-05	19.19	BX648831	BX648831	Homo sapiens mRNA; cDNA DKFZp686J06116 (from clone DKFZp686J06116). [BX648831]	XR_000195
A_32_P89352	4.86E-05	4.626	AK125899	AK125899	Homo sapiens cDNA FLJ43911 fis, clone TESTI4010928. [AK125899]	
A_32_P134209	4.86E-05	3.868	THC2403913		AV752763 AV752763 NPD Homo sapiens cDNA clone NPDBCA03 5', mRNA sequence [AV752763]	
A_23_P391506	4.86E-05	3.809	NM_016389	NM_016389	Homo sapiens influenza virus NS1A binding protein (IVNS1ABP), transcript variant 2, mRNA [NM_016389]	NM_016389
A_24_P410797	4.89E-05	14.29	AK125979	AK125979	Homo sapiens cDNA FLJ43991 fis, clone TESTI4019843, highly similar to Rattus norvegicus huntingtin-associated protein interacting protein (duo) (Hapip). [AK125979]	
A_23_P63379	4.90E-05	7.552	NM_012113	NM_012113	Homo sapiens carbonic anhydrase XIV (CA14), mRNA [NM_012113]	NM_012113
A_23_P44295	4.90E-05	2.047	NM_015097	NM_015097	Homo sapiens cytoplasmic linker associated protein 2 (CLASP2), mRNA [NM_015097]	NM_015097
A_23_P140884	4.92E-05	11.01	BC014971	BC014971	Homo sapiens, Similar to tubulin, beta, 2, clone IMAGE:4873024, mRNA. [BC014971]	XM_371684
A_23_P417383	4.92E-05	4.205	NM_152792	NM_152792	Homo sapiens hypothetical protein FLJ25084 (FLJ25084), mRNA [NM_152792]	NM_152792
A_23_P250404	4.92E-05	2.063	NM_005732	NM_005732	Homo sapiens RAD50 homolog (S. cerevisiae) (RAD50), transcript variant 1, mRNA [NM_005732]	NM_005732
A_24_P935491	4.94E-05	33.1	NM_000090	NM_000090	Homo sapiens collagen, type III, alpha 1 (Ehlers-Danlos syndrome type IV, autosomal dominant) (COL3A1), mRNA [NM_000090]	NM_000090
A_23_P21376	4.94E-05	3.433	NM_012301	NM_012301	Homo sapiens membrane associated guanylate kinase, WW and PDZ domain containing 2 (MAGI2), mRNA [NM_012301]	NM_012301
A_32_P208424	4.97E-05	2.504	NM_002107	NM_002107	Homo sapiens H3 histone, family 3A (H3F3A), mRNA [NM_002107]	NM_002107
A_23_P211039	5.02E-05	10.96	NM_006988	NM_006988	Homo sapiens a disintegrin-like and metalloprotease (reprolysin type) with thrombospondin type 1 motif, 1 (ADAMTS1), mRNA [NM_006988]	NM_006988
A_24_P304636	5.02E-05	9.615	NM_004755	NM_004755	Homo sapiens ribosomal protein S6 kinase, 90kDa, polypeptide 5 (RPS6KA5), transcript variant 1, mRNA [NM_004755]	NM_004755
A_32_P34003	5.02E-05	5.817	THC2313453			
A_24_P47681	5.02E-05	4.187	NM_018448	NM_018448	Homo sapiens cullin-associated and neddylation-dissociated 1 (CAND1), mRNA [NM_018448]	NM_018448
A_32_P97496	5.02E-05	3.692	NM_181722	NM_181722	Homo sapiens hypothetical protein LOC285908 (LOC285908), mRNA [NM_181722]	NM_181722
A_24_P179183	5.04E-05	4.722	NM_015208	NM_015208	Homo sapiens ankyrin repeat domain 12 (ANKRD12), mRNA [NM_015208]	NM_015208
A_24_P149023	5.06E-05	3.9	NM_004866	NM_004866	Homo sapiens secretory carrier membrane protein 1 (SCAMP1), transcript variant 1, mRNA [NM_004866]	NM_004866
A_23_P75299	5.06E-05	2.093	NM_022126	NM_022126	Homo sapiens phospholysine phosphohistidine inorganic pyrophosphate phosphatase (LHPP), mRNA [NM_022126]	NM_022126
A_23_P86021	5.07E-05	2.827	NM_003944	NM_003944	Homo sapiens selenium binding protein 1 (SELENBP1), mRNA [NM_003944]	NM_003944

A_23_P65278	5.15E-05	6.745	NM_015678	NM_015678	Homo sapiens neurobeachin (NBEA), mRNA [NM_015678]	NM_015678
A_23_P22422	5.19E-05	3.306	NM_013364	NM_013364	Homo sapiens paraneoplastic antigen MA3 (PNMA3), mRNA [NM_013364]	NM_013364
A_24_P2584	5.20E-05	5.102	BC001892	BC001892	Homo sapiens chromosome 1 open reading frame 63, transcript variant 2, mRNA (cDNA clone MGC:1827 IMAGE:3534629), complete cds. [BC001892]	
A_23_P218068	5.20E-05	4.503	NM_019012	NM_019012	Homo sapiens pleckstrin homology domain containing, family A member 5 (PLEKHA5), mRNA [NM_019012]	NM_019012
A_32_P228124	5.20E-05	2.635	AL832747	AL832747	Homo sapiens mRNA; cDNA DKFZp686D0521 (from clone DKFZp686D0521). [AL832747]	
A_23_P154875	5.22E-05	3.369	NM_012105	NM_012105	Homo sapiens beta-site APP-cleaving enzyme 2 (BACE2), transcript variant a, mRNA [NM_012105]	NM_012105
A_32_P68533	5.24E-05	4.334	ENST00000307507		Homo sapiens mRNA; cDNA DKFZp686O21143 (from clone DKFZp686O21143). [BX648834]	XM_291019
A_32_P130265	5.24E-05	2.327	NM_014827	NM_014827	Homo sapiens zinc finger CCCH-type containing 11A (ZC3H11A), mRNA [NM_014827]	NM_014827
A_24_P706312	5.28E-05	3.342	ENST00000306311		full-length cDNA clone CS0DF004YO22 of Fetal brain of Homo sapiens (human). [CR603865]	
A_23_P250735	5.30E-05	2.275	NM_175709	NM_175709	Homo sapiens chromobox homolog 7 (CBX7), mRNA [NM_175709]	NM_175709
A_23_P103601	5.32E-05	8.987	NM_020379	NM_020379	Homo sapiens mannosidase, alpha, class 1C, member 1 (MAN1C1), mRNA [NM_020379]	NM_020379
A_23_P111797	5.33E-05	2.719	AL136837	AL136837	Homo sapiens mRNA; cDNA DKFZp434F142 (from clone DKFZp434F142). [AL136837]	
A_23_P42397	5.34E-05	27.77	NM_153362	NM_153362	Homo sapiens protease, serine, 35 (PRSS35), mRNA [NM_153362]	NM_153362
A_23_P25475	5.35E-05	29.72	NM_003578	NM_003578	Homo sapiens sterol O-acyltransferase 2 (SOAT2), mRNA [NM_003578]	NM_003578
A_23_P217319	5.38E-05	33.51	NM_004114	NM_004114	Homo sapiens fibroblast growth factor 13 (FGF13), transcript variant 1A, mRNA [NM_004114]	NM_004114
A_32_P95067	5.38E-05	6.108	A_32_P95067			
A_24_P383609	5.40E-05	4.403	NM_199461	NM_199461	Homo sapiens nanos homolog 1 (Drosophila) (NANOS1), transcript variant 1, mRNA [NM_199461]	NM_199461
A_23_P94911	5.40E-05	2.599	AK098175	AK098175	Homo sapiens cDNA FLJ40856 fis, clone TRACH2016498, moderately similar to ZINC FINGER PROTEIN 184. [AK098175]	XM_371174
A_23_P2683	5.41E-05	2.409	NM_024604	NM_024604	Homo sapiens hypothetical protein FLJ21908 (FLJ21908), mRNA [NM_024604]	NM_024604
A_23_P217901	5.42E-05	17.44	BC038219	BC038219	Homo sapiens, clone IMAGE:3634113, mRNA. [BC038219]	
A_32_P201979	5.43E-05	2.682	NM_182765	NM_182765	Homo sapiens HECT domain containing 2 (HECTD2), transcript variant 1, mRNA [NM_182765]	NM_182765
A_23_P82941	5.43E-05	2.455	NM_006421	NM_006421	Homo sapiens ADP-ribosylation factor guanine nucleotide-exchange factor 1(brefeldin A-inhibited) (ARFGEF1), mRNA [NM_006421]	NM_006421
A_23_P202071	5.45E-05	102.8	NM_006561	NM_006561	Homo sapiens CUG triplet repeat, RNA binding protein 2 (CUGBP2), transcript variant 2, mRNA [NM_006561]	NM_006561
A_23_P2705	5.54E-05	2.862	NM_005767	NM_005767	Homo sapiens purinergic receptor P2Y, G-protein coupled, 5 (P2RY5), mRNA [NM_005767]	NM_005767
A_24_P412486	5.56E-05	6.349	CR601315	CR601315	full-length cDNA clone CS0DC001YJ02 of Neuroblastoma Cot 25-normalized of Homo sapiens (human). [CR601315]	
A_23_P121657	5.60E-05	12.23	NM_005114	NM_005114	Homo sapiens heparan sulfate (glucosamine) 3-O-sulfotransferase 1 (HS3ST1), mRNA [NM_005114]	NM_005114
A_24_P166663	5.60E-05	5.092	NM_001259	NM_001259	Homo sapiens cyclin-dependent kinase 6 (CDK6), mRNA [NM_001259]	NM_001259
A_23_P147397	5.60E-05	3.282	AB082528	AB082528	Homo sapiens mRNA for KIAA1997 protein. [AB082528]	XM_370652
A_23_P19020	5.61E-05	23.04	NM_005460	NM_005460	Homo sapiens synuclein, alpha interacting protein (synphilin) (SNCAIP), mRNA [NM_005460]	NM_005460
A_23_P500936	5.61E-05	9.841	NM_021784	NM_021784	Homo sapiens forkhead box A2 (FOXA2), transcript variant 1, mRNA [NM_021784]	NM_021784
A_32_P9468	5.64E-05	2.354	AK130822	AK130822	Homo sapiens cDNA FLJ27312 fis, clone TMS06371. [AK130822]	
A_23_P10182	5.71E-05	5.309	NM_003500	NM_003500	Homo sapiens acyl-Coenzyme A oxidase 2, branched chain (ACOX2), mRNA [NM_003500]	NM_003500
A_24_P66125	5.72E-05	8.055	NM_006603	NM_006603	Homo sapiens stromal antigen 2 (STAG2), mRNA [NM_006603]	NM_006603
A_32_P2392	5.72E-05	4.051	NM_181076	NM_181076	Homo sapiens 88-kDa golgi protein (GM88), transcript variant 2, mRNA [NM_181076]	NM_181076
A_23_P98483	5.72E-05	3.724	NM_021211	NM_021211	Homo sapiens transposon-derived Buster1 transposase-like protein gene (LOC58486), mRNA [NM_021211]	NM_021211
A_23_P255104	5.72E-05	2.204	NM_005779	NM_005779	Homo sapiens lipoma HMGIC fusion partner-like 2 (LHFPL2), mRNA [NM_005779]	NM_005779
A_24_P756494	5.77E-05	12.98	AK057923	AK057923	Homo sapiens cDNA FLJ25194 fis, clone REC04095. [AK057923]	
A_23_P114662	5.77E-05	4.996	NM_001889	NM_001889	Homo sapiens crystallin, zeta (quinone reductase) (CRYZ), mRNA [NM_001889]	NM_001889
A_32_P108254	5.78E-05	6.79	NM_017565	NM_017565	Homo sapiens family with sequence similarity 20, member A (FAM20A), mRNA [NM_017565]	NM_017565
A_24_P78540	5.78E-05	2.524	NM_003297	NM_003297	Homo sapiens nuclear receptor subfamily 2, group C, member 1 (NR2C1), mRNA [NM_003297]	NM_003297
A_24_P916364	5.82E-05	9.444	AK025613	AK025613	Homo sapiens cDNA: FLJ21960 fis, clone HEP05517. [AK025613]	

A_23_P156748	5.84E-05	5.348	NM_015245	NM_015245	Homo sapiens ankyrin repeat and sterile alpha motif domain containing 1 (ANKS1), mRNA [NM_015245]	NM_015245
A_24_P551067	5.84E-05	4.969	CR603951	CR603951	full-length cDNA clone CS0DM011YC22 of Fetal liver of Homo sapiens (human). [CR603951]	XM_499585
A_23_P133284	5.84E-05	2.065	NM_018343	NM_018343	Homo sapiens RIO kinase 2 (yeast) (RIOK2), mRNA [NM_018343]	NM_018343
A_23_P110122	5.84E-05	2.009	NM_004354	NM_004354	Homo sapiens cyclin G2 (CCNG2), mRNA [NM_004354]	NM_004354
A_23_P306500	5.85E-05	2.538	NM_033360	NM_033360	Homo sapiens v-Ki-ras2 Kirsten rat sarcoma viral oncogene homolog (KRAS), transcript variant a, mRNA [NM_033360]	NM_033360
A_24_P268993	5.95E-05	8.23	NM_052971	NM_052971	Homo sapiens liver-expressed antimicrobial peptide 2 (LEAP-2), mRNA [NM_052971]	NM_052971
A_23_P18493	5.96E-05	8.938	NM_080685	NM_080685	Homo sapiens protein tyrosine phosphatase, non-receptor type 13 (APO-1/CD95 (Fas)-associated phosphatase) (PTPN13), transcript variant 4, mRNA [NM_080685]	NM_080685
A_32_P123629	5.96E-05	4.321	THC2373876		BM069797 ie89f02.y1 Melton Normalized Human Islet 4 N4-HIS 1 Homo sapiens cDNA clone IMAGE:5674130 5', mRNA sequence [BM069797]	
A_23_P375281	5.96E-05	3.682	NM_080706	NM_080706	Homo sapiens transient receptor potential cation channel, subfamily V, member 1 (TRPV1), transcript variant 3, mRNA [NM_080706]	NM_080706
A_23_P208210	5.96E-05	2.864	NM_014650	NM_014650	Homo sapiens zinc finger protein 432 (ZNF432), mRNA [NM_014650]	NM_014650
A_32_P195401	5.97E-05	7.96	NM_173511	NM_173511	Homo sapiens amyotrophic lateral sclerosis 2 (juvenile) chromosome region, candidate 13 (ALS2CR13), mRNA [NM_173511]	NM_173511
A_23_P102950	6.00E-05	6.356	NM_080860	NM_080860	Homo sapiens testis specific A2 homolog (mouse) (TSGA2), mRNA [NM_080860]	NM_080860
A_32_P63858	6.00E-05	5.609	THC2279910		ALU6_HUMAN (P39193) Alu subfamily SP sequence contamination warning entry, partial (5%) [THC2279910]	
A_32_P127248	6.00E-05	2.91	CR936742	CR936742	Homo sapiens mRNA; cDNA DKFZp686G1442 (from clone DKFZp686G1442). [CR936742]	
A_23_P16648	6.00E-05	2.222	NM_017573	NM_017573	Homo sapiens proprotein convertase subtilisin/kexin type 4 (PCSK4), mRNA [NM_017573]	NM_017573
A_23_P357936	6.00E-05	2.058	NM_014010	NM_014010	Homo sapiens astrotactin 2 (ASTN2), transcript variant 1, mRNA [NM_014010]	NM_014010
A_23_P143348	6.01E-05	10.06	NM_021220	NM_021220	Homo sapiens ovo-like 2 (Drosophila) (OVOL2), mRNA [NM_021220]	NM_021220
A_23_P360754	6.02E-05	12.42	NM_005099	NM_005099	Homo sapiens a disintegrin-like and metalloprotease (reprolysin type) with thrombospondin type 1 motif, 4 (ADAMTS4), mRNA [NM_005099]	NM_005099
A_23_P157736	6.02E-05	8.406	NM_032728	NM_032728	Homo sapiens phosphatidic acid phosphatase type 2 domain containing 3 (PPAPDC3), mRNA [NM_032728]	NM_032728
A_32_P87074	6.03E-05	2.612	AK022150	AK022150	Homo sapiens cDNA FLJ12088 fis, clone HEMBB1002545. [AK022150]	
A_23_P305060	6.05E-05	2.013	NM_005746	NM_005746	Homo sapiens pre-B-cell colony enhancing factor 1 (PBEF1), transcript variant 1, mRNA [NM_005746]	NM_005746
A_24_P310630	6.10E-05	3.12	NM_080632	NM_080632	Homo sapiens UPF3 regulator of nonsense transcripts homolog B (yeast) (UPF3B), transcript variant 1, mRNA [NM_080632]	NM_080632
A_32_P23125	6.15E-05	17.36	THC2435906		Q8BU53 (Q8BU53) Mus musculus 2 days pregnant adult female oviduct cDNA, RIKEN full-length enriched library, clone:E230038K10 product:procollagen-proline, 2-oxoglutarate 4-dioxygenase (proline 4-hydroxylase), alpha II polypeptide, full insert sequence,...	
A_32_P44808	6.15E-05	6.723	AK124941	AK124941	Homo sapiens cDNA FLJ42951 fis, clone BRSTN2007765. [AK124941]	
A_23_P4628	6.15E-05	4.373	NM_025027	NM_025027	Homo sapiens zinc finger protein 606 (ZNF606), mRNA [NM_025027]	NM_025027
A_23_P416965	6.16E-05	5.064	NM_015398	NM_015398	Homo sapiens DKFZP564J102 protein (DKFZP564J102), transcript variant 1, mRNA [NM_015398]	NM_015398
A_23_P328836	6.16E-05	3.762	NM_032440	NM_032440	Homo sapiens ligand-dependent corepressor (MLR2), mRNA [NM_032440]	NM_032440
A_32_P35433	6.16E-05	3.31	CR600638	CR600638	full-length cDNA clone CS0DI053YD12 of Placenta Cot 25-normalized of Homo sapiens (human). [CR600638]	
A_23_P371966	6.18E-05	4	AB075826	AB075826	Homo sapiens mRNA for KIAA1946 protein. [AB075826]	
A_23_P134827	6.19E-05	2.923	NM_004674	NM_004674	Homo sapiens ash2 (absent, small, or homeotic)-like (Drosophila) (ASH2L), mRNA [NM_004674]	NM_004674
A_24_P868583	6.20E-05	5.55	NM_001012421	NM_001012421	Homo sapiens OTTHUMP00000064580 (LOC441430), mRNA [NM_001012421]	NM_001012421
A_23_P121095	6.21E-05	4.149	NM_001068	NM_001068	Homo sapiens topoisomerase (DNA) II beta 180kDa (TOP2B), mRNA [NM_001068]	NM_001068
A_24_P191833	6.21E-05	4.083	NM_139168	NM_139168	Homo sapiens splicing factor, arginine/serine-rich 12 (SFRS12), mRNA [NM_139168]	NM_139168
A_24_P388528	6.21E-05	3.994	NM_173216	NM_173216	Homo sapiens ST6 beta-galactosamide alpha-2,6-sialyltranferase 1 (ST6GAL1), transcript variant 1, mRNA [NM_173216]	NM_173216
A_23_P27795	6.23E-05	12.82	NM_021102	NM_021102	Homo sapiens serine protease inhibitor, Kunitz type, 2 (SPINT2), mRNA [NM_021102]	NM_021102
A_23_P28307	6.23E-05	3.462	NP1165618		GB|AL832534.1|AL832534.1 Homo sapiens mRNA; cDNA DKFZp547I2016 (from clone DKFZp547I2016) [NP1165618]	
A_32_P4814	6.24E-05	2.302	NM_032508	NM_032508	Homo sapiens family with sequence similarity 11, member A (FAM11A), mRNA [NM_032508]	NM_032508

A_24_P478940	6.25E-05	6.676	A_24_P478940			
A_23_P102202	6.25E-05	3.995	NM_000179	NM_000179	Homo sapiens mutS homolog 6 (E. coli) (MSH6), mRNA [NM_000179]	NM_000179
A_24_P650482	6.25E-05	3.08	AK056822	AK056822	Homo sapiens cDNA FLJ32260 fis, clone PROST1000334. [AK056822]	
A_24_P372625	6.25E-05	2.899	NM_016422	NM_016422	Homo sapiens ring finger protein 141 (RNF141), mRNA [NM_016422]	NM_016422
A_24_P923142	6.26E-05	4.634	BC020784	BC020784	Homo sapiens similar to RIKEN cDNA 1200014N16 gene, mRNA (cDNA clone IMAGE:4773508), complete cds. [BC020784]	
A_23_P367610	6.26E-05	3.417	NM_178123	NM_178123	Homo sapiens SEC14 and spectrin domains 1 (SESTD1), mRNA [NM_178123]	NM_178123
A_24_P166794	6.27E-05	3.731	BC047111	BC047111	Homo sapiens, clone IMAGE:5314178, mRNA. [BC047111]	
A_24_P331882	6.28E-05	6.279	AL133028	AL133028	Homo sapiens mRNA; cDNA DKFZp434F117 (from clone DKFZp434F117). [AL133028]	XM_044178
A_32_P46817	6.28E-05	2.878	THC2280638		RL2A_HUMAN (P46776) 60S ribosomal protein L27a, partial (24%) [THC2280638]	
A_23_P359738	6.28E-05	2.1	NM_015630	NM_015630	Homo sapiens enhancer of polycomb homolog 2 (Drosophila) (EPC2), mRNA [NM_015630]	NM_015630
A_23_P144959	6.29E-05	8.024	NM_004385	NM_004385	Homo sapiens chondroitin sulfate proteoglycan 2 (versican) (CSPG2), mRNA [NM_004385]	NM_004385
A_32_P35232	6.29E-05	6.906	THC2404169			
A_24_P917015	6.29E-05	5.507	AF288405	AF288405	Homo sapiens G protein interaction factor 1-like mRNA sequence. [AF288405]	XM_370652
A_23_P254165	6.29E-05	5.185	NM_021785	NM_021785	Homo sapiens retinoic acid induced 2 (RAI2), mRNA [NM_021785]	NM_021785
A_24_P246573	6.29E-05	4.005	NM_001018000	NM_001018000	Homo sapiens kazrin (KIAA1026), transcript variant B, mRNA [NM_001018000]	NM_001018000
A_23_P252541	6.31E-05	6.804	NM_177403	NM_177403	Homo sapiens RAB7B, member RAS oncogene family (RAB7B), mRNA [NM_177403]	NM_177403
A_23_P388900	6.34E-05	8.012	NM_018420	NM_018420	Homo sapiens solute carrier family 22 (organic cation transporter), member 15 (SLC22A15), mRNA [NM_018420]	NM_018420
A_23_P202004	6.38E-05	2.819	NM_020200	NM_020200	Homo sapiens phosphoribosyl transferase domain containing 1 (PRTFDC1), mRNA [NM_020200]	NM_020200
A_23_P7212	6.40E-05	6.142	NM_000204	NM_000204	Homo sapiens I factor (complement) (IF), mRNA [NM_000204]	NM_000204
A_23_P213661	6.41E-05	3.61	NM_015216	NM_015216	Homo sapiens KIAA0433 protein (KIAA0433), mRNA [NM_015216]	NM_015216
A_24_P620456	6.41E-05	3.184	AK024921	AK024921	Homo sapiens cDNA: FLJ21268 fis, clone COL01718. [AK024921]	
A_24_P57631	6.43E-05	79.54	NM_004484	NM_004484	Homo sapiens glypican 3 (GPC3), mRNA [NM_004484]	NM_004484
A_23_P215060	6.43E-05	21.39	NM_005397	NM_005397	Homo sapiens podocalyxin-like (PODXL), transcript variant 2, mRNA [NM_005397]	NM_005397
A_24_P344961	6.43E-05	14.21	NM_133265	NM_133265	Homo sapiens angiomotin (AMOT), mRNA [NM_133265]	NM_133265
A_23_P169494	6.47E-05	25.62	NM_000607	NM_000607	Homo sapiens orosomucoid 1 (ORM1), mRNA [NM_000607]	NM_000607
A_23_P121234	6.47E-05	16.11	A_23_P121234			
A_23_P81721	6.47E-05	8.371	NM_004277	NM_004277	Homo sapiens solute carrier family 25, member 27 (SLC25A27), nuclear gene encoding mitochondrial protein, mRNA [NM_004277]	NM_004277
A_23_P139123	6.47E-05	4.46	NM_000062	NM_000062	Homo sapiens serine (or cysteine) proteinase inhibitor, clade G (C1 inhibitor), member 1, (angioedema, hereditary) (SERPING1), mRNA [NM_000062]	NM_000062
A_23_P55682	6.47E-05	3.091	NM_023926	NM_023926	Homo sapiens zinc finger protein 447 (ZNF447), mRNA [NM_023926]	NM_023926
A_24_P823708	6.49E-05	3.616	BC021732	BC021732	Homo sapiens, clone IMAGE:4795401, mRNA. [BC021732]	
A_23_P1286	6.52E-05	11.59	NM_144661	NM_144661	Homo sapiens chromosome 10 open reading frame 82 (C10orf82), mRNA [NM_144661]	NM_144661
A_24_P942250	6.53E-05	5.449	NM_025134	NM_025134	Homo sapiens chromodomain helicase DNA binding protein 9 (CHD9), mRNA [NM_025134]	NM_025134
A_23_P304682	6.55E-05	11.54	ENST00000320378		Homo sapiens cDNA FLJ39084 fis, clone NT2RP7018871. [AK096403]	
A_32_P194182	6.55E-05	4.644	A_32_P194182			
A_32_P161262	6.55E-05	2.238	THC2377294			
A_23_P134347	6.57E-05	25.01	NM_031311	NM_031311	Homo sapiens carboxypeptidase, vitellogenic-like (CPVL), transcript variant 1, mRNA [NM_031311]	NM_031311
A_23_P54006	6.58E-05	3.375	NM_015382	NM_015382	Homo sapiens HECT domain containing 1 (HECTD1), mRNA [NM_015382]	NM_015382
A_24_P79631	6.58E-05	3.249	CR598503	CR598503	full-length cDNA clone CS0DF014YO17 of Fetal brain of Homo sapiens (human). [CR598503]	
A_24_P941487	6.60E-05	6.932	NM_001008401	NM_001008401	Homo sapiens FLJ16231 protein (FLJ16231), mRNA [NM_001008401]	NM_001008401
A_23_P396804	6.60E-05	4.535	NM_198893	NM_198893	Homo sapiens zinc finger protein 160 (ZNF160), transcript variant 2, mRNA [NM_198893]	NM_198893
A_23_P381979	6.60E-05	3.097	NM_003605	NM_003605	Homo sapiens O-linked N-acetylglucosamine (GlcNAc) transferase (UDP-N-acetylglucosamine:polypeptide-N-acetylglucosaminyl transferase) (OGT), transcript variant 3, mRNA [NM_003605]	NM_003605

A_23_P107552	6.60E-05	2.057	NM_014939	NM_014939	Homo sapiens KIAA1012 (KIAA1012), mRNA [NM_014939]	NM_014939
A_24_P602168	6.62E-05	37.29	THC2314177		ALU1_HUMAN (P39188) Alu subfamily J sequence contamination warning entry, partial (9%) [THC2314177]	
A_23_P7342	6.64E-05	20.01	NM_001075	NM_001075	Homo sapiens UDP glucuronosyltransferase 2 family, polypeptide B10 (UGT2B10), mRNA [NM_001075]	NM_001075
A_24_P122921	6.64E-05	8.734	THC2305303		Q6JTU6 (Q6JTU6) BCL2-like 11 transcript variant 9, complete [THC2305303]	
A_24_P662177	6.64E-05	4.94	THC2448843		O57150 (O57150) H88, partial (32%) [THC2448843]	
A_23_P98446	6.64E-05	3.999	NM_001024956	NM_001024956	Homo sapiens sterol-C5-desaturase (ERG3 delta-5-desaturase homolog, fungal)-like (SC5DL), transcript variant 2, mRNA [NM_001024956]	NM_001024956
A_23_P85969	6.64E-05	3.971	NM_182976	NM_182976	Homo sapiens zinc finger protein 326 (ZNF326), transcript variant 1, mRNA [NM_182976]	NM_182976
A_24_P277934	6.66E-05	7.474	NM_000089	NM_000089	Homo sapiens collagen, type I, alpha 2 (COL1A2), mRNA [NM_000089]	NM_000089
A_23_P114084	6.66E-05	5.719	NM_000444	NM_000444	Homo sapiens phosphate regulating endopeptidase homolog, X-linked (hypophosphatemia, vitamin D resistant rickets) (PHEX), transcript variant 1843529, mRNA [NM_000444]	NM_000444
A_23_P217409	6.66E-05	3.746	NM_004652	NM_004652	Homo sapiens ubiquitin specific protease 9, X-linked (fat facets-like, Drosophila) (USP9X), transcript variant 1, mRNA [NM_004652]	NM_004652
A_23_P118571	6.75E-05	17.49	NM_025237	NM_025237	Homo sapiens sclerosteosis (SOST), mRNA [NM_025237]	NM_025237
A_23_P159952	6.76E-05	61.14	NM_018476	NM_018476	Homo sapiens brain expressed, X-linked 1 (BEX1), mRNA [NM_018476]	NM_018476
A_23_P25069	6.76E-05	8.582	BC039117	BC039117	Homo sapiens ovostatin 2, mRNA (cDNA clone IMAGE:4827636), with apparent retained intron. [BC039117]	XM_495907
A_24_P315120	6.77E-05	5.415	NM_001855	NM_001855	Homo sapiens collagen, type XV, alpha 1 (COL15A1), mRNA [NM_001855]	NM_001855
A_24_P320699	6.80E-05	21.72	NM_001013398	NM_001013398	Homo sapiens insulin-like growth factor binding protein 3 (IGFBP3), transcript variant 1, mRNA [NM_001013398]	NM_001013398
A_24_P419039	6.80E-05	12.34	NM_020766	NM_020766	Homo sapiens protocadherin 19 (PCDH19), mRNA [NM_020766]	NM_020766
A_23_P130333	6.82E-05	344.3	NM_000371	NM_000371	Homo sapiens transthyretin (prealbumin, amyloidosis type I) (TTR), mRNA [NM_000371]	NM_000371
A_24_P296808	6.82E-05	4.408	NM_018215	NM_018215	Homo sapiens hypothetical protein FLJ10781 (FLJ10781), mRNA [NM_018215]	NM_018215
A_23_P161719	6.82E-05	2.273	NM_152434	NM_152434	Homo sapiens CWF19-like 2, cell cycle control (S. pombe) (CWF19L2), mRNA [NM_152434]	NM_152434
A_23_P411723	6.83E-05	3.1	NM_002655	NM_002655	Homo sapiens pleiomorphic adenoma gene 1 (PLAG1), mRNA [NM_002655]	NM_002655
A_23_P31721	6.88E-05	2.236	NM_001951	NM_001951	Homo sapiens E2F transcription factor 5, p130-binding (E2F5), mRNA [NM_001951]	NM_001951
A_24_P454313	6.88E-05	2.221	NM_031157	NM_031157	Homo sapiens heterogeneous nuclear ribonucleoprotein A1 (HNRPA1), transcript variant 2, mRNA [NM_031157]	NM_031157
A_23_P44505	6.89E-05	3.691	NM_003597	NM_003597	Homo sapiens Kruppel-like factor 11 (KLF11), mRNA [NM_003597]	NM_003597
A_23_P214281	6.89E-05	2.132	NM_133367	NM_133367	Homo sapiens progestin and adipoQ receptor family member VIII (PAQR8), mRNA [NM_133367]	NM_133367
A_23_P111402	6.90E-05	44.5	NM_032784	NM_032784	Homo sapiens thrombospondin, type I, domain containing 2 (THSD2), mRNA [NM_032784]	NM_032784
A_23_P64617	6.90E-05	4.951	NM_012193	NM_012193	Homo sapiens frizzled homolog 4 (Drosophila) (FZD4), mRNA [NM_012193]	NM_012193
A_23_P390032	6.91E-05	3.179	NM_153226	NM_153226	Homo sapiens transmembrane protein 20 (TMEM20), mRNA [NM_153226]	NM_153226
A_23_P67198	6.93E-05	7.647	NM_015692	NM_015692	Homo sapiens C3 and PZP-like, alpha-2-macroglobulin domain containing 8 (CPAMD8), mRNA [NM_015692]	NM_015692
A_24_P926580	6.94E-05	6.568	AK001808	AK001808	Homo sapiens cDNA FLJ10946 fis, clone PLACE1000005. [AK001808]	
A_23_P258944	6.94E-05	2.618	NM_012328	NM_012328	Homo sapiens DnaJ (Hsp40) homolog, subfamily B, member 9 (DNAJB9), mRNA [NM_012328]	NM_012328
A_23_P208198	6.96E-05	3.491	AK093036	AK093036	Homo sapiens cDNA FLJ35717 fis, clone TESTI2000598. [AK093036]	
A_32_P106615	7.02E-05	23	AW268902	AW268902	AW268902 xv48h10.x1 Soares_NFL_T_GBC_S1 Homo sapiens cDNA clone IMAGE:2816419 3', mRNA sequence [AW268902]	
A_32_P71796	7.02E-05	6.695	THC2369196		ALU1_HUMAN (P39188) Alu subfamily J sequence contamination warning entry, partial (5%) [THC2369196]	
A_32_P148047	7.02E-05	4.445	A_32_P148047			
A_23_P158096	7.02E-05	3.91	AK021957	AK021957	Homo sapiens cDNA FLJ11895 fis, clone HEMBA1007301, weakly similar to COLLAGEN ALPHA 1(III) CHAIN. [AK021957]	
A_24_P132787	7.02E-05	3.137	NM_021252	NM_021252	Homo sapiens RAB18, member RAS oncogene family (RAB18), mRNA [NM_021252]	NM_021252
A_24_P88763	7.02E-05	2.359	NM_032603	NM_032603	Homo sapiens lysyl oxidase-like 3 (LOXL3), mRNA [NM_032603]	NM_032603
A_23_P408675	7.03E-05	3.005	NM_003972	NM_003972	Homo sapiens BTAF1 RNA polymerase II, B-TFIID transcription factor-associated, 170kDa (Mot1 homolog, S. cerevisiae) (BTAF1), mRNA [NM_003972]	NM_003972
A_23_P169278	7.07E-05	9.825	NM_015239	NM_015239	Homo sapiens ATP/GTP binding protein 1 (AGTPBP1), mRNA [NM_015239]	NM_015239

A_23_P57760	7.07E-05	5.148	NM_152282	NM_152282	Homo sapiens acid phosphatase-like 2 (ACPL2), mRNA [NM_152282]	NM_152282
A_24_P379413	7.09E-05	37.38	NM_000565	NM_000565	Homo sapiens interleukin 6 receptor (IL6R), transcript variant 1, mRNA [NM_000565]	NM_000565
A_32_P6344	7.12E-05	3.028	NM_003010	NM_003010	Homo sapiens mitogen-activated protein kinase kinase 4 (MAP2K4), mRNA [NM_003010]	NM_003010
A_23_P70660	7.13E-05	4.748	NM_017633	NM_017633	Homo sapiens family with sequence similarity 46, member A (FAM46A), mRNA [NM_017633]	NM_017633
A_23_P100420	7.13E-05	2.584	NM_015144	NM_015144	Homo sapiens zinc finger, CCHC domain containing 14 (ZCCHC14), mRNA [NM_015144]	NM_015144
A_23_P400373	7.17E-05	2.613	NM_152519	NM_152519	Homo sapiens hypothetical protein FLJ23861 (FLJ23861), mRNA [NM_152519]	NM_152519
A_24_P914817	7.21E-05	3.314	NM_013255	NM_013255	Homo sapiens muskelin 1, intracellular mediator containing kelch motifs (MKLN1), mRNA [NM_013255]	NM_013255
A_23_P381431	7.21E-05	2.45	NM_030769	NM_030769	Homo sapiens N-acetylneuraminate pyruvate lyase (dihydrodipicolinate synthase) (NPL), mRNA [NM_030769]	NM_030769
A_32_P77742	7.24E-05	24.64	THC2338229			
A_23_P46396	7.30E-05	6.489	NM_021190	NM_021190	Homo sapiens polypyrimidine tract binding protein 2 (PTBP2), mRNA [NM_021190]	NM_021190
A_32_P38228	7.30E-05	4.474	A_32_P38228			
A_23_P44768	7.30E-05	2.234	NM_013254	NM_013254	Homo sapiens TANK-binding kinase 1 (TBK1), mRNA [NM_013254]	NM_013254
A_24_P56317	7.35E-05	2.548	NM_144778	NM_144778	Homo sapiens muscleblind-like 2 (Drosophila) (MBNL2), transcript variant 1, mRNA [NM_144778]	NM_144778
A_23_P397635	7.35E-05	2.023	NM_007131	NM_007131	Homo sapiens zinc finger protein 75 (D8C6) (ZNF75), mRNA [NM_007131]	NM_007131
A_24_P167877	7.36E-05	5.038	BC036263	BC036263	Homo sapiens KIAA0220-like protein, mRNA (cDNA clone MGC:9515 IMAGE:3903371), complete cds. [BC036263]	XM_370939
A_23_P119464	7.36E-05	4.39	NM_033103	NM_033103	Homo sapiens rhophilin, Rho GTPase binding protein 2 (RHPN2), mRNA [NM_033103]	NM_033103
A_23_P256542	7.36E-05	3.963	NM_014367	NM_014367	Homo sapiens growth and transformation-dependent protein (E2IG5), mRNA [NM_014367]	NM_014367
A_32_P147969	7.36E-05	3.076	AL080232	AL080232	Homo sapiens mRNA; cDNA DKFZp586A061 (from clone DKFZp586A061) [AL080232]	
A_23_P107903	7.36E-05	2.578	AK126263	AK126263	Homo sapiens cDNA FLJ44275 fis, clone TOVAR2002800. [AK126263]	XM_290867
A_23_P398637	7.36E-05	2.476	NM_152292	NM_152292	Homo sapiens RNA (guanine-9-) methyltransferase domain containing 2 (RG9MTD2), mRNA [NM_152292]	NM_152292
A_32_P54186	7.36E-05	2.393	THC2343551			
A_23_P28213	7.36E-05	2.335	NM_131916	NM_131916	Homo sapiens peptidylprolyl isomerase (cyclophilin)-like 3 (PPIL3), transcript variant PPIL3c, mRNA [NM_131916]	NM_131916
A_23_P215051	7.36E-05	2.022	NM_018479	NM_018479	Homo sapiens enoyl Coenzyme A hydratase domain containing 1 (ECHDC1), transcript variant 2, mRNA [NM_018479]	NM_018479
A_24_P343233	7.37E-05	12.29	NM_002124	NM_002124	Homo sapiens major histocompatibility complex, class II, DR beta 1 (HLA-DRB1), mRNA [NM_002124]	NM_002124
A_23_P50081	7.37E-05	10.26	NM_014214	NM_014214	Homo sapiens inositol(myo)-1(or 4)-monophosphatase 2 (IMPA2), mRNA [NM_014214]	NM_014214
A_24_P895528	7.37E-05	8.676	AK022035	AK022035	Homo sapiens cDNA FLJ11973 fis, clone HEMBB1001221. [AK022035]	
A_32_P147622	7.37E-05	7.023	BC064982	BC064982	Homo sapiens cDNA clone IMAGE:2959727, partial cds. [BC064982]	XM_498568
A_23_P114947	7.37E-05	2.991	NM_002923	NM_002923	Homo sapiens regulator of G-protein signalling 2, 24kDa (RGS2), mRNA [NM_002923]	NM_002923
A_23_P335039	7.39E-05	7.73	NM_133474	NM_133474	Homo sapiens KIAA1982 protein (KIAA1982), mRNA [NM_133474]	NM_133474
A_23_P341537	7.41E-05	3.643	NM_031915	NM_031915	Homo sapiens SET domain, bifurcated 2 (SETDB2), mRNA [NM_031915]	NM_031915
A_32_P201958	7.44E-05	6.398	AK090739	AK090739	Homo sapiens cDNA FLJ33420 fis, clone BRACE2020028. [AK090739]	
A_23_P206733	7.44E-05	2.916	NM_001266	NM_001266	Homo sapiens carboxylesterase 1 (monocyte/macrophage serine esterase 1) (CES1), transcript variant 3, mRNA [NM_001266]	NM_001266
A_23_P207400	7.45E-05	2.095	NM_007295	NM_007295	Homo sapiens breast cancer 1, early onset (BRCA1), transcript variant BRCA1b, mRNA [NM_007295]	NM_007295
A_23_P91081	7.48E-05	79	NM_002354	NM_002354	Homo sapiens tumor-associated calcium signal transducer 1 (TACSTD1), mRNA [NM_002354]	NM_002354
A_23_P250164	7.51E-05	7.297	NM_000187	NM_000187	Homo sapiens homogentisate 1,2-dioxygenase (homogentisate oxidase) (HGD), mRNA [NM_000187]	NM_000187
A_32_P223551	7.51E-05	5.246	THC2374771		LPXK_RHIME (Q92RP7) Tetraacyldisaccharide 4'-kinase (Lipid A 4'-kinase) , partial (5%) [THC2374771]	
A_23_P138435	7.51E-05	3.533	NM_020338	NM_020338	Homo sapiens retinoic acid induced 17 (RAI17), mRNA [NM_020338]	NM_020338
A_24_P265274	7.51E-05	2.661	NM_000089	NM_000089	Homo sapiens collagen, type I, alpha 2 (COL1A2), mRNA [NM_000089]	NM_000089
A_24_P174341	7.55E-05	3.682	NM_058241	NM_058241	Homo sapiens cyclin T2 (CCNT2), transcript variant b, mRNA [NM_058241]	NM_058241
A_23_P148969	7.57E-05	2.51	NM_017768	NM_017768	Homo sapiens leucine rich repeat containing 40 (LRRC40), mRNA [NM_017768]	NM_017768
A_32_P127153	7.58E-05	2.427	NM_003104	NM_003104	Homo sapiens sorbitol dehydrogenase (SORD), mRNA [NM_003104]	NM_003104

A_23_P73297	7.59E-05	7.544	NM_004742	NM_004742	Homo sapiens membrane associated guanylate kinase, WW and PDZ domain containing 1 (MAGI1), mRNA [NM_004742]	NM_004742
A_24_P152649	7.59E-05	2.714	AK092522	AK092522	Homo sapiens cDNA FLJ35203 fis, clone PLACE6018441, moderately similar to Mus musculus peroxisomal long chain acyl-CoA thioesterase Ib (Pte1b) gene. [AK092522]	
A_23_P44244	7.62E-05	7.346	NM_003069	NM_003069	Homo sapiens SWI/SNF related, matrix associated, actin dependent regulator of chromatin, subfamily a, member 1 (SMARCA1), transcript variant 1, mRNA [NM_003069]	NM_003069
A_23_P115824	7.65E-05	4.821	NM_012207	NM_012207	Homo sapiens heterogeneous nuclear ribonucleoprotein H3 (2H9) (HNRPH3), transcript variant 2H9, mRNA [NM_012207]	NM_012207
A_23_P204579	7.66E-05	2.72	NM_003211	NM_003211	Homo sapiens thymine-DNA glycosylase (TDG), transcript variant 1, mRNA [NM_003211]	NM_003211
A_23_P46238	7.67E-05	5.881	NM_033440	NM_033440	Homo sapiens elastase 2A (ELA2A), mRNA [NM_033440]	NM_033440
A_32_P167631	7.67E-05	3.179	THC2320257			
A_24_P240338	7.67E-05	2.774	NM_001812	NM_001812	Homo sapiens centromere protein C 1 (CENPC1), mRNA [NM_001812]	NM_001812
A_23_P346048	7.69E-05	2.884	NM_001024681	NM_001024681	Homo sapiens D15F37 gene (D15F37), mRNA [NM_001024681]	NM_001024681
A_24_P360206	7.71E-05	3.721	NM_018902	NM_018902	Homo sapiens protocadherin alpha 11 (PCDHA11), transcript variant 1, mRNA [NM_018902]	NM_018902
A_23_P408455	7.72E-05	6.432	AL049246	AL049246	Homo sapiens mRNA; cDNA DKFZp564C053 (from clone DKFZp564C053). [AL049246]	
A_23_P256391	7.76E-05	4.474	NM_002078	NM_002078	Homo sapiens golgi autoantigen, golgin subfamily a, 4 (GOLGA4), mRNA [NM_002078]	NM_002078
A_23_P82651	7.82E-05	6.511	NM_002523	NM_002523	Homo sapiens neuronal pentraxin II (NPTX2), mRNA [NM_002523]	NM_002523
A_23_P72117	7.82E-05	5.244	NM_006714	NM_006714	Homo sapiens sphingomyelin phosphodiesterase, acid-like 3A (SMPDL3A), mRNA [NM_006714]	NM_006714
A_23_P345887	7.83E-05	5.706	AF445027	AF445027	Homo sapiens clone 114 tumor rejection antigen mRNA, complete cds. [AF445027]	
A_32_P168605	7.84E-05	37.3	BC039411	BC039411	Homo sapiens, clone IMAGE:5301690, mRNA. [BC039411]	
A_23_P167818	7.85E-05	3.215	NM_024581	NM_024581	Homo sapiens chromosome 6 open reading frame 60 (C6orf60), mRNA [NM_024581]	NM_024581
A_23_P115842	7.85E-05	2.335	NM_018237	NM_018237	Homo sapiens cell division cycle and apoptosis regulator 1 (CCAR1), mRNA [NM_018237]	NM_018237
A_23_P93027	7.86E-05	22.23	NM_003862	NM_003862	Homo sapiens fibroblast growth factor 18 (FGF18), transcript variant 1, mRNA [NM_003862]	NM_003862
A_23_P34827	7.88E-05	2.026	NM_020897	NM_020897	Homo sapiens hyperpolarization activated cyclic nucleotide-gated potassium channel 3 (HCN3), mRNA [NM_020897]	NM_020897
A_24_P13083	7.89E-05	10.46	NM_130783	NM_130783	Homo sapiens tetraspanin 18 (TSPAN18), mRNA [NM_130783]	NM_130783
A_23_P142974	7.89E-05	4.137	NM_001007231	NM_001007231	Homo sapiens Rho GTPase activating protein 25 (ARHGAP25), transcript variant 1, mRNA [NM_001007231]	NM_001007231
A_24_P303454	7.89E-05	3.933	NM_012454	NM_012454	Homo sapiens T-cell lymphoma invasion and metastasis 2 (TIAM2), transcript variant 1, mRNA [NM_012454]	NM_012454
A_24_P213161	7.89E-05	2.328	NM_017852	NM_017852	Homo sapiens NACHT, leucine rich repeat and PYD containing 2 (NALP2), mRNA [NM_017852]	NM_017852
A_32_P192922	7.91E-05	4.667	AL050061	AL050061	Homo sapiens mRNA; cDNA DKFZp566J123 (from clone DKFZp566J123). [AL050061]	
A_23_P428548	7.91E-05	3.255	NM_182641	NM_182641	Homo sapiens fetal Alzheimer antigen (FALZ), transcript variant 1, mRNA [NM_182641]	NM_182641
A_24_P941166	7.92E-05	2.992	NM_001001661	NM_001001661	Homo sapiens zinc finger protein 425 (ZNF425), mRNA [NM_001001661]	NM_001001661
A_24_P217572	7.93E-05	4.613	NM_001957	NM_001957	Homo sapiens endothelin receptor type A (EDNRA), mRNA [NM_001957]	NM_001957
A_23_P212159	7.94E-05	5.293	NM_024923	NM_024923	Homo sapiens nucleoporin 210kDa (NUP210), mRNA [NM_024923]	NM_024923
A_24_P649327	7.96E-05	3.292	AK124953	AK124953	Homo sapiens cDNA FLJ42963 fis, clone BRSTN2012380. [AK124953]	XM_372556
A_23_P373799	7.99E-05	2.383	NM_020943	NM_020943	Homo sapiens KIAA1604 protein (KIAA1604), mRNA [NM_020943]	NM_020943
A_24_P913005	7.99E-05	2.053	THC2372128		MMFXR1H9 fragile-X-related protein 1 isoform f {Mus musculus;} , partial (12%) [THC2372128]	
A_23_P125253	8.01E-05	3.783	NM_001290	NM_001290	Homo sapiens LIM domain binding 2 (LDB2), mRNA [NM_001290]	NM_001290
A_32_P117354	8.04E-05	7.104	NM_014988	NM_014988	Homo sapiens KIAA1102 protein (KIAA1102), mRNA [NM_014988]	NM_014988
A_23_P18692	8.06E-05	2.055	NM_000671	NM_000671	Homo sapiens alcohol dehydrogenase 5 (class III), chi polypeptide (ADH5), mRNA [NM_000671]	NM_000671
A_32_P146635	8.07E-05	112.8	CR603982	CR603982	full-length cDNA clone CS0DF021YL03 of Fetal brain of Homo sapiens (human). [CR603982]	
A_23_P64051	8.07E-05	5.916	A_23_P64051			
A_23_P364465	8.07E-05	4.974	NM_022913	NM_022913	Homo sapiens GC-rich promoter binding protein 1 (GPBP1), mRNA [NM_022913]	NM_022913
A_32_P202134	8.10E-05	11.11	AB051463	AB051463	Homo sapiens mRNA for KIAA1676 protein, partial cds. [AB051463]	
A_23_P67367	8.13E-05	6.288	NM_014475	NM_014475	Homo sapiens dihydrodiol dehydrogenase (dimeric) (DHDH), mRNA [NM_014475]	NM_014475
A_32_P125338	8.13E-05	2.557	NM_207334	NM_207334	Homo sapiens family with sequence similarity 43, member B (FAM43B), mRNA [NM_207334]	NM_207334

A_23_P416711	8.16E-05	18.73	NM_152996	NM_152996	Homo sapiens ST6 (alpha-N-acetyl-neuraminyl-2,3-beta-galactosyl-1,3)-N-acetylgalactosaminide alpha-2,6-sialyltransferase 3 (ST6GALNAC3), mRNA [NM_152996]	NM_152996
A_23_P20502	8.16E-05	7.352	NM_130849	NM_130849	Homo sapiens solute carrier family 39 (zinc transporter), member 4 (SLC39A4), transcript variant 2, mRNA [NM_130849]	NM_130849
A_23_P404606	8.16E-05	5.482	NM_153607	NM_153607	Homo sapiens adult retina protein (LOC153222), mRNA [NM_153607]	NM_153607
A_23_P357365	8.16E-05	2.11	NM_005862	NM_005862	Homo sapiens stromal antigen 1 (STAG1), mRNA [NM_005862]	NM_005862
A_23_P23526	8.17E-05	2.989	NM_033020	NM_033020	Homo sapiens tripartite motif-containing 33 (TRIM33), transcript variant beta, mRNA [NM_033020]	NM_033020
A_24_P34476	8.22E-05	3.315	NM_018151	NM_018151	Homo sapiens RAP1 interacting factor homolog (yeast) (RIF1), mRNA [NM_018151]	NM_018151
A_23_P143143	8.28E-05	16.09	NM_002166	NM_002166	Homo sapiens inhibitor of DNA binding 2, dominant negative helix-loop-helix protein (ID2), mRNA [NM_002166]	NM_002166
A_24_P648880	8.28E-05	3.771	NR_002211	NR_002211	Homo sapiens Meis1, myeloid ecotropic viral integration site 1 homolog 4 (mouse) (MEIS4) on chromosome 17 [NR_002211]	NR_002211
A_23_P7642	8.31E-05	5.089	NM_003118	NM_003118	Homo sapiens secreted protein, acidic, cysteine-rich (osteonectin) (SPARC), mRNA [NM_003118]	NM_003118
A_32_P167471	8.32E-05	36.08	AK125038	AK125038	Homo sapiens cDNA FLJ43048 fis, clone BRTHA3004502. [AK125038]	
A_24_P576174	8.32E-05	3.037	AK025344	AK025344	Homo sapiens cDNA: FLJ21691 fis, clone COL09555. [AK025344]	
A_24_P940921	8.33E-05	3.339	AK125448	AK125448	Homo sapiens cDNA FLJ43459 fis, clone OCBBF2035564. [AK125448]	
A_24_P753161	8.34E-05	2.691	NM_001204	NM_001204	Homo sapiens bone morphogenetic protein receptor, type II (serine/threonine kinase) (BMPR2), transcript variant 1, mRNA [NM_001204]	NM_001204
A_23_P113462	8.40E-05	14.31	NM_017641	NM_017641	Homo sapiens kinesin family member 21A (KIF21A), mRNA [NM_017641]	NM_017641
A_23_P70371	8.40E-05	4.444	NM_015153	NM_015153	Homo sapiens PHD finger protein 3 (PHF3), mRNA [NM_015153]	NM_015153
A_24_P929818	8.43E-05	3.144	S73202	S73202	argininosuccinate synthetase [human, Japanese classical citrullinemia patient A82, mRNA Partial Mutant, 91 nt]. [S73202]	
A_23_P96590	8.45E-05	22.24	NM_014710	NM_014710	Homo sapiens G protein-coupled receptor associated sorting protein 1 (GPRASP1), mRNA [NM_014710]	NM_014710
A_23_P22143	8.48E-05	9.385	NM_000283	NM_000283	Homo sapiens phosphodiesterase 6B, cGMP-specific, rod, beta (congenital stationary night blindness 3, autosomal dominant) (PDE6B), mRNA [NM_000283]	NM_000283
A_23_P73114	8.48E-05	7.882	NM_000313	NM_000313	Homo sapiens protein S (alpha) (PROS1), mRNA [NM_000313]	NM_000313
A_23_P31399	8.48E-05	2.784	NM_000305	NM_000305	Homo sapiens paraoxonase 2 (PON2), transcript variant 1, mRNA [NM_000305]	NM_000305
A_32_P85593	8.50E-05	2.181	NM_194324	NM_194324	Homo sapiens hypothetical protein MGC39900 (MGC39900), mRNA [NM_194324]	NM_194324
A_23_P145336	8.52E-05	11.84	V00522	V00522	Human mRNA encoding major histocompatibility complex gene HLA-DR beta-I. [V00522]	
A_23_P349615	8.52E-05	4.323	ENST00000361262		Homo sapiens mRNA for KIAA0594 protein, partial cds. [AB011166]	
A_23_P128728	8.52E-05	4.064	NM_001172	NM_001172	Homo sapiens arginase, type II (ARG2), nuclear gene encoding mitochondrial protein, mRNA [NM_001172]	NM_001172
A_32_P224888	8.53E-05	7.252	AK054895	AK054895	Homo sapiens cDNA FLJ30333 fis, clone BRACE2007262. [AK054895]	
A_23_P51317	8.53E-05	2.898	NM_019083	NM_019083	Homo sapiens hypothetical protein FLJ10287 (FLJ10287), mRNA [NM_019083]	NM_019083
A_23_P73192	8.56E-05	12.04	NM_024726	NM_024726	Homo sapiens IQ motif containing with AAA domain (IQCA), mRNA [NM_024726]	NM_024726
A_24_P23625	8.57E-05	3.457	ENST00000245519		Homo sapiens heparan sulfate (glucosamine) 3-O-sulfotransferase 3B1, mRNA (cDNA clone MGC:71688 IMAGE:30343352), complete cds. [BC063301]	
A_23_P148204	8.57E-05	2.319	NM_017944	NM_017944	Homo sapiens ubiquitin specific protease 47 (USP47), mRNA [NM_017944]	NM_017944
A_23_P410965	8.58E-05	6.532	NM_020888	NM_020888	Homo sapiens KIAA1522 (KIAA1522), mRNA [NM_020888]	NM_020888
A_23_P65262	8.59E-05	4.454	NM_033111	NM_033111	Homo sapiens CG016 (LOC88523), mRNA [NM_033111]	NM_033111
A_23_P115902	8.68E-05	3.834	A_23_P115902			
A_23_P367628	8.73E-05	3.152	NM_014802	NM_014802	Homo sapiens KIAA0528 gene product (KIAA0528), mRNA [NM_014802]	NM_014802
A_32_P4581	8.74E-05	13.72	AK130118	AK130118	Homo sapiens cDNA FLJ26608 fis, clone LVR00914. [AK130118]	
A_23_P120048	8.74E-05	5.132	NM_013450	NM_013450	Homo sapiens bromodomain adjacent to zinc finger domain, 2B (BAZ2B), mRNA [NM_013450]	NM_013450
A_24_P110558	8.74E-05	4.779	NM_001007189	NM_001007189	Homo sapiens similar to bovine IgA regulatory protein (LOC492311), mRNA [NM_001007189]	NM_001007189
A_23_P217704	8.74E-05	3.976	NM_003918	NM_003918	Homo sapiens glycogenin 2 (GYG2), mRNA [NM_003918]	NM_003918
A_24_P337546	8.74E-05	3.748	NM_014895	NM_014895	Homo sapiens chromosome 6 open reading frame 84 (C6orf84), mRNA [NM_014895]	NM_014895
A_32_P211708	8.76E-05	18.76	THC2277716			

A_23_P325411	8.76E-05	4.639	THC2310302		Q9Y474 (Q9Y474) DNA-binding protein, complete [THC2310302]	
A_32_P211353	8.76E-05	4.08	THC2272851		Q7V9T5 (Q7V9T5) Uncharacterized membrane protein, partial (10%) [THC2272851]	
A_23_P161424	8.76E-05	2.473	NM_032812	NM_032812	Homo sapiens plexin domain containing 2 (PLXDC2), mRNA [NM_032812]	NM_032812
A_32_P190049	8.85E-05	2.596	AK056809	AK056809	Homo sapiens cDNA FLJ32247 fis, clone PROST1000120. [AK056809]	
A_23_P89921	8.89E-05	3.706	NM_013256	NM_013256	Homo sapiens zinc finger protein 180 (HHZ168) (ZNF180), mRNA [NM_013256]	NM_013256
A_23_P65699	8.89E-05	2.747	NM_025137	NM_025137	Homo sapiens hypothetical protein FLJ21439 (FLJ21439), mRNA [NM_025137]	NM_025137
A_23_P158976	8.90E-05	7.482	NM_000392	NM_000392	Homo sapiens ATP-binding cassette, sub-family C (CFTR/MRP), member 2 (ABCC2), mRNA [NM_000392]	NM_000392
A_23_P301530	8.97E-05	11.82	NM_020987	NM_020987	Homo sapiens ankyrin 3, node of Ranvier (ankyrin G) (ANK3), transcript variant 1, mRNA [NM_020987]	NM_020987
A_32_P16989	9.12E-05	4.952	A_32_P16989			
A_23_P375165	9.20E-05	3.361	NM_207459	NM_207459	Homo sapiens FLJ35767 protein (FLJ35767), mRNA [NM_207459]	NM_207459
A_23_P32577	9.21E-05	8.41	NM_080759	NM_080759	Homo sapiens dachshund homolog 1 (Drosophila) (DACH1), transcript variant 1, mRNA [NM_080759]	NM_080759
A_24_P921260	9.21E-05	3.412	AK022030	AK022030	Homo sapiens cDNA FLJ11968 fis, clone HEMBB1001133. [AK022030]	
A_23_P389102	9.22E-05	9.63	NM_015194	NM_015194	Homo sapiens myosin ID (MYO1D), mRNA [NM_015194]	NM_015194
A_24_P807031	9.22E-05	4.411	NM_001017971	NM_001017971	Homo sapiens hypothetical protein LOC92270 (LOC92270), mRNA [NM_001017971]	NM_001017971
A_32_P152767	9.24E-05	15.26	NM_207371	NM_207371	Homo sapiens FLJ45187 protein (FLJ45187), mRNA [NM_207371]	NM_207371
A_24_P226700	9.24E-05	4.198	BX648950	BX648950	Homo sapiens mRNA; cDNA DKFZp686E1648 (from clone DKFZp686E1648). [BX648950]	
A_23_P157460	9.24E-05	3.768	NM_018660	NM_018660	Homo sapiens zinc finger protein 395 (ZNF395), mRNA [NM_018660]	NM_018660
A_24_P365327	9.24E-05	2.604	NM_015578	NM_015578	Homo sapiens family with sequence similarity 61, member A (FAM61A), mRNA [NM_015578]	NM_015578
A_23_P151805	9.24E-05	2.441	NM_006329	NM_006329	Homo sapiens fibulin 5 (FBLN5), mRNA [NM_006329]	NM_006329
A_24_P110564	9.24E-05	2.372	NM_006985	NM_006985	Homo sapiens nuclear pore complex interacting protein (NPIP), mRNA [NM_006985]	NM_006985
A_23_P339954	9.24E-05	2.323	NM_001018059	NM_001018059	Homo sapiens similar to nuclear pore complex interacting protein (LOC440348), mRNA [NM_001018059]	NM_001018059
A_24_P854964	9.25E-05	2.474	BC037740	BC037740	Homo sapiens cDNA clone IMAGE:5263531, partial cds. [BC037740]	
A_32_P9127	9.28E-05	3.704	THC2406727			
A_32_P70724	9.28E-05	3.123	NM_006618	NM_006618	Homo sapiens Jumonji, AT rich interactive domain 1B (RBP2-like) (JARID1B), mRNA [NM_006618]	NM_006618
A_23_P151565	9.37E-05	3.808	NM_014990	NM_014990	Homo sapiens GTPase activating Rap/RanGAP domain-like 1 (GARNL1), transcript variant 1, mRNA [NM_014990]	NM_014990
A_23_P257649	9.38E-05	15.27	NM_002899	NM_002899	Homo sapiens retinol binding protein 1, cellular (RBP1), mRNA [NM_002899]	NM_002899
A_23_P207221	9.38E-05	8.368	NM_018242	NM_018242	Homo sapiens hypothetical protein FLJ10847 (FLJ10847), mRNA [NM_018242]	NM_018242
A_23_P148584	9.41E-05	8.141	NM_144658	NM_144658	Homo sapiens dedicator of cytokinesis 11 (DOCK11), mRNA [NM_144658]	NM_144658
A_23_P252155	9.41E-05	4.11	NM_018387	NM_018387	Homo sapiens spermatid perinuclear RNA binding protein (STRBP), mRNA [NM_018387]	NM_018387
A_24_P541576	9.43E-05	3.41	AK026938	AK026938	Homo sapiens cDNA: FLJ23285 fis, clone HEP09071. [AK026938]	XM_374317
A_23_P147423	9.44E-05	19.96	NM_182920	NM_182920	Homo sapiens a disintegrin-like and metalloprotease (reprolysin type) with thrombospondin type 1 motif, 9 (ADAMTS9), mRNA [NM_182920]	NM_182920
A_23_P303087	9.47E-05	34.21	NM_002825	NM_002825	Homo sapiens pleiotrophin (heparin binding growth factor 8, neurite growth-promoting factor 1) (PTN), mRNA [NM_002825]	NM_002825
A_24_P321525	9.47E-05	21.92	NM_032918	NM_032918	Homo sapiens RAS-like, estrogen-regulated, growth inhibitor (RERG), mRNA [NM_032918]	NM_032918
A_23_P120435	9.47E-05	2.114	NM_080614	NM_080614	Homo sapiens WAP four-disulfide core domain 3 (WFDC3), transcript variant 1, mRNA [NM_080614]	NM_080614
A_23_P78518	9.52E-05	3.445	AK128234	AK128234	Homo sapiens cDNA FLJ46368 fis, clone TESTI4051504. [AK128234]	
A_24_P53778	9.55E-05	19.37	NM_080878	NM_080878	Homo sapiens intelectin 2 (ITLN2), mRNA [NM_080878]	NM_080878
A_23_P69537	9.56E-05	17.55	NM_006681	NM_006681	Homo sapiens neuromedin U (NMU), mRNA [NM_006681]	NM_006681
A_23_P106241	9.57E-05	2.882	NM_004239	NM_004239	Homo sapiens thyroid hormone receptor interactor 11 (TRIP11), mRNA [NM_004239]	NM_004239
A_24_P940803	9.57E-05	2.252	NM_033505	NM_033505	Homo sapiens selenoprotein I (SELI), mRNA [NM_033505]	NM_033505
A_23_P127279	9.57E-05	2.172	NM_019054	NM_019054	Homo sapiens family with sequence similarity 35, member A (FAM35A), mRNA [NM_019054]	NM_019054
A_32_P140656	9.58E-05	5.451	CR621148	CR621148	full-length cDNA clone CS0DC029YJ02 of Neuroblastoma Cot 25-normalized of Homo sapiens (human). [CR621148]	
A_23_P97181	9.67E-05	29.32	NM_022469	NM_022469	Homo sapiens gremlin 2, cysteine knot superfamily, homolog (Xenopus laevis) (GREM2), mRNA [NM_022469]	NM_022469

A_23_P100660	9.67E-05	15.3	NM_002615	NM_002615	Homo sapiens serine (or cysteine) proteinase inhibitor, clade F (alpha-2 antiplasmin, pigment epithelium derived factor), member 1 (SERPINF1), mRNA [NM_002615]	NM_002615
A_23_P200930	9.67E-05	2.025	NM_000254	NM_000254	Homo sapiens 5-methyltetrahydrofolate-homocysteine methyltransferase (MTR), mRNA [NM_000254]	NM_000254
A_24_P286054	9.68E-05	8.181	AB002303	AB002303	Homo sapiens mRNA for KIAA0305 gene, partial cds. [AB002303]	
A_23_P5983	9.68E-05	7.029	NM_006227	NM_006227	Homo sapiens phospholipid transfer protein (PLTP), transcript variant 1, mRNA [NM_006227]	NM_006227
A_24_P177353	9.68E-05	4.263	BC054050	BC054050	Homo sapiens THO complex 2, mRNA (cDNA clone IMAGE:5556338), partial cds. [BC054050]	
A_23_P87810	9.73E-05	2.846	THC2373936		O19057 (O19057) Fertilin alpha protein, partial (18%) [THC2373936]	
A_32_P91107	9.73E-05	2.501	BC089156	BC089156	Homo sapiens cDNA clone IMAGE:6576427. [BC089156]	
A_23_P155556	9.79E-05	2.194	NM_019895	NM_019895	Homo sapiens chromosome 3 open reading frame 4 (C3orf4), mRNA [NM_019895]	NM_019895
A_23_P357504	9.80E-05	4.159	ENST00000328520		Q765F0 (Q765F0) PreC/core protein, partial (13%) [THC2251776]	
A_23_P128919	9.80E-05	3.274	NM_002306	NM_002306	Homo sapiens lectin, galactoside-binding, soluble, 3 (galectin 3) (LGALS3), mRNA [NM_002306]	NM_002306
A_23_P88209	9.80E-05	2.825	NM_016106	NM_016106	Homo sapiens sec1 family domain containing 1 (SCFD1), transcript variant 1, mRNA [NM_016106]	NM_016106
A_23_P157926	9.83E-05	6.573	NM_152570	NM_152570	Homo sapiens leucine rich repeat neuronal 6C (LRRN6C), mRNA [NM_152570]	NM_152570
A_23_P144684	9.84E-05	4.175	NM_032290	NM_032290	Homo sapiens ankyrin repeat domain 32 (ANKRD32), mRNA [NM_032290]	NM_032290
A_32_P156851	9.85E-05	12.62	NM_005822	NM_005822	Homo sapiens Down syndrome critical region gene 1-like 1 (DSCR1L1), mRNA [NM_005822]	NM_005822
A_23_P91350	9.89E-05	3.746	ENST00000265183		Homo sapiens mRNA for KIAA1434 protein, partial cds. [AB037855]	
A_23_P258698	9.89E-05	3.039	NM_005908	NM_005908	Homo sapiens mannosidase, beta A, lysosomal (MANBA), mRNA [NM_005908]	NM_005908
A_23_P141415	9.91E-05	4.165	NM_032133	NM_032133	Homo sapiens MYCBP associated protein (MYCBPAP), mRNA [NM_032133]	NM_032133
A_24_P396720	9.91E-05	2.092	NM_002709	NM_002709	Homo sapiens protein phosphatase 1, catalytic subunit, beta isoform (PPP1CB), transcript variant 1, mRNA [NM_002709]	NM_002709
A_23_P27306	9.98E-05	142.7	NM_030781	NM_030781	Homo sapiens collectin sub-family member 12 (COLEC12), transcript variant II, mRNA [NM_030781]	NM_030781
A_24_P142503	9.98E-05	35.38	NM_018242	NM_018242	Homo sapiens hypothetical protein FLJ10847 (FLJ10847), mRNA [NM_018242]	NM_018242
A_23_P361841	1.00E-04	3.355	ENST00000357529		Homo sapiens mRNA for KIAA1238 protein, partial cds. [AB033064]	
A_32_P167904	0.000101	8.911	CR624679	CR624679	full-length cDNA clone CS0DF003YC20 of Fetal brain of Homo sapiens (human). [CR624679]	
A_24_P14584	0.000101	3.348	NM_012105	NM_012105	Homo sapiens beta-site APP-cleaving enzyme 2 (BACE2), transcript variant a, mRNA [NM_012105]	NM_012105
A_23_P66454	0.000101	2.984	NM_018530	NM_018530	Homo sapiens gasdermin-like (GSDML), mRNA [NM_018530]	NM_018530
A_32_P218806	0.000101	2.919	AK024516	AK024516	Homo sapiens cDNA: FLJ20863 fis, clone ADKA01804. [AK024516]	
A_23_P102391	0.000102	183.3	NM_014585	NM_014585	Homo sapiens solute carrier family 40 (iron-regulated transporter), member 1 (SLC40A1), mRNA [NM_014585]	NM_014585
A_32_P4626	0.000102	25.41	AF143325	AF143325	Homo sapiens clone IMAGE:110436 mRNA sequence. [AF143325]	
A_23_P356216	0.000102	4.795	BC026965	BC026965	Homo sapiens cDNA clone MGC:34901 IMAGE:5103002, complete cds. [BC026965]	
A_24_P13041	0.000102	4.116	NM_145307	NM_145307	Homo sapiens pleckstrin homology domain containing, family K member 1 (PLEKHK1), mRNA [NM_145307]	NM_145307
A_23_P113005	0.000102	3.822	NM_004428	NM_004428	Homo sapiens ephrin-A1 (EFNA1), transcript variant 1, mRNA [NM_004428]	NM_004428
A_24_P82200	0.000102	3.061	NM_170676	NM_170676	Homo sapiens Meis1, myeloid ecotropic viral integration site 1 homolog 2 (mouse) (MEIS2), transcript variant d, mRNA [NM_170676]	NM_170676
A_23_P20443	0.000102	2.298	NM_021020	NM_021020	Homo sapiens leucine zipper, putative tumor suppressor 1 (LZTS1), mRNA [NM_021020]	NM_021020
A_24_P399174	0.000102	2.243	NM_004703	NM_004703	Homo sapiens rabaptin, RAB GTPase binding effector protein 1 (RABEP1), mRNA [NM_004703]	NM_004703
A_23_P202334	0.000103	92.02	NM_023028	NM_023028	Homo sapiens fibroblast growth factor receptor 2 (bacteria-expressed kinase, keratinocyte growth factor receptor, craniofacial dysostosis 1, Crouzon syndrome, Pfeiffer syndrome, Jackson-Weiss syndrome) (FGFR2), transcript variant 10, mRNA [NM_023028]	NM_023028
A_23_P204286	0.000103	19.1	NM_000900	NM_000900	Homo sapiens matrix Gla protein (MGP), mRNA [NM_000900]	NM_000900
A_32_P67303	0.000103	4.926	THC2328806			
A_32_P22750	0.000103	2.83	CA421238	CA421238	CA421238 UI-H-FG0-bct-h-02-0-UI.s1 NCI_CGAP_EN1_2 Homo sapiens cDNA clone UI-H-FG0-bct-h-02-0-UI 3', mRNA sequence [CA421238]	
A_24_P213478	0.000103	2.54	NM_020796	NM_020796	Homo sapiens sema domain, transmembrane domain (TM), and cytoplasmic domain, (semaphorin) 6A (SEMA6A), mRNA [NM_020796]	NM_020796
A_23_P12044	0.000103	2.485	NM_018022	NM_018022	Homo sapiens transmembrane protein 51 (TMEM51), mRNA [NM_018022]	NM_018022

A_24_P30923	0.000103	2.469	NM_003498	NM_003498	Homo sapiens stannin (SNN), mRNA [NM_003498]	NM_003498
A_23_P314145	0.000103	2.189	NM_005715	NM_005715	Homo sapiens uronyl-2-sulfotransferase (UST), mRNA [NM_005715]	NM_005715
A_32_P45844	0.000104	6.673	THC2312104		NAC2_HUMAN (Q9UPR5) Sodium/calcium exchanger 2 precursor (Na(+)/Ca(2+)-exchange protein 2), partial (82%) [THC2312104]	
A_32_P79584	0.000104	4.565	NM_001011553	NM_001011553	Homo sapiens septin 7 (SEPT7), transcript variant 2, mRNA [NM_001011553]	NM_001011553
A_24_P149124	0.000104	3.875	NM_004772	NM_004772	Homo sapiens chromosome 5 open reading frame 13 (C5orf13), mRNA [NM_004772]	NM_004772
A_23_P332439	0.000104	3.615	AB007870	AB007870	Homo sapiens KIAA0410 mRNA, partial cds. [AB007870]	
A_23_P82738	0.000104	3.604	NM_012415	NM_012415	Homo sapiens RAD54 homolog B (S. cerevisiae) (RAD54B), transcript variant 1, mRNA [NM_012415]	NM_012415
A_23_P101811	0.000104	3.505	NM_021030	NM_021030	Homo sapiens zinc finger protein 14 (KOX 6) (ZNF14), mRNA [NM_021030]	NM_021030
A_23_P424002	0.000104	3.244	NM_002697	NM_002697	Homo sapiens POU domain, class 2, transcription factor 1 (POU2F1), mRNA [NM_002697]	NM_002697
A_24_P388433	0.000104	2.913	NM_002718	NM_002718	Homo sapiens protein phosphatase 2 (formerly 2A), regulatory subunit B'', alpha (PPP2R3A), transcript variant 1, mRNA [NM_002718]	NM_002718
A_23_P251785	0.000104	2.572	NM_024561	NM_024561	Homo sapiens NMDA receptor regulated 1-like (NARG1L), transcript variant 1, mRNA [NM_024561]	NM_024561
A_24_P362904	0.000105	5.006	NM_004567	NM_004567	Homo sapiens 6-phosphofructo-2-kinase/fructose-2,6-biphosphatase 4 (PFKFB4), mRNA [NM_004567]	NM_004567
A_24_P300777	0.000105	3.327	NM_001109	NM_001109	Homo sapiens a disintegrin and metalloproteinase domain 8 (ADAM8), mRNA [NM_001109]	NM_001109
A_32_P207436	0.000105	3.245	BC007307	BC007307	Homo sapiens, Similar to zinc finger protein 268, clone IMAGE:3352268, mRNA, partial cds. [BC007307]	XM_039908
A_24_P102053	0.000106	10.29	NM_002538	NM_002538	Homo sapiens occludin (OCLN), mRNA [NM_002538]	NM_002538
A_23_P8913	0.000106	5.244	NM_000067	NM_000067	Homo sapiens carbonic anhydrase II (CA2), mRNA [NM_000067]	NM_000067
A_24_P627984	0.000106	5.154	THC2437914		ALU1_HUMAN (P39188) Alu subfamily J sequence contamination warning entry, partial (15%) [THC2437914]	
A_24_P14464	0.000106	3.847	NM_080736	NM_080736	Homo sapiens WAP four-disulfide core domain 2 (WFDC2), transcript variant 2, mRNA [NM_080736]	NM_080736
A_24_P406693	0.000107	5.927	NM_000917	NM_000917	Homo sapiens procollagen-proline, 2-oxoglutarate 4-dioxygenase (proline 4-hydroxylase), alpha polypeptide I (P4HA1), transcript variant 1, mRNA [NM_000917]	NM_000917
A_24_P716394	0.000107	4.116	THC2360841		ALU7_HUMAN (P39194) Alu subfamily SQ sequence contamination warning entry, partial (10%) [THC2360844]	
A_23_P372888	0.000108	4.808	NM_006918	NM_006918	Homo sapiens sterol-C5-desaturase (ERG3 delta-5-desaturase homolog, fungal)-like (SC5DL), transcript variant 1, mRNA [NM_006918]	NM_006918
A_23_P40059	0.000108	3.319	NM_000534	NM_000534	Homo sapiens PMS1 postmeiotic segregation increased 1 (S. cerevisiae) (PMS1), mRNA [NM_000534]	NM_000534
A_23_P50276	0.000109	14.58	NM_031917	NM_031917	Homo sapiens angiopoietin-like 6 (ANGPTL6), mRNA [NM_031917]	NM_031917
A_23_P360797	0.000109	4.816	NM_002527	NM_002527	Homo sapiens neurotrophin 3 (NTF3), mRNA [NM_002527]	NM_002527
A_24_P238525	0.000109	3.031	ENST00000324709			
A_24_P54847	0.000109	2.602	CR603201	CR603201	full-length cDNA clone CS0DF008YB18 of Fetal brain of Homo sapiens (human). [CR603201]	
A_23_P22548	0.000109	2.566	NM_152579	NM_152579	Homo sapiens hypothetical protein FLJ38564 (FLJ38564), mRNA [NM_152579]	NM_152579
A_32_P6253	0.000109	2.459	THC2279919			
A_32_P55427	0.00011	5.056	THC2319816		Q9F8M7 (Q9F8M7) DTDP-glucose 4,6-dehydratase (Fragment), partial (11%) [THC2319816]	
A_24_P942694	0.00011	3.986	NM_018017	NM_018017	Homo sapiens chromosome 10 open reading frame 118 (C10orf118), mRNA [NM_018017]	NM_018017
A_32_P192480	0.000111	47.68	CR627122	CR627122	Homo sapiens mRNA; cDNA DKFZp779M2422 (from clone DKFZp779M2422). [CR627122]	
A_23_P22013	0.000111	30.98	NM_173640	NM_173640	Homo sapiens roof plate-specific spondin (RSPONDIN), mRNA [NM_173640]	NM_173640
A_32_P232647	0.000111	27.97	A_32_P232647			
A_23_P70359	0.000111	6.304	NM_024929	NM_024929	Homo sapiens chromosome 6 open reading frame 59 (C6orf59), mRNA [NM_024929]	NM_024929
A_23_P3532	0.000111	5.176	NM_004862	NM_004862	Homo sapiens lipopolysaccharide-induced TNF factor (LITAF), mRNA [NM_004862]	NM_004862
A_32_P59673	0.000111	3.744	NM_199136	NM_199136	Homo sapiens hypothetical protein MGC72075 (MGC72075), mRNA [NM_199136]	NM_199136
A_23_P115064	0.000111	3.588	NM_001878	NM_001878	Homo sapiens cellular retinoic acid binding protein 2 (CRABP2), mRNA [NM_001878]	NM_001878
A_32_P211080	0.000111	2.593	NM_001024681	NM_001024681	Homo sapiens D15F37 gene (D15F37), mRNA [NM_001024681]	NM_001024681
A_23_P156431	0.000112	5.844	NM_005907	NM_005907	Homo sapiens mannosidase, alpha, class 1A, member 1 (MAN1A1), mRNA [NM_005907]	NM_005907
A_32_P122136	0.000112	5.525	AK057596	AK057596	Homo sapiens cDNA FLJ33034 fis, clone THYMU2000236. [AK057596]	XM_498456
A_23_P19894	0.000113	50.35	NM_198098	NM_198098	Homo sapiens aquaporin 1 (channel-forming integral protein, 28kDa) (AQP1), transcript variant 1, mRNA [NM_198098]	NM_198098

A_23_P9402	0.000113	9.339	NM_147164	NM_147164	Homo sapiens ciliary neurotrophic factor receptor (CNTFR), transcript variant 1, mRNA [NM_147164]	NM_147164
A_23_P395555	0.000113	5.763	NM_016444	NM_016444	Homo sapiens zinc finger protein 226 (ZNF226), mRNA [NM_016444]	NM_016444
A_24_P504050	0.000113	4.504	BX648484	BX648484	Homo sapiens mRNA; cDNA DKFZp686O1555 (from clone DKFZp686O1555). [BX648484]	
A_24_P323072	0.000113	4.144	NM_178332	NM_178332	Homo sapiens gonadotropin-releasing hormone 2 (GNRH2), transcript variant 2, mRNA [NM_178332]	NM_178332
A_23_P7543	0.000113	3.111	NM_014733	NM_014733	Homo sapiens zinc finger, FYVE domain containing 16 (ZFYVE16), mRNA [NM_014733]	NM_014733
A_24_P844995	0.000113	2.258	AK054826	AK054826	Homo sapiens cDNA FLJ30264 fis, clone BRACE2002613, moderately similar to Rattus norvegicus rsly1p mRNA. [AK054826]	
A_23_P152066	0.000113	2.247	AK054969	AK054969	Homo sapiens cDNA FLJ30407 fis, clone BRACE2008553. [AK054969]	
A_23_P98304	0.000114	7.997	NM_018043	NM_018043	Homo sapiens transmembrane protein 16A (TMEM16A), mRNA [NM_018043]	NM_018043
A_23_P15226	0.000114	4.036	A_23_P15226			
A_23_P127426	0.000114	2.826	NM_003273	NM_003273	Homo sapiens transmembrane 7 superfamily member 2 (TM7SF2), mRNA [NM_003273]	NM_003273
A_23_P353704	0.000114	2.252	NM_019593	NM_019593	Homo sapiens hypothetical protein KIAA1434 (KIAA1434), mRNA [NM_019593]	NM_019593
A_23_P144348	0.000115	17.02	NM_004787	NM_004787	Homo sapiens slit homolog 2 (Drosophila) (SLIT2), mRNA [NM_004787]	NM_004787
A_23_P133000	0.000115	5.329	AK021601	AK021601	Homo sapiens cDNA FLJ11539 fis, clone HEMBA1002748. [AK021601]	XM_496724
A_32_P23854	0.000115	2.265	NM_020946	NM_020946	Homo sapiens KIAA1608 (KIAA1608), transcript variant 1, mRNA [NM_020946]	NM_020946
A_23_P399265	0.000116	28.59	S82024	S82024	SCG10=neuron-specific growth-associated protein/stathmin homolog [human, embryo, mRNA, 696 nt]. [S82024]	
A_24_P402222	0.000116	10.33	NM_022555	NM_022555	Homo sapiens major histocompatibility complex, class II, DR beta 3 (HLA-DRB3), mRNA [NM_022555]	NM_022555
A_23_P105251	0.000116	6.855	NM_005269	NM_005269	Homo sapiens glioma-associated oncogene homolog 1 (zinc finger protein) (GLI1), mRNA [NM_005269]	NM_005269
A_32_P181166	0.000116	5.429	NM_174896	NM_174896	Homo sapiens chromosome 1 open reading frame 162 (C1orf162), mRNA [NM_174896]	NM_174896
A_23_P69877	0.000116	2.962	AK091550	AK091550	Homo sapiens cDNA FLJ34231 fis, clone FCBBF3025905, highly similar to Mus musculus (clone pMLZ-1) zinc finger protein (Zfp) mRNA. [AK091550]	
A_23_P127220	0.000117	11.05	NM_021800	NM_021800	Homo sapiens DnaJ (Hsp40) homolog, subfamily C, member 12 (DNAJC12), transcript variant 1, mRNA [NM_021800]	NM_021800
A_24_P119141	0.000117	7.671	NM_000313	NM_000313	Homo sapiens protein S (alpha) (PROS1), mRNA [NM_000313]	NM_000313
A_23_P156425	0.000117	6.612	NM_005907	NM_005907	Homo sapiens mannosidase, alpha, class 1A, member 1 (MAN1A1), mRNA [NM_005907]	NM_005907
A_32_P128586	0.000117	5.987	BI086245	BI086245	602849648F1 NIH_MGC_10 Homo sapiens cDNA clone IMAGE:4991459 5', mRNA sequence [BI086245]	
A_23_P70719	0.000117	4.3	NM_000426	NM_000426	Homo sapiens laminin, alpha 2 (merosin, congenital muscular dystrophy) (LAMA2), mRNA [NM_000426]	NM_000426
A_23_P306105	0.000117	4.248	NM_020474	NM_020474	Homo sapiens UDP-N-acetyl-alpha-D-galactosamine:polypeptide N-acetylgalactosaminyltransferase 1 (GalNAc-T1) (GALNT1), mRNA [NM_020474]	NM_020474
A_24_P261470	0.000117	3.023	NM_138732	NM_138732	Homo sapiens neurexin 2 (NRXN2), transcript variant alpha-2, mRNA [NM_138732]	NM_138732
A_23_P154349	0.000117	2.908	NM_014362	NM_014362	Homo sapiens 3-hydroxyisobutyryl-Coenzyme A hydrolase (HIBCH), transcript variant 1, mRNA [NM_014362]	NM_014362
A_23_P125233	0.000117	2.885	NM_001299	NM_001299	Homo sapiens calponin 1, basic, smooth muscle (CNN1), mRNA [NM_001299]	NM_001299
A_23_P393401	0.000117	2.842	BC008178	BC008178	Homo sapiens hypothetical protein LOC339047, mRNA (cDNA clone IMAGE:4184431), complete cds. [BC008178]	
A_24_P268210	0.000117	2.744	CR614186	CR614186	full-length cDNA clone CS0DC007YG11 of Neuroblastoma Cot 25-normalized of Homo sapiens (human). [CR614186]	
A_24_P165595	0.000117	2.577	THC2310678		Q82VR6 (Q82VR6) Phosphate transport system permease protein, partial (6%) [THC2310678]	
A_24_P225468	0.000117	2.405	NM_030920	NM_030920	Homo sapiens acidic (leucine-rich) nuclear phosphoprotein 32 family, member E (ANP32E), mRNA [NM_030920]	NM_030920
A_32_P193792	0.000117	2.38	AF161353	AF161353	Homo sapiens HSPC090 mRNA, partial cds. [AF161353]	
A_24_P324814	0.000117	2.293	NM_138363	NM_138363	Homo sapiens hypothetical protein BC009518 (LOC90799), mRNA [NM_138363]	NM_138363
A_23_P212034	0.000117	2.2	AL831922	AL831922	Homo sapiens mRNA; cDNA DKFZp761P0818 (from clone DKFZp761P0818). [AL831922]	
A_24_P317907	0.000118	29.16	NM_015385	NM_015385	Homo sapiens sorbin and SH3 domain containing 1 (SORBS1), transcript variant 2, mRNA [NM_015385]	NM_015385
A_24_P143171	0.000118	7.242	NM_031442	NM_031442	Homo sapiens transmembrane protein 47 (TMEM47), mRNA [NM_031442]	NM_031442
A_23_P349966	0.000118	7.02	NM_152913	NM_152913	Homo sapiens hypothetical protein DKFZp761L1417 (DKFZp761L1417), mRNA [NM_152913]	NM_152913
A_23_P205177	0.000118	5.789	NM_000504	NM_000504	Homo sapiens coagulation factor X (F10), mRNA [NM_000504]	NM_000504
A_23_P148807	0.000118	5.722	NM_003503	NM_003503	Homo sapiens CDC7 cell division cycle 7 (S. cerevisiae) (CDC7), mRNA [NM_003503]	NM_003503

A_24_P109082	0.000118	5.514	NM_020935	NM_020935	Homo sapiens ubiquitin specific protease 37 (USP37), mRNA [NM_020935]	NM_020935
A_23_P23279	0.000118	3.643	NM_052862	NM_052862	Homo sapiens RCSD domain containing 1 (RCSD1), mRNA [NM_052862]	NM_052862
A_23_P6066	0.000118	3.452	NM_019609	NM_019609	Homo sapiens carboxypeptidase X (M14 family) (CPXM), mRNA [NM_019609]	NM_019609
A_23_P333498	0.000118	3.128	NM_030636	NM_030636	Homo sapiens KIAA1706 protein (KIAA1706), mRNA [NM_030636]	NM_030636
A_23_P66798	0.000119	22.75	NM_002276	NM_002276	Homo sapiens keratin 19 (KRT19), mRNA [NM_002276]	NM_002276
A_23_P66948	0.000119	15.79	NM_022751	NM_022751	Homo sapiens family with sequence similarity 59, member A (FAM59A), mRNA [NM_022751]	NM_022751
A_23_P252163	0.000119	13.58	NM_004938	NM_004938	Homo sapiens death-associated protein kinase 1 (DAPK1), mRNA [NM_004938]	NM_004938
A_23_P345081	0.000119	2.964	NM_001009956	NM_001009956	Homo sapiens zinc finger protein 655 (ZNF655), transcript variant 6, mRNA [NM_001009956]	NM_001009956
A_23_P76823	0.000119	2.944	NM_199165	NM_199165	Homo sapiens adenylosuccinate synthase like 1 (ADSSL1), transcript variant 1, mRNA [NM_199165]	NM_199165
A_24_P365975	0.00012	34.35	NM_005202	NM_005202	Homo sapiens collagen, type VIII, alpha 2 (COL8A2), mRNA [NM_005202]	NM_005202
A_23_P257003	0.00012	33.96	NM_006200	NM_006200	Homo sapiens proprotein convertase subtilisin/kexin type 5 (PCSK5), mRNA [NM_006200]	NM_006200
A_23_P258463	0.00012	29.35	NM_006017	NM_006017	Homo sapiens prominin 1 (PROM1), mRNA [NM_006017]	NM_006017
A_23_P392384	0.00012	4.634	NM_001002260	NM_001002260	Homo sapiens chromosome 9 open reading frame 58 (C9orf58), transcript variant 2, mRNA [NM_001002260]	NM_001002260
A_23_P202484	0.00012	4.167	NM_032772	NM_032772	Homo sapiens zinc finger protein 503 (ZNF503), mRNA [NM_032772]	NM_032772
A_23_P114172	0.00012	3.754	NM_017752	NM_017752	Homo sapiens FLJ20298 protein (FLJ20298), transcript variant 1, mRNA [NM_017752]	NM_017752
A_23_P29096	0.00012	3.523	NM_002606	NM_002606	Homo sapiens phosphodiesterase 9A (PDE9A), transcript variant 1, mRNA [NM_002606]	NM_002606
A_23_P143374	0.00012	2.336	NM_025176	NM_025176	Homo sapiens KIAA0980 protein (KIAA0980), mRNA [NM_025176]	NM_025176
A_23_P258048	0.00012	2.264	AK056630	AK056630	Homo sapiens cDNA FLJ32068 fis, clone OCBBF1000114. [AK056630]	
A_23_P254179	0.00012	2.194	NM_015339	NM_015339	Homo sapiens activity-dependent neuroprotector (ADNP), transcript variant 1, mRNA [NM_015339]	NM_015339
A_24_P302249	0.000121	82.73	NM_001643	NM_001643	Homo sapiens apolipoprotein A-II (APOA2), mRNA [NM_001643]	NM_001643
A_24_P84668	0.000121	26.44	NM_015687	NM_015687	Homo sapiens filamin A interacting protein 1 (FILIP1), mRNA [NM_015687]	NM_015687
A_23_P163567	0.000121	17.78	NM_018667	NM_018667	Homo sapiens sphingomyelin phosphodiesterase 3, neutral membrane (neutral sphingomyelinase II) (SMPD3), mRNA [NM_018667]	NM_018667
A_23_P320261	0.000121	13.08	NM_033317	NM_033317	Homo sapiens dermokine (ZD52F10), mRNA [NM_033317]	NM_033317
A_23_P153146	0.000121	12.81	NM_004361	NM_004361	Homo sapiens cadherin 7, type 2 (CDH7), transcript variant b, mRNA [NM_004361]	NM_004361
A_24_P178602	0.000121	2.456	NM_198457	NM_198457	Homo sapiens zinc finger protein 600 (ZNF600), mRNA [NM_198457]	NM_198457
A_23_P500353	0.000121	2.372	NM_021614	NM_021614	Homo sapiens potassium intermediate/small conductance calcium-activated channel, subfamily N, member 2 (KCNN2), transcript variant 1, mRNA [NM_021614]	NM_021614
A_23_P204847	0.000122	32.01	NM_002298	NM_002298	Homo sapiens lymphocyte cytosolic protein 1 (L-plastin) (LCP1), mRNA [NM_002298]	NM_002298
A_32_P87631	0.000122	13	BC017507	BC017507	Homo sapiens, clone IMAGE:4850148, mRNA. [BC017507]	
A_24_P856722	0.000122	7.943	AI791206	AI791206	nu30f08.y5 NCI_CGAP_Ov5 Homo sapiens cDNA clone IMAGE:1212231 similar to contains Alu repetitive element;contains element MER37 repetitive element ;, mRNA sequence [AI791206]	
A_32_P146826	0.000122	6.296	THC2441367			
A_32_P221590	0.000122	3.395	A_32_P221590			
A_23_P161706	0.000122	2.758	THC2317900		Q9H2Q1 (Q9H2Q1) AD031, partial (69%) [THC2317900]	
A_24_P314351	0.000122	2.616	NM_006777	NM_006777	Homo sapiens zinc finger and BTB domain containing 33 (ZBTB33), mRNA [NM_006777]	NM_006777
A_24_P332623	0.000122	2.199	AL133018	AL133018	Homo sapiens mRNA; cDNA DKFZp434F0327 (from clone DKFZp434F0327). [AL133018]	
A_23_P155514	0.000123	74.06	NM_001622	NM_001622	Homo sapiens alpha-2-HS-glycoprotein (AHSG), mRNA [NM_001622]	NM_001622
A_32_P126259	0.000123	20.15	THC2443836			
A_23_P17620	0.000123	13.7	NM_052954	NM_052954	Homo sapiens cysteine/tyrosine-rich 1 (CYYR1), mRNA [NM_052954]	NM_052954
A_23_P206760	0.000123	7.732	NM_005143	NM_005143	Homo sapiens haptoglobin (HP), mRNA [NM_005143]	NM_005143
A_24_P282043	0.000123	4.879	NM_006969	NM_006969	Homo sapiens zinc finger protein 28 (KOX 24) (ZNF28), mRNA [NM_006969]	NM_006969
A_24_P246351	0.000123	3.6	CR615589	CR615589	full-length cDNA clone CS0DC026YJ18 of Neuroblastoma Cot 25-normalized of Homo sapiens (human). [CR615589]	
A_23_P25638	0.000123	3.343	NM_024546	NM_024546	Homo sapiens chromosome 13 open reading frame 7 (C13orf7), mRNA [NM_024546]	NM_024546

A_23_P168847	0.000123	2.783	NM_172366	NM_172366	Homo sapiens F-box protein 16 (FBXO16), mRNA [NM_172366]	NM_172366
A_23_P212756	0.000123	2.604	NM_001004057	NM_001004057	Homo sapiens G protein-coupled receptor kinase 4 (GRK4), transcript variant 3, mRNA [NM_001004057]	NM_001004057
A_23_P124742	0.000123	2.469	NM_001277	NM_001277	Homo sapiens choline kinase alpha (CHKA), transcript variant 1, mRNA [NM_001277]	NM_001277
A_23_P39465	0.000124	3.972	NM_004335	NM_004335	Homo sapiens bone marrow stromal cell antigen 2 (BST2), mRNA [NM_004335]	NM_004335
A_23_P212715	0.000124	2.253	NM_170662	NM_170662	Homo sapiens Cas-Br-M (murine) ecotropic retroviral transforming sequence b (CBLB), mRNA [NM_170662]	NM_170662
A_23_P161091	0.000124	2.084	NM_024772	NM_024772	Homo sapiens zinc finger, MYM domain containing 1 (ZMYM1), mRNA [NM_024772]	NM_024772
A_23_P202988	0.000124	2.076	NM_015423	NM_015423	Homo sapiens aminoadipate-semialdehyde dehydrogenase-phosphopantetheinyl transferase (AASDHPPT), mRNA [NM_015423]	NM_015423
A_24_P286114	0.000125	21.52	NM_004172	NM_004172	Homo sapiens solute carrier family 1 (glial high affinity glutamate transporter), member 3 (SLC1A3), mRNA [NM_004172]	NM_004172
A_32_P209702	0.000125	7.6	THC2283850			
A_24_P16214	0.000125	2.526	AK090827	AK090827	Homo sapiens cDNA FLJ33508 fis, clone BRAMY2005094. [AK090827]	
A_23_P10194	0.000126	4.695	NM_201575	NM_201575	Homo sapiens seizure related 6 homolog (mouse)-like 2 (SEZ6L2), transcript variant 2, mRNA [NM_201575]	NM_201575
A_23_P26854	0.000127	26.39	NM_014859	NM_014859	Homo sapiens KIAA0672 gene product (KIAA0672), mRNA [NM_014859]	NM_014859
A_24_P940725	0.000128	10.04	AL080186	AL080186	Homo sapiens mRNA; cDNA DKFZp564B0769 (from clone DKFZp564B0769); partial cds. [AL080186]	
A_24_P133288	0.000128	7.159	X97675	X97675	H.sapiens mRNA for plakophilin 2a and b. [X97675]	
A_23_P215751	0.000128	3.078	NM_005000	NM_005000	Homo sapiens NADH dehydrogenase (ubiquinone) 1 alpha subcomplex, 5, 13kDa (NDUFA5), nuclear gene encoding mitochondrial protein, mRNA [NM_005000]	NM_005000
A_23_P110430	0.000128	2.623	NM_002448	NM_002448	Homo sapiens msh homeo box homolog 1 (Drosophila) (MSX1), mRNA [NM_002448]	NM_002448
A_23_P144145	0.000128	2.491	AF292100	AF292100	Homo sapiens RP42 protein mRNA, complete cds. [AF292100]	
A_23_P8281	0.000128	2.308	NM_000416	NM_000416	Homo sapiens interferon gamma receptor 1 (IFNGR1), mRNA [NM_000416]	NM_000416
A_24_P788772	0.000129	16.03	THC2373524		APE_HUMAN (P02649) Apolipoprotein E precursor (Apo-E), partial (50%) [THC2373524]	
A_23_P58770	0.000129	14.76	NM_004821	NM_004821	Homo sapiens heart and neural crest derivatives expressed 1 (HAND1), mRNA [NM_004821]	NM_004821
A_23_P368484	0.000129	4.831	NM_207387	NM_207387	Homo sapiens FLJ35696 protein (FLJ35696), mRNA [NM_207387]	NM_207387
A_32_P18723	0.000129	4.223	AK095472	AK095472	Homo sapiens cDNA FLJ38153 fis, clone DFNES1000083. [AK095472]	
A_23_P58898	0.000129	3.714	NM_012115	NM_012115	Homo sapiens CASP8 associated protein 2 (CASP8AP2), mRNA [NM_012115]	NM_012115
A_32_P142077	0.000129	2.993	THC2300892		A46461 T-cell receptor eta chain - human (fragment) {Homo sapiens;} , complete [THC2300892]	
A_24_P151834	0.000129	2.658	NM_020740	NM_020740	Homo sapiens ankyrin repeat and FYVE domain containing 1 (ANKFY1), transcript variant 2, mRNA [NM_020740]	NM_020740
A_23_P79591	0.00013	107	NM_000384	NM_000384	Homo sapiens apolipoprotein B (including Ag(x) antigen) (APOB), mRNA [NM_000384]	NM_000384
A_23_P323943	0.00013	7.931	NM_178498	NM_178498	Homo sapiens solute carrier family 5 (sodium/glucose cotransporter), member 12 (SLC5A12), mRNA [NM_178498]	NM_178498
A_23_P335695	0.00013	5.49	AK023854	AK023854	Homo sapiens cDNA FLJ13792 fis, clone THYRO1000072, weakly similar to MYOSIN LIGHT CHAIN KINASE, SMOOTH MUSCLE AND NON-MUSCLE ISOZYMES (EC 2.7.1.117). [AK023854]	
A_24_P89872	0.00013	3.676	NM_197977	NM_197977	Homo sapiens zinc finger protein 189 (ZNF189), mRNA [NM_197977]	NM_197977
A_24_P255645	0.00013	2.495	NM_020800	NM_020800	Homo sapiens WD repeat domain 56 (WDR56), mRNA [NM_020800]	NM_020800
A_23_P337168	0.00013	2.442	NM_153013	NM_153013	Homo sapiens hypothetical protein FLJ30596 (FLJ30596), mRNA [NM_153013]	NM_153013
A_23_P389118	0.00013	2.161	NM_001025356	NM_001025356	Homo sapiens transmembrane protein 16F (TMEM16F), mRNA [NM_001025356]	NM_001025356
A_23_P105276	0.000131	9.051	NM_003428	NM_003428	Homo sapiens zinc finger protein 84 (HPF2) (ZNF84), mRNA [NM_003428]	NM_003428
A_23_P73787	0.000131	4.342	NM_153183	NM_153183	Homo sapiens nudix (nucleoside diphosphate linked moiety X)-type motif 10 (NUDT10), mRNA [NM_153183]	NM_153183
A_23_P90419	0.000132	6.38	NM_025245	NM_025245	Homo sapiens pre-B-cell leukemia transcription factor 4 (PBX4), mRNA [NM_025245]	NM_025245
A_24_P108291	0.000132	2.802	NM_018439	NM_018439	Homo sapiens hypothetical protein IMPACT (IMPACT), mRNA [NM_018439]	NM_018439
A_23_P73220	0.000132	2.464	NM_018351	NM_018351	Homo sapiens FYVE, RhoGEF and PH domain containing 6 (FGD6), mRNA [NM_018351]	NM_018351
A_23_P73012	0.000132	2.201	NM_032823	NM_032823	Homo sapiens chromosome 9 open reading frame 3 (C9orf3), mRNA [NM_032823]	NM_032823
A_24_P406754	0.000133	8.042	NM_032211	NM_032211	Homo sapiens lysyl oxidase-like 4 (LOXL4), mRNA [NM_032211]	NM_032211

A_24_P926960	0.000133	5.864	AF086414	AF086414	Homo sapiens full length insert cDNA clone ZD77F06. [AF086414]	
A_23_P138492	0.000133	3.933	NM_004210	NM_004210	Homo sapiens neuralized-like (Drosophila) (NEURL), mRNA [NM_004210]	NM_004210
A_23_P54556	0.000133	3.125	NM_014048	NM_014048	Homo sapiens MKL/myocardin-like 2 (MKL2), mRNA [NM_014048]	NM_014048
A_32_P118372	0.000133	2.905	NM_015693	NM_015693	Homo sapiens PDZ domain containing 6 (PDZK6), mRNA [NM_015693]	NM_015693
A_24_P134392	0.000133	2.359	NM_006948	NM_006948	Homo sapiens stress 70 protein chaperone, microsome-associated, 60kDa (STCH), mRNA [NM_006948]	NM_006948
A_23_P200493	0.000133	2.09	NM_002296	NM_002296	Homo sapiens lamin B receptor (LBR), transcript variant 1, mRNA [NM_002296]	NM_002296
A_23_P135239	0.000134	5.545	NM_005077	NM_005077	Homo sapiens transducin-like enhancer of split 1 (E(sp1) homolog, Drosophila) (TLE1), mRNA [NM_005077]	NM_005077
A_23_P41917	0.000134	5.467	NM_004272	NM_004272	Homo sapiens homer homolog 1 (Drosophila) (HOMER1), mRNA [NM_004272]	NM_004272
A_32_P76853	0.000134	5.357	XM_370839	XM_370839	PREDICTED: Homo sapiens similar to hypothetical protein (LOC440234), mRNA [XM_370839]	XM_370839
A_23_P143331	0.000134	4.382	NM_001200	NM_001200	Homo sapiens bone morphogenetic protein 2 (BMP2), mRNA [NM_001200]	NM_001200
A_23_P91390	0.000134	4.046	NM_000361	NM_000361	Homo sapiens thrombomodulin (THBD), mRNA [NM_000361]	NM_000361
A_23_P254472	0.000134	3.113	NM_024573	NM_024573	Homo sapiens chromosome 6 open reading frame 211 (C6orf211), mRNA [NM_024573]	NM_024573
A_32_P13612	0.000134	2.547	THC2347074			
A_32_P3290	0.000134	2.391	NM_017645	NM_017645	Homo sapiens family with sequence similarity 29, member A (FAM29A), mRNA [NM_017645]	NM_017645
A_24_P116766	0.000135	10.41	AL834501	AL834501	Homo sapiens mRNA; cDNA DKFZp761N202 (from clone DKFZp761N202). [AL834501]	
A_23_P25746	0.000135	6.251	BC007251	BC007251	Homo sapiens chromosome 14 open reading frame 128, mRNA (cDNA clone MGC:15504 IMAGE:2990071), complete cds. [BC007251]	
A_23_P300220	0.000135	4.112	NM_145008	NM_145008	Homo sapiens yippee-like 4 (Drosophila) (YPEL4), mRNA [NM_145008]	NM_145008
A_23_P217304	0.000135	2.862	NM_021140	NM_021140	Homo sapiens ubiquitously transcribed tetratricopeptide repeat, X chromosome (UTX), mRNA [NM_021140]	NM_021140
A_23_P57697	0.000135	2.427	NM_020865	NM_020865	Homo sapiens DEAH (Asp-Glu-Ala-His) box polypeptide 36 (DHX36), mRNA [NM_020865]	NM_020865
A_23_P157580	0.000135	2.064	NM_005625	NM_005625	Homo sapiens syndecan binding protein (syntenin) (SDCBP), transcript variant 1, mRNA [NM_005625]	NM_005625
A_24_P154868	0.000136	17.17	NM_005588	NM_005588	Homo sapiens meprin A, alpha (PABA peptide hydrolase) (MEP1A), mRNA [NM_005588]	NM_005588
A_32_P187009	0.000136	13.15	AK026295	AK026295	Homo sapiens cDNA: FLJ22642 fis, clone HSI06970. [AK026295]	
A_23_P120667	0.000136	9.041	NM_021219	NM_021219	Homo sapiens junctional adhesion molecule 2 (JAM2), mRNA [NM_021219]	NM_021219
A_24_P450596	0.000136	7.019	CR627133	CR627133	Homo sapiens mRNA; cDNA DKFZp686J154 (from clone DKFZp686J154). [CR627133]	
A_23_P30495	0.000136	3.777	NM_000859	NM_000859	Homo sapiens 3-hydroxy-3-methylglutaryl-Coenzyme A reductase (HMGCR), mRNA [NM_000859]	NM_000859
A_23_P29855	0.000136	3.77	NM_003715	NM_003715	Homo sapiens vesicle docking protein p115 (VDP), mRNA [NM_003715]	NM_003715
A_23_P382584	0.000137	7.839	NM_001819	NM_001819	Homo sapiens chromogranin B (secretogranin 1) (CHGB), mRNA [NM_001819]	NM_001819
A_24_P942850	0.000137	5.308	NM_024989	NM_024989	Homo sapiens GPI deacylase (PGAP1), mRNA [NM_024989]	NM_024989
A_24_P242357	0.000137	3.361	NM_012262	NM_012262	Homo sapiens heparan sulfate 2-O-sulfotransferase 1 (HS2ST1), mRNA [NM_012262]	NM_012262
A_23_P76658	0.000138	39.65	NM_052818	NM_052818	Homo sapiens hypothetical gene CG018 (CG018), mRNA [NM_052818]	NM_052818
A_24_P265856	0.000138	9.144	NM_020654	NM_020654	Homo sapiens SUMO1/sentrin specific protease 7 (SENP7), mRNA [NM_020654]	NM_020654
A_23_P68198	0.000138	2.781	NM_015677	NM_015677	Homo sapiens SH3 domain containing, Ysc84-like 1 (S. cerevisiae) (SH3YL1), mRNA [NM_015677]	NM_015677
A_23_P210608	0.000138	2.672	NM_006526	NM_006526	Homo sapiens zinc finger protein 217 (ZNF217), mRNA [NM_006526]	NM_006526
A_23_P58251	0.000139	26.08	NM_001014448	NM_001014448	Homo sapiens carboxypeptidase Z (CPZ), transcript variant 3, mRNA [NM_001014448]	NM_001014448
A_24_P10214	0.000139	11.25	NM_014178	NM_014178	Homo sapiens syntaxin binding protein 6 (amisyn) (STXBP6), mRNA [NM_014178]	NM_014178
A_23_P21976	0.000139	4.165	NM_001897	NM_001897	Homo sapiens chondroitin sulfate proteoglycan 4 (melanoma-associated) (CSPG4), mRNA [NM_001897]	NM_001897
A_32_P138178	0.000139	3.699	BE835321	BE835321	BE835321 RC5-FN0022-300600-022-G12 FN0022 Homo sapiens cDNA, mRNA sequence [BE835321]	
A_23_P5370	0.000139	2.707	NM_019845	NM_019845	Homo sapiens reprimo, TP53 dependant G2 arrest mediator candidate (RPRM), mRNA [NM_019845]	NM_019845
A_24_P936758	0.000139	2.428	AK074614	AK074614	Homo sapiens cDNA FLJ90133 fis, clone HEMBB1000567. [AK074614]	
A_32_P51237	0.00014	57.63	NM_181712	NM_181712	Homo sapiens ankyrin repeat domain 38 (ANKRD38), mRNA [NM_181712]	NM_181712
A_23_P8640	0.00014	9.944	NM_001505	NM_001505	Homo sapiens G protein-coupled receptor 30 (GPR30), mRNA [NM_001505]	NM_001505
A_32_P148914	0.00014	3.018	BC037255	BC037255	Homo sapiens hypothetical LOC389634, mRNA (cDNA clone IMAGE:4157715). [BC037255]	

A_32_P67544	0.00014	2.561	BX647764	BX647764	Homo sapiens mRNA; cDNA DKFZp686E0352 (from clone DKFZp686E0352). [BX647764]	
A_32_P205431	0.000141	10.7	AF085962	AF085962	Homo sapiens full length insert cDNA clone YS02G11. [AF085962]	
A_24_P8892	0.000141	2.945	NM_006037	NM_006037	Homo sapiens histone deacetylase 4 (HDAC4), mRNA [NM_006037]	NM_006037
A_23_P139143	0.000141	2.494	NM_004177	NM_004177	Homo sapiens syntaxin 3A (STX3A), mRNA [NM_004177]	NM_004177
A_24_P166807	0.000142	9.978	NM_005079	NM_005079	Homo sapiens tumor protein D52 (TPD52), transcript variant 3, mRNA [NM_005079]	NM_005079
A_23_P91334	0.000142	6.161	NM_052970	NM_052970	Homo sapiens heat shock 70kD protein 12B (HSPA12B), mRNA [NM_052970]	NM_052970
A_23_P396981	0.000142	2.869	NM_001012506	NM_001012506	Homo sapiens hypothetical protein LOC285331 (LOC285331), mRNA [NM_001012506]	NM_001012506
A_23_P107724	0.000142	2.823	NM_013380	NM_013380	Homo sapiens zinc finger protein 228 (ZNF228), mRNA [NM_013380]	NM_013380
A_24_P131752	0.000142	2.454	AL831922	AL831922	Homo sapiens mRNA; cDNA DKFZp761P0818 (from clone DKFZp761P0818). [AL831922]	
A_32_P25397	0.000143	5.968	ENST00000328681		Homo sapiens cDNA FLJ12900 fis, clone NT2RP2004321. [AK022962]	
A_24_P348660	0.000143	4.761	NM_138271	NM_138271	Homo sapiens alpha thalassemia/mental retardation syndrome X-linked (RAD54 homolog, S. cerevisiae) (ATRX), transcript variant 3, mRNA [NM_138271]	NM_138271
A_23_P71480	0.000144	15.27	NM_005218	NM_005218	Homo sapiens defensin, beta 1 (DEFB1), mRNA [NM_005218]	NM_005218
A_23_P152559	0.000144	8.298	NM_004758	NM_004758	Homo sapiens benzodiazapine receptor (peripheral) associated protein 1 (BZRAP1), mRNA [NM_004758]	NM_004758
A_32_P20912	0.000144	2.891	AK025669	AK025669	Homo sapiens cDNA: FLJ22016 fis, clone HEP07422. [AK025669]	
A_32_P226801	0.000145	16.76	THC2453346		AA890297 aj94e10.s1 Soares_parathyroid_tumor_NbHPA Homo sapiens cDNA clone IMAGE:1404138 3', mRNA sequence [AA890297]	
A_23_P148015	0.000145	10.46	NM_004655	NM_004655	Homo sapiens axin 2 (conductin, axil) (AXIN2), mRNA [NM_004655]	NM_004655
A_23_P390445	0.000145	3.683	THC2427890		Q8TD54 (Q8TD54) Protein phosphatase 1 regulatory subunit 1A, partial (53%) [THC2427890]	
A_24_P414376	0.000145	3.521	THC2371729			
A_23_P20494	0.000146	6.909	NM_006096	NM_006096	Homo sapiens N-myc downstream regulated gene 1 (NDRG1), mRNA [NM_006096]	NM_006096
A_32_P73071	0.000146	4.875	THC2453866			
A_32_P207789	0.000148	5.988	BQ017638	BQ017638	BQ017638 UI-H-DI0-auv-p-03-0-UI.s1 NCI_CGAP_DI0 Homo sapiens cDNA clone IMAGE:5875058 3', mRNA sequence [BQ017638]	
A_23_P48585	0.000148	3.873	NM_005407	NM_005407	Homo sapiens sal-like 2 (Drosophila) (SALL2), mRNA [NM_005407]	NM_005407
A_23_P107283	0.000149	3.276	NM_002145	NM_002145	Homo sapiens homeo box B2 (HOXB2), mRNA [NM_002145]	NM_002145
A_23_P212914	0.000149	3.15	CR598922	CR598922	full-length cDNA clone CS0DD007YM02 of Neuroblastoma Cot 50-normalized of Homo sapiens (human). [CR598922]	
A_23_P404667	0.000149	2.993	NM_001197	NM_001197	Homo sapiens BCL2-interacting killer (apoptosis-inducing) (BIK), mRNA [NM_001197]	NM_001197
A_32_P148824	0.00015	4.636	NM_017847	NM_017847	Homo sapiens chromosome 1 open reading frame 27 (C1orf27), mRNA [NM_017847]	NM_017847
A_24_P221668	0.000151	3.546	AA359500	AA359500	AA359500 EST68526 Fetal lung II Homo sapiens cDNA 5' end, mRNA sequence [AA359500]	
A_23_P136693	0.000151	2.364	AL832747	AL832747	Homo sapiens mRNA; cDNA DKFZp686D0521 (from clone DKFZp686D0521). [AL832747]	
A_23_P162861	0.000152	69.9	ENST00000332273		Sequence 1 from Patent WO03046006. [AX772926]	
A_23_P374104	0.000152	3.803	NM_012098	NM_012098	Homo sapiens angiopoietin-like 2 (ANGPTL2), mRNA [NM_012098]	NM_012098
A_23_P383601	0.000152	3.165	ENST00000316535		Homo sapiens hypothetical protein FLJ31306, mRNA (cDNA clone IMAGE:4838556), partial cds. [BC034618]	
A_24_P415959	0.000152	2.576	NM_001969	NM_001969	Homo sapiens eukaryotic translation initiation factor 5 (EIF5), transcript variant 1, mRNA [NM_001969]	NM_001969
A_23_P71855	0.000153	8.901	NM_001735	NM_001735	Homo sapiens complement component 5 (C5), mRNA [NM_001735]	NM_001735
A_32_P24832	0.000153	8.329	NM_020190	NM_020190	Homo sapiens olfactomedin-like 3 (OLFML3), mRNA [NM_020190]	NM_020190
A_32_P205792	0.000154	6.286	A_32_P205792			
A_23_P75071	0.000154	2.857	NM_016195	NM_016195	Homo sapiens M-phase phosphoprotein 1 (MPHOSPH1), mRNA [NM_016195]	NM_016195
A_23_P88095	0.000154	2.808	NM_014832	NM_014832	Homo sapiens TBC1 domain family, member 4 (TBC1D4), mRNA [NM_014832]	NM_014832
A_24_P928522	0.000154	2.752	AK025142	AK025142	Homo sapiens cDNA: FLJ21489 fis, clone COL05450. [AK025142]	
A_23_P412059	0.000154	2.505	BC064616	BC064616	Homo sapiens zinc finger, RAN-binding domain containing 3, mRNA (cDNA clone IMAGE:5575956), complete cds. [BC064616]	
A_23_P255714	0.000154	2.362	NM_025103	NM_025103	Homo sapiens coiled-coil domain containing 2 (CCDC2), mRNA [NM_025103]	NM_025103
A_23_P83098	0.000155	48.68	NM_000689	NM_000689	Homo sapiens aldehyde dehydrogenase 1 family, member A1 (ALDH1A1), mRNA [NM_000689]	NM_000689

A_24_P920715	0.000155	33.42	A_24_P920715			
A_23_P34433	0.000155	4.812	NM_001009881	NM_001009881	Homo sapiens zinc finger, CCHC domain containing 11 (ZCCHC11), transcript variant 1, mRNA [NM_001009881]	NM_001009881
A_32_P187160	0.000155	2.784	A_32_P187160			
A_23_P157299	0.000155	2.389	NM_001129	NM_001129	Homo sapiens AE binding protein 1 (AEBP1), mRNA [NM_001129]	NM_001129
A_24_P917833	0.000155	2.057	NM_006827	NM_006827	Homo sapiens transmembrane trafficking protein (TMP21), mRNA [NM_006827]	NM_006827
A_23_P384748	0.000156	48.23	NM_172069	NM_172069	Homo sapiens pleckstrin homology domain containing, family H (with MyTH4 domain) member 2 (PLEKHH2), mRNA [NM_172069]	NM_172069
A_23_P32414	0.000156	19.55	NM_016542	NM_016542	Homo sapiens Mst3 and SOK1-related kinase (MASK), mRNA [NM_016542]	NM_016542
A_23_P156970	0.000156	9.351	NM_002402	NM_002402	Homo sapiens mesoderm specific transcript homolog (mouse) (MEST), transcript variant 1, mRNA [NM_002402]	NM_002402
A_32_P159023	0.000156	6.299	THC2285720		BX114329 BX114329 Soares_NhHMPu_S1 Homo sapiens cDNA clone IMAGp998G064741 ; IMAGE:1932317, mRNA sequence [BX114329]	
A_32_P41924	0.000156	5.489	AF086011	AF086011	Homo sapiens full length insert cDNA clone YW18A11. [AF086011]	
A_24_P890536	0.000156	4.646	CR627148	CR627148	Homo sapiens mRNA; cDNA DKFZp779F2127 (from clone DKFZp779F2127). [CR627148]	
A_24_P922948	0.000156	2.319	ENST00000216214		Homo sapiens cDNA FLJ43037 fis, clone BRTHA3002933, highly similar to Homo sapiens uroplakin 3 (UPK3). [AK125027]	
A_23_P154447	0.000157	2.335	NM_015934	NM_015934	Homo sapiens nucleolar protein NOP5/NOP58 (NOP5/NOP58), mRNA [NM_015934]	NM_015934
A_23_P34915	0.000158	4.703	NM_004024	NM_004024	Homo sapiens activating transcription factor 3 (ATF3), mRNA [NM_004024]	NM_004024
A_23_P216307	0.000159	116.4	NM_004349	NM_004349	Homo sapiens runt-related transcription factor 1; translocated to, 1 (cyclin D-related) (RUNX1T1), transcript variant 1, mRNA [NM_004349]	NM_004349
A_24_P623814	0.000159	17.84	AK023526	AK023526	Homo sapiens cDNA FLJ13464 fis, clone PLACE1003478. [AK023526]	
A_32_P221305	0.000159	4.749	BC073935	BC073935	Homo sapiens cDNA clone IMAGE:5219247, partial cds. [BC073935]	XM_498535
A_24_P56270	0.000159	3.912	CR612226	CR612226	full-length cDNA clone CS0DF019YP13 of Fetal brain of Homo sapiens (human). [CR612226]	
A_23_P19702	0.000159	2.494	NM_145342	NM_145342	Homo sapiens mitogen-activated protein kinase kinase kinase 7 interacting protein 2 (MAP3K7IP2), transcript variant 2, mRNA [NM_145342]	NM_145342
A_23_P78458	0.00016	3.036	NM_021632	NM_021632	Homo sapiens zinc finger protein 350 (ZNF350), mRNA [NM_021632]	NM_021632
A_24_P337058	0.00016	2.066	NM_020307	NM_020307	Homo sapiens cyclin L1 (CCNL1), mRNA [NM_020307]	NM_020307
A_23_P78543	0.000161	4.396	NM_005498	NM_005498	Homo sapiens adaptor-related protein complex 1, mu 2 subunit (AP1M2), mRNA [NM_005498]	NM_005498
A_23_P52697	0.000161	3.107	NM_020404	NM_020404	Homo sapiens CD248 antigen, endosialin (CD248), mRNA [NM_020404]	NM_020404
A_32_P42989	0.000162	9.75	A_32_P42989			
A_24_P715719	0.000162	7.795	AL049443	AL049443	Homo sapiens mRNA; cDNA DKFZp586N2020 (from clone DKFZp586N2020). [AL049443]	
A_23_P379864	0.000162	6.365	BC006267	BC006267	Homo sapiens asparaginase like 1, mRNA (cDNA clone IMAGE:3952485), complete cds. [BC006267]	
A_32_P19752	0.000162	3.234	NM_144664	NM_144664	Homo sapiens hypothetical protein MGC33371 (MGC33371), mRNA [NM_144664]	NM_144664
A_24_P323932	0.000162	2.372	ENST00000319822		Homo sapiens, clone IMAGE:2820942, mRNA, partial cds. [BC006474]	XM_031553
A_24_P166613	0.000163	5.765	NM_017549	NM_017549	Homo sapiens ependymin related protein 1 (zebrafish) (EPDR1), mRNA [NM_017549]	NM_017549
A_24_P295709	0.000163	3.053	THC2310930			
A_24_P938614	0.000164	7.382	AK026697	AK026697	Homo sapiens cDNA: FLJ23044 fis, clone LNG02454. [AK026697]	
A_24_P37903	0.000164	3.278	THC2371798			
A_23_P408376	0.000165	23.06	AB007877	AB007877	Homo sapiens KIAA0417 mRNA, complete cds. [AB007877]	XM_048898
A_23_P121374	0.000165	4.405	NM_018987	NM_018987	Homo sapiens sema domain, seven thrombospondin repeats (type 1 and type 1-like), transmembrane domain (TM) and short cytoplasmic domain, (semaphorin) 5B (SEMA5B), mRNA [NM_018987]	NM_018987
A_32_P174908	0.000166	3.015	NM_015045	NM_015045	Homo sapiens KIAA0261 (KIAA0261), mRNA [NM_015045]	NM_015045
A_23_P104451	0.000166	2.084	NM_024045	NM_024045	Homo sapiens DEAD (Asp-Glu-Ala-Asp) box polypeptide 50 (DDX50), mRNA [NM_024045]	NM_024045
A_24_P761130	0.000167	6.178	AK097080	AK097080	Homo sapiens cDNA FLJ39761 fis, clone SPLEN1000083. [AK097080]	XM_496251
A_23_P209879	0.000167	5.891	AK128731	AK128731	Homo sapiens cDNA FLJ46899 fis, clone UTERU3022588, highly similar to Cyclic-AMP-dependent transcription factor ATF-2. [AK128731]	
A_24_P52168	0.000167	4.216	A_24_P52168			

A_23_P35456	0.000167	2.658	NM_014631	NM_014631	Homo sapiens SH3 multiple domains 1 (SH3MD1), mRNA [NM_014631]	NM_014631
A_23_P422911	0.000168	39.34	NM_153456	NM_153456	Homo sapiens heparan sulfate 6-O-sulfotransferase 3 (HS6ST3), mRNA [NM_153456]	NM_153456
A_23_P204296	0.000168	8.099	NM_032918	NM_032918	Homo sapiens RAS-like, estrogen-regulated, growth inhibitor (RERG), mRNA [NM_032918]	NM_032918
A_32_P89679	0.000168	3.754	THC2311626		ALU1_HUMAN (P39188) Alu subfamily J sequence contamination warning entry, partial (10%) [THC2311626]	
A_32_P107876	0.000169	103.4	NM_025074	NM_025074	Homo sapiens Fraser syndrome 1 (FRAS1), transcript variant 1, mRNA [NM_025074]	NM_025074
A_32_P205624	0.000169	6.055	ENST00000264554		Homo sapiens mRNA for Sck, partial cds. [AB001451]	
A_23_P122304	0.000169	2.538	NM_001527	NM_001527	Homo sapiens histone deacetylase 2 (HDAC2), mRNA [NM_001527]	NM_001527
A_32_P25050	0.000169	2.456	NM_172037	NM_172037	Homo sapiens retinol dehydrogenase 10 (all-trans) (RDH10), mRNA [NM_172037]	NM_172037
A_24_P109214	0.000172	26.73	NM_001645	NM_001645	Homo sapiens apolipoprotein C-I (APOC1), mRNA [NM_001645]	NM_001645
A_23_P252817	0.000173	53.72	NM_001048	NM_001048	Homo sapiens somatostatin (SST), mRNA [NM_001048]	NM_001048
A_24_P180243	0.000173	7.965	NM_053039	NM_053039	Homo sapiens UDP glucuronosyltransferase 2 family, polypeptide B28 (UGT2B28), mRNA [NM_053039]	NM_053039
A_32_P232237	0.000173	6.587	THC2409354			
A_23_P343104	0.000173	5.086	AK055463	AK055463	Homo sapiens cDNA FLJ30901 fis, clone FEBRA2005778, weakly similar to INTEGUMENTARY MUCIN A.1 PRECURSOR. [AK055463]	
A_24_P269814	0.000173	4.007	NM_001001974	NM_001001974	Homo sapiens pleckstrin homology domain containing, family A (phosphoinositide binding specific) member 1 (PLEKHA1), transcript variant 2, mRNA [NM_001001974]	NM_001001974
A_23_P256504	0.000174	19.87	NM_001633	NM_001633	Homo sapiens alpha-1-microglobulin/bikunin precursor (AMBP), mRNA [NM_001633]	NM_001633
A_32_P214665	0.000174	3.69	ENST00000309878		Homo sapiens cDNA clone IMAGE:4792407, partial cds. [BC067884]	
A_24_P4816	0.000174	2.906	NM_031412	NM_031412	Homo sapiens GABA(A) receptor-associated protein like 1 (GABARAPL1), mRNA [NM_031412]	NM_031412
A_23_P146347	0.000174	2.752	NM_017645	NM_017645	Homo sapiens family with sequence similarity 29, member A (FAM29A), mRNA [NM_017645]	NM_017645
A_23_P94380	0.000175	18.01	NM_001002260	NM_001002260	Homo sapiens chromosome 9 open reading frame 58 (C9orf58), transcript variant 2, mRNA [NM_001002260]	NM_001002260
A_24_P372913	0.000175	7.727	NM_000545	NM_000545	Homo sapiens transcription factor 1, hepatic; LF-B1, hepatic nuclear factor (HNF1), albumin proximal factor (TCF1), mRNA [NM_000545]	NM_000545
A_23_P83403	0.000175	3.392	NM_014988	NM_014988	Homo sapiens KIAA1102 protein (KIAA1102), mRNA [NM_014988]	NM_014988
A_23_P500244	0.000175	3.215	NM_080706	NM_080706	Homo sapiens transient receptor potential cation channel, subfamily V, member 1 (TRPV1), transcript variant 3, mRNA [NM_080706]	NM_080706
A_32_P211414	0.000176	7.347	THC2280849			
A_23_P304489	0.000176	5.103	NM_015381	NM_015381	Homo sapiens family with sequence similarity 19 (chemokine (C-C motif)-like), member A5 (FAM19A5), mRNA [NM_015381]	NM_015381
A_23_P426021	0.000176	3.853	NM_015187	NM_015187	Homo sapiens KIAA0746 protein (KIAA0746), mRNA [NM_015187]	NM_015187
A_23_P58967	0.000176	2.917	NM_014827	NM_014827	Homo sapiens zinc finger CCCH-type containing 11A (ZC3H11A), mRNA [NM_014827]	NM_014827
A_32_P127818	0.000176	2.017	NM_014761	NM_014761	Homo sapiens KIAA0174 (KIAA0174), mRNA [NM_014761]	NM_014761
A_23_P24260	0.000177	7.659	THC2435513		ENP1_HUMAN (P49961) Ectonucleoside triphosphate diphosphohydrolase 1 (NTPDase1) (Ecto-ATP diphosphohydrolase) (ATPDase) (Lymphoid cell activation antigen) (Ecto-apyrase) (CD39 antigen) , partial (92%) [THC2435513]	
A_23_P132454	0.000177	2.785	NM_020307	NM_020307	Homo sapiens cyclin L1 (CCNL1), mRNA [NM_020307]	NM_020307
A_23_P169351	0.000178	26.85	NM_003026	NM_003026	Homo sapiens SH3-domain GRB2-like 2 (SH3GL2), mRNA [NM_003026]	NM_003026
A_32_P174025	0.000178	6.143	NM_182704	NM_182704	Homo sapiens selenoprotein V (SELV), mRNA [NM_182704]	NM_182704
A_23_P302005	0.000178	6.108	NM_006873	NM_006873	Homo sapiens stoned B-like factor (SBLF), mRNA [NM_006873]	NM_006873
A_24_P870509	0.000178	4.721	AF086261	AF086261	Homo sapiens full length insert cDNA clone ZD42A11. [AF086261]	
A_32_P118657	0.000179	10.09	AK022044	AK022044	Homo sapiens cDNA FLJ11982 fis, clone HEMBB1001335. [AK022044]	
A_23_P213745	0.00018	104.5	NM_004887	NM_004887	Homo sapiens chemokine (C-X-C motif) ligand 14 (CXCL14), mRNA [NM_004887]	NM_004887
A_32_P194372	0.00018	4.166	AK129547	AK129547	Homo sapiens cDNA FLJ26036 fis, clone PRS00145. [AK129547]	
A_32_P121908	0.00018	3.088	BE973568	BE973568	601680932F1 NIH_MGC_83 Homo sapiens cDNA clone IMAGE:3951222 5', mRNA sequence [BE973568]	
A_23_P101258	0.00018	2.386	NM_025040	NM_025040	Homo sapiens zinc finger protein 614 (ZNF614), mRNA [NM_025040]	NM_025040
A_23_P42045	0.00018	2.321	NM_181837	NM_181837	Homo sapiens origin recognition complex, subunit 3-like (yeast) (ORC3L), transcript variant 1, mRNA [NM_181837]	NM_181837

A_24_P75158	0.000181	4.304	AL832683	AL832683	Homo sapiens mRNA; cDNA DKFZp313P0917 (from clone DKFZp313P0917). [AL832683]	
A_23_P93348	0.000181	3.197	NM_002341	NM_002341	Homo sapiens lymphotoxin beta (TNF superfamily, member 3) (LTB), transcript variant 1, mRNA [NM_002341]	NM_002341
A_24_P204675	0.000181	2.263	BC094882	BC094882	Homo sapiens cDNA clone MGC:105000 IMAGE:3093162, complete cds. [BC094882]	XM_290670
A_24_P368575	0.000181	2.142	NM_003615	NM_003615	Homo sapiens solute carrier family 4, sodium bicarbonate cotransporter, member 7 (SLC4A7), mRNA [NM_003615]	NM_003615
A_23_P155509	0.000182	283.8	NM_001622	NM_001622	Homo sapiens alpha-2-HS-glycoprotein (AHSG), mRNA [NM_001622]	NM_001622
A_32_P154911	0.000182	46.77	NM_175887	NM_175887	Homo sapiens hypothetical protein LOC222171 (LOC222171), mRNA [NM_175887]	NM_175887
A_24_P145009	0.000182	27.71	NM_001013723	NM_001013723	Homo sapiens hypothetical gene supported by AK094370 (LOC441208), mRNA [NM_001013723]	NM_001013723
A_23_P127911	0.000182	2.525	NM_015430	NM_015430	Homo sapiens regeneration associated muscle protease (DKFZP586H2123), transcript variant 1, mRNA [NM_015430]	NM_015430
A_23_P254573	0.000182	2.222	CR598046	CR598046	full-length cDNA clone CS0DI043YJ07 of Placenta Cot 25-normalized of Homo sapiens (human). [CR598046]	XM_168590
A_24_P334402	0.000182	2.131	NM_024920	NM_024920	Homo sapiens DnaJ (Hsp40) homolog, subfamily B, member 14 (DNAJB14), mRNA [NM_024920]	NM_024920
A_24_P930963	0.000183	5.813	CR616309	CR616309	full-length cDNA clone CS0DF015YK23 of Fetal brain of Homo sapiens (human). [CR616309]	
A_23_P161686	0.000183	2.996	NM_014715	NM_014715	Homo sapiens Rho GTPase-activating protein (RICS), mRNA [NM_014715]	NM_014715
A_32_P93736	0.000184	6.124	THC2379106		Q93NK8 (Q93NK8) YsaW, partial (7%) [THC2379106]	
A_23_P120863	0.000184	4.49	NM_004861	NM_004861	Homo sapiens galactose-3-O-sulfotransferase 1 (GAL3ST1), mRNA [NM_004861]	NM_004861
A_24_P32920	0.000184	3.259	CR597240	CR597240	full-length cDNA clone CS0DI085YF14 of Placenta Cot 25-normalized of Homo sapiens (human). [CR597240]	
A_24_P99984	0.000185	9.792	THC2338942			
A_24_P326511	0.000185	3.848	NM_015385	NM_015385	Homo sapiens sorbin and SH3 domain containing 1 (SORBS1), transcript variant 2, mRNA [NM_015385]	NM_015385
A_32_P127105	0.000185	3.683	BC037740	BC037740	Homo sapiens cDNA clone IMAGE:5263531, partial cds. [BC037740]	
A_23_P309865	0.000185	3.328	NM_152695	NM_152695	Homo sapiens zinc finger protein 449 (ZNF449), mRNA [NM_152695]	NM_152695
A_23_P203009	0.000185	3.094	NM_003478	NM_003478	Homo sapiens cullin 5 (CUL5), mRNA [NM_003478]	NM_003478
A_23_P50195	0.000185	2.618	A_23_P50195			
A_23_P142616	0.000185	2.316	NM_016252	NM_016252	Homo sapiens baculoviral IAP repeat-containing 6 (apollon) (BIRC6), mRNA [NM_016252]	NM_016252
A_32_P94160	0.000186	52.63	BC043195	BC043195	Homo sapiens cDNA clone IMAGE:5288757, partial cds. [BC043195]	
A_32_P129752	0.000186	16.62	NM_001017970	NM_001017970	Homo sapiens transmembrane protein 30B (TMEM30B), mRNA [NM_001017970]	NM_001017970
A_32_P82650	0.000186	11.09	BM701175	BM701175	UI-E-EJ0-ahj-e-13-0-UI.r1 UI-E-EJ0 Homo sapiens cDNA clone UI-E-EJ0-ahj-e-13-0-UI 5', mRNA sequence [BM701175]	
A_32_P146844	0.000186	2.787	THC2406576		ALU2_HUMAN (P39189) Alu subfamily SB sequence contamination warning entry, partial (4%) [THC2406576]	
A_23_P387184	0.000186	2.622	ENST00000343505		Homo sapiens mRNA for KIAA1357 protein, partial cds. [AB037778]	XM_496826
A_24_P171041	0.000186	2.252	AL136922	AL136922	Homo sapiens mRNA; cDNA DKFZp586J151 (from clone DKFZp586J151). [AL136922]	
A_23_P52597	0.000187	11.89	NM_031909	NM_031909	Homo sapiens C1q and tumor necrosis factor related protein 4 (C1QTNF4), mRNA [NM_031909]	NM_031909
A_32_P144999	0.000187	7.425	XM_374169	XM_374169	PREDICTED: Homo sapiens hypothetical LOC389393 (LOC389393), mRNA [XM_374169]	XM_374169
A_24_P288116	0.000187	3.792	BC040982	BC040982	Homo sapiens, clone IMAGE:4798675, mRNA. [BC040982]	
A_23_P108871	0.000187	2.738	NM_032494	NM_032494	Homo sapiens zinc finger CCCH-type containing 8 (ZC3H8), mRNA [NM_032494]	NM_032494
A_24_P62505	0.000188	3.848	NM_015101	NM_015101	Homo sapiens glycosyltransferase 25 domain containing 2 (GLT25D2), mRNA [NM_015101]	NM_015101
A_23_P94879	0.00019	45.59	NM_000506	NM_000506	Homo sapiens coagulation factor II (thrombin) (F2), mRNA [NM_000506]	NM_000506
A_23_P250444	0.00019	21.56	NM_000166	NM_000166	Homo sapiens gap junction protein, beta 1, 32kDa (connexin 32, Charcot-Marie-Tooth neuropathy, X-linked) (GJB1), mRNA [NM_000166]	NM_000166
A_32_P35486	0.00019	6.04	THC2317058			
A_23_P112201	0.00019	2.297	NM_015061	NM_015061	Homo sapiens jumonji domain containing 2C (JMJD2C), mRNA [NM_015061]	NM_015061
A_32_P10495	0.000191	12.83	THC2274697		ALU7_HUMAN (P39194) Alu subfamily SQ sequence contamination warning entry, partial (31%) [THC2274697]	
A_23_P168771	0.000191	4.522	NM_020879	NM_020879	Homo sapiens KIAA1505 protein (KIAA1505), mRNA [NM_020879]	NM_020879
A_24_P286898	0.000191	3.107	AK125150	AK125150	Homo sapiens cDNA FLJ43160 fis, clone FCBBF2000199. [AK125150]	
A_32_P100109	0.000191	2.856	AK097484	AK097484	Homo sapiens cDNA FLJ40165 fis, clone TESTI2015962. [AK097484]	

A_24_P23979	0.000192	6.605	NM_016379	NM_016379	Homo sapiens variable charge, X-linked 3A (VCX3A), mRNA [NM_016379]	NM_016379
A_23_P254512	0.000193	11.33	NM_004428	NM_004428	Homo sapiens ephrin-A1 (EFNA1), transcript variant 1, mRNA [NM_004428]	NM_004428
A_32_P9931	0.000193	6.193	A_32_P9931			
A_23_P111267	0.000193	6.128	NM_031469	NM_031469	Homo sapiens SH3 domain binding glutamic acid-rich protein like 2 (SH3BGRL2), mRNA [NM_031469]	NM_031469
A_23_P383132	0.000193	4.143	NM_015094	NM_015094	Homo sapiens hypermethylated in cancer 2 (HIC2), mRNA [NM_015094]	NM_015094
A_23_P218827	0.000193	2.248	NM_006596	NM_006596	Homo sapiens polymerase (DNA directed), theta (POLQ), transcript variant 1, mRNA [NM_006596]	NM_006596
A_23_P95130	0.000193	2.192	NM_207113	NM_207113	Homo sapiens solute carrier family 37 (glycerol-3-phosphate transporter), member 3 (SLC37A3), transcript variant 1, mRNA [NM_207113]	NM_207113
A_24_P32085	0.000194	24.68	NM_024761	NM_024761	Homo sapiens MOB1, Mps One Binder kinase activator-like 2B (yeast) (MOBKL2B), mRNA [NM_024761]	NM_024761
A_23_P213255	0.000194	2.681	NM_020159	NM_020159	Homo sapiens SWI/SNF-related, matrix-associated actin-dependent regulator of chromatin, subfamily a, containing DEAD/H box 1 (SMARCAD1), mRNA [NM_020159]	NM_020159
A_32_P75399	0.000195	4.55	THC2308675		HUMSEF21A SEF2-1A protein {Homo sapiens;} , partial (5%) [THC2308675]	
A_23_P361014	0.000195	3.541	NM_020856	NM_020856	Homo sapiens zinc finger protein 537 (ZNF537), mRNA [NM_020856]	NM_020856
A_24_P114339	0.000195	3.145	CR604908	CR604908	full-length cDNA clone CS0DF020YB09 of Fetal brain of Homo sapiens (human). [CR604908]	
A_23_P212500	0.000196	95.7	NM_001063	NM_001063	Homo sapiens transferrin (TF), mRNA [NM_001063]	NM_001063
A_24_P929807	0.000196	5.482	AB052759	AB052759	Homo sapiens hAWMS1 mRNA, complete cds. [AB052759]	
A_23_P19369	0.000197	8.255	NM_017640	NM_017640	Homo sapiens leucine rich repeat containing 16 (LRRC16), mRNA [NM_017640]	NM_017640
A_23_P212204	0.000197	6.336	BX648380	BX648380	Homo sapiens mRNA; cDNA DKFZp686A20205 (from clone DKFZp686A20205). [BX648380]	
A_32_P482979	0.000197	2.27	NM_203390	NM_203390	Homo sapiens similar to RIKEN cDNA 3000004N20 (LOC389677), mRNA [NM_203390]	NM_203390
A_23_P30377	0.000197	2.059	NM_014829	NM_014829	Homo sapiens DEAD (Asp-Glu-Ala-Asp) box polypeptide 46 (DDX46), mRNA [NM_014829]	NM_014829
A_24_P295010	0.000198	25.78	NM_004155	NM_004155	Homo sapiens serine (or cysteine) proteinase inhibitor, clade B (ovalbumin), member 9 (SERPINB9), mRNA [NM_004155]	NM_004155
A_24_P289260	0.000199	9.756	NM_214462	NM_214462	Homo sapiens dapper, antagonist of beta-catenin, homolog 2 (Xenopus laevis) (DACT2), mRNA [NM_214462]	NM_214462
A_23_P144677	0.000199	4.927	AF178574	AF178574	Homo sapiens MSTP146 (MST146) mRNA, complete cds. [AF178574]	
A_23_P34142	0.000199	2.852	NM_016303	NM_016303	Homo sapiens WW domain binding protein 5 (WBP5), transcript variant 1, mRNA [NM_016303]	NM_016303
A_24_P367100	0.000199	2.721	A_24_P367100			
A_23_P153026	0.000199	2.167	NM_000152	NM_000152	Homo sapiens glucosidase, alpha; acid (Pompe disease, glycogen storage disease type II) (GAA), mRNA [NM_000152]	NM_000152
A_23_P53417	0.0002	24.24	NM_006741	NM_006741	Homo sapiens protein phosphatase 1, regulatory (inhibitor) subunit 1A (PPP1R1A), mRNA [NM_006741]	NM_006741
A_32_P55438	0.0002	13.35	A_32_P55438			
A_32_P5480	0.0002	9.589	NM_203463	NM_203463	Homo sapiens LAG1 longevity assurance homolog 6 (S. cerevisiae) (LASS6), mRNA [NM_203463]	NM_203463
A_32_P104478	0.0002	4.338	AK026881	AK026881	Homo sapiens cDNA: FLJ23228 fis, clone CAE06654. [AK026881]	
A_23_P102160	0.0002	3.262	AF435956	AF435956	Homo sapiens unknown mRNA. [AF435956]	
A_23_P16817	0.000201	3.398	NM_004071	NM_004071	Homo sapiens CDC-like kinase 1 (CLK1), transcript variant 1, mRNA [NM_004071]	NM_004071
A_23_P350070	0.000202	2.49	NM_015436	NM_015436	Homo sapiens ring finger and CHY zinc finger domain containing 1 (RCHY1), transcript variant 1, mRNA [NM_015436]	NM_015436
A_23_P29784	0.000202	2.375	AL096748	AL096748	Homo sapiens mRNA; cDNA DKFZp434A043 (from clone DKFZp434A043); partial cds. [AL096748]	
A_23_P385114	0.000202	2.317	NM_023929	NM_023929	Homo sapiens zinc finger and BTB domain containing 10 (ZBTB10), mRNA [NM_023929]	NM_023929
A_24_P898915	0.000203	3.966	NM_001004321	NM_001004321	Homo sapiens FLJ45445 protein (FLJ45445), mRNA [NM_001004321]	NM_001004321
A_23_P314642	0.000203	3.514	NM_017666	NM_017666	Homo sapiens suppressor of hairy wing homolog 3 (Drosophila) (SUHW3), mRNA [NM_017666]	NM_017666
A_24_P702813	0.000203	2.188	AK023131	AK023131	Homo sapiens cDNA FLJ13069 fis, clone NT2RP3001752. [AK023131]	
A_23_P115417	0.000204	2.352	NM_015149	NM_015149	Homo sapiens ral guanine nucleotide dissociation stimulator-like 1 (RGL1), mRNA [NM_015149]	NM_015149
A_23_P99540	0.000204	2.116	NM_004926	NM_004926	Homo sapiens zinc finger protein 36, C3H type-like 1 (ZFP36L1), mRNA [NM_004926]	NM_004926
A_23_P134426	0.000205	22.28	NM_001005340	NM_001005340	Homo sapiens glycoprotein (transmembrane) nmb (GPNMB), transcript variant 1, mRNA [NM_001005340]	NM_001005340
A_23_P400580	0.000205	3.907	AB040883	AB040883	Homo sapiens mRNA for KIAA1450 protein, partial cds. [AB040883]	

A_24_P369898	0.000205	2.659	ENST00000293201		Homo sapiens mRNA for KIAA1783 protein, partial cds. [AB058686]	XM_496245
A_24_P334726	0.000206	10.96	NM_015689	NM_015689	Homo sapiens KIAA1277 (KIAA1277), mRNA [NM_015689]	NM_015689
A_23_P106602	0.000206	5.614	NM_031476	NM_031476	Homo sapiens cysteine-rich secretory protein LCCL domain containing 2 (CRISPLD2), mRNA [NM_031476]	NM_031476
A_24_P159837	0.000206	5.156	ENST00000328088		Homo sapiens mRNA for PREDICTED: similar to regulator of sex-limitation candidate 11 variant protein. [AB209226]	
A_23_P339601	0.000206	3.018	NM_152472	NM_152472	Homo sapiens zinc finger protein 578 (ZNF578), mRNA [NM_152472]	NM_152472
A_23_P170649	0.000207	32.35	NM_153225	NM_153225	Homo sapiens RPE-spondin (RPESP), mRNA [NM_153225]	NM_153225
A_32_P83845	0.000207	3.116	NM_012258	NM_012258	Homo sapiens hairy/enhancer-of-split related with YRPW motif 1 (HEY1), mRNA [NM_012258]	NM_012258
A_24_P77432	0.000207	2.806	NM_133631	NM_133631	Homo sapiens roundabout, axon guidance receptor, homolog 1 (Drosophila) (ROBO1), transcript variant 2, mRNA [NM_133631]	NM_133631
A_32_P76602	0.000207	2.559	THC2281350			
A_23_P93641	0.000208	45.74	NM_020299	NM_020299	Homo sapiens aldo-keto reductase family 1, member B10 (aldose reductase) (AKR1B10), mRNA [NM_020299]	NM_020299
A_32_P181131	0.000208	18.33	AW467174	AW467174	AW467174 ha35g06.x1 NCI_CGAP_Kid12 Homo sapiens cDNA clone IMAGE:2875738 3' similar to gb:X60673_rna1 GTP:AMP PHOSPHOTRANSFERASE MITOCHONDRIAL (HUMAN);, mRNA sequence [AW467174]	
A_23_P121637	0.000208	8.651	NM_003619	NM_003619	Homo sapiens protease, serine, 12 (neurotrypsin, motopsin) (PRSS12), mRNA [NM_003619]	NM_003619
A_32_P46214	0.000209	28.21	NM_173653	NM_173653	Homo sapiens solute carrier family 9 (sodium/hydrogen exchanger), isoform 9 (SLC9A9), mRNA [NM_173653]	NM_173653
A_32_P154445	0.000209	7.363	CR590573	CR590573	full-length cDNA clone CS0DI042YD07 of Placenta Cot 25-normalized of Homo sapiens (human). [CR590573]	
A_24_P20327	0.000209	6.013	NM_014079	NM_014079	Homo sapiens Kruppel-like factor 15 (KLF15), mRNA [NM_014079]	NM_014079
A_24_P746314	0.000209	4.759	THC2403712		aspartate aminotranferase {Xylella fastidiosa Temecula1;} , partial (4%) [THC2403712]	
A_23_P4551	0.00021	33.63	NM_015559	NM_015559	Homo sapiens SET binding protein 1 (SETBP1), mRNA [NM_015559]	NM_015559
A_24_P944714	0.00021	6.713	AL390129	AL390129	Homo sapiens mRNA; cDNA DKFZp761K0912 (from clone DKFZp761K0912). [AL390129]	
A_24_P414999	0.00021	4.396	NM_018407	NM_018407	Homo sapiens lysosomal associated protein transmembrane 4 beta (LAPTM4B), mRNA [NM_018407]	NM_018407
A_24_P200848	0.00021	3.02	THC2337268			
A_23_P92410	0.00021	2.428	NM_004346	NM_004346	Homo sapiens caspase 3, apoptosis-related cysteine protease (CASP3), transcript variant alpha, mRNA [NM_004346]	NM_004346
A_32_P218249	0.00021	2.247	NM_003211	NM_003211	Homo sapiens thymine-DNA glycosylase (TDG), transcript variant 1, mRNA [NM_003211]	NM_003211
A_23_P16866	0.000211	38.42	NM_007127	NM_007127	Homo sapiens villin 1 (VIL1), mRNA [NM_007127]	NM_007127
A_23_P24903	0.000211	2.765	NM_176072	NM_176072	Homo sapiens purinergic receptor P2Y, G-protein coupled, 2 (P2RY2), transcript variant 1, mRNA [NM_176072]	NM_176072
A_23_P30687	0.000212	12.13	NM_004155	NM_004155	Homo sapiens serine (or cysteine) proteinase inhibitor, clade B (ovalbumin), member 9 (SERPINB9), mRNA [NM_004155]	NM_004155
A_23_P407628	0.000213	2.076	NM_006766	NM_006766	Homo sapiens MYST histone acetyltransferase (monocytic leukemia) 3 (MYST3), mRNA [NM_006766]	NM_006766
A_23_P118392	0.000214	7.7	NM_016084	NM_016084	Homo sapiens RAS, dexamethasone-induced 1 (RASD1), mRNA [NM_016084]	NM_016084
A_23_P102000	0.000215	21.6	NM_001008540	NM_001008540	Homo sapiens chemokine (C-X-C motif) receptor 4 (CXCR4), transcript variant 1, mRNA [NM_001008540]	NM_001008540
A_23_P25503	0.000215	3.739	NM_014923	NM_014923	Homo sapiens fibronectin type III domain containing 3A (FNDC3A), mRNA [NM_014923]	NM_014923
A_32_P163472	0.000216	3.907	A_32_P163472			
A_23_P145957	0.000216	2.391	NM_022445	NM_022445	Homo sapiens thiamin pyrophosphokinase 1 (TPK1), mRNA [NM_022445]	NM_022445
A_23_P71017	0.000217	12.67	NM_001306	NM_001306	Homo sapiens claudin 3 (CLDN3), mRNA [NM_001306]	NM_001306
A_23_P202219	0.000217	12.29	NM_015916	NM_015916	Homo sapiens family with sequence similarity 26, member B (FAM26B), mRNA [NM_015916]	NM_015916
A_23_P1083	0.000217	11.22	NM_002060	NM_002060	Homo sapiens gap junction protein, alpha 4, 37kDa (connexin 37) (GJA4), mRNA [NM_002060]	NM_002060
A_23_P134085	0.000217	4.758	NM_173515	NM_173515	Homo sapiens CNKSR family member 3 (CNKSR3), mRNA [NM_173515]	NM_173515
A_32_P167122	0.000217	2.943	NM_018254	NM_018254	Homo sapiens REST corepressor 3 (RCOR3), mRNA [NM_018254]	NM_018254
A_32_P226858	0.000217	2.3	XM_378898	XM_378898	PREDICTED: Homo sapiens hypothetical LOC400782 (LOC400782), mRNA [XM_378898]	XM_378898
A_23_P93737	0.000218	7.414	NM_004411	NM_004411	Homo sapiens dynein, cytoplasmic, intermediate polypeptide 1 (DNCI1), mRNA [NM_004411]	NM_004411
A_23_P331700	0.000218	4.424	NM_153043	NM_153043	Homo sapiens hypothetical protein FLJ37078 (FLJ37078), mRNA [NM_153043]	NM_153043
A_23_P50735	0.000218	3.947	BC043228	BC043228	Homo sapiens, Similar to zinc finger protein 302, clone IMAGE:5295602, mRNA. [BC043228]	XM_290835

A_23_P3204	0.000218	3.444	NM_002748	NM_002748	Homo sapiens mitogen-activated protein kinase 6 (MAPK6), mRNA [NM_002748]	NM_002748
A_23_P58205	0.000219	231.4	NM_001134	NM_001134	Homo sapiens alpha-fetoprotein (AFP), mRNA [NM_001134]	NM_001134
A_23_P322756	0.000219	17.98	AB051463	AB051463	Homo sapiens mRNA for KIAA1676 protein, partial cds. [AB051463]	
A_23_P70648	0.000219	11.57	NM_000865	NM_000865	Homo sapiens 5-hydroxytryptamine (serotonin) receptor 1E (HTR1E), mRNA [NM_000865]	NM_000865
A_23_P22672	0.000219	2.912	NM_024810	NM_024810	Homo sapiens chromosome X open reading frame 45 (CXorf45), mRNA [NM_024810]	NM_024810
A_23_P120973	0.000219	2.72	NM_017911	NM_017911	Homo sapiens chromosome 22 open reading frame 8 (C22orf8), mRNA [NM_017911]	NM_017911
A_24_P920188	0.000219	2.57	AF230200	AF230200	Homo sapiens OVN6-2 mRNA, partial cds. [AF230200]	
A_24_P123347	0.000219	2.396	NM_002703	NM_002703	Homo sapiens phosphoribosyl pyrophosphate amidotransferase (PPAT), mRNA [NM_002703]	NM_002703
A_23_P56898	0.00022	3.053	NM_003937	NM_003937	Homo sapiens kynureninase (L-kynurenine hydrolase) (KYNU), mRNA [NM_003937]	NM_003937
A_23_P59960	0.00022	2.821	NM_031461	NM_031461	Homo sapiens cysteine-rich secretory protein LCCL domain containing 1 (CRISPLD1), mRNA [NM_031461]	NM_031461
A_23_P99473	0.00022	2.141	NM_023011	NM_023011	Homo sapiens UPF3 regulator of nonsense transcripts homolog A (yeast) (UPF3A), transcript variant 1, mRNA [NM_023011]	NM_023011
A_23_P154025	0.00022	2.082	NM_003142	NM_003142	Homo sapiens Sjogren syndrome antigen B (autoantigen La) (SSB), mRNA [NM_003142]	NM_003142
A_23_P41804	0.000221	7.323	NM_033120	NM_033120	Homo sapiens naked cuticle homolog 2 (Drosophila) (NKD2), mRNA [NM_033120]	NM_033120
A_23_P143885	0.000222	4.99	NM_019555	NM_019555	Homo sapiens Rho guanine nucleotide exchange factor (GEF) 3 (ARHGEF3), mRNA [NM_019555]	NM_019555
A_23_P336342	0.000223	4.699	ENST00000316708		Homo sapiens cDNA FLJ42585 fis, clone BRACE3009237. [AK124576]	
A_24_P358606	0.000223	2.825	A_24_P358606			
A_23_P159974	0.000224	12.67	NM_033495	NM_033495	Homo sapiens kelch-like 13 (Drosophila) (KLHL13), mRNA [NM_033495]	NM_033495
A_23_P17345	0.000224	8.869	NM_005461	NM_005461	Homo sapiens v-maf musculoaponeurotic fibrosarcoma oncogene homolog B (avian) (MAFB), mRNA [NM_005461]	NM_005461
A_24_P738859	0.000224	7.636	AK075186	AK075186	Homo sapiens cDNA FLJ90705 fis, clone PLACE1007591. [AK075186]	
A_23_P140427	0.000224	3.757	NM_016337	NM_016337	Homo sapiens Enah/Vasp-like (EVL), mRNA [NM_016337]	NM_016337
A_23_P136460	0.000224	3.177	NM_016603	NM_016603	Homo sapiens chromosome 5 open reading frame 5 (C5orf5), mRNA [NM_016603]	NM_016603
A_23_P433676	0.000224	2.519	NM_020951	NM_020951	Homo sapiens zinc finger protein 529 (ZNF529), mRNA [NM_020951]	NM_020951
A_23_P151506	0.000224	2.493	NM_016445	NM_016445	Homo sapiens pleckstrin 2 (PLEK2), mRNA [NM_016445]	NM_016445
A_24_P328524	0.000224	2.376	NM_003947	NM_003947	Homo sapiens kalirin, RhoGEF kinase (KALRN), transcript variant 2, mRNA [NM_003947]	NM_003947
A_23_P15621	0.000224	2.371	NM_032391	NM_032391	Homo sapiens small nuclear protein PRAC (PRAC), mRNA [NM_032391]	NM_032391
A_23_P500892	0.000225	5.139	NM_003320	NM_003320	Homo sapiens tubby homolog (mouse) (TUB), transcript variant 1, mRNA [NM_003320]	NM_003320
A_23_P393051	0.000226	8.012	NM_152365	NM_152365	Homo sapiens chromosome 1 open reading frame 172 (C1orf172), mRNA [NM_152365]	NM_152365
A_23_P108342	0.000226	4.223	NM_016536	NM_016536	Homo sapiens zinc finger protein 571 (ZNF571), mRNA [NM_016536]	NM_016536
A_23_P208325	0.000226	3.362	NM_004234	NM_004234	Homo sapiens zinc finger protein 235 (ZNF235), mRNA [NM_004234]	NM_004234
A_23_P66739	0.000227	21.93	NM_177550	NM_177550	Homo sapiens solute carrier family 13 (sodium-dependent citrate transporter), member 5 (SLC13A5), mRNA [NM_177550]	NM_177550
A_24_P319364	0.000227	9.782	NM_144503	NM_144503	Homo sapiens F11 receptor (F11R), transcript variant 4, mRNA [NM_144503]	NM_144503
A_23_P1722	0.000227	6.139	NM_080659	NM_080659	Homo sapiens similar to RIKEN cDNA 2310030G06 gene (MGC14839), mRNA [NM_080659]	NM_080659
A_23_P81392	0.000227	5.664	NM_015238	NM_015238	Homo sapiens KIBRA protein (KIBRA), mRNA [NM_015238]	NM_015238
A_24_P224488	0.000227	2.431	AK055986	AK055986	Homo sapiens cDNA FLJ31424 fis, clone NT2NE2000392. [AK055986]	
A_23_P54389	0.000227	2.1	NM_024611	NM_024611	Homo sapiens NMDA receptor regulated 2 (NARG2), transcript variant 1, mRNA [NM_024611]	NM_024611
A_23_P150394	0.000228	5.348	NM_022003	NM_022003	Homo sapiens FXYD domain containing ion transport regulator 6 (FXYD6), mRNA [NM_022003]	NM_022003
A_24_P201879	0.000228	2.491	NM_021645	NM_021645	Homo sapiens UTP14, U3 small nucleolar ribonucleoprotein, homolog C (yeast) (UTP14C), mRNA [NM_021645]	NM_021645
A_24_P123521	0.000228	2.435	NM_020666	NM_020666	Homo sapiens CDC-like kinase 4 (CLK4), mRNA [NM_020666]	NM_020666
A_23_P403955	0.000228	2.359	NM_007375	NM_007375	Homo sapiens TAR DNA binding protein (TARDBP), mRNA [NM_007375]	NM_007375
A_23_P334218	0.000228	2.307	NM_145647	NM_145647	Homo sapiens WD repeat domain 67 (WDR67), mRNA [NM_145647]	NM_145647
A_23_P316511	0.000228	2.115	NM_002146	NM_002146	Homo sapiens homeo box B3 (HOXB3), mRNA [NM_002146]	NM_002146

A_23_P104224	0.000229	7.069	NM_138933	NM_138933	Homo sapiens apobec-1 complementation factor (ACF), transcript variant 3, mRNA [NM_138933]	NM_138933
A_32_P184039	0.000229	5.3	A_32_P184039			
A_23_P24004	0.000229	4.079	NM_001547	NM_001547	Homo sapiens interferon-induced protein with tetratricopeptide repeats 2 (IFIT2), mRNA [NM_001547]	NM_001547
A_23_P26865	0.000229	3.543	NM_002470	NM_002470	Homo sapiens myosin, heavy polypeptide 3, skeletal muscle, embryonic (MYH3), mRNA [NM_002470]	NM_002470
A_23_P134684	0.000229	3.538	NM_024567	NM_024567	Homo sapiens hypothetical protein FLJ21616 (FLJ21616), mRNA [NM_024567]	NM_024567
A_23_P149818	0.000229	2.31	NM_080599	NM_080599	Homo sapiens UPF2 regulator of nonsense transcripts homolog (yeast) (UPF2), transcript variant 1, mRNA [NM_080599]	NM_080599
A_32_P106315	0.00023	4.463	BX641009	BX641009	Homo sapiens mRNA; cDNA DKFZp686D13227 (from clone DKFZp686D13227) [BX641009]	
A_23_P201790	0.00023	3.149	NM_032105	NM_032105	Homo sapiens protein phosphatase 1, regulatory (inhibitor) subunit 12B (PPP1R12B), transcript variant 2, mRNA [NM_032105]	NM_032105
A_23_P27035	0.000231	6.239	NM_015544	NM_015544	Homo sapiens DKFZP564K1964 protein (DKFZP564K1964), mRNA [NM_015544]	NM_015544
A_23_P112004	0.000231	6.211	NM_012472	NM_012472	Homo sapiens leucine rich repeat containing 6 (LRRC6), mRNA [NM_012472]	NM_012472
A_24_P63522	0.000232	8.03	NM_002130	NM_002130	Homo sapiens 3-hydroxy-3-methylglutaryl-Coenzyme A synthase 1 (soluble) (HMGCS1), mRNA [NM_002130]	NM_002130
A_32_P164203	0.000232	4.567	THC2436337			
A_32_P192376	0.000232	2.074	THC2312617			
A_24_P180680	0.000233	8.023	NM_018407	NM_018407	Homo sapiens lysosomal associated protein transmembrane 4 beta (LAPTM4B), mRNA [NM_018407]	NM_018407
A_32_P47200	0.000233	2.174	AU121805	AU121805	AU121805 MAMMA1 Homo sapiens cDNA clone MAMMA1001009 5', mRNA sequence [AU121805]	
A_32_P220472	0.000233	2.09	NM_019006	NM_019006	Homo sapiens zinc finger, A20 domain containing 3 (ZA20D3), mRNA [NM_019006]	NM_019006
A_23_P67453	0.000234	3.695	NM_000363	NM_000363	Homo sapiens troponin I, cardiac (TNNI3), mRNA [NM_000363]	NM_000363
A_32_P106732	0.000234	3.168	ENST00000267430		Homo sapiens mRNA for KIAA1596 protein, partial cds. [AB046816]	XM_048128
A_24_P116909	0.000235	4.461	NM_006785	NM_006785	Homo sapiens mucosa associated lymphoid tissue lymphoma translocation gene 1 (MALT1), transcript variant 1, mRNA [NM_006785]	NM_006785
A_24_P15965	0.000235	4.194	NM_203282	NM_203282	Homo sapiens zinc finger protein 539 (ZNF539), mRNA [NM_203282]	NM_203282
A_23_P37327	0.000235	2.312	NM_020326	NM_020326	Homo sapiens ATP-binding cassette, sub-family D (ALD), member 4 (ABCD4), transcript variant 5, mRNA [NM_020326]	NM_020326
A_23_P320530	0.000236	8.834	AK091274	AK091274	Homo sapiens cDNA FLJ33955 fis, clone CTONG2018652, moderately similar to ZINC FINGER PROTEIN MFG-3. [AK091274]	
A_32_P219116	0.000236	3.291	NM_018451	NM_018451	Homo sapiens centromere protein J (CENPJ), mRNA [NM_018451]	NM_018451
A_23_P212508	0.000237	52.34	NM_001063	NM_001063	Homo sapiens transferrin (TF), mRNA [NM_001063]	NM_001063
A_23_P203972	0.000237	25.05	NM_007197	NM_007197	Homo sapiens frizzled homolog 10 (Drosophila) (FZD10), mRNA [NM_007197]	NM_007197
A_23_P121564	0.000237	7.904	NM_000857	NM_000857	Homo sapiens guanylate cyclase 1, soluble, beta 3 (GUCY1B3), mRNA [NM_000857]	NM_000857
A_23_P336198	0.000238	7.542	AK093655	AK093655	Homo sapiens cDNA FLJ36336 fis, clone THYMU2006303. [AK093655]	
A_23_P40415	0.000238	5.129	NM_007038	NM_007038	Homo sapiens a disintegrin-like and metalloprotease (reprolysin type) with thrombospondin type 1 motif, 5 (aggrecanase-2) (ADAMTS5), mRNA [NM_007038]	NM_007038
A_23_P5234	0.000238	4.218	NM_003423	NM_003423	Homo sapiens zinc finger protein 43 (HTF6) (ZNF43), mRNA [NM_003423]	NM_003423
A_32_P79763	0.000238	3.441	AK023134	AK023134	Homo sapiens cDNA FLJ13072 fis, clone NT2RP3001844. [AK023134]	XM_117117
A_23_P212728	0.000238	2.345	BC020955	BC020955	Homo sapiens hypothetical protein FLJ11046, mRNA (cDNA clone MGC:8800 IMAGE:3847561), complete cds. [BC020955]	
A_32_P174151	0.000239	2.279	BC036263	BC036263	Homo sapiens KIAA0220-like protein, mRNA (cDNA clone MGC:9515 IMAGE:3903371), complete cds. [BC036263]	XM_496136
A_23_P252132	0.00024	18.69	NM_002839	NM_002839	Homo sapiens protein tyrosine phosphatase, receptor type, D (PTPRD), transcript variant 1, mRNA [NM_002839]	NM_002839
A_23_P58419	0.00024	5.682	NM_002253	NM_002253	Homo sapiens kinase insert domain receptor (a type III receptor tyrosine kinase) (KDR), mRNA [NM_002253]	NM_002253
A_23_P431360	0.000241	4.314	NM_016423	NM_016423	Homo sapiens zinc finger protein 219 (ZNF219), mRNA [NM_016423]	NM_016423
A_24_P338648	0.000241	3	NM_001177	NM_001177	Homo sapiens ADP-ribosylation factor-like 1 (ARL1), mRNA [NM_001177]	NM_001177
A_32_P42976	0.000242	7.034	THC2381319		BC022074 C6orf102 protein {Homo sapiens;} , partial (6%) [THC2381319]	
A_24_P254101	0.000242	4.677	AK125846	AK125846	Homo sapiens cDNA FLJ43858 fis, clone TESTI4007373. [AK125846]	
A_32_P92445	0.000242	4.027	AK092715	AK092715	Homo sapiens cDNA FLJ35396 fis, clone SKNSH2003483. [AK092715]	

A_23_P325017	0.000242	2.662	NM_147128	NM_147128	Homo sapiens zinc and ring finger 2 (ZNRF2), mRNA [NM_147128]	NM_147128
A_32_P140823	0.000242	2.475	NM_006985	NM_006985	Homo sapiens nuclear pore complex interacting protein (NPIP), mRNA [NM_006985]	NM_006985
A_23_P256334	0.000242	2.358	NM_181501	NM_181501	Homo sapiens integrin, alpha 1 (ITGA1), mRNA [NM_181501]	NM_181501
A_23_P212511	0.000243	2.128	NM_133462	NM_133462	Homo sapiens tetratricopeptide repeat domain 14 (TTC14), mRNA [NM_133462]	NM_133462
A_32_P217051	0.000244	47.01	THC2330484			
A_24_P3140	0.000244	4.566	NM_014497	NM_014497	Homo sapiens zinc finger protein 638 (ZNF638), transcript variant 1, mRNA [NM_014497]	NM_014497
A_23_P209619	0.000244	3.38	CR617774	CR617774	full-length cDNA clone CS0DC002YP09 of Neuroblastoma Cot 25-normalized of Homo sapiens (human). [CR617774]	
A_32_P112493	0.000245	12.97	NM_138370	NM_138370	Homo sapiens hypothetical protein BC007901 (LOC91461), mRNA [NM_138370]	NM_138370
A_23_P163666	0.000245	12.11	NM_053284	NM_053284	Homo sapiens WAP, follistatin/kazal, immunoglobulin, kunitz and netrin domain containing 1 (WFIKKN1), mRNA [NM_053284]	NM_053284
A_23_P203013	0.000245	3.183	NM_002519	NM_002519	Homo sapiens nuclear protein, ataxia-telangiectasia locus (NPAT), mRNA [NM_002519]	NM_002519
A_32_P50834	0.000246	3.519	NM_006729	NM_006729	Homo sapiens diaphanous homolog 2 (Drosophila) (DIAPH2), transcript variant 156, mRNA [NM_006729]	NM_006729
A_32_P216872	0.000246	2.865	BX647358	BX647358	Homo sapiens mRNA; cDNA DKFZp686B0962 (from clone DKFZp686B0962). [BX647358]	
A_24_P6381	0.000247	8.812	NM_178276	NM_178276	Homo sapiens chromosome 5 open reading frame 12 (C5orf12), mRNA [NM_178276]	NM_178276
A_32_P33213	0.000247	8.313	THC2358845			
A_24_P114334	0.000247	5.374	AL050139	AL050139	Homo sapiens mRNA; cDNA DKFZp586M141 (from clone DKFZp586M141). [AL050139]	
A_24_P34344	0.000247	3.043	NM_033020	NM_033020	Homo sapiens tripartite motif-containing 33 (TRIM33), transcript variant beta, mRNA [NM_033020]	NM_033020
A_24_P115774	0.000247	2.559	NM_001166	NM_001166	Homo sapiens baculoviral IAP repeat-containing 2 (BIRC2), mRNA [NM_001166]	NM_001166
A_24_P382113	0.000247	2.415	NM_006107	NM_006107	Homo sapiens cisplatin resistance-associated overexpressed protein (CROP), transcript variant 2, mRNA [NM_006107]	NM_006107
A_32_P20240	0.000248	2.793	BX648857	BX648857	Homo sapiens mRNA; cDNA DKFZp686N17231 (from clone DKFZp686N17231). [BX648857]	
A_23_P2601	0.000249	3.295	NM_003299	NM_003299	Homo sapiens tumor rejection antigen (gp96) 1 (TRA1), mRNA [NM_003299]	NM_003299
A_32_P220739	0.000249	3.169	NM_033160	NM_033160	Homo sapiens zinc finger protein 658 (ZNF658), mRNA [NM_033160]	NM_033160
A_23_P145644	0.00025	95.23	NM_000790	NM_000790	Homo sapiens dopa decarboxylase (aromatic L-amino acid decarboxylase) (DDC), mRNA [NM_000790]	NM_000790
A_23_P28927	0.00025	5.899	A_23_P28927			
A_32_P69296	0.00025	4.992	AK130071	AK130071	Homo sapiens cDNA FLJ26561 fis, clone LNF03981. [AK130071]	
A_24_P915361	0.000251	2.874	AF086536	AF086536	Homo sapiens full length insert cDNA clone ZE08A03. [AF086536]	
A_23_P47991	0.000252	3.117	NM_015335	NM_015335	Homo sapiens thyroid hormone receptor associated protein 2 (THRAP2), mRNA [NM_015335]	NM_015335
A_23_P138805	0.000252	2.361	NM_012124	NM_012124	Homo sapiens cysteine and histidine-rich domain (CHORD)-containing, zinc binding protein 1 (CHORDC1), mRNA [NM_012124]	NM_012124
A_23_P123596	0.000253	12.15	NM_000170	NM_000170	Homo sapiens glycine dehydrogenase (decarboxylating; glycine decarboxylase, glycine cleavage system protein P) (GLDC), mRNA [NM_000170]	NM_000170
A_32_P177685	0.000253	9	AA665072	AA665072	AA665072 nu76b01.s1 NCI_CGAP_Alv1 Homo sapiens cDNA clone IMAGE:1216585, mRNA sequence [AA665072]	
A_24_P303080	0.000253	4.157	THC2370211			
A_24_P332683	0.000253	3.651	NM_001008401	NM_001008401	Homo sapiens FLJ16231 protein (FLJ16231), mRNA [NM_001008401]	NM_001008401
A_24_P363408	0.000255	16.56	NM_012259	NM_012259	Homo sapiens hairy/enhancer-of-split related with YRPW motif 2 (HEY2), mRNA [NM_012259]	NM_012259
A_23_P373100	0.000255	14.31	BC020879	BC020879	Homo sapiens hypothetical protein MGC24103, mRNA (cDNA clone MGC:24103 IMAGE:4613905), complete cds. [BC020879]	
A_23_P37375	0.000255	11.49	NM_004755	NM_004755	Homo sapiens ribosomal protein S6 kinase, 90kDa, polypeptide 5 (RPS6KA5), transcript variant 1, mRNA [NM_004755]	NM_004755
A_23_P45087	0.000255	6.807	NM_016220	NM_016220	Homo sapiens zinc finger protein 588 (ZNF588), transcript variant 1, mRNA [NM_016220]	NM_016220
A_24_P35891	0.000255	3.278	NM_016423	NM_016423	Homo sapiens zinc finger protein 219 (ZNF219), mRNA [NM_016423]	NM_016423
A_32_P84342	0.000255	2.875	THC2315140		predicted protein {Methanosarcina acetivorans C2A;} , partial (11%) [THC2315140]	
A_23_P254702	0.000255	2.388	NM_003472	NM_003472	Homo sapiens DEK oncogene (DNA binding) (DEK), mRNA [NM_003472]	NM_003472
A_23_P124855	0.000255	2.251	NM_032226	NM_032226	Homo sapiens zinc finger, CCHC domain containing 7 (ZCCHC7), mRNA [NM_032226]	NM_032226
A_23_P137097	0.000256	3.939	NM_006517	NM_006517	Homo sapiens solute carrier family 16 (monocarboxylic acid transporters), member 2 (SLC16A2), mRNA [NM_006517]	NM_006517

A_24_P405002	0.000256	3.344	NM_152835	NM_152835	Homo sapiens PDLIM1 interacting kinase 1 like (PDIK1L), mRNA [NM_152835]	NM_152835
A_23_P45409	0.000256	3.105	NM_080632	NM_080632	Homo sapiens UPF3 regulator of nonsense transcripts homolog B (yeast) (UPF3B), transcript variant 1, mRNA [NM_080632]	NM_080632
A_24_P322474	0.000256	2.473	NM_006202	NM_006202	Homo sapiens phosphodiesterase 4A, cAMP-specific (phosphodiesterase E2 dunce homolog, Drosophila) (PDE4A), mRNA [NM_006202]	NM_006202
A_32_P190737	0.000257	5.027	AK057981	AK057981	Homo sapiens cDNA FLJ25252 fis, clone STM03814. [AK057981]	
A_32_P115505	0.000257	2.887	NM_015565	NM_015565	Homo sapiens zinc finger protein 294 (ZNF294), mRNA [NM_015565]	NM_015565
A_23_P391344	0.000257	2.661	NM_145313	NM_145313	Homo sapiens RasGEF domain family, member 1A (RASGEF1A), mRNA [NM_145313]	NM_145313
A_23_P164081	0.000257	2.509	NM_005964	NM_005964	Homo sapiens myosin, heavy polypeptide 10, non-muscle (MYH10), mRNA [NM_005964]	NM_005964
A_23_P26916	0.000257	2.204	NM_001015053	NM_001015053	Homo sapiens histone deacetylase 5 (HDAC5), transcript variant 3, mRNA [NM_001015053]	NM_001015053
A_32_P177955	0.000258	5.467	BC030123	BC030123	Homo sapiens, clone IMAGE:4815474, mRNA. [BC030123]	XM_499157
A_23_P104138	0.000258	3.356	BC007286	BC007286	Homo sapiens hypothetical protein MGC15634, mRNA (cDNA clone MGC:15634 IMAGE:3344302), complete cds. [BC007286]	
A_24_P371303	0.000258	2.236	NM_015224	NM_015224	Homo sapiens retinoblastoma-associated protein 140 (RAP140), mRNA [NM_015224]	NM_015224
A_24_P356916	0.000259	20.25	NM_001011554	NM_001011554	Homo sapiens solute carrier family 13 (sodium-dependent dicarboxylate transporter), member 3 (SLC13A3), transcript variant 2, mRNA [NM_001011554]	NM_001011554
A_23_P214079	0.00026	9.923	NM_003122	NM_003122	Homo sapiens serine protease inhibitor, Kazal type 1 (SPINK1), mRNA [NM_003122]	NM_003122
A_23_P402000	0.00026	2.875	AK091585	AK091585	Homo sapiens cDNA FLJ34266 fis, clone FEBRA2002682, moderately similar to ZINC FINGER PROTEIN 91. [AK091585]	XM_030378
A_23_P31006	0.000261	31.04	NM_002125	NM_002125	Homo sapiens major histocompatibility complex, class II, DR beta 5 (HLA-DRB5), mRNA [NM_002125]	NM_002125
A_32_P29632	0.000261	16.28	NM_001338	NM_001338	Homo sapiens coxsackie virus and adenovirus receptor (CXADR), mRNA [NM_001338]	NM_001338
A_24_P936393	0.000261	2.671	A_24_P936393			
A_24_P406714	0.000261	2.598	NM_004641	NM_004641	Homo sapiens myeloid/lymphoid or mixed-lineage leukemia (trithorax homolog, Drosophila); translocated to, 10 (MLLT10), transcript variant 1, mRNA [NM_004641]	NM_004641
A_32_P206541	0.000262	4.806	AK128714	AK128714	Homo sapiens cDNA FLJ46881 fis, clone UTERU3015647, moderately similar to Embigin precursor. [AK128714]	
A_23_P357207	0.000263	14.58	NM_138409	NM_138409	Homo sapiens chromosome 6 open reading frame 117 (C6orf117), mRNA [NM_138409]	NM_138409
A_32_P113887	0.000264	11.52	THC2446900			
A_32_P11372	0.000266	17.45	NM_174950	NM_174950	Homo sapiens hypothetical protein FLJ30435 (FLJ30435), mRNA [NM_174950]	NM_174950
A_23_P40174	0.000267	6.283	NM_004994	NM_004994	Homo sapiens matrix metalloproteinase 9 (gelatinase B, 92kDa gelatinase, 92kDa type IV collagenase) (MMP9), mRNA [NM_004994]	NM_004994
A_24_P45446	0.000267	4.794	NM_052941	NM_052941	Homo sapiens guanylate binding protein 4 (GBP4), mRNA [NM_052941]	NM_052941
A_23_P76743	0.000267	3.723	AX781433	AX781433	Sequence 16 from Patent EP1321519. [AX781433]	
A_32_P42197	0.000268	2.381	NM_031157	NM_031157	Homo sapiens heterogeneous nuclear ribonucleoprotein A1 (HNRPA1), transcript variant 2, mRNA [NM_031157]	NM_031157
A_23_P52031	0.000268	2.25	NM_002633	NM_002633	Homo sapiens phosphoglucomutase 1 (PGM1), mRNA [NM_002633]	NM_002633
A_32_P140489	0.000269	4.028	NM_001001557	NM_001001557	Homo sapiens growth differentiation factor 6 (GDF6), mRNA [NM_001001557]	NM_001001557
A_24_P222237	0.000269	2.166	NM_001024681	NM_001024681	Homo sapiens D15F37 gene (D15F37), mRNA [NM_001024681]	NM_001024681
A_32_P161855	0.00027	7.928	NM_018689	NM_018689	Homo sapiens KIAA1199 (KIAA1199), mRNA [NM_018689]	NM_018689
A_24_P260300	0.00027	3.888	CR622342	CR622342	full-length cDNA clone CS0DJ006YC05 of T cells (Jurkat cell line) Cot 10-normalized of Homo sapiens (human). [CR622342]	
A_23_P382081	0.00027	2.881	NM_144722	NM_144722	Homo sapiens KPL2 protein (FLJ23577), transcript variant 2, mRNA [NM_144722]	NM_144722
A_23_P114903	0.00027	2.562	NM_002155	NM_002155	Homo sapiens heat shock 70kDa protein 6 (HSP70B') (HSPA6), mRNA [NM_002155]	NM_002155
A_23_P205355	0.000272	2.997	NM_000624	NM_000624	Homo sapiens serine (or cysteine) proteinase inhibitor, clade A (alpha-1 antiproteinase, antitrypsin), member 5 (SERPINA5), mRNA [NM_000624]	NM_000624
A_23_P23947	0.000273	7.086	NM_005204	NM_005204	Homo sapiens mitogen-activated protein kinase kinase kinase 8 (MAP3K8), mRNA [NM_005204]	NM_005204
A_32_P145385	0.000273	5.746	AK001118	AK001118	Homo sapiens cDNA FLJ10256 fis, clone HEMBB1000870. [AK001118]	
A_24_P284093	0.000273	3.513	NM_016651	NM_016651	Homo sapiens dapper, antagonist of beta-catenin, homolog 1 (Xenopus laevis) (DACT1), mRNA [NM_016651]	NM_016651
A_23_P328323	0.000275	5.034	NM_018211	NM_018211	Homo sapiens hypothetical protein FLJ10770 (KIAA1579), mRNA [NM_018211]	NM_018211

A_23_P38723	0.000275	2.946	AK126324	AK126324	Homo sapiens cDNA FLJ44350 fis, clone TRACH3006228. [AK126324]	
A_24_P497843	0.000276	6.562	THC2290002			
A_24_P71973	0.000277	10.97	NM_002253	NM_002253	Homo sapiens kinase insert domain receptor (a type III receptor tyrosine kinase) (KDR), mRNA [NM_002253]	NM_002253
A_24_P733308	0.000277	8.664	THC2309960		Q7ZX66 (Q7ZX66) RNPC7 protein (Fragment), partial (9%) [THC2309960]	
A_23_P88880	0.000277	7.334	NM_015069	NM_015069	Homo sapiens zinc finger protein 423 (ZNF423), mRNA [NM_015069]	NM_015069
A_24_P303647	0.000277	3.154	ENST00000297145		Homo sapiens mRNA; cDNA DKFZp762M126 (from clone DKFZp762M126). [AL834437]	
A_23_P333998	0.000278	2.827	AF090919	AF090919	Homo sapiens clone HQ0327 PRO0327 mRNA, complete cds. [AF090919]	
A_23_P82402	0.000279	5.831	NM_138426	NM_138426	Homo sapiens glucocorticoid induced transcript 1 (GLCCI1), mRNA [NM_138426]	NM_138426
A_23_P37654	0.000279	3.07	ENST00000310492		Homo sapiens mRNA for KIAA0518 protein, partial cds. [AB011090]	XM_031689
A_23_P354217	0.000279	2.346	NM_153266	NM_153266	Homo sapiens hypothetical protein MGC33486 (MGC33486), mRNA [NM_153266]	NM_153266
A_24_P141995	0.00028	2.415	NM_032632	NM_032632	Homo sapiens poly(A) polymerase alpha (PAPOLA), mRNA [NM_032632]	NM_032632
A_24_P325992	0.000281	16.09	NM_002310	NM_002310	Homo sapiens leukemia inhibitory factor receptor (LIFR), mRNA [NM_002310]	NM_002310
A_23_P120794	0.000282	7.884	NM_004173	NM_004173	Homo sapiens solute carrier family 7 (cationic amino acid transporter, y+ system), member 4 (SLC7A4), mRNA [NM_004173]	NM_004173
A_23_P401076	0.000283	6.405	NM_145006	NM_145006	Homo sapiens sushi domain containing 3 (SUSD3), mRNA [NM_145006]	NM_145006
A_23_P154379	0.000283	4.52	NM_003960	NM_003960	Homo sapiens N-acetyltransferase 8 (camello like) (NAT8), mRNA [NM_003960]	NM_003960
A_23_P57709	0.000284	19.41	NM_013363	NM_013363	Homo sapiens procollagen C-endopeptidase enhancer 2 (PCOLCE2), mRNA [NM_013363]	NM_013363
A_23_P152995	0.000284	4.56	NM_001045	NM_001045	Homo sapiens solute carrier family 6 (neurotransmitter transporter, serotonin), member 4 (SLC6A4), mRNA [NM_001045]	NM_001045
A_32_P1772	0.000284	2.892	NM_020207	NM_020207	Homo sapiens chromosome 9 open reading frame 102 (C9orf102), mRNA [NM_020207]	NM_020207
A_23_P318581	0.000284	2.296	AB037851	AB037851	Homo sapiens mRNA for KIAA1430 protein, partial cds. [AB037851]	
A_24_P923102	0.000285	3.857	BC016950	BC016950	Homo sapiens kelch-like 23 (Drosophila), mRNA (cDNA clone IMAGE:3854163), complete cds. [BC016950]	
A_23_P312851	0.000286	4.627	NM_006928	NM_006928	Homo sapiens silver homolog (mouse) (SILV), mRNA [NM_006928]	NM_006928
A_23_P501193	0.000287	14.28	NM_170741	NM_170741	Homo sapiens potassium inwardly-rectifying channel, subfamily J, member 16 (KCNJ16), transcript variant 2, mRNA [NM_170741]	NM_170741
A_23_P107775	0.000287	5.113	NM_139172	NM_139172	Homo sapiens MDAC1 (MDAC1), mRNA [NM_139172]	NM_139172
A_23_P61854	0.000287	2.804	ENST00000358746		Homo sapiens mRNA for KIAA0372 gene, partial cds. [AB002370]	
A_23_P86195	0.000288	12.35	NM_152369	NM_152369	Homo sapiens hypothetical protein MGC45474 (MGC45474), mRNA [NM_152369]	NM_152369
A_24_P168398	0.000288	4.648	NM_003451	NM_003451	Homo sapiens zinc finger protein 177 (ZNF177), mRNA [NM_003451]	NM_003451
A_23_P108932	0.000288	4.364	NR_002229	NR_002229	Homo sapiens ribosomal protein L23a pseudogene 13 (RPL23AP13) on chromosome 2 [NR_002229]	NR_002229
A_24_P387869	0.000288	3.233	BC029749	BC029749	Homo sapiens, clone IMAGE:4513287, mRNA, partial cds. [BC029749]	
A_23_P100539	0.000288	2.115	CR609146	CR609146	full-length cDNA clone CS0DM005YF09 of Fetal liver of Homo sapiens (human). [CR609146]	
A_23_P18223	0.000288	2.015	NM_002215	NM_002215	Homo sapiens inter-alpha (globulin) inhibitor H1 (ITIH1), mRNA [NM_002215]	NM_002215
A_24_P395610	0.000289	5.595	NM_201433	NM_201433	Homo sapiens growth arrest-specific 7 (GAS7), transcript variant c, mRNA [NM_201433]	NM_201433
A_23_P215111	0.000289	3.911	NM_020632	NM_020632	Homo sapiens ATPase, H+ transporting, lysosomal V0 subunit a isoform 4 (ATP6V0A4), transcript variant 1, mRNA [NM_020632]	NM_020632
A_24_P380734	0.000289	2.974	NM_002998	NM_002998	Homo sapiens syndecan 2 (heparan sulfate proteoglycan 1, cell surface-associated, fibroglycan) (SDC2), mRNA [NM_002998]	NM_002998
A_23_P316150	0.000289	2.962	NM_020800	NM_020800	Homo sapiens WD repeat domain 56 (WDR56), mRNA [NM_020800]	NM_020800
A_24_P325035	0.000289	2.19	AK092090	AK092090	Homo sapiens cDNA FLJ34771 fis, clone NT2NE2003150. [AK092090]	
A_23_P89710	0.000289	2.003	NM_018069	NM_018069	Homo sapiens centrosomal protein 192 kDa (Cep192), transcript variant 2, mRNA [NM_018069]	NM_018069
A_32_P168326	0.00029	4.642	NM_001012421	NM_001012421	Homo sapiens OTTHUMP00000064580 (LOC441430), mRNA [NM_001012421]	NM_001012421
A_24_P940776	0.00029	3.796	CR749289	CR749289	Homo sapiens mRNA; cDNA DKFZp686L02246 (from clone DKFZp686L02246). [CR749289]	
A_23_P301051	0.00029	3.739	ENST00000244221		Homo sapiens mRNA for KIAA1155 protein, partial cds. [AB032981]	XM_376062
A_23_P4462	0.00029	3.408	A_23_P4462			

A_23_P18684	0.000291	23.43	NM_004362	NM_004362	Homo sapiens calmegin (CLGN), mRNA [NM_004362]	NM_004362
A_23_P217379	0.000291	5.741	NM_033641	NM_033641	Homo sapiens collagen, type IV, alpha 6 (COL4A6), transcript variant B, mRNA [NM_033641]	NM_033641
A_24_P538708	0.000291	3.289	AK124263	AK124263	Homo sapiens cDNA FLJ42269 fis, clone TKIDN2015285. [AK124263]	
A_23_P9485	0.000292	10.38	NM_000608	NM_000608	Homo sapiens orosomucoid 2 (ORM2), mRNA [NM_000608]	NM_000608
A_23_P162466	0.000293	13.15	NM_004572	NM_004572	Homo sapiens plakophilin 2 (PKP2), transcript variant 2b, mRNA [NM_004572]	NM_004572
A_23_P203023	0.000293	3.275	NM_002906	NM_002906	Homo sapiens radixin (RDX), mRNA [NM_002906]	NM_002906
A_32_P119096	0.000293	2.078	AF052107	AF052107	Homo sapiens clone 23620 mRNA sequence. [AF052107]	
A_23_P141447	0.000294	8.443	NM_145654	NM_145654	Homo sapiens RAD52 homolog B (S. cerevisiae) (RAD52B), mRNA [NM_145654]	NM_145654
A_24_P416370	0.000294	4.438	NM_024015	NM_024015	Homo sapiens homeo box B4 (HOXB4), mRNA [NM_024015]	NM_024015
A_32_P41035	0.000294	3.431	AK092942	AK092942	Homo sapiens cDNA FLJ35623 fis, clone SPLEN2010986. [AK092942]	XM_496251
A_23_P500734	0.000294	2.175	NM_015100	NM_015100	Homo sapiens pogo transposable element with ZNF domain (POGZ), transcript variant 1, mRNA [NM_015100]	NM_015100
A_24_P943106	0.000295	6.368	AB002330	AB002330	Human mRNA for KIAA0332 gene, partial cds. [AB002330]	XM_031553
A_23_P7582	0.000295	3.224	NM_003202	NM_003202	Homo sapiens transcription factor 7 (T-cell specific, HMG-box) (TCF7), transcript variant 1, mRNA [NM_003202]	NM_003202
A_23_P132175	0.000295	2.07	NM_023004	NM_023004	Homo sapiens reticulon 4 receptor (RTN4R), mRNA [NM_023004]	NM_023004
A_32_P106523	0.000296	6.838	THC2441641		B40201 artifact-warning sequence (translated ALU class B) - human {Homo sapiens;} , partial (13%) [THC2441641]	
A_24_P510047	0.000296	6.225	AK022086	AK022086	Homo sapiens cDNA FLJ12024 fis, clone HEMBB1001797. [AK022086]	
A_23_P363647	0.000296	5.543	NM_182540	NM_182540	Homo sapiens DEAD/H (Asp-Glu-Ala-Asp/His) box polypeptide 26B (DDX26B), mRNA [NM_182540]	NM_182540
A_23_P110957	0.000297	22.05	NM_001452	NM_001452	Homo sapiens forkhead box F2 (FOXF2), mRNA [NM_001452]	NM_001452
A_23_P48535	0.000297	5.576	NM_001173	NM_001173	Homo sapiens Rho GTPase activating protein 5 (ARHGAP5), mRNA [NM_001173]	NM_001173
A_24_P384588	0.000297	4.831	NM_001012452	NM_001012452	Homo sapiens hypothetical protein FLJ32679 (FLJ32679), mRNA [NM_001012452]	NM_001012452
A_24_P416257	0.000297	2.38	NM_015044	NM_015044	Homo sapiens golgi associated, gamma adaptin ear containing, ARF binding protein 2 (GGA2), transcript variant 1, mRNA [NM_015044]	NM_015044
A_32_P136033	0.000298	2.648	AK090477	AK090477	Homo sapiens mRNA for FLJ00399 protein. [AK090477]	
A_23_P63584	0.000298	2.162	NM_015446	NM_015446	Homo sapiens AT hook containing transcription factor 1 (AHCTF1), mRNA [NM_015446]	NM_015446
A_32_P109029	0.000299	27.11	NM_022036	NM_022036	Homo sapiens G protein-coupled receptor, family C, group 5, member C (GPRC5C), transcript variant 1, mRNA [NM_022036]	NM_022036
A_32_P131640	0.000299	6.294	NM_173078	NM_173078	Homo sapiens SLIT and NTRK-like family, member 4 (SLITRK4), mRNA [NM_173078]	NM_173078
A_23_P23705	0.0003	4.932	BC040655	BC040655	Homo sapiens cDNA clone IMAGE:4798227, partial cds. [BC040655]	
A_23_P53856	0.0003	3.312	NM_014887	NM_014887	Homo sapiens phosphonoformate immuno-associated protein 5 (PFAAP5), mRNA [NM_014887]	NM_014887
A_24_P943575	0.0003	2.047	NM_032221	NM_032221	Homo sapiens chromodomain helicase DNA binding protein 6 (CHD6), mRNA [NM_032221]	NM_032221
A_23_P377141	0.000301	5.256	NM_015186	NM_015186	Homo sapiens vacuolar protein sorting 13A (yeast) (VPS13A), transcript variant B, mRNA [NM_015186]	NM_015186
A_23_P129005	0.000301	3.034	BC008219	BC008219	Homo sapiens KIAA1305, mRNA (cDNA clone MGC:17689 IMAGE:3865984), complete cds. [BC008219]	XM_370756
A_23_P431252	0.000303	7.948	NM_032505	NM_032505	Homo sapiens T-cell activation kelch repeat protein (TA-KRP), mRNA [NM_032505]	NM_032505
A_23_P143068	0.000304	32.72	NM_024726	NM_024726	Homo sapiens IQ motif containing with AAA domain (IQCA), mRNA [NM_024726]	NM_024726
A_24_P173823	0.000304	10.73	BC044624	BC044624	Homo sapiens cDNA clone IMAGE:5288080, partial cds. [BC044624]	
A_24_P203964	0.000304	4.218	NM_207331	NM_207331	Homo sapiens hypothetical protein LOC153561 (LOC153561), mRNA [NM_207331]	NM_207331
A_24_P270728	0.000304	2.225	NM_012385	NM_012385	Homo sapiens p8 protein (candidate of metastasis 1) (P8), mRNA [NM_012385]	NM_012385
A_23_P45025	0.000305	5.214	NM_138980	NM_138980	Homo sapiens mitogen-activated protein kinase 10 (MAPK10), transcript variant 3, mRNA [NM_138980]	NM_138980
A_32_P67610	0.000305	3.952	CR605444	CR605444	full-length cDNA clone CS0DK008YC22 of HeLa cells Cot 25-normalized of Homo sapiens (human). [CR605444]	
A_23_P379034	0.000305	3.675	NM_025045	NM_025045	Homo sapiens BAI1-associated protein 2-like 2 (BAIAP2L2), mRNA [NM_025045]	NM_025045
A_23_P162734	0.000305	2.047	NM_005977	NM_005977	Homo sapiens ring finger protein (C3H2C3 type) 6 (RNF6), transcript variant 1, mRNA [NM_005977]	NM_005977
A_32_P161554	0.000306	5.043	THC2280139			
A_23_P52657	0.000306	3.866	AK056607	AK056607	Homo sapiens cDNA FLJ32045 fis, clone NTONG2001014, highly similar to GLYCOGEN PHOSPHORYLASE, MUSCLE FORM (EC 2.4.1.1). [AK056607]	

A_23_P131614	0.000306	3.185	NM_004369	NM_004369	Homo sapiens collagen, type VI, alpha 3 (COL6A3), transcript variant 1, mRNA [NM_004369]	NM_004369
A_24_P197196	0.000306	2.039	NM_005689	NM_005689	Homo sapiens ATP-binding cassette, sub-family B (MDR/TAP), member 6 (ABCB6), nuclear gene encoding mitochondrial protein, mRNA [NM_005689]	NM_005689
A_24_P79040	0.000307	12.07	NM_144691	NM_144691	Homo sapiens calpain 12 (CAPN12), mRNA [NM_144691]	NM_144691
A_32_P42780	0.000307	2.171	THC2338292			
A_32_P3214	0.000308	2.321	THC2433340		Q93KJ6 (Q93KJ6) Dissimilatory (Bi-)sulfite reductase beta subunit (Fragment), partial (5%) [THC2433340]	
A_32_P101799	0.000309	2.424	THC2282944			
A_23_P44569	0.000311	4.382	NM_000392	NM_000392	Homo sapiens ATP-binding cassette, sub-family C (CFTR/MRP), member 2 (ABCC2), mRNA [NM_000392]	NM_000392
A_23_P141866	0.000311	2.102	NM_003435	NM_003435	Homo sapiens zinc finger protein 134 (clone pHZ-15) (ZNF134), mRNA [NM_003435]	NM_003435
A_23_P337262	0.000312	6.881	NM_153000	NM_153000	Homo sapiens adenomatosis polyposis coli down-regulated 1 (APCDD1), mRNA [NM_153000]	NM_153000
A_32_P76060	0.000312	2.557	CR627421	CR627421	Homo sapiens mRNA; cDNA DKFZp781F2227 (from clone DKFZp781F2227). [CR627421]	
A_24_P57977	0.000313	5.397	NM_025248	NM_025248	Homo sapiens SNAP25-interacting protein (SNIP), mRNA [NM_025248]	NM_025248
A_23_P33856	0.000313	5.129	NM_006603	NM_006603	Homo sapiens stromal antigen 2 (STAG2), mRNA [NM_006603]	NM_006603
A_32_P143000	0.000313	4.078	ENST00000261275		Homo sapiens mRNA for KIAA0574 protein, partial cds. [AB011146]	
A_24_P398016	0.000313	3.903	NM_018387	NM_018387	Homo sapiens spermatid perinuclear RNA binding protein (STRBP), mRNA [NM_018387]	NM_018387
A_23_P98876	0.000313	3.353	NM_173596	NM_173596	Homo sapiens solute carrier family 39 (metal ion transporter), member 5 (SLC39A5), mRNA [NM_173596]	NM_173596
A_32_P44210	0.000313	3.222	BC037328	BC037328	Homo sapiens cDNA clone IMAGE:5263455, partial cds. [BC037328]	
A_24_P6808	0.000313	3.044	NM_004713	NM_004713	Homo sapiens serologically defined colon cancer antigen 1 (SDCCAG1), mRNA [NM_004713]	NM_004713
A_24_P21831	0.000313	2.042	NM_019591	NM_019591	Homo sapiens zinc finger protein 26 (KOX 20) (ZNF26), mRNA [NM_019591]	NM_019591
A_23_P371835	0.000314	15.75	NM_080473	NM_080473	Homo sapiens GATA binding protein 5 (GATA5), mRNA [NM_080473]	NM_080473
A_23_P83368	0.000314	5.027	NM_016373	NM_016373	Homo sapiens WW domain containing oxidoreductase (WWOX), transcript variant 1, mRNA [NM_016373]	NM_016373
A_23_P100883	0.000314	2.981	NM_015355	NM_015355	Homo sapiens suppressor of zeste 12 homolog (Drosophila) (SUZ12), mRNA [NM_015355]	NM_015355
A_32_P199217	0.000315	3.696	THC2458577			
A_23_P4649	0.000316	24.92	NM_001645	NM_001645	Homo sapiens apolipoprotein C-I (APOC1), mRNA [NM_001645]	NM_001645
A_24_P791669	0.000316	3.759	ENST00000356555		PREDICTED: Homo sapiens similar to hypothetical protein 9630041N07 (LOC345462), mRNA [XM_293828]	XM_293828
A_23_P111860	0.000316	2.804	NM_018059	NM_018059	Homo sapiens hypothetical protein FLJ10324 (FLJ10324), mRNA [NM_018059]	NM_018059
A_23_P502832	0.000316	2.385	NM_006047	NM_006047	Homo sapiens RNA binding motif protein 12 (RBM12), transcript variant 1, mRNA [NM_006047]	NM_006047
A_23_P5550	0.000316	2.096	NM_015317	NM_015317	Homo sapiens pumilio homolog 2 (Drosophila) (PUM2), mRNA [NM_015317]	NM_015317
A_23_P152420	0.000316	2.027	NM_014615	NM_014615	Homo sapiens KIAA0182 protein (KIAA0182), mRNA [NM_014615]	NM_014615
A_32_P15288	0.000317	5.883	THC2285720		BX114329 BX114329 Soares_NhHMPu_S1 Homo sapiens cDNA clone IMAGp998G064741 ; IMAGE:1932317, mRNA sequence [BX114329]	
A_24_P385313	0.000317	3.025	NM_002840	NM_002840	Homo sapiens protein tyrosine phosphatase, receptor type, F (PTPRF), transcript variant 1, mRNA [NM_002840]	NM_002840
A_23_P28434	0.000317	2.575	NM_003761	NM_003761	Homo sapiens vesicle-associated membrane protein 8 (endobrevin) (VAMP8), mRNA [NM_003761]	NM_003761
A_23_P81581	0.000318	7.43	NM_001713	NM_001713	Homo sapiens betaine-homocysteine methyltransferase (BHMT), mRNA [NM_001713]	NM_001713
A_32_P154121	0.000318	4.89	THC2340670			
A_23_P167005	0.000318	4.384	NM_014373	NM_014373	Homo sapiens G protein-coupled receptor 160 (GPR160), mRNA [NM_014373]	NM_014373
A_24_P678418	0.000318	3.741	NM_207443	NM_207443	Homo sapiens FLJ45244 protein (FLJ45244), mRNA [NM_207443]	NM_207443
A_23_P20852	0.000318	2.205	NM_001698	NM_001698	Homo sapiens AU RNA binding protein/enoyl-Coenzyme A hydratase (AUH), nuclear gene encoding mitochondrial protein, mRNA [NM_001698]	NM_001698
A_24_P273799	0.000319	17.51	AL713659	AL713659	Homo sapiens mRNA; cDNA DKFZp667D1012 (from clone DKFZp667D1012). [AL713659]	
A_23_P121926	0.00032	293.6	NM_005410	NM_005410	Homo sapiens selenoprotein P, plasma, 1 (SEPP1), mRNA [NM_005410]	NM_005410
A_23_P254816	0.00032	5.865	NM_004609	NM_004609	Homo sapiens transcription factor 15 (basic helix-loop-helix) (TCF15), mRNA [NM_004609]	NM_004609
A_23_P384085	0.00032	4.953	NM_181453	NM_181453	Homo sapiens GRIP and coiled-coil domain containing 2 (GCC2), transcript variant 1, mRNA [NM_181453]	NM_181453
A_23_P364107	0.00032	2.536	NM_018353	NM_018353	Homo sapiens chromosome 14 open reading frame 106 (C14orf106), mRNA [NM_018353]	NM_018353

A_23_P201731	0.00032	2.136	NM_004619	NM_004619	Homo sapiens TNF receptor-associated factor 5 (TRAF5), transcript variant 1, mRNA [NM_004619]	NM_004619
A_32_P137266	0.00032	2.103	NM_032437	NM_032437	Homo sapiens KIAA1799 protein (KIAA1799), mRNA [NM_032437]	NM_032437
A_24_P205045	0.000321	27.88	NM_015576	NM_015576	Homo sapiens CAZ-associated structural protein (CAST), mRNA [NM_015576]	NM_015576
A_24_P330518	0.000321	5.427	NM_001218	NM_001218	Homo sapiens carbonic anhydrase XII (CA12), transcript variant 1, mRNA [NM_001218]	NM_001218
A_23_P129188	0.000321	3.882	NM_033429	NM_033429	Homo sapiens calmodulin-like 4 (CALML4), mRNA [NM_033429]	NM_033429
A_24_P103264	0.000321	3.144	NM_003360	NM_003360	Homo sapiens UDP glycosyltransferase 8 (UDP-galactose ceramide galactosyltransferase) (UGT8), mRNA [NM_003360]	NM_003360
A_32_P66934	0.000322	7.437	BX641014	BX641014	Homo sapiens mRNA; cDNA DKFZp686I19109 (from clone DKFZp686I19109) [BX641014]	
A_23_P385911	0.000322	6.025	NM_030633	NM_030633	Homo sapiens KIAA1712 (KIAA1712), mRNA [NM_030633]	NM_030633
A_32_P211363	0.000322	3.01	NM_133446	NM_133446	Homo sapiens centaurin, gamma-like family, member 1 (CTGLF1), mRNA [NM_133446]	NM_133446
A_23_P124427	0.000322	2.884	NM_012224	NM_012224	Homo sapiens NIMA (never in mitosis gene a)-related kinase 1 (NEK1), mRNA [NM_012224]	NM_012224
A_32_P38404	0.000323	3.175	BC015133	BC015133	Homo sapiens cDNA clone IMAGE:3934193, partial cds. [BC015133]	
A_32_P15211	0.000324	12.09	THC2373821		Q14966 (Q14966) Nuclear protein, NP220, partial (5%) [THC2373821]	
A_23_P164706	0.000324	3.907	NM_003451	NM_003451	Homo sapiens zinc finger protein 177 (ZNF177), mRNA [NM_003451]	NM_003451
A_23_P32444	0.000324	2.33	NM_032348	NM_032348	Homo sapiens matrix-remodelling associated 8 (MXRA8), mRNA [NM_032348]	NM_032348
A_23_P135616	0.000324	2.001	NM_016930	NM_016930	Homo sapiens syntaxin 18 (STX18), mRNA [NM_016930]	NM_016930
A_24_P45728	0.000325	5.7	NM_020770	NM_020770	Homo sapiens cingulin (CGN), mRNA [NM_020770]	NM_020770
A_23_P337422	0.000325	2.773	NM_001018067	NM_001018067	Homo sapiens PAI-1 mRNA binding protein (PAI-RBP1), transcript variant 1, mRNA [NM_001018067]	NM_001018067
A_24_P889070	0.000326	12.6	AK023647	AK023647	Homo sapiens cDNA FLJ13585 fis, clone PLACE1009150. [AK023647]	
A_24_P649357	0.000326	4.15	NM_021652	NM_021652	Homo sapiens SMA4 (SMA4), mRNA [NM_021652]	NM_021652
A_23_P146554	0.000327	8.831	NM_000954	NM_000954	Homo sapiens prostaglandin D2 synthase 21kDa (brain) (PTGDS), mRNA [NM_000954]	NM_000954
A_32_P203300	0.000327	2.87	THC2306884			
A_23_P327069	0.000327	2.14	NM_014743	NM_014743	Homo sapiens KIAA0232 gene product (KIAA0232), mRNA [NM_014743]	NM_014743
A_23_P89145	0.000328	5.908	NM_018300	NM_018300	Homo sapiens zinc finger protein 83 (HPF1) (ZNF83), mRNA [NM_018300]	NM_018300
A_23_P92590	0.000328	2.313	NM_133370	NM_133370	Homo sapiens splicing factor YT521-B (YT521), mRNA [NM_133370]	NM_133370
A_23_P207493	0.000329	8.124	NM_016424	NM_016424	Homo sapiens cisplatin resistance-associated overexpressed protein (CROP), transcript variant 1, mRNA [NM_016424]	NM_016424
A_32_P50066	0.000329	4.581	CR749274	CR749274	Homo sapiens mRNA; cDNA DKFZp781M044 (from clone DKFZp781M044). [CR749274]	
A_24_P886096	0.00033	6.619	AK094413	AK094413	Homo sapiens cDNA FLJ37094 fis, clone BRACE2018337. [AK094413]	
A_23_P25706	0.00033	2.878	NM_024734	NM_024734	Homo sapiens calmin (calponin-like, transmembrane) (CLMN), mRNA [NM_024734]	NM_024734
A_23_P427148	0.00033	2.158	NM_152465	NM_152465	Homo sapiens proline-rich cyclin A1-interacting protein (PROCA1), mRNA [NM_152465]	NM_152465
A_32_P20221	0.000331	2.794	AK095738	AK095738	Homo sapiens cDNA FLJ38419 fis, clone FEBRA2009846. [AK095738]	
A_24_P335202	0.000332	6.373	NM_015424	NM_015424	Homo sapiens chordin-like 2 (CHRDL2), mRNA [NM_015424]	NM_015424
A_32_P199002	0.000333	3.869	BX478538	BX478538	DKFZp686P07204_r1 686 (synonym: hlcc3) Homo sapiens cDNA clone DKFZp686P07204 5', mRNA sequence [BX478538]	
A_32_P213678	0.000333	2.631	AK123439	AK123439	Homo sapiens cDNA FLJ41445 fis, clone BRSTN2002105. [AK123439]	
A_23_P167367	0.000334	10.61	NM_153426	NM_153426	Homo sapiens paired-like homeodomain transcription factor 2 (PITX2), transcript variant 2, mRNA [NM_153426]	NM_153426
A_23_P209032	0.000334	6.468	NM_018443	NM_018443	Homo sapiens zinc finger protein 302 (ZNF302), transcript variant 1, mRNA [NM_018443]	NM_018443
A_23_P27180	0.000334	2.105	NM_032141	NM_032141	Homo sapiens hypothetical protein DKFZp434K1421 (DKFZP434K1421), mRNA [NM_032141]	NM_032141
A_23_P59999	0.000335	4.781	THC2437580		Q300_MOUSE (Q02722) Protein Q300, partial (14%) [THC2437580]	
A_24_P505790	0.000337	31.79	AK131274	AK131274	Homo sapiens cDNA FLJ16218 fis, clone CTONG3001501, highly similar to Mus musculus glucocorticoid-induced gene 1 mRNA. [AK131274]	
A_23_P213050	0.000337	8.58	NM_000860	NM_000860	Homo sapiens hydroxyprostaglandin dehydrogenase 15-(NAD) (HPGD), mRNA [NM_000860]	NM_000860
A_23_P121106	0.000337	5.077	NM_003865	NM_003865	Homo sapiens homeo box (expressed in ES cells) 1 (HESX1), mRNA [NM_003865]	NM_003865
A_23_P52797	0.000337	3.39	NM_030770	NM_030770	Homo sapiens transmembrane protease, serine 5 (spinesin) (TMPRSS5), mRNA [NM_030770]	NM_030770

A_32_P104063	0.000337	2.853	AF275804	AF275804	Homo sapiens PNAS-108 mRNA, partial sequence. [AF275804]	
A_23_P145824	0.000338	23.53	AK000075	AK000075	Homo sapiens cDNA FLJ20068 fis, clone COL01755. [AK000075]	XM_371933
A_32_P67747	0.000338	4.241	THC2436814			
A_23_P130187	0.000338	2.926	A_23_P130187			
A_23_P91230	0.000339	39.37	NM_003064	NM_003064	Homo sapiens secretory leukocyte protease inhibitor (antileukoproteinase) (SLPI), mRNA [NM_003064]	NM_003064
A_23_P31798	0.000339	3.851	NM_000015	NM_000015	Homo sapiens N-acetyltransferase 2 (arylamine N-acetyltransferase) (NAT2), mRNA [NM_000015]	NM_000015
A_24_P529168	0.00034	4.354	NM_213724	NM_213724	Homo sapiens chromosome 13 open reading frame 25 (C13orf25), transcript variant 1, mRNA [NM_213724]	NM_213724
A_24_P931955	0.000341	6.477	THC2278676			
A_23_P138635	0.000341	5.801	NM_004052	NM_004052	Homo sapiens BCL2/adenovirus E1B 19kDa interacting protein 3 (BNIP3), nuclear gene encoding mitochondrial protein, mRNA [NM_004052]	NM_004052
A_23_P46390	0.000341	3.603	NM_032872	NM_032872	Homo sapiens synaptotagmin-like 1 (SYTL1), mRNA [NM_032872]	NM_032872
A_32_P125832	0.000344	14.61	AK123079	AK123079	Homo sapiens cDNA FLJ41084 fis, clone ADRGL2010974. [AK123079]	
A_23_P23996	0.000345	25.16	NM_000429	NM_000429	Homo sapiens methionine adenosyltransferase I, alpha (MAT1A), mRNA [NM_000429]	NM_000429
A_24_P481375	0.000345	5.765	AK021668	AK021668	Homo sapiens cDNA FLJ11606 fis, clone HEMBA1003942. [AK021668]	
A_23_P202939	0.000345	5.525	NM_001642	NM_001642	Homo sapiens amyloid beta (A4) precursor-like protein 2 (APLP2), mRNA [NM_001642]	NM_001642
A_24_P942648	0.000345	5.334	AL133642	AL133642	Homo sapiens mRNA; cDNA DKFZp586G1721 (from clone DKFZp586G1721). [AL133642]	
A_23_P363163	0.000345	2.045	NM_001008390	NM_001008390	Homo sapiens CGG triplet repeat binding protein 1 (CGGBP1), transcript variant 1, mRNA [NM_001008390]	NM_001008390
A_23_P203686	0.000345	2.013	ENST00000260045		Homo sapiens cDNA FLJ10660 fis, clone NT2RP2006100, highly similar to Homo sapiens mRNA; cDNA DKFZp564B102. [AK001522]	
A_24_P772488	0.000346	9.88	THC2311602			
A_24_P236935	0.000346	2.354	NM_001012964	NM_001012964	Homo sapiens kallikrein 6 (neurosin, zyme) (KLK6), transcript variant B, mRNA [NM_001012964]	NM_001012964
A_23_P72680	0.000346	2.214	NM_014055	NM_014055	Homo sapiens carnitine deficiency-associated, expressed in ventricle 1 (CDV1), transcript variant 1, mRNA [NM_014055]	NM_014055
A_23_P78265	0.000347	3.388	NM_004138	NM_004138	Homo sapiens keratin, hair, acidic, 3A (KRTHA3A), mRNA [NM_004138]	NM_004138
A_24_P380330	0.000347	2.898	NM_024594	NM_024594	Homo sapiens pantothenate kinase 3 (PANK3), mRNA [NM_024594]	NM_024594
A_23_P201951	0.000347	2.711	NM_016374	NM_016374	Homo sapiens AT rich interactive domain 4B (RBP1- like) (ARID4B), transcript variant 1, mRNA [NM_016374]	NM_016374
A_23_P3663	0.000348	2.29	NM_138418	NM_138418	Homo sapiens hypothetical protein MGC15416 (MGC15416), transcript variant 2, mRNA [NM_138418]	NM_138418
A_23_P69179	0.000348	2.159	NM_018192	NM_018192	Homo sapiens leprecan-like 1 (LEPREL1), mRNA [NM_018192]	NM_018192
A_24_P678620	0.000349	2.595	THC2405710			
A_32_P53558	0.000349	2.478	ENST00000341569		PREDICTED: Homo sapiens similar to Nedd-4-like E3 ubiquitin-protein ligase WWP1 (WW domain-containing protein 1) (LOC339843), mRNA [XM_290351]	XM_290351
A_23_P45099	0.000351	62.69	NM_002125	NM_002125	Homo sapiens major histocompatibility complex, class II, DR beta 5 (HLA-DRB5), mRNA [NM_002125]	NM_002125
A_24_P67806	0.000352	3.392	ENST00000333549		Homo sapiens, clone IMAGE:3909623, mRNA, partial cds. [BC015894]	
A_24_P385012	0.000353	2.183	BC030084	BC030084	Homo sapiens cDNA clone IMAGE:4791887, partial cds. [BC030084]	
A_23_P256008	0.000354	29.19	NM_024786	NM_024786	Homo sapiens zinc finger, DHHC-type containing 11 (ZDHHC11), mRNA [NM_024786]	NM_024786
A_32_P80610	0.000357	2.613	AK124080	AK124080	Homo sapiens cDNA FLJ42086 fis, clone TESOP1000127. [AK124080]	
A_23_P73135	0.000357	2.031	NM_031941	NM_031941	Homo sapiens Usher syndrome 1C binding protein 1 (USHBP1), mRNA [NM_031941]	NM_031941
A_32_P79345	0.000358	6.896	THC2410202		ALU6_HUMAN (P39193) Alu subfamily SP sequence contamination warning entry, partial (11%) [THC2410202]	
A_32_P13337	0.00036	30.65	THC2408277			
A_23_P216468	0.00036	15.17	NM_004170	NM_004170	Homo sapiens solute carrier family 1 (neuronal/epithelial high affinity glutamate transporter, system Xag), member 1 (SLC1A1), mRNA [NM_004170]	NM_004170
A_24_P326398	0.00036	6.365	NM_173689	NM_173689	Homo sapiens crumbs homolog 2 (Drosophila) (CRB2), mRNA [NM_173689]	NM_173689
A_32_P228804	0.00036	4.232	BX649112	BX649112	Homo sapiens mRNA; cDNA DKFZp686E02109 (from clone DKFZp686E02109). [BX649112]	
A_23_P33511	0.00036	3.405	AX721087	AX721087	Sequence 47 from Patent WO0220754. [AX721087]	
A_32_P50587	0.00036	3.363	THC2448178			
A_24_P9090	0.00036	3.269	NM_005463	NM_005463	Homo sapiens heterogeneous nuclear ribonucleoprotein D-like (HNRPDL), transcript variant 1, mRNA [NM_005463]	NM_005463

A_32_P144421	0.00036	2.768	NM_053042	NM_053042	Homo sapiens KIAA1729 protein (KIAA1729), mRNA [NM_053042]	NM_053042
A_23_P200524	0.000362	16.72	NM_001002921	NM_001002921	Homo sapiens adenylate kinase 3-like 2 (AK3L2), mRNA [NM_001002921]	NM_001002921
A_23_P436336	0.000362	2.712	NM_198284	NM_198284	Homo sapiens hypothetical protein LOC349114 (LOC349114), mRNA [NM_198284]	NM_198284
A_23_P100341	0.000362	2.292	NM_014321	NM_014321	Homo sapiens origin recognition complex, subunit 6 homolog-like (yeast) (ORC6L), mRNA [NM_014321]	NM_014321
A_23_P115785	0.000362	2.157	NM_145235	NM_145235	Homo sapiens fibronectin type III and ankyrin repeat domains 1 (FANK1), mRNA [NM_145235]	NM_145235
A_24_P113144	0.000363	4.092	NM_024857	NM_024857	Homo sapiens chromosome 17 open reading frame 41 (C17orf41), mRNA [NM_024857]	NM_024857
A_32_P27991	0.000364	3.927	CR610181	CR610181	full-length cDNA clone CS0DM006YA12 of Fetal liver of Homo sapiens (human). [CR610181]	
A_24_P784846	0.000364	3.771	AK021906	AK021906	Homo sapiens cDNA FLJ11844 fis, clone HEMBA1006665. [AK021906]	
A_23_P128967	0.000364	2.137	NM_005589	NM_005589	Homo sapiens aldehyde dehydrogenase 6 family, member A1 (ALDH6A1), nuclear gene encoding mitochondrial protein, mRNA [NM_005589]	NM_005589
A_23_P132948	0.000365	2.289	NM_201999	NM_201999	Homo sapiens E74-like factor 2 (ets domain transcription factor) (ELF2), transcript variant 1, mRNA [NM_201999]	NM_201999
A_23_P116898	0.000366	49.1	NM_000014	NM_000014	Homo sapiens alpha-2-macroglobulin (A2M), mRNA [NM_000014]	NM_000014
A_23_P138524	0.000366	5.372	NM_198148	NM_198148	Homo sapiens carboxypeptidase X (M14 family), member 2 (CPXM2), mRNA [NM_198148]	NM_198148
A_23_P205228	0.000368	11.87	NM_000053	NM_000053	Homo sapiens ATPase, Cu++ transporting, beta polypeptide (Wilson disease) (ATP7B), transcript variant 1, mRNA [NM_000053]	NM_000053
A_23_P90510	0.000369	5.934	NM_138393	NM_138393	Homo sapiens chromosome 19 open reading frame 32 (C19orf32), mRNA [NM_138393]	NM_138393
A_23_P390148	0.000369	5.442	NM_014636	NM_014636	Homo sapiens Ral GEF with PH domain and SH3 binding motif 1 (RALGPS1), mRNA [NM_014636]	NM_014636
A_24_P350644	0.000369	2.187	AB011087	AB011087	Homo sapiens mRNA for KIAA0515 protein, partial cds. [AB011087]	XM_497080
A_23_P26124	0.00037	16.76	NM_134260	NM_134260	Homo sapiens RAR-related orphan receptor A (RORA), transcript variant 2, mRNA [NM_134260]	NM_134260
A_24_P157926	0.00037	14.58	NM_006290	NM_006290	Homo sapiens tumor necrosis factor, alpha-induced protein 3 (TNFAIP3), mRNA [NM_006290]	NM_006290
A_32_P175739	0.00037	4.107	NM_000189	NM_000189	Homo sapiens hexokinase 2 (HK2), mRNA [NM_000189]	NM_000189
A_24_P943193	0.00037	3.251	AB007896	AB007896	Homo sapiens KIAA0436 mRNA, partial cds. [AB007896]	
A_23_P355536	0.00037	3.19	AK127213	AK127213	Homo sapiens cDNA FLJ45280 fis, clone BRHIP3001360. [AK127213]	
A_23_P144531	0.00037	2.873	AK026323	AK026323	Homo sapiens cDNA: FLJ22670 fis, clone HSI08684. [AK026323]	
A_23_P5405	0.00037	2.536	NM_172070	NM_172070	Homo sapiens zinc finger protein 650 (ZNF650), mRNA [NM_172070]	NM_172070
A_24_P916614	0.00037	2.287	CR749471	CR749471	Homo sapiens mRNA; cDNA DKFZp781I1117 (from clone DKFZp781I1117). [CR749471]	
A_23_P121533	0.00037	2.22	NM_012445	NM_012445	Homo sapiens spondin 2, extracellular matrix protein (SPON2), mRNA [NM_012445]	NM_012445
A_24_P4426	0.000371	2.198	NM_014937	NM_014937	Homo sapiens inositol polyphosphate-5-phosphatase F (INPP5F), transcript variant 1, mRNA [NM_014937]	NM_014937
A_23_P114423	0.000372	78.72	NM_004683	NM_004683	Homo sapiens regucalcin (senescence marker protein-30) (RGN), transcript variant 1, mRNA [NM_004683]	NM_004683
A_24_P405430	0.000372	4.018	CR607569	CR607569	full-length cDNA clone CS0DF027YA11 of Fetal brain of Homo sapiens (human). [CR607569]	
A_23_P9932	0.000372	3.469	NM_145341	NM_145341	Homo sapiens programmed cell death 4 (neoplastic transformation inhibitor) (PDCD4), transcript variant 2, mRNA [NM_145341]	NM_145341
A_24_P6517	0.000373	7.14	NM_001029884	NM_001029884	Homo sapiens pleckstrin homology domain containing, family G (with RhoGef domain) member 1 (PLEKHG1), mRNA [NM_001029884]	NM_001029884
A_32_P190682	0.000373	5.312	THC2347318			
A_32_P170444	0.000373	3.79	CR625990	CR625990	full-length cDNA clone CS0DI074YB14 of Placenta Cot 25-normalized of Homo sapiens (human). [CR625990]	
A_24_P344087	0.000373	3.106	NM_005132	NM_005132	Homo sapiens REC8-like 1 (yeast) (REC8L1), mRNA [NM_005132]	NM_005132
A_24_P239076	0.000373	2.114	NM_001013618	NM_001013618	Homo sapiens similar to omega protein (LOC91353), mRNA [NM_001013618]	NM_001013618
A_23_P37727	0.000374	11.92	NM_002996	NM_002996	Homo sapiens chemokine (C-X3-C motif) ligand 1 (CX3CL1), mRNA [NM_002996]	NM_002996
A_32_P83326	0.000374	7.025	AK130705	AK130705	Homo sapiens cDNA FLJ27195 fis, clone SYN02786. [AK130705]	
A_23_P255591	0.000374	4.028	X78926	X78926	H.sapiens HZF3 mRNA for zinc finger protein. [X78926]	
A_32_P155043	0.000374	2.734	A_32_P155043			
A_23_P142389	0.000374	2.368	NM_205834	NM_205834	Homo sapiens liver-specific bHLH-Zip transcription factor (LISCH7), transcript variant 2, mRNA [NM_205834]	NM_205834
A_23_P308924	0.000374	2.361	AB051487	AB051487	Homo sapiens mRNA for KIAA1700 protein, partial cds. [AB051487]	
A_32_P168464	0.000375	4.311	AK056476	AK056476	Homo sapiens cDNA FLJ31914 fis, clone NT2RP7004884. [AK056476]	

A_32_P26895	0.000375	2.035	ENST00000281244		Homo sapiens mRNA for KIAA1600 protein, partial cds. [AB046820]	
A_24_P369232	0.000376	15.15	NM_031455	NM_031455	Homo sapiens coiled-coil domain containing 3 (CCDC3), mRNA [NM_031455]	NM_031455
A_23_P26094	0.000376	3.34	NM_024755	NM_024755	Homo sapiens modulator of estrogen induced transcription (FLJ13213), transcript variant 1, mRNA [NM_024755]	NM_024755
A_23_P257971	0.000377	4.338	NM_001353	NM_001353	Homo sapiens aldo-keto reductase family 1, member C1 (dihydrodiol dehydrogenase 1; 20-alpha (3-alpha)-hydroxysteroid dehydrogenase) (AKR1C1), mRNA [NM_001353]	NM_001353
A_23_P60002	0.000377	3.598	NM_014673	NM_014673	Homo sapiens KIAA0103 (KIAA0103), mRNA [NM_014673]	NM_014673
A_24_P116587	0.000377	3.413	NM_201575	NM_201575	Homo sapiens seizure related 6 homolog (mouse)-like 2 (SEZ6L2), transcript variant 2, mRNA [NM_201575]	NM_201575
A_24_P229728	0.000377	2.875	A_24_P229728			
A_24_P945194	0.000377	2.014	NM_013374	NM_013374	Homo sapiens programmed cell death 6 interacting protein (PDCD6IP), mRNA [NM_013374]	NM_013374
A_23_P28906	0.000378	4.982	AB046810	AB046810	Homo sapiens mRNA for KIAA1590 protein, partial cds. [AB046810]	
A_23_P105803	0.000378	4.952	NM_002010	NM_002010	Homo sapiens fibroblast growth factor 9 (glia-activating factor) (FGF9), mRNA [NM_002010]	NM_002010
A_32_P219520	0.000379	4.663	NM_014350	NM_014350	Homo sapiens tumor necrosis factor, alpha-induced protein 8 (TNFAIP8), mRNA [NM_014350]	NM_014350
A_23_P72127	0.000379	3.04	NM_020836	NM_020836	Homo sapiens likely ortholog of rat brain-enriched guanylate kinase-associated protein (KIAA1446), mRNA [NM_020836]	NM_020836
A_23_P168130	0.000379	2.151	NM_054111	NM_054111	Homo sapiens inositol hexaphosphate kinase 3 (IHPK3), mRNA [NM_054111]	NM_054111
A_23_P215634	0.00038	63.56	NM_001013398	NM_001013398	Homo sapiens insulin-like growth factor binding protein 3 (IGFBP3), transcript variant 1, mRNA [NM_001013398]	NM_001013398
A_23_P156890	0.00038	8.38	NM_003206	NM_003206	Homo sapiens transcription factor 21 (TCF21), transcript variant 2, mRNA [NM_003206]	NM_003206
A_32_P159289	0.00038	6.851	A_32_P159289			
A_23_P132718	0.00038	3.82	NM_004636	NM_004636	Homo sapiens sema domain, immunoglobulin domain (Ig), short basic domain, secreted, (semaphorin) 3B (SEMA3B), transcript variant 1, mRNA [NM_004636]	NM_004636
A_24_P920880	0.00038	2.443	AY279352	AY279352	Homo sapiens truncated zinc finger protein 447 isoform (ZNF447) mRNA, complete cds. [AY279352]	
A_24_P941643	0.00038	2.293	NM_182734	NM_182734	Homo sapiens phospholipase C, beta 1 (phosphoinositide-specific) (PLCB1), transcript variant 2, mRNA [NM_182734]	NM_182734
A_23_P211233	0.000383	10.04	NM_001849	NM_001849	Homo sapiens collagen, type VI, alpha 2 (COL6A2), transcript variant 2C2, mRNA [NM_001849]	NM_001849
A_23_P127033	0.000383	5.524	NM_024693	NM_024693	Homo sapiens enoyl Coenzyme A hydratase domain containing 3 (ECHDC3), mRNA [NM_024693]	NM_024693
A_32_P6682	0.000383	4.732	THC2436690		ALU1_HUMAN (P39188) Alu subfamily J sequence contamination warning entry, partial (6%) [THC2436690]	
A_23_P89755	0.000383	2.401	NM_016271	NM_016271	Homo sapiens ring finger protein 138 (RNF138), transcript variant 1, mRNA [NM_016271]	NM_016271
A_24_P787914	0.000385	5.2	U25029	U25029	Human glucocorticoid receptor alpha mRNA, variant 3' UTR. [U25029]	
A_23_P136817	0.000386	2.642	NM_014708	NM_014708	Homo sapiens kinetochore associated 1 (KNTC1), mRNA [NM_014708]	NM_014708
A_24_P354689	0.000387	3.158	NM_004598	NM_004598	Homo sapiens sparc/osteonectin, cwcv and kazal-like domains proteoglycan (testican) (SPOCK), mRNA [NM_004598]	NM_004598
A_23_P94338	0.000388	26.5	NM_006209	NM_006209	Homo sapiens ectonucleotide pyrophosphatase/phosphodiesterase 2 (autotaxin) (ENPP2), mRNA [NM_006209]	NM_006209
A_24_P178444	0.000388	2.393	A_24_P178444			
A_23_P402952	0.000389	2.153	NM_001008747	NM_001008747	Homo sapiens similar to CTAGE6 (LOC441294), mRNA [NM_001008747]	NM_001008747
A_32_P194779	0.00039	3.706	AB082524	AB082524	Homo sapiens mRNA for KIAA1993 protein. [AB082524]	
A_32_P213637	0.00039	3.661	AK092942	AK092942	Homo sapiens cDNA FLJ35623 fis, clone SPLEN2010986. [AK092942]	
A_24_P724807	0.00039	3.026	A_24_P724807			
A_24_P124325	0.00039	2.653	CR749800	CR749800	Homo sapiens mRNA; cDNA DKFZp781B0249 (from clone DKFZp781B0249). [CR749800]	
A_32_P191746	0.000391	11.08	THC2455550			
A_32_P132317	0.000391	6.494	A_32_P132317			
A_32_P164758	0.000391	2.309	NM_001013685	NM_001013685	Homo sapiens hypothetical LOC401357 (LOC401357), mRNA [NM_001013685]	NM_001013685
A_24_P339858	0.000392	3.206	NM_153204	NM_153204	Homo sapiens chromosome 21 open reading frame 90 (C21orf90), mRNA [NM_153204]	NM_153204
A_32_P203592	0.000394	3.242	NM_015092	NM_015092	Homo sapiens PI-3-kinase-related kinase SMG-1 (SMG1), mRNA [NM_015092]	NM_015092
A_23_P376591	0.000394	3.202	NM_206808	NM_206808	Homo sapiens citrate lyase beta like (CLYBL), transcript variant 2, mRNA [NM_206808]	NM_206808

A_24_P927650	0.000396	4.499	AK056744	AK056744	Homo sapiens cDNA FLJ32182 fis, clone PLACE6001823. [AK056744]	
A_23_P422831	0.000398	8.053	NM_004816	NM_004816	Homo sapiens chromosome 9 open reading frame 61 (C9orf61), mRNA [NM_004816]	NM_004816
A_32_P12580	0.000398	2.091	AY134745	AY134745	Homo sapiens medulloblastoma antigen MU-MB-50.13 mRNA, complete cds. [AY134745]	
A_32_P193378	0.000399	11.46	AK055370	AK055370	Homo sapiens cDNA FLJ30808 fis, clone FEBRA2001383. [AK055370]	
A_32_P509964	0.000399	2.924	AF136408	AF136408	Homo sapiens unknown mRNA. [AF136408]	
A_32_P179239	0.0004	2.534	AK123297	AK123297	Homo sapiens cDNA FLJ41303 fis, clone BRAMY2042131. [AK123297]	
A_23_P207280	0.0004	2.28	NM_006380	NM_006380	Homo sapiens amyloid beta precursor protein (cytoplasmic tail) binding protein 2 (APPBP2), mRNA [NM_006380]	NM_006380
A_32_P23731	0.000401	23.89	THC2309960		Q7ZX66 (Q7ZX66) RNPC7 protein (Fragment), partial (9%) [THC2309960]	
A_24_P246361	0.000402	85.85	NM_006633	NM_006633	Homo sapiens IQ motif containing GTPase activating protein 2 (IQGAP2), mRNA [NM_006633]	NM_006633
A_23_P93269	0.000402	5.441	NM_003447	NM_003447	Homo sapiens zinc finger protein 165 (ZNF165), mRNA [NM_003447]	NM_003447
A_23_P435051	0.000402	2.631	AB014575	AB014575	Homo sapiens mRNA for KIAA0675 protein, partial cds. [AB014575]	
A_23_P78018	0.000403	4.433	NM_018672	NM_018672	Homo sapiens ATP-binding cassette, sub-family A (ABC1), member 5 (ABCA5), transcript variant 1, mRNA [NM_018672]	NM_018672
A_23_P209449	0.000404	4.668	NM_003507	NM_003507	Homo sapiens frizzled homolog 7 (Drosophila) (FZD7), mRNA [NM_003507]	NM_003507
A_23_P133648	0.000404	2.32	NM_016255	NM_016255	Homo sapiens family with sequence similarity 8, member A1 (FAM8A1), mRNA [NM_016255]	NM_016255
A_23_P77731	0.000405	13.63	NM_001888	NM_001888	Homo sapiens crystallin, mu (CRYM), transcript variant 1, mRNA [NM_001888]	NM_001888
A_23_P154627	0.000407	3.545	AF230201	AF230201	Homo sapiens OVC10-2 mRNA, complete cds. [AF230201]	
A_23_P27638	0.000407	2.623	NM_144566	NM_144566	Homo sapiens hypothetical protein DKFZp434I1610 (DKFZp434I1610), mRNA [NM_144566]	NM_144566
A_24_P418908	0.000408	56.63	AK021798	AK021798	Homo sapiens cDNA FLJ11736 fis, clone HEMBA1005468. [AK021798]	
A_23_P426663	0.000408	3.597	NM_198159	NM_198159	Homo sapiens microphthalmia-associated transcription factor (MITF), transcript variant 1, mRNA [NM_198159]	NM_198159
A_24_P150466	0.000408	3.065	ENST00000361956		Homo sapiens mRNA for secreted modular calcium-binding protein (smoc1 gene). [AJ249900]	
A_23_P214139	0.000409	3.659	NM_002912	NM_002912	Homo sapiens REV3-like, catalytic subunit of DNA polymerase zeta (yeast) (REV3L), mRNA [NM_002912]	NM_002912
A_23_P71591	0.000409	2.244	NM_017948	NM_017948	Homo sapiens nucleolar protein 8 (NOL8), mRNA [NM_017948]	NM_017948
A_23_P398836	0.00041	5.945	NM_020784	NM_020784	Homo sapiens KIAA1344 (KIAA1344), mRNA [NM_020784]	NM_020784
A_23_P35564	0.00041	2.986	NM_015490	NM_015490	Homo sapiens SEC31-like 2 (S. cerevisiae) (SEC31L2), transcript variant 1, mRNA [NM_015490]	NM_015490
A_32_P221958	0.000411	2.944	NM_133446	NM_133446	Homo sapiens centaurin, gamma-like family, member 1 (CTGLF1), mRNA [NM_133446]	NM_133446
A_23_P21473	0.000411	2.623	NM_024491	NM_024491	Homo sapiens p10-binding protein (Cep70), mRNA [NM_024491]	NM_024491
A_23_P143867	0.000411	2.016	NM_007114	NM_007114	Homo sapiens TATA element modulatory factor 1 (TMF1), mRNA [NM_007114]	NM_007114
A_24_P306063	0.000412	6.714	NM_020819	NM_020819	Homo sapiens KIAA1411 (KIAA1411), mRNA [NM_020819]	NM_020819
A_23_P309261	0.000412	5.871	NM_147171	NM_147171	Homo sapiens A kinase (PRKA) anchor protein (yotiao) 9 (AKAP9), transcript variant 1, mRNA [NM_147171]	NM_147171
A_24_P340813	0.000412	5.097	NM_032870	NM_032870	Homo sapiens chromosome 6 open reading frame 111 (C6orf111), mRNA [NM_032870]	NM_032870
A_32_P36942	0.000412	2.224	A_32_P36942			
A_23_P379649	0.000413	4.719	NM_001003940	NM_001003940	Homo sapiens Bcl2 modifying factor (BMF), transcript variant 1, mRNA [NM_001003940]	NM_001003940
A_23_P105794	0.000413	3.63	NM_033255	NM_033255	Homo sapiens epithelial stromal interaction 1 (breast) (EPSTI1), mRNA [NM_033255]	NM_033255
A_32_P167856	0.000413	2.534	AF338232	AF338232	Homo sapiens CTAGE-4 protein mRNA, complete cds. [AF338232]	XM_496933
A_23_P205046	0.000414	4.632	NM_017664	NM_017664	Homo sapiens ankyrin repeat domain 10 (ANKRD10), mRNA [NM_017664]	NM_017664
A_32_P162537	0.000414	2.1	THC2288797		Q753E4 (Q753E4) AFR372Wp, partial (3%) [THC2288797]	
A_23_P125656	0.000415	2.358	NM_001356	NM_001356	Homo sapiens DEAD (Asp-Glu-Ala-Asp) box polypeptide 3, X-linked (DDX3X), transcript variant 2, mRNA [NM_001356]	NM_001356
A_32_P198731	0.000415	2.204	ENST00000315447		Homo sapiens cDNA FLJ39342 fis, clone OCBBF2018873. [AK096661]	
A_23_P85371	0.000416	2.006	NM_004582	NM_004582	Homo sapiens Rab geranylgeranyltransferase, beta subunit (RABGGTB), mRNA [NM_004582]	NM_004582
A_23_P126037	0.000417	5.069	NM_012421	NM_012421	Homo sapiens rearranged L-myc fusion sequence (RLF), mRNA [NM_012421]	NM_012421
A_23_P205894	0.000417	3.929	NM_005159	NM_005159	Homo sapiens actin, alpha, cardiac muscle (ACTC), mRNA [NM_005159]	NM_005159
A_23_P320578	0.000419	21.69	NM_002928	NM_002928	Homo sapiens regulator of G-protein signalling 16 (RGS16), mRNA [NM_002928]	NM_002928

A_32_P191004	0.000419	6.815	ENST00000238789		Homo sapiens mRNA for KIAA1240 protein, partial cds. [AB033066]	XM_039676
A_23_P393686	0.000419	2.317	NM_175075	NM_175075	Homo sapiens chromosome 8 open reading frame 42 (C8orf42), mRNA [NM_175075]	NM_175075
A_23_P64217	0.00042	4.226	NM_024891	NM_024891	Homo sapiens hypothetical protein FLJ11783 (FLJ11783), mRNA [NM_024891]	NM_024891
A_23_P11922	0.000421	4.357	AK024925	AK024925	Homo sapiens cDNA: FLJ21272 fis, clone COL01753. [AK024925]	
A_24_P237389	0.000421	3.121	NM_001412	NM_001412	Homo sapiens eukaryotic translation initiation factor 1A, X-linked (EIF1AX), mRNA [NM_001412]	NM_001412
A_24_P119685	0.000422	5.971	NM_052843	NM_052843	Homo sapiens obscurin, cytoskeletal calmodulin and titin-interacting RhoGEF (OBSCN), mRNA [NM_052843]	NM_052843
A_32_P475642	0.000422	2.025	AK025166	AK025166	Homo sapiens cDNA: FLJ21513 fis, clone COL05778. [AK025166]	
A_23_P420442	0.000423	86.35	NM_153618	NM_153618	Homo sapiens sema domain, transmembrane domain (TM), and cytoplasmic domain, (semaphorin) 6D (SEMA6D), transcript variant 4, mRNA [NM_153618]	NM_153618
A_23_P45999	0.000423	10.09	NM_012168	NM_012168	Homo sapiens F-box protein 2 (FBXO2), mRNA [NM_012168]	NM_012168
A_24_P59471	0.000423	2.919	AK094159	AK094159	Homo sapiens cDNA FLJ36840 fis, clone ASTRO2011461. [AK094159]	
A_32_P133038	0.000424	6.94	THC2358845			
A_23_P139418	0.000424	6.166	NM_198516	NM_198516	Homo sapiens UDP-N-acetyl-alpha-D-galactosamine:polypeptide N-acetylgalactosaminyltransferase-like 4 (GALNTL4), mRNA [NM_198516]	NM_198516
A_23_P501831	0.000425	6.87	NM_032385	NM_032385	Homo sapiens chromosome 5 open reading frame 4 (C5orf4), transcript variant 2, mRNA [NM_032385]	NM_032385
A_23_P209298	0.000427	8.941	BC032822	BC032822	Homo sapiens erythrocyte membrane protein band 4.1 like 5, mRNA (cDNA clone MGC:26029 IMAGE:4827274), complete cds. [BC032822]	
A_32_P48043	0.000428	3.039	AK026980	AK026980	Homo sapiens cDNA: FLJ23327 fis, clone HEP12630, highly similar to HSZNF37 Homo sapiens ZNF37A mRNA for zinc finger protein. [AK026980]	
A_23_P387031	0.00043	14.05	NM_173465	NM_173465	Homo sapiens collagen, type XXIII, alpha 1 (COL23A1), mRNA [NM_173465]	NM_173465
A_23_P59855	0.00043	4.255	NM_006524	NM_006524	Homo sapiens zinc finger protein 138 (ZNF138), mRNA [NM_006524]	NM_006524
A_23_P108437	0.000431	10	AK024850	AK024850	Homo sapiens cDNA: FLJ21197 fis, clone COL00201. [AK024850]	
A_32_P103678	0.000435	28.89	THC2374684			
A_23_P92672	0.000435	8.002	NM_002538	NM_002538	Homo sapiens occludin (OCLN), mRNA [NM_002538]	NM_002538
A_23_P145114	0.000435	4.121	NM_001498	NM_001498	Homo sapiens glutamate-cysteine ligase, catalytic subunit (GCLC), mRNA [NM_001498]	NM_001498
A_23_P52727	0.000435	2.764	NM_182964	NM_182964	Homo sapiens neuron navigator 2 (NAV2), transcript variant 1, mRNA [NM_182964]	NM_182964
A_23_P31064	0.000435	2.736	NM_015529	NM_015529	Homo sapiens monooxygenase, DBH-like 1 (MOXD1), mRNA [NM_015529]	NM_015529
A_24_P665504	0.000435	2.48	BC092421	BC092421	Homo sapiens cDNA clone IMAGE:30378758. [BC092421]	XM_373704
A_23_P412980	0.000435	2.138	NM_015132	NM_015132	Homo sapiens sorting nexin 13 (SNX13), mRNA [NM_015132]	NM_015132
A_23_P401718	0.000436	2.731	NM_207310	NM_207310	Homo sapiens hypothetical protein DKFZp434E2321 (DKFZp434E2321), mRNA [NM_207310]	NM_207310
A_23_P5131	0.000436	2.349	NM_016368	NM_016368	Homo sapiens myo-inositol 1-phosphate synthase A1 (ISYNA1), mRNA [NM_016368]	NM_016368
A_23_P69617	0.000438	4.961	NM_003728	NM_003728	Homo sapiens unc-5 homolog C (C. elegans) (UNC5C), mRNA [NM_003728]	NM_003728
A_23_P88439	0.000438	4.642	NM_152332	NM_152332	Homo sapiens membrane targeting (tandem) C2 domain containing 1 (MTAC2D1), mRNA [NM_152332]	NM_152332
A_32_P66364	0.000438	2.405	BC017943	BC017943	Homo sapiens protein phosphatase 1, regulatory (inhibitor) subunit 1C, mRNA (cDNA clone MGC:24041 IMAGE:4288919), complete cds. [BC017943]	
A_23_P14062	0.000438	2.099	NM_020401	NM_020401	Homo sapiens nucleoporin 107kDa (NUP107), mRNA [NM_020401]	NM_020401
A_23_P259442	0.000439	25.28	NM_001873	NM_001873	Homo sapiens carboxypeptidase E (CPE), mRNA [NM_001873]	NM_001873
A_23_P317347	0.000439	4.261	NM_052911	NM_052911	Homo sapiens establishment of cohesion 1 homolog 1 (S. cerevisiae) (ESCO1), mRNA [NM_052911]	NM_052911
A_23_P70670	0.00044	18.86	NM_004233	NM_004233	Homo sapiens CD83 antigen (activated B lymphocytes, immunoglobulin superfamily) (CD83), mRNA [NM_004233]	NM_004233
A_23_P134854	0.00044	4.291	NM_194284	NM_194284	Homo sapiens claudin 23 (CLDN23), mRNA [NM_194284]	NM_194284
A_23_P211493	0.00044	3.122	NM_153609	NM_153609	Homo sapiens transmembrane protease, serine 6 (TMPRSS6), mRNA [NM_153609]	NM_153609
A_24_P265051	0.000441	25.26	AK095606	AK095606	Homo sapiens cDNA FLJ38287 fis, clone FCBBF3008362, moderately similar to PLEXIN 4 PRECURSOR. [AK095606]	XM_039393
A_23_P13969	0.000442	2.878	BC052611	BC052611	Homo sapiens cDNA clone MGC:59724 IMAGE:6252827, complete cds. [BC052611]	
A_23_P94546	0.000442	2.465	NM_025211	NM_025211	Homo sapiens G kinase anchoring protein 1 (GKAP1), mRNA [NM_025211]	NM_025211

A_23_P2884	0.000442	2.101	NM_004569	NM_004569	Homo sapiens phosphatidylinositol glycan, class H (PIGH), mRNA [NM_004569]	NM_004569
A_32_P161455	0.000443	3.436	AL831999	AL831999	Homo sapiens mRNA; cDNA DKFZp451K063 (from clone DKFZp451K063). [AL831999]	
A_24_P62783	0.000443	2.857	NM_004102	NM_004102	Homo sapiens fatty acid binding protein 3, muscle and heart (mammary-derived growth inhibitor) (FABP3), mRNA [NM_004102]	NM_004102
A_23_P50646	0.000443	2.257	BC071811	BC071811	Homo sapiens cDNA clone IMAGE:6452513, partial cds. [BC071811]	
A_32_P114235	0.000443	2.088	BX112278	BX112278	BX112278 BX112278 Soares fetal liver spleen 1NFLS Homo sapiens cDNA clone IMAGp998K20129 ; IMAGE:127243, mRNA sequence [BX112278]	
A_23_P433132	0.000444	4.71	NM_173853	NM_173853	Homo sapiens keratinocyte associated protein 3 (KRTCAP3), mRNA [NM_173853]	NM_173853
A_32_P136967	0.000445	6.68	ENST00000328046		Homo sapiens mRNA for KIAA1677 protein, partial cds. [AB051464]	
A_23_P426511	0.000445	2.949	NM_138698	NM_138698	Homo sapiens prematurely terminated mRNA decay factor-like (LOC91431), mRNA [NM_138698]	NM_138698
A_23_P78782	0.000445	2.726	NM_001217	NM_001217	Homo sapiens carbonic anhydrase XI (CA11), mRNA [NM_001217]	NM_001217
A_23_P16409	0.000446	11.83	NM_144691	NM_144691	Homo sapiens calpain 12 (CAPN12), mRNA [NM_144691]	NM_144691
A_23_P4096	0.000447	23.28	NM_000717	NM_000717	Homo sapiens carbonic anhydrase IV (CA4), mRNA [NM_000717]	NM_000717
A_32_P198295	0.000447	2.482	THC2446045			
A_23_P3274	0.000448	24.97	NM_004884	NM_004884	Homo sapiens putative neuronal cell adhesion molecule (PUNC), mRNA [NM_004884]	NM_004884
A_23_P420692	0.000449	10.64	NM_015053	NM_015053	Homo sapiens protein tyrosine phosphatase, receptor type, f polypeptide (PTPRF), interacting protein (liprin), alpha 4 (PPFIA4), mRNA [NM_015053]	NM_015053
A_23_P211007	0.000449	4.516	NM_003489	NM_003489	Homo sapiens nuclear receptor interacting protein 1 (NRIP1), mRNA [NM_003489]	NM_003489
A_23_P14649	0.000449	2.554	AK021784	AK021784	Homo sapiens cDNA FLJ11722 fis, clone HEMBA1005311. [AK021784]	
A_23_P313640	0.000449	2.332	NM_006609	NM_006609	Homo sapiens mitogen-activated protein kinase kinase kinase 2 (MAP3K2), mRNA [NM_006609]	NM_006609
A_23_P339480	0.000449	2.083	NM_003642	NM_003642	Homo sapiens histone acetyltransferase 1 (HAT1), mRNA [NM_003642]	NM_003642
A_24_P365515	0.00045	5.836	NM_021784	NM_021784	Homo sapiens forkhead box A2 (FOXA2), transcript variant 1, mRNA [NM_021784]	NM_021784
A_23_P115597	0.00045	2.191	NM_014915	NM_014915	Homo sapiens ankyrin repeat domain 26 (ANKRD26), mRNA [NM_014915]	NM_014915
A_23_P101246	0.000451	4.877	BC016993	BC016993	Homo sapiens, clone IMAGE:4401841, mRNA. [BC016993]	XM_085831
A_32_P524014	0.000451	2.84	AK023675	AK023675	Homo sapiens cDNA FLJ13613 fis, clone PLACE1010856. [AK023675]	
A_24_P214556	0.000451	2.41	THC2380706		Q6UXG1 (Q6UXG1) YVTM2421, partial (3%) [THC2380706]	
A_24_P568190	0.000452	6.402	NM_207331	NM_207331	Homo sapiens hypothetical protein LOC153561 (LOC153561), mRNA [NM_207331]	NM_207331
A_23_P113161	0.000453	4.062	NM_030806	NM_030806	Homo sapiens chromosome 1 open reading frame 21 (C1orf21), mRNA [NM_030806]	NM_030806
A_23_P209408	0.000453	4.059	NM_032977	NM_032977	Homo sapiens caspase 10, apoptosis-related cysteine protease (CASP10), transcript variant D, mRNA [NM_032977]	NM_032977
A_23_P301855	0.000453	3.292	NM_002338	NM_002338	Homo sapiens limbic system-associated membrane protein (LSAMP), mRNA [NM_002338]	NM_002338
A_23_P336023	0.000453	3.207	NM_015928	NM_015928	Homo sapiens androgen-induced proliferation inhibitor (APRIN), transcript variant 2, mRNA [NM_015928]	NM_015928
A_23_P95221	0.000454	87.78	NM_005577	NM_005577	Homo sapiens lipoprotein, Lp(a) (LPA), mRNA [NM_005577]	NM_005577
A_24_P201404	0.000454	4.927	NM_014039	NM_014039	Homo sapiens PTD012 protein (PTD012), mRNA [NM_014039]	NM_014039
A_23_P14105	0.000454	3.304	NM_001268	NM_001268	Homo sapiens regulator of chromosome condensation (RCC1) and BTB (POZ) domain containing protein 2 (RCBTB2), mRNA [NM_001268]	NM_001268
A_23_P118834	0.000455	3.067	NM_001067	NM_001067	Homo sapiens topoisomerase (DNA) II alpha 170kDa (TOP2A), mRNA [NM_001067]	NM_001067
A_23_P253495	0.000456	27.74	NM_000847	NM_000847	Homo sapiens glutathione S-transferase A3 (GSTA3), mRNA [NM_000847]	NM_000847
A_24_P40721	0.000456	5.265	ENST00000217204		Q5TD12 (Q5TD12) OTTHUMP00000030291 (Fragment), partial (46%) [THC2403720]	
A_23_P340382	0.000456	3.004	ENST00000295031		Homo sapiens mRNA for KIAA1841 protein, partial cds. [AB058744]	
A_23_P201816	0.000458	3.696	NM_014810	NM_014810	Homo sapiens centrosome-associated protein 350 (CAP350), mRNA [NM_014810]	NM_014810
A_32_P31744	0.000459	5.74	AK021664	AK021664	Homo sapiens cDNA FLJ11602 fis, clone HEMBA1003908. [AK021664]	
A_23_P254688	0.000459	2.441	NM_023943	NM_023943	Homo sapiens hypothetical protein MGC3040 (MGC3040), mRNA [NM_023943]	NM_023943
A_23_P428129	0.00046	35.9	NM_000076	NM_000076	Homo sapiens cyclin-dependent kinase inhibitor 1C (p57, Kip2) (CDKN1C), mRNA [NM_000076]	NM_000076
A_23_P145984	0.000461	18	NM_012338	NM_012338	Homo sapiens tetraspanin 12 (TSPAN12), mRNA [NM_012338]	NM_012338

A_23_P250571	0.000462	8.623	NM_005509	NM_005509	Homo sapiens Dmx-like 1 (DMXL1), mRNA [NM_005509]	NM_005509
A_23_P346900	0.000462	2.653	NM_001005505	NM_001005505	Homo sapiens calcium channel, voltage-dependent, alpha 2/delta subunit 2 (CACNA2D2), transcript variant 1, mRNA [NM_001005505]	NM_001005505
A_24_P803885	0.000463	7.227	NM_207326	NM_207326	Homo sapiens hypothetical protein LOC149134 (LOC149134), mRNA [NM_207326]	NM_207326
A_24_P192627	0.000464	8.788	NM_004529	NM_004529	Homo sapiens myeloid/lymphoid or mixed-lineage leukemia (trithorax homolog, Drosophila); translocated to, 3 (MLLT3), mRNA [NM_004529]	NM_004529
A_32_P310335	0.000465	14.73	AK056079	AK056079	Homo sapiens cDNA FLJ31517 fis, clone NT2RI2000007. [AK056079]	
A_24_P48204	0.000465	2.911	NM_003004	NM_003004	Homo sapiens secreted and transmembrane 1 (SECTM1), mRNA [NM_003004]	NM_003004
A_32_P214471	0.000467	3.365	THC2439581		Q73HM5 (Q73HM5) Phosphatidate cytidylyltransferase , partial (6%) [THC2439581]	
A_24_P82142	0.000468	2.456	NM_207038	NM_207038	Homo sapiens transcription factor 12 (HTF4, helix-loop-helix transcription factors 4) (TCF12), transcript variant 4, mRNA [NM_207038]	NM_207038
A_23_P91697	0.000471	5.586	NM_004737	NM_004737	Homo sapiens like-glycosyltransferase (LARGE), transcript variant 1, mRNA [NM_004737]	NM_004737
A_23_P154338	0.000472	9.791	NM_025202	NM_025202	Homo sapiens EF-hand domain family, member D1 (EFHD1), mRNA [NM_025202]	NM_025202
A_32_P228341	0.000472	3.893	AK055783	AK055783	Homo sapiens cDNA FLJ31221 fis, clone KIDNE2004279. [AK055783]	
A_23_P18082	0.000472	2.585	NM_032806	NM_032806	Homo sapiens glycosyltransferase (AGO61), mRNA [NM_032806]	NM_032806
A_23_P133123	0.000472	2.34	NM_032117	NM_032117	Homo sapiens GAJ protein (GAJ), mRNA [NM_032117]	NM_032117
A_23_P211673	0.000473	3.237	AL512711	AL512711	Homo sapiens mRNA; cDNA DKFZp761B039 (from clone DKFZp761B039). [AL512711]	
A_23_P54517	0.000475	3.263	NM_006293	NM_006293	Homo sapiens TYRO3 protein tyrosine kinase (TYRO3), mRNA [NM_006293]	NM_006293
A_24_P325176	0.000477	3.275	AB029032	AB029032	Homo sapiens mRNA for KIAA1109 protein, partial cds. [AB029032]	XM_371706
A_23_P259090	0.000477	3.235	NM_031438	NM_031438	Homo sapiens nudix (nucleoside diphosphate linked moiety X)-type motif 12 (NUDT12), mRNA [NM_031438]	NM_031438
A_32_P166372	0.000477	3.187	THC2294276		ALU2_HUMAN (P39189) Alu subfamily SB sequence contamination warning entry, partial (17%) [THC2294276]	
A_24_P402242	0.000478	16.99	NM_000090	NM_000090	Homo sapiens collagen, type III, alpha 1 (Ehlers-Danlos syndrome type IV, autosomal dominant) (COL3A1), mRNA [NM_000090]	NM_000090
A_24_P372012	0.000478	5.449	NM_004968	NM_004968	Homo sapiens islet cell autoantigen 1, 69kDa (ICA1), transcript variant 2, mRNA [NM_004968]	NM_004968
A_24_P380679	0.000478	4.249	NM_182597	NM_182597	Homo sapiens hypothetical protein FLJ39575 (FLJ39575), mRNA [NM_182597]	NM_182597
A_23_P51797	0.000478	4.183	THC2349786		Q5T9S5 (Q5T9S5) Sarcoma antigen NY-SAR-41 (NY-SAR-41), partial (9%) [THC2349786]	
A_23_P205336	0.000478	2.545	NM_016472	NM_016472	Homo sapiens chromosome 14 open reading frame 129 (C14orf129), mRNA [NM_016472]	NM_016472
A_24_P182539	0.000478	2.097	ENST00000317868		Homo sapiens cDNA FLJ14867 fis, clone PLACE1002319. [AK027773]	
A_24_P106910	0.00048	5.157	ENST00000331920		Homo sapiens cDNA FLJ42602 fis, clone BRACE3011271, moderately similar to Patched protein. [AK124593]	
A_23_P101253	0.00048	2.501	NM_025040	NM_025040	Homo sapiens zinc finger protein 614 (ZNF614), mRNA [NM_025040]	NM_025040
A_24_P686247	0.000481	13.17	THC2435579		ALU6_HUMAN (P39193) Alu subfamily SP sequence contamination warning entry, partial (22%) [THC2435579]	
A_23_P78975	0.000482	2.67	A_23_P78975			
A_24_P677734	0.000482	2.118	BX648343	BX648343	Homo sapiens mRNA; cDNA DKFZp686I16170 (from clone DKFZp686I16170). [BX648343]	
A_23_P36305	0.000482	2.056	NM_033388	NM_033388	Homo sapiens FLJ00012 protein (FLJ00012), mRNA [NM_033388]	NM_033388
A_32_P204330	0.000483	3.762	AK093982	AK093982	Homo sapiens cDNA FLJ36663 fis, clone UTERU2002826. [AK093982]	
A_23_P323774	0.000484	9.901	NM_001001484	NM_001001484	Homo sapiens phosphotriesterase related (PTER), transcript variant 1, mRNA [NM_001001484]	NM_001001484
A_23_P9056	0.000484	2.459	NM_014781	NM_014781	Homo sapiens RB1-inducible coiled-coil 1 (RB1CC1), mRNA [NM_014781]	NM_014781
A_32_P177300	0.000485	5.343	BQ186377	BQ186377	UI-E-EJ1-ajr-a-19-0-UI.r1 UI-E-EJ1 Homo sapiens cDNA clone UI-E-EJ1-ajr-a-19-0-UI 5', mRNA sequence [BQ186377]	
A_32_P54242	0.000487	3.555	NM_153686	NM_153686	Homo sapiens transcription factor MLR1 (MLR1), mRNA [NM_153686]	NM_153686
A_23_P144999	0.000487	3.316	NM_016340	NM_016340	Homo sapiens Rap guanine nucleotide exchange factor (GEF) 6 (RAPGEF6), mRNA [NM_016340]	NM_016340
A_23_P259103	0.000487	3.192	NM_022090	NM_022090	Homo sapiens transposon-derived Buster3 transposase-like (LOC63920), mRNA [NM_022090]	NM_022090
A_32_P183022	0.000487	2.913	NM_001008493	NM_001008493	Homo sapiens enabled homolog (Drosophila) (ENAH), transcript variant 1, mRNA [NM_001008493]	NM_001008493
A_23_P395582	0.000488	11.07	NM_174900	NM_174900	Homo sapiens zinc finger protein 42 (ZFP42), mRNA [NM_174900]	NM_174900
A_23_P76538	0.000488	6.676	NM_017899	NM_017899	Homo sapiens hypothetical protein FLJ20607 (TSC), mRNA [NM_017899]	NM_017899

A_32_P215700	0.000488	5.805	NM_181643	NM_181643	Homo sapiens chromosome 1 open reading frame 88 (C1orf88), mRNA [NM_181643]	NM_181643
A_24_P233560	0.000489	5.037	A_24_P233560			
A_23_P374250	0.000489	2.385	NM_173812	NM_173812	Homo sapiens dpy-19-like 2 (C. elegans) (DPY19L2), mRNA [NM_173812]	NM_173812
A_32_P202703	0.000489	2.145	AL713796	AL713796	Homo sapiens mRNA; cDNA DKFZp667B1610 (from clone DKFZp667B1610). [AL713796]	XM_374317
A_23_P100001	0.000493	5.076	NM_207446	NM_207446	Homo sapiens hypothetical gene supported by AK075564; BC060873 (LOC400451), mRNA [NM_207446]	NM_207446
A_24_P282343	0.000493	4.42	NM_003159	NM_003159	Homo sapiens cyclin-dependent kinase-like 5 (CDKL5), mRNA [NM_003159]	NM_003159
A_23_P29023	0.000493	2.359	NM_032910	NM_032910	Homo sapiens chromosome 21 open reading frame 119 (C21orf119), mRNA [NM_032910]	NM_032910
A_23_P253524	0.000494	2.577	NM_001813	NM_001813	Homo sapiens centromere protein E, 312kDa (CENPE), mRNA [NM_001813]	NM_001813
A_23_P70047	0.000496	3.41	NM_199189	NM_199189	Homo sapiens matrin 3 (MATR3), transcript variant 1, mRNA [NM_199189]	NM_199189
A_23_P43846	0.000496	2.959	NM_001445	NM_001445	Homo sapiens fatty acid binding protein 6, ileal (gastrotropin) (FABP6), mRNA [NM_001445]	NM_001445
A_23_P254741	0.000497	3.149	NM_003102	NM_003102	Homo sapiens superoxide dismutase 3, extracellular (SOD3), mRNA [NM_003102]	NM_003102
A_23_P129577	0.000497	2.422	NM_033208	NM_033208	Homo sapiens tigger transposable element derived 7 (TIGD7), mRNA [NM_033208]	NM_033208
A_23_P72001	0.000498	4.409	NM_174933	NM_174933	Homo sapiens phytanoyl-CoA dioxygenase domain containing 1 (PHYHD1), mRNA [NM_174933]	NM_174933
A_32_P89827	0.000499	2.949	AL137411	AL137411	Homo sapiens mRNA; cDNA DKFZp434M082 (from clone DKFZp434M082). [AL137411]	XR_000221
A_32_P158892	0.0005	2.315	THC2340773			
A_24_P129341	0.000501	43.44	NM_020299	NM_020299	Homo sapiens aldo-keto reductase family 1, member B10 (aldose reductase) (AKR1B10), mRNA [NM_020299]	NM_020299
A_23_P110624	0.000501	4.835	NM_001332	NM_001332	Homo sapiens catenin (cadherin-associated protein), delta 2 (neural plakophilin-related arm-repeat protein) (CTNND2), mRNA [NM_001332]	NM_001332
A_32_P491904	0.000501	4.156	AK091057	AK091057	Homo sapiens cDNA FLJ33738 fis, clone BRAWH2018527. [AK091057]	
A_32_P77933	0.000501	2.865	AF316855	AF316855	Homo sapiens colon cancer-associated antigen AgSK1-2HT-ECS mRNA, complete cds. [AF316855]	
A_24_P230691	0.000501	2.513	AK025303	AK025303	Homo sapiens cDNA: FLJ21650 fis, clone COL08516. [AK025303]	
A_23_P166109	0.000502	17.25	NM_198391	NM_198391	Homo sapiens fibronectin leucine rich transmembrane protein 3 (FLRT3), transcript variant 2, mRNA [NM_198391]	NM_198391
A_32_P104053	0.000502	4.388	THC2372182		O13102 (O13102) Activin type IIB receptor precursor, partial (5%) [THC2372182]	
A_23_P202501	0.000502	2.478	NM_018363	NM_018363	Homo sapiens chromosome 10 open reading frame 59 (C10orf59), mRNA [NM_018363]	NM_018363
A_24_P36285	0.000503	2.18	NM_013398	NM_013398	Homo sapiens zinc finger protein 224 (ZNF224), mRNA [NM_013398]	NM_013398
A_23_P93722	0.000503	2.031	AK074077	AK074077	Homo sapiens mRNA for FLJ00148 protein. [AK074077]	
A_32_P208403	0.000504	2.537	NM_053064	NM_053064	Homo sapiens guanine nucleotide binding protein (G protein), gamma 2 (GNG2), mRNA [NM_053064]	NM_053064
A_24_P366315	0.000505	10.06	NM_017641	NM_017641	Homo sapiens kinesin family member 21A (KIF21A), mRNA [NM_017641]	NM_017641
A_23_P12928	0.000506	37.13	ENST00000340797		Homo sapiens solute carrier family 5 (sodium/glucose cotransporter), member 12, mRNA (cDNA clone IMAGE:5187504), partial cds. [BC041454]	
A_23_P71598	0.000508	2.782	NM_003829	NM_003829	Homo sapiens multiple PDZ domain protein (MPDZ), mRNA [NM_003829]	NM_003829
A_23_P46045	0.000509	17.58	NM_003617	NM_003617	Homo sapiens regulator of G-protein signalling 5 (RGS5), mRNA [NM_003617]	NM_003617
A_32_P227921	0.000509	10.69	THC2283605			
A_23_P211363	0.000509	4.581	NM_002073	NM_002073	Homo sapiens guanine nucleotide binding protein (G protein), alpha z polypeptide (GNAZ), mRNA [NM_002073]	NM_002073
A_23_P38388	0.000509	4.054	NM_153229	NM_153229	Homo sapiens hypothetical protein FLJ33318 (FLJ33318), mRNA [NM_153229]	NM_153229
A_24_P712350	0.000509	3.282	NM_001821	NM_001821	Homo sapiens choroideremia-like (Rab escort protein 2) (CHML), mRNA [NM_001821]	NM_001821
A_32_P51894	0.000509	2.18	CR624054	CR624054	full-length cDNA clone CS0DC029YL12 of Neuroblastoma Cot 25-normalized of Homo sapiens (human). [CR624054]	
A_24_P521994	0.00051	4.775	NM_017644	NM_017644	Homo sapiens DRE1 protein (DRE1), mRNA [NM_017644]	NM_017644
A_24_P19993	0.000511	2.759	NM_014827	NM_014827	Homo sapiens zinc finger CCCH-type containing 11A (ZC3H11A), mRNA [NM_014827]	NM_014827
A_23_P121480	0.000512	5.111	NM_001004196	NM_001004196	Homo sapiens CD200 antigen (CD200), transcript variant 2, mRNA [NM_001004196]	NM_001004196
A_23_P128060	0.000512	2.718	NM_019591	NM_019591	Homo sapiens zinc finger protein 26 (KOX 20) (ZNF26), mRNA [NM_019591]	NM_019591
A_32_P313405	0.000514	62.29	NM_005559	NM_005559	Homo sapiens laminin, alpha 1 (LAMA1), mRNA [NM_005559]	NM_005559
A_23_P215024	0.000515	12.23	NM_018214	NM_018214	Homo sapiens leucine rich repeat containing 1 (LRRC1), mRNA [NM_018214]	NM_018214

A_24_P608330	0.000515	4.833	AK095242	AK095242	Homo sapiens cDNA FLJ37923 fis, clone CTONG1000283, weakly similar to BETA-CATENIN. [AK095242]	
A_24_P171058	0.000516	3.664	AL834364	AL834364	Homo sapiens mRNA; cDNA DKFZp762C1112 (from clone DKFZp762C1112). [AL834364]	
A_23_P500464	0.000517	11.08	NM_001844	NM_001844	Homo sapiens collagen, type II, alpha 1 (primary osteoarthritis, spondyloepiphyseal dysplasia, congenital) (COL2A1), transcript variant 1, mRNA [NM_001844]	NM_001844
A_23_P154411	0.000517	2.837	NM_004792	NM_004792	Homo sapiens peptidyl-prolyl isomerase G (cyclophilin G) (PPIG), mRNA [NM_004792]	NM_004792
A_24_P48723	0.000519	107.9	NM_000961	NM_000961	Homo sapiens prostaglandin I2 (prostacyclin) synthase (PTGIS), mRNA [NM_000961]	NM_000961
A_32_P8813	0.000519	3.785	AK090515	AK090515	Homo sapiens cDNA FLJ33196 fis, clone ADRGL2006034. [AK090515]	XM_378514
A_32_P90709	0.000521	7.329	BC045657	BC045657	Homo sapiens, clone IMAGE:5270591, mRNA. [BC045657]	
A_23_P421032	0.000521	4.286	NM_174977	NM_174977	Homo sapiens SEC14-like 4 (S. cerevisiae) (SEC14L4), mRNA [NM_174977]	NM_174977
A_32_P86400	0.000521	3.567	CR933624	CR933624	Homo sapiens mRNA; cDNA DKFZp686F0735 (from clone DKFZp686F0735). [CR933624]	XM_371760
A_23_P252981	0.000521	3.193	NM_021804	NM_021804	Homo sapiens angiotensin I converting enzyme (peptidyl-dipeptidase A) 2 (ACE2), mRNA [NM_021804]	NM_021804
A_23_P20363	0.000521	3.137	NM_016010	NM_016010	Homo sapiens CGI-62 protein (CGI-62), mRNA [NM_016010]	NM_016010
A_23_P56654	0.000521	2.874	NM_032601	NM_032601	Homo sapiens methylmalonyl CoA epimerase (MCEE), mRNA [NM_032601]	NM_032601
A_23_P16652	0.000521	2.082	NM_152791	NM_152791	Homo sapiens zinc finger protein 555 (ZNF555), mRNA [NM_152791]	NM_152791
A_23_P120883	0.000521	2.006	NM_002133	NM_002133	Homo sapiens heme oxygenase (decycling) 1 (HMOX1), mRNA [NM_002133]	NM_002133
A_23_P319792	0.000522	3.142	NM_019001	NM_019001	Homo sapiens 5'-3' exoribonuclease 1 (XRN1), mRNA [NM_019001]	NM_019001
A_23_P46333	0.000523	3.621	NM_007358	NM_007358	Homo sapiens metal response element binding transcription factor 2 (MTF2), mRNA [NM_007358]	NM_007358
A_23_P142533	0.000524	51.59	NM_000090	NM_000090	Homo sapiens collagen, type III, alpha 1 (Ehlers-Danlos syndrome type IV, autosomal dominant) (COL3A1), mRNA [NM_000090]	NM_000090
A_32_P186364	0.000525	15.29	BC031314	BC031314	Homo sapiens, clone IMAGE:5276765, mRNA. [BC031314]	
A_23_P33914	0.000526	7.402	NM_017681	NM_017681	Homo sapiens hypothetical protein FLJ20130 (FLJ20130), mRNA [NM_017681]	NM_017681
A_23_P212522	0.000526	2.307	NM_014616	NM_014616	Homo sapiens ATPase, Class VI, type 11B (ATP11B), mRNA [NM_014616]	NM_014616
A_23_P58228	0.000528	9.483	NM_017855	NM_017855	Homo sapiens APin protein (APIN), mRNA [NM_017855]	NM_017855
A_32_P208120	0.000528	5.85	NM_153498	NM_153498	Homo sapiens calcium/calmodulin-dependent protein kinase ID (CAMK1D), transcript variant 2, mRNA [NM_153498]	NM_153498
A_32_P67577	0.000529	4.334	THC2401493			
A_32_P174258	0.00053	7.362	BQ719988	BQ719988	AGENCOURT_8103790 Lupski_sympathetic_trunk Homo sapiens cDNA clone IMAGE:6190912 5', mRNA sequence [BQ719988]	
A_23_P251412	0.00053	4.292	NM_006998	NM_006998	Homo sapiens secretagogin, EF-hand calcium binding protein (SCGN), mRNA [NM_006998]	NM_006998
A_23_P254025	0.00053	3.246	NM_003408	NM_003408	Homo sapiens zinc finger protein 37 homolog (mouse) (ZFP37), mRNA [NM_003408]	NM_003408
A_24_P261383	0.00053	2.212	NM_024116	NM_024116	Homo sapiens hypothetical protein MGC5306 (MGC5306), mRNA [NM_024116]	NM_024116
A_23_P436353	0.000531	6.199	ENST00000351017		Homo sapiens mRNA; cDNA DKFZp761K2213 (from clone DKFZp761K2213); partial cds. [AL161973]	
A_32_P220161	0.000531	3.949	BI497361	BI497361	df135e07.y1 Morton Fetal Cochlea Homo sapiens cDNA clone IMAGE:2537964 5', mRNA sequence [BI497361]	
A_23_P13822	0.000532	3.407	NM_018423	NM_018423	Homo sapiens serine/threonine/tyrosine kinase 1 (STYK1), mRNA [NM_018423]	NM_018423
A_32_P4985	0.000533	30.42	NM_015215	NM_015215	Homo sapiens calmodulin binding transcription activator 1 (CAMTA1), mRNA [NM_015215]	NM_015215
A_32_P193322	0.000533	4.465	NM_152756	NM_152756	Homo sapiens TORC2-specific protein AVO3 (AVO3), mRNA [NM_152756]	NM_152756
A_23_P41166	0.000533	3.11	NM_003781	NM_003781	Homo sapiens UDP-Gal:betaGlcNAc beta 1,3-galactosyltransferase, polypeptide 3 (B3GALT3), transcript variant 1, mRNA [NM_003781]	NM_003781
A_23_P80048	0.000533	2.984	NR_001442	NR_001442	Homo sapiens fer-1-like 4 (C. elegans) (FER1L4) on chromosome 20 [NR_001442]	NR_001442
A_23_P99260	0.000533	2.333	NM_006838	NM_006838	Homo sapiens methionyl aminopeptidase 2 (METAP2), mRNA [NM_006838]	NM_006838
A_23_P341700	0.000534	2.092	NM_001007101	NM_001007101	Homo sapiens zinc finger protein 484 (ZNF484), transcript variant 2, mRNA [NM_001007101]	NM_001007101
A_32_P86739	0.000535	4.251	NM_001010911	NM_001010911	Homo sapiens chromosome 10 open reading frame 114 (C10orf114), mRNA [NM_001010911]	NM_001010911
A_23_P258037	0.000536	3.672	NM_018433	NM_018433	Homo sapiens jumonji domain containing 1A (JMJD1A), mRNA [NM_018433]	NM_018433
A_32_P195850	0.000537	2.283	NM_173812	NM_173812	Homo sapiens dpy-19-like 2 (C. elegans) (DPY19L2), mRNA [NM_173812]	NM_173812
A_32_P231493	0.000537	2.22	AF339771	AF339771	Homo sapiens clone IMAGE:1257951, mRNA sequence. [AF339771]	
A_23_P372834	0.000538	36.92	NM_000385	NM_000385	Homo sapiens aquaporin 1 (channel-forming integral protein, 28kDa) (AQP1), transcript variant 2, mRNA [NM_000385]	NM_000385

A_32_P109755	0.000538	3.592	AF462446	AF462446	Homo sapiens unknown mRNA. [AF462446]	
A_24_P341089	0.000538	3.271	ENST00000321577		Homo sapiens, clone IMAGE:5167446, mRNA. [BC031698]	
A_23_P367899	0.000538	2.1	NM_000121	NM_000121	Homo sapiens erythropoietin receptor (EPOR), mRNA [NM_000121]	NM_000121
A_23_P37111	0.000539	3.013	NM_177438	NM_177438	Homo sapiens Dicer1, Dcr-1 homolog (Drosophila) (DICER1), transcript variant 1, mRNA [NM_177438]	NM_177438
A_23_P135634	0.000539	2.019	AF217963	AF217963	Homo sapiens NRAGE mRNA, complete cds. [AF217963]	
A_23_P69738	0.00054	4.683	NM_023940	NM_023940	Homo sapiens RAS-like, family 11, member B (RASL11B), mRNA [NM_023940]	NM_023940
A_24_P217804	0.000542	3.119	THC2368225			
A_23_P120354	0.000542	2.027	NM_023016	NM_023016	Homo sapiens chromosome 2 open reading frame 26 (C2orf26), mRNA [NM_023016]	NM_023016
A_23_P385126	0.000543	3.994	NM_139160	NM_139160	Homo sapiens novel 58.3 KDA protein (LOC91614), mRNA [NM_139160]	NM_139160
A_32_P220700	0.000545	8.077	THC2440162			
A_23_P212792	0.000545	2.713	NM_025009	NM_025009	Homo sapiens centrosomal protein 4 (CEP4), mRNA [NM_025009]	NM_025009
A_23_P122068	0.000548	7.427	NM_181435	NM_181435	Homo sapiens C1q and tumor necrosis factor related protein 3 (C1QTNF3), transcript variant 2, mRNA [NM_181435]	NM_181435
A_23_P360804	0.000551	5.72	NM_020939	NM_020939	Homo sapiens copine V (CPNE5), mRNA [NM_020939]	NM_020939
A_32_P176902	0.000551	2.922	A_32_P176902			
A_24_P23546	0.000552	26.71	NM_198956	NM_198956	Homo sapiens Sp8 transcription factor (SP8), transcript variant 2, mRNA [NM_198956]	NM_198956
A_24_P345290	0.000553	37.27	A_24_P345290			
A_23_P79360	0.000556	10.33	NM_052946	NM_052946	Homo sapiens nitric oxide synthase trafficker (NOSTRIN), mRNA [NM_052946]	NM_052946
A_24_P242361	0.000556	3.563	NM_012262	NM_012262	Homo sapiens heparan sulfate 2-O-sulfotransferase 1 (HS2ST1), mRNA [NM_012262]	NM_012262
A_23_P424	0.000558	12.31	NM_018650	NM_018650	Homo sapiens MAP/microtubule affinity-regulating kinase 1 (MARK1), mRNA [NM_018650]	NM_018650
A_24_P548453	0.000558	2.692	CR620400	CR620400	full-length cDNA clone CS0DJ015YA13 of T cells (Jurkat cell line) Cot 10-normalized of Homo sapiens (human). [CR620400]	
A_24_P240487	0.000559	4.362	NM_000301	NM_000301	Homo sapiens plasminogen (PLG), mRNA [NM_000301]	NM_000301
A_23_P69573	0.000561	16.17	NM_000856	NM_000856	Homo sapiens guanylate cyclase 1, soluble, alpha 3 (GUCY1A3), mRNA [NM_000856]	NM_000856
A_24_P181422	0.000561	2.467	NM_172097	NM_172097	Homo sapiens cation channel, sperm associated 2 (CATSPER2), transcript variant 4, mRNA [NM_172097]	NM_172097
A_23_P94591	0.000564	4.143	NM_032928	NM_032928	Homo sapiens hypothetical protein MGC14141 (MGC14141), mRNA [NM_032928]	NM_032928
A_24_P307759	0.000565	6.228	NM_015180	NM_015180	Homo sapiens spectrin repeat containing, nuclear envelope 2 (SYNE2), transcript variant 1, mRNA [NM_015180]	NM_015180
A_23_P109907	0.000565	2.756	NM_175924	NM_175924	Homo sapiens immunoglobulin-like domain containing receptor 1 (ILDR1), mRNA [NM_175924]	NM_175924
A_32_P56661	0.000567	7.119	AK074614	AK074614	Homo sapiens cDNA FLJ90133 fis, clone HEMBB1000567. [AK074614]	
A_23_P2233	0.000567	4.573	NM_006928	NM_006928	Homo sapiens silver homolog (mouse) (SILV), mRNA [NM_006928]	NM_006928
A_23_P33154	0.000568	2.201	NM_014393	NM_014393	Homo sapiens staufen, RNA binding protein, homolog 2 (Drosophila) (STAU2), mRNA [NM_014393]	NM_014393
A_23_P128174	0.000569	4.098	NM_175623	NM_175623	Homo sapiens RAB3A interacting protein (rabin3) (RAB3IP), transcript variant alpha 2, mRNA [NM_175623]	NM_175623
A_32_P127542	0.000569	2.479	AK024516	AK024516	Homo sapiens cDNA: FLJ20863 fis, clone ADKA01804. [AK024516]	
A_23_P34700	0.000571	2.491	NM_000364	NM_000364	Homo sapiens troponin T2, cardiac (TNNT2), transcript variant 1, mRNA [NM_000364]	NM_000364
A_23_P103201	0.000571	2.392	NM_017761	NM_017761	Homo sapiens proline-rich nuclear receptor coactivator 2 (PNRC2), mRNA [NM_017761]	NM_017761
A_23_P156402	0.000572	3.162	NM_003551	NM_003551	Homo sapiens non-metastatic cells 5, protein expressed in (nucleoside-diphosphate kinase) (NME5), mRNA [NM_003551]	NM_003551
A_24_P682550	0.000572	2.647	A_24_P682550			
A_23_P375517	0.000573	4.497	NM_138286	NM_138286	Homo sapiens zinc finger protein 681 (ZNF681), mRNA [NM_138286]	NM_138286
A_24_P321766	0.000574	4.247	NM_000624	NM_000624	Homo sapiens serine (or cysteine) proteinase inhibitor, clade A (alpha-1 antiproteinase, antitrypsin), member 5 (SERPINA5), mRNA [NM_000624]	NM_000624
A_23_P85742	0.000574	3.838	NM_003851	NM_003851	Homo sapiens cellular repressor of E1A-stimulated genes 1 (CREG1), mRNA [NM_003851]	NM_003851
A_23_P149938	0.000574	3.375	THC2378635			
A_23_P61580	0.000574	2.615	NM_182543	NM_182543	Homo sapiens NOL1/NOP2/Sun domain family, member 6 (NSUN6), mRNA [NM_182543]	NM_182543
A_23_P212383	0.000574	2.015	NM_014016	NM_014016	Homo sapiens SAC1 suppressor of actin mutations 1-like (yeast) (SACM1L), mRNA [NM_014016]	NM_014016

A_23_P84018	0.000575	3.934	NM_000943	NM_000943	Homo sapiens peptidylprolyl isomerase C (cyclophilin C) (PPIC), mRNA [NM_000943]	NM_000943
A_32_P165990	0.000576	3.172	AK094623	AK094623	Homo sapiens cDNA FLJ37304 fis, clone BRAMY2016070. [AK094623]	
A_24_P105564	0.000576	2.645	NM_005399	NM_005399	Homo sapiens protein kinase, AMP-activated, beta 2 non-catalytic subunit (PRKAB2), mRNA [NM_005399]	NM_005399
A_32_P132589	0.000576	2.522	NM_001004339	NM_001004339	Homo sapiens zyg-11 homolog A (C. elegans) (ZYG11A), mRNA [NM_001004339]	NM_001004339
A_32_P183904	0.000577	2.58	ENST00000361989		Homo sapiens Src homology 2 domain containing F, mRNA (cDNA clone IMAGE:3162799), partial cds. [BC007586]	
A_32_P57013	0.000578	8.475	BU540282	BU540282	BU540282 AGENCOURT_10326456 NIH_MGC_141 Homo sapiens cDNA clone IMAGE:6571686 5', mRNA sequence [BU540282]	
A_23_P338919	0.000578	5.187	NM_005876	NM_005876	Homo sapiens aortic preferentially expressed gene 1 (APEG1), mRNA [NM_005876]	NM_005876
A_23_P5586	0.000578	2.037	NM_005791	NM_005791	Homo sapiens M-phase phosphoprotein 10 (U3 small nucleolar ribonucleoprotein) (MPHOSPH10), mRNA [NM_005791]	NM_005791
A_23_P74895	0.000579	9.141	NM_024674	NM_024674	Homo sapiens lin-28 homolog (C. elegans) (LIN28), mRNA [NM_024674]	NM_024674
A_32_P223777	0.00058	6.892	THC2315176		IL6B_HUMAN (P40189) Interleukin-6 receptor beta chain precursor (IL-6R-beta) (Interleukin 6 signal transducer) (Membrane glycoprotein 130) (gp130) (Oncostatin M receptor) (CDw130) (CD130 antigen), partial (27%) [THC2315176]	
A_24_P277155	0.00058	3.927	NM_003071	NM_003071	Homo sapiens SWI/SNF related, matrix associated, actin dependent regulator of chromatin, subfamily a, member 3 (SMARCA3), transcript variant 1, mRNA [NM_003071]	NM_003071
A_24_P713729	0.00058	2.723	THC2373625		ALU8_HUMAN (P39195) Alu subfamily SX sequence contamination warning entry, partial (6%) [THC2373624]	
A_23_P141785	0.00058	2.456	A_23_P141785			
A_24_P333306	0.000581	4.114	AK023737	AK023737	Homo sapiens cDNA FLJ13675 fis, clone PLACE1011875, highly similar to Homo sapiens mRNA for KIAA0580 protein. [AK023737]	
A_32_P61439	0.000582	8.258	THC2416008		U74612 forkhead box M1A {Homo sapiens;} , partial (3%) [THC2416008]	
A_24_P54808	0.000582	2.707	NM_020803	NM_020803	Homo sapiens kelch-like 8 (Drosophila) (KLHL8), mRNA [NM_020803]	NM_020803
A_23_P168419	0.000582	2.2	BX640742	BX640742	Homo sapiens mRNA; cDNA DKFZp686C08112 (from clone DKFZp686C08112). [BX640742]	
A_32_P46191	0.000583	5.476	ENST00000356194		PREDICTED: Homo sapiens similar to zinc finger protein 91 (HPF7, HTF10) (LOC442695), mRNA [XM_499440]	XM_498210
A_32_P149298	0.000583	3.032	ENST00000295031		Homo sapiens mRNA for KIAA1841 protein, partial cds. [AB058744]	
A_24_P183094	0.000583	2.348	AJ306929	AJ306929	Homo sapiens ORF for hypothetical protein. [AJ306929]	
A_23_P99642	0.000584	23.66	NM_003982	NM_003982	Homo sapiens solute carrier family 7 (cationic amino acid transporter, y+ system), member 7 (SLC7A7), mRNA [NM_003982]	NM_003982
A_32_P206123	0.000585	80.86	NM_000301	NM_000301	Homo sapiens plasminogen (PLG), mRNA [NM_000301]	NM_000301
A_24_P288149	0.000585	3.213	THC2339776			
A_23_P169197	0.000585	2.836	NM_032303	NM_032303	Homo sapiens hydroxysteroid dehydrogenase like 2 (HSDL2), mRNA [NM_032303]	NM_032303
A_24_P208737	0.000585	2.384	THC2306718			
A_24_P11061	0.000586	6.766	NM_153479	NM_153479	Homo sapiens chondrosarcoma associated gene 1 (CSAG1), transcript variant b, mRNA [NM_153479]	NM_153479
A_32_P116323	0.000587	2.268	NM_015092	NM_015092	Homo sapiens PI-3-kinase-related kinase SMG-1 (SMG1), mRNA [NM_015092]	NM_015092
A_23_P148088	0.000589	141.3	NM_000509	NM_000509	Homo sapiens fibrinogen gamma chain (FGG), transcript variant gamma-A, mRNA [NM_000509]	NM_000509
A_23_P904	0.000589	6.644	NM_024603	NM_024603	Homo sapiens chromosome 1 open reading frame 165 (C1orf165), mRNA [NM_024603]	NM_024603
A_23_P122805	0.000589	2.177	NM_032842	NM_032842	Homo sapiens hypothetical protein FLJ14803 (FLJ14803), mRNA [NM_032842]	NM_032842
A_24_P336931	0.00059	2.954	NM_020970	NM_020970	Homo sapiens KIAA1641 (KIAA1641), mRNA [NM_020970]	NM_020970
A_23_P25605	0.00059	2.796	NM_032138	NM_032138	Homo sapiens kelch repeat and BTB (POZ) domain containing 7 (KBTBD7), mRNA [NM_032138]	NM_032138
A_32_P30600	0.000591	6.214	THC2284350			
A_32_P224040	0.000591	3.795	THC2274685		AF326941 5H1 {Caenorhabditis elegans;} , partial (16%) [THC2274685]	
A_23_P107763	0.000592	2.387	NM_006631	NM_006631	Homo sapiens zinc finger protein 266 (ZNF266), mRNA [NM_006631]	NM_006631
A_23_P397120	0.000593	3.857	ENST00000211092		Homo sapiens HSPC323 mRNA, partial cds. [AF161441]	XM_209196
A_23_P149798	0.000594	4.09	AK027209	AK027209	Homo sapiens cDNA: FLJ23556 fis, clone LNG09443. [AK027209]	
A_32_P111587	0.000594	2.554	AF086098	AF086098	Homo sapiens full length insert cDNA clone YZ88E12. [AF086098]	
A_23_P163347	0.000595	2.019	NM_016166	NM_016166	Homo sapiens protein inhibitor of activated STAT, 1 (PIAS1), mRNA [NM_016166]	NM_016166

A_23_P26281	0.000596	2.754	NR_001565	NR_001565	Homo sapiens pseudogene MGC10997 (MGC10997) on chromosome 15 [NR_001565]	NR_001565
A_23_P39294	0.000597	4.376	ENST00000312785		Homo sapiens mRNA full length insert cDNA clone EUROIMAGE 1759349. [AL365412]	
A_23_P102364	0.000597	4.34	NM_019850	NM_019850	Homo sapiens neuronal guanine nucleotide exchange factor (NGEF), mRNA [NM_019850]	NM_019850
A_23_P215208	0.000598	2.361	NM_022116	NM_022116	Homo sapiens fidgetin-like 1 (FIGNL1), mRNA [NM_022116]	NM_022116
A_24_P333421	0.000599	2.056	AB011115	AB011115	Homo sapiens mRNA for KIAA0543 protein, partial cds. [AB011115]	XM_376720
A_23_P62684	0.000602	2.313	AF217973	AF217973	Homo sapiens clone PP1345 unknown mRNA. [AF217973]	
A_24_P826348	0.000603	4.249	AB111887	AB111887	Homo sapiens mRNA for KIAA2035 protein. [AB111887]	
A_24_P48057	0.000605	5.732	NM_005853	NM_005853	Homo sapiens iroquois homeobox protein 5 (IRX5), mRNA [NM_005853]	NM_005853
A_23_P72014	0.000605	4.424	A_23_P72014			
A_23_P48339	0.000607	2.576	NM_175605	NM_175605	Homo sapiens tetratricopeptide repeat domain 10 (TTC10), transcript variant 1, mRNA [NM_175605]	NM_175605
A_23_P204158	0.000607	2.16	NM_032814	NM_032814	Homo sapiens hypothetical protein FLJ14627 (FLJ14627), mRNA [NM_032814]	NM_032814
A_23_P254353	0.000608	3.403	NM_006647	NM_006647	Homo sapiens NADPH oxidase activator 1 (NOXA1), mRNA [NM_006647]	NM_006647
A_23_P11286	0.000608	2.271	NM_019597	NM_019597	Homo sapiens heterogeneous nuclear ribonucleoprotein H2 (H') (HNRPH2), mRNA [NM_019597]	NM_019597
A_24_P659980	0.000609	4.037	THC2281837			
A_23_P432034	0.000609	3.662	NM_173510	NM_173510	Homo sapiens hypothetical protein FLJ33814 (FLJ33814), mRNA [NM_173510]	NM_173510
A_24_P307869	0.000609	3.266	NM_001015002	NM_001015002	Homo sapiens lethal giant larvae homolog 2 (Drosophila) (LLGL2), transcript variant 2, mRNA [NM_001015002]	NM_001015002
A_23_P12113	0.000609	2.341	NM_014053	NM_014053	Homo sapiens feline leukemia virus subgroup C cellular receptor (FLVCR), mRNA [NM_014053]	NM_014053
A_24_P535219	0.00061	3.021	AJ420510	AJ420510	Homo sapiens mRNA full length insert cDNA clone EUROIMAGE 2120537. [AJ420510]	
A_23_P33356	0.000612	86	AF261918	AF261918	Homo sapiens disintegrin metalloproteinase with thrombospondin repeats (ADAMTS9) mRNA, complete cds. [AF261918]	
A_24_P744818	0.000612	2.076	AK021546	AK021546	Homo sapiens cDNA FLJ11484 fis, clone HEMBA1001835. [AK021546]	
A_23_P385246	0.000614	4.237	NM_153331	NM_153331	Homo sapiens potassium channel tetramerisation domain containing 6 (KCTD6), mRNA [NM_153331]	NM_153331
A_32_P159651	0.000614	3.276	NM_003884	NM_003884	Homo sapiens p300/CBP-associated factor (PCAF), mRNA [NM_003884]	NM_003884
A_32_P88415	0.000616	3.704	NM_133371	NM_133371	Homo sapiens myozenin 3 (MYOZ3), mRNA [NM_133371]	NM_133371
A_23_P138541	0.000618	2.486	NM_003739	NM_003739	Homo sapiens aldo-keto reductase family 1, member C3 (3-alpha hydroxysteroid dehydrogenase, type II) (AKR1C3), mRNA [NM_003739]	NM_003739
A_24_P506726	0.000618	2.278	AK022936	AK022936	Homo sapiens cDNA FLJ12874 fis, clone NT2RP2003769. [AK022936]	
A_23_P309619	0.00062	2.074	AK123704	AK123704	Homo sapiens cDNA FLJ41710 fis, clone HLUNG2011041, weakly similar to Basic proline-rich peptide IB-8a. [AK123704]	XM_371461
A_24_P20120	0.000624	2.291	NM_018084	NM_018084	Homo sapiens KIAA1212 (KIAA1212), mRNA [NM_018084]	NM_018084
A_23_P307310	0.000626	4.414	NM_013227	NM_013227	Homo sapiens aggrecan 1 (chondroitin sulfate proteoglycan 1, large aggregating proteoglycan, antigen identified by monoclonal antibody A0122) (AGC1), transcript variant 2, mRNA [NM_013227]	NM_013227
A_23_P334864	0.000626	3.449	NM_173822	NM_173822	Homo sapiens hypothetical protein MGC39518 (MGC39518), mRNA [NM_173822]	NM_173822
A_24_P602871	0.000627	23.29	THC2278542			
A_23_P70069	0.000627	4.818	AK000420	AK000420	Homo sapiens cDNA FLJ20413 fis, clone KAT02170. [AK000420]	
A_24_P120537	0.000627	3.1	NM_152550	NM_152550	Homo sapiens SH3 domain containing ring finger 2 (SH3RF2), mRNA [NM_152550]	NM_152550
A_23_P5616	0.000629	2.515	NM_018151	NM_018151	Homo sapiens RAP1 interacting factor homolog (yeast) (RIF1), mRNA [NM_018151]	NM_018151
A_23_P123228	0.00063	3.948	NM_000111	NM_000111	Homo sapiens solute carrier family 26, member 3 (SLC26A3), mRNA [NM_000111]	NM_000111
A_24_P601972	0.000632	2.023	ENST00000344142		Homo sapiens cDNA clone IMAGE:3950925, partial cds. [BC014578]	XM_291016
A_24_P358667	0.000633	2.945	AF075112	AF075112	Homo sapiens full length insert cDNA YU76E12. [AF075112]	
A_23_P114814	0.000634	17.94	NM_021205	NM_021205	Homo sapiens ras homolog gene family, member U (RHOU), mRNA [NM_021205]	NM_021205
A_23_P143964	0.000634	5.72	NM_004844	NM_004844	Homo sapiens SH3-domain binding protein 5 (BTK-associated) (SH3BP5), transcript variant 1, mRNA [NM_004844]	NM_004844
A_23_P63447	0.000634	2.938	THC2373845			
A_23_P361448	0.000635	7.797	NM_144665	NM_144665	Homo sapiens sestrin 3 (SESN3), mRNA [NM_144665]	NM_144665

A_32_P162374	0.000635	3.798	NM_173529	NM_173529	Homo sapiens chromosome 18 open reading frame 54 (C18orf54), mRNA [NM_173529]	NM_173529
A_23_P121250	0.000637	2.278	NM_001967	NM_001967	Homo sapiens eukaryotic translation initiation factor 4A, isoform 2 (EIF4A2), mRNA [NM_001967]	NM_001967
A_24_P226241	0.000638	21.49	NM_153234	NM_153234	Homo sapiens Lix1 homolog (mouse) (LIX1), mRNA [NM_153234]	NM_153234
A_24_P649507	0.000638	3.396	THC2438003		Q9BVX4 (Q9BVX4) MGC5566 protein, partial (23%) [THC2438003]	
A_23_P387000	0.000638	2.684	NM_173683	NM_173683	Homo sapiens X Kell blood group precursor-related family, member 6 (XKR6), transcript variant 2, mRNA [NM_173683]	NM_173683
A_23_P104464	0.000642	11.63	NM_000698	NM_000698	Homo sapiens arachidonate 5-lipoxygenase (ALOX5), mRNA [NM_000698]	NM_000698
A_24_P163237	0.000642	3.728	NM_020225	NM_020225	Homo sapiens storkhead box 2 (STOX2), mRNA [NM_020225]	NM_020225
A_23_P11664	0.000643	2.798	NM_004768	NM_004768	Homo sapiens splicing factor, arginine/serine-rich 11 (SFRS11), mRNA [NM_004768]	NM_004768
A_24_P13390	0.000644	2.436	NM_032814	NM_032814	Homo sapiens hypothetical protein FLJ14627 (FLJ14627), mRNA [NM_032814]	NM_032814
A_32_P75425	0.000645	4.406	NM_001013665	NM_001013665	Homo sapiens hypothetical LOC399744 (LOC399744), mRNA [NM_001013665]	NM_001013665
A_23_P168306	0.000646	2.808	NM_003931	NM_003931	Homo sapiens WAS protein family, member 1 (WASF1), transcript variant 1, mRNA [NM_003931]	NM_003931
A_24_P257579	0.000647	8.5	NM_022140	NM_022140	Homo sapiens erythrocyte membrane protein band 4.1 like 4A (EPB41L4A), mRNA [NM_022140]	NM_022140
A_24_P944588	0.000647	2.742	AK074843	AK074843	Homo sapiens cDNA FLJ90362 fis, clone NT2RP2003940, weakly similar to ZINC FINGER PROTEIN 43. [AK074843]	
A_23_P58960	0.000647	2.662	NM_020133	NM_020133	Homo sapiens 1-acylglycerol-3-phosphate O-acyltransferase 4 (lysophosphatidic acid acyltransferase, delta) (AGPAT4), transcript variant 1, mRNA [NM_020133]	NM_020133
A_24_P889103	0.000648	3.01	CR597846	CR597846	full-length cDNA clone CS0DC012YL18 of Neuroblastoma Cot 25-normalized of Homo sapiens (human). [CR597846]	
A_23_P166823	0.000649	2.653	NM_003280	NM_003280	Homo sapiens troponin C, slow (TNNC1), mRNA [NM_003280]	NM_003280
A_32_P385587	0.00065	3.408	NM_000032	NM_000032	Homo sapiens aminolevulinate, delta-, synthase 2 (sideroblastic/hypochromic anemia) (ALAS2), nuclear gene encoding mitochondrial protein, mRNA [NM_000032]	NM_000032
A_23_P83931	0.000651	3.248	NM_005863	NM_005863	Homo sapiens neuroepithelial cell transforming gene 1 (NET1), mRNA [NM_005863]	NM_005863
A_23_P123727	0.000651	2.079	NM_024617	NM_024617	Homo sapiens zinc finger, CCHC domain containing 6 (ZCCHC6), mRNA [NM_024617]	NM_024617
A_24_P100613	0.000652	6.879	NM_005559	NM_005559	Homo sapiens laminin, alpha 1 (LAMA1), mRNA [NM_005559]	NM_005559
A_23_P161481	0.000652	6.63	NM_014431	NM_014431	Homo sapiens KIAA1274 (KIAA1274), mRNA [NM_014431]	NM_014431
A_24_P490857	0.000655	2.588	BC037535	BC037535	Homo sapiens cDNA clone IMAGE:5274219, partial cds. [BC037535]	
A_24_P337774	0.000656	2.908	CR618686	CR618686	full-length cDNA clone CS0DC012YO16 of Neuroblastoma Cot 25-normalized of Homo sapiens (human). [CR618686]	
A_23_P72568	0.000658	4.054	NM_003794	NM_003794	Homo sapiens sorting nexin 4 (SNX4), mRNA [NM_003794]	NM_003794
A_32_P196837	0.00066	13.63	AA449494	AA449494	AA449494 zx08h11.s1 Soares_total_fetus_Nb2HF8_9w Homo sapiens cDNA clone IMAGE:785925 3' similar to gb:U15981 ADRENAL SPECIFIC 30 KD PROTEIN (HUMAN);, mRNA sequence [AA449494]	
A_23_P59375	0.00066	12.27	NM_001546	NM_001546	Homo sapiens inhibitor of DNA binding 4, dominant negative helix-loop-helix protein (ID4), mRNA [NM_001546]	NM_001546
A_23_P20864	0.00066	5.867	NM_012098	NM_012098	Homo sapiens angiopoietin-like 2 (ANGPTL2), mRNA [NM_012098]	NM_012098
A_23_P423074	0.000661	8.478	CR936791	CR936791	Homo sapiens mRNA; cDNA DKFZp781C2356 (from clone DKFZp781C2356). [CR936791]	XM_032571
A_23_P33984	0.000661	5.465	NM_020665	NM_020665	Homo sapiens transmembrane protein 27 (TMEM27), mRNA [NM_020665]	NM_020665
A_23_P209232	0.000661	3.557	NM_024692	NM_024692	Homo sapiens restin-like 2 (RSNL2), mRNA [NM_024692]	NM_024692
A_32_P217773	0.000661	2.963	NM_032872	NM_032872	Homo sapiens synaptotagmin-like 1 (SYTL1), mRNA [NM_032872]	NM_032872
A_24_P405981	0.000661	2.473	CR598481	CR598481	full-length cDNA clone CS0DD001YH15 of Neuroblastoma Cot 50-normalized of Homo sapiens (human). [CR598481]	
A_24_P926783	0.000661	2.248	AK001357	AK001357	Homo sapiens cDNA FLJ10495 fis, clone NT2RP2000297, moderately similar to ZINC FINGER PROTEIN 184. [AK001357]	XR_000217
A_24_P630916	0.000663	4.582	THC2366591		ALU7_HUMAN (P39194) Alu subfamily SQ sequence contamination warning entry, partial (12%) [THC2366591]	
A_23_P147822	0.000663	2.623	NM_022772	NM_022772	Homo sapiens EPS8-like 2 (EPS8L2), mRNA [NM_022772]	NM_022772
A_23_P127964	0.000663	2.116	NM_199418	NM_199418	Homo sapiens prolylcarboxypeptidase (angiotensinase C) (PRCP), transcript variant 2, mRNA [NM_199418]	NM_199418
A_32_P452655	0.000665	3.347	NM_009587	NM_009587	Homo sapiens lectin, galactoside-binding, soluble, 9 (galectin 9) (LGALS9), transcript variant long, mRNA [NM_009587]	NM_009587
A_32_P121079	0.000666	3.325	AK090467	AK090467	Homo sapiens mRNA for FLJ00388 protein. [AK090467]	
A_24_P300952	0.000666	2.973	NM_001642	NM_001642	Homo sapiens amyloid beta (A4) precursor-like protein 2 (APLP2), mRNA [NM_001642]	NM_001642

A_23_P421379	0.000667	6.552	NM_000612	NM_000612	Homo sapiens insulin-like growth factor 2 (somatomedin A) (IGF2), mRNA [NM_000612]	NM_000612
A_23_P335069	0.000667	2.157	AL832613	AL832613	Homo sapiens mRNA; cDNA DKFZp451K1917 (from clone DKFZp451K1917). [AL832613]	
A_23_P87049	0.000669	14.68	NM_003105	NM_003105	Homo sapiens sortilin-related receptor, L(DLR class) A repeats-containing (SORL1), mRNA [NM_003105]	NM_003105
A_24_P209171	0.000669	4.458	NM_031469	NM_031469	Homo sapiens SH3 domain binding glutamic acid-rich protein like 2 (SH3BGRL2), mRNA [NM_031469]	NM_031469
A_24_P126325	0.000669	3.117	NM_207035	NM_207035	Homo sapiens chromosome 1 open reading frame 63 (C1orf63), transcript variant 1, mRNA [NM_207035]	NM_207035
A_23_P58877	0.00067	2.164	NM_020399	NM_020399	Homo sapiens golgi associated PDZ and coiled-coil motif containing (GOPC), transcript variant 1, mRNA [NM_020399]	NM_020399
A_23_P35871	0.000672	2.512	NM_024680	NM_024680	Homo sapiens E2F transcription factor 8 (E2F8), mRNA [NM_024680]	NM_024680
A_24_P710730	0.000673	4.697	NM_207331	NM_207331	Homo sapiens hypothetical protein LOC153561 (LOC153561), mRNA [NM_207331]	NM_207331
A_23_P37785	0.000673	3.315	BC070103	BC070103	Homo sapiens potassium channel tetramerisation domain containing 19, mRNA (cDNA clone IMAGE:5268205). [BC070103]	XM_085367
A_23_P125748	0.000674	13.66	NM_032441	NM_032441	Homo sapiens zinc finger, matrin type 1 (ZMAT1), transcript variant 3, mRNA [NM_032441]	NM_032441
A_23_P340148	0.000674	6.892	NM_021998	NM_021998	Homo sapiens zinc finger protein 6 (CMPX1) (ZNF6), mRNA [NM_021998]	NM_021998
A_23_P210176	0.000674	4.2	NM_000210	NM_000210	Homo sapiens integrin, alpha 6 (ITGA6), mRNA [NM_000210]	NM_000210
A_23_P48717	0.000675	2.255	NM_006432	NM_006432	Homo sapiens Niemann-Pick disease, type C2 (NPC2), mRNA [NM_006432]	NM_006432
A_23_P321160	0.000676	6.366	AB058774	AB058774	Homo sapiens mRNA for KIAA1871 protein, partial cds. [AB058774]	XM_290737
A_24_P232763	0.000676	2.28	ENST00000242848		Homo sapiens mRNA for KIAA0853 protein, partial cds. [AB020660]	
A_32_P92505	0.000677	2.644	NM_182551	NM_182551	Homo sapiens lysocardiolipin acyltransferase (LYCAT), transcript variant 1, mRNA [NM_182551]	NM_182551
A_23_P413180	0.000678	6.19	NM_017440	NM_017440	Homo sapiens Mdm4, transformed 3T3 cell double minute 1, p53 binding protein (mouse) (MDM1), transcript variant 1, mRNA [NM_017440]	NM_017440
A_24_P818010	0.000681	6.022	AK097080	AK097080	Homo sapiens cDNA FLJ39761 fis, clone SPLEN1000083. [AK097080]	XM_496672
A_23_P42848	0.000681	2.957	NM_194455	NM_194455	Homo sapiens KRIT1, ankyrin repeat containing (KRIT1), transcript variant 4, mRNA [NM_194455]	NM_194455
A_32_P75661	0.000681	2.531	THC2309312			
A_32_P229618	0.000683	3.833	NM_175892	NM_175892	Homo sapiens hypothetical protein FLJ37266 (FLJ37266), mRNA [NM_175892]	NM_175892
A_23_P419202	0.000683	2.92	NM_033160	NM_033160	Homo sapiens zinc finger protein 658 (ZNF658), mRNA [NM_033160]	NM_033160
A_23_P434268	0.000684	3.787	AB029040	AB029040	Homo sapiens mRNA for KIAA1117 protein, partial cds. [AB029040]	
A_23_P416751	0.000684	2.043	NM_173530	NM_173530	Homo sapiens zinc finger protein 610 (ZNF610), mRNA [NM_173530]	NM_173530
A_24_P67898	0.000687	4.532	AF307332	AF307332	Homo sapiens meningioma-expressed antigen 5s splice variant mRNA, complete cds. [AF307332]	
A_24_P72518	0.000687	2.161	NM_015328	NM_015328	Homo sapiens KIAA0828 protein (KIAA0828), mRNA [NM_015328]	NM_015328
A_32_P189845	0.000689	4.635	NM_178558	NM_178558	Homo sapiens zinc finger protein 680 (ZNF680), mRNA [NM_178558]	NM_178558
A_24_P268893	0.000691	2.33	NM_144721	NM_144721	Homo sapiens THAP domain containing 6 (THAP6), mRNA [NM_144721]	NM_144721
A_23_P201376	0.000693	9.426	NM_014021	NM_014021	Homo sapiens synovial sarcoma, X breakpoint 2 interacting protein (SSX2IP), mRNA [NM_014021]	NM_014021
A_23_P69329	0.000693	7.07	NM_007312	NM_007312	Homo sapiens hyaluronoglucosaminidase 1 (HYAL1), transcript variant 1, mRNA [NM_007312]	NM_007312
A_23_P205098	0.000693	5.538	NM_015032	NM_015032	Homo sapiens androgen-induced proliferation inhibitor (APRIN), transcript variant 1, mRNA [NM_015032]	NM_015032
A_24_P153713	0.000693	2.11	BC005052	BC005052	Homo sapiens MARVEL domain containing 3, mRNA (cDNA clone IMAGE:2820819), partial cds. [BC005052]	
A_24_P312417	0.000694	2.13	AB046792	AB046792	Homo sapiens mRNA for KIAA1572 protein, partial cds. [AB046792]	
A_23_P214739	0.000695	2.16	NM_012160	NM_012160	Homo sapiens F-box and leucine-rich repeat protein 4 (FBXL4), mRNA [NM_012160]	NM_012160
A_24_P187706	0.000698	5.376	NM_002078	NM_002078	Homo sapiens golgi autoantigen, golgin subfamily a, 4 (GOLGA4), mRNA [NM_002078]	NM_002078
A_24_P153511	0.000698	2.228	NM_020841	NM_020841	Homo sapiens oxysterol binding protein-like 8 (OSBPL8), transcript variant 1, mRNA [NM_020841]	NM_020841
A_23_P90223	0.0007	3.008	NM_152279	NM_152279	Homo sapiens zinc finger protein 585B (ZNF585B), mRNA [NM_152279]	NM_152279
A_24_P114255	0.0007	2.029	NM_138799	NM_138799	Homo sapiens O-acyltransferase (membrane bound) domain containing 2 (OACT2), mRNA [NM_138799]	NM_138799
A_23_P110196	0.000701	30.34	NM_016323	NM_016323	Homo sapiens hect domain and RLD 5 (HERC5), mRNA [NM_016323]	NM_016323
A_24_P307695	0.000701	2.556	NM_033402	NM_033402	Homo sapiens KIAA1764 protein (KIAA1764), mRNA [NM_033402]	NM_033402
A_24_P267523	0.000702	9.319	NM_144613	NM_144613	Homo sapiens cytochrome c oxidase subunit VIb polypeptide 2 (testis) (COX6B2), mRNA [NM_144613]	NM_144613

A_23_P7033	0.000702	2.062	NM_022149	NM_022149	Homo sapiens melanoma antigen family F, 1 (MAGEF1), mRNA [NM_022149]	NM_022149
A_23_P123242	0.000703	4.615	NM_021163	NM_021163	Homo sapiens RB-associated KRAB repressor (RBAK), mRNA [NM_021163]	NM_021163
A_24_P417036	0.000703	2.552	NM_001001930	NM_001001930	Homo sapiens peroxisome proliferative activated receptor, alpha (PPARA), transcript variant 6, mRNA [NM_001001930]	NM_001001930
A_32_P86578	0.000704	13.95	BC032913	BC032913	Homo sapiens hypothetical gene supported by BC032913; BC048425, mRNA (cDNA clone IMAGE:5265535). [BC032913]	XM_374002
A_23_P78099	0.000706	10.02	NM_000638	NM_000638	Homo sapiens vitronectin (serum spreading factor, somatomedin B, complement S-protein) (VTN), mRNA [NM_000638]	NM_000638
A_24_P206427	0.000706	3.931	NM_001568	NM_001568	Homo sapiens eukaryotic translation initiation factor 3, subunit 6 48kDa (EIF3S6), mRNA [NM_001568]	NM_001568
A_23_P208991	0.000706	2.47	NM_002579	NM_002579	Homo sapiens paralemmin (PALM), mRNA [NM_002579]	NM_002579
A_23_P118544	0.000707	2.068	NM_004859	NM_004859	Homo sapiens clathrin, heavy polypeptide (Hc) (CLTC), mRNA [NM_004859]	NM_004859
A_24_P413941	0.000708	2.553	NM_153689	NM_153689	Homo sapiens hypothetical protein FLJ38973 (FLJ38973), mRNA [NM_153689]	NM_153689
A_24_P914513	0.000708	2.118	NM_183050	NM_183050	Homo sapiens branched chain keto acid dehydrogenase E1, beta polypeptide (maple syrup urine disease) (BCKDHB), nuclear gene encoding mitochondrial protein, transcript variant 1, mRNA [NM_183050]	NM_183050
A_23_P34710	0.000708	2.043	ENST00000357180		full-length cDNA clone CS0DF032YO23 of Fetal brain of Homo sapiens (human). [CR604521]	
A_32_P22989	0.00071	2.668	A_32_P22989			
A_32_P74120	0.000716	2.382	BC070363	BC070363	Homo sapiens cDNA clone IMAGE:3960708, partial cds. [BC070363]	XM_378841
A_23_P153037	0.000717	3.304	NM_020787	NM_020787	Homo sapiens zinc finger protein 624 (ZNF624), mRNA [NM_020787]	NM_020787
A_23_P171074	0.000718	31.22	NM_004867	NM_004867	Homo sapiens integral membrane protein 2A (ITM2A), mRNA [NM_004867]	NM_004867
A_24_P128255	0.000718	7.536	THC2275950			
A_23_P434212	0.000718	4.806	NM_177529	NM_177529	Homo sapiens sulfotransferase family, cytosolic, 1A, phenol-preferring, member 1 (SULT1A1), transcript variant 2, mRNA [NM_177529]	NM_177529
A_24_P19268	0.000718	4.572	NM_021269	NM_021269	Homo sapiens zinc finger protein 15-like 1 (KOX 8) (ZNF15L1), mRNA [NM_021269]	NM_021269
A_32_P128974	0.000718	3.543	AW971123	AW971123	EST383210 MAGE resequences, MAGL Homo sapiens cDNA, mRNA sequence [AW971123]	
A_23_P101905	0.000718	3.506	NM_005883	NM_005883	Homo sapiens adenomatosis polyposis coli 2 (APC2), mRNA [NM_005883]	NM_005883
A_23_P109733	0.000718	2.511	NM_144718	NM_144718	Homo sapiens hypothetical protein AY099107 (LOC152185), mRNA [NM_144718]	NM_144718
A_23_P431410	0.000718	2.355	BC065192	BC065192	Homo sapiens chromosome 2 open reading frame 12, mRNA (cDNA clone IMAGE:5205269), partial cds. [BC065192]	
A_24_P871940	0.000719	17.16	BX537788	BX537788	Homo sapiens mRNA; cDNA DKFZp686P1449 (from clone DKFZp686P1449) [BX537788]	
A_23_P88404	0.000719	8.932	NM_003239	NM_003239	Homo sapiens transforming growth factor, beta 3 (TGFB3), mRNA [NM_003239]	NM_003239
A_23_P121441	0.000719	6.766	NM_014893	NM_014893	Homo sapiens neuroligin 4, Y-linked (NLGN4Y), mRNA [NM_014893]	NM_014893
A_23_P88470	0.00072	3.925	NM_017672	NM_017672	Homo sapiens transient receptor potential cation channel, subfamily M, member 7 (TRPM7), mRNA [NM_017672]	NM_017672
A_23_P206092	0.00072	2.46	NM_032808	NM_032808	Homo sapiens leucine rich repeat neuronal 6A (LRRN6A), mRNA [NM_032808]	NM_032808
A_24_P123601	0.000722	4.188	NM_013994	NM_013994	Homo sapiens discoidin domain receptor family, member 1 (DDR1), transcript variant 3, mRNA [NM_013994]	NM_013994
A_23_P119916	0.000724	5.543	NM_006522	NM_006522	Homo sapiens wingless-type MMTV integration site family, member 6 (WNT6), mRNA [NM_006522]	NM_006522
A_32_P5276	0.000726	2.55	AB073386	AB073386	Homo sapiens infant liver cDNA, clone:HMFN1864, full insert sequence. [AB073386]	
A_32_P31827	0.000727	4.718	THC2279918		Cobalt transport protein cbiQ {Bacillus cereus ATCC 14579;} , partial (10%) [THC2279918]	
A_24_P64653	0.000728	3.104	NM_152637	NM_152637	Homo sapiens hypothetical protein MGC17301 (MGC17301), mRNA [NM_152637]	NM_152637
A_24_P397386	0.000731	25.99	BX648635	BX648635	Homo sapiens mRNA; cDNA DKFZp686C03120 (from clone DKFZp686C03120). [BX648635]	
A_23_P2831	0.000732	29.13	NM_003991	NM_003991	Homo sapiens endothelin receptor type B (EDNRB), transcript variant 2, mRNA [NM_003991]	NM_003991
A_32_P132276	0.000733	2.67	BE091362	BE091362	BE091362 PM1-BT0728-220300-001-f09 BT0728 Homo sapiens cDNA, mRNA sequence [BE091362]	
A_24_P234792	0.000733	2.006	THC2277727		KC13_RAT (Q62763) Casein kinase I, gamma 3 isoform (CKI-gamma 3) , partial (38%) [THC2277727]	
A_24_P158314	0.000734	6.805	NM_032293	NM_032293	Homo sapiens GTPase activating Rap/RanGAP domain-like 3 (GARNL3), mRNA [NM_032293]	NM_032293
A_23_P160214	0.000735	10.66	BC028374	BC028374	Homo sapiens chromosome 1 open reading frame 34, mRNA (cDNA clone IMAGE:4827153), partial cds. [BC028374]	XM_375729
A_23_P29153	0.000735	2.662	NM_014433	NM_014433	Homo sapiens rhabdoid tumor deletion region gene 1 (RTDR1), mRNA [NM_014433]	NM_014433
A_32_P169179	0.000736	5.289	NR_002307	NR_002307	Homo sapiens msh homeobox homolog 2 (Drosophila) pseudogene (MSX2P) on chromosome 17 [NR_002307]	NR_002307

A_23_P133470	0.000736	4.335	NM_014819	NM_014819	Homo sapiens praja 2, RING-H2 motif containing (PJA2), mRNA [NM_014819]	NM_014819
A_32_P82863	0.000738	3.756	BX538288	BX538288	Homo sapiens mRNA; cDNA DKFZp779O175 (from clone DKFZp779O175). [BX538288]	XM_375606
A_23_P16006	0.000738	2.869	NM_198457	NM_198457	Homo sapiens zinc finger protein 600 (ZNF600), mRNA [NM_198457]	NM_198457
A_23_P150950	0.000739	2.769	NM_144982	NM_144982	Homo sapiens hypothetical protein MGC23401 (MGC23401), mRNA [NM_144982]	NM_144982
A_23_P94063	0.000739	2.671	AY260738	AY260738	Homo sapiens truncated zinc finger protein 36 mRNA, complete cds. [AY260738]	
A_24_P876522	0.000739	2.428	AK022110	AK022110	Homo sapiens cDNA FLJ12048 fis, clone HEMBB1001990. [AK022110]	
A_23_P380526	0.000743	6.278	NM_018189	NM_018189	Homo sapiens developmental pluripotency associated 4 (DPPA4), mRNA [NM_018189]	NM_018189
A_24_P208567	0.000743	3.497	NM_003855	NM_003855	Homo sapiens interleukin 18 receptor 1 (IL18R1), mRNA [NM_003855]	NM_003855
A_23_P203183	0.000746	53.72	NM_000040	NM_000040	Homo sapiens apolipoprotein C-III (APOC3), mRNA [NM_000040]	NM_000040
A_23_P98350	0.000746	2.577	NM_001165	NM_001165	Homo sapiens baculoviral IAP repeat-containing 3 (BIRC3), transcript variant 1, mRNA [NM_001165]	NM_001165
A_23_P33759	0.000747	5.731	NM_004753	NM_004753	Homo sapiens dehydrogenase/reductase (SDR family) member 3 (DHRS3), mRNA [NM_004753]	NM_004753
A_24_P8371	0.000748	2.33	ENST00000329078		Homo sapiens, Similar to spinster-like protein, clone IMAGE:4814561, mRNA, partial cds. [BC041772]	
A_24_P781846	0.000749	6.039	AK024092	AK024092	Homo sapiens cDNA FLJ14030 fis, clone HEMBA1004086. [AK024092]	
A_23_P259328	0.000749	2.047	NM_003630	NM_003630	Homo sapiens peroxisomal biogenesis factor 3 (PEX3), mRNA [NM_003630]	NM_003630
A_32_P194423	0.00075	11.71	THC2408398			
A_24_P460763	0.000751	3.547	AK022443	AK022443	Homo sapiens cDNA FLJ12381 fis, clone MAMMA1002566. [AK022443]	
A_32_P64570	0.000751	2.143	NM_153186	NM_153186	Homo sapiens ankyrin repeat domain 15 (ANKRD15), transcript variant 2, mRNA [NM_153186]	NM_153186
A_24_P923011	0.000756	9.774	NM_000301	NM_000301	Homo sapiens plasminogen (PLG), mRNA [NM_000301]	NM_000301
A_23_P86550	0.000756	2.484	NM_003750	NM_003750	Homo sapiens eukaryotic translation initiation factor 3, subunit 10 theta, 150/170kDa (EIF3S10), mRNA [NM_003750]	NM_003750
A_32_P71113	0.000757	6.824	A_32_P71113			
A_24_P345679	0.000758	3.612	ENST00000355893		Homo sapiens mRNA; cDNA DKFZp686A04129 (from clone DKFZp686A04129) [BX641078]	
A_23_P146417	0.000758	2.486	NM_032012	NM_032012	Homo sapiens chromosome 9 open reading frame 5 (C9orf5), mRNA [NM_032012]	NM_032012
A_23_P166663	0.00076	3.336	NM_012096	NM_012096	Homo sapiens adaptor protein containing pH domain, PTB domain and leucine zipper motif 1 (APPL), mRNA [NM_012096]	NM_012096
A_23_P216068	0.00076	2.968	NM_014109	NM_014109	Homo sapiens ATPase family, AAA domain containing 2 (ATAD2), mRNA [NM_014109]	NM_014109
A_24_P365322	0.00076	2.264	AK095600	AK095600	Homo sapiens cDNA FLJ38281 fis, clone FCBBF3005729, moderately similar to Homo sapiens GIOT-4 mRNA for gonadotropin inducible transcription repressor-4. [AK095600]	
A_23_P89780	0.00076	2.144	NM_198129	NM_198129	Homo sapiens laminin, alpha 3 (LAMA3), transcript variant 1, mRNA [NM_198129]	NM_198129
A_23_P140434	0.000764	10.32	NM_018728	NM_018728	Homo sapiens myosin VC (MYO5C), mRNA [NM_018728]	NM_018728
A_32_P94801	0.000764	7.033	THC2308876			
A_32_P185701	0.000764	5.934	BC041959	BC041959	Homo sapiens, clone IMAGE:5302136, mRNA. [BC041959]	
A_32_P153725	0.000764	3.55	NM_015275	NM_015275	Homo sapiens KIAA1033 (KIAA1033), mRNA [NM_015275]	NM_015275
A_23_P48713	0.000764	2.23	NM_152444	NM_152444	Homo sapiens zinc binding alcohol dehydrogenase, domain containing 1 (ZADH1), mRNA [NM_152444]	NM_152444
A_23_P106682	0.000765	8.247	NM_001424	NM_001424	Homo sapiens epithelial membrane protein 2 (EMP2), mRNA [NM_001424]	NM_001424
A_23_P6561	0.000768	4.31	NM_018029	NM_018029	Homo sapiens hypothetical protein FLJ10213 (FLJ10213), mRNA [NM_018029]	NM_018029
A_32_P158786	0.000768	2.587	ENST00000333722		Homo sapiens, clone IMAGE:5271073, mRNA, partial cds. [BC039369]	XM_056680
A_32_P183983	0.000771	2.996	BC037838	BC037838	Homo sapiens, clone IMAGE:4813920, mRNA. [BC037838]	
A_23_P210100	0.000772	4.379	NM_019885	NM_019885	Homo sapiens cytochrome P450, family 26, subfamily B, polypeptide 1 (CYP26B1), mRNA [NM_019885]	NM_019885
A_23_P103588	0.000773	5.948	NM_005518	NM_005518	Homo sapiens 3-hydroxy-3-methylglutaryl-Coenzyme A synthase 2 (mitochondrial) (HMGCS2), mRNA [NM_005518]	NM_005518
A_24_P208961	0.000773	2.856	NM_133370	NM_133370	Homo sapiens splicing factor YT521-B (YT521), mRNA [NM_133370]	NM_133370
A_24_P165423	0.000774	3.358	NM_052960	NM_052960	Homo sapiens retinol binding protein 7, cellular (RBP7), mRNA [NM_052960]	NM_052960
A_32_P84772	0.000778	2.988	NM_001008401	NM_001008401	Homo sapiens FLJ16231 protein (FLJ16231), mRNA [NM_001008401]	NM_001008401

A_32_P157471	0.000781	2.979	CF143262	CF143262	CF143262 UI-HF-BR0p-aqt-a-11-0-UI.r1 NIH_MGC_52 Homo sapiens cDNA clone IMAGE:3101469 5', mRNA sequence [CF143262]	
A_24_P791829	0.000783	3.473	THC2305590			
A_23_P391396	0.000784	3.1	NM_001005463	NM_001005463	Homo sapiens early B-cell factor 3 (EBF3), mRNA [NM_001005463]	NM_001005463
A_23_P216596	0.000785	11.57	AK075235	AK075235	Homo sapiens cDNA FLJ90754 fis, clone PLACE4000354, weakly similar to COMPLEMENT RECEPTOR TYPE 1 PRECURSOR. [AK075235]	
A_23_P19102	0.000785	3.325	AK021777	AK021777	Homo sapiens cDNA FLJ11715 fis, clone HEMBA1005223. [AK021777]	
A_32_P200947	0.000786	2.744	BC035518	BC035518	Homo sapiens, clone IMAGE:4214654, mRNA. [BC035518]	
A_23_P209459	0.000787	3.07	NM_004482	NM_004482	Homo sapiens UDP-N-acetyl-alpha-D-galactosamine:polypeptide N-acetylgalactosaminyltransferase 3 (GalNAc-T3) (GALNT3), mRNA [NM_004482]	NM_004482
A_24_P456452	0.000788	3.121	AK074562	AK074562	Homo sapiens cDNA FLJ90081 fis, clone HEMBA1004952. [AK074562]	
A_23_P256342	0.000791	2.276	BC045667	BC045667	Homo sapiens sorting nexin 13, mRNA (cDNA clone MGC:44494 IMAGE:5298570), complete cds. [BC045667]	
A_23_P106773	0.000795	7.384	NM_177528	NM_177528	Homo sapiens sulfotransferase family, cytosolic, 1A, phenol-preferring, member 2 (SULT1A2), transcript variant 2, mRNA [NM_177528]	NM_177528
A_23_P308150	0.000795	2.87	NM_152424	NM_152424	Homo sapiens hypothetical protein FLJ39827 (FLJ39827), mRNA [NM_152424]	NM_152424
A_23_P401774	0.000798	21.51	NM_018712	NM_018712	Homo sapiens ELMO domain containing 1 (ELMOD1), mRNA [NM_018712]	NM_018712
A_32_P55241	0.0008	8.554	NM_001007538	NM_001007538	Homo sapiens transmembrane protein 46 (TMEM46), mRNA [NM_001007538]	NM_001007538
A_23_P30666	0.000801	3.958	NM_014452	NM_014452	Homo sapiens tumor necrosis factor receptor superfamily, member 21 (TNFRSF21), mRNA [NM_014452]	NM_014452
A_23_P37914	0.000802	8.071	NM_052944	NM_052944	Homo sapiens solute carrier family 5 (sodium/glucose cotransporter), member 11 (SLC5A11), mRNA [NM_052944]	NM_052944
A_23_P56734	0.000802	2.895	NM_006895	NM_006895	Homo sapiens histamine N-methyltransferase (HNMT), transcript variant 1, mRNA [NM_006895]	NM_006895
A_23_P430140	0.000803	2.578	AF090930	AF090930	Homo sapiens clone HQ0478 PRO0478 mRNA, complete cds. [AF090930]	
A_23_P93311	0.000804	2.86	NM_013994	NM_013994	Homo sapiens discoidin domain receptor family, member 1 (DDR1), transcript variant 3, mRNA [NM_013994]	NM_013994
A_23_P373054	0.000805	2.274	NM_173826	NM_173826	Homo sapiens chromosome 3 open reading frame 23 (C3orf23), transcript variant 1, mRNA [NM_173826]	NM_173826
A_23_P55601	0.000806	2.134	NM_007345	NM_007345	Homo sapiens zinc finger protein 236 (ZNF236), mRNA [NM_007345]	NM_007345
A_23_P429977	0.000808	13.54	NM_000218	NM_000218	Homo sapiens potassium voltage-gated channel, KQT-like subfamily, member 1 (KCNQ1), transcript variant 1, mRNA [NM_000218]	NM_000218
A_24_P491923	0.000809	2.441	THC2273623			
A_23_P4082	0.000809	2.073	NM_006584	NM_006584	Homo sapiens chaperonin containing TCP1, subunit 6B (zeta 2) (CCT6B), mRNA [NM_006584]	NM_006584
A_32_P72477	0.00081	6.62	A_32_P72477			
A_32_P221774	0.000811	12.91	THC2283319			
A_32_P188860	0.000812	15.88	AK125591	AK125591	Homo sapiens cDNA FLJ43603 fis, clone SPLEN2005767. [AK125591]	
A_32_P209735	0.000813	6.187	BC048193	BC048193	Homo sapiens, clone IMAGE:4590099, mRNA. [BC048193]	XM_496741
A_24_P167642	0.000813	3.764	NM_000161	NM_000161	Homo sapiens GTP cyclohydrolase 1 (dopa-responsive dystonia) (GCH1), transcript variant 1, mRNA [NM_000161]	NM_000161
A_23_P74042	0.000815	6.952	NM_012302	NM_012302	Homo sapiens latrophilin 2 (LPHN2), mRNA [NM_012302]	NM_012302
A_24_P675731	0.000815	5.505	THC2316878			
A_23_P77328	0.000815	3.794	NM_005258	NM_005258	Homo sapiens GTP cyclohydrolase I feedback regulator (GCHFR), mRNA [NM_005258]	NM_005258
A_24_P691775	0.000815	2.373	ENST00000340510		PREDICTED: Homo sapiens hypothetical gene supported by AK097404; NM_198284 (LOC440726), mRNA [XM_498835]	XM_498835
A_23_P95810	0.000815	2.325	NM_007183	NM_007183	Homo sapiens plakophilin 3 (PKP3), mRNA [NM_007183]	NM_007183
A_24_P886966	0.000815	2.211	AK123483	AK123483	Homo sapiens cDNA FLJ41489 fis, clone BRTHA2004582. [AK123483]	
A_23_P117363	0.000816	11.23	NM_001756	NM_001756	Homo sapiens serine (or cysteine) proteinase inhibitor, clade A (alpha-1 antiproteinase, antitrypsin), member 6 (SERPINA6), mRNA [NM_001756]	NM_001756
A_24_P97145	0.000817	3.504	NM_020738	NM_020738	Homo sapiens kinase D-interacting substance of 220 kDa (KIDINS220), mRNA [NM_020738]	NM_020738
A_24_P688133	0.000817	2.901	AK124299	AK124299	Homo sapiens cDNA FLJ42306 fis, clone TRACH2001646. [AK124299]	
A_32_P228618	0.000819	2.35	NM_001003793	NM_001003793	Homo sapiens RNA binding motif, single stranded interacting protein (RBMS3), transcript variant 1, mRNA [NM_001003793]	NM_001003793
A_23_P101642	0.000821	5.516	NM_002842	NM_002842	Homo sapiens protein tyrosine phosphatase, receptor type, H (PTPRH), mRNA [NM_002842]	NM_002842

A_23_P140316	0.000822	6.135	ENST00000321662		Homo sapiens mRNA; cDNA DKFZp762F0713 (from clone DKFZp762F0713). [AL834372]	XM_290615
A_24_P22746	0.000824	3.122	THC2336861		Q96FJ6 (Q96FJ6) Zinc binding alcohol dehydrogenase, domain containing 2, complete [THC2336861]	
A_24_P76898	0.000824	2.2	ENST00000328724		Homo sapiens mRNA; cDNA DKFZp761H0317 (from clone DKFZp761H0317). [AL834350]	
A_24_P90216	0.000827	9.259	NM_018490	NM_018490	Homo sapiens leucine-rich repeat-containing G protein-coupled receptor 4 (LGR4), mRNA [NM_018490]	NM_018490
A_23_P308954	0.00083	3.738	NM_030639	NM_030639	Homo sapiens basic helix-loop-helix domain containing, class B, 9 (BHLHB9), mRNA [NM_030639]	NM_030639
A_24_P524462	0.000831	2.526	A_24_P524462			
A_23_P46769	0.000832	2.784	AK126751	AK126751	Homo sapiens cDNA FLJ44798 fis, clone BRACE3040863. [AK126751]	
A_24_P165656	0.000833	2.398	NM_005813	NM_005813	Homo sapiens protein kinase D3 (PRKD3), mRNA [NM_005813]	NM_005813
A_23_P259462	0.000834	7.293	NM_206841	NM_206841	Homo sapiens Fraser syndrome 1 (FRAS1), transcript variant 2, mRNA [NM_206841]	NM_206841
A_24_P150791	0.000834	3.117	NM_020655	NM_020655	Homo sapiens junctophilin 3 (JPH3), mRNA [NM_020655]	NM_020655
A_24_P934800	0.000834	2.226	AB040937	AB040937	Homo sapiens mRNA for KIAA1504 protein, partial cds. [AB040937]	
A_24_P46953	0.000835	3.764	NM_013257	NM_013257	Homo sapiens serum/glucocorticoid regulated kinase-like (SGKL), transcript variant 1, mRNA [NM_013257]	NM_013257
A_32_P16323	0.000836	2.952	ENST00000355077		Homo sapiens cDNA clone IMAGE:30389199, with apparent retained intron. [BC063888]	
A_23_P91414	0.000837	8.181	NM_080625	NM_080625	Homo sapiens chromosome 20 open reading frame 160 (C20orf160), mRNA [NM_080625]	NM_080625
A_23_P78053	0.000837	4.389	NM_030802	NM_030802	Homo sapiens C/EBP-induced protein (LOC81558), mRNA [NM_030802]	NM_030802
A_24_P570049	0.000837	2.377	NM_001001930	NM_001001930	Homo sapiens peroxisome proliferative activated receptor, alpha (PPARA), transcript variant 6, mRNA [NM_001001930]	NM_001001930
A_23_P92222	0.000842	12.21	NM_152673	NM_152673	Homo sapiens mucin 20 (MUC20), mRNA [NM_152673]	NM_152673
A_23_P79488	0.000842	3.848	NM_172311	NM_172311	Homo sapiens stoned B/TFIIA-alpha/beta-like factor (SALF), mRNA [NM_172311]	NM_172311
A_24_P722068	0.000842	3.06	THC2268343			
A_24_P298077	0.000842	2.79	NM_017664	NM_017664	Homo sapiens ankyrin repeat domain 10 (ANKRD10), mRNA [NM_017664]	NM_017664
A_32_P46495	0.000845	2.674	BX090412	BX090412	BX090412 Soares_testis_NHT Homo sapiens cDNA clone IMAGp998J221862 ; IMAGE:757365, mRNA sequence [BX090412]	
A_23_P395595	0.000846	2.942	NM_015308	NM_015308	Homo sapiens formin binding protein 4 (FNBP4), mRNA [NM_015308]	NM_015308
A_23_P58117	0.000848	2.496	NM_020859	NM_020859	Homo sapiens shroom (SHRM), mRNA [NM_020859]	NM_020859
A_24_P28657	0.000849	3.833	NM_015446	NM_015446	Homo sapiens AT hook containing transcription factor 1 (AHCTF1), mRNA [NM_015446]	NM_015446
A_24_P257151	0.000852	2.472	NM_004071	NM_004071	Homo sapiens CDC-like kinase 1 (CLK1), transcript variant 1, mRNA [NM_004071]	NM_004071
A_23_P500130	0.000855	2.991	NM_153186	NM_153186	Homo sapiens ankyrin repeat domain 15 (ANKRD15), transcript variant 2, mRNA [NM_153186]	NM_153186
A_32_P187817	0.000856	6.622	AW967501	AW967501	EST379576 MAGE resequences, MAGJ Homo sapiens cDNA, mRNA sequence [AW967501]	
A_23_P351204	0.000856	4.549	NM_172109	NM_172109	Homo sapiens potassium voltage-gated channel, KQT-like subfamily, member 2 (KCNQ2), transcript variant 5, mRNA [NM_172109]	NM_172109
A_24_P69379	0.000858	3.463	NM_022900	NM_022900	Homo sapiens O-acetyltransferase (CAS1), mRNA [NM_022900]	NM_022900
A_23_P132784	0.000858	2.411	NM_001013439	NM_001013439	Homo sapiens fragile X mental retardation, autosomal homolog 1 (FXR1), transcript variant 3, mRNA [NM_001013439]	NM_001013439
A_23_P378690	0.00086	3.74	CR610885	CR610885	full-length cDNA clone CS0DC019YC18 of Neuroblastoma Cot 25-normalized of Homo sapiens (human). [CR610885]	
A_24_P134653	0.00086	2.324	NM_003611	NM_003611	Homo sapiens oral-facial-digital syndrome 1 (OFD1), mRNA [NM_003611]	NM_003611
A_24_P173234	0.000862	2.804	NM_024840	NM_024840	Homo sapiens zinc finger protein 613 (ZNF613), mRNA [NM_024840]	NM_024840
A_24_P126628	0.000863	2.387	NM_015257	NM_015257	Homo sapiens KIAA0286 protein (KIAA0286), mRNA [NM_015257]	NM_015257
A_32_P47538	0.000865	14.79	BC037919	BC037919	Homo sapiens, clone IMAGE:5278089, mRNA. [BC037919]	
A_23_P125303	0.000868	12.23	NM_001555	NM_001555	Homo sapiens immunoglobulin superfamily, member 1 (IGSF1), transcript variant 1, mRNA [NM_001555]	NM_001555
A_24_P320880	0.000869	2.109	NM_022133	NM_022133	Homo sapiens sorting nexin 16 (SNX16), transcript variant 1, mRNA [NM_022133]	NM_022133
A_23_P93141	0.000871	82.07	NM_153699	NM_153699	Homo sapiens glutathione S-transferase A5 (GSTA5), mRNA [NM_153699]	NM_153699
A_23_P204689	0.000871	2.81	NM_001004419	NM_001004419	Homo sapiens C-type lectin superfamily 2, member D (CLEC2D), transcript variant 2, mRNA [NM_001004419]	NM_001004419
A_23_P254442	0.000871	2.577	NM_001004302	NM_001004302	Homo sapiens hypothetical protein LOC155060 (LOC155060), mRNA [NM_001004302]	NM_001004302
A_23_P119923	0.000871	2.242	NM_020184	NM_020184	Homo sapiens cyclin M4 (CNNM4), mRNA [NM_020184]	NM_020184

A_32_P179686	0.000873	4.93	BX641027	BX641027	Homo sapiens mRNA; cDNA DKFZp686O10247 (from clone DKFZp686O10247). [BX641027]	
A_23_P90359	0.000874	20.83	NM_004558	NM_004558	Homo sapiens neurturin (NRTN), mRNA [NM_004558]	NM_004558
A_24_P943957	0.000874	3.832	NM_015040	NM_015040	Homo sapiens phosphatidylinositol-3-phosphate/phosphatidylinositol 5-kinase, type III (PIP5K3), transcript variant 2, mRNA [NM_015040]	NM_015040
A_24_P159227	0.000877	2.753	NM_020168	NM_020168	Homo sapiens p21(CDKN1A)-activated kinase 6 (PAK6), mRNA [NM_020168]	NM_020168
A_24_P491087	0.000879	2.186	AK092180	AK092180	Homo sapiens cDNA FLJ34861 fis, clone NT2NE2012847. [AK092180]	
A_24_P403959	0.000881	2.58	NM_198232	NM_198232	Homo sapiens ribonuclease, RNase A family, 1 (pancreatic) (RNASE1), transcript variant 3, mRNA [NM_198232]	NM_198232
A_23_P393099	0.000882	4.201	NM_003226	NM_003226	Homo sapiens trefoil factor 3 (intestinal) (TFF3), mRNA [NM_003226]	NM_003226
A_23_P356122	0.000882	3.319	NM_015555	NM_015555	Homo sapiens zinc finger protein 451 (ZNF451), mRNA [NM_015555]	NM_015555
A_23_P101208	0.000882	2.748	NM_001914	NM_001914	Homo sapiens cytochrome b-5 (CYB5), transcript variant 2, mRNA [NM_001914]	NM_001914
A_23_P395075	0.000885	3.094	NM_018433	NM_018433	Homo sapiens jumonji domain containing 1A (JMJD1A), mRNA [NM_018433]	NM_018433
A_23_P317756	0.000886	4.09	NM_202000	NM_202000	Homo sapiens SA hypertension-associated homolog (rat) (SAH), transcript variant 2, mRNA [NM_202000]	NM_202000
A_23_P104471	0.000886	3.518	NM_001007271	NM_001007271	Homo sapiens dual specificity phosphatase 13 (DUSP13), transcript variant 1, mRNA [NM_001007271]	NM_001007271
A_24_P917123	0.000888	3.662	NM_013262	NM_013262	Homo sapiens myosin regulatory light chain interacting protein (MYLIP), mRNA [NM_013262]	NM_013262
A_32_P83453	0.000889	3.663	XM_208438	XM_208438	PREDICTED: Homo sapiens similar to Tetratricopeptide repeat protein 3 (TPR repeat protein D) (LOC286495), mRNA [XM_208438]	XM_208438
A_24_P456723	0.000889	3.068	BC039374	BC039374	Homo sapiens, clone IMAGE:5271446, mRNA. [BC039374]	
A_24_P284584	0.00089	2.129	NM_032497	NM_032497	Homo sapiens zinc finger protein 559 (ZNF559), mRNA [NM_032497]	NM_032497
A_23_P428468	0.000891	7.461	BC063594	BC063594	Homo sapiens zinc finger protein 292, mRNA (cDNA clone IMAGE:4526801), partial cds. [BC063594]	XM_048070
A_24_P246406	0.000891	2.367	CR600872	CR600872	full-length cDNA clone CS0DF015YL14 of Fetal brain of Homo sapiens (human). [CR600872]	
A_23_P30693	0.000892	73.89	NM_000301	NM_000301	Homo sapiens plasminogen (PLG), mRNA [NM_000301]	NM_000301
A_23_P164650	0.000892	28.78	NM_000041	NM_000041	Homo sapiens apolipoprotein E (APOE), mRNA [NM_000041]	NM_000041
A_24_P930707	0.000892	2.137	THC2368606		BX098637 BX098637 Soares fetal liver spleen 1NFLS Homo sapiens cDNA clone IMAGp998F16386 ; IMAGE:200847, mRNA sequence [BX098637]	
A_24_P360078	0.000893	6.139	NM_006726	NM_006726	Homo sapiens LPS-responsive vesicle trafficking, beach and anchor containing (LRBA), mRNA [NM_006726]	NM_006726
A_23_P217570	0.000898	11.03	NM_014289	NM_014289	Homo sapiens calpain 6 (CAPN6), mRNA [NM_014289]	NM_014289
A_23_P421175	0.000899	2.726	BC033256	BC033256	Homo sapiens FLJ46072 protein, mRNA (cDNA clone IMAGE:5455669), partial cds. [BC033256]	
A_32_P49764	0.000901	5.041	THC2394165		DBP_HUMAN (Q10586) D-site-binding protein (Albumin D box-binding protein) (TAXREB302), partial (6%) [THC2394165]	
A_23_P24176	0.000905	2.352	NM_019084	NM_019084	Homo sapiens cyclin J (CCNJ), mRNA [NM_019084]	NM_019084
A_23_P89841	0.00091	3.573	A_23_P89841			
A_24_P114739	0.00091	3.468	NM_172127	NM_172127	Homo sapiens calcium/calmodulin-dependent protein kinase (CaM kinase) II delta (CAMK2D), transcript variant 1, mRNA [NM_172127]	NM_172127
A_32_P34522	0.00091	3.109	BC010544	BC010544	Homo sapiens cDNA clone IMAGE:3462401, partial cds. [BC010544]	
A_23_P134953	0.00091	2.394	NM_001122	NM_001122	Homo sapiens adipose differentiation-related protein (ADFP), mRNA [NM_001122]	NM_001122
A_23_P214300	0.000911	35.91	NM_000846	NM_000846	Homo sapiens glutathione S-transferase A2 (GSTA2), mRNA [NM_000846]	NM_000846
A_23_P202435	0.000911	4.406	NM_016824	NM_016824	Homo sapiens adducin 3 (gamma) (ADD3), transcript variant 1, mRNA [NM_016824]	NM_016824
A_24_P123720	0.000911	2.275	A_24_P123720			
A_23_P116235	0.000912	3.903	NM_001012334	NM_001012334	Homo sapiens midkine (neurite growth-promoting factor 2) (MDK), transcript variant 1, mRNA [NM_001012334]	NM_001012334
A_23_P25873	0.000912	2.872	NM_007086	NM_007086	Homo sapiens WD repeat and HMG-box DNA binding protein 1 (WDHD1), transcript variant 1, mRNA [NM_007086]	NM_007086
A_24_P532212	0.000912	2.031	AK026896	AK026896	Homo sapiens cDNA: FLJ23243 fis, clone COL01757. [AK026896]	
A_24_P262201	0.000913	3.824	NM_001017389	NM_001017389	Homo sapiens sulfotransferase family, cytosolic, 1A, phenol-preferring, member 4 (SULT1A4), transcript variant 1, mRNA [NM_001017389]	NM_001017389
A_23_P12363	0.000916	3.345	NM_005012	NM_005012	Homo sapiens receptor tyrosine kinase-like orphan receptor 1 (ROR1), mRNA [NM_005012]	NM_005012
A_23_P43175	0.00092	2.481	NM_144710	NM_144710	Homo sapiens septin 10 (SEPT10), transcript variant 1, mRNA [NM_144710]	NM_144710

A_32_P134427	0.000923	2.382	BC048201	BC048201	Homo sapiens, clone IMAGE:3660074, mRNA. [BC048201]	
A_23_P164284	0.000923	2.178	NM_001307	NM_001307	Homo sapiens claudin 7 (CLDN7), mRNA [NM_001307]	NM_001307
A_23_P126613	0.000926	4.527	NM_080429	NM_080429	Homo sapiens aquaporin 10 (AQP10), mRNA [NM_080429]	NM_080429
A_23_P347198	0.000928	4.34	NM_003111	NM_003111	Homo sapiens Sp3 transcription factor (SP3), transcript variant 1, mRNA [NM_003111]	NM_003111
A_24_P414556	0.000929	2.537	NM_012382	NM_012382	Homo sapiens osmosis responsive factor (OSRF), mRNA [NM_012382]	NM_012382
A_23_P98022	0.000929	2.258	NM_012238	NM_012238	Homo sapiens sirtuin (silent mating type information regulation 2 homolog) 1 (S. cerevisiae) (SIRT1), mRNA [NM_012238]	NM_012238
A_23_P59657	0.000929	2.022	NM_003592	NM_003592	Homo sapiens cullin 1 (CUL1), mRNA [NM_003592]	NM_003592
A_24_P101651	0.00093	2.39	NM_001025306	NM_001025306	Homo sapiens CSAG family, member 4 (CSAG4), mRNA [NM_001025306]	NM_001025306
A_23_P114057	0.000932	3.198	NM_017789	NM_017789	Homo sapiens sema domain, immunoglobulin domain (Ig), transmembrane domain (TM) and short cytoplasmic domain, (semaphorin) 4C (SEMA4C), mRNA [NM_017789]	NM_017789
A_23_P61674	0.000933	5.692	NM_020666	NM_020666	Homo sapiens CDC-like kinase 4 (CLK4), mRNA [NM_020666]	NM_020666
A_32_P10936	0.000935	14.8	NM_004061	NM_004061	Homo sapiens cadherin 12, type 2 (N-cadherin 2) (CDH12), mRNA [NM_004061]	NM_004061
A_32_P109835	0.000935	2.575	THC2316236			
A_32_P115606	0.000935	2.232	AK131385	AK131385	Homo sapiens cDNA FLJ16460 fis, clone BRCAN2018240. [AK131385]	
A_23_P150325	0.000937	4.163	NM_032021	NM_032021	Homo sapiens AD031 protein (AD031), mRNA [NM_032021]	NM_032021
A_32_P136800	0.000937	2.776	A_32_P136800			
A_24_P79529	0.000939	4.544	NM_203311	NM_203311	Homo sapiens CSAG family, member 3A (CSAG3A), mRNA [NM_203311]	NM_203311
A_24_P42495	0.000939	2.831	BC010544	BC010544	Homo sapiens cDNA clone IMAGE:3462401, partial cds. [BC010544]	
A_32_P204048	0.000941	28.1	CR601260	CR601260	full-length cDNA clone CS0DM001YA20 of Fetal liver of Homo sapiens (human). [CR601260]	
A_23_P43476	0.000941	5.068	NM_003383	NM_003383	Homo sapiens very low density lipoprotein receptor (VLDLR), transcript variant 1, mRNA [NM_003383]	NM_003383
A_24_P106794	0.000941	2.654	NM_032869	NM_032869	Homo sapiens NudC domain containing 1 (NUDCD1), mRNA [NM_032869]	NM_032869
A_23_P90220	0.000942	3.442	NM_152655	NM_152655	Homo sapiens zinc finger protein 585A (ZNF585A), transcript variant 1, mRNA [NM_152655]	NM_152655
A_32_P14243	0.000942	2.578	THC2439490		Q835K0 (Q835K0) ATP-dependent RNA helicase, DEAD/DEAH box family, partial (4%) [THC2439490]	
A_23_P317465	0.000943	2.195	NM_016530	NM_016530	Homo sapiens RAB8B, member RAS oncogene family (RAB8B), mRNA [NM_016530]	NM_016530
A_32_P34750	0.000945	3.076	AV702101	AV702101	AV702101 ADB Homo sapiens cDNA clone ADBCGB06 5', mRNA sequence [AV702101]	
A_23_P61623	0.000946	5.057	AL109695	AL109695	Homo sapiens mRNA full length insert cDNA clone EUROIMAGE 39820. [AL109695]	
A_23_P303810	0.000946	3.871	AK098569	AK098569	Homo sapiens cDNA FLJ25703 fis, clone TST04744. [AK098569]	XM_376254
A_23_P164814	0.000946	2.256	NM_024323	NM_024323	Homo sapiens hypothetical protein MGC11271 (MGC11271), mRNA [NM_024323]	NM_024323
A_24_P270452	0.00095	4.342	THC2275804		CB133932 K-EST0185164 L9SNU354 Homo sapiens cDNA clone L9SNU354-10-C01 5', mRNA sequence [CB133932]	
A_24_P222516	0.00095	2.234	AK126014	AK126014	Homo sapiens cDNA FLJ44026 fis, clone TESTI4026762. [AK126014]	XM_044178
A_23_P314191	0.000951	6.119	NM_015336	NM_015336	Homo sapiens zinc finger, DHHC-type containing 17 (ZDHHC17), mRNA [NM_015336]	NM_015336
A_32_P43855	0.000951	3.433	BX476711	BX476711	DKFZp686M05188_r1 686 (synonym: hlcc3) Homo sapiens cDNA clone DKFZp686M05188 5', mRNA sequence [BX476711]	
A_24_P260443	0.000952	4.698	NM_003248	NM_003248	Homo sapiens thrombospondin 4 (THBS4), mRNA [NM_003248]	NM_003248
A_32_P126222	0.000952	2.391	AW302758	AW302758	AW302758 xr55g08.x1 NCI_CGAP_Ov26 Homo sapiens cDNA clone IMAGE:2764094 3', mRNA sequence [AW302758]	
A_24_P914649	0.000954	2.839	BX108121	BX108121	BX108121 Soares_testis_NHT Homo sapiens cDNA clone IMAGp998B051795, mRNA sequence [BX108121]	
A_24_P762886	0.000955	6.685	AB007954	AB007954	Homo sapiens mRNA, chromosome 1 specific transcript KIAA0485. [AB007954]	
A_24_P79403	0.000955	4.529	NM_002619	NM_002619	Homo sapiens platelet factor 4 (chemokine (C-X-C motif) ligand 4) (PF4), mRNA [NM_002619]	NM_002619
A_23_P163113	0.000956	3.185	NM_017922	NM_017922	Homo sapiens PRP39 pre-mRNA processing factor 39 homolog (yeast) (PRPF39), mRNA [NM_017922]	NM_017922
A_23_P13364	0.000961	2.123	NM_005013	NM_005013	Homo sapiens nucleobindin 2 (NUCB2), mRNA [NM_005013]	NM_005013
A_24_P274615	0.000962	3.914	NM_020801	NM_020801	Homo sapiens arrestin domain containing 3 (ARRDC3), mRNA [NM_020801]	NM_020801
A_24_P642771	0.000963	4.895	AK024956	AK024956	Homo sapiens cDNA: FLJ21303 fis, clone COL02107. [AK024956]	
A_23_P381954	0.000965	2.388	NM_000121	NM_000121	Homo sapiens erythropoietin receptor (EPOR), mRNA [NM_000121]	NM_000121

A_23_P415510	0.000966	4.855	NM_005558	NM_005558	Homo sapiens ladinin 1 (LAD1), mRNA [NM_005558]	NM_005558
A_23_P108415	0.000967	2.27	NM_001349	NM_001349	Homo sapiens aspartyl-tRNA synthetase (DARS), mRNA [NM_001349]	NM_001349
A_24_P307896	0.000968	4.302	BC012484	BC012484	Homo sapiens cDNA clone IMAGE:4472100, partial cds. [BC012484]	
A_32_P125496	0.000968	2.25	BX647641	BX647641	Homo sapiens mRNA; cDNA DKFZp451L096 (from clone DKFZp451L096). [BX647641]	
A_23_P39602	0.000968	2.102	NM_147233	NM_147233	Homo sapiens nuclear receptor coactivator 1 (NCOA1), transcript variant 3, mRNA [NM_147233]	NM_147233
A_24_P941649	0.000969	2.281	AL834189	AL834189	Homo sapiens mRNA; cDNA DKFZp762I185 (from clone DKFZp762I185). [AL834189]	
A_23_P326691	0.00097	2.195	NM_005610	NM_005610	Homo sapiens retinoblastoma binding protein 4 (RBBP4), mRNA [NM_005610]	NM_005610
A_32_P139196	0.000972	5.045	NM_213723	NM_213723	Homo sapiens chromosome 13 open reading frame 25 (C13orf25), transcript variant 2, mRNA [NM_213723]	NM_213723
A_24_P316305	0.000974	3.269	ENST00000156471		Homo sapiens cDNA FLJ10311 fis, clone NT2RM2000359, highly similar to Homo sapiens mRNA for KIAA0560 protein. [AK001173]	
A_32_P129968	0.000976	2.438	CR936662	CR936662	Homo sapiens mRNA; cDNA DKFZp781F1775 (from clone DKFZp781F1775). [CR936662]	
A_23_P148255	0.000978	6.083	NM_153488	NM_153488	Homo sapiens melanoma antigen family A, 2B (MAGEA2B), mRNA [NM_153488]	NM_153488
A_23_P163408	0.00098	3.108	NM_020843	NM_020843	Homo sapiens zinc finger protein 291 (ZNF291), mRNA [NM_020843]	NM_020843
A_23_P98910	0.000982	2.33	NM_006152	NM_006152	Homo sapiens lymphoid-restricted membrane protein (LRMP), mRNA [NM_006152]	NM_006152
A_32_P229299	0.000983	2.616	NM_032458	NM_032458	Homo sapiens PHD finger protein 6 (PHF6), transcript variant 2, mRNA [NM_032458]	NM_032458
A_24_P944049	0.000986	2.113	AB011154	AB011154	Homo sapiens mRNA for KIAA0582 protein, partial cds. [AB011154]	
A_23_P48988	0.000988	14.59	NM_003027	NM_003027	Homo sapiens SH3-domain GRB2-like 3 (SH3GL3), mRNA [NM_003027]	NM_003027
A_24_P392991	0.000989	7.653	NM_005557	NM_005557	Homo sapiens keratin 16 (focal non-epidermolytic palmoplantar keratoderma) (KRT16), mRNA [NM_005557]	NM_005557
A_23_P127840	0.000991	2.2	NM_013249	NM_013249	Homo sapiens zinc finger protein 214 (ZNF214), mRNA [NM_013249]	NM_013249
A_24_P165816	0.000992	2.583	AK023645	AK023645	Homo sapiens cDNA FLJ13583 fis, clone PLACE1009050. [AK023645]	
A_23_P80902	0.000993	2.832	NM_020242	NM_020242	Homo sapiens kinesin family member 15 (KIF15), mRNA [NM_020242]	NM_020242
A_32_P43812	0.000993	2.477	NM_015115	NM_015115	Homo sapiens KIAA0276 protein (KIAA0276), mRNA [NM_015115]	NM_015115
A_23_P396328	0.000998	3.324	AK098775	AK098775	Homo sapiens cDNA FLJ25909 fis, clone CBR04667, highly similar to Homo sapiens multi PDZ domain protein MUPP1 (MUPP1) mRNA. [AK098775]	
A_24_P179585	0.000999	18.04	NM_018650	NM_018650	Homo sapiens MAP/microtubule affinity-regulating kinase 1 (MARK1), mRNA [NM_018650]	NM_018650
A_23_P75800	0.001	5.08	NM_013401	NM_013401	Homo sapiens RAB3A interacting protein (rabin3)-like 1 (RAB3IL1), mRNA [NM_013401]	NM_013401
A_24_P816844	0.001	3.887	THC2433066			
A_23_P217151	0.001	2.996	NM_017776	NM_017776	Homo sapiens zinc finger protein 673 (ZNF673), mRNA [NM_017776]	NM_017776
A_23_P100693	0.001	2.623	NM_014877	NM_014877	Homo sapiens helicase with zinc finger (HELZ), mRNA [NM_014877]	NM_014877
A_23_P435697	0.001	2.605	NM_015608	NM_015608	Homo sapiens chromosome 10 open reading frame 137 (C10orf137), mRNA [NM_015608]	NM_015608
A_24_P94402	0.00101	63.87	NM_005378	NM_005378	Homo sapiens v-myc myelocytomatosis viral related oncogene, neuroblastoma derived (avian) (MYCN), mRNA [NM_005378]	NM_005378
A_23_P101193	0.00101	15.62	AK025336	AK025336	Homo sapiens cDNA: FLJ21683 fis, clone COL09335. [AK025336]	XM_371116
A_24_P237757	0.00101	9.57	AL136621	AL136621	Homo sapiens mRNA; cDNA DKFZp564B162 (from clone DKFZp564B162). [AL136621]	
A_32_P10083	0.00101	6.16	BC040293	BC040293	Homo sapiens, clone IMAGE:4820330, mRNA. [BC040293]	
A_24_P261169	0.00101	4.671	NM_006378	NM_006378	Homo sapiens sema domain, immunoglobulin domain (Ig), transmembrane domain (TM) and short cytoplasmic domain, (semaphorin) 4D (SEMA4D), mRNA [NM_006378]	NM_006378
A_23_P164022	0.00101	2.996	NM_033375	NM_033375	Homo sapiens myosin IC (MYO1C), mRNA [NM_033375]	NM_033375
A_23_P209689	0.00101	2.758	NM_004850	NM_004850	Homo sapiens Rho-associated, coiled-coil containing protein kinase 2 (ROCK2), mRNA [NM_004850]	NM_004850
A_23_P310022	0.00101	2.432	NM_019590	NM_019590	Homo sapiens KIAA1217 (KIAA1217), mRNA [NM_019590]	NM_019590
A_23_P98310	0.00101	2.051	NM_001326	NM_001326	Homo sapiens cleavage stimulation factor, 3' pre-RNA, subunit 3, 77kDa (CSTF3), mRNA [NM_001326]	NM_001326
A_23_P116187	0.00102	25.1	NM_031938	NM_031938	Homo sapiens beta-carotene dioxygenase 2 (BCDO2), mRNA [NM_031938]	NM_031938
A_32_P748131	0.00102	5.207	AB075828	AB075828	Homo sapiens mRNA for KIAA1948 protein. [AB075828]	
A_24_P25326	0.00102	2.721	AB037774	AB037774	Homo sapiens mRNA for KIAA1353 protein, partial cds. [AB037774]	
A_24_P320254	0.00102	2.135	NM_005520	NM_005520	Homo sapiens heterogeneous nuclear ribonucleoprotein H1 (H) (HNRPH1), mRNA [NM_005520]	NM_005520

A_23_P79416	0.00102	2.122	NM_025133	NM_025133	Homo sapiens F-box protein 11 (FBXO11), transcript variant 1, mRNA [NM_025133]	NM_025133
A_24_P376139	0.00103	7.899	AK057798	AK057798	Homo sapiens cDNA FLJ25069 fis, clone CBL05145. [AK057798]	
A_23_P422212	0.00103	7.744	NM_173508	NM_173508	Homo sapiens solute carrier family 35, member F3 (SLC35F3), mRNA [NM_173508]	NM_173508
A_23_P97283	0.00103	4.553	NM_024897	NM_024897	Homo sapiens progestin and adipoQ receptor family member VI (PAQR6), transcript variant 1, mRNA [NM_024897]	NM_024897
A_23_P32684	0.00103	4.208	AF116619	AF116619	Homo sapiens PRO1051 mRNA, complete cds. [AF116619]	
A_23_P417261	0.00103	3.8	NM_144715	NM_144715	Homo sapiens EF-hand domain family, member B (EFHB), mRNA [NM_144715]	NM_144715
A_24_P186342	0.00103	3.164	NM_020917	NM_020917	Homo sapiens mouse zinc finger protein 14-like (KIAA1559), mRNA [NM_020917]	NM_020917
A_24_P348806	0.00103	2.999	NM_175058	NM_175058	Homo sapiens pleckstrin homology domain containing, family A member 2 (PLEKHA7), mRNA [NM_175058]	NM_175058
A_23_P117971	0.00103	2.73	M15530	M15530	Human B-cell growth factor (BCGF1) mRNA, complete cds. [M15530]	
A_32_P120484	0.00103	2.581	THC2404072		A45981 peptidylprolyl isomerase CyP-40 - human {Homo sapiens;} , partial (12%) [THC2404072]	
A_32_P178842	0.00103	2.449	BM906215	BM906215	AGENCOURT_6621270 NIH_MGC_125 Homo sapiens cDNA clone IMAGE:5590136 5', mRNA sequence [BM906215]	
A_23_P147495	0.00103	2.15	NM_021946	NM_021946	Homo sapiens BCL6 co-repressor-like 1 (BCORL1), mRNA [NM_021946]	NM_021946
A_23_P360964	0.00104	14.04	NM_145056	NM_145056	Homo sapiens thymus expressed gene 3-like (MGC15476), mRNA [NM_145056]	NM_145056
A_23_P415021	0.00104	9.008	NM_014033	NM_014033	Homo sapiens DKFZP586A0522 protein (DKFZP586A0522), mRNA [NM_014033]	NM_014033
A_23_P333138	0.00104	5.579	ENST00000261188		O99981 (O99981) NADH dehydrogenase subunit 4 , partial (5%) [THC2339600]	
A_23_P67569	0.00104	5.404	NM_024888	NM_024888	Homo sapiens plasticity-related gene 2 (PRG2), mRNA [NM_024888]	NM_024888
A_24_P119201	0.00104	5.068	NM_015832	NM_015832	Homo sapiens methyl-CpG binding domain protein 2 (MBD2), transcript variant testis-specific, mRNA [NM_015832]	NM_015832
A_23_P145786	0.00104	4.828	NM_032951	NM_032951	Homo sapiens Williams Beuren syndrome chromosome region 14 (WBSCR14), transcript variant 1, mRNA [NM_032951]	NM_032951
A_23_P11244	0.00104	3.545	NM_145119	NM_145119	Homo sapiens praja 1 (PJA1), mRNA [NM_145119]	NM_145119
A_23_P134734	0.00104	3.19	NM_017786	NM_017786	Homo sapiens hypothetical protein FLJ20366 (FLJ20366), mRNA [NM_017786]	NM_017786
A_24_P299308	0.00104	2.917	AK097149	AK097149	Homo sapiens cDNA FLJ39830 fis, clone SPLEN2012846. [AK097149]	
A_23_P112341	0.00104	2.751	NM_024945	NM_024945	Homo sapiens chromosome 9 open reading frame 76 (C9orf76), mRNA [NM_024945]	NM_024945
A_23_P205841	0.00104	2.175	NM_006901	NM_006901	Homo sapiens myosin IXA (MYO9A), mRNA [NM_006901]	NM_006901
A_23_P164387	0.00104	2.153	NM_003079	NM_003079	Homo sapiens SWI/SNF related, matrix associated, actin dependent regulator of chromatin, subfamily e, member 1 (SMARCE1), mRNA [NM_003079]	NM_003079
A_23_P166087	0.00104	2.11	NM_014737	NM_014737	Homo sapiens Ras association (RalGDS/AF-6) domain family 2 (RASSF2), transcript variant 1, mRNA [NM_014737]	NM_014737
A_32_P167883	0.00105	4.499	THC2344420		ALU7_HUMAN (P39194) Alu subfamily SQ sequence contamination warning entry, partial (21%) [THC2344420]	
A_23_P5611	0.00105	3.366	NM_018151	NM_018151	Homo sapiens RAP1 interacting factor homolog (yeast) (RIF1), mRNA [NM_018151]	NM_018151
A_32_P80587	0.00105	2.499	A_32_P80587			
A_24_P208909	0.00105	2.377	NM_015271	NM_015271	Homo sapiens tripartite motif-containing 2 (TRIM2), mRNA [NM_015271]	NM_015271
A_23_P30567	0.00106	8.604	NM_001882	NM_001882	Homo sapiens corticotropin releasing hormone binding protein (CRHBP), mRNA [NM_001882]	NM_001882
A_24_P37441	0.00106	6.388	NM_002610	NM_002610	Homo sapiens pyruvate dehydrogenase kinase, isoenzyme 1 (PDK1), nuclear gene encoding mitochondrial protein, mRNA [NM_002610]	NM_002610
A_23_P138655	0.00106	3.016	NM_057157	NM_057157	Homo sapiens cytochrome P450, family 26, subfamily A, polypeptide 1 (CYP26A1), transcript variant 2, mRNA [NM_057157]	NM_057157
A_23_P48029	0.00106	2.679	NM_016184	NM_016184	Homo sapiens C-type lectin domain family 4, member A (CLEC4A), transcript variant 1, mRNA [NM_016184]	NM_016184
A_23_P412389	0.00106	2.508	NM_033649	NM_033649	Homo sapiens fibroblast growth factor 18 (FGF18), transcript variant 2, mRNA [NM_033649]	NM_033649
A_23_P73150	0.00106	2.192	NM_031421	NM_031421	Homo sapiens hypothetical protein DKFZp434H0115 (DKFZP434H0115), mRNA [NM_031421]	NM_031421
A_23_P254917	0.00106	2.044	NM_006149	NM_006149	Homo sapiens lectin, galactoside-binding, soluble, 4 (galectin 4) (LGALS4), mRNA [NM_006149]	NM_006149
A_23_P395609	0.00107	67.24	NM_147189	NM_147189	Homo sapiens hypothetical protein MGC39325 (MGC39325), mRNA [NM_147189]	NM_147189
A_24_P643776	0.00107	42.19	THC2437143			
A_32_P156237	0.00107	20.78	A_32_P156237			

A_23_P117298	0.00107	17.06	NM_000131	NM_000131	Homo sapiens coagulation factor VII (serum prothrombin conversion accelerator) (F7), transcript variant 1, mRNA [NM_000131]	NM_000131
A_23_P421811	0.00107	4.068	NM_152503	NM_152503	Homo sapiens chromosome 20 open reading frame 132 (C20orf132), transcript variant 1, mRNA [NM_152503]	NM_152503
A_32_P875758	0.00107	3.291	AB046850	AB046850	Homo sapiens mRNA for KIAA1630 protein, partial cds. [AB046850]	
A_23_P336796	0.00107	2.943	CR590862	CR590862	full-length cDNA clone CS0DI036YC04 of Placenta Cot 25-normalized of Homo sapiens (human). [CR590862]	XM_290597
A_23_P200030	0.00107	2.552	NM_003838	NM_003838	Homo sapiens fucose-1-phosphate guanylyltransferase (FPGT), mRNA [NM_003838]	NM_003838
A_23_P127054	0.00107	2.515	NM_148977	NM_148977	Homo sapiens pantothenate kinase 1 (PANK1), transcript variant alpha, mRNA [NM_148977]	NM_148977
A_23_P163087	0.00108	11.6	NM_007361	NM_007361	Homo sapiens nidogen 2 (osteonidogen) (NID2), mRNA [NM_007361]	NM_007361
A_32_P38323	0.00108	9.478	NM_004155	NM_004155	Homo sapiens serine (or cysteine) proteinase inhibitor, clade B (ovalbumin), member 9 (SERPINB9), mRNA [NM_004155]	NM_004155
A_23_P401675	0.00108	5.75	NM_144724	NM_144724	Homo sapiens MARVEL domain containing 2 (MARVELD2), mRNA [NM_144724]	NM_144724
A_23_P34045	0.00108	5.225	NM_001399	NM_001399	Homo sapiens ectodysplasin A (EDA), transcript variant 1, mRNA [NM_001399]	NM_001399
A_23_P418083	0.00108	4.447	NM_181714	NM_181714	Homo sapiens chromosome 6 open reading frame 152 (C6orf152), mRNA [NM_181714]	NM_181714
A_23_P31896	0.00108	4.087	NM_003033	NM_003033	Homo sapiens ST3 beta-galactoside alpha-2,3-sialyltransferase 1 (ST3GAL1), transcript variant 1, mRNA [NM_003033]	NM_003033
A_23_P114983	0.00108	3.91	NM_032588	NM_032588	Homo sapiens tripartite motif-containing 63 (TRIM63), mRNA [NM_032588]	NM_032588
A_24_P126417	0.00108	2.501	NM_014709	NM_014709	Homo sapiens ubiquitin specific protease 34 (USP34), mRNA [NM_014709]	NM_014709
A_24_P920181	0.00108	2.438	AK026979	AK026979	Homo sapiens cDNA: FLJ23326 fis, clone HEP12573. [AK026979]	
A_32_P9963	0.00108	2.431	NM_004506	NM_004506	Homo sapiens heat shock transcription factor 2 (HSF2), mRNA [NM_004506]	NM_004506
A_23_P40315	0.00108	2.149	NM_018152	NM_018152	Homo sapiens chromosome 20 open reading frame 12 (C20orf12), mRNA [NM_018152]	NM_018152
A_23_P36364	0.00108	2.123	NM_033209	NM_033209	Homo sapiens Thy-1 co-transcribed (LOC94105), mRNA [NM_033209]	NM_033209
A_23_P375372	0.00109	77.89	NM_021871	NM_021871	Homo sapiens fibrinogen alpha chain (FGA), transcript variant alpha, mRNA [NM_021871]	NM_021871
A_32_P69849	0.00109	33.39	NM_001012978	NM_001012978	Homo sapiens NGFRAP1-like 1 (NGFRAP1L1), mRNA [NM_001012978]	NM_001012978
A_23_P66137	0.00109	6.306	NM_014587	NM_014587	Homo sapiens SRY (sex determining region Y)-box 8 (SOX8), mRNA [NM_014587]	NM_014587
A_32_P210168	0.00109	5.933	XM_370873	XM_370873	PREDICTED: Homo sapiens similar to RIKEN cDNA 6030419C18 gene (LOC388135), mRNA [XM_370873]	XM_370873
A_23_P53267	0.00109	3.238	NM_198261	NM_198261	Homo sapiens similar to splicing factor, arginine/serine-rich 4 (FLJ11021), transcript variant 2, mRNA [NM_198261]	NM_198261
A_23_P209200	0.00109	2.868	NM_001238	NM_001238	Homo sapiens cyclin E1 (CCNE1), transcript variant 1, mRNA [NM_001238]	NM_001238
A_23_P158593	0.00109	2.674	NM_000093	NM_000093	Homo sapiens collagen, type V, alpha 1 (COL5A1), mRNA [NM_000093]	NM_000093
A_32_P141612	0.00109	2.67	NM_002552	NM_002552	Homo sapiens origin recognition complex, subunit 4-like (yeast) (ORC4L), transcript variant 2, mRNA [NM_002552]	NM_002552
A_23_P167194	0.00109	2.48	NM_001812	NM_001812	Homo sapiens centromere protein C 1 (CENPC1), mRNA [NM_001812]	NM_001812
A_23_P33673	0.00109	2.471	NM_001003818	NM_001003818	Homo sapiens tripartite motif-containing 6 (TRIM6), transcript variant 1, mRNA [NM_001003818]	NM_001003818
A_23_P114232	0.00109	2.371	NM_006406	NM_006406	Homo sapiens peroxiredoxin 4 (PRDX4), mRNA [NM_006406]	NM_006406
A_23_P85598	0.00109	2.033	NM_020247	NM_020247	Homo sapiens chaperone, ABC1 activity of bc1 complex like (S. pombe) (CABC1), mRNA [NM_020247]	NM_020247
A_32_P211141	0.00109	2.024	BC022429	BC022429	Homo sapiens cDNA clone MGC:24679 IMAGE:4270959, complete cds. [BC022429]	
A_23_P76901	0.0011	8.566	NM_015549	NM_015549	Homo sapiens pleckstrin homology domain containing, family G (with RhoGef domain) member 3 (PLEKHG3), mRNA [NM_015549]	NM_015549
A_23_P121851	0.0011	3.853	NM_018935	NM_018935	Homo sapiens protocadherin beta 15 (PCDHB15), mRNA [NM_018935]	NM_018935
A_24_P83102	0.0011	2.513	NM_020070	NM_020070	Homo sapiens immunoglobulin lambda-like polypeptide 1 (IGLL1), transcript variant 1, mRNA [NM_020070]	NM_020070
A_24_P180383	0.0011	2.489	NM_015384	NM_015384	Homo sapiens Nipped-B homolog (Drosophila) (NIPBL), transcript variant B, mRNA [NM_015384]	NM_015384
A_23_P96761	0.0011	2.337	NM_000016	NM_000016	Homo sapiens acyl-Coenzyme A dehydrogenase, C-4 to C-12 straight chain (ACADM), nuclear gene encoding mitochondrial protein, mRNA [NM_000016]	NM_000016
A_23_P136964	0.0011	2.069	NM_000328	NM_000328	Homo sapiens retinitis pigmentosa GTPase regulator (RPGR), transcript variant A, mRNA [NM_000328]	NM_000328
A_24_P300394	0.00111	92.81	NM_000846	NM_000846	Homo sapiens glutathione S-transferase A2 (GSTA2), mRNA [NM_000846]	NM_000846
A_23_P31893	0.00111	4.267	NM_003033	NM_003033	Homo sapiens ST3 beta-galactoside alpha-2,3-sialyltransferase 1 (ST3GAL1), transcript variant 1, mRNA [NM_003033]	NM_003033

A_23_P337424	0.00111	2.912	NM_001018067	NM_001018067	Homo sapiens PAI-1 mRNA binding protein (PAI-RBP1), transcript variant 1, mRNA [NM_001018067]	NM_001018067
A_24_P68019	0.00111	2.712	NM_138347	NM_138347	Homo sapiens zinc finger protein 551 (ZNF551), mRNA [NM_138347]	NM_138347
A_24_P190379	0.00111	2.525	NM_018443	NM_018443	Homo sapiens zinc finger protein 302 (ZNF302), transcript variant 1, mRNA [NM_018443]	NM_018443
A_24_P252705	0.00111	2.085	NM_004412	NM_004412	Homo sapiens DNA (cytosine-5-)-methyltransferase 2 (DNMT2), transcript variant a, mRNA [NM_004412]	NM_004412
A_23_P205900	0.00112	5.235	NM_001012338	NM_001012338	Homo sapiens neurotrophic tyrosine kinase, receptor, type 3 (NTRK3), transcript variant 1, mRNA [NM_001012338]	NM_001012338
A_24_P128880	0.00112	3.468	BC048298	BC048298	Homo sapiens transducin (beta)-like 1X-linked receptor 1, mRNA (cDNA clone IMAGE:5527421), complete cds. [BC048298]	
A_32_P96807	0.00112	3.436	AY726570	AY726570	Homo sapiens clone TESTIS-724 mRNA sequence. [AY726570]	
A_23_P56150	0.00112	3.243	NM_032753	NM_032753	Homo sapiens hypothetical protein MGC15631 (MGC15631), mRNA [NM_032753]	NM_032753
A_23_P428738	0.00112	3.071	NM_001145	NM_001145	Homo sapiens angiogenin, ribonuclease, RNase A family, 5 (ANG), mRNA [NM_001145]	NM_001145
A_23_P134113	0.00112	2.603	NM_052831	NM_052831	Homo sapiens chromosome 6 open reading frame 192 (C6orf192), mRNA [NM_052831]	NM_052831
A_24_P284959	0.00112	2.602	NM_019035	NM_019035	Homo sapiens protocadherin 18 (PCDH18), mRNA [NM_019035]	NM_019035
A_23_P257296	0.00112	2.311	NM_003226	NM_003226	Homo sapiens trefoil factor 3 (intestinal) (TFF3), mRNA [NM_003226]	NM_003226
A_24_P178631	0.00112	2.131	AK027667	AK027667	Homo sapiens cDNA FLJ14761 fis, clone NT2RP3003302. [AK027667]	
A_24_P332739	0.00112	2.126	NM_206908	NM_206908	Homo sapiens chromosome 6 open reading frame 216 (C6orf216), transcript variant 2, mRNA [NM_206908]	NM_206908
A_23_P94141	0.00112	2.108	NM_006550	NM_006550	Homo sapiens fibrinogen silencer binding protein (FSBP), mRNA [NM_006550]	NM_006550
A_32_P75141	0.00113	7.39	A_32_P75141			
A_32_P146898	0.00113	6.523	AA353695	AA353695	AA353695 EST62114 Jurkat T-cells V Homo sapiens cDNA 5' end, mRNA sequence [AA353695]	
A_23_P39154	0.00113	4.942	NM_003419	NM_003419	Homo sapiens zinc finger protein 345 (ZNF345), mRNA [NM_003419]	NM_003419
A_24_P943997	0.00113	4.908	NM_178815	NM_178815	Homo sapiens ADP-ribosylation factor-like 8 (ARL8), mRNA [NM_178815]	NM_178815
A_32_P73507	0.00113	4.465	THC2339389			
A_23_P206228	0.00113	3.936	NM_017684	NM_017684	Homo sapiens vacuolar protein sorting 13C (yeast) (VPS13C), transcript variant 1A, mRNA [NM_017684]	NM_017684
A_24_P22079	0.00113	3.4	NM_002015	NM_002015	Homo sapiens forkhead box O1A (rhabdomyosarcoma) (FOXO1A), mRNA [NM_002015]	NM_002015
A_23_P87839	0.00113	3.18	NM_004719	NM_004719	Homo sapiens splicing factor, arginine/serine-rich 2, interacting protein (SFRS2IP), mRNA [NM_004719]	NM_004719
A_23_P332399	0.00113	3.171	NM_016315	NM_016315	Homo sapiens GULP, engulfment adaptor PTB domain containing 1 (GULP1), mRNA [NM_016315]	NM_016315
A_23_P422724	0.00113	2.951	NM_000943	NM_000943	Homo sapiens peptidylprolyl isomerase C (cyclophilin C) (PPIC), mRNA [NM_000943]	NM_000943
A_24_P247512	0.00113	2.665	AK098175	AK098175	Homo sapiens cDNA FLJ40856 fis, clone TRACH2016498, moderately similar to ZINC FINGER PROTEIN 184. [AK098175]	XM_371174
A_32_P61657	0.00113	2.491	NM_006955	NM_006955	Homo sapiens zinc finger protein 11B (ZNF11B), mRNA [NM_006955]	NM_006955
A_23_P75283	0.00114	38.26	NM_006744	NM_006744	Homo sapiens retinol binding protein 4, plasma (RBP4), mRNA [NM_006744]	NM_006744
A_23_P75529	0.00114	28.88	NM_022062	NM_022062	Homo sapiens PBX/knotted 1 homeobox 2 (PKNOX2), mRNA [NM_022062]	NM_022062
A_23_P43141	0.00114	3.67	NM_001568	NM_001568	Homo sapiens eukaryotic translation initiation factor 3, subunit 6 48kDa (EIF3S6), mRNA [NM_001568]	NM_001568
A_23_P156809	0.00114	3.263	NM_001018022	NM_001018022	Homo sapiens similar to FKSG62 (LOC389286), mRNA [NM_001018022]	NM_001018022
A_23_P152505	0.00114	3.075	NM_020686	NM_020686	Homo sapiens 4-aminobutyrate aminotransferase (ABAT), nuclear gene encoding mitochondrial protein, transcript variant 1, mRNA [NM_020686]	NM_020686
A_23_P312174	0.00114	2.885	NM_015120	NM_015120	Homo sapiens Alstrom syndrome 1 (ALMS1), mRNA [NM_015120]	NM_015120
A_24_P347447	0.00114	2.743	NM_014992	NM_014992	Homo sapiens dishevelled associated activator of morphogenesis 1 (DAAM1), mRNA [NM_014992]	NM_014992
A_32_P45894	0.00114	2.17	NM_018991	NM_018991	Homo sapiens DKFZp434A0131 protein (DKFZP434A0131), transcript variant 1, mRNA [NM_018991]	NM_018991
A_32_P35800	0.00114	2.092	CR749441	CR749441	Homo sapiens mRNA; cDNA DKFZp781J2344 (from clone DKFZp781J2344). [CR749441]	XM_029101
A_23_P342910	0.00115	6.564	ENST00000328046		Homo sapiens mRNA for KIAA1677 protein, partial cds. [AB051464]	
A_24_P220947	0.00115	4.472	NM_001353	NM_001353	Homo sapiens aldo-keto reductase family 1, member C1 (dihydrodiol dehydrogenase 1; 20-alpha (3-alpha)-hydroxysteroid dehydrogenase) (AKR1C1), mRNA [NM_001353]	NM_001353
A_23_P167256	0.00115	2.492	AK127326	AK127326	Homo sapiens cDNA FLJ45397 fis, clone BRHIP3027651, weakly similar to Bromodomain-containing protein 1. [AK127326]	

A_24_P98251	0.00115	2.386	NM_017890	NM_017890	Homo sapiens vacuolar protein sorting 13B (yeast) (VPS13B), transcript variant 5, mRNA [NM_017890]	NM_017890
A_23_P258088	0.00116	4.549	NM_020804	NM_020804	Homo sapiens protein kinase C and casein kinase substrate in neurons 1 (PACSIN1), mRNA [NM_020804]	NM_020804
A_32_P34516	0.00116	2.384	AK056119	AK056119	Homo sapiens cDNA FLJ31557 fis, clone NT2RI2001307. [AK056119]	
A_23_P258887	0.00116	2.278	NM_012190	NM_012190	Homo sapiens aldehyde dehydrogenase 1 family, member L1 (ALDH1L1), mRNA [NM_012190]	NM_012190
A_23_P18713	0.00117	6.974	NM_004827	NM_004827	Homo sapiens ATP-binding cassette, sub-family G (WHITE), member 2 (ABCG2), mRNA [NM_004827]	NM_004827
A_24_P330234	0.00117	3.98	NM_007106	NM_007106	Homo sapiens ubiquitin-like 3 (UBL3), mRNA [NM_007106]	NM_007106
A_23_P21644	0.00117	3.567	NM_016245	NM_016245	Homo sapiens dehydrogenase/reductase (SDR family) member 8 (DHRS8), mRNA [NM_016245]	NM_016245
A_23_P75260	0.00117	2.525	NM_032023	NM_032023	Homo sapiens Ras association (RalGDS/AF-6) domain family 4 (RASSF4), transcript variant 1, mRNA [NM_032023]	NM_032023
A_23_P398275	0.00117	2.522	AF316855	AF316855	Homo sapiens colon cancer-associated antigen AgSK1-2HT-ECS mRNA, complete cds. [AF316855]	
A_24_P192183	0.00117	2.367	NM_007214	NM_007214	Homo sapiens SEC63-like (S. cerevisiae) (SEC63), mRNA [NM_007214]	NM_007214
A_24_P192586	0.00117	2.333	ENST00000337863		full-length cDNA clone CS0DF005YF01 of Fetal brain of Homo sapiens (human). [CR596783]	
A_23_P85008	0.00118	27.12	NM_000898	NM_000898	Homo sapiens monoamine oxidase B (MAOB), nuclear gene encoding mitochondrial protein, mRNA [NM_000898]	NM_000898
A_32_P166356	0.00118	7.488	THC2350463			
A_23_P313603	0.00118	3.704	AK056855	AK056855	Homo sapiens cDNA FLJ32293 fis, clone PROST2001739. [AK056855]	XM_374902
A_23_P99762	0.00119	2.921	NM_021239	NM_021239	Homo sapiens RNA binding motif protein 25 (RBM25), mRNA [NM_021239]	NM_021239
A_32_P34881	0.00119	2.752	THC2305677		HMG1_HUMAN (P09429) High mobility group protein 1 (HMG-1), partial (7%) [THC2305677]	
A_23_P152262	0.0012	13.35	NM_004413	NM_004413	Homo sapiens dipeptidase 1 (renal) (DPEP1), mRNA [NM_004413]	NM_004413
A_24_P484699	0.0012	5.67	AK021467	AK021467	Homo sapiens cDNA FLJ11405 fis, clone HEMBA1000769. [AK021467]	
A_32_P155035	0.0012	4.602	AK096500	AK096500	Homo sapiens cDNA FLJ39181 fis, clone OCBBF2004235. [AK096500]	
A_23_P304511	0.0012	3.701	NM_032347	NM_032347	Homo sapiens zinc finger protein 397 (ZNF397), mRNA [NM_032347]	NM_032347
A_23_P386450	0.0012	3.551	NM_016218	NM_016218	Homo sapiens polymerase (DNA directed) kappa (POLK), mRNA [NM_016218]	NM_016218
A_32_P540407	0.0012	3.078	NM_207333	NM_207333	Homo sapiens zinc finger protein like (ZFPL), mRNA [NM_207333]	NM_207333
A_23_P167227	0.0012	2.152	NM_005327	NM_005327	Homo sapiens L-3-hydroxyacyl-Coenzyme A dehydrogenase, short chain (HADHSC), mRNA [NM_005327]	NM_005327
A_32_P53107	0.0012	2.042	CR609342	CR609342	full-length cDNA clone CS0DA002YO22 of Neuroblastoma of Homo sapiens (human). [CR609342]	
A_23_P84070	0.0012	2.01	NM_016648	NM_016648	Homo sapiens HDCMA18P protein (HDCMA18P), mRNA [NM_016648]	NM_016648
A_23_P361419	0.0012	2.003	NM_018369	NM_018369	Homo sapiens DEP domain containing 1B (DEPDC1B), mRNA [NM_018369]	NM_018369
A_23_P101407	0.00121	8.017	NM_000064	NM_000064	Homo sapiens complement component 3 (C3), mRNA [NM_000064]	NM_000064
A_23_P3424	0.00121	4.501	NM_017793	NM_017793	Homo sapiens ribonuclease P 25kDa subunit (RPP25), mRNA [NM_017793]	NM_017793
A_23_P16806	0.00121	3.308	BC004487	BC004487	Homo sapiens hypothetical protein MGC10701, mRNA (cDNA clone MGC:10701 IMAGE:3832541), complete cds. [BC004487]	
A_24_P928574	0.00121	2.9	AK023400	AK023400	Homo sapiens cDNA FLJ13338 fis, clone OVARC1001883. [AK023400]	
A_23_P325501	0.00121	2.661	NM_015358	NM_015358	Homo sapiens MORC family CW-type zinc finger 3 (MORC3), mRNA [NM_015358]	NM_015358
A_24_P229726	0.00121	2.634	A_24_P229726			
A_23_P422732	0.00121	2.514	NM_145172	NM_145172	Homo sapiens WD repeat domain 63 (WDR63), mRNA [NM_145172]	NM_145172
A_24_P139993	0.00121	2.406	NM_032977	NM_032977	Homo sapiens caspase 10, apoptosis-related cysteine protease (CASP10), transcript variant D, mRNA [NM_032977]	NM_032977
A_32_P37974	0.00121	2.322	AK056809	AK056809	Homo sapiens cDNA FLJ32247 fis, clone PROST1000120. [AK056809]	
A_23_P95599	0.00121	2.297	NM_001012731	NM_001012731	Homo sapiens hypothetical protein LOC283874 (LOC283874), mRNA [NM_001012731]	NM_001012731
A_23_P135857	0.00121	2.042	NM_004836	NM_004836	Homo sapiens eukaryotic translation initiation factor 2-alpha kinase 3 (EIF2AK3), mRNA [NM_004836]	NM_004836
A_23_P360329	0.00122	7.489	NM_017977	NM_017977	Homo sapiens absent in melanoma 1-like (AIM1L), mRNA [NM_017977]	NM_017977
A_32_P144908	0.00122	5.815	NM_203282	NM_203282	Homo sapiens zinc finger protein 539 (ZNF539), mRNA [NM_203282]	NM_203282
A_23_P8175	0.00122	3.434	NM_006718	NM_006718	Homo sapiens pleiomorphic adenoma gene-like 1 (PLAGL1), transcript variant 2, mRNA [NM_006718]	NM_006718

A_23_P307536	0.00122	3.212	NM_024676	NM_024676	Homo sapiens chromosome 1 open reading frame 113 (C1orf113), mRNA [NM_024676]	NM_024676
A_32_P83547	0.00122	2.58	THC2399645			
A_24_P251661	0.00122	2.539	NM_171828	NM_171828	Homo sapiens potassium large conductance calcium-activated channel, subfamily M beta member 3 (KCNMB3), transcript variant 1, mRNA [NM_171828]	NM_171828
A_24_P285880	0.00122	2.347	NM_003262	NM_003262	Homo sapiens translocation protein 1 (TLOC1), mRNA [NM_003262]	NM_003262
A_23_P127140	0.00122	2.273	NM_014904	NM_014904	Homo sapiens RAB11 family interacting protein 2 (class I) (RAB11FIP2), mRNA [NM_014904]	NM_014904
A_23_P208143	0.00123	5.34	AF533250	AF533250	Homo sapiens zinc finger protein (ZNF397) mRNA, complete cds. [AF533250]	
A_23_P82979	0.00123	4.767	NM_006059	NM_006059	Homo sapiens laminin, gamma 3 (LAMC3), mRNA [NM_006059]	NM_006059
A_23_P131074	0.00123	3.185	NM_016585	NM_016585	Homo sapiens Theg homolog (mouse) (THEG), transcript variant 1, mRNA [NM_016585]	NM_016585
A_24_P380132	0.00123	2.843	NM_203505	NM_203505	Homo sapiens Ras-GTPase activating protein SH3 domain-binding protein 2 (G3BP2), transcript variant 1, mRNA [NM_203505]	NM_203505
A_23_P69100	0.00123	2.567	NM_015595	NM_015595	Homo sapiens Src homology 3 domain-containing guanine nucleotide exchange factor (SGEF), mRNA [NM_015595]	NM_015595
A_32_P190294	0.00123	2.47	ENST00000327721		PREDICTED: Homo sapiens hypothetical protein MGC22265 (MGC22265), mRNA [XM_380175]	XM_380175
A_24_P398092	0.00123	2.123	NM_002874	NM_002874	Homo sapiens RAD23 homolog B (S. cerevisiae) (RAD23B), mRNA [NM_002874]	NM_002874
A_24_P926972	0.00123	2.097	AK000923	AK000923	Homo sapiens cDNA FLJ10061 fis, clone HEMBA1001413. [AK000923]	
A_23_P390504	0.00123	2.074	NM_001453	NM_001453	Homo sapiens forkhead box C1 (FOXC1), mRNA [NM_001453]	NM_001453
A_23_P375494	0.00124	24.68	NM_004364	NM_004364	Homo sapiens CCAAT/enhancer binding protein (C/EBP), alpha (CEBPA), mRNA [NM_004364]	NM_004364
A_23_P7727	0.00124	12.32	NM_001884	NM_001884	Homo sapiens hyaluronan and proteoglycan link protein 1 (HAPLN1), mRNA [NM_001884]	NM_001884
A_32_P104841	0.00124	4.942	AF087980	AF087980	Homo sapiens full length insert cDNA clone YW27H10. [AF087980]	
A_23_P429670	0.00124	4.26	NM_032440	NM_032440	Homo sapiens ligand-dependent corepressor (MLR2), mRNA [NM_032440]	NM_032440
A_23_P49279	0.00124	2.467	NM_001001436	NM_001001436	Homo sapiens similar to RIKEN cDNA 4921524J17 (LOC388272), mRNA [NM_001001436]	NM_001001436
A_32_P36582	0.00124	2.347	AK123649	AK123649	Homo sapiens cDNA FLJ41655 fis, clone FEBRA2025477. [AK123649]	
A_23_P202170	0.00124	2.308	NM_012215	NM_012215	Homo sapiens meningioma expressed antigen 5 (hyaluronidase) (MGEA5), mRNA [NM_012215]	NM_012215
A_23_P50638	0.00124	2.267	NM_052972	NM_052972	Homo sapiens leucine-rich alpha-2-glycoprotein 1 (LRG1), mRNA [NM_052972]	NM_052972
A_32_P105195	0.00124	2.257	NM_014829	NM_014829	Homo sapiens DEAD (Asp-Glu-Ala-Asp) box polypeptide 46 (DDX46), mRNA [NM_014829]	NM_014829
A_23_P35684	0.00124	2.191	NM_014937	NM_014937	Homo sapiens inositol polyphosphate-5-phosphatase F (INPP5F), transcript variant 1, mRNA [NM_014937]	NM_014937
A_32_P124580	0.00124	2.12	AA490192	AA490192	AA490192 aa43f10.r1 Soares_NhHMPu_S1 Homo sapiens cDNA clone IMAGE:823723 5', mRNA sequence [AA490192]	
A_23_P211126	0.00124	2.008	NM_130436	NM_130436	Homo sapiens dual-specificity tyrosine-(Y)-phosphorylation regulated kinase 1A (DYRK1A), transcript variant 2, mRNA [NM_130436]	NM_130436
A_23_P77859	0.00125	13.82	NM_203411	NM_203411	Homo sapiens similar to RIKEN cDNA 2600017H02 (LOC92162), mRNA [NM_203411]	NM_203411
A_24_P40417	0.00125	6.277	NM_002024	NM_002024	Homo sapiens fragile X mental retardation 1 (FMR1), mRNA [NM_002024]	NM_002024
A_23_P86461	0.00125	3.98	THC2435505			
A_23_P151570	0.00125	3.694	NM_014990	NM_014990	Homo sapiens GTPase activating Rap/RanGAP domain-like 1 (GARNL1), transcript variant 1, mRNA [NM_014990]	NM_014990
A_23_P12755	0.00125	3.131	NM_032211	NM_032211	Homo sapiens lysyl oxidase-like 4 (LOXL4), mRNA [NM_032211]	NM_032211
A_24_P80571	0.00125	2.22	CN284574	CN284574	CN284574 17000531534200 GRN_EB Homo sapiens cDNA 5', mRNA sequence [CN284574]	
A_23_P332584	0.00126	13	ENST00000176186		Homo sapiens mRNA for KIAA1107 protein, partial cds. [AB029030]	XM_034086
A_24_P329152	0.00126	2.845	NM_012120	NM_012120	Homo sapiens CD2-associated protein (CD2AP), mRNA [NM_012120]	NM_012120
A_23_P357794	0.00126	2.456	NM_022045	NM_022045	Homo sapiens Mdm2, transformed 3T3 cell double minute 2, p53 binding protein (mouse) binding protein, 104kDa (MTBP), mRNA [NM_022045]	NM_022045
A_23_P423457	0.00126	2.439	NM_178276	NM_178276	Homo sapiens chromosome 5 open reading frame 12 (C5orf12), mRNA [NM_178276]	NM_178276
A_23_P324633	0.00126	2.352	NM_018325	NM_018325	Homo sapiens chromosome 9 open reading frame 72 (C9orf72), transcript variant 1, mRNA [NM_018325]	NM_018325
A_32_P126375	0.00127	20.58	NM_198270	NM_198270	Homo sapiens Nance-Horan syndrome (congenital cataracts and dental anomalies) (NHS), mRNA [NM_198270]	NM_198270
A_23_P4653	0.00127	4.056	NM_145275	NM_145275	Homo sapiens kinesin light chain 2-like (KLC2L), transcript variant 2, mRNA [NM_145275]	NM_145275
A_23_P255194	0.00127	3.465	NM_014827	NM_014827	Homo sapiens zinc finger CCCH-type containing 11A (ZC3H11A), mRNA [NM_014827]	NM_014827

A_23_P401098	0.00127	2.175	NM_153211	NM_153211	Homo sapiens chromosome 18 open reading frame 17 (C18orf17), mRNA [NM_153211]	NM_153211
A_23_P30956	0.00128	3.827	NM_015323	NM_015323	Homo sapiens KIAA0776 (KIAA0776), mRNA [NM_015323]	NM_015323
A_23_P135787	0.00128	3.272	BX537520	BX537520	Homo sapiens mRNA; cDNA DKFZp686F09142 (from clone DKFZp686F09142). [BX537520]	
A_32_P232192	0.00128	2.65	AK094293	AK094293	Homo sapiens cDNA FLJ36974 fis, clone BRACE2006264. [AK094293]	
A_23_P102994	0.00128	2.418	NM_002650	NM_002650	Homo sapiens phosphatidylinositol 4-kinase, catalytic, alpha polypeptide (PIK4CA), transcript variant 1, mRNA [NM_002650]	NM_002650
A_32_P119348	0.00129	5.145	AK130049	AK130049	Homo sapiens cDNA FLJ26539 fis, clone KDN09310. [AK130049]	
A_23_P30175	0.00129	2.758	NM_018695	NM_018695	Homo sapiens erbb2 interacting protein (ERBB2IP), transcript variant 2, mRNA [NM_018695]	NM_018695
A_23_P379945	0.00129	2.608	AB040942	AB040942	Homo sapiens mRNA for KIAA1509 protein, partial cds. [AB040942]	XM_029353
A_23_P426305	0.0013	7.765	NM_003734	NM_003734	Homo sapiens amine oxidase, copper containing 3 (vascular adhesion protein 1) (AOC3), mRNA [NM_003734]	NM_003734
A_23_P354942	0.0013	4.518	NM_175910	NM_175910	Homo sapiens zinc finger protein 493 (ZNF493), mRNA [NM_175910]	NM_175910
A_23_P35943	0.0013	3.68	NM_178510	NM_178510	Homo sapiens ankyrin repeat and kinase domain containing 1 (ANKK1), mRNA [NM_178510]	NM_178510
A_24_P920319	0.0013	2.347	NM_021211	NM_021211	Homo sapiens transposon-derived Buster1 transposase-like protein gene (LOC58486), mRNA [NM_021211]	NM_021211
A_23_P258221	0.0013	2.104	NM_005688	NM_005688	Homo sapiens ATP-binding cassette, sub-family C (CFTR/MRP), member 5 (ABCC5), transcript variant 1, mRNA [NM_005688]	NM_005688
A_32_P183367	0.00131	5.306	A_32_P183367			
A_32_P226786	0.00131	4.801	BC045174	BC045174	Homo sapiens, clone IMAGE:5273245, mRNA. [BC045174]	
A_24_P513262	0.00131	3.002	AK026485	AK026485	Homo sapiens cDNA: FLJ22832 fis, clone KAIA4195. [AK026485]	
A_23_P53390	0.00131	2.485	NM_002837	NM_002837	Homo sapiens protein tyrosine phosphatase, receptor type, B (PTPRB), mRNA [NM_002837]	NM_002837
A_24_P295791	0.00132	8.643	NM_032564	NM_032564	Homo sapiens diacylglycerol O-acyltransferase homolog 2 (mouse) (DGAT2), mRNA [NM_032564]	NM_032564
A_23_P167920	0.00132	7.573	NM_005618	NM_005618	Homo sapiens delta-like 1 (Drosophila) (DLL1), mRNA [NM_005618]	NM_005618
A_24_P389612	0.00132	7.358	CR625971	CR625971	full-length cDNA clone CS0DL002YL19 of B cells (Ramos cell line) Cot 25-normalized of Homo sapiens (human). [CR625971]	
A_23_P4353	0.00132	6.376	NM_134264	NM_134264	Homo sapiens WD repeat and SOCS box-containing 1 (WSB1), transcript variant 3, mRNA [NM_134264]	NM_134264
A_23_P141730	0.00132	4.631	NM_001943	NM_001943	Homo sapiens desmoglein 2 (DSG2), mRNA [NM_001943]	NM_001943
A_32_P206612	0.00132	3.869	BX089851	BX089851	BX089851 NCI_CGAP_Kid11 Homo sapiens cDNA clone IMAGp998K245260 ; IMAGE:2131727, mRNA sequence [BX089851]	
A_24_P215475	0.00132	3.376	NM_015394	NM_015394	Homo sapiens zinc finger protein 10 (ZNF10), mRNA [NM_015394]	NM_015394
A_23_P26895	0.00132	2.691	NM_016261	NM_016261	Homo sapiens tubulin, delta 1 (TUBD1), mRNA [NM_016261]	NM_016261
A_24_P335901	0.00132	2.66	NM_006631	NM_006631	Homo sapiens zinc finger protein 266 (ZNF266), mRNA [NM_006631]	NM_006631
A_23_P8311	0.00132	2.569	NM_016614	NM_016614	Homo sapiens TRAF and TNF receptor associated protein (TTRAP), mRNA [NM_016614]	NM_016614
A_23_P319572	0.00132	2.292	NM_005122	NM_005122	Homo sapiens nuclear receptor subfamily 1, group I, member 3 (NR1I3), mRNA [NM_005122]	NM_005122
A_23_P55911	0.00132	2.154	NM_152357	NM_152357	Homo sapiens zinc finger protein 440 (ZNF440), mRNA [NM_152357]	NM_152357
A_23_P15402	0.00132	2.046	NM_133491	NM_133491	Homo sapiens spermidine/spermine N1-acetyltransferase 2 (SAT2), mRNA [NM_133491]	NM_133491
A_23_P69941	0.00133	7.042	AK002097	AK002097	Homo sapiens cDNA FLJ11235 fis, clone PLACE1008488. [AK002097]	XM_496773
A_32_P133780	0.00133	5.417	BQ189538	BQ189538	UI-E-EJ1-aka-o-17-0-UI.r1 UI-E-EJ1 Homo sapiens cDNA clone UI-E-EJ1-aka-o-17-0-UI 5', mRNA sequence [BQ189538]	
A_23_P215931	0.00133	3.387	NM_015344	NM_015344	Homo sapiens leptin receptor overlapping transcript-like 1 (LEPROTL1), mRNA [NM_015344]	NM_015344
A_24_P344516	0.00133	2.788	NM_024924	NM_024924	Homo sapiens hypothetical protein FLJ12985 (FLJ12985), mRNA [NM_024924]	NM_024924
A_24_P585004	0.00133	2.287	A_24_P585004			
A_23_P60565	0.00133	2.154	NM_005649	NM_005649	Homo sapiens zinc finger protein 354A (ZNF354A), mRNA [NM_005649]	NM_005649
A_23_P383009	0.00133	2	NM_000599	NM_000599	Homo sapiens insulin-like growth factor binding protein 5 (IGFBP5), mRNA [NM_000599]	NM_000599
A_32_P144381	0.00134	13.39	A_32_P144381			
A_32_P62963	0.00134	4.97	ENST00000323509			
A_32_P141938	0.00134	3.778	THC2453775			

A_32_P117313	0.00134	3.632	AF130048	AF130048	Homo sapiens clone FLB3344 PRO0845 mRNA, complete cds. [AF130048]	
A_23_P433690	0.00134	3.39	NM_018555	NM_018555	Homo sapiens zinc finger protein 331 (ZNF331), mRNA [NM_018555]	NM_018555
A_23_P407695	0.00135	44.55	NM_176782	NM_176782	Homo sapiens chromosome 1 open reading frame 179 (C1orf179), mRNA [NM_176782]	NM_176782
A_23_P145096	0.00135	39.11	NM_005084	NM_005084	Homo sapiens phospholipase A2, group VII (platelet-activating factor acetylhydrolase, plasma) (PLA2G7), mRNA [NM_005084]	NM_005084
A_23_P347059	0.00135	3.36	NM_173468	NM_173468	Homo sapiens MOB1, Mps One Binder kinase activator-like 1A (yeast) (MOBKL1A), mRNA [NM_173468]	NM_173468
A_23_P32955	0.00136	13.26	U08023	U08023	Human cellular proto-oncogene (c-mer) mRNA, complete cds. [U08023]	
A_24_P364066	0.00136	6.817	BC030112	BC030112	Homo sapiens, clone IMAGE:4799578, mRNA. [BC030112]	
A_23_P94095	0.00136	5.759	NM_198401	NM_198401	Homo sapiens ankyrin repeat domain 46 (ANKRD46), mRNA [NM_198401]	NM_198401
A_32_P115749	0.00136	5.216	CD104030	CD104030	AGENCOURT_14006900 NIH_MGC_186 Homo sapiens cDNA clone IMAGE:30372058 5', mRNA sequence [CD104030]	
A_24_P15114	0.00136	4.485	NM_020935	NM_020935	Homo sapiens ubiquitin specific protease 37 (USP37), mRNA [NM_020935]	NM_020935
A_23_P217737	0.00136	3.282	L06133	L06133	Human putative Cu++-transporting P-type ATPase mRNA, complete cds. [L06133]	
A_24_P205008	0.00136	3.28	AK094466	AK094466	Homo sapiens cDNA FLJ37147 fis, clone BRACE2025316, weakly similar to tRNA-splicing endonuclease subunit. [AK094466]	
A_32_P93036	0.00136	2.119	CA307890	CA307890	CA307890 UI-H-FT1-bib-a-05-0-UI.s1 NCI_CGAP_FT1 Homo sapiens cDNA clone UI-H-FT1-bib-a-05-0-UI 3', mRNA sequence [CA307890]	
A_23_P53668	0.00136	2.102	NM_006166	NM_006166	Homo sapiens nuclear transcription factor Y, beta (NFYB), mRNA [NM_006166]	NM_006166
A_32_P183765	0.00137	65.56	AK126298	AK126298	Homo sapiens cDNA FLJ44318 fis, clone TRACH3000780. [AK126298]	
A_23_P145054	0.00137	4.489	BC038997	BC038997	Homo sapiens chromosome 6 open reading frame 189, mRNA (cDNA clone IMAGE:6059932), partial cds. [BC038997]	
A_23_P324453	0.00137	4.342	NM_004876	NM_004876	Homo sapiens zinc finger protein 254 (ZNF254), mRNA [NM_004876]	NM_004876
A_32_P60343	0.00137	4.047	AK090778	AK090778	Homo sapiens cDNA FLJ33459 fis, clone BRAMY2000585. [AK090778]	XM_294765
A_23_P384965	0.00137	3.337	THC2314039		NAH9_HUMAN (Q8IVB4) Sodium/hydrogen exchanger 9 (Na(+)/H(+) exchanger 9) (NHE-9), partial (9%) [THC2314039]	
A_32_P180958	0.00137	3.034	THC2401540		ALU8_HUMAN (P39195) Alu subfamily SX sequence contamination warning entry, partial (27%) [THC2401540]	
A_24_P927537	0.00137	2.072	THC2269654			
A_23_P217528	0.00138	14.8	NM_007250	NM_007250	Homo sapiens Kruppel-like factor 8 (KLF8), mRNA [NM_007250]	NM_007250
A_23_P149613	0.00138	12.09	NM_002021	NM_002021	Homo sapiens flavin containing monooxygenase 1 (FMO1), mRNA [NM_002021]	NM_002021
A_23_P340263	0.00138	4.886	NM_173662	NM_173662	Homo sapiens ring finger protein 175 (RNF175), mRNA [NM_173662]	NM_173662
A_24_P73730	0.00138	2.828	NM_014157	NM_014157	Homo sapiens HSPC065 protein (HSPC065), mRNA [NM_014157]	NM_014157
A_23_P201963	0.00138	2.679	AF196185	AF196185	Homo sapiens atypical PKC isotype-specific interacting protein long variant mRNA, complete cds. [AF196185]	
A_23_P10743	0.00138	2.638	NM_000283	NM_000283	Homo sapiens phosphodiesterase 6B, cGMP-specific, rod, beta (congenital stationary night blindness 3, autosomal dominant) (PDE6B), mRNA [NM_000283]	NM_000283
A_23_P151405	0.00138	2.145	NM_018204	NM_018204	Homo sapiens cytoskeleton associated protein 2 (CKAP2), mRNA [NM_018204]	NM_018204
A_23_P213326	0.00138	2.102	NM_017747	NM_017747	Homo sapiens ankyrin repeat and KH domain containing 1 (ANKHD1), transcript variant 1, mRNA [NM_017747]	NM_017747
A_32_P42236	0.00139	3.798	XM_370839	XM_370839	PREDICTED: Homo sapiens similar to hypothetical protein (LOC440234), mRNA [XM_370839]	XM_370839
A_23_P132378	0.00139	3.297	NM_014246	NM_014246	Homo sapiens cadherin, EGF LAG seven-pass G-type receptor 1 (flamingo homolog, Drosophila) (CELSR1), mRNA [NM_014246]	NM_014246
A_32_P162524	0.00139	2.711	THC2339079		Q89FH7 (Q89FH7) Bll6723 protein, partial (3%) [THC2339079]	
A_24_P127928	0.00139	2.594	NM_012414	NM_012414	Homo sapiens rab3 GTPase-activating protein, non-catalytic subunit (150kD) (RAB3-GAP150), mRNA [NM_012414]	NM_012414
A_24_P90097	0.0014	6.474	NM_016824	NM_016824	Homo sapiens adducin 3 (gamma) (ADD3), transcript variant 1, mRNA [NM_016824]	NM_016824
A_23_P208737	0.0014	4.167	NM_004497	NM_004497	Homo sapiens forkhead box A3 (FOXA3), mRNA [NM_004497]	NM_004497
A_32_P41099	0.0014	3.86	THC2445411		Q5W952 (Q5W952) NADH dehydrogenase subunit 1 (Fragment), partial (6%) [THC2445411]	
A_23_P143285	0.0014	2.522	AK000809	AK000809	Homo sapiens cDNA FLJ20802 fis, clone ADSU01223. [AK000809]	
A_23_P206424	0.0014	2.343	NM_025134	NM_025134	Homo sapiens chromodomain helicase DNA binding protein 9 (CHD9), mRNA [NM_025134]	NM_025134
A_23_P70998	0.0014	2.221	ENST00000297227		full-length cDNA clone CS0DF028YG12 of Fetal brain of Homo sapiens (human). [CR590623]	

A_23_P129157	0.0014	2.174	NM_024608	NM_024608	Homo sapiens nei endonuclease VIII-like 1 (E. coli) (NEIL1), mRNA [NM_024608]	NM_024608
A_32_P133090	0.00141	3.925	XM_379210	XM_379210	PREDICTED: Homo sapiens hypothetical LOC401085 (LOC401085), mRNA [XM_379210]	XM_379210
A_23_P312246	0.00141	2.26	NM_024725	NM_024725	Homo sapiens hypothetical protein FLJ23518 (FLJ23518), mRNA [NM_024725]	NM_024725
A_23_P354805	0.00141	2.026	NM_007249	NM_007249	Homo sapiens Kruppel-like factor 12 (KLF12), transcript variant 1, mRNA [NM_007249]	NM_007249
A_24_P106542	0.00142	9.101	NM_032784	NM_032784	Homo sapiens thrombospondin, type I, domain containing 2 (THSD2), mRNA [NM_032784]	NM_032784
A_23_P259251	0.00142	6.918	NM_012281	NM_012281	Homo sapiens potassium voltage-gated channel, Shal-related subfamily, member 2 (KCND2), mRNA [NM_012281]	NM_012281
A_23_P145424	0.00142	4.429	NM_014895	NM_014895	Homo sapiens chromosome 6 open reading frame 84 (C6orf84), mRNA [NM_014895]	NM_014895
A_23_P211631	0.00142	4.21	NM_006486	NM_006486	Homo sapiens fibulin 1 (FBLN1), transcript variant D, mRNA [NM_006486]	NM_006486
A_23_P47135	0.00142	2.4	NM_005693	NM_005693	Homo sapiens nuclear receptor subfamily 1, group H, member 3 (NR1H3), mRNA [NM_005693]	NM_005693
A_23_P16242	0.00142	2.289	NM_021143	NM_021143	Homo sapiens zinc finger protein 20 (KOX 13) (ZNF20), mRNA [NM_021143]	NM_021143
A_24_P158421	0.00142	2.138	NM_020150	NM_020150	Homo sapiens SAR1a gene homolog 1 (S. cerevisiae) (SARA1), mRNA [NM_020150]	NM_020150
A_24_P113572	0.00143	9.888	NM_138415	NM_138415	Homo sapiens PHD finger protein 21B (PHF21B), mRNA [NM_138415]	NM_138415
A_32_P171386	0.00143	7.288	THC2455681			
A_24_P650011	0.00143	6.977	A_24_P650011			
A_24_P928830	0.00143	6.534	A_24_P928830			
A_23_P54781	0.00143	4.39	BC051317	BC051317	Homo sapiens retinoblastoma binding protein 6, mRNA (cDNA clone IMAGE:6214974), complete cds. [BC051317]	
A_23_P146444	0.00143	2.622	BX648086	BX648086	Homo sapiens mRNA; cDNA DKFZp686G19226 (from clone DKFZp686G19226). [BX648086]	
A_23_P388433	0.00143	2.348	ENST00000296496		full-length cDNA clone CS0DI016YF21 of Placenta Cot 25-normalized of Homo sapiens (human). [CR597270]	
A_32_P184488	0.00143	2.089	ENST00000292140		Homo sapiens cDNA clone IMAGE:4299555, partial cds. [BC007947]	
A_32_P58201	0.00144	9.483	AK021570	AK021570	Homo sapiens cDNA FLJ11508 fis, clone HEMBA1002162. [AK021570]	
A_24_P391230	0.00145	48.27	NM_052954	NM_052954	Homo sapiens cysteine/tyrosine-rich 1 (CYYR1), mRNA [NM_052954]	NM_052954
A_24_P724984	0.00145	4.76	AK001846	AK001846	Homo sapiens cDNA FLJ10984 fis, clone PLACE1001810. [AK001846]	
A_23_P46639	0.00145	4.431	NM_000562	NM_000562	Homo sapiens complement component 8, alpha polypeptide (C8A), mRNA [NM_000562]	NM_000562
A_23_P31376	0.00146	5.468	NM_018334	NM_018334	Homo sapiens leucine rich repeat neuronal 3 (LRRN3), mRNA [NM_018334]	NM_018334
A_32_P11230	0.00146	4.801	NM_001013665	NM_001013665	Homo sapiens hypothetical LOC399744 (LOC399744), mRNA [NM_001013665]	NM_001013665
A_24_P8220	0.00146	4.195	BC001196	BC001196	Homo sapiens, Similar to heparan sulfate 6-O-sulfotransferase, clone IMAGE:3355592, mRNA, partial cds. [BC001196]	
A_32_P32406	0.00146	3.809	X15675	X15675	Human pTR7 mRNA for repetitive sequence. [X15675]	
A_32_P101689	0.00146	2.761	NM_014888	NM_014888	Homo sapiens family with sequence similarity 3, member C (FAM3C), mRNA [NM_014888]	NM_014888
A_32_P24741	0.00146	2.701	AK093729	AK093729	Homo sapiens cDNA FLJ36410 fis, clone THYMU2010637. [AK093729]	XM_496251
A_23_P39814	0.00146	2.105	NM_004882	NM_004882	Homo sapiens CBF1 interacting corepressor (CIR), transcript variant 1, mRNA [NM_004882]	NM_004882
A_24_P42446	0.00147	5.974	NM_001015508	NM_001015508	Homo sapiens purine-rich element binding protein G (PURG), transcript variant B, mRNA [NM_001015508]	NM_001015508
A_23_P110412	0.00147	5.73	AK023055	AK023055	Homo sapiens cDNA FLJ12993 fis, clone NT2RP3000197. [AK023055]	
A_24_P937095	0.00147	4.852	U68494	U68494	Human hbc647 mRNA sequence. [U68494]	
A_23_P356731	0.00147	3.251	NM_152509	NM_152509	Homo sapiens hypothetical protein FLJ31568 (FLJ31568), mRNA [NM_152509]	NM_152509
A_24_P139208	0.00147	2.928	NM_013396	NM_013396	Homo sapiens ubiquitin specific protease 25 (USP25), mRNA [NM_013396]	NM_013396
A_24_P860842	0.00147	2.314	BC027988	BC027988	Homo sapiens hypothetical protein LOC253842, mRNA (cDNA clone IMAGE:4993690), partial cds. [BC027988]	
A_32_P171856	0.00147	2.057	THC2341414			
A_23_P135417	0.00148	95.08	NM_145740	NM_145740	Homo sapiens glutathione S-transferase A1 (GSTA1), mRNA [NM_145740]	NM_145740
A_32_P11670	0.00148	2.872	BC030083	BC030083	Homo sapiens, clone IMAGE:4791783, mRNA. [BC030083]	
A_23_P52362	0.00148	2.736	NM_003055	NM_003055	Homo sapiens solute carrier family 18 (vesicular acetylcholine), member 3 (SLC18A3), mRNA [NM_003055]	NM_003055
A_32_P62137	0.00148	2.51	A_32_P62137			
A_23_P83277	0.00148	2.118	NM_004512	NM_004512	Homo sapiens interleukin 11 receptor, alpha (IL11RA), transcript variant 1, mRNA [NM_004512]	NM_004512

A_23_P305120	0.00148	2.033	NM_144994	NM_144994	Homo sapiens ankyrin repeat domain 23 (ANKRD23), mRNA [NM_144994]	NM_144994
A_23_P217428	0.00149	15.77	NM_001174	NM_001174	Homo sapiens Rho GTPase activating protein 6 (ARHGAP6), transcript variant 2, mRNA [NM_001174]	NM_001174
A_32_P40673	0.00149	10.74	THC2281732			
A_24_P476086	0.00149	6.185	THC2339518		Q9P2R9 (Q9P2R9) SRp25 nuclear protein (ARL6IP4 protein), partial (6%) [THC2339518]	
A_23_P46812	0.00149	4.318	NM_014912	NM_014912	Homo sapiens cytoplasmic polyadenylation element binding protein 3 (CPEB3), mRNA [NM_014912]	NM_014912
A_32_P179526	0.0015	7.667	BC010934	BC010934	Homo sapiens zinc finger and BTB domain containing 20, mRNA (cDNA clone IMAGE:4291354), partial cds. [BC010934]	
A_23_P73809	0.0015	5.21	NM_020871	NM_020871	Homo sapiens leucine-rich repeats and calponin homology (CH) domain containing 2 (LRCH2), mRNA [NM_020871]	NM_020871
A_32_P6015	0.0015	4.812	NM_005515	NM_005515	Homo sapiens homeo box HB9 (HLXB9), mRNA [NM_005515]	NM_005515
A_23_P254756	0.0015	3.066	NM_006016	NM_006016	Homo sapiens CD164 antigen, sialomucin (CD164), mRNA [NM_006016]	NM_006016
A_23_P252664	0.00151	3.083	NM_000150	NM_000150	Homo sapiens fucosyltransferase 6 (alpha (1,3) fucosyltransferase) (FUT6), mRNA [NM_000150]	NM_000150
A_23_P259594	0.00151	2.501	NM_016377	NM_016377	Homo sapiens A kinase (PRKA) anchor protein 7 (AKAP7), transcript variant gamma, mRNA [NM_016377]	NM_016377
A_23_P208238	0.00151	2.375	NM_003438	NM_003438	Homo sapiens zinc finger protein 137 (clone pHZ-30) (ZNF137), mRNA [NM_003438]	NM_003438
A_24_P586390	0.00151	2.166	AK123446	AK123446	Homo sapiens cDNA FLJ41452 fis, clone BRSTN2010363. [AK123446]	XM_379275
A_23_P52121	0.00152	59.95	NM_002614	NM_002614	Homo sapiens PDZ domain containing 1 (PDZK1), mRNA [NM_002614]	NM_002614
A_23_P91552	0.00152	21.72	NM_206965	NM_206965	Homo sapiens formiminotransferase cyclodeaminase (FTCD), transcript variant A, mRNA [NM_206965]	NM_206965
A_24_P56240	0.00152	11.44	NM_153634	NM_153634	Homo sapiens copine VIII (CPNE8), mRNA [NM_153634]	NM_153634
A_23_P79032	0.00152	3.911	BC004943	BC004943	Homo sapiens hypothetical protein MGC10814, mRNA (cDNA clone MGC:10814 IMAGE:3613095), complete cds. [BC004943]	
A_24_P839530	0.00152	3.192	A_24_P839530			
A_23_P115645	0.00153	12.1	NM_006561	NM_006561	Homo sapiens CUG triplet repeat, RNA binding protein 2 (CUGBP2), transcript variant 2, mRNA [NM_006561]	NM_006561
A_23_P21804	0.00153	6.363	A_23_P21804			
A_24_P97785	0.00153	3.847	CR611332	CR611332	full-length cDNA clone CS0DF014YA22 of Fetal brain of Homo sapiens (human). [CR611332]	
A_23_P373927	0.00153	2.895	NM_015051	NM_015051	Homo sapiens thioredoxin domain containing 4 (endoplasmic reticulum) (TXNDC4), mRNA [NM_015051]	NM_015051
A_32_P197720	0.00153	2.776	CR738137	CR738137	CR738137 Soares_testis_NHT Homo sapiens cDNA clone IMAGp971D1846 ; IMAGE:731424 5', mRNA sequence [CR738137]	
A_24_P839239	0.00153	2.625	AK002036	AK002036	Homo sapiens cDNA FLJ11174 fis, clone PLACE1007367. [AK002036]	
A_24_P391991	0.00153	2.598	AK124132	AK124132	Homo sapiens cDNA FLJ42138 fis, clone TESTI2036684. [AK124132]	XM_295200
A_23_P77468	0.00153	2.132	AK024389	AK024389	Homo sapiens cDNA FLJ14327 fis, clone PLACE4000250. [AK024389]	
A_24_P341538	0.00154	8.651	NM_201286	NM_201286	Homo sapiens ubiquitin specific protease 51 (USP51), mRNA [NM_201286]	NM_201286
A_23_P130359	0.00154	8.638	NM_030672	NM_030672	Homo sapiens Rho GTPase activating protein 28 (ARHGAP28), transcript variant 2, mRNA [NM_030672]	NM_030672
A_23_P143952	0.00154	2.689	NM_024548	NM_024548	Homo sapiens leucine-rich repeats and IQ motif containing 2 (LRRIQ2), mRNA [NM_024548]	NM_024548
A_23_P201521	0.00155	3.604	A_23_P201521			
A_32_P209208	0.00156	10.59	AK091839	AK091839	Homo sapiens cDNA FLJ34520 fis, clone HLUNG2006935, weakly similar to CALCYPHOSINE. [AK091839]	
A_32_P68504	0.00156	9.76	ENST00000356022		Homo sapiens mRNA for KIAA1571 protein, partial cds. [AB046791]	XM_371590
A_32_P226356	0.00156	5.344	THC2317058			
A_23_P113212	0.00156	2.617	NM_018004	NM_018004	Homo sapiens transmembrane protein 45A (TMEM45A), mRNA [NM_018004]	NM_018004
A_24_P14634	0.00156	2.424	NM_133455	NM_133455	Homo sapiens EMI domain containing 1 (EMID1), mRNA [NM_133455]	NM_133455
A_23_P348146	0.00157	29.77	NM_144595	NM_144595	Homo sapiens hypothetical protein FLJ30046 (FLJ30046), mRNA [NM_144595]	NM_144595
A_32_P179646	0.00157	4.647	A_32_P179646			
A_23_P258612	0.00157	3.854	NM_016529	NM_016529	Homo sapiens ATPase, aminophospholipid transporter-like, Class I, type 8A, member 2 (ATP8A2), mRNA [NM_016529]	NM_016529
A_24_P942469	0.00157	2.844	AK129652	AK129652	Homo sapiens cDNA FLJ26141 fis, clone TST03911. [AK129652]	
A_24_P166311	0.00157	2.704	NM_022090	NM_022090	Homo sapiens transposon-derived Buster3 transposase-like (LOC63920), mRNA [NM_022090]	NM_022090
A_32_P58029	0.00157	2.464	A_32_P58029			

A_23_P91910	0.00158	4.486	NM_020353	NM_020353	Homo sapiens phospholipid scramblase 4 (PLSCR4), mRNA [NM_020353]	NM_020353
A_23_P410613	0.00158	2.874	NM_152261	NM_152261	Homo sapiens hypothetical protein MGC17943 (MGC17943), mRNA [NM_152261]	NM_152261
A_23_P35970	0.00158	2.296	NM_001467	NM_001467	Homo sapiens solute carrier family 37 (glycerol-6-phosphate transporter), member 4 (SLC37A4), mRNA [NM_001467]	NM_001467
A_24_P129232	0.00158	2.203	NM_020755	NM_020755	Homo sapiens tumor differentially expressed 2 (TDE2), mRNA [NM_020755]	NM_020755
A_24_P278234	0.00159	7.124	AB075864	AB075864	Homo sapiens mRNA for KIAA1984 protein. [AB075864]	
A_24_P367576	0.00159	2.98	AK125170	AK125170	Homo sapiens cDNA FLJ43180 fis, clone FCBBF3013846. [AK125170]	
A_23_P93818	0.00159	2.697	ENST00000305954		Homo sapiens mRNA; cDNA DKFZp434A0131 (from clone DKFZp434A0131). [AL137492]	
A_23_P54447	0.0016	12.09	NM_030944	NM_030944	Homo sapiens chromosome 15 open reading frame 5 (C15orf5), mRNA [NM_030944]	NM_030944
A_32_P167459	0.0016	2.995	AK021744	AK021744	Homo sapiens cDNA FLJ11682 fis, clone HEMBA1004880. [AK021744]	
A_23_P29994	0.0016	2.738	NM_203284	NM_203284	Homo sapiens recombining binding protein suppressor of hairless (Drosophila) (RBPSUH), transcript variant 4, mRNA [NM_203284]	NM_203284
A_23_P335958	0.0016	2.646	NM_152529	NM_152529	Homo sapiens G protein-coupled receptor 155 (GPR155), mRNA [NM_152529]	NM_152529
A_24_P222114	0.00161	10.29	NM_018177	NM_018177	Homo sapiens Nedd4 binding protein 2 (N4BP2), mRNA [NM_018177]	NM_018177
A_23_P379327	0.00161	5.058	AB032990	AB032990	Homo sapiens mRNA for KIAA1164 protein, partial cds. [AB032990]	
A_32_P233256	0.00161	4.151	THC2412206			
A_23_P320897	0.00161	3.935	NM_019049	NM_019049	Homo sapiens hypothetical protein FLJ20054 (FLJ20054), mRNA [NM_019049]	NM_019049
A_24_P105761	0.00161	3.445	NM_018433	NM_018433	Homo sapiens jumonji domain containing 1A (JMJD1A), mRNA [NM_018433]	NM_018433
A_24_P944458	0.00161	2.807	NM_016133	NM_016133	Homo sapiens insulin induced gene 2 (INSIG2), mRNA [NM_016133]	NM_016133
A_32_P103508	0.00161	2.474	AK096022	AK096022	Homo sapiens cDNA FLJ38703 fis, clone KIDNE2002265. [AK096022]	
A_23_P259506	0.00161	2.378	NM_032412	NM_032412	Homo sapiens putative nuclear protein ORF1-FL49 (ORF1-FL49), mRNA [NM_032412]	NM_032412
A_32_P10311	0.00161	2.035	AF305816	AF305816	Homo sapiens PRO0633 mRNA, complete cds. [AF305816]	
A_24_P148750	0.00162	2.815	NM_004844	NM_004844	Homo sapiens SH3-domain binding protein 5 (BTK-associated) (SH3BP5), transcript variant 1, mRNA [NM_004844]	NM_004844
A_32_P192594	0.00162	2.761	CR624390	CR624390	full-length cDNA clone CS0DC018YB19 of Neuroblastoma Cot 25-normalized of Homo sapiens (human). [CR624390]	XM_378399
A_23_P151059	0.00162	2.583	NM_018088	NM_018088	Homo sapiens hypothetical protein FLJ10408 (FLJ10408), mRNA [NM_018088]	NM_018088
A_23_P213754	0.00162	2.527	NM_016480	NM_016480	Homo sapiens poly(A) binding protein interacting protein 2 (PAIP2), mRNA [NM_016480]	NM_016480
A_23_P213137	0.00163	2.557	NM_032622	NM_032622	Homo sapiens ligand of numb-protein X (LNX), mRNA [NM_032622]	NM_032622
A_23_P61945	0.00163	2.481	NM_198159	NM_198159	Homo sapiens microphthalmia-associated transcription factor (MITF), transcript variant 1, mRNA [NM_198159]	NM_198159
A_23_P120062	0.00163	2.117	NM_003203	NM_003203	Homo sapiens chromosome 2 open reading frame 3 (C2orf3), mRNA [NM_003203]	NM_003203
A_24_P924484	0.00164	4.676	K03200	K03200	Human melanoma-associated antigen p97 (melanotransferrin) mRNA, 3' flank. [K03200]	
A_24_P174613	0.00164	3.652	NM_033632	NM_033632	Homo sapiens F-box and WD-40 domain protein 7 (archipelago homolog, Drosophila) (FBXW7), transcript variant 1, mRNA [NM_033632]	NM_033632
A_32_P213692	0.00164	2.348	ENST00000216468		Homo sapiens cDNA: FLJ22042 fis, clone HEP09065. [AK025695]	
A_23_P148602	0.00164	2.169	NM_003669	NM_003669	Homo sapiens inactivation escape 1 (INE1), mRNA [NM_003669]	NM_003669
A_24_P23258	0.00164	2.044	NM_015124	NM_015124	Homo sapiens death-inducing-protein (DIP), mRNA [NM_015124]	NM_015124
A_23_P65532	0.00165	6.653	NM_021255	NM_021255	Homo sapiens pellino homolog 2 (Drosophila) (PELI2), mRNA [NM_021255]	NM_021255
A_32_P49116	0.00165	4.615	CR617865	CR617865	full-length cDNA clone CS0DF021YD16 of Fetal brain of Homo sapiens (human). [CR617865]	
A_23_P156811	0.00165	4.465	NM_001018022	NM_001018022	Homo sapiens similar to FKSG62 (LOC389286), mRNA [NM_001018022]	NM_001018022
A_32_P184746	0.00165	4.323	THC2378401			
A_24_P892402	0.00165	4.206	AK057652	AK057652	Homo sapiens cDNA FLJ33090 fis, clone TRACH2000559. [AK057652]	
A_23_P358995	0.00165	4.051	NM_015525	NM_015525	Homo sapiens inhibitor of Bruton agammaglobulinemia tyrosine kinase (IBTK), mRNA [NM_015525]	NM_015525
A_23_P346670	0.00165	3.073	AK000249	AK000249	Homo sapiens cDNA FLJ20242 fis, clone COLF6369. [AK000249]	
A_32_P141708	0.00165	2.433	THC2374166		BM091097 ig22c10.y1 Human Fetal Pancreas 1B Homo sapiens cDNA 5', mRNA sequence [BM091097]	
A_32_P43878	0.00166	6.801	THC2379275			

A_32_P185530	0.00166	4.976	THC2316492			
A_23_P413888	0.00166	3.037	NM_001029858	NM_001029858	Homo sapiens solute carrier family 35, member F1 (SLC35F1), mRNA [NM_001029858]	NM_001029858
A_24_P576191	0.00166	2.289	CR593388	CR593388	full-length cDNA clone CS0DI067YA03 of Placenta Cot 25-normalized of Homo sapiens (human). [CR593388]	
A_23_P212497	0.00166	2.158	NM_032169	NM_032169	Homo sapiens putative acyl-CoA dehydrogenase (FLJ12592), mRNA [NM_032169]	NM_032169
A_23_P29985	0.00166	2.131	AK022953	AK022953	Homo sapiens cDNA FLJ12891 fis, clone NT2RP2004142. [AK022953]	
A_23_P167417	0.00166	2.014	NM_015455	NM_015455	Homo sapiens CCR4-NOT transcription complex, subunit 6 (CNOT6), mRNA [NM_015455]	NM_015455
A_23_P77103	0.00166	2.007	NM_003104	NM_003104	Homo sapiens sorbitol dehydrogenase (SORD), mRNA [NM_003104]	NM_003104
A_24_P394408	0.00167	2.364	NM_014423	NM_014423	Homo sapiens AF4/FMR2 family, member 4 (AFF4), mRNA [NM_014423]	NM_014423
A_23_P369210	0.00167	2.155	NM_000084	NM_000084	Homo sapiens chloride channel 5 (nephrolithiasis 2, X-linked, Dent disease) (CLCN5), mRNA [NM_000084]	NM_000084
A_23_P408271	0.00168	2.546	NM_016245	NM_016245	Homo sapiens dehydrogenase/reductase (SDR family) member 8 (DHRS8), mRNA [NM_016245]	NM_016245
A_32_P129950	0.00169	5.659	NM_001012754	NM_001012754	Homo sapiens similar to RIKEN cDNA 8030451K01 (LOC387921), transcript variant 1, mRNA [NM_001012754]	NM_001012754
A_23_P4604	0.00169	3.346	NM_198457	NM_198457	Homo sapiens zinc finger protein 600 (ZNF600), mRNA [NM_198457]	NM_198457
A_23_P125117	0.00169	3.16	NM_173546	NM_173546	Homo sapiens hypothetical protein MGC35097 (MGC35097), mRNA [NM_173546]	NM_173546
A_24_P357576	0.00169	2.621	NM_019600	NM_019600	Homo sapiens hypothetical protein FLJ10980 (FLJ10980), mRNA [NM_019600]	NM_019600
A_23_P34396	0.00169	2.149	NM_020317	NM_020317	Homo sapiens chromosome 1 open reading frame 63 (C1orf63), transcript variant 2, mRNA [NM_020317]	NM_020317
A_23_P150609	0.0017	22.24	NM_001007139	NM_001007139	Homo sapiens putative insulin-like growth factor II associated protein (LOC492304), mRNA [NM_001007139]	NM_001007139
A_24_P589028	0.0017	18.65	AK026750	AK026750	Homo sapiens cDNA: FLJ23097 fis, clone LNG07418. [AK026750]	
A_32_P113736	0.0017	3.658	THC2282618			
A_32_P179807	0.0017	3.479	A_32_P179807			
A_24_P172768	0.0017	2.378	NM_004124	NM_004124	Homo sapiens glia maturation factor, beta (GMFB), mRNA [NM_004124]	NM_004124
A_24_P196592	0.0017	2.282	NM_024302	NM_024302	Homo sapiens matrix metalloproteinase 28 (MMP28), transcript variant 1, mRNA [NM_024302]	NM_024302
A_23_P50418	0.0017	2.063	NM_153358	NM_153358	Homo sapiens hypothetical protein FLJ90396 (FLJ90396), mRNA [NM_153358]	NM_153358
A_32_P69475	0.00171	2.846	AK023633	AK023633	Homo sapiens cDNA FLJ13571 fis, clone PLACE1008405. [AK023633]	
A_23_P217611	0.00171	2.109	NM_016607	NM_016607	Homo sapiens armadillo repeat containing, X-linked 3 (ARMCX3), transcript variant 1, mRNA [NM_016607]	NM_016607
A_23_P56659	0.00172	4.11	NM_133637	NM_133637	Homo sapiens DEAQ box polypeptide 1 (RNA-dependent ATPase) (DQX1), mRNA [NM_133637]	NM_133637
A_23_P43150	0.00172	3.486	NM_001017926	NM_001017926	Homo sapiens zinc fingers and homeoboxes 1 (ZHX1), transcript variant 1, mRNA [NM_001017926]	NM_001017926
A_23_P422933	0.00172	3.478	NM_020809	NM_020809	Homo sapiens Rho GTPase activating protein 20 (ARHGAP20), mRNA [NM_020809]	NM_020809
A_32_P2883	0.00172	3.152	THC2269190		Q7PWY5 (Q7PWY5) ENSANGP00000012061, partial (5%) [THC2269190]	
A_24_P277456	0.00172	2.706	CR605719	CR605719	full-length cDNA clone CS0DK002YG10 of HeLa cells Cot 25-normalized of Homo sapiens (human). [CR605719]	
A_23_P375147	0.00172	2.565	AK125196	AK125196	Homo sapiens cDNA FLJ43206 fis, clone FEBRA2009419. [AK125196]	
A_32_P136982	0.00172	2.193	XM_088677	XM_088677	PREDICTED: Homo sapiens similar to UPF3 regulator of nonsense transcripts homolog B isoform 2 (LOC158796), mRNA [XM_088677]	XM_088677
A_23_P129258	0.00172	2.162	A_23_P129258			
A_23_P123315	0.00172	2.047	BC067244	BC067244	Homo sapiens cDNA clone IMAGE:4807381, partial cds. [BC067244]	
A_32_P52046	0.00173	4.669	A_32_P52046			
A_24_P551028	0.00173	2.978	NM_001001664	NM_001001664	Homo sapiens hypothetical protein LOC339745 (LOC339745), mRNA [NM_001001664]	NM_001001664
A_32_P20703	0.00174	10.69	AK026418	AK026418	Homo sapiens cDNA: FLJ22765 fis, clone KAIA1180. [AK026418]	
A_23_P317919	0.00174	6.202	NM_021148	NM_021148	Homo sapiens zinc finger protein 273 (ZNF273), mRNA [NM_021148]	NM_021148
A_24_P371399	0.00174	4.027	NM_173552	NM_173552	Homo sapiens hypothetical protein MGC33365 (MGC33365), mRNA [NM_173552]	NM_173552
A_23_P252642	0.00174	3.999	NM_152384	NM_152384	Homo sapiens Bardet-Biedl syndrome 5 (BBS5), mRNA [NM_152384]	NM_152384
A_23_P156355	0.00174	2.156	NM_153354	NM_153354	Homo sapiens hypothetical protein MGC33214 (MGC33214), mRNA [NM_153354]	NM_153354
A_23_P211603	0.00174	2.06	A_23_P211603			
A_32_P217750	0.00175	5.029	NM_002183	NM_002183	Homo sapiens interleukin 3 receptor, alpha (low affinity) (IL3RA), mRNA [NM_002183]	NM_002183

A_24_P923934	0.00175	3.924	AF085846	AF085846	Homo sapiens full length insert cDNA clone YI46F09. [AF085846]	
A_23_P132611	0.00175	3.712	AF088066	AF088066	Homo sapiens full length insert cDNA clone ZD86C03. [AF088066]	
A_23_P423197	0.00175	2.296	NM_002957	NM_002957	Homo sapiens retinoid X receptor, alpha (RXRA), mRNA [NM_002957]	NM_002957
A_23_P5422	0.00175	2.277	AB082523	AB082523	Homo sapiens mRNA for KIAA1992 protein. [AB082523]	
A_24_P81965	0.00175	2.002	NM_021033	NM_021033	Homo sapiens RAP2A, member of RAS oncogene family (RAP2A), mRNA [NM_021033]	NM_021033
A_23_P254212	0.00176	3.511	NM_013347	NM_013347	Homo sapiens replication protein A4, 34kDa (RPA4), mRNA [NM_013347]	NM_013347
A_23_P372308	0.00176	2.755	NM_020211	NM_020211	Homo sapiens RGM domain family, member A (RGMA), mRNA [NM_020211]	NM_020211
A_32_P155811	0.00176	2.707	THC2276639			
A_23_P161399	0.00177	2.674	NM_130439	NM_130439	Homo sapiens MAX interactor 1 (MXI1), transcript variant 2, mRNA [NM_130439]	NM_130439
A_23_P47220	0.00177	2.245	A_23_P47220			
A_23_P356139	0.00177	2.01	NM_018121	NM_018121	Homo sapiens chromosome 10 open reading frame 6 (C10orf6), mRNA [NM_018121]	NM_018121
A_23_P23611	0.00179	26.36	NM_001008219	NM_001008219	Homo sapiens amylase, alpha 1C; salivary (AMY1C), mRNA [NM_001008219]	NM_001008219
A_23_P209578	0.00179	3.998	NM_013445	NM_013445	Homo sapiens glutamate decarboxylase 1 (brain, 67kDa) (GAD1), transcript variant GAD25, mRNA [NM_013445]	NM_013445
A_23_P150919	0.00179	3.175	ENST00000330079		Homo sapiens cDNA FLJ14466 fis, clone MAMMA1000416. [AK027372]	
A_23_P51996	0.00179	2.23	NM_007269	NM_007269	Homo sapiens syntaxin binding protein 3 (STXBP3), mRNA [NM_007269]	NM_007269
A_23_P156117	0.0018	2.209	NM_014376	NM_014376	Homo sapiens cytoplasmic FMR1 interacting protein 2 (CYFIP2), mRNA [NM_014376]	NM_014376
A_24_P636390	0.0018	2.132	AF009267	AF009267	Homo sapiens clone FBA1 Cri-du-chat region mRNA. [AF009267]	
A_32_P41526	0.00181	2.085	NM_133446	NM_133446	Homo sapiens centaurin, gamma-like family, member 1 (CTGLF1), mRNA [NM_133446]	NM_133446
A_23_P98282	0.00182	3.452	NM_006946	NM_006946	Homo sapiens spectrin, beta, non-erythrocytic 2 (SPTBN2), mRNA [NM_006946]	NM_006946
A_24_P291231	0.00182	2.971	NM_016831	NM_016831	Homo sapiens period homolog 3 (Drosophila) (PER3), mRNA [NM_016831]	NM_016831
A_24_P95273	0.00182	2.713	NM_015046	NM_015046	Homo sapiens amyotrophic lateral sclerosis 4 (ALS4), mRNA [NM_015046]	NM_015046
A_32_P180315	0.00183	5.827	NM_020893	NM_020893	Homo sapiens KIAA1529 (KIAA1529), mRNA [NM_020893]	NM_020893
A_23_P8482	0.00183	3.589	NM_001011553	NM_001011553	Homo sapiens septin 7 (SEPT7), transcript variant 2, mRNA [NM_001011553]	NM_001011553
A_24_P244952	0.00183	2.213	NM_015092	NM_015092	Homo sapiens PI-3-kinase-related kinase SMG-1 (SMG1), mRNA [NM_015092]	NM_015092
A_23_P122937	0.00184	13.11	NM_014800	NM_014800	Homo sapiens engulfment and cell motility 1 (ced-12 homolog, C. elegans) (ELMO1), transcript variant 1, mRNA [NM_014800]	NM_014800
A_24_P630490	0.00184	5.169	BC020859	BC020859	Homo sapiens, Similar to deafness, autosomal dominant 5 homolog (human), clone IMAGE:4551670, mRNA. [BC020859]	
A_24_P148503	0.00184	4.546	AK024850	AK024850	Homo sapiens cDNA: FLJ21197 fis, clone COL00201. [AK024850]	
A_23_P314250	0.00184	4.257	NM_033387	NM_033387	Homo sapiens family with sequence similarity 78, member A (FAM78A), mRNA [NM_033387]	NM_033387
A_23_P202587	0.00184	4.006	NM_018330	NM_018330	Homo sapiens KIAA1598 (KIAA1598), mRNA [NM_018330]	NM_018330
A_24_P923676	0.00184	3.663	X15674	X15674	Human pTR5 mRNA for repetitive sequence. [X15674]	
A_23_P326893	0.00184	3.515	NM_145045	NM_145045	Homo sapiens hypothetical protein MGC20983 (MGC20983), mRNA [NM_145045]	NM_145045
A_23_P157569	0.00184	3.247	NM_144650	NM_144650	Homo sapiens alcohol dehydrogenase, iron containing, 1 (ADHFE1), mRNA [NM_144650]	NM_144650
A_23_P255812	0.00184	2.816	NM_016127	NM_016127	Homo sapiens hypothetical protein MGC8721 (MGC8721), mRNA [NM_016127]	NM_016127
A_23_P164958	0.00184	2.315	NM_032040	NM_032040	Homo sapiens coiled-coil domain containing 8 (CCDC8), mRNA [NM_032040]	NM_032040
A_23_P35546	0.00185	12.5	THC2344956			
A_23_P3602	0.00185	2.165	THC2303868		Q99P30 (Q99P30) Coenzyme A diphosphatase, partial (64%) [THC2303868]	
A_23_P119794	0.00185	2.087	CR608907	CR608907	full-length cDNA clone CS0DM002YA18 of Fetal liver of Homo sapiens (human). [CR608907]	
A_23_P68487	0.00186	5.013	NM_001719	NM_001719	Homo sapiens bone morphogenetic protein 7 (osteogenic protein 1) (BMP7), mRNA [NM_001719]	NM_001719
A_23_P149270	0.00186	3.544	THC2249196		CICL_HUMAN (P51801) Chloride channel protein ClC-Kb (Chloride channel Kb) (ClC-K2), complete [THC2249196]	
A_24_P450372	0.00186	3.481	L40520	L40520	Homo sapiens (clone DR3) mRNA fragment. [L40520]	
A_23_P347632	0.00186	3.104	NM_014751	NM_014751	Homo sapiens metastasis suppressor 1 (MTSS1), mRNA [NM_014751]	NM_014751
A_23_P210074	0.00186	2.488	NM_032788	NM_032788	Homo sapiens zinc finger protein 514 (ZNF514), mRNA [NM_032788]	NM_032788

A_23_P121869	0.00186	2.227	NM_021036	NM_021036	Homo sapiens SMA5 (SMA5), mRNA [NM_021036]	NM_021036
A_23_P8981	0.00187	8.026	NM_000349	NM_000349	Homo sapiens steroidogenic acute regulator (STAR), nuclear gene encoding mitochondrial protein, transcript variant 1, mRNA [NM_000349]	NM_000349
A_32_P159535	0.00187	7.769	THC2303047		RS24_HUMAN (P62847) 40S ribosomal protein S24, partial (77%) [THC2303047]	
A_23_P125643	0.00187	7.629	NM_024087	NM_024087	Homo sapiens ankyrin repeat and SOCS box-containing 9 (ASB9), mRNA [NM_024087]	NM_024087
A_24_P307395	0.00187	4.87	ENST00000332097			
A_23_P6651	0.00187	3.063	NM_015224	NM_015224	Homo sapiens retinoblastoma-associated protein 140 (RAP140), mRNA [NM_015224]	NM_015224
A_24_P128187	0.00187	2.876	BC034499	BC034499	Homo sapiens hypothetical protein LOC148203, mRNA (cDNA clone MGC:26923 IMAGE:4838161), complete cds. [BC034499]	
A_32_P110485	0.00188	4.872	THC2315024			
A_32_P165297	0.00189	18.87	CR627122	CR627122	Homo sapiens mRNA; cDNA DKFZp779M2422 (from clone DKFZp779M2422). [CR627122]	
A_32_P23209	0.00189	9.071	THC2448537		ALU6_HUMAN (P39193) Alu subfamily SP sequence contamination warning entry, partial (8%) [THC2448537]	
A_23_P94840	0.00189	5.684	NM_130897	NM_130897	Homo sapiens dynein, cytoplasmic, light polypeptide 2B (DNCL2B), mRNA [NM_130897]	NM_130897
A_23_P342709	0.00189	4.791	NM_152676	NM_152676	Homo sapiens F-box protein 15 (FBXO15), mRNA [NM_152676]	NM_152676
A_24_P203630	0.00189	3.054	NM_001010914	NM_001010914	Homo sapiens protein immuno-reactive with anti-PTH polyclonal antibodies (LOC400986), mRNA [NM_001010914]	NM_001010914
A_23_P390172	0.00189	2.57	NM_021133	NM_021133	Homo sapiens ribonuclease L (2',5'-oligoisoadenylate synthetase-dependent) (RNASEL), mRNA [NM_021133]	NM_021133
A_24_P240187	0.0019	11.63	NM_020873	NM_020873	Homo sapiens leucine rich repeat neuronal 1 (LRRN1), mRNA [NM_020873]	NM_020873
A_23_P125705	0.0019	4.568	NM_021963	NM_021963	Homo sapiens nucleosome assembly protein 1-like 2 (NAP1L2), mRNA [NM_021963]	NM_021963
A_23_P45821	0.0019	4.511	THC2281747		Q96KY5 (Q96KY5) MGC27169 protein, complete [THC2281747]	
A_32_P181826	0.0019	2.558	THC2342112			
A_23_P25615	0.00191	4.851	NM_017826	NM_017826	Homo sapiens hypothetical protein FLJ20449 (FLJ20449), mRNA [NM_017826]	NM_017826
A_24_P33989	0.00191	4.378	NM_022351	NM_022351	Homo sapiens EF-hand calcium binding protein 1 (EFCBP1), mRNA [NM_022351]	NM_022351
A_24_P488352	0.00191	3.95	THC2280913		ALU1_HUMAN (P39188) Alu subfamily J sequence contamination warning entry, partial (12%) [THC2280913]	
A_23_P436369	0.00191	2.545	NM_015687	NM_015687	Homo sapiens filamin A interacting protein 1 (FILIP1), mRNA [NM_015687]	NM_015687
A_32_P92654	0.00191	2.035	NM_021036	NM_021036	Homo sapiens SMA5 (SMA5), mRNA [NM_021036]	NM_021036
A_32_P123966	0.00191	2.029	NM_015272	NM_015272	Homo sapiens KIAA1005 protein (KIAA1005), mRNA [NM_015272]	NM_015272
A_32_P232214	0.00192	17.21	THC2316753		Q91TG6 (Q91TG6) T130, partial (7%) [THC2316753]	
A_23_P74993	0.00192	6.059	AK096104	AK096104	Homo sapiens cDNA FLJ38785 fis, clone LIVER2001329. [AK096104]	
A_24_P399980	0.00192	4.866	NM_014799	NM_014799	Homo sapiens hephaestin (HEPH), transcript variant 2, mRNA [NM_014799]	NM_014799
A_23_P9875	0.00192	2.85	NM_007170	NM_007170	Homo sapiens testis-specific kinase 2 (TESK2), mRNA [NM_007170]	NM_007170
A_23_P55666	0.00192	2.544	AK023047	AK023047	Homo sapiens cDNA FLJ12985 fis, clone NT2RP3000050, moderately similar to ZINC FINGER PROTEIN 91. [AK023047]	
A_23_P139558	0.00192	2.524	NM_022771	NM_022771	Homo sapiens TBC1 domain family, member 15 (TBC1D15), mRNA [NM_022771]	NM_022771
A_23_P352291	0.00192	2.485	NM_054016	NM_054016	Homo sapiens FUS interacting protein (serine/arginine-rich) 1 (FUSIP1), transcript variant 2, mRNA [NM_054016]	NM_054016
A_24_P940426	0.00193	4.889	NM_206855	NM_206855	Homo sapiens quaking homolog, KH domain RNA binding (mouse) (QKI), transcript variant 4, mRNA [NM_206855]	NM_206855
A_23_P147199	0.00193	2.604	NM_006629	NM_006629	Homo sapiens zinc finger protein 271 (ZNF271), mRNA [NM_006629]	NM_006629
A_24_P573533	0.00193	2.504	NM_172003	NM_172003	Homo sapiens COBW domain containing 2 (CBWD2), mRNA [NM_172003]	NM_172003
A_23_P374695	0.00194	4.504	NM_000459	NM_000459	Homo sapiens TEK tyrosine kinase, endothelial (venous malformations, multiple cutaneous and mucosal) (TEK), mRNA [NM_000459]	NM_000459
A_32_P219148	0.00194	3.203	BC030211	BC030211	Homo sapiens, clone IMAGE:5199989, mRNA. [BC030211]	
A_23_P216693	0.00195	2.653	NM_004529	NM_004529	Homo sapiens myeloid/lymphoid or mixed-lineage leukemia (trithorax homolog, Drosophila); translocated to, 3 (MLLT3), mRNA [NM_004529]	NM_004529
A_32_P116488	0.00196	4.88	THC2283842			
A_23_P158885	0.00196	3.251	AK096342	AK096342	Homo sapiens cDNA FLJ39023 fis, clone NT2RP7004348, highly similar to ZINC FINGER PROTEIN 93. [AK096342]	

A_32_P117170	0.00196	2.77	ENST00000341533		Homo sapiens hypothetical metallo-hydrolase/oxidoreductase structure-containing protein (C7orf18) mRNA, complete cds. [AY357337]	
A_32_P38623	0.00197	38.21	BC037849	BC037849	Homo sapiens cDNA clone IMAGE:4815736, partial cds. [BC037849]	
A_23_P203391	0.00197	3.546	BC006267	BC006267	Homo sapiens asparaginase like 1, mRNA (cDNA clone IMAGE:3952485), complete cds. [BC006267]	
A_23_P161998	0.00199	12.56	NM_000613	NM_000613	Homo sapiens hemopexin (HPX), mRNA [NM_000613]	NM_000613
A_23_P256158	0.00199	3.487	NM_000683	NM_000683	Homo sapiens adrenergic, alpha-2C-, receptor (ADRA2C), mRNA [NM_000683]	NM_000683
A_23_P65217	0.00199	2.29	NM_014305	NM_014305	Homo sapiens TDP-glucose 4,6-dehydratase (TGDS), mRNA [NM_014305]	NM_014305
A_32_P231226	0.00199	2.203	AK095046	AK095046	Homo sapiens cDNA FLJ37727 fis, clone BRHIP2019972. [AK095046]	
A_23_P11017	0.002	20.2	AK021866	AK021866	Homo sapiens cDNA FLJ11804 fis, clone HEMBA1006272, moderately similar to RETROVIRUS-RELATED PROTEASE (EC 3.4.23.-). [AK021866]	XM_496515
A_24_P122940	0.002	4.897	THC2440296			
A_23_P361049	0.002	2.495	NM_012223	NM_012223	Homo sapiens myosin IB (MYO1B), mRNA [NM_012223]	NM_012223
A_23_P300033	0.00201	5.361	NM_006206	NM_006206	Homo sapiens platelet-derived growth factor receptor, alpha polypeptide (PDGFRA), mRNA [NM_006206]	NM_006206
A_23_P133359	0.00201	4.895	NM_030613	NM_030613	Homo sapiens zinc finger protein 2 homolog (mouse) (ZFP2), mRNA [NM_030613]	NM_030613
A_23_P57268	0.00202	8.94	NM_001338	NM_001338	Homo sapiens coxsackie virus and adenovirus receptor (CXADR), mRNA [NM_001338]	NM_001338
A_23_P392541	0.00202	4.848	NM_015087	NM_015087	Homo sapiens spastic paraplegia 20, spartin (Troyer syndrome) (SPG20), mRNA [NM_015087]	NM_015087
A_23_P205959	0.00202	2.226	NM_000693	NM_000693	Homo sapiens aldehyde dehydrogenase 1 family, member A3 (ALDH1A3), mRNA [NM_000693]	NM_000693
A_24_P382130	0.00202	2.099	NM_032141	NM_032141	Homo sapiens hypothetical protein DKFZp434K1421 (DKFZP434K1421), mRNA [NM_032141]	NM_032141
A_23_P208551	0.00203	2.63	NM_001008701	NM_001008701	Homo sapiens latrophilin 1 (LPHN1), transcript variant 1, mRNA [NM_001008701]	NM_001008701
A_23_P203645	0.00203	2.04	NM_021212	NM_021212	Homo sapiens HCF-binding transcription factor Zhangfei (ZF), mRNA [NM_021212]	NM_021212
A_24_P668351	0.00204	40.3	THC2405936			
A_23_P341938	0.00204	3.009	NM_005450	NM_005450	Homo sapiens noggin (NOG), mRNA [NM_005450]	NM_005450
A_23_P435407	0.00205	5.876	NM_001448	NM_001448	Homo sapiens glypican 4 (GPC4), mRNA [NM_001448]	NM_001448
A_32_P334492	0.00205	5.155	AK021554	AK021554	Homo sapiens cDNA FLJ11492 fis, clone HEMBA1001939. [AK021554]	
A_23_P146855	0.00205	3.616	NM_001585	NM_001585	Homo sapiens chromosome 22 open reading frame 1 (C22orf1), mRNA [NM_001585]	NM_001585
A_23_P91001	0.00205	2.507	NM_019048	NM_019048	Homo sapiens HCV NS3-transactivated protein 1 (NS3TP1), mRNA [NM_019048]	NM_019048
A_24_P28622	0.00205	2.21	NM_144567	NM_144567	Homo sapiens similar to RIKEN cDNA 2610307I21 (LOC90806), mRNA [NM_144567]	NM_144567
A_32_P26721	0.00205	2.134	A_32_P26721			
A_23_P47282	0.00205	2.117	NM_021978	NM_021978	Homo sapiens suppression of tumorigenicity 14 (colon carcinoma, matriptase, epithin) (ST14), mRNA [NM_021978]	NM_021978
A_32_P32254	0.00206	5.136	NM_001848	NM_001848	Homo sapiens collagen, type VI, alpha 1 (COL6A1), mRNA [NM_001848]	NM_001848
A_23_P368794	0.00206	4.02	NM_174937	NM_174937	Homo sapiens transcription elongation regulator 1-like (TCERG1L), mRNA [NM_174937]	NM_174937
A_23_P73577	0.00206	2.841	ENST00000297871		Homo sapiens t-complex-associated-testis-expressed 1-like, mRNA (cDNA clone MGC:5007 IMAGE:3448623), complete cds. [BC000968]	
A_24_P290153	0.00206	2.545	NM_000495	NM_000495	Homo sapiens collagen, type IV, alpha 5 (Alport syndrome) (COL4A5), transcript variant 1, mRNA [NM_000495]	NM_000495
A_23_P408167	0.00206	2.327	NM_001004051	NM_001004051	Homo sapiens G protein-coupled receptor associated sorting protein 2 (GPRASP2), transcript variant 1, mRNA [NM_001004051]	NM_001004051
A_23_P60166	0.00207	8.546	NM_022783	NM_022783	Homo sapiens DEP domain containing 6 (DEPDC6), mRNA [NM_022783]	NM_022783
A_23_P30634	0.00207	7.652	NM_021813	NM_021813	Homo sapiens BTB and CNC homology 1, basic leucine zipper transcription factor 2 (BACH2), mRNA [NM_021813]	NM_021813
A_23_P171117	0.00207	2.528	NM_024657	NM_024657	Homo sapiens MORC family CW-type zinc finger 4 (MORC4), mRNA [NM_024657]	NM_024657
A_23_P303815	0.00207	2.504	AF451988	AF451988	Homo sapiens chromosome 3 unknown mRNA. [AF451988]	XM_376254
A_32_P169500	0.00208	5.676	THC2433217		ALU1_HUMAN (P39188) Alu subfamily J sequence contamination warning entry, partial (14%) [THC2433217]	
A_32_P191285	0.00208	4.528	THC2319894			
A_32_P116840	0.00208	3.437	NM_203356	NM_203356	Homo sapiens CTAGE family, member 5 (CTAGE5), transcript variant 4, mRNA [NM_203356]	NM_203356
A_32_P108826	0.00208	2.992	NM_194314	NM_194314	Homo sapiens zinc finger and BTB domain containing 41 (ZBTB41), mRNA [NM_194314]	NM_194314

A_24_P598516	0.00208	2.789	AK021595	AK021595	Homo sapiens cDNA FLJ11533 fis, clone HEMBA1002678. [AK021595]	
A_32_P42149	0.00208	2.765	AA521057	AA521057	aa71e06.s1 NCI_CGAP_GCB1 Homo sapiens cDNA clone IMAGE:826402 3' similar to contains Alu repetitive element;contains element LTR5 repetitive element ;, mRNA sequence [AA521057]	
A_23_P215318	0.00208	2.314	NM_014396	NM_014396	Homo sapiens vacuolar protein sorting 41 (yeast) (VPS41), transcript variant 1, mRNA [NM_014396]	NM_014396
A_23_P142738	0.00208	2.149	NM_152390	NM_152390	Homo sapiens hypothetical protein MGC33926 (MGC33926), mRNA [NM_152390]	NM_152390
A_23_P13713	0.00209	11.03	NM_006262	NM_006262	Homo sapiens peripherin (PRPH), mRNA [NM_006262]	NM_006262
A_23_P51690	0.00209	7.197	NM_020407	NM_020407	Homo sapiens Rhesus blood group, B glycoprotein (RHBG), mRNA [NM_020407]	NM_020407
A_23_P170037	0.00209	2.432	NM_033290	NM_033290	Homo sapiens midline 1 (Opitz/BBB syndrome) (MID1), transcript variant 3, mRNA [NM_033290]	NM_033290
A_23_P203191	0.0021	43.53	NM_000039	NM_000039	Homo sapiens apolipoprotein A-I (APOA1), mRNA [NM_000039]	NM_000039
A_23_P216556	0.0021	6.444	NM_018424	NM_018424	Homo sapiens erythrocyte membrane protein band 4.1 like 4B (EPB41L4B), transcript variant 1, mRNA [NM_018424]	NM_018424
A_23_P70201	0.0021	2.518	NM_001270	NM_001270	Homo sapiens chromodomain helicase DNA binding protein 1 (CHD1), mRNA [NM_001270]	NM_001270
A_23_P208302	0.0021	2.391	NM_000483	NM_000483	Homo sapiens apolipoprotein C-II (APOC2), mRNA [NM_000483]	NM_000483
A_23_P20328	0.00211	2.795	CX165016	CX165016	HESC2_23_G02.g1_A035 NIH_MGC_258 Homo sapiens cDNA clone IMAGE:7468613 5', mRNA sequence [CX165016]	
A_24_P166168	0.00211	2.189	AK096319	AK096319	Homo sapiens cDNA FLJ39000 fis, clone NT2RI2022468. [AK096319]	
A_23_P207520	0.00212	3.697	NM_000088	NM_000088	Homo sapiens collagen, type I, alpha 1 (COL1A1), mRNA [NM_000088]	NM_000088
A_23_P257993	0.00212	3.267	NM_004944	NM_004944	Homo sapiens deoxyribonuclease I-like 3 (DNASE1L3), mRNA [NM_004944]	NM_004944
A_24_P95029	0.00212	2.62	NM_006024	NM_006024	Homo sapiens Tax1 (human T-cell leukemia virus type I) binding protein 1 (TAX1BP1), mRNA [NM_006024]	NM_006024
A_32_P67837	0.00212	2.067	CR590573	CR590573	full-length cDNA clone CS0DI042YD07 of Placenta Cot 25-normalized of Homo sapiens (human). [CR590573]	
A_23_P57199	0.00213	3.04	NM_080920	NM_080920	Homo sapiens gamma-glutamyltransferase-like activity 4 (GGTLA4), transcript variant C, mRNA [NM_080920]	NM_080920
A_32_P8604	0.00213	2.261	AK093691	AK093691	Homo sapiens cDNA FLJ36372 fis, clone THYMU2008072. [AK093691]	
A_23_P358662	0.00214	3.526	ENST00000296519		Homo sapiens mRNA for KIAA1712 protein, partial cds. [AB051499]	
A_24_P278747	0.00214	3.016	NM_001759	NM_001759	Homo sapiens cyclin D2 (CCND2), mRNA [NM_001759]	NM_001759
A_23_P50320	0.00214	2.721	A_23_P50320			
A_23_P150827	0.00214	2.456	THC2284017		Q8QQ03 (Q8QQ03) CMP190.5bL, partial (11%) [THC2284017]	
A_23_P148916	0.00214	2.314	NM_148909	NM_148909	Homo sapiens oxysterol binding protein-like 9 (OSBPL9), transcript variant 7, mRNA [NM_148909]	NM_148909
A_23_P39799	0.00214	2.201	NM_032603	NM_032603	Homo sapiens lysyl oxidase-like 3 (LOXL3), mRNA [NM_032603]	NM_032603
A_23_P66017	0.00215	5.114	NM_145239	NM_145239	Homo sapiens similar to lymphocyte antigen 6 complex, locus G5B; G5b protein; open reading frame 31 (LOC112476), mRNA [NM_145239]	NM_145239
A_32_P213946	0.00215	2.855	A_32_P213946			
A_23_P381102	0.00215	2.519	NM_207310	NM_207310	Homo sapiens hypothetical protein DKFZp434E2321 (DKFZp434E2321), mRNA [NM_207310]	NM_207310
A_23_P106412	0.00216	13.09	AK130644	AK130644	Homo sapiens cDNA FLJ27134 fis, clone SPL08315. [AK130644]	
A_32_P142881	0.00216	2.58	NM_015092	NM_015092	Homo sapiens PI-3-kinase-related kinase SMG-1 (SMG1), mRNA [NM_015092]	NM_015092
A_32_P38745	0.00216	2.557	THC2350949			
A_24_P355568	0.00217	8.098	NM_005330	NM_005330	Homo sapiens hemoglobin, epsilon 1 (HBE1), mRNA [NM_005330]	NM_005330
A_23_P38427	0.00217	4.563	NM_032932	NM_032932	Homo sapiens RAB11 family interacting protein 4 (class II) (RAB11FIP4), mRNA [NM_032932]	NM_032932
A_23_P66774	0.00217	3.093	NM_153338	NM_153338	Homo sapiens hypothetical protein FLJ90165 (FLJ90165), mRNA [NM_153338]	NM_153338
A_23_P124384	0.00217	2.448	NM_006884	NM_006884	Homo sapiens short stature homeobox 2 (SHOX2), transcript variant SHOX2a, mRNA [NM_006884]	NM_006884
A_23_P342668	0.00217	2.112	NM_005088	NM_005088	Homo sapiens DNA segment on chromosome X and Y (unique) 155 expressed sequence (DXYS155E), transcript variant 1, mRNA [NM_005088]	NM_005088
A_23_P200936	0.00217	2.012	NM_000254	NM_000254	Homo sapiens 5-methyltetrahydrofolate-homocysteine methyltransferase (MTR), mRNA [NM_000254]	NM_000254
A_23_P258862	0.00218	7.927	NM_145341	NM_145341	Homo sapiens programmed cell death 4 (neoplastic transformation inhibitor) (PDCD4), transcript variant 2, mRNA [NM_145341]	NM_145341
A_32_P212343	0.00218	4.257	AK057710	AK057710	Homo sapiens cDNA FLJ33148 fis, clone UTERU2000238. [AK057710]	
A_32_P51005	0.00218	2.15	AL834342	AL834342	Homo sapiens mRNA; cDNA DKFZp761P2314 (from clone DKFZp761P2314). [AL834342]	

A_23_P66311	0.00219	4.267	NM_005223	NM_005223	Homo sapiens deoxyribonuclease I (DNASE1), mRNA [NM_005223]	NM_005223
A_32_P163125	0.00219	3.312	NM_147156	NM_147156	Homo sapiens transmembrane protein 23 (TMEM23), mRNA [NM_147156]	NM_147156
A_24_P136438	0.00219	2.647	NM_014915	NM_014915	Homo sapiens ankyrin repeat domain 26 (ANKRD26), mRNA [NM_014915]	NM_014915
A_23_P259357	0.0022	4.029	ENST00000343605		full-length cDNA clone CS0DD009YN24 of Neuroblastoma Cot 50-normalized of Homo sapiens (human). [CR614052]	
A_24_P303852	0.0022	2.676	AK055619	AK055619	Homo sapiens cDNA FLJ31057 fis, clone HSYRA2000787. [AK055619]	
A_32_P63086	0.0022	2.259	BC041913	BC041913	Homo sapiens, clone IMAGE:5299642, mRNA. [BC041913]	
A_32_P6415	0.0022	2.029	NM_015088	NM_015088	Homo sapiens trinucleotide repeat containing 6B (TNRC6B), transcript variant 1, mRNA [NM_015088]	NM_015088
A_24_P54390	0.00221	8.538	NM_170672	NM_170672	Homo sapiens RAS guanyl releasing protein 3 (calcium and DAG-regulated) (RASGRP3), mRNA [NM_170672]	NM_170672
A_23_P324327	0.00221	3.424	NM_016235	NM_016235	Homo sapiens G protein-coupled receptor, family C, group 5, member B (GPRC5B), mRNA [NM_016235]	NM_016235
A_24_P943263	0.00221	2.421	NM_006989	NM_006989	Homo sapiens RAS p21 protein activator 4 (RASA4), mRNA [NM_006989]	NM_006989
A_24_P256674	0.00221	2.234	BC040474	BC040474	Homo sapiens Rho guanine nucleotide exchange factor (GEF) 10, mRNA (cDNA clone IMAGE:4250879), complete cds. [BC040474]	
A_23_P95790	0.00222	3.804	NM_017625	NM_017625	Homo sapiens intelectin 1 (galactofuranose binding) (ITLN1), mRNA [NM_017625]	NM_017625
A_32_P139260	0.00223	6.663	THC2410817		ALU1_HUMAN (P39188) Alu subfamily J sequence contamination warning entry, partial (6%) [THC2410817]	
A_24_P18621	0.00223	2.695	NM_153207	NM_153207	Homo sapiens AE binding protein 2 (AEBP2), mRNA [NM_153207]	NM_153207
A_23_P120710	0.00223	2.6	NM_003316	NM_003316	Homo sapiens tetratricopeptide repeat domain 3 (TTC3), transcript variant 1, mRNA [NM_003316]	NM_003316
A_24_P142983	0.00223	2.062	NM_002650	NM_002650	Homo sapiens phosphatidylinositol 4-kinase, catalytic, alpha polypeptide (PIK4CA), transcript variant 1, mRNA [NM_002650]	NM_002650
A_23_P215505	0.00224	9.671	D87467	D87467	Homo sapiens mRNA for KIAA0277 gene, partial cds. [D87467]	
A_32_P153833	0.00224	2.708	AK098597	AK098597	Homo sapiens cDNA FLJ25731 fis, clone TST05584. [AK098597]	
A_24_P48408	0.00225	2.219	NM_003799	NM_003799	Homo sapiens RNA (guanine-7-) methyltransferase (RNMT), mRNA [NM_003799]	NM_003799
A_23_P412409	0.00226	3.13	NM_015172	NM_015172	Homo sapiens BAT2 domain containing 1 (BAT2D1), mRNA [NM_015172]	NM_015172
A_24_P175176	0.00226	3.007	NM_020432	NM_020432	Homo sapiens putative homeodomain transcription factor 2 (PHTF2), mRNA [NM_020432]	NM_020432
A_23_P330461	0.00226	2.379	NM_144686	NM_144686	Homo sapiens transmembrane channel-like 4 (TMC4), mRNA [NM_144686]	NM_144686
A_23_P83388	0.00227	4.964	NM_031308	NM_031308	Homo sapiens epiplakin 1 (EPPK1), mRNA [NM_031308]	NM_031308
A_23_P363344	0.00227	2.211	NM_000366	NM_000366	Homo sapiens tropomyosin 1 (alpha) (TPM1), transcript variant 5, mRNA [NM_000366]	NM_000366
A_32_P162150	0.00228	2.935	AK096786	AK096786	Homo sapiens cDNA FLJ39467 fis, clone PROST2012448. [AK096786]	
A_32_P203430	0.00228	2.165	NM_194325	NM_194325	Homo sapiens zinc finger protein 30 (KOX 28) (ZNF30), mRNA [NM_194325]	NM_194325
A_24_P274987	0.00229	5.577	NM_003692	NM_003692	Homo sapiens transmembrane protein with EGF-like and two follistatin-like domains 1 (TMEFF1), mRNA [NM_003692]	NM_003692
A_24_P349151	0.00229	4.174	NM_194292	NM_194292	Homo sapiens spindle assembly abnormal protein 6 (SAS-6), mRNA [NM_194292]	NM_194292
A_23_P99226	0.00229	2.276	NM_012240	NM_012240	Homo sapiens sirtuin (silent mating type information regulation 2 homolog) 4 (S. cerevisiae) (SIRT4), mRNA [NM_012240]	NM_012240
A_32_P178635	0.00229	2.103	BC033590	BC033590	Homo sapiens, clone IMAGE:4344826, mRNA. [BC033590]	
A_32_P122579	0.0023	2.484	THC2284074		HSU52965 ENX-1 {Homo sapiens;} , partial (9%) [THC2284074]	
A_23_P167017	0.0023	2.464	NM_022135	NM_022135	Homo sapiens popeye domain containing 2 (POPDC2), mRNA [NM_022135]	NM_022135
A_24_P52004	0.0023	2.306	NM_015200	NM_015200	Homo sapiens SCC-112 protein (SCC-112), mRNA [NM_015200]	NM_015200
A_23_P403588	0.00231	3.1	BC000226	BC000226	Homo sapiens ubiquitin specific protease 47, mRNA (cDNA clone IMAGE:3350895), complete cds. [BC000226]	
A_23_P405707	0.00231	2.837	NM_020926	NM_020926	Homo sapiens BCL6 co-repressor (BCOR), transcript variant 2, mRNA [NM_020926]	NM_020926
A_23_P165707	0.00231	2.475	NM_174898	NM_174898	Homo sapiens hypothetical protein LOC129530 (LOC129530), mRNA [NM_174898]	NM_174898
A_32_P61298	0.00232	3.216	AK054921	AK054921	Homo sapiens cDNA FLJ30359 fis, clone BRACE2007760, highly similar to 40S RIBOSOMAL PROTEIN S15A. [AK054921]	
A_24_P295620	0.00232	2.333	NM_015423	NM_015423	Homo sapiens aminoadipate-semialdehyde dehydrogenase-phosphopantetheinyl transferase (AASDHPPT), mRNA [NM_015423]	NM_015423
A_23_P314101	0.00233	7.783	NM_019601	NM_019601	Homo sapiens sushi domain containing 2 (SUSD2), mRNA [NM_019601]	NM_019601
A_23_P393425	0.00233	2.921	NM_173797	NM_173797	Homo sapiens PAP associated domain containing 4 (PAPD4), mRNA [NM_173797]	NM_173797

A_32_P196047	0.00233	2.416	NM_181787	NM_181787	Homo sapiens dpy-19-like 4 (C. elegans) (DPY19L4), mRNA [NM_181787]	NM_181787
A_23_P218717	0.00234	2.614	NM_014948	NM_014948	Homo sapiens U-box domain containing 5 (UBOX5), transcript variant 1, mRNA [NM_014948]	NM_014948
A_23_P400378	0.00234	2.491	NM_170699	NM_170699	Homo sapiens G protein-coupled bile acid receptor 1 (GPBAR1), mRNA [NM_170699]	NM_170699
A_32_P125558	0.00234	2.315	THC2397609		AA411302 zv24g06.r1 Soares_NhHMPu_S1 Homo sapiens cDNA clone IMAGE:754618 5', mRNA sequence [AA411302]	
A_23_P204269	0.00234	2.255	NM_006313	NM_006313	Homo sapiens ubiquitin specific protease 15 (USP15), mRNA [NM_006313]	NM_006313
A_23_P52058	0.00235	2.575	NM_005646	NM_005646	Homo sapiens TAR (HIV) RNA binding protein 1 (TARBP1), mRNA [NM_005646]	NM_005646
A_23_P130876	0.00235	2.034	NM_178544	NM_178544	Homo sapiens zinc finger protein 546 (ZNF546), mRNA [NM_178544]	NM_178544
A_32_P47754	0.00236	8.931	BC060766	BC060766	Homo sapiens solute carrier family 2 (facilitated glucose transporter), member 14, mRNA (cDNA clone MGC:71510 IMAGE:5297510), complete cds. [BC060766]	
A_23_P157527	0.00236	3.038	NM_033402	NM_033402	Homo sapiens KIAA1764 protein (KIAA1764), mRNA [NM_033402]	NM_033402
A_23_P13137	0.00236	2.274	AY358815	AY358815	Homo sapiens clone DNA108923 SFVP2550 (UNQ2550) mRNA, complete cds. [AY358815]	
A_23_P140277	0.00237	4.881	NM_015180	NM_015180	Homo sapiens spectrin repeat containing, nuclear envelope 2 (SYNE2), transcript variant 1, mRNA [NM_015180]	NM_015180
A_24_P256337	0.00237	2.283	NM_201269	NM_201269	Homo sapiens zinc finger protein 644 (ZNF644), transcript variant 1, mRNA [NM_201269]	NM_201269
A_32_P489130	0.00239	2.133	NM_153252	NM_153252	Homo sapiens bromodomain and WD repeat domain containing 3 (BRWD3), mRNA [NM_153252]	NM_153252
A_32_P104448	0.0024	3.495	THC2397633			
A_32_P191290	0.0024	3.429	BM045853	BM045853	603624848F1 NIH_MGC_40 Homo sapiens cDNA clone IMAGE:5451514 5', mRNA sequence [BM045853]	
A_24_P99046	0.0024	2.83	NM_015000	NM_015000	Homo sapiens serine/threonine kinase 38 like (STK38L), mRNA [NM_015000]	NM_015000
A_24_P81298	0.0024	2.442	THC2336427		PPP6_HUMAN (O00743) Serine/threonine protein phosphatase 6 (PP6) , partial (9%) [THC2336427]	
A_32_P133916	0.00241	3.767	THC2405066		predicted protein {Methanosarcina acetivorans C2A;} , partial (13%) [THC2405066]	
A_23_P103756	0.00242	3.117	NM_002557	NM_002557	Homo sapiens oviductal glycoprotein 1, 120kDa (mucin 9, oviductin) (OVGP1), mRNA [NM_002557]	NM_002557
A_23_P210445	0.00243	4.444	NM_015478	NM_015478	Homo sapiens l(3)mbt-like (Drosophila) (L3MBTL), transcript variant I, mRNA [NM_015478]	NM_015478
A_24_P278460	0.00243	2.258	NM_032228	NM_032228	Homo sapiens male sterility domain containing 2 (MLSTD2), mRNA [NM_032228]	NM_032228
A_24_P183664	0.00243	2.124	AB014544	AB014544	Homo sapiens mRNA for KIAA0644 protein, partial cds. [AB014544]	XM_376588
A_23_P69918	0.00244	2.167	AK022657	AK022657	Homo sapiens cDNA FLJ12595 fis, clone NT2RM4001344, weakly similar to HYPOTHETICAL GTP-BINDING PROTEIN IN POP2-HOL1 INTERGENIC REGION. [AK022657]	
A_23_P124619	0.00245	48.3	NM_020672	NM_020672	Homo sapiens S100 calcium binding protein A14 (S100A14), mRNA [NM_020672]	NM_020672
A_24_P713312	0.00245	8.071	THC2314371		ALU8_HUMAN (P39195) Alu subfamily SX sequence contamination warning entry, partial (10%) [THC2314371]	
A_32_P184417	0.00245	5.281	BE181102	BE181102	CM3-HT0629-260400-161-b05 HT0629 Homo sapiens cDNA, mRNA sequence [BE181102]	
A_23_P56304	0.00245	4.016	NM_025189	NM_025189	Homo sapiens zinc finger protein 430 (ZNF430), mRNA [NM_025189]	NM_025189
A_24_P729905	0.00245	3.112	NM_133446	NM_133446	Homo sapiens centaurin, gamma-like family, member 1 (CTGLF1), mRNA [NM_133446]	NM_133446
A_24_P376902	0.00245	2.155	NM_138468	NM_138468	Homo sapiens amyotrophic lateral sclerosis 2 (juvenile) chromosome region, candidate 15 (ALS2CR15), mRNA [NM_138468]	NM_138468
A_23_P399001	0.00245	2.137	NM_016463	NM_016463	Homo sapiens CXXC finger 5 (CXXC5), mRNA [NM_016463]	NM_016463
A_23_P430044	0.00245	2.099	NM_032701	NM_032701	Homo sapiens suppressor of variegation 4-20 homolog 2 (Drosophila) (SUV420H2), mRNA [NM_032701]	NM_032701
A_23_P410408	0.00246	2.973	NM_175883	NM_175883	Homo sapiens olfactory receptor, family 7, subfamily D, member 2 (OR7D2), mRNA [NM_175883]	NM_175883
A_24_P699896	0.00246	2.062	BC036637	BC036637	Homo sapiens cDNA clone IMAGE:5296862. [BC036637]	
A_24_P762613	0.00247	8.783	AK021543	AK021543	Homo sapiens cDNA FLJ11481 fis, clone HEMBA1001803. [AK021543]	
A_23_P53866	0.00247	3.621	NM_020751	NM_020751	Homo sapiens component of oligomeric golgi complex 6 (COG6), mRNA [NM_020751]	NM_020751
A_23_P216869	0.00247	2.592	NM_197977	NM_197977	Homo sapiens zinc finger protein 189 (ZNF189), mRNA [NM_197977]	NM_197977
A_23_P110569	0.00248	5.805	NM_018700	NM_018700	Homo sapiens tripartite motif-containing 36 (TRIM36), transcript variant 1, mRNA [NM_018700]	NM_018700
A_23_P359052	0.00249	4.277	NM_148894	NM_148894	Homo sapiens family with sequence similarity 44, member A (FAM44A), mRNA [NM_148894]	NM_148894
A_23_P60753	0.00249	3.709	THC2337941			
A_23_P433016	0.00249	3.181	NM_001996	NM_001996	Homo sapiens fibulin 1 (FBLN1), transcript variant C, mRNA [NM_001996]	NM_001996
A_32_P11451	0.00249	2.793	NM_015938	NM_015938	Homo sapiens NMD3 homolog (S. cerevisiae) (NMD3), mRNA [NM_015938]	NM_015938

A_24_P203000	0.00249	2.778	NM_000878	NM_000878	Homo sapiens interleukin 2 receptor, beta (IL2RB), mRNA [NM_000878]	NM_000878
A_23_P61688	0.0025	6.156	NM_006598	NM_006598	Homo sapiens solute carrier family 12 (potassium/chloride transporters), member 7 (SLC12A7), mRNA [NM_006598]	NM_006598
A_32_P162709	0.0025	3.464	CR624517	CR624517	full-length cDNA clone CS0DC002YA18 of Neuroblastoma Cot 25-normalized of Homo sapiens (human). [CR624517]	
A_24_P190877	0.0025	2.507	NM_030934	NM_030934	Homo sapiens chromosome 1 open reading frame 25 (C1orf25), mRNA [NM_030934]	NM_030934
A_23_P157365	0.0025	2.286	NM_014888	NM_014888	Homo sapiens family with sequence similarity 3, member C (FAM3C), mRNA [NM_014888]	NM_014888
A_32_P213103	0.00251	72.56	CA414006	CA414006	CA414006 UI-H-EZ0-ban-f-19-0-UI.s1 NCI_CGAP_Ch1 Homo sapiens cDNA clone UI-H-EZ0-ban-f-19-0-UI 3', mRNA sequence [CA414006]	
A_24_P179611	0.00251	4.124	NM_003292	NM_003292	Homo sapiens translocated promoter region (to activated MET oncogene) (TPR), mRNA [NM_003292]	NM_003292
A_32_P224449	0.00252	2.908	THC2375353			
A_32_P226186	0.00252	2.709	THC2266474			
A_24_P80915	0.00254	5.811	THC2428671			
A_24_P228228	0.00254	3.76	NM_004775	NM_004775	Homo sapiens UDP-Gal:betaGlcNAc beta 1,4- galactosyltransferase, polypeptide 6 (B4GALT6), mRNA [NM_004775]	NM_004775
A_23_P340218	0.00255	6.089	NM_152599	NM_152599	Homo sapiens hypothetical protein FLJ35773 (FLJ35773), mRNA [NM_152599]	NM_152599
A_24_P726336	0.00255	3.809	THC2304714			
A_24_P341909	0.00256	3.253	NM_020121	NM_020121	Homo sapiens UDP-glucose ceramide glucosyltransferase-like 2 (UGCGL2), mRNA [NM_020121]	NM_020121
A_32_P24651	0.00256	2.739	AK095707	AK095707	Homo sapiens cDNA FLJ38388 fis, clone FEBRA2004485. [AK095707]	
A_32_P49959	0.00256	2.325	BX111592	BX111592	BX111592 Soares_testis_NHT Homo sapiens cDNA clone IMAGp998D162621, mRNA sequence [BX111592]	
A_23_P79302	0.00256	2.053	NM_177964	NM_177964	Homo sapiens hypothetical protein LOC130576 (LOC130576), mRNA [NM_177964]	NM_177964
A_32_P200697	0.00257	8.866	NM_181709	NM_181709	Homo sapiens hypothetical protein LOC144347 (LOC144347), mRNA [NM_181709]	NM_181709
A_23_P94762	0.00257	2.27	NM_058230	NM_058230	Homo sapiens zinc finger protein 354B (ZNF354B), mRNA [NM_058230]	NM_058230
A_23_P97339	0.00257	2.27	NM_004696	NM_004696	Homo sapiens solute carrier family 16 (monocarboxylic acid transporters), member 4 (SLC16A4), mRNA [NM_004696]	NM_004696
A_23_P156620	0.00258	2.254	NM_007149	NM_007149	Homo sapiens zinc finger protein 184 (Kruppel-like) (ZNF184), mRNA [NM_007149]	NM_007149
A_24_P81900	0.00259	4.247	NM_006931	NM_006931	Homo sapiens solute carrier family 2 (facilitated glucose transporter), member 3 (SLC2A3), mRNA [NM_006931]	NM_006931
A_24_P174755	0.00259	3.938	NM_003060	NM_003060	Homo sapiens solute carrier family 22 (organic cation transporter), member 5 (SLC22A5), mRNA [NM_003060]	NM_003060
A_23_P350074	0.00259	3.588	NM_153233	NM_153233	Homo sapiens hypothetical protein FLJ36445 (FLJ36445), mRNA [NM_153233]	NM_153233
A_23_P41021	0.00259	2.359	NM_007184	NM_007184	Homo sapiens nischarin (NISCH), mRNA [NM_007184]	NM_007184
A_24_P666795	0.00259	2.174	A_24_P666795			
A_23_P259641	0.00259	2.065	NM_004456	NM_004456	Homo sapiens enhancer of zeste homolog 2 (Drosophila) (EZH2), transcript variant 1, mRNA [NM_004456]	NM_004456
A_23_P53530	0.00259	2.02	NM_025198	NM_025198	Homo sapiens MTERF domain containing 3 (MTERFD3), mRNA [NM_025198]	NM_025198
A_23_P413456	0.0026	2	AK128423	AK128423	Homo sapiens cDNA FLJ46566 fis, clone THYMU3040829, moderately similar to Cold-inducible RNA-binding protein. [AK128423]	
A_23_P216340	0.00262	12.11	NM_006748	NM_006748	Homo sapiens Src-like-adaptor (SLA), mRNA [NM_006748]	NM_006748
A_32_P226205	0.00262	4.264	NM_033400	NM_033400	Homo sapiens zinc finger homeobox 2 (ZFHX2), mRNA [NM_033400]	NM_033400
A_24_P401241	0.00262	3.105	NM_198893	NM_198893	Homo sapiens zinc finger protein 160 (ZNF160), transcript variant 2, mRNA [NM_198893]	NM_198893
A_23_P353905	0.00262	2.74	AB051487	AB051487	Homo sapiens mRNA for KIAA1700 protein, partial cds. [AB051487]	
A_24_P917934	0.00262	2.226	AL832665	AL832665	Homo sapiens mRNA; cDNA DKFZp313J1712 (from clone DKFZp313J1712). [AL832665]	
A_23_P406071	0.00262	2.102	NM_207442	NM_207442	Homo sapiens FLJ39779 protein (FLJ39779), mRNA [NM_207442]	NM_207442
A_32_P154361	0.00263	5.631	THC2415390			
A_23_P254654	0.00263	4.939	NM_004669	NM_004669	Homo sapiens chloride intracellular channel 3 (CLIC3), mRNA [NM_004669]	NM_004669
A_23_P78608	0.00263	2.597	NM_024898	NM_024898	Homo sapiens family with sequence similarity 31, member C (FAM31C), mRNA [NM_024898]	NM_024898
A_24_P538403	0.00263	2.542	BC041849	BC041849	Homo sapiens, Similar to Rho-associated, coiled-coil containing protein kinase 1, clone IMAGE:5269982, mRNA. [BC041849]	
A_23_P148609	0.00264	2.785	NM_021796	NM_021796	Homo sapiens placenta-specific 1 (PLAC1), mRNA [NM_021796]	NM_021796
A_23_P74309	0.00265	2.677	NM_014697	NM_014697	Homo sapiens nitric oxide synthase 1 (neuronal) adaptor protein (NOS1AP), mRNA [NM_014697]	NM_014697

A_24_P396702	0.00266	4.223	NM_014880	NM_014880	Homo sapiens CD302 antigen (CD302), mRNA [NM_014880]	NM_014880
A_32_P114246	0.00266	3.42	BC000226	BC000226	Homo sapiens ubiquitin specific protease 47, mRNA (cDNA clone IMAGE:3350895), complete cds. [BC000226]	
A_23_P119583	0.00266	2.354	NM_000923	NM_000923	Homo sapiens phosphodiesterase 4C, cAMP-specific (phosphodiesterase E1 dunce homolog, Drosophila) (PDE4C), mRNA [NM_000923]	NM_000923
A_32_P227027	0.00267	2.002	THC2288599			
A_24_P194881	0.00269	5.856	ENST00000262795		Homo sapiens mRNA for KIAA1650 protein, partial cds. [AB051437]	
A_24_P399220	0.00269	4.268	THC2372144		O46887 (O46887) ATP phosphoribosyltransferase , partial (5%) [THC2372144]	
A_32_P68148	0.00269	2.996	XM_497613	XM_497613	PREDICTED: Homo sapiens similar to zinc finger protein 91 (HPF7, HTF10) (LOC401905), mRNA [XM_497613]	XM_497613
A_23_P48387	0.00269	2.359	NM_015928	NM_015928	Homo sapiens androgen-induced proliferation inhibitor (APRIN), transcript variant 2, mRNA [NM_015928]	NM_015928
A_23_P255750	0.0027	2.868	NM_024857	NM_024857	Homo sapiens chromosome 17 open reading frame 41 (C17orf41), mRNA [NM_024857]	NM_024857
A_23_P51711	0.00271	2.929	NM_015849	NM_015849	Homo sapiens elastase 2B (ELA2B), mRNA [NM_015849]	NM_015849
A_32_P53524	0.00271	2.34	BC092429	BC092429	Homo sapiens cDNA clone IMAGE:30530513. [BC092429]	
A_23_P168974	0.00272	2.749	AK128645	AK128645	Homo sapiens cDNA FLJ46804 fis, clone TRACH3032570, highly similar to Homo sapiens syndecan binding protein (syntenin) (SDCBP). [AK128645]	
A_24_P224727	0.00274	21.2	NM_004364	NM_004364	Homo sapiens CCAAT/enhancer binding protein (C/EBP), alpha (CEBPA), mRNA [NM_004364]	NM_004364
A_24_P418744	0.00275	2.435	A_24_P418744			
A_23_P92954	0.00275	2.195	NM_000414	NM_000414	Homo sapiens hydroxysteroid (17-beta) dehydrogenase 4 (HSD17B4), mRNA [NM_000414]	NM_000414
A_23_P218225	0.00276	2.035	NM_014298	NM_014298	Homo sapiens quinolinate phosphoribosyltransferase (nicotinate-nucleotide pyrophosphorylase (carboxylating)) (QPRT), mRNA [NM_014298]	NM_014298
A_24_P114671	0.00276	2.017	AL117599	AL117599	Homo sapiens mRNA; cDNA DKFZp564I0463 (from clone DKFZp564I0463). [AL117599]	
A_23_P100220	0.00277	4.06	NM_024939	NM_024939	Homo sapiens hypothetical protein FLJ21918 (FLJ21918), mRNA [NM_024939]	NM_024939
A_23_P153441	0.00277	2.277	NM_013312	NM_013312	Homo sapiens hook homolog 2 (Drosophila) (HOOK2), mRNA [NM_013312]	NM_013312
A_32_P228886	0.00277	2.008	BX115350	BX115350	BX115350 Soares fetal liver spleen 1NFLS Homo sapiens cDNA clone IMAGp998L11127, mRNA sequence [BX115350]	
A_23_P111919	0.00278	4.466	NM_013357	NM_013357	Homo sapiens purine-rich element binding protein G (PURG), transcript variant A, mRNA [NM_013357]	NM_013357
A_23_P64980	0.00278	3.218	NM_016615	NM_016615	Homo sapiens solute carrier family 6 (neurotransmitter transporter, GABA), member 13 (SLC6A13), mRNA [NM_016615]	NM_016615
A_23_P3483	0.00278	2.23	NM_021098	NM_021098	Homo sapiens calcium channel, voltage-dependent, alpha 1H subunit (CACNA1H), transcript variant 1, mRNA [NM_021098]	NM_021098
A_24_P257022	0.00279	2.286	NM_000364	NM_000364	Homo sapiens troponin T2, cardiac (TNNT2), transcript variant 1, mRNA [NM_000364]	NM_000364
A_23_P3651	0.0028	5.329	NM_005332	NM_005332	Homo sapiens hemoglobin, zeta (HBZ), mRNA [NM_005332]	NM_005332
A_23_P53198	0.00281	9.902	NM_032564	NM_032564	Homo sapiens diacylglycerol O-acyltransferase homolog 2 (mouse) (DGAT2), mRNA [NM_032564]	NM_032564
A_32_P186921	0.00281	3.208	BC032805	BC032805	Homo sapiens cDNA clone MGC:45556 IMAGE:4186857, complete cds. [BC032805]	
A_24_P484894	0.00281	2.812	BC045718	BC045718	Homo sapiens, clone IMAGE:4797078, mRNA. [BC045718]	
A_32_P122285	0.00282	3.048	A_32_P122285			
A_23_P160406	0.00283	3.127	NM_016121	NM_016121	Homo sapiens potassium channel tetramerisation domain containing 3 (KCTD3), mRNA [NM_016121]	NM_016121
A_23_P114929	0.00283	3.004	NM_015415	NM_015415	Homo sapiens brain protein 44 (BRP44), mRNA [NM_015415]	NM_015415
A_24_P330309	0.00283	2.254	NM_002892	NM_002892	Homo sapiens AT rich interactive domain 4A (RBP1-like) (ARID4A), transcript variant 1, mRNA [NM_002892]	NM_002892
A_23_P113380	0.00286	2.847	BC000226	BC000226	Homo sapiens ubiquitin specific protease 47, mRNA (cDNA clone IMAGE:3350895), complete cds. [BC000226]	
A_24_P381199	0.00286	2.279	NM_001003818	NM_001003818	Homo sapiens tripartite motif-containing 6 (TRIM6), transcript variant 1, mRNA [NM_001003818]	NM_001003818
A_23_P79108	0.00287	5.259	NM_138813	NM_138813	Homo sapiens ATPase, Class I, type 8B, member 3 (ATP8B3), mRNA [NM_138813]	NM_138813
A_23_P383118	0.00287	4.69	AB040944	AB040944	Homo sapiens mRNA for KIAA1511 protein, partial cds. [AB040944]	XM_046581
A_23_P125423	0.00287	3.941	NM_001733	NM_001733	Homo sapiens complement component 1, r subcomponent (C1R), mRNA [NM_001733]	NM_001733
A_23_P19723	0.00288	13.39	NM_021073	NM_021073	Homo sapiens bone morphogenetic protein 5 (BMP5), mRNA [NM_021073]	NM_021073
A_24_P190541	0.00288	6.183	NM_018963	NM_018963	Homo sapiens bromodomain and WD repeat domain containing 1 (BRWD1), transcript variant 1, mRNA [NM_018963]	NM_018963
A_32_P64716	0.00288	4.678	BC035106	BC035106	Homo sapiens cDNA clone IMAGE:5262677, partial cds. [BC035106]	

A_24_P786172	0.00288	2.714	NM_015088	NM_015088	Homo sapiens trinucleotide repeat containing 6B (TNRC6B), transcript variant 1, mRNA [NM_015088]	NM_015088
A_23_P67971	0.00288	2.191	NM_138801	NM_138801	Homo sapiens galactose mutarotase (aldose 1-epimerase) (GALM), mRNA [NM_138801]	NM_138801
A_23_P408108	0.00288	2.076	NM_006980	NM_006980	Homo sapiens mitochondrial transcription termination factor (MTERF), nuclear gene encoding mitochondrial protein, mRNA [NM_006980]	NM_006980
A_23_P160377	0.00289	3.465	NM_003462	NM_003462	Homo sapiens dynein, axonemal, light intermediate polypeptide 1 (DNALI1), mRNA [NM_003462]	NM_003462
A_32_P42253	0.0029	3.597	THC2356023			
A_24_P937119	0.0029	3.589	AK123473	AK123473	Homo sapiens cDNA FLJ41479 fis, clone BRTHA2002442. [AK123473]	XM_048070
A_23_P64173	0.0029	2.83	NM_001017534	NM_001017534	Homo sapiens CARD only protein (COPl), transcript variant 1, mRNA [NM_001017534]	NM_001017534
A_32_P472968	0.00291	7.002	BC047110	BC047110	Homo sapiens, clone IMAGE:5312754, mRNA. [BC047110]	
A_24_P311845	0.00291	4.756	CR612518	CR612518	full-length cDNA clone CS0DF004YF08 of Fetal brain of Homo sapiens (human). [CR612518]	
A_23_P142560	0.00292	4.14	NM_014795	NM_014795	Homo sapiens zinc finger homeobox 1b (ZFHX1B), mRNA [NM_014795]	NM_014795
A_24_P181295	0.00292	3.001	NM_001001872	NM_001001872	Homo sapiens chromosome 14 open reading frame 37 (C14orf37), mRNA [NM_001001872]	NM_001001872
A_24_P409904	0.00293	6.826	AK130705	AK130705	Homo sapiens cDNA FLJ27195 fis, clone SYN02786. [AK130705]	
A_32_P205303	0.00293	4.988	AF070595	AF070595	Homo sapiens clone 24583 mRNA sequence. [AF070595]	
A_24_P364072	0.00293	4.261	THC2364724			
A_32_P134109	0.00293	2.458	AF086375	AF086375	Homo sapiens full length insert cDNA clone ZD68B12. [AF086375]	
A_24_P943156	0.00293	2.221	NM_020774	NM_020774	Homo sapiens mindbomb homolog 1 (Drosophila) (MIB1), mRNA [NM_020774]	NM_020774
A_23_P55107	0.00293	2.131	NM_014683	NM_014683	Homo sapiens unc-51-like kinase 2 (C. elegans) (ULK2), mRNA [NM_014683]	NM_014683
A_23_P250002	0.00294	4.069	NM_020771	NM_020771	Homo sapiens HECT domain and ankyrin repeat containing, E3 ubiquitin protein ligase 1 (HACE1), mRNA [NM_020771]	NM_020771
A_23_P45365	0.00294	3.534	NM_033380	NM_033380	Homo sapiens collagen, type IV, alpha 5 (Alport syndrome) (COL4A5), transcript variant 2, mRNA [NM_033380]	NM_033380
A_23_P203475	0.00294	2.487	NM_145040	NM_145040	Homo sapiens protein kinase C, delta binding protein (PRKCDBP), mRNA [NM_145040]	NM_145040
A_32_P86	0.00295	3.167	BC048193	BC048193	Homo sapiens, clone IMAGE:4590099, mRNA. [BC048193]	
A_23_P82169	0.00295	2.927	NM_003107	NM_003107	Homo sapiens SRY (sex determining region Y)-box 4 (SOX4), mRNA [NM_003107]	NM_003107
A_32_P179317	0.00296	3.508	BF761348	BF761348	BF761348 RC2-CS0018-041000-015-g01 CS0018 Homo sapiens cDNA, mRNA sequence [BF761348]	
A_23_P40295	0.00296	2.918	NM_012261	NM_012261	Homo sapiens chromosome 20 open reading frame 103 (C20orf103), mRNA [NM_012261]	NM_012261
A_24_P29733	0.00296	2.853	NM_012395	NM_012395	Homo sapiens PFTAIRE protein kinase 1 (PFTK1), mRNA [NM_012395]	NM_012395
A_23_P116902	0.00297	4.314	NM_021071	NM_021071	Homo sapiens Dombrock blood group (DO), mRNA [NM_021071]	NM_021071
A_32_P118522	0.00297	3.33	AI571129	AI571129	AI571129 tn85e01.x1 NCI_CGAP_Ut2 Homo sapiens cDNA clone IMAGE:2176344 3', mRNA sequence [AI571129]	
A_24_P191417	0.00297	2.929	NM_005966	NM_005966	Homo sapiens NGFI-A binding protein 1 (EGR1 binding protein 1) (NAB1), mRNA [NM_005966]	NM_005966
A_24_P690273	0.00297	2.378	AK024900	AK024900	Homo sapiens cDNA: FLJ21247 fis, clone COL01205. [AK024900]	
A_24_P414205	0.00297	2.023	NM_017541	NM_017541	Homo sapiens crystallin, gamma S (CRYGS), mRNA [NM_017541]	NM_017541
A_24_P576219	0.00298	3.013	BC035091	BC035091	Homo sapiens, Similar to hypothetical protein FLJ20489, clone IMAGE:5261717, mRNA. [BC035091]	
A_24_P548264	0.00299	5.046	A_24_P548264			
A_24_P210406	0.00299	3.55	NM_001730	NM_001730	Homo sapiens Kruppel-like factor 5 (intestinal) (KLF5), mRNA [NM_001730]	NM_001730
A_24_P808100	0.00299	3.346	THC2334551			
A_24_P394569	0.003	3.605	AK056855	AK056855	Homo sapiens cDNA FLJ32293 fis, clone PROST2001739. [AK056855]	XM_374902
A_23_P365614	0.003	2.314	NM_004557	NM_004557	Homo sapiens Notch homolog 4 (Drosophila) (NOTCH4), mRNA [NM_004557]	NM_004557
A_32_P141257	0.003	2.099	BX647750	BX647750	Homo sapiens mRNA; cDNA DKFZp779H233 (from clone DKFZp779H233). [BX647750]	
A_23_P15174	0.00301	3.417	NM_005949	NM_005949	Homo sapiens metallothionein 1F (functional) (MT1F), mRNA [NM_005949]	NM_005949
A_23_P33723	0.00301	3.154	NM_004244	NM_004244	Homo sapiens CD163 antigen (CD163), transcript variant 1, mRNA [NM_004244]	NM_004244
A_23_P161563	0.00301	2.616	NM_022337	NM_022337	Homo sapiens RAB38, member RAS oncogene family (RAB38), mRNA [NM_022337]	NM_022337
A_32_P98136	0.00302	3.744	BC037317	BC037317	Homo sapiens KIAA1107, mRNA (cDNA clone IMAGE:5259498), partial cds. [BC037317]	
A_23_P205646	0.00302	3.152	NM_198794	NM_198794	Homo sapiens mitogen-activated protein kinase kinase kinase kinase 5 (MAP4K5), transcript variant 2, mRNA [NM_198794]	NM_198794

A_24_P933514	0.00302	2.579	AK094334	AK094334	Homo sapiens cDNA FLJ37015 fis, clone BRACE2010208. [AK094334]	
A_32_P132766	0.00303	3.384	THC2406017		Q6C1M3 (Q6C1M3) Similarity, partial (6%) [THC2406017]	
A_24_P652033	0.00304	2.743	AK026768	AK026768	Homo sapiens cDNA: FLJ23115 fis, clone LNG07933. [AK026768]	
A_23_P339818	0.00305	4.776	NM_183376	NM_183376	Homo sapiens arrestin domain containing 4 (ARRDC4), mRNA [NM_183376]	NM_183376
A_23_P10206	0.00305	3.959	NM_005328	NM_005328	Homo sapiens hyaluronan synthase 2 (HAS2), mRNA [NM_005328]	NM_005328
A_24_P273561	0.00305	3.842	NM_018169	NM_018169	Homo sapiens hypothetical protein FLJ10652 (FLJ10652), mRNA [NM_018169]	NM_018169
A_24_P910490	0.00306	3.871	THC2340757		Q96AZ2 (Q96AZ2) C2orf18 protein, partial (69%) [THC2340757]	
A_23_P95736	0.00306	3.675	NM_001001415	NM_001001415	Homo sapiens zinc finger protein 429 (ZNF429), mRNA [NM_001001415]	NM_001001415
A_24_P306814	0.00306	2.569	A_24_P306814			
A_23_P212552	0.00306	2.052	NM_024665	NM_024665	Homo sapiens transducin (beta)-like 1X-linked receptor 1 (TBL1XR1), mRNA [NM_024665]	NM_024665
A_32_P126609	0.00307	3.742	ENST00000361262		Homo sapiens mRNA for KIAA0594 protein, partial cds. [AB011166]	
A_32_P223859	0.00307	2.03	NM_006714	NM_006714	Homo sapiens sphingomyelin phosphodiesterase, acid-like 3A (SMPDL3A), mRNA [NM_006714]	NM_006714
A_24_P225719	0.00308	2.724	NM_015387	NM_015387	Homo sapiens preimplantation protein 3 (PREI3), transcript variant 1, mRNA [NM_015387]	NM_015387
A_23_P61149	0.00308	2.437	NM_001017915	NM_001017915	Homo sapiens inositol polyphosphate-5-phosphatase, 145kDa (INPP5D), transcript variant 1, mRNA [NM_001017915]	NM_001017915
A_24_P910580	0.00309	3.784	NM_181077	NM_181077	Homo sapiens 88-kDa golgi protein (GM88), transcript variant 3, mRNA [NM_181077]	NM_181077
A_23_P151915	0.00309	2.953	ENST00000313774		Homo sapiens glucosaminyl (N-acetyl) transferase 3, mucin type, mRNA (cDNA clone MGC:9086 IMAGE:3851937), complete cds. [BC017032]	
A_24_P307665	0.0031	3.834	AK021848	AK021848	Homo sapiens cDNA FLJ11786 fis, clone HEMBA1006036. [AK021848]	
A_23_P67278	0.0031	2.657	NM_005815	NM_005815	Homo sapiens zinc finger protein 443 (ZNF443), mRNA [NM_005815]	NM_005815
A_32_P96752	0.00311	3.619	AW946823	AW946823	AW946823 RC2-ET0022-080500-012-b10 ET0022 Homo sapiens cDNA, mRNA sequence [AW946823]	
A_32_P146450	0.00312	3.918	AW948903	AW948903	AW948903 QV4-FT0004-130500-212-h09 FT0004 Homo sapiens cDNA, mRNA sequence [AW948903]	
A_24_P941188	0.00312	2.766	AB007928	AB007928	Homo sapiens mRNA for KIAA0459 protein, partial cds. [AB007928]	XM_375697
A_23_P80382	0.00312	2.737	NM_015366	NM_015366	Homo sapiens proline rich protein 5 (PRR5), transcript variant 2, mRNA [NM_015366]	NM_015366
A_32_P212802	0.00313	2.222	THC2267456			
A_23_P19510	0.00313	2.035	NM_182549	NM_182549	Homo sapiens major histocompatibility complex, class II, DQ beta 2 (HLA-DQB2), mRNA [NM_182549]	NM_182549
A_23_P208172	0.00314	2.659	AK023652	AK023652	Homo sapiens cDNA FLJ13590 fis, clone PLACE1009398, moderately similar to ZINC FINGER PROTEIN 135. [AK023652]	
A_24_P341292	0.00314	2.41	NM_024733	NM_024733	Homo sapiens zinc finger protein 665 (ZNF665), mRNA [NM_024733]	NM_024733
A_23_P309599	0.00315	3.481	AK026760	AK026760	Homo sapiens cDNA: FLJ23107 fis, clone LNG07738. [AK026760]	
A_23_P214876	0.00316	4.432	NM_004973	NM_004973	Homo sapiens Jumonji, AT rich interactive domain 2 (JARID2), mRNA [NM_004973]	NM_004973
A_24_P169634	0.00316	3.497	AF019382	AF019382	Homo sapiens mannose-binding protein-A pseudogene (MBL1P1) mRNA sequence. [AF019382]	XR_000276
A_23_P28898	0.00317	7.217	NM_000933	NM_000933	Homo sapiens phospholipase C, beta 4 (PLCB4), transcript variant 1, mRNA [NM_000933]	NM_000933
A_24_P592012	0.00318	2.875	AK126720	AK126720	Homo sapiens cDNA FLJ44766 fis, clone BRACE3032537. [AK126720]	
A_24_P303160	0.00318	2.386	NM_016218	NM_016218	Homo sapiens polymerase (DNA directed) kappa (POLK), mRNA [NM_016218]	NM_016218
A_24_P398898	0.00319	2.19	NM_206839	NM_206839	Homo sapiens mortality factor 4 like 1 (MORF4L1), transcript variant 2, mRNA [NM_206839]	NM_206839
A_24_P227069	0.0032	5.917	NM_020918	NM_020918	Homo sapiens glycerol-3-phosphate acyltransferase, mitochondrial (GPAM), mRNA [NM_020918]	NM_020918
A_32_P117127	0.0032	2.83	ENST00000330336		Homo sapiens ODZ3 (ODZ3) mRNA, partial cds. [AF195420]	XM_497983
A_23_P64962	0.00321	5.202	A_23_P64962			
A_24_P847326	0.00321	2.392	ENST00000335282			
A_23_P27734	0.00321	2.376	NM_002517	NM_002517	Homo sapiens neuronal PAS domain protein 1 (NPAS1), mRNA [NM_002517]	NM_002517
A_23_P394605	0.00322	3.572	ENST00000265341		Homo sapiens mRNA; cDNA DKFZp564I062 (from clone DKFZp564I062). [AL080064]	
A_23_P42695	0.00322	2.149	NM_024051	NM_024051	Homo sapiens chromosome 7 open reading frame 24 (C7orf24), mRNA [NM_024051]	NM_024051
A_23_P101121	0.00323	2.341	THC2404912		Q7XXR9 (Q7XXR9) Katanin, partial (44%) [THC2404912]	

A_23_P210829	0.00324	2.68	NM_018257	NM_018257	Homo sapiens chromosome 20 open reading frame 36 (C20orf36), mRNA [NM_018257]	NM_018257
A_24_P873263	0.00325	2.418	NM_015355	NM_015355	Homo sapiens suppressor of zeste 12 homolog (Drosophila) (SUZ12), mRNA [NM_015355]	NM_015355
A_32_P194891	0.00325	2.334	THC2282954		Q6CQJ2 (Q6CQJ2) Similarity, partial (13%) [THC2323726]	
A_24_P854913	0.00325	2.122	THC2275676			
A_23_P30163	0.00326	21.58	NM_024614	NM_024614	Homo sapiens hypothetical protein FLJ13197 (FLJ13197), mRNA [NM_024614]	NM_024614
A_24_P870620	0.00326	9.67	NM_002825	NM_002825	Homo sapiens pleiotrophin (heparin binding growth factor 8, neurite growth-promoting factor 1) (PTN), mRNA [NM_002825]	NM_002825
A_24_P497226	0.00326	2.991	NM_003161	NM_003161	Homo sapiens ribosomal protein S6 kinase, 70kDa, polypeptide 1 (RPS6KB1), mRNA [NM_003161]	NM_003161
A_24_P814872	0.00327	3.663	A_24_P814872			
A_32_P147696	0.00327	3.245	AK090602	AK090602	Homo sapiens cDNA FLJ33283 fis, clone ASTRO2009177. [AK090602]	XM_378312
A_32_P69333	0.00327	3.187	BX956036	BX956036	DKFZp781J1775_r1 781 (synonym: hlcc4) Homo sapiens cDNA clone DKFZp781J1775 5', mRNA sequence [BX956036]	XM_373489
A_24_P409013	0.00328	6.281	NM_024763	NM_024763	Homo sapiens WD repeat domain 78 (WDR78), transcript variant 1, mRNA [NM_024763]	NM_024763
A_24_P934435	0.0033	2.856	AF086077	AF086077	Homo sapiens full length insert cDNA clone YZ60H05. [AF086077]	
A_23_P218190	0.0033	2.212	NM_000070	NM_000070	Homo sapiens calpain 3, (p94) (CAPN3), transcript variant 1, mRNA [NM_000070]	NM_000070
A_23_P6481	0.00331	2.415	NM_015088	NM_015088	Homo sapiens trinucleotide repeat containing 6B (TNRC6B), transcript variant 1, mRNA [NM_015088]	NM_015088
A_24_P295590	0.00332	3.619	NM_032023	NM_032023	Homo sapiens Ras association (RalGDS/AF-6) domain family 4 (RASSF4), transcript variant 1, mRNA [NM_032023]	NM_032023
A_24_P273423	0.00334	3.476	AK023542	AK023542	Homo sapiens cDNA FLJ13480 fis, clone PLACE1003768. [AK023542]	
A_23_P132738	0.00335	2.21	NM_017541	NM_017541	Homo sapiens crystallin, gamma S (CRYGS), mRNA [NM_017541]	NM_017541
A_32_P12430	0.00336	4.528	NM_015087	NM_015087	Homo sapiens spastic paraplegia 20, spartin (Troyer syndrome) (SPG20), mRNA [NM_015087]	NM_015087
A_23_P77612	0.00337	4.231	NM_145348	NM_145348	Homo sapiens kringle containing transmembrane protein 2 (KREMEN2), transcript variant 3, mRNA [NM_145348]	NM_145348
A_23_P128084	0.00337	2.824	NM_002206	NM_002206	Homo sapiens integrin, alpha 7 (ITGA7), mRNA [NM_002206]	NM_002206
A_24_P285179	0.00338	2.542	THC2337957		Q6IHS4 (Q6IHS4) HDC01261, partial (9%) [THC2337957]	
A_23_P48358	0.00338	2.059	NM_000282	NM_000282	Homo sapiens propionyl Coenzyme A carboxylase, alpha polypeptide (PCCA), mRNA [NM_000282]	NM_000282
A_24_P255524	0.00339	2.606	AF247820	AF247820	Homo sapiens NAG22 protein mRNA, complete cds. [AF247820]	
A_32_P139708	0.00342	5.434	THC2290220			
A_32_P197585	0.00342	4.952	THC2377770		Q5SVL1 (Q5SVL1) Caspase 7, apoptosis-related cysteine protease, partial (12%) [THC2377770]	
A_23_P29816	0.00342	3.771	NM_015900	NM_015900	Homo sapiens phospholipase A1 member A (PLA1A), mRNA [NM_015900]	NM_015900
A_23_P39050	0.00342	3.004	ENST00000341191		Homo sapiens ZFP-36 for a zinc finger protein, mRNA (cDNA clone IMAGE:4992175), partial cds. [BC063560]	XM_375568
A_32_P169353	0.00343	2.575	A_32_P169353			
A_23_P37505	0.00343	2.209	NM_130810	NM_130810	Homo sapiens dyslexia susceptibility 1 candidate 1 (DYX1C1), mRNA [NM_130810]	NM_130810
A_32_P213861	0.00343	2.199	NM_205857	NM_205857	Homo sapiens chromosome 4 open reading frame 12 (C4orf12), mRNA [NM_205857]	NM_205857
A_24_P68079	0.00344	3.209	ENST00000301807		Homo sapiens mRNA for KIAA0342 protein, partial cds. [AB002340]	XM_047357
A_24_P255516	0.00346	2.009	AY359091	AY359091	Homo sapiens clone DNA150157 AAAP6077 (UNQ6077) mRNA, complete cds. [AY359091]	XM_496436
A_23_P8108	0.00347	7.286	NM_002123	NM_002123	Homo sapiens major histocompatibility complex, class II, DQ beta 1 (HLA-DQB1), mRNA [NM_002123]	NM_002123
A_23_P256084	0.00349	6.06	NM_000047	NM_000047	Homo sapiens arylsulfatase E (chondrodysplasia punctata 1) (ARSE), mRNA [NM_000047]	NM_000047
A_24_P231132	0.00349	4.177	NM_001106	NM_001106	Homo sapiens activin A receptor, type IIB (ACVR2B), mRNA [NM_001106]	NM_001106
A_24_P16950	0.0035	2.801	NM_198893	NM_198893	Homo sapiens zinc finger protein 160 (ZNF160), transcript variant 2, mRNA [NM_198893]	NM_198893
A_24_P194971	0.00352	3.622	ENST00000355095			XM_047550
A_23_P92614	0.00352	2.429	NM_133636	NM_133636	Homo sapiens DNA helicase HEL308 (HEL308), mRNA [NM_133636]	NM_133636
A_23_P218817	0.00353	2.081	NM_152246	NM_152246	Homo sapiens carnitine palmitoyltransferase 1B (muscle) (CPT1B), nuclear gene encoding mitochondrial protein, transcript variant 3, mRNA [NM_152246]	NM_152246
A_23_P138631	0.00354	4.01	NM_005445	NM_005445	Homo sapiens chondroitin sulfate proteoglycan 6 (bamacan) (CSPG6), mRNA [NM_005445]	NM_005445
A_23_P358542	0.00354	2.401	NM_145754	NM_145754	Homo sapiens kinesin family member C2 (KIFC2), mRNA [NM_145754]	NM_145754

A_24_P884376	0.00354	2.242	XM_499519	XM_499519	PREDICTED: Homo sapiens hypothetical gene supported by AK092942; NM_198284 (LOC442539), mRNA [XM_499519]	XM_499519
A_23_P99604	0.00354	2.012	NM_017769	NM_017769	Homo sapiens KIAA1333 (KIAA1333), mRNA [NM_017769]	NM_017769
A_32_P151133	0.00355	2.027	CR601496	CR601496	full-length cDNA clone CS0DI062YF05 of Placenta Cot 25-normalized of Homo sapiens (human). [CR601496]	
A_23_P99853	0.00358	2.783	NM_019600	NM_019600	Homo sapiens hypothetical protein FLJ10980 (FLJ10980), mRNA [NM_019600]	NM_019600
A_24_P606239	0.00358	2.246	AK056073	AK056073	Homo sapiens cDNA FLJ31511 fis, clone NT2RI1000035. [AK056073]	
A_23_P326142	0.00359	3.001	NM_014411	NM_014411	Homo sapiens nasopharyngeal carcinoma associated gene protein-8 (NAG8), mRNA [NM_014411]	NM_014411
A_24_P15563	0.0036	2.655	AB032978	AB032978	Homo sapiens mRNA for KIAA1152 protein, partial cds. [AB032978]	
A_24_P131392	0.00362	2.885	NM_144713	NM_144713	Homo sapiens family with sequence similarity 82, member A (FAM82A), mRNA [NM_144713]	NM_144713
A_24_P110213	0.00362	2.711	BX647750	BX647750	Homo sapiens mRNA; cDNA DKFZp779H233 (from clone DKFZp779H233). [BX647750]	
A_32_P78131	0.00362	2.565	THC2305027			
A_23_P112481	0.00363	9.056	NM_004925	NM_004925	Homo sapiens aquaporin 3 (AQP3), mRNA [NM_004925]	NM_004925
A_24_P919283	0.00363	3.56	A_24_P919283			
A_23_P373031	0.00363	3.081	NM_000719	NM_000719	Homo sapiens calcium channel, voltage-dependent, L type, alpha 1C subunit (CACNA1C), mRNA [NM_000719]	NM_000719
A_24_P706752	0.00364	2.558	A_24_P706752			
A_23_P310956	0.00365	4.852	NM_058175	NM_058175	Homo sapiens collagen, type VI, alpha 2 (COL6A2), transcript variant 2C2a', mRNA [NM_058175]	NM_058175
A_23_P2032	0.00366	4.628	A_23_P2032			
A_24_P192434	0.00366	2.343	NM_017489	NM_017489	Homo sapiens telomeric repeat binding factor (NIMA-interacting) 1 (TERF1), transcript variant 1, mRNA [NM_017489]	NM_017489
A_24_P285501	0.00367	3.219	NM_172070	NM_172070	Homo sapiens zinc finger protein 650 (ZNF650), mRNA [NM_172070]	NM_172070
A_24_P125273	0.00367	2.593	NM_001661	NM_001661	Homo sapiens ADP-ribosylation factor 4-like (ARF4L), mRNA [NM_001661]	NM_001661
A_23_P88099	0.00367	2.472	AK092739	AK092739	Homo sapiens cDNA FLJ35420 fis, clone SMINT2001183. [AK092739]	
A_23_P98402	0.00367	2.121	NM_015996	NM_015996	Homo sapiens SID1 transmembrane family, member 2 (SIDT2), mRNA [NM_015996]	NM_015996
A_32_P105469	0.00368	2.628	AK074696	AK074696	Homo sapiens cDNA FLJ90215 fis, clone MAMMA1002205. [AK074696]	
A_32_P222961	0.0037	4.63	NM_001012968	NM_001012968	Homo sapiens hypothetical protein LOC139886 (LOC139886), mRNA [NM_001012968]	NM_001012968
A_23_P6909	0.00371	7.813	NM_178445	NM_178445	Homo sapiens chemokine (C-C motif) receptor-like 1 (CCRL1), transcript variant 1, mRNA [NM_178445]	NM_178445
A_24_P233995	0.00372	3.926	NM_022746	NM_022746	Homo sapiens MOCO sulphurase C-terminal domain containing 1 (MOSC1), mRNA [NM_022746]	NM_022746
A_32_P172453	0.00374	4.579	THC2442819			
A_23_P56938	0.00374	2.661	NM_002908	NM_002908	Homo sapiens v-rel reticuloendotheliosis viral oncogene homolog (avian) (REL), mRNA [NM_002908]	NM_002908
A_24_P153207	0.00374	2.168	CR627133	CR627133	Homo sapiens mRNA; cDNA DKFZp686J154 (from clone DKFZp686J154). [CR627133]	XM_292740
A_24_P766716	0.00375	2.357	AK126405	AK126405	Homo sapiens cDNA FLJ44441 fis, clone UTERU2020242. [AK126405]	
A_23_P94030	0.00376	2.483	NM_002291	NM_002291	Homo sapiens laminin, beta 1 (LAMB1), mRNA [NM_002291]	NM_002291
A_24_P778649	0.0038	3.39	BX640923	BX640923	Homo sapiens mRNA; cDNA DKFZp686B01123 (from clone DKFZp686B01123). [BX640923]	
A_23_P397543	0.0038	2.488	NM_181722	NM_181722	Homo sapiens hypothetical protein LOC285908 (LOC285908), mRNA [NM_181722]	NM_181722
A_23_P213620	0.00381	6.654	NM_004576	NM_004576	Homo sapiens protein phosphatase 2 (formerly 2A), regulatory subunit B (PR 52), beta isoform (PPP2R2B), transcript variant 1, mRNA [NM_004576]	NM_004576
A_32_P74243	0.00381	2.913	BF366211	BF366211	BF366211 IL2-NT0099-300500-096-H08 NT0099 Homo sapiens cDNA, mRNA sequence [BF366211]	
A_32_P87568	0.00381	2.568	NM_001008493	NM_001008493	Homo sapiens enabled homolog (Drosophila) (ENAH), transcript variant 1, mRNA [NM_001008493]	NM_001008493
A_23_P98786	0.00381	2.383	NM_000260	NM_000260	Homo sapiens myosin VIIA (Usher syndrome 1B (autosomal recessive, severe)) (MYO7A), mRNA [NM_000260]	NM_000260
A_24_P151032	0.00381	2.059	NM_002476	NM_002476	Homo sapiens myosin, light polypeptide 4, alkali; atrial, embryonic (MYL4), transcript variant 2, mRNA [NM_002476]	NM_002476
A_23_P31335	0.00382	3.275	AF505656	AF505656	Homo sapiens zinc finger protein 325 (ZNF325) mRNA, complete cds. [AF505656]	
A_32_P180920	0.00382	2.946	AY685922	AY685922	Homo sapiens glioma amplified sequence 64 mRNA, complete cds. [AY685922]	
A_32_P77225	0.00385	4.265	THC2386194			
A_23_P39441	0.00385	2.848	NM_133473	NM_133473	Homo sapiens zinc finger protein 431 (ZNF431), mRNA [NM_133473]	NM_133473

A_23_P49865	0.00385	2.649	NM_015982	NM_015982	Homo sapiens germ cell specific Y-box binding protein (YBX2), mRNA [NM_015982]	NM_015982
A_32_P222684	0.00386	7.161	ENST00000261364		Homo sapiens PR-domain zinc finger protein 6 isoform A (PRDM6) mRNA, partial cds; alternatively spliced. [AF272898]	
A_23_P254594	0.00386	2.376	NM_000825	NM_000825	Homo sapiens gonadotropin-releasing hormone 1 (luteinizing-releasing hormone) (GNRH1), mRNA [NM_000825]	NM_000825
A_23_P96285	0.00387	7.78	NM_022912	NM_022912	Homo sapiens chromosome 2 open reading frame 23 (C2orf23), mRNA [NM_022912]	NM_022912
A_24_P410516	0.00387	3.5	NM_198079	NM_198079	Homo sapiens golgi autoantigen, golgin subfamily a-like (FLJ40113), mRNA [NM_198079]	NM_198079
A_32_P22702	0.00387	2.643	NM_033222	NM_033222	Homo sapiens PC4 and SFRS1 interacting protein 1 (PSIP1), transcript variant 2, mRNA [NM_033222]	NM_033222
A_23_P36166	0.00387	2.495	NM_002645	NM_002645	Homo sapiens phosphoinositide-3-kinase, class 2, alpha polypeptide (PIK3C2A), mRNA [NM_002645]	NM_002645
A_23_P156880	0.0039	3.102	NM_006208	NM_006208	Homo sapiens ectonucleotide pyrophosphatase/phosphodiesterase 1 (ENPP1), mRNA [NM_006208]	NM_006208
A_24_P166789	0.0039	2.842	THC2361388			
A_24_P264909	0.0039	2.13	NM_019080	NM_019080	Homo sapiens Nedd4 family interacting protein 2 (NDFIP2), mRNA [NM_019080]	NM_019080
A_32_P53338	0.00391	3.284	CA312250	CA312250	CA312250 UI-CF-FN0-afm-d-23-0-UI.s1 UI-CF-FN0 Homo sapiens cDNA clone UI-CF-FN0-afm-d-23-0-UI 3', mRNA sequence [CA312250]	
A_32_P61145	0.00391	2.543	AK096154	AK096154	Homo sapiens cDNA FLJ38835 fis, clone MESAN2002424. [AK096154]	
A_23_P328766	0.00392	4.652	NM_145287	NM_145287	Homo sapiens zinc finger protein 519 (ZNF519), mRNA [NM_145287]	NM_145287
A_24_P405621	0.00393	2.25	NM_007184	NM_007184	Homo sapiens nischarin (NISCH), mRNA [NM_007184]	NM_007184
A_24_P938403	0.00394	7.396	BC008690	BC008690	Homo sapiens junctophilin 3, mRNA (cDNA clone IMAGE:3867947), complete cds. [BC008690]	
A_23_P149019	0.00395	2.283	NM_001703	NM_001703	Homo sapiens brain-specific angiogenesis inhibitor 2 (BAI2), mRNA [NM_001703]	NM_001703
A_23_P26890	0.00398	5.135	NM_032950	NM_032950	Homo sapiens matrix metalloproteinase 28 (MMP28), transcript variant 2, mRNA [NM_032950]	NM_032950
A_23_P106024	0.00398	2.029	NM_002226	NM_002226	Homo sapiens jagged 2 (JAG2), transcript variant 1, mRNA [NM_002226]	NM_002226
A_24_P605190	0.00399	2.602	THC2278663		BX491310 DKFZp686K2197_r1 686 (synonym: hlcc3) Homo sapiens cDNA clone DKFZp686K2197 5', mRNA sequence [BX491310]	
A_32_P218671	0.00399	2.33	BG575983	BG575983	602597328F1 NIH_MGC_87 Homo sapiens cDNA clone IMAGE:4705974 5', mRNA sequence [BG575983]	
A_23_P83838	0.004	2.435	NM_004056	NM_004056	Homo sapiens carbonic anhydrase VIII (CA8), mRNA [NM_004056]	NM_004056
A_24_P52697	0.00401	22	NR_002196	NR_002196	Homo sapiens H19, imprinted maternally expressed untranslated mRNA (H19) on chromosome 11 [NR_002196]	NR_002196
A_24_P31275	0.00401	4.571	NM_001678	NM_001678	Homo sapiens ATPase, Na+/K+ transporting, beta 2 polypeptide (ATP1B2), mRNA [NM_001678]	NM_001678
A_23_P51397	0.00401	2.288	NM_001008493	NM_001008493	Homo sapiens enabled homolog (Drosophila) (ENAH), transcript variant 1, mRNA [NM_001008493]	NM_001008493
A_32_P170406	0.00402	6.671	NM_015059	NM_015059	Homo sapiens talin 2 (TLN2), mRNA [NM_015059]	NM_015059
A_32_P9368	0.00403	4.132	THC2339078			
A_32_P13370	0.00403	2.867	AK091672	AK091672	Homo sapiens cDNA FLJ34353 fis, clone FEBRA2011665. [AK091672]	
A_32_P55144	0.00403	2.316	BX537816	BX537816	Homo sapiens mRNA; cDNA DKFZp686M03111 (from clone DKFZp686M03111) [BX537816]	
A_32_P60065	0.00404	5.925	NM_004101	NM_004101	Homo sapiens coagulation factor II (thrombin) receptor-like 2 (F2RL2), mRNA [NM_004101]	NM_004101
A_23_P427083	0.00405	3.026	NM_173622	NM_173622	Homo sapiens hypothetical protein FLJ36674 (FLJ36674), mRNA [NM_173622]	NM_173622
A_32_P142440	0.00406	3.747	NM_174936	NM_174936	Homo sapiens proprotein convertase subtilisin/kexin type 9 (PCSK9), mRNA [NM_174936]	NM_174936
A_23_P363472	0.00408	4.176	AB032991	AB032991	Homo sapiens mRNA for KIAA1165 protein, partial cds. [AB032991]	
A_32_P55414	0.00411	4.907	A_32_P55414			
A_23_P215806	0.00411	3.427	NM_024498	NM_024498	Homo sapiens zinc finger protein 117 (HPF9) (ZNF117), mRNA [NM_024498]	NM_024498
A_23_P161507	0.00411	2.413	NM_004923	NM_004923	Homo sapiens metallothionein-like 5, testis-specific (tesmin) (MTL5), mRNA [NM_004923]	NM_004923
A_23_P136013	0.00412	4.5	A_23_P136013			
A_23_P9779	0.00412	4.298	NM_005853	NM_005853	Homo sapiens iroquois homeobox protein 5 (IRX5), mRNA [NM_005853]	NM_005853
A_24_P185854	0.00414	10.84	NM_004010	NM_004010	Homo sapiens dystrophin (muscular dystrophy, Duchenne and Becker types) (DMD), transcript variant Dp427p2, mRNA [NM_004010]	NM_004010
A_24_P151325	0.00414	3.947	NM_173531	NM_173531	Homo sapiens zinc finger protein 100 (ZNF100), mRNA [NM_173531]	NM_173531
A_32_P110016	0.00414	3.901	A_32_P110016			

A_23_P378288	0.00414	2.826	BX647761	BX647761	Homo sapiens mRNA; cDNA DKFZp686K2231 (from clone DKFZp686K2231). [BX647761]	
A_32_P90740	0.00414	2.11	AF086427	AF086427	Homo sapiens full length insert cDNA clone ZD79D11. [AF086427]	
A_32_P94176	0.00415	2.801	THC2427266			
A_32_P776626	0.00416	2.998	NM_001024457	NM_001024457	Homo sapiens Ran binding protein 2-like 2 (RANBP2L2), mRNA [NM_001024457]	NM_001024457
A_32_P154601	0.00418	2.117	AK125850	AK125850	Homo sapiens cDNA FLJ43862 fis, clone TESTI4007775. [AK125850]	
A_24_P911676	0.00422	2.028	NM_003107	NM_003107	Homo sapiens SRY (sex determining region Y)-box 4 (SOX4), mRNA [NM_003107]	NM_003107
A_24_P896373	0.00423	2.087	THC2300907			
A_24_P136155	0.00424	5.473	A_24_P136155			
A_23_P388798	0.00426	4.508	AF467442	AF467442	Homo sapiens Smith-Magenis syndrome chromosome region candidate 5 protein (SMCR5) mRNA, complete cds. [AF467442]	
A_24_P586264	0.00426	4.281	AK092450	AK092450	Homo sapiens cDNA FLJ35131 fis, clone PLACE6008824. [AK092450]	
A_24_P204639	0.00426	2.893	ENST00000331425			XM_496879
A_23_P78108	0.00426	2.347	NM_005165	NM_005165	Homo sapiens aldolase C, fructose-bisphosphate (ALDOC), mRNA [NM_005165]	NM_005165
A_23_P55256	0.00426	2.22	NM_014897	NM_014897	Homo sapiens zinc finger protein 652 (ZNF652), mRNA [NM_014897]	NM_014897
A_23_P37514	0.00426	2.151	BC011905	BC011905	Homo sapiens DKFZP434H132 protein, mRNA (cDNA clone IMAGE:4121832), complete cds. [BC011905]	
A_23_P150379	0.00427	3.503	NM_144765	NM_144765	Homo sapiens epithelial V-like antigen 1 (EVA1), transcript variant 2, mRNA [NM_144765]	NM_144765
A_23_P168388	0.00428	4.375	NM_175571	NM_175571	Homo sapiens GTPase, IMAP family member 8 (GIMAP8), mRNA [NM_175571]	NM_175571
A_23_P75097	0.0043	3.04	NM_024895	NM_024895	Homo sapiens PDZ domain containing 7 (PDZK7), mRNA [NM_024895]	NM_024895
A_23_P350187	0.00431	2.252	ENST00000265149		Homo sapiens mRNA for KIAA1546 protein, partial cds. [AB046766]	XM_042301
A_23_P301336	0.00434	2.718	NM_014472	NM_014472	Homo sapiens chromosome 10 open reading frame 28 (C10orf28), mRNA [NM_014472]	NM_014472
A_24_P373286	0.00434	2.566	NM_014711	NM_014711	Homo sapiens CP110 protein (CP110), mRNA [NM_014711]	NM_014711
A_24_P700052	0.00435	6.555	AK098638	AK098638	Homo sapiens cDNA FLJ25772 fis, clone TST06461. [AK098638]	
A_32_P41375	0.00435	2.98	AK093729	AK093729	Homo sapiens cDNA FLJ36410 fis, clone THYMU2010637. [AK093729]	XM_373704
A_23_P308731	0.00435	2.522	NM_138328	NM_138328	Homo sapiens rhomboid, veinlet-like 4 (Drosophila) (RHBDL4), mRNA [NM_138328]	NM_138328
A_23_P34637	0.0044	9.031	NM_000537	NM_000537	Homo sapiens renin (REN), mRNA [NM_000537]	NM_000537
A_32_P101264	0.0044	3.64	THC2375782			
A_32_P7721	0.00442	16.95	BC048264	BC048264	Homo sapiens hypothetical protein LOC283666, mRNA (cDNA clone IMAGE:4415549), partial cds. [BC048264]	
A_32_P207802	0.00443	4.579	THC2405783			
A_24_P193435	0.00443	2.501	NM_003257	NM_003257	Homo sapiens tight junction protein 1 (zona occludens 1) (TJP1), transcript variant 1, mRNA [NM_003257]	NM_003257
A_23_P169978	0.00443	2.243	NM_020747	NM_020747	Homo sapiens zinc finger protein 608 (ZNF608), mRNA [NM_020747]	NM_020747
A_23_P33196	0.00443	2.172	NM_000393	NM_000393	Homo sapiens collagen, type V, alpha 2 (COL5A2), mRNA [NM_000393]	NM_000393
A_24_P272761	0.00444	3.221	NM_020946	NM_020946	Homo sapiens KIAA1608 (KIAA1608), transcript variant 1, mRNA [NM_020946]	NM_020946
A_24_P179816	0.00444	3.008	NM_024330	NM_024330	Homo sapiens solute carrier family 27 (fatty acid transporter), member 3 (SLC27A3), mRNA [NM_024330]	NM_024330
A_23_P22134	0.00446	4.478	NM_001717	NM_001717	Homo sapiens basonuclin 1 (BNC1), mRNA [NM_001717]	NM_001717
A_23_P165408	0.00446	2.451	NM_144711	NM_144711	Homo sapiens kelch-like 23 (Drosophila) (KLHL23), mRNA [NM_144711]	NM_144711
A_24_P15083	0.00446	2.436	A_24_P15083			
A_23_P85441	0.00447	3.685	NM_020789	NM_020789	Homo sapiens immunoglobulin superfamily, member 9 (IGSF9), mRNA [NM_020789]	NM_020789
A_32_P7308	0.00448	2.358	THC2407640			
A_32_P197870	0.00448	2.245	AB067506	AB067506	Homo sapiens mRNA for KIAA1919 protein, partial cds. [AB067506]	
A_32_P150748	0.00448	2.134	G31710	G31710	sWSS2257 Eric D. Green Homo sapiens STS cDNA, sequence tagged site. [G31710]	
A_24_P218814	0.00449	2.302	NM_002905	NM_002905	Homo sapiens retinol dehydrogenase 5 (11-cis and 9-cis) (RDH5), mRNA [NM_002905]	NM_002905
A_23_P4536	0.0045	5.953	NM_012307	NM_012307	Homo sapiens erythrocyte membrane protein band 4.1-like 3 (EPB41L3), mRNA [NM_012307]	NM_012307
A_23_P28507	0.00451	2.831	NM_012214	NM_012214	Homo sapiens mannosyl (alpha-1,3-)-glycoprotein beta-1,4-N-acetylglucosaminyltransferase, isoenzyme A (MGAT4A), mRNA [NM_012214]	NM_012214

A_24_P129277	0.00453	2.242	NM_006092	NM_006092	Homo sapiens caspase recruitment domain family, member 4 (CARD4), mRNA [NM_006092]	NM_006092
A_23_P123848	0.00454	2.703	NM_032552	NM_032552	Homo sapiens DAB2 interacting protein (DAB2IP), transcript variant 1, mRNA [NM_032552]	NM_032552
A_23_P147431	0.00455	2.479	NM_002350	NM_002350	Homo sapiens v-yes-1 Yamaguchi sarcoma viral related oncogene homolog (LYN), mRNA [NM_002350]	NM_002350
A_23_P6914	0.00455	2.229	NM_022776	NM_022776	Homo sapiens oxysterol binding protein-like 11 (OSBPL11), mRNA [NM_022776]	NM_022776
A_23_P376686	0.00456	2.021	NM_019602	NM_019602	Homo sapiens butyrophilin-like 2 (MHC class II associated) (BTNL2), mRNA [NM_019602]	NM_019602
A_23_P10081	0.00459	2.077	NM_182896	NM_182896	Homo sapiens ADP-ribosylation factor-like 2-like 1 (ARL2L1), transcript variant 1, mRNA [NM_182896]	NM_182896
A_24_P146188	0.0046	4.823	NM_014942	NM_014942	Homo sapiens ankyrin repeat domain 6 (ANKRD6), mRNA [NM_014942]	NM_014942
A_23_P128050	0.0046	3.866	NM_030766	NM_030766	Homo sapiens BCL2-like 14 (apoptosis facilitator) (BCL2L14), transcript variant 2, mRNA [NM_030766]	NM_030766
A_23_P135826	0.00461	3.181	AK096342	AK096342	Homo sapiens cDNA FLJ39023 fis, clone NT2RP7004348, highly similar to ZINC FINGER PROTEIN 93. [AK096342]	
A_23_P147786	0.00461	2.979	NM_014677	NM_014677	Homo sapiens regulating synaptic membrane exocytosis 2 (RIMS2), mRNA [NM_014677]	NM_014677
A_23_P256641	0.00462	2.024	NM_012282	NM_012282	Homo sapiens KCNE1-like (KCNE1L), mRNA [NM_012282]	NM_012282
A_24_P717305	0.00464	3.427	A_24_P717305			
A_23_P108534	0.00465	3.385	A_23_P108534			
A_24_P914102	0.00466	7.498	A_24_P914102			
A_32_P181107	0.00466	7.201	A_32_P181107			
A_32_P117812	0.00466	2.07	BX640928	BX640928	Homo sapiens mRNA; cDNA DKFZp779J2370 (from clone DKFZp779J2370). [BX640928]	
A_23_P255952	0.00467	5.509	NM_004999	NM_004999	Homo sapiens myosin VI (MYO6), mRNA [NM_004999]	NM_004999
A_24_P187119	0.00467	2.409	ENST00000355247			
A_23_P8653	0.00469	3.087	NM_024788	NM_024788	Homo sapiens hypothetical protein FLJ21062 (FLJ21062), mRNA [NM_024788]	NM_024788
A_24_P387321	0.00469	2.421	NM_016264	NM_016264	Homo sapiens zinc finger protein 44 (KOX 7) (ZNF44), mRNA [NM_016264]	NM_016264
A_32_P143216	0.00469	2.117	AK026322	AK026322	Homo sapiens cDNA: FLJ22669 fis, clone HSI08594. [AK026322]	
A_23_P309779	0.00471	5.505	NM_018177	NM_018177	Homo sapiens Nedd4 binding protein 2 (N4BP2), mRNA [NM_018177]	NM_018177
A_23_P49060	0.00471	2.806	NM_003710	NM_003710	Homo sapiens serine protease inhibitor, Kunitz type 1 (SPINT1), transcript variant 2, mRNA [NM_003710]	NM_003710
A_23_P38860	0.00472	2.784	ENST00000252482		Q96GV2 (Q96GV2) XTP7, complete [THC2262919]	
A_23_P429082	0.00475	2.753	NM_194285	NM_194285	Homo sapiens hypothetical protein FLJ39441 (FLJ39441), mRNA [NM_194285]	NM_194285
A_32_P8881	0.00476	2.606	NM_133446	NM_133446	Homo sapiens centaurin, gamma-like family, member 1 (CTGLF1), mRNA [NM_133446]	NM_133446
A_23_P167237	0.00476	2.452	NM_031911	NM_031911	Homo sapiens C1q and tumor necrosis factor related protein 7 (C1QTNF7), mRNA [NM_031911]	NM_031911
A_23_P29365	0.00476	2.055	ENST00000295859		Homo sapiens YEAF1 mRNA for YY1 and E4TF1 associated factor 1, complete cds. [AB029551]	
A_23_P165848	0.00478	2.44	NM_007046	NM_007046	Homo sapiens elastin microfibril interfacer 1 (EMILIN1), mRNA [NM_007046]	NM_007046
A_32_P79483	0.00478	2.288	BC033993	BC033993	Homo sapiens hypothetical protein LOC283481, mRNA (cDNA clone IMAGE:5296747), partial cds. [BC033993]	
A_32_P517749	0.00479	7.301	NM_004586	NM_004586	Homo sapiens ribosomal protein S6 kinase, 90kDa, polypeptide 3 (RPS6KA3), mRNA [NM_004586]	NM_004586
A_32_P32413	0.0048	64.39	AK123972	AK123972	Homo sapiens cDNA FLJ41978 fis, clone SKNSH2000482. [AK123972]	
A_32_P94976	0.0048	2.011	AA968804	AA968804	oq91d03.s1 NCI_CGAP_Kid6 Homo sapiens cDNA clone IMAGE:1593701 3', mRNA sequence [AA968804]	
A_32_P111266	0.00487	2.317	THC2453189		PEBB_HUMAN (Q13951) Core-binding factor, beta subunit (CBF-beta) (Polyomavirus enhancer binding protein 2 beta subunit) (PEBP2-beta) (PEA2-beta) (SL3-3 enhancer factor 1 beta subunit) (SL3/AKV core-binding factor beta subunit), partial (43%) [THC2453189]	
A_23_P387630	0.00488	2.422	NM_014725	NM_014725	Homo sapiens START domain containing 8 (STARD8), mRNA [NM_014725]	NM_014725
A_23_P76159	0.00488	2.157	BC041930	BC041930	Homo sapiens, clone IMAGE:5300538, mRNA. [BC041930]	
A_24_P68229	0.00488	2.05	BC021803	BC021803	Homo sapiens KIAA0826, mRNA (cDNA clone MGC:25069 IMAGE:4498553), complete cds. [BC021803]	XM_093839
A_32_P199049	0.00489	17.44	THC2314201		Q7QYY0 (Q7QYY0) GLP_164_21502_20957, partial (9%) [THC2314201]	
A_24_P383480	0.00489	2.341	NM_000313	NM_000313	Homo sapiens protein S (alpha) (PROS1), mRNA [NM_000313]	NM_000313
A_24_P260440	0.00491	2.096	AK124768	AK124768	Homo sapiens cDNA FLJ42778 fis, clone BRAWH3005146. [AK124768]	
A_23_P130642	0.00491	2.061	NM_178514	NM_178514	Homo sapiens hypothetical protein LOC283487 (LOC283487), mRNA [NM_178514]	NM_178514

A_32_P164573	0.00492	4.414	THC2314215		RR12_SPIMX (P42344) Chloroplast 30S ribosomal protein S12, partial (11%) [THC2314215]	
A_23_P3302	0.00492	2.515	NM_018365	NM_018365	Homo sapiens meiosis-specific nuclear structural 1 (MNS1), mRNA [NM_018365]	NM_018365
A_32_P2354	0.00492	2.361	BX508036	BX508036	BX508036 DKFZp686E08263_r1 686 (synonym: hlcc3) Homo sapiens cDNA clone DKFZp686E08263 5', mRNA sequence [BX508036]	
A_24_P19410	0.00492	2.324	NM_175709	NM_175709	Homo sapiens chromobox homolog 7 (CBX7), mRNA [NM_175709]	NM_175709
A_32_P141969	0.00494	3.95	AK094929	AK094929	Homo sapiens cDNA FLJ37610 fis, clone BRCOC2011398. [AK094929]	
A_23_P121987	0.00495	9.566	NM_033035	NM_033035	Homo sapiens thymic stromal lymphopoietin (TSLP), transcript variant 1, mRNA [NM_033035]	NM_033035
A_24_P796652	0.00495	2.095	U69195	U69195	U69195 Soares infant brain 1NIB Homo sapiens cDNA clone 32996, mRNA sequence [U69195]	
A_23_P101871	0.00495	2.034	NM_024907	NM_024907	Homo sapiens F-box protein 17 (FBXO17), transcript variant 2, mRNA [NM_024907]	NM_024907
A_24_P510502	0.00496	5.552	N53226	N53226	N53226 yv57e10.s1 Soares fetal liver spleen 1NFLS Homo sapiens cDNA clone IMAGE:246858 3' similar to gb:X07868_rna1 PUTATIVE INSULIN-LIKE GROWTH FACTOR II ASSOCIATED (HUMAN);, mRNA sequence [N53226]	
A_23_P153930	0.00496	2.809	NM_001616	NM_001616	Homo sapiens activin A receptor, type IIA (ACVR2A), mRNA [NM_001616]	NM_001616
A_32_P37584	0.00498	4.173	AW340352	AW340352	AW340352 hd03g08.x1 Soares_NFL_T_GBC_S1 Homo sapiens cDNA clone IMAGE:2908478 3' similar to TR:P70413 P70413 CYCLIN D-INTERACTING MYB-LIKE PROTEIN. ;, mRNA sequence [AW340352]	
A_23_P339687	0.00498	3.617	NM_138330	NM_138330	Homo sapiens zinc finger protein 675 (ZNF675), mRNA [NM_138330]	NM_138330
A_24_P375322	0.00498	2.78	ENST00000322259		Homo sapiens cDNA FLJ31276 fis, clone KIDNE2006376, weakly similar to GAMMA-GLUTAMYLTRANSPEPTIDASE 1 PRECURSOR (EC 2.3.2.2). [AK055838]	
A_32_P192354	0.005	2.992	A_32_P192354			
A_32_P61708	0.005	2.765	A_32_P61708			
A_23_P406702	0.005	2.457	NM_203459	NM_203459	Homo sapiens calmodulin regulated spectrin-associated protein 1-like 1 (CAMSAP1L1), mRNA [NM_203459]	NM_203459
A_23_P88630	0.005	2.133	NM_000057	NM_000057	Homo sapiens Bloom syndrome (BLM), mRNA [NM_000057]	NM_000057
A_23_P25463	0.00501	3.344	NM_025114	NM_025114	Homo sapiens centrosome protein cep290 (Cep290), mRNA [NM_025114]	NM_025114
A_24_P275984	0.00501	2.173	AK025434	AK025434	Homo sapiens cDNA: FLJ21781 fis, clone HEP00223. [AK025434]	
A_23_P10025	0.00502	8.093	NM_006159	NM_006159	Homo sapiens NEL-like 2 (chicken) (NELL2), mRNA [NM_006159]	NM_006159
A_23_P59691	0.00502	2.852	NM_006193	NM_006193	Homo sapiens paired box gene 4 (PAX4), mRNA [NM_006193]	NM_006193
A_23_P71741	0.00502	2.664	NM_017576	NM_017576	Homo sapiens kinesin family member 27 (KIF27), mRNA [NM_017576]	NM_017576
A_23_P97218	0.00503	3.443	CR596670	CR596670	full-length cDNA clone CS0DF018YM18 of Fetal brain of Homo sapiens (human). [CR596670]	
A_24_P84822	0.00503	2.298	NM_006780	NM_006780	Homo sapiens SMA3 (SMA3), mRNA [NM_006780]	NM_006780
A_24_P28977	0.00505	2.587	NM_003304	NM_003304	Homo sapiens transient receptor potential cation channel, subfamily C, member 1 (TRPC1), mRNA [NM_003304]	NM_003304
A_32_P34	0.00507	2.312	AK092544	AK092544	Homo sapiens cDNA FLJ35225 fis, clone PROST2001116. [AK092544]	
A_23_P128230	0.00508	3.109	NM_002135	NM_002135	Homo sapiens nuclear receptor subfamily 4, group A, member 1 (NR4A1), transcript variant 1, mRNA [NM_002135]	NM_002135
A_23_P64661	0.0051	3.351	NM_032496	NM_032496	Homo sapiens Rho GTPase activating protein 9 (ARHGAP9), mRNA [NM_032496]	NM_032496
A_24_P199500	0.00512	2.126	NM_007212	NM_007212	Homo sapiens ring finger protein 2 (RNF2), mRNA [NM_007212]	NM_007212
A_23_P64539	0.00513	19.43	NM_000559	NM_000559	Homo sapiens hemoglobin, gamma A (HBG1), mRNA [NM_000559]	NM_000559
A_23_P215913	0.00513	2.38	NM_203339	NM_203339	Homo sapiens clusterin (complement lysis inhibitor, SP-40,40, sulfated glycoprotein 2, testosterone-repressed prostate message 2, apolipoprotein J) (CLU), transcript variant 2, mRNA [NM_203339]	NM_203339
A_24_P310256	0.00514	2.124	NM_139284	NM_139284	Homo sapiens leucine-rich repeat LGI family, member 4 (LGI4), mRNA [NM_139284]	NM_139284
A_24_P204290	0.00516	2.444	ENST00000342830			XM_496049
A_24_P807883	0.00516	2.43	THC2279305			
A_24_P288890	0.00517	3.166	NM_181709	NM_181709	Homo sapiens hypothetical protein LOC144347 (LOC144347), mRNA [NM_181709]	NM_181709
A_32_P74409	0.00518	2.945	ENST00000339446		Homo sapiens hypothetical LOC387763, mRNA (cDNA clone IMAGE:6272440), partial cds. [BC052560]	XM_373497
A_23_P167030	0.0052	3.264	NM_000316	NM_000316	Homo sapiens parathyroid hormone receptor 1 (PTHR1), mRNA [NM_000316]	NM_000316
A_23_P120809	0.0052	2.509	NM_080839	NM_080839	Homo sapiens gamma-glutamyltransferase-like 4 (GGTL4), transcript variant 2, mRNA [NM_080839]	NM_080839

A_24_P160263	0.0052	2.059	NM_201222	NM_201222	Homo sapiens melanoma antigen family D, 2 (MAGED2), transcript variant 3, mRNA [NM_201222]	NM_201222
A_24_P410605	0.00521	2.055	BC080541	BC080541	Homo sapiens receptor tyrosine kinase-like orphan receptor 1, mRNA (cDNA clone IMAGE:5477978), complete cds. [BC080541]	
A_23_P3312	0.00522	5.875	NM_005545	NM_005545	Homo sapiens immunoglobulin superfamily containing leucine-rich repeat (ISLR), transcript variant 1, mRNA [NM_005545]	NM_005545
A_24_P310224	0.00524	3.085	NM_032584	NM_032584	Homo sapiens zinc finger protein 347 (ZNF347), mRNA [NM_032584]	NM_032584
A_23_P127915	0.00524	2.224	NM_030906	NM_030906	Homo sapiens serine/threonine kinase 33 (STK33), mRNA [NM_030906]	NM_030906
A_23_P118327	0.00524	2.019	NM_017736	NM_017736	Homo sapiens THUMP domain containing 1 (THUMPD1), mRNA [NM_017736]	NM_017736
A_24_P281988	0.00525	4.751	NM_015184	NM_015184	Homo sapiens phospholipase C-like 2 (PLCL2), mRNA [NM_015184]	NM_015184
A_24_P83379	0.00525	2.62	NM_178583	NM_178583	Homo sapiens WD repeat and FYVE domain containing 3 (WDFY3), transcript variant 2, mRNA [NM_178583]	NM_178583
A_32_P49508	0.00526	4.41	NM_019036	NM_019036	Homo sapiens 3-hydroxymethyl-3-methylglutaryl-Coenzyme A lyase-like 1 (HMGCLL1), mRNA [NM_019036]	NM_019036
A_23_P405885	0.00526	3.76	NM_138815	NM_138815	Homo sapiens developmental pluripotency associated 2 (DPPA2), mRNA [NM_138815]	NM_138815
A_23_P204929	0.00526	2.796	NM_016248	NM_016248	Homo sapiens A kinase (PRKA) anchor protein 11 (AKAP11), transcript variant 1, mRNA [NM_016248]	NM_016248
A_23_P54918	0.00528	2.34	NM_153486	NM_153486	Homo sapiens lactate dehydrogenase D (LDHD), nuclear gene encoding mitochondrial protein, transcript variant 1, mRNA [NM_153486]	NM_153486
A_24_P20814	0.00529	7.338	NM_030568	NM_030568	Homo sapiens chromosome 6 open reading frame 148 (C6orf148), mRNA [NM_030568]	NM_030568
A_24_P170327	0.00529	3.802	NM_013255	NM_013255	Homo sapiens muskelin 1, intracellular mediator containing kelch motifs (MKLN1), mRNA [NM_013255]	NM_013255
A_32_P152255	0.00531	3.017	AK094623	AK094623	Homo sapiens cDNA FLJ37304 fis, clone BRAMY2016070. [AK094623]	
A_23_P112162	0.00531	2.176	NM_012079	NM_012079	Homo sapiens diacylglycerol O-acyltransferase homolog 1 (mouse) (DGAT1), mRNA [NM_012079]	NM_012079
A_23_P51082	0.00532	2.93	THC2242915		ALU1_HUMAN (P39188) Alu subfamily J sequence contamination warning entry, partial (4%) [THC2242915]	
A_23_P25003	0.00532	2.081	NM_002956	NM_002956	Homo sapiens restin (Reed-Steinberg cell-expressed intermediate filament-associated protein) (RSN), transcript variant 1, mRNA [NM_002956]	NM_002956
A_24_P236008	0.00533	2.443	AB037781	AB037781	Homo sapiens mRNA for KIAA1360 protein, partial cds. [AB037781]	
A_24_P778607	0.00533	2.408	AK090897	AK090897	Homo sapiens cDNA FLJ33578 fis, clone BRAMY2011639. [AK090897]	
A_23_P124585	0.00533	2.371	AK096342	AK096342	Homo sapiens cDNA FLJ39023 fis, clone NT2RP7004348, highly similar to ZINC FINGER PROTEIN 93. [AK096342]	
A_23_P89762	0.00535	2.892	NM_194449	NM_194449	Homo sapiens PH domain and leucine rich repeat protein phosphatase (PHLPP), mRNA [NM_194449]	NM_194449
A_24_P943205	0.00537	2.823	ENST00000313624		Homo sapiens mRNA; cDNA DKFZp667P0410 (from clone DKFZp667P0410). [AL831953]	
A_23_P429020	0.00537	2.258	NM_181786	NM_181786	Homo sapiens GLI-Kruppel family member HKR1 (HKR1), mRNA [NM_181786]	NM_181786
A_24_P374943	0.0054	8.81	NM_001338	NM_001338	Homo sapiens coxsackie virus and adenovirus receptor (CXADR), mRNA [NM_001338]	NM_001338
A_23_P359647	0.0054	2.742	NM_138714	NM_138714	Homo sapiens nuclear factor of activated T-cells 5, tonicity-responsive (NFAT5), transcript variant 1, mRNA [NM_138714]	NM_138714
A_23_P53137	0.00541	19.48	NM_000559	NM_000559	Homo sapiens hemoglobin, gamma A (HBG1), mRNA [NM_000559]	NM_000559
A_23_P74629	0.00544	2.019	NM_001030	NM_001030	Homo sapiens ribosomal protein S27 (metallopanstimulin 1) (RPS27), mRNA [NM_001030]	NM_001030
A_23_P148879	0.00546	11.28	NM_000702	NM_000702	Homo sapiens ATPase, Na+/K+ transporting, alpha 2 (+) polypeptide (ATP1A2), mRNA [NM_000702]	NM_000702
A_23_P51518	0.00546	3.654	NM_003617	NM_003617	Homo sapiens regulator of G-protein signalling 5 (RGS5), mRNA [NM_003617]	NM_003617
A_23_P52176	0.00546	3.075	NM_003431	NM_003431	Homo sapiens zinc finger protein 124 (HZF-16) (ZNF124), mRNA [NM_003431]	NM_003431
A_23_P115246	0.00547	2.275	NM_003665	NM_003665	Homo sapiens ficolin (collagen/fibrinogen domain containing) 3 (Hakata antigen) (FCN3), transcript variant 1, mRNA [NM_003665]	NM_003665
A_23_P27994	0.00549	2.797	NM_003332	NM_003332	Homo sapiens TYRO protein tyrosine kinase binding protein (TYROBP), transcript variant 1, mRNA [NM_003332]	NM_003332
A_32_P93493	0.00551	8.558	BC036018	BC036018	Homo sapiens, Similar to protein tyrosine phosphatase, receptor type, G, clone IMAGE:5278250, mRNA. [BC036018]	
A_23_P131435	0.00552	3.136	NM_014880	NM_014880	Homo sapiens CD302 antigen (CD302), mRNA [NM_014880]	NM_014880
A_32_P181077	0.00553	14.03	NM_203447	NM_203447	Homo sapiens dedicator of cytokinesis 8 (DOCK8), mRNA [NM_203447]	NM_203447
A_24_P741023	0.00553	3.055	BC008476	BC008476	Homo sapiens, clone IMAGE:4290767, mRNA. [BC008476]	
A_23_P253921	0.00556	2.004	NM_018651	NM_018651	Homo sapiens zinc finger protein 167 (ZNF167), transcript variant 1, mRNA [NM_018651]	NM_018651
A_23_P60499	0.00558	3.928	NM_021224	NM_021224	Homo sapiens zinc finger protein 462 (ZNF462), mRNA [NM_021224]	NM_021224
A_32_P41026	0.00559	3.912	THC2270160			

A_23_P16516	0.00559	2.166	NM_005224	NM_005224	Homo sapiens AT rich interactive domain 3A (BRIGHT- like) (ARID3A), mRNA [NM_005224]	NM_005224
A_23_P368145	0.00559	2.116	NM_152281	NM_152281	Homo sapiens SCY1-like 1 binding protein 1 (SCYL1BP1), mRNA [NM_152281]	NM_152281
A_23_P62857	0.0056	5.431	A_23_P62857			
A_23_P110473	0.0056	3.424	NM_004536	NM_004536	Homo sapiens baculoviral IAP repeat-containing 1 (BIRC1), mRNA [NM_004536]	NM_004536
A_23_P211468	0.00563	3.634	AA837799	AA837799	AA837799 oe06h09.s1 NCI_CGAP_Ov2 Homo sapiens cDNA clone IMAGE:1385153, mRNA sequence [AA837799]	
A_23_P343382	0.00564	6.095	AB011086	AB011086	Homo sapiens mRNA for KIAA0514 protein, partial cds. [AB011086]	
A_24_P453740	0.00565	2.362	NM_194283	NM_194283	Homo sapiens DnaJ homology subfamily A member 5 (DNAJA5), transcript variant 1, mRNA [NM_194283]	NM_194283
A_23_P124252	0.00567	2.574	NM_020397	NM_020397	Homo sapiens calcium/calmodulin-dependent protein kinase ID (CAMK1D), transcript variant 1, mRNA [NM_020397]	NM_020397
A_24_P283225	0.00568	5.491	NM_004999	NM_004999	Homo sapiens myosin VI (MYO6), mRNA [NM_004999]	NM_004999
A_23_P125164	0.00568	4.183	NM_002012	NM_002012	Homo sapiens fragile histidine triad gene (FHIT), mRNA [NM_002012]	NM_002012
A_32_P162494	0.0057	2.569	THC2375981		Q5SSW7 (Q5SSW7) CAMP responsive elment binding protein-like 1, partial (5%) [THC2375981]	
A_23_P39682	0.00572	4.319	NM_001079	NM_001079	Homo sapiens zeta-chain (TCR) associated protein kinase 70kDa (ZAP70), transcript variant 1, mRNA [NM_001079]	NM_001079
A_23_P330578	0.00572	3.644	NM_144620	NM_144620	Homo sapiens leucine rich repeat containing 39 (LRRC39), mRNA [NM_144620]	NM_144620
A_23_P109322	0.00574	3.373	NM_006198	NM_006198	Homo sapiens Purkinje cell protein 4 (PCP4), mRNA [NM_006198]	NM_006198
A_23_P62583	0.00574	3.128	NM_001409	NM_001409	Homo sapiens EGF-like-domain, multiple 3 (EGFL3), mRNA [NM_001409]	NM_001409
A_32_P7015	0.00574	2.153	NM_012339	NM_012339	Homo sapiens tetraspanin 15 (TSPAN15), mRNA [NM_012339]	NM_012339
A_32_P15017	0.00576	2.66	NM_152756	NM_152756	Homo sapiens TORC2-specific protein AVO3 (AVO3), mRNA [NM_152756]	NM_152756
A_24_P238499	0.00576	2.004	NM_001012716	NM_001012716	Homo sapiens chromosome 18 open reading frame 56 (C18orf56), mRNA [NM_001012716]	NM_001012716
A_24_P196400	0.00583	3.597	NM_017934	NM_017934	Homo sapiens pleckstrin homology domain interacting protein (PHIP), mRNA [NM_017934]	NM_017934
A_23_P56356	0.00583	2.712	BC042674	BC042674	Homo sapiens phospholipase B1, mRNA (cDNA clone MGC:35447 IMAGE:5191712), complete cds. [BC042674]	
A_32_P160216	0.00584	2.919	THC2442020		Q6ILN9 (Q6ILN9) HDC08824, partial (15%) [THC2442020]	
A_23_P50834	0.00585	2.956	NM_182515	NM_182515	Homo sapiens hypothetical protein LOC148206 (LOC148206), mRNA [NM_182515]	NM_182515
A_24_P365807	0.00586	3.296	NM_004429	NM_004429	Homo sapiens ephrin-B1 (EFNB1), mRNA [NM_004429]	NM_004429
A_23_P208259	0.00588	2.769	NM_032584	NM_032584	Homo sapiens zinc finger protein 347 (ZNF347), mRNA [NM_032584]	NM_032584
A_23_P65890	0.00588	2.228	NM_006383	NM_006383	Homo sapiens calcium and integrin binding family member 2 (CIB2), mRNA [NM_006383]	NM_006383
A_24_P40306	0.00588	2.22	NM_000185	NM_000185	Homo sapiens serine (or cysteine) proteinase inhibitor, clade D (heparin cofactor), member 1 (SERPIND1), mRNA [NM_000185]	NM_000185
A_32_P206549	0.0059	2.52	BC035156	BC035156	Homo sapiens, clone IMAGE:5265210, mRNA. [BC035156]	
A_32_P118942	0.00591	3.385	THC2277556		Q9B8U2 (Q9B8U2) NADH dehydrogenase subunit 6, partial (10%) [THC2277556]	
A_32_P88987	0.00593	4.435	AK022346	AK022346	Homo sapiens cDNA FLJ12284 fis, clone MAMMA1001757. [AK022346]	
A_24_P145333	0.00593	2.987	THC2262874			
A_23_P422766	0.00593	2.245	NM_172193	NM_172193	Homo sapiens kelch domain containing 1 (KLHDC1), mRNA [NM_172193]	NM_172193
A_23_P382065	0.00596	7.207	NM_016242	NM_016242	Homo sapiens endomucin (EMCN), mRNA [NM_016242]	NM_016242
A_32_P12703	0.00598	2.22	THC2411678			
A_32_P171793	0.00599	2.261	THC2317110			
A_23_P343963	0.00601	2.144	NM_138435	NM_138435	Homo sapiens family with sequence similarity 83, member F (FAM83F), mRNA [NM_138435]	NM_138435
A_24_P161725	0.00602	4.743	BC089388	BC089388	Homo sapiens cDNA clone IMAGE:30390722, containing frame-shift errors. [BC089388]	XM_496028
A_23_P3911	0.00603	2.884	NM_020405	NM_020405	Homo sapiens plexin domain containing 1 (PLXDC1), mRNA [NM_020405]	NM_020405
A_23_P217596	0.00603	2.315	AF022789	AF022789	Homo sapiens ubiquitin hydrolyzing enzyme I (UBH1) mRNA, partial cds. [AF022789]	
A_32_P65530	0.00608	2.172	A_32_P65530			
A_32_P196287	0.00611	3.828	THC2316688		MUSHOX222 homeobox mh22b-related protein {Mus musculus;} , partial (14%) [THC2316688]	
A_24_P108311	0.00611	2.15	NM_015277	NM_015277	Homo sapiens neural precursor cell expressed, developmentally down-regulated 4-like (NEDD4L), mRNA [NM_015277]	NM_015277
A_23_P211047	0.00612	2.14	NM_206866	NM_206866	Homo sapiens BTB and CNC homology 1, basic leucine zipper transcription factor 1 (BACH1), transcript variant 1, mRNA [NM_206866]	NM_206866

A_23_P67771	0.00613	2.4	NM_000465	NM_000465	Homo sapiens BRCA1 associated RING domain 1 (BARD1), mRNA [NM_000465]	NM_000465
A_23_P166779	0.00616	4.21	NM_013343	NM_013343	Homo sapiens loss of heterozygosity, 3, chromosomal region 2, gene A (LOH3CR2A), mRNA [NM_013343]	NM_013343
A_24_P150874	0.00616	2.133	CR611712	CR611712	full-length cDNA clone CS0DB005YH06 of Neuroblastoma Cot 10-normalized of Homo sapiens (human). [CR611712]	
A_24_P912228	0.00618	2.116	AK095564	AK095564	Homo sapiens cDNA FLJ38245 fis, clone FCBBF2007186. [AK095564]	
A_24_P328969	0.0062	2.582	NM_030799	NM_030799	Homo sapiens Yip1 domain family, member 5 (YIPF5), transcript variant 2, mRNA [NM_030799]	NM_030799
A_23_P210224	0.00621	4.165	AL713706	AL713706	Homo sapiens mRNA; cDNA DKFZp761O1810 (from clone DKFZp761O1810). [AL713706]	
A_32_P35969	0.00621	2.871	CR605947	CR605947	full-length cDNA clone CS0DD004YC02 of Neuroblastoma Cot 50-normalized of Homo sapiens (human). [CR605947]	
A_23_P354908	0.00622	4.019	NM_015039	NM_015039	Homo sapiens nicotinamide nucleotide adenylyltransferase 2 (NMNAT2), transcript variant 1, mRNA [NM_015039]	NM_015039
A_24_P205427	0.00622	2.445	AY129023	AY129023	Homo sapiens clone FP18821 unknown mRNA. [AY129023]	
A_23_P10559	0.00624	2.682	AK131529	AK131529	Homo sapiens cDNA FLJ16758 fis, clone BRACE3038687, moderately similar to Homo sapiens apoptosis-associated tyrosine kinase (AATK). [AK131529]	
A_23_P125788	0.00624	2.531	NM_152278	NM_152278	Homo sapiens transcription elongation factor A (SII)-like 7 (TCEAL7), mRNA [NM_152278]	NM_152278
A_24_P102920	0.00627	5.269	AK002156	AK002156	Homo sapiens cDNA FLJ11294 fis, clone PLACE1009708. [AK002156]	
A_32_P132928	0.00629	2.27	A_32_P132928			
A_24_P402690	0.0063	2.378	NM_030926	NM_030926	Homo sapiens integral membrane protein 2C (ITM2C), transcript variant 1, mRNA [NM_030926]	NM_030926
A_32_P212258	0.00631	3.778	THC2445005			
A_32_P39003	0.00631	2.025	NM_006780	NM_006780	Homo sapiens SMA3 (SMA3), mRNA [NM_006780]	NM_006780
A_23_P320242	0.00631	2.011	NM_152748	NM_152748	Homo sapiens KIAA1324-like (KIAA1324L), mRNA [NM_152748]	NM_152748
A_23_P109072	0.00632	5.546	NM_020436	NM_020436	Homo sapiens sal-like 4 (Drosophila) (SALL4), mRNA [NM_020436]	NM_020436
A_23_P35576	0.00632	3.83	NM_014803	NM_014803	Homo sapiens zinc finger protein 518 (ZNF518), mRNA [NM_014803]	NM_014803
A_23_P130158	0.00632	2.358	NM_030753	NM_030753	Homo sapiens wingless-type MMTV integration site family, member 3 (WNT3), mRNA [NM_030753]	NM_030753
A_24_P14328	0.00632	2.068	AL834146	AL834146	Homo sapiens mRNA; cDNA DKFZp761F06121 (from clone DKFZp761F06121). [AL834146]	
A_32_P50406	0.00633	2.798	AK093729	AK093729	Homo sapiens cDNA FLJ36410 fis, clone THYMU2010637. [AK093729]	XM_373704
A_24_P772330	0.00633	2.657	AK093903	AK093903	Homo sapiens cDNA FLJ36584 fis, clone TRACH2013450. [AK093903]	
A_32_P71212	0.00635	5.696	A_32_P71212			
A_24_P179407	0.00636	2.374	NM_018351	NM_018351	Homo sapiens FYVE, RhoGEF and PH domain containing 6 (FGD6), mRNA [NM_018351]	NM_018351
A_23_P349083	0.0064	2.59	NM_138782	NM_138782	Homo sapiens FCH domain only 2 (FCHO2), mRNA [NM_138782]	NM_138782
A_32_P98752	0.0064	2.334	THC2446811			
A_23_P399146	0.00641	2.084	NM_153263	NM_153263	Homo sapiens zinc finger protein 549 (ZNF549), mRNA [NM_153263]	NM_153263
A_23_P212258	0.00648	7.306	NM_000893	NM_000893	Homo sapiens kininogen 1 (KNG1), mRNA [NM_000893]	NM_000893
A_24_P562369	0.00648	3.676	A_24_P562369			
A_24_P192301	0.00648	2.826	THC2373802		Q89P93 (Q89P93) Bll3590 protein, partial (4%) [THC2373802]	
A_24_P12865	0.00648	2.003	NM_000155	NM_000155	Homo sapiens galactose-1-phosphate uridylyltransferase (GALT), transcript variant 1, mRNA [NM_000155]	NM_000155
A_23_P168812	0.00649	2.133	NM_145111	NM_145111	Homo sapiens hypothetical protein DKFZp727G131 (DKFZp727G131), mRNA [NM_145111]	NM_145111
A_23_P89062	0.00652	4.528	NM_024048	NM_024048	Homo sapiens hypothetical protein MGC3020 (MGC3020), mRNA [NM_024048]	NM_024048
A_23_P120243	0.00652	2.829	NM_024501	NM_024501	Homo sapiens homeo box D1 (HOXD1), mRNA [NM_024501]	NM_024501
A_23_P200477	0.00652	2.013	AB043587	AB043587	Homo sapiens gcp60 mRNA for golgi resident protein GCP60, complete cds. [AB043587]	
A_32_P183718	0.00653	31.09	NM_001003845	NM_001003845	Homo sapiens Sp5 transcription factor (SP5), mRNA [NM_001003845]	NM_001003845
A_23_P258582	0.00656	2.269	BX648681	BX648681	Homo sapiens mRNA; cDNA DKFZp686M0154 (from clone DKFZp686M0154). [BX648681]	
A_32_P171253	0.00661	2.253	THC2442210		Q9F8M7 (Q9F8M7) DTDP-glucose 4,6-dehydratase (Fragment), partial (11%) [THC2442210]	
A_24_P394533	0.00661	2.195	NM_000434	NM_000434	Homo sapiens sialidase 1 (lysosomal sialidase) (NEU1), mRNA [NM_000434]	NM_000434
A_23_P102890	0.00662	2.21	NM_032476	NM_032476	Homo sapiens mitochondrial ribosomal protein S6 (MRPS6), nuclear gene encoding mitochondrial protein, mRNA [NM_032476]	NM_032476

A_32_P179854	0.00666	2.8	CR607153	CR607153	full-length cDNA clone CS0DJ012YN17 of T cells (Jurkat cell line) Cot 10-normalized of Homo sapiens (human). [CR607153]	
A_24_P887092	0.00669	4.386	THC2339791			
A_24_P550411	0.00669	2.776	BX387925	BX387925	BX387925 BX387925 Homo sapiens T CELLS (JURKAT CELL LINE) COT 10-NORMALIZED Homo sapiens cDNA clone CS0DJ009YB07 3-PRIME, mRNA sequence [BX387925]	
A_24_P141520	0.00675	6.215	AK022297	AK022297	Homo sapiens cDNA FLJ12235 fis, clone MAMMA1001243. [AK022297]	
A_23_P317620	0.00675	2.432	NM_005737	NM_005737	Homo sapiens ADP-ribosylation factor-like 7 (ARL7), mRNA [NM_005737]	NM_005737
A_23_P377434	0.00675	2.148	AK027341	AK027341	Homo sapiens cDNA FLJ14435 fis, clone HEMBA1007085. [AK027341]	XM_376567
A_24_P240166	0.00677	2.087	NM_145753	NM_145753	Homo sapiens pleckstrin homology-like domain, family B, member 2 (PHLDB2), mRNA [NM_145753]	NM_145753
A_23_P328237	0.00678	2.442	NM_194285	NM_194285	Homo sapiens hypothetical protein FLJ39441 (FLJ39441), mRNA [NM_194285]	NM_194285
A_32_P229221	0.00682	2.627	AB209318	AB209318	Homo sapiens mRNA for glutamate receptor, ionotropic, delta 2 variant protein. [AB209318]	
A_24_P938006	0.00684	2.763	A_24_P938006			
A_24_P135344	0.00684	2.489	NM_014715	NM_014715	Homo sapiens Rho GTPase-activating protein (RICS), mRNA [NM_014715]	NM_014715
A_24_P28165	0.00684	2.487	NM_015071	NM_015071	Homo sapiens Rho GTPase activating protein 26 (ARHGAP26), mRNA [NM_015071]	NM_015071
A_24_P247044	0.00685	6.202	ENST00000355095		Homo sapiens mRNA for KIAA1473 protein, partial cds. [AB040906]	XM_047554
A_24_P101314	0.00685	2.27	NM_138356	NM_138356	Homo sapiens Src homology 2 domain containing F (SHF), mRNA [NM_138356]	NM_138356
A_24_P271149	0.00686	2.861	CR592222	CR592222	full-length cDNA clone CS0DB003YO14 of Neuroblastoma Cot 10-normalized of Homo sapiens (human). [CR592222]	
A_24_P53976	0.00687	4.305	NM_002065	NM_002065	Homo sapiens glutamate-ammonia ligase (glutamine synthase) (GLUL), mRNA [NM_002065]	NM_002065
A_32_P2303	0.00687	3.762	A_32_P2303			
A_23_P28953	0.00691	7.778	NM_175850	NM_175850	Homo sapiens DNA (cytosine-5-)-methyltransferase 3 beta (DNMT3B), transcript variant 6, mRNA [NM_175850]	NM_175850
A_32_P353072	0.00692	2.6	NM_018374	NM_018374	Homo sapiens hypothetical protein FLJ11273 (FLJ11273), mRNA [NM_018374]	NM_018374
A_24_P256692	0.00694	2.37	D87742	D87742	Human mRNA for KIAA0268 gene, partial cds. [D87742]	XM_371369
A_23_P63999	0.00695	2.021	CR614032	CR614032	full-length cDNA clone CS0DL003YC18 of B cells (Ramos cell line) Cot 25-normalized of Homo sapiens (human). [CR614032]	
A_32_P209582	0.00697	4.043	THC2318614		ALU1_HUMAN (P39188) Alu subfamily J sequence contamination warning entry, partial (5%) [THC2318614]	
A_32_P94618	0.00697	3.768	THC2295157			
A_23_P372638	0.00698	3.299	NM_025134	NM_025134	Homo sapiens chromodomain helicase DNA binding protein 9 (CHD9), mRNA [NM_025134]	NM_025134
A_23_P60259	0.00701	2.857	NM_018112	NM_018112	Homo sapiens transmembrane protein 38B (TMEM38B), mRNA [NM_018112]	NM_018112
A_24_P58381	0.00703	4.153	NM_017780	NM_017780	Homo sapiens chromodomain helicase DNA binding protein 7 (CHD7), mRNA [NM_017780]	NM_017780
A_23_P81898	0.00704	5.861	NM_006398	NM_006398	Homo sapiens ubiquitin D (UBD), mRNA [NM_006398]	NM_006398
A_32_P200934	0.00705	2.257	AK021443	AK021443	Homo sapiens cDNA FLJ11381 fis, clone HEMBA1000501. [AK021443]	
A_23_P66347	0.00706	2.462	A_23_P66347			
A_23_P115726	0.00708	3.655	NM_194298	NM_194298	Homo sapiens solute carrier family 16 (monocarboxylic acid transporters), member 9 (SLC16A9), mRNA [NM_194298]	NM_194298
A_23_P76136	0.00708	2.894	AY358804	AY358804	Homo sapiens clone DNA100312 VSSW1971 (UNQ1971) mRNA, complete cds. [AY358804]	
A_23_P500601	0.00709	5.979	NM_033017	NM_033017	Homo sapiens tripartite motif-containing 4 (TRIM4), transcript variant alpha, mRNA [NM_033017]	NM_033017
A_23_P146943	0.00709	4.958	NM_001677	NM_001677	Homo sapiens ATPase, Na+/K+ transporting, beta 1 polypeptide (ATP1B1), transcript variant 1, mRNA [NM_001677]	NM_001677
A_23_P139408	0.00711	2.232	NM_021964	NM_021964	Homo sapiens zinc finger protein 148 (pHZ-52) (ZNF148), mRNA [NM_021964]	NM_021964
A_23_P30614	0.00714	6.563	NM_002667	NM_002667	Homo sapiens phospholamban (PLN), mRNA [NM_002667]	NM_002667
A_32_P129269	0.00714	2.64	ENST00000283426		Homo sapiens mRNA for KIAA1909 protein, partial cds. [AB067496]	
A_23_P216149	0.00718	2.917	NM_017489	NM_017489	Homo sapiens telomeric repeat binding factor (NIMA-interacting) 1 (TERF1), transcript variant 1, mRNA [NM_017489]	NM_017489
A_24_P489649	0.0072	2.366	XM_067448	XM_067448	PREDICTED: Homo sapiens similar to MEST (LOC131572), mRNA [XM_067448]	XM_067448
A_23_P7752	0.00722	9.166	NM_020796	NM_020796	Homo sapiens sema domain, transmembrane domain (TM), and cytoplasmic domain, (semaphorin) 6A (SEMA6A), mRNA [NM_020796]	NM_020796

A_32_P69783	0.00724	3.527	THC2445784		Q9F8M7 (Q9F8M7) DTDP-glucose 4,6-dehydratase (Fragment), partial (11%) [THC2445784]	
A_23_P312179	0.00727	2.251	NM_015120	NM_015120	Homo sapiens Alstrom syndrome 1 (ALMS1), mRNA [NM_015120]	NM_015120
A_32_P98435	0.00728	4.51	AF293366	AF293366	Homo sapiens clone 122482 unknown mRNA. [AF293366]	
A_23_P315131	0.00728	2.285	AK024388	AK024388	Homo sapiens cDNA FLJ14326 fis, clone PLACE4000247. [AK024388]	
A_23_P256470	0.0073	4.73	NM_000905	NM_000905	Homo sapiens neuropeptide Y (NPY), mRNA [NM_000905]	NM_000905
A_32_P40999	0.0073	2.263	A_32_P40999			
A_32_P108203	0.00731	5.162	THC2403644		AF042857 lung cancer antigen NY-LU-12 variant A {Homo sapiens;} , partial (9%) [THC2403644]	
A_23_P202737	0.00731	2.219	NM_003772	NM_003772	Homo sapiens jerky homolog-like (mouse) (JRKL), mRNA [NM_003772]	NM_003772
A_23_P114616	0.00733	2.356	ENST00000262346		Homo sapiens mRNA; cDNA DKFZp566D1346 (from clone DKFZp566D1346). [AL136717]	
A_24_P247699	0.00735	2.313	NM_015172	NM_015172	Homo sapiens BAT2 domain containing 1 (BAT2D1), mRNA [NM_015172]	NM_015172
A_23_P140928	0.00739	2.739	NM_024847	NM_024847	Homo sapiens transmembrane channel-like 7 (TMC7), mRNA [NM_024847]	NM_024847
A_23_P62752	0.00739	2.34	NM_002521	NM_002521	Homo sapiens natriuretic peptide precursor B (NPPB), mRNA [NM_002521]	NM_002521
A_23_P70297	0.0074	40.66	AB023174	AB023174	Homo sapiens mRNA for KIAA0957 protein, partial cds. [AB023174]	
A_23_P17269	0.0074	2.435	NM_018084	NM_018084	Homo sapiens KIAA1212 (KIAA1212), mRNA [NM_018084]	NM_018084
A_23_P352799	0.00743	2.933	ENST00000329610		Homo sapiens prepro-NPW mRNA for prepro-Neuropeptide W polypeptide, partial cds. [AB084276]	XM_496111
A_23_P119943	0.00743	2.766	NM_000597	NM_000597	Homo sapiens insulin-like growth factor binding protein 2, 36kDa (IGFBP2), mRNA [NM_000597]	NM_000597
A_23_P169558	0.00743	2.656	NM_024790	NM_024790	Homo sapiens centrosome spindle pole associated protein (CSPP), mRNA [NM_024790]	NM_024790
A_23_P17593	0.00743	2.315	NM_001794	NM_001794	Homo sapiens cadherin 4, type 1, R-cadherin (retinal) (CDH4), mRNA [NM_001794]	NM_001794
A_23_P80068	0.00743	2.005	NM_006806	NM_006806	Homo sapiens BTG family, member 3 (BTG3), mRNA [NM_006806]	NM_006806
A_23_P370544	0.00747	4.768	AJ549812	AJ549812	Homo sapiens mRNA for hypothetical protein (FL25415 gene). [AJ549812]	XM_371586
A_23_P150053	0.00749	2.17	NM_001613	NM_001613	Homo sapiens actin, alpha 2, smooth muscle, aorta (ACTA2), mRNA [NM_001613]	NM_001613
A_23_P108042	0.00751	4.941	NM_004829	NM_004829	Homo sapiens natural cytotoxicity triggering receptor 1 (NCR1), mRNA [NM_004829]	NM_004829
A_24_P367282	0.00751	3.298	ENST00000323012			
A_23_P41487	0.00751	2.086	NM_015130	NM_015130	Homo sapiens KIAA0882 protein (KIAA0882), mRNA [NM_015130]	NM_015130
A_23_P60627	0.00757	3.299	NM_001141	NM_001141	Homo sapiens arachidonate 15-lipoxygenase, second type (ALOX15B), mRNA [NM_001141]	NM_001141
A_23_P85140	0.00758	3.303	NM_080390	NM_080390	Homo sapiens transcription elongation factor A (SII)-like 2 (TCEAL2), mRNA [NM_080390]	NM_080390
A_23_P377957	0.00759	2.141	NM_138444	NM_138444	Homo sapiens potassium channel tetramerisation domain containing 12 (KCTD12), mRNA [NM_138444]	NM_138444
A_24_P607880	0.00762	2.046	AL832758	AL832758	Homo sapiens mRNA; cDNA DKFZp686C0927 (from clone DKFZp686C0927). [AL832758]	
A_24_P588897	0.00767	6.923	THC2277195			
A_23_P130753	0.0077	2.755	NM_001352	NM_001352	Homo sapiens D site of albumin promoter (albumin D-box) binding protein (DBP), mRNA [NM_001352]	NM_001352
A_23_P34144	0.00772	2.921	NM_014061	NM_014061	Homo sapiens melanoma antigen family H, 1 (MAGEH1), mRNA [NM_014061]	NM_014061
A_23_P11051	0.00774	2.73	BC072400	BC072400	Homo sapiens THO complex 2, mRNA (cDNA clone IMAGE:6043316), complete cds. [BC072400]	
A_23_P157963	0.00776	2.719	NM_017738	NM_017738	Homo sapiens chromosome 9 open reading frame 39 (C9orf39), mRNA [NM_017738]	NM_017738
A_32_P220567	0.00776	2.165	A_32_P220567			
A_23_P20484	0.00777	5.517	NM_201553	NM_201553	Homo sapiens fibrinogen-like 1 (FGL1), transcript variant 4, mRNA [NM_201553]	NM_201553
A_24_P243749	0.00778	6.454	NM_002612	NM_002612	Homo sapiens pyruvate dehydrogenase kinase, isoenzyme 4 (PDK4), mRNA [NM_002612]	NM_002612
A_23_P67312	0.00779	2.847	NM_003437	NM_003437	Homo sapiens zinc finger protein 136 (clone pHZ-20) (ZNF136), mRNA [NM_003437]	NM_003437
A_23_P206568	0.00783	2.181	A_23_P206568			
A_23_P116430	0.00788	4.242	NM_005709	NM_005709	Homo sapiens Usher syndrome 1C (autosomal recessive, severe) (USH1C), transcript variant 1, mRNA [NM_005709]	NM_005709
A_32_P70875	0.00788	2.701	THC2289574			
A_24_P405298	0.00789	2.067	NM_002709	NM_002709	Homo sapiens protein phosphatase 1, catalytic subunit, beta isoform (PPP1CB), transcript variant 1, mRNA [NM_002709]	NM_002709
A_32_P30966	0.00795	13.66	THC2297943			

A_23_P359897	0.00797	3.004	AB051505	AB051505	Homo sapiens mRNA for KIAA1718 protein, partial cds. [AB051505]	XM_376680
A_23_P415015	0.00802	2.992	NM_022374	NM_022374	Homo sapiens ADP-ribosylation factor-like 6 interacting protein 2 (ARL6IP2), mRNA [NM_022374]	NM_022374
A_23_P210253	0.00802	2.286	NM_152879	NM_152879	Homo sapiens diacylglycerol kinase, delta 130kDa (DGKD), transcript variant 2, mRNA [NM_152879]	NM_152879
A_32_P105281	0.00803	2.91	AW935085	AW935085	AW935085 RC2-DT0002-290200-017-e02_1 DT0002 Homo sapiens cDNA, mRNA sequence [AW935085]	
A_24_P335263	0.00803	2.26	NM_199040	NM_199040	Homo sapiens nudix (nucleoside diphosphate linked moiety X)-type motif 4 (NUDT4), transcript variant 2, mRNA [NM_199040]	NM_199040
A_23_P69055	0.00806	2.192	NM_006991	NM_006991	Homo sapiens zinc finger protein 197 (ZNF197), transcript variant 1, mRNA [NM_006991]	NM_006991
A_24_P35400	0.00807	3.012	NM_007101	NM_007101	Homo sapiens sarcosine dehydrogenase (SARDH), mRNA [NM_007101]	NM_007101
A_23_P370651	0.00807	2.155	NM_014883	NM_014883	Homo sapiens family with sequence similarity 13, member A1 (FAM13A1), transcript variant 1, mRNA [NM_014883]	NM_014883
A_23_P259707	0.00809	6.892	NM_207299	NM_207299	Homo sapiens plasticity related gene 3 (PRG-3), transcript variant 1, mRNA [NM_207299]	NM_207299
A_23_P204609	0.00809	2.117	NM_017599	NM_017599	Homo sapiens transmembrane protein vezatin (VEZATIN), mRNA [NM_017599]	NM_017599
A_23_P217845	0.00811	5.073	NM_002928	NM_002928	Homo sapiens regulator of G-protein signalling 16 (RGS16), mRNA [NM_002928]	NM_002928
A_24_P192209	0.00817	3.471	NM_007139	NM_007139	Homo sapiens zinc finger protein 92 (HTF12) (ZNF92), mRNA [NM_007139]	NM_007139
A_24_P33895	0.00817	2.15	NM_004024	NM_004024	Homo sapiens activating transcription factor 3 (ATF3), mRNA [NM_004024]	NM_004024
A_23_P136683	0.0082	5.474	M20432	M20432	Human MHC class II HLA-DQ-beta (DR2-DQw1/DR4 DQw3) mRNA, complete cds, clone ROF2D. [M20432]	
A_32_P127599	0.00822	2.247	BM668210	BM668210	BM668210 UI-E-CK1-afk-b-22-0-UI.s2 UI-E-CK1 Homo sapiens cDNA clone UI-E-CK1-afk-b-22-0-UI 3', mRNA sequence [BM668210]	
A_23_P162010	0.00823	2.729	NM_176875	NM_176875	Homo sapiens cholecystokinin B receptor (CCKBR), mRNA [NM_176875]	NM_176875
A_23_P349416	0.00826	2.005	NM_001982	NM_001982	Homo sapiens v-erb-b2 erythroblastic leukemia viral oncogene homolog 3 (avian) (ERBB3), transcript variant 1, mRNA [NM_001982]	NM_001982
A_23_P322593	0.00829	3.16	NM_152684	NM_152684	Homo sapiens hypothetical protein FLJ39653 (FLJ39653), mRNA [NM_152684]	NM_152684
A_24_P231104	0.0083	6.339	NM_001003679	NM_001003679	Homo sapiens leptin receptor (LEPR), transcript variant 2, mRNA [NM_001003679]	NM_001003679
A_32_P214565	0.00832	2.896	A_32_P214565			
A_24_P320970	0.00836	2.095	BX538061	BX538061	Homo sapiens mRNA; cDNA DKFZp686J16172 (from clone DKFZp686J16172). [BX538061]	
A_24_P79855	0.00837	3.348	ENST00000341963		Homo sapiens, hypothetical protein DKFZp566H0824, clone IMAGE:4607730, mRNA. [BC029383]	
A_23_P340131	0.00838	3.21	NM_005865	NM_005865	Homo sapiens protease, serine, 16 (thymus) (PRSS16), mRNA [NM_005865]	NM_005865
A_24_P637257	0.0084	2.138	BX537532	BX537532	Homo sapiens mRNA; cDNA DKFZp686L07201 (from clone DKFZp686L07201). [BX537532]	
A_23_P402437	0.00844	2.094	AL832190	AL832190	Homo sapiens mRNA; cDNA DKFZp686A202 (from clone DKFZp686A202). [AL832190]	
A_23_P9280	0.00847	2.762	ENST00000297820		full-length cDNA clone CS0DH005YC19 of T cells (Jurkat cell line) of Homo sapiens (human). [CR613032]	XM_372122
A_23_P58642	0.00847	2.646	NM_002653	NM_002653	Homo sapiens paired-like homeodomain transcription factor 1 (PITX1), mRNA [NM_002653]	NM_002653
A_23_P255273	0.00859	2.362	NM_002874	NM_002874	Homo sapiens RAD23 homolog B (S. cerevisiae) (RAD23B), mRNA [NM_002874]	NM_002874
A_23_P64058	0.00859	2.273	NM_005825	NM_005825	Homo sapiens RAS guanyl releasing protein 2 (calcium and DAG-regulated) (RASGRP2), transcript variant 1, mRNA [NM_005825]	NM_005825
A_32_P86533	0.00869	3.423	AF038185	AF038185	Homo sapiens clone 23700 mRNA sequence. [AF038185]	
A_24_P171601	0.00873	4.62	NM_001012651	NM_001012651	Homo sapiens natural killer-tumor recognition sequence (NKTR), transcript variant 2, mRNA [NM_001012651]	NM_001012651
A_32_P80850	0.00873	3.593	NM_021110	NM_021110	Homo sapiens collagen, type XIV, alpha 1 (undulin) (COL14A1), mRNA [NM_021110]	NM_021110
A_24_P202637	0.00878	3.597	NM_138372	NM_138372	Homo sapiens hypothetical protein BC001610 (LOC91661), mRNA [NM_138372]	NM_138372
A_24_P316270	0.00883	2.62	AK094969	AK094969	Homo sapiens cDNA FLJ37650 fis, clone BRHIP2000553, weakly similar to UBIQUITIN-ACTIVATING ENZYME E1 1. [AK094969]	
A_32_P128701	0.00883	2.212	AF085848	AF085848	Homo sapiens full length insert cDNA clone YI48C03. [AF085848]	
A_23_P115021	0.00885	2.668	NM_001103	NM_001103	Homo sapiens actinin, alpha 2 (ACTN2), mRNA [NM_001103]	NM_001103
A_24_P128741	0.00887	3.443	THC2432288		Q15694 (Q15694) Protein immuno-reactive with anti-PTH polyclonal antibodies (Fragment), partial (59%) [THC2432288]	
A_32_P150449	0.00889	3.713	THC2342544			
A_32_P106864	0.0089	2.724	AA303143	AA303143	AA303143 EST13031 Uterus tumor I Homo sapiens cDNA 5' end similar to hypothetical protein KIAA0222, mRNA sequence [AA303143]	

A_23_P452	0.0089	2.431	NM_001014796	NM_001014796	Homo sapiens discoidin domain receptor family, member 2 (DDR2), transcript variant 1, mRNA [NM_001014796]	NM_001014796
A_24_P104538	0.0089	2.097	BC000924	BC000924	Homo sapiens neighbor of BRCA1 gene 2, mRNA (cDNA clone MGC:5031 IMAGE:3446931), complete cds. [BC000924]	XM_496240
A_23_P100056	0.00894	2.415	NM_194272	NM_194272	Homo sapiens RNA binding protein with multiple splicing 2 (RBPMS2), mRNA [NM_194272]	NM_194272
A_24_P922684	0.00897	2.151	BC020539	BC020539	Homo sapiens, clone IMAGE:3048919, mRNA, partial cds. [BC020539]	
A_24_P332862	0.00898	2.535	NM_175910	NM_175910	Homo sapiens zinc finger protein 493 (ZNF493), mRNA [NM_175910]	NM_175910
A_23_P160940	0.009	3.013	NM_000350	NM_000350	Homo sapiens ATP-binding cassette, sub-family A (ABC1), member 4 (ABCA4), mRNA [NM_000350]	NM_000350
A_24_P184799	0.00917	2.806	NM_004086	NM_004086	Homo sapiens coagulation factor C homolog, cochlin (Limulus polyphemus) (COCH), mRNA [NM_004086]	NM_004086
A_24_P282060	0.00919	4.156	AK093006	AK093006	Homo sapiens cDNA FLJ35687 fis, clone SPLEN2019349. [AK093006]	
A_32_P11262	0.00924	2.64	AK124396	AK124396	Homo sapiens cDNA FLJ42405 fis, clone ASTRO3000474. [AK124396]	
A_24_P396231	0.00924	2.239	BC040653	BC040653	Homo sapiens cDNA clone IMAGE:4797120, partial cds. [BC040653]	
A_24_P940524	0.00925	2.58	NM_003410	NM_003410	Homo sapiens zinc finger protein, X-linked (ZFX), mRNA [NM_003410]	NM_003410
A_32_P106194	0.00926	2.048	NM_153229	NM_153229	Homo sapiens hypothetical protein FLJ33318 (FLJ33318), mRNA [NM_153229]	NM_153229
A_23_P251937	0.00927	3.759	NM_030627	NM_030627	Homo sapiens cytoplasmic polyadenylation element binding protein 4 (CPEB4), mRNA [NM_030627]	NM_030627
A_24_P399942	0.00927	2.376	NM_173694	NM_173694	Homo sapiens ATPase, Class VI, type 11C (ATP11C), transcript variant 1, mRNA [NM_173694]	NM_173694
A_23_P104522	0.00938	2.322	NM_006393	NM_006393	Homo sapiens nebulette (NEBL), transcript variant 1, mRNA [NM_006393]	NM_006393
A_23_P45955	0.00942	2.436	NM_014466	NM_014466	Homo sapiens tektin 2 (testicular) (TEKT2), mRNA [NM_014466]	NM_014466
A_24_P607904	0.00944	22.49	AK026826	AK026826	Homo sapiens cDNA: FLJ23173 fis, clone LNG10019. [AK026826]	
A_23_P112482	0.0095	6.356	NM_004925	NM_004925	Homo sapiens aquaporin 3 (AQP3), mRNA [NM_004925]	NM_004925
A_32_P178945	0.0095	3.93	NM_018566	NM_018566	Homo sapiens YOD1 OTU deubiquinating enzyme 1 homolog (yeast) (YOD1), mRNA [NM_018566]	NM_018566
A_23_P138352	0.00954	2.138	NM_004185	NM_004185	Homo sapiens wingless-type MMTV integration site family, member 2B (WNT2B), transcript variant WNT-2B1, mRNA [NM_004185]	NM_004185
A_23_P319423	0.00956	3.39	NM_003740	NM_003740	Homo sapiens potassium channel, subfamily K, member 5 (KCNK5), mRNA [NM_003740]	NM_003740
A_32_P160615	0.00957	3.304	A_32_P160615			
A_23_P501515	0.00958	2.244	NM_031844	NM_031844	Homo sapiens heterogeneous nuclear ribonucleoprotein U (scaffold attachment factor A) (HNRPU), transcript variant 1, mRNA [NM_031844]	NM_031844
A_24_P71938	0.00959	2.48	NM_005900	NM_005900	Homo sapiens SMAD, mothers against DPP homolog 1 (Drosophila) (SMAD1), transcript variant 1, mRNA [NM_005900]	NM_005900
A_23_P117163	0.00961	2.114	NM_018191	NM_018191	Homo sapiens regulator of chromosome condensation (RCC1) and BTB (POZ) domain containing protein 1 (RCBTB1), mRNA [NM_018191]	NM_018191
A_23_P53176	0.00963	3.7	NM_016725	NM_016725	Homo sapiens folate receptor 1 (adult) (FOLR1), transcript variant 1, mRNA [NM_016725]	NM_016725
A_23_P392575	0.00965	9.935	NM_080671	NM_080671	Homo sapiens potassium voltage-gated channel, Isk-related family, member 4 (KCNE4), mRNA [NM_080671]	NM_080671
A_24_P29401	0.00967	2.381	NM_181523	NM_181523	Homo sapiens phosphoinositide-3-kinase, regulatory subunit 1 (p85 alpha) (PIK3R1), transcript variant 1, mRNA [NM_181523]	NM_181523
A_32_P199923	0.0097	3.061	CD653123	CD653123	CD653123 AGENCOURT_14552349 NIA Human H1 Embryonic Stem Cell cDNA Library (Long) Homo sapiens cDNA clone IMAGE:30425925 5', mRNA sequence [CD653123]	
A_23_P129738	0.00976	2.905	NM_004524	NM_004524	Homo sapiens lethal giant larvae homolog 2 (Drosophila) (LLGL2), transcript variant 1, mRNA [NM_004524]	NM_004524
A_23_P251855	0.00976	2.099	NM_006598	NM_006598	Homo sapiens solute carrier family 12 (potassium/chloride transporters), member 7 (SLC12A7), mRNA [NM_006598]	NM_006598
A_23_P73835	0.00976	2.037	NM_019556	NM_019556	Homo sapiens motile sperm domain containing 1 (MOSPD1), mRNA [NM_019556]	NM_019556
A_23_P429449	0.00978	2.414	NM_033119	NM_033119	Homo sapiens naked cuticle homolog 1 (Drosophila) (NKD1), mRNA [NM_033119]	NM_033119
A_24_P941988	0.00979	2.091	NM_014946	NM_014946	Homo sapiens spastin (SPAST), transcript variant 1, mRNA [NM_014946]	NM_014946
A_23_P320739	0.00981	2.904	NM_002397	NM_002397	Homo sapiens MADS box transcription enhancer factor 2, polypeptide C (myocyte enhancer factor 2C) (MEF2C), mRNA [NM_002397]	NM_002397
A_23_P164047	0.00987	2.387	NM_012329	NM_012329	Homo sapiens monocyte to macrophage differentiation-associated (MMD), mRNA [NM_012329]	NM_012329
A_32_P102383	0.0099	3.192	THC2323059			
A_23_P253622	0.00995	5.249	NM_020970	NM_020970	Homo sapiens KIAA1641 (KIAA1641), mRNA [NM_020970]	NM_020970

A_24_P131646	0.00995	3.223	NM_000258	NM_000258	Homo sapiens myosin, light polypeptide 3, alkali; ventricular, skeletal, slow (MYL3), mRNA [NM_000258]	NM_000258
A_23_P163402	0.00999	2.995	NM_000499	NM_000499	Homo sapiens cytochrome P450, family 1, subfamily A, polypeptide 1 (CYP1A1), mRNA [NM_000499]	NM_000499
A_32_P150012	0.01	4.01	NM_017780	NM_017780	Homo sapiens chromodomain helicase DNA binding protein 7 (CHD7), mRNA [NM_017780]	NM_017780
A_23_P371011	0.01	2.888	NM_182490	NM_182490	Homo sapiens zinc finger protein 227 (ZNF227), mRNA [NM_182490]	NM_182490
A_32_P196918	0.01	2.532	AK057956	AK057956	Homo sapiens cDNA FLJ25227 fis, clone STM01429. [AK057956]	
A_32_P174832	0.0101	4.61	BI523298	BI523298	BI523298 603175578T1 NIH_MGC_121 Homo sapiens cDNA clone IMAGE:5239842 3', mRNA sequence [BI523298]	
A_32_P104619	0.0101	3.831	AK094156	AK094156	Homo sapiens cDNA FLJ36837 fis, clone ASTRO2011422. [AK094156]	
A_23_P20022	0.0101	2.566	NM_013332	NM_013332	Homo sapiens hypoxia-inducible protein 2 (HIG2), mRNA [NM_013332]	NM_013332
A_23_P20427	0.0101	2.201	NM_015178	NM_015178	Homo sapiens Rho-related BTB domain containing 2 (RHOBTB2), mRNA [NM_015178]	NM_015178
A_24_P328333	0.0101	2.114	NM_004850	NM_004850	Homo sapiens Rho-associated, coiled-coil containing protein kinase 2 (ROCK2), mRNA [NM_004850]	NM_004850
A_23_P103877	0.0102	2.903	CR622769	CR622769	full-length cDNA clone CS0DI025YD24 of Placenta Cot 25-normalized of Homo sapiens (human). [CR622769]	
A_32_P55979	0.0102	2.236	THC2439252		BC018029 6-pyruvoyltetrahydropterin synthase {Homo sapiens;} , partial (25%) [THC2439252]	
A_23_P140830	0.0102	2.214	NM_024712	NM_024712	Homo sapiens engulfment and cell motility 3 (ced-12 homolog, C. elegans) (ELMO3), mRNA [NM_024712]	NM_024712
A_23_P83818	0.0102	2.03	NM_000093	NM_000093	Homo sapiens collagen, type V, alpha 1 (COL5A1), mRNA [NM_000093]	NM_000093
A_23_P106922	0.0103	2.816	NM_021615	NM_021615	Homo sapiens carbohydrate (N-acetylglucosamine 6-O) sulfotransferase 6 (CHST6), mRNA [NM_021615]	NM_021615
A_32_P214054	0.0104	2.808	THC2411106			
A_32_P220696	0.0104	2.452	NM_017489	NM_017489	Homo sapiens telomeric repeat binding factor (NIMA-interacting) 1 (TERF1), transcript variant 1, mRNA [NM_017489]	NM_017489
A_24_P389415	0.0105	8.794	NM_007257	NM_007257	Homo sapiens paraneoplastic antigen MA2 (PNMA2), mRNA [NM_007257]	NM_007257
A_23_P64611	0.0105	2.471	NM_176798	NM_176798	Homo sapiens pyrimidinergic receptor P2Y, G-protein coupled, 6 (P2RY6), transcript variant 2, mRNA [NM_176798]	NM_176798
A_23_P301372	0.0105	2.049	NM_153365	NM_153365	Homo sapiens hypothetical protein FLJ90013 (FLJ90013), mRNA [NM_153365]	NM_153365
A_23_P328545	0.0106	4.253	NM_014211	NM_014211	Homo sapiens gamma-aminobutyric acid (GABA) A receptor, pi (GABRP), mRNA [NM_014211]	NM_014211
A_24_P556328	0.0106	4.183	A_24_P556328			
A_24_P940135	0.0107	2.341	BC024007	BC024007	Homo sapiens chitobiase, di-N-acetyl-, mRNA (cDNA clone IMAGE:4823479), complete cds. [BC024007]	
A_23_P50376	0.0107	2.331	NM_018555	NM_018555	Homo sapiens zinc finger protein 331 (ZNF331), mRNA [NM_018555]	NM_018555
A_23_P129587	0.0107	2.068	NM_153028	NM_153028	Homo sapiens zinc finger protein 75a (ZNF75A), mRNA [NM_153028]	NM_153028
A_32_P171200	0.0108	5.155	BU607658	BU607658	BU607658 UI-CF-FN0-aev-j-05-0-UI.s1 UI-CF-FN0 Homo sapiens cDNA clone UI-CF-FN0-aev-j-05-0-UI 3', mRNA sequence [BU607658]	
A_24_P917044	0.0108	2.668	AF483645	AF483645	Homo sapiens capacitative calcium channel protein Trp1 mRNA, partial cds; alternatively spliced. [AF483645]	
A_24_P84540	0.0108	2.107	NM_001009569	NM_001009569	Homo sapiens myeloid/lymphoid or mixed-lineage leukemia (trithorax homolog, Drosophila); translocated to, 10 (MLLT10), transcript variant 2, mRNA [NM_001009569]	NM_001009569
A_32_P222474	0.0109	2.272	THC2373518		Q6X1Z6 (Q6X1Z6) BICP22 transcription factor, partial (7%) [THC2373518]	
A_24_P206604	0.011	2.518	NM_004566	NM_004566	Homo sapiens 6-phosphofructo-2-kinase/fructose-2,6-biphosphatase 3 (PFKFB3), mRNA [NM_004566]	NM_004566
A_24_P208045	0.011	2.1	NM_025191	NM_025191	Homo sapiens chromosome 1 open reading frame 22 (C1orf22), mRNA [NM_025191]	NM_025191
A_24_P32790	0.0111	2.849	NM_018566	NM_018566	Homo sapiens YOD1 OTU deubiquinating enzyme 1 homolog (yeast) (YOD1), mRNA [NM_018566]	NM_018566
A_32_P47543	0.0111	2.468	BG112935	BG112935	602283549F1 NIH_MGC_86 Homo sapiens cDNA clone IMAGE:4370964 5', mRNA sequence [BG112935]	
A_24_P829261	0.0112	7.944	BX538238	BX538238	Homo sapiens mRNA; cDNA DKFZp686B0790 (from clone DKFZp686B0790); complete cds. [BX538238]	
A_32_P6452	0.0112	3.34	A_32_P6452			
A_32_P222060	0.0112	3.281	THC2345721			
A_24_P145316	0.0113	2.405	NM_183040	NM_183040	Homo sapiens dystrobrevin binding protein 1 (DTNBP1), transcript variant 2, mRNA [NM_183040]	NM_183040
A_24_P366390	0.0114	2.828	NM_198079	NM_198079	Homo sapiens golgi autoantigen, golgin subfamily a-like (FLJ40113), mRNA [NM_198079]	NM_198079
A_23_P382705	0.0115	4.908	NM_152588	NM_152588	Homo sapiens hypothetical protein DKFZp762A217 (DKFZp762A217), mRNA [NM_152588]	NM_152588
A_24_P354849	0.0115	2.835	THC2403680			

A_32_P125808	0.0115	2.061	THC2241453			
A_24_P360529	0.0116	4.87	NM_002604	NM_002604	Homo sapiens phosphodiesterase 7A (PDE7A), transcript variant 2, mRNA [NM_002604]	NM_002604
A_23_P106194	0.0116	2.489	NM_005252	NM_005252	Homo sapiens v-fos FBJ murine osteosarcoma viral oncogene homolog (FOS), mRNA [NM_005252]	NM_005252
A_24_P920693	0.0116	2.412	NM_006107	NM_006107	Homo sapiens cisplatin resistance-associated overexpressed protein (CROP), transcript variant 2, mRNA [NM_006107]	NM_006107
A_24_P136484	0.0116	2.109	AK090397	AK090397	Homo sapiens mRNA for FLJ00272 protein. [AK090397]	
A_23_P113453	0.0117	3.326	A_23_P113453			
A_23_P349463	0.0117	3.103	NM_022097	NM_022097	Homo sapiens hepatocellular carcinoma antigen gene 520 (LOC63928), mRNA [NM_022097]	NM_022097
A_23_P363896	0.0117	2.351	NM_152350	NM_152350	Homo sapiens hypothetical protein MGC40157 (MGC40157), mRNA [NM_152350]	NM_152350
A_23_P48705	0.0117	2.093	NM_152444	NM_152444	Homo sapiens zinc binding alcohol dehydrogenase, domain containing 1 (ZADH1), mRNA [NM_152444]	NM_152444
A_23_P36882	0.0118	8.401	NM_006183	NM_006183	Homo sapiens neurotensin (NTS), mRNA [NM_006183]	NM_006183
A_32_P85273	0.0119	2.393	NM_173613	NM_173613	Homo sapiens hypothetical protein FLJ35785 (FLJ35785), mRNA [NM_173613]	NM_173613
A_32_P186865	0.0119	2.307	THC2406944		Q9F8M7 (Q9F8M7) DTDP-glucose 4,6-dehydratase (Fragment), partial (11%) [THC2406944]	
A_32_P199801	0.012	2.984	NM_052885	NM_052885	Homo sapiens solute carrier family 2 (facilitated glucose transporter), member 13 (SLC2A13), mRNA [NM_052885]	NM_052885
A_24_P236091	0.012	2.547	NM_001975	NM_001975	Homo sapiens enolase 2 (gamma, neuronal) (ENO2), mRNA [NM_001975]	NM_001975
A_24_P392925	0.0121	5.241	NM_001014985	NM_001014985	Homo sapiens hypothetical LOC388323 (LOC388323), mRNA [NM_001014985]	NM_001014985
A_24_P68153	0.0121	4.716	BC005136	BC005136	Homo sapiens solute carrier family 35 (UDP-N-acetylglucosamine (UDP-GlcNAc) transporter), member A3, mRNA (cDNA clone MGC:1421 IMAGE:3505772), complete cds. [BC005136]	
A_24_P346725	0.0121	3.655	AK001094	AK001094	Homo sapiens cDNA FLJ10232 fis, clone HEMBB1000244. [AK001094]	
A_23_P67702	0.0121	3.091	NM_003429	NM_003429	Homo sapiens zinc finger protein 85 (HPF4, HTF1) (ZNF85), mRNA [NM_003429]	NM_003429
A_23_P395703	0.0121	2.792	NM_016578	NM_016578	Homo sapiens hepatitis B virus x associated protein (HBXAP), mRNA [NM_016578]	NM_016578
A_23_P99285	0.0121	2.769	NM_006143	NM_006143	Homo sapiens G protein-coupled receptor 19 (GPR19), mRNA [NM_006143]	NM_006143
A_23_P121545	0.0122	4.606	NM_201591	NM_201591	Homo sapiens glycoprotein M6A (GPM6A), transcript variant 2, mRNA [NM_201591]	NM_201591
A_32_P59737	0.0122	2.179	THC2438324		ALU1_HUMAN (P39188) Alu subfamily J sequence contamination warning entry, partial (12%) [THC2438324]	
A_24_P200854	0.0122	2.116	NM_006735	NM_006735	Homo sapiens homeo box A2 (HOXA2), mRNA [NM_006735]	NM_006735
A_32_P351277	0.0122	2.017	BC004968	BC004968	Homo sapiens, clone IMAGE:3543963, mRNA. [BC004968]	XM_496109
A_23_P363206	0.0123	2.467	NM_006910	NM_006910	Homo sapiens retinoblastoma binding protein 6 (RBBP6), transcript variant 1, mRNA [NM_006910]	NM_006910
A_23_P202206	0.0123	2.181	NM_183239	NM_183239	Homo sapiens glutathione S-transferase omega 2 (GSTO2), mRNA [NM_183239]	NM_183239
A_23_P147711	0.0124	2.15	NM_000906	NM_000906	Homo sapiens natriuretic peptide receptor A/guanylate cyclase A (atrionatriuretic peptide receptor A) (NPR1), mRNA [NM_000906]	NM_000906
A_24_P544882	0.0124	2.147	AK026687	AK026687	Homo sapiens cDNA: FLJ23034 fis, clone LNG02018. [AK026687]	
A_32_P180435	0.0125	2.748	NM_175064	NM_175064	Homo sapiens Williams Beuren syndrome chromosome region 19 (WBSCR19), mRNA [NM_175064]	NM_175064
A_32_P14669	0.0125	2.581	NM_001009883	NM_001009883	Homo sapiens zinc finger protein 66 (ZNF66), mRNA [NM_001009883]	NM_001009883
A_24_P928272	0.0126	2.419	AF086376	AF086376	Homo sapiens full length insert cDNA clone ZD68D12. [AF086376]	
A_32_P118556	0.0126	2.38	A_32_P118556			
A_24_P331918	0.0126	2.376	NM_001848	NM_001848	Homo sapiens collagen, type VI, alpha 1 (COL6A1), mRNA [NM_001848]	NM_001848
A_32_P77665	0.0126	2.191	THC2384975		ALU7_HUMAN (P39194) Alu subfamily SQ sequence contamination warning entry, partial (6%) [THC2384975]	
A_24_P290709	0.0126	2.089	CR593166	CR593166	full-length cDNA clone CS0DD005YN08 of Neuroblastoma Cot 50-normalized of Homo sapiens (human). [CR593166]	
A_32_P224566	0.0127	3.842	NM_001004317	NM_001004317	Homo sapiens FLJ16517 protein (FLJ16517), mRNA [NM_001004317]	NM_001004317
A_23_P86171	0.0127	2.534	NM_004474	NM_004474	Homo sapiens forkhead box D2 (FOXD2), mRNA [NM_004474]	NM_004474
A_23_P51376	0.0128	4.823	NM_024522	NM_024522	Homo sapiens hypothetical protein FLJ12650 (FLJ12650), mRNA [NM_024522]	NM_024522
A_32_P220580	0.0128	3.243	AK124352	AK124352	Homo sapiens cDNA FLJ42361 fis, clone UTERU2025366. [AK124352]	
A_24_P68008	0.0128	3.17	NM_198545	NM_198545	Homo sapiens hypothetical gene supported by AK075558; BC021286 (LOC374946), mRNA [NM_198545]	NM_198545
A_32_P85539	0.0128	2.281	THC2364429			

A_32_P71437	0.0128	2.225	THC2270648			
A_23_P73239	0.0128	2.2	NM_205842	NM_205842	Homo sapiens NCK-associated protein 1 (NCKAP1), transcript variant 2, mRNA [NM_205842]	NM_205842
A_24_P237270	0.0129	2.673	NM_000675	NM_000675	Homo sapiens adenosine A2a receptor (ADORA2A), mRNA [NM_000675]	NM_000675
A_32_P134679	0.0131	12.74	A_32_P134679			
A_23_P108501	0.0131	6.032	NM_004438	NM_004438	Homo sapiens EPH receptor A4 (EPHA4), mRNA [NM_004438]	NM_004438
A_24_P933675	0.0131	3.001	CR616640	CR616640	full-length cDNA clone CS0DE011YE22 of Placenta of Homo sapiens (human). [CR616640]	
A_23_P148737	0.0132	5.817	NM_004997	NM_004997	Homo sapiens myosin binding protein H (MYBPH), mRNA [NM_004997]	NM_004997
A_23_P160433	0.0132	3.315	NM_024709	NM_024709	Homo sapiens chromosome 1 open reading frame 115 (C1orf115), mRNA [NM_024709]	NM_024709
A_24_P221414	0.0132	2.757	NM_004411	NM_004411	Homo sapiens dynein, cytoplasmic, intermediate polypeptide 1 (DNCI1), mRNA [NM_004411]	NM_004411
A_32_P156006	0.0133	2.289	BC018099	BC018099	Homo sapiens, clone IMAGE:4796019, mRNA. [BC018099]	
A_24_P335048	0.0135	3.199	NM_018017	NM_018017	Homo sapiens chromosome 10 open reading frame 118 (C10orf118), mRNA [NM_018017]	NM_018017
A_23_P148753	0.0135	2.691	NM_014935	NM_014935	Homo sapiens pleckstrin homology domain containing, family A member 6 (PLEKHA6), mRNA [NM_014935]	NM_014935
A_23_P365119	0.0135	2.445	NM_152675	NM_152675	Homo sapiens hypothetical protein FLJ23754 (FLJ23754), mRNA [NM_152675]	NM_152675
A_23_P70318	0.0135	2.41	NM_014936	NM_014936	Homo sapiens ectonucleotide pyrophosphatase/phosphodiesterase 4 (putative function) (ENPP4), mRNA [NM_014936]	NM_014936
A_32_P101002	0.0136	4.486	THC2448203		ALU1_HUMAN (P39188) Alu subfamily J sequence contamination warning entry, partial (5%) [THC2448203]	
A_32_P88817	0.0136	2.858	THC2267456		ALU6_HUMAN (P39193) Alu subfamily SP sequence contamination warning entry, partial (20%) [THC2266190]	
A_23_P81153	0.0136	2.054	NM_033632	NM_033632	Homo sapiens F-box and WD-40 domain protein 7 (archipelago homolog, Drosophila) (FBXW7), transcript variant 1, mRNA [NM_033632]	NM_033632
A_23_P70448	0.0137	3.714	NM_005325	NM_005325	Homo sapiens histone 1, H1a (HIST1H1A), mRNA [NM_005325]	NM_005325
A_23_P155463	0.0137	2.839	NM_024512	NM_024512	Homo sapiens leucine rich repeat containing 2 (LRRC2), mRNA [NM_024512]	NM_024512
A_32_P111524	0.0138	3.267	BC033539	BC033539	Homo sapiens, clone IMAGE:4819052, mRNA. [BC033539]	
A_24_P358619	0.0139	2.143	ENST00000355650		PREDICTED: Homo sapiens similar to zinc finger protein 91 (HPF7, HTF10) (LOC126502), mRNA [XM_065124]	XM_065124
A_23_P332713	0.014	3.347	ENST00000262961		Homo sapiens mRNA for KIAA1086 protein, partial cds. [AB029009]	XM_047610
A_23_P49900	0.014	2.144	NM_014389	NM_014389	Homo sapiens proline-, glutamic acid-, leucine-rich protein 1 (PELP1), mRNA [NM_014389]	NM_014389
A_23_P340188	0.0141	9.071	NM_138819	NM_138819	Homo sapiens hypothetical protein BC017868 (RP3-473B4.1), mRNA [NM_138819]	NM_138819
A_32_P127492	0.0141	5.026	AA873311	AA873311	AA873311 oh76g08.s1 NCI_CGAP_Kid3 Homo sapiens cDNA clone IMAGE:1472990 3' similar to gb:J05021 EZRIN (HUMAN);, mRNA sequence [AA873311]	
A_23_P40896	0.0141	3.555	NM_018155	NM_018155	Homo sapiens solute carrier family 25, member 36 (SLC25A36), mRNA [NM_018155]	NM_018155
A_23_P59738	0.0141	3.259	NM_021223	NM_021223	Homo sapiens myosin, light polypeptide 7, regulatory (MYL7), mRNA [NM_021223]	NM_021223
A_24_P115183	0.0141	2.522	NM_001305	NM_001305	Homo sapiens claudin 4 (CLDN4), mRNA [NM_001305]	NM_001305
A_23_P138706	0.0141	2.478	NM_000681	NM_000681	Homo sapiens adrenergic, alpha-2A-, receptor (ADRA2A), mRNA [NM_000681]	NM_000681
A_32_P224586	0.0142	2.93	THC2342537		ALU1_HUMAN (P39188) Alu subfamily J sequence contamination warning entry, partial (5%) [THC2342537]	
A_23_P89981	0.0142	2.736	NM_000774	NM_000774	Homo sapiens cytochrome P450, family 2, subfamily F, polypeptide 1 (CYP2F1), mRNA [NM_000774]	NM_000774
A_32_P124245	0.0142	2.27	NM_175747	NM_175747	Homo sapiens oligodendrocyte transcription factor 3 (OLIG3), mRNA [NM_175747]	NM_175747
A_24_P645914	0.0143	4.953	AK025909	AK025909	Homo sapiens cDNA: FLJ22256 fis, clone HRC02860. [AK025909]	
A_23_P23227	0.0144	3.255	NM_006256	NM_006256	Homo sapiens protein kinase N2 (PKN2), mRNA [NM_006256]	NM_006256
A_24_P291973	0.0144	2.273	NM_001011553	NM_001011553	Homo sapiens septin 7 (SEPT7), transcript variant 2, mRNA [NM_001011553]	NM_001011553
A_23_P128319	0.0145	2.855	NM_001682	NM_001682	Homo sapiens ATPase, Ca++ transporting, plasma membrane 1 (ATP2B1), transcript variant 2, mRNA [NM_001682]	NM_001682
A_23_P502470	0.0145	2.784	NM_002184	NM_002184	Homo sapiens interleukin 6 signal transducer (gp130, oncostatin M receptor) (IL6ST), transcript variant 1, mRNA [NM_002184]	NM_002184
A_23_P300301	0.0145	2.742	NM_004241	NM_004241	Homo sapiens jumonji domain containing 1C (JMJD1C), mRNA [NM_004241]	NM_004241
A_23_P38630	0.0145	2.373	NM_001050	NM_001050	Homo sapiens somatostatin receptor 2 (SSTR2), mRNA [NM_001050]	NM_001050
A_24_P54797	0.0145	2.203	NM_203284	NM_203284	Homo sapiens recombining binding protein suppressor of hairless (Drosophila) (RBPSUH), transcript variant 4, mRNA [NM_203284]	NM_203284

A_24_P266466	0.0145	2.054	NM_005293	NM_005293	Homo sapiens G protein-coupled receptor 20 (GPR20), mRNA [NM_005293]	NM_005293
A_32_P201150	0.0146	2.553	THC2399007		Q96FS0 (Q96FS0) KRAS2 protein, partial (24%) [THC2399007]	
A_32_P68050	0.0146	2.28	NM_012224	NM_012224	Homo sapiens NIMA (never in mitosis gene a)-related kinase 1 (NEK1), mRNA [NM_012224]	NM_012224
A_23_P218784	0.0147	2.447	NM_006386	NM_006386	Homo sapiens DEAD (Asp-Glu-Ala-Asp) box polypeptide 17 (DDX17), transcript variant 1, mRNA [NM_006386]	NM_006386
A_32_P81092	0.0147	2.023	THC2373083			
A_24_P42316	0.0149	2.442	NM_018429	NM_018429	Homo sapiens B double prime 1, subunit of RNA polymerase III transcription initiation factor IIIB (BDP1), mRNA [NM_018429]	NM_018429

Supplemental Table S1D

Genes upregulated in hESC-EC vs. EB

P-Value ≤ 0.015 with multiple testing correction (Benjamini and Hochberg), Fold Change ≥ 2.0

Gene Name	*P* -value	Fold change	Common	Genbank	Description	RefSeq
A_23_P53345	7.07E-07	12.17	NM_020183	NM_020183	Homo sapiens aryl hydrocarbon receptor nuclear translocator-like 2 (ARNTL2), mRNA [NM_020183]	NM_020183
A_23_P151710	9.67E-07	28.74	NM_000956	NM_000956	Homo sapiens prostaglandin E receptor 2 (subtype EP2), 53kDa (PTGER2), mRNA [NM_000956]	NM_000956
A_32_P74983	1.99E-06	6.85	ENST00000252229			
A_23_P421423	2.98E-06	3.76	NM_006291	NM_006291	Homo sapiens tumor necrosis factor, alpha-induced protein 2 (TNFAIP2), mRNA [NM_006291]	NM_006291
A_23_P374782	2.98E-06	4.46	NM_031892	NM_031892	Homo sapiens SH3-domain kinase binding protein 1 (SH3KBP1), transcript variant 1, mRNA [NM_031892]	NM_031892
A_24_P359205	2.98E-06	4.46	BC010094	BC010094	Homo sapiens phosphatidylinositol glycan, class Q, mRNA (cDNA clone IMAGE:3357878), partial cds. [BC010094]	
A_23_P4561	2.98E-06	9.35	NM_198833	NM_198833	Homo sapiens serine (or cysteine) proteinase inhibitor, clade B (ovalbumin), member 8 (SERPINB8), transcript variant 2, mRNA [NM_198833]	NM_198833
A_24_P261417	2.98E-06	16.31	NM_015881	NM_015881	Homo sapiens dickkopf homolog 3 (Xenopus laevis) (DKK3), transcript variant 1, mRNA [NM_015881]	NM_015881
A_23_P405129	4.11E-06	21.19	NM_000428	NM_000428	Homo sapiens latent transforming growth factor beta binding protein 2 (LTBP2), mRNA [NM_000428]	NM_000428
A_23_P162425	4.45E-06	3.64	NM_016072	NM_016072	Homo sapiens golgi transport 1 homolog B (S. cerevisiae) (GOLT1B), mRNA [NM_016072]	NM_016072
A_24_P385611	4.45E-06	8.93	NM_003113	NM_003113	Homo sapiens nuclear antigen Sp100 (SP100), mRNA [NM_003113]	NM_003113
A_23_P16469	4.45E-06	11.66	NM_001005377	NM_001005377	Homo sapiens plasminogen activator, urokinase receptor (PLAUR), transcript variant 3, mRNA [NM_001005377]	NM_001005377
A_24_P156490	4.81E-06	8.40	NM_002247	NM_002247	Homo sapiens potassium large conductance calcium-activated channel, subfamily M, alpha member 1 (KCNMA1), transcript variant 2, mRNA [NM_002247]	NM_002247
A_23_P157051	5.03E-06	2.54	NM_004722	NM_004722	Homo sapiens adaptor-related protein complex 4, mu 1 subunit (AP4M1), mRNA [NM_004722]	NM_004722
A_32_P50522	5.33E-06	5.65	NM_000801	NM_000801	Homo sapiens FK506 binding protein 1A, 12kDa (FKBP1A), transcript variant 12B, mRNA [NM_000801]	NM_000801
A_23_P82449	5.33E-06	7.94	NM_004403	NM_004403	Homo sapiens deafness, autosomal dominant 5 (DFNA5), mRNA [NM_004403]	NM_004403
A_23_P211561	5.33E-06	13.44	AK093341	AK093341	Homo sapiens cDNA FLJ36022 fis, clone TESTI2016599. [AK093341]	
A_23_P165360	5.56E-06	3.89	NM_016114	NM_016114	Homo sapiens ankyrin repeat and SOCS box-containing 1 (ASB1), mRNA [NM_016114]	NM_016114
A_24_P114124	5.76E-06	2.99	NM_006762	NM_006762	Homo sapiens lysosomal associated multispanning membrane protein 5 (LAPTM5), mRNA [NM_006762]	NM_006762
A_23_P77401	5.76E-06	5.26	NM_018340	NM_018340	Homo sapiens hypothetical protein FLJ11151 (FLJ11151), mRNA [NM_018340]	NM_018340
A_23_P55544	5.76E-06	6.54	NM_133459	NM_133459	Homo sapiens collagen and calcium binding EGF domains 1 (CCBE1), mRNA [NM_133459]	NM_133459
A_23_P165778	5.93E-06	45.45	NM_024101	NM_024101	Homo sapiens melanophilin (MLPH), mRNA [NM_024101]	NM_024101
A_24_P916686	6.14E-06	5.78	BC015962	BC015962	Homo sapiens, clone IMAGE:4081125, mRNA, partial cds. [BC015962]	
A_24_P59667	6.16E-06	2.75	BC028068	BC028068	Homo sapiens Janus kinase 3 (a protein tyrosine kinase, leukocyte), mRNA (cDNA clone MGC:39993 IMAGE:5212575), complete cds. [BC028068]	
A_24_P283189	6.18E-06	3.80	NM_000591	NM_000591	Homo sapiens CD14 antigen (CD14), mRNA [NM_000591]	NM_000591
A_23_P421843	6.20E-06	4.08	ENST00000285206		Homo sapiens hypothetical protein LOC201191, mRNA (cDNA clone IMAGE:5296646), partial cds. [BC047081]	
A_23_P47614	6.20E-06	21.98	NM_003311	NM_003311	Homo sapiens pleckstrin homology-like domain, family A, member 2 (PHLDA2), mRNA [NM_003311]	NM_003311
A_24_P734060	6.32E-06	3.77	AL832183	AL832183	Homo sapiens mRNA; cDNA DKFZp686D0720 (from clone DKFZp686D0720). [AL832183]	
A_23_P170498	6.39E-06	2.28	NM_173474	NM_173474	Homo sapiens N-terminal asparagine amidase (NTAN1), mRNA [NM_173474]	NM_173474
A_23_P211659	6.39E-06	2.82	NM_022766	NM_022766	Homo sapiens ceramide kinase (CERK), transcript variant 1, mRNA [NM_022766]	NM_022766
A_23_P138417	6.39E-06	3.39	NM_012425	NM_012425	Homo sapiens Ras suppressor protein 1 (RSU1), transcript variant 1, mRNA [NM_012425]	NM_012425
A_23_P120270	6.50E-06	7.14	NM_139279	NM_139279	Homo sapiens multiple coagulation factor deficiency 2 (MCFD2), mRNA [NM_139279]	NM_139279
A_23_P41075	6.54E-06	2.79	NM_032355	NM_032355	Homo sapiens MON1 homolog A (yeast) (MON1A), mRNA [NM_032355]	NM_032355
A_24_P342880	6.65E-06	3.45	NM_016598	NM_016598	Homo sapiens zinc finger, DHHC-type containing 3 (ZDHHC3), mRNA [NM_016598]	NM_016598
A_23_P63503	6.65E-06	3.61	NM_002871	NM_002871	Homo sapiens RAB interacting factor (RABIF), mRNA [NM_002871]	NM_002871
A_23_P117727	6.82E-06	2.38	NM_139242	NM_139242	Homo sapiens mitochondrial methionyl-tRNA formyltransferase (MTFMT), mRNA [NM_139242]	NM_139242
A_23_P70968	6.82E-06	6.71	NM_006896	NM_006896	Homo sapiens homeo box A7 (HOXA7), mRNA [NM_006896]	NM_006896

A_23_P73097	7.31E-06	14.27	NM_170587	NM_170587	Homo sapiens regulator of G-protein signalling 20 (RGS20), transcript variant 1, mRNA [NM_170587]	NM_170587
A_32_P34920	7.35E-06	6.54	NM_004472	NM_004472	Homo sapiens forkhead box D1 (FOXD1), mRNA [NM_004472]	NM_004472
A_23_P33187	7.36E-06	2.95	NM_032318	NM_032318	Homo sapiens hippocampus abundant gene transcript-like 2 (HIATL2), mRNA [NM_032318]	NM_032318
A_24_P107528	7.36E-06	3.24	NM_024738	NM_024738	Homo sapiens hypothetical protein FLJ21415 (FLJ21415), mRNA [NM_024738]	NM_024738
A_23_P57137	7.36E-06	4.52	NM_018347	NM_018347	Homo sapiens chromosome 20 open reading frame 29 (C20orf29), mRNA [NM_018347]	NM_018347
A_23_P216742	7.36E-06	6.62	NM_002581	NM_002581	Homo sapiens pregnancy-associated plasma protein A, pappalysin 1 (PAPPA), mRNA [NM_002581]	NM_002581
A_24_P924697	7.36E-06	7.69	AK055915	AK055915	Homo sapiens cDNA FLJ31353 fis, clone MESAN2000264. [AK055915]	
A_23_P162047	7.36E-06	14.66	NM_015881	NM_015881	Homo sapiens dickkopf homolog 3 (Xenopus laevis) (DKK3), transcript variant 1, mRNA [NM_015881]	NM_015881
A_23_P364024	7.36E-06	18.42	NM_006851	NM_006851	Homo sapiens GLI pathogenesis-related 1 (glioma) (GLIPR1), mRNA [NM_006851]	NM_006851
A_23_P92517	7.47E-06	6.90	NM_031956	NM_031956	Homo sapiens NYD-SP14 protein (NYD-SP14), mRNA [NM_031956]	NM_031956
A_24_P193582	7.73E-06	4.98	NM_017702	NM_017702	Homo sapiens hypothetical protein FLJ20186 (FLJ20186), transcript variant 2, mRNA [NM_017702]	NM_017702
A_23_P78405	7.80E-06	2.86	NM_006033	NM_006033	Homo sapiens lipase, endothelial (LIPG), mRNA [NM_006033]	NM_006033
A_32_P164494	8.14E-06	4.24	H11348	H11348	H11348 ym13h04.s1 Soares infant brain 1NIB Homo sapiens cDNA clone IMAGE:47855 3' similar to gb:X63657_rna1 FOLLICULAR VARIANT TRANSLOCATION PROTEIN 1 PRECURSOR (HUMAN);, mRNA sequence [H11348]	
A_24_P169688	8.14E-06	9.26	NM_005931	NM_005931	Homo sapiens MHC class I polypeptide-related sequence B (MICB), mRNA [NM_005931]	NM_005931
A_23_P121196	8.40E-06	2.16	NM_024334	NM_024334	Homo sapiens transmembrane protein 43 (TMEM43), mRNA [NM_024334]	NM_024334
A_23_P137848	8.40E-06	2.56	NM_145729	NM_145729	Homo sapiens mitochondrial ribosomal protein L24 (MRPL24), nuclear gene encoding mitochondrial protein, transcript variant 1, mRNA [NM_145729]	NM_145729
A_24_P265135	8.68E-06	6.99	NM_014671	NM_014671	Homo sapiens ubiquitin protein ligase E3C (UBE3C), mRNA [NM_014671]	NM_014671
A_23_P24843	8.69E-06	3.30	NM_014632	NM_014632	Homo sapiens microtubule associated monoxygenase, calponin and LIM domain containing 2 (MICAL2), mRNA [NM_014632]	NM_014632
A_23_P163467	8.75E-06	6.25	NM_207380	NM_207380	Homo sapiens FLJ43339 protein (FLJ43339), mRNA [NM_207380]	NM_207380
A_23_P165927	8.91E-06	10.46	NM_015894	NM_015894	Homo sapiens stathmin-like 3 (STMN3), mRNA [NM_015894]	NM_015894
A_23_P215819	9.03E-06	4.61	NM_001009957	NM_001009957	Homo sapiens zinc finger protein 655 (ZNF655), transcript variant 5, mRNA [NM_001009957]	NM_001009957
A_23_P161918	9.16E-06	4.27	NM_024098	NM_024098	Homo sapiens hypothetical protein MGC2574 (MGC2574), mRNA [NM_024098]	NM_024098
A_24_P917612	9.27E-06	3.07	BC009627	BC009627	Homo sapiens within bgcn homolog (Drosophila), mRNA (cDNA clone IMAGE:3897762), partial cds. [BC009627]	
A_32_P228348	9.78E-06	5.99	NM_207505	NM_207505	Homo sapiens FLJ45248 protein (FLJ45248), mRNA [NM_207505]	NM_207505
A_23_P3792	9.91E-06	2.15	NM_003486	NM_003486	Homo sapiens solute carrier family 7 (cationic amino acid transporter, y+ system), member 5 (SLC7A5), mRNA [NM_003486]	NM_003486
A_32_P218707	9.91E-06	6.37	XM_379108	XM_379108	PREDICTED: Homo sapiens similar to CDNA sequence BC012256 (LOC400969), mRNA [XM_379108]	XM_379108
A_24_P95439	1.03E-05	4.39	NM_001014438	NM_001014438	Homo sapiens cysteinyl-tRNA synthetase (CARS), transcript variant 4, mRNA [NM_001014438]	NM_001014438
A_23_P15326	1.04E-05	3.39	NM_005082	NM_005082	Homo sapiens tripartite motif-containing 25 (TRIM25), mRNA [NM_005082]	NM_005082
A_24_P273726	1.04E-05	10.56	NM_198080	NM_198080	Homo sapiens methionine sulfoxide reductase B3 (MSRB3), mRNA [NM_198080]	NM_198080
A_23_P379071	1.04E-05	14.79	NM_145032	NM_145032	Homo sapiens F-box and leucine-rich repeat protein 13 (FBXL13), mRNA [NM_145032]	NM_145032
A_23_P59718	1.05E-05	2.44	NM_003130	NM_003130	Homo sapiens sorcin (SRI), transcript variant 1, mRNA [NM_003130]	NM_003130
A_24_P264597	1.05E-05	5.03	A_24_P264597			
A_23_P47304	1.07E-05	20.24	NM_004347	NM_004347	Homo sapiens caspase 5, apoptosis-related cysteine protease (CASP5), mRNA [NM_004347]	NM_004347
A_24_P349606	1.08E-05	2.34	A_24_P349606			
A_23_P95672	1.13E-05	3.34	NM_006548	NM_006548	Homo sapiens IGF-II mRNA-binding protein 2 (IMP-2), transcript variant 1, mRNA [NM_006548]	NM_006548
A_23_P206612	1.17E-05	3.04	AK126447	AK126447	Homo sapiens cDNA FLJ44483 fis, clone UTERU2033375. [AK126447]	
A_23_P416305	1.27E-05	3.97	ENST00000356931		Homo sapiens cDNA FLJ43464 fis, clone OCBBF2036225. [AK125453]	
A_24_P212715	1.28E-05	3.76	A_24_P212715			
A_23_P65678	1.28E-05	5.81	NM_000138	NM_000138	Homo sapiens fibrillin 1 (Marfan syndrome) (FBN1), mRNA [NM_000138]	NM_000138
A_23_P487	1.31E-05	2.65	NM_012474	NM_012474	Homo sapiens uridine-cytidine kinase 2 (UCK2), mRNA [NM_012474]	NM_012474

A_23_P135294	1.31E-05	3.23	NM_000692	NM_000692	Homo sapiens aldehyde dehydrogenase 1 family, member B1 (ALDH1B1), nuclear gene encoding mitochondrial protein, mRNA [NM_000692]	NM_000692
A_23_P86818	1.31E-05	3.53	NM_004813	NM_004813	Homo sapiens peroxisomal biogenesis factor 16 (PEX16), transcript variant 1, mRNA [NM_004813]	NM_004813
A_23_P65584	1.35E-05	2.02	NM_182476	NM_182476	Homo sapiens coenzyme Q6 homolog (yeast) (COQ6), transcript variant 1, mRNA [NM_182476]	NM_182476
A_24_P134488	1.35E-05	2.85	NM_052880	NM_052880	Homo sapiens HGFL gene (MGC17330), mRNA [NM_052880]	NM_052880
A_24_P274607	1.36E-05	2.21	AK054960	AK054960	Homo sapiens cDNA FLJ30398 fis, clone BRACE2008402, highly similar to Homo sapiens steroid receptor RNA activator isoform 3 mRNA. [AK054960]	XR_000132
A_23_P154086	1.36E-05	2.34	NM_004328	NM_004328	Homo sapiens BCS1-like (yeast) (BCS1L), mRNA [NM_004328]	NM_004328
A_23_P40975	1.36E-05	2.48	NM_014674	NM_014674	Homo sapiens ER degradation enhancer, mannosidase alpha-like 1 (EDEM1), mRNA [NM_014674]	NM_014674
A_23_P14863	1.36E-05	3.05	NM_024111	NM_024111	Homo sapiens hypothetical protein MGC4504 (MGC4504), mRNA [NM_024111]	NM_024111
A_24_P410246	1.36E-05	4.03	A_24_P410246			
A_32_P119197	1.36E-05	5.08	BF686720	BF686720	602143863F1 NIH_MGC_46 Homo sapiens cDNA clone IMAGE:4304846 5', mRNA sequence [BF686720]	
A_23_P61371	1.36E-05	7.52	NM_198282	NM_198282	Homo sapiens hypothetical protein LOC340061 (LOC340061), mRNA [NM_198282]	NM_198282
A_23_P29237	1.36E-05	8.13	NM_145641	NM_145641	Homo sapiens apolipoprotein L, 3 (APOL3), transcript variant beta/a, mRNA [NM_145641]	NM_145641
A_32_P82462	1.36E-05	9.52	BC021861	BC021861	Homo sapiens hypothetical LOC554202, mRNA (cDNA clone MGC:30053 IMAGE:5139119), complete cds. [BC021861]	
A_23_P43490	1.36E-05	10.55	NM_058197	NM_058197	Homo sapiens cyclin-dependent kinase inhibitor 2A (melanoma, p16, inhibits CDK4) (CDKN2A), transcript variant 3, mRNA [NM_058197]	NM_058197
A_24_P269687	1.40E-05	3.55	NM_000113	NM_000113	Homo sapiens torsin family 1, member A (torsin A) (TOR1A), mRNA [NM_000113]	NM_000113
A_23_P340333	1.40E-05	3.66	NM_033397	NM_033397	Homo sapiens KIAA1754 (KIAA1754), mRNA [NM_033397]	NM_033397
A_23_P127891	1.40E-05	28.01	NM_170735	NM_170735	Homo sapiens brain-derived neurotrophic factor (BDNF), transcript variant 1, mRNA [NM_170735]	NM_170735
A_24_P186986	1.42E-05	2.57	ENST00000322426		PREDICTED: Homo sapiens similar to ATP synthase lipid-binding protein, mitochondrial precursor (ATP synthase proteolipid P1) (ATPase protein 9) (ATPase subunit C) (LOC390424), mRNA [XM_497383]	XM_497383
A_24_P253251	1.50E-05	3.25	NM_003045	NM_003045	Homo sapiens solute carrier family 7 (cationic amino acid transporter, y+ system), member 1 (SLC7A1), mRNA [NM_003045]	NM_003045
A_23_P318039	1.53E-05	3.30	NM_005444	NM_005444	Homo sapiens RCD1 required for cell differentiation1 homolog (S. pombe) (RQCD1), mRNA [NM_005444]	NM_005444
A_23_P47818	1.54E-05	3.17	NM_198324	NM_198324	Homo sapiens citrate synthase (CS), nuclear gene encoding mitochondrial protein, transcript variant 2, mRNA [NM_198324]	NM_198324
A_23_P104493	1.54E-05	4.88	NM_001015880	NM_001015880	Homo sapiens 3'-phosphoadenosine 5'-phosphosulfate synthase 2 (PAPSS2), transcript variant 2, mRNA [NM_001015880]	NM_001015880
A_23_P404494	1.55E-05	28.33	NM_002185	NM_002185	Homo sapiens interleukin 7 receptor (IL7R), mRNA [NM_002185]	NM_002185
A_24_P286687	1.56E-05	5.99	NM_016353	NM_016353	Homo sapiens zinc finger, DHHC-type containing 2 (ZDHHC2), mRNA [NM_016353]	NM_016353
A_24_P323114	1.56E-05	15.17	NR_001446	NR_001446	Homo sapiens annexin A2 pseudogene 3 (ANXA2P3) on chromosome 10 [NR_001446]	NR_001446
A_23_P257578	1.58E-05	2.33	AK054960	AK054960	Homo sapiens cDNA FLJ30398 fis, clone BRACE2008402, highly similar to Homo sapiens steroid receptor RNA activator isoform 3 mRNA. [AK054960]	XR_000132
A_23_P134527	1.58E-05	3.94	NM_006555	NM_006555	Homo sapiens SNARE protein Ykt6 (YKT6), mRNA [NM_006555]	NM_006555
A_23_P29953	1.58E-05	8.47	NM_172174	NM_172174	Homo sapiens interleukin 15 (IL15), transcript variant 1, mRNA [NM_172174]	NM_172174
A_24_P251734	1.58E-05	13.99	NM_020116	NM_020116	Homo sapiens follistatin-like 5 (FSTL5), mRNA [NM_020116]	NM_020116
A_32_P143850	1.59E-05	3.04	BC024198	BC024198	Homo sapiens, clone IMAGE:4821649, mRNA. [BC024198]	XR_000273
A_24_P62615	1.59E-05	3.80	NM_006367	NM_006367	Homo sapiens CAP, adenylate cyclase-associated protein 1 (yeast) (CAP1), mRNA [NM_006367]	NM_006367
A_24_P217489	1.59E-05	6.33	NM_000824	NM_000824	Homo sapiens glycine receptor, beta (GLRB), mRNA [NM_000824]	NM_000824
A_23_P123071	1.59E-05	8.47	NM_001233	NM_001233	Homo sapiens caveolin 2 (CAV2), transcript variant 1, mRNA [NM_001233]	NM_001233
A_32_P123729	1.59E-05	9.52	BX394228	BX394228	BX394228 BX394228 Homo sapiens NEUROBLASTOMA COT 25-NORMALIZED Homo sapiens cDNA clone CS0DC012YM19 3-PRIME, mRNA sequence [BX394228]	
A_23_P501933	1.59E-05	10.88	NM_145814	NM_145814	Homo sapiens calcium channel, voltage-dependent, gamma subunit 6 (CACNG6), transcript variant 1, mRNA [NM_145814]	NM_145814
A_23_P46455	1.60E-05	2.07	CR619944	CR619944	full-length cDNA clone CS0DF007YK17 of Fetal brain of Homo sapiens (human). [CR619944]	

A_23_P48964	1.60E-05	2.11	NM_018668	NM_018668	Homo sapiens vacuolar protein sorting 33B (yeast) (VPS33B), mRNA [NM_018668]	NM_018668
A_24_P125469	1.60E-05	3.76	NM_006033	NM_006033	Homo sapiens lipase, endothelial (LIPG), mRNA [NM_006033]	NM_006033
A_23_P257516	1.60E-05	4.42	NM_000247	NM_000247	Homo sapiens MHC class I polypeptide-related sequence A (MICA), mRNA [NM_000247]	NM_000247
A_23_P369966	1.60E-05	5.71	NM_152426	NM_152426	Homo sapiens apolipoprotein B mRNA editing enzyme, catalytic polypeptide-like 3D (APOBEC3D), mRNA [NM_152426]	NM_152426
A_23_P47034	1.60E-05	6.29	NM_002729	NM_002729	Homo sapiens hematopoietically expressed homeobox (HHEX), mRNA [NM_002729]	NM_002729
A_23_P311912	1.60E-05	10.52	BC090889	BC090889	Homo sapiens chromosome 14 open reading frame 78, mRNA (cDNA clone MGC:102983 IMAGE:30387958), complete cds. [BC090889]	XM_290629
A_24_P140608	1.61E-05	5.75	NM_001945	NM_001945	Homo sapiens heparin-binding EGF-like growth factor (HBEGF), mRNA [NM_001945]	NM_001945
A_23_P417372	1.63E-05	3.86	NM_017894	NM_017894	Homo sapiens zinc finger and SCAN domain containing 2 (ZSCAN2), transcript variant 2, mRNA [NM_017894]	NM_017894
A_23_P353035	1.66E-05	8.85	NM_001553	NM_001553	Homo sapiens insulin-like growth factor binding protein 7 (IGFBP7), mRNA [NM_001553]	NM_001553
A_23_P214821	1.68E-05	15.97	NM_001955	NM_001955	Homo sapiens endothelin 1 (EDN1), mRNA [NM_001955]	NM_001955
A_24_P224926	1.70E-05	2.56	NM_002405	NM_002405	Homo sapiens manic fringe homolog (Drosophila) (MFNG), mRNA [NM_002405]	NM_002405
A_23_P47247	1.70E-05	2.65	NM_018393	NM_018393	Homo sapiens hypothetical protein FLJ11336 (FLJ11336), mRNA [NM_018393]	NM_018393
A_23_P345942	1.72E-05	2.70	NM_174889	NM_174889	Homo sapiens Myc-induced mitochondria protein (mimitin), mRNA [NM_174889]	NM_174889
A_23_P43484	1.72E-05	27.93	NM_058197	NM_058197	Homo sapiens cyclin-dependent kinase inhibitor 2A (melanoma, p16, inhibits CDK4) (CDKN2A), transcript variant 3, mRNA [NM_058197]	NM_058197
A_23_P203882	1.77E-05	4.65	NM_022791	NM_022791	Homo sapiens matrix metalloproteinase 19 (MMP19), transcript variant rasi-6, mRNA [NM_022791]	NM_022791
A_24_P940166	1.77E-05	5.00	NM_001015880	NM_001015880	Homo sapiens 3'-phosphoadenosine 5'-phosphosulfate synthase 2 (PAPSS2), transcript variant 2, mRNA [NM_001015880]	NM_001015880
A_23_P46429	1.77E-05	10.19	NM_001554	NM_001554	Homo sapiens cysteine-rich, angiogenic inducer, 61 (CYR61), mRNA [NM_001554]	NM_001554
A_23_P435394	1.78E-05	5.26	NM_000958	NM_000958	Homo sapiens prostaglandin E receptor 4 (subtype EP4) (PTGER4), mRNA [NM_000958]	NM_000958
A_23_P138099	1.80E-05	4.39	NM_007314	NM_007314	Homo sapiens v-abl Abelson murine leukemia viral oncogene homolog 2 (arg, Abelson-related gene) (ABL2), transcript variant b, mRNA [NM_007314]	NM_007314
A_23_P630	1.81E-05	2.16	NM_018116	NM_018116	Homo sapiens misato homolog 1 (Drosophila) (MSTO1), mRNA [NM_018116]	NM_018116
A_23_P129246	1.84E-05	2.74	NM_025201	NM_025201	Homo sapiens PH domain-containing protein (pp9099), mRNA [NM_025201]	NM_025201
A_23_P209183	1.85E-05	2.25	NM_024656	NM_024656	Homo sapiens glycosyltransferase 25 domain containing 1 (GLT25D1), mRNA [NM_024656]	NM_024656
A_23_P26759	1.85E-05	2.53	NM_138793	NM_138793	Homo sapiens calcium activated nucleotidase 1 (CANT1), mRNA [NM_138793]	NM_138793
A_23_P89798	1.87E-05	2.30	NM_001012515	NM_001012515	Homo sapiens ferrochelatase (protoporphyria) (FECH), nuclear gene encoding mitochondrial protein, transcript variant 1, mRNA [NM_001012515]	NM_001012515
A_32_P193771	1.87E-05	3.37	A_32_P193771			
A_24_P942893	1.88E-05	2.81	NM_144582	NM_144582	Homo sapiens testis expressed sequence 261 (TEX261), mRNA [NM_144582]	NM_144582
A_23_P417404	1.88E-05	6.99	AF151783	AF151783	Homo sapiens MEG3 (MEG3) mRNA, complete cds. [AF151783]	
A_23_P252062	1.88E-05	12.15	NM_138711	NM_138711	Homo sapiens peroxisome proliferative activated receptor, gamma (PPARG), transcript variant 3, mRNA [NM_138711]	NM_138711
A_23_P154037	1.94E-05	5.38	NM_001159	NM_001159	Homo sapiens aldehyde oxidase 1 (AOX1), mRNA [NM_001159]	NM_001159
A_23_P17512	1.98E-05	3.41	NM_080820	NM_080820	Homo sapiens histidyl-tRNA synthetase 2 (HARS2), mRNA [NM_080820]	NM_080820
A_24_P194199	2.01E-05	2.31	NM_020433	NM_020433	Homo sapiens junctophilin 2 (JPH2), transcript variant 1, mRNA [NM_020433]	NM_020433
A_23_P116512	2.03E-05	3.21	NM_024841	NM_024841	Homo sapiens hypothetical protein FLJ14213 (FLJ14213), mRNA [NM_024841]	NM_024841
A_23_P259071	2.03E-05	3.70	NM_001657	NM_001657	Homo sapiens amphiregulin (schwannoma-derived growth factor) (AREG), mRNA [NM_001657]	NM_001657
A_23_P90790	2.05E-05	2.65	NM_199227	NM_199227	Homo sapiens methionine aminopeptidase 1D (MAP1D), mRNA [NM_199227]	NM_199227
A_23_P207719	2.06E-05	2.04	NM_004287	NM_004287	Homo sapiens golgi SNAP receptor complex member 2 (GOSR2), transcript variant A, mRNA [NM_004287]	NM_004287
A_23_P129786	2.06E-05	3.33	NM_001005291	NM_001005291	Homo sapiens sterol regulatory element binding transcription factor 1 (SREBF1), transcript variant 1, mRNA [NM_001005291]	NM_001005291
A_24_P501520	2.09E-05	3.52	AK098185	AK098185	Homo sapiens cDNA FLJ40866 fis, clone TRACH2018718. [AK098185]	
A_23_P209394	2.09E-05	4.03	AF009619	AF009619	Homo sapiens FLAME-1-delta mRNA, alternatively spliced, complete cds. [AF009619]	
A_24_P380629	2.12E-05	3.97	NM_007189	NM_007189	Homo sapiens ATP-binding cassette, sub-family F (GCN20), member 2 (ABCF2), nuclear gene encoding mitochondrial protein, transcript variant 1, mRNA [NM_007189]	NM_007189

A_32_P149288	2.14E-05	2.19	BC040307	BC040307	Homo sapiens cDNA clone IMAGE:4830091, partial cds. [BC040307]	
A_23_P15233	2.14E-05	2.96	A_23_P15233			
A_23_P67169	2.14E-05	3.62	NM_000641	NM_000641	Homo sapiens interleukin 11 (IL11), mRNA [NM_000641]	NM_000641
A_24_P84608	2.14E-05	3.88	A_24_P84608			
A_24_P391229	2.14E-05	4.29	NM_015666	NM_015666	Homo sapiens GTP binding protein 5 (putative) (GTPBP5), mRNA [NM_015666]	NM_015666
A_24_P212990	2.14E-05	8.70	AL365511	AL365511	Novel human gene mapping to chomosome 22. [AL365511]	
A_24_P130962	2.15E-05	2.47	NM_022371	NM_022371	Homo sapiens torsin family 3, member A (TOR3A), mRNA [NM_022371]	NM_022371
A_24_P83738	2.15E-05	3.50	NM_198188	NM_198188	Homo sapiens astrotactin 2 (ASTN2), transcript variant 4, mRNA [NM_198188]	NM_198188
A_32_P41127	2.16E-05	4.13	NM_002622	NM_002622	Homo sapiens prefoldin 1 (PFDN1), mRNA [NM_002622]	NM_002622
A_24_P918317	2.17E-05	8.62	NM_015881	NM_015881	Homo sapiens dickkopf homolog 3 (Xenopus laevis) (DKK3), transcript variant 1, mRNA [NM_015881]	NM_015881
A_23_P143817	2.17E-05	18.90	NM_053025	NM_053025	Homo sapiens myosin, light polypeptide kinase (MYLK), transcript variant 1, mRNA [NM_053025]	NM_053025
A_23_P82420	2.18E-05	2.80	NM_004603	NM_004603	Homo sapiens syntaxin 1A (brain) (STX1A), mRNA [NM_004603]	NM_004603
A_23_P348524	2.18E-05	3.69	NM_001007125	NM_001007125	Homo sapiens bA299N6.3 (LOC198437), mRNA [NM_001007125]	NM_001007125
A_23_P206724	2.18E-05	5.59	NM_175617	NM_175617	Homo sapiens metallothionein 1E (functional) (MT1E), mRNA [NM_175617]	NM_175617
A_23_P168556	2.19E-05	2.51	NM_004603	NM_004603	Homo sapiens syntaxin 1A (brain) (STX1A), mRNA [NM_004603]	NM_004603
A_24_P156113	2.19E-05	2.98	NM_014601	NM_014601	Homo sapiens EH-domain containing 2 (EHD2), mRNA [NM_014601]	NM_014601
A_32_P135890	2.19E-05	4.83	NM_001013680	NM_001013680	Homo sapiens similar to HIV TAT specific factor 1; cofactor required for Tat activation of HIV-1 transcription (LOC401233), mRNA [NM_001013680]	NM_001013680
A_23_P88893	2.19E-05	5.24	NM_017702	NM_017702	Homo sapiens hypothetical protein FLJ20186 (FLJ20186), transcript variant 2, mRNA [NM_017702]	NM_017702
A_24_P333525	2.19E-05	5.32	NM_014857	NM_014857	Homo sapiens RAB GTPase activating protein 1-like (RABGAP1L), mRNA [NM_014857]	NM_014857
A_23_P97402	2.19E-05	7.58	NM_020439	NM_020439	Homo sapiens calcium/calmodulin-dependent protein kinase IG (CAMK1G), mRNA [NM_020439]	NM_020439
A_32_P32061	2.20E-05	4.41	NM_013310	NM_013310	Homo sapiens chromosome 2 open reading frame 27 (C2orf27), mRNA [NM_013310]	NM_013310
A_24_P354900	2.21E-05	8.33	AK098194	AK098194	Homo sapiens cDNA FLJ40875 fis, clone UMVEN2000069. [AK098194]	
A_23_P105028	2.28E-05	6.62	NM_015459	NM_015459	Homo sapiens DKFZP564J0863 protein (DKFZP564J0863), mRNA [NM_015459]	NM_015459
A_24_P876864	2.32E-05	3.68	BC061590	BC061590	Homo sapiens cDNA clone MGC:75203 IMAGE:6502529, complete cds. [BC061590]	
A_23_P111260	2.32E-05	25.77	NM_002526	NM_002526	Homo sapiens 5'-nucleotidase, ecto (CD73) (NT5E), mRNA [NM_002526]	NM_002526
A_24_P154948	2.33E-05	2.71	NM_002047	NM_002047	Homo sapiens glycyl-tRNA synthetase (GARS), mRNA [NM_002047]	NM_002047
A_23_P98884	2.35E-05	2.56	NM_194358	NM_194358	Homo sapiens ring finger protein 41 (RNF41), transcript variant 2, mRNA [NM_194358]	NM_194358
A_23_P58002	2.38E-05	2.74	NM_022171	NM_022171	Homo sapiens T-cell leukemia translocation altered gene (TCTA), mRNA [NM_022171]	NM_022171
A_23_P202837	2.38E-05	6.90	NM_053056	NM_053056	Homo sapiens cyclin D1 (PRAD1: parathyroid adenomatosis 1) (CCND1), mRNA [NM_053056]	NM_053056
A_24_P43884	2.39E-05	2.44	NM_001006617	NM_001006617	Homo sapiens mitogen-activated protein kinase associated protein 1 (MAPKAP1), transcript variant 1, mRNA [NM_001006617]	NM_001006617
A_23_P33015	2.40E-05	3.05	NM_021128	NM_021128	Homo sapiens polymerase (RNA) II (DNA directed) polypeptide L, 7.6kDa (POLR2L), mRNA [NM_021128]	NM_021128
A_32_P144920	2.41E-05	2.35	NM_015509	NM_015509	Homo sapiens adaptin-ear-binding coat-associated protein 1 (NECAP1), mRNA [NM_015509]	NM_015509
A_23_P1691	2.48E-05	3.17	NM_002421	NM_002421	Homo sapiens matrix metalloproteinase 1 (interstitial collagenase) (MMP1), mRNA [NM_002421]	NM_002421
A_23_P85164	2.49E-05	2.51	NM_006730	NM_006730	Homo sapiens deoxyribonuclease I-like 1 (DNASE1L1), transcript variant 1, mRNA [NM_006730]	NM_006730
A_23_P157117	2.49E-05	2.72	NM_182898	NM_182898	Homo sapiens cAMP responsive element binding protein 5 (CREB5), transcript variant 1, mRNA [NM_182898]	NM_182898
A_24_P40928	2.49E-05	3.69	ENST00000281228			
A_32_P63886	2.49E-05	11.81	A_32_P63886			
A_23_P216836	2.52E-05	3.88	BC070091	BC070091	Homo sapiens caspase recruitment domain family, member 9, mRNA (cDNA clone MGC:87491 IMAGE:30343821), complete cds. [BC070091]	
A_23_P351270	2.53E-05	11.88	NM_002581	NM_002581	Homo sapiens pregnancy-associated plasma protein A, pappalysin 1 (PAPPA), mRNA [NM_002581]	NM_002581
A_32_P135243	2.55E-05	4.26	NM_015440	NM_015440	Homo sapiens methylenetetrahydrofolate dehydrogenase (NADP+ dependent) 1-like (MTHFD1L), mRNA [NM_015440]	NM_015440
A_23_P53152	2.56E-05	4.37	NM_020642	NM_020642	Homo sapiens chromosome 11 open reading frame 17 (C11orf17), transcript variant 2, mRNA [NM_020642]	NM_020642

A_23_P205159	2.56E-05	4.78	NM_005358	NM_005358	Homo sapiens LIM domain 7 (LMO7), mRNA [NM_005358]	NM_005358
A_32_P6769	2.60E-05	2.07	BC071598	BC071598	Homo sapiens hypothetical protein MGC25181, mRNA (cDNA clone MGC:87530 IMAGE:30334929), complete cds. [BC071598]	
A_24_P181585	2.60E-05	2.33	NM_018509	NM_018509	Homo sapiens hypothetical protein PRO1855 (PRO1855), mRNA [NM_018509]	NM_018509
A_23_P372096	2.60E-05	3.65	AK097804	AK097804	Homo sapiens cDNA FLJ40485 fis, clone TESTI2043857, moderately similar to Homo sapiens nolp mRNA. [AK097804]	
A_23_P202978	2.60E-05	4.61	NM_033292	NM_033292	Homo sapiens caspase 1, apoptosis-related cysteine protease (interleukin 1, beta, convertase) (CASP1), transcript variant alpha, mRNA [NM_033292]	NM_033292
A_23_P344719	2.60E-05	7.58	NM_003898	NM_003898	Homo sapiens synaptojanin 2 (SYNJ2), mRNA [NM_003898]	NM_003898
A_24_P406060	2.60E-05	13.05	NM_182757	NM_182757	Homo sapiens IBR domain containing 2 (IBRDC2), mRNA [NM_182757]	NM_182757
A_23_P12463	2.63E-05	3.44	NM_002826	NM_002826	Homo sapiens quiescin Q6 (QSCN6), transcript variant 1, mRNA [NM_002826]	NM_002826
A_23_P141405	2.64E-05	2.16	NM_002512	NM_002512	Homo sapiens non-metastatic cells 2, protein (NM23B) expressed in (NME2), transcript variant 2, mRNA [NM_002512]	NM_002512
A_24_P533142	2.64E-05	2.54	A_24_P533142			
A_32_P225916	2.66E-05	3.73	BM994413	BM994413	UI-H-DH0-aul-n-17-0-UI.s1 NCI_CGAP_DH0 Homo sapiens cDNA clone IMAGE:5871184 3', mRNA sequence [BM994413]	
A_24_P932887	2.66E-05	25.64	AK097227	AK097227	Homo sapiens cDNA FLJ39908 fis, clone SPLEN2017620. [AK097227]	
A_23_P63371	2.67E-05	10.05	NM_003189	NM_003189	Homo sapiens T-cell acute lymphocytic leukemia 1 (TAL1), mRNA [NM_003189]	NM_003189
A_23_P65870	2.68E-05	2.75	NM_012170	NM_012170	Homo sapiens F-box protein 22 (FBXO22), transcript variant 2, mRNA [NM_012170]	NM_012170
A_23_P150789	2.68E-05	11.24	NM_007173	NM_007173	Homo sapiens protease, serine, 23 (PRSS23), mRNA [NM_007173]	NM_007173
A_24_P371758	2.71E-05	2.24	NM_032479	NM_032479	Homo sapiens mitochondrial ribosomal protein L36 (MRPL36), nuclear gene encoding mitochondrial protein, mRNA [NM_032479]	NM_032479
A_32_P209230	2.72E-05	6.17	NM_133467	NM_133467	Homo sapiens Cbp/p300-interacting transactivator, with Glu/Asp-rich carboxy-terminal domain, 4 (CITED4), mRNA [NM_133467]	NM_133467
A_23_P111000	2.72E-05	22.08	NM_002800	NM_002800	Homo sapiens proteasome (prosome, macropain) subunit, beta type, 9 (large multifunctional protease 2) (PSMB9), transcript variant 1, mRNA [NM_002800]	NM_002800
A_24_P204515	2.73E-05	2.57	ENST00000342275		RST6901 Athersys RAGE Library Homo sapiens cDNA, mRNA sequence [BG187898]	XM_497067
A_23_P75500	2.73E-05	2.62	NM_015399	NM_015399	Homo sapiens breast cancer metastasis suppressor 1 (BRMS1), transcript variant 1, mRNA [NM_015399]	NM_015399
A_32_P201496	2.73E-05	3.17	NM_001018052	NM_001018052	Homo sapiens polymerase (RNA) III (DNA directed) polypeptide H (22.9kD) (POLR3H), transcript variant 2, mRNA [NM_001018052]	NM_001018052
A_23_P109171	2.73E-05	6.67	NM_001195	NM_001195	Homo sapiens beaded filament structural protein 1, filensin (BFSP1), mRNA [NM_001195]	NM_001195
A_24_P30141	2.74E-05	2.03	NM_203438	NM_203438	Homo sapiens chromosome 10 open reading frame 4 (C10orf4), transcript variant FRA10AC1-3.3, mRNA [NM_203438]	NM_203438
A_23_P50426	2.74E-05	2.27	NM_015493	NM_015493	Homo sapiens ankyrin repeat domain 25 (ANKRD25), mRNA [NM_015493]	NM_015493
A_23_P43810	2.75E-05	7.87	NM_206943	NM_206943	Homo sapiens latent transforming growth factor beta binding protein 1 (LTBP1), transcript variant 1, mRNA [NM_206943]	NM_206943
A_23_P46426	2.75E-05	18.38	NM_001554	NM_001554	Homo sapiens cysteine-rich, angiogenic inducer, 61 (CYR61), mRNA [NM_001554]	NM_001554
A_23_P201386	2.79E-05	3.24	NM_012137	NM_012137	Homo sapiens dimethylarginine dimethylaminohydrolase 1 (DDAH1), mRNA [NM_012137]	NM_012137
A_32_P118847	2.79E-05	5.95	BC044619	BC044619	Homo sapiens hypothetical gene supported by AK124699, mRNA (cDNA clone IMAGE:5270292). [BC044619]	XM_499008
A_24_P416660	2.80E-05	2.62	NM_080665	NM_080665	Homo sapiens similar to RIKEN cDNA B230118G17 gene (MGC19604), mRNA [NM_080665]	NM_080665
A_23_P419624	2.84E-05	2.29	NM_006698	NM_006698	Homo sapiens bladder cancer associated protein (BLCAP), mRNA [NM_006698]	NM_006698
A_23_P125624	2.84E-05	2.88	NM_012332	NM_012332	Homo sapiens acyl-Coenzyme A thioesterase 2, mitochondrial (ACATE2), mRNA [NM_012332]	NM_012332
A_24_P753476	2.84E-05	4.03	BC043374	BC043374	Homo sapiens hypothetical protein LOC340508, mRNA (cDNA clone IMAGE:5172009), partial cds. [BC043374]	
A_23_P204630	2.87E-05	25.19	NM_021229	NM_021229	Homo sapiens netrin 4 (NTN4), mRNA [NM_021229]	NM_021229
A_23_P64121	2.95E-05	17.73	NM_152314	NM_152314	Homo sapiens hypothetical protein MGC34830 (MGC34830), mRNA [NM_152314]	NM_152314
A_23_P10858	2.97E-05	2.02	ENST00000318139		Homo sapiens KIAA0692 protein, mRNA (cDNA clone IMAGE:5473979), partial cds. [BC062373]	
A_23_P1981	2.97E-05	3.85	NM_000207	NM_000207	Homo sapiens insulin (INS), mRNA [NM_000207]	NM_000207
A_23_P401904	2.98E-05	2.43	NM_001009936	NM_001009936	Homo sapiens PHD finger protein 19 (PHF19), transcript variant 2, mRNA [NM_001009936]	NM_001009936

A_24_P42501	2.98E-05	4.48	NM_012332	NM_012332	Homo sapiens acyl-Coenzyme A thioesterase 2, mitochondrial (ACATE2), mRNA [NM_012332]	NM_012332
A_23_P210619	2.99E-05	2.69	NM_016407	NM_016407	Homo sapiens chromosome 20 open reading frame 43 (C20orf43), mRNA [NM_016407]	NM_016407
A_23_P17307	3.00E-05	2.58	NM_018270	NM_018270	Homo sapiens chromosome 20 open reading frame 20 (C20orf20), mRNA [NM_018270]	NM_018270
A_24_P13991	3.02E-05	2.59	NM_170607	NM_170607	Homo sapiens MAX-like protein X (MLX), transcript variant 3, mRNA [NM_170607]	NM_170607
A_24_P404822	3.07E-05	13.28	NM_017413	NM_017413	Homo sapiens apelin, AGTRL1 ligand (APLN), mRNA [NM_017413]	NM_017413
A_24_P389038	3.08E-05	2.42	NM_005452	NM_005452	Homo sapiens WD repeat domain 46 (WDR46), mRNA [NM_005452]	NM_005452
A_23_P100074	3.08E-05	3.18	NM_020371	NM_020371	Homo sapiens apoptosis, caspase activation inhibitor (AVEN), mRNA [NM_020371]	NM_020371
A_24_P187459	3.10E-05	2.56	ENST00000336230			
A_23_P501583	3.10E-05	2.95	NM_002505	NM_002505	Homo sapiens nuclear transcription factor Y, alpha (NFYA), transcript variant 1, mRNA [NM_002505]	NM_002505
A_32_P194848	3.12E-05	12.25	NM_003564	NM_003564	Homo sapiens transgelin 2 (TAGLN2), mRNA [NM_003564]	NM_003564
A_23_P63017	3.13E-05	2.37	NM_006499	NM_006499	Homo sapiens lectin, galactoside-binding, soluble, 8 (galectin 8) (LGALS8), transcript variant 1, mRNA [NM_006499]	NM_006499
A_24_P115762	3.15E-05	8.40	NM_148170	NM_148170	Homo sapiens cathepsin C (CTSC), transcript variant 2, mRNA [NM_148170]	NM_148170
A_24_P15823	3.19E-05	3.05	A_24_P15823			
A_32_P170736	3.27E-05	2.88	AK098422	AK098422	Homo sapiens cDNA FLJ25556 fis, clone JTH02629. [AK098422]	
A_23_P53081	3.28E-05	2.15	NM_020896	NM_020896	Homo sapiens oxysterol binding protein-like 5 (OSBPL5), transcript variant 1, mRNA [NM_020896]	NM_020896
A_23_P39074	3.28E-05	2.43	NM_006270	NM_006270	Homo sapiens related RAS viral (r-ras) oncogene homolog (RRAS), mRNA [NM_006270]	NM_006270
A_23_P323751	3.28E-05	2.63	NM_030919	NM_030919	Homo sapiens chromosome 20 open reading frame 129 (C20orf129), mRNA [NM_030919]	NM_030919
A_23_P422178	3.28E-05	4.20	NM_003588	NM_003588	Homo sapiens cullin 4B (CUL4B), mRNA [NM_003588]	NM_003588
A_24_P102411	3.28E-05	4.41	BC016743	BC016743	Homo sapiens BRF1 homolog, subunit of RNA polymerase III transcription initiation factor IIIB (S. cerevisiae), mRNA (cDNA clone IMAGE:4830425), complete cds. [BC016743]	
A_24_P884915	3.30E-05	2.75	A_24_P884915			
A_24_P201739	3.30E-05	5.92	NM_005475	NM_005475	Homo sapiens lymphocyte adaptor protein (LNK), mRNA [NM_005475]	NM_005475
A_24_P212764	3.34E-05	3.08	A_24_P212764			
A_24_P245646	3.35E-05	2.07	NM_033550	NM_033550	Homo sapiens TP53 regulating kinase (TP53RK), mRNA [NM_033550]	NM_033550
A_24_P352864	3.37E-05	4.78	NM_005789	NM_005789	Homo sapiens proteasome (prosome, macropain) activator subunit 3 (PA28 gamma; Ki) (PSME3), transcript variant 1, mRNA [NM_005789]	NM_005789
A_24_P16913	3.37E-05	7.52	NM_005845	NM_005845	Homo sapiens ATP-binding cassette, sub-family C (CFTR/MRP), member 4 (ABCC4), mRNA [NM_005845]	NM_005845
A_24_P719579	3.38E-05	3.24	ENST00000343626		Homo sapiens cDNA clone IMAGE:5797600, partial cds. [BC062294]	XM_375449
A_23_P326319	3.41E-05	3.08	NM_033201	NM_033201	Homo sapiens chromosome 16 open reading frame 45 (C16orf45), mRNA [NM_033201]	NM_033201
A_23_P210690	3.41E-05	6.85	NM_021158	NM_021158	Homo sapiens tribbles homolog 3 (Drosophila) (TRIB3), mRNA [NM_021158]	NM_021158
A_24_P113295	3.42E-05	2.33	NM_004069	NM_004069	Homo sapiens adaptor-related protein complex 2, sigma 1 subunit (AP2S1), transcript variant AP17, mRNA [NM_004069]	NM_004069
A_23_P54649	3.42E-05	2.34	NM_153425	NM_153425	Homo sapiens TNFRSF1A-associated via death domain (TRADD), transcript variant 2, mRNA [NM_153425]	NM_153425
A_24_P57730	3.42E-05	4.76	NM_181304	NM_181304	Homo sapiens mitochondrial ribosomal protein L52 (MRPL52), nuclear gene encoding mitochondrial protein, transcript variant 4, mRNA [NM_181304]	NM_181304
A_24_P245767	3.43E-05	2.54	NM_006936	NM_006936	Homo sapiens SMT3 suppressor of mif two 3 homolog 3 (yeast) (SUMO3), mRNA [NM_006936]	NM_006936
A_32_P122754	3.44E-05	3.13	NM_080655	NM_080655	Homo sapiens similar to RIKEN cDNA 5730528L13 gene (MGC17337), mRNA [NM_080655]	NM_080655
A_24_P206121	3.44E-05	3.32	NM_004137	NM_004137	Homo sapiens potassium large conductance calcium-activated channel, subfamily M, beta member 1 (KCNMB1), mRNA [NM_004137]	NM_004137
A_24_P349590	3.44E-05	4.52	A_24_P349590			
A_32_P186138	3.45E-05	2.30	BX647075	BX647075	Homo sapiens mRNA; cDNA DKFZp781B0241 (from clone DKFZp781B0241). [BX647075]	XM_376522
A_23_P39402	3.45E-05	3.11	NM_198867	NM_198867	Homo sapiens hypothetical protein MGC15677 (MGC15677), transcript variant 1, mRNA [NM_198867]	NM_198867
A_23_P206212	3.45E-05	14.37	NM_003246	NM_003246	Homo sapiens thrombospondin 1 (THBS1), mRNA [NM_003246]	NM_003246
A_24_P289818	3.50E-05	10.94	A_24_P289818			

A_24_P39654	3.51E-05	2.54	NM_002086	NM_002086	Homo sapiens growth factor receptor-bound protein 2 (GRB2), transcript variant 1, mRNA [NM_002086]	NM_002086
A_24_P59607	3.54E-05	4.55	BC019938	BC019938	Homo sapiens hypothetical protein LOC339263, mRNA (cDNA clone IMAGE:3924994), partial cds. [BC019938]	XM_378661
A_24_P205874	3.57E-05	2.04	NM_004206	NM_004206	Homo sapiens SEC22 vesicle trafficking protein-like 3 (S. cerevisiae) (SEC22L3), transcript variant 2, mRNA [NM_004206]	NM_004206
A_24_P708161	3.68E-05	2.07	A_24_P708161			
A_23_P409093	3.68E-05	7.35	NM_178826	NM_178826	Homo sapiens transmembrane protein 16D (TMEM16D), mRNA [NM_178826]	NM_178826
A_24_P42603	3.69E-05	4.33	NM_007118	NM_007118	Homo sapiens triple functional domain (PTPRF interacting) (TRIO), mRNA [NM_007118]	NM_007118
A_23_P160968	3.69E-05	4.67	NM_018891	NM_018891	Homo sapiens laminin, gamma 2 (LAMC2), transcript variant 2, mRNA [NM_018891]	NM_018891
A_24_P825942	3.74E-05	6.67	AK001796	AK001796	Homo sapiens cDNA FLJ10934 fis, clone OVARC1000640. [AK001796]	
A_23_P22027	3.76E-05	3.12	NM_198336	NM_198336	Homo sapiens insulin induced gene 1 (INSIG1), transcript variant 2, mRNA [NM_198336]	NM_198336
A_23_P210643	3.80E-05	2.54	NM_016397	NM_016397	Homo sapiens TH1-like (Drosophila) (TH1L), transcript variant 2, mRNA [NM_016397]	NM_016397
A_24_P467449	3.80E-05	3.37	THC2437930		ALU7_HUMAN (P39194) Alu subfamily SQ sequence contamination warning entry, partial (4%) [THC2437930]	
A_23_P3885	3.83E-05	4.42	NM_024109	NM_024109	Homo sapiens hypothetical protein MGC2654 (MGC2654), mRNA [NM_024109]	NM_024109
A_24_P187948	3.84E-05	3.53	NM_197966	NM_197966	Homo sapiens BH3 interacting domain death agonist (BID), transcript variant 1, mRNA [NM_197966]	NM_197966
A_24_P103004	3.88E-05	5.21	NM_005415	NM_005415	Homo sapiens solute carrier family 20 (phosphate transporter), member 1 (SLC20A1), mRNA [NM_005415]	NM_005415
A_23_P316974	3.89E-05	5.13	NM_003898	NM_003898	Homo sapiens synaptojanin 2 (SYNJ2), mRNA [NM_003898]	NM_003898
A_32_P36313	3.95E-05	2.75	THC2310680		AF262221 SNARE Vti1a protein {Rattus norvegicus;} , complete [THC2310680]	
A_23_P1819	3.97E-05	2.95	NM_012378	NM_012378	Homo sapiens olfactory receptor, family 8, subfamily B, member 8 (OR8B8), mRNA [NM_012378]	NM_012378
A_23_P391264	4.08E-05	3.46	NM_007047	NM_007047	Homo sapiens butyrophilin, subfamily 3, member A2 (BTN3A2), mRNA [NM_007047]	NM_007047
A_23_P32938	4.14E-05	3.10	NM_004398	NM_004398	Homo sapiens DEAD (Asp-Glu-Ala-Asp) box polypeptide 10 (DDX10), mRNA [NM_004398]	NM_004398
A_23_P100392	4.16E-05	2.42	NM_007108	NM_007108	Homo sapiens transcription elongation factor B (SIII), polypeptide 2 (18kDa, elongin B) (TCEB2), transcript variant 1, mRNA [NM_007108]	NM_007108
A_24_P38081	4.18E-05	8.26	NM_004117	NM_004117	Homo sapiens FK506 binding protein 5 (FKBP5), mRNA [NM_004117]	NM_004117
A_24_P261083	4.19E-05	3.40	NM_004337	NM_004337	Homo sapiens chromosome 8 open reading frame 1 (C8orf1), mRNA [NM_004337]	NM_004337
A_23_P95027	4.21E-05	2.03	NM_016466	NM_016466	Homo sapiens ankyrin repeat domain 39 (ANKRD39), mRNA [NM_016466]	NM_016466
A_23_P54728	4.21E-05	2.04	NM_023933	NM_023933	Homo sapiens chromosome 16 open reading frame 24 (C16orf24), mRNA [NM_023933]	NM_023933
A_24_P219769	4.21E-05	2.64	NM_018035	NM_018035	Homo sapiens hypothetical protein FLJ10241 (FLJ10241), mRNA [NM_018035]	NM_018035
A_23_P120467	4.23E-05	7.41	NM_199427	NM_199427	Homo sapiens zinc finger protein 64 homolog (mouse) (ZFP64), transcript variant 4, mRNA [NM_199427]	NM_199427
A_23_P256445	4.26E-05	3.13	NM_025054	NM_025054	Homo sapiens valosin containing protein (p97)/p47 complex interacting protein 1 (VCPIP1), mRNA [NM_025054]	NM_025054
A_23_P44831	4.26E-05	4.15	NM_018482	NM_018482	Homo sapiens development and differentiation enhancing factor 1 (DDEF1), mRNA [NM_018482]	NM_018482
A_23_P19673	4.26E-05	12.22	NM_005627	NM_005627	Homo sapiens serum/glucocorticoid regulated kinase (SGK), mRNA [NM_005627]	NM_005627
A_23_P164691	4.27E-05	2.14	NM_002162	NM_002162	Homo sapiens intercellular adhesion molecule 3 (ICAM3), mRNA [NM_002162]	NM_002162
A_32_P209989	4.28E-05	2.35	NM_022163	NM_022163	Homo sapiens mitochondrial ribosomal protein L46 (MRPL46), nuclear gene encoding mitochondrial protein, mRNA [NM_022163]	NM_022163
A_23_P257668	4.28E-05	3.72	NM_024926	NM_024926	Homo sapiens hypothetical protein FLJ12571 (FLJ12571), mRNA [NM_024926]	NM_024926
A_23_P89941	4.28E-05	4.85	NM_001800	NM_001800	Homo sapiens cyclin-dependent kinase inhibitor 2D (p19, inhibits CDK4) (CDKN2D), transcript variant 1, mRNA [NM_001800]	NM_001800
A_32_P91902	4.32E-05	3.09	A_32_P91902			
A_23_P146584	4.34E-05	3.40	NM_080655	NM_080655	Homo sapiens similar to RIKEN cDNA 5730528L13 gene (MGC17337), mRNA [NM_080655]	NM_080655
A_23_P70785	4.42E-05	6.80	NM_001624	NM_001624	Homo sapiens absent in melanoma 1 (AIM1), mRNA [NM_001624]	NM_001624
A_23_P168188	4.47E-05	2.58	NM_001009991	NM_001009991	Homo sapiens synaptotagmin-like 3 (SYTL3), mRNA [NM_001009991]	NM_001009991
A_23_P24723	4.47E-05	2.74	NM_016464	NM_016464	Homo sapiens hypothetical protein HSPC196 (HSPC196), mRNA [NM_016464]	NM_016464
A_24_P91238	4.50E-05	2.52	NM_032837	NM_032837	Homo sapiens hypothetical protein FLJ14775 (FLJ14775), mRNA [NM_032837]	NM_032837
A_23_P52634	4.50E-05	4.13	NM_015368	NM_015368	Homo sapiens pannexin 1 (PANX1), mRNA [NM_015368]	NM_015368

A_23_P75523	4.50E-05	6.49	NM_203330	NM_203330	Homo sapiens CD59 antigen p18-20 (antigen identified by monoclonal antibodies 16.3A5, EJ16, EJ30, EL32 and G344) (CD59), transcript variant 1, mRNA [NM_203330]	NM_203330
A_23_P61268	4.52E-05	2.31	NM_016458	NM_016458	Homo sapiens brain protein 16 (LOC51236), mRNA [NM_016458]	NM_016458
A_23_P57033	4.58E-05	4.08	NM_006809	NM_006809	Homo sapiens translocase of outer mitochondrial membrane 34 (TOMM34), nuclear gene encoding mitochondrial protein, mRNA [NM_006809]	NM_006809
A_24_P699737	4.60E-05	3.55	AK022339	AK022339	Homo sapiens cDNA FLJ12277 fis, clone MAMMA1001711. [AK022339]	
A_23_P131816	4.64E-05	2.02	NM_052951	NM_052951	Homo sapiens deoxynucleotidyltransferase, terminal, interacting protein 1 (DNTTIP1), mRNA [NM_052951]	NM_052951
A_23_P79122	4.64E-05	2.25	NM_018467	NM_018467	Homo sapiens uncharacterized hematopoietic stem/progenitor cells protein MDS032 (MDS032), mRNA [NM_018467]	NM_018467
A_23_P116840	4.64E-05	2.46	NM_021934	NM_021934	Homo sapiens hypothetical protein FLJ11773 (FLJ11773), mRNA [NM_021934]	NM_021934
A_24_P233786	4.64E-05	3.01	NM_052966	NM_052966	Homo sapiens chromosome 1 open reading frame 24 (C1orf24), transcript variant 2, mRNA [NM_052966]	NM_052966
A_24_P145134	4.64E-05	3.02	BC010943	BC010943	Homo sapiens oncostatin M receptor, mRNA (cDNA clone IMAGE:4043935), complete cds. [BC010943]	
A_24_P51777	4.64E-05	3.03	NM_178335	NM_178335	Homo sapiens chromosome 3 open reading frame 6 (C3orf6), transcript variant 2, mRNA [NM_178335]	NM_178335
A_23_P154667	4.64E-05	5.49	NM_000801	NM_000801	Homo sapiens FK506 binding protein 1A, 12kDa (FKBP1A), transcript variant 12B, mRNA [NM_000801]	NM_000801
A_32_P94798	4.64E-05	5.65	NM_001002857	NM_001002857	Homo sapiens annexin A2 (ANXA2), transcript variant 2, mRNA [NM_001002857]	NM_001002857
A_32_P163089	4.64E-05	5.88	NM_207376	NM_207376	Homo sapiens hypothetical protein (LOC387882), mRNA [NM_207376]	NM_207376
A_23_P165783	4.64E-05	28.41	NM_024101	NM_024101	Homo sapiens melanophilin (MLPH), mRNA [NM_024101]	NM_024101
A_23_P502710	4.69E-05	2.54	NM_152237	NM_152237	Homo sapiens growth arrest-specific 2 like 1 (GAS2L1), transcript variant 3, mRNA [NM_152237]	NM_152237
A_23_P326963	4.70E-05	5.56	NM_178496	NM_178496	Homo sapiens similar to BcDNA:GH11415 gene product (LOC151963), mRNA [NM_178496]	NM_178496
A_23_P102731	4.72E-05	2.25	NM_175839	NM_175839	Homo sapiens spermine oxidase (SMOX), transcript variant 1, mRNA [NM_175839]	NM_175839
A_23_P80032	4.72E-05	2.98	NM_005225	NM_005225	Homo sapiens E2F transcription factor 1 (E2F1), mRNA [NM_005225]	NM_005225
A_23_P105044	4.79E-05	2.63	NM_021134	NM_021134	Homo sapiens mitochondrial ribosomal protein L23 (MRPL23), nuclear gene encoding mitochondrial protein, mRNA [NM_021134]	NM_021134
A_23_P3866	4.85E-05	4.35	NM_021149	NM_021149	Homo sapiens coactosin-like 1 (Dictyostelium) (COTL1), mRNA [NM_021149]	NM_021149
A_23_P61551	4.86E-05	2.42	NM_006110	NM_006110	Homo sapiens CD2 antigen (cytoplasmic tail) binding protein 2 (CD2BP2), mRNA [NM_006110]	NM_006110
A_23_P111593	4.86E-05	4.31	NM_004227	NM_004227	Homo sapiens pleckstrin homology, Sec7 and coiled-coil domains 3 (PSCD3), mRNA [NM_004227]	NM_004227
A_23_P58796	4.86E-05	4.78	NM_001012761	NM_001012761	Homo sapiens RGM domain family, member B (RGMB), transcript variant 1, mRNA [NM_001012761]	NM_001012761
A_24_P167668	4.86E-05	5.05	NM_000428	NM_000428	Homo sapiens latent transforming growth factor beta binding protein 2 (LTBP2), mRNA [NM_000428]	NM_000428
A_24_P225448	4.86E-05	6.67	NM_004078	NM_004078	Homo sapiens cysteine and glycine-rich protein 1 (CSRP1), mRNA [NM_004078]	NM_004078
A_23_P132763	4.86E-05	16.39	NM_016206	NM_016206	Homo sapiens vestigial-like 3 (VGL-3), mRNA [NM_016206]	NM_016206
A_23_P111413	4.89E-05	2.67	NM_018718	NM_018718	Homo sapiens testis specific, 14 (TSGA14), mRNA [NM_018718]	NM_018718
A_23_P136978	4.90E-05	8.26	NM_014467	NM_014467	Homo sapiens sushi-repeat-containing protein, X-linked 2 (SRPX2), mRNA [NM_014467]	NM_014467
A_24_P349547	4.92E-05	4.95	A_24_P349547			
A_32_P66881	4.92E-05	9.52	THC2280549			
A_24_P910733	4.98E-05	3.30	NM_178335	NM_178335	Homo sapiens chromosome 3 open reading frame 6 (C3orf6), transcript variant 2, mRNA [NM_178335]	NM_178335
A_23_P164702	4.99E-05	2.68	NM_015956	NM_015956	Homo sapiens mitochondrial ribosomal protein L4 (MRPL4), nuclear gene encoding mitochondrial protein, transcript variant 1, mRNA [NM_015956]	NM_015956
A_23_P259189	4.99E-05	2.99	NM_013943	NM_013943	Homo sapiens chloride intracellular channel 4 (CLIC4), mRNA [NM_013943]	NM_013943
A_32_P206839	4.99E-05	4.05	AF075027	AF075027	Homo sapiens full length insert cDNA YI37C01. [AF075027]	
A_23_P86283	5.02E-05	3.62	NM_006762	NM_006762	Homo sapiens lysosomal associated multispanning membrane protein 5 (LAPTM5), mRNA [NM_006762]	NM_006762
A_24_P135193	5.02E-05	4.39	NM_018314	NM_018314	Homo sapiens ubiquitin-conjugating enzyme E2-like (UEV3), mRNA [NM_018314]	NM_018314
A_23_P11729	5.05E-05	3.66	NM_015872	NM_015872	Homo sapiens zinc finger and BTB domain containing 7B (ZBTB7B), mRNA [NM_015872]	NM_015872
A_32_P96719	5.07E-05	2.39	NM_024745	NM_024745	Homo sapiens SHC SH2-domain binding protein 1 (SHCBP1), mRNA [NM_024745]	NM_024745
A_23_P30024	5.08E-05	2.34	NM_003998	NM_003998	Homo sapiens nuclear factor of kappa light polypeptide gene enhancer in B-cells 1 (p105) (NFKB1), mRNA [NM_003998]	NM_003998
A_24_P376339	5.12E-05	2.72	BC016333	BC016333	Homo sapiens cyclin L2, mRNA (cDNA clone MGC:23944 IMAGE:4076039), complete cds. [BC016333]	

A_23_P29303	5.14E-05	4.24	NM_015703	NM_015703	Homo sapiens CGI-96 protein (CGI-96), mRNA [NM_015703]	NM_015703
A_24_P168510	5.15E-05	2.43	NM_024069	NM_024069	Homo sapiens hypothetical protein MGC2749 (MGC2749), mRNA [NM_024069]	NM_024069
A_32_P215002	5.15E-05	9.71	AI342696	AI342696	AI342696 qo35e02.x1 NCI_CGAP_Lu5 Homo sapiens cDNA clone IMAGE:1910522 3' similar to gb:M59040 CD44 ANTIGEN, EPITHELIAL FORM PRECURSOR (HUMAN);, mRNA sequence [AI342696]	
A_24_P143189	5.20E-05	5.41	NM_183049	NM_183049	Homo sapiens thymosin-like 3 (TMSL3), mRNA [NM_183049]	NM_183049
A_24_P636882	5.20E-05	5.92	THC2337176		ALU1_HUMAN (P39188) Alu subfamily J sequence contamination warning entry, partial (30%) [THC2337176]	
A_23_P59099	5.25E-05	2.56	NM_013937	NM_013937	Homo sapiens olfactory receptor, family 11, subfamily A, member 1 (OR11A1), mRNA [NM_013937]	NM_013937
A_23_P201706	5.25E-05	11.79	NM_005978	NM_005978	Homo sapiens S100 calcium binding protein A2 (S100A2), mRNA [NM_005978]	NM_005978
A_23_P132248	5.28E-05	3.03	NM_019103	NM_019103	Homo sapiens zinc finger, matrin type 5 (ZMAT5), transcript variant 1, mRNA [NM_019103]	NM_019103
A_23_P95417	5.32E-05	14.27	NM_003840	NM_003840	Homo sapiens tumor necrosis factor receptor superfamily, member 10d, decoy with truncated death domain (TNFRSF10D), mRNA [NM_003840]	NM_003840
A_32_P102300	5.34E-05	2.44	THC2338243		Q6PGA0 (Q6PGA0) Rcor3 protein (Fragment), partial (8%) [THC2338243]	
A_23_P118061	5.35E-05	2.39	NM_181641	NM_181641	Homo sapiens chemokine-like factor (CKLF), transcript variant 4, mRNA [NM_181641]	NM_181641
A_23_P144113	5.35E-05	3.89	NM_002070	NM_002070	Homo sapiens guanine nucleotide binding protein (G protein), alpha inhibiting activity polypeptide 2 (GNAI2), mRNA [NM_002070]	NM_002070
A_23_P151075	5.40E-05	6.13	NM_001175	NM_001175	Homo sapiens Rho GDP dissociation inhibitor (GDI) beta (ARHGDIB), mRNA [NM_001175]	NM_001175
A_24_P340866	5.41E-05	2.30	A_24_P340866			
A_23_P131771	5.41E-05	3.73	NM_199360	NM_199360	Homo sapiens tumor protein D52-like 2 (TPD52L2), transcript variant 1, mRNA [NM_199360]	NM_199360
A_32_P192970	5.41E-05	13.02	NM_170726	NM_170726	Homo sapiens aldehyde dehydrogenase 4 family, member A1 (ALDH4A1), nuclear gene encoding mitochondrial protein, transcript variant P5CDhS, mRNA [NM_170726]	NM_170726
A_23_P108751	5.41E-05	36.76	NM_201555	NM_201555	Homo sapiens four and a half LIM domains 2 (FHL2), transcript variant 2, mRNA [NM_201555]	NM_201555
A_23_P140807	5.43E-05	3.31	NM_002801	NM_002801	Homo sapiens proteasome (prosome, macropain) subunit, beta type, 10 (PSMB10), mRNA [NM_002801]	NM_002801
A_24_P17719	5.45E-05	3.85	NM_015990	NM_015990	Homo sapiens kelch-like 5 (Drosophila) (KLHL5), mRNA [NM_015990]	NM_015990
A_23_P206272	5.50E-05	2.40	NM_024598	NM_024598	Homo sapiens hypothetical protein FLJ13154 (FLJ13154), mRNA [NM_024598]	NM_024598
A_23_P144357	5.54E-05	2.13	NM_172250	NM_172250	Homo sapiens methylmalonic aciduria (cobalamin deficiency) cblA type (MMAA), mRNA [NM_172250]	NM_172250
A_23_P120325	5.56E-05	3.27	NM_002254	NM_002254	Homo sapiens kinesin family member 3C (KIF3C), mRNA [NM_002254]	NM_002254
A_23_P13946	5.56E-05	10.00	NM_005475	NM_005475	Homo sapiens lymphocyte adaptor protein (LNK), mRNA [NM_005475]	NM_005475
A_24_P80204	5.59E-05	6.17	NM_005434	NM_005434	Homo sapiens BENE protein (BENE), mRNA [NM_005434]	NM_005434
A_23_P24375	5.60E-05	2.17	NM_017670	NM_017670	Homo sapiens OTU domain, ubiquitin aldehyde binding 1 (OTUB1), mRNA [NM_017670]	NM_017670
A_23_P77590	5.60E-05	2.29	NM_018052	NM_018052	Homo sapiens Vac14 homolog (S. cerevisiae) (VAC14), mRNA [NM_018052]	NM_018052
A_23_P144244	5.60E-05	3.39	THC2286151		T09533 COX17 protein - human {Homo sapiens;} , partial (98%) [THC2286151]	
A_24_P795371	5.60E-05	3.53	AK000872	AK000872	Homo sapiens cDNA FLJ10010 fis, clone HEMBA1000302. [AK000872]	XM_498625
A_23_P354187	5.61E-05	2.56	NM_144628	NM_144628	Homo sapiens TBC1 domain family, member 20 (TBC1D20), mRNA [NM_144628]	NM_144628
A_24_P322741	5.63E-05	2.84	NM_000628	NM_000628	Homo sapiens interleukin 10 receptor, beta (IL10RB), mRNA [NM_000628]	NM_000628
A_23_P314760	5.65E-05	5.38	NM_016203	NM_016203	Homo sapiens protein kinase, AMP-activated, gamma 2 non-catalytic subunit (PRKAG2), mRNA [NM_016203]	NM_016203
A_24_P77904	5.72E-05	4.42	NM_018951	NM_018951	Homo sapiens homeo box A10 (HOXA10), transcript variant 1, mRNA [NM_018951]	NM_018951
A_23_P92073	5.77E-05	3.51	NM_001003935	NM_001003935	Homo sapiens poly (ADP-ribose) polymerase family, member 3 (PARP3), transcript variant 3, mRNA [NM_001003935]	NM_001003935
A_23_P355075	5.78E-05	3.23	AK023669	AK023669	Homo sapiens cDNA FLJ13607 fis, clone PLACE1010624. [AK023669]	
A_23_P380266	5.78E-05	3.52	AK074447	AK074447	Homo sapiens cDNA FLJ23867 fis, clone LNG09729. [AK074447]	
A_23_P74391	5.78E-05	4.00	NM_014322	NM_014322	Homo sapiens opsin 3 (encephalopsin, panopsin) (OPN3), mRNA [NM_014322]	NM_014322
A_23_P168211	5.79E-05	3.51	NM_030796	NM_030796	Homo sapiens EGFR-coamplified and overexpressed protein (ECOP), mRNA [NM_030796]	NM_030796
A_24_P153803	5.79E-05	3.83	NM_020663	NM_020663	Homo sapiens ras homolog gene family, member J (RHOJ), mRNA [NM_020663]	NM_020663
A_24_P256513	5.84E-05	2.75	BC002828	BC002828	Homo sapiens angiogenic factor with G patch and FHA domains 1, mRNA (cDNA clone IMAGE:3659316), complete cds. [BC002828]	

A_23_P256297	5.84E-05	3.06	NM_015056	NM_015056	Homo sapiens KIAA0179 (KIAA0179), mRNA [NM_015056]	NM_015056
A_24_P68342	5.84E-05	3.23	NM_001845	NM_001845	Homo sapiens collagen, type IV, alpha 1 (COL4A1), mRNA [NM_001845]	NM_001845
A_32_P129540	5.84E-05	3.46	XM_495933	XM_495933	PREDICTED: Homo sapiens similar to POM121-like protein (LOC440110), mRNA [XM_495933]	XM_495933
A_23_P29769	5.84E-05	3.64	NM_015472	NM_015472	Homo sapiens WW domain containing transcription regulator 1 (WWTR1), mRNA [NM_015472]	NM_015472
A_24_P354488	5.84E-05	4.65	THC2370530		ASAL_HUMAN (Q02083) N-acylsphingosine amidohydrolase-like precursor (Acid ceramidase-like protein) (ASAH-like protein) , partial (81%) [THC2370530]	
A_23_P145485	5.84E-05	13.28	NM_025217	NM_025217	Homo sapiens UL16 binding protein 2 (ULBP2), mRNA [NM_025217]	NM_025217
A_24_P91232	5.88E-05	2.38	NM_032837	NM_032837	Homo sapiens hypothetical protein FLJ14775 (FLJ14775), mRNA [NM_032837]	NM_032837
A_24_P307184	5.91E-05	2.43	A_24_P307184			
A_23_P416034	5.92E-05	3.13	NM_207106	NM_207106	Homo sapiens 26S proteasome-associated UCH interacting protein 1 (UIP1), transcript variant 3, mRNA [NM_207106]	NM_207106
A_23_P39019	5.95E-05	2.09	NM_052850	NM_052850	Homo sapiens growth arrest and DNA-damage-inducible, gamma interacting protein 1 (GADD45GIP1), mRNA [NM_052850]	NM_052850
A_23_P134946	5.96E-05	2.56	NM_014665	NM_014665	Homo sapiens leucine rich repeat containing 14 (LRRC14), mRNA [NM_014665]	NM_014665
A_23_P87759	5.96E-05	3.00	NM_006331	NM_006331	Homo sapiens C2f protein (C2F), mRNA [NM_006331]	NM_006331
A_24_P239606	5.96E-05	7.35	NM_015675	NM_015675	Homo sapiens growth arrest and DNA-damage-inducible, beta (GADD45B), mRNA [NM_015675]	NM_015675
A_24_P402438	5.96E-05	7.69	NM_003238	NM_003238	Homo sapiens transforming growth factor, beta 2 (TGFB2), mRNA [NM_003238]	NM_003238
A_24_P244096	5.98E-05	2.20	NM_001002762	NM_001002762	Homo sapiens DnaJ (Hsp40) homolog, subfamily B, member 12 (DNAJB12), transcript variant 1, mRNA [NM_001002762]	NM_001002762
A_23_P427703	5.98E-05	5.75	X97261	X97261	H.sapiens mRNA for metallothionein isoform 1R. [X97261]	
A_23_P63026	5.99E-05	3.16	NM_006499	NM_006499	Homo sapiens lectin, galactoside-binding, soluble, 8 (galectin 8) (LGALS8), transcript variant 1, mRNA [NM_006499]	NM_006499
A_24_P332151	5.99E-05	3.38	A_24_P332151			
A_23_P154507	6.01E-05	3.37	NM_022334	NM_022334	Homo sapiens integrin beta 1 binding protein 1 (ITGB1BP1), transcript variant 2, mRNA [NM_022334]	NM_022334
A_24_P234701	6.12E-05	2.60	CR621132	CR621132	full-length cDNA clone CS0DC015YK09 of Neuroblastoma Cot 25-normalized of Homo sapiens (human). [CR621132]	
A_23_P326204	6.13E-05	4.10	NM_152621	NM_152621	Homo sapiens hypothetical protein MGC26963 (MGC26963), mRNA [NM_152621]	NM_152621
A_23_P353056	6.15E-05	2.31	NM_014807	NM_014807	Homo sapiens transmembrane protein 24 (TMEM24), mRNA [NM_014807]	NM_014807
A_24_P345336	6.15E-05	3.14	NM_002871	NM_002871	Homo sapiens RAB interacting factor (RABIF), mRNA [NM_002871]	NM_002871
A_23_P256051	6.16E-05	3.41	NM_203302	NM_203302	Homo sapiens similar to RPL23AP7 protein (MGC70863), transcript variant 2, mRNA [NM_203302]	NM_203302
A_23_P55251	6.18E-05	7.58	NM_002204	NM_002204	Homo sapiens integrin, alpha 3 (antigen CD49C, alpha 3 subunit of VLA-3 receptor) (ITGA3), transcript variant a, mRNA [NM_002204]	NM_002204
A_24_P332364	6.23E-05	2.71	A_24_P332364			
A_23_P367026	6.23E-05	3.47	NM_015600	NM_015600	Homo sapiens chromosome 20 open reading frame 22 (C20orf22), mRNA [NM_015600]	NM_015600
A_23_P23074	6.23E-05	14.86	NM_006417	NM_006417	Homo sapiens interferon-induced protein 44 (IFI44), mRNA [NM_006417]	NM_006417
A_24_P652609	6.25E-05	15.46	AK054645	AK054645	Homo sapiens cDNA FLJ30083 fis, clone BGGI12001097, weakly similar to Homo sapiens contactin associated protein (Caspr) mRNA. [AK054645]	XM_372097
A_23_P406424	6.26E-05	2.45	NM_175744	NM_175744	Homo sapiens ras homolog gene family, member C (RHOC), mRNA [NM_175744]	NM_175744
A_23_P162476	6.26E-05	2.54	NM_031299	NM_031299	Homo sapiens cell division cycle associated 3 (CDCA3), mRNA [NM_031299]	NM_031299
A_23_P344392	6.28E-05	2.58	NM_002542	NM_002542	Homo sapiens 8-oxoguanine DNA glycosylase (OGG1), nuclear gene encoding mitochondrial protein, transcript variant 1a, mRNA [NM_002542]	NM_002542
A_23_P209636	6.28E-05	4.74	NM_006449	NM_006449	Homo sapiens CDC42 effector protein (Rho GTPase binding) 3 (CDC42EP3), mRNA [NM_006449]	NM_006449
A_23_P49597	6.29E-05	2.36	NM_020360	NM_020360	Homo sapiens phospholipid scramblase 3 (PLSCR3), mRNA [NM_020360]	NM_020360
A_24_P288954	6.29E-05	2.56	NM_001013694	NM_001013694	Homo sapiens similar to SRR1-like protein (LOC402055), mRNA [NM_001013694]	NM_001013694
A_23_P110108	6.31E-05	6.29	NM_024628	NM_024628	Homo sapiens solute carrier family 12 (potassium/chloride transporters), member 8 (SLC12A8), mRNA [NM_024628]	NM_024628
A_23_P47155	6.31E-05	6.80	NM_015368	NM_015368	Homo sapiens pannexin 1 (PANX1), mRNA [NM_015368]	NM_015368
A_23_P159956	6.32E-05	3.57	NM_012216	NM_012216	Homo sapiens midline 2 (MID2), transcript variant 1, mRNA [NM_012216]	NM_012216
A_23_P256890	6.32E-05	3.70	CR611847	CR611847	full-length cDNA clone CS0DI077YC19 of Placenta Cot 25-normalized of Homo sapiens (human). [CR611847]	

A_23_P366394	6.36E-05	2.95	NM_016653	NM_016653	Homo sapiens sterile alpha motif and leucine zipper containing kinase AZK (ZAK), transcript variant 1, mRNA [NM_016653]	NM_016653
A_24_P172481	6.36E-05	14.22	NM_006074	NM_006074	Homo sapiens tripartite motif-containing 22 (TRIM22), mRNA [NM_006074]	NM_006074
A_24_P596251	6.39E-05	4.74	A_24_P596251			
A_23_P154235	6.41E-05	3.33	NM_004688	NM_004688	Homo sapiens N-myc (and STAT) interactor (NMI), mRNA [NM_004688]	NM_004688
A_23_P384024	6.47E-05	2.63	NM_178335	NM_178335	Homo sapiens chromosome 3 open reading frame 6 (C3orf6), transcript variant 2, mRNA [NM_178335]	NM_178335
A_23_P50338	6.47E-05	4.88	NM_006087	NM_006087	Homo sapiens tubulin, beta 4 (TUBB4), mRNA [NM_006087]	NM_006087
A_23_P428842	6.48E-05	2.58	NM_138399	NM_138399	Homo sapiens transmembrane protein 44 (TMEM44), transcript variant 1, mRNA [NM_138399]	NM_138399
A_32_P85433	6.53E-05	30.77	BQ130701	BQ130701	BQ130701 ij86c06.y1 Human insulinoma Homo sapiens cDNA clone IMAGE:5778418 5' similar to SW:CTGF_HUMAN P29279 CONNECTIVE TISSUE GROWTH FACTOR PRECURSOR. [1] ;, mRNA sequence [BQ130701]	
A_23_P320113	6.57E-05	3.16	NM_080725	NM_080725	Homo sapiens sulfiredoxin 1 homolog (S. cerevisiae) (SRXN1), mRNA [NM_080725]	NM_080725
A_23_P102582	6.60E-05	2.28	NM_199484	NM_199484	Homo sapiens chromosome 20 open reading frame 24 (C20orf24), transcript variant 3, mRNA [NM_199484]	NM_199484
A_23_P211627	6.60E-05	2.84	NM_153684	NM_153684	Homo sapiens nucleoporin 50kDa (NUP50), transcript variant 1, mRNA [NM_153684]	NM_153684
A_23_P250294	6.60E-05	2.90	NM_016006	NM_016006	Homo sapiens abhydrolase domain containing 5 (ABHD5), mRNA [NM_016006]	NM_016006
A_23_P127584	6.60E-05	18.05	NM_006169	NM_006169	Homo sapiens nicotinamide N-methyltransferase (NNMT), mRNA [NM_006169]	NM_006169
A_23_P79259	6.64E-05	3.95	NM_014521	NM_014521	Homo sapiens SH3-domain binding protein 4 (SH3BP4), mRNA [NM_014521]	NM_014521
A_24_P272653	6.74E-05	6.94	A_24_P272653			
A_24_P71661	6.76E-05	2.41	NM_006371	NM_006371	Homo sapiens cartilage associated protein (CRTAP), mRNA [NM_006371]	NM_006371
A_24_P126741	6.76E-05	3.25	ENST00000309178		PREDICTED: Homo sapiens similar to Glutaredoxin (Thioltransferase) (TTase) (LOC441113), mRNA [XM_496782]	XM_496782
A_32_P132748	6.76E-05	5.59	BC018626	BC018626	Homo sapiens, Similar to hect domain and RLD 2, clone IMAGE:4581928, mRNA. [BC018626]	
A_24_P185604	6.77E-05	2.66	NM_032013	NM_032013	Homo sapiens NDRG family member 3 (NDRG3), transcript variant 1, mRNA [NM_032013]	NM_032013
A_23_P25224	6.80E-05	2.24	NM_003651	NM_003651	Homo sapiens cold shock domain protein A (CSDA), mRNA [NM_003651]	NM_003651
A_23_P374767	6.80E-05	3.62	NM_015983	NM_015983	Homo sapiens ubiquitin-conjugating enzyme E2D 4 (putative) (UBE2D4), mRNA [NM_015983]	NM_015983
A_23_P319617	6.81E-05	2.37	NM_019886	NM_019886	Homo sapiens carbohydrate (N-acetylglucosamine 6-O) sulfotransferase 7 (CHST7), mRNA [NM_019886]	NM_019886
A_23_P166068	6.82E-05	2.60	NM_174856	NM_174856	Homo sapiens isocitrate dehydrogenase 3 (NAD+) beta (IDH3B), nuclear gene encoding mitochondrial protein, transcript variant 3, mRNA [NM_174856]	NM_174856
A_24_P128140	6.83E-05	2.39	NM_015476	NM_015476	Homo sapiens chromosome 18 open reading frame 10 (C18orf10), mRNA [NM_015476]	NM_015476
A_24_P211064	6.86E-05	3.46	NM_020648	NM_020648	Homo sapiens twisted gastrulation homolog 1 (Drosophila) (TWSG1), mRNA [NM_020648]	NM_020648
A_24_P187750	6.88E-05	2.67	NM_001659	NM_001659	Homo sapiens ADP-ribosylation factor 3 (ARF3), mRNA [NM_001659]	NM_001659
A_23_P92552	6.89E-05	2.04	NM_004564	NM_004564	Homo sapiens PET112-like (yeast) (PET112L), mRNA [NM_004564]	NM_004564
A_23_P164228	6.96E-05	2.27	NM_005175	NM_005175	Homo sapiens ATP synthase, H+ transporting, mitochondrial F0 complex, subunit c (subunit 9), isoform 1 (ATP5G1), nuclear gene encoding mitochondrial protein, transcript variant 1, mRNA [NM_005175]	NM_005175
A_23_P8432	6.97E-05	2.33	NM_006833	NM_006833	Homo sapiens COP9 constitutive photomorphogenic homolog subunit 6 (Arabidopsis) (COPS6), mRNA [NM_006833]	NM_006833
A_23_P207537	7.02E-05	2.38	NM_007026	NM_007026	Homo sapiens dual specificity phosphatase 14 (DUSP14), mRNA [NM_007026]	NM_007026
A_23_P359043	7.02E-05	5.18	NM_007203	NM_007203	Homo sapiens PALM2-AKAP2 protein (PALM2-AKAP2), transcript variant 1, mRNA [NM_007203]	NM_007203
A_32_P38806	7.05E-05	2.09	A_32_P38806			
A_23_P360605	7.08E-05	6.33	BC040542	BC040542	Homo sapiens KIAA0802, mRNA (cDNA clone MGC:39663 IMAGE:5268201), complete cds. [BC040542]	
A_23_P156977	7.11E-05	4.35	NM_004507	NM_004507	Homo sapiens HUS1 checkpoint homolog (S. pombe) (HUS1), mRNA [NM_004507]	NM_004507
A_24_P137434	7.12E-05	2.82	NM_080927	NM_080927	Homo sapiens discoidin, CUB and LCCL domain containing 2 (DCBLD2), mRNA [NM_080927]	NM_080927
A_23_P103631	7.14E-05	2.94	NM_006824	NM_006824	Homo sapiens EBNA1 binding protein 2 (EBNA1BP2), mRNA [NM_006824]	NM_006824
A_23_P96965	7.14E-05	3.79	NM_030786	NM_030786	Homo sapiens syncoilin, intermediate filament 1 (SYNC1), mRNA [NM_030786]	NM_030786
A_23_P214185	7.17E-05	2.16	NM_016021	NM_016021	Homo sapiens ubiquitin-conjugating enzyme E2, J1 (UBC6 homolog, yeast) (UBE2J1), mRNA [NM_016021]	NM_016021

A_23_P7896	7.20E-05	4.20	NM_020185	NM_020185	Homo sapiens dual specificity phosphatase 22 (DUSP22), mRNA [NM_020185]	NM_020185
A_23_P15285	7.21E-05	2.82	NM_024109	NM_024109	Homo sapiens hypothetical protein MGC2654 (MGC2654), mRNA [NM_024109]	NM_024109
A_24_P406132	7.21E-05	2.85	NM_002754	NM_002754	Homo sapiens mitogen-activated protein kinase 13 (MAPK13), mRNA [NM_002754]	NM_002754
A_24_P767222	7.24E-05	4.44	THC2404909			
A_23_P134454	7.24E-05	22.83	NM_001753	NM_001753	Homo sapiens caveolin 1, caveolae protein, 22kDa (CAV1), mRNA [NM_001753]	NM_001753
A_24_P846738	7.28E-05	2.05	A_24_P846738			
A_23_P109794	7.30E-05	3.06	NM_015199	NM_015199	Homo sapiens ankyrin repeat domain 28 (ANKRD28), mRNA [NM_015199]	NM_015199
A_23_P42257	7.31E-05	4.10	NM_003897	NM_003897	Homo sapiens immediate early response 3 (IER3), transcript variant short, mRNA [NM_003897]	NM_003897
A_23_P13725	7.33E-05	6.71	NM_182767	NM_182767	Homo sapiens solute carrier family 6, member 15 (SLC6A15), transcript variant 1, mRNA [NM_182767]	NM_182767
A_24_P143440	7.35E-05	2.14	NM_177953	NM_177953	Homo sapiens dynein, cytoplasmic, light polypeptide 2A (DNCL2A), transcript variant 2, mRNA [NM_177953]	NM_177953
A_23_P125348	7.35E-05	2.70	NM_021210	NM_021210	Homo sapiens trafficking protein particle complex 1 (TRAPPC1), mRNA [NM_021210]	NM_021210
A_23_P108294	7.36E-05	2.13	NM_177543	NM_177543	Homo sapiens phosphatidic acid phosphatase type 2C (PPAP2C), transcript variant 3, mRNA [NM_177543]	NM_177543
A_23_P79818	7.36E-05	2.18	NM_016470	NM_016470	Homo sapiens chromosome 20 open reading frame 111 (C20orf111), mRNA [NM_016470]	NM_016470
A_23_P360983	7.36E-05	2.66	NM_174889	NM_174889	Homo sapiens Myc-induced mitochondria protein (mimitin), mRNA [NM_174889]	NM_174889
A_24_P915784	7.36E-05	10.55	AK125207	AK125207	Homo sapiens cDNA FLJ43217 fis, clone FEBRA2022388. [AK125207]	
A_23_P71904	7.37E-05	3.03	NM_014064	NM_014064	Homo sapiens AD-003 protein (AD-003), mRNA [NM_014064]	NM_014064
A_23_P133438	7.37E-05	5.88	NM_019018	NM_019018	Homo sapiens hypothetical protein FLJ11127 (FLJ11127), mRNA [NM_019018]	NM_019018
A_32_P760762	7.41E-05	6.62	A_32_P760762			
A_24_P118341	7.44E-05	3.64	BF869497	BF869497	BF869497 IL3-ET0115-091000-286-D05 ET0115 Homo sapiens cDNA, mRNA sequence [BF869497]	
A_23_P54373	7.44E-05	4.27	NM_004580	NM_004580	Homo sapiens RAB27A, member RAS oncogene family (RAB27A), transcript variant 1, mRNA [NM_004580]	NM_004580
A_23_P151150	7.45E-05	2.11	NM_202002	NM_202002	Homo sapiens forkhead box M1 (FOXM1), transcript variant 1, mRNA [NM_202002]	NM_202002
A_32_P52911	7.45E-05	2.65	NM_018269	NM_018269	Homo sapiens membrane-type 1 matrix metalloproteinase cytoplasmic tail binding protein-1 (MTCBP-1), mRNA [NM_018269]	NM_018269
A_23_P99731	7.46E-05	2.82	A_23_P99731			
A_23_P166677	7.50E-05	2.36	NM_022736	NM_022736	Homo sapiens major facilitator superfamily domain containing 1 (MFSD1), mRNA [NM_022736]	NM_022736
A_24_P4212	7.51E-05	2.18	NM_023078	NM_023078	Homo sapiens pyrroline-5-carboxylate reductase-like (PYCRL), mRNA [NM_023078]	NM_023078
A_32_P69399	7.51E-05	3.38	BQ072652	BQ072652	AGENCOURT_6763016 NIH_MGC_118 Homo sapiens cDNA clone IMAGE:5756116 5', mRNA sequence [BQ072652]	XM_373922
A_24_P150160	7.51E-05	4.35	NM_004265	NM_004265	Homo sapiens fatty acid desaturase 2 (FADS2), mRNA [NM_004265]	NM_004265
A_24_P753638	7.51E-05	5.05	A_24_P753638			
A_23_P138567	7.53E-05	5.00	NM_032797	NM_032797	Homo sapiens apoptosis-inducing factor (AIF)-like mitochondrion-associated inducer of death (AMID), mRNA [NM_032797]	NM_032797
A_32_P44453	7.56E-05	4.85	NM_002194	NM_002194	Homo sapiens inositol polyphosphate-1-phosphatase (INPP1), mRNA [NM_002194]	NM_002194
A_24_P211151	7.58E-05	3.09	NM_020158	NM_020158	Homo sapiens exosome component 5 (EXOSC5), mRNA [NM_020158]	NM_020158
A_23_P163475	7.58E-05	4.55	NM_207380	NM_207380	Homo sapiens FLJ43339 protein (FLJ43339), mRNA [NM_207380]	NM_207380
A_23_P210920	7.62E-05	2.36	NM_000178	NM_000178	Homo sapiens glutathione synthetase (GSS), mRNA [NM_000178]	NM_000178
A_23_P345139	7.62E-05	3.83	NM_174983	NM_174983	Homo sapiens chromosome 19 open reading frame 28 (C19orf28), mRNA [NM_174983]	NM_174983
A_23_P135357	7.63E-05	2.06	NM_018146	NM_018146	Homo sapiens RNA methyltransferase like 1 (RNMTL1), mRNA [NM_018146]	NM_018146
A_23_P46604	7.63E-05	3.88	NM_020448	NM_020448	Homo sapiens NIPA-like domain containing 3 (NPAL3), mRNA [NM_020448]	NM_020448
A_23_P86682	7.65E-05	2.39	NM_013451	NM_013451	Homo sapiens fer-1-like 3, myoferlin (C. elegans) (FER1L3), transcript variant 1, mRNA [NM_013451]	NM_013451
A_23_P145024	7.65E-05	5.85	NM_000024	NM_000024	Homo sapiens adrenergic, beta-2-, receptor, surface (ADRB2), mRNA [NM_000024]	NM_000024
A_24_P944519	7.68E-05	2.64	NM_006203	NM_006203	Homo sapiens phosphodiesterase 4D, cAMP-specific (phosphodiesterase E3 dunce homolog, Drosophila) (PDE4D), mRNA [NM_006203]	NM_006203
A_32_P300427	7.71E-05	10.93	NM_153360	NM_153360	Homo sapiens hypothetical protein FLJ90166 (FLJ90166), mRNA [NM_153360]	NM_153360

A_24_P273245	7.74E-05	4.90	A_24_P273245			
A_23_P134935	7.75E-05	3.77	NM_001394	NM_001394	Homo sapiens dual specificity phosphatase 4 (DUSP4), transcript variant 1, mRNA [NM_001394]	NM_001394
A_23_P200160	7.75E-05	24.88	NM_001014975	NM_001014975	Homo sapiens complement factor H (CFH), transcript variant 2, mRNA [NM_001014975]	NM_001014975
A_23_P215265	7.76E-05	2.55	NM_007353	NM_007353	Homo sapiens guanine nucleotide binding protein (G protein) alpha 12 (GNA12), mRNA [NM_007353]	NM_007353
A_24_P332263	7.76E-05	4.10	A_24_P332263			
A_23_P61569	7.79E-05	2.35	NM_003762	NM_003762	Homo sapiens vesicle-associated membrane protein 4 (VAMP4), transcript variant 2, mRNA [NM_003762]	NM_003762
A_24_P89457	7.79E-05	6.41	NM_078467	NM_078467	Homo sapiens cyclin-dependent kinase inhibitor 1A (p21, Cip1) (CDKN1A), transcript variant 2, mRNA [NM_078467]	NM_078467
A_23_P149494	7.80E-05	3.47	NM_015600	NM_015600	Homo sapiens chromosome 20 open reading frame 22 (C20orf22), mRNA [NM_015600]	NM_015600
A_23_P15284	7.81E-05	2.51	NM_024109	NM_024109	Homo sapiens hypothetical protein MGC2654 (MGC2654), mRNA [NM_024109]	NM_024109
A_23_P23221	7.82E-05	2.47	NM_001924	NM_001924	Homo sapiens growth arrest and DNA-damage-inducible, alpha (GADD45A), mRNA [NM_001924]	NM_001924
A_23_P127467	7.83E-05	2.73	NM_032325	NM_032325	Homo sapiens hypothetical protein MGC11102 (MGC11102), mRNA [NM_032325]	NM_032325
A_24_P201702	7.83E-05	5.18	NM_005127	NM_005127	Homo sapiens C-type lectin domain family 2, member B (CLEC2B), mRNA [NM_005127]	NM_005127
A_23_P26954	7.84E-05	2.02	NM_006373	NM_006373	Homo sapiens vesicle amine transport protein 1 homolog (T californica) (VAT1), mRNA [NM_006373]	NM_006373
A_23_P162982	7.85E-05	2.29	NM_021004	NM_021004	Homo sapiens dehydrogenase/reductase (SDR family) member 4 (DHRS4), mRNA [NM_021004]	NM_021004
A_24_P829789	7.85E-05	4.78	THC2433033		PSA7_HUMAN (O14818) Proteasome subunit alpha type 7 (Proteasome subunit RC6-1) (Proteasome subunit XAPC7) , partial (22%) [THC2433033]	
A_23_P435604	7.86E-05	2.39	NM_015047	NM_015047	Homo sapiens KIAA0090 (KIAA0090), mRNA [NM_015047]	NM_015047
A_24_P600036	7.86E-05	29.59	A_24_P600036			
A_24_P165965	7.88E-05	2.99	NM_007275	NM_007275	Homo sapiens tumor suppressor candidate 2 (TUSC2), mRNA [NM_007275]	NM_007275
A_23_P76480	7.90E-05	5.32	BF213738	BF213738	BF213738 601847628F1 NIH_MGC_55 Homo sapiens cDNA clone IMAGE:4078519 5', mRNA sequence [BF213738]	
A_24_P97770	7.93E-05	5.08	NM_020462	NM_020462	Homo sapiens endoplasmic reticulum-golgi intermediate compartment 32 kDa protein (KIAA1181), mRNA [NM_020462]	NM_020462
A_24_P187954	7.94E-05	2.30	NM_213654	NM_213654	Homo sapiens armadillo repeat containing 8 (ARMC8), mRNA [NM_213654]	NM_213654
A_23_P78383	8.10E-05	2.78	NM_013326	NM_013326	Homo sapiens chromosome 18 open reading frame 8 (C18orf8), mRNA [NM_013326]	NM_013326
A_24_P250176	8.13E-05	2.78	NM_170607	NM_170607	Homo sapiens MAX-like protein X (MLX), transcript variant 3, mRNA [NM_170607]	NM_170607
A_23_P873	8.13E-05	5.18	NM_004848	NM_004848	Homo sapiens chromosome 1 open reading frame 38 (C1orf38), mRNA [NM_004848]	NM_004848
A_23_P65674	8.16E-05	3.77	NM_014547	NM_014547	Homo sapiens tropomodulin 3 (ubiquitous) (TMOD3), mRNA [NM_014547]	NM_014547
A_23_P28733	8.28E-05	3.68	NM_002895	NM_002895	Homo sapiens retinoblastoma-like 1 (p107) (RBL1), transcript variant 1, mRNA [NM_002895]	NM_002895
A_24_P349783	8.30E-05	2.08	A_24_P349783			
A_23_P87150	8.30E-05	14.04	NM_004811	NM_004811	Homo sapiens leupaxin (LPXN), mRNA [NM_004811]	NM_004811
A_24_P484797	8.32E-05	2.21	AF279614	AF279614	Homo sapiens cell death-inducing CIDE-like effector (CICE) pseudogene, mRNA sequence. [AF279614]	XR_000206
A_24_P377499	8.32E-05	5.88	NM_145323	NM_145323	Homo sapiens oxysterol binding protein-like 3 (OSBPL3), transcript variant 5, mRNA [NM_145323]	NM_145323
A_24_P937405	8.34E-05	11.60	NM_007173	NM_007173	Homo sapiens protease, serine, 23 (PRSS23), mRNA [NM_007173]	NM_007173
A_24_P913016	8.36E-05	2.43	BC004338	BC004338	Homo sapiens, clone IMAGE:3631945, mRNA, partial cds. [BC004338]	
A_23_P331908	8.38E-05	2.72	AK097878	AK097878	Homo sapiens cDNA FLJ40559 fis, clone THYMU2002910. [AK097878]	
A_23_P28450	8.40E-05	2.38	NM_015425	NM_015425	Homo sapiens polymerase (RNA) I polypeptide A, 194kDa (POLR1A), mRNA [NM_015425]	NM_015425
A_23_P35989	8.40E-05	3.72	NM_139178	NM_139178	Homo sapiens prostate cancer antigen-1 (DEPC-1), mRNA [NM_139178]	NM_139178
A_24_P147461	8.40E-05	11.95	NM_198833	NM_198833	Homo sapiens serine (or cysteine) proteinase inhibitor, clade B (ovalbumin), member 8 (SERPINB8), transcript variant 2, mRNA [NM_198833]	NM_198833
A_23_P120594	8.44E-05	3.31	NM_032501	NM_032501	Homo sapiens acetyl-Coenzyme A synthetase 2 (AMP forming)-like (ACAS2L), nuclear gene encoding mitochondrial protein, mRNA [NM_032501]	NM_032501
A_24_P315754	8.45E-05	2.40	ENST00000343233			
A_23_P46852	8.45E-05	2.51	NM_024928	NM_024928	Homo sapiens oligonucleotide/oligosaccharide-binding fold containing 1 (OBFC1), mRNA [NM_024928]	NM_024928
A_23_P201636	8.45E-05	4.98	NM_005562	NM_005562	Homo sapiens laminin, gamma 2 (LAMC2), transcript variant 1, mRNA [NM_005562]	NM_005562

A_24_P931443	8.45E-05	8.13	BC098567	BC098567	Homo sapiens G protein-coupled receptor 68, mRNA (cDNA clone MGC:111379 IMAGE:30925589), complete cds [BC098567]	
A_24_P7510	8.48E-05	2.36	A_24_P7510			
A_24_P218757	8.48E-05	4.35	NM_018172	NM_018172	Homo sapiens hypothetical protein FLJ10661 (FLJ10661), transcript variant 1, mRNA [NM_018172]	NM_018172
A_23_P61222	8.52E-05	2.48	AJ002425	AJ002425	Homo sapiens mRNA for p65 protein. [AJ002425]	
A_32_P217261	8.55E-05	2.06	AK023660	AK023660	Homo sapiens cDNA FLJ13598 fis, clone PLACE1009921. [AK023660]	
A_24_P74932	8.55E-05	5.95	NM_002668	NM_002668	Homo sapiens proteolipid protein 2 (colonic epithelium-enriched) (PLP2), mRNA [NM_002668]	NM_002668
A_23_P41854	8.55E-05	9.26	NM_032587	NM_032587	Homo sapiens caspase recruitment domain family, member 6 (CARD6), mRNA [NM_032587]	NM_032587
A_23_P218463	8.57E-05	2.02	NM_013376	NM_013376	Homo sapiens SERTA domain containing 1 (SERTAD1), mRNA [NM_013376]	NM_013376
A_24_P222126	8.59E-05	2.92	NM_016397	NM_016397	Homo sapiens TH1-like (Drosophila) (TH1L), transcript variant 2, mRNA [NM_016397]	NM_016397
A_24_P795230	8.68E-05	3.06	AK027541	AK027541	Homo sapiens cDNA FLJ14635 fis, clone NT2RP2001196. [AK027541]	
A_24_P65910	8.68E-05	5.32	NM_018244	NM_018244	Homo sapiens chromosome 20 open reading frame 44 (C20orf44), transcript variant 1, mRNA [NM_018244]	NM_018244
A_24_P936605	8.69E-05	3.66	U87972	U87972	Human NAD+-isocitrate dehydrogenase mRNA, partial cds. [U87972]	
A_24_P124032	8.74E-05	2.15	NM_003821	NM_003821	Homo sapiens receptor-interacting serine-threonine kinase 2 (RIPK2), mRNA [NM_003821]	NM_003821
A_24_P65864	8.74E-05	2.63	NM_016143	NM_016143	Homo sapiens NSFL1 (p97) cofactor (p47) (NSFL1C), transcript variant 1, mRNA [NM_016143]	NM_016143
A_23_P133656	8.74E-05	2.72	NM_002290	NM_002290	Homo sapiens laminin, alpha 4 (LAMA4), mRNA [NM_002290]	NM_002290
A_23_P203364	8.74E-05	3.01	NM_024092	NM_024092	Homo sapiens hypothetical protein MGC5508 (MGC5508), mRNA [NM_024092]	NM_024092
A_23_P132560	8.74E-05	3.22	NM_145037	NM_145037	Homo sapiens family with sequence similarity 55, member C (FAM55C), mRNA [NM_145037]	NM_145037
A_24_P341019	8.74E-05	3.36	NM_001009924	NM_001009924	Homo sapiens chromosome 20 open reading frame 30 (C20orf30), transcript variant 2, mRNA [NM_001009924]	NM_001009924
A_23_P90601	8.74E-05	6.10	NM_182915	NM_182915	Homo sapiens STEAP family member 3 (STEAP3), transcript variant 1, mRNA [NM_182915]	NM_182915
A_23_P118203	8.91E-05	5.21	NM_145252	NM_145252	Homo sapiens similar to common salivary protein 1 (LOC124220), mRNA [NM_145252]	NM_145252
A_23_P150009	8.98E-05	2.12	NM_198046	NM_198046	Homo sapiens zinc finger, DHHC-type containing 16 (ZDHHC16), transcript variant 5, mRNA [NM_198046]	NM_198046
A_24_P415208	8.99E-05	2.82	BC060806	BC060806	Homo sapiens cDNA clone MGC:71628 IMAGE:30336414, complete cds. [BC060806]	
A_23_P49924	9.08E-05	2.16	NM_052935	NM_052935	Homo sapiens hypothetical protein MGC20781 (MGC20781), mRNA [NM_052935]	NM_052935
A_24_P361006	9.08E-05	2.65	NM_005002	NM_005002	Homo sapiens NADH dehydrogenase (ubiquinone) 1 alpha subcomplex, 9, 39kDa (NDUFA9), mRNA [NM_005002]	NM_005002
A_23_P84063	9.16E-05	6.67	NM_016522	NM_016522	Homo sapiens neurotrimin (HNT), mRNA [NM_016522]	NM_016522
A_24_P810074	9.16E-05	7.14	A_24_P810074			
A_23_P161194	9.18E-05	3.57	NM_003380	NM_003380	Homo sapiens vimentin (VIM), mRNA [NM_003380]	NM_003380
A_24_P290856	9.22E-05	2.99	NM_130853	NM_130853	Homo sapiens protein tyrosine phosphatase, receptor type, S (PTPRS), transcript variant 3, mRNA [NM_130853]	NM_130853
A_23_P52402	9.22E-05	3.60	NM_014889	NM_014889	Homo sapiens pitrilysin metalloproteinase 1 (PITRM1), mRNA [NM_014889]	NM_014889
A_23_P2271	9.22E-05	6.13	NM_198965	NM_198965	Homo sapiens parathyroid hormone-like hormone (PTHLH), transcript variant 1, mRNA [NM_198965]	NM_198965
A_24_P205316	9.24E-05	2.48	AK074041	AK074041	Homo sapiens mRNA for FLJ00095 protein. [AK074041]	
A_24_P164838	9.25E-05	2.21	NM_006038	NM_006038	Homo sapiens spermatogenesis associated 2 (SPATA2), mRNA [NM_006038]	NM_006038
A_23_P208310	9.27E-05	2.38	NM_012099	NM_012099	Homo sapiens CD3E antigen, epsilon polypeptide associated protein (CD3EAP), mRNA [NM_012099]	NM_012099
A_23_P117694	9.27E-05	3.57	NM_006091	NM_006091	Homo sapiens coronin, actin binding protein, 2B (CORO2B), mRNA [NM_006091]	NM_006091
A_32_P148345	9.27E-05	9.26	NM_001002857	NM_001002857	Homo sapiens annexin A2 (ANXA2), transcript variant 2, mRNA [NM_001002857]	NM_001002857
A_32_P99171	9.37E-05	3.21	AF131762	AF131762	Homo sapiens clone 25218 mRNA sequence. [AF131762]	
A_23_P75978	9.37E-05	3.70	NM_030813	NM_030813	Homo sapiens suppressor of potassium transport defect 3 (SKD3), mRNA [NM_030813]	NM_030813
A_23_P252413	9.37E-05	5.92	ENST00000245185		Homo sapiens metallothionein 2A, mRNA (cDNA clone MGC:12397 IMAGE:4051220), complete cds. [BC007034]	
A_23_P122947	9.38E-05	2.49	NM_015060	NM_015060	Homo sapiens KIAA0241 protein (KIAA0241), mRNA [NM_015060]	NM_015060
A_24_P322354	9.44E-05	2.09	NM_145060	NM_145060	Homo sapiens chromosome 18 open reading frame 24 (C18orf24), mRNA [NM_145060]	NM_145060
A_24_P46417	9.44E-05	2.71	NM_005990	NM_005990	Homo sapiens serine/threonine kinase 10 (STK10), mRNA [NM_005990]	NM_005990
A_24_P935881	9.45E-05	2.11	NM_022978	NM_022978	Homo sapiens small EDRK-rich factor 1B (centromeric) (SERF1B), mRNA [NM_022978]	NM_022978

A_23_P160240	9.54E-05	3.18	NM_016361	NM_016361	Homo sapiens acid phosphatase 6, lysophosphatidic (ACP6), mRNA [NM_016361]	NM_016361
A_24_P639629	9.57E-05	3.18	AK056245	AK056245	Homo sapiens cDNA FLJ31683 fis, clone NT2RI2005353. [AK056245]	
A_23_P356598	9.63E-05	3.62	NM_018057	NM_018057	Homo sapiens solute carrier family 6, member 15 (SLC6A15), transcript variant 2, mRNA [NM_018057]	NM_018057
A_23_P101707	9.64E-05	2.10	NM_005817	NM_005817	Homo sapiens mannose-6-phosphate receptor binding protein 1 (M6PRBP1), mRNA [NM_005817]	NM_005817
A_23_P71526	9.67E-05	3.79	NM_018482	NM_018482	Homo sapiens development and differentiation enhancing factor 1 (DDEF1), mRNA [NM_018482]	NM_018482
A_32_P30693	9.68E-05	3.31	NM_003707	NM_003707	Homo sapiens RuvB-like 1 (E. coli) (RUVBL1), mRNA [NM_003707]	NM_003707
A_23_P406187	9.73E-05	2.99	NM_153006	NM_153006	Homo sapiens N-acetylglutamate synthase (NAGS), mRNA [NM_153006]	NM_153006
A_32_P7193	9.76E-05	4.63	A_32_P7193			
A_23_P258234	9.79E-05	8.62	AK098194	AK098194	Homo sapiens cDNA FLJ40875 fis, clone UMVEN2000069. [AK098194]	
A_23_P337753	9.80E-05	4.22	NM_138402	NM_138402	Homo sapiens hypothetical protein BC004921 (LOC93349), mRNA [NM_138402]	NM_138402
A_23_P51646	9.80E-05	5.81	NM_004073	NM_004073	Homo sapiens polo-like kinase 3 (Drosophila) (PLK3), mRNA [NM_004073]	NM_004073
A_24_P530977	9.80E-05	5.88	A_24_P530977			
A_24_P27644	9.91E-05	2.77	THC2364223		Q28610 (Q28610) Oryctolagus cuniculus Na+/glucose cotransporter-related protein, partial (22%) [THC2364223]	
A_23_P205449	0.0001	2.28	NM_017955	NM_017955	Homo sapiens cell division cycle associated 4 (CDCA4), transcript variant 1, mRNA [NM_017955]	NM_017955
A_24_P227831	0.0001	2.82	NM_019862	NM_019862	Homo sapiens ATP-binding cassette, sub-family C (CFTR/MRP), member 1 (ABCC1), transcript variant 2, mRNA [NM_019862]	NM_019862
A_23_P92140	0.0001	4.35	NM_002468	NM_002468	Homo sapiens myeloid differentiation primary response gene (88) (MYD88), mRNA [NM_002468]	NM_002468
A_24_P803809	0.0001	4.90	A_24_P803809			
A_24_P784765	1.00E-04	11.01	NM_203330	NM_203330	Homo sapiens CD59 antigen p18-20 (antigen identified by monoclonal antibodies 16.3A5, EJ16, EJ30, EL32 and G344) (CD59), transcript variant 1, mRNA [NM_203330]	NM_203330
A_23_P161041	0.000101	2.53	NM_021633	NM_021633	Homo sapiens kelch-like 12 (Drosophila) (KLHL12), mRNA [NM_021633]	NM_021633
A_24_P316939	0.000101	2.74	BC010662	BC010662	Homo sapiens cDNA clone IMAGE:3855382, complete cds. [BC010662]	
A_23_P100469	0.000101	3.05	NM_017853	NM_017853	Homo sapiens thioredoxin-like 4B (TXNL4B), mRNA [NM_017853]	NM_017853
A_23_P112275	0.000101	14.16	NM_005458	NM_005458	Homo sapiens G protein-coupled receptor 51 (GPR51), mRNA [NM_005458]	NM_005458
A_23_P117654	0.000103	2.79	NM_000259	NM_000259	Homo sapiens myosin VA (heavy polypeptide 12, myoxin) (MYO5A), mRNA [NM_000259]	NM_000259
A_23_P351667	0.000103	4.72	NM_003812	NM_003812	Homo sapiens a disintegrin and metalloproteinase domain 23 (ADAM23), mRNA [NM_003812]	NM_003812
A_23_P83624	0.000103	5.99	NM_021149	NM_021149	Homo sapiens coactosin-like 1 (Dictyostelium) (COTL1), mRNA [NM_021149]	NM_021149
A_23_P118536	0.000103	6.41	NM_018042	NM_018042	Homo sapiens hypothetical protein FLJ10260 (FLJ10260), mRNA [NM_018042]	NM_018042
A_23_P334955	0.000103	7.87	NM_053279	NM_053279	Homo sapiens chromosome 8 open reading frame 13 (C8orf13), mRNA [NM_053279]	NM_053279
A_23_P63190	0.000104	2.42	NM_002524	NM_002524	Homo sapiens neuroblastoma RAS viral (v-ras) oncogene homolog (NRAS), mRNA [NM_002524]	NM_002524
A_23_P91151	0.000104	2.49	NM_003038	NM_003038	Homo sapiens solute carrier family 1 (glutamate/neutral amino acid transporter), member 4 (SLC1A4), mRNA [NM_003038]	NM_003038
A_32_P172848	0.000104	2.71	NM_000167	NM_000167	Homo sapiens glycerol kinase (GK), transcript variant 2, mRNA [NM_000167]	NM_000167
A_23_P150931	0.000105	2.02	NM_018113	NM_018113	Homo sapiens limb region 1 homolog (mouse)-like (LMBR1L), mRNA [NM_018113]	NM_018113
A_24_P48791	0.000105	2.86	BX648379	BX648379	Homo sapiens mRNA; cDNA DKFZp686L18198 (from clone DKFZp686L18198). [BX648379]	
A_24_P925422	0.000105	9.01	THC2308587		1AK7 Destrin, Nmr, 20 Structures. {Sus scrofa;} , partial (37%) [THC2308587]	
A_32_P17525	0.000105	11.09	THC2438621			
A_23_P132619	0.000105	16.75	NM_000916	NM_000916	Homo sapiens oxytocin receptor (OXTR), mRNA [NM_000916]	NM_000916
A_23_P9458	0.000106	2.13	NM_022490	NM_022490	Homo sapiens polymerase (RNA) I associated factor 1 (PRAF1), mRNA [NM_022490]	NM_022490
A_23_P25336	0.000106	3.42	NM_016433	NM_016433	Homo sapiens glycolipid transfer protein (GLTP), mRNA [NM_016433]	NM_016433
A_24_P385739	0.000106	6.33	NM_020390	NM_020390	Homo sapiens eukaryotic translation initiation factor 5A2 (EIF5A2), mRNA [NM_020390]	NM_020390
A_24_P305541	0.000106	7.41	NM_021158	NM_021158	Homo sapiens tribbles homolog 3 (Drosophila) (TRIB3), mRNA [NM_021158]	NM_021158
A_23_P88904	0.000107	2.13	NM_002528	NM_002528	Homo sapiens nth endonuclease III-like 1 (E. coli) (NTHL1), mRNA [NM_002528]	NM_002528
A_23_P160025	0.000107	2.27	NM_005531	NM_005531	Homo sapiens interferon, gamma-inducible protein 16 (IFI16), mRNA [NM_005531]	NM_005531

A_23_P339053	0.000107	4.37	NM_173501	NM_173501	Homo sapiens chromosome 16 open reading frame 52 (C16orf52), mRNA [NM_173501]	NM_173501
A_23_P93677	0.000108	2.03	NM_017994	NM_017994	Homo sapiens hypothetical protein FLJ10099 (FLJ10099), mRNA [NM_017994]	NM_017994
A_23_P434890	0.000108	2.29	NM_014550	NM_014550	Homo sapiens caspase recruitment domain family, member 10 (CARD10), mRNA [NM_014550]	NM_014550
A_23_P164427	0.000109	2.92	NM_024052	NM_024052	Homo sapiens chromosome 17 open reading frame 39 (C17orf39), mRNA [NM_024052]	NM_024052
A_23_P354387	0.000109	3.04	NM_013451	NM_013451	Homo sapiens fer-1-like 3, myoferlin (C. elegans) (FER1L3), transcript variant 1, mRNA [NM_013451]	NM_013451
A_23_P153320	0.000109	6.67	NM_000201	NM_000201	Homo sapiens intercellular adhesion molecule 1 (CD54), human rhinovirus receptor (ICAM1), mRNA [NM_000201]	NM_000201
A_23_P384517	0.00011	2.67	NM_004130	NM_004130	Homo sapiens glycogenin (GYG), mRNA [NM_004130]	NM_004130
A_32_P39394	0.00011	4.46	AB209404	AB209404	Homo sapiens mRNA for GLIS family zinc finger 3 variant protein. [AB209404]	
A_23_P106844	0.00011	6.13	NM_005953	NM_005953	Homo sapiens metallothionein 2A (MT2A), mRNA [NM_005953]	NM_005953
A_32_P169785	0.000111	2.10	A_32_P169785			
A_24_P937169	0.000111	2.11	BG944303	BG944303	BG944303 ax48h12.x2 Hembase; Erythroid Progenitor Cells (LCB:ax library) Homo sapiens cDNA clone ax48h12 random, mRNA sequence [BG944303]	
A_23_P370005	0.000111	2.18	NM_003804	NM_003804	Homo sapiens receptor (TNFRSF)-interacting serine-threonine kinase 1 (RIPK1), mRNA [NM_003804]	NM_003804
A_24_P124370	0.000111	2.58	NM_018222	NM_018222	Homo sapiens parvin, alpha (PARVA), mRNA [NM_018222]	NM_018222
A_32_P99533	0.000111	2.87	AI423557	AI423557	AI423557 tf73d12.x1 NCI_CGAP_Brn23 Homo sapiens cDNA clone IMAGE:2104919 3' similar to gb:M13755 INTERFERON-INDUCED 17 KD PROTEIN (HUMAN);contains TAR1.t3 TAR1 repetitive element ;, mRNA sequence [AI423557]	
A_24_P409250	0.000111	3.11	ENST00000308436		GB|AK074061.1|BAB84887.1 FLJ00132 protein [Homo sapiens] [NP434181]	
A_24_P106145	0.000111	3.17	NM_017491	NM_017491	Homo sapiens WD repeat domain 1 (WDR1), transcript variant 1, mRNA [NM_017491]	NM_017491
A_24_P253215	0.000111	3.47	NM_006331	NM_006331	Homo sapiens C2f protein (C2F), mRNA [NM_006331]	NM_006331
A_24_P808838	0.000111	3.62	A_24_P808838			
A_23_P68529	0.000111	3.65	NM_033453	NM_033453	Homo sapiens inosine triphosphatase (nucleoside triphosphate pyrophosphatase) (ITPA), transcript variant 1, mRNA [NM_033453]	NM_033453
A_23_P160559	0.000111	3.82	NM_004425	NM_004425	Homo sapiens extracellular matrix protein 1 (ECM1), transcript variant 1, mRNA [NM_004425]	NM_004425
A_23_P83175	0.000111	5.26	NM_001010915	NM_001010915	Homo sapiens similar to RIKEN 4933428I03 (LOC401494), mRNA [NM_001010915]	NM_001010915
A_24_P52801	0.000112	3.29	NM_017670	NM_017670	Homo sapiens OTU domain, ubiquitin aldehyde binding 1 (OTUB1), mRNA [NM_017670]	NM_017670
A_24_P36745	0.000113	2.19	NM_144970	NM_144970	Homo sapiens chromosome X open reading frame 38 (CXorf38), mRNA [NM_144970]	NM_144970
A_23_P345460	0.000113	2.70	NM_015432	NM_015432	Homo sapiens pleckstrin homology domain containing, family G (with RhoGef domain) member 4 (PLEKHG4), mRNA [NM_015432]	NM_015432
A_23_P216610	0.000113	2.99	NM_022486	NM_022486	Homo sapiens sushi domain containing 1 (SUSD1), mRNA [NM_022486]	NM_022486
A_23_P144796	0.000114	2.13	NM_003687	NM_003687	Homo sapiens PDZ and LIM domain 4 (PDLIM4), mRNA [NM_003687]	NM_003687
A_24_P942068	0.000114	2.62	ENST00000313631		Homo sapiens mRNA for KIAA1636 protein, partial cds. [AB046856]	XM_371074
A_32_P109653	0.000114	3.37	THC2377128			
A_23_P330895	0.000115	2.44	NM_001001716	NM_001001716	Homo sapiens nuclear factor of kappa light polypeptide gene enhancer in B-cells inhibitor, beta (NFKBIB), transcript variant 2, mRNA [NM_001001716]	NM_001001716
A_32_P59302	0.000115	2.51	BC080552	BC080552	Homo sapiens cDNA clone IMAGE:6254031. [BC080552]	
A_24_P280868	0.000115	3.00	NM_032916	NM_032916	Homo sapiens hypothetical protein MGC16279 (MGC16279), mRNA [NM_032916]	NM_032916
A_23_P211997	0.000115	4.31	NM_032970	NM_032970	Homo sapiens SEC22 vesicle trafficking protein-like 3 (S. cerevisiae) (SEC22L3), transcript variant 1, mRNA [NM_032970]	NM_032970
A_24_P277576	0.000115	4.83	NM_004237	NM_004237	Homo sapiens thyroid hormone receptor interactor 13 (TRIP13), mRNA [NM_004237]	NM_004237
A_23_P84576	0.000115	6.10	NM_053034	NM_053034	Homo sapiens anthrax toxin receptor 1 (ANTXR1), transcript variant 2, mRNA [NM_053034]	NM_053034
A_32_P28309	0.000116	2.22	AI916036	AI916036	AI916036 wi44c02.x1 NCI_CGAP_Co16 Homo sapiens cDNA clone IMAGE:2393090 3' similar to TR:O75300 O75300 ES/130. ;, mRNA sequence [AI916036]	
A_23_P130182	0.000116	2.36	NM_004217	NM_004217	Homo sapiens aurora kinase B (AURKB), mRNA [NM_004217]	NM_004217
A_24_P251381	0.000116	2.69	NM_024782	NM_024782	Homo sapiens hypothetical protein FLJ12610 (FLJ12610), mRNA [NM_024782]	NM_024782
A_32_P141724	0.000116	2.71	NM_053041	NM_053041	Homo sapiens COMM domain containing 7 (COMMD7), mRNA [NM_053041]	NM_053041

A_23_P33720	0.000116	3.05	NM_006567	NM_006567	Homo sapiens phenylalanine-tRNA synthetase 2 (mitochondrial) (FARS2), nuclear gene encoding mitochondrial protein, mRNA [NM_006567]	NM_006567
A_23_P102071	0.000116	3.37	AK027315	AK027315	Homo sapiens cDNA FLJ14409 fis, clone HEMBA1004408, moderately similar to PEPTIDYL-PROLYL CIS-TRANS ISOMERASE 10 (EC 5.2.1.8). [AK027315]	
A_24_P65803	0.000116	3.65	NM_018270	NM_018270	Homo sapiens chromosome 20 open reading frame 20 (C20orf20), mRNA [NM_018270]	NM_018270
A_24_P131522	0.000117	2.25	NM_032208	NM_032208	Homo sapiens anthrax toxin receptor 1 (ANTXR1), transcript variant 1, mRNA [NM_032208]	NM_032208
A_23_P9465	0.000117	2.26	NM_004957	NM_004957	Homo sapiens folylpolyglutamate synthase (FPGS), nuclear gene encoding mitochondrial protein, transcript variant 1, mRNA [NM_004957]	NM_004957
A_23_P1602	0.000117	2.43	NM_006779	NM_006779	Homo sapiens CDC42 effector protein (Rho GTPase binding) 2 (CDC42EP2), mRNA [NM_006779]	NM_006779
A_24_P60441	0.000117	2.53	NM_153717	NM_153717	Homo sapiens Ellis van Creveld syndrome (EVC), transcript variant 2, mRNA [NM_153717]	NM_153717
A_23_P311616	0.000117	2.66	NM_015167	NM_015167	Homo sapiens phosphatidylserine receptor (PTDSR), mRNA [NM_015167]	NM_015167
A_24_P297078	0.000117	3.92	NM_020531	NM_020531	Homo sapiens chromosome 20 open reading frame 3 (C20orf3), mRNA [NM_020531]	NM_020531
A_23_P374862	0.000117	4.07	NM_000574	NM_000574	Homo sapiens decay accelerating factor for complement (CD55, Cromer blood group system) (DAF), mRNA [NM_000574]	NM_000574
A_24_P13533	0.000117	4.10	NM_203467	NM_203467	Homo sapiens peptidylprolyl isomerase (cyclophilin)-like 5 (PPIL5), transcript variant 3, mRNA [NM_203467]	NM_203467
A_24_P316005	0.000117	5.49	NM_014857	NM_014857	Homo sapiens RAB GTPase activating protein 1-like (RABGAP1L), mRNA [NM_014857]	NM_014857
A_23_P138693	0.000118	2.09	NM_004808	NM_004808	Homo sapiens N-myristoyltransferase 2 (NMT2), mRNA [NM_004808]	NM_004808
A_23_P431381	0.000118	2.38	NM_173608	NM_173608	Homo sapiens chromosome 14 open reading frame 80 (C14orf80), mRNA [NM_173608]	NM_173608
A_32_P182511	0.000118	2.55	THC2281182			
A_23_P165657	0.000118	2.69	NM_005415	NM_005415	Homo sapiens solute carrier family 20 (phosphate transporter), member 1 (SLC20A1), mRNA [NM_005415]	NM_005415
A_23_P338325	0.000118	3.18	NM_005230	NM_005230	Homo sapiens ELK3, ETS-domain protein (SRF accessory protein 2) (ELK3), mRNA [NM_005230]	NM_005230
A_32_P226338	0.000118	3.25	A_32_P226338			
A_23_P35256	0.000119	2.03	NM_006468	NM_006468	Homo sapiens polymerase (RNA) III (DNA directed) polypeptide C (62kD) (POLR3C), mRNA [NM_006468]	NM_006468
A_23_P102517	0.000119	2.10	NM_002601	NM_002601	Homo sapiens phosphodiesterase 6D, cGMP-specific, rod, delta (PDE6D), mRNA [NM_002601]	NM_002601
A_23_P251186	0.000119	2.87	NM_007198	NM_007198	Homo sapiens proline synthetase co-transcribed homolog (bacterial) (PROSC), mRNA [NM_007198]	NM_007198
A_23_P19004	0.000119	2.99	NM_023924	NM_023924	Homo sapiens bromodomain containing 9 (BRD9), transcript variant 1, mRNA [NM_023924]	NM_023924
A_23_P53588	0.000119	3.61	NM_030775	NM_030775	Homo sapiens wingless-type MMTV integration site family, member 5B (WNT5B), transcript variant 2, mRNA [NM_030775]	NM_030775
A_24_P323395	0.000119	12.45	NM_016441	NM_016441	Homo sapiens cysteine rich transmembrane BMP regulator 1 (chordin-like) (CRIM1), mRNA [NM_016441]	NM_016441
A_24_P110471	0.00012	2.25	THC2276423		Q6IFN5 (Q6IFN5) Olfactory receptor OR19-14, partial (67%) [THC2276423]	
A_23_P435610	0.00012	2.51	NM_015047	NM_015047	Homo sapiens KIAA0090 (KIAA0090), mRNA [NM_015047]	NM_015047
A_23_P28811	0.00012	2.90	NM_014484	NM_014484	Homo sapiens molybdenum cofactor synthesis 3 (MOCS3), mRNA [NM_014484]	NM_014484
A_24_P290013	0.00012	4.74	ENST00000338711		ALU7_HUMAN (P39194) Alu subfamily SQ sequence contamination warning entry, partial (14%) [THC2432443]	
A_24_P12626	0.00012	30.58	NM_001753	NM_001753	Homo sapiens caveolin 1, caveolae protein, 22kDa (CAV1), mRNA [NM_001753]	NM_001753
A_23_P43557	0.000121	2.00	NM_024820	NM_024820	Homo sapiens KIAA1608 (KIAA1608), transcript variant 2, mRNA [NM_024820]	NM_024820
A_24_P98613	0.000121	2.16	NM_030927	NM_030927	Homo sapiens tetraspanin 14 (TSPAN14), mRNA [NM_030927]	NM_030927
A_23_P352022	0.000121	2.20	ENST00000315255		Human PVT-IGLC fusion protein mRNA, 5' end. [M34432]	XM_372058
A_24_P810828	0.000121	2.66	BC044637	BC044637	Homo sapiens hypothetical protein LOC348926, mRNA (cDNA clone IMAGE:4546485), partial cds. [BC044637]	
A_23_P160354	0.000121	2.68	NM_181690	NM_181690	Homo sapiens v-akt murine thymoma viral oncogene homolog 3 (protein kinase B, gamma) (AKT3), transcript variant 2, mRNA [NM_181690]	NM_181690
A_23_P255869	0.000121	3.13	NM_018465	NM_018465	Homo sapiens chromosome 9 open reading frame 46 (C9orf46), mRNA [NM_018465]	NM_018465
A_23_P418031	0.000121	3.45	ENST00000304963		Homo sapiens mRNA for FLJ00074 protein, partial cds. [AK024480]	
A_23_P217755	0.000121	3.55	NM_001649	NM_001649	Homo sapiens apical protein-like (Xenopus laevis) (APXL), mRNA [NM_001649]	NM_001649
A_23_P323783	0.000121	3.72	NM_144703	NM_144703	Homo sapiens family with sequence similarity 61, member B (FAM61B), mRNA [NM_144703]	NM_144703
A_23_P218144	0.000121	11.57	NM_000428	NM_000428	Homo sapiens latent transforming growth factor beta binding protein 2 (LTBP2), mRNA [NM_000428]	NM_000428

A_23_P144896	0.000122	3.37	NM_203353	NM_203353	Homo sapiens PDZ and LIM domain 7 (enigma) (PDLIM7), transcript variant 3, mRNA [NM_203353]	NM_203353
A_32_P4199	0.000122	3.55	NM_173557	NM_173557	Homo sapiens ring finger protein 152 (RNF152), mRNA [NM_173557]	NM_173557
A_24_P122050	0.000122	3.68	NM_006814	NM_006814	Homo sapiens proteasome (prosome, macropain) inhibitor subunit 1 (PI31) (PSMF1), transcript variant 1, mRNA [NM_006814]	NM_006814
A_23_P215566	0.000122	4.59	NM_001621	NM_001621	Homo sapiens aryl hydrocarbon receptor (AHR), mRNA [NM_001621]	NM_001621
A_32_P93144	0.000123	2.33	XM_372108	XM_372108	PREDICTED: Homo sapiens similar to Ribosome biogenesis protein BMS1 homolog (LOC389748), mRNA [XM_372108]	XM_372108
A_24_P17909	0.000123	2.35	NM_015950	NM_015950	Homo sapiens mitochondrial ribosomal protein L2 (MRPL2), nuclear gene encoding mitochondrial protein, mRNA [NM_015950]	NM_015950
A_23_P211207	0.000123	2.42	NM_015833	NM_015833	Homo sapiens adenosine deaminase, RNA-specific, B1 (RED1 homolog rat) (ADARB1), transcript variant DRABA2b, mRNA [NM_015833]	NM_015833
A_23_P61426	0.000123	2.61	NM_012331	NM_012331	Homo sapiens methionine sulfoxide reductase A (MSRA), mRNA [NM_012331]	NM_012331
A_23_P383385	0.000123	2.99	NM_019896	NM_019896	Homo sapiens polymerase (DNA-directed), epsilon 4 (p12 subunit) (POLE4), mRNA [NM_019896]	NM_019896
A_23_P384044	0.000123	3.25	NM_152495	NM_152495	Homo sapiens cornichon homolog 3 (Drosophila) (CNIH3), mRNA [NM_152495]	NM_152495
A_23_P47682	0.000123	8.77	NM_020645	NM_020645	Homo sapiens nuclear receptor interacting protein 3 (NRIP3), mRNA [NM_020645]	NM_020645
A_24_P399630	0.000124	2.39	NM_002730	NM_002730	Homo sapiens protein kinase, cAMP-dependent, catalytic, alpha (PRKACA), transcript variant 1, mRNA [NM_002730]	NM_002730
A_23_P64404	0.000124	3.95	NM_021727	NM_021727	Homo sapiens fatty acid desaturase 3 (FADS3), mRNA [NM_021727]	NM_021727
A_24_P915227	0.000124	5.99	AY129015	AY129015	Homo sapiens clone FP17889 unknown mRNA. [AY129015]	
A_24_P649747	0.000125	2.02	NM_014753	NM_014753	Homo sapiens BMS1-like, ribosome assembly protein (yeast) (BMS1L), mRNA [NM_014753]	NM_014753
A_23_P502915	0.000125	2.96	NM_017491	NM_017491	Homo sapiens WD repeat domain 1 (WDR1), transcript variant 1, mRNA [NM_017491]	NM_017491
A_23_P257945	0.000126	2.87	NM_005333	NM_005333	Homo sapiens holocytochrome c synthase (cytochrome c heme-lyase) (HCCS), mRNA [NM_005333]	NM_005333
A_24_P68222	0.000128	2.05	BC019893	BC019893	Homo sapiens hypothetical gene supported by AK125149, mRNA (cDNA clone IMAGE:5001663), partial cds. [BC019893]	XM_379694
A_23_P157943	0.000128	2.43	NM_016219	NM_016219	Homo sapiens mannosidase, alpha, class 1B, member 1 (MAN1B1), mRNA [NM_016219]	NM_016219
A_23_P150903	0.000128	3.15	NM_018099	NM_018099	Homo sapiens male sterility domain containing 1 (MLSTD1), mRNA [NM_018099]	NM_018099
A_24_P357183	0.000128	3.39	NM_177439	NM_177439	Homo sapiens FtsJ homolog 1 (E. coli) (FTSJ1), transcript variant 3, mRNA [NM_177439]	NM_177439
A_24_P13649	0.000128	3.40	NM_032714	NM_032714	Homo sapiens chromosome 14 open reading frame 151 (C14orf151), mRNA [NM_032714]	NM_032714
A_23_P162037	0.000128	4.07	NM_001178	NM_001178	Homo sapiens aryl hydrocarbon receptor nuclear translocator-like (ARNTL), mRNA [NM_001178]	NM_001178
A_23_P252681	0.000128	4.42	NM_005017	NM_005017	Homo sapiens phosphate cytidylyltransferase 1, choline, alpha isoform (PCYT1A), mRNA [NM_005017]	NM_005017
A_32_P214925	0.000128	4.78	NM_173678	NM_173678	Homo sapiens hypothetical protein FLJ40722 (FLJ40722), mRNA [NM_173678]	NM_173678
A_23_P76488	0.000128	29.94	NM_001423	NM_001423	Homo sapiens epithelial membrane protein 1 (EMP1), mRNA [NM_001423]	NM_001423
A_23_P52382	0.000129	2.23	NM_017626	NM_017626	Homo sapiens DnaJ (Hsp40) homolog, subfamily B, member 12 (DNAJB12), transcript variant 2, mRNA [NM_017626]	NM_017626
A_23_P398294	0.000129	2.51	NM_003959	NM_003959	Homo sapiens huntingtin interacting protein-1-related (HIP1R), mRNA [NM_003959]	NM_003959
A_24_P131697	0.000129	2.55	NM_022899	NM_022899	Homo sapiens ARP8 actin-related protein 8 homolog (yeast) (ACTR8), mRNA [NM_022899]	NM_022899
A_24_P13715	0.000129	3.29	NM_147188	NM_147188	Homo sapiens F-box protein 22 (FBXO22), transcript variant 1, mRNA [NM_147188]	NM_147188
A_23_P200396	0.00013	2.08	NM_019118	NM_019118	Homo sapiens chromosome 1 open reading frame 91 (C1orf91), mRNA [NM_019118]	NM_019118
A_23_P210719	0.00013	2.11	NM_016143	NM_016143	Homo sapiens NSFL1 (p97) cofactor (p47) (NSFL1C), transcript variant 1, mRNA [NM_016143]	NM_016143
A_23_P6381	0.00013	2.48	NM_002430	NM_002430	Homo sapiens meningioma (disrupted in balanced translocation) 1 (MN1), mRNA [NM_002430]	NM_002430
A_23_P251548	0.00013	2.66	NM_144582	NM_144582	Homo sapiens testis expressed sequence 261 (TEX261), mRNA [NM_144582]	NM_144582
A_23_P128486	0.00013	4.48	NM_020375	NM_020375	Homo sapiens chromosome 12 open reading frame 5 (C12orf5), mRNA [NM_020375]	NM_020375
A_23_P79911	0.000132	2.05	NM_006814	NM_006814	Homo sapiens proteasome (prosome, macropain) inhibitor subunit 1 (PI31) (PSMF1), transcript variant 1, mRNA [NM_006814]	NM_006814
A_24_P833097	0.000132	2.92	BE243669	BE243669	BE243669 TCBAP1D1375 Pediatric pre-B cell acute lymphoblastic leukemia Baylor-HGSC project=TCBA Homo sapiens cDNA clone TCBAP1375, mRNA sequence [BE243669]	
A_23_P127186	0.000133	2.21	NM_206862	NM_206862	Homo sapiens transforming, acidic coiled-coil containing protein 2 (TACC2), transcript variant 1, mRNA [NM_206862]	NM_206862

A_24_P387839	0.000133	2.49	NM_144780	NM_144780	Homo sapiens degenerative spermatocyte homolog 1, lipid desaturase (Drosophila) (DEGS1), transcript variant 2, mRNA [NM_144780]	NM_144780
A_32_P210223	0.000133	2.71	THC2345565			
A_32_P208915	0.000133	3.04	CA421315	CA421315	CA421315 UI-H-FG0-bdb-f-18-0-UI.s1 NCI_CGAP_EN1_2 Homo sapiens cDNA clone UI-H-FG0-bdb-f-18-0-UI 3', mRNA sequence [CA421315]	
A_24_P357465	0.000133	3.33	NM_021202	NM_021202	Homo sapiens tumor protein p53 inducible nuclear protein 2 (TP53INP2), mRNA [NM_021202]	NM_021202
A_24_P126731	0.000133	3.41	ENST00000327665			
A_23_P122896	0.000134	2.10	NM_003227	NM_003227	Homo sapiens transferrin receptor 2 (TFR2), mRNA [NM_003227]	NM_003227
A_24_P927474	0.000134	2.54	ENST00000361500			
A_32_P101235	0.000134	3.52	NM_013282	NM_013282	Homo sapiens ubiquitin-like, containing PHD and RING finger domains, 1 (UHRF1), mRNA [NM_013282]	NM_013282
A_23_P213766	0.000134	4.05	NM_054027	NM_054027	Homo sapiens ankylosis, progressive homolog (mouse) (ANKH), mRNA [NM_054027]	NM_054027
A_23_P25559	0.000135	4.85	NM_005845	NM_005845	Homo sapiens ATP-binding cassette, sub-family C (CFTR/MRP), member 4 (ABCC4), mRNA [NM_005845]	NM_005845
A_23_P211957	0.000135	6.21	NM_001024847	NM_001024847	Homo sapiens transforming growth factor, beta receptor II (70/80kDa) (TGFBR2), transcript variant 1, mRNA [NM_001024847]	NM_001024847
A_24_P196688	0.000135	8.26	BC056891	BC056891	Homo sapiens caspase recruitment domain family, member 8, mRNA (cDNA clone MGC:65033 IMAGE:6144772), complete cds. [BC056891]	
A_24_P126181	0.000136	2.22	NM_176880	NM_176880	Homo sapiens TR4 orphan receptor associated protein TRA16 (TRA16), mRNA [NM_176880]	NM_176880
A_23_P165651	0.000136	2.35	NM_145644	NM_145644	Homo sapiens mitochondrial ribosomal protein L35 (MRPL35), nuclear gene encoding mitochondrial protein, transcript variant 2, mRNA [NM_145644]	NM_145644
A_23_P250054	0.000136	2.75	NM_022064	NM_022064	Homo sapiens ring finger protein 123 (RNF123), mRNA [NM_022064]	NM_022064
A_32_P61693	0.000136	2.79	AK131287	AK131287	Homo sapiens cDNA FLJ16240 fis, clone HCASM2003357. [AK131287]	
A_23_P64837	0.000136	2.92	NM_020467	NM_020467	Homo sapiens hypothetical protein from clone 643 (LOC57228), mRNA [NM_020467]	NM_020467
A_24_P159948	0.000136	3.92	NM_003224	NM_003224	Homo sapiens ADP-ribosylation factor related protein 1 (ARFRP1), mRNA [NM_003224]	NM_003224
A_24_P375510	0.000136	4.61	ENST00000327893			
A_24_P12932	0.000137	2.08	NM_016065	NM_016065	Homo sapiens mitochondrial ribosomal protein S16 (MRPS16), nuclear gene encoding mitochondrial protein, mRNA [NM_016065]	NM_016065
A_24_P327815	0.000137	2.40	NM_006819	NM_006819	Homo sapiens stress-induced-phosphoprotein 1 (Hsp70/Hsp90-organizing protein) (STIP1), mRNA [NM_006819]	NM_006819
A_24_P85365	0.000137	3.88	ENST00000309979		Homo sapiens N-deacetylase/N-sulfotransferase (heparan glucosaminyl) 2, mRNA (cDNA clone IMAGE:4665687), partial cds. [BC018681]	
A_23_P211252	0.000138	2.04	NM_001001438	NM_001001438	Homo sapiens lanosterol synthase (2,3-oxidosqualene-lanosterol cyclase) (LSS), transcript variant 2, mRNA [NM_001001438]	NM_001001438
A_23_P165952	0.000138	2.55	NM_024855	NM_024855	Homo sapiens ARP5 actin-related protein 5 homolog (yeast) (ACTR5), mRNA [NM_024855]	NM_024855
A_24_P76759	0.000138	3.56	BC021301	BC021301	Homo sapiens FERM, RhoGEF and pleckstrin domain protein 2, mRNA (cDNA clone MGC:29593 IMAGE:5013180), complete cds. [BC021301]	
A_24_P921086	0.000138	6.80	THC2376213		Q78EJ8 (Q78EJ8) Calpain , partial (78%) [THC2376213]	
A_24_P213144	0.000139	2.58	A_24_P213144			
A_23_P138819	0.000139	3.05	NM_004927	NM_004927	Homo sapiens mitochondrial ribosomal protein L49 (MRPL49), nuclear gene encoding mitochondrial protein, mRNA [NM_004927]	NM_004927
A_23_P76731	0.000139	3.15	NM_014226	NM_014226	Homo sapiens renal tumor antigen (RAGE), mRNA [NM_014226]	NM_014226
A_24_P337867	0.000139	3.17	NM_032831	NM_032831	Homo sapiens chromosome 7 open reading frame 19 (C7orf19), mRNA [NM_032831]	NM_032831
A_23_P430785	0.000139	4.46	NM_006654	NM_006654	Homo sapiens fibroblast growth factor receptor substrate 2 (FRS2), mRNA [NM_006654]	NM_006654
A_23_P110022	0.000139	4.59	NM_032638	NM_032638	Homo sapiens GATA binding protein 2 (GATA2), mRNA [NM_032638]	NM_032638
A_24_P763655	0.000139	5.49	THC2271508		BOVCALP calpactin I heavy chain (p36) {Bos taurus;} , partial (53%) [THC2271508]	
A_23_P218405	0.00014	2.31	NM_004489	NM_004489	Homo sapiens G protein pathway suppressor 2 (GPS2), mRNA [NM_004489]	NM_004489
A_24_P922808	0.00014	4.95	ENST00000346571		Homo sapiens chromosome 1 open reading frame 121, mRNA (cDNA clone IMAGE:4718788), partial cds. [BC020640]	
A_23_P54953	0.000141	2.30	NM_013260	NM_013260	Homo sapiens transcriptional regulator protein (HCNGP), mRNA [NM_013260]	NM_013260
A_23_P31550	0.000141	2.72	AK021933	AK021933	Homo sapiens cDNA FLJ11871 fis, clone HEMBA1007052. [AK021933]	

A_23_P91640	0.000141	3.80	NM_020437	NM_020437	Homo sapiens similar to aspartate beta hydroxylase (ASPH) (LOC57168), mRNA [NM_020437]	NM_020437
A_23_P4435	0.000142	2.61	NM_015229	NM_015229	Homo sapiens KIAA0664 protein (KIAA0664), mRNA [NM_015229]	NM_015229
A_24_P703830	0.000142	2.98	THC2375654		NNS3_HUMAN (P60323) Nanos homolog 3, partial (31%) [THC2375654]	
A_32_P225768	0.000142	3.00	A_32_P225768			
A_24_P194845	0.000143	2.07	NM_198829	NM_198829	Homo sapiens ras-related C3 botulinum toxin substrate 1 (rho family, small GTP binding protein Rac1) (RAC1), transcript variant Rac1c, mRNA [NM_198829]	NM_198829
A_23_P59481	0.000143	2.26	NM_014671	NM_014671	Homo sapiens ubiquitin protein ligase E3C (UBE3C), mRNA [NM_014671]	NM_014671
A_32_P505133	0.000143	2.80	NM_178830	NM_178830	Homo sapiens hypothetical protein FLJ36888 (FLJ36888), mRNA [NM_178830]	NM_178830
A_24_P915692	0.000143	5.78	BC047362	BC047362	Homo sapiens cDNA clone IMAGE:5315177, partial cds. [BC047362]	
A_32_P516818	0.000143	8.93	ENST00000312275		Homo sapiens, Similar to hypothetical protein BC010062, clone IMAGE:5197576, mRNA, partial cds. [BC029129]	XM_496664
A_23_P155351	0.000144	3.45	NM_000060	NM_000060	Homo sapiens biotinidase (BTD), mRNA [NM_000060]	NM_000060
A_23_P76435	0.000144	3.77	NM_176818	NM_176818	Homo sapiens hypothetical protein 15E1.2 (15E1.2), mRNA [NM_176818]	NM_176818
A_23_P57036	0.000144	3.97	NM_001250	NM_001250	Homo sapiens CD40 antigen (TNF receptor superfamily member 5) (CD40), transcript variant 1, mRNA [NM_001250]	NM_001250
A_23_P86931	0.000144	4.57	NM_198277	NM_198277	Homo sapiens solute carrier family 37 (glycerol-3-phosphate transporter), member 2 (SLC37A2), mRNA [NM_198277]	NM_198277
A_23_P218131	0.000144	4.76	NM_032714	NM_032714	Homo sapiens chromosome 14 open reading frame 151 (C14orf151), mRNA [NM_032714]	NM_032714
A_32_P210202	0.000144	6.33	NM_203394	NM_203394	Homo sapiens E2F transcription factor 7 (E2F7), mRNA [NM_203394]	NM_203394
A_23_P316850	0.000145	2.86	NM_182577	NM_182577	Homo sapiens chromosome 19 open reading frame 19 (C19orf19), mRNA [NM_182577]	NM_182577
A_24_P128085	0.000146	4.65	BC044642	BC044642	Homo sapiens membrane associated DNA binding protein, mRNA (cDNA clone MGC:52176 IMAGE:5455191), complete cds. [BC044642]	
A_24_P85775	0.000146	5.68	NM_004848	NM_004848	Homo sapiens chromosome 1 open reading frame 38 (C1orf38), mRNA [NM_004848]	NM_004848
A_23_P316612	0.000146	17.76	NM_147193	NM_147193	Homo sapiens GLIS family zinc finger 1 (GLIS1), mRNA [NM_147193]	NM_147193
A_23_P97780	0.000147	2.84	BC043009	BC043009	Homo sapiens hypothetical protein LOC338620, mRNA (cDNA clone IMAGE:6023208), partial cds. [BC043009]	
A_24_P56330	0.000147	3.95	NM_152324	NM_152324	Homo sapiens hypothetical protein MGC35169 (MGC35169), mRNA [NM_152324]	NM_152324
A_23_P202361	0.000148	2.55	NM_182639	NM_182639	Homo sapiens Hermansky-Pudlak syndrome 1 (HPS1), transcript variant 3, mRNA [NM_182639]	NM_182639
A_24_P942321	0.000148	3.11	NM_018027	NM_018027	Homo sapiens FERM domain containing 4A (FRMD4A), mRNA [NM_018027]	NM_018027
A_23_P329353	0.000148	11.29	NM_015463	NM_015463	Homo sapiens chromosome 2 open reading frame 32 (C2orf32), mRNA [NM_015463]	NM_015463
A_24_P118472	0.000149	2.23	A_24_P118472			
A_23_P143089	0.000149	2.47	NM_002437	NM_002437	Homo sapiens MpV17 transgene, murine homolog, glomerulosclerosis (MPV17), mRNA [NM_002437]	NM_002437
A_24_P340696	0.000149	2.53	NM_170694	NM_170694	Homo sapiens serine hydrolase-like (SERHL), mRNA [NM_170694]	NM_170694
A_32_P192804	0.000149	2.91	BF195626	BF195626	BF195626 7n86a01.x1 NCI_CGAP_Ov18 Homo sapiens cDNA clone IMAGE:3571464 3' similar to SW:ER72_HUMAN P13667 PROTEIN DISULFIDE ISOMERASE-RELATED PROTEIN ERP-72 PRECURSOR ;, mRNA sequence [BF195626]	
A_23_P60306	0.000149	8.70	NM_003266	NM_003266	Homo sapiens toll-like receptor 4 (TLR4), transcript variant 3, mRNA [NM_003266]	NM_003266
A_23_P217763	0.000149	13.30	AK055976	AK055976	Homo sapiens cDNA FLJ31414 fis, clone NT2NE2000260, weakly similar to THYMOSIN BETA-4. [AK055976]	
A_24_P535256	0.000149	29.94	AK001903	AK001903	Homo sapiens cDNA FLJ11041 fis, clone PLACE1004405. [AK001903]	
A_23_P85392	0.00015	2.02	NM_007033	NM_007033	Homo sapiens RER1 retention in endoplasmic reticulum 1 homolog (S. cerevisiae) (RER1), mRNA [NM_007033]	NM_007033
A_24_P38944	0.00015	4.55	NM_024098	NM_024098	Homo sapiens hypothetical protein MGC2574 (MGC2574), mRNA [NM_024098]	NM_024098
A_32_P184937	0.00015	7.35	BU678941	BU678941	BU678941 UI-CF-DU1-aat-a-09-0-UI.s1 UI-CF-DU1 Homo sapiens cDNA clone UI-CF-DU1-aat-a-09-0-UI 3', mRNA sequence [BU678941]	
A_23_P204484	0.000151	2.30	NM_006861	NM_006861	Homo sapiens RAB35, member RAS oncogene family (RAB35), mRNA [NM_006861]	NM_006861
A_24_P212565	0.000151	2.32	A_24_P212565			
A_23_P18372	0.000151	2.45	NM_032047	NM_032047	Homo sapiens UDP-GlcNAc:betaGal beta-1,3-N-acetylglucosaminyltransferase 5 (B3GNT5), mRNA [NM_032047]	NM_032047
A_23_P210939	0.000151	2.56	NM_181468	NM_181468	Homo sapiens integrin beta 4 binding protein (ITGB4BP), transcript variant 2, mRNA [NM_181468]	NM_181468
A_23_P251051	0.000151	2.68	NM_181826	NM_181826	Homo sapiens neurofibromin 2 (bilateral acoustic neuroma) (NF2), transcript variant 3, mRNA [NM_181826]	NM_181826

A_23_P255317	0.000151	2.72	NM_004290	NM_004290	Homo sapiens ring finger protein 14 (RNF14), transcript variant 1, mRNA [NM_004290]	NM_004290
A_23_P6456	0.000151	3.10	NM_024313	NM_024313	Homo sapiens hypothetical protein MGC3731 (MGC3731), mRNA [NM_024313]	NM_024313
A_24_P127063	0.000151	3.76	ENST00000321482			
A_23_P96542	0.000152	2.53	NM_001017980	NM_001017980	Homo sapiens hypothetical protein LOC203547 (LOC203547), mRNA [NM_001017980]	NM_001017980
A_23_P380076	0.000152	3.30	BC072415	BC072415	Homo sapiens cDNA clone IMAGE:6168734, partial cds. [BC072415]	
A_24_P374513	0.000152	5.78	THC2242927		Q5TD55 (Q5TD55) OTTHUMP00000031270 (Fragment), complete [THC2242927]	
A_23_P167389	0.000154	2.56	NM_022481	NM_022481	Homo sapiens centaurin, delta 3 (CENTD3), mRNA [NM_022481]	NM_022481
A_23_P154234	0.000154	2.82	NM_019896	NM_019896	Homo sapiens polymerase (DNA-directed), epsilon 4 (p12 subunit) (POLE4), mRNA [NM_019896]	NM_019896
A_24_P330796	0.000154	2.82	NM_017777	NM_017777	Homo sapiens hypothetical protein FLJ20345 (FLJ20345), mRNA [NM_017777]	NM_017777
A_24_P102726	0.000155	2.11	NM_000309	NM_000309	Homo sapiens protoporphyrinogen oxidase (PPOX), nuclear gene encoding mitochondrial protein, mRNA [NM_000309]	NM_000309
A_23_P2873	0.000155	2.16	NM_182923	NM_182923	Homo sapiens kinesin 2 60/70kDa (KNS2), transcript variant 2, mRNA [NM_182923]	NM_182923
A_23_P341275	0.000155	2.62	NM_015029	NM_015029	Homo sapiens processing of precursor 1, ribonuclease P/MRP subunit (S. cerevisiae) (POP1), mRNA [NM_015029]	NM_015029
A_24_P255123	0.000155	3.91	A_24_P255123			
A_24_P318656	0.000155	11.30	NM_000212	NM_000212	Homo sapiens integrin, beta 3 (platelet glycoprotein IIIa, antigen CD61) (ITGB3), mRNA [NM_000212]	NM_000212
A_23_P256205	0.000155	20.49	NM_014945	NM_014945	Homo sapiens actin binding LIM protein family, member 3 (ABLIM3), mRNA [NM_014945]	NM_014945
A_23_P123234	0.000155	20.88	A_23_P123234			
A_23_P16096	0.000156	2.07	NM_020230	NM_020230	Homo sapiens peter pan homolog (Drosophila) (PPAN), mRNA [NM_020230]	NM_020230
A_23_P19517	0.000156	2.17	NM_002224	NM_002224	Homo sapiens inositol 1,4,5-triphosphate receptor, type 3 (ITPR3), mRNA [NM_002224]	NM_002224
A_24_P65779	0.000156	2.33	THC2370154		AF348700 ubiquitin A-52 residue ribosomal protein fusion product 1 {Homo sapiens;} , complete [THC2370154]	
A_24_P334130	0.000156	3.25	NM_054034	NM_054034	Homo sapiens fibronectin 1 (FN1), transcript variant 7, mRNA [NM_054034]	NM_054034
A_24_P935318	0.000156	3.29	NM_022839	NM_022839	Homo sapiens mitochondrial ribosomal protein S11 (MRPS11), nuclear gene encoding mitochondrial protein, transcript variant 1, mRNA [NM_022839]	NM_022839
A_32_P201620	0.000156	5.95	A_32_P201620			
A_23_P140994	0.000157	2.02	NM_007242	NM_007242	Homo sapiens DEAD (Asp-Glu-Ala-As) box polypeptide 19 (DDX19), transcript variant 1, mRNA [NM_007242]	NM_007242
A_24_P73920	0.000157	2.29	CR609948	CR609948	full-length cDNA clone CS0DH006YD11 of T cells (Jurkat cell line) of Homo sapiens (human). [CR609948]	
A_23_P25313	0.000157	2.40	NM_080626	NM_080626	Homo sapiens BRI3 binding protein (BRI3BP), mRNA [NM_080626]	NM_080626
A_32_P135902	0.000157	2.55	NM_001416	NM_001416	Homo sapiens eukaryotic translation initiation factor 4A, isoform 1 (EIF4A1), mRNA [NM_001416]	NM_001416
A_23_P218726	0.000157	2.79	NM_152930	NM_152930	Homo sapiens copine I (CPNE1), transcript variant 7, mRNA [NM_152930]	NM_152930
A_24_P913431	0.000157	3.14	AK131423	AK131423	Homo sapiens cDNA FLJ16543 fis, clone OCBBF3002654, highly similar to Triple functional domain protein. [AK131423]	
A_23_P100754	0.000157	4.07	NM_022739	NM_022739	Homo sapiens SMAD specific E3 ubiquitin protein ligase 2 (SMURF2), mRNA [NM_022739]	NM_022739
A_24_P698759	0.000158	2.16	A_24_P698759			
A_23_P146908	0.000158	2.23	NM_004853	NM_004853	Homo sapiens syntaxin 8 (STX8), mRNA [NM_004853]	NM_004853
A_23_P68966	0.000158	2.30	CR626044	CR626044	full-length cDNA clone CS0DI082YE05 of Placenta Cot 25-normalized of Homo sapiens (human). [CR626044]	
A_24_P572229	0.000158	2.36	A_24_P572229			
A_23_P110345	0.000158	2.50	NM_012110	NM_012110	Homo sapiens cysteine-rich hydrophobic domain 2 (CHIC2), mRNA [NM_012110]	NM_012110
A_24_P693986	0.000158	2.96	NM_001013642	NM_001013642	Homo sapiens hypothetical LOC388610 (LOC388610), mRNA [NM_001013642]	NM_001013642
A_24_P92823	0.000158	8.13	A_24_P92823			
A_24_P632160	0.000159	2.55	A_24_P632160			
A_32_P108387	0.000159	3.37	CF528315	CF528315	CF528315 UI-1-BC0-aen-f-04-0-UI.s1 NCI_CGAP_Pl1 Homo sapiens cDNA clone UI-1-BC0-aen-f-04-0-UI 3', mRNA sequence [CF528315]	
A_24_P327181	0.000159	4.88	NM_032387	NM_032387	Homo sapiens WNK lysine deficient protein kinase 4 (WNK4), mRNA [NM_032387]	NM_032387
A_24_P66027	0.000159	6.54	NM_004900	NM_004900	Homo sapiens apolipoprotein B mRNA editing enzyme, catalytic polypeptide-like 3B (APOBEC3B), mRNA [NM_004900]	NM_004900

A_32_P16204	0.00016	2.93	ENST00000295549		Homo sapiens hypothetical gene supported by BC013438, mRNA (cDNA clone IMAGE:3899073), partial cds. [BC013438]	XM_374020
A_23_P338890	0.00016	2.95	NM_002827	NM_002827	Homo sapiens protein tyrosine phosphatase, non-receptor type 1 (PTPN1), mRNA [NM_002827]	NM_002827
A_24_P156295	0.00016	4.10	NM_018677	NM_018677	Homo sapiens acetyl-Coenzyme A synthetase 2 (ADP forming) (ACAS2), transcript variant 1, mRNA [NM_018677]	NM_018677
A_32_P85813	0.000161	2.33	BF326020	BF326020	BF326020 QV1-AN0059-040800-295-e05 AN0059 Homo sapiens cDNA, mRNA sequence [BF326020]	
A_32_P6917	0.000163	2.60	NM_025207	NM_025207	Homo sapiens FAD-synthetase (PP591), transcript variant 1, mRNA [NM_025207]	NM_025207
A_23_P214756	0.000163	2.74	NM_020455	NM_020455	Homo sapiens G protein-coupled receptor 126 (GPR126), mRNA [NM_020455]	NM_020455
A_24_P282237	0.000163	3.60	NM_000947	NM_000947	Homo sapiens primase, polypeptide 2A, 58kDa (PRIM2A), mRNA [NM_000947]	NM_000947
A_23_P214907	0.000163	5.13	NM_015440	NM_015440	Homo sapiens methylenetetrahydrofolate dehydrogenase (NADP+ dependent) 1-like (MTHFD1L), mRNA [NM_015440]	NM_015440
A_23_P112726	0.000163	5.88	NM_002977	NM_002977	Homo sapiens sodium channel, voltage-gated, type IX, alpha (SCN9A), mRNA [NM_002977]	NM_002977
A_23_P33376	0.000164	5.46	NM_002595	NM_002595	Homo sapiens PCTAIRE protein kinase 2 (PCTK2), mRNA [NM_002595]	NM_002595
A_23_P210708	0.000165	3.08	NM_080792	NM_080792	Homo sapiens protein tyrosine phosphatase, non-receptor type substrate 1 (PTPNS1), mRNA [NM_080792]	NM_080792
A_24_P160001	0.000165	3.36	NM_054014	NM_054014	Homo sapiens FK506 binding protein 1A, 12kDa (FKBP1A), transcript variant 12A, mRNA [NM_054014]	NM_054014
A_23_P73593	0.000165	3.69	NM_002444	NM_002444	Homo sapiens moesin (MSN), mRNA [NM_002444]	NM_002444
A_23_P215175	0.000167	3.37	NM_005692	NM_005692	Homo sapiens ATP-binding cassette, sub-family F (GCN20), member 2 (ABCF2), nuclear gene encoding mitochondrial protein, transcript variant 2, mRNA [NM_005692]	NM_005692
A_32_P109057	0.000168	2.00	NM_173825	NM_173825	Homo sapiens RAB, member of RAS oncogene family-like 3 (RABL3), mRNA [NM_173825]	NM_173825
A_24_P416837	0.000168	2.05	NM_024958	NM_024958	Homo sapiens chromosome 20 open reading frame 98 (C20orf98), mRNA [NM_024958]	NM_024958
A_24_P391960	0.000168	2.16	ENST00000361845		PREDICTED: Homo sapiens similar to Eukaryotic translation initiation factor 1 (eIF1) (Protein translation factor SUI1 homolog) (Sui1iso1) (LOC441916), mRNA [XM_497726]	XM_497726
A_24_P24332	0.000168	2.70	A_24_P24332			
A_32_P166693	0.000168	2.79	THC2265769		Q9GEJ2 (Q9GEJ2) NADH dehydrogenase subunit F (Fragment), partial (5%) [THC2265769]	
A_24_P416131	0.000168	3.61	NM_021149	NM_021149	Homo sapiens coactosin-like 1 (Dictyostelium) (COTL1), mRNA [NM_021149]	NM_021149
A_23_P126803	0.000168	3.88	NM_005717	NM_005717	Homo sapiens actin related protein 2/3 complex, subunit 5, 16kDa (ARPC5), mRNA [NM_005717]	NM_005717
A_32_P100464	0.000168	4.46	THC2340639		Q6MX26 (Q6MX26) PE-PGRS FAMILY PROTEIN, partial (6%) [THC2340639]	
A_24_P82032	0.000168	4.63	NM_020663	NM_020663	Homo sapiens ras homolog gene family, member J (RHOJ), mRNA [NM_020663]	NM_020663
A_24_P636451	0.000169	2.26	AK023559	AK023559	Homo sapiens cDNA FLJ13497 fis, clone PLACE1004518. [AK023559]	
A_23_P414913	0.000169	2.41	NM_022343	NM_022343	Homo sapiens chromosome 9 open reading frame 19 (C9orf19), mRNA [NM_022343]	NM_022343
A_23_P416036	0.000169	2.59	NM_207107	NM_207107	Homo sapiens 26S proteasome-associated UCH interacting protein 1 (UIP1), transcript variant 2, mRNA [NM_207107]	NM_207107
A_23_P350574	0.000169	2.74	NM_152378	NM_152378	Homo sapiens Fc receptor-like and mucin-like 2 (FCRLM2), mRNA [NM_152378]	NM_152378
A_24_P393470	0.000169	2.88	BC004449	BC004449	Homo sapiens MADS box transcription enhancer factor 2, polypeptide B (myocyte enhancer factor 2B), mRNA (cDNA clone IMAGE:2819918), complete cds. [BC004449]	
A_23_P15603	0.000169	3.12	NM_024864	NM_024864	Homo sapiens hypothetical protein FLJ22578 (FLJ22578), mRNA [NM_024864]	NM_024864
A_24_P71244	0.000169	4.03	NM_005026	NM_005026	Homo sapiens phosphoinositide-3-kinase, catalytic, delta polypeptide (PIK3CD), mRNA [NM_005026]	NM_005026
A_23_P352861	0.000169	24.45	BC028083	BC028083	Homo sapiens T cell receptor beta variable 5-4, mRNA (cDNA clone MGC:40031 IMAGE:5217067), complete cds. [BC028083]	
A_24_P103060	0.00017	2.18	NM_139279	NM_139279	Homo sapiens multiple coagulation factor deficiency 2 (MCFD2), mRNA [NM_139279]	NM_139279
A_23_P205293	0.00017	2.94	NM_018199	NM_018199	Homo sapiens chromosome 14 open reading frame 114 (C14orf114), mRNA [NM_018199]	NM_018199
A_24_P50390	0.00017	3.16	A_24_P50390			
A_24_P183150	0.00017	4.00	NM_002090	NM_002090	Homo sapiens chemokine (C-X-C motif) ligand 3 (CXCL3), mRNA [NM_002090]	NM_002090
A_23_P406425	0.000172	2.59	NM_173359	NM_173359	Homo sapiens eukaryotic translation initiation factor 4E member 3 (EIF4E3), mRNA [NM_173359]	NM_173359
A_24_P416656	0.000172	2.78	NM_080665	NM_080665	Homo sapiens similar to RIKEN cDNA B230118G17 gene (MGC19604), mRNA [NM_080665]	NM_080665
A_23_P24870	0.000172	4.88	NM_000610	NM_000610	Homo sapiens CD44 antigen (homing function and Indian blood group system) (CD44), transcript variant 1, mRNA [NM_000610]	NM_000610
A_23_P410159	0.000172	5.26	NM_178422	NM_178422	Homo sapiens progestin and adipoQ receptor family member VII (PAQR7), mRNA [NM_178422]	NM_178422

A_23_P37983	0.000172	6.02	NM_005947	NM_005947	Homo sapiens metallothionein 1B (functional) (MT1B), mRNA [NM_005947]	NM_005947
A_23_P86653	0.000172	27.78	NM_002727	NM_002727	Homo sapiens proteoglycan 1, secretory granule (PRG1), mRNA [NM_002727]	NM_002727
A_24_P126851	0.000173	2.08	A_24_P126851			
A_23_P75889	0.000173	2.25	NM_175932	NM_175932	Homo sapiens proteasome (prosome, macropain) 26S subunit, non-ATPase, 13 (PSMD13), transcript variant 2, mRNA [NM_175932]	NM_175932
A_32_P128572	0.000173	2.26	NM_020948	NM_020948	Homo sapiens mesoderm induction early response 1 homolog (Xenopus laevis) (MIER1), mRNA [NM_020948]	NM_020948
A_23_P77887	0.000173	2.54	NM_006224	NM_006224	Homo sapiens phosphatidylinositol transfer protein, alpha (PITPNA), mRNA [NM_006224]	NM_006224
A_23_P145153	0.000173	2.86	NM_002598	NM_002598	Homo sapiens programmed cell death 2 (PDCD2), transcript variant 1, mRNA [NM_002598]	NM_002598
A_24_P356830	0.000173	4.22	AK129956	AK129956	Homo sapiens cDNA FLJ26446 fis, clone KDN02743. [AK129956]	
A_23_P216448	0.000174	5.81	NM_005596	NM_005596	Homo sapiens nuclear factor I/B (NFIB), mRNA [NM_005596]	NM_005596
A_32_P104809	0.000175	3.56	A_32_P104809			
A_24_P116805	0.000176	2.09	NM_213662	NM_213662	Homo sapiens signal transducer and activator of transcription 3 (acute-phase response factor) (STAT3), transcript variant 3, mRNA [NM_213662]	NM_213662
A_23_P202080	0.000176	3.21	NM_030927	NM_030927	Homo sapiens tetraspanin 14 (TSPAN14), mRNA [NM_030927]	NM_030927
A_23_P150249	0.000176	3.38	NM_006848	NM_006848	Homo sapiens hepatitis delta antigen-interacting protein A (DIPA), mRNA [NM_006848]	NM_006848
A_24_P89284	0.000177	2.31	NM_002109	NM_002109	Homo sapiens histidyl-tRNA synthetase (HARS), mRNA [NM_002109]	NM_002109
A_24_P322635	0.000177	2.45	NM_182764	NM_182764	Homo sapiens engulfment and cell motility 2 (ced-12 homolog, C. elegans) (ELMO2), transcript variant 3, mRNA [NM_182764]	NM_182764
A_24_P166045	0.000177	3.01	AF070525	AF070525	Homo sapiens clone 24706 mRNA sequence. [AF070525]	
A_32_P64936	0.000177	4.27	X05126	X05126	Human fibroblast mRNA fragment with Alu sequence (pRHF11). [X05126]	
A_32_P32315	0.000177	39.53	A_32_P32315			
A_23_P345820	0.000178	2.35	NM_014991	NM_014991	Homo sapiens WD repeat and FYVE domain containing 3 (WDFY3), transcript variant 1, mRNA [NM_014991]	NM_014991
A_24_P928217	0.000178	2.75	AK026155	AK026155	Homo sapiens cDNA: FLJ22502 fis, clone HRC11383. [AK026155]	
A_24_P528213	0.000179	3.45	BF593129	BF593129	BF593129 7o49h09.x1 NCI_CGAP_Kid11 Homo sapiens cDNA clone IMAGE:3577600 3', mRNA sequence [BF593129]	
A_23_P132159	0.00018	2.22	NM_017414	NM_017414	Homo sapiens ubiquitin specific protease 18 (USP18), mRNA [NM_017414]	NM_017414
A_23_P319153	0.00018	3.00	AL512694	AL512694	Homo sapiens mRNA; cDNA DKFZp761J2423 (from clone DKFZp761J2423). [AL512694]	
A_24_P194420	0.000181	2.38	NM_024821	NM_024821	Homo sapiens hypothetical protein FLJ22349 (FLJ22349), mRNA [NM_024821]	NM_024821
A_24_P332595	0.000181	2.59	A_24_P332595			
A_23_P200001	0.000181	2.61	NM_144573	NM_144573	Homo sapiens nexilin (F actin binding protein) (NEXN), mRNA [NM_144573]	NM_144573
A_32_P158181	0.000181	3.65	THC2437757			
A_23_P26223	0.000182	2.08	NM_000048	NM_000048	Homo sapiens argininosuccinate lyase (ASL), transcript variant 2, mRNA [NM_000048]	NM_000048
A_23_P34741	0.000182	2.09	NM_015871	NM_015871	Homo sapiens zinc finger protein 593 (ZNF593), mRNA [NM_015871]	NM_015871
A_23_P118712	0.000182	7.30	BC011636	BC011636	Homo sapiens slingshot homolog 2 (Drosophila), mRNA (cDNA clone IMAGE:4101583), complete cds. [BC011636]	
A_23_P103923	0.000184	2.03	NM_014864	NM_014864	Homo sapiens family with sequence similarity 20, member B (FAM20B), mRNA [NM_014864]	NM_014864
A_23_P78170	0.000184	2.91	NM_014520	NM_014520	Homo sapiens MYB binding protein (P160) 1a (MYBBP1A), mRNA [NM_014520]	NM_014520
A_23_P129606	0.000185	2.48	NM_001015047	NM_001015047	Homo sapiens DDX19-DDX19L protein (DDX19-DDX19L), mRNA [NM_001015047]	NM_001015047
A_24_P676216	0.000186	2.33	ENST00000342859		PREDICTED: Homo sapiens similar to Glyceraldehyde 3-phosphate dehydrogenase, liver (GAPDH) (LOC392549), mRNA [XM_373373]	XM_373373
A_32_P228917	0.000186	2.73	BC002350	BC002350	Homo sapiens cDNA clone IMAGE:2820510. [BC002350]	
A_24_P922475	0.000186	4.08	BC042363	BC042363	Homo sapiens cDNA clone IMAGE:5285571, complete cds. [BC042363]	
A_23_P250629	0.000186	9.26	NM_004159	NM_004159	Homo sapiens proteasome (prosome, macropain) subunit, beta type, 8 (large multifunctional protease 7) (PSMB8), transcript variant 1, mRNA [NM_004159]	NM_004159
A_23_P35912	0.000186	14.97	NM_033306	NM_033306	Homo sapiens caspase 4, apoptosis-related cysteine protease (CASP4), transcript variant gamma, mRNA [NM_033306]	NM_033306
A_24_P331537	0.000187	2.16	NM_021049	NM_021049	Homo sapiens melanoma antigen family A, 5 (MAGEA5), mRNA [NM_021049]	NM_021049

A_23_P170337	0.000188	2.11	NM_003748	NM_003748	Homo sapiens aldehyde dehydrogenase 4 family, member A1 (ALDH4A1), nuclear gene encoding mitochondrial protein, transcript variant P5CDhL, mRNA [NM_003748]	NM_003748
A_23_P86390	0.000188	5.15	NM_003873	NM_003873	Homo sapiens neuropilin 1 (NRP1), transcript variant 1, mRNA [NM_003873]	NM_003873
A_23_P130926	0.000189	2.36	NM_017914	NM_017914	Homo sapiens chromosome 19 open reading frame 24 (C19orf24), mRNA [NM_017914]	NM_017914
A_24_P38572	0.000189	2.46	NM_130793	NM_130793	Homo sapiens nucleolar protein family 6 (RNA-associated) (NOL6), transcript variant beta, mRNA [NM_130793]	NM_130793
A_23_P218597	0.000189	4.27	NM_002518	NM_002518	Homo sapiens neuronal PAS domain protein 2 (NPAS2), mRNA [NM_002518]	NM_002518
A_23_P208880	0.00019	2.24	NM_013282	NM_013282	Homo sapiens ubiquitin-like, containing PHD and RING finger domains, 1 (UHRF1), mRNA [NM_013282]	NM_013282
A_23_P425752	0.00019	2.69	NM_033219	NM_033219	Homo sapiens tripartite motif-containing 14 (TRIM14), transcript variant 2, mRNA [NM_033219]	NM_033219
A_23_P110151	0.00019	2.70	NM_031305	NM_031305	Homo sapiens Rho GTPase activating protein 24 (ARHGAP24), mRNA [NM_031305]	NM_031305
A_24_P354300	0.00019	2.70	NM_015426	NM_015426	Homo sapiens WD repeat domain 51A (WDR51A), mRNA [NM_015426]	NM_015426
A_23_P162540	0.00019	3.01	NM_031954	NM_031954	Homo sapiens potassium channel tetramerisation domain containing 10 (KCTD10), mRNA [NM_031954]	NM_031954
A_23_P210538	0.00019	3.44	NM_022086	NM_022086	Homo sapiens engulfment and cell motility 2 (ced-12 homolog, C. elegans) (ELMO2), transcript variant 2, mRNA [NM_022086]	NM_022086
A_24_P83118	0.00019	4.08	NM_152511	NM_152511	Homo sapiens dual specificity phosphatase 18 (DUSP18), mRNA [NM_152511]	NM_152511
A_23_P119390	0.00019	5.18	NM_014601	NM_014601	Homo sapiens EH-domain containing 2 (EHD2), mRNA [NM_014601]	NM_014601
A_23_P112798	0.00019	5.59	NM_001312	NM_001312	Homo sapiens cysteine-rich protein 2 (CRIP2), mRNA [NM_001312]	NM_001312
A_23_P102770	0.000191	2.30	NM_016100	NM_016100	Homo sapiens N-acetyltransferase 5 (ARD1 homolog, S. cerevisiae) (NAT5), transcript variant 1, mRNA [NM_016100]	NM_016100
A_23_P77761	0.000192	2.31	NM_004035	NM_004035	Homo sapiens acyl-Coenzyme A oxidase 1, palmitoyl (ACOX1), transcript variant 1, mRNA [NM_004035]	NM_004035
A_23_P76499	0.000192	2.38	NM_005002	NM_005002	Homo sapiens NADH dehydrogenase (ubiquinone) 1 alpha subcomplex, 9, 39kDa (NDUFA9), mRNA [NM_005002]	NM_005002
A_24_P306614	0.000192	3.76	A_24_P306614			
A_23_P49338	0.000192	6.17	NM_016639	NM_016639	Homo sapiens tumor necrosis factor receptor superfamily, member 12A (TNFRSF12A), mRNA [NM_016639]	NM_016639
A_24_P330303	0.000192	7.35	NM_152330	NM_152330	Homo sapiens FERM domain containing 6 (FRMD6), mRNA [NM_152330]	NM_152330
A_23_P131866	0.000193	2.53	NM_198433	NM_198433	Homo sapiens serine/threonine kinase 6 (STK6), transcript variant 1, mRNA [NM_198433]	NM_198433
A_24_P263672	0.000193	2.72	NM_080926	NM_080926	Homo sapiens hypothetical protein similar to KIAA0187 gene product (LOC96610), mRNA [NM_080926]	NM_080926
A_23_P49610	0.000193	3.21	NM_032895	NM_032895	Homo sapiens hypothetical protein MGC14376 (MGC14376), transcript variant 1, mRNA [NM_032895]	NM_032895
A_24_P123052	0.000194	2.12	NM_002886	NM_002886	Homo sapiens RAP2B, member of RAS oncogene family (RAP2B), mRNA [NM_002886]	NM_002886
A_24_P88554	0.000194	2.13	NM_003846	NM_003846	Homo sapiens peroxisomal biogenesis factor 11B (PEX11B), mRNA [NM_003846]	NM_003846
A_24_P341006	0.000194	2.16	A_24_P341006			
A_24_P96961	0.000194	2.42	NM_025106	NM_025106	Homo sapiens SPRY domain-containing SOCS box protein SSB-1 (SSB1), mRNA [NM_025106]	NM_025106
A_24_P914590	0.000194	5.92	ENST00000279013		Homo sapiens chromosome 20 open reading frame 43, mRNA (cDNA clone IMAGE:3615348), complete cds. [BC002769]	
A_23_P146644	0.000194	7.09	NM_001002857	NM_001002857	Homo sapiens annexin A2 (ANXA2), transcript variant 2, mRNA [NM_001002857]	NM_001002857
A_23_P26426	0.000195	2.13	NM_014427	NM_014427	Homo sapiens copine VII (CPNE7), transcript variant 2, mRNA [NM_014427]	NM_014427
A_23_P205724	0.000195	2.40	NM_199043	NM_199043	Homo sapiens chromosome 14 open reading frame 102 (C14orf102), transcript variant 2, mRNA [NM_199043]	NM_199043
A_23_P204581	0.000195	6.76	NM_003330	NM_003330	Homo sapiens thioredoxin reductase 1 (TXNRD1), transcript variant 1, mRNA [NM_003330]	NM_003330
A_23_P417363	0.000197	2.55	AK057267	AK057267	Homo sapiens cDNA FLJ32705 fis, clone TESTI2000600, weakly similar to RESTIN. [AK057267]	
A_23_P96556	0.000197	4.41	NM_203391	NM_203391	Homo sapiens glycerol kinase (GK), transcript variant 1, mRNA [NM_203391]	NM_203391
A_23_P9415	0.000198	2.20	NM_002197	NM_002197	Homo sapiens aconitase 1, soluble (ACO1), mRNA [NM_002197]	NM_002197
A_32_P15169	0.000199	2.20	A_32_P15169			
A_23_P1615	0.000199	2.81	NM_004214	NM_004214	Homo sapiens fibroblast growth factor (acidic) intracellular binding protein (FIBP), transcript variant 2, mRNA [NM_004214]	NM_004214
A_24_P698141	0.000199	4.78	AK125299	AK125299	Homo sapiens cDNA FLJ43309 fis, clone NT2RI2004618, highly similar to Cytosolic acyl coenzyme A thioester hydrolase (EC 3.1.2.2). [AK125299]	XR_000194
A_23_P65651	0.0002	2.02	NM_004184	NM_004184	Homo sapiens tryptophanyl-tRNA synthetase (WARS), transcript variant 1, mRNA [NM_004184]	NM_004184

A_23_P10927	0.0002	2.52	NM_031940	NM_031940	Homo sapiens TM2 domain containing 2 (TM2D2), transcript variant 2, mRNA [NM_031940]	NM_031940
A_23_P22214	0.0002	2.60	NM_004157	NM_004157	Homo sapiens protein kinase, cAMP-dependent, regulatory, type II, alpha (PRKAR2A), mRNA [NM_004157]	NM_004157
A_23_P120899	0.0002	2.85	NM_000395	NM_000395	Homo sapiens colony stimulating factor 2 receptor, beta, low-affinity (granulocyte-macrophage) (CSF2RB), mRNA [NM_000395]	NM_000395
A_24_P134340	0.0002	3.45	NM_182483	NM_182483	Homo sapiens NSFL1 (p97) cofactor (p47) (NSFL1C), transcript variant 3, mRNA [NM_182483]	NM_182483
A_23_P9166	0.0002	3.62	NM_006289	NM_006289	Homo sapiens talin 1 (TLN1), mRNA [NM_006289]	NM_006289
A_23_P121215	0.0002	5.05	NM_003656	NM_003656	Homo sapiens calcium/calmodulin-dependent protein kinase I (CAMK1), mRNA [NM_003656]	NM_003656
A_23_P59005	0.0002	6.10	NM_000593	NM_000593	Homo sapiens transporter 1, ATP-binding cassette, sub-family B (MDR/TAP) (TAP1), mRNA [NM_000593]	NM_000593
A_24_P370946	0.0002	9.01	NM_001554	NM_001554	Homo sapiens cysteine-rich, angiogenic inducer, 61 (CYR61), mRNA [NM_001554]	NM_001554
A_24_P280803	0.000201	2.15	BC018140	BC018140	Homo sapiens ribosomal protein S21, mRNA (cDNA clone MGC:9438 IMAGE:3903320), complete cds. [BC018140]	
A_23_P387471	0.000201	3.83	NM_005931	NM_005931	Homo sapiens MHC class I polypeptide-related sequence B (MICB), mRNA [NM_005931]	NM_005931
A_23_P58529	0.000202	2.04	NM_014473	NM_014473	Homo sapiens dimethyladenosine transferase (HSA9761), mRNA [NM_014473]	NM_014473
A_32_P48842	0.000202	2.12	CR595668	CR595668	full-length cDNA clone CS0DB003YC18 of Neuroblastoma Cot 10-normalized of Homo sapiens (human). [CR595668]	
A_24_P320796	0.000202	2.17	NM_182827	NM_182827	Homo sapiens FK506 binding protein 9-like (FKBP9L), mRNA [NM_182827]	NM_182827
A_23_P168479	0.000202	5.32	NM_005402	NM_005402	Homo sapiens v-ral simian leukemia viral oncogene homolog A (ras related) (RALA), mRNA [NM_005402]	NM_005402
A_24_P64233	0.000203	2.12	NM_000692	NM_000692	Homo sapiens aldehyde dehydrogenase 1 family, member B1 (ALDH1B1), nuclear gene encoding mitochondrial protein, mRNA [NM_000692]	NM_000692
A_32_P918263	0.000203	2.76	A_32_P918263			
A_23_P103110	0.000203	3.11	NM_012323	NM_012323	Homo sapiens v-maf musculoaponeurotic fibrosarcoma oncogene homolog F (avian) (MAFF), transcript variant 1, mRNA [NM_012323]	NM_012323
A_24_P123166	0.000204	2.49	NM_022171	NM_022171	Homo sapiens T-cell leukemia translocation altered gene (TCTA), mRNA [NM_022171]	NM_022171
A_23_P126908	0.000204	3.12	NM_003820	NM_003820	Homo sapiens tumor necrosis factor receptor superfamily, member 14 (herpesvirus entry mediator) (TNFRSF14), mRNA [NM_003820]	NM_003820
A_24_P255786	0.000205	2.25	A_24_P255786			
A_24_P152833	0.000205	2.36	CV323793	CV323793	CV323793 CM4-CN0090-130201-722-e04 CN0090 Homo sapiens cDNA, mRNA sequence [CV323793]	
A_23_P251293	0.000205	3.28	NM_003087	NM_003087	Homo sapiens synuclein, gamma (breast cancer-specific protein 1) (SNCG), mRNA [NM_003087]	NM_003087
A_23_P333420	0.000205	3.42	NM_002883	NM_002883	Homo sapiens Ran GTPase activating protein 1 (RANGAP1), mRNA [NM_002883]	NM_002883
A_23_P23380	0.000205	4.39	NM_022821	NM_022821	Homo sapiens elongation of very long chain fatty acids (FEN1/Elo2, SUR4/Elo3, yeast)-like 1 (ELOVL1), mRNA [NM_022821]	NM_022821
A_23_P200067	0.000206	2.49	NM_004442	NM_004442	Homo sapiens EPH receptor B2 (EPHB2), transcript variant 2, mRNA [NM_004442]	NM_004442
A_24_P237686	0.000206	4.10	ENST00000236199			
A_23_P214026	0.000206	4.59	NM_001999	NM_001999	Homo sapiens fibrillin 2 (congenital contractural arachnodactyly) (FBN2), mRNA [NM_001999]	NM_001999
A_23_P420428	0.000207	2.06	NM_053041	NM_053041	Homo sapiens COMM domain containing 7 (COMMD7), mRNA [NM_053041]	NM_053041
A_24_P252078	0.000208	2.74	BC067086	BC067086	Homo sapiens cDNA clone MGC:71335 IMAGE:6088873, complete cds. [BC067086]	
A_32_P149432	0.000209	2.16	NM_001416	NM_001416	Homo sapiens eukaryotic translation initiation factor 4A, isoform 1 (EIF4A1), mRNA [NM_001416]	NM_001416
A_23_P145711	0.000209	2.87	NM_024728	NM_024728	Homo sapiens chromosome 7 open reading frame 10 (C7orf10), mRNA [NM_024728]	NM_024728
A_23_P51339	0.000209	3.19	NM_007034	NM_007034	Homo sapiens DnaJ (Hsp40) homolog, subfamily B, member 4 (DNAJB4), mRNA [NM_007034]	NM_007034
A_24_P347378	0.000209	12.12	NM_001629	NM_001629	Homo sapiens arachidonate 5-lipoxygenase-activating protein (ALOX5AP), mRNA [NM_001629]	NM_001629
A_32_P28528	0.00021	2.01	A_32_P28528			
A_24_P253755	0.00021	2.34	NM_004278	NM_004278	Homo sapiens phosphatidylinositol glycan, class L (PIGL), mRNA [NM_004278]	NM_004278
A_24_P237265	0.00021	3.56	NM_002745	NM_002745	Homo sapiens mitogen-activated protein kinase 1 (MAPK1), transcript variant 1, mRNA [NM_002745]	NM_002745
A_23_P83857	0.00021	3.58	NM_000240	NM_000240	Homo sapiens monoamine oxidase A (MAOA), nuclear gene encoding mitochondrial protein, mRNA [NM_000240]	NM_000240
A_23_P217049	0.000211	2.44	NM_014286	NM_014286	Homo sapiens frequenin homolog (Drosophila) (FREQ), mRNA [NM_014286]	NM_014286
A_23_P91468	0.000211	2.80	NM_002792	NM_002792	Homo sapiens proteasome (prosome, macropain) subunit, alpha type, 7 (PSMA7), transcript variant 1, mRNA [NM_002792]	NM_002792
A_24_P252602	0.000211	3.10	NM_014286	NM_014286	Homo sapiens frequenin homolog (Drosophila) (FREQ), mRNA [NM_014286]	NM_014286

A_23_P337934	0.000211	3.14	NM_017556	NM_017556	Homo sapiens filamin binding LIM protein 1 (FBLIM1), transcript variant 1, mRNA [NM_017556]	NM_017556
A_24_P594094	0.000211	4.57	A_24_P594094			
A_23_P155316	0.000212	2.38	NM_007362	NM_007362	Homo sapiens nuclear cap binding protein subunit 2, 20kDa (NCBP2), mRNA [NM_007362]	NM_007362
A_24_P324886	0.000212	3.10	AK098050	AK098050	Homo sapiens cDNA FLJ40731 fis, clone TKIDN2000521. [AK098050]	
A_23_P344973	0.000212	3.97	NM_079423	NM_079423	Homo sapiens myosin, light polypeptide 6, alkali, smooth muscle and non-muscle (MYL6), transcript variant 2, mRNA [NM_079423]	NM_079423
A_23_P156861	0.000212	12.35	NM_012419	NM_012419	Homo sapiens regulator of G-protein signalling 17 (RGS17), mRNA [NM_012419]	NM_012419
A_23_P393531	0.000214	2.11	NM_004027	NM_004027	Homo sapiens inositol polyphosphate-4-phosphatase, type I, 107kDa (INPP4A), transcript variant a, mRNA [NM_004027]	NM_004027
A_23_P312224	0.000214	3.23	NM_019022	NM_019022	Homo sapiens thioredoxin domain containing 10 (TXNDC10), mRNA [NM_019022]	NM_019022
A_23_P62741	0.000214	7.69	BC025721	BC025721	Homo sapiens EGF, latrophilin and seven transmembrane domain containing 1, mRNA (cDNA clone MGC:34204 IMAGE:5229055), complete cds. [BC025721]	XM_371262
A_23_P111635	0.000215	2.05	NM_016086	NM_016086	Homo sapiens dual specificity phosphatase 24 (putative) (DUSP24), mRNA [NM_016086]	NM_016086
A_23_P147950	0.000215	2.15	AK057293	AK057293	Homo sapiens cDNA FLJ32731 fis, clone TESTI2001134. [AK057293]	XM_372038
A_32_P186038	0.000215	2.23	THC2359128		RS13_CHICK (Q6ITC7) 40S ribosomal protein S13, partial (48%) [THC2359128]	
A_23_P334870	0.000215	4.12	NM_145316	NM_145316	Homo sapiens chromosome 6 open reading frame 128 (C6orf128), mRNA [NM_145316]	NM_145316
A_32_P24762	0.000215	7.58	THC2272599		CA438893 UI-H-DH0-aut-f-13-0-UI.s1 NCI_CGAP_DH0 Homo sapiens cDNA clone UI-H-DH0-aut-f-13-0-UI 3', mRNA sequence [CA438893]	
A_24_P60680	0.000216	2.45	NM_013941	NM_013941	Homo sapiens olfactory receptor, family 10, subfamily C, member 1 (OR10C1), mRNA [NM_013941]	NM_013941
A_23_P87279	0.000218	2.08	NM_014555	NM_014555	Homo sapiens transient receptor potential cation channel, subfamily M, member 5 (TRPM5), mRNA [NM_014555]	NM_014555
A_23_P206661	0.000218	2.27	NM_000903	NM_000903	Homo sapiens NAD(P)H dehydrogenase, quinone 1 (NQO1), transcript variant 1, mRNA [NM_000903]	NM_000903
A_23_P397856	0.000218	2.29	NM_052887	NM_052887	Homo sapiens toll-interleukin 1 receptor (TIR) domain containing adaptor protein (TIRAP), transcript variant 1, mRNA [NM_052887]	NM_052887
A_23_P93442	0.000218	2.33	NM_015278	NM_015278	Homo sapiens SAM and SH3 domain containing 1 (SASH1), mRNA [NM_015278]	NM_015278
A_23_P42375	0.000218	3.21	NM_006834	NM_006834	Homo sapiens RAB32, member RAS oncogene family (RAB32), mRNA [NM_006834]	NM_006834
A_23_P54840	0.000218	4.18	NM_005946	NM_005946	Homo sapiens metallothionein 1A (functional) (MT1A), mRNA [NM_005946]	NM_005946
A_24_P34155	0.000218	4.37	D43967	D43967	Homo sapiens mRNA for AML1a protein, complete cds. [D43967]	
A_23_P255111	0.000218	7.69	A_23_P255111			
A_23_P30784	0.000219	2.26	NM_013375	NM_013375	Homo sapiens activator of basal transcription 1 (ABT1), mRNA [NM_013375]	NM_013375
A_32_P119998	0.000219	3.28	AI608782	AI608782	AI608782 tw94g05.x1 NCI_CGAP_HN6 Homo sapiens cDNA clone IMAGE:2267384 3' similar to gb:K00558 TUBULIN ALPHA-1 CHAIN (HUMAN);, mRNA sequence [AI608782]	
A_23_P200015	0.000219	42.19	NM_174858	NM_174858	Homo sapiens adenylate kinase 5 (AK5), transcript variant 1, mRNA [NM_174858]	NM_174858
A_24_P222139	0.00022	3.34	AK025047	AK025047	Homo sapiens cDNA: FLJ21394 fis, clone COL03536. [AK025047]	
A_24_P52921	0.000221	3.53	NM_005504	NM_005504	Homo sapiens branched chain aminotransferase 1, cytosolic (BCAT1), mRNA [NM_005504]	NM_005504
A_23_P91829	0.000222	6.94	NM_080927	NM_080927	Homo sapiens discoidin, CUB and LCCL domain containing 2 (DCBLD2), mRNA [NM_080927]	NM_080927
A_24_P915371	0.000224	2.15	AF487338	AF487338	Homo sapiens multiple myeloma overexpression gene 2 (MYEOV2) mRNA, complete cds. [AF487338]	
A_23_P256540	0.000224	2.18	NM_004891	NM_004891	Homo sapiens mitochondrial ribosomal protein L33 (MRPL33), nuclear gene encoding mitochondrial protein, transcript variant 1, mRNA [NM_004891]	NM_004891
A_23_P157628	0.000224	2.27	NM_004942	NM_004942	Homo sapiens defensin, beta 4 (DEFB4), mRNA [NM_004942]	NM_004942
A_24_P49421	0.000224	2.50	ENST00000271124			
A_24_P247303	0.000224	2.60	A_24_P247303			
A_23_P148162	0.000224	2.79	NM_018269	NM_018269	Homo sapiens membrane-type 1 matrix metalloproteinase cytoplasmic tail binding protein-1 (MTCBP-1), mRNA [NM_018269]	NM_018269
A_32_P181891	0.000224	3.79	AK097571	AK097571	Homo sapiens cDNA FLJ40252 fis, clone TESTI2024299. [AK097571]	
A_23_P111206	0.000224	7.87	NM_004117	NM_004117	Homo sapiens FK506 binding protein 5 (FKBP5), mRNA [NM_004117]	NM_004117
A_32_P232883	0.000224	9.17	BC089451	BC089451	Homo sapiens cDNA clone IMAGE:30554612. [BC089451]	

A_23_P339582	0.000225	2.21	NM_144970	NM_144970	Homo sapiens chromosome X open reading frame 38 (CXorf38), mRNA [NM_144970]	NM_144970
A_23_P80086	0.000225	2.78	NM_013240	NM_013240	Homo sapiens chromosome 21 open reading frame 127 (C21orf127), transcript variant 1, mRNA [NM_013240]	NM_013240
A_23_P5831	0.000225	3.94	NM_134421	NM_134421	Homo sapiens hippocalcin-like 1 (HPCAL1), transcript variant 2, mRNA [NM_134421]	NM_134421
A_23_P119362	0.000226	2.89	NM_001425	NM_001425	Homo sapiens epithelial membrane protein 3 (EMP3), mRNA [NM_001425]	NM_001425
A_23_P161624	0.000226	7.35	NM_005438	NM_005438	Homo sapiens FOS-like antigen 1 (FOSL1), mRNA [NM_005438]	NM_005438
A_23_P369983	0.000227	2.17	NM_174905	NM_174905	Homo sapiens hypothetical protein LOC147965 (LOC147965), mRNA [NM_174905]	NM_174905
A_23_P137361	0.000227	2.21	NM_017818	NM_017818	Homo sapiens WD repeat domain 8 (WDR8), mRNA [NM_017818]	NM_017818
A_24_P207139	0.000227	2.29	NM_033238	NM_033238	Homo sapiens promyelocytic leukemia (PML), transcript variant 1, mRNA [NM_033238]	NM_033238
A_23_P57877	0.000227	2.36	NM_000688	NM_000688	Homo sapiens aminolevulinate, delta-, synthase 1 (ALAS1), transcript variant 1, mRNA [NM_000688]	NM_000688
A_23_P330209	0.000227	2.48	NM_057178	NM_057178	Homo sapiens rififylin (RFFL), transcript variant 1, mRNA [NM_057178]	NM_057178
A_23_P133606	0.000227	3.40	NM_001046	NM_001046	Homo sapiens solute carrier family 12 (sodium/potassium/chloride transporters), member 2 (SLC12A2), mRNA [NM_001046]	NM_001046
A_24_P358305	0.000227	3.47	A_24_P358305			
A_32_P140289	0.000228	2.05	CR601567	CR601567	full-length cDNA clone CS0DN003YI06 of Adult brain of Homo sapiens (human). [CR601567]	
A_23_P336040	0.000228	3.03	NM_020399	NM_020399	Homo sapiens golgi associated PDZ and coiled-coil motif containing (GOPC), transcript variant 1, mRNA [NM_020399]	NM_020399
A_24_P152404	0.000229	2.52	BC032118	BC032118	Homo sapiens cDNA clone IMAGE:5016307, partial cds. [BC032118]	
A_23_P155755	0.000229	56.18	NM_002993	NM_002993	Homo sapiens chemokine (C-X-C motif) ligand 6 (granulocyte chemotactic protein 2) (CXCL6), mRNA [NM_002993]	NM_002993
A_23_P35848	0.00023	2.01	NM_018161	NM_018161	Homo sapiens NAD synthetase 1 (NADSYN1), mRNA [NM_018161]	NM_018161
A_24_P73389	0.00023	2.65	NM_003576	NM_003576	Homo sapiens serine/threonine kinase 24 (STE20 homolog, yeast) (STK24), mRNA [NM_003576]	NM_003576
A_23_P362659	0.000231	3.05	NM_002468	NM_002468	Homo sapiens myeloid differentiation primary response gene (88) (MYD88), mRNA [NM_002468]	NM_002468
A_23_P4572	0.000231	3.07	NM_006471	NM_006471	Homo sapiens myosin regulatory light chain MRCL3 (MRCL3), mRNA [NM_006471]	NM_006471
A_23_P203498	0.000231	11.79	NM_006074	NM_006074	Homo sapiens tripartite motif-containing 22 (TRIM22), mRNA [NM_006074]	NM_006074
A_32_P486693	0.000232	6.76	AK093229	AK093229	Homo sapiens cDNA FLJ35910 fis, clone TESTI2009987. [AK093229]	
A_23_P141974	0.000233	2.10	NM_003290	NM_003290	Homo sapiens tropomyosin 4 (TPM4), mRNA [NM_003290]	NM_003290
A_23_P27256	0.000233	2.53	NM_020648	NM_020648	Homo sapiens twisted gastrulation homolog 1 (Drosophila) (TWSG1), mRNA [NM_020648]	NM_020648
A_23_P4662	0.000233	2.77	NM_005178	NM_005178	Homo sapiens B-cell CLL/lymphoma 3 (BCL3), mRNA [NM_005178]	NM_005178
A_24_P470754	0.000233	2.79	A_24_P470754			
A_23_P154929	0.000233	3.95	NM_197966	NM_197966	Homo sapiens BH3 interacting domain death agonist (BID), transcript variant 1, mRNA [NM_197966]	NM_197966
A_23_P34176	0.000234	2.08	NM_015691	NM_015691	Homo sapiens KIAA1280 protein (KIAA1280), mRNA [NM_015691]	NM_015691
A_32_P53486	0.000234	2.13	BC022832	BC022832	Homo sapiens bolA-like 2 (E. coli), mRNA (cDNA clone IMAGE:4647797), with apparent retained intron. [BC022832]	
A_24_P161393	0.000234	2.24	A_24_P161393			
A_23_P135364	0.000234	2.82	NM_012145	NM_012145	Homo sapiens deoxythymidylate kinase (thymidylate kinase) (DTYMK), mRNA [NM_012145]	NM_012145
A_24_P692030	0.000234	3.16	A_24_P692030			
A_24_P402080	0.000234	4.12	NM_001025100	NM_001025100	Homo sapiens myelin basic protein (MBP), transcript variant 8, mRNA [NM_001025100]	NM_001025100
A_24_P326660	0.000235	5.08	NM_006500	NM_006500	Homo sapiens melanoma cell adhesion molecule (MCAM), mRNA [NM_006500]	NM_006500
A_23_P134125	0.000235	10.01	NM_005923	NM_005923	Homo sapiens mitogen-activated protein kinase kinase kinase 5 (MAP3K5), mRNA [NM_005923]	NM_005923
A_24_P298013	0.000236	2.51	NM_019096	NM_019096	Homo sapiens GTP binding protein 2 (GTPBP2), mRNA [NM_019096]	NM_019096
A_23_P57413	0.000236	7.04	NM_014634	NM_014634	Homo sapiens protein phosphatase 1F (PP2C domain containing) (PPM1F), mRNA [NM_014634]	NM_014634
A_23_P115407	0.000237	2.35	NM_146421	NM_146421	Homo sapiens glutathione S-transferase M1 (GSTM1), transcript variant 2, mRNA [NM_146421]	NM_146421
A_23_P432583	0.000237	4.52	NM_016178	NM_016178	Homo sapiens ornithine decarboxylase antizyme 3 (OAZ3), mRNA [NM_016178]	NM_016178
A_24_P267997	0.000238	2.21	NM_015602	NM_015602	Homo sapiens torsin A interacting protein 1 (TOR1AIP1), mRNA [NM_015602]	NM_015602

A_23_P206280	0.000238	2.48	NM_201525	NM_201525	Homo sapiens G protein-coupled receptor 56 (GPR56), transcript variant 3, mRNA [NM_201525]	NM_201525
A_24_P93901	0.000238	3.45	BC063531	BC063531	Homo sapiens SIN3 homolog B, transcription regulator (yeast), mRNA (cDNA clone IMAGE:4417458), complete cds. [BC063531]	
A_32_P226078	0.000238	3.47	NM_016178	NM_016178	Homo sapiens ornithine decarboxylase antizyme 3 (OAZ3), mRNA [NM_016178]	NM_016178
A_24_P337796	0.000238	5.38	NM_004760	NM_004760	Homo sapiens serine/threonine kinase 17a (apoptosis-inducing) (STK17A), mRNA [NM_004760]	NM_004760
A_23_P50146	0.000238	5.59	NM_213602	NM_213602	Homo sapiens CD33 antigen-like 3 (CD33L3), mRNA [NM_213602]	NM_213602
A_24_P53051	0.000239	2.18	NM_171846	NM_171846	Homo sapiens lactamase, beta (LACTB), nuclear gene encoding mitochondrial protein, transcript variant 2, mRNA [NM_171846]	NM_171846
A_24_P521662	0.000239	2.35	A_24_P521662			
A_24_P367329	0.000239	2.83	ENST00000332813			XM_496078
A_23_P89550	0.000239	7.63	NM_033004	NM_033004	Homo sapiens NACHT, leucine rich repeat and PYD (pyrin domain) containing 1 (NALP1), transcript variant 1, mRNA [NM_033004]	NM_033004
A_23_P397238	0.00024	2.60	NM_054014	NM_054014	Homo sapiens FK506 binding protein 1A, 12kDa (FKBP1A), transcript variant 12A, mRNA [NM_054014]	NM_054014
A_24_P669216	0.00024	3.09	THC2314754		O99381 (O99381) Cytochrome C oxidase subunit I (Fragment), partial (5%) [THC2314754]	
A_23_P7655	0.000241	2.23	NM_013978	NM_013978	Homo sapiens BCL2/adenovirus E1B 19kDa interacting protein 1 (BNIP1), transcript variant BNIP1-a, mRNA [NM_013978]	NM_013978
A_24_P943301	0.000241	2.47	ENST00000292357		Homo sapiens cDNA FLJ25943 fis, clone JTH10559. [AK098809]	
A_23_P154698	0.000241	2.69	NM_003818	NM_003818	Homo sapiens CDP-diacylglycerol synthase (phosphatidate cytidylyltransferase) 2 (CDS2), mRNA [NM_003818]	NM_003818
A_23_P502196	0.000241	3.10	NM_174855	NM_174855	Homo sapiens isocitrate dehydrogenase 3 (NAD+) beta (IDH3B), nuclear gene encoding mitochondrial protein, transcript variant 2, mRNA [NM_174855]	NM_174855
A_32_P44139	0.000241	31.55	AA627222	AA627222	AA627222 nq63b01.s1 NCI_CGAP_Ov6 Homo sapiens cDNA clone IMAGE:1154665 similar to gb:J04456 GALECTIN-1 (HUMAN);, mRNA sequence [AA627222]	
A_23_P13914	0.000242	2.20	NM_032656	NM_032656	Homo sapiens DEAH (Asp-Glu-Ala-His) box polypeptide 37 (DHX37), mRNA [NM_032656]	NM_032656
A_32_P184916	0.000242	2.54	NM_021629	NM_021629	Homo sapiens guanine nucleotide binding protein (G protein), beta polypeptide 4 (GNB4), mRNA [NM_021629]	NM_021629
A_23_P126782	0.000243	2.16	NM_001993	NM_001993	Homo sapiens coagulation factor III (thromboplastin, tissue factor) (F3), mRNA [NM_001993]	NM_001993
A_24_P247660	0.000243	2.89	NM_001002033	NM_001002033	Homo sapiens hematological and neurological expressed 1 (HN1), transcript variant 3, mRNA [NM_001002033]	NM_001002033
A_24_P393958	0.000243	2.89	NM_007034	NM_007034	Homo sapiens DnaJ (Hsp40) homolog, subfamily B, member 4 (DNAJB4), mRNA [NM_007034]	NM_007034
A_24_P301146	0.000243	5.59	NM_005358	NM_005358	Homo sapiens LIM domain 7 (LMO7), mRNA [NM_005358]	NM_005358
A_24_P319923	0.000243	24.33	NM_053025	NM_053025	Homo sapiens myosin, light polypeptide kinase (MYLK), transcript variant 1, mRNA [NM_053025]	NM_053025
A_24_P252057	0.000244	2.44	NM_153020	NM_153020	Homo sapiens RNA binding motif protein 24 (RBM24), mRNA [NM_153020]	NM_153020
A_23_P18186	0.000244	3.92	NM_021629	NM_021629	Homo sapiens guanine nucleotide binding protein (G protein), beta polypeptide 4 (GNB4), mRNA [NM_021629]	NM_021629
A_23_P99216	0.000245	2.79	NM_015918	NM_015918	Homo sapiens processing of precursor 5, ribonuclease P/MRP subunit (S. cerevisiae) (POP5), transcript variant 1, mRNA [NM_015918]	NM_015918
A_23_P158083	0.000246	2.97	NM_001261	NM_001261	Homo sapiens cyclin-dependent kinase 9 (CDC2-related kinase) (CDK9), mRNA [NM_001261]	NM_001261
A_23_P87421	0.000246	23.81	BC063022	BC063022	Homo sapiens cDNA clone IMAGE:5246259, partial cds. [BC063022]	
A_23_P167674	0.000247	2.03	NM_000505	NM_000505	Homo sapiens coagulation factor XII (Hageman factor) (F12), mRNA [NM_000505]	NM_000505
A_23_P100868	0.000247	2.31	AK026518	AK026518	Homo sapiens cDNA: FLJ22865 fis, clone KAT02171. [AK026518]	
A_32_P122925	0.000247	2.44	CR594732	CR594732	full-length cDNA clone CS0DE010YB08 of Placenta of Homo sapiens (human). [CR594732]	
A_23_P206960	0.000247	2.47	NM_003003	NM_003003	Homo sapiens SEC14-like 1 (S. cerevisiae) (SEC14L1), mRNA [NM_003003]	NM_003003
A_24_P96171	0.000247	2.51	NM_030578	NM_030578	Homo sapiens hypothetical protein MGC4093 (MGC4093), mRNA [NM_030578]	NM_030578
A_32_P512061	0.000247	2.56	NR_002188	NR_002188	Homo sapiens glucosidase, beta; acid, pseudogene (GBAP) on chromosome 1 [NR_002188]	NR_002188
A_23_P319719	0.000247	3.80	NM_138575	NM_138575	Homo sapiens Bcl-XL-binding protein v68 (MGC5352), mRNA [NM_138575]	NM_138575
A_24_P371281	0.000247	3.80	NM_025246	NM_025246	Homo sapiens transmembrane protein 22 (TMEM22), mRNA [NM_025246]	NM_025246
A_23_P148308	0.000247	3.91	NM_001017430	NM_001017430	Homo sapiens RNA binding motif (RNP1, RRM) protein 3 (RBM3), transcript variant 2, mRNA [NM_001017430]	NM_001017430
A_23_P23346	0.000248	2.13	NM_006818	NM_006818	Homo sapiens myeloid/lymphoid or mixed-lineage leukemia (trithorax homolog, Drosophila); translocated to, 11 (MLLT11), mRNA [NM_006818]	NM_006818

A_24_P134526	0.000248	4.31	NM_007311	NM_007311	Homo sapiens benzodiazapine receptor (peripheral) (BZRP), transcript variant PBR-S, mRNA [NM_007311]	NM_007311
A_24_P313397	0.00025	3.14	CR621534	CR621534	full-length cDNA clone CS0DI061YF17 of Placenta Cot 25-normalized of Homo sapiens (human). [CR621534]	
A_23_P108673	0.00025	5.99	NM_032181	NM_032181	Homo sapiens hypothetical protein FLJ13391 (FLJ13391), mRNA [NM_032181]	NM_032181
A_23_P113393	0.00025	19.53	NM_017413	NM_017413	Homo sapiens apelin, AGTRL1 ligand (APLN), mRNA [NM_017413]	NM_017413
A_23_P200940	0.000251	2.42	NM_006347	NM_006347	Homo sapiens peptidyl prolyl isomerase H (cyclophilin H) (PPIH), mRNA [NM_006347]	NM_006347
A_23_P129492	0.000251	2.56	NM_016332	NM_016332	Homo sapiens selenoprotein X, 1 (SEPX1), mRNA [NM_016332]	NM_016332
A_24_P929083	0.000251	3.33	THC2275899		BC073800 BTB (POZ) domain containing 4 {Homo sapiens;} , partial (3%) [THC2275899]	
A_23_P151159	0.000251	3.47	NM_032300	NM_032300	Homo sapiens hypothetical protein MGC10854 (MGC10854), mRNA [NM_032300]	NM_032300
A_23_P405088	0.000251	3.51	NM_005602	NM_005602	Homo sapiens claudin 11 (oligodendrocyte transmembrane protein) (CLDN11), mRNA [NM_005602]	NM_005602
A_32_P217655	0.000251	6.90	XM_496405	XM_496405	PREDICTED: Homo sapiens similar to D(1B) dopamine receptor (D(5) dopamine receptor) (D1beta dopamine receptor) (LOC440684), mRNA [XM_496405]	XM_496405
A_23_P71981	0.000253	2.11	NM_005702	NM_005702	Homo sapiens Era G-protein-like 1 (E. coli) (ERAL1), mRNA [NM_005702]	NM_005702
A_24_P287780	0.000253	2.44	NM_002768	NM_002768	Homo sapiens procollagen (type III) N-endopeptidase (PCOLN3), mRNA [NM_002768]	NM_002768
A_32_P191527	0.000253	2.96	NM_020382	NM_020382	Homo sapiens PR/SET domain containing protein 8 (SET8), mRNA [NM_020382]	NM_020382
A_23_P64129	0.000253	4.20	NM_006410	NM_006410	Homo sapiens HIV-1 Tat interactive protein 2, 30kDa (HTATIP2), mRNA [NM_006410]	NM_006410
A_23_P15202	0.000255	2.06	NM_001361	NM_001361	Homo sapiens dihydroorotate dehydrogenase (DHODH), nuclear gene encoding mitochondrial protein, transcript variant 1, mRNA [NM_001361]	NM_001361
A_23_P166193	0.000255	2.40	NM_018217	NM_018217	Homo sapiens chromosome 20 open reading frame 31 (C20orf31), mRNA [NM_018217]	NM_018217
A_24_P593733	0.000256	2.14	XM_497005	XM_497005	PREDICTED: Homo sapiens similar to G protein pathway suppressor 2 (LOC392281), mRNA [XM_497005]	XM_497005
A_24_P348892	0.000256	2.79	NM_052849	NM_052849	Homo sapiens hypothetical protein MGC20481 (MGC20481), mRNA [NM_052849]	NM_052849
A_24_P621434	0.000256	3.08	THC2266610		BC007276 HSPA8 protein {Homo sapiens;} , partial (17%) [THC2266610]	
A_24_P919304	0.000256	3.65	BC012204	BC012204	Homo sapiens fibronectin type III domain containing 3B, mRNA (cDNA clone IMAGE:3882800), complete cds. [BC012204]	
A_23_P65174	0.000256	3.94	NM_016119	NM_016119	Homo sapiens PHD finger protein 11 (PHF11), mRNA [NM_016119]	NM_016119
A_24_P1545	0.000257	3.31	NM_015633	NM_015633	Homo sapiens FGFR1 oncogene partner 2 (FGFR1OP2), mRNA [NM_015633]	NM_015633
A_23_P151415	0.000257	3.58	NM_032116	NM_032116	Homo sapiens katanin p60 subunit A-like 1 (KATNAL1), transcript variant 1, mRNA [NM_032116]	NM_032116
A_23_P210849	0.000257	3.82	NM_002862	NM_002862	Homo sapiens phosphorylase, glycogen; brain (PYGB), mRNA [NM_002862]	NM_002862
A_24_P23034	0.000257	4.22	NM_021035	NM_021035	Homo sapiens KIAA1404 protein (KIAA1404), mRNA [NM_021035]	NM_021035
A_24_P235049	0.000258	3.14	NM_015440	NM_015440	Homo sapiens methylenetetrahydrofolate dehydrogenase (NADP+ dependent) 1-like (MTHFD1L), mRNA [NM_015440]	NM_015440
A_23_P111228	0.000258	3.73	NM_017421	NM_017421	Homo sapiens coenzyme Q3 homolog, methyltransferase (yeast) (COQ3), mRNA [NM_017421]	NM_017421
A_32_P207494	0.000259	2.07	NM_025115	NM_025115	Homo sapiens chromosome 8 open reading frame 41 (C8orf41), mRNA [NM_025115]	NM_025115
A_23_P12423	0.000259	2.14	NM_022089	NM_022089	Homo sapiens ATPase type 13A2 (ATP13A2), mRNA [NM_022089]	NM_022089
A_23_P154675	0.000259	2.65	NM_198216	NM_198216	Homo sapiens small nuclear ribonucleoprotein polypeptides B and B1 (SNRPB), transcript variant 1, mRNA [NM_198216]	NM_198216
A_24_P293120	0.000261	2.22	NM_024327	NM_024327	Homo sapiens zinc finger protein 576 (ZNF576), mRNA [NM_024327]	NM_024327
A_24_P610375	0.000261	2.31	CA306742	CA306742	CA306742 UI-H-FT1-bht-f-03-0-UI.s1 NCI_CGAP_FT1 Homo sapiens cDNA clone UI-H-FT1-bht-f-03-0-UI 3', mRNA sequence [CA306742]	
A_24_P71834	0.000261	2.68	NM_016141	NM_016141	Homo sapiens dynein, cytoplasmic, light intermediate polypeptide 1 (DNCLI1), mRNA [NM_016141]	NM_016141
A_23_P357929	0.000261	4.42	NM_015139	NM_015139	Homo sapiens solute carrier family 35 (UDP-glucuronic acid/UDP-N-acetylgalactosamine dual transporter), member D1 (SLC35D1), mRNA [NM_015139]	NM_015139
A_23_P102706	0.000261	4.57	NM_014723	NM_014723	Homo sapiens syntaphilin (SNPH), mRNA [NM_014723]	NM_014723
A_23_P72651	0.000261	10.92	THC2337324		EOMD_HUMAN (O95936) Eomesodermin homolog, partial (3%) [THC2337324]	
A_23_P50990	0.000262	2.23	NM_024322	NM_024322	Homo sapiens hypothetical protein MGC11266 (MGC11266), mRNA [NM_024322]	NM_024322
A_23_P23924	0.000262	2.73	NM_001748	NM_001748	Homo sapiens calpain 2, (m/II) large subunit (CAPN2), mRNA [NM_001748]	NM_001748
A_24_P186370	0.000262	7.09	NM_002444	NM_002444	Homo sapiens moesin (MSN), mRNA [NM_002444]	NM_002444

A_23_P137103	0.000263	2.01	NM_001416	NM_001416	Homo sapiens eukaryotic translation initiation factor 4A, isoform 1 (EIF4A1), mRNA [NM_001416]	NM_001416
A_23_P145863	0.000263	2.69	NM_005620	NM_005620	Homo sapiens S100 calcium binding protein A11 (calgizzarin) (S100A11), mRNA [NM_005620]	NM_005620
A_24_P54863	0.000263	6.13	NM_152400	NM_152400	Homo sapiens hypothetical protein FLJ39370 (FLJ39370), mRNA [NM_152400]	NM_152400
A_23_P102842	0.000264	2.14	NM_021100	NM_021100	Homo sapiens NFS1 nitrogen fixation 1 (S. cerevisiae) (NFS1), nuclear gene encoding mitochondrial protein, transcript variant 1, mRNA [NM_021100]	NM_021100
A_23_P218079	0.000264	3.60	NM_018976	NM_018976	Homo sapiens solute carrier family 38, member 2 (SLC38A2), mRNA [NM_018976]	NM_018976
A_24_P238855	0.000264	3.97	NM_020382	NM_020382	Homo sapiens PR/SET domain containing protein 8 (SET8), mRNA [NM_020382]	NM_020382
A_23_P36825	0.000265	10.80	NM_003979	NM_003979	Homo sapiens G protein-coupled receptor, family C, group 5, member A (GPRC5A), mRNA [NM_003979]	NM_003979
A_24_P930327	0.000266	2.04	A_24_P930327			
A_23_P6119	0.000267	2.19	NM_032986	NM_032986	Homo sapiens Sec23 homolog B (S. cerevisiae) (SEC23B), transcript variant 3, mRNA [NM_032986]	NM_032986
A_24_P332341	0.000267	2.60	ENST00000306744		Homo sapiens hypothetical protein MGC13098, mRNA (cDNA clone IMAGE:4074285), partial cds. [BC062722]	XM_379819
A_23_P87013	0.000267	6.25	NM_001001522	NM_001001522	Homo sapiens transgelin (TAGLN), transcript variant 1, mRNA [NM_001001522]	NM_001001522
A_23_P154199	0.000268	2.11	NM_002712	NM_002712	Homo sapiens protein phosphatase 1, regulatory subunit 7 (PPP1R7), mRNA [NM_002712]	NM_002712
A_23_P341567	0.000269	3.53	NM_178833	NM_178833	Homo sapiens hypothetical protein BC009732 (LOC133308), mRNA [NM_178833]	NM_178833
A_32_P117464	0.000269	3.68	NM_178496	NM_178496	Homo sapiens similar to BcDNA:GH11415 gene product (LOC151963), mRNA [NM_178496]	NM_178496
A_24_P827794	0.00027	2.50	ENST00000333401			
A_32_P4505	0.000273	3.06	BQ007298	BQ007298	UI-1-BC0-afm-c-04-0-UI.s1 NCI_CGAP_Pl1 Homo sapiens cDNA clone UI-1-BC0-afm-c-04-0-UI 3', mRNA sequence [BQ007298]	
A_24_P928052	0.000273	3.33	NM_003873	NM_003873	Homo sapiens neuropilin 1 (NRP1), transcript variant 1, mRNA [NM_003873]	NM_003873
A_23_P123666	0.000274	3.91	NM_018421	NM_018421	Homo sapiens TBC1 domain family, member 2 (TBC1D2), mRNA [NM_018421]	NM_018421
A_23_P216920	0.000274	5.35	NM_014397	NM_014397	Homo sapiens NIMA (never in mitosis gene a)-related kinase 6 (NEK6), mRNA [NM_014397]	NM_014397
A_23_P80763	0.000275	2.09	BC017572	BC017572	Homo sapiens poliovirus receptor-related 3, mRNA (cDNA clone IMAGE:4066485), complete cds. [BC017572]	
A_24_P262738	0.000275	2.82	NM_024050	NM_024050	Homo sapiens cross-immune reaction antigen PCIA1 (PCIA1), mRNA [NM_024050]	NM_024050
A_24_P243278	0.000275	3.77	NM_004394	NM_004394	Homo sapiens death-associated protein (DAP), mRNA [NM_004394]	NM_004394
A_23_P164141	0.000276	2.17	NM_176863	NM_176863	Homo sapiens proteasome (prosome, macropain) activator subunit 3 (PA28 gamma; Ki) (PSME3), transcript variant 2, mRNA [NM_176863]	NM_176863
A_23_P2843	0.000276	2.64	NM_017905	NM_017905	Homo sapiens transmembrane and coiled-coil domains 3 (TMCO3), mRNA [NM_017905]	NM_017905
A_23_P34366	0.000276	4.29	NM_000864	NM_000864	Homo sapiens 5-hydroxytryptamine (serotonin) receptor 1D (HTR1D), mRNA [NM_000864]	NM_000864
A_24_P727884	0.000277	19.08	U10991	U10991	Human G2 protein mRNA, partial cds. [U10991]	XM_039515
A_23_P392897	0.000278	2.09	A_23_P392897			
A_32_P123176	0.000278	2.48	AA573471	AA573471	AA573471 nm53f10.s1 NCI_CGAP_Br2 Homo sapiens cDNA clone IMAGE:1071979 3', mRNA sequence [AA573471]	
A_24_P799048	0.000278	9.35	AX721193	AX721193	Sequence 153 from Patent WO0220754. [AX721193]	
A_23_P122924	0.000278	13.95	NM_002192	NM_002192	Homo sapiens inhibin, beta A (activin A, activin AB alpha polypeptide) (INHBA), mRNA [NM_002192]	NM_002192
A_24_P390793	0.00028	2.05	NM_001416	NM_001416	Homo sapiens eukaryotic translation initiation factor 4A, isoform 1 (EIF4A1), mRNA [NM_001416]	NM_001416
A_32_P29118	0.00028	2.16	NM_152754	NM_152754	Homo sapiens sema domain, immunoglobulin domain (Ig), short basic domain, secreted, (semaphorin) 3D (SEMA3D), mRNA [NM_152754]	NM_152754
A_23_P254363	0.00028	3.29	NM_000950	NM_000950	Homo sapiens proline rich Gla (G-carboxyglutamic acid) 1 (PRRG1), mRNA [NM_000950]	NM_000950
A_32_P142625	0.000281	2.08	AA292852	AA292852	zt64e12.r1 Soares_testis_NHT Homo sapiens cDNA clone IMAGE:727150 5', mRNA sequence [AA292852]	
A_23_P202280	0.000281	2.61	NM_014000	NM_014000	Homo sapiens vinculin (VCL), transcript variant 1, mRNA [NM_014000]	NM_014000
A_23_P259621	0.000282	2.28	NM_032463	NM_032463	Homo sapiens linker for activation of T cells family, member 2 (LAT2), transcript variant 2, mRNA [NM_032463]	NM_032463
A_32_P29784	0.000283	2.21	NM_153649	NM_153649	Homo sapiens tropomyosin 3 (TPM3), transcript variant 2, mRNA [NM_153649]	NM_153649
A_23_P354193	0.000283	2.49	NM_144628	NM_144628	Homo sapiens TBC1 domain family, member 20 (TBC1D20), mRNA [NM_144628]	NM_144628
A_23_P342744	0.000283	3.04	NM_153713	NM_153713	Homo sapiens Lix1 homolog (mouse) like (LIX1L), mRNA [NM_153713]	NM_153713
A_32_P174385	0.000283	3.22	A_32_P174385			

A_23_P25150	0.000283	3.48	NM_006897	NM_006897	Homo sapiens homeo box C9 (HOXC9), mRNA [NM_006897]	NM_006897
A_32_P115358	0.000283	6.10	NM_023075	NM_023075	Homo sapiens metallophosphoesterase 1 (MPPE1), transcript variant 1, mRNA [NM_023075]	NM_023075
A_24_P144620	0.000284	3.28	NM_018452	NM_018452	Homo sapiens chromosome 6 open reading frame 35 (C6orf35), mRNA [NM_018452]	NM_018452
A_24_P887768	0.000284	3.57	THC2303783		BC007276 HSPA8 protein {Homo sapiens;} , partial (6%) [THC2303783]	
A_23_P407614	0.000284	4.05	NM_152901	NM_152901	Homo sapiens pyrin domain containing 1 (PYDC1), mRNA [NM_152901]	NM_152901
A_23_P203729	0.000288	2.24	NM_002869	NM_002869	Homo sapiens RAB6A, member RAS oncogene family (RAB6A), transcript variant 1, mRNA [NM_002869]	NM_002869
A_23_P116587	0.000289	2.23	NM_006189	NM_006189	Homo sapiens olfactory marker protein (OMP), mRNA [NM_006189]	NM_006189
A_23_P126752	0.000289	2.35	NM_004930	NM_004930	Homo sapiens capping protein (actin filament) muscle Z-line, beta (CAPZB), mRNA [NM_004930]	NM_004930
A_23_P350059	0.000289	2.36	BC063642	BC063642	Homo sapiens phosphodiesterase 4D interacting protein (myomegalin), mRNA (cDNA clone IMAGE:4746110), complete cds. [BC063642]	
A_23_P216043	0.000289	3.08	NM_024295	NM_024295	Homo sapiens Der1-like domain family, member 1 (DERL1), mRNA [NM_024295]	NM_024295
A_23_P37624	0.000289	3.34	AF140675	AF140675	Homo sapiens zinc metalloprotease ADAMTS7 (ADAMTS7) mRNA, complete cds. [AF140675]	
A_24_P218979	0.00029	2.21	NM_031299	NM_031299	Homo sapiens cell division cycle associated 3 (CDCA3), mRNA [NM_031299]	NM_031299
A_24_P69150	0.00029	2.84	NM_004290	NM_004290	Homo sapiens ring finger protein 14 (RNF14), transcript variant 1, mRNA [NM_004290]	NM_004290
A_23_P338912	0.00029	11.30	NM_007350	NM_007350	Homo sapiens pleckstrin homology-like domain, family A, member 1 (PHLDA1), mRNA [NM_007350]	NM_007350
A_23_P24104	0.00029	17.09	NM_002658	NM_002658	Homo sapiens plasminogen activator, urokinase (PLAU), mRNA [NM_002658]	NM_002658
A_24_P934162	0.000291	2.72	AK128413	AK128413	Homo sapiens cDNA FLJ46556 fis, clone THYMU3039807. [AK128413]	
A_32_P167176	0.000291	2.86	THC2343936			
A_23_P53458	0.000291	2.95	NM_014325	NM_014325	Homo sapiens coronin, actin binding protein, 1C (CORO1C), mRNA [NM_014325]	NM_014325
A_24_P250535	0.000292	2.92	NM_021156	NM_021156	Homo sapiens hypothetical protein DJ971N18.2 (DJ971N18.2), mRNA [NM_021156]	NM_021156
A_23_P200325	0.000292	5.08	NM_014857	NM_014857	Homo sapiens RAB GTPase activating protein 1-like (RABGAP1L), mRNA [NM_014857]	NM_014857
A_23_P394064	0.000292	5.62	NM_012232	NM_012232	Homo sapiens polymerase I and transcript release factor (PTRF), mRNA [NM_012232]	NM_012232
A_24_P162287	0.000292	6.21	NM_007186	NM_007186	Homo sapiens centrosomal protein 2 (CEP2), mRNA [NM_007186]	NM_007186
A_23_P16722	0.000292	15.31	NM_014689	NM_014689	Homo sapiens dedicator of cytokinesis 10 (DOCK10), mRNA [NM_014689]	NM_014689
A_23_P216655	0.000293	4.81	NM_014788	NM_014788	Homo sapiens tripartite motif-containing 14 (TRIM14), transcript variant 1, mRNA [NM_014788]	NM_014788
A_23_P392222	0.000294	2.08	ENST00000320188		Homo sapiens chromosome 9 open reading frame 119, mRNA (cDNA clone IMAGE:5168627), partial cds. [BC029911]	XM_372143
A_23_P81717	0.000294	2.19	NM_024919	NM_024919	Homo sapiens FERM domain containing 1 (FRMD1), mRNA [NM_024919]	NM_024919
A_24_P106057	0.000294	2.19	AK129838	AK129838	Homo sapiens cDNA FLJ26328 fis, clone HRT01493. [AK129838]	
A_24_P15702	0.000294	2.62	A_24_P15702			
A_24_P108779	0.000294	2.70	NM_024681	NM_024681	Homo sapiens potassium channel tetramerisation domain containing 17 (KCTD17), mRNA [NM_024681]	NM_024681
A_32_P111235	0.000294	2.92	BG612665	BG612665	602641001F1 NIH_MGC_61 Homo sapiens cDNA clone IMAGE:4771873 5', mRNA sequence [BG612665]	
A_23_P30464	0.000294	3.16	NM_030567	NM_030567	Homo sapiens proline rich 7 (synaptic) (PRR7), mRNA [NM_030567]	NM_030567
A_32_P202092	0.000294	3.53	THC2406182			
A_32_P224327	0.000294	4.50	AK056401	AK056401	Homo sapiens cDNA FLJ31839 fis, clone NT2RP7000086. [AK056401]	
A_23_P259413	0.000295	2.12	NM_017548	NM_017548	Homo sapiens hypothetical protein H41 (H41), mRNA [NM_017548]	NM_017548
A_24_P5935	0.000295	2.77	NM_199203	NM_199203	Homo sapiens ubiquitin-conjugating enzyme E2 variant 1 (Kua-UEV), transcript variant 1, mRNA [NM_199203]	NM_199203
A_32_P3400	0.000295	2.82	BF754999	BF754999	BF754999 QV0-CT0583-181000-428-a09 CT0583 Homo sapiens cDNA, mRNA sequence [BF754999]	
A_23_P359588	0.000295	2.86	NM_032098	NM_032098	Homo sapiens protocadherin gamma subfamily B, 4 (PCDHGB4), transcript variant 2, mRNA [NM_032098]	NM_032098
A_23_P55127	0.000296	2.11	NM_020233	NM_020233	Homo sapiens x 006 protein (MDS006), mRNA [NM_020233]	NM_020233
A_24_P201089	0.000296	3.12	NM_016612	NM_016612	Homo sapiens solute carrier family 25, member 37 (SLC25A37), transcript variant 1, mRNA [NM_016612]	NM_016612
A_32_P69492	0.000296	3.31	AK025631	AK025631	Homo sapiens cDNA: FLJ21978 fis, clone HEP06046, highly similar to AF158185 Homo sapiens xeroderma pigmentosum variant RAD30 (RAD30) mRNA. [AK025631]	
A_23_P76450	0.000296	10.33	NM_007350	NM_007350	Homo sapiens pleckstrin homology-like domain, family A, member 1 (PHLDA1), mRNA [NM_007350]	NM_007350

A_32_P399546	0.000297	10.86	AF256215	AF256215	Homo sapiens cycle-like factor CLIF mRNA, complete cds. [AF256215]	
A_24_P84021	0.000298	2.18	XM_496693	XM_496693	PREDICTED: Homo sapiens similar to hypertension-related calcium-regulated gene (LOC441016), mRNA [XM_496693]	XM_496693
A_24_P912751	0.000299	2.90	AY358619	AY358619	Homo sapiens clone DNA107443 AGLW2560 (UNQ2560) mRNA, complete cds. [AY358619]	
A_24_P315405	0.0003	2.25	A_24_P315405			
A_24_P499215	0.0003	2.31	XM_495808	XM_495808	PREDICTED: Homo sapiens similar to KIAA0592 protein (LOC439961), mRNA [XM_495808]	XM_495808
A_24_P204664	0.000301	2.07	A_24_P204664			
A_32_P191141	0.000301	5.85	A_32_P191141			
A_23_P206396	0.000302	2.80	NM_016951	NM_016951	Homo sapiens chemokine-like factor (CKLF), transcript variant 1, mRNA [NM_016951]	NM_016951
A_23_P126186	0.000303	2.03	NM_003676	NM_003676	Homo sapiens degenerative spermatocyte homolog 1, lipid desaturase (Drosophila) (DEGS1), transcript variant 1, mRNA [NM_003676]	NM_003676
A_23_P124003	0.000303	2.26	NM_170683	NM_170683	Homo sapiens purinergic receptor P2X, ligand-gated ion channel, 2 (P2RX2), transcript variant 4, mRNA [NM_170683]	NM_170683
A_23_P399201	0.000303	5.71	NM_183049	NM_183049	Homo sapiens thymosin-like 3 (TMSL3), mRNA [NM_183049]	NM_183049
A_23_P137765	0.000305	2.11	NM_000437	NM_000437	Homo sapiens platelet-activating factor acetylhydrolase 2, 40kDa (PAFAH2), mRNA [NM_000437]	NM_000437
A_23_P120002	0.000305	3.76	NM_004510	NM_004510	Homo sapiens SP110 nuclear body protein (SP110), transcript variant b, mRNA [NM_004510]	NM_004510
A_23_P126727	0.000306	4.44	NM_024654	NM_024654	Homo sapiens hypothetical protein FLJ23323 (FLJ23323), mRNA [NM_024654]	NM_024654
A_23_P125772	0.000307	2.04	NM_014370	NM_014370	Homo sapiens serine/threonine kinase 23 (STK23), mRNA [NM_014370]	NM_014370
A_23_P73142	0.000308	2.39	NM_016360	NM_016360	Homo sapiens clone HQ0477 PRO0477p (LOC51204), mRNA [NM_016360]	NM_016360
A_32_P49555	0.000308	2.77	NM_174889	NM_174889	Homo sapiens Myc-induced mitochondria protein (mimitin), mRNA [NM_174889]	NM_174889
A_23_P166333	0.000308	3.01	NM_005659	NM_005659	Homo sapiens ubiquitin fusion degradation 1-like (UFD1L), mRNA [NM_005659]	NM_005659
A_23_P117767	0.000309	2.83	NM_017828	NM_017828	Homo sapiens COMM domain containing 4 (COMMD4), mRNA [NM_017828]	NM_017828
A_23_P148194	0.000309	2.93	NM_018269	NM_018269	Homo sapiens membrane-type 1 matrix metalloproteinase cytoplasmic tail binding protein-1 (MTCBP-1), mRNA [NM_018269]	NM_018269
A_24_P74374	0.00031	2.00	NM_000308	NM_000308	Homo sapiens protective protein for beta-galactosidase (galactosialidosis) (PPGB), mRNA [NM_000308]	NM_000308
A_32_P144953	0.00031	2.86	ENST00000316429		PREDICTED: Homo sapiens similar to seven transmembrane helix receptor (LOC442105), mRNA [XM_497958]	XM_497958
A_24_P236680	0.00031	4.15	THC2287766			
A_24_P658427	0.000311	7.04	CR619603	CR619603	full-length cDNA clone CS0DF007YC14 of Fetal brain of Homo sapiens (human). [CR619603]	
A_23_P69030	0.000311	26.25	NM_001850	NM_001850	Homo sapiens collagen, type VIII, alpha 1 (COL8A1), transcript variant 1, mRNA [NM_001850]	NM_001850
A_23_P141434	0.000312	2.66	NM_000664	NM_000664	Homo sapiens acetyl-Coenzyme A carboxylase alpha (ACACA), transcript variant 6, mRNA [NM_000664]	NM_000664
A_24_P266035	0.000312	2.75	NM_014473	NM_014473	Homo sapiens dimethyladenosine transferase (HSA9761), mRNA [NM_014473]	NM_014473
A_23_P114839	0.000313	2.03	NM_004468	NM_004468	Homo sapiens four and a half LIM domains 3 (FHL3), mRNA [NM_004468]	NM_004468
A_32_P40611	0.000313	2.55	A_32_P40611			
A_23_P331479	0.000313	3.44	BC066644	BC066644	Homo sapiens KIAA1949, mRNA (cDNA clone MGC:71094 IMAGE:5575643), complete cds. [BC066644]	
A_23_P66100	0.000313	4.05	CR607189	CR607189	full-length cDNA clone CS0DK007YF11 of HeLa cells Cot 25-normalized of Homo sapiens (human). [CR607189]	
A_24_P204244	0.000313	6.76	NR_001562	NR_001562	Homo sapiens annexin A2 pseudogene 1 (ANXA2P1) on chromosome 4 [NR_001562]	NR_001562
A_24_P687085	0.000314	2.01	AK024933	AK024933	Homo sapiens cDNA: FLJ21280 fis, clone COL01884. [AK024933]	
A_23_P143559	0.000314	2.02	NM_007098	NM_007098	Homo sapiens clathrin, heavy polypeptide-like 1 (CLTCL1), transcript variant 2, mRNA [NM_007098]	NM_007098
A_23_P154874	0.000314	2.30	NM_006052	NM_006052	Homo sapiens Down syndrome critical region gene 3 (DSCR3), mRNA [NM_006052]	NM_006052
A_24_P255654	0.000314	2.59	A_24_P255654			
A_32_P195065	0.000314	3.14	NM_004263	NM_004263	Homo sapiens sema domain, immunoglobulin domain (Ig), transmembrane domain (TM) and short cytoplasmic domain, (semaphorin) 4F (SEMA4F), mRNA [NM_004263]	NM_004263
A_32_P19135	0.000314	4.12	NM_016154	NM_016154	Homo sapiens RAB4B, member RAS oncogene family (RAB4B), mRNA [NM_016154]	NM_016154
A_23_P145197	0.000315	2.51	NM_004053	NM_004053	Homo sapiens bystin-like (BYSL), mRNA [NM_004053]	NM_004053
A_24_P337657	0.000315	2.67	NM_003131	NM_003131	Homo sapiens serum response factor (c-fos serum response element-binding transcription factor) (SRF), mRNA [NM_003131]	NM_003131

A_24_P115450	0.000315	2.78	NM_018688	NM_018688	Homo sapiens bridging integrator 3 (BIN3), mRNA [NM_018688]	NM_018688
A_23_P163782	0.000315	4.42	NM_005951	NM_005951	Homo sapiens metallothionein 1H (MT1H), mRNA [NM_005951]	NM_005951
A_23_P100556	0.000316	2.03	NM_001013839	NM_001013839	Homo sapiens exocyst complex component 7 (EXOC7), transcript variant 1, mRNA [NM_001013839]	NM_001013839
A_23_P68401	0.000316	2.03	NM_030877	NM_030877	Homo sapiens catenin, beta like 1 (CTNNBL1), mRNA [NM_030877]	NM_030877
A_23_P201086	0.000316	2.08	NM_001658	NM_001658	Homo sapiens ADP-ribosylation factor 1 (ARF1), transcript variant 4, mRNA [NM_001658]	NM_001658
A_32_P69930	0.000316	2.44	NM_153376	NM_153376	Homo sapiens hypothetical protein FLJ90575 (FLJ90575), mRNA [NM_153376]	NM_153376
A_23_P94689	0.000316	3.91	NM_030914	NM_030914	Homo sapiens chromosome 9 open reading frame 74 (C9orf74), mRNA [NM_030914]	NM_030914
A_23_P29939	0.000317	6.25	NM_007308	NM_007308	Homo sapiens synuclein, alpha (non A4 component of amyloid precursor) (SNCA), transcript variant NACP112, mRNA [NM_007308]	NM_007308
A_24_P460195	0.000319	3.64	A_24_P460195			
A_24_P263036	0.00032	2.50	NM_003748	NM_003748	Homo sapiens aldehyde dehydrogenase 4 family, member A1 (ALDH4A1), nuclear gene encoding mitochondrial protein, transcript variant P5CDhL, mRNA [NM_003748]	NM_003748
A_23_P252052	0.00032	2.58	NM_182909	NM_182909	Homo sapiens downregulated in ovarian cancer 1 (DOC1), transcript variant 1, mRNA [NM_182909]	NM_182909
A_24_P148811	0.00032	3.58	NM_003707	NM_003707	Homo sapiens RuvB-like 1 (E. coli) (RUVBL1), mRNA [NM_003707]	NM_003707
A_32_P92642	0.00032	3.77	BE710245	BE710245	BE710245 IL3-HT0619-120700-212-G05 HT0619 Homo sapiens cDNA, mRNA sequence [BE710245]	
A_24_P494807	0.000321	2.24	ENST00000338548		PSDA_HUMAN (O75832) 26S proteasome non-ATPase regulatory subunit 10 (26S proteasome regulatory subunit p28) (Gankyrin), partial (60%) [THC2294587]	
A_23_P161152	0.000322	2.43	NM_014317	NM_014317	Homo sapiens trans-prenyltransferase (TPRT), mRNA [NM_014317]	NM_014317
A_23_P311608	0.000323	2.51	NM_054022	NM_054022	Homo sapiens golgi SNAP receptor complex member 2 (GOSR2), transcript variant B, mRNA [NM_054022]	NM_054022
A_23_P91850	0.000323	2.60	NM_144717	NM_144717	Homo sapiens fibronectin type III domain containing 6 (FNDC6), mRNA [NM_144717]	NM_144717
A_24_P76210	0.000323	2.64	THC2365247		Q6QAQ1 (Q6QAQ1) Cytoskeletal beta actin (Fragment), partial (76%) [THC2365247]	
A_24_P210420	0.000323	8.33	NM_178006	NM_178006	Homo sapiens START domain containing 13 (STARD13), transcript variant alpha, mRNA [NM_178006]	NM_178006
A_23_P164307	0.000324	2.11	NM_153827	NM_153827	Homo sapiens misshapen-like kinase 1 (zebrafish) (MINK1), transcript variant 3, mRNA [NM_153827]	NM_153827
A_24_P417996	0.000324	2.12	A_24_P417996			
A_32_P29806	0.000324	2.22	NM_003805	NM_003805	Homo sapiens CASP2 and RIPK1 domain containing adaptor with death domain (CRADD), mRNA [NM_003805]	NM_003805
A_23_P30884	0.000324	2.30	NM_001288	NM_001288	Homo sapiens chloride intracellular channel 1 (CLIC1), mRNA [NM_001288]	NM_001288
A_24_P609382	0.000324	3.30	AK123143	AK123143	Homo sapiens cDNA FLJ41148 fis, clone BRACE2037310. [AK123143]	
A_23_P142878	0.000325	3.17	NM_032827	NM_032827	Homo sapiens atonal homolog 8 (Drosophila) (ATOH8), mRNA [NM_032827]	NM_032827
A_23_P102832	0.000325	3.86	NM_007186	NM_007186	Homo sapiens centrosomal protein 2 (CEP2), mRNA [NM_007186]	NM_007186
A_23_P102494	0.000326	2.09	NM_017877	NM_017877	Homo sapiens chromosome 2 open reading frame 18 (C2orf18), mRNA [NM_017877]	NM_017877
A_23_P81993	0.000326	2.48	NM_178508	NM_178508	Homo sapiens chromosome 6 open reading frame 1 (C6orf1), transcript variant 1, mRNA [NM_178508]	NM_178508
A_24_P169343	0.000326	2.99	NM_153698	NM_153698	Homo sapiens chromosome 9 open reading frame 21 (C9orf21), mRNA [NM_153698]	NM_153698
A_24_P15906	0.000326	3.17	A_24_P15906			
A_32_P82251	0.000326	4.33	CA420826	CA420826	CA420826 UI-H-FG0-bcr-c-23-0-UI.s1 NCI_CGAP_EN1_2 Homo sapiens cDNA clone UI-H-FG0-bcr-c-23-0-UI 3', mRNA sequence [CA420826]	
A_23_P373598	0.000327	2.18	AK056767	AK056767	Homo sapiens cDNA FLJ32205 fis, clone PLACE6003094. [AK056767]	
A_23_P215956	0.000327	2.83	NM_002467	NM_002467	Homo sapiens v-myc myelocytomatosis viral oncogene homolog (avian) (MYC), mRNA [NM_002467]	NM_002467
A_23_P161231	0.000327	3.45	NM_030971	NM_030971	Homo sapiens sideroflexin 3 (SFXN3), mRNA [NM_030971]	NM_030971
A_32_P80532	0.000327	3.89	THC2304788		BF733908 PM2-AN0089-181000-007-h04 AN0089 Homo sapiens cDNA, mRNA sequence [BF733908]	
A_23_P103775	0.000327	4.13	NM_032270	NM_032270	Homo sapiens leucine rich repeat containing 8 family, member C (LRRC8C), mRNA [NM_032270]	NM_032270
A_23_P126159	0.000327	5.26	NM_002143	NM_002143	Homo sapiens hippocalcin (HPCA), mRNA [NM_002143]	NM_002143
A_24_P25354	0.000327	9.01	NM_006283	NM_006283	Homo sapiens transforming, acidic coiled-coil containing protein 1 (TACC1), mRNA [NM_006283]	NM_006283
A_23_P139722	0.000328	2.17	NM_001065	NM_001065	Homo sapiens tumor necrosis factor receptor superfamily, member 1A (TNFRSF1A), mRNA [NM_001065]	NM_001065
A_23_P155837	0.000328	2.61	NM_183075	NM_183075	Homo sapiens cytochrome P450, family 2, subfamily U, polypeptide 1 (CYP2U1), mRNA [NM_183075]	NM_183075

A_23_P24234	0.000329	2.21	NM_033282	NM_033282	Homo sapiens opsin 4 (melanopsin) (OPN4), mRNA [NM_033282]	NM_033282
A_24_P59569	0.000329	2.82	BC000922	BC000922	Homo sapiens hypothetical gene supported by BC000922, mRNA (cDNA clone IMAGE:3447073), partial cds. [BC000922]	XM_378793
A_24_P698136	0.000329	4.22	AK125299	AK125299	Homo sapiens cDNA FLJ43309 fis, clone NT2RI2004618, highly similar to Cytosolic acyl coenzyme A thioester hydrolase (EC 3.1.2.2). [AK125299]	XR_000194
A_32_P210691	0.000333	2.06	A_32_P210691			
A_32_P935688	0.000333	2.48	AK095331	AK095331	Homo sapiens cDNA FLJ38012 fis, clone CTONG2012554. [AK095331]	
A_24_P196024	0.000333	3.66	A_24_P196024			
A_32_P222335	0.000334	2.19	THC2274391		RIFK_HUMAN (Q969G6) Riboflavin kinase (ATP:riboflavin 5'-phosphotransferase) (Flavokinase) , complete [THC2274391]	
A_23_P333330	0.000334	2.25	BC005015	BC005015	Homo sapiens zinc finger, DHHC-type containing 24, mRNA (cDNA clone IMAGE:3636082), partial cds. [BC005015]	
A_23_P257895	0.000334	2.28	NM_138957	NM_138957	Homo sapiens mitogen-activated protein kinase 1 (MAPK1), transcript variant 2, mRNA [NM_138957]	NM_138957
A_23_P92132	0.000334	2.61	NM_006764	NM_006764	Homo sapiens interferon-related developmental regulator 2 (IFRD2), mRNA [NM_006764]	NM_006764
A_23_P92202	0.000334	3.04	NM_021971	NM_021971	Homo sapiens GDP-mannose pyrophosphorylase B (GMPPB), transcript variant 2, mRNA [NM_021971]	NM_021971
A_24_P116017	0.000334	4.10	NM_002813	NM_002813	Homo sapiens proteasome (prosome, macropain) 26S subunit, non-ATPase, 9 (PSMD9), mRNA [NM_002813]	NM_002813
A_32_P38093	0.000334	4.20	THC2343678		Q6E5T4 (Q6E5T4) Claudin 2, partial (5%) [THC2343678]	
A_23_P113825	0.000335	2.18	NM_144653	NM_144653	Homo sapiens BTB (POZ) domain containing 14A (BTBD14A), mRNA [NM_144653]	NM_144653
A_24_P357809	0.000335	2.27	NM_020642	NM_020642	Homo sapiens chromosome 11 open reading frame 17 (C11orf17), transcript variant 2, mRNA [NM_020642]	NM_020642
A_23_P68610	0.000335	2.37	NM_012112	NM_012112	Homo sapiens TPX2, microtubule-associated, homolog (Xenopus laevis) (TPX2), mRNA [NM_012112]	NM_012112
A_24_P196851	0.000335	2.96	NM_006289	NM_006289	Homo sapiens talin 1 (TLN1), mRNA [NM_006289]	NM_006289
A_24_P272967	0.000335	3.02	NM_015060	NM_015060	Homo sapiens KIAA0241 protein (KIAA0241), mRNA [NM_015060]	NM_015060
A_24_P142118	0.000335	22.03	NM_003246	NM_003246	Homo sapiens thrombospondin 1 (THBS1), mRNA [NM_003246]	NM_003246
A_24_P852082	0.000336	2.08	AK125829	AK125829	Homo sapiens cDNA FLJ43841 fis, clone TESTI4006137. [AK125829]	XM_378250
A_23_P359762	0.000336	2.66	NM_152914	NM_152914	Homo sapiens transcript expressed during hematopoiesis 2 (MGC33894), mRNA [NM_152914]	NM_152914
A_23_P157545	0.000336	2.87	NM_006167	NM_006167	Homo sapiens NK3 transcription factor related, locus 1 (Drosophila) (NKX3-1), mRNA [NM_006167]	NM_006167
A_32_P220715	0.000336	3.36	NM_022818	NM_022818	Homo sapiens microtubule-associated protein 1 light chain 3 beta (MAP1LC3B), mRNA [NM_022818]	NM_022818
A_23_P399726	0.000336	5.43	NM_024627	NM_024627	Homo sapiens hypothetical protein FLJ21125 (FLJ21125), mRNA [NM_024627]	NM_024627
A_23_P11025	0.000337	2.99	NM_007150	NM_007150	Homo sapiens zinc finger protein 185 (LIM domain) (ZNF185), mRNA [NM_007150]	NM_007150
A_23_P163458	0.000338	3.94	NM_139265	NM_139265	Homo sapiens EH-domain containing 4 (EHD4), mRNA [NM_139265]	NM_139265
A_24_P58037	0.000338	4.90	ENST00000276938		PREDICTED: Homo sapiens similar to microtubule-associated proteins 1A [XM_373277]	XM_373277
A_23_P29723	0.000339	2.14	NM_001012410	NM_001012410	Homo sapiens shugoshin-like 1 (S. pombe) (SGOL1), transcript variant A2, mRNA [NM_001012410]	NM_001012410
A_23_P79931	0.000339	2.87	NM_139322	NM_139322	Homo sapiens attractin (ATRN), transcript variant 2, mRNA [NM_139322]	NM_139322
A_23_P9392	0.000342	2.56	NM_016390	NM_016390	Homo sapiens chromosome 9 open reading frame 114 (C9orf114), mRNA [NM_016390]	NM_016390
A_24_P179013	0.000342	2.76	ENST00000333462		GB|AL031963.40|CAD70623.1 dJ40E16.3 (novel gene similar to D. melanogaster CG5327) [Homo sapiens] [NP1083521]	
A_23_P300056	0.000342	3.31	NM_044472	NM_044472	Homo sapiens cell division cycle 42 (GTP binding protein, 25kDa) (CDC42), transcript variant 2, mRNA [NM_044472]	NM_044472
A_23_P151870	0.000343	2.30	NM_015554	NM_015554	Homo sapiens glucuronyl C5-epimerase (GLCE), mRNA [NM_015554]	NM_015554
A_23_P71037	0.000343	2.68	NM_000600	NM_000600	Homo sapiens interleukin 6 (interferon, beta 2) (IL6), mRNA [NM_000600]	NM_000600
A_24_P398319	0.000343	3.13	NM_020644	NM_020644	Homo sapiens chromosome 11 open reading frame 15 (C11orf15), mRNA [NM_020644]	NM_020644
A_23_P406330	0.000344	2.67	NM_022733	NM_022733	Homo sapiens stromal membrane-associated protein 1-like (SMAP1L), mRNA [NM_022733]	NM_022733
A_24_P330009	0.000345	2.18	NM_021128	NM_021128	Homo sapiens polymerase (RNA) II (DNA directed) polypeptide L, 7.6kDa (POLR2L), mRNA [NM_021128]	NM_021128
A_24_P930088	0.000345	2.77	AK123264	AK123264	Homo sapiens cDNA FLJ41270 fis, clone BRAMY2036387. [AK123264]	
A_24_P83158	0.000345	2.96	NR_002184	NR_002184	Homo sapiens similar to CGI-96 (dJ222E13.2) on chromosome 22 [NR_002184]	NR_002184
A_24_P194184	0.000345	3.13	NM_199484	NM_199484	Homo sapiens chromosome 20 open reading frame 24 (C20orf24), transcript variant 3, mRNA [NM_199484]	NM_199484

A_24_P84698	0.000345	3.45	A_24_P84698			
A_23_P201887	0.000345	3.68	NM_005057	NM_005057	Homo sapiens retinoblastoma binding protein 5 (RBBP5), mRNA [NM_005057]	NM_005057
A_32_P210744	0.000345	6.33	A_32_P210744			
A_24_P170514	0.000346	2.53	BC042060	BC042060	Homo sapiens cDNA clone IMAGE:5590288, partial cds. [BC042060]	
A_24_P75979	0.000347	5.52	ENST00000341372		PREDICTED: Homo sapiens similar to tropomyosin 3 (LOC221875), mRNA [XM_167254]	XM_167254
A_23_P67829	0.000348	2.33	NM_025076	NM_025076	Homo sapiens UDP-glucuronate decarboxylase 1 (UXS1), mRNA [NM_025076]	NM_025076
A_23_P65442	0.000349	2.07	NM_006084	NM_006084	Homo sapiens interferon-stimulated transcription factor 3, gamma 48kDa (ISGF3G), mRNA [NM_006084]	NM_006084
A_23_P6786	0.000349	2.33	BC005184	BC005184	Homo sapiens tRNA nucleotidyl transferase, CCA-adding, 1, mRNA (cDNA clone IMAGE:3686608), complete cds. [BC005184]	
A_24_P225616	0.000349	2.33	NM_001034	NM_001034	Homo sapiens ribonucleotide reductase M2 polypeptide (RRM2), mRNA [NM_001034]	NM_001034
A_24_P381975	0.000349	2.54	BC010609	BC010609	Homo sapiens carbohydrate (N-acetylglucosamine 6-O) sulfotransferase 5, mRNA (cDNA clone MGC:16805 IMAGE:4214587), complete cds. [BC010609]	XM_370992
A_24_P223518	0.00035	2.27	NM_007270	NM_007270	Homo sapiens FK506 binding protein 9, 63 kDa (FKBP9), mRNA [NM_007270]	NM_007270
A_24_P49800	0.00035	2.36	A_24_P49800			
A_23_P149200	0.00035	2.38	NM_001255	NM_001255	Homo sapiens CDC20 cell division cycle 20 homolog (S. cerevisiae) (CDC20), mRNA [NM_001255]	NM_001255
A_23_P328206	0.00035	2.68	BC041628	BC041628	Homo sapiens dynamin binding protein, mRNA (cDNA clone MGC:52388 IMAGE:4329464), complete cds. [BC041628]	
A_23_P213527	0.00035	5.52	AW901755	AW901755	AW901755 QV0-NN1020-170400-192-f12 NN1020 Homo sapiens cDNA, mRNA sequence [AW901755]	
A_24_P100535	0.000351	2.10	NM_181519	NM_181519	Homo sapiens synaptotagmin XV (SYT15), transcript variant b, mRNA [NM_181519]	NM_181519
A_23_P87482	0.000351	3.14	NM_024068	NM_024068	Homo sapiens hypothetical protein MGC2731 (MGC2731), mRNA [NM_024068]	NM_024068
A_23_P126899	0.000352	2.53	NM_003846	NM_003846	Homo sapiens peroxisomal biogenesis factor 11B (PEX11B), mRNA [NM_003846]	NM_003846
A_24_P622186	0.000352	3.18	NM_080926	NM_080926	Homo sapiens hypothetical protein similar to KIAA0187 gene product (LOC96610), mRNA [NM_080926]	NM_080926
A_23_P354953	0.000353	2.53	NM_013433	NM_013433	Homo sapiens transportin 2 (importin 3, karyopherin beta 2b) (TNPO2), mRNA [NM_013433]	NM_013433
A_23_P217688	0.000353	2.60	NM_004089	NM_004089	Homo sapiens TSC22 domain family, member 3 (TSC22D3), transcript variant 2, mRNA [NM_004089]	NM_004089
A_23_P400217	0.000354	2.08	NM_032853	NM_032853	Homo sapiens melanoma associated antigen (mutated) 1 (MUM1), mRNA [NM_032853]	NM_032853
A_23_P151267	0.000354	2.68	NM_016357	NM_016357	Homo sapiens epithelial protein lost in neoplasm beta (EPLIN), mRNA [NM_016357]	NM_016357
A_23_P20196	0.000354	4.31	NM_005720	NM_005720	Homo sapiens actin related protein 2/3 complex, subunit 1B, 41kDa (ARPC1B), mRNA [NM_005720]	NM_005720
A_24_P107291	0.000357	2.97	NM_181699	NM_181699	Homo sapiens protein phosphatase 2 (formerly 2A), regulatory subunit A (PR 65), beta isoform (PPP2R1B), transcript variant 2, mRNA [NM_181699]	NM_181699
A_24_P707530	0.000357	5.68	THC2336549		S10030 urease 11K chain - Ureaplasma urealyticum {Ureaplasma urealyticum;} , partial (10%) [THC2336549]	
A_23_P215525	0.000357	6.33	NM_145323	NM_145323	Homo sapiens oxysterol binding protein-like 3 (OSBPL3), transcript variant 5, mRNA [NM_145323]	NM_145323
A_23_P391637	0.000358	2.28	ENST00000293970		Homo sapiens mRNA for KIAA1171 protein, partial cds. [AB032997]	XM_370928
A_32_P135385	0.000359	2.29	NM_001013694	NM_001013694	Homo sapiens similar to SRR1-like protein (LOC402055), mRNA [NM_001013694]	NM_001013694
A_23_P405761	0.000359	2.78	NM_012250	NM_012250	Homo sapiens related RAS viral (r-ras) oncogene homolog 2 (RRAS2), mRNA [NM_012250]	NM_012250
A_23_P318115	0.000359	2.93	NM_020467	NM_020467	Homo sapiens hypothetical protein from clone 643 (LOC57228), mRNA [NM_020467]	NM_020467
A_23_P72387	0.00036	4.46	NM_021638	NM_021638	Homo sapiens actin filament associated protein (AFAP), transcript variant 1, mRNA [NM_021638]	NM_021638
A_32_P212886	0.000361	2.20	BC014117	BC014117	Homo sapiens thromboxane A synthase 1 (platelet, cytochrome P450, family 5, subfamily A), mRNA (cDNA clone MGC:20885 IMAGE:4548935), complete cds. [BC014117]	XM_374236
A_24_P110306	0.000361	2.79	ENST00000330140		Homo sapiens cDNA FLJ26697 fis, clone PCD00618. [AK130207]	
A_23_P61406	0.000361	24.45	NM_016848	NM_016848	Homo sapiens SHC (Src homology 2 domain containing) transforming protein 3 (SHC3), mRNA [NM_016848]	NM_016848
A_24_P102343	0.000362	2.08	NM_017957	NM_017957	Homo sapiens epsin 3 (EPN3), mRNA [NM_017957]	NM_017957
A_23_P58266	0.000362	2.17	NM_005980	NM_005980	Homo sapiens S100 calcium binding protein P (S100P), mRNA [NM_005980]	NM_005980
A_23_P143987	0.000362	2.83	NM_006395	NM_006395	Homo sapiens APG7 autophagy 7-like (S. cerevisiae) (APG7L), mRNA [NM_006395]	NM_006395
A_24_P135483	0.000362	2.88	AK074558	AK074558	Homo sapiens cDNA FLJ90077 fis, clone HEMBA1004454. [AK074558]	

A_24_P273143	0.000362	4.88	NM_052871	NM_052871	Homo sapiens hypothetical protein MGC4677 (MGC4677), mRNA [NM_052871]	NM_052871
A_32_P148407	0.000362	5.46	A_32_P148407			
A_24_P188116	0.000363	2.30	NM_020349	NM_020349	Homo sapiens ankyrin repeat domain 2 (stretch responsive muscle) (ANKRD2), mRNA [NM_020349]	NM_020349
A_32_P49631	0.000363	2.48	CR594811	CR594811	full-length cDNA clone CS0DL006YD08 of B cells (Ramos cell line) Cot 25-normalized of Homo sapiens (human). [CR594811]	
A_23_P117274	0.000363	3.52	NM_005932	NM_005932	Homo sapiens mitochondrial intermediate peptidase (MIPEP), nuclear gene encoding mitochondrial protein, mRNA [NM_005932]	NM_005932
A_23_P61050	0.000363	13.30	NM_152649	NM_152649	Homo sapiens mixed lineage kinase domain-like (MLKL), mRNA [NM_152649]	NM_152649
A_23_P252106	0.000364	3.06	NM_003821	NM_003821	Homo sapiens receptor-interacting serine-threonine kinase 2 (RIPK2), mRNA [NM_003821]	NM_003821
A_23_P97532	0.000364	4.03	NM_004781	NM_004781	Homo sapiens vesicle-associated membrane protein 3 (cellubrevin) (VAMP3), mRNA [NM_004781]	NM_004781
A_24_P85099	0.000364	12.29	NM_003483	NM_003483	Homo sapiens high mobility group AT-hook 2 (HMGA2), transcript variant 1, mRNA [NM_003483]	NM_003483
A_23_P360874	0.000365	2.03	NM_152892	NM_152892	Homo sapiens hypothetical protein DKFZp434K1815 (DKFZp434K1815), mRNA [NM_152892]	NM_152892
A_24_P345948	0.000365	2.08	NM_001012732	NM_001012732	Homo sapiens dCMP deaminase (DCTD), transcript variant 1, mRNA [NM_001012732]	NM_001012732
A_23_P257278	0.000366	2.90	NM_016082	NM_016082	Homo sapiens CDK5 regulatory subunit associated protein 1 (CDK5RAP1), transcript variant 2, mRNA [NM_016082]	NM_016082
A_23_P99253	0.000366	4.90	NM_004664	NM_004664	Homo sapiens lin-7 homolog A (C. elegans) (LIN7A), mRNA [NM_004664]	NM_004664
A_23_P33022	0.000367	2.60	NM_021128	NM_021128	Homo sapiens polymerase (RNA) II (DNA directed) polypeptide L, 7.6kDa (POLR2L), mRNA [NM_021128]	NM_021128
A_23_P51105	0.000367	8.20	NM_016441	NM_016441	Homo sapiens cysteine rich transmembrane BMP regulator 1 (chordin-like) (CRIM1), mRNA [NM_016441]	NM_016441
A_24_P208595	0.000368	4.81	NM_053034	NM_053034	Homo sapiens anthrax toxin receptor 1 (ANTXR1), transcript variant 2, mRNA [NM_053034]	NM_053034
A_24_P63118	0.000369	2.43	NM_013374	NM_013374	Homo sapiens programmed cell death 6 interacting protein (PDCD6IP), mRNA [NM_013374]	NM_013374
A_23_P82642	0.000369	3.86	NM_018697	NM_018697	Homo sapiens LanC lantibiotic synthetase component C-like 2 (bacterial) (LANCL2), mRNA [NM_018697]	NM_018697
A_23_P51958	0.000369	4.07	NM_182744	NM_182744	Homo sapiens neuroblastoma, suppression of tumorigenicity 1 (NBL1), transcript variant 1, mRNA [NM_182744]	NM_182744
A_23_P48977	0.00037	2.10	NM_022839	NM_022839	Homo sapiens mitochondrial ribosomal protein S11 (MRPS11), nuclear gene encoding mitochondrial protein, transcript variant 1, mRNA [NM_022839]	NM_022839
A_24_P37319	0.00037	2.43	NM_004457	NM_004457	Homo sapiens acyl-CoA synthetase long-chain family member 3 (ACSL3), transcript variant 1, mRNA [NM_004457]	NM_004457
A_32_P36884	0.00037	3.31	ENST00000356170			
A_23_P63798	0.000372	2.18	NM_001300	NM_001300	Homo sapiens Kruppel-like factor 6 (KLF6), transcript variant 2, mRNA [NM_001300]	NM_001300
A_24_P692198	0.000373	3.08	NM_002080	NM_002080	Homo sapiens glutamic-oxaloacetic transaminase 2, mitochondrial (aspartate aminotransferase 2) (GOT2), mRNA [NM_002080]	NM_002080
A_24_P349580	0.000374	2.28	A_24_P349580			
A_24_P413884	0.000374	2.33	NM_001809	NM_001809	Homo sapiens centromere protein A, 17kDa (CENPA), mRNA [NM_001809]	NM_001809
A_23_P43273	0.000374	2.65	NM_000127	NM_000127	Homo sapiens exostoses (multiple) 1 (EXT1), mRNA [NM_000127]	NM_000127
A_24_P56130	0.000374	4.13	NM_079423	NM_079423	Homo sapiens myosin, light polypeptide 6, alkali, smooth muscle and non-muscle (MYL6), transcript variant 2, mRNA [NM_079423]	NM_079423
A_23_P111995	0.000375	3.79	NM_002318	NM_002318	Homo sapiens lysyl oxidase-like 2 (LOXL2), mRNA [NM_002318]	NM_002318
A_23_P57379	0.000376	2.87	NM_003504	NM_003504	Homo sapiens CDC45 cell division cycle 45-like (S. cerevisiae) (CDC45L), mRNA [NM_003504]	NM_003504
A_32_P64928	0.000376	6.58	XM_496978	XM_496978	PREDICTED: Homo sapiens similar to Destrin (Actin-depolymerizing factor) (ADF) (LOC441354), mRNA [XM_496978]	XM_496978
A_32_P88719	0.000377	2.29	NM_080879	NM_080879	Homo sapiens RAB40A, member RAS oncogene family (RAB40A), mRNA [NM_080879]	NM_080879
A_32_P184888	0.000377	2.71	THC2343253			
A_24_P690983	0.000377	2.96	NM_080926	NM_080926	Homo sapiens hypothetical protein similar to KIAA0187 gene product (LOC96610), mRNA [NM_080926]	NM_080926
A_24_P215653	0.000377	12.63	NM_175060	NM_175060	Homo sapiens C-type lectin domain family 14, member A (CLEC14A), mRNA [NM_175060]	NM_175060
A_32_P196142	0.000378	36.76	THC2400010			
A_24_P923749	0.000379	2.04	NM_032740	NM_032740	Homo sapiens SFT2 domain containing 3 (SFT2D3), mRNA [NM_032740]	NM_032740
A_23_P82316	0.000379	2.16	NM_024653	NM_024653	Homo sapiens PRKR interacting protein 1 (IL11 inducible) (PRKRIP1), mRNA [NM_024653]	NM_024653
A_24_P377144	0.000379	3.26	NM_058172	NM_058172	Homo sapiens anthrax toxin receptor 2 (ANTXR2), mRNA [NM_058172]	NM_058172

A_24_P219686	0.000379	3.28	NM_006788	NM_006788	Homo sapiens ralA binding protein 1 (RALBP1), mRNA [NM_006788]	NM_006788
A_24_P184692	0.000379	4.52	NM_003317	NM_003317	Homo sapiens thyroid transcription factor 1 (TITF1), mRNA [NM_003317]	NM_003317
A_24_P193011	0.000379	11.01	NM_053056	NM_053056	Homo sapiens cyclin D1 (PRAD1: parathyroid adenomatosis 1) (CCND1), mRNA [NM_053056]	NM_053056
A_24_P690235	0.00038	2.29	ENST00000312412		Homo sapiens cDNA FLJ43844 fis, clone TESTI4006308, highly similar to Puromycin-sensitive aminopeptidase (EC 3.4.11.-). [AK125832]	
A_24_P112377	0.00038	2.39	NM_138620	NM_138620	Homo sapiens DEAD (Asp-Glu-Ala-Asp) box polypeptide 31 (DDX31), transcript variant 2, mRNA [NM_138620]	NM_138620
A_23_P302094	0.00038	2.93	NM_033416	NM_033416	Homo sapiens IMP4, U3 small nucleolar ribonucleoprotein, homolog (yeast) (IMP4), mRNA [NM_033416]	NM_033416
A_24_P307046	0.00038	2.99	A_24_P307046			
A_24_P186779	0.00038	4.18	A_24_P186779			
A_24_P393844	0.000381	2.10	NM_001384	NM_001384	Homo sapiens DPH2 homolog (S. cerevisiae) (DPH2), transcript variant 1, mRNA [NM_001384]	NM_001384
A_32_P436884	0.000381	2.31	NM_001010867	NM_001010867	Homo sapiens chromosome 1 open reading frame 69 (C1orf69), mRNA [NM_001010867]	NM_001010867
A_24_P385341	0.000381	2.45	NM_014388	NM_014388	Homo sapiens chromosome 1 open reading frame 107 (C1orf107), mRNA [NM_014388]	NM_014388
A_32_P10003	0.000381	2.59	NM_080386	NM_080386	Homo sapiens alpha-tubulin isotype H2-alpha (H2-ALPHA), mRNA [NM_080386]	NM_080386
A_24_P195454	0.000381	3.79	A_24_P195454			
A_23_P410059	0.000382	2.27	NM_004804	NM_004804	Homo sapiens WD repeat domain 39 (WDR39), mRNA [NM_004804]	NM_004804
A_23_P115046	0.000382	3.91	NM_020365	NM_020365	Homo sapiens eukaryotic translation initiation factor 2B, subunit 3 gamma, 58kDa (EIF2B3), mRNA [NM_020365]	NM_020365
A_23_P13797	0.000383	2.19	NM_024738	NM_024738	Homo sapiens hypothetical protein FLJ21415 (FLJ21415), mRNA [NM_024738]	NM_024738
A_32_P228570	0.000383	2.95	A_32_P228570			
A_32_P29016	0.000383	2.95	THC2320434			
A_24_P919989	0.000383	4.88	BC015334	BC015334	Homo sapiens, clone IMAGE:4391654, mRNA, partial cds. [BC015334]	
A_23_P141055	0.000383	6.62	NM_015927	NM_015927	Homo sapiens transforming growth factor beta 1 induced transcript 1 (TGFB1I1), mRNA [NM_015927]	NM_015927
A_24_P269853	0.000384	3.94	NM_005736	NM_005736	Homo sapiens ARP1 actin-related protein 1 homolog A, centractin alpha (yeast) (ACTR1A), mRNA [NM_005736]	NM_005736
A_24_P363679	0.000385	2.04	NM_015941	NM_015941	Homo sapiens ATPase, H+ transporting, lysosomal 50/57kDa, V1 subunit H (ATP6V1H), transcript variant 1, mRNA [NM_015941]	NM_015941
A_24_P125096	0.000385	4.78	NM_005952	NM_005952	Homo sapiens metallothionein 1X (MT1X), mRNA [NM_005952]	NM_005952
A_23_P250042	0.000386	2.04	NM_016275	NM_016275	Homo sapiens selenoprotein T (SELT), mRNA [NM_016275]	NM_016275
A_23_P216501	0.000386	4.59	NM_213674	NM_213674	Homo sapiens tropomyosin 2 (beta) (TPM2), transcript variant 2, mRNA [NM_213674]	NM_213674
A_23_P251075	0.000387	2.80	NM_005491	NM_005491	Homo sapiens chromosome X open reading frame 6 (CXorf6), mRNA [NM_005491]	NM_005491
A_23_P156180	0.000387	3.21	NM_003059	NM_003059	Homo sapiens solute carrier family 22 (organic cation transporter), member 4 (SLC22A4), mRNA [NM_003059]	NM_003059
A_24_P213956	0.000388	2.31	U05597	U05597	Human anion exchanger 3 cardiac isoform (cAE3) mRNA, partial cds. [U05597]	
A_24_P363100	0.000388	4.15	NM_173670	NM_173670	Homo sapiens RGM domain family, member B (RGMB), transcript variant 2, mRNA [NM_173670]	NM_173670
A_24_P681563	0.000389	2.35	A_24_P681563			
A_23_P30223	0.00039	2.20	NM_001047	NM_001047	Homo sapiens steroid-5-alpha-reductase, alpha polypeptide 1 (3-oxo-5 alpha-steroid delta 4-dehydrogenase alpha 1) (SRD5A1), mRNA [NM_001047]	NM_001047
A_23_P90533	0.00039	2.42	NM_006627	NM_006627	Homo sapiens processing of precursor 4, ribonuclease P/MRP subunit (S. cerevisiae) (POP4), mRNA [NM_006627]	NM_006627
A_24_P625382	0.00039	2.54	NM_003651	NM_003651	Homo sapiens cold shock domain protein A (CSDA), mRNA [NM_003651]	NM_003651
A_23_P138495	0.00039	6.80	NM_006504	NM_006504	Homo sapiens protein tyrosine phosphatase, receptor type, E (PTPRE), transcript variant 1, mRNA [NM_006504]	NM_006504
A_23_P143535	0.00039	7.94	NM_033661	NM_033661	Homo sapiens WD repeat domain 4 (WDR4), transcript variant 2, mRNA [NM_033661]	NM_033661
A_23_P110725	0.000391	2.28	NM_206907	NM_206907	Homo sapiens protein kinase, AMP-activated, alpha 1 catalytic subunit (PRKAA1), transcript variant 2, mRNA [NM_206907]	NM_206907
A_23_P14458	0.000391	4.20	NM_144581	NM_144581	Homo sapiens chromosome 14 open reading frame 149 (C14orf149), mRNA [NM_144581]	NM_144581
A_32_P159574	0.000392	2.05	BC028022	BC028022	Homo sapiens, clone IMAGE:5218412, mRNA. [BC028022]	
A_23_P332937	0.000392	2.82	NM_152480	NM_152480	Homo sapiens chromosome 19 open reading frame 23 (C19orf23), mRNA [NM_152480]	NM_152480
A_23_P145376	0.000392	2.87	NM_002754	NM_002754	Homo sapiens mitogen-activated protein kinase 13 (MAPK13), mRNA [NM_002754]	NM_002754

A_23_P142714	0.000394	2.39	NM_003705	NM_003705	Homo sapiens solute carrier family 25 (mitochondrial carrier, Aralar), member 12 (SLC25A12), mRNA [NM_003705]	NM_003705
A_23_P30315	0.000394	75.76	NM_033342	NM_033342	Homo sapiens tripartite motif-containing 7 (TRIM7), transcript variant 6, mRNA [NM_033342]	NM_033342
A_24_P116871	0.000395	2.18	NM_006701	NM_006701	Homo sapiens thioredoxin-like 4A (TXNL4A), mRNA [NM_006701]	NM_006701
A_23_P95930	0.000395	2.40	NM_001015886	NM_001015886	Homo sapiens high mobility group AT-hook 2 (HMGA2), transcript variant 3, mRNA [NM_001015886]	NM_001015886
A_23_P65427	0.000396	2.12	NM_002818	NM_002818	Homo sapiens proteasome (prosome, macropain) activator subunit 2 (PA28 beta) (PSME2), mRNA [NM_002818]	NM_002818
A_23_P218743	0.000398	4.02	NM_020132	NM_020132	Homo sapiens 1-acylglycerol-3-phosphate O-acyltransferase 3 (AGPAT3), mRNA [NM_020132]	NM_020132
A_24_P246863	0.000398	4.42	A_24_P246863			
A_23_P67980	0.000398	5.41	NM_003709	NM_003709	Homo sapiens Kruppel-like factor 7 (ubiquitous) (KLF7), mRNA [NM_003709]	NM_003709
A_32_P219845	0.000399	2.65	THC2343880			
A_23_P378526	0.000399	3.40	NM_016434	NM_016434	Homo sapiens regulator of telomere elongation helicase 1 (RTEL1), transcript variant 1, mRNA [NM_016434]	NM_016434
A_23_P60047	0.0004	6.71	NM_006283	NM_006283	Homo sapiens transforming, acidic coiled-coil containing protein 1 (TACC1), mRNA [NM_006283]	NM_006283
A_24_P209772	0.000401	2.93	NM_007234	NM_007234	Homo sapiens dynactin 3 (p22) (DCTN3), transcript variant 1, mRNA [NM_007234]	NM_007234
A_32_P23659	0.000401	7.14	BC056883	BC056883	Homo sapiens breast carcinoma amplified sequence 4, mRNA (cDNA clone IMAGE:5764497), partial cds. [BC056883]	
A_23_P365817	0.000402	2.07	NM_138689	NM_138689	Homo sapiens protein phosphatase 1, regulatory (inhibitor) subunit 14B (PPP1R14B), mRNA [NM_138689]	NM_138689
A_23_P27627	0.000402	2.54	NM_004317	NM_004317	Homo sapiens arsA arsenite transporter, ATP-binding, homolog 1 (bacterial) (ASNA1), mRNA [NM_004317]	NM_004317
A_23_P54991	0.000404	2.13	NM_080677	NM_080677	Homo sapiens dynein light chain 2 (Dlc2), mRNA [NM_080677]	NM_080677
A_23_P25525	0.000404	2.15	NM_002097	NM_002097	Homo sapiens general transcription factor IIIA (GTF3A), mRNA [NM_002097]	NM_002097
A_23_P130689	0.000404	2.16	NM_032377	NM_032377	Homo sapiens elongation factor 1 homolog (ELF1, S. cerevisiae) (ELOF1), mRNA [NM_032377]	NM_032377
A_23_P126790	0.000404	2.31	NM_006513	NM_006513	Homo sapiens seryl-tRNA synthetase (SARS), mRNA [NM_006513]	NM_006513
A_24_P69654	0.000404	2.94	NM_001300	NM_001300	Homo sapiens Kruppel-like factor 6 (KLF6), transcript variant 2, mRNA [NM_001300]	NM_001300
A_24_P226008	0.000404	16.78	NM_007283	NM_007283	Homo sapiens monoglyceride lipase (MGLL), transcript variant 1, mRNA [NM_007283]	NM_007283
A_24_P565556	0.000407	2.40	NM_019109	NM_019109	Homo sapiens asparagine-linked glycosylation 1 homolog (yeast, beta-1,4-mannosyltransferase) (ALG1), mRNA [NM_019109]	NM_019109
A_23_P20814	0.000407	2.43	NM_014314	NM_014314	Homo sapiens DEAD (Asp-Glu-Ala-Asp) box polypeptide 58 (DDX58), mRNA [NM_014314]	NM_014314
A_24_P98249	0.000407	4.50	NM_006283	NM_006283	Homo sapiens transforming, acidic coiled-coil containing protein 1 (TACC1), mRNA [NM_006283]	NM_006283
A_32_P192692	0.000407	6.85	NM_001014797	NM_001014797	Homo sapiens potassium large conductance calcium-activated channel, subfamily M, alpha member 1 (KCNMA1), transcript variant 1, mRNA [NM_001014797]	NM_001014797
A_23_P17490	0.000408	2.05	NM_001009924	NM_001009924	Homo sapiens chromosome 20 open reading frame 30 (C20orf30), transcript variant 2, mRNA [NM_001009924]	NM_001009924
A_32_P229965	0.000408	2.21	AA662695	AA662695	AA662695 nv06h02.s1 NCI_CGAP_Pr22 Homo sapiens cDNA clone IMAGE:1219443 3', mRNA sequence [AA662695]	
A_32_P201677	0.000409	2.17	BE730710	BE730710	601570665F1 NIH_MGC_21 Homo sapiens cDNA clone IMAGE:3845272 5', mRNA sequence [BE730710]	
A_24_P239689	0.000409	3.72	NM_019072	NM_019072	Homo sapiens small glutamine-rich tetratricopeptide repeat (TPR)-containing, beta (SGTB), mRNA [NM_019072]	NM_019072
A_23_P204436	0.000411	2.28	NM_139201	NM_139201	Homo sapiens G protein-coupled receptor kinase interactor 2 (GIT2), transcript variant 4, mRNA [NM_139201]	NM_139201
A_24_P333019	0.000411	2.33	NM_007219	NM_007219	Homo sapiens ring finger protein 24 (RNF24), mRNA [NM_007219]	NM_007219
A_32_P199301	0.000412	2.23	NM_007111	NM_007111	Homo sapiens transcription factor Dp-1 (TFDP1), mRNA [NM_007111]	NM_007111
A_23_P135769	0.000413	2.39	NM_001101	NM_001101	Homo sapiens actin, beta (ACTB), mRNA [NM_001101]	NM_001101
A_23_P213000	0.000413	3.79	NM_017491	NM_017491	Homo sapiens WD repeat domain 1 (WDR1), transcript variant 1, mRNA [NM_017491]	NM_017491
A_23_P120660	0.000414	2.30	NM_001024	NM_001024	Homo sapiens ribosomal protein S21 (RPS21), mRNA [NM_001024]	NM_001024
A_23_P168692	0.000414	3.15	NM_001014436	NM_001014436	Homo sapiens drebrin-like (DBNL), transcript variant 2, mRNA [NM_001014436]	NM_001014436
A_32_P75094	0.000415	2.32	NM_032797	NM_032797	Homo sapiens apoptosis-inducing factor (AIF)-like mitochondrion-associated inducer of death (AMID), mRNA [NM_032797]	NM_032797
A_23_P98261	0.000416	2.27	NM_006396	NM_006396	Homo sapiens Sjogren's syndrome/scleroderma autoantigen 1 (SSSCA1), mRNA [NM_006396]	NM_006396
A_24_P393787	0.000416	2.60	NM_017846	NM_017846	Homo sapiens tRNA selenocysteine associated protein (SECP43), mRNA [NM_017846]	NM_017846
A_23_P171034	0.000417	2.34	NM_015922	NM_015922	Homo sapiens NAD(P) dependent steroid dehydrogenase-like (NSDHL), mRNA [NM_015922]	NM_015922

A_23_P91487	0.000417	2.90	NM_016940	NM_016940	Homo sapiens chromosome 21 open reading frame 6 (C21orf6), mRNA [NM_016940]	NM_016940
A_23_P329133	0.000419	2.29	NM_002080	NM_002080	Homo sapiens glutamic-oxaloacetic transaminase 2, mitochondrial (aspartate aminotransferase 2) (GOT2), mRNA [NM_002080]	NM_002080
A_24_P401582	0.000419	3.00	A_24_P401582			
A_23_P2793	0.000419	10.33	NM_001629	NM_001629	Homo sapiens arachidonate 5-lipoxygenase-activating protein (ALOX5AP), mRNA [NM_001629]	NM_001629
A_24_P653603	0.00042	3.04	BC079831	BC079831	Homo sapiens hypothetical LOC284184, mRNA (cDNA clone IMAGE:6572580). [BC079831]	XM_211367
A_23_P41645	0.000421	2.01	NM_012081	NM_012081	Homo sapiens elongation factor, RNA polymerase II, 2 (ELL2), mRNA [NM_012081]	NM_012081
A_24_P392022	0.000421	2.57	NM_201400	NM_201400	Homo sapiens hypothetical protein SB153 (SB153), transcript variant 1, mRNA [NM_201400]	NM_201400
A_24_P658584	0.000421	2.80	NM_015278	NM_015278	Homo sapiens SAM and SH3 domain containing 1 (SASH1), mRNA [NM_015278]	NM_015278
A_24_P306214	0.000421	3.17	NM_020947	NM_020947	Homo sapiens KIAA1609 protein (KIAA1609), mRNA [NM_020947]	NM_020947
A_32_P220938	0.000421	3.32	NM_006916	NM_006916	Homo sapiens ribulose-5-phosphate-3-epimerase (RPE), transcript variant 2, mRNA [NM_006916]	NM_006916
A_23_P254888	0.000421	4.41	NM_003461	NM_003461	Homo sapiens zyxin (ZYX), transcript variant 1, mRNA [NM_003461]	NM_003461
A_23_P256724	0.000423	2.01	NM_003841	NM_003841	Homo sapiens tumor necrosis factor receptor superfamily, member 10c, decoy without an intracellular domain (TNFRSF10C), mRNA [NM_003841]	NM_003841
A_24_P56884	0.000423	3.09	NM_152464	NM_152464	Homo sapiens chromosome 17 open reading frame 32 (C17orf32), mRNA [NM_152464]	NM_152464
A_23_P156327	0.000423	3.56	NM_000358	NM_000358	Homo sapiens transforming growth factor, beta-induced, 68kDa (TGFBI), mRNA [NM_000358]	NM_000358
A_23_P369479	0.000428	2.14	NM_170721	NM_170721	Homo sapiens musashi homolog 2 (Drosophila) (MSI2), transcript variant 2, mRNA [NM_170721]	NM_170721
A_32_P28872	0.000429	2.27	NM_006755	NM_006755	Homo sapiens transaldolase 1 (TALDO1), mRNA [NM_006755]	NM_006755
A_24_P323997	0.000429	2.55	NM_198479	NM_198479	Homo sapiens tetra-peptide repeat homeobox (TPRX), mRNA [NM_198479]	NM_198479
A_23_P27167	0.00043	3.37	NM_002936	NM_002936	Homo sapiens ribonuclease H1 (RNASEH1), mRNA [NM_002936]	NM_002936
A_32_P111274	0.000431	2.12	CD671824	CD671824	CD671824 fg07f07.x1 Human Iris cDNA (Normalized): fg Homo sapiens cDNA clone fg07f07 3', mRNA sequence [CD671824]	
A_24_P74487	0.000431	2.21	NM_058182	NM_058182	Homo sapiens chromosome 21 open reading frame 51 (C21orf51), mRNA [NM_058182]	NM_058182
A_23_P19663	0.000431	30.86	NM_001901	NM_001901	Homo sapiens connective tissue growth factor (CTGF), mRNA [NM_001901]	NM_001901
A_23_P258321	0.000433	2.04	NM_015969	NM_015969	Homo sapiens mitochondrial ribosomal protein S17 (MRPS17), nuclear gene encoding mitochondrial protein, mRNA [NM_015969]	NM_015969
A_23_P167841	0.000433	2.05	NM_019842	NM_019842	Homo sapiens potassium voltage-gated channel, KQT-like subfamily, member 5 (KCNQ5), mRNA [NM_019842]	NM_019842
A_32_P98975	0.000433	2.89	NM_052849	NM_052849	Homo sapiens hypothetical protein MGC20481 (MGC20481), mRNA [NM_052849]	NM_052849
A_23_P112602	0.000433	4.52	NM_018207	NM_018207	Homo sapiens tripartite motif-containing 62 (TRIM62), mRNA [NM_018207]	NM_018207
A_23_P358928	0.000434	2.22	NM_003130	NM_003130	Homo sapiens sorcin (SRI), transcript variant 1, mRNA [NM_003130]	NM_003130
A_23_P96812	0.000435	2.13	NM_017739	NM_017739	Homo sapiens protein O-linked mannose beta1,2-N-acetylglucosaminyltransferase (POMGNT1), mRNA [NM_017739]	NM_017739
A_32_P99432	0.000435	2.18	BC042161	BC042161	Homo sapiens trafficking protein particle complex 5, mRNA (cDNA clone MGC:52424 IMAGE:4869377), complete cds. [BC042161]	XM_058961
A_23_P364504	0.000435	2.44	ENST00000344233		Homo sapiens cDNA FLJ37034 fis, clone BRACE2011478. [AK094353]	
A_24_P273716	0.000435	2.53	NM_014797	NM_014797	Homo sapiens zinc finger and BTB domain containing 24 (ZBTB24), mRNA [NM_014797]	NM_014797
A_23_P210872	0.000437	2.51	NM_014742	NM_014742	Homo sapiens transmembrane 9 superfamily protein member 4 (TM9SF4), mRNA [NM_014742]	NM_014742
A_24_P264416	0.000438	2.26	A_24_P264416			
A_23_P149812	0.000438	4.61	NM_012425	NM_012425	Homo sapiens Ras suppressor protein 1 (RSU1), transcript variant 1, mRNA [NM_012425]	NM_012425
A_24_P124875	0.000439	2.57	NM_023112	NM_023112	Homo sapiens OTU domain, ubiquitin aldehyde binding 2 (OTUB2), mRNA [NM_023112]	NM_023112
A_24_P164894	0.000439	2.99	NM_006052	NM_006052	Homo sapiens Down syndrome critical region gene 3 (DSCR3), mRNA [NM_006052]	NM_006052
A_32_P86763	0.000439	15.08	NM_004613	NM_004613	Homo sapiens transglutaminase 2 (C polypeptide, protein-glutamine-gamma-glutamyltransferase) (TGM2), transcript variant 1, mRNA [NM_004613]	NM_004613
A_23_P36562	0.00044	3.62	NM_002205	NM_002205	Homo sapiens integrin, alpha 5 (fibronectin receptor, alpha polypeptide) (ITGA5), mRNA [NM_002205]	NM_002205
A_24_P237766	0.00044	4.18	NM_003003	NM_003003	Homo sapiens SEC14-like 1 (S. cerevisiae) (SEC14L1), mRNA [NM_003003]	NM_003003
A_23_P397293	0.00044	4.41	NM_017527	NM_017527	Homo sapiens lymphocyte antigen 6 complex, locus K (LY6K), mRNA [NM_017527]	NM_017527

A_24_P633575	0.00044	4.72	AF086329	AF086329	Homo sapiens full length insert cDNA clone ZD54C08. [AF086329]	
A_24_P244100	0.000441	2.30	NM_001002762	NM_001002762	Homo sapiens DnaJ (Hsp40) homolog, subfamily B, member 12 (DNAJB12), transcript variant 1, mRNA [NM_001002762]	NM_001002762
A_23_P103070	0.000441	2.60	NM_003405	NM_003405	Homo sapiens tyrosine 3-monooxygenase/tryptophan 5-monooxygenase activation protein, eta polypeptide (YWHAH), mRNA [NM_003405]	NM_003405
A_32_P47616	0.000441	11.03	AW994037	AW994037	AW994037 RC3-BN0036-090200-011-f03 BN0036 Homo sapiens cDNA, mRNA sequence [AW994037]	
A_23_P97394	0.000442	2.37	NM_003567	NM_003567	Homo sapiens breast cancer anti-estrogen resistance 3 (BCAR3), mRNA [NM_003567]	NM_003567
A_24_P73669	0.000443	2.40	NM_002094	NM_002094	Homo sapiens G1 to S phase transition 1 (GSPT1), mRNA [NM_002094]	NM_002094
A_23_P253368	0.000443	5.56	NM_018951	NM_018951	Homo sapiens homeo box A10 (HOXA10), transcript variant 1, mRNA [NM_018951]	NM_018951
A_23_P89601	0.000444	2.10	NM_002278	NM_002278	Homo sapiens keratin, hair, acidic, 2 (KRTHA2), mRNA [NM_002278]	NM_002278
A_24_P405054	0.000444	2.92	NM_015609	NM_015609	Homo sapiens chromosome 1 open reading frame 144 (C1orf144), mRNA [NM_015609]	NM_015609
A_23_P255376	0.000444	4.61	NM_017918	NM_017918	Homo sapiens hypothetical protein FLJ20647 (FLJ20647), mRNA [NM_017918]	NM_017918
A_23_P209215	0.000445	2.17	NM_005843	NM_005843	Homo sapiens signal transducing adaptor molecule (SH3 domain and ITAM motif) 2 (STAM2), mRNA [NM_005843]	NM_005843
A_23_P151497	0.000445	2.48	NM_152307	NM_152307	Homo sapiens chromosome 14 open reading frame 172 (C14orf172), mRNA [NM_152307]	NM_152307
A_24_P50543	0.000445	2.67	BC031266	BC031266	Homo sapiens, clone IMAGE:5277162, mRNA. [BC031266]	XM_378511
A_32_P49035	0.000445	3.76	A_32_P49035			
A_23_P404481	0.000445	4.05	NM_001400	NM_001400	Homo sapiens endothelial differentiation, sphingolipid G-protein-coupled receptor, 1 (EDG1), mRNA [NM_001400]	NM_001400
A_23_P316012	0.000446	4.83	NM_020663	NM_020663	Homo sapiens ras homolog gene family, member J (RHOJ), mRNA [NM_020663]	NM_020663
A_23_P433071	0.000447	2.08	NM_003766	NM_003766	Homo sapiens beclin 1 (coiled-coil, myosin-like BCL2 interacting protein) (BECN1), mRNA [NM_003766]	NM_003766
A_24_P181108	0.000448	2.03	NM_018093	NM_018093	Homo sapiens WD repeat domain 74 (WDR74), mRNA [NM_018093]	NM_018093
A_24_P315474	0.000449	2.30	THC2275974		EF1B_HUMAN (P24534) Elongation factor 1-beta (EF-1-beta), complete [THC2275974]	
A_32_P143824	0.000449	6.67	A_32_P143824			
A_24_P59220	0.000451	2.54	ENST00000303830		PREDICTED: Homo sapiens similar to POTE2A (LOC344227), mRNA [XM_292982]	XM_292982
A_24_P139901	0.000451	2.87	NM_002101	NM_002101	Homo sapiens glycophorin C (Gerbich blood group) (GYPC), transcript variant 1, mRNA [NM_002101]	NM_002101
A_24_P6903	0.000451	2.87	NM_001017992	NM_001017992	Homo sapiens similar to RIKEN cDNA 4732495G21 gene (DKFZp686D0972), mRNA [NM_001017992]	NM_001017992
A_32_P221832	0.000451	3.31	CD630738	CD630738	CD630738 56066364H1 FLP Homo sapiens cDNA, mRNA sequence [CD630738]	
A_23_P329924	0.000453	2.13	NM_177551	NM_177551	Homo sapiens G protein-coupled receptor 109A (GPR109A), mRNA [NM_177551]	NM_177551
A_23_P426501	0.000453	4.15	BC007329	BC007329	Homo sapiens transducin-like enhancer of split 6 (E(sp1) homolog, Drosophila), mRNA (cDNA clone IMAGE:3687767). [BC007329]	
A_24_P324783	0.000455	5.15	NM_000020	NM_000020	Homo sapiens activin A receptor type II-like 1 (ACVRL1), mRNA [NM_000020]	NM_000020
A_32_P78395	0.000458	2.23	THC2374967		BX842574 POSSIBLE RESUSCITATION-PROMOTING FACTOR RPFA {Mycobacterium tuberculosis H37Rv;} , partial (6%) [THC2374967]	
A_24_P487766	0.000459	2.10	A_24_P487766			
A_23_P416112	0.000459	2.35	NM_152617	NM_152617	Homo sapiens ring finger protein 168 (RNF168), mRNA [NM_152617]	NM_152617
A_23_P111020	0.00046	2.27	NM_003131	NM_003131	Homo sapiens serum response factor (c-fos serum response element-binding transcription factor) (SRF), mRNA [NM_003131]	NM_003131
A_23_P211973	0.00046	2.46	NM_024800	NM_024800	Homo sapiens NIMA (never in mitosis gene a)- related kinase 11 (NEK11), transcript variant 1, mRNA [NM_024800]	NM_024800
A_23_P407142	0.00046	2.80	NM_033631	NM_033631	Homo sapiens leucine zipper protein 1 (LUZP1), mRNA [NM_033631]	NM_033631
A_24_P850336	0.00046	2.83	A_24_P850336			
A_24_P209765	0.000461	2.14	NM_007234	NM_007234	Homo sapiens dynactin 3 (p22) (DCTN3), transcript variant 1, mRNA [NM_007234]	NM_007234
A_23_P63870	0.000462	2.18	NM_144660	NM_144660	Homo sapiens sterile alpha motif domain containing 8 (SAMD8), mRNA [NM_144660]	NM_144660
A_23_P207911	0.000462	2.44	NM_016113	NM_016113	Homo sapiens transient receptor potential cation channel, subfamily V, member 2 (TRPV2), mRNA [NM_016113]	NM_016113
A_23_P32294	0.000463	2.41	NM_014612	NM_014612	Homo sapiens chromosome 9 open reading frame 10 (C9orf10), mRNA [NM_014612]	NM_014612
A_23_P20566	0.000463	4.41	NM_213674	NM_213674	Homo sapiens tropomyosin 2 (beta) (TPM2), transcript variant 2, mRNA [NM_213674]	NM_213674
A_24_P280983	0.000463	6.71	BC025338	BC025338	Homo sapiens, clone IMAGE:4865533, mRNA. [BC025338]	

A_23_P118693	0.000464	2.04	NM_003593	NM_003593	Homo sapiens forkhead box N1 (FOXN1), mRNA [NM_003593]	NM_003593
A_32_P808	0.000464	2.17	BC040993	BC040993	Homo sapiens KIAA1458 protein, mRNA (cDNA clone MGC:43785 IMAGE:5272534), complete cds. [BC040993]	XM_044434
A_23_P154566	0.000464	21.10	NM_032883	NM_032883	Homo sapiens chromosome 20 open reading frame 100 (C20orf100), mRNA [NM_032883]	NM_032883
A_24_P155502	0.000466	2.58	NM_005538	NM_005538	Homo sapiens inhibin, beta C (INHBC), mRNA [NM_005538]	NM_005538
A_23_P57089	0.000467	2.30	NM_020182	NM_020182	Homo sapiens transmembrane, prostate androgen induced RNA (TMEPAI), transcript variant 1, mRNA [NM_020182]	NM_020182
A_23_P105957	0.000467	3.79	NM_001102	NM_001102	Homo sapiens actinin, alpha 1 (ACTN1), mRNA [NM_001102]	NM_001102
A_24_P280953	0.000469	2.01	A_24_P280953			
A_23_P255503	0.000469	3.45	NM_002572	NM_002572	Homo sapiens platelet-activating factor acetylhydrolase, isoform Ib, beta subunit 30kDa (PAFAH1B2), mRNA [NM_002572]	NM_002572
A_23_P126593	0.00047	3.09	NM_005620	NM_005620	Homo sapiens S100 calcium binding protein A11 (calgizzarin) (S100A11), mRNA [NM_005620]	NM_005620
A_24_P228717	0.00047	5.52	NM_002872	NM_002872	Homo sapiens ras-related C3 botulinum toxin substrate 2 (rho family, small GTP binding protein Rac2) (RAC2), mRNA [NM_002872]	NM_002872
A_23_P206484	0.000472	2.22	NM_005262	NM_005262	Homo sapiens growth factor, augmenter of liver regeneration (ERV1 homolog, S. cerevisiae) (GFER), mRNA [NM_005262]	NM_005262
A_24_P135921	0.000474	2.12	ENST00000329070		PREDICTED: Homo sapiens similar to PROHIBITIN (B-CELL RECEPTOR ASSOCIATED PROTEIN 32) (BAP 32) (LOC441510), mRNA [XM_497140]	XM_497140
A_23_P62335	0.000474	2.44	NM_018196	NM_018196	Homo sapiens trimethyllysine hydroxylase, epsilon (TMLHE), mRNA [NM_018196]	NM_018196
A_24_P186354	0.000475	2.10	A_24_P186354			
A_23_P391725	0.000476	2.28	NM_001938	NM_001938	Homo sapiens down-regulator of transcription 1, TBP-binding (negative cofactor 2) (DR1), mRNA [NM_001938]	NM_001938
A_24_P810290	0.000476	4.00	AK098668	AK098668	Homo sapiens cDNA FLJ25802 fis, clone TST07145. [AK098668]	XM_113641
A_23_P38334	0.000476	5.38	NM_032387	NM_032387	Homo sapiens WNK lysine deficient protein kinase 4 (WNK4), mRNA [NM_032387]	NM_032387
A_24_P124550	0.000476	12.92	NM_053056	NM_053056	Homo sapiens cyclin D1 (PRAD1: parathyroid adenomatosis 1) (CCND1), mRNA [NM_053056]	NM_053056
A_32_P84846	0.000477	2.01	NM_001005851	NM_001005851	Homo sapiens hypothetical BC331191_1 (LOC163131), mRNA [NM_001005851]	NM_001005851
A_32_P141338	0.000478	3.62	BE930053	BE930053	BE930053 CM2-GN0054-230800-344-a10 GN0054 Homo sapiens cDNA, mRNA sequence [BE930053]	
A_23_P93046	0.000479	2.07	NM_007277	NM_007277	Homo sapiens SEC6-like 1 (S. cerevisiae) (SEC6L1), mRNA [NM_007277]	NM_007277
A_23_P26629	0.000479	3.44	NM_013258	NM_013258	Homo sapiens PYD and CARD domain containing (PYCARD), transcript variant 1, mRNA [NM_013258]	NM_013258
A_24_P388570	0.00048	3.72	NM_032778	NM_032778	Homo sapiens MYC induced nuclear antigen (MINA), transcript variant 2, mRNA [NM_032778]	NM_032778
A_32_P30905	0.00048	4.12	BC032420	BC032420	Homo sapiens WDFY family member 4, mRNA (cDNA clone MGC:40604 IMAGE:5221804), complete cds. [BC032420]	
A_24_P162211	0.000481	2.14	NM_001001438	NM_001001438	Homo sapiens lanosterol synthase (2,3-oxidosqualene-lanosterol cyclase) (LSS), transcript variant 2, mRNA [NM_001001438]	NM_001001438
A_23_P204364	0.000481	2.15	NM_006170	NM_006170	Homo sapiens nucleolar protein 1, 120kDa (NOL1), mRNA [NM_006170]	NM_006170
A_23_P9416	0.000482	2.11	NM_002197	NM_002197	Homo sapiens aconitase 1, soluble (ACO1), mRNA [NM_002197]	NM_002197
A_24_P307025	0.000482	2.39	NR_000029	NR_000029	Homo sapiens ribosomal protein L23a pseudogene 7 (RPL23AP7) on chromosome 2 [NR_000029]	NR_000029
A_23_P33303	0.000482	3.37	NM_005026	NM_005026	Homo sapiens phosphoinositide-3-kinase, catalytic, delta polypeptide (PIK3CD), mRNA [NM_005026]	NM_005026
A_32_P74643	0.000483	2.86	NM_001627	NM_001627	Homo sapiens activated leukocyte cell adhesion molecule (ALCAM), mRNA [NM_001627]	NM_001627
A_23_P90311	0.000484	2.27	NM_182919	NM_182919	Homo sapiens toll-like receptor adaptor molecule 1 (TICAM1), transcript variant 2, mRNA [NM_182919]	NM_182919
A_23_P77031	0.000484	2.54	NM_007039	NM_007039	Homo sapiens protein tyrosine phosphatase, non-receptor type 21 (PTPN21), mRNA [NM_007039]	NM_007039
A_24_P116132	0.000487	2.28	NM_015509	NM_015509	Homo sapiens adaptin-ear-binding coat-associated protein 1 (NECAP1), mRNA [NM_015509]	NM_015509
A_23_P42042	0.000487	2.63	NM_020466	NM_020466	Homo sapiens hypothetical protein dJ122O8.2 (DJ122O8.2), mRNA [NM_020466]	NM_020466
A_32_P201723	0.000487	3.97	BU624603	BU624603	BU624603 UI-H-FG1-bgk-p-15-0-UI.s1 NCI_CGAP_FG1 Homo sapiens cDNA clone UI-H-FG1-bgk-p-15-0-UI 3', mRNA sequence [BU624603]	
A_23_P73429	0.000487	33.56	NM_005335	NM_005335	Homo sapiens hematopoietic cell-specific Lyn substrate 1 (HCLS1), mRNA [NM_005335]	NM_005335
A_23_P379550	0.000488	2.44	NM_003680	NM_003680	Homo sapiens tyrosyl-tRNA synthetase (YARS), mRNA [NM_003680]	NM_003680
A_24_P301846	0.000488	3.47	NM_175085	NM_175085	Homo sapiens phosphoribosylglycinamide formyltransferase, phosphoribosylglycinamide synthetase, phosphoribosylaminoimidazole synthetase (GART), transcript variant 2, mRNA [NM_175085]	NM_175085

A_23_P48691	0.000489	2.08	NM_001663	NM_001663	Homo sapiens ADP-ribosylation factor 6 (ARF6), mRNA [NM_001663]	NM_001663
A_32_P233019	0.000489	2.20	NM_012430	NM_012430	Homo sapiens SEC22 vesicle trafficking protein-like 2 (S. cerevisiae) (SEC22L2), mRNA [NM_012430]	NM_012430
A_24_P116233	0.000489	3.22	NM_020456	NM_020456	Homo sapiens chromosome 13 open reading frame 1 (C13orf1), mRNA [NM_020456]	NM_020456
A_24_P93371	0.00049	2.45	NM_017828	NM_017828	Homo sapiens COMM domain containing 4 (COMMD4), mRNA [NM_017828]	NM_017828
A_23_P58137	0.000491	6.10	BM930307	BM930307	UI-E-EJ1-ajh-l-11-0-UI.r1 UI-E-EJ1 Homo sapiens cDNA clone UI-E-EJ1-ajh-l-11-0-UI 5', mRNA sequence [BM930307]	
A_32_P127052	0.000494	2.66	NM_152395	NM_152395	Homo sapiens nudix (nucleoside diphosphate linked moiety X)-type motif 16 (NUDT16), mRNA [NM_152395]	NM_152395
A_24_P349965	0.000494	3.09	BC033086	BC033086	Homo sapiens transcription factor 19 (SC1), mRNA (cDNA clone MGC:45652 IMAGE:3160434), complete cds. [BC033086]	
A_23_P150718	0.000495	2.03	NM_001665	NM_001665	Homo sapiens ras homolog gene family, member G (rho G) (RHOG), mRNA [NM_001665]	NM_001665
A_23_P73589	0.000495	3.91	NM_002444	NM_002444	Homo sapiens moesin (MSN), mRNA [NM_002444]	NM_002444
A_23_P252471	0.000496	3.13	NM_000442	NM_000442	Homo sapiens platelet/endothelial cell adhesion molecule (CD31 antigen) (PECAM1), mRNA [NM_000442]	NM_000442
A_24_P108005	0.000497	2.04	NM_022818	NM_022818	Homo sapiens microtubule-associated protein 1 light chain 3 beta (MAP1LC3B), mRNA [NM_022818]	NM_022818
A_24_P51786	0.000497	3.10	NM_174908	NM_174908	Homo sapiens chromosome 3 open reading frame 6 (C3orf6), transcript variant 1, mRNA [NM_174908]	NM_174908
A_23_P124542	0.000498	2.14	M26004	M26004	Human CR2/CD21/C3d/Epstein-Barr virus receptor mRNA, complete cds. [M26004]	
A_23_P82051	0.000498	2.26	NM_058182	NM_058182	Homo sapiens chromosome 21 open reading frame 51 (C21orf51), mRNA [NM_058182]	NM_058182
A_32_P32739	0.000498	2.62	NM_153006	NM_153006	Homo sapiens N-acetylglutamate synthase (NAGS), mRNA [NM_153006]	NM_153006
A_23_P124905	0.000498	4.63	NM_002522	NM_002522	Homo sapiens neuronal pentraxin I (NPTX1), mRNA [NM_002522]	NM_002522
A_23_P250485	0.000498	4.76	NM_018207	NM_018207	Homo sapiens tripartite motif-containing 62 (TRIM62), mRNA [NM_018207]	NM_018207
A_24_P152527	0.0005	2.41	NM_006373	NM_006373	Homo sapiens vesicle amine transport protein 1 homolog (T californica) (VAT1), mRNA [NM_006373]	NM_006373
A_23_P9135	0.0005	7.94	NM_033655	NM_033655	Homo sapiens contactin associated protein-like 3 (CNTNAP3), mRNA [NM_033655]	NM_033655
A_23_P109055	0.000502	2.24	NM_033550	NM_033550	Homo sapiens TP53 regulating kinase (TP53RK), mRNA [NM_033550]	NM_033550
A_24_P306896	0.000502	2.55	A_24_P306896			
A_24_P54879	0.000503	2.01	NM_005506	NM_005506	Homo sapiens scavenger receptor class B, member 2 (SCARB2), mRNA [NM_005506]	NM_005506
A_24_P110780	0.000503	3.58	NM_199343	NM_199343	Homo sapiens chromosome 1 open reading frame 118 (C1orf118), mRNA [NM_199343]	NM_199343
A_23_P67321	0.000504	2.70	NM_138334	NM_138334	Homo sapiens hypothetical transmembrane protein SBBI54 (SBBI54), mRNA [NM_138334]	NM_138334
A_24_P307974	0.000504	3.40	BC033728	BC033728	Homo sapiens cDNA clone MGC:45470 IMAGE:5166848, complete cds. [BC033728]	
A_24_P155058	0.000506	2.14	NM_001556	NM_001556	Homo sapiens inhibitor of kappa light polypeptide gene enhancer in B-cells, kinase beta (IKBKB), mRNA [NM_001556]	NM_001556
A_23_P94998	0.000506	2.91	NM_012318	NM_012318	Homo sapiens leucine zipper-EF-hand containing transmembrane protein 1 (LETM1), mRNA [NM_012318]	NM_012318
A_24_P213494	0.000506	4.93	NM_006504	NM_006504	Homo sapiens protein tyrosine phosphatase, receptor type, E (PTPRE), transcript variant 1, mRNA [NM_006504]	NM_006504
A_24_P331704	0.000507	11.36	NM_182507	NM_182507	Homo sapiens hypothetical protein LOC144501 (LOC144501), mRNA [NM_182507]	NM_182507
A_23_P88848	0.000509	2.07	NM_017803	NM_017803	Homo sapiens dihydrouridine synthase 2-like (SMM1, S. cerevisiae) (DUS2L), mRNA [NM_017803]	NM_017803
A_23_P399255	0.000509	3.48	NM_152737	NM_152737	Homo sapiens ring finger protein 182 (RNF182), mRNA [NM_152737]	NM_152737
A_23_P360777	0.000509	8.70	NM_013957	NM_013957	Homo sapiens neuregulin 1 (NRG1), transcript variant HRG-beta2, mRNA [NM_013957]	NM_013957
A_23_P110712	0.000512	5.52	NM_004417	NM_004417	Homo sapiens dual specificity phosphatase 1 (DUSP1), mRNA [NM_004417]	NM_004417
A_23_P202696	0.000513	2.17	NM_016506	NM_016506	Homo sapiens kelch repeat and BTB (POZ) domain containing 4 (KBTBD4), transcript variant 2, mRNA [NM_016506]	NM_016506
A_32_P221437	0.000513	3.25	A_32_P221437			
A_23_P152782	0.000515	2.06	NM_005533	NM_005533	Homo sapiens interferon-induced protein 35 (IFI35), mRNA [NM_005533]	NM_005533
A_23_P255331	0.000515	5.68	NM_032623	NM_032623	Homo sapiens ovary-specific acidic protein (OSAP), mRNA [NM_032623]	NM_032623
A_32_P191859	0.000519	2.36	NM_020382	NM_020382	Homo sapiens PR/SET domain containing protein 8 (SET8), mRNA [NM_020382]	NM_020382
A_23_P250714	0.000519	2.99	NM_005107	NM_005107	Homo sapiens endonuclease G-like 1 (ENDOGL1), mRNA [NM_005107]	NM_005107
A_23_P8196	0.00052	2.67	CR591849	CR591849	full-length cDNA clone CS0DC003YO01 of Neuroblastoma Cot 25-normalized of Homo sapiens (human). [CR591849]	
A_24_P42071	0.00052	3.16	ENST00000332925			

A_32_P159726	0.000521	2.26	BC014227	BC014227	Homo sapiens KIAA1244, mRNA (cDNA clone IMAGE:4547495), partial cds. [BC014227]	
A_24_P279760	0.000521	2.36	A_24_P279760			
A_24_P392146	0.000521	2.52	BC036911	BC036911	Homo sapiens KIAA1632, mRNA (cDNA clone IMAGE:5139756), complete cds. [BC036911]	
A_24_P57700	0.000523	4.12	NM_015035	NM_015035	Homo sapiens zinc fingers and homeoboxes 3 (ZHX3), mRNA [NM_015035]	NM_015035
A_32_P120330	0.000523	7.52	THC2329478			
A_24_P259276	0.000524	2.05	NM_207340	NM_207340	Homo sapiens zinc finger, DHHC-type containing 24 (ZDHHC24), mRNA [NM_207340]	NM_207340
A_23_P346206	0.000524	2.11	NM_001015885	NM_001015885	Homo sapiens RAE1 RNA export 1 homolog (S. pombe) (RAE1), transcript variant 2, mRNA [NM_001015885]	NM_001015885
A_24_P291814	0.000524	8.40	NM_004370	NM_004370	Homo sapiens collagen, type XII, alpha 1 (COL12A1), transcript variant long, mRNA [NM_004370]	NM_004370
A_23_P212119	0.000526	4.61	NM_054110	NM_054110	Homo sapiens UDP-N-acetyl-alpha-D-galactosamine:polypeptide N-acetylgalactosaminyltransferase-like 2 (GALNTL2), mRNA [NM_054110]	NM_054110
A_23_P218646	0.000527	4.10	NM_032945	NM_032945	Homo sapiens tumor necrosis factor receptor superfamily, member 6b, decoy (TNFRSF6B), transcript variant M68C, mRNA [NM_032945]	NM_032945
A_24_P327499	0.000528	2.04	NM_014657	NM_014657	Homo sapiens KIAA0406 gene product (KIAA0406), mRNA [NM_014657]	NM_014657
A_23_P68059	0.000528	2.58	NM_032673	NM_032673	Homo sapiens polycomb group ring finger 1 (PCGF1), mRNA [NM_032673]	NM_032673
A_24_P483083	0.000528	3.56	NM_007111	NM_007111	Homo sapiens transcription factor Dp-1 (TFDP1), mRNA [NM_007111]	NM_007111
A_24_P198844	0.000529	2.03	NM_004870	NM_004870	Homo sapiens mannose-P-dolichol utilization defect 1 (MPDU1), mRNA [NM_004870]	NM_004870
A_23_P103276	0.000529	2.04	NM_138417	NM_138417	Homo sapiens hypothetical protein BC012173 (MGC20419), mRNA [NM_138417]	NM_138417
A_24_P413126	0.000532	2.51	NM_020182	NM_020182	Homo sapiens transmembrane, prostate androgen induced RNA (TMEPAI), transcript variant 1, mRNA [NM_020182]	NM_020182
A_24_P145229	0.000532	3.41	NM_032816	NM_032816	Homo sapiens hypothetical protein FLJ14640 (FLJ14640), mRNA [NM_032816]	NM_032816
A_24_P835500	0.000533	2.03	AK026647	AK026647	Homo sapiens cDNA: FLJ22994 fis, clone KAT11918. [AK026647]	
A_23_P83328	0.000533	4.13	NM_000118	NM_000118	Homo sapiens endoglin (Osler-Rendu-Weber syndrome 1) (ENG), mRNA [NM_000118]	NM_000118
A_23_P303242	0.000533	4.65	NM_005952	NM_005952	Homo sapiens metallothionein 1X (MT1X), mRNA [NM_005952]	NM_005952
A_23_P37244	0.000534	2.04	NM_003082	NM_003082	Homo sapiens small nuclear RNA activating complex, polypeptide 1, 43kDa (SNAPC1), mRNA [NM_003082]	NM_003082
A_24_P228667	0.000535	2.04	NM_003776	NM_003776	Homo sapiens mitochondrial ribosomal protein L40 (MRPL40), nuclear gene encoding mitochondrial protein, mRNA [NM_003776]	NM_003776
A_24_P274842	0.000537	2.46	NM_007233	NM_007233	Homo sapiens TP53 activated protein 1 (TP53AP1), mRNA [NM_007233]	NM_007233
A_24_P221198	0.000539	2.05	NM_203397	NM_203397	Homo sapiens similar to hypothetical protein MGC49416 (LOC255374), mRNA [NM_203397]	NM_203397
A_24_P404033	0.00054	2.06	NM_182616	NM_182616	Homo sapiens hypothetical protein MGC61550 (MGC61550), mRNA [NM_182616]	NM_182616
A_24_P379765	0.00054	2.48	NM_018221	NM_018221	Homo sapiens MOB1, Mps One Binder kinase activator-like 1B (yeast) (MOBK1B), mRNA [NM_018221]	NM_018221
A_23_P402787	0.000541	2.11	NM_145253	NM_145253	Homo sapiens LOC124402 (LOC124402), mRNA [NM_145253]	NM_145253
A_23_P31602	0.000542	2.30	NM_003910	NM_003910	Homo sapiens maternal G10 transcript (G10), mRNA [NM_003910]	NM_003910
A_24_P57611	0.000542	2.95	NM_021637	NM_021637	Homo sapiens transmembrane protein 35 (TMEM35), mRNA [NM_021637]	NM_021637
A_23_P67042	0.000542	5.92	NM_017947	NM_017947	Homo sapiens molybdenum cofactor sulfurase (MOCOS), mRNA [NM_017947]	NM_017947
A_23_P389465	0.000543	2.35	NM_173598	NM_173598	Homo sapiens kinase suppressor of ras 2 (KSR2), mRNA [NM_173598]	NM_173598
A_32_P151577	0.000543	2.37	AF116719	AF116719	Homo sapiens PRO2987 mRNA, complete cds. [AF116719]	
A_23_P155301	0.000543	2.54	NM_145910	NM_145910	Homo sapiens NIMA (never in mitosis gene a)- related kinase 11 (NEK11), transcript variant 2, mRNA [NM_145910]	NM_145910
A_23_P79069	0.000545	2.43	AK024488	AK024488	Homo sapiens mRNA for FLJ00087 protein, partial cds. [AK024488]	XM_029084
A_32_P137399	0.000545	4.05	ENST00000335142		human full-length cDNA clone CS0DK001YK16 of HeLa cells of Homo sapiens (human). [BX248296]	XM_375081
A_24_P201171	0.000546	3.33	NM_003165	NM_003165	Homo sapiens syntaxin binding protein 1 (STXBP1), mRNA [NM_003165]	NM_003165
A_24_P85300	0.000549	5.49	AB033063	AB033063	Homo sapiens mRNA for KIAA1237 protein, partial cds. [AB033063]	XM_087386
A_23_P145895	0.00055	2.17	NM_007233	NM_007233	Homo sapiens TP53 activated protein 1 (TP53AP1), mRNA [NM_007233]	NM_007233
A_23_P74609	0.000551	3.02	NM_015714	NM_015714	Homo sapiens putative lymphocyte G0/G1 switch gene (G0S2), mRNA [NM_015714]	NM_015714
A_23_P109420	0.000552	2.79	NM_080926	NM_080926	Homo sapiens hypothetical protein similar to KIAA0187 gene product (LOC96610), mRNA [NM_080926]	NM_080926

A_24_P850172	0.000553	2.18	A_24_P850172			
A_23_P65000	0.000553	2.22	NM_013300	NM_013300	Homo sapiens protein predicted by clone 23733 (HSU79274), mRNA [NM_013300]	NM_013300
A_32_P191665	0.000555	2.04	BF088423	BF088423	BF088423 CM1-HT0877-190900-426-c05 HT0877 Homo sapiens cDNA, mRNA sequence [BF088423]	
A_24_P461497	0.000556	3.28	ENST00000338852		PREDICTED: Homo sapiens similar to actin 3 - fruit fly (Drosophila melanogaster) (fragments) (LOC441836), mRNA [XM_497605]	XM_497605
A_23_P168259	0.000556	12.42	NM_025217	NM_025217	Homo sapiens UL16 binding protein 2 (ULBP2), mRNA [NM_025217]	NM_025217
A_32_P85999	0.000557	4.83	NM_001257	NM_001257	Homo sapiens cadherin 13, H-cadherin (heart) (CDH13), mRNA [NM_001257]	NM_001257
A_32_P25775	0.00056	2.39	BQ339588	BQ339588	BQ339588 QV3-NN0200-111100-376-b09 NN0200 Homo sapiens cDNA, mRNA sequence [BQ339588]	
A_23_P79889	0.00056	2.39	NM_020673	NM_020673	Homo sapiens RAB22A, member RAS oncogene family (RAB22A), mRNA [NM_020673]	NM_020673
A_24_P372901	0.00056	2.41	NM_000431	NM_000431	Homo sapiens mevalonate kinase (mevalonic aciduria) (MVK), mRNA [NM_000431]	NM_000431
A_23_P214681	0.00056	2.42	NM_177435	NM_177435	Homo sapiens peroxisome proliferative activated receptor, delta (PPARD), transcript variant 2, mRNA [NM_177435]	NM_177435
A_23_P47691	0.00056	2.64	NM_003141	NM_003141	Homo sapiens tripartite motif-containing 21 (TRIM21), mRNA [NM_003141]	NM_003141
A_24_P67432	0.000562	2.33	A_24_P67432			
A_24_P376287	0.000564	3.56	NM_019594	NM_019594	Homo sapiens leucine rich repeat containing 8 family, member A (LRRC8A), mRNA [NM_019594]	NM_019594
A_23_P46351	0.000565	2.23	NM_006862	NM_006862	Homo sapiens tudor and KH domain containing (TDRKH), mRNA [NM_006862]	NM_006862
A_23_P315872	0.000565	2.26	NM_022340	NM_022340	Homo sapiens zinc finger, FYVE domain containing 20 (ZFYVE20), mRNA [NM_022340]	NM_022340
A_23_P112478	0.000566	2.18	NM_001161	NM_001161	Homo sapiens nudix (nucleoside diphosphate linked moiety X)-type motif 2 (NUDT2), transcript variant 1, mRNA [NM_001161]	NM_001161
A_23_P205499	0.000566	2.46	NM_014045	NM_014045	Homo sapiens low density lipoprotein receptor-related protein 10 (LRP10), mRNA [NM_014045]	NM_014045
A_32_P118896	0.000566	2.54	THC2345956		ALU8_HUMAN (P39195) Alu subfamily SX sequence contamination warning entry, partial (10%) [THC2345956]	
A_23_P344531	0.000566	3.00	ENST00000307662		Homo sapiens mRNA for KIAA1029 protein, partial cds. [AB028952]	
A_23_P121702	0.000566	3.82	NM_001014446	NM_001014446	Homo sapiens OCIA domain containing 2 (OCIAD2), transcript variant 1, mRNA [NM_001014446]	NM_001014446
A_23_P24318	0.000567	2.30	NM_001007071	NM_001007071	Homo sapiens ribosomal protein S6 kinase, 70kDa, polypeptide 2 (RPS6KB2), transcript variant 2, mRNA [NM_001007071]	NM_001007071
A_23_P210658	0.000568	2.17	NM_016045	NM_016045	Homo sapiens chromosome 20 open reading frame 45 (C20orf45), mRNA [NM_016045]	NM_016045
A_32_P5800	0.000568	3.52	CR602075	CR602075	full-length cDNA clone CS0DD009YF23 of Neuroblastoma Cot 50-normalized of Homo sapiens (human). [CR602075]	
A_32_P163392	0.000568	3.98	CR608961	CR608961	full-length cDNA clone CS0DI034YJ14 of Placenta Cot 25-normalized of Homo sapiens (human). [CR608961]	
A_23_P55064	0.000569	2.04	NM_018081	NM_018081	Homo sapiens hypothetical protein FLJ10385 (FLJ10385), mRNA [NM_018081]	NM_018081
A_23_P48198	0.000569	2.49	NM_031302	NM_031302	Homo sapiens glycosyltransferase 8 domain containing 2 (GLT8D2), mRNA [NM_031302]	NM_031302
A_24_P323715	0.000571	2.36	XM_498088	XM_498088	PREDICTED: Homo sapiens similar to myosin:SUBUNIT=regulatory light chain (LOC442204), mRNA [XM_498088]	XM_498088
A_24_P203678	0.000571	3.08	NM_000019	NM_000019	Homo sapiens acetyl-Coenzyme A acetyltransferase 1 (acetoacetyl Coenzyme A thiolase) (ACAT1), nuclear gene encoding mitochondrial protein, mRNA [NM_000019]	NM_000019
A_23_P73096	0.000571	3.70	AW993939	AW993939	AW993939 RC1-BN0035-130400-013-b06 BN0035 Homo sapiens cDNA, mRNA sequence [AW993939]	
A_23_P165061	0.000572	2.37	NM_198969	NM_198969	Homo sapiens amino-terminal enhancer of split (AES), transcript variant 1, mRNA [NM_198969]	NM_198969
A_23_P500998	0.000572	3.77	NM_152739	NM_152739	Homo sapiens homeo box A9 (HOXA9), transcript variant 1, mRNA [NM_152739]	NM_152739
A_23_P76109	0.000573	2.10	NM_145058	NM_145058	Homo sapiens hypothetical protein MGC7036 (MGC7036), mRNA [NM_145058]	NM_145058
A_24_P7470	0.000573	3.28	A_24_P7470			
A_23_P407115	0.000574	3.53	NM_138687	NM_138687	Homo sapiens phosphatidylinositol-4-phosphate 5-kinase, type II, beta (PIP5K2B), transcript variant 2, mRNA [NM_138687]	NM_138687
A_23_P127957	0.000574	9.17	NM_182904	NM_182904	Homo sapiens procollagen-proline, 2-oxoglutarate 4-dioxygenase (proline 4-hydroxylase), alpha polypeptide III (P4HA3), mRNA [NM_182904]	NM_182904
A_24_P511686	0.000576	2.82	CR616845	CR616845	full-length cDNA clone CS0DF020YJ04 of Fetal brain of Homo sapiens (human). [CR616845]	XM_378309
A_23_P60933	0.000576	5.46	NM_005950	NM_005950	Homo sapiens metallothionein 1G (MT1G), mRNA [NM_005950]	NM_005950
A_32_P161681	0.000578	2.23	NM_001008528	NM_001008528	Homo sapiens matrix-remodelling associated 7 (MXRA7), transcript variant 1, mRNA [NM_001008528]	NM_001008528
A_23_P92025	0.000578	2.27	NM_022094	NM_022094	Homo sapiens cell death-inducing DFFA-like effector c (CIDEC), mRNA [NM_022094]	NM_022094

A_24_P461389	0.000578	2.94	THC2365641		BT007382 ubiquitin-conjugating enzyme E2 variant 1 {Homo sapiens;} , partial (93%) [THC2365641]	
A_24_P244410	0.000578	3.36	NM_014042	NM_014042	Homo sapiens DKFZP564M082 protein (DKFZP564M082), mRNA [NM_014042]	NM_014042
A_24_P622468	0.000578	3.89	BU076424	BU076424	BU076424 im51d09.x1 HR85 islet Homo sapiens cDNA clone IMAGE:6038465 3' similar to SW:MIC2_HUMAN P14209 T-CELL SURFACE GLYCOPROTEIN E2 PRECURSOR ;, mRNA sequence [BU076424]	
A_24_P247464	0.000579	2.27	A_24_P247464			
A_32_P2730	0.00058	2.06	BC005081	BC005081	Homo sapiens brevican, mRNA (cDNA clone IMAGE:3618761), partial cds. [BC005081]	
A_32_P104263	0.00058	2.56	THC2432735			
A_23_P158024	0.00058	2.69	NM_007234	NM_007234	Homo sapiens dynactin 3 (p22) (DCTN3), transcript variant 1, mRNA [NM_007234]	NM_007234
A_23_P46170	0.000581	2.01	NM_201542	NM_201542	Homo sapiens mediator of RNA polymerase II transcription, subunit 8 homolog (yeast) (MED8), transcript variant 1, mRNA [NM_201542]	NM_201542
A_23_P4308	0.000581	6.58	NM_002663	NM_002663	Homo sapiens phospholipase D2 (PLD2), mRNA [NM_002663]	NM_002663
A_23_P131706	0.000583	2.66	NM_212552	NM_212552	Homo sapiens bolA-like 3 (E. coli) (BOLA3), mRNA [NM_212552]	NM_212552
A_24_P409971	0.000583	2.69	NM_144573	NM_144573	Homo sapiens nexilin (F actin binding protein) (NEXN), mRNA [NM_144573]	NM_144573
A_23_P411379	0.000583	3.05	NM_178508	NM_178508	Homo sapiens chromosome 6 open reading frame 1 (C6orf1), transcript variant 1, mRNA [NM_178508]	NM_178508
A_24_P385148	0.000583	3.33	NM_006991	NM_006991	Homo sapiens zinc finger protein 197 (ZNF197), transcript variant 1, mRNA [NM_006991]	NM_006991
A_24_P382187	0.000584	4.46	NM_001552	NM_001552	Homo sapiens insulin-like growth factor binding protein 4 (IGFBP4), mRNA [NM_001552]	NM_001552
A_24_P83586	0.000585	2.58	NM_015506	NM_015506	Homo sapiens DKFZp564I122 protein (DKFZp564I122), mRNA [NM_015506]	NM_015506
A_24_P178523	0.000585	2.93	A_24_P178523			
A_24_P303480	0.000585	3.75	NM_006834	NM_006834	Homo sapiens RAB32, member RAS oncogene family (RAB32), mRNA [NM_006834]	NM_006834
A_32_P72351	0.000585	6.94	AK026140	AK026140	Homo sapiens cDNA: FLJ22487 fis, clone HRC10931. [AK026140]	
A_23_P502913	0.000586	2.43	NM_017491	NM_017491	Homo sapiens WD repeat domain 1 (WDR1), transcript variant 1, mRNA [NM_017491]	NM_017491
A_23_P103104	0.000586	2.60	NM_002405	NM_002405	Homo sapiens manic fringe homolog (Drosophila) (MFNG), mRNA [NM_002405]	NM_002405
A_24_P834110	0.000588	3.82	XM_373538	XM_373538	PREDICTED: Homo sapiens similar to microtubule-associated proteins 1A/1B light chain 3 (LOC387869), mRNA [XM_373538]	XM_373538
A_32_P45087	0.000589	2.86	A_32_P45087			
A_32_P165102	0.000589	2.91	N21624	N21624	N21624 yx60a10.s1 Soares melanocyte 2NbHM Homo sapiens cDNA clone IMAGE:266106 3' similar to gb:X56468_rna1 14-3-3 PROTEIN THETA (HUMAN);, mRNA sequence [N21624]	
A_32_P129010	0.000589	3.38	NM_001013615	NM_001013615	Homo sapiens hypothetical LOC541468 protein (LOC541468), mRNA [NM_001013615]	NM_001013615
A_24_P69538	0.000589	14.73	NM_138557	NM_138557	Homo sapiens toll-like receptor 4 (TLR4), transcript variant 4, mRNA [NM_138557]	NM_138557
A_24_P910833	0.00059	2.83	AF086305	AF086305	Homo sapiens full length insert cDNA clone ZD50H12. [AF086305]	
A_23_P14369	0.000591	2.02	AB028979	AB028979	Homo sapiens mRNA for KIAA1056 protein, partial cds. [AB028979]	XM_375065
A_24_P259799	0.000591	2.47	NM_001002234	NM_001002234	Homo sapiens sodium channel modifier 1 (SCNM1), transcript variant 2, mRNA [NM_001002234]	NM_001002234
A_32_P63162	0.000592	2.38	THC2267053		HLAF_HUMAN (P30511) HLA class I histocompatibility antigen, alpha chain F precursor (HLA F antigen) (Leukocyte antigen F) (CDA12), partial (19%) [THC2267053]	
A_32_P108748	0.000593	2.35	A_32_P108748			
A_23_P166459	0.000593	14.73	NM_002305	NM_002305	Homo sapiens lectin, galactoside-binding, soluble, 1 (galectin 1) (LGALS1), mRNA [NM_002305]	NM_002305
A_23_P103795	0.000594	2.28	BC000907	BC000907	Homo sapiens cDNA clone IMAGE:2984856, complete cds. [BC000907]	
A_23_P56933	0.000594	2.80	NM_020532	NM_020532	Homo sapiens reticulon 4 (RTN4), transcript variant 1, mRNA [NM_020532]	NM_020532
A_24_P170309	0.000596	2.45	A_24_P170309			
A_24_P83615	0.000596	4.61	NM_014922	NM_014922	Homo sapiens NACHT, leucine rich repeat and PYD (pyrin domain) containing 1 (NALP1), transcript variant 2, mRNA [NM_014922]	NM_014922
A_23_P165608	0.000597	3.28	NM_004263	NM_004263	Homo sapiens sema domain, immunoglobulin domain (Ig), transmembrane domain (TM) and short cytoplasmic domain, (semaphorin) 4F (SEMA4F), mRNA [NM_004263]	NM_004263
A_23_P330999	0.000599	2.35	NM_015979	NM_015979	Homo sapiens cofactor required for Sp1 transcriptional activation, subunit 3, 130kDa (CRSP3), transcript variant 2, mRNA [NM_015979]	NM_015979
A_24_P107695	0.000599	4.42	NM_001102	NM_001102	Homo sapiens actinin, alpha 1 (ACTN1), mRNA [NM_001102]	NM_001102

A_32_P219279	0.000599	7.63	NM_052906	NM_052906	Homo sapiens KIAA1904 protein (KIAA1904), mRNA [NM_052906]	NM_052906
A_23_P88802	0.0006	2.14	NM_024731	NM_024731	Homo sapiens chromosome 16 open reading frame 44 (C16orf44), mRNA [NM_024731]	NM_024731
A_23_P129556	0.0006	2.59	NM_000418	NM_000418	Homo sapiens interleukin 4 receptor (IL4R), transcript variant 1, mRNA [NM_000418]	NM_000418
A_24_P410389	0.000603	3.53	NM_015367	NM_015367	Homo sapiens BCL2-like 13 (apoptosis facilitator) (BCL2L13), nuclear gene encoding mitochondrial protein, mRNA [NM_015367]	NM_015367
A_23_P105307	0.000603	4.02	NM_201444	NM_201444	Homo sapiens diacylglycerol kinase, alpha 80kDa (DGKA), transcript variant 1, mRNA [NM_201444]	NM_201444
A_24_P42633	0.000604	2.39	NM_044472	NM_044472	Homo sapiens cell division cycle 42 (GTP binding protein, 25kDa) (CDC42), transcript variant 2, mRNA [NM_044472]	NM_044472
A_23_P136504	0.000604	2.79	NM_030631	NM_030631	Homo sapiens solute carrier family 25 (mitochondrial oxodicarboxylate carrier), member 21 (SLC25A21), mRNA [NM_030631]	NM_030631
A_24_P302038	0.000604	3.13	NM_014824	NM_014824	Homo sapiens FCH and double SH3 domains 2 (FCHSD2), mRNA [NM_014824]	NM_014824
A_24_P158385	0.000605	2.06	NM_138462	NM_138462	Homo sapiens zinc finger, MYND-type containing 19 (ZMYND19), mRNA [NM_138462]	NM_138462
A_24_P383762	0.000606	2.16	NM_175062	NM_175062	Homo sapiens RasGEF domain family, member 1C (RASGEF1C), mRNA [NM_175062]	NM_175062
A_32_P223422	0.00061	3.40	BF887921	BF887921	BF887921 PM2-TN0027-081100-017-h01 TN0027 Homo sapiens cDNA, mRNA sequence [BF887921]	
A_23_P208523	0.000612	2.87	NM_024316	NM_024316	Homo sapiens leukocyte receptor cluster (LRC) member 1 (LENG1), mRNA [NM_024316]	NM_024316
A_23_P346311	0.000612	3.69	NM_138762	NM_138762	Homo sapiens BCL2-associated X protein (BAX), transcript variant gamma, mRNA [NM_138762]	NM_138762
A_24_P928415	0.000614	2.10	BC032462	BC032462	Homo sapiens vacuolar protein sorting 29 (yeast), mRNA (cDNA clone MGC:40428 IMAGE:5197243), complete cds. [BC032462]	
A_23_P53838	0.000614	2.24	NM_003749	NM_003749	Homo sapiens insulin receptor substrate 2 (IRS2), mRNA [NM_003749]	NM_003749
A_23_P23443	0.000615	2.07	NM_024329	NM_024329	Homo sapiens EF-hand domain family, member D2 (EFHD2), mRNA [NM_024329]	NM_024329
A_23_P214464	0.000615	3.38	NM_001760	NM_001760	Homo sapiens cyclin D3 (CCND3), mRNA [NM_001760]	NM_001760
A_23_P431388	0.000615	6.58	NM_144569	NM_144569	Homo sapiens SPOC domain containing 1 (SPOCD1), mRNA [NM_144569]	NM_144569
A_23_P34597	0.000616	2.25	NM_001785	NM_001785	Homo sapiens cytidine deaminase (CDA), mRNA [NM_001785]	NM_001785
A_24_P154080	0.000616	2.25	NM_001397	NM_001397	Homo sapiens endothelin converting enzyme 1 (ECE1), mRNA [NM_001397]	NM_001397
A_24_P342127	0.000616	2.55	NM_014997	NM_014997	Homo sapiens KIAA0265 protein (KIAA0265), mRNA [NM_014997]	NM_014997
A_23_P147238	0.000616	3.52	NM_018639	NM_018639	Homo sapiens WD repeat and SOCS box-containing 2 (WSB2), mRNA [NM_018639]	NM_018639
A_32_P82560	0.000618	2.40	THC2360885			
A_24_P230288	0.000618	2.53	BG009439	BG009439	BG009439 PM4-GN0183-021200-001-h08 GN0183 Homo sapiens cDNA, mRNA sequence [BG009439]	
A_23_P502641	0.000618	2.89	NM_032970	NM_032970	Homo sapiens SEC22 vesicle trafficking protein-like 3 (S. cerevisiae) (SEC22L3), transcript variant 1, mRNA [NM_032970]	NM_032970
A_23_P356330	0.000619	2.11	NM_015704	NM_015704	Homo sapiens DNA segment, Chr 15, Wayne State University 75, expressed (D15Wsu75e), mRNA [NM_015704]	NM_015704
A_32_P98927	0.000619	2.53	ENST00000352103		Homo sapiens cDNA FLJ13195 fis, clone NT2RP3004424, weakly similar to Homo sapiens mRNA for stromal antigen 3 (STAG3 gene). [AK023257]	XM_498429
A_23_P99942	0.000619	3.07	NM_024652	NM_024652	Homo sapiens leucine-rich repeat kinase 1 (LRRK1), mRNA [NM_024652]	NM_024652
A_23_P40354	0.00062	2.28	NM_012325	NM_012325	Homo sapiens microtubule-associated protein, RP/EB family, member 1 (MAPRE1), mRNA [NM_012325]	NM_012325
A_23_P64712	0.000621	2.48	NM_024809	NM_024809	Homo sapiens hypothetical protein FLJ12975 (FLJ12975), mRNA [NM_024809]	NM_024809
A_24_P125353	0.000622	2.14	NM_007359	NM_007359	Homo sapiens cancer susceptibility candidate 3 (CASC3), mRNA [NM_007359]	NM_007359
A_24_P230938	0.000622	2.94	BC022054	BC022054	Homo sapiens chromosome 10 open reading frame 83, mRNA (cDNA clone IMAGE:4690584), complete cds. [BC022054]	
A_24_P139152	0.000622	215.52	AL359062	AL359062	Homo sapiens mRNA full length insert cDNA clone EUROIMAGE 1913076. [AL359062]	
A_32_P207420	0.000623	2.28	THC2282652		Q9RW53 (Q9RW53) Mg(2+) transport ATPase-related protein, partial (8%) [THC2282652]	
A_23_P124733	0.000623	2.56	NM_015697	NM_015697	Homo sapiens coenzyme Q2 homolog, prenyltransferase (yeast) (COQ2), mRNA [NM_015697]	NM_015697
A_23_P358597	0.000623	8.20	NM_022361	NM_022361	Homo sapiens popeye domain containing 3 (POPDC3), mRNA [NM_022361]	NM_022361
A_23_P73801	0.000624	2.20	NM_001006640	NM_001006640	Homo sapiens transcription elongation factor A (SII)-like 1 (TCEAL1), transcript variant 3, mRNA [NM_001006640]	NM_001006640
A_32_P171348	0.000624	3.46	A_32_P171348			
A_23_P26994	0.000624	4.46	NM_031498	NM_031498	Homo sapiens guanine nucleotide binding protein (G protein), gamma transducing activity polypeptide 2 (GNGT2), mRNA [NM_031498]	NM_031498

A_23_P217886	0.000625	2.34	NM_194458	NM_194458	Homo sapiens ubiquitin-conjugating enzyme E2, J2 (UBC6 homolog, yeast) (UBE2J2), transcript variant 3, mRNA [NM_194458]	NM_194458
A_24_P27412	0.000626	2.32	NM_005701	NM_005701	Homo sapiens RNA, U transporter 1 (RNUT1), mRNA [NM_005701]	NM_005701
A_23_P257423	0.000626	2.54	NM_144988	NM_144988	Homo sapiens hypothetical protein MGC19780 (MGC19780), mRNA [NM_144988]	NM_144988
A_23_P139864	0.00063	2.39	NM_031289	NM_031289	Homo sapiens germ cell associated 1 (GSG1), transcript variant 1, mRNA [NM_031289]	NM_031289
A_24_P100664	0.00063	2.46	NM_170784	NM_170784	Homo sapiens McKusick-Kaufman syndrome (MKKS), transcript variant 2, mRNA [NM_170784]	NM_170784
A_24_P353964	0.00063	3.68	NM_000182	NM_000182	Homo sapiens hydroxyacyl-Coenzyme A dehydrogenase/3-ketoacyl-Coenzyme A thiolase/enoyl-Coenzyme A hydratase (trifunctional protein), alpha subunit (HADHA), mRNA [NM_000182]	NM_000182
A_23_P91168	0.000632	2.48	NM_144710	NM_144710	Homo sapiens septin 10 (SEPT10), transcript variant 1, mRNA [NM_144710]	NM_144710
A_23_P200096	0.000632	2.74	NM_025106	NM_025106	Homo sapiens SPRY domain-containing SOCS box protein SSB-1 (SSB1), mRNA [NM_025106]	NM_025106
A_23_P414855	0.000633	2.39	NM_033224	NM_033224	Homo sapiens purine-rich element binding protein B (PURB), mRNA [NM_033224]	NM_033224
A_24_P131293	0.000633	2.39	NM_002699	NM_002699	Homo sapiens POU domain, class 3, transcription factor 1 (POU3F1), mRNA [NM_002699]	NM_002699
A_23_P415984	0.000633	4.90	NM_002518	NM_002518	Homo sapiens neuronal PAS domain protein 2 (NPAS2), mRNA [NM_002518]	NM_002518
A_24_P307580	0.000633	7.87	AF092095	AF092095	Homo sapiens alternatively spliced product of metastasis-suppressor gene CC3 (TC3) mRNA, complete cds. [AF092095]	
A_24_P372862	0.000634	3.47	NM_001987	NM_001987	Homo sapiens ets variant gene 6 (TEL oncogene) (ETV6), mRNA [NM_001987]	NM_001987
A_23_P90895	0.000635	2.27	NM_152994	NM_152994	Homo sapiens smooth muscle myosin heavy chain 11 isoform SM1-like (LOC129285), mRNA [NM_152994]	NM_152994
A_23_P65129	0.000636	2.28	NM_032840	NM_032840	Homo sapiens hypothetical protein FLJ14800 (FLJ14800), mRNA [NM_032840]	NM_032840
A_23_P329198	0.000636	3.15	NM_022837	NM_022837	Homo sapiens hypothetical protein FLJ22833 (FLJ22833), mRNA [NM_022837]	NM_022837
A_23_P123544	0.00064	2.37	NM_017444	NM_017444	Homo sapiens chromatin accessibility complex 1 (CHRAC1), mRNA [NM_017444]	NM_017444
A_24_P49597	0.000642	2.29	ENST00000299756		PREDICTED: Homo sapiens similar to Chain A, Crystal Structure Of The R463a Mutant Of Human Glutamate Dehydrogenase (LOC390859), mRNA [XM_372695]	XM_372695
A_32_P1614	0.000643	2.79	AK023018	AK023018	Homo sapiens cDNA FLJ12956 fis, clone NT2RP2005501. [AK023018]	
A_24_P68585	0.000643	2.87	NM_183001	NM_183001	Homo sapiens SHC (Src homology 2 domain containing) transforming protein 1 (SHC1), transcript variant 1, mRNA [NM_183001]	NM_183001
A_23_P9086	0.000644	2.27	NM_016353	NM_016353	Homo sapiens zinc finger, DHHC-type containing 2 (ZDHHC2), mRNA [NM_016353]	NM_016353
A_23_P139486	0.000644	2.40	NM_004642	NM_004642	Homo sapiens CDK2-associated protein 1 (CDK2AP1), mRNA [NM_004642]	NM_004642
A_24_P390096	0.000644	18.08	U16307	U16307	Homo sapiens glioma pathogenesis-related protein (GliPR) mRNA, complete cds. [U16307]	
A_23_P130141	0.000646	2.97	NM_020162	NM_020162	Homo sapiens DEAH (Asp-Glu-Ala-His) box polypeptide 33 (DHX33), mRNA [NM_020162]	NM_020162
A_32_P104572	0.000646	3.52	BM670971	BM670971	UI-E-DW1-ahe-d-15-0-UI.s1 UI-E-DW1 Homo sapiens cDNA clone UI-E-DW1-ahe-d-15-0-UI 3', mRNA sequence [BM670971]	
A_24_P98948	0.000646	34.01	THC2272264			
A_24_P393864	0.000647	2.13	NM_006608	NM_006608	Homo sapiens putative homeodomain transcription factor 1 (PHTF1), mRNA [NM_006608]	NM_006608
A_23_P425750	0.000648	2.12	NM_033415	NM_033415	Homo sapiens armadillo repeat containing 6 (ARMC6), mRNA [NM_033415]	NM_033415
A_23_P64343	0.000648	2.73	NM_012456	NM_012456	Homo sapiens translocase of inner mitochondrial membrane 10 homolog (yeast) (TIMM10), mRNA [NM_012456]	NM_012456
A_24_P115443	0.00065	2.13	NM_018688	NM_018688	Homo sapiens bridging integrator 3 (BIN3), mRNA [NM_018688]	NM_018688
A_24_P142151	0.00065	2.14	NM_144598	NM_144598	Homo sapiens leucine rich repeat containing 28 (LRRC28), mRNA [NM_144598]	NM_144598
A_24_P416728	0.000651	2.88	NM_198970	NM_198970	Homo sapiens amino-terminal enhancer of split (AES), transcript variant 3, mRNA [NM_198970]	NM_198970
A_23_P130553	0.000651	3.32	NM_138392	NM_138392	Homo sapiens SH3KBP1 binding protein 1 (SHKBP1), mRNA [NM_138392]	NM_138392
A_23_P143845	0.000652	2.16	NM_015508	NM_015508	Homo sapiens TCDD-inducible poly(ADP-ribose) polymerase (TIPARP), mRNA [NM_015508]	NM_015508
A_24_P330625	0.000652	2.30	NM_032815	NM_032815	Homo sapiens nuclear factor of activated T-cells, cytoplasmic, calcineurin-dependent 2 interacting protein (NFATC2IP), mRNA [NM_032815]	NM_032815
A_23_P95172	0.000653	2.61	NM_020914	NM_020914	Homo sapiens chromosome 17 open reading frame 27 (C17orf27), mRNA [NM_020914]	NM_020914
A_23_P34835	0.000653	4.05	NM_005572	NM_005572	Homo sapiens lamin A/C (LMNA), transcript variant 2, mRNA [NM_005572]	NM_005572
A_24_P367752	0.000655	3.48	BC012888	BC012888	Homo sapiens N-deacetylase/N-sulfotransferase (heparan glucosaminyl) 1, mRNA (cDNA clone MGC:9410 IMAGE:3882074), complete cds. [BC012888]	

A_24_P914479	0.000656	2.84	BC002724	BC002724	Homo sapiens sorting nexin 5, mRNA (cDNA clone IMAGE:3629947), complete cds. [BC002724]	
A_23_P132644	0.000656	8.70	NM_020792	NM_020792	Homo sapiens arylacetamide deacetylase-like 1 (AADACL1), mRNA [NM_020792]	NM_020792
A_23_P204745	0.000657	3.11	NM_139071	NM_139071	Homo sapiens SWI/SNF related, matrix associated, actin dependent regulator of chromatin, subfamily d, member 1 (SMARCD1), transcript variant 2, mRNA [NM_139071]	NM_139071
A_24_P96403	0.000657	4.31	NM_001001890	NM_001001890	Homo sapiens runt-related transcription factor 1 (acute myeloid leukemia 1; aml1 oncogene) (RUNX1), transcript variant 2, mRNA [NM_001001890]	NM_001001890
A_23_P81298	0.000657	47.39	AK025816	AK025816	Homo sapiens cDNA: FLJ22163 fis, clone HRC00430. [AK025816]	XM_371755
A_24_P940149	0.000658	2.12	NM_199050	NM_199050	Homo sapiens chromosome 21 open reading frame 25 (C21orf25), mRNA [NM_199050]	NM_199050
A_23_P37676	0.000658	5.65	NM_007223	NM_007223	Homo sapiens putative G protein coupled receptor (GPR), mRNA [NM_007223]	NM_007223
A_23_P152804	0.000659	2.18	NM_198175	NM_198175	Homo sapiens non-metastatic cells 1, protein (NM23A) expressed in (NME1), transcript variant 1, mRNA [NM_198175]	NM_198175
A_23_P51718	0.000659	2.37	NM_003819	NM_003819	Homo sapiens poly(A) binding protein, cytoplasmic 4 (inducible form) (PABPC4), mRNA [NM_003819]	NM_003819
A_24_P717609	0.000659	2.42	A_24_P717609			
A_24_P475115	0.000659	2.62	A_24_P475115			
A_23_P74138	0.000659	3.62	NM_003564	NM_003564	Homo sapiens transgelin 2 (TAGLN2), mRNA [NM_003564]	NM_003564
A_24_P135322	0.000661	2.88	NM_003873	NM_003873	Homo sapiens neuropilin 1 (NRP1), transcript variant 1, mRNA [NM_003873]	NM_003873
A_24_P199097	0.000661	4.12	NM_006809	NM_006809	Homo sapiens translocase of outer mitochondrial membrane 34 (TOMM34), nuclear gene encoding mitochondrial protein, mRNA [NM_006809]	NM_006809
A_23_P208389	0.000662	3.91	NM_021913	NM_021913	Homo sapiens AXL receptor tyrosine kinase (AXL), transcript variant 1, mRNA [NM_021913]	NM_021913
A_23_P201035	0.000663	2.53	NM_001005749	NM_001005749	Homo sapiens glucosidase, beta; acid (includes glucosylceramidase) (GBA), transcript variant 4, mRNA [NM_001005749]	NM_001005749
A_23_P250122	0.000664	2.35	NM_020223	NM_020223	Homo sapiens family with sequence similarity 20, member C (FAM20C), mRNA [NM_020223]	NM_020223
A_24_P89257	0.000665	2.38	BC012766	BC012766	Homo sapiens endoplasmic reticulum-golgi intermediate compartment 32 kDa protein, mRNA (cDNA clone MGC:16233 IMAGE:3677787), complete cds. [BC012766]	
A_23_P18901	0.000666	2.18	NM_004707	NM_004707	Homo sapiens APG12 autophagy 12-like (S. cerevisiae) (APG12L), mRNA [NM_004707]	NM_004707
A_23_P40718	0.000666	3.60	NM_001003828	NM_001003828	Homo sapiens parvin, beta (PARVB), transcript variant 1, mRNA [NM_001003828]	NM_001003828
A_23_P210811	0.000666	4.10	NM_012072	NM_012072	Homo sapiens complement component 1, q subcomponent, receptor 1 (C1QR1), mRNA [NM_012072]	NM_012072
A_23_P77493	0.000666	6.85	NM_006086	NM_006086	Homo sapiens tubulin, beta 3 (TUBB3), mRNA [NM_006086]	NM_006086
A_23_P203888	0.000667	2.59	NM_022791	NM_022791	Homo sapiens matrix metalloproteinase 19 (MMP19), transcript variant rasi-6, mRNA [NM_022791]	NM_022791
A_24_P58579	0.000667	2.83	NM_153649	NM_153649	Homo sapiens tropomyosin 3 (TPM3), transcript variant 2, mRNA [NM_153649]	NM_153649
A_23_P217866	0.00067	2.07	AK094968	AK094968	Homo sapiens cDNA FLJ37649 fis, clone BRHIP2000534, moderately similar to GAMMA-INTERFERON-INDUCIBLE PROTEIN IFI-16. [AK094968]	
A_23_P21706	0.000671	3.40	NM_001905	NM_001905	Homo sapiens CTP synthase (CTPS), mRNA [NM_001905]	NM_001905
A_23_P52373	0.000672	2.03	NM_003635	NM_003635	Homo sapiens N-deacetylase/N-sulfotransferase (heparan glucosaminyl) 2 (NDST2), mRNA [NM_003635]	NM_003635
A_24_P41781	0.000672	2.22	A_24_P41781			
A_24_P361896	0.000672	6.10	NM_005953	NM_005953	Homo sapiens metallothionein 2A (MT2A), mRNA [NM_005953]	NM_005953
A_24_P219324	0.000673	2.92	NM_004614	NM_004614	Homo sapiens thymidine kinase 2, mitochondrial (TK2), mRNA [NM_004614]	NM_004614
A_24_P17710	0.000674	4.98	NM_015990	NM_015990	Homo sapiens kelch-like 5 (Drosophila) (KLHL5), mRNA [NM_015990]	NM_015990
A_24_P505255	0.000675	2.44	A_24_P505255			
A_24_P68162	0.000677	2.20	NM_173602	NM_173602	Homo sapiens KIAA1463 protein (KIAA1463), mRNA [NM_173602]	NM_173602
A_23_P95470	0.000677	3.31	NM_004357	NM_004357	Homo sapiens CD151 antigen (CD151), transcript variant 1, mRNA [NM_004357]	NM_004357
A_24_P49349	0.000677	6.41	NM_014857	NM_014857	Homo sapiens RAB GTPase activating protein 1-like (RABGAP1L), mRNA [NM_014857]	NM_014857
A_24_P186414	0.000678	2.01	NM_021943	NM_021943	Homo sapiens testis expressed sequence 27 (TEX27), mRNA [NM_021943]	NM_021943
A_24_P200549	0.000678	2.62	NM_017706	NM_017706	Homo sapiens WD repeat domain 55 (WDR55), mRNA [NM_017706]	NM_017706
A_23_P148249	0.000678	20.92	ENST00000261862		Homo sapiens cDNA FLJ27224 fis, clone SYN04819. [AK130734]	
A_32_P151387	0.00068	2.06	THC2382871			

A_24_P221485	0.000682	5.81	A_24_P221485			
A_23_P380208	0.000683	30.77	NM_024621	NM_024621	Homo sapiens ventricular zone expressed PH domain homolog 1 (zebrafish) (VEPH1), mRNA [NM_024621]	NM_024621
A_32_P139894	0.000684	2.16	THC2307212			
A_24_P247987	0.000684	3.62	BC010099	BC010099	Homo sapiens hypothetical protein FLJ13154, mRNA (cDNA clone IMAGE:3506308), complete cds. [BC010099]	
A_23_P39202	0.000685	2.10	NM_033520	NM_033520	Homo sapiens chromosome 19 open reading frame 33 (C19orf33), mRNA [NM_033520]	NM_033520
A_32_P218785	0.000686	2.14	BC042649	BC042649	Homo sapiens, clone IMAGE:4826012, mRNA. [BC042649]	
A_23_P59045	0.000686	2.85	NM_021052	NM_021052	Homo sapiens histone 1, H2ae (HIST1H2AE), mRNA [NM_021052]	NM_021052
A_24_P117029	0.000687	4.90	NM_000527	NM_000527	Homo sapiens low density lipoprotein receptor (familial hypercholesterolemia) (LDLR), mRNA [NM_000527]	NM_000527
A_23_P210977	0.000688	2.22	NM_016558	NM_016558	Homo sapiens SCAN domain containing 1 (SCAND1), transcript variant 1, mRNA [NM_016558]	NM_016558
A_32_P113533	0.000688	2.65	A_32_P113533			
A_24_P58597	0.000688	3.97	A_24_P58597			
A_23_P430818	0.000691	2.04	NM_014181	NM_014181	Homo sapiens HSPC159 protein (HSPC159), mRNA [NM_014181]	NM_014181
A_32_P117884	0.000691	2.29	A_32_P117884			
A_23_P163801	0.000691	2.79	NM_017566	NM_017566	Homo sapiens kelch domain containing 4 (KLHDC4), mRNA [NM_017566]	NM_017566
A_32_P24122	0.000691	6.76	NM_015894	NM_015894	Homo sapiens stathmin-like 3 (STMN3), mRNA [NM_015894]	NM_015894
A_24_P273253	0.000691	8.20	BC090889	BC090889	Homo sapiens chromosome 14 open reading frame 78, mRNA (cDNA clone MGC:102983 IMAGE:30387958), complete cds. [BC090889]	XM_290629
A_24_P474188	0.000692	6.17	A_24_P474188			
A_23_P381449	0.000693	2.04	NM_003110	NM_003110	Homo sapiens Sp2 transcription factor (SP2), mRNA [NM_003110]	NM_003110
A_23_P340922	0.000693	2.62	NM_032370	NM_032370	Homo sapiens zinc finger protein 414 (ZNF414), mRNA [NM_032370]	NM_032370
A_23_P27265	0.000693	4.03	NM_032160	NM_032160	Homo sapiens chromosome 18 open reading frame 4 (C18orf4), mRNA [NM_032160]	NM_032160
A_23_P14841	0.000694	2.18	NM_001011664	NM_001011664	Homo sapiens casein kinase 1, gamma 1 (CSNK1G1), transcript variant 1, mRNA [NM_001011664]	NM_001011664
A_32_P99902	0.000694	2.39	NM_144597	NM_144597	Homo sapiens hypothetical protein MGC29937 (MGC29937), mRNA [NM_144597]	NM_144597
A_23_P12965	0.000695	3.30	NM_002033	NM_002033	Homo sapiens fucosyltransferase 4 (alpha (1,3) fucosyltransferase, myeloid-specific) (FUT4), mRNA [NM_002033]	NM_002033
A_24_P378019	0.000698	2.84	NM_004031	NM_004031	Homo sapiens interferon regulatory factor 7 (IRF7), transcript variant d, mRNA [NM_004031]	NM_004031
A_24_P255836	0.000699	2.17	A_24_P255836			
A_23_P11461	0.000699	2.47	NM_199203	NM_199203	Homo sapiens ubiquitin-conjugating enzyme E2 variant 1 (Kua-UEV), transcript variant 1, mRNA [NM_199203]	NM_199203
A_24_P408424	0.0007	3.52	NM_002473	NM_002473	Homo sapiens myosin, heavy polypeptide 9, non-muscle (MYH9), mRNA [NM_002473]	NM_002473
A_24_P247749	0.000702	2.26	NM_014999	NM_014999	Homo sapiens RAB21, member RAS oncogene family (RAB21), mRNA [NM_014999]	NM_014999
A_23_P217475	0.000703	2.53	NM_006123	NM_006123	Homo sapiens iduronate 2-sulfatase (Hunter syndrome) (IDS), transcript variant 2, mRNA [NM_006123]	NM_006123
A_23_P139099	0.000706	2.08	NM_001005275	NM_001005275	Homo sapiens olfactory receptor, family 4, subfamily A, member 15 (OR4A15), mRNA [NM_001005275]	NM_001005275
A_32_P122891	0.000706	2.37	AK092163	AK092163	Homo sapiens cDNA FLJ34844 fis, clone NT2NE2011154, highly similar to Homo sapiens mRNA for TIP49. [AK092163]	
A_23_P502783	0.000706	3.18	NM_147147	NM_147147	Homo sapiens blood vessel epicardial substance (BVES), transcript variant B, mRNA [NM_147147]	NM_147147
A_24_P923251	0.000707	14.93	NM_198951	NM_198951	Homo sapiens transglutaminase 2 (C polypeptide, protein-glutamine-gamma-glutamyltransferase) (TGM2), transcript variant 2, mRNA [NM_198951]	NM_198951
A_32_P213330	0.000708	6.02	BC012946	BC012946	Homo sapiens Rho-guanine nucleotide exchange factor, mRNA (cDNA clone MGC:21017 IMAGE:4454960), complete cds. [BC012946]	XM_371755
A_32_P160039	0.000711	2.28	ENST00000359244			
A_23_P138717	0.000712	3.28	NM_001005339	NM_001005339	Homo sapiens regulator of G-protein signalling 10 (RGS10), transcript variant 1, mRNA [NM_001005339]	NM_001005339
A_32_P226620	0.000712	3.79	THC2441546			
A_24_P195276	0.000713	2.07	XM_296315	XM_296315	PREDICTED: Homo sapiens similar to 60S ribosomal protein L35 (LOC341604), mRNA [XM_296315]	XM_296315
A_24_P275723	0.000713	2.35	AK126622	AK126622	Homo sapiens cDNA FLJ44660 fis, clone BRACE3002344, weakly similar to Vegetatible incompatibility protein HET-E-1. [AK126622]	
A_23_P317324	0.000715	2.33	NM_005241	NM_005241	Homo sapiens ecotropic viral integration site 1 (EVI1), mRNA [NM_005241]	NM_005241

A_23_P154786	0.000718	2.99	NM_032514	NM_032514	Homo sapiens microtubule-associated protein 1 light chain 3 alpha (MAP1LC3A), transcript variant 1, mRNA [NM_032514]	NM_032514
A_24_P391531	0.000719	2.21	NM_004375	NM_004375	Homo sapiens COX11 homolog, cytochrome c oxidase assembly protein (yeast) (COX11), nuclear gene encoding mitochondrial protein, mRNA [NM_004375]	NM_004375
A_24_P417546	0.000719	2.56	BC004936	BC004936	Homo sapiens stearoyl-CoA desaturase 5, mRNA (cDNA clone MGC:10777 IMAGE:3607979), complete cds. [BC004936]	
A_32_P54594	0.000719	3.69	BI050742	BI050742	BI050742 RC6-GN0071-080101-025-H04 GN0071 Homo sapiens cDNA, mRNA sequence [BI050742]	
A_32_P864453	0.00072	2.02	AF086139	AF086139	Homo sapiens full length insert cDNA clone ZA91F08. [AF086139]	
A_23_P425240	0.00072	2.13	NM_001508	NM_001508	Homo sapiens G protein-coupled receptor 39 (GPR39), mRNA [NM_001508]	NM_001508
A_24_P201552	0.000721	3.41	NM_005103	NM_005103	Homo sapiens fasciculation and elongation protein zeta 1 (zygin I) (FEZ1), transcript variant 1, mRNA [NM_005103]	NM_005103
A_24_P943597	0.000721	10.34	AF220656	AF220656	Homo sapiens apoptosis-associated nuclear protein PHLDA1 (PHLDA1) mRNA, partial cds. [AF220656]	
A_23_P368645	0.000722	2.35	NM_003659	NM_003659	Homo sapiens alkylglycerone phosphate synthase (AGPS), mRNA [NM_003659]	NM_003659
A_23_P205101	0.000724	2.35	NM_025138	NM_025138	Homo sapiens chromosome 13 open reading frame 23 (C13orf23), transcript variant 1, mRNA [NM_025138]	NM_025138
A_23_P374082	0.000725	2.53	NM_033274	NM_033274	Homo sapiens a disintegrin and metalloproteinase domain 19 (meltrin beta) (ADAM19), transcript variant 2, mRNA [NM_033274]	NM_033274
A_23_P205393	0.000726	2.15	NM_175748	NM_175748	Homo sapiens chromosome 14 open reading frame 130 (C14orf130), transcript variant 2, mRNA [NM_175748]	NM_175748
A_32_P175321	0.000727	2.20	AK057740	AK057740	Homo sapiens cDNA FLJ25011 fis, clone CBL01244. [AK057740]	
A_24_P335358	0.000728	2.51	NM_025215	NM_025215	Homo sapiens pseudouridylate synthase 1 (PUS1), transcript variant 1, mRNA [NM_025215]	NM_025215
A_24_P202581	0.000729	2.44	NM_199002	NM_199002	Homo sapiens Rho guanine nucleotide exchange factor (GEF) 1 (ARHGEF1), transcript variant 1, mRNA [NM_199002]	NM_199002
A_23_P35330	0.000729	2.98	NM_024503	NM_024503	Homo sapiens human immunodeficiency virus type I enhancer binding protein 3 (HIVEP3), mRNA [NM_024503]	NM_024503
A_24_P221770	0.000729	3.25	NM_001011713	NM_001011713	Homo sapiens chromosome 14 open reading frame 35 (C14orf35), mRNA [NM_001011713]	NM_001011713
A_24_P73408	0.000731	2.13	NM_145293	NM_145293	Homo sapiens similar to hypothetical protein FLJ20897 (LOC196549), mRNA [NM_145293]	NM_145293
A_23_P202905	0.000731	3.31	NM_148910	NM_148910	Homo sapiens toll-interleukin 1 receptor (TIR) domain containing adaptor protein (TIRAP), transcript variant 2, mRNA [NM_148910]	NM_148910
A_23_P210726	0.000732	2.07	NM_021874	NM_021874	Homo sapiens cell division cycle 25B (CDC25B), transcript variant 4, mRNA [NM_021874]	NM_021874
A_24_P151498	0.000732	2.62	NM_206962	NM_206962	Homo sapiens HMT1 hnRNP methyltransferase-like 1 (S. cerevisiae) (HRMT1L1), transcript variant 1, mRNA [NM_206962]	NM_206962
A_23_P16337	0.000733	2.16	NM_005500	NM_005500	Homo sapiens SUMO-1 activating enzyme subunit 1 (SAE1), mRNA [NM_005500]	NM_005500
A_24_P753760	0.000734	2.06	A_24_P753760			
A_24_P339611	0.000735	2.16	NM_004708	NM_004708	Homo sapiens programmed cell death 5 (PDCD5), mRNA [NM_004708]	NM_004708
A_24_P118271	0.000738	2.17	A_24_P118271			
A_23_P431638	0.000738	2.54	BC029662	BC029662	Homo sapiens chromosome 20 open reading frame 142, mRNA (cDNA clone IMAGE:4933017), with apparent retained intron. [BC029662]	
A_24_P26177	0.000738	3.28	NM_014703	NM_014703	Homo sapiens Vpr-binding protein (VprBP), mRNA [NM_014703]	NM_014703
A_23_P216278	0.000739	3.97	NM_018482	NM_018482	Homo sapiens development and differentiation enhancing factor 1 (DDEF1), mRNA [NM_018482]	NM_018482
A_23_P214168	0.00074	7.52	NM_004370	NM_004370	Homo sapiens collagen, type XII, alpha 1 (COL12A1), transcript variant long, mRNA [NM_004370]	NM_004370
A_24_P59297	0.000742	2.13	AF161344	AF161344	Homo sapiens HSPC081 mRNA, partial cds. [AF161344]	
A_24_P348326	0.000743	2.25	NM_004843	NM_004843	Homo sapiens interleukin 27 receptor, alpha (IL27RA), mRNA [NM_004843]	NM_004843
A_23_P101796	0.000743	2.32	NM_033025	NM_033025	Homo sapiens synapse defective 1, Rho GTPase, homolog 1 (C. elegans) (SYDE1), mRNA [NM_033025]	NM_033025
A_23_P165301	0.000743	2.88	NM_006916	NM_006916	Homo sapiens ribulose-5-phosphate-3-epimerase (RPE), transcript variant 2, mRNA [NM_006916]	NM_006916
A_23_P3221	0.000743	10.09	NM_021199	NM_021199	Homo sapiens sulfide quinone reductase-like (yeast) (SQRDL), mRNA [NM_021199]	NM_021199
A_24_P57847	0.000744	2.93	NM_199052	NM_199052	Homo sapiens chromosome 20 open reading frame 7 (C20orf7), transcript variant 2, mRNA [NM_199052]	NM_199052
A_23_P58207	0.000744	3.52	NM_021159	NM_021159	Homo sapiens RAP1, GTP-GDP dissociation stimulator 1 (RAP1GDS1), mRNA [NM_021159]	NM_021159
A_23_P16225	0.000746	2.06	NM_017682	NM_017682	Homo sapiens vitelliform macular dystrophy 2-like 1 (VMD2L1), mRNA [NM_017682]	NM_017682
A_23_P29036	0.000746	2.07	NM_005534	NM_005534	Homo sapiens interferon gamma receptor 2 (interferon gamma transducer 1) (IFNGR2), mRNA [NM_005534]	NM_005534

A_24_P135841	0.000746	2.39	ENST00000328043		Homo sapiens low density lipoprotein receptor-related protein 11, mRNA (cDNA clone IMAGE:4072521), partial cds. [BC016788]	
A_24_P233915	0.000746	2.72	NM_001001653	NM_001001653	Homo sapiens mediator of RNA polymerase II transcription, subunit 8 homolog (yeast) (MED8), transcript variant 4, mRNA [NM_001001653]	NM_001001653
A_24_P244470	0.000746	2.92	NM_194358	NM_194358	Homo sapiens ring finger protein 41 (RNF41), transcript variant 2, mRNA [NM_194358]	NM_194358
A_23_P39561	0.000749	2.22	NM_080678	NM_080678	Homo sapiens NEDD8-conjugating enzyme (NCE2), mRNA [NM_080678]	NM_080678
A_32_P101653	0.00075	2.00	XM_499110	XM_499110	PREDICTED: Homo sapiens LOC441345 (LOC441345), mRNA [XM_499110]	XM_499110
A_32_P37721	0.00075	4.72	NM_153451	NM_153451	Homo sapiens oral cancer overexpressed 1 (ORAOV1), mRNA [NM_153451]	NM_153451
A_23_P501996	0.000752	2.17	NM_199203	NM_199203	Homo sapiens ubiquitin-conjugating enzyme E2 variant 1 (Kua-UEV), transcript variant 1, mRNA [NM_199203]	NM_199203
A_23_P16116	0.000752	3.17	NM_016154	NM_016154	Homo sapiens RAB4B, member RAS oncogene family (RAB4B), mRNA [NM_016154]	NM_016154
A_23_P92543	0.000753	3.45	NM_152540	NM_152540	Homo sapiens sec1 family domain containing 2 (SCFD2), mRNA [NM_152540]	NM_152540
A_24_P196519	0.000755	3.23	NM_002451	NM_002451	Homo sapiens methylthioadenosine phosphorylase (MTAP), mRNA [NM_002451]	NM_002451
A_23_P209726	0.000756	2.78	NM_007237	NM_007237	Homo sapiens SP140 nuclear body protein (SP140), transcript variant 1, mRNA [NM_007237]	NM_007237
A_32_P120014	0.000758	2.03	A_32_P120014			
A_32_P99804	0.000761	2.79	THC2377845		Q6PEL1 (Q6PEL1) ZCWCC3 protein (Fragment), partial (17%) [THC2377845]	
A_24_P221335	0.000761	4.03	A_24_P221335			
A_23_P162238	0.000763	2.20	X54871	X54871	H.sapiens mRNA for ras-related protein Rab5b. [X54871]	
A_24_P370557	0.000763	2.57	NM_000950	NM_000950	Homo sapiens proline rich Gla (G-carboxyglutamic acid) 1 (PRRG1), mRNA [NM_000950]	NM_000950
A_23_P254801	0.000764	2.00	NM_002660	NM_002660	Homo sapiens phospholipase C, gamma 1 (PLCG1), transcript variant 1, mRNA [NM_002660]	NM_002660
A_23_P250380	0.000764	2.25	NM_015274	NM_015274	Homo sapiens mannosidase, alpha, class 2B, member 2 (MAN2B2), mRNA [NM_015274]	NM_015274
A_32_P147241	0.000764	2.93	NM_182470	NM_182470	Homo sapiens pyruvate kinase, muscle (PKM2), transcript variant 2, mRNA [NM_182470]	NM_182470
A_23_P129925	0.000764	21.37	NM_152270	NM_152270	Homo sapiens hypothetical protein FLJ34922 (FLJ34922), mRNA [NM_152270]	NM_152270
A_23_P34527	0.000765	2.09	NM_025207	NM_025207	Homo sapiens FAD-synthetase (PP591), transcript variant 1, mRNA [NM_025207]	NM_025207
A_23_P39364	0.000768	2.07	NM_004838	NM_004838	Homo sapiens homer homolog 3 (Drosophila) (HOMER3), mRNA [NM_004838]	NM_004838
A_24_P197964	0.000768	4.98	NM_014788	NM_014788	Homo sapiens tripartite motif-containing 14 (TRIM14), transcript variant 1, mRNA [NM_014788]	NM_014788
A_23_P93499	0.000771	2.43	NM_016020	NM_016020	Homo sapiens transcription factor B1, mitochondrial (TFB1M), mRNA [NM_016020]	NM_016020
A_24_P158089	0.000771	32.47	NM_000602	NM_000602	Homo sapiens serine (or cysteine) proteinase inhibitor, clade E (nexin, plasminogen activator inhibitor type 1), member 1 (SERPINE1), mRNA [NM_000602]	NM_000602
A_23_P365189	0.000772	2.61	NM_015099	NM_015099	Homo sapiens calmodulin binding transcription activator 2 (CAMTA2), mRNA [NM_015099]	NM_015099
A_24_P221105	0.000772	3.19	A_24_P221105			
A_24_P212860	0.000778	2.64	NM_016481	NM_016481	Homo sapiens chromosome 9 open reading frame 156 (C9orf156), mRNA [NM_016481]	NM_016481
A_24_P205589	0.000778	4.42	NM_181864	NM_181864	Homo sapiens brain acyl-CoA hydrolase (BACH), transcript variant hBACHb, mRNA [NM_181864]	NM_181864
A_23_P123413	0.000778	43.10	NM_014729	NM_014729	Homo sapiens thymus high mobility group box protein TOX (TOX), mRNA [NM_014729]	NM_014729
A_23_P165162	0.000779	2.29	NM_021175	NM_021175	Homo sapiens hepcidin antimicrobial peptide (HAMP), mRNA [NM_021175]	NM_021175
A_23_P119102	0.000779	3.03	NM_003370	NM_003370	Homo sapiens vasodilator-stimulated phosphoprotein (VASP), transcript variant 1, mRNA [NM_003370]	NM_003370
A_23_P431521	0.000779	3.51	NM_021814	NM_021814	Homo sapiens ELOVL family member 5, elongation of long chain fatty acids (FEN1/Elo2, SUR4/Elo3-like, yeast) (ELOVL5), mRNA [NM_021814]	NM_021814
A_24_P205458	0.000781	2.01	NM_003607	NM_003607	Homo sapiens CDC42 binding protein kinase alpha (DMPK-like) (CDC42BPA), transcript variant B, mRNA [NM_003607]	NM_003607
A_32_P125525	0.000781	2.46	AA334114	AA334114	AA334114 EST38270 Embryo, 9 week Homo sapiens cDNA 5' end similar to similar to keratin 18, mRNA sequence [AA334114]	
A_23_P80136	0.000783	2.19	NM_003683	NM_003683	Homo sapiens DNA segment on chromosome 21 (unique) 2056 expressed sequence (D21S2056E), mRNA [NM_003683]	NM_003683
A_24_P315873	0.000785	2.29	ENST00000352815			XM_171032
A_23_P136724	0.000786	5.32	BX640843	BX640843	Homo sapiens mRNA; cDNA DKFZp686B14224 (from clone DKFZp686B14224). [BX640843]	
A_23_P106433	0.000789	2.13	NM_033544	NM_033544	Homo sapiens similar to cyclin-E binding protein 1 (H. sapiens) (MGC14386), transcript variant 1, mRNA [NM_033544]	NM_033544

A_23_P404120	0.000789	2.26	NM_173802	NM_173802	Homo sapiens hypothetical protein MGC50559 (MGC50559), mRNA [NM_173802]	NM_173802
A_32_P209148	0.000789	2.35	BC044616	BC044616	Homo sapiens, clone IMAGE:5267190, mRNA. [BC044616]	
A_23_P130919	0.000791	2.54	NM_130807	NM_130807	Homo sapiens MOB1, Mps One Binder kinase activator-like 2A (yeast) (MOBKL2A), mRNA [NM_130807]	NM_130807
A_24_P79054	0.000791	2.73	NM_000660	NM_000660	Homo sapiens transforming growth factor, beta 1 (Camurati-Engelmann disease) (TGFB1), mRNA [NM_000660]	NM_000660
A_23_P124486	0.000792	2.15	NM_002833	NM_002833	Homo sapiens protein tyrosine phosphatase, non-receptor type 9 (PTPN9), mRNA [NM_002833]	NM_002833
A_24_P382661	0.000792	2.59	NM_005239	NM_005239	Homo sapiens v-ets erythroblastosis virus E26 oncogene homolog 2 (avian) (ETS2), mRNA [NM_005239]	NM_005239
A_24_P936767	0.000795	2.16	NM_005082	NM_005082	Homo sapiens tripartite motif-containing 25 (TRIM25), mRNA [NM_005082]	NM_005082
A_23_P38041	0.000795	2.33	NM_016256	NM_016256	Homo sapiens N-acetylglucosamine-1-phosphodiester alpha-N-acetylglucosaminidase (NAGPA), mRNA [NM_016256]	NM_016256
A_23_P23522	0.000795	2.47	CR619489	CR619489	full-length cDNA clone CS0DC011YP01 of Neuroblastoma Cot 25-normalized of Homo sapiens (human). [CR619489]	XM_375838
A_24_P380061	0.000795	2.58	NM_031305	NM_031305	Homo sapiens Rho GTPase activating protein 24 (ARHGAP24), mRNA [NM_031305]	NM_031305
A_23_P44993	0.000799	2.06	NM_006755	NM_006755	Homo sapiens transaldolase 1 (TALDO1), mRNA [NM_006755]	NM_006755
A_24_P683013	0.000801	2.79	BM696546	BM696546	UI-E-DW0-agk-i-01-0-UI.r1 UI-E-DW0 Homo sapiens cDNA clone UI-E-DW0-agk-i-01-0-UI 5', mRNA sequence [BM696546]	
A_24_P245815	0.000801	5.00	AL161993	AL161993	Homo sapiens mRNA; cDNA DKFZp761P039 (from clone DKFZp761P039); partial cds. [AL161993]	
A_23_P64232	0.000802	2.01	NM_003904	NM_003904	Homo sapiens zinc finger protein 259 (ZNF259), mRNA [NM_003904]	NM_003904
A_32_P150735	0.000802	2.49	NM_001008407	NM_001008407	Homo sapiens B-cell receptor-associated protein 29 (BCAP29), transcript variant 4, mRNA [NM_001008407]	NM_001008407
A_23_P414343	0.000804	4.22	NM_005951	NM_005951	Homo sapiens metallothionein 1H (MT1H), mRNA [NM_005951]	NM_005951
A_32_P140501	0.000806	4.69	A_32_P140501			
A_23_P27353	0.000809	2.08	NM_007163	NM_007163	Homo sapiens solute carrier family 14 (urea transporter), member 2 (SLC14A2), mRNA [NM_007163]	NM_007163
A_23_P35820	0.000809	2.79	NM_005507	NM_005507	Homo sapiens cofilin 1 (non-muscle) (CFL1), mRNA [NM_005507]	NM_005507
A_23_P154367	0.000812	7.35	NM_004226	NM_004226	Homo sapiens serine/threonine kinase 17b (apoptosis-inducing) (STK17B), mRNA [NM_004226]	NM_004226
A_24_P153448	0.000814	2.72	AF218008	AF218008	Homo sapiens clone PP3501 unknown mRNA. [AF218008]	
A_23_P46924	0.000815	2.82	NM_001007793	NM_001007793	Homo sapiens BUB3 budding uninhibited by benzimidazoles 3 homolog (yeast) (BUB3), transcript variant 2, mRNA [NM_001007793]	NM_001007793
A_24_P255965	0.000815	8.77	A_24_P255965			
A_24_P179033	0.000817	2.13	THC2270926		RS2_HUMAN (P15880) 40S ribosomal protein S2 (S4) (LLRep3 protein), partial (85%) [THC2270926]	
A_23_P124024	0.000819	2.06	NM_032286	NM_032286	Homo sapiens hypothetical protein MGC5309 (TRG20), mRNA [NM_032286]	NM_032286
A_32_P137939	0.000819	2.43	NM_001101	NM_001101	Homo sapiens actin, beta (ACTB), mRNA [NM_001101]	NM_001101
A_23_P9192	0.000822	3.27	NM_005156	NM_005156	Homo sapiens ROD1 regulator of differentiation 1 (S. pombe) (ROD1), mRNA [NM_005156]	NM_005156
A_24_P614148	0.000823	2.32	NM_206910	NM_206910	Homo sapiens chromosome 6 open reading frame 216 (C6orf216), transcript variant 3, mRNA [NM_206910]	NM_206910
A_23_P110557	0.000824	2.44	NM_133369	NM_133369	Homo sapiens unc-5 homolog A (C. elegans) (UNC5A), mRNA [NM_133369]	NM_133369
A_24_P198598	0.000825	2.78	NM_002675	NM_002675	Homo sapiens promyelocytic leukemia (PML), transcript variant 6, mRNA [NM_002675]	NM_002675
A_23_P66525	0.000826	2.06	NM_006042	NM_006042	Homo sapiens heparan sulfate (glucosamine) 3-O-sulfotransferase 3A1 (HS3ST3A1), mRNA [NM_006042]	NM_006042
A_23_P209933	0.000826	2.83	NM_006464	NM_006464	Homo sapiens trans-golgi network protein 2 (TGOLN2), mRNA [NM_006464]	NM_006464
A_24_P241995	0.000829	2.72	NM_021075	NM_021075	Homo sapiens NADH dehydrogenase (ubiquinone) flavoprotein 3, 10kDa (NDUFV3), nuclear gene encoding mitochondrial protein, transcript variant 1, mRNA [NM_021075]	NM_021075
A_24_P278367	0.000834	2.41	NM_005736	NM_005736	Homo sapiens ARP1 actin-related protein 1 homolog A, centractin alpha (yeast) (ACTR1A), mRNA [NM_005736]	NM_005736
A_23_P218835	0.000834	3.30	NM_002268	NM_002268	Homo sapiens karyopherin alpha 4 (importin alpha 3) (KPNA4), mRNA [NM_002268]	NM_002268
A_24_P392496	0.000834	4.85	ENST00000315293			XM_372099
A_23_P40156	0.000835	2.40	NM_024331	NM_024331	Homo sapiens chromosome 20 open reading frame 121 (C20orf121), mRNA [NM_024331]	NM_024331
A_24_P190190	0.000835	2.87	NM_000723	NM_000723	Homo sapiens calcium channel, voltage-dependent, beta 1 subunit (CACNB1), transcript variant 1, mRNA [NM_000723]	NM_000723
A_23_P37966	0.000836	2.07	A_23_P37966			
A_24_P364954	0.000836	2.32	NM_024648	NM_024648	Homo sapiens hypothetical protein FLJ22222 (FLJ22222), transcript variant 1, mRNA [NM_024648]	NM_024648

A_24_P384018	0.000837	2.06	NR_002171	NR_002171	Homo sapiens olfactory receptor, family 7, subfamily E, member 156 pseudogene (OR7E156P) on chromosome 13 [NR_002171]	NR_002171
A_23_P251342	0.000837	2.12	NM_002093	NM_002093	Homo sapiens glycogen synthase kinase 3 beta (GSK3B), mRNA [NM_002093]	NM_002093
A_32_P117453	0.000837	6.90	BE764339	BE764339	BE764339 IL5-NT0071-180700-116-h04 NT0071 Homo sapiens cDNA, mRNA sequence [BE764339]	
A_24_P254965	0.000839	2.27	NM_018486	NM_018486	Homo sapiens histone deacetylase 8 (HDAC8), mRNA [NM_018486]	NM_018486
A_32_P186710	0.000839	3.56	THC2248354			
A_24_P6921	0.000839	4.98	NM_001013744	NM_001013744	Homo sapiens hypothetical LOC541471 protein (LOC541471), mRNA [NM_001013744]	NM_001013744
A_23_P135123	0.00084	3.95	BG216229	BG216229	BG216229 RST35803 Athersys RAGE Library Homo sapiens cDNA, mRNA sequence [BG216229]	
A_32_P493225	0.000842	2.20	NM_198988	NM_198988	Homo sapiens leukocyte receptor cluster (LRC) member 9 (LENG9), mRNA [NM_198988]	NM_198988
A_24_P409494	0.000842	2.54	NM_015367	NM_015367	Homo sapiens BCL2-like 13 (apoptosis facilitator) (BCL2L13), nuclear gene encoding mitochondrial protein, mRNA [NM_015367]	NM_015367
A_24_P205025	0.000842	2.60	AK096925	AK096925	Homo sapiens cDNA FLJ39606 fis, clone SKNSH2006234. [AK096925]	
A_24_P181055	0.000842	3.12	NM_006278	NM_006278	Homo sapiens ST3 beta-galactoside alpha-2,3-sialyltransferase 4 (ST3GAL4), mRNA [NM_006278]	NM_006278
A_23_P215517	0.000844	2.72	BC009555	BC009555	Homo sapiens kelch-like 7 (Drosophila), mRNA (cDNA clone IMAGE:3899090), complete cds. [BC009555]	
A_23_P79155	0.000848	2.71	ENST00000329321		Homo sapiens cDNA FLJ16071 fis, clone HSYRA2001003, highly similar to PUTATIVE G PROTEIN-COUPLED RECEPTOR GPR39. [AK122643]	
A_23_P342131	0.000848	3.33	NM_153611	NM_153611	Homo sapiens cytochrome b, ascorbate dependent 3 (CYBASC3), mRNA [NM_153611]	NM_153611
A_23_P51699	0.000856	2.10	NM_004723	NM_004723	Homo sapiens rho/rac guanine nucleotide exchange factor (GEF) 2 (ARHGEF2), mRNA [NM_004723]	NM_004723
A_24_P349274	0.000857	2.11	NM_001004727	NM_001004727	Homo sapiens olfactory receptor, family 4, subfamily X, member 2 (OR4X2), mRNA [NM_001004727]	NM_001004727
A_24_P283294	0.000858	2.91	NM_018141	NM_018141	Homo sapiens mitochondrial ribosomal protein S10 (MRPS10), nuclear gene encoding mitochondrial protein, mRNA [NM_018141]	NM_018141
A_23_P214176	0.000859	2.98	NM_133493	NM_133493	Homo sapiens CD109 antigen (Gov platelet alloantigens) (CD109), mRNA [NM_133493]	NM_133493
A_24_P491643	0.00086	2.07	THC2365017		HSU02032 ribosomal protein L23a {Homo sapiens;} , partial (89%) [THC2365017]	
A_23_P206077	0.000865	2.37	NM_022767	NM_022767	Homo sapiens interferon stimulated exonuclease gene 20kDa-like 1 (ISG20L1), mRNA [NM_022767]	NM_022767
A_23_P88819	0.000865	7.14	NM_017458	NM_017458	Homo sapiens major vault protein (MVP), transcript variant 1, mRNA [NM_017458]	NM_017458
A_23_P151614	0.000866	2.10	NM_006263	NM_006263	Homo sapiens proteasome (prosome, macropain) activator subunit 1 (PA28 alpha) (PSME1), transcript variant 1, mRNA [NM_006263]	NM_006263
A_24_P703462	0.000866	2.34	THC2308747		Q969M7 (Q969M7) NEDD8-conjugating enzyme NCE2, partial (70%) [THC2308747]	
A_23_P302914	0.000866	2.65	NM_020972	NM_020972	Homo sapiens zinc finger, FYVE domain containing 28 (ZFYVE28), mRNA [NM_020972]	NM_020972
A_23_P214533	0.000867	2.37	NM_030899	NM_030899	Homo sapiens zinc finger protein 323 (ZNF323), transcript variant 1, mRNA [NM_030899]	NM_030899
A_24_P412943	0.000869	2.60	NM_174905	NM_174905	Homo sapiens hypothetical protein LOC147965 (LOC147965), mRNA [NM_174905]	NM_174905
A_24_P599225	0.000869	5.52	A_24_P599225			
A_32_P203184	0.000871	2.82	CR606809	CR606809	full-length cDNA clone CS0DI054YJ04 of Placenta Cot 25-normalized of Homo sapiens (human). [CR606809]	
A_24_P942945	0.000871	3.57	NM_198569	NM_198569	Homo sapiens G protein-coupled receptor 126 (GPR126), mRNA [NM_198569]	NM_198569
A_23_P431591	0.000871	4.31	NM_001014279	NM_001014279	Homo sapiens similar to annexin II receptor (LOC389289), mRNA [NM_001014279]	NM_001014279
A_32_P72553	0.000871	4.31	THC2430771		ALU1_HUMAN (P39188) Alu subfamily J sequence contamination warning entry, partial (5%) [THC2430771]	
A_24_P200000	0.000871	4.98	NM_182915	NM_182915	Homo sapiens STEAP family member 3 (STEAP3), transcript variant 1, mRNA [NM_182915]	NM_182915
A_23_P94795	0.000873	2.25	NM_003213	NM_003213	Homo sapiens TEA domain family member 4 (TEAD4), transcript variant 1, mRNA [NM_003213]	NM_003213
A_23_P65388	0.000874	17.42	NM_175060	NM_175060	Homo sapiens C-type lectin domain family 14, member A (CLEC14A), mRNA [NM_175060]	NM_175060
A_24_P266285	0.000876	4.83	NM_152558	NM_152558	Homo sapiens IQ motif containing E (IQCE), mRNA [NM_152558]	NM_152558
A_23_P142506	0.000876	6.54	NM_015675	NM_015675	Homo sapiens growth arrest and DNA-damage-inducible, beta (GADD45B), mRNA [NM_015675]	NM_015675
A_23_P213319	0.000877	3.01	NM_197941	NM_197941	Homo sapiens a disintegrin-like and metalloprotease (reprolysin type) with thrombospondin type 1 motif, 6 (ADAMTS6), mRNA [NM_197941]	NM_197941
A_24_P867201	0.000879	2.16	CR613944	CR613944	full-length cDNA clone CS0CAP007YK21 of Thymus of Homo sapiens (human). [CR613944]	
A_23_P7030	0.000881	2.16	NM_007208	NM_007208	Homo sapiens mitochondrial ribosomal protein L3 (MRPL3), nuclear gene encoding mitochondrial protein, mRNA [NM_007208]	NM_007208

A_23_P121265	0.000882	2.94	NM_007284	NM_007284	Homo sapiens PTK9L protein tyrosine kinase 9-like (A6-related protein) (PTK9L), mRNA [NM_007284]	NM_007284
A_23_P328600	0.000882	3.24	NM_024959	NM_024959	Homo sapiens solute carrier family 24 (sodium/potassium/calcium exchanger), member 6 (SLC24A6), mRNA [NM_024959]	NM_024959
A_23_P255653	0.000884	11.06	NM_003844	NM_003844	Homo sapiens tumor necrosis factor receptor superfamily, member 10a (TNFRSF10A), mRNA [NM_003844]	NM_003844
A_24_P295877	0.000886	3.30	NM_014704	NM_014704	Homo sapiens glycine-, glutamate-, thienylcyclohexylpiperidine-binding protein (GlyBP), mRNA [NM_014704]	NM_014704
A_23_P357811	0.000887	2.09	NM_021038	NM_021038	Homo sapiens muscleblind-like (Drosophila) (MBNL1), transcript variant 1, mRNA [NM_021038]	NM_021038
A_24_P32560	0.000888	2.43	NM_198573	NM_198573	Homo sapiens GAAI470 (UNQ470), mRNA [NM_198573]	NM_198573
A_32_P63342	0.000889	3.95	AA903587	AA903587	AA903587 ok63d11.s1 NCI_CGAP_GC4 Homo sapiens cDNA clone IMAGE:1518645 3' similar to gb:L23805 ALPHA-CATENIN (HUMAN);, mRNA sequence [AA903587]	
A_23_P126426	0.00089	2.44	NM_001384	NM_001384	Homo sapiens DPH2 homolog (S. cerevisiae) (DPH2), transcript variant 1, mRNA [NM_001384]	NM_001384
A_24_P411749	0.000891	2.43	NM_198569	NM_198569	Homo sapiens G protein-coupled receptor 126 (GPR126), mRNA [NM_198569]	NM_198569
A_32_P208076	0.000891	9.43	A_32_P208076			
A_24_P324506	0.000896	2.49	A_24_P324506			
A_23_P52219	0.000897	3.46	NM_006459	NM_006459	Homo sapiens SPFH domain family, member 1 (SPFH1), mRNA [NM_006459]	NM_006459
A_23_P22614	0.000898	2.18	NM_145802	NM_145802	Homo sapiens septin 6 (SEPT6), transcript variant V, mRNA [NM_145802]	NM_145802
A_23_P142590	0.000898	2.62	NM_176799	NM_176799	Homo sapiens integrin-linked kinase-associated serine/threonine phosphatase 2C (ILKAP), transcript variant 2, mRNA [NM_176799]	NM_176799
A_23_P502747	0.000899	3.28	NM_170692	NM_170692	Homo sapiens RAS protein activator like 2 (RASAL2), transcript variant 2, mRNA [NM_170692]	NM_170692
A_24_P405313	0.0009	5.18	NM_003705	NM_003705	Homo sapiens solute carrier family 25 (mitochondrial carrier, Aralar), member 12 (SLC25A12), mRNA [NM_003705]	NM_003705
A_24_P229766	0.000901	2.09	ENST00000318527			
A_23_P310068	0.000902	2.31	NM_001009894	NM_001009894	Homo sapiens hypothetical protein DKFZp434N2030 (DKFZp434N2030), mRNA [NM_001009894]	NM_001009894
A_23_P79927	0.000904	2.17	NM_006392	NM_006392	Homo sapiens nucleolar protein 5A (56kDa with KKE/D repeat) (NOL5A), mRNA [NM_006392]	NM_006392
A_24_P58944	0.000907	2.17	ENST00000352815			XM_171032
A_24_P261326	0.000908	2.61	NM_005736	NM_005736	Homo sapiens ARP1 actin-related protein 1 homolog A, centractin alpha (yeast) (ACTR1A), mRNA [NM_005736]	NM_005736
A_32_P231617	0.000909	4.48	NM_014220	NM_014220	Homo sapiens transmembrane 4 L six family member 1 (TM4SF1), mRNA [NM_014220]	NM_014220
A_24_P409560	0.00091	2.60	A_24_P409560			
A_24_P652318	0.000911	3.41	AK098360	AK098360	Homo sapiens cDNA FLJ25494 fis, clone CBR01476. [AK098360]	
A_23_P717	0.000912	2.61	NM_018252	NM_018252	Homo sapiens chromosome 1 open reading frame 75 (C1orf75), mRNA [NM_018252]	NM_018252
A_32_P95176	0.000912	2.63	AK127378	AK127378	Homo sapiens cDNA FLJ45454 fis, clone BRSTN2008475, moderately similar to Homo sapiens growth arrest-specific 8 (GAS8). [AK127378]	
A_23_P325562	0.000912	5.95	NM_006671	NM_006671	Homo sapiens solute carrier family 1 (glutamate transporter), member 7 (SLC1A7), mRNA [NM_006671]	NM_006671
A_24_P75230	0.000913	2.14	NM_033546	NM_033546	Homo sapiens myosin regulatory light chain MRLC2 (MRLC2), mRNA [NM_033546]	NM_033546
A_24_P9454	0.000915	2.02	NM_002541	NM_002541	Homo sapiens oxoglutarate (alpha-ketoglutarate) dehydrogenase (lipoamide) (OGDH), nuclear gene encoding mitochondrial protein, transcript variant 1, mRNA [NM_002541]	NM_002541
A_23_P143461	0.000915	2.04	AJ457067	AJ457067	Homo sapiens partial mRNA for high tyrosine glycine keratin associated protein 19.1 (KRTAP19.1 gene). [AJ457067]	
A_32_P2452	0.000915	3.65	AK125140	AK125140	Homo sapiens cDNA FLJ43150 fis, clone CTONG3009239. [AK125140]	
A_23_P117778	0.000918	2.03	NM_032907	NM_032907	Homo sapiens ubiquitin-like 7 (bone marrow stromal cell-derived) (UBL7), transcript variant 1, mRNA [NM_032907]	NM_032907
A_24_P279328	0.000926	2.48	NM_130766	NM_130766	Homo sapiens skeletal muscle and kidney enriched inositol phosphatase (SKIP), transcript variant 2, mRNA [NM_130766]	NM_130766
A_24_P175427	0.000926	3.05	NM_199177	NM_199177	Homo sapiens mitochondrial ribosome recycling factor (MRRF), nuclear gene encoding mitochondrial protein, transcript variant 2, mRNA [NM_199177]	NM_199177
A_32_P25357	0.000928	2.02	NM_004933	NM_004933	Homo sapiens cadherin 15, M-cadherin (myotubule) (CDH15), mRNA [NM_004933]	NM_004933
A_24_P120115	0.000929	2.25	NM_003879	NM_003879	Homo sapiens CASP8 and FADD-like apoptosis regulator (CFLAR), mRNA [NM_003879]	NM_003879
A_23_P253958	0.00093	2.38	NM_005824	NM_005824	Homo sapiens leucine rich repeat containing 17 (LRRC17), mRNA [NM_005824]	NM_005824
A_24_P578641	0.00093	3.57	A_24_P578641			

A_24_P382591	0.000933	2.09	NM_001009924	NM_001009924	Homo sapiens chromosome 20 open reading frame 30 (C20orf30), transcript variant 2, mRNA [NM_001009924]	NM_001009924
A_24_P7965	0.000934	2.17	NM_206594	NM_206594	Homo sapiens estrogen-related receptor gamma (ESRRG), transcript variant 2, mRNA [NM_206594]	NM_206594
A_24_P134372	0.000936	2.42	NM_004798	NM_004798	Homo sapiens kinesin family member 3B (KIF3B), mRNA [NM_004798]	NM_004798
A_23_P333227	0.000938	2.35	BC012766	BC012766	Homo sapiens endoplasmic reticulum-golgi intermediate compartment 32 kDa protein, mRNA (cDNA clone MGC:16233 IMAGE:3677787), complete cds. [BC012766]	
A_32_P143880	0.000939	2.25	NM_015955	NM_015955	Homo sapiens chromosome 2 open reading frame 4 (C2orf4), mRNA [NM_015955]	NM_015955
A_23_P28153	0.00094	3.48	AF175767	AF175767	Homo sapiens putative selenocysteine lyase (SCLY) mRNA, complete cds. [AF175767]	
A_24_P749042	0.000941	5.62	A_24_P749042			
A_23_P434040	0.000942	2.08	BC029609	BC029609	Homo sapiens hypothetical LOC554206, mRNA (cDNA clone MGC:39831 IMAGE:5302675), complete cds. [BC029609]	
A_24_P323941	0.000942	2.43	NM_001012971	NM_001012971	Homo sapiens chromosome 20 open reading frame 106 (C20orf106), mRNA [NM_001012971]	NM_001012971
A_23_P16671	0.000942	2.80	NM_024050	NM_024050	Homo sapiens cross-immune reaction antigen PCIA1 (PCIA1), mRNA [NM_024050]	NM_024050
A_23_P71148	0.000943	2.11	NM_000712	NM_000712	Homo sapiens biliverdin reductase A (BLVRA), mRNA [NM_000712]	NM_000712
A_23_P122228	0.000945	2.00	NM_004553	NM_004553	Homo sapiens NADH dehydrogenase (ubiquinone) Fe-S protein 6, 13kDa (NADH-coenzyme Q reductase) (NDUFS6), mRNA [NM_004553]	NM_004553
A_24_P256579	0.000945	2.81	AB051533	AB051533	Homo sapiens mRNA for KIAA1746 protein, partial cds. [AB051533]	
A_24_P323795	0.000946	2.67	A_24_P323795			
A_23_P22915	0.000947	2.57	NM_133496	NM_133496	Homo sapiens solute carrier family 30 (zinc transporter), member 7 (SLC30A7), mRNA [NM_133496]	NM_133496
A_24_P564462	0.000947	3.55	CN430223	CN430223	17000600171867 GRN_PREHEP Homo sapiens cDNA 5', mRNA sequence [CN430223]	
A_23_P143650	0.000947	8.93	NM_152511	NM_152511	Homo sapiens dual specificity phosphatase 18 (DUSP18), mRNA [NM_152511]	NM_152511
A_24_P6889	0.000948	2.02	NM_006099	NM_006099	Homo sapiens protein inhibitor of activated STAT, 3 (PIAS3), mRNA [NM_006099]	NM_006099
A_23_P203505	0.000949	2.22	NM_033034	NM_033034	Homo sapiens tripartite motif-containing 5 (TRIM5), transcript variant alpha, mRNA [NM_033034]	NM_033034
A_23_P158239	0.000949	2.35	NM_005412	NM_005412	Homo sapiens serine hydroxymethyltransferase 2 (mitochondrial) (SHMT2), mRNA [NM_005412]	NM_005412
A_32_P10643	0.00095	2.16	A_32_P10643			
A_32_P139021	0.00095	2.70	A_32_P139021			
A_23_P80008	0.000951	2.20	NM_033118	NM_033118	Homo sapiens myosin light chain kinase 2, skeletal muscle (MYLK2), mRNA [NM_033118]	NM_033118
A_23_P69908	0.000951	2.26	NM_002064	NM_002064	Homo sapiens glutaredoxin (thioltransferase) (GLRX), mRNA [NM_002064]	NM_002064
A_24_P647163	0.000951	2.49	THC2437037		ALU1_HUMAN (P39188) Alu subfamily J sequence contamination warning entry, partial (12%) [THC2437037]	
A_24_P416346	0.000951	2.55	NM_001986	NM_001986	Homo sapiens ets variant gene 4 (E1A enhancer binding protein, E1AF) (ETV4), mRNA [NM_001986]	NM_001986
A_23_P350234	0.000951	3.05	NM_001012989	NM_001012989	Homo sapiens ubiquitin-conjugating enzyme E2N-like (UBE2NL), mRNA [NM_001012989]	NM_001012989
A_32_P144629	0.000952	2.02	THC2339084		Q93HV7 (Q93HV7) Probable dihydrolipoamide acyltransferase (Fragment), partial (5%) [THC2339084]	
A_32_P53852	0.000953	2.03	BC050563	BC050563	Homo sapiens hypothetical protein LOC202051, mRNA (cDNA clone MGC:57735 IMAGE:5751687), complete cds. [BC050563]	XM_114430
A_24_P332504	0.000954	3.47	A_24_P332504			
A_24_P478556	0.000955	2.13	BC030102	BC030102	Homo sapiens cDNA clone IMAGE:4796690, partial cds. [BC030102]	
A_23_P364537	0.000958	2.10	NM_175066	NM_175066	Homo sapiens DEAD (Asp-Glu-Ala-Asp) box polypeptide 51 (DDX51), mRNA [NM_175066]	NM_175066
A_23_P29422	0.000958	5.85	NM_004130	NM_004130	Homo sapiens glycogenin (GYG), mRNA [NM_004130]	NM_004130
A_24_P235012	0.00096	2.39	NM_014341	NM_014341	Homo sapiens mitochondrial carrier homolog 1 (C. elegans) (MTCH1), nuclear gene encoding mitochondrial protein, mRNA [NM_014341]	NM_014341
A_23_P218108	0.00096	4.63	NM_052950	NM_052950	Homo sapiens WD repeat and FYVE domain containing 2 (WDFY2), mRNA [NM_052950]	NM_052950
A_24_P926770	0.000961	2.21	NM_014045	NM_014045	Homo sapiens low density lipoprotein receptor-related protein 10 (LRP10), mRNA [NM_014045]	NM_014045
A_24_P328320	0.000961	2.38	NM_015530	NM_015530	Homo sapiens golgi reassembly stacking protein 2, 55kDa (GORASP2), mRNA [NM_015530]	NM_015530
A_23_P213298	0.000964	26.25	NM_024590	NM_024590	Homo sapiens arylsulfatase J (ARSJ), mRNA [NM_024590]	NM_024590
A_23_P359091	0.000966	2.66	U68019	U68019	Homo sapiens mad protein homolog (hMAD-3) mRNA, complete cds. [U68019]	
A_23_P111297	0.000966	2.75	NM_006638	NM_006638	Homo sapiens ribonuclease P 40kDa subunit (RPP40), mRNA [NM_006638]	NM_006638

A_24_P358381	0.000966	3.22	NM_012227	NM_012227	Homo sapiens GTP binding protein 6 (putative) (GTPBP6), mRNA [NM_012227]	NM_012227
A_24_P922606	0.000967	2.26	NM_015231	NM_015231	Homo sapiens nucleoporin 160kDa (NUP160), mRNA [NM_015231]	NM_015231
A_24_P307653	0.000967	5.65	NM_182767	NM_182767	Homo sapiens solute carrier family 6, member 15 (SLC6A15), transcript variant 1, mRNA [NM_182767]	NM_182767
A_24_P272523	0.000968	2.18	A_24_P272523			
A_24_P20383	0.000968	5.95	NM_005718	NM_005718	Homo sapiens actin related protein 2/3 complex, subunit 4, 20kDa (ARPC4), transcript variant 1, mRNA [NM_005718]	NM_005718
A_24_P931476	0.000969	2.74	BC043603	BC043603	Homo sapiens, Similar to hypothetical protein FLJ33215, clone IMAGE:5503684, mRNA. [BC043603]	
A_32_P19806	0.000972	2.65	NM_003777	NM_003777	Homo sapiens dynein, axonemal, heavy polypeptide 11 (DNAH11), mRNA [NM_003777]	NM_003777
A_32_P194115	0.000972	3.36	NM_015374	NM_015374	Homo sapiens unc-84 homolog B (C. elegans) (UNC84B), mRNA [NM_015374]	NM_015374
A_24_P151305	0.000972	3.44	NM_016270	NM_016270	Homo sapiens Kruppel-like factor 2 (lung) (KLF2), mRNA [NM_016270]	NM_016270
A_23_P146849	0.000972	3.46	NM_005503	NM_005503	Homo sapiens amyloid beta (A4) precursor protein-binding, family A, member 2 (X11-like) (APBA2), mRNA [NM_005503]	NM_005503
A_23_P149111	0.000972	3.46	NM_005401	NM_005401	Homo sapiens protein tyrosine phosphatase, non-receptor type 14 (PTPN14), mRNA [NM_005401]	NM_005401
A_23_P82550	0.000972	6.33	NM_004760	NM_004760	Homo sapiens serine/threonine kinase 17a (apoptosis-inducing) (STK17A), mRNA [NM_004760]	NM_004760
A_23_P12514	0.000973	2.14	NM_175744	NM_175744	Homo sapiens ras homolog gene family, member C (RHOC), mRNA [NM_175744]	NM_175744
A_23_P80040	0.000973	2.30	NM_006404	NM_006404	Homo sapiens protein C receptor, endothelial (EPCR) (PROCR), mRNA [NM_006404]	NM_006404
A_23_P94494	0.000974	3.21	NM_007005	NM_007005	Homo sapiens transducin-like enhancer of split 4 (E(sp1) homolog, Drosophila) (TLE4), mRNA [NM_007005]	NM_007005
A_24_P29595	0.000974	3.48	NM_139244	NM_139244	Homo sapiens syntaxin binding protein 5 (tomosyn) (STXBP5), mRNA [NM_139244]	NM_139244
A_23_P202316	0.000976	2.46	NM_001007793	NM_001007793	Homo sapiens BUB3 budding uninhibited by benzimidazoles 3 homolog (yeast) (BUB3), transcript variant 2, mRNA [NM_001007793]	NM_001007793
A_23_P44366	0.000976	4.22	BC068598	BC068598	Homo sapiens protein kinase, AMP-activated, gamma 2 non-catalytic subunit, mRNA (cDNA clone MGC:87635 IMAGE:5259756), complete cds. [BC068598]	
A_23_P254271	0.000976	4.57	NM_032525	NM_032525	Homo sapiens tubulin, beta 6 (TUBB6), mRNA [NM_032525]	NM_032525
A_23_P113111	0.000976	6.29	NM_000044	NM_000044	Homo sapiens androgen receptor (dihydrotestosterone receptor; testicular feminization; spinal and bulbar muscular atrophy; Kennedy disease) (AR), transcript variant 1, mRNA [NM_000044]	NM_000044
A_24_P180654	0.000977	2.07	BC063666	BC063666	Homo sapiens cAMP responsive element binding protein 3-like 2, mRNA (cDNA clone IMAGE:4185677), complete cds. [BC063666]	
A_24_P50381	0.000981	2.48	A_24_P50381			
A_23_P88134	0.000981	2.54	NM_032630	NM_032630	Homo sapiens cyclin-dependent kinase 2-interacting protein (CINP), mRNA [NM_032630]	NM_032630
A_24_P418203	0.000981	18.94	NM_033655	NM_033655	Homo sapiens contactin associated protein-like 3 (CNTNAP3), mRNA [NM_033655]	NM_033655
A_24_P116571	0.000982	3.75	NM_016256	NM_016256	Homo sapiens N-acetylglucosamine-1-phosphodiester alpha-N-acetylglucosaminidase (NAGPA), mRNA [NM_016256]	NM_016256
A_24_P271014	0.000983	2.21	NM_005134	NM_005134	Homo sapiens protein phosphatase 4, regulatory subunit 1 (PPP4R1), mRNA [NM_005134]	NM_005134
A_23_P51410	0.000983	2.47	NM_022743	NM_022743	Homo sapiens SET and MYND domain containing 3 (SMYD3), mRNA [NM_022743]	NM_022743
A_24_P138022	0.000984	2.69	NM_014612	NM_014612	Homo sapiens chromosome 9 open reading frame 10 (C9orf10), mRNA [NM_014612]	NM_014612
A_24_P355693	0.000984	3.53	NM_018367	NM_018367	Homo sapiens phytoceramidase, alkaline (PHCA), mRNA [NM_018367]	NM_018367
A_32_P54475	0.000985	2.04	THC2307560			
A_24_P75708	0.000988	4.39	ENST00000321005			
A_23_P132308	0.000993	2.44	NM_032561	NM_032561	Homo sapiens chromosome 22 open reading frame 23 (C22orf23), mRNA [NM_032561]	NM_032561
A_23_P37503	0.000995	2.78	NM_004998	NM_004998	Homo sapiens myosin IE (MYO1E), mRNA [NM_004998]	NM_004998
A_23_P128154	0.000996	2.13	NM_032704	NM_032704	Homo sapiens tubulin alpha 6 (TUBA6), mRNA [NM_032704]	NM_032704
A_24_P76078	0.000997	2.21	ENST00000330044		PREDICTED: Homo sapiens similar to NADH-ubiquinone oxidoreductase 15 kDa subunit (Complex I-15 kDa) (CI-15 kDa) (LOC342659), mRNA [XM_292664]	XM_292664
A_24_P101629	0.000999	2.34	NM_003928	NM_003928	Homo sapiens CAAX box 1 (CXX1), mRNA [NM_003928]	NM_003928
A_23_P358709	0.001	2.07	AB033060	AB033060	Homo sapiens mRNA for KIAA1234 protein, partial cds. [AB033060]	
A_23_P111492	0.001	2.08	NM_023948	NM_023948	Homo sapiens motile sperm domain containing 3 (MOSPD3), mRNA [NM_023948]	NM_023948
A_23_P158059	0.001	2.18	NM_017873	NM_017873	Homo sapiens ankyrin repeat and SOCS box-containing 6 (ASB6), transcript variant 1, mRNA [NM_017873]	NM_017873

A_24_P400751	0.001	2.52	ENST00000312398			
A_23_P4808	0.001	6.25	NM_000955	NM_000955	Homo sapiens prostaglandin E receptor 1 (subtype EP1), 42kDa (PTGER1), mRNA [NM_000955]	NM_000955
A_24_P182494	0.00101	2.20	NM_007207	NM_007207	Homo sapiens dual specificity phosphatase 10 (DUSP10), transcript variant 1, mRNA [NM_007207]	NM_007207
A_24_P131222	0.00101	2.23	NM_022089	NM_022089	Homo sapiens ATPase type 13A2 (ATP13A2), mRNA [NM_022089]	NM_022089
A_24_P82880	0.00101	2.35	NM_003290	NM_003290	Homo sapiens tropomyosin 4 (TPM4), mRNA [NM_003290]	NM_003290
A_23_P393856	0.00101	2.42	AK021929	AK021929	Homo sapiens cDNA FLJ11867 fis, clone HEMBA1006976, weakly similar to H.sapiens mRNA for Gal-beta(1-3/1-4)GlcNAc alpha-2.3-sialyltransferase. [AK021929]	
A_23_P357147	0.00101	2.60	NM_199424	NM_199424	Homo sapiens WW domain containing E3 ubiquitin protein ligase 2 (WWP2), transcript variant 2, mRNA [NM_199424]	NM_199424
A_23_P14734	0.00101	2.79	NM_015920	NM_015920	Homo sapiens ribosomal protein S27-like (RPS27L), mRNA [NM_015920]	NM_015920
A_23_P41674	0.00101	2.87	NM_152407	NM_152407	Homo sapiens GrpE-like 2, mitochondrial (E. coli) (GRPEL2), mRNA [NM_152407]	NM_152407
A_23_P128036	0.00101	2.92	NM_004990	NM_004990	Homo sapiens methionine-tRNA synthetase (MARS), mRNA [NM_004990]	NM_004990
A_32_P221569	0.00101	2.92	BM989272	BM989272	BM989272 UI-H-DP0-ats-f-05-0-UI.s1 NCI_CGAP_Fs1 Homo sapiens cDNA clone IMAGE:5863684 3', mRNA sequence [BM989272]	
A_24_P256583	0.00101	2.93	NM_194291	NM_194291	Homo sapiens hypothetical protein BC017881 (LOC157378), mRNA [NM_194291]	NM_194291
A_23_P83289	0.00101	2.96	NM_016174	NM_016174	Homo sapiens cerebral endothelial cell adhesion molecule 1 (CEECAM1), mRNA [NM_016174]	NM_016174
A_24_P256539	0.00101	3.08	NM_016534	NM_016534	Homo sapiens apoptosis-related protein PNAS-1 (FLJ39616), mRNA [NM_016534]	NM_016534
A_23_P200535	0.00101	3.26	NM_014408	NM_014408	Homo sapiens trafficking protein particle complex 3 (TRAPPC3), mRNA [NM_014408]	NM_014408
A_32_P235727	0.00101	3.52	A_32_P235727			
A_24_P302332	0.00101	4.44	NM_018090	NM_018090	Homo sapiens adaptin-ear-binding coat-associated protein 2 (NECAP2), mRNA [NM_018090]	NM_018090
A_24_P409985	0.00101	4.93	NM_001011655	NM_001011655	Homo sapiens transmembrane protein 44 (TMEM44), transcript variant 2, mRNA [NM_001011655]	NM_001011655
A_23_P365685	0.00101	7.87	NM_033514	NM_033514	Homo sapiens LIM and senescent cell antigen-like domains 3 (LIMS3), mRNA [NM_033514]	NM_033514
A_24_P251053	0.00102	2.07	NM_024329	NM_024329	Homo sapiens EF-hand domain family, member D2 (EFHD2), mRNA [NM_024329]	NM_024329
A_32_P169754	0.00102	2.36	NM_004559	NM_004559	Homo sapiens nuclease sensitive element binding protein 1 (NSEP1), mRNA [NM_004559]	NM_004559
A_24_P410952	0.00102	3.66	NM_003768	NM_003768	Homo sapiens phosphoprotein enriched in astrocytes 15 (PEA15), mRNA [NM_003768]	NM_003768
A_23_P155666	0.00102	4.08	NM_014435	NM_014435	Homo sapiens N-acylsphingosine amidohydrolase (acid ceramidase)-like (ASAHL), mRNA [NM_014435]	NM_014435
A_32_P34696	0.00102	4.55	THC2285122			
A_23_P500364	0.00103	2.16	NM_138707	NM_138707	Homo sapiens B-cell CLL/lymphoma 7B (BCL7B), transcript variant 2, mRNA [NM_138707]	NM_138707
A_24_P360763	0.00103	2.21	NM_001881	NM_001881	Homo sapiens cAMP responsive element modulator (CREM), transcript variant 2, mRNA [NM_001881]	NM_001881
A_23_P214444	0.00103	2.82	NM_000544	NM_000544	Homo sapiens transporter 2, ATP-binding cassette, sub-family B (MDR/TAP) (TAP2), transcript variant 1, mRNA [NM_000544]	NM_000544
A_24_P84482	0.00103	3.21	A_24_P84482			
A_24_P137713	0.00103	3.30	NM_030899	NM_030899	Homo sapiens zinc finger protein 323 (ZNF323), transcript variant 1, mRNA [NM_030899]	NM_030899
A_24_P255763	0.00103	3.42	A_24_P255763			
A_23_P10785	0.00103	4.37	NM_145206	NM_145206	Homo sapiens vesicle transport through interaction with t-SNAREs homolog 1A (yeast) (VTI1A), mRNA [NM_145206]	NM_145206
A_24_P418106	0.00103	4.42	A_24_P418106			
A_23_P22200	0.00104	2.03	NM_024095	NM_024095	Homo sapiens ankyrin repeat and SOCS box-containing 8 (ASB8), mRNA [NM_024095]	NM_024095
A_24_P711050	0.00104	2.07	A_24_P711050			
A_23_P77980	0.00104	2.07	NM_000342	NM_000342	Homo sapiens solute carrier family 4, anion exchanger, member 1 (erythrocyte membrane protein band 3, Diego blood group) (SLC4A1), mRNA [NM_000342]	NM_000342
A_23_P153489	0.00104	2.28	NM_002975	NM_002975	Homo sapiens C-type lectin domain family 11, member A (CLEC11A), mRNA [NM_002975]	NM_002975
A_23_P214882	0.00104	2.44	NM_019041	NM_019041	Homo sapiens mitochondrial translational release factor 1-like (MTRF1L), mRNA [NM_019041]	NM_019041
A_23_P15857	0.00104	2.58	NM_005134	NM_005134	Homo sapiens protein phosphatase 4, regulatory subunit 1 (PPP4R1), mRNA [NM_005134]	NM_005134
A_23_P165698	0.00104	2.61	NM_024093	NM_024093	Homo sapiens hypothetical protein MGC5509 (MGC5509), mRNA [NM_024093]	NM_024093
A_32_P119744	0.00104	2.72	BC039550	BC039550	Homo sapiens, clone IMAGE:5760770, mRNA. [BC039550]	XM_298151

A_23_P139704	0.00104	2.79	NM_001946	NM_001946	Homo sapiens dual specificity phosphatase 6 (DUSP6), transcript variant 1, mRNA [NM_001946]	NM_001946
A_24_P920484	0.00104	3.02	AW169007	AW169007	AW169007 xj16f07.x1 NCI_CGAP_Ut2 Homo sapiens cDNA clone IMAGE:2657413 3' similar to WP:Y55F3B_743.B CE22557 ;, mRNA sequence [AW169007]	
A_23_P142325	0.00104	3.16	AB013462	AB013462	Homo sapiens mRNA for Fzr1, complete cds. [AB013462]	
A_24_P853366	0.00104	3.68	A_24_P853366			
A_23_P117540	0.00104	3.97	NM_145251	NM_145251	Homo sapiens serine/threonine/tyrosine interacting protein (STYX), mRNA [NM_145251]	NM_145251
A_23_P116195	0.00104	4.39	THC2277082		Q7PKG0 (Q7PKG0) ENSANGP00000024462 (Fragment), partial (45%) [THC2277082]	
A_32_P163937	0.00105	2.13	BQ064481	BQ064481	AGENCOURT_6853651 NIH_MGC_99 Homo sapiens cDNA clone IMAGE:5926217 5', mRNA sequence [BQ064481]	
A_24_P127462	0.00105	2.49	A_24_P127462			
A_23_P103951	0.00105	2.62	A_23_P103951			
A_23_P119266	0.00105	2.63	NM_001375	NM_001375	Homo sapiens deoxyribonuclease II, lysosomal (DNASE2), mRNA [NM_001375]	NM_001375
A_23_P361773	0.00105	2.82	NM_001760	NM_001760	Homo sapiens cyclin D3 (CCND3), mRNA [NM_001760]	NM_001760
A_23_P60146	0.00105	2.86	NM_006207	NM_006207	Homo sapiens platelet-derived growth factor receptor-like (PDGFRL), mRNA [NM_006207]	NM_006207
A_32_P202977	0.00105	4.85	THC2383225		Q7PV63 (Q7PV63) ENSANGP00000020166, partial (6%) [THC2383225]	
A_24_P530690	0.00106	2.02	A_24_P530690			
A_23_P169030	0.00106	2.49	NM_003842	NM_003842	Homo sapiens tumor necrosis factor receptor superfamily, member 10b (TNFRSF10B), transcript variant 1, mRNA [NM_003842]	NM_003842
A_24_P139867	0.00106	2.72	NM_018207	NM_018207	Homo sapiens tripartite motif-containing 62 (TRIM62), mRNA [NM_018207]	NM_018207
A_24_P20506	0.00106	2.75	NM_001014809	NM_001014809	Homo sapiens collapsin response mediator protein 1 (CRMP1), transcript variant 1, mRNA [NM_001014809]	NM_001014809
A_24_P876862	0.00106	3.51	BC061590	BC061590	Homo sapiens cDNA clone MGC:75203 IMAGE:6502529, complete cds. [BC061590]	
A_23_P377982	0.00106	3.61	NM_175884	NM_175884	Homo sapiens hypothetical protein FLJ36031 (FLJ36031), mRNA [NM_175884]	NM_175884
A_32_P197109	0.00107	2.36	AI559980	AI559980	AI559980 tq77f07.x1 NCI_CGAP_Ut1 Homo sapiens cDNA clone IMAGE:2214853 3', mRNA sequence [AI559980]	
A_23_P125001	0.00107	2.73	NM_005052	NM_005052	Homo sapiens ras-related C3 botulinum toxin substrate 3 (rho family, small GTP binding protein Rac3) (RAC3), mRNA [NM_005052]	NM_005052
A_32_P6868	0.00107	2.87	NM_015472	NM_015472	Homo sapiens WW domain containing transcription regulator 1 (WWTR1), mRNA [NM_015472]	NM_015472
A_23_P45913	0.00107	3.00	NM_004814	NM_004814	Homo sapiens WD repeat domain 57 (U5 snRNP specific) (WDR57), mRNA [NM_004814]	NM_004814
A_24_P918762	0.00107	4.39	THC2436839		ALU1_HUMAN (P39188) Alu subfamily J sequence contamination warning entry, partial (5%) [THC2436839]	
A_23_P141894	0.00107	6.45	NM_006505	NM_006505	Homo sapiens poliovirus receptor (PVR), mRNA [NM_006505]	NM_006505
A_23_P148984	0.00108	2.11	NM_018122	NM_018122	Homo sapiens aspartyl-tRNA synthetase 2 (mitochondrial) (DARS2), mRNA [NM_018122]	NM_018122
A_24_P50328	0.00108	2.74	A_24_P50328			
A_23_P315843	0.00108	2.92	NM_020967	NM_020967	Homo sapiens nuclear receptor coactivator 5 (NCOA5), mRNA [NM_020967]	NM_020967
A_23_P218770	0.00108	3.70	NM_002872	NM_002872	Homo sapiens ras-related C3 botulinum toxin substrate 2 (rho family, small GTP binding protein Rac2) (RAC2), mRNA [NM_002872]	NM_002872
A_23_P121064	0.00108	38.91	NM_002852	NM_002852	Homo sapiens pentraxin-related gene, rapidly induced by IL-1 beta (PTX3), mRNA [NM_002852]	NM_002852
A_24_P941505	0.00109	2.03	NM_014612	NM_014612	Homo sapiens chromosome 9 open reading frame 10 (C9orf10), mRNA [NM_014612]	NM_014612
A_23_P148546	0.00109	2.04	NM_003491	NM_003491	Homo sapiens ARD1 homolog A, N-acetyltransferase (S. cerevisiae) (ARD1A), mRNA [NM_003491]	NM_003491
A_24_P132039	0.00109	2.15	NM_004290	NM_004290	Homo sapiens ring finger protein 14 (RNF14), transcript variant 1, mRNA [NM_004290]	NM_004290
A_24_P842962	0.00109	2.21	THC2379232			
A_24_P75019	0.00109	2.23	AF274938	AF274938	Homo sapiens PNAS-13 mRNA, complete cds. [AF274938]	XM_496861
A_23_P155890	0.00109	2.26	ENST00000286794		Homo sapiens hypothetical protein MGC10646, mRNA (cDNA clone IMAGE:4025308), partial cds. [BC004552]	XM_496704
A_23_P417113	0.00109	2.30	BC011923	BC011923	Homo sapiens proline rich 8, mRNA (cDNA clone MGC:20460 IMAGE:4542314), complete cds. [BC011923]	
A_23_P127525	0.00109	5.41	NM_005238	NM_005238	Homo sapiens v-ets erythroblastosis virus E26 oncogene homolog 1 (avian) (ETS1), mRNA [NM_005238]	NM_005238
A_24_P110591	0.0011	2.13	A_24_P110591			

A_23_P72643	0.0011	2.14	NM_003816	NM_003816	Homo sapiens a disintegrin and metalloproteinase domain 9 (meltrin gamma) (ADAM9), transcript variant 1, mRNA [NM_003816]	NM_003816
A_24_P91701	0.0011	2.18	NM_016449	NM_016449	Homo sapiens hypothetical protein LOC51233 (LOC51233), mRNA [NM_016449]	NM_016449
A_23_P407112	0.0011	2.34	NM_145263	NM_145263	Homo sapiens spermatogenesis associated 18 homolog (rat) (SPATA18), mRNA [NM_145263]	NM_145263
A_23_P207967	0.0011	2.59	NM_014772	NM_014772	Homo sapiens KIAA0427 (KIAA0427), mRNA [NM_014772]	NM_014772
A_23_P436407	0.0011	2.71	NM_005333	NM_005333	Homo sapiens holocytochrome c synthase (cytochrome c heme-lyase) (HCCS), mRNA [NM_005333]	NM_005333
A_23_P99249	0.0011	2.83	NM_003848	NM_003848	Homo sapiens succinate-CoA ligase, GDP-forming, beta subunit (SUCLG2), mRNA [NM_003848]	NM_003848
A_23_P259892	0.0011	2.96	X66610	X66610	H.sapiens mRNA for enolase. [X66610]	
A_23_P100788	0.0011	3.77	NM_012448	NM_012448	Homo sapiens signal transducer and activator of transcription 5B (STAT5B), mRNA [NM_012448]	NM_012448
A_23_P68624	0.0011	4.27	NM_172236	NM_172236	Homo sapiens protein O-fucosyltransferase 1 (POFUT1), transcript variant 2, mRNA [NM_172236]	NM_172236
A_24_P316363	0.00111	2.11	NM_001521	NM_001521	Homo sapiens general transcription factor IIIC, polypeptide 2, beta 110kDa (GTF3C2), mRNA [NM_001521]	NM_001521
A_32_P137408	0.00111	2.12	THC2317808		Q8CFL1 (Q8CFL1) Stxbp4 protein (Syntaxin binding protein 4), partial (3%) [THC2317808]	
A_23_P155106	0.00111	2.19	NM_024821	NM_024821	Homo sapiens hypothetical protein FLJ22349 (FLJ22349), mRNA [NM_024821]	NM_024821
A_24_P359191	0.00111	2.35	NM_003043	NM_003043	Homo sapiens solute carrier family 6 (neurotransmitter transporter, taurine), member 6 (SLC6A6), mRNA [NM_003043]	NM_003043
A_32_P157531	0.00111	2.75	NM_024065	NM_024065	Homo sapiens phosducin-like 3 (PDCL3), mRNA [NM_024065]	NM_024065
A_32_P99715	0.00111	3.27	BU731317	BU731317	BU731317 UI-E-CI1-afr-o-04-0-UI.s1 UI-E-CI1 Homo sapiens cDNA clone UI-E-CI1-afr-o-04-0-UI 3', mRNA sequence [BU731317]	
A_23_P113283	0.00111	3.37	NM_022470	NM_022470	Homo sapiens p53 target zinc finger protein (WIG1), transcript variant 1, mRNA [NM_022470]	NM_022470
A_23_P322196	0.00111	3.48	NM_138400	NM_138400	Homo sapiens nucleolar protein with MIF4G domain 1 (NOM1), mRNA [NM_138400]	NM_138400
A_23_P47208	0.00112	2.02	NM_003860	NM_003860	Homo sapiens barrier to autointegration factor 1 (BANF1), mRNA [NM_003860]	NM_003860
A_23_P34115	0.00112	2.07	NM_004135	NM_004135	Homo sapiens isocitrate dehydrogenase 3 (NAD+) gamma (IDH3G), nuclear gene encoding mitochondrial protein, transcript variant 1, mRNA [NM_004135]	NM_004135
A_24_P375421	0.00112	2.13	NM_024798	NM_024798	Homo sapiens sorting nexin 22 (SNX22), mRNA [NM_024798]	NM_024798
A_24_P370887	0.00112	2.15	NM_004781	NM_004781	Homo sapiens vesicle-associated membrane protein 3 (cellubrevin) (VAMP3), mRNA [NM_004781]	NM_004781
A_32_P104518	0.00112	2.41	AF194971	AF194971	Homo sapiens unknown (NAG12) mRNA, complete cds. [AF194971]	XM_379656
A_23_P16089	0.00112	2.43	NM_004230	NM_004230	Homo sapiens endothelial differentiation, sphingolipid G-protein-coupled receptor, 5 (EDG5), mRNA [NM_004230]	NM_004230
A_23_P103996	0.00112	2.72	NM_002061	NM_002061	Homo sapiens glutamate-cysteine ligase, modifier subunit (GCLM), mRNA [NM_002061]	NM_002061
A_23_P13679	0.00112	3.12	NM_020782	NM_020782	Homo sapiens kelch domain containing 5 (KLHDC5), mRNA [NM_020782]	NM_020782
A_23_P96568	0.00112	3.18	NM_001456	NM_001456	Homo sapiens filamin A, alpha (actin binding protein 280) (FLNA), mRNA [NM_001456]	NM_001456
A_24_P141481	0.00112	3.77	NM_203330	NM_203330	Homo sapiens CD59 antigen p18-20 (antigen identified by monoclonal antibodies 16.3A5, EJ16, EJ30, EL32 and G344) (CD59), transcript variant 1, mRNA [NM_203330]	NM_203330
A_23_P38468	0.00112	4.48	NM_014604	NM_014604	Homo sapiens Tax1 (human T-cell leukemia virus type I) binding protein 3 (TAX1BP3), mRNA [NM_014604]	NM_014604
A_24_P334005	0.00113	2.03	NM_017887	NM_017887	Homo sapiens chromosome 1 open reading frame 123 (C1orf123), mRNA [NM_017887]	NM_017887
A_23_P94118	0.00113	2.23	NM_002095	NM_002095	Homo sapiens general transcription factor IIE, polypeptide 2, beta 34kDa (GTF2E2), mRNA [NM_002095]	NM_002095
A_23_P29263	0.00113	2.31	NM_152221	NM_152221	Homo sapiens casein kinase 1, epsilon (CSNK1E), transcript variant 1, mRNA [NM_152221]	NM_152221
A_23_P59210	0.00113	2.71	NM_000389	NM_000389	Homo sapiens cyclin-dependent kinase inhibitor 1A (p21, Cip1) (CDKN1A), transcript variant 1, mRNA [NM_000389]	NM_000389
A_24_P238896	0.00113	3.57	A_24_P238896			
A_23_P168167	0.00113	3.61	NM_032511	NM_032511	Homo sapiens chromosome 6 open reading frame 168 (C6orf168), mRNA [NM_032511]	NM_032511
A_24_P65616	0.00113	3.68	NM_006505	NM_006505	Homo sapiens poliovirus receptor (PVR), mRNA [NM_006505]	NM_006505
A_32_P22622	0.00113	3.75	NM_003703	NM_003703	Homo sapiens chromosome 4 open reading frame 9 (C4orf9), mRNA [NM_003703]	NM_003703
A_24_P255914	0.00114	2.16	A_24_P255914			
A_32_P141296	0.00114	2.20	BC019356	BC019356	Homo sapiens cDNA clone IMAGE:3503646. [BC019356]	
A_24_P9321	0.00114	2.62	NM_003533	NM_003533	Homo sapiens histone 1, H3i (HIST1H3I), mRNA [NM_003533]	NM_003533

A_32_P143980	0.00114	2.70	AA464246	AA464246	AA464246 zx81a12.s1 Soares ovary tumor NbHOT Homo sapiens cDNA clone IMAGE:810142 3' similar to gb:X58536 HLA CLASS I HISTOCOMPATIBILITY ANTIGEN, CW-4 CW*0401 ALPHA (HUMAN);, mRNA sequence [AA464246]	
A_32_P42213	0.00114	3.58	A_32_P42213			
A_23_P57588	0.00115	2.02	NM_016426	NM_016426	Homo sapiens G-2 and S-phase expressed 1 (GTSE1), mRNA [NM_016426]	NM_016426
A_23_P118370	0.00115	2.14	AK022252	AK022252	Homo sapiens cDNA FLJ12190 fis, clone MAMMA1000842. [AK022252]	
A_23_P156739	0.00115	2.26	NM_032340	NM_032340	Homo sapiens chromosome 6 open reading frame 125 (C6orf125), mRNA [NM_032340]	NM_032340
A_32_P359110	0.00115	2.30	NM_198317	NM_198317	Homo sapiens kelch-like 17 (Drosophila) (KLHL17), mRNA [NM_198317]	NM_198317
A_23_P3775	0.00115	2.34	NM_018233	NM_018233	Homo sapiens hypothetical protein FLJ10826 (FLJ10826), mRNA [NM_018233]	NM_018233
A_24_P350186	0.00115	2.39	NM_030630	NM_030630	Homo sapiens chromosome 17 open reading frame 28 (C17orf28), mRNA [NM_030630]	NM_030630
A_24_P33705	0.00115	2.59	NM_198432	NM_198432	Homo sapiens regulator of G-protein signalling 12 (RGS12), transcript variant 5, mRNA [NM_198432]	NM_198432
A_24_P349869	0.00115	2.85	A_24_P349869			
A_23_P311732	0.00115	2.92	NM_018841	NM_018841	Homo sapiens guanine nucleotide binding protein (G protein), gamma 12 (GNG12), mRNA [NM_018841]	NM_018841
A_32_P202042	0.00115	3.13	BC020340	BC020340	Homo sapiens anaphase promoting complex subunit 5, mRNA (cDNA clone IMAGE:3882030). [BC020340]	
A_23_P162171	0.00115	3.17	NM_006500	NM_006500	Homo sapiens melanoma cell adhesion molecule (MCAM), mRNA [NM_006500]	NM_006500
A_24_P161244	0.00115	4.27	NM_153649	NM_153649	Homo sapiens tropomyosin 3 (TPM3), transcript variant 2, mRNA [NM_153649]	NM_153649
A_32_P71858	0.00115	5.78	AK124776	AK124776	Homo sapiens cDNA FLJ42786 fis, clone BRAWH3006761. [AK124776]	
A_23_P41942	0.00115	10.14	NM_006467	NM_006467	Homo sapiens polymerase (RNA) III (DNA directed) polypeptide G (32kD) (POLR3G), mRNA [NM_006467]	NM_006467
A_23_P63402	0.00116	2.14	NM_013296	NM_013296	Homo sapiens G-protein signalling modulator 2 (AGS3-like, C. elegans) (GPSM2), mRNA [NM_013296]	NM_013296
A_24_P94222	0.00116	3.26	NM_017556	NM_017556	Homo sapiens filamin binding LIM protein 1 (FBLIM1), transcript variant 1, mRNA [NM_017556]	NM_017556
A_23_P49041	0.00116	4.18	NM_024956	NM_024956	Homo sapiens transmembrane protein 62 (TMEM62), mRNA [NM_024956]	NM_024956
A_23_P210948	0.00116	4.50	NM_018244	NM_018244	Homo sapiens chromosome 20 open reading frame 44 (C20orf44), transcript variant 1, mRNA [NM_018244]	NM_018244
A_23_P46964	0.00117	2.02	NM_017902	NM_017902	Homo sapiens hypoxia-inducible factor 1, alpha subunit inhibitor (HIF1AN), mRNA [NM_017902]	NM_017902
A_32_P200600	0.00117	2.10	AJ711693	AJ711693	AJ711693 AJ711693 CMPD01 Homo sapiens cDNA clone CMPD09562, mRNA sequence [AJ711693]	
A_23_P9443	0.00117	2.16	NM_016520	NM_016520	Homo sapiens chromosome 9 open reading frame 78 (C9orf78), transcript variant 2, mRNA [NM_016520]	NM_016520
A_24_P324986	0.00117	2.39	NM_001007234	NM_001007234	Homo sapiens excision repair cross-complementing rodent repair deficiency, complementation group 8 (ERCC8), transcript variant 3, mRNA [NM_001007234]	NM_001007234
A_23_P35099	0.00117	2.79	NM_016183	NM_016183	Homo sapiens chromosome 1 open reading frame 33 (C1orf33), mRNA [NM_016183]	NM_016183
A_32_P171043	0.00117	3.53	BI090438	BI090438	602853723F1 NIH_MGC_10 Homo sapiens cDNA clone IMAGE:4995269 5', mRNA sequence [BI090438]	
A_32_P192545	0.00117	4.10	NM_001006938	NM_001006938	Homo sapiens LOC158931 protein (LOC158931), mRNA [NM_001006938]	NM_001006938
A_23_P201655	0.00118	2.25	NM_012333	NM_012333	Homo sapiens c-myc binding protein (MYCBP), mRNA [NM_012333]	NM_012333
A_24_P188071	0.00118	2.36	NM_032704	NM_032704	Homo sapiens tubulin alpha 6 (TUBA6), mRNA [NM_032704]	NM_032704
A_24_P348203	0.00118	2.46	NM_025061	NM_025061	Homo sapiens leucine rich repeat containing 8 family, member E (LRRC8E), mRNA [NM_025061]	NM_025061
A_23_P256487	0.00118	26.67	THC2372784			
A_24_P76313	0.00119	2.16	NM_207334	NM_207334	Homo sapiens family with sequence similarity 43, member B (FAM43B), mRNA [NM_207334]	NM_207334
A_23_P48910	0.00119	2.53	NM_004809	NM_004809	Homo sapiens stomatin (EPB72)-like 1 (STOML1), mRNA [NM_004809]	NM_004809
A_32_P72968	0.00119	3.94	BG989839	BG989839	BG989839 PM0-HT0913-200101-004-f03 HT0913 Homo sapiens cDNA, mRNA sequence [BG989839]	
A_24_P583040	0.00119	4.17	BC041467	BC041467	Homo sapiens hypothetical protein LOC339210, mRNA (cDNA clone IMAGE:5215233), partial cds. [BC041467]	XM_378687
A_32_P80678	0.00119	4.95	NM_001005845	NM_001005845	Homo sapiens a disintegrin and metalloproteinase domain 9 (meltrin gamma) (ADAM9), transcript variant 2, mRNA [NM_001005845]	NM_001005845
A_23_P38876	0.0012	2.06	NM_005357	NM_005357	Homo sapiens lipase, hormone-sensitive (LIPE), mRNA [NM_005357]	NM_005357
A_24_P76805	0.0012	2.06	NM_205861	NM_205861	Homo sapiens dehydrodolichyl diphosphate synthase (DHDDS), transcript variant 2, mRNA [NM_205861]	NM_205861
A_23_P421465	0.0012	2.12	NM_015409	NM_015409	Homo sapiens E1A binding protein p400 (EP400), mRNA [NM_015409]	NM_015409
A_23_P165668	0.0012	2.22	AK025657	AK025657	Homo sapiens cDNA: FLJ22004 fis, clone HEP06871. [AK025657]	

A_24_P413735	0.0012	2.28	NM_018198	NM_018198	Homo sapiens DnaJ (Hsp40) homolog, subfamily C, member 11 (DNAJC11), mRNA [NM_018198]	NM_018198
A_24_P707102	0.0012	2.29	THC2283468		Q8WC13 (Q8WC13) ATPase 6 (Fragment), partial (7%) [THC2283468]	
A_24_P170869	0.0012	2.43	BC011136	BC011136	Homo sapiens, clone IMAGE:3448872, mRNA, partial cds. [BC011136]	
A_32_P79719	0.0012	2.58	THC2364880		Q9BYX7 (Q9BYX7) FKSG30, partial (41%) [THC2364880]	
A_24_P107277	0.0012	2.64	NM_006442	NM_006442	Homo sapiens DR1-associated protein 1 (negative cofactor 2 alpha) (DRAP1), mRNA [NM_006442]	NM_006442
A_23_P23457	0.0012	2.72	NM_017556	NM_017556	Homo sapiens filamin binding LIM protein 1 (FBLIM1), transcript variant 1, mRNA [NM_017556]	NM_017556
A_23_P98580	0.0012	2.75	NM_004265	NM_004265	Homo sapiens fatty acid desaturase 2 (FADS2), mRNA [NM_004265]	NM_004265
A_23_P416894	0.0012	2.77	ENST00000257626		Homo sapiens mRNA full length insert cDNA clone EUROIMAGE 293605. [AL079277]	
A_32_P57717	0.0012	2.83	AA292106	AA292106	AA292106 zr58h03.s1 Soares_NhHMPu_S1 Homo sapiens cDNA clone IMAGE:667637 3', mRNA sequence [AA292106]	
A_32_P86456	0.0012	3.06	BU616890	BU616890	UI-H-FH1-bfj-m-22-0-UI.s1 NCI_CGAP_FH1 Homo sapiens cDNA clone UI-H-FH1-bfj-m-22-0-UI 3', mRNA sequence [BU616890]	
A_24_P256050	0.0012	7.30	A_24_P256050			
A_32_P156564	0.00121	2.42	THC2312748			
A_32_P214441	0.00121	2.79	BM994377	BM994377	UI-H-DH0-aul-h-01-0-UI.s1 NCI_CGAP_DH0 Homo sapiens cDNA clone IMAGE:5871024 3', mRNA sequence [BM994377]	
A_24_P75700	0.00121	3.18	A_24_P75700			
A_23_P141429	0.00121	3.89	NM_016428	NM_016428	Homo sapiens ABI gene family, member 3 (ABI3), mRNA [NM_016428]	NM_016428
A_23_P117623	0.00122	2.07	NM_018319	NM_018319	Homo sapiens tyrosyl-DNA phosphodiesterase 1 (TDP1), transcript variant 1, mRNA [NM_018319]	NM_018319
A_24_P239731	0.00122	2.07	NM_004776	NM_004776	Homo sapiens UDP-Gal:betaGlcNAc beta 1,4- galactosyltransferase, polypeptide 5 (B4GALT5), mRNA [NM_004776]	NM_004776
A_23_P3681	0.00122	2.11	NM_018092	NM_018092	Homo sapiens neuropilin (NRP) and tolloid (TLL)-like 2 (NETO2), mRNA [NM_018092]	NM_018092
A_24_P903378	0.00122	2.14	AV762338	AV762338	AV762338 MDS Homo sapiens cDNA clone MDSBKA11 5', mRNA sequence [AV762338]	
A_23_P133227	0.00122	2.20	NM_002109	NM_002109	Homo sapiens histidyl-tRNA synthetase (HARS), mRNA [NM_002109]	NM_002109
A_23_P131463	0.00122	2.27	NM_016552	NM_016552	Homo sapiens ankyrin repeat and MYND domain containing 1 (ANKMY1), transcript variant 1, mRNA [NM_016552]	NM_016552
A_23_P206822	0.00122	2.31	NM_015171	NM_015171	Homo sapiens exportin 6 (XPO6), mRNA [NM_015171]	NM_015171
A_23_P257905	0.00122	2.36	NM_001810	NM_001810	Homo sapiens centromere protein B, 80kDa (CENPB), mRNA [NM_001810]	NM_001810
A_23_P254648	0.00122	2.39	NM_012164	NM_012164	Homo sapiens F-box and WD-40 domain protein 2 (FBXW2), mRNA [NM_012164]	NM_012164
A_24_P383523	0.00122	2.75	NM_015589	NM_015589	Homo sapiens sterile alpha motif domain containing 4 (SAMD4), mRNA [NM_015589]	NM_015589
A_24_P457912	0.00122	3.12	NM_080476	NM_080476	Homo sapiens CDC91 cell division cycle 91-like 1 (S. cerevisiae) (CDC91L1), mRNA [NM_080476]	NM_080476
A_32_P154091	0.00122	3.13	AK124295	AK124295	Homo sapiens cDNA FLJ42301 fis, clone TOVAR2002514. [AK124295]	XM_374260
A_23_P65401	0.00122	3.58	NM_021914	NM_021914	Homo sapiens cofilin 2 (muscle) (CFL2), transcript variant 1, mRNA [NM_021914]	NM_021914
A_32_P223644	0.00123	2.18	NM_025083	NM_025083	Homo sapiens hypothetical protein FLJ21128 (FLJ21128), mRNA [NM_025083]	NM_025083
A_23_P54064	0.00123	2.39	NM_019852	NM_019852	Homo sapiens methyltransferase like 3 (METTL3), mRNA [NM_019852]	NM_019852
A_23_P12950	0.00123	2.73	NM_016506	NM_016506	Homo sapiens kelch repeat and BTB (POZ) domain containing 4 (KBTBD4), transcript variant 2, mRNA [NM_016506]	NM_016506
A_24_P266037	0.00123	2.92	NM_014473	NM_014473	Homo sapiens dimethyladenosine transferase (HSA9761), mRNA [NM_014473]	NM_014473
A_32_P33802	0.00123	3.83	AA219130	AA219130	AA219130 zq99g05.s1 Stratagene NT2 neuronal precursor 937230 Homo sapiens cDNA clone IMAGE:650168 3', mRNA sequence [AA219130]	
A_23_P119196	0.00123	4.90	NM_016270	NM_016270	Homo sapiens Kruppel-like factor 2 (lung) (KLF2), mRNA [NM_016270]	NM_016270
A_23_P211302	0.00123	6.80	NM_033661	NM_033661	Homo sapiens WD repeat domain 4 (WDR4), transcript variant 2, mRNA [NM_033661]	NM_033661
A_32_P25889	0.00123	71.43	BC036938	BC036938	Homo sapiens, clone IMAGE:5404753, mRNA. [BC036938]	
A_24_P344711	0.00124	2.11	NM_020132	NM_020132	Homo sapiens 1-acylglycerol-3-phosphate O-acyltransferase 3 (AGPAT3), mRNA [NM_020132]	NM_020132
A_32_P97149	0.00124	2.33	THC2282995		AF055376 short form transcription factor C-MAF {Homo sapiens;} , partial (7%) [THC2282995]	
A_32_P159939	0.00124	2.56	THC2378276		Q7QA91 (Q7QA91) ENSANGP00000012641 (Fragment), partial (4%) [THC2378276]	
A_23_P330486	0.00124	2.68	NM_152416	NM_152416	Homo sapiens chromosome 8 open reading frame 38 (C8orf38), mRNA [NM_152416]	NM_152416

A_24_P919633	0.00124	2.85	AF281279	AF281279	Homo sapiens unknown mRNA, complete cds. [AF281279]	
A_24_P313597	0.00124	3.58	NM_003766	NM_003766	Homo sapiens beclin 1 (coiled-coil, myosin-like BCL2 interacting protein) (BECN1), mRNA [NM_003766]	NM_003766
A_23_P385673	0.00124	3.82	X89399	X89399	Homo sapiens mRNA for Ins(1,3,4,5)P4-binding protein. [X89399]	
A_23_P409462	0.00124	14.71	NM_173674	NM_173674	Homo sapiens discoidin, CUB and LCCL domain containing 1 (DCBLD1), mRNA [NM_173674]	NM_173674
A_23_P218685	0.00125	2.49	NM_199203	NM_199203	Homo sapiens ubiquitin-conjugating enzyme E2 variant 1 (Kua-UEV), transcript variant 1, mRNA [NM_199203]	NM_199203
A_23_P218523	0.00125	2.66	AF218008	AF218008	Homo sapiens clone PP3501 unknown mRNA. [AF218008]	
A_24_P228579	0.00125	2.72	NM_199052	NM_199052	Homo sapiens chromosome 20 open reading frame 7 (C20orf7), transcript variant 2, mRNA [NM_199052]	NM_199052
A_23_P319492	0.00126	2.03	NM_024556	NM_024556	Homo sapiens hypothetical protein FLJ21103 (FLJ21103), mRNA [NM_024556]	NM_024556
A_23_P36397	0.00126	2.23	NM_000785	NM_000785	Homo sapiens cytochrome P450, family 27, subfamily B, polypeptide 1 (CYP27B1), nuclear gene encoding mitochondrial protein, mRNA [NM_000785]	NM_000785
A_23_P109201	0.00127	2.15	NM_020531	NM_020531	Homo sapiens chromosome 20 open reading frame 3 (C20orf3), mRNA [NM_020531]	NM_020531
A_23_P70409	0.00127	2.16	NM_004875	NM_004875	Homo sapiens polymerase (RNA) I polypeptide C, 30kDa (POLR1C), transcript variant 2, mRNA [NM_004875]	NM_004875
A_23_P89824	0.00127	2.44	NM_032933	NM_032933	Homo sapiens chromosome 18 open reading frame 45 (C18orf45), mRNA [NM_032933]	NM_032933
A_24_P391260	0.00127	2.48	NM_004339	NM_004339	Homo sapiens pituitary tumor-transforming 1 interacting protein (PTTG1IP), mRNA [NM_004339]	NM_004339
A_23_P24555	0.00127	2.89	NM_015157	NM_015157	Homo sapiens pleckstrin homology-like domain, family B, member 1 (PHLDB1), mRNA [NM_015157]	NM_015157
A_24_P311917	0.00127	2.94	NM_006994	NM_006994	Homo sapiens butyrophilin, subfamily 3, member A3 (BTN3A3), transcript variant 1, mRNA [NM_006994]	NM_006994
A_23_P413641	0.00127	3.07	NM_020820	NM_020820	Homo sapiens phosphatidylinositol 3,4,5-trisphosphate-dependent RAC exchanger 1 (PREX1), mRNA [NM_020820]	NM_020820
A_32_P98534	0.00127	3.09	NM_021633	NM_021633	Homo sapiens kelch-like 12 (Drosophila) (KLHL12), mRNA [NM_021633]	NM_021633
A_32_P181222	0.00127	12.95	NM_002247	NM_002247	Homo sapiens potassium large conductance calcium-activated channel, subfamily M, alpha member 1 (KCNMA1), transcript variant 2, mRNA [NM_002247]	NM_002247
A_24_P608468	0.00128	2.03	AK056793	AK056793	Homo sapiens cDNA FLJ32231 fis, clone PLACE6004491. [AK056793]	
A_23_P5995	0.00128	2.30	NM_006420	NM_006420	Homo sapiens ADP-ribosylation factor guanine nucleotide-exchange factor 2 (brefeldin A-inhibited) (ARFGEF2), mRNA [NM_006420]	NM_006420
A_23_P138856	0.00128	2.32	NM_006442	NM_006442	Homo sapiens DR1-associated protein 1 (negative cofactor 2 alpha) (DRAP1), mRNA [NM_006442]	NM_006442
A_23_P47863	0.00128	2.35	NM_016551	NM_016551	Homo sapiens transmembrane 7 superfamily member 3 (TM7SF3), mRNA [NM_016551]	NM_016551
A_23_P349203	0.00128	2.54	AK027172	AK027172	Homo sapiens cDNA: FLJ23519 fis, clone LNG04908. [AK027172]	
A_23_P47867	0.00128	2.61	NM_003622	NM_003622	Homo sapiens PTPRF interacting protein, binding protein 1 (liprin beta 1) (PPFIBP1), transcript variant 1, mRNA [NM_003622]	NM_003622
A_23_P409626	0.00128	2.84	ENST00000360796		Homo sapiens mRNA; cDNA DKFZp762N1910 (from clone DKFZp762N1910). [AL834470]	XM_495877
A_23_P102058	0.00128	3.61	NM_002381	NM_002381	Homo sapiens matrilin 3 (MATN3), mRNA [NM_002381]	NM_002381
A_23_P15073	0.00129	2.14	NM_001005920	NM_001005920	Homo sapiens hypothetical LOC339123 (LOC339123), mRNA [NM_001005920]	NM_001005920
A_24_P925062	0.00129	2.44	NM_001008528	NM_001008528	Homo sapiens matrix-remodelling associated 7 (MXRA7), transcript variant 1, mRNA [NM_001008528]	NM_001008528
A_24_P242204	0.00129	2.59	NM_145799	NM_145799	Homo sapiens septin 6 (SEPT6), transcript variant I, mRNA [NM_145799]	NM_145799
A_23_P27721	0.00129	2.67	NM_014959	NM_014959	Homo sapiens caspase recruitment domain family, member 8 (CARD8), mRNA [NM_014959]	NM_014959
A_24_P375340	0.0013	2.10	A_24_P375340			
A_24_P61150	0.0013	2.18	NM_002247	NM_002247	Homo sapiens potassium large conductance calcium-activated channel, subfamily M, alpha member 1 (KCNMA1), transcript variant 2, mRNA [NM_002247]	NM_002247
A_23_P64184	0.0013	2.29	A_23_P64184			
A_23_P399501	0.0013	2.44	NM_182470	NM_182470	Homo sapiens pyruvate kinase, muscle (PKM2), transcript variant 2, mRNA [NM_182470]	NM_182470
A_24_P126471	0.0013	2.68	NM_020246	NM_020246	Homo sapiens solute carrier family 12 (potassium/chloride transporters), member 9 (SLC12A9), mRNA [NM_020246]	NM_020246
A_24_P264004	0.0013	2.92	A_24_P264004			
A_23_P258531	0.0013	2.95	NM_002637	NM_002637	Homo sapiens phosphorylase kinase, alpha 1 (muscle) (PHKA1), mRNA [NM_002637]	NM_002637
A_23_P327519	0.0013	3.38	NM_139164	NM_139164	Homo sapiens START domain containing 4, sterol regulated (STARD4), mRNA [NM_139164]	NM_139164
A_32_P59475	0.0013	3.88	BQ019626	BQ019626	BQ019626 UI-H-ED0-axd-f-18-0-UI.s1 NCI_CGAP_ED0 Homo sapiens cDNA clone IMAGE:5827217 3', mRNA sequence [BQ019626]	

A_23_P124456	0.0013	3.98	NM_006203	NM_006203	Homo sapiens phosphodiesterase 4D, cAMP-specific (phosphodiesterase E3 dunce homolog, Drosophila) (PDE4D), mRNA [NM_006203]	NM_006203
A_24_P90349	0.00131	2.29	NM_174940	NM_174940	Homo sapiens hypothetical protein LOC283232 (LOC283232), mRNA [NM_174940]	NM_174940
A_23_P21560	0.00131	2.36	NM_030797	NM_030797	Homo sapiens family with sequence similarity 49, member A (FAM49A), mRNA [NM_030797]	NM_030797
A_23_P58937	0.00131	2.46	NM_012288	NM_012288	Homo sapiens translocation associated membrane protein 2 (TRAM2), mRNA [NM_012288]	NM_012288
A_23_P230	0.00131	2.53	NM_004623	NM_004623	Homo sapiens tetratricopeptide repeat domain 4 (TTC4), mRNA [NM_004623]	NM_004623
A_23_P66787	0.00131	2.60	U18197	U18197	Human ATP:citrate lyase mRNA, complete cds. [U18197]	
A_24_P383769	0.00131	2.92	A_24_P383769			
A_24_P84880	0.00131	3.09	ENST00000332444		Homo sapiens cDNA FLJ31247 fis, clone KIDNE2005296, weakly similar to ACTIN, CYTOPLASMIC 1. [AK055809]	
A_24_P75994	0.00131	3.48	A_24_P75994			
A_24_P754999	0.00132	2.23	BU622073	BU622073	BU622073 UI-H-FH1-bfu-a-21-0-UI.s1 NCI_CGAP_FH1 Homo sapiens cDNA clone UI-H-FH1-bfu-a-21-0-UI 3', mRNA sequence [BU622073]	
A_23_P356526	0.00132	2.43	NM_033092	NM_033092	Homo sapiens tripartite motif-containing 5 (TRIM5), transcript variant gamma, mRNA [NM_033092]	NM_033092
A_32_P18668	0.00132	2.62	NM_002867	NM_002867	Homo sapiens RAB3B, member RAS oncogene family (RAB3B), mRNA [NM_002867]	NM_002867
A_32_P120567	0.00132	2.79	NM_199072	NM_199072	Homo sapiens MyoD family inhibitor domain containing (MDFIC), mRNA [NM_199072]	NM_199072
A_24_P184931	0.00132	2.98	NM_016561	NM_016561	Homo sapiens bifunctional apoptosis regulator (BFAR), mRNA [NM_016561]	NM_016561
A_24_P2648	0.00132	4.93	NM_005401	NM_005401	Homo sapiens protein tyrosine phosphatase, non-receptor type 14 (PTPN14), mRNA [NM_005401]	NM_005401
A_23_P116614	0.00132	6.45	NM_001014811	NM_001014811	Homo sapiens malic enzyme 3, NADP(+)-dependent, mitochondrial (ME3), nuclear gene encoding mitochondrial protein, transcript variant 2, mRNA [NM_001014811]	NM_001014811
A_32_P456537	0.00133	2.25	NM_030752	NM_030752	Homo sapiens t-complex 1 (TCP1), transcript variant 1, mRNA [NM_030752]	NM_030752
A_24_P139861	0.00133	2.28	NM_018207	NM_018207	Homo sapiens tripartite motif-containing 62 (TRIM62), mRNA [NM_018207]	NM_018207
A_24_P7330	0.00133	2.45	A_24_P7330			
A_24_P109351	0.00133	3.10	NM_001009814	NM_001009814	Homo sapiens KIAA0564 protein (KIAA0564), transcript variant 2, mRNA [NM_001009814]	NM_001009814
A_32_P101110	0.00133	5.85	AF086187	AF086187	Homo sapiens full length insert cDNA clone ZC30H06. [AF086187]	
A_23_P207999	0.00133	7.30	NM_021127	NM_021127	Homo sapiens phorbol-12-myristate-13-acetate-induced protein 1 (PMAIP1), mRNA [NM_021127]	NM_021127
A_24_P211351	0.00134	2.13	A_24_P211351			
A_24_P336577	0.00134	2.35	NM_019099	NM_019099	Homo sapiens hypothetical protein LOC55924 (LOC55924), transcript variant 1, mRNA [NM_019099]	NM_019099
A_24_P187154	0.00134	2.59	A_24_P187154			
A_23_P96985	0.00134	2.66	NM_012486	NM_012486	Homo sapiens presenilin 2 (Alzheimer disease 4) (PSEN2), transcript variant 2, mRNA [NM_012486]	NM_012486
A_23_P6878	0.00134	2.81	NM_004186	NM_004186	Homo sapiens sema domain, immunoglobulin domain (Ig), short basic domain, secreted, (semaphorin) 3F (SEMA3F), mRNA [NM_004186]	NM_004186
A_23_P65041	0.00134	3.00	AF334184	AF334184	Homo sapiens FKSG42 (FKSG42) mRNA, complete cds. [AF334184]	
A_32_P61936	0.00134	8.93	BC039676	BC039676	Homo sapiens, clone IMAGE:5173389, mRNA. [BC039676]	XM_373518
A_23_P329971	0.00135	2.44	NM_032483	NM_032483	Homo sapiens phosphatidic acid phosphatase type 2 domain containing 1B (PPAPDC1B), mRNA [NM_032483]	NM_032483
A_32_P117758	0.00135	2.68	BG208131	BG208131	RST27621 Athersys RAGE Library Homo sapiens cDNA, mRNA sequence [BG208131]	
A_24_P127641	0.00135	3.01	AK128882	AK128882	Homo sapiens cDNA FLJ46914 fis, clone SPLEN2027852. [AK128882]	XM_499014
A_24_P20200	0.00135	3.70	NM_017958	NM_017958	Homo sapiens pleckstrin homology domain containing, family B (evectins) member 2 (PLEKHB2), mRNA [NM_017958]	NM_017958
A_23_P208595	0.00135	4.20	NM_000527	NM_000527	Homo sapiens low density lipoprotein receptor (familial hypercholesterolemia) (LDLR), mRNA [NM_000527]	NM_000527
A_23_P212688	0.00136	2.02	NM_005241	NM_005241	Homo sapiens ecotropic viral integration site 1 (EVI1), mRNA [NM_005241]	NM_005241
A_23_P206684	0.00136	2.03	NM_199424	NM_199424	Homo sapiens WW domain containing E3 ubiquitin protein ligase 2 (WWP2), transcript variant 2, mRNA [NM_199424]	NM_199424
A_32_P145010	0.00136	2.39	CR594811	CR594811	full-length cDNA clone CS0DL006YD08 of B cells (Ramos cell line) Cot 25-normalized of Homo sapiens (human). [CR594811]	
A_23_P5359	0.00136	3.24	NM_015650	NM_015650	Homo sapiens TNF receptor-associated factor 3 interacting protein 1 (TRAF3IP1), mRNA [NM_015650]	NM_015650
A_32_P162250	0.00136	3.26	NM_033515	NM_033515	Homo sapiens Rho GTPase activating protein 18 (ARHGAP18), mRNA [NM_033515]	NM_033515

A_23_P133386	0.00136	12.35	NM_006909	NM_006909	Homo sapiens Ras protein-specific guanine nucleotide-releasing factor 2 (RASGRF2), mRNA [NM_006909]	NM_006909
A_23_P502274	0.00137	3.15	NM_002751	NM_002751	Homo sapiens mitogen-activated protein kinase 11 (MAPK11), transcript variant 1, mRNA [NM_002751]	NM_002751
A_24_P234196	0.00137	4.83	NM_001034	NM_001034	Homo sapiens ribonucleotide reductase M2 polypeptide (RRM2), mRNA [NM_001034]	NM_001034
A_23_P58396	0.00137	5.38	NM_016205	NM_016205	Homo sapiens platelet derived growth factor C (PDGFC), mRNA [NM_016205]	NM_016205
A_23_P29566	0.00137	6.13	NM_005718	NM_005718	Homo sapiens actin related protein 2/3 complex, subunit 4, 20kDa (ARPC4), transcript variant 1, mRNA [NM_005718]	NM_005718
A_32_P58361	0.00137	13.83	THC2276504		Q9UID7 (Q9UID7) CYR61 protein, partial (87%) [THC2276504]	
A_24_P288993	0.00138	2.33	ENST00000244249		PREDICTED: Homo sapiens similar to NADH dehydrogenase (ubiquinone) 1 beta subcomplex, 4, 15kDa; ADH-ubiquinone oxidoreductase B15 subunit; NADH dehydrogenase (ubiquinone) 1 beta subcomplex, 4 (15kD, B15) (LOC402175), mRNA [XM_497963]	XM_497963
A_23_P355244	0.00138	2.55	NM_017654	NM_017654	Homo sapiens sterile alpha motif domain containing 9 (SAMD9), mRNA [NM_017654]	NM_017654
A_24_P192978	0.00138	2.67	NM_173583	NM_173583	Homo sapiens hypothetical protein FLJ33790 (FLJ33790), mRNA [NM_173583]	NM_173583
A_24_P926314	0.00138	3.23	NM_199133	NM_199133	Homo sapiens hypothetical protein LOC134145 (LOC134145), mRNA [NM_199133]	NM_199133
A_23_P5392	0.00138	3.89	NM_004881	NM_004881	Homo sapiens tumor protein p53 inducible protein 3 (TP53I3), transcript variant 1, mRNA [NM_004881]	NM_004881
A_23_P97457	0.00139	2.03	NM_022831	NM_022831	Homo sapiens chromosome 1 open reading frame 80 (C1orf80), mRNA [NM_022831]	NM_022831
A_24_P401381	0.00139	3.12	A_24_P401381			
A_24_P81695	0.0014	2.05	NM_014758	NM_014758	Homo sapiens sorting nexin 19 (SNX19), mRNA [NM_014758]	NM_014758
A_23_P25534	0.0014	2.15	THC2310223		Q5VW08 (Q5VW08) OTTHUMP00000042245, partial (19%) [THC2310223]	
A_24_P39508	0.0014	2.17	NM_144601	NM_144601	Homo sapiens chemokine-like factor super family 3 (CKLFSF3), transcript variant 1, mRNA [NM_144601]	NM_144601
A_32_P195456	0.0014	2.17	AI263083	AI263083	AI263083 qz35a02.x1 NCI_CGAP_Kid11 Homo sapiens cDNA clone IMAGE:2028842 3', mRNA sequence [AI263083]	
A_23_P94736	0.0014	2.27	NM_175039	NM_175039	Homo sapiens ST6 (alpha-N-acetyl-neuraminyl-2,3-beta-galactosyl-1,3)-N-acetylgalactosaminide alpha-2,6-sialyltransferase 4 (ST6GALNAC4), transcript variant 2, mRNA [NM_175039]	NM_175039
A_23_P143274	0.0014	2.66	NM_024958	NM_024958	Homo sapiens chromosome 20 open reading frame 98 (C20orf98), mRNA [NM_024958]	NM_024958
A_24_P295245	0.0014	2.74	NM_032467	NM_032467	Homo sapiens aspartate beta-hydroxylase (ASPH), transcript variant 4, mRNA [NM_032467]	NM_032467
A_23_P129301	0.0014	2.84	NM_018097	NM_018097	Homo sapiens chromosome 15 open reading frame 25 (C15orf25), mRNA [NM_018097]	NM_018097
A_23_P109143	0.0014	3.04	NM_000311	NM_000311	Homo sapiens prion protein (p27-30) (Creutzfeld-Jakob disease, Gerstmann-Strausler-Scheinker syndrome, fatal familial insomnia) (PRNP), transcript variant 1, mRNA [NM_000311]	NM_000311
A_32_P214795	0.00141	2.48	AK128072	AK128072	Homo sapiens cDNA FLJ46193 fis, clone TESTI4006234. [AK128072]	
A_23_P502553	0.00142	2.01	NM_171982	NM_171982	Homo sapiens tripartite motif-containing 35 (TRIM35), transcript variant 2, mRNA [NM_171982]	NM_171982
A_23_P253571	0.00142	2.41	NM_032758	NM_032758	Homo sapiens PHD finger protein 5A (PHF5A), mRNA [NM_032758]	NM_032758
A_24_P204257	0.00142	2.42	A_24_P204257			
A_24_P100277	0.00142	2.50	NM_007326	NM_007326	Homo sapiens cytochrome b5 reductase 3 (CYB5R3), transcript variant S, mRNA [NM_007326]	NM_007326
A_24_P79808	0.00142	2.58	NM_020524	NM_020524	Homo sapiens pre-B-cell leukemia transcription factor interacting protein 1 (PBXIP1), mRNA [NM_020524]	NM_020524
A_24_P135769	0.00142	3.79	NM_198315	NM_198315	Homo sapiens loss of heterozygosity, 11, chromosomal region 2, gene A (LOH11CR2A), transcript variant 2, mRNA [NM_198315]	NM_198315
A_32_P58280	0.00142	3.91	NM_001013848	NM_001013848	Homo sapiens SEC15-like 1 (S. cerevisiae) (SEC15L1), transcript variant 2, mRNA [NM_001013848]	NM_001013848
A_24_P945113	0.00142	5.81	CR596233	CR596233	full-length cDNA clone CS0DF012YN06 of Fetal brain of Homo sapiens (human). [CR596233]	
A_23_P105227	0.00142	10.62	NM_001014811	NM_001014811	Homo sapiens malic enzyme 3, NADP(+)-dependent, mitochondrial (ME3), nuclear gene encoding mitochondrial protein, transcript variant 2, mRNA [NM_001014811]	NM_001014811
A_32_P18547	0.00143	2.40	NM_058181	NM_058181	Homo sapiens chromosome 21 open reading frame 57 (C21orf57), transcript variant 1, mRNA [NM_058181]	NM_058181
A_23_P311468	0.00143	2.44	NM_182498	NM_182498	Homo sapiens hypothetical protein MGC51082 (MGC51082), mRNA [NM_182498]	NM_182498
A_24_P42681	0.00143	3.27	NM_002808	NM_002808	Homo sapiens proteasome (prosome, macropain) 26S subunit, non-ATPase, 2 (PSMD2), mRNA [NM_002808]	NM_002808
A_24_P15292	0.00143	3.70	NM_032018	NM_032018	Homo sapiens chromosome 1 open reading frame 124 (C1orf124), transcript variant 1, mRNA [NM_032018]	NM_032018
A_24_P332647	0.00143	3.85	AK095421	AK095421	Homo sapiens cDNA FLJ38102 fis, clone D3OST2000618, moderately similar to Drosophila melanogaster slingshot mRNA. [AK095421]	

A_23_P21324	0.00143	6.25	NM_057179	NM_057179	Homo sapiens twist homolog 2 (Drosophila) (TWIST2), mRNA [NM_057179]	NM_057179
A_24_P881527	0.00144	2.00	CR749275	CR749275	Homo sapiens mRNA; cDNA DKFZp781O2021 (from clone DKFZp781O2021). [CR749275]	
A_24_P913947	0.00144	2.07	ENST00000291567		Human glucose-6-phosphate dehydrogenase variant A-, partial cds. [M19866]	
A_23_P25644	0.00144	2.11	NM_018386	NM_018386	Homo sapiens hypothetical protein FLJ11305 (FLJ11305), mRNA [NM_018386]	NM_018386
A_23_P28105	0.00144	2.23	NM_004622	NM_004622	Homo sapiens translin (TSN), mRNA [NM_004622]	NM_004622
A_24_P354715	0.00144	20.37	NM_002526	NM_002526	Homo sapiens 5'-nucleotidase, ecto (CD73) (NT5E), mRNA [NM_002526]	NM_002526
A_24_P83544	0.00145	2.24	ENST00000242916		GB|AL136318.13|CAC10197.1 dJ254P11.1 (novel protein similar to RNA polymerase II elongation factor SIII, subunit P15) [NP239768]	
A_24_P378368	0.00145	2.39	NM_003791	NM_003791	Homo sapiens membrane-bound transcription factor protease, site 1 (MBTPS1), transcript variant 1, mRNA [NM_003791]	NM_003791
A_23_P126388	0.00145	2.42	NM_031286	NM_031286	Homo sapiens SH3 domain binding glutamic acid-rich protein like 3 (SH3BGRL3), mRNA [NM_031286]	NM_031286
A_24_P306136	0.00145	3.14	AL136875	AL136875	Homo sapiens mRNA; cDNA DKFZp434D105 (from clone DKFZp434D105). [AL136875]	
A_24_P176173	0.00145	5.43	NM_000428	NM_000428	Homo sapiens latent transforming growth factor beta binding protein 2 (LTBP2), mRNA [NM_000428]	NM_000428
A_23_P156289	0.00145	6.41	NM_003999	NM_003999	Homo sapiens oncostatin M receptor (OSMR), mRNA [NM_003999]	NM_003999
A_24_P396340	0.00146	3.75	NM_138428	NM_138428	Homo sapiens hypothetical protein BC011880 (LOC113444), mRNA [NM_138428]	NM_138428
A_24_P168726	0.00146	4.74	NM_181831	NM_181831	Homo sapiens neurofibromin 2 (bilateral acoustic neuroma) (NF2), transcript variant 13, mRNA [NM_181831]	NM_181831
A_23_P425073	0.00147	2.02	NM_002898	NM_002898	Homo sapiens RNA binding motif, single stranded interacting protein 2 (RBMS2), mRNA [NM_002898]	NM_002898
A_24_P346431	0.00147	2.16	AK092864	AK092864	Homo sapiens cDNA FLJ35545 fis, clone SPLEN2003918, moderately similar to TENSIN. [AK092864]	
A_24_P313678	0.00147	2.40	NM_016060	NM_016060	Homo sapiens mediator of RNA polymerase II transcription, subunit 31 homolog (yeast) (MED31), mRNA [NM_016060]	NM_016060
A_23_P74178	0.00148	2.01	NM_001525	NM_001525	Homo sapiens hypocretin (orexin) receptor 1 (HCRTR1), mRNA [NM_001525]	NM_001525
A_24_P136683	0.00148	2.05	BC021816	BC021816	Homo sapiens carbonic anhydrase VB-like, mRNA (cDNA clone IMAGE:3460742), partial cds. [BC021816]	XM_291346
A_24_P43391	0.00148	2.13	NM_018475	NM_018475	Homo sapiens TPA regulated locus (TPARL), mRNA [NM_018475]	NM_018475
A_24_P569294	0.00148	2.14	NM_021107	NM_021107	Homo sapiens mitochondrial ribosomal protein S12 (MRPS12), nuclear gene encoding mitochondrial protein, transcript variant 1, mRNA [NM_021107]	NM_021107
A_23_P152487	0.00148	2.20	NM_005548	NM_005548	Homo sapiens lysyl-tRNA synthetase (KARS), mRNA [NM_005548]	NM_005548
A_32_P93792	0.00148	2.67	A_32_P93792			
A_23_P157240	0.00148	2.75	J05272	J05272	Human IMP dehydrogenase type 1 mRNA complete cds. [J05272]	
A_24_P342086	0.00148	2.98	NM_199423	NM_199423	Homo sapiens WW domain containing E3 ubiquitin protein ligase 2 (WWP2), transcript variant 3, mRNA [NM_199423]	NM_199423
A_24_P315306	0.00148	3.11	AK096327	AK096327	Homo sapiens cDNA FLJ39008 fis, clone NT2RI2025255. [AK096327]	XM_091331
A_24_P578445	0.00148	3.62	A_24_P578445			
A_23_P434442	0.00148	3.73	NM_001006933	NM_001006933	Homo sapiens transcription elongation factor A (SII)-like 3 (TCEAL3), transcript variant 1, mRNA [NM_001006933]	NM_001006933
A_23_P213944	0.00148	4.05	NM_001945	NM_001945	Homo sapiens heparin-binding EGF-like growth factor (HBEGF), mRNA [NM_001945]	NM_001945
A_24_P16071	0.00148	5.59	A_24_P16071			
A_24_P414446	0.00149	2.11	NM_138348	NM_138348	Homo sapiens hypothetical protein BC007706 (LOC90268), mRNA [NM_138348]	NM_138348
A_24_P71021	0.00149	2.15	NM_002074	NM_002074	Homo sapiens guanine nucleotide binding protein (G protein), beta polypeptide 1 (GNB1), mRNA [NM_002074]	NM_002074
A_32_P91273	0.00149	2.16	AK000085	AK000085	Homo sapiens cDNA FLJ20078 fis, clone COL02974. [AK000085]	
A_24_P399680	0.00149	2.44	NM_080821	NM_080821	Homo sapiens chromosome 20 open reading frame 108 (C20orf108), mRNA [NM_080821]	NM_080821
A_23_P18887	0.00149	2.46	NM_022132	NM_022132	Homo sapiens methylcrotonoyl-Coenzyme A carboxylase 2 (beta) (MCCC2), mRNA [NM_022132]	NM_022132
A_32_P46351	0.00149	2.57	THC2436745		RSF1_HUMAN (Q9NS23) Ras association domain family 1 (Ras association, RalGDS/AF-6, domain family 1), partial (11%) [THC2436745]	
A_32_P224840	0.00149	3.50	NM_001012398	NM_001012398	Homo sapiens fused toes homolog (mouse) (FTS), transcript variant 1, mRNA [NM_001012398]	NM_001012398
A_32_P120638	0.0015	2.07	NM_033547	NM_033547	Homo sapiens hypothetical gene MGC16733 similar to CG12113 (MGC16733), mRNA [NM_033547]	NM_033547
A_23_P139547	0.0015	2.07	NM_006009	NM_006009	Homo sapiens tubulin, alpha 3 (TUBA3), mRNA [NM_006009]	NM_006009
A_24_P195621	0.0015	2.42	A_24_P195621			

A_24_P29975	0.0015	2.53	NM_030978	NM_030978	Homo sapiens actin related protein 2/3 complex, subunit 5-like (ARPC5L), mRNA [NM_030978]	NM_030978
A_23_P325932	0.0015	2.62	NM_004371	NM_004371	Homo sapiens coatomer protein complex, subunit alpha (COPA), mRNA [NM_004371]	NM_004371
A_23_P167692	0.0015	2.73	NM_002752	NM_002752	Homo sapiens mitogen-activated protein kinase 9 (MAPK9), transcript variant 1, mRNA [NM_002752]	NM_002752
A_23_P58321	0.0015	2.73	NM_001237	NM_001237	Homo sapiens cyclin A2 (CCNA2), mRNA [NM_001237]	NM_001237
A_24_P142743	0.0015	3.23	NM_004368	NM_004368	Homo sapiens calponin 2 (CNN2), transcript variant 1, mRNA [NM_004368]	NM_004368
A_32_P163169	0.0015	3.42	NM_003374	NM_003374	Homo sapiens voltage-dependent anion channel 1 (VDAC1), mRNA [NM_003374]	NM_003374
A_23_P147106	0.0015	3.44	NM_017723	NM_017723	Homo sapiens hypothetical protein FLJ20245 (FLJ20245), mRNA [NM_017723]	NM_017723
A_32_P82807	0.0015	3.89	NM_020382	NM_020382	Homo sapiens PR/SET domain containing protein 8 (SET8), mRNA [NM_020382]	NM_020382
A_24_P407742	0.0015	4.07	A_24_P407742			
A_23_P304524	0.0015	4.15	NM_152619	NM_152619	Homo sapiens doublecortin and CaM kinase-like 2 (DCAMKL2), mRNA [NM_152619]	NM_152619
A_23_P84344	0.00151	2.05	NM_021805	NM_021805	Homo sapiens single Ig IL-1R-related molecule (SIGIRR), mRNA [NM_021805]	NM_021805
A_23_P45864	0.00151	2.13	NM_003285	NM_003285	Homo sapiens tenascin R (restrictin, janusin) (TNR), mRNA [NM_003285]	NM_003285
A_24_P532180	0.00151	2.31	AK023635	AK023635	Homo sapiens cDNA FLJ13573 fis, clone PLACE1008584. [AK023635]	
A_32_P213831	0.00151	3.80	NM_020704	NM_020704	Homo sapiens family with sequence similarity 40, member B (FAM40B), mRNA [NM_020704]	NM_020704
A_32_P17182	0.00151	7.41	N48043	N48043	N48043 yy23h06.r1 Soares melanocyte 2NbHM Homo sapiens cDNA clone IMAGE:272123 5' similar to gb:X04665_cds1 THROMBOSPONDIN 1 PRECURSOR (HUMAN);, mRNA sequence [N48043]	
A_23_P87461	0.00152	2.07	NM_001478	NM_001478	Homo sapiens UDP-N-acetyl-alpha-D-galactosamine:(N-acetylneuraminyl)-galactosylglucosylceramide N-acetylgalactosaminyltransferase (GalNAc-T) (GALGT), mRNA [NM_001478]	NM_001478
A_24_P925241	0.00152	2.20	AK000422	AK000422	Homo sapiens cDNA FLJ20415 fis, clone KAT02189. [AK000422]	
A_24_P174550	0.00152	2.64	NM_001664	NM_001664	Homo sapiens ras homolog gene family, member A (RHOA), mRNA [NM_001664]	NM_001664
A_24_P634530	0.00152	3.06	THC2281463			
A_23_P101655	0.00152	3.61	NM_004924	NM_004924	Homo sapiens actinin, alpha 4 (ACTN4), mRNA [NM_004924]	NM_004924
A_24_P364296	0.00152	4.33	NM_001980	NM_001980	Homo sapiens epimorphin (EPIM), transcript variant 1, mRNA [NM_001980]	NM_001980
A_24_P471121	0.00152	4.74	XM_497677	XM_497677	PREDICTED: Homo sapiens similar to GYG protein (LOC441885), mRNA [XM_497677]	XM_497677
A_23_P212781	0.00152	5.03	THC2364375		BC008083 SEPT11 protein {Homo sapiens;} , partial (17%) [THC2364375]	
A_32_P77989	0.00152	9.52	NM_018092	NM_018092	Homo sapiens neuropilin (NRP) and tolloid (TLL)-like 2 (NETO2), mRNA [NM_018092]	NM_018092
A_24_P258633	0.00153	2.10	NM_006086	NM_006086	Homo sapiens tubulin, beta 3 (TUBB3), mRNA [NM_006086]	NM_006086
A_23_P395493	0.00153	2.40	BC031631	BC031631	Homo sapiens cofilin pseudogene 1, mRNA (cDNA clone IMAGE:5168640). [BC031631]	
A_24_P257416	0.00153	3.29	NM_002089	NM_002089	Homo sapiens chemokine (C-X-C motif) ligand 2 (CXCL2), mRNA [NM_002089]	NM_002089
A_23_P121051	0.00154	2.15	NM_000532	NM_000532	Homo sapiens propionyl Coenzyme A carboxylase, beta polypeptide (PCCB), mRNA [NM_000532]	NM_000532
A_23_P29083	0.00154	2.26	NM_003056	NM_003056	Homo sapiens solute carrier family 19 (folate transporter), member 1 (SLC19A1), transcript variant 1, mRNA [NM_003056]	NM_003056
A_23_P259955	0.00154	2.27	NM_000557	NM_000557	Homo sapiens growth differentiation factor 5 (cartilage-derived morphogenetic protein-1) (GDF5), mRNA [NM_000557]	NM_000557
A_23_P218879	0.00154	2.35	NM_016381	NM_016381	Homo sapiens three prime repair exonuclease 1 (TREX1), transcript variant 1, mRNA [NM_016381]	NM_016381
A_23_P208706	0.00154	2.88	NM_138764	NM_138764	Homo sapiens BCL2-associated X protein (BAX), transcript variant epsilon, mRNA [NM_138764]	NM_138764
A_23_P138835	0.00155	2.33	NM_005186	NM_005186	Homo sapiens calpain 1, (mu/I) large subunit (CAPN1), mRNA [NM_005186]	NM_005186
A_24_P232500	0.00155	2.40	NM_004927	NM_004927	Homo sapiens mitochondrial ribosomal protein L49 (MRPL49), nuclear gene encoding mitochondrial protein, mRNA [NM_004927]	NM_004927
A_24_P353740	0.00155	2.60	NM_018704	NM_018704	Homo sapiens hypothetical protein DKFZp547A023 (DKFZp547A023), mRNA [NM_018704]	NM_018704
A_23_P101742	0.00155	2.74	NM_172251	NM_172251	Homo sapiens mitochondrial ribosomal protein L54 (MRPL54), nuclear gene encoding mitochondrial protein, mRNA [NM_172251]	NM_172251
A_23_P50241	0.00156	2.07	NM_001294	NM_001294	Homo sapiens cleft lip and palate associated transmembrane protein 1 (CLPTM1), mRNA [NM_001294]	NM_001294
A_23_P333552	0.00156	2.59	NM_182477	NM_182477	Homo sapiens gametogenetin (GGN), transcript variant 2, mRNA [NM_182477]	NM_182477

A_24_P582241	0.00157	2.07	THC2268341		HSU18930 MHC class I antigen HLA-A2 {Homo sapiens;} , partial (24%) [THC2268341]	
A_23_P408095	0.00157	2.42	NM_001011546	NM_001011546	Homo sapiens destrin (actin depolymerizing factor) (DSTN), transcript variant 2, mRNA [NM_001011546]	NM_001011546
A_23_P128161	0.00157	2.43	NM_032704	NM_032704	Homo sapiens tubulin alpha 6 (TUBA6), mRNA [NM_032704]	NM_032704
A_32_P91821	0.00157	2.52	BM041657	BM041657	BM041657 603614719T1 NIH_MGC_108 Homo sapiens cDNA clone IMAGE:5556485 3', mRNA sequence [BM041657]	
A_23_P206741	0.00157	9.26	A_23_P206741			
A_24_P517918	0.00158	2.03	ENST00000338567		Human IgG anti-platelet GPIIb/IIIa antibody heavy chain (ha4g1) mRNA, partial cds. [U38663]	XM_370835
A_24_P63827	0.00158	2.18	NM_005494	NM_005494	Homo sapiens DnaJ (Hsp40) homolog, subfamily B, member 6 (DNAJB6), transcript variant 2, mRNA [NM_005494]	NM_005494
A_24_P237117	0.00158	2.30	NM_172318	NM_172318	Homo sapiens potassium voltage-gated channel, subfamily G, member 1 (KCNG1), transcript variant 2, mRNA [NM_172318]	NM_172318
A_32_P210572	0.00159	2.34	NM_001024594	NM_001024594	Homo sapiens chromosome 1 open reading frame 53 (C1orf53), mRNA [NM_001024594]	NM_001024594
A_23_P266	0.00159	2.38	U82382	U82382	Homo sapiens PIN1 peptidyl-prolyl cis/trans isomerase-like (PIN1L) mRNA, complete cds. [U82382]	
A_24_P456884	0.00159	3.28	BC047952	BC047952	Homo sapiens, clone IMAGE:5785547, mRNA. [BC047952]	
A_23_P23616	0.00159	5.26	NM_032129	NM_032129	Homo sapiens pleckstrin homology domain containing, family N member 1 (PLEKHN1), mRNA [NM_032129]	NM_032129
A_23_P27133	0.00159	6.58	NM_002275	NM_002275	Homo sapiens keratin 15 (KRT15), mRNA [NM_002275]	NM_002275
A_32_P158019	0.00159	10.72	BX095305	BX095305	BX095305 Soares_pregnant_uterus_NbHPU Homo sapiens cDNA clone IMAGp998M221116, mRNA sequence [BX095305]	
A_24_P315256	0.0016	2.83	A_24_P315256			
A_32_P232327	0.0016	3.38	BG993059	BG993059	BG993059 RC4-HT1109-060201-013-f11 HT1109 Homo sapiens cDNA, mRNA sequence [BG993059]	
A_24_P594683	0.0016	3.57	THC2301371		Q864S5 (Q864S5) Peptidylprolyl isomerase A, partial (91%) [THC2301371]	
A_23_P52978	0.0016	4.90	NM_014502	NM_014502	Homo sapiens PRP19/PSO4 pre-mRNA processing factor 19 homolog (S. cerevisiae) (PRPF19), mRNA [NM_014502]	NM_014502
A_24_P916496	0.0016	4.93	NM_002737	NM_002737	Homo sapiens protein kinase C, alpha (PRKCA), mRNA [NM_002737]	NM_002737
A_23_P140668	0.00161	2.05	NM_005530	NM_005530	Homo sapiens isocitrate dehydrogenase 3 (NAD+) alpha (IDH3A), nuclear gene encoding mitochondrial protein, mRNA [NM_005530]	NM_005530
A_32_P216004	0.00161	2.10	AF130091	AF130091	Homo sapiens clone FLB9630 PRO2603 mRNA, complete cds. [AF130091]	
A_32_P88635	0.00161	2.12	NM_032264	NM_032264	Homo sapiens hypothetical protein AE2 (AE2), mRNA [NM_032264]	NM_032264
A_23_P211488	0.00161	2.15	NM_145637	NM_145637	Homo sapiens apolipoprotein L, 2 (APOL2), transcript variant beta, mRNA [NM_145637]	NM_145637
A_24_P136299	0.00161	2.19	A_24_P136299			
A_32_P47874	0.00161	2.19	THC2440787			
A_23_P90089	0.00161	2.34	NM_013976	NM_013976	Homo sapiens glutaryl-Coenzyme A dehydrogenase (GCDH), nuclear gene encoding mitochondrial protein, transcript variant 2, mRNA [NM_013976]	NM_013976
A_23_P170088	0.00161	2.48	BC065002	BC065002	Homo sapiens hypothetical protein FLJ20433, mRNA (cDNA clone IMAGE:6023681), complete cds. [BC065002]	
A_24_P414282	0.00161	2.67	THC2277620		Q9NQR4 (Q9NQR4) Nit protein 2 (CUA002), partial (92%) [THC2277620]	
A_23_P341312	0.00161	2.70	NM_198969	NM_198969	Homo sapiens amino-terminal enhancer of split (AES), transcript variant 1, mRNA [NM_198969]	NM_198969
A_24_P213794	0.00161	2.75	NM_012118	NM_012118	Homo sapiens CCR4 carbon catabolite repression 4-like (S. cerevisiae) (CCRN4L), mRNA [NM_012118]	NM_012118
A_23_P425516	0.00161	2.86	NM_012171	NM_012171	Homo sapiens tetraspanin 17 (TSPAN17), transcript variant 1, mRNA [NM_012171]	NM_012171
A_24_P290764	0.00161	3.21	BC007879	BC007879	Homo sapiens chromosome 9 open reading frame 10, mRNA (cDNA clone IMAGE:3938062), partial cds. [BC007879]	
A_23_P315241	0.00161	3.36	NM_004924	NM_004924	Homo sapiens actinin, alpha 4 (ACTN4), mRNA [NM_004924]	NM_004924
A_23_P335920	0.00162	2.18	NM_021135	NM_021135	Homo sapiens ribosomal protein S6 kinase, 90kDa, polypeptide 2 (RPS6KA2), transcript variant 1, mRNA [NM_021135]	NM_021135
A_23_P7941	0.00162	2.19	NM_015950	NM_015950	Homo sapiens mitochondrial ribosomal protein L2 (MRPL2), nuclear gene encoding mitochondrial protein, mRNA [NM_015950]	NM_015950
A_23_P59441	0.00162	2.21	NM_032826	NM_032826	Homo sapiens solute carrier family 35, member B4 (SLC35B4), mRNA [NM_032826]	NM_032826
A_23_P124164	0.00162	2.54	NM_020701	NM_020701	Homo sapiens KIAA1160 protein (KIAA1160), mRNA [NM_020701]	NM_020701
A_32_P148476	0.00162	2.61	ENST00000304245		PREDICTED: Homo sapiens similar to Ran-specific GTPase-activating protein (Ran binding protein 1) (RanBP1) (LOC389842), mRNA [XM_372200]	XM_372200
A_23_P149221	0.00162	2.63	NM_024602	NM_024602	Homo sapiens HECT domain containing 3 (HECTD3), mRNA [NM_024602]	NM_024602

A_24_P138713	0.00162	3.41	NM_182922	NM_182922	Homo sapiens hypothetical protein FLJ20718 (FLJ20718), transcript variant 2, mRNA [NM_182922]	NM_182922
A_24_P272910	0.00163	2.07	A_24_P272910			
A_32_P201498	0.00163	2.11	NM_001009924	NM_001009924	Homo sapiens chromosome 20 open reading frame 30 (C20orf30), transcript variant 2, mRNA [NM_001009924]	NM_001009924
A_23_P400255	0.00163	2.12	NM_004090	NM_004090	Homo sapiens dual specificity phosphatase 3 (vaccinia virus phosphatase VH1-related) (DUSP3), mRNA [NM_004090]	NM_004090
A_23_P87011	0.00163	2.46	NM_001001522	NM_001001522	Homo sapiens transgelin (TAGLN), transcript variant 1, mRNA [NM_001001522]	NM_001001522
A_23_P104881	0.00164	2.57	NM_019055	NM_019055	Homo sapiens roundabout homolog 4, magic roundabout (Drosophila) (ROBO4), mRNA [NM_019055]	NM_019055
A_23_P39076	0.00165	2.33	NM_006270	NM_006270	Homo sapiens related RAS viral (r-ras) oncogene homolog (RRAS), mRNA [NM_006270]	NM_006270
A_23_P93321	0.00165	3.09	NM_007109	NM_007109	Homo sapiens transcription factor 19 (SC1) (TCF19), mRNA [NM_007109]	NM_007109
A_24_P853302	0.00165	3.88	A_24_P853302			
A_32_P107372	0.00165	11.39	NM_002053	NM_002053	Homo sapiens guanylate binding protein 1, interferon-inducible, 67kDa (GBP1), mRNA [NM_002053]	NM_002053
A_32_P155776	0.00166	2.10	NM_001017421	NM_001017421	Homo sapiens actin-like protein (FKSG30), mRNA [NM_001017421]	NM_001017421
A_23_P74914	0.00167	2.21	NM_014777	NM_014777	Homo sapiens KIAA0133 (KIAA0133), mRNA [NM_014777]	NM_014777
A_24_P350124	0.00167	2.32	NM_020954	NM_020954	Homo sapiens KIAA1618 (KIAA1618), mRNA [NM_020954]	NM_020954
A_24_P307572	0.00167	3.28	NM_033121	NM_033121	Homo sapiens ankyrin repeat domain 13 (ANKRD13), mRNA [NM_033121]	NM_033121
A_24_P393880	0.00168	2.25	NM_153649	NM_153649	Homo sapiens tropomyosin 3 (TPM3), transcript variant 2, mRNA [NM_153649]	NM_153649
A_23_P36521	0.00168	2.32	NM_001659	NM_001659	Homo sapiens ADP-ribosylation factor 3 (ARF3), mRNA [NM_001659]	NM_001659
A_23_P61112	0.00168	2.54	AF089746	AF089746	Homo sapiens Bcl-2 related ovarian killer (BOK) mRNA, complete cds. [AF089746]	
A_23_P89056	0.00169	2.05	NM_014669	NM_014669	Homo sapiens nucleoporin 93kDa (NUP93), mRNA [NM_014669]	NM_014669
A_24_P210888	0.00169	2.19	AK002019	AK002019	Homo sapiens cDNA FLJ11157 fis, clone PLACE1006961. [AK002019]	
A_23_P79251	0.00169	2.36	NM_014600	NM_014600	Homo sapiens EH-domain containing 3 (EHD3), mRNA [NM_014600]	NM_014600
A_32_P211621	0.00169	2.39	BC053880	BC053880	Homo sapiens cDNA clone IMAGE:5753455. [BC053880]	
A_24_P176131	0.0017	2.35	NM_052950	NM_052950	Homo sapiens WD repeat and FYVE domain containing 2 (WDFY2), mRNA [NM_052950]	NM_052950
A_23_P107214	0.0017	2.46	NM_201434	NM_201434	Homo sapiens RAB5C, member RAS oncogene family (RAB5C), transcript variant 1, mRNA [NM_201434]	NM_201434
A_23_P97005	0.0017	2.48	NM_002227	NM_002227	Homo sapiens Janus kinase 1 (a protein tyrosine kinase) (JAK1), mRNA [NM_002227]	NM_002227
A_23_P143958	0.0017	3.41	BC049823	BC049823	Homo sapiens ribosomal protein L22-like 1, mRNA (cDNA clone IMAGE:4865966). [BC049823]	XM_114317
A_23_P81017	0.00171	2.65	NM_020337	NM_020337	Homo sapiens KIAA1223 protein (KIAA1223), mRNA [NM_020337]	NM_020337
A_23_P55926	0.00171	2.70	NM_007121	NM_007121	Homo sapiens nuclear receptor subfamily 1, group H, member 2 (NR1H2), mRNA [NM_007121]	NM_007121
A_24_P221724	0.00171	2.73	ENST00000330567			
A_24_P238131	0.00171	2.95	ENST00000309558		Homo sapiens mRNA for KIAA0527 protein, partial cds. [AB011099]	XM_171054
A_23_P34093	0.00171	4.74	NM_000402	NM_000402	Homo sapiens glucose-6-phosphate dehydrogenase (G6PD), nuclear gene encoding mitochondrial protein, mRNA [NM_000402]	NM_000402
A_24_P277615	0.00171	5.32	NM_203297	NM_203297	Homo sapiens tripartite motif-containing 7 (TRIM7), transcript variant 2, mRNA [NM_203297]	NM_203297
A_24_P160874	0.00172	2.02	NM_001948	NM_001948	Homo sapiens dUTP pyrophosphatase (DUT), nuclear gene encoding mitochondrial protein, transcript variant 2, mRNA [NM_001948]	NM_001948
A_23_P54055	0.00172	2.16	NM_032876	NM_032876	Homo sapiens jub, ajuba homolog (Xenopus laevis) (JUB), transcript variant 1, mRNA [NM_032876]	NM_032876
A_32_P84526	0.00172	2.45	BM994363	BM994363	UI-H-DH0-aul-d-13-0-UI.s1 NCI_CGAP_DH0 Homo sapiens cDNA clone IMAGE:5870940 3', mRNA sequence [BM994363]	
A_23_P200443	0.00172	2.51	NM_003029	NM_003029	Homo sapiens SHC (Src homology 2 domain containing) transforming protein 1 (SHC1), transcript variant 2, mRNA [NM_003029]	NM_003029
A_32_P71768	0.00172	3.17	BM504117	BM504117	BM504117 ih21d06.x1 Human insulinoma Homo sapiens cDNA 3' similar to SW:HMG1_CRIGR P07156 HIGH MOBILITY GROUP PROTEIN HMG1 ;, mRNA sequence [BM504117]	
A_24_P330030	0.00172	4.59	NM_153451	NM_153451	Homo sapiens oral cancer overexpressed 1 (ORAOV1), mRNA [NM_153451]	NM_153451
A_24_P940115	0.00173	2.16	NM_182643	NM_182643	Homo sapiens deleted in liver cancer 1 (DLC1), transcript variant 1, mRNA [NM_182643]	NM_182643
A_23_P412554	0.00173	2.21	NM_145055	NM_145055	Homo sapiens chromosome 18 open reading frame 25 (C18orf25), transcript variant 1, mRNA [NM_145055]	NM_145055

A_23_P54834	0.00173	2.84	NM_016101	NM_016101	Homo sapiens comparative gene identification transcript 37 (CGI-37), mRNA [NM_016101]	NM_016101
A_24_P58894	0.00173	3.08	XM_378054	XM_378054	PREDICTED: Homo sapiens similar to hypothetical protein (LOC402360), mRNA [XM_378054]	XM_378054
A_23_P152768	0.00174	2.12	NM_001070	NM_001070	Homo sapiens tubulin, gamma 1 (TUBG1), mRNA [NM_001070]	NM_001070
A_23_P52939	0.00174	2.13	NM_003627	NM_003627	Homo sapiens solute carrier family 43, member 1 (SLC43A1), mRNA [NM_003627]	NM_003627
A_24_P274270	0.00174	2.25	NM_139266	NM_139266	Homo sapiens signal transducer and activator of transcription 1, 91kDa (STAT1), transcript variant beta, mRNA [NM_139266]	NM_139266
A_23_P33444	0.00174	2.46	NM_006750	NM_006750	Homo sapiens syntrophin, beta 2 (dystrophin-associated protein A1, 59kDa, basic component 2) (SNTB2), transcript variant 1, mRNA [NM_006750]	NM_006750
A_23_P94703	0.00174	2.49	NM_014506	NM_014506	Homo sapiens torsin family 1, member B (torsin B) (TOR1B), mRNA [NM_014506]	NM_014506
A_24_P239140	0.00174	4.18	NM_000819	NM_000819	Homo sapiens phosphoribosylglycinamide formyltransferase, phosphoribosylglycinamide synthetase, phosphoribosylaminoimidazole synthetase (GART), transcript variant 1, mRNA [NM_000819]	NM_000819
A_24_P219474	0.00174	7.46	NM_144677	NM_144677	Homo sapiens mannosyl (alpha-1,6-)-glycoprotein beta-1,6-N-acetyl-glucosaminyltransferase, isoenzyme B (MGAT5B), transcript variant 1, mRNA [NM_144677]	NM_144677
A_23_P33277	0.00175	2.16	NM_006128	NM_006128	Homo sapiens bone morphogenetic protein 1 (BMP1), transcript variant BMP1-2, mRNA [NM_006128]	NM_006128
A_24_P579780	0.00175	2.18	CR622844	CR622844	full-length cDNA clone CS0DF026YC16 of Fetal brain of Homo sapiens (human). [CR622844]	
A_32_P89976	0.00175	2.19	BC017766	BC017766	Homo sapiens RNA polymerase I associated factor 53, mRNA (cDNA clone IMAGE:4695468), partial cds. [BC017766]	
A_23_P362712	0.00175	2.39	NM_148914	NM_148914	Homo sapiens abhydrolase domain containing 11 (ABHD11), transcript variant 3, mRNA [NM_148914]	NM_148914
A_23_P86054	0.00175	2.56	NM_015023	NM_015023	Homo sapiens WD and tetratricopeptide repeats 1 (WDTC1), mRNA [NM_015023]	NM_015023
A_23_P303523	0.00175	3.76	NM_002254	NM_002254	Homo sapiens kinesin family member 3C (KIF3C), mRNA [NM_002254]	NM_002254
A_32_P60687	0.00175	3.77	AI278811	AI278811	AI278811 qo50a11.x1 NCI_CGAP_Co8 Homo sapiens cDNA clone IMAGE:1911932 3' similar to gb:K02276 MYC PROTO-ONCOGENE PROTEIN (HUMAN);, mRNA sequence [AI278811]	
A_24_P222432	0.00175	4.02	BC032585	BC032585	Homo sapiens cDNA clone IMAGE:5500261, partial cds. [BC032585]	
A_23_P325991	0.00175	4.95	NM_181831	NM_181831	Homo sapiens neurofibromin 2 (bilateral acoustic neuroma) (NF2), transcript variant 13, mRNA [NM_181831]	NM_181831
A_23_P85460	0.00175	5.46	NM_001262	NM_001262	Homo sapiens cyclin-dependent kinase inhibitor 2C (p18, inhibits CDK4) (CDKN2C), transcript variant 1, mRNA [NM_001262]	NM_001262
A_24_P229531	0.00175	9.52	NM_022837	NM_022837	Homo sapiens hypothetical protein FLJ22833 (FLJ22833), mRNA [NM_022837]	NM_022837
A_24_P682601	0.00176	2.12	A_24_P682601			
A_23_P126120	0.00176	2.98	NM_033319	NM_033319	Homo sapiens chromosome 1 open reading frame 155 (C1orf155), mRNA [NM_033319]	NM_033319
A_23_P380271	0.00176	3.41	AK074447	AK074447	Homo sapiens cDNA FLJ23867 fis, clone LNG09729. [AK074447]	
A_23_P43898	0.00176	4.15	NM_173567	NM_173567	Homo sapiens abhydrolase domain containing 7 (ABHD7), mRNA [NM_173567]	NM_173567
A_24_P152094	0.00176	4.31	XM_062890	XM_062890	PREDICTED: Homo sapiens similar to peptidyl-Pro cis trans isomerase (LOC121981), mRNA [XM_062890]	XM_062890
A_23_P71530	0.00177	8.47	NM_002546	NM_002546	Homo sapiens tumor necrosis factor receptor superfamily, member 11b (osteoprotegerin) (TNFRSF11B), mRNA [NM_002546]	NM_002546
A_23_P28090	0.00178	2.14	NM_003765	NM_003765	Homo sapiens syntaxin 10 (STX10), mRNA [NM_003765]	NM_003765
A_24_P129417	0.00178	2.16	NM_006131	NM_006131	Homo sapiens bone morphogenetic protein 1 (BMP1), transcript variant BMP1-5, mRNA [NM_006131]	NM_006131
A_23_P135015	0.00178	2.54	BC001725	BC001725	Homo sapiens KIAA0258, mRNA (cDNA clone MGC:755 IMAGE:3533857), complete cds. [BC001725]	
A_24_P154914	0.00178	3.15	NM_016220	NM_016220	Homo sapiens zinc finger protein 588 (ZNF588), transcript variant 1, mRNA [NM_016220]	NM_016220
A_24_P33444	0.00179	2.17	NM_006761	NM_006761	Homo sapiens tyrosine 3-monooxygenase/tryptophan 5-monooxygenase activation protein, epsilon polypeptide (YWHAE), mRNA [NM_006761]	NM_006761
A_23_P369746	0.00179	2.23	ENST00000269290		Homo sapiens HSPC254 mRNA, partial cds. [AF161372]	
A_23_P259797	0.00179	2.49	NM_174917	NM_174917	Homo sapiens hypothetical protein LOC197322 (LOC197322), mRNA [NM_174917]	NM_174917
A_24_P623631	0.00179	2.59	ENST00000330227			
A_32_P39324	0.00179	2.69	CB993898	CB993898	AGENCOURT_13617208 NIH_MGC_148 Homo sapiens cDNA clone IMAGE:30333013 5', mRNA sequence [CB993898]	
A_23_P315571	0.00179	5.85	NM_015150	NM_015150	Homo sapiens raft-linking protein (RAFTLIN), mRNA [NM_015150]	NM_015150

A_24_P605233	0.0018	2.03	THC2282717		Q87726 (Q87726) Glycoprotein 120 (Fragment), partial (13%) [THC2282717]	
A_23_P104346	0.0018	2.32	NM_005028	NM_005028	Homo sapiens phosphatidylinositol-4-phosphate 5-kinase, type II, alpha (PIP5K2A), mRNA [NM_005028]	NM_005028
A_23_P112512	0.0018	3.06	NM_033412	NM_033412	Homo sapiens mitochondrial carrier triple repeat 1 (MCART1), mRNA [NM_033412]	NM_033412
A_32_P148710	0.0018	3.07	NM_005507	NM_005507	Homo sapiens cofilin 1 (non-muscle) (CFL1), mRNA [NM_005507]	NM_005507
A_24_P218151	0.0018	6.33	NM_016352	NM_016352	Homo sapiens carboxypeptidase A4 (CPA4), mRNA [NM_016352]	NM_016352
A_24_P298737	0.00181	2.01	ENST00000357592			
A_23_P216200	0.00181	2.17	NM_018361	NM_018361	Homo sapiens 1-acylglycerol-3-phosphate O-acyltransferase 5 (lysophosphatidic acid acyltransferase, epsilon) (AGPAT5), mRNA [NM_018361]	NM_018361
A_23_P300124	0.00181	2.18	NM_144775	NM_144775	Homo sapiens Smith-Magenis syndrome chromosome region, candidate 8 (SMCR8), mRNA [NM_144775]	NM_144775
A_23_P162879	0.00181	2.33	NM_032374	NM_032374	Homo sapiens chromosome 14 open reading frame 153 (C14orf153), mRNA [NM_032374]	NM_032374
A_24_P213206	0.00181	3.28	CR627448	CR627448	Homo sapiens mRNA; cDNA DKFZp781A1422 (from clone DKFZp781A1422). [CR627448]	
A_32_P48498	0.00181	9.52	BF197320	BF197320	BF197320 hr78g02.x1 NCI_CGAP_Kid11 Homo sapiens cDNA clone IMAGE:3134642 3', mRNA sequence [BF197320]	
A_24_P71153	0.00182	2.02	AK129536	AK129536	Homo sapiens cDNA FLJ26025 fis, clone PCD08250. [AK129536]	
A_24_P101879	0.00182	2.14	A_24_P101879			
A_23_P129144	0.00182	2.78	NM_152451	NM_152451	Homo sapiens GRINL1A combined protein (Gcom1), transcript variant 8, mRNA [NM_152451]	NM_152451
A_23_P200439	0.00183	2.07	NM_001111	NM_001111	Homo sapiens adenosine deaminase, RNA-specific (ADAR), transcript variant 1, mRNA [NM_001111]	NM_001111
A_23_P102965	0.00183	2.16	NM_015367	NM_015367	Homo sapiens BCL2-like 13 (apoptosis facilitator) (BCL2L13), nuclear gene encoding mitochondrial protein, mRNA [NM_015367]	NM_015367
A_24_P179044	0.00183	3.08	NM_016224	NM_016224	Homo sapiens sorting nexin 9 (SNX9), mRNA [NM_016224]	NM_016224
A_24_P350245	0.00183	3.70	NM_024940	NM_024940	Homo sapiens dedicator of cytokinesis 5 (DOCK5), mRNA [NM_024940]	NM_024940
A_24_P290286	0.00183	7.87	NM_182904	NM_182904	Homo sapiens procollagen-proline, 2-oxoglutarate 4-dioxygenase (proline 4-hydroxylase), alpha polypeptide III (P4HA3), mRNA [NM_182904]	NM_182904
A_23_P100441	0.00184	2.03	NM_024946	NM_024946	Homo sapiens NEFA-interacting nuclear protein NIP30 (NIP30), mRNA [NM_024946]	NM_024946
A_23_P427039	0.00184	2.19	NM_152667	NM_152667	Homo sapiens haloacid dehalogenase-like hydrolase domain containing 4 (HDHD4), mRNA [NM_152667]	NM_152667
A_24_P41662	0.00184	2.21	A_24_P41662			
A_24_P41975	0.00184	2.58	AK056402	AK056402	Homo sapiens cDNA FLJ31840 fis, clone NT2RP7000109, highly similar to Homo sapiens putative RNA binding protein mRNA. [AK056402]	
A_23_P255637	0.00184	2.92	A_23_P255637			
A_23_P104563	0.00184	5.15	NM_001876	NM_001876	Homo sapiens carnitine palmitoyltransferase 1A (liver) (CPT1A), nuclear gene encoding mitochondrial protein, mRNA [NM_001876]	NM_001876
A_23_P501007	0.00184	14.86	NM_004105	NM_004105	Homo sapiens EGF-containing fibulin-like extracellular matrix protein 1 (EFEMP1), transcript variant 1, mRNA [NM_004105]	NM_004105
A_23_P12866	0.00185	2.02	NM_153336	NM_153336	Homo sapiens chromosome 10 open reading frame 89 (C10orf89), mRNA [NM_153336]	NM_153336
A_23_P94174	0.00185	2.17	NM_014175	NM_014175	Homo sapiens mitochondrial ribosomal protein L15 (MRPL15), nuclear gene encoding mitochondrial protein, mRNA [NM_014175]	NM_014175
A_23_P146367	0.00185	2.30	NM_032310	NM_032310	Homo sapiens chromosome 9 open reading frame 89 (C9orf89), mRNA [NM_032310]	NM_032310
A_23_P147109	0.00185	2.82	NM_017723	NM_017723	Homo sapiens hypothetical protein FLJ20245 (FLJ20245), mRNA [NM_017723]	NM_017723
A_32_P30649	0.00185	4.42	NM_004454	NM_004454	Homo sapiens ets variant gene 5 (ets-related molecule) (ETV5), mRNA [NM_004454]	NM_004454
A_23_P205778	0.00185	4.83	NM_016194	NM_016194	Homo sapiens guanine nucleotide binding protein (G protein), beta 5 (GNB5), transcript variant 2, mRNA [NM_016194]	NM_016194
A_32_P83795	0.00186	2.03	A_32_P83795			
A_23_P422851	0.00186	2.04	NM_138375	NM_138375	Homo sapiens Cdk5 and Abl enzyme substrate 1 (CABLES1), mRNA [NM_138375]	NM_138375
A_24_P137582	0.00186	2.16	NM_170681	NM_170681	Homo sapiens G elongation factor, mitochondrial 2 (GFM2), nuclear gene encoding mitochondrial protein, transcript variant 3, mRNA [NM_170681]	NM_170681
A_24_P42539	0.00186	2.25	BC020978	BC020978	Homo sapiens chromosome 4 open reading frame 10, mRNA (cDNA clone IMAGE:3847615), partial cds. [BC020978]	
A_23_P211926	0.00186	2.40	NM_003392	NM_003392	Homo sapiens wingless-type MMTV integration site family, member 5A (WNT5A), mRNA [NM_003392]	NM_003392

A_24_P188377	0.00186	2.70	NM_000574	NM_000574	Homo sapiens decay accelerating factor for complement (CD55, Cromer blood group system) (DAF), mRNA [NM_000574]	NM_000574
A_23_P432056	0.00186	2.89	NM_178568	NM_178568	Homo sapiens reticulon 4 receptor-like 1 (RTN4RL1), mRNA [NM_178568]	NM_178568
A_24_P126425	0.00186	3.15	NM_032222	NM_032222	Homo sapiens hypothetical protein FLJ22374 (FLJ22374), mRNA [NM_032222]	NM_032222
A_32_P194264	0.00186	3.64	NM_001008708	NM_001008708	Homo sapiens similar to RIKEN cDNA 2510006C20 gene (LOC494143), mRNA [NM_001008708]	NM_001008708
A_24_P64895	0.00187	2.05	THC2338707			
A_23_P344421	0.00187	8.13	NM_019055	NM_019055	Homo sapiens roundabout homolog 4, magic roundabout (Drosophila) (ROBO4), mRNA [NM_019055]	NM_019055
A_23_P133332	0.00188	2.15	NM_015084	NM_015084	Homo sapiens mitochondrial ribosomal protein S27 (MRPS27), nuclear gene encoding mitochondrial protein, mRNA [NM_015084]	NM_015084
A_32_P119869	0.00188	2.34	THC2314566		Q8LQA6 (Q8LQA6) OJ1125_C04.6 protein, partial (21%) [THC2314566]	
A_23_P155807	0.00188	2.53	AY629351	AY629351	Homo sapiens DHHC domain-containing zinc finger protein mRNA, complete cds. [AY629351]	XM_376310
A_23_P319520	0.00189	2.02	NM_145003	NM_145003	Homo sapiens hypothetical protein FLJ31164 (FLJ31164), mRNA [NM_145003]	NM_145003
A_23_P321349	0.00189	2.14	NM_138421	NM_138421	Homo sapiens hypothetical protein BC012010 (LOC113174), mRNA [NM_138421]	NM_138421
A_24_P326635	0.00189	2.14	NM_001619	NM_001619	Homo sapiens adrenergic, beta, receptor kinase 1 (ADRBK1), mRNA [NM_001619]	NM_001619
A_23_P22086	0.00189	2.20	A_23_P22086			
A_32_P214395	0.00189	2.26	THC2279825			
A_23_P214908	0.0019	2.07	AY374131	AY374131	Homo sapiens truncated C1-tetrahydrofolate synthase mRNA, complete cds; nuclear gene for mitochondrial product; alternatively spliced. [AY374131]	
A_23_P307392	0.0019	2.54	NM_012074	NM_012074	Homo sapiens D4, zinc and double PHD fingers, family 3 (DPF3), mRNA [NM_012074]	NM_012074
A_23_P109655	0.0019	3.16	NM_025246	NM_025246	Homo sapiens transmembrane protein 22 (TMEM22), mRNA [NM_025246]	NM_025246
A_32_P187176	0.00191	2.00	NM_199176	NM_199176	Homo sapiens mitochondrial ribosome recycling factor (MRRF), nuclear gene encoding mitochondrial protein, transcript variant 3, mRNA [NM_199176]	NM_199176
A_23_P125990	0.00191	2.06	NM_004091	NM_004091	Homo sapiens E2F transcription factor 2 (E2F2), mRNA [NM_004091]	NM_004091
A_24_P400970	0.00191	2.11	A_24_P400970			
A_24_P286190	0.00191	2.80	NM_033342	NM_033342	Homo sapiens tripartite motif-containing 7 (TRIM7), transcript variant 6, mRNA [NM_033342]	NM_033342
A_24_P109644	0.00192	2.17	AF229166	AF229166	Homo sapiens docking protein 1-like protein mRNA, partial cds. [AF229166]	
A_23_P27606	0.00192	2.45	NM_004843	NM_004843	Homo sapiens interleukin 27 receptor, alpha (IL27RA), mRNA [NM_004843]	NM_004843
A_24_P308628	0.00192	2.65	NM_016173	NM_016173	Homo sapiens HemK methyltransferase family member 1 (HEMK1), mRNA [NM_016173]	NM_016173
A_23_P205789	0.00192	2.75	NM_002041	NM_002041	Homo sapiens GA binding protein transcription factor, beta subunit 2 (GABPB2), transcript variant gamma-1, mRNA [NM_002041]	NM_002041
A_23_P109488	0.00192	3.79	NM_052880	NM_052880	Homo sapiens HGFL gene (MGC17330), mRNA [NM_052880]	NM_052880
A_23_P44636	0.00192	4.00	NM_016238	NM_016238	Homo sapiens anaphase promoting complex subunit 7 (ANAPC7), mRNA [NM_016238]	NM_016238
A_23_P404685	0.00193	2.03	NM_178348	NM_178348	Homo sapiens late cornified envelope 1A (LCE1A), mRNA [NM_178348]	NM_178348
A_24_P409390	0.00193	2.08	ENST00000314123			
A_24_P628384	0.00193	2.39	NM_001001655	NM_001001655	Homo sapiens similar to hypothetical protein 9530023G02 (MGC90512), mRNA [NM_001001655]	NM_001001655
A_23_P205296	0.00193	2.43	NM_018199	NM_018199	Homo sapiens chromosome 14 open reading frame 114 (C14orf114), mRNA [NM_018199]	NM_018199
A_24_P924721	0.00193	2.78	NM_001018008	NM_001018008	Homo sapiens tropomyosin 1 (alpha) (TPM1), transcript variant 6, mRNA [NM_001018008]	NM_001018008
A_23_P38519	0.00193	6.76	NM_000212	NM_000212	Homo sapiens integrin, beta 3 (platelet glycoprotein IIIa, antigen CD61) (ITGB3), mRNA [NM_000212]	NM_000212
A_23_P63209	0.00193	56.50	NM_181755	NM_181755	Homo sapiens hydroxysteroid (11-beta) dehydrogenase 1 (HSD11B1), transcript variant 2, mRNA [NM_181755]	NM_181755
A_23_P121945	0.00194	2.01	NM_001001502	NM_001001502	Homo sapiens synuclein, beta (SNCB), transcript variant 1, mRNA [NM_001001502]	NM_001001502
A_23_P160449	0.00194	2.20	NM_032833	NM_032833	Homo sapiens protein phosphatase 1, regulatory (inhibitor) subunit 15B (PPP1R15B), mRNA [NM_032833]	NM_032833
A_23_P89410	0.00194	2.43	NM_003766	NM_003766	Homo sapiens beclin 1 (coiled-coil, myosin-like BCL2 interacting protein) (BECN1), mRNA [NM_003766]	NM_003766
A_32_P146286	0.00194	2.69	AL122093	AL122093	Homo sapiens mRNA; cDNA DKFZp434B2115 (from clone DKFZp434B2115). [AL122093]	
A_23_P317184	0.00194	2.82	NM_006309	NM_006309	Homo sapiens leucine rich repeat (in FLII) interacting protein 2 (LRRFIP2), transcript variant 1, mRNA [NM_006309]	NM_006309
A_32_P120818	0.00194	3.28	BI061130	BI061130	BI061130 IL3-UT0115-050201-434-A08 UT0115 Homo sapiens cDNA, mRNA sequence [BI061130]	

A_24_P118452	0.00194	3.56	NM_012247	NM_012247	Homo sapiens selenophosphate synthetase 1 (SEPHS1), mRNA [NM_012247]	NM_012247
A_24_P187626	0.00196	2.15	A_24_P187626			
A_32_P171903	0.00196	2.19	THC2404941		HUMGSTE beta 1,4-galactosyl-transferase precursor {Homo sapiens;} , partial (6%) [THC2404941]	
A_24_P922378	0.00196	2.33	NM_004449	NM_004449	Homo sapiens v-ets erythroblastosis virus E26 oncogene like (avian) (ERG), transcript variant 2, mRNA [NM_004449]	NM_004449
A_32_P141445	0.00197	2.06	BC034822	BC034822	Homo sapiens SPR pseudogene, mRNA (cDNA clone MGC:34796 IMAGE:5166892), complete cds. [BC034822]	
A_23_P127565	0.00197	2.28	NM_178834	NM_178834	Homo sapiens layilin (LOC143903), mRNA [NM_178834]	NM_178834
A_23_P77552	0.00197	2.41	NM_198490	NM_198490	Homo sapiens RAB43, member RAS oncogene family (RAB43), mRNA [NM_198490]	NM_198490
A_24_P649582	0.00197	2.78	BC052374	BC052374	Homo sapiens hypothetical protein LOC253039, mRNA (cDNA clone IMAGE:5749939), partial cds. [BC052374]	
A_23_P369899	0.00197	3.06	NM_015444	NM_015444	Homo sapiens Ras-induced senescence 1 (RIS1), mRNA [NM_015444]	NM_015444
A_24_P398370	0.00197	9.90	A_24_P398370			
A_24_P355493	0.00198	2.06	NM_022126	NM_022126	Homo sapiens phospholysine phosphohistidine inorganic pyrophosphate phosphatase (LHPP), mRNA [NM_022126]	NM_022126
A_23_P82047	0.00198	2.77	THC2273080			
A_24_P41890	0.00199	2.57	A_24_P41890			
A_23_P142333	0.00199	2.69	NM_016263	NM_016263	Homo sapiens fizzy/cell division cycle 20 related 1 (Drosophila) (FZR1), mRNA [NM_016263]	NM_016263
A_32_P26017	0.00199	2.87	AA112254	AA112254	AA112254 zm65a11.s1 Stratagene fibroblast (#937212) Homo sapiens cDNA clone IMAGE:530492 3', mRNA sequence [AA112254]	
A_23_P64102	0.00199	4.07	NM_004292	NM_004292	Homo sapiens Ras and Rab interactor 1 (RIN1), mRNA [NM_004292]	NM_004292
A_23_P129075	0.002	2.13	NM_024908	NM_024908	Homo sapiens WD repeat domain 76 (WDR76), mRNA [NM_024908]	NM_024908
A_23_P91379	0.002	2.17	NM_018993	NM_018993	Homo sapiens Ras and Rab interactor 2 (RIN2), mRNA [NM_018993]	NM_018993
A_23_P54929	0.002	2.35	NM_020424	NM_020424	Homo sapiens hypothetical protein A-211C6.1 (LOC57149), mRNA [NM_020424]	NM_020424
A_24_P383802	0.002	2.66	A_24_P383802			
A_32_P529165	0.002	2.99	AB040895	AB040895	Homo sapiens mRNA for KIAA1462 protein, partial cds. [AB040895]	XM_166132
A_23_P138308	0.002	3.19	NM_001779	NM_001779	Homo sapiens CD58 antigen, (lymphocyte function-associated antigen 3) (CD58), mRNA [NM_001779]	NM_001779
A_23_P113701	0.00201	2.17	X06374	X06374	Human mRNA for platelet-derived growth factor PDGF-A. [X06374]	
A_24_P721699	0.00201	2.22	AK027222	AK027222	Homo sapiens cDNA: FLJ23569 fis, clone LNG11861. [AK027222]	
A_24_P220897	0.00201	2.49	ENST00000302091		Homo sapiens cDNA FLJ25874 fis, clone CBR02446. [AK098740]	XM_114430
A_24_P418762	0.00201	2.62	NM_020870	NM_020870	Homo sapiens SH3 multiple domains 2 (SH3MD2), mRNA [NM_020870]	NM_020870
A_23_P215642	0.00201	2.84	NM_022748	NM_022748	Homo sapiens tensin 3 (TNS3), mRNA [NM_022748]	NM_022748
A_24_P921446	0.00202	4.50	BC017854	BC017854	Homo sapiens epithelial membrane protein 1, mRNA (cDNA clone IMAGE:4691099), partial cds. [BC017854]	
A_23_P74359	0.00202	6.13	NM_004078	NM_004078	Homo sapiens cysteine and glycine-rich protein 1 (CSRP1), mRNA [NM_004078]	NM_004078
A_24_P328231	0.00203	2.03	NM_032179	NM_032179	Homo sapiens cleavage and polyadenylation specific factor 3-like (CPSF3L), transcript variant 2, mRNA [NM_032179]	NM_032179
A_32_P96692	0.00204	2.03	THC2340838		Q9BX12 (Q9BX12) GTP binding protein 2 (Fragment), partial (26%) [THC2340838]	
A_24_P31165	0.00204	2.22	NM_002055	NM_002055	Homo sapiens glial fibrillary acidic protein (GFAP), mRNA [NM_002055]	NM_002055
A_32_P13417	0.00204	2.75	AK091942	AK091942	Homo sapiens cDNA FLJ34623 fis, clone KIDNE2015073. [AK091942]	
A_23_P218476	0.00204	2.83	NM_030578	NM_030578	Homo sapiens hypothetical protein MGC4093 (MGC4093), mRNA [NM_030578]	NM_030578
A_32_P178696	0.00204	3.53	BC046172	BC046172	Homo sapiens cDNA clone IMAGE:3463889, partial cds. [BC046172]	
A_23_P313389	0.00204	8.00	NM_003358	NM_003358	Homo sapiens UDP-glucose ceramide glucosyltransferase (UGCG), mRNA [NM_003358]	NM_003358
A_23_P19164	0.00205	2.28	NM_003314	NM_003314	Homo sapiens tetratricopeptide repeat domain 1 (TTC1), mRNA [NM_003314]	NM_003314
A_23_P103041	0.00205	2.57	NM_152236	NM_152236	Homo sapiens growth arrest-specific 2 like 1 (GAS2L1), transcript variant 2, mRNA [NM_152236]	NM_152236
A_32_P179771	0.00205	3.62	AI088710	AI088710	AI088710 qa12c10.x1 NCI_CGAP_Brn23 Homo sapiens cDNA clone IMAGE:1686546 3' similar to gb:L06132 OUTER MITOCHONDRIAL MEMBRANE PROTEIN PORIN (HUMAN);, mRNA sequence [AI088710]	
A_23_P15864	0.00206	2.09	NM_003927	NM_003927	Homo sapiens methyl-CpG binding domain protein 2 (MBD2), transcript variant 1, mRNA [NM_003927]	NM_003927
A_23_P144697	0.00206	2.12	NM_133377	NM_133377	Homo sapiens RAD1 homolog (S. pombe) (RAD1), transcript variant 3, mRNA [NM_133377]	NM_133377

A_24_P230176	0.00206	2.12	BC009369	BC009369	Homo sapiens similar to RIKEN cDNA 3110023B02, mRNA (cDNA clone MGC:16597 IMAGE:4110481), complete cds. [BC009369]	XM_375500
A_32_P103220	0.00206	2.75	NM_030955	NM_030955	Homo sapiens a disintegrin-like and metalloprotease (reprolysin type) with thrombospondin type 1 motif, 12 (ADAMTS12), mRNA [NM_030955]	NM_030955
A_32_P18470	0.00206	2.86	NM_001012979	NM_001012979	Homo sapiens transcription elongation factor A (SII)-like 5 (TCEAL5), mRNA [NM_001012979]	NM_001012979
A_23_P347169	0.00206	2.95	NM_001001927	NM_001001927	Homo sapiens mitochondrial tumor suppressor 1 (MTUS1), nuclear gene encoding mitochondrial protein, transcript variant 3, mRNA [NM_001001927]	NM_001001927
A_24_P185186	0.00206	4.37	NM_174920	NM_174920	Homo sapiens hypothetical protein LOC201191 (LOC201191), mRNA [NM_174920]	NM_174920
A_32_P34444	0.00206	4.72	NM_025135	NM_025135	Homo sapiens formin homology 2 domain containing 3 (FHOD3), mRNA [NM_025135]	NM_025135
A_32_P94521	0.00207	2.49	A_32_P94521			
A_24_P406986	0.00208	2.07	NM_199329	NM_199329	Homo sapiens solute carrier family 43, member 3 (SLC43A3), mRNA [NM_199329]	NM_199329
A_23_P427634	0.00208	2.26	NM_080871	NM_080871	Homo sapiens ankyrin repeat and SOCS box-containing 10 (ASB10), mRNA [NM_080871]	NM_080871
A_23_P76705	0.00208	2.30	NM_006322	NM_006322	Homo sapiens tubulin, gamma complex associated protein 3 (TUBGCP3), mRNA [NM_006322]	NM_006322
A_24_P134235	0.00208	2.46	NM_003685	NM_003685	Homo sapiens KH-type splicing regulatory protein (FUSE binding protein 2) (KHSRP), mRNA [NM_003685]	NM_003685
A_32_P172779	0.00208	3.69	THC2336812		BF930236 IL5-NT0227-111200-319-g04 NT0227 Homo sapiens cDNA, mRNA sequence [BF930236]	
A_32_P201976	0.00208	4.52	AI860683	AI860683	AI860683 wl15a10.x1 NCI_CGAP_Ut1 Homo sapiens cDNA clone IMAGE:2424954 3', mRNA sequence [AI860683]	
A_32_P7823	0.00209	2.15	NM_144578	NM_144578	Homo sapiens chromosome 14 open reading frame 32 (C14orf32), mRNA [NM_144578]	NM_144578
A_23_P91827	0.00209	2.72	NM_032778	NM_032778	Homo sapiens MYC induced nuclear antigen (MINA), transcript variant 2, mRNA [NM_032778]	NM_032778
A_23_P19624	0.00209	3.46	NM_001718	NM_001718	Homo sapiens bone morphogenetic protein 6 (BMP6), mRNA [NM_001718]	NM_001718
A_24_P299794	0.0021	3.53	NM_005057	NM_005057	Homo sapiens retinoblastoma binding protein 5 (RBBP5), mRNA [NM_005057]	NM_005057
A_23_P9209	0.00211	2.15	NM_018376	NM_018376	Homo sapiens nipsnap homolog 3B (C. elegans) (NIPSNAP3B), mRNA [NM_018376]	NM_018376
A_23_P258367	0.00211	2.39	NM_001499	NM_001499	Homo sapiens GLE1 RNA export mediator-like (yeast) (GLE1L), transcript variant 2, mRNA [NM_001499]	NM_001499
A_23_P209944	0.00212	3.26	NM_017750	NM_017750	Homo sapiens all-trans-13,14-dihydroretinol saturase (RetSat), mRNA [NM_017750]	NM_017750
A_24_P7594	0.00212	3.80	AK097266	AK097266	Homo sapiens cDNA FLJ39947 fis, clone SPLEN2024232. [AK097266]	
A_23_P26037	0.00212	12.47	NM_032892	NM_032892	Homo sapiens FERM domain containing 5 (FRMD5), mRNA [NM_032892]	NM_032892
A_23_P250035	0.00213	2.02	NM_004168	NM_004168	Homo sapiens succinate dehydrogenase complex, subunit A, flavoprotein (Fp) (SDHA), nuclear gene encoding mitochondrial protein, mRNA [NM_004168]	NM_004168
A_23_P33927	0.00213	2.04	AF334945	AF334945	Homo sapiens FKSG43 (FKSG43) mRNA, complete cds. [AF334945]	
A_23_P39008	0.00213	2.19	NM_080864	NM_080864	Homo sapiens relaxin 3 (RLN3), mRNA [NM_080864]	NM_080864
A_24_P398323	0.00213	2.21	NM_001003827	NM_001003827	Homo sapiens tripartite motif-containing 34 (TRIM34), transcript variant 4, mRNA [NM_001003827]	NM_001003827
A_23_P96955	0.00213	2.43	NM_032648	NM_032648	Homo sapiens chromosome 1 open reading frame 90 (C1orf90), mRNA [NM_032648]	NM_032648
A_32_P157846	0.00213	2.73	AK092260	AK092260	Homo sapiens cDNA FLJ34941 fis, clone NT2RP7007480. [AK092260]	
A_23_P40453	0.00213	39.06	NM_001236	NM_001236	Homo sapiens carbonyl reductase 3 (CBR3), mRNA [NM_001236]	NM_001236
A_23_P168576	0.00214	2.06	NM_152992	NM_152992	Homo sapiens POM (POM121 homolog, rat) and ZP3 fusion (POMZP3), transcript variant 2, mRNA [NM_152992]	NM_152992
A_23_P257538	0.00214	2.19	NM_006606	NM_006606	Homo sapiens retinoblastoma binding protein 9 (RBBP9), transcript variant 1, mRNA [NM_006606]	NM_006606
A_23_P59444	0.00214	2.30	NM_032826	NM_032826	Homo sapiens solute carrier family 35, member B4 (SLC35B4), mRNA [NM_032826]	NM_032826
A_23_P11032	0.00214	2.42	NM_032591	NM_032591	Homo sapiens solute carrier family 9 (sodium/hydrogen exchanger), isoform 7 (SLC9A7), mRNA [NM_032591]	NM_032591
A_23_P250564	0.00214	2.43	NM_005400	NM_005400	Homo sapiens protein kinase C, epsilon (PRKCE), mRNA [NM_005400]	NM_005400
A_32_P147790	0.00214	2.48	BC071732	BC071732	Homo sapiens cDNA clone IMAGE:3862422, partial cds. [BC071732]	
A_32_P41327	0.00214	2.71	AA910154	AA910154	AA910154 od92h01.s1 NCI_CGAP_Br5 Homo sapiens cDNA clone IMAGE:1377745, mRNA sequence [AA910154]	
A_23_P71014	0.00214	2.83	NM_017528	NM_017528	Homo sapiens Williams Beuren syndrome chromosome region 22 (WBSCR22), mRNA [NM_017528]	NM_017528
A_24_P273132	0.00214	3.64	NM_013296	NM_013296	Homo sapiens G-protein signalling modulator 2 (AGS3-like, C. elegans) (GPSM2), mRNA [NM_013296]	NM_013296
A_24_P76008	0.00215	2.26	XM_373004	XM_373004	PREDICTED: Homo sapiens similar to KIAA1552 protein (LOC391594), mRNA [XM_373004]	XM_373004
A_24_P127042	0.00215	2.27	A_24_P127042			

A_24_P73962	0.00215	2.39	THC2400647		PSDB_HUMAN 26S proteasome non-ATPase regulatory subunit 11 (26S proteasomeregulatory subunit S9) (26S proteasome regulatory subunit p44.5). {Homo sapiens;} , partial (39%) [THC2400647]	
A_23_P159920	0.00215	2.53	NM_003639	NM_003639	Homo sapiens inhibitor of kappa light polypeptide gene enhancer in B-cells, kinase gamma (IKBKG), mRNA [NM_003639]	NM_003639
A_23_P37068	0.00215	2.77	NM_145725	NM_145725	Homo sapiens TNF receptor-associated factor 3 (TRAF3), transcript variant 1, mRNA [NM_145725]	NM_145725
A_32_P63166	0.00215	3.61	W45382	W45382	W45382 zc80e10.s1 Pancreatic Islet Homo sapiens cDNA clone IMAGE:328650 3' similar to gb:D13748 EUKARYOTIC INITIATION FACTOR 4A-I (HUMAN);, mRNA sequence [W45382]	
A_23_P20337	0.00215	4.15	NM_001386	NM_001386	Homo sapiens dihydropyrimidinase-like 2 (DPYSL2), mRNA [NM_001386]	NM_001386
A_23_P141376	0.00215	7.58	NM_012232	NM_012232	Homo sapiens polymerase I and transcript release factor (PTRF), mRNA [NM_012232]	NM_012232
A_24_P14485	0.00216	2.04	NM_017495	NM_017495	Homo sapiens RNA-binding region (RNP1, RRM) containing 1 (RNPC1), transcript variant 1, mRNA [NM_017495]	NM_017495
A_24_P324314	0.00216	2.53	NM_001008529	NM_001008529	Homo sapiens matrix-remodelling associated 7 (MXRA7), transcript variant 2, mRNA [NM_001008529]	NM_001008529
A_23_P114740	0.00216	12.58	NM_002113	NM_002113	Homo sapiens complement factor H-related 1 (CFHL1), mRNA [NM_002113]	NM_002113
A_23_P132115	0.00217	2.06	NM_173354	NM_173354	Homo sapiens SNF1-like kinase (SNF1LK), mRNA [NM_173354]	NM_173354
A_24_P164388	0.00217	2.22	NM_031211	NM_031211	Homo sapiens LAT1-3TM protein (LAT1-3TM), mRNA [NM_031211]	NM_031211
A_24_P159323	0.00217	2.59	NM_022770	NM_022770	Homo sapiens hypothetical protein FLJ13912 (FLJ13912), mRNA [NM_022770]	NM_022770
A_23_P16673	0.00217	3.02	NM_004368	NM_004368	Homo sapiens calponin 2 (CNN2), transcript variant 1, mRNA [NM_004368]	NM_004368
A_24_P387158	0.00217	3.41	NM_005782	NM_005782	Homo sapiens THO complex 4 (THOC4), mRNA [NM_005782]	NM_005782
A_24_P325333	0.00217	4.12	NM_002600	NM_002600	Homo sapiens phosphodiesterase 4B, cAMP-specific (phosphodiesterase E4 dunce homolog, Drosophila) (PDE4B), mRNA [NM_002600]	NM_002600
A_24_P198217	0.00218	2.36	NM_147777	NM_147777	Homo sapiens sorting nexin 15 (SNX15), transcript variant B, mRNA [NM_147777]	NM_147777
A_23_P37497	0.00218	2.64	U14391	U14391	Human myosin-IC mRNA, complete cds. [U14391]	
A_32_P17163	0.00218	2.91	AK002195	AK002195	Homo sapiens cDNA FLJ11333 fis, clone PLACE1010616. [AK002195]	
A_24_P653354	0.00218	3.03	THC2440229			
A_24_P190424	0.00218	3.32	NM_005370	NM_005370	Homo sapiens RAB8A, member RAS oncogene family (RAB8A), mRNA [NM_005370]	NM_005370
A_23_P203957	0.00218	5.05	NM_031920	NM_031920	Homo sapiens ARG99 protein (ARG99), transcript variant 1, mRNA [NM_031920]	NM_031920
A_24_P123408	0.00218	15.55	NM_014945	NM_014945	Homo sapiens actin binding LIM protein family, member 3 (ABLIM3), mRNA [NM_014945]	NM_014945
A_24_P306034	0.00219	2.23	AK098736	AK098736	Homo sapiens cDNA FLJ25870 fis, clone CBR02141. [AK098736]	
A_23_P309967	0.00219	2.27	NM_032486	NM_032486	Homo sapiens dynactin 4 (MGC3248), mRNA [NM_032486]	NM_032486
A_23_P62890	0.00219	19.80	NM_002053	NM_002053	Homo sapiens guanylate binding protein 1, interferon-inducible, 67kDa (GBP1), mRNA [NM_002053]	NM_002053
A_23_P103371	0.0022	2.16	NM_052998	NM_052998	Homo sapiens arginine decarboxylase (ADC), mRNA [NM_052998]	NM_052998
A_32_P91250	0.0022	2.29	NM_003347	NM_003347	Homo sapiens ubiquitin-conjugating enzyme E2L 3 (UBE2L3), transcript variant 1, mRNA [NM_003347]	NM_003347
A_23_P169249	0.0022	4.55	NM_017585	NM_017585	Homo sapiens solute carrier family 2 (facilitated glucose transporter), member 6 (SLC2A6), mRNA [NM_017585]	NM_017585
A_23_P253752	0.00221	2.07	NM_138419	NM_138419	Homo sapiens family with sequence similarity 54, member A (FAM54A), mRNA [NM_138419]	NM_138419
A_24_P71373	0.00221	2.17	NM_003047	NM_003047	Homo sapiens solute carrier family 9 (sodium/hydrogen exchanger), isoform 1 (antiporter, Na+/H+, amiloride sensitive) (SLC9A1), mRNA [NM_003047]	NM_003047
A_23_P5863	0.00221	2.21	CR600305	CR600305	full-length cDNA clone CS0DL001YI02 of B cells (Ramos cell line) Cot 25-normalized of Homo sapiens (human). [CR600305]	
A_23_P3963	0.00221	2.65	NM_014603	NM_014603	Homo sapiens paraneoplastic antigen (HUMPPA), mRNA [NM_014603]	NM_014603
A_23_P204801	0.00221	2.98	NM_032148	NM_032148	Homo sapiens solute carrier family 41, member 2 (SLC41A2), mRNA [NM_032148]	NM_032148
A_24_P683917	0.00222	2.04	AK022486	AK022486	Homo sapiens cDNA FLJ12424 fis, clone MAMMA1003099. [AK022486]	
A_23_P111480	0.00222	2.18	ENST00000352103		Homo sapiens cDNA FLJ13195 fis, clone NT2RP3004424, weakly similar to Homo sapiens mRNA for stromal antigen 3 (STAG3 gene). [AK023257]	XM_498429
A_23_P11279	0.00223	2.04	NM_018466	NM_018466	Homo sapiens glycosyltransferase 28 domain containing 1 (GLT28D1), mRNA [NM_018466]	NM_018466
A_23_P423480	0.00223	2.06	NM_173555	NM_173555	Homo sapiens trypsin domain containing 1 (TYSND1), mRNA [NM_173555]	NM_173555
A_24_P87579	0.00223	2.10	NM_001017916	NM_001017916	Homo sapiens cytochrome b-561 (CYB561), transcript variant 2, mRNA [NM_001017916]	NM_001017916

A_32_P101313	0.00223	2.16	NM_001010915	NM_001010915	Homo sapiens similar to RIKEN 4933428I03 (LOC401494), mRNA [NM_001010915]	NM_001010915
A_23_P39356	0.00223	2.17	NM_005303	NM_005303	Homo sapiens G protein-coupled receptor 40 (GPR40), mRNA [NM_005303]	NM_005303
A_24_P86968	0.00223	2.20	NM_000733	NM_000733	Homo sapiens CD3E antigen, epsilon polypeptide (TiT3 complex) (CD3E), mRNA [NM_000733]	NM_000733
A_23_P89343	0.00223	2.26	NM_152244	NM_152244	Homo sapiens sorting nexin 11 (SNX11), transcript variant 1, mRNA [NM_152244]	NM_152244
A_23_P159227	0.00223	5.88	NM_207191	NM_207191	Homo sapiens a disintegrin and metalloproteinase domain 15 (metargidin) (ADAM15), transcript variant 1, mRNA [NM_207191]	NM_207191
A_23_P102351	0.00224	2.12	NM_004854	NM_004854	Homo sapiens carbohydrate sulfotransferase 10 (CHST10), mRNA [NM_004854]	NM_004854
A_32_P70372	0.00224	2.51	BM995362	BM995362	UI-H-ED0-axb-n-22-0-UI.s1 NCI_CGAP_ED0 Homo sapiens cDNA clone IMAGE:5826645 3', mRNA sequence [BM995362]	
A_32_P117908	0.00224	3.62	A_32_P117908			
A_23_P109427	0.00224	3.66	NM_000854	NM_000854	Homo sapiens glutathione S-transferase theta 2 (GSTT2), mRNA [NM_000854]	NM_000854
A_23_P160167	0.00225	2.09	NM_005727	NM_005727	Homo sapiens tetraspanin 1 (TSPAN1), mRNA [NM_005727]	NM_005727
A_32_P10589	0.00226	2.08	A_32_P10589			
A_24_P764598	0.00226	2.17	NM_080392	NM_080392	Homo sapiens protein tyrosine phosphatase type IVA, member 2 (PTP4A2), transcript variant 3, mRNA [NM_080392]	NM_080392
A_24_P191047	0.00226	2.18	NM_019060	NM_019060	Homo sapiens chromosome 1 open reading frame 42 (C1orf42), mRNA [NM_019060]	NM_019060
A_24_P29885	0.00226	2.21	NM_002514	NM_002514	Homo sapiens nephroblastoma overexpressed gene (NOV), mRNA [NM_002514]	NM_002514
A_23_P59637	0.00227	2.29	AB018259	AB018259	Homo sapiens mRNA for KIAA0716 protein, partial cds. [AB018259]	
A_23_P310222	0.00227	2.87	NM_152263	NM_152263	Homo sapiens tropomyosin 3 (TPM3), transcript variant 1, mRNA [NM_152263]	NM_152263
A_23_P57784	0.00228	2.19	NM_021101	NM_021101	Homo sapiens claudin 1 (CLDN1), mRNA [NM_021101]	NM_021101
A_32_P216841	0.00228	2.98	NM_145263	NM_145263	Homo sapiens spermatogenesis associated 18 homolog (rat) (SPATA18), mRNA [NM_145263]	NM_145263
A_24_P222043	0.00228	3.19	NM_002131	NM_002131	Homo sapiens high mobility group AT-hook 1 (HMGA1), transcript variant 2, mRNA [NM_002131]	NM_002131
A_23_P251104	0.00228	3.27	NM_000232	NM_000232	Homo sapiens sarcoglycan, beta (43kDa dystrophin-associated glycoprotein) (SGCB), mRNA [NM_000232]	NM_000232
A_32_P213755	0.00229	2.46	AL555100	AL555100	AL555100 AL555100 Homo sapiens HELA CELLS COT 25-NORMALIZED Homo sapiens cDNA clone CS0DK007YP02 3-PRIME, mRNA sequence [AL555100]	
A_23_P52738	0.00229	2.67	NM_014026	NM_014026	Homo sapiens decapping enzyme, scavenger (DCPS), mRNA [NM_014026]	NM_014026
A_24_P366994	0.00229	2.82	NM_004309	NM_004309	Homo sapiens Rho GDP dissociation inhibitor (GDI) alpha (ARHGDIA), mRNA [NM_004309]	NM_004309
A_23_P22473	0.00229	3.17	NM_012280	NM_012280	Homo sapiens FtsJ homolog 1 (E. coli) (FTSJ1), transcript variant 1, mRNA [NM_012280]	NM_012280
A_23_P118113	0.0023	2.06	NM_005632	NM_005632	Homo sapiens small optic lobes homolog (Drosophila) (SOLH), mRNA [NM_005632]	NM_005632
A_23_P149649	0.0023	2.54	NM_003000	NM_003000	Homo sapiens succinate dehydrogenase complex, subunit B, iron sulfur (Ip) (SDHB), mRNA [NM_003000]	NM_003000
A_24_P338788	0.0023	2.87	NM_145203	NM_145203	Homo sapiens casein kinase 1, alpha 1-like (CSNK1A1L), mRNA [NM_145203]	NM_145203
A_24_P231513	0.0023	3.12	NM_005721	NM_005721	Homo sapiens ARP3 actin-related protein 3 homolog (yeast) (ACTR3), mRNA [NM_005721]	NM_005721
A_23_P207014	0.00231	2.39	NM_002876	NM_002876	Homo sapiens RAD51 homolog C (S. cerevisiae) (RAD51C), transcript variant 2, mRNA [NM_002876]	NM_002876
A_23_P165962	0.00231	2.92	NM_016004	NM_016004	Homo sapiens chromosome 20 open reading frame 9 (C20orf9), mRNA [NM_016004]	NM_016004
A_32_P228331	0.00231	3.50	A_32_P228331			
A_32_P220152	0.00232	2.15	NM_005898	NM_005898	Homo sapiens membrane component, chromosome 11, surface marker 1 (M11S1), transcript variant 1, mRNA [NM_005898]	NM_005898
A_24_P882666	0.00232	2.55	A_24_P882666			
A_24_P339944	0.00232	2.64	NM_002608	NM_002608	Homo sapiens platelet-derived growth factor beta polypeptide (simian sarcoma viral (v-sis) oncogene homolog) (PDGFB), transcript variant 1, mRNA [NM_002608]	NM_002608
A_24_P633902	0.00233	2.39	NM_014455	NM_014455	Homo sapiens zinc finger protein 364 (ZNF364), mRNA [NM_014455]	NM_014455
A_23_P66211	0.00233	2.72	NM_152341	NM_152341	Homo sapiens progestin and adipoQ receptor family member IV (PAQR4), mRNA [NM_152341]	NM_152341
A_32_P233049	0.00233	3.33	G36726	G36726	SHGC-52890 Human Homo sapiens STS cDNA, sequence tagged site. [G36726]	
A_24_P755169	0.00233	3.52	A_24_P755169			
A_24_P203407	0.00233	4.65	BC029662	BC029662	Homo sapiens chromosome 20 open reading frame 142, mRNA (cDNA clone IMAGE:4933017), with apparent retained intron. [BC029662]	XM_371399

A_23_P113441	0.00234	2.02	NM_018130	NM_018130	Homo sapiens SHQ1 homolog (S. cerevisiae) (SHQ1), mRNA [NM_018130]	NM_018130
A_23_P94301	0.00234	2.07	NM_003313	NM_003313	Homo sapiens tissue specific transplantation antigen P35B (TSTA3), mRNA [NM_003313]	NM_003313
A_24_P290770	0.00234	2.48	NM_001003941	NM_001003941	Homo sapiens oxoglutarate (alpha-ketoglutarate) dehydrogenase (lipoamide) (OGDH), nuclear gene encoding mitochondrial protein, transcript variant 2, mRNA [NM_001003941]	NM_001003941
A_24_P102043	0.00234	2.55	THC2307660		P70670 (P70670) Alpha-NAC, muscle-specific form gp220, partial (14%) [THC2307660]	
A_24_P155016	0.00234	3.13	NM_018718	NM_018718	Homo sapiens testis specific, 14 (TSGA14), mRNA [NM_018718]	NM_018718
A_23_P357284	0.00234	9.01	NM_005282	NM_005282	Homo sapiens G protein-coupled receptor 4 (GPR4), mRNA [NM_005282]	NM_005282
A_24_P349039	0.00235	2.78	NM_020754	NM_020754	Homo sapiens Cdc42 GTPase-activating protein (CDGAP), mRNA [NM_020754]	NM_020754
A_32_P186889	0.00235	3.13	NM_153649	NM_153649	Homo sapiens tropomyosin 3 (TPM3), transcript variant 2, mRNA [NM_153649]	NM_153649
A_23_P338479	0.00235	27.55	NM_014143	NM_014143	Homo sapiens CD274 antigen (CD274), mRNA [NM_014143]	NM_014143
A_23_P5845	0.00236	2.07	NM_000221	NM_000221	Homo sapiens ketohexokinase (fructokinase) (KHK), transcript variant a, mRNA [NM_000221]	NM_000221
A_23_P86917	0.00236	2.09	NM_003824	NM_003824	Homo sapiens Fas (TNFRSF6)-associated via death domain (FADD), mRNA [NM_003824]	NM_003824
A_24_P378506	0.00236	2.16	NM_023079	NM_023079	Homo sapiens hypothetical protein FLJ13855 (FLJ13855), mRNA [NM_023079]	NM_023079
A_23_P58298	0.00236	2.19	NM_138386	NM_138386	Homo sapiens hypothetical protein BC008207 (LOC92345), mRNA [NM_138386]	NM_138386
A_23_P22926	0.00236	2.20	NM_002074	NM_002074	Homo sapiens guanine nucleotide binding protein (G protein), beta polypeptide 1 (GNB1), mRNA [NM_002074]	NM_002074
A_23_P362261	0.00236	3.51	ENST00000260257		Homo sapiens, clone IMAGE:3677165, mRNA, partial cds. [BC006136]	
A_32_P177267	0.00236	3.70	BC056262	BC056262	Homo sapiens similar to mitochondrial carrier triple repeat 1, mRNA (cDNA clone MGC:62031 IMAGE:6618254), complete cds. [BC056262]	XM_084000
A_23_P77630	0.00237	2.67	NM_022818	NM_022818	Homo sapiens microtubule-associated protein 1 light chain 3 beta (MAP1LC3B), mRNA [NM_022818]	NM_022818
A_23_P95213	0.00237	2.75	BC005913	BC005913	Homo sapiens surfactant, pulmonary-associated protein C, mRNA (cDNA clone MGC:14509 IMAGE:4043169), complete cds. [BC005913]	
A_23_P204133	0.00237	2.86	NM_007210	NM_007210	Homo sapiens UDP-N-acetyl-alpha-D-galactosamine:polypeptide N-acetylgalactosaminyltransferase 6 (GalNAc-T6) (GALNT6), mRNA [NM_007210]	NM_007210
A_23_P126031	0.00237	3.07	NM_001004469	NM_001004469	Homo sapiens olfactory receptor, family 10, subfamily J, member 5 (OR10J5), mRNA [NM_001004469]	NM_001004469
A_23_P51051	0.00238	2.14	NM_005081	NM_005081	Homo sapiens zinc finger protein 142 (clone pHZ-49) (ZNF142), mRNA [NM_005081]	NM_005081
A_23_P398254	0.00238	2.81	NM_020429	NM_020429	Homo sapiens SMAD specific E3 ubiquitin protein ligase 1 (SMURF1), transcript variant 1, mRNA [NM_020429]	NM_020429
A_32_P42913	0.00238	2.84	AI051172	AI051172	AI051172 oy94a06.x1 Soares_fetal_liver_spleen_1NFLS_S1 Homo sapiens cDNA clone IMAGE:1673458 3' similar to SW:SSRG_RAT Q08013 TRANSLOCON-ASSOCIATED PROTEIN, GAMMA SUBUNIT ;, mRNA sequence [AI051172]	
A_24_P489399	0.00238	3.18	A_24_P489399			
A_24_P156922	0.00238	3.36	NM_001007098	NM_001007098	Homo sapiens sterol carrier protein 2 (SCP2), transcript variant 2, mRNA [NM_001007098]	NM_001007098
A_23_P57570	0.00238	4.46	NM_017436	NM_017436	Homo sapiens alpha 1,4-galactosyltransferase (globotriaosylceramide synthase) (A4GALT), mRNA [NM_017436]	NM_017436
A_24_P311604	0.00239	2.39	NM_145048	NM_145048	Homo sapiens hypothetical protein MGC29898 (MGC29898), mRNA [NM_145048]	NM_145048
A_23_P339557	0.00239	2.48	NM_032228	NM_032228	Homo sapiens male sterility domain containing 2 (MLSTD2), mRNA [NM_032228]	NM_032228
A_24_P678104	0.00239	6.76	NM_015894	NM_015894	Homo sapiens stathmin-like 3 (STMN3), mRNA [NM_015894]	NM_015894
A_32_P120895	0.0024	2.57	NM_153374	NM_153374	Homo sapiens LysM, putative peptidoglycan-binding, domain containing 2 (LYSMD2), mRNA [NM_153374]	NM_153374
A_32_P143048	0.0024	2.64	NM_004799	NM_004799	Homo sapiens zinc finger, FYVE domain containing 9 (ZFYVE9), transcript variant 3, mRNA [NM_004799]	NM_004799
A_23_P166100	0.00241	2.09	NM_021156	NM_021156	Homo sapiens hypothetical protein DJ971N18.2 (DJ971N18.2), mRNA [NM_021156]	NM_021156
A_23_P394014	0.00241	2.26	NM_015134	NM_015134	Homo sapiens myosin phosphatase-Rho interacting protein (M-RIP), mRNA [NM_015134]	NM_015134
A_23_P44643	0.00241	2.38	NM_016238	NM_016238	Homo sapiens anaphase promoting complex subunit 7 (ANAPC7), mRNA [NM_016238]	NM_016238
A_23_P102183	0.00241	2.42	L48692	L48692	Homo sapiens (clone p5-23-3) mRNA. [L48692]	
A_23_P102192	0.00241	2.84	NM_145686	NM_145686	Homo sapiens mitogen-activated protein kinase kinase kinase kinase 4 (MAP4K4), transcript variant 2, mRNA [NM_145686]	NM_145686
A_32_P85676	0.00241	4.46	NM_018401	NM_018401	Homo sapiens serine/threonine kinase 32B (STK32B), mRNA [NM_018401]	NM_018401

A_23_P348264	0.00241	9.17	NM_144652	NM_144652	Homo sapiens leucine zipper-EF-hand containing transmembrane protein 2 (LETM2), mRNA [NM_144652]	NM_144652
A_32_P53822	0.00242	2.04	BQ359867	BQ359867	BQ359867 PM0-HN0132-090501-006-C04 HN0132 Homo sapiens cDNA, mRNA sequence [BQ359867]	
A_24_P136641	0.00242	2.04	BC013655	BC013655	Homo sapiens, clone IMAGE:4152599, mRNA, partial cds. [BC013655]	
A_23_P143016	0.00242	2.31	NM_006673	NM_006673	Homo sapiens AT rich interactive domain 5A (MRF1-like) (ARID5A), transcript variant 2, mRNA [NM_006673]	NM_006673
A_23_P353574	0.00242	2.85	NM_133494	NM_133494	Homo sapiens NIMA (never in mitosis gene a)-related kinase 7 (NEK7), mRNA [NM_133494]	NM_133494
A_24_P278637	0.00242	4.22	NM_003824	NM_003824	Homo sapiens Fas (TNFRSF6)-associated via death domain (FADD), mRNA [NM_003824]	NM_003824
A_24_P362646	0.00243	2.05	NM_005783	NM_005783	Homo sapiens thioredoxin domain containing 9 (TXNDC9), mRNA [NM_005783]	NM_005783
A_23_P63281	0.00243	2.08	NM_001029885	NM_001029885	Homo sapiens hypothetical protein MGC10334 (MGC10334), mRNA [NM_001029885]	NM_001029885
A_24_P120251	0.00243	2.33	NM_138786	NM_138786	Homo sapiens transmembrane 4 L six family member 18 (TM4SF18), mRNA [NM_138786]	NM_138786
A_24_P916718	0.00243	2.58	NM_207336	NM_207336	Homo sapiens zinc finger protein 467 (ZNF467), mRNA [NM_207336]	NM_207336
A_23_P212025	0.00243	5.00	AL833148	AL833148	Homo sapiens mRNA; cDNA DKFZp313H1440 (from clone DKFZp313H1440). [AL833148]	XM_172341
A_24_P354468	0.00244	2.13	NM_002106	NM_002106	Homo sapiens H2A histone family, member Z (H2AFZ), mRNA [NM_002106]	NM_002106
A_23_P149281	0.00244	3.62	NM_004431	NM_004431	Homo sapiens EPH receptor A2 (EPHA2), mRNA [NM_004431]	NM_004431
A_24_P901084	0.00245	2.20	XM_374965	XM_374965	PREDICTED: Homo sapiens similar to BC004636 protein (LOC400010), mRNA [XM_374965]	XM_374965
A_32_P143689	0.00245	2.29	BQ008507	BQ008507	BQ008507 UI-H-ED1-ayk-d-19-0-UI.s1 NCI_CGAP_ED1 Homo sapiens cDNA clone IMAGE:5839842 3', mRNA sequence [BQ008507]	
A_32_P230838	0.00245	2.48	BF085047	BF085047	BF085047 PM0-GN0018-130900-003-c12 GN0018 Homo sapiens cDNA, mRNA sequence [BF085047]	
A_23_P19182	0.00245	2.53	NM_016606	NM_016606	Homo sapiens chromosome 5 open reading frame 19 (C5orf19), mRNA [NM_016606]	NM_016606
A_24_P297166	0.00245	2.88	NM_001009939	NM_001009939	Homo sapiens septin 5 (SEPT5), transcript variant 2, mRNA [NM_001009939]	NM_001009939
A_23_P128008	0.00245	5.59	NM_021827	NM_021827	Homo sapiens hypothetical protein FLJ23514 (FLJ23514), mRNA [NM_021827]	NM_021827
A_24_P212314	0.00246	2.19	A_24_P212314			
A_23_P403745	0.00246	2.25	ENST00000254951		Homo sapiens cDNA clone IMAGE:5123182, partial cds. [BC024752]	
A_23_P358417	0.00246	2.30	NM_004085	NM_004085	Homo sapiens translocase of inner mitochondrial membrane 8 homolog A (yeast) (TIMM8A), nuclear gene encoding mitochondrial protein, mRNA [NM_004085]	NM_004085
A_32_P144619	0.00246	2.86	BF827074	BF827074	BF827074 MR2-HN0035-241100-009-e09 HN0035 Homo sapiens cDNA, mRNA sequence [BF827074]	
A_23_P35906	0.00246	17.12	NM_033306	NM_033306	Homo sapiens caspase 4, apoptosis-related cysteine protease (CASP4), transcript variant gamma, mRNA [NM_033306]	NM_033306
A_24_P212559	0.00247	2.07	NM_003482	NM_003482	Homo sapiens myeloid/lymphoid or mixed-lineage leukemia 2 (MLL2), mRNA [NM_003482]	NM_003482
A_23_P163580	0.00247	2.13	NM_025082	NM_025082	Homo sapiens hypothetical protein FLJ13111 (FLJ13111), mRNA [NM_025082]	NM_025082
A_23_P29655	0.00247	2.94	NM_020685	NM_020685	Homo sapiens chromosome 3 open reading frame 14 (C3orf14), mRNA [NM_020685]	NM_020685
A_24_P384573	0.00248	2.62	NM_013986	NM_013986	Homo sapiens Ewing sarcoma breakpoint region 1 (EWSR1), transcript variant EWS-b, mRNA [NM_013986]	NM_013986
A_24_P304549	0.00248	3.75	NM_005561	NM_005561	Homo sapiens lysosomal-associated membrane protein 1 (LAMP1), mRNA [NM_005561]	NM_005561
A_24_P340771	0.00249	2.18	A_24_P340771			
A_24_P106306	0.00249	2.29	NM_016093	NM_016093	Homo sapiens ribosomal protein L26-like 1 (RPL26L1), mRNA [NM_016093]	NM_016093
A_24_P410320	0.00249	2.72	AF091089	AF091089	Homo sapiens clone 666 unknown mRNA, partial sequence. [AF091089]	
A_24_P357056	0.0025	3.10	NM_152906	NM_152906	Homo sapiens hypothetical protein DKFZp761P1121 (DKFZp761P1121), mRNA [NM_152906]	NM_152906
A_24_P388659	0.0025	3.19	NM_031305	NM_031305	Homo sapiens Rho GTPase activating protein 24 (ARHGAP24), mRNA [NM_031305]	NM_031305
A_23_P24129	0.0025	26.18	NM_012242	NM_012242	Homo sapiens dickkopf homolog 1 (Xenopus laevis) (DKK1), mRNA [NM_012242]	NM_012242
A_24_P139742	0.00251	2.06	NM_022787	NM_022787	Homo sapiens nicotinamide nucleotide adenylyltransferase 1 (NMNAT1), mRNA [NM_022787]	NM_022787
A_24_P238616	0.00251	2.19	XM_498380	XM_498380	PREDICTED: Homo sapiens similar to Chain O, Crystal Structure Of The Rabbit Muscle Glyceraldehyde-3- Phosphate Dehydrogenase (Gapdh) (LOC402418), mRNA [XM_498380]	XM_498380
A_24_P112447	0.00251	2.21	NM_020354	NM_020354	Homo sapiens ectonucleoside triphosphate diphosphohydrolase 7 (ENTPD7), mRNA [NM_020354]	NM_020354
A_24_P419132	0.00251	4.22	NM_006733	NM_006733	Homo sapiens FSH primary response (LRPR1 homolog, rat) 1 (FSHPRH1), mRNA [NM_006733]	NM_006733

A_24_P639441	0.00251	4.52	NM_203330	NM_203330	Homo sapiens CD59 antigen p18-20 (antigen identified by monoclonal antibodies 16.3A5, EJ16, EJ30, EL32 and G344) (CD59), transcript variant 1, mRNA [NM_203330]	NM_203330
A_24_P944827	0.00252	2.42	AL122075	AL122075	Homo sapiens mRNA; cDNA DKFZp434N0735 (from clone DKFZp434N0735); partial cds. [AL122075]	
A_32_P70519	0.00252	2.76	BP420721	BP420721	BP420721 Homo sapiens small intestine Homo sapiens cDNA clone HIE04034r 3', mRNA sequence [BP420721]	
A_23_P170030	0.00252	5.59	NM_016564	NM_016564	Homo sapiens BM88 antigen (BM88), mRNA [NM_016564]	NM_016564
A_24_P282309	0.00252	6.99	NM_133337	NM_133337	Homo sapiens fer-1-like 3, myoferlin (C. elegans) (FER1L3), transcript variant 2, mRNA [NM_133337]	NM_133337
A_23_P311192	0.00253	2.16	NM_178324	NM_178324	Homo sapiens serine palmitoyltransferase, long chain base subunit 1 (SPTLC1), transcript variant 2, mRNA [NM_178324]	NM_178324
A_24_P129483	0.00253	2.40	NM_173492	NM_173492	Homo sapiens phosphatidylinositol-4-phosphate 5-kinase-like 1 (PIP5KL1), mRNA [NM_173492]	NM_173492
A_23_P9509	0.00253	2.82	A_23_P9509			
A_23_P16423	0.00254	2.08	AL390151	AL390151	Homo sapiens mRNA; cDNA DKFZp547O146 (from clone DKFZp547O146). [AL390151]	
A_24_P556030	0.00254	2.12	A_24_P556030			
A_23_P123466	0.00254	2.19	NM_153332	NM_153332	Homo sapiens three prime histone mRNA exonuclease 1 (THEX1), mRNA [NM_153332]	NM_153332
A_24_P255798	0.00254	2.22	NM_030974	NM_030974	Homo sapiens shank-interacting protein-like 1 (Sharpin), mRNA [NM_030974]	NM_030974
A_24_P324214	0.00254	2.22	A_24_P324214			
A_32_P831181	0.00254	2.25	NM_080626	NM_080626	Homo sapiens BRI3 binding protein (BRI3BP), mRNA [NM_080626]	NM_080626
A_24_P50281	0.00254	2.81	A_24_P50281			
A_32_P118686	0.00254	4.17	XM_498911	XM_498911	PREDICTED: Homo sapiens LOC440909 (LOC440909), mRNA [XM_498911]	XM_498911
A_23_P54147	0.00255	2.03	NM_016350	NM_016350	Homo sapiens ninein (GSK3B interacting protein) (NIN), transcript variant 4, mRNA [NM_016350]	NM_016350
A_23_P88351	0.00255	2.64	NM_015915	NM_015915	Homo sapiens spastic paraplegia 3A (autosomal dominant) (SPG3A), mRNA [NM_015915]	NM_015915
A_23_P164112	0.00255	2.74	NM_014798	NM_014798	Homo sapiens pleckstrin homology domain containing, family M (with RUN domain) member 1 (PLEKHM1), mRNA [NM_014798]	NM_014798
A_23_P433785	0.00255	4.44	NM_002561	NM_002561	Homo sapiens purinergic receptor P2X, ligand-gated ion channel, 5 (P2RX5), transcript variant 1, mRNA [NM_002561]	NM_002561
A_23_P206284	0.00256	2.44	NM_201525	NM_201525	Homo sapiens G protein-coupled receptor 56 (GPR56), transcript variant 3, mRNA [NM_201525]	NM_201525
A_24_P213503	0.00256	5.35	NM_006504	NM_006504	Homo sapiens protein tyrosine phosphatase, receptor type, E (PTPRE), transcript variant 1, mRNA [NM_006504]	NM_006504
A_24_P324074	0.00257	2.01	A_24_P324074			
A_24_P141786	0.00257	2.08	NM_001681	NM_001681	Homo sapiens ATPase, Ca++ transporting, cardiac muscle, slow twitch 2 (ATP2A2), transcript variant 2, mRNA [NM_001681]	NM_001681
A_24_P19983	0.00257	2.54	THC2437888		Q6XZE5 (Q6XZE5) CINCIN1, partial (5%) [THC2437888]	
A_23_P15414	0.00257	5.35	NM_145351	NM_145351	Homo sapiens scavenger receptor class F, member 1 (SCARF1), transcript variant 4, mRNA [NM_145351]	NM_145351
A_24_P223384	0.00258	2.00	NM_003513	NM_003513	Homo sapiens histone 1, H2ab (HIST1H2AB), mRNA [NM_003513]	NM_003513
A_23_P377717	0.00258	2.35	NM_002516	NM_002516	Homo sapiens neuro-oncological ventral antigen 2 (NOVA2), mRNA [NM_002516]	NM_002516
A_24_P388786	0.00258	3.48	AK026756	AK026756	Homo sapiens cDNA: FLJ23103 fis, clone LNG07621. [AK026756]	
A_23_P210015	0.00259	2.02	CR616748	CR616748	full-length cDNA clone CS0DK007YM08 of HeLa cells Cot 25-normalized of Homo sapiens (human). [CR616748]	
A_24_P216681	0.00259	2.29	NM_032881	NM_032881	Homo sapiens LSM10, U7 small nuclear RNA associated (LSM10), mRNA [NM_032881]	NM_032881
A_23_P342727	0.00259	6.80	NM_178006	NM_178006	Homo sapiens START domain containing 13 (STARD13), transcript variant alpha, mRNA [NM_178006]	NM_178006
A_32_P207169	0.0026	3.33	BC094794	BC094794	Homo sapiens cDNA clone IMAGE:30520069. [BC094794]	
A_23_P10701	0.0026	3.75	NM_173075	NM_173075	Homo sapiens amyloid beta (A4) precursor protein-binding, family B, member 2 (Fe65-like) (APBB2), mRNA [NM_173075]	NM_173075
A_23_P71889	0.00261	2.11	NM_153437	NM_153437	Homo sapiens outer dense fiber of sperm tails 2 (ODF2), transcript variant 2, mRNA [NM_153437]	NM_153437
A_24_P683011	0.00261	2.43	BM696546	BM696546	UI-E-DW0-agk-i-01-0-UI.r1 UI-E-DW0 Homo sapiens cDNA clone UI-E-DW0-agk-i-01-0-UI 5', mRNA sequence [BM696546]	
A_24_P255082	0.00261	3.70	NM_198563	NM_198563	Homo sapiens Similar to RIKEN cDNA 1810038N08 gene (MGC52022), mRNA [NM_198563]	NM_198563
A_23_P66241	0.00261	7.81	NM_176870	NM_176870	Homo sapiens metallothionein 1K (MT1K), mRNA [NM_176870]	NM_176870
A_24_P393322	0.00262	2.09	AK091963	AK091963	Homo sapiens cDNA FLJ34644 fis, clone KIDNE2017040. [AK091963]	

A_24_P319635	0.00262	3.70	NM_021960	NM_021960	Homo sapiens myeloid cell leukemia sequence 1 (BCL2-related) (MCL1), transcript variant 1, mRNA [NM_021960]	NM_021960
A_23_P104073	0.00262	6.49	NM_002960	NM_002960	Homo sapiens S100 calcium binding protein A3 (S100A3), mRNA [NM_002960]	NM_002960
A_23_P402899	0.00262	16.69	NM_144975	NM_144975	Homo sapiens hypothetical protein MGC19764 (MGC19764), mRNA [NM_144975]	NM_144975
A_24_P337104	0.00263	2.25	NM_000916	NM_000916	Homo sapiens oxytocin receptor (OXTR), mRNA [NM_000916]	NM_000916
A_24_P289178	0.00264	2.37	BC009078	BC009078	Homo sapiens MGC17624 protein, mRNA (cDNA clone MGC:17624 IMAGE:3855543), complete cds. [BC009078]	
A_23_P388168	0.00264	2.56	NM_002867	NM_002867	Homo sapiens RAB3B, member RAS oncogene family (RAB3B), mRNA [NM_002867]	NM_002867
A_24_P105771	0.00264	3.51	NM_005721	NM_005721	Homo sapiens ARP3 actin-related protein 3 homolog (yeast) (ACTR3), mRNA [NM_005721]	NM_005721
A_32_P161762	0.00264	6.58	NM_004348	NM_004348	Homo sapiens runt-related transcription factor 2 (RUNX2), transcript variant 3, mRNA [NM_004348]	NM_004348
A_23_P314712	0.00265	4.76	NM_012189	NM_012189	Homo sapiens calcium binding tyrosine-(Y)-phosphorylation regulated (fibrousheathin 2) (CABYR), transcript variant 1, mRNA [NM_012189]	NM_012189
A_32_P45875	0.00266	3.02	AW009873	AW009873	AW009873 ws88f04.x1 NCI_CGAP_Co3 Homo sapiens cDNA clone IMAGE:2505055 3' similar to TR:Q62084 Q62084 PHOSPHOLIPASE C NEIGHBORING ;, mRNA sequence [AW009873]	
A_32_P82448	0.00266	3.30	H78340	H78340	H78340 yu79e09.s1 Soares fetal liver spleen 1NFLS Homo sapiens cDNA clone IMAGE:240040 3', mRNA sequence [H78340]	
A_32_P97536	0.00267	2.27	BQ185350	BQ185350	BQ185350 UI-E-EJ1-aju-n-07-0-UI.s1 UI-E-EJ1 Homo sapiens cDNA clone UI-E-EJ1-aju-n-07-0-UI 3', mRNA sequence [BQ185350]	
A_24_P80152	0.00267	2.87	NM_144582	NM_144582	Homo sapiens testis expressed sequence 261 (TEX261), mRNA [NM_144582]	NM_144582
A_23_P500251	0.00267	2.96	NM_003404	NM_003404	Homo sapiens tyrosine 3-monooxygenase/tryptophan 5-monooxygenase activation protein, beta polypeptide (YWHAB), transcript variant 1, mRNA [NM_003404]	NM_003404
A_24_P184295	0.00267	3.16	AF099011	AF099011	Homo sapiens EH-domain containing protein testilin mRNA, complete cds. [AF099011]	
A_24_P242138	0.00267	3.61	NM_182614	NM_182614	Homo sapiens hypothetical protein MGC20579 (MGC20579), mRNA [NM_182614]	NM_182614
A_23_P345650	0.00268	2.07	NM_015691	NM_015691	Homo sapiens KIAA1280 protein (KIAA1280), mRNA [NM_015691]	NM_015691
A_23_P404871	0.00268	2.09	BC012766	BC012766	Homo sapiens endoplasmic reticulum-golgi intermediate compartment 32 kDa protein, mRNA (cDNA clone MGC:16233 IMAGE:3677787), complete cds. [BC012766]	
A_23_P217326	0.00268	2.24	NM_001449	NM_001449	Homo sapiens four and a half LIM domains 1 (FHL1), mRNA [NM_001449]	NM_001449
A_24_P269779	0.0027	2.02	NM_004273	NM_004273	Homo sapiens carbohydrate (chondroitin 6) sulfotransferase 3 (CHST3), mRNA [NM_004273]	NM_004273
A_23_P32558	0.0027	2.14	NM_017588	NM_017588	Homo sapiens WD repeat domain 5 (WDR5), transcript variant 1, mRNA [NM_017588]	NM_017588
A_32_P106512	0.0027	2.35	THC2308340			
A_24_P362394	0.0027	2.39	NM_007259	NM_007259	Homo sapiens vacuolar protein sorting 45A (yeast) (VPS45A), mRNA [NM_007259]	NM_007259
A_23_P51136	0.0027	2.57	NM_004040	NM_004040	Homo sapiens ras homolog gene family, member B (RHOB), mRNA [NM_004040]	NM_004040
A_23_P2203	0.00271	2.31	NM_016281	NM_016281	Homo sapiens TAO kinase 3 (TAOK3), mRNA [NM_016281]	NM_016281
A_32_P179177	0.00271	2.46	NM_181519	NM_181519	Homo sapiens synaptotagmin XV (SYT15), transcript variant b, mRNA [NM_181519]	NM_181519
A_24_P305597	0.00271	2.56	NM_007002	NM_007002	Homo sapiens adhesion regulating molecule 1 (ADRM1), transcript variant 1, mRNA [NM_007002]	NM_007002
A_24_P148653	0.00271	2.72	NM_018385	NM_018385	Homo sapiens hypothetical protein FLJ11301 (FLJ11301), mRNA [NM_018385]	NM_018385
A_23_P329870	0.00272	2.67	NM_024599	NM_024599	Homo sapiens rhomboid, veinlet-like 6 (Drosophila) (RHBDL6), transcript variant 1, mRNA [NM_024599]	NM_024599
A_23_P45324	0.00272	2.82	NM_021637	NM_021637	Homo sapiens transmembrane protein 35 (TMEM35), mRNA [NM_021637]	NM_021637
A_23_P16047	0.00272	4.24	NM_005282	NM_005282	Homo sapiens G protein-coupled receptor 4 (GPR4), mRNA [NM_005282]	NM_005282
A_23_P371495	0.00272	5.95	AK125140	AK125140	Homo sapiens cDNA FLJ43150 fis, clone CTONG3009239. [AK125140]	
A_24_P375002	0.00273	2.11	NM_004559	NM_004559	Homo sapiens nuclease sensitive element binding protein 1 (NSEP1), mRNA [NM_004559]	NM_004559
A_24_P236949	0.00273	2.91	NM_018381	NM_018381	Homo sapiens hypothetical protein FLJ11286 (FLJ11286), mRNA [NM_018381]	NM_018381
A_23_P115286	0.00274	2.90	NM_003921	NM_003921	Homo sapiens B-cell CLL/lymphoma 10 (BCL10), mRNA [NM_003921]	NM_003921
A_24_P933180	0.00275	2.38	NM_000821	NM_000821	Homo sapiens gamma-glutamyl carboxylase (GGCX), mRNA [NM_000821]	NM_000821
A_24_P203658	0.00275	2.60	ENST00000297544		PREDICTED: Homo sapiens similar to 60S acidic ribosomal protein P1 (LOC133609), mRNA [XM_068430]	XM_068430
A_23_P357101	0.00275	5.43	NM_145298	NM_145298	Homo sapiens apolipoprotein B mRNA editing enzyme, catalytic polypeptide-like 3F (APOBEC3F), transcript variant 1, mRNA [NM_145298]	NM_145298
A_32_P221723	0.00276	2.26	THC2350968		AB100453 YY1 associated factor-2-b {Mus musculus;} , partial (63%) [THC2350968]	

A_23_P216894	0.00276	2.95	NM_001006617	NM_001006617	Homo sapiens mitogen-activated protein kinase associated protein 1 (MAPKAP1), transcript variant 1, mRNA [NM_001006617]	NM_001006617
A_23_P101774	0.00277	2.19	NM_000453	NM_000453	Homo sapiens solute carrier family 5 (sodium iodide symporter), member 5 (SLC5A5), mRNA [NM_000453]	NM_000453
A_24_P167683	0.00277	2.38	NM_014216	NM_014216	Homo sapiens inositol 1,3,4-triphosphate 5/6 kinase (ITPK1), mRNA [NM_014216]	NM_014216
A_24_P359856	0.00277	2.38	NM_006037	NM_006037	Homo sapiens histone deacetylase 4 (HDAC4), mRNA [NM_006037]	NM_006037
A_32_P188	0.00277	2.39	BM458245	BM458245	AGENCOURT_6411506 NIH_MGC_71 Homo sapiens cDNA clone IMAGE:5530621 5', mRNA sequence [BM458245]	XM_373700
A_23_P28878	0.00277	3.72	AK022713	AK022713	Homo sapiens cDNA FLJ12651 fis, clone NT2RM4002062, moderately similar to ASPARTYL-TRNA SYNTHETASE (EC 6.1.1.12). [AK022713]	
A_32_P65571	0.00278	2.31	BX648855	BX648855	Homo sapiens mRNA; cDNA DKFZp686L05231 (from clone DKFZp686L05231). [BX648855]	
A_23_P146981	0.00278	3.31	A_23_P146981			
A_23_P81369	0.00278	4.63	NM_023038	NM_023038	Homo sapiens a disintegrin and metalloproteinase domain 19 (meltrin beta) (ADAM19), transcript variant 1, mRNA [NM_023038]	NM_023038
A_24_P314681	0.00278	4.65	ENST00000248179		BX281397 Soares_NFL_T_GBC_S1 Homo sapiens cDNA clone IMAGp998G075145 ; IMAGE:2087454, mRNA sequence [BX281397]	XM_370585
A_23_P323749	0.00279	2.11	NM_025049	NM_025049	Homo sapiens chromosome 15 open reading frame 20 (C15orf20), mRNA [NM_025049]	NM_025049
A_23_P2262	0.00279	2.27	NM_004264	NM_004264	Homo sapiens SRB7 suppressor of RNA polymerase B homolog (yeast) (SURB7), mRNA [NM_004264]	NM_004264
A_32_P182246	0.00279	2.54	A_32_P182246			
A_23_P134295	0.0028	2.38	NM_198949	NM_198949	Homo sapiens nudix (nucleoside diphosphate linked moiety X)-type motif 1 (NUDT1), transcript variant 2B, mRNA [NM_198949]	NM_198949
A_24_P110201	0.0028	2.40	A_24_P110201			
A_24_P922631	0.0028	2.43	BC092511	BC092511	Homo sapiens cDNA clone IMAGE:5271968. [BC092511]	XM_059672
A_24_P258235	0.0028	2.54	NM_001004739	NM_001004739	Homo sapiens olfactory receptor, family 5, subfamily L, member 2 (OR5L2), mRNA [NM_001004739]	NM_001004739
A_32_P166921	0.0028	2.76	AV692191	AV692191	AV692191 AV692191 GKC Homo sapiens cDNA clone GKCFFD09 5', mRNA sequence [AV692191]	
A_23_P55099	0.0028	7.69	NM_002737	NM_002737	Homo sapiens protein kinase C, alpha (PRKCA), mRNA [NM_002737]	NM_002737
A_32_P144220	0.00281	2.03	XM_498088	XM_498088	PREDICTED: Homo sapiens similar to myosin:SUBUNIT=regulatory light chain (LOC442204), mRNA [XM_498088]	XM_498088
A_24_P759747	0.00281	2.11	BC029479	BC029479	Homo sapiens, clone IMAGE:4734409, mRNA. [BC029479]	XM_211090
A_24_P46689	0.00281	2.45	NM_015278	NM_015278	Homo sapiens SAM and SH3 domain containing 1 (SASH1), mRNA [NM_015278]	NM_015278
A_23_P126416	0.00281	4.15	NM_005424	NM_005424	Homo sapiens tyrosine kinase with immunoglobulin-like and EGF-like domains 1 (TIE1), mRNA [NM_005424]	NM_005424
A_23_P158627	0.00282	2.31	NM_024078	NM_024078	Homo sapiens hypothetical protein MGC3162 (MGC3162), mRNA [NM_024078]	NM_024078
A_24_P58177	0.00282	2.31	BC009182	BC009182	Homo sapiens chromosome 14 open reading frame 159, mRNA (cDNA clone IMAGE:3943486), complete cds. [BC009182]	
A_23_P321388	0.00282	2.77	NM_153341	NM_153341	Homo sapiens IBR domain containing 3 (IBRDC3), mRNA [NM_153341]	NM_153341
A_24_P76666	0.00282	3.05	NM_177559	NM_177559	Homo sapiens casein kinase 2, alpha 1 polypeptide (CSNK2A1), transcript variant 1, mRNA [NM_177559]	NM_177559
A_32_P70626	0.00282	8.40	THC2379184		AF030177 N-acetylglucosaminyl transferase component Gpi1 {Homo sapiens;} , partial (4%) [THC2379184]	
A_32_P45588	0.00283	2.31	BM474343	BM474343	BM474343 AGENCOURT_6489981 NIH_MGC_71 Homo sapiens cDNA clone IMAGE:5521645 5', mRNA sequence [BM474343]	
A_23_P357571	0.00283	2.52	NM_000854	NM_000854	Homo sapiens glutathione S-transferase theta 2 (GSTT2), mRNA [NM_000854]	NM_000854
A_23_P376096	0.00283	2.87	NM_014261	NM_014261	Homo sapiens toll-like receptor adaptor molecule 1 (TICAM1), transcript variant 1, mRNA [NM_014261]	NM_014261
A_23_P97541	0.00283	2.92	NM_000715	NM_000715	Homo sapiens complement component 4 binding protein, alpha (C4BPA), mRNA [NM_000715]	NM_000715
A_23_P104876	0.00283	3.07	NM_017425	NM_017425	Homo sapiens sperm autoantigenic protein 17 (SPA17), mRNA [NM_017425]	NM_017425
A_24_P55092	0.00283	3.80	NM_133493	NM_133493	Homo sapiens CD109 antigen (Gov platelet alloantigens) (CD109), mRNA [NM_133493]	NM_133493
A_23_P60016	0.00284	2.10	AF095289	AF095289	Homo sapiens pituitary tumor transforming gene protein 3 (PTTG3) mRNA, complete cds. [AF095289]	
A_24_P75840	0.00284	2.56	ENST00000325635			
A_23_P85585	0.00284	3.47	NM_022164	NM_022164	Homo sapiens lipocalin 7 (LCN7), mRNA [NM_022164]	NM_022164
A_24_P178093	0.00285	2.21	NM_006114	NM_006114	Homo sapiens translocase of outer mitochondrial membrane 40 homolog (yeast) (TOMM40), mRNA [NM_006114]	NM_006114

A_23_P144257	0.00285	2.29	NM_198189	NM_198189	Homo sapiens COP9 constitutive photomorphogenic homolog subunit 8 (Arabidopsis) (COPS8), transcript variant 2, mRNA [NM_198189]	NM_198189
A_23_P343914	0.00285	3.30	NM_177966	NM_177966	Homo sapiens 2'-phosphodiesterase (2'-PDE), mRNA [NM_177966]	NM_177966
A_23_P426398	0.00285	4.55	NM_138575	NM_138575	Homo sapiens Bcl-XL-binding protein v68 (MGC5352), mRNA [NM_138575]	NM_138575
A_23_P170687	0.00286	2.22	NM_012267	NM_012267	Homo sapiens hsp70-interacting protein (HSPBP1), mRNA [NM_012267]	NM_012267
A_32_P501400	0.00286	2.24	NM_030752	NM_030752	Homo sapiens t-complex 1 (TCP1), transcript variant 1, mRNA [NM_030752]	NM_030752
A_23_P105264	0.00286	2.97	NM_001987	NM_001987	Homo sapiens ets variant gene 6 (TEL oncogene) (ETV6), mRNA [NM_001987]	NM_001987
A_23_P117238	0.00287	3.22	NM_024537	NM_024537	Homo sapiens hypothetical protein FLJ12118 (FLJ12118), mRNA [NM_024537]	NM_024537
A_23_P120931	0.00287	4.98	NM_014508	NM_014508	Homo sapiens apolipoprotein B mRNA editing enzyme, catalytic polypeptide-like 3C (APOBEC3C), mRNA [NM_014508]	NM_014508
A_23_P45712	0.00288	2.15	XM_372785	XM_372785	PREDICTED: Homo sapiens similar to peptidylprolyl isomerase A isoform 1; cyclophilin A; peptidyl-prolyl cis-trans isomerase A; T cell cyclophilin; rotamase; cyclosporin A-binding protein (LOC391062), mRNA [XM_372785]	XM_372785
A_24_P900721	0.00288	2.55	A_24_P900721			
A_32_P117977	0.00288	2.81	BX360933	BX360933	BX360933 BX360933 Homo sapiens PLACENTA COT 25-NORMALIZED Homo sapiens cDNA clone CS0DI077YB17 3-PRIME, mRNA sequence [BX360933]	
A_24_P58727	0.00289	3.25	A_24_P58727			
A_23_P16733	0.00289	3.41	NM_002881	NM_002881	Homo sapiens v-ral simian leukemia viral oncogene homolog B (ras related; GTP binding protein) (RALB), mRNA [NM_002881]	NM_002881
A_23_P431776	0.0029	2.72	NM_001986	NM_001986	Homo sapiens ets variant gene 4 (E1A enhancer binding protein, E1AF) (ETV4), mRNA [NM_001986]	NM_001986
A_23_P24192	0.0029	2.87	NM_015179	NM_015179	Homo sapiens KIAA0690 (KIAA0690), mRNA [NM_015179]	NM_015179
A_32_P191541	0.00291	2.07	BU853136	BU853136	AGENCOURT_10411121 NIH_MGC_82 Homo sapiens cDNA clone IMAGE:6619843 5', mRNA sequence [BU853136]	
A_32_P416583	0.00291	2.40	NM_032206	NM_032206	Homo sapiens nucleotide-binding oligomerization domains 27 (NOD27), mRNA [NM_032206]	NM_032206
A_24_P340679	0.00291	2.70	NM_001008741	NM_001008741	Homo sapiens peptidylprolyl isomerase A-like (LOC388817), mRNA [NM_001008741]	NM_001008741
A_24_P305345	0.00292	2.07	NM_021155	NM_021155	Homo sapiens CD209 antigen (CD209), mRNA [NM_021155]	NM_021155
A_23_P89708	0.00292	2.33	NM_018129	NM_018129	Homo sapiens pyridoxine 5'-phosphate oxidase (PNPO), mRNA [NM_018129]	NM_018129
A_23_P90032	0.00292	3.08	NM_025061	NM_025061	Homo sapiens leucine rich repeat containing 8 family, member E (LRRC8E), mRNA [NM_025061]	NM_025061
A_32_P24382	0.00293	3.37	BC063625	BC063625	Homo sapiens keratin associated protein 2-4, mRNA (cDNA clone MGC:74790 IMAGE:3907481), complete cds. [BC063625]	
A_24_P11384	0.00293	3.68	NM_018948	NM_018948	Homo sapiens mitogen-inducible gene 6 (MIG-6), mRNA [NM_018948]	NM_018948
A_24_P174563	0.00293	3.73	NM_025196	NM_025196	Homo sapiens GrpE-like 1, mitochondrial (E. coli) (GRPEL1), mRNA [NM_025196]	NM_025196
A_24_P75456	0.00293	4.39	ENST00000330189		PREDICTED: Homo sapiens similar to peptidylprolyl isomerase A (LOC402252), mRNA [XM_377934]	XM_377934
A_23_P25913	0.00294	3.02	NM_031427	NM_031427	Homo sapiens chromosome 14 open reading frame 168 (C14orf168), mRNA [NM_031427]	NM_031427
A_24_P345314	0.00295	2.00	NM_003189	NM_003189	Homo sapiens T-cell acute lymphocytic leukemia 1 (TAL1), mRNA [NM_003189]	NM_003189
A_23_P86330	0.00295	2.07	NM_016545	NM_016545	Homo sapiens immediate early response 5 (IER5), mRNA [NM_016545]	NM_016545
A_24_P68631	0.00295	2.23	NM_175065	NM_175065	Homo sapiens histone 2, H2ab (HIST2H2AB), mRNA [NM_175065]	NM_175065
A_23_P430120	0.00295	2.33	NM_001430	NM_001430	Homo sapiens endothelial PAS domain protein 1 (EPAS1), mRNA [NM_001430]	NM_001430
A_32_P150632	0.00296	2.04	THC2360823			
A_23_P214111	0.00296	2.15	NM_022113	NM_022113	Homo sapiens kinesin family member 13A (KIF13A), mRNA [NM_022113]	NM_022113
A_32_P2634	0.00296	2.15	CR604707	CR604707	full-length cDNA clone CS0DI009YC01 of Placenta Cot 25-normalized of Homo sapiens (human). [CR604707]	XM_498482
A_23_P144807	0.00296	2.62	AF440762	AF440762	Homo sapiens septin SEPT8_v2 (KIAA0202) mRNA, partial cds, alternatively spliced. [AF440762]	XM_034872
A_23_P256279	0.00296	2.72	NM_145267	NM_145267	Homo sapiens chromosome 6 open reading frame 57 (C6orf57), mRNA [NM_145267]	NM_145267
A_24_P41483	0.00296	2.77	ENST00000321952			
A_24_P118231	0.00296	3.01	NM_001003803	NM_001003803	Homo sapiens ATP synthase, H+ transporting, mitochondrial F0 complex, subunit s (factor B) (ATP5S), nuclear gene encoding mitochondrial protein, transcript variant 1, mRNA [NM_001003803]	NM_001003803

A_23_P42718	0.00296	6.54	NM_004289	NM_004289	Homo sapiens nuclear factor (erythroid-derived 2)-like 3 (NFE2L3), mRNA [NM_004289]	NM_004289
A_23_P53557	0.00298	4.22	NM_002342	NM_002342	Homo sapiens lymphotoxin beta receptor (TNFR superfamily, member 3) (LTBR), mRNA [NM_002342]	NM_002342
A_23_P433504	0.00299	2.00	AK091315	AK091315	Homo sapiens cDNA FLJ33996 fis, clone DFNES2008881. [AK091315]	
A_23_P370569	0.00299	3.00	NM_152440	NM_152440	Homo sapiens hypothetical protein FLJ32549 (FLJ32549), mRNA [NM_152440]	NM_152440
A_23_P406616	0.00299	3.06	NM_175884	NM_175884	Homo sapiens hypothetical protein FLJ36031 (FLJ36031), mRNA [NM_175884]	NM_175884
A_24_P169976	0.00299	3.37	A_24_P169976			
A_23_P344400	0.00299	3.47	NM_020731	NM_020731	Homo sapiens aryl-hydrocarbon receptor repressor (AHRR), mRNA [NM_020731]	NM_020731
A_23_P210425	0.00299	3.82	NM_181526	NM_181526	Homo sapiens myosin, light polypeptide 9, regulatory (MYL9), transcript variant 2, mRNA [NM_181526]	NM_181526
A_32_P182662	0.003	2.87	NM_022831	NM_022831	Homo sapiens chromosome 1 open reading frame 80 (C1orf80), mRNA [NM_022831]	NM_022831
A_32_P205550	0.00301	2.24	NM_016093	NM_016093	Homo sapiens ribosomal protein L26-like 1 (RPL26L1), mRNA [NM_016093]	NM_016093
A_32_P752551	0.00301	2.62	AF130109	AF130109	Homo sapiens clone FLB5740 PRO1512 mRNA, complete cds. [AF130109]	
A_23_P306804	0.00301	7.09	NM_133467	NM_133467	Homo sapiens Cbp/p300-interacting transactivator, with Glu/Asp-rich carboxy-terminal domain, 4 (CITED4), mRNA [NM_133467]	NM_133467
A_32_P102252	0.00302	2.26	CR591764	CR591764	full-length cDNA clone CS0DF026YI01 of Fetal brain of Homo sapiens (human). [CR591764]	
A_24_P179504	0.00302	3.69	NM_144668	NM_144668	Homo sapiens WD repeat domain 66 (WDR66), mRNA [NM_144668]	NM_144668
A_23_P202773	0.00304	2.11	NM_016611	NM_016611	Homo sapiens potassium channel, subfamily K, member 4 (KCNK4), transcript variant 1, mRNA [NM_016611]	NM_016611
A_24_P55148	0.00304	2.27	NM_021058	NM_021058	Homo sapiens histone 1, H2bj (HIST1H2BJ), mRNA [NM_021058]	NM_021058
A_23_P77593	0.00304	2.52	NM_018052	NM_018052	Homo sapiens Vac14 homolog (S. cerevisiae) (VAC14), mRNA [NM_018052]	NM_018052
A_24_P360674	0.00304	6.33	NM_078487	NM_078487	Homo sapiens cyclin-dependent kinase inhibitor 2B (p15, inhibits CDK4) (CDKN2B), transcript variant 2, mRNA [NM_078487]	NM_078487
A_32_P157671	0.00305	2.43	A_32_P157671			
A_23_P401361	0.00305	2.60	ENST00000280562		Homo sapiens mRNA for KIAA1457 protein, partial cds. [AB040890]	
A_23_P206707	0.00305	4.33	NM_005950	NM_005950	Homo sapiens metallothionein 1G (MT1G), mRNA [NM_005950]	NM_005950
A_24_P75748	0.00306	2.96	A_24_P75748			
A_23_P210969	0.00307	2.13	NM_016436	NM_016436	Homo sapiens PHD finger protein 20 (PHF20), mRNA [NM_016436]	NM_016436
A_32_P121234	0.00307	2.25	A_32_P121234			
A_23_P210886	0.00307	2.68	NM_138578	NM_138578	Homo sapiens BCL2-like 1 (BCL2L1), nuclear gene encoding mitochondrial protein, transcript variant 1, mRNA [NM_138578]	NM_138578
A_24_P626920	0.00308	2.61	NM_005063	NM_005063	Homo sapiens stearoyl-CoA desaturase (delta-9-desaturase) (SCD), mRNA [NM_005063]	NM_005063
A_23_P116091	0.00308	2.86	NM_130443	NM_130443	Homo sapiens dipeptidylpeptidase 3 (DPP3), transcript variant 2, mRNA [NM_130443]	NM_130443
A_32_P48086	0.00308	3.13	BC015434	BC015434	Homo sapiens, clone IMAGE:4414697, mRNA. [BC015434]	
A_24_P54485	0.00309	2.39	NM_032357	NM_032357	Homo sapiens hypothetical protein MGC12981 (MGC12981), mRNA [NM_032357]	NM_032357
A_32_P47497	0.00309	3.56	N29747	N29747	N29747 yw90e10.s1 Soares_placenta_8to9weeks_2NbHP8to9W Homo sapiens cDNA clone IMAGE:259530 3' similar to SP:SW:IPK1_BOVIN P16436 PROTEIN KINASE C INHIBITOR 1 ;, mRNA sequence [N29747]	
A_23_P118174	0.00311	2.06	NM_005030	NM_005030	Homo sapiens polo-like kinase 1 (Drosophila) (PLK1), mRNA [NM_005030]	NM_005030
A_24_P81789	0.00311	2.08	NM_019034	NM_019034	Homo sapiens ras homolog gene family, member F (in filopodia) (RHOF), mRNA [NM_019034]	NM_019034
A_23_P170058	0.00311	2.14	NM_002794	NM_002794	Homo sapiens proteasome (prosome, macropain) subunit, beta type, 2 (PSMB2), mRNA [NM_002794]	NM_002794
A_24_P272073	0.00311	2.33	ENST00000335078			
A_23_P62361	0.00311	2.44	NM_014235	NM_014235	Homo sapiens ubiquitin-like 4 (UBL4), mRNA [NM_014235]	NM_014235
A_23_P131502	0.00311	3.55	NM_153712	NM_153712	Homo sapiens tubulin tyrosine ligase (TTL), mRNA [NM_153712]	NM_153712
A_32_P49654	0.00312	2.70	BC020336	BC020336	Homo sapiens ribosomal protein S2, mRNA (cDNA clone IMAGE:3857902). [BC020336]	
A_23_P32233	0.00312	2.71	NM_004235	NM_004235	Homo sapiens Kruppel-like factor 4 (gut) (KLF4), mRNA [NM_004235]	NM_004235
A_24_P264772	0.00312	3.28	NM_178230	NM_178230	Homo sapiens peptidylprolyl isomerase A (cyclophilin A)-like 4 (PPIAL4), mRNA [NM_178230]	NM_178230
A_23_P106425	0.00313	2.13	NM_182616	NM_182616	Homo sapiens hypothetical protein MGC61550 (MGC61550), mRNA [NM_182616]	NM_182616

A_23_P161064	0.00313	2.21	NM_014455	NM_014455	Homo sapiens zinc finger protein 364 (ZNF364), mRNA [NM_014455]	NM_014455
A_23_P40657	0.00313	2.32	NM_014291	NM_014291	Homo sapiens glycine C-acetyltransferase (2-amino-3-ketobutyrate coenzyme A ligase) (GCAT), nuclear gene encoding mitochondrial protein, mRNA [NM_014291]	NM_014291
A_24_P181672	0.00313	2.33	NM_001009905	NM_001009905	Homo sapiens UDP-GlcNAc:betaGal beta-1,3-N-acetylglucosaminyltransferase-like 1 (B3GNTL1), mRNA [NM_001009905]	NM_001009905
A_32_P95757	0.00313	2.95	NM_145316	NM_145316	Homo sapiens chromosome 6 open reading frame 128 (C6orf128), mRNA [NM_145316]	NM_145316
A_24_P99216	0.00313	3.30	NM_014045	NM_014045	Homo sapiens low density lipoprotein receptor-related protein 10 (LRP10), mRNA [NM_014045]	NM_014045
A_32_P98098	0.00314	2.07	BE719098	BE719098	BE719098 RC6-HT0840-280700-011-E09 HT0840 Homo sapiens cDNA, mRNA sequence [BE719098]	
A_23_P164733	0.00314	2.46	NM_004756	NM_004756	Homo sapiens numb homolog (Drosophila)-like (NUMBL), mRNA [NM_004756]	NM_004756
A_23_P347040	0.00314	2.79	NM_138287	NM_138287	Homo sapiens deltex 3-like (Drosophila) (DTX3L), mRNA [NM_138287]	NM_138287
A_24_P401036	0.00314	4.05	XM_373301	XM_373301	PREDICTED: Homo sapiens similar to peptidyl-Pro cis trans isomerase (LOC392352), mRNA [XM_373301]	XM_373301
A_32_P215304	0.00314	8.77	THC2305888			
A_24_P284420	0.00315	2.07	NM_001005862	NM_001005862	Homo sapiens v-erb-b2 erythroblastic leukemia viral oncogene homolog 2, neuro/glioblastoma derived oncogene homolog (avian) (ERBB2), transcript variant 2, mRNA [NM_001005862]	NM_001005862
A_24_P338971	0.00315	2.48	NM_030922	NM_030922	Homo sapiens non imprinted in Prader-Willi/Angelman syndrome 2 (NIPA2), transcript variant 1, mRNA [NM_030922]	NM_030922
A_24_P283928	0.00315	3.79	NM_024068	NM_024068	Homo sapiens hypothetical protein MGC2731 (MGC2731), mRNA [NM_024068]	NM_024068
A_23_P56630	0.00316	2.07	NM_007315	NM_007315	Homo sapiens signal transducer and activator of transcription 1, 91kDa (STAT1), transcript variant alpha, mRNA [NM_007315]	NM_007315
A_24_P329065	0.00316	2.61	NM_007048	NM_007048	Homo sapiens butyrophilin, subfamily 3, member A1 (BTN3A1), transcript variant 1, mRNA [NM_007048]	NM_007048
A_23_P42241	0.00317	2.07	NM_030876	NM_030876	Homo sapiens olfactory receptor, family 5, subfamily V, member 1 (OR5V1), mRNA [NM_030876]	NM_030876
A_23_P166248	0.00318	2.55	NM_004414	NM_004414	Homo sapiens Down syndrome critical region gene 1 (DSCR1), transcript variant 1, mRNA [NM_004414]	NM_004414
A_24_P127159	0.00318	2.83	M69012	M69012	Human guanine nucleotide-binding regulatory protein (G-y-2-alpha) mRNA, partial cds. [M69012]	XR_000184
A_23_P140792	0.00318	2.96	NM_013302	NM_013302	Homo sapiens eukaryotic elongation factor-2 kinase (EEF2K), mRNA [NM_013302]	NM_013302
A_24_P252739	0.00318	4.59	NM_001008490	NM_001008490	Homo sapiens Kruppel-like factor 6 (KLF6), transcript variant 1, mRNA [NM_001008490]	NM_001008490
A_23_P422144	0.00318	5.38	NM_153690	NM_153690	Homo sapiens family with sequence similarity 43, member A (FAM43A), mRNA [NM_153690]	NM_153690
A_24_P254285	0.0032	2.09	NM_052865	NM_052865	Homo sapiens chromosome 20 open reading frame 72 (C20orf72), mRNA [NM_052865]	NM_052865
A_24_P272389	0.0032	4.67	AK092228	AK092228	Homo sapiens cDNA FLJ34909 fis, clone NT2RI2009301, moderately similar to BIFUNCTIONAL METHYLENETETRAHYDROFOLATE DEHYDROGENASE/CYCLOHYDROLASE, MITOCHONDRIAL PRECURSOR. [AK092228]	XM_496652
A_32_P42684	0.0032	6.71	THC2310563			
A_23_P330788	0.00321	2.04	NM_015075	NM_015075	Homo sapiens IQ motif and Sec7 domain 2 (IQSEC2), mRNA [NM_015075]	NM_015075
A_23_P78302	0.00321	2.67	NM_003204	NM_003204	Homo sapiens nuclear factor (erythroid-derived 2)-like 1 (NFE2L1), mRNA [NM_003204]	NM_003204
A_23_P152406	0.00322	2.12	NM_032330	NM_032330	Homo sapiens calpain, small subunit 2 (CAPNS2), mRNA [NM_032330]	NM_032330
A_24_P213487	0.00322	2.15	AF271775	AF271775	Homo sapiens DC49 mRNA, complete cds. [AF271775]	
A_23_P435501	0.00322	2.69	NM_198941	NM_198941	Homo sapiens tumor differentially expressed 1 (TDE1), transcript variant 2, mRNA [NM_198941]	NM_198941
A_23_P29199	0.00322	4.08	NM_181831	NM_181831	Homo sapiens neurofibromin 2 (bilateral acoustic neuroma) (NF2), transcript variant 13, mRNA [NM_181831]	NM_181831
A_24_P406870	0.00323	2.01	NM_001014795	NM_001014795	Homo sapiens integrin-linked kinase (ILK), transcript variant 3, mRNA [NM_001014795]	NM_001014795
A_23_P212595	0.00323	2.02	NM_014703	NM_014703	Homo sapiens Vpr-binding protein (VprBP), mRNA [NM_014703]	NM_014703
A_24_P288323	0.00323	4.42	NM_139159	NM_139159	Homo sapiens dipeptidylpeptidase 9 (DPP9), mRNA [NM_139159]	NM_139159
A_23_P350249	0.00323	4.95	AB065086	AB065086	Homo sapiens mRNA for OK/KNS-CL.4, complete cds. [AB065086]	
A_24_P902313	0.00324	2.30	BF129169	BF129169	601811439F1 NIH_MGC_48 Homo sapiens cDNA clone IMAGE:4054473 5', mRNA sequence [BF129169]	
A_23_P56949	0.00324	2.97	NM_014755	NM_014755	Homo sapiens SERTA domain containing 2 (SERTAD2), mRNA [NM_014755]	NM_014755
A_24_P136905	0.00324	2.97	AF116713	AF116713	Homo sapiens PRO2769 mRNA, complete cds. [AF116713]	
A_23_P258418	0.00325	2.30	NM_024309	NM_024309	Homo sapiens TNFAIP3 interacting protein 2 (TNIP2), mRNA [NM_024309]	NM_024309

A_24_P217365	0.00325	2.39	NM_015199	NM_015199	Homo sapiens ankyrin repeat domain 28 (ANKRD28), mRNA [NM_015199]	NM_015199
A_23_P156284	0.00326	2.16	NM_080881	NM_080881	Homo sapiens drebrin 1 (DBN1), transcript variant 2, mRNA [NM_080881]	NM_080881
A_23_P79086	0.00326	2.62	NM_002503	NM_002503	Homo sapiens nuclear factor of kappa light polypeptide gene enhancer in B-cells inhibitor, beta (NFKBIB), transcript variant 1, mRNA [NM_002503]	NM_002503
A_23_P123645	0.00326	6.94	THC2427833			
A_23_P50887	0.00327	2.46	NM_006055	NM_006055	Homo sapiens LanC lantibiotic synthetase component C-like 1 (bacterial) (LANCL1), mRNA [NM_006055]	NM_006055
A_23_P324538	0.00328	2.21	NM_178433	NM_178433	Homo sapiens late cornified envelope 3B (LCE3B), mRNA [NM_178433]	NM_178433
A_23_P46844	0.00329	2.10	NM_030912	NM_030912	Homo sapiens tripartite motif-containing 8 (TRIM8), mRNA [NM_030912]	NM_030912
A_32_P147149	0.0033	2.12	AF088062	AF088062	Homo sapiens full length insert cDNA clone ZD74E10. [AF088062]	
A_23_P144726	0.0033	2.44	NM_016391	NM_016391	Homo sapiens hypothetical protein HSPC111 (HSPC111), mRNA [NM_016391]	NM_016391
A_23_P67271	0.0033	2.58	NM_002741	NM_002741	Homo sapiens protein kinase N1 (PKN1), transcript variant 2, mRNA [NM_002741]	NM_002741
A_23_P164035	0.0033	2.83	NM_002805	NM_002805	Homo sapiens proteasome (prosome, macropain) 26S subunit, ATPase, 5 (PSMC5), mRNA [NM_002805]	NM_002805
A_24_P911571	0.0033	14.45	BC011671	BC011671	Homo sapiens guanine nucleotide binding protein (G protein), beta 5, mRNA (cDNA clone IMAGE:4131809), complete cds. [BC011671]	
A_23_P252362	0.00331	2.14	NM_016640	NM_016640	Homo sapiens mitochondrial ribosomal protein S30 (MRPS30), nuclear gene encoding mitochondrial protein, mRNA [NM_016640]	NM_016640
A_23_P203323	0.00331	2.24	BC013255	BC013255	Homo sapiens hypothetical protein MGC10485, mRNA (cDNA clone IMAGE:3871805). [BC013255]	
A_24_P84558	0.00332	2.03	A_24_P84558			
A_24_P324787	0.00332	4.72	NM_015493	NM_015493	Homo sapiens ankyrin repeat domain 25 (ANKRD25), mRNA [NM_015493]	NM_015493
A_24_P777185	0.00333	2.19	A_24_P777185			
A_32_P133464	0.00333	2.72	AI885257	AI885257	AI885257 wl91f02.x1 NCI_CGAP_Brn25 Homo sapiens cDNA clone IMAGE:2432283 3', mRNA sequence [AI885257]	
A_24_P245298	0.00333	3.53	NM_153012	NM_153012	Homo sapiens tumor necrosis factor (ligand) superfamily, member 12 (TNFSF12), transcript variant 2, mRNA [NM_153012]	NM_153012
A_24_P400690	0.00334	2.24	ENST00000309333			
A_23_P30411	0.00334	2.46	A_23_P30411			
A_24_P300483	0.00334	2.99	NM_024054	NM_024054	Homo sapiens chromosome 7 open reading frame 25 (C7orf25), mRNA [NM_024054]	NM_024054
A_24_P923381	0.00334	4.61	NR_002219	NR_002219	Homo sapiens effector cell protease receptor 1 (EPR1) on chromosome 17 [NR_002219]	NR_002219
A_32_P69536	0.00334	5.75	THC2381210		Q5T2R2 (Q5T2R2) OTTHUMP00000046079, partial (28%) [THC2381210]	
A_24_P246626	0.00335	2.25	ENST00000308384		PREDICTED: Homo sapiens similar to MHC HLA-SX-alpha (LOC442203), mRNA [XM_498087]	XM_498087
A_23_P258814	0.00335	2.45	ENST00000328644			
A_32_P163908	0.00335	3.40	NM_014669	NM_014669	Homo sapiens nucleoporin 93kDa (NUP93), mRNA [NM_014669]	NM_014669
A_23_P139919	0.00337	2.79	NM_018413	NM_018413	Homo sapiens carbohydrate (chondroitin 4) sulfotransferase 11 (CHST11), mRNA [NM_018413]	NM_018413
A_23_P210419	0.00337	3.11	NM_014902	NM_014902	Homo sapiens discs, large (Drosophila) homolog-associated protein 4 (DLGAP4), transcript variant 1, mRNA [NM_014902]	NM_014902
A_24_P229164	0.00337	4.12	NM_003959	NM_003959	Homo sapiens huntingtin interacting protein-1-related (HIP1R), mRNA [NM_003959]	NM_003959
A_24_P942112	0.00338	2.15	NM_019014	NM_019014	Homo sapiens polymerase (RNA) I polypeptide B, 128kDa (POLR1B), mRNA [NM_019014]	NM_019014
A_32_P164238	0.00338	3.42	BE709763	BE709763	BE709763 IL3-HT0618-290600-128-C08 HT0618 Homo sapiens cDNA, mRNA sequence [BE709763]	
A_24_P79591	0.00339	2.28	BX433547	BX433547	BX433547 Homo sapiens PLACENTA Homo sapiens cDNA clone CL0BA005ZH09 3-PRIME, mRNA sequence [BX433547]	
A_24_P66908	0.00339	2.43	A_24_P66908			
A_23_P75325	0.00339	2.50	NM_003901	NM_003901	Homo sapiens sphingosine-1-phosphate lyase 1 (SGPL1), mRNA [NM_003901]	NM_003901
A_23_P156788	0.00339	2.87	NM_003764	NM_003764	Homo sapiens syntaxin 11 (STX11), mRNA [NM_003764]	NM_003764
A_23_P330727	0.00339	3.33	NM_182612	NM_182612	Homo sapiens hypothetical protein FLJ34283 (FLJ34283), mRNA [NM_182612]	NM_182612
A_23_P57497	0.00339	3.98	NM_002473	NM_002473	Homo sapiens myosin, heavy polypeptide 9, non-muscle (MYH9), mRNA [NM_002473]	NM_002473
A_24_P63608	0.0034	2.92	NM_017838	NM_017838	Homo sapiens nucleolar protein family A, member 2 (H/ACA small nucleolar RNPs) (NOLA2), mRNA [NM_017838]	NM_017838

A_24_P145629	0.00341	2.05	NM_178865	NM_178865	Homo sapiens tumor differentially expressed 2-like (TDE2L), mRNA [NM_178865]	NM_178865
A_23_P68106	0.00341	4.46	NM_021103	NM_021103	Homo sapiens thymosin, beta 10 (TMSB10), mRNA [NM_021103]	NM_021103
A_32_P38689	0.00342	2.07	N52008	N52008	N52008 yz08b03.s1 Soares_multiple_sclerosis_2NbHMSP Homo sapiens cDNA clone IMAGE:282413 3', mRNA sequence [N52008]	
A_23_P123463	0.00342	2.13	NM_153332	NM_153332	Homo sapiens three prime histone mRNA exonuclease 1 (THEX1), mRNA [NM_153332]	NM_153332
A_32_P207243	0.00342	2.26	AF132973	AF132973	Homo sapiens CGI-39 protein mRNA, complete cds. [AF132973]	
A_32_P223918	0.00342	2.80	BF858887	BF858887	BF858887 RC5-FT0194-071200-023-E10 FT0194 Homo sapiens cDNA, mRNA sequence [BF858887]	
A_23_P146187	0.00342	3.31	NM_015169	NM_015169	Homo sapiens RRS1 ribosome biogenesis regulator homolog (S. cerevisiae) (RRS1), mRNA [NM_015169]	NM_015169
A_23_P108604	0.00343	2.08	NM_145686	NM_145686	Homo sapiens mitogen-activated protein kinase kinase kinase kinase 4 (MAP4K4), transcript variant 2, mRNA [NM_145686]	NM_145686
A_23_P87257	0.00343	2.26	NM_022061	NM_022061	Homo sapiens mitochondrial ribosomal protein L17 (MRPL17), nuclear gene encoding mitochondrial protein, mRNA [NM_022061]	NM_022061
A_23_P1552	0.00343	2.92	NM_001814	NM_001814	Homo sapiens cathepsin C (CTSC), transcript variant 1, mRNA [NM_001814]	NM_001814
A_32_P45204	0.00343	3.60	AI752947	AI752947	AI752947 cr02f10.x2 Human bone marrow stromal cells Homo sapiens cDNA clone HBMSC_cr02f10 3', mRNA sequence [AI752947]	
A_23_P420942	0.00344	3.86	NM_175617	NM_175617	Homo sapiens metallothionein 1E (functional) (MT1E), mRNA [NM_175617]	NM_175617
A_24_P167012	0.00344	3.94	NM_005118	NM_005118	Homo sapiens tumor necrosis factor (ligand) superfamily, member 15 (TNFSF15), mRNA [NM_005118]	NM_005118
A_24_P152278	0.00344	5.35	A_24_P152278			
A_23_P215461	0.00345	2.29	NM_002314	NM_002314	Homo sapiens LIM domain kinase 1 (LIMK1), transcript variant 1, mRNA [NM_002314]	NM_002314
A_32_P42705	0.00345	7.94	BC017721	BC017721	Homo sapiens, clone IMAGE:4429392, mRNA, partial cds. [BC017721]	
A_23_P40088	0.00346	13.14	NM_003887	NM_003887	Homo sapiens development and differentiation enhancing factor 2 (DDEF2), mRNA [NM_003887]	NM_003887
A_23_P112159	0.00348	2.22	NM_012154	NM_012154	Homo sapiens eukaryotic translation initiation factor 2C, 2 (EIF2C2), mRNA [NM_012154]	NM_012154
A_24_P244706	0.00348	9.43	NM_001049	NM_001049	Homo sapiens somatostatin receptor 1 (SSTR1), mRNA [NM_001049]	NM_001049
A_24_P409346	0.00349	2.92	NM_006310	NM_006310	Homo sapiens aminopeptidase puromycin sensitive (NPEPPS), mRNA [NM_006310]	NM_006310
A_24_P339201	0.00349	3.40	NM_024042	NM_024042	Homo sapiens meteorin, glial cell differentiation regulator (METRN), mRNA [NM_024042]	NM_024042
A_23_P10699	0.00349	3.50	CR624966	CR624966	full-length cDNA clone CS0DK008YO11 of HeLa cells Cot 25-normalized of Homo sapiens (human). [CR624966]	
A_23_P90357	0.00349	5.43	NM_201636	NM_201636	Homo sapiens thromboxane A2 receptor (TBXA2R), transcript variant 1, mRNA [NM_201636]	NM_201636
A_23_P17330	0.0035	2.44	NM_017859	NM_017859	Homo sapiens uridine-cytidine kinase 1-like 1 (UCKL1), mRNA [NM_017859]	NM_017859
A_23_P70417	0.0035	2.88	NM_018141	NM_018141	Homo sapiens mitochondrial ribosomal protein S10 (MRPS10), nuclear gene encoding mitochondrial protein, mRNA [NM_018141]	NM_018141
A_23_P202117	0.0035	3.94	BC007377	BC007377	Homo sapiens polycomb group ring finger 5, mRNA (cDNA clone IMAGE:3640258), complete cds. [BC007377]	
A_23_P128532	0.00351	2.01	NM_152318	NM_152318	Homo sapiens hypothetical protein MGC40397 (MGC40397), mRNA [NM_152318]	NM_152318
A_23_P151662	0.00351	2.82	NM_197957	NM_197957	Homo sapiens MYC associated factor X (MAX), transcript variant 6, mRNA [NM_197957]	NM_197957
A_23_P301247	0.00351	3.47	NM_003517	NM_003517	Homo sapiens histone 2, H2ac (HIST2H2AC), mRNA [NM_003517]	NM_003517
A_24_P263623	0.00352	2.37	NM_006601	NM_006601	Homo sapiens prostaglandin E synthase 3 (cytosolic) (PTGES3), mRNA [NM_006601]	NM_006601
A_24_P205120	0.00352	2.69	NM_199367	NM_199367	Homo sapiens spastic paraplegia 7, paraplegin (pure and complicated autosomal recessive) (SPG7), nuclear gene encoding mitochondrial protein, transcript variant 2, mRNA [NM_199367]	NM_199367
A_24_P75408	0.00353	4.27	A_24_P75408			
A_32_P53538	0.00354	2.11	ENST00000314571		Homo sapiens cDNA FLJ14915 fis, clone PLACE1006878, weakly similar to TRNA-SPLICING ENDONUCLEASE SUBUNIT SEN2 (EC 3.1.27.9). [AK027821]	
A_32_P176594	0.00354	2.33	AB046834	AB046834	Homo sapiens mRNA for KIAA1614 protein, partial cds. [AB046834]	XM_046531
A_23_P404902	0.00354	2.54	AB075840	AB075840	Homo sapiens mRNA for KIAA1960 protein. [AB075840]	
A_23_P426279	0.00354	3.02	BU944300	BU944300	AGENCOURT_10545189 NIH_MGC_107 Homo sapiens cDNA clone IMAGE:6728554 5', mRNA sequence [BU944300]	
A_24_P328504	0.00354	4.17	NM_007237	NM_007237	Homo sapiens SP140 nuclear body protein (SP140), transcript variant 1, mRNA [NM_007237]	NM_007237
A_23_P154688	0.00355	3.73	NM_032034	NM_032034	Homo sapiens solute carrier family 4, sodium bicarbonate transporter-like, member 11 (SLC4A11), mRNA [NM_032034]	NM_032034

A_24_P917866	0.00357	2.09	NM_003011	NM_003011	Homo sapiens SET translocation (myeloid leukemia-associated) (SET), mRNA [NM_003011]	NM_003011
A_24_P79712	0.00357	2.39	AK125259	AK125259	Homo sapiens cDNA FLJ43269 fis, clone KIDNE2000665. [AK125259]	
A_32_P89709	0.00358	2.11	NM_001018004	NM_001018004	Homo sapiens tropomyosin 1 (alpha) (TPM1), transcript variant 3, mRNA [NM_001018004]	NM_001018004
A_24_P347065	0.00358	2.99	ENST00000265641		Homo sapiens partial mRNA for carnitine palmitoyltransferase I (CPT1A gene). [AJ420378]	
A_23_P123336	0.00359	3.25	NM_018444	NM_018444	Homo sapiens protein phosphatase 2C, magnesium-dependent, catalytic subunit (PPM2C), nuclear gene encoding mitochondrial protein, mRNA [NM_018444]	NM_018444
A_24_P316489	0.00359	3.82	NM_004544	NM_004544	Homo sapiens NADH dehydrogenase (ubiquinone) 1 alpha subcomplex, 10, 42kDa (NDUFA10), nuclear gene encoding mitochondrial protein, mRNA [NM_004544]	NM_004544
A_23_P68665	0.0036	2.63	NM_007002	NM_007002	Homo sapiens adhesion regulating molecule 1 (ADRM1), transcript variant 1, mRNA [NM_007002]	NM_007002
A_23_P338981	0.0036	2.79	NM_134268	NM_134268	Homo sapiens cytoglobin (CYGB), mRNA [NM_134268]	NM_134268
A_24_P873598	0.00361	2.18	AL050122	AL050122	Homo sapiens mRNA; cDNA DKFZp586E121 (from clone DKFZp586E121) [AL050122]	
A_24_P928639	0.00361	2.20	BC032648	BC032648	Homo sapiens small optic lobes homolog (Drosophila), mRNA (cDNA clone IMAGE:5518093), partial cds. [BC032648]	
A_23_P259823	0.00361	2.69	NM_017910	NM_017910	Homo sapiens hypothetical protein FLJ20628 (FLJ20628), mRNA [NM_017910]	NM_017910
A_24_P175989	0.00362	2.13	NM_057180	NM_057180	Homo sapiens vacuolar protein sorting 29 (yeast) (VPS29), transcript variant 2, mRNA [NM_057180]	NM_057180
A_23_P335486	0.00363	2.06	NM_178837	NM_178837	Homo sapiens similar to hypothetical testis protein from macaque (LOC352909), mRNA [NM_178837]	NM_178837
A_24_P75778	0.00363	2.11	A_24_P75778			
A_24_P40827	0.00363	2.15	NM_133627	NM_133627	Homo sapiens RAD51-like 3 (S. cerevisiae) (RAD51L3), transcript variant 2, mRNA [NM_133627]	NM_133627
A_24_P195400	0.00363	2.17	A_24_P195400			
A_23_P315815	0.00363	3.92	NM_013961	NM_013961	Homo sapiens neuregulin 1 (NRG1), transcript variant GGF, mRNA [NM_013961]	NM_013961
A_23_P76364	0.00364	2.32	NM_001769	NM_001769	Homo sapiens CD9 antigen (p24) (CD9), mRNA [NM_001769]	NM_001769
A_23_P206877	0.00366	2.21	NM_018427	NM_018427	Homo sapiens RRN3 RNA polymerase I transcription factor homolog (yeast) (RRN3), mRNA [NM_018427]	NM_018427
A_23_P7528	0.00366	5.29	NM_001387	NM_001387	Homo sapiens dihydropyrimidinase-like 3 (DPYSL3), mRNA [NM_001387]	NM_001387
A_23_P146572	0.00367	2.12	NM_015392	NM_015392	Homo sapiens neural proliferation, differentiation and control, 1 (NPDC1), mRNA [NM_015392]	NM_015392
A_23_P25515	0.00367	2.30	NM_004685	NM_004685	Homo sapiens myotubularin related protein 6 (MTMR6), mRNA [NM_004685]	NM_004685
A_32_P189093	0.00367	2.79	AI090167	AI090167	AI090167 qb33g12.x1 Soares_pregnant_uterus_NbHPU Homo sapiens cDNA clone IMAGE:1698118 3', mRNA sequence [AI090167]	
A_23_P66664	0.00367	3.08	NM_133439	NM_133439	Homo sapiens transcriptional adaptor 2 (ADA2 homolog, yeast)-like (TADA2L), transcript variant 2, mRNA [NM_133439]	NM_133439
A_32_P158524	0.00368	2.17	THC2304851		HSA132583 puromycin sensitive aminopeptidase {Homo sapiens;} , partial (3%) [THC2304851]	
A_24_P393336	0.00368	2.27	AK093583	AK093583	Homo sapiens cDNA FLJ36264 fis, clone THYMU2002815, weakly similar to AXONIN-1 PRECURSOR. [AK093583]	
A_23_P404129	0.00368	2.43	NM_145207	NM_145207	Homo sapiens spermatogenesis associated 5 (SPATA5), mRNA [NM_145207]	NM_145207
A_24_P344307	0.00368	3.56	NM_176863	NM_176863	Homo sapiens proteasome (prosome, macropain) activator subunit 3 (PA28 gamma; Ki) (PSME3), transcript variant 2, mRNA [NM_176863]	NM_176863
A_32_P163147	0.00369	2.86	NM_182607	NM_182607	Homo sapiens V-set and immunoglobulin domain containing 1 (VSIG1), mRNA [NM_182607]	NM_182607
A_23_P360240	0.0037	3.53	NM_138768	NM_138768	Homo sapiens myeloma overexpressed gene (in a subset of t(11;14) positive multiple myelomas) (MYEOV), mRNA [NM_138768]	NM_138768
A_23_P135548	0.0037	5.81	NM_000110	NM_000110	Homo sapiens dihydropyrimidine dehydrogenase (DPYD), mRNA [NM_000110]	NM_000110
A_23_P100103	0.00371	2.21	NM_015289	NM_015289	Homo sapiens vacuolar protein sorting 39 (yeast) (VPS39), mRNA [NM_015289]	NM_015289
A_23_P399851	0.00371	2.49	NM_198334	NM_198334	Homo sapiens glucosidase, alpha; neutral AB (GANAB), mRNA [NM_198334]	NM_198334
A_24_P837537	0.00371	4.27	A_24_P837537			
A_32_P57877	0.00372	3.05	BX433326	BX433326	BX433326 BX433326 Homo sapiens FETAL LIVER Homo sapiens cDNA clone CS0DM004YL09 3-PRIME, mRNA sequence [BX433326]	
A_32_P42104	0.00373	2.17	BC011998	BC011998	Homo sapiens, clone IMAGE:4479080, mRNA, partial cds. [BC011998]	
A_23_P130488	0.00373	2.78	NM_000400	NM_000400	Homo sapiens excision repair cross-complementing rodent repair deficiency, complementation group 2 (xeroderma pigmentosum D) (ERCC2), mRNA [NM_000400]	NM_000400
A_24_P55437	0.00373	3.01	NM_152829	NM_152829	Homo sapiens testis derived transcript (3 LIM domains) (TES), transcript variant 2, mRNA [NM_152829]	NM_152829

A_23_P218774	0.00373	3.68	NM_002872	NM_002872	Homo sapiens ras-related C3 botulinum toxin substrate 2 (rho family, small GTP binding protein Rac2) (RAC2), mRNA [NM_002872]	NM_002872
A_24_P192485	0.00373	9.26	NM_002546	NM_002546	Homo sapiens tumor necrosis factor receptor superfamily, member 11b (osteoprotegerin) (TNFRSF11B), mRNA [NM_002546]	NM_002546
A_24_P937319	0.00374	2.15	AK097197	AK097197	Homo sapiens cDNA FLJ39878 fis, clone SPLEN2016045, moderately similar to Homo sapiens mRNA for Hrs. [AK097197]	
A_23_P79803	0.00374	2.90	NM_080607	NM_080607	Homo sapiens chromosome 20 open reading frame 102 (C20orf102), mRNA [NM_080607]	NM_080607
A_23_P258504	0.00375	2.39	BC063385	BC063385	Homo sapiens T cell receptor alpha locus, mRNA (cDNA clone MGC:71411 IMAGE:4853814), complete cds. [BC063385]	
A_23_P75083	0.00375	2.54	NM_212479	NM_212479	Homo sapiens zinc finger, MYND domain containing 11 (ZMYND11), transcript variant 2, mRNA [NM_212479]	NM_212479
A_32_P107033	0.00375	3.46	AW939027	AW939027	AW939027 QV1-DT0068-240200-085-b05 DT0068 Homo sapiens cDNA, mRNA sequence [AW939027]	
A_32_P54553	0.00375	5.21	ENST00000292729		Homo sapiens partial mRNA for ubiquitin-specific protease 41 (USP41 gene). [AJ586979]	XM_036729
A_24_P162718	0.00376	3.72	NM_005572	NM_005572	Homo sapiens lamin A/C (LMNA), transcript variant 2, mRNA [NM_005572]	NM_005572
A_24_P383901	0.00378	2.22	A_24_P383901			
A_23_P54963	0.00379	2.10	NM_032478	NM_032478	Homo sapiens mitochondrial ribosomal protein L38 (MRPL38), nuclear gene encoding mitochondrial protein, mRNA [NM_032478]	NM_032478
A_24_P253723	0.00379	2.40	NM_032895	NM_032895	Homo sapiens hypothetical protein MGC14376 (MGC14376), transcript variant 1, mRNA [NM_032895]	NM_032895
A_24_P631594	0.0038	2.14	BC012360	BC012360	Homo sapiens, clone IMAGE:4641261, mRNA. [BC012360]	
A_23_P436138	0.0038	4.63	NM_145114	NM_145114	Homo sapiens MYC associated factor X (MAX), transcript variant 4, mRNA [NM_145114]	NM_145114
A_23_P119789	0.00381	2.03	NR_000034	NR_000034	Homo sapiens family with sequence similarity 11, member B (FAM11B) on chromosome 2 [NR_000034]	NR_000034
A_23_P300076	0.00381	2.08	NM_032263	NM_032263	Homo sapiens IQ motif containing G (IQCG), mRNA [NM_032263]	NM_032263
A_24_P370372	0.00382	2.08	NM_014292	NM_014292	Homo sapiens chromobox homolog 6 (CBX6), mRNA [NM_014292]	NM_014292
A_32_P53884	0.00382	3.68	BC040307	BC040307	Homo sapiens cDNA clone IMAGE:4830091, partial cds. [BC040307]	
A_24_P389994	0.00383	2.42	NM_023018	NM_023018	Homo sapiens NAD kinase (NADK), mRNA [NM_023018]	NM_023018
A_23_P36795	0.00383	3.39	NM_005639	NM_005639	Homo sapiens synaptotagmin I (SYT1), mRNA [NM_005639]	NM_005639
A_32_P206136	0.00383	6.58	AW178774	AW178774	AW178774 PM0-HT0123-310899-001-b08 HT0123 Homo sapiens cDNA, mRNA sequence [AW178774]	
A_32_P46526	0.00384	2.43	NM_173491	NM_173491	Homo sapiens LSM11, U7 small nuclear RNA associated (LSM11), mRNA [NM_173491]	NM_173491
A_24_P247879	0.00384	2.45	AK097244	AK097244	Homo sapiens cDNA FLJ39925 fis, clone SPLEN2021122. [AK097244]	
A_23_P22119	0.00384	3.08	NM_201380	NM_201380	Homo sapiens plectin 1, intermediate filament binding protein 500kDa (PLEC1), transcript variant 6, mRNA [NM_201380]	NM_201380
A_23_P157914	0.00384	5.92	NM_153267	NM_153267	Homo sapiens MAM domain containing 2 (MAMDC2), mRNA [NM_153267]	NM_153267
A_32_P200490	0.00385	2.19	A_32_P200490			
A_24_P624182	0.00385	3.09	CB528527	CB528527	CB528527 UI-H-FT2-bjd-k-24-0-UI.s1 NCI_CGAP_FT2 Homo sapiens cDNA clone UI-H-FT2-bjd-k-24-0-UI 3', mRNA sequence [CB528527]	
A_24_P810697	0.00385	3.29	NM_001008529	NM_001008529	Homo sapiens matrix-remodelling associated 7 (MXRA7), transcript variant 2, mRNA [NM_001008529]	NM_001008529
A_23_P96017	0.00386	2.00	A_23_P96017			
A_23_P60227	0.00386	2.28	NM_005893	NM_005893	Homo sapiens calicin (CCIN), mRNA [NM_005893]	NM_005893
A_23_P417237	0.00387	3.61	NM_020177	NM_020177	Homo sapiens fem-1 homolog c (C.elegans) (FEM1C), mRNA [NM_020177]	NM_020177
A_24_P167806	0.00388	3.91	NM_005530	NM_005530	Homo sapiens isocitrate dehydrogenase 3 (NAD+) alpha (IDH3A), nuclear gene encoding mitochondrial protein, mRNA [NM_005530]	NM_005530
A_32_P63324	0.00389	2.07	THC2284306		Q9F8M7 (Q9F8M7) DTDP-glucose 4,6-dehydratase (Fragment), partial (11%) [THC2284306]	
A_24_P48139	0.0039	2.55	NM_012091	NM_012091	Homo sapiens adenosine deaminase, tRNA-specific 1 (ADAT1), mRNA [NM_012091]	NM_012091
A_23_P331670	0.00391	3.56	NM_002862	NM_002862	Homo sapiens phosphorylase, glycogen; brain (PYGB), mRNA [NM_002862]	NM_002862
A_23_P123010	0.00391	3.94	NM_138707	NM_138707	Homo sapiens B-cell CLL/lymphoma 7B (BCL7B), transcript variant 2, mRNA [NM_138707]	NM_138707
A_23_P3562	0.00393	2.09	NM_032178	NM_032178	Homo sapiens hypothetical protein FLJ13291 (FLJ13291), mRNA [NM_032178]	NM_032178
A_23_P371933	0.00394	3.02	BC012536	BC012536	Homo sapiens hydroxysteroid (17-beta) dehydrogenase 12, mRNA (cDNA clone IMAGE:4281565), complete cds. [BC012536]	
A_32_P101031	0.00394	3.06	NM_144586	NM_144586	Homo sapiens LY6/PLAUR domain containing 1 (LYPDC1), mRNA [NM_144586]	NM_144586

A_24_P307854	0.00395	3.00	BC006426	BC006426	Homo sapiens F-box and leucine-rich repeat protein 18, mRNA (cDNA clone MGC:12908 IMAGE:4026501), complete cds. [BC006426]	
A_23_P43820	0.00396	3.15	NM_032793	NM_032793	Homo sapiens major facilitator superfamily domain containing 2 (MFSD2), mRNA [NM_032793]	NM_032793
A_23_P301877	0.00396	4.10	NM_004269	NM_004269	Homo sapiens cofactor required for Sp1 transcriptional activation, subunit 8, 34kDa (CRSP8), mRNA [NM_004269]	NM_004269
A_24_P23190	0.00398	2.64	NM_173510	NM_173510	Homo sapiens hypothetical protein FLJ33814 (FLJ33814), mRNA [NM_173510]	NM_173510
A_23_P153583	0.00398	3.08	NM_006247	NM_006247	Homo sapiens protein phosphatase 5, catalytic subunit (PPP5C), mRNA [NM_006247]	NM_006247
A_32_P233735	0.00398	3.13	NM_020704	NM_020704	Homo sapiens family with sequence similarity 40, member B (FAM40B), mRNA [NM_020704]	NM_020704
A_23_P66658	0.004	2.29	NM_001488	NM_001488	Homo sapiens transcriptional adaptor 2 (ADA2 homolog, yeast)-like (TADA2L), transcript variant 1, mRNA [NM_001488]	NM_001488
A_24_P39101	0.004	2.36	NM_031954	NM_031954	Homo sapiens potassium channel tetramerisation domain containing 10 (KCTD10), mRNA [NM_031954]	NM_031954
A_23_P129801	0.00401	2.06	NM_006822	NM_006822	Homo sapiens RAB40B, member RAS oncogene family (RAB40B), mRNA [NM_006822]	NM_006822
A_32_P923011	0.00401	2.12	AF346307	AF346307	Homo sapiens hypothetical protein (F379) mRNA, complete cds. [AF346307]	
A_24_P341489	0.00401	2.58	A_24_P341489			
A_24_P7179	0.00402	2.20	NM_206890	NM_206890	Homo sapiens chromosome 21 open reading frame 106 (C21orf106), transcript variant 3, mRNA [NM_206890]	NM_206890
A_24_P362540	0.00402	2.28	NM_003887	NM_003887	Homo sapiens development and differentiation enhancing factor 2 (DDEF2), mRNA [NM_003887]	NM_003887
A_24_P283320	0.00402	2.46	NM_005389	NM_005389	Homo sapiens protein-L-isoaspartate (D-aspartate) O-methyltransferase (PCMT1), mRNA [NM_005389]	NM_005389
A_24_P187174	0.00402	3.22	A_24_P187174			
A_24_P122337	0.00402	5.62	NM_080737	NM_080737	Homo sapiens synaptotagmin-like 4 (granuphilin-a) (SYTL4), mRNA [NM_080737]	NM_080737
A_23_P78311	0.00403	2.07	NM_031216	NM_031216	Homo sapiens SEH1-like (S. cerevisiae) (SEH1L), transcript variant 2, mRNA [NM_031216]	NM_031216
A_24_P133542	0.00403	2.46	NM_002675	NM_002675	Homo sapiens promyelocytic leukemia (PML), transcript variant 6, mRNA [NM_002675]	NM_002675
A_23_P166453	0.00403	2.60	NM_152243	NM_152243	Homo sapiens CDC42 effector protein (Rho GTPase binding) 1 (CDC42EP1), transcript variant 1, mRNA [NM_152243]	NM_152243
A_24_P93012	0.00403	2.75	ENST00000294573			
A_23_P350467	0.00404	2.02	NM_004759	NM_004759	Homo sapiens mitogen-activated protein kinase-activated protein kinase 2 (MAPKAPK2), transcript variant 1, mRNA [NM_004759]	NM_004759
A_24_P323916	0.00404	2.37	A_24_P323916			
A_23_P106998	0.00404	3.03	NM_016070	NM_016070	Homo sapiens mitochondrial ribosomal protein S23 (MRPS23), nuclear gene encoding mitochondrial protein, mRNA [NM_016070]	NM_016070
A_23_P201538	0.00405	3.68	NM_002228	NM_002228	Homo sapiens v-jun sarcoma virus 17 oncogene homolog (avian) (JUN), mRNA [NM_002228]	NM_002228
A_23_P73609	0.00405	6.17	NM_000266	NM_000266	Homo sapiens Norrie disease (pseudoglioma) (NDP), mRNA [NM_000266]	NM_000266
A_24_P418536	0.00407	2.00	XM_497700	XM_497700	PREDICTED: Homo sapiens similar to ribosomal protein L6 (LOC343495), mRNA [XM_497700]	XM_497700
A_24_P551302	0.00407	2.21	BC038432	BC038432	Homo sapiens cDNA clone IMAGE:5162874, partial cds. [BC038432]	
A_23_P56228	0.00408	2.21	NM_016573	NM_016573	Homo sapiens GEM interacting protein (GMIP), mRNA [NM_016573]	NM_016573
A_24_P285032	0.00408	2.60	NM_006123	NM_006123	Homo sapiens iduronate 2-sulfatase (Hunter syndrome) (IDS), transcript variant 2, mRNA [NM_006123]	NM_006123
A_32_P171695	0.00408	2.61	BC075861	BC075861	Homo sapiens chromosome 10 open reading frame 130, mRNA (cDNA clone MGC:90420 IMAGE:5171414), complete cds. [BC075861]	
A_23_P22444	0.00409	2.05	NM_002621	NM_002621	Homo sapiens properdin P factor, complement (PFC), mRNA [NM_002621]	NM_002621
A_23_P73982	0.00409	2.23	NM_018087	NM_018087	Homo sapiens transmembrane protein 48 (TMEM48), mRNA [NM_018087]	NM_018087
A_23_P109677	0.00409	3.28	A_23_P109677			
A_23_P79130	0.0041	2.01	NM_024310	NM_024310	Homo sapiens pleckstrin homology domain containing, family F (with FYVE domain) member 1 (PLEKHF1), mRNA [NM_024310]	NM_024310
A_24_P469746	0.0041	2.30	ENST00000334973		Homo sapiens chromosome 2 unknown mRNA. [AF451986]	XM_371605
A_23_P104199	0.00411	2.42	NM_133376	NM_133376	Homo sapiens integrin, beta 1 (fibronectin receptor, beta polypeptide, antigen CD29 includes MDF2, MSK12) (ITGB1), transcript variant 1E, mRNA [NM_133376]	NM_133376
A_23_P207766	0.00411	2.60	NM_004309	NM_004309	Homo sapiens Rho GDP dissociation inhibitor (GDI) alpha (ARHGDIA), mRNA [NM_004309]	NM_004309
A_32_P71032	0.00411	20.53	THC2407039			

A_24_P169574	0.00412	2.28	ENST00000309081			
A_24_P933704	0.00413	2.10	NM_138822	NM_138822	Homo sapiens peptidylglycine alpha-amidating monooxygenase (PAM), transcript variant 4, mRNA [NM_138822]	NM_138822
A_23_P209167	0.00414	3.60	NM_005860	NM_005860	Homo sapiens follistatin-like 3 (secreted glycoprotein) (FSTL3), mRNA [NM_005860]	NM_005860
A_32_P228254	0.00415	2.34	BX456116	BX456116	BX456116 Homo sapiens T CELLS (JURKAT CELL LINE) Homo sapiens cDNA clone CS0DH006YI03 3-PRIME, mRNA sequence [BX456116]	
A_23_P336015	0.00415	2.57	NM_015658	NM_015658	Homo sapiens DKFZP564C186 protein (DKFZP564C186), mRNA [NM_015658]	NM_015658
A_24_P362805	0.00415	3.92	NM_152776	NM_152776	Homo sapiens hypothetical protein MGC40579 (MGC40579), mRNA [NM_152776]	NM_152776
A_32_P64418	0.00416	2.03	NM_152383	NM_152383	Homo sapiens hypothetical protein MGC42174 (MGC42174), mRNA [NM_152383]	NM_152383
A_23_P163622	0.00416	3.04	NM_001012398	NM_001012398	Homo sapiens fused toes homolog (mouse) (FTS), transcript variant 1, mRNA [NM_001012398]	NM_001012398
A_32_P123440	0.00417	2.00	AW572218	AW572218	AW572218 xk92f04.x2 NCI_CGAP_Ut4 Homo sapiens cDNA clone IMAGE:2674207 3', mRNA sequence [AW572218]	
A_23_P65555	0.00417	2.29	NM_021818	NM_021818	Homo sapiens salvador homolog 1 (Drosophila) (SAV1), mRNA [NM_021818]	NM_021818
A_23_P55477	0.00417	2.80	NM_000676	NM_000676	Homo sapiens adenosine A2b receptor (ADORA2B), mRNA [NM_000676]	NM_000676
A_32_P141262	0.00419	2.00	NM_054026	NM_054026	Homo sapiens CCR4-NOT transcription complex, subunit 7 (CNOT7), transcript variant 2, mRNA [NM_054026]	NM_054026
A_23_P3146	0.00419	2.03	NM_004863	NM_004863	Homo sapiens serine palmitoyltransferase, long chain base subunit 2 (SPTLC2), mRNA [NM_004863]	NM_004863
A_23_P110403	0.00419	2.50	NM_014476	NM_014476	Homo sapiens PDZ and LIM domain 3 (PDLIM3), mRNA [NM_014476]	NM_014476
A_24_P259083	0.00419	3.85	NM_080792	NM_080792	Homo sapiens protein tyrosine phosphatase, non-receptor type substrate 1 (PTPNS1), mRNA [NM_080792]	NM_080792
A_23_P19176	0.00419	8.20	NM_206966	NM_206966	Homo sapiens similar to AVLV472 (MGC23985), mRNA [NM_206966]	NM_206966
A_23_P97265	0.0042	2.07	NM_017725	NM_017725	Homo sapiens G patch domain containing 4 (GPATC4), transcript variant 3, mRNA [NM_017725]	NM_017725
A_32_P212373	0.0042	2.23	BM473515	BM473515	AGENCOURT_6466667 NIH_MGC_88 Homo sapiens cDNA clone IMAGE:5561951 5', mRNA sequence [BM473515]	
A_24_P297888	0.00421	4.18	NM_002451	NM_002451	Homo sapiens methylthioadenosine phosphorylase (MTAP), mRNA [NM_002451]	NM_002451
A_32_P134634	0.00422	2.00	THC2271582		ALU5_HUMAN (P39192) Alu subfamily SC sequence contamination warning entry, partial (9%) [THC2271582]	
A_24_P205137	0.00422	2.32	BC011498	BC011498	Homo sapiens histone deacetylase 6, mRNA (cDNA clone IMAGE:4179066), complete cds. [BC011498]	
A_24_P268015	0.00422	2.58	NM_022756	NM_022756	Homo sapiens chromosome 1 open reading frame 149 (C1orf149), mRNA [NM_022756]	NM_022756
A_32_P179295	0.00423	2.14	BC020462	BC020462	Homo sapiens ribosomal protein S9, mRNA (cDNA clone IMAGE:3856008). [BC020462]	
A_23_P136493	0.00423	4.48	NM_013962	NM_013962	Homo sapiens neuregulin 1 (NRG1), transcript variant GGF2, mRNA [NM_013962]	NM_013962
A_23_P122674	0.00424	2.16	NM_017906	NM_017906	Homo sapiens PAK1 interacting protein 1 (PAK1IP1), mRNA [NM_017906]	NM_017906
A_23_P204482	0.00426	2.34	NM_006861	NM_006861	Homo sapiens RAB35, member RAS oncogene family (RAB35), mRNA [NM_006861]	NM_006861
A_24_P263803	0.00427	2.80	ENST00000308819		PREDICTED: Homo sapiens similar to Chloride intracellular channel protein 1 (Nuclear chloride ion channel 27) (NCC27) (p64 CLCP) (Chloride channel ABP) (LOC390363), mRNA [XM_495936]	XM_495936
A_23_P114105	0.00427	3.38	NM_012317	NM_012317	Homo sapiens leucine zipper, down-regulated in cancer 1 (LDOC1), mRNA [NM_012317]	NM_012317
A_23_P337242	0.00429	9.26	NM_003242	NM_003242	Homo sapiens transforming growth factor, beta receptor II (70/80kDa) (TGFBR2), transcript variant 2, mRNA [NM_003242]	NM_003242
A_24_P324644	0.0043	3.30	A_24_P324644			
A_32_P151077	0.00431	2.48	BC063441	BC063441	Homo sapiens cDNA clone IMAGE:5527329, partial cds. [BC063441]	
A_23_P366908	0.00431	3.01	NM_145257	NM_145257	Homo sapiens chromosome 1 open reading frame 96 (C1orf96), mRNA [NM_145257]	NM_145257
A_24_P307486	0.00431	3.57	ENST00000309556		PREDICTED: Homo sapiens similar to peptidyl-Pro cis trans isomerase (LOC128192), mRNA [XM_060887]	XM_060887
A_23_P314526	0.00432	2.11	NM_005803	NM_005803	Homo sapiens flotillin 1 (FLOT1), mRNA [NM_005803]	NM_005803
A_23_P132260	0.00433	2.01	NM_014303	NM_014303	Homo sapiens pescadillo homolog 1, containing BRCT domain (zebrafish) (PES1), mRNA [NM_014303]	NM_014303
A_32_P106466	0.00433	3.09	A_32_P106466			
A_24_P205213	0.00435	2.16	NM_198709	NM_198709	Homo sapiens arylsulfatase B (ARSB), transcript variant 2, mRNA [NM_198709]	NM_198709
A_24_P47547	0.00435	2.23	NM_006325	NM_006325	Homo sapiens RAN, member RAS oncogene family (RAN), mRNA [NM_006325]	NM_006325
A_23_P41697	0.00435	2.72	NM_020177	NM_020177	Homo sapiens fem-1 homolog c (C.elegans) (FEM1C), mRNA [NM_020177]	NM_020177

A_32_P963	0.00436	2.05	THC2301567			
A_32_P5673	0.00436	2.18	BG336702	BG336702	602405126F1 NIH_MGC_21 Homo sapiens cDNA clone IMAGE:4542655 5', mRNA sequence [BG336702]	
A_24_P486503	0.00436	2.46	ENST00000343567			
A_24_P409410	0.00436	2.68	A_24_P409410			
A_23_P31046	0.00437	2.80	NM_025107	NM_025107	Homo sapiens myc target 1 (MYCT1), mRNA [NM_025107]	NM_025107
A_32_P17133	0.00438	2.03	NM_006241	NM_006241	Homo sapiens protein phosphatase 1, regulatory (inhibitor) subunit 2 (PPP1R2), mRNA [NM_006241]	NM_006241
A_24_P84834	0.00439	4.74	NM_201444	NM_201444	Homo sapiens diacylglycerol kinase, alpha 80kDa (DGKA), transcript variant 1, mRNA [NM_201444]	NM_201444
A_23_P431933	0.0044	2.04	NM_032294	NM_032294	Homo sapiens calcium/calmodulin-dependent protein kinase kinase 1, alpha (CAMKK1), transcript variant 1, mRNA [NM_032294]	NM_032294
A_32_P206751	0.00441	2.19	AA903779	AA903779	AA903779 ok66c08.s1 NCI_CGAP_GC4 Homo sapiens cDNA clone IMAGE:1518926 3' similar to gb:M29536 TRANSLATIONAL INITIATION FACTOR 2 BETA SUBUNIT (HUMAN);, mRNA sequence [AA903779]	
A_32_P227525	0.00441	2.38	AL522622	AL522622	AL522622 AL522622 Homo sapiens NEUROBLASTOMA COT 10-NORMALIZED Homo sapiens cDNA clone CS0DB009YE01 3-PRIME, mRNA sequence [AL522622]	
A_32_P161755	0.00441	2.75	BI058698	BI058698	BI058698 PM1-GN0499-060401-006-h10 GN0499 Homo sapiens cDNA, mRNA sequence [BI058698]	
A_24_P794447	0.00441	5.00	AK021552	AK021552	Homo sapiens cDNA FLJ11490 fis, clone HEMBA1001918. [AK021552]	XM_378316
A_23_P381017	0.00442	2.00	NM_152559	NM_152559	Homo sapiens Williams Beuren syndrome chromosome region 27 (WBSCR27), mRNA [NM_152559]	NM_152559
A_32_P43711	0.00442	2.43	NM_014598	NM_014598	Homo sapiens suppressor of cytokine signaling 7 (SOCS7), mRNA [NM_014598]	NM_014598
A_24_P274795	0.00443	2.01	NM_018719	NM_018719	Homo sapiens cell division cycle associated 7-like (CDCA7L), mRNA [NM_018719]	NM_018719
A_23_P207143	0.00444	2.07	A_23_P207143			
A_24_P246943	0.00444	2.89	NM_001008741	NM_001008741	Homo sapiens peptidylprolyl isomerase A-like (LOC388817), mRNA [NM_001008741]	NM_001008741
A_23_P208915	0.00445	4.98	NM_139159	NM_139159	Homo sapiens dipeptidylpeptidase 9 (DPP9), mRNA [NM_139159]	NM_139159
A_23_P326844	0.00446	2.56	NM_174913	NM_174913	Homo sapiens chromosome 14 open reading frame 21 (C14orf21), mRNA [NM_174913]	NM_174913
A_24_P358205	0.00447	2.06	A_24_P358205			
A_23_P115573	0.00449	2.02	NM_198149	NM_198149	Homo sapiens transmembrane protein 58 (TMEM58), mRNA [NM_198149]	NM_198149
A_23_P251259	0.00449	2.10	NM_001517	NM_001517	Homo sapiens general transcription factor IIH, polypeptide 4, 52kDa (GTF2H4), mRNA [NM_001517]	NM_001517
A_23_P412927	0.00449	2.12	A_23_P412927			
A_23_P116264	0.00449	3.06	NM_006176	NM_006176	Homo sapiens neurogranin (protein kinase C substrate, RC3) (NRGN), mRNA [NM_006176]	NM_006176
A_24_P392540	0.00449	3.15	XM_116396	XM_116396	PREDICTED: Homo sapiens similar to peptidyl-Pro cis trans isomerase (LOC202227), mRNA [XM_116396]	XM_116396
A_23_P87528	0.00449	5.35	NM_005504	NM_005504	Homo sapiens branched chain aminotransferase 1, cytosolic (BCAT1), mRNA [NM_005504]	NM_005504
A_23_P76622	0.0045	2.07	NM_001922	NM_001922	Homo sapiens dopachrome tautomerase (dopachrome delta-isomerase, tyrosine-related protein 2) (DCT), mRNA [NM_001922]	NM_001922
A_23_P422193	0.00453	2.73	NM_003173	NM_003173	Homo sapiens suppressor of variegation 3-9 homolog 1 (Drosophila) (SUV39H1), mRNA [NM_003173]	NM_003173
A_23_P32500	0.00454	2.86	NM_015136	NM_015136	Homo sapiens stabilin 1 (STAB1), mRNA [NM_015136]	NM_015136
A_23_P350005	0.00454	4.65	NM_173553	NM_173553	Homo sapiens hypothetical protein FLJ25801 (FLJ25801), mRNA [NM_173553]	NM_173553
A_23_P342138	0.00455	4.31	NM_152702	NM_152702	Homo sapiens chromosome 9 open reading frame 94 (C9orf94), mRNA [NM_152702]	NM_152702
A_24_P226755	0.00455	25.77	NM_014729	NM_014729	Homo sapiens thymus high mobility group box protein TOX (TOX), mRNA [NM_014729]	NM_014729
A_24_P312072	0.00456	2.88	NM_020408	NM_020408	Homo sapiens chromosome 6 open reading frame 149 (C6orf149), mRNA [NM_020408]	NM_020408
A_24_P294191	0.00456	3.32	NM_002881	NM_002881	Homo sapiens v-ral simian leukemia viral oncogene homolog B (ras related; GTP binding protein) (RALB), mRNA [NM_002881]	NM_002881
A_23_P395172	0.00457	3.34	AK095685	AK095685	Homo sapiens cDNA FLJ38366 fis, clone FEBRA2001134. [AK095685]	
A_24_P541483	0.00457	3.83	ENST00000329854			
A_24_P377394	0.00458	2.34	NM_003449	NM_003449	Homo sapiens tripartite motif-containing 26 (TRIM26), mRNA [NM_003449]	NM_003449
A_32_P72394	0.00459	2.43	NM_002735	NM_002735	Homo sapiens protein kinase, cAMP-dependent, regulatory, type I, beta (PRKAR1B), mRNA [NM_002735]	NM_002735
A_24_P247608	0.00459	2.45	NM_005389	NM_005389	Homo sapiens protein-L-isoaspartate (D-aspartate) O-methyltransferase (PCMT1), mRNA [NM_005389]	NM_005389
A_23_P148047	0.00459	2.62	NM_000958	NM_000958	Homo sapiens prostaglandin E receptor 4 (subtype EP4) (PTGER4), mRNA [NM_000958]	NM_000958

A_23_P149690	0.0046	2.08	NM_018216	NM_018216	Homo sapiens pantothenate kinase 4 (PANK4), mRNA [NM_018216]	NM_018216
A_24_P409750	0.0046	2.33	A_24_P409750			
A_24_P392622	0.0046	2.46	A_24_P392622			
A_23_P200901	0.0046	2.65	NM_004436	NM_004436	Homo sapiens endosulfine alpha (ENSA), transcript variant 3, mRNA [NM_004436]	NM_004436
A_23_P37296	0.0046	2.92	NM_015684	NM_015684	Homo sapiens ATP synthase, H+ transporting, mitochondrial F0 complex, subunit s (factor B) (ATP5S), nuclear gene encoding mitochondrial protein, transcript variant 3, mRNA [NM_015684]	NM_015684
A_32_P49284	0.00461	2.60	XM_372040	XM_372040	PREDICTED: Homo sapiens similar to asparagine synthetase; glutamine-dependent asparagine synthetase; TS11 cell cycle control protein (LOC389652), mRNA [XM_372040]	XM_372040
A_24_P67494	0.00462	2.17	XM_496705	XM_496705	PREDICTED: Homo sapiens similar to karyopherin alpha 2 (LOC339991), mRNA [XM_496705]	XM_496705
A_23_P29046	0.00462	5.43	NM_001757	NM_001757	Homo sapiens carbonyl reductase 1 (CBR1), mRNA [NM_001757]	NM_001757
A_24_P51061	0.00462	7.81	NM_080927	NM_080927	Homo sapiens discoidin, CUB and LCCL domain containing 2 (DCBLD2), mRNA [NM_080927]	NM_080927
A_23_P10077	0.00463	2.30	NM_020376	NM_020376	Homo sapiens patatin-like phospholipase domain containing 2 (PNPLA2), mRNA [NM_020376]	NM_020376
A_24_P151084	0.00464	2.24	NM_018235	NM_018235	Homo sapiens CNDP dipeptidase 2 (metallopeptidase M20 family) (CNDP2), mRNA [NM_018235]	NM_018235
A_24_P920016	0.00464	2.35	AK124651	AK124651	Homo sapiens cDNA FLJ42660 fis, clone BRAMY2010808. [AK124651]	XM_209656
A_24_P191847	0.00464	2.51	NM_024754	NM_024754	Homo sapiens pentatricopeptide repeat domain 2 (PTCD2), mRNA [NM_024754]	NM_024754
A_24_P313744	0.00465	2.20	NM_005151	NM_005151	Homo sapiens ubiquitin specific protease 14 (tRNA-guanine transglycosylase) (USP14), mRNA [NM_005151]	NM_005151
A_23_P35809	0.00465	2.22	NM_144585	NM_144585	Homo sapiens solute carrier family 22 (organic anion/cation transporter), member 12 (SLC22A12), transcript variant 1, mRNA [NM_144585]	NM_144585
A_23_P117599	0.00465	2.24	NM_012111	NM_012111	Homo sapiens AHA1, activator of heat shock 90kDa protein ATPase homolog 1 (yeast) (AHSA1), mRNA [NM_012111]	NM_012111
A_32_P82907	0.00466	2.06	A_32_P82907			
A_23_P102769	0.00468	2.53	NM_052865	NM_052865	Homo sapiens chromosome 20 open reading frame 72 (C20orf72), mRNA [NM_052865]	NM_052865
A_32_P144852	0.00468	2.56	CR622110	CR622110	full-length cDNA clone CS0DC025YP03 of Neuroblastoma Cot 25-normalized of Homo sapiens (human). [CR622110]	
A_24_P287826	0.00469	2.22	NM_018124	NM_018124	Homo sapiens ring finger and WD repeat domain 3 (RFWD3), mRNA [NM_018124]	NM_018124
A_24_P945293	0.00469	2.33	NM_016079	NM_016079	Homo sapiens vacuolar protein sorting 24 (yeast) (VPS24), transcript variant 1, mRNA [NM_016079]	NM_016079
A_32_P725218	0.00469	2.99	NM_001005372	NM_001005372	Homo sapiens hypothetical protein FLJ12760 (FLJ12760), mRNA [NM_001005372]	NM_001005372
A_24_P24982	0.00469	3.48	A_24_P24982			
A_23_P212307	0.0047	2.07	NM_182903	NM_182903	Homo sapiens kinesin family member 9 (KIF9), transcript variant 3, mRNA [NM_182903]	NM_182903
A_24_P323434	0.0047	2.46	NM_152562	NM_152562	Homo sapiens cell division cycle associated 2 (CDCA2), mRNA [NM_152562]	NM_152562
A_24_P924602	0.00471	2.08	NM_199293	NM_199293	Homo sapiens tyrosine hydroxylase (TH), transcript variant 3, mRNA [NM_199293]	NM_199293
A_24_P418126	0.00471	2.16	ENST00000321619		full-length cDNA clone CS0DI012YN13 of Placenta Cot 25-normalized of Homo sapiens (human). [CR594829]	
A_23_P208866	0.00471	2.42	NM_004877	NM_004877	Homo sapiens glia maturation factor, gamma (GMFG), mRNA [NM_004877]	NM_004877
A_32_P158376	0.00471	5.88	BF210146	BF210146	601874052F1 NIH_MGC_54 Homo sapiens cDNA clone IMAGE:4098852 5', mRNA sequence [BF210146]	
A_24_P161581	0.00472	2.12	ENST00000344339			
A_24_P388622	0.00472	2.16	NM_016930	NM_016930	Homo sapiens syntaxin 18 (STX18), mRNA [NM_016930]	NM_016930
A_23_P102523	0.00473	2.09	NM_003128	NM_003128	Homo sapiens spectrin, beta, non-erythrocytic 1 (SPTBN1), transcript variant 1, mRNA [NM_003128]	NM_003128
A_23_P351837	0.00473	2.22	NM_173583	NM_173583	Homo sapiens hypothetical protein FLJ33790 (FLJ33790), mRNA [NM_173583]	NM_173583
A_23_P162589	0.00473	2.65	NM_001017535	NM_001017535	Homo sapiens vitamin D (1,25- dihydroxyvitamin D3) receptor (VDR), transcript variant 2, mRNA [NM_001017535]	NM_001017535
A_23_P9836	0.00473	7.04	NM_004454	NM_004454	Homo sapiens ets variant gene 5 (ets-related molecule) (ETV5), mRNA [NM_004454]	NM_004454
A_23_P425880	0.00477	2.59	NM_007118	NM_007118	Homo sapiens triple functional domain (PTPRF interacting) (TRIO), mRNA [NM_007118]	NM_007118
A_32_P117760	0.00477	2.76	BG208131	BG208131	RST27621 Athersys RAGE Library Homo sapiens cDNA, mRNA sequence [BG208131]	
A_24_P101786	0.00477	3.12	NM_198554	NM_198554	Homo sapiens thyroid adenoma associated (THADA), transcript variant 2, mRNA [NM_198554]	NM_198554
A_32_P233860	0.0048	3.46	NM_145690	NM_145690	Homo sapiens tyrosine 3-monooxygenase/tryptophan 5-monooxygenase activation protein, zeta polypeptide (YWHAZ), transcript variant 2, mRNA [NM_145690]	NM_145690

A_32_P15389	0.00481	2.25	AA427821	AA427821	AA427821 zw53a02.s1 Soares_total_fetus_Nb2HF8_9w Homo sapiens cDNA clone IMAGE:773738 3', mRNA sequence [AA427821]	
A_23_P206177	0.00481	2.42	NM_003825	NM_003825	Homo sapiens synaptosomal-associated protein, 23kDa (SNAP23), transcript variant 1, mRNA [NM_003825]	NM_003825
A_23_P342118	0.00484	2.08	NM_138442	NM_138442	Homo sapiens hypothetical protein BC013949 (LOC115098), mRNA [NM_138442]	NM_138442
A_23_P127948	0.00484	5.13	NM_001124	NM_001124	Homo sapiens adrenomedullin (ADM), mRNA [NM_001124]	NM_001124
A_32_P151102	0.00484	6.94	NM_002444	NM_002444	Homo sapiens moesin (MSN), mRNA [NM_002444]	NM_002444
A_24_P15754	0.00486	2.25	NM_006114	NM_006114	Homo sapiens translocase of outer mitochondrial membrane 40 homolog (yeast) (TOMM40), mRNA [NM_006114]	NM_006114
A_24_P211206	0.00486	2.29	NM_001010856	NM_001010856	Homo sapiens hypothetical protein LOC147804 (LOC147804), mRNA [NM_001010856]	NM_001010856
A_32_P468522	0.00486	4.93	AF031138	AF031138	Homo sapiens 1C7 precursor, mRNA, alternatively spliced, complete cds. [AF031138]	
A_23_P89018	0.00489	2.27	NM_032296	NM_032296	Homo sapiens FLYWCH-type zinc finger 1 (FLYWCH1), transcript variant 1, mRNA [NM_032296]	NM_032296
A_24_P399622	0.00491	2.02	NM_199444	NM_199444	Homo sapiens coatomer protein complex, subunit epsilon (COPE), transcript variant 3, mRNA [NM_199444]	NM_199444
A_23_P74278	0.00491	2.31	AB209081	AB209081	Homo sapiens mRNA for phosphodiesterase 4B, cAMP-specific (phosphodiesterase E4 dunce homolog, Drosophila) variant protein. [AB209081]	
A_24_P381455	0.00491	6.25	BC018435	BC018435	Homo sapiens hypothetical protein FLJ11259, mRNA (cDNA clone MGC:21716 IMAGE:4474297), complete cds. [BC018435]	
A_23_P123440	0.00492	2.23	NM_007175	NM_007175	Homo sapiens SPFH domain family, member 2 (SPFH2), transcript variant 1, mRNA [NM_007175]	NM_007175
A_23_P35230	0.00492	2.45	NM_172350	NM_172350	Homo sapiens membrane cofactor protein (CD46, trophoblast-lymphocyte cross-reactive antigen) (MCP), transcript variant n, mRNA [NM_172350]	NM_172350
A_23_P61466	0.00492	4.44	NM_174941	NM_174941	Homo sapiens scavenger receptor cysteine-rich type 1 protein M160 (M160), mRNA [NM_174941]	NM_174941
A_24_P89718	0.00493	3.14	NM_024926	NM_024926	Homo sapiens hypothetical protein FLJ12571 (FLJ12571), mRNA [NM_024926]	NM_024926
A_32_P51855	0.00493	6.85	BC038556	BC038556	Homo sapiens, clone IMAGE:3446976, mRNA. [BC038556]	
A_24_P52189	0.00494	2.22	AK055407	AK055407	Homo sapiens cDNA FLJ30845 fis, clone FEBRA2002727. [AK055407]	
A_24_P773539	0.00494	3.92	XM_372128	XM_372128	PREDICTED: Homo sapiens similar to Osteotesticular phosphatase; protein tyrosine phosphatase receptor type V; protein tyrosine phosphatase receptor type W; protein tyrosine phosphatase, receptor type, V (LOC389772), mRNA [XM_372128]	XM_372128
A_23_P210361	0.00495	2.22	NM_022082	NM_022082	Homo sapiens chromosome 20 open reading frame 59 (C20orf59), mRNA [NM_022082]	NM_022082
A_23_P102973	0.00496	2.04	NM_022719	NM_022719	Homo sapiens DiGeorge syndrome critical region gene 14 (DGCR14), mRNA [NM_022719]	NM_022719
A_24_P203226	0.00496	2.59	NM_178230	NM_178230	Homo sapiens peptidylprolyl isomerase A (cyclophilin A)-like 4 (PPIAL4), mRNA [NM_178230]	NM_178230
A_23_P416656	0.00497	2.37	NM_033375	NM_033375	Homo sapiens myosin IC (MYO1C), mRNA [NM_033375]	NM_033375
A_24_P226278	0.00497	2.70	NM_015288	NM_015288	Homo sapiens PHD finger protein 15 (PHF15), mRNA [NM_015288]	NM_015288
A_24_P366989	0.00498	3.38	THC2307535		GDIR_HUMAN (P52565) Rho GDP-dissociation inhibitor 1 (Rho GDI 1) (Rho-GDI alpha), partial (67%) [THC2307535]	
A_24_P366555	0.00498	4.10	BC032715	BC032715	Homo sapiens parathyroid hormone-responsive B1 gene, mRNA (cDNA clone IMAGE:5519851), complete cds. [BC032715]	
A_23_P393656	0.00499	2.48	AK055893	AK055893	Homo sapiens cDNA FLJ31331 fis, clone MAMGL1000056. [AK055893]	
A_24_P336759	0.00499	3.14	NM_021960	NM_021960	Homo sapiens myeloid cell leukemia sequence 1 (BCL2-related) (MCL1), transcript variant 1, mRNA [NM_021960]	NM_021960
A_24_P332532	0.005	2.74	A_24_P332532			
A_32_P330000	0.005	3.24	NM_207477	NM_207477	Homo sapiens FLJ27365 protein (FLJ27365), mRNA [NM_207477]	NM_207477
A_23_P136573	0.00501	2.04	NM_003896	NM_003896	Homo sapiens ST3 beta-galactoside alpha-2,3-sialyltransferase 5 (ST3GAL5), mRNA [NM_003896]	NM_003896
A_24_P794833	0.00501	2.15	A_24_P794833			
A_32_P327810	0.00501	2.17	AF116624	AF116624	Homo sapiens PRO1146 mRNA, complete cds. [AF116624]	
A_24_P930764	0.00501	3.07	BC039241	BC039241	Homo sapiens chromosome 1 open reading frame 96, mRNA (cDNA clone MGC:32857 IMAGE:4731004), complete cds. [BC039241]	
A_24_P933618	0.00502	2.32	THC2423318		AF399755 SNX29 {Mus musculus;} , partial (28%) [THC2423318]	
A_23_P253301	0.00502	2.77	NM_053024	NM_053024	Homo sapiens profilin 2 (PFN2), transcript variant 1, mRNA [NM_053024]	NM_053024
A_24_P74828	0.00502	6.90	NM_175622	NM_175622	Homo sapiens metallothionein 1J (MT1J), mRNA [NM_175622]	NM_175622
A_24_P384210	0.00504	2.04	A_24_P384210			

A_23_P28238	0.00505	2.26	NM_014748	NM_014748	Homo sapiens sorting nexin 17 (SNX17), mRNA [NM_014748]	NM_014748
A_32_P195181	0.00506	2.82	AI380795	AI380795	AI380795 tg04c07.x1 NCI_CGAP_CLL1 Homo sapiens cDNA clone IMAGE:2107788 3' similar to gb:M16462 NADH-CYTOCHROME B5 REDUCTASE (HUMAN);, mRNA sequence [AI380795]	
A_23_P98275	0.00507	2.22	NM_002419	NM_002419	Homo sapiens mitogen-activated protein kinase kinase kinase 11 (MAP3K11), mRNA [NM_002419]	NM_002419
A_32_P165407	0.00507	3.97	AK024926	AK024926	Homo sapiens cDNA: FLJ21273 fis, clone COL01778. [AK024926]	
A_23_P52610	0.00508	2.15	NM_000107	NM_000107	Homo sapiens damage-specific DNA binding protein 2, 48kDa (DDB2), mRNA [NM_000107]	NM_000107
A_24_P274928	0.00508	3.04	NM_025069	NM_025069	Homo sapiens hypothetical protein FLJ14299 (FLJ14299), mRNA [NM_025069]	NM_025069
A_23_P138680	0.00508	5.81	NM_172200	NM_172200	Homo sapiens interleukin 15 receptor, alpha (IL15RA), transcript variant 2, mRNA [NM_172200]	NM_172200
A_24_P118247	0.0051	2.47	NM_001012762	NM_001012762	Homo sapiens hypothetical protein LOC348180 (LOC348180), transcript variant 2, mRNA [NM_001012762]	NM_001012762
A_23_P32722	0.00512	2.14	NM_024741	NM_024741	Homo sapiens zinc finger protein 408 (ZNF408), mRNA [NM_024741]	NM_024741
A_24_P2093	0.00512	2.31	NM_020196	NM_020196	Homo sapiens XPA binding protein 2 (XAB2), mRNA [NM_020196]	NM_020196
A_24_P222054	0.00513	2.54	AF271776	AF271776	Homo sapiens DC48 mRNA, complete cds. [AF271776]	
A_24_P278839	0.00513	4.31	NM_194271	NM_194271	Homo sapiens ring finger protein 34 (RNF34), transcript variant 1, mRNA [NM_194271]	NM_194271
A_24_P410678	0.00514	4.15	NM_002227	NM_002227	Homo sapiens Janus kinase 1 (a protein tyrosine kinase) (JAK1), mRNA [NM_002227]	NM_002227
A_23_P211878	0.00517	2.17	NM_001457	NM_001457	Homo sapiens filamin B, beta (actin binding protein 278) (FLNB), mRNA [NM_001457]	NM_001457
A_23_P311869	0.00517	2.32	NM_003896	NM_003896	Homo sapiens ST3 beta-galactoside alpha-2,3-sialyltransferase 5 (ST3GAL5), mRNA [NM_003896]	NM_003896
A_23_P395585	0.00518	2.10	NM_152533	NM_152533	Homo sapiens chromosome 3 open reading frame 22 (C3orf22), mRNA [NM_152533]	NM_152533
A_32_P18072	0.00518	2.16	THC2319687		Q9S729 (Q9S729) GlsA, partial (3%) [THC2319687]	
A_24_P367242	0.00518	2.18	NM_181607	NM_181607	Homo sapiens keratin associated protein 19-1 (KRTAP19-1), mRNA [NM_181607]	NM_181607
A_24_P411121	0.00519	2.23	NM_148901	NM_148901	Homo sapiens tumor necrosis factor receptor superfamily, member 18 (TNFRSF18), transcript variant 2, mRNA [NM_148901]	NM_148901
A_32_P55045	0.00519	2.58	AK091722	AK091722	Homo sapiens cDNA FLJ34403 fis, clone HCHON2001607. [AK091722]	
A_32_P59811	0.0052	2.07	THC2430346		PR18_HUMAN (Q99633) Pre-mRNA splicing factor 18 (PRP18 homolog) (hPRP18), partial (31%) [THC2430346]	
A_24_P196175	0.00521	2.02	A_24_P196175			
A_24_P117866	0.00522	2.88	NM_001119	NM_001119	Homo sapiens adducin 1 (alpha) (ADD1), transcript variant 1, mRNA [NM_001119]	NM_001119
A_24_P373126	0.00524	3.05	NM_152307	NM_152307	Homo sapiens chromosome 14 open reading frame 172 (C14orf172), mRNA [NM_152307]	NM_152307
A_23_P105066	0.00525	2.02	NM_001014795	NM_001014795	Homo sapiens integrin-linked kinase (ILK), transcript variant 3, mRNA [NM_001014795]	NM_001014795
A_23_P145694	0.00526	2.38	NM_001673	NM_001673	Homo sapiens asparagine synthetase (ASNS), transcript variant 2, mRNA [NM_001673]	NM_001673
A_23_P343719	0.00526	2.67	NM_000932	NM_000932	Homo sapiens phospholipase C, beta 3 (phosphatidylinositol-specific) (PLCB3), mRNA [NM_000932]	NM_000932
A_23_P318396	0.00527	2.27	NM_198700	NM_198700	Homo sapiens CUG triplet repeat, RNA binding protein 1 (CUGBP1), transcript variant 2, mRNA [NM_198700]	NM_198700
A_23_P152949	0.00527	2.62	NM_033413	NM_033413	Homo sapiens leucine rich repeat containing 46 (LRRC46), mRNA [NM_033413]	NM_033413
A_32_P226498	0.00528	2.61	BC047717	BC047717	Homo sapiens, clone IMAGE:5734530, mRNA. [BC047717]	
A_23_P102109	0.00529	2.03	NM_025019	NM_025019	Homo sapiens tubulin, alpha 4 (TUBA4), mRNA [NM_025019]	NM_025019
A_23_P405794	0.00529	2.60	NM_153006	NM_153006	Homo sapiens N-acetylglutamate synthase (NAGS), mRNA [NM_153006]	NM_153006
A_23_P28638	0.00529	3.75	NM_017958	NM_017958	Homo sapiens pleckstrin homology domain containing, family B (evectins) member 2 (PLEKHB2), mRNA [NM_017958]	NM_017958
A_32_P110390	0.0053	3.53	NM_173490	NM_173490	Homo sapiens proline-rich protein PRP2 (PRP2), mRNA [NM_173490]	NM_173490
A_23_P408830	0.00531	2.33	NM_172215	NM_172215	Homo sapiens calcium/calmodulin-dependent protein kinase kinase 2, beta (CAMKK2), transcript variant 6, mRNA [NM_172215]	NM_172215
A_24_P275386	0.00531	2.55	ENST00000332107		Homo sapiens I3 binding protein (BRI3BP) mRNA, complete cds. [AF284094]	
A_32_P149416	0.00531	4.50	BG001037	BG001037	BG001037 RC5-GN0132-131100-012-E05 GN0132 Homo sapiens cDNA, mRNA sequence [BG001037]	
A_24_P148261	0.00531	4.67	AK021874	AK021874	Homo sapiens cDNA FLJ11812 fis, clone HEMBA1006364. [AK021874]	
A_32_P228414	0.00532	2.03	NM_015635	NM_015635	Homo sapiens GTPase activating protein and VPS9 domains 1 (GAPVD1), mRNA [NM_015635]	NM_015635

A_32_P215078	0.00532	2.95	BF857817	BF857817	BF857817 QV1-FT0202-071100-465-d08 FT0202 Homo sapiens cDNA, mRNA sequence [BF857817]	
A_23_P306148	0.00533	2.46	NM_002675	NM_002675	Homo sapiens promyelocytic leukemia (PML), transcript variant 6, mRNA [NM_002675]	NM_002675
A_23_P331049	0.00534	3.03	NM_006426	NM_006426	Homo sapiens dihydropyrimidinase-like 4 (DPYSL4), mRNA [NM_006426]	NM_006426
A_23_P56559	0.00534	10.96	NM_005771	NM_005771	Homo sapiens dehydrogenase/reductase (SDR family) member 9 (DHRS9), transcript variant 1, mRNA [NM_005771]	NM_005771
A_24_P886960	0.00535	2.43	AK026213	AK026213	Homo sapiens cDNA: FLJ22560 fis, clone HSI01610. [AK026213]	
A_24_P210244	0.00535	2.47	U09088	U09088	Human thymopoietin gamma mRNA, complete cds. [U09088]	
A_24_P75621	0.00536	2.51	ENST00000318117			
A_24_P464540	0.00536	2.59	BC047940	BC047940	Homo sapiens family with sequence similarity 41, member C, mRNA (cDNA clone IMAGE:5201580), partial cds. [BC047940]	
A_23_P57323	0.00537	2.76	NM_004449	NM_004449	Homo sapiens v-ets erythroblastosis virus E26 oncogene like (avian) (ERG), transcript variant 2, mRNA [NM_004449]	NM_004449
A_24_P941912	0.00539	2.33	NM_138287	NM_138287	Homo sapiens deltex 3-like (Drosophila) (DTX3L), mRNA [NM_138287]	NM_138287
A_23_P49009	0.0054	2.02	NM_153613	NM_153613	Homo sapiens PLSC domain containing protein (LOC254531), mRNA [NM_153613]	NM_153613
A_24_P390784	0.0054	2.66	NM_001005291	NM_001005291	Homo sapiens sterol regulatory element binding transcription factor 1 (SREBF1), transcript variant 1, mRNA [NM_001005291]	NM_001005291
A_24_P367602	0.00544	2.97	AK092260	AK092260	Homo sapiens cDNA FLJ34941 fis, clone NT2RP7007480. [AK092260]	
A_32_P227400	0.00545	2.70	A_32_P227400			
A_24_P599453	0.00547	2.45	CR610404	CR610404	full-length cDNA clone CS0DA008YA11 of Neuroblastoma of Homo sapiens (human). [CR610404]	
A_24_P669220	0.00549	3.46	THC2314754		O99381 (O99381) Cytochrome C oxidase subunit I (Fragment), partial (5%) [THC2314754]	
A_24_P59099	0.0055	2.11	A_24_P59099			
A_24_P15821	0.0055	2.55	A_24_P15821			
A_24_P339305	0.00551	2.40	NM_023079	NM_023079	Homo sapiens hypothetical protein FLJ13855 (FLJ13855), mRNA [NM_023079]	NM_023079
A_32_P209325	0.00551	2.82	AK130530	AK130530	Homo sapiens cDNA FLJ27020 fis, clone SLV06144. [AK130530]	
A_32_P187050	0.00553	2.26	A_32_P187050			
A_23_P75310	0.00553	3.57	NM_021226	NM_021226	Homo sapiens Rho GTPase activating protein 22 (ARHGAP22), mRNA [NM_021226]	NM_021226
A_24_P354337	0.00554	2.10	NM_032146	NM_032146	Homo sapiens ADP-ribosylation factor-like 6 (ARL6), transcript variant 1, mRNA [NM_032146]	NM_032146
A_24_P372123	0.00554	2.17	NM_018396	NM_018396	Homo sapiens methyltransferase like 2 (METTL2), mRNA [NM_018396]	NM_018396
A_32_P162338	0.00554	3.72	NM_153649	NM_153649	Homo sapiens tropomyosin 3 (TPM3), transcript variant 2, mRNA [NM_153649]	NM_153649
A_24_P408206	0.00555	4.26	NM_002730	NM_002730	Homo sapiens protein kinase, cAMP-dependent, catalytic, alpha (PRKACA), transcript variant 1, mRNA [NM_002730]	NM_002730
A_24_P289795	0.00557	2.04	ENST00000323057		Homo sapiens skin-specific protein (xp33) mRNA, partial cds. [AF005082]	XM_371314
A_24_P84711	0.00557	2.07	A_24_P84711			
A_23_P116387	0.00557	2.21	NM_020238	NM_020238	Homo sapiens inner centromere protein antigens 135/155kDa (INCENP), mRNA [NM_020238]	NM_020238
A_24_P41570	0.00557	2.23	NM_002106	NM_002106	Homo sapiens H2A histone family, member Z (H2AFZ), mRNA [NM_002106]	NM_002106
A_23_P416242	0.00557	2.45	NM_178841	NM_178841	Homo sapiens ring finger protein 166 (RNF166), mRNA [NM_178841]	NM_178841
A_24_P96234	0.00557	4.39	NM_031209	NM_031209	Homo sapiens queuine tRNA-ribosyltransferase 1 (tRNA-guanine transglycosylase) (QTRT1), mRNA [NM_031209]	NM_031209
A_24_P383680	0.00558	2.00	A_24_P383680			
A_24_P581618	0.00558	2.12	A_24_P581618			
A_32_P113472	0.00559	5.08	CA314451	CA314451	CA314451 UI-CF-FN0-afh-a-09-0-UI.s1 UI-CF-FN0 Homo sapiens cDNA clone UI-CF-FN0-afh-a-09-0-UI 3', mRNA sequence [CA314451]	
A_24_P359838	0.0056	3.94	NM_153712	NM_153712	Homo sapiens tubulin tyrosine ligase (TTL), mRNA [NM_153712]	NM_153712
A_24_P13190	0.00561	3.13	NM_138961	NM_138961	Homo sapiens endothelial cell adhesion molecule (ESAM), mRNA [NM_138961]	NM_138961
A_24_P231250	0.00562	2.20	NM_020451	NM_020451	Homo sapiens selenoprotein N, 1 (SEPN1), transcript variant 1, mRNA [NM_020451]	NM_020451
A_24_P358976	0.00563	2.10	NM_018279	NM_018279	Homo sapiens transmembrane protein 19 (TMEM19), mRNA [NM_018279]	NM_018279
A_23_P160503	0.00563	2.85	NM_016066	NM_016066	Homo sapiens glutaredoxin 2 (GLRX2), transcript variant 1, mRNA [NM_016066]	NM_016066

A_23_P46748	0.00564	2.07	NM_001278	NM_001278	Homo sapiens conserved helix-loop-helix ubiquitous kinase (CHUK), mRNA [NM_001278]	NM_001278
A_23_P126836	0.00565	2.20	NM_003326	NM_003326	Homo sapiens tumor necrosis factor (ligand) superfamily, member 4 (tax-transcriptionally activated glycoprotein 1, 34kDa) (TNFSF4), mRNA [NM_003326]	NM_003326
A_32_P41662	0.00566	2.01	NM_019041	NM_019041	Homo sapiens mitochondrial translational release factor 1-like (MTRF1L), mRNA [NM_019041]	NM_019041
A_23_P210686	0.00566	2.60	NM_033089	NM_033089	Homo sapiens zinc finger, CCHC domain containing 3 (ZCCHC3), mRNA [NM_033089]	NM_033089
A_23_P64090	0.00567	2.46	NM_030981	NM_030981	Homo sapiens RAB1B, member RAS oncogene family (RAB1B), mRNA [NM_030981]	NM_030981
A_24_P101722	0.00567	2.82	ENST00000355049		PREDICTED: Homo sapiens similar to peptidyl-Pro cis trans isomerase (LOC126170), mRNA [XM_497621]	XM_497621
A_23_P84648	0.00569	2.62	ENST00000340855		Homo sapiens cDNA FLJ31038 fis, clone HSYRA2000159. [AK055600]	
A_23_P35148	0.00569	4.52	NM_005645	NM_005645	Homo sapiens TAF13 RNA polymerase II, TATA box binding protein (TBP)-associated factor, 18kDa (TAF13), mRNA [NM_005645]	NM_005645
A_23_P157170	0.0057	2.02	NM_032317	NM_032317	Homo sapiens Williams Beuren syndrome chromosome region 18 (WBSCR18), mRNA [NM_032317]	NM_032317
A_24_P98277	0.0057	2.21	NM_001002296	NM_001002296	Homo sapiens golgi autoantigen, golgin subfamily a, 7 (GOLGA7), transcript variant 2, mRNA [NM_001002296]	NM_001002296
A_23_P163455	0.0057	3.25	NM_002373	NM_002373	Homo sapiens microtubule-associated protein 1A (MAP1A), mRNA [NM_002373]	NM_002373
A_23_P152984	0.00572	2.11	NM_005782	NM_005782	Homo sapiens THO complex 4 (THOC4), mRNA [NM_005782]	NM_005782
A_23_P137830	0.00572	2.75	XM_060887	XM_060887	PREDICTED: Homo sapiens similar to peptidyl-Pro cis trans isomerase (LOC128192), mRNA [XM_060887]	XM_060887
A_23_P53603	0.00573	2.19	NM_001681	NM_001681	Homo sapiens ATPase, Ca++ transporting, cardiac muscle, slow twitch 2 (ATP2A2), transcript variant 2, mRNA [NM_001681]	NM_001681
A_24_P213763	0.00573	3.82	CR625942	CR625942	full-length cDNA clone CS0DL007YI18 of B cells (Ramos cell line) Cot 25-normalized of Homo sapiens (human). [CR625942]	
A_24_P303145	0.00576	2.45	AK054966	AK054966	Homo sapiens cDNA FLJ30404 fis, clone BRACE2008481. [AK054966]	
A_24_P918436	0.00577	2.32	NM_152221	NM_152221	Homo sapiens casein kinase 1, epsilon (CSNK1E), transcript variant 1, mRNA [NM_152221]	NM_152221
A_32_P109401	0.00578	2.16	AF130102	AF130102	Homo sapiens clone FLB2205 PRO0522 mRNA, complete cds. [AF130102]	
A_23_P422872	0.0058	2.13	NM_175892	NM_175892	Homo sapiens hypothetical protein FLJ37266 (FLJ37266), mRNA [NM_175892]	NM_175892
A_23_P317056	0.0058	2.15	ENST00000361681			
A_24_P229616	0.00581	3.21	A_24_P229616			
A_24_P250227	0.00582	2.27	NM_021724	NM_021724	Homo sapiens nuclear receptor subfamily 1, group D, member 1 (NR1D1), mRNA [NM_021724]	NM_021724
A_24_P282434	0.00582	2.65	NM_002843	NM_002843	Homo sapiens protein tyrosine phosphatase, receptor type, J (PTPRJ), mRNA [NM_002843]	NM_002843
A_24_P502652	0.00582	4.12	A_24_P502652			
A_23_P211345	0.00583	3.80	NM_080647	NM_080647	Homo sapiens T-box 1 (TBX1), transcript variant C, mRNA [NM_080647]	NM_080647
A_23_P147755	0.00584	2.01	NM_017413	NM_017413	Homo sapiens apelin, AGTRL1 ligand (APLN), mRNA [NM_017413]	NM_017413
A_23_P79015	0.00584	2.43	NM_199037	NM_199037	Homo sapiens sodium channel, voltage-gated, type I, beta (SCN1B), transcript variant b, mRNA [NM_199037]	NM_199037
A_24_P492562	0.00584	3.21	NM_145690	NM_145690	Homo sapiens tyrosine 3-monooxygenase/tryptophan 5-monooxygenase activation protein, zeta polypeptide (YWHAZ), transcript variant 2, mRNA [NM_145690]	NM_145690
A_23_P213350	0.00585	2.19	A_23_P213350			
A_23_P98183	0.00585	2.64	NM_005343	NM_005343	Homo sapiens v-Ha-ras Harvey rat sarcoma viral oncogene homolog (HRAS), transcript variant 1, mRNA [NM_005343]	NM_005343
A_24_P913227	0.00587	2.09	BC010944	BC010944	Homo sapiens CDC23 (cell division cycle 23, yeast, homolog), mRNA (cDNA clone IMAGE:4281800), complete cds. [BC010944]	
A_23_P321377	0.00587	3.28	NM_152374	NM_152374	Homo sapiens hypothetical protein FLJ38984 (FLJ38984), mRNA [NM_152374]	NM_152374
A_24_P59147	0.00588	10.70	ENST00000332560			
A_23_P86012	0.00589	2.59	NM_001017402	NM_001017402	Homo sapiens laminin, beta 3 (LAMB3), transcript variant 2, mRNA [NM_001017402]	NM_001017402
A_24_P266273	0.0059	2.47	NM_172020	NM_172020	Homo sapiens POM121 membrane glycoprotein (rat) (POM121), mRNA [NM_172020]	NM_172020
A_23_P137630	0.00592	2.34	NM_016287	NM_016287	Homo sapiens heterochromatin protein 1, binding protein 3 (HP1BP3), mRNA [NM_016287]	NM_016287
A_23_P17811	0.00594	2.70	NM_012429	NM_012429	Homo sapiens SEC14-like 2 (S. cerevisiae) (SEC14L2), mRNA [NM_012429]	NM_012429
A_32_P120120	0.00595	2.20	T19827	T19827	T19827 B601F Heart Homo sapiens cDNA clone B601 similar to Hypothetical protein upstream of N-ras, mRNA sequence [T19827]	
A_24_P135515	0.00596	2.61	A_24_P135515			

A_23_P88626	0.00596	3.13	NM_001150	NM_001150	Homo sapiens alanyl (membrane) aminopeptidase (aminopeptidase N, aminopeptidase M, microsomal aminopeptidase, CD13, p150) (ANPEP), mRNA [NM_001150]	NM_001150
A_24_P666340	0.00598	2.07	THC2408022			
A_23_P392383	0.00599	2.05	NM_033551	NM_033551	Homo sapiens La ribonucleoprotein domain family, member 1 (LARP1), mRNA [NM_033551]	NM_033551
A_23_P369471	0.00599	2.06	NM_178431	NM_178431	Homo sapiens late cornified envelope 3A (LCE3A), mRNA [NM_178431]	NM_178431
A_24_P643587	0.00599	2.07	NM_024095	NM_024095	Homo sapiens ankyrin repeat and SOCS box-containing 8 (ASB8), mRNA [NM_024095]	NM_024095
A_23_P162449	0.00601	9.26	NM_020762	NM_020762	Homo sapiens SLIT-ROBO Rho GTPase activating protein 1 (SRGAP1), mRNA [NM_020762]	NM_020762
A_23_P356466	0.00602	2.28	BC004219	BC004219	Homo sapiens cDNA clone IMAGE:3354271, complete cds. [BC004219]	
A_23_P68824	0.00602	2.31	NM_003073	NM_003073	Homo sapiens SWI/SNF related, matrix associated, actin dependent regulator of chromatin, subfamily b, member 1 (SMARCB1), transcript variant 1, mRNA [NM_003073]	NM_003073
A_24_P706340	0.00602	3.51	L10374	L10374	Human (clone CTG-A4) mRNA sequence. [L10374]	
A_24_P117803	0.00603	2.37	ENST00000252509		GB|AL589786.8|CAD13460.1 bA161I19.3 (similar to ribosomal protein L36) [Homo sapiens] [NP414444]	
A_24_P4334	0.00604	2.07	NM_194328	NM_194328	Homo sapiens ring finger protein 38 (RNF38), transcript variant 2, mRNA [NM_194328]	NM_194328
A_24_P194748	0.00606	2.06	ENST00000095634		Homo sapiens, Similar to zinc finger protein 275, clone IMAGE:3453569, mRNA. [BC001545]	XM_372267
A_32_P146552	0.00606	2.24	BQ021969	BQ021969	UI-1-BB0-acw-h-09-0-UI.s1 NCI_CGAP_Pl4 Homo sapiens cDNA clone UI-1-BB0-acw-h-09-0-UI 3', mRNA sequence [BQ021969]	
A_23_P216215	0.00609	3.14	NM_014637	NM_014637	Homo sapiens chondrocyte protein with a poly-proline region (CHPPR), mRNA [NM_014637]	NM_014637
A_32_P220109	0.0061	2.44	BE924986	BE924986	BE924986 MR1-AN0036-280800-002-f08 AN0036 Homo sapiens cDNA, mRNA sequence [BE924986]	
A_24_P255663	0.00611	2.88	A_24_P255663			
A_24_P50139	0.00611	10.46	A_24_P50139			
A_23_P37685	0.00612	2.05	NM_024600	NM_024600	Homo sapiens chromosome 16 open reading frame 30 (C16orf30), mRNA [NM_024600]	NM_024600
A_23_P34325	0.00612	5.32	NM_033300	NM_033300	Homo sapiens low density lipoprotein receptor-related protein 8, apolipoprotein e receptor (LRP8), transcript variant 2, mRNA [NM_033300]	NM_033300
A_23_P47410	0.00615	2.51	NM_138961	NM_138961	Homo sapiens endothelial cell adhesion molecule (ESAM), mRNA [NM_138961]	NM_138961
A_24_P944291	0.00617	2.28	NM_016263	NM_016263	Homo sapiens fizzy/cell division cycle 20 related 1 (Drosophila) (FZR1), mRNA [NM_016263]	NM_016263
A_24_P238836	0.00617	2.70	A_24_P238836			
A_24_P67189	0.00618	2.15	NM_199339	NM_199339	Homo sapiens hypothetical protein LOC374768 (LOC374768), mRNA [NM_199339]	NM_199339
A_24_P39724	0.0062	2.08	NM_144609	NM_144609	Homo sapiens hypothetical protein FLJ31795 (FLJ31795), mRNA [NM_144609]	NM_144609
A_23_P92349	0.00621	2.21	NM_001004356	NM_001004356	Homo sapiens fibroblast growth factor receptor-like 1 (FGFRL1), transcript variant 1, mRNA [NM_001004356]	NM_001004356
A_23_P142518	0.00622	2.39	NM_052876	NM_052876	Homo sapiens BTB (POZ) domain containing 14B (BTBD14B), mRNA [NM_052876]	NM_052876
A_24_P160466	0.00622	3.69	NM_052899	NM_052899	Homo sapiens G protein-regulated inducer of neurite outgrowth 1 (KIAA1893), mRNA [NM_052899]	NM_052899
A_24_P366415	0.00623	3.29	XM_292963	XM_292963	PREDICTED: Homo sapiens similar to peptidylprolyl isomerase A (LOC344178), mRNA [XM_292963]	XM_292963
A_23_P116679	0.00624	2.70	NM_006191	NM_006191	Homo sapiens proliferation-associated 2G4, 38kDa (PA2G4), mRNA [NM_006191]	NM_006191
A_23_P334664	0.00626	3.01	NM_033247	NM_033247	Homo sapiens promyelocytic leukemia (PML), transcript variant 8, mRNA [NM_033247]	NM_033247
A_23_P58815	0.00627	2.15	NM_012176	NM_012176	Homo sapiens F-box protein 4 (FBXO4), transcript variant 1, mRNA [NM_012176]	NM_012176
A_32_P221352	0.00631	2.87	BG943680	BG943680	BG943680 ax40g03.x1 Hembase; Erythroid Progenitor Cells (LCB:ax library) Homo sapiens cDNA clone ax40g03 random, mRNA sequence [BG943680]	
A_32_P149755	0.00632	2.00	A_32_P149755			
A_24_P92168	0.00632	2.29	ENST00000339367			
A_32_P78943	0.00632	3.19	CA419778	CA419778	CA419778 UI-H-FH0-bcm-e-20-0-UI.s1 NCI_CGAP_FH0 Homo sapiens cDNA clone UI-H-FH0-bcm-e-20-0-UI 3', mRNA sequence [CA419778]	
A_32_P217428	0.00632	5.71	CA772440	CA772440	CA772440 io96d01.y1 HR85 islet Homo sapiens cDNA clone IMAGE:6134065 5', mRNA sequence [CA772440]	
A_23_P120146	0.00635	2.27	NM_004257	NM_004257	Homo sapiens transforming growth factor, beta receptor associated protein 1 (TGFBRAP1), mRNA [NM_004257]	NM_004257
A_23_P107257	0.00635	2.43	NM_001007226	NM_001007226	Homo sapiens speckle-type POZ protein (SPOP), transcript variant 1, mRNA [NM_001007226]	NM_001007226
A_24_P375728	0.00636	2.20	NM_138383	NM_138383	Homo sapiens hypothetical protein BC002770 (LOC92154), mRNA [NM_138383]	NM_138383

A_23_P167767	0.00636	2.27	ENST00000360515		full-length cDNA clone CS0DI056YF21 of Placenta Cot 25-normalized of Homo sapiens (human). [CR626635]	
A_23_P97553	0.00636	2.52	BX648708	BX648708	Homo sapiens mRNA; cDNA DKFZp686M0947 (from clone DKFZp686M0947). [BX648708]	
A_23_P7144	0.00636	2.56	NM_001511	NM_001511	Homo sapiens chemokine (C-X-C motif) ligand 1 (melanoma growth stimulating activity, alpha) (CXCL1), mRNA [NM_001511]	NM_001511
A_24_P178415	0.00636	2.59	A_24_P178415			
A_23_P142239	0.00637	2.07	NM_001013617	NM_001013617	Homo sapiens hypothetical LOC541469 protein (LOC541469), mRNA [NM_001013617]	NM_001013617
A_23_P10640	0.00637	2.23	NM_178543	NM_178543	Homo sapiens ectonucleotide pyrophosphatase/phosphodiesterase 7 (ENPP7), mRNA [NM_178543]	NM_178543
A_23_P13740	0.00638	10.60	NM_014903	NM_014903	Homo sapiens neuron navigator 3 (NAV3), mRNA [NM_014903]	NM_014903
A_32_P158253	0.00639	2.11	A_32_P158253			
A_24_P274814	0.0064	2.19	NM_030984	NM_030984	Homo sapiens thromboxane A synthase 1 (platelet, cytochrome P450, family 5, subfamily A) (TBXAS1), transcript variant TXS-II, mRNA [NM_030984]	NM_030984
A_23_P153651	0.0064	3.01	NM_024333	NM_024333	Homo sapiens fibronectin type III and SPRY domain containing 1 (FSD1), mRNA [NM_024333]	NM_024333
A_24_P89708	0.00641	2.65	NM_000883	NM_000883	Homo sapiens IMP (inosine monophosphate) dehydrogenase 1 (IMPDH1), transcript variant 1, mRNA [NM_000883]	NM_000883
A_32_P64263	0.00642	2.25	BQ029628	BQ029628	BQ029628 UI-H-DT0-aua-h-07-0-UI.s1 NCI_CGAP_DT0 Homo sapiens cDNA clone IMAGE:5866806 3', mRNA sequence [BQ029628]	
A_32_P35855	0.00642	2.63	BE612944	BE612944	BE612944 601451889T1 NIH_MGC_66 Homo sapiens cDNA clone IMAGE:3855646 3', mRNA sequence [BE612944]	
A_24_P418138	0.00644	2.26	AF218021	AF218021	Homo sapiens clone PP552 unknown mRNA. [AF218021]	
A_24_P934541	0.00644	2.83	A_24_P934541			
A_23_P388170	0.00645	3.05	NM_213568	NM_213568	Homo sapiens solute carrier family 39 (zinc transporter), member 3 (SLC39A3), transcript variant 2, mRNA [NM_213568]	NM_213568
A_24_P298616	0.00646	2.08	A_24_P298616			
A_32_P136130	0.00646	2.24	A_32_P136130			
A_23_P130856	0.00647	2.19	NM_032816	NM_032816	Homo sapiens hypothetical protein FLJ14640 (FLJ14640), mRNA [NM_032816]	NM_032816
A_24_P31527	0.00648	2.09	NM_003926	NM_003926	Homo sapiens methyl-CpG binding domain protein 3 (MBD3), mRNA [NM_003926]	NM_003926
A_24_P7211	0.00649	2.38	NM_033551	NM_033551	Homo sapiens La ribonucleoprotein domain family, member 1 (LARP1), mRNA [NM_033551]	NM_033551
A_24_P75688	0.00652	2.48	A_24_P75688			
A_23_P405216	0.00654	2.01	NM_173656	NM_173656	Homo sapiens zinc finger protein 619 (ZNF619), mRNA [NM_173656]	NM_173656
A_24_P917449	0.00654	2.46	ENST00000360796		Homo sapiens mRNA; cDNA DKFZp762N1910 (from clone DKFZp762N1910). [AL834470]	XM_495877
A_32_P165836	0.00654	2.58	NM_177966	NM_177966	Homo sapiens 2'-phosphodiesterase (2'-PDE), mRNA [NM_177966]	NM_177966
A_32_P171488	0.00654	3.82	BG950086	BG950086	BG950086 CM1-CT0377-020101-710-g05 CT0377 Homo sapiens cDNA, mRNA sequence [BG950086]	
A_23_P80353	0.00655	2.23	NM_001003689	NM_001003689	Homo sapiens l(3)mbt-like 2 (Drosophila) (L3MBTL2), transcript variant 2, mRNA [NM_001003689]	NM_001003689
A_23_P211816	0.00656	2.04	NM_002375	NM_002375	Homo sapiens microtubule-associated protein 4 (MAP4), transcript variant 1, mRNA [NM_002375]	NM_002375
A_23_P258714	0.00656	2.32	NM_006295	NM_006295	Homo sapiens valyl-tRNA synthetase (VARS), mRNA [NM_006295]	NM_006295
A_24_P677642	0.0066	2.05	AK056375	AK056375	Homo sapiens cDNA FLJ31813 fis, clone NT2RI2009517. [AK056375]	
A_32_P138348	0.00661	3.82	NM_017527	NM_017527	Homo sapiens lymphocyte antigen 6 complex, locus K (LY6K), mRNA [NM_017527]	NM_017527
A_23_P211985	0.00662	2.26	NM_017719	NM_017719	Homo sapiens SNF related kinase (SNRK), mRNA [NM_017719]	NM_017719
A_23_P152024	0.00664	3.09	NM_004383	NM_004383	Homo sapiens c-src tyrosine kinase (CSK), mRNA [NM_004383]	NM_004383
A_23_P202307	0.00664	3.12	NM_005539	NM_005539	Homo sapiens inositol polyphosphate-5-phosphatase, 40kDa (INPP5A), mRNA [NM_005539]	NM_005539
A_24_P116669	0.00665	2.92	NM_138793	NM_138793	Homo sapiens calcium activated nucleotidase 1 (CANT1), mRNA [NM_138793]	NM_138793
A_23_P63980	0.00667	2.72	NM_024036	NM_024036	Homo sapiens leucine rich repeat and fibronectin type III domain containing 4 (LRFN4), mRNA [NM_024036]	NM_024036
A_23_P147918	0.00669	2.60	NM_080388	NM_080388	Homo sapiens S100 calcium binding protein A16 (S100A16), mRNA [NM_080388]	NM_080388
A_23_P134366	0.0067	3.08	NM_004956	NM_004956	Homo sapiens ets variant gene 1 (ETV1), mRNA [NM_004956]	NM_004956
A_32_P80198	0.00673	2.53	A_32_P80198			

A_32_P194072	0.00674	2.69	NM_015395	NM_015395	Homo sapiens DKFZP434B0335 protein (DKFZP434B0335), mRNA [NM_015395]	NM_015395
A_23_P164752	0.00674	3.42	NM_005370	NM_005370	Homo sapiens RAB8A, member RAS oncogene family (RAB8A), mRNA [NM_005370]	NM_005370
A_24_P267452	0.00674	3.44	NM_012099	NM_012099	Homo sapiens CD3E antigen, epsilon polypeptide associated protein (CD3EAP), mRNA [NM_012099]	NM_012099
A_32_P12050	0.00675	2.04	AI198465	AI198465	AI198465 qf88b03.x1 Soares_placenta_8to9weeks_2NbHP8to9W Homo sapiens cDNA clone IMAGE:1757069 3', mRNA sequence [AI198465]	
A_23_P20532	0.00675	2.29	AK024257	AK024257	Homo sapiens cDNA FLJ14195 fis, clone NT2RP3001723, weakly similar to Homo sapiens cell recognition molecule Caspr2 (CASPR2) mRNA. [AK024257]	XM_497089
A_23_P303329	0.00675	2.44	NM_145232	NM_145232	Homo sapiens LOC90353 (LOC90353), mRNA [NM_145232]	NM_145232
A_23_P127522	0.00675	2.74	NM_145014	NM_145014	Homo sapiens hydrolethalus syndrome 1 (HYLS1), mRNA [NM_145014]	NM_145014
A_23_P55990	0.00678	2.08	NM_003827	NM_003827	Homo sapiens N-ethylmaleimide-sensitive factor attachment protein, alpha (NAPA), mRNA [NM_003827]	NM_003827
A_24_P673786	0.00678	2.23	CR616529	CR616529	full-length cDNA clone CS0DC028YI02 of Neuroblastoma Cot 25-normalized of Homo sapiens (human). [CR616529]	
A_23_P108514	0.00679	2.08	NM_003691	NM_003691	Homo sapiens serine/threonine kinase 16 (STK16), transcript variant 1, mRNA [NM_003691]	NM_003691
A_23_P63618	0.00679	2.16	NM_005063	NM_005063	Homo sapiens stearoyl-CoA desaturase (delta-9-desaturase) (SCD), mRNA [NM_005063]	NM_005063
A_32_P205006	0.00679	2.19	AI613259	AI613259	AI613259 ty35c04.x1 NCI_CGAP_Ut2 Homo sapiens cDNA clone IMAGE:2281062 3' similar to gb:X69391 60S RIBOSOMAL PROTEIN L6 (HUMAN);, mRNA sequence [AI613259]	
A_24_P686965	0.00679	2.66	AK124869	AK124869	Homo sapiens cDNA FLJ42879 fis, clone BRHIP3001283. [AK124869]	XM_375698
A_23_P136787	0.00684	2.55	NM_032336	NM_032336	Homo sapiens SLD5 homolog (SLD5), mRNA [NM_032336]	NM_032336
A_23_P205500	0.00685	3.36	A_23_P205500			
A_24_P930487	0.00686	2.24	A_24_P930487			
A_23_P430764	0.00686	2.67	NM_020240	NM_020240	Homo sapiens CDC42 small effector 2 (CDC42SE2), mRNA [NM_020240]	NM_020240
A_24_P63950	0.00687	3.27	NM_001283	NM_001283	Homo sapiens adaptor-related protein complex 1, sigma 1 subunit (AP1S1), transcript variant 1, mRNA [NM_001283]	NM_001283
A_24_P751082	0.00688	2.75	THC2364015		CLS1_HUMAN (O94985) Calsyntenin-1 precursor, partial (20%) [THC2364015]	
A_23_P41227	0.00689	2.59	NM_001627	NM_001627	Homo sapiens activated leukocyte cell adhesion molecule (ALCAM), mRNA [NM_001627]	NM_001627
A_24_P101271	0.0069	2.30	A_24_P101271			
A_23_P6474	0.0069	2.46	NM_014876	NM_014876	Homo sapiens KIAA0063 gene product (KIAA0063), mRNA [NM_014876]	NM_014876
A_24_P133933	0.0069	3.11	NM_004309	NM_004309	Homo sapiens Rho GDP dissociation inhibitor (GDI) alpha (ARHGDIA), mRNA [NM_004309]	NM_004309
A_23_P43800	0.00692	3.62	NM_015201	NM_015201	Homo sapiens block of proliferation 1 (BOP1), mRNA [NM_015201]	NM_015201
A_24_P37300	0.00693	2.30	AF052115	AF052115	Homo sapiens clone 23688 mRNA sequence. [AF052115]	
A_23_P123503	0.00694	2.39	NM_025195	NM_025195	Homo sapiens tribbles homolog 1 (Drosophila) (TRIB1), mRNA [NM_025195]	NM_025195
A_23_P101427	0.00699	2.20	NM_145245	NM_145245	Homo sapiens ecotropic viral integration site 5-like (EVI5L), mRNA [NM_145245]	NM_145245
A_23_P26810	0.00699	3.48	NM_000546	NM_000546	Homo sapiens tumor protein p53 (Li-Fraumeni syndrome) (TP53), mRNA [NM_000546]	NM_000546
A_23_P84202	0.007	2.21	NM_024515	NM_024515	Homo sapiens pre-mRNA splicing factor-like (MGC4645), mRNA [NM_024515]	NM_024515
A_32_P26299	0.007	2.24	THC2333984			
A_32_P26330	0.007	2.74	NM_005153	NM_005153	Homo sapiens ubiquitin specific protease 10 (USP10), mRNA [NM_005153]	NM_005153
A_23_P348257	0.007	2.75	NM_014840	NM_014840	Homo sapiens NUAK family, SNF1-like kinase, 1 (NUAK1), mRNA [NM_014840]	NM_014840
A_24_P844470	0.00701	2.28	A_24_P844470			
A_32_P191354	0.00701	3.07	AY117690	AY117690	Homo sapiens lung cancer oncogene 5 (HLC5) mRNA, complete cds. [AY117690]	
A_24_P5153	0.00702	2.37	NM_024817	NM_024817	Homo sapiens hypothetical protein FLJ13710 (FLJ13710), mRNA [NM_024817]	NM_024817
A_23_P140256	0.00703	2.04	NM_000270	NM_000270	Homo sapiens nucleoside phosphorylase (NP), mRNA [NM_000270]	NM_000270
A_32_P210717	0.00703	2.29	Z28739	Z28739	Z28739 HSB86F111 STRATAGENE Human skeletal muscle cDNA library, cat. #936215. Homo sapiens cDNA clone 86F11, mRNA sequence [Z28739]	
A_24_P40978	0.00704	2.43	ENST00000292543		Homo sapiens mRNA for FLJ00352 protein. [AK160381]	
A_23_P385500	0.00706	3.23	NM_178841	NM_178841	Homo sapiens ring finger protein 166 (RNF166), mRNA [NM_178841]	NM_178841
A_23_P129496	0.00708	2.28	NM_001795	NM_001795	Homo sapiens cadherin 5, type 2, VE-cadherin (vascular epithelium) (CDH5), mRNA [NM_001795]	NM_001795

A_32_P226907	0.00708	2.73	AK098506	AK098506	Homo sapiens cDNA FLJ25640 fis, clone STM04823. [AK098506]	
A_23_P107211	0.00709	3.03	NM_201434	NM_201434	Homo sapiens RAB5C, member RAS oncogene family (RAB5C), transcript variant 1, mRNA [NM_201434]	NM_201434
A_24_P902052	0.00711	2.30	CR611723	CR611723	full-length cDNA clone CS0DK002YP11 of HeLa cells Cot 25-normalized of Homo sapiens (human). [CR611723]	
A_23_P171296	0.00712	2.26	NM_002436	NM_002436	Homo sapiens membrane protein, palmitoylated 1, 55kDa (MPP1), mRNA [NM_002436]	NM_002436
A_24_P254705	0.00712	3.09	NM_020394	NM_020394	Homo sapiens zinc finger protein 695 (ZNF695), mRNA [NM_020394]	NM_020394
A_24_P417935	0.00712	3.39	CR590071	CR590071	full-length cDNA clone CS0DC010YD15 of Neuroblastoma Cot 25-normalized of Homo sapiens (human). [CR590071]	
A_32_P69987	0.00713	3.50	A_32_P69987			
A_23_P200222	0.00715	4.13	NM_033300	NM_033300	Homo sapiens low density lipoprotein receptor-related protein 8, apolipoprotein e receptor (LRP8), transcript variant 2, mRNA [NM_033300]	NM_033300
A_32_P148507	0.00716	3.31	BE707436	BE707436	BE707436 QV2-HT0496-120700-272-e03 HT0496 Homo sapiens cDNA, mRNA sequence [BE707436]	
A_32_P204722	0.00718	2.03	BM723328	BM723328	UI-E-EJ0-aio-o-14-0-UI.r1 UI-E-EJ0 Homo sapiens cDNA clone UI-E-EJ0-aio-o-14-0-UI 5', mRNA sequence [BM723328]	
A_24_P375609	0.00718	2.67	NM_001970	NM_001970	Homo sapiens eukaryotic translation initiation factor 5A (EIF5A), mRNA [NM_001970]	NM_001970
A_32_P213210	0.0072	2.22	ENST00000306733		PREDICTED: Homo sapiens similar to U1 snRNP-specific protein C (LOC391800), mRNA [XM_498006]	XM_498006
A_32_P78208	0.00721	2.04	AK123627	AK123627	Homo sapiens cDNA FLJ41633 fis, clone FCBBF3003435. [AK123627]	
A_23_P430758	0.00722	2.45	NM_020240	NM_020240	Homo sapiens CDC42 small effector 2 (CDC42SE2), mRNA [NM_020240]	NM_020240
A_24_P257201	0.00722	3.36	NM_016503	NM_016503	Homo sapiens mitochondrial ribosomal protein L30 (MRPL30), nuclear gene encoding mitochondrial protein, transcript variant 2, mRNA [NM_016503]	NM_016503
A_23_P217820	0.00724	2.16	NM_014762	NM_014762	Homo sapiens 24-dehydrocholesterol reductase (DHCR24), mRNA [NM_014762]	NM_014762
A_24_P161086	0.00725	2.01	NM_001001716	NM_001001716	Homo sapiens nuclear factor of kappa light polypeptide gene enhancer in B-cells inhibitor, beta (NFKBIB), transcript variant 2, mRNA [NM_001001716]	NM_001001716
A_24_P38702	0.0073	2.02	NM_145285	NM_145285	Homo sapiens NK2 transcription factor related, locus 3 (Drosophila) (NKX2-3), mRNA [NM_145285]	NM_145285
A_24_P194081	0.0073	2.67	NM_144779	NM_144779	Homo sapiens FXYD domain containing ion transport regulator 5 (FXYD5), transcript variant 1, mRNA [NM_144779]	NM_144779
A_23_P76034	0.00732	2.00	NM_203286	NM_203286	Homo sapiens poliovirus receptor-related 1 (herpesvirus entry mediator C; nectin) (PVRL1), transcript variant 3, mRNA [NM_203286]	NM_203286
A_32_P84289	0.00732	2.39	NM_152371	NM_152371	Homo sapiens chromosome 1 open reading frame 93 (C1orf93), mRNA [NM_152371]	NM_152371
A_23_P859	0.00733	2.20	NM_001126	NM_001126	Homo sapiens adenylosuccinate synthase (ADSS), mRNA [NM_001126]	NM_001126
A_23_P127153	0.00734	4.18	U88048	U88048	Human clone KiSS-16 unknown product mRNA, complete cds. [U88048]	
A_23_P207138	0.00738	2.10	NM_203351	NM_203351	Homo sapiens mitogen-activated protein kinase kinase kinase 3 (MAP3K3), transcript variant 1, mRNA [NM_203351]	NM_203351
A_23_P165959	0.00738	2.49	NM_016004	NM_016004	Homo sapiens chromosome 20 open reading frame 9 (C20orf9), mRNA [NM_016004]	NM_016004
A_32_P12327	0.00738	2.58	THC2405057			
A_23_P153461	0.0074	2.16	NM_022737	NM_022737	Homo sapiens lipid phosphate phosphatase-related protein type 2 (LPPR2), mRNA [NM_022737]	NM_022737
A_23_P168683	0.00742	2.06	NM_019082	NM_019082	Homo sapiens DEAD (Asp-Glu-Ala-Asp) box polypeptide 56 (DDX56), mRNA [NM_019082]	NM_019082
A_24_P107208	0.00746	2.27	NM_203364	NM_203364	Homo sapiens membrane component, chromosome 11, surface marker 1 (M11S1), transcript variant 2, mRNA [NM_203364]	NM_203364
A_23_P410982	0.00749	2.38	ENST00000342337		PREDICTED: Homo sapiens similar to dJ834A16.1 (similar to PGAM) (LOC392473), mRNA [XM_373351]	XM_373351
A_23_P139950	0.0075	2.54	NM_002267	NM_002267	Homo sapiens karyopherin alpha 3 (importin alpha 4) (KPNA3), mRNA [NM_002267]	NM_002267
A_24_P930647	0.00751	2.07	S75896	S75896	clone 39-1 [human, brain, mRNA Mitochondrial Partial Mutant, 201 nt]. [S75896]	
A_24_P314571	0.00751	2.68	NM_182513	NM_182513	Homo sapiens spindle pole body component 24 homolog (S. cerevisiae) (SPBC24), mRNA [NM_182513]	NM_182513
A_23_P387722	0.00753	2.28	NM_213649	NM_213649	Homo sapiens sideroflexin 4 (SFXN4), transcript variant 1, mRNA [NM_213649]	NM_213649
A_24_P143543	0.00758	2.68	ENST00000273340			
A_24_P101181	0.00758	2.93	A_24_P101181			
A_23_P76145	0.00759	11.40	AF256215	AF256215	Homo sapiens cycle-like factor CLIF mRNA, complete cds. [AF256215]	
A_23_P206697	0.00765	2.60	NM_030579	NM_030579	Homo sapiens outer mitochondrial membrane cytochrome b5 (CYB5-M), mRNA [NM_030579]	NM_030579
A_23_P102743	0.00765	4.13	NM_015939	NM_015939	Homo sapiens CGI-09 protein (CGI-09), mRNA [NM_015939]	NM_015939

A_24_P916314	0.00766	2.36	NM_005338	NM_005338	Homo sapiens huntingtin interacting protein 1 (HIP1), mRNA [NM_005338]	NM_005338
A_23_P46309	0.00766	3.66	NM_001269	NM_001269	Homo sapiens regulator of chromosome condensation 1 (RCC1), mRNA [NM_001269]	NM_001269
A_23_P113645	0.00767	3.76	BC043166	BC043166	Homo sapiens potassium voltage-gated channel, shaker-related subfamily, beta member 1, transcript variant 3, mRNA (cDNA clone MGC:44147 IMAGE:5286833), complete cds. [BC043166]	
A_23_P100632	0.00768	2.72	NM_001002033	NM_001002033	Homo sapiens hematological and neurological expressed 1 (HN1), transcript variant 3, mRNA [NM_001002033]	NM_001002033
A_23_P325690	0.00768	3.34	NM_144698	NM_144698	Homo sapiens ankyrin repeat domain 35 (ANKRD35), mRNA [NM_144698]	NM_144698
A_23_P167711	0.00769	2.35	NM_005927	NM_005927	Homo sapiens microfibrillar-associated protein 3 (MFAP3), mRNA [NM_005927]	NM_005927
A_23_P361584	0.00769	20.28	NM_152680	NM_152680	Homo sapiens hypothetical protein FLJ32028 (FLJ32028), mRNA [NM_152680]	NM_152680
A_24_P170753	0.00772	2.11	NM_182613	NM_182613	Homo sapiens hypothetical protein FLJ33915 (FLJ33915), mRNA [NM_182613]	NM_182613
A_24_P916853	0.00772	2.38	A_24_P916853			
A_23_P348323	0.00776	2.07	NM_007144	NM_007144	Homo sapiens polycomb group ring finger 2 (PCGF2), mRNA [NM_007144]	NM_007144
A_23_P166051	0.00776	2.11	NM_031229	NM_031229	Homo sapiens chromosome 20 open reading frame 18 (C20orf18), transcript variant 2, mRNA [NM_031229]	NM_031229
A_24_P263767	0.00776	2.23	ENST00000355804		GB|M80470.1|AAA66955.1 MHC class I HLA-J antigen [NP111779]	
A_32_P36005	0.00779	2.66	THC2265453		AA601920 np02d02.s1 NCI_CGAP_Pr2 Homo sapiens cDNA clone IMAGE:1115139 similar to gb:M90356_cds1 TRANSCRIPTION FACTOR BTF3 (HUMAN);, mRNA sequence [AA601920]	
A_32_P95672	0.00782	2.37	AW875618	AW875618	AW875618 QV2-PT0012-040400-124-b11 PT0012 Homo sapiens cDNA, mRNA sequence [AW875618]	
A_24_P75308	0.00783	2.03	A_24_P75308			
A_24_P221123	0.00783	2.91	NM_006191	NM_006191	Homo sapiens proliferation-associated 2G4, 38kDa (PA2G4), mRNA [NM_006191]	NM_006191
A_24_P927072	0.00783	3.42	A_24_P927072			
A_23_P205986	0.00787	2.56	NM_000875	NM_000875	Homo sapiens insulin-like growth factor 1 receptor (IGF1R), mRNA [NM_000875]	NM_000875
A_24_P161403	0.00787	2.80	BG190769	BG190769	RST9844 Athersys RAGE Library Homo sapiens cDNA, mRNA sequence [BG190769]	XM_067176
A_32_P80231	0.0079	12.27	BM973227	BM973227	UI-CF-EC1-abw-d-20-0-UI.s1 UI-CF-EC1 Homo sapiens cDNA clone UI-CF-EC1-abw-d-20-0-UI 3', mRNA sequence [BM973227]	
A_23_P99579	0.00791	2.48	NM_032490	NM_032490	Homo sapiens chromosome 14 open reading frame 142 (C14orf142), mRNA [NM_032490]	NM_032490
A_24_P576677	0.00792	2.00	BC040420	BC040420	Homo sapiens cDNA clone IMAGE:4215236. [BC040420]	
A_24_P418637	0.00792	2.88	NM_012090	NM_012090	Homo sapiens microtubule-actin crosslinking factor 1 (MACF1), transcript variant 1, mRNA [NM_012090]	NM_012090
A_24_P323778	0.00794	2.72	ENST00000338221			
A_24_P256668	0.00795	2.09	NM_014153	NM_014153	Homo sapiens zinc finger CCCH-type containing 7A (ZC3H7A), mRNA [NM_014153]	NM_014153
A_23_P340296	0.00799	3.10	BC009979	BC009979	Homo sapiens G-protein signalling modulator 1 (AGS3-like, C. elegans), mRNA (cDNA clone MGC:16636 IMAGE:4121647), complete cds. [BC009979]	
A_32_P167723	0.00802	3.15	A_32_P167723			
A_23_P384857	0.00803	2.13	ENST00000244491		PREDICTED: Homo sapiens similar to dJ408B20.3 (novel protein similar to 60S acidic ribosomal protein P2 (RPLP2)) (LOC442175), mRNA [XM_498057]	XM_498057
A_23_P407551	0.00803	4.52	NM_015250	NM_015250	Homo sapiens bicaudal D homolog 2 (Drosophila) (BICD2), transcript variant 2, mRNA [NM_015250]	NM_015250
A_23_P161269	0.00804	2.16	NM_018590	NM_018590	Homo sapiens chondroitin sulfate GalNAcT-2 (GALNACT-2), mRNA [NM_018590]	NM_018590
A_23_P253451	0.00804	5.92	NM_020949	NM_020949	Homo sapiens solute carrier family 7 (cationic amino acid transporter, y+ system), member 14 (SLC7A14), mRNA [NM_020949]	NM_020949
A_23_P361482	0.00805	4.74	NM_153712	NM_153712	Homo sapiens tubulin tyrosine ligase (TTL), mRNA [NM_153712]	NM_153712
A_32_P114896	0.00809	2.32	NM_006601	NM_006601	Homo sapiens prostaglandin E synthase 3 (cytosolic) (PTGES3), mRNA [NM_006601]	NM_006601
A_24_P216253	0.00809	2.97	NM_014902	NM_014902	Homo sapiens discs, large (Drosophila) homolog-associated protein 4 (DLGAP4), transcript variant 1, mRNA [NM_014902]	NM_014902
A_24_P945059	0.00811	2.55	BC022556	BC022556	Homo sapiens myc target 1, mRNA (cDNA clone MGC:26933 IMAGE:4793564), complete cds. [BC022556]	
A_24_P210637	0.00812	2.19	NM_130468	NM_130468	Homo sapiens dermatan 4 sulfotransferase 1 (D4ST1), mRNA [NM_130468]	NM_130468
A_24_P829181	0.00813	3.94	BC034002	BC034002	Homo sapiens, clone IMAGE:5092955, mRNA, partial cds. [BC034002]	
A_23_P97481	0.00814	2.77	AK126792	AK126792	Homo sapiens cDNA FLJ44842 fis, clone BRACE3049714, weakly similar to Homo sapiens NYD-TSPG protein (NYD-TSPG). [AK126792]	

A_24_P218056	0.00815	2.08	NM_015332	NM_015332	Homo sapiens NudC domain containing 3 (NUDCD3), mRNA [NM_015332]	NM_015332
A_32_P172608	0.00815	2.78	AI961400	AI961400	AI961400 wt22a12.x1 NCI_CGAP_Ut1 Homo sapiens cDNA clone IMAGE:2508190 3' similar to gb:X60036 MITOCHONDRIAL PHOSPHATE CARRIER PROTEIN PRECURSOR (HUMAN);, mRNA sequence [AI961400]	
A_24_P141707	0.00816	2.52	NM_031479	NM_031479	Homo sapiens inhibin, beta E (INHBE), mRNA [NM_031479]	NM_031479
A_23_P34510	0.00819	2.24	NM_198040	NM_198040	Homo sapiens polyhomeotic-like 2 (Drosophila) (PHC2), transcript variant 1, mRNA [NM_198040]	NM_198040
A_24_P341427	0.0082	2.52	A_24_P341427			
A_23_P66038	0.0082	2.59	NM_013304	NM_013304	Homo sapiens zinc finger, DHHC-type containing 1 (ZDHHC1), mRNA [NM_013304]	NM_013304
A_23_P19084	0.0082	4.39	NM_004499	NM_004499	Homo sapiens heterogeneous nuclear ribonucleoprotein A/B (HNRPAB), transcript variant 2, mRNA [NM_004499]	NM_004499
A_23_P66063	0.00821	2.66	NM_020786	NM_020786	Homo sapiens pyruvate dehydrogenase phosphatase isoenzyme 2 (PDP2), mRNA [NM_020786]	NM_020786
A_24_P67534	0.00823	4.59	ENST00000330288		Homo sapiens, clone IMAGE:3868989, mRNA, partial cds. [BC008642]	
A_23_P33072	0.00826	2.10	NM_032272	NM_032272	Homo sapiens MAF1 homolog (S. cerevisiae) (MAF1), mRNA [NM_032272]	NM_032272
A_23_P61140	0.00827	2.14	NM_006480	NM_006480	Homo sapiens regulator of G-protein signalling 14 (RGS14), mRNA [NM_006480]	NM_006480
A_23_P217998	0.00828	2.21	NM_032801	NM_032801	Homo sapiens junctional adhesion molecule 3 (JAM3), mRNA [NM_032801]	NM_032801
A_23_P347508	0.00829	2.36	NM_023077	NM_023077	Homo sapiens chromosome 1 open reading frame 163 (C1orf163), mRNA [NM_023077]	NM_023077
A_23_P133739	0.00829	3.06	NM_148959	NM_148959	Homo sapiens HUS1 checkpoint homolog b (S. pombe) (HUS1B), mRNA [NM_148959]	NM_148959
A_24_P118946	0.0083	2.53	A_24_P118946			
A_23_P15394	0.00835	2.15	NM_001251	NM_001251	Homo sapiens CD68 antigen (CD68), mRNA [NM_001251]	NM_001251
A_23_P64010	0.00837	2.04	NM_014342	NM_014342	Homo sapiens mitochondrial carrier homolog 2 (C. elegans) (MTCH2), nuclear gene encoding mitochondrial protein, mRNA [NM_014342]	NM_014342
A_23_P99163	0.00837	2.43	NM_018370	NM_018370	Homo sapiens hypothetical protein FLJ11259 (FLJ11259), mRNA [NM_018370]	NM_018370
A_23_P343237	0.00839	3.73	NM_144567	NM_144567	Homo sapiens similar to RIKEN cDNA 2610307I21 (LOC90806), mRNA [NM_144567]	NM_144567
A_24_P341476	0.0084	2.56	THC2301753		AF139893 cyclophilin 18 {Oryctolagus cuniculus;} , partial (84%) [THC2301753]	
A_24_P358462	0.00841	2.43	NM_198576	NM_198576	Homo sapiens agrin (AGRN), mRNA [NM_198576]	NM_198576
A_24_P306469	0.00842	2.46	ENST00000305319		PREDICTED: Homo sapiens similar to ribosomal protein S17 (LOC257039), mRNA [XM_172230]	XM_172230
A_32_P96272	0.00842	2.50	NM_001008741	NM_001008741	Homo sapiens peptidylprolyl isomerase A-like (LOC388817), mRNA [NM_001008741]	NM_001008741
A_24_P230708	0.00842	3.85	NM_177966	NM_177966	Homo sapiens 2'-phosphodiesterase (2'-PDE), mRNA [NM_177966]	NM_177966
A_23_P130995	0.00843	3.13	NM_144779	NM_144779	Homo sapiens FXYD domain containing ion transport regulator 5 (FXYD5), transcript variant 1, mRNA [NM_144779]	NM_144779
A_23_P162256	0.00846	2.04	NM_003677	NM_003677	Homo sapiens density-regulated protein (DENR), mRNA [NM_003677]	NM_003677
A_24_P84008	0.00848	3.72	ENST00000311061		PREDICTED: Homo sapiens similar to Splicing factor U2AF 35 kDa subunit (U2 auxiliary factor 35 kDa subunit) (U2 snRNP auxiliary factor small subunit) (LOC441722), mRNA [XM_497450]	XM_497450
A_23_P161218	0.00848	5.78	NM_014391	NM_014391	Homo sapiens ankyrin repeat domain 1 (cardiac muscle) (ANKRD1), mRNA [NM_014391]	NM_014391
A_23_P432554	0.0085	2.14	NM_174899	NM_174899	Homo sapiens F-box protein 36 (FBXO36), mRNA [NM_174899]	NM_174899
A_24_P148657	0.0085	2.82	NM_018385	NM_018385	Homo sapiens hypothetical protein FLJ11301 (FLJ11301), mRNA [NM_018385]	NM_018385
A_23_P364428	0.00852	4.95	NM_020439	NM_020439	Homo sapiens calcium/calmodulin-dependent protein kinase IG (CAMK1G), mRNA [NM_020439]	NM_020439
A_23_P67529	0.00853	2.31	NM_002250	NM_002250	Homo sapiens potassium intermediate/small conductance calcium-activated channel, subfamily N, member 4 (KCNN4), mRNA [NM_002250]	NM_002250
A_24_P32895	0.00853	2.65	CA848728	CA848728	CA848728 ir24f10.y1 HR85 islet Homo sapiens cDNA clone IMAGE:6546379 5' similar to SW:CYPH_HUMAN P05092 PEPTIDYL-PROLYL CIS-TRANS ISOMERASE A ;, mRNA sequence [CA848728]	
A_23_P250385	0.00857	2.54	NM_005322	NM_005322	Homo sapiens histone 1, H1b (HIST1H1B), mRNA [NM_005322]	NM_005322
A_32_P35220	0.00859	2.14	NM_172003	NM_172003	Homo sapiens COBW domain containing 2 (CBWD2), mRNA [NM_172003]	NM_172003
A_23_P48001	0.00861	2.51	ENST00000355675		PREDICTED: Homo sapiens similar to peptidyl-Pro cis trans isomerase (LOC341457), mRNA [XM_292085]	XM_292085
A_23_P104607	0.00863	2.02	NM_002804	NM_002804	Homo sapiens proteasome (prosome, macropain) 26S subunit, ATPase, 3 (PSMC3), mRNA [NM_002804]	NM_002804
A_23_P4885	0.00864	2.26	NM_014203	NM_014203	Homo sapiens adaptor-related protein complex 2, alpha 1 subunit (AP2A1), transcript variant 1, mRNA [NM_014203]	NM_014203

A_32_P56874	0.00865	2.83	BG502322	BG502322	BG502322 602550205F1 NIH_MGC_61 Homo sapiens cDNA clone IMAGE:4657992 5', mRNA sequence [BG502322]	
A_24_P43681	0.00868	2.18	NM_014063	NM_014063	Homo sapiens drebrin-like (DBNL), transcript variant 1, mRNA [NM_014063]	NM_014063
A_24_P920555	0.00868	2.25	BM994983	BM994983	BM994983 UI-H-ED0-awz-n-15-0-UI.s1 NCI_CGAP_ED0 Homo sapiens cDNA clone IMAGE:5825870 3', mRNA sequence [BM994983]	
A_32_P36182	0.0087	2.73	BE935032	BE935032	BE935032 PM4-MT0105-180800-003-c08 MT0105 Homo sapiens cDNA, mRNA sequence [BE935032]	
A_23_P54460	0.00871	2.66	NM_152594	NM_152594	Homo sapiens sprouty-related, EVH1 domain containing 1 (SPRED1), mRNA [NM_152594]	NM_152594
A_32_P176036	0.00876	3.27	AK127258	AK127258	Homo sapiens cDNA FLJ45325 fis, clone BRHIP3006717. [AK127258]	
A_23_P100408	0.00877	2.15	NM_006181	NM_006181	Homo sapiens netrin 2-like (chicken) (NTN2L), mRNA [NM_006181]	NM_006181
A_32_P188193	0.00877	2.16	THC2445517			
A_32_P215318	0.00878	3.94	NM_198839	NM_198839	Homo sapiens acetyl-Coenzyme A carboxylase alpha (ACACA), transcript variant 2, mRNA [NM_198839]	NM_198839
A_24_P307126	0.0088	2.44	A_24_P307126			
A_24_P228637	0.00882	2.01	NM_001007260	NM_001007260	Homo sapiens chromosome 21 open reading frame 18 (C21orf18), transcript variant 4, mRNA [NM_001007260]	NM_001007260
A_23_P2990	0.00883	2.24	NM_001805	NM_001805	Homo sapiens CCAAT/enhancer binding protein (C/EBP), epsilon (CEBPE), mRNA [NM_001805]	NM_001805
A_23_P8004	0.00884	2.23	ENST00000356177		Homo sapiens histone 1, H3h, mRNA (cDNA clone MGC:4577 IMAGE:3030790), complete cds. [BC007518]	
A_32_P63643	0.00889	3.36	AW007825	AW007825	AW007825 wt03f01.x1 NCI_CGAP_Co3 Homo sapiens cDNA clone IMAGE:2506393 3' similar to gb:Y00282 RIBOPHORIN II PRECURSOR (HUMAN);, mRNA sequence [AW007825]	
A_23_P379794	0.0089	2.61	NM_178517	NM_178517	Homo sapiens phosphatidylinositol glycan, class W (PIGW), mRNA [NM_178517]	NM_178517
A_24_P391748	0.00891	2.12	A_24_P391748			
A_24_P923807	0.00892	2.34	THC2406669			
A_24_P105721	0.00893	2.07	NM_134442	NM_134442	Homo sapiens cAMP responsive element binding protein 1 (CREB1), transcript variant B, mRNA [NM_134442]	NM_134442
A_23_P110304	0.00894	2.01	ENST00000264903		Homo sapiens mRNA; cDNA DKFZp434K098 (from clone DKFZp434K098); partial cds. [AL133112]	
A_23_P376557	0.00894	2.18	NM_022718	NM_022718	Homo sapiens matrix metalloproteinase 25 (MMP25), transcript variant 2, mRNA [NM_022718]	NM_022718
A_24_P32203	0.00894	2.35	ENST00000230559		Human mucin (MUC8) mRNA, partial cds. [U14383]	
A_32_P207054	0.00894	2.54	NM_013986	NM_013986	Homo sapiens Ewing sarcoma breakpoint region 1 (EWSR1), transcript variant EWS-b, mRNA [NM_013986]	NM_013986
A_23_P2960	0.00895	2.08	NM_005163	NM_005163	Homo sapiens v-akt murine thymoma viral oncogene homolog 1 (AKT1), transcript variant 1, mRNA [NM_005163]	NM_005163
A_24_P940310	0.00896	2.02	ENST00000270201		Homo sapiens chromosome 21 C21orf108 mRNA, partial cds. [AF231919]	
A_23_P48740	0.00897	2.17	NM_013989	NM_013989	Homo sapiens deiodinase, iodothyronine, type II (DIO2), transcript variant 1, mRNA [NM_013989]	NM_013989
A_23_P42563	0.00898	3.02	NM_012470	NM_012470	Homo sapiens transportin 3 (TNPO3), mRNA [NM_012470]	NM_012470
A_24_P331373	0.00899	2.12	NM_031444	NM_031444	Homo sapiens chromosome 22 open reading frame 13 (C22orf13), mRNA [NM_031444]	NM_031444
A_23_P312415	0.00899	5.75	NM_052866	NM_052866	Homo sapiens ADAMTS-like 1 (ADAMTSL1), transcript variant 2, mRNA [NM_052866]	NM_052866
A_24_P396980	0.00902	4.93	NM_053024	NM_053024	Homo sapiens profilin 2 (PFN2), transcript variant 1, mRNA [NM_053024]	NM_053024
A_23_P401547	0.00903	2.37	NM_015480	NM_015480	Homo sapiens poliovirus receptor-related 3 (PVRL3), mRNA [NM_015480]	NM_015480
A_24_P684119	0.00904	4.29	BC043212	BC043212	Homo sapiens cDNA clone IMAGE:5295205, with apparent retained intron. [BC043212]	
A_24_P375132	0.00905	2.02	A_24_P375132			
A_32_P135450	0.00905	2.15	A_32_P135450			
A_24_P259490	0.00909	2.05	NM_001658	NM_001658	Homo sapiens ADP-ribosylation factor 1 (ARF1), transcript variant 4, mRNA [NM_001658]	NM_001658
A_24_P409697	0.00909	2.06	BG206642	BG206642	RST26093 Athersys RAGE Library Homo sapiens cDNA, mRNA sequence [BG206642]	XM_377820
A_23_P78244	0.0091	2.00	BC053363	BC053363	Homo sapiens cDNA clone IMAGE:6141118, partial cds. [BC053363]	
A_23_P369316	0.00915	3.05	NM_138959	NM_138959	Homo sapiens vang-like 1 (van gogh, Drosophila) (VANGL1), mRNA [NM_138959]	NM_138959
A_24_P195629	0.00917	2.29	THC2301132		BC069050 PPIA protein {Homo sapiens;} , partial (86%) [THC2301132]	
A_32_P155786	0.00922	2.51	BC020336	BC020336	Homo sapiens ribosomal protein S2, mRNA (cDNA clone IMAGE:3857902). [BC020336]	
A_24_P117528	0.00922	2.54	NM_002765	NM_002765	Homo sapiens phosphoribosyl pyrophosphate synthetase 2 (PRPS2), mRNA [NM_002765]	NM_002765
A_24_P116606	0.00924	2.31	NM_130791	NM_130791	Homo sapiens WW domain containing oxidoreductase (WWOX), transcript variant 6, mRNA [NM_130791]	NM_130791

A_24_P278621	0.00925	3.07	NM_012193	NM_012193	Homo sapiens frizzled homolog 4 (Drosophila) (FZD4), mRNA [NM_012193]	NM_012193
A_32_P184092	0.00927	2.07	THC2430394		BQ339129 CM0-NN1155-251000-621-e12 NN1155 Homo sapiens cDNA, mRNA sequence [BQ339129]	
A_24_P812018	0.00927	2.31	A_24_P812018			
A_24_P737815	0.00927	2.38	U01925	U01925	Human BTK region clone 2f10-rpi mRNA. [U01925]	
A_23_P54576	0.00929	2.28	NM_005550	NM_005550	Homo sapiens kinesin family member C3 (KIFC3), mRNA [NM_005550]	NM_005550
A_23_P146922	0.0093	3.41	NM_000820	NM_000820	Homo sapiens growth arrest-specific 6 (GAS6), mRNA [NM_000820]	NM_000820
A_24_P397515	0.00932	2.08	NM_006353	NM_006353	Homo sapiens high mobility group nucleosomal binding domain 4 (HMGN4), mRNA [NM_006353]	NM_006353
A_24_P123133	0.00932	2.90	NM_020231	NM_020231	Homo sapiens x 010 protein (MDS010), mRNA [NM_020231]	NM_020231
A_24_P206305	0.00933	2.07	NM_182924	NM_182924	Homo sapiens MICAL-like 2 (MICAL-L2), transcript variant 1, mRNA [NM_182924]	NM_182924
A_23_P309278	0.00933	2.14	NM_173158	NM_173158	Homo sapiens nuclear receptor subfamily 4, group A, member 1 (NR4A1), transcript variant 3, mRNA [NM_173158]	NM_173158
A_24_P827491	0.00933	4.41	NM_006191	NM_006191	Homo sapiens proliferation-associated 2G4, 38kDa (PA2G4), mRNA [NM_006191]	NM_006191
A_32_P225328	0.00933	4.93	A_32_P225328			
A_32_P135558	0.00934	2.24	NM_153261	NM_153261	Homo sapiens hypothetical protein FLJ38101 (FLJ38101), mRNA [NM_153261]	NM_153261
A_23_P213508	0.00934	17.42	NM_000439	NM_000439	Homo sapiens proprotein convertase subtilisin/kexin type 1 (PCSK1), mRNA [NM_000439]	NM_000439
A_23_P2431	0.00936	3.94	NM_004054	NM_004054	Homo sapiens complement component 3a receptor 1 (C3AR1), mRNA [NM_004054]	NM_004054
A_23_P143926	0.00942	3.60	BC014794	BC014794	Homo sapiens unc-51-like kinase 4 (C. elegans), mRNA (cDNA clone MGC:16994 IMAGE:4343362), complete cds. [BC014794]	
A_23_P133728	0.00943	2.07	NM_003643	NM_003643	Homo sapiens glial cells missing homolog 1 (Drosophila) (GCM1), mRNA [NM_003643]	NM_003643
A_32_P150300	0.00947	3.13	CR992331	CR992331	CR992331 RZPD no.9016 Homo sapiens cDNA clone RZPDp9016A0141 5', mRNA sequence [CR992331]	
A_24_P911928	0.00948	2.93	BC032233	BC032233	Homo sapiens, clone IMAGE:5222754, mRNA, partial cds. [BC032233]	XM_034274
A_24_P166012	0.00952	2.44	NM_020640	NM_020640	Homo sapiens RP42 homolog (RP42), mRNA [NM_020640]	NM_020640
A_24_P31235	0.00953	2.49	NM_001970	NM_001970	Homo sapiens eukaryotic translation initiation factor 5A (EIF5A), mRNA [NM_001970]	NM_001970
A_32_P198923	0.00956	3.77	NM_145690	NM_145690	Homo sapiens tyrosine 3-monooxygenase/tryptophan 5-monooxygenase activation protein, zeta polypeptide (YWHAZ), transcript variant 2, mRNA [NM_145690]	NM_145690
A_32_P20492	0.00957	2.00	THC2410448		BB1_HUMAN (P50290) Growth-regulating protein BB1, partial (98%) [THC2410448]	
A_24_P25040	0.00959	2.27	A_24_P25040			
A_24_P101146	0.00959	3.73	ENST00000312024			
A_24_P564396	0.0096	2.75	THC2335868		ALU5_HUMAN (P39192) Alu subfamily SC sequence contamination warning entry, partial (8%) [THC2335868]	
A_32_P165477	0.00962	4.41	BC041925	BC041925	Homo sapiens solute carrier family 7, (cationic amino acid transporter, y+ system) member 11, mRNA (cDNA clone IMAGE:5300264), partial cds. [BC041925]	
A_24_P402779	0.00965	2.98	NM_001003935	NM_001003935	Homo sapiens poly (ADP-ribose) polymerase family, member 3 (PARP3), transcript variant 3, mRNA [NM_001003935]	NM_001003935
A_24_P384578	0.00971	2.64	NM_013986	NM_013986	Homo sapiens Ewing sarcoma breakpoint region 1 (EWSR1), transcript variant EWS-b, mRNA [NM_013986]	NM_013986
A_24_P392742	0.00972	2.07	A_24_P392742			
A_23_P318300	0.00972	2.18	NM_133646	NM_133646	Homo sapiens sterile alpha motif and leucine zipper containing kinase AZK (ZAK), transcript variant 2, mRNA [NM_133646]	NM_133646
A_32_P136450	0.00974	2.94	THC2449500		AF220206 Nedd4 WW domain-binding protein 2 {Mus musculus;} , partial (52%) [THC2449500]	
A_24_P364838	0.00975	2.18	U82108	U82108	Human SIP-1 mRNA, complete cds. [U82108]	
A_24_P891276	0.00976	4.18	A_24_P891276			
A_24_P119002	0.00977	2.74	NM_015475	NM_015475	Homo sapiens DKFZP564F0522 protein (DKFZP564F0522), mRNA [NM_015475]	NM_015475
A_24_P75558	0.00979	2.12	A_24_P75558			
A_24_P149036	0.0098	2.23	NM_001387	NM_001387	Homo sapiens dihydropyrimidinase-like 3 (DPYSL3), mRNA [NM_001387]	NM_001387
A_32_P98979	0.0098	4.85	THC2281332			
A_24_P135406	0.0099	3.02	NM_017634	NM_017634	Homo sapiens potassium channel tetramerisation domain containing 9 (KCTD9), mRNA [NM_017634]	NM_017634
A_24_P18146	0.00997	2.15	NM_015310	NM_015310	Homo sapiens pleckstrin and Sec7 domain containing 3 (PSD3), transcript variant 1, mRNA [NM_015310]	NM_015310

A_23_P120182	0.01	2.06	NM_004544	NM_004544	Homo sapiens NADH dehydrogenase (ubiquinone) 1 alpha subcomplex, 10, 42kDa (NDUFA10), nuclear gene encoding mitochondrial protein, mRNA [NM_004544]	NM_004544
A_23_P402157	0.01	2.15	NM_024681	NM_024681	Homo sapiens potassium channel tetramerisation domain containing 17 (KCTD17), mRNA [NM_024681]	NM_024681
A_24_P101561	0.01	2.31	A_24_P101561			
A_32_P107746	0.01	2.74	NM_207042	NM_207042	Homo sapiens endosulfine alpha (ENSA), transcript variant 1, mRNA [NM_207042]	NM_207042
A_24_P919399	0.01	3.27	NM_020453	NM_020453	Homo sapiens ATPase, Class V, type 10D (ATP10D), mRNA [NM_020453]	NM_020453
A_23_P348737	0.01	5.68	NM_005654	NM_005654	Homo sapiens nuclear receptor subfamily 2, group F, member 1 (NR2F1), mRNA [NM_005654]	NM_005654
A_24_P363023	0.0101	2.70	NM_018569	NM_018569	Homo sapiens chromosome 4 open reading frame 16 (C4orf16), mRNA [NM_018569]	NM_018569
A_32_P117860	0.0101	2.92	W05707	W05707	W05707 za87h03.r1 Soares_fetal_lung_NbHL19W Homo sapiens cDNA clone IMAGE:299573 5' similar to SW:TCPD_MOUSE P80315 T-COMPLEX PROTEIN 1, DELTA SUBUNIT ;, mRNA sequence [W05707]	
A_24_P290373	0.0101	4.55	NM_022101	NM_022101	Homo sapiens hypothetical protein FLJ22965 (FLJ22965), mRNA [NM_022101]	NM_022101
A_24_P97129	0.0102	2.11	NM_001006946	NM_001006946	Homo sapiens syndecan 1 (SDC1), transcript variant 1, mRNA [NM_001006946]	NM_001006946
A_32_P22338	0.0102	2.49	NM_013374	NM_013374	Homo sapiens programmed cell death 6 interacting protein (PDCD6IP), mRNA [NM_013374]	NM_013374
A_24_P384755	0.0102	2.99	NM_024894	NM_024894	Homo sapiens hypothetical LOC79954 (FLJ14075), mRNA [NM_024894]	NM_024894
A_24_P928798	0.0103	2.09	AK056260	AK056260	Homo sapiens cDNA FLJ31698 fis, clone NT2RI2005966. [AK056260]	
A_24_P96325	0.0103	2.21	NM_181484	NM_181484	Homo sapiens zinc finger, CCCH-type with G patch domain (ZGPAT), transcript variant 2, mRNA [NM_181484]	NM_181484
A_23_P19291	0.0103	2.28	NM_001069	NM_001069	Homo sapiens tubulin, beta 2 (TUBB2), mRNA [NM_001069]	NM_001069
A_24_P924957	0.0103	2.78	L32537	L32537	Homo sapiens (clone XP6G6B) mRNA, partial EST. [L32537]	
A_32_P114146	0.0104	2.11	A_32_P114146			
A_23_P106720	0.0104	2.65	NM_006453	NM_006453	Homo sapiens transducin (beta)-like 3 (TBL3), mRNA [NM_006453]	NM_006453
A_32_P175979	0.0104	2.86	THC2277837			
A_32_P233091	0.0105	2.07	NR_002202	NR_002202	Homo sapiens ferritin, heavy polypeptide-like 7 (FTHL7) on chromosome 13 [NR_002202]	NR_002202
A_23_P68922	0.0105	2.10	NM_033386	NM_033386	Homo sapiens MICAL-like 1 (MICAL-L1), mRNA [NM_033386]	NM_033386
A_24_P413920	0.0105	2.26	NM_145175	NM_145175	Homo sapiens family with sequence similarity 84, member A (FAM84A), mRNA [NM_145175]	NM_145175
A_24_P298946	0.0105	2.29	A_24_P298946			
A_24_P170283	0.0105	3.79	A_24_P170283			
A_24_P571864	0.0106	2.13	A_24_P571864			
A_23_P386514	0.0106	2.17	NM_005506	NM_005506	Homo sapiens scavenger receptor class B, member 2 (SCARB2), mRNA [NM_005506]	NM_005506
A_23_P82324	0.0106	2.38	NM_032415	NM_032415	Homo sapiens caspase recruitment domain family, member 11 (CARD11), mRNA [NM_032415]	NM_032415
A_24_P230130	0.0106	2.43	ENST00000328558		PREDICTED: Homo sapiens similar to peptidylprolyl isomerase A isoform 1; cyclophilin A; peptidyl-prolyl cis-trans isomerase A; T cell cyclophilin; rotamase; cyclosporin A-binding protein (LOC401859), mRNA [XM_377444]	XM_377444
A_24_P626812	0.0106	2.58	A_24_P626812			
A_23_P22378	0.0106	3.01	NM_003108	NM_003108	Homo sapiens SRY (sex determining region Y)-box 11 (SOX11), mRNA [NM_003108]	NM_003108
A_24_P41882	0.0107	2.06	NM_213636	NM_213636	Homo sapiens PDZ and LIM domain 7 (enigma) (PDLIM7), transcript variant 4, mRNA [NM_213636]	NM_213636
A_32_P139414	0.0107	2.29	AW804491	AW804491	AW804491 QV0-UM0093-170400-191-d05 UM0093 Homo sapiens cDNA, mRNA sequence [AW804491]	
A_24_P714707	0.0107	2.48	A_24_P714707			
A_23_P201687	0.0107	2.56	BC012091	BC012091	Homo sapiens hairy and enhancer of split 2 (Drosophila), mRNA (cDNA clone IMAGE:4634002), complete cds. [BC012091]	
A_24_P365349	0.0107	2.70	A_24_P365349			
A_23_P354270	0.0107	2.84	NM_007353	NM_007353	Homo sapiens guanine nucleotide binding protein (G protein) alpha 12 (GNA12), mRNA [NM_007353]	NM_007353
A_23_P5785	0.0107	3.75	NM_152384	NM_152384	Homo sapiens Bardet-Biedl syndrome 5 (BBS5), mRNA [NM_152384]	NM_152384
A_23_P82959	0.0108	2.02	NM_003923	NM_003923	Homo sapiens forkhead box H1 (FOXH1), mRNA [NM_003923]	NM_003923
A_23_P66732	0.0108	2.08	NM_031965	NM_031965	Homo sapiens germ cell associated 2 (haspin) (GSG2), mRNA [NM_031965]	NM_031965
A_23_P29594	0.0108	2.11	NM_052969	NM_052969	Homo sapiens ribosomal protein L39-like (RPL39L), mRNA [NM_052969]	NM_052969

A_24_P914000	0.0108	2.54	BC001437	BC001437	Homo sapiens hypothetical protein BC001437, mRNA (cDNA clone MGC:1013 IMAGE:3138594), complete cds. [BC001437]	
A_24_P213321	0.0108	2.79	A_24_P213321			
A_24_P156781	0.0108	5.41	NM_003629	NM_003629	Homo sapiens phosphoinositide-3-kinase, regulatory subunit 3 (p55, gamma) (PIK3R3), mRNA [NM_003629]	NM_003629
A_24_P358215	0.0109	2.02	A_24_P358215			
A_24_P455060	0.0109	2.06	CR616058	CR616058	full-length cDNA clone CS0DB009YD15 of Neuroblastoma Cot 10-normalized of Homo sapiens (human). [CR616058]	
A_32_P34552	0.0109	2.06	NM_002690	NM_002690	Homo sapiens polymerase (DNA directed), beta (POLB), mRNA [NM_002690]	NM_002690
A_24_P346762	0.0109	2.11	NM_025182	NM_025182	Homo sapiens KIAA1539 (KIAA1539), mRNA [NM_025182]	NM_025182
A_23_P103099	0.0109	2.72	NM_014309	NM_014309	Homo sapiens RNA binding motif protein 9 (RBM9), mRNA [NM_014309]	NM_014309
A_32_P183987	0.0109	3.21	AA431401	AA431401	AA431401 zw79a05.s1 Soares_testis_NHT Homo sapiens cDNA clone IMAGE:782384 3', mRNA sequence [AA431401]	
A_23_P41455	0.0109	5.41	NM_003305	NM_003305	Homo sapiens transient receptor potential cation channel, subfamily C, member 3 (TRPC3), mRNA [NM_003305]	NM_003305
A_24_P13376	0.011	2.02	NM_024551	NM_024551	Homo sapiens adiponectin receptor 2 (ADIPOR2), mRNA [NM_024551]	NM_024551
A_23_P133956	0.011	2.18	NM_002263	NM_002263	Homo sapiens kinesin family member C1 (KIFC1), mRNA [NM_002263]	NM_002263
A_32_P36292	0.011	2.70	THC2267175		AI573267 tn03f02.x1 NCI_CGAP_Brn25 Homo sapiens cDNA clone IMAGE:2166555 3', mRNA sequence [AI573267]	
A_23_P77415	0.011	2.84	NM_013370	NM_013370	Homo sapiens pregnancy-induced growth inhibitor (OKL38), transcript variant 1, mRNA [NM_013370]	NM_013370
A_32_P147189	0.011	2.89	BC071732	BC071732	Homo sapiens cDNA clone IMAGE:3862422, partial cds. [BC071732]	
A_24_P147190	0.011	2.94	NM_002582	NM_002582	Homo sapiens poly(A)-specific ribonuclease (deadenylation nuclease) (PARN), mRNA [NM_002582]	NM_002582
A_23_P97161	0.0111	2.02	NM_003594	NM_003594	Homo sapiens transcription termination factor, RNA polymerase II (TTF2), mRNA [NM_003594]	NM_003594
A_32_P2050	0.0111	2.02	THC2373975		BM561501 AGENCOURT_6567312 NIH_MGC_88 Homo sapiens cDNA clone IMAGE:5739715 5', mRNA sequence [BM561501]	
A_23_P16058	0.0111	2.13	NM_145288	NM_145288	Homo sapiens zinc finger protein 342 (ZNF342), mRNA [NM_145288]	NM_145288
A_24_P936953	0.0111	2.16	A_24_P936953			
A_23_P19036	0.0111	2.24	NM_006058	NM_006058	Homo sapiens TNFAIP3 interacting protein 1 (TNIP1), mRNA [NM_006058]	NM_006058
A_32_P61061	0.0111	2.38	NM_001008741	NM_001008741	Homo sapiens peptidylprolyl isomerase A-like (LOC388817), mRNA [NM_001008741]	NM_001008741
A_24_P119456	0.0111	7.81	NM_015136	NM_015136	Homo sapiens stabilin 1 (STAB1), mRNA [NM_015136]	NM_015136
A_32_P16662	0.0112	2.03	F10881	F10881	F10881 HSC3LC012 normalized infant brain cDNA Homo sapiens cDNA clone c-3lc01 3', mRNA sequence [F10881]	
A_23_P158380	0.0112	2.06	A_23_P158380			
A_24_P126931	0.0112	2.08	A_24_P126931			
A_23_P3131	0.0112	2.09	NM_033116	NM_033116	Homo sapiens NIMA (never in mitosis gene a)- related kinase 9 (NEK9), mRNA [NM_033116]	NM_033116
A_23_P423331	0.0112	2.18	NM_032536	NM_032536	Homo sapiens netrin G2 (NTNG2), mRNA [NM_032536]	NM_032536
A_32_P195853	0.0112	2.35	AK001565	AK001565	Homo sapiens cDNA FLJ10703 fis, clone NT2RP3000836. [AK001565]	
A_24_P136653	0.0112	11.67	NM_004289	NM_004289	Homo sapiens nuclear factor (erythroid-derived 2)-like 3 (NFE2L3), mRNA [NM_004289]	NM_004289
A_24_P632230	0.0113	2.10	A_24_P632230			
A_24_P932451	0.0113	2.47	BX641037	BX641037	Homo sapiens mRNA; cDNA DKFZp686H10114 (from clone DKFZp686H10114). [BX641037]	
A_23_P132889	0.0113	2.48	NM_001014809	NM_001014809	Homo sapiens collapsin response mediator protein 1 (CRMP1), transcript variant 1, mRNA [NM_001014809]	NM_001014809
A_23_P156198	0.0113	2.65	NM_015288	NM_015288	Homo sapiens PHD finger protein 15 (PHF15), mRNA [NM_015288]	NM_015288
A_24_P186030	0.0113	2.71	NM_002760	NM_002760	Homo sapiens protein kinase, Y-linked (PRKY), mRNA [NM_002760]	NM_002760
A_32_P164661	0.0113	2.99	AI302935	AI302935	AI302935 qn53c07.x1 NCI_CGAP_Kid5 Homo sapiens cDNA clone IMAGE:1901964 3', mRNA sequence [AI302935]	
A_32_P37474	0.0114	2.02	ENST00000340836		PREDICTED: Homo sapiens similar to supervillin isoform 2; membrane-associated F-actin binding protein p205; archvillin (LOC387648), mRNA [XM_370542]	XM_370542
A_24_P521544	0.0114	2.98	A_24_P521544			
A_24_P289139	0.0114	4.20	NM_031892	NM_031892	Homo sapiens SH3-domain kinase binding protein 1 (SH3KBP1), transcript variant 1, mRNA [NM_031892]	NM_031892
A_23_P26729	0.0115	2.04	NM_004645	NM_004645	Homo sapiens coilin (COIL), mRNA [NM_004645]	NM_004645

A_24_P152775	0.0115	2.54	A_24_P152775			
A_23_P2307	0.0115	2.77	NM_144593	NM_144593	Homo sapiens Ras homolog enriched in brain like 1 (RHEBL1), mRNA [NM_144593]	NM_144593
A_23_P148121	0.0115	3.17	ENST00000309295		Homo sapiens mRNA; cDNA DKFZp762C186 (from clone DKFZp762C186). [AL834433]	XM_170658
A_32_P459849	0.0116	2.03	NM_153453	NM_153453	Homo sapiens vestigial like 2 (Drosophila) (VGLL2), transcript variant 2, mRNA [NM_153453]	NM_153453
A_24_P88690	0.0116	2.10	NM_000578	NM_000578	Homo sapiens solute carrier family 11 (proton-coupled divalent metal ion transporters), member 1 (SLC11A1), mRNA [NM_000578]	NM_000578
A_24_P17945	0.0116	2.18	NM_003309	NM_003309	Homo sapiens TSPY-like 1 (TSPYL1), mRNA [NM_003309]	NM_003309
A_32_P50275	0.0116	5.26	AK095453	AK095453	Homo sapiens cDNA FLJ38134 fis, clone D9OST2000440. [AK095453]	
A_23_P169879	0.0117	2.15	NM_001009937	NM_001009937	Homo sapiens solute carrier family 25, member 26 (SLC25A26), transcript variant 1, mRNA [NM_001009937]	NM_001009937
A_24_P169258	0.0117	2.35	NM_005993	NM_005993	Homo sapiens tubulin-specific chaperone d (TBCD), mRNA [NM_005993]	NM_005993
A_32_P110942	0.0117	3.32	CA432213	CA432213	CA432213 UI-H-FG1-bgr-l-15-0-UI.s1 NCI_CGAP_FG1 Homo sapiens cDNA clone UI-H-FG1-bgr-l-15-0-UI 3', mRNA sequence [CA432213]	
A_23_P91657	0.0118	2.18	NM_013986	NM_013986	Homo sapiens Ewing sarcoma breakpoint region 1 (EWSR1), transcript variant EWS-b, mRNA [NM_013986]	NM_013986
A_32_P202778	0.0118	2.21	A_32_P202778			
A_24_P313445	0.0118	2.37	NM_020912	NM_020912	Homo sapiens FLYWCH-type zinc finger 1 (FLYWCH1), transcript variant 2, mRNA [NM_020912]	NM_020912
A_23_P29773	0.0118	3.70	NM_014398	NM_014398	Homo sapiens lysosomal-associated membrane protein 3 (LAMP3), mRNA [NM_014398]	NM_014398
A_23_P36513	0.0118	3.76	NM_212461	NM_212461	Homo sapiens protein kinase, AMP-activated, gamma 1 non-catalytic subunit (PRKAG1), transcript variant 2, mRNA [NM_212461]	NM_212461
A_24_P24806	0.0119	2.06	A_24_P24806			
A_24_P106953	0.0119	2.24	NM_198939	NM_198939	Homo sapiens prostaglandin E synthase 2 (PTGES2), transcript variant 3, mRNA [NM_198939]	NM_198939
A_23_P408328	0.012	2.11	NM_138632	NM_138632	Homo sapiens Tara-like protein (HRIHFB2122), transcript variant 2, mRNA [NM_138632]	NM_138632
A_24_P313895	0.012	2.12	BC005365	BC005365	Homo sapiens mitogen-activated protein kinase kinase 7, mRNA (cDNA clone IMAGE:3829438). [BC005365]	
A_23_P145555	0.012	2.22	NM_138569	NM_138569	Homo sapiens chromosome 6 open reading frame 142 (C6orf142), mRNA [NM_138569]	NM_138569
A_23_P34126	0.012	2.45	NM_001711	NM_001711	Homo sapiens biglycan (BGN), mRNA [NM_001711]	NM_001711
A_24_P772061	0.012	2.51	NM_001008741	NM_001008741	Homo sapiens peptidylprolyl isomerase A-like (LOC388817), mRNA [NM_001008741]	NM_001008741
A_23_P122563	0.012	2.81	NM_014260	NM_014260	Homo sapiens HLA class II region expressed gene KE2 (HKE2), mRNA [NM_014260]	NM_014260
A_24_P548966	0.012	3.29	THC2308059			
A_32_P152696	0.0121	2.15	A_32_P152696			
A_32_P102575	0.0121	2.25	THC2428070		RL35_HUMAN (P42766) 60S ribosomal protein L35, partial (67%) [THC2428070]	
A_23_P5813	0.0121	2.91	NM_144949	NM_144949	Homo sapiens suppressor of cytokine signaling 5 (SOCS5), transcript variant 2, mRNA [NM_144949]	NM_144949
A_32_P30785	0.0121	3.05	THC2430759		BM069569 ie89g07.x1 Melton Normalized Human Islet 4 N4-HIS 1 Homo sapiens cDNA clone IMAGE:5674188 3', mRNA sequence [BM069569]	
A_24_P734953	0.0121	3.06	NM_001013642	NM_001013642	Homo sapiens hypothetical LOC388610 (LOC388610), mRNA [NM_001013642]	NM_001013642
A_32_P115515	0.0122	2.04	AK026131	AK026131	Homo sapiens cDNA: FLJ22478 fis, clone HRC10816. [AK026131]	
A_23_P213288	0.0122	2.07	AF389338	AF389338	Homo sapiens acyl-CoA-desaturase mRNA, complete cds. [AF389338]	
A_24_P916378	0.0122	2.11	BC008217	BC008217	Homo sapiens heterogeneous nuclear ribonucleoprotein L-like, mRNA (cDNA clone MGC:5437 IMAGE:3449607), complete cds. [BC008217]	
A_24_P689479	0.0122	4.26	BU738278	BU738278	UI-E-DW1-ahe-f-18-0-UI.s1 UI-E-DW1 Homo sapiens cDNA clone UI-E-DW1-ahe-f-18-0-UI 3', mRNA sequence [BU738278]	
A_32_P76441	0.0123	2.22	AV699150	AV699150	AV699150 AV699150 GKD Homo sapiens cDNA clone GKDABC12 5', mRNA sequence [AV699150]	
A_32_P30558	0.0123	2.70	CV416790	CV416790	CV416790 RC3-GN0269-170101-013-c05 GN0269 Homo sapiens cDNA, mRNA sequence [CV416790]	
A_24_P12413	0.0123	2.76	NM_012288	NM_012288	Homo sapiens translocation associated membrane protein 2 (TRAM2), mRNA [NM_012288]	NM_012288
A_24_P348925	0.0124	2.17	NM_003858	NM_003858	Homo sapiens cyclin K (CCNK), mRNA [NM_003858]	NM_003858
A_24_P218688	0.0124	2.67	NM_000694	NM_000694	Homo sapiens aldehyde dehydrogenase 3 family, member B1 (ALDH3B1), mRNA [NM_000694]	NM_000694
A_32_P132959	0.0125	2.20	BG055107	BG055107	BG055107 nac99g04.x1 NCI_CGAP_Pr28 Homo sapiens cDNA clone IMAGE:3432366 3' similar to TR:Q9Y4G5 Q9Y4G5 KIAA0325 PROTEIN ;, mRNA sequence [BG055107]	

A_24_P221885	0.0125	2.21	AK096634	AK096634	Homo sapiens cDNA FLJ39315 fis, A-OCBBF2013926. [AK096634]	
A_23_P146004	0.0125	2.36	NM_024814	NM_024814	Homo sapiens Cas-Br-M (murine) ecotropic retroviral transforming sequence-like 1 (CBLL1), mRNA [NM_024814]	NM_024814
A_23_P151297	0.0125	2.76	NM_015319	NM_015319	Homo sapiens tensin like C1 domain containing phosphatase (tensin 2) (TENC1), transcript variant 1, mRNA [NM_015319]	NM_015319
A_23_P357104	0.0125	3.21	NM_001155	NM_001155	Homo sapiens annexin A6 (ANXA6), transcript variant 1, mRNA [NM_001155]	NM_001155
A_23_P388762	0.0125	3.61	AK026244	AK026244	Homo sapiens cDNA: FLJ22591 fis, clone HSI03124. [AK026244]	
A_23_P123256	0.0125	4.02	NM_014891	NM_014891	Homo sapiens PDGFA associated protein 1 (PDAP1), mRNA [NM_014891]	NM_014891
A_23_P356109	0.0126	2.11	NM_152749	NM_152749	Homo sapiens ataxin 7-like 4 (ATXN7L4), mRNA [NM_152749]	NM_152749
A_23_P332316	0.0126	2.26	NM_152285	NM_152285	Homo sapiens arrestin domain containing 1 (ARRDC1), mRNA [NM_152285]	NM_152285
A_32_P201378	0.0127	2.03	AL137476	AL137476	Homo sapiens mRNA; cDNA DKFZp434I0623 (from clone DKFZp434I0623). [AL137476]	
A_23_P363769	0.0127	2.10	NM_002284	NM_002284	Homo sapiens keratin, hair, basic, 6 (monilethrix) (KRTHB6), mRNA [NM_002284]	NM_002284
A_32_P33937	0.0127	2.58	ENST00000355190		Homo sapiens mRNA; cDNA DKFZp686L01145 (from clone DKFZp686L01145). [BX538108]	
A_24_P349428	0.0127	2.85	A_24_P349428			
A_23_P97871	0.0128	2.11	BX641020	BX641020	Homo sapiens mRNA; cDNA DKFZp686G23148 (from clone DKFZp686G23148). [BX641020]	
A_24_P301547	0.0128	2.18	NM_017941	NM_017941	Homo sapiens lung cancer-related protein 8 (HLC-8), mRNA [NM_017941]	NM_017941
A_23_P52410	0.0128	2.69	NM_145307	NM_145307	Homo sapiens pleckstrin homology domain containing, family K member 1 (PLEKHK1), mRNA [NM_145307]	NM_145307
A_32_P8529	0.0129	2.08	AF130050	AF130050	Homo sapiens clone FLB3442 PRO0872 mRNA, complete cds. [AF130050]	
A_23_P142424	0.0129	2.29	NM_024660	NM_024660	Homo sapiens hypothetical protein FLJ22573 (FLJ22573), mRNA [NM_024660]	NM_024660
A_23_P304237	0.0129	2.56	NM_198679	NM_198679	Homo sapiens Rap guanine nucleotide exchange factor (GEF) 1 (RAPGEF1), transcript variant 2, mRNA [NM_198679]	NM_198679
A_23_P80566	0.0129	2.93	NM_006506	NM_006506	Homo sapiens RAS p21 protein activator 2 (RASA2), mRNA [NM_006506]	NM_006506
A_23_P71492	0.0129	3.18	NM_006158	NM_006158	Homo sapiens neurofilament, light polypeptide 68kDa (NEFL), mRNA [NM_006158]	NM_006158
A_23_P37545	0.013	2.15	NM_024666	NM_024666	Homo sapiens hypothetical protein FLJ11506 (FLJ11506), mRNA [NM_024666]	NM_024666
A_24_P453544	0.0131	2.22	AY239294	AY239294	Homo sapiens proliferation-inducing protein 9 mRNA, complete cds. [AY239294]	
A_24_P392842	0.0131	2.35	A_24_P392842			
A_24_P316059	0.0131	2.52	ENST00000333604			
A_24_P355816	0.0132	2.57	NM_018370	NM_018370	Homo sapiens hypothetical protein FLJ11259 (FLJ11259), mRNA [NM_018370]	NM_018370
A_23_P216094	0.0132	4.07	NM_004318	NM_004318	Homo sapiens aspartate beta-hydroxylase (ASPH), transcript variant 1, mRNA [NM_004318]	NM_004318
A_24_P141005	0.0133	2.10	NM_182715	NM_182715	Homo sapiens synaptophysin-like 1 (SYPL1), transcript variant 2, mRNA [NM_182715]	NM_182715
A_24_P396489	0.0133	2.61	CR601391	CR601391	full-length cDNA clone CS0DL008YF05 of B cells (Ramos cell line) Cot 25-normalized of Homo sapiens (human). [CR601391]	
A_24_P64329	0.0133	3.30	NM_173575	NM_173575	Homo sapiens serine/threonine kinase 32C (STK32C), mRNA [NM_173575]	NM_173575
A_24_P942370	0.0134	2.03	NM_003774	NM_003774	Homo sapiens UDP-N-acetyl-alpha-D-galactosamine:polypeptide N-acetylgalactosaminyltransferase 4 (GalNAc-T4) (GALNT4), mRNA [NM_003774]	NM_003774
A_24_P126682	0.0134	2.13	NM_022877	NM_022877	Homo sapiens survival of motor neuron 2, centromeric (SMN2), transcript variant c, mRNA [NM_022877]	NM_022877
A_23_P416395	0.0134	2.86	NM_003714	NM_003714	Homo sapiens stanniocalcin 2 (STC2), mRNA [NM_003714]	NM_003714
A_24_P20806	0.0134	3.13	NM_030567	NM_030567	Homo sapiens proline rich 7 (synaptic) (PRR7), mRNA [NM_030567]	NM_030567
A_24_P177631	0.0135	2.04	AL832120	AL832120	Homo sapiens mRNA; cDNA DKFZp686B2110 (from clone DKFZp686B2110). [AL832120]	XM_291344
A_24_P204067	0.0135	2.57	THC2304728		N1L4_HUMAN (Q99733) Nucleosome assembly protein 1-like 4 (Nucleosome assembly protein 2) (NAP2), partial (59%) [THC2304728]	
A_23_P252636	0.0135	2.79	NM_022048	NM_022048	Homo sapiens casein kinase 1, gamma 1 (CSNK1G1), transcript variant 2, mRNA [NM_022048]	NM_022048
A_23_P104583	0.0136	2.22	NM_020441	NM_020441	Homo sapiens coronin, actin binding protein, 1B (CORO1B), transcript variant 1, mRNA [NM_020441]	NM_020441
A_24_P927142	0.0136	2.96	BQ334425	BQ334425	BQ334425 RC2-MT0142-151100-017-a11 MT0142 Homo sapiens cDNA, mRNA sequence [BQ334425]	
A_23_P77117	0.0136	5.95	NM_023003	NM_023003	Homo sapiens transmembrane 6 superfamily member 1 (TM6SF1), mRNA [NM_023003]	NM_023003

A_24_P46484	0.0137	2.25	NM_018047	NM_018047	Homo sapiens RNA binding motif protein 22 (RBM22), mRNA [NM_018047]	NM_018047
A_23_P207632	0.0137	2.29	NM_174953	NM_174953	Homo sapiens ATPase, Ca++ transporting, ubiquitous (ATP2A3), transcript variant 5, mRNA [NM_174953]	NM_174953
A_23_P93938	0.0137	2.51	ENST00000258775		Human mRNA for KIAA0363 gene, partial cds. [AB002361]	XM_374432
A_32_P147090	0.0137	3.33	NM_199357	NM_199357	Homo sapiens Rho GTPase activating protein 11A (ARHGAP11A), mRNA [NM_199357]	NM_199357
A_23_P62642	0.0137	5.08	NM_012337	NM_012337	Homo sapiens coiled-coil domain containing 19 (CCDC19), mRNA [NM_012337]	NM_012337
A_24_P830690	0.0138	2.01	NM_002613	NM_002613	Homo sapiens 3-phosphoinositide dependent protein kinase-1 (PDPK1), mRNA [NM_002613]	NM_002613
A_23_P21363	0.0139	2.38	NM_024060	NM_024060	Homo sapiens AHNAK nucleoprotein (desmoyokin) (AHNAK), transcript variant 2, mRNA [NM_024060]	NM_024060
A_32_P157415	0.0139	2.68	BE714413	BE714413	BE714413 PM1-HT0724-140600-001-e06 HT0724 Homo sapiens cDNA, mRNA sequence [BE714413]	
A_32_P104746	0.0139	2.70	NM_020972	NM_020972	Homo sapiens zinc finger, FYVE domain containing 28 (ZFYVE28), mRNA [NM_020972]	NM_020972
A_24_P801197	0.0139	4.17	AK131396	AK131396	Homo sapiens cDNA FLJ16488 fis, clone BRTHA3008826. [AK131396]	
A_23_P373687	0.014	2.09	NM_144709	NM_144709	Homo sapiens hypothetical protein FLJ32312 (FLJ32312), mRNA [NM_144709]	NM_144709
A_23_P88691	0.014	2.19	NM_000745	NM_000745	Homo sapiens cholinergic receptor, nicotinic, alpha polypeptide 5 (CHRNA5), mRNA [NM_000745]	NM_000745
A_24_P63109	0.0141	2.05	NM_006241	NM_006241	Homo sapiens protein phosphatase 1, regulatory (inhibitor) subunit 2 (PPP1R2), mRNA [NM_006241]	NM_006241
A_24_P122874	0.0141	2.20	NM_015049	NM_015049	Homo sapiens amyotrophic lateral sclerosis 2 (juvenile) chromosome region, candidate 3 (ALS2CR3), mRNA [NM_015049]	NM_015049
A_32_P67355	0.0141	2.23	A_32_P67355			
A_23_P81094	0.0141	3.65	THC2338051		BC047447 LOC133308 protein {Homo sapiens;} , partial (25%) [THC2338051]	
A_24_P633686	0.0142	2.07	A_24_P633686			
A_24_P612921	0.0142	2.12	A_24_P612921			
A_24_P166073	0.0143	3.31	NM_153757	NM_153757	Homo sapiens nucleosome assembly protein 1-like 5 (NAP1L5), mRNA [NM_153757]	NM_153757
A_24_P926450	0.0143	8.20	THC2274524			
A_23_P343954	0.0144	2.07	NM_006546	NM_006546	Homo sapiens IGF-II mRNA-binding protein 1 (IMP-1), mRNA [NM_006546]	NM_006546
A_23_P502314	0.0145	2.00	NM_078481	NM_078481	Homo sapiens CD97 antigen (CD97), transcript variant 1, mRNA [NM_078481]	NM_078481
A_24_P134568	0.0145	2.22	NM_006915	NM_006915	Homo sapiens retinitis pigmentosa 2 (X-linked recessive) (RP2), mRNA [NM_006915]	NM_006915
A_23_P90780	0.0145	2.38	THC2348230		Q80YT6 (Q80YT6) Malate dehydrogenase 1B, NAD (Soluble), partial (41%) [THC2348230]	
A_23_P62021	0.0145	4.59	L12350	L12350	Human thrombospondin 2 (THBS2) mRNA, complete cds. [L12350]	
A_24_P138556	0.0146	2.33	NM_004094	NM_004094	Homo sapiens eukaryotic translation initiation factor 2, subunit 1 alpha, 35kDa (EIF2S1), mRNA [NM_004094]	NM_004094
A_24_P300076	0.0147	2.02	NM_004186	NM_004186	Homo sapiens sema domain, immunoglobulin domain (Ig), short basic domain, secreted, (semaphorin) 3F (SEMA3F), mRNA [NM_004186]	NM_004186
A_23_P429624	0.0147	2.14	NM_178545	NM_178545	Homo sapiens transmembrane protein 52 (TMEM52), mRNA [NM_178545]	NM_178545
A_23_P156919	0.0147	2.16	NM_005284	NM_005284	Homo sapiens G protein-coupled receptor 6 (GPR6), mRNA [NM_005284]	NM_005284
A_23_P58390	0.0147	3.25	NM_152400	NM_152400	Homo sapiens hypothetical protein FLJ39370 (FLJ39370), mRNA [NM_152400]	NM_152400
A_23_P110791	0.0147	3.34	NM_005211	NM_005211	Homo sapiens colony stimulating factor 1 receptor, formerly McDonough feline sarcoma viral (v-fms) oncogene homolog (CSF1R), mRNA [NM_005211]	NM_005211
A_24_P293114	0.0148	2.13	NM_020971	NM_020971	Homo sapiens spectrin, beta, non-erythrocytic 4 (SPTBN4), transcript variant 1, mRNA [NM_020971]	NM_020971
A_32_P54260	0.0148	2.21	A_32_P54260			
A_24_P255218	0.0148	3.69	NM_000259	NM_000259	Homo sapiens myosin VA (heavy polypeptide 12, myoxin) (MYO5A), mRNA [NM_000259]	NM_000259
A_24_P700210	0.0149	2.25	THC2436815		Q8R3T9 (Q8R3T9) Dnm3 protein, partial (7%) [THC2436815]	
A_32_P149111	0.015	2.06	AK125015	AK125015	Homo sapiens cDNA FLJ43025 fis, clone BRTHA2018707. [AK125015]	
A_32_P12388	0.015	2.47	AA974271	AA974271	AA974271 oq14c05.s1 NCI_CGAP_GC4 Homo sapiens cDNA clone IMAGE:1586312 3', mRNA sequence [AA974271]	

Supplemental Table S1E

Genes upregulated in hESC-EC vs. HUVEC

P-Value ≤ 0.015 with multiple testing correction (Benjamini and Hochberg), Fold Change ≥ 2.0

Gene Name	*P*-value	Fold change	Common	Genbank	Description	RefSeq
A_23_P140207	3.37E-07	7.09	NM_004563	NM_004563	Homo sapiens phosphoenolpyruvate carboxykinase 2 (mitochondrial) (PCK2), nuclear gene encoding mitochondrial protein, transcript variant 1, mRNA [NM_004563]	NM_004563
A_32_P121549	3.37E-07	42.55	BC034319	BC034319	Homo sapiens, clone IMAGE:4837650, mRNA, partial cds. [BC034319]	
A_23_P214080	4.19E-07	13.23	NM_001964	NM_001964	Homo sapiens early growth response 1 (EGR1), mRNA [NM_001964]	NM_001964
A_23_P131614	4.19E-07	61.35	NM_004369	NM_004369	Homo sapiens collagen, type VI, alpha 3 (COL6A3), transcript variant 1, mRNA [NM_004369]	NM_004369
A_23_P429950	4.74E-07	18.94	NM_000216	NM_000216	Homo sapiens Kallmann syndrome 1 sequence (KAL1), mRNA [NM_000216]	NM_000216
A_23_P68851	5.19E-07	13.25	AK172861	AK172861	Homo sapiens cDNA FLJ24091 fis, clone COL10314. [AK172861]	
A_23_P211631	5.19E-07	80.00	NM_006486	NM_006486	Homo sapiens fibulin 1 (FBLN1), transcript variant D, mRNA [NM_006486]	NM_006486
A_23_P145529	5.56E-07	192.31	NM_181795	NM_181795	Homo sapiens protein kinase (cAMP-dependent, catalytic) inhibitor beta (PKIB), transcript variant 1, mRNA [NM_181795]	NM_181795
A_23_P152876	6.96E-07	10.80	NM_031934	NM_031934	Homo sapiens RAB34, member RAS oncogene family (RAB34), mRNA [NM_031934]	NM_031934
A_23_P163087	7.57E-07	7.09	NM_007361	NM_007361	Homo sapiens nidogen 2 (osteonidogen) (NID2), mRNA [NM_007361]	NM_007361
A_23_P82000	8.03E-07	3.73	NM_003214	NM_003214	Homo sapiens TEA domain family member 3 (TEAD3), mRNA [NM_003214]	NM_003214
A_32_P34920	8.03E-07	38.31	NM_004472	NM_004472	Homo sapiens forkhead box D1 (FOXD1), mRNA [NM_004472]	NM_004472
A_23_P306215	8.03E-07	115.74	NM_145175	NM_145175	Homo sapiens family with sequence similarity 84, member A (FAM84A), mRNA [NM_145175]	NM_145175
A_32_P209230	8.55E-07	6.58	NM_133467	NM_133467	Homo sapiens Cbp/p300-interacting transactivator, with Glu/Asp-rich carboxy-terminal domain, 4 (CITED4), mRNA [NM_133467]	NM_133467
A_24_P945283	8.55E-07	7.35	AB033058	AB033058	Homo sapiens mRNA for KIAA1232 protein, partial cds. [AB033058]	
A_23_P145694	8.55E-07	10.71	NM_001673	NM_001673	Homo sapiens asparagine synthetase (ASNS), transcript variant 2, mRNA [NM_001673]	NM_001673
A_23_P52336	9.27E-07	15.92	NM_170744	NM_170744	Homo sapiens unc-5 homolog B (C. elegans) (UNC5B), mRNA [NM_170744]	NM_170744
A_24_P802145	9.27E-07	124.69	THC2339772			
A_24_P108311	9.29E-07	5.92	NM_015277	NM_015277	Homo sapiens neural precursor cell expressed, developmentally down-regulated 4-like (NEDD4L), mRNA [NM_015277]	NM_015277
A_23_P345118	9.36E-07	4.08	NM_002648	NM_002648	Homo sapiens pim-1 oncogene (PIM1), mRNA [NM_002648]	NM_002648
A_23_P75299	9.63E-07	4.98	NM_022126	NM_022126	Homo sapiens phospholysine phosphohistidine inorganic pyrophosphate phosphatase (LHPP), mRNA [NM_022126]	NM_022126
A_23_P362228	1.06E-06	3.51	NM_138479	NM_138479	Homo sapiens hypothetical protein BC007899 (LOC148898), transcript variant 1, mRNA [NM_138479]	NM_138479
A_24_P301146	1.06E-06	5.21	NM_005358	NM_005358	Homo sapiens LIM domain 7 (LMO7), mRNA [NM_005358]	NM_005358
A_23_P86731	1.06E-06	5.43	NM_005674	NM_005674	Homo sapiens zinc finger protein 239 (ZNF239), mRNA [NM_005674]	NM_005674
A_23_P216742	1.06E-06	28.33	NM_002581	NM_002581	Homo sapiens pregnancy-associated plasma protein A, pappalysin 1 (PAPPA), mRNA [NM_002581]	NM_002581
A_23_P399078	1.06E-06	31.15	NM_000362	NM_000362	Homo sapiens tissue inhibitor of metalloproteinase 3 (Sorsby fundus dystrophy, pseudoinflammatory) (TIMP3), mRNA [NM_000362]	NM_000362
A_24_P131522	1.10E-06	16.08	NM_032208	NM_032208	Homo sapiens anthrax toxin receptor 1 (ANTXR1), transcript variant 1, mRNA [NM_032208]	NM_032208
A_23_P202327	1.10E-06	26.67	NM_003474	NM_003474	Homo sapiens a disintegrin and metalloproteinase domain 12 (meltrin alpha) (ADAM12), transcript variant 1, mRNA [NM_003474]	NM_003474
A_23_P139260	1.16E-06	7.87	NM_183233	NM_183233	Homo sapiens solute carrier family 22 (organic cation transporter), member 18 (SLC22A18), transcript variant 2, mRNA [NM_183233]	NM_183233
A_23_P107351	1.16E-06	8.77	BC051787	BC051787	Homo sapiens NACHT, leucine rich repeat and PYD (pyrin domain) containing 1, mRNA (cDNA clone MGC:57544 IMAGE:5756099), complete cds. [BC051787]	
A_23_P67042	1.23E-06	6.06	NM_017947	NM_017947	Homo sapiens molybdenum cofactor sulfurase (MOCOS), mRNA [NM_017947]	NM_017947
A_23_P334955	1.23E-06	84.75	NM_053279	NM_053279	Homo sapiens chromosome 8 open reading frame 13 (C8orf13), mRNA [NM_053279]	NM_053279
A_23_P200015	1.26E-06	6.37	NM_174858	NM_174858	Homo sapiens adenylate kinase 5 (AK5), transcript variant 1, mRNA [NM_174858]	NM_174858
A_23_P135061	1.26E-06	8.70	NM_003389	NM_003389	Homo sapiens coronin, actin binding protein, 2A (CORO2A), transcript variant 1, mRNA [NM_003389]	NM_003389

A_23_P165927	1.26E-06	42.02	NM_015894	NM_015894	Homo sapiens stathmin-like 3 (STMN3), mRNA [NM_015894]	NM_015894
A_23_P74609	1.29E-06	16.84	NM_015714	NM_015714	Homo sapiens putative lymphocyte G0/G1 switch gene (G0S2), mRNA [NM_015714]	NM_015714
A_23_P328074	1.29E-06	41.49	NM_002968	NM_002968	Homo sapiens sal-like 1 (Drosophila) (SALL1), mRNA [NM_002968]	NM_002968
A_24_P795230	1.29E-06	78.13	AK027541	AK027541	Homo sapiens cDNA FLJ14635 fis, clone NT2RP2001196. [AK027541]	
A_23_P154806	1.33E-06	8.47	NM_012156	NM_012156	Homo sapiens erythrocyte membrane protein band 4.1-like 1 (EPB41L1), transcript variant 1, mRNA [NM_012156]	NM_012156
A_23_P165778	1.42E-06	188.32	NM_024101	NM_024101	Homo sapiens melanophilin (MLPH), mRNA [NM_024101]	NM_024101
A_24_P418408	1.55E-06	3.12	NM_198552	NM_198552	Homo sapiens chromosome 1 open reading frame 153 (C1orf153), mRNA [NM_198552]	NM_198552
A_32_P217655	1.59E-06	3.57	XM_496405	XM_496405	PREDICTED: Homo sapiens similar to D(1B) dopamine receptor (D(5) dopamine receptor) (D1beta dopamine receptor) (LOC440684), mRNA [XM_496405]	XM_496405
A_23_P411296	1.59E-06	6.94	NM_005194	NM_005194	Homo sapiens CCAAT/enhancer binding protein (C/EBP), beta (CEBPB), mRNA [NM_005194]	NM_005194
A_24_P346431	1.59E-06	10.98	AK092864	AK092864	Homo sapiens cDNA FLJ35545 fis, clone SPLEN2003918, moderately similar to TENSIN. [AK092864]	
A_23_P90357	1.65E-06	2.99	NM_201636	NM_201636	Homo sapiens thromboxane A2 receptor (TBXA2R), transcript variant 1, mRNA [NM_201636]	NM_201636
A_23_P79518	1.65E-06	57.80	NM_000576	NM_000576	Homo sapiens interleukin 1, beta (IL1B), mRNA [NM_000576]	NM_000576
A_24_P156490	1.68E-06	6.80	NM_002247	NM_002247	Homo sapiens potassium large conductance calcium-activated channel, subfamily M, alpha member 1 (KCNMA1), transcript variant 2, mRNA [NM_002247]	NM_002247
A_23_P71530	1.86E-06	7.58	NM_002546	NM_002546	Homo sapiens tumor necrosis factor receptor superfamily, member 11b (osteoprotegerin) (TNFRSF11B), mRNA [NM_002546]	NM_002546
A_23_P138885	1.86E-06	40.82	NM_203371	NM_203371	Homo sapiens similar to RIKEN cDNA 1110018M03 (LOC387758), mRNA [NM_203371]	NM_203371
A_23_P94800	1.88E-06	8.93	NM_002961	NM_002961	Homo sapiens S100 calcium binding protein A4 (calcium protein, calvasculin, metastasin, murine placental homolog) (S100A4), transcript variant 1, mRNA [NM_002961]	NM_002961
A_23_P332399	1.96E-06	5.05	NM_016315	NM_016315	Homo sapiens GULP, engulfment adaptor PTB domain containing 1 (GULP1), mRNA [NM_016315]	NM_016315
A_23_P167401	1.99E-06	4.12	NM_018931	NM_018931	Homo sapiens protocadherin beta 11 (PCDHB11), mRNA [NM_018931]	NM_018931
A_32_P103945	1.99E-06	4.22	NM_212543	NM_212543	Homo sapiens UDP-Gal:betaGlcNAc beta 1,4- galactosyltransferase, polypeptide 4 (B4GALT4), transcript variant 1, mRNA [NM_212543]	NM_212543
A_23_P168167	1.99E-06	5.75	NM_032511	NM_032511	Homo sapiens chromosome 6 open reading frame 168 (C6orf168), mRNA [NM_032511]	NM_032511
A_23_P145965	2.00E-06	3.14	NM_003596	NM_003596	Homo sapiens tyrosylprotein sulfotransferase 1 (TPST1), mRNA [NM_003596]	NM_003596
A_23_P126103	2.00E-06	5.88	NM_001902	NM_001902	Homo sapiens cystathionase (cystathionine gamma-lyase) (CTH), transcript variant 1, mRNA [NM_001902]	NM_001902
A_24_P59667	2.00E-06	9.43	BC028068	BC028068	Homo sapiens Janus kinase 3 (a protein tyrosine kinase, leukocyte), mRNA (cDNA clone MGC:39993 IMAGE:5212575), complete cds. [BC028068]	
A_23_P137097	2.16E-06	5.18	NM_006517	NM_006517	Homo sapiens solute carrier family 16 (monocarboxylic acid transporters), member 2 (SLC16A2), mRNA [NM_006517]	NM_006517
A_23_P76731	2.25E-06	7.69	NM_014226	NM_014226	Homo sapiens renal tumor antigen (RAGE), mRNA [NM_014226]	NM_014226
A_24_P398370	2.28E-06	23.87	A_24_P398370			
A_24_P924697	2.29E-06	6.85	AK055915	AK055915	Homo sapiens cDNA FLJ31353 fis, clone MESAN2000264. [AK055915]	
A_32_P49284	2.29E-06	10.64	XM_372040	XM_372040	PREDICTED: Homo sapiens similar to asparagine synthetase; glutamine-dependent asparagine synthetase; TS11 cell cycle control protein (LOC389652), mRNA [XM_372040]	XM_372040
A_23_P52697	2.29E-06	16.84	NM_020404	NM_020404	Homo sapiens CD248 antigen, endosialin (CD248), mRNA [NM_020404]	NM_020404
A_24_P18137	2.29E-06	24.27	BC066952	BC066952	Homo sapiens neurofilament, light polypeptide 68kDa, mRNA (cDNA clone MGC:87388 IMAGE:5264373), complete cds. [BC066952]	
A_24_P23625	2.36E-06	46.51	ENST00000245519		Homo sapiens heparan sulfate (glucosamine) 3-O-sulfotransferase 3B1, mRNA (cDNA clone MGC:71688 IMAGE:30343352), complete cds. [BC063301]	
A_23_P170186	2.47E-06	5.99	NM_017570	NM_017570	Homo sapiens 5-oxoprolinase (ATP-hydrolysing) (OPLAH), mRNA [NM_017570]	NM_017570
A_23_P102571	2.48E-06	2.24	NM_020062	NM_020062	Homo sapiens SLC2A4 regulator (SLC2A4RG), mRNA [NM_020062]	NM_020062
A_23_P127565	2.53E-06	16.64	NM_178834	NM_178834	Homo sapiens layilin (LOC143903), mRNA [NM_178834]	NM_178834
A_24_P274607	2.54E-06	2.14	AK054960	AK054960	Homo sapiens cDNA FLJ30398 fis, clone BRACE2008402, highly similar to Homo sapiens steroid receptor RNA activator isoform 3 mRNA. [AK054960]	XR_000132
A_24_P109661	2.55E-06	3.52	A_24_P109661			
A_24_P944588	2.55E-06	47.85	AK074843	AK074843	Homo sapiens cDNA FLJ90362 fis, clone NT2RP2003940, weakly similar to ZINC FINGER PROTEIN 43. [AK074843]	

A_23_P204751	2.59E-06	10.18	NM_020039	NM_020039	Homo sapiens amiloride-sensitive cation channel 2, neuronal (ACCN2), transcript variant 1, mRNA [NM_020039]	NM_020039
A_32_P114284	2.59E-06	16.05	THC2338091		NQRB_CHLMU (Q9PKB6) Probable Na(+)-translocating NADH-quinone reductase subunit B (Na(+)-translocating NQR subunit B) (Na(+)-NQR subunit B) (NQR complex subunit B) (NQR-1 subunit B) , partial (4%) [THC2338091]	
A_23_P301133	2.61E-06	3.03	NM_001005912	NM_001005912	Homo sapiens inositol hexaphosphate kinase 2 (IHPK2), transcript variant 5, mRNA [NM_001005912]	NM_001005912
A_23_P24129	2.61E-06	3.13	NM_012242	NM_012242	Homo sapiens dickkopf homolog 1 (Xenopus laevis) (DKK1), mRNA [NM_012242]	NM_012242
A_23_P211522	2.61E-06	4.63	NM_145738	NM_145738	Homo sapiens synaptogyrin 1 (SYNGR1), transcript variant 1c, mRNA [NM_145738]	NM_145738
A_23_P259071	2.61E-06	112.61	NM_001657	NM_001657	Homo sapiens amphiregulin (schwannoma-derived growth factor) (AREG), mRNA [NM_001657]	NM_001657
A_23_P9523	2.63E-06	5.78	NM_022128	NM_022128	Homo sapiens ribokinase (RBKS), mRNA [NM_022128]	NM_022128
A_23_P421423	2.70E-06	4.55	NM_006291	NM_006291	Homo sapiens tumor necrosis factor, alpha-induced protein 2 (TNFAIP2), mRNA [NM_006291]	NM_006291
A_24_P277934	2.72E-06	21.79	NM_000089	NM_000089	Homo sapiens collagen, type I, alpha 2 (COL1A2), mRNA [NM_000089]	NM_000089
A_23_P151710	2.81E-06	502.51	NM_000956	NM_000956	Homo sapiens prostaglandin E receptor 2 (subtype EP2), 53kDa (PTGER2), mRNA [NM_000956]	NM_000956
A_23_P117582	2.82E-06	8.70	NM_130469	NM_130469	Homo sapiens jun dimerization protein 2 (JDP2), mRNA [NM_130469]	NM_130469
A_32_P32254	2.82E-06	14.58	NM_001848	NM_001848	Homo sapiens collagen, type VI, alpha 1 (COL6A1), mRNA [NM_001848]	NM_001848
A_23_P165360	2.85E-06	5.85	NM_016114	NM_016114	Homo sapiens ankyrin repeat and SOCS box-containing 1 (ASB1), mRNA [NM_016114]	NM_016114
A_23_P129695	2.85E-06	98.04	NM_138440	NM_138440	Homo sapiens slit-like 2 (Drosophila) (SLITL2), mRNA [NM_138440]	NM_138440
A_23_P124084	2.92E-06	14.79	NM_005576	NM_005576	Homo sapiens lysyl oxidase-like 1 (LOXL1), mRNA [NM_005576]	NM_005576
A_24_P244706	2.93E-06	74.07	NM_001049	NM_001049	Homo sapiens somatostatin receptor 1 (SSTR1), mRNA [NM_001049]	NM_001049
A_23_P360964	2.99E-06	2.25	NM_145056	NM_145056	Homo sapiens thymus expressed gene 3-like (MGC15476), mRNA [NM_145056]	NM_145056
A_23_P216023	2.99E-06	13.50	NM_001146	NM_001146	Homo sapiens angiopoietin 1 (ANGPT1), transcript variant 1, mRNA [NM_001146]	NM_001146
A_24_P211565	3.08E-06	3.22	NM_031910	NM_031910	Homo sapiens C1q and tumor necrosis factor related protein 6 (C1QTNF6), transcript variant 1, mRNA [NM_031910]	NM_031910
A_24_P88763	3.08E-06	5.52	NM_032603	NM_032603	Homo sapiens lysyl oxidase-like 3 (LOXL3), mRNA [NM_032603]	NM_032603
A_23_P87013	3.08E-06	8.62	NM_001001522	NM_001001522	Homo sapiens transgelin (TAGLN), transcript variant 1, mRNA [NM_001001522]	NM_001001522
A_23_P32444	3.08E-06	53.19	NM_032348	NM_032348	Homo sapiens matrix-remodelling associated 8 (MXRA8), mRNA [NM_032348]	NM_032348
A_23_P211926	3.08E-06	256.41	NM_003392	NM_003392	Homo sapiens wingless-type MMTV integration site family, member 5A (WNT5A), mRNA [NM_003392]	NM_003392
A_23_P409093	3.20E-06	17.79	NM_178826	NM_178826	Homo sapiens transmembrane protein 16D (TMEM16D), mRNA [NM_178826]	NM_178826
A_23_P218225	3.23E-06	12.58	NM_014298	NM_014298	Homo sapiens quinolinate phosphoribosyltransferase (nicotinate-nucleotide pyrophosphorylase (carboxylating)) (QPRT), mRNA [NM_014298]	NM_014298
A_23_P166508	3.26E-06	5.81	BC038245	BC038245	Homo sapiens, clone IMAGE:5241654, mRNA. [BC038245]	XM_086879
A_24_P310864	3.29E-06	2.53	NM_152499	NM_152499	Homo sapiens coiled-coil domain containing 24 (CCDC24), mRNA [NM_152499]	NM_152499
A_23_P76901	3.46E-06	6.02	NM_015549	NM_015549	Homo sapiens pleckstrin homology domain containing, family G (with RhoGef domain) member 3 (PLEKHG3), mRNA [NM_015549]	NM_015549
A_32_P18440	3.51E-06	6.49	BX641020	BX641020	Homo sapiens mRNA; cDNA DKFZp686G23148 (from clone DKFZp686G23148). [BX641020]	
A_23_P391906	3.51E-06	13.89	BC044246	BC044246	Homo sapiens KIAA1913, mRNA (cDNA clone MGC:50847 IMAGE:5760073), complete cds. [BC044246]	
A_23_P414273	3.51E-06	24.63	NM_032947	NM_032947	Homo sapiens putative small membrane protein NID67 (NID67), mRNA [NM_032947]	NM_032947
A_23_P91870	3.89E-06	2.25	NM_032343	NM_032343	Homo sapiens coiled-coil-helix-coiled-coil-helix domain containing 6 (CHCHD6), mRNA [NM_032343]	NM_032343
A_23_P300033	3.92E-06	59.17	NM_006206	NM_006206	Homo sapiens platelet-derived growth factor receptor, alpha polypeptide (PDGFRA), mRNA [NM_006206]	NM_006206
A_24_P780319	4.06E-06	3.25	A_24_P780319			
A_24_P208595	4.31E-06	11.82	NM_053034	NM_053034	Homo sapiens anthrax toxin receptor 1 (ANTXR1), transcript variant 2, mRNA [NM_053034]	NM_053034
A_24_P227230	4.66E-06	31.25	NM_014333	NM_014333	Homo sapiens immunoglobulin superfamily, member 4 (IGSF4), mRNA [NM_014333]	NM_014333
A_23_P11071	4.68E-06	2.16	NM_203473	NM_203473	Homo sapiens porcupine homolog (Drosophila) (PORCN), transcript variant B, mRNA [NM_203473]	NM_203473
A_23_P384044	4.68E-06	32.47	NM_152495	NM_152495	Homo sapiens cornichon homolog 3 (Drosophila) (CNIH3), mRNA [NM_152495]	NM_152495
A_23_P122650	4.78E-06	3.75	A_23_P122650			
A_23_P151805	4.78E-06	4.18	NM_006329	NM_006329	Homo sapiens fibulin 5 (FBLN5), mRNA [NM_006329]	NM_006329
A_23_P97700	4.78E-06	5.52	NM_006472	NM_006472	Homo sapiens thioredoxin interacting protein (TXNIP), mRNA [NM_006472]	NM_006472

A_23_P92517	4.83E-06	13.62	NM_031956	NM_031956	Homo sapiens NYD-SP14 protein (NYD-SP14), mRNA [NM_031956]	NM_031956
A_23_P209246	4.91E-06	6.25	AB209354	AB209354	Homo sapiens mRNA for GLI-Kruppel family member GLI2 isoform beta variant protein. [AB209354]	
A_23_P22143	4.91E-06	7.25	NM_000283	NM_000283	Homo sapiens phosphodiesterase 6B, cGMP-specific, rod, beta (congenital stationary night blindness 3, autosomal dominant) (PDE6B), mRNA [NM_000283]	NM_000283
A_24_P471242	4.92E-06	3.52	A_24_P471242			
A_23_P339119	4.94E-06	9.62	NM_024560	NM_024560	Homo sapiens FLJ21963 protein (FLJ21963), mRNA [NM_024560]	NM_024560
A_32_P196142	5.12E-06	5.95	THC2400010			
A_23_P325562	5.19E-06	8.62	NM_006671	NM_006671	Homo sapiens solute carrier family 1 (glutamate transporter), member 7 (SLC1A7), mRNA [NM_006671]	NM_006671
A_32_P215866	5.19E-06	12.84	THC2312367			
A_32_P47874	5.23E-06	8.93	THC2440787			
A_23_P252062	5.28E-06	53.48	NM_138711	NM_138711	Homo sapiens peroxisome proliferative activated receptor, gamma (PPARG), transcript variant 3, mRNA [NM_138711]	NM_138711
A_32_P34444	5.32E-06	4.88	NM_025135	NM_025135	Homo sapiens formin homology 2 domain containing 3 (FHOD3), mRNA [NM_025135]	NM_025135
A_23_P118203	5.34E-06	18.98	NM_145252	NM_145252	Homo sapiens similar to common salivary protein 1 (LOC124220), mRNA [NM_145252]	NM_145252
A_24_P213161	5.37E-06	3.15	NM_017852	NM_017852	Homo sapiens NACHT, leucine rich repeat and PYD containing 2 (NALP2), mRNA [NM_017852]	NM_017852
A_23_P125233	5.59E-06	5.92	NM_001299	NM_001299	Homo sapiens calponin 1, basic, smooth muscle (CNN1), mRNA [NM_001299]	NM_001299
A_23_P106405	5.69E-06	5.38	NM_002487	NM_002487	Homo sapiens necdin homolog (mouse) (NDN), mRNA [NM_002487]	NM_002487
A_23_P371824	5.69E-06	10.36	NM_020127	NM_020127	Homo sapiens tuftelin 1 (TUFT1), mRNA [NM_020127]	NM_020127
A_23_P80759	5.74E-06	4.31	BC017572	BC017572	Homo sapiens poliovirus receptor-related 3, mRNA (cDNA clone IMAGE:4066485), complete cds. [BC017572]	
A_23_P99661	5.81E-06	29.24	NM_018071	NM_018071	Homo sapiens hypothetical protein FLJ10357 (FLJ10357), mRNA [NM_018071]	NM_018071
A_24_P18105	5.82E-06	2.41	NM_032466	NM_032466	Homo sapiens aspartate beta-hydroxylase (ASPH), transcript variant 3, mRNA [NM_032466]	NM_032466
A_32_P77252	5.84E-06	5.78	THC2375310			
A_23_P154962	5.84E-06	9.09	BC035246	BC035246	Homo sapiens KIAA1666 protein, mRNA (cDNA clone MGC:42740 IMAGE:4827837), complete cds. [BC035246]	XM_036936
A_23_P203957	5.84E-06	12.92	NM_031920	NM_031920	Homo sapiens ARG99 protein (ARG99), transcript variant 1, mRNA [NM_031920]	NM_031920
A_23_P120103	5.84E-06	16.26	NM_002252	NM_002252	Homo sapiens potassium voltage-gated channel, delayed-rectifier, subfamily S, member 3 (KCNS3), mRNA [NM_002252]	NM_002252
A_23_P31873	5.84E-06	20.04	NM_001002233	NM_001002233	Homo sapiens RAB11 family interacting protein 1 (class I) (RAB11FIP1), transcript variant 2, mRNA [NM_001002233]	NM_001002233
A_23_P301138	6.03E-06	2.24	NM_001005912	NM_001005912	Homo sapiens inositol hexaphosphate kinase 2 (IHPK2), transcript variant 5, mRNA [NM_001005912]	NM_001005912
A_23_P160968	6.03E-06	6.76	NM_018891	NM_018891	Homo sapiens laminin, gamma 2 (LAMC2), transcript variant 2, mRNA [NM_018891]	NM_018891
A_23_P137173	6.03E-06	8.77	NM_021992	NM_021992	Homo sapiens thymosin-like 8 (TMSL8), mRNA [NM_021992]	NM_021992
A_23_P160318	6.06E-06	4.27	NM_001856	NM_001856	Homo sapiens collagen, type XVI, alpha 1 (COL16A1), mRNA [NM_001856]	NM_001856
A_24_P219094	6.11E-06	5.49	CR936651	CR936651	Homo sapiens mRNA; cDNA DKFZp686G1344 (from clone DKFZp686G1344). [CR936651]	
A_23_P217379	6.18E-06	3.92	NM_033641	NM_033641	Homo sapiens collagen, type IV, alpha 6 (COL4A6), transcript variant B, mRNA [NM_033641]	NM_033641
A_23_P136355	6.18E-06	5.62	NM_018194	NM_018194	Homo sapiens hedgehog acyltransferase (HHAT), mRNA [NM_018194]	NM_018194
A_32_P117185	6.18E-06	8.20	THC2406981		Q9LCV5 (Q9LCV5) Atp operon (Fragment), partial (3%) [THC2406981]	
A_24_P62530	6.18E-06	11.99	NM_021205	NM_021205	Homo sapiens ras homolog gene family, member U (RHOU), mRNA [NM_021205]	NM_021205
A_32_P20523	6.32E-06	3.31	NM_145715	NM_145715	Homo sapiens tigger transposable element derived 2 (TIGD2), mRNA [NM_145715]	NM_145715
A_32_P123255	6.34E-06	6.85	CR593492	CR593492	full-length cDNA clone CS0DI054YC18 of Placenta Cot 25-normalized of Homo sapiens (human). [CR593492]	XM_293380
A_24_P213370	6.35E-06	3.38	AK129982	AK129982	Homo sapiens cDNA FLJ26472 fis, clone KDN04506. [AK129982]	
A_23_P416395	6.35E-06	4.61	NM_003714	NM_003714	Homo sapiens stanniocalcin 2 (STC2), mRNA [NM_003714]	NM_003714
A_23_P63128	6.35E-06	5.08	AK024186	AK024186	Homo sapiens cDNA FLJ14124 fis, clone MAMMA1002498. [AK024186]	
A_24_P327886	6.35E-06	5.56	NM_003196	NM_003196	Homo sapiens transcription elongation factor A (SII), 3 (TCEA3), mRNA [NM_003196]	NM_003196
A_32_P198731	6.48E-06	6.17	ENST00000315447		Homo sapiens cDNA FLJ39342 fis, clone OCBBF2018873. [AK096661]	
A_23_P200239	6.52E-06	2.19	NM_018982	NM_018982	Homo sapiens Yip1 domain family, member 1 (YIPF1), mRNA [NM_018982]	NM_018982

A_23_P155755	6.52E-06	7.75	NM_002993	NM_002993	Homo sapiens chemokine (C-X-C motif) ligand 6 (granulocyte chemotactic protein 2) (CXCL6), mRNA [NM_002993]	NM_002993
A_23_P22378	6.75E-06	21.05	NM_003108	NM_003108	Homo sapiens SRY (sex determining region Y)-box 11 (SOX11), mRNA [NM_003108]	NM_003108
A_24_P166443	6.82E-06	2.42	NM_002121	NM_002121	Homo sapiens major histocompatibility complex, class II, DP beta 1 (HLA-DPB1), mRNA [NM_002121]	NM_002121
A_23_P35205	6.87E-06	3.50	ENST00000236261		Homo sapiens Down syndrome critical region gene 1-like 2, mRNA (cDNA clone MGC:46109 IMAGE:5770066), complete cds. [BC035854]	
A_32_P201329	6.89E-06	33.56	BC062758	BC062758	Homo sapiens cDNA clone IMAGE:4081583, partial cds. [BC062758]	
A_23_P57784	6.89E-06	50.51	NM_021101	NM_021101	Homo sapiens claudin 1 (CLDN1), mRNA [NM_021101]	NM_021101
A_32_P40288	6.95E-06	13.26	NM_052913	NM_052913	Homo sapiens KIAA1913 (KIAA1913), mRNA [NM_052913]	NM_052913
A_23_P360340	7.12E-06	2.33	NM_001008224	NM_001008224	Homo sapiens uveal autoantigen with coiled-coil domains and ankyrin repeats (UACA), transcript variant 2, mRNA [NM_001008224]	NM_001008224
A_23_P44724	7.14E-06	3.95	NM_001321	NM_001321	Homo sapiens cysteine and glycine-rich protein 2 (CSRP2), mRNA [NM_001321]	NM_001321
A_23_P14863	7.30E-06	3.44	NM_024111	NM_024111	Homo sapiens hypothetical protein MGC4504 (MGC4504), mRNA [NM_024111]	NM_024111
A_23_P83007	7.38E-06	3.72	NM_203403	NM_203403	Homo sapiens chromosome 9 open reading frame 150 (C9orf150), mRNA [NM_203403]	NM_203403
A_23_P84063	7.43E-06	5.92	NM_016522	NM_016522	Homo sapiens neurotrimin (HNT), mRNA [NM_016522]	NM_016522
A_23_P62999	7.65E-06	6.67	NM_004455	NM_004455	Homo sapiens exostoses (multiple)-like 1 (EXTL1), mRNA [NM_004455]	NM_004455
A_23_P84576	7.66E-06	12.08	NM_053034	NM_053034	Homo sapiens anthrax toxin receptor 1 (ANTXR1), transcript variant 2, mRNA [NM_053034]	NM_053034
A_23_P250102	7.98E-06	3.69	ENST00000295989		Homo sapiens mRNA for KIAA0667 protein, partial cds. [AB014567]	XM_371617
A_24_P703642	8.07E-06	5.81	THC2406981		Q9LCV5 (Q9LCV5) Atp operon (Fragment), partial (3%) [THC2406981]	
A_32_P300427	8.09E-06	10.26	NM_153360	NM_153360	Homo sapiens hypothetical protein FLJ90166 (FLJ90166), mRNA [NM_153360]	NM_153360
A_23_P339818	8.28E-06	10.10	NM_183376	NM_183376	Homo sapiens arrestin domain containing 4 (ARRDC4), mRNA [NM_183376]	NM_183376
A_24_P412156	8.35E-06	32.79	NM_000609	NM_000609	Homo sapiens chemokine (C-X-C motif) ligand 12 (stromal cell-derived factor 1) (CXCL12), transcript variant 1, mRNA [NM_000609]	NM_000609
A_23_P207811	8.38E-06	3.46	NM_006451	NM_006451	Homo sapiens poly(A) binding protein interacting protein 1 (PAIP1), transcript variant 1, mRNA [NM_006451]	NM_006451
A_23_P121533	8.38E-06	99.01	NM_012445	NM_012445	Homo sapiens spondin 2, extracellular matrix protein (SPON2), mRNA [NM_012445]	NM_012445
A_23_P83277	8.52E-06	3.86	NM_004512	NM_004512	Homo sapiens interleukin 11 receptor, alpha (IL11RA), transcript variant 1, mRNA [NM_004512]	NM_004512
A_23_P71067	8.53E-06	18.59	NM_000474	NM_000474	Homo sapiens twist homolog 1 (acrocephalosyndactyly 3; Saethre-Chotzen syndrome) (Drosophila) (TWIST1), mRNA [NM_000474]	NM_000474
A_32_P148407	8.62E-06	4.20	A_32_P148407			
A_23_P57199	8.62E-06	4.72	NM_080920	NM_080920	Homo sapiens gamma-glutamyltransferase-like activity 4 (GGTLA4), transcript variant C, mRNA [NM_080920]	NM_080920
A_23_P169039	8.73E-06	63.29	NM_003068	NM_003068	Homo sapiens snail homolog 2 (Drosophila) (SNAI2), mRNA [NM_003068]	NM_003068
A_23_P4662	9.07E-06	2.37	NM_005178	NM_005178	Homo sapiens B-cell CLL/lymphoma 3 (BCL3), mRNA [NM_005178]	NM_005178
A_23_P132619	9.07E-06	34.01	NM_000916	NM_000916	Homo sapiens oxytocin receptor (OXTR), mRNA [NM_000916]	NM_000916
A_23_P52207	9.16E-06	3.34	NM_012342	NM_012342	Homo sapiens BMP and activin membrane-bound inhibitor homolog (Xenopus laevis) (BAMBI), mRNA [NM_012342]	NM_012342
A_23_P132718	9.19E-06	2.29	NM_004636	NM_004636	Homo sapiens sema domain, immunoglobulin domain (Ig), short basic domain, secreted, (semaphorin) 3B (SEMA3B), transcript variant 1, mRNA [NM_004636]	NM_004636
A_23_P10127	9.19E-06	4.22	NM_003012	NM_003012	Homo sapiens secreted frizzled-related protein 1 (SFRP1), mRNA [NM_003012]	NM_003012
A_23_P52410	9.19E-06	4.61	NM_145307	NM_145307	Homo sapiens pleckstrin homology domain containing, family K member 1 (PLEKHK1), mRNA [NM_145307]	NM_145307
A_24_P233786	9.19E-06	5.78	NM_052966	NM_052966	Homo sapiens chromosome 1 open reading frame 24 (C1orf24), transcript variant 2, mRNA [NM_052966]	NM_052966
A_24_P760368	9.19E-06	26.18	THC2434152		Q9HBV3 (Q9HBV3) PP3111, partial (17%) [THC2434152]	
A_23_P79155	9.40E-06	6.33	ENST00000329321		Homo sapiens cDNA FLJ16071 fis, clone HSYRA2001003, highly similar to PUTATIVE G PROTEIN-COUPLED RECEPTOR GPR39. [AK122643]	
A_23_P143817	9.40E-06	9.43	NM_053025	NM_053025	Homo sapiens myosin, light polypeptide kinase (MYLK), transcript variant 1, mRNA [NM_053025]	NM_053025
A_24_P416131	9.52E-06	2.20	NM_021149	NM_021149	Homo sapiens coactosin-like 1 (Dictyostelium) (COTL1), mRNA [NM_021149]	NM_021149
A_23_P421175	9.59E-06	7.09	BC033256	BC033256	Homo sapiens FLJ46072 protein, mRNA (cDNA clone IMAGE:5455669), partial cds. [BC033256]	
A_23_P1431	9.59E-06	15.24	NM_004329	NM_004329	Homo sapiens bone morphogenetic protein receptor, type IA (BMPR1A), mRNA [NM_004329]	NM_004329

A_24_P143492	1.01E-05	7.19	NM_001010974	NM_001010974	Homo sapiens breast carcinoma amplified sequence 4 (BCAS4), transcript variant 3, mRNA [NM_001010974]	NM_001010974
A_24_P235429	1.03E-05	7.87	NM_005502	NM_005502	Homo sapiens ATP-binding cassette, sub-family A (ABC1), member 1 (ABCA1), mRNA [NM_005502]	NM_005502
A_23_P63541	1.03E-05	34.13	AK001019	AK001019	Homo sapiens cDNA FLJ10157 fis, clone HEMBA1003461. [AK001019]	XM_371354
A_23_P420417	1.04E-05	2.66	NM_138463	NM_138463	Homo sapiens hypothetical protein BC014072 (LOC116238), mRNA [NM_138463]	NM_138463
A_23_P43276	1.04E-05	2.88	NM_032777	NM_032777	Homo sapiens G protein-coupled receptor 124 (GPR124), mRNA [NM_032777]	NM_032777
A_23_P51986	1.04E-05	3.30	NM_006697	NM_006697	Homo sapiens myotubularin related protein 11 (MTMR11), transcript variant 1, mRNA [NM_006697]	NM_006697
A_32_P194891	1.04E-05	4.69	THC2282954		Q6CQJ2 (Q6CQJ2) Similarity, partial (13%) [THC2323726]	
A_23_P364504	1.04E-05	11.04	ENST00000344233		Homo sapiens cDNA FLJ37034 fis, clone BRACE2011478. [AK094353]	
A_23_P202448	1.04E-05	81.97	NM_199168	NM_199168	Homo sapiens chemokine (C-X-C motif) ligand 12 (stromal cell-derived factor 1) (CXCL12), transcript variant 2, mRNA [NM_199168]	NM_199168
A_24_P110914	1.05E-05	2.13	AK127569	AK127569	Homo sapiens cDNA FLJ45662 fis, clone CTONG2027150. [AK127569]	
A_23_P53247	1.06E-05	3.50	NM_178502	NM_178502	Homo sapiens deltex 3 homolog (Drosophila) (DTX3), mRNA [NM_178502]	NM_178502
A_23_P164451	1.06E-05	12.44	NM_005994	NM_005994	Homo sapiens T-box 2 (TBX2), mRNA [NM_005994]	NM_005994
A_23_P139500	1.07E-05	7.58	NM_030762	NM_030762	Homo sapiens basic helix-loop-helix domain containing, class B, 3 (BHLHB3), mRNA [NM_030762]	NM_030762
A_24_P320545	1.08E-05	5.21	NM_002821	NM_002821	Homo sapiens PTK7 protein tyrosine kinase 7 (PTK7), transcript variant PTK7-1, mRNA [NM_002821]	NM_002821
A_23_P203882	1.09E-05	5.68	NM_022791	NM_022791	Homo sapiens matrix metalloproteinase 19 (MMP19), transcript variant rasi-6, mRNA [NM_022791]	NM_022791
A_32_P72351	1.09E-05	7.87	AK026140	AK026140	Homo sapiens cDNA: FLJ22487 fis, clone HRC10931. [AK026140]	
A_23_P68529	1.10E-05	2.13	NM_033453	NM_033453	Homo sapiens inosine triphosphatase (nucleoside triphosphate pyrophosphatase) (ITPA), transcript variant 1, mRNA [NM_033453]	NM_033453
A_23_P15174	1.10E-05	2.24	NM_005949	NM_005949	Homo sapiens metallothionein 1F (functional) (MT1F), mRNA [NM_005949]	NM_005949
A_32_P827528	1.10E-05	4.08	BI089613	BI089613	602854489F1 NIH_MGC_10 Homo sapiens cDNA clone IMAGE:4995743 5', mRNA sequence [BI089613]	
A_24_P360674	1.10E-05	5.68	NM_078487	NM_078487	Homo sapiens cyclin-dependent kinase inhibitor 2B (p15, inhibits CDK4) (CDKN2B), transcript variant 2, mRNA [NM_078487]	NM_078487
A_23_P25150	1.10E-05	55.87	NM_006897	NM_006897	Homo sapiens homeo box C9 (HOXC9), mRNA [NM_006897]	NM_006897
A_23_P95930	1.12E-05	5.52	NM_001015886	NM_001015886	Homo sapiens high mobility group AT-hook 2 (HMGA2), transcript variant 3, mRNA [NM_001015886]	NM_001015886
A_23_P132889	1.12E-05	11.92	NM_001014809	NM_001014809	Homo sapiens collapsin response mediator protein 1 (CRMP1), transcript variant 1, mRNA [NM_001014809]	NM_001014809
A_23_P147514	1.14E-05	2.36	ENST00000305099		Homo sapiens hypothetical protein DKFZp547E052, mRNA (cDNA clone IMAGE:5493862), partial cds. [BC062636]	
A_23_P257043	1.14E-05	4.39	NM_005261	NM_005261	Homo sapiens GTP binding protein overexpressed in skeletal muscle (GEM), transcript variant 1, mRNA [NM_005261]	NM_005261
A_23_P4611	1.15E-05	2.70	NM_012254	NM_012254	Homo sapiens solute carrier family 27 (fatty acid transporter), member 5 (SLC27A5), mRNA [NM_012254]	NM_012254
A_32_P92642	1.15E-05	3.73	BE710245	BE710245	BE710245 IL3-HT0619-120700-212-G05 HT0619 Homo sapiens cDNA, mRNA sequence [BE710245]	
A_23_P57667	1.15E-05	4.46	NM_032242	NM_032242	Homo sapiens plexin A1 (PLXNA1), mRNA [NM_032242]	NM_032242
A_23_P358597	1.15E-05	6.67	NM_022361	NM_022361	Homo sapiens popeye domain containing 3 (POPDC3), mRNA [NM_022361]	NM_022361
A_23_P373521	1.15E-05	6.67	NM_021973	NM_021973	Homo sapiens heart and neural crest derivatives expressed 2 (HAND2), mRNA [NM_021973]	NM_021973
A_32_P319200	1.16E-05	2.86	NM_199127	NM_199127	Homo sapiens gamma-glutamyltransferase-like 4 (GGTL4), transcript variant 1, mRNA [NM_199127]	NM_199127
A_32_P16323	1.16E-05	39.06	ENST00000355077		Homo sapiens cDNA clone IMAGE:30389199, with apparent retained intron. [BC063888]	
A_24_P330303	1.17E-05	2.31	NM_152330	NM_152330	Homo sapiens FERM domain containing 6 (FRMD6), mRNA [NM_152330]	NM_152330
A_24_P319923	1.19E-05	13.66	NM_053025	NM_053025	Homo sapiens myosin, light polypeptide kinase (MYLK), transcript variant 1, mRNA [NM_053025]	NM_053025
A_23_P99996	1.21E-05	3.98	NM_019066	NM_019066	Homo sapiens MAGE-like 2 (MAGEL2), mRNA [NM_019066]	NM_019066
A_23_P253451	1.21E-05	13.66	NM_020949	NM_020949	Homo sapiens solute carrier family 7 (cationic amino acid transporter, y+ system), member 14 (SLC7A14), mRNA [NM_020949]	NM_020949
A_23_P14798	1.22E-05	17.67	NM_002499	NM_002499	Homo sapiens neogenin homolog 1 (chicken) (NEO1), mRNA [NM_002499]	NM_002499
A_23_P6771	1.23E-05	9.01	NM_014583	NM_014583	Homo sapiens LIM and cysteine-rich domains 1 (LMCD1), mRNA [NM_014583]	NM_014583
A_23_P65518	1.24E-05	9.62	NM_016651	NM_016651	Homo sapiens dapper, antagonist of beta-catenin, homolog 1 (Xenopus laevis) (DACT1), mRNA [NM_016651]	NM_016651
A_23_P52161	1.25E-05	4.95	NM_182901	NM_182901	Homo sapiens chromosome 11 open reading frame 17 (C11orf17), transcript variant 1, mRNA [NM_182901]	NM_182901

A_24_P923676	1.25E-05	27.03	X15674	X15674	Human pTR5 mRNA for repetitive sequence. [X15674]	
A_23_P166248	1.27E-05	2.41	NM_004414	NM_004414	Homo sapiens Down syndrome critical region gene 1 (DSCR1), transcript variant 1, mRNA [NM_004414]	NM_004414
A_23_P113613	1.28E-05	3.14	NM_022842	NM_022842	Homo sapiens CUB domain containing protein 1 (CDCP1), transcript variant 1, mRNA [NM_022842]	NM_022842
A_23_P110791	1.28E-05	30.12	NM_005211	NM_005211	Homo sapiens colony stimulating factor 1 receptor, formerly McDonough feline sarcoma viral (v-fms) oncogene homolog (CSF1R), mRNA [NM_005211]	NM_005211
A_23_P382	1.29E-05	2.39	NM_018125	NM_018125	Homo sapiens Rho guanine nucleotide exchange factor (GEF) 10-like (ARHGEF10L), transcript variant 1, mRNA [NM_018125]	NM_018125
A_24_P256063	1.29E-05	2.93	ENST00000332292			
A_32_P161855	1.29E-05	7.75	NM_018689	NM_018689	Homo sapiens KIAA1199 (KIAA1199), mRNA [NM_018689]	NM_018689
A_23_P97952	1.31E-05	2.16	NM_198472	NM_198472	Homo sapiens chromosome 10 open reading frame 125 (C10orf125), mRNA [NM_198472]	NM_198472
A_23_P133438	1.31E-05	31.55	NM_019018	NM_019018	Homo sapiens hypothetical protein FLJ11127 (FLJ11127), mRNA [NM_019018]	NM_019018
A_23_P218086	1.32E-05	2.59	AB032995	AB032995	Homo sapiens mRNA for KIAA1169 protein, partial cds. [AB032995]	
A_24_P924484	1.33E-05	2.35	K03200	K03200	Human melanoma-associated antigen p97 (melanotransferrin) mRNA, 3' flank. [K03200]	
A_24_P384369	1.36E-05	3.11	ENST00000330875			
A_23_P142533	1.36E-05	22.12	NM_000090	NM_000090	Homo sapiens collagen, type III, alpha 1 (Ehlers-Danlos syndrome type IV, autosomal dominant) (COL3A1), mRNA [NM_000090]	NM_000090
A_24_P383660	1.38E-05	2.88	ENST00000312751			
A_32_P134427	1.38E-05	13.61	BC048201	BC048201	Homo sapiens, clone IMAGE:3660074, mRNA. [BC048201]	
A_23_P50146	1.38E-05	15.29	NM_213602	NM_213602	Homo sapiens CD33 antigen-like 3 (CD33L3), mRNA [NM_213602]	NM_213602
A_23_P63209	1.39E-05	42.92	NM_181755	NM_181755	Homo sapiens hydroxysteroid (11-beta) dehydrogenase 1 (HSD11B1), transcript variant 2, mRNA [NM_181755]	NM_181755
A_32_P109516	1.41E-05	4.00	XM_374029	XM_374029	PREDICTED: Homo sapiens hypothetical LOC389089 (LOC389089), mRNA [XM_374029]	XM_374029
A_23_P106194	1.41E-05	6.02	NM_005252	NM_005252	Homo sapiens v-fos FBJ murine osteosarcoma viral oncogene homolog (FOS), mRNA [NM_005252]	NM_005252
A_23_P209449	1.42E-05	12.17	NM_003507	NM_003507	Homo sapiens frizzled homolog 7 (Drosophila) (FZD7), mRNA [NM_003507]	NM_003507
A_24_P173823	1.46E-05	2.51	BC044624	BC044624	Homo sapiens cDNA clone IMAGE:5288080, partial cds. [BC044624]	
A_23_P32253	1.46E-05	3.11	NM_005384	NM_005384	Homo sapiens nuclear factor, interleukin 3 regulated (NFIL3), mRNA [NM_005384]	NM_005384
A_23_P128574	1.46E-05	8.06	NM_017993	NM_017993	Homo sapiens hypothetical protein FLJ10094 (FLJ10094), mRNA [NM_017993]	NM_017993
A_23_P316612	1.46E-05	58.82	NM_147193	NM_147193	Homo sapiens GLIS family zinc finger 1 (GLIS1), mRNA [NM_147193]	NM_147193
A_23_P62890	1.48E-05	2.24	NM_002053	NM_002053	Homo sapiens guanylate binding protein 1, interferon-inducible, 67kDa (GBP1), mRNA [NM_002053]	NM_002053
A_24_P247233	1.48E-05	2.91	XM_498018	XM_498018	PREDICTED: Homo sapiens similar to Keratin, type I cytoskeletal 18 (Cytokeratin 18) (K18) (CK 18) (LOC391827), mRNA [XM_498018]	XM_498018
A_23_P14083	1.48E-05	3.34	NM_181847	NM_181847	Homo sapiens adhesion molecule with Ig-like domain 2 (AMIGO2), mRNA [NM_181847]	NM_181847
A_24_P402080	1.48E-05	4.17	NM_001025100	NM_001025100	Homo sapiens myelin basic protein (MBP), transcript variant 8, mRNA [NM_001025100]	NM_001025100
A_32_P3385	1.50E-05	2.07	CR611098	CR611098	full-length cDNA clone CS0DI060YD22 of Placenta Cot 25-normalized of Homo sapiens (human). [CR611098]	XM_379417
A_24_P139901	1.50E-05	9.01	NM_002101	NM_002101	Homo sapiens glycophorin C (Gerbich blood group) (GYPC), transcript variant 1, mRNA [NM_002101]	NM_002101
A_23_P78405	1.52E-05	3.83	NM_006033	NM_006033	Homo sapiens lipase, endothelial (LIPG), mRNA [NM_006033]	NM_006033
A_23_P21324	1.54E-05	3.03	NM_057179	NM_057179	Homo sapiens twist homolog 2 (Drosophila) (TWIST2), mRNA [NM_057179]	NM_057179
A_23_P75790	1.56E-05	2.61	NM_013279	NM_013279	Homo sapiens chromosome 11 open reading frame 9 (C11orf9), mRNA [NM_013279]	NM_013279
A_23_P161647	1.56E-05	3.23	NM_000920	NM_000920	Homo sapiens pyruvate carboxylase (PC), nuclear gene encoding mitochondrial protein, transcript variant A, mRNA [NM_000920]	NM_000920
A_32_P129010	1.58E-05	4.72	NM_001013615	NM_001013615	Homo sapiens hypothetical LOC541468 protein (LOC541468), mRNA [NM_001013615]	NM_001013615
A_23_P501183	1.61E-05	3.13	NM_148980	NM_148980	Homo sapiens Williams Beuren syndrome chromosome region 20C (WBSCR20C), transcript variant 3, mRNA [NM_148980]	NM_148980
A_23_P97871	1.61E-05	3.37	BX641020	BX641020	Homo sapiens mRNA; cDNA DKFZp686G23148 (from clone DKFZp686G23148). [BX641020]	
A_24_P110471	1.62E-05	2.30	THC2276423		Q6IFN5 (Q6IFN5) Olfactory receptor OR19-14, partial (67%) [THC2276423]	
A_23_P157865	1.62E-05	3.23	NM_002160	NM_002160	Homo sapiens tenascin C (hexabrachion) (TNC), mRNA [NM_002160]	NM_002160
A_23_P359214	1.62E-05	12.38	AK096715	AK096715	Homo sapiens cDNA FLJ39396 fis, clone PLACE6008640. [AK096715]	

A_23_P102058	1.63E-05	4.12	NM_002381	NM_002381	Homo sapiens matrilin 3 (MATN3), mRNA [NM_002381]	NM_002381
A_23_P372234	1.63E-05	4.24	NM_001218	NM_001218	Homo sapiens carbonic anhydrase XII (CA12), transcript variant 1, mRNA [NM_001218]	NM_001218
A_23_P212500	1.64E-05	4.27	NM_001063	NM_001063	Homo sapiens transferrin (TF), mRNA [NM_001063]	NM_001063
A_32_P104432	1.65E-05	4.00	NM_203306	NM_203306	Homo sapiens hypothetical protein MGC39606 (MGC39606), mRNA [NM_203306]	NM_203306
A_23_P4212	1.65E-05	22.42	NM_006361	NM_006361	Homo sapiens homeo box B13 (HOXB13), mRNA [NM_006361]	NM_006361
A_23_P154585	1.72E-05	2.03	NM_033421	NM_033421	Homo sapiens chromosome 20 open reading frame 161 (C20orf161), transcript variant 1, mRNA [NM_033421]	NM_033421
A_24_P753592	1.72E-05	8.20	BC018589	BC018589	Homo sapiens cDNA clone IMAGE:3863301. [BC018589]	
A_23_P215070	1.74E-05	3.52	NM_018718	NM_018718	Homo sapiens testis specific, 14 (TSGA14), mRNA [NM_018718]	NM_018718
A_32_P62145	1.74E-05	5.92	BC013423	BC013423	Homo sapiens, clone IMAGE:3892140, mRNA. [BC013423]	
A_32_P115606	1.77E-05	2.49	AK131385	AK131385	Homo sapiens cDNA FLJ16460 fis, clone BRCAN2018240. [AK131385]	
A_24_P230486	1.77E-05	2.93	A_24_P230486			
A_24_P148261	1.77E-05	8.20	AK021874	AK021874	Homo sapiens cDNA FLJ11812 fis, clone HEMBA1006364. [AK021874]	
A_23_P79302	1.77E-05	9.01	NM_177964	NM_177964	Homo sapiens hypothetical protein LOC130576 (LOC130576), mRNA [NM_177964]	NM_177964
A_24_P56310	1.77E-05	30.03	NM_148957	NM_148957	Homo sapiens tumor necrosis factor receptor superfamily, member 19 (TNFRSF19), transcript variant 2, mRNA [NM_148957]	NM_148957
A_23_P35414	1.80E-05	5.52	NM_005398	NM_005398	Homo sapiens protein phosphatase 1, regulatory (inhibitor) subunit 3C (PPP1R3C), mRNA [NM_005398]	NM_005398
A_32_P218355	1.80E-05	13.87	AK074567	AK074567	Homo sapiens cDNA FLJ90086 fis, clone HEMBA1005145. [AK074567]	XM_371820
A_24_P100535	1.81E-05	5.18	NM_181519	NM_181519	Homo sapiens synaptotagmin XV (SYT15), transcript variant b, mRNA [NM_181519]	NM_181519
A_23_P94216	1.85E-05	2.62	NM_152271	NM_152271	Homo sapiens hypothetical protein FLJ23749 (FLJ23749), mRNA [NM_152271]	NM_152271
A_23_P30776	1.86E-05	3.05	NM_003523	NM_003523	Homo sapiens histone 1, H2be (HIST1H2BE), mRNA [NM_003523]	NM_003523
A_23_P422115	1.87E-05	2.04	NM_144654	NM_144654	Homo sapiens chromosome 9 open reading frame 116 (C9orf116), mRNA [NM_144654]	NM_144654
A_24_P109214	1.87E-05	3.62	NM_001645	NM_001645	Homo sapiens apolipoprotein C-I (APOC1), mRNA [NM_001645]	NM_001645
A_32_P67303	1.87E-05	11.61	THC2328806			
A_24_P154037	1.87E-05	13.87	NM_003749	NM_003749	Homo sapiens insulin receptor substrate 2 (IRS2), mRNA [NM_003749]	NM_003749
A_24_P816844	1.87E-05	50.76	THC2433066			
A_23_P205031	1.89E-05	6.45	NM_001846	NM_001846	Homo sapiens collagen, type IV, alpha 2 (COL4A2), mRNA [NM_001846]	NM_001846
A_23_P109143	1.90E-05	3.57	NM_000311	NM_000311	Homo sapiens prion protein (p27-30) (Creutzfeld-Jakob disease, Gerstmann-Strausler-Scheinker syndrome, fatal familial insomnia) (PRNP), transcript variant 1, mRNA [NM_000311]	NM_000311
A_24_P392060	1.91E-05	2.25	NM_198567	NM_198567	Homo sapiens FLJ44216 protein (FLJ44216), mRNA [NM_198567]	NM_198567
A_23_P91520	1.92E-05	2.44	NM_007341	NM_007341	Homo sapiens SH3 domain binding glutamic acid-rich protein (SH3BGR), transcript variant 1, mRNA [NM_007341]	NM_007341
A_23_P131816	1.94E-05	2.04	NM_052951	NM_052951	Homo sapiens deoxynucleotidyltransferase, terminal, interacting protein 1 (DNTTIP1), mRNA [NM_052951]	NM_052951
A_23_P215744	1.94E-05	3.61	NM_033427	NM_033427	Homo sapiens cortactin binding protein 2 (CTTNBP2), mRNA [NM_033427]	NM_033427
A_23_P56559	1.94E-05	6.37	NM_005771	NM_005771	Homo sapiens dehydrogenase/reductase (SDR family) member 9 (DHRS9), transcript variant 1, mRNA [NM_005771]	NM_005771
A_24_P294124	2.02E-05	2.10	NM_014755	NM_014755	Homo sapiens SERTA domain containing 2 (SERTAD2), mRNA [NM_014755]	NM_014755
A_24_P90774	2.02E-05	3.89	NM_001008224	NM_001008224	Homo sapiens uveal autoantigen with coiled-coil domains and ankyrin repeats (UACA), transcript variant 2, mRNA [NM_001008224]	NM_001008224
A_23_P433820	2.08E-05	2.14	NM_152658	NM_152658	Homo sapiens THAP domain containing 8 (THAP8), mRNA [NM_152658]	NM_152658
A_23_P258814	2.08E-05	2.22	ENST00000328644			
A_24_P24645	2.08E-05	3.17	XM_497978	XM_497978	PREDICTED: Homo sapiens similar to Keratin, type I cytoskeletal 18 (Cytokeratin 18) (K18) (CK 18) (LOC132391), mRNA [XM_497978]	XM_497978
A_23_P57036	2.08E-05	3.41	NM_001250	NM_001250	Homo sapiens CD40 antigen (TNF receptor superfamily member 5) (CD40), transcript variant 1, mRNA [NM_001250]	NM_001250
A_23_P20743	2.08E-05	4.00	NM_032342	NM_032342	Homo sapiens chromosome 9 open reading frame 125 (C9orf125), mRNA [NM_032342]	NM_032342
A_23_P14072	2.08E-05	5.29	NM_002273	NM_002273	Homo sapiens keratin 8 (KRT8), mRNA [NM_002273]	NM_002273

A_23_P500353	2.08E-05	10.14	NM_021614	NM_021614	Homo sapiens potassium intermediate/small conductance calcium-activated channel, subfamily N, member 2 (KCNN2), transcript variant 1, mRNA [NM_021614]	NM_021614
A_23_P123172	2.08E-05	11.74	NR_002157	NR_002157	Homo sapiens olfactory receptor, family 2, subfamily A, member 9 pseudogene (OR2A9P) on chromosome 7 [NR_002157]	NR_002157
A_32_P58977	2.09E-05	10.85	AW068592	AW068592	AW068592 cn21d01.x1 Normal Human Trabecular Bone Cells Homo sapiens cDNA clone NHTBC_cn21d01 random, mRNA sequence [AW068592]	
A_23_P96041	2.11E-05	2.61	NM_032227	NM_032227	Homo sapiens hypothetical protein FLJ22679 (RP13-360B22.2), mRNA [NM_032227]	NM_032227
A_23_P114883	2.17E-05	2.07	NM_002023	NM_002023	Homo sapiens fibromodulin (FMOD), mRNA [NM_002023]	NM_002023
A_24_P12401	2.17E-05	3.34	NM_001025366	NM_001025366	Homo sapiens vascular endothelial growth factor (VEGF), transcript variant 1, mRNA [NM_001025366]	NM_001025366
A_23_P95810	2.17E-05	3.85	NM_007183	NM_007183	Homo sapiens plakophilin 3 (PKP3), mRNA [NM_007183]	NM_007183
A_24_P785894	2.17E-05	4.02	XM_373881	XM_373881	PREDICTED: Homo sapiens hypothetical LOC388727 (LOC388727), mRNA [XM_373881]	XM_373881
A_24_P230057	2.20E-05	3.07	ENST00000327707			
A_23_P50426	2.23E-05	2.67	NM_015493	NM_015493	Homo sapiens ankyrin repeat domain 25 (ANKRD25), mRNA [NM_015493]	NM_015493
A_24_P365571	2.24E-05	2.02	NM_032476	NM_032476	Homo sapiens mitochondrial ribosomal protein S6 (MRPS6), nuclear gene encoding mitochondrial protein, mRNA [NM_032476]	NM_032476
A_32_P88262	2.25E-05	9.17	AK094547	AK094547	Homo sapiens cDNA FLJ37228 fis, clone BRAMY2000411. [AK094547]	
A_23_P154367	2.26E-05	3.91	NM_004226	NM_004226	Homo sapiens serine/threonine kinase 17b (apoptosis-inducing) (STK17B), mRNA [NM_004226]	NM_004226
A_24_P248185	2.27E-05	3.18	NM_153685	NM_153685	Homo sapiens hypothetical protein DKFZp547D2210 (DKFZp547D2210), mRNA [NM_153685]	NM_153685
A_23_P10858	2.28E-05	2.13	ENST00000318139		Homo sapiens KIAA0692 protein, mRNA (cDNA clone IMAGE:5473979), partial cds. [BC062373]	
A_24_P59062	2.28E-05	3.69	NM_080839	NM_080839	Homo sapiens gamma-glutamyltransferase-like 4 (GGTL4), transcript variant 2, mRNA [NM_080839]	NM_080839
A_32_P71032	2.33E-05	4.74	THC2407039			
A_23_P417415	2.37E-05	2.34	NM_147161	NM_147161	Homo sapiens thioesterase, adipose associated (THEA), transcript variant 2, mRNA [NM_147161]	NM_147161
A_23_P42178	2.37E-05	3.02	NM_003522	NM_003522	Homo sapiens histone 1, H2bf (HIST1H2BF), mRNA [NM_003522]	NM_003522
A_24_P281443	2.38E-05	2.98	A_24_P281443			
A_23_P91850	2.41E-05	4.41	NM_144717	NM_144717	Homo sapiens fibronectin type III domain containing 6 (FNDC6), mRNA [NM_144717]	NM_144717
A_23_P55477	2.41E-05	4.46	NM_000676	NM_000676	Homo sapiens adenosine A2b receptor (ADORA2B), mRNA [NM_000676]	NM_000676
A_23_P87150	2.42E-05	5.35	NM_004811	NM_004811	Homo sapiens leupaxin (LPXN), mRNA [NM_004811]	NM_004811
A_32_P18547	2.44E-05	2.89	NM_058181	NM_058181	Homo sapiens chromosome 21 open reading frame 57 (C21orf57), transcript variant 1, mRNA [NM_058181]	NM_058181
A_24_P192485	2.44E-05	9.01	NM_002546	NM_002546	Homo sapiens tumor necrosis factor receptor superfamily, member 11b (osteoprotegerin) (TNFRSF11B), mRNA [NM_002546]	NM_002546
A_24_P930062	2.45E-05	4.59	NM_016463	NM_016463	Homo sapiens CXXC finger 5 (CXXC5), mRNA [NM_016463]	NM_016463
A_24_P66027	2.46E-05	2.28	NM_004900	NM_004900	Homo sapiens apolipoprotein B mRNA editing enzyme, catalytic polypeptide-like 3B (APOBEC3B), mRNA [NM_004900]	NM_004900
A_32_P151544	2.46E-05	3.97	NM_000224	NM_000224	Homo sapiens keratin 18 (KRT18), transcript variant 1, mRNA [NM_000224]	NM_000224
A_23_P338919	2.46E-05	11.57	NM_005876	NM_005876	Homo sapiens aortic preferentially expressed gene 1 (APEG1), mRNA [NM_005876]	NM_005876
A_32_P71744	2.48E-05	2.98	THC2370609			
A_23_P171117	2.50E-05	2.39	NM_024657	NM_024657	Homo sapiens MORC family CW-type zinc finger 4 (MORC4), mRNA [NM_024657]	NM_024657
A_23_P111635	2.52E-05	2.23	NM_016086	NM_016086	Homo sapiens dual specificity phosphatase 24 (putative) (DUSP24), mRNA [NM_016086]	NM_016086
A_23_P31721	2.52E-05	2.43	NM_001951	NM_001951	Homo sapiens E2F transcription factor 5, p130-binding (E2F5), mRNA [NM_001951]	NM_001951
A_23_P20752	2.52E-05	6.76	NM_178432	NM_178432	Homo sapiens cell cycle related kinase (CCRK), transcript variant 1, mRNA [NM_178432]	NM_178432
A_24_P350838	2.52E-05	11.39	X62009	X62009	Homo sapiens partial mRNA for fibrillin 5. [X62009]	
A_23_P138717	2.54E-05	2.80	NM_001005339	NM_001005339	Homo sapiens regulator of G-protein signalling 10 (RGS10), transcript variant 1, mRNA [NM_001005339]	NM_001005339
A_23_P26426	2.55E-05	4.13	NM_014427	NM_014427	Homo sapiens copine VII (CPNE7), transcript variant 2, mRNA [NM_014427]	NM_014427
A_23_P111000	2.57E-05	5.78	NM_002800	NM_002800	Homo sapiens proteasome (prosome, macropain) subunit, beta type, 9 (large multifunctional protease 2) (PSMB9), transcript variant 1, mRNA [NM_002800]	NM_002800
A_23_P399001	2.57E-05	7.35	NM_016463	NM_016463	Homo sapiens CXXC finger 5 (CXXC5), mRNA [NM_016463]	NM_016463

A_24_P639671	2.57E-05	100.00	AK095831	AK095831	Homo sapiens cDNA FLJ38512 fis, clone HCHON2000503. [AK095831]	
A_23_P110531	2.58E-05	24.39	NM_013409	NM_013409	Homo sapiens follistatin (FST), transcript variant FST344, mRNA [NM_013409]	NM_013409
A_23_P27584	2.61E-05	2.08	NM_001020818	NM_001020818	Homo sapiens myeloid-associated differentiation marker (MYADM), transcript variant 1, mRNA [NM_001020818]	NM_001020818
A_23_P217704	2.61E-05	7.30	NM_003918	NM_003918	Homo sapiens glycogenin 2 (GYG2), mRNA [NM_003918]	NM_003918
A_24_P241318	2.70E-05	2.39	NM_181340	NM_181340	Homo sapiens WD repeat domain 21A (WDR21A), transcript variant 2, mRNA [NM_181340]	NM_181340
A_32_P2452	2.70E-05	14.97	AK125140	AK125140	Homo sapiens cDNA FLJ43150 fis, clone CTONG3009239. [AK125140]	
A_23_P156025	2.70E-05	39.53	ENST00000302057		Homo sapiens homeodomain protein IRXA2 (IRX2) mRNA, complete cds. [AY335940]	
A_23_P7033	2.72E-05	3.25	NM_022149	NM_022149	Homo sapiens melanoma antigen family F, 1 (MAGEF1), mRNA [NM_022149]	NM_022149
A_23_P157247	2.72E-05	6.94	NM_152689	NM_152689	Homo sapiens hypothetical protein MGC9712 (MGC9712), mRNA [NM_152689]	NM_152689
A_23_P24870	2.73E-05	2.16	NM_000610	NM_000610	Homo sapiens CD44 antigen (homing function and Indian blood group system) (CD44), transcript variant 1, mRNA [NM_000610]	NM_000610
A_23_P324340	2.73E-05	2.67	NM_033510	NM_033510	Homo sapiens dispatched homolog 2 (Drosophila) (DISP2), mRNA [NM_033510]	NM_033510
A_24_P306704	2.73E-05	2.80	A_24_P306704			
A_23_P59069	2.73E-05	3.02	NM_003527	NM_003527	Homo sapiens histone 1, H2bo (HIST1H2BO), mRNA [NM_003527]	NM_003527
A_23_P24884	2.73E-05	3.09	NM_005418	NM_005418	Homo sapiens suppression of tumorigenicity 5 (ST5), transcript variant 1, mRNA [NM_005418]	NM_005418
A_23_P88849	2.73E-05	7.09	NM_004165	NM_004165	Homo sapiens Ras-related associated with diabetes (RRAD), mRNA [NM_004165]	NM_004165
A_32_P190404	2.75E-05	4.63	AY030238	AY030238	Homo sapiens LQK1 hypothetical protein long isoform (LQK1) mRNA, complete cds, alternatively spliced. [AY030238]	
A_23_P153745	2.77E-05	4.93	NM_006332	NM_006332	Homo sapiens interferon, gamma-inducible protein 30 (IFI30), mRNA [NM_006332]	NM_006332
A_23_P392126	2.81E-05	2.90	AK055808	AK055808	Homo sapiens cDNA FLJ31246 fis, clone KIDNE2005266. [AK055808]	XM_375430
A_23_P211047	2.82E-05	2.91	NM_206866	NM_206866	Homo sapiens BTB and CNC homology 1, basic leucine zipper transcription factor 1 (BACH1), transcript variant 1, mRNA [NM_206866]	NM_206866
A_23_P27023	2.83E-05	5.10	NM_032932	NM_032932	Homo sapiens RAB11 family interacting protein 4 (class II) (RAB11FIP4), mRNA [NM_032932]	NM_032932
A_32_P32391	2.84E-05	2.22	NR_002171	NR_002171	Homo sapiens olfactory receptor, family 7, subfamily E, member 156 pseudogene (OR7E156P) on chromosome 13 [NR_002171]	NR_002171
A_23_P383986	2.84E-05	2.85	NM_015892	NM_015892	Homo sapiens B cell RAG associated protein (GALNAC4S-6ST), mRNA [NM_015892]	NM_015892
A_23_P58137	2.84E-05	4.18	BM930307	BM930307	UI-E-EJ1-ajh-l-11-0-UI.r1 UI-E-EJ1 Homo sapiens cDNA clone UI-E-EJ1-ajh-l-11-0-UI 5', mRNA sequence [BM930307]	
A_23_P170667	2.84E-05	11.30	NM_181718	NM_181718	Homo sapiens hypothetical protein LOC253982 (LOC253982), mRNA [NM_181718]	NM_181718
A_23_P160559	2.85E-05	5.08	NM_004425	NM_004425	Homo sapiens extracellular matrix protein 1 (ECM1), transcript variant 1, mRNA [NM_004425]	NM_004425
A_23_P159237	2.85E-05	10.55	AB209066	AB209066	Homo sapiens mRNA for G protein-coupled receptor 20 variant protein. [AB209066]	
A_23_P325093	2.86E-05	4.07	NM_178026	NM_178026	Homo sapiens gamma-glutamyltransferase-like 3 (GGTL3), transcript variant 3, mRNA [NM_178026]	NM_178026
A_23_P62081	2.86E-05	6.41	NM_003020	NM_003020	Homo sapiens secretory granule, neuroendocrine protein 1 (7B2 protein) (SGNE1), mRNA [NM_003020]	NM_003020
A_23_P54055	2.87E-05	2.76	NM_032876	NM_032876	Homo sapiens jub, ajuba homolog (Xenopus laevis) (JUB), transcript variant 1, mRNA [NM_032876]	NM_032876
A_23_P253484	2.87E-05	3.56	NM_016228	NM_016228	Homo sapiens aminoadipate aminotransferase (AADAT), transcript variant 1, mRNA [NM_016228]	NM_016228
A_24_P896205	2.87E-05	7.09	XM_498676	XM_498676	PREDICTED: Homo sapiens LOC440450 (LOC440450), mRNA [XM_498676]	XM_498676
A_23_P57137	2.93E-05	2.42	NM_018347	NM_018347	Homo sapiens chromosome 20 open reading frame 29 (C20orf29), mRNA [NM_018347]	NM_018347
A_24_P183664	2.93E-05	4.72	AB014544	AB014544	Homo sapiens mRNA for KIAA0644 protein, partial cds. [AB014544]	XM_376588
A_24_P354900	2.94E-05	2.40	AK098194	AK098194	Homo sapiens cDNA FLJ40875 fis, clone UMVEN2000069. [AK098194]	
A_24_P230466	2.94E-05	3.16	A_24_P230466			
A_23_P89249	2.95E-05	5.88	NM_001005862	NM_001005862	Homo sapiens v-erb-b2 erythroblastic leukemia viral oncogene homolog 2, neuro/glioblastoma derived oncogene homolog (avian) (ERBB2), transcript variant 2, mRNA [NM_001005862]	NM_001005862
A_24_P218151	2.95E-05	9.62	NM_016352	NM_016352	Homo sapiens carboxypeptidase A4 (CPA4), mRNA [NM_016352]	NM_016352
A_32_P108544	3.01E-05	2.93	NM_001024457	NM_001024457	Homo sapiens Ran binding protein 2-like 2 (RANBP2L2), mRNA [NM_001024457]	NM_001024457
A_24_P354715	3.01E-05	3.73	NM_002526	NM_002526	Homo sapiens 5'-nucleotidase, ecto (CD73) (NT5E), mRNA [NM_002526]	NM_002526

A_23_P377214	3.08E-05	2.11	NM_144608	NM_144608	Homo sapiens hexamthylene bis-acetamide inducible 2 (HEXIM2), mRNA [NM_144608]	NM_144608
A_23_P31073	3.14E-05	5.99	NM_005375	NM_005375	Homo sapiens v-myb myeloblastosis viral oncogene homolog (avian) (MYB), mRNA [NM_005375]	NM_005375
A_23_P20443	3.15E-05	34.25	NM_021020	NM_021020	Homo sapiens leucine zipper, putative tumor suppressor 1 (LZTS1), mRNA [NM_021020]	NM_021020
A_23_P41455	3.16E-05	26.04	NM_003305	NM_003305	Homo sapiens transient receptor potential cation channel, subfamily C, member 3 (TRPC3), mRNA [NM_003305]	NM_003305
A_24_P358406	3.17E-05	2.85	A_24_P358406			
A_23_P103011	3.19E-05	2.26	NM_004914	NM_004914	Homo sapiens RAB36, member RAS oncogene family (RAB36), mRNA [NM_004914]	NM_004914
A_23_P391586	3.22E-05	3.09	NM_001018004	NM_001018004	Homo sapiens tropomyosin 1 (alpha) (TPM1), transcript variant 3, mRNA [NM_001018004]	NM_001018004
A_23_P391344	3.22E-05	4.69	NM_145313	NM_145313	Homo sapiens RasGEF domain family, member 1A (RASGEF1A), mRNA [NM_145313]	NM_145313
A_23_P211598	3.24E-05	4.78	NM_002676	NM_002676	Homo sapiens phosphomannomutase 1 (PMM1), mRNA [NM_002676]	NM_002676
A_23_P27265	3.26E-05	2.84	NM_032160	NM_032160	Homo sapiens chromosome 18 open reading frame 4 (C18orf4), mRNA [NM_032160]	NM_032160
A_23_P257578	3.31E-05	2.03	AK054960	AK054960	Homo sapiens cDNA FLJ30398 fis, clone BRACE2008402, highly similar to Homo sapiens steroid receptor RNA activator isoform 3 mRNA. [AK054960]	XR_000132
A_23_P212968	3.31E-05	11.45	NM_001073	NM_001073	Homo sapiens UDP glucuronosyltransferase 2 family, polypeptide B11 (UGT2B11), mRNA [NM_001073]	NM_001073
A_32_P156171	3.32E-05	3.38	THC2341051			
A_23_P87011	3.33E-05	3.95	NM_001001522	NM_001001522	Homo sapiens transgelin (TAGLN), transcript variant 1, mRNA [NM_001001522]	NM_001001522
A_23_P314145	3.35E-05	6.85	NM_005715	NM_005715	Homo sapiens uronyl-2-sulfotransferase (UST), mRNA [NM_005715]	NM_005715
A_24_P182494	3.35E-05	13.81	NM_007207	NM_007207	Homo sapiens dual specificity phosphatase 10 (DUSP10), transcript variant 1, mRNA [NM_007207]	NM_007207
A_23_P164958	3.36E-05	18.90	NM_032040	NM_032040	Homo sapiens coiled-coil domain containing 8 (CCDC8), mRNA [NM_032040]	NM_032040
A_32_P210202	3.41E-05	2.66	NM_203394	NM_203394	Homo sapiens E2F transcription factor 7 (E2F7), mRNA [NM_203394]	NM_203394
A_23_P65918	3.42E-05	6.76	NM_002220	NM_002220	Homo sapiens inositol 1,4,5-trisphosphate 3-kinase A (ITPKA), mRNA [NM_002220]	NM_002220
A_24_P57528	3.43E-05	3.34	NM_139177	NM_139177	Homo sapiens solute carrier family 39 (metal ion transporter), member 11 (SLC39A11), mRNA [NM_139177]	NM_139177
A_23_P59261	3.43E-05	4.05	NM_006670	NM_006670	Homo sapiens trophoblast glycoprotein (TPBG), mRNA [NM_006670]	NM_006670
A_23_P218523	3.44E-05	2.11	AF218008	AF218008	Homo sapiens clone PP3501 unknown mRNA. [AF218008]	
A_32_P117026	3.50E-05	2.56	AK094796	AK094796	Homo sapiens cDNA FLJ37477 fis, clone BRAWH2013047. [AK094796]	
A_23_P122443	3.56E-05	2.48	NM_005319	NM_005319	Homo sapiens histone 1, H1c (HIST1H1C), mRNA [NM_005319]	NM_005319
A_23_P416581	3.56E-05	5.38	NM_002073	NM_002073	Homo sapiens guanine nucleotide binding protein (G protein), alpha z polypeptide (GNAZ), mRNA [NM_002073]	NM_002073
A_23_P203920	3.57E-05	4.52	NM_005086	NM_005086	Homo sapiens sarcospan (Kras oncogene-associated gene) (SSPN), mRNA [NM_005086]	NM_005086
A_23_P156970	3.57E-05	12.66	NM_002402	NM_002402	Homo sapiens mesoderm specific transcript homolog (mouse) (MEST), transcript variant 1, mRNA [NM_002402]	NM_002402
A_23_P428184	3.63E-05	3.40	NM_021065	NM_021065	Homo sapiens histone 1, H2ad (HIST1H2AD), mRNA [NM_021065]	NM_021065
A_24_P648880	3.72E-05	6.90	NR_002211	NR_002211	Homo sapiens Meis1, myeloid ecotropic viral integration site 1 homolog 4 (mouse) (MEIS4) on chromosome 17 [NR_002211]	NR_002211
A_23_P211468	3.73E-05	19.27	AA837799	AA837799	AA837799 oe06h09.s1 NCI_CGAP_Ov2 Homo sapiens cDNA clone IMAGE:1385153, mRNA sequence [AA837799]	
A_23_P30784	3.74E-05	2.07	NM_013375	NM_013375	Homo sapiens activator of basal transcription 1 (ABT1), mRNA [NM_013375]	NM_013375
A_23_P423331	3.74E-05	4.57	NM_032536	NM_032536	Homo sapiens netrin G2 (NTNG2), mRNA [NM_032536]	NM_032536
A_23_P209636	3.74E-05	5.95	NM_006449	NM_006449	Homo sapiens CDC42 effector protein (Rho GTPase binding) 3 (CDC42EP3), mRNA [NM_006449]	NM_006449
A_23_P103981	3.75E-05	4.76	NM_003516	NM_003516	Homo sapiens histone 2, H2aa (HIST2H2AA), mRNA [NM_003516]	NM_003516
A_32_P116556	3.77E-05	2.57	AB058761	AB058761	Homo sapiens mRNA for KIAA1858 protein, partial cds. [AB058761]	XM_040592
A_32_P162537	3.78E-05	3.45	THC2288797		Q753E4 (Q753E4) AFR372Wp, partial (3%) [THC2288797]	
A_23_P24104	3.78E-05	3.61	NM_002658	NM_002658	Homo sapiens plasminogen activator, urokinase (PLAU), mRNA [NM_002658]	NM_002658
A_23_P326160	3.85E-05	2.02	NM_025079	NM_025079	Homo sapiens zinc finger CCCH-type containing 12A (ZC3H12A), mRNA [NM_025079]	NM_025079
A_23_P212061	3.85E-05	5.75	NM_007289	NM_007289	Homo sapiens membrane metallo-endopeptidase (neutral endopeptidase, enkephalinase, CALLA, CD10) (MME), transcript variant 2b, mRNA [NM_007289]	NM_007289
A_32_P195924	3.89E-05	2.33	THC2378147		Q755F9 (Q755F9) AFL136Wp, partial (4%) [THC2378147]	
A_24_P810084	3.92E-05	2.20	BC033983	BC033983	Homo sapiens, clone IMAGE:5295564, mRNA. [BC033983]	

A_24_P229911	3.92E-05	2.25	A_24_P229911			
A_23_P155857	3.95E-05	2.56	NM_198041	NM_198041	Homo sapiens nudix (nucleoside diphosphate linked moiety X)-type motif 6 (NUDT6), transcript variant 2, mRNA [NM_198041]	NM_198041
A_23_P215819	3.96E-05	2.53	NM_001009957	NM_001009957	Homo sapiens zinc finger protein 655 (ZNF655), transcript variant 5, mRNA [NM_001009957]	NM_001009957
A_24_P65910	3.98E-05	2.35	NM_018244	NM_018244	Homo sapiens chromosome 20 open reading frame 44 (C20orf44), transcript variant 1, mRNA [NM_018244]	NM_018244
A_24_P867201	3.99E-05	2.26	CR613944	CR613944	full-length cDNA clone CS0CAP007YK21 of Thymus of Homo sapiens (human). [CR613944]	
A_24_P920447	4.00E-05	2.60	NM_020215	NM_020215	Homo sapiens chromosome 14 open reading frame 132 (C14orf132), mRNA [NM_020215]	NM_020215
A_23_P164912	4.00E-05	2.61	NM_022165	NM_022165	Homo sapiens lin-7 homolog B (C. elegans) (LIN7B), mRNA [NM_022165]	NM_022165
A_32_P105549	4.00E-05	3.02	NM_001630	NM_001630	Homo sapiens annexin A8 (ANXA8), mRNA [NM_001630]	NM_001630
A_23_P154566	4.00E-05	5.78	NM_032883	NM_032883	Homo sapiens chromosome 20 open reading frame 100 (C20orf100), mRNA [NM_032883]	NM_032883
A_23_P213518	4.02E-05	2.08	NM_173060	NM_173060	Homo sapiens calpastatin (CAST), transcript variant 2, mRNA [NM_173060]	NM_173060
A_23_P76969	4.03E-05	3.42	NM_015556	NM_015556	Homo sapiens signal-induced proliferation-associated 1 like 1 (SIPA1L1), mRNA [NM_015556]	NM_015556
A_23_P216325	4.03E-05	3.73	NM_004315	NM_004315	Homo sapiens N-acylsphingosine amidohydrolase (acid ceramidase) 1 (ASAH1), transcript variant 2, mRNA [NM_004315]	NM_004315
A_23_P58506	4.11E-05	2.76	BX538289	BX538289	Homo sapiens mRNA; cDNA DKFZp779C185 (from clone DKFZp779C185). [BX538289]	
A_24_P409420	4.13E-05	3.17	A_24_P409420			
A_23_P302672	4.14E-05	2.28	NM_145244	NM_145244	Homo sapiens DNA-damage-inducible transcript 4-like (DDIT4L), mRNA [NM_145244]	NM_145244
A_23_P207911	4.14E-05	11.49	NM_016113	NM_016113	Homo sapiens transient receptor potential cation channel, subfamily V, member 2 (TRPV2), mRNA [NM_016113]	NM_016113
A_24_P125469	4.20E-05	3.36	NM_006033	NM_006033	Homo sapiens lipase, endothelial (LIPG), mRNA [NM_006033]	NM_006033
A_24_P92183	4.23E-05	3.15	ENST00000255136		Homo sapiens, clone IMAGE:4745538, mRNA. [BC039151]	XM_114158
A_23_P156289	4.23E-05	3.92	NM_003999	NM_003999	Homo sapiens oncostatin M receptor (OSMR), mRNA [NM_003999]	NM_003999
A_23_P141362	4.23E-05	4.72	NM_001466	NM_001466	Homo sapiens frizzled homolog 2 (Drosophila) (FZD2), mRNA [NM_001466]	NM_001466
A_24_P401601	4.25E-05	3.34	A_24_P401601			
A_24_P271323	4.28E-05	2.65	NM_030777	NM_030777	Homo sapiens solute carrier family 2 (facilitated glucose transporter), member 10 (SLC2A10), mRNA [NM_030777]	NM_030777
A_24_P3783	4.40E-05	2.48	NM_003521	NM_003521	Homo sapiens histone 1, H2bm (HIST1H2BM), mRNA [NM_003521]	NM_003521
A_24_P542608	4.44E-05	4.90	THC2279230		AL563955 AL563955 Homo sapiens FETAL LIVER Homo sapiens cDNA clone CS0DM001YO03 3-PRIME, mRNA sequence [AL563955]	
A_23_P306730	4.45E-05	2.60	L02932	L02932	Human peroxisome proliferator activated receptor mRNA, complete cds. [L02932]	
A_23_P347070	4.45E-05	76.34	NM_018440	NM_018440	Homo sapiens phosphoprotein associated with glycosphingolipid microdomains 1 (PAG1), mRNA [NM_018440]	NM_018440
A_32_P93144	4.46E-05	6.21	XM_372108	XM_372108	PREDICTED: Homo sapiens similar to Ribosome biogenesis protein BMS1 homolog (LOC389748), mRNA [XM_372108]	XM_372108
A_23_P421401	4.46E-05	183.15	NM_002609	NM_002609	Homo sapiens platelet-derived growth factor receptor, beta polypeptide (PDGFRB), mRNA [NM_002609]	NM_002609
A_32_P194032	4.47E-05	2.87	NM_152271	NM_152271	Homo sapiens hypothetical protein FLJ23749 (FLJ23749), mRNA [NM_152271]	NM_152271
A_23_P395054	4.50E-05	8.55	BC008813	BC008813	Homo sapiens annexin A8, mRNA (cDNA clone MGC:10405 IMAGE:3958020), complete cds. [BC008813]	
A_23_P167983	4.51E-05	6.06	ENST00000314088		Homo sapiens histone 1, H2ac, mRNA (cDNA clone MGC:1730 IMAGE:2988620), complete cds. [BC017379]	
A_23_P256473	4.52E-05	115.61	NM_006379	NM_006379	Homo sapiens sema domain, immunoglobulin domain (Ig), short basic domain, secreted, (semaphorin) 3C (SEMA3C), mRNA [NM_006379]	NM_006379
A_32_P42224	4.53E-05	5.78	BX097190	BX097190	BX097190 Soares placenta Nb2HP Homo sapiens cDNA clone IMAGp998G19212, mRNA sequence [BX097190]	
A_23_P111054	4.54E-05	2.71	NM_021062	NM_021062	Homo sapiens histone 1, H2bb (HIST1H2BB), mRNA [NM_021062]	NM_021062
A_24_P307653	4.54E-05	4.17	NM_182767	NM_182767	Homo sapiens solute carrier family 6, member 15 (SLC6A15), transcript variant 1, mRNA [NM_182767]	NM_182767
A_24_P375360	4.56E-05	2.60	A_24_P375360			
A_23_P162589	4.56E-05	7.04	NM_001017535	NM_001017535	Homo sapiens vitamin D (1,25- dihydroxyvitamin D3) receptor (VDR), transcript variant 2, mRNA [NM_001017535]	NM_001017535
A_32_P12183	4.59E-05	2.35	NM_198284	NM_198284	Homo sapiens hypothetical protein LOC349114 (LOC349114), mRNA [NM_198284]	NM_198284
A_32_P169550	4.59E-05	8.33	BX099371	BX099371	BX099371 BX099371 NCI_CGAP_Lu24 Homo sapiens cDNA clone IMAGp998O205798 ; IMAGE:2337259, mRNA sequence [BX099371]	

A_23_P429998	4.61E-05	6.94	NM_006732	NM_006732	Homo sapiens FBJ murine osteosarcoma viral oncogene homolog B (FOSB), mRNA [NM_006732]	NM_006732
A_24_P62668	4.65E-05	2.90	AK021425	AK021425	Homo sapiens cDNA FLJ11363 fis, clone HEMBA1000251. [AK021425]	
A_23_P121527	4.70E-05	2.10	NM_015990	NM_015990	Homo sapiens kelch-like 5 (Drosophila) (KLHL5), mRNA [NM_015990]	NM_015990
A_23_P400449	4.70E-05	23.26	NM_020927	NM_020927	Homo sapiens KIAA1576 protein (KIAA1576), mRNA [NM_020927]	NM_020927
A_24_P16230	4.76E-05	2.76	ENST00000331037			
A_23_P118493	4.77E-05	6.10	NM_005486	NM_005486	Homo sapiens target of myb1-like 1 (chicken) (TOM1L1), mRNA [NM_005486]	NM_005486
A_32_P89709	4.81E-05	3.21	NM_001018004	NM_001018004	Homo sapiens tropomyosin 1 (alpha) (TPM1), transcript variant 3, mRNA [NM_001018004]	NM_001018004
A_23_P38816	4.81E-05	6.17	NM_130786	NM_130786	Homo sapiens alpha-1-B glycoprotein (A1BG), mRNA [NM_130786]	NM_130786
A_24_P59569	4.81E-05	6.41	BC000922	BC000922	Homo sapiens hypothetical gene supported by BC000922, mRNA (cDNA clone IMAGE:3447073), partial cds. [BC000922]	XM_378793
A_24_P912799	4.81E-05	157.48	AF056434	AF056434	Homo sapiens clone FBD8 Cri-du-chat critical region mRNA. [AF056434]	
A_24_P13390	4.83E-05	3.10	NM_032814	NM_032814	Homo sapiens hypothetical protein FLJ14627 (FLJ14627), mRNA [NM_032814]	NM_032814
A_23_P93722	4.83E-05	4.50	AK074077	AK074077	Homo sapiens mRNA for FLJ00148 protein. [AK074077]	
A_32_P161762	4.83E-05	320.51	NM_004348	NM_004348	Homo sapiens runt-related transcription factor 2 (RUNX2), transcript variant 3, mRNA [NM_004348]	NM_004348
A_23_P8834	4.86E-05	3.12	NM_001979	NM_001979	Homo sapiens epoxide hydrolase 2, cytoplasmic (EPHX2), mRNA [NM_001979]	NM_001979
A_24_P415012	4.89E-05	2.22	NM_013437	NM_013437	Homo sapiens low density lipoprotein-related protein 12 (LRP12), mRNA [NM_013437]	NM_013437
A_23_P146444	4.91E-05	15.53	BX648086	BX648086	Homo sapiens mRNA; cDNA DKFZp686G19226 (from clone DKFZp686G19226). [BX648086]	
A_24_P915196	4.99E-05	2.15	AF038174	AF038174	Homo sapiens clone 23579 mRNA sequence. [AF038174]	
A_24_P186746	5.01E-05	2.97	A_24_P186746			
A_23_P10206	5.03E-05	23.20	NM_005328	NM_005328	Homo sapiens hyaluronan synthase 2 (HAS2), mRNA [NM_005328]	NM_005328
A_23_P99320	5.05E-05	2.92	NM_000224	NM_000224	Homo sapiens keratin 18 (KRT18), transcript variant 1, mRNA [NM_000224]	NM_000224
A_23_P9485	5.11E-05	3.39	NM_000608	NM_000608	Homo sapiens orosomucoid 2 (ORM2), mRNA [NM_000608]	NM_000608
A_23_P95221	5.11E-05	4.98	NM_005577	NM_005577	Homo sapiens lipoprotein, Lp(a) (LPA), mRNA [NM_005577]	NM_005577
A_23_P369634	5.13E-05	2.48	AF289596	AF289596	Homo sapiens clone pp7882 unknown mRNA. [AF289596]	
A_23_P56197	5.13E-05	9.80	NM_004750	NM_004750	Homo sapiens cytokine receptor-like factor 1 (CRLF1), mRNA [NM_004750]	NM_004750
A_23_P147025	5.15E-05	3.22	NM_004794	NM_004794	Homo sapiens RAB33A, member RAS oncogene family (RAB33A), mRNA [NM_004794]	NM_004794
A_23_P125717	5.20E-05	48.78	NM_004538	NM_004538	Homo sapiens nucleosome assembly protein 1-like 3 (NAP1L3), mRNA [NM_004538]	NM_004538
A_23_P41645	5.21E-05	2.27	NM_012081	NM_012081	Homo sapiens elongation factor, RNA polymerase II, 2 (ELL2), mRNA [NM_012081]	NM_012081
A_24_P76300	5.23E-05	4.46	NM_003444	NM_003444	Homo sapiens zinc finger protein 154 (pHZ-92) (ZNF154), mRNA [NM_003444]	NM_003444
A_24_P919460	5.26E-05	2.20	AB065089	AB065089	Homo sapiens OK/KNS-cl.7 mRNA for ribosomal protein S2, complete cds. [AB065089]	
A_23_P205159	5.26E-05	3.60	NM_005358	NM_005358	Homo sapiens LIM domain 7 (LMO7), mRNA [NM_005358]	NM_005358
A_23_P421843	5.26E-05	3.79	ENST00000285206		Homo sapiens hypothetical protein LOC201191, mRNA (cDNA clone IMAGE:5296646), partial cds. [BC047081]	
A_24_P358131	5.34E-05	2.99	ENST00000351766			
A_23_P22350	5.37E-05	2.63	NM_023927	NM_023927	Homo sapiens HCV NS3-transactivated protein 2 (NS3TP2), mRNA [NM_023927]	NM_023927
A_24_P595223	5.37E-05	4.31	AK098753	AK098753	Homo sapiens cDNA FLJ25887 fis, clone CBR02996. [AK098753]	
A_23_P126782	5.39E-05	59.88	NM_001993	NM_001993	Homo sapiens coagulation factor III (thromboplastin, tissue factor) (F3), mRNA [NM_001993]	NM_001993
A_23_P77493	5.40E-05	2.53	NM_006086	NM_006086	Homo sapiens tubulin, beta 3 (TUBB3), mRNA [NM_006086]	NM_006086
A_24_P782308	5.42E-05	3.48	CR596491	CR596491	full-length cDNA clone CS0DF038YD07 of Fetal brain of Homo sapiens (human). [CR596491]	
A_23_P73311	5.42E-05	9.71	NM_052958	NM_052958	Homo sapiens chromosome 8 open reading frame 34 (C8orf34), mRNA [NM_052958]	NM_052958
A_24_P11462	5.51E-05	4.83	NM_052998	NM_052998	Homo sapiens arginine decarboxylase (ADC), mRNA [NM_052998]	NM_052998
A_24_P686014	5.52E-05	3.17	A_24_P686014			
A_32_P135091	5.64E-05	2.37	NM_018301	NM_018301	Homo sapiens hypothetical protein FLJ11016 (FLJ11016), mRNA [NM_018301]	NM_018301

A_23_P335695	5.64E-05	7.41	AK023854	AK023854	Homo sapiens cDNA FLJ13792 fis, clone THYRO1000072, weakly similar to MYOSIN LIGHT CHAIN KINASE, SMOOTH MUSCLE AND NON-MUSCLE ISOZYMES (EC 2.7.1.117). [AK023854]	
A_32_P59302	5.65E-05	2.52	BC080552	BC080552	Homo sapiens cDNA clone IMAGE:6254031. [BC080552]	
A_23_P46378	5.66E-05	2.25	NM_017837	NM_017837	Homo sapiens phosphatidylinositol glycan, class V (PIGV), mRNA [NM_017837]	NM_017837
A_23_P500381	5.66E-05	2.57	NM_019859	NM_019859	Homo sapiens 5-hydroxytryptamine (serotonin) receptor 7 (adenylate cyclase-coupled) (HTR7), transcript variant d, mRNA [NM_019859]	NM_019859
A_23_P7313	5.66E-05	4.22	NM_000582	NM_000582	Homo sapiens secreted phosphoprotein 1 (osteopontin, bone sialoprotein I, early T-lymphocyte activation 1) (SPP1), mRNA [NM_000582]	NM_000582
A_32_P224094	5.66E-05	19.05	U09850	U09850	Human zinc finger protein (ZNF143) mRNA, complete cds. [U09850]	
A_23_P139965	5.67E-05	3.89	NM_001010897	NM_001010897	Homo sapiens chromosome 13 open reading frame 21 (C13orf21), mRNA [NM_001010897]	NM_001010897
A_24_P66001	5.80E-05	2.20	NM_001003684	NM_001003684	Homo sapiens ubiquinol-cytochrome c reductase complex (7.2 kD) (UCRC), transcript variant 2, mRNA [NM_001003684]	NM_001003684
[truncated: 605,135 more chars]
