# Supplementary material for: Functional and Transcriptional Characterization of Human Embryonic Stem Cell-Derived Endothelial Cells for Treatment of Myocardial Infarction
Source: PLoS One. 2009 Dec 31;4(12):e8443. doi: 10.1371/journal.pone.0008443 (PMC2795856; doi:10.1371/journal.pone.0008443)
Supplement: Table S2 — Over-represented gene ontology (GO) terms in significant gene lists from microarray data. (0.90 MB PDF) [file pone.0008443.s008.pdf]

Supplemental Table S2A

GO Biological Process Categories Overrepresented in hESC vs. EB

GO Category	Genes in Category	% of Genes in Category	Genes in List in Category	% of Genes in List in Category	*P* -Value
GO:6139: nucleobase, nucleoside, nucleotide and nucleic acid metabolism	5227	25.43	482	37.05	6.91E-22
GO:278: mitotic cell cycle	339	1.649	74	5.688	2.34E-21
GO:7049: cell cycle	1151	5.6	158	12.14	5.44E-21
GO:279: M phase	321	1.562	71	5.457	6.97E-21
GO:7067: mitosis	259	1.26	60	4.612	7.09E-19
GO:87: M phase of mitotic cell cycle	264	1.284	60	4.612	1.91E-18
GO:6260: DNA replication	257	1.25	57	4.381	4.52E-17
GO:51301: cell division	301	1.465	61	4.689	3.41E-16
GO:6259: DNA metabolism	971	4.724	129	9.915	4.35E-16
GO:6396: RNA processing	656	3.192	91	6.995	1.05E-12
GO:16070: RNA metabolism	786	3.824	102	7.84	2.73E-12
GO:6261: DNA-dependent DNA replication	129	0.628	33	2.537	2.91E-12
GO:7046: ribosome biogenesis	102	0.496	29	2.229	3.49E-12
GO:43283: biopolymer metabolism	4478	21.79	386	29.67	3.85E-12
GO:44238: primary metabolism	11033	53.68	811	62.34	4.55E-11
GO:50875: cellular physiological process	15952	77.61	1100	84.55	7.24E-11
GO:6364: rRNA processing	71	0.345	22	1.691	2.23E-10
GO:75: cell cycle checkpoint	55	0.268	19	1.46	4.65E-10
GO:44237: cellular metabolism	11473	55.82	832	63.95	4.67E-10
GO:6270: DNA replication initiation	29	0.141	14	1.076	4.88E-10
GO:8152: metabolism	12150	59.12	872	67.03	7.97E-10
GO:16072: rRNA metabolism	76	0.37	22	1.691	9.60E-10
GO:6996: organelle organization and biogenesis	1378	6.705	144	11.07	1.09E-09
GO:74: regulation of progression through cell cycle	732	3.562	89	6.841	2.03E-09
GO:42254: ribosome biogenesis and assembly	131	0.637	29	2.229	2.40E-09
GO:7051: spindle organization and biogenesis	25	0.122	12	0.922	9.46E-09
GO:7028: cytoplasm organization and biogenesis	148	0.72	30	2.306	1.14E-08
GO:6188: IMP biosynthesis	8	0.0389	7	0.538	3.03E-08
GO:6189: 'de novo' IMP biosynthesis	8	0.0389	7	0.538	3.03E-08
GO:46040: IMP metabolism	8	0.0389	7	0.538	3.03E-08
GO:67: DNA replication and chromosome cycle	19	0.0924	10	0.769	5.44E-08
GO:7088: regulation of mitosis	59	0.287	17	1.307	8.28E-08
GO:46112: nucleobase biosynthesis	16	0.0778	9	0.692	1.21E-07
GO:9113: purine base biosynthesis	9	0.0438	7	0.538	1.29E-07
GO:46483: heterocycle metabolism	85	0.414	20	1.537	2.47E-07
GO:51052: regulation of DNA metabolism	50	0.243	15	1.153	2.66E-07
GO:6144: purine base metabolism	10	0.0487	7	0.538	4.06E-07
GO:9112: nucleobase metabolism	18	0.0876	9	0.692	4.58E-07
GO:6275: regulation of DNA replication	23	0.112	10	0.769	5.34E-07
GO:70: mitotic sister chromatid segregation	29	0.141	11	0.846	7.51E-07
GO:31570: DNA integrity checkpoint	19	0.0924	9	0.692	8.21E-07
GO:7093: mitotic checkpoint	15	0.073	8	0.615	1.09E-06

GO:819: sister chromatid segregation	30	0.146	11	0.846	1.12E-06
GO:7059: chromosome segregation	56	0.272	15	1.153	1.34E-06
GO:6520: amino acid metabolism	348	1.693	46	3.536	1.76E-06
GO:43170: macromolecule metabolism	6957	33.85	518	39.82	1.95E-06
GO:51325: interphase	81	0.394	18	1.384	2.37E-06
GO:7017: microtubule-based process	241	1.173	35	2.69	3.68E-06
GO:7094: mitotic spindle checkpoint	6	0.0292	5	0.384	5.74E-06
GO:31577: spindle checkpoint	6	0.0292	5	0.384	5.74E-06
GO:6974: response to DNA damage stimulus	355	1.727	45	3.459	6.95E-06
GO:6519: amino acid and derivative metabolism	411	2	50	3.843	7.05E-06
GO:51329: interphase of mitotic cell cycle	79	0.384	17	1.307	7.14E-06
GO:7582: physiological process	17631	85.78	1167	89.7	8.62E-06
GO:9987: cellular process	18591	90.45	1218	93.62	1.54E-05
GO:6562: proline catabolism	7	0.0341	5	0.384	1.90E-05
GO:6400: tRNA modification	7	0.0341	5	0.384	1.90E-05
GO:30705: cytoskeleton-dependent intracellular transport	149	0.725	24	1.845	2.16E-05
GO:7018: microtubule-based movement	149	0.725	24	1.845	2.16E-05
GO:6399: tRNA metabolism	132	0.642	22	1.691	2.73E-05
GO:9719: response to endogenous stimulus	375	1.825	45	3.459	2.80E-05
GO:16043: cell organization and biogenesis	2519	12.26	207	15.91	3.52E-05
GO:8380: RNA splicing	300	1.46	38	2.921	3.56E-05
GO:6397: mRNA processing	379	1.844	45	3.459	3.64E-05
GO:7052: mitotic spindle organization and biogenesis	17	0.0827	7	0.538	4.44E-05
GO:6725: aromatic compound metabolism	146	0.71	23	1.768	4.56E-05
GO:51276: chromosome organization and biogenesis	506	2.462	55	4.228	6.37E-05
GO:6760: folic acid and derivative metabolism	18	0.0876	7	0.538	6.86E-05
GO:1932: regulation of protein amino acid phosphorylation	24	0.117	8	0.615	7.43E-05
GO:85: G2 phase of mitotic cell cycle	5	0.0243	4	0.307	7.59E-05
GO:51319: G2 phase	5	0.0243	4	0.307	7.59E-05
GO:9064: glutamine family amino acid metabolism	60	0.292	13	0.999	7.79E-05
GO:398: nuclear mRNA splicing, via spliceosome	204	0.993	28	2.152	9.22E-05
GO:375: RNA splicing, via transesterification reactions	204	0.993	28	2.152	9.22E-05
GO:377: RNA splicing, via transesterification reactions with bulged adenosine as nucleophile	204	0.993	28	2.152	9.22E-05
GO:9127: purine nucleoside monophosphate biosynthesis	25	0.122	8	0.615	0.000103
GO:9168: purine ribonucleoside monophosphate biosynthesis	25	0.122	8	0.615	0.000103
GO:9126: purine nucleoside monophosphate metabolism	25	0.122	8	0.615	0.000103
GO:9167: purine ribonucleoside monophosphate metabolism	25	0.122	8	0.615	0.000103
GO:9396: folic acid and derivative biosynthesis	14	0.0681	6	0.461	0.000123
GO:6521: regulation of amino acid metabolism	26	0.127	8	0.615	0.000141
GO:9308: amine metabolism	500	2.433	53	4.074	0.000163
GO:6281: DNA repair	312	1.518	37	2.844	0.000178
GO:6541: glutamine metabolism	21	0.102	7	0.538	0.000212
GO:76: DNA replication checkpoint	6	0.0292	4	0.307	0.000216
GO:6560: proline metabolism	16	0.0778	6	0.461	0.000293
GO:16071: mRNA metabolism	427	2.078	46	3.536	0.0003
GO:42770: DNA damage response, signal transduction	29	0.141	8	0.615	0.000327

GO:42325: regulation of phosphorylation	30	0.146	8	0.615	0.000421
GO:51244: regulation of cellular physiological process	5009	24.37	368	28.29	0.000454
GO:31497: chromatin assembly	149	0.725	21	1.614	0.000464
GO:6626: protein targeting to mitochondrion	31	0.151	8	0.615	0.000536
GO:9156: ribonucleoside monophosphate biosynthesis	32	0.156	8	0.615	0.000676
GO:51174: regulation of phosphorus metabolism	32	0.156	8	0.615	0.000676
GO:19220: regulation of phosphate metabolism	32	0.156	8	0.615	0.000676
GO:9161: ribonucleoside monophosphate metabolism	32	0.156	8	0.615	0.000676
GO:6807: nitrogen compound metabolism	532	2.588	53	4.074	0.000726
GO:19219: regulation of nucleobase, nucleoside, nucleotide and nucleic acid metabolism	3417	16.63	259	19.91	0.000731
GO:6334: nucleosome assembly	134	0.652	19	1.46	0.000785
GO:7001: chromosome organization and biogenesis (sensu Eukaryota)	485	2.36	49	3.766	0.000835
GO:9124: nucleoside monophosphate biosynthesis	33	0.161	8	0.615	0.000843
GO:9123: nucleoside monophosphate metabolism	33	0.161	8	0.615	0.000843
GO:723: telomere maintenance	41	0.199	9	0.692	0.000879
GO:17038: protein import	125	0.608	18	1.384	0.000888
GO:6350: transcription	3517	17.11	265	20.37	0.000892
GO:6913: nucleocytoplasmic transport	203	0.988	25	1.922	0.0011
GO:6403: RNA localization	98	0.477	15	1.153	0.00124
GO:6351: transcription, DNA-dependent	3244	15.78	245	18.83	0.00128
GO:19752: carboxylic acid metabolism	639	3.109	60	4.612	0.00145
GO:9451: RNA modification	28	0.136	7	0.538	0.00146
GO:50791: regulation of physiological process	5180	25.2	374	28.75	0.00147
GO:8033: tRNA processing	71	0.345	12	0.922	0.00156
GO:6777: Mo-molybdopterin cofactor biosynthesis	9	0.0438	4	0.307	0.00156
GO:6271: DNA strand elongation	9	0.0438	4	0.307	0.00156
GO:19720: Mo-molybdopterin cofactor metabolism	9	0.0438	4	0.307	0.00156
GO:6082: organic acid metabolism	641	3.119	60	4.612	0.00156
GO:18193: peptidyl-amino acid modification	62	0.302	11	0.846	0.00161
GO:77: DNA damage checkpoint	15	0.073	5	0.384	0.00177
GO:30032: lamellipodium biogenesis	5	0.0243	3	0.231	0.0023
GO:7164: establishment of tissue polarity	5	0.0243	3	0.231	0.0023
GO:8295: spermidine biosynthesis	5	0.0243	3	0.231	0.0023
GO:9649: entrainment of circadian clock	5	0.0243	3	0.231	0.0023
GO:7010: cytoskeleton organization and biogenesis	602	2.929	56	4.304	0.00252
GO:31323: regulation of cellular metabolism	3642	17.72	269	20.68	0.00254
GO:51327: M phase of meiotic cell cycle	66	0.321	11	0.846	0.0027
GO:7126: meiosis	66	0.321	11	0.846	0.0027
GO:51321: meiotic cell cycle	66	0.321	11	0.846	0.0027
GO:6333: chromatin assembly or disassembly	221	1.075	25	1.922	0.00352
GO:6535: cysteine biosynthesis from serine	2	0.00973	2	0.154	0.004
GO:19343: cysteine biosynthesis via cystathione	2	0.00973	2	0.154	0.004
GO:6597: spermine biosynthesis	2	0.00973	2	0.154	0.004
GO:8215: spermine metabolism	2	0.00973	2	0.154	0.004
GO:45817: positive regulation of global transcription from RNA polymerase II promoter	2	0.00973	2	0.154	0.004
GO:6335: DNA replication-dependent nucleosome assembly	2	0.00973	2	0.154	0.004

GO:46080: dUTP metabolism	2	0.00973	2	0.154	0.004
GO:51169: nuclear transport	189	0.92	22	1.691	0.00424
GO:30879: mammary gland development	6	0.0292	3	0.231	0.00438
GO:8216: spermidine metabolism	6	0.0292	3	0.231	0.00438
GO:6273: lagging strand elongation	6	0.0292	3	0.231	0.00438
GO:6269: DNA replication, synthesis of RNA primer	6	0.0292	3	0.231	0.00438
GO:45449: regulation of transcription	3356	16.33	247	18.99	0.00469
GO:6355: regulation of transcription, DNA-dependent	3150	15.33	233	17.91	0.00482
GO:6323: DNA packaging	427	2.078	41	3.151	0.00522
GO:6561: proline biosynthesis	12	0.0584	4	0.307	0.00525
GO:9262: deoxyribonucleotide metabolism	12	0.0584	4	0.307	0.00525
GO:48015: phosphoinositide-mediated signaling	105	0.511	14	1.076	0.00639
GO:50794: regulation of cellular process	5371	26.13	379	29.13	0.00641
GO:50657: nucleic acid transport	95	0.462	13	0.999	0.00678
GO:50658: RNA transport	95	0.462	13	0.999	0.00678
GO:51236: establishment of RNA localization	95	0.462	13	0.999	0.00678
GO:7004: telomerase-dependent telomere maintenance	20	0.0973	5	0.384	0.00703
GO:8625: induction of apoptosis via death domain receptors	13	0.0633	4	0.307	0.0072
GO:79: regulation of cyclin dependent protein kinase activity	55	0.268	9	0.692	0.00722
GO:16246: RNA interference	7	0.0341	3	0.231	0.0073
GO:6474: N-terminal protein amino acid acetylation	7	0.0341	3	0.231	0.0073
GO:45039: protein import into mitochondrial inner membrane	7	0.0341	3	0.231	0.0073
GO:8652: amino acid biosynthesis	75	0.365	11	0.846	0.00733
GO:51028: mRNA transport	75	0.365	11	0.846	0.00733
GO:6606: protein import into nucleus	108	0.525	14	1.076	0.00817
GO:15931: nucleobase, nucleoside, nucleotide and nucleic acid transport	108	0.525	14	1.076	0.00817
GO:51170: nuclear import	108	0.525	14	1.076	0.00817
GO:9309: amine biosynthesis	109	0.53	14	1.076	0.00884
GO:44271: nitrogen compound biosynthesis	109	0.53	14	1.076	0.00884
GO:6325: establishment and/or maintenance of chromatin architecture	415	2.019	39	2.998	0.00892
GO:45893: positive regulation of transcription, DNA-dependent	143	0.696	17	1.307	0.00903
GO:7076: mitotic chromosome condensation	14	0.0681	4	0.307	0.00958
GO:40029: regulation of gene expression, epigenetic	78	0.38	11	0.846	0.00981

Supplemental Table S2B

GO Biological Process Categories Overrepresented in EB vs. hESC

GO Category	Genes in Category	% of Genes in Category	Genes in List in Category	% of Genes in List in Category	*P* -Value
GO:6817: phosphate transport	146	0.71	41	2.692	4.18E-14
GO:48513: organ development	907	4.413	129	8.47	3.40E-13
GO:50878: regulation of body fluids	157	0.764	40	2.626	2.68E-12
GO:7596: blood coagulation	132	0.642	36	2.364	3.81E-12
GO:50817: coagulation	139	0.676	37	2.429	4.27E-12
GO:7599: hemostasis	140	0.681	37	2.429	5.39E-12
GO:42060: wound healing	145	0.705	37	2.429	1.67E-11
GO:1944: vasculature development	115	0.56	30	1.97	7.62E-10
GO:15698: inorganic anion transport	229	1.114	44	2.889	4.30E-09
GO:48514: blood vessel morphogenesis	113	0.55	28	1.838	9.64E-09
GO:1568: blood vessel development	113	0.55	28	1.838	9.64E-09
GO:1501: skeletal development	204	0.993	40	2.626	1.18E-08
GO:7275: development	2960	14.4	294	19.3	2.96E-08
GO:6820: anion transport	271	1.319	47	3.086	3.76E-08
GO:1525: angiogenesis	108	0.525	26	1.707	6.07E-08
GO:7169: transmembrane receptor protein tyrosine kinase signaling pathway	228	1.109	39	2.561	7.62E-07
GO:48523: negative regulation of cellular process	1122	5.459	124	8.142	3.95E-06
GO:48519: negative regulation of biological process	1223	5.95	133	8.733	4.02E-06
GO:9887: organ morphogenesis	310	1.508	46	3.02	4.90E-06
GO:7167: enzyme linked receptor protein signaling pathway	310	1.508	46	3.02	4.90E-06
GO:9888: tissue development	263	1.28	39	2.561	2.55E-05
GO:50819: negative regulation of coagulation	30	0.146	10	0.657	3.68E-05
GO:50818: regulation of coagulation	30	0.146	10	0.657	3.68E-05
GO:9653: morphogenesis	940	4.574	103	6.763	3.74E-05
GO:43118: negative regulation of physiological process	1056	5.138	113	7.42	4.37E-05
GO:9611: response to wounding	554	2.695	67	4.399	4.91E-05
GO:31214: biomineral formation	58	0.282	14	0.919	6.49E-05
GO:1503: ossification	58	0.282	14	0.919	6.49E-05
GO:7264: small GTPase mediated signal transduction	520	2.53	63	4.137	7.75E-05
GO:46849: bone remodeling	60	0.292	14	0.919	9.65E-05
GO:6508: proteolysis	950	4.622	101	6.632	0.000141
GO:6888: ER to Golgi transport	55	0.268	13	0.854	0.000148
GO:7154: cell communication	5496	26.74	468	30.73	0.000172
GO:6953: acute-phase response	36	0.175	10	0.657	0.000207
GO:30195: negative regulation of blood coagulation	19	0.0924	7	0.46	0.000275
GO:30193: regulation of blood coagulation	19	0.0924	7	0.46	0.000275
GO:8015: circulation	171	0.832	26	1.707	0.000361
GO:30323: respiratory tube development	10	0.0487	5	0.328	0.000408
GO:51243: negative regulation of cellular physiological process	1016	4.943	104	6.829	0.000455
GO:16477: cell migration	147	0.715	23	1.51	0.00051
GO:42730: fibrinolysis	16	0.0778	6	0.394	0.000686

GO:8283: cell proliferation	810	3.941	85	5.581	0.0007
GO:9605: response to external stimulus	1052	5.118	106	6.96	0.000701
GO:30154: cell differentiation	820	3.99	85	5.581	0.00101
GO:51181: cofactor transport	12	0.0584	5	0.328	0.00113
GO:6879: iron ion homeostasis	38	0.185	9	0.591	0.0015
GO:46329: negative regulation of JNK cascade	4	0.0195	3	0.197	0.00153
GO:15781: pyrimidine nucleotide-sugar transport	8	0.0389	4	0.263	0.00165
GO:50874: organismal physiological process	2704	13.16	238	15.63	0.00207
GO:6040: amino sugar metabolism	40	0.195	9	0.591	0.0022
GO:9968: negative regulation of signal transduction	98	0.477	16	1.051	0.0022
GO:46916: transition metal ion homeostasis	48	0.234	10	0.657	0.00236
GO:30324: lung development	9	0.0438	4	0.263	0.0028
GO:6349: imprinting	9	0.0438	4	0.263	0.0028
GO:6041: glucosamine metabolism	34	0.165	8	0.525	0.00284
GO:8277: regulation of G-protein coupled receptor protein signaling pathway	42	0.204	9	0.591	0.00314
GO:6030: chitin metabolism	15	0.073	5	0.328	0.00355
GO:30279: negative regulation of ossification	5	0.0243	3	0.197	0.00362
GO:46851: negative regulation of bone remodeling	5	0.0243	3	0.197	0.00362
GO:1570: vasculogenesis	10	0.0487	4	0.263	0.00439
GO:35295: tube development	29	0.141	7	0.46	0.00443
GO:51180: vitamin transport	16	0.0778	5	0.328	0.00486
GO:43067: regulation of programmed cell death	476	2.316	51	3.349	0.00508
GO:6629: lipid metabolism	870	4.233	85	5.581	0.00525
GO:51216: cartilage development	30	0.146	7	0.46	0.00542
GO:30100: regulation of endocytosis	30	0.146	7	0.46	0.00542
GO:7155: cell adhesion	1002	4.875	96	6.303	0.00546
GO:1946: lymphangiogenesis	2	0.00973	2	0.131	0.00549
GO:7499: ectoderm and mesoderm interaction	2	0.00973	2	0.131	0.00549
GO:48699: neurogenesis	2	0.00973	2	0.131	0.00549
GO:1945: lymph vessel development	2	0.00973	2	0.131	0.00549
GO:19471: 4-hydroxyproline metabolism	2	0.00973	2	0.131	0.00549
GO:18401: peptidyl-proline hydroxylation to 4-hydroxy-L-proline	2	0.00973	2	0.131	0.00549
GO:1574: ganglioside biosynthesis	2	0.00973	2	0.131	0.00549
GO:18208: peptidyl-proline modification	2	0.00973	2	0.131	0.00549
GO:19511: peptidyl-proline hydroxylation	2	0.00973	2	0.131	0.00549
GO:15782: CMP-sialic acid transport	2	0.00973	2	0.131	0.00549
GO:15789: UDP-N-acetylgalactosamine transport	2	0.00973	2	0.131	0.00549
GO:6907: pinocytosis	2	0.00973	2	0.131	0.00549
GO:46627: negative regulation of insulin receptor signaling pathway	2	0.00973	2	0.131	0.00549
GO:7411: axon guidance	54	0.263	10	0.657	0.00576
GO:7165: signal transduction	4419	21.5	367	24.1	0.00613
GO:8219: cell death	840	4.087	82	5.384	0.00617
GO:8285: negative regulation of cell proliferation	240	1.168	29	1.904	0.00635
GO:6575: amino acid derivative metabolism	81	0.394	13	0.854	0.00636
GO:6662: glycerol ether metabolism	17	0.0827	5	0.328	0.00646
GO:6639: acylglycerol metabolism	17	0.0827	5	0.328	0.00646
GO:6638: neutral lipid metabolism	17	0.0827	5	0.328	0.00646

GO:46486: glycerolipid metabolism	17	0.0827	5	0.328	0.00646
GO:30278: regulation of ossification	11	0.0535	4	0.263	0.00649
GO:46850: regulation of bone remodeling	11	0.0535	4	0.263	0.00649
GO:15674: di-, tri-valent inorganic cation transport	209	1.017	26	1.707	0.0065
GO:6869: lipid transport	119	0.579	17	1.116	0.0067
GO:50766: positive regulation of phagocytosis	6	0.0292	3	0.197	0.00685
GO:50764: regulation of phagocytosis	6	0.0292	3	0.197	0.00685
GO:16265: death	844	4.106	82	5.384	0.00694
GO:42127: regulation of cell proliferation	439	2.136	47	3.086	0.00704
GO:41: transition metal ion transport	82	0.399	13	0.854	0.00706
GO:6044: N-acetylglucosamine metabolism	32	0.156	7	0.46	0.00787
GO:9880: embryonic pattern specification	18	0.0876	5	0.328	0.00841
GO:44255: cellular lipid metabolism	687	3.343	68	4.465	0.00884
GO:9628: response to abiotic stimulus	617	3.002	62	4.071	0.00886
GO:15780: nucleotide-sugar transport	12	0.0584	4	0.263	0.00917
GO:42981: regulation of apoptosis	469	2.282	49	3.217	0.00941

Supplemental Table S2C

GO Biological Process Categories Overrepresented in EB vs. hESC-EC

GO Category	Genes in Category	% of Genes in Category	Genes in List in Category	% of Genes in List in Category	*P*-Value
GO:6355: regulation of transcription, DNA-dependent	3150	15.33	453	19.92	2.45E-10
GO:50789: regulation of biological process	5781	28.13	766	33.69	4.36E-10
GO:6351: transcription, DNA-dependent	3244	15.78	459	20.18	1.80E-09
GO:19219: regulation of nucleobase, nucleoside, nucleotide and nucleic acid metabolism	3417	16.63	479	21.06	2.64E-09
GO:45449: regulation of transcription	3356	16.33	471	20.71	3.21E-09
GO:50794: regulation of cellular process	5371	26.13	709	31.18	6.33E-09
GO:50791: regulation of physiological process	5180	25.2	686	30.17	7.56E-09
GO:31323: regulation of cellular metabolism	3642	17.72	500	21.99	2.03E-08
GO:6350: transcription	3517	17.11	482	21.2	5.07E-08
GO:51244: regulation of cellular physiological process	5009	24.37	659	28.98	5.36E-08
GO:19222: regulation of metabolism	3747	18.23	508	22.34	8.31E-08
GO:6817: phosphate transport	146	0.71	37	1.627	9.71E-07
GO:48513: organ development	907	4.413	145	6.376	2.75E-06
GO:8015: circulation	171	0.832	40	1.759	3.21E-06
GO:50878: regulation of body fluids	157	0.764	36	1.583	1.56E-05
GO:6139: nucleobase, nucleoside, nucleotide and nucleic acid metabolism	5227	25.43	661	29.07	1.69E-05
GO:7596: blood coagulation	132	0.642	31	1.363	3.62E-05
GO:7599: hemostasis	140	0.681	32	1.407	4.83E-05
GO:7167: enzyme linked receptor protein signaling pathway	310	1.508	57	2.507	8.05E-05
GO:7169: transmembrane receptor protein tyrosine kinase signaling pathway	228	1.109	45	1.979	8.11E-05
GO:42060: wound healing	145	0.705	32	1.407	9.86E-05
GO:50817: coagulation	139	0.676	31	1.363	0.000102
GO:15698: inorganic anion transport	229	1.114	44	1.935	0.000183
GO:1501: skeletal development	204	0.993	40	1.759	0.000226
GO:7275: development	2960	14.4	381	16.75	0.000482
GO:6930: substrate-bound cell migration, cell extension	5	0.0243	4	0.176	0.000681
GO:6402: mRNA catabolism	36	0.175	11	0.484	0.00128
GO:184: mRNA catabolism, nonsense-mediated decay	31	0.151	10	0.44	0.00134
GO:8286: insulin receptor signaling pathway	31	0.151	10	0.44	0.00134
GO:16477: cell migration	147	0.715	29	1.275	0.0014
GO:30324: lung development	9	0.0438	5	0.22	0.00142
GO:51260: protein homooligomerization	22	0.107	8	0.352	0.0017
GO:46834: lipid phosphorylation	6	0.0292	4	0.176	0.00186
GO:46854: phosphoinositide phosphorylation	6	0.0292	4	0.176	0.00186
GO:46928: regulation of neurotransmitter secretion	6	0.0292	4	0.176	0.00186
GO:7155: cell adhesion	1002	4.875	140	6.157	0.00203
GO:48731: system development	739	3.596	107	4.705	0.00214
GO:7399: nervous system development	735	3.576	106	4.661	0.00254
GO:30323: respiratory tube development	10	0.0487	5	0.22	0.00258
GO:30195: negative regulation of blood coagulation	19	0.0924	7	0.308	0.00304
GO:30193: regulation of blood coagulation	19	0.0924	7	0.308	0.00304

GO:31214: biomineral formation	58	0.282	14	0.616	0.00364
GO:1503: ossification	58	0.282	14	0.616	0.00364
GO:30902: hindbrain development	7	0.0341	4	0.176	0.00397
GO:50819: negative regulation of coagulation	30	0.146	9	0.396	0.00401
GO:50818: regulation of coagulation	30	0.146	9	0.396	0.00401
GO:30048: actin filament-based movement	20	0.0973	7	0.308	0.00423
GO:30278: regulation of ossification	11	0.0535	5	0.22	0.00429
GO:46850: regulation of bone remodeling	11	0.0535	5	0.22	0.00429
GO:48523: negative regulation of cellular process	1122	5.459	152	6.684	0.00446
GO:48519: negative regulation of biological process	1223	5.95	164	7.212	0.00479
GO:30147: natriuresis	4	0.0195	3	0.132	0.00496
GO:30146: diuresis	4	0.0195	3	0.132	0.00496
GO:46849: bone remodeling	60	0.292	14	0.616	0.00505
GO:6869: lipid transport	119	0.579	23	1.011	0.00538
GO:42730: fibrinolysis	16	0.0778	6	0.264	0.00546
GO:1508: regulation of action potential	16	0.0778	6	0.264	0.00546
GO:6820: anion transport	271	1.319	44	1.935	0.00598
GO:19884: antigen presentation, exogenous antigen	32	0.156	9	0.396	0.00642
GO:6928: cell motility	356	1.732	55	2.419	0.00669
GO:51674: localization of cell	356	1.732	55	2.419	0.00669
GO:40011: locomotion	356	1.732	55	2.419	0.00669
GO:51181: cofactor transport	12	0.0584	5	0.22	0.0067
GO:6909: phagocytosis	38	0.185	10	0.44	0.00693
GO:6929: substrate-bound cell migration	8	0.0389	4	0.176	0.00724
GO:43118: negative regulation of physiological process	1056	5.138	142	6.245	0.00757
GO:7588: excretion	64	0.311	14	0.616	0.00917
GO:50954: sensory perception of mechanical stimulus	174	0.847	30	1.319	0.00917
GO:7605: sensory perception of sound	174	0.847	30	1.319	0.00917
GO:6939: smooth muscle contraction	34	0.165	9	0.396	0.00981
GO:19886: antigen processing, exogenous antigen via MHC class II	34	0.165	9	0.396	0.00981
GO:16525: negative regulation of angiogenesis	13	0.0633	5	0.22	0.0099

Supplemental Table S2D

GO Biological Process Categories Overrepresented in hESC-EC vs. EB

GO Category	Genes in Category	% of Genes in Category	Genes in List in Category	% of Genes in List in Category	*P*-Value
GO:48522: positive regulation of cellular process	815	3.965	154	6.121	1.72E-08
GO:7243: protein kinase cascade	482	2.345	101	4.014	3.23E-08
GO:48518: positive regulation of biological process	975	4.744	175	6.955	8.00E-08
GO:6915: apoptosis	790	3.844	147	5.843	9.83E-08
GO:6917: induction of apoptosis	213	1.036	54	2.146	1.10E-07
GO:12502: induction of programmed cell death	214	1.041	54	2.146	1.29E-07
GO:12501: programmed cell death	796	3.873	147	5.843	1.58E-07
GO:42981: regulation of apoptosis	469	2.282	96	3.816	2.23E-07
GO:43065: positive regulation of apoptosis	235	1.143	57	2.266	2.48E-07
GO:43068: positive regulation of programmed cell death	238	1.158	57	2.266	3.89E-07
GO:43067: regulation of programmed cell death	476	2.316	96	3.816	4.54E-07
GO:16265: death	844	4.106	152	6.041	4.73E-07
GO:7249: I-kappaB kinase/NF-kappaB cascade	200	0.973	50	1.987	5.04E-07
GO:8219: cell death	840	4.087	151	6.002	5.78E-07
GO:43123: positive regulation of I-kappaB kinase/NF-kappaB cascade	150	0.73	40	1.59	1.18E-06
GO:43122: regulation of I-kappaB kinase/NF-kappaB cascade	158	0.769	41	1.63	1.88E-06
GO:16043: cell organization and biogenesis	2519	12.26	380	15.1	3.35E-06
GO:30029: actin filament-based process	246	1.197	55	2.186	5.87E-06
GO:74: regulation of progression through cell cycle	732	3.562	130	5.167	6.62E-06
GO:51242: positive regulation of cellular physiological process	653	3.177	118	4.69	7.70E-06
GO:7049: cell cycle	1151	5.6	188	7.472	1.63E-05
GO:8632: apoptotic program	95	0.462	27	1.073	1.78E-05
GO:9967: positive regulation of signal transduction	172	0.837	41	1.63	1.78E-05
GO:43119: positive regulation of physiological process	681	3.313	120	4.769	2.14E-05
GO:7010: cytoskeleton organization and biogenesis	602	2.929	108	4.293	2.53E-05
GO:7046: ribosome biogenesis	102	0.496	28	1.113	2.54E-05
GO:30036: actin cytoskeleton organization and biogenesis	229	1.114	50	1.987	2.97E-05
GO:8624: induction of apoptosis by extracellular signals	45	0.219	16	0.636	4.74E-05
GO:9451: RNA modification	28	0.136	12	0.477	5.04E-05
GO:7242: intracellular signaling cascade	1818	8.845	275	10.93	7.41E-05
GO:7178: transmembrane receptor protein serine/threonine kinase signaling pathway	64	0.311	19	0.755	0.00016
GO:48519: negative regulation of biological process	1223	5.95	191	7.591	0.000187
GO:48523: negative regulation of cellular process	1122	5.459	177	7.035	0.000191
GO:45210: FasL biosynthesis	4	0.0195	4	0.159	0.000224
GO:6858: extracellular transport	4	0.0195	4	0.159	0.000224
GO:6364: rRNA processing	71	0.345	20	0.795	0.000239
GO:9605: response to external stimulus	1052	5.118	166	6.598	0.000297
GO:43085: positive regulation of enzyme activity	170	0.827	37	1.471	0.000324
GO:8283: cell proliferation	810	3.941	132	5.246	0.000324
GO:6919: caspase activation	52	0.253	16	0.636	0.000329
GO:43280: positive regulation of caspase activity	52	0.253	16	0.636	0.000329

GO:43281: regulation of caspase activity	52	0.253	16	0.636	0.000329
GO:16192: vesicle-mediated transport	580	2.822	99	3.935	0.000357
GO:8625: induction of apoptosis via death domain receptors	13	0.0633	7	0.278	0.000357
GO:51338: regulation of transferase activity	197	0.958	41	1.63	0.000429
GO:45859: regulation of protein kinase activity	191	0.929	40	1.59	0.000439
GO:7595: lactation	10	0.0487	6	0.238	0.000452
GO:154: rRNA modification	7	0.0341	5	0.199	0.000464
GO:6636: fatty acid desaturation	7	0.0341	5	0.199	0.000464
GO:51345: positive regulation of hydrolase activity	54	0.263	16	0.636	0.000528
GO:44260: cellular macromolecule metabolism	5196	25.28	703	27.94	0.000629
GO:16072: rRNA metabolism	76	0.37	20	0.795	0.000633
GO:19538: protein metabolism	5170	25.15	699	27.78	0.000709
GO:6626: protein targeting to mitochondrion	31	0.151	11	0.437	0.000734
GO:7015: actin filament organization	46	0.224	14	0.556	0.000862
GO:45860: positive regulation of protein kinase activity	78	0.38	20	0.795	0.000904
GO:51258: protein polymerization	62	0.302	17	0.676	0.000956
GO:51347: positive regulation of transferase activity	84	0.409	21	0.835	0.000971
GO:7162: negative regulation of cell adhesion	23	0.112	9	0.358	0.000992
GO:42254: ribosome biogenesis and assembly	131	0.637	29	1.153	0.00103
GO:50790: regulation of enzyme activity	377	1.834	67	2.663	0.00105
GO:44267: cellular protein metabolism	5126	24.94	691	27.46	0.00106
GO:6099: tricarboxylic acid cycle	37	0.18	12	0.477	0.00107
GO:8637: apoptotic mitochondrial changes	19	0.0924	8	0.318	0.00108
GO:6996: organelle organization and biogenesis	1378	6.705	206	8.188	0.00111
GO:51649: establishment of cellular localization	1034	5.031	159	6.32	0.00125
GO:9966: regulation of signal transduction	442	2.151	76	3.021	0.00132
GO:7165: signal transduction	4419	21.5	600	23.85	0.00133
GO:46356: acetyl-CoA catabolism	38	0.185	12	0.477	0.00139
GO:7264: small GTPase mediated signal transduction	520	2.53	87	3.458	0.00146
GO:15031: protein transport	1009	4.909	155	6.161	0.0015
GO:51641: cellular localization	1047	5.094	160	6.359	0.00158
GO:46907: intracellular transport	1025	4.987	157	6.24	0.00158
GO:9615: response to virus	82	0.399	20	0.795	0.00175
GO:7179: transforming growth factor beta receptor signaling pathway	44	0.214	13	0.517	0.00177
GO:19509: methionine salvage	3	0.0146	3	0.119	0.00183
GO:43102: amino acid salvage	3	0.0146	3	0.119	0.00183
GO:18345: protein palmitoylation	3	0.0146	3	0.119	0.00183
GO:43405: regulation of MAPK activity	83	0.404	20	0.795	0.00205
GO:187: activation of MAPK activity	45	0.219	13	0.517	0.00221
GO:7154: cell communication	5496	26.74	732	29.09	0.00252
GO:1525: angiogenesis	108	0.525	24	0.954	0.00253
GO:42060: wound healing	145	0.705	30	1.192	0.0026
GO:902: cellular morphogenesis	453	2.204	76	3.021	0.0026
GO:45786: negative regulation of progression through cell cycle	255	1.241	47	1.868	0.00263
GO:50654: chondroitin sulfate proteoglycan metabolism	13	0.0633	6	0.238	0.00266
GO:30166: proteoglycan biosynthesis	26	0.127	9	0.358	0.00269
GO:6893: Golgi to plasma membrane transport	6	0.0292	4	0.159	0.00274

GO:9408: response to heat	6	0.0292	4	0.159	0.00274
GO:43406: positive regulation of MAPK activity	46	0.224	13	0.517	0.00274
GO:9109: coenzyme catabolism	41	0.199	12	0.477	0.00286
GO:8361: regulation of cell size	245	1.192	45	1.789	0.00342
GO:16049: cell growth	245	1.192	45	1.789	0.00342
GO:7028: cytoplasm organization and biogenesis	148	0.72	30	1.192	0.00358
GO:8104: protein localization	1076	5.235	161	6.399	0.00362
GO:9396: folic acid and derivative biosynthesis	14	0.0681	6	0.238	0.00418
GO:6897: endocytosis	248	1.207	45	1.789	0.00432
GO:9058: biosynthesis	1974	9.604	279	11.09	0.0044
GO:51243: negative regulation of cellular physiological process	1016	4.943	152	6.041	0.00464
GO:48514: blood vessel morphogenesis	113	0.55	24	0.954	0.00468
GO:1568: blood vessel development	113	0.55	24	0.954	0.00468
GO:6399: tRNA metabolism	132	0.642	27	1.073	0.00485
GO:6937: regulation of muscle contraction	49	0.238	13	0.517	0.00498
GO:30148: sphingolipid biosynthesis	19	0.0924	7	0.278	0.00539
GO:9266: response to temperature stimulus	19	0.0924	7	0.278	0.00539
GO:6790: sulfur metabolism	102	0.496	22	0.874	0.00544
GO:9186: deoxyribonucleoside diphosphate metabolism	7	0.0341	4	0.159	0.00577
GO:1944: vasculature development	115	0.56	24	0.954	0.00589
GO:45184: establishment of protein localization	1045	5.084	155	6.161	0.00589
GO:6914: autophagy	34	0.165	10	0.397	0.00604
GO:9247: glycolipid biosynthesis	15	0.073	6	0.238	0.00626
GO:6633: fatty acid biosynthesis	79	0.384	18	0.715	0.00628
GO:44272: sulfur compound biosynthesis	56	0.272	14	0.556	0.00645
GO:51187: cofactor catabolism	45	0.219	12	0.477	0.00656
GO:9060: aerobic respiration	45	0.219	12	0.477	0.00656
GO:6944: membrane fusion	45	0.219	12	0.477	0.00656
GO:43118: negative regulation of physiological process	1056	5.138	156	6.2	0.00666
GO:50650: chondroitin sulfate proteoglycan biosynthesis	11	0.0535	5	0.199	0.00668
GO:43037: translation	289	1.406	50	1.987	0.00715
GO:8610: lipid biosynthesis	319	1.552	54	2.146	0.00831
GO:42157: lipoprotein metabolism	94	0.457	20	0.795	0.0091
GO:6084: acetyl-CoA metabolism	47	0.229	12	0.477	0.00946
GO:8037: cell recognition	31	0.151	9	0.358	0.00988

Supplemental Table S2E

GO Biological Process Categories Overrepresented in hESC-EC vs. HUVEC

GO Category	Genes in Category	% of Genes in Category	Genes in List in Category	% of Genes in List in Category	*P* -Value
GO:7275: development	2960	14.4	317	22.64	7.05E-18
GO:48513: organ development	907	4.413	125	8.929	1.93E-14
GO:1501: skeletal development	204	0.993	42	3	8.43E-11
GO:6817: phosphate transport	146	0.71	33	2.357	7.08E-10
GO:7154: cell communication	5496	26.74	470	33.57	2.86E-09
GO:7155: cell adhesion	1002	4.875	116	8.286	1.03E-08
GO:9653: morphogenesis	940	4.574	110	7.857	1.42E-08
GO:50874: organismal physiological process	2704	13.16	252	18	6.00E-08
GO:7399: nervous system development	735	3.576	89	6.357	7.74E-08
GO:48731: system development	739	3.596	89	6.357	9.91E-08
GO:42445: hormone metabolism	70	0.341	17	1.214	3.29E-06
GO:6334: nucleosome assembly	134	0.652	25	1.786	3.59E-06
GO:8015: circulation	171	0.832	29	2.071	4.58E-06
GO:6349: imprinting	9	0.0438	6	0.429	6.94E-06
GO:51239: regulation of organismal physiological process	193	0.939	31	2.214	6.94E-06
GO:6936: muscle contraction	232	1.129	35	2.5	7.72E-06
GO:31497: chromatin assembly	149	0.725	26	1.857	8.27E-06
GO:7169: transmembrane receptor protein tyrosine kinase signaling pathway	228	1.109	34	2.429	1.33E-05
GO:9887: organ morphogenesis	310	1.508	42	3	1.57E-05
GO:51056: regulation of small GTPase mediated signal transduction	94	0.457	19	1.357	1.59E-05
GO:6750: glutathione biosynthesis	19	0.0924	8	0.571	1.74E-05
GO:9605: response to external stimulus	1052	5.118	107	7.643	1.79E-05
GO:48010: vascular endothelial growth factor receptor signaling pathway	4	0.0195	4	0.286	2.14E-05
GO:30947: regulation of vascular endothelial growth factor receptor signaling pathway	4	0.0195	4	0.286	2.14E-05
GO:30949: positive regulation of vascular endothelial growth factor receptor signaling pathway	4	0.0195	4	0.286	2.14E-05
GO:51241: negative regulation of organismal physiological process	32	0.156	10	0.714	3.36E-05
GO:7167: enzyme linked receptor protein signaling pathway	310	1.508	41	2.929	3.47E-05
GO:15698: inorganic anion transport	229	1.114	33	2.357	3.60E-05
GO:16055: Wnt receptor signaling pathway	162	0.788	26	1.857	3.76E-05
GO:35023: regulation of Rho protein signal transduction	46	0.224	12	0.857	4.22E-05
GO:6954: inflammatory response	294	1.43	39	2.786	4.99E-05
GO:43062: extracellular structure organization and biogenesis	78	0.38	16	1.143	6.04E-05
GO:30198: extracellular matrix organization and biogenesis	78	0.38	16	1.143	6.04E-05
GO:50918: positive chemotaxis	8	0.0389	5	0.357	6.85E-05
GO:50926: regulation of positive chemotaxis	8	0.0389	5	0.357	6.85E-05
GO:50927: positive regulation of positive chemotaxis	8	0.0389	5	0.357	6.85E-05
GO:50930: induction of positive chemotaxis	8	0.0389	5	0.357	6.85E-05
GO:44272: sulfur compound biosynthesis	56	0.272	13	0.929	7.69E-05
GO:9611: response to wounding	554	2.695	62	4.429	7.95E-05
GO:6749: glutathione metabolism	23	0.112	8	0.571	8.83E-05
GO:16051: carbohydrate biosynthesis	115	0.56	20	1.429	9.24E-05

GO:9308: amine metabolism	500	2.433	57	4.071	9.27E-05
GO:6820: anion transport	271	1.319	36	2.571	9.28E-05
GO:30279: negative regulation of ossification	5	0.0243	4	0.286	0.000101
GO:46851: negative regulation of bone remodeling	5	0.0243	4	0.286	0.000101
GO:6953: acute-phase response	36	0.175	10	0.714	0.000103
GO:42446: hormone biosynthesis	43	0.209	11	0.786	0.000106
GO:31214: biomineral formation	58	0.282	13	0.929	0.000113
GO:1503: ossification	58	0.282	13	0.929	0.000113
GO:9309: amine biosynthesis	109	0.53	19	1.357	0.000133
GO:44271: nitrogen compound biosynthesis	109	0.53	19	1.357	0.000133
GO:7517: muscle development	255	1.241	34	2.429	0.000133
GO:50920: regulation of chemotaxis	9	0.0438	5	0.357	0.000145
GO:50921: positive regulation of chemotaxis	9	0.0438	5	0.357	0.000145
GO:9966: regulation of signal transduction	442	2.151	51	3.643	0.000158
GO:46849: bone remodeling	60	0.292	13	0.929	0.000163
GO:6790: sulfur metabolism	102	0.496	18	1.286	0.000168
GO:6519: amino acid and derivative metabolism	411	2	48	3.429	0.000183
GO:45807: positive regulation of endocytosis	14	0.0681	6	0.429	0.000184
GO:7417: central nervous system development	169	0.822	25	1.786	0.000201
GO:6807: nitrogen compound metabolism	532	2.588	58	4.143	0.000266
GO:1944: vasculature development	115	0.56	19	1.357	0.000273
GO:7267: cell-cell signaling	750	3.649	76	5.429	0.000327
GO:19886: antigen processing, exogenous antigen via MHC class II	34	0.165	9	0.643	0.00034
GO:1525: angiogenesis	108	0.525	18	1.286	0.000351
GO:6937: regulation of muscle contraction	49	0.238	11	0.786	0.00037
GO:16337: cell-cell adhesion	302	1.469	37	2.643	0.000386
GO:30278: regulation of ossification	11	0.0535	5	0.357	0.000475
GO:46850: regulation of bone remodeling	11	0.0535	5	0.357	0.000475
GO:7156: homophilic cell adhesion	181	0.881	25	1.786	0.000579
GO:48514: blood vessel morphogenesis	113	0.55	18	1.286	0.000615
GO:1568: blood vessel development	113	0.55	18	1.286	0.000615
GO:6730: one-carbon compound metabolism	60	0.292	12	0.857	0.000626
GO:42553: cellular nerve ensheathment	7	0.0341	4	0.286	0.000635
GO:7272: ionic insulation of neurons by glial cells	7	0.0341	4	0.286	0.000635
GO:42552: myelination	7	0.0341	4	0.286	0.000635
GO:30104: water homeostasis	7	0.0341	4	0.286	0.000635
GO:30154: cell differentiation	820	3.99	80	5.714	0.000728
GO:8286: insulin receptor signaling pathway	31	0.151	8	0.571	0.000871
GO:6575: amino acid derivative metabolism	81	0.394	14	1	0.00106
GO:19884: antigen presentation, exogenous antigen	32	0.156	8	0.571	0.00109
GO:30502: negative regulation of bone mineralization	4	0.0195	3	0.214	0.0012
GO:45210: FasL biosynthesis	4	0.0195	3	0.214	0.0012
GO:6883: sodium ion homeostasis	4	0.0195	3	0.214	0.0012
GO:8652: amino acid biosynthesis	75	0.365	13	0.929	0.00154
GO:15669: gas transport	35	0.17	8	0.571	0.00204
GO:15671: oxygen transport	35	0.17	8	0.571	0.00204
GO:9888: tissue development	263	1.28	31	2.214	0.00204

GO:30500: regulation of bone mineralization	9	0.0438	4	0.286	0.00205
GO:6725: aromatic compound metabolism	146	0.71	20	1.429	0.00217
GO:9064: glutamine family amino acid metabolism	60	0.292	11	0.786	0.00219
GO:6541: glutamine metabolism	21	0.102	6	0.429	0.0022
GO:50878: regulation of body fluids	157	0.764	21	1.5	0.00229
GO:6955: immune response	1148	5.586	103	7.357	0.00235
GO:16477: cell migration	147	0.715	20	1.429	0.00235
GO:30282: bone mineralization	15	0.073	5	0.357	0.00245
GO:8209: androgen metabolism	5	0.0243	3	0.214	0.00284
GO:19752: carboxylic acid metabolism	639	3.109	62	4.429	0.00311
GO:6952: defense response	1276	6.208	112	8	0.00315
GO:1570: vasculogenesis	10	0.0487	4	0.286	0.00323
GO:6082: organic acid metabolism	641	3.119	62	4.429	0.00334
GO:42730: fibrinolysis	16	0.0778	5	0.357	0.00337
GO:1508: regulation of action potential	16	0.0778	5	0.357	0.00337
GO:50819: negative regulation of coagulation	30	0.146	7	0.5	0.0034
GO:50818: regulation of coagulation	30	0.146	7	0.5	0.0034
GO:7165: signal transduction	4419	21.5	342	24.43	0.00351
GO:6629: lipid metabolism	870	4.233	80	5.714	0.00371
GO:50776: regulation of immune response	94	0.457	14	1	0.00447
GO:9084: glutamine family amino acid biosynthesis	17	0.0827	5	0.357	0.00451
GO:46578: regulation of Ras protein signal transduction	17	0.0827	5	0.357	0.00451
GO:7223: frizzled-2 signaling pathway	24	0.117	6	0.429	0.00458
GO:6333: chromatin assembly or disassembly	221	1.075	26	1.857	0.00459
GO:7499: ectoderm and mesoderm interaction	2	0.00973	2	0.143	0.00464
GO:46716: muscle maintenance	2	0.00973	2	0.143	0.00464
GO:9128: purine nucleoside monophosphate catabolism	2	0.00973	2	0.143	0.00464
GO:9169: purine ribonucleoside monophosphate catabolism	2	0.00973	2	0.143	0.00464
GO:6196: AMP catabolism	2	0.00973	2	0.143	0.00464
GO:9125: nucleoside monophosphate catabolism	2	0.00973	2	0.143	0.00464
GO:9158: ribonucleoside monophosphate catabolism	2	0.00973	2	0.143	0.00464
GO:6702: androgen biosynthesis	2	0.00973	2	0.143	0.00464
GO:42448: progesterone metabolism	2	0.00973	2	0.143	0.00464
GO:42271: susceptibility to natural killer cell mediated cytotoxicity	2	0.00973	2	0.143	0.00464
GO:51495: positive regulation of cytoskeleton organization and biogenesis	2	0.00973	2	0.143	0.00464
GO:51496: positive regulation of stress fiber formation	2	0.00973	2	0.143	0.00464
GO:51492: regulation of stress fiber formation	2	0.00973	2	0.143	0.00464
GO:6907: pinocytosis	2	0.00973	2	0.143	0.00464
GO:46627: negative regulation of insulin receptor signaling pathway	2	0.00973	2	0.143	0.00464
GO:9613: response to pest, pathogen or parasite	726	3.532	68	4.857	0.00469
GO:6520: amino acid metabolism	348	1.693	37	2.643	0.00482
GO:6576: biogenic amine metabolism	67	0.326	11	0.786	0.00531
GO:50766: positive regulation of phagocytosis	6	0.0292	3	0.214	0.00539
GO:50764: regulation of phagocytosis	6	0.0292	3	0.214	0.00539
GO:43149: stress fiber formation	6	0.0292	3	0.214	0.00539
GO:50777: negative regulation of immune response	25	0.122	6	0.429	0.00569
GO:51050: positive regulation of transport	25	0.122	6	0.429	0.00569

GO:8016: regulation of heart contraction rate	41	0.199	8	0.571	0.00576
GO:8207: C21-steroid hormone metabolism	18	0.0876	5	0.357	0.0059
GO:8283: cell proliferation	810	3.941	74	5.286	0.00599
GO:7166: cell surface receptor linked signal transduction	1812	8.816	150	10.71	0.00642
GO:9968: negative regulation of signal transduction	98	0.477	14	1	0.0065
GO:42127: regulation of cell proliferation	439	2.136	44	3.143	0.00662
GO:43207: response to external biotic stimulus	763	3.712	70	5	0.00672
GO:7266: Rho protein signal transduction	79	0.384	12	0.857	0.00697
GO:8366: nerve ensheathment	19	0.0924	5	0.357	0.00757
GO:30195: negative regulation of blood coagulation	19	0.0924	5	0.357	0.00757
GO:30193: regulation of blood coagulation	19	0.0924	5	0.357	0.00757
GO:42330: taxis	177	0.861	21	1.5	0.00924
GO:6935: chemotaxis	177	0.861	21	1.5	0.00924
GO:30182: neuron differentiation	166	0.808	20	1.429	0.00927
GO:46580: negative regulation of Ras protein signal transduction	13	0.0633	4	0.286	0.00932
GO:51058: negative regulation of small GTPase mediated signal transduction	13	0.0633	4	0.286	0.00932
GO:6584: catecholamine metabolism	20	0.0973	5	0.357	0.00953
GO:44255: cellular lipid metabolism	687	3.343	63	4.5	0.00985

Supplemental Table S2F

GO Biological Process Categories Overrepresented in HUVEC vs. hESC-EC

GO Category	Genes in Category	% of Genes in Category	Genes in List in Category	% of Genes in List in Category	*P*-Value
GO:6468: protein amino acid phosphorylation	905	4.403	103	7.984	2.57E-09
GO:7154: cell communication	5496	26.74	421	32.64	7.24E-07
GO:16310: phosphorylation	1069	5.201	106	8.217	1.66E-06
GO:6793: phosphorus metabolism	1321	6.427	123	9.535	5.72E-06
GO:6796: phosphate metabolism	1321	6.427	123	9.535	5.72E-06
GO:7599: hemostasis	140	0.681	24	1.86	6.35E-06
GO:7596: blood coagulation	132	0.642	23	1.783	7.44E-06
GO:1525: angiogenesis	108	0.525	20	1.55	1.13E-05
GO:42060: wound healing	145	0.705	24	1.86	1.18E-05
GO:50878: regulation of body fluids	157	0.764	25	1.938	1.56E-05
GO:50817: coagulation	139	0.676	23	1.783	1.78E-05
GO:48514: blood vessel morphogenesis	113	0.55	20	1.55	2.27E-05
GO:1568: blood vessel development	113	0.55	20	1.55	2.27E-05
GO:7165: signal transduction	4419	21.5	337	26.12	2.50E-05
GO:1944: vasculature development	115	0.56	20	1.55	2.95E-05
GO:7167: enzyme linked receptor protein signaling pathway	310	1.508	38	2.946	6.09E-05
GO:9249: protein-lipoylation	6	0.0292	4	0.31	0.000209
GO:46834: lipid phosphorylation	6	0.0292	4	0.31	0.000209
GO:46854: phosphoinositide phosphorylation	6	0.0292	4	0.31	0.000209
GO:46928: regulation of neurotransmitter secretion	6	0.0292	4	0.31	0.000209
GO:7275: development	2960	14.4	230	17.83	0.000241
GO:50434: positive regulation of viral transcription	3	0.0146	3	0.233	0.000247
GO:43392: negative regulation of DNA binding	3	0.0146	3	0.233	0.000247
GO:30319: di-, tri-valent inorganic anion homeostasis	3	0.0146	3	0.233	0.000247
GO:51100: negative regulation of binding	3	0.0146	3	0.233	0.000247
GO:7155: cell adhesion	1002	4.875	90	6.977	0.000361
GO:6880: intracellular sequestering of iron ion	7	0.0341	4	0.31	0.000464
GO:9653: morphogenesis	940	4.574	84	6.512	0.000657
GO:6516: glycoprotein catabolism	25	0.122	7	0.543	0.000666
GO:50789: regulation of biological process	5781	28.13	414	32.09	0.000676
GO:50794: regulation of cellular process	5371	26.13	386	29.92	0.000875
GO:51238: sequestering of metal ion	8	0.0389	4	0.31	0.000881
GO:46782: regulation of viral transcription	4	0.0195	3	0.233	0.000941
GO:16584: nucleosome spacing	4	0.0195	3	0.233	0.000941
GO:9299: mRNA transcription	4	0.0195	3	0.233	0.000941
GO:19083: viral transcription	4	0.0195	3	0.233	0.000941
GO:30002: anion homeostasis	4	0.0195	3	0.233	0.000941
GO:19080: viral genome expression	4	0.0195	3	0.233	0.000941
GO:9887: organ morphogenesis	310	1.508	34	2.636	0.00111
GO:6826: iron ion transport	52	0.253	10	0.775	0.00129
GO:50791: regulation of physiological process	5180	25.2	371	28.76	0.00149

GO:18065: protein-cofactor linkage	9	0.0438	4	0.31	0.00151
GO:51244: regulation of cellular physiological process	5009	24.37	359	27.83	0.00176
GO:6879: iron ion homeostasis	38	0.185	8	0.62	0.00212
GO:16477: cell migration	147	0.715	19	1.473	0.00216
GO:51101: regulation of DNA binding	5	0.0243	3	0.233	0.00224
GO:50906: detection of stimulus during sensory perception	5	0.0243	3	0.233	0.00224
GO:48524: positive regulation of viral life cycle	5	0.0243	3	0.233	0.00224
GO:16197: endosome transport	47	0.229	9	0.698	0.00229
GO:7169: transmembrane receptor protein tyrosine kinase signaling pathway	228	1.109	26	2.016	0.00237
GO:7595: lactation	10	0.0487	4	0.31	0.00239
GO:1709: cell fate determination	23	0.112	6	0.465	0.00242
GO:6355: regulation of transcription, DNA-dependent	3150	15.33	234	18.14	0.00252
GO:30334: regulation of cell migration	31	0.151	7	0.543	0.00262
GO:6351: transcription, DNA-dependent	3244	15.78	240	18.6	0.00271
GO:35315: hair cell differentiation	2	0.00973	2	0.155	0.00394
GO:42491: auditory receptor cell differentiation	2	0.00973	2	0.155	0.00394
GO:9912: auditory receptor cell fate commitment	2	0.00973	2	0.155	0.00394
GO:45602: negative regulation of endothelial cell differentiation	2	0.00973	2	0.155	0.00394
GO:16139: glycoside catabolism	2	0.00973	2	0.155	0.00394
GO:16142: O-glycoside catabolism	2	0.00973	2	0.155	0.00394
GO:16137: glycoside metabolism	2	0.00973	2	0.155	0.00394
GO:16140: O-glycoside metabolism	2	0.00973	2	0.155	0.00394
GO:9880: embryonic pattern specification	18	0.0876	5	0.388	0.00416
GO:1569: patterning of blood vessels	6	0.0292	3	0.233	0.00427
GO:6895: Golgi to endosome transport	12	0.0584	4	0.31	0.00509
GO:30031: cell projection biogenesis	19	0.0924	5	0.388	0.00536
GO:7179: transforming growth factor beta receptor signaling pathway	44	0.214	8	0.62	0.00552
GO:6928: cell motility	356	1.732	35	2.713	0.00578
GO:51674: localization of cell	356	1.732	35	2.713	0.00578
GO:40011: locomotion	356	1.732	35	2.713	0.00578
GO:7032: endosome organization and biogenesis	54	0.263	9	0.698	0.00605
GO:48513: organ development	907	4.413	76	5.891	0.00608
GO:7178: transmembrane receptor protein serine/threonine kinase signaling pathway	64	0.311	10	0.775	0.00625
GO:30048: actin filament-based movement	20	0.0973	5	0.388	0.00678
GO:8360: regulation of cell shape	55	0.268	9	0.698	0.00684
GO:48488: synaptic vesicle endocytosis	7	0.0341	3	0.233	0.00713
GO:45449: regulation of transcription	3356	16.33	243	18.84	0.00729
GO:7242: intracellular signaling cascade	1818	8.845	139	10.78	0.00788
GO:45165: cell fate commitment	29	0.141	6	0.465	0.00824
GO:6350: transcription	3517	17.11	253	19.61	0.00838
GO:30030: cell projection organization and biogenesis	21	0.102	5	0.388	0.00845
GO:51270: regulation of cell motility	38	0.185	7	0.543	0.00858
GO:40012: regulation of locomotion	38	0.185	7	0.543	0.00858
GO:50795: regulation of behavior	38	0.185	7	0.543	0.00858
GO:19219: regulation of nucleobase, nucleoside, nucleotide and nucleic acid metabolism	3417	16.63	246	19.07	0.00904
GO:46916: transition metal ion homeostasis	48	0.234	8	0.62	0.00942

GO:7626: locomotory behavior	369	1.795	35	2.713	0.00988
