# Supplementary material for: Functional and Transcriptional Characterization of Human Embryonic Stem Cell-Derived Endothelial Cells for Treatment of Myocardial Infarction
Source: PLoS One. 2009 Dec 31;4(12):e8443. doi: 10.1371/journal.pone.0008443 (PMC2795856; doi:10.1371/journal.pone.0008443)
Supplement: Table S3 — K-means clustering significant gene lists. (4.69 MB PDF) [file pone.0008443.s009.pdf]

Supplemental Table S3A

Kmeans Cluster 1 - 5,089 Genes

Gene Name	*P*-value	Common	Genbank	Description	RefSeq
A_32_P178696	1.60E-07	BC046172	BC046172	Homo sapiens cDNA clone IMAGE:3463889, partial cds. [BC046172]	
A_23_P53345	1.60E-07	NM_020183	NM_020183	Homo sapiens aryl hydrocarbon receptor nuclear translocator-like 2 (ARNTL2), mRNA [NM_020183]	NM_020183
A_23_P30243	1.60E-07	NM_022350	NM_022350	Homo sapiens leukocyte-derived arginine aminopeptidase (LRAP), mRNA [NM_022350]	NM_022350
A_23_P129925	1.60E-07	NM_152270	NM_152270	Homo sapiens hypothetical protein FLJ34922 (FLJ34922), mRNA [NM_152270]	NM_152270
A_23_P401106	1.60E-07	NM_002599	NM_002599	Homo sapiens phosphodiesterase 2A, cGMP-stimulated (PDE2A), mRNA [NM_002599]	NM_002599
A_24_P224926	1.60E-07	NM_002405	NM_002405	Homo sapiens manic fringe homolog (Drosophila) (MFNG), mRNA [NM_002405]	NM_002405
A_23_P103104	1.71E-07	NM_002405	NM_002405	Homo sapiens manic fringe homolog (Drosophila) (MFNG), mRNA [NM_002405]	NM_002405
A_24_P272993	1.71E-07	BX161452	BX161452	human full-length cDNA clone CS0DI033YL14 of Placenta of Homo sapiens (human). [BX161452]	XR_000167
A_23_P48513	1.72E-07	NM_005532	NM_005532	Homo sapiens interferon, alpha-inducible protein 27 (IFI27), transcript variant a, mRNA [NM_005532]	NM_005532
A_23_P253602	1.87E-07	NM_001721	NM_001721	Homo sapiens BMX non-receptor tyrosine kinase (BMX), mRNA [NM_001721]	NM_001721
A_23_P405129	2.04E-07	NM_000428	NM_000428	Homo sapiens latent transforming growth factor beta binding protein 2 (LTBP2), mRNA [NM_000428]	NM_000428
A_24_P182929	2.04E-07	NM_003471	NM_003471	Homo sapiens potassium voltage-gated channel, shaker-related subfamily, beta member 1 (KCNAB1), transcript variant 2, mRNA [NM_003471]	NM_003471
A_24_P380629	2.18E-07	NM_007189	NM_007189	Homo sapiens ATP-binding cassette, sub-family F (GCN20), member 2 (ABCF2), nuclear gene encoding mitochondrial protein, transcript variant 1, mRNA [NM_007189]	NM_007189
A_23_P374782	2.18E-07	NM_031892	NM_031892	Homo sapiens SH3-domain kinase binding protein 1 (SH3KBP1), transcript variant 1, mRNA [NM_031892]	NM_031892
A_23_P167389	2.18E-07	NM_022481	NM_022481	Homo sapiens centaurin, delta 3 (CENTD3), mRNA [NM_022481]	NM_022481
A_23_P113393	2.19E-07	NM_017413	NM_017413	Homo sapiens apelin, AGTRL1 ligand (APLN), mRNA [NM_017413]	NM_017413
A_23_P39525	2.23E-07	NM_024785	NM_024785	Homo sapiens hypothetical protein FLJ22746 (FLJ22746), mRNA [NM_024785]	NM_024785
A_24_P318656	2.25E-07	NM_000212	NM_000212	Homo sapiens integrin, beta 3 (platelet glycoprotein IIIa, antigen CD61) (ITGB3), mRNA [NM_000212]	NM_000212
A_23_P93442	2.25E-07	NM_015278	NM_015278	Homo sapiens SAM and SH3 domain containing 1 (SASH1), mRNA [NM_015278]	NM_015278
A_24_P261417	2.29E-07	NM_015881	NM_015881	Homo sapiens dickkopf homolog 3 (Xenopus laevis) (DKK3), transcript variant 1, mRNA [NM_015881]	NM_015881
A_23_P105562	2.29E-07	NM_000552	NM_000552	Homo sapiens von Willebrand factor (VWF), mRNA [NM_000552]	NM_000552
A_23_P150057	2.32E-07	NM_024756	NM_024756	Homo sapiens multimerin 2 (MMRN2), mRNA [NM_024756]	NM_024756
A_23_P7325	2.34E-07	NM_004334	NM_004334	Homo sapiens bone marrow stromal cell antigen 1 (BST1), mRNA [NM_004334]	NM_004334
A_23_P140876	2.82E-07	NM_001089	NM_001089	Homo sapiens ATP-binding cassette, sub-family A (ABC1), member 3 (ABCA3), mRNA [NM_001089]	NM_001089
A_32_P74983	2.82E-07	ENST00000252229			
A_24_P82032	2.84E-07	NM_020663	NM_020663	Homo sapiens ras homolog gene family, member J (RHOJ), mRNA [NM_020663]	NM_020663
A_24_P910733	2.84E-07	NM_178335	NM_178335	Homo sapiens chromosome 3 open reading frame 6 (C3orf6), transcript variant 2, mRNA [NM_178335]	NM_178335
A_23_P206284	2.90E-07	NM_201525	NM_201525	Homo sapiens G protein-coupled receptor 56 (GPR56), transcript variant 3, mRNA [NM_201525]	NM_201525
A_23_P4561	3.00E-07	NM_198833	NM_198833	Homo sapiens serine (or cysteine) proteinase inhibitor, clade B (ovalbumin), member 8 (SERPINB8), transcript variant 2, mRNA [NM_198833]	NM_198833
A_32_P209230	3.00E-07	NM_133467	NM_133467	Homo sapiens Cbp/p300-interacting transactivator, with Glu/Asp-rich carboxy-terminal domain, 4 (CITED4), mRNA [NM_133467]	NM_133467
A_23_P162047	3.00E-07	NM_015881	NM_015881	Homo sapiens dickkopf homolog 3 (Xenopus laevis) (DKK3), transcript variant 1, mRNA [NM_015881]	NM_015881
A_23_P161439	3.09E-07	NM_006829	NM_006829	Homo sapiens chromosome 10 open reading frame 116 (C10orf116), mRNA [NM_006829]	NM_006829
A_32_P69149	3.15E-07	NM_012449	NM_012449	Homo sapiens six transmembrane epithelial antigen of the prostate 1 (STEAP1), mRNA [NM_012449]	NM_012449
A_23_P132159	3.29E-07	NM_017414	NM_017414	Homo sapiens ubiquitin specific protease 18 (USP18), mRNA [NM_017414]	NM_017414
A_24_P363802	3.32E-07	NM_005047	NM_005047	Homo sapiens proteasome (prosome, macropain) 26S subunit, non-ATPase, 5 (PSMD5), mRNA [NM_005047]	NM_005047
A_24_P263144	3.32E-07	NM_001721	NM_001721	Homo sapiens BMX non-receptor tyrosine kinase (BMX), mRNA [NM_001721]	NM_001721
A_23_P251680	3.32E-07	NM_000754	NM_000754	Homo sapiens catechol-O-methyltransferase (COMT), transcript variant MB-COMT, mRNA [NM_000754]	NM_000754
A_32_P218707	3.48E-07	XM_379108	XM_379108	PREDICTED: Homo sapiens similar to CDNA sequence BC012256 (LOC400969), mRNA [XM_379108]	XM_379108
A_23_P19624	3.58E-07	NM_001718	NM_001718	Homo sapiens bone morphogenetic protein 6 (BMP6), mRNA [NM_001718]	NM_001718
A_23_P250294	3.72E-07	NM_016006	NM_016006	Homo sapiens abhydrolase domain containing 5 (ABHD5), mRNA [NM_016006]	NM_016006
A_24_P213763	3.74E-07	CR625942	CR625942	full-length cDNA clone CS0DL007YI18 of B cells (Ramos cell line) Cot 25-normalized of Homo sapiens (human). [CR625942]	

A_23_P145631	3.74E-07	NM_024711	NM_024711	Homo sapiens GTPase, IMAP family member 6 (GIMAP6), transcript variant 1, mRNA [NM_024711]	NM_024711
A_23_P133656	3.74E-07	NM_002290	NM_002290	Homo sapiens laminin, alpha 4 (LAMA4), mRNA [NM_002290]	NM_002290
A_23_P344421	4.00E-07	NM_019055	NM_019055	Homo sapiens roundabout homolog 4, magic roundabout (Drosophila) (ROBO4), mRNA [NM_019055]	NM_019055
A_24_P319923	4.29E-07	NM_053025	NM_053025	Homo sapiens myosin, light polypeptide kinase (MYLK), transcript variant 1, mRNA [NM_053025]	NM_053025
A_24_P932887	4.39E-07	AK097227	AK097227	Homo sapiens cDNA FLJ39908 fis, clone SPLEN2017620. [AK097227]	
A_32_P30905	4.39E-07	BC032420	BC032420	Homo sapiens WDFY family member 4, mRNA (cDNA clone MGC:40604 IMAGE:5221804), complete cds. [BC032420]	
A_32_P101031	4.39E-07	NM_144586	NM_144586	Homo sapiens LY6/PLAUR domain containing 1 (LYPDC1), mRNA [NM_144586]	NM_144586
A_24_P332263	4.74E-07	A_24_P332263			
A_24_P359205	4.76E-07	BC010094	BC010094	Homo sapiens phosphatidylinositol glycan, class Q, mRNA (cDNA clone IMAGE:3357878), partial cds. [BC010094]	
A_32_P194072	4.87E-07	NM_015395	NM_015395	Homo sapiens DKFZP434B0335 protein (DKFZP434B0335), mRNA [NM_015395]	NM_015395
A_23_P57413	5.07E-07	NM_014634	NM_014634	Homo sapiens protein phosphatase 1F (PP2C domain containing) (PPM1F), mRNA [NM_014634]	NM_014634
A_23_P120325	5.07E-07	NM_002254	NM_002254	Homo sapiens kinesin family member 3C (KIF3C), mRNA [NM_002254]	NM_002254
A_23_P204630	5.07E-07	NM_021229	NM_021229	Homo sapiens netrin 4 (NTN4), mRNA [NM_021229]	NM_021229
A_24_P342096	5.42E-07	BC002886	BC002886	Homo sapiens cDNA clone IMAGE:3939857. [BC002886]	XM_499139
A_24_P80204	5.49E-07	NM_005434	NM_005434	Homo sapiens BENE protein (BENE), mRNA [NM_005434]	NM_005434
A_23_P150903	5.56E-07	NM_018099	NM_018099	Homo sapiens male sterility domain containing 1 (MLSTD1), mRNA [NM_018099]	NM_018099
A_23_P203475	5.56E-07	NM_145040	NM_145040	Homo sapiens protein kinase C, delta binding protein (PRKCDBP), mRNA [NM_145040]	NM_145040
A_23_P209394	5.63E-07	AF009619	AF009619	Homo sapiens FLAME-1-delta mRNA, alternatively spliced, complete cds. [AF009619]	
A_23_P67042	5.72E-07	NM_017947	NM_017947	Homo sapiens molybdenum cofactor sulfurase (MOCOS), mRNA [NM_017947]	NM_017947
A_23_P217168	5.84E-07	NM_024689	NM_024689	Homo sapiens chromosome X open reading frame 36 (CXorf36), mRNA [NM_024689]	NM_024689
A_23_P29939	5.86E-07	NM_007308	NM_007308	Homo sapiens synuclein, alpha (non A4 component of amyloid precursor) (SNCA), transcript variant NACP112, mRNA [NM_007308]	NM_007308
A_23_P351667	6.08E-07	NM_003812	NM_003812	Homo sapiens a disintegrin and metalloproteinase domain 23 (ADAM23), mRNA [NM_003812]	NM_003812
A_32_P82462	6.39E-07	BC021861	BC021861	Homo sapiens hypothetical LOC554202, mRNA (cDNA clone MGC:30053 IMAGE:5139119), complete cds. [BC021861]	
A_23_P214756	6.39E-07	NM_020455	NM_020455	Homo sapiens G protein-coupled receptor 126 (GPR126), mRNA [NM_020455]	NM_020455
A_23_P24433	6.39E-07	NM_003793	NM_003793	Homo sapiens cathepsin F (CTSF), mRNA [NM_003793]	NM_003793
A_32_P122754	6.39E-07	NM_080655	NM_080655	Homo sapiens similar to RIKEN cDNA 5730528L13 gene (MGC17337), mRNA [NM_080655]	NM_080655
A_23_P105028	6.68E-07	NM_015459	NM_015459	Homo sapiens DKFZP564J0863 protein (DKFZP564J0863), mRNA [NM_015459]	NM_015459
A_32_P16204	6.78E-07	ENST00000295549		Homo sapiens hypothetical gene supported by BC013438, mRNA (cDNA clone IMAGE:3899073), partial cds. [BC013438]	XM_374020
A_23_P138099	6.89E-07	NM_007314	NM_007314	Homo sapiens v-abl Abelson murine leukemia viral oncogene homolog 2 (arg, Abelson-related gene) (ABL2), transcript variant b, mRNA [NM_007314]	NM_007314
A_24_P404822	6.93E-07	NM_017413	NM_017413	Homo sapiens apelin, AGTRL1 ligand (APLN), mRNA [NM_017413]	NM_017413
A_24_P753476	6.94E-07	BC043374	BC043374	Homo sapiens hypothetical protein LOC340508, mRNA (cDNA clone IMAGE:5172009), partial cds. [BC043374]	
A_23_P200015	7.01E-07	NM_174858	NM_174858	Homo sapiens adenylate kinase 5 (AK5), transcript variant 1, mRNA [NM_174858]	NM_174858
A_24_P248240	7.22E-07	NM_152280	NM_152280	Homo sapiens synaptotagmin XI (SYT11), mRNA [NM_152280]	NM_152280
A_24_P301146	7.22E-07	NM_005358	NM_005358	Homo sapiens LIM domain 7 (LMO7), mRNA [NM_005358]	NM_005358
A_32_P50522	7.22E-07	NM_000801	NM_000801	Homo sapiens FK506 binding protein 1A, 12kDa (FKBP1A), transcript variant 12B, mRNA [NM_000801]	NM_000801
A_24_P51777	7.43E-07	NM_178335	NM_178335	Homo sapiens chromosome 3 open reading frame 6 (C3orf6), transcript variant 2, mRNA [NM_178335]	NM_178335
A_23_P258151	7.67E-07	THC2247947		Q8IYP3 (Q8IYP3) FGD5 protein, complete [THC2247947]	
A_23_P211659	7.97E-07	NM_022766	NM_022766	Homo sapiens ceramide kinase (CERK), transcript variant 1, mRNA [NM_022766]	NM_022766
A_23_P35848	7.98E-07	NM_018161	NM_018161	Homo sapiens NAD synthetase 1 (NADSYN1), mRNA [NM_018161]	NM_018161
A_24_P274607	8.08E-07	AK054960	AK054960	Homo sapiens cDNA FLJ30398 fis, clone BRACE2008402, highly similar to Homo sapiens steroid receptor RNA activator isoform 3 mRNA. [AK054960]	XR_000132
A_23_P16469	8.25E-07	NM_001005377	NM_001005377	Homo sapiens plasminogen activator, urokinase receptor (PLAUR), transcript variant 3, mRNA [NM_001005377]	NM_001005377
A_23_P374767	8.46E-07	NM_015983	NM_015983	Homo sapiens ubiquitin-conjugating enzyme E2D 4 (putative) (UBE2D4), mRNA [NM_015983]	NM_015983
A_23_P154875	8.55E-07	NM_012105	NM_012105	Homo sapiens beta-site APP-cleaving enzyme 2 (BACE2), transcript variant a, mRNA [NM_012105]	NM_012105
A_24_P153803	8.82E-07	NM_020663	NM_020663	Homo sapiens ras homolog gene family, member J (RHOJ), mRNA [NM_020663]	NM_020663

A_23_P32175	8.97E-07	NM_014368	NM_014368	Homo sapiens LIM homeobox 6 (LHX6), transcript variant 1, mRNA [NM_014368]	NM_014368
A_23_P71530	8.97E-07	NM_002546	NM_002546	Homo sapiens tumor necrosis factor receptor superfamily, member 11b (osteoprotegerin) (TNFRSF11B), mRNA [NM_002546]	NM_002546
A_23_P324813	8.97E-07	NM_181844	NM_181844	Homo sapiens B-cell CLL/lymphoma 6, member B (zinc finger protein) (BCL6B), mRNA [NM_181844]	NM_181844
A_23_P17287	8.97E-07	ENST00000351760		Homo sapiens hypothetical protein LOC285148, mRNA (cDNA clone IMAGE:5303287), partial cds. [BC067865]	
A_23_P117654	9.48E-07	NM_000259	NM_000259	Homo sapiens myosin VA (heavy polypeptide 12, myoxin) (MYO5A), mRNA [NM_000259]	NM_000259
A_32_P101110	9.60E-07	AF086187	AF086187	Homo sapiens full length insert cDNA clone ZC30H06. [AF086187]	
A_23_P169576	9.70E-07	NM_001013848	NM_001013848	Homo sapiens SEC15-like 1 (S. cerevisiae) (SEC15L1), transcript variant 2, mRNA [NM_001013848]	NM_001013848
A_23_P63503	9.73E-07	NM_002871	NM_002871	Homo sapiens RAB interacting factor (RABIF), mRNA [NM_002871]	NM_002871
A_23_P108673	9.73E-07	NM_032181	NM_032181	Homo sapiens hypothetical protein FLJ13391 (FLJ13391), mRNA [NM_032181]	NM_032181
A_24_P12626	9.73E-07	NM_001753	NM_001753	Homo sapiens caveolin 1, caveolae protein, 22kDa (CAV1), mRNA [NM_001753]	NM_001753
A_23_P88893	9.73E-07	NM_017702	NM_017702	Homo sapiens hypothetical protein FLJ20186 (FLJ20186), transcript variant 2, mRNA [NM_017702]	NM_017702
A_23_P50974	9.91E-07	NM_020760	NM_020760	Homo sapiens HECT, C2 and WW domain containing E3 ubiquitin protein ligase 2 (HECW2), mRNA [NM_020760]	NM_020760
A_23_P49638	9.96E-07	NM_006613	NM_006613	Homo sapiens GRB2-related adaptor protein (GRAP), mRNA [NM_006613]	NM_006613
A_23_P121447	1.02E-06	NM_032487	NM_032487	Homo sapiens actin related protein M1 (ARPM1), mRNA [NM_032487]	NM_032487
A_23_P121253	1.02E-06	NM_003810	NM_003810	Homo sapiens tumor necrosis factor (ligand) superfamily, member 10 (TNFSF10), mRNA [NM_003810]	NM_003810
A_23_P29800	1.02E-06	NM_005602	NM_005602	Homo sapiens claudin 11 (oligodendrocyte transmembrane protein) (CLDN11), mRNA [NM_005602]	NM_005602
A_23_P167129	1.02E-06	NM_022475	NM_022475	Homo sapiens hedgehog interacting protein (HHIP), mRNA [NM_022475]	NM_022475
A_23_P214176	1.03E-06	NM_133493	NM_133493	Homo sapiens CD109 antigen (Gov platelet alloantigens) (CD109), mRNA [NM_133493]	NM_133493
A_32_P76247	1.03E-06	NM_001540	NM_001540	Homo sapiens heat shock 27kDa protein 1 (HSPB1), mRNA [NM_001540]	NM_001540
A_23_P38519	1.04E-06	NM_000212	NM_000212	Homo sapiens integrin, beta 3 (platelet glycoprotein IIIa, antigen CD61) (ITGB3), mRNA [NM_000212]	NM_000212
A_24_P283189	1.06E-06	NM_000591	NM_000591	Homo sapiens CD14 antigen (CD14), mRNA [NM_000591]	NM_000591
A_23_P258234	1.06E-06	AK098194	AK098194	Homo sapiens cDNA FLJ40875 fis, clone UMVEN2000069. [AK098194]	
A_23_P64121	1.06E-06	NM_152314	NM_152314	Homo sapiens hypothetical protein MGC34830 (MGC34830), mRNA [NM_152314]	NM_152314
A_23_P211627	1.06E-06	NM_153684	NM_153684	Homo sapiens nucleoporin 50kDa (NUP50), transcript variant 1, mRNA [NM_153684]	NM_153684
A_24_P916686	1.06E-06	BC015962	BC015962	Homo sapiens, clone IMAGE:4081125, mRNA, partial cds. [BC015962]	
A_23_P36562	1.06E-06	NM_002205	NM_002205	Homo sapiens integrin, alpha 5 (fibronectin receptor, alpha polypeptide) (ITGA5), mRNA [NM_002205]	NM_002205
A_23_P422851	1.06E-06	NM_138375	NM_138375	Homo sapiens Cdk5 and Abl enzyme substrate 1 (CABLES1), mRNA [NM_138375]	NM_138375
A_23_P103232	1.06E-06	NM_017823	NM_017823	Homo sapiens dual specificity phosphatase 23 (DUSP23), mRNA [NM_017823]	NM_017823
A_23_P201711	1.06E-06	NM_014624	NM_014624	Homo sapiens S100 calcium binding protein A6 (calcyclin) (S100A6), mRNA [NM_014624]	NM_014624
A_23_P168259	1.07E-06	NM_025217	NM_025217	Homo sapiens UL16 binding protein 2 (ULBP2), mRNA [NM_025217]	NM_025217
A_24_P46689	1.08E-06	NM_015278	NM_015278	Homo sapiens SAM and SH3 domain containing 1 (SASH1), mRNA [NM_015278]	NM_015278
A_24_P270460	1.10E-06	NM_005532	NM_005532	Homo sapiens interferon, alpha-inducible protein 27 (IFI27), transcript variant a, mRNA [NM_005532]	NM_005532
A_23_P379071	1.12E-06	NM_145032	NM_145032	Homo sapiens F-box and leucine-rich repeat protein 13 (FBXL13), mRNA [NM_145032]	NM_145032
A_23_P160354	1.13E-06	NM_181690	NM_181690	Homo sapiens v-akt murine thymoma viral oncogene homolog 3 (protein kinase B, gamma) (AKT3), transcript variant 2, mRNA [NM_181690]	NM_181690
A_23_P137238	1.13E-06	NM_004653	NM_004653	Homo sapiens Smcy homolog, Y-linked (mouse) (SMCY), mRNA [NM_004653]	NM_004653
A_23_P170498	1.13E-06	NM_173474	NM_173474	Homo sapiens N-terminal asparagine amidase (NTAN1), mRNA [NM_173474]	NM_173474
A_23_P35597	1.15E-06	NM_007021	NM_007021	Homo sapiens chromosome 10 open reading frame 10 (C10orf10), mRNA [NM_007021]	NM_007021
A_23_P121702	1.15E-06	NM_001014446	NM_001014446	Homo sapiens OCIA domain containing 2 (OCIAD2), transcript variant 1, mRNA [NM_001014446]	NM_001014446
A_23_P161624	1.17E-06	NM_005438	NM_005438	Homo sapiens FOS-like antigen 1 (FOSL1), mRNA [NM_005438]	NM_005438
A_23_P41075	1.18E-06	NM_032355	NM_032355	Homo sapiens MON1 homolog A (yeast) (MON1A), mRNA [NM_032355]	NM_032355
A_23_P77401	1.20E-06	NM_018340	NM_018340	Homo sapiens hypothetical protein FLJ11151 (FLJ11151), mRNA [NM_018340]	NM_018340
A_23_P47034	1.20E-06	NM_002729	NM_002729	Homo sapiens hematopoietically expressed homeobox (HHEX), mRNA [NM_002729]	NM_002729
A_23_P257704	1.21E-06	NM_001540	NM_001540	Homo sapiens heat shock 27kDa protein 1 (HSPB1), mRNA [NM_001540]	NM_001540
A_23_P89431	1.22E-06	NM_002982	NM_002982	Homo sapiens chemokine (C-C motif) ligand 2 (CCL2), mRNA [NM_002982]	NM_002982

A_23_P345820	1.23E-06	NM_014991	NM_014991	Homo sapiens WD repeat and FYVE domain containing 3 (WDFY3), transcript variant 1, mRNA [NM_014991]	NM_014991
A_24_P83118	1.24E-06	NM_152511	NM_152511	Homo sapiens dual specificity phosphatase 18 (DUSP18), mRNA [NM_152511]	NM_152511
A_23_P3963	1.24E-06	NM_014603	NM_014603	Homo sapiens paraneoplastic antigen (HUMPPA), mRNA [NM_014603]	NM_014603
A_24_P265135	1.25E-06	NM_014671	NM_014671	Homo sapiens ubiquitin protein ligase E3C (UBE3C), mRNA [NM_014671]	NM_014671
A_23_P304716	1.26E-06	NM_019089	NM_019089	Homo sapiens hairy and enhancer of split 2 (Drosophila) (HES2), mRNA [NM_019089]	NM_019089
A_24_P172481	1.26E-06	NM_006074	NM_006074	Homo sapiens tripartite motif-containing 22 (TRIM22), mRNA [NM_006074]	NM_006074
A_24_P169688	1.30E-06	NM_005931	NM_005931	Homo sapiens MHC class I polypeptide-related sequence B (MICB), mRNA [NM_005931]	NM_005931
A_23_P381945	1.31E-06	NM_005556	NM_005556	Homo sapiens keratin 7 (KRT7), mRNA [NM_005556]	NM_005556
A_23_P256890	1.31E-06	CR611847	CR611847	full-length cDNA clone CS0DI077YC19 of Placenta Cot 25-normalized of Homo sapiens (human). [CR611847]	
A_24_P22976	1.32E-06	NM_015683	NM_015683	Homo sapiens arrestin domain containing 2 (ARRDC2), transcript variant 1, mRNA [NM_015683]	NM_015683
A_24_P342880	1.32E-06	NM_016598	NM_016598	Homo sapiens zinc finger, DHHC-type containing 3 (ZDHHC3), mRNA [NM_016598]	NM_016598
A_23_P23669	1.32E-06	NM_017734	NM_017734	Homo sapiens palmdelphin (PALMD), mRNA [NM_017734]	NM_017734
A_23_P94819	1.33E-06	NM_006987	NM_006987	Homo sapiens rabphilin 3A-like (without C2 domains) (RPH3AL), mRNA [NM_006987]	NM_006987
A_24_P403959	1.35E-06	NM_198232	NM_198232	Homo sapiens ribonuclease, RNase A family, 1 (pancreatic) (RNASE1), transcript variant 3, mRNA [NM_198232]	NM_198232
A_23_P39925	1.35E-06	NM_003494	NM_003494	Homo sapiens dysferlin, limb girdle muscular dystrophy 2B (autosomal recessive) (DYSF), mRNA [NM_003494]	NM_003494
A_24_P125096	1.35E-06	NM_005952	NM_005952	Homo sapiens metallothionein 1X (MT1X), mRNA [NM_005952]	NM_005952
A_23_P73135	1.36E-06	NM_031941	NM_031941	Homo sapiens Usher syndrome 1C binding protein 1 (USHBP1), mRNA [NM_031941]	NM_031941
A_24_P114124	1.36E-06	NM_006762	NM_006762	Homo sapiens lysosomal associated multispanning membrane protein 5 (LAPTM5), mRNA [NM_006762]	NM_006762
A_24_P942493	1.36E-06	NM_015253	NM_015253	Homo sapiens KIAA0523 protein (KIAA0523), mRNA [NM_015253]	NM_015253
A_23_P146584	1.36E-06	NM_080655	NM_080655	Homo sapiens similar to RIKEN cDNA 5730528L13 gene (MGC17337), mRNA [NM_080655]	NM_080655
A_23_P150789	1.39E-06	NM_007173	NM_007173	Homo sapiens protease, serine, 23 (PRSS23), mRNA [NM_007173]	NM_007173
A_23_P338325	1.40E-06	NM_005230	NM_005230	Homo sapiens ELK3, ETS-domain protein (SRF accessory protein 2) (ELK3), mRNA [NM_005230]	NM_005230
A_23_P218879	1.40E-06	NM_016381	NM_016381	Homo sapiens three prime repair exonuclease 1 (TREX1), transcript variant 1, mRNA [NM_016381]	NM_016381
A_24_P55092	1.41E-06	NM_133493	NM_133493	Homo sapiens CD109 antigen (Gov platelet alloantigens) (CD109), mRNA [NM_133493]	NM_133493
A_23_P168479	1.42E-06	NM_005402	NM_005402	Homo sapiens v-ral simian leukemia viral oncogene homolog A (ras related) (RALA), mRNA [NM_005402]	NM_005402
A_23_P329133	1.43E-06	NM_002080	NM_002080	Homo sapiens glutamic-oxaloacetic transaminase 2, mitochondrial (aspartate aminotransferase 2) (GOT2), mRNA [NM_002080]	NM_002080
A_23_P216448	1.45E-06	NM_005596	NM_005596	Homo sapiens nuclear factor I/B (NFIB), mRNA [NM_005596]	NM_005596
A_23_P99515	1.47E-06	NM_032849	NM_032849	Homo sapiens hypothetical protein FLJ14834 (FLJ14834), mRNA [NM_032849]	NM_032849
A_32_P139367	1.52E-06	BC038749	BC038749	Homo sapiens cDNA clone IMAGE:5269084, partial cds. [BC038749]	
A_24_P140608	1.53E-06	NM_001945	NM_001945	Homo sapiens heparin-binding EGF-like growth factor (HBEGF), mRNA [NM_001945]	NM_001945
A_23_P135474	1.54E-06	NM_016491	NM_016491	Homo sapiens mitochondrial ribosomal protein L37 (MRPL37), nuclear gene encoding mitochondrial protein, mRNA [NM_016491]	NM_016491
A_23_P29237	1.57E-06	NM_145641	NM_145641	Homo sapiens apolipoprotein L, 3 (APOL3), transcript variant beta/a, mRNA [NM_145641]	NM_145641
A_23_P23074	1.62E-06	NM_006417	NM_006417	Homo sapiens interferon-induced protein 44 (IFI44), mRNA [NM_006417]	NM_006417
A_23_P250629	1.62E-06	NM_004159	NM_004159	Homo sapiens proteasome (prosome, macropain) subunit, beta type, 8 (large multifunctional protease 7) (PSMB8), transcript variant 1, mRNA [NM_004159]	NM_004159
A_32_P148407	1.62E-06	A_32_P148407			
A_23_P138693	1.63E-06	NM_004808	NM_004808	Homo sapiens N-myristoyltransferase 2 (NMT2), mRNA [NM_004808]	NM_004808
A_23_P218131	1.63E-06	NM_032714	NM_032714	Homo sapiens chromosome 14 open reading frame 151 (C14orf151), mRNA [NM_032714]	NM_032714
A_23_P206612	1.64E-06	AK126447	AK126447	Homo sapiens cDNA FLJ44483 fis, clone UTERU2033375. [AK126447]	
A_23_P157051	1.64E-06	NM_004722	NM_004722	Homo sapiens adaptor-related protein complex 4, mu 1 subunit (AP4M1), mRNA [NM_004722]	NM_004722
A_23_P20814	1.64E-06	NM_014314	NM_014314	Homo sapiens DEAD (Asp-Glu-Ala-Asp) box polypeptide 58 (DDX58), mRNA [NM_014314]	NM_014314
A_23_P132644	1.64E-06	NM_020792	NM_020792	Homo sapiens arylacetamide deacetylase-like 1 (AADACL1), mRNA [NM_020792]	NM_020792
A_23_P67661	1.64E-06	NM_001864	NM_001864	Homo sapiens cytochrome c oxidase subunit VIIa polypeptide 1 (muscle) (COX7A1), mRNA [NM_001864]	NM_001864
A_23_P47955	1.65E-06	NM_006187	NM_006187	Homo sapiens 2'-5'-oligoadenylate synthetase 3, 100kDa (OAS3), mRNA [NM_006187]	NM_006187
A_32_P2050	1.66E-06	THC2373975		BM561501 AGENCOURT_6567312 NIH_MGC_88 Homo sapiens cDNA clone IMAGE:5739715 5', mRNA sequence [BM561501]	

A_32_P213330	1.66E-06	BC012946	BC012946	Homo sapiens Rho-guanine nucleotide exchange factor, mRNA (cDNA clone MGC:21017 IMAGE:4454960), complete cds. [BC012946]	XM_371755
A_23_P21155	1.66E-06	NM_025083	NM_025083	Homo sapiens hypothetical protein FLJ21128 (FLJ21128), mRNA [NM_025083]	NM_025083
A_23_P166087	1.66E-06	NM_014737	NM_014737	Homo sapiens Ras association (RalGDS/AF-6) domain family 2 (RASSF2), transcript variant 1, mRNA [NM_014737]	NM_014737
A_23_P8812	1.69E-06	W60781	W60781	W60781 zd26f05.r1 Soares_fetal_heart_NbHH19W Homo sapiens cDNA clone IMAGE:341793 5' similar to gb:J02874 FATTY ACID-BINDING PROTEIN, ADIPOCYTE (HUMAN);, mRNA sequence [W60781]	
A_23_P47304	1.69E-06	NM_004347	NM_004347	Homo sapiens caspase 5, apoptosis-related cysteine protease (CASP5), mRNA [NM_004347]	NM_004347
A_24_P14584	1.69E-06	NM_012105	NM_012105	Homo sapiens beta-site APP-cleaving enzyme 2 (BACE2), transcript variant a, mRNA [NM_012105]	NM_012105
A_23_P254271	1.70E-06	NM_032525	NM_032525	Homo sapiens tubulin, beta 6 (TUBB6), mRNA [NM_032525]	NM_032525
A_24_P140475	1.71E-06	NM_021069	NM_021069	Homo sapiens Arg/Abl-interacting protein ArgBP2 (ARGBP2), transcript variant 2, mRNA [NM_021069]	NM_021069
A_23_P24129	1.71E-06	NM_012242	NM_012242	Homo sapiens dickkopf homolog 1 (Xenopus laevis) (DKK1), mRNA [NM_012242]	NM_012242
A_23_P255376	1.73E-06	NM_017918	NM_017918	Homo sapiens hypothetical protein FLJ20647 (FLJ20647), mRNA [NM_017918]	NM_017918
A_23_P117727	1.74E-06	NM_139242	NM_139242	Homo sapiens mitochondrial methionyl-tRNA formyltransferase (MTFMT), mRNA [NM_139242]	NM_139242
A_24_P188377	1.74E-06	NM_000574	NM_000574	Homo sapiens decay accelerating factor for complement (CD55, Cromer blood group system) (DAF), mRNA [NM_000574]	NM_000574
A_24_P658584	1.74E-06	NM_015278	NM_015278	Homo sapiens SAM and SH3 domain containing 1 (SASH1), mRNA [NM_015278]	NM_015278
A_23_P18539	1.74E-06	NM_007351	NM_007351	Homo sapiens multimerin 1 (MMRN1), mRNA [NM_007351]	NM_007351
A_23_P143817	1.74E-06	NM_053025	NM_053025	Homo sapiens myosin, light polypeptide kinase (MYLK), transcript variant 1, mRNA [NM_053025]	NM_053025
A_23_P63209	1.76E-06	NM_181755	NM_181755	Homo sapiens hydroxysteroid (11-beta) dehydrogenase 1 (HSD11B1), transcript variant 2, mRNA [NM_181755]	NM_181755
A_24_P916547	1.76E-06	THC2336837		Q7R1L5 (Q7R1L5) GLP_28_68912_68484, partial (9%) [THC2336837]	
A_23_P150064	1.76E-06	NM_024756	NM_024756	Homo sapiens multimerin 2 (MMRN2), mRNA [NM_024756]	NM_024756
A_23_P206396	1.77E-06	NM_016951	NM_016951	Homo sapiens chemokine-like factor (CKLF), transcript variant 1, mRNA [NM_016951]	NM_016951
A_32_P152696	1.78E-06	A_32_P152696			
A_23_P126908	1.83E-06	NM_003820	NM_003820	Homo sapiens tumor necrosis factor receptor superfamily, member 14 (herpesvirus entry mediator) (TNFRSF14), mRNA [NM_003820]	NM_003820
A_24_P413126	1.85E-06	NM_020182	NM_020182	Homo sapiens transmembrane, prostate androgen induced RNA (TMEPAI), transcript variant 1, mRNA [NM_020182]	NM_020182
A_24_P142118	1.90E-06	NM_003246	NM_003246	Homo sapiens thrombospondin 1 (THBS1), mRNA [NM_003246]	NM_003246
A_23_P334870	1.91E-06	NM_145316	NM_145316	Homo sapiens chromosome 6 open reading frame 128 (C6orf128), mRNA [NM_145316]	NM_145316
A_24_P652609	1.91E-06	AK054645	AK054645	Homo sapiens cDNA FLJ30083 fis, clone BGGI12001097, weakly similar to Homo sapiens contactin associated protein (Caspr) mRNA. [AK054645]	XM_372097
A_23_P4536	1.91E-06	NM_012307	NM_012307	Homo sapiens erythrocyte membrane protein band 4.1-like 3 (EPB41L3), mRNA [NM_012307]	NM_012307
A_24_P86537	1.91E-06	NM_001540	NM_001540	Homo sapiens heat shock 27kDa protein 1 (HSPB1), mRNA [NM_001540]	NM_001540
A_23_P331908	1.92E-06	AK097878	AK097878	Homo sapiens cDNA FLJ40559 fis, clone THYMU2002910. [AK097878]	
A_23_P201687	1.92E-06	BC012091	BC012091	Homo sapiens hairy and enhancer of split 2 (Drosophila), mRNA (cDNA clone IMAGE:4634002), complete cds. [BC012091]	
A_32_P149288	1.92E-06	BC040307	BC040307	Homo sapiens cDNA clone IMAGE:4830091, partial cds. [BC040307]	
A_23_P144113	1.92E-06	NM_002070	NM_002070	Homo sapiens guanine nucleotide binding protein (G protein), alpha inhibiting activity polypeptide 2 (GNAI2), mRNA [NM_002070]	NM_002070
A_23_P428842	1.92E-06	NM_138399	NM_138399	Homo sapiens transmembrane protein 44 (TMEM44), transcript variant 1, mRNA [NM_138399]	NM_138399
A_23_P214821	1.92E-06	NM_001955	NM_001955	Homo sapiens endothelin 1 (EDN1), mRNA [NM_001955]	NM_001955
A_23_P48596	1.95E-06	NM_198232	NM_198232	Homo sapiens ribonuclease, RNase A family, 1 (pancreatic) (RNASE1), transcript variant 3, mRNA [NM_198232]	NM_198232
A_23_P138686	1.96E-06	NM_004808	NM_004808	Homo sapiens N-myristoyltransferase 2 (NMT2), mRNA [NM_004808]	NM_004808
A_23_P501007	1.96E-06	NM_004105	NM_004105	Homo sapiens EGF-containing fibulin-like extracellular matrix protein 1 (EFEMP1), transcript variant 1, mRNA [NM_004105]	NM_004105
A_23_P253958	1.99E-06	NM_005824	NM_005824	Homo sapiens leucine rich repeat containing 17 (LRRC17), mRNA [NM_005824]	NM_005824
A_23_P148629	1.99E-06	NM_004681	NM_004681	Homo sapiens eukaryotic translation initiation factor 1A, Y-linked (EIF1AY), mRNA [NM_004681]	NM_004681
A_23_P215819	2.00E-06	NM_001009957	NM_001009957	Homo sapiens zinc finger protein 655 (ZNF655), transcript variant 5, mRNA [NM_001009957]	NM_001009957
A_23_P214533	2.04E-06	NM_030899	NM_030899	Homo sapiens zinc finger protein 323 (ZNF323), transcript variant 1, mRNA [NM_030899]	NM_030899
A_23_P59718	2.04E-06	NM_003130	NM_003130	Homo sapiens sorcin (SRI), transcript variant 1, mRNA [NM_003130]	NM_003130
A_23_P138417	2.04E-06	NM_012425	NM_012425	Homo sapiens Ras suppressor protein 1 (RSU1), transcript variant 1, mRNA [NM_012425]	NM_012425
A_23_P170238	2.06E-06	NM_020815	NM_020815	Homo sapiens protocadherin 10 (PCDH10), transcript variant 2, mRNA [NM_020815]	NM_020815

A_24_P326660	2.06E-06	NM_006500	NM_006500	Homo sapiens melanoma cell adhesion molecule (MCAM), mRNA [NM_006500]	NM_006500
A_23_P116533	2.06E-06	NM_015055	NM_015055	Homo sapiens SWAP-70 protein (SWAP70), mRNA [NM_015055]	NM_015055
A_24_P201739	2.09E-06	NM_005475	NM_005475	Homo sapiens lymphocyte adaptor protein (LNK), mRNA [NM_005475]	NM_005475
A_23_P418015	2.09E-06	NM_014268	NM_014268	Homo sapiens microtubule-associated protein, RP/EB family, member 2 (MAPRE2), mRNA [NM_014268]	NM_014268
A_23_P111766	2.14E-06	A_23_P111766			
A_23_P218743	2.16E-06	NM_020132	NM_020132	Homo sapiens 1-acylglycerol-3-phosphate O-acyltransferase 3 (AGPAT3), mRNA [NM_020132]	NM_020132
A_23_P411113	2.17E-06	NM_003632	NM_003632	Homo sapiens contactin associated protein 1 (CNTNAP1), mRNA [NM_003632]	NM_003632
A_23_P316601	2.19E-06	NM_006912	NM_006912	Homo sapiens Ras-like without CAAX 1 (RIT1), mRNA [NM_006912]	NM_006912
A_23_P105138	2.21E-06	NM_001752	NM_001752	Homo sapiens catalase (CAT), mRNA [NM_001752]	NM_001752
A_23_P358597	2.23E-06	NM_022361	NM_022361	Homo sapiens popeye domain containing 3 (POPDC3), mRNA [NM_022361]	NM_022361
A_23_P218597	2.25E-06	NM_002518	NM_002518	Homo sapiens neuronal PAS domain protein 2 (NPAS2), mRNA [NM_002518]	NM_002518
A_24_P942893	2.26E-06	NM_144582	NM_144582	Homo sapiens testis expressed sequence 261 (TEX261), mRNA [NM_144582]	NM_144582
A_23_P170464	2.30E-06	NM_031297	NM_031297	Homo sapiens hypothetical protein DKFZp761H1710 (DKFZP761H1710), mRNA [NM_031297]	NM_031297
A_24_P281395	2.30E-06	A_24_P281395			
A_23_P303242	2.30E-06	NM_005952	NM_005952	Homo sapiens metallothionein 1X (MT1X), mRNA [NM_005952]	NM_005952
A_23_P255331	2.31E-06	NM_032623	NM_032623	Homo sapiens ovary-specific acidic protein (OSAP), mRNA [NM_032623]	NM_032623
A_24_P280868	2.32E-06	NM_032916	NM_032916	Homo sapiens hypothetical protein MGC16279 (MGC16279), mRNA [NM_032916]	NM_032916
A_24_P262395	2.32E-06	NM_012138	NM_012138	Homo sapiens apoptosis antagonizing transcription factor (AATF), mRNA [NM_012138]	NM_012138
A_23_P154929	2.34E-06	NM_197966	NM_197966	Homo sapiens BH3 interacting domain death agonist (BID), transcript variant 1, mRNA [NM_197966]	NM_197966
A_23_P46447	2.35E-06	BX538051	BX538051	Homo sapiens mRNA; cDNA DKFZp686F09156 (from clone DKFZp686F09156). [BX538051]	
A_23_P216836	2.35E-06	BC070091	BC070091	Homo sapiens caspase recruitment domain family, member 9, mRNA (cDNA clone MGC:87491 IMAGE:30343821), complete cds. [BC070091]	
A_23_P73097	2.37E-06	NM_170587	NM_170587	Homo sapiens regulator of G-protein signalling 20 (RGS20), transcript variant 1, mRNA [NM_170587]	NM_170587
A_23_P143016	2.41E-06	NM_006673	NM_006673	Homo sapiens AT rich interactive domain 5A (MRF1-like) (ARID5A), transcript variant 2, mRNA [NM_006673]	NM_006673
A_32_P139894	2.43E-06	THC2307212			
A_24_P330303	2.43E-06	NM_152330	NM_152330	Homo sapiens FERM domain containing 6 (FRMD6), mRNA [NM_152330]	NM_152330
A_24_P290286	2.44E-06	NM_182904	NM_182904	Homo sapiens procollagen-proline, 2-oxoglutarate 4-dioxygenase (proline 4-hydroxylase), alpha polypeptide III (P4HA3), mRNA [NM_182904]	NM_182904
A_24_P416660	2.44E-06	NM_080665	NM_080665	Homo sapiens similar to RIKEN cDNA B230118G17 gene (MGC19604), mRNA [NM_080665]	NM_080665
A_24_P168726	2.45E-06	NM_181831	NM_181831	Homo sapiens neurofibromin 2 (bilateral acoustic neuroma) (NF2), transcript variant 13, mRNA [NM_181831]	NM_181831
A_23_P155755	2.45E-06	NM_002993	NM_002993	Homo sapiens chemokine (C-X-C motif) ligand 6 (granulocyte chemotactic protein 2) (CXCL6), mRNA [NM_002993]	NM_002993
A_32_P300427	2.47E-06	NM_153360	NM_153360	Homo sapiens hypothetical protein FLJ90166 (FLJ90166), mRNA [NM_153360]	NM_153360
A_23_P156788	2.49E-06	NM_003764	NM_003764	Homo sapiens syntaxin 11 (STX11), mRNA [NM_003764]	NM_003764
A_24_P649582	2.50E-06	BC052374	BC052374	Homo sapiens hypothetical protein LOC253039, mRNA (cDNA clone IMAGE:5749939), partial cds. [BC052374]	
A_23_P63026	2.50E-06	NM_006499	NM_006499	Homo sapiens lectin, galactoside-binding, soluble, 8 (galectin 8) (LGALS8), transcript variant 1, mRNA [NM_006499]	NM_006499
A_23_P339053	2.50E-06	NM_173501	NM_173501	Homo sapiens chromosome 16 open reading frame 52 (C16orf52), mRNA [NM_173501]	NM_173501
A_23_P123071	2.50E-06	NM_001233	NM_001233	Homo sapiens caveolin 2 (CAV2), transcript variant 1, mRNA [NM_001233]	NM_001233
A_23_P259314	2.50E-06	NM_001008	NM_001008	Homo sapiens ribosomal protein S4, Y-linked 1 (RPS4Y1), mRNA [NM_001008]	NM_001008
A_23_P60933	2.50E-06	NM_005950	NM_005950	Homo sapiens metallothionein 1G (MT1G), mRNA [NM_005950]	NM_005950
A_24_P264597	2.53E-06	A_24_P264597			
A_24_P175347	2.53E-06	NM_024295	NM_024295	Homo sapiens Der1-like domain family, member 1 (DERL1), mRNA [NM_024295]	NM_024295
A_32_P225759	2.54E-06	THC2346166			
A_23_P29769	2.54E-06	NM_015472	NM_015472	Homo sapiens WW domain containing transcription regulator 1 (WWTR1), mRNA [NM_015472]	NM_015472
A_24_P354900	2.55E-06	AK098194	AK098194	Homo sapiens cDNA FLJ40875 fis, clone UMVEN2000069. [AK098194]	
A_32_P164494	2.56E-06	H11348	H11348	H11348 ym13h04.s1 Soares infant brain 1NIB Homo sapiens cDNA clone IMAGE:47855 3' similar to gb:X63657_rna1 FOLLICULAR VARIANT TRANSLOCATION PROTEIN 1 PRECURSOR (HUMAN);, mRNA sequence [H11348]	

A_23_P321972	2.56E-06	NM_152536	NM_152536	Homo sapiens FYVE, RhoGEF and PH domain containing 5 (FGD5), mRNA [NM_152536]	NM_152536
A_24_P357936	2.57E-06	ENST00000308894		Homo sapiens HSPC324 mRNA, partial cds. [AF161442]	
A_23_P141429	2.57E-06	NM_016428	NM_016428	Homo sapiens ABI gene family, member 3 (ABI3), mRNA [NM_016428]	NM_016428
A_32_P32061	2.59E-06	NM_013310	NM_013310	Homo sapiens chromosome 2 open reading frame 27 (C2orf27), mRNA [NM_013310]	NM_013310
A_23_P30024	2.59E-06	NM_003998	NM_003998	Homo sapiens nuclear factor of kappa light polypeptide gene enhancer in B-cells 1 (p105) (NFKB1), mRNA [NM_003998]	NM_003998
A_23_P70849	2.60E-06	NM_000603	NM_000603	Homo sapiens nitric oxide synthase 3 (endothelial cell) (NOS3), mRNA [NM_000603]	NM_000603
A_23_P154667	2.62E-06	NM_000801	NM_000801	Homo sapiens FK506 binding protein 1A, 12kDa (FKBP1A), transcript variant 12B, mRNA [NM_000801]	NM_000801
A_24_P12435	2.63E-06	NM_181782	NM_181782	Homo sapiens nuclear receptor coactivator 7 (NCOA7), mRNA [NM_181782]	NM_181782
A_32_P118896	2.63E-06	THC2345956		ALU8_HUMAN (P39195) Alu subfamily SX sequence contamination warning entry, partial (10%) [THC2345956]	
A_23_P120270	2.63E-06	NM_139279	NM_139279	Homo sapiens multiple coagulation factor deficiency 2 (MCFD2), mRNA [NM_139279]	NM_139279
A_23_P324384	2.64E-06	ENST00000288666		Homo sapiens ribosomal protein S4 mRNA, complete cds. [AF497481]	
A_23_P203743	2.65E-06	NM_012296	NM_012296	Homo sapiens GRB2-associated binding protein 2 (GAB2), transcript variant 2, mRNA [NM_012296]	NM_012296
A_24_P364838	2.65E-06	U82108	U82108	Human SIP-1 mRNA, complete cds. [U82108]	
A_23_P140748	2.66E-06	NM_022910	NM_022910	Homo sapiens NDRG family member 4 (NDRG4), mRNA [NM_022910]	NM_022910
A_23_P204133	2.68E-06	NM_007210	NM_007210	Homo sapiens UDP-N-acetyl-alpha-D-galactosamine:polypeptide N-acetylgalactosaminyltransferase 6 (GalNAc-T6) (GALNT6), mRNA [NM_007210]	NM_007210
A_23_P326760	2.68E-06	NM_015460	NM_015460	Homo sapiens myosin VIIA and Rab interacting protein (MYRIP), mRNA [NM_015460]	NM_015460
A_24_P89708	2.69E-06	NM_000883	NM_000883	Homo sapiens IMP (inosine monophosphate) dehydrogenase 1 (IMPDH1), transcript variant 1, mRNA [NM_000883]	NM_000883
A_23_P91430	2.72E-06	NM_007238	NM_007238	Homo sapiens peroxisomal membrane protein 4, 24kDa (PXMP4), transcript variant 1, mRNA [NM_007238]	NM_007238
A_23_P49041	2.75E-06	NM_024956	NM_024956	Homo sapiens transmembrane protein 62 (TMEM62), mRNA [NM_024956]	NM_024956
A_24_P286687	2.75E-06	NM_016353	NM_016353	Homo sapiens zinc finger, DHHC-type containing 2 (ZDHHC2), mRNA [NM_016353]	NM_016353
A_23_P163782	2.75E-06	NM_005951	NM_005951	Homo sapiens metallothionein 1H (MT1H), mRNA [NM_005951]	NM_005951
A_24_P383523	2.78E-06	NM_015589	NM_015589	Homo sapiens sterile alpha motif domain containing 4 (SAMD4), mRNA [NM_015589]	NM_015589
A_23_P257516	2.80E-06	NM_000247	NM_000247	Homo sapiens MHC class I polypeptide-related sequence A (MICA), mRNA [NM_000247]	NM_000247
A_32_P196142	2.80E-06	THC2400010			
A_23_P163458	2.80E-06	NM_139265	NM_139265	Homo sapiens EH-domain containing 4 (EHD4), mRNA [NM_139265]	NM_139265
A_24_P410246	2.81E-06	A_24_P410246			
A_23_P111000	2.81E-06	NM_002800	NM_002800	Homo sapiens proteasome (prosome, macropain) subunit, beta type, 9 (large multifunctional protease 2) (PSMB9), transcript variant 1, mRNA [NM_002800]	NM_002800
A_23_P58796	2.82E-06	NM_001012761	NM_001012761	Homo sapiens RGM domain family, member B (RGMB), transcript variant 1, mRNA [NM_001012761]	NM_001012761
A_24_P535256	2.83E-06	AK001903	AK001903	Homo sapiens cDNA FLJ11041 fis, clone PLACE1004405. [AK001903]	
A_32_P188193	2.84E-06	THC2445517			
A_24_P234701	2.84E-06	CR621132	CR621132	full-length cDNA clone CS0DC015YK09 of Neuroblastoma Cot 25-normalized of Homo sapiens (human). [CR621132]	
A_23_P128486	2.84E-06	NM_020375	NM_020375	Homo sapiens chromosome 12 open reading frame 5 (C12orf5), mRNA [NM_020375]	NM_020375
A_23_P208310	2.86E-06	NM_012099	NM_012099	Homo sapiens CD3E antigen, epsilon polypeptide associated protein (CD3EAP), mRNA [NM_012099]	NM_012099
A_23_P77117	2.87E-06	NM_023003	NM_023003	Homo sapiens transmembrane 6 superfamily member 1 (TM6SF1), mRNA [NM_023003]	NM_023003
A_24_P658427	2.89E-06	CR619603	CR619603	full-length cDNA clone CS0DF007YC14 of Fetal brain of Homo sapiens (human). [CR619603]	
A_23_P152807	2.89E-06	NM_018346	NM_018346	Homo sapiens radical S-adenosyl methionine domain containing 1 (RSAD1), mRNA [NM_018346]	NM_018346
A_24_P354689	2.89E-06	NM_004598	NM_004598	Homo sapiens sparc/osteonectin, cwcv and kazal-like domains proteoglycan (testican) (SPOCK), mRNA [NM_004598]	NM_004598
A_24_P247987	2.90E-06	BC010099	BC010099	Homo sapiens hypothetical protein FLJ13154, mRNA (cDNA clone IMAGE:3506308), complete cds. [BC010099]	
A_23_P118536	2.92E-06	NM_018042	NM_018042	Homo sapiens hypothetical protein FLJ10260 (FLJ10260), mRNA [NM_018042]	NM_018042
A_23_P250042	2.92E-06	NM_016275	NM_016275	Homo sapiens selenoprotein T (SELT), mRNA [NM_016275]	NM_016275
A_23_P28507	2.95E-06	NM_012214	NM_012214	Homo sapiens mannosyl (alpha-1,3-)-glycoprotein beta-1,4-N-acetylglucosaminyltransferase, isoenzyme A (MGAT4A), mRNA [NM_012214]	NM_012214
A_23_P64898	2.97E-06	NM_005810	NM_005810	Homo sapiens killer cell lectin-like receptor subfamily G, member 1 (KLRG1), mRNA [NM_005810]	NM_005810
A_24_P300076	3.00E-06	NM_004186	NM_004186	Homo sapiens sema domain, immunoglobulin domain (Ig), short basic domain, secreted, (semaphorin) 3F (SEMA3F), mRNA [NM_004186]	NM_004186

A_23_P22027	3.00E-06	NM_198336	NM_198336	Homo sapiens insulin induced gene 1 (INSIG1), transcript variant 2, mRNA [NM_198336]	NM_198336
A_32_P330000	3.01E-06	NM_207477	NM_207477	Homo sapiens FLJ27365 protein (FLJ27365), mRNA [NM_207477]	NM_207477
A_23_P156852	3.01E-06	NM_206836	NM_206836	Homo sapiens peroxisomal D3,D2-enoyl-CoA isomerase (PECI), transcript variant 2, mRNA [NM_206836]	NM_206836
A_23_P362261	3.02E-06	ENST00000260257		Homo sapiens, clone IMAGE:3677165, mRNA, partial cds. [BC006136]	
A_23_P162211	3.02E-06	NM_018050	NM_018050	Homo sapiens MANSC domain containing 1 (MANSC1), mRNA [NM_018050]	NM_018050
A_23_P148372	3.03E-06	NM_001325	NM_001325	Homo sapiens cleavage stimulation factor, 3' pre-RNA, subunit 2, 64kDa (CSTF2), mRNA [NM_001325]	NM_001325
A_23_P42588	3.04E-06	NM_018384	NM_018384	Homo sapiens GTPase, IMAP family member 5 (GIMAP5), mRNA [NM_018384]	NM_018384
A_23_P135257	3.08E-06	NM_002771	NM_002771	Homo sapiens protease, serine, 3 (mesotrypsin) (PRSS3), mRNA [NM_002771]	NM_002771
A_23_P146572	3.08E-06	NM_015392	NM_015392	Homo sapiens neural proliferation, differentiation and control, 1 (NPDC1), mRNA [NM_015392]	NM_015392
A_32_P105465	3.09E-06	AK093713	AK093713	Homo sapiens cDNA FLJ36394 fis, clone THYMU2009104. [AK093713]	
A_23_P50508	3.10E-06	NM_003706	NM_003706	Homo sapiens phospholipase A2, group IVC (cytosolic, calcium-independent) (PLA2G4C), mRNA [NM_003706]	NM_003706
A_23_P61050	3.10E-06	NM_152649	NM_152649	Homo sapiens mixed lineage kinase domain-like (MLKL), mRNA [NM_152649]	NM_152649
A_24_P154948	3.10E-06	NM_002047	NM_002047	Homo sapiens glycyl-tRNA synthetase (GARS), mRNA [NM_002047]	NM_002047
A_23_P205164	3.11E-06	NM_006237	NM_006237	Homo sapiens POU domain, class 4, transcription factor 1 (POU4F1), mRNA [NM_006237]	NM_006237
A_23_P94998	3.11E-06	NM_012318	NM_012318	Homo sapiens leucine zipper-EF-hand containing transmembrane protein 1 (LETM1), mRNA [NM_012318]	NM_012318
A_23_P340333	3.11E-06	NM_033397	NM_033397	Homo sapiens KIAA1754 (KIAA1754), mRNA [NM_033397]	NM_033397
A_32_P70626	3.12E-06	THC2379184		AF030177 N-acetylglucosaminyl transferase component Gpi1 {Homo sapiens;} , partial (4%) [THC2379184]	
A_23_P24104	3.12E-06	NM_002658	NM_002658	Homo sapiens plasminogen activator, urokinase (PLAU), mRNA [NM_002658]	NM_002658
A_32_P63886	3.13E-06	A_32_P63886			
A_23_P352535	3.13E-06	NM_015568	NM_015568	Homo sapiens protein phosphatase 1, regulatory (inhibitor) subunit 16B (PPP1R16B), mRNA [NM_015568]	NM_015568
A_23_P14863	3.14E-06	NM_024111	NM_024111	Homo sapiens hypothetical protein MGC4504 (MGC4504), mRNA [NM_024111]	NM_024111
A_23_P134527	3.16E-06	NM_006555	NM_006555	Homo sapiens SNARE protein Ykt6 (YKT6), mRNA [NM_006555]	NM_006555
A_23_P19517	3.16E-06	NM_002224	NM_002224	Homo sapiens inositol 1,4,5-triphosphate receptor, type 3 (ITPR3), mRNA [NM_002224]	NM_002224
A_23_P25929	3.17E-06	NM_014239	NM_014239	Homo sapiens eukaryotic translation initiation factor 2B, subunit 2 beta, 39kDa (EIF2B2), mRNA [NM_014239]	NM_014239
A_23_P113645	3.21E-06	BC043166	BC043166	Homo sapiens potassium voltage-gated channel, shaker-related subfamily, beta member 1, transcript variant 3, mRNA (cDNA clone MGC:44147 IMAGE:5286833), complete cds. [BC043166]	
A_23_P328600	3.23E-06	NM_024959	NM_024959	Homo sapiens solute carrier family 24 (sodium/potassium/calcium exchanger), member 6 (SLC24A6), mRNA [NM_024959]	NM_024959
A_23_P95417	3.24E-06	NM_003840	NM_003840	Homo sapiens tumor necrosis factor receptor superfamily, member 10d, decoy with truncated death domain (TNFRSF10D), mRNA [NM_003840]	NM_003840
A_23_P15414	3.24E-06	NM_145351	NM_145351	Homo sapiens scavenger receptor class F, member 1 (SCARF1), transcript variant 4, mRNA [NM_145351]	NM_145351
A_23_P137035	3.24E-06	NM_003662	NM_003662	Homo sapiens pirin (iron-binding nuclear protein) (PIR), transcript variant 1, mRNA [NM_003662]	NM_003662
A_23_P422178	3.28E-06	NM_003588	NM_003588	Homo sapiens cullin 4B (CUL4B), mRNA [NM_003588]	NM_003588
A_23_P51105	3.29E-06	NM_016441	NM_016441	Homo sapiens cysteine rich transmembrane BMP regulator 1 (chordin-like) (CRIM1), mRNA [NM_016441]	NM_016441
A_23_P71928	3.30E-06	NM_005489	NM_005489	Homo sapiens SH2 domain containing 3C (SH2D3C), transcript variant 1, mRNA [NM_005489]	NM_005489
A_23_P56590	3.30E-06	NM_006333	NM_006333	Homo sapiens nuclear DNA-binding protein (C1D), transcript variant 1, mRNA [NM_006333]	NM_006333
A_23_P206212	3.30E-06	NM_003246	NM_003246	Homo sapiens thrombospondin 1 (THBS1), mRNA [NM_003246]	NM_003246
A_32_P94444	3.30E-06	NM_002770	NM_002770	Homo sapiens protease, serine, 2 (trypsin 2) (PRSS2), transcript variant 1, mRNA [NM_002770]	NM_002770
A_23_P15326	3.31E-06	NM_005082	NM_005082	Homo sapiens tripartite motif-containing 25 (TRIM25), mRNA [NM_005082]	NM_005082
A_24_P295877	3.32E-06	NM_014704	NM_014704	Homo sapiens glycine-, glutamate-, thienylcyclohexylpiperidine-binding protein (GlyBP), mRNA [NM_014704]	NM_014704
A_23_P91640	3.32E-06	NM_020437	NM_020437	Homo sapiens similar to aspartate beta hydroxylase (ASPH) (LOC57168), mRNA [NM_020437]	NM_020437
A_24_P54863	3.32E-06	NM_152400	NM_152400	Homo sapiens hypothetical protein FLJ39370 (FLJ39370), mRNA [NM_152400]	NM_152400
A_32_P118847	3.33E-06	BC044619	BC044619	Homo sapiens hypothetical gene supported by AK124699, mRNA (cDNA clone IMAGE:5270292). [BC044619]	XM_499008
A_23_P31046	3.35E-06	NM_025107	NM_025107	Homo sapiens myc target 1 (MYCT1), mRNA [NM_025107]	NM_025107
A_23_P138856	3.35E-06	NM_006442	NM_006442	Homo sapiens DR1-associated protein 1 (negative cofactor 2 alpha) (DRAP1), mRNA [NM_006442]	NM_006442
A_23_P131183	3.38E-06	NM_001485	NM_001485	Homo sapiens gastrulation brain homeo box 2 (GBX2), mRNA [NM_001485]	NM_001485

A_24_P290013	3.40E-06	ENST00000338711		ALU7_HUMAN (P39194) Alu subfamily SQ sequence contamination warning entry, partial (14%) [THC2432443]	
A_24_P165965	3.40E-06	NM_007275	NM_007275	Homo sapiens tumor suppressor candidate 2 (TUSC2), mRNA [NM_007275]	NM_007275
A_23_P56228	3.41E-06	NM_016573	NM_016573	Homo sapiens GEM interacting protein (GMIP), mRNA [NM_016573]	NM_016573
A_23_P310274	3.41E-06	NM_002770	NM_002770	Homo sapiens protease, serine, 2 (trypsin 2) (PRSS2), transcript variant 1, mRNA [NM_002770]	NM_002770
A_23_P487	3.41E-06	NM_012474	NM_012474	Homo sapiens uridine-cytidine kinase 2 (UCK2), mRNA [NM_012474]	NM_012474
A_24_P917612	3.42E-06	BC009627	BC009627	Homo sapiens within bgcn homolog (Drosophila), mRNA (cDNA clone IMAGE:3897762), partial cds. [BC009627]	
A_23_P64404	3.42E-06	NM_021727	NM_021727	Homo sapiens fatty acid desaturase 3 (FADS3), mRNA [NM_021727]	NM_021727
A_24_P136454	3.44E-06	NM_022648	NM_022648	Homo sapiens tensin 1 (TNS1), mRNA [NM_022648]	NM_022648
A_24_P333525	3.45E-06	NM_014857	NM_014857	Homo sapiens RAB GTPase activating protein 1-like (RABGAP1L), mRNA [NM_014857]	NM_014857
A_23_P26954	3.46E-06	NM_006373	NM_006373	Homo sapiens vesicle amine transport protein 1 homolog (T californica) (VAT1), mRNA [NM_006373]	NM_006373
A_23_P374862	3.47E-06	NM_000574	NM_000574	Homo sapiens decay accelerating factor for complement (CD55, Cromer blood group system) (DAF), mRNA [NM_000574]	NM_000574
A_23_P144827	3.47E-06	NM_012304	NM_012304	Homo sapiens F-box and leucine-rich repeat protein 7 (FBXL7), mRNA [NM_012304]	NM_012304
A_23_P371039	3.48E-06	NM_002531	NM_002531	Homo sapiens neurotensin receptor 1 (high affinity) (NTSR1), mRNA [NM_002531]	NM_002531
A_23_P47148	3.48E-06	NM_016931	NM_016931	Homo sapiens NADPH oxidase 4 (NOX4), mRNA [NM_016931]	NM_016931
A_24_P15043	3.51E-06	NM_025010	NM_025010	Homo sapiens kelch-like 18 (Drosophila) (KLHL18), mRNA [NM_025010]	NM_025010
A_23_P64837	3.53E-06	NM_020467	NM_020467	Homo sapiens hypothetical protein from clone 643 (LOC57228), mRNA [NM_020467]	NM_020467
A_23_P47410	3.53E-06	NM_138961	NM_138961	Homo sapiens endothelial cell adhesion molecule (ESAM), mRNA [NM_138961]	NM_138961
A_24_P354488	3.61E-06	THC2370530		ASAL_HUMAN (Q02083) N-acylsphingosine amidohydrolase-like precursor (Acid ceramidase-like protein) (ASAH-like protein) , partial (81%) [THC2370530]	
A_24_P252785	3.61E-06	NM_000314	NM_000314	Homo sapiens phosphatase and tensin homolog (mutated in multiple advanced cancers 1) (PTEN), mRNA [NM_000314]	NM_000314
A_23_P317184	3.62E-06	NM_006309	NM_006309	Homo sapiens leucine rich repeat (in FLII) interacting protein 2 (LRRFIP2), transcript variant 1, mRNA [NM_006309]	NM_006309
A_24_P639441	3.62E-06	NM_203330	NM_203330	Homo sapiens CD59 antigen p18-20 (antigen identified by monoclonal antibodies 16.3A5, EJ16, EJ30, EL32 and G344) (CD59), transcript variant 1, mRNA [NM_203330]	NM_203330
A_24_P416131	3.62E-06	NM_021149	NM_021149	Homo sapiens coactosin-like 1 (Dictyostelium) (COTL1), mRNA [NM_021149]	NM_021149
A_23_P210643	3.63E-06	NM_016397	NM_016397	Homo sapiens TH1-like (Drosophila) (TH1L), transcript variant 2, mRNA [NM_016397]	NM_016397
A_24_P192485	3.68E-06	NM_002546	NM_002546	Homo sapiens tumor necrosis factor receptor superfamily, member 11b (osteoprotegerin) (TNFRSF11B), mRNA [NM_002546]	NM_002546
A_23_P13725	3.69E-06	NM_182767	NM_182767	Homo sapiens solute carrier family 6, member 15 (SLC6A15), transcript variant 1, mRNA [NM_182767]	NM_182767
A_23_P312415	3.71E-06	NM_052866	NM_052866	Homo sapiens ADAMTS-like 1 (ADAMTSL1), transcript variant 2, mRNA [NM_052866]	NM_052866
A_23_P161918	3.72E-06	NM_024098	NM_024098	Homo sapiens hypothetical protein MGC2574 (MGC2574), mRNA [NM_024098]	NM_024098
A_23_P216188	3.77E-06	NM_001556	NM_001556	Homo sapiens inhibitor of kappa light polypeptide gene enhancer in B-cells, kinase beta (IKBKB), mRNA [NM_001556]	NM_001556
A_23_P65910	3.80E-06	NM_153260	NM_153260	Homo sapiens hypothetical protein FLJ36812 (FLJ36812), mRNA [NM_153260]	NM_153260
A_24_P649388	3.80E-06	NM_058187	NM_058187	Homo sapiens chromosome 21 open reading frame 63 (C21orf63), mRNA [NM_058187]	NM_058187
A_23_P67339	3.80E-06	NM_020650	NM_020650	Homo sapiens reticulocalbin 3, EF-hand calcium binding domain (RCN3), mRNA [NM_020650]	NM_020650
A_23_P45166	3.80E-06	NM_007198	NM_007198	Homo sapiens proline synthetase co-transcribed homolog (bacterial) (PROSC), mRNA [NM_007198]	NM_007198
A_24_P212764	3.80E-06	A_24_P212764			
A_24_P181108	3.80E-06	NM_018093	NM_018093	Homo sapiens WD repeat domain 74 (WDR74), mRNA [NM_018093]	NM_018093
A_23_P106575	3.80E-06	ENST00000245206		Homo sapiens cDNA FLJ26346 fis, clone HRT04038, highly similar to Homo sapiens titin (TTN), transcript variant N2-B. [AK129856]	
A_24_P193582	3.80E-06	NM_017702	NM_017702	Homo sapiens hypothetical protein FLJ20186 (FLJ20186), transcript variant 2, mRNA [NM_017702]	NM_017702
A_23_P154566	3.80E-06	NM_032883	NM_032883	Homo sapiens chromosome 20 open reading frame 100 (C20orf100), mRNA [NM_032883]	NM_032883
A_32_P104746	3.84E-06	NM_020972	NM_020972	Homo sapiens zinc finger, FYVE domain containing 28 (ZFYVE28), mRNA [NM_020972]	NM_020972
A_23_P46426	3.84E-06	NM_001554	NM_001554	Homo sapiens cysteine-rich, angiogenic inducer, 61 (CYR61), mRNA [NM_001554]	NM_001554
A_24_P25354	3.85E-06	NM_006283	NM_006283	Homo sapiens transforming, acidic coiled-coil containing protein 1 (TACC1), mRNA [NM_006283]	NM_006283
A_23_P210939	3.85E-06	NM_181468	NM_181468	Homo sapiens integrin beta 4 binding protein (ITGB4BP), transcript variant 2, mRNA [NM_181468]	NM_181468
A_23_P159227	3.87E-06	NM_207191	NM_207191	Homo sapiens a disintegrin and metalloproteinase domain 15 (metargidin) (ADAM15), transcript variant 1, mRNA [NM_207191]	NM_207191
A_23_P9293	3.87E-06	NM_004817	NM_004817	Homo sapiens tight junction protein 2 (zona occludens 2) (TJP2), transcript variant 1, mRNA [NM_004817]	NM_004817

A_23_P157117	3.89E-06	NM_182898	NM_182898	Homo sapiens cAMP responsive element binding protein 5 (CREB5), transcript variant 1, mRNA [NM_182898]	NM_182898
A_23_P135294	3.89E-06	NM_000692	NM_000692	Homo sapiens aldehyde dehydrogenase 1 family, member B1 (ALDH1B1), nuclear gene encoding mitochondrial protein, mRNA [NM_000692]	NM_000692
A_24_P382533	3.92E-06	NM_005386	NM_005386	Homo sapiens neuronatin (NNAT), transcript variant 1, mRNA [NM_005386]	NM_005386
A_23_P35912	3.92E-06	NM_033306	NM_033306	Homo sapiens caspase 4, apoptosis-related cysteine protease (CASP4), transcript variant gamma, mRNA [NM_033306]	NM_033306
A_23_P141894	3.93E-06	NM_006505	NM_006505	Homo sapiens poliovirus receptor (PVR), mRNA [NM_006505]	NM_006505
A_23_P145024	3.93E-06	NM_000024	NM_000024	Homo sapiens adrenergic, beta-2-, receptor, surface (ADRB2), mRNA [NM_000024]	NM_000024
A_23_P215525	3.93E-06	NM_145323	NM_145323	Homo sapiens oxysterol binding protein-like 3 (OSBPL3), transcript variant 5, mRNA [NM_145323]	NM_145323
A_32_P96719	3.94E-06	NM_024745	NM_024745	Homo sapiens SHC SH2-domain binding protein 1 (SHCBP1), mRNA [NM_024745]	NM_024745
A_23_P134946	3.94E-06	NM_014665	NM_014665	Homo sapiens leucine rich repeat containing 14 (LRRC14), mRNA [NM_014665]	NM_014665
A_23_P24375	3.94E-06	NM_017670	NM_017670	Homo sapiens OTU domain, ubiquitin aldehyde binding 1 (OTUB1), mRNA [NM_017670]	NM_017670
A_24_P186986	3.94E-06	ENST00000322426		PREDICTED: Homo sapiens similar to ATP synthase lipid-binding protein, mitochondrial precursor (ATP synthase proteolipid P1) (ATPase protein 9) (ATPase subunit C) (LOC390424), mRNA [XM_497383]	XM_497383
A_23_P256487	3.95E-06	THC2372784			
A_23_P202720	4.00E-06	NM_018389	NM_018389	Homo sapiens solute carrier family 35, member C1 (SLC35C1), mRNA [NM_018389]	NM_018389
A_32_P42925	4.02E-06	NM_033546	NM_033546	Homo sapiens myosin regulatory light chain MRLC2 (MRLC2), mRNA [NM_033546]	NM_033546
A_24_P199774	4.03E-06	NM_022164	NM_022164	Homo sapiens lipocalin 7 (LCN7), mRNA [NM_022164]	NM_022164
A_23_P63825	4.04E-06	NM_002079	NM_002079	Homo sapiens glutamic-oxaloacetic transaminase 1, soluble (aspartate aminotransferase 1) (GOT1), mRNA [NM_002079]	NM_002079
A_23_P111413	4.07E-06	NM_018718	NM_018718	Homo sapiens testis specific, 14 (TSGA14), mRNA [NM_018718]	NM_018718
A_23_P401176	4.08E-06	U77706	U77706	Human laminin alpha 4 chain (LAMA4*-1) mRNA, complete cds. [U77706]	
A_24_P7594	4.12E-06	AK097266	AK097266	Homo sapiens cDNA FLJ39947 fis, clone SPLEN2024232. [AK097266]	
A_24_P76759	4.12E-06	BC021301	BC021301	Homo sapiens FERM, RhoGEF and pleckstrin domain protein 2, mRNA (cDNA clone MGC:29593 IMAGE:5013180), complete cds. [BC021301]	
A_24_P636451	4.12E-06	AK023559	AK023559	Homo sapiens cDNA FLJ13497 fis, clone PLACE1004518. [AK023559]	
A_24_P107528	4.14E-06	NM_024738	NM_024738	Homo sapiens hypothetical protein FLJ21415 (FLJ21415), mRNA [NM_024738]	NM_024738
A_23_P218646	4.14E-06	NM_032945	NM_032945	Homo sapiens tumor necrosis factor receptor superfamily, member 6b, decoy (TNFRSF6B), transcript variant M68C, mRNA [NM_032945]	NM_032945
A_24_P891276	4.16E-06	A_24_P891276			
A_23_P86818	4.16E-06	NM_004813	NM_004813	Homo sapiens peroxisomal biogenesis factor 16 (PEX16), transcript variant 1, mRNA [NM_004813]	NM_004813
A_24_P349547	4.16E-06	A_24_P349547			
A_23_P53152	4.20E-06	NM_020642	NM_020642	Homo sapiens chromosome 11 open reading frame 17 (C11orf17), transcript variant 2, mRNA [NM_020642]	NM_020642
A_23_P71037	4.23E-06	NM_000600	NM_000600	Homo sapiens interleukin 6 (interferon, beta 2) (IL6), mRNA [NM_000600]	NM_000600
A_23_P47247	4.27E-06	NM_018393	NM_018393	Homo sapiens hypothetical protein FLJ11336 (FLJ11336), mRNA [NM_018393]	NM_018393
A_24_P166073	4.28E-06	NM_153757	NM_153757	Homo sapiens nucleosome assembly protein 1-like 5 (NAP1L5), mRNA [NM_153757]	NM_153757
A_23_P259292	4.28E-06	NM_015645	NM_015645	Homo sapiens C1q and tumor necrosis factor related protein 5 (C1QTNF5), mRNA [NM_015645]	NM_015645
A_23_P104881	4.28E-06	NM_019055	NM_019055	Homo sapiens roundabout homolog 4, magic roundabout (Drosophila) (ROBO4), mRNA [NM_019055]	NM_019055
A_23_P74778	4.28E-06	NM_024579	NM_024579	Homo sapiens chromosome 1 open reading frame 54 (C1orf54), mRNA [NM_024579]	NM_024579
A_23_P33187	4.31E-06	NM_032318	NM_032318	Homo sapiens hippocampus abundant gene transcript-like 2 (HIATL2), mRNA [NM_032318]	NM_032318
A_23_P144668	4.31E-06	NM_138809	NM_138809	Homo sapiens similar to mouse 2310016A09Rik gene (LOC134147), mRNA [NM_138809]	NM_138809
A_23_P302125	4.32E-06	NM_178566	NM_178566	Homo sapiens zinc finger, DHHC-type containing 21 (ZDHHC21), mRNA [NM_178566]	NM_178566
A_24_P137713	4.33E-06	NM_030899	NM_030899	Homo sapiens zinc finger protein 323 (ZNF323), transcript variant 1, mRNA [NM_030899]	NM_030899
A_23_P68529	4.33E-06	NM_033453	NM_033453	Homo sapiens inosine triphosphatase (nucleoside triphosphate pyrophosphatase) (ITPA), transcript variant 1, mRNA [NM_033453]	NM_033453
A_24_P186370	4.35E-06	NM_002444	NM_002444	Homo sapiens moesin (MSN), mRNA [NM_002444]	NM_002444
A_23_P207138	4.35E-06	NM_203351	NM_203351	Homo sapiens mitogen-activated protein kinase kinase kinase 3 (MAP3K3), transcript variant 1, mRNA [NM_203351]	NM_203351
A_23_P23191	4.37E-06	NM_005529	NM_005529	Homo sapiens heparan sulfate proteoglycan 2 (perlecan) (HSPG2), mRNA [NM_005529]	NM_005529
A_23_P24903	4.39E-06	NM_176072	NM_176072	Homo sapiens purinergic receptor P2Y, G-protein coupled, 2 (P2RY2), transcript variant 1, mRNA [NM_176072]	NM_176072
A_24_P287075	4.43E-06	NM_004579	NM_004579	Homo sapiens mitogen-activated protein kinase kinase kinase kinase 2 (MAP4K2), mRNA [NM_004579]	NM_004579

A_32_P194848	4.44E-06	NM_003564	NM_003564	Homo sapiens transgelin 2 (TAGLN2), mRNA [NM_003564]	NM_003564
A_23_P86653	4.44E-06	NM_002727	NM_002727	Homo sapiens proteoglycan 1, secretory granule (PRG1), mRNA [NM_002727]	NM_002727
A_24_P471121	4.52E-06	XM_497677	XM_497677	PREDICTED: Homo sapiens similar to GYG protein (LOC441885), mRNA [XM_497677]	XM_497677
A_23_P389250	4.53E-06	XM_292021	XM_292021	PREDICTED: Homo sapiens similar to hypothetical protein (LOC341346), mRNA [XM_292021]	XM_292021
A_24_P41781	4.53E-06	A_24_P41781			
A_23_P422144	4.55E-06	NM_153690	NM_153690	Homo sapiens family with sequence similarity 43, member A (FAM43A), mRNA [NM_153690]	NM_153690
A_24_P245767	4.56E-06	NM_006936	NM_006936	Homo sapiens SMT3 suppressor of mif two 3 homolog 3 (yeast) (SUMO3), mRNA [NM_006936]	NM_006936
A_32_P168727	4.57E-06	A_32_P168727			
A_23_P52382	4.57E-06	NM_017626	NM_017626	Homo sapiens DnaJ (Hsp40) homolog, subfamily B, member 12 (DNAJB12), transcript variant 2, mRNA [NM_017626]	NM_017626
A_24_P274270	4.57E-06	NM_139266	NM_139266	Homo sapiens signal transducer and activator of transcription 1, 91kDa (STAT1), transcript variant beta, mRNA [NM_139266]	NM_139266
A_24_P13991	4.57E-06	NM_170607	NM_170607	Homo sapiens MAX-like protein X (MLX), transcript variant 3, mRNA [NM_170607]	NM_170607
A_24_P337796	4.57E-06	NM_004760	NM_004760	Homo sapiens serine/threonine kinase 17a (apoptosis-inducing) (STK17A), mRNA [NM_004760]	NM_004760
A_23_P502641	4.58E-06	NM_032970	NM_032970	Homo sapiens SEC22 vesicle trafficking protein-like 3 (S. cerevisiae) (SEC22L3), transcript variant 1, mRNA [NM_032970]	NM_032970
A_24_P57730	4.58E-06	NM_181304	NM_181304	Homo sapiens mitochondrial ribosomal protein L52 (MRPL52), nuclear gene encoding mitochondrial protein, transcript variant 4, mRNA [NM_181304]	NM_181304
A_24_P344711	4.59E-06	NM_020132	NM_020132	Homo sapiens 1-acylglycerol-3-phosphate O-acyltransferase 3 (AGPAT3), mRNA [NM_020132]	NM_020132
A_23_P121141	4.61E-06	NM_014065	NM_014065	Homo sapiens HT001 protein (HT001), mRNA [NM_014065]	NM_014065
A_23_P203505	4.61E-06	NM_033034	NM_033034	Homo sapiens tripartite motif-containing 5 (TRIM5), transcript variant alpha, mRNA [NM_033034]	NM_033034
A_23_P302914	4.62E-06	NM_020972	NM_020972	Homo sapiens zinc finger, FYVE domain containing 28 (ZFYVE28), mRNA [NM_020972]	NM_020972
A_32_P115050	4.62E-06	CR620567	CR620567	full-length cDNA clone CS0DH007YA24 of T cells (Jurkat cell line) of Homo sapiens (human). [CR620567]	
A_24_P269687	4.62E-06	NM_000113	NM_000113	Homo sapiens torsin family 1, member A (torsin A) (TOR1A), mRNA [NM_000113]	NM_000113
A_23_P141376	4.64E-06	NM_012232	NM_012232	Homo sapiens polymerase I and transcript release factor (PTRF), mRNA [NM_012232]	NM_012232
A_23_P111260	4.67E-06	NM_002526	NM_002526	Homo sapiens 5'-nucleotidase, ecto (CD73) (NT5E), mRNA [NM_002526]	NM_002526
A_23_P208523	4.68E-06	NM_024316	NM_024316	Homo sapiens leukocyte receptor cluster (LRC) member 1 (LENG1), mRNA [NM_024316]	NM_024316
A_24_P62615	4.68E-06	NM_006367	NM_006367	Homo sapiens CAP, adenylate cyclase-associated protein 1 (yeast) (CAP1), mRNA [NM_006367]	NM_006367
A_24_P349783	4.74E-06	A_24_P349783			
A_23_P154874	4.74E-06	NM_006052	NM_006052	Homo sapiens Down syndrome critical region gene 3 (DSCR3), mRNA [NM_006052]	NM_006052
A_23_P154037	4.74E-06	NM_001159	NM_001159	Homo sapiens aldehyde oxidase 1 (AOX1), mRNA [NM_001159]	NM_001159
A_23_P214168	4.74E-06	NM_004370	NM_004370	Homo sapiens collagen, type XII, alpha 1 (COL12A1), transcript variant long, mRNA [NM_004370]	NM_004370
A_23_P27721	4.75E-06	NM_014959	NM_014959	Homo sapiens caspase recruitment domain family, member 8 (CARD8), mRNA [NM_014959]	NM_014959
A_23_P28772	4.80E-06	NM_018478	NM_018478	Homo sapiens chromosome 20 open reading frame 35 (C20orf35), mRNA [NM_018478]	NM_018478
A_32_P180265	4.81E-06	THC2336839		Q7QV00 (Q7QV00) GLP_61_1203_1451, partial (17%) [THC2336839]	
A_23_P47790	4.81E-06	NM_005371	NM_005371	Homo sapiens methyltransferase like 1 (METTL1), transcript variant 1, mRNA [NM_005371]	NM_005371
A_23_P129496	4.82E-06	NM_001795	NM_001795	Homo sapiens cadherin 5, type 2, VE-cadherin (vascular epithelium) (CDH5), mRNA [NM_001795]	NM_001795
A_24_P264143	4.82E-06	XM_377109	XM_377109	PREDICTED: Homo sapiens similar to 40S ribosomal protein SA (p40) (34/67 kDa laminin receptor) (Colon carcinoma laminin-binding protein) (NEM/1CHD4) (Multidrug resistance-associated protein MGr1-Ag) (LOC401638), mRNA [XM_377109]	XM_377109
A_23_P365817	4.83E-06	NM_138689	NM_138689	Homo sapiens protein phosphatase 1, regulatory (inhibitor) subunit 14B (PPP1R14B), mRNA [NM_138689]	NM_138689
A_24_P600036	4.84E-06	A_24_P600036			
A_23_P89550	4.84E-06	NM_033004	NM_033004	Homo sapiens NACHT, leucine rich repeat and PYD (pyrin domain) containing 1 (NALP1), transcript variant 1, mRNA [NM_033004]	NM_033004
A_23_P154367	4.84E-06	NM_004226	NM_004226	Homo sapiens serine/threonine kinase 17b (apoptosis-inducing) (STK17B), mRNA [NM_004226]	NM_004226
A_24_P324886	4.84E-06	AK098050	AK098050	Homo sapiens cDNA FLJ40731 fis, clone TKIDN2000521. [AK098050]	
A_23_P121795	4.84E-06	NM_021069	NM_021069	Homo sapiens Arg/Abl-interacting protein ArgBP2 (ARGBP2), transcript variant 2, mRNA [NM_021069]	NM_021069
A_32_P516818	4.88E-06	ENST00000312275		Homo sapiens, Similar to hypothetical protein BC010062, clone IMAGE:5197576, mRNA, partial cds. [BC029129]	XM_496664
A_23_P70785	4.89E-06	NM_001624	NM_001624	Homo sapiens absent in melanoma 1 (AIM1), mRNA [NM_001624]	NM_001624

A_23_P369966	4.92E-06	NM_152426	NM_152426	Homo sapiens apolipoprotein B mRNA editing enzyme, catalytic polypeptide-like 3D (APOBEC3D), mRNA [NM_152426]	NM_152426
A_23_P873	4.92E-06	NM_004848	NM_004848	Homo sapiens chromosome 1 open reading frame 38 (C1orf38), mRNA [NM_004848]	NM_004848
A_23_P46429	4.92E-06	NM_001554	NM_001554	Homo sapiens cysteine-rich, angiogenic inducer, 61 (CYR61), mRNA [NM_001554]	NM_001554
A_23_P311885	4.93E-06	NM_032438	NM_032438	Homo sapiens l(3)mbt-like 3 (Drosophila) (L3MBTL3), transcript variant 1, mRNA [NM_032438]	NM_032438
A_23_P129246	4.93E-06	NM_025201	NM_025201	Homo sapiens PH domain-containing protein (pp9099), mRNA [NM_025201]	NM_025201
A_23_P352861	4.93E-06	BC028083	BC028083	Homo sapiens T cell receptor beta variable 5-4, mRNA (cDNA clone MGC:40031 IMAGE:5217067), complete cds. [BC028083]	
A_24_P72479	4.93E-06	NM_006409	NM_006409	Homo sapiens actin related protein 2/3 complex, subunit 1A, 41kDa (ARPC1A), mRNA [NM_006409]	NM_006409
A_23_P218476	4.94E-06	NM_030578	NM_030578	Homo sapiens hypothetical protein MGC4093 (MGC4093), mRNA [NM_030578]	NM_030578
A_32_P143824	5.02E-06	A_32_P143824			
A_23_P95672	5.04E-06	NM_006548	NM_006548	Homo sapiens IGF-II mRNA-binding protein 2 (IMP-2), transcript variant 1, mRNA [NM_006548]	NM_006548
A_24_P66027	5.04E-06	NM_004900	NM_004900	Homo sapiens apolipoprotein B mRNA editing enzyme, catalytic polypeptide-like 3B (APOBEC3B), mRNA [NM_004900]	NM_004900
A_23_P89835	5.07E-06	CR597075	CR597075	full-length cDNA clone CS0DF037YI18 of Fetal brain of Homo sapiens (human). [CR597075]	
A_24_P42603	5.10E-06	NM_007118	NM_007118	Homo sapiens triple functional domain (PTPRF interacting) (TRIO), mRNA [NM_007118]	NM_007118
A_23_P6878	5.13E-06	NM_004186	NM_004186	Homo sapiens sema domain, immunoglobulin domain (Ig), short basic domain, secreted, (semaphorin) 3F (SEMA3F), mRNA [NM_004186]	NM_004186
A_23_P5831	5.13E-06	NM_134421	NM_134421	Homo sapiens hippocalcin-like 1 (HPCAL1), transcript variant 2, mRNA [NM_134421]	NM_134421
A_23_P130995	5.14E-06	NM_144779	NM_144779	Homo sapiens FXYD domain containing ion transport regulator 5 (FXYD5), transcript variant 1, mRNA [NM_144779]	NM_144779
A_23_P168229	5.14E-06	NM_022085	NM_022085	Homo sapiens thioredoxin domain containing 5 (TXNDC5), transcript variant 2, mRNA [NM_022085]	NM_022085
A_24_P218757	5.15E-06	NM_018172	NM_018172	Homo sapiens hypothetical protein FLJ10661 (FLJ10661), transcript variant 1, mRNA [NM_018172]	NM_018172
A_32_P34444	5.16E-06	NM_025135	NM_025135	Homo sapiens formin homology 2 domain containing 3 (FHOD3), mRNA [NM_025135]	NM_025135
A_23_P216949	5.18E-06	NM_005388	NM_005388	Homo sapiens phosducin-like (PDCL), mRNA [NM_005388]	NM_005388
A_23_P61466	5.19E-06	NM_174941	NM_174941	Homo sapiens scavenger receptor cysteine-rich type 1 protein M160 (M160), mRNA [NM_174941]	NM_174941
A_32_P119197	5.19E-06	BF686720	BF686720	602143863F1 NIH_MGC_46 Homo sapiens cDNA clone IMAGE:4304846 5', mRNA sequence [BF686720]	
A_24_P250176	5.19E-06	NM_170607	NM_170607	Homo sapiens MAX-like protein X (MLX), transcript variant 3, mRNA [NM_170607]	NM_170607
A_23_P62840	5.19E-06	NM_024640	NM_024640	Homo sapiens ischemia/reperfusion inducible protein (YRDC), mRNA [NM_024640]	NM_024640
A_24_P95439	5.20E-06	NM_001014438	NM_001014438	Homo sapiens cysteinyl-tRNA synthetase (CARS), transcript variant 4, mRNA [NM_001014438]	NM_001014438
A_32_P57160	5.21E-06	NM_207507	NM_207507	Homo sapiens FLJ45202 protein (FLJ45202), mRNA [NM_207507]	NM_207507
A_23_P107963	5.22E-06	NM_000148	NM_000148	Homo sapiens fucosyltransferase 1 (galactoside 2-alpha-L-fucosyltransferase) (FUT1), mRNA [NM_000148]	NM_000148
A_23_P115316	5.24E-06	NM_022371	NM_022371	Homo sapiens torsin family 3, member A (TOR3A), mRNA [NM_022371]	NM_022371
A_24_P803809	5.25E-06	A_24_P803809			
A_23_P139486	5.26E-06	NM_004642	NM_004642	Homo sapiens CDK2-associated protein 1 (CDK2AP1), mRNA [NM_004642]	NM_004642
A_23_P503200	5.31E-06	NM_018288	NM_018288	Homo sapiens PHD finger protein 10 (PHF10), transcript variant 1, mRNA [NM_018288]	NM_018288
A_24_P416656	5.32E-06	NM_080665	NM_080665	Homo sapiens similar to RIKEN cDNA B230118G17 gene (MGC19604), mRNA [NM_080665]	NM_080665
A_23_P26375	5.34E-06	NM_022914	NM_022914	Homo sapiens adrenocortical dysplasia homolog (mouse) (ACD), mRNA [NM_022914]	NM_022914
A_23_P24555	5.38E-06	NM_015157	NM_015157	Homo sapiens pleckstrin homology-like domain, family B, member 1 (PHLDB1), mRNA [NM_015157]	NM_015157
A_23_P127891	5.39E-06	NM_170735	NM_170735	Homo sapiens brain-derived neurotrophic factor (BDNF), transcript variant 1, mRNA [NM_170735]	NM_170735
A_24_P918317	5.40E-06	NM_015881	NM_015881	Homo sapiens dickkopf homolog 3 (Xenopus laevis) (DKK3), transcript variant 1, mRNA [NM_015881]	NM_015881
A_23_P69310	5.42E-06	NM_003965	NM_003965	Homo sapiens chemokine (C-C motif) receptor-like 2 (CCRL2), mRNA [NM_003965]	NM_003965
A_24_P406060	5.42E-06	NM_182757	NM_182757	Homo sapiens IBR domain containing 2 (IBRDC2), mRNA [NM_182757]	NM_182757
A_23_P154086	5.42E-06	NM_004328	NM_004328	Homo sapiens BCS1-like (yeast) (BCS1L), mRNA [NM_004328]	NM_004328
A_23_P149200	5.46E-06	NM_001255	NM_001255	Homo sapiens CDC20 cell division cycle 20 homolog (S. cerevisiae) (CDC20), mRNA [NM_001255]	NM_001255
A_24_P247660	5.46E-06	NM_001002033	NM_001002033	Homo sapiens hematological and neurological expressed 1 (HN1), transcript variant 3, mRNA [NM_001002033]	NM_001002033
A_23_P73577	5.49E-06	ENST00000297871		Homo sapiens t-complex-associated-testis-expressed 1-like, mRNA (cDNA clone MGC:5007 IMAGE:3448623), complete cds. [BC000968]	
A_32_P99171	5.50E-06	AF131762	AF131762	Homo sapiens clone 25218 mRNA sequence. [AF131762]	

A_24_P398323	5.51E-06	NM_001003827	NM_001003827	Homo sapiens tripartite motif-containing 34 (TRIM34), transcript variant 4, mRNA [NM_001003827]	NM_001003827
A_23_P142849	5.52E-06	NM_005168	NM_005168	Homo sapiens Rho family GTPase 3 (RND3), mRNA [NM_005168]	NM_005168
A_24_P120115	5.59E-06	NM_003879	NM_003879	Homo sapiens CASP8 and FADD-like apoptosis regulator (CFLAR), mRNA [NM_003879]	NM_003879
A_24_P307854	5.60E-06	BC006426	BC006426	Homo sapiens F-box and leucine-rich repeat protein 18, mRNA (cDNA clone MGC:12908 IMAGE:4026501), complete cds. [BC006426]	
A_23_P330895	5.60E-06	NM_001001716	NM_001001716	Homo sapiens nuclear factor of kappa light polypeptide gene enhancer in B-cells inhibitor, beta (NFKBIB), transcript variant 2, mRNA [NM_001001716]	NM_001001716
A_24_P196688	5.60E-06	BC056891	BC056891	Homo sapiens caspase recruitment domain family, member 8, mRNA (cDNA clone MGC:65033 IMAGE:6144772), complete cds. [BC056891]	
A_23_P122662	5.60E-06	NM_018988	NM_018988	Homo sapiens glucose-fructose oxidoreductase domain containing 1 (GFOD1), mRNA [NM_018988]	NM_018988
A_23_P417372	5.61E-06	NM_017894	NM_017894	Homo sapiens zinc finger and SCAN domain containing 2 (ZSCAN2), transcript variant 2, mRNA [NM_017894]	NM_017894
A_23_P358548	5.64E-06	NM_199329	NM_199329	Homo sapiens solute carrier family 43, member 3 (SLC43A3), mRNA [NM_199329]	NM_199329
A_23_P109393	5.64E-06	NM_001670	NM_001670	Homo sapiens armadillo repeat gene deletes in velocardiofacial syndrome (ARVCF), mRNA [NM_001670]	NM_001670
A_32_P117758	5.65E-06	BG208131	BG208131	RST27621 Athersys RAGE Library Homo sapiens cDNA, mRNA sequence [BG208131]	
A_23_P127957	5.67E-06	NM_182904	NM_182904	Homo sapiens procollagen-proline, 2-oxoglutarate 4-dioxygenase (proline 4-hydroxylase), alpha polypeptide III (P4HA3), mRNA [NM_182904]	NM_182904
A_23_P57588	5.70E-06	NM_016426	NM_016426	Homo sapiens G-2 and S-phase expressed 1 (GTSE1), mRNA [NM_016426]	NM_016426
A_24_P349489	5.70E-06	A_24_P349489			
A_23_P144807	5.72E-06	AF440762	AF440762	Homo sapiens septin SEPT8_v2 (KIAA0202) mRNA, partial cds, alternatively spliced. [AF440762]	XM_034872
A_24_P371758	5.72E-06	NM_032479	NM_032479	Homo sapiens mitochondrial ribosomal protein L36 (MRPL36), nuclear gene encoding mitochondrial protein, mRNA [NM_032479]	NM_032479
A_23_P356330	5.75E-06	NM_015704	NM_015704	Homo sapiens DNA segment, Chr 15, Wayne State University 75, expressed (D15Wsu75e), mRNA [NM_015704]	NM_015704
A_23_P21436	5.76E-06	NM_015651	NM_015651	Homo sapiens PHD finger protein 19 (PHF19), transcript variant 1, mRNA [NM_015651]	NM_015651
A_32_P132206	5.76E-06	NM_017414	NM_017414	Homo sapiens ubiquitin specific protease 18 (USP18), mRNA [NM_017414]	NM_017414
A_23_P163475	5.80E-06	NM_207380	NM_207380	Homo sapiens FLJ43339 protein (FLJ43339), mRNA [NM_207380]	NM_207380
A_24_P120251	5.80E-06	NM_138786	NM_138786	Homo sapiens transmembrane 4 L six family member 18 (TM4SF18), mRNA [NM_138786]	NM_138786
A_23_P13065	5.80E-06	NM_019028	NM_019028	Homo sapiens zinc finger, DHHC-type containing 13 (ZDHHC13), transcript variant 1, mRNA [NM_019028]	NM_019028
A_23_P149111	5.81E-06	NM_005401	NM_005401	Homo sapiens protein tyrosine phosphatase, non-receptor type 14 (PTPN14), mRNA [NM_005401]	NM_005401
A_32_P226078	5.83E-06	NM_016178	NM_016178	Homo sapiens ornithine decarboxylase antizyme 3 (OAZ3), mRNA [NM_016178]	NM_016178
A_23_P326204	5.83E-06	NM_152621	NM_152621	Homo sapiens hypothetical protein MGC26963 (MGC26963), mRNA [NM_152621]	NM_152621
A_23_P398294	5.86E-06	NM_003959	NM_003959	Homo sapiens huntingtin interacting protein-1-related (HIP1R), mRNA [NM_003959]	NM_003959
A_32_P135385	5.87E-06	NM_001013694	NM_001013694	Homo sapiens similar to SRR1-like protein (LOC402055), mRNA [NM_001013694]	NM_001013694
A_23_P100868	5.87E-06	AK026518	AK026518	Homo sapiens cDNA: FLJ22865 fis, clone KAT02171. [AK026518]	
A_23_P43800	5.90E-06	NM_015201	NM_015201	Homo sapiens block of proliferation 1 (BOP1), mRNA [NM_015201]	NM_015201
A_24_P229164	5.91E-06	NM_003959	NM_003959	Homo sapiens huntingtin interacting protein-1-related (HIP1R), mRNA [NM_003959]	NM_003959
A_24_P261083	5.91E-06	NM_004337	NM_004337	Homo sapiens chromosome 8 open reading frame 1 (C8orf1), mRNA [NM_004337]	NM_004337
A_23_P206960	5.91E-06	NM_003003	NM_003003	Homo sapiens SEC14-like 1 (S. cerevisiae) (SEC14L1), mRNA [NM_003003]	NM_003003
A_23_P125624	5.91E-06	NM_012332	NM_012332	Homo sapiens acyl-Coenzyme A thioesterase 2, mitochondrial (ACATE2), mRNA [NM_012332]	NM_012332
A_24_P235870	5.94E-06	NM_018320	NM_018320	Homo sapiens ring finger protein 121 (RNF121), transcript variant 1, mRNA [NM_018320]	NM_018320
A_24_P801197	5.96E-06	AK131396	AK131396	Homo sapiens cDNA FLJ16488 fis, clone BRTHA3008826. [AK131396]	
A_23_P212870	5.99E-06	NM_005900	NM_005900	Homo sapiens SMAD, mothers against DPP homolog 1 (Drosophila) (SMAD1), transcript variant 1, mRNA [NM_005900]	NM_005900
A_23_P162171	6.04E-06	NM_006500	NM_006500	Homo sapiens melanoma cell adhesion molecule (MCAM), mRNA [NM_006500]	NM_006500
A_24_P227831	6.05E-06	NM_019862	NM_019862	Homo sapiens ATP-binding cassette, sub-family C (CFTR/MRP), member 1 (ABCC1), transcript variant 2, mRNA [NM_019862]	NM_019862
A_24_P708161	6.06E-06	A_24_P708161			
A_23_P205296	6.06E-06	NM_018199	NM_018199	Homo sapiens chromosome 14 open reading frame 114 (C14orf114), mRNA [NM_018199]	NM_018199
A_23_P630	6.06E-06	NM_018116	NM_018116	Homo sapiens misato homolog 1 (Drosophila) (MSTO1), mRNA [NM_018116]	NM_018116
A_24_P108779	6.06E-06	NM_024681	NM_024681	Homo sapiens potassium channel tetramerisation domain containing 17 (KCTD17), mRNA [NM_024681]	NM_024681
A_24_P225448	6.06E-06	NM_004078	NM_004078	Homo sapiens cysteine and glycine-rich protein 1 (CSRP1), mRNA [NM_004078]	NM_004078
A_32_P147949	6.08E-06	THC2441537			

A_24_P212715	6.10E-06	A_24_P212715			
A_24_P194017	6.12E-06	NM_015953	NM_015953	Homo sapiens nitric oxide synthase interacting protein (NOSIP), mRNA [NM_015953]	NM_015953
A_23_P68610	6.12E-06	NM_012112	NM_012112	Homo sapiens TPX2, microtubule-associated, homolog (Xenopus laevis) (TPX2), mRNA [NM_012112]	NM_012112
A_24_P68162	6.23E-06	NM_173602	NM_173602	Homo sapiens KIAA1463 protein (KIAA1463), mRNA [NM_173602]	NM_173602
A_23_P42257	6.23E-06	NM_003897	NM_003897	Homo sapiens immediate early response 3 (IER3), transcript variant short, mRNA [NM_003897]	NM_003897
A_23_P311912	6.26E-06	BC090889	BC090889	Homo sapiens chromosome 14 open reading frame 78, mRNA (cDNA clone MGC:102983 IMAGE:30387958), complete cds. [BC090889]	XM_290629
A_23_P401904	6.33E-06	NM_001009936	NM_001009936	Homo sapiens PHD finger protein 19 (PHF19), transcript variant 2, mRNA [NM_001009936]	NM_001009936
A_24_P833097	6.36E-06	BE243669	BE243669	BE243669 TCBAP1D1375 Pediatric pre-B cell acute lymphoblastic leukemia Baylor-HGSC project=TCBA Homo sapiens cDNA clone TCBAP1375, mRNA sequence [BE243669]	
A_23_P64044	6.37E-06	NM_178443	NM_178443	Homo sapiens UNC-112 related protein 2 (URP2), transcript variant URP2LF, mRNA [NM_178443]	NM_178443
A_23_P138495	6.40E-06	NM_006504	NM_006504	Homo sapiens protein tyrosine phosphatase, receptor type, E (PTPRE), transcript variant 1, mRNA [NM_006504]	NM_006504
A_23_P47818	6.41E-06	NM_198324	NM_198324	Homo sapiens citrate synthase (CS), nuclear gene encoding mitochondrial protein, transcript variant 2, mRNA [NM_198324]	NM_198324
A_32_P143850	6.42E-06	BC024198	BC024198	Homo sapiens, clone IMAGE:4821649, mRNA. [BC024198]	XR_000273
A_23_P355075	6.45E-06	AK023669	AK023669	Homo sapiens cDNA FLJ13607 fis, clone PLACE1010624. [AK023669]	
A_23_P111804	6.47E-06	NM_022750	NM_022750	Homo sapiens poly (ADP-ribose) polymerase family, member 12 (PARP12), mRNA [NM_022750]	NM_022750
A_32_P86962	6.48E-06	NM_203448	NM_203448	Homo sapiens hypothetical protein MGC21881 (MGC21881), mRNA [NM_203448]	NM_203448
A_24_P246710	6.48E-06	NM_017954	NM_017954	Homo sapiens Ca2+-dependent activator protein for secretion 2 (CADPS2), transcript variant 1, mRNA [NM_017954]	NM_017954
A_32_P201496	6.49E-06	NM_001018052	NM_001018052	Homo sapiens polymerase (RNA) III (DNA directed) polypeptide H (22.9kD) (POLR3H), transcript variant 2, mRNA [NM_001018052]	NM_001018052
A_24_P205589	6.49E-06	NM_181864	NM_181864	Homo sapiens brain acyl-CoA hydrolase (BACH), transcript variant hBACHb, mRNA [NM_181864]	NM_181864
A_23_P59005	6.50E-06	NM_000593	NM_000593	Homo sapiens transporter 1, ATP-binding cassette, sub-family B (MDR/TAP) (TAP1), mRNA [NM_000593]	NM_000593
A_23_P376704	6.51E-06	NM_198289	NM_198289	Homo sapiens cell death-inducing DFFA-like effector a (CIDEA), transcript variant 2, mRNA [NM_198289]	NM_198289
A_23_P16722	6.51E-06	NM_014689	NM_014689	Homo sapiens dedicator of cytokinesis 10 (DOCK10), mRNA [NM_014689]	NM_014689
A_23_P202837	6.51E-06	NM_053056	NM_053056	Homo sapiens cyclin D1 (PRAD1: parathyroid adenomatosis 1) (CCND1), mRNA [NM_053056]	NM_053056
A_24_P84099	6.53E-06	THC2372384		Q9Y5L6 (Q9Y5L6) Apoptosis related protein APR-5 (Fragment), partial (86%) [THC2372384]	
A_24_P390583	6.56E-06	THC2336533			
A_23_P359245	6.57E-06	NM_000245	NM_000245	Homo sapiens met proto-oncogene (hepatocyte growth factor receptor) (MET), mRNA [NM_000245]	NM_000245
A_23_P93772	6.58E-06	NM_019102	NM_019102	Homo sapiens homeo box A5 (HOXA5), mRNA [NM_019102]	NM_019102
A_23_P33015	6.58E-06	NM_021128	NM_021128	Homo sapiens polymerase (RNA) II (DNA directed) polypeptide L, 7.6kDa (POLR2L), mRNA [NM_021128]	NM_021128
A_23_P256205	6.58E-06	NM_014945	NM_014945	Homo sapiens actin binding LIM protein family, member 3 (ABLIM3), mRNA [NM_014945]	NM_014945
A_32_P486693	6.63E-06	AK093229	AK093229	Homo sapiens cDNA FLJ35910 fis, clone TESTI2009987. [AK093229]	
A_23_P252874	6.63E-06	NM_015234	NM_015234	Homo sapiens G protein-coupled receptor 116 (GPR116), mRNA [NM_015234]	NM_015234
A_23_P18186	6.63E-06	NM_021629	NM_021629	Homo sapiens guanine nucleotide binding protein (G protein), beta polypeptide 4 (GNB4), mRNA [NM_021629]	NM_021629
A_23_P257945	6.63E-06	NM_005333	NM_005333	Homo sapiens holocytochrome c synthase (cytochrome c heme-lyase) (HCCS), mRNA [NM_005333]	NM_005333
A_23_P105957	6.63E-06	NM_001102	NM_001102	Homo sapiens actinin, alpha 1 (ACTN1), mRNA [NM_001102]	NM_001102
A_23_P103996	6.66E-06	NM_002061	NM_002061	Homo sapiens glutamate-cysteine ligase, modifier subunit (GCLM), mRNA [NM_002061]	NM_002061
A_23_P134935	6.66E-06	NM_001394	NM_001394	Homo sapiens dual specificity phosphatase 4 (DUSP4), transcript variant 1, mRNA [NM_001394]	NM_001394
A_23_P80008	6.67E-06	NM_033118	NM_033118	Homo sapiens myosin light chain kinase 2, skeletal muscle (MYLK2), mRNA [NM_033118]	NM_033118
A_32_P56874	6.71E-06	BG502322	BG502322	BG502322 602550205F1 NIH_MGC_61 Homo sapiens cDNA clone IMAGE:4657992 5', mRNA sequence [BG502322]	
A_24_P253251	6.72E-06	NM_003045	NM_003045	Homo sapiens solute carrier family 7 (cationic amino acid transporter, y+ system), member 1 (SLC7A1), mRNA [NM_003045]	NM_003045
A_23_P47614	6.76E-06	NM_003311	NM_003311	Homo sapiens pleckstrin homology-like domain, family A, member 2 (PHLDA2), mRNA [NM_003311]	NM_003311
A_24_P63118	6.77E-06	NM_013374	NM_013374	Homo sapiens programmed cell death 6 interacting protein (PDCD6IP), mRNA [NM_013374]	NM_013374
A_24_P359295	6.77E-06	NM_174925	NM_174925	Homo sapiens hypothetical protein LOC205251 (LOC205251), mRNA [NM_174925]	NM_174925
A_24_P115762	6.86E-06	NM_148170	NM_148170	Homo sapiens cathepsin C (CTSC), transcript variant 2, mRNA [NM_148170]	NM_148170
A_24_P915784	6.88E-06	AK125207	AK125207	Homo sapiens cDNA FLJ43217 fis, clone FEBRA2022388. [AK125207]	

A_23_P372255	6.91E-06	NM_002221	NM_002221	Homo sapiens inositol 1,4,5-trisphosphate 3-kinase B (ITPKB), mRNA [NM_002221]	NM_002221
A_23_P845	6.91E-06	NM_032174	NM_032174	Homo sapiens hypothetical protein FLJ12770 (FLJ12770), mRNA [NM_032174]	NM_032174
A_24_P84608	6.92E-06	A_24_P84608			
A_23_P256956	6.92E-06	NM_005733	NM_005733	Homo sapiens kinesin family member 20A (KIF20A), mRNA [NM_005733]	NM_005733
A_23_P35989	6.93E-06	NM_139178	NM_139178	Homo sapiens prostate cancer antigen-1 (DEPC-1), mRNA [NM_139178]	NM_139178
A_24_P130066	6.97E-06	NM_017743	NM_017743	Homo sapiens dipeptidylpeptidase 8 (DPP8), transcript variant 2, mRNA [NM_017743]	NM_017743
A_23_P257578	6.98E-06	AK054960	AK054960	Homo sapiens cDNA FLJ30398 fis, clone BRACE2008402, highly similar to Homo sapiens steroid receptor RNA activator isoform 3 mRNA. [AK054960]	XR_000132
A_32_P7823	7.01E-06	NM_144578	NM_144578	Homo sapiens chromosome 14 open reading frame 32 (C14orf32), mRNA [NM_144578]	NM_144578
A_23_P66347	7.01E-06	A_23_P66347			
A_23_P49924	7.03E-06	NM_052935	NM_052935	Homo sapiens hypothetical protein MGC20781 (MGC20781), mRNA [NM_052935]	NM_052935
A_23_P360777	7.04E-06	NM_013957	NM_013957	Homo sapiens neuregulin 1 (NRG1), transcript variant HRG-beta2, mRNA [NM_013957]	NM_013957
A_24_P42501	7.04E-06	NM_012332	NM_012332	Homo sapiens acyl-Coenzyme A thioesterase 2, mitochondrial (ACATE2), mRNA [NM_012332]	NM_012332
A_32_P123729	7.09E-06	BX394228	BX394228	BX394228 BX394228 Homo sapiens NEUROBLASTOMA COT 25-NORMALIZED Homo sapiens cDNA clone CS0DC012YM19 3-PRIME, mRNA sequence [BX394228]	
A_23_P27133	7.12E-06	NM_002275	NM_002275	Homo sapiens keratin 15 (KRT15), mRNA [NM_002275]	NM_002275
A_24_P113295	7.12E-06	NM_004069	NM_004069	Homo sapiens adaptor-related protein complex 2, sigma 1 subunit (AP2S1), transcript variant AP17, mRNA [NM_004069]	NM_004069
A_24_P922378	7.16E-06	NM_004449	NM_004449	Homo sapiens v-ets erythroblastosis virus E26 oncogene like (avian) (ERG), transcript variant 2, mRNA [NM_004449]	NM_004449
A_23_P161634	7.16E-06	NM_025128	NM_025128	Homo sapiens MUS81 endonuclease homolog (yeast) (MUS81), mRNA [NM_025128]	NM_025128
A_32_P34696	7.19E-06	THC2285122			
A_23_P98884	7.25E-06	NM_194358	NM_194358	Homo sapiens ring finger protein 41 (RNF41), transcript variant 2, mRNA [NM_194358]	NM_194358
A_24_P698136	7.26E-06	AK125299	AK125299	Homo sapiens cDNA FLJ43309 fis, clone NT2RI2004618, highly similar to Cytosolic acyl coenzyme A thioester hydrolase (EC 3.1.2.2). [AK125299]	XR_000194
A_23_P256724	7.26E-06	NM_003841	NM_003841	Homo sapiens tumor necrosis factor receptor superfamily, member 10c, decoy without an intracellular domain (TNFRSF10C), mRNA [NM_003841]	NM_003841
A_23_P29953	7.27E-06	NM_172174	NM_172174	Homo sapiens interleukin 15 (IL15), transcript variant 1, mRNA [NM_172174]	NM_172174
A_24_P345336	7.27E-06	NM_002871	NM_002871	Homo sapiens RAB interacting factor (RABIF), mRNA [NM_002871]	NM_002871
A_24_P259799	7.30E-06	NM_001002234	NM_001002234	Homo sapiens sodium channel modifier 1 (SCNM1), transcript variant 2, mRNA [NM_001002234]	NM_001002234
A_24_P65910	7.38E-06	NM_018244	NM_018244	Homo sapiens chromosome 20 open reading frame 44 (C20orf44), transcript variant 1, mRNA [NM_018244]	NM_018244
A_24_P168510	7.40E-06	NM_024069	NM_024069	Homo sapiens hypothetical protein MGC2749 (MGC2749), mRNA [NM_024069]	NM_024069
A_23_P116840	7.41E-06	NM_021934	NM_021934	Homo sapiens hypothetical protein FLJ11773 (FLJ11773), mRNA [NM_021934]	NM_021934
A_23_P384024	7.43E-06	NM_178335	NM_178335	Homo sapiens chromosome 3 open reading frame 6 (C3orf6), transcript variant 2, mRNA [NM_178335]	NM_178335
A_24_P194081	7.44E-06	NM_144779	NM_144779	Homo sapiens FXYD domain containing ion transport regulator 5 (FXYD5), transcript variant 1, mRNA [NM_144779]	NM_144779
A_23_P48964	7.45E-06	NM_018668	NM_018668	Homo sapiens vacuolar protein sorting 33B (yeast) (VPS33B), mRNA [NM_018668]	NM_018668
A_32_P182388	7.45E-06	NM_021217	NM_021217	Homo sapiens zinc finger protein 77 (pT1) (ZNF77), mRNA [NM_021217]	NM_021217
A_32_P505133	7.46E-06	NM_178830	NM_178830	Homo sapiens hypothetical protein FLJ36888 (FLJ36888), mRNA [NM_178830]	NM_178830
A_23_P338912	7.47E-06	NM_007350	NM_007350	Homo sapiens pleckstrin homology-like domain, family A, member 1 (PHLDA1), mRNA [NM_007350]	NM_007350
A_23_P206707	7.50E-06	NM_005950	NM_005950	Homo sapiens metallothionein 1G (MT1G), mRNA [NM_005950]	NM_005950
A_23_P8240	7.51E-06	NM_012135	NM_012135	Homo sapiens family with sequence similarity 50, member B (FAM50B), mRNA [NM_012135]	NM_012135
A_23_P132763	7.52E-06	NM_016206	NM_016206	Homo sapiens vestigial-like 3 (VGL-3), mRNA [NM_016206]	NM_016206
A_24_P366656	7.52E-06	NM_001009555	NM_001009555	Homo sapiens SH3 domain protein D19 (SH3D19), mRNA [NM_001009555]	NM_001009555
A_24_P349606	7.52E-06	A_24_P349606			
A_23_P314760	7.52E-06	NM_016203	NM_016203	Homo sapiens protein kinase, AMP-activated, gamma 2 non-catalytic subunit (PRKAG2), mRNA [NM_016203]	NM_016203
A_32_P129288	7.52E-06	NM_030981	NM_030981	Homo sapiens RAB1B, member RAS oncogene family (RAB1B), mRNA [NM_030981]	NM_030981
A_23_P161628	7.53E-06	NM_025128	NM_025128	Homo sapiens MUS81 endonuclease homolog (yeast) (MUS81), mRNA [NM_025128]	NM_025128
A_23_P57036	7.55E-06	NM_001250	NM_001250	Homo sapiens CD40 antigen (TNF receptor superfamily member 5) (CD40), transcript variant 1, mRNA [NM_001250]	NM_001250
A_24_P251734	7.56E-06	NM_020116	NM_020116	Homo sapiens follistatin-like 5 (FSTL5), mRNA [NM_020116]	NM_020116

A_24_P153853	7.66E-06	NM_001005207	NM_001005207	Homo sapiens tripartite motif-containing 37 (TRIM37), transcript variant 2, mRNA [NM_001005207]	NM_001005207
A_23_P304171	7.66E-06	ENST00000273582		Homo sapiens mRNA for KIAA0226 protein, partial cds. [D86979]	XM_032901
A_23_P75500	7.75E-06	NM_015399	NM_015399	Homo sapiens breast cancer metastasis suppressor 1 (BRMS1), transcript variant 1, mRNA [NM_015399]	NM_015399
A_23_P251293	7.79E-06	NM_003087	NM_003087	Homo sapiens synuclein, gamma (breast cancer-specific protein 1) (SNCG), mRNA [NM_003087]	NM_003087
A_32_P91902	7.79E-06	A_32_P91902			
A_23_P124044	7.80E-06	NM_021008	NM_021008	Homo sapiens deformed epidermal autoregulatory factor 1 (Drosophila) (DEAF1), mRNA [NM_021008]	NM_021008
A_23_P105313	7.80E-06	NM_001414	NM_001414	Homo sapiens eukaryotic translation initiation factor 2B, subunit 1 alpha, 26kDa (EIF2B1), mRNA [NM_001414]	NM_001414
A_23_P47857	7.82E-06	NM_016551	NM_016551	Homo sapiens transmembrane 7 superfamily member 3 (TM7SF3), mRNA [NM_016551]	NM_016551
A_23_P43434	7.83E-06	NM_005047	NM_005047	Homo sapiens proteasome (prosome, macropain) 26S subunit, non-ATPase, 5 (PSMD5), mRNA [NM_005047]	NM_005047
A_24_P218979	7.83E-06	NM_031299	NM_031299	Homo sapiens cell division cycle associated 3 (CDCA3), mRNA [NM_031299]	NM_031299
A_23_P327519	7.84E-06	NM_139164	NM_139164	Homo sapiens START domain containing 4, sterol regulated (STARD4), mRNA [NM_139164]	NM_139164
A_23_P344719	7.85E-06	NM_003898	NM_003898	Homo sapiens synaptojanin 2 (SYNJ2), mRNA [NM_003898]	NM_003898
A_23_P3792	7.85E-06	NM_003486	NM_003486	Homo sapiens solute carrier family 7 (cationic amino acid transporter, y+ system), member 5 (SLC7A5), mRNA [NM_003486]	NM_003486
A_23_P103110	7.86E-06	NM_012323	NM_012323	Homo sapiens v-maf musculoaponeurotic fibrosarcoma oncogene homolog F (avian) (MAFF), transcript variant 1, mRNA [NM_012323]	NM_012323
A_23_P2271	7.90E-06	NM_198965	NM_198965	Homo sapiens parathyroid hormone-like hormone (PTHLH), transcript variant 1, mRNA [NM_198965]	NM_198965
A_23_P97780	7.91E-06	BC043009	BC043009	Homo sapiens hypothetical protein LOC338620, mRNA (cDNA clone IMAGE:6023208), partial cds. [BC043009]	
A_23_P103276	7.92E-06	NM_138417	NM_138417	Homo sapiens hypothetical protein BC012173 (MGC20419), mRNA [NM_138417]	NM_138417
A_24_P377499	7.96E-06	NM_145323	NM_145323	Homo sapiens oxysterol binding protein-like 3 (OSBPL3), transcript variant 5, mRNA [NM_145323]	NM_145323
A_23_P35256	7.96E-06	NM_006468	NM_006468	Homo sapiens polymerase (RNA) III (DNA directed) polypeptide C (62kD) (POLR3C), mRNA [NM_006468]	NM_006468
A_23_P15202	7.96E-06	NM_001361	NM_001361	Homo sapiens dihydroorotate dehydrogenase (DHODH), nuclear gene encoding mitochondrial protein, transcript variant 1, mRNA [NM_001361]	NM_001361
A_23_P37910	8.02E-06	NM_002746	NM_002746	Homo sapiens mitogen-activated protein kinase 3 (MAPK3), mRNA [NM_002746]	NM_002746
A_23_P323751	8.03E-06	NM_030919	NM_030919	Homo sapiens chromosome 20 open reading frame 129 (C20orf129), mRNA [NM_030919]	NM_030919
A_23_P431381	8.07E-06	NM_173608	NM_173608	Homo sapiens chromosome 14 open reading frame 80 (C14orf80), mRNA [NM_173608]	NM_173608
A_23_P150009	8.07E-06	NM_198046	NM_198046	Homo sapiens zinc finger, DHHC-type containing 16 (ZDHHC16), transcript variant 5, mRNA [NM_198046]	NM_198046
A_23_P102706	8.08E-06	NM_014723	NM_014723	Homo sapiens syntaphilin (SNPH), mRNA [NM_014723]	NM_014723
A_23_P213766	8.10E-06	NM_054027	NM_054027	Homo sapiens ankylosis, progressive homolog (mouse) (ANKH), mRNA [NM_054027]	NM_054027
A_32_P182395	8.14E-06	THC2434943		Q8WUP8 (Q8WUP8) MGC21881 protein (Fragment), partial (36%) [THC2434943]	
A_23_P144896	8.14E-06	NM_203353	NM_203353	Homo sapiens PDZ and LIM domain 7 (enigma) (PDLIM7), transcript variant 3, mRNA [NM_203353]	NM_203353
A_23_P326319	8.14E-06	NM_033201	NM_033201	Homo sapiens chromosome 16 open reading frame 45 (C16orf45), mRNA [NM_033201]	NM_033201
A_32_P135243	8.17E-06	NM_015440	NM_015440	Homo sapiens methylenetetrahydrofolate dehydrogenase (NADP+ dependent) 1-like (MTHFD1L), mRNA [NM_015440]	NM_015440
A_32_P17182	8.18E-06	N48043	N48043	N48043 yy23h06.r1 Soares melanocyte 2NbHM Homo sapiens cDNA clone IMAGE:272123 5' similar to gb:X04665_cds1 THROMBOSPONDIN 1 PRECURSOR (HUMAN);, mRNA sequence [N48043]	
A_23_P51958	8.19E-06	NM_182744	NM_182744	Homo sapiens neuroblastoma, suppression of tumorigenicity 1 (NBL1), transcript variant 1, mRNA [NM_182744]	NM_182744
A_32_P210202	8.24E-06	NM_203394	NM_203394	Homo sapiens E2F transcription factor 7 (E2F7), mRNA [NM_203394]	NM_203394
A_23_P65870	8.26E-06	NM_012170	NM_012170	Homo sapiens F-box protein 22 (FBXO22), transcript variant 2, mRNA [NM_012170]	NM_012170
A_32_P197109	8.33E-06	AI559980	AI559980	AI559980 tq77f07.x1 NCI_CGAP_Ut1 Homo sapiens cDNA clone IMAGE:2214853 3', mRNA sequence [AI559980]	
A_24_P38081	8.33E-06	NM_004117	NM_004117	Homo sapiens FK506 binding protein 5 (FKBP5), mRNA [NM_004117]	NM_004117
A_24_P415208	8.33E-06	BC060806	BC060806	Homo sapiens cDNA clone MGC:71628 IMAGE:30336414, complete cds. [BC060806]	
A_23_P169629	8.33E-06	NM_005412	NM_005412	Homo sapiens serine hydroxymethyltransferase 2 (mitochondrial) (SHMT2), mRNA [NM_005412]	NM_005412
A_32_P39324	8.36E-06	CB993898	CB993898	AGENCOURT_13617208 NIH_MGC_148 Homo sapiens cDNA clone IMAGE:30333013 5', mRNA sequence [CB993898]	
A_23_P329772	8.36E-06	NM_013245	NM_013245	Homo sapiens vacuolar protein sorting 4A (yeast) (VPS4A), mRNA [NM_013245]	NM_013245
A_23_P43490	8.42E-06	NM_058197	NM_058197	Homo sapiens cyclin-dependent kinase inhibitor 2A (melanoma, p16, inhibits CDK4) (CDKN2A), transcript variant 3, mRNA [NM_058197]	NM_058197
A_23_P19673	8.46E-06	NM_005627	NM_005627	Homo sapiens serum/glucocorticoid regulated kinase (SGK), mRNA [NM_005627]	NM_005627
A_23_P1981	8.47E-06	NM_000207	NM_000207	Homo sapiens insulin (INS), mRNA [NM_000207]	NM_000207

A_24_P916251	8.48E-06	NM_000991	NM_000991	Homo sapiens ribosomal protein L28 (RPL28), mRNA [NM_000991]	NM_000991
A_23_P165355	8.53E-06	NM_005687	NM_005687	Homo sapiens phenylalanine-tRNA synthetase-like, beta subunit (FARSLB), mRNA [NM_005687]	NM_005687
A_23_P110022	8.56E-06	NM_032638	NM_032638	Homo sapiens GATA binding protein 2 (GATA2), mRNA [NM_032638]	NM_032638
A_23_P60306	8.57E-06	NM_003266	NM_003266	Homo sapiens toll-like receptor 4 (TLR4), transcript variant 3, mRNA [NM_003266]	NM_003266
A_23_P406187	8.60E-06	NM_153006	NM_153006	Homo sapiens N-acetylglutamate synthase (NAGS), mRNA [NM_153006]	NM_153006
A_23_P57293	8.61E-06	ENST00000270201		Homo sapiens chromosome 21 C21orf108 mRNA, partial cds. [AF231919]	
A_23_P73593	8.64E-06	NM_002444	NM_002444	Homo sapiens moesin (MSN), mRNA [NM_002444]	NM_002444
A_23_P363936	8.67E-06	NM_014278	NM_014278	Homo sapiens heat shock 70kDa protein 4-like (HSPA4L), mRNA [NM_014278]	NM_014278
A_23_P85585	8.75E-06	NM_022164	NM_022164	Homo sapiens lipocalin 7 (LCN7), mRNA [NM_022164]	NM_022164
A_23_P404494	8.76E-06	NM_002185	NM_002185	Homo sapiens interleukin 7 receptor (IL7R), mRNA [NM_002185]	NM_002185
A_23_P52531	8.78E-06	NM_152644	NM_152644	Homo sapiens family with sequence similarity 24, member B (FAM24B), mRNA [NM_152644]	NM_152644
A_23_P368909	8.78E-06	NM_178514	NM_178514	Homo sapiens hypothetical protein LOC283487 (LOC283487), mRNA [NM_178514]	NM_178514
A_23_P168211	8.84E-06	NM_030796	NM_030796	Homo sapiens EGFR-coamplified and overexpressed protein (ECOP), mRNA [NM_030796]	NM_030796
A_23_P207719	8.89E-06	NM_004287	NM_004287	Homo sapiens golgi SNAP receptor complex member 2 (GOSR2), transcript variant A, mRNA [NM_004287]	NM_004287
A_24_P359165	8.93E-06	BC000134	BC000134	Homo sapiens SWAP-70 protein, mRNA (cDNA clone IMAGE:2900736), containing frame-shift errors. [BC000134]	
A_24_P299794	8.93E-06	NM_005057	NM_005057	Homo sapiens retinoblastoma binding protein 5 (RBBP5), mRNA [NM_005057]	NM_005057
A_32_P135890	8.93E-06	NM_001013680	NM_001013680	Homo sapiens similar to HIV TAT specific factor 1; cofactor required for Tat activation of HIV-1 transcription (LOC401233), mRNA [NM_001013680]	NM_001013680
A_24_P257259	8.93E-06	NM_006501	NM_006501	Homo sapiens myelin-associated oligodendrocyte basic protein (MOBP), transcript variant 2, mRNA [NM_006501]	NM_006501
A_32_P49631	8.93E-06	CR594811	CR594811	full-length cDNA clone CS0DL006YD08 of B cells (Ramos cell line) Cot 25-normalized of Homo sapiens (human). [CR594811]	
A_23_P18604	8.93E-06	NM_015907	NM_015907	Homo sapiens leucine aminopeptidase 3 (LAP3), mRNA [NM_015907]	NM_015907
A_23_P207131	8.93E-06	NM_203351	NM_203351	Homo sapiens mitogen-activated protein kinase kinase kinase 3 (MAP3K3), transcript variant 1, mRNA [NM_203351]	NM_203351
A_23_P419624	8.95E-06	NM_006698	NM_006698	Homo sapiens bladder cancer associated protein (BLCAP), mRNA [NM_006698]	NM_006698
A_23_P47682	8.96E-06	NM_020645	NM_020645	Homo sapiens nuclear receptor interacting protein 3 (NRIP3), mRNA [NM_020645]	NM_020645
A_23_P13914	8.97E-06	NM_032656	NM_032656	Homo sapiens DEAH (Asp-Glu-Ala-His) box polypeptide 37 (DHX37), mRNA [NM_032656]	NM_032656
A_23_P26759	8.99E-06	NM_138793	NM_138793	Homo sapiens calcium activated nucleotidase 1 (CANT1), mRNA [NM_138793]	NM_138793
A_23_P421465	9.00E-06	NM_015409	NM_015409	Homo sapiens E1A binding protein p400 (EP400), mRNA [NM_015409]	NM_015409
A_24_P167668	9.01E-06	NM_000428	NM_000428	Homo sapiens latent transforming growth factor beta binding protein 2 (LTBP2), mRNA [NM_000428]	NM_000428
A_24_P107695	9.02E-06	NM_001102	NM_001102	Homo sapiens actinin, alpha 1 (ACTN1), mRNA [NM_001102]	NM_001102
A_23_P253081	9.04E-06	NM_002183	NM_002183	Homo sapiens interleukin 3 receptor, alpha (low affinity) (IL3RA), mRNA [NM_002183]	NM_002183
A_23_P57393	9.06E-06	THC2377764			
A_23_P348524	9.07E-06	NM_001007125	NM_001007125	Homo sapiens bA299N6.3 (LOC198437), mRNA [NM_001007125]	NM_001007125
A_32_P128572	9.09E-06	NM_020948	NM_020948	Homo sapiens mesoderm induction early response 1 homolog (Xenopus laevis) (MIER1), mRNA [NM_020948]	NM_020948
A_24_P323395	9.10E-06	NM_016441	NM_016441	Homo sapiens cysteine rich transmembrane BMP regulator 1 (chordin-like) (CRIM1), mRNA [NM_016441]	NM_016441
A_23_P23457	9.10E-06	NM_017556	NM_017556	Homo sapiens filamin binding LIM protein 1 (FBLIM1), transcript variant 1, mRNA [NM_017556]	NM_017556
A_23_P76364	9.10E-06	NM_001769	NM_001769	Homo sapiens CD9 antigen (p24) (CD9), mRNA [NM_001769]	NM_001769
A_24_P784765	9.12E-06	NM_203330	NM_203330	Homo sapiens CD59 antigen p18-20 (antigen identified by monoclonal antibodies 16.3A5, EJ16, EJ30, EL32 and G344) (CD59), transcript variant 1, mRNA [NM_203330]	NM_203330
A_23_P433785	9.14E-06	NM_002561	NM_002561	Homo sapiens purinergic receptor P2X, ligand-gated ion channel, 5 (P2RX5), transcript variant 1, mRNA [NM_002561]	NM_002561
A_23_P216278	9.14E-06	NM_018482	NM_018482	Homo sapiens development and differentiation enhancing factor 1 (DDEF1), mRNA [NM_018482]	NM_018482
A_24_P928217	9.20E-06	AK026155	AK026155	Homo sapiens cDNA: FLJ22502 fis, clone HRC11383. [AK026155]	
A_24_P402080	9.20E-06	NM_001025100	NM_001025100	Homo sapiens myelin basic protein (MBP), transcript variant 8, mRNA [NM_001025100]	NM_001025100
A_23_P49674	9.28E-06	NM_173728	NM_173728	Homo sapiens Rho guanine nucleotide exchange factor (GEF) 15 (ARHGEF15), mRNA [NM_173728]	NM_173728
A_23_P115872	9.29E-06	NM_018131	NM_018131	Homo sapiens chromosome 10 open reading frame 3 (C10orf3), mRNA [NM_018131]	NM_018131
A_24_P272313	9.32E-06	NM_207362	NM_207362	Homo sapiens similar to 2010300C02Rik protein (MGC42367), mRNA [NM_207362]	NM_207362

A_24_P876864	9.32E-06	BC061590	BC061590	Homo sapiens cDNA clone MGC:75203 IMAGE:6502529, complete cds. [BC061590]	
A_24_P110471	9.35E-06	THC2276423		Q6IFN5 (Q6IFN5) Olfactory receptor OR19-14, partial (67%) [THC2276423]	
A_23_P72651	9.37E-06	THC2337324		EOMD_HUMAN (O95936) Eomesodermin homolog, partial (3%) [THC2337324]	
A_32_P10432	9.39E-06	THC2346713		Q9NY59 (Q9NY59) Neutral sphingomyelinase II , partial (3%) [THC2346713]	
A_24_P186379	9.39E-06	NM_198472	NM_198472	Homo sapiens chromosome 10 open reading frame 125 (C10orf125), mRNA [NM_198472]	NM_198472
A_23_P28878	9.40E-06	AK022713	AK022713	Homo sapiens cDNA FLJ12651 fis, clone NT2RM4002062, moderately similar to ASPARTYL-TRNA SYNTHETASE (EC 6.1.1.12). [AK022713]	
A_32_P172848	9.45E-06	NM_000167	NM_000167	Homo sapiens glycerol kinase (GK), transcript variant 2, mRNA [NM_000167]	NM_000167
A_23_P217763	9.47E-06	AK055976	AK055976	Homo sapiens cDNA FLJ31414 fis, clone NT2NE2000260, weakly similar to THYMOSIN BETA-4. [AK055976]	
A_32_P193771	9.48E-06	A_32_P193771			
A_24_P269398	9.50E-06	NM_032982	NM_032982	Homo sapiens caspase 2, apoptosis-related cysteine protease (neural precursor cell expressed, developmentally down-regulated 2) (CASP2), transcript variant 1, mRNA [NM_032982]	NM_032982
A_24_P372134	9.51E-06	NM_018295	NM_018295	Homo sapiens hypothetical protein FLJ11000 (FLJ11000), mRNA [NM_018295]	NM_018295
A_23_P1936	9.56E-06	NM_025080	NM_025080	Homo sapiens asparaginase like 1 (ASRGL1), mRNA [NM_025080]	NM_025080
A_24_P335305	9.64E-06	NM_006187	NM_006187	Homo sapiens 2'-5'-oligoadenylate synthetase 3, 100kDa (OAS3), mRNA [NM_006187]	NM_006187
A_32_P80231	9.65E-06	BM973227	BM973227	UI-CF-EC1-abw-d-20-0-UI.s1 UI-CF-EC1 Homo sapiens cDNA clone UI-CF-EC1-abw-d-20-0-UI 3', mRNA sequence [BM973227]	
A_24_P160413	9.65E-06	NM_178546	NM_178546	Homo sapiens hypothetical protein LOC339483 (LOC339483), mRNA [NM_178546]	NM_178546
A_24_P914479	9.68E-06	BC002724	BC002724	Homo sapiens sorting nexin 5, mRNA (cDNA clone IMAGE:3629947), complete cds. [BC002724]	
A_23_P83328	9.69E-06	NM_000118	NM_000118	Homo sapiens endoglin (Osler-Rendu-Weber syndrome 1) (ENG), mRNA [NM_000118]	NM_000118
A_23_P159839	9.69E-06	NM_152692	NM_152692	Homo sapiens C1GALT1-specific chaperone 1 (C1GALT1C1), transcript variant 1, mRNA [NM_152692]	NM_152692
A_23_P24723	9.70E-06	NM_016464	NM_016464	Homo sapiens hypothetical protein HSPC196 (HSPC196), mRNA [NM_016464]	NM_016464
A_32_P108474	9.74E-06	NM_002940	NM_002940	Homo sapiens ATP-binding cassette, sub-family E (OABP), member 1 (ABCE1), mRNA [NM_002940]	NM_002940
A_23_P370625	9.74E-06	NM_020451	NM_020451	Homo sapiens selenoprotein N, 1 (SEPN1), transcript variant 1, mRNA [NM_020451]	NM_020451
A_23_P166333	9.74E-06	NM_005659	NM_005659	Homo sapiens ubiquitin fusion degradation 1-like (UFD1L), mRNA [NM_005659]	NM_005659
A_32_P30004	9.79E-06	AF086044	AF086044	Homo sapiens full length insert cDNA clone YX74D05. [AF086044]	
A_23_P138680	9.79E-06	NM_172200	NM_172200	Homo sapiens interleukin 15 receptor, alpha (IL15RA), transcript variant 2, mRNA [NM_172200]	NM_172200
A_24_P58037	9.79E-06	ENST00000276938		PREDICTED: Homo sapiens similar to microtubule-associated proteins 1A [XM_373277]	XM_373277
A_23_P138567	9.79E-06	NM_032797	NM_032797	Homo sapiens apoptosis-inducing factor (AIF)-like mitochondrion-associated inducer of death (AMID), mRNA [NM_032797]	NM_032797
A_23_P3574	9.79E-06	NM_030819	NM_030819	Homo sapiens hypothetical protein MGC11335 (MGC11335), mRNA [NM_030819]	NM_030819
A_32_P6868	9.81E-06	NM_015472	NM_015472	Homo sapiens WW domain containing transcription regulator 1 (WWTR1), mRNA [NM_015472]	NM_015472
A_23_P55468	9.83E-06	NM_013337	NM_013337	Homo sapiens translocase of inner mitochondrial membrane 22 homolog (yeast) (TIMM22), mRNA [NM_013337]	NM_013337
A_23_P89460	9.83E-06	NM_012138	NM_012138	Homo sapiens apoptosis antagonizing transcription factor (AATF), mRNA [NM_012138]	NM_012138
A_24_P583040	9.87E-06	BC041467	BC041467	Homo sapiens hypothetical protein LOC339210, mRNA (cDNA clone IMAGE:5215233), partial cds. [BC041467]	XM_378687
A_32_P154731	9.87E-06	AK124192	AK124192	Homo sapiens cDNA FLJ42198 fis, clone THYMU2034338. [AK124192]	
A_23_P137248	9.87E-06	NM_002760	NM_002760	Homo sapiens protein kinase, Y-linked (PRKY), mRNA [NM_002760]	NM_002760
A_23_P166459	9.90E-06	NM_002305	NM_002305	Homo sapiens lectin, galactoside-binding, soluble, 1 (galectin 1) (LGALS1), mRNA [NM_002305]	NM_002305
A_23_P70020	9.95E-06	NM_002622	NM_002622	Homo sapiens prefoldin 1 (PFDN1), mRNA [NM_002622]	NM_002622
A_24_P324783	9.97E-06	NM_000020	NM_000020	Homo sapiens activin A receptor type II-like 1 (ACVRL1), mRNA [NM_000020]	NM_000020
A_23_P104493	9.97E-06	NM_001015880	NM_001015880	Homo sapiens 3'-phosphoadenosine 5'-phosphosulfate synthase 2 (PAPSS2), transcript variant 2, mRNA [NM_001015880]	NM_001015880
A_23_P43898	9.99E-06	NM_173567	NM_173567	Homo sapiens abhydrolase domain containing 7 (ABHD7), mRNA [NM_173567]	NM_173567
A_23_P84448	1.00E-05	NM_025019	NM_025019	Homo sapiens tubulin, alpha 4 (TUBA4), mRNA [NM_025019]	NM_025019
A_23_P46455	1.00E-05	CR619944	CR619944	full-length cDNA clone CS0DF007YK17 of Fetal brain of Homo sapiens (human). [CR619944]	
A_32_P99432	1.00E-05	BC042161	BC042161	Homo sapiens trafficking protein particle complex 5, mRNA (cDNA clone MGC:52424 IMAGE:4869377), complete cds. [BC042161]	XM_058961
A_23_P216655	1.01E-05	NM_014788	NM_014788	Homo sapiens tripartite motif-containing 14 (TRIM14), transcript variant 1, mRNA [NM_014788]	NM_014788
A_23_P25313	1.01E-05	NM_080626	NM_080626	Homo sapiens BRI3 binding protein (BRI3BP), mRNA [NM_080626]	NM_080626
A_23_P25224	1.01E-05	NM_003651	NM_003651	Homo sapiens cold shock domain protein A (CSDA), mRNA [NM_003651]	NM_003651

A_23_P51906	1.01E-05	NM_012394	NM_012394	Homo sapiens prefoldin 2 (PFDN2), mRNA [NM_012394]	NM_012394
A_32_P191527	1.02E-05	NM_020382	NM_020382	Homo sapiens PR/SET domain containing protein 8 (SET8), mRNA [NM_020382]	NM_020382
A_23_P204436	1.02E-05	NM_139201	NM_139201	Homo sapiens G protein-coupled receptor kinase interactor 2 (GIT2), transcript variant 4, mRNA [NM_139201]	NM_139201
A_23_P17663	1.02E-05	NM_002462	NM_002462	Homo sapiens myxovirus (influenza virus) resistance 1, interferon-inducible protein p78 (mouse) (MX1), mRNA [NM_002462]	NM_002462
A_23_P345139	1.02E-05	NM_174983	NM_174983	Homo sapiens chromosome 19 open reading frame 28 (C19orf28), mRNA [NM_174983]	NM_174983
A_23_P502913	1.02E-05	NM_017491	NM_017491	Homo sapiens WD repeat domain 1 (WDR1), transcript variant 1, mRNA [NM_017491]	NM_017491
A_32_P124833	1.04E-05	BX100088	BX100088	BX100088 BX100088 Soares_testis_NHT Homo sapiens cDNA clone IMAGp998K133560 ; IMAGE:1409412, mRNA sequence [BX100088]	
A_23_P203498	1.04E-05	NM_006074	NM_006074	Homo sapiens tripartite motif-containing 22 (TRIM22), mRNA [NM_006074]	NM_006074
A_23_P13438	1.04E-05	NM_032320	NM_032320	Homo sapiens K+ channel tetramerization protein (GMRP-1), mRNA [NM_032320]	NM_032320
A_32_P5976	1.04E-05	NM_177533	NM_177533	Homo sapiens nudix (nucleoside diphosphate linked moiety X)-type motif 14 (NUDT14), mRNA [NM_177533]	NM_177533
A_23_P344451	1.04E-05	NM_016073	NM_016073	Homo sapiens hepatoma-derived growth factor, related protein 3 (HDGFRP3), mRNA [NM_016073]	NM_016073
A_23_P127948	1.04E-05	NM_001124	NM_001124	Homo sapiens adrenomedullin (ADM), mRNA [NM_001124]	NM_001124
A_24_P135193	1.05E-05	NM_018314	NM_018314	Homo sapiens ubiquitin-conjugating enzyme E2-like (UEV3), mRNA [NM_018314]	NM_018314
A_23_P215265	1.05E-05	NM_007353	NM_007353	Homo sapiens guanine nucleotide binding protein (G protein) alpha 12 (GNA12), mRNA [NM_007353]	NM_007353
A_24_P134526	1.05E-05	NM_007311	NM_007311	Homo sapiens benzodiazapine receptor (peripheral) (BZRP), transcript variant PBR-S, mRNA [NM_007311]	NM_007311
A_23_P205293	1.06E-05	NM_018199	NM_018199	Homo sapiens chromosome 14 open reading frame 114 (C14orf114), mRNA [NM_018199]	NM_018199
A_32_P41127	1.06E-05	NM_002622	NM_002622	Homo sapiens prefoldin 1 (PFDN1), mRNA [NM_002622]	NM_002622
A_24_P922475	1.06E-05	BC042363	BC042363	Homo sapiens cDNA clone IMAGE:5285571, complete cds. [BC042363]	
A_23_P143987	1.06E-05	NM_006395	NM_006395	Homo sapiens APG7 autophagy 7-like (S. cerevisiae) (APG7L), mRNA [NM_006395]	NM_006395
A_24_P38944	1.06E-05	NM_024098	NM_024098	Homo sapiens hypothetical protein MGC2574 (MGC2574), mRNA [NM_024098]	NM_024098
A_23_P60517	1.06E-05	NM_181425	NM_181425	Homo sapiens frataxin (FXN), nuclear gene encoding mitochondrial protein, transcript variant 2, mRNA [NM_181425]	NM_181425
A_24_P219769	1.07E-05	NM_018035	NM_018035	Homo sapiens hypothetical protein FLJ10241 (FLJ10241), mRNA [NM_018035]	NM_018035
A_24_P940166	1.07E-05	NM_001015880	NM_001015880	Homo sapiens 3'-phosphoadenosine 5'-phosphosulfate synthase 2 (PAPSS2), transcript variant 2, mRNA [NM_001015880]	NM_001015880
A_23_P75540	1.07E-05	NM_032795	NM_032795	Homo sapiens RNA pseudouridylate synthase domain containing 4 (RPUSD4), mRNA [NM_032795]	NM_032795
A_23_P65388	1.07E-05	NM_175060	NM_175060	Homo sapiens C-type lectin domain family 14, member A (CLEC14A), mRNA [NM_175060]	NM_175060
A_23_P126388	1.07E-05	NM_031286	NM_031286	Homo sapiens SH3 domain binding glutamic acid-rich protein like 3 (SH3BGRL3), mRNA [NM_031286]	NM_031286
A_23_P318039	1.08E-05	NM_005444	NM_005444	Homo sapiens RCD1 required for cell differentiation1 homolog (S. pombe) (RQCD1), mRNA [NM_005444]	NM_005444
A_23_P12965	1.08E-05	NM_002033	NM_002033	Homo sapiens fucosyltransferase 4 (alpha (1,3) fucosyltransferase, myeloid-specific) (FUT4), mRNA [NM_002033]	NM_002033
A_23_P257895	1.08E-05	NM_138957	NM_138957	Homo sapiens mitogen-activated protein kinase 1 (MAPK1), transcript variant 2, mRNA [NM_138957]	NM_138957
A_24_P101617	1.08E-05	ENST00000328135		PREDICTED: Homo sapiens similar to 60S ribosomal protein L17 (L23) (Amino acid starvation-induced protein) (ASI) (LOC402695), mRNA [XM_380044]	XM_380044
A_23_P154235	1.08E-05	NM_004688	NM_004688	Homo sapiens N-myc (and STAT) interactor (NMI), mRNA [NM_004688]	NM_004688
A_24_P307466	1.08E-05	ENST00000332917			
A_23_P54840	1.09E-05	NM_005946	NM_005946	Homo sapiens metallothionein 1A (functional) (MT1A), mRNA [NM_005946]	NM_005946
A_24_P389843	1.10E-05	NM_014956	NM_014956	Homo sapiens KIAA1052 protein (Cep164), mRNA [NM_014956]	NM_014956
A_23_P39656	1.10E-05	NM_022453	NM_022453	Homo sapiens ring finger protein 25 (RNF25), mRNA [NM_022453]	NM_022453
A_24_P204515	1.10E-05	ENST00000342275		RST6901 Athersys RAGE Library Homo sapiens cDNA, mRNA sequence [BG187898]	XM_497067
A_23_P201706	1.10E-05	NM_005978	NM_005978	Homo sapiens S100 calcium binding protein A2 (S100A2), mRNA [NM_005978]	NM_005978
A_23_P92727	1.10E-05	NM_015577	NM_015577	Homo sapiens retinoic acid induced 14 (RAI14), mRNA [NM_015577]	NM_015577
A_23_P46141	1.11E-05	NM_004079	NM_004079	Homo sapiens cathepsin S (CTSS), mRNA [NM_004079]	NM_004079
A_23_P213350	1.11E-05	A_23_P213350			
A_23_P145218	1.11E-05	NM_024018	NM_024018	Homo sapiens butyrophilin, subfamily 2, member A3 (BTN2A3), mRNA [NM_024018]	NM_024018
A_23_P73589	1.11E-05	NM_002444	NM_002444	Homo sapiens moesin (MSN), mRNA [NM_002444]	NM_002444
A_24_P307580	1.12E-05	AF092095	AF092095	Homo sapiens alternatively spliced product of metastasis-suppressor gene CC3 (TC3) mRNA, complete cds. [AF092095]	
A_24_P186779	1.12E-05	A_24_P186779			

A_23_P83175	1.12E-05	NM_001010915	NM_001010915	Homo sapiens similar to RIKEN 4933428I03 (LOC401494), mRNA [NM_001010915]	NM_001010915
A_23_P142878	1.12E-05	NM_032827	NM_032827	Homo sapiens atonal homolog 8 (Drosophila) (ATOH8), mRNA [NM_032827]	NM_032827
A_24_P252364	1.12E-05	NM_005010	NM_005010	Homo sapiens neuronal cell adhesion molecule (NRCAM), mRNA [NM_005010]	NM_005010
A_23_P206140	1.12E-05	NM_018602	NM_018602	Homo sapiens DnaJ (Hsp40) homolog, subfamily A, member 4 (DNAJA4), mRNA [NM_018602]	NM_018602
A_24_P212677	1.13E-05	BC032393	BC032393	Homo sapiens ring finger protein 170, mRNA (cDNA clone MGC:40251 IMAGE:5200847), complete cds. [BC032393]	
A_32_P95757	1.13E-05	NM_145316	NM_145316	Homo sapiens chromosome 6 open reading frame 128 (C6orf128), mRNA [NM_145316]	NM_145316
A_23_P256784	1.13E-05	NM_002457	NM_002457	Homo sapiens mucin 2, intestinal/tracheal (MUC2), mRNA [NM_002457]	NM_002457
A_24_P349274	1.13E-05	NM_001004727	NM_001004727	Homo sapiens olfactory receptor, family 4, subfamily X, member 2 (OR4X2), mRNA [NM_001004727]	NM_001004727
A_23_P96556	1.13E-05	NM_203391	NM_203391	Homo sapiens glycerol kinase (GK), transcript variant 1, mRNA [NM_203391]	NM_203391
A_23_P410159	1.13E-05	NM_178422	NM_178422	Homo sapiens progestin and adipoQ receptor family member VII (PAQR7), mRNA [NM_178422]	NM_178422
A_32_P139021	1.13E-05	A_32_P139021			
A_24_P15823	1.13E-05	A_24_P15823			
A_24_P235049	1.13E-05	NM_015440	NM_015440	Homo sapiens methylenetetrahydrofolate dehydrogenase (NADP+ dependent) 1-like (MTHFD1L), mRNA [NM_015440]	NM_015440
A_23_P207537	1.13E-05	NM_007026	NM_007026	Homo sapiens dual specificity phosphatase 14 (DUSP14), mRNA [NM_007026]	NM_007026
A_23_P370097	1.13E-05	NM_152388	NM_152388	Homo sapiens amyotrophic lateral sclerosis 2 (juvenile) chromosome region, candidate 4 (ALS2CR4), mRNA [NM_152388]	NM_152388
A_23_P126031	1.14E-05	NM_001004469	NM_001004469	Homo sapiens olfactory receptor, family 10, subfamily J, member 5 (OR10J5), mRNA [NM_001004469]	NM_001004469
A_23_P357284	1.14E-05	NM_005282	NM_005282	Homo sapiens G protein-coupled receptor 4 (GPR4), mRNA [NM_005282]	NM_005282
A_23_P118061	1.14E-05	NM_181641	NM_181641	Homo sapiens chemokine-like factor (CKLF), transcript variant 4, mRNA [NM_181641]	NM_181641
A_23_P415882	1.14E-05	NM_001001563	NM_001001563	Homo sapiens translocase of inner mitochondrial membrane 50 homolog (yeast) (TIMM50), mRNA [NM_001001563]	NM_001001563
A_24_P187948	1.14E-05	NM_197966	NM_197966	Homo sapiens BH3 interacting domain death agonist (BID), transcript variant 1, mRNA [NM_197966]	NM_197966
A_23_P9416	1.14E-05	NM_002197	NM_002197	Homo sapiens aconitase 1, soluble (ACO1), mRNA [NM_002197]	NM_002197
A_23_P253752	1.15E-05	NM_138419	NM_138419	Homo sapiens family with sequence similarity 54, member A (FAM54A), mRNA [NM_138419]	NM_138419
A_24_P215653	1.15E-05	NM_175060	NM_175060	Homo sapiens C-type lectin domain family 14, member A (CLEC14A), mRNA [NM_175060]	NM_175060
A_23_P257668	1.16E-05	NM_024926	NM_024926	Homo sapiens hypothetical protein FLJ12571 (FLJ12571), mRNA [NM_024926]	NM_024926
A_32_P109057	1.16E-05	NM_173825	NM_173825	Homo sapiens RAB, member of RAS oncogene family-like 3 (RABL3), mRNA [NM_173825]	NM_173825
A_23_P213359	1.16E-05	NM_002587	NM_002587	Homo sapiens protocadherin 1 (cadherin-like 1) (PCDH1), transcript variant 1, mRNA [NM_002587]	NM_002587
A_24_P289818	1.16E-05	A_24_P289818			
A_32_P225916	1.16E-05	BM994413	BM994413	UI-H-DH0-aul-n-17-0-UI.s1 NCI_CGAP_DH0 Homo sapiens cDNA clone IMAGE:5871184 3', mRNA sequence [BM994413]	
A_23_P393531	1.17E-05	NM_004027	NM_004027	Homo sapiens inositol polyphosphate-4-phosphatase, type I, 107kDa (INPP4A), transcript variant a, mRNA [NM_004027]	NM_004027
A_23_P142424	1.17E-05	NM_024660	NM_024660	Homo sapiens hypothetical protein FLJ22573 (FLJ22573), mRNA [NM_024660]	NM_024660
A_32_P70220	1.17E-05	NM_152272	NM_152272	Homo sapiens CHMP family, member 7 (CHMP7), mRNA [NM_152272]	NM_152272
A_23_P17307	1.17E-05	NM_018270	NM_018270	Homo sapiens chromosome 20 open reading frame 20 (C20orf20), mRNA [NM_018270]	NM_018270
A_32_P299	1.18E-05	THC2308403		CA307538 UI-H-FT1-bic-o-24-0-UI.s1 NCI_CGAP_FT1 Homo sapiens cDNA clone UI-H-FT1-bic-o-24-0-UI 3', mRNA sequence [CA307538]	
A_23_P256445	1.18E-05	NM_025054	NM_025054	Homo sapiens valosin containing protein (p97)/p47 complex interacting protein 1 (VCPIP1), mRNA [NM_025054]	NM_025054
A_23_P6381	1.18E-05	NM_002430	NM_002430	Homo sapiens meningioma (disrupted in balanced translocation) 1 (MN1), mRNA [NM_002430]	NM_002430
A_24_P150160	1.18E-05	NM_004265	NM_004265	Homo sapiens fatty acid desaturase 2 (FADS2), mRNA [NM_004265]	NM_004265
A_32_P32315	1.18E-05	A_32_P32315			
A_24_P273245	1.18E-05	A_24_P273245			
A_24_P243278	1.18E-05	NM_004394	NM_004394	Homo sapiens death-associated protein (DAP), mRNA [NM_004394]	NM_004394
A_23_P358957	1.18E-05	NM_006549	NM_006549	Homo sapiens calcium/calmodulin-dependent protein kinase kinase 2, beta (CAMKK2), transcript variant 1, mRNA [NM_006549]	NM_006549
A_23_P256107	1.19E-05	NM_006665	NM_006665	Homo sapiens heparanase (HPSE), mRNA [NM_006665]	NM_006665
A_23_P145485	1.19E-05	NM_025217	NM_025217	Homo sapiens UL16 binding protein 2 (ULBP2), mRNA [NM_025217]	NM_025217
A_23_P15146	1.19E-05	NM_001012631	NM_001012631	Homo sapiens interleukin 32 (IL32), transcript variant 1, mRNA [NM_001012631]	NM_001012631
A_32_P206839	1.19E-05	AF075027	AF075027	Homo sapiens full length insert cDNA YI37C01. [AF075027]	

A_24_P703462	1.20E-05	THC2308747		Q969M7 (Q969M7) NEDD8-conjugating enzyme NCE2, partial (70%) [THC2308747]	
A_24_P184692	1.20E-05	NM_003317	NM_003317	Homo sapiens thyroid transcription factor 1 (TITF1), mRNA [NM_003317]	NM_003317
A_24_P314681	1.20E-05	ENST00000248179		BX281397 Soares_NFL_T_GBC_S1 Homo sapiens cDNA clone IMAGp998G075145 ; IMAGE:2087454, mRNA sequence [BX281397]	XM_370585
A_24_P390096	1.20E-05	U16307	U16307	Homo sapiens glioma pathogenesis-related protein (GliPR) mRNA, complete cds. [U16307]	
A_24_P829181	1.20E-05	BC034002	BC034002	Homo sapiens, clone IMAGE:5092955, mRNA, partial cds. [BC034002]	
A_24_P122050	1.20E-05	NM_006814	NM_006814	Homo sapiens proteasome (prosome, macropain) inhibitor subunit 1 (PI31) (PSMF1), transcript variant 1, mRNA [NM_006814]	NM_006814
A_32_P32739	1.20E-05	NM_153006	NM_153006	Homo sapiens N-acetylglutamate synthase (NAGS), mRNA [NM_153006]	NM_153006
A_23_P413815	1.20E-05	NM_173517	NM_173517	Homo sapiens vitamin K epoxide reductase complex, subunit 1-like 1 (VKORC1L1), mRNA [NM_173517]	NM_173517
A_23_P345942	1.20E-05	NM_174889	NM_174889	Homo sapiens Myc-induced mitochondria protein (mimitin), mRNA [NM_174889]	NM_174889
A_23_P134454	1.20E-05	NM_001753	NM_001753	Homo sapiens caveolin 1, caveolae protein, 22kDa (CAV1), mRNA [NM_001753]	NM_001753
A_23_P155666	1.21E-05	NM_014435	NM_014435	Homo sapiens N-acylsphingosine amidohydrolase (acid ceramidase)-like (ASAHL), mRNA [NM_014435]	NM_014435
A_23_P152527	1.21E-05	NM_001088	NM_001088	Homo sapiens arylalkylamine N-acetyltransferase (AANAT), mRNA [NM_001088]	NM_001088
A_23_P23380	1.21E-05	NM_022821	NM_022821	Homo sapiens elongation of very long chain fatty acids (FEN1/Elo2, SUR4/Elo3, yeast)-like 1 (ELOVL1), mRNA [NM_022821]	NM_022821
A_23_P350005	1.21E-05	NM_173553	NM_173553	Homo sapiens hypothetical protein FLJ25801 (FLJ25801), mRNA [NM_173553]	NM_173553
A_32_P134098	1.21E-05	BC042539	BC042539	Homo sapiens similar to prostaglandin E receptor 4, subtype EP4; PGE receptor, EP4 subtype; prostaglandin E2 receptor, mRNA (cDNA clone IMAGE:4830816), partial cds. [BC042539]	XM_376869
A_23_P134809	1.21E-05	NM_003580	NM_003580	Homo sapiens neutral sphingomyelinase (N-SMase) activation associated factor (NSMAF), mRNA [NM_003580]	NM_003580
A_32_P399546	1.22E-05	AF256215	AF256215	Homo sapiens cycle-like factor CLIF mRNA, complete cds. [AF256215]	
A_23_P151150	1.22E-05	NM_202002	NM_202002	Homo sapiens forkhead box M1 (FOXM1), transcript variant 1, mRNA [NM_202002]	NM_202002
A_23_P33364	1.22E-05	NM_001009555	NM_001009555	Homo sapiens SH3 domain protein D19 (SH3D19), mRNA [NM_001009555]	NM_001009555
A_24_P349965	1.23E-05	BC033086	BC033086	Homo sapiens transcription factor 19 (SC1), mRNA (cDNA clone MGC:45652 IMAGE:3160434), complete cds. [BC033086]	
A_23_P211985	1.23E-05	NM_017719	NM_017719	Homo sapiens SNF related kinase (SNRK), mRNA [NM_017719]	NM_017719
A_32_P209989	1.23E-05	NM_022163	NM_022163	Homo sapiens mitochondrial ribosomal protein L46 (MRPL46), nuclear gene encoding mitochondrial protein, mRNA [NM_022163]	NM_022163
A_23_P63980	1.23E-05	NM_024036	NM_024036	Homo sapiens leucine rich repeat and fibronectin type III domain containing 4 (LRFN4), mRNA [NM_024036]	NM_024036
A_24_P323715	1.23E-05	XM_498088	XM_498088	PREDICTED: Homo sapiens similar to myosin:SUBUNIT=regulatory light chain (LOC442204), mRNA [XM_498088]	XM_498088
A_23_P99942	1.24E-05	NM_024652	NM_024652	Homo sapiens leucine-rich repeat kinase 1 (LRRK1), mRNA [NM_024652]	NM_024652
A_23_P106024	1.24E-05	NM_002226	NM_002226	Homo sapiens jagged 2 (JAG2), transcript variant 1, mRNA [NM_002226]	NM_002226
A_23_P208595	1.24E-05	NM_000527	NM_000527	Homo sapiens low density lipoprotein receptor (familial hypercholesterolemia) (LDLR), mRNA [NM_000527]	NM_000527
A_24_P93901	1.24E-05	BC063531	BC063531	Homo sapiens SIN3 homolog B, transcription regulator (yeast), mRNA (cDNA clone IMAGE:4417458), complete cds. [BC063531]	
A_24_P128140	1.24E-05	NM_015476	NM_015476	Homo sapiens chromosome 18 open reading frame 10 (C18orf10), mRNA [NM_015476]	NM_015476
A_23_P150249	1.24E-05	NM_006848	NM_006848	Homo sapiens hepatitis delta antigen-interacting protein A (DIPA), mRNA [NM_006848]	NM_006848
A_24_P33705	1.25E-05	NM_198432	NM_198432	Homo sapiens regulator of G-protein signalling 12 (RGS12), transcript variant 5, mRNA [NM_198432]	NM_198432
A_24_P66233	1.25E-05	NR_001543	NR_001543	Homo sapiens testis-specific transcript, Y-linked 14 (TTTY14) on chromosome Y [NR_001543]	NR_001543
A_24_P384239	1.25E-05	A_24_P384239			
A_32_P117464	1.25E-05	NM_178496	NM_178496	Homo sapiens similar to BcDNA:GH11415 gene product (LOC151963), mRNA [NM_178496]	NM_178496
A_24_P39654	1.25E-05	NM_002086	NM_002086	Homo sapiens growth factor receptor-bound protein 2 (GRB2), transcript variant 1, mRNA [NM_002086]	NM_002086
A_23_P95470	1.25E-05	NM_004357	NM_004357	Homo sapiens CD151 antigen (CD151), transcript variant 1, mRNA [NM_004357]	NM_004357
A_32_P69938	1.26E-05	NM_203448	NM_203448	Homo sapiens hypothetical protein MGC21881 (MGC21881), mRNA [NM_203448]	NM_203448
A_23_P130130	1.26E-05	NM_181844	NM_181844	Homo sapiens B-cell CLL/lymphoma 6, member B (zinc finger protein) (BCL6B), mRNA [NM_181844]	NM_181844
A_23_P61268	1.26E-05	NM_016458	NM_016458	Homo sapiens brain protein 16 (LOC51236), mRNA [NM_016458]	NM_016458
A_24_P40928	1.26E-05	ENST00000281228			
A_23_P102965	1.26E-05	NM_015367	NM_015367	Homo sapiens BCL2-like 13 (apoptosis facilitator) (BCL2L13), nuclear gene encoding mitochondrial protein, mRNA [NM_015367]	NM_015367
A_24_P42633	1.26E-05	NM_044472	NM_044472	Homo sapiens cell division cycle 42 (GTP binding protein, 25kDa) (CDC42), transcript variant 2, mRNA [NM_044472]	NM_044472
A_24_P77364	1.26E-05	NM_212552	NM_212552	Homo sapiens bolA-like 3 (E. coli) (BOLA3), mRNA [NM_212552]	NM_212552

A_24_P460195	1.27E-05	A_24_P460195			
A_23_P43484	1.28E-05	NM_058197	NM_058197	Homo sapiens cyclin-dependent kinase inhibitor 2A (melanoma, p16, inhibits CDK4) (CDKN2A), transcript variant 3, mRNA [NM_058197]	NM_058197
A_23_P348264	1.29E-05	NM_144652	NM_144652	Homo sapiens leucine zipper-EF-hand containing transmembrane protein 2 (LETM2), mRNA [NM_144652]	NM_144652
A_23_P102973	1.29E-05	NM_022719	NM_022719	Homo sapiens DiGeorge syndrome critical region gene 14 (DGCR14), mRNA [NM_022719]	NM_022719
A_23_P321855	1.29E-05	NM_003899	NM_003899	Homo sapiens Rho guanine nucleotide exchange factor (GEF) 7 (ARHGEF7), transcript variant 1, mRNA [NM_003899]	NM_003899
A_23_P59481	1.29E-05	NM_014671	NM_014671	Homo sapiens ubiquitin protein ligase E3C (UBE3C), mRNA [NM_014671]	NM_014671
A_23_P149626	1.29E-05	NM_198681	NM_198681	Homo sapiens pleckstrin homology domain containing, family G (with RhoGef domain) member 5 (PLEKHG5), transcript variant 2, mRNA [NM_198681]	NM_198681
A_23_P380881	1.30E-05	NM_152345	NM_152345	Homo sapiens ankyrin repeat domain 13B (ANKRD13B), mRNA [NM_152345]	NM_152345
A_32_P6769	1.30E-05	BC071598	BC071598	Homo sapiens hypothetical protein MGC25181, mRNA (cDNA clone MGC:87530 IMAGE:30334929), complete cds. [BC071598]	
A_24_P94222	1.30E-05	NM_017556	NM_017556	Homo sapiens filamin binding LIM protein 1 (FBLIM1), transcript variant 1, mRNA [NM_017556]	NM_017556
A_23_P35820	1.30E-05	NM_005507	NM_005507	Homo sapiens cofilin 1 (non-muscle) (CFL1), mRNA [NM_005507]	NM_005507
A_23_P119095	1.31E-05	NM_006663	NM_006663	Homo sapiens protein phosphatase 1, regulatory (inhibitor) subunit 13 like (PPP1R13L), mRNA [NM_006663]	NM_006663
A_23_P2366	1.31E-05	NM_199040	NM_199040	Homo sapiens nudix (nucleoside diphosphate linked moiety X)-type motif 4 (NUDT4), transcript variant 2, mRNA [NM_199040]	NM_199040
A_23_P337242	1.32E-05	NM_003242	NM_003242	Homo sapiens transforming growth factor, beta receptor II (70/80kDa) (TGFBR2), transcript variant 2, mRNA [NM_003242]	NM_003242
A_23_P90790	1.32E-05	NM_199227	NM_199227	Homo sapiens methionine aminopeptidase 1D (MAP1D), mRNA [NM_199227]	NM_199227
A_23_P205159	1.32E-05	NM_005358	NM_005358	Homo sapiens LIM domain 7 (LMO7), mRNA [NM_005358]	NM_005358
A_23_P409168	1.32E-05	AK131104	AK131104	Homo sapiens mRNA for FLJ00341 protein. [AK131104]	XM_291064
A_23_P218144	1.32E-05	NM_000428	NM_000428	Homo sapiens latent transforming growth factor beta binding protein 2 (LTBP2), mRNA [NM_000428]	NM_000428
A_24_P74932	1.32E-05	NM_002668	NM_002668	Homo sapiens proteolipid protein 2 (colonic epithelium-enriched) (PLP2), mRNA [NM_002668]	NM_002668
A_23_P18372	1.32E-05	NM_032047	NM_032047	Homo sapiens UDP-GlcNAc:betaGal beta-1,3-N-acetylglucosaminyltransferase 5 (B3GNT5), mRNA [NM_032047]	NM_032047
A_24_P259276	1.32E-05	NM_207340	NM_207340	Homo sapiens zinc finger, DHHC-type containing 24 (ZDHHC24), mRNA [NM_207340]	NM_207340
A_24_P315754	1.32E-05	ENST00000343233			
A_23_P77493	1.32E-05	NM_006086	NM_006086	Homo sapiens tubulin, beta 3 (TUBB3), mRNA [NM_006086]	NM_006086
A_32_P216841	1.33E-05	NM_145263	NM_145263	Homo sapiens spermatogenesis associated 18 homolog (rat) (SPATA18), mRNA [NM_145263]	NM_145263
A_24_P75708	1.33E-05	ENST00000321005			
A_23_P11729	1.34E-05	NM_015872	NM_015872	Homo sapiens zinc finger and BTB domain containing 7B (ZBTB7B), mRNA [NM_015872]	NM_015872
A_24_P834110	1.34E-05	XM_373538	XM_373538	PREDICTED: Homo sapiens similar to microtubule-associated proteins 1A/1B light chain 3 (LOC387869), mRNA [XM_373538]	XM_373538
A_24_P205874	1.34E-05	NM_004206	NM_004206	Homo sapiens SEC22 vesicle trafficking protein-like 3 (S. cerevisiae) (SEC22L3), transcript variant 2, mRNA [NM_004206]	NM_004206
A_23_P5785	1.35E-05	NM_152384	NM_152384	Homo sapiens Bardet-Biedl syndrome 5 (BBS5), mRNA [NM_152384]	NM_152384
A_32_P184916	1.35E-05	NM_021629	NM_021629	Homo sapiens guanine nucleotide binding protein (G protein), beta polypeptide 4 (GNB4), mRNA [NM_021629]	NM_021629
A_23_P217049	1.35E-05	NM_014286	NM_014286	Homo sapiens frequenin homolog (Drosophila) (FREQ), mRNA [NM_014286]	NM_014286
A_24_P305597	1.35E-05	NM_007002	NM_007002	Homo sapiens adhesion regulating molecule 1 (ADRM1), transcript variant 1, mRNA [NM_007002]	NM_007002
A_24_P102411	1.36E-05	BC016743	BC016743	Homo sapiens BRF1 homolog, subunit of RNA polymerase III transcription initiation factor IIIB (S. cerevisiae), mRNA (cDNA clone IMAGE:4830425), complete cds. [BC016743]	
A_23_P164702	1.36E-05	NM_015956	NM_015956	Homo sapiens mitochondrial ribosomal protein L4 (MRPL4), nuclear gene encoding mitochondrial protein, transcript variant 1, mRNA [NM_015956]	NM_015956
A_24_P316939	1.36E-05	BC010662	BC010662	Homo sapiens cDNA clone IMAGE:3855382, complete cds. [BC010662]	
A_23_P13946	1.38E-05	NM_005475	NM_005475	Homo sapiens lymphocyte adaptor protein (LNK), mRNA [NM_005475]	NM_005475
A_23_P38346	1.38E-05	NM_024119	NM_024119	Homo sapiens likely ortholog of mouse D11lgp2 (LGP2), mRNA [NM_024119]	NM_024119
A_23_P57927	1.38E-05	NM_003773	NM_003773	Homo sapiens hyaluronoglucosaminidase 2 (HYAL2), transcript variant 1, mRNA [NM_003773]	NM_003773
A_23_P218523	1.38E-05	AF218008	AF218008	Homo sapiens clone PP3501 unknown mRNA. [AF218008]	
A_23_P151307	1.39E-05	NM_006105	NM_006105	Homo sapiens Rap guanine nucleotide exchange factor (GEF) 3 (RAPGEF3), mRNA [NM_006105]	NM_006105
A_24_P85775	1.39E-05	NM_004848	NM_004848	Homo sapiens chromosome 1 open reading frame 38 (C1orf38), mRNA [NM_004848]	NM_004848
A_23_P147755	1.39E-05	NM_017413	NM_017413	Homo sapiens apelin, AGTRL1 ligand (APLN), mRNA [NM_017413]	NM_017413
A_23_P210920	1.39E-05	NM_000178	NM_000178	Homo sapiens glutathione synthetase (GSS), mRNA [NM_000178]	NM_000178

A_23_P436138	1.40E-05	NM_145114	NM_145114	Homo sapiens MYC associated factor X (MAX), transcript variant 4, mRNA [NM_145114]	NM_145114
A_23_P68966	1.40E-05	CR626044	CR626044	full-length cDNA clone CS0DI082YE05 of Placenta Cot 25-normalized of Homo sapiens (human). [CR626044]	
A_24_P17909	1.40E-05	NM_015950	NM_015950	Homo sapiens mitochondrial ribosomal protein L2 (MRPL2), nuclear gene encoding mitochondrial protein, mRNA [NM_015950]	NM_015950
A_23_P416305	1.40E-05	ENST00000356931		Homo sapiens cDNA FLJ43464 fis, clone OCBBF2036225. [AK125453]	
A_23_P362659	1.40E-05	NM_002468	NM_002468	Homo sapiens myeloid differentiation primary response gene (88) (MYD88), mRNA [NM_002468]	NM_002468
A_24_P578445	1.40E-05	A_24_P578445			
A_23_P406425	1.41E-05	NM_173359	NM_173359	Homo sapiens eukaryotic translation initiation factor 4E member 3 (EIF4E3), mRNA [NM_173359]	NM_173359
A_23_P202080	1.41E-05	NM_030927	NM_030927	Homo sapiens tetraspanin 14 (TSPAN14), mRNA [NM_030927]	NM_030927
A_23_P131139	1.41E-05	NM_052952	NM_052952	Homo sapiens disrupted in renal carcinoma 1 (DIRC1), mRNA [NM_052952]	NM_052952
A_23_P53162	1.42E-05	NM_003156	NM_003156	Homo sapiens stromal interaction molecule 1 (STIM1), mRNA [NM_003156]	NM_003156
A_23_P15233	1.42E-05	A_23_P15233			
A_23_P126727	1.42E-05	NM_024654	NM_024654	Homo sapiens hypothetical protein FLJ23323 (FLJ23323), mRNA [NM_024654]	NM_024654
A_24_P269853	1.42E-05	NM_005736	NM_005736	Homo sapiens ARP1 actin-related protein 1 homolog A, centractin alpha (yeast) (ACTR1A), mRNA [NM_005736]	NM_005736
A_24_P482009	1.43E-05	THC2361927		FRG1_HUMAN (Q14331) FRG1 protein (FSHD region gene 1 protein), partial (33%) [THC2361927]	
A_23_P341275	1.43E-05	NM_015029	NM_015029	Homo sapiens processing of precursor 1, ribonuclease P/MRP subunit (S. cerevisiae) (POP1), mRNA [NM_015029]	NM_015029
A_23_P145153	1.44E-05	NM_002598	NM_002598	Homo sapiens programmed cell death 2 (PDCD2), transcript variant 1, mRNA [NM_002598]	NM_002598
A_23_P34176	1.44E-05	NM_015691	NM_015691	Homo sapiens KIAA1280 protein (KIAA1280), mRNA [NM_015691]	NM_015691
A_23_P380010	1.45E-05	ENST00000290607		Homo sapiens mRNA; cDNA DKFZp781J069 (from clone DKFZp781J069). [CR749416]	
A_24_P931443	1.45E-05	BC098567	BC098567	Homo sapiens G protein-coupled receptor 68, mRNA (cDNA clone MGC:111379 IMAGE:30925589), complete cds [BC098567]	
A_23_P56559	1.45E-05	NM_005771	NM_005771	Homo sapiens dehydrogenase/reductase (SDR family) member 9 (DHRS9), transcript variant 1, mRNA [NM_005771]	NM_005771
A_23_P39402	1.45E-05	NM_198867	NM_198867	Homo sapiens hypothetical protein MGC15677 (MGC15677), transcript variant 1, mRNA [NM_198867]	NM_198867
A_24_P239176	1.45E-05	AF177925	AF177925	Homo sapiens mucin 4 (MUC4) mRNA, partial cds. [AF177925]	
A_32_P191859	1.45E-05	NM_020382	NM_020382	Homo sapiens PR/SET domain containing protein 8 (SET8), mRNA [NM_020382]	NM_020382
A_23_P201386	1.45E-05	NM_012137	NM_012137	Homo sapiens dimethylarginine dimethylaminohydrolase 1 (DDAH1), mRNA [NM_012137]	NM_012137
A_23_P33613	1.45E-05	NM_024096	NM_024096	Homo sapiens XTP3-transactivated protein A (XTP3TPA), mRNA [NM_024096]	NM_024096
A_24_P532180	1.46E-05	AK023635	AK023635	Homo sapiens cDNA FLJ13573 fis, clone PLACE1008584. [AK023635]	
A_32_P64475	1.46E-05	NM_152608	NM_152608	Homo sapiens chromosome 1 open reading frame 55 (C1orf55), mRNA [NM_152608]	NM_152608
A_32_P23659	1.46E-05	BC056883	BC056883	Homo sapiens breast carcinoma amplified sequence 4, mRNA (cDNA clone IMAGE:5764497), partial cds. [BC056883]	
A_24_P130962	1.46E-05	NM_022371	NM_022371	Homo sapiens torsin family 3, member A (TOR3A), mRNA [NM_022371]	NM_022371
A_23_P3355	1.46E-05	NM_002693	NM_002693	Homo sapiens polymerase (DNA directed), gamma (POLG), mRNA [NM_002693]	NM_002693
A_24_P916816	1.47E-05	L79989	L79989	Homo sapiens nuclear autoantigen mRNA, partial cds; alternatively spliced. [L79989]	
A_23_P121573	1.47E-05	BC035666	BC035666	Homo sapiens cDNA clone MGC:46019 IMAGE:5724430, complete cds. [BC035666]	
A_23_P103923	1.47E-05	NM_014864	NM_014864	Homo sapiens family with sequence similarity 20, member B (FAM20B), mRNA [NM_014864]	NM_014864
A_23_P118712	1.47E-05	BC011636	BC011636	Homo sapiens slingshot homolog 2 (Drosophila), mRNA (cDNA clone IMAGE:4101583), complete cds. [BC011636]	
A_23_P44831	1.47E-05	NM_018482	NM_018482	Homo sapiens development and differentiation enhancing factor 1 (DDEF1), mRNA [NM_018482]	NM_018482
A_24_P391229	1.47E-05	NM_015666	NM_015666	Homo sapiens GTP binding protein 5 (putative) (GTPBP5), mRNA [NM_015666]	NM_015666
A_23_P123622	1.47E-05	NM_000907	NM_000907	Homo sapiens natriuretic peptide receptor B/guanylate cyclase B (atrionatriuretic peptide receptor B) (NPR2), mRNA [NM_000907]	NM_000907
A_23_P65674	1.47E-05	NM_014547	NM_014547	Homo sapiens tropomodulin 3 (ubiquitous) (TMOD3), mRNA [NM_014547]	NM_014547
A_24_P303815	1.47E-05	NM_152306	NM_152306	Homo sapiens ubiquitin-like, containing PHD and RING finger domains, 2 (UHRF2), transcript variant 1, mRNA [NM_152306]	NM_152306
A_23_P11025	1.47E-05	NM_007150	NM_007150	Homo sapiens zinc finger protein 185 (LIM domain) (ZNF185), mRNA [NM_007150]	NM_007150
A_23_P15285	1.47E-05	NM_024109	NM_024109	Homo sapiens hypothetical protein MGC2654 (MGC2654), mRNA [NM_024109]	NM_024109
A_23_P251104	1.47E-05	NM_000232	NM_000232	Homo sapiens sarcoglycan, beta (43kDa dystrophin-associated glycoprotein) (SGCB), mRNA [NM_000232]	NM_000232
A_23_P115105	1.47E-05	NM_015609	NM_015609	Homo sapiens chromosome 1 open reading frame 144 (C1orf144), mRNA [NM_015609]	NM_015609
A_23_P211488	1.48E-05	NM_145637	NM_145637	Homo sapiens apolipoprotein L, 2 (APOL2), transcript variant beta, mRNA [NM_145637]	NM_145637

A_32_P462013	1.48E-05	AK021694	AK021694	Homo sapiens cDNA FLJ11632 fis, clone HEMBA1004272. [AK021694]	
A_23_P34527	1.49E-05	NM_025207	NM_025207	Homo sapiens FAD-synthetase (PP591), transcript variant 1, mRNA [NM_025207]	NM_025207
A_24_P236949	1.50E-05	NM_018381	NM_018381	Homo sapiens hypothetical protein FLJ11286 (FLJ11286), mRNA [NM_018381]	NM_018381
A_24_P250666	1.50E-05	NM_003018	NM_003018	Homo sapiens surfactant, pulmonary-associated protein C (SFTPC), mRNA [NM_003018]	NM_003018
A_24_P15292	1.50E-05	NM_032018	NM_032018	Homo sapiens chromosome 1 open reading frame 124 (C1orf124), transcript variant 1, mRNA [NM_032018]	NM_032018
A_24_P399630	1.50E-05	NM_002730	NM_002730	Homo sapiens protein kinase, cAMP-dependent, catalytic, alpha (PRKACA), transcript variant 1, mRNA [NM_002730]	NM_002730
A_24_P533142	1.50E-05	A_24_P533142			
A_24_P71834	1.50E-05	NM_016141	NM_016141	Homo sapiens dynein, cytoplasmic, light intermediate polypeptide 1 (DNCLI1), mRNA [NM_016141]	NM_016141
A_24_P206305	1.50E-05	NM_182924	NM_182924	Homo sapiens MICAL-like 2 (MICAL-L2), transcript variant 1, mRNA [NM_182924]	NM_182924
A_23_P345460	1.50E-05	NM_015432	NM_015432	Homo sapiens pleckstrin homology domain containing, family G (with RhoGef domain) member 4 (PLEKHG4), mRNA [NM_015432]	NM_015432
A_23_P431388	1.50E-05	NM_144569	NM_144569	Homo sapiens SPOC domain containing 1 (SPOCD1), mRNA [NM_144569]	NM_144569
A_23_P307002	1.50E-05	AK023110	AK023110	Homo sapiens cDNA FLJ13048 fis, clone NT2RP3001399, weakly similar to SSU72 PROTEIN. [AK023110]	
A_24_P194199	1.51E-05	NM_020433	NM_020433	Homo sapiens junctophilin 2 (JPH2), transcript variant 1, mRNA [NM_020433]	NM_020433
A_23_P41267	1.51E-05	BC010526	BC010526	Homo sapiens hypothetical LOC401127, mRNA (cDNA clone MGC:18091 IMAGE:4151462), complete cds. [BC010526]	
A_23_P108751	1.51E-05	NM_201555	NM_201555	Homo sapiens four and a half LIM domains 2 (FHL2), transcript variant 2, mRNA [NM_201555]	NM_201555
A_24_P83738	1.52E-05	NM_198188	NM_198188	Homo sapiens astrotactin 2 (ASTN2), transcript variant 4, mRNA [NM_198188]	NM_198188
A_32_P110390	1.53E-05	NM_173490	NM_173490	Homo sapiens proline-rich protein PRP2 (PRP2), mRNA [NM_173490]	NM_173490
A_23_P128384	1.53E-05	NM_057180	NM_057180	Homo sapiens vacuolar protein sorting 29 (yeast) (VPS29), transcript variant 2, mRNA [NM_057180]	NM_057180
A_23_P29199	1.54E-05	NM_181831	NM_181831	Homo sapiens neurofibromin 2 (bilateral acoustic neuroma) (NF2), transcript variant 13, mRNA [NM_181831]	NM_181831
A_23_P112275	1.54E-05	NM_005458	NM_005458	Homo sapiens G protein-coupled receptor 51 (GPR51), mRNA [NM_005458]	NM_005458
A_24_P189112	1.54E-05	NM_173830	NM_173830	Homo sapiens chromosome 6 open reading frame 182 (C6orf182), mRNA [NM_173830]	NM_173830
A_23_P102058	1.55E-05	NM_002381	NM_002381	Homo sapiens matrilin 3 (MATN3), mRNA [NM_002381]	NM_002381
A_23_P52939	1.55E-05	NM_003627	NM_003627	Homo sapiens solute carrier family 43, member 1 (SLC43A1), mRNA [NM_003627]	NM_003627
A_23_P83917	1.55E-05	NM_012308	NM_012308	Homo sapiens F-box and leucine-rich repeat protein 11 (FBXL11), mRNA [NM_012308]	NM_012308
A_23_P112602	1.55E-05	NM_018207	NM_018207	Homo sapiens tripartite motif-containing 62 (TRIM62), mRNA [NM_018207]	NM_018207
A_23_P108604	1.55E-05	NM_145686	NM_145686	Homo sapiens mitogen-activated protein kinase kinase kinase kinase 4 (MAP4K4), transcript variant 2, mRNA [NM_145686]	NM_145686
A_23_P343826	1.56E-05	NM_033449	NM_033449	Homo sapiens FCH and double SH3 domains 1 (FCHSD1), mRNA [NM_033449]	NM_033449
A_23_P203364	1.56E-05	NM_024092	NM_024092	Homo sapiens hypothetical protein MGC5508 (MGC5508), mRNA [NM_024092]	NM_024092
A_23_P501933	1.56E-05	NM_145814	NM_145814	Homo sapiens calcium channel, voltage-dependent, gamma subunit 6 (CACNG6), transcript variant 1, mRNA [NM_145814]	NM_145814
A_23_P142796	1.56E-05	NM_017980	NM_017980	Homo sapiens LIM and senescent cell antigen-like domains 2 (LIMS2), mRNA [NM_017980]	NM_017980
A_23_P214907	1.56E-05	NM_015440	NM_015440	Homo sapiens methylenetetrahydrofolate dehydrogenase (NADP+ dependent) 1-like (MTHFD1L), mRNA [NM_015440]	NM_015440
A_23_P36018	1.57E-05	NM_014312	NM_014312	Homo sapiens V-set and immunoglobulin domain containing 2 (VSIG2), mRNA [NM_014312]	NM_014312
A_23_P36825	1.57E-05	NM_003979	NM_003979	Homo sapiens G protein-coupled receptor, family C, group 5, member A (GPRC5A), mRNA [NM_003979]	NM_003979
A_24_P221770	1.57E-05	NM_001011713	NM_001011713	Homo sapiens chromosome 14 open reading frame 35 (C14orf35), mRNA [NM_001011713]	NM_001011713
A_23_P7655	1.57E-05	NM_013978	NM_013978	Homo sapiens BCL2/adenovirus E1B 19kDa interacting protein 1 (BNIP1), transcript variant BNIP1-a, mRNA [NM_013978]	NM_013978
A_23_P360874	1.57E-05	NM_152892	NM_152892	Homo sapiens hypothetical protein DKFZp434K1815 (DKFZp434K1815), mRNA [NM_152892]	NM_152892
A_24_P352864	1.58E-05	NM_005789	NM_005789	Homo sapiens proteasome (prosome, macropain) activator subunit 3 (PA28 gamma; Ki) (PSME3), transcript variant 1, mRNA [NM_005789]	NM_005789
A_24_P944299	1.58E-05	ENST00000334218		Homo sapiens mRNA for KIAA0451 protein, partial cds. [AB007920]	
A_24_P349590	1.58E-05	A_24_P349590			
A_23_P150510	1.58E-05	NM_153450	NM_153450	Homo sapiens mediator of RNA polymerase II transcription, subunit 19 homolog (yeast) (MED19), mRNA [NM_153450]	NM_153450
A_23_P165783	1.59E-05	NM_024101	NM_024101	Homo sapiens melanophilin (MLPH), mRNA [NM_024101]	NM_024101
A_23_P388244	1.59E-05	NM_173793	NM_173793	Homo sapiens hypothetical protein LOC128977 (LOC128977), mRNA [NM_173793]	NM_173793
A_23_P380266	1.60E-05	AK074447	AK074447	Homo sapiens cDNA FLJ23867 fis, clone LNG09729. [AK074447]	
A_24_P206121	1.60E-05	NM_004137	NM_004137	Homo sapiens potassium large conductance calcium-activated channel, subfamily M, beta member 1 (KCNMB1), mRNA [NM_004137]	NM_004137

A_23_P145197	1.60E-05	NM_004053	NM_004053	Homo sapiens bystin-like (BYSL), mRNA [NM_004053]	NM_004053
A_23_P395111	1.61E-05	AK025947	AK025947	Homo sapiens cDNA: FLJ22294 fis, clone HRC04426. [AK025947]	
A_23_P54900	1.61E-05	NM_016936	NM_016936	Homo sapiens ubinuclein 1 (UBN1), mRNA [NM_016936]	NM_016936
A_24_P116805	1.62E-05	NM_213662	NM_213662	Homo sapiens signal transducer and activator of transcription 3 (acute-phase response factor) (STAT3), transcript variant 3, mRNA [NM_213662]	NM_213662
A_23_P16834	1.62E-05	NM_022823	NM_022823	Homo sapiens fibronectin type III domain containing 4 (FNDC4), mRNA [NM_022823]	NM_022823
A_23_P391778	1.62E-05	NM_033044	NM_033044	Homo sapiens microtubule-actin crosslinking factor 1 (MACF1), transcript variant 2, mRNA [NM_033044]	NM_033044
A_23_P94736	1.63E-05	NM_175039	NM_175039	Homo sapiens ST6 (alpha-N-acetyl-neuraminyl-2,3-beta-galactosyl-1,3)-N-acetylgalactosaminide alpha-2,6-sialyltransferase 4 (ST6GALNAC4), transcript variant 2, mRNA [NM_175039]	NM_175039
A_23_P316974	1.63E-05	NM_003898	NM_003898	Homo sapiens synaptojanin 2 (SYNJ2), mRNA [NM_003898]	NM_003898
A_23_P91468	1.63E-05	NM_002792	NM_002792	Homo sapiens proteasome (prosome, macropain) subunit, alpha type, 7 (PSMA7), transcript variant 1, mRNA [NM_002792]	NM_002792
A_32_P98534	1.64E-05	NM_021633	NM_021633	Homo sapiens kelch-like 12 (Drosophila) (KLHL12), mRNA [NM_021633]	NM_021633
A_24_P267997	1.64E-05	NM_015602	NM_015602	Homo sapiens torsin A interacting protein 1 (TOR1AIP1), mRNA [NM_015602]	NM_015602
A_23_P210948	1.64E-05	NM_018244	NM_018244	Homo sapiens chromosome 20 open reading frame 44 (C20orf44), transcript variant 1, mRNA [NM_018244]	NM_018244
A_23_P203392	1.64E-05	NM_025080	NM_025080	Homo sapiens asparaginase like 1 (ASRGL1), mRNA [NM_025080]	NM_025080
A_23_P502274	1.64E-05	NM_002751	NM_002751	Homo sapiens mitogen-activated protein kinase 11 (MAPK11), transcript variant 1, mRNA [NM_002751]	NM_002751
A_23_P66306	1.64E-05	NM_019109	NM_019109	Homo sapiens asparagine-linked glycosylation 1 homolog (yeast, beta-1,4-mannosyltransferase) (ALG1), mRNA [NM_019109]	NM_019109
A_23_P122924	1.64E-05	NM_002192	NM_002192	Homo sapiens inhibin, beta A (activin A, activin AB alpha polypeptide) (INHBA), mRNA [NM_002192]	NM_002192
A_23_P103631	1.64E-05	NM_006824	NM_006824	Homo sapiens EBNA1 binding protein 2 (EBNA1BP2), mRNA [NM_006824]	NM_006824
A_23_P32500	1.65E-05	NM_015136	NM_015136	Homo sapiens stabilin 1 (STAB1), mRNA [NM_015136]	NM_015136
A_23_P122863	1.65E-05	NM_001001555	NM_001001555	Homo sapiens growth factor receptor-bound protein 10 (GRB10), transcript variant 4, mRNA [NM_001001555]	NM_001001555
A_32_P117860	1.66E-05	W05707	W05707	W05707 za87h03.r1 Soares_fetal_lung_NbHL19W Homo sapiens cDNA clone IMAGE:299573 5' similar to SW:TCPD_MOUSE P80315 T-COMPLEX PROTEIN 1, DELTA SUBUNIT ;, mRNA sequence [W05707]	
A_24_P73408	1.66E-05	NM_145293	NM_145293	Homo sapiens similar to hypothetical protein FLJ20897 (LOC196549), mRNA [NM_145293]	NM_145293
A_23_P34126	1.67E-05	NM_001711	NM_001711	Homo sapiens biglycan (BGN), mRNA [NM_001711]	NM_001711
A_24_P226008	1.67E-05	NM_007283	NM_007283	Homo sapiens monoglyceride lipase (MGLL), transcript variant 1, mRNA [NM_007283]	NM_007283
A_23_P21706	1.67E-05	NM_001905	NM_001905	Homo sapiens CTP synthase (CTPS), mRNA [NM_001905]	NM_001905
A_32_P115358	1.68E-05	NM_023075	NM_023075	Homo sapiens metallophosphoesterase 1 (MPPE1), transcript variant 1, mRNA [NM_023075]	NM_023075
A_24_P273253	1.68E-05	BC090889	BC090889	Homo sapiens chromosome 14 open reading frame 78, mRNA (cDNA clone MGC:102983 IMAGE:30387958), complete cds. [BC090889]	XM_290629
A_23_P361405	1.68E-05	NM_003549	NM_003549	Homo sapiens hyaluronoglucosaminidase 3 (HYAL3), mRNA [NM_003549]	NM_003549
A_24_P609382	1.69E-05	AK123143	AK123143	Homo sapiens cDNA FLJ41148 fis, clone BRACE2037310. [AK123143]	
A_23_P51051	1.69E-05	NM_005081	NM_005081	Homo sapiens zinc finger protein 142 (clone pHZ-49) (ZNF142), mRNA [NM_005081]	NM_005081
A_23_P121499	1.69E-05	NM_006005	NM_006005	Homo sapiens Wolfram syndrome 1 (wolframin) (WFS1), mRNA [NM_006005]	NM_006005
A_24_P418138	1.70E-05	AF218021	AF218021	Homo sapiens clone PP552 unknown mRNA. [AF218021]	
A_23_P15580	1.70E-05	NM_052855	NM_052855	Homo sapiens ankyrin repeat domain 40 (ANKRD40), mRNA [NM_052855]	NM_052855
A_32_P54553	1.70E-05	ENST00000292729		Homo sapiens partial mRNA for ubiquitin-specific protease 41 (USP41 gene). [AJ586979]	XM_036729
A_24_P788878	1.70E-05	THC2371963		AIP1_HUMAN (Q86UL8) Atrophin-1 interacting protein 1 (Atrophin-1 interacting protein A) (Membrane associated guanylate kinase inverted-2) (MAGI-2), partial (3%) [THC2371963]	
A_23_P29303	1.70E-05	NM_015703	NM_015703	Homo sapiens CGI-96 protein (CGI-96), mRNA [NM_015703]	NM_015703
A_32_P200595	1.72E-05	A_32_P200595			
A_23_P82088	1.72E-05	NM_016588	NM_016588	Homo sapiens neuritin 1 (NRN1), mRNA [NM_016588]	NM_016588
A_23_P58137	1.73E-05	BM930307	BM930307	UI-E-EJ1-ajh-l-11-0-UI.r1 UI-E-EJ1 Homo sapiens cDNA clone UI-E-EJ1-ajh-l-11-0-UI 5', mRNA sequence [BM930307]	
A_23_P395493	1.73E-05	BC031631	BC031631	Homo sapiens cofilin pseudogene 1, mRNA (cDNA clone IMAGE:5168640). [BC031631]	
A_32_P141445	1.75E-05	BC034822	BC034822	Homo sapiens SPR pseudogene, mRNA (cDNA clone MGC:34796 IMAGE:5166892), complete cds. [BC034822]	
A_23_P139066	1.75E-05	NM_016422	NM_016422	Homo sapiens ring finger protein 141 (RNF141), mRNA [NM_016422]	NM_016422
A_23_P89798	1.76E-05	NM_001012515	NM_001012515	Homo sapiens ferrochelatase (protoporphyria) (FECH), nuclear gene encoding mitochondrial protein, transcript variant 1, mRNA [NM_001012515]	NM_001012515
A_24_P719579	1.76E-05	ENST00000343626		Homo sapiens cDNA clone IMAGE:5797600, partial cds. [BC062294]	XM_375449

A_23_P251387	1.77E-05	NM_004726	NM_004726	Homo sapiens RALBP1 associated Eps domain containing 2 (REPS2), mRNA [NM_004726]	NM_004726
A_24_P73920	1.77E-05	CR609948	CR609948	full-length cDNA clone CS0DH006YD11 of T cells (Jurkat cell line) of Homo sapiens (human). [CR609948]	
A_23_P104372	1.77E-05	NM_015190	NM_015190	Homo sapiens DnaJ (Hsp40) homolog, subfamily C, member 9 (DNAJC9), mRNA [NM_015190]	NM_015190
A_24_P724886	1.78E-05	THC2334650		BQ437598 AGENCOURT_7826771 NIH_MGC_67 Homo sapiens cDNA clone IMAGE:6153024 5', mRNA sequence [BQ437598]	
A_23_P65129	1.78E-05	NM_032840	NM_032840	Homo sapiens hypothetical protein FLJ14800 (FLJ14800), mRNA [NM_032840]	NM_032840
A_23_P14734	1.78E-05	NM_015920	NM_015920	Homo sapiens ribosomal protein S27-like (RPS27L), mRNA [NM_015920]	NM_015920
A_23_P213527	1.79E-05	AW901755	AW901755	AW901755 QV0-NN1020-170400-192-f12 NN1020 Homo sapiens cDNA, mRNA sequence [AW901755]	
A_23_P251232	1.79E-05	NR_001543	NR_001543	Homo sapiens testis-specific transcript, Y-linked 14 (TTTY14) on chromosome Y [NR_001543]	NR_001543
A_23_P102439	1.80E-05	NM_001002036	NM_001002036	Homo sapiens astacin-like metalloendopeptidase (M12 family) (ASTL), mRNA [NM_001002036]	NM_001002036
A_24_P75748	1.80E-05	A_24_P75748			
A_23_P346390	1.81E-05	AK094877	AK094877	Homo sapiens cDNA FLJ37558 fis, clone BRCOC1000087. [AK094877]	
A_24_P799048	1.81E-05	AX721193	AX721193	Sequence 153 from Patent WO0220754. [AX721193]	
A_24_P68222	1.81E-05	BC019893	BC019893	Homo sapiens hypothetical gene supported by AK125149, mRNA (cDNA clone IMAGE:5001663), partial cds. [BC019893]	XM_379694
A_23_P258814	1.81E-05	ENST00000328644			
A_23_P116694	1.81E-05	NM_001029	NM_001029	Homo sapiens ribosomal protein S26 (RPS26), mRNA [NM_001029]	NM_001029
A_24_P921446	1.82E-05	BC017854	BC017854	Homo sapiens epithelial membrane protein 1, mRNA (cDNA clone IMAGE:4691099), partial cds. [BC017854]	
A_24_P913598	1.83E-05	S66917	S66917	EWS...FLI-1 {junction, translocation} [human, Ewing's sarcoma and malignant melanoma, mRNA PartialMutant, 3 genes, 78 nt]. [S66917]	
A_24_P37873	1.83E-05	NM_007036	NM_007036	Homo sapiens endothelial cell-specific molecule 1 (ESM1), mRNA [NM_007036]	NM_007036
A_32_P25889	1.83E-05	BC036938	BC036938	Homo sapiens, clone IMAGE:5404753, mRNA. [BC036938]	
A_23_P105227	1.85E-05	NM_001014811	NM_001014811	Homo sapiens malic enzyme 3, NADP(+)-dependent, mitochondrial (ME3), nuclear gene encoding mitochondrial protein, transcript variant 2, mRNA [NM_001014811]	NM_001014811
A_24_P57611	1.85E-05	NM_021637	NM_021637	Homo sapiens transmembrane protein 35 (TMEM35), mRNA [NM_021637]	NM_021637
A_23_P356526	1.85E-05	NM_033092	NM_033092	Homo sapiens tripartite motif-containing 5 (TRIM5), transcript variant gamma, mRNA [NM_033092]	NM_033092
A_24_P261326	1.85E-05	NM_005736	NM_005736	Homo sapiens ARP1 actin-related protein 1 homolog A, centractin alpha (yeast) (ACTR1A), mRNA [NM_005736]	NM_005736
A_24_P106145	1.85E-05	NM_017491	NM_017491	Homo sapiens WD repeat domain 1 (WDR1), transcript variant 1, mRNA [NM_017491]	NM_017491
A_23_P26810	1.86E-05	NM_000546	NM_000546	Homo sapiens tumor protein p53 (Li-Fraumeni syndrome) (TP53), mRNA [NM_000546]	NM_000546
A_24_P109351	1.86E-05	NM_001009814	NM_001009814	Homo sapiens KIAA0564 protein (KIAA0564), transcript variant 2, mRNA [NM_001009814]	NM_001009814
A_24_P206758	1.87E-05	NM_013306	NM_013306	Homo sapiens sorting nexin 15 (SNX15), transcript variant A, mRNA [NM_013306]	NM_013306
A_23_P141405	1.87E-05	NM_002512	NM_002512	Homo sapiens non-metastatic cells 2, protein (NM23B) expressed in (NME2), transcript variant 2, mRNA [NM_002512]	NM_002512
A_24_P384018	1.88E-05	NR_002171	NR_002171	Homo sapiens olfactory receptor, family 7, subfamily E, member 156 pseudogene (OR7E156P) on chromosome 13 [NR_002171]	NR_002171
A_23_P3885	1.88E-05	NM_024109	NM_024109	Homo sapiens hypothetical protein MGC2654 (MGC2654), mRNA [NM_024109]	NM_024109
A_23_P72068	1.88E-05	NM_001500	NM_001500	Homo sapiens GDP-mannose 4,6-dehydratase (GMDS), mRNA [NM_001500]	NM_001500
A_23_P26037	1.88E-05	NM_032892	NM_032892	Homo sapiens FERM domain containing 5 (FRMD5), mRNA [NM_032892]	NM_032892
A_23_P2843	1.88E-05	NM_017905	NM_017905	Homo sapiens transmembrane and coiled-coil domains 3 (TMCO3), mRNA [NM_017905]	NM_017905
A_23_P58443	1.88E-05	NM_020690	NM_020690	Homo sapiens MASK-4E-BP3 alternate reading frame gene (MASK-BP3), mRNA [NM_020690]	NM_020690
A_23_P149529	1.88E-05	NM_002353	NM_002353	Homo sapiens tumor-associated calcium signal transducer 2 (TACSTD2), mRNA [NM_002353]	NM_002353
A_24_P348090	1.88E-05	NM_002396	NM_002396	Homo sapiens malic enzyme 2, NAD(+)-dependent, mitochondrial (ME2), nuclear gene encoding mitochondrial protein, mRNA [NM_002396]	NM_002396
A_23_P14340	1.88E-05	NM_014672	NM_014672	Homo sapiens KIAA0391 (KIAA0391), mRNA [NM_014672]	NM_014672
A_24_P727884	1.88E-05	U10991	U10991	Human G2 protein mRNA, partial cds. [U10991]	XM_039515
A_23_P141315	1.88E-05	NM_001014445	NM_001014445	Homo sapiens notchless homolog 1 (Drosophila) (NLE1), transcript variant 2, mRNA [NM_001014445]	NM_001014445
A_23_P15305	1.88E-05	NM_002766	NM_002766	Homo sapiens phosphoribosyl pyrophosphate synthetase-associated protein 1 (PRPSAP1), mRNA [NM_002766]	NM_002766
A_23_P129786	1.88E-05	NM_001005291	NM_001005291	Homo sapiens sterol regulatory element binding transcription factor 1 (SREBF1), transcript variant 1, mRNA [NM_001005291]	NM_001005291
A_32_P167176	1.89E-05	THC2343936			
A_23_P130965	1.89E-05	NM_015683	NM_015683	Homo sapiens arrestin domain containing 2 (ARRDC2), transcript variant 1, mRNA [NM_015683]	NM_015683
A_23_P73420	1.89E-05	NM_031466	NM_031466	Homo sapiens IKK2 binding protein (T1), mRNA [NM_031466]	NM_031466

A_23_P162476	1.89E-05	NM_031299	NM_031299	Homo sapiens cell division cycle associated 3 (CDCA3), mRNA [NM_031299]	NM_031299
A_23_P205828	1.89E-05	NM_003257	NM_003257	Homo sapiens tight junction protein 1 (zona occludens 1) (TJP1), transcript variant 1, mRNA [NM_003257]	NM_003257
A_24_P305541	1.91E-05	NM_021158	NM_021158	Homo sapiens tribbles homolog 3 (Drosophila) (TRIB3), mRNA [NM_021158]	NM_021158
A_23_P140705	1.91E-05	ENST00000249776		Homo sapiens chromosome 15 open reading frame 23, mRNA (cDNA clone IMAGE:3952251), partial cds. [BC004543]	
A_23_P103951	1.92E-05	A_23_P103951			
A_23_P78191	1.93E-05	NM_025099	NM_025099	Homo sapiens hypothetical protein FLJ22170 (FLJ22170), mRNA [NM_025099]	NM_025099
A_23_P250910	1.93E-05	NM_053067	NM_053067	Homo sapiens ubiquilin 1 (UBQLN1), transcript variant 2, mRNA [NM_053067]	NM_053067
A_23_P112798	1.93E-05	NM_001312	NM_001312	Homo sapiens cysteine-rich protein 2 (CRIP2), mRNA [NM_001312]	NM_001312
A_32_P92783	1.93E-05	NM_006819	NM_006819	Homo sapiens stress-induced-phosphoprotein 1 (Hsp70/Hsp90-organizing protein) (STIP1), mRNA [NM_006819]	NM_006819
A_32_P182941	1.93E-05	NM_001005	NM_001005	Homo sapiens ribosomal protein S3 (RPS3), mRNA [NM_001005]	NM_001005
A_23_P58983	1.94E-05	NM_017772	NM_017772	Homo sapiens TBC1 domain family, member 22B (TBC1D22B), mRNA [NM_017772]	NM_017772
A_23_P160177	1.94E-05	NM_144699	NM_144699	Homo sapiens ATPase, Na+/K+ transporting, alpha 4 polypeptide (ATP1A4), transcript variant 1, mRNA [NM_144699]	NM_144699
A_23_P100074	1.94E-05	NM_020371	NM_020371	Homo sapiens apoptosis, caspase activation inhibitor (AVEN), mRNA [NM_020371]	NM_020371
A_32_P10003	1.94E-05	NM_080386	NM_080386	Homo sapiens alpha-tubulin isotype H2-alpha (H2-ALPHA), mRNA [NM_080386]	NM_080386
A_23_P335813	1.96E-05	NM_016272	NM_016272	Homo sapiens transducer of ERBB2, 2 (TOB2), mRNA [NM_016272]	NM_016272
A_23_P112103	1.96E-05	NM_024736	NM_024736	Homo sapiens gasdermin domain containing 1 (GSDMDC1), mRNA [NM_024736]	NM_024736
A_23_P164228	1.96E-05	NM_005175	NM_005175	Homo sapiens ATP synthase, H+ transporting, mitochondrial F0 complex, subunit c (subunit 9), isoform 1 (ATP5G1), nuclear gene encoding mitochondrial protein, transcript variant 1, mRNA [NM_005175]	NM_005175
A_24_P810074	1.97E-05	A_24_P810074			
A_24_P97770	1.97E-05	NM_020462	NM_020462	Homo sapiens endoplasmic reticulum-golgi intermediate compartment 32 kDa protein (KIAA1181), mRNA [NM_020462]	NM_020462
A_23_P138776	1.97E-05	NM_001610	NM_001610	Homo sapiens acid phosphatase 2, lysosomal (ACP2), mRNA [NM_001610]	NM_001610
A_23_P97532	1.97E-05	NM_004781	NM_004781	Homo sapiens vesicle-associated membrane protein 3 (cellubrevin) (VAMP3), mRNA [NM_004781]	NM_004781
A_23_P135357	1.97E-05	NM_018146	NM_018146	Homo sapiens RNA methyltransferase like 1 (RNMTL1), mRNA [NM_018146]	NM_018146
A_23_P52402	1.97E-05	NM_014889	NM_014889	Homo sapiens pitrilysin metalloproteinase 1 (PITRM1), mRNA [NM_014889]	NM_014889
A_24_P278621	1.98E-05	NM_012193	NM_012193	Homo sapiens frizzled homolog 4 (Drosophila) (FZD4), mRNA [NM_012193]	NM_012193
A_23_P66355	1.98E-05	NM_000213	NM_000213	Homo sapiens integrin, beta 4 (ITGB4), transcript variant 1, mRNA [NM_000213]	NM_000213
A_23_P218405	1.98E-05	NM_004489	NM_004489	Homo sapiens G protein pathway suppressor 2 (GPS2), mRNA [NM_004489]	NM_004489
A_23_P132248	1.98E-05	NM_019103	NM_019103	Homo sapiens zinc finger, matrin type 5 (ZMAT5), transcript variant 1, mRNA [NM_019103]	NM_019103
A_23_P343954	1.98E-05	NM_006546	NM_006546	Homo sapiens IGF-II mRNA-binding protein 1 (IMP-1), mRNA [NM_006546]	NM_006546
A_24_P263036	1.98E-05	NM_003748	NM_003748	Homo sapiens aldehyde dehydrogenase 4 family, member A1 (ALDH4A1), nuclear gene encoding mitochondrial protein, transcript variant P5CDhL, mRNA [NM_003748]	NM_003748
A_24_P389038	1.98E-05	NM_005452	NM_005452	Homo sapiens WD repeat domain 46 (WDR46), mRNA [NM_005452]	NM_005452
A_24_P187459	1.99E-05	ENST00000336230			
A_24_P205458	1.99E-05	NM_003607	NM_003607	Homo sapiens CDC42 binding protein kinase alpha (DMPK-like) (CDC42BPA), transcript variant B, mRNA [NM_003607]	NM_003607
A_23_P20804	1.99E-05	NM_147202	NM_147202	Homo sapiens chromosome 9 open reading frame 25 (C9orf25), mRNA [NM_147202]	NM_147202
A_23_P60816	1.99E-05	NM_013374	NM_013374	Homo sapiens programmed cell death 6 interacting protein (PDCD6IP), mRNA [NM_013374]	NM_013374
A_23_P380208	2.00E-05	NM_024621	NM_024621	Homo sapiens ventricular zone expressed PH domain homolog 1 (zebrafish) (VEPH1), mRNA [NM_024621]	NM_024621
A_24_P372672	2.00E-05	NM_001931	NM_001931	Homo sapiens dihydrolipoamide S-acetyltransferase (E2 component of pyruvate dehydrogenase complex) (DLAT), mRNA [NM_001931]	NM_001931
A_24_P43884	2.00E-05	NM_001006617	NM_001006617	Homo sapiens mitogen-activated protein kinase associated protein 1 (MAPKAP1), transcript variant 1, mRNA [NM_001006617]	NM_001006617
A_23_P90601	2.00E-05	NM_182915	NM_182915	Homo sapiens STEAP family member 3 (STEAP3), transcript variant 1, mRNA [NM_182915]	NM_182915
A_23_P206901	2.00E-05	NM_017668	NM_017668	Homo sapiens nudE nuclear distribution gene E homolog 1 (A. nidulans) (NDE1), mRNA [NM_017668]	NM_017668
A_23_P76488	2.00E-05	NM_001423	NM_001423	Homo sapiens epithelial membrane protein 1 (EMP1), mRNA [NM_001423]	NM_001423
A_24_P256570	2.01E-05	BC014215	BC014215	Homo sapiens cDNA clone IMAGE:4110098, partial cds. [BC014215]	
A_32_P67533	2.01E-05	NM_032438	NM_032438	Homo sapiens l(3)mbt-like 3 (Drosophila) (L3MBTL3), transcript variant 1, mRNA [NM_032438]	NM_032438
A_24_P699737	2.02E-05	AK022339	AK022339	Homo sapiens cDNA FLJ12277 fis, clone MAMMA1001711. [AK022339]	

A_24_P67585	2.02E-05	NM_016206	NM_016206	Homo sapiens vestigial-like 3 (VGL-3), mRNA [NM_016206]	NM_016206
A_24_P912751	2.02E-05	AY358619	AY358619	Homo sapiens clone DNA107443 AGLW2560 (UNQ2560) mRNA, complete cds. [AY358619]	
A_24_P378019	2.02E-05	NM_004031	NM_004031	Homo sapiens interferon regulatory factor 7 (IRF7), transcript variant d, mRNA [NM_004031]	NM_004031
A_23_P302094	2.02E-05	NM_033416	NM_033416	Homo sapiens IMP4, U3 small nucleolar ribonucleoprotein, homolog (yeast) (IMP4), mRNA [NM_033416]	NM_033416
A_23_P501460	2.03E-05	NM_033489	NM_033489	Homo sapiens cell division cycle 2-like 1 (PITSLRE proteins) (CDC2L1), transcript variant 5, mRNA [NM_033489]	NM_033489
A_23_P106433	2.04E-05	NM_033544	NM_033544	Homo sapiens similar to cyclin-E binding protein 1 (H. sapiens) (MGC14386), transcript variant 1, mRNA [NM_033544]	NM_033544
A_24_P412238	2.04E-05	NM_025128	NM_025128	Homo sapiens MUS81 endonuclease homolog (yeast) (MUS81), mRNA [NM_025128]	NM_025128
A_23_P88904	2.04E-05	NM_002528	NM_002528	Homo sapiens nth endonuclease III-like 1 (E. coli) (NTHL1), mRNA [NM_002528]	NM_002528
A_24_P30141	2.05E-05	NM_203438	NM_203438	Homo sapiens chromosome 10 open reading frame 4 (C10orf4), transcript variant FRA10AC1-3.3, mRNA [NM_203438]	NM_203438
A_24_P134235	2.05E-05	NM_003685	NM_003685	Homo sapiens KH-type splicing regulatory protein (FUSE binding protein 2) (KHSRP), mRNA [NM_003685]	NM_003685
A_23_P119502	2.05E-05	NM_003775	NM_003775	Homo sapiens endothelial differentiation, G-protein-coupled receptor 6 (EDG6), mRNA [NM_003775]	NM_003775
A_32_P221437	2.05E-05	A_32_P221437			
A_23_P74391	2.05E-05	NM_014322	NM_014322	Homo sapiens opsin 3 (encephalopsin, panopsin) (OPN3), mRNA [NM_014322]	NM_014322
A_24_P876862	2.05E-05	BC061590	BC061590	Homo sapiens cDNA clone MGC:75203 IMAGE:6502529, complete cds. [BC061590]	
A_23_P213298	2.05E-05	NM_024590	NM_024590	Homo sapiens arylsulfatase J (ARSJ), mRNA [NM_024590]	NM_024590
A_23_P17512	2.06E-05	NM_080820	NM_080820	Homo sapiens histidyl-tRNA synthetase 2 (HARS2), mRNA [NM_080820]	NM_080820
A_23_P60405	2.07E-05	NM_022779	NM_022779	Homo sapiens DEAD (Asp-Glu-Ala-Asp) box polypeptide 31 (DDX31), transcript variant 1, mRNA [NM_022779]	NM_022779
A_32_P117322	2.07E-05	BG192275	BG192275	RST11383 Athersys RAGE Library Homo sapiens cDNA, mRNA sequence [BG192275]	
A_24_P188975	2.07E-05	AK002097	AK002097	Homo sapiens cDNA FLJ11235 fis, clone PLACE1008488. [AK002097]	XM_496773
A_24_P734892	2.07E-05	THC2406285		Q9FH26 (Q9FH26) Arabidopsis thaliana genomic DNA, chromosome 5, TAC clone:K20J1, partial (3%) [THC2406285]	
A_23_P61569	2.08E-05	NM_003762	NM_003762	Homo sapiens vesicle-associated membrane protein 4 (VAMP4), transcript variant 2, mRNA [NM_003762]	NM_003762
A_24_P917492	2.08E-05	BC009479	BC009479	Homo sapiens tubulin tyrosine ligase-like family, member 3, mRNA (cDNA clone IMAGE:3841498), complete cds. [BC009479]	
A_24_P110062	2.08E-05	BC014048	BC014048	Homo sapiens protein tyrosine phosphatase, mitochondrial 1, mRNA (cDNA clone IMAGE:3348134), partial cds. [BC014048]	
A_23_P92552	2.08E-05	NM_004564	NM_004564	Homo sapiens PET112-like (yeast) (PET112L), mRNA [NM_004564]	NM_004564
A_23_P412526	2.08E-05	AF126020	AF126020	Homo sapiens B-cell receptor-associated protein BAP29 mRNA, complete cds. [AF126020]	
A_24_P160466	2.10E-05	NM_052899	NM_052899	Homo sapiens G protein-regulated inducer of neurite outgrowth 1 (KIAA1893), mRNA [NM_052899]	NM_052899
A_32_P49555	2.10E-05	NM_174889	NM_174889	Homo sapiens Myc-induced mitochondria protein (mimitin), mRNA [NM_174889]	NM_174889
A_24_P327181	2.11E-05	NM_032387	NM_032387	Homo sapiens WNK lysine deficient protein kinase 4 (WNK4), mRNA [NM_032387]	NM_032387
A_24_P418203	2.11E-05	NM_033655	NM_033655	Homo sapiens contactin associated protein-like 3 (CNTNAP3), mRNA [NM_033655]	NM_033655
A_24_P687131	2.11E-05	CR619653	CR619653	full-length cDNA clone CS0DC014YO10 of Neuroblastoma Cot 25-normalized of Homo sapiens (human). [CR619653]	XM_379111
A_23_P206741	2.11E-05	A_23_P206741			
A_23_P87323	2.11E-05	NM_014186	NM_014186	Homo sapiens COMM domain containing 9 (COMMD9), mRNA [NM_014186]	NM_014186
A_32_P186138	2.12E-05	BX647075	BX647075	Homo sapiens mRNA; cDNA DKFZp781B0241 (from clone DKFZp781B0241). [BX647075]	XM_376522
A_23_P95027	2.12E-05	NM_016466	NM_016466	Homo sapiens ankyrin repeat domain 39 (ANKRD39), mRNA [NM_016466]	NM_016466
A_24_P594683	2.13E-05	THC2301371		Q864S5 (Q864S5) Peptidylprolyl isomerase A, partial (91%) [THC2301371]	
A_24_P272300	2.13E-05	NM_199044	NM_199044	Homo sapiens NOL1/NOP2/Sun domain family, member 4 (NSUN4), mRNA [NM_199044]	NM_199044
A_23_P204364	2.13E-05	NM_006170	NM_006170	Homo sapiens nucleolar protein 1, 120kDa (NOL1), mRNA [NM_006170]	NM_006170
A_23_P53630	2.13E-05	NM_014868	NM_014868	Homo sapiens ring finger protein 10 (RNF10), mRNA [NM_014868]	NM_014868
A_23_P432583	2.14E-05	NM_016178	NM_016178	Homo sapiens ornithine decarboxylase antizyme 3 (OAZ3), mRNA [NM_016178]	NM_016178
A_23_P1615	2.14E-05	NM_004214	NM_004214	Homo sapiens fibroblast growth factor (acidic) intracellular binding protein (FIBP), transcript variant 2, mRNA [NM_004214]	NM_004214
A_23_P116264	2.15E-05	NM_006176	NM_006176	Homo sapiens neurogranin (protein kinase C substrate, RC3) (NRGN), mRNA [NM_006176]	NM_006176
A_23_P138000	2.15E-05	NM_030913	NM_030913	Homo sapiens sema domain, transmembrane domain (TM), and cytoplasmic domain, (semaphorin) 6C (SEMA6C), mRNA [NM_030913]	NM_030913
A_23_P73429	2.15E-05	NM_005335	NM_005335	Homo sapiens hematopoietic cell-specific Lyn substrate 1 (HCLS1), mRNA [NM_005335]	NM_005335
A_23_P123974	2.16E-05	NM_012145	NM_012145	Homo sapiens deoxythymidylate kinase (thymidylate kinase) (DTYMK), mRNA [NM_012145]	NM_012145

A_23_P342131	2.17E-05	NM_153611	NM_153611	Homo sapiens cytochrome b, ascorbate dependent 3 (CYBASC3), mRNA [NM_153611]	NM_153611
A_23_P259094	2.17E-05	NM_025231	NM_025231	Homo sapiens zinc finger protein 435 (ZNF435), mRNA [NM_025231]	NM_025231
A_23_P337934	2.17E-05	NM_017556	NM_017556	Homo sapiens filamin binding LIM protein 1 (FBLIM1), transcript variant 1, mRNA [NM_017556]	NM_017556
A_23_P142506	2.18E-05	NM_015675	NM_015675	Homo sapiens growth arrest and DNA-damage-inducible, beta (GADD45B), mRNA [NM_015675]	NM_015675
A_23_P76450	2.18E-05	NM_007350	NM_007350	Homo sapiens pleckstrin homology-like domain, family A, member 1 (PHLDA1), mRNA [NM_007350]	NM_007350
A_23_P314666	2.18E-05	NM_138428	NM_138428	Homo sapiens hypothetical protein BC011880 (LOC113444), mRNA [NM_138428]	NM_138428
A_24_P201171	2.19E-05	NM_003165	NM_003165	Homo sapiens syntaxin binding protein 1 (STXBP1), mRNA [NM_003165]	NM_003165
A_23_P131176	2.19E-05	AK021716	AK021716	Homo sapiens cDNA FLJ11654 fis, clone HEMBA1004542. [AK021716]	
A_23_P109794	2.20E-05	NM_015199	NM_015199	Homo sapiens ankyrin repeat domain 28 (ANKRD28), mRNA [NM_015199]	NM_015199
A_23_P134014	2.20E-05	NM_182503	NM_182503	Homo sapiens deaminase domain containing 1 (DEADC1), mRNA [NM_182503]	NM_182503
A_23_P167509	2.20E-05	AK172724	AK172724	Homo sapiens cDNA PSEC0002 fis, clone: NT2RM1000295. [AK172724]	
A_23_P254888	2.20E-05	NM_003461	NM_003461	Homo sapiens zyxin (ZYX), transcript variant 1, mRNA [NM_003461]	NM_003461
A_24_P395042	2.20E-05	NM_003754	NM_003754	Homo sapiens eukaryotic translation initiation factor 3, subunit 5 epsilon, 47kDa (EIF3S5), mRNA [NM_003754]	NM_003754
A_23_P28733	2.21E-05	NM_002895	NM_002895	Homo sapiens retinoblastoma-like 1 (p107) (RBL1), transcript variant 1, mRNA [NM_002895]	NM_002895
A_32_P58796	2.21E-05	NM_145809	NM_145809	Homo sapiens TL132 protein (LOC220594), mRNA [NM_145809]	NM_145809
A_24_P371281	2.21E-05	NM_025246	NM_025246	Homo sapiens transmembrane protein 22 (TMEM22), mRNA [NM_025246]	NM_025246
A_24_P198598	2.21E-05	NM_002675	NM_002675	Homo sapiens promyelocytic leukemia (PML), transcript variant 6, mRNA [NM_002675]	NM_002675
A_32_P111235	2.23E-05	BG612665	BG612665	602641001F1 NIH_MGC_61 Homo sapiens cDNA clone IMAGE:4771873 5', mRNA sequence [BG612665]	
A_23_P119311	2.23E-05	NM_012315	NM_012315	Homo sapiens kallikrein 9 (KLK9), mRNA [NM_012315]	NM_012315
A_24_P92823	2.23E-05	A_24_P92823			
A_23_P153651	2.24E-05	NM_024333	NM_024333	Homo sapiens fibronectin type III and SPRY domain containing 1 (FSD1), mRNA [NM_024333]	NM_024333
A_23_P359043	2.26E-05	NM_007203	NM_007203	Homo sapiens PALM2-AKAP2 protein (PALM2-AKAP2), transcript variant 1, mRNA [NM_007203]	NM_007203
A_23_P83266	2.26E-05	NM_004435	NM_004435	Homo sapiens endonuclease G (ENDOG), nuclear gene encoding mitochondrial protein, mRNA [NM_004435]	NM_004435
A_24_P349756	2.26E-05	A_24_P349756			
A_32_P226009	2.27E-05	NM_023073	NM_023073	Homo sapiens hypothetical protein FLJ13231 (FLJ13231), mRNA [NM_023073]	NM_023073
A_24_P307653	2.27E-05	NM_182767	NM_182767	Homo sapiens solute carrier family 6, member 15 (SLC6A15), transcript variant 1, mRNA [NM_182767]	NM_182767
A_32_P182394	2.27E-05	NM_021217	NM_021217	Homo sapiens zinc finger protein 77 (pT1) (ZNF77), mRNA [NM_021217]	NM_021217
A_23_P210811	2.27E-05	NM_012072	NM_012072	Homo sapiens complement component 1, q subcomponent, receptor 1 (C1QR1), mRNA [NM_012072]	NM_012072
A_23_P11160	2.27E-05	AK022711	AK022711	Homo sapiens cDNA FLJ12649 fis, clone NT2RM4002044. [AK022711]	
A_23_P24192	2.27E-05	NM_015179	NM_015179	Homo sapiens KIAA0690 (KIAA0690), mRNA [NM_015179]	NM_015179
A_23_P259189	2.27E-05	NM_013943	NM_013943	Homo sapiens chloride intracellular channel 4 (CLIC4), mRNA [NM_013943]	NM_013943
A_23_P24515	2.27E-05	NM_000019	NM_000019	Homo sapiens acetyl-Coenzyme A acetyltransferase 1 (acetoacetyl Coenzyme A thiolase) (ACAT1), nuclear gene encoding mitochondrial protein, mRNA [NM_000019]	NM_000019
A_24_P418762	2.28E-05	NM_020870	NM_020870	Homo sapiens SH3 multiple domains 2 (SH3MD2), mRNA [NM_020870]	NM_020870
A_23_P41470	2.28E-05	NM_017631	NM_017631	Homo sapiens hypothetical protein FLJ20035 (FLJ20035), mRNA [NM_017631]	NM_017631
A_23_P27265	2.28E-05	NM_032160	NM_032160	Homo sapiens chromosome 18 open reading frame 4 (C18orf4), mRNA [NM_032160]	NM_032160
A_23_P59637	2.28E-05	AB018259	AB018259	Homo sapiens mRNA for KIAA0716 protein, partial cds. [AB018259]	
A_24_P186030	2.29E-05	NM_002760	NM_002760	Homo sapiens protein kinase, Y-linked (PRKY), mRNA [NM_002760]	NM_002760
A_23_P156977	2.29E-05	NM_004507	NM_004507	Homo sapiens HUS1 checkpoint homolog (S. pombe) (HUS1), mRNA [NM_004507]	NM_004507
A_24_P237265	2.29E-05	NM_002745	NM_002745	Homo sapiens mitogen-activated protein kinase 1 (MAPK1), transcript variant 1, mRNA [NM_002745]	NM_002745
A_23_P425752	2.30E-05	NM_033219	NM_033219	Homo sapiens tripartite motif-containing 14 (TRIM14), transcript variant 2, mRNA [NM_033219]	NM_033219
A_23_P387471	2.30E-05	NM_005931	NM_005931	Homo sapiens MHC class I polypeptide-related sequence B (MICB), mRNA [NM_005931]	NM_005931
A_23_P319719	2.31E-05	NM_138575	NM_138575	Homo sapiens Bcl-XL-binding protein v68 (MGC5352), mRNA [NM_138575]	NM_138575
A_23_P258272	2.31E-05	NM_006643	NM_006643	Homo sapiens serologically defined colon cancer antigen 3 (SDCCAG3), mRNA [NM_006643]	NM_006643
A_24_P201153	2.32E-05	NM_201629	NM_201629	Homo sapiens tight junction protein 2 (zona occludens 2) (TJP2), transcript variant 2, mRNA [NM_201629]	NM_201629

A_23_P34496	2.33E-05	NM_018056	NM_018056	Homo sapiens transmembrane protein 39B (TMEM39B), mRNA [NM_018056]	NM_018056
A_23_P164691	2.33E-05	NM_002162	NM_002162	Homo sapiens intercellular adhesion molecule 3 (ICAM3), mRNA [NM_002162]	NM_002162
A_24_P884915	2.33E-05	A_24_P884915			
A_23_P203629	2.34E-05	NM_173044	NM_173044	Homo sapiens interleukin 18 binding protein (IL18BP), transcript variant D, mRNA [NM_173044]	NM_173044
A_24_P13649	2.34E-05	NM_032714	NM_032714	Homo sapiens chromosome 14 open reading frame 151 (C14orf151), mRNA [NM_032714]	NM_032714
A_24_P89457	2.34E-05	NM_078467	NM_078467	Homo sapiens cyclin-dependent kinase inhibitor 1A (p21, Cip1) (CDKN1A), transcript variant 2, mRNA [NM_078467]	NM_078467
A_23_P405088	2.34E-05	NM_005602	NM_005602	Homo sapiens claudin 11 (oligodendrocyte transmembrane protein) (CLDN11), mRNA [NM_005602]	NM_005602
A_23_P134991	2.35E-05	AF090921	AF090921	Homo sapiens clone HQ0365 PRO0365 mRNA, complete cds. [AF090921]	
A_32_P51855	2.35E-05	BC038556	BC038556	Homo sapiens, clone IMAGE:3446976, mRNA. [BC038556]	
A_23_P311616	2.35E-05	NM_015167	NM_015167	Homo sapiens phosphatidylserine receptor (PTDSR), mRNA [NM_015167]	NM_015167
A_23_P115167	2.36E-05	NM_015350	NM_015350	Homo sapiens leucine rich repeat containing 8 family, member B (LRRC8B), mRNA [NM_015350]	NM_015350
A_23_P215175	2.36E-05	NM_005692	NM_005692	Homo sapiens ATP-binding cassette, sub-family F (GCN20), member 2 (ABCF2), nuclear gene encoding mitochondrial protein, transcript variant 2, mRNA [NM_005692]	NM_005692
A_23_P335920	2.36E-05	NM_021135	NM_021135	Homo sapiens ribosomal protein S6 kinase, 90kDa, polypeptide 2 (RPS6KA2), transcript variant 1, mRNA [NM_021135]	NM_021135
A_23_P68665	2.36E-05	NM_007002	NM_007002	Homo sapiens adhesion regulating molecule 1 (ADRM1), transcript variant 1, mRNA [NM_007002]	NM_007002
A_24_P60441	2.37E-05	NM_153717	NM_153717	Homo sapiens Ellis van Creveld syndrome (EVC), transcript variant 2, mRNA [NM_153717]	NM_153717
A_23_P128408	2.37E-05	NM_016399	NM_016399	Homo sapiens p53-inducible cell-survival factor (P53CSV), mRNA [NM_016399]	NM_016399
A_23_P327307	2.39E-05	NM_002577	NM_002577	Homo sapiens p21 (CDKN1A)-activated kinase 2 (PAK2), mRNA [NM_002577]	NM_002577
A_23_P90484	2.39E-05	NM_017827	NM_017827	Homo sapiens seryl-tRNA synthetase 2 (SARS2), mRNA [NM_017827]	NM_017827
A_23_P7896	2.40E-05	NM_020185	NM_020185	Homo sapiens dual specificity phosphatase 22 (DUSP22), mRNA [NM_020185]	NM_020185
A_24_P307184	2.40E-05	A_24_P307184			
A_24_P278367	2.40E-05	NM_005736	NM_005736	Homo sapiens ARP1 actin-related protein 1 homolog A, centractin alpha (yeast) (ACTR1A), mRNA [NM_005736]	NM_005736
A_23_P20196	2.40E-05	NM_005720	NM_005720	Homo sapiens actin related protein 2/3 complex, subunit 1B, 41kDa (ARPC1B), mRNA [NM_005720]	NM_005720
A_23_P78664	2.41E-05	NM_138998	NM_138998	Homo sapiens DEAD (Asp-Glu-Ala-Asp) box polypeptide 39 (DDX39), transcript variant 2, mRNA [NM_138998]	NM_138998
A_24_P83615	2.43E-05	NM_014922	NM_014922	Homo sapiens NACHT, leucine rich repeat and PYD (pyrin domain) containing 1 (NALP1), transcript variant 2, mRNA [NM_014922]	NM_014922
A_24_P353300	2.43E-05	NM_016733	NM_016733	Homo sapiens LIM domain kinase 2 (LIMK2), transcript variant 2b, mRNA [NM_016733]	NM_016733
A_23_P12178	2.44E-05	NM_181715	NM_181715	Homo sapiens transducer of regulated cAMP response element-binding protein (CREB) 2 (TORC2), mRNA [NM_181715]	NM_181715
A_24_P308628	2.44E-05	NM_016173	NM_016173	Homo sapiens HemK methyltransferase family member 1 (HEMK1), mRNA [NM_016173]	NM_016173
A_23_P121423	2.46E-05	NM_001789	NM_001789	Homo sapiens cell division cycle 25A (CDC25A), transcript variant 1, mRNA [NM_001789]	NM_001789
A_24_P846738	2.46E-05	A_24_P846738			
A_24_P313504	2.46E-05	NM_005030	NM_005030	Homo sapiens polo-like kinase 1 (Drosophila) (PLK1), mRNA [NM_005030]	NM_005030
A_24_P87931	2.46E-05	NM_145343	NM_145343	Homo sapiens apolipoprotein L, 1 (APOL1), transcript variant 2, mRNA [NM_145343]	NM_145343
A_24_P29595	2.47E-05	NM_139244	NM_139244	Homo sapiens syntaxin binding protein 5 (tomosyn) (STXBP5), mRNA [NM_139244]	NM_139244
A_23_P55990	2.47E-05	NM_003827	NM_003827	Homo sapiens N-ethylmaleimide-sensitive factor attachment protein, alpha (NAPA), mRNA [NM_003827]	NM_003827
A_23_P397341	2.47E-05	NM_152341	NM_152341	Homo sapiens progestin and adipoQ receptor family member IV (PAQR4), mRNA [NM_152341]	NM_152341
A_23_P50338	2.47E-05	NM_006087	NM_006087	Homo sapiens tubulin, beta 4 (TUBB4), mRNA [NM_006087]	NM_006087
A_23_P37778	2.48E-05	NM_013241	NM_013241	Homo sapiens formin homology 2 domain containing 1 (FHOD1), mRNA [NM_013241]	NM_013241
A_23_P107959	2.48E-05	NM_015953	NM_015953	Homo sapiens nitric oxide synthase interacting protein (NOSIP), mRNA [NM_015953]	NM_015953
A_23_P34741	2.49E-05	NM_015871	NM_015871	Homo sapiens zinc finger protein 593 (ZNF593), mRNA [NM_015871]	NM_015871
A_23_P502915	2.49E-05	NM_017491	NM_017491	Homo sapiens WD repeat domain 1 (WDR1), transcript variant 1, mRNA [NM_017491]	NM_017491
A_23_P140807	2.50E-05	NM_002801	NM_002801	Homo sapiens proteasome (prosome, macropain) subunit, beta type, 10 (PSMB10), mRNA [NM_002801]	NM_002801
A_24_P127159	2.51E-05	M69012	M69012	Human guanine nucleotide-binding regulatory protein (G-y-2-alpha) mRNA, partial cds. [M69012]	XR_000184
A_24_P255965	2.52E-05	A_24_P255965			
A_32_P162095	2.52E-05	THC2381061			

A_23_P75978	2.52E-05	NM_030813	NM_030813	Homo sapiens suppressor of potassium transport defect 3 (SKD3), mRNA [NM_030813]	NM_030813
A_23_P47863	2.52E-05	NM_016551	NM_016551	Homo sapiens transmembrane 7 superfamily member 3 (TM7SF3), mRNA [NM_016551]	NM_016551
A_23_P33444	2.52E-05	NM_006750	NM_006750	Homo sapiens syntrophin, beta 2 (dystrophin-associated protein A1, 59kDa, basic component 2) (SNTB2), transcript variant 1, mRNA [NM_006750]	NM_006750
A_32_P32250	2.52E-05	NM_022063	NM_022063	Homo sapiens chromosome 10 open reading frame 84 (C10orf84), mRNA [NM_022063]	NM_022063
A_23_P207766	2.52E-05	NM_004309	NM_004309	Homo sapiens Rho GDP dissociation inhibitor (GDI) alpha (ARHGDIA), mRNA [NM_004309]	NM_004309
A_24_P184295	2.52E-05	AF099011	AF099011	Homo sapiens EH-domain containing protein testilin mRNA, complete cds. [AF099011]	
A_23_P20832	2.53E-05	NM_003127	NM_003127	Homo sapiens spectrin, alpha, non-erythrocytic 1 (alpha-fodrin) (SPTAN1), mRNA [NM_003127]	NM_003127
A_23_P166353	2.53E-05	NM_198157	NM_198157	Homo sapiens ubiquitin-conjugating enzyme E2L 3 (UBE2L3), transcript variant 2, mRNA [NM_198157]	NM_198157
A_23_P77761	2.54E-05	NM_004035	NM_004035	Homo sapiens acyl-Coenzyme A oxidase 1, palmitoyl (ACOX1), transcript variant 1, mRNA [NM_004035]	NM_004035
A_23_P19663	2.54E-05	NM_001901	NM_001901	Homo sapiens connective tissue growth factor (CTGF), mRNA [NM_001901]	NM_001901
A_24_P808838	2.55E-05	A_24_P808838			
A_24_P405054	2.55E-05	NM_015609	NM_015609	Homo sapiens chromosome 1 open reading frame 144 (C1orf144), mRNA [NM_015609]	NM_015609
A_24_P625382	2.55E-05	NM_003651	NM_003651	Homo sapiens cold shock domain protein A (CSDA), mRNA [NM_003651]	NM_003651
A_23_P320113	2.55E-05	NM_080725	NM_080725	Homo sapiens sulfiredoxin 1 homolog (S. cerevisiae) (SRXN1), mRNA [NM_080725]	NM_080725
A_23_P325991	2.56E-05	NM_181831	NM_181831	Homo sapiens neurofibromin 2 (bilateral acoustic neuroma) (NF2), transcript variant 13, mRNA [NM_181831]	NM_181831
A_23_P259098	2.56E-05	NM_025231	NM_025231	Homo sapiens zinc finger protein 435 (ZNF435), mRNA [NM_025231]	NM_025231
A_24_P357809	2.56E-05	NM_020642	NM_020642	Homo sapiens chromosome 11 open reading frame 17 (C11orf17), transcript variant 2, mRNA [NM_020642]	NM_020642
A_23_P131771	2.57E-05	NM_199360	NM_199360	Homo sapiens tumor protein D52-like 2 (TPD52L2), transcript variant 1, mRNA [NM_199360]	NM_199360
A_24_P158089	2.57E-05	NM_000602	NM_000602	Homo sapiens serine (or cysteine) proteinase inhibitor, clade E (nexin, plasminogen activator inhibitor type 1), member 1 (SERPINE1), mRNA [NM_000602]	NM_000602
A_23_P8432	2.58E-05	NM_006833	NM_006833	Homo sapiens COP9 constitutive photomorphogenic homolog subunit 6 (Arabidopsis) (COPS6), mRNA [NM_006833]	NM_006833
A_24_P340866	2.59E-05	A_24_P340866			
A_24_P52801	2.59E-05	NM_017670	NM_017670	Homo sapiens OTU domain, ubiquitin aldehyde binding 1 (OTUB1), mRNA [NM_017670]	NM_017670
A_24_P44596	2.60E-05	NM_014940	NM_014940	Homo sapiens MON1 homolog B (yeast) (MON1B), mRNA [NM_014940]	NM_014940
A_24_P102151	2.61E-05	AK000231	AK000231	Homo sapiens cDNA FLJ20224 fis, clone COLF5039. [AK000231]	
A_23_P64499	2.61E-05	NM_053005	NM_053005	Homo sapiens HCCA2 protein (HCCA2), mRNA [NM_053005]	NM_053005
A_24_P116571	2.63E-05	NM_016256	NM_016256	Homo sapiens N-acetylglucosamine-1-phosphodiester alpha-N-acetylglucosaminidase (NAGPA), mRNA [NM_016256]	NM_016256
A_23_P345564	2.63E-05	NM_182647	NM_182647	Homo sapiens opiate receptor-like 1 (OPRL1), transcript variant 1, mRNA [NM_182647]	NM_182647
A_23_P87379	2.63E-05	AK131525	AK131525	Homo sapiens cDNA FLJ16750 fis, clone ADRGL2011190, highly similar to cGMP-dependent 3',5'-cyclic phosphodiesterase (EC 3.1.4.17). [AK131525]	
A_24_P176044	2.63E-05	NM_012063	NM_012063	Homo sapiens dynamin 1-like (DNM1L), transcript variant 2, mRNA [NM_012063]	NM_012063
A_23_P46604	2.64E-05	NM_020448	NM_020448	Homo sapiens NIPA-like domain containing 3 (NPAL3), mRNA [NM_020448]	NM_020448
A_23_P23438	2.64E-05	NM_022367	NM_022367	Homo sapiens sema domain, immunoglobulin domain (Ig), transmembrane domain (TM) and short cytoplasmic domain, (semaphorin) 4A (SEMA4A), mRNA [NM_022367]	NM_022367
A_23_P83624	2.64E-05	NM_021149	NM_021149	Homo sapiens coactosin-like 1 (Dictyostelium) (COTL1), mRNA [NM_021149]	NM_021149
A_23_P258340	2.64E-05	NM_001008741	NM_001008741	Homo sapiens peptidylprolyl isomerase A-like (LOC388817), mRNA [NM_001008741]	NM_001008741
A_23_P25559	2.65E-05	NM_005845	NM_005845	Homo sapiens ATP-binding cassette, sub-family C (CFTR/MRP), member 4 (ABCC4), mRNA [NM_005845]	NM_005845
A_24_P257416	2.65E-05	NM_002089	NM_002089	Homo sapiens chemokine (C-X-C motif) ligand 2 (CXCL2), mRNA [NM_002089]	NM_002089
A_23_P117540	2.65E-05	NM_145251	NM_145251	Homo sapiens serine/threonine/tyrosine interacting protein (STYX), mRNA [NM_145251]	NM_145251
A_23_P63402	2.65E-05	NM_013296	NM_013296	Homo sapiens G-protein signalling modulator 2 (AGS3-like, C. elegans) (GPSM2), mRNA [NM_013296]	NM_013296
A_23_P212329	2.66E-05	NM_015466	NM_015466	Homo sapiens protein tyrosine phosphatase, non-receptor type 23 (PTPN23), mRNA [NM_015466]	NM_015466
A_23_P16671	2.67E-05	NM_024050	NM_024050	Homo sapiens cross-immune reaction antigen PCIA1 (PCIA1), mRNA [NM_024050]	NM_024050
A_23_P152082	2.68E-05	NM_016642	NM_016642	Homo sapiens spectrin, beta, non-erythrocytic 5 (SPTBN5), mRNA [NM_016642]	NM_016642
A_32_P129540	2.68E-05	XM_495933	XM_495933	PREDICTED: Homo sapiens similar to POM121-like protein (LOC440110), mRNA [XM_495933]	XM_495933
A_23_P96331	2.68E-05	NM_000377	NM_000377	Homo sapiens Wiskott-Aldrich syndrome (eczema-thrombocytopenia) (WAS), mRNA [NM_000377]	NM_000377
A_23_P24318	2.68E-05	NM_001007071	NM_001007071	Homo sapiens ribosomal protein S6 kinase, 70kDa, polypeptide 2 (RPS6KB2), transcript variant 2, mRNA [NM_001007071]	NM_001007071

A_24_P23034	2.68E-05	NM_021035	NM_021035	Homo sapiens KIAA1404 protein (KIAA1404), mRNA [NM_021035]	NM_021035
A_23_P37535	2.68E-05	NM_016530	NM_016530	Homo sapiens RAB8B, member RAS oncogene family (RAB8B), mRNA [NM_016530]	NM_016530
A_23_P146084	2.69E-05	NM_000637	NM_000637	Homo sapiens glutathione reductase (GSR), mRNA [NM_000637]	NM_000637
A_23_P434890	2.69E-05	NM_014550	NM_014550	Homo sapiens caspase recruitment domain family, member 10 (CARD10), mRNA [NM_014550]	NM_014550
A_23_P64762	2.70E-05	NM_003566	NM_003566	Homo sapiens early endosome antigen 1, 162kD (EEA1), mRNA [NM_003566]	NM_003566
A_23_P57033	2.70E-05	NM_006809	NM_006809	Homo sapiens translocase of outer mitochondrial membrane 34 (TOMM34), nuclear gene encoding mitochondrial protein, mRNA [NM_006809]	NM_006809
A_32_P42684	2.70E-05	THC2310563			
A_24_P214556	2.71E-05	THC2380706		Q6UXG1 (Q6UXG1) YVTM2421, partial (3%) [THC2380706]	
A_24_P945181	2.71E-05	NM_013286	NM_013286	Homo sapiens RNA binding motif protein 15B (RBM15B), mRNA [NM_013286]	NM_013286
A_24_P366735	2.71E-05	NM_175863	NM_175863	Homo sapiens AT rich interactive domain 1B (SWI1-like) (ARID1B), transcript variant 3, mRNA [NM_175863]	NM_175863
A_32_P70519	2.72E-05	BP420721	BP420721	BP420721 Homo sapiens small intestine Homo sapiens cDNA clone HIE04034r 3', mRNA sequence [BP420721]	
A_32_P163392	2.72E-05	CR608961	CR608961	full-length cDNA clone CS0DI034YJ14 of Placenta Cot 25-normalized of Homo sapiens (human). [CR608961]	
A_23_P15603	2.72E-05	NM_024864	NM_024864	Homo sapiens hypothetical protein FLJ22578 (FLJ22578), mRNA [NM_024864]	NM_024864
A_23_P416034	2.73E-05	NM_207106	NM_207106	Homo sapiens 26S proteasome-associated UCH interacting protein 1 (UIP1), transcript variant 3, mRNA [NM_207106]	NM_207106
A_23_P77965	2.73E-05	NM_022070	NM_022070	Homo sapiens amplified in breast cancer 1 (ABC1), mRNA [NM_022070]	NM_022070
A_23_P92202	2.73E-05	NM_021971	NM_021971	Homo sapiens GDP-mannose pyrophosphorylase B (GMPPB), transcript variant 2, mRNA [NM_021971]	NM_021971
A_23_P206272	2.74E-05	NM_024598	NM_024598	Homo sapiens hypothetical protein FLJ13154 (FLJ13154), mRNA [NM_024598]	NM_024598
A_24_P765569	2.74E-05	AI650285	AI650285	AI650285 wa18a08.x1 NCI_CGAP_Kid11 Homo sapiens cDNA clone IMAGE:2298422 3', mRNA sequence [AI650285]	
A_24_P71244	2.74E-05	NM_005026	NM_005026	Homo sapiens phosphoinositide-3-kinase, catalytic, delta polypeptide (PIK3CD), mRNA [NM_005026]	NM_005026
A_23_P152024	2.74E-05	NM_004383	NM_004383	Homo sapiens c-src tyrosine kinase (CSK), mRNA [NM_004383]	NM_004383
A_23_P30655	2.74E-05	NM_004556	NM_004556	Homo sapiens nuclear factor of kappa light polypeptide gene enhancer in B-cells inhibitor, epsilon (NFKBIE), mRNA [NM_004556]	NM_004556
A_23_P96853	2.74E-05	NM_007051	NM_007051	Homo sapiens Fas (TNFRSF6) associated factor 1 (FAF1), transcript variant 1, mRNA [NM_007051]	NM_007051
A_23_P42884	2.74E-05	NM_032014	NM_032014	Homo sapiens mitochondrial ribosomal protein S24 (MRPS24), nuclear gene encoding mitochondrial protein, mRNA [NM_032014]	NM_032014
A_23_P161231	2.75E-05	NM_030971	NM_030971	Homo sapiens sideroflexin 3 (SFXN3), mRNA [NM_030971]	NM_030971
A_23_P16047	2.76E-05	NM_005282	NM_005282	Homo sapiens G protein-coupled receptor 4 (GPR4), mRNA [NM_005282]	NM_005282
A_24_P936767	2.76E-05	NM_005082	NM_005082	Homo sapiens tripartite motif-containing 25 (TRIM25), mRNA [NM_005082]	NM_005082
A_23_P344392	2.76E-05	NM_002542	NM_002542	Homo sapiens 8-oxoguanine DNA glycosylase (OGG1), nuclear gene encoding mitochondrial protein, transcript variant 1a, mRNA [NM_002542]	NM_002542
A_24_P381455	2.78E-05	BC018435	BC018435	Homo sapiens hypothetical protein FLJ11259, mRNA (cDNA clone MGC:21716 IMAGE:4474297), complete cds. [BC018435]	
A_23_P390518	2.78E-05	NM_003839	NM_003839	Homo sapiens tumor necrosis factor receptor superfamily, member 11a, NFKB activator (TNFRSF11A), mRNA [NM_003839]	NM_003839
A_23_P203729	2.78E-05	NM_002869	NM_002869	Homo sapiens RAB6A, member RAS oncogene family (RAB6A), transcript variant 1, mRNA [NM_002869]	NM_002869
A_23_P54728	2.78E-05	NM_023933	NM_023933	Homo sapiens chromosome 16 open reading frame 24 (C16orf24), mRNA [NM_023933]	NM_023933
A_23_P394448	2.79E-05	ENST00000310974		Homo sapiens mRNA for KIAA0877 protein, partial cds. [AB020684]	XM_371891
A_23_P89018	2.80E-05	NM_032296	NM_032296	Homo sapiens FLYWCH-type zinc finger 1 (FLYWCH1), transcript variant 1, mRNA [NM_032296]	NM_032296
A_32_P64936	2.80E-05	X05126	X05126	Human fibroblast mRNA fragment with Alu sequence (pRHF11). [X05126]	
A_23_P76684	2.80E-05	NM_006054	NM_006054	Homo sapiens reticulon 3 (RTN3), transcript variant 1, mRNA [NM_006054]	NM_006054
A_23_P208779	2.80E-05	NM_133180	NM_133180	Homo sapiens EPS8-like 1 (EPS8L1), transcript variant 1, mRNA [NM_133180]	NM_133180
A_23_P45712	2.80E-05	XM_372785	XM_372785	PREDICTED: Homo sapiens similar to peptidylprolyl isomerase A isoform 1; cyclophilin A; peptidyl-prolyl cis-trans isomerase A; T cell cyclophilin; rotamase; cyclosporin A-binding protein (LOC391062), mRNA [XM_372785]	XM_372785
A_24_P406986	2.82E-05	NM_199329	NM_199329	Homo sapiens solute carrier family 43, member 3 (SLC43A3), mRNA [NM_199329]	NM_199329
A_32_P57728	2.82E-05	ENST00000222396		Homo sapiens PMS2L13 mRNA, partial cds. [AB017004]	
A_23_P164000	2.82E-05	NM_015670	NM_015670	Homo sapiens SUMO1/sentrin/SMT3 specific protease 3 (SENP3), mRNA [NM_015670]	NM_015670
A_32_P148710	2.82E-05	NM_005507	NM_005507	Homo sapiens cofilin 1 (non-muscle) (CFL1), mRNA [NM_005507]	NM_005507
A_23_P422044	2.83E-05	NM_032009	NM_032009	Homo sapiens protocadherin gamma subfamily A, 2 (PCDHGA2), transcript variant 2, mRNA [NM_032009]	NM_032009
A_24_P91701	2.83E-05	NM_016449	NM_016449	Homo sapiens hypothetical protein LOC51233 (LOC51233), mRNA [NM_016449]	NM_016449

A_23_P203420	2.83E-05	NM_004739	NM_004739	Homo sapiens metastasis associated 1 family, member 2 (MTA2), mRNA [NM_004739]	NM_004739
A_32_P18258	2.83E-05	NM_006755	NM_006755	Homo sapiens transaldolase 1 (TALDO1), mRNA [NM_006755]	NM_006755
A_23_P86390	2.84E-05	NM_003873	NM_003873	Homo sapiens neuropilin 1 (NRP1), transcript variant 1, mRNA [NM_003873]	NM_003873
A_23_P9135	2.84E-05	NM_033655	NM_033655	Homo sapiens contactin associated protein-like 3 (CNTNAP3), mRNA [NM_033655]	NM_033655
A_24_P335656	2.85E-05	NM_003004	NM_003004	Homo sapiens secreted and transmembrane 1 (SECTM1), mRNA [NM_003004]	NM_003004
A_23_P333420	2.85E-05	NM_002883	NM_002883	Homo sapiens Ran GTPase activating protein 1 (RANGAP1), mRNA [NM_002883]	NM_002883
A_32_P79719	2.85E-05	THC2364880		Q9BYX7 (Q9BYX7) FKSG30, partial (41%) [THC2364880]	
A_23_P212339	2.86E-05	NM_024513	NM_024513	Homo sapiens FYVE and coiled-coil domain containing 1 (FYCO1), mRNA [NM_024513]	NM_024513
A_24_P393844	2.86E-05	NM_001384	NM_001384	Homo sapiens DPH2 homolog (S. cerevisiae) (DPH2), transcript variant 1, mRNA [NM_001384]	NM_001384
A_24_P137434	2.87E-05	NM_080927	NM_080927	Homo sapiens discoidin, CUB and LCCL domain containing 2 (DCBLD2), mRNA [NM_080927]	NM_080927
A_23_P218726	2.88E-05	NM_152930	NM_152930	Homo sapiens copine I (CPNE1), transcript variant 7, mRNA [NM_152930]	NM_152930
A_23_P104689	2.88E-05	BC014095	BC014095	Homo sapiens v-rel reticuloendotheliosis viral oncogene homolog A, nuclear factor of kappa light polypeptide gene enhancer in B-cells 3, p65 (avian), mRNA (cDNA clone IMAGE:4547184), complete cds. [BC014095]	
A_24_P139094	2.88E-05	NM_003025	NM_003025	Homo sapiens SH3-domain GRB2-like 1 (SH3GL1), mRNA [NM_003025]	NM_003025
A_23_P31550	2.89E-05	AK021933	AK021933	Homo sapiens cDNA FLJ11871 fis, clone HEMBA1007052. [AK021933]	
A_24_P937546	2.89E-05	AL832450	AL832450	Homo sapiens mRNA; cDNA DKFZp434I2129 (from clone DKFZp434I2129). [AL832450]	
A_23_P19176	2.90E-05	NM_206966	NM_206966	Homo sapiens similar to AVLV472 (MGC23985), mRNA [NM_206966]	NM_206966
A_23_P78383	2.90E-05	NM_013326	NM_013326	Homo sapiens chromosome 18 open reading frame 8 (C18orf8), mRNA [NM_013326]	NM_013326
A_24_P126181	2.90E-05	NM_176880	NM_176880	Homo sapiens TR4 orphan receptor associated protein TRA16 (TRA16), mRNA [NM_176880]	NM_176880
A_23_P132388	2.90E-05	NM_005138	NM_005138	Homo sapiens SCO cytochrome oxidase deficient homolog 2 (yeast) (SCO2), nuclear gene encoding mitochondrial protein, mRNA [NM_005138]	NM_005138
A_32_P188	2.91E-05	BM458245	BM458245	AGENCOURT_6411506 NIH_MGC_71 Homo sapiens cDNA clone IMAGE:5530621 5', mRNA sequence [BM458245]	XM_373700
A_24_P288954	2.91E-05	NM_001013694	NM_001013694	Homo sapiens similar to SRR1-like protein (LOC402055), mRNA [NM_001013694]	NM_001013694
A_24_P91232	2.91E-05	NM_032837	NM_032837	Homo sapiens hypothetical protein FLJ14775 (FLJ14775), mRNA [NM_032837]	NM_032837
A_23_P128008	2.92E-05	NM_021827	NM_021827	Homo sapiens hypothetical protein FLJ23514 (FLJ23514), mRNA [NM_021827]	NM_021827
A_23_P256682	2.92E-05	NM_014481	NM_014481	Homo sapiens APEX nuclease (apurinic/apyrimidinic endonuclease) 2 (APEX2), nuclear gene encoding mitochondrial protein, mRNA [NM_014481]	NM_014481
A_24_P126851	2.93E-05	A_24_P126851			
A_24_P75008	2.93E-05	ENST00000255741		PREDICTED: Homo sapiens similar to Guanine nucleotide-binding protein, alpha-11 subunit (GL2) (LOC346329), mRNA [XR_000184]	XR_000184
A_23_P201854	2.94E-05	AF052692	AF052692	Homo sapiens connexin 31 (GJB3) mRNA, complete cds. [AF052692]	
A_23_P148410	2.94E-05	NM_031894	NM_031894	Homo sapiens ferritin, heavy polypeptide-like 17 (FTHL17), mRNA [NM_031894]	NM_031894
A_23_P356565	2.94E-05	NM_015324	NM_015324	Homo sapiens KIAA0409 protein (KIAA0409), mRNA [NM_015324]	NM_015324
A_24_P181585	2.94E-05	NM_018509	NM_018509	Homo sapiens hypothetical protein PRO1855 (PRO1855), mRNA [NM_018509]	NM_018509
A_24_P176173	2.95E-05	NM_000428	NM_000428	Homo sapiens latent transforming growth factor beta binding protein 2 (LTBP2), mRNA [NM_000428]	NM_000428
A_23_P127522	2.96E-05	NM_145014	NM_145014	Homo sapiens hydrolethalus syndrome 1 (HYLS1), mRNA [NM_145014]	NM_145014
A_23_P204581	2.96E-05	NM_003330	NM_003330	Homo sapiens thioredoxin reductase 1 (TXNRD1), transcript variant 1, mRNA [NM_003330]	NM_003330
A_23_P318115	2.97E-05	NM_020467	NM_020467	Homo sapiens hypothetical protein from clone 643 (LOC57228), mRNA [NM_020467]	NM_020467
A_23_P37897	2.98E-05	NM_020314	NM_020314	Homo sapiens esophageal cancer associated protein (MGC16824), mRNA [NM_020314]	NM_020314
A_23_P90089	2.98E-05	NM_013976	NM_013976	Homo sapiens glutaryl-Coenzyme A dehydrogenase (GCDH), nuclear gene encoding mitochondrial protein, transcript variant 2, mRNA [NM_013976]	NM_013976
A_23_P415984	2.99E-05	NM_002518	NM_002518	Homo sapiens neuronal PAS domain protein 2 (NPAS2), mRNA [NM_002518]	NM_002518
A_23_P40718	2.99E-05	NM_001003828	NM_001003828	Homo sapiens parvin, beta (PARVB), transcript variant 1, mRNA [NM_001003828]	NM_001003828
A_23_P319617	2.99E-05	NM_019886	NM_019886	Homo sapiens carbohydrate (N-acetylglucosamine 6-O) sulfotransferase 7 (CHST7), mRNA [NM_019886]	NM_019886
A_24_P692030	3.00E-05	A_24_P692030			
A_24_P306614	3.00E-05	A_24_P306614			
A_23_P107206	3.02E-05	NM_213662	NM_213662	Homo sapiens signal transducer and activator of transcription 3 (acute-phase response factor) (STAT3), transcript variant 3, mRNA [NM_213662]	NM_213662
A_23_P13502	3.02E-05	NM_016565	NM_016565	Homo sapiens E2IG2 protein (E2IG2), mRNA [NM_016565]	NM_016565
A_24_P13381	3.03E-05	NM_147204	NM_147204	Homo sapiens transient receptor potential cation channel, subfamily V, member 4 (TRPV4), transcript variant 2, mRNA [NM_147204]	NM_147204

A_23_P321388	3.03E-05	NM_153341	NM_153341	Homo sapiens IBR domain containing 3 (IBRDC3), mRNA [NM_153341]	NM_153341
A_23_P201587	3.03E-05	NM_002959	NM_002959	Homo sapiens sortilin 1 (SORT1), mRNA [NM_002959]	NM_002959
A_23_P211785	3.03E-05	NM_003420	NM_003420	Homo sapiens zinc finger protein 35 (clone HF.10) (ZNF35), mRNA [NM_003420]	NM_003420
A_32_P102300	3.03E-05	THC2338243		Q6PGA0 (Q6PGA0) Rcor3 protein (Fragment), partial (8%) [THC2338243]	
A_23_P30315	3.03E-05	NM_033342	NM_033342	Homo sapiens tripartite motif-containing 7 (TRIM7), transcript variant 6, mRNA [NM_033342]	NM_033342
A_24_P381838	3.03E-05	NM_006122	NM_006122	Homo sapiens mannosidase, alpha, class 2A, member 2 (MAN2A2), mRNA [NM_006122]	NM_006122
A_24_P65779	3.03E-05	THC2370154		AF348700 ubiquitin A-52 residue ribosomal protein fusion product 1 {Homo sapiens;} , complete [THC2370154]	
A_23_P205778	3.05E-05	NM_016194	NM_016194	Homo sapiens guanine nucleotide binding protein (G protein), beta 5 (GNB5), transcript variant 2, mRNA [NM_016194]	NM_016194
A_24_P121956	3.05E-05	NM_173804	NM_173804	Homo sapiens hypothetical protein MGC30208 (MGC30208), mRNA [NM_173804]	NM_173804
A_23_P354193	3.09E-05	NM_144628	NM_144628	Homo sapiens TBC1 domain family, member 20 (TBC1D20), mRNA [NM_144628]	NM_144628
A_23_P1926	3.10E-05	NM_017841	NM_017841	Homo sapiens hypothetical protein FLJ20487 (FLJ20487), mRNA [NM_017841]	NM_017841
A_23_P143774	3.11E-05	NM_018995	NM_018995	Homo sapiens Mov10l1, Moloney leukemia virus 10-like 1, homolog (mouse) (MOV10L1), mRNA [NM_018995]	NM_018995
A_24_P23190	3.11E-05	NM_173510	NM_173510	Homo sapiens hypothetical protein FLJ33814 (FLJ33814), mRNA [NM_173510]	NM_173510
A_23_P353652	3.11E-05	NM_005587	NM_005587	Homo sapiens MADS box transcription enhancer factor 2, polypeptide A (myocyte enhancer factor 2A) (MEF2A), mRNA [NM_005587]	NM_005587
A_24_P253078	3.11E-05	NM_206914	NM_206914	Homo sapiens hepatocellularcarcinoma-associated antigen HCA557a (DKFZP586D0919), transcript variant 2, mRNA [NM_206914]	NM_206914
A_23_P96955	3.11E-05	NM_032648	NM_032648	Homo sapiens chromosome 1 open reading frame 90 (C1orf90), mRNA [NM_032648]	NM_032648
A_32_P232883	3.11E-05	BC089451	BC089451	Homo sapiens cDNA clone IMAGE:30554612. [BC089451]	
A_23_P139277	3.11E-05	NM_018362	NM_018362	Homo sapiens lin-7 homolog C (C. elegans) (LIN7C), mRNA [NM_018362]	NM_018362
A_23_P146339	3.11E-05	NM_005309	NM_005309	Homo sapiens glutamic-pyruvate transaminase (alanine aminotransferase) (GPT), mRNA [NM_005309]	NM_005309
A_24_P56884	3.11E-05	NM_152464	NM_152464	Homo sapiens chromosome 17 open reading frame 32 (C17orf32), mRNA [NM_152464]	NM_152464
A_24_P348892	3.11E-05	NM_052849	NM_052849	Homo sapiens hypothetical protein MGC20481 (MGC20481), mRNA [NM_052849]	NM_052849
A_24_P751082	3.12E-05	THC2364015		CLS1_HUMAN (O94985) Calsyntenin-1 precursor, partial (20%) [THC2364015]	
A_32_P40547	3.12E-05	CR615599	CR615599	full-length cDNA clone CS0DJ010YG18 of T cells (Jurkat cell line) Cot 10-normalized of Homo sapiens (human). [CR615599]	XR_000167
A_24_P187954	3.12E-05	NM_213654	NM_213654	Homo sapiens armadillo repeat containing 8 (ARMC8), mRNA [NM_213654]	NM_213654
A_24_P239606	3.12E-05	NM_015675	NM_015675	Homo sapiens growth arrest and DNA-damage-inducible, beta (GADD45B), mRNA [NM_015675]	NM_015675
A_24_P256513	3.13E-05	BC002828	BC002828	Homo sapiens angiogenic factor with G patch and FHA domains 1, mRNA (cDNA clone IMAGE:3659316), complete cds. [BC002828]	
A_23_P10927	3.13E-05	NM_031940	NM_031940	Homo sapiens TM2 domain containing 2 (TM2D2), transcript variant 2, mRNA [NM_031940]	NM_031940
A_23_P126159	3.14E-05	NM_002143	NM_002143	Homo sapiens hippocalcin (HPCA), mRNA [NM_002143]	NM_002143
A_24_P861099	3.14E-05	AK094718	AK094718	Homo sapiens cDNA FLJ37399 fis, clone BRAMY2027587. [AK094718]	
A_23_P152160	3.15E-05	NM_003498	NM_003498	Homo sapiens stannin (SNN), mRNA [NM_003498]	NM_003498
A_23_P107933	3.15E-05	NM_016440	NM_016440	Homo sapiens vaccinia related kinase 3 (VRK3), transcript variant 1, mRNA [NM_016440]	NM_016440
A_24_P374154	3.16E-05	NM_005877	NM_005877	Homo sapiens splicing factor 3a, subunit 1, 120kDa (SF3A1), transcript variant 1, mRNA [NM_005877]	NM_005877
A_24_P923251	3.16E-05	NM_198951	NM_198951	Homo sapiens transglutaminase 2 (C polypeptide, protein-glutamine-gamma-glutamyltransferase) (TGM2), transcript variant 2, mRNA [NM_198951]	NM_198951
A_23_P67980	3.17E-05	NM_003709	NM_003709	Homo sapiens Kruppel-like factor 7 (ubiquitous) (KLF7), mRNA [NM_003709]	NM_003709
A_24_P913016	3.17E-05	BC004338	BC004338	Homo sapiens, clone IMAGE:3631945, mRNA, partial cds. [BC004338]	
A_24_P48318	3.17E-05	NM_017748	NM_017748	Homo sapiens hypothetical protein FLJ20291 (FLJ20291), mRNA [NM_017748]	NM_017748
A_23_P135364	3.17E-05	NM_012145	NM_012145	Homo sapiens deoxythymidylate kinase (thymidylate kinase) (DTYMK), mRNA [NM_012145]	NM_012145
A_24_P124957	3.17E-05	NM_004663	NM_004663	Homo sapiens RAB11A, member RAS oncogene family (RAB11A), mRNA [NM_004663]	NM_004663
A_32_P117145	3.18E-05	NM_018494	NM_018494	Homo sapiens leucine-rich repeats and death domain containing (LRDD), transcript variant 2, mRNA [NM_018494]	NM_018494
A_24_P231250	3.19E-05	NM_020451	NM_020451	Homo sapiens selenoprotein N, 1 (SEPN1), transcript variant 1, mRNA [NM_020451]	NM_020451
A_32_P120895	3.19E-05	NM_153374	NM_153374	Homo sapiens LysM, putative peptidoglycan-binding, domain containing 2 (LYSMD2), mRNA [NM_153374]	NM_153374
A_23_P202374	3.19E-05	NM_078470	NM_078470	Homo sapiens COX15 homolog, cytochrome c oxidase assembly protein (yeast) (COX15), nuclear gene encoding mitochondrial protein, transcript variant 1, mRNA [NM_078470]	NM_078470
A_24_P255836	3.20E-05	A_24_P255836			

A_23_P213944	3.20E-05	NM_001945	NM_001945	Homo sapiens heparin-binding EGF-like growth factor (HBEGF), mRNA [NM_001945]	NM_001945
A_23_P207014	3.21E-05	NM_002876	NM_002876	Homo sapiens RAD51 homolog C (S. cerevisiae) (RAD51C), transcript variant 2, mRNA [NM_002876]	NM_002876
A_23_P204801	3.22E-05	NM_032148	NM_032148	Homo sapiens solute carrier family 41, member 2 (SLC41A2), mRNA [NM_032148]	NM_032148
A_23_P416112	3.23E-05	NM_152617	NM_152617	Homo sapiens ring finger protein 168 (RNF168), mRNA [NM_152617]	NM_152617
A_23_P300867	3.24E-05	NM_138731	NM_138731	Homo sapiens mirror-image polydactyly 1 (MIPOL1), mRNA [NM_138731]	NM_138731
A_23_P92132	3.24E-05	NM_006764	NM_006764	Homo sapiens interferon-related developmental regulator 2 (IFRD2), mRNA [NM_006764]	NM_006764
A_24_P49349	3.25E-05	NM_014857	NM_014857	Homo sapiens RAB GTPase activating protein 1-like (RABGAP1L), mRNA [NM_014857]	NM_014857
A_23_P44942	3.25E-05	XM_370664	XM_370664	PREDICTED: Homo sapiens similar to hypothetical protein (LOC387816), mRNA [XM_370664]	XM_370664
A_23_P9166	3.25E-05	NM_006289	NM_006289	Homo sapiens talin 1 (TLN1), mRNA [NM_006289]	NM_006289
A_23_P407142	3.25E-05	NM_033631	NM_033631	Homo sapiens leucine zipper protein 1 (LUZP1), mRNA [NM_033631]	NM_033631
A_23_P75889	3.25E-05	NM_175932	NM_175932	Homo sapiens proteasome (prosome, macropain) 26S subunit, non-ATPase, 13 (PSMD13), transcript variant 2, mRNA [NM_175932]	NM_175932
A_23_P98631	3.26E-05	NM_181507	NM_181507	Homo sapiens Hermansky-Pudlak syndrome 5 (HPS5), transcript variant 1, mRNA [NM_181507]	NM_181507
A_24_P298228	3.27E-05	A_24_P298228			
A_23_P149398	3.28E-05	CR614330	CR614330	full-length cDNA clone CS0DB003YM09 of Neuroblastoma Cot 10-normalized of Homo sapiens (human). [CR614330]	
A_23_P425516	3.29E-05	NM_012171	NM_012171	Homo sapiens tetraspanin 17 (TSPAN17), transcript variant 1, mRNA [NM_012171]	NM_012171
A_23_P117767	3.29E-05	NM_017828	NM_017828	Homo sapiens COMM domain containing 4 (COMMD4), mRNA [NM_017828]	NM_017828
A_23_P13018	3.29E-05	NM_198897	NM_198897	Homo sapiens fibroblast growth factor (acidic) intracellular binding protein (FIBP), transcript variant 1, mRNA [NM_198897]	NM_198897
A_24_P221206	3.29E-05	BC042161	BC042161	Homo sapiens trafficking protein particle complex 5, mRNA (cDNA clone MGC:52424 IMAGE:4869377), complete cds. [BC042161]	XM_058961
A_23_P211302	3.30E-05	NM_033661	NM_033661	Homo sapiens WD repeat domain 4 (WDR4), transcript variant 2, mRNA [NM_033661]	NM_033661
A_24_P385341	3.31E-05	NM_014388	NM_014388	Homo sapiens chromosome 1 open reading frame 107 (C1orf107), mRNA [NM_014388]	NM_014388
A_32_P19797	3.32E-05	BX115897	BX115897	BX115897 BX115897 Soares_multiple_sclerosis_2NbHMSP Homo sapiens cDNA clone IMAGp998J16611 ; IMAGE:276975, mRNA sequence [BX115897]	
A_23_P17880	3.33E-05	NM_005740	NM_005740	Homo sapiens dynein, axonemal, light polypeptide 4 (DNAL4), mRNA [NM_005740]	NM_005740
A_24_P316489	3.33E-05	NM_004544	NM_004544	Homo sapiens NADH dehydrogenase (ubiquinone) 1 alpha subcomplex, 10, 42kDa (NDUFA10), nuclear gene encoding mitochondrial protein, mRNA [NM_004544]	NM_004544
A_23_P400181	3.33E-05	NM_023939	NM_023939	Homo sapiens hypothetical protein MGC2752 (MGC2752), mRNA [NM_023939]	NM_023939
A_24_P222126	3.33E-05	NM_016397	NM_016397	Homo sapiens TH1-like (Drosophila) (TH1L), transcript variant 2, mRNA [NM_016397]	NM_016397
A_23_P133694	3.33E-05	NM_004955	NM_004955	Homo sapiens solute carrier family 29 (nucleoside transporters), member 1 (SLC29A1), mRNA [NM_004955]	NM_004955
A_23_P251548	3.34E-05	NM_144582	NM_144582	Homo sapiens testis expressed sequence 261 (TEX261), mRNA [NM_144582]	NM_144582
A_24_P366415	3.34E-05	XM_292963	XM_292963	PREDICTED: Homo sapiens similar to peptidylprolyl isomerase A (LOC344178), mRNA [XM_292963]	XM_292963
A_23_P89824	3.35E-05	NM_032933	NM_032933	Homo sapiens chromosome 18 open reading frame 45 (C18orf45), mRNA [NM_032933]	NM_032933
A_23_P12849	3.35E-05	NM_178150	NM_178150	Homo sapiens F-box protein, helicase, 18 (FBXO18), transcript variant 2, mRNA [NM_178150]	NM_178150
A_32_P93391	3.36E-05	XM_372723	XM_372723	PREDICTED: Homo sapiens similar to RP2 protein, testosterone-regulated - ricefield mouse (Mus caroli) (LOC390916), mRNA [XM_372723]	XM_372723
A_23_P314120	3.36E-05	NM_005198	NM_005198	Homo sapiens choline kinase beta (CHKB), transcript variant 1, mRNA [NM_005198]	NM_005198
A_23_P19004	3.36E-05	NM_023924	NM_023924	Homo sapiens bromodomain containing 9 (BRD9), transcript variant 1, mRNA [NM_023924]	NM_023924
A_23_P33022	3.36E-05	NM_021128	NM_021128	Homo sapiens polymerase (RNA) II (DNA directed) polypeptide L, 7.6kDa (POLR2L), mRNA [NM_021128]	NM_021128
A_23_P141434	3.37E-05	NM_000664	NM_000664	Homo sapiens acetyl-Coenzyme A carboxylase alpha (ACACA), transcript variant 6, mRNA [NM_000664]	NM_000664
A_24_P935881	3.38E-05	NM_022978	NM_022978	Homo sapiens small EDRK-rich factor 1B (centromeric) (SERF1B), mRNA [NM_022978]	NM_022978
A_32_P74643	3.39E-05	NM_001627	NM_001627	Homo sapiens activated leukocyte cell adhesion molecule (ALCAM), mRNA [NM_001627]	NM_001627
A_23_P61202	3.39E-05	NM_058190	NM_058190	Homo sapiens chromosome 21 open reading frame 70 (C21orf70), mRNA [NM_058190]	NM_058190
A_23_P100469	3.40E-05	NM_017853	NM_017853	Homo sapiens thioredoxin-like 4B (TXNL4B), mRNA [NM_017853]	NM_017853
A_23_P250054	3.40E-05	NM_022064	NM_022064	Homo sapiens ring finger protein 123 (RNF123), mRNA [NM_022064]	NM_022064
A_23_P162037	3.40E-05	NM_001178	NM_001178	Homo sapiens aryl hydrocarbon receptor nuclear translocator-like (ARNTL), mRNA [NM_001178]	NM_001178
A_24_P341426	3.40E-05	A_24_P341426			
A_23_P379026	3.40E-05	NM_019096	NM_019096	Homo sapiens GTP binding protein 2 (GTPBP2), mRNA [NM_019096]	NM_019096

A_23_P131706	3.40E-05	NM_212552	NM_212552	Homo sapiens bolA-like 3 (E. coli) (BOLA3), mRNA [NM_212552]	NM_212552
A_24_P139152	3.41E-05	AL359062	AL359062	Homo sapiens mRNA full length insert cDNA clone EUROIMAGE 1913076. [AL359062]	
A_23_P210872	3.41E-05	NM_014742	NM_014742	Homo sapiens transmembrane 9 superfamily protein member 4 (TM9SF4), mRNA [NM_014742]	NM_014742
A_23_P116614	3.41E-05	NM_001014811	NM_001014811	Homo sapiens malic enzyme 3, NADP(+)-dependent, mitochondrial (ME3), nuclear gene encoding mitochondrial protein, transcript variant 2, mRNA [NM_001014811]	NM_001014811
A_32_P179771	3.42E-05	AI088710	AI088710	AI088710 qa12c10.x1 NCI_CGAP_Brn23 Homo sapiens cDNA clone IMAGE:1686546 3' similar to gb:L06132 OUTER MITOCHONDRIAL MEMBRANE PROTEIN PORIN (HUMAN);, mRNA sequence [AI088710]	
A_23_P352957	3.42E-05	NM_025090	NM_025090	Homo sapiens ubiquitin specific protease 36 (USP36), mRNA [NM_025090]	NM_025090
A_24_P141522	3.42E-05	NM_016320	NM_016320	Homo sapiens nucleoporin 98kDa (NUP98), transcript variant 1, mRNA [NM_016320]	NM_016320
A_23_P391764	3.43E-05	NM_198679	NM_198679	Homo sapiens Rap guanine nucleotide exchange factor (GEF) 1 (RAPGEF1), transcript variant 2, mRNA [NM_198679]	NM_198679
A_24_P103004	3.43E-05	NM_005415	NM_005415	Homo sapiens solute carrier family 20 (phosphate transporter), member 1 (SLC20A1), mRNA [NM_005415]	NM_005415
A_24_P118341	3.43E-05	BF869497	BF869497	BF869497 IL3-ET0115-091000-286-D05 ET0115 Homo sapiens cDNA, mRNA sequence [BF869497]	
A_23_P111593	3.44E-05	NM_004227	NM_004227	Homo sapiens pleckstrin homology, Sec7 and coiled-coil domains 3 (PSCD3), mRNA [NM_004227]	NM_004227
A_24_P409985	3.44E-05	NM_001011655	NM_001011655	Homo sapiens transmembrane protein 44 (TMEM44), transcript variant 2, mRNA [NM_001011655]	NM_001011655
A_32_P61936	3.44E-05	BC039676	BC039676	Homo sapiens, clone IMAGE:5173389, mRNA. [BC039676]	XM_373518
A_23_P371933	3.46E-05	BC012536	BC012536	Homo sapiens hydroxysteroid (17-beta) dehydrogenase 12, mRNA (cDNA clone IMAGE:4281565), complete cds. [BC012536]	
A_24_P26554	3.46E-05	NM_020408	NM_020408	Homo sapiens chromosome 6 open reading frame 149 (C6orf149), mRNA [NM_020408]	NM_020408
A_23_P372386	3.46E-05	NM_173833	NM_173833	Homo sapiens hypothetical protein MGC45780 (MGC45780), mRNA [NM_173833]	NM_173833
A_23_P79259	3.46E-05	NM_014521	NM_014521	Homo sapiens SH3-domain binding protein 4 (SH3BP4), mRNA [NM_014521]	NM_014521
A_23_P60047	3.46E-05	NM_006283	NM_006283	Homo sapiens transforming, acidic coiled-coil containing protein 1 (TACC1), mRNA [NM_006283]	NM_006283
A_24_P277576	3.48E-05	NM_004237	NM_004237	Homo sapiens thyroid hormone receptor interactor 13 (TRIP13), mRNA [NM_004237]	NM_004237
A_32_P141724	3.48E-05	NM_053041	NM_053041	Homo sapiens COMM domain containing 7 (COMMD7), mRNA [NM_053041]	NM_053041
A_23_P51853	3.52E-05	NM_032283	NM_032283	Homo sapiens zinc finger, DHHC-type containing 18 (ZDHHC18), mRNA [NM_032283]	NM_032283
A_23_P360983	3.52E-05	NM_174889	NM_174889	Homo sapiens Myc-induced mitochondria protein (mimitin), mRNA [NM_174889]	NM_174889
A_23_P402787	3.53E-05	NM_145253	NM_145253	Homo sapiens LOC124402 (LOC124402), mRNA [NM_145253]	NM_145253
A_24_P65864	3.54E-05	NM_016143	NM_016143	Homo sapiens NSFL1 (p97) cofactor (p47) (NSFL1C), transcript variant 1, mRNA [NM_016143]	NM_016143
A_32_P221832	3.55E-05	CD630738	CD630738	CD630738 56066364H1 FLP Homo sapiens cDNA, mRNA sequence [CD630738]	
A_24_P385326	3.56E-05	ENST00000292357		Homo sapiens mRNA for FLJ00193 protein. [AK074121]	XM_371320
A_23_P151159	3.56E-05	NM_032300	NM_032300	Homo sapiens hypothetical protein MGC10854 (MGC10854), mRNA [NM_032300]	NM_032300
A_24_P136683	3.58E-05	BC021816	BC021816	Homo sapiens carbonic anhydrase VB-like, mRNA (cDNA clone IMAGE:3460742), partial cds. [BC021816]	XM_291346
A_23_P30264	3.58E-05	NM_016338	NM_016338	Homo sapiens importin 11 (IPO11), mRNA [NM_016338]	NM_016338
A_23_P5281	3.58E-05	NM_005583	NM_005583	Homo sapiens lymphoblastic leukemia derived sequence 1 (LYL1), mRNA [NM_005583]	NM_005583
A_23_P366394	3.59E-05	NM_016653	NM_016653	Homo sapiens sterile alpha motif and leucine zipper containing kinase AZK (ZAK), transcript variant 1, mRNA [NM_016653]	NM_016653
A_24_P212990	3.59E-05	AL365511	AL365511	Novel human gene mapping to chomosome 22. [AL365511]	
A_23_P500251	3.59E-05	NM_003404	NM_003404	Homo sapiens tyrosine 3-monooxygenase/tryptophan 5-monooxygenase activation protein, beta polypeptide (YWHAB), transcript variant 1, mRNA [NM_003404]	NM_003404
A_23_P426398	3.60E-05	NM_138575	NM_138575	Homo sapiens Bcl-XL-binding protein v68 (MGC5352), mRNA [NM_138575]	NM_138575
A_23_P165636	3.60E-05	NM_001747	NM_001747	Homo sapiens capping protein (actin filament), gelsolin-like (CAPG), mRNA [NM_001747]	NM_001747
A_23_P318420	3.62E-05	NM_018704	NM_018704	Homo sapiens hypothetical protein DKFZp547A023 (DKFZp547A023), mRNA [NM_018704]	NM_018704
A_24_P943040	3.63E-05	NM_198066	NM_198066	Homo sapiens glucosamine-phosphate N-acetyltransferase 1 (GNPNAT1), mRNA [NM_198066]	NM_198066
A_24_P187750	3.64E-05	NM_001659	NM_001659	Homo sapiens ADP-ribosylation factor 3 (ARF3), mRNA [NM_001659]	NM_001659
A_23_P66813	3.64E-05	NM_016538	NM_016538	Homo sapiens sirtuin (silent mating type information regulation 2 homolog) 7 (S. cerevisiae) (SIRT7), mRNA [NM_016538]	NM_016538
A_23_P161041	3.64E-05	NM_021633	NM_021633	Homo sapiens kelch-like 12 (Drosophila) (KLHL12), mRNA [NM_021633]	NM_021633
A_23_P139704	3.64E-05	NM_001946	NM_001946	Homo sapiens dual specificity phosphatase 6 (DUSP6), transcript variant 1, mRNA [NM_001946]	NM_001946
A_32_P183918	3.65E-05	BC025340	BC025340	Homo sapiens hypothetical protein MGC39372, mRNA (cDNA clone MGC:39372 IMAGE:5089466), complete cds. [BC025340]	XM_376463
A_23_P380766	3.65E-05	NM_006035	NM_006035	Homo sapiens CDC42 binding protein kinase beta (DMPK-like) (CDC42BPB), mRNA [NM_006035]	NM_006035

A_24_P541489	3.66E-05	X83412	X83412	H.sapiens B1 mRNA for mucin. [X83412]	XM_499249
A_23_P22214	3.66E-05	NM_004157	NM_004157	Homo sapiens protein kinase, cAMP-dependent, regulatory, type II, alpha (PRKAR2A), mRNA [NM_004157]	NM_004157
A_24_P64233	3.66E-05	NM_000692	NM_000692	Homo sapiens aldehyde dehydrogenase 1 family, member B1 (ALDH1B1), nuclear gene encoding mitochondrial protein, mRNA [NM_000692]	NM_000692
A_24_P7470	3.67E-05	A_24_P7470			
A_32_P235727	3.68E-05	A_32_P235727			
A_24_P196024	3.68E-05	A_24_P196024			
A_23_P160598	3.68E-05	CR591540	CR591540	full-length cDNA clone CS0DE004YG04 of Placenta of Homo sapiens (human). [CR591540]	
A_23_P24594	3.70E-05	NM_006597	NM_006597	Homo sapiens heat shock 70kDa protein 8 (HSPA8), transcript variant 1, mRNA [NM_006597]	NM_006597
A_23_P81298	3.71E-05	AK025816	AK025816	Homo sapiens cDNA: FLJ22163 fis, clone HRC00430. [AK025816]	XM_371755
A_23_P4572	3.73E-05	NM_006471	NM_006471	Homo sapiens myosin regulatory light chain MRCL3 (MRCL3), mRNA [NM_006471]	NM_006471
A_32_P208076	3.74E-05	A_32_P208076			
A_23_P215517	3.74E-05	BC009555	BC009555	Homo sapiens kelch-like 7 (Drosophila), mRNA (cDNA clone IMAGE:3899090), complete cds. [BC009555]	
A_23_P133386	3.76E-05	NM_006909	NM_006909	Homo sapiens Ras protein-specific guanine nucleotide-releasing factor 2 (RASGRF2), mRNA [NM_006909]	NM_006909
A_23_P151179	3.76E-05	NM_022895	NM_022895	Homo sapiens hypothetical protein FLJ12448 (FLJ12448), mRNA [NM_022895]	NM_022895
A_23_P67228	3.76E-05	NM_003083	NM_003083	Homo sapiens small nuclear RNA activating complex, polypeptide 2, 45kDa (SNAPC2), mRNA [NM_003083]	NM_003083
A_23_P158083	3.78E-05	NM_001261	NM_001261	Homo sapiens cyclin-dependent kinase 9 (CDC2-related kinase) (CDK9), mRNA [NM_001261]	NM_001261
A_24_P179769	3.78E-05	NM_205861	NM_205861	Homo sapiens dehydrodolichyl diphosphate synthase (DHDDS), transcript variant 2, mRNA [NM_205861]	NM_205861
A_23_P1552	3.78E-05	NM_001814	NM_001814	Homo sapiens cathepsin C (CTSC), transcript variant 1, mRNA [NM_001814]	NM_001814
A_24_P277615	3.80E-05	NM_203297	NM_203297	Homo sapiens tripartite motif-containing 7 (TRIM7), transcript variant 2, mRNA [NM_203297]	NM_203297
A_32_P22883	3.80E-05	NM_058190	NM_058190	Homo sapiens chromosome 21 open reading frame 70 (C21orf70), mRNA [NM_058190]	NM_058190
A_23_P358928	3.80E-05	NM_003130	NM_003130	Homo sapiens sorcin (SRI), transcript variant 1, mRNA [NM_003130]	NM_003130
A_23_P165651	3.82E-05	NM_145644	NM_145644	Homo sapiens mitochondrial ribosomal protein L35 (MRPL35), nuclear gene encoding mitochondrial protein, transcript variant 2, mRNA [NM_145644]	NM_145644
A_24_P337867	3.83E-05	NM_032831	NM_032831	Homo sapiens chromosome 7 open reading frame 19 (C7orf19), mRNA [NM_032831]	NM_032831
A_23_P202361	3.84E-05	NM_182639	NM_182639	Homo sapiens Hermansky-Pudlak syndrome 1 (HPS1), transcript variant 3, mRNA [NM_182639]	NM_182639
A_24_P84880	3.85E-05	ENST00000332444		Homo sapiens cDNA FLJ31247 fis, clone KIDNE2005296, weakly similar to ACTIN, CYTOPLASMIC 1. [AK055809]	
A_23_P47155	3.86E-05	NM_015368	NM_015368	Homo sapiens pannexin 1 (PANX1), mRNA [NM_015368]	NM_015368
A_24_P354300	3.87E-05	NM_015426	NM_015426	Homo sapiens WD repeat domain 51A (WDR51A), mRNA [NM_015426]	NM_015426
A_32_P143880	3.87E-05	NM_015955	NM_015955	Homo sapiens chromosome 2 open reading frame 4 (C2orf4), mRNA [NM_015955]	NM_015955
A_24_P914940	3.88E-05	BC010931	BC010931	Homo sapiens MADS box transcription enhancer factor 2, polypeptide B (myocyte enhancer factor 2B), mRNA (cDNA clone IMAGE:4280324), complete cds. [BC010931]	
A_23_P397856	3.89E-05	NM_052887	NM_052887	Homo sapiens toll-interleukin 1 receptor (TIR) domain containing adaptor protein (TIRAP), transcript variant 1, mRNA [NM_052887]	NM_052887
A_23_P103041	3.89E-05	NM_152236	NM_152236	Homo sapiens growth arrest-specific 2 like 1 (GAS2L1), transcript variant 2, mRNA [NM_152236]	NM_152236
A_32_P224840	3.89E-05	NM_001012398	NM_001012398	Homo sapiens fused toes homolog (mouse) (FTS), transcript variant 1, mRNA [NM_001012398]	NM_001012398
A_32_P202042	3.90E-05	BC020340	BC020340	Homo sapiens anaphase promoting complex subunit 5, mRNA (cDNA clone IMAGE:3882030). [BC020340]	
A_24_P343621	3.90E-05	NM_024693	NM_024693	Homo sapiens enoyl Coenzyme A hydratase domain containing 3 (ECHDC3), mRNA [NM_024693]	NM_024693
A_23_P102842	3.91E-05	NM_021100	NM_021100	Homo sapiens NFS1 nitrogen fixation 1 (S. cerevisiae) (NFS1), nuclear gene encoding mitochondrial protein, transcript variant 1, mRNA [NM_021100]	NM_021100
A_24_P135483	3.92E-05	AK074558	AK074558	Homo sapiens cDNA FLJ90077 fis, clone HEMBA1004454. [AK074558]	
A_23_P210886	3.92E-05	NM_138578	NM_138578	Homo sapiens BCL2-like 1 (BCL2L1), nuclear gene encoding mitochondrial protein, transcript variant 1, mRNA [NM_138578]	NM_138578
A_23_P87482	3.92E-05	NM_024068	NM_024068	Homo sapiens hypothetical protein MGC2731 (MGC2731), mRNA [NM_024068]	NM_024068
A_23_P91829	3.92E-05	NM_080927	NM_080927	Homo sapiens discoidin, CUB and LCCL domain containing 2 (DCBLD2), mRNA [NM_080927]	NM_080927
A_24_P338145	3.92E-05	NM_013442	NM_013442	Homo sapiens stomatin (EPB72)-like 2 (STOML2), mRNA [NM_013442]	NM_013442
A_24_P374513	3.92E-05	THC2242927		Q5TD55 (Q5TD55) OTTHUMP00000031270 (Fragment), complete [THC2242927]	
A_23_P143650	3.93E-05	NM_152511	NM_152511	Homo sapiens dual specificity phosphatase 18 (DUSP18), mRNA [NM_152511]	NM_152511
A_23_P50807	3.93E-05	NM_021107	NM_021107	Homo sapiens mitochondrial ribosomal protein S12 (MRPS12), nuclear gene encoding mitochondrial protein, transcript variant 1, mRNA [NM_021107]	NM_021107

A_32_P17525	3.95E-05	THC2438621			
A_23_P108574	3.95E-05	NM_005813	NM_005813	Homo sapiens protein kinase D3 (PRKD3), mRNA [NM_005813]	NM_005813
A_23_P326963	3.96E-05	NM_178496	NM_178496	Homo sapiens similar to BcDNA:GH11415 gene product (LOC151963), mRNA [NM_178496]	NM_178496
A_24_P375485	3.96E-05	NM_207381	NM_207381	Homo sapiens tumor necrosis factor, alpha-induced protein 8-like 3 (TNFAIP8L3), mRNA [NM_207381]	NM_207381
A_24_P87579	3.96E-05	NM_001017916	NM_001017916	Homo sapiens cytochrome b-561 (CYB561), transcript variant 2, mRNA [NM_001017916]	NM_001017916
A_23_P16915	3.96E-05	NM_012413	NM_012413	Homo sapiens glutaminyl-peptide cyclotransferase (glutaminyl cyclase) (QPCT), mRNA [NM_012413]	NM_012413
A_23_P82324	3.96E-05	NM_032415	NM_032415	Homo sapiens caspase recruitment domain family, member 11 (CARD11), mRNA [NM_032415]	NM_032415
A_23_P119214	3.96E-05	NM_006351	NM_006351	Homo sapiens translocase of inner mitochondrial membrane 44 homolog (yeast) (TIMM44), mRNA [NM_006351]	NM_006351
A_24_P361006	3.96E-05	NM_005002	NM_005002	Homo sapiens NADH dehydrogenase (ubiquinone) 1 alpha subcomplex, 9, 39kDa (NDUFA9), mRNA [NM_005002]	NM_005002
A_23_P92140	3.97E-05	NM_002468	NM_002468	Homo sapiens myeloid differentiation primary response gene (88) (MYD88), mRNA [NM_002468]	NM_002468
A_23_P46903	3.97E-05	NM_172171	NM_172171	Homo sapiens calcium/calmodulin-dependent protein kinase (CaM kinase) II gamma (CAMK2G), transcript variant 1, mRNA [NM_172171]	NM_172171
A_23_P105307	4.00E-05	NM_201444	NM_201444	Homo sapiens diacylglycerol kinase, alpha 80kDa (DGKA), transcript variant 1, mRNA [NM_201444]	NM_201444
A_24_P925361	4.00E-05	AJ227863	AJ227863	Homo sapiens partial mRNA; ID YG39-2B. [AJ227863]	
A_24_P272653	4.01E-05	A_24_P272653			
A_23_P128396	4.01E-05	NM_194271	NM_194271	Homo sapiens ring finger protein 34 (RNF34), transcript variant 1, mRNA [NM_194271]	NM_194271
A_23_P131909	4.02E-05	BC021178	BC021178	Homo sapiens cDNA clone MGC:33216 IMAGE:5265299, complete cds. [BC021178]	
A_24_P341019	4.02E-05	NM_001009924	NM_001009924	Homo sapiens chromosome 20 open reading frame 30 (C20orf30), transcript variant 2, mRNA [NM_001009924]	NM_001009924
A_24_P237766	4.03E-05	NM_003003	NM_003003	Homo sapiens SEC14-like 1 (S. cerevisiae) (SEC14L1), mRNA [NM_003003]	NM_003003
A_23_P51136	4.03E-05	NM_004040	NM_004040	Homo sapiens ras homolog gene family, member B (RHOB), mRNA [NM_004040]	NM_004040
A_23_P109593	4.03E-05	NM_014346	NM_014346	Homo sapiens TBC1 domain family, member 22A (TBC1D22A), mRNA [NM_014346]	NM_014346
A_23_P343719	4.05E-05	NM_000932	NM_000932	Homo sapiens phospholipase C, beta 3 (phosphatidylinositol-specific) (PLCB3), mRNA [NM_000932]	NM_000932
A_23_P151653	4.05E-05	NM_080649	NM_080649	Homo sapiens APEX nuclease (multifunctional DNA repair enzyme) 1 (APEX1), transcript variant 3, mRNA [NM_080649]	NM_080649
A_23_P154698	4.06E-05	NM_003818	NM_003818	Homo sapiens CDP-diacylglycerol synthase (phosphatidate cytidylyltransferase) 2 (CDS2), mRNA [NM_003818]	NM_003818
A_23_P39277	4.07E-05	A_23_P39277			
A_23_P141100	4.07E-05	NM_012091	NM_012091	Homo sapiens adenosine deaminase, tRNA-specific 1 (ADAT1), mRNA [NM_012091]	NM_012091
A_24_P397584	4.07E-05	NM_003192	NM_003192	Homo sapiens tubulin-specific chaperone c (TBCC), mRNA [NM_003192]	NM_003192
A_24_P97931	4.07E-05	NM_001320	NM_001320	Homo sapiens casein kinase 2, beta polypeptide (CSNK2B), mRNA [NM_001320]	NM_001320
A_23_P259413	4.08E-05	NM_017548	NM_017548	Homo sapiens hypothetical protein H41 (H41), mRNA [NM_017548]	NM_017548
A_23_P29723	4.09E-05	NM_001012410	NM_001012410	Homo sapiens shugoshin-like 1 (S. pombe) (SGOL1), transcript variant A2, mRNA [NM_001012410]	NM_001012410
A_23_P352022	4.09E-05	ENST00000315255		Human PVT-IGLC fusion protein mRNA, 5' end. [M34432]	XM_372058
A_24_P24332	4.09E-05	A_24_P24332			
A_23_P431543	4.09E-05	NM_182612	NM_182612	Homo sapiens hypothetical protein FLJ34283 (FLJ34283), mRNA [NM_182612]	NM_182612
A_23_P304237	4.12E-05	NM_198679	NM_198679	Homo sapiens Rap guanine nucleotide exchange factor (GEF) 1 (RAPGEF1), transcript variant 2, mRNA [NM_198679]	NM_198679
A_24_P152468	4.12E-05	AK096229	AK096229	Homo sapiens cDNA FLJ38910 fis, clone NT2NE2006813, weakly similar to CELL SURFACE GLYCOPROTEIN 1 PRECURSOR. [AK096229]	
A_23_P150718	4.12E-05	NM_001665	NM_001665	Homo sapiens ras homolog gene family, member G (rho G) (RHOG), mRNA [NM_001665]	NM_001665
A_23_P125348	4.13E-05	NM_021210	NM_021210	Homo sapiens trafficking protein particle complex 1 (TRAPPC1), mRNA [NM_021210]	NM_021210
A_23_P399201	4.13E-05	NM_183049	NM_183049	Homo sapiens thymosin-like 3 (TMSL3), mRNA [NM_183049]	NM_183049
A_23_P79911	4.14E-05	NM_006814	NM_006814	Homo sapiens proteasome (prosome, macropain) inhibitor subunit 1 (PI31) (PSMF1), transcript variant 1, mRNA [NM_006814]	NM_006814
A_23_P65851	4.15E-05	AB082533	AB082533	Homo sapiens mRNA for KIAA2002 protein. [AB082533]	XM_370878
A_24_P187448	4.15E-05	A_24_P187448			
A_32_P227673	4.15E-05	THC2305334			
A_23_P52634	4.15E-05	NM_015368	NM_015368	Homo sapiens pannexin 1 (PANX1), mRNA [NM_015368]	NM_015368
A_24_P332151	4.16E-05	A_24_P332151			
A_24_P321752	4.16E-05	NM_015072	NM_015072	Homo sapiens KIAA0998 (KIAA0998), mRNA [NM_015072]	NM_015072

A_23_P56010	4.16E-05	NM_004943	NM_004943	Homo sapiens dystrophia myotonica-containing WD repeat motif (DMWD), mRNA [NM_004943]	NM_004943
A_24_P273143	4.16E-05	NM_052871	NM_052871	Homo sapiens hypothetical protein MGC4677 (MGC4677), mRNA [NM_052871]	NM_052871
A_32_P137035	4.17E-05	NM_006510	NM_006510	Homo sapiens ret finger protein (RFP), transcript variant alpha, mRNA [NM_006510]	NM_006510
A_24_P210420	4.19E-05	NM_178006	NM_178006	Homo sapiens START domain containing 13 (STARD13), transcript variant alpha, mRNA [NM_178006]	NM_178006
A_23_P85853	4.19E-05	NM_024587	NM_024587	Homo sapiens transmembrane protein 53 (TMEM53), mRNA [NM_024587]	NM_024587
A_24_P245646	4.19E-05	NM_033550	NM_033550	Homo sapiens TP53 regulating kinase (TP53RK), mRNA [NM_033550]	NM_033550
A_24_P918762	4.20E-05	THC2436839		ALU1_HUMAN (P39188) Alu subfamily J sequence contamination warning entry, partial (5%) [THC2436839]	
A_23_P385267	4.21E-05	NM_173618	NM_173618	Homo sapiens hypothetical protein FLJ90652 (FLJ90652), mRNA [NM_173618]	NM_173618
A_23_P3681	4.23E-05	NM_018092	NM_018092	Homo sapiens neuropilin (NRP) and tolloid (TLL)-like 2 (NETO2), mRNA [NM_018092]	NM_018092
A_23_P168443	4.24E-05	NM_004444	NM_004444	Homo sapiens EPH receptor B4 (EPHB4), mRNA [NM_004444]	NM_004444
A_24_P474188	4.26E-05	A_24_P474188			
A_32_P130788	4.27E-05	NM_001010971	NM_001010971	Homo sapiens dnaj-like protein (LOC148418), mRNA [NM_001010971]	NM_001010971
A_23_P319153	4.27E-05	AL512694	AL512694	Homo sapiens mRNA; cDNA DKFZp761J2423 (from clone DKFZp761J2423). [AL512694]	
A_24_P178523	4.27E-05	A_24_P178523			
A_23_P150741	4.27E-05	ENST00000334126		Homo sapiens mRNA; cDNA DKFZp586P0123 (from clone DKFZp586P0123). [AL080220]	
A_24_P934583	4.28E-05	AY358688	AY358688	Homo sapiens clone DNA62312 GALI1870 (UNQ1870) mRNA, complete cds. [AY358688]	
A_23_P100754	4.28E-05	NM_022739	NM_022739	Homo sapiens SMAD specific E3 ubiquitin protein ligase 2 (SMURF2), mRNA [NM_022739]	NM_022739
A_23_P250714	4.29E-05	NM_005107	NM_005107	Homo sapiens endonuclease G-like 1 (ENDOGL1), mRNA [NM_005107]	NM_005107
A_23_P76051	4.29E-05	NM_005538	NM_005538	Homo sapiens inhibin, beta C (INHBC), mRNA [NM_005538]	NM_005538
A_24_P84021	4.29E-05	XM_496693	XM_496693	PREDICTED: Homo sapiens similar to hypertension-related calcium-regulated gene (LOC441016), mRNA [XM_496693]	XM_496693
A_23_P97005	4.29E-05	NM_002227	NM_002227	Homo sapiens Janus kinase 1 (a protein tyrosine kinase) (JAK1), mRNA [NM_002227]	NM_002227
A_23_P1819	4.30E-05	NM_012378	NM_012378	Homo sapiens olfactory receptor, family 8, subfamily B, member 8 (OR8B8), mRNA [NM_012378]	NM_012378
A_23_P306148	4.30E-05	NM_002675	NM_002675	Homo sapiens promyelocytic leukemia (PML), transcript variant 6, mRNA [NM_002675]	NM_002675
A_32_P353677	4.31E-05	AF343078	AF343078	Homo sapiens TOB3 mRNA, complete cds. [AF343078]	
A_24_P327815	4.32E-05	NM_006819	NM_006819	Homo sapiens stress-induced-phosphoprotein 1 (Hsp70/Hsp90-organizing protein) (STIP1), mRNA [NM_006819]	NM_006819
A_23_P66100	4.33E-05	CR607189	CR607189	full-length cDNA clone CS0DK007YF11 of HeLa cells Cot 25-normalized of Homo sapiens (human). [CR607189]	
A_23_P216402	4.33E-05	NM_031432	NM_031432	Homo sapiens uridine-cytidine kinase 1 (UCK1), mRNA [NM_031432]	NM_031432
A_24_P141005	4.34E-05	NM_182715	NM_182715	Homo sapiens synaptophysin-like 1 (SYPL1), transcript variant 2, mRNA [NM_182715]	NM_182715
A_32_P181020	4.34E-05	NM_005510	NM_005510	Homo sapiens dom-3 homolog Z (C. elegans) (DOM3Z), mRNA [NM_005510]	NM_005510
A_23_P252962	4.34E-05	NM_001001132	NM_001001132	Homo sapiens intersectin 1 (SH3 domain protein) (ITSN1), transcript variant 2, mRNA [NM_001001132]	NM_001001132
A_23_P27571	4.35E-05	NM_020533	NM_020533	Homo sapiens mucolipin 1 (MCOLN1), mRNA [NM_020533]	NM_020533
A_23_P363826	4.36E-05	NM_033406	NM_033406	Homo sapiens F-box protein 3 (FBXO3), transcript variant 2, mRNA [NM_033406]	NM_033406
A_23_P37624	4.37E-05	AF140675	AF140675	Homo sapiens zinc metalloprotease ADAMTS7 (ADAMTS7) mRNA, complete cds. [AF140675]	
A_32_P540991	4.37E-05	AK057591	AK057591	Homo sapiens cDNA FLJ33029 fis, clone THYMU2000162. [AK057591]	
A_32_P194115	4.37E-05	NM_015374	NM_015374	Homo sapiens unc-84 homolog B (C. elegans) (UNC84B), mRNA [NM_015374]	NM_015374
A_23_P202696	4.37E-05	NM_016506	NM_016506	Homo sapiens kelch repeat and BTB (POZ) domain containing 4 (KBTBD4), transcript variant 2, mRNA [NM_016506]	NM_016506
A_23_P9144	4.37E-05	NM_152306	NM_152306	Homo sapiens ubiquitin-like, containing PHD and RING finger domains, 2 (UHRF2), transcript variant 1, mRNA [NM_152306]	NM_152306
A_24_P143189	4.38E-05	NM_183049	NM_183049	Homo sapiens thymosin-like 3 (TMSL3), mRNA [NM_183049]	NM_183049
A_23_P218751	4.40E-05	NM_053004	NM_053004	Homo sapiens guanine nucleotide binding protein (G protein), beta polypeptide 1-like (GNB1L), mRNA [NM_053004]	NM_053004
A_23_P59099	4.41E-05	NM_013937	NM_013937	Homo sapiens olfactory receptor, family 11, subfamily A, member 1 (OR11A1), mRNA [NM_013937]	NM_013937
A_24_P287526	4.41E-05	A_24_P287526			
A_23_P92082	4.41E-05	NM_001064	NM_001064	Homo sapiens transketolase (Wernicke-Korsakoff syndrome) (TKT), mRNA [NM_001064]	NM_001064
A_32_P192970	4.42E-05	NM_170726	NM_170726	Homo sapiens aldehyde dehydrogenase 4 family, member A1 (ALDH4A1), nuclear gene encoding mitochondrial protein, transcript variant P5CDhS, mRNA [NM_170726]	NM_170726

A_32_P44139	4.42E-05	AA627222	AA627222	AA627222 nq63b01.s1 NCI_CGAP_Ov6 Homo sapiens cDNA clone IMAGE:1154665 similar to gb:J04456 GALECTIN-1 (HUMAN);, mRNA sequence [AA627222]	
A_23_P54373	4.42E-05	NM_004580	NM_004580	Homo sapiens RAB27A, member RAS oncogene family (RAB27A), transcript variant 1, mRNA [NM_004580]	NM_004580
A_32_P186710	4.42E-05	THC2248354			
A_24_P578641	4.43E-05	A_24_P578641			
A_23_P23346	4.43E-05	NM_006818	NM_006818	Homo sapiens myeloid/lymphoid or mixed-lineage leukemia (trithorax homolog, Drosophila); translocated to, 11 (MLLT11), mRNA [NM_006818]	NM_006818
A_24_P134229	4.44E-05	AK098427	AK098427	Homo sapiens cDNA FLJ25561 fis, clone JTH02933. [AK098427]	
A_23_P203586	4.44E-05	NM_016320	NM_016320	Homo sapiens nucleoporin 98kDa (NUP98), transcript variant 1, mRNA [NM_016320]	NM_016320
A_24_P229616	4.44E-05	A_24_P229616			
A_23_P163178	4.44E-05	NM_006888	NM_006888	Homo sapiens calmodulin 1 (phosphorylase kinase, delta) (CALM1), mRNA [NM_006888]	NM_006888
A_32_P155030	4.46E-05	AK096483	AK096483	Homo sapiens cDNA FLJ39164 fis, clone OCBBF2002656. [AK096483]	
A_23_P201887	4.47E-05	NM_005057	NM_005057	Homo sapiens retinoblastoma binding protein 5 (RBBP5), mRNA [NM_005057]	NM_005057
A_24_P80152	4.47E-05	NM_144582	NM_144582	Homo sapiens testis expressed sequence 261 (TEX261), mRNA [NM_144582]	NM_144582
A_32_P170749	4.47E-05	NM_001013739	NM_001013739	Homo sapiens similar to Cohesin subunit SA-3 (Stromal antigen 3) (Stromalin 3) (SCC3 homolog 3) (LOC442578), mRNA [NM_001013739]	NM_001013739
A_24_P108351	4.48E-05	NM_007040	NM_007040	Homo sapiens heterogeneous nuclear ribonucleoprotein U-like 1 (HNRPUL1), transcript variant 1, mRNA [NM_007040]	NM_007040
A_23_P108785	4.48E-05	NM_005721	NM_005721	Homo sapiens ARP3 actin-related protein 3 homolog (yeast) (ACTR3), mRNA [NM_005721]	NM_005721
A_23_P76034	4.50E-05	NM_203286	NM_203286	Homo sapiens poliovirus receptor-related 1 (herpesvirus entry mediator C; nectin) (PVRL1), transcript variant 3, mRNA [NM_203286]	NM_203286
A_23_P148249	4.50E-05	ENST00000261862		Homo sapiens cDNA FLJ27224 fis, clone SYN04819. [AK130734]	
A_23_P142154	4.50E-05	NM_031485	NM_031485	Homo sapiens glutamate-rich WD repeat containing 1 (GRWD1), mRNA [NM_031485]	NM_031485
A_23_P308581	4.50E-05	NM_033112	NM_033112	Homo sapiens chromosome 6 open reading frame 153 (C6orf153), mRNA [NM_033112]	NM_033112
A_24_P124831	4.51E-05	NM_002028	NM_002028	Homo sapiens farnesyltransferase, CAAX box, beta (FNTB), mRNA [NM_002028]	NM_002028
A_24_P91238	4.52E-05	NM_032837	NM_032837	Homo sapiens hypothetical protein FLJ14775 (FLJ14775), mRNA [NM_032837]	NM_032837
A_32_P58280	4.53E-05	NM_001013848	NM_001013848	Homo sapiens SEC15-like 1 (S. cerevisiae) (SEC15L1), transcript variant 2, mRNA [NM_001013848]	NM_001013848
A_24_P333019	4.53E-05	NM_007219	NM_007219	Homo sapiens ring finger protein 24 (RNF24), mRNA [NM_007219]	NM_007219
A_23_P26777	4.53E-05	NM_024844	NM_024844	Homo sapiens pericentrin 1 (PCNT1), mRNA [NM_024844]	NM_024844
A_32_P72447	4.53E-05	NM_014501	NM_014501	Homo sapiens ubiquitin-conjugating enzyme E2S (UBE2S), mRNA [NM_014501]	NM_014501
A_23_P82550	4.54E-05	NM_004760	NM_004760	Homo sapiens serine/threonine kinase 17a (apoptosis-inducing) (STK17A), mRNA [NM_004760]	NM_004760
A_24_P48791	4.54E-05	BX648379	BX648379	Homo sapiens mRNA; cDNA DKFZp686L18198 (from clone DKFZp686L18198). [BX648379]	
A_23_P1452	4.55E-05	NM_022146	NM_022146	Homo sapiens G protein-coupled receptor 147 (GPR147), mRNA [NM_022146]	NM_022146
A_23_P211997	4.57E-05	NM_032970	NM_032970	Homo sapiens SEC22 vesicle trafficking protein-like 3 (S. cerevisiae) (SEC22L3), transcript variant 1, mRNA [NM_032970]	NM_032970
A_24_P218587	4.57E-05	NM_004268	NM_004268	Homo sapiens cofactor required for Sp1 transcriptional activation, subunit 6, 77kDa (CRSP6), mRNA [NM_004268]	NM_004268
A_24_P370946	4.57E-05	NM_001554	NM_001554	Homo sapiens cysteine-rich, angiogenic inducer, 61 (CYR61), mRNA [NM_001554]	NM_001554
A_23_P353478	4.59E-05	NM_000246	NM_000246	Homo sapiens MHC class II transactivator (MHC2TA), mRNA [NM_000246]	NM_000246
A_23_P354953	4.59E-05	NM_013433	NM_013433	Homo sapiens transportin 2 (importin 3, karyopherin beta 2b) (TNPO2), mRNA [NM_013433]	NM_013433
A_24_P375421	4.60E-05	NM_024798	NM_024798	Homo sapiens sorting nexin 22 (SNX22), mRNA [NM_024798]	NM_024798
A_23_P123463	4.60E-05	NM_153332	NM_153332	Homo sapiens three prime histone mRNA exonuclease 1 (THEX1), mRNA [NM_153332]	NM_153332
A_23_P102832	4.60E-05	NM_007186	NM_007186	Homo sapiens centrosomal protein 2 (CEP2), mRNA [NM_007186]	NM_007186
A_24_P319675	4.60E-05	NM_016131	NM_016131	Homo sapiens RAB10, member RAS oncogene family (RAB10), mRNA [NM_016131]	NM_016131
A_23_P1505	4.61E-05	NM_002335	NM_002335	Homo sapiens low density lipoprotein receptor-related protein 5 (LRP5), mRNA [NM_002335]	NM_002335
A_23_P78685	4.61E-05	NM_004461	NM_004461	Homo sapiens phenylalanine-tRNA synthetase-like, alpha subunit (FARSLA), mRNA [NM_004461]	NM_004461
A_24_P242138	4.62E-05	NM_182614	NM_182614	Homo sapiens hypothetical protein MGC20579 (MGC20579), mRNA [NM_182614]	NM_182614
A_23_P377830	4.62E-05	NM_016237	NM_016237	Homo sapiens anaphase promoting complex subunit 5 (ANAPC5), mRNA [NM_016237]	NM_016237
A_23_P107036	4.62E-05	NM_003876	NM_003876	Homo sapiens chromosome 17 open reading frame 35 (C17orf35), mRNA [NM_003876]	NM_003876
A_23_P203445	4.63E-05	NM_018314	NM_018314	Homo sapiens ubiquitin-conjugating enzyme E2-like (UEV3), mRNA [NM_018314]	NM_018314

A_24_P65803	4.64E-05	NM_018270	NM_018270	Homo sapiens chromosome 20 open reading frame 20 (C20orf20), mRNA [NM_018270]	NM_018270
A_23_P98261	4.64E-05	NM_006396	NM_006396	Homo sapiens Sjogren's syndrome/scleroderma autoantigen 1 (SSSCA1), mRNA [NM_006396]	NM_006396
A_24_P101114	4.65E-05	NM_206999	NM_206999	Homo sapiens CCR4-NOT transcription complex, subunit 1 (CNOT1), transcript variant 2, mRNA [NM_206999]	NM_206999
A_23_P29684	4.65E-05	NM_015873	NM_015873	Homo sapiens villin-like (VILL), mRNA [NM_015873]	NM_015873
A_23_P140821	4.66E-05	NM_016948	NM_016948	Homo sapiens par-6 partitioning defective 6 homolog alpha (C.elegans) (PARD6A), mRNA [NM_016948]	NM_016948
A_24_P238855	4.66E-05	NM_020382	NM_020382	Homo sapiens PR/SET domain containing protein 8 (SET8), mRNA [NM_020382]	NM_020382
A_23_P103486	4.66E-05	NM_000775	NM_000775	Homo sapiens cytochrome P450, family 2, subfamily J, polypeptide 2 (CYP2J2), mRNA [NM_000775]	NM_000775
A_23_P211436	4.66E-05	NM_005877	NM_005877	Homo sapiens splicing factor 3a, subunit 1, 120kDa (SF3A1), transcript variant 1, mRNA [NM_005877]	NM_005877
A_23_P160849	4.67E-05	NM_004106	NM_004106	Homo sapiens Fc fragment of IgE, high affinity I, receptor for; gamma polypeptide (FCER1G), mRNA [NM_004106]	NM_004106
A_24_P264772	4.67E-05	NM_178230	NM_178230	Homo sapiens peptidylprolyl isomerase A (cyclophilin A)-like 4 (PPIAL4), mRNA [NM_178230]	NM_178230
A_24_P302038	4.68E-05	NM_014824	NM_014824	Homo sapiens FCH and double SH3 domains 2 (FCHSD2), mRNA [NM_014824]	NM_014824
A_24_P211151	4.68E-05	NM_020158	NM_020158	Homo sapiens exosome component 5 (EXOSC5), mRNA [NM_020158]	NM_020158
A_24_P289178	4.68E-05	BC009078	BC009078	Homo sapiens MGC17624 protein, mRNA (cDNA clone MGC:17624 IMAGE:3855543), complete cds. [BC009078]	
A_23_P300770	4.69E-05	NM_174916	NM_174916	Homo sapiens ubiquitin protein ligase E3 component n-recognin 1 (UBR1), mRNA [NM_174916]	NM_174916
A_23_P69030	4.69E-05	NM_001850	NM_001850	Homo sapiens collagen, type VIII, alpha 1 (COL8A1), transcript variant 1, mRNA [NM_001850]	NM_001850
A_23_P202881	4.69E-05	NM_005103	NM_005103	Homo sapiens fasciculation and elongation protein zeta 1 (zygin I) (FEZ1), transcript variant 1, mRNA [NM_005103]	NM_005103
A_23_P421513	4.70E-05	NM_145809	NM_145809	Homo sapiens TL132 protein (LOC220594), mRNA [NM_145809]	NM_145809
A_23_P369641	4.70E-05	AF289596	AF289596	Homo sapiens clone pp7882 unknown mRNA. [AF289596]	
A_23_P20255	4.70E-05	NM_014066	NM_014066	Homo sapiens COMM domain containing 5 (COMMD5), mRNA [NM_014066]	NM_014066
A_23_P214882	4.71E-05	NM_019041	NM_019041	Homo sapiens mitochondrial translational release factor 1-like (MTRF1L), mRNA [NM_019041]	NM_019041
A_24_P389415	4.71E-05	NM_007257	NM_007257	Homo sapiens paraneoplastic antigen MA2 (PNMA2), mRNA [NM_007257]	NM_007257
A_32_P210223	4.72E-05	THC2345565			
A_23_P252681	4.72E-05	NM_005017	NM_005017	Homo sapiens phosphate cytidylyltransferase 1, choline, alpha isoform (PCYT1A), mRNA [NM_005017]	NM_005017
A_24_P222139	4.73E-05	AK025047	AK025047	Homo sapiens cDNA: FLJ21394 fis, clone COL03536. [AK025047]	
A_24_P176484	4.73E-05	NM_015144	NM_015144	Homo sapiens zinc finger, CCHC domain containing 14 (ZCCHC14), mRNA [NM_015144]	NM_015144
A_24_P415168	4.73E-05	NM_030914	NM_030914	Homo sapiens chromosome 9 open reading frame 74 (C9orf74), mRNA [NM_030914]	NM_030914
A_32_P32923	4.73E-05	A_32_P32923			
A_24_P330009	4.73E-05	NM_021128	NM_021128	Homo sapiens polymerase (RNA) II (DNA directed) polypeptide L, 7.6kDa (POLR2L), mRNA [NM_021128]	NM_021128
A_23_P71148	4.74E-05	NM_000712	NM_000712	Homo sapiens biliverdin reductase A (BLVRA), mRNA [NM_000712]	NM_000712
A_23_P131518	4.75E-05	CR621399	CR621399	full-length cDNA clone CS0DI010YB22 of Placenta Cot 25-normalized of Homo sapiens (human). [CR621399]	
A_24_P362646	4.75E-05	NM_005783	NM_005783	Homo sapiens thioredoxin domain containing 9 (TXNDC9), mRNA [NM_005783]	NM_005783
A_23_P408239	4.76E-05	AL117400	AL117400	Homo sapiens mRNA; cDNA DKFZp434O051 (from clone DKFZp434O051). [AL117400]	
A_23_P399255	4.76E-05	NM_152737	NM_152737	Homo sapiens ring finger protein 182 (RNF182), mRNA [NM_152737]	NM_152737
A_23_P86570	4.76E-05	NM_004034	NM_004034	Homo sapiens annexin A7 (ANXA7), transcript variant 2, mRNA [NM_004034]	NM_004034
A_32_P52816	4.77E-05	AK092083	AK092083	Homo sapiens cDNA FLJ34764 fis, clone NT2NE2002311. [AK092083]	
A_24_P100517	4.77E-05	NM_178448	NM_178448	Homo sapiens chromosome 9 open reading frame 140 (C9orf140), mRNA [NM_178448]	NM_178448
A_24_P408206	4.78E-05	NM_002730	NM_002730	Homo sapiens protein kinase, cAMP-dependent, catalytic, alpha (PRKACA), transcript variant 1, mRNA [NM_002730]	NM_002730
A_23_P217637	4.78E-05	NM_004085	NM_004085	Homo sapiens translocase of inner mitochondrial membrane 8 homolog A (yeast) (TIMM8A), nuclear gene encoding mitochondrial protein, mRNA [NM_004085]	NM_004085
A_23_P4286	4.79E-05	NM_017523	NM_017523	Homo sapiens XIAP associated factor-1 (BIRC4BP), transcript variant 1, mRNA [NM_017523]	NM_017523
A_23_P502553	4.79E-05	NM_171982	NM_171982	Homo sapiens tripartite motif-containing 35 (TRIM35), transcript variant 2, mRNA [NM_171982]	NM_171982
A_23_P53458	4.79E-05	NM_014325	NM_014325	Homo sapiens coronin, actin binding protein, 1C (CORO1C), mRNA [NM_014325]	NM_014325
A_24_P128085	4.81E-05	BC044642	BC044642	Homo sapiens membrane associated DNA binding protein, mRNA (cDNA clone MGC:52176 IMAGE:5455191), complete cds. [BC044642]	
A_23_P209933	4.81E-05	NM_006464	NM_006464	Homo sapiens trans-golgi network protein 2 (TGOLN2), mRNA [NM_006464]	NM_006464
A_23_P168692	4.82E-05	NM_001014436	NM_001014436	Homo sapiens drebrin-like (DBNL), transcript variant 2, mRNA [NM_001014436]	NM_001014436

A_23_P132019	4.82E-05	NM_031483	NM_031483	Homo sapiens itchy homolog E3 ubiquitin protein ligase (mouse) (ITCH), mRNA [NM_031483]	NM_031483
A_24_P228579	4.82E-05	NM_199052	NM_199052	Homo sapiens chromosome 20 open reading frame 7 (C20orf7), transcript variant 2, mRNA [NM_199052]	NM_199052
A_32_P158355	4.83E-05	NM_001015050	NM_001015050	Homo sapiens similar to beta-1,4-mannosyltransferase; beta-1,4 mannosyltransferase (LOC200810), mRNA [NM_001015050]	NM_001015050
A_23_P62139	4.84E-05	NM_005710	NM_005710	Homo sapiens polyglutamine binding protein 1 (PQBP1), mRNA [NM_005710]	NM_005710
A_23_P39019	4.84E-05	NM_052850	NM_052850	Homo sapiens growth arrest and DNA-damage-inducible, gamma interacting protein 1 (GADD45GIP1), mRNA [NM_052850]	NM_052850
A_23_P256297	4.85E-05	NM_015056	NM_015056	Homo sapiens KIAA0179 (KIAA0179), mRNA [NM_015056]	NM_015056
A_23_P325726	4.86E-05	NM_015547	NM_015547	Homo sapiens thioesterase, adipose associated (THEA), transcript variant 1, mRNA [NM_015547]	NM_015547
A_24_P36745	4.86E-05	NM_144970	NM_144970	Homo sapiens chromosome X open reading frame 38 (CXorf38), mRNA [NM_144970]	NM_144970
A_24_P399888	4.88E-05	NM_001002876	NM_001002876	Homo sapiens chromosome 22 open reading frame 18 (C22orf18), transcript variant 2, mRNA [NM_001002876]	NM_001002876
A_23_P91512	4.89E-05	NM_144492	NM_144492	Homo sapiens claudin 14 (CLDN14), transcript variant 1, mRNA [NM_144492]	NM_144492
A_23_P322196	4.91E-05	NM_138400	NM_138400	Homo sapiens nucleolar protein with MIF4G domain 1 (NOM1), mRNA [NM_138400]	NM_138400
A_24_P928415	4.92E-05	BC032462	BC032462	Homo sapiens vacuolar protein sorting 29 (yeast), mRNA (cDNA clone MGC:40428 IMAGE:5197243), complete cds. [BC032462]	
A_23_P430785	4.92E-05	NM_006654	NM_006654	Homo sapiens fibroblast growth factor receptor substrate 2 (FRS2), mRNA [NM_006654]	NM_006654
A_23_P122947	4.92E-05	NM_015060	NM_015060	Homo sapiens KIAA0241 protein (KIAA0241), mRNA [NM_015060]	NM_015060
A_23_P76799	4.92E-05	NM_013448	NM_013448	Homo sapiens bromodomain adjacent to zinc finger domain, 1A (BAZ1A), transcript variant 1, mRNA [NM_013448]	NM_013448
A_23_P26994	4.93E-05	NM_031498	NM_031498	Homo sapiens guanine nucleotide binding protein (G protein), gamma transducing activity polypeptide 2 (GNGT2), mRNA [NM_031498]	NM_031498
A_23_P127793	4.93E-05	NM_153265	NM_153265	Homo sapiens echinoderm microtubule associated protein like 3 (EML3), mRNA [NM_153265]	NM_153265
A_24_P347185	4.94E-05	NM_003717	NM_003717	Homo sapiens neuropeptide FF-amide peptide precursor (NPFF), mRNA [NM_003717]	NM_003717
A_32_P225768	4.94E-05	A_32_P225768			
A_23_P96812	4.94E-05	NM_017739	NM_017739	Homo sapiens protein O-linked mannose beta1,2-N-acetylglucosaminyltransferase (POMGNT1), mRNA [NM_017739]	NM_017739
A_23_P3562	4.94E-05	NM_032178	NM_032178	Homo sapiens hypothetical protein FLJ13291 (FLJ13291), mRNA [NM_032178]	NM_032178
A_24_P98948	4.96E-05	THC2272264			
A_24_P38572	4.97E-05	NM_130793	NM_130793	Homo sapiens nucleolar protein family 6 (RNA-associated) (NOL6), transcript variant beta, mRNA [NM_130793]	NM_130793
A_23_P4308	4.98E-05	NM_002663	NM_002663	Homo sapiens phospholipase D2 (PLD2), mRNA [NM_002663]	NM_002663
A_24_P151305	5.00E-05	NM_016270	NM_016270	Homo sapiens Kruppel-like factor 2 (lung) (KLF2), mRNA [NM_016270]	NM_016270
A_23_P100556	5.00E-05	NM_001013839	NM_001013839	Homo sapiens exocyst complex component 7 (EXOC7), transcript variant 1, mRNA [NM_001013839]	NM_001013839
A_32_P718498	5.01E-05	NM_005937	NM_005937	Homo sapiens myeloid/lymphoid or mixed-lineage leukemia (trithorax homolog, Drosophila); translocated to, 6 (MLLT6), mRNA [NM_005937]	NM_005937
A_23_P342891	5.01E-05	BC002421	BC002421	Homo sapiens neurofilament 3 (150kDa medium), mRNA (cDNA clone IMAGE:3163440), partial cds. [BC002421]	
A_32_P190864	5.04E-05	A_32_P190864			
A_24_P257971	5.06E-05	NM_005094	NM_005094	Homo sapiens solute carrier family 27 (fatty acid transporter), member 4 (SLC27A4), mRNA [NM_005094]	NM_005094
A_32_P80897	5.07E-05	BC033110	BC033110	Homo sapiens, clone IMAGE:5443970, mRNA, partial cds. [BC033110]	
A_23_P14026	5.07E-05	NM_001310	NM_001310	Homo sapiens cAMP responsive element binding protein-like 2 (CREBL2), mRNA [NM_001310]	NM_001310
A_23_P216017	5.09E-05	NM_004901	NM_004901	Homo sapiens ectonucleoside triphosphate diphosphohydrolase 4 (ENTPD4), mRNA [NM_004901]	NM_004901
A_24_P167806	5.09E-05	NM_005530	NM_005530	Homo sapiens isocitrate dehydrogenase 3 (NAD+) alpha (IDH3A), nuclear gene encoding mitochondrial protein, mRNA [NM_005530]	NM_005530
A_32_P144220	5.09E-05	XM_498088	XM_498088	PREDICTED: Homo sapiens similar to myosin:SUBUNIT=regulatory light chain (LOC442204), mRNA [XM_498088]	XM_498088
A_23_P27627	5.10E-05	NM_004317	NM_004317	Homo sapiens arsA arsenite transporter, ATP-binding, homolog 1 (bacterial) (ASNA1), mRNA [NM_004317]	NM_004317
A_24_P357914	5.12E-05	ENST00000307198		Homo sapiens clone pp7517 unknown mRNA. [AF289588]	
A_24_P126471	5.12E-05	NM_020246	NM_020246	Homo sapiens solute carrier family 12 (potassium/chloride transporters), member 9 (SLC12A9), mRNA [NM_020246]	NM_020246
A_24_P914590	5.12E-05	ENST00000279013		Homo sapiens chromosome 20 open reading frame 43, mRNA (cDNA clone IMAGE:3615348), complete cds. [BC002769]	
A_23_P372096	5.12E-05	AK097804	AK097804	Homo sapiens cDNA FLJ40485 fis, clone TESTI2043857, moderately similar to Homo sapiens nolp mRNA. [AK097804]	
A_24_P332364	5.14E-05	A_24_P332364			
A_23_P44195	5.14E-05	NM_138962	NM_138962	Homo sapiens musashi homolog 2 (Drosophila) (MSI2), transcript variant 1, mRNA [NM_138962]	NM_138962
A_32_P64928	5.15E-05	XM_496978	XM_496978	PREDICTED: Homo sapiens similar to Destrin (Actin-depolymerizing factor) (ADF) (LOC441354), mRNA [XM_496978]	XM_496978

A_23_P102582	5.15E-05	NM_199484	NM_199484	Homo sapiens chromosome 20 open reading frame 24 (C20orf24), transcript variant 3, mRNA [NM_199484]	NM_199484
A_24_P316005	5.16E-05	NM_014857	NM_014857	Homo sapiens RAB GTPase activating protein 1-like (RABGAP1L), mRNA [NM_014857]	NM_014857
A_23_P57089	5.16E-05	NM_020182	NM_020182	Homo sapiens transmembrane, prostate androgen induced RNA (TMEPAI), transcript variant 1, mRNA [NM_020182]	NM_020182
A_23_P200325	5.17E-05	NM_014857	NM_014857	Homo sapiens RAB GTPase activating protein 1-like (RABGAP1L), mRNA [NM_014857]	NM_014857
A_23_P416036	5.17E-05	NM_207107	NM_207107	Homo sapiens 26S proteasome-associated UCH interacting protein 1 (UIP1), transcript variant 2, mRNA [NM_207107]	NM_207107
A_23_P152406	5.18E-05	NM_032330	NM_032330	Homo sapiens calpain, small subunit 2 (CAPNS2), mRNA [NM_032330]	NM_032330
A_24_P170869	5.18E-05	BC011136	BC011136	Homo sapiens, clone IMAGE:3448872, mRNA, partial cds. [BC011136]	
A_23_P364537	5.18E-05	NM_175066	NM_175066	Homo sapiens DEAD (Asp-Glu-Ala-Asp) box polypeptide 51 (DDX51), mRNA [NM_175066]	NM_175066
A_23_P102759	5.19E-05	NM_014962	NM_014962	Homo sapiens BTB (POZ) domain containing 3 (BTBD3), transcript variant 1, mRNA [NM_014962]	NM_014962
A_32_P760762	5.19E-05	A_32_P760762			
A_24_P850172	5.19E-05	A_24_P850172			
A_24_P765053	5.20E-05	THC2370447		ALU5_HUMAN (P39192) Alu subfamily SC sequence contamination warning entry, partial (13%) [THC2370447]	
A_23_P303523	5.20E-05	NM_002254	NM_002254	Homo sapiens kinesin family member 3C (KIF3C), mRNA [NM_002254]	NM_002254
A_23_P100632	5.20E-05	NM_001002033	NM_001002033	Homo sapiens hematological and neurological expressed 1 (HN1), transcript variant 3, mRNA [NM_001002033]	NM_001002033
A_23_P356070	5.21E-05	NM_002020	NM_002020	Homo sapiens fms-related tyrosine kinase 4 (FLT4), transcript variant 2, mRNA [NM_002020]	NM_002020
A_23_P117302	5.21E-05	NM_031943	NM_031943	Homo sapiens IFP38 (IFP38), mRNA [NM_031943]	NM_031943
A_24_P364845	5.21E-05	NM_004220	NM_004220	Homo sapiens zinc finger protein 213 (ZNF213), mRNA [NM_004220]	NM_004220
A_32_P201868	5.21E-05	BG178211	BG178211	602329933F1 NIH_MGC_91 Homo sapiens cDNA clone IMAGE:4431248 5', mRNA sequence [BG178211]	
A_23_P150876	5.22E-05	NM_024667	NM_024667	Homo sapiens hypothetical protein FLJ12750 (FLJ12750), mRNA [NM_024667]	NM_024667
A_24_P26177	5.23E-05	NM_014703	NM_014703	Homo sapiens Vpr-binding protein (VprBP), mRNA [NM_014703]	NM_014703
A_23_P9465	5.23E-05	NM_004957	NM_004957	Homo sapiens folylpolyglutamate synthase (FPGS), nuclear gene encoding mitochondrial protein, transcript variant 1, mRNA [NM_004957]	NM_004957
A_24_P272886	5.23E-05	ENST00000328703			
A_23_P29422	5.26E-05	NM_004130	NM_004130	Homo sapiens glycogenin (GYG), mRNA [NM_004130]	NM_004130
A_23_P38046	5.27E-05	NM_024535	NM_024535	Homo sapiens coronin 7 (CORO7), mRNA [NM_024535]	NM_024535
A_32_P131998	5.28E-05	A_32_P131998			
A_24_P152335	5.28E-05	NM_001001349	NM_001001349	Homo sapiens NFKB inhibitor interacting Ras-like 2 (NKIRAS2), transcript variant 1, mRNA [NM_001001349]	NM_001001349
A_23_P413788	5.28E-05	NM_173540	NM_173540	Homo sapiens fucosyltransferase 11 (alpha (1,3) fucosyltransferase) (FUT11), mRNA [NM_173540]	NM_173540
A_32_P38806	5.29E-05	A_32_P38806			
A_23_P79628	5.29E-05	NM_014614	NM_014614	Homo sapiens proteasome (prosome, macropain) activator subunit 4 (PSME4), mRNA [NM_014614]	NM_014614
A_23_P23017	5.29E-05	NM_017887	NM_017887	Homo sapiens chromosome 1 open reading frame 123 (C1orf123), mRNA [NM_017887]	NM_017887
A_32_P516342	5.30E-05	NM_182608	NM_182608	Homo sapiens ankyrin repeat domain 33 (ANKRD33), mRNA [NM_182608]	NM_182608
A_23_P360179	5.32E-05	NM_032442	NM_032442	Homo sapiens KIAA1787 protein (KIAA1787), transcript variant 1, mRNA [NM_032442]	NM_032442
A_24_P367329	5.32E-05	ENST00000332813			XM_496078
A_23_P65651	5.32E-05	NM_004184	NM_004184	Homo sapiens tryptophanyl-tRNA synthetase (WARS), transcript variant 1, mRNA [NM_004184]	NM_004184
A_24_P246863	5.33E-05	A_24_P246863			
A_24_P271014	5.34E-05	NM_005134	NM_005134	Homo sapiens protein phosphatase 4, regulatory subunit 1 (PPP4R1), mRNA [NM_005134]	NM_005134
A_23_P365189	5.35E-05	NM_015099	NM_015099	Homo sapiens calmodulin binding transcription activator 2 (CAMTA2), mRNA [NM_015099]	NM_015099
A_23_P3775	5.35E-05	NM_018233	NM_018233	Homo sapiens hypothetical protein FLJ10826 (FLJ10826), mRNA [NM_018233]	NM_018233
A_23_P371876	5.36E-05	NM_138775	NM_138775	Homo sapiens hypothetical protein BC015183 (LOC91801), mRNA [NM_138775]	NM_138775
A_23_P255869	5.36E-05	NM_018465	NM_018465	Homo sapiens chromosome 9 open reading frame 46 (C9orf46), mRNA [NM_018465]	NM_018465
A_23_P5392	5.37E-05	NM_004881	NM_004881	Homo sapiens tumor protein p53 inducible protein 3 (TP53I3), transcript variant 1, mRNA [NM_004881]	NM_004881
A_24_P65199	5.39E-05	NM_052987	NM_052987	Homo sapiens cyclin-dependent kinase (CDC2-like) 10 (CDK10), transcript variant 2, mRNA [NM_052987]	NM_052987
A_32_P24382	5.40E-05	BC063625	BC063625	Homo sapiens keratin associated protein 2-4, mRNA (cDNA clone MGC:74790 IMAGE:3907481), complete cds. [BC063625]	
A_23_P9392	5.40E-05	NM_016390	NM_016390	Homo sapiens chromosome 9 open reading frame 114 (C9orf114), mRNA [NM_016390]	NM_016390

A_23_P19437	5.40E-05	NM_021959	NM_021959	Homo sapiens protein phosphatase 1, regulatory (inhibitor) subunit 11 (PPP1R11), transcript variant 1, mRNA [NM_021959]	NM_021959
A_24_P159948	5.40E-05	NM_003224	NM_003224	Homo sapiens ADP-ribosylation factor related protein 1 (ARFRP1), mRNA [NM_003224]	NM_003224
A_23_P126426	5.40E-05	NM_001384	NM_001384	Homo sapiens DPH2 homolog (S. cerevisiae) (DPH2), transcript variant 1, mRNA [NM_001384]	NM_001384
A_24_P6921	5.40E-05	NM_001013744	NM_001013744	Homo sapiens hypothetical LOC541471 protein (LOC541471), mRNA [NM_001013744]	NM_001013744
A_24_P370557	5.41E-05	NM_000950	NM_000950	Homo sapiens proline rich Gla (G-carboxyglutamic acid) 1 (PRRG1), mRNA [NM_000950]	NM_000950
A_32_P210691	5.42E-05	A_32_P210691			
A_24_P698141	5.42E-05	AK125299	AK125299	Homo sapiens cDNA FLJ43309 fis, clone NT2RI2004618, highly similar to Cytosolic acyl coenzyme A thioester hydrolase (EC 3.1.2.2). [AK125299]	XR_000194
A_23_P102192	5.42E-05	NM_145686	NM_145686	Homo sapiens mitogen-activated protein kinase kinase kinase kinase 4 (MAP4K4), transcript variant 2, mRNA [NM_145686]	NM_145686
A_32_P84289	5.42E-05	NM_152371	NM_152371	Homo sapiens chromosome 1 open reading frame 93 (C1orf93), mRNA [NM_152371]	NM_152371
A_23_P104563	5.43E-05	NM_001876	NM_001876	Homo sapiens carnitine palmitoyltransferase 1A (liver) (CPT1A), nuclear gene encoding mitochondrial protein, mRNA [NM_001876]	NM_001876
A_23_P308673	5.43E-05	NM_004783	NM_004783	Homo sapiens TAO kinase 2 (TAOK2), mRNA [NM_004783]	NM_004783
A_23_P37954	5.43E-05	NM_001761	NM_001761	Homo sapiens cyclin F (CCNF), mRNA [NM_001761]	NM_001761
A_23_P123413	5.43E-05	NM_014729	NM_014729	Homo sapiens thymus high mobility group box protein TOX (TOX), mRNA [NM_014729]	NM_014729
A_23_P310068	5.43E-05	NM_001009894	NM_001009894	Homo sapiens hypothetical protein DKFZp434N2030 (DKFZp434N2030), mRNA [NM_001009894]	NM_001009894
A_23_P16337	5.43E-05	NM_005500	NM_005500	Homo sapiens SUMO-1 activating enzyme subunit 1 (SAE1), mRNA [NM_005500]	NM_005500
A_23_P208900	5.45E-05	NM_032108	NM_032108	Homo sapiens sema domain, transmembrane domain (TM), and cytoplasmic domain, (semaphorin) 6B (SEMA6B), transcript variant SEMA6B.3, mRNA [NM_032108]	NM_032108
A_23_P211445	5.45E-05	NM_016733	NM_016733	Homo sapiens LIM domain kinase 2 (LIMK2), transcript variant 2b, mRNA [NM_016733]	NM_016733
A_24_P412943	5.47E-05	NM_174905	NM_174905	Homo sapiens hypothetical protein LOC147965 (LOC147965), mRNA [NM_174905]	NM_174905
A_23_P318208	5.48E-05	NM_001128	NM_001128	Homo sapiens adaptor-related protein complex 1, gamma 1 subunit (AP1G1), mRNA [NM_001128]	NM_001128
A_23_P34366	5.49E-05	NM_000864	NM_000864	Homo sapiens 5-hydroxytryptamine (serotonin) receptor 1D (HTR1D), mRNA [NM_000864]	NM_000864
A_32_P37721	5.49E-05	NM_153451	NM_153451	Homo sapiens oral cancer overexpressed 1 (ORAOV1), mRNA [NM_153451]	NM_153451
A_23_P99579	5.49E-05	NM_032490	NM_032490	Homo sapiens chromosome 14 open reading frame 142 (C14orf142), mRNA [NM_032490]	NM_032490
A_23_P48676	5.49E-05	NM_002863	NM_002863	Homo sapiens phosphorylase, glycogen; liver (Hers disease, glycogen storage disease type VI) (PYGL), mRNA [NM_002863]	NM_002863
A_24_P200000	5.50E-05	NM_182915	NM_182915	Homo sapiens STEAP family member 3 (STEAP3), transcript variant 1, mRNA [NM_182915]	NM_182915
A_23_P47839	5.50E-05	NM_020936	NM_020936	Homo sapiens DEAD (Asp-Glu-Ala-Asp) box polypeptide 55 (DDX55), mRNA [NM_020936]	NM_020936
A_23_P164313	5.52E-05	NM_153827	NM_153827	Homo sapiens misshapen-like kinase 1 (zebrafish) (MINK1), transcript variant 3, mRNA [NM_153827]	NM_153827
A_23_P104555	5.52E-05	NM_020349	NM_020349	Homo sapiens ankyrin repeat domain 2 (stretch responsive muscle) (ANKRD2), mRNA [NM_020349]	NM_020349
A_24_P586523	5.52E-05	NM_019109	NM_019109	Homo sapiens asparagine-linked glycosylation 1 homolog (yeast, beta-1,4-mannosyltransferase) (ALG1), mRNA [NM_019109]	NM_019109
A_23_P201461	5.56E-05	NM_016831	NM_016831	Homo sapiens period homolog 3 (Drosophila) (PER3), mRNA [NM_016831]	NM_016831
A_23_P112512	5.56E-05	NM_033412	NM_033412	Homo sapiens mitochondrial carrier triple repeat 1 (MCART1), mRNA [NM_033412]	NM_033412
A_23_P80032	5.59E-05	NM_005225	NM_005225	Homo sapiens E2F transcription factor 1 (E2F1), mRNA [NM_005225]	NM_005225
A_32_P228570	5.60E-05	A_32_P228570			
A_24_P647146	5.60E-05	NM_015401	NM_015401	Homo sapiens histone deacetylase 7A (HDAC7A), transcript variant 1, mRNA [NM_015401]	NM_015401
A_23_P52610	5.60E-05	NM_000107	NM_000107	Homo sapiens damage-specific DNA binding protein 2, 48kDa (DDB2), mRNA [NM_000107]	NM_000107
A_23_P119266	5.61E-05	NM_001375	NM_001375	Homo sapiens deoxyribonuclease II, lysosomal (DNASE2), mRNA [NM_001375]	NM_001375
A_23_P258982	5.61E-05	NM_013355	NM_013355	Homo sapiens protein kinase N3 (PKN3), mRNA [NM_013355]	NM_013355
A_23_P384517	5.61E-05	NM_004130	NM_004130	Homo sapiens glycogenin (GYG), mRNA [NM_004130]	NM_004130
A_32_P206136	5.63E-05	AW178774	AW178774	AW178774 PM0-HT0123-310899-001-b08 HT0123 Homo sapiens cDNA, mRNA sequence [AW178774]	
A_23_P67391	5.63E-05	NM_007059	NM_007059	Homo sapiens kaptin (actin binding protein) (KPTN), mRNA [NM_007059]	NM_007059
A_24_P279220	5.63E-05	NM_001694	NM_001694	Homo sapiens ATPase, H+ transporting, lysosomal 16kDa, V0 subunit c (ATP6V0C), mRNA [NM_001694]	NM_001694
A_23_P418031	5.63E-05	ENST00000304963		Homo sapiens mRNA for FLJ00074 protein, partial cds. [AK024480]	
A_32_P104809	5.64E-05	A_32_P104809			
A_23_P141194	5.64E-05	NM_001545	NM_001545	Homo sapiens immature colon carcinoma transcript 1 (ICT1), mRNA [NM_001545]	NM_001545

A_23_P250767	5.65E-05	NM_019061	NM_019061	Homo sapiens myotubularin related protein 12 (MTMR12), mRNA [NM_019061]	NM_019061
A_24_P583350	5.65E-05	NM_001015050	NM_001015050	Homo sapiens similar to beta-1,4-mannosyltransferase; beta-1,4 mannosyltransferase (LOC200810), mRNA [NM_001015050]	NM_001015050
A_23_P133606	5.66E-05	NM_001046	NM_001046	Homo sapiens solute carrier family 12 (sodium/potassium/chloride transporters), member 2 (SLC12A2), mRNA [NM_001046]	NM_001046
A_24_P53150	5.66E-05	NM_032271	NM_032271	Homo sapiens TNF receptor-associated factor 7 (TRAF7), transcript variant 1, mRNA [NM_032271]	NM_032271
A_32_P227400	5.67E-05	A_32_P227400			
A_24_P378788	5.67E-05	NM_024881	NM_024881	Homo sapiens solute carrier family 35, member E1 (SLC35E1), mRNA [NM_024881]	NM_024881
A_24_P130792	5.67E-05	NM_199044	NM_199044	Homo sapiens NOL1/NOP2/Sun domain family, member 4 (NSUN4), mRNA [NM_199044]	NM_199044
A_32_P93328	5.68E-05	AK129584	AK129584	Homo sapiens cDNA FLJ26073 fis, clone RCT01314. [AK129584]	
A_23_P168928	5.68E-05	NM_000497	NM_000497	Homo sapiens cytochrome P450, family 11, subfamily B, polypeptide 1 (CYP11B1), nuclear gene encoding mitochondrial protein, transcript variant 1, mRNA [NM_000497]	NM_000497
A_23_P351275	5.68E-05	NM_181597	NM_181597	Homo sapiens uridine phosphorylase 1 (UPP1), transcript variant 2, mRNA [NM_181597]	NM_181597
A_23_P332937	5.69E-05	NM_152480	NM_152480	Homo sapiens chromosome 19 open reading frame 23 (C19orf23), mRNA [NM_152480]	NM_152480
A_23_P127805	5.72E-05	THC2438100		ALU2_HUMAN (P39189) Alu subfamily SB sequence contamination warning entry, partial (6%) [THC2438100]	
A_23_P1117	5.72E-05	NM_198328	NM_198328	Homo sapiens suppression of tumorigenicity 7 like (ST7L), transcript variant 6, mRNA [NM_198328]	NM_198328
A_24_P713185	5.72E-05	BF576096	BF576096	BF576096 602132409F1 NIH_MGC_81 Homo sapiens cDNA clone IMAGE:4271522 5', mRNA sequence [BF576096]	
A_23_P339582	5.73E-05	NM_144970	NM_144970	Homo sapiens chromosome X open reading frame 38 (CXorf38), mRNA [NM_144970]	NM_144970
A_24_P534290	5.73E-05	A_24_P534290			
A_24_P239689	5.74E-05	NM_019072	NM_019072	Homo sapiens small glutamine-rich tetratricopeptide repeat (TPR)-containing, beta (SGTB), mRNA [NM_019072]	NM_019072
A_24_P927474	5.74E-05	ENST00000361500			
A_23_P102071	5.75E-05	AK027315	AK027315	Homo sapiens cDNA FLJ14409 fis, clone HEMBA1004408, moderately similar to PEPTIDYL-PROLYL CIS-TRANS ISOMERASE 10 (EC 5.2.1.8). [AK027315]	
A_24_P355693	5.76E-05	NM_018367	NM_018367	Homo sapiens phytoceramidase, alkaline (PHCA), mRNA [NM_018367]	NM_018367
A_24_P633575	5.76E-05	AF086329	AF086329	Homo sapiens full length insert cDNA clone ZD54C08. [AF086329]	
A_24_P118326	5.76E-05	THC2423311		VATL_HUMAN (P27449) Vacuolar ATP synthase 16 kDa proteolipid subunit , partial (39%) [THC2423311]	
A_23_P20532	5.80E-05	AK024257	AK024257	Homo sapiens cDNA FLJ14195 fis, clone NT2RP3001723, weakly similar to Homo sapiens cell recognition molecule Caspr2 (CASPR2) mRNA. [AK024257]	XM_497089
A_24_P150486	5.81E-05	NM_004863	NM_004863	Homo sapiens serine palmitoyltransferase, long chain base subunit 2 (SPTLC2), mRNA [NM_004863]	NM_004863
A_23_P106544	5.84E-05	NM_020188	NM_020188	Homo sapiens DC13 protein (DC13), mRNA [NM_020188]	NM_020188
A_32_P108387	5.85E-05	CF528315	CF528315	CF528315 UI-1-BC0-aen-f-04-0-UI.s1 NCI_CGAP_Pl1 Homo sapiens cDNA clone UI-1-BC0-aen-f-04-0-UI 3', mRNA sequence [CF528315]	
A_24_P376287	5.85E-05	NM_019594	NM_019594	Homo sapiens leucine rich repeat containing 8 family, member A (LRRC8A), mRNA [NM_019594]	NM_019594
A_24_P530977	5.85E-05	A_24_P530977			
A_24_P6135	5.86E-05	NM_001003689	NM_001003689	Homo sapiens l(3)mbt-like 2 (Drosophila) (L3MBTL2), transcript variant 2, mRNA [NM_001003689]	NM_001003689
A_23_P109171	5.86E-05	NM_001195	NM_001195	Homo sapiens beaded filament structural protein 1, filensin (BFSP1), mRNA [NM_001195]	NM_001195
A_24_P125353	5.87E-05	NM_007359	NM_007359	Homo sapiens cancer susceptibility candidate 3 (CASC3), mRNA [NM_007359]	NM_007359
A_23_P214779	5.87E-05	NM_007124	NM_007124	Homo sapiens utrophin (homologous to dystrophin) (UTRN), mRNA [NM_007124]	NM_007124
A_32_P207420	5.88E-05	THC2282652		Q9RW53 (Q9RW53) Mg(2+) transport ATPase-related protein, partial (8%) [THC2282652]	
A_23_P9415	5.89E-05	NM_002197	NM_002197	Homo sapiens aconitase 1, soluble (ACO1), mRNA [NM_002197]	NM_002197
A_23_P399726	5.91E-05	NM_024627	NM_024627	Homo sapiens hypothetical protein FLJ21125 (FLJ21125), mRNA [NM_024627]	NM_024627
A_23_P252704	5.91E-05	NM_016096	NM_016096	Homo sapiens HSPC038 protein (LOC51123), mRNA [NM_016096]	NM_016096
A_23_P51679	5.92E-05	NM_005920	NM_005920	Homo sapiens MADS box transcription enhancer factor 2, polypeptide D (myocyte enhancer factor 2D) (MEF2D), mRNA [NM_005920]	NM_005920
A_23_P147326	5.94E-05	NM_178865	NM_178865	Homo sapiens tumor differentially expressed 2-like (TDE2L), mRNA [NM_178865]	NM_178865
A_23_P425073	5.95E-05	NM_002898	NM_002898	Homo sapiens RNA binding motif, single stranded interacting protein 2 (RBMS2), mRNA [NM_002898]	NM_002898
A_23_P36408	5.95E-05	NM_022916	NM_022916	Homo sapiens vacuolar protein sorting 33A (yeast) (VPS33A), mRNA [NM_022916]	NM_022916
A_23_P211878	5.95E-05	NM_001457	NM_001457	Homo sapiens filamin B, beta (actin binding protein 278) (FLNB), mRNA [NM_001457]	NM_001457
A_24_P116669	5.96E-05	NM_138793	NM_138793	Homo sapiens calcium activated nucleotidase 1 (CANT1), mRNA [NM_138793]	NM_138793
A_32_P207124	5.96E-05	NM_173571	NM_173571	Homo sapiens hypothetical protein LOC255313 (LOC255313), mRNA [NM_173571]	NM_173571

A_24_P167012	5.97E-05	NM_005118	NM_005118	Homo sapiens tumor necrosis factor (ligand) superfamily, member 15 (TNFSF15), mRNA [NM_005118]	NM_005118
A_24_P528213	5.97E-05	BF593129	BF593129	BF593129 7o49h09.x1 NCI_CGAP_Kid11 Homo sapiens cDNA clone IMAGE:3577600 3', mRNA sequence [BF593129]	
A_23_P4435	5.98E-05	NM_015229	NM_015229	Homo sapiens KIAA0664 protein (KIAA0664), mRNA [NM_015229]	NM_015229
A_24_P19828	5.98E-05	NM_025077	NM_025077	Homo sapiens target of EGR1, member 1 (nuclear) (TOE1), mRNA [NM_025077]	NM_025077
A_23_P134125	5.98E-05	NM_005923	NM_005923	Homo sapiens mitogen-activated protein kinase kinase kinase 5 (MAP3K5), mRNA [NM_005923]	NM_005923
A_24_P187154	6.00E-05	A_24_P187154			
A_24_P219474	6.01E-05	NM_144677	NM_144677	Homo sapiens mannosyl (alpha-1,6-)-glycoprotein beta-1,6-N-acetyl-glucosaminyltransferase, isoenzyme B (MGAT5B), transcript variant 1, mRNA [NM_144677]	NM_144677
A_24_P357056	6.03E-05	NM_152906	NM_152906	Homo sapiens hypothetical protein DKFZp761P1121 (DKFZp761P1121), mRNA [NM_152906]	NM_152906
A_24_P217834	6.03E-05	NM_003530	NM_003530	Homo sapiens histone 1, H3d (HIST1H3D), mRNA [NM_003530]	NM_003530
A_23_P32938	6.03E-05	NM_004398	NM_004398	Homo sapiens DEAD (Asp-Glu-Ala-Asp) box polypeptide 10 (DDX10), mRNA [NM_004398]	NM_004398
A_24_P776784	6.05E-05	BM687208	BM687208	UI-E-CQ1-adb-g-05-0-UI.r1 UI-E-CQ1 Homo sapiens cDNA clone UI-E-CQ1-adb-g-05-0-UI 5', mRNA sequence [BM687208]	
A_24_P366535	6.06E-05	ENST00000312855			
A_23_P127460	6.07E-05	NM_153253	NM_153253	Homo sapiens signal-induced proliferation-associated gene 1 (SIPA1), transcript variant 1, mRNA [NM_153253]	NM_153253
A_24_P126731	6.07E-05	ENST00000327665			
A_23_P163630	6.08E-05	NM_152287	NM_152287	Homo sapiens zinc finger protein 276 homolog (mouse) (ZFP276), mRNA [NM_152287]	NM_152287
A_23_P70249	6.08E-05	NM_001790	NM_001790	Homo sapiens cell division cycle 25C (CDC25C), transcript variant 1, mRNA [NM_001790]	NM_001790
A_23_P40453	6.09E-05	NM_001236	NM_001236	Homo sapiens carbonyl reductase 3 (CBR3), mRNA [NM_001236]	NM_001236
A_23_P73142	6.10E-05	NM_016360	NM_016360	Homo sapiens clone HQ0477 PRO0477p (LOC51204), mRNA [NM_016360]	NM_016360
A_23_P19691	6.10E-05	NM_014320	NM_014320	Homo sapiens heme binding protein 2 (HEBP2), mRNA [NM_014320]	NM_014320
A_32_P132194	6.11E-05	BX110985	BX110985	BX110985 Soares_testis_NHT Homo sapiens cDNA clone IMAGp998B074410, mRNA sequence [BX110985]	
A_24_P133542	6.11E-05	NM_002675	NM_002675	Homo sapiens promyelocytic leukemia (PML), transcript variant 6, mRNA [NM_002675]	NM_002675
A_23_P253395	6.11E-05	NM_006377	NM_006377	Homo sapiens unc-13 homolog B (C. elegans) (UNC13B), mRNA [NM_006377]	NM_006377
A_23_P77590	6.11E-05	NM_018052	NM_018052	Homo sapiens Vac14 homolog (S. cerevisiae) (VAC14), mRNA [NM_018052]	NM_018052
A_24_P219686	6.12E-05	NM_006788	NM_006788	Homo sapiens ralA binding protein 1 (RALBP1), mRNA [NM_006788]	NM_006788
A_23_P63289	6.12E-05	NM_014188	NM_014188	Homo sapiens Ssu72 RNA polymerase II CTD phosphatase homolog (yeast) (SSU72), mRNA [NM_014188]	NM_014188
A_23_P352435	6.14E-05	NM_002926	NM_002926	Homo sapiens regulator of G-protein signalling 12 (RGS12), transcript variant 2, mRNA [NM_002926]	NM_002926
A_24_P408424	6.14E-05	NM_002473	NM_002473	Homo sapiens myosin, heavy polypeptide 9, non-muscle (MYH9), mRNA [NM_002473]	NM_002473
A_23_P205449	6.15E-05	NM_017955	NM_017955	Homo sapiens cell division cycle associated 4 (CDCA4), transcript variant 1, mRNA [NM_017955]	NM_017955
A_23_P310532	6.15E-05	NM_138358	NM_138358	Homo sapiens hypothetical protein BC011833 (LOC90580), mRNA [NM_138358]	NM_138358
A_23_P66421	6.15E-05	NM_015654	NM_015654	Homo sapiens N-acetyltransferase 9 (NAT9), mRNA [NM_015654]	NM_015654
A_24_P690983	6.16E-05	NM_080926	NM_080926	Homo sapiens hypothetical protein similar to KIAA0187 gene product (LOC96610), mRNA [NM_080926]	NM_080926
A_24_P928798	6.18E-05	AK056260	AK056260	Homo sapiens cDNA FLJ31698 fis, clone NT2RI2005966. [AK056260]	
A_24_P222516	6.20E-05	AK126014	AK126014	Homo sapiens cDNA FLJ44026 fis, clone TESTI4026762. [AK126014]	XM_044178
A_23_P358531	6.20E-05	NM_178034	NM_178034	Homo sapiens phospholipase A2, group IVD (cytosolic) (PLA2G4D), mRNA [NM_178034]	NM_178034
A_23_P352365	6.20E-05	BC015239	BC015239	Homo sapiens zinc finger and BTB domain containing 8, mRNA (cDNA clone MGC:17919 IMAGE:3914043), complete cds. [BC015239]	
A_24_P915692	6.20E-05	BC047362	BC047362	Homo sapiens cDNA clone IMAGE:5315177, partial cds. [BC047362]	
A_32_P7193	6.21E-05	A_32_P7193			
A_24_P935318	6.22E-05	NM_022839	NM_022839	Homo sapiens mitochondrial ribosomal protein S11 (MRPS11), nuclear gene encoding mitochondrial protein, transcript variant 1, mRNA [NM_022839]	NM_022839
A_23_P121215	6.23E-05	NM_003656	NM_003656	Homo sapiens calcium/calmodulin-dependent protein kinase I (CAMK1), mRNA [NM_003656]	NM_003656
A_24_P125067	6.23E-05	NM_013302	NM_013302	Homo sapiens eukaryotic elongation factor-2 kinase (EEF2K), mRNA [NM_013302]	NM_013302
A_23_P407115	6.27E-05	NM_138687	NM_138687	Homo sapiens phosphatidylinositol-4-phosphate 5-kinase, type II, beta (PIP5K2B), transcript variant 2, mRNA [NM_138687]	NM_138687
A_23_P71727	6.27E-05	NM_001827	NM_001827	Homo sapiens CDC28 protein kinase regulatory subunit 2 (CKS2), mRNA [NM_001827]	NM_001827
A_23_P16096	6.28E-05	NM_020230	NM_020230	Homo sapiens peter pan homolog (Drosophila) (PPAN), mRNA [NM_020230]	NM_020230

A_24_P330625	6.29E-05	NM_032815	NM_032815	Homo sapiens nuclear factor of activated T-cells, cytoplasmic, calcineurin-dependent 2 interacting protein (NFATC2IP), mRNA [NM_032815]	NM_032815
A_24_P98783	6.29E-05	NM_031433	NM_031433	Homo sapiens membrane frizzled-related protein (MFRP), mRNA [NM_031433]	NM_031433
A_23_P160729	6.29E-05	NM_006594	NM_006594	Homo sapiens adaptor-related protein complex 4, beta 1 subunit (AP4B1), mRNA [NM_006594]	NM_006594
A_23_P31536	6.29E-05	NM_003143	NM_003143	Homo sapiens single-stranded DNA binding protein 1 (SSBP1), mRNA [NM_003143]	NM_003143
A_24_P280762	6.31E-05	NM_030568	NM_030568	Homo sapiens chromosome 6 open reading frame 148 (C6orf148), mRNA [NM_030568]	NM_030568
A_24_P170309	6.31E-05	A_24_P170309			
A_23_P54953	6.31E-05	NM_013260	NM_013260	Homo sapiens transcriptional regulator protein (HCNGP), mRNA [NM_013260]	NM_013260
A_24_P13533	6.31E-05	NM_203467	NM_203467	Homo sapiens peptidylprolyl isomerase (cyclophilin)-like 5 (PPIL5), transcript variant 3, mRNA [NM_203467]	NM_203467
A_23_P218579	6.32E-05	NM_024506	NM_024506	Homo sapiens galactosidase, beta 1-like (GLB1L), mRNA [NM_024506]	NM_024506
A_24_P331704	6.32E-05	NM_182507	NM_182507	Homo sapiens hypothetical protein LOC144501 (LOC144501), mRNA [NM_182507]	NM_182507
A_24_P347067	6.32E-05	NM_025164	NM_025164	Homo sapiens KIAA0999 protein (KIAA0999), mRNA [NM_025164]	NM_025164
A_23_P142289	6.32E-05	NM_002067	NM_002067	Homo sapiens guanine nucleotide binding protein (G protein), alpha 11 (Gq class) (GNA11), mRNA [NM_002067]	NM_002067
A_32_P123176	6.33E-05	AA573471	AA573471	AA573471 nm53f10.s1 NCI_CGAP_Br2 Homo sapiens cDNA clone IMAGE:1071979 3', mRNA sequence [AA573471]	
A_23_P77721	6.33E-05	AK127004	AK127004	Homo sapiens cDNA FLJ45059 fis, clone BRAWH3023274. [AK127004]	
A_24_P234196	6.33E-05	NM_001034	NM_001034	Homo sapiens ribonucleotide reductase M2 polypeptide (RRM2), mRNA [NM_001034]	NM_001034
A_24_P391960	6.33E-05	ENST00000361845		PREDICTED: Homo sapiens similar to Eukaryotic translation initiation factor 1 (eIF1) (Protein translation factor SUI1 homolog) (Sui1iso1) (LOC441916), mRNA [XM_497726]	XM_497726
A_23_P155351	6.36E-05	NM_000060	NM_000060	Homo sapiens biotinidase (BTD), mRNA [NM_000060]	NM_000060
A_24_P107303	6.37E-05	NM_001558	NM_001558	Homo sapiens interleukin 10 receptor, alpha (IL10RA), mRNA [NM_001558]	NM_001558
A_24_P58477	6.38E-05	A_24_P58477			
A_32_P62863	6.38E-05	NM_014575	NM_014575	Homo sapiens schwannomin interacting protein 1 (SCHIP1), mRNA [NM_014575]	NM_014575
A_23_P251771	6.38E-05	NM_025215	NM_025215	Homo sapiens pseudouridylate synthase 1 (PUS1), transcript variant 1, mRNA [NM_025215]	NM_025215
A_23_P143535	6.39E-05	NM_033661	NM_033661	Homo sapiens WD repeat domain 4 (WDR4), transcript variant 2, mRNA [NM_033661]	NM_033661
A_23_P100779	6.40E-05	NM_014233	NM_014233	Homo sapiens upstream binding transcription factor, RNA polymerase I (UBTF), mRNA [NM_014233]	NM_014233
A_24_P385739	6.40E-05	NM_020390	NM_020390	Homo sapiens eukaryotic translation initiation factor 5A2 (EIF5A2), mRNA [NM_020390]	NM_020390
A_23_P92543	6.41E-05	NM_152540	NM_152540	Homo sapiens sec1 family domain containing 2 (SCFD2), mRNA [NM_152540]	NM_152540
A_23_P8185	6.41E-05	NM_006519	NM_006519	Homo sapiens t-complex-associated-testis-expressed 1-like 1 (TCTEL1), mRNA [NM_006519]	NM_006519
A_24_P272515	6.42E-05	XM_293018	XM_293018	PREDICTED: Homo sapiens similar to Fatty acid-binding protein, epidermal (E-FABP) (Psoriasis-associated fatty acid-binding protein homolog) (PA-FABP) (LOC344332), mRNA [XM_293018]	XM_293018
A_24_P393470	6.42E-05	BC004449	BC004449	Homo sapiens MADS box transcription enhancer factor 2, polypeptide B (myocyte enhancer factor 2B), mRNA (cDNA clone IMAGE:2819918), complete cds. [BC004449]	
A_32_P159820	6.42E-05	THC2341688		Q86UA3 (Q86UA3) MYG1 protein, partial (11%) [THC2341688]	
A_24_P81740	6.42E-05	NM_006755	NM_006755	Homo sapiens transaldolase 1 (TALDO1), mRNA [NM_006755]	NM_006755
A_23_P43557	6.44E-05	NM_024820	NM_024820	Homo sapiens KIAA1608 (KIAA1608), transcript variant 2, mRNA [NM_024820]	NM_024820
A_23_P88831	6.45E-05	NM_003983	NM_003983	Homo sapiens solute carrier family 7 (cationic amino acid transporter, y+ system), member 6 (SLC7A6), mRNA [NM_003983]	NM_003983
A_23_P24234	6.46E-05	NM_033282	NM_033282	Homo sapiens opsin 4 (melanopsin) (OPN4), mRNA [NM_033282]	NM_033282
A_23_P251186	6.46E-05	NM_007198	NM_007198	Homo sapiens proline synthetase co-transcribed homolog (bacterial) (PROSC), mRNA [NM_007198]	NM_007198
A_23_P78734	6.46E-05	NM_024729	NM_024729	Homo sapiens myosin, heavy polypeptide 14 (MYH14), mRNA [NM_024729]	NM_024729
A_23_P324461	6.49E-05	NM_177401	NM_177401	Homo sapiens midnolin (MIDN), mRNA [NM_177401]	NM_177401
A_24_P353740	6.51E-05	NM_018704	NM_018704	Homo sapiens hypothetical protein DKFZp547A023 (DKFZp547A023), mRNA [NM_018704]	NM_018704
A_23_P251730	6.51E-05	NM_001010986	NM_001010986	Homo sapiens ATPase, Class VI, type 11C (ATP11C), transcript variant 2, mRNA [NM_001010986]	NM_001010986
A_24_P178723	6.51E-05	A_24_P178723			
A_23_P46351	6.52E-05	NM_006862	NM_006862	Homo sapiens tudor and KH domain containing (TDRKH), mRNA [NM_006862]	NM_006862
A_32_P216241	6.52E-05	AF269287	AF269287	Homo sapiens PP1416 mRNA, complete cds. [AF269287]	
A_23_P94795	6.55E-05	NM_003213	NM_003213	Homo sapiens TEA domain family member 4 (TEAD4), transcript variant 1, mRNA [NM_003213]	NM_003213
A_24_P927886	6.55E-05	L40630	L40630	Homo sapiens GTP-binding protein alpha 11 (GA11) mRNA, partial cds. [L40630]	

A_24_P932293	6.56E-05	CR610954	CR610954	full-length cDNA clone CS0DI059YG13 of Placenta Cot 25-normalized of Homo sapiens (human). [CR610954]	
A_23_P133227	6.56E-05	NM_002109	NM_002109	Homo sapiens histidyl-tRNA synthetase (HARS), mRNA [NM_002109]	NM_002109
A_23_P368645	6.57E-05	NM_003659	NM_003659	Homo sapiens alkylglycerone phosphate synthase (AGPS), mRNA [NM_003659]	NM_003659
A_23_P58647	6.57E-05	NM_001903	NM_001903	Homo sapiens catenin (cadherin-associated protein), alpha 1, 102kDa (CTNNA1), mRNA [NM_001903]	NM_001903
A_23_P42975	6.58E-05	NM_002736	NM_002736	Homo sapiens protein kinase, cAMP-dependent, regulatory, type II, beta (PRKAR2B), mRNA [NM_002736]	NM_002736
A_23_P79818	6.58E-05	NM_016470	NM_016470	Homo sapiens chromosome 20 open reading frame 111 (C20orf111), mRNA [NM_016470]	NM_016470
A_24_P392022	6.62E-05	NM_201400	NM_201400	Homo sapiens hypothetical protein SB153 (SB153), transcript variant 1, mRNA [NM_201400]	NM_201400
A_24_P199097	6.62E-05	NM_006809	NM_006809	Homo sapiens translocase of outer mitochondrial membrane 34 (TOMM34), nuclear gene encoding mitochondrial protein, mRNA [NM_006809]	NM_006809
A_32_P207494	6.62E-05	NM_025115	NM_025115	Homo sapiens chromosome 8 open reading frame 41 (C8orf41), mRNA [NM_025115]	NM_025115
A_32_P118686	6.63E-05	XM_498911	XM_498911	PREDICTED: Homo sapiens LOC440909 (LOC440909), mRNA [XM_498911]	XM_498911
A_23_P54540	6.63E-05	NM_001013703	NM_001013703	Homo sapiens eukaryotic translation initiation factor 2 alpha kinase 4 (EIF2AK4), mRNA [NM_001013703]	NM_001013703
A_23_P251051	6.63E-05	NM_181826	NM_181826	Homo sapiens neurofibromin 2 (bilateral acoustic neuroma) (NF2), transcript variant 3, mRNA [NM_181826]	NM_181826
A_23_P213204	6.65E-05	NM_133334	NM_133334	Homo sapiens Wolf-Hirschhorn syndrome candidate 1 (WHSC1), transcript variant 7, mRNA [NM_133334]	NM_133334
A_24_P215352	6.65E-05	NM_145040	NM_145040	Homo sapiens protein kinase C, delta binding protein (PRKCDBP), mRNA [NM_145040]	NM_145040
A_32_P144953	6.66E-05	ENST00000316429		PREDICTED: Homo sapiens similar to seven transmembrane helix receptor (LOC442105), mRNA [XM_497958]	XM_497958
A_23_P96542	6.66E-05	NM_001017980	NM_001017980	Homo sapiens hypothetical protein LOC203547 (LOC203547), mRNA [NM_001017980]	NM_001017980
A_23_P23616	6.68E-05	NM_032129	NM_032129	Homo sapiens pleckstrin homology domain containing, family N member 1 (PLEKHN1), mRNA [NM_032129]	NM_032129
A_23_P135123	6.68E-05	BG216229	BG216229	BG216229 RST35803 Athersys RAGE Library Homo sapiens cDNA, mRNA sequence [BG216229]	
A_32_P143245	6.68E-05	NM_001012507	NM_001012507	Homo sapiens chromosome 6 open reading frame 173 (C6orf173), mRNA [NM_001012507]	NM_001012507
A_24_P375761	6.71E-05	NM_001006608	NM_001006608	Homo sapiens hypothetical STGC3 (STGC3), mRNA [NM_001006608]	NM_001006608
A_23_P164148	6.71E-05	NM_170607	NM_170607	Homo sapiens MAX-like protein X (MLX), transcript variant 3, mRNA [NM_170607]	NM_170607
A_23_P34093	6.74E-05	NM_000402	NM_000402	Homo sapiens glucose-6-phosphate dehydrogenase (G6PD), nuclear gene encoding mitochondrial protein, mRNA [NM_000402]	NM_000402
A_23_P258321	6.76E-05	NM_015969	NM_015969	Homo sapiens mitochondrial ribosomal protein S17 (MRPS17), nuclear gene encoding mitochondrial protein, mRNA [NM_015969]	NM_015969
A_23_P136724	6.77E-05	BX640843	BX640843	Homo sapiens mRNA; cDNA DKFZp686B14224 (from clone DKFZp686B14224). [BX640843]	
A_32_P120726	6.77E-05	THC2386267			
A_24_P20806	6.77E-05	NM_030567	NM_030567	Homo sapiens proline rich 7 (synaptic) (PRR7), mRNA [NM_030567]	NM_030567
A_32_P69399	6.78E-05	BQ072652	BQ072652	AGENCOURT_6763016 NIH_MGC_118 Homo sapiens cDNA clone IMAGE:5756116 5', mRNA sequence [BQ072652]	XM_373922
A_23_P371239	6.80E-05	NM_198390	NM_198390	Homo sapiens c-Maf-inducing protein (CMIP), transcript variant C-mip, mRNA [NM_198390]	NM_198390
A_24_P22887	6.80E-05	NM_005053	NM_005053	Homo sapiens RAD23 homolog A (S. cerevisiae) (RAD23A), mRNA [NM_005053]	NM_005053
A_24_P213206	6.84E-05	CR627448	CR627448	Homo sapiens mRNA; cDNA DKFZp781A1422 (from clone DKFZp781A1422). [CR627448]	
A_24_P196851	6.84E-05	NM_006289	NM_006289	Homo sapiens talin 1 (TLN1), mRNA [NM_006289]	NM_006289
A_23_P503115	6.85E-05	NM_021574	NM_021574	Homo sapiens breakpoint cluster region (BCR), transcript variant 2, mRNA [NM_021574]	NM_021574
A_24_P339944	6.86E-05	NM_002608	NM_002608	Homo sapiens platelet-derived growth factor beta polypeptide (simian sarcoma viral (v-sis) oncogene homolog) (PDGFB), transcript variant 1, mRNA [NM_002608]	NM_002608
A_24_P264059	6.86E-05	AF318342	AF318342	Homo sapiens pp14571 mRNA, complete cds. [AF318342]	
A_23_P213832	6.86E-05	NM_032566	NM_032566	Homo sapiens esophagus cancer-related gene-2 (ECG2), mRNA [NM_032566]	NM_032566
A_23_P6119	6.88E-05	NM_032986	NM_032986	Homo sapiens Sec23 homolog B (S. cerevisiae) (SEC23B), transcript variant 3, mRNA [NM_032986]	NM_032986
A_23_P207387	6.89E-05	NM_032484	NM_032484	Homo sapiens homolog of mouse LGP1 (LGP1), mRNA [NM_032484]	NM_032484
A_24_P799580	6.89E-05	A_24_P799580			
A_24_P419132	6.91E-05	NM_006733	NM_006733	Homo sapiens FSH primary response (LRPR1 homolog, rat) 1 (FSHPRH1), mRNA [NM_006733]	NM_006733
A_24_P164838	6.92E-05	NM_006038	NM_006038	Homo sapiens spermatogenesis associated 2 (SPATA2), mRNA [NM_006038]	NM_006038
A_24_P134789	6.92E-05	NM_181688	NM_181688	Homo sapiens keratin associated protein 10-10 (KRTAP10-10), mRNA [NM_181688]	NM_181688
A_24_P753638	6.93E-05	A_24_P753638			
A_23_P84344	6.93E-05	NM_021805	NM_021805	Homo sapiens single Ig IL-1R-related molecule (SIGIRR), mRNA [NM_021805]	NM_021805

A_24_P937169	6.95E-05	BG944303	BG944303	BG944303 ax48h12.x2 Hembase; Erythroid Progenitor Cells (LCB:ax library) Homo sapiens cDNA clone ax48h12 random, mRNA sequence [BG944303]	
A_23_P250072	6.96E-05	A_23_P250072			
A_23_P338890	6.96E-05	NM_002827	NM_002827	Homo sapiens protein tyrosine phosphatase, non-receptor type 1 (PTPN1), mRNA [NM_002827]	NM_002827
A_24_P169258	6.97E-05	NM_005993	NM_005993	Homo sapiens tubulin-specific chaperone d (TBCD), mRNA [NM_005993]	NM_005993
A_24_P118231	6.97E-05	NM_001003803	NM_001003803	Homo sapiens ATP synthase, H+ transporting, mitochondrial F0 complex, subunit s (factor B) (ATP5S), nuclear gene encoding mitochondrial protein, transcript variant 1, mRNA [NM_001003803]	NM_001003803
A_24_P405313	6.98E-05	NM_003705	NM_003705	Homo sapiens solute carrier family 25 (mitochondrial carrier, Aralar), member 12 (SLC25A12), mRNA [NM_003705]	NM_003705
A_23_P214411	6.98E-05	NM_006708	NM_006708	Homo sapiens glyoxalase I (GLO1), mRNA [NM_006708]	NM_006708
A_23_P6802	6.98E-05	NM_004704	NM_004704	Homo sapiens RNA, U3 small nucleolar interacting protein 2 (RNU3IP2), mRNA [NM_004704]	NM_004704
A_23_P162106	6.98E-05	NM_016055	NM_016055	Homo sapiens mitochondrial ribosomal protein L48 (MRPL48), nuclear gene encoding mitochondrial protein, mRNA [NM_016055]	NM_016055
A_24_P195629	6.99E-05	THC2301132		BC069050 PPIA protein {Homo sapiens;} , partial (86%) [THC2301132]	
A_24_P149645	7.00E-05	NM_014281	NM_014281	Homo sapiens fuse-binding protein-interacting repressor (SIAHBP1), transcript variant 2, mRNA [NM_014281]	NM_014281
A_23_P140256	7.00E-05	NM_000270	NM_000270	Homo sapiens nucleoside phosphorylase (NP), mRNA [NM_000270]	NM_000270
A_32_P29784	7.00E-05	NM_153649	NM_153649	Homo sapiens tropomyosin 3 (TPM3), transcript variant 2, mRNA [NM_153649]	NM_153649
A_24_P156295	7.01E-05	NM_018677	NM_018677	Homo sapiens acetyl-Coenzyme A synthetase 2 (ADP forming) (ACAS2), transcript variant 1, mRNA [NM_018677]	NM_018677
A_23_P170761	7.01E-05	NM_006457	NM_006457	Homo sapiens PDZ and LIM domain 5 (PDLIM5), transcript variant 1, mRNA [NM_006457]	NM_006457
A_23_P122896	7.03E-05	NM_003227	NM_003227	Homo sapiens transferrin receptor 2 (TFR2), mRNA [NM_003227]	NM_003227
A_23_P203888	7.06E-05	NM_022791	NM_022791	Homo sapiens matrix metalloproteinase 19 (MMP19), transcript variant rasi-6, mRNA [NM_022791]	NM_022791
A_24_P135579	7.06E-05	ENST00000361165		PREDICTED: Homo sapiens similar to UNR-interacting protein (WD-40 repeat protein PT-WD) (MAP activator with WD repeats) (LOC344382), mRNA [XM_293026]	XM_293026
A_24_P307486	7.06E-05	ENST00000309556		PREDICTED: Homo sapiens similar to peptidyl-Pro cis trans isomerase (LOC128192), mRNA [XM_060887]	XM_060887
A_32_P13728	7.06E-05	NM_006597	NM_006597	Homo sapiens heat shock 70kDa protein 8 (HSPA8), transcript variant 1, mRNA [NM_006597]	NM_006597
A_23_P210361	7.07E-05	NM_022082	NM_022082	Homo sapiens chromosome 20 open reading frame 59 (C20orf59), mRNA [NM_022082]	NM_022082
A_24_P81695	7.08E-05	NM_014758	NM_014758	Homo sapiens sorting nexin 19 (SNX19), mRNA [NM_014758]	NM_014758
A_23_P10870	7.08E-05	NM_014908	NM_014908	Homo sapiens transmembrane protein 15 (TMEM15), mRNA [NM_014908]	NM_014908
A_32_P86763	7.09E-05	NM_004613	NM_004613	Homo sapiens transglutaminase 2 (C polypeptide, protein-glutamine-gamma-glutamyltransferase) (TGM2), transcript variant 1, mRNA [NM_004613]	NM_004613
A_23_P420942	7.10E-05	NM_175617	NM_175617	Homo sapiens metallothionein 1E (functional) (MT1E), mRNA [NM_175617]	NM_175617
A_24_P136470	7.10E-05	NM_024018	NM_024018	Homo sapiens butyrophilin, subfamily 2, member A3 (BTN2A3), mRNA [NM_024018]	NM_024018
A_23_P76435	7.11E-05	NM_176818	NM_176818	Homo sapiens hypothetical protein 15E1.2 (15E1.2), mRNA [NM_176818]	NM_176818
A_24_P59239	7.13E-05	A_24_P59239			
A_23_P149812	7.13E-05	NM_012425	NM_012425	Homo sapiens Ras suppressor protein 1 (RSU1), transcript variant 1, mRNA [NM_012425]	NM_012425
A_23_P99771	7.15E-05	NM_006029	NM_006029	Homo sapiens paraneoplastic antigen MA1 (PNMA1), mRNA [NM_006029]	NM_006029
A_23_P18641	7.16E-05	NM_031953	NM_031953	Homo sapiens sorting nexin 25 (SNX25), mRNA [NM_031953]	NM_031953
A_23_P342744	7.17E-05	NM_153713	NM_153713	Homo sapiens Lix1 homolog (mouse) like (LIX1L), mRNA [NM_153713]	NM_153713
A_24_P7510	7.18E-05	A_24_P7510			
A_24_P913431	7.18E-05	AK131423	AK131423	Homo sapiens cDNA FLJ16543 fis, clone OCBBF3002654, highly similar to Triple functional domain protein. [AK131423]	
A_24_P40978	7.18E-05	ENST00000292543		Homo sapiens mRNA for FLJ00352 protein. [AK160381]	
A_23_P139895	7.18E-05	BC006795	BC006795	Homo sapiens hypothetical protein MGC5370, mRNA (cDNA clone IMAGE:3049213), partial cds. [BC006795]	
A_23_P122579	7.19E-05	NM_001350	NM_001350	Homo sapiens death-associated protein 6 (DAXX), mRNA [NM_001350]	NM_001350
A_32_P53486	7.19E-05	BC022832	BC022832	Homo sapiens bolA-like 2 (E. coli), mRNA (cDNA clone IMAGE:4647797), with apparent retained intron. [BC022832]	
A_24_P330796	7.20E-05	NM_017777	NM_017777	Homo sapiens hypothetical protein FLJ20345 (FLJ20345), mRNA [NM_017777]	NM_017777
A_32_P132748	7.20E-05	BC018626	BC018626	Homo sapiens, Similar to hect domain and RLD 2, clone IMAGE:4581928, mRNA. [BC018626]	
A_32_P209325	7.20E-05	AK130530	AK130530	Homo sapiens cDNA FLJ27020 fis, clone SLV06144. [AK130530]	
A_23_P405794	7.21E-05	NM_153006	NM_153006	Homo sapiens N-acetylglutamate synthase (NAGS), mRNA [NM_153006]	NM_153006
A_24_P392496	7.22E-05	ENST00000315293			XM_372099

A_24_P363100	7.24E-05	NM_173670	NM_173670	Homo sapiens RGM domain family, member B (RGMB), transcript variant 2, mRNA [NM_173670]	NM_173670
A_23_P214111	7.24E-05	NM_022113	NM_022113	Homo sapiens kinesin family member 13A (KIF13A), mRNA [NM_022113]	NM_022113
A_23_P67648	7.24E-05	NM_001749	NM_001749	Homo sapiens calpain, small subunit 1 (CAPNS1), transcript variant 1, mRNA [NM_001749]	NM_001749
A_23_P347040	7.25E-05	NM_138287	NM_138287	Homo sapiens deltex 3-like (Drosophila) (DTX3L), mRNA [NM_138287]	NM_138287
A_23_P200685	7.25E-05	NM_017898	NM_017898	Homo sapiens MOCO sulphurase C-terminal domain containing 2 (MOSC2), mRNA [NM_017898]	NM_017898
A_24_P313397	7.25E-05	CR621534	CR621534	full-length cDNA clone CS0DI061YF17 of Placenta Cot 25-normalized of Homo sapiens (human). [CR621534]	
A_32_P28309	7.30E-05	AI916036	AI916036	AI916036 wi44c02.x1 NCI_CGAP_Co16 Homo sapiens cDNA clone IMAGE:2393090 3' similar to TR:O75300 O75300 ES/130. ;, mRNA sequence [AI916036]	
A_23_P72840	7.30E-05	NM_018973	NM_018973	Homo sapiens dolichyl-phosphate mannosyltransferase polypeptide 3 (DPM3), transcript variant 1, mRNA [NM_018973]	NM_018973
A_23_P317657	7.31E-05	NM_024005	NM_024005	Homo sapiens DEAD (Asp-Glu-Ala-Asp) box polypeptide 3, X-linked (DDX3X), transcript variant 1, mRNA [NM_024005]	NM_024005
A_23_P98183	7.31E-05	NM_005343	NM_005343	Homo sapiens v-Ha-ras Harvey rat sarcoma viral oncogene homolog (HRAS), transcript variant 1, mRNA [NM_005343]	NM_005343
A_23_P15284	7.32E-05	NM_024109	NM_024109	Homo sapiens hypothetical protein MGC2654 (MGC2654), mRNA [NM_024109]	NM_024109
A_23_P69513	7.35E-05	NM_198587	NM_198587	Homo sapiens regulator of G-protein signalling 12 (RGS12), transcript variant 7, mRNA [NM_198587]	NM_198587
A_32_P703	7.35E-05	THC2318640		BX104049 BX104049 NCI_CGAP_GC6 Homo sapiens cDNA clone IMAGp998I144942 ; IMAGE:2009557, mRNA sequence [BX104049]	
A_23_P88802	7.36E-05	NM_024731	NM_024731	Homo sapiens chromosome 16 open reading frame 44 (C16orf44), mRNA [NM_024731]	NM_024731
A_23_P161218	7.36E-05	NM_014391	NM_014391	Homo sapiens ankyrin repeat domain 1 (cardiac muscle) (ANKRD1), mRNA [NM_014391]	NM_014391
A_23_P60387	7.38E-05	NM_017617	NM_017617	Homo sapiens Notch homolog 1, translocation-associated (Drosophila) (NOTCH1), mRNA [NM_017617]	NM_017617
A_23_P165657	7.38E-05	NM_005415	NM_005415	Homo sapiens solute carrier family 20 (phosphate transporter), member 1 (SLC20A1), mRNA [NM_005415]	NM_005415
A_23_P131924	7.41E-05	NM_000678	NM_000678	Homo sapiens adrenergic, alpha-1D-, receptor (ADRA1D), mRNA [NM_000678]	NM_000678
A_24_P516215	7.41E-05	NM_014062	NM_014062	Homo sapiens nin one binding protein (NOB1P), mRNA [NM_014062]	NM_014062
A_23_P8142	7.42E-05	NM_001832	NM_001832	Homo sapiens colipase, pancreatic (CLPS), mRNA [NM_001832]	NM_001832
A_24_P245298	7.42E-05	NM_153012	NM_153012	Homo sapiens tumor necrosis factor (ligand) superfamily, member 12 (TNFSF12), transcript variant 2, mRNA [NM_153012]	NM_153012
A_23_P9214	7.43E-05	NM_018946	NM_018946	Homo sapiens N-acetylneuraminic acid synthase (sialic acid synthase) (NANS), mRNA [NM_018946]	NM_018946
A_23_P63870	7.44E-05	NM_144660	NM_144660	Homo sapiens sterile alpha motif domain containing 8 (SAMD8), mRNA [NM_144660]	NM_144660
A_23_P139983	7.46E-05	NM_022459	NM_022459	Homo sapiens exportin 4 (XPO4), mRNA [NM_022459]	NM_022459
A_23_P154688	7.47E-05	NM_032034	NM_032034	Homo sapiens solute carrier family 4, sodium bicarbonate transporter-like, member 11 (SLC4A11), mRNA [NM_032034]	NM_032034
A_23_P148194	7.47E-05	NM_018269	NM_018269	Homo sapiens membrane-type 1 matrix metalloproteinase cytoplasmic tail binding protein-1 (MTCBP-1), mRNA [NM_018269]	NM_018269
A_23_P377717	7.48E-05	NM_002516	NM_002516	Homo sapiens neuro-oncological ventral antigen 2 (NOVA2), mRNA [NM_002516]	NM_002516
A_23_P68998	7.50E-05	NM_017584	NM_017584	Homo sapiens myo-inositol oxygenase (MIOX), mRNA [NM_017584]	NM_017584
A_23_P145068	7.50E-05	NM_012123	NM_012123	Homo sapiens mitochondrial translation optimization 1 homolog (S. cerevisiae) (MTO1), mRNA [NM_012123]	NM_012123
A_24_P134372	7.51E-05	NM_004798	NM_004798	Homo sapiens kinesin family member 3B (KIF3B), mRNA [NM_004798]	NM_004798
A_32_P186038	7.53E-05	THC2359128		RS13_CHICK (Q6ITC7) 40S ribosomal protein S13, partial (48%) [THC2359128]	
A_23_P357101	7.54E-05	NM_145298	NM_145298	Homo sapiens apolipoprotein B mRNA editing enzyme, catalytic polypeptide-like 3F (APOBEC3F), transcript variant 1, mRNA [NM_145298]	NM_145298
A_23_P130731	7.54E-05	NM_024682	NM_024682	Homo sapiens TBC1 domain family, member 17 (TBC1D17), mRNA [NM_024682]	NM_024682
A_23_P109488	7.55E-05	NM_052880	NM_052880	Homo sapiens HGFL gene (MGC17330), mRNA [NM_052880]	NM_052880
A_23_P94689	7.55E-05	NM_030914	NM_030914	Homo sapiens chromosome 9 open reading frame 74 (C9orf74), mRNA [NM_030914]	NM_030914
A_23_P36157	7.56E-05	NM_018093	NM_018093	Homo sapiens WD repeat domain 74 (WDR74), mRNA [NM_018093]	NM_018093
A_23_P395524	7.56E-05	NM_007062	NM_007062	Homo sapiens PWP1 homolog (S. cerevisiae) (PWP1), mRNA [NM_007062]	NM_007062
A_24_P9454	7.57E-05	NM_002541	NM_002541	Homo sapiens oxoglutarate (alpha-ketoglutarate) dehydrogenase (lipoamide) (OGDH), nuclear gene encoding mitochondrial protein, transcript variant 1, mRNA [NM_002541]	NM_002541
A_23_P153461	7.58E-05	NM_022737	NM_022737	Homo sapiens lipid phosphate phosphatase-related protein type 2 (LPPR2), mRNA [NM_022737]	NM_022737
A_23_P217054	7.59E-05	NM_024345	NM_024345	Homo sapiens WD repeat domain 32 (WDR32), mRNA [NM_024345]	NM_024345
A_23_P170337	7.60E-05	NM_003748	NM_003748	Homo sapiens aldehyde dehydrogenase 4 family, member A1 (ALDH4A1), nuclear gene encoding mitochondrial protein, transcript variant P5CDhL, mRNA [NM_003748]	NM_003748
A_24_P185604	7.61E-05	NM_032013	NM_032013	Homo sapiens NDRG family member 3 (NDRG3), transcript variant 1, mRNA [NM_032013]	NM_032013

A_23_P35591	7.61E-05	NM_016046	NM_016046	Homo sapiens exosome component 1 (EXOSC1), mRNA [NM_016046]	NM_016046
A_32_P56713	7.63E-05	ENST00000359540		Homo sapiens cDNA FLJ36027 fis, clone TESTI2016888, weakly similar to BREAKPOINT CLUSTER REGION PROTEIN (EC 2.7.1.-). [AK093346]	XM_496519
A_23_P51646	7.63E-05	NM_004073	NM_004073	Homo sapiens polo-like kinase 3 (Drosophila) (PLK3), mRNA [NM_004073]	NM_004073
A_23_P255653	7.65E-05	NM_003844	NM_003844	Homo sapiens tumor necrosis factor receptor superfamily, member 10a (TNFRSF10A), mRNA [NM_003844]	NM_003844
A_23_P119337	7.67E-05	NM_012068	NM_012068	Homo sapiens activating transcription factor 5 (ATF5), mRNA [NM_012068]	NM_012068
A_24_P339664	7.68E-05	NM_020170	NM_020170	Homo sapiens nicalin homolog (zebrafish) (NCLN), mRNA [NM_020170]	NM_020170
A_23_P34115	7.69E-05	NM_004135	NM_004135	Homo sapiens isocitrate dehydrogenase 3 (NAD+) gamma (IDH3G), nuclear gene encoding mitochondrial protein, transcript variant 1, mRNA [NM_004135]	NM_004135
A_24_P902091	7.70E-05	AF321617	AF321617	Homo sapiens unknown mRNA. [AF321617]	
A_23_P50990	7.71E-05	NM_024322	NM_024322	Homo sapiens hypothetical protein MGC11266 (MGC11266), mRNA [NM_024322]	NM_024322
A_23_P154894	7.71E-05	NM_000100	NM_000100	Homo sapiens cystatin B (stefin B) (CSTB), mRNA [NM_000100]	NM_000100
A_23_P143559	7.74E-05	NM_007098	NM_007098	Homo sapiens clathrin, heavy polypeptide-like 1 (CLTCL1), transcript variant 2, mRNA [NM_007098]	NM_007098
A_23_P156957	7.75E-05	NM_181782	NM_181782	Homo sapiens nuclear receptor coactivator 7 (NCOA7), mRNA [NM_181782]	NM_181782
A_32_P214925	7.75E-05	NM_173678	NM_173678	Homo sapiens hypothetical protein FLJ40722 (FLJ40722), mRNA [NM_173678]	NM_173678
A_24_P236680	7.79E-05	THC2287766			
A_24_P262738	7.79E-05	NM_024050	NM_024050	Homo sapiens cross-immune reaction antigen PCIA1 (PCIA1), mRNA [NM_024050]	NM_024050
A_23_P140035	7.79E-05	NM_007187	NM_007187	Homo sapiens WW domain binding protein 4 (formin binding protein 21) (WBP4), mRNA [NM_007187]	NM_007187
A_23_P10785	7.81E-05	NM_145206	NM_145206	Homo sapiens vesicle transport through interaction with t-SNAREs homolog 1A (yeast) (VTI1A), mRNA [NM_145206]	NM_145206
A_23_P253774	7.84E-05	A_23_P253774			
A_23_P98722	7.85E-05	NM_005898	NM_005898	Homo sapiens membrane component, chromosome 11, surface marker 1 (M11S1), transcript variant 1, mRNA [NM_005898]	NM_005898
A_23_P373598	7.88E-05	AK056767	AK056767	Homo sapiens cDNA FLJ32205 fis, clone PLACE6003094. [AK056767]	
A_24_P112377	7.90E-05	NM_138620	NM_138620	Homo sapiens DEAD (Asp-Glu-Ala-Asp) box polypeptide 31 (DDX31), transcript variant 2, mRNA [NM_138620]	NM_138620
A_23_P40088	7.92E-05	NM_003887	NM_003887	Homo sapiens development and differentiation enhancing factor 2 (DDEF2), mRNA [NM_003887]	NM_003887
A_24_P470754	7.92E-05	A_24_P470754			
A_23_P138819	7.92E-05	NM_004927	NM_004927	Homo sapiens mitochondrial ribosomal protein L49 (MRPL49), nuclear gene encoding mitochondrial protein, mRNA [NM_004927]	NM_004927
A_23_P255503	7.92E-05	NM_002572	NM_002572	Homo sapiens platelet-activating factor acetylhydrolase, isoform Ib, beta subunit 30kDa (PAFAH1B2), mRNA [NM_002572]	NM_002572
A_23_P256773	7.92E-05	NM_003310	NM_003310	Homo sapiens tumor suppressing subtransferable candidate 1 (TSSC1), mRNA [NM_003310]	NM_003310
A_23_P501877	7.93E-05	NM_018197	NM_018197	Homo sapiens zinc finger protein 64 homolog (mouse) (ZFP64), transcript variant 1, mRNA [NM_018197]	NM_018197
A_23_P115046	7.94E-05	NM_020365	NM_020365	Homo sapiens eukaryotic translation initiation factor 2B, subunit 3 gamma, 58kDa (EIF2B3), mRNA [NM_020365]	NM_020365
A_23_P27493	7.94E-05	NM_020230	NM_020230	Homo sapiens peter pan homolog (Drosophila) (PPAN), mRNA [NM_020230]	NM_020230
A_23_P5903	7.95E-05	NM_016354	NM_016354	Homo sapiens solute carrier organic anion transporter family, member 4A1 (SLCO4A1), mRNA [NM_016354]	NM_016354
A_24_P312915	7.96E-05	NM_033489	NM_033489	Homo sapiens cell division cycle 2-like 1 (PITSLRE proteins) (CDC2L1), transcript variant 5, mRNA [NM_033489]	NM_033489
A_32_P201620	7.97E-05	A_32_P201620			
A_23_P120467	7.97E-05	NM_199427	NM_199427	Homo sapiens zinc finger protein 64 homolog (mouse) (ZFP64), transcript variant 4, mRNA [NM_199427]	NM_199427
A_23_P413641	7.97E-05	NM_020820	NM_020820	Homo sapiens phosphatidylinositol 3,4,5-trisphosphate-dependent RAC exchanger 1 (PREX1), mRNA [NM_020820]	NM_020820
A_23_P12079	7.99E-05	NM_153763	NM_153763	Homo sapiens potassium voltage-gated channel, Shaw-related subfamily, member 4 (KCNC4), transcript variant 2, mRNA [NM_153763]	NM_153763
A_23_P338479	7.99E-05	NM_014143	NM_014143	Homo sapiens CD274 antigen (CD274), mRNA [NM_014143]	NM_014143
A_23_P217208	8.00E-05	NM_005660	NM_005660	Homo sapiens solute carrier family 35 (UDP-galactose transporter), member A2 (SLC35A2), mRNA [NM_005660]	NM_005660
A_23_P86931	8.00E-05	NM_198277	NM_198277	Homo sapiens solute carrier family 37 (glycerol-3-phosphate transporter), member 2 (SLC37A2), mRNA [NM_198277]	NM_198277
A_23_P16166	8.00E-05	NM_006702	NM_006702	Homo sapiens neuropathy target esterase (NTE), mRNA [NM_006702]	NM_006702
A_23_P100156	8.03E-05	NM_017849	NM_017849	Homo sapiens hypothetical protein FLJ20507 (FLJ20507), mRNA [NM_017849]	NM_017849
A_23_P55149	8.03E-05	NM_001282	NM_001282	Homo sapiens adaptor-related protein complex 2, beta 1 subunit (AP2B1), mRNA [NM_001282]	NM_001282
A_24_P213794	8.04E-05	NM_012118	NM_012118	Homo sapiens CCR4 carbon catabolite repression 4-like (S. cerevisiae) (CCRN4L), mRNA [NM_012118]	NM_012118
A_23_P39931	8.04E-05	NM_003494	NM_003494	Homo sapiens dysferlin, limb girdle muscular dystrophy 2B (autosomal recessive) (DYSF), mRNA [NM_003494]	NM_003494
A_23_P44366	8.04E-05	BC068598	BC068598	Homo sapiens protein kinase, AMP-activated, gamma 2 non-catalytic subunit, mRNA (cDNA clone MGC:87635 IMAGE:5259756), complete cds. [BC068598]	

A_24_P183150	8.05E-05	NM_002090	NM_002090	Homo sapiens chemokine (C-X-C motif) ligand 3 (CXCL3), mRNA [NM_002090]	NM_002090
A_24_P917783	8.05E-05	X90978	X90978	H.sapiens mRNA for an acute myeloid leukaemia protein (1793bp). [X90978]	
A_32_P109653	8.05E-05	THC2377128			
A_23_P5171	8.05E-05	A_23_P5171			
A_32_P4364	8.08E-05	NM_005745	NM_005745	Homo sapiens B-cell receptor-associated protein 31 (BCAP31), mRNA [NM_005745]	NM_005745
A_24_P340696	8.10E-05	NM_170694	NM_170694	Homo sapiens serine hydrolase-like (SERHL), mRNA [NM_170694]	NM_170694
A_32_P30693	8.11E-05	NM_003707	NM_003707	Homo sapiens RuvB-like 1 (E. coli) (RUVBL1), mRNA [NM_003707]	NM_003707
A_23_P15108	8.11E-05	NM_031477	NM_031477	Homo sapiens yippee-like 3 (Drosophila) (YPEL3), mRNA [NM_031477]	NM_031477
A_32_P85433	8.11E-05	BQ130701	BQ130701	BQ130701 ij86c06.y1 Human insulinoma Homo sapiens cDNA clone IMAGE:5778418 5' similar to SW:CTGF_HUMAN P29279 CONNECTIVE TISSUE GROWTH FACTOR PRECURSOR. [1] ;, mRNA sequence [BQ130701]	
A_23_P167841	8.12E-05	NM_019842	NM_019842	Homo sapiens potassium voltage-gated channel, KQT-like subfamily, member 5 (KCNQ5), mRNA [NM_019842]	NM_019842
A_24_P58579	8.12E-05	NM_153649	NM_153649	Homo sapiens tropomyosin 3 (TPM3), transcript variant 2, mRNA [NM_153649]	NM_153649
A_23_P89910	8.15E-05	NM_024707	NM_024707	Homo sapiens gem (nuclear organelle) associated protein 7 (GEMIN7), transcript variant 1, mRNA [NM_024707]	NM_024707
A_23_P90032	8.16E-05	NM_025061	NM_025061	Homo sapiens leucine rich repeat containing 8 family, member E (LRRC8E), mRNA [NM_025061]	NM_025061
A_32_P226338	8.17E-05	A_32_P226338			
A_23_P119102	8.18E-05	NM_003370	NM_003370	Homo sapiens vasodilator-stimulated phosphoprotein (VASP), transcript variant 1, mRNA [NM_003370]	NM_003370
A_24_P394368	8.19E-05	NM_025132	NM_025132	Homo sapiens WD repeat domain 19 (WDR19), mRNA [NM_025132]	NM_025132
A_23_P213247	8.19E-05	NM_033535	NM_033535	Homo sapiens F-box and leucine-rich repeat protein 5 (FBXL5), transcript variant 2, mRNA [NM_033535]	NM_033535
A_23_P48550	8.19E-05	NM_015005	NM_015005	Homo sapiens KIAA0284 (KIAA0284), mRNA [NM_015005]	NM_015005
A_23_P315815	8.20E-05	NM_013961	NM_013961	Homo sapiens neuregulin 1 (NRG1), transcript variant GGF, mRNA [NM_013961]	NM_013961
A_23_P78170	8.20E-05	NM_014520	NM_014520	Homo sapiens MYB binding protein (P160) 1a (MYBBP1A), mRNA [NM_014520]	NM_014520
A_24_P213144	8.21E-05	A_24_P213144			
A_23_P132560	8.22E-05	NM_145037	NM_145037	Homo sapiens family with sequence similarity 55, member C (FAM55C), mRNA [NM_145037]	NM_145037
A_23_P17471	8.23E-05	NM_022760	NM_022760	Homo sapiens chromosome 20 open reading frame 81 (C20orf81), mRNA [NM_022760]	NM_022760
A_32_P806841	8.23E-05	NM_005738	NM_005738	Homo sapiens ADP-ribosylation factor-like 4 (ARL4), transcript variant 1, mRNA [NM_005738]	NM_005738
A_24_P599225	8.25E-05	A_24_P599225			
A_23_P114839	8.25E-05	NM_004468	NM_004468	Homo sapiens four and a half LIM domains 3 (FHL3), mRNA [NM_004468]	NM_004468
A_32_P12372	8.25E-05	THC2355348		ALU1_HUMAN (P39188) Alu subfamily J sequence contamination warning entry, partial (7%) [THC2355348]	
A_23_P117778	8.25E-05	NM_032907	NM_032907	Homo sapiens ubiquitin-like 7 (bone marrow stromal cell-derived) (UBL7), transcript variant 1, mRNA [NM_032907]	NM_032907
A_23_P82461	8.26E-05	THC2253738		I46880 T-cell receptor beta chain - rabbit (fragment) {Oryctolagus cuniculus;} , partial (9%) [THC2253738]	
A_24_P936605	8.27E-05	U87972	U87972	Human NAD+-isocitrate dehydrogenase mRNA, partial cds. [U87972]	
A_24_P409661	8.27E-05	A_24_P409661			
A_23_P330209	8.29E-05	NM_057178	NM_057178	Homo sapiens rififylin (RFFL), transcript variant 1, mRNA [NM_057178]	NM_057178
A_23_P316850	8.29E-05	NM_182577	NM_182577	Homo sapiens chromosome 19 open reading frame 19 (C19orf19), mRNA [NM_182577]	NM_182577
A_23_P323783	8.31E-05	NM_144703	NM_144703	Homo sapiens family with sequence similarity 61, member B (FAM61B), mRNA [NM_144703]	NM_144703
A_23_P98580	8.32E-05	NM_004265	NM_004265	Homo sapiens fatty acid desaturase 2 (FADS2), mRNA [NM_004265]	NM_004265
A_24_P94419	8.33E-05	NM_015147	NM_015147	Homo sapiens KIAA0582 (KIAA0582), mRNA [NM_015147]	NM_015147
A_23_P49559	8.34E-05	NM_181790	NM_181790	Homo sapiens G protein-coupled receptor 142 (GPR142), mRNA [NM_181790]	NM_181790
A_32_P34876	8.35E-05	NM_020212	NM_020212	Homo sapiens hypothetical protein from EUROIMAGE 384293 (LOC56964), mRNA [NM_020212]	NM_020212
A_24_P95059	8.35E-05	NM_007155	NM_007155	Homo sapiens zona pellucida glycoprotein 3 (sperm receptor) (ZP3), mRNA [NM_007155]	NM_007155
A_24_P372562	8.35E-05	BC020242	BC020242	Homo sapiens protein tyrosine phosphatase, mitochondrial 1, mRNA (cDNA clone MGC:31981 IMAGE:4591438), complete cds. [BC020242]	XM_374879
A_23_P56810	8.36E-05	NM_018158	NM_018158	Homo sapiens solute carrier family 4 (anion exchanger), member 1, adaptor protein (SLC4A1AP), mRNA [NM_018158]	NM_018158
A_24_P42681	8.37E-05	NM_002808	NM_002808	Homo sapiens proteasome (prosome, macropain) 26S subunit, non-ATPase, 2 (PSMD2), mRNA [NM_002808]	NM_002808
A_23_P114155	8.41E-05	NM_015698	NM_015698	Homo sapiens G patch domain and KOW motifs (GPKOW), mRNA [NM_015698]	NM_015698
A_23_P144639	8.43E-05	NM_018502	NM_018502	Homo sapiens hypothetical protein PRO1580 (PRO1580), mRNA [NM_018502]	NM_018502

A_23_P10911	8.46E-05	NM_173542	NM_173542	Homo sapiens hypothetical protein LOC196463 (LOC196463), mRNA [NM_173542]	NM_173542
A_24_P375510	8.47E-05	ENST00000327893			
A_23_P205724	8.47E-05	NM_199043	NM_199043	Homo sapiens chromosome 14 open reading frame 102 (C14orf102), transcript variant 2, mRNA [NM_199043]	NM_199043
A_23_P52373	8.47E-05	NM_003635	NM_003635	Homo sapiens N-deacetylase/N-sulfotransferase (heparan glucosaminyl) 2 (NDST2), mRNA [NM_003635]	NM_003635
A_32_P190959	8.48E-05	ENST00000343496		Human rearranged immunoglobulin heavy chain (IGH@) gene, 5' end. [L06610]	
A_23_P15493	8.48E-05	NM_001015509	NM_001015509	Homo sapiens Bcl-2 inhibitor of transcription (BIT1), nuclear gene encoding mitochondrial protein, transcript variant 1, mRNA [NM_001015509]	NM_001015509
A_24_P419109	8.49E-05	NM_004381	NM_004381	Homo sapiens cAMP responsive element binding protein-like 1 (CREBL1), mRNA [NM_004381]	NM_004381
A_24_P4212	8.51E-05	NM_023078	NM_023078	Homo sapiens pyrroline-5-carboxylate reductase-like (PYCRL), mRNA [NM_023078]	NM_023078
A_23_P100103	8.52E-05	NM_015289	NM_015289	Homo sapiens vacuolar protein sorting 39 (yeast) (VPS39), mRNA [NM_015289]	NM_015289
A_32_P99533	8.53E-05	AI423557	AI423557	AI423557 tf73d12.x1 NCI_CGAP_Brn23 Homo sapiens cDNA clone IMAGE:2104919 3' similar to gb:M13755 INTERFERON-INDUCED 17 KD PROTEIN (HUMAN);contains TAR1.t3 TAR1 repetitive element ;, mRNA sequence [AI423557]	
A_24_P324301	8.53E-05	ENST00000329367		Homo sapiens mRNA for hypothetical protein, partial. [AJ011409]	
A_23_P28090	8.53E-05	NM_003765	NM_003765	Homo sapiens syntaxin 10 (STX10), mRNA [NM_003765]	NM_003765
A_23_P138058	8.53E-05	NM_015658	NM_015658	Homo sapiens DKFZP564C186 protein (DKFZP564C186), mRNA [NM_015658]	NM_015658
A_24_P915227	8.54E-05	AY129015	AY129015	Homo sapiens clone FP17889 unknown mRNA. [AY129015]	
A_23_P109655	8.54E-05	NM_025246	NM_025246	Homo sapiens transmembrane protein 22 (TMEM22), mRNA [NM_025246]	NM_025246
A_23_P54758	8.54E-05	NM_016641	NM_016641	Homo sapiens membrane interacting protein of RGS16 (MIR16), mRNA [NM_016641]	NM_016641
A_23_P32199	8.55E-05	NM_000755	NM_000755	Homo sapiens carnitine acetyltransferase (CRAT), transcript variant 1, mRNA [NM_000755]	NM_000755
A_24_P480722	8.55E-05	ENST00000355855			
A_23_P420256	8.55E-05	NM_021925	NM_021925	Homo sapiens hypothetical protein FLJ21820 (FLJ21820), mRNA [NM_021925]	NM_021925
A_24_P313678	8.56E-05	NM_016060	NM_016060	Homo sapiens mediator of RNA polymerase II transcription, subunit 31 homolog (yeast) (MED31), mRNA [NM_016060]	NM_016060
A_32_P115015	8.56E-05	NM_001791	NM_001791	Homo sapiens cell division cycle 42 (GTP binding protein, 25kDa) (CDC42), transcript variant 1, mRNA [NM_001791]	NM_001791
A_23_P129313	8.57E-05	ENST00000249760		Homo sapiens isovaleryl Coenzyme A dehydrogenase, mRNA (cDNA clone MGC:3496 IMAGE:3627787), complete cds. [BC017202]	
A_23_P207614	8.58E-05	NM_014680	NM_014680	Homo sapiens KIAA0100 gene product (KIAA0100), mRNA [NM_014680]	NM_014680
A_24_P228717	8.60E-05	NM_002872	NM_002872	Homo sapiens ras-related C3 botulinum toxin substrate 2 (rho family, small GTP binding protein Rac2) (RAC2), mRNA [NM_002872]	NM_002872
A_24_P925491	8.61E-05	AF402619	AF402619	Homo sapiens synovial sarcoma SYT/SSX1 fusion protein (SYT/SSX1 fusion) mRNA, partial cds. [AF402619]	
A_24_P267552	8.63E-05	NM_198533	NM_198533	Homo sapiens short-chain dehydrogenase/reductase 10 (SCDR10), transcript variant e, mRNA [NM_198533]	NM_198533
A_24_P220897	8.64E-05	ENST00000302091		Homo sapiens cDNA FLJ25874 fis, clone CBR02446. [AK098740]	XM_114430
A_24_P410809	8.64E-05	NM_014709	NM_014709	Homo sapiens ubiquitin specific protease 34 (USP34), mRNA [NM_014709]	NM_014709
A_23_P106439	8.64E-05	NM_033544	NM_033544	Homo sapiens similar to cyclin-E binding protein 1 (H. sapiens) (MGC14386), transcript variant 1, mRNA [NM_033544]	NM_033544
A_23_P64184	8.65E-05	A_23_P64184			
A_23_P64650	8.65E-05	NM_005726	NM_005726	Homo sapiens Ts translation elongation factor, mitochondrial (TSFM), mRNA [NM_005726]	NM_005726
A_23_P159920	8.66E-05	NM_003639	NM_003639	Homo sapiens inhibitor of kappa light polypeptide gene enhancer in B-cells, kinase gamma (IKBKG), mRNA [NM_003639]	NM_003639
A_32_P80089	8.66E-05	ENST00000346061		Homo sapiens, Similar to FSHD region gene 1, clone IMAGE:4297267, mRNA. [BC017921]	
A_24_P690924	8.69E-05	NM_032986	NM_032986	Homo sapiens Sec23 homolog B (S. cerevisiae) (SEC23B), transcript variant 3, mRNA [NM_032986]	NM_032986
A_23_P107401	8.72E-05	NM_003255	NM_003255	Homo sapiens tissue inhibitor of metalloproteinase 2 (TIMP2), mRNA [NM_003255]	NM_003255
A_23_P163787	8.73E-05	NM_004530	NM_004530	Homo sapiens matrix metalloproteinase 2 (gelatinase A, 72kDa gelatinase, 72kDa type IV collagenase) (MMP2), mRNA [NM_004530]	NM_004530
A_23_P103795	8.73E-05	BC000907	BC000907	Homo sapiens cDNA clone IMAGE:2984856, complete cds. [BC000907]	
A_32_P144710	8.74E-05	NM_176880	NM_176880	Homo sapiens TR4 orphan receptor associated protein TRA16 (TRA16), mRNA [NM_176880]	NM_176880
A_32_P26017	8.75E-05	AA112254	AA112254	AA112254 zm65a11.s1 Stratagene fibroblast (#937212) Homo sapiens cDNA clone IMAGE:530492 3', mRNA sequence [AA112254]	
A_24_P79199	8.75E-05	NM_033405	NM_033405	Homo sapiens peroxisomal proliferator-activated receptor A interacting complex 285 (PRIC285), mRNA [NM_033405]	NM_033405
A_23_P80891	8.75E-05	NM_003363	NM_003363	Homo sapiens ubiquitin specific protease 4 (proto-oncogene) (USP4), transcript variant 1, mRNA [NM_003363]	NM_003363
A_23_P215658	8.75E-05	NM_030900	NM_030900	Homo sapiens transforming growth factor beta regulator 4 (TBRG4), transcript variant 2, mRNA [NM_030900]	NM_030900

A_23_P33720	8.76E-05	NM_006567	NM_006567	Homo sapiens phenylalanine-tRNA synthetase 2 (mitochondrial) (FARS2), nuclear gene encoding mitochondrial protein, mRNA [NM_006567]	NM_006567
A_23_P26629	8.76E-05	NM_013258	NM_013258	Homo sapiens PYD and CARD domain containing (PYCARD), transcript variant 1, mRNA [NM_013258]	NM_013258
A_23_P131365	8.77E-05	NM_005006	NM_005006	Homo sapiens NADH dehydrogenase (ubiquinone) Fe-S protein 1, 75kDa (NADH-coenzyme Q reductase) (NDUFS1), nuclear gene encoding mitochondrial protein, mRNA [NM_005006]	NM_005006
A_23_P138835	8.79E-05	NM_005186	NM_005186	Homo sapiens calpain 1, (mu/I) large subunit (CAPN1), mRNA [NM_005186]	NM_005186
A_23_P131646	8.79E-05	NM_144563	NM_144563	Homo sapiens ribose 5-phosphate isomerase A (ribose 5-phosphate epimerase) (RPIA), mRNA [NM_144563]	NM_144563
A_23_P331092	8.80E-05	NM_152571	NM_152571	Homo sapiens hypothetical protein FLJ36779 (FLJ36779), mRNA [NM_152571]	NM_152571
A_23_P141208	8.80E-05	NM_015510	NM_015510	Homo sapiens DKFZP566O084 protein (DKFZp566O084), mRNA [NM_015510]	NM_015510
A_24_P187197	8.81E-05	BX648930	BX648930	Homo sapiens mRNA; cDNA DKFZp686A023 (from clone DKFZp686A023). [BX648930]	
A_24_P155502	8.83E-05	NM_005538	NM_005538	Homo sapiens inhibin, beta C (INHBC), mRNA [NM_005538]	NM_005538
A_24_P245815	8.84E-05	AL161993	AL161993	Homo sapiens mRNA; cDNA DKFZp761P039 (from clone DKFZp761P039); partial cds. [AL161993]	
A_24_P114183	8.84E-05	NM_002004	NM_002004	Homo sapiens farnesyl diphosphate synthase (farnesyl pyrophosphate synthetase, dimethylallyltranstransferase, geranyltranstransferase) (FDPS), mRNA [NM_002004]	NM_002004
A_32_P224327	8.87E-05	AK056401	AK056401	Homo sapiens cDNA FLJ31839 fis, clone NT2RP7000086. [AK056401]	
A_23_P371129	8.87E-05	NM_032444	NM_032444	Homo sapiens BTB (POZ) domain containing 12 (BTBD12), mRNA [NM_032444]	NM_032444
A_24_P347065	8.89E-05	ENST00000265641		Homo sapiens partial mRNA for carnitine palmitoyltransferase I (CPT1A gene). [AJ420378]	
A_23_P80136	8.89E-05	NM_003683	NM_003683	Homo sapiens DNA segment on chromosome 21 (unique) 2056 expressed sequence (D21S2056E), mRNA [NM_003683]	NM_003683
A_24_P4661	8.89E-05	NM_004879	NM_004879	Homo sapiens etoposide induced 2.4 mRNA (EI24), transcript variant 1, mRNA [NM_004879]	NM_004879
A_23_P28450	8.91E-05	NM_015425	NM_015425	Homo sapiens polymerase (RNA) I polypeptide A, 194kDa (POLR1A), mRNA [NM_015425]	NM_015425
A_23_P130926	8.93E-05	NM_017914	NM_017914	Homo sapiens chromosome 19 open reading frame 24 (C19orf24), mRNA [NM_017914]	NM_017914
A_24_P467449	8.94E-05	THC2437930		ALU7_HUMAN (P39194) Alu subfamily SQ sequence contamination warning entry, partial (4%) [THC2437930]	
A_32_P220938	8.95E-05	NM_006916	NM_006916	Homo sapiens ribulose-5-phosphate-3-epimerase (RPE), transcript variant 2, mRNA [NM_006916]	NM_006916
A_24_P279328	8.97E-05	NM_130766	NM_130766	Homo sapiens skeletal muscle and kidney enriched inositol phosphatase (SKIP), transcript variant 2, mRNA [NM_130766]	NM_130766
A_24_P93371	8.97E-05	NM_017828	NM_017828	Homo sapiens COMM domain containing 4 (COMMD4), mRNA [NM_017828]	NM_017828
A_23_P10077	8.98E-05	NM_020376	NM_020376	Homo sapiens patatin-like phospholipase domain containing 2 (PNPLA2), mRNA [NM_020376]	NM_020376
A_23_P71904	9.00E-05	NM_014064	NM_014064	Homo sapiens AD-003 protein (AD-003), mRNA [NM_014064]	NM_014064
A_23_P153320	9.01E-05	NM_000201	NM_000201	Homo sapiens intercellular adhesion molecule 1 (CD54), human rhinovirus receptor (ICAM1), mRNA [NM_000201]	NM_000201
A_32_P512500	9.01E-05	NM_024742	NM_024742	Homo sapiens armadillo repeat containing 5 (ARMC5), mRNA [NM_024742]	NM_024742
A_32_P15035	9.02E-05	AK127555	AK127555	Homo sapiens cDNA FLJ45648 fis, clone CTONG2009033. [AK127555]	
A_24_P924681	9.03E-05	BQ717518	BQ717518	AGENCOURT_8478230 Lupski_sympathetic_trunk Homo sapiens cDNA clone IMAGE:6196840 5', mRNA sequence [BQ717518]	
A_24_P357183	9.03E-05	NM_177439	NM_177439	Homo sapiens FtsJ homolog 1 (E. coli) (FTSJ1), transcript variant 3, mRNA [NM_177439]	NM_177439
A_23_P57379	9.03E-05	NM_003504	NM_003504	Homo sapiens CDC45 cell division cycle 45-like (S. cerevisiae) (CDC45L), mRNA [NM_003504]	NM_003504
A_24_P27644	9.04E-05	THC2364223		Q28610 (Q28610) Oryctolagus cuniculus Na+/glucose cotransporter-related protein, partial (22%) [THC2364223]	
A_23_P77031	9.04E-05	NM_007039	NM_007039	Homo sapiens protein tyrosine phosphatase, non-receptor type 21 (PTPN21), mRNA [NM_007039]	NM_007039
A_24_P293120	9.09E-05	NM_024327	NM_024327	Homo sapiens zinc finger protein 576 (ZNF576), mRNA [NM_024327]	NM_024327
A_23_P69868	9.09E-05	NM_022978	NM_022978	Homo sapiens small EDRK-rich factor 1B (centromeric) (SERF1B), mRNA [NM_022978]	NM_022978
A_23_P166196	9.11E-05	NM_015511	NM_015511	Homo sapiens chromosome 20 open reading frame 4 (C20orf4), mRNA [NM_015511]	NM_015511
A_32_P120977	9.14E-05	NM_013237	NM_013237	Homo sapiens px19-like protein (PX19), mRNA [NM_013237]	NM_013237
A_23_P168449	9.16E-05	NM_006989	NM_006989	Homo sapiens RAS p21 protein activator 4 (RASA4), mRNA [NM_006989]	NM_006989
A_23_P64232	9.16E-05	NM_003904	NM_003904	Homo sapiens zinc finger protein 259 (ZNF259), mRNA [NM_003904]	NM_003904
A_24_P653603	9.18E-05	BC079831	BC079831	Homo sapiens hypothetical LOC284184, mRNA (cDNA clone IMAGE:6572580). [BC079831]	XM_211367
A_24_P15906	9.19E-05	A_24_P15906			
A_24_P79808	9.19E-05	NM_020524	NM_020524	Homo sapiens pre-B-cell leukemia transcription factor interacting protein 1 (PBXIP1), mRNA [NM_020524]	NM_020524
A_23_P80752	9.20E-05	NM_015103	NM_015103	Homo sapiens plexin D1 (PLXND1), mRNA [NM_015103]	NM_015103
A_23_P160167	9.20E-05	NM_005727	NM_005727	Homo sapiens tetraspanin 1 (TSPAN1), mRNA [NM_005727]	NM_005727

A_23_P254363	9.22E-05	NM_000950	NM_000950	Homo sapiens proline rich Gla (G-carboxyglutamic acid) 1 (PRRG1), mRNA [NM_000950]	NM_000950
A_23_P254288	9.22E-05	A_23_P254288			
A_23_P160240	9.24E-05	NM_016361	NM_016361	Homo sapiens acid phosphatase 6, lysophosphatidic (ACP6), mRNA [NM_016361]	NM_016361
A_24_P641673	9.26E-05	A_24_P641673			
A_24_P159323	9.31E-05	NM_022770	NM_022770	Homo sapiens hypothetical protein FLJ13912 (FLJ13912), mRNA [NM_022770]	NM_022770
A_23_P204187	9.31E-05	NM_024854	NM_024854	Homo sapiens hypothetical protein FLJ22028 (FLJ22028), mRNA [NM_024854]	NM_024854
A_24_P226755	9.32E-05	NM_014729	NM_014729	Homo sapiens thymus high mobility group box protein TOX (TOX), mRNA [NM_014729]	NM_014729
A_24_P298013	9.33E-05	NM_019096	NM_019096	Homo sapiens GTP binding protein 2 (GTPBP2), mRNA [NM_019096]	NM_019096
A_24_P128683	9.34E-05	NM_015680	NM_015680	Homo sapiens chromosome 2 open reading frame 24 (C2orf24), mRNA [NM_015680]	NM_015680
A_23_P151415	9.34E-05	NM_032116	NM_032116	Homo sapiens katanin p60 subunit A-like 1 (KATNAL1), transcript variant 1, mRNA [NM_032116]	NM_032116
A_23_P169529	9.34E-05	NM_004504	NM_004504	Homo sapiens HIV-1 Rev binding protein (HRB), mRNA [NM_004504]	NM_004504
A_23_P117694	9.35E-05	NM_006091	NM_006091	Homo sapiens coronin, actin binding protein, 2B (CORO2B), mRNA [NM_006091]	NM_006091
A_23_P142022	9.36E-05	NM_015629	NM_015629	Homo sapiens PRP31 pre-mRNA processing factor 31 homolog (yeast) (PRPF31), mRNA [NM_015629]	NM_015629
A_23_P56213	9.37E-05	NM_020895	NM_020895	Homo sapiens KIAA1533 (KIAA1533), mRNA [NM_020895]	NM_020895
A_23_P107369	9.40E-05	U91329	U91329	Human kinesin-like motor protein KIF1C mRNA, complete cds. [U91329]	
A_32_P223422	9.42E-05	BF887921	BF887921	BF887921 PM2-TN0027-081100-017-h01 TN0027 Homo sapiens cDNA, mRNA sequence [BF887921]	
A_24_P573978	9.42E-05	THC2371907		CCHU cytochrome c [validated] - human {Homo sapiens;} , partial (85%) [THC2371907]	
A_23_P144816	9.43E-05	NM_003374	NM_003374	Homo sapiens voltage-dependent anion channel 1 (VDAC1), mRNA [NM_003374]	NM_003374
A_23_P164421	9.46E-05	BC066344	BC066344	Homo sapiens cDNA clone IMAGE:5312898, partial cds. [BC066344]	
A_23_P200096	9.47E-05	NM_025106	NM_025106	Homo sapiens SPRY domain-containing SOCS box protein SSB-1 (SSB1), mRNA [NM_025106]	NM_025106
A_23_P105044	9.47E-05	NM_021134	NM_021134	Homo sapiens mitochondrial ribosomal protein L23 (MRPL23), nuclear gene encoding mitochondrial protein, mRNA [NM_021134]	NM_021134
A_23_P433791	9.50E-05	NM_001010984	NM_001010984	Homo sapiens chromosome 1 open reading frame 124 (C1orf124), transcript variant 2, mRNA [NM_001010984]	NM_001010984
A_32_P63342	9.52E-05	AA903587	AA903587	AA903587 ok63d11.s1 NCI_CGAP_GC4 Homo sapiens cDNA clone IMAGE:1518645 3' similar to gb:L23805 ALPHA-CATENIN (HUMAN);, mRNA sequence [AA903587]	
A_23_P168062	9.52E-05	NM_003587	NM_003587	Homo sapiens DEAH (Asp-Glu-Ala-His) box polypeptide 16 (DHX16), mRNA [NM_003587]	NM_003587
A_23_P318604	9.53E-05	BC004544	BC004544	Homo sapiens cDNA clone IMAGE:3945559, partial cds. [BC004544]	
A_32_P71447	9.53E-05	NM_015261	NM_015261	Homo sapiens KIAA0056 protein (hCAP-D3), mRNA [NM_015261]	NM_015261
A_32_P98966	9.54E-05	CR620010	CR620010	full-length cDNA clone CS0DK001YG07 of HeLa cells Cot 25-normalized of Homo sapiens (human). [CR620010]	
A_24_P829789	9.57E-05	THC2433033		PSA7_HUMAN (O14818) Proteasome subunit alpha type 7 (Proteasome subunit RC6-1) (Proteasome subunit XAPC7) , partial (22%) [THC2433033]	
A_24_P65507	9.58E-05	NM_144998	NM_144998	Homo sapiens stimulated by retinoic acid 13 homolog (mouse) (STRA13), mRNA [NM_144998]	NM_144998
A_23_P217741	9.59E-05	NM_001830	NM_001830	Homo sapiens chloride channel 4 (CLCN4), mRNA [NM_001830]	NM_001830
A_23_P84860	9.59E-05	NM_007177	NM_007177	Homo sapiens TU3A protein (TU3A), mRNA [NM_007177]	NM_007177
A_23_P206474	9.60E-05	NM_032271	NM_032271	Homo sapiens TNF receptor-associated factor 7 (TRAF7), transcript variant 1, mRNA [NM_032271]	NM_032271
A_23_P150852	9.60E-05	NM_015292	NM_015292	Homo sapiens family with sequence similarity 62 (C2 domain containing), member A (FAM62A), mRNA [NM_015292]	NM_015292
A_23_P89283	9.61E-05	NM_006224	NM_006224	Homo sapiens phosphatidylinositol transfer protein, alpha (PITPNA), mRNA [NM_006224]	NM_006224
A_24_P203226	9.61E-05	NM_178230	NM_178230	Homo sapiens peptidylprolyl isomerase A (cyclophilin A)-like 4 (PPIAL4), mRNA [NM_178230]	NM_178230
A_23_P161615	9.62E-05	NM_002689	NM_002689	Homo sapiens polymerase (DNA directed), alpha 2 (70kD subunit) (POLA2), mRNA [NM_002689]	NM_002689
A_24_P913075	9.63E-05	AV647560	AV647560	AV647560 GLC Homo sapiens cDNA clone GLCAZE12 3', mRNA sequence [AV647560]	
A_23_P60227	9.64E-05	NM_005893	NM_005893	Homo sapiens calicin (CCIN), mRNA [NM_005893]	NM_005893
A_23_P37415	9.65E-05	NM_014701	NM_014701	Homo sapiens KIAA0256 gene product (KIAA0256), mRNA [NM_014701]	NM_014701
A_24_P274795	9.66E-05	NM_018719	NM_018719	Homo sapiens cell division cycle associated 7-like (CDCA7L), mRNA [NM_018719]	NM_018719
A_23_P40611	9.67E-05	NM_000355	NM_000355	Homo sapiens transcobalamin II; macrocytic anemia (TCN2), mRNA [NM_000355]	NM_000355
A_23_P215976	9.68E-05	NM_057749	NM_057749	Homo sapiens cyclin E2 (CCNE2), transcript variant 1, mRNA [NM_057749]	NM_057749
A_23_P206484	9.68E-05	NM_005262	NM_005262	Homo sapiens growth factor, augmenter of liver regeneration (ERV1 homolog, S. cerevisiae) (GFER), mRNA [NM_005262]	NM_005262

A_23_P94636	9.69E-05	NM_018835	NM_018835	Homo sapiens membrane associated DNA binding protein (MNAB), mRNA [NM_018835]	NM_018835
A_23_P2873	9.69E-05	NM_182923	NM_182923	Homo sapiens kinesin 2 60/70kDa (KNS2), transcript variant 2, mRNA [NM_182923]	NM_182923
A_23_P149834	9.70E-05	NM_007055	NM_007055	Homo sapiens polymerase (RNA) III (DNA directed) polypeptide A, 155kDa (POLR3A), mRNA [NM_007055]	NM_007055
A_23_P209477	9.70E-05	NM_177983	NM_177983	Homo sapiens protein phosphatase 1G (formerly 2C), magnesium-dependent, gamma isoform (PPM1G), transcript variant 1, mRNA [NM_177983]	NM_177983
A_23_P79161	9.70E-05	NM_013237	NM_013237	Homo sapiens px19-like protein (PX19), mRNA [NM_013237]	NM_013237
A_32_P393316	9.71E-05	U79275	U79275	Human clone 23947 mRNA, partial cds. [U79275]	
A_23_P29083	9.71E-05	NM_003056	NM_003056	Homo sapiens solute carrier family 19 (folate transporter), member 1 (SLC19A1), transcript variant 1, mRNA [NM_003056]	NM_003056
A_24_P71373	9.72E-05	NM_003047	NM_003047	Homo sapiens solute carrier family 9 (sodium/hydrogen exchanger), isoform 1 (antiporter, Na+/H+, amiloride sensitive) (SLC9A1), mRNA [NM_003047]	NM_003047
A_24_P15270	9.72E-05	NM_021574	NM_021574	Homo sapiens breakpoint cluster region (BCR), transcript variant 2, mRNA [NM_021574]	NM_021574
A_24_P203814	9.74E-05	A_24_P203814			
A_23_P120931	9.76E-05	NM_014508	NM_014508	Homo sapiens apolipoprotein B mRNA editing enzyme, catalytic polypeptide-like 3C (APOBEC3C), mRNA [NM_014508]	NM_014508
A_23_P367405	9.77E-05	NM_000281	NM_000281	Homo sapiens 6-pyruvoyl-tetrahydropterin synthase/dimerization cofactor of hepatocyte nuclear factor 1 alpha (TCF1) (PCBD1), transcript variant 1, mRNA [NM_000281]	NM_000281
A_23_P258393	9.79E-05	NM_144641	NM_144641	Homo sapiens protein phosphatase 1M (PP2C domain containing) (PPM1M), mRNA [NM_144641]	NM_144641
A_23_P120254	9.79E-05	NM_020185	NM_020185	Homo sapiens dual specificity phosphatase 22 (DUSP22), mRNA [NM_020185]	NM_020185
A_24_P934135	9.82E-05	AK092791	AK092791	Homo sapiens cDNA FLJ35472 fis, clone SMINT2007062. [AK092791]	
A_23_P210849	9.83E-05	NM_002862	NM_002862	Homo sapiens phosphorylase, glycogen; brain (PYGB), mRNA [NM_002862]	NM_002862
A_32_P738377	9.84E-05	NM_194310	NM_194310	Homo sapiens hypothetical protein LOC284837 (LOC284837), mRNA [NM_194310]	NM_194310
A_23_P24751	9.84E-05	NM_173810	NM_173810	Homo sapiens hypothetical protein MGC29649 (MGC29649), mRNA [NM_173810]	NM_173810
A_23_P319492	9.85E-05	NM_024556	NM_024556	Homo sapiens hypothetical protein FLJ21103 (FLJ21103), mRNA [NM_024556]	NM_024556
A_32_P120567	9.87E-05	NM_199072	NM_199072	Homo sapiens MyoD family inhibitor domain containing (MDFIC), mRNA [NM_199072]	NM_199072
A_23_P47691	9.87E-05	NM_003141	NM_003141	Homo sapiens tripartite motif-containing 21 (TRIM21), mRNA [NM_003141]	NM_003141
A_23_P35066	9.90E-05	NM_015976	NM_015976	Homo sapiens sorting nexin 7 (SNX7), transcript variant 1, mRNA [NM_015976]	NM_015976
A_32_P63166	9.91E-05	W45382	W45382	W45382 zc80e10.s1 Pancreatic Islet Homo sapiens cDNA clone IMAGE:328650 3' similar to gb:D13748 EUKARYOTIC INITIATION FACTOR 4A-I (HUMAN);, mRNA sequence [W45382]	
A_24_P263595	9.93E-05	BX648280	BX648280	Homo sapiens mRNA; cDNA DKFZp686E09125 (from clone DKFZp686E09125). [BX648280]	XM_496134
A_24_P89284	9.93E-05	NM_002109	NM_002109	Homo sapiens histidyl-tRNA synthetase (HARS), mRNA [NM_002109]	NM_002109
A_23_P316531	9.95E-05	NM_005298	NM_005298	Homo sapiens G protein-coupled receptor 25 (GPR25), mRNA [NM_005298]	NM_005298
A_24_P209285	9.95E-05	NM_018141	NM_018141	Homo sapiens mitochondrial ribosomal protein S10 (MRPS10), nuclear gene encoding mitochondrial protein, mRNA [NM_018141]	NM_018141
A_23_P315336	9.96E-05	NM_172209	NM_172209	Homo sapiens TAP binding protein (tapasin) (TAPBP), transcript variant 3, mRNA [NM_172209]	NM_172209
A_23_P410059	1.00E-04	NM_004804	NM_004804	Homo sapiens WD repeat domain 39 (WDR39), mRNA [NM_004804]	NM_004804
A_23_P2582	0.0001	NM_015401	NM_015401	Homo sapiens histone deacetylase 7A (HDAC7A), transcript variant 1, mRNA [NM_015401]	NM_015401
A_24_P366445	0.0001	A_24_P366445			
A_23_P129606	0.000101	NM_001015047	NM_001015047	Homo sapiens DDX19-DDX19L protein (DDX19-DDX19L), mRNA [NM_001015047]	NM_001015047
A_24_P620648	0.000101	AK025276	AK025276	Homo sapiens cDNA: FLJ21623 fis, clone COL07915. [AK025276]	
A_24_P177653	0.000101	ENST00000239725		PREDICTED: Homo sapiens similar to bA508N22.1 (HSPC025) (LOC340947), mRNA [XM_291763]	XM_291763
A_23_P502142	0.000101	NM_002037	NM_002037	Homo sapiens FYN oncogene related to SRC, FGR, YES (FYN), transcript variant 1, mRNA [NM_002037]	NM_002037
A_23_P110686	0.000101	NM_003714	NM_003714	Homo sapiens stanniocalcin 2 (STC2), mRNA [NM_003714]	NM_003714
A_23_P57877	0.000102	NM_000688	NM_000688	Homo sapiens aminolevulinate, delta-, synthase 1 (ALAS1), transcript variant 1, mRNA [NM_000688]	NM_000688
A_24_P290783	0.000102	BC011942	BC011942	Homo sapiens cDNA clone IMAGE:4336144, partial cds. [BC011942]	
A_24_P676216	0.000102	ENST00000342859		PREDICTED: Homo sapiens similar to Glyceraldehyde 3-phosphate dehydrogenase, liver (GAPDH) (LOC392549), mRNA [XM_373373]	XM_373373
A_23_P12601	0.000102	NM_018706	NM_018706	Homo sapiens dehydrogenase E1 and transketolase domain containing 1 (DHTKD1), mRNA [NM_018706]	NM_018706
A_23_P142776	0.000102	NM_003754	NM_003754	Homo sapiens eukaryotic translation initiation factor 3, subunit 5 epsilon, 47kDa (EIF3S5), mRNA [NM_003754]	NM_003754
A_32_P28872	0.000102	NM_006755	NM_006755	Homo sapiens transaldolase 1 (TALDO1), mRNA [NM_006755]	NM_006755
A_23_P427114	0.000103	NM_031899	NM_031899	Homo sapiens golgi reassembly stacking protein 1, 65kDa (GORASP1), mRNA [NM_031899]	NM_031899

A_24_P36944	0.000103	NM_014812	NM_014812	Homo sapiens KIAA0470 (KIAA0470), mRNA [NM_014812]	NM_014812
A_23_P47735	0.000103	NM_032871	NM_032871	Homo sapiens tumor necrosis factor receptor superfamily, member 19-like (TNFRSF19L), transcript variant 1, mRNA [NM_032871]	NM_032871
A_24_P935652	0.000103	CR606629	CR606629	full-length cDNA clone CS0DJ007YF12 of T cells (Jurkat cell line) Cot 10-normalized of Homo sapiens (human). [CR606629]	
A_32_P159574	0.000103	BC028022	BC028022	Homo sapiens, clone IMAGE:5218412, mRNA. [BC028022]	
A_24_P838947	0.000103	BC037244	BC037244	Homo sapiens, clone IMAGE:5168266, mRNA. [BC037244]	
A_24_P283294	0.000103	NM_018141	NM_018141	Homo sapiens mitochondrial ribosomal protein S10 (MRPS10), nuclear gene encoding mitochondrial protein, mRNA [NM_018141]	NM_018141
A_23_P416656	0.000103	NM_033375	NM_033375	Homo sapiens myosin IC (MYO1C), mRNA [NM_033375]	NM_033375
A_23_P218331	0.000103	NM_001017916	NM_001017916	Homo sapiens cytochrome b-561 (CYB561), transcript variant 2, mRNA [NM_001017916]	NM_001017916
A_23_P202199	0.000103	NM_014720	NM_014720	Homo sapiens STE20-like kinase (yeast) (SLK), mRNA [NM_014720]	NM_014720
A_23_P66241	0.000103	NM_176870	NM_176870	Homo sapiens metallothionein 1K (MT1K), mRNA [NM_176870]	NM_176870
A_24_P31235	0.000103	NM_001970	NM_001970	Homo sapiens eukaryotic translation initiation factor 5A (EIF5A), mRNA [NM_001970]	NM_001970
A_23_P162238	0.000104	X54871	X54871	H.sapiens mRNA for ras-related protein Rab5b. [X54871]	
A_23_P129075	0.000104	NM_024908	NM_024908	Homo sapiens WD repeat domain 76 (WDR76), mRNA [NM_024908]	NM_024908
A_23_P100788	0.000104	NM_012448	NM_012448	Homo sapiens signal transducer and activator of transcription 5B (STAT5B), mRNA [NM_012448]	NM_012448
A_23_P320887	0.000104	NM_020239	NM_020239	Homo sapiens CDC42 small effector 1 (CDC42SE1), mRNA [NM_020239]	NM_020239
A_23_P145247	0.000104	NM_152736	NM_152736	Homo sapiens zinc finger protein 187 (ZNF187), transcript variant 3, mRNA [NM_152736]	NM_152736
A_24_P85099	0.000104	NM_003483	NM_003483	Homo sapiens high mobility group AT-hook 2 (HMGA2), transcript variant 1, mRNA [NM_003483]	NM_003483
A_24_P143032	0.000104	NM_007061	NM_007061	Homo sapiens CDC42 effector protein (Rho GTPase binding) 1 (CDC42EP1), transcript variant 2, mRNA [NM_007061]	NM_007061
A_23_P111311	0.000104	NM_144497	NM_144497	Homo sapiens A kinase (PRKA) anchor protein (gravin) 12 (AKAP12), transcript variant 2, mRNA [NM_144497]	NM_144497
A_23_P111228	0.000104	NM_017421	NM_017421	Homo sapiens coenzyme Q3 homolog, methyltransferase (yeast) (COQ3), mRNA [NM_017421]	NM_017421
A_32_P184937	0.000104	BU678941	BU678941	BU678941 UI-CF-DU1-aat-a-09-0-UI.s1 UI-CF-DU1 Homo sapiens cDNA clone UI-CF-DU1-aat-a-09-0-UI 3', mRNA sequence [BU678941]	
A_23_P65963	0.000104	NM_016561	NM_016561	Homo sapiens bifunctional apoptosis regulator (BFAR), mRNA [NM_016561]	NM_016561
A_32_P186018	0.000104	THC2289056			
A_24_P290314	0.000104	ENST00000332498			
A_23_P357780	0.000104	NM_004109	NM_004109	Homo sapiens ferredoxin 1 (FDX1), nuclear gene encoding mitochondrial protein, mRNA [NM_004109]	NM_004109
A_32_P104825	0.000104	NM_080678	NM_080678	Homo sapiens NEDD8-conjugating enzyme (NCE2), mRNA [NM_080678]	NM_080678
A_23_P123234	0.000104	A_23_P123234			
A_23_P70213	0.000105	NM_000038	NM_000038	Homo sapiens adenomatosis polyposis coli (APC), mRNA [NM_000038]	NM_000038
A_23_P72697	0.000105	NM_178172	NM_178172	Homo sapiens high density lipoprotein-binding protein (LOC338328), mRNA [NM_178172]	NM_178172
A_24_P220575	0.000105	XM_292197	XM_292197	PREDICTED: Homo sapiens similar to bA215B13.2 (fumarate hydratase (FH) pseudogene) (LOC341651), mRNA [XM_292197]	XM_292197
A_23_P87528	0.000105	NM_005504	NM_005504	Homo sapiens branched chain aminotransferase 1, cytosolic (BCAT1), mRNA [NM_005504]	NM_005504
A_24_P33156	0.000105	NM_001010982	NM_001010982	Homo sapiens arylformamidase (AFMID), mRNA [NM_001010982]	NM_001010982
A_23_P113728	0.000105	NM_138384	NM_138384	Homo sapiens GTP_binding protein (GTP), mRNA [NM_138384]	NM_138384
A_32_P3127	0.000105	AF038199	AF038199	Homo sapiens clone 23728 mRNA sequence. [AF038199]	
A_24_P266037	0.000105	NM_014473	NM_014473	Homo sapiens dimethyladenosine transferase (HSA9761), mRNA [NM_014473]	NM_014473
A_23_P100392	0.000105	NM_007108	NM_007108	Homo sapiens transcription elongation factor B (SIII), polypeptide 2 (18kDa, elongin B) (TCEB2), transcript variant 1, mRNA [NM_007108]	NM_007108
A_24_P915371	0.000105	AF487338	AF487338	Homo sapiens multiple myeloma overexpression gene 2 (MYEOV2) mRNA, complete cds. [AF487338]	
A_32_P218228	0.000106	NM_001002034	NM_001002034	Homo sapiens LOC150368 protein (LOC150368), mRNA [NM_001002034]	NM_001002034
A_32_P224850	0.000106	THC2378694			
A_23_P215566	0.000106	NM_001621	NM_001621	Homo sapiens aryl hydrocarbon receptor (AHR), mRNA [NM_001621]	NM_001621
A_24_P21752	0.000106	NM_032811	NM_032811	Homo sapiens transforming growth factor beta regulator 1 (TBRG1), mRNA [NM_032811]	NM_032811
A_23_P90533	0.000106	NM_006627	NM_006627	Homo sapiens processing of precursor 4, ribonuclease P/MRP subunit (S. cerevisiae) (POP4), mRNA [NM_006627]	NM_006627
A_23_P35906	0.000106	NM_033306	NM_033306	Homo sapiens caspase 4, apoptosis-related cysteine protease (CASP4), transcript variant gamma, mRNA [NM_033306]	NM_033306
A_24_P201089	0.000107	NM_016612	NM_016612	Homo sapiens solute carrier family 25, member 37 (SLC25A37), transcript variant 1, mRNA [NM_016612]	NM_016612

A_32_P147790	0.000107	BC071732	BC071732	Homo sapiens cDNA clone IMAGE:3862422, partial cds. [BC071732]	
A_23_P47980	0.000107	NM_033045	NM_033045	Homo sapiens keratin, hair, basic, 4 (KRTHB4), mRNA [NM_033045]	NM_033045
A_24_P632160	0.000107	A_24_P632160			
A_32_P6917	0.000107	NM_025207	NM_025207	Homo sapiens FAD-synthetase (PP591), transcript variant 1, mRNA [NM_025207]	NM_025207
A_23_P420873	0.000107	NM_021724	NM_021724	Homo sapiens nuclear receptor subfamily 1, group D, member 1 (NR1D1), mRNA [NM_021724]	NM_021724
A_23_P214354	0.000107	NM_018303	NM_018303	Homo sapiens SEC5-like 1 (S. cerevisiae) (SEC5L1), mRNA [NM_018303]	NM_018303
A_23_P380076	0.000107	BC072415	BC072415	Homo sapiens cDNA clone IMAGE:6168734, partial cds. [BC072415]	
A_23_P210726	0.000107	NM_021874	NM_021874	Homo sapiens cell division cycle 25B (CDC25B), transcript variant 4, mRNA [NM_021874]	NM_021874
A_24_P100664	0.000107	NM_170784	NM_170784	Homo sapiens McKusick-Kaufman syndrome (MKKS), transcript variant 2, mRNA [NM_170784]	NM_170784
A_24_P39843	0.000107	NM_001017916	NM_001017916	Homo sapiens cytochrome b-561 (CYB561), transcript variant 2, mRNA [NM_001017916]	NM_001017916
A_23_P362046	0.000107	NM_138779	NM_138779	Homo sapiens hypothetical protein BC015148 (LOC93081), mRNA [NM_138779]	NM_138779
A_23_P15113	0.000107	NM_001006634	NM_001006634	Homo sapiens Rho GTPase activating protein 17 (ARHGAP17), transcript variant 1, mRNA [NM_001006634]	NM_001006634
A_24_P287129	0.000107	NM_006597	NM_006597	Homo sapiens heat shock 70kDa protein 8 (HSPA8), transcript variant 1, mRNA [NM_006597]	NM_006597
A_24_P324577	0.000108	AB040899	AB040899	Homo sapiens mRNA for KIAA1466 protein, partial cds. [AB040899]	XM_374529
A_24_P934162	0.000108	AK128413	AK128413	Homo sapiens cDNA FLJ46556 fis, clone THYMU3039807. [AK128413]	
A_24_P194730	0.000108	NM_181846	NM_181846	Homo sapiens GLI-Kruppel family member HKR2 (HKR2), mRNA [NM_181846]	NM_181846
A_23_P1248	0.000108	NM_017787	NM_017787	Homo sapiens chromosome 10 open reading frame 26 (C10orf26), mRNA [NM_017787]	NM_017787
A_23_P146379	0.000108	NM_012416	NM_012416	Homo sapiens RAN binding protein 6 (RANBP6), mRNA [NM_012416]	NM_012416
A_24_P102726	0.000108	NM_000309	NM_000309	Homo sapiens protoporphyrinogen oxidase (PPOX), nuclear gene encoding mitochondrial protein, mRNA [NM_000309]	NM_000309
A_23_P400255	0.000108	NM_004090	NM_004090	Homo sapiens dual specificity phosphatase 3 (vaccinia virus phosphatase VH1-related) (DUSP3), mRNA [NM_004090]	NM_004090
A_23_P161152	0.000108	NM_014317	NM_014317	Homo sapiens trans-prenyltransferase (TPRT), mRNA [NM_014317]	NM_014317
A_23_P204484	0.000108	NM_006861	NM_006861	Homo sapiens RAB35, member RAS oncogene family (RAB35), mRNA [NM_006861]	NM_006861
A_23_P55190	0.000108	NM_004247	NM_004247	Homo sapiens elongation factor Tu GTP binding domain containing 2 (EFTUD2), mRNA [NM_004247]	NM_004247
A_23_P81017	0.000109	NM_020337	NM_020337	Homo sapiens KIAA1223 protein (KIAA1223), mRNA [NM_020337]	NM_020337
A_24_P203407	0.000109	BC029662	BC029662	Homo sapiens chromosome 20 open reading frame 142, mRNA (cDNA clone IMAGE:4933017), with apparent retained intron. [BC029662]	XM_371399
A_32_P154091	0.000109	AK124295	AK124295	Homo sapiens cDNA FLJ42301 fis, clone TOVAR2002514. [AK124295]	XM_374260
A_23_P114105	0.000109	NM_012317	NM_012317	Homo sapiens leucine zipper, down-regulated in cancer 1 (LDOC1), mRNA [NM_012317]	NM_012317
A_23_P38115	0.000109	NM_017748	NM_017748	Homo sapiens hypothetical protein FLJ20291 (FLJ20291), mRNA [NM_017748]	NM_017748
A_23_P309224	0.000109	NM_018238	NM_018238	Homo sapiens multiple substrate lipid kinase (MULK), mRNA [NM_018238]	NM_018238
A_24_P572229	0.000109	A_24_P572229			
A_23_P87432	0.000109	BC025767	BC025767	Homo sapiens rhophilin, Rho GTPase binding protein 1, mRNA (cDNA clone MGC:34714 IMAGE:5211354), complete cds. [BC025767]	
A_23_P103837	0.000109	D63478	D63478	Human mRNA for KIAA0144 gene, complete cds. [D63478]	
A_24_P152094	0.000109	XM_062890	XM_062890	PREDICTED: Homo sapiens similar to peptidyl-Pro cis trans isomerase (LOC121981), mRNA [XM_062890]	XM_062890
A_23_P63190	0.000109	NM_002524	NM_002524	Homo sapiens neuroblastoma RAS viral (v-ras) oncogene homolog (NRAS), mRNA [NM_002524]	NM_002524
A_32_P113508	0.000109	NM_030803	NM_030803	Homo sapiens APG16 autophagy 16-like (S. cerevisiae) (APG16L), transcript variant 1, mRNA [NM_030803]	NM_030803
A_32_P99902	0.000109	NM_144597	NM_144597	Homo sapiens hypothetical protein MGC29937 (MGC29937), mRNA [NM_144597]	NM_144597
A_23_P254997	0.000109	NM_020408	NM_020408	Homo sapiens chromosome 6 open reading frame 149 (C6orf149), mRNA [NM_020408]	NM_020408
A_23_P54991	0.000109	NM_080677	NM_080677	Homo sapiens dynein light chain 2 (Dlc2), mRNA [NM_080677]	NM_080677
A_24_P202306	0.000109	NM_001383	NM_001383	Homo sapiens DPH1 homolog (S. cerevisiae) (DPH1), mRNA [NM_001383]	NM_001383
A_32_P96776	0.000109	THC2311248		Q8WVI0 (Q8WVI0) LOC440957 protein (Fragment), partial (16%) [THC2311248]	
A_24_P161403	0.000109	BG190769	BG190769	RST9844 Athersys RAGE Library Homo sapiens cDNA, mRNA sequence [BG190769]	XM_067176
A_32_P24762	0.00011	THC2272599		CA438893 UI-H-DH0-aut-f-13-0-UI.s1 NCI_CGAP_DH0 Homo sapiens cDNA clone UI-H-DH0-aut-f-13-0-UI 3', mRNA sequence [CA438893]	
A_32_P177267	0.00011	BC056262	BC056262	Homo sapiens similar to mitochondrial carrier triple repeat 1, mRNA (cDNA clone MGC:62031 IMAGE:6618254), complete cds. [BC056262]	XM_084000
A_32_P72968	0.00011	BG989839	BG989839	BG989839 PM0-HT0913-200101-004-f03 HT0913 Homo sapiens cDNA, mRNA sequence [BG989839]	

A_24_P414446	0.00011	NM_138348	NM_138348	Homo sapiens hypothetical protein BC007706 (LOC90268), mRNA [NM_138348]	NM_138348
A_23_P14754	0.00011	NM_178232	NM_178232	Homo sapiens hyaluronan and proteoglycan link protein 3 (HAPLN3), mRNA [NM_178232]	NM_178232
A_24_P76805	0.00011	NM_205861	NM_205861	Homo sapiens dehydrodolichyl diphosphate synthase (DHDDS), transcript variant 2, mRNA [NM_205861]	NM_205861
A_24_P340286	0.00011	BC068590	BC068590	Homo sapiens hypothetical protein BC012882, mRNA (cDNA clone IMAGE:4819656), partial cds. [BC068590]	
A_23_P55045	0.00011	NM_021734	NM_021734	Homo sapiens solute carrier family 25 (mitochondrial deoxynucleotide carrier), member 19 (SLC25A19), mRNA [NM_021734]	NM_021734
A_23_P164427	0.00011	NM_024052	NM_024052	Homo sapiens chromosome 17 open reading frame 39 (C17orf39), mRNA [NM_024052]	NM_024052
A_23_P98275	0.00011	NM_002419	NM_002419	Homo sapiens mitogen-activated protein kinase kinase kinase 11 (MAP3K11), mRNA [NM_002419]	NM_002419
A_23_P135778	0.00011	NM_018461	NM_018461	Homo sapiens protein phosphatase 2, regulatory subunit B, delta isoform (PPP2R2D), transcript variant 1, mRNA [NM_018461]	NM_018461
A_24_P335358	0.00011	NM_025215	NM_025215	Homo sapiens pseudouridylate synthase 1 (PUS1), transcript variant 1, mRNA [NM_025215]	NM_025215
A_23_P10685	0.00011	NM_012267	NM_012267	Homo sapiens hsp70-interacting protein (HSPBP1), mRNA [NM_012267]	NM_012267
A_23_P213000	0.00011	NM_017491	NM_017491	Homo sapiens WD repeat domain 1 (WDR1), transcript variant 1, mRNA [NM_017491]	NM_017491
A_23_P55174	0.000111	NM_138387	NM_138387	Homo sapiens glucose 6 phosphatase, catalytic, 3 (G6PC3), mRNA [NM_138387]	NM_138387
A_23_P350172	0.000111	ENST00000299563		Homo sapiens mRNA; cDNA DKFZp686K10126 (from clone DKFZp686K10126). [BX640750]	XM_495886
A_24_P134340	0.000111	NM_182483	NM_182483	Homo sapiens NSFL1 (p97) cofactor (p47) (NSFL1C), transcript variant 3, mRNA [NM_182483]	NM_182483
A_23_P134176	0.000111	NM_000636	NM_000636	Homo sapiens superoxide dismutase 2, mitochondrial (SOD2), nuclear gene encoding mitochondrial protein, transcript variant 1, mRNA [NM_000636]	NM_000636
A_24_P340491	0.000111	ENST00000278194		Homo sapiens ribosomal protein L36a pseudogene, mRNA (cDNA clone MGC:71312 IMAGE:6259908), complete cds. [BC058160]	
A_24_P925314	0.000112	AK127910	AK127910	Homo sapiens cDNA FLJ46017 fis, clone SPLEN2016139. [AK127910]	
A_23_P205216	0.000112	NM_006649	NM_006649	Homo sapiens UTP14, U3 small nucleolar ribonucleoprotein, homolog A (yeast) (UTP14A), mRNA [NM_006649]	NM_006649
A_32_P208915	0.000112	CA421315	CA421315	CA421315 UI-H-FG0-bdb-f-18-0-UI.s1 NCI_CGAP_EN1_2 Homo sapiens cDNA clone UI-H-FG0-bdb-f-18-0-UI 3', mRNA sequence [CA421315]	
A_23_P143089	0.000112	NM_002437	NM_002437	Homo sapiens MpV17 transgene, murine homolog, glomerulosclerosis (MPV17), mRNA [NM_002437]	NM_002437
A_23_P110504	0.000112	NM_030782	NM_030782	Homo sapiens cisplatin resistance related protein CRR9p (CRR9), mRNA [NM_030782]	NM_030782
A_23_P66038	0.000113	NM_013304	NM_013304	Homo sapiens zinc finger, DHHC-type containing 1 (ZDHHC1), mRNA [NM_013304]	NM_013304
A_24_P110306	0.000113	ENST00000330140		Homo sapiens cDNA FLJ26697 fis, clone PCD00618. [AK130207]	
A_24_P15702	0.000113	A_24_P15702			
A_24_P417526	0.000113	NM_207350	NM_207350	Homo sapiens similar to FRG1 protein (FSHD region gene 1 protein) (MGC72104), mRNA [NM_207350]	NM_207350
A_24_P152404	0.000113	BC032118	BC032118	Homo sapiens cDNA clone IMAGE:5016307, partial cds. [BC032118]	
A_24_P131697	0.000113	NM_022899	NM_022899	Homo sapiens ARP8 actin-related protein 8 homolog (yeast) (ACTR8), mRNA [NM_022899]	NM_022899
A_23_P213344	0.000113	NM_005219	NM_005219	Homo sapiens diaphanous homolog 1 (Drosophila) (DIAPH1), mRNA [NM_005219]	NM_005219
A_24_P202527	0.000113	NM_005901	NM_005901	Homo sapiens SMAD, mothers against DPP homolog 2 (Drosophila) (SMAD2), transcript variant 1, mRNA [NM_005901]	NM_005901
A_24_P282237	0.000114	NM_000947	NM_000947	Homo sapiens primase, polypeptide 2A, 58kDa (PRIM2A), mRNA [NM_000947]	NM_000947
A_24_P48284	0.000114	NM_017928	NM_017928	Homo sapiens hypothetical protein FLJ20694 (FLJ20694), mRNA [NM_017928]	NM_017928
A_23_P381203	0.000114	NM_015202	NM_015202	Homo sapiens KIAA0556 protein (KIAA0556), mRNA [NM_015202]	NM_015202
A_24_P326708	0.000114	NM_003957	NM_003957	Homo sapiens BR serine/threonine kinase 2 (BRSK2), mRNA [NM_003957]	NM_003957
A_24_P50381	0.000115	A_24_P50381			
A_24_P199121	0.000115	NM_004609	NM_004609	Homo sapiens transcription factor 15 (basic helix-loop-helix) (TCF15), mRNA [NM_004609]	NM_004609
A_24_P341279	0.000115	NM_014965	NM_014965	Homo sapiens OGT(O-Glc-NAc transferase)-interacting protein 106 KDa (OIP106), mRNA [NM_014965]	NM_014965
A_23_P208915	0.000115	NM_139159	NM_139159	Homo sapiens dipeptidylpeptidase 9 (DPP9), mRNA [NM_139159]	NM_139159
A_23_P312224	0.000115	NM_019022	NM_019022	Homo sapiens thioredoxin domain containing 10 (TXNDC10), mRNA [NM_019022]	NM_019022
A_24_P593733	0.000115	XM_497005	XM_497005	PREDICTED: Homo sapiens similar to G protein pathway suppressor 2 (LOC392281), mRNA [XM_497005]	XM_497005
A_32_P147297	0.000115	CR592318	CR592318	full-length cDNA clone CS0DC003YI17 of Neuroblastoma Cot 25-normalized of Homo sapiens (human). [CR592318]	
A_24_P322354	0.000115	NM_145060	NM_145060	Homo sapiens chromosome 18 open reading frame 24 (C18orf24), mRNA [NM_145060]	NM_145060
A_23_P146361	0.000115	NM_007171	NM_007171	Homo sapiens protein-O-mannosyltransferase 1 (POMT1), mRNA [NM_007171]	NM_007171
A_23_P90634	0.000115	NM_152523	NM_152523	Homo sapiens hypothetical protein FLJ40432 (FLJ40432), mRNA [NM_152523]	NM_152523

A_32_P95223	0.000115	ENST00000358551		PREDICTED: Homo sapiens similar to Farnesyl pyrophosphate synthetase (FPP synthetase) (FPS) (Farnesyl diphosphate synthetase) (LOC441261), mRNA [XM_496902]	XM_496902
A_23_P157943	0.000116	NM_016219	NM_016219	Homo sapiens mannosidase, alpha, class 1B, member 1 (MAN1B1), mRNA [NM_016219]	NM_016219
A_23_P99253	0.000116	NM_004664	NM_004664	Homo sapiens lin-7 homolog A (C. elegans) (LIN7A), mRNA [NM_004664]	NM_004664
A_23_P98232	0.000116	NM_006782	NM_006782	Homo sapiens zinc finger protein-like 1 (ZFPL1), mRNA [NM_006782]	NM_006782
A_32_P86009	0.000116	A_32_P86009			
A_24_P50972	0.000117	AK097517	AK097517	Homo sapiens cDNA FLJ40198 fis, clone TESTI2019975, weakly similar to TRICHOHYALIN. [AK097517]	XM_498421
A_24_P312519	0.000117	NM_022129	NM_022129	Homo sapiens MAWD binding protein (MAWBP), mRNA [NM_022129]	NM_022129
A_23_P152199	0.000117	NM_001907	NM_001907	Homo sapiens chymotrypsin-like (CTRL), mRNA [NM_001907]	NM_001907
A_24_P302802	0.000117	NM_000532	NM_000532	Homo sapiens propionyl Coenzyme A carboxylase, beta polypeptide (PCCB), mRNA [NM_000532]	NM_000532
A_24_P873598	0.000118	AL050122	AL050122	Homo sapiens mRNA; cDNA DKFZp586E121 (from clone DKFZp586E121) [AL050122]	
A_24_P349782	0.000118	AK092305	AK092305	Homo sapiens cDNA FLJ34986 fis, clone OCBBF2010945. [AK092305]	
A_32_P191665	0.000118	BF088423	BF088423	BF088423 CM1-HT0877-190900-426-c05 HT0877 Homo sapiens cDNA, mRNA sequence [BF088423]	
A_24_P229531	0.000118	NM_022837	NM_022837	Homo sapiens hypothetical protein FLJ22833 (FLJ22833), mRNA [NM_022837]	NM_022837
A_24_P200549	0.000118	NM_017706	NM_017706	Homo sapiens WD repeat domain 55 (WDR55), mRNA [NM_017706]	NM_017706
A_32_P101235	0.000118	NM_013282	NM_013282	Homo sapiens ubiquitin-like, containing PHD and RING finger domains, 1 (UHRF1), mRNA [NM_013282]	NM_013282
A_24_P191656	0.000118	NM_014049	NM_014049	Homo sapiens acyl-Coenzyme A dehydrogenase family, member 9 (ACAD9), mRNA [NM_014049]	NM_014049
A_23_P102517	0.000118	NM_002601	NM_002601	Homo sapiens phosphodiesterase 6D, cGMP-specific, rod, delta (PDE6D), mRNA [NM_002601]	NM_002601
A_32_P234604	0.000118	NM_005022	NM_005022	Homo sapiens profilin 1 (PFN1), mRNA [NM_005022]	NM_005022
A_24_P367571	0.000119	NM_017794	NM_017794	Homo sapiens KIAA1797 (KIAA1797), mRNA [NM_017794]	NM_017794
A_24_P353709	0.000119	BC002431	BC002431	Homo sapiens UDP-Gal:betaGlcNAc beta 1,4- galactosyltransferase, polypeptide 2, mRNA (cDNA clone MGC:2008 IMAGE:3347310), complete cds. [BC002431]	
A_32_P61729	0.000119	NM_201430	NM_201430	Homo sapiens reticulon 3 (RTN3), transcript variant 4, mRNA [NM_201430]	NM_201430
A_24_P107291	0.000119	NM_181699	NM_181699	Homo sapiens protein phosphatase 2 (formerly 2A), regulatory subunit A (PR 65), beta isoform (PPP2R1B), transcript variant 2, mRNA [NM_181699]	NM_181699
A_23_P152768	0.000119	NM_001070	NM_001070	Homo sapiens tubulin, gamma 1 (TUBG1), mRNA [NM_001070]	NM_001070
A_23_P58529	0.000119	NM_014473	NM_014473	Homo sapiens dimethyladenosine transferase (HSA9761), mRNA [NM_014473]	NM_014473
A_24_P354468	0.000119	NM_002106	NM_002106	Homo sapiens H2A histone family, member Z (H2AFZ), mRNA [NM_002106]	NM_002106
A_23_P119006	0.00012	THC2438889		ALU5_HUMAN (P39192) Alu subfamily SC sequence contamination warning entry, partial (4%) [THC2438889]	
A_23_P170088	0.00012	BC065002	BC065002	Homo sapiens hypothetical protein FLJ20433, mRNA (cDNA clone IMAGE:6023681), complete cds. [BC065002]	
A_23_P359497	0.00012	BC010609	BC010609	Homo sapiens carbohydrate (N-acetylglucosamine 6-O) sulfotransferase 5, mRNA (cDNA clone MGC:16805 IMAGE:4214587), complete cds. [BC010609]	
A_23_P13885	0.00012	NM_001007026	NM_001007026	Homo sapiens atrophin 1 (ATN1), transcript variant 1, mRNA [NM_001007026]	NM_001007026
A_23_P204958	0.00012	NM_014572	NM_014572	Homo sapiens LATS, large tumor suppressor, homolog 2 (Drosophila) (LATS2), mRNA [NM_014572]	NM_014572
A_32_P25176	0.00012	NM_207474	NM_207474	Homo sapiens FLJ42953 protein (FLJ42953), mRNA [NM_207474]	NM_207474
A_23_P117683	0.00012	NM_016400	NM_016400	Homo sapiens Huntingtin interacting protein K (HYPK), mRNA [NM_016400]	NM_016400
A_23_P370682	0.000121	NM_138456	NM_138456	Homo sapiens hypothetical protein BC012330 (MGC20410), mRNA [NM_138456]	NM_138456
A_24_P944291	0.000121	NM_016263	NM_016263	Homo sapiens fizzy/cell division cycle 20 related 1 (Drosophila) (FZR1), mRNA [NM_016263]	NM_016263
A_24_P416059	0.000121	NM_006715	NM_006715	Homo sapiens mannosidase, alpha, class 2C, member 1 (MAN2C1), mRNA [NM_006715]	NM_006715
A_23_P120146	0.000121	NM_004257	NM_004257	Homo sapiens transforming growth factor, beta receptor associated protein 1 (TGFBRAP1), mRNA [NM_004257]	NM_004257
A_23_P28538	0.000121	NM_031902	NM_031902	Homo sapiens mitochondrial ribosomal protein S5 (MRPS5), nuclear gene encoding mitochondrial protein, mRNA [NM_031902]	NM_031902
A_24_P933319	0.000121	AK002107	AK002107	Homo sapiens cDNA FLJ11245 fis, clone PLACE1008629. [AK002107]	
A_32_P213831	0.000122	NM_020704	NM_020704	Homo sapiens family with sequence similarity 40, member B (FAM40B), mRNA [NM_020704]	NM_020704
A_23_P334664	0.000122	NM_033247	NM_033247	Homo sapiens promyelocytic leukemia (PML), transcript variant 8, mRNA [NM_033247]	NM_033247
A_24_P63397	0.000122	L10123	L10123	Homo sapiens surfactant protein A mRNA, complete cds. [L10123]	
A_23_P165952	0.000122	NM_024855	NM_024855	Homo sapiens ARP5 actin-related protein 5 homolog (yeast) (ACTR5), mRNA [NM_024855]	NM_024855
A_23_P1775	0.000122	NM_001382	NM_001382	Homo sapiens dolichyl-phosphate (UDP-N-acetylglucosamine) N-acetylglucosaminephosphotransferase 1 (GlcNAc-1-P transferase) (DPAGT1), transcript variant 1, mRNA [NM_001382]	NM_001382

A_23_P107373	0.000122	NM_021079	NM_021079	Homo sapiens N-myristoyltransferase 1 (NMT1), mRNA [NM_021079]	NM_021079
A_23_P218170	0.000122	NM_018671	NM_018671	Homo sapiens smooth muscle cell associated protein-1 (SMAP-1), transcript variant 2, mRNA [NM_018671]	NM_018671
A_32_P18822	0.000123	BM054818	BM054818	BM054818 ie86e05.x1 Melton Normalized Human Islet 4 N4-HIS 1 Homo sapiens cDNA clone IMAGE:5673705 3', mRNA sequence [BM054818]	
A_32_P187851	0.000123	BF689038	BF689038	BF689038 602185294T1 NIH_MGC_43 Homo sapiens cDNA clone IMAGE:4299791 3', mRNA sequence [BF689038]	
A_23_P206077	0.000123	NM_022767	NM_022767	Homo sapiens interferon stimulated exonuclease gene 20kDa-like 1 (ISG20L1), mRNA [NM_022767]	NM_022767
A_32_P138330	0.000123	NM_017592	NM_017592	Homo sapiens intersex-like (Drosophila) (IXL), mRNA [NM_017592]	NM_017592
A_23_P99731	0.000123	A_23_P99731			
A_24_P226782	0.000124	NM_031439	NM_031439	Homo sapiens SRY (sex determining region Y)-box 7 (SOX7), mRNA [NM_031439]	NM_031439
A_23_P217755	0.000124	NM_001649	NM_001649	Homo sapiens apical protein-like (Xenopus laevis) (APXL), mRNA [NM_001649]	NM_001649
A_23_P54064	0.000124	NM_019852	NM_019852	Homo sapiens methyltransferase like 3 (METTL3), mRNA [NM_019852]	NM_019852
A_24_P266035	0.000124	NM_014473	NM_014473	Homo sapiens dimethyladenosine transferase (HSA9761), mRNA [NM_014473]	NM_014473
A_23_P14273	0.000124	NM_024071	NM_024071	Homo sapiens zinc finger, FYVE domain containing 21 (ZFYVE21), mRNA [NM_024071]	NM_024071
A_24_P121846	0.000125	NM_003433	NM_003433	Homo sapiens zinc finger protein 132 (clone pHZ-12) (ZNF132), mRNA [NM_003433]	NM_003433
A_24_P372862	0.000125	NM_001987	NM_001987	Homo sapiens ets variant gene 6 (TEL oncogene) (ETV6), mRNA [NM_001987]	NM_001987
A_23_P120899	0.000125	NM_000395	NM_000395	Homo sapiens colony stimulating factor 2 receptor, beta, low-affinity (granulocyte-macrophage) (CSF2RB), mRNA [NM_000395]	NM_000395
A_23_P140698	0.000125	NM_018163	NM_018163	Homo sapiens DnaJ (Hsp40) homolog, subfamily C, member 17 (DNAJC17), mRNA [NM_018163]	NM_018163
A_23_P43549	0.000125	BC042539	BC042539	Homo sapiens similar to prostaglandin E receptor 4, subtype EP4; PGE receptor, EP4 subtype; prostaglandin E2 receptor, mRNA (cDNA clone IMAGE:4830816), partial cds. [BC042539]	
A_23_P74914	0.000125	NM_014777	NM_014777	Homo sapiens KIAA0133 (KIAA0133), mRNA [NM_014777]	NM_014777
A_32_P918263	0.000125	A_32_P918263			
A_23_P5266	0.000125	NM_023937	NM_023937	Homo sapiens mitochondrial ribosomal protein L34 (MRPL34), nuclear gene encoding mitochondrial protein, mRNA [NM_023937]	NM_023937
A_24_P1731	0.000126	NM_005587	NM_005587	Homo sapiens MADS box transcription enhancer factor 2, polypeptide A (myocyte enhancer factor 2A) (MEF2A), mRNA [NM_005587]	NM_005587
A_23_P432056	0.000126	NM_178568	NM_178568	Homo sapiens reticulon 4 receptor-like 1 (RTN4RL1), mRNA [NM_178568]	NM_178568
A_32_P193695	0.000126	ENST00000329833		GB|AF130088.1|AAG35514.1 PRO2474 [NP297887]	
A_32_P62090	0.000126	ENST00000299415			
A_24_P50801	0.000126	NM_201264	NM_201264	Homo sapiens neuropilin 2 (NRP2), transcript variant 6, mRNA [NM_201264]	NM_201264
A_24_P58894	0.000126	XM_378054	XM_378054	PREDICTED: Homo sapiens similar to hypothetical protein (LOC402360), mRNA [XM_378054]	XM_378054
A_23_P136325	0.000126	NM_133264	NM_133264	Homo sapiens WIRE protein (WIRE), mRNA [NM_133264]	NM_133264
A_23_P9458	0.000126	NM_022490	NM_022490	Homo sapiens polymerase (RNA) I associated factor 1 (PRAF1), mRNA [NM_022490]	NM_022490
A_23_P335198	0.000126	NM_001410	NM_001410	Homo sapiens EGF-like-domain, multiple 4 (EGFL4), mRNA [NM_001410]	NM_001410
A_24_P350576	0.000126	ENST00000284483		Homo sapiens mRNA for KIAA0551 protein, partial cds. [AB011123]	
A_23_P119344	0.000126	NM_003598	NM_003598	Homo sapiens TEA domain family member 2 (TEAD2), mRNA [NM_003598]	NM_003598
A_32_P115258	0.000126	THC2313022		C40201 artifact-warning sequence (translated ALU class C) - human {Homo sapiens;} , partial (4%) [THC2313022]	
A_24_P749042	0.000126	A_24_P749042			
A_23_P258689	0.000126	NM_017802	NM_017802	Homo sapiens hypothetical protein FLJ20397 (FLJ20397), mRNA [NM_017802]	NM_017802
A_32_P104000	0.000126	NM_173475	NM_173475	Homo sapiens hypothetical protein MGC48972 (MGC48972), mRNA [NM_173475]	NM_173475
A_24_P708363	0.000126	XM_170597	XM_170597	PREDICTED: Homo sapiens similar to peptidylprolyl isomerase A (LOC256374), mRNA [XM_170597]	XM_170597
A_24_P31527	0.000127	NM_003926	NM_003926	Homo sapiens methyl-CpG binding domain protein 3 (MBD3), mRNA [NM_003926]	NM_003926
A_24_P345209	0.000127	NM_001004023	NM_001004023	Homo sapiens dual-specificity tyrosine-(Y)-phosphorylation regulated kinase 3 (DYRK3), transcript variant 2, mRNA [NM_001004023]	NM_001004023
A_23_P130182	0.000127	NM_004217	NM_004217	Homo sapiens aurora kinase B (AURKB), mRNA [NM_004217]	NM_004217
A_24_P161655	0.000127	A_24_P161655			
A_23_P97328	0.000127	NM_024319	NM_024319	Homo sapiens chromosome 1 open reading frame 35 (C1orf35), mRNA [NM_024319]	NM_024319
A_24_P752279	0.000127	A_24_P752279			
A_32_P202859	0.000128	NM_181788	NM_181788	Homo sapiens HANP1 (H1T2), mRNA [NM_181788]	NM_181788

A_23_P350467	0.000128	NM_004759	NM_004759	Homo sapiens mitogen-activated protein kinase-activated protein kinase 2 (MAPKAPK2), transcript variant 1, mRNA [NM_004759]	NM_004759
A_23_P12950	0.000128	NM_016506	NM_016506	Homo sapiens kelch repeat and BTB (POZ) domain containing 4 (KBTBD4), transcript variant 2, mRNA [NM_016506]	NM_016506
A_24_P85365	0.000128	ENST00000309979		Homo sapiens N-deacetylase/N-sulfotransferase (heparan glucosaminyl) 2, mRNA (cDNA clone IMAGE:4665687), partial cds. [BC018681]	
A_23_P157170	0.000128	NM_032317	NM_032317	Homo sapiens Williams Beuren syndrome chromosome region 18 (WBSCR18), mRNA [NM_032317]	NM_032317
A_24_P298946	0.000128	A_24_P298946			
A_24_P359838	0.000129	NM_153712	NM_153712	Homo sapiens tubulin tyrosine ligase (TTL), mRNA [NM_153712]	NM_153712
A_24_P407742	0.000129	A_24_P407742			
A_24_P252602	0.000129	NM_014286	NM_014286	Homo sapiens frequenin homolog (Drosophila) (FREQ), mRNA [NM_014286]	NM_014286
A_24_P605233	0.000129	THC2282717		Q87726 (Q87726) Glycoprotein 120 (Fragment), partial (13%) [THC2282717]	
A_24_P669216	0.000129	THC2314754		O99381 (O99381) Cytochrome C oxidase subunit I (Fragment), partial (5%) [THC2314754]	
A_23_P53614	0.000129	NM_006768	NM_006768	Homo sapiens BRCA1 associated protein (BRAP), mRNA [NM_006768]	NM_006768
A_23_P82316	0.000129	NM_024653	NM_024653	Homo sapiens PRKR interacting protein 1 (IL11 inducible) (PRKRIP1), mRNA [NM_024653]	NM_024653
A_23_P171034	0.000129	NM_015922	NM_015922	Homo sapiens NAD(P) dependent steroid dehydrogenase-like (NSDHL), mRNA [NM_015922]	NM_015922
A_24_P363745	0.000129	NM_014665	NM_014665	Homo sapiens leucine rich repeat containing 14 (LRRC14), mRNA [NM_014665]	NM_014665
A_24_P182122	0.000129	ENST00000361390		Homo sapiens NADH dehydrogenase subnit 1 mRNA, partial cds; mitochondrial gene for mitochondrial product. [AF216862]	
A_23_P55136	0.000129	NM_016492	NM_016492	Homo sapiens RAN guanine nucleotide release factor (RANGNRF), mRNA [NM_016492]	NM_016492
A_24_P417984	0.000129	NM_138689	NM_138689	Homo sapiens protein phosphatase 1, regulatory (inhibitor) subunit 14B (PPP1R14B), mRNA [NM_138689]	NM_138689
A_24_P887768	0.00013	THC2303783		BC007276 HSPA8 protein {Homo sapiens;} , partial (6%) [THC2303783]	
A_32_P228917	0.00013	BC002350	BC002350	Homo sapiens cDNA clone IMAGE:2820510. [BC002350]	
A_23_P205818	0.00013	NM_014659	NM_014659	Homo sapiens KIAA0377 gene product (KIAA0377), transcript variant 2, mRNA [NM_014659]	NM_014659
A_23_P130141	0.000131	NM_020162	NM_020162	Homo sapiens DEAH (Asp-Glu-Ala-His) box polypeptide 33 (DHX33), mRNA [NM_020162]	NM_020162
A_23_P35782	0.000131	NM_024771	NM_024771	Homo sapiens hypothetical protein FLJ13848 (FLJ13848), mRNA [NM_024771]	NM_024771
A_23_P380271	0.000131	AK074447	AK074447	Homo sapiens cDNA FLJ23867 fis, clone LNG09729. [AK074447]	
A_23_P157205	0.000131	NM_016116	NM_016116	Homo sapiens ankyrin repeat and SOCS box-containing 4 (ASB4), transcript variant 1, mRNA [NM_016116]	NM_016116
A_23_P161183	0.000131	NM_022494	NM_022494	Homo sapiens zinc finger, DHHC-type containing 6 (ZDHHC6), mRNA [NM_022494]	NM_022494
A_23_P218835	0.000131	NM_002268	NM_002268	Homo sapiens karyopherin alpha 4 (importin alpha 3) (KPNA4), mRNA [NM_002268]	NM_002268
A_23_P118888	0.000131	NM_000430	NM_000430	Homo sapiens platelet-activating factor acetylhydrolase, isoform Ib, alpha subunit 45kDa (PAFAH1B1), mRNA [NM_000430]	NM_000430
A_24_P199905	0.000131	NM_006826	NM_006826	Homo sapiens tyrosine 3-monooxygenase/tryptophan 5-monooxygenase activation protein, theta polypeptide (YWHAQ), mRNA [NM_006826]	NM_006826
A_32_P196694	0.000132	THC2353419			
A_24_P900721	0.000132	A_24_P900721			
A_24_P75456	0.000132	ENST00000330189		PREDICTED: Homo sapiens similar to peptidylprolyl isomerase A (LOC402252), mRNA [XM_377934]	XM_377934
A_23_P115636	0.000132	NM_004412	NM_004412	Homo sapiens DNA (cytosine-5-)-methyltransferase 2 (DNMT2), transcript variant a, mRNA [NM_004412]	NM_004412
A_23_P77887	0.000132	NM_006224	NM_006224	Homo sapiens phosphatidylinositol transfer protein, alpha (PITPNA), mRNA [NM_006224]	NM_006224
A_23_P64129	0.000132	NM_006410	NM_006410	Homo sapiens HIV-1 Tat interactive protein 2, 30kDa (HTATIP2), mRNA [NM_006410]	NM_006410
A_24_P258800	0.000133	NM_032484	NM_032484	Homo sapiens homolog of mouse LGP1 (LGP1), mRNA [NM_032484]	NM_032484
A_24_P910833	0.000133	AF086305	AF086305	Homo sapiens full length insert cDNA clone ZD50H12. [AF086305]	
A_23_P408473	0.000133	AL834145	AL834145	Homo sapiens mRNA; cDNA DKFZp434P167 (from clone DKFZp434P167). [AL834145]	XM_378044
A_23_P60146	0.000133	NM_006207	NM_006207	Homo sapiens platelet-derived growth factor receptor-like (PDGFRL), mRNA [NM_006207]	NM_006207
A_23_P107587	0.000133	NM_000271	NM_000271	Homo sapiens Niemann-Pick disease, type C1 (NPC1), mRNA [NM_000271]	NM_000271
A_24_P83158	0.000133	NR_002184	NR_002184	Homo sapiens similar to CGI-96 (dJ222E13.2) on chromosome 22 [NR_002184]	NR_002184
A_24_P409971	0.000133	NM_144573	NM_144573	Homo sapiens nexilin (F actin binding protein) (NEXN), mRNA [NM_144573]	NM_144573
A_24_P213175	0.000133	A_24_P213175			
A_24_P255663	0.000133	A_24_P255663			
A_24_P255082	0.000134	NM_198563	NM_198563	Homo sapiens Similar to RIKEN cDNA 1810038N08 gene (MGC52022), mRNA [NM_198563]	NM_198563

A_24_P97825	0.000134	NM_015621	NM_015621	Homo sapiens DKFZP434C171 protein (DKFZP434C171), mRNA [NM_015621]	NM_015621
A_32_P436884	0.000134	NM_001010867	NM_001010867	Homo sapiens chromosome 1 open reading frame 69 (C1orf69), mRNA [NM_001010867]	NM_001010867
A_23_P111492	0.000134	NM_023948	NM_023948	Homo sapiens motile sperm domain containing 3 (MOSPD3), mRNA [NM_023948]	NM_023948
A_23_P501010	0.000134	NM_000494	NM_000494	Homo sapiens collagen, type XVII, alpha 1 (COL17A1), transcript variant long, mRNA [NM_000494]	NM_000494
A_32_P82807	0.000134	NM_020382	NM_020382	Homo sapiens PR/SET domain containing protein 8 (SET8), mRNA [NM_020382]	NM_020382
A_23_P155106	0.000134	NM_024821	NM_024821	Homo sapiens hypothetical protein FLJ22349 (FLJ22349), mRNA [NM_024821]	NM_024821
A_23_P169249	0.000134	NM_017585	NM_017585	Homo sapiens solute carrier family 2 (facilitated glucose transporter), member 6 (SLC2A6), mRNA [NM_017585]	NM_017585
A_23_P46907	0.000134	NM_006077	NM_006077	Homo sapiens calcium binding atopy-related autoantigen 1 (CBARA1), mRNA [NM_006077]	NM_006077
A_24_P298846	0.000134	NM_001029863	NM_001029863	Homo sapiens chromosome 6 open reading frame 120 (C6orf120), mRNA [NM_001029863]	NM_001029863
A_23_P72044	0.000134	NM_017921	NM_017921	Homo sapiens nuclear protein localization 4 (NPL4), mRNA [NM_017921]	NM_017921
A_23_P155837	0.000135	NM_183075	NM_183075	Homo sapiens cytochrome P450, family 2, subfamily U, polypeptide 1 (CYP2U1), mRNA [NM_183075]	NM_183075
A_24_P266285	0.000135	NM_152558	NM_152558	Homo sapiens IQ motif containing E (IQCE), mRNA [NM_152558]	NM_152558
A_24_P239988	0.000135	NM_006277	NM_006277	Homo sapiens intersectin 2 (ITSN2), transcript variant 1, mRNA [NM_006277]	NM_006277
A_24_P202581	0.000135	NM_199002	NM_199002	Homo sapiens Rho guanine nucleotide exchange factor (GEF) 1 (ARHGEF1), transcript variant 1, mRNA [NM_199002]	NM_199002
A_24_P62708	0.000135	NM_002731	NM_002731	Homo sapiens protein kinase, cAMP-dependent, catalytic, beta (PRKACB), transcript variant 2, mRNA [NM_002731]	NM_002731
A_23_P314712	0.000135	NM_012189	NM_012189	Homo sapiens calcium binding tyrosine-(Y)-phosphorylation regulated (fibrousheathin 2) (CABYR), transcript variant 1, mRNA [NM_012189]	NM_012189
A_23_P17430	0.000135	NM_017495	NM_017495	Homo sapiens RNA-binding region (RNP1, RRM) containing 1 (RNPC1), transcript variant 1, mRNA [NM_017495]	NM_017495
A_23_P66319	0.000135	NM_014567	NM_014567	Homo sapiens breast cancer anti-estrogen resistance 1 (BCAR1), mRNA [NM_014567]	NM_014567
A_23_P201179	0.000136	NM_006608	NM_006608	Homo sapiens putative homeodomain transcription factor 1 (PHTF1), mRNA [NM_006608]	NM_006608
A_23_P37676	0.000136	NM_007223	NM_007223	Homo sapiens putative G protein coupled receptor (GPR), mRNA [NM_007223]	NM_007223
A_23_P167905	0.000136	AK000383	AK000383	Homo sapiens cDNA FLJ20376 fis, clone HUV01087. [AK000383]	
A_23_P10135	0.000136	NM_001001349	NM_001001349	Homo sapiens NFKB inhibitor interacting Ras-like 2 (NKIRAS2), transcript variant 1, mRNA [NM_001001349]	NM_001001349
A_23_P169428	0.000136	NM_015679	NM_015679	Homo sapiens TruB pseudouridine (psi) synthase homolog 2 (E. coli) (TRUB2), mRNA [NM_015679]	NM_015679
A_24_P193011	0.000136	NM_053056	NM_053056	Homo sapiens cyclin D1 (PRAD1: parathyroid adenomatosis 1) (CCND1), mRNA [NM_053056]	NM_053056
A_23_P155229	0.000136	NM_007107	NM_007107	Homo sapiens signal sequence receptor, gamma (translocon-associated protein gamma) (SSR3), mRNA [NM_007107]	NM_007107
A_24_P288323	0.000137	NM_139159	NM_139159	Homo sapiens dipeptidylpeptidase 9 (DPP9), mRNA [NM_139159]	NM_139159
A_24_P123408	0.000137	NM_014945	NM_014945	Homo sapiens actin binding LIM protein family, member 3 (ABLIM3), mRNA [NM_014945]	NM_014945
A_23_P129492	0.000137	NM_016332	NM_016332	Homo sapiens selenoprotein X, 1 (SEPX1), mRNA [NM_016332]	NM_016332
A_23_P82913	0.000137	NM_017956	NM_017956	Homo sapiens homolog of yeast tRNA methyltransferase (TRM12), mRNA [NM_017956]	NM_017956
A_24_P30206	0.000137	NM_078468	NM_078468	Homo sapiens BRCA2 and CDKN1A interacting protein (BCCIP), transcript variant B, mRNA [NM_078468]	NM_078468
A_23_P157513	0.000138	NM_005372	NM_005372	Homo sapiens v-mos Moloney murine sarcoma viral oncogene homolog (MOS), mRNA [NM_005372]	NM_005372
A_23_P3450	0.000138	NM_014444	NM_014444	Homo sapiens gamma tubulin ring complex protein (76p gene) (76P), mRNA [NM_014444]	NM_014444
A_24_P614148	0.000138	NM_206910	NM_206910	Homo sapiens chromosome 6 open reading frame 216 (C6orf216), transcript variant 3, mRNA [NM_206910]	NM_206910
A_24_P692198	0.000138	NM_002080	NM_002080	Homo sapiens glutamic-oxaloacetic transaminase 2, mitochondrial (aspartate aminotransferase 2) (GOT2), mRNA [NM_002080]	NM_002080
A_23_P6456	0.000138	NM_024313	NM_024313	Homo sapiens hypothetical protein MGC3731 (MGC3731), mRNA [NM_024313]	NM_024313
A_24_P335221	0.000139	NM_201430	NM_201430	Homo sapiens reticulon 3 (RTN3), transcript variant 4, mRNA [NM_201430]	NM_201430
A_23_P37296	0.000139	NM_015684	NM_015684	Homo sapiens ATP synthase, H+ transporting, mitochondrial F0 complex, subunit s (factor B) (ATP5S), nuclear gene encoding mitochondrial protein, transcript variant 3, mRNA [NM_015684]	NM_015684
A_24_P341616	0.000139	A_24_P341616			
A_23_P44993	0.000139	NM_006755	NM_006755	Homo sapiens transaldolase 1 (TALDO1), mRNA [NM_006755]	NM_006755
A_32_P193908	0.00014	BM968705	BM968705	BM968705 UI-CF-DU1-aak-f-20-0-UI.s1 UI-CF-DU1 Homo sapiens cDNA clone UI-CF-DU1-aak-f-20-0-UI 3', mRNA sequence [BM968705]	
A_24_P330030	0.00014	NM_153451	NM_153451	Homo sapiens oral cancer overexpressed 1 (ORAOV1), mRNA [NM_153451]	NM_153451
A_32_P214340	0.00014	THC2281903		CXAA_HUMAN Gap junction alpha-10 protein (Connexin 59) (Cx59). {Homo sapiens;} , partial (10%) [THC2281903]	
A_23_P124559	0.00014	NM_031300	NM_031300	Homo sapiens MAX dimerization protein 3 (MXD3), mRNA [NM_031300]	NM_031300

A_23_P71981	0.00014	NM_005702	NM_005702	Homo sapiens Era G-protein-like 1 (E. coli) (ERAL1), mRNA [NM_005702]	NM_005702
A_23_P42198	0.00014	NM_003534	NM_003534	Homo sapiens histone 1, H3g (HIST1H3G), mRNA [NM_003534]	NM_003534
A_24_P371628	0.000141	NM_054027	NM_054027	Homo sapiens ankylosis, progressive homolog (mouse) (ANKH), mRNA [NM_054027]	NM_054027
A_23_P28811	0.000141	NM_014484	NM_014484	Homo sapiens molybdenum cofactor synthesis 3 (MOCS3), mRNA [NM_014484]	NM_014484
A_24_P100277	0.000141	NM_007326	NM_007326	Homo sapiens cytochrome b5 reductase 3 (CYB5R3), transcript variant S, mRNA [NM_007326]	NM_007326
A_23_P257057	0.000141	NM_016647	NM_016647	Homo sapiens mesenchymal stem cell protein DSCD75 (LOC51337), mRNA [NM_016647]	NM_016647
A_23_P47086	0.000141	NM_021046	NM_021046	Homo sapiens keratin associated protein 5-8 (KRTAP5-8), mRNA [NM_021046]	NM_021046
A_23_P14716	0.000141	NM_031284	NM_031284	Homo sapiens ADP-dependent glucokinase (ADPGK), mRNA [NM_031284]	NM_031284
A_24_P160001	0.000141	NM_054014	NM_054014	Homo sapiens FK506 binding protein 1A, 12kDa (FKBP1A), transcript variant 12A, mRNA [NM_054014]	NM_054014
A_24_P235012	0.000141	NM_014341	NM_014341	Homo sapiens mitochondrial carrier homolog 1 (C. elegans) (MTCH1), nuclear gene encoding mitochondrial protein, mRNA [NM_014341]	NM_014341
A_23_P111273	0.000141	NM_016495	NM_016495	Homo sapiens TBC1 domain family, member 7 (TBC1D7), mRNA [NM_016495]	NM_016495
A_24_P324011	0.000142	NM_015353	NM_015353	Homo sapiens potassium channel tetramerisation domain containing 2 (KCTD2), mRNA [NM_015353]	NM_015353
A_23_P49269	0.000142	NM_058192	NM_058192	Homo sapiens RNA pseudouridylate synthase domain containing 1 (RPUSD1), mRNA [NM_058192]	NM_058192
A_24_P679796	0.000142	A_24_P679796			
A_24_P185186	0.000143	NM_174920	NM_174920	Homo sapiens hypothetical protein LOC201191 (LOC201191), mRNA [NM_174920]	NM_174920
A_23_P98159	0.000143	NM_139075	NM_139075	Homo sapiens two pore segment channel 2 (TPCN2), mRNA [NM_139075]	NM_139075
A_24_P272073	0.000143	ENST00000335078			
A_24_P410952	0.000143	NM_003768	NM_003768	Homo sapiens phosphoprotein enriched in astrocytes 15 (PEA15), mRNA [NM_003768]	NM_003768
A_32_P232865	0.000143	NM_021574	NM_021574	Homo sapiens breakpoint cluster region (BCR), transcript variant 2, mRNA [NM_021574]	NM_021574
A_23_P56553	0.000143	NM_024770	NM_024770	Homo sapiens hypothetical protein FLJ13984 (FLJ13984), mRNA [NM_024770]	NM_024770
A_24_P290764	0.000144	BC007879	BC007879	Homo sapiens chromosome 9 open reading frame 10, mRNA (cDNA clone IMAGE:3938062), partial cds. [BC007879]	
A_23_P116682	0.000144	NM_139067	NM_139067	Homo sapiens SWI/SNF related, matrix associated, actin dependent regulator of chromatin, subfamily c, member 2 (SMARCC2), transcript variant 2, mRNA [NM_139067]	NM_139067
A_24_P51683	0.000144	NM_003936	NM_003936	Homo sapiens cyclin-dependent kinase 5, regulatory subunit 2 (p39) (CDK5R2), mRNA [NM_003936]	NM_003936
A_24_P367326	0.000144	ENST00000332687			
A_24_P31583	0.000144	NM_005919	NM_005919	Homo sapiens MADS box transcription enhancer factor 2, polypeptide B (myocyte enhancer factor 2B) (MEF2B), mRNA [NM_005919]	NM_005919
A_23_P432512	0.000144	NM_148175	NM_148175	Homo sapiens peptidylprolyl isomerase (cyclophilin)-like 2 (PPIL2), transcript variant 2, mRNA [NM_148175]	NM_148175
A_23_P162540	0.000144	NM_031954	NM_031954	Homo sapiens potassium channel tetramerisation domain containing 10 (KCTD10), mRNA [NM_031954]	NM_031954
A_23_P319970	0.000144	BC034271	BC034271	Homo sapiens Fanconi anemia, complementation group C, mRNA (cDNA clone IMAGE:4777682), with apparent retained intron. [BC034271]	
A_23_P159039	0.000144	NM_182706	NM_182706	Homo sapiens scribbled homolog (Drosophila) (SCRIB), transcript variant 1, mRNA [NM_182706]	NM_182706
A_24_P247014	0.000144	A_24_P247014			
A_24_P919452	0.000145	THC2281539		Q7PY94 (Q7PY94) ENSANGP00000018352, partial (14%) [THC2281539]	
A_24_P926314	0.000145	NM_199133	NM_199133	Homo sapiens hypothetical protein LOC134145 (LOC134145), mRNA [NM_199133]	NM_199133
A_23_P61100	0.000145	NM_024816	NM_024816	Homo sapiens rabaptin, RAB GTPase binding effector protein 2 (RABEP2), mRNA [NM_024816]	NM_024816
A_24_P23951	0.000145	NM_207474	NM_207474	Homo sapiens FLJ42953 protein (FLJ42953), mRNA [NM_207474]	NM_207474
A_32_P123990	0.000146	BC015835	BC015835	Homo sapiens cDNA clone IMAGE:4293096, partial cds. [BC015835]	
A_24_P925422	0.000146	THC2308587		1AK7 Destrin, Nmr, 20 Structures. {Sus scrofa;} , partial (37%) [THC2308587]	
A_23_P407614	0.000146	NM_152901	NM_152901	Homo sapiens pyrin domain containing 1 (PYDC1), mRNA [NM_152901]	NM_152901
A_23_P19036	0.000146	NM_006058	NM_006058	Homo sapiens TNFAIP3 interacting protein 1 (TNIP1), mRNA [NM_006058]	NM_006058
A_23_P154158	0.000146	NM_032718	NM_032718	Homo sapiens hypothetical protein MGC11332 (MGC11332), mRNA [NM_032718]	NM_032718
A_23_P308073	0.000146	NM_014363	NM_014363	Homo sapiens spastic ataxia of Charlevoix-Saguenay (sacsin) (SACS), mRNA [NM_014363]	NM_014363
A_23_P396541	0.000146	ENST00000310074		Homo sapiens mRNA; cDNA DKFZp686A15231 (from clone DKFZp686A15231). [BX641147]	XM_291105
A_23_P336015	0.000146	NM_015658	NM_015658	Homo sapiens DKFZP564C186 protein (DKFZP564C186), mRNA [NM_015658]	NM_015658
A_32_P206401	0.000146	NM_181701	NM_181701	Homo sapiens quiescin Q6-like 1 (QSCN6L1), mRNA [NM_181701]	NM_181701
A_24_P419309	0.000146	NM_004814	NM_004814	Homo sapiens WD repeat domain 57 (U5 snRNP specific) (WDR57), mRNA [NM_004814]	NM_004814

A_23_P44037	0.000146	NM_001614	NM_001614	Homo sapiens actin, gamma 1 (ACTG1), mRNA [NM_001614]	NM_001614
A_24_P167338	0.000147	NM_014488	NM_014488	Homo sapiens RAB30, member RAS oncogene family (RAB30), mRNA [NM_014488]	NM_014488
A_23_P97541	0.000147	NM_000715	NM_000715	Homo sapiens complement component 4 binding protein, alpha (C4BPA), mRNA [NM_000715]	NM_000715
A_24_P891899	0.000147	AI369525	AI369525	AI369525 ta68h08.x1 Soares_total_fetus_Nb2HF8_9w Homo sapiens cDNA clone IMAGE:2049279 3' similar to contains element MIR repetitive element ;, mRNA sequence [AI369525]	
A_24_P123052	0.000147	NM_002886	NM_002886	Homo sapiens RAP2B, member of RAS oncogene family (RAP2B), mRNA [NM_002886]	NM_002886
A_23_P75973	0.000147	NM_018320	NM_018320	Homo sapiens ring finger protein 121 (RNF121), transcript variant 1, mRNA [NM_018320]	NM_018320
A_23_P127467	0.000147	NM_032325	NM_032325	Homo sapiens hypothetical protein MGC11102 (MGC11102), mRNA [NM_032325]	NM_032325
A_24_P124032	0.000147	NM_003821	NM_003821	Homo sapiens receptor-interacting serine-threonine kinase 2 (RIPK2), mRNA [NM_003821]	NM_003821
A_24_P163477	0.000147	NM_018292	NM_018292	Homo sapiens glutaminyl-tRNA synthase (glutamine-hydrolyzing)-like 1 (QRSL1), mRNA [NM_018292]	NM_018292
A_23_P117943	0.000147	A_23_P117943			
A_23_P103398	0.000147	NM_000447	NM_000447	Homo sapiens presenilin 2 (Alzheimer disease 4) (PSEN2), transcript variant 1, mRNA [NM_000447]	NM_000447
A_24_P67268	0.000147	ENST00000326693			
A_24_P31003	0.000148	NM_005262	NM_005262	Homo sapiens growth factor, augmenter of liver regeneration (ERV1 homolog, S. cerevisiae) (GFER), mRNA [NM_005262]	NM_005262
A_23_P137361	0.000148	NM_017818	NM_017818	Homo sapiens WD repeat domain 8 (WDR8), mRNA [NM_017818]	NM_017818
A_23_P53346	0.000149	NM_015257	NM_015257	Homo sapiens KIAA0286 protein (KIAA0286), mRNA [NM_015257]	NM_015257
A_24_P315474	0.000149	THC2275974		EF1B_HUMAN (P24534) Elongation factor 1-beta (EF-1-beta), complete [THC2275974]	
A_23_P9086	0.000149	NM_016353	NM_016353	Homo sapiens zinc finger, DHHC-type containing 2 (ZDHHC2), mRNA [NM_016353]	NM_016353
A_24_P621434	0.000149	THC2266610		BC007276 HSPA8 protein {Homo sapiens;} , partial (17%) [THC2266610]	
A_32_P223852	0.000149	THC2350529			
A_23_P501770	0.000149	NM_032166	NM_032166	Homo sapiens three prime repair exonuclease 1 (TREX1), transcript variant 5, mRNA [NM_032166]	NM_032166
A_24_P55971	0.000149	NM_003377	NM_003377	Homo sapiens vascular endothelial growth factor B (VEGFB), mRNA [NM_003377]	NM_003377
A_24_P273865	0.000149	NM_004275	NM_004275	Homo sapiens Trf (TATA binding protein-related factor)-proximal homolog (Drosophila) (TRFP), mRNA [NM_004275]	NM_004275
A_23_P35467	0.000149	NM_018464	NM_018464	Homo sapiens chromosome 10 open reading frame 70 (C10orf70), mRNA [NM_018464]	NM_018464
A_24_P305312	0.00015	NM_014417	NM_014417	Homo sapiens BCL2 binding component 3 (BBC3), mRNA [NM_014417]	NM_014417
A_24_P318073	0.00015	NM_032795	NM_032795	Homo sapiens RNA pseudouridylate synthase domain containing 4 (RPUSD4), mRNA [NM_032795]	NM_032795
A_24_P257099	0.00015	NM_018410	NM_018410	Homo sapiens hypothetical protein DKFZp762E1312 (DKFZp762E1312), mRNA [NM_018410]	NM_018410
A_23_P170352	0.00015	NM_002949	NM_002949	Homo sapiens mitochondrial ribosomal protein L12 (MRPL12), nuclear gene encoding mitochondrial protein, mRNA [NM_002949]	NM_002949
A_24_P127701	0.000151	XM_378015	XM_378015	PREDICTED: Homo sapiens similar to chromosome 11 open reading frame2; chromosome 11 open reading frame2 (LOC402330), mRNA [XM_378015]	XM_378015
A_24_P287780	0.000151	NM_002768	NM_002768	Homo sapiens procollagen (type III) N-endopeptidase (PCOLN3), mRNA [NM_002768]	NM_002768
A_24_P73389	0.000151	NM_003576	NM_003576	Homo sapiens serine/threonine kinase 24 (STE20 homolog, yeast) (STK24), mRNA [NM_003576]	NM_003576
A_24_P246963	0.000151	ENST00000321887			
A_24_P2463	0.000151	NM_133336	NM_133336	Homo sapiens Wolf-Hirschhorn syndrome candidate 1 (WHSC1), transcript variant 9, mRNA [NM_133336]	NM_133336
A_24_P170365	0.000152	A_24_P170365			
A_32_P58601	0.000152	AF086468	AF086468	Homo sapiens full length insert cDNA clone ZD86H05. [AF086468]	
A_24_P771278	0.000152	A_24_P771278			
A_32_P72553	0.000152	THC2430771		ALU1_HUMAN (P39188) Alu subfamily J sequence contamination warning entry, partial (5%) [THC2430771]	
A_24_P106728	0.000152	NM_019042	NM_019042	Homo sapiens hypothetical protein FLJ20485 (FLJ20485), mRNA [NM_019042]	NM_019042
A_24_P106953	0.000152	NM_198939	NM_198939	Homo sapiens prostaglandin E synthase 2 (PTGES2), transcript variant 3, mRNA [NM_198939]	NM_198939
A_24_P404593	0.000152	NM_177405	NM_177405	Homo sapiens cat eye syndrome chromosome region, candidate 1 (CECR1), transcript variant 2, mRNA [NM_177405]	NM_177405
A_23_P230	0.000152	NM_004623	NM_004623	Homo sapiens tetratricopeptide repeat domain 4 (TTC4), mRNA [NM_004623]	NM_004623
A_23_P27827	0.000152	NM_015898	NM_015898	Homo sapiens zinc finger and BTB domain containing 7A (ZBTB7A), mRNA [NM_015898]	NM_015898
A_23_P168629	0.000152	NM_018077	NM_018077	Homo sapiens RNA binding motif protein 28 (RBM28), mRNA [NM_018077]	NM_018077
A_24_P50328	0.000153	A_24_P50328			

A_24_P66685	0.000153	NM_001011713	NM_001011713	Homo sapiens chromosome 14 open reading frame 35 (C14orf35), mRNA [NM_001011713]	NM_001011713
A_23_P55064	0.000153	NM_018081	NM_018081	Homo sapiens hypothetical protein FLJ10385 (FLJ10385), mRNA [NM_018081]	NM_018081
A_23_P337729	0.000153	NM_024789	NM_024789	Homo sapiens chromosome 10 open reading frame 77 (C10orf77), mRNA [NM_024789]	NM_024789
A_24_P51786	0.000153	NM_174908	NM_174908	Homo sapiens chromosome 3 open reading frame 6 (C3orf6), transcript variant 1, mRNA [NM_174908]	NM_174908
A_32_P144238	0.000153	XM_499133	XM_499133	PREDICTED: Homo sapiens LOC441392 (LOC441392), mRNA [XM_499133]	XM_499133
A_24_P161494	0.000153	ENST00000327591		PREDICTED: Homo sapiens similar to 40S ribosomal protein S26 (LOC401470), mRNA [XM_376787]	XM_376787
A_23_P206059	0.000153	NM_003981	NM_003981	Homo sapiens protein regulator of cytokinesis 1 (PRC1), transcript variant 1, mRNA [NM_003981]	NM_003981
A_24_P205137	0.000154	BC011498	BC011498	Homo sapiens histone deacetylase 6, mRNA (cDNA clone IMAGE:4179066), complete cds. [BC011498]	
A_24_P856176	0.000154	A_24_P856176			
A_23_P68401	0.000154	NM_030877	NM_030877	Homo sapiens catenin, beta like 1 (CTNNBL1), mRNA [NM_030877]	NM_030877
A_23_P155441	0.000154	NM_052859	NM_052859	Homo sapiens RFT1 homolog (S. cerevisiae) (RFT1), mRNA [NM_052859]	NM_052859
A_23_P20248	0.000154	NM_002755	NM_002755	Homo sapiens mitogen-activated protein kinase kinase 1 (MAP2K1), mRNA [NM_002755]	NM_002755
A_32_P163147	0.000155	NM_182607	NM_182607	Homo sapiens V-set and immunoglobulin domain containing 1 (VSIG1), mRNA [NM_182607]	NM_182607
A_23_P83599	0.000155	NM_002735	NM_002735	Homo sapiens protein kinase, cAMP-dependent, regulatory, type I, beta (PRKAR1B), mRNA [NM_002735]	NM_002735
A_24_P336577	0.000155	NM_019099	NM_019099	Homo sapiens hypothetical protein LOC55924 (LOC55924), transcript variant 1, mRNA [NM_019099]	NM_019099
A_23_P218269	0.000155	NM_016151	NM_016151	Homo sapiens TAO kinase 2 (TAOK2), mRNA [NM_016151]	NM_016151
A_24_P298545	0.000155	NM_153363	NM_153363	Homo sapiens zinc finger protein 679 (ZNF679), mRNA [NM_153363]	NM_153363
A_23_P71433	0.000155	NM_001001481	NM_001001481	Homo sapiens hypothetical protein FLJ11011 (FLJ11011), transcript variant 1, mRNA [NM_001001481]	NM_001001481
A_23_P93321	0.000156	NM_007109	NM_007109	Homo sapiens transcription factor 19 (SC1) (TCF19), mRNA [NM_007109]	NM_007109
A_24_P922808	0.000156	ENST00000346571		Homo sapiens chromosome 1 open reading frame 121, mRNA (cDNA clone IMAGE:4718788), partial cds. [BC020640]	
A_23_P91168	0.000156	NM_144710	NM_144710	Homo sapiens septin 10 (SEPT10), transcript variant 1, mRNA [NM_144710]	NM_144710
A_23_P136493	0.000156	NM_013962	NM_013962	Homo sapiens neuregulin 1 (NRG1), transcript variant GGF2, mRNA [NM_013962]	NM_013962
A_23_P215491	0.000156	NM_002991	NM_002991	Homo sapiens chemokine (C-C motif) ligand 24 (CCL24), mRNA [NM_002991]	NM_002991
A_23_P49467	0.000156	AF116698	AF116698	Homo sapiens PRO2289 mRNA, complete cds. [AF116698]	
A_32_P80532	0.000157	THC2304788		BF733908 PM2-AN0089-181000-007-h04 AN0089 Homo sapiens cDNA, mRNA sequence [BF733908]	
A_24_P37939	0.000157	CR592161	CR592161	full-length cDNA clone CS0DK011YL05 of HeLa cells Cot 25-normalized of Homo sapiens (human). [CR592161]	
A_24_P940149	0.000157	NM_199050	NM_199050	Homo sapiens chromosome 21 open reading frame 25 (C21orf25), mRNA [NM_199050]	NM_199050
A_23_P352402	0.000157	NM_153256	NM_153256	Homo sapiens chromosome 10 open reading frame 47 (C10orf47), mRNA [NM_153256]	NM_153256
A_23_P63696	0.000157	NM_032112	NM_032112	Homo sapiens mitochondrial ribosomal protein L43 (MRPL43), nuclear gene encoding mitochondrial protein, transcript variant 1, mRNA [NM_032112]	NM_032112
A_23_P89708	0.000158	NM_018129	NM_018129	Homo sapiens pyridoxine 5'-phosphate oxidase (PNPO), mRNA [NM_018129]	NM_018129
A_32_P220897	0.000158	A_32_P220897			
A_23_P143461	0.000158	AJ457067	AJ457067	Homo sapiens partial mRNA for high tyrosine glycine keratin associated protein 19.1 (KRTAP19.1 gene). [AJ457067]	
A_24_P128068	0.000158	BC007822	BC007822	Homo sapiens likely ortholog of mouse ubiquitin-conjugating enzyme E2-230K, mRNA (cDNA clone IMAGE:4303741), partial cds. [BC007822]	
A_23_P123666	0.000158	NM_018421	NM_018421	Homo sapiens TBC1 domain family, member 2 (TBC1D2), mRNA [NM_018421]	NM_018421
A_23_P87141	0.000158	NM_006831	NM_006831	Homo sapiens ATP/GTP-binding protein (HEAB), mRNA [NM_006831]	NM_006831
A_32_P53670	0.000158	AA442488	AA442488	zv59a10.r1 Soares_testis_NHT Homo sapiens cDNA clone IMAGE:757914 5' similar to contains Alu repetitive element;, mRNA sequence [AA442488]	
A_23_P383385	0.000158	NM_019896	NM_019896	Homo sapiens polymerase (DNA-directed), epsilon 4 (p12 subunit) (POLE4), mRNA [NM_019896]	NM_019896
A_24_P49800	0.000158	A_24_P49800			
A_24_P29885	0.000159	NM_002514	NM_002514	Homo sapiens nephroblastoma overexpressed gene (NOV), mRNA [NM_002514]	NM_002514
A_23_P355311	0.000159	NM_003738	NM_003738	Homo sapiens patched homolog 2 (Drosophila) (PTCH2), mRNA [NM_003738]	NM_003738
A_24_P125894	0.000159	NM_014634	NM_014634	Homo sapiens protein phosphatase 1F (PP2C domain containing) (PPM1F), mRNA [NM_014634]	NM_014634
A_32_P18668	0.000159	NM_002867	NM_002867	Homo sapiens RAB3B, member RAS oncogene family (RAB3B), mRNA [NM_002867]	NM_002867
A_23_P13740	0.000159	NM_014903	NM_014903	Homo sapiens neuron navigator 3 (NAV3), mRNA [NM_014903]	NM_014903
A_23_P406616	0.000159	NM_175884	NM_175884	Homo sapiens hypothetical protein FLJ36031 (FLJ36031), mRNA [NM_175884]	NM_175884

A_24_P67408	0.000159	ENST00000331606			
A_24_P215882	0.00016	NM_001176	NM_001176	Homo sapiens Rho GDP dissociation inhibitor (GDI) gamma (ARHGDIG), mRNA [NM_001176]	NM_001176
A_24_P273132	0.00016	NM_013296	NM_013296	Homo sapiens G-protein signalling modulator 2 (AGS3-like, C. elegans) (GPSM2), mRNA [NM_013296]	NM_013296
A_24_P289477	0.00016	ENST00000311232			
A_23_P5845	0.00016	NM_000221	NM_000221	Homo sapiens ketohexokinase (fructokinase) (KHK), transcript variant a, mRNA [NM_000221]	NM_000221
A_23_P397899	0.00016	NM_021218	NM_021218	Homo sapiens chromosome 9 open reading frame 80 (C9orf80), mRNA [NM_021218]	NM_021218
A_32_P100830	0.00016	A_32_P100830			
A_23_P210719	0.00016	NM_016143	NM_016143	Homo sapiens NSFL1 (p97) cofactor (p47) (NSFL1C), transcript variant 1, mRNA [NM_016143]	NM_016143
A_23_P254498	0.00016	NM_016492	NM_016492	Homo sapiens RAN guanine nucleotide release factor (RANGNRF), mRNA [NM_016492]	NM_016492
A_23_P30884	0.00016	NM_001288	NM_001288	Homo sapiens chloride intracellular channel 1 (CLIC1), mRNA [NM_001288]	NM_001288
A_23_P371885	0.000161	NM_016632	NM_016632	Homo sapiens ADP-ribosylation factor-like (LOC51326), mRNA [NM_016632]	NM_016632
A_23_P339557	0.000161	NM_032228	NM_032228	Homo sapiens male sterility domain containing 2 (MLSTD2), mRNA [NM_032228]	NM_032228
A_24_P153448	0.000161	AF218008	AF218008	Homo sapiens clone PP3501 unknown mRNA. [AF218008]	
A_23_P5974	0.000161	NM_080603	NM_080603	Homo sapiens zinc finger, SWIM-type containing 1 (ZSWIM1), mRNA [NM_080603]	NM_080603
A_23_P200741	0.000161	NM_001937	NM_001937	Homo sapiens dermatopontin (DPT), mRNA [NM_001937]	NM_001937
A_23_P88848	0.000161	NM_017803	NM_017803	Homo sapiens dihydrouridine synthase 2-like (SMM1, S. cerevisiae) (DUS2L), mRNA [NM_017803]	NM_017803
A_24_P179033	0.000161	THC2270926		RS2_HUMAN (P15880) 40S ribosomal protein S2 (S4) (LLRep3 protein), partial (85%) [THC2270926]	
A_24_P406870	0.000161	NM_001014795	NM_001014795	Homo sapiens integrin-linked kinase (ILK), transcript variant 3, mRNA [NM_001014795]	NM_001014795
A_23_P129556	0.000161	NM_000418	NM_000418	Homo sapiens interleukin 4 receptor (IL4R), transcript variant 1, mRNA [NM_000418]	NM_000418
A_23_P102884	0.000162	NM_203446	NM_203446	Homo sapiens synaptojanin 1 (SYNJ1), transcript variant 2, mRNA [NM_203446]	NM_203446
A_24_P126425	0.000162	NM_032222	NM_032222	Homo sapiens hypothetical protein FLJ22374 (FLJ22374), mRNA [NM_032222]	NM_032222
A_23_P218108	0.000162	NM_052950	NM_052950	Homo sapiens WD repeat and FYVE domain containing 2 (WDFY2), mRNA [NM_052950]	NM_052950
A_24_P130352	0.000162	NM_015510	NM_015510	Homo sapiens DKFZP566O084 protein (DKFZp566O084), mRNA [NM_015510]	NM_015510
A_23_P328206	0.000162	BC041628	BC041628	Homo sapiens dynamin binding protein, mRNA (cDNA clone MGC:52388 IMAGE:4329464), complete cds. [BC041628]	
A_24_P810828	0.000162	BC044637	BC044637	Homo sapiens hypothetical protein LOC348926, mRNA (cDNA clone IMAGE:4546485), partial cds. [BC044637]	
A_32_P78395	0.000163	THC2374967		BX842574 POSSIBLE RESUSCITATION-PROMOTING FACTOR RPFA {Mycobacterium tuberculosis H37Rv;} , partial (6%) [THC2374967]	
A_32_P42705	0.000163	BC017721	BC017721	Homo sapiens, clone IMAGE:4429392, mRNA, partial cds. [BC017721]	
A_23_P205875	0.000163	NM_033640	NM_033640	Homo sapiens SCAN domain containing 2 (SCAND2), transcript variant 6, mRNA [NM_033640]	NM_033640
A_23_P33027	0.000163	AB020674	AB020674	Homo sapiens mRNA for KIAA0867 protein, partial cds. [AB020674]	
A_23_P111092	0.000163	NM_030883	NM_030883	Homo sapiens olfactory receptor, family 2, subfamily H, member 1 (OR2H1), mRNA [NM_030883]	NM_030883
A_24_P693986	0.000163	NM_001013642	NM_001013642	Homo sapiens hypothetical LOC388610 (LOC388610), mRNA [NM_001013642]	NM_001013642
A_24_P341427	0.000163	A_24_P341427			
A_24_P926450	0.000164	THC2274524			
A_24_P13715	0.000164	NM_147188	NM_147188	Homo sapiens F-box protein 22 (FBXO22), transcript variant 1, mRNA [NM_147188]	NM_147188
A_32_P19806	0.000164	NM_003777	NM_003777	Homo sapiens dynein, axonemal, heavy polypeptide 11 (DNAH11), mRNA [NM_003777]	NM_003777
A_32_P200237	0.000164	AJ291676	AJ291676	Homo sapiens mRNA for chromosome 8 open reading frame 9 (c8ORF9). [AJ291676]	
A_23_P68922	0.000164	NM_033386	NM_033386	Homo sapiens MICAL-like 1 (MICAL-L1), mRNA [NM_033386]	NM_033386
A_24_P239177	0.000164	AF177925	AF177925	Homo sapiens mucin 4 (MUC4) mRNA, partial cds. [AF177925]	
A_23_P266	0.000164	U82382	U82382	Homo sapiens PIN1 peptidyl-prolyl cis/trans isomerase-like (PIN1L) mRNA, complete cds. [U82382]	
A_23_P323563	0.000164	NM_022835	NM_022835	Homo sapiens pleckstrin homology domain containing, family G (with RhoGef domain) member 2 (PLEKHG2), mRNA [NM_022835]	NM_022835
A_32_P128209	0.000165	NM_175616	NM_175616	Homo sapiens FIS (FIS), mRNA [NM_175616]	NM_175616
A_24_P155791	0.000165	NM_024708	NM_024708	Homo sapiens ankyrin repeat and SOCS box-containing 7 (ASB7), transcript variant 1, mRNA [NM_024708]	NM_024708
A_24_P357709	0.000165	NM_001009921	NM_001009921	Homo sapiens KIAA0804 (KIAA0804), transcript variant 1, mRNA [NM_001009921]	NM_001009921
A_23_P208369	0.000165	NM_025194	NM_025194	Homo sapiens inositol 1,4,5-trisphosphate 3-kinase C (ITPKC), mRNA [NM_025194]	NM_025194

A_23_P9192	0.000165	NM_005156	NM_005156	Homo sapiens ROD1 regulator of differentiation 1 (S. pombe) (ROD1), mRNA [NM_005156]	NM_005156
A_23_P258837	0.000165	NM_032790	NM_032790	Homo sapiens hypothetical protein FLJ14466 (FLJ14466), mRNA [NM_032790]	NM_032790
A_23_P96209	0.000165	NM_025232	NM_025232	Homo sapiens chromosome 8 open reading frame 20 (C8orf20), mRNA [NM_025232]	NM_025232
A_23_P1280	0.000165	NM_015947	NM_015947	Homo sapiens activating signal cointegrator 1 complex subunit 1 (ASCC1), mRNA [NM_015947]	NM_015947
A_24_P375609	0.000165	NM_001970	NM_001970	Homo sapiens eukaryotic translation initiation factor 5A (EIF5A), mRNA [NM_001970]	NM_001970
A_23_P5995	0.000166	NM_006420	NM_006420	Homo sapiens ADP-ribosylation factor guanine nucleotide-exchange factor 2 (brefeldin A-inhibited) (ARFGEF2), mRNA [NM_006420]	NM_006420
A_23_P16116	0.000166	NM_016154	NM_016154	Homo sapiens RAB4B, member RAS oncogene family (RAB4B), mRNA [NM_016154]	NM_016154
A_23_P95172	0.000166	NM_020914	NM_020914	Homo sapiens chromosome 17 open reading frame 27 (C17orf27), mRNA [NM_020914]	NM_020914
A_32_P30075	0.000166	THC2321023		BPAP_BOVIN (P84291) Pregnancy associated protein bPAP (Fragments), partial (10%) [THC2321023]	
A_24_P392540	0.000166	XM_116396	XM_116396	PREDICTED: Homo sapiens similar to peptidyl-Pro cis trans isomerase (LOC202227), mRNA [XM_116396]	XM_116396
A_23_P120839	0.000167	NM_001008697	NM_001008697	Homo sapiens tuftelin interacting protein 11 (TFIP11), transcript variant 1, mRNA [NM_001008697]	NM_001008697
A_23_P124760	0.000167	NM_014815	NM_014815	Homo sapiens thyroid hormone receptor associated protein 4 (THRAP4), mRNA [NM_014815]	NM_014815
A_23_P115215	0.000167	NM_005997	NM_005997	Homo sapiens transcription factor-like 1 (TCFL1), mRNA [NM_005997]	NM_005997
A_23_P78289	0.000167	NM_032837	NM_032837	Homo sapiens hypothetical protein FLJ14775 (FLJ14775), mRNA [NM_032837]	NM_032837
A_23_P344973	0.000167	NM_079423	NM_079423	Homo sapiens myosin, light polypeptide 6, alkali, smooth muscle and non-muscle (MYL6), transcript variant 2, mRNA [NM_079423]	NM_079423
A_32_P9575	0.000168	NM_032351	NM_032351	Homo sapiens mitochondrial ribosomal protein L45 (MRPL45), nuclear gene encoding mitochondrial protein, mRNA [NM_032351]	NM_032351
A_24_P91222	0.000168	NM_001003725	NM_001003725	Homo sapiens WD repeat domain 68 (WDR68), transcript variant 2, mRNA [NM_001003725]	NM_001003725
A_24_P304549	0.000168	NM_005561	NM_005561	Homo sapiens lysosomal-associated membrane protein 1 (LAMP1), mRNA [NM_005561]	NM_005561
A_24_P614940	0.000168	A_24_P614940			
A_23_P148308	0.000168	NM_001017430	NM_001017430	Homo sapiens RNA binding motif (RNP1, RRM) protein 3 (RBM3), transcript variant 2, mRNA [NM_001017430]	NM_001017430
A_23_P137103	0.000168	NM_001416	NM_001416	Homo sapiens eukaryotic translation initiation factor 4A, isoform 1 (EIF4A1), mRNA [NM_001416]	NM_001416
A_24_P653354	0.000169	THC2440229			
A_23_P168576	0.000169	NM_152992	NM_152992	Homo sapiens POM (POM121 homolog, rat) and ZP3 fusion (POMZP3), transcript variant 2, mRNA [NM_152992]	NM_152992
A_23_P160449	0.000169	NM_032833	NM_032833	Homo sapiens protein phosphatase 1, regulatory (inhibitor) subunit 15B (PPP1R15B), mRNA [NM_032833]	NM_032833
A_32_P137399	0.000169	ENST00000335142		human full-length cDNA clone CS0DK001YK16 of HeLa cells of Homo sapiens (human). [BX248296]	XM_375081
A_24_P417935	0.000169	CR590071	CR590071	full-length cDNA clone CS0DC010YD15 of Neuroblastoma Cot 25-normalized of Homo sapiens (human). [CR590071]	
A_24_P478556	0.000169	BC030102	BC030102	Homo sapiens cDNA clone IMAGE:4796690, partial cds. [BC030102]	
A_24_P161244	0.000169	NM_153649	NM_153649	Homo sapiens tropomyosin 3 (TPM3), transcript variant 2, mRNA [NM_153649]	NM_153649
A_23_P112159	0.000169	NM_012154	NM_012154	Homo sapiens eukaryotic translation initiation factor 2C, 2 (EIF2C2), mRNA [NM_012154]	NM_012154
A_24_P229903	0.000169	A_24_P229903			
A_23_P110062	0.000169	NM_003907	NM_003907	Homo sapiens eukaryotic translation initiation factor 2B, subunit 5 epsilon, 82kDa (EIF2B5), mRNA [NM_003907]	NM_003907
A_23_P132936	0.000169	NM_021928	NM_021928	Homo sapiens signal peptidase complex subunit 3 homolog (S. cerevisiae) (SPCS3), mRNA [NM_021928]	NM_021928
A_24_P374516	0.000169	NM_021109	NM_021109	Homo sapiens thymosin, beta 4, X-linked (TMSB4X), mRNA [NM_021109]	NM_021109
A_24_P933135	0.00017	A_24_P933135			
A_23_P93046	0.00017	NM_007277	NM_007277	Homo sapiens SEC6-like 1 (S. cerevisiae) (SEC6L1), mRNA [NM_007277]	NM_007277
A_24_P647682	0.00017	A_24_P647682			
A_23_P209778	0.00017	NM_004805	NM_004805	Homo sapiens polymerase (RNA) II (DNA directed) polypeptide D (POLR2D), mRNA [NM_004805]	NM_004805
A_23_P103885	0.00017	NM_017900	NM_017900	Homo sapiens aurora kinase A interacting protein 1 (AURKAIP1), mRNA [NM_017900]	NM_017900
A_24_P290961	0.000171	NM_015291	NM_015291	Homo sapiens DnaJ (Hsp40) homolog, subfamily C, member 16 (DNAJC16), mRNA [NM_015291]	NM_015291
A_24_P942112	0.000171	NM_019014	NM_019014	Homo sapiens polymerase (RNA) I polypeptide B, 128kDa (POLR1B), mRNA [NM_019014]	NM_019014
A_23_P81926	0.000171	NM_014069	NM_014069	Homo sapiens psoriasis susceptibility 1 candidate 2 (PSORS1C2), mRNA [NM_014069]	NM_014069
A_23_P157628	0.000171	NM_004942	NM_004942	Homo sapiens defensin, beta 4 (DEFB4), mRNA [NM_004942]	NM_004942
A_24_P418126	0.000171	ENST00000321619		full-length cDNA clone CS0DI012YN13 of Placenta Cot 25-normalized of Homo sapiens (human). [CR594829]	

A_24_P59722	0.000171	NM_147152	NM_147152	Homo sapiens intersectin 2 (ITSN2), transcript variant 2, mRNA [NM_147152]	NM_147152
A_24_P20383	0.000171	NM_005718	NM_005718	Homo sapiens actin related protein 2/3 complex, subunit 4, 20kDa (ARPC4), transcript variant 1, mRNA [NM_005718]	NM_005718
A_23_P109026	0.000171	NM_022358	NM_022358	Homo sapiens potassium channel, subfamily K, member 15 (KCNK15), mRNA [NM_022358]	NM_022358
A_23_P123866	0.000171	NM_016525	NM_016525	Homo sapiens ubiquitin associated protein 1 (UBAP1), mRNA [NM_016525]	NM_016525
A_24_P272403	0.000171	BE816155	BE816155	BE816155 RC0-BN0198-220500-021-f04 BN0198 Homo sapiens cDNA, mRNA sequence [BE816155]	
A_23_P89410	0.000171	NM_003766	NM_003766	Homo sapiens beclin 1 (coiled-coil, myosin-like BCL2 interacting protein) (BECN1), mRNA [NM_003766]	NM_003766
A_32_P220715	0.000171	NM_022818	NM_022818	Homo sapiens microtubule-associated protein 1 light chain 3 beta (MAP1LC3B), mRNA [NM_022818]	NM_022818
A_23_P142018	0.000171	NM_015629	NM_015629	Homo sapiens PRP31 pre-mRNA processing factor 31 homolog (yeast) (PRPF31), mRNA [NM_015629]	NM_015629
A_23_P50722	0.000171	NM_024069	NM_024069	Homo sapiens hypothetical protein MGC2749 (MGC2749), mRNA [NM_024069]	NM_024069
A_24_P818529	0.000172	Z48511	Z48511	H.sapiens XG mRNA (clone PEP11). [Z48511]	
A_23_P118693	0.000172	NM_003593	NM_003593	Homo sapiens forkhead box N1 (FOXN1), mRNA [NM_003593]	NM_003593
A_32_P49999	0.000172	BE379139	BE379139	601238109F1 NIH_MGC_44 Homo sapiens cDNA clone IMAGE:3609890 5', mRNA sequence [BE379139]	
A_23_P13482	0.000172	NM_020643	NM_020643	Homo sapiens chromosome 11 open reading frame 16 (C11orf16), mRNA [NM_020643]	NM_020643
A_23_P74138	0.000172	NM_003564	NM_003564	Homo sapiens transgelin 2 (TAGLN2), mRNA [NM_003564]	NM_003564
A_23_P98923	0.000173	NM_006390	NM_006390	Homo sapiens importin 8 (IPO8), mRNA [NM_006390]	NM_006390
A_23_P49310	0.000173	NM_033266	NM_033266	Homo sapiens endoplasmic reticulum to nucleus signalling 2 (ERN2), mRNA [NM_033266]	NM_033266
A_24_P235360	0.000173	NM_017884	NM_017884	Homo sapiens PIN2-interacting protein 1 (PINX1), mRNA [NM_017884]	NM_017884
A_32_P122925	0.000173	CR594732	CR594732	full-length cDNA clone CS0DE010YB08 of Placenta of Homo sapiens (human). [CR594732]	
A_23_P151043	0.000173	NM_024854	NM_024854	Homo sapiens hypothetical protein FLJ22028 (FLJ22028), mRNA [NM_024854]	NM_024854
A_24_P406132	0.000173	NM_002754	NM_002754	Homo sapiens mitogen-activated protein kinase 13 (MAPK13), mRNA [NM_002754]	NM_002754
A_23_P428827	0.000173	NM_018064	NM_018064	Homo sapiens chromosome 6 open reading frame 166 (C6orf166), mRNA [NM_018064]	NM_018064
A_24_P315405	0.000173	A_24_P315405			
A_24_P600603	0.000173	A_24_P600603			
A_23_P29897	0.000174	NM_005935	NM_005935	Homo sapiens AF4/FMR2 family, member 1 (AFF1), mRNA [NM_005935]	NM_005935
A_24_P930327	0.000174	A_24_P930327			
A_23_P119789	0.000174	NR_000034	NR_000034	Homo sapiens family with sequence similarity 11, member B (FAM11B) on chromosome 2 [NR_000034]	NR_000034
A_24_P690235	0.000174	ENST00000312412		Homo sapiens cDNA FLJ43844 fis, clone TESTI4006308, highly similar to Puromycin-sensitive aminopeptidase (EC 3.4.11.-). [AK125832]	
A_23_P69840	0.000174	NM_080670	NM_080670	Homo sapiens solute carrier family 35, member A4 (SLC35A4), mRNA [NM_080670]	NM_080670
A_23_P400217	0.000174	NM_032853	NM_032853	Homo sapiens melanoma associated antigen (mutated) 1 (MUM1), mRNA [NM_032853]	NM_032853
A_24_P247626	0.000175	NM_001004432	NM_001004432	Homo sapiens leucine rich repeat neuronal 6D (LRRN6D), mRNA [NM_001004432]	NM_001004432
A_32_P26034	0.000175	THC2282095		ALU5_HUMAN (P39192) Alu subfamily SC sequence contamination warning entry, partial (15%) [THC2282095]	
A_24_P698759	0.000175	A_24_P698759			
A_23_P15375	0.000175	NM_024585	NM_024585	Homo sapiens armadillo repeat containing 7 (ARMC7), mRNA [NM_024585]	NM_024585
A_23_P205789	0.000175	NM_002041	NM_002041	Homo sapiens GA binding protein transcription factor, beta subunit 2 (GABPB2), transcript variant gamma-1, mRNA [NM_002041]	NM_002041
A_23_P169470	0.000175	NM_001261	NM_001261	Homo sapiens cyclin-dependent kinase 9 (CDC2-related kinase) (CDK9), mRNA [NM_001261]	NM_001261
A_24_P638294	0.000175	NM_001029	NM_001029	Homo sapiens ribosomal protein S26 (RPS26), mRNA [NM_001029]	NM_001029
A_23_P49338	0.000175	NM_016639	NM_016639	Homo sapiens tumor necrosis factor receptor superfamily, member 12A (TNFRSF12A), mRNA [NM_016639]	NM_016639
A_24_P368748	0.000176	A_24_P368748			
A_23_P22557	0.000176	AL832120	AL832120	Homo sapiens mRNA; cDNA DKFZp686B2110 (from clone DKFZp686B2110). [AL832120]	XM_291344
A_23_P48237	0.000176	NM_006337	NM_006337	Homo sapiens microspherule protein 1 (MCRS1), transcript variant 1, mRNA [NM_006337]	NM_006337
A_24_P216681	0.000176	NM_032881	NM_032881	Homo sapiens LSM10, U7 small nuclear RNA associated (LSM10), mRNA [NM_032881]	NM_032881
A_23_P37718	0.000177	NM_001297	NM_001297	Homo sapiens cyclic nucleotide gated channel beta 1 (CNGB1), mRNA [NM_001297]	NM_001297
A_24_P594094	0.000177	A_24_P594094			
A_24_P252780	0.000178	NM_198514	NM_198514	Homo sapiens NHL repeat containing 2 (NHLRC2), mRNA [NM_198514]	NM_198514

A_23_P109864	0.000178	NM_004122	NM_004122	Homo sapiens growth hormone secretagogue receptor (GHSR), transcript variant 1b, mRNA [NM_004122]	NM_004122
A_23_P256540	0.000178	NM_004891	NM_004891	Homo sapiens mitochondrial ribosomal protein L33 (MRPL33), nuclear gene encoding mitochondrial protein, transcript variant 1, mRNA [NM_004891]	NM_004891
A_23_P38154	0.000178	NM_024417	NM_024417	Homo sapiens ferredoxin reductase (FDXR), nuclear gene encoding mitochondrial protein, transcript variant 1, mRNA [NM_024417]	NM_024417
A_32_P232381	0.000179	THC2401087			
A_23_P62642	0.000179	NM_012337	NM_012337	Homo sapiens coiled-coil domain containing 19 (CCDC19), mRNA [NM_012337]	NM_012337
A_24_P56130	0.000179	NM_079423	NM_079423	Homo sapiens myosin, light polypeptide 6, alkali, smooth muscle and non-muscle (MYL6), transcript variant 2, mRNA [NM_079423]	NM_079423
A_24_P252739	0.00018	NM_001008490	NM_001008490	Homo sapiens Kruppel-like factor 6 (KLF6), transcript variant 1, mRNA [NM_001008490]	NM_001008490
A_24_P272845	0.00018	NM_004947	NM_004947	Homo sapiens dedicator of cytokinesis 3 (DOCK3), mRNA [NM_004947]	NM_004947
A_24_P295412	0.00018	NM_007126	NM_007126	Homo sapiens valosin-containing protein (VCP), mRNA [NM_007126]	NM_007126
A_32_P168431	0.00018	ENST00000358081		PREDICTED: Homo sapiens similar to 40S ribosomal protein S26 (LOC441486), mRNA [XM_497095]	XM_497095
A_24_P824592	0.000181	NM_002139	NM_002139	Homo sapiens RNA binding motif protein, X-linked (RBMX), mRNA [NM_002139]	NM_002139
A_23_P149678	0.000181	NM_006099	NM_006099	Homo sapiens protein inhibitor of activated STAT, 3 (PIAS3), mRNA [NM_006099]	NM_006099
A_23_P250485	0.000182	NM_018207	NM_018207	Homo sapiens tripartite motif-containing 62 (TRIM62), mRNA [NM_018207]	NM_018207
A_24_P159548	0.000182	NM_080669	NM_080669	Homo sapiens similar to RIKEN cDNA 1110002C08 gene (MGC9564), mRNA [NM_080669]	NM_080669
A_23_P54223	0.000182	NM_004581	NM_004581	Homo sapiens Rab geranylgeranyltransferase, alpha subunit (RABGGTA), transcript variant 2, mRNA [NM_004581]	NM_004581
A_24_P204664	0.000182	A_24_P204664			
A_24_P244410	0.000182	NM_014042	NM_014042	Homo sapiens DKFZP564M082 protein (DKFZP564M082), mRNA [NM_014042]	NM_014042
A_23_P37877	0.000182	NM_022493	NM_022493	Homo sapiens nuclear prelamin A recognition factor-like (NARFL), mRNA [NM_022493]	NM_022493
A_23_P87759	0.000182	NM_006331	NM_006331	Homo sapiens C2f protein (C2F), mRNA [NM_006331]	NM_006331
A_24_P499215	0.000183	XM_495808	XM_495808	PREDICTED: Homo sapiens similar to KIAA0592 protein (LOC439961), mRNA [XM_495808]	XM_495808
A_32_P198601	0.000183	A_32_P198601			
A_32_P191860	0.000183	AK093495	AK093495	Homo sapiens cDNA FLJ36176 fis, clone TESTI2026491. [AK093495]	
A_23_P141917	0.000183	NM_003331	NM_003331	Homo sapiens tyrosine kinase 2 (TYK2), mRNA [NM_003331]	NM_003331
A_23_P74178	0.000183	NM_001525	NM_001525	Homo sapiens hypocretin (orexin) receptor 1 (HCRTR1), mRNA [NM_001525]	NM_001525
A_23_P139207	0.000183	AJ243950	AJ243950	Homo sapiens mRNA for deafness locus associated putative guanine nucleotide exchange factor (DelGEF gene, splice variant DelGEF1). [AJ243950]	
A_32_P83203	0.000184	NM_205863	NM_205863	Homo sapiens amyotrophic lateral sclerosis 2 (juvenile) chromosome region, candidate 19 (ALS2CR19), transcript variant a, mRNA [NM_205863]	NM_205863
A_23_P348857	0.000184	NM_014812	NM_014812	Homo sapiens KIAA0470 (KIAA0470), mRNA [NM_014812]	NM_014812
A_23_P253723	0.000184	NM_006583	NM_006583	Homo sapiens retinal pigment epithelium-derived rhodopsin homolog (RRH), mRNA [NM_006583]	NM_006583
A_23_P26117	0.000184	NM_006715	NM_006715	Homo sapiens mannosidase, alpha, class 2C, member 1 (MAN2C1), mRNA [NM_006715]	NM_006715
A_24_P238616	0.000184	XM_498380	XM_498380	PREDICTED: Homo sapiens similar to Chain O, Crystal Structure Of The Rabbit Muscle Glyceraldehyde-3- Phosphate Dehydrogenase (Gapdh) (LOC402418), mRNA [XM_498380]	XM_498380
A_24_P298029	0.000184	ENST00000308836			
A_23_P377664	0.000185	AB053306	AB053306	Homo sapiens ALS2 mRNA, complete cds, short form. [AB053306]	
A_23_P99710	0.000185	NM_014749	NM_014749	Homo sapiens KIAA0586 (KIAA0586), mRNA [NM_014749]	NM_014749
A_24_P188325	0.000185	NM_015984	NM_015984	Homo sapiens ubiquitin carboxyl-terminal hydrolase L5 (UCHL5), mRNA [NM_015984]	NM_015984
A_24_P255786	0.000185	A_24_P255786			
A_24_P930314	0.000186	AK056698	AK056698	Homo sapiens cDNA FLJ32136 fis, clone PEBLM2000395, moderately similar to ZINC FINGER PROTEIN 165. [AK056698]	
A_24_P9321	0.000186	NM_003533	NM_003533	Homo sapiens histone 1, H3i (HIST1H3I), mRNA [NM_003533]	NM_003533
A_23_P147238	0.000186	NM_018639	NM_018639	Homo sapiens WD repeat and SOCS box-containing 2 (WSB2), mRNA [NM_018639]	NM_018639
A_23_P155835	0.000186	NM_183075	NM_183075	Homo sapiens cytochrome P450, family 2, subfamily U, polypeptide 1 (CYP2U1), mRNA [NM_183075]	NM_183075
A_23_P100602	0.000186	NM_005993	NM_005993	Homo sapiens tubulin-specific chaperone d (TBCD), mRNA [NM_005993]	NM_005993
A_23_P54963	0.000186	NM_032478	NM_032478	Homo sapiens mitochondrial ribosomal protein L38 (MRPL38), nuclear gene encoding mitochondrial protein, mRNA [NM_032478]	NM_032478
A_23_P70417	0.000186	NM_018141	NM_018141	Homo sapiens mitochondrial ribosomal protein S10 (MRPS10), nuclear gene encoding mitochondrial protein, mRNA [NM_018141]	NM_018141
A_24_P334005	0.000186	NM_017887	NM_017887	Homo sapiens chromosome 1 open reading frame 123 (C1orf123), mRNA [NM_017887]	NM_017887

A_23_P44581	0.000186	NM_014287	NM_014287	Homo sapiens NODAL modulator 1 (NOMO1), mRNA [NM_014287]	NM_014287
A_23_P212706	0.000186	NM_022488	NM_022488	Homo sapiens APG3 autophagy 3-like (S. cerevisiae) (APG3L), mRNA [NM_022488]	NM_022488
A_23_P48977	0.000186	NM_022839	NM_022839	Homo sapiens mitochondrial ribosomal protein S11 (MRPS11), nuclear gene encoding mitochondrial protein, transcript variant 1, mRNA [NM_022839]	NM_022839
A_23_P117955	0.000186	NM_000485	NM_000485	Homo sapiens adenine phosphoribosyltransferase (APRT), mRNA [NM_000485]	NM_000485
A_24_P256579	0.000187	AB051533	AB051533	Homo sapiens mRNA for KIAA1746 protein, partial cds. [AB051533]	
A_23_P200535	0.000187	NM_014408	NM_014408	Homo sapiens trafficking protein particle complex 3 (TRAPPC3), mRNA [NM_014408]	NM_014408
A_23_P5976	0.000188	NM_080603	NM_080603	Homo sapiens zinc finger, SWIM-type containing 1 (ZSWIM1), mRNA [NM_080603]	NM_080603
A_24_P34944	0.000188	NM_032094	NM_032094	Homo sapiens protocadherin gamma subfamily A, 12 (PCDHGA12), transcript variant 2, mRNA [NM_032094]	NM_032094
A_24_P286190	0.000188	NM_033342	NM_033342	Homo sapiens tripartite motif-containing 7 (TRIM7), transcript variant 6, mRNA [NM_033342]	NM_033342
A_24_P359191	0.000188	NM_003043	NM_003043	Homo sapiens solute carrier family 6 (neurotransmitter transporter, taurine), member 6 (SLC6A6), mRNA [NM_003043]	NM_003043
A_24_P278853	0.000188	NM_032813	NM_032813	Homo sapiens hypothetical protein FLJ14624 (FLJ14624), mRNA [NM_032813]	NM_032813
A_23_P67583	0.000188	NM_018174	NM_018174	Homo sapiens BPY2 interacting protein 1 (BPY2IP1), mRNA [NM_018174]	NM_018174
A_23_P80086	0.000188	NM_013240	NM_013240	Homo sapiens chromosome 21 open reading frame 127 (C21orf127), transcript variant 1, mRNA [NM_013240]	NM_013240
A_23_P130900	0.000188	NM_020170	NM_020170	Homo sapiens nicalin homolog (zebrafish) (NCLN), mRNA [NM_020170]	NM_020170
A_24_P178415	0.000188	A_24_P178415			
A_24_P237613	0.000189	NM_182488	NM_182488	Homo sapiens ubiquitin specific protease 12 (USP12), mRNA [NM_182488]	NM_182488
A_32_P117760	0.000189	BG208131	BG208131	RST27621 Athersys RAGE Library Homo sapiens cDNA, mRNA sequence [BG208131]	
A_24_P178093	0.000189	NM_006114	NM_006114	Homo sapiens translocase of outer mitochondrial membrane 40 homolog (yeast) (TOMM40), mRNA [NM_006114]	NM_006114
A_24_P203678	0.000189	NM_000019	NM_000019	Homo sapiens acetyl-Coenzyme A acetyltransferase 1 (acetoacetyl Coenzyme A thiolase) (ACAT1), nuclear gene encoding mitochondrial protein, mRNA [NM_000019]	NM_000019
A_23_P2998	0.000189	NM_005015	NM_005015	Homo sapiens oxidase (cytochrome c) assembly 1-like (OXA1L), mRNA [NM_005015]	NM_005015
A_24_P346604	0.00019	NM_001001481	NM_001001481	Homo sapiens hypothetical protein FLJ11011 (FLJ11011), transcript variant 1, mRNA [NM_001001481]	NM_001001481
A_24_P483083	0.00019	NM_007111	NM_007111	Homo sapiens transcription factor Dp-1 (TFDP1), mRNA [NM_007111]	NM_007111
A_23_P135465	0.00019	NM_173811	NM_173811	Homo sapiens hypothetical protein FLJ32675 (FLJ32675), mRNA [NM_173811]	NM_173811
A_23_P152782	0.00019	NM_005533	NM_005533	Homo sapiens interferon-induced protein 35 (IFI35), mRNA [NM_005533]	NM_005533
A_24_P276888	0.00019	AK027859	AK027859	Homo sapiens cDNA FLJ14953 fis, clone PLACE3000160. [AK027859]	
A_24_P804263	0.00019	NM_001320	NM_001320	Homo sapiens casein kinase 2, beta polypeptide (CSNK2B), mRNA [NM_001320]	NM_001320
A_23_P138426	0.000191	BC042943	BC042943	Homo sapiens USP6 N-terminal like, mRNA (cDNA clone MGC:41831 IMAGE:5296060), complete cds. [BC042943]	XM_374768
A_32_P53908	0.000192	NM_015252	NM_015252	Homo sapiens EH domain binding protein 1 (EHBP1), mRNA [NM_015252]	NM_015252
A_23_P82474	0.000192	NM_001002926	NM_001002926	Homo sapiens TWIST neighbor (TWISTNB), mRNA [NM_001002926]	NM_001002926
A_24_P251688	0.000192	NM_018358	NM_018358	Homo sapiens ATP-binding cassette, sub-family F (GCN20), member 3 (ABCF3), mRNA [NM_018358]	NM_018358
A_23_P123343	0.000192	NM_032869	NM_032869	Homo sapiens NudC domain containing 1 (NUDCD1), mRNA [NM_032869]	NM_032869
A_23_P106822	0.000192	NM_014062	NM_014062	Homo sapiens nin one binding protein (NOB1P), mRNA [NM_014062]	NM_014062
A_23_P116091	0.000192	NM_130443	NM_130443	Homo sapiens dipeptidylpeptidase 3 (DPP3), transcript variant 2, mRNA [NM_130443]	NM_130443
A_24_P76313	0.000193	NM_207334	NM_207334	Homo sapiens family with sequence similarity 43, member B (FAM43B), mRNA [NM_207334]	NM_207334
A_23_P360605	0.000193	BC040542	BC040542	Homo sapiens KIAA0802, mRNA (cDNA clone MGC:39663 IMAGE:5268201), complete cds. [BC040542]	
A_23_P149419	0.000193	NM_022078	NM_022078	Homo sapiens G patch domain containing 3 (GPATC3), mRNA [NM_022078]	NM_022078
A_23_P74097	0.000193	NM_003198	NM_003198	Homo sapiens transcription elongation factor B (SIII), polypeptide 3 (110kDa, elongin A) (TCEB3), mRNA [NM_003198]	NM_003198
A_24_P198844	0.000193	NM_004870	NM_004870	Homo sapiens mannose-P-dolichol utilization defect 1 (MPDU1), mRNA [NM_004870]	NM_004870
A_23_P256413	0.000193	NM_138410	NM_138410	Homo sapiens chemokine-like factor super family 7 (CKLFSF7), transcript variant 1, mRNA [NM_138410]	NM_138410
A_23_P120414	0.000193	NM_003404	NM_003404	Homo sapiens tyrosine 3-monooxygenase/tryptophan 5-monooxygenase activation protein, beta polypeptide (YWHAB), transcript variant 1, mRNA [NM_003404]	NM_003404
A_23_P14841	0.000194	NM_001011664	NM_001011664	Homo sapiens casein kinase 1, gamma 1 (CSNK1G1), transcript variant 1, mRNA [NM_001011664]	NM_001011664
A_24_P222432	0.000194	BC032585	BC032585	Homo sapiens cDNA clone IMAGE:5500261, partial cds. [BC032585]	
A_32_P88719	0.000194	NM_080879	NM_080879	Homo sapiens RAB40A, member RAS oncogene family (RAB40A), mRNA [NM_080879]	NM_080879
A_23_P154022	0.000194	NM_025203	NM_025203	Homo sapiens hypothetical protein FLJ21945 (FLJ21945), mRNA [NM_025203]	NM_025203

A_23_P161501	0.000194	NM_024954	NM_024954	Homo sapiens ubiquitin domain containing 1 (UBTD1), mRNA [NM_024954]	NM_024954
A_24_P194420	0.000195	NM_024821	NM_024821	Homo sapiens hypothetical protein FLJ22349 (FLJ22349), mRNA [NM_024821]	NM_024821
A_24_P7179	0.000195	NM_206890	NM_206890	Homo sapiens chromosome 21 open reading frame 106 (C21orf106), transcript variant 3, mRNA [NM_206890]	NM_206890
A_23_P428875	0.000195	NM_152362	NM_152362	Homo sapiens tumor necrosis factor, alpha-induced protein 8-like 1 (TNFAIP8L1), mRNA [NM_152362]	NM_152362
A_23_P166193	0.000195	NM_018217	NM_018217	Homo sapiens chromosome 20 open reading frame 31 (C20orf31), mRNA [NM_018217]	NM_018217
A_24_P401739	0.000195	NM_001006634	NM_001006634	Homo sapiens Rho GTPase activating protein 17 (ARHGAP17), transcript variant 1, mRNA [NM_001006634]	NM_001006634
A_23_P89455	0.000195	NM_005827	NM_005827	Homo sapiens solute carrier family 35, member B1 (SLC35B1), mRNA [NM_005827]	NM_005827
A_32_P156963	0.000195	NM_001614	NM_001614	Homo sapiens actin, gamma 1 (ACTG1), mRNA [NM_001614]	NM_001614
A_24_P76105	0.000196	NM_207354	NM_207354	Homo sapiens ankyrin repeat domain 13 family, member D (ANKRD13D), mRNA [NM_207354]	NM_207354
A_23_P333227	0.000196	BC012766	BC012766	Homo sapiens endoplasmic reticulum-golgi intermediate compartment 32 kDa protein, mRNA (cDNA clone MGC:16233 IMAGE:3677787), complete cds. [BC012766]	
A_24_P358305	0.000196	A_24_P358305			
A_32_P174292	0.000196	AK128192	AK128192	Homo sapiens cDNA FLJ46319 fis, clone TESTI4042420. [AK128192]	XM_498884
A_23_P140994	0.000196	NM_007242	NM_007242	Homo sapiens DEAD (Asp-Glu-Ala-As) box polypeptide 19 (DDX19), transcript variant 1, mRNA [NM_007242]	NM_007242
A_23_P123393	0.000197	NM_004519	NM_004519	Homo sapiens potassium voltage-gated channel, KQT-like subfamily, member 3 (KCNQ3), mRNA [NM_004519]	NM_004519
A_23_P61406	0.000197	NM_016848	NM_016848	Homo sapiens SHC (Src homology 2 domain containing) transforming protein 3 (SHC3), mRNA [NM_016848]	NM_016848
A_32_P90080	0.000197	ENST00000323716		full-length cDNA clone CS0DF023YN08 of Fetal brain of Homo sapiens (human). [CR620954]	XM_371655
A_23_P26799	0.000197	NM_004870	NM_004870	Homo sapiens mannose-P-dolichol utilization defect 1 (MPDU1), mRNA [NM_004870]	NM_004870
A_24_P195749	0.000198	A_24_P195749			
A_24_P652318	0.000198	AK098360	AK098360	Homo sapiens cDNA FLJ25494 fis, clone CBR01476. [AK098360]	
A_24_P280029	0.000198	NM_020315	NM_020315	Homo sapiens pyridoxal (pyridoxine, vitamin B6) phosphatase (PDXP), mRNA [NM_020315]	NM_020315
A_23_P92012	0.000198	NM_025265	NM_025265	Homo sapiens tRNA splicing endonuclease 2 homolog (SEN2, S. cerevisiae) (TSEN2), mRNA [NM_025265]	NM_025265
A_24_P152833	0.000198	CV323793	CV323793	CV323793 CM4-CN0090-130201-722-e04 CN0090 Homo sapiens cDNA, mRNA sequence [CV323793]	
A_23_P353574	0.000199	NM_133494	NM_133494	Homo sapiens NIMA (never in mitosis gene a)-related kinase 7 (NEK7), mRNA [NM_133494]	NM_133494
A_23_P252292	0.000199	NM_006733	NM_006733	Homo sapiens FSH primary response (LRPR1 homolog, rat) 1 (FSHPRH1), mRNA [NM_006733]	NM_006733
A_23_P65174	0.000199	NM_016119	NM_016119	Homo sapiens PHD finger protein 11 (PHF11), mRNA [NM_016119]	NM_016119
A_32_P19135	0.0002	NM_016154	NM_016154	Homo sapiens RAB4B, member RAS oncogene family (RAB4B), mRNA [NM_016154]	NM_016154
A_24_P117896	0.0002	NM_020452	NM_020452	Homo sapiens ATPase, Class I, type 8B, member 2 (ATP8B2), transcript variant 1, mRNA [NM_020452]	NM_020452
A_23_P13679	0.0002	NM_020782	NM_020782	Homo sapiens kelch domain containing 5 (KLHDC5), mRNA [NM_020782]	NM_020782
A_24_P84698	0.0002	A_24_P84698			
A_23_P323743	0.0002	NM_025049	NM_025049	Homo sapiens chromosome 15 open reading frame 20 (C15orf20), mRNA [NM_025049]	NM_025049
A_24_P50390	0.000201	A_24_P50390			
A_32_P2377	0.000201	U04815	U04815	Human protein kinase PITSLRE alpha 1 mRNA, complete cds. [U04815]	
A_24_P227065	0.000201	NM_005029	NM_005029	Homo sapiens paired-like homeodomain transcription factor 3 (PITX3), mRNA [NM_005029]	NM_005029
A_23_P162314	0.000201	NM_021044	NM_021044	Homo sapiens desert hedgehog homolog (Drosophila) (DHH), mRNA [NM_021044]	NM_021044
A_24_P485219	0.000201	NM_032121	NM_032121	Homo sapiens implantation-associated protein (DKFZp564K142), mRNA [NM_032121]	NM_032121
A_24_P186216	0.000201	BC041361	BC041361	Homo sapiens SCC-112 protein, mRNA (cDNA clone IMAGE:5273075), complete cds. [BC041361]	
A_23_P145761	0.000201	NM_005738	NM_005738	Homo sapiens ADP-ribosylation factor-like 4 (ARL4), transcript variant 1, mRNA [NM_005738]	NM_005738
A_23_P384499	0.000201	NM_020761	NM_020761	Homo sapiens raptor (raptor), mRNA [NM_020761]	NM_020761
A_23_P109677	0.000201	A_23_P109677			
A_23_P20970	0.000201	NM_004707	NM_004707	Homo sapiens APG12 autophagy 12-like (S. cerevisiae) (APG12L), mRNA [NM_004707]	NM_004707
A_24_P267748	0.000202	NM_014434	NM_014434	Homo sapiens NADPH dependent diflavin oxidoreductase 1 (NDOR1), mRNA [NM_014434]	NM_014434
A_24_P16815	0.000202	NM_005096	NM_005096	Homo sapiens zinc finger protein 261 (ZNF261), transcript variant 1, mRNA [NM_005096]	NM_005096
A_23_P110725	0.000202	NM_206907	NM_206907	Homo sapiens protein kinase, AMP-activated, alpha 1 catalytic subunit (PRKAA1), transcript variant 2, mRNA [NM_206907]	NM_206907

A_24_P916195	0.000202	NM_016426	NM_016426	Homo sapiens G-2 and S-phase expressed 1 (GTSE1), mRNA [NM_016426]	NM_016426
A_23_P397371	0.000202	NM_013284	NM_013284	Homo sapiens polymerase (DNA directed), mu (POLM), mRNA [NM_013284]	NM_013284
A_24_P911420	0.000202	AK001336	AK001336	Homo sapiens cDNA FLJ10474 fis, clone NT2RP2000067. [AK001336]	XM_371717
A_24_P50639	0.000202	A_24_P50639			
A_23_P48001	0.000202	ENST00000355675		PREDICTED: Homo sapiens similar to peptidyl-Pro cis trans isomerase (LOC341457), mRNA [XM_292085]	XM_292085
A_23_P34767	0.000202	NM_004559	NM_004559	Homo sapiens nuclease sensitive element binding protein 1 (NSEP1), mRNA [NM_004559]	NM_004559
A_32_P28528	0.000203	A_32_P28528			
A_23_P100413	0.000203	NM_058219	NM_058219	Homo sapiens exosome component 6 (EXOSC6), mRNA [NM_058219]	NM_058219
A_24_P302332	0.000203	NM_018090	NM_018090	Homo sapiens adaptin-ear-binding coat-associated protein 2 (NECAP2), mRNA [NM_018090]	NM_018090
A_23_P142380	0.000203	NM_005858	NM_005858	Homo sapiens A kinase (PRKA) anchor protein 8 (AKAP8), mRNA [NM_005858]	NM_005858
A_23_P132956	0.000203	NM_004181	NM_004181	Homo sapiens ubiquitin carboxyl-terminal esterase L1 (ubiquitin thiolesterase) (UCHL1), mRNA [NM_004181]	NM_004181
A_23_P380839	0.000204	NM_020193	NM_020193	Homo sapiens chromosome 11 open reading frame 30 (C11orf30), mRNA [NM_020193]	NM_020193
A_23_P209726	0.000204	NM_007237	NM_007237	Homo sapiens SP140 nuclear body protein (SP140), transcript variant 1, mRNA [NM_007237]	NM_007237
A_23_P333552	0.000204	NM_182477	NM_182477	Homo sapiens gametogenetin (GGN), transcript variant 2, mRNA [NM_182477]	NM_182477
A_24_P850336	0.000204	A_24_P850336			
A_23_P104046	0.000204	NM_006085	NM_006085	Homo sapiens 3'(2'), 5'-bisphosphate nucleotidase 1 (BPNT1), mRNA [NM_006085]	NM_006085
A_32_P122891	0.000204	AK092163	AK092163	Homo sapiens cDNA FLJ34844 fis, clone NT2NE2011154, highly similar to Homo sapiens mRNA for TIP49. [AK092163]	
A_23_P151614	0.000204	NM_006263	NM_006263	Homo sapiens proteasome (prosome, macropain) activator subunit 1 (PA28 alpha) (PSME1), transcript variant 1, mRNA [NM_006263]	NM_006263
A_24_P633902	0.000204	NM_014455	NM_014455	Homo sapiens zinc finger protein 364 (ZNF364), mRNA [NM_014455]	NM_014455
A_32_P233735	0.000205	NM_020704	NM_020704	Homo sapiens family with sequence similarity 40, member B (FAM40B), mRNA [NM_020704]	NM_020704
A_24_P287392	0.000205	NM_019858	NM_019858	Homo sapiens gene rich cluster, A gene (GRCA), transcript variant A-2, mRNA [NM_019858]	NM_019858
A_24_P340853	0.000205	NM_032789	NM_032789	Homo sapiens poly (ADP-ribose) polymerase family, member 10 (PARP10), mRNA [NM_032789]	NM_032789
A_23_P158190	0.000205	NM_015627	NM_015627	Homo sapiens low density lipoprotein receptor adaptor protein 1 (LDLRAP1), mRNA [NM_015627]	NM_015627
A_23_P58937	0.000205	NM_012288	NM_012288	Homo sapiens translocation associated membrane protein 2 (TRAM2), mRNA [NM_012288]	NM_012288
A_23_P41204	0.000205	NM_144635	NM_144635	Homo sapiens hypothetical protein MGC21688 (MGC21688), mRNA [NM_144635]	NM_144635
A_23_P46063	0.000205	NM_013330	NM_013330	Homo sapiens non-metastatic cells 7, protein expressed in (nucleoside-diphosphate kinase) (NME7), transcript variant 1, mRNA [NM_013330]	NM_013330
A_23_P202316	0.000205	NM_001007793	NM_001007793	Homo sapiens BUB3 budding uninhibited by benzimidazoles 3 homolog (yeast) (BUB3), transcript variant 2, mRNA [NM_001007793]	NM_001007793
A_32_P202977	0.000206	THC2383225		Q7PV63 (Q7PV63) ENSANGP00000020166, partial (6%) [THC2383225]	
A_23_P164112	0.000206	NM_014798	NM_014798	Homo sapiens pleckstrin homology domain containing, family M (with RUN domain) member 1 (PLEKHM1), mRNA [NM_014798]	NM_014798
A_23_P123133	0.000206	NM_002541	NM_002541	Homo sapiens oxoglutarate (alpha-ketoglutarate) dehydrogenase (lipoamide) (OGDH), nuclear gene encoding mitochondrial protein, transcript variant 1, mRNA [NM_002541]	NM_002541
A_24_P942370	0.000207	NM_003774	NM_003774	Homo sapiens UDP-N-acetyl-alpha-D-galactosamine:polypeptide N-acetylgalactosaminyltransferase 4 (GalNAc-T4) (GALNT4), mRNA [NM_003774]	NM_003774
A_23_P10701	0.000207	NM_173075	NM_173075	Homo sapiens amyloid beta (A4) precursor protein-binding, family B, member 2 (Fe65-like) (APBB2), mRNA [NM_173075]	NM_173075
A_23_P39110	0.000207	NM_006666	NM_006666	Homo sapiens RuvB-like 2 (E. coli) (RUVBL2), mRNA [NM_006666]	NM_006666
A_23_P409462	0.000208	NM_173674	NM_173674	Homo sapiens discoidin, CUB and LCCL domain containing 1 (DCBLD1), mRNA [NM_173674]	NM_173674
A_24_P936376	0.000208	X68990	X68990	Homo sapiens CR2 mRNA for complement receptor. [X68990]	
A_24_P222043	0.000208	NM_002131	NM_002131	Homo sapiens high mobility group AT-hook 1 (HMGA1), transcript variant 2, mRNA [NM_002131]	NM_002131
A_23_P149042	0.000208	NM_013328	NM_013328	Homo sapiens pyrroline-5-carboxylate reductase family, member 2 (PYCR2), mRNA [NM_013328]	NM_013328
A_23_P165301	0.000208	NM_006916	NM_006916	Homo sapiens ribulose-5-phosphate-3-epimerase (RPE), transcript variant 2, mRNA [NM_006916]	NM_006916
A_23_P39561	0.000208	NM_080678	NM_080678	Homo sapiens NEDD8-conjugating enzyme (NCE2), mRNA [NM_080678]	NM_080678
A_32_P135902	0.000208	NM_001416	NM_001416	Homo sapiens eukaryotic translation initiation factor 4A, isoform 1 (EIF4A1), mRNA [NM_001416]	NM_001416
A_23_P84782	0.000208	NM_015963	NM_015963	Homo sapiens THAP domain containing 4 (THAP4), mRNA [NM_015963]	NM_015963
A_23_P126790	0.000208	NM_006513	NM_006513	Homo sapiens seryl-tRNA synthetase (SARS), mRNA [NM_006513]	NM_006513
A_24_P349869	0.000208	A_24_P349869			

A_24_P149704	0.000209	NM_138709	NM_138709	Homo sapiens DAB2 interacting protein (DAB2IP), transcript variant 2, mRNA [NM_138709]	NM_138709
A_32_P63162	0.000209	THC2267053		HLAF_HUMAN (P30511) HLA class I histocompatibility antigen, alpha chain F precursor (HLA F antigen) (Leukocyte antigen F) (CDA12), partial (19%) [THC2267053]	
A_23_P50887	0.000209	NM_006055	NM_006055	Homo sapiens LanC lantibiotic synthetase component C-like 1 (bacterial) (LANCL1), mRNA [NM_006055]	NM_006055
A_23_P54147	0.000209	NM_016350	NM_016350	Homo sapiens ninein (GSK3B interacting protein) (NIN), transcript variant 4, mRNA [NM_016350]	NM_016350
A_23_P17706	0.000209	NM_014339	NM_014339	Homo sapiens interleukin 17 receptor (IL17R), mRNA [NM_014339]	NM_014339
A_24_P213110	0.000209	NM_004375	NM_004375	Homo sapiens COX11 homolog, cytochrome c oxidase assembly protein (yeast) (COX11), nuclear gene encoding mitochondrial protein, mRNA [NM_004375]	NM_004375
A_23_P378526	0.000209	NM_016434	NM_016434	Homo sapiens regulator of telomere elongation helicase 1 (RTEL1), transcript variant 1, mRNA [NM_016434]	NM_016434
A_32_P71788	0.00021	NM_002014	NM_002014	Homo sapiens FK506 binding protein 4, 59kDa (FKBP4), mRNA [NM_002014]	NM_002014
A_23_P336513	0.00021	NM_015465	NM_015465	Homo sapiens gem (nuclear organelle) associated protein 5 (GEMIN5), mRNA [NM_015465]	NM_015465
A_23_P78526	0.00021	NM_020219	NM_020219	Homo sapiens carcinoembryonic antigen-related cell adhesion molecule 19 (CEACAM19), mRNA [NM_020219]	NM_020219
A_23_P97423	0.00021	NM_017582	NM_017582	Homo sapiens ubiquitin-conjugating enzyme E2Q (putative) (UBE2Q), mRNA [NM_017582]	NM_017582
A_23_P138465	0.00021	NM_004741	NM_004741	Homo sapiens nucleolar and coiled-body phosphoprotein 1 (NOLC1), mRNA [NM_004741]	NM_004741
A_24_P365469	0.00021	NM_004776	NM_004776	Homo sapiens UDP-Gal:betaGlcNAc beta 1,4- galactosyltransferase, polypeptide 5 (B4GALT5), mRNA [NM_004776]	NM_004776
A_23_P204745	0.000211	NM_139071	NM_139071	Homo sapiens SWI/SNF related, matrix associated, actin dependent regulator of chromatin, subfamily d, member 1 (SMARCD1), transcript variant 2, mRNA [NM_139071]	NM_139071
A_23_P309967	0.000211	NM_032486	NM_032486	Homo sapiens dynactin 4 (MGC3248), mRNA [NM_032486]	NM_032486
A_24_P168760	0.000211	BC046350	BC046350	Homo sapiens cDNA clone IMAGE:4513243, complete cds. [BC046350]	
A_23_P385500	0.000212	NM_178841	NM_178841	Homo sapiens ring finger protein 166 (RNF166), mRNA [NM_178841]	NM_178841
A_23_P412321	0.000212	NM_000579	NM_000579	Homo sapiens chemokine (C-C motif) receptor 5 (CCR5), mRNA [NM_000579]	NM_000579
A_23_P45871	0.000212	NM_006820	NM_006820	Homo sapiens interferon-induced protein 44-like (IFI44L), mRNA [NM_006820]	NM_006820
A_24_P238666	0.000212	A_24_P238666			
A_23_P44132	0.000212	NM_004104	NM_004104	Homo sapiens fatty acid synthase (FASN), mRNA [NM_004104]	NM_004104
A_23_P153185	0.000212	ENST00000299502		Homo sapiens serine (or cysteine) proteinase inhibitor, clade B (ovalbumin), member 2, mRNA (cDNA clone MGC:13616 IMAGE:4281085), complete cds. [BC012609]	
A_24_P396105	0.000213	NM_153273	NM_153273	Homo sapiens inositol hexaphosphate kinase 1 (IHPK1), transcript variant 1, mRNA [NM_153273]	NM_153273
A_23_P20225	0.000213	NM_015713	NM_015713	Homo sapiens ribonucleotide reductase M2 B (TP53 inducible) (RRM2B), mRNA [NM_015713]	NM_015713
A_23_P202280	0.000213	NM_014000	NM_014000	Homo sapiens vinculin (VCL), transcript variant 1, mRNA [NM_014000]	NM_014000
A_23_P65830	0.000213	NM_198527	NM_198527	Homo sapiens Similar to RIKEN cDNA 1110033O09 gene (MGC45386), mRNA [NM_198527]	NM_198527
A_23_P156824	0.000214	NM_000863	NM_000863	Homo sapiens 5-hydroxytryptamine (serotonin) receptor 1B (HTR1B), mRNA [NM_000863]	NM_000863
A_24_P52921	0.000214	NM_005504	NM_005504	Homo sapiens branched chain aminotransferase 1, cytosolic (BCAT1), mRNA [NM_005504]	NM_005504
A_24_P66908	0.000214	A_24_P66908			
A_23_P91293	0.000214	NM_004738	NM_004738	Homo sapiens VAMP (vesicle-associated membrane protein)-associated protein B and C (VAPB), mRNA [NM_004738]	NM_004738
A_23_P502747	0.000215	NM_170692	NM_170692	Homo sapiens RAS protein activator like 2 (RASAL2), transcript variant 2, mRNA [NM_170692]	NM_170692
A_24_P253755	0.000215	NM_004278	NM_004278	Homo sapiens phosphatidylinositol glycan, class L (PIGL), mRNA [NM_004278]	NM_004278
A_24_P932253	0.000215	A_24_P932253			
A_23_P251841	0.000215	NM_004796	NM_004796	Homo sapiens neurexin 3 (NRXN3), transcript variant alpha, mRNA [NM_004796]	NM_004796
A_23_P122233	0.000215	NM_001014990	NM_001014990	Homo sapiens mitochondrial ribosomal protein L22 (MRPL22), nuclear gene encoding mitochondrial protein, transcript variant 2, mRNA [NM_001014990]	NM_001014990
A_23_P82068	0.000215	NM_016167	NM_016167	Homo sapiens nucleolar protein 7, 27kDa (NOL7), mRNA [NM_016167]	NM_016167
A_24_P649735	0.000216	THC2281176		Q6PJX0 (Q6PJX0) MADP-1 protein (Fragment), partial (64%) [THC2281176]	
A_23_P87279	0.000216	NM_014555	NM_014555	Homo sapiens transient receptor potential cation channel, subfamily M, member 5 (TRPM5), mRNA [NM_014555]	NM_014555
A_23_P111206	0.000216	NM_004117	NM_004117	Homo sapiens FK506 binding protein 5 (FKBP5), mRNA [NM_004117]	NM_004117
A_23_P150238	0.000216	NM_031450	NM_031450	Homo sapiens basophilic leukemia expressed protein BLES03 (Bles03), mRNA [NM_031450]	NM_031450
A_23_P138253	0.000216	NM_015607	NM_015607	Homo sapiens chromosome 1 open reading frame 77 (C1orf77), mRNA [NM_015607]	NM_015607
A_23_P87500	0.000216	NM_014182	NM_014182	Homo sapiens ORM1-like 2 (S. cerevisiae) (ORMDL2), mRNA [NM_014182]	NM_014182

A_24_P334361	0.000217	NM_017631	NM_017631	Homo sapiens hypothetical protein FLJ20035 (FLJ20035), mRNA [NM_017631]	NM_017631
A_23_P124456	0.000217	NM_006203	NM_006203	Homo sapiens phosphodiesterase 4D, cAMP-specific (phosphodiesterase E3 dunce homolog, Drosophila) (PDE4D), mRNA [NM_006203]	NM_006203
A_24_P382637	0.000217	NM_015666	NM_015666	Homo sapiens GTP binding protein 5 (putative) (GTPBP5), mRNA [NM_015666]	NM_015666
A_32_P214795	0.000217	AK128072	AK128072	Homo sapiens cDNA FLJ46193 fis, clone TESTI4006234. [AK128072]	
A_23_P151497	0.000217	NM_152307	NM_152307	Homo sapiens chromosome 14 open reading frame 172 (C14orf172), mRNA [NM_152307]	NM_152307
A_23_P154058	0.000217	NM_172195	NM_172195	Homo sapiens eukaryotic translation initiation factor 2B, subunit 4 delta, 67kDa (EIF2B4), transcript variant 2, mRNA [NM_172195]	NM_172195
A_23_P405761	0.000217	NM_012250	NM_012250	Homo sapiens related RAS viral (r-ras) oncogene homolog 2 (RRAS2), mRNA [NM_012250]	NM_012250
A_32_P114574	0.000217	NM_014412	NM_014412	Homo sapiens calcyclin binding protein (CACYBP), transcript variant 1, mRNA [NM_014412]	NM_014412
A_24_P101722	0.000217	ENST00000355049		PREDICTED: Homo sapiens similar to peptidyl-Pro cis trans isomerase (LOC126170), mRNA [XM_497621]	XM_497621
A_24_P772061	0.000217	NM_001008741	NM_001008741	Homo sapiens peptidylprolyl isomerase A-like (LOC388817), mRNA [NM_001008741]	NM_001008741
A_23_P30223	0.000218	NM_001047	NM_001047	Homo sapiens steroid-5-alpha-reductase, alpha polypeptide 1 (3-oxo-5 alpha-steroid delta 4-dehydrogenase alpha 1) (SRD5A1), mRNA [NM_001047]	NM_001047
A_24_P318457	0.000218	NM_001950	NM_001950	Homo sapiens E2F transcription factor 4, p107/p130-binding (E2F4), mRNA [NM_001950]	NM_001950
A_23_P214681	0.000219	NM_177435	NM_177435	Homo sapiens peroxisome proliferative activated receptor, delta (PPARD), transcript variant 2, mRNA [NM_177435]	NM_177435
A_23_P329375	0.000219	NM_002702	NM_002702	Homo sapiens POU domain, class 6, transcription factor 1 (POU6F1), mRNA [NM_002702]	NM_002702
A_23_P85980	0.000219	NM_020965	NM_020965	Homo sapiens membrane associated guanylate kinase, WW and PDZ domain containing 3 (MAGI3), transcript variant 1, mRNA [NM_020965]	NM_020965
A_24_P173475	0.000219	AK055306	AK055306	Homo sapiens cDNA FLJ30744 fis, clone FEBRA2000378. [AK055306]	
A_23_P82642	0.000219	NM_018697	NM_018697	Homo sapiens LanC lantibiotic synthetase component C-like 2 (bacterial) (LANCL2), mRNA [NM_018697]	NM_018697
A_24_P51037	0.000219	BC000853	BC000853	Homo sapiens chromosome 2 open reading frame 3, mRNA (cDNA clone IMAGE:3459069), complete cds. [BC000853]	
A_23_P89343	0.000219	NM_152244	NM_152244	Homo sapiens sorting nexin 11 (SNX11), transcript variant 1, mRNA [NM_152244]	NM_152244
A_23_P203702	0.000219	CA976180	CA976180	AGENCOURT_8955062 Lupski_sciatic_nerve Homo sapiens cDNA clone IMAGE:6202605 5', mRNA sequence [CA976180]	
A_24_P67432	0.000219	A_24_P67432			
A_32_P215304	0.00022	THC2305888			
A_24_P135489	0.00022	NM_030927	NM_030927	Homo sapiens tetraspanin 14 (TSPAN14), mRNA [NM_030927]	NM_030927
A_23_P81717	0.00022	NM_024919	NM_024919	Homo sapiens FERM domain containing 1 (FRMD1), mRNA [NM_024919]	NM_024919
A_23_P399851	0.00022	NM_198334	NM_198334	Homo sapiens glucosidase, alpha; neutral AB (GANAB), mRNA [NM_198334]	NM_198334
A_23_P97573	0.00022	NM_004698	NM_004698	Homo sapiens PRP3 pre-mRNA processing factor 3 homolog (yeast) (PRPF3), mRNA [NM_004698]	NM_004698
A_23_P163622	0.00022	NM_001012398	NM_001012398	Homo sapiens fused toes homolog (mouse) (FTS), transcript variant 1, mRNA [NM_001012398]	NM_001012398
A_23_P154282	0.00022	NM_032309	NM_032309	Homo sapiens coiled-coil-helix-coiled-coil-helix domain containing 5 (CHCHD5), mRNA [NM_032309]	NM_032309
A_23_P90589	0.00022	NM_022915	NM_022915	Homo sapiens mitochondrial ribosomal protein L44 (MRPL44), nuclear gene encoding mitochondrial protein, mRNA [NM_022915]	NM_022915
A_24_P107277	0.00022	NM_006442	NM_006442	Homo sapiens DR1-associated protein 1 (negative cofactor 2 alpha) (DRAP1), mRNA [NM_006442]	NM_006442
A_24_P175427	0.000221	NM_199177	NM_199177	Homo sapiens mitochondrial ribosome recycling factor (MRRF), nuclear gene encoding mitochondrial protein, transcript variant 2, mRNA [NM_199177]	NM_199177
A_24_P684119	0.000221	BC043212	BC043212	Homo sapiens cDNA clone IMAGE:5295205, with apparent retained intron. [BC043212]	
A_23_P17773	0.000221	NM_031444	NM_031444	Homo sapiens chromosome 22 open reading frame 13 (C22orf13), mRNA [NM_031444]	NM_031444
A_23_P22499	0.000221	NM_019067	NM_019067	Homo sapiens guanine nucleotide binding protein-like 3 (nucleolar)-like (GNL3L), mRNA [NM_019067]	NM_019067
A_23_P377982	0.000221	NM_175884	NM_175884	Homo sapiens hypothetical protein FLJ36031 (FLJ36031), mRNA [NM_175884]	NM_175884
A_23_P37966	0.000221	A_23_P37966			
A_23_P105066	0.000221	NM_001014795	NM_001014795	Homo sapiens integrin-linked kinase (ILK), transcript variant 3, mRNA [NM_001014795]	NM_001014795
A_23_P103070	0.000221	NM_003405	NM_003405	Homo sapiens tyrosine 3-monooxygenase/tryptophan 5-monooxygenase activation protein, eta polypeptide (YWHAH), mRNA [NM_003405]	NM_003405
A_24_P381975	0.000222	BC010609	BC010609	Homo sapiens carbohydrate (N-acetylglucosamine 6-O) sulfotransferase 5, mRNA (cDNA clone MGC:16805 IMAGE:4214587), complete cds. [BC010609]	XM_370992
A_23_P51761	0.000222	NM_001005279	NM_001005279	Homo sapiens olfactory receptor, family 6, subfamily K, member 2 (OR6K2), mRNA [NM_001005279]	NM_001005279
A_24_P8454	0.000222	NM_172345	NM_172345	Homo sapiens sperm associated antigen 9 (SPAG9), transcript variant 2, mRNA [NM_172345]	NM_172345
A_32_P227764	0.000222	BC034962	BC034962	Homo sapiens, clone IMAGE:4822098, mRNA, partial cds. [BC034962]	
A_23_P125990	0.000222	NM_004091	NM_004091	Homo sapiens E2F transcription factor 2 (E2F2), mRNA [NM_004091]	NM_004091
A_24_P76546	0.000222	NM_006990	NM_006990	Homo sapiens WAS protein family, member 2 (WASF2), mRNA [NM_006990]	NM_006990

A_32_P231302	0.000222	CR602702	CR602702	full-length cDNA clone CS0DI063YN06 of Placenta Cot 25-normalized of Homo sapiens (human). [CR602702]	XM_498557
A_24_P36425	0.000222	NM_017798	NM_017798	Homo sapiens YTH domain family, member 1 (YTHDF1), mRNA [NM_017798]	NM_017798
A_24_P102636	0.000223	NM_000321	NM_000321	Homo sapiens retinoblastoma 1 (including osteosarcoma) (RB1), mRNA [NM_000321]	NM_000321
A_24_P8888	0.000223	NM_013310	NM_013310	Homo sapiens chromosome 2 open reading frame 27 (C2orf27), mRNA [NM_013310]	NM_013310
A_23_P25187	0.000223	NM_002281	NM_002281	Homo sapiens keratin, hair, basic, 1 (KRTHB1), mRNA [NM_002281]	NM_002281
A_32_P113533	0.000223	A_32_P113533			
A_24_P332647	0.000224	AK095421	AK095421	Homo sapiens cDNA FLJ38102 fis, clone D3OST2000618, moderately similar to Drosophila melanogaster slingshot mRNA. [AK095421]	
A_24_P49597	0.000224	ENST00000299756		PREDICTED: Homo sapiens similar to Chain A, Crystal Structure Of The R463a Mutant Of Human Glutamate Dehydrogenase (LOC390859), mRNA [XM_372695]	XM_372695
A_23_P213069	0.000224	NM_018126	NM_018126	Homo sapiens transmembrane protein 33 (TMEM33), mRNA [NM_018126]	NM_018126
A_24_P213325	0.000224	A_24_P213325			
A_24_P174924	0.000224	NM_003537	NM_003537	Homo sapiens histone 1, H3b (HIST1H3B), mRNA [NM_003537]	NM_003537
A_24_P12932	0.000224	NM_016065	NM_016065	Homo sapiens mitochondrial ribosomal protein S16 (MRPS16), nuclear gene encoding mitochondrial protein, mRNA [NM_016065]	NM_016065
A_32_P129810	0.000225	AK098511	AK098511	Homo sapiens cDNA FLJ25645 fis, clone SYN00113. [AK098511]	
A_24_P41975	0.000225	AK056402	AK056402	Homo sapiens cDNA FLJ31840 fis, clone NT2RP7000109, highly similar to Homo sapiens putative RNA binding protein mRNA. [AK056402]	
A_24_P932451	0.000226	BX641037	BX641037	Homo sapiens mRNA; cDNA DKFZp686H10114 (from clone DKFZp686H10114). [BX641037]	
A_32_P78904	0.000226	THC2312785			
A_24_P921232	0.000226	BC017848	BC017848	Homo sapiens, clone IMAGE:4696946, mRNA, partial cds. [BC017848]	
A_23_P362207	0.000226	NM_003559	NM_003559	Homo sapiens phosphatidylinositol-4-phosphate 5-kinase, type II, beta (PIP5K2B), transcript variant 1, mRNA [NM_003559]	NM_003559
A_24_P307974	0.000226	BC033728	BC033728	Homo sapiens cDNA clone MGC:45470 IMAGE:5166848, complete cds. [BC033728]	
A_24_P179615	0.000226	U44954	U44954	Human NMDA receptor glutamate-binding chain (hnrgw) mRNA, partial cds. [U44954]	
A_23_P64712	0.000227	NM_024809	NM_024809	Homo sapiens hypothetical protein FLJ12975 (FLJ12975), mRNA [NM_024809]	NM_024809
A_23_P33303	0.000227	NM_005026	NM_005026	Homo sapiens phosphoinositide-3-kinase, catalytic, delta polypeptide (PIK3CD), mRNA [NM_005026]	NM_005026
A_24_P413735	0.000227	NM_018198	NM_018198	Homo sapiens DnaJ (Hsp40) homolog, subfamily C, member 11 (DNAJC11), mRNA [NM_018198]	NM_018198
A_23_P373079	0.000227	NM_152716	NM_152716	Homo sapiens hypothetical protein FLJ36874 (FLJ36874), mRNA [NM_152716]	NM_152716
A_23_P105583	0.000227	NM_031465	NM_031465	Homo sapiens hypothetical protein MGC13204 (MGC13204), mRNA [NM_031465]	NM_031465
A_24_P331904	0.000227	NM_017828	NM_017828	Homo sapiens COMM domain containing 4 (COMMD4), mRNA [NM_017828]	NM_017828
A_24_P212024	0.000228	ENST00000259219		Homo sapiens clone FD2K myosin-reactive immunoglobulin light chain variable region mRNA, partial cds. [AF035044]	XM_372952
A_23_P29773	0.000228	NM_014398	NM_014398	Homo sapiens lysosomal-associated membrane protein 3 (LAMP3), mRNA [NM_014398]	NM_014398
A_23_P219176	0.000228	NM_002957	NM_002957	Homo sapiens retinoid X receptor, alpha (RXRA), mRNA [NM_002957]	NM_002957
A_23_P422268	0.000228	NM_001003725	NM_001003725	Homo sapiens WD repeat domain 68 (WDR68), transcript variant 2, mRNA [NM_001003725]	NM_001003725
A_23_P22614	0.000228	NM_145802	NM_145802	Homo sapiens septin 6 (SEPT6), transcript variant V, mRNA [NM_145802]	NM_145802
A_23_P103532	0.000228	NM_007369	NM_007369	Homo sapiens G protein-coupled receptor 161 (GPR161), transcript variant 1, mRNA [NM_007369]	NM_007369
A_23_P155316	0.000228	NM_007362	NM_007362	Homo sapiens nuclear cap binding protein subunit 2, 20kDa (NCBP2), mRNA [NM_007362]	NM_007362
A_24_P345314	0.000229	NM_003189	NM_003189	Homo sapiens T-cell acute lymphocytic leukemia 1 (TAL1), mRNA [NM_003189]	NM_003189
A_23_P313476	0.000229	NM_032448	NM_032448	Homo sapiens KIAA1838 (KIAA1838), mRNA [NM_032448]	NM_032448
A_24_P374863	0.000229	ENST00000300639		Homo sapiens mRNA for FLJ00330 protein. [AK090421]	
A_23_P53018	0.000229	NM_054108	NM_054108	Homo sapiens H-rev107-like protein 5 (HRLP5), mRNA [NM_054108]	NM_054108
A_24_P303974	0.000229	NM_006023	NM_006023	Homo sapiens chromosome 10 open reading frame 7 (C10orf7), mRNA [NM_006023]	NM_006023
A_23_P166068	0.000229	NM_174856	NM_174856	Homo sapiens isocitrate dehydrogenase 3 (NAD+) beta (IDH3B), nuclear gene encoding mitochondrial protein, transcript variant 3, mRNA [NM_174856]	NM_174856
A_24_P280803	0.000229	BC018140	BC018140	Homo sapiens ribosomal protein S21, mRNA (cDNA clone MGC:9438 IMAGE:3903320), complete cds. [BC018140]	
A_24_P39759	0.00023	NM_197939	NM_197939	Homo sapiens ring finger protein 135 (RNF135), transcript variant 2, mRNA [NM_197939]	NM_197939
A_23_P52219	0.00023	NM_006459	NM_006459	Homo sapiens SPFH domain family, member 1 (SPFH1), mRNA [NM_006459]	NM_006459
A_23_P94301	0.00023	NM_003313	NM_003313	Homo sapiens tissue specific transplantation antigen P35B (TSTA3), mRNA [NM_003313]	NM_003313
A_24_P6294	0.00023	AK000180	AK000180	Homo sapiens cDNA FLJ20173 fis, clone COL09814. [AK000180]	

A_24_P356015	0.000231	NM_004094	NM_004094	Homo sapiens eukaryotic translation initiation factor 2, subunit 1 alpha, 35kDa (EIF2S1), mRNA [NM_004094]	NM_004094
A_23_P412554	0.000231	NM_145055	NM_145055	Homo sapiens chromosome 18 open reading frame 25 (C18orf25), transcript variant 1, mRNA [NM_145055]	NM_145055
A_24_P113086	0.000231	NM_000430	NM_000430	Homo sapiens platelet-activating factor acetylhydrolase, isoform Ib, alpha subunit 45kDa (PAFAH1B1), mRNA [NM_000430]	NM_000430
A_23_P104579	0.000231	NM_005851	NM_005851	Homo sapiens CDK2-associated protein 2 (CDK2AP2), mRNA [NM_005851]	NM_005851
A_24_P381029	0.000231	NM_006541	NM_006541	Homo sapiens thioredoxin-like 2 (TXNL2), mRNA [NM_006541]	NM_006541
A_23_P165691	0.000231	NM_005805	NM_005805	Homo sapiens proteasome (prosome, macropain) 26S subunit, non-ATPase, 14 (PSMD14), mRNA [NM_005805]	NM_005805
A_24_P308096	0.000232	NM_000215	NM_000215	Homo sapiens Janus kinase 3 (a protein tyrosine kinase, leukocyte) (JAK3), mRNA [NM_000215]	NM_000215
A_23_P388681	0.000232	NM_001419	NM_001419	Homo sapiens ELAV (embryonic lethal, abnormal vision, Drosophila)-like 1 (Hu antigen R) (ELAVL1), mRNA [NM_001419]	NM_001419
A_23_P201676	0.000232	NM_015935	NM_015935	Homo sapiens KIAA0859 (KIAA0859), transcript variant 1, mRNA [NM_015935]	NM_015935
A_23_P16944	0.000232	NM_001006946	NM_001006946	Homo sapiens syndecan 1 (SDC1), transcript variant 1, mRNA [NM_001006946]	NM_001006946
A_23_P89601	0.000233	NM_002278	NM_002278	Homo sapiens keratin, hair, acidic, 2 (KRTHA2), mRNA [NM_002278]	NM_002278
A_24_P210888	0.000233	AK002019	AK002019	Homo sapiens cDNA FLJ11157 fis, clone PLACE1006961. [AK002019]	
A_23_P63281	0.000233	NM_001029885	NM_001029885	Homo sapiens hypothetical protein MGC10334 (MGC10334), mRNA [NM_001029885]	NM_001029885
A_24_P678104	0.000233	NM_015894	NM_015894	Homo sapiens stathmin-like 3 (STMN3), mRNA [NM_015894]	NM_015894
A_23_P75310	0.000233	NM_021226	NM_021226	Homo sapiens Rho GTPase activating protein 22 (ARHGAP22), mRNA [NM_021226]	NM_021226
A_24_P457912	0.000234	NM_080476	NM_080476	Homo sapiens CDC91 cell division cycle 91-like 1 (S. cerevisiae) (CDC91L1), mRNA [NM_080476]	NM_080476
A_23_P105197	0.000234	NM_004055	NM_004055	Homo sapiens calpain 5 (CAPN5), mRNA [NM_004055]	NM_004055
A_24_P383080	0.000234	NM_182800	NM_182800	Homo sapiens arsenate resistance protein ARS2 (ARS2), transcript variant 2, mRNA [NM_182800]	NM_182800
A_24_P213321	0.000234	A_24_P213321			
A_32_P169131	0.000234	NM_019109	NM_019109	Homo sapiens asparagine-linked glycosylation 1 homolog (yeast, beta-1,4-mannosyltransferase) (ALG1), mRNA [NM_019109]	NM_019109
A_23_P312863	0.000234	NM_006088	NM_006088	Homo sapiens tubulin, beta, 2 (TUBB2), mRNA [NM_006088]	NM_006088
A_24_P928281	0.000235	AK024456	AK024456	Homo sapiens mRNA for FLJ00048 protein, partial cds. [AK024456]	
A_24_P60680	0.000235	NM_013941	NM_013941	Homo sapiens olfactory receptor, family 10, subfamily C, member 1 (OR10C1), mRNA [NM_013941]	NM_013941
A_24_P687085	0.000235	AK024933	AK024933	Homo sapiens cDNA: FLJ21280 fis, clone COL01884. [AK024933]	
A_24_P153926	0.000235	NM_018322	NM_018322	Homo sapiens chromosome 6 open reading frame 64 (C6orf64), mRNA [NM_018322]	NM_018322
A_23_P130531	0.000235	NM_007065	NM_007065	Homo sapiens CDC37 cell division cycle 37 homolog (S. cerevisiae) (CDC37), mRNA [NM_007065]	NM_007065
A_23_P68624	0.000236	NM_172236	NM_172236	Homo sapiens protein O-fucosyltransferase 1 (POFUT1), transcript variant 2, mRNA [NM_172236]	NM_172236
A_32_P889903	0.000236	AF086288	AF086288	Homo sapiens full length insert cDNA clone ZD48A05. [AF086288]	
A_23_P11752	0.000236	NM_000911	NM_000911	Homo sapiens opioid receptor, delta 1 (OPRD1), mRNA [NM_000911]	NM_000911
A_23_P317796	0.000236	NM_178176	NM_178176	Homo sapiens monoacylglycerol O-acyltransferase 3 (MOGAT3), mRNA [NM_178176]	NM_178176
A_32_P200787	0.000236	NM_145702	NM_145702	Homo sapiens tigger transposable element derived 1 (TIGD1), mRNA [NM_145702]	NM_145702
A_23_P87082	0.000236	NM_022370	NM_022370	Homo sapiens roundabout, axon guidance receptor, homolog 3 (Drosophila) (ROBO3), mRNA [NM_022370]	NM_022370
A_23_P48691	0.000236	NM_001663	NM_001663	Homo sapiens ADP-ribosylation factor 6 (ARF6), mRNA [NM_001663]	NM_001663
A_24_P340679	0.000236	NM_001008741	NM_001008741	Homo sapiens peptidylprolyl isomerase A-like (LOC388817), mRNA [NM_001008741]	NM_001008741
A_23_P210538	0.000237	NM_022086	NM_022086	Homo sapiens engulfment and cell motility 2 (ced-12 homolog, C. elegans) (ELMO2), transcript variant 2, mRNA [NM_022086]	NM_022086
A_24_P765715	0.000237	THC2280475		AI565054 tq79a09.x1 NCI_CGAP_Ut1 Homo sapiens cDNA clone IMAGE:2215000 3', mRNA sequence [AI565054]	
A_32_P1614	0.000237	AK023018	AK023018	Homo sapiens cDNA FLJ12956 fis, clone NT2RP2005501. [AK023018]	
A_23_P94703	0.000237	NM_014506	NM_014506	Homo sapiens torsin family 1, member B (torsin B) (TOR1B), mRNA [NM_014506]	NM_014506
A_24_P76644	0.000237	NM_031211	NM_031211	Homo sapiens LAT1-3TM protein (LAT1-3TM), mRNA [NM_031211]	NM_031211
A_24_P320171	0.000238	NM_152544	NM_152544	Homo sapiens hypothetical protein FLJ35725 (FLJ35725), mRNA [NM_152544]	NM_152544
A_23_P67271	0.000238	NM_002741	NM_002741	Homo sapiens protein kinase N1 (PKN1), transcript variant 2, mRNA [NM_002741]	NM_002741
A_23_P38041	0.000238	NM_016256	NM_016256	Homo sapiens N-acetylglucosamine-1-phosphodiester alpha-N-acetylglucosaminidase (NAGPA), mRNA [NM_016256]	NM_016256
A_32_P184933	0.000238	NM_014501	NM_014501	Homo sapiens ubiquitin-conjugating enzyme E2S (UBE2S), mRNA [NM_014501]	NM_014501
A_23_P160631	0.000238	NM_005998	NM_005998	Homo sapiens chaperonin containing TCP1, subunit 3 (gamma) (CCT3), transcript variant 1, mRNA [NM_005998]	NM_005998

A_24_P372901	0.000239	NM_000431	NM_000431	Homo sapiens mevalonate kinase (mevalonic aciduria) (MVK), mRNA [NM_000431]	NM_000431
A_23_P77797	0.000239	NM_001004707	NM_001004707	Homo sapiens olfactory receptor, family 4, subfamily D, member 2 (OR4D2), mRNA [NM_001004707]	NM_001004707
A_23_P193	0.000239	NM_022774	NM_022774	Homo sapiens chromosome 1 open reading frame 176 (C1orf176), mRNA [NM_022774]	NM_022774
A_24_P313597	0.000239	NM_003766	NM_003766	Homo sapiens beclin 1 (coiled-coil, myosin-like BCL2 interacting protein) (BECN1), mRNA [NM_003766]	NM_003766
A_24_P328675	0.000239	NM_015466	NM_015466	Homo sapiens protein tyrosine phosphatase, non-receptor type 23 (PTPN23), mRNA [NM_015466]	NM_015466
A_24_P75979	0.000241	ENST00000341372		PREDICTED: Homo sapiens similar to tropomyosin 3 (LOC221875), mRNA [XM_167254]	XM_167254
A_24_P302785	0.000241	NM_173825	NM_173825	Homo sapiens RAB, member of RAS oncogene family-like 3 (RABL3), mRNA [NM_173825]	NM_173825
A_24_P67494	0.000241	XM_496705	XM_496705	PREDICTED: Homo sapiens similar to karyopherin alpha 2 (LOC339991), mRNA [XM_496705]	XM_496705
A_23_P502783	0.000242	NM_147147	NM_147147	Homo sapiens blood vessel epicardial substance (BVES), transcript variant B, mRNA [NM_147147]	NM_147147
A_32_P89073	0.000242	NM_138352	NM_138352	Homo sapiens sterile alpha motif domain containing 1 (SAMD1), mRNA [NM_138352]	NM_138352
A_32_P88626	0.000242	NM_023923	NM_023923	Homo sapiens phosphatase and actin regulator 4 (PHACTR4), mRNA [NM_023923]	NM_023923
A_23_P25215	0.000242	NM_032941	NM_032941	Homo sapiens RecQ protein-like (DNA helicase Q1-like) (RECQL), transcript variant 2, mRNA [NM_032941]	NM_032941
A_23_P38235	0.000243	NM_000789	NM_000789	Homo sapiens angiotensin I converting enzyme (peptidyl-dipeptidase A) 1 (ACE), transcript variant 1, mRNA [NM_000789]	NM_000789
A_24_P315638	0.000243	XM_171536	XM_171536	PREDICTED: Homo sapiens MAS-related GPR, member E (MRGPRE), mRNA [XM_171536]	XM_171536
A_23_P22444	0.000244	NM_002621	NM_002621	Homo sapiens properdin P factor, complement (PFC), mRNA [NM_002621]	NM_002621
A_24_P45251	0.000244	NM_020137	NM_020137	Homo sapiens GRIP1 associated protein 1 (GRIPAP1), transcript variant 1, mRNA [NM_020137]	NM_020137
A_23_P435610	0.000244	NM_015047	NM_015047	Homo sapiens KIAA0090 (KIAA0090), mRNA [NM_015047]	NM_015047
A_24_P78526	0.000245	NM_199188	NM_199188	Homo sapiens La ribonucleoprotein domain family, member 4 (LARP4), transcript variant 2, mRNA [NM_199188]	NM_199188
A_32_P102581	0.000245	BG708379	BG708379	602672332F1 NIH_MGC_96 Homo sapiens cDNA clone IMAGE:4795231 5', mRNA sequence [BG708379]	
A_24_P243373	0.000245	NM_018940	NM_018940	Homo sapiens protocadherin beta 7 (PCDHB7), mRNA [NM_018940]	NM_018940
A_23_P129301	0.000245	NM_018097	NM_018097	Homo sapiens chromosome 15 open reading frame 25 (C15orf25), mRNA [NM_018097]	NM_018097
A_23_P145408	0.000245	NM_032020	NM_032020	Homo sapiens fucosidase, alpha-L- 2, plasma (FUCA2), mRNA [NM_032020]	NM_032020
A_24_P263443	0.000245	ENST00000259550			
A_24_P919989	0.000246	BC015334	BC015334	Homo sapiens, clone IMAGE:4391654, mRNA, partial cds. [BC015334]	
A_24_P853302	0.000246	A_24_P853302			
A_23_P127339	0.000246	NM_018425	NM_018425	Homo sapiens phosphatidylinositol 4-kinase type II (PI4KII), mRNA [NM_018425]	NM_018425
A_32_P41662	0.000246	NM_019041	NM_019041	Homo sapiens mitochondrial translational release factor 1-like (MTRF1L), mRNA [NM_019041]	NM_019041
A_32_P149111	0.000247	AK125015	AK125015	Homo sapiens cDNA FLJ43025 fis, clone BRTHA2018707. [AK125015]	
A_24_P341593	0.000247	ENST00000319587			
A_32_P11786	0.000247	NM_006275	NM_006275	Homo sapiens splicing factor, arginine/serine-rich 6 (SFRS6), mRNA [NM_006275]	NM_006275
A_24_P873688	0.000247	BC039021	BC039021	Homo sapiens cDNA clone IMAGE:6043059, partial cds. [BC039021]	
A_23_P398254	0.000248	NM_020429	NM_020429	Homo sapiens SMAD specific E3 ubiquitin protein ligase 1 (SMURF1), transcript variant 1, mRNA [NM_020429]	NM_020429
A_23_P208158	0.000248	NM_001392	NM_001392	Homo sapiens dystrobrevin, alpha (DTNA), transcript variant 7, mRNA [NM_001392]	NM_001392
A_23_P103672	0.000248	NM_006617	NM_006617	Homo sapiens nestin (NES), mRNA [NM_006617]	NM_006617
A_24_P124875	0.000249	NM_023112	NM_023112	Homo sapiens OTU domain, ubiquitin aldehyde binding 2 (OTUB2), mRNA [NM_023112]	NM_023112
A_24_P58283	0.000249	NM_003111	NM_003111	Homo sapiens Sp3 transcription factor (SP3), transcript variant 1, mRNA [NM_003111]	NM_003111
A_24_P246943	0.000249	NM_001008741	NM_001008741	Homo sapiens peptidylprolyl isomerase A-like (LOC388817), mRNA [NM_001008741]	NM_001008741
A_24_P913947	0.00025	ENST00000291567		Human glucose-6-phosphate dehydrogenase variant A-, partial cds. [M19866]	
A_23_P371794	0.00025	NM_000725	NM_000725	Homo sapiens calcium channel, voltage-dependent, beta 3 subunit (CACNB3), mRNA [NM_000725]	NM_000725
A_24_P381945	0.00025	NM_002134	NM_002134	Homo sapiens heme oxygenase (decycling) 2 (HMOX2), mRNA [NM_002134]	NM_002134
A_23_P32294	0.00025	NM_014612	NM_014612	Homo sapiens chromosome 9 open reading frame 10 (C9orf10), mRNA [NM_014612]	NM_014612
A_23_P100326	0.00025	NM_012075	NM_012075	Homo sapiens chromosome 16 open reading frame 35 (C16orf35), mRNA [NM_012075]	NM_012075
A_24_P243086	0.000251	NM_144635	NM_144635	Homo sapiens hypothetical protein MGC21688 (MGC21688), mRNA [NM_144635]	NM_144635

A_32_P831181	0.000251	NM_080626	NM_080626	Homo sapiens BRI3 binding protein (BRI3BP), mRNA [NM_080626]	NM_080626
A_23_P45913	0.000251	NM_004814	NM_004814	Homo sapiens WD repeat domain 57 (U5 snRNP specific) (WDR57), mRNA [NM_004814]	NM_004814
A_23_P257278	0.000251	NM_016082	NM_016082	Homo sapiens CDK5 regulatory subunit associated protein 1 (CDK5RAP1), transcript variant 2, mRNA [NM_016082]	NM_016082
A_23_P46924	0.000251	NM_001007793	NM_001007793	Homo sapiens BUB3 budding uninhibited by benzimidazoles 3 homolog (yeast) (BUB3), transcript variant 2, mRNA [NM_001007793]	NM_001007793
A_24_P678741	0.000252	XM_371461	XM_371461	PREDICTED: Homo sapiens KIAA1671 protein (KIAA1671), mRNA [XM_371461]	XM_371461
A_24_P221485	0.000253	A_24_P221485			
A_23_P315843	0.000253	NM_020967	NM_020967	Homo sapiens nuclear receptor coactivator 5 (NCOA5), mRNA [NM_020967]	NM_020967
A_24_P259083	0.000253	NM_080792	NM_080792	Homo sapiens protein tyrosine phosphatase, non-receptor type substrate 1 (PTPNS1), mRNA [NM_080792]	NM_080792
A_23_P416686	0.000253	NM_020155	NM_020155	Homo sapiens chromosome 11 hypothetical protein ORF4 (C11ORF4), mRNA [NM_020155]	NM_020155
A_23_P346969	0.000253	NM_006219	NM_006219	Homo sapiens phosphoinositide-3-kinase, catalytic, beta polypeptide (PIK3CB), mRNA [NM_006219]	NM_006219
A_23_P213471	0.000253	NM_024830	NM_024830	Homo sapiens hypothetical protein FLJ12443 (FLJ12443), mRNA [NM_024830]	NM_024830
A_23_P127525	0.000253	NM_005238	NM_005238	Homo sapiens v-ets erythroblastosis virus E26 oncogene homolog 1 (avian) (ETS1), mRNA [NM_005238]	NM_005238
A_23_P37503	0.000253	NM_004998	NM_004998	Homo sapiens myosin IE (MYO1E), mRNA [NM_004998]	NM_004998
A_32_P177797	0.000253	BC053632	BC053632	Homo sapiens cDNA clone IMAGE:6500775, partial cds. [BC053632]	
A_23_P218079	0.000253	NM_018976	NM_018976	Homo sapiens solute carrier family 38, member 2 (SLC38A2), mRNA [NM_018976]	NM_018976
A_23_P300255	0.000253	NM_002484	NM_002484	Homo sapiens nucleotide binding protein 1 (MinD homolog, E. coli) (NUBP1), mRNA [NM_002484]	NM_002484
A_24_P307424	0.000253	A_24_P307424			
A_24_P76210	0.000253	THC2365247		Q6QAQ1 (Q6QAQ1) Cytoskeletal beta actin (Fragment), partial (76%) [THC2365247]	
A_24_P416837	0.000254	NM_024958	NM_024958	Homo sapiens chromosome 20 open reading frame 98 (C20orf98), mRNA [NM_024958]	NM_024958
A_23_P423957	0.000255	NM_024963	NM_024963	Homo sapiens F-box and leucine-rich repeat protein 18 (FBXL18), mRNA [NM_024963]	NM_024963
A_24_P346762	0.000255	NM_025182	NM_025182	Homo sapiens KIAA1539 (KIAA1539), mRNA [NM_025182]	NM_025182
A_23_P105264	0.000255	NM_001987	NM_001987	Homo sapiens ets variant gene 6 (TEL oncogene) (ETV6), mRNA [NM_001987]	NM_001987
A_23_P3584	0.000255	NM_012320	NM_012320	Homo sapiens lysophospholipase 3 (lysosomal phospholipase A2) (LYPLA3), mRNA [NM_012320]	NM_012320
A_24_P79153	0.000256	NM_079834	NM_079834	Homo sapiens secretory carrier membrane protein 4 (SCAMP4), mRNA [NM_079834]	NM_079834
A_24_P409697	0.000256	BG206642	BG206642	RST26093 Athersys RAGE Library Homo sapiens cDNA, mRNA sequence [BG206642]	XM_377820
A_23_P254934	0.000257	NM_004796	NM_004796	Homo sapiens neurexin 3 (NRXN3), transcript variant alpha, mRNA [NM_004796]	NM_004796
A_24_P196519	0.000257	NM_002451	NM_002451	Homo sapiens methylthioadenosine phosphorylase (MTAP), mRNA [NM_002451]	NM_002451
A_32_P158019	0.000258	BX095305	BX095305	BX095305 Soares_pregnant_uterus_NbHPU Homo sapiens cDNA clone IMAGp998M221116, mRNA sequence [BX095305]	
A_32_P58361	0.000258	THC2276504		Q9UID7 (Q9UID7) CYR61 protein, partial (87%) [THC2276504]	
A_24_P251381	0.000258	NM_024782	NM_024782	Homo sapiens hypothetical protein FLJ12610 (FLJ12610), mRNA [NM_024782]	NM_024782
A_23_P339554	0.000258	NM_032228	NM_032228	Homo sapiens male sterility domain containing 2 (MLSTD2), mRNA [NM_032228]	NM_032228
A_24_P324488	0.000258	A_24_P324488			
A_23_P342185	0.000258	NM_015045	NM_015045	Homo sapiens KIAA0261 (KIAA0261), mRNA [NM_015045]	NM_015045
A_24_P42308	0.000259	AK056449	AK056449	Homo sapiens cDNA FLJ31887 fis, clone NT2RP7003050. [AK056449]	
A_23_P6272	0.000259	NM_015259	NM_015259	Homo sapiens inducible T-cell co-stimulator ligand (ICOSLG), mRNA [NM_015259]	NM_015259
A_23_P153502	0.000259	NM_032375	NM_032375	Homo sapiens AKT1 substrate 1 (proline-rich) (AKT1S1), mRNA [NM_032375]	NM_032375
A_23_P86855	0.000259	NM_014067	NM_014067	Homo sapiens LRP16 protein (LRP16), mRNA [NM_014067]	NM_014067
A_23_P116557	0.00026	NM_009587	NM_009587	Homo sapiens lectin, galactoside-binding, soluble, 9 (galectin 9) (LGALS9), transcript variant long, mRNA [NM_009587]	NM_009587
A_23_P306479	0.00026	ENST00000309829		Homo sapiens cDNA clone IMAGE:2989953, partial cds. [BC003066]	
A_23_P163801	0.00026	NM_017566	NM_017566	Homo sapiens kelch domain containing 4 (KLHDC4), mRNA [NM_017566]	NM_017566
A_23_P52903	0.00026	NM_052875	NM_052875	Homo sapiens hypothetical protein MGC10485 (MGC10485), mRNA [NM_052875]	NM_052875
A_32_P201677	0.00026	BE730710	BE730710	601570665F1 NIH_MGC_21 Homo sapiens cDNA clone IMAGE:3845272 5', mRNA sequence [BE730710]	
A_23_P23151	0.00026	NM_014812	NM_014812	Homo sapiens KIAA0470 (KIAA0470), mRNA [NM_014812]	NM_014812
A_32_P187327	0.00026	NM_006088	NM_006088	Homo sapiens tubulin, beta, 2 (TUBB2), mRNA [NM_006088]	NM_006088

A_24_P110780	0.000261	NM_199343	NM_199343	Homo sapiens chromosome 1 open reading frame 118 (C1orf118), mRNA [NM_199343]	NM_199343
A_32_P210744	0.000261	A_32_P210744			
A_24_P342178	0.000261	BC020868	BC020868	Homo sapiens signal transducer and activator of transcription 5B, mRNA (cDNA clone IMAGE:4605440), complete cds. [BC020868]	
A_32_P150300	0.000261	CR992331	CR992331	CR992331 RZPD no.9016 Homo sapiens cDNA clone RZPDp9016A0141 5', mRNA sequence [CR992331]	
A_23_P13797	0.000261	NM_024738	NM_024738	Homo sapiens hypothetical protein FLJ21415 (FLJ21415), mRNA [NM_024738]	NM_024738
A_23_P14543	0.000261	NM_006020	NM_006020	Homo sapiens alkB, alkylation repair homolog (E. coli) (ALKBH), mRNA [NM_006020]	NM_006020
A_23_P12423	0.000261	NM_022089	NM_022089	Homo sapiens ATPase type 13A2 (ATP13A2), mRNA [NM_022089]	NM_022089
A_24_P201552	0.000261	NM_005103	NM_005103	Homo sapiens fasciculation and elongation protein zeta 1 (zygin I) (FEZ1), transcript variant 1, mRNA [NM_005103]	NM_005103
A_23_P200984	0.000261	NM_002840	NM_002840	Homo sapiens protein tyrosine phosphatase, receptor type, F (PTPRF), transcript variant 1, mRNA [NM_002840]	NM_002840
A_23_P111745	0.000261	NM_031449	NM_031449	Homo sapiens hypothetical protein DKFZp761I2123 (DKFZp761I2123), transcript variant 1, mRNA [NM_031449]	NM_031449
A_24_P937319	0.000262	AK097197	AK097197	Homo sapiens cDNA FLJ39878 fis, clone SPLEN2016045, moderately similar to Homo sapiens mRNA for Hrs. [AK097197]	
A_24_P296280	0.000262	NM_018145	NM_018145	Homo sapiens family with sequence similarity 82, member C (FAM82C), mRNA [NM_018145]	NM_018145
A_23_P385673	0.000263	X89399	X89399	Homo sapiens mRNA for Ins(1,3,4,5)P4-binding protein. [X89399]	
A_32_P149432	0.000263	NM_001416	NM_001416	Homo sapiens eukaryotic translation initiation factor 4A, isoform 1 (EIF4A1), mRNA [NM_001416]	NM_001416
A_23_P431521	0.000264	NM_021814	NM_021814	Homo sapiens ELOVL family member 5, elongation of long chain fatty acids (FEN1/Elo2, SUR4/Elo3-like, yeast) (ELOVL5), mRNA [NM_021814]	NM_021814
A_23_P77980	0.000264	NM_000342	NM_000342	Homo sapiens solute carrier family 4, anion exchanger, member 1 (erythrocyte membrane protein band 3, Diego blood group) (SLC4A1), mRNA [NM_000342]	NM_000342
A_23_P152218	0.000264	NM_001950	NM_001950	Homo sapiens E2F transcription factor 4, p107/p130-binding (E2F4), mRNA [NM_001950]	NM_001950
A_24_P38951	0.000264	NM_032871	NM_032871	Homo sapiens tumor necrosis factor receptor superfamily, member 19-like (TNFRSF19L), transcript variant 1, mRNA [NM_032871]	NM_032871
A_23_P343914	0.000265	NM_177966	NM_177966	Homo sapiens 2'-phosphodiesterase (2'-PDE), mRNA [NM_177966]	NM_177966
A_24_P521662	0.000265	A_24_P521662			
A_32_P143048	0.000265	NM_004799	NM_004799	Homo sapiens zinc finger, FYVE domain containing 9 (ZFYVE9), transcript variant 3, mRNA [NM_004799]	NM_004799
A_32_P73222	0.000265	AA631847	AA631847	np61b02.s1 NCI_CGAP_Br2 Homo sapiens cDNA clone IMAGE:1130763 3' similar to TR:G971838 G971838 60S RIBOSOMAL PROTEIN L34 ;, mRNA sequence [AA631847]	
A_24_P102043	0.000265	THC2307660		P70670 (P70670) Alpha-NAC, muscle-specific form gp220, partial (14%) [THC2307660]	
A_24_P211351	0.000266	A_24_P211351			
A_24_P313576	0.000267	NM_014232	NM_014232	Homo sapiens vesicle-associated membrane protein 2 (synaptobrevin 2) (VAMP2), mRNA [NM_014232]	NM_014232
A_23_P89155	0.000267	NM_001258	NM_001258	Homo sapiens cyclin-dependent kinase 3 (CDK3), mRNA [NM_001258]	NM_001258
A_24_P462899	0.000267	NM_001012507	NM_001012507	Homo sapiens chromosome 6 open reading frame 173 (C6orf173), mRNA [NM_001012507]	NM_001012507
A_24_P41890	0.000267	A_24_P41890			
A_23_P158239	0.000267	NM_005412	NM_005412	Homo sapiens serine hydroxymethyltransferase 2 (mitochondrial) (SHMT2), mRNA [NM_005412]	NM_005412
A_32_P218636	0.000267	BC014228	BC014228	Homo sapiens prohibitin pseudogene, mRNA (cDNA clone MGC:20874 IMAGE:4547239), complete cds. [BC014228]	
A_32_P218332	0.000268	NM_174917	NM_174917	Homo sapiens hypothetical protein LOC197322 (LOC197322), mRNA [NM_174917]	NM_174917
A_23_P77876	0.000268	NM_002816	NM_002816	Homo sapiens proteasome (prosome, macropain) 26S subunit, non-ATPase, 12 (PSMD12), transcript variant 1, mRNA [NM_002816]	NM_002816
A_24_P622186	0.000269	NM_080926	NM_080926	Homo sapiens hypothetical protein similar to KIAA0187 gene product (LOC96610), mRNA [NM_080926]	NM_080926
A_24_P158385	0.000269	NM_138462	NM_138462	Homo sapiens zinc finger, MYND-type containing 19 (ZMYND19), mRNA [NM_138462]	NM_138462
A_23_P91814	0.000269	NM_017875	NM_017875	Homo sapiens hypothetical protein FLJ20551 (FLJ20551), mRNA [NM_017875]	NM_017875
A_24_P203658	0.000269	ENST00000297544		PREDICTED: Homo sapiens similar to 60S acidic ribosomal protein P1 (LOC133609), mRNA [XM_068430]	XM_068430
A_23_P164507	0.00027	NM_005433	NM_005433	Homo sapiens v-yes-1 Yamaguchi sarcoma viral oncogene homolog 1 (YES1), mRNA [NM_005433]	NM_005433
A_24_P401582	0.00027	A_24_P401582			
A_24_P133822	0.00027	NM_018127	NM_018127	Homo sapiens elaC homolog 2 (E. coli) (ELAC2), mRNA [NM_018127]	NM_018127
A_23_P138782	0.000271	NM_001610	NM_001610	Homo sapiens acid phosphatase 2, lysosomal (ACP2), mRNA [NM_001610]	NM_001610
A_24_P666340	0.000271	THC2408022			
A_24_P90349	0.000271	NM_174940	NM_174940	Homo sapiens hypothetical protein LOC283232 (LOC283232), mRNA [NM_174940]	NM_174940
A_24_P147407	0.000271	NM_153335	NM_153335	Homo sapiens protein kinase LYK5 (LYK5), transcript variant 3, mRNA [NM_153335]	NM_153335

A_24_P476386	0.000271	BQ432816	BQ432816	AGENCOURT_7838565 NIH_MGC_82 Homo sapiens cDNA clone IMAGE:6101456 5', mRNA sequence [BQ432816]	
A_23_P91702	0.000271	NM_003753	NM_003753	Homo sapiens eukaryotic translation initiation factor 3, subunit 7 zeta, 66/67kDa (EIF3S7), mRNA [NM_003753]	NM_003753
A_23_P433504	0.000272	AK091315	AK091315	Homo sapiens cDNA FLJ33996 fis, clone DFNES2008881. [AK091315]	
A_24_P527716	0.000272	BC094740	BC094740	Homo sapiens cDNA clone IMAGE:30531558, containing frame-shift errors. [BC094740]	
A_23_P6344	0.000272	NM_022044	NM_022044	Homo sapiens stromal cell-derived factor 2-like 1 (SDF2L1), mRNA [NM_022044]	NM_022044
A_23_P137765	0.000273	NM_000437	NM_000437	Homo sapiens platelet-activating factor acetylhydrolase 2, 40kDa (PAFAH2), mRNA [NM_000437]	NM_000437
A_32_P82560	0.000273	THC2360885			
A_32_P30649	0.000273	NM_004454	NM_004454	Homo sapiens ets variant gene 5 (ets-related molecule) (ETV5), mRNA [NM_004454]	NM_004454
A_23_P256868	0.000273	NM_152624	NM_152624	Homo sapiens DCP2 decapping enzyme homolog (S. cerevisiae) (DCP2), mRNA [NM_152624]	NM_152624
A_32_P42913	0.000274	AI051172	AI051172	AI051172 oy94a06.x1 Soares_fetal_liver_spleen_1NFLS_S1 Homo sapiens cDNA clone IMAGE:1673458 3' similar to SW:SSRG_RAT Q08013 TRANSLOCON-ASSOCIATED PROTEIN, GAMMA SUBUNIT ;, mRNA sequence [AI051172]	
A_24_P306136	0.000274	AL136875	AL136875	Homo sapiens mRNA; cDNA DKFZp434D105 (from clone DKFZp434D105). [AL136875]	
A_32_P195853	0.000274	AK001565	AK001565	Homo sapiens cDNA FLJ10703 fis, clone NT2RP3000836. [AK001565]	
A_23_P76557	0.000274	NM_018838	NM_018838	Homo sapiens NADH dehydrogenase (ubiquinone) 1 alpha subcomplex, 12 (NDUFA12), mRNA [NM_018838]	NM_018838
A_23_P208482	0.000275	NM_214677	NM_214677	Homo sapiens C-type lectin domain family 4, member M (CLEC4M), transcript variant 4, mRNA [NM_214677]	NM_214677
A_32_P15706	0.000275	A_32_P15706			
A_23_P341312	0.000275	NM_198969	NM_198969	Homo sapiens amino-terminal enhancer of split (AES), transcript variant 1, mRNA [NM_198969]	NM_198969
A_24_P192262	0.000275	NM_005402	NM_005402	Homo sapiens v-ral simian leukemia viral oncogene homolog A (ras related) (RALA), mRNA [NM_005402]	NM_005402
A_24_P926770	0.000276	NM_014045	NM_014045	Homo sapiens low density lipoprotein receptor-related protein 10 (LRP10), mRNA [NM_014045]	NM_014045
A_23_P202117	0.000276	BC007377	BC007377	Homo sapiens polycomb group ring finger 5, mRNA (cDNA clone IMAGE:3640258), complete cds. [BC007377]	
A_23_P8339	0.000276	NM_014161	NM_014161	Homo sapiens mitochondrial ribosomal protein L18 (MRPL18), nuclear gene encoding mitochondrial protein, mRNA [NM_014161]	NM_014161
A_24_P191884	0.000277	ENST00000297540		Homo sapiens cDNA FLJ13193 fis, clone NT2RP3004348, moderately similar to R.norvegicus mRNA for cytosolic resiniferatoxin-binding protein. [AK023255]	
A_24_P318134	0.000277	NM_032786	NM_032786	Homo sapiens zinc finger CCCH-type containing 10 (ZC3H10), mRNA [NM_032786]	NM_032786
A_23_P419714	0.000277	NM_152322	NM_152322	Homo sapiens BTB (POZ) domain containing 11 (BTBD11), transcript variant 1, mRNA [NM_152322]	NM_152322
A_32_P202092	0.000277	THC2406182			
A_24_P213628	0.000277	AK095727	AK095727	Homo sapiens cDNA FLJ38408 fis, clone FEBRA2009029. [AK095727]	
A_32_P148476	0.000277	ENST00000304245		PREDICTED: Homo sapiens similar to Ran-specific GTPase-activating protein (Ran binding protein 1) (RanBP1) (LOC389842), mRNA [XM_372200]	XM_372200
A_24_P943597	0.000277	AF220656	AF220656	Homo sapiens apoptosis-associated nuclear protein PHLDA1 (PHLDA1) mRNA, partial cds. [AF220656]	
A_23_P37497	0.000277	U14391	U14391	Human myosin-IC mRNA, complete cds. [U14391]	
A_23_P78014	0.000277	NM_016437	NM_016437	Homo sapiens tubulin, gamma 2 (TUBG2), mRNA [NM_016437]	NM_016437
A_32_P57717	0.000277	AA292106	AA292106	AA292106 zr58h03.s1 Soares_NhHMPu_S1 Homo sapiens cDNA clone IMAGE:667637 3', mRNA sequence [AA292106]	
A_23_P118909	0.000278	NM_020232	NM_020232	Homo sapiens tumor necrosis factor superfamily, member 5-induced protein 1 (TNFSF5IP1), mRNA [NM_020232]	NM_020232
A_24_P569294	0.000278	NM_021107	NM_021107	Homo sapiens mitochondrial ribosomal protein S12 (MRPS12), nuclear gene encoding mitochondrial protein, transcript variant 1, mRNA [NM_021107]	NM_021107
A_24_P176445	0.000278	NM_005326	NM_005326	Homo sapiens hydroxyacylglutathione hydrolase (HAGH), mRNA [NM_005326]	NM_005326
A_32_P100464	0.000279	THC2340639		Q6MX26 (Q6MX26) PE-PGRS FAMILY PROTEIN, partial (6%) [THC2340639]	
A_32_P194264	0.000279	NM_001008708	NM_001008708	Homo sapiens similar to RIKEN cDNA 2510006C20 gene (LOC494143), mRNA [NM_001008708]	NM_001008708
A_23_P500873	0.000279	NM_133627	NM_133627	Homo sapiens RAD51-like 3 (S. cerevisiae) (RAD51L3), transcript variant 2, mRNA [NM_133627]	NM_133627
A_32_P86028	0.000279	NM_001017	NM_001017	Homo sapiens ribosomal protein S13 (RPS13), mRNA [NM_001017]	NM_001017
A_23_P53324	0.00028	NM_024623	NM_024623	Homo sapiens hypothetical protein FLJ13491 (FLJ13491), mRNA [NM_024623]	NM_024623
A_24_P99152	0.00028	NM_004926	NM_004926	Homo sapiens zinc finger protein 36, C3H type-like 1 (ZFP36L1), mRNA [NM_004926]	NM_004926
A_23_P201432	0.00028	NM_001126	NM_001126	Homo sapiens adenylosuccinate synthase (ADSS), mRNA [NM_001126]	NM_001126
A_24_P331373	0.000281	NM_031444	NM_031444	Homo sapiens chromosome 22 open reading frame 13 (C22orf13), mRNA [NM_031444]	NM_031444
A_24_P324644	0.000281	A_24_P324644			
A_23_P66658	0.000281	NM_001488	NM_001488	Homo sapiens transcriptional adaptor 2 (ADA2 homolog, yeast)-like (TADA2L), transcript variant 1, mRNA [NM_001488]	NM_001488

A_23_P21425	0.000281	NM_178471	NM_178471	Homo sapiens G protein-coupled receptor 119 (GPR119), mRNA [NM_178471]	NM_178471
A_23_P100676	0.000282	NM_017575	NM_017575	Homo sapiens chromosome 17 open reading frame 31 (C17orf31), mRNA [NM_017575]	NM_017575
A_23_P215154	0.000282	NM_016118	NM_016118	Homo sapiens NEDD8 ultimate buster-1 (NYREN18), mRNA [NM_016118]	NM_016118
A_24_P132950	0.000282	NM_005969	NM_005969	Homo sapiens nucleosome assembly protein 1-like 4 (NAP1L4), mRNA [NM_005969]	NM_005969
A_24_P48617	0.000283	NM_006830	NM_006830	Homo sapiens ubiquinol-cytochrome c reductase, 6.4kDa subunit (UQCR), mRNA [NM_006830]	NM_006830
A_24_P195454	0.000283	A_24_P195454			
A_24_P289404	0.000283	NM_001029	NM_001029	Homo sapiens ribosomal protein S26 (RPS26), mRNA [NM_001029]	NM_001029
A_24_P22562	0.000284	BC069097	BC069097	Homo sapiens apoptosis related protein, mRNA (cDNA clone MGC:95372 IMAGE:7216911), complete cds. [BC069097]	XM_498424
A_23_P136986	0.000284	NM_198450	NM_198450	Homo sapiens chromosome X open reading frame 33 (CXorf33), mRNA [NM_198450]	NM_198450
A_32_P136130	0.000284	A_32_P136130			
A_23_P308021	0.000284	NM_145200	NM_145200	Homo sapiens calcium binding protein 4 (CABP4), mRNA [NM_145200]	NM_145200
A_23_P79323	0.000284	NM_003936	NM_003936	Homo sapiens cyclin-dependent kinase 5, regulatory subunit 2 (p39) (CDK5R2), mRNA [NM_003936]	NM_003936
A_23_P433071	0.000284	NM_003766	NM_003766	Homo sapiens beclin 1 (coiled-coil, myosin-like BCL2 interacting protein) (BECN1), mRNA [NM_003766]	NM_003766
A_23_P121265	0.000284	NM_007284	NM_007284	Homo sapiens PTK9L protein tyrosine kinase 9-like (A6-related protein) (PTK9L), mRNA [NM_007284]	NM_007284
A_23_P216501	0.000284	NM_213674	NM_213674	Homo sapiens tropomyosin 2 (beta) (TPM2), transcript variant 2, mRNA [NM_213674]	NM_213674
A_23_P207301	0.000285	NM_018304	NM_018304	Homo sapiens hypothetical protein FLJ11029 (FLJ11029), mRNA [NM_018304]	NM_018304
A_32_P209148	0.000286	BC044616	BC044616	Homo sapiens, clone IMAGE:5267190, mRNA. [BC044616]	
A_32_P52590	0.000286	AA887631	AA887631	AA887631 nq96b08.s1 NCI_CGAP_Co10 Homo sapiens cDNA clone IMAGE:1160151 3', mRNA sequence [AA887631]	
A_23_P96985	0.000286	NM_012486	NM_012486	Homo sapiens presenilin 2 (Alzheimer disease 4) (PSEN2), transcript variant 2, mRNA [NM_012486]	NM_012486
A_24_P286868	0.000286	CR592483	CR592483	full-length cDNA clone CS0DL004YD15 of B cells (Ramos cell line) Cot 25-normalized of Homo sapiens (human). [CR592483]	
A_24_P641406	0.000286	XM_377558	XM_377558	PREDICTED: Homo sapiens similar to elongation factor 1 delta (LOC401937), mRNA [XM_377558]	XM_377558
A_23_P76499	0.000286	NM_005002	NM_005002	Homo sapiens NADH dehydrogenase (ubiquinone) 1 alpha subcomplex, 9, 39kDa (NDUFA9), mRNA [NM_005002]	NM_005002
A_23_P500364	0.000287	NM_138707	NM_138707	Homo sapiens B-cell CLL/lymphoma 7B (BCL7B), transcript variant 2, mRNA [NM_138707]	NM_138707
A_24_P124550	0.000287	NM_053056	NM_053056	Homo sapiens cyclin D1 (PRAD1: parathyroid adenomatosis 1) (CCND1), mRNA [NM_053056]	NM_053056
A_23_P23356	0.000287	NM_016052	NM_016052	Homo sapiens CGI-115 protein (CGI-115), mRNA [NM_016052]	NM_016052
A_23_P252106	0.000287	NM_003821	NM_003821	Homo sapiens receptor-interacting serine-threonine kinase 2 (RIPK2), mRNA [NM_003821]	NM_003821
A_32_P67259	0.000287	NM_004168	NM_004168	Homo sapiens succinate dehydrogenase complex, subunit A, flavoprotein (Fp) (SDHA), nuclear gene encoding mitochondrial protein, mRNA [NM_004168]	NM_004168
A_23_P368624	0.000288	NM_000748	NM_000748	Homo sapiens cholinergic receptor, nicotinic, beta polypeptide 2 (neuronal) (CHRNB2), mRNA [NM_000748]	NM_000748
A_32_P117977	0.000288	BX360933	BX360933	BX360933 BX360933 Homo sapiens PLACENTA COT 25-NORMALIZED Homo sapiens cDNA clone CS0DI077YB17 3-PRIME, mRNA sequence [BX360933]	
A_24_P339611	0.000288	NM_004708	NM_004708	Homo sapiens programmed cell death 5 (PDCD5), mRNA [NM_004708]	NM_004708
A_24_P323778	0.000288	ENST00000338221			
A_32_P176594	0.000289	AB046834	AB046834	Homo sapiens mRNA for KIAA1614 protein, partial cds. [AB046834]	XM_046531
A_24_P212096	0.000289	NM_020443	NM_020443	Homo sapiens neuron navigator 1 (NAV1), mRNA [NM_020443]	NM_020443
A_24_P549518	0.000289	A_24_P549518			
A_32_P28712	0.00029	THC2441398		Q13860 (Q13860) BGP protein (Fragment), partial (19%) [THC2441398]	
A_24_P181055	0.00029	NM_006278	NM_006278	Homo sapiens ST3 beta-galactoside alpha-2,3-sialyltransferase 4 (ST3GAL4), mRNA [NM_006278]	NM_006278
A_23_P40354	0.00029	NM_012325	NM_012325	Homo sapiens microtubule-associated protein, RP/EB family, member 1 (MAPRE1), mRNA [NM_012325]	NM_012325
A_24_P45651	0.00029	NM_001005464	NM_001005464	Homo sapiens histone H3/o (H3/o), mRNA [NM_001005464]	NM_001005464
A_32_P10100	0.00029	A_32_P10100			
A_23_P79015	0.000291	NM_199037	NM_199037	Homo sapiens sodium channel, voltage-gated, type I, beta (SCN1B), transcript variant b, mRNA [NM_199037]	NM_199037
A_24_P213956	0.000291	U05597	U05597	Human anion exchanger 3 cardiac isoform (cAE3) mRNA, partial cds. [U05597]	
A_23_P326844	0.000291	NM_174913	NM_174913	Homo sapiens chromosome 14 open reading frame 21 (C14orf21), mRNA [NM_174913]	NM_174913
A_23_P29263	0.000291	NM_152221	NM_152221	Homo sapiens casein kinase 1, epsilon (CSNK1E), transcript variant 1, mRNA [NM_152221]	NM_152221
A_23_P7172	0.000291	NM_018290	NM_018290	Homo sapiens phosphoglucomutase 2 (PGM2), mRNA [NM_018290]	NM_018290

A_24_P263672	0.000291	NM_080926	NM_080926	Homo sapiens hypothetical protein similar to KIAA0187 gene product (LOC96610), mRNA [NM_080926]	NM_080926
A_23_P71972	0.000292	NM_018560	NM_018560	Homo sapiens WW domain containing oxidoreductase (WWOX), transcript variant 2, mRNA [NM_018560]	NM_018560
A_23_P433218	0.000292	NR_002185	NR_002185	Homo sapiens olfactory receptor, family 7, subfamily E, member 91 pseudogene (OR7E91P) on chromosome 2 [NR_002185]	NR_002185
A_24_P118452	0.000292	NM_012247	NM_012247	Homo sapiens selenophosphate synthetase 1 (SEPHS1), mRNA [NM_012247]	NM_012247
A_24_P230416	0.000292	A_24_P230416			
A_24_P84834	0.000293	NM_201444	NM_201444	Homo sapiens diacylglycerol kinase, alpha 80kDa (DGKA), transcript variant 1, mRNA [NM_201444]	NM_201444
A_23_P155711	0.000293	NM_018248	NM_018248	Homo sapiens nei endonuclease VIII-like 3 (E. coli) (NEIL3), mRNA [NM_018248]	NM_018248
A_24_P238427	0.000293	A_24_P238427			
A_23_P97481	0.000294	AK126792	AK126792	Homo sapiens cDNA FLJ44842 fis, clone BRACE3049714, weakly similar to Homo sapiens NYD-TSPG protein (NYD-TSPG). [AK126792]	
A_23_P158627	0.000294	NM_024078	NM_024078	Homo sapiens hypothetical protein MGC3162 (MGC3162), mRNA [NM_024078]	NM_024078
A_32_P68459	0.000294	NM_203430	NM_203430	Homo sapiens peptidylprolyl isomerase A (cyclophilin A) (PPIA), transcript variant 2, mRNA [NM_203430]	NM_203430
A_32_P214441	0.000295	BM994377	BM994377	UI-H-DH0-aul-h-01-0-UI.s1 NCI_CGAP_DH0 Homo sapiens cDNA clone IMAGE:5871024 3', mRNA sequence [BM994377]	
A_24_P889462	0.000295	BX640624	BX640624	Homo sapiens mRNA; cDNA DKFZp686K18196 (from clone DKFZp686K18196). [BX640624]	
A_23_P330486	0.000295	NM_152416	NM_152416	Homo sapiens chromosome 8 open reading frame 38 (C8orf38), mRNA [NM_152416]	NM_152416
A_32_P188178	0.000295	NM_004238	NM_004238	Homo sapiens thyroid hormone receptor interactor 12 (TRIP12), mRNA [NM_004238]	NM_004238
A_24_P148811	0.000295	NM_003707	NM_003707	Homo sapiens RuvB-like 1 (E. coli) (RUVBL1), mRNA [NM_003707]	NM_003707
A_23_P77020	0.000295	NM_012461	NM_012461	Homo sapiens TERF1 (TRF1)-interacting nuclear factor 2 (TINF2), mRNA [NM_012461]	NM_012461
A_23_P154675	0.000295	NM_198216	NM_198216	Homo sapiens small nuclear ribonucleoprotein polypeptides B and B1 (SNRPB), transcript variant 1, mRNA [NM_198216]	NM_198216
A_23_P336728	0.000296	AL049980	AL049980	Homo sapiens mRNA; cDNA DKFZp564C152 (from clone DKFZp564C152) [AL049980]	
A_23_P332374	0.000296	NM_007147	NM_007147	Homo sapiens zinc finger protein 175 (ZNF175), mRNA [NM_007147]	NM_007147
A_23_P47426	0.000296	NM_014384	NM_014384	Homo sapiens acyl-Coenzyme A dehydrogenase family, member 8 (ACAD8), mRNA [NM_014384]	NM_014384
A_23_P96568	0.000296	NM_001456	NM_001456	Homo sapiens filamin A, alpha (actin binding protein 280) (FLNA), mRNA [NM_001456]	NM_001456
A_23_P36397	0.000297	NM_000785	NM_000785	Homo sapiens cytochrome P450, family 27, subfamily B, polypeptide 1 (CYP27B1), nuclear gene encoding mitochondrial protein, mRNA [NM_000785]	NM_000785
A_23_P7535	0.000297	NM_022304	NM_022304	Homo sapiens histamine receptor H2 (HRH2), mRNA [NM_022304]	NM_022304
A_23_P26439	0.000297	NM_024043	NM_024043	Homo sapiens hypothetical protein MGC3101 (MGC3101), mRNA [NM_024043]	NM_024043
A_23_P315122	0.000297	NM_004097	NM_004097	Homo sapiens empty spiracles homolog 1 (Drosophila) (EMX1), mRNA [NM_004097]	NM_004097
A_24_P375340	0.000298	A_24_P375340			
A_24_P217365	0.000298	NM_015199	NM_015199	Homo sapiens ankyrin repeat domain 28 (ANKRD28), mRNA [NM_015199]	NM_015199
A_23_P36183	0.000298	NM_005316	NM_005316	Homo sapiens general transcription factor IIH, polypeptide 1 (62kD subunit) (GTF2H1), mRNA [NM_005316]	NM_005316
A_23_P168541	0.000298	NM_024067	NM_024067	Homo sapiens chromosome 7 open reading frame 26 (C7orf26), mRNA [NM_024067]	NM_024067
A_23_P59772	0.000299	NM_000083	NM_000083	Homo sapiens chloride channel 1, skeletal muscle (Thomsen disease, autosomal dominant) (CLCN1), mRNA [NM_000083]	NM_000083
A_23_P151595	0.000299	NM_006032	NM_006032	Homo sapiens copine VI (neuronal) (CPNE6), mRNA [NM_006032]	NM_006032
A_23_P37076	0.000299	NM_001015049	NM_001015049	Homo sapiens BCL2-associated athanogene 5 (BAG5), transcript variant 1, mRNA [NM_001015049]	NM_001015049
A_23_P87603	0.000299	NM_004178	NM_004178	Homo sapiens TAR (HIV) RNA binding protein 2 (TARBP2), transcript variant 3, mRNA [NM_004178]	NM_004178
A_24_P393880	0.000299	NM_153649	NM_153649	Homo sapiens tropomyosin 3 (TPM3), transcript variant 2, mRNA [NM_153649]	NM_153649
A_23_P36700	0.0003	NM_018009	NM_018009	Homo sapiens TAP binding protein-like (TAPBPL), mRNA [NM_018009]	NM_018009
A_32_P138348	0.0003	NM_017527	NM_017527	Homo sapiens lymphocyte antigen 6 complex, locus K (LY6K), mRNA [NM_017527]	NM_017527
A_23_P502808	0.0003	NM_178013	NM_178013	Homo sapiens proline rich membrane anchor 1 (PRIMA1), mRNA [NM_178013]	NM_178013
A_23_P138461	0.0003	NM_021830	NM_021830	Homo sapiens progressive external ophthalmoplegia 1 (PEO1), mRNA [NM_021830]	NM_021830
A_23_P73511	0.0003	NM_001654	NM_001654	Homo sapiens v-raf murine sarcoma 3611 viral oncogene homolog (ARAF), mRNA [NM_001654]	NM_001654
A_23_P252491	0.0003	NM_016484	NM_016484	Homo sapiens PDZ domain containing 11 (PDZK11), mRNA [NM_016484]	NM_016484
A_24_P161393	0.0003	A_24_P161393			
A_32_P359110	0.000301	NM_198317	NM_198317	Homo sapiens kelch-like 17 (Drosophila) (KLHL17), mRNA [NM_198317]	NM_198317
A_23_P82823	0.000301	NM_017884	NM_017884	Homo sapiens PIN2-interacting protein 1 (PINX1), mRNA [NM_017884]	NM_017884

A_32_P58163	0.000301	NM_024713	NM_024713	Homo sapiens chromosome 15 open reading frame 29 (C15orf29), mRNA [NM_024713]	NM_024713
A_24_P43391	0.000301	NM_018475	NM_018475	Homo sapiens TPA regulated locus (TPARL), mRNA [NM_018475]	NM_018475
A_23_P154234	0.000301	NM_019896	NM_019896	Homo sapiens polymerase (DNA-directed), epsilon 4 (p12 subunit) (POLE4), mRNA [NM_019896]	NM_019896
A_24_P82957	0.000302	NM_018683	NM_018683	Homo sapiens zinc finger protein 313 (ZNF313), mRNA [NM_018683]	NM_018683
A_24_P357037	0.000302	NM_182688	NM_182688	Homo sapiens ubiquitin-conjugating enzyme E2G 2 (UBC7 homolog, yeast) (UBE2G2), transcript variant 2, mRNA [NM_182688]	NM_182688
A_32_P506600	0.000302	NM_006325	NM_006325	Homo sapiens RAN, member RAS oncogene family (RAN), mRNA [NM_006325]	NM_006325
A_23_P42042	0.000303	NM_020466	NM_020466	Homo sapiens hypothetical protein dJ122O8.2 (DJ122O8.2), mRNA [NM_020466]	NM_020466
A_24_P632230	0.000303	A_24_P632230			
A_32_P182662	0.000303	NM_022831	NM_022831	Homo sapiens chromosome 1 open reading frame 80 (C1orf80), mRNA [NM_022831]	NM_022831
A_23_P74269	0.000303	NM_003132	NM_003132	Homo sapiens spermidine synthase (SRM), mRNA [NM_003132]	NM_003132
A_23_P17144	0.000303	NM_007266	NM_007266	Homo sapiens XPA binding protein 1, GTPase (XAB1), mRNA [NM_007266]	NM_007266
A_32_P103726	0.000304	THC2335352		OXIDOREDUCTASE {Brucella melitensis;} , partial (5%) [THC2335352]	
A_24_P48139	0.000304	NM_012091	NM_012091	Homo sapiens adenosine deaminase, tRNA-specific 1 (ADAT1), mRNA [NM_012091]	NM_012091
A_23_P31477	0.000304	NM_015332	NM_015332	Homo sapiens NudC domain containing 3 (NUDCD3), mRNA [NM_015332]	NM_015332
A_24_P127063	0.000304	ENST00000321482			
A_24_P261724	0.000304	NM_014868	NM_014868	Homo sapiens ring finger protein 10 (RNF10), mRNA [NM_014868]	NM_014868
A_23_P416894	0.000305	ENST00000257626		Homo sapiens mRNA full length insert cDNA clone EUROIMAGE 293605. [AL079277]	
A_23_P200001	0.000305	NM_144573	NM_144573	Homo sapiens nexilin (F actin binding protein) (NEXN), mRNA [NM_144573]	NM_144573
A_32_P218989	0.000305	ENST00000163282		Human nuclease-sensitive element DNA-binding protein mRNA, complete cds. [M83234]	
A_23_P214444	0.000306	NM_000544	NM_000544	Homo sapiens transporter 2, ATP-binding cassette, sub-family B (MDR/TAP) (TAP2), transcript variant 1, mRNA [NM_000544]	NM_000544
A_23_P48320	0.000306	NM_005288	NM_005288	Homo sapiens G protein-coupled receptor 12 (GPR12), mRNA [NM_005288]	NM_005288
A_23_P49254	0.000306	NM_005331	NM_005331	Homo sapiens hemoglobin, theta 1 (HBQ1), mRNA [NM_005331]	NM_005331
A_24_P901084	0.000306	XM_374965	XM_374965	PREDICTED: Homo sapiens similar to BC004636 protein (LOC400010), mRNA [XM_374965]	XM_374965
A_23_P409541	0.000306	NM_152705	NM_152705	Homo sapiens hypothetical protein MGC9850 (MGC9850), mRNA [NM_152705]	NM_152705
A_23_P402157	0.000306	NM_024681	NM_024681	Homo sapiens potassium channel tetramerisation domain containing 17 (KCTD17), mRNA [NM_024681]	NM_024681
A_23_P930	0.000306	NM_016022	NM_016022	Homo sapiens anterior pharynx defective 1 homolog A (C. elegans) (APH1A), mRNA [NM_016022]	NM_016022
A_23_P158059	0.000306	NM_017873	NM_017873	Homo sapiens ankyrin repeat and SOCS box-containing 6 (ASB6), transcript variant 1, mRNA [NM_017873]	NM_017873
A_23_P114282	0.000306	NM_014060	NM_014060	Homo sapiens malignant T cell amplified sequence 1 (MCTS1), mRNA [NM_014060]	NM_014060
A_23_P137830	0.000306	XM_060887	XM_060887	PREDICTED: Homo sapiens similar to peptidyl-Pro cis trans isomerase (LOC128192), mRNA [XM_060887]	XM_060887
A_23_P124733	0.000307	NM_015697	NM_015697	Homo sapiens coenzyme Q2 homolog, prenyltransferase (yeast) (COQ2), mRNA [NM_015697]	NM_015697
A_24_P176255	0.000309	NM_005254	NM_005254	Homo sapiens GA binding protein transcription factor, beta subunit 2 (GABPB2), transcript variant beta-1, mRNA [NM_005254]	NM_005254
A_32_P192033	0.00031	AK055918	AK055918	Homo sapiens cDNA FLJ31356 fis, clone MESAN2000337. [AK055918]	
A_23_P154786	0.00031	NM_032514	NM_032514	Homo sapiens microtubule-associated protein 1 light chain 3 alpha (MAP1LC3A), transcript variant 1, mRNA [NM_032514]	NM_032514
A_24_P42071	0.00031	ENST00000332925			
A_32_P63848	0.00031	NM_000436	NM_000436	Homo sapiens 3-oxoacid CoA transferase 1 (OXCT1), nuclear gene encoding mitochondrial protein, mRNA [NM_000436]	NM_000436
A_23_P20566	0.00031	NM_213674	NM_213674	Homo sapiens tropomyosin 2 (beta) (TPM2), transcript variant 2, mRNA [NM_213674]	NM_213674
A_23_P430764	0.000311	NM_020240	NM_020240	Homo sapiens CDC42 small effector 2 (CDC42SE2), mRNA [NM_020240]	NM_020240
A_23_P166502	0.000311	ENST00000361204		Homo sapiens sterol regulatory element binding transcription factor 2, mRNA (cDNA clone IMAGE:5498684), partial cds. [BC051799]	
A_23_P41674	0.000311	NM_152407	NM_152407	Homo sapiens GrpE-like 2, mitochondrial (E. coli) (GRPEL2), mRNA [NM_152407]	NM_152407
A_23_P78802	0.000311	NM_016457	NM_016457	Homo sapiens protein kinase D2 (PRKD2), mRNA [NM_016457]	NM_016457
A_24_P364087	0.000311	NM_012139	NM_012139	Homo sapiens deafness locus associated putative guanine nucleotide exchange factor (DELGEF), mRNA [NM_012139]	NM_012139
A_23_P158406	0.000312	BC008390	BC008390	Homo sapiens phosphodiesterase 4D, cAMP-specific (phosphodiesterase E3 dunce homolog, Drosophila), mRNA (cDNA clone IMAGE:4280941), complete cds. [BC008390]	
A_23_P61826	0.000312	NM_020993	NM_020993	Homo sapiens B-cell CLL/lymphoma 7A (BCL7A), transcript variant 1, mRNA [NM_020993]	NM_020993

A_23_P141055	0.000312	NM_015927	NM_015927	Homo sapiens transforming growth factor beta 1 induced transcript 1 (TGFB1I1), mRNA [NM_015927]	NM_015927
A_24_P392109	0.000312	NM_018455	NM_018455	Homo sapiens uncharacterized bone marrow protein BM039 (BM039), mRNA [NM_018455]	NM_018455
A_24_P927432	0.000313	BC035592	BC035592	Homo sapiens T cell receptor gamma variable 5, mRNA (cDNA clone MGC:45453 IMAGE:5575279), complete cds. [BC035592]	
A_24_P904903	0.000313	NM_004640	NM_004640	Homo sapiens HLA-B associated transcript 1 (BAT1), transcript variant 1, mRNA [NM_004640]	NM_004640
A_23_P163143	0.000313	NM_203488	NM_203488	Homo sapiens acylphosphatase 1, erythrocyte (common) type (ACYP1), transcript variant 2, mRNA [NM_203488]	NM_203488
A_24_P486503	0.000314	ENST00000343567			
A_23_P86943	0.000314	NM_003139	NM_003139	Homo sapiens signal recognition particle receptor ('docking protein') (SRPR), mRNA [NM_003139]	NM_003139
A_23_P381449	0.000315	NM_003110	NM_003110	Homo sapiens Sp2 transcription factor (SP2), mRNA [NM_003110]	NM_003110
A_23_P211816	0.000315	NM_002375	NM_002375	Homo sapiens microtubule-associated protein 4 (MAP4), transcript variant 1, mRNA [NM_002375]	NM_002375
A_23_P9152	0.000315	NM_005772	NM_005772	Homo sapiens RNA terminal phosphate cyclase-like 1 (RCL1), mRNA [NM_005772]	NM_005772
A_23_P363313	0.000316	NM_153357	NM_153357	Homo sapiens solute carrier family 16 (monocarboxylic acid transporters), member 11 (SLC16A11), mRNA [NM_153357]	NM_153357
A_24_P49421	0.000316	ENST00000271124			
A_23_P126623	0.000316	NM_002631	NM_002631	Homo sapiens phosphogluconate dehydrogenase (PGD), mRNA [NM_002631]	NM_002631
A_23_P11237	0.000317	NM_004606	NM_004606	Homo sapiens TAF1 RNA polymerase II, TATA box binding protein (TBP)-associated factor, 250kDa (TAF1), transcript variant 1, mRNA [NM_004606]	NM_004606
A_32_P34941	0.000317	THC2378378			
A_32_P468522	0.000317	AF031138	AF031138	Homo sapiens 1C7 precursor, mRNA, alternatively spliced, complete cds. [AF031138]	
A_24_P136094	0.000317	AB040942	AB040942	Homo sapiens mRNA for KIAA1509 protein, partial cds. [AB040942]	XM_029353
A_23_P14369	0.000317	AB028979	AB028979	Homo sapiens mRNA for KIAA1056 protein, partial cds. [AB028979]	XM_375065
A_32_P151387	0.000317	THC2382871			
A_23_P64743	0.000317	NM_015000	NM_015000	Homo sapiens serine/threonine kinase 38 like (STK38L), mRNA [NM_015000]	NM_015000
A_32_P175321	0.000317	AK057740	AK057740	Homo sapiens cDNA FLJ25011 fis, clone CBL01244. [AK057740]	
A_32_P46899	0.000318	THC2339675		ALU2_HUMAN (P39189) Alu subfamily SB sequence contamination warning entry, partial (3%) [THC2339675]	
A_24_P86240	0.000318	NM_198892	NM_198892	Homo sapiens BMP2 inducible kinase (BMP2K), transcript variant 1, mRNA [NM_198892]	NM_198892
A_24_P350245	0.000318	NM_024940	NM_024940	Homo sapiens dedicator of cytokinesis 5 (DOCK5), mRNA [NM_024940]	NM_024940
A_23_P321377	0.000318	NM_152374	NM_152374	Homo sapiens hypothetical protein FLJ38984 (FLJ38984), mRNA [NM_152374]	NM_152374
A_32_P88635	0.000318	NM_032264	NM_032264	Homo sapiens hypothetical protein AE2 (AE2), mRNA [NM_032264]	NM_032264
A_23_P43679	0.000318	NM_133374	NM_133374	Homo sapiens zinc finger protein 618 (ZNF618), mRNA [NM_133374]	NM_133374
A_32_P105773	0.000318	NM_015640	NM_015640	Homo sapiens PAI-1 mRNA binding protein (PAI-RBP1), transcript variant 4, mRNA [NM_015640]	NM_015640
A_24_P306425	0.000319	NM_002956	NM_002956	Homo sapiens restin (Reed-Steinberg cell-expressed intermediate filament-associated protein) (RSN), transcript variant 1, mRNA [NM_002956]	NM_002956
A_23_P41697	0.00032	NM_020177	NM_020177	Homo sapiens fem-1 homolog c (C.elegans) (FEM1C), mRNA [NM_020177]	NM_020177
A_23_P255968	0.00032	NM_003967	NM_003967	Homo sapiens trace amine associated receptor 5 (TAAR5), mRNA [NM_003967]	NM_003967
A_32_P501400	0.00032	NM_030752	NM_030752	Homo sapiens t-complex 1 (TCP1), transcript variant 1, mRNA [NM_030752]	NM_030752
A_24_P175989	0.00032	NM_057180	NM_057180	Homo sapiens vacuolar protein sorting 29 (yeast) (VPS29), transcript variant 2, mRNA [NM_057180]	NM_057180
A_32_P75299	0.00032	NM_001001790	NM_001001790	Homo sapiens chromosome 9 open reading frame 105 (C9orf105), mRNA [NM_001001790]	NM_001001790
A_24_P581618	0.000321	A_24_P581618			
A_24_P144620	0.000321	NM_018452	NM_018452	Homo sapiens chromosome 6 open reading frame 35 (C6orf35), mRNA [NM_018452]	NM_018452
A_23_P411379	0.000321	NM_178508	NM_178508	Homo sapiens chromosome 6 open reading frame 1 (C6orf1), transcript variant 1, mRNA [NM_178508]	NM_178508
A_32_P8365	0.000321	NM_015329	NM_015329	Homo sapiens KIAA0892 (KIAA0892), mRNA [NM_015329]	NM_015329
A_24_P43681	0.000321	NM_014063	NM_014063	Homo sapiens drebrin-like (DBNL), transcript variant 1, mRNA [NM_014063]	NM_014063
A_23_P20777	0.000321	NM_033117	NM_033117	Homo sapiens RNA binding motif protein 18 (RBM18), mRNA [NM_033117]	NM_033117
A_23_P81094	0.000322	THC2338051		BC047447 LOC133308 protein {Homo sapiens;} , partial (25%) [THC2338051]	
A_24_P364516	0.000322	NM_018191	NM_018191	Homo sapiens regulator of chromosome condensation (RCC1) and BTB (POZ) domain containing protein 1 (RCBTB1), mRNA [NM_018191]	NM_018191
A_24_P229669	0.000322	NM_173613	NM_173613	Homo sapiens hypothetical protein FLJ35785 (FLJ35785), mRNA [NM_173613]	NM_173613
A_23_P97157	0.000322	NM_018085	NM_018085	Homo sapiens importin 9 (IPO9), mRNA [NM_018085]	NM_018085

A_23_P155890	0.000322	ENST00000286794		Homo sapiens hypothetical protein MGC10646, mRNA (cDNA clone IMAGE:4025308), partial cds. [BC004552]	XM_496704
A_23_P425750	0.000322	NM_033415	NM_033415	Homo sapiens armadillo repeat containing 6 (ARMC6), mRNA [NM_033415]	NM_033415
A_23_P396867	0.000323	NM_178582	NM_178582	Homo sapiens histocompatibility (minor) 13 (HM13), transcript variant 4, mRNA [NM_178582]	NM_178582
A_32_P14386	0.000323	THC2307122		Q76LB7 (Q76LB7) Calmodulin, partial (47%) [THC2307122]	
A_23_P347131	0.000323	AK094181	AK094181	Homo sapiens cDNA FLJ36862 fis, clone ASTRO2015529. [AK094181]	
A_24_P204204	0.000324	A_24_P204204			
A_24_P323997	0.000324	NM_198479	NM_198479	Homo sapiens tetra-peptide repeat homeobox (TPRX), mRNA [NM_198479]	NM_198479
A_32_P218785	0.000324	BC042649	BC042649	Homo sapiens, clone IMAGE:4826012, mRNA. [BC042649]	
A_23_P431587	0.000324	NM_001009921	NM_001009921	Homo sapiens KIAA0804 (KIAA0804), transcript variant 1, mRNA [NM_001009921]	NM_001009921
A_23_P15857	0.000324	NM_005134	NM_005134	Homo sapiens protein phosphatase 4, regulatory subunit 1 (PPP4R1), mRNA [NM_005134]	NM_005134
A_23_P152087	0.000324	NM_018145	NM_018145	Homo sapiens family with sequence similarity 82, member C (FAM82C), mRNA [NM_018145]	NM_018145
A_23_P156842	0.000324	NM_004280	NM_004280	Homo sapiens eukaryotic translation elongation factor 1 epsilon 1 (EEF1E1), mRNA [NM_004280]	NM_004280
A_24_P59247	0.000324	ENST00000333731		PREDICTED: Homo sapiens similar to eukaryotic translation initiation factor 3, subunit 5 (epsilon) (LOC390282), mRNA [XM_372447]	XM_372447
A_32_P123788	0.000325	NM_024298	NM_024298	Homo sapiens leukocyte receptor cluster (LRC) member 4 (LENG4), mRNA [NM_024298]	NM_024298
A_24_P926115	0.000325	A_24_P926115			
A_23_P208847	0.000325	NM_016539	NM_016539	Homo sapiens sirtuin (silent mating type information regulation 2 homolog) 6 (S. cerevisiae) (SIRT6), mRNA [NM_016539]	NM_016539
A_32_P125525	0.000325	AA334114	AA334114	AA334114 EST38270 Embryo, 9 week Homo sapiens cDNA 5' end similar to similar to keratin 18, mRNA sequence [AA334114]	
A_23_P32558	0.000325	NM_017588	NM_017588	Homo sapiens WD repeat domain 5 (WDR5), transcript variant 1, mRNA [NM_017588]	NM_017588
A_24_P340966	0.000325	A_24_P340966			
A_24_P291814	0.000326	NM_004370	NM_004370	Homo sapiens collagen, type XII, alpha 1 (COL12A1), transcript variant long, mRNA [NM_004370]	NM_004370
A_32_P170178	0.000326	THC2343350			
A_23_P213394	0.000326	BC027302	BC027302	Homo sapiens hypothetical protein LOC285682, mRNA (cDNA clone IMAGE:4212883), partial cds. [BC027302]	
A_24_P468331	0.000326	H01893	H01893	H01893 yj32e12.s1 Soares placenta Nb2HP Homo sapiens cDNA clone IMAGE:150478 3' similar to contains Alu repetitive element;contains MER4 repetitive element ;, mRNA sequence [H01893]	
A_24_P933180	0.000326	NM_000821	NM_000821	Homo sapiens gamma-glutamyl carboxylase (GGCX), mRNA [NM_000821]	NM_000821
A_23_P429184	0.000326	NM_198066	NM_198066	Homo sapiens glucosamine-phosphate N-acetyltransferase 1 (GNPNAT1), mRNA [NM_198066]	NM_198066
A_23_P3753	0.000327	NM_152457	NM_152457	Homo sapiens zinc finger protein 597 (ZNF597), mRNA [NM_152457]	NM_152457
A_32_P195756	0.000327	THC2438041		ALU5_HUMAN (P39192) Alu subfamily SC sequence contamination warning entry, partial (11%) [THC2438041]	
A_32_P191084	0.000327	NM_018992	NM_018992	Homo sapiens potassium channel tetramerisation domain containing 5 (KCTD5), mRNA [NM_018992]	NM_018992
A_23_P211814	0.000327	NM_002375	NM_002375	Homo sapiens microtubule-associated protein 4 (MAP4), transcript variant 1, mRNA [NM_002375]	NM_002375
A_23_P134637	0.000328	BC037399	BC037399	Homo sapiens KIAA0415 protein, mRNA (cDNA clone MGC:35238 IMAGE:5172434), complete cds. [BC037399]	XM_166527
A_24_P58944	0.000328	ENST00000352815			XM_171032
A_23_P345650	0.000328	NM_015691	NM_015691	Homo sapiens KIAA1280 protein (KIAA1280), mRNA [NM_015691]	NM_015691
A_32_P120014	0.000329	A_32_P120014			
A_23_P128541	0.000329	NM_006700	NM_006700	Homo sapiens TRAF-type zinc finger domain containing 1 (TRAFD1), mRNA [NM_006700]	NM_006700
A_32_P108748	0.000329	A_32_P108748			
A_23_P73982	0.00033	NM_018087	NM_018087	Homo sapiens transmembrane protein 48 (TMEM48), mRNA [NM_018087]	NM_018087
A_23_P201357	0.00033	NM_018188	NM_018188	Homo sapiens ATPase family, AAA domain containing 3A (ATAD3A), mRNA [NM_018188]	NM_018188
A_32_P195456	0.000331	AI263083	AI263083	AI263083 qz35a02.x1 NCI_CGAP_Kid11 Homo sapiens cDNA clone IMAGE:2028842 3', mRNA sequence [AI263083]	
A_32_P47616	0.000331	AW994037	AW994037	AW994037 RC3-BN0036-090200-011-f03 BN0036 Homo sapiens cDNA, mRNA sequence [AW994037]	
A_24_P227211	0.000331	NM_022822	NM_022822	Homo sapiens likely ortholog of kinesin light chain 2 (KLC2), mRNA [NM_022822]	NM_022822
A_23_P92349	0.000331	NM_001004356	NM_001004356	Homo sapiens fibroblast growth factor receptor-like 1 (FGFRL1), transcript variant 1, mRNA [NM_001004356]	NM_001004356
A_23_P158533	0.000331	NM_021008	NM_021008	Homo sapiens deformed epidermal autoregulatory factor 1 (Drosophila) (DEAF1), mRNA [NM_021008]	NM_021008

A_24_P32895	0.000331	CA848728	CA848728	CA848728 ir24f10.y1 HR85 islet Homo sapiens cDNA clone IMAGE:6546379 5' similar to SW:CYPH_HUMAN P05092 PEPTIDYL-PROLYL CIS-TRANS ISOMERASE A ;, mRNA sequence [CA848728]	
A_24_P122874	0.000332	NM_015049	NM_015049	Homo sapiens amyotrophic lateral sclerosis 2 (juvenile) chromosome region, candidate 3 (ALS2CR3), mRNA [NM_015049]	NM_015049
A_24_P881527	0.000332	CR749275	CR749275	Homo sapiens mRNA; cDNA DKFZp781O2021 (from clone DKFZp781O2021). [CR749275]	
A_23_P13524	0.000332	NM_032273	NM_032273	Homo sapiens hypothetical protein DKFZp586C1924 (DKFZp586C1924), mRNA [NM_032273]	NM_032273
A_24_P551842	0.000332	ENST00000361789		Human mitochondrial cytochrome b gene, partial cds. [M28016]	
A_23_P16089	0.000333	NM_004230	NM_004230	Homo sapiens endothelial differentiation, sphingolipid G-protein-coupled receptor, 5 (EDG5), mRNA [NM_004230]	NM_004230
A_24_P239183	0.000333	AF177925	AF177925	Homo sapiens mucin 4 (MUC4) mRNA, partial cds. [AF177925]	
A_23_P111487	0.000333	NM_182800	NM_182800	Homo sapiens arsenate resistance protein ARS2 (ARS2), transcript variant 2, mRNA [NM_182800]	NM_182800
A_24_P315873	0.000333	ENST00000352815			XM_171032
A_24_P87498	0.000334	NM_024107	NM_024107	Homo sapiens hypothetical protein MGC3123 (MGC3123), transcript variant 1, mRNA [NM_024107]	NM_024107
A_23_P371410	0.000334	NM_207578	NM_207578	Homo sapiens protein kinase, cAMP-dependent, catalytic, beta (PRKACB), transcript variant 3, mRNA [NM_207578]	NM_207578
A_24_P367752	0.000334	BC012888	BC012888	Homo sapiens N-deacetylase/N-sulfotransferase (heparan glucosaminyl) 1, mRNA (cDNA clone MGC:9410 IMAGE:3882074), complete cds. [BC012888]	
A_24_P243396	0.000334	NM_001008657	NM_001008657	Homo sapiens Treacher Collins-Franceschetti syndrome 1 (TCOF1), transcript variant 3, mRNA [NM_001008657]	NM_001008657
A_23_P254801	0.000334	NM_002660	NM_002660	Homo sapiens phospholipase C, gamma 1 (PLCG1), transcript variant 1, mRNA [NM_002660]	NM_002660
A_23_P397293	0.000334	NM_017527	NM_017527	Homo sapiens lymphocyte antigen 6 complex, locus K (LY6K), mRNA [NM_017527]	NM_017527
A_24_P253215	0.000334	NM_006331	NM_006331	Homo sapiens C2f protein (C2F), mRNA [NM_006331]	NM_006331
A_24_P922631	0.000335	BC092511	BC092511	Homo sapiens cDNA clone IMAGE:5271968. [BC092511]	XM_059672
A_23_P107483	0.000336	NM_012373	NM_012373	Homo sapiens olfactory receptor, family 3, subfamily A, member 3 (OR3A3), mRNA [NM_012373]	NM_012373
A_23_P30913	0.000336	NM_033554	NM_033554	Homo sapiens major histocompatibility complex, class II, DP alpha 1 (HLA-DPA1), mRNA [NM_033554]	NM_033554
A_23_P127186	0.000336	NM_206862	NM_206862	Homo sapiens transforming, acidic coiled-coil containing protein 2 (TACC2), transcript variant 1, mRNA [NM_206862]	NM_206862
A_23_P131866	0.000336	NM_198433	NM_198433	Homo sapiens serine/threonine kinase 6 (STK6), transcript variant 1, mRNA [NM_198433]	NM_198433
A_23_P148484	0.000337	NM_016120	NM_016120	Homo sapiens ring finger protein 12 (RNF12), transcript variant 1, mRNA [NM_016120]	NM_016120
A_24_P393864	0.000337	NM_006608	NM_006608	Homo sapiens putative homeodomain transcription factor 1 (PHTF1), mRNA [NM_006608]	NM_006608
A_24_P302797	0.000337	NM_000532	NM_000532	Homo sapiens propionyl Coenzyme A carboxylase, beta polypeptide (PCCB), mRNA [NM_000532]	NM_000532
A_23_P30464	0.000337	NM_030567	NM_030567	Homo sapiens proline rich 7 (synaptic) (PRR7), mRNA [NM_030567]	NM_030567
A_23_P18579	0.000337	NM_006607	NM_006607	Homo sapiens pituitary tumor-transforming 2 (PTTG2), mRNA [NM_006607]	NM_006607
A_24_P929137	0.000338	THC2361427			
A_32_P151102	0.000338	NM_002444	NM_002444	Homo sapiens moesin (MSN), mRNA [NM_002444]	NM_002444
A_24_P944827	0.000338	AL122075	AL122075	Homo sapiens mRNA; cDNA DKFZp434N0735 (from clone DKFZp434N0735); partial cds. [AL122075]	
A_24_P64329	0.000338	NM_173575	NM_173575	Homo sapiens serine/threonine kinase 32C (STK32C), mRNA [NM_173575]	NM_173575
A_23_P217998	0.000338	NM_032801	NM_032801	Homo sapiens junctional adhesion molecule 3 (JAM3), mRNA [NM_032801]	NM_032801
A_23_P21234	0.000338	BM725480	BM725480	BM725480 UI-E-EJ0-aie-p-22-0-UI.r1 UI-E-EJ0 Homo sapiens cDNA clone UI-E-EJ0-aie-p-22-0-UI 5', mRNA sequence [BM725480]	
A_23_P212307	0.000338	NM_182903	NM_182903	Homo sapiens kinesin family member 9 (KIF9), transcript variant 3, mRNA [NM_182903]	NM_182903
A_24_P933565	0.000339	NM_024989	NM_024989	Homo sapiens GPI deacylase (PGAP1), mRNA [NM_024989]	NM_024989
A_24_P144337	0.00034	ENST00000327691			
A_23_P77415	0.00034	NM_013370	NM_013370	Homo sapiens pregnancy-induced growth inhibitor (OKL38), transcript variant 1, mRNA [NM_013370]	NM_013370
A_24_P162073	0.00034	NM_021574	NM_021574	Homo sapiens breakpoint cluster region (BCR), transcript variant 2, mRNA [NM_021574]	NM_021574
A_23_P129486	0.00034	NM_016332	NM_016332	Homo sapiens selenoprotein X, 1 (SEPX1), mRNA [NM_016332]	NM_016332
A_24_P250227	0.000341	NM_021724	NM_021724	Homo sapiens nuclear receptor subfamily 1, group D, member 1 (NR1D1), mRNA [NM_021724]	NM_021724
A_24_P409410	0.000341	A_24_P409410			
A_24_P116351	0.000341	NM_006427	NM_006427	Homo sapiens CD27-binding (Siva) protein (SIVA), transcript variant 1, mRNA [NM_006427]	NM_006427
A_23_P302116	0.000342	NM_177423	NM_177423	Homo sapiens protein tyrosine phosphatase, receptor type, f polypeptide (PTPRF), interacting protein (liprin), alpha 1 (PPFIA1), transcript variant 1, mRNA [NM_177423]	NM_177423

A_23_P164307	0.000342	NM_153827	NM_153827	Homo sapiens misshapen-like kinase 1 (zebrafish) (MINK1), transcript variant 3, mRNA [NM_153827]	NM_153827
A_23_P217958	0.000342	NM_033500	NM_033500	Homo sapiens hexokinase 1 (HK1), nuclear gene encoding mitochondrial protein, transcript variant 5, mRNA [NM_033500]	NM_033500
A_24_P230288	0.000343	BG009439	BG009439	BG009439 PM4-GN0183-021200-001-h08 GN0183 Homo sapiens cDNA, mRNA sequence [BG009439]	
A_24_P350186	0.000343	NM_030630	NM_030630	Homo sapiens chromosome 17 open reading frame 28 (C17orf28), mRNA [NM_030630]	NM_030630
A_24_P116132	0.000343	NM_015509	NM_015509	Homo sapiens adaptin-ear-binding coat-associated protein 1 (NECAP1), mRNA [NM_015509]	NM_015509
A_23_P104065	0.000343	NM_170705	NM_170705	Homo sapiens isoprenylcysteine carboxyl methyltransferase (ICMT), transcript variant 2, mRNA [NM_170705]	NM_170705
A_23_P2129	0.000343	NM_018480	NM_018480	Homo sapiens uncharacterized hypothalamus protein HT007 (HT007), mRNA [NM_018480]	NM_018480
A_23_P74653	0.000343	NM_006600	NM_006600	Homo sapiens nuclear distribution gene C homolog (A. nidulans) (NUDC), mRNA [NM_006600]	NM_006600
A_23_P86037	0.000344	NM_017582	NM_017582	Homo sapiens ubiquitin-conjugating enzyme E2Q (putative) (UBE2Q), mRNA [NM_017582]	NM_017582
A_24_P560909	0.000344	THC2277661		ALU1_HUMAN (P39188) Alu subfamily J sequence contamination warning entry, partial (22%) [THC2277661]	
A_23_P259272	0.000344	NM_018639	NM_018639	Homo sapiens WD repeat and SOCS box-containing 2 (WSB2), mRNA [NM_018639]	NM_018639
A_24_P940576	0.000345	NM_020782	NM_020782	Homo sapiens kelch domain containing 5 (KLHDC5), mRNA [NM_020782]	NM_020782
A_24_P669220	0.000346	THC2314754		O99381 (O99381) Cytochrome C oxidase subunit I (Fragment), partial (5%) [THC2314754]	
A_24_P103922	0.000346	NM_001329	NM_001329	Homo sapiens C-terminal binding protein 2 (CTBP2), transcript variant 1, mRNA [NM_001329]	NM_001329
A_24_P135515	0.000346	A_24_P135515			
A_32_P13417	0.000347	AK091942	AK091942	Homo sapiens cDNA FLJ34623 fis, clone KIDNE2015073. [AK091942]	
A_32_P6221	0.000347	ENST00000359244		Homo sapiens cDNA FLJ31209 fis, clone KIDNE2003377. [AK055771]	
A_24_P244420	0.000347	NM_018367	NM_018367	Homo sapiens phytoceramidase, alkaline (PHCA), mRNA [NM_018367]	NM_018367
A_23_P161552	0.000347	NM_032389	NM_032389	Homo sapiens zinc finger protein 289, ID1 regulated (ZNF289), mRNA [NM_032389]	NM_032389
A_23_P61960	0.000348	NM_145230	NM_145230	Homo sapiens ATPase, H+ transporting V0 subunit E isoform 2-like (rat) (ATP6V0E2L), mRNA [NM_145230]	NM_145230
A_23_P6786	0.000348	BC005184	BC005184	Homo sapiens tRNA nucleotidyl transferase, CCA-adding, 1, mRNA (cDNA clone IMAGE:3686608), complete cds. [BC005184]	
A_24_P852082	0.000348	AK125829	AK125829	Homo sapiens cDNA FLJ43841 fis, clone TESTI4006137. [AK125829]	XM_378250
A_23_P146187	0.000348	NM_015169	NM_015169	Homo sapiens RRS1 ribosome biogenesis regulator homolog (S. cerevisiae) (RRS1), mRNA [NM_015169]	NM_015169
A_23_P154199	0.000348	NM_002712	NM_002712	Homo sapiens protein phosphatase 1, regulatory subunit 7 (PPP1R7), mRNA [NM_002712]	NM_002712
A_24_P300483	0.000349	NM_024054	NM_024054	Homo sapiens chromosome 7 open reading frame 25 (C7orf25), mRNA [NM_024054]	NM_024054
A_23_P117274	0.000349	NM_005932	NM_005932	Homo sapiens mitochondrial intermediate peptidase (MIPEP), nuclear gene encoding mitochondrial protein, mRNA [NM_005932]	NM_005932
A_23_P148798	0.000349	NM_015984	NM_015984	Homo sapiens ubiquitin carboxyl-terminal hydrolase L5 (UCHL5), mRNA [NM_015984]	NM_015984
A_23_P83045	0.000349	NM_007126	NM_007126	Homo sapiens valosin-containing protein (VCP), mRNA [NM_007126]	NM_007126
A_23_P39684	0.00035	NM_012290	NM_012290	Homo sapiens tousled-like kinase 1 (TLK1), mRNA [NM_012290]	NM_012290
A_23_P210658	0.00035	NM_016045	NM_016045	Homo sapiens chromosome 20 open reading frame 45 (C20orf45), mRNA [NM_016045]	NM_016045
A_23_P110557	0.000351	NM_133369	NM_133369	Homo sapiens unc-5 homolog A (C. elegans) (UNC5A), mRNA [NM_133369]	NM_133369
A_24_P283834	0.000351	NM_000795	NM_000795	Homo sapiens dopamine receptor D2 (DRD2), transcript variant 1, mRNA [NM_000795]	NM_000795
A_23_P251342	0.000351	NM_002093	NM_002093	Homo sapiens glycogen synthase kinase 3 beta (GSK3B), mRNA [NM_002093]	NM_002093
A_23_P149281	0.000352	NM_004431	NM_004431	Homo sapiens EPH receptor A2 (EPHA2), mRNA [NM_004431]	NM_004431
A_23_P127195	0.000352	NM_003675	NM_003675	Homo sapiens PRP18 pre-mRNA processing factor 18 homolog (yeast) (PRPF18), mRNA [NM_003675]	NM_003675
A_32_P192865	0.000353	THC2376823			
A_32_P42426	0.000353	AA126789	AA126789	AA126789 zn87e10.s1 Stratagene lung carcinoma 937218 Homo sapiens cDNA clone IMAGE:565194 3', mRNA sequence [AA126789]	
A_23_P125772	0.000353	NM_014370	NM_014370	Homo sapiens serine/threonine kinase 23 (STK23), mRNA [NM_014370]	NM_014370
A_32_P66261	0.000353	AA282192	AA282192	AA282192 zs89b10.r1 NCI_CGAP_GCB1 Homo sapiens cDNA clone IMAGE:704635 5', mRNA sequence [AA282192]	
A_24_P373768	0.000353	NM_000164	NM_000164	Homo sapiens gastric inhibitory polypeptide receptor (GIPR), mRNA [NM_000164]	NM_000164
A_24_P173088	0.000354	NM_003152	NM_003152	Homo sapiens signal transducer and activator of transcription 5A (STAT5A), mRNA [NM_003152]	NM_003152
A_32_P160875	0.000354	BG547692	BG547692	602575538F1 NIH_MGC_77 Homo sapiens cDNA clone IMAGE:4703745 5', mRNA sequence [BG547692]	
A_24_P392480	0.000354	NM_001012643	NM_001012643	Homo sapiens hypothetical protein LOC339344 (LOC339344), mRNA [NM_001012643]	NM_001012643

A_24_P289029	0.000354	NM_014744	NM_014744	Homo sapiens TBC1 domain family, member 5 (TBC1D5), mRNA [NM_014744]	NM_014744
A_23_P204324	0.000354	NM_012062	NM_012062	Homo sapiens dynamin 1-like (DNM1L), transcript variant 1, mRNA [NM_012062]	NM_012062
A_23_P259797	0.000355	NM_174917	NM_174917	Homo sapiens hypothetical protein LOC197322 (LOC197322), mRNA [NM_174917]	NM_174917
A_24_P27412	0.000355	NM_005701	NM_005701	Homo sapiens RNA, U transporter 1 (RNUT1), mRNA [NM_005701]	NM_005701
A_24_P229766	0.000355	ENST00000318527			
A_23_P433063	0.000355	NM_033064	NM_033064	Homo sapiens ataxia, cerebellar, Cayman type (caytaxin) (ATCAY), mRNA [NM_033064]	NM_033064
A_32_P150541	0.000356	BI830189	BI830189	BI830189 603072855F1 NIH_MGC_119 Homo sapiens cDNA clone IMAGE:5164981 5', mRNA sequence [BI830189]	
A_23_P124003	0.000356	NM_170683	NM_170683	Homo sapiens purinergic receptor P2X, ligand-gated ion channel, 2 (P2RX2), transcript variant 4, mRNA [NM_170683]	NM_170683
A_32_P198412	0.000356	BC017972	BC017972	Homo sapiens, clone IMAGE:4693260, mRNA. [BC017972]	
A_23_P54626	0.000356	NM_032830	NM_032830	Homo sapiens cirrhosis, autosomal recessive 1A (cirhin) (CIRH1A), mRNA [NM_032830]	NM_032830
A_32_P127052	0.000356	NM_152395	NM_152395	Homo sapiens nudix (nucleoside diphosphate linked moiety X)-type motif 16 (NUDT16), mRNA [NM_152395]	NM_152395
A_32_P60709	0.000357	THC2406514			
A_23_P25097	0.000357	NM_032338	NM_032338	Homo sapiens hypothetical protein MGC14817 (MGC14817), mRNA [NM_032338]	NM_032338
A_23_P88781	0.000357	NM_020313	NM_020313	Homo sapiens cytokine induced apoptosis inhibitor 1 (CIAPIN1), mRNA [NM_020313]	NM_020313
A_23_P340296	0.000358	BC009979	BC009979	Homo sapiens G-protein signalling modulator 1 (AGS3-like, C. elegans), mRNA (cDNA clone MGC:16636 IMAGE:4121647), complete cds. [BC009979]	
A_24_P390315	0.000358	ENST00000321130		Homo sapiens mRNA for KIAA0391 gene, partial cds. [AB002389]	
A_24_P353486	0.000358	NM_057162	NM_057162	Homo sapiens kelch-like 4 (Drosophila) (KLHL4), transcript variant 2, mRNA [NM_057162]	NM_057162
A_23_P140630	0.000358	NM_021819	NM_021819	Homo sapiens lectin, mannose-binding, 1 like (LMAN1L), mRNA [NM_021819]	NM_021819
A_23_P360240	0.000358	NM_138768	NM_138768	Homo sapiens myeloma overexpressed gene (in a subset of t(11;14) positive multiple myelomas) (MYEOV), mRNA [NM_138768]	NM_138768
A_32_P3400	0.000359	BF754999	BF754999	BF754999 QV0-CT0583-181000-428-a09 CT0583 Homo sapiens cDNA, mRNA sequence [BF754999]	
A_23_P216215	0.000359	NM_014637	NM_014637	Homo sapiens chondrocyte protein with a poly-proline region (CHPPR), mRNA [NM_014637]	NM_014637
A_24_P923749	0.000359	NM_032740	NM_032740	Homo sapiens SFT2 domain containing 3 (SFT2D3), mRNA [NM_032740]	NM_032740
A_23_P53603	0.000359	NM_001681	NM_001681	Homo sapiens ATPase, Ca++ transporting, cardiac muscle, slow twitch 2 (ATP2A2), transcript variant 2, mRNA [NM_001681]	NM_001681
A_24_P101101	0.000359	ENST00000310218		PREDICTED: Homo sapiens similar to large subunit ribosomal protein L36a (LOC441727), mRNA [XM_497458]	XM_497458
A_32_P99804	0.00036	THC2377845		Q6PEL1 (Q6PEL1) ZCWCC3 protein (Fragment), partial (17%) [THC2377845]	
A_24_P386323	0.00036	NM_005833	NM_005833	Homo sapiens Rab9 effector protein with kelch motifs (RABEPK), mRNA [NM_005833]	NM_005833
A_24_P152144	0.000361	A_24_P152144			
A_24_P62237	0.000361	NM_022766	NM_022766	Homo sapiens ceramide kinase (CERK), transcript variant 1, mRNA [NM_022766]	NM_022766
A_23_P15348	0.000361	NM_015134	NM_015134	Homo sapiens myosin phosphatase-Rho interacting protein (M-RIP), mRNA [NM_015134]	NM_015134
A_23_P106481	0.000361	NM_016454	NM_016454	Homo sapiens hypothetical protein LOC51234 (LOC51234), mRNA [NM_016454]	NM_016454
A_24_P417546	0.000362	BC004936	BC004936	Homo sapiens stearoyl-CoA desaturase 5, mRNA (cDNA clone MGC:10777 IMAGE:3607979), complete cds. [BC004936]	
A_23_P130689	0.000362	NM_032377	NM_032377	Homo sapiens elongation factor 1 homolog (ELF1, S. cerevisiae) (ELOF1), mRNA [NM_032377]	NM_032377
A_23_P206103	0.000362	NM_015518	NM_015518	Homo sapiens unc-51-like kinase 3 (C. elegans) (ULK3), mRNA [NM_015518]	NM_015518
A_32_P45087	0.000363	A_32_P45087			
A_24_P162287	0.000363	NM_007186	NM_007186	Homo sapiens centrosomal protein 2 (CEP2), mRNA [NM_007186]	NM_007186
A_24_P390784	0.000363	NM_001005291	NM_001005291	Homo sapiens sterol regulatory element binding transcription factor 1 (SREBF1), transcript variant 1, mRNA [NM_001005291]	NM_001005291
A_23_P200222	0.000363	NM_033300	NM_033300	Homo sapiens low density lipoprotein receptor-related protein 8, apolipoprotein e receptor (LRP8), transcript variant 2, mRNA [NM_033300]	NM_033300
A_24_P5153	0.000364	NM_024817	NM_024817	Homo sapiens hypothetical protein FLJ13710 (FLJ13710), mRNA [NM_024817]	NM_024817
A_23_P73548	0.000364	NM_020137	NM_020137	Homo sapiens GRIP1 associated protein 1 (GRIPAP1), transcript variant 1, mRNA [NM_020137]	NM_020137
A_23_P145844	0.000365	NM_000245	NM_000245	Homo sapiens met proto-oncogene (hepatocyte growth factor receptor) (MET), mRNA [NM_000245]	NM_000245
A_23_P167711	0.000365	NM_005927	NM_005927	Homo sapiens microfibrillar-associated protein 3 (MFAP3), mRNA [NM_005927]	NM_005927
A_23_P379550	0.000365	NM_003680	NM_003680	Homo sapiens tyrosyl-tRNA synthetase (YARS), mRNA [NM_003680]	NM_003680
A_24_P945293	0.000366	NM_016079	NM_016079	Homo sapiens vacuolar protein sorting 24 (yeast) (VPS24), transcript variant 1, mRNA [NM_016079]	NM_016079
A_23_P336678	0.000366	NM_145171	NM_145171	Homo sapiens glycoprotein hormone beta 5 (GPHB5), mRNA [NM_145171]	NM_145171

A_23_P218317	0.000366	NM_012336	NM_012336	Homo sapiens nuclear prelamin A recognition factor (NARF), transcript variant 1, mRNA [NM_012336]	NM_012336
A_23_P76145	0.000367	AF256215	AF256215	Homo sapiens cycle-like factor CLIF mRNA, complete cds. [AF256215]	
A_23_P325625	0.000367	NM_173690	NM_173690	Homo sapiens chromosome 9 open reading frame 126 (C9orf126), mRNA [NM_173690]	NM_173690
A_23_P124542	0.000367	M26004	M26004	Human CR2/CD21/C3d/Epstein-Barr virus receptor mRNA, complete cds. [M26004]	
A_23_P115762	0.000367	NM_007265	NM_007265	Homo sapiens suppressor of S. cerevisiae gcr2 (HSGT1), mRNA [NM_007265]	NM_007265
A_23_P357360	0.000368	NM_018344	NM_018344	Homo sapiens solute carrier family 29 (nucleoside transporters), member 3 (SLC29A3), mRNA [NM_018344]	NM_018344
A_23_P48910	0.000368	NM_004809	NM_004809	Homo sapiens stomatin (EPB72)-like 1 (STOML1), mRNA [NM_004809]	NM_004809
A_23_P152245	0.000368	NM_052987	NM_052987	Homo sapiens cyclin-dependent kinase (CDC2-like) 10 (CDK10), transcript variant 2, mRNA [NM_052987]	NM_052987
A_23_P142325	0.000368	AB013462	AB013462	Homo sapiens mRNA for Fzr1, complete cds. [AB013462]	
A_23_P258418	0.000368	NM_024309	NM_024309	Homo sapiens TNFAIP3 interacting protein 2 (TNIP2), mRNA [NM_024309]	NM_024309
A_24_P5935	0.000368	NM_199203	NM_199203	Homo sapiens ubiquitin-conjugating enzyme E2 variant 1 (Kua-UEV), transcript variant 1, mRNA [NM_199203]	NM_199203
A_24_P135921	0.000368	ENST00000329070		PREDICTED: Homo sapiens similar to PROHIBITIN (B-CELL RECEPTOR ASSOCIATED PROTEIN 32) (BAP 32) (LOC441510), mRNA [XM_497140]	XM_497140
A_23_P98477	0.000369	NM_025004	NM_025004	Homo sapiens coiled-coil domain containing 15 (CCDC15), mRNA [NM_025004]	NM_025004
A_24_P921103	0.000369	AK057576	AK057576	Homo sapiens cDNA FLJ33014 fis, clone THYMU1000382. [AK057576]	
A_23_P213508	0.000369	NM_000439	NM_000439	Homo sapiens proprotein convertase subtilisin/kexin type 1 (PCSK1), mRNA [NM_000439]	NM_000439
A_23_P431776	0.000369	NM_001986	NM_001986	Homo sapiens ets variant gene 4 (E1A enhancer binding protein, E1AF) (ETV4), mRNA [NM_001986]	NM_001986
A_23_P77813	0.000369	NM_024619	NM_024619	Homo sapiens fructosamine-3-kinase-related protein (FN3KRP), mRNA [NM_024619]	NM_024619
A_23_P409945	0.000369	NM_004152	NM_004152	Homo sapiens ornithine decarboxylase antizyme 1 (OAZ1), mRNA [NM_004152]	NM_004152
A_23_P417237	0.00037	NM_020177	NM_020177	Homo sapiens fem-1 homolog c (C.elegans) (FEM1C), mRNA [NM_020177]	NM_020177
A_23_P9509	0.00037	A_23_P9509			
A_23_P95594	0.00037	NM_000662	NM_000662	Homo sapiens N-acetyltransferase 1 (arylamine N-acetyltransferase) (NAT1), mRNA [NM_000662]	NM_000662
A_24_P68585	0.00037	NM_183001	NM_183001	Homo sapiens SHC (Src homology 2 domain containing) transforming protein 1 (SHC1), transcript variant 1, mRNA [NM_183001]	NM_183001
A_23_P73096	0.00037	AW993939	AW993939	AW993939 RC1-BN0035-130400-013-b06 BN0035 Homo sapiens cDNA, mRNA sequence [AW993939]	
A_24_P793228	0.00037	XR_000292	XR_000292	PREDICTED: Homo sapiens similar to ATP-binding cassette sub-family E member 1 (RNase L inhibitor) (Ribonuclease 4 inhibitor) (RNS4I) (LOC442517), mRNA [XR_000292]	XR_000292
A_24_P916853	0.000371	A_24_P916853			
A_24_P56837	0.000371	NM_080863	NM_080863	Homo sapiens ankyrin repeat and SOCS box-containing 16 (ASB16), mRNA [NM_080863]	NM_080863
A_23_P309361	0.000371	NM_144584	NM_144584	Homo sapiens chromosome 1 open reading frame 59 (C1orf59), mRNA [NM_144584]	NM_144584
A_24_P299996	0.000373	NM_018130	NM_018130	Homo sapiens SHQ1 homolog (S. cerevisiae) (SHQ1), mRNA [NM_018130]	NM_018130
A_32_P219279	0.000373	NM_052906	NM_052906	Homo sapiens KIAA1904 protein (KIAA1904), mRNA [NM_052906]	NM_052906
A_24_P131222	0.000373	NM_022089	NM_022089	Homo sapiens ATPase type 13A2 (ATP13A2), mRNA [NM_022089]	NM_022089
A_24_P58727	0.000373	A_24_P58727			
A_23_P133146	0.000373	NM_002106	NM_002106	Homo sapiens H2A histone family, member Z (H2AFZ), mRNA [NM_002106]	NM_002106
A_24_P4334	0.000374	NM_194328	NM_194328	Homo sapiens ring finger protein 38 (RNF38), transcript variant 2, mRNA [NM_194328]	NM_194328
A_23_P42718	0.000374	NM_004289	NM_004289	Homo sapiens nuclear factor (erythroid-derived 2)-like 3 (NFE2L3), mRNA [NM_004289]	NM_004289
A_32_P146286	0.000374	AL122093	AL122093	Homo sapiens mRNA; cDNA DKFZp434B2115 (from clone DKFZp434B2115). [AL122093]	
A_23_P397238	0.000375	NM_054014	NM_054014	Homo sapiens FK506 binding protein 1A, 12kDa (FKBP1A), transcript variant 12A, mRNA [NM_054014]	NM_054014
A_24_P279263	0.000375	NM_022452	NM_022452	Homo sapiens fibrosin 1 (FBS1), mRNA [NM_022452]	NM_022452
A_23_P207170	0.000376	NM_022640	NM_022640	Homo sapiens chorionic somatomammotropin hormone 1 (placental lactogen) (CSH1), transcript variant 2, mRNA [NM_022640]	NM_022640
A_23_P254384	0.000377	XM_498241	XM_498241	PREDICTED: Homo sapiens similar to heat shock 70kD protein binding protein; progesterone receptor-associated p48 protein; putative tumor suppressor ST13; Hsp70-interacting protein; suppression of tumorigenicity 13 (colon carcinoma) (Hsp70-interacting...	XM_498241
A_23_P29566	0.000377	NM_005718	NM_005718	Homo sapiens actin related protein 2/3 complex, subunit 4, 20kDa (ARPC4), transcript variant 1, mRNA [NM_005718]	NM_005718
A_24_P393787	0.000377	NM_017846	NM_017846	Homo sapiens tRNA selenocysteine associated protein (SECP43), mRNA [NM_017846]	NM_017846
A_23_P146637	0.000377	NM_005866	NM_005866	Homo sapiens opioid receptor, sigma 1 (OPRS1), transcript variant 1, mRNA [NM_005866]	NM_005866

A_23_P22915	0.000378	NM_133496	NM_133496	Homo sapiens solute carrier family 30 (zinc transporter), member 7 (SLC30A7), mRNA [NM_133496]	NM_133496
A_32_P165477	0.000378	BC041925	BC041925	Homo sapiens solute carrier family 7, (cationic amino acid transporter, y+ system) member 11, mRNA (cDNA clone IMAGE:5300264), partial cds. [BC041925]	
A_32_P202588	0.000379	BU620016	BU620016	UI-H-FH1-bfr-e-10-0-UI.s1 NCI_CGAP_FH1 Homo sapiens cDNA clone UI-H-FH1-bfr-e-10-0-UI 3', mRNA sequence [BU620016]	
A_24_P322313	0.00038	AK055561	AK055561	Homo sapiens cDNA FLJ30999 fis, clone HLUNG1000110, weakly similar to PISTIL-SPECIFIC EXTENSIN-LIKE PROTEIN PRECURSOR. [AK055561]	
A_32_P935688	0.000381	AK095331	AK095331	Homo sapiens cDNA FLJ38012 fis, clone CTONG2012554. [AK095331]	
A_23_P319013	0.000382	NM_152604	NM_152604	Homo sapiens zinc finger protein 383 (ZNF383), mRNA [NM_152604]	NM_152604
A_23_P369987	0.000382	AB002315	AB002315	Homo sapiens mRNA for KIAA0317 gene, partial cds. [AB002315]	
A_23_P1948	0.000382	NM_024099	NM_024099	Homo sapiens hypothetical protein MGC2477 (MGC2477), mRNA [NM_024099]	NM_024099
A_24_P255914	0.000383	A_24_P255914			
A_24_P289170	0.000383	ENST00000282163		full-length cDNA clone CS0DK012YL12 of HeLa cells Cot 25-normalized of Homo sapiens (human). [CR606047]	XM_496003
A_32_P136450	0.000383	THC2449500		AF220206 Nedd4 WW domain-binding protein 2 {Mus musculus;} , partial (52%) [THC2449500]	
A_32_P171348	0.000383	A_32_P171348			
A_23_P426989	0.000383	NM_030645	NM_030645	Homo sapiens KIAA1720 protein (KIAA1720), mRNA [NM_030645]	NM_030645
A_24_P33461	0.000384	NM_001013718	NM_001013718	Homo sapiens similar to LOC285679 protein (LOC441120), mRNA [NM_001013718]	NM_001013718
A_24_P903378	0.000384	AV762338	AV762338	AV762338 MDS Homo sapiens cDNA clone MDSBKA11 5', mRNA sequence [AV762338]	
A_23_P130553	0.000384	NM_138392	NM_138392	Homo sapiens SH3KBP1 binding protein 1 (SHKBP1), mRNA [NM_138392]	NM_138392
A_23_P94942	0.000384	NM_030974	NM_030974	Homo sapiens shank-interacting protein-like 1 (Sharpin), mRNA [NM_030974]	NM_030974
A_23_P47208	0.000384	NM_003860	NM_003860	Homo sapiens barrier to autointegration factor 1 (BANF1), mRNA [NM_003860]	NM_003860
A_24_P739075	0.000384	A_24_P739075			
A_32_P15169	0.000384	A_32_P15169			
A_23_P257250	0.000385	NM_015196	NM_015196	Homo sapiens KIAA0922 protein (KIAA0922), mRNA [NM_015196]	NM_015196
A_24_P44891	0.000386	NM_013433	NM_013433	Homo sapiens transportin 2 (importin 3, karyopherin beta 2b) (TNPO2), mRNA [NM_013433]	NM_013433
A_32_P219845	0.000386	THC2343880			
A_23_P34877	0.000386	NM_022768	NM_022768	Homo sapiens RNA binding motif protein 15 (RBM15), mRNA [NM_022768]	NM_022768
A_23_P22086	0.000386	A_23_P22086			
A_24_P324214	0.000386	A_24_P324214			
A_32_P179177	0.000387	NM_181519	NM_181519	Homo sapiens synaptotagmin XV (SYT15), transcript variant b, mRNA [NM_181519]	NM_181519
A_23_P323749	0.000387	NM_025049	NM_025049	Homo sapiens chromosome 15 open reading frame 20 (C15orf20), mRNA [NM_025049]	NM_025049
A_23_P38987	0.000387	NM_014516	NM_014516	Homo sapiens CCR4-NOT transcription complex, subunit 3 (CNOT3), mRNA [NM_014516]	NM_014516
A_23_P74668	0.000387	NM_152290	NM_152290	Homo sapiens chromosome 1 open reading frame 158 (C1orf158), mRNA [NM_152290]	NM_152290
A_23_P103476	0.000387	NM_013319	NM_013319	Homo sapiens UbiA prenyltransferase domain containing 1 (UBIAD1), mRNA [NM_013319]	NM_013319
A_23_P141715	0.000387	NM_015476	NM_015476	Homo sapiens chromosome 18 open reading frame 10 (C18orf10), mRNA [NM_015476]	NM_015476
A_23_P101783	0.000389	NM_005535	NM_005535	Homo sapiens interleukin 12 receptor, beta 1 (IL12RB1), transcript variant 1, mRNA [NM_005535]	NM_005535
A_32_P153505	0.000389	THC2342907			
A_23_P35427	0.000389	NM_033637	NM_033637	Homo sapiens beta-transducin repeat containing (BTRC), transcript variant 1, mRNA [NM_033637]	NM_033637
A_23_P257988	0.000389	AF151697	AF151697	Homo sapiens sentrin-specific protease (SENP2) mRNA, complete cds. [AF151697]	
A_23_P150069	0.000389	NM_001009943	NM_001009943	Homo sapiens ankyrin repeat domain 16 (ANKRD16), transcript variant 4, mRNA [NM_001009943]	NM_001009943
A_23_P52978	0.00039	NM_014502	NM_014502	Homo sapiens PRP19/PSO4 pre-mRNA processing factor 19 homolog (S. cerevisiae) (PRPF19), mRNA [NM_014502]	NM_014502
A_24_P230938	0.00039	BC022054	BC022054	Homo sapiens chromosome 10 open reading frame 83, mRNA (cDNA clone IMAGE:4690584), complete cds. [BC022054]	
A_23_P142714	0.00039	NM_003705	NM_003705	Homo sapiens solute carrier family 25 (mitochondrial carrier, Aralar), member 12 (SLC25A12), mRNA [NM_003705]	NM_003705
A_23_P138308	0.000391	NM_001779	NM_001779	Homo sapiens CD58 antigen, (lymphocyte function-associated antigen 3) (CD58), mRNA [NM_001779]	NM_001779
A_24_P150149	0.000392	THC2315472			
A_24_P931711	0.000392	A_24_P931711			
A_23_P98864	0.000392	NM_005895	NM_005895	Homo sapiens golgi autoantigen, golgin subfamily a, 3 (GOLGA3), mRNA [NM_005895]	NM_005895

A_24_P935310	0.000393	AK027340	AK027340	Homo sapiens cDNA FLJ14434 fis, clone HEMBA1006921. [AK027340]	
A_23_P388168	0.000393	NM_002867	NM_002867	Homo sapiens RAB3B, member RAS oncogene family (RAB3B), mRNA [NM_002867]	NM_002867
A_23_P255016	0.000393	NM_020120	NM_020120	Homo sapiens UDP-glucose ceramide glucosyltransferase-like 1 (UGCGL1), transcript variant 1, mRNA [NM_020120]	NM_020120
A_32_P157415	0.000394	BE714413	BE714413	BE714413 PM1-HT0724-140600-001-e06 HT0724 Homo sapiens cDNA, mRNA sequence [BE714413]	
A_24_P221105	0.000394	A_24_P221105			
A_23_P209356	0.000394	NM_014946	NM_014946	Homo sapiens spastin (SPAST), transcript variant 1, mRNA [NM_014946]	NM_014946
A_23_P39185	0.000394	NM_138412	NM_138412	Homo sapiens retinol dehydrogenase 13 (all-trans and 9-cis) (RDH13), mRNA [NM_138412]	NM_138412
A_23_P94494	0.000395	NM_007005	NM_007005	Homo sapiens transducin-like enhancer of split 4 (E(sp1) homolog, Drosophila) (TLE4), mRNA [NM_007005]	NM_007005
A_24_P258846	0.000395	NM_172390	NM_172390	Homo sapiens nuclear factor of activated T-cells, cytoplasmic, calcineurin-dependent 1 (NFATC1), transcript variant 1, mRNA [NM_172390]	NM_172390
A_23_P204448	0.000395	NM_016237	NM_016237	Homo sapiens anaphase promoting complex subunit 5 (ANAPC5), mRNA [NM_016237]	NM_016237
A_23_P140792	0.000396	NM_013302	NM_013302	Homo sapiens eukaryotic elongation factor-2 kinase (EEF2K), mRNA [NM_013302]	NM_013302
A_24_P530690	0.000397	A_24_P530690			
A_24_P375405	0.000397	XM_497610	XM_497610	PREDICTED: Homo sapiens similar to 60S ribosomal protein L12 (LOC441840), mRNA [XM_497610]	XM_497610
A_24_P790909	0.000398	AI652176	AI652176	AI652176 wb47h05.x1 NCI_CGAP_GC6 Homo sapiens cDNA clone IMAGE:2308857 3', mRNA sequence [AI652176]	
A_23_P59358	0.000398	NM_173830	NM_173830	Homo sapiens chromosome 6 open reading frame 182 (C6orf182), mRNA [NM_173830]	NM_173830
A_23_P11461	0.000398	NM_199203	NM_199203	Homo sapiens ubiquitin-conjugating enzyme E2 variant 1 (Kua-UEV), transcript variant 1, mRNA [NM_199203]	NM_199203
A_23_P300797	0.000399	NM_173529	NM_173529	Homo sapiens chromosome 18 open reading frame 54 (C18orf54), mRNA [NM_173529]	NM_173529
A_23_P112820	0.000399	NM_005226	NM_005226	Homo sapiens endothelial differentiation, sphingolipid G-protein-coupled receptor, 3 (EDG3), mRNA [NM_005226]	NM_005226
A_24_P238769	0.000399	A_24_P238769			
A_24_P923832	0.0004	NM_139027	NM_139027	Homo sapiens a disintegrin-like and metalloprotease (reprolysin type) with thrombospondin type 1 motif, 13 (ADAMTS13), transcript variant 2, mRNA [NM_139027]	NM_139027
A_23_P300905	0.0004	NM_145012	NM_145012	Homo sapiens chromosome 10 open reading frame 9 (C10orf9), transcript variant 1, mRNA [NM_145012]	NM_145012
A_23_P87329	0.0004	NM_024662	NM_024662	Homo sapiens N-acetyltransferase-like protein (FLJ10774), mRNA [NM_024662]	NM_024662
A_24_P396340	0.000401	NM_138428	NM_138428	Homo sapiens hypothetical protein BC011880 (LOC113444), mRNA [NM_138428]	NM_138428
A_23_P302709	0.000401	NM_005126	NM_005126	Homo sapiens nuclear receptor subfamily 1, group D, member 2 (NR1D2), mRNA [NM_005126]	NM_005126
A_23_P258367	0.000401	NM_001499	NM_001499	Homo sapiens GLE1 RNA export mediator-like (yeast) (GLE1L), transcript variant 2, mRNA [NM_001499]	NM_001499
A_32_P23748	0.000401	BC037911	BC037911	Homo sapiens, clone IMAGE:5277449, mRNA. [BC037911]	
A_24_P152527	0.000401	NM_006373	NM_006373	Homo sapiens vesicle amine transport protein 1 homolog (T californica) (VAT1), mRNA [NM_006373]	NM_006373
A_24_P73669	0.000401	NM_002094	NM_002094	Homo sapiens G1 to S phase transition 1 (GSPT1), mRNA [NM_002094]	NM_002094
A_23_P119543	0.000401	NM_006339	NM_006339	Homo sapiens high-mobility group 20B (HMG20B), mRNA [NM_006339]	NM_006339
A_24_P323941	0.000402	NM_001012971	NM_001012971	Homo sapiens chromosome 20 open reading frame 106 (C20orf106), mRNA [NM_001012971]	NM_001012971
A_32_P146552	0.000402	BQ021969	BQ021969	UI-1-BB0-acw-h-09-0-UI.s1 NCI_CGAP_Pl4 Homo sapiens cDNA clone UI-1-BB0-acw-h-09-0-UI 3', mRNA sequence [BQ021969]	
A_23_P71091	0.000402	NM_024523	NM_024523	Homo sapiens GRIP and coiled-coil domain containing 1 (GCC1), mRNA [NM_024523]	NM_024523
A_23_P208880	0.000402	NM_013282	NM_013282	Homo sapiens ubiquitin-like, containing PHD and RING finger domains, 1 (UHRF1), mRNA [NM_013282]	NM_013282
A_23_P15937	0.000402	NM_005901	NM_005901	Homo sapiens SMAD, mothers against DPP homolog 2 (Drosophila) (SMAD2), transcript variant 1, mRNA [NM_005901]	NM_005901
A_24_P388502	0.000403	NM_022485	NM_022485	Homo sapiens hypothetical protein FLJ22405 (FLJ22405), mRNA [NM_022485]	NM_022485
A_24_P398082	0.000403	AK098728	AK098728	Homo sapiens cDNA FLJ25862 fis, clone CBR01781. [AK098728]	
A_24_P278637	0.000403	NM_003824	NM_003824	Homo sapiens Fas (TNFRSF6)-associated via death domain (FADD), mRNA [NM_003824]	NM_003824
A_23_P38262	0.000403	NM_018127	NM_018127	Homo sapiens elaC homolog 2 (E. coli) (ELAC2), mRNA [NM_018127]	NM_018127
A_23_P94579	0.000404	ENST00000361063		Homo sapiens cDNA FLJ33266 fis, clone ASTRO2007047. [AK090585]	
A_32_P218493	0.000404	THC2281165		AY491776 alpha 1A adrenoceptor isoform 2c {Homo sapiens;} , partial (11%) [THC2281165]	
A_32_P12639	0.000404	NM_002816	NM_002816	Homo sapiens proteasome (prosome, macropain) 26S subunit, non-ATPase, 12 (PSMD12), transcript variant 1, mRNA [NM_002816]	NM_002816
A_24_P16071	0.000405	A_24_P16071			
A_23_P17633	0.000405	NM_000629	NM_000629	Homo sapiens interferon (alpha, beta and omega) receptor 1 (IFNAR1), mRNA [NM_000629]	NM_000629

A_24_P418106	0.000405	A_24_P418106			
A_24_P414183	0.000405	NM_016819	NM_016819	Homo sapiens 8-oxoguanine DNA glycosylase (OGG1), nuclear gene encoding mitochondrial protein, transcript variant 1b, mRNA [NM_016819]	NM_016819
A_24_P338788	0.000406	NM_145203	NM_145203	Homo sapiens casein kinase 1, alpha 1-like (CSNK1A1L), mRNA [NM_145203]	NM_145203
A_24_P118376	0.000406	NM_198444	NM_198444	Homo sapiens carcinoembryonic antigen-related cell adhesion molecule 20 (CEACAM20), mRNA [NM_198444]	NM_198444
A_24_P372643	0.000406	NM_007105	NM_007105	Homo sapiens solute carrier family 22 (organic cation transporter), member 1-like antisense (SLC22A1LS), mRNA [NM_007105]	NM_007105
A_24_P513764	0.000407	A_24_P513764			
A_32_P111098	0.000408	W72519	W72519	W72519 zd64h01.s1 Soares_fetal_heart_NbHH19W Homo sapiens cDNA clone IMAGE:345457 3' similar to gb:M33552 LYMPHOCYTE-SPECIFIC PROTEIN LSP1 (HUMAN);, mRNA sequence [W72519]	
A_23_P391637	0.000409	ENST00000293970		Homo sapiens mRNA for KIAA1171 protein, partial cds. [AB032997]	XM_370928
A_23_P151662	0.000409	NM_197957	NM_197957	Homo sapiens MYC associated factor X (MAX), transcript variant 6, mRNA [NM_197957]	NM_197957
A_23_P92057	0.000409	NM_006218	NM_006218	Homo sapiens phosphoinositide-3-kinase, catalytic, alpha polypeptide (PIK3CA), mRNA [NM_006218]	NM_006218
A_24_P127042	0.00041	A_24_P127042			
A_23_P122197	0.00041	NM_031966	NM_031966	Homo sapiens cyclin B1 (CCNB1), mRNA [NM_031966]	NM_031966
A_23_P156284	0.00041	NM_080881	NM_080881	Homo sapiens drebrin 1 (DBN1), transcript variant 2, mRNA [NM_080881]	NM_080881
A_23_P76705	0.000411	NM_006322	NM_006322	Homo sapiens tubulin, gamma complex associated protein 3 (TUBGCP3), mRNA [NM_006322]	NM_006322
A_24_P244447	0.000411	NM_145308	NM_145308	Homo sapiens hypothetical protein BC004224 (LOC220070), mRNA [NM_145308]	NM_145308
A_23_P5731	0.000411	NM_016044	NM_016044	Homo sapiens fumarylacetoacetate hydrolase domain containing 2A (FAHD2A), mRNA [NM_016044]	NM_016044
A_24_P353964	0.000412	NM_000182	NM_000182	Homo sapiens hydroxyacyl-Coenzyme A dehydrogenase/3-ketoacyl-Coenzyme A thiolase/enoyl-Coenzyme A hydratase (trifunctional protein), alpha subunit (HADHA), mRNA [NM_000182]	NM_000182
A_23_P206435	0.000412	NM_001481	NM_001481	Homo sapiens growth arrest-specific 8 (GAS8), mRNA [NM_001481]	NM_001481
A_23_P56894	0.000412	NM_177538	NM_177538	Homo sapiens cytochrome P450, family 20, subfamily A, polypeptide 1 (CYP20A1), transcript variant 1, mRNA [NM_177538]	NM_177538
A_23_P59179	0.000412	NM_021976	NM_021976	Homo sapiens retinoid X receptor, beta (RXRB), mRNA [NM_021976]	NM_021976
A_23_P434421	0.000412	NM_181698	NM_181698	Homo sapiens chromosome 10 open reading frame 9 (C10orf9), transcript variant 2, mRNA [NM_181698]	NM_181698
A_23_P407684	0.000412	NM_178167	NM_178167	Homo sapiens zinc finger protein 598 (ZNF598), mRNA [NM_178167]	NM_178167
A_24_P115971	0.000412	NM_021932	NM_021932	Homo sapiens likely ortholog of mouse synembryn (RIC8), mRNA [NM_021932]	NM_021932
A_24_P59297	0.000413	AF161344	AF161344	Homo sapiens HSPC081 mRNA, partial cds. [AF161344]	
A_23_P78571	0.000413	NM_144613	NM_144613	Homo sapiens cytochrome c oxidase subunit VIb polypeptide 2 (testis) (COX6B2), mRNA [NM_144613]	NM_144613
A_32_P464568	0.000413	NM_020944	NM_020944	Homo sapiens glucosidase, beta (bile acid) 2 (GBA2), mRNA [NM_020944]	NM_020944
A_23_P93499	0.000413	NM_016020	NM_016020	Homo sapiens transcription factor B1, mitochondrial (TFB1M), mRNA [NM_016020]	NM_016020
A_32_P128310	0.000414	NM_017774	NM_017774	Homo sapiens CDK5 regulatory subunit associated protein 1-like 1 (CDKAL1), mRNA [NM_017774]	NM_017774
A_32_P199301	0.000414	NM_007111	NM_007111	Homo sapiens transcription factor Dp-1 (TFDP1), mRNA [NM_007111]	NM_007111
A_23_P97274	0.000414	NM_052837	NM_052837	Homo sapiens secretory carrier membrane protein 3 (SCAMP3), transcript variant 2, mRNA [NM_052837]	NM_052837
A_24_P80138	0.000414	NM_024065	NM_024065	Homo sapiens phosducin-like 3 (PDCL3), mRNA [NM_024065]	NM_024065
A_23_P9756	0.000414	NM_014281	NM_014281	Homo sapiens fuse-binding protein-interacting repressor (SIAHBP1), transcript variant 2, mRNA [NM_014281]	NM_014281
A_24_P15821	0.000414	A_24_P15821			
A_23_P126766	0.000415	NM_006697	NM_006697	Homo sapiens myotubularin related protein 11 (MTMR11), transcript variant 1, mRNA [NM_006697]	NM_006697
A_32_P212520	0.000415	BC013679	BC013679	Homo sapiens cDNA clone IMAGE:3857956, partial cds. [BC013679]	
A_24_P147849	0.000415	A_24_P147849			
A_32_P212886	0.000415	BC014117	BC014117	Homo sapiens thromboxane A synthase 1 (platelet, cytochrome P450, family 5, subfamily A), mRNA (cDNA clone MGC:20885 IMAGE:4548935), complete cds. [BC014117]	XM_374236
A_23_P132260	0.000415	NM_014303	NM_014303	Homo sapiens pescadillo homolog 1, containing BRCT domain (zebrafish) (PES1), mRNA [NM_014303]	NM_014303
A_24_P370372	0.000415	NM_014292	NM_014292	Homo sapiens chromobox homolog 6 (CBX6), mRNA [NM_014292]	NM_014292
A_23_P161064	0.000415	NM_014455	NM_014455	Homo sapiens zinc finger protein 364 (ZNF364), mRNA [NM_014455]	NM_014455
A_32_P104518	0.000416	AF194971	AF194971	Homo sapiens unknown (NAG12) mRNA, complete cds. [AF194971]	XM_379656
A_24_P884506	0.000416	AW028759	AW028759	AW028759 wv34f02.x1 NCI_CGAP_Ov18 Homo sapiens cDNA clone IMAGE:2531451 3', mRNA sequence [AW028759]	
A_32_P96419	0.000416	THC2290313			

A_32_P53852	0.000416	BC050563	BC050563	Homo sapiens hypothetical protein LOC202051, mRNA (cDNA clone MGC:57735 IMAGE:5751687), complete cds. [BC050563]	XM_114430
A_24_P40229	0.000416	NM_016652	NM_016652	Homo sapiens Crn, crooked neck-like 1 (Drosophila) (CRNKL1), mRNA [NM_016652]	NM_016652
A_24_P144499	0.000416	NM_178230	NM_178230	Homo sapiens peptidylprolyl isomerase A (cyclophilin A)-like 4 (PPIAL4), mRNA [NM_178230]	NM_178230
A_23_P155288	0.000417	NM_024996	NM_024996	Homo sapiens G elongation factor, mitochondrial 1 (GFM1), nuclear gene encoding mitochondrial protein, mRNA [NM_024996]	NM_024996
A_23_P215956	0.000418	NM_002467	NM_002467	Homo sapiens v-myc myelocytomatosis viral oncogene homolog (avian) (MYC), mRNA [NM_002467]	NM_002467
A_24_P827794	0.000419	ENST00000333401			
A_24_P918527	0.000419	M74720	M74720	Human SEF2-1D protein (SEF2-1D) mRNA, partial cds. [M74720]	
A_23_P57643	0.000419	NM_016372	NM_016372	Homo sapiens G protein-coupled receptor 175 (GPR175), mRNA [NM_016372]	NM_016372
A_23_P212400	0.000419	NM_012191	NM_012191	Homo sapiens N-acetyltransferase 6 (NAT6), mRNA [NM_012191]	NM_012191
A_23_P393656	0.00042	AK055893	AK055893	Homo sapiens cDNA FLJ31331 fis, clone MAMGL1000056. [AK055893]	
A_23_P90997	0.00042	NM_148961	NM_148961	Homo sapiens otospiralin (OTOS), mRNA [NM_148961]	NM_148961
A_24_P316363	0.00042	NM_001521	NM_001521	Homo sapiens general transcription factor IIIC, polypeptide 2, beta 110kDa (GTF3C2), mRNA [NM_001521]	NM_001521
A_23_P123578	0.00042	ENST00000292015		Homo sapiens KIAA0515, mRNA (cDNA clone MGC:10526 IMAGE:3944379), complete cds. [BC002872]	
A_23_P155079	0.00042	NM_013356	NM_013356	Homo sapiens solute carrier 16 (monocarboxylic acid transporters), member 8 (SLC16A8), mRNA [NM_013356]	NM_013356
A_23_P117238	0.00042	NM_024537	NM_024537	Homo sapiens hypothetical protein FLJ12118 (FLJ12118), mRNA [NM_024537]	NM_024537
A_23_P310560	0.00042	NM_152395	NM_152395	Homo sapiens nudix (nucleoside diphosphate linked moiety X)-type motif 16 (NUDT16), mRNA [NM_152395]	NM_152395
A_24_P898583	0.00042	NM_003449	NM_003449	Homo sapiens tripartite motif-containing 26 (TRIM26), mRNA [NM_003449]	NM_003449
A_24_P341476	0.00042	THC2301753		AF139893 cyclophilin 18 {Oryctolagus cuniculus;} , partial (84%) [THC2301753]	
A_23_P133338	0.000421	NM_017675	NM_017675	Homo sapiens protocadherin LKC (PC-LKC), mRNA [NM_017675]	NM_017675
A_32_P55045	0.000421	AK091722	AK091722	Homo sapiens cDNA FLJ34403 fis, clone HCHON2001607. [AK091722]	
A_23_P347720	0.000421	BC080577	BC080577	Homo sapiens cDNA clone MGC:99667 IMAGE:6597328, complete cds. [BC080577]	
A_23_P214908	0.000421	AY374131	AY374131	Homo sapiens truncated C1-tetrahydrofolate synthase mRNA, complete cds; nuclear gene for mitochondrial product; alternatively spliced. [AY374131]	
A_23_P127613	0.000421	NM_016146	NM_016146	Homo sapiens trafficking protein particle complex 4 (TRAPPC4), mRNA [NM_016146]	NM_016146
A_24_P711050	0.000422	A_24_P711050			
A_24_P349580	0.000424	A_24_P349580			
A_23_P34066	0.000424	NM_176786	NM_176786	Homo sapiens interleukin 9 receptor (IL9R), transcript variant 2, mRNA [NM_176786]	NM_176786
A_23_P210015	0.000424	CR616748	CR616748	full-length cDNA clone CS0DK007YM08 of HeLa cells Cot 25-normalized of Homo sapiens (human). [CR616748]	
A_23_P395365	0.000424	NM_001246	NM_001246	Homo sapiens ectonucleoside triphosphate diphosphohydrolase 2 (ENTPD2), transcript variant 2, mRNA [NM_001246]	NM_001246
A_23_P90855	0.000425	NM_014808	NM_014808	Homo sapiens FERM, RhoGEF and pleckstrin domain protein 2 (FARP2), mRNA [NM_014808]	NM_014808
A_24_P119456	0.000425	NM_015136	NM_015136	Homo sapiens stabilin 1 (STAB1), mRNA [NM_015136]	NM_015136
A_24_P456884	0.000425	BC047952	BC047952	Homo sapiens, clone IMAGE:5785547, mRNA. [BC047952]	
A_23_P66732	0.000425	NM_031965	NM_031965	Homo sapiens germ cell associated 2 (haspin) (GSG2), mRNA [NM_031965]	NM_031965
A_32_P22622	0.000425	NM_003703	NM_003703	Homo sapiens chromosome 4 open reading frame 9 (C4orf9), mRNA [NM_003703]	NM_003703
A_23_P101427	0.000425	NM_145245	NM_145245	Homo sapiens ecotropic viral integration site 5-like (EVI5L), mRNA [NM_145245]	NM_145245
A_23_P109427	0.000425	NM_000854	NM_000854	Homo sapiens glutathione S-transferase theta 2 (GSTT2), mRNA [NM_000854]	NM_000854
A_24_P285480	0.000425	NM_004891	NM_004891	Homo sapiens mitochondrial ribosomal protein L33 (MRPL33), nuclear gene encoding mitochondrial protein, transcript variant 1, mRNA [NM_004891]	NM_004891
A_23_P7229	0.000425	NM_033625	NM_033625	Homo sapiens ribosomal protein L34 (RPL34), transcript variant 2, mRNA [NM_033625]	NM_033625
A_24_P390793	0.000425	NM_001416	NM_001416	Homo sapiens eukaryotic translation initiation factor 4A, isoform 1 (EIF4A1), mRNA [NM_001416]	NM_001416
A_24_P221335	0.000426	A_24_P221335			
A_24_P505255	0.000426	A_24_P505255			
A_23_P7941	0.000426	NM_015950	NM_015950	Homo sapiens mitochondrial ribosomal protein L2 (MRPL2), nuclear gene encoding mitochondrial protein, mRNA [NM_015950]	NM_015950
A_23_P258714	0.000426	NM_006295	NM_006295	Homo sapiens valyl-tRNA synthetase (VARS), mRNA [NM_006295]	NM_006295
A_23_P58298	0.000427	NM_138386	NM_138386	Homo sapiens hypothetical protein BC008207 (LOC92345), mRNA [NM_138386]	NM_138386
A_23_P410110	0.000427	AK055129	AK055129	Homo sapiens cDNA FLJ30567 fis, clone BRAWH2005225. [AK055129]	XM_499566

A_23_P100011	0.000427	NM_005829	NM_005829	Homo sapiens adaptor-related protein complex 3, sigma 2 subunit (AP3S2), mRNA [NM_005829]	NM_005829
A_24_P417007	0.000428	THC2278244		RPB6_CRIGR (P61217) DNA-directed RNA polymerases I, II, and III 14.4 kDa polypeptide (RPB6) (RPABC14.4) (RPB14.4) , complete [THC2278244]	
A_24_P332341	0.000428	ENST00000306744		Homo sapiens hypothetical protein MGC13098, mRNA (cDNA clone IMAGE:4074285), partial cds. [BC062722]	XM_379819
A_23_P335988	0.000428	NM_014235	NM_014235	Homo sapiens ubiquitin-like 4 (UBL4), mRNA [NM_014235]	NM_014235
A_23_P54576	0.000428	NM_005550	NM_005550	Homo sapiens kinesin family member C3 (KIFC3), mRNA [NM_005550]	NM_005550
A_23_P51884	0.000429	NM_153339	NM_153339	Homo sapiens pseudouridylate synthase-like 1 (PUSL1), mRNA [NM_153339]	NM_153339
A_23_P131723	0.000429	NM_006826	NM_006826	Homo sapiens tyrosine 3-monooxygenase/tryptophan 5-monooxygenase activation protein, theta polypeptide (YWHAQ), mRNA [NM_006826]	NM_006826
A_32_P99715	0.00043	BU731317	BU731317	BU731317 UI-E-CI1-afr-o-04-0-UI.s1 UI-E-CI1 Homo sapiens cDNA clone UI-E-CI1-afr-o-04-0-UI 3', mRNA sequence [BU731317]	
A_32_P885123	0.00043	A_32_P885123			
A_23_P165698	0.00043	NM_024093	NM_024093	Homo sapiens hypothetical protein MGC5509 (MGC5509), mRNA [NM_024093]	NM_024093
A_24_P364954	0.00043	NM_024648	NM_024648	Homo sapiens hypothetical protein FLJ22222 (FLJ22222), transcript variant 1, mRNA [NM_024648]	NM_024648
A_23_P25989	0.00043	NM_032233	NM_032233	Homo sapiens chromosome 14 open reading frame 154 (C14orf154), transcript variant 1, mRNA [NM_032233]	NM_032233
A_24_P198217	0.000431	NM_147777	NM_147777	Homo sapiens sorting nexin 15 (SNX15), transcript variant B, mRNA [NM_147777]	NM_147777
A_24_P115450	0.000431	NM_018688	NM_018688	Homo sapiens bridging integrator 3 (BIN3), mRNA [NM_018688]	NM_018688
A_24_P167377	0.000432	NM_206899	NM_206899	Homo sapiens olfactory receptor, family 10, subfamily P, member 1 (OR10P1), mRNA [NM_206899]	NM_206899
A_24_P218056	0.000432	NM_015332	NM_015332	Homo sapiens NudC domain containing 3 (NUDCD3), mRNA [NM_015332]	NM_015332
A_23_P42241	0.000432	NM_030876	NM_030876	Homo sapiens olfactory receptor, family 5, subfamily V, member 1 (OR5V1), mRNA [NM_030876]	NM_030876
A_23_P75453	0.000432	NM_130803	NM_130803	Homo sapiens multiple endocrine neoplasia I (MEN1), transcript variant e1E, mRNA [NM_130803]	NM_130803
A_23_P52569	0.000432	NM_181843	NM_181843	Homo sapiens nudix (nucleoside diphosphate linked moiety X)-type motif 8 (NUDT8), mRNA [NM_181843]	NM_181843
A_23_P167674	0.000432	NM_000505	NM_000505	Homo sapiens coagulation factor XII (Hageman factor) (F12), mRNA [NM_000505]	NM_000505
A_24_P127235	0.000432	NM_021574	NM_021574	Homo sapiens breakpoint cluster region (BCR), transcript variant 2, mRNA [NM_021574]	NM_021574
A_23_P53298	0.000432	NM_001487	NM_001487	Homo sapiens biogenesis of lysosome-related organelles complex-1, subunit 1 (BLOC1S1), mRNA [NM_001487]	NM_001487
A_24_P342127	0.000434	NM_014997	NM_014997	Homo sapiens KIAA0265 protein (KIAA0265), mRNA [NM_014997]	NM_014997
A_23_P25644	0.000434	NM_018386	NM_018386	Homo sapiens hypothetical protein FLJ11305 (FLJ11305), mRNA [NM_018386]	NM_018386
A_24_P494807	0.000435	ENST00000338548		PSDA_HUMAN (O75832) 26S proteasome non-ATPase regulatory subunit 10 (26S proteasome regulatory subunit p28) (Gankyrin), partial (60%) [THC2294587]	
A_23_P116587	0.000435	NM_006189	NM_006189	Homo sapiens olfactory marker protein (OMP), mRNA [NM_006189]	NM_006189
A_24_P297537	0.000435	NM_182574	NM_182574	Homo sapiens hypothetical protein FLJ36070 (FLJ36070), mRNA [NM_182574]	NM_182574
A_24_P123133	0.000435	NM_020231	NM_020231	Homo sapiens x 010 protein (MDS010), mRNA [NM_020231]	NM_020231
A_24_P102895	0.000435	NM_015023	NM_015023	Homo sapiens WD and tetratricopeptide repeats 1 (WDTC1), mRNA [NM_015023]	NM_015023
A_23_P418493	0.000435	NM_145691	NM_145691	Homo sapiens ATP synthase mitochondrial F1 complex assembly factor 2 (ATPAF2), nuclear gene encoding mitochondrial protein, mRNA [NM_145691]	NM_145691
A_23_P376599	0.000435	NM_006788	NM_006788	Homo sapiens ralA binding protein 1 (RALBP1), mRNA [NM_006788]	NM_006788
A_23_P58072	0.000435	A_23_P58072			
A_24_P190190	0.000436	NM_000723	NM_000723	Homo sapiens calcium channel, voltage-dependent, beta 1 subunit (CACNB1), transcript variant 1, mRNA [NM_000723]	NM_000723
A_32_P4505	0.000437	BQ007298	BQ007298	UI-1-BC0-afm-c-04-0-UI.s1 NCI_CGAP_Pl1 Homo sapiens cDNA clone UI-1-BC0-afm-c-04-0-UI 3', mRNA sequence [BQ007298]	
A_23_P349203	0.000437	AK027172	AK027172	Homo sapiens cDNA: FLJ23519 fis, clone LNG04908. [AK027172]	
A_24_P327499	0.000437	NM_014657	NM_014657	Homo sapiens KIAA0406 gene product (KIAA0406), mRNA [NM_014657]	NM_014657
A_23_P78717	0.000437	NM_022046	NM_022046	Homo sapiens kallikrein 14 (KLK14), mRNA [NM_022046]	NM_022046
A_32_P84846	0.000437	NM_001005851	NM_001005851	Homo sapiens hypothetical BC331191_1 (LOC163131), mRNA [NM_001005851]	NM_001005851
A_23_P430758	0.000437	NM_020240	NM_020240	Homo sapiens CDC42 small effector 2 (CDC42SE2), mRNA [NM_020240]	NM_020240
A_24_P140788	0.000438	NM_173558	NM_173558	Homo sapiens FYVE, RhoGEF and PH domain containing 2 (FGD2), mRNA [NM_173558]	NM_173558
A_32_P142625	0.000438	AA292852	AA292852	zt64e12.r1 Soares_testis_NHT Homo sapiens cDNA clone IMAGE:727150 5', mRNA sequence [AA292852]	
A_32_P98298	0.000438	NM_004420	NM_004420	Homo sapiens dual specificity phosphatase 8 (DUSP8), mRNA [NM_004420]	NM_004420
A_24_P410678	0.000439	NM_002227	NM_002227	Homo sapiens Janus kinase 1 (a protein tyrosine kinase) (JAK1), mRNA [NM_002227]	NM_002227
A_23_P315872	0.000441	NM_022340	NM_022340	Homo sapiens zinc finger, FYVE domain containing 20 (ZFYVE20), mRNA [NM_022340]	NM_022340

A_23_P213541	0.000441	NM_052870	NM_052870	Homo sapiens sorting nexin associated golgi protein 1 (SNAG1), mRNA [NM_052870]	NM_052870
A_24_P392622	0.000441	A_24_P392622			
A_32_P137849	0.000441	THC2448998		Q75CH7 (Q75CH7) ACL058Wp, partial (3%) [THC2448998]	
A_24_P337657	0.000441	NM_003131	NM_003131	Homo sapiens serum response factor (c-fos serum response element-binding transcription factor) (SRF), mRNA [NM_003131]	NM_003131
A_23_P14928	0.000441	NM_020312	NM_020312	Homo sapiens chromosome 16 open reading frame 49 (C16orf49), mRNA [NM_020312]	NM_020312
A_23_P200940	0.000442	NM_006347	NM_006347	Homo sapiens peptidyl prolyl isomerase H (cyclophilin H) (PPIH), mRNA [NM_006347]	NM_006347
A_24_P306896	0.000443	A_24_P306896			
A_24_P59220	0.000443	ENST00000303830		PREDICTED: Homo sapiens similar to POTE2A (LOC344227), mRNA [XM_292982]	XM_292982
A_23_P300136	0.000444	AK074445	AK074445	Homo sapiens cDNA FLJ23865 fis, clone LNG09225. [AK074445]	
A_24_P256929	0.000444	NM_024869	NM_024869	Homo sapiens glycine/arginine rich protein 1 (GRRP1), mRNA [NM_024869]	NM_024869
A_24_P157698	0.000444	NM_173828	NM_173828	Homo sapiens chromosome 5 open reading frame 16 (C5orf16), mRNA [NM_173828]	NM_173828
A_24_P878419	0.000444	A_24_P878419			
A_32_P456537	0.000444	NM_030752	NM_030752	Homo sapiens t-complex 1 (TCP1), transcript variant 1, mRNA [NM_030752]	NM_030752
A_23_P32913	0.000444	NM_031905	NM_031905	Homo sapiens SVH protein (SVH), mRNA [NM_031905]	NM_031905
A_23_P117623	0.000444	NM_018319	NM_018319	Homo sapiens tyrosyl-DNA phosphodiesterase 1 (TDP1), transcript variant 1, mRNA [NM_018319]	NM_018319
A_24_P936953	0.000445	A_24_P936953			
A_24_P410510	0.000445	AK095213	AK095213	Homo sapiens cDNA FLJ37894 fis, clone BRTHA2004639. [AK095213]	
A_24_P386622	0.000445	NM_004041	NM_004041	Homo sapiens arrestin, beta 1 (ARRB1), transcript variant 1, mRNA [NM_004041]	NM_004041
A_23_P61398	0.000445	NM_001001852	NM_001001852	Homo sapiens pim-3 oncogene (PIM3), mRNA [NM_001001852]	NM_001001852
A_24_P410389	0.000446	NM_015367	NM_015367	Homo sapiens BCL2-like 13 (apoptosis facilitator) (BCL2L13), nuclear gene encoding mitochondrial protein, mRNA [NM_015367]	NM_015367
A_24_P32935	0.000446	NM_000803	NM_000803	Homo sapiens folate receptor 2 (fetal) (FOLR2), mRNA [NM_000803]	NM_000803
A_24_P352116	0.000446	ENST00000361063		Homo sapiens cDNA FLJ33266 fis, clone ASTRO2007047. [AK090585]	
A_23_P130865	0.000446	NM_018074	NM_018074	Homo sapiens hypothetical protein FLJ10374 (FLJ10374), mRNA [NM_018074]	NM_018074
A_24_P829541	0.000446	NM_001012626	NM_001012626	Homo sapiens hypothetical protein LOC285074 (LOC285074), mRNA [NM_001012626]	NM_001012626
A_23_P152919	0.000447	NM_002532	NM_002532	Homo sapiens nucleoporin 88kDa (NUP88), mRNA [NM_002532]	NM_002532
A_24_P252057	0.000448	NM_153020	NM_153020	Homo sapiens RNA binding motif protein 24 (RBM24), mRNA [NM_153020]	NM_153020
A_24_P371425	0.000448	NM_032166	NM_032166	Homo sapiens three prime repair exonuclease 1 (TREX1), transcript variant 5, mRNA [NM_032166]	NM_032166
A_23_P124224	0.000448	NM_004941	NM_004941	Homo sapiens DEAH (Asp-Glu-Ala-His) box polypeptide 8 (DHX8), mRNA [NM_004941]	NM_004941
A_24_P135384	0.000449	BC021276	BC021276	Homo sapiens immunoglobulin heavy constant delta, mRNA (cDNA clone MGC:29633 IMAGE:4855067), complete cds. [BC021276]	
A_24_P519817	0.000449	BC022562	BC022562	Homo sapiens cDNA clone IMAGE:4794059, partial cds. [BC022562]	
A_24_P203689	0.000449	NM_198525	NM_198525	Homo sapiens kinesin family member 7 (KIF7), mRNA [NM_198525]	NM_198525
A_23_P146908	0.000449	NM_004853	NM_004853	Homo sapiens syntaxin 8 (STX8), mRNA [NM_004853]	NM_004853
A_23_P424734	0.000452	NM_178516	NM_178516	Homo sapiens hypothetical protein LOC283849 (LOC283849), mRNA [NM_178516]	NM_178516
A_23_P152753	0.000452	AY358101	AY358101	Homo sapiens clone DNA108695 Wpep3002 (UNQ3002) mRNA, complete cds. [AY358101]	
A_32_P219368	0.000452	NM_152858	NM_152858	Homo sapiens Wilms tumor 1 associated protein (WTAP), transcript variant 3, mRNA [NM_152858]	NM_152858
A_24_P111134	0.000452	NM_013382	NM_013382	Homo sapiens protein-O-mannosyltransferase 2 (POMT2), mRNA [NM_013382]	NM_013382
A_23_P426565	0.000453	NM_178519	NM_178519	Homo sapiens hypothetical protein FLJ39421 (FLJ39421), mRNA [NM_178519]	NM_178519
A_32_P96272	0.000453	NM_001008741	NM_001008741	Homo sapiens peptidylprolyl isomerase A-like (LOC388817), mRNA [NM_001008741]	NM_001008741
A_23_P215479	0.000454	NM_003388	NM_003388	Homo sapiens cytoplasmic linker 2 (CYLN2), transcript variant 1, mRNA [NM_003388]	NM_003388
A_24_P300841	0.000454	NM_001008938	NM_001008938	Homo sapiens cytoskeleton associated protein 5 (CKAP5), transcript variant 1, mRNA [NM_001008938]	NM_001008938
A_23_P132417	0.000454	NM_018385	NM_018385	Homo sapiens hypothetical protein FLJ11301 (FLJ11301), mRNA [NM_018385]	NM_018385
A_23_P394014	0.000454	NM_015134	NM_015134	Homo sapiens myosin phosphatase-Rho interacting protein (M-RIP), mRNA [NM_015134]	NM_015134
A_23_P126752	0.000454	NM_004930	NM_004930	Homo sapiens capping protein (actin filament) muscle Z-line, beta (CAPZB), mRNA [NM_004930]	NM_004930
A_23_P87257	0.000454	NM_022061	NM_022061	Homo sapiens mitochondrial ribosomal protein L17 (MRPL17), nuclear gene encoding mitochondrial protein, mRNA [NM_022061]	NM_022061

A_24_P242688	0.000455	NM_000182	NM_000182	Homo sapiens hydroxyacyl-Coenzyme A dehydrogenase/3-ketoacyl-Coenzyme A thiolase/enoyl-Coenzyme A hydratase (trifunctional protein), alpha subunit (HADHA), mRNA [NM_000182]	NM_000182
A_23_P121051	0.000457	NM_000532	NM_000532	Homo sapiens propionyl Coenzyme A carboxylase, beta polypeptide (PCCB), mRNA [NM_000532]	NM_000532
A_24_P326635	0.000458	NM_001619	NM_001619	Homo sapiens adrenergic, beta, receptor kinase 1 (ADRBK1), mRNA [NM_001619]	NM_001619
A_32_P141238	0.000458	NM_020373	NM_020373	Homo sapiens transmembrane protein 16B (TMEM16B), mRNA [NM_020373]	NM_020373
A_23_P250825	0.000458	NM_014504	NM_014504	Homo sapiens RAB guanine nucleotide exchange factor (GEF) 1 (RABGEF1), mRNA [NM_014504]	NM_014504
A_24_P190402	0.000458	NM_014931	NM_014931	Homo sapiens KIAA1115 (KIAA1115), mRNA [NM_014931]	NM_014931
A_23_P34968	0.000458	NM_001002234	NM_001002234	Homo sapiens sodium channel modifier 1 (SCNM1), transcript variant 2, mRNA [NM_001002234]	NM_001002234
A_23_P408822	0.00046	NM_145663	NM_145663	Homo sapiens Dbf4-related factor 1 (DRF1), transcript variant 1, mRNA [NM_145663]	NM_145663
A_23_P63050	0.00046	NM_000374	NM_000374	Homo sapiens uroporphyrinogen decarboxylase (UROD), mRNA [NM_000374]	NM_000374
A_23_P330537	0.000461	NM_080861	NM_080861	Homo sapiens SPRY domain-containing SOCS box protein SSB-3 (SSB3), mRNA [NM_080861]	NM_080861
A_32_P65395	0.000461	THC2234672		AB019038 beta-1,4 mannosyltransferase {Homo sapiens;} , complete [THC2234672]	
A_23_P218398	0.000462	AF028825	AF028825	Homo sapiens Tax interaction protein 15 mRNA, complete cds. [AF028825]	
A_23_P200999	0.000462	NM_174963	NM_174963	Homo sapiens ST3 beta-galactoside alpha-2,3-sialyltransferase 3 (ST3GAL3), transcript variant 1, mRNA [NM_174963]	NM_174963
A_23_P353436	0.000463	BC091515	BC091515	Homo sapiens chromosome 9 open reading frame 81, mRNA (cDNA clone MGC:111492 IMAGE:6194709), complete cds. [BC091515]	
A_24_P837537	0.000464	A_24_P837537			
A_32_P53884	0.000464	BC040307	BC040307	Homo sapiens cDNA clone IMAGE:4830091, partial cds. [BC040307]	
A_32_P181722	0.000464	CR606587	CR606587	full-length cDNA clone CS0DI044YA04 of Placenta Cot 25-normalized of Homo sapiens (human). [CR606587]	
A_23_P254648	0.000464	NM_012164	NM_012164	Homo sapiens F-box and WD-40 domain protein 2 (FBXW2), mRNA [NM_012164]	NM_012164
A_24_P306355	0.000465	ENST00000295608			
A_23_P130876	0.000466	NM_178544	NM_178544	Homo sapiens zinc finger protein 546 (ZNF546), mRNA [NM_178544]	NM_178544
A_23_P45864	0.000466	NM_003285	NM_003285	Homo sapiens tenascin R (restrictin, janusin) (TNR), mRNA [NM_003285]	NM_003285
A_23_P67529	0.000466	NM_002250	NM_002250	Homo sapiens potassium intermediate/small conductance calcium-activated channel, subfamily N, member 4 (KCNN4), mRNA [NM_002250]	NM_002250
A_23_P217886	0.000466	NM_194458	NM_194458	Homo sapiens ubiquitin-conjugating enzyme E2, J2 (UBC6 homolog, yeast) (UBE2J2), transcript variant 3, mRNA [NM_194458]	NM_194458
A_23_P5626	0.000467	A_23_P5626			
A_23_P146237	0.000467	NM_015024	NM_015024	Homo sapiens exportin 7 (XPO7), mRNA [NM_015024]	NM_015024
A_24_P391260	0.000467	NM_004339	NM_004339	Homo sapiens pituitary tumor-transforming 1 interacting protein (PTTG1IP), mRNA [NM_004339]	NM_004339
A_32_P97149	0.000468	THC2282995		AF055376 short form transcription factor C-MAF {Homo sapiens;} , partial (7%) [THC2282995]	
A_23_P141520	0.000468	NM_174893	NM_174893	Homo sapiens hypothetical protein MGC49942 (MGC49942), mRNA [NM_174893]	NM_174893
A_23_P158349	0.000469	NM_173825	NM_173825	Homo sapiens RAB, member of RAS oncogene family-like 3 (RABL3), mRNA [NM_173825]	NM_173825
A_23_P376096	0.000469	NM_014261	NM_014261	Homo sapiens toll-like receptor adaptor molecule 1 (TICAM1), transcript variant 1, mRNA [NM_014261]	NM_014261
A_23_P128246	0.000469	NM_007076	NM_007076	Homo sapiens Huntingtin interacting protein E (HYPE), mRNA [NM_007076]	NM_007076
A_23_P134295	0.000469	NM_198949	NM_198949	Homo sapiens nudix (nucleoside diphosphate linked moiety X)-type motif 1 (NUDT1), transcript variant 2B, mRNA [NM_198949]	NM_198949
A_23_P126803	0.000469	NM_005717	NM_005717	Homo sapiens actin related protein 2/3 complex, subunit 5, 16kDa (ARPC5), mRNA [NM_005717]	NM_005717
A_24_P304987	0.00047	NM_013260	NM_013260	Homo sapiens transcriptional regulator protein (HCNGP), mRNA [NM_013260]	NM_013260
A_23_P400884	0.000472	NM_012279	NM_012279	Homo sapiens zinc finger protein 346 (ZNF346), mRNA [NM_012279]	NM_012279
A_24_P152385	0.000472	THC2425829		ATPN_BOVIN (Q28852) ATP synthase g chain, mitochondrial (ATPase subunit G) , partial (92%) [THC2425829]	
A_24_P400751	0.000472	ENST00000312398			
A_32_P174999	0.000472	BM564463	BM564463	AGENCOURT_6563581 NIH_MGC_88 Homo sapiens cDNA clone IMAGE:5737328 5', mRNA sequence [BM564463]	
A_24_P477102	0.000472	XM_209097	XM_209097	PREDICTED: Homo sapiens similar to FLJ10101 protein (LOC284269), mRNA [XM_209097]	XM_209097
A_23_P717	0.000472	NM_018252	NM_018252	Homo sapiens chromosome 1 open reading frame 75 (C1orf75), mRNA [NM_018252]	NM_018252
A_24_P146892	0.000472	NM_032790	NM_032790	Homo sapiens hypothetical protein FLJ14466 (FLJ14466), mRNA [NM_032790]	NM_032790
A_23_P369479	0.000473	NM_170721	NM_170721	Homo sapiens musashi homolog 2 (Drosophila) (MSI2), transcript variant 2, mRNA [NM_170721]	NM_170721
A_24_P412512	0.000474	NM_016350	NM_016350	Homo sapiens ninein (GSK3B interacting protein) (NIN), transcript variant 4, mRNA [NM_016350]	NM_016350

A_23_P6869	0.000474	NM_007024	NM_007024	Homo sapiens placental protein 6 (PL6), mRNA [NM_007024]	NM_007024
A_23_P322845	0.000474	BC033025	BC033025	Homo sapiens HTPAP protein, mRNA (cDNA clone MGC:32924 IMAGE:5267610), complete cds. [BC033025]	
A_24_P32151	0.000475	NM_182705	NM_182705	Homo sapiens hypothetical protein MGC45871 (MGC45871), mRNA [NM_182705]	NM_182705
A_23_P64343	0.000475	NM_012456	NM_012456	Homo sapiens translocase of inner mitochondrial membrane 10 homolog (yeast) (TIMM10), mRNA [NM_012456]	NM_012456
A_23_P127153	0.000476	U88048	U88048	Human clone KiSS-16 unknown product mRNA, complete cds. [U88048]	
A_24_P341626	0.000477	ENST00000332079			
A_23_P379746	0.000477	BC020855	BC020855	Homo sapiens hypothetical protein MGC24039, mRNA (cDNA clone IMAGE:4286826), complete cds. [BC020855]	
A_23_P320717	0.000477	NM_145818	NM_145818	Homo sapiens component of oligomeric golgi complex 4 (COG4), transcript variant Cog4S, mRNA [NM_145818]	NM_145818
A_24_P105283	0.000477	NM_005066	NM_005066	Homo sapiens splicing factor proline/glutamine-rich (polypyrimidine tract binding protein associated) (SFPQ), mRNA [NM_005066]	NM_005066
A_23_P70534	0.000478	NM_001003693	NM_001003693	Homo sapiens chromosome 6 open reading frame 21 (C6orf21), mRNA [NM_001003693]	NM_001003693
A_23_P157240	0.000478	J05272	J05272	Human IMP dehydrogenase type 1 mRNA complete cds. [J05272]	
A_24_P74896	0.000478	NM_182611	NM_182611	Homo sapiens G protein-coupled receptor 144 (GPR144), mRNA [NM_182611]	NM_182611
A_32_P229965	0.000478	AA662695	AA662695	AA662695 nv06h02.s1 NCI_CGAP_Pr22 Homo sapiens cDNA clone IMAGE:1219443 3', mRNA sequence [AA662695]	
A_24_P376294	0.000478	NM_007040	NM_007040	Homo sapiens heterogeneous nuclear ribonucleoprotein U-like 1 (HNRPUL1), transcript variant 1, mRNA [NM_007040]	NM_007040
A_32_P135558	0.000479	NM_153261	NM_153261	Homo sapiens hypothetical protein FLJ38101 (FLJ38101), mRNA [NM_153261]	NM_153261
A_24_P41570	0.00048	NM_002106	NM_002106	Homo sapiens H2A histone family, member Z (H2AFZ), mRNA [NM_002106]	NM_002106
A_32_P70483	0.000481	THC2322041			
A_23_P55926	0.000481	NM_007121	NM_007121	Homo sapiens nuclear receptor subfamily 1, group H, member 2 (NR1H2), mRNA [NM_007121]	NM_007121
A_23_P90296	0.000481	NM_004714	NM_004714	Homo sapiens dual-specificity tyrosine-(Y)-phosphorylation regulated kinase 1B (DYRK1B), transcript variant a, mRNA [NM_004714]	NM_004714
A_24_P910169	0.000481	A_24_P910169			
A_24_P272967	0.000482	NM_015060	NM_015060	Homo sapiens KIAA0241 protein (KIAA0241), mRNA [NM_015060]	NM_015060
A_23_P15073	0.000482	NM_001005920	NM_001005920	Homo sapiens hypothetical LOC339123 (LOC339123), mRNA [NM_001005920]	NM_001005920
A_24_P638453	0.000482	A_24_P638453			
A_23_P51781	0.000482	NM_017846	NM_017846	Homo sapiens tRNA selenocysteine associated protein (SECP43), mRNA [NM_017846]	NM_017846
A_23_P85560	0.000482	NM_003757	NM_003757	Homo sapiens eukaryotic translation initiation factor 3, subunit 2 beta, 36kDa (EIF3S2), mRNA [NM_003757]	NM_003757
A_23_P99216	0.000483	NM_015918	NM_015918	Homo sapiens processing of precursor 5, ribonuclease P/MRP subunit (S. cerevisiae) (POP5), transcript variant 1, mRNA [NM_015918]	NM_015918
A_23_P77807	0.000484	NM_030665	NM_030665	Homo sapiens retinoic acid induced 1 (RAI1), mRNA [NM_030665]	NM_030665
A_24_P341489	0.000484	A_24_P341489			
A_23_P130648	0.000484	NM_005716	NM_005716	Homo sapiens GIPC PDZ domain containing family, member 1 (GIPC1), transcript variant 1, mRNA [NM_005716]	NM_005716
A_23_P250035	0.000486	NM_004168	NM_004168	Homo sapiens succinate dehydrogenase complex, subunit A, flavoprotein (Fp) (SDHA), nuclear gene encoding mitochondrial protein, mRNA [NM_004168]	NM_004168
A_24_P612020	0.000487	AK096241	AK096241	Homo sapiens cDNA FLJ38922 fis, clone NT2NE2011691. [AK096241]	
A_24_P358381	0.000488	NM_012227	NM_012227	Homo sapiens GTP binding protein 6 (putative) (GTPBP6), mRNA [NM_012227]	NM_012227
A_32_P41203	0.000489	A_32_P41203			
A_24_P200297	0.000489	NM_172027	NM_172027	Homo sapiens ankyrin repeat and BTB (POZ) domain containing 1 (ABTB1), transcript variant 2, mRNA [NM_172027]	NM_172027
A_23_P361584	0.000489	NM_152680	NM_152680	Homo sapiens hypothetical protein FLJ32028 (FLJ32028), mRNA [NM_152680]	NM_152680
A_32_P88605	0.00049	A_32_P88605			
A_23_P117743	0.00049	NM_024708	NM_024708	Homo sapiens ankyrin repeat and SOCS box-containing 7 (ASB7), transcript variant 1, mRNA [NM_024708]	NM_024708
A_24_P50281	0.00049	A_24_P50281			
A_23_P57570	0.00049	NM_017436	NM_017436	Homo sapiens alpha 1,4-galactosyltransferase (globotriaosylceramide synthase) (A4GALT), mRNA [NM_017436]	NM_017436
A_23_P60718	0.000491	NM_024105	NM_024105	Homo sapiens asparagine-linked glycosylation 12 homolog (yeast, alpha-1,6-mannosyltransferase) (ALG12), mRNA [NM_024105]	NM_024105
A_24_P11737	0.000492	AK095678	AK095678	Homo sapiens cDNA FLJ38359 fis, clone FEBRA2000321. [AK095678]	XM_379121
A_24_P916522	0.000493	U62539	U62539	Human B cell specific activator protein BSAP (PAX5) mRNA, partial cds. [U62539]	
A_23_P108200	0.000493	NM_002866	NM_002866	Homo sapiens RAB3A, member RAS oncogene family (RAB3A), mRNA [NM_002866]	NM_002866
A_24_P375573	0.000494	ENST00000328613			

A_23_P321261	0.000494	NM_002396	NM_002396	Homo sapiens malic enzyme 2, NAD(+)-dependent, mitochondrial (ME2), nuclear gene encoding mitochondrial protein, mRNA [NM_002396]	NM_002396
A_23_P212844	0.000494	NM_006342	NM_006342	Homo sapiens transforming, acidic coiled-coil containing protein 3 (TACC3), mRNA [NM_006342]	NM_006342
A_23_P338168	0.000495	NM_019085	NM_019085	Homo sapiens F-box and leucine-rich repeat protein 19 (FBXL19), mRNA [NM_019085]	NM_019085
A_23_P141656	0.000495	NM_001388	NM_001388	Homo sapiens developmentally regulated GTP binding protein 2 (DRG2), mRNA [NM_001388]	NM_001388
A_23_P56971	0.000496	NM_017896	NM_017896	Homo sapiens chromosome 20 open reading frame 11 (C20orf11), mRNA [NM_017896]	NM_017896
A_23_P161338	0.000496	NM_021129	NM_021129	Homo sapiens pyrophosphatase (inorganic) (PP), mRNA [NM_021129]	NM_021129
A_23_P66787	0.000497	U18197	U18197	Human ATP:citrate lyase mRNA, complete cds. [U18197]	
A_32_P66974	0.000498	NM_004716	NM_004716	Homo sapiens proprotein convertase subtilisin/kexin type 7 (PCSK7), mRNA [NM_004716]	NM_004716
A_32_P201498	0.000498	NM_001009924	NM_001009924	Homo sapiens chromosome 20 open reading frame 30 (C20orf30), transcript variant 2, mRNA [NM_001009924]	NM_001009924
A_24_P752496	0.0005	AK095886	AK095886	Homo sapiens cDNA FLJ38567 fis, clone HCHON2005166. [AK095886]	
A_32_P98776	0.0005	THC2375957			
A_23_P88134	0.0005	NM_032630	NM_032630	Homo sapiens cyclin-dependent kinase 2-interacting protein (CINP), mRNA [NM_032630]	NM_032630
A_32_P130437	0.0005	T47335	T47335	T47335 yb10e12.s1 Stratagene placenta (#937225) Homo sapiens cDNA clone IMAGE:70798 3', mRNA sequence [T47335]	
A_23_P164141	0.0005	NM_176863	NM_176863	Homo sapiens proteasome (prosome, macropain) activator subunit 3 (PA28 gamma; Ki) (PSME3), transcript variant 2, mRNA [NM_176863]	NM_176863
A_23_P114826	0.0005	NM_031280	NM_031280	Homo sapiens mitochondrial ribosomal protein S15 (MRPS15), nuclear gene encoding mitochondrial protein, mRNA [NM_031280]	NM_031280
A_24_P409330	0.0005	NM_181304	NM_181304	Homo sapiens mitochondrial ribosomal protein L52 (MRPL52), nuclear gene encoding mitochondrial protein, transcript variant 4, mRNA [NM_181304]	NM_181304
A_23_P38468	0.000501	NM_014604	NM_014604	Homo sapiens Tax1 (human T-cell leukemia virus type I) binding protein 3 (TAX1BP3), mRNA [NM_014604]	NM_014604
A_24_P46159	0.000501	NM_016479	NM_016479	Homo sapiens scotin (SCOTIN), mRNA [NM_016479]	NM_016479
A_23_P38334	0.000502	NM_032387	NM_032387	Homo sapiens WNK lysine deficient protein kinase 4 (WNK4), mRNA [NM_032387]	NM_032387
A_23_P210419	0.000502	NM_014902	NM_014902	Homo sapiens discs, large (Drosophila) homolog-associated protein 4 (DLGAP4), transcript variant 1, mRNA [NM_014902]	NM_014902
A_23_P383977	0.000502	NM_006049	NM_006049	Homo sapiens small nuclear RNA activating complex, polypeptide 5, 19kDa (SNAPC5), mRNA [NM_006049]	NM_006049
A_23_P334186	0.000503	NM_005920	NM_005920	Homo sapiens MADS box transcription enhancer factor 2, polypeptide D (myocyte enhancer factor 2D) (MEF2D), mRNA [NM_005920]	NM_005920
A_23_P139864	0.000503	NM_031289	NM_031289	Homo sapiens germ cell associated 1 (GSG1), transcript variant 1, mRNA [NM_031289]	NM_031289
A_24_P134195	0.000503	NM_138373	NM_138373	Homo sapiens myeloid-associated differentiation marker (MYADM), transcript variant 2, mRNA [NM_138373]	NM_138373
A_32_P140262	0.000503	A_32_P140262			
A_23_P337033	0.000504	NM_012384	NM_012384	Homo sapiens glucocorticoid modulatory element binding protein 2 (GMEB2), mRNA [NM_012384]	NM_012384
A_32_P159726	0.000504	BC014227	BC014227	Homo sapiens KIAA1244, mRNA (cDNA clone IMAGE:4547495), partial cds. [BC014227]	
A_23_P17490	0.000504	NM_001009924	NM_001009924	Homo sapiens chromosome 20 open reading frame 30 (C20orf30), transcript variant 2, mRNA [NM_001009924]	NM_001009924
A_23_P432360	0.000505	NM_000303	NM_000303	Homo sapiens phosphomannomutase 2 (PMM2), mRNA [NM_000303]	NM_000303
A_24_P323795	0.000506	A_24_P323795			
A_23_P21346	0.000506	NM_006315	NM_006315	Homo sapiens polycomb group ring finger 3 (PCGF3), mRNA [NM_006315]	NM_006315
A_23_P303203	0.000507	NM_152272	NM_152272	Homo sapiens CHMP family, member 7 (CHMP7), mRNA [NM_152272]	NM_152272
A_23_P154457	0.000508	NM_024894	NM_024894	Homo sapiens hypothetical LOC79954 (FLJ14075), mRNA [NM_024894]	NM_024894
A_32_P98927	0.000508	ENST00000352103		Homo sapiens cDNA FLJ13195 fis, clone NT2RP3004424, weakly similar to Homo sapiens mRNA for stromal antigen 3 (STAG3 gene). [AK023257]	XM_498429
A_23_P123010	0.000508	NM_138707	NM_138707	Homo sapiens B-cell CLL/lymphoma 7B (BCL7B), transcript variant 2, mRNA [NM_138707]	NM_138707
A_24_P382591	0.000508	NM_001009924	NM_001009924	Homo sapiens chromosome 20 open reading frame 30 (C20orf30), transcript variant 2, mRNA [NM_001009924]	NM_001009924
A_23_P143120	0.000509	NM_003183	NM_003183	Homo sapiens a disintegrin and metalloproteinase domain 17 (tumor necrosis factor, alpha, converting enzyme) (ADAM17), transcript variant 1, mRNA [NM_003183]	NM_003183
A_23_P124190	0.00051	NM_130390	NM_130390	Homo sapiens tripartite motif-containing 34 (TRIM34), transcript variant 3, mRNA [NM_130390]	NM_130390
A_23_P162449	0.00051	NM_020762	NM_020762	Homo sapiens SLIT-ROBO Rho GTPase activating protein 1 (SRGAP1), mRNA [NM_020762]	NM_020762
A_23_P212025	0.00051	AL833148	AL833148	Homo sapiens mRNA; cDNA DKFZp313H1440 (from clone DKFZp313H1440). [AL833148]	XM_172341
A_23_P145146	0.00051	NM_002598	NM_002598	Homo sapiens programmed cell death 2 (PDCD2), transcript variant 1, mRNA [NM_002598]	NM_002598
A_23_P200507	0.00051	NM_014184	NM_014184	Homo sapiens cornichon homolog 4 (Drosophila) (CNIH4), mRNA [NM_014184]	NM_014184
A_23_P10156	0.000511	NM_024591	NM_024591	Homo sapiens chromatin modifying protein 6 (CHMP6), mRNA [NM_024591]	NM_024591

A_32_P29016	0.000512	THC2320434			
A_24_P22892	0.000512	NM_024076	NM_024076	Homo sapiens potassium channel tetramerisation domain containing 15 (KCTD15), mRNA [NM_024076]	NM_024076
A_32_P54594	0.000512	BI050742	BI050742	BI050742 RC6-GN0071-080101-025-H04 GN0071 Homo sapiens cDNA, mRNA sequence [BI050742]	
A_23_P251248	0.000512	NM_003192	NM_003192	Homo sapiens tubulin-specific chaperone c (TBCC), mRNA [NM_003192]	NM_003192
A_23_P167313	0.000513	NM_002938	NM_002938	Homo sapiens ring finger protein 4 (RNF4), mRNA [NM_002938]	NM_002938
A_23_P86054	0.000514	NM_015023	NM_015023	Homo sapiens WD and tetratricopeptide repeats 1 (WDTC1), mRNA [NM_015023]	NM_015023
A_32_P117884	0.000514	A_32_P117884			
A_23_P149221	0.000514	NM_024602	NM_024602	Homo sapiens HECT domain containing 3 (HECTD3), mRNA [NM_024602]	NM_024602
A_23_P35114	0.000514	NM_016274	NM_016274	Homo sapiens CK2 interacting protein 1; HQ0024c protein (CKIP-1), mRNA [NM_016274]	NM_016274
A_23_P64238	0.000515	NM_006760	NM_006760	Homo sapiens uroplakin 2 (UPK2), mRNA [NM_006760]	NM_006760
A_32_P206175	0.000515	THC2429183		PTNI_HUMAN (Q99952) Tyrosine-protein phosphatase, non-receptor type 18 (Brain-derived phosphatase) , partial (6%) [THC2429183]	
A_23_P75220	0.000515	NM_031212	NM_031212	Homo sapiens solute carrier family 25, member 28 (SLC25A28), mRNA [NM_031212]	NM_031212
A_24_P417757	0.000516	ENST00000306575			
A_24_P138022	0.000516	NM_014612	NM_014612	Homo sapiens chromosome 9 open reading frame 10 (C9orf10), mRNA [NM_014612]	NM_014612
A_24_P413884	0.000516	NM_001809	NM_001809	Homo sapiens centromere protein A, 17kDa (CENPA), mRNA [NM_001809]	NM_001809
A_32_P63365	0.000518	BE697496	BE697496	BE697496 RC0-CT0428-310700-031-g12 CT0428 Homo sapiens cDNA, mRNA sequence [BE697496]	
A_24_P914062	0.000518	BE644757	BE644757	BE644757 7e39h04.x1 NCI_CGAP_Lu24 Homo sapiens cDNA clone IMAGE:3284887 3' similar to gb:U04897 NUCLEAR RECEPTOR ROR-ALPHA-1 (HUMAN);, mRNA sequence [BE644757]	
A_24_P401381	0.000518	A_24_P401381			
A_23_P77360	0.000518	NM_004804	NM_004804	Homo sapiens WD repeat domain 39 (WDR39), mRNA [NM_004804]	NM_004804
A_24_P170514	0.000519	BC042060	BC042060	Homo sapiens cDNA clone IMAGE:5590288, partial cds. [BC042060]	
A_23_P359762	0.000519	NM_152914	NM_152914	Homo sapiens transcript expressed during hematopoiesis 2 (MGC33894), mRNA [NM_152914]	NM_152914
A_24_P69053	0.000519	AK001336	AK001336	Homo sapiens cDNA FLJ10474 fis, clone NT2RP2000067. [AK001336]	XM_371717
A_24_P780353	0.000519	XM_377933	XM_377933	PREDICTED: Homo sapiens similar to Hypothetical protein MGC76216 (LOC402251), mRNA [XM_377933]	XM_377933
A_24_P152635	0.000519	NM_015959	NM_015959	Homo sapiens thioredoxin-related transmembrane protein 2 (TMX2), mRNA [NM_015959]	NM_015959
A_23_P57347	0.000519	NM_006031	NM_006031	Homo sapiens pericentrin 2 (kendrin) (PCNT2), mRNA [NM_006031]	NM_006031
A_23_P215088	0.000519	NM_016478	NM_016478	Homo sapiens zinc finger, C3HC-type containing 1 (ZC3HC1), mRNA [NM_016478]	NM_016478
A_24_P75840	0.00052	ENST00000325635			
A_24_P451992	0.00052	A_24_P451992			
A_23_P67321	0.00052	NM_138334	NM_138334	Homo sapiens hypothetical transmembrane protein SBBI54 (SBBI54), mRNA [NM_138334]	NM_138334
A_24_P76854	0.000521	AJ296345	AJ296345	Homo sapiens partial mRNA for keratin associated protein (KRTAP2.1A gene). [AJ296345]	
A_23_P108823	0.000521	NM_032523	NM_032523	Homo sapiens oxysterol binding protein-like 6 (OSBPL6), transcript variant 1, mRNA [NM_032523]	NM_032523
A_23_P130836	0.000522	NM_005317	NM_005317	Homo sapiens granzyme M (lymphocyte met-ase 1) (GZMM), mRNA [NM_005317]	NM_005317
A_23_P148546	0.000523	NM_003491	NM_003491	Homo sapiens ARD1 homolog A, N-acetyltransferase (S. cerevisiae) (ARD1A), mRNA [NM_003491]	NM_003491
A_23_P393713	0.000524	NM_002180	NM_002180	Homo sapiens immunoglobulin mu binding protein 2 (IGHMBP2), mRNA [NM_002180]	NM_002180
A_23_P119395	0.000524	NM_000164	NM_000164	Homo sapiens gastric inhibitory polypeptide receptor (GIPR), mRNA [NM_000164]	NM_000164
A_23_P402610	0.000524	NM_012393	NM_012393	Homo sapiens phosphoribosylformylglycinamidine synthase (FGAR amidotransferase) (PFAS), mRNA [NM_012393]	NM_012393
A_23_P119377	0.000524	NM_004228	NM_004228	Homo sapiens pleckstrin homology, Sec7 and coiled-coil domains 2 (cytohesin-2) (PSCD2), transcript variant 2, mRNA [NM_004228]	NM_004228
A_23_P162970	0.000524	NM_024658	NM_024658	Homo sapiens importin 4 (IPO4), mRNA [NM_024658]	NM_024658
A_23_P384405	0.000524	NM_001440	NM_001440	Homo sapiens exostoses (multiple)-like 3 (EXTL3), mRNA [NM_001440]	NM_001440
A_23_P40574	0.000525	THC2365004		CRB2_HUMAN (P43320) Beta crystallin B2 (BP), partial (72%) [THC2365004]	
A_24_P197964	0.000525	NM_014788	NM_014788	Homo sapiens tripartite motif-containing 14 (TRIM14), transcript variant 1, mRNA [NM_014788]	NM_014788
A_23_P205393	0.000525	NM_175748	NM_175748	Homo sapiens chromosome 14 open reading frame 130 (C14orf130), transcript variant 2, mRNA [NM_175748]	NM_175748
A_24_P461389	0.000526	THC2365641		BT007382 ubiquitin-conjugating enzyme E2 variant 1 {Homo sapiens;} , partial (93%) [THC2365641]	
A_24_P231494	0.000526	NM_012100	NM_012100	Homo sapiens aspartyl aminopeptidase (DNPEP), mRNA [NM_012100]	NM_012100

A_23_P387374	0.000526	BC033110	BC033110	Homo sapiens, clone IMAGE:5443970, mRNA, partial cds. [BC033110]	
A_23_P366455	0.000526	NM_133264	NM_133264	Homo sapiens WIRE protein (WIRE), mRNA [NM_133264]	NM_133264
A_23_P430558	0.000527	NM_000751	NM_000751	Homo sapiens cholinergic receptor, nicotinic, delta polypeptide (CHRND), mRNA [NM_000751]	NM_000751
A_23_P334282	0.000527	NM_017593	NM_017593	Homo sapiens BMP2 inducible kinase (BMP2K), transcript variant 2, mRNA [NM_017593]	NM_017593
A_23_P138025	0.000527	NM_018066	NM_018066	Homo sapiens hypothetical protein FLJ10349 (FLJ10349), mRNA [NM_018066]	NM_018066
A_32_P186889	0.000527	NM_153649	NM_153649	Homo sapiens tropomyosin 3 (TPM3), transcript variant 2, mRNA [NM_153649]	NM_153649
A_24_P366555	0.000528	BC032715	BC032715	Homo sapiens parathyroid hormone-responsive B1 gene, mRNA (cDNA clone IMAGE:5519851), complete cds. [BC032715]	
A_23_P407551	0.000528	NM_015250	NM_015250	Homo sapiens bicaudal D homolog 2 (Drosophila) (BICD2), transcript variant 2, mRNA [NM_015250]	NM_015250
A_23_P56127	0.000529	NM_019108	NM_019108	Homo sapiens hypothetical protein FLJ12886 (FLJ12886), mRNA [NM_019108]	NM_019108
A_23_P123539	0.000529	NM_002717	NM_002717	Homo sapiens protein phosphatase 2 (formerly 2A), regulatory subunit B (PR 52), alpha isoform (PPP2R2A), mRNA [NM_002717]	NM_002717
A_32_P147241	0.000529	NM_182470	NM_182470	Homo sapiens pyruvate kinase, muscle (PKM2), transcript variant 2, mRNA [NM_182470]	NM_182470
A_23_P11032	0.00053	NM_032591	NM_032591	Homo sapiens solute carrier family 9 (sodium/hydrogen exchanger), isoform 7 (SLC9A7), mRNA [NM_032591]	NM_032591
A_23_P654	0.00053	NM_003443	NM_003443	Homo sapiens zinc finger and BTB domain containing 17 (ZBTB17), mRNA [NM_003443]	NM_003443
A_24_P143076	0.000531	NM_144671	NM_144671	Homo sapiens hypothetical protein FLJ32356 (FLJ32356), mRNA [NM_144671]	NM_144671
A_24_P6889	0.000532	NM_006099	NM_006099	Homo sapiens protein inhibitor of activated STAT, 3 (PIAS3), mRNA [NM_006099]	NM_006099
A_32_P126410	0.000532	AK000901	AK000901	Homo sapiens cDNA FLJ10039 fis, clone HEMBA1000975. [AK000901]	
A_23_P13202	0.000533	A_23_P13202			
A_23_P123544	0.000534	NM_017444	NM_017444	Homo sapiens chromatin accessibility complex 1 (CHRAC1), mRNA [NM_017444]	NM_017444
A_24_P201491	0.000535	NM_004927	NM_004927	Homo sapiens mitochondrial ribosomal protein L49 (MRPL49), nuclear gene encoding mitochondrial protein, mRNA [NM_004927]	NM_004927
A_24_P461497	0.000536	ENST00000338852		PREDICTED: Homo sapiens similar to actin 3 - fruit fly (Drosophila melanogaster) (fragments) (LOC441836), mRNA [XM_497605]	XM_497605
A_23_P104607	0.000536	NM_002804	NM_002804	Homo sapiens proteasome (prosome, macropain) 26S subunit, ATPase, 3 (PSMC3), mRNA [NM_002804]	NM_002804
A_23_P154330	0.000536	NM_005783	NM_005783	Homo sapiens thioredoxin domain containing 9 (TXNDC9), mRNA [NM_005783]	NM_005783
A_24_P282309	0.000537	NM_133337	NM_133337	Homo sapiens fer-1-like 3, myoferlin (C. elegans) (FER1L3), transcript variant 2, mRNA [NM_133337]	NM_133337
A_23_P88280	0.000537	NM_138376	NM_138376	Homo sapiens tetratricopeptide repeat domain 5 (TTC5), mRNA [NM_138376]	NM_138376
A_23_P2114	0.000537	NM_017907	NM_017907	Homo sapiens hypothetical protein FLJ20625 (FLJ20625), mRNA [NM_017907]	NM_017907
A_23_P122782	0.000537	NM_030752	NM_030752	Homo sapiens t-complex 1 (TCP1), transcript variant 1, mRNA [NM_030752]	NM_030752
A_24_P41662	0.000538	A_24_P41662			
A_32_P808	0.000538	BC040993	BC040993	Homo sapiens KIAA1458 protein, mRNA (cDNA clone MGC:43785 IMAGE:5272534), complete cds. [BC040993]	XM_044434
A_23_P64954	0.000538	NM_003481	NM_003481	Homo sapiens ubiquitin specific protease 5 (isopeptidase T) (USP5), mRNA [NM_003481]	NM_003481
A_32_P758423	0.000539	AL833908	AL833908	Homo sapiens mRNA; cDNA DKFZp434L2319 (from clone DKFZp434L2319). [AL833908]	XM_062871
A_23_P46740	0.00054	NM_183058	NM_183058	Homo sapiens lysozyme-like 2 (LYZL2), mRNA [NM_183058]	NM_183058
A_24_P264154	0.00054	NM_014157	NM_014157	Homo sapiens HSPC065 protein (HSPC065), mRNA [NM_014157]	NM_014157
A_24_P348326	0.00054	NM_004843	NM_004843	Homo sapiens interleukin 27 receptor, alpha (IL27RA), mRNA [NM_004843]	NM_004843
A_24_P196562	0.000541	NM_021603	NM_021603	Homo sapiens FXYD domain containing ion transport regulator 2 (FXYD2), transcript variant b, mRNA [NM_021603]	NM_021603
A_24_P109644	0.000541	AF229166	AF229166	Homo sapiens docking protein 1-like protein mRNA, partial cds. [AF229166]	
A_24_P406591	0.000543	THC2339144		Q8GUS1 (Q8GUS1) NADPH:P450 reductase, partial (3%) [THC2339144]	
A_24_P247608	0.000544	NM_005389	NM_005389	Homo sapiens protein-L-isoaspartate (D-aspartate) O-methyltransferase (PCMT1), mRNA [NM_005389]	NM_005389
A_23_P357104	0.000544	NM_001155	NM_001155	Homo sapiens annexin A6 (ANXA6), transcript variant 1, mRNA [NM_001155]	NM_001155
A_24_P100742	0.000545	NM_014189	NM_014189	Homo sapiens adducin 1 (alpha) (ADD1), transcript variant 2, mRNA [NM_014189]	NM_014189
A_24_P777185	0.000545	A_24_P777185			
A_24_P331711	0.000545	NM_182578	NM_182578	Homo sapiens hypothetical protein FLJ37964 (FLJ37964), mRNA [NM_182578]	NM_182578
A_23_P135769	0.000545	NM_001101	NM_001101	Homo sapiens actin, beta (ACTB), mRNA [NM_001101]	NM_001101
A_23_P110896	0.000546	NM_012434	NM_012434	Homo sapiens solute carrier family 17 (anion/sugar transporter), member 5 (SLC17A5), mRNA [NM_012434]	NM_012434
A_24_P145229	0.000546	NM_032816	NM_032816	Homo sapiens hypothetical protein FLJ14640 (FLJ14640), mRNA [NM_032816]	NM_032816
A_32_P26443	0.000546	THC2258710			

A_23_P77630	0.000547	NM_022818	NM_022818	Homo sapiens microtubule-associated protein 1 light chain 3 beta (MAP1LC3B), mRNA [NM_022818]	NM_022818
A_24_P401150	0.000547	XM_373367	XM_373367	PREDICTED: Homo sapiens similar to chromosome 14 open reading frame 87 (LOC392531), mRNA [XM_373367]	XM_373367
A_24_P264416	0.000547	A_24_P264416			
A_24_P258633	0.000547	NM_006086	NM_006086	Homo sapiens tubulin, beta 3 (TUBB3), mRNA [NM_006086]	NM_006086
A_23_P153583	0.000548	NM_006247	NM_006247	Homo sapiens protein phosphatase 5, catalytic subunit (PPP5C), mRNA [NM_006247]	NM_006247
A_24_P316127	0.000548	AF255792	AF255792	Homo sapiens DC30 mRNA, complete cds. [AF255792]	
A_24_P88079	0.000549	AK096772	AK096772	Homo sapiens cDNA FLJ39453 fis, clone PROST2010046, highly similar to Homo sapiens secretory mucin MUC6 (MUC6) mRNA. [AK096772]	XM_290540
A_23_P218486	0.000549	NM_016145	NM_016145	Homo sapiens PTD008 protein (PTD008), mRNA [NM_016145]	NM_016145
A_32_P511713	0.00055	NM_173525	NM_173525	Homo sapiens hypothetical protein MGC34805 (MGC34805), mRNA [NM_173525]	NM_173525
A_23_P23646	0.000551	NM_007122	NM_007122	Homo sapiens upstream transcription factor 1 (USF1), transcript variant 1, mRNA [NM_007122]	NM_007122
A_32_P79115	0.000551	BX093077	BX093077	BX093077 BX093077 Soares breast 2NbHBst Homo sapiens cDNA clone IMAGp998O23240 ; IMAGE:153838, mRNA sequence [BX093077]	
A_24_P44931	0.000551	NM_032868	NM_032868	Homo sapiens hypothetical protein FLJ14981 (FLJ14981), mRNA [NM_032868]	NM_032868
A_24_P346587	0.000552	NM_014637	NM_014637	Homo sapiens chondrocyte protein with a poly-proline region (CHPPR), mRNA [NM_014637]	NM_014637
A_23_P44533	0.000553	NM_153268	NM_153268	Homo sapiens phosphatidylinositol-specific phospholipase C, X domain containing 2 (PLCXD2), mRNA [NM_153268]	NM_153268
A_23_P417113	0.000553	BC011923	BC011923	Homo sapiens proline rich 8, mRNA (cDNA clone MGC:20460 IMAGE:4542314), complete cds. [BC011923]	
A_23_P363196	0.000553	NM_014418	NM_014418	Homo sapiens T-cell leukemia/lymphoma 6 (TCL6), transcript variant TCL6a2, mRNA [NM_014418]	NM_014418
A_24_P189458	0.000553	NM_017615	NM_017615	Homo sapiens chromosome 10 open reading frame 86 (C10orf86), mRNA [NM_017615]	NM_017615
A_23_P9836	0.000554	NM_004454	NM_004454	Homo sapiens ets variant gene 5 (ets-related molecule) (ETV5), mRNA [NM_004454]	NM_004454
A_23_P213468	0.000554	A_23_P213468			
A_23_P143662	0.000554	NM_014338	NM_014338	Homo sapiens phosphatidylserine decarboxylase (PISD), mRNA [NM_014338]	NM_014338
A_23_P128706	0.000554	NM_001376	NM_001376	Homo sapiens dynein, cytoplasmic, heavy polypeptide 1 (DNCH1), mRNA [NM_001376]	NM_001376
A_24_P375728	0.000555	NM_138383	NM_138383	Homo sapiens hypothetical protein BC002770 (LOC92154), mRNA [NM_138383]	NM_138383
A_32_P493225	0.000557	NM_198988	NM_198988	Homo sapiens leukocyte receptor cluster (LRC) member 9 (LENG9), mRNA [NM_198988]	NM_198988
A_24_P289573	0.000557	A_24_P289573			
A_23_P25913	0.000557	NM_031427	NM_031427	Homo sapiens chromosome 14 open reading frame 168 (C14orf168), mRNA [NM_031427]	NM_031427
A_24_P204334	0.000557	A_24_P204334			
A_23_P154070	0.000557	NM_006000	NM_006000	Homo sapiens tubulin, alpha 1 (testis specific) (TUBA1), mRNA [NM_006000]	NM_006000
A_24_P179013	0.000558	ENST00000333462		GB|AL031963.40|CAD70623.1 dJ40E16.3 (novel gene similar to D. melanogaster CG5327) [Homo sapiens] [NP1083521]	
A_24_P121171	0.000558	NM_015517	NM_015517	Homo sapiens MBD2 (methyl-CpG-binding protein)-interacting zinc finger protein (MIZF), transcript variant 1, mRNA [NM_015517]	NM_015517
A_23_P118306	0.000558	NM_005147	NM_005147	Homo sapiens DnaJ (Hsp40) homolog, subfamily A, member 3 (DNAJA3), mRNA [NM_005147]	NM_005147
A_23_P118038	0.000558	NM_005796	NM_005796	Homo sapiens nuclear transport factor 2 (NUTF2), mRNA [NM_005796]	NM_005796
A_24_P226267	0.000559	NM_000806	NM_000806	Homo sapiens gamma-aminobutyric acid (GABA) A receptor, alpha 1 (GABRA1), mRNA [NM_000806]	NM_000806
A_32_P163233	0.000559	THC2444653		BC031068 AADAT protein {Homo sapiens;} , partial (8%) [THC2444653]	
A_24_P349356	0.00056	NM_207354	NM_207354	Homo sapiens ankyrin repeat domain 13 family, member D (ANKRD13D), mRNA [NM_207354]	NM_207354
A_24_P127462	0.00056	A_24_P127462			
A_23_P109420	0.00056	NM_080926	NM_080926	Homo sapiens hypothetical protein similar to KIAA0187 gene product (LOC96610), mRNA [NM_080926]	NM_080926
A_23_P48266	0.000561	NM_153348	NM_153348	Homo sapiens F-box and WD-40 domain protein 8 (FBXW8), transcript variant 1, mRNA [NM_153348]	NM_153348
A_23_P132468	0.000562	NM_003615	NM_003615	Homo sapiens solute carrier family 4, sodium bicarbonate cotransporter, member 7 (SLC4A7), mRNA [NM_003615]	NM_003615
A_23_P139919	0.000562	NM_018413	NM_018413	Homo sapiens carbohydrate (chondroitin 4) sulfotransferase 11 (CHST11), mRNA [NM_018413]	NM_018413
A_24_P235316	0.000562	NM_005662	NM_005662	Homo sapiens voltage-dependent anion channel 3 (VDAC3), mRNA [NM_005662]	NM_005662
A_32_P40611	0.000563	A_32_P40611			
A_24_P81473	0.000563	NM_002079	NM_002079	Homo sapiens glutamic-oxaloacetic transaminase 1, soluble (aspartate aminotransferase 1) (GOT1), mRNA [NM_002079]	NM_002079
A_24_P409560	0.000564	A_24_P409560			

A_32_P99753	0.000564	NM_207307	NM_207307	Homo sapiens hypothetical protein LOC90288 (LOC90288), mRNA [NM_207307]	NM_207307
A_23_P97161	0.000564	NM_003594	NM_003594	Homo sapiens transcription termination factor, RNA polymerase II (TTF2), mRNA [NM_003594]	NM_003594
A_23_P69188	0.000564	NM_206831	NM_206831	Homo sapiens zinc finger, CSL-type containing 2 (ZCSL2), mRNA [NM_206831]	NM_206831
A_24_P100695	0.000566	ENST00000255145		Homo sapiens mRNA; cDNA DKFZp686H07111 (from clone DKFZp686H07111). [BX647082]	XM_372866
A_24_P350228	0.000566	NM_021945	NM_021945	Homo sapiens chromosome 6 open reading frame 85 (C6orf85), mRNA [NM_021945]	NM_021945
A_23_P414884	0.000566	NM_004382	NM_004382	Homo sapiens corticotropin releasing hormone receptor 1 (CRHR1), mRNA [NM_004382]	NM_004382
A_23_P165380	0.000567	NM_014617	NM_014617	Homo sapiens crystallin, gamma A (CRYGA), mRNA [NM_014617]	NM_014617
A_32_P72466	0.000567	NM_024771	NM_024771	Homo sapiens hypothetical protein FLJ13848 (FLJ13848), mRNA [NM_024771]	NM_024771
A_23_P82588	0.000567	NM_197964	NM_197964	Homo sapiens hypothetical protein HSPC268 (HSPC268), mRNA [NM_197964]	NM_197964
A_24_P902052	0.000567	CR611723	CR611723	full-length cDNA clone CS0DK002YP11 of HeLa cells Cot 25-normalized of Homo sapiens (human). [CR611723]	
A_23_P152284	0.000567	NM_024571	NM_024571	Homo sapiens chromosome 16 open reading frame 33 (C16orf33), mRNA [NM_024571]	NM_024571
A_23_P97265	0.000567	NM_017725	NM_017725	Homo sapiens G patch domain containing 4 (GPATC4), transcript variant 3, mRNA [NM_017725]	NM_017725
A_32_P65943	0.000568	NM_032378	NM_032378	Homo sapiens eukaryotic translation elongation factor 1 delta (guanine nucleotide exchange protein) (EEF1D), transcript variant 1, mRNA [NM_032378]	NM_032378
A_24_P410320	0.000569	AF091089	AF091089	Homo sapiens clone 666 unknown mRNA, partial sequence. [AF091089]	
A_23_P111188	0.000569	NM_005453	NM_005453	Homo sapiens zinc finger protein 297 (ZNF297), mRNA [NM_005453]	NM_005453
A_24_P379765	0.000569	NM_018221	NM_018221	Homo sapiens MOB1, Mps One Binder kinase activator-like 1B (yeast) (MOBK1B), mRNA [NM_018221]	NM_018221
A_24_P202154	0.000569	NM_024986	NM_024986	Homo sapiens hypothetical protein FLJ12331 (FLJ12331), mRNA [NM_024986]	NM_024986
A_32_P137939	0.000569	NM_001101	NM_001101	Homo sapiens actin, beta (ACTB), mRNA [NM_001101]	NM_001101
A_24_P759955	0.00057	THC2280373			
A_32_P181822	0.000571	R88965	R88965	R88965 ym98g05.r1 Soares adult brain N2b4HB55Y Homo sapiens cDNA clone IMAGE:167000 5', mRNA sequence [R88965]	
A_24_P232500	0.000571	NM_004927	NM_004927	Homo sapiens mitochondrial ribosomal protein L49 (MRPL49), nuclear gene encoding mitochondrial protein, mRNA [NM_004927]	NM_004927
A_23_P56868	0.000572	A_23_P56868			
A_23_P40936	0.000572	NM_003298	NM_003298	Homo sapiens nuclear receptor subfamily 2, group C, member 2 (NR2C2), mRNA [NM_003298]	NM_003298
A_23_P60240	0.000572	NM_032634	NM_032634	Homo sapiens phosphatidylinositol glycan, class O (PIGO), transcript variant 1, mRNA [NM_032634]	NM_032634
A_24_P305467	0.000573	AK024670	AK024670	Homo sapiens cDNA: FLJ21017 fis, clone CAE05907. [AK024670]	
A_23_P107214	0.000574	NM_201434	NM_201434	Homo sapiens RAB5C, member RAS oncogene family (RAB5C), transcript variant 1, mRNA [NM_201434]	NM_201434
A_23_P128154	0.000574	NM_032704	NM_032704	Homo sapiens tubulin alpha 6 (TUBA6), mRNA [NM_032704]	NM_032704
A_23_P57497	0.000575	NM_002473	NM_002473	Homo sapiens myosin, heavy polypeptide 9, non-muscle (MYH9), mRNA [NM_002473]	NM_002473
A_32_P48498	0.000576	BF197320	BF197320	BF197320 hr78g02.x1 NCI_CGAP_Kid11 Homo sapiens cDNA clone IMAGE:3134642 3', mRNA sequence [BF197320]	
A_24_P270814	0.000576	NM_016823	NM_016823	Homo sapiens v-crk sarcoma virus CT10 oncogene homolog (avian) (CRK), transcript variant II, mRNA [NM_016823]	NM_016823
A_23_P40039	0.000576	NM_031445	NM_031445	Homo sapiens hypothetical protein MGC4268 (MGC4268), mRNA [NM_031445]	NM_031445
A_23_P147331	0.000577	NM_178865	NM_178865	Homo sapiens tumor differentially expressed 2-like (TDE2L), mRNA [NM_178865]	NM_178865
A_23_P334635	0.000578	NM_003724	NM_003724	Homo sapiens jerky homolog (mouse) (JRK), mRNA [NM_003724]	NM_003724
A_23_P72330	0.000578	A_23_P72330			
A_24_P239364	0.000578	AB004064	AB004064	Homo sapiens mRNA for tomoregulin, complete cds. [AB004064]	
A_23_P118607	0.000578	NM_025267	NM_025267	Homo sapiens hypothetical protein MGC2744 (MGC2744), mRNA [NM_025267]	NM_025267
A_32_P192586	0.000579	A_32_P192586			
A_24_P23995	0.00058	BC012758	BC012758	Homo sapiens ring finger protein 187, mRNA (cDNA clone IMAGE:3633225), partial cds. [BC012758]	
A_32_P133244	0.00058	NM_002582	NM_002582	Homo sapiens poly(A)-specific ribonuclease (deadenylation nuclease) (PARN), mRNA [NM_002582]	NM_002582
A_23_P395911	0.000581	AK021860	AK021860	Homo sapiens cDNA FLJ11798 fis, clone HEMBA1006198, weakly similar to PROLINE-RICH PROTEIN MP-2 PRECURSOR. [AK021860]	
A_23_P425932	0.000582	NM_145206	NM_145206	Homo sapiens vesicle transport through interaction with t-SNAREs homolog 1A (yeast) (VTI1A), mRNA [NM_145206]	NM_145206
A_32_P220881	0.000582	AA807922	AA807922	AA807922 nu98d12.s1 NCI_CGAP_Pr22 Homo sapiens cDNA clone IMAGE:1218743 3', mRNA sequence [AA807922]	
A_24_P341938	0.000584	NM_024625	NM_024625	Homo sapiens zinc finger CCCH-type, antiviral 1 (ZC3HAV1), transcript variant 2, mRNA [NM_024625]	NM_024625
A_23_P151791	0.000584	NM_181657	NM_181657	Homo sapiens leukotriene B4 receptor (LTB4R), mRNA [NM_181657]	NM_181657

A_23_P2960	0.000584	NM_005163	NM_005163	Homo sapiens v-akt murine thymoma viral oncogene homolog 1 (AKT1), transcript variant 1, mRNA [NM_005163]	NM_005163
A_23_P48669	0.000584	NM_005192	NM_005192	Homo sapiens cyclin-dependent kinase inhibitor 3 (CDK2-associated dual specificity phosphatase) (CDKN3), mRNA [NM_005192]	NM_005192
A_32_P190316	0.000585	THC2377297			
A_23_P11279	0.000586	NM_018466	NM_018466	Homo sapiens glycosyltransferase 28 domain containing 1 (GLT28D1), mRNA [NM_018466]	NM_018466
A_32_P222335	0.000587	THC2274391		RIFK_HUMAN (Q969G6) Riboflavin kinase (ATP:riboflavin 5'-phosphotransferase) (Flavokinase) , complete [THC2274391]	
A_23_P65427	0.000587	NM_002818	NM_002818	Homo sapiens proteasome (prosome, macropain) activator subunit 2 (PA28 beta) (PSME2), mRNA [NM_002818]	NM_002818
A_24_P255218	0.000589	NM_000259	NM_000259	Homo sapiens myosin VA (heavy polypeptide 12, myoxin) (MYO5A), mRNA [NM_000259]	NM_000259
A_23_P144054	0.000589	NM_006254	NM_006254	Homo sapiens protein kinase C, delta (PRKCD), transcript variant 1, mRNA [NM_006254]	NM_006254
A_24_P319635	0.00059	NM_021960	NM_021960	Homo sapiens myeloid cell leukemia sequence 1 (BCL2-related) (MCL1), transcript variant 1, mRNA [NM_021960]	NM_021960
A_23_P2431	0.000591	NM_004054	NM_004054	Homo sapiens complement component 3a receptor 1 (C3AR1), mRNA [NM_004054]	NM_004054
A_32_P45153	0.000591	THC2441937			
A_24_P354800	0.000591	NM_002119	NM_002119	Homo sapiens major histocompatibility complex, class II, DO alpha (HLA-DOA), mRNA [NM_002119]	NM_002119
A_23_P16214	0.000591	NM_032301	NM_032301	Homo sapiens F-box and WD-40 domain protein 9 (FBXW9), mRNA [NM_032301]	NM_032301
A_23_P26745	0.000592	AK026675	AK026675	Homo sapiens cDNA: FLJ23022 fis, clone LNG01117. [AK026675]	
A_23_P427754	0.000592	NM_152219	NM_152219	Homo sapiens gap junction protein, chi 1, 31.9kDa (connexin 31.9) (GJC1), mRNA [NM_152219]	NM_152219
A_24_P854199	0.000592	NM_002634	NM_002634	Homo sapiens prohibitin (PHB), mRNA [NM_002634]	NM_002634
A_23_P351314	0.000593	NM_015891	NM_015891	Homo sapiens cell division cycle 40 homolog (yeast) (CDC40), mRNA [NM_015891]	NM_015891
A_24_P298928	0.000594	A_24_P298928			
A_24_P285378	0.000594	NM_019118	NM_019118	Homo sapiens chromosome 1 open reading frame 91 (C1orf91), mRNA [NM_019118]	NM_019118
A_24_P348203	0.000594	NM_025061	NM_025061	Homo sapiens leucine rich repeat containing 8 family, member E (LRRC8E), mRNA [NM_025061]	NM_025061
A_24_P362394	0.000594	NM_007259	NM_007259	Homo sapiens vacuolar protein sorting 45A (yeast) (VPS45A), mRNA [NM_007259]	NM_007259
A_23_P97892	0.000597	NM_003893	NM_003893	Homo sapiens LIM domain binding 1 (LDB1), mRNA [NM_003893]	NM_003893
A_24_P392271	0.000597	A_24_P392271			
A_23_P64567	0.000598	NM_016147	NM_016147	Homo sapiens protein phosphatase methylesterase-1 (PME-1), mRNA [NM_016147]	NM_016147
A_24_P910121	0.000598	THC2286456		MRIP_HUMAN (Q8NFW9) Rab effector MyRIP (Myosin-VIIa- and Rab-interacting protein) (Exophilin 8) (Slp homolog lacking C2 domains-c), partial (5%) [THC2286456]	
A_24_P917866	0.000598	NM_003011	NM_003011	Homo sapiens SET translocation (myeloid leukemia-associated) (SET), mRNA [NM_003011]	NM_003011
A_23_P301995	0.000598	NM_173083	NM_173083	Homo sapiens lin-9 homolog (C. elegans) (LIN9), mRNA [NM_173083]	NM_173083
A_24_P690285	0.000598	AK027214	AK027214	Homo sapiens cDNA: FLJ23561 fis, clone LNG09974. [AK027214]	
A_23_P371613	0.000598	NM_203298	NM_203298	Homo sapiens coiled-coil-helix-coiled-coil-helix domain containing 1 (CHCHD1), mRNA [NM_203298]	NM_203298
A_24_P80633	0.000599	NM_001903	NM_001903	Homo sapiens catenin (cadherin-associated protein), alpha 1, 102kDa (CTNNA1), mRNA [NM_001903]	NM_001903
A_32_P60687	0.000601	AI278811	AI278811	AI278811 qo50a11.x1 NCI_CGAP_Co8 Homo sapiens cDNA clone IMAGE:1911932 3' similar to gb:K02276 MYC PROTO-ONCOGENE PROTEIN (HUMAN);, mRNA sequence [AI278811]	
A_24_P912372	0.000601	AB018297	AB018297	Homo sapiens mRNA for KIAA0754 protein, partial cds. [AB018297]	
A_24_P75778	0.000601	A_24_P75778			
A_23_P1523	0.000601	NM_014578	NM_014578	Homo sapiens ras homolog gene family, member D (RHOD), mRNA [NM_014578]	NM_014578
A_24_P230808	0.000601	NM_001002860	NM_001002860	Homo sapiens BTB (POZ) domain containing 7 (BTBD7), transcript variant 1, mRNA [NM_001002860]	NM_001002860
A_23_P316960	0.000602	NM_000837	NM_000837	Homo sapiens glutamate receptor, ionotropic, N-methyl D-asparate-associated protein 1 (glutamate binding) (GRINA), transcript variant 1, mRNA [NM_000837]	NM_000837
A_23_P115286	0.000602	NM_003921	NM_003921	Homo sapiens B-cell CLL/lymphoma 10 (BCL10), mRNA [NM_003921]	NM_003921
A_23_P165346	0.000602	NM_005687	NM_005687	Homo sapiens phenylalanine-tRNA synthetase-like, beta subunit (FARSLB), mRNA [NM_005687]	NM_005687
A_24_P332504	0.000603	A_24_P332504			
A_23_P429383	0.000603	NM_014213	NM_014213	Homo sapiens homeo box D9 (HOXD9), mRNA [NM_014213]	NM_014213
A_24_P914057	0.000603	M14755	M14755	Homo sapiens ABL1 protein (ABL1) mRNA, partial cds. [M14755]	
A_24_P351304	0.000604	NM_006839	NM_006839	Homo sapiens inner membrane protein, mitochondrial (mitofilin) (IMMT), mRNA [NM_006839]	NM_006839

A_24_P125993	0.000605	NM_004712	NM_004712	Homo sapiens hepatocyte growth factor-regulated tyrosine kinase substrate (HGS), mRNA [NM_004712]	NM_004712
A_23_P68824	0.000606	NM_003073	NM_003073	Homo sapiens SWI/SNF related, matrix associated, actin dependent regulator of chromatin, subfamily b, member 1 (SMARCB1), transcript variant 1, mRNA [NM_003073]	NM_003073
A_24_P7642	0.000606	NM_001444	NM_001444	Homo sapiens fatty acid binding protein 5 (psoriasis-associated) (FABP5), mRNA [NM_001444]	NM_001444
A_23_P340922	0.000607	NM_032370	NM_032370	Homo sapiens zinc finger protein 414 (ZNF414), mRNA [NM_032370]	NM_032370
A_32_P233155	0.000607	BX105253	BX105253	BX105253 Soares_testis_NHT Homo sapiens cDNA clone IMAGp998E224410, mRNA sequence [BX105253]	
A_24_P942805	0.000607	CR607189	CR607189	full-length cDNA clone CS0DK007YF11 of HeLa cells Cot 25-normalized of Homo sapiens (human). [CR607189]	
A_24_P212851	0.000607	NM_003072	NM_003072	Homo sapiens SWI/SNF related, matrix associated, actin dependent regulator of chromatin, subfamily a, member 4 (SMARCA4), mRNA [NM_003072]	NM_003072
A_24_P290263	0.000608	A_24_P290263			
A_23_P161488	0.000608	NM_003252	NM_003252	Homo sapiens TIA1 cytotoxic granule-associated RNA binding protein-like 1 (TIAL1), transcript variant 1, mRNA [NM_003252]	NM_003252
A_24_P117803	0.000609	ENST00000252509		GB|AL589786.8|CAD13460.1 bA161I19.3 (similar to ribosomal protein L36) [Homo sapiens] [NP414444]	
A_24_P334445	0.000609	NM_001017405	NM_001017405	Homo sapiens macrophage erythroblast attacher (MAEA), transcript variant 1, mRNA [NM_001017405]	NM_001017405
A_23_P257905	0.00061	NM_001810	NM_001810	Homo sapiens centromere protein B, 80kDa (CENPB), mRNA [NM_001810]	NM_001810
A_24_P379727	0.00061	NM_032309	NM_032309	Homo sapiens coiled-coil-helix-coiled-coil-helix domain containing 5 (CHCHD5), mRNA [NM_032309]	NM_032309
A_24_P720202	0.00061	NM_022170	NM_022170	Homo sapiens Williams-Beuren syndrome chromosome region 1 (WBSCR1), transcript variant 1, mRNA [NM_022170]	NM_022170
A_32_P171921	0.000611	THC2265989		BI333945 602997212F1 NIH_MGC_12 Homo sapiens cDNA clone IMAGE:5139483 5', mRNA sequence [BI333945]	
A_23_P75441	0.000611	NM_032344	NM_032344	Homo sapiens nudix (nucleoside diphosphate linked moiety X)-type motif 22 (NUDT22), mRNA [NM_032344]	NM_032344
A_23_P10699	0.000612	CR624966	CR624966	full-length cDNA clone CS0DK008YO11 of HeLa cells Cot 25-normalized of Homo sapiens (human). [CR624966]	
A_24_P263524	0.000612	NM_005783	NM_005783	Homo sapiens thioredoxin domain containing 9 (TXNDC9), mRNA [NM_005783]	NM_005783
A_23_P427634	0.000613	NM_080871	NM_080871	Homo sapiens ankyrin repeat and SOCS box-containing 10 (ASB10), mRNA [NM_080871]	NM_080871
A_24_P416301	0.000613	NM_004514	NM_004514	Homo sapiens forkhead box K2 (FOXK2), transcript variant 1, mRNA [NM_004514]	NM_004514
A_23_P54692	0.000614	AK022609	AK022609	Homo sapiens cDNA FLJ12547 fis, clone NT2RM4000634. [AK022609]	
A_32_P87145	0.000615	A_32_P87145			
A_24_P837085	0.000615	A_24_P837085			
A_32_P111274	0.000615	CD671824	CD671824	CD671824 fg07f07.x1 Human Iris cDNA (Normalized): fg Homo sapiens cDNA clone fg07f07 3', mRNA sequence [CD671824]	
A_23_P95292	0.000615	NM_014741	NM_014741	Homo sapiens KIAA0652 gene product (KIAA0652), mRNA [NM_014741]	NM_014741
A_24_P242440	0.000615	NM_003780	NM_003780	Homo sapiens UDP-Gal:betaGlcNAc beta 1,4- galactosyltransferase, polypeptide 2 (B4GALT2), transcript variant 2, mRNA [NM_003780]	NM_003780
A_24_P255628	0.000616	NM_015197	NM_015197	Homo sapiens phosphofurin acidic cluster sorting protein 2 (PACS2), mRNA [NM_015197]	NM_015197
A_23_P64010	0.000616	NM_014342	NM_014342	Homo sapiens mitochondrial carrier homolog 2 (C. elegans) (MTCH2), nuclear gene encoding mitochondrial protein, mRNA [NM_014342]	NM_014342
A_32_P41496	0.000617	THC2457445		Q8NFZ5 (Q8NFZ5) Fetal liver LKB1-interacting protein, partial (28%) [THC2457445]	
A_24_P41250	0.000617	A_24_P41250			
A_24_P247464	0.000619	A_24_P247464			
A_23_P158817	0.00062	AK130614	AK130614	Homo sapiens cDNA FLJ27104 fis, clone SPL04981, highly similar to Ig gamma-2 chain C region. [AK130614]	
A_24_P255654	0.00062	A_24_P255654			
A_23_P12147	0.000622	NM_152485	NM_152485	Homo sapiens chromosome 1 open reading frame 74 (C1orf74), mRNA [NM_152485]	NM_152485
A_23_P315206	0.000622	NM_004059	NM_004059	Homo sapiens cysteine conjugate-beta lyase; cytoplasmic (glutamine transaminase K, kyneurenine aminotransferase) (CCBL1), mRNA [NM_004059]	NM_004059
A_23_P219207	0.000622	NM_031407	NM_031407	Homo sapiens HECT, UBA and WWE domain containing 1 (HUWE1), mRNA [NM_031407]	NM_031407
A_23_P111020	0.000623	NM_003131	NM_003131	Homo sapiens serum response factor (c-fos serum response element-binding transcription factor) (SRF), mRNA [NM_003131]	NM_003131
A_23_P366253	0.000623	NM_032051	NM_032051	Homo sapiens zinc finger protein 278 (ZNF278), transcript variant 4, mRNA [NM_032051]	NM_032051
A_23_P3767	0.000624	NM_173215	NM_173215	Homo sapiens nuclear factor of activated T-cells 5, tonicity-responsive (NFAT5), transcript variant 5, mRNA [NM_173215]	NM_173215
A_24_P364296	0.000625	NM_001980	NM_001980	Homo sapiens epimorphin (EPIM), transcript variant 1, mRNA [NM_001980]	NM_001980
A_23_P3146	0.000626	NM_004863	NM_004863	Homo sapiens serine palmitoyltransferase, long chain base subunit 2 (SPTLC2), mRNA [NM_004863]	NM_004863
A_32_P364462	0.000627	NM_017805	NM_017805	Homo sapiens Ras interacting protein 1 (RASIP1), mRNA [NM_017805]	NM_017805
A_23_P103571	0.000628	NM_006699	NM_006699	Homo sapiens mannosidase, alpha, class 1A, member 2 (MAN1A2), mRNA [NM_006699]	NM_006699

A_23_P48080	0.000628	XR_000220	XR_000220	PREDICTED: Homo sapiens RPL13-2 pseudogene (LOC283345), misc RNA [XR_000220]	XR_000220
A_23_P214344	0.000629	AF218313	AF218313	Homo sapiens putative helicase RUVBL mRNA, complete cds. [AF218313]	
A_23_P414855	0.000629	NM_033224	NM_033224	Homo sapiens purine-rich element binding protein B (PURB), mRNA [NM_033224]	NM_033224
A_23_P1641	0.000629	NM_005133	NM_005133	Homo sapiens RCE1 homolog, prenyl protein protease (S. cerevisiae) (RCE1), mRNA [NM_005133]	NM_005133
A_24_P32560	0.00063	NM_198573	NM_198573	Homo sapiens GAAI470 (UNQ470), mRNA [NM_198573]	NM_198573
A_23_P68059	0.00063	NM_032673	NM_032673	Homo sapiens polycomb group ring finger 1 (PCGF1), mRNA [NM_032673]	NM_032673
A_23_P156739	0.00063	NM_032340	NM_032340	Homo sapiens chromosome 6 open reading frame 125 (C6orf125), mRNA [NM_032340]	NM_032340
A_23_P436407	0.000631	NM_005333	NM_005333	Homo sapiens holocytochrome c synthase (cytochrome c heme-lyase) (HCCS), mRNA [NM_005333]	NM_005333
A_24_P193961	0.000631	NM_004823	NM_004823	Homo sapiens potassium channel, subfamily K, member 6 (KCNK6), mRNA [NM_004823]	NM_004823
A_24_P262355	0.000631	NM_002634	NM_002634	Homo sapiens prohibitin (PHB), mRNA [NM_002634]	NM_002634
A_23_P420831	0.000632	NM_052828	NM_052828	Homo sapiens tripartite motif-containing 10 (TRIM10), transcript variant 2, mRNA [NM_052828]	NM_052828
A_24_P32118	0.000632	NM_015346	NM_015346	Homo sapiens zinc finger, FYVE domain containing 26 (ZFYVE26), mRNA [NM_015346]	NM_015346
A_32_P203184	0.000633	CR606809	CR606809	full-length cDNA clone CS0DI054YJ04 of Placenta Cot 25-normalized of Homo sapiens (human). [CR606809]	
A_24_P148653	0.000633	NM_018385	NM_018385	Homo sapiens hypothetical protein FLJ11301 (FLJ11301), mRNA [NM_018385]	NM_018385
A_24_P396980	0.000633	NM_053024	NM_053024	Homo sapiens profilin 2 (PFN2), transcript variant 1, mRNA [NM_053024]	NM_053024
A_23_P372151	0.000634	ENST00000248420		Homo sapiens NY-REN-24 antigen mRNA, partial cds. [AF155102]	
A_24_P409750	0.000634	A_24_P409750			
A_23_P18901	0.000634	NM_004707	NM_004707	Homo sapiens APG12 autophagy 12-like (S. cerevisiae) (APG12L), mRNA [NM_004707]	NM_004707
A_23_P164733	0.000635	NM_004756	NM_004756	Homo sapiens numb homolog (Drosophila)-like (NUMBL), mRNA [NM_004756]	NM_004756
A_32_P233091	0.000635	NR_002202	NR_002202	Homo sapiens ferritin, heavy polypeptide-like 7 (FTHL7) on chromosome 13 [NR_002202]	NR_002202
A_24_P106306	0.000635	NM_016093	NM_016093	Homo sapiens ribosomal protein L26-like 1 (RPL26L1), mRNA [NM_016093]	NM_016093
A_23_P23960	0.000635	NM_001001342	NM_001001342	Homo sapiens biogenesis of lysosome-related organelles complex-1, subunit 2 (BLOC1S2), transcript variant 2, mRNA [NM_001001342]	NM_001001342
A_24_P388703	0.000636	BC030111	BC030111	Homo sapiens hypothetical locus MGC42157, mRNA (cDNA clone MGC:42157 IMAGE:4799398), complete cds. [BC030111]	XM_498443
A_23_P399112	0.000636	NM_138373	NM_138373	Homo sapiens myeloid-associated differentiation marker (MYADM), transcript variant 2, mRNA [NM_138373]	NM_138373
A_23_P60069	0.000636	NM_005662	NM_005662	Homo sapiens voltage-dependent anion channel 3 (VDAC3), mRNA [NM_005662]	NM_005662
A_23_P56673	0.000637	NM_031288	NM_031288	Homo sapiens high mobility group AT-hook 1-like 4 (HMGA1L4), mRNA [NM_031288]	NM_031288
A_24_P285032	0.000637	NM_006123	NM_006123	Homo sapiens iduronate 2-sulfatase (Hunter syndrome) (IDS), transcript variant 2, mRNA [NM_006123]	NM_006123
A_32_P148507	0.000638	BE707436	BE707436	BE707436 QV2-HT0496-120700-272-e03 HT0496 Homo sapiens cDNA, mRNA sequence [BE707436]	
A_23_P420373	0.000638	NM_175630	NM_175630	Homo sapiens DNA (cytosine-5-)-methyltransferase 3 alpha (DNMT3A), transcript variant 4, mRNA [NM_175630]	NM_175630
A_24_P188116	0.000638	NM_020349	NM_020349	Homo sapiens ankyrin repeat domain 2 (stretch responsive muscle) (ANKRD2), mRNA [NM_020349]	NM_020349
A_32_P116813	0.000639	NM_020408	NM_020408	Homo sapiens chromosome 6 open reading frame 149 (C6orf149), mRNA [NM_020408]	NM_020408
A_23_P86917	0.000639	NM_003824	NM_003824	Homo sapiens Fas (TNFRSF6)-associated via death domain (FADD), mRNA [NM_003824]	NM_003824
A_23_P402899	0.000641	NM_144975	NM_144975	Homo sapiens hypothetical protein MGC19764 (MGC19764), mRNA [NM_144975]	NM_144975
A_24_P291401	0.000641	NM_153342	NM_153342	Homo sapiens fasting-inducible integral membrane protein TM6P1 (FLJ90024), mRNA [NM_153342]	NM_153342
A_23_P158007	0.000641	NM_032799	NM_032799	Homo sapiens zinc finger, DHHC-type containing 12 (ZDHHC12), mRNA [NM_032799]	NM_032799
A_24_P64524	0.000642	NM_173578	NM_173578	Homo sapiens hypothetical protein FLJ90834 (FLJ90834), mRNA [NM_173578]	NM_173578
A_24_P306788	0.000643	A_24_P306788			
A_23_P205101	0.000643	NM_025138	NM_025138	Homo sapiens chromosome 13 open reading frame 23 (C13orf23), transcript variant 1, mRNA [NM_025138]	NM_025138
A_24_P794515	0.000644	BC031316	BC031316	Homo sapiens cDNA clone IMAGE:5277380, partial cds. [BC031316]	
A_24_P102343	0.000645	NM_017957	NM_017957	Homo sapiens epsin 3 (EPN3), mRNA [NM_017957]	NM_017957
A_23_P334751	0.000645	NM_152490	NM_152490	Homo sapiens UDP-GalNAc:betaGlcNAc beta 1,3-galactosaminyltransferase, polypeptide 2 (B3GALNT2), mRNA [NM_152490]	NM_152490
A_23_P501745	0.000645	NM_153818	NM_153818	Homo sapiens peroxisome biogenesis factor 10 (PEX10), transcript variant 1, mRNA [NM_153818]	NM_153818
A_24_P230282	0.000646	NM_016378	NM_016378	Homo sapiens variable charge, X-linked 2 (VCX2), mRNA [NM_016378]	NM_016378

A_23_P16063	0.000646	NM_002088	NM_002088	Homo sapiens glutamate receptor, ionotropic, kainate 5 (GRIK5), mRNA [NM_002088]	NM_002088
A_24_P63608	0.000647	NM_017838	NM_017838	Homo sapiens nucleolar protein family A, member 2 (H/ACA small nucleolar RNPs) (NOLA2), mRNA [NM_017838]	NM_017838
A_23_P73702	0.000648	NM_005120	NM_005120	Homo sapiens mediator of RNA polymerase II transcription, subunit 12 homolog (yeast) (MED12), mRNA [NM_005120]	NM_005120
A_24_P16420	0.000649	NP106190		GB|X76785.1|CAA54180.1 hypothetical protein [NP106190]	
A_24_P93012	0.000649	ENST00000294573			
A_32_P191541	0.000649	BU853136	BU853136	AGENCOURT_10411121 NIH_MGC_82 Homo sapiens cDNA clone IMAGE:6619843 5', mRNA sequence [BU853136]	
A_23_P4885	0.000649	NM_014203	NM_014203	Homo sapiens adaptor-related protein complex 2, alpha 1 subunit (AP2A1), transcript variant 1, mRNA [NM_014203]	NM_014203
A_24_P218765	0.000649	THC2265991		RS3_HUMAN (P23396) 40S ribosomal protein S3, partial (96%) [THC2265991]	
A_32_P18523	0.000649	AI064892	AI064892	AI064892 HA0699 Human fetal liver cDNA library Homo sapiens cDNA, mRNA sequence [AI064892]	
A_24_P307046	0.000649	A_24_P307046			
A_24_P924602	0.00065	NM_199293	NM_199293	Homo sapiens tyrosine hydroxylase (TH), transcript variant 3, mRNA [NM_199293]	NM_199293
A_23_P54809	0.00065	NM_020982	NM_020982	Homo sapiens claudin 9 (CLDN9), mRNA [NM_020982]	NM_020982
A_23_P108265	0.000651	NM_012377	NM_012377	Homo sapiens olfactory receptor, family 7, subfamily C, member 2 (OR7C2), mRNA [NM_012377]	NM_012377
A_23_P396062	0.000655	NM_021168	NM_021168	Homo sapiens RAB40C, member RAS oncogene family (RAB40C), mRNA [NM_021168]	NM_021168
A_23_P502196	0.000655	NM_174855	NM_174855	Homo sapiens isocitrate dehydrogenase 3 (NAD+) beta (IDH3B), nuclear gene encoding mitochondrial protein, transcript variant 2, mRNA [NM_174855]	NM_174855
A_32_P7521	0.000656	AA065042	AA065042	AA065042 zm12g12.s1 Stratagene pancreas (#937208) Homo sapiens cDNA clone IMAGE:525478 3', mRNA sequence [AA065042]	
A_23_P27392	0.000658	NM_207324	NM_207324	Homo sapiens hypothetical protein LOC147650 (LOC147650), mRNA [NM_207324]	NM_207324
A_24_P161086	0.000658	NM_001001716	NM_001001716	Homo sapiens nuclear factor of kappa light polypeptide gene enhancer in B-cells inhibitor, beta (NFKBIB), transcript variant 2, mRNA [NM_001001716]	NM_001001716
A_32_P119998	0.000658	AI608782	AI608782	AI608782 tw94g05.x1 NCI_CGAP_HN6 Homo sapiens cDNA clone IMAGE:2267384 3' similar to gb:K00558 TUBULIN ALPHA-1 CHAIN (HUMAN);, mRNA sequence [AI608782]	
A_23_P149649	0.000658	NM_003000	NM_003000	Homo sapiens succinate dehydrogenase complex, subunit B, iron sulfur (Ip) (SDHB), mRNA [NM_003000]	NM_003000
A_23_P251562	0.000658	NM_007275	NM_007275	Homo sapiens tumor suppressor candidate 2 (TUSC2), mRNA [NM_007275]	NM_007275
A_23_P139297	0.000659	NM_017583	NM_017583	Homo sapiens tripartite motif-containing 44 (TRIM44), mRNA [NM_017583]	NM_017583
A_24_P256415	0.000661	AK094860	AK094860	Homo sapiens cDNA FLJ37541 fis, clone BRCAN2026340. [AK094860]	
A_24_P140621	0.000661	NM_024565	NM_024565	Homo sapiens hypothetical protein FLJ14166 (FLJ14166), mRNA [NM_024565]	NM_024565
A_23_P253301	0.000662	NM_053024	NM_053024	Homo sapiens profilin 2 (PFN2), transcript variant 1, mRNA [NM_053024]	NM_053024
A_24_P33105	0.000664	ENST00000327271			
A_32_P16258	0.000664	AK023791	AK023791	Homo sapiens cDNA FLJ13729 fis, clone PLACE3000121, weakly similar to VESICULAR TRAFFIC CONTROL PROTEIN SEC15. [AK023791]	XM_039570
A_23_P154801	0.000664	NM_015638	NM_015638	Homo sapiens transient receptor potential cation channel, subfamily C, member 4 associated protein (TRPC4AP), transcript variant 1, mRNA [NM_015638]	NM_015638
A_23_P126212	0.000666	NM_022111	NM_022111	Homo sapiens claspin homolog (Xenopus laevis) (CLSPN), mRNA [NM_022111]	NM_022111
A_23_P300056	0.000668	NM_044472	NM_044472	Homo sapiens cell division cycle 42 (GTP binding protein, 25kDa) (CDC42), transcript variant 2, mRNA [NM_044472]	NM_044472
A_32_P112279	0.000668	NM_017804	NM_017804	Homo sapiens decreased expression in renal and prostate (DERPC), transcript variant 1, mRNA [NM_017804]	NM_017804
A_24_P67534	0.000669	ENST00000330288		Homo sapiens, clone IMAGE:3868989, mRNA, partial cds. [BC008642]	
A_23_P301247	0.000673	NM_003517	NM_003517	Homo sapiens histone 2, H2ac (HIST2H2AC), mRNA [NM_003517]	NM_003517
A_24_P916718	0.000674	NM_207336	NM_207336	Homo sapiens zinc finger protein 467 (ZNF467), mRNA [NM_207336]	NM_207336
A_24_P941505	0.000674	NM_014612	NM_014612	Homo sapiens chromosome 9 open reading frame 10 (C9orf10), mRNA [NM_014612]	NM_014612
A_23_P63618	0.000676	NM_005063	NM_005063	Homo sapiens stearoyl-CoA desaturase (delta-9-desaturase) (SCD), mRNA [NM_005063]	NM_005063
A_32_P2177	0.000677	THC2283618			
A_23_P400945	0.000677	NM_005240	NM_005240	Homo sapiens ets variant gene 3 (ETV3), mRNA [NM_005240]	NM_005240
A_23_P70991	0.000677	NM_006303	NM_006303	Homo sapiens JTV1 gene (JTV1), mRNA [NM_006303]	NM_006303
A_23_P5610	0.000679	NM_001381	NM_001381	Homo sapiens docking protein 1, 62kDa (downstream of tyrosine kinase 1) (DOK1), mRNA [NM_001381]	NM_001381
A_23_P144311	0.000679	NM_000938	NM_000938	Homo sapiens polymerase (RNA) II (DNA directed) polypeptide B, 140kDa (POLR2B), mRNA [NM_000938]	NM_000938
A_23_P130107	0.00068	AK001683	AK001683	Homo sapiens cDNA FLJ10821 fis, clone NT2RP4001057. [AK001683]	
A_32_P160348	0.000682	THC2292718			

A_24_P145633	0.000682	NM_004767	NM_004767	Homo sapiens G-protein coupled receptor 37 like 1 (GPR37L1), mRNA [NM_004767]	NM_004767
A_24_P628384	0.000682	NM_001001655	NM_001001655	Homo sapiens similar to hypothetical protein 9530023G02 (MGC90512), mRNA [NM_001001655]	NM_001001655
A_23_P250948	0.000682	NM_001003694	NM_001003694	Homo sapiens bromodomain and PHD finger containing, 1 (BRPF1), transcript variant 1, mRNA [NM_001003694]	NM_001003694
A_24_P476533	0.000683	THC2381848			
A_24_P415624	0.000684	NM_014938	NM_014938	Homo sapiens Mlx interactor (MONDOA), mRNA [NM_014938]	NM_014938
A_23_P104876	0.000684	NM_017425	NM_017425	Homo sapiens sperm autoantigenic protein 17 (SPA17), mRNA [NM_017425]	NM_017425
A_23_P43086	0.000684	NM_018688	NM_018688	Homo sapiens bridging integrator 3 (BIN3), mRNA [NM_018688]	NM_018688
A_23_P81212	0.000684	NM_016067	NM_016067	Homo sapiens mitochondrial ribosomal protein S18C (MRPS18C), nuclear gene encoding mitochondrial protein, mRNA [NM_016067]	NM_016067
A_23_P325932	0.000685	NM_004371	NM_004371	Homo sapiens coatomer protein complex, subunit alpha (COPA), mRNA [NM_004371]	NM_004371
A_24_P917886	0.000685	ENST00000358378		H.sapiens (MAR11) MUC5AC mRNA for mucin (partial). [Z34282]	
A_24_P267452	0.000685	NM_012099	NM_012099	Homo sapiens CD3E antigen, epsilon polypeptide associated protein (CD3EAP), mRNA [NM_012099]	NM_012099
A_24_P89718	0.000687	NM_024926	NM_024926	Homo sapiens hypothetical protein FLJ12571 (FLJ12571), mRNA [NM_024926]	NM_024926
A_23_P415533	0.000687	AK054879	AK054879	Homo sapiens cDNA FLJ30317 fis, clone BRACE2003594. [AK054879]	
A_24_P532420	0.000687	AL080233	AL080233	Homo sapiens mRNA; cDNA DKFZp586L111 (from clone DKFZp586L111). [AL080233]	
A_24_P743806	0.000687	AJ420454	AJ420454	Homo sapiens mRNA full length insert cDNA clone EUROIMAGE 1517766. [AJ420454]	
A_24_P289753	0.000688	A_24_P289753			
A_24_P24453	0.000689	A_24_P24453			
A_24_P232049	0.000689	NM_014504	NM_014504	Homo sapiens RAB guanine nucleotide exchange factor (GEF) 1 (RABGEF1), mRNA [NM_014504]	NM_014504
A_23_P73609	0.00069	NM_000266	NM_000266	Homo sapiens Norrie disease (pseudoglioma) (NDP), mRNA [NM_000266]	NM_000266
A_24_P922606	0.00069	NM_015231	NM_015231	Homo sapiens nucleoporin 160kDa (NUP160), mRNA [NM_015231]	NM_015231
A_23_P111297	0.00069	NM_006638	NM_006638	Homo sapiens ribonuclease P 40kDa subunit (RPP40), mRNA [NM_006638]	NM_006638
A_23_P124164	0.000693	NM_020701	NM_020701	Homo sapiens KIAA1160 protein (KIAA1160), mRNA [NM_020701]	NM_020701
A_24_P187626	0.000694	A_24_P187626			
A_24_P383762	0.000695	NM_175062	NM_175062	Homo sapiens RasGEF domain family, member 1C (RASGEF1C), mRNA [NM_175062]	NM_175062
A_23_P37475	0.000695	NM_030800	NM_030800	Homo sapiens hypothetical protein DKFZp564O1664 (DKFZP564O1664), mRNA [NM_030800]	NM_030800
A_24_P133933	0.000696	NM_004309	NM_004309	Homo sapiens Rho GDP dissociation inhibitor (GDI) alpha (ARHGDIA), mRNA [NM_004309]	NM_004309
A_23_P38497	0.000697	NM_003562	NM_003562	Homo sapiens solute carrier family 25 (mitochondrial carrier; oxoglutarate carrier), member 11 (SLC25A11), mRNA [NM_003562]	NM_003562
A_23_P160438	0.000699	NM_002479	NM_002479	Homo sapiens myogenin (myogenic factor 4) (MYOG), mRNA [NM_002479]	NM_002479
A_32_P163469	0.000699	ENST00000323642		Homo sapiens mRNA; cDNA DKFZp686K2237 (from clone DKFZp686K2237). [AL833530]	
A_24_P283928	0.0007	NM_024068	NM_024068	Homo sapiens hypothetical protein MGC2731 (MGC2731), mRNA [NM_024068]	NM_024068
A_32_P194164	0.0007	AK092875	AK092875	Homo sapiens cDNA FLJ35556 fis, clone SPLEN2004844. [AK092875]	
A_23_P218126	0.0007	BC024289	BC024289	Homo sapiens cDNA clone MGC:39273 IMAGE:5440834, complete cds. [BC024289]	
A_24_P770494	0.000701	A_24_P770494			
A_23_P107048	0.000702	BC002811	BC002811	Homo sapiens cDNA clone IMAGE:3636371, partial cds. [BC002811]	
A_32_P11499	0.000702	NM_003348	NM_003348	Homo sapiens ubiquitin-conjugating enzyme E2N (UBC13 homolog, yeast) (UBE2N), mRNA [NM_003348]	NM_003348
A_24_P487766	0.000703	A_24_P487766			
A_23_P49082	0.000703	NM_012225	NM_012225	Homo sapiens nucleotide binding protein 2 (MinD homolog, E. coli) (NUBP2), mRNA [NM_012225]	NM_012225
A_23_P369471	0.000704	NM_178431	NM_178431	Homo sapiens late cornified envelope 3A (LCE3A), mRNA [NM_178431]	NM_178431
A_23_P42331	0.000704	NM_145904	NM_145904	Homo sapiens high mobility group AT-hook 1 (HMGA1), transcript variant 6, mRNA [NM_145904]	NM_145904
A_24_P384851	0.000707	NM_001007559	NM_001007559	Homo sapiens synovial sarcoma translocation, chromosome 18 (SS18), transcript variant 1, mRNA [NM_001007559]	NM_001007559
A_24_P313445	0.000708	NM_020912	NM_020912	Homo sapiens FLYWCH-type zinc finger 1 (FLYWCH1), transcript variant 2, mRNA [NM_020912]	NM_020912
A_24_P416728	0.000708	NM_198970	NM_198970	Homo sapiens amino-terminal enhancer of split (AES), transcript variant 3, mRNA [NM_198970]	NM_198970
A_24_P194184	0.000709	NM_199484	NM_199484	Homo sapiens chromosome 20 open reading frame 24 (C20orf24), transcript variant 3, mRNA [NM_199484]	NM_199484
A_24_P254965	0.00071	NM_018486	NM_018486	Homo sapiens histone deacetylase 8 (HDAC8), mRNA [NM_018486]	NM_018486

A_23_P68452	0.00071	NM_173179	NM_173179	Homo sapiens solute carrier family 35, member C2 (SLC35C2), transcript variant 1, mRNA [NM_173179]	NM_173179
A_32_P178503	0.000711	BC032064	BC032064	Homo sapiens, clone IMAGE:5163879, mRNA. [BC032064]	
A_23_P203323	0.000711	BC013255	BC013255	Homo sapiens hypothetical protein MGC10485, mRNA (cDNA clone IMAGE:3871805). [BC013255]	
A_32_P216004	0.000711	AF130091	AF130091	Homo sapiens clone FLB9630 PRO2603 mRNA, complete cds. [AF130091]	
A_23_P8539	0.000711	NM_139179	NM_139179	Homo sapiens KCCR13L (LOC221955), mRNA [NM_139179]	NM_139179
A_32_P91250	0.000711	NM_003347	NM_003347	Homo sapiens ubiquitin-conjugating enzyme E2L 3 (UBE2L3), transcript variant 1, mRNA [NM_003347]	NM_003347
A_23_P130293	0.000712	NM_015208	NM_015208	Homo sapiens ankyrin repeat domain 12 (ANKRD12), mRNA [NM_015208]	NM_015208
A_24_P220058	0.000712	NM_012325	NM_012325	Homo sapiens microtubule-associated protein, RP/EB family, member 1 (MAPRE1), mRNA [NM_012325]	NM_012325
A_32_P812442	0.000713	NM_181774	NM_181774	Homo sapiens solute carrier family 36 (proton/amino acid symporter), member 3 (SLC36A3), mRNA [NM_181774]	NM_181774
A_23_P51361	0.000713	NM_175852	NM_175852	Homo sapiens taxilin (DKFZp451J0118), mRNA [NM_175852]	NM_175852
A_23_P77833	0.000713	NM_024419	NM_024419	Homo sapiens phosphatidylglycerophosphate synthase (PGS1), mRNA [NM_024419]	NM_024419
A_23_P133174	0.000713	NM_018475	NM_018475	Homo sapiens TPA regulated locus (TPARL), mRNA [NM_018475]	NM_018475
A_32_P52076	0.000716	BX537551	BX537551	Homo sapiens mRNA; cDNA DKFZp686M0346 (from clone DKFZp686M0346). [BX537551]	
A_24_P194508	0.000716	NM_001006683	NM_001006683	Homo sapiens spindlin-like protein 2 (SPIN-2), transcript variant 3, mRNA [NM_001006683]	NM_001006683
A_24_P477516	0.000718	A_24_P477516			
A_23_P155807	0.000718	AY629351	AY629351	Homo sapiens DHHC domain-containing zinc finger protein mRNA, complete cds. [AY629351]	XM_376310
A_32_P121855	0.000718	THC2378865			
A_32_P318086	0.000718	NM_025207	NM_025207	Homo sapiens FAD-synthetase (PP591), transcript variant 1, mRNA [NM_025207]	NM_025207
A_32_P8396	0.00072	THC2374342		ALU7_HUMAN (P39194) Alu subfamily SQ sequence contamination warning entry, partial (15%) [THC2374342]	
A_24_P85322	0.000721	BC008927	BC008927	Homo sapiens zinc finger, FYVE domain containing 26, mRNA (cDNA clone IMAGE:3836316), partial cds. [BC008927]	
A_23_P98763	0.000721	NM_145309	NM_145309	Homo sapiens leucine rich repeat containing 51 (LRRC51), mRNA [NM_145309]	NM_145309
A_24_P911612	0.000722	A_24_P911612			
A_32_P72394	0.000722	NM_002735	NM_002735	Homo sapiens protein kinase, cAMP-dependent, regulatory, type I, beta (PRKAR1B), mRNA [NM_002735]	NM_002735
A_23_P106720	0.000722	NM_006453	NM_006453	Homo sapiens transducin (beta)-like 3 (TBL3), mRNA [NM_006453]	NM_006453
A_32_P61061	0.000722	NM_001008741	NM_001008741	Homo sapiens peptidylprolyl isomerase A-like (LOC388817), mRNA [NM_001008741]	NM_001008741
A_24_P182182	0.000722	NM_001152	NM_001152	Homo sapiens solute carrier family 25 (mitochondrial carrier; adenine nucleotide translocator), member 5 (SLC25A5), mRNA [NM_001152]	NM_001152
A_23_P98173	0.000725	NM_005608	NM_005608	Homo sapiens protein tyrosine phosphatase, receptor type, C-associated protein (PTPRCAP), mRNA [NM_005608]	NM_005608
A_24_P41149	0.000725	ENST00000308118		PREDICTED: Homo sapiens similar to 40S ribosomal protein S6 (Phosphoprotein NP33) (LOC440086), mRNA [XM_495912]	XM_495912
A_23_P7030	0.000725	NM_007208	NM_007208	Homo sapiens mitochondrial ribosomal protein L3 (MRPL3), nuclear gene encoding mitochondrial protein, mRNA [NM_007208]	NM_007208
A_23_P54477	0.000725	NM_018648	NM_018648	Homo sapiens nucleolar protein family A, member 3 (H/ACA small nucleolar RNPs) (NOLA3), mRNA [NM_018648]	NM_018648
A_24_P310667	0.000726	NM_031407	NM_031407	Homo sapiens HECT, UBA and WWE domain containing 1 (HUWE1), mRNA [NM_031407]	NM_031407
A_32_P115130	0.000727	NM_032477	NM_032477	Homo sapiens mitochondrial ribosomal protein L41 (MRPL41), nuclear gene encoding mitochondrial protein, mRNA [NM_032477]	NM_032477
A_24_P393322	0.000728	AK091963	AK091963	Homo sapiens cDNA FLJ34644 fis, clone KIDNE2017040. [AK091963]	
A_23_P100795	0.000728	NM_213662	NM_213662	Homo sapiens signal transducer and activator of transcription 3 (acute-phase response factor) (STAT3), transcript variant 3, mRNA [NM_213662]	NM_213662
A_23_P252362	0.000728	NM_016640	NM_016640	Homo sapiens mitochondrial ribosomal protein S30 (MRPS30), nuclear gene encoding mitochondrial protein, mRNA [NM_016640]	NM_016640
A_24_P58597	0.000728	A_24_P58597			
A_23_P350234	0.000728	NM_001012989	NM_001012989	Homo sapiens ubiquitin-conjugating enzyme E2N-like (UBE2NL), mRNA [NM_001012989]	NM_001012989
A_23_P300124	0.00073	NM_144775	NM_144775	Homo sapiens Smith-Magenis syndrome chromosome region, candidate 8 (SMCR8), mRNA [NM_144775]	NM_144775
A_24_P409361	0.00073	A_24_P409361			
A_23_P73559	0.00073	NM_004463	NM_004463	Homo sapiens FYVE, RhoGEF and PH domain containing 1 (faciogenital dysplasia) (FGD1), mRNA [NM_004463]	NM_004463
A_24_P24972	0.000731	ENST00000332804		full-length cDNA clone CS0DI026YJ08 of Placenta Cot 25-normalized of Homo sapiens (human). [CR590757]	XM_377760
A_23_P116890	0.000731	NM_006249	NM_006249	Homo sapiens proline-rich protein BstNI subfamily 3 (PRB3), mRNA [NM_006249]	NM_006249
A_23_P49009	0.000731	NM_153613	NM_153613	Homo sapiens PLSC domain containing protein (LOC254531), mRNA [NM_153613]	NM_153613
A_24_P348885	0.000731	NM_182580	NM_182580	Homo sapiens cytochrome b-561 domain containing 1 (CYB561D1), mRNA [NM_182580]	NM_182580

A_24_P324074	0.000731	A_24_P324074			
A_23_P56949	0.000732	NM_014755	NM_014755	Homo sapiens SERTA domain containing 2 (SERTAD2), mRNA [NM_014755]	NM_014755
A_32_P101623	0.000732	BG191459	BG191459	RST10553 Athersys RAGE Library Homo sapiens cDNA, mRNA sequence [BG191459]	
A_24_P15856	0.000733	ENST00000323662		Homo sapiens mRNA for KIAA1875 protein, partial cds. [AB058778]	
A_24_P916314	0.000734	NM_005338	NM_005338	Homo sapiens huntingtin interacting protein 1 (HIP1), mRNA [NM_005338]	NM_005338
A_24_P178963	0.000734	ENST00000332844		PREDICTED: Homo sapiens similar to unc-93 homolog B1; unc93 (C.elegans) homolog B; unc-93 related protein; unc93 (C. elegans) homolog B1 (LOC285479), mRNA [XM_211908]	XM_211908
A_23_P18887	0.000735	NM_022132	NM_022132	Homo sapiens methylcrotonoyl-Coenzyme A carboxylase 2 (beta) (MCCC2), mRNA [NM_022132]	NM_022132
A_24_P853366	0.000735	A_24_P853366			
A_23_P120660	0.000735	NM_001024	NM_001024	Homo sapiens ribosomal protein S21 (RPS21), mRNA [NM_001024]	NM_001024
A_24_P323916	0.000736	A_24_P323916			
A_24_P934755	0.000736	AB209519	AB209519	Homo sapiens mRNA for eukaryotic translation initiation factor 4E member 2 variant protein. [AB209519]	
A_23_P310552	0.000737	NM_147195	NM_147195	Homo sapiens FLJ35740 protein (FLJ35740), mRNA [NM_147195]	NM_147195
A_24_P328231	0.000737	NM_032179	NM_032179	Homo sapiens cleavage and polyadenylation specific factor 3-like (CPSF3L), transcript variant 2, mRNA [NM_032179]	NM_032179
A_24_P24244	0.000737	NM_001007026	NM_001007026	Homo sapiens atrophin 1 (ATN1), transcript variant 1, mRNA [NM_001007026]	NM_001007026
A_24_P193592	0.000738	NM_001761	NM_001761	Homo sapiens cyclin F (CCNF), mRNA [NM_001761]	NM_001761
A_23_P66063	0.000739	NM_020786	NM_020786	Homo sapiens pyruvate dehydrogenase phosphatase isoenzyme 2 (PDP2), mRNA [NM_020786]	NM_020786
A_24_P374962	0.000739	NM_018991	NM_018991	Homo sapiens DKFZp434A0131 protein (DKFZP434A0131), transcript variant 1, mRNA [NM_018991]	NM_018991
A_32_P35855	0.000739	BE612944	BE612944	BE612944 601451889T1 NIH_MGC_66 Homo sapiens cDNA clone IMAGE:3855646 3', mRNA sequence [BE612944]	
A_24_P384210	0.000739	A_24_P384210			
A_24_P660811	0.00074	NM_001013651	NM_001013651	Homo sapiens hypothetical gene supported by AK128318 (LOC389607), mRNA [NM_001013651]	NM_001013651
A_23_P303390	0.00074	NM_005378	NM_005378	Homo sapiens v-myc myelocytomatosis viral related oncogene, neuroblastoma derived (avian) (MYCN), mRNA [NM_005378]	NM_005378
A_24_P315066	0.000742	NM_031449	NM_031449	Homo sapiens hypothetical protein DKFZp761I2123 (DKFZp761I2123), transcript variant 1, mRNA [NM_031449]	NM_031449
A_23_P157449	0.000742	NM_005034	NM_005034	Homo sapiens polymerase (RNA) II (DNA directed) polypeptide K, 7.0kDa (POLR2K), mRNA [NM_005034]	NM_005034
A_23_P258531	0.000743	NM_002637	NM_002637	Homo sapiens phosphorylase kinase, alpha 1 (muscle) (PHKA1), mRNA [NM_002637]	NM_002637
A_23_P27606	0.000743	NM_004843	NM_004843	Homo sapiens interleukin 27 receptor, alpha (IL27RA), mRNA [NM_004843]	NM_004843
A_32_P165836	0.000744	NM_177966	NM_177966	Homo sapiens 2'-phosphodiesterase (2'-PDE), mRNA [NM_177966]	NM_177966
A_23_P136961	0.000744	A_23_P136961			
A_24_P391531	0.000745	NM_004375	NM_004375	Homo sapiens COX11 homolog, cytochrome c oxidase assembly protein (yeast) (COX11), nuclear gene encoding mitochondrial protein, mRNA [NM_004375]	NM_004375
A_24_P84898	0.000745	NM_004111	NM_004111	Homo sapiens flap structure-specific endonuclease 1 (FEN1), mRNA [NM_004111]	NM_004111
A_23_P50241	0.000746	NM_001294	NM_001294	Homo sapiens cleft lip and palate associated transmembrane protein 1 (CLPTM1), mRNA [NM_001294]	NM_001294
A_23_P253571	0.000746	NM_032758	NM_032758	Homo sapiens PHD finger protein 5A (PHF5A), mRNA [NM_032758]	NM_032758
A_23_P17624	0.000747	NM_003253	NM_003253	Homo sapiens T-cell lymphoma invasion and metastasis 1 (TIAM1), mRNA [NM_003253]	NM_003253
A_24_P307065	0.000747	A_24_P307065			
A_32_P155016	0.000749	NM_206833	NM_206833	Homo sapiens cortexin 1 (CTXN1), mRNA [NM_206833]	NM_206833
A_23_P51699	0.000749	NM_004723	NM_004723	Homo sapiens rho/rac guanine nucleotide exchange factor (GEF) 2 (ARHGEF2), mRNA [NM_004723]	NM_004723
A_24_P475814	0.00075	A_24_P475814			
A_23_P122387	0.00075	NM_018135	NM_018135	Homo sapiens mitochondrial ribosomal protein S18A (MRPS18A), nuclear gene encoding mitochondrial protein, mRNA [NM_018135]	NM_018135
A_24_P734720	0.000751	NM_152523	NM_152523	Homo sapiens hypothetical protein FLJ40432 (FLJ40432), mRNA [NM_152523]	NM_152523
A_23_P348383	0.000752	AB037766	AB037766	Homo sapiens mRNA for KIAA1345 protein, partial cds. [AB037766]	XM_106386
A_24_P20200	0.000752	NM_017958	NM_017958	Homo sapiens pleckstrin homology domain containing, family B (evectins) member 2 (PLEKHB2), mRNA [NM_017958]	NM_017958
A_23_P43613	0.000752	NM_182739	NM_182739	Homo sapiens NADH dehydrogenase (ubiquinone) 1 beta subcomplex, 6, 17kDa (NDUFB6), nuclear gene encoding mitochondrial protein, transcript variant 2, mRNA [NM_182739]	NM_182739
A_23_P67399	0.000753	NM_013403	NM_013403	Homo sapiens striatin, calmodulin binding protein 4 (STRN4), mRNA [NM_013403]	NM_013403
A_23_P215549	0.000754	NM_000940	NM_000940	Homo sapiens paraoxonase 3 (PON3), mRNA [NM_000940]	NM_000940

A_24_P155058	0.000754	NM_001556	NM_001556	Homo sapiens inhibitor of kappa light polypeptide gene enhancer in B-cells, kinase beta (IKBKB), mRNA [NM_001556]	NM_001556
A_24_P119405	0.000754	AK096566	AK096566	Homo sapiens cDNA FLJ39247 fis, clone OCBBF2008520. [AK096566]	
A_23_P106945	0.000754	NM_001114	NM_001114	Homo sapiens adenylate cyclase 7 (ADCY7), mRNA [NM_001114]	NM_001114
A_24_P13663	0.000754	BG530163	BG530163	602558656F1 NIH_MGC_61 Homo sapiens cDNA clone IMAGE:4696932 5', mRNA sequence [BG530163]	
A_32_P220127	0.000754	NM_033625	NM_033625	Homo sapiens ribosomal protein L34 (RPL34), transcript variant 2, mRNA [NM_033625]	NM_033625
A_24_P239731	0.000755	NM_004776	NM_004776	Homo sapiens UDP-Gal:betaGlcNAc beta 1,4- galactosyltransferase, polypeptide 5 (B4GALT5), mRNA [NM_004776]	NM_004776
A_23_P409626	0.000756	ENST00000360796		Homo sapiens mRNA; cDNA DKFZp762N1910 (from clone DKFZp762N1910). [AL834470]	XM_495877
A_23_P346405	0.000757	NM_138357	NM_138357	Homo sapiens chromosome 10 open reading frame 42 (C10orf42), mRNA [NM_138357]	NM_138357
A_23_P153827	0.000758	NM_005934	NM_005934	Homo sapiens myeloid/lymphoid or mixed-lineage leukemia (trithorax homolog, Drosophila); translocated to, 1 (MLLT1), mRNA [NM_005934]	NM_005934
A_24_P6903	0.000758	NM_001017992	NM_001017992	Homo sapiens similar to RIKEN cDNA 4732495G21 gene (DKFZp686D0972), mRNA [NM_001017992]	NM_001017992
A_23_P377318	0.000759	NM_153221	NM_153221	Homo sapiens cartilage intermediate layer protein 2 (CILP2), mRNA [NM_153221]	NM_153221
A_23_P121345	0.000759	NM_018138	NM_018138	Homo sapiens hypothetical protein FLJ10560 (FLJ10560), mRNA [NM_018138]	NM_018138
A_23_P375	0.000759	NM_018101	NM_018101	Homo sapiens cell division cycle associated 8 (CDCA8), mRNA [NM_018101]	NM_018101
A_23_P396626	0.00076	NM_007247	NM_007247	Homo sapiens AP1 gamma subunit binding protein 1 (AP1GBP1), transcript variant 1, mRNA [NM_007247]	NM_007247
A_24_P117672	0.00076	NM_021228	NM_021228	Homo sapiens serine arginine-rich pre-mRNA splicing factor SR-A1 (SR-A1), mRNA [NM_021228]	NM_021228
A_23_P36513	0.00076	NM_212461	NM_212461	Homo sapiens protein kinase, AMP-activated, gamma 1 non-catalytic subunit (PRKAG1), transcript variant 2, mRNA [NM_212461]	NM_212461
A_32_P185089	0.000761	A_32_P185089			
A_32_P195181	0.000762	AI380795	AI380795	AI380795 tg04c07.x1 NCI_CGAP_CLL1 Homo sapiens cDNA clone IMAGE:2107788 3' similar to gb:M16462 NADH-CYTOCHROME B5 REDUCTASE (HUMAN);, mRNA sequence [AI380795]	
A_32_P162797	0.000762	THC2283658			
A_24_P290799	0.000762	NM_024419	NM_024419	Homo sapiens phosphatidylglycerophosphate synthase (PGS1), mRNA [NM_024419]	NM_024419
A_24_P259490	0.000762	NM_001658	NM_001658	Homo sapiens ADP-ribosylation factor 1 (ARF1), transcript variant 4, mRNA [NM_001658]	NM_001658
A_24_P389409	0.000763	NM_006129	NM_006129	Homo sapiens bone morphogenetic protein 1 (BMP1), transcript variant BMP1-3, mRNA [NM_006129]	NM_006129
A_24_P116606	0.000764	NM_130791	NM_130791	Homo sapiens WW domain containing oxidoreductase (WWOX), transcript variant 6, mRNA [NM_130791]	NM_130791
A_24_P17945	0.000764	NM_003309	NM_003309	Homo sapiens TSPY-like 1 (TSPYL1), mRNA [NM_003309]	NM_003309
A_23_P116872	0.000764	NM_002723	NM_002723	Homo sapiens proline-rich protein BstNI subfamily 4 (PRB4), mRNA [NM_002723]	NM_002723
A_32_P198518	0.000766	THC2304443		HSU36501 SP100-B {Homo sapiens;} , partial (65%) [THC2304443]	
A_23_P363769	0.000766	NM_002284	NM_002284	Homo sapiens keratin, hair, basic, 6 (monilethrix) (KRTHB6), mRNA [NM_002284]	NM_002284
A_23_P25525	0.000766	NM_002097	NM_002097	Homo sapiens general transcription factor IIIA (GTF3A), mRNA [NM_002097]	NM_002097
A_24_P83544	0.000767	ENST00000242916		GB|AL136318.13|CAC10197.1 dJ254P11.1 (novel protein similar to RNA polymerase II elongation factor SIII, subunit P15) [NP239768]	
A_23_P254970	0.000767	ENST00000361355		Homo sapiens cDNA FLJ25070 fis, clone CBL05164. [AK057799]	
A_23_P137046	0.000767	NM_022567	NM_022567	Homo sapiens nyctalopin (NYX), mRNA [NM_022567]	NM_022567
A_23_P350059	0.000768	BC063642	BC063642	Homo sapiens phosphodiesterase 4D interacting protein (myomegalin), mRNA (cDNA clone IMAGE:4746110), complete cds. [BC063642]	
A_23_P128532	0.000768	NM_152318	NM_152318	Homo sapiens hypothetical protein MGC40397 (MGC40397), mRNA [NM_152318]	NM_152318
A_24_P332902	0.000769	A_24_P332902			
A_24_P240242	0.000771	NM_002375	NM_002375	Homo sapiens microtubule-associated protein 4 (MAP4), transcript variant 1, mRNA [NM_002375]	NM_002375
A_32_P135664	0.000771	AK129849	AK129849	Homo sapiens cDNA FLJ26339 fis, clone HRT02975. [AK129849]	
A_23_P61487	0.000772	NM_018205	NM_018205	Homo sapiens leucine rich repeat containing 20 (LRRC20), transcript variant 3, mRNA [NM_018205]	NM_018205
A_23_P359904	0.000773	NM_182612	NM_182612	Homo sapiens hypothetical protein FLJ34283 (FLJ34283), mRNA [NM_182612]	NM_182612
A_24_P376787	0.000774	NM_032752	NM_032752	Homo sapiens zinc finger protein 496 (ZNF496), mRNA [NM_032752]	NM_032752
A_23_P66289	0.000774	NM_024706	NM_024706	Homo sapiens zinc finger protein 668 (ZNF668), mRNA [NM_024706]	NM_024706
A_24_P382026	0.000775	NM_020679	NM_020679	Homo sapiens AD023 protein (AD023), mRNA [NM_020679]	NM_020679
A_24_P564462	0.000776	CN430223	CN430223	17000600171867 GRN_PREHEP Homo sapiens cDNA 5', mRNA sequence [CN430223]	
A_23_P6196	0.000776	NM_016558	NM_016558	Homo sapiens SCAN domain containing 1 (SCAND1), transcript variant 1, mRNA [NM_016558]	NM_016558

A_24_P83586	0.000777	NM_015506	NM_015506	Homo sapiens DKFZp564I122 protein (DKFZp564I122), mRNA [NM_015506]	NM_015506
A_24_P920016	0.000778	AK124651	AK124651	Homo sapiens cDNA FLJ42660 fis, clone BRAMY2010808. [AK124651]	XM_209656
A_24_P358462	0.000778	NM_198576	NM_198576	Homo sapiens agrin (AGRN), mRNA [NM_198576]	NM_198576
A_24_P38446	0.000778	NM_021174	NM_021174	Homo sapiens KIAA1967 (KIAA1967), transcript variant 1, mRNA [NM_021174]	NM_021174
A_24_P911571	0.000779	BC011671	BC011671	Homo sapiens guanine nucleotide binding protein (G protein), beta 5, mRNA (cDNA clone IMAGE:4131809), complete cds. [BC011671]	
A_24_P641130	0.000781	NM_080686	NM_080686	Homo sapiens HLA-B associated transcript 2 (BAT2), transcript variant 1, mRNA [NM_080686]	NM_080686
A_23_P329924	0.000782	NM_177551	NM_177551	Homo sapiens G protein-coupled receptor 109A (GPR109A), mRNA [NM_177551]	NM_177551
A_23_P346311	0.000782	NM_138762	NM_138762	Homo sapiens BCL2-associated X protein (BAX), transcript variant gamma, mRNA [NM_138762]	NM_138762
A_23_P382775	0.000783	NM_014417	NM_014417	Homo sapiens BCL2 binding component 3 (BBC3), mRNA [NM_014417]	NM_014417
A_23_P36521	0.000784	NM_001659	NM_001659	Homo sapiens ADP-ribosylation factor 3 (ARF3), mRNA [NM_001659]	NM_001659
A_23_P60016	0.000784	AF095289	AF095289	Homo sapiens pituitary tumor transforming gene protein 3 (PTTG3) mRNA, complete cds. [AF095289]	
A_23_P107087	0.000784	NM_018128	NM_018128	Homo sapiens hypothetical protein FLJ10534 (FLJ10534), mRNA [NM_018128]	NM_018128
A_24_P298700	0.000785	AF161340	AF161340	Homo sapiens HSPC077 mRNA, partial cds. [AF161340]	
A_32_P151087	0.000785	A_32_P151087			
A_23_P119295	0.000786	NM_016581	NM_016581	Homo sapiens signaling intermediate in Toll pathway, evolutionarily conserved (SITPEC), mRNA [NM_016581]	NM_016581
A_23_P145376	0.000787	NM_002754	NM_002754	Homo sapiens mitogen-activated protein kinase 13 (MAPK13), mRNA [NM_002754]	NM_002754
A_23_P9903	0.000792	NM_178326	NM_178326	Homo sapiens APG4 autophagy 4 homolog B (S. cerevisiae) (APG4B), transcript variant 2, mRNA [NM_178326]	NM_178326
A_23_P27332	0.000792	NM_003199	NM_003199	Homo sapiens transcription factor 4 (TCF4), mRNA [NM_003199]	NM_003199
A_24_P152793	0.000793	ENST00000324925			
A_24_P524519	0.000794	ENST00000334207			
A_24_P393087	0.000795	ENST00000342911			
A_23_P39542	0.000795	NM_001017927	NM_001017927	Homo sapiens hypothetical protein LOC130355 (LOC130355), mRNA [NM_001017927]	NM_001017927
A_23_P387943	0.000796	NM_032982	NM_032982	Homo sapiens caspase 2, apoptosis-related cysteine protease (neural precursor cell expressed, developmentally down-regulated 2) (CASP2), transcript variant 1, mRNA [NM_032982]	NM_032982
A_23_P70384	0.000796	NM_003958	NM_003958	Homo sapiens ring finger protein 8 (RNF8), transcript variant 1, mRNA [NM_003958]	NM_003958
A_24_P290354	0.000796	NM_006007	NM_006007	Homo sapiens zinc finger, A20 domain containing 2 (ZA20D2), mRNA [NM_006007]	NM_006007
A_23_P39008	0.000797	NM_080864	NM_080864	Homo sapiens relaxin 3 (RLN3), mRNA [NM_080864]	NM_080864
A_24_P130026	0.000797	NM_001015049	NM_001015049	Homo sapiens BCL2-associated athanogene 5 (BAG5), transcript variant 1, mRNA [NM_001015049]	NM_001015049
A_24_P71021	0.000797	NM_002074	NM_002074	Homo sapiens guanine nucleotide binding protein (G protein), beta polypeptide 1 (GNB1), mRNA [NM_002074]	NM_002074
A_23_P36795	0.000799	NM_005639	NM_005639	Homo sapiens synaptotagmin I (SYT1), mRNA [NM_005639]	NM_005639
A_23_P44781	0.0008	AK128546	AK128546	Homo sapiens cDNA FLJ46705 fis, clone TRACH3015346. [AK128546]	
A_32_P155776	0.0008	NM_001017421	NM_001017421	Homo sapiens actin-like protein (FKSG30), mRNA [NM_001017421]	NM_001017421
A_23_P217820	0.000801	NM_014762	NM_014762	Homo sapiens 24-dehydrocholesterol reductase (DHCR24), mRNA [NM_014762]	NM_014762
A_23_P28153	0.000801	AF175767	AF175767	Homo sapiens putative selenocysteine lyase (SCLY) mRNA, complete cds. [AF175767]	
A_23_P300826	0.000802	NM_145029	NM_145029	Homo sapiens chromosome 6 open reading frame 136 (C6orf136), mRNA [NM_145029]	NM_145029
A_23_P165162	0.000804	NM_021175	NM_021175	Homo sapiens hepcidin antimicrobial peptide (HAMP), mRNA [NM_021175]	NM_021175
A_24_P100605	0.000804	ENST00000202677			XM_046600
A_24_P75688	0.000805	A_24_P75688			
A_23_P204482	0.000805	NM_006861	NM_006861	Homo sapiens RAB35, member RAS oncogene family (RAB35), mRNA [NM_006861]	NM_006861
A_24_P595369	0.000806	AK023465	AK023465	Homo sapiens cDNA FLJ13403 fis, clone PLACE1001517, moderately similar to Homo sapiens gene for glycosylphosphatidylinositol anchor attachment 1 (GPAA1). [AK023465]	XM_496582
A_24_P280953	0.000807	A_24_P280953			
A_24_P674118	0.000809	A_24_P674118			
A_24_P362805	0.000809	NM_152776	NM_152776	Homo sapiens hypothetical protein MGC40579 (MGC40579), mRNA [NM_152776]	NM_152776
A_24_P238609	0.000809	NM_006674	NM_006674	Homo sapiens HLA complex P5 (HCP5), mRNA [NM_006674]	NM_006674

A_24_P556030	0.000809	A_24_P556030			
A_32_P98979	0.000809	THC2281332			
A_24_P117954	0.00081	NM_194281	NM_194281	Homo sapiens chromosome 18 open reading frame 37 (C18orf37), mRNA [NM_194281]	NM_194281
A_32_P230838	0.000811	BF085047	BF085047	BF085047 PM0-GN0018-130900-003-c12 GN0018 Homo sapiens cDNA, mRNA sequence [BF085047]	
A_32_P137461	0.000812	AA780485	AA780485	ac65f02.s1 Stratagene fetal retina 937202 Homo sapiens cDNA clone IMAGE:867483 3' similar to gb:X69392 60S RIBOSOMAL PROTEIN L26 (HUMAN);, mRNA sequence [AA780485]	
A_23_P141335	0.000812	NM_199282	NM_199282	Homo sapiens Rho GTPase activating protein 27 (ARHGAP27), mRNA [NM_199282]	NM_199282
A_24_P50767	0.000812	AF161342	AF161342	Homo sapiens HSPC079 mRNA, partial cds. [AF161342]	
A_24_P73848	0.000814	AK057261	AK057261	Homo sapiens cDNA FLJ32699 fis, clone TESTI2000435, weakly similar to CALDESMON. [AK057261]	
A_24_P7192	0.000815	NM_032446	NM_032446	Homo sapiens MEGF10 protein (MEGF10), mRNA [NM_032446]	NM_032446
A_24_P116871	0.000817	NM_006701	NM_006701	Homo sapiens thioredoxin-like 4A (TXNL4A), mRNA [NM_006701]	NM_006701
A_23_P157380	0.000818	NM_153033	NM_153033	Homo sapiens potassium channel tetramerisation domain containing 7 (KCTD7), mRNA [NM_153033]	NM_153033
A_24_P67618	0.00082	XM_374273	XM_374273	PREDICTED: Homo sapiens similar to Heat shock protein HSP 90-beta (HSP 84) (Tumor specific transplantation 84 kDa antigen) (TSTA) (LOC389669), mRNA [XM_374273]	XM_374273
A_32_P66523	0.000821	BM908037	BM908037	AGENCOURT_6707579 NIH_MGC_119 Homo sapiens cDNA clone IMAGE:5745013 5', mRNA sequence [BM908037]	
A_23_P82839	0.000821	NM_178819	NM_178819	Homo sapiens putative lysophosphatidic acid acyltransferase (DKFZp586M1819), mRNA [NM_178819]	NM_178819
A_24_P63642	0.000823	NM_003181	NM_003181	Homo sapiens T, brachyury homolog (mouse) (T), mRNA [NM_003181]	NM_003181
A_23_P119714	0.000823	NM_014173	NM_014173	Homo sapiens HSPC142 protein (HSPC142), mRNA [NM_014173]	NM_014173
A_23_P131502	0.000824	NM_153712	NM_153712	Homo sapiens tubulin tyrosine ligase (TTL), mRNA [NM_153712]	NM_153712
A_32_P75772	0.000824	THC2424993		ALU7_HUMAN (P39194) Alu subfamily SQ sequence contamination warning entry, partial (5%) [THC2424993]	
A_24_P292020	0.000824	NM_006234	NM_006234	Homo sapiens polymerase (RNA) II (DNA directed) polypeptide J, 13.3kDa (POLR2J), mRNA [NM_006234]	NM_006234
A_23_P100441	0.000825	NM_024946	NM_024946	Homo sapiens NEFA-interacting nuclear protein NIP30 (NIP30), mRNA [NM_024946]	NM_024946
A_23_P416242	0.000827	NM_178841	NM_178841	Homo sapiens ring finger protein 166 (RNF166), mRNA [NM_178841]	NM_178841
A_23_P9894	0.000827	NM_005788	NM_005788	Homo sapiens HMT1 hnRNP methyltransferase-like 3 (S. cerevisiae) (HRMT1L3), mRNA [NM_005788]	NM_005788
A_23_P112429	0.000827	NM_007209	NM_007209	Homo sapiens ribosomal protein L35 (RPL35), mRNA [NM_007209]	NM_007209
A_23_P366125	0.000828	ENST00000307394		Homo sapiens mRNA for FLJ00151 protein. [AK074080]	
A_24_P414045	0.000829	NM_006302	NM_006302	Homo sapiens glucosidase I (GCS1), mRNA [NM_006302]	NM_006302
A_23_P250122	0.000829	NM_020223	NM_020223	Homo sapiens family with sequence similarity 20, member C (FAM20C), mRNA [NM_020223]	NM_020223
A_23_P201845	0.00083	NM_007167	NM_007167	Homo sapiens zinc finger protein 258 (ZNF258), transcript variant 1, mRNA [NM_007167]	NM_007167
A_23_P65041	0.000831	AF334184	AF334184	Homo sapiens FKSG42 (FKSG42) mRNA, complete cds. [AF334184]	
A_24_P57847	0.000831	NM_199052	NM_199052	Homo sapiens chromosome 20 open reading frame 7 (C20orf7), transcript variant 2, mRNA [NM_199052]	NM_199052
A_23_P102351	0.000831	NM_004854	NM_004854	Homo sapiens carbohydrate sulfotransferase 10 (CHST10), mRNA [NM_004854]	NM_004854
A_24_P230176	0.000831	BC009369	BC009369	Homo sapiens similar to RIKEN cDNA 3110023B02, mRNA (cDNA clone MGC:16597 IMAGE:4110481), complete cds. [BC009369]	XM_375500
A_23_P30338	0.000834	NM_001239	NM_001239	Homo sapiens cyclin H (CCNH), mRNA [NM_001239]	NM_001239
A_23_P27353	0.000835	NM_007163	NM_007163	Homo sapiens solute carrier family 14 (urea transporter), member 2 (SLC14A2), mRNA [NM_007163]	NM_007163
A_23_P257014	0.000835	NM_005873	NM_005873	Homo sapiens regulator of G-protein signalling 19 (RGS19), mRNA [NM_005873]	NM_005873
A_24_P61753	0.000835	NM_015229	NM_015229	Homo sapiens KIAA0664 protein (KIAA0664), mRNA [NM_015229]	NM_015229
A_24_P67189	0.000839	NM_199339	NM_199339	Homo sapiens hypothetical protein LOC374768 (LOC374768), mRNA [NM_199339]	NM_199339
A_23_P200874	0.000839	NM_022778	NM_022778	Homo sapiens coiled-coil domain containing 21 (CCDC21), mRNA [NM_022778]	NM_022778
A_23_P341471	0.000839	NM_003348	NM_003348	Homo sapiens ubiquitin-conjugating enzyme E2N (UBC13 homolog, yeast) (UBE2N), mRNA [NM_003348]	NM_003348
A_23_P251118	0.00084	NM_005578	NM_005578	Homo sapiens LIM domain containing preferred translocation partner in lipoma (LPP), mRNA [NM_005578]	NM_005578
A_23_P113656	0.000841	NM_006087	NM_006087	Homo sapiens tubulin, beta 4 (TUBB4), mRNA [NM_006087]	NM_006087
A_24_P190424	0.000841	NM_005370	NM_005370	Homo sapiens RAB8A, member RAS oncogene family (RAB8A), mRNA [NM_005370]	NM_005370
A_23_P32328	0.000841	NM_020408	NM_020408	Homo sapiens chromosome 6 open reading frame 149 (C6orf149), mRNA [NM_020408]	NM_020408
A_23_P119698	0.000842	NM_016579	NM_016579	Homo sapiens CD320 antigen (CD320), mRNA [NM_016579]	NM_016579

A_24_P397204	0.000843	AK054756	AK054756	Homo sapiens cDNA FLJ30194 fis, clone BRACE2001352. [AK054756]	
A_24_P39454	0.000844	NM_032196	NM_032196	Homo sapiens homolog of yeast INO80 (INO80), transcript variant 2, mRNA [NM_032196]	NM_032196
A_24_P717609	0.000845	A_24_P717609			
A_24_P409402	0.000845	A_24_P409402			
A_24_P253723	0.000845	NM_032895	NM_032895	Homo sapiens hypothetical protein MGC14376 (MGC14376), transcript variant 1, mRNA [NM_032895]	NM_032895
A_23_P94174	0.000846	NM_014175	NM_014175	Homo sapiens mitochondrial ribosomal protein L15 (MRPL15), nuclear gene encoding mitochondrial protein, mRNA [NM_014175]	NM_014175
A_24_P115443	0.000847	NM_018688	NM_018688	Homo sapiens bridging integrator 3 (BIN3), mRNA [NM_018688]	NM_018688
A_23_P411612	0.000848	ENST00000338146		Homo sapiens clone pp6781 unknown mRNA. [AF289571]	
A_23_P404685	0.000848	NM_178348	NM_178348	Homo sapiens late cornified envelope 1A (LCE1A), mRNA [NM_178348]	NM_178348
A_23_P18590	0.000849	NM_152544	NM_152544	Homo sapiens hypothetical protein FLJ35725 (FLJ35725), mRNA [NM_152544]	NM_152544
A_23_P143958	0.000849	BC049823	BC049823	Homo sapiens ribosomal protein L22-like 1, mRNA (cDNA clone IMAGE:4865966). [BC049823]	XM_114317
A_24_P233878	0.000851	NM_017686	NM_017686	Homo sapiens ganglioside induced differentiation associated protein 2 (GDAP2), mRNA [NM_017686]	NM_017686
A_23_P6688	0.000851	NM_018075	NM_018075	Homo sapiens hypothetical protein FLJ10375 (FLJ10375), mRNA [NM_018075]	NM_018075
A_24_P464489	0.000854	XM_497719	XM_497719	PREDICTED: Homo sapiens similar to ribosomal protein L27a; ribosomal protein L29 homolog (yeast) (LOC391124), mRNA [XM_497719]	XM_497719
A_23_P130488	0.000854	NM_000400	NM_000400	Homo sapiens excision repair cross-complementing rodent repair deficiency, complementation group 2 (xeroderma pigmentosum D) (ERCC2), mRNA [NM_000400]	NM_000400
A_23_P44636	0.000855	NM_016238	NM_016238	Homo sapiens anaphase promoting complex subunit 7 (ANAPC7), mRNA [NM_016238]	NM_016238
A_24_P309317	0.000855	NM_002778	NM_002778	Homo sapiens prosaposin (variant Gaucher disease and variant metachromatic leukodystrophy) (PSAP), mRNA [NM_002778]	NM_002778
A_23_P165280	0.000856	NM_002695	NM_002695	Homo sapiens polymerase (RNA) II (DNA directed) polypeptide E, 25kDa (POLR2E), mRNA [NM_002695]	NM_002695
A_23_P123125	0.000857	NM_031443	NM_031443	Homo sapiens cerebral cavernous malformation 2 (CCM2), transcript variant 2, mRNA [NM_031443]	NM_031443
A_24_P294931	0.000858	NM_180976	NM_180976	Homo sapiens protein phosphatase 2, regulatory subunit B (B56), delta isoform (PPP2R5D), transcript variant 2, mRNA [NM_180976]	NM_180976
A_23_P95080	0.00086	CR749275	CR749275	Homo sapiens mRNA; cDNA DKFZp781O2021 (from clone DKFZp781O2021). [CR749275]	
A_23_P148984	0.00086	NM_018122	NM_018122	Homo sapiens aspartyl-tRNA synthetase 2 (mitochondrial) (DARS2), mRNA [NM_018122]	NM_018122
A_24_P740705	0.000862	NM_194326	NM_194326	Homo sapiens hypothetical protein MGC52010 (MGC52010), mRNA [NM_194326]	NM_194326
A_32_P87491	0.000863	U79293	U79293	Human clone 23948 mRNA sequence. [U79293]	
A_32_P95176	0.000863	AK127378	AK127378	Homo sapiens cDNA FLJ45454 fis, clone BRSTN2008475, moderately similar to Homo sapiens growth arrest-specific 8 (GAS8). [AK127378]	
A_23_P345710	0.000863	NM_152531	NM_152531	Homo sapiens chromosome 3 open reading frame 21 (C3orf21), mRNA [NM_152531]	NM_152531
A_23_P251765	0.000863	NM_017869	NM_017869	Homo sapiens BTG3 associated nuclear protein (BANP), transcript variant 1, mRNA [NM_017869]	NM_017869
A_23_P17444	0.000864	BG680979	BG680979	BG680979 602628792F1 NCI_CGAP_Skn4 Homo sapiens cDNA clone IMAGE:4753583 5', mRNA sequence [BG680979]	
A_23_P66110	0.000865	NM_000548	NM_000548	Homo sapiens tuberous sclerosis 2 (TSC2), transcript variant 1, mRNA [NM_000548]	NM_000548
A_23_P140241	0.000866	NM_020920	NM_020920	Homo sapiens chromodomain helicase DNA binding protein 8 (CHD8), mRNA [NM_020920]	NM_020920
A_23_P77623	0.000866	NM_006927	NM_006927	Homo sapiens ST3 beta-galactoside alpha-2,3-sialyltransferase 2 (ST3GAL2), mRNA [NM_006927]	NM_006927
A_23_P21063	0.000867	NM_198056	NM_198056	Homo sapiens sodium channel, voltage-gated, type V, alpha (long QT syndrome 3) (SCN5A), transcript variant 1, mRNA [NM_198056]	NM_198056
A_24_P859967	0.000868	BC085019	BC085019	Homo sapiens hypothetical LOC554250, mRNA (cDNA clone MGC:99835 IMAGE:6650156), complete cds. [BC085019]	
A_24_P19195	0.000868	NM_016145	NM_016145	Homo sapiens PTD008 protein (PTD008), mRNA [NM_016145]	NM_016145
A_23_P156996	0.000869	AF086442	AF086442	Homo sapiens full length insert cDNA clone ZD81B04. [AF086442]	XM_499391
A_24_P397150	0.000869	NM_005255	NM_005255	Homo sapiens cyclin G associated kinase (GAK), mRNA [NM_005255]	NM_005255
A_23_P149992	0.00087	NM_020992	NM_020992	Homo sapiens PDZ and LIM domain 1 (elfin) (PDLIM1), mRNA [NM_020992]	NM_020992
A_23_P79927	0.000871	NM_006392	NM_006392	Homo sapiens nucleolar protein 5A (56kDa with KKE/D repeat) (NOL5A), mRNA [NM_006392]	NM_006392
A_23_P5938	0.000872	NM_152257	NM_152257	Homo sapiens KIAA0889 protein (KIAA0889), mRNA [NM_152257]	NM_152257
A_23_P35230	0.000872	NM_172350	NM_172350	Homo sapiens membrane cofactor protein (CD46, trophoblast-lymphocyte cross-reactive antigen) (MCP), transcript variant n, mRNA [NM_172350]	NM_172350
A_24_P408083	0.000872	NM_001379	NM_001379	Homo sapiens DNA (cytosine-5-)-methyltransferase 1 (DNMT1), mRNA [NM_001379]	NM_001379
A_24_P557534	0.000872	ENST00000361789		Homo sapiens clone 35w unknown mRNA; mitochondrial gene for mitochondrial product. [AF391805]	
A_23_P16225	0.000873	NM_017682	NM_017682	Homo sapiens vitelliform macular dystrophy 2-like 1 (VMD2L1), mRNA [NM_017682]	NM_017682

A_24_P75408	0.000873	A_24_P75408			
A_24_P270166	0.000873	NM_001017956	NM_001017956	Homo sapiens amplified in osteosarcoma (OS9), transcript variant 2, mRNA [NM_001017956]	NM_001017956
A_32_P110505	0.000874	A_32_P110505			
A_24_P48587	0.000875	NM_172341	NM_172341	Homo sapiens presenilin enhancer 2 homolog (C. elegans) (PSENEN), mRNA [NM_172341]	NM_172341
A_23_P55551	0.000877	NM_020412	NM_020412	Homo sapiens chromatin modifying protein 1B (CHMP1B), mRNA [NM_020412]	NM_020412
A_24_P873414	0.000879	CR603184	CR603184	full-length cDNA clone CS0DM012YE16 of Fetal liver of Homo sapiens (human). [CR603184]	
A_32_P65934	0.00088	BF802139	BF802139	BF802139 CM0-CI0095-301000-648-e04 CI0095 Homo sapiens cDNA, mRNA sequence [BF802139]	
A_23_P411277	0.00088	NM_003041	NM_003041	Homo sapiens solute carrier family 5 (sodium/glucose cotransporter), member 2 (SLC5A2), mRNA [NM_003041]	NM_003041
A_24_P592591	0.000881	THC2270231		PBEF_HUMAN (P43490) Pre-B cell enhancing factor precursor, partial (20%) [THC2270231]	
A_32_P923011	0.000882	AF346307	AF346307	Homo sapiens hypothetical protein (F379) mRNA, complete cds. [AF346307]	
A_23_P101742	0.000882	NM_172251	NM_172251	Homo sapiens mitochondrial ribosomal protein L54 (MRPL54), nuclear gene encoding mitochondrial protein, mRNA [NM_172251]	NM_172251
A_24_P256050	0.000883	A_24_P256050			
A_24_P133162	0.000883	NM_178122	NM_178122	Homo sapiens hypothetical protein LOC90529 (LOC90529), mRNA [NM_178122]	NM_178122
A_32_P36884	0.000883	ENST00000356170			
A_23_P125001	0.000884	NM_005052	NM_005052	Homo sapiens ras-related C3 botulinum toxin substrate 3 (rho family, small GTP binding protein Rac3) (RAC3), mRNA [NM_005052]	NM_005052
A_24_P50294	0.000885	A_24_P50294			
A_23_P399501	0.000886	NM_182470	NM_182470	Homo sapiens pyruvate kinase, muscle (PKM2), transcript variant 2, mRNA [NM_182470]	NM_182470
A_23_P325802	0.000887	BC020688	BC020688	Homo sapiens ubiquitin specific protease 15, mRNA (cDNA clone IMAGE:4689787), complete cds. [BC020688]	
A_24_P2648	0.000887	NM_005401	NM_005401	Homo sapiens protein tyrosine phosphatase, non-receptor type 14 (PTPN14), mRNA [NM_005401]	NM_005401
A_23_P331177	0.000888	NM_177455	NM_177455	Homo sapiens basic helix-loop-helix domain containing, class B, 8 (BHLHB8), mRNA [NM_177455]	NM_177455
A_24_P191067	0.00089	NM_001009566	NM_001009566	Homo sapiens calsyntenin 1 (CLSTN1), transcript variant 1, mRNA [NM_001009566]	NM_001009566
A_24_P230130	0.00089	ENST00000328558		PREDICTED: Homo sapiens similar to peptidylprolyl isomerase A isoform 1; cyclophilin A; peptidyl-prolyl cis-trans isomerase A; T cell cyclophilin; rotamase; cyclosporin A-binding protein (LOC401859), mRNA [XM_377444]	XM_377444
A_32_P15070	0.000892	AY429544	AY429544	Homo sapiens HMGCRv_4 mRNA sequence; alternatively spliced. [AY429544]	
A_32_P114483	0.000893	NM_153344	NM_153344	Homo sapiens chromosome 6 open reading frame 141 (C6orf141), mRNA [NM_153344]	NM_153344
A_24_P269779	0.000894	NM_004273	NM_004273	Homo sapiens carbohydrate (chondroitin 6) sulfotransferase 3 (CHST3), mRNA [NM_004273]	NM_004273
A_23_P38876	0.000896	NM_005357	NM_005357	Homo sapiens lipase, hormone-sensitive (LIPE), mRNA [NM_005357]	NM_005357
A_23_P128036	0.000897	NM_004990	NM_004990	Homo sapiens methionine-tRNA synthetase (MARS), mRNA [NM_004990]	NM_004990
A_23_P409988	0.000899	NM_024947	NM_024947	Homo sapiens polyhomeotic like 3 (Drosophila) (PHC3), mRNA [NM_024947]	NM_024947
A_24_P417606	0.000899	ENST00000294915		PREDICTED: Homo sapiens similar to 60S ribosomal protein L35 (LOC440737), mRNA [XM_496446]	XM_496446
A_24_P154914	0.0009	NM_016220	NM_016220	Homo sapiens zinc finger protein 588 (ZNF588), transcript variant 1, mRNA [NM_016220]	NM_016220
A_23_P89113	0.0009	NM_024845	NM_024845	Homo sapiens hypothetical protein FLJ14154 (FLJ14154), mRNA [NM_024845]	NM_024845
A_24_P205242	0.000901	BC034378	BC034378	Homo sapiens ribosomal protein L23, mRNA (cDNA clone MGC:34067 IMAGE:5186030), complete cds. [BC034378]	
A_23_P206382	0.000901	NM_025187	NM_025187	Homo sapiens lin-10 protein homolog (Lin10), mRNA [NM_025187]	NM_025187
A_24_P192978	0.000902	NM_173583	NM_173583	Homo sapiens hypothetical protein FLJ33790 (FLJ33790), mRNA [NM_173583]	NM_173583
A_24_P341187	0.000902	NM_020944	NM_020944	Homo sapiens glucosidase, beta (bile acid) 2 (GBA2), mRNA [NM_020944]	NM_020944
A_23_P315286	0.000902	NM_138774	NM_138774	Homo sapiens chromosome 19 open reading frame 22 (C19orf22), mRNA [NM_138774]	NM_138774
A_32_P160398	0.000903	AI613259	AI613259	AI613259 ty35c04.x1 NCI_CGAP_Ut2 Homo sapiens cDNA clone IMAGE:2281062 3' similar to gb:X69391 60S RIBOSOMAL PROTEIN L6 (HUMAN);, mRNA sequence [AI613259]	
A_32_P70372	0.000903	BM995362	BM995362	UI-H-ED0-axb-n-22-0-UI.s1 NCI_CGAP_ED0 Homo sapiens cDNA clone IMAGE:5826645 3', mRNA sequence [BM995362]	
A_24_P117029	0.000903	NM_000527	NM_000527	Homo sapiens low density lipoprotein receptor (familial hypercholesterolemia) (LDLR), mRNA [NM_000527]	NM_000527
A_24_P101786	0.000905	NM_198554	NM_198554	Homo sapiens thyroid adenoma associated (THADA), transcript variant 2, mRNA [NM_198554]	NM_198554
A_23_P201508	0.000905	NM_006212	NM_006212	Homo sapiens 6-phosphofructo-2-kinase/fructose-2,6-biphosphatase 2 (PFKFB2), transcript variant 1, mRNA [NM_006212]	NM_006212
A_32_P191120	0.000905	THC2287925			
A_24_P416346	0.000905	NM_001986	NM_001986	Homo sapiens ets variant gene 4 (E1A enhancer binding protein, E1AF) (ETV4), mRNA [NM_001986]	NM_001986

A_23_P28057	0.000905	NM_003249	NM_003249	Homo sapiens thimet oligopeptidase 1 (THOP1), mRNA [NM_003249]	NM_003249
A_24_P369154	0.000906	NM_002721	NM_002721	Homo sapiens protein phosphatase 6, catalytic subunit (PPP6C), mRNA [NM_002721]	NM_002721
A_32_P213755	0.000906	AL555100	AL555100	AL555100 AL555100 Homo sapiens HELA CELLS COT 25-NORMALIZED Homo sapiens cDNA clone CS0DK007YP02 3-PRIME, mRNA sequence [AL555100]	
A_24_P32672	0.000906	NM_016050	NM_016050	Homo sapiens mitochondrial ribosomal protein L11 (MRPL11), nuclear gene encoding mitochondrial protein, transcript variant 1, mRNA [NM_016050]	NM_016050
A_24_P73158	0.000906	NM_004111	NM_004111	Homo sapiens flap structure-specific endonuclease 1 (FEN1), mRNA [NM_004111]	NM_004111
A_23_P152804	0.000906	NM_198175	NM_198175	Homo sapiens non-metastatic cells 1, protein (NM23A) expressed in (NME1), transcript variant 1, mRNA [NM_198175]	NM_198175
A_23_P158231	0.000908	NM_005503	NM_005503	Homo sapiens amyloid beta (A4) precursor protein-binding, family A, member 2 (X11-like) (APBA2), mRNA [NM_005503]	NM_005503
A_23_P214587	0.000911	NM_003449	NM_003449	Homo sapiens tripartite motif-containing 26 (TRIM26), mRNA [NM_003449]	NM_003449
A_32_P123	0.000911	THC2295911		ADT2_HUMAN ADP,ATP carrier protein, fibroblast isoform (ADP/ATP translocase 2)(Adenine nucleotide translocator 2) (ANT 2). {Homo sapiens;} , partial (62%) [THC2295911]	
A_24_P565556	0.000912	NM_019109	NM_019109	Homo sapiens asparagine-linked glycosylation 1 homolog (yeast, beta-1,4-mannosyltransferase) (ALG1), mRNA [NM_019109]	NM_019109
A_32_P35441	0.000912	BX104176	BX104176	BX104176 BX104176 NCI_CGAP_CLL1 Homo sapiens cDNA clone IMAGp998J025109 ; IMAGE:2073697, mRNA sequence [BX104176]	
A_23_P50374	0.000914	NM_130771	NM_130771	Homo sapiens osteoclast-associated receptor (OSCAR), transcript variant 3, mRNA [NM_130771]	NM_130771
A_24_P195276	0.000915	XM_296315	XM_296315	PREDICTED: Homo sapiens similar to 60S ribosomal protein L35 (LOC341604), mRNA [XM_296315]	XM_296315
A_32_P221452	0.000915	BI753995	BI753995	BI753995 603027430F1 NIH_MGC_114 Homo sapiens cDNA clone IMAGE:5197951 5', mRNA sequence [BI753995]	
A_32_P79396	0.000916	NM_005746	NM_005746	Homo sapiens pre-B-cell colony enhancing factor 1 (PBEF1), transcript variant 1, mRNA [NM_005746]	NM_005746
A_24_P409390	0.000917	ENST00000314123			
A_24_P41593	0.000917	A_24_P41593			
A_23_P141847	0.000919	NM_003969	NM_003969	Homo sapiens ubiquitin-conjugating enzyme E2M (UBC12 homolog, yeast) (UBE2M), mRNA [NM_003969]	NM_003969
A_24_P7232	0.000921	NM_024519	NM_024519	Homo sapiens family with sequence similarity 65, member A (FAM65A), mRNA [NM_024519]	NM_024519
A_32_P143377	0.000921	NM_001017970	NM_001017970	Homo sapiens transmembrane protein 30B (TMEM30B), mRNA [NM_001017970]	NM_001017970
A_23_P66664	0.000921	NM_133439	NM_133439	Homo sapiens transcriptional adaptor 2 (ADA2 homolog, yeast)-like (TADA2L), transcript variant 2, mRNA [NM_133439]	NM_133439
A_24_P40827	0.000923	NM_133627	NM_133627	Homo sapiens RAD51-like 3 (S. cerevisiae) (RAD51L3), transcript variant 2, mRNA [NM_133627]	NM_133627
A_23_P322043	0.000924	NM_015327	NM_015327	Homo sapiens Est1p-like protein B (EST1B), mRNA [NM_015327]	NM_015327
A_23_P113523	0.000926	NM_012227	NM_012227	Homo sapiens GTP binding protein 6 (putative) (GTPBP6), mRNA [NM_012227]	NM_012227
A_24_P540560	0.000927	A_24_P540560			
A_32_P69536	0.000928	THC2381210		Q5T2R2 (Q5T2R2) OTTHUMP00000046079, partial (28%) [THC2381210]	
A_24_P75245	0.000929	ENST00000328270		Homo sapiens CAGL79 mRNA, partial cds. [U80753]	XM_376618
A_24_P348989	0.000936	NM_006863	NM_006863	Homo sapiens leukocyte immunoglobulin-like receptor, subfamily A (with TM domain), member 1 (LILRA1), mRNA [NM_006863]	NM_006863
A_24_P327011	0.00094	NM_021823	NM_021823	Homo sapiens phosphopantothenoylcysteine decarboxylase (PPCDC), mRNA [NM_021823]	NM_021823
A_24_P920484	0.00094	AW169007	AW169007	AW169007 xj16f07.x1 NCI_CGAP_Ut2 Homo sapiens cDNA clone IMAGE:2657413 3' similar to WP:Y55F3B_743.B CE22557 ;, mRNA sequence [AW169007]	
A_23_P84350	0.00094	NM_024596	NM_024596	Homo sapiens microcephaly, primary autosomal recessive 1 (MCPH1), mRNA [NM_024596]	NM_024596
A_24_P919995	0.00094	L17326	L17326	Human pre-T/NK cell associated protein (1F6) mRNA, 3' end. [L17326]	
A_24_P250614	0.00094	NM_005877	NM_005877	Homo sapiens splicing factor 3a, subunit 1, 120kDa (SF3A1), transcript variant 1, mRNA [NM_005877]	NM_005877
A_32_P223644	0.000941	NM_025083	NM_025083	Homo sapiens hypothetical protein FLJ21128 (FLJ21128), mRNA [NM_025083]	NM_025083
A_24_P290068	0.000941	A_24_P290068			
A_23_P5325	0.000941	NM_000122	NM_000122	Homo sapiens excision repair cross-complementing rodent repair deficiency, complementation group 3 (xeroderma pigmentosum group B complementing) (ERCC3), mRNA [NM_000122]	NM_000122
A_24_P203984	0.000941	A_24_P203984			
A_23_P314086	0.000941	NM_194460	NM_194460	Homo sapiens ring finger protein 126 (RNF126), transcript variant 2, mRNA [NM_194460]	NM_194460
A_24_P278602	0.000943	NM_173044	NM_173044	Homo sapiens interleukin 18 binding protein (IL18BP), transcript variant D, mRNA [NM_173044]	NM_173044
A_23_P218793	0.000944	NM_022098	NM_022098	Homo sapiens hypothetical protein LOC63929 (LOC63929), mRNA [NM_022098]	NM_022098
A_24_P131293	0.000945	NM_002699	NM_002699	Homo sapiens POU domain, class 3, transcription factor 1 (POU3F1), mRNA [NM_002699]	NM_002699
A_23_P156861	0.000945	NM_012419	NM_012419	Homo sapiens regulator of G-protein signalling 17 (RGS17), mRNA [NM_012419]	NM_012419

A_23_P337726	0.000945	AK131288	AK131288	Homo sapiens cDNA FLJ16241 fis, clone HCASM2008536, weakly similar to DNA-REPAIR PROTEIN XRCC1. [AK131288]	XM_496332
A_32_P835626	0.000946	AF531436	AF531436	Homo sapiens CGI-301 protein mRNA, complete cds. [AF531436]	
A_32_P220666	0.000949	THC2379706		Q86XQ1 (Q86XQ1) XTP9, partial (3%) [THC2379706]	
A_23_P27096	0.000949	NM_005022	NM_005022	Homo sapiens profilin 1 (PFN1), mRNA [NM_005022]	NM_005022
A_23_P206684	0.00095	NM_199424	NM_199424	Homo sapiens WW domain containing E3 ubiquitin protein ligase 2 (WWP2), transcript variant 2, mRNA [NM_199424]	NM_199424
A_23_P104583	0.000951	NM_020441	NM_020441	Homo sapiens coronin, actin binding protein, 1B (CORO1B), transcript variant 1, mRNA [NM_020441]	NM_020441
A_32_P220762	0.000952	AK123248	AK123248	Homo sapiens cDNA FLJ41254 fis, clone BRAMY2033594. [AK123248]	
A_24_P23400	0.000952	NM_005629	NM_005629	Homo sapiens solute carrier family 6 (neurotransmitter transporter, creatine), member 8 (SLC6A8), mRNA [NM_005629]	NM_005629
A_23_P2397	0.000953	NM_178169	NM_178169	Homo sapiens Ras association (RalGDS/AF-6) domain family 3 (RASSF3), mRNA [NM_178169]	NM_178169
A_24_P95038	0.000953	NM_203430	NM_203430	Homo sapiens peptidylprolyl isomerase A (cyclophilin A) (PPIA), transcript variant 2, mRNA [NM_203430]	NM_203430
A_23_P322076	0.000954	BC033126	BC033126	Homo sapiens zinc finger protein 697, mRNA (cDNA clone MGC:45731 IMAGE:3050333), complete cds. [BC033126]	XM_371286
A_23_P255418	0.000954	NM_018419	NM_018419	Homo sapiens SRY (sex determining region Y)-box 18 (SOX18), mRNA [NM_018419]	NM_018419
A_23_P412577	0.000955	NM_173505	NM_173505	Homo sapiens ankyrin repeat domain 29 (ANKRD29), mRNA [NM_173505]	NM_173505
A_23_P168584	0.000955	NM_152992	NM_152992	Homo sapiens POM (POM121 homolog, rat) and ZP3 fusion (POMZP3), transcript variant 2, mRNA [NM_152992]	NM_152992
A_23_P168868	0.000955	NM_014754	NM_014754	Homo sapiens phosphatidylserine synthase 1 (PTDSS1), mRNA [NM_014754]	NM_014754
A_23_P22473	0.000957	NM_012280	NM_012280	Homo sapiens FtsJ homolog 1 (E. coli) (FTSJ1), transcript variant 1, mRNA [NM_012280]	NM_012280
A_32_P188752	0.000958	AK129584	AK129584	Homo sapiens cDNA FLJ26073 fis, clone RCT01314. [AK129584]	
A_23_P118135	0.000959	THC2308359		ALU8_HUMAN (P39195) Alu subfamily SX sequence contamination warning entry, partial (13%) [THC2308359]	
A_24_P173865	0.000959	AK027396	AK027396	Homo sapiens cDNA FLJ14490 fis, clone MAMMA1002886. [AK027396]	
A_24_P229304	0.000959	ENST00000281893			
A_32_P165102	0.000962	N21624	N21624	N21624 yx60a10.s1 Soares melanocyte 2NbHM Homo sapiens cDNA clone IMAGE:266106 3' similar to gb:X56468_rna1 14-3-3 PROTEIN THETA (HUMAN);, mRNA sequence [N21624]	
A_24_P21044	0.000962	NM_032302	NM_032302	Homo sapiens hypothetical protein MGC10911 (MGC10911), mRNA [NM_032302]	NM_032302
A_24_P930764	0.000964	BC039241	BC039241	Homo sapiens chromosome 1 open reading frame 96, mRNA (cDNA clone MGC:32857 IMAGE:4731004), complete cds. [BC039241]	
A_32_P115043	0.000964	A_32_P115043			
A_23_P102622	0.000964	NM_003833	NM_003833	Homo sapiens matrilin 4 (MATN4), transcript variant 1, mRNA [NM_003833]	NM_003833
A_23_P80353	0.000964	NM_001003689	NM_001003689	Homo sapiens l(3)mbt-like 2 (Drosophila) (L3MBTL2), transcript variant 2, mRNA [NM_001003689]	NM_001003689
A_24_P33217	0.000964	A_24_P33217			
A_23_P200901	0.000965	NM_004436	NM_004436	Homo sapiens endosulfine alpha (ENSA), transcript variant 3, mRNA [NM_004436]	NM_004436
A_24_P391853	0.000966	ENST00000272762			
A_23_P29081	0.000967	NM_001002021	NM_001002021	Homo sapiens phosphofructokinase, liver (PFKL), transcript variant 1, mRNA [NM_001002021]	NM_001002021
A_24_P264685	0.000968	A_24_P264685			
A_24_P403403	0.000968	NM_007209	NM_007209	Homo sapiens ribosomal protein L35 (RPL35), mRNA [NM_007209]	NM_007209
A_23_P52082	0.000972	NM_015434	NM_015434	Homo sapiens chromosome 1 open reading frame 73 (C1orf73), mRNA [NM_015434]	NM_015434
A_32_P32463	0.000973	A_32_P32463			
A_23_P208706	0.000974	NM_138764	NM_138764	Homo sapiens BCL2-associated X protein (BAX), transcript variant epsilon, mRNA [NM_138764]	NM_138764
A_23_P309278	0.000977	NM_173158	NM_173158	Homo sapiens nuclear receptor subfamily 4, group A, member 1 (NR4A1), transcript variant 3, mRNA [NM_173158]	NM_173158
A_24_P916407	0.00098	A_24_P916407			
A_24_P295745	0.00098	NM_153201	NM_153201	Homo sapiens heat shock 70kDa protein 8 (HSPA8), transcript variant 2, mRNA [NM_153201]	NM_153201
A_23_P56736	0.000981	NM_080386	NM_080386	Homo sapiens alpha-tubulin isotype H2-alpha (H2-ALPHA), mRNA [NM_080386]	NM_080386
A_24_P919876	0.000982	Y14488	Y14488	Homo sapiens mRNA for putative 14kD protein containing SHMT homology, clone pUS1215. [Y14488]	
A_24_P324565	0.000982	NM_005505	NM_005505	Homo sapiens scavenger receptor class B, member 1 (SCARB1), mRNA [NM_005505]	NM_005505
A_24_P167030	0.000982	NM_207012	NM_207012	Homo sapiens adaptor-related protein complex 3, mu 1 subunit (AP3M1), transcript variant 1, mRNA [NM_207012]	NM_207012
A_24_P29260	0.000982	NM_054013	NM_054013	Homo sapiens mannosyl (alpha-1,3-)-glycoprotein beta-1,4-N-acetylglucosaminyltransferase, isoenzyme B (MGAT4B), transcript variant 2, mRNA [NM_054013]	NM_054013

A_24_P145629	0.000983	NM_178865	NM_178865	Homo sapiens tumor differentially expressed 2-like (TDE2L), mRNA [NM_178865]	NM_178865
A_23_P68752	0.000984	NM_015151	NM_015151	Homo sapiens chromosome 21 open reading frame 106 (C21orf106), transcript variant 1, mRNA [NM_015151]	NM_015151
A_24_P603890	0.000984	XM_498140	XM_498140	PREDICTED: Homo sapiens similar to ADP,ATP carrier protein, fibroblast isoform (ADP/ATP translocase 2) (Adenine nucleotide translocator 2) (ANT 2) (LOC442255), mRNA [XM_498140]	XM_498140
A_23_P49351	0.000988	NM_015944	NM_015944	Homo sapiens CGI-14 protein (CGI-14), mRNA [NM_015944]	NM_015944
A_23_P27781	0.000989	NM_024317	NM_024317	Homo sapiens leukocyte immunoglobulin-like receptor pseudogene 2 (LILRP2), mRNA [NM_024317]	NM_024317
A_24_P143543	0.000989	ENST00000273340			
A_24_P366989	0.000989	THC2307535		GDIR_HUMAN (P52565) Rho GDP-dissociation inhibitor 1 (Rho GDI 1) (Rho-GDI alpha), partial (67%) [THC2307535]	
A_24_P202748	0.000989	NM_024074	NM_024074	Homo sapiens transmembrane protein 38A (TMEM38A), mRNA [NM_024074]	NM_024074
A_24_P399362	0.00099	NM_020232	NM_020232	Homo sapiens tumor necrosis factor superfamily, member 5-induced protein 1 (TNFSF5IP1), mRNA [NM_020232]	NM_020232
A_24_P927072	0.000992	A_24_P927072			
A_24_P717462	0.000992	A_24_P717462			
A_24_P296457	0.000992	NM_000294	NM_000294	Homo sapiens phosphorylase kinase, gamma 2 (testis) (PHKG2), mRNA [NM_000294]	NM_000294
A_24_P323628	0.000992	BC064938	BC064938	Homo sapiens cDNA clone IMAGE:6158500, partial cds. [BC064938]	XM_371204
A_23_P125157	0.000993	NM_032815	NM_032815	Homo sapiens nuclear factor of activated T-cells, cytoplasmic, calcineurin-dependent 2 interacting protein (NFATC2IP), mRNA [NM_032815]	NM_032815
A_23_P436179	0.000994	NM_001152	NM_001152	Homo sapiens solute carrier family 25 (mitochondrial carrier; adenine nucleotide translocator), member 5 (SLC25A5), mRNA [NM_001152]	NM_001152
A_24_P940310	0.000995	ENST00000270201		Homo sapiens chromosome 21 C21orf108 mRNA, partial cds. [AF231919]	
A_23_P357147	0.000995	NM_199424	NM_199424	Homo sapiens WW domain containing E3 ubiquitin protein ligase 2 (WWP2), transcript variant 2, mRNA [NM_199424]	NM_199424
A_23_P214594	0.000995	NM_024839	NM_024839	Homo sapiens ribonuclease P 21kDa subunit (RPP21), mRNA [NM_024839]	NM_024839
A_24_P916378	0.000996	BC008217	BC008217	Homo sapiens heterogeneous nuclear ribonucleoprotein L-like, mRNA (cDNA clone MGC:5437 IMAGE:3449607), complete cds. [BC008217]	
A_24_P41483	0.000996	ENST00000321952			
A_24_P75994	0.000996	A_24_P75994			
A_23_P56249	0.000996	NM_001281	NM_001281	Homo sapiens cytoskeleton associated protein 1 (CKAP1), mRNA [NM_001281]	NM_001281
A_24_P126931	0.000997	A_24_P126931			
A_24_P313756	0.000998	NM_032525	NM_032525	Homo sapiens tubulin, beta 6 (TUBB6), mRNA [NM_032525]	NM_032525

Supplemental Table S3B

Kmeans Cluster 2 - 3,998 Genes

Gene Name	*P* -value	Common	Genbank	Description	RefSeq
A_23_P19134	1.60E-07	NM_032119	NM_032119	Homo sapiens monogenic, audiogenic seizure susceptibility 1 homolog (mouse) (MASS1), mRNA [NM_032119]	NM_032119
A_23_P166508	1.60E-07	BC038245	BC038245	Homo sapiens, clone IMAGE:5241654, mRNA. [BC038245]	XM_086879
A_32_P313405	1.60E-07	NM_005559	NM_005559	Homo sapiens laminin, alpha 1 (LAMA1), mRNA [NM_005559]	NM_005559
A_23_P159952	1.60E-07	NM_018476	NM_018476	Homo sapiens brain expressed, X-linked 1 (BEX1), mRNA [NM_018476]	NM_018476
A_32_P107876	1.60E-07	NM_025074	NM_025074	Homo sapiens Fraser syndrome 1 (FRAS1), transcript variant 1, mRNA [NM_025074]	NM_025074
A_23_P22735	1.60E-07	NM_032621	NM_032621	Homo sapiens brain expressed X-linked 2 (BEX2), mRNA [NM_032621]	NM_032621
A_23_P430068	1.65E-07	NM_006474	NM_006474	Homo sapiens podoplanin (PDPN), transcript variant 1, mRNA [NM_006474]	NM_006474
A_24_P789425	1.71E-07	AK026966	AK026966	Homo sapiens cDNA: FLJ23313 fis, clone HEP11919. [AK026966]	
A_23_P8513	1.71E-07	NM_013322	NM_013322	Homo sapiens sorting nexin 10 (SNX10), mRNA [NM_013322]	NM_013322
A_23_P117190	1.71E-07	NM_013238	NM_013238	Homo sapiens DnaJ (Hsp40) homolog, subfamily C, member 15 (DNAJC15), mRNA [NM_013238]	NM_013238
A_23_P319423	1.72E-07	NM_003740	NM_003740	Homo sapiens potassium channel, subfamily K, member 5 (KCNK5), mRNA [NM_003740]	NM_003740
A_23_P500501	1.72E-07	NM_000142	NM_000142	Homo sapiens fibroblast growth factor receptor 3 (achondroplasia, thanatophoric dwarfism) (FGFR3), transcript variant 1, mRNA [NM_000142]	NM_000142
A_32_P27917	1.94E-07	BC009415	BC009415	Homo sapiens kinesin family member 26A, mRNA (cDNA clone MGC:14884 IMAGE:3502885), complete cds. [BC009415]	XM_050278
A_32_P154473	2.08E-07	ENST00000334436		Homo sapiens mRNA for KIAA0531 protein, partial cds. [AB011103]	
A_32_P167904	2.11E-07	CR624679	CR624679	full-length cDNA clone CS0DF003YC20 of Fetal brain of Homo sapiens (human). [CR624679]	
A_23_P145437	2.11E-07	NM_017934	NM_017934	Homo sapiens pleckstrin homology domain interacting protein (PHIP), mRNA [NM_017934]	NM_017934
A_23_P209449	2.11E-07	NM_003507	NM_003507	Homo sapiens frizzled homolog 7 (Drosophila) (FZD7), mRNA [NM_003507]	NM_003507
A_23_P7873	2.11E-07	NM_002388	NM_002388	Homo sapiens MCM3 minichromosome maintenance deficient 3 (S. cerevisiae) (MCM3), mRNA [NM_002388]	NM_002388
A_23_P369328	2.18E-07	NM_145306	NM_145306	Homo sapiens chromosome 10 open reading frame 35 (C10orf35), mRNA [NM_145306]	NM_145306
A_23_P91081	2.18E-07	NM_002354	NM_002354	Homo sapiens tumor-associated calcium signal transducer 1 (TACSTD1), mRNA [NM_002354]	NM_002354
A_32_P12183	2.18E-07	NM_198284	NM_198284	Homo sapiens hypothetical protein LOC349114 (LOC349114), mRNA [NM_198284]	NM_198284
A_23_P110253	2.25E-07	NM_000222	NM_000222	Homo sapiens v-kit Hardy-Zuckerman 4 feline sarcoma viral oncogene homolog (KIT), mRNA [NM_000222]	NM_000222
A_23_P2181	2.29E-07	NM_001001336	NM_001001336	Homo sapiens cytochrome b5 reductase 2 (CYB5R2), transcript variant 2, mRNA [NM_001001336]	NM_001001336
A_23_P138655	2.29E-07	NM_057157	NM_057157	Homo sapiens cytochrome P450, family 26, subfamily A, polypeptide 1 (CYP26A1), transcript variant 2, mRNA [NM_057157]	NM_057157
A_24_P180680	2.29E-07	NM_018407	NM_018407	Homo sapiens lysosomal associated protein transmembrane 4 beta (LAPTM4B), mRNA [NM_018407]	NM_018407
A_24_P32085	2.35E-07	NM_024761	NM_024761	Homo sapiens MOB1, Mps One Binder kinase activator-like 2B (yeast) (MOBKL2B), mRNA [NM_024761]	NM_024761
A_24_P167063	2.70E-07	NM_014803	NM_014803	Homo sapiens zinc finger protein 518 (ZNF518), mRNA [NM_014803]	NM_014803
A_23_P217319	2.73E-07	NM_004114	NM_004114	Homo sapiens fibroblast growth factor 13 (FGF13), transcript variant 1A, mRNA [NM_004114]	NM_004114
A_23_P429950	2.77E-07	NM_000216	NM_000216	Homo sapiens Kallmann syndrome 1 sequence (KAL1), mRNA [NM_000216]	NM_000216
A_23_P5435	2.77E-07	AY358993	AY358993	Homo sapiens clone DNA129535 MRV222 (UNQ3066) mRNA, complete cds. [AY358993]	
A_32_P129660	2.77E-07	NM_181453	NM_181453	Homo sapiens GRIP and coiled-coil domain containing 2 (GCC2), transcript variant 1, mRNA [NM_181453]	NM_181453
A_23_P131676	2.78E-07	NM_020311	NM_020311	Homo sapiens chemokine orphan receptor 1 (CMKOR1), mRNA [NM_020311]	NM_020311
A_23_P100220	2.82E-07	NM_024939	NM_024939	Homo sapiens hypothetical protein FLJ21918 (FLJ21918), mRNA [NM_024939]	NM_024939
A_24_P945283	2.82E-07	AB033058	AB033058	Homo sapiens mRNA for KIAA1232 protein, partial cds. [AB033058]	
A_23_P128084	2.82E-07	NM_002206	NM_002206	Homo sapiens integrin, alpha 7 (ITGA7), mRNA [NM_002206]	NM_002206
A_23_P20743	2.84E-07	NM_032342	NM_032342	Homo sapiens chromosome 9 open reading frame 125 (C9orf125), mRNA [NM_032342]	NM_032342
A_24_P105191	2.90E-07	NM_147175	NM_147175	Homo sapiens heparan sulfate 6-O-sulfotransferase 2 (HS6ST2), transcript variant S, mRNA [NM_147175]	NM_147175
A_23_P213166	3.00E-07	NM_138698	NM_138698	Homo sapiens prematurely terminated mRNA decay factor-like (LOC91431), mRNA [NM_138698]	NM_138698
A_24_P182461	3.00E-07	NM_001542	NM_001542	Homo sapiens immunoglobulin superfamily, member 3 (IGSF3), transcript variant 1, mRNA [NM_001542]	NM_001542
A_23_P26854	3.00E-07	NM_014859	NM_014859	Homo sapiens KIAA0672 gene product (KIAA0672), mRNA [NM_014859]	NM_014859
A_23_P60130	3.00E-07	NM_052886	NM_052886	Homo sapiens mal, T-cell differentiation protein 2 (MAL2), mRNA [NM_052886]	NM_052886

A_23_P98930	3.00E-07	NM_018169	NM_018169	Homo sapiens hypothetical protein FLJ10652 (FLJ10652), mRNA [NM_018169]	NM_018169
A_23_P27023	3.02E-07	NM_032932	NM_032932	Homo sapiens RAB11 family interacting protein 4 (class II) (RAB11FIP4), mRNA [NM_032932]	NM_032932
A_23_P51187	3.15E-07	NM_002744	NM_002744	Homo sapiens protein kinase C, zeta (PRKCZ), mRNA [NM_002744]	NM_002744
A_23_P217901	3.28E-07	BC038219	BC038219	Homo sapiens, clone IMAGE:3634113, mRNA. [BC038219]	
A_23_P137173	3.29E-07	NM_021992	NM_021992	Homo sapiens thymosin-like 8 (TMSL8), mRNA [NM_021992]	NM_021992
A_23_P40174	3.37E-07	NM_004994	NM_004994	Homo sapiens matrix metalloproteinase 9 (gelatinase B, 92kDa gelatinase, 92kDa type IV collagenase) (MMP9), mRNA [NM_004994]	NM_004994
A_23_P114670	3.69E-07	NM_014448	NM_014448	Homo sapiens Rho guanine exchange factor (GEF) 16 (ARHGEF16), mRNA [NM_014448]	NM_014448
A_23_P202269	3.73E-07	NM_020987	NM_020987	Homo sapiens ankyrin 3, node of Ranvier (ankyrin G) (ANK3), transcript variant 1, mRNA [NM_020987]	NM_020987
A_23_P137484	3.74E-07	NM_019079	NM_019079	Homo sapiens hypothetical protein FLJ10884 (ECAT11), mRNA [NM_019079]	NM_019079
A_23_P31721	3.87E-07	NM_001951	NM_001951	Homo sapiens E2F transcription factor 5, p130-binding (E2F5), mRNA [NM_001951]	NM_001951
A_24_P916586	3.93E-07	BC010091	BC010091	Homo sapiens bicaudal D homolog 1 (Drosophila), mRNA (cDNA clone IMAGE:3050215), with apparent retained intron. [BC010091]	
A_23_P156025	3.98E-07	ENST00000302057		Homo sapiens homeodomain protein IRXA2 (IRX2) mRNA, complete cds. [AY335940]	
A_24_P98371	3.98E-07	NM_033222	NM_033222	Homo sapiens PC4 and SFRS1 interacting protein 1 (PSIP1), transcript variant 2, mRNA [NM_033222]	NM_033222
A_23_P7282	4.00E-07	ENST00000323570		Homo sapiens cDNA FLJ38038 fis, clone CTONG2013907. [AK095357]	
A_23_P146456	4.00E-07	NM_001333	NM_001333	Homo sapiens cathepsin L2 (CTSL2), mRNA [NM_001333]	NM_001333
A_23_P102202	4.00E-07	NM_000179	NM_000179	Homo sapiens mutS homolog 6 (E. coli) (MSH6), mRNA [NM_000179]	NM_000179
A_24_P339429	4.27E-07	AK024229	AK024229	Homo sapiens cDNA FLJ14167 fis, clone NT2RP2001214. [AK024229]	
A_32_P195401	4.28E-07	NM_173511	NM_173511	Homo sapiens amyotrophic lateral sclerosis 2 (juvenile) chromosome region, candidate 13 (ALS2CR13), mRNA [NM_173511]	NM_173511
A_23_P161297	4.33E-07	NM_018245	NM_018245	Homo sapiens oxoglutarate dehydrogenase-like (OGDHL), mRNA [NM_018245]	NM_018245
A_23_P415652	4.39E-07	NM_024642	NM_024642	Homo sapiens UDP-N-acetyl-alpha-D-galactosamine:polypeptide N-acetylgalactosaminyltransferase 12 (GalNAc-T12) (GALNT12), mRNA [NM_024642]	NM_024642
A_23_P99405	4.39E-07	NM_003453	NM_003453	Homo sapiens zinc finger protein 198 (ZNF198), mRNA [NM_003453]	NM_003453
A_23_P94319	4.50E-07	NM_014867	NM_014867	Homo sapiens KIAA0711 gene product (KIAA0711), mRNA [NM_014867]	NM_014867
A_23_P88630	4.58E-07	NM_000057	NM_000057	Homo sapiens Bloom syndrome (BLM), mRNA [NM_000057]	NM_000057
A_23_P77422	4.68E-07	NM_024516	NM_024516	Homo sapiens chromosome 16 open reading frame 53 (C16orf53), mRNA [NM_024516]	NM_024516
A_32_P151875	4.76E-07	NM_020781	NM_020781	Homo sapiens zinc finger protein 398 (ZNF398), transcript variant 2, mRNA [NM_020781]	NM_020781
A_32_P108655	4.76E-07	NM_001005353	NM_001005353	Homo sapiens adenylate kinase 3-like 1 (AK3L1), nuclear gene encoding mitochondrial protein, transcript variant 1, mRNA [NM_001005353]	NM_001005353
A_23_P115922	4.76E-07	NM_004096	NM_004096	Homo sapiens eukaryotic translation initiation factor 4E binding protein 2 (EIF4EBP2), mRNA [NM_004096]	NM_004096
A_23_P401055	4.80E-07	NM_003106	NM_003106	Homo sapiens SRY (sex determining region Y)-box 2 (SOX2), mRNA [NM_003106]	NM_003106
A_23_P106131	4.85E-07	NM_182926	NM_182926	Homo sapiens kinectin 1 (kinesin receptor) (KTN1), mRNA [NM_182926]	NM_182926
A_24_P886336	4.90E-07	BC029907	BC029907	Homo sapiens, clone IMAGE:5175565, mRNA. [BC029907]	
A_23_P345118	4.94E-07	NM_002648	NM_002648	Homo sapiens pim-1 oncogene (PIM1), mRNA [NM_002648]	NM_002648
A_23_P64792	4.95E-07	NM_014505	NM_014505	Homo sapiens potassium large conductance calcium-activated channel, subfamily M, beta member 4 (KCNMB4), mRNA [NM_014505]	NM_014505
A_24_P418250	5.07E-07	NM_203306	NM_203306	Homo sapiens hypothetical protein MGC39606 (MGC39606), mRNA [NM_203306]	NM_203306
A_24_P388528	5.07E-07	NM_173216	NM_173216	Homo sapiens ST6 beta-galactosamide alpha-2,6-sialyltranferase 1 (ST6GAL1), transcript variant 1, mRNA [NM_173216]	NM_173216
A_23_P111373	5.32E-07	NM_020662	NM_020662	Homo sapiens MRS2-like, magnesium homeostasis factor (S. cerevisiae) (MRS2L), mRNA [NM_020662]	NM_020662
A_32_P232647	5.77E-07	A_32_P232647			
A_32_P221429	5.80E-07	THC2374165			
A_23_P432034	5.84E-07	NM_173510	NM_173510	Homo sapiens hypothetical protein FLJ33814 (FLJ33814), mRNA [NM_173510]	NM_173510
A_23_P140884	5.88E-07	BC014971	BC014971	Homo sapiens, Similar to tubulin, beta, 2, clone IMAGE:4873024, mRNA. [BC014971]	XM_371684
A_23_P87709	6.01E-07	NM_024829	NM_024829	Homo sapiens hypothetical protein FLJ22662 (FLJ22662), mRNA [NM_024829]	NM_024829
A_32_P82895	6.06E-07	AB075837	AB075837	Homo sapiens mRNA for KIAA1957 protein. [AB075837]	XM_065166
A_32_P154911	6.17E-07	NM_175887	NM_175887	Homo sapiens hypothetical protein LOC222171 (LOC222171), mRNA [NM_175887]	NM_175887
A_32_P86578	6.17E-07	BC032913	BC032913	Homo sapiens hypothetical gene supported by BC032913; BC048425, mRNA (cDNA clone IMAGE:5265535). [BC032913]	XM_374002
A_23_P50195	6.35E-07	A_23_P50195			
A_23_P1043	6.39E-07	NM_018265	NM_018265	Homo sapiens chromosome 1 open reading frame 106 (C1orf106), mRNA [NM_018265]	NM_018265

A_24_P607880	6.39E-07	AL832758	AL832758	Homo sapiens mRNA; cDNA DKFZp686C0927 (from clone DKFZp686C0927). [AL832758]	
A_23_P93629	6.39E-07	NM_015905	NM_015905	Homo sapiens tripartite motif-containing 24 (TRIM24), transcript variant 1, mRNA [NM_015905]	NM_015905
A_23_P398172	6.65E-07	NM_020819	NM_020819	Homo sapiens KIAA1411 (KIAA1411), mRNA [NM_020819]	NM_020819
A_23_P500614	6.65E-07	NM_001243	NM_001243	Homo sapiens tumor necrosis factor receptor superfamily, member 8 (TNFRSF8), transcript variant 1, mRNA [NM_001243]	NM_001243
A_23_P104054	6.67E-07	NM_016227	NM_016227	Homo sapiens chromosome 1 open reading frame 9 (C1orf9), transcript variant 2, mRNA [NM_016227]	NM_016227
A_24_P923102	6.73E-07	BC016950	BC016950	Homo sapiens kelch-like 23 (Drosophila), mRNA (cDNA clone IMAGE:3854163), complete cds. [BC016950]	
A_23_P105276	6.85E-07	NM_003428	NM_003428	Homo sapiens zinc finger protein 84 (HPF2) (ZNF84), mRNA [NM_003428]	NM_003428
A_32_P178966	6.89E-07	THC2373940			
A_23_P416965	6.94E-07	NM_015398	NM_015398	Homo sapiens DKFZP564J102 protein (DKFZP564J102), transcript variant 1, mRNA [NM_015398]	NM_015398
A_24_P309415	6.94E-07	NM_052932	NM_052932	Homo sapiens pro-oncosis receptor inducing membrane injury gene (PORIMIN), mRNA [NM_052932]	NM_052932
A_23_P168761	7.01E-07	ENST00000265220		PTPZ_HUMAN (P23471) Receptor-type tyrosine-protein phosphatase zeta precursor (R-PTP-zeta) , complete [THC2235743]	
A_32_P106117	7.12E-07	NM_015044	NM_015044	Homo sapiens golgi associated, gamma adaptin ear containing, ARF binding protein 2 (GGA2), transcript variant 1, mRNA [NM_015044]	NM_015044
A_23_P57709	7.12E-07	NM_013363	NM_013363	Homo sapiens procollagen C-endopeptidase enhancer 2 (PCOLCE2), mRNA [NM_013363]	NM_013363
A_32_P229493	7.22E-07	BC004287	BC004287	Homo sapiens, clone IMAGE:3618365, mRNA. [BC004287]	
A_23_P100056	7.22E-07	NM_194272	NM_194272	Homo sapiens RNA binding protein with multiple splicing 2 (RBPMS2), mRNA [NM_194272]	NM_194272
A_23_P324327	7.22E-07	NM_016235	NM_016235	Homo sapiens G protein-coupled receptor, family C, group 5, member B (GPRC5B), mRNA [NM_016235]	NM_016235
A_32_P69368	7.22E-07	NM_002166	NM_002166	Homo sapiens inhibitor of DNA binding 2, dominant negative helix-loop-helix protein (ID2), mRNA [NM_002166]	NM_002166
A_23_P417942	7.22E-07	NM_001024948	NM_001024948	Homo sapiens formin binding protein 1-like (FNBP1L), transcript variant 1, mRNA [NM_001024948]	NM_001024948
A_23_P320261	7.22E-07	NM_033317	NM_033317	Homo sapiens dermokine (ZD52F10), mRNA [NM_033317]	NM_033317
A_23_P51376	7.38E-07	NM_024522	NM_024522	Homo sapiens hypothetical protein FLJ12650 (FLJ12650), mRNA [NM_024522]	NM_024522
A_23_P257164	7.41E-07	NM_000481	NM_000481	Homo sapiens aminomethyltransferase (glycine cleavage system protein T) (AMT), mRNA [NM_000481]	NM_000481
A_32_P194032	7.60E-07	NM_152271	NM_152271	Homo sapiens hypothetical protein FLJ23749 (FLJ23749), mRNA [NM_152271]	NM_152271
A_23_P50081	7.63E-07	NM_014214	NM_014214	Homo sapiens inositol(myo)-1(or 4)-monophosphatase 2 (IMPA2), mRNA [NM_014214]	NM_014214
A_23_P110569	8.55E-07	NM_018700	NM_018700	Homo sapiens tripartite motif-containing 36 (TRIM36), transcript variant 1, mRNA [NM_018700]	NM_018700
A_23_P122906	8.55E-07	NM_015570	NM_015570	Homo sapiens autism susceptibility candidate 2 (AUTS2), mRNA [NM_015570]	NM_015570
A_23_P48585	8.55E-07	NM_005407	NM_005407	Homo sapiens sal-like 2 (Drosophila) (SALL2), mRNA [NM_005407]	NM_005407
A_23_P89249	8.55E-07	NM_001005862	NM_001005862	Homo sapiens v-erb-b2 erythroblastic leukemia viral oncogene homolog 2, neuro/glioblastoma derived oncogene homolog (avian) (ERBB2), transcript variant 2, mRNA [NM_001005862]	NM_001005862
A_23_P138631	8.60E-07	NM_005445	NM_005445	Homo sapiens chondroitin sulfate proteoglycan 6 (bamacan) (CSPG6), mRNA [NM_005445]	NM_005445
A_23_P99582	8.60E-07	NM_002687	NM_002687	Homo sapiens pinin, desmosome associated protein (PNN), mRNA [NM_002687]	NM_002687
A_23_P254816	8.72E-07	NM_004609	NM_004609	Homo sapiens transcription factor 15 (basic helix-loop-helix) (TCF15), mRNA [NM_004609]	NM_004609
A_32_P32413	8.82E-07	AK123972	AK123972	Homo sapiens cDNA FLJ41978 fis, clone SKNSH2000482. [AK123972]	
A_23_P169278	8.82E-07	NM_015239	NM_015239	Homo sapiens ATP/GTP binding protein 1 (AGTPBP1), mRNA [NM_015239]	NM_015239
A_32_P24059	8.97E-07	BX640861	BX640861	Homo sapiens mRNA; cDNA DKFZp686C20250 (from clone DKFZp686C20250) [BX640861]	
A_23_P77731	8.97E-07	NM_001888	NM_001888	Homo sapiens crystallin, mu (CRYM), transcript variant 1, mRNA [NM_001888]	NM_001888
A_23_P128543	9.05E-07	NM_014166	NM_014166	Homo sapiens mediator of RNA polymerase II transcription, subunit 4 homolog (yeast) (MED4), mRNA [NM_014166]	NM_014166
A_32_P88415	9.22E-07	NM_133371	NM_133371	Homo sapiens myozenin 3 (MYOZ3), mRNA [NM_133371]	NM_133371
A_24_P579356	9.56E-07	NM_001010000	NM_001010000	Homo sapiens Rho GTPase activating protein 28 (ARHGAP28), transcript variant 1, mRNA [NM_001010000]	NM_001010000
A_23_P202071	9.56E-07	NM_006561	NM_006561	Homo sapiens CUG triplet repeat, RNA binding protein 2 (CUGBP2), transcript variant 2, mRNA [NM_006561]	NM_006561
A_23_P211909	9.67E-07	NM_002670	NM_002670	Homo sapiens plastin 1 (I isoform) (PLS1), mRNA [NM_002670]	NM_002670
A_23_P252163	9.73E-07	NM_004938	NM_004938	Homo sapiens death-associated protein kinase 1 (DAPK1), mRNA [NM_004938]	NM_004938
A_32_P58937	9.73E-07	AL133577	AL133577	Homo sapiens mRNA; cDNA DKFZp434G0972 (from clone DKFZp434G0972). [AL133577]	
A_23_P157736	9.73E-07	NM_032728	NM_032728	Homo sapiens phosphatidic acid phosphatase type 2 domain containing 3 (PPAPDC3), mRNA [NM_032728]	NM_032728
A_23_P40059	9.73E-07	NM_000534	NM_000534	Homo sapiens PMS1 postmeiotic segregation increased 1 (S. cerevisiae) (PMS1), mRNA [NM_000534]	NM_000534

A_24_P94402	9.73E-07	NM_005378	NM_005378	Homo sapiens v-myc myelocytomatosis viral related oncogene, neuroblastoma derived (avian) (MYCN), mRNA [NM_005378]	NM_005378
A_23_P56050	9.73E-07	M19308	M19308	Human slow skeletal muscle troponin T mRNA, clone M1. [M19308]	
A_24_P456944	9.91E-07	AK123446	AK123446	Homo sapiens cDNA FLJ41452 fis, clone BRSTN2010363. [AK123446]	
A_23_P208238	1.00E-06	NM_003438	NM_003438	Homo sapiens zinc finger protein 137 (clone pHZ-30) (ZNF137), mRNA [NM_003438]	NM_003438
A_23_P85250	1.01E-06	NM_013230	NM_013230	Homo sapiens CD24 antigen (small cell lung carcinoma cluster 4 antigen) (CD24), mRNA [NM_013230]	NM_013230
A_23_P91250	1.02E-06	NM_199441	NM_199441	Homo sapiens zinc finger protein 334 (ZNF334), transcript variant 2, mRNA [NM_199441]	NM_199441
A_23_P408455	1.02E-06	AL049246	AL049246	Homo sapiens mRNA; cDNA DKFZp564C053 (from clone DKFZp564C053). [AL049246]	
A_23_P121234	1.02E-06	A_23_P121234			
A_32_P42574	1.02E-06	NM_032800	NM_032800	Homo sapiens hypothetical protein FLJ14525 (FLJ14525), mRNA [NM_032800]	NM_032800
A_23_P412764	1.03E-06	AB075838	AB075838	Homo sapiens mRNA for KIAA1958 protein. [AB075838]	
A_23_P18493	1.03E-06	NM_080685	NM_080685	Homo sapiens protein tyrosine phosphatase, non-receptor type 13 (APO-1/CD95 (Fas)-associated phosphatase) (PTPN13), transcript variant 4, mRNA [NM_080685]	NM_080685
A_23_P25674	1.03E-06	NM_001823	NM_001823	Homo sapiens creatine kinase, brain (CKB), mRNA [NM_001823]	NM_001823
A_23_P211136	1.04E-06	NM_018963	NM_018963	Homo sapiens bromodomain and WD repeat domain containing 1 (BRWD1), transcript variant 1, mRNA [NM_018963]	NM_018963
A_24_P686247	1.04E-06	THC2435579		ALU6_HUMAN (P39193) Alu subfamily SP sequence contamination warning entry, partial (22%) [THC2435579]	
A_23_P384056	1.04E-06	NM_022757	NM_022757	Homo sapiens coiled-coil domain containing 14 (CCDC14), mRNA [NM_022757]	NM_022757
A_23_P344481	1.05E-06	NM_152709	NM_152709	Homo sapiens storkhead box 1 (STOX1), mRNA [NM_152709]	NM_152709
A_23_P333640	1.06E-06	NM_173462	NM_173462	Homo sapiens papilin, proteoglycan-like sulfated glycoprotein (PAPLN), mRNA [NM_173462]	NM_173462
A_32_P121226	1.06E-06	A_32_P121226			
A_24_P122921	1.06E-06	THC2305303		Q6JTU6 (Q6JTU6) BCL2-like 11 transcript variant 9, complete [THC2305303]	
A_24_P932632	1.06E-06	CR617018	CR617018	full-length cDNA clone CS0DG001YH13 of B cells (Ramos cell line) of Homo sapiens (human). [CR617018]	
A_23_P27795	1.06E-06	NM_021102	NM_021102	Homo sapiens serine protease inhibitor, Kunitz type, 2 (SPINT2), mRNA [NM_021102]	NM_021102
A_23_P31073	1.07E-06	NM_005375	NM_005375	Homo sapiens v-myb myeloblastosis viral oncogene homolog (avian) (MYB), mRNA [NM_005375]	NM_005375
A_24_P402588	1.08E-06	NM_138553	NM_138553	Homo sapiens B-cell CLL/lymphoma 11A (zinc finger protein) (BCL11A), transcript variant 5, mRNA [NM_138553]	NM_138553
A_23_P162010	1.09E-06	NM_176875	NM_176875	Homo sapiens cholecystokinin B receptor (CCKBR), mRNA [NM_176875]	NM_176875
A_24_P555510	1.10E-06	THC2437881		Q9NBA9 (Q9NBA9) Stretchin-MLCK (Fragment), partial (5%) [THC2437881]	
A_32_P74955	1.11E-06	NM_152641	NM_152641	Homo sapiens AT rich interactive domain 2 (ARID, RFX-like) (ARID2), mRNA [NM_152641]	NM_152641
A_24_P942786	1.12E-06	AK024870	AK024870	Homo sapiens cDNA: FLJ21217 fis, clone COL00536. [AK024870]	
A_23_P123448	1.12E-06	AL136588	AL136588	Homo sapiens mRNA; cDNA DKFZp761D112 (from clone DKFZp761D112). [AL136588]	
A_23_P360329	1.12E-06	NM_017977	NM_017977	Homo sapiens absent in melanoma 1-like (AIM1L), mRNA [NM_017977]	NM_017977
A_24_P289383	1.12E-06	NM_017780	NM_017780	Homo sapiens chromodomain helicase DNA binding protein 7 (CHD7), mRNA [NM_017780]	NM_017780
A_24_P213161	1.12E-06	NM_017852	NM_017852	Homo sapiens NACHT, leucine rich repeat and PYD containing 2 (NALP2), mRNA [NM_017852]	NM_017852
A_32_P129269	1.15E-06	ENST00000283426		Homo sapiens mRNA for KIAA1909 protein, partial cds. [AB067496]	
A_32_P45375	1.16E-06	AF037219	AF037219	Homo sapiens PIX1 mRNA sequence. [AF037219]	
A_32_P89371	1.20E-06	NM_005054	NM_005054	Homo sapiens RAN binding protein 2-like 1 (RANBP2L1), transcript variant 1, mRNA [NM_005054]	NM_005054
A_23_P126075	1.20E-06	NM_002245	NM_002245	Homo sapiens potassium channel, subfamily K, member 1 (KCNK1), mRNA [NM_002245]	NM_002245
A_23_P41917	1.21E-06	NM_004272	NM_004272	Homo sapiens homer homolog 1 (Drosophila) (HOMER1), mRNA [NM_004272]	NM_004272
A_23_P206359	1.21E-06	NM_004360	NM_004360	Homo sapiens cadherin 1, type 1, E-cadherin (epithelial) (CDH1), mRNA [NM_004360]	NM_004360
A_23_P7783	1.22E-06	NM_016604	NM_016604	Homo sapiens jumonji domain containing 1B (JMJD1B), mRNA [NM_016604]	NM_016604
A_23_P49155	1.22E-06	NM_001793	NM_001793	Homo sapiens cadherin 3, type 1, P-cadherin (placental) (CDH3), mRNA [NM_001793]	NM_001793
A_32_P226205	1.23E-06	NM_033400	NM_033400	Homo sapiens zinc finger homeobox 2 (ZFHX2), mRNA [NM_033400]	NM_033400
A_23_P75529	1.24E-06	NM_022062	NM_022062	Homo sapiens PBX/knotted 1 homeobox 2 (PKNOX2), mRNA [NM_022062]	NM_022062
A_23_P201551	1.24E-06	NM_006113	NM_006113	Homo sapiens vav 3 oncogene (VAV3), mRNA [NM_006113]	NM_006113
A_24_P85942	1.24E-06	NM_181453	NM_181453	Homo sapiens GRIP and coiled-coil domain containing 2 (GCC2), transcript variant 1, mRNA [NM_181453]	NM_181453
A_32_P211752	1.24E-06	THC2315973			
A_24_P299685	1.26E-06	NM_198389	NM_198389	Homo sapiens podoplanin (PDPN), transcript variant 2, mRNA [NM_198389]	NM_198389

A_32_P192376	1.28E-06	THC2312617			
A_23_P134237	1.28E-06	NM_002889	NM_002889	Homo sapiens retinoic acid receptor responder (tazarotene induced) 2 (RARRES2), mRNA [NM_002889]	NM_002889
A_24_P26073	1.28E-06	NM_133259	NM_133259	Homo sapiens leucine-rich PPR-motif containing (LRPPRC), mRNA [NM_133259]	NM_133259
A_23_P168167	1.29E-06	NM_032511	NM_032511	Homo sapiens chromosome 6 open reading frame 168 (C6orf168), mRNA [NM_032511]	NM_032511
A_23_P167818	1.30E-06	NM_024581	NM_024581	Homo sapiens chromosome 6 open reading frame 60 (C6orf60), mRNA [NM_024581]	NM_024581
A_23_P25121	1.30E-06	NM_016594	NM_016594	Homo sapiens FK506 binding protein 11, 19 kDa (FKBP11), mRNA [NM_016594]	NM_016594
A_24_P52168	1.31E-06	A_24_P52168			
A_23_P103511	1.31E-06	AK125122	AK125122	Homo sapiens cDNA FLJ43132 fis, clone CTONG3005813. [AK125122]	XM_378908
A_23_P256008	1.31E-06	NM_024786	NM_024786	Homo sapiens zinc finger, DHHC-type containing 11 (ZDHHC11), mRNA [NM_024786]	NM_024786
A_23_P335039	1.31E-06	NM_133474	NM_133474	Homo sapiens KIAA1982 protein (KIAA1982), mRNA [NM_133474]	NM_133474
A_24_P153456	1.32E-06	NM_024786	NM_024786	Homo sapiens zinc finger, DHHC-type containing 11 (ZDHHC11), mRNA [NM_024786]	NM_024786
A_24_P13083	1.35E-06	NM_130783	NM_130783	Homo sapiens tetraspanin 18 (TSPAN18), mRNA [NM_130783]	NM_130783
A_23_P93269	1.35E-06	NM_003447	NM_003447	Homo sapiens zinc finger protein 165 (ZNF165), mRNA [NM_003447]	NM_003447
A_23_P213883	1.35E-06	NM_133433	NM_133433	Homo sapiens Nipped-B homolog (Drosophila) (NIPBL), transcript variant A, mRNA [NM_133433]	NM_133433
A_23_P314691	1.36E-06	NM_152382	NM_152382	Homo sapiens hypothetical protein FLJ37953 (FLJ37953), mRNA [NM_152382]	NM_152382
A_32_P127501	1.36E-06	AV753543	AV753543	AV753543 AV753543 NPD Homo sapiens cDNA clone NPDBEC03 5', mRNA sequence [AV753543]	
A_32_P114918	1.36E-06	BM665043	BM665043	UI-E-CQ1-aev-p-07-0-UI.s1 UI-E-CQ1 Homo sapiens cDNA clone UI-E-CQ1-aev-p-07-0-UI 3', mRNA sequence [BM665043]	
A_24_P712350	1.36E-06	NM_001821	NM_001821	Homo sapiens choroideremia-like (Rab escort protein 2) (CHML), mRNA [NM_001821]	NM_001821
A_23_P119593	1.36E-06	NM_024794	NM_024794	Homo sapiens abhydrolase domain containing 9 (ABHD9), mRNA [NM_024794]	NM_024794
A_23_P213620	1.39E-06	NM_004576	NM_004576	Homo sapiens protein phosphatase 2 (formerly 2A), regulatory subunit B (PR 52), beta isoform (PPP2R2B), transcript variant 1, mRNA [NM_004576]	NM_004576
A_23_P51660	1.39E-06	NM_012222	NM_012222	Homo sapiens mutY homolog (E. coli) (MUTYH), mRNA [NM_012222]	NM_012222
A_23_P127915	1.41E-06	NM_030906	NM_030906	Homo sapiens serine/threonine kinase 33 (STK33), mRNA [NM_030906]	NM_030906
A_23_P426021	1.41E-06	NM_015187	NM_015187	Homo sapiens KIAA0746 protein (KIAA0746), mRNA [NM_015187]	NM_015187
A_23_P380526	1.41E-06	NM_018189	NM_018189	Homo sapiens developmental pluripotency associated 4 (DPPA4), mRNA [NM_018189]	NM_018189
A_23_P52017	1.46E-06	NM_018136	NM_018136	Homo sapiens asp (abnormal spindle)-like, microcephaly associated (Drosophila) (ASPM), mRNA [NM_018136]	NM_018136
A_23_P200298	1.47E-06	NM_000028	NM_000028	Homo sapiens amylo-1, 6-glucosidase, 4-alpha-glucanotransferase (glycogen debranching enzyme, glycogen storage disease type III) (AGL), transcript variant 4, mRNA [NM_000028]	NM_000028
A_24_P383640	1.47E-06	AF268617	AF268617	Homo sapiens POU 5 domain protein (POU5FLC12) mRNA, complete cds. [AF268617]	XR_000266
A_24_P173823	1.49E-06	BC044624	BC044624	Homo sapiens cDNA clone IMAGE:5288080, partial cds. [BC044624]	
A_32_P47538	1.51E-06	BC037919	BC037919	Homo sapiens, clone IMAGE:5278089, mRNA. [BC037919]	
A_23_P215419	1.51E-06	NM_004968	NM_004968	Homo sapiens islet cell autoantigen 1, 69kDa (ICA1), transcript variant 2, mRNA [NM_004968]	NM_004968
A_24_P356916	1.52E-06	NM_001011554	NM_001011554	Homo sapiens solute carrier family 13 (sodium-dependent dicarboxylate transporter), member 3 (SLC13A3), transcript variant 2, mRNA [NM_001011554]	NM_001011554
A_23_P306105	1.52E-06	NM_020474	NM_020474	Homo sapiens UDP-N-acetyl-alpha-D-galactosamine:polypeptide N-acetylgalactosaminyltransferase 1 (GalNAc-T1) (GALNT1), mRNA [NM_020474]	NM_020474
A_24_P706314	1.52E-06	ENST00000306311		full-length cDNA clone CS0DJ006YK13 of T cells (Jurkat cell line) Cot 10-normalized of Homo sapiens (human). [CR620336]	
A_23_P210330	1.54E-06	ENST00000238875		full-length cDNA clone CS0DL009YB17 of B cells (Ramos cell line) Cot 25-normalized of Homo sapiens (human). [CR593568]	
A_23_P145984	1.55E-06	NM_012338	NM_012338	Homo sapiens tetraspanin 12 (TSPAN12), mRNA [NM_012338]	NM_012338
A_23_P30395	1.55E-06	AL161991	AL161991	Homo sapiens mRNA; cDNA DKFZp761C169 (from clone DKFZp761C169). [AL161991]	
A_23_P367676	1.55E-06	NM_015477	NM_015477	Homo sapiens SIN3 homolog A, transcription regulator (yeast) (SIN3A), mRNA [NM_015477]	NM_015477
A_24_P136711	1.56E-06	BC030757	BC030757	Homo sapiens, clone IMAGE:4797534, mRNA, partial cds. [BC030757]	
A_23_P133123	1.57E-06	NM_032117	NM_032117	Homo sapiens GAJ protein (GAJ), mRNA [NM_032117]	NM_032117
A_23_P251043	1.58E-06	NM_024893	NM_024893	Homo sapiens chromosome 20 open reading frame 39 (C20orf39), mRNA [NM_024893]	NM_024893
A_24_P926960	1.59E-06	AF086414	AF086414	Homo sapiens full length insert cDNA clone ZD77F06. [AF086414]	
A_23_P106675	1.60E-06	NM_002661	NM_002661	Homo sapiens phospholipase C, gamma 2 (phosphatidylinositol-specific) (PLCG2), mRNA [NM_002661]	NM_002661
A_23_P40108	1.61E-06	NM_001853	NM_001853	Homo sapiens collagen, type IX, alpha 3 (COL9A3), mRNA [NM_001853]	NM_001853
A_32_P186364	1.62E-06	BC031314	BC031314	Homo sapiens, clone IMAGE:5276765, mRNA. [BC031314]	

A_23_P154526	1.62E-06	NM_004490	NM_004490	Homo sapiens growth factor receptor-bound protein 14 (GRB14), mRNA [NM_004490]	NM_004490
A_23_P53417	1.62E-06	NM_006741	NM_006741	Homo sapiens protein phosphatase 1, regulatory (inhibitor) subunit 1A (PPP1R1A), mRNA [NM_006741]	NM_006741
A_24_P392661	1.62E-06	A_24_P392661			
A_23_P28015	1.63E-06	NM_144693	NM_144693	Homo sapiens zinc finger protein 558 (ZNF558), mRNA [NM_144693]	NM_144693
A_23_P10127	1.63E-06	NM_003012	NM_003012	Homo sapiens secreted frizzled-related protein 1 (SFRP1), mRNA [NM_003012]	NM_003012
A_32_P233799	1.64E-06	AB007953	AB007953	Homo sapiens mRNA, chromosome 1 specific transcript KIAA0484. [AB007953]	
A_32_P109755	1.64E-06	AF462446	AF462446	Homo sapiens unknown mRNA. [AF462446]	
A_32_P88349	1.64E-06	CR620599	CR620599	full-length cDNA clone CS0DF021YI14 of Fetal brain of Homo sapiens (human). [CR620599]	XM_498811
A_32_P49959	1.64E-06	BX111592	BX111592	BX111592 Soares_testis_NHT Homo sapiens cDNA clone IMAGp998D162621, mRNA sequence [BX111592]	
A_32_P132563	1.64E-06	NM_002701	NM_002701	Homo sapiens POU domain, class 5, transcription factor 1 (POU5F1), transcript variant 1, mRNA [NM_002701]	NM_002701
A_23_P88580	1.64E-06	NM_006465	NM_006465	Homo sapiens AT rich interactive domain 3B (BRIGHT- like) (ARID3B), mRNA [NM_006465]	NM_006465
A_23_P41424	1.66E-06	NM_022154	NM_022154	Homo sapiens solute carrier family 39 (zinc transporter), member 8 (SLC39A8), mRNA [NM_022154]	NM_022154
A_32_P223319	1.68E-06	NM_052911	NM_052911	Homo sapiens establishment of cohesion 1 homolog 1 (S. cerevisiae) (ESCO1), mRNA [NM_052911]	NM_052911
A_23_P142738	1.69E-06	NM_152390	NM_152390	Homo sapiens hypothetical protein MGC33926 (MGC33926), mRNA [NM_152390]	NM_152390
A_23_P30294	1.69E-06	NM_001801	NM_001801	Homo sapiens cysteine dioxygenase, type I (CDO1), mRNA [NM_001801]	NM_001801
A_24_P91852	1.71E-06	NM_006520	NM_006520	Homo sapiens t-complex-associated-testis-expressed 1-like (TCTE1L), mRNA [NM_006520]	NM_006520
A_23_P32414	1.71E-06	NM_016542	NM_016542	Homo sapiens Mst3 and SOK1-related kinase (MASK), mRNA [NM_016542]	NM_016542
A_23_P20852	1.73E-06	NM_001698	NM_001698	Homo sapiens AU RNA binding protein/enoyl-Coenzyme A hydratase (AUH), nuclear gene encoding mitochondrial protein, mRNA [NM_001698]	NM_001698
A_24_P921260	1.75E-06	AK022030	AK022030	Homo sapiens cDNA FLJ11968 fis, clone HEMBB1001133. [AK022030]	
A_23_P123596	1.76E-06	NM_000170	NM_000170	Homo sapiens glycine dehydrogenase (decarboxylating; glycine decarboxylase, glycine cleavage system protein P) (GLDC), mRNA [NM_000170]	NM_000170
A_24_P48403	1.77E-06	NM_005433	NM_005433	Homo sapiens v-yes-1 Yamaguchi sarcoma viral oncogene homolog 1 (YES1), mRNA [NM_005433]	NM_005433
A_32_P25397	1.78E-06	ENST00000328681		Homo sapiens cDNA FLJ12900 fis, clone NT2RP2004321. [AK022962]	
A_23_P54144	1.78E-06	NM_001202	NM_001202	Homo sapiens bone morphogenetic protein 4 (BMP4), transcript variant 1, mRNA [NM_001202]	NM_001202
A_23_P212617	1.78E-06	NM_003234	NM_003234	Homo sapiens transferrin receptor (p90, CD71) (TFRC), mRNA [NM_003234]	NM_003234
A_23_P337790	1.79E-06	NM_173082	NM_173082	Homo sapiens SNF2 histone linker PHD RING helicase (SHPRH), mRNA [NM_173082]	NM_173082
A_23_P156748	1.79E-06	NM_015245	NM_015245	Homo sapiens ankyrin repeat and sterile alpha motif domain containing 1 (ANKS1), mRNA [NM_015245]	NM_015245
A_23_P10025	1.80E-06	NM_006159	NM_006159	Homo sapiens NEL-like 2 (chicken) (NELL2), mRNA [NM_006159]	NM_006159
A_32_P134209	1.80E-06	THC2403913		AV752763 AV752763 NPD Homo sapiens cDNA clone NPDBCA03 5', mRNA sequence [AV752763]	
A_23_P391443	1.81E-06	AB032983	AB032983	Homo sapiens mRNA for KIAA1157 protein, partial cds. [AB032983]	XM_350880
A_23_P108437	1.81E-06	AK024850	AK024850	Homo sapiens cDNA: FLJ21197 fis, clone COL00201. [AK024850]	
A_23_P13753	1.82E-06	NM_006163	NM_006163	Homo sapiens nuclear factor (erythroid-derived 2), 45kDa (NFE2), mRNA [NM_006163]	NM_006163
A_23_P307400	1.82E-06	NM_138363	NM_138363	Homo sapiens hypothetical protein BC009518 (LOC90799), mRNA [NM_138363]	NM_138363
A_23_P66719	1.85E-06	NM_144683	NM_144683	Homo sapiens hypothetical protein MGC23280 (MGC23280), mRNA [NM_144683]	NM_144683
A_23_P391344	1.85E-06	NM_145313	NM_145313	Homo sapiens RasGEF domain family, member 1A (RASGEF1A), mRNA [NM_145313]	NM_145313
A_23_P40078	1.86E-06	NM_003400	NM_003400	Homo sapiens exportin 1 (CRM1 homolog, yeast) (XPO1), mRNA [NM_003400]	NM_003400
A_23_P92093	1.87E-06	NM_001407	NM_001407	Homo sapiens cadherin, EGF LAG seven-pass G-type receptor 3 (flamingo homolog, Drosophila) (CELSR3), mRNA [NM_001407]	NM_001407
A_32_P97169	1.89E-06	BX640888	BX640888	Homo sapiens mRNA; cDNA DKFZp686H20120 (from clone DKFZp686H20120). [BX640888]	
A_24_P169773	1.90E-06	AF338232	AF338232	Homo sapiens CTAGE-4 protein mRNA, complete cds. [AF338232]	XM_496933
A_23_P312174	1.90E-06	NM_015120	NM_015120	Homo sapiens Alstrom syndrome 1 (ALMS1), mRNA [NM_015120]	NM_015120
A_23_P429689	1.91E-06	BC016987	BC016987	Homo sapiens KIAA1327 protein, mRNA (cDNA clone MGC:21978 IMAGE:4395670), complete cds. [BC016987]	
A_24_P38316	1.92E-06	THC2374505			
A_24_P411186	1.92E-06	NM_022893	NM_022893	Homo sapiens B-cell CLL/lymphoma 11A (zinc finger protein) (BCL11A), transcript variant 1, mRNA [NM_022893]	NM_022893
A_23_P63379	1.92E-06	NM_012113	NM_012113	Homo sapiens carbonic anhydrase XIV (CA14), mRNA [NM_012113]	NM_012113
A_23_P131383	1.93E-06	NM_018062	NM_018062	Homo sapiens Fanconi anemia, complementation group L (FANCL), mRNA [NM_018062]	NM_018062

A_24_P55295	1.93E-06	NM_000165	NM_000165	Homo sapiens gap junction protein, alpha 1, 43kDa (connexin 43) (GJA1), mRNA [NM_000165]	NM_000165
A_23_P29680	1.94E-06	NM_024046	NM_024046	Homo sapiens CaM kinase-like vesicle-associated (CAMKV), mRNA [NM_024046]	NM_024046
A_23_P39574	1.94E-06	NM_173466	NM_173466	Homo sapiens hypothetical protein DKFZp434P055 (DKFZp434P055), mRNA [NM_173466]	NM_173466
A_23_P15174	1.94E-06	NM_005949	NM_005949	Homo sapiens metallothionein 1F (functional) (MT1F), mRNA [NM_005949]	NM_005949
A_23_P144165	1.94E-06	NM_014648	NM_014648	Homo sapiens zinc finger DAZ interacting protein 3 (DZIP3), mRNA [NM_014648]	NM_014648
A_24_P340659	1.94E-06	AF268613	AF268613	Homo sapiens POU 5 domain protein (POU5FLC1) mRNA, complete cds. [AF268613]	
A_23_P106922	1.95E-06	NM_021615	NM_021615	Homo sapiens carbohydrate (N-acetylglucosamine 6-O) sulfotransferase 6 (CHST6), mRNA [NM_021615]	NM_021615
A_32_P405942	1.96E-06	CR620977	CR620977	full-length cDNA clone CS0CAP004YK15 of Thymus of Homo sapiens (human). [CR620977]	
A_23_P217297	1.96E-06	NM_021998	NM_021998	Homo sapiens zinc finger protein 6 (CMPX1) (ZNF6), mRNA [NM_021998]	NM_021998
A_32_P129752	1.96E-06	NM_001017970	NM_001017970	Homo sapiens transmembrane protein 30B (TMEM30B), mRNA [NM_001017970]	NM_001017970
A_23_P12363	1.96E-06	NM_005012	NM_005012	Homo sapiens receptor tyrosine kinase-like orphan receptor 1 (ROR1), mRNA [NM_005012]	NM_005012
A_23_P308924	1.99E-06	AB051487	AB051487	Homo sapiens mRNA for KIAA1700 protein, partial cds. [AB051487]	
A_23_P213592	2.00E-06	NM_014901	NM_014901	Homo sapiens ring finger protein 44 (RNF44), mRNA [NM_014901]	NM_014901
A_23_P335329	2.02E-06	NM_004485	NM_004485	Homo sapiens guanine nucleotide binding protein (G protein), gamma 4 (GNG4), mRNA [NM_004485]	NM_004485
A_23_P209879	2.06E-06	AK128731	AK128731	Homo sapiens cDNA FLJ46899 fis, clone UTERU3022588, highly similar to Cyclic-AMP-dependent transcription factor ATF-2. [AK128731]	
A_32_P153725	2.06E-06	NM_015275	NM_015275	Homo sapiens KIAA1033 (KIAA1033), mRNA [NM_015275]	NM_015275
A_32_P221305	2.07E-06	BC073935	BC073935	Homo sapiens cDNA clone IMAGE:5219247, partial cds. [BC073935]	XM_498535
A_23_P132175	2.07E-06	NM_023004	NM_023004	Homo sapiens reticulon 4 receptor (RTN4R), mRNA [NM_023004]	NM_023004
A_24_P525749	2.07E-06	AK091547	AK091547	Homo sapiens cDNA FLJ34228 fis, clone FCBBF3025417. [AK091547]	
A_23_P154065	2.07E-06	NM_006000	NM_006000	Homo sapiens tubulin, alpha 1 (testis specific) (TUBA1), mRNA [NM_006000]	NM_006000
A_32_P146635	2.07E-06	CR603982	CR603982	full-length cDNA clone CS0DF021YL03 of Fetal brain of Homo sapiens (human). [CR603982]	
A_32_P35232	2.08E-06	THC2404169			
A_23_P319583	2.08E-06	NM_014747	NM_014747	Homo sapiens regulating synaptic membrane exocytosis 3 (RIMS3), mRNA [NM_014747]	NM_014747
A_32_P219116	2.08E-06	NM_018451	NM_018451	Homo sapiens centromere protein J (CENPJ), mRNA [NM_018451]	NM_018451
A_23_P28485	2.08E-06	NM_012198	NM_012198	Homo sapiens grancalcin, EF-hand calcium binding protein (GCA), mRNA [NM_012198]	NM_012198
A_24_P246361	2.09E-06	NM_006633	NM_006633	Homo sapiens IQ motif containing GTPase activating protein 2 (IQGAP2), mRNA [NM_006633]	NM_006633
A_23_P382043	2.09E-06	NM_152729	NM_152729	Homo sapiens 5'-nucleotidase, cytosolic II-like 1 (NT5C2L1), mRNA [NM_152729]	NM_152729
A_23_P205489	2.10E-06	NM_182728	NM_182728	Homo sapiens solute carrier family 7 (cationic amino acid transporter, y+ system), member 8 (SLC7A8), transcript variant 2, mRNA [NM_182728]	NM_182728
A_23_P203332	2.10E-06	NM_022074	NM_022074	Homo sapiens FLJ22794 protein (FLJ22794), transcript variant 1, mRNA [NM_022074]	NM_022074
A_23_P48561	2.10E-06	NM_005864	NM_005864	Homo sapiens embryonal Fyn-associated substrate (EFS), transcript variant 1, mRNA [NM_005864]	NM_005864
A_32_P52046	2.12E-06	A_32_P52046			
A_24_P886040	2.12E-06	NM_152624	NM_152624	Homo sapiens DCP2 decapping enzyme homolog (S. cerevisiae) (DCP2), mRNA [NM_152624]	NM_152624
A_24_P116535	2.15E-06	NM_002428	NM_002428	Homo sapiens matrix metalloproteinase 15 (membrane-inserted) (MMP15), mRNA [NM_002428]	NM_002428
A_23_P361419	2.16E-06	NM_018369	NM_018369	Homo sapiens DEP domain containing 1B (DEPDC1B), mRNA [NM_018369]	NM_018369
A_23_P386	2.16E-06	NM_018125	NM_018125	Homo sapiens Rho guanine nucleotide exchange factor (GEF) 10-like (ARHGEF10L), transcript variant 1, mRNA [NM_018125]	NM_018125
A_24_P166473	2.17E-06	AK023682	AK023682	Homo sapiens cDNA FLJ13620 fis, clone PLACE1010947. [AK023682]	
A_23_P74042	2.17E-06	NM_012302	NM_012302	Homo sapiens latrophilin 2 (LPHN2), mRNA [NM_012302]	NM_012302
A_23_P218608	2.18E-06	NM_015904	NM_015904	Homo sapiens eukaryotic translation initiation factor 5B (EIF5B), mRNA [NM_015904]	NM_015904
A_24_P13041	2.19E-06	NM_145307	NM_145307	Homo sapiens pleckstrin homology domain containing, family K member 1 (PLEKHK1), mRNA [NM_145307]	NM_145307
A_24_P92183	2.19E-06	ENST00000255136		Homo sapiens, clone IMAGE:4745538, mRNA. [BC039151]	XM_114158
A_23_P128728	2.20E-06	NM_001172	NM_001172	Homo sapiens arginase, type II (ARG2), nuclear gene encoding mitochondrial protein, mRNA [NM_001172]	NM_001172
A_24_P191833	2.21E-06	NM_139168	NM_139168	Homo sapiens splicing factor, arginine/serine-rich 12 (SFRS12), mRNA [NM_139168]	NM_139168
A_24_P371962	2.21E-06	NM_001634	NM_001634	Homo sapiens adenosylmethionine decarboxylase 1 (AMD1), mRNA [NM_001634]	NM_001634
A_23_P148807	2.22E-06	NM_003503	NM_003503	Homo sapiens CDC7 cell division cycle 7 (S. cerevisiae) (CDC7), mRNA [NM_003503]	NM_003503

A_23_P100001	2.22E-06	NM_207446	NM_207446	Homo sapiens hypothetical gene supported by AK075564; BC060873 (LOC400451), mRNA [NM_207446]	NM_207446
A_23_P364465	2.22E-06	NM_022913	NM_022913	Homo sapiens GC-rich promoter binding protein 1 (GPBP1), mRNA [NM_022913]	NM_022913
A_23_P118722	2.23E-06	NM_001671	NM_001671	Homo sapiens asialoglycoprotein receptor 1 (ASGR1), mRNA [NM_001671]	NM_001671
A_24_P706312	2.25E-06	ENST00000306311		full-length cDNA clone CS0DF004YO22 of Fetal brain of Homo sapiens (human). [CR603865]	
A_23_P154447	2.25E-06	NM_015934	NM_015934	Homo sapiens nucleolar protein NOP5/NOP58 (NOP5/NOP58), mRNA [NM_015934]	NM_015934
A_23_P33173	2.26E-06	NM_004487	NM_004487	Homo sapiens golgi autoantigen, golgin subfamily b, macrogolgin (with transmembrane signal), 1 (GOLGB1), mRNA [NM_004487]	NM_004487
A_24_P823708	2.27E-06	BC021732	BC021732	Homo sapiens, clone IMAGE:4795401, mRNA. [BC021732]	
A_32_P3998	2.28E-06	NM_001004301	NM_001004301	Homo sapiens FLJ16542 protein (FLJ16542), mRNA [NM_001004301]	NM_001004301
A_23_P202170	2.30E-06	NM_012215	NM_012215	Homo sapiens meningioma expressed antigen 5 (hyaluronidase) (MGEA5), mRNA [NM_012215]	NM_012215
A_23_P214876	2.32E-06	NM_004973	NM_004973	Homo sapiens Jumonji, AT rich interactive domain 2 (JARID2), mRNA [NM_004973]	NM_004973
A_32_P137336	2.34E-06	BC013077	BC013077	Homo sapiens, clone IMAGE:3459334, mRNA. [BC013077]	
A_23_P154266	2.34E-06	NM_017952	NM_017952	Homo sapiens FLJ20758 protein (FLJ20758), mRNA [NM_017952]	NM_017952
A_23_P424	2.35E-06	NM_018650	NM_018650	Homo sapiens MAP/microtubule affinity-regulating kinase 1 (MARK1), mRNA [NM_018650]	NM_018650
A_32_P72351	2.35E-06	AK026140	AK026140	Homo sapiens cDNA: FLJ22487 fis, clone HRC10931. [AK026140]	
A_23_P319133	2.40E-06	NM_018981	NM_018981	Homo sapiens DnaJ (Hsp40) homolog, subfamily C, member 10 (DNAJC10), mRNA [NM_018981]	NM_018981
A_23_P201998	2.42E-06	THC2372371			
A_23_P166566	2.43E-06	NM_024768	NM_024768	Homo sapiens hypothetical protein FLJ12057 (FLJ12057), mRNA [NM_024768]	NM_024768
A_23_P204246	2.43E-06	NM_004426	NM_004426	Homo sapiens polyhomeotic-like 1 (Drosophila) (PHC1), mRNA [NM_004426]	NM_004426
A_23_P435697	2.43E-06	NM_015608	NM_015608	Homo sapiens chromosome 10 open reading frame 137 (C10orf137), mRNA [NM_015608]	NM_015608
A_23_P28307	2.43E-06	NP1165618		GB|AL832534.1|AL832534.1 Homo sapiens mRNA; cDNA DKFZp547I2016 (from clone DKFZp547I2016) [NP1165618]	
A_23_P63232	2.43E-06	NM_130898	NM_130898	Homo sapiens cAMP responsive element binding protein 3-like 4 (CREB3L4), mRNA [NM_130898]	NM_130898
A_23_P110433	2.46E-06	NM_015342	NM_015342	Homo sapiens peptidylprolyl isomerase domain and WD repeat containing 1 (PPWD1), mRNA [NM_015342]	NM_015342
A_23_P383118	2.49E-06	AB040944	AB040944	Homo sapiens mRNA for KIAA1511 protein, partial cds. [AB040944]	XM_046581
A_23_P80594	2.49E-06	NM_015184	NM_015184	Homo sapiens phospholipase C-like 2 (PLCL2), mRNA [NM_015184]	NM_015184
A_32_P226858	2.49E-06	XM_378898	XM_378898	PREDICTED: Homo sapiens hypothetical LOC400782 (LOC400782), mRNA [XM_378898]	XM_378898
A_24_P105102	2.49E-06	NM_182687	NM_182687	Homo sapiens protein kinase, membrane associated tyrosine/threonine 1 (PKMYT1), transcript variant 2, mRNA [NM_182687]	NM_182687
A_32_P209778	2.50E-06	AA151106	AA151106	zl48e07.s1 Soares_pregnant_uterus_NbHPU Homo sapiens cDNA clone IMAGE:505188 3', mRNA sequence [AA151106]	
A_23_P415510	2.51E-06	NM_005558	NM_005558	Homo sapiens ladinin 1 (LAD1), mRNA [NM_005558]	NM_005558
A_32_P202134	2.51E-06	AB051463	AB051463	Homo sapiens mRNA for KIAA1676 protein, partial cds. [AB051463]	
A_23_P121095	2.52E-06	NM_001068	NM_001068	Homo saipens topoisomerase (DNA) II beta 180kDa (TOP2B), mRNA [NM_001068]	NM_001068
A_24_P303454	2.53E-06	NM_012454	NM_012454	Homo sapiens T-cell lymphoma invasion and metastasis 2 (TIAM2), transcript variant 1, mRNA [NM_012454]	NM_012454
A_23_P111797	2.55E-06	AL136837	AL136837	Homo sapiens mRNA; cDNA DKFZp434F142 (from clone DKFZp434F142). [AL136837]	
A_24_P940725	2.56E-06	AL080186	AL080186	Homo sapiens mRNA; cDNA DKFZp564B0769 (from clone DKFZp564B0769); partial cds. [AL080186]	
A_23_P427217	2.58E-06	NM_004241	NM_004241	Homo sapiens jumonji domain containing 1C (JMJD1C), mRNA [NM_004241]	NM_004241
A_23_P338952	2.58E-06	NM_022753	NM_022753	Homo sapiens S100P binding protein Riken (S100PBPR), transcript variant 1, mRNA [NM_022753]	NM_022753
A_23_P122615	2.60E-06	NM_032870	NM_032870	Homo sapiens chromosome 6 open reading frame 111 (C6orf111), mRNA [NM_032870]	NM_032870
A_32_P152767	2.60E-06	NM_207371	NM_207371	Homo sapiens FLJ45187 protein (FLJ45187), mRNA [NM_207371]	NM_207371
A_32_P211708	2.60E-06	THC2277716			
A_23_P6561	2.60E-06	NM_018029	NM_018029	Homo sapiens hypothetical protein FLJ10213 (FLJ10213), mRNA [NM_018029]	NM_018029
A_23_P252371	2.60E-06	NM_002894	NM_002894	Homo sapiens retinoblastoma binding protein 8 (RBBP8), transcript variant 1, mRNA [NM_002894]	NM_002894
A_23_P30495	2.60E-06	NM_000859	NM_000859	Homo sapiens 3-hydroxy-3-methylglutaryl-Coenzyme A reductase (HMGCR), mRNA [NM_000859]	NM_000859
A_23_P22378	2.63E-06	NM_003108	NM_003108	Homo sapiens SRY (sex determining region Y)-box 11 (SOX11), mRNA [NM_003108]	NM_003108
A_23_P154740	2.63E-06	NM_018474	NM_018474	Homo sapiens chromosome 20 open reading frame 19 (C20orf19), mRNA [NM_018474]	NM_018474
A_32_P108254	2.63E-06	NM_017565	NM_017565	Homo sapiens family with sequence similarity 20, member A (FAM20A), mRNA [NM_017565]	NM_017565

A_23_P216556	2.64E-06	NM_018424	NM_018424	Homo sapiens erythrocyte membrane protein band 4.1 like 4B (EPB41L4B), transcript variant 1, mRNA [NM_018424]	NM_018424
A_32_P139196	2.65E-06	NM_213723	NM_213723	Homo sapiens chromosome 13 open reading frame 25 (C13orf25), transcript variant 2, mRNA [NM_213723]	NM_213723
A_32_P84242	2.65E-06	CR936791	CR936791	Homo sapiens mRNA; cDNA DKFZp781C2356 (from clone DKFZp781C2356). [CR936791]	XM_032571
A_24_P144601	2.65E-06	NM_002701	NM_002701	Homo sapiens POU domain, class 5, transcription factor 1 (POU5F1), transcript variant 1, mRNA [NM_002701]	NM_002701
A_23_P96420	2.67E-06	NM_002139	NM_002139	Homo sapiens RNA binding motif protein, X-linked (RBMX), mRNA [NM_002139]	NM_002139
A_23_P130149	2.68E-06	NM_001976	NM_001976	Homo sapiens enolase 3 (beta, muscle) (ENO3), transcript variant 1, mRNA [NM_001976]	NM_001976
A_32_P95914	2.69E-06	CR749603	CR749603	Homo sapiens mRNA; cDNA DKFZp686C20164 (from clone DKFZp686C20164). [CR749603]	
A_23_P8664	2.69E-06	NM_021145	NM_021145	Homo sapiens cyclin D binding myb-like transcription factor 1 (DMTF1), mRNA [NM_021145]	NM_021145
A_23_P125705	2.70E-06	NM_021963	NM_021963	Homo sapiens nucleosome assembly protein 1-like 2 (NAP1L2), mRNA [NM_021963]	NM_021963
A_24_P131752	2.70E-06	AL831922	AL831922	Homo sapiens mRNA; cDNA DKFZp761P0818 (from clone DKFZp761P0818). [AL831922]	
A_24_P90216	2.72E-06	NM_018490	NM_018490	Homo sapiens leucine-rich repeat-containing G protein-coupled receptor 4 (LGR4), mRNA [NM_018490]	NM_018490
A_23_P154025	2.73E-06	NM_003142	NM_003142	Homo sapiens Sjogren syndrome antigen B (autoantigen La) (SSB), mRNA [NM_003142]	NM_003142
A_24_P235248	2.74E-06	NM_002851	NM_002851	Homo sapiens protein tyrosine phosphatase, receptor-type, Z polypeptide 1 (PTPRZ1), mRNA [NM_002851]	NM_002851
A_24_P158193	2.75E-06	A_24_P158193			
A_24_P109082	2.75E-06	NM_020935	NM_020935	Homo sapiens ubiquitin specific protease 37 (USP37), mRNA [NM_020935]	NM_020935
A_23_P218637	2.75E-06	NM_005054	NM_005054	Homo sapiens RAN binding protein 2-like 1 (RANBP2L1), transcript variant 1, mRNA [NM_005054]	NM_005054
A_23_P202520	2.75E-06	NM_001003408	NM_001003408	Homo sapiens actin binding LIM protein 1 (ABLIM1), transcript variant 3, mRNA [NM_001003408]	NM_001003408
A_23_P250813	2.78E-06	NM_000553	NM_000553	Homo sapiens Werner syndrome (WRN), mRNA [NM_000553]	NM_000553
A_24_P132099	2.80E-06	NM_138730	NM_138730	Homo sapiens high mobility group nucleosomal binding domain 3 (HMGN3), transcript variant 2, mRNA [NM_138730]	NM_138730
A_23_P205098	2.81E-06	NM_015032	NM_015032	Homo sapiens androgen-induced proliferation inhibitor (APRIN), transcript variant 1, mRNA [NM_015032]	NM_015032
A_23_P82950	2.81E-06	NM_006197	NM_006197	Homo sapiens pericentriolar material 1 (PCM1), mRNA [NM_006197]	NM_006197
A_23_P147431	2.83E-06	NM_002350	NM_002350	Homo sapiens v-yes-1 Yamaguchi sarcoma viral related oncogene homolog (LYN), mRNA [NM_002350]	NM_002350
A_23_P426511	2.84E-06	NM_138698	NM_138698	Homo sapiens prematurely terminated mRNA decay factor-like (LOC91431), mRNA [NM_138698]	NM_138698
A_24_P942250	2.84E-06	NM_025134	NM_025134	Homo sapiens chromodomain helicase DNA binding protein 9 (CHD9), mRNA [NM_025134]	NM_025134
A_24_P296808	2.84E-06	NM_018215	NM_018215	Homo sapiens hypothetical protein FLJ10781 (FLJ10781), mRNA [NM_018215]	NM_018215
A_23_P217409	2.84E-06	NM_004652	NM_004652	Homo sapiens ubiquitin specific protease 9, X-linked (fat facets-like, Drosophila) (USP9X), transcript variant 1, mRNA [NM_004652]	NM_004652
A_32_P4018	2.85E-06	AK000776	AK000776	Homo sapiens cDNA FLJ20769 fis, clone COL06674. [AK000776]	
A_23_P151426	2.85E-06	NM_002015	NM_002015	Homo sapiens forkhead box O1A (rhabdomyosarcoma) (FOXO1A), mRNA [NM_002015]	NM_002015
A_23_P19702	2.85E-06	NM_145342	NM_145342	Homo sapiens mitogen-activated protein kinase kinase kinase 7 interacting protein 2 (MAP3K7IP2), transcript variant 2, mRNA [NM_145342]	NM_145342
A_23_P70371	2.87E-06	NM_015153	NM_015153	Homo sapiens PHD finger protein 3 (PHF3), mRNA [NM_015153]	NM_015153
A_23_P22422	2.87E-06	NM_013364	NM_013364	Homo sapiens paraneoplastic antigen MA3 (PNMA3), mRNA [NM_013364]	NM_013364
A_23_P18196	2.87E-06	NM_002916	NM_002916	Homo sapiens replication factor C (activator 1) 4, 37kDa (RFC4), transcript variant 1, mRNA [NM_002916]	NM_002916
A_23_P251505	2.89E-06	NM_148956	NM_148956	Homo sapiens NOL1/NOP2/Sun domain family, member 5 (NSUN5), transcript variant 1, mRNA [NM_148956]	NM_148956
A_23_P216038	2.90E-06	NM_032205	NM_032205	Homo sapiens PHD finger protein 20-like 1 (PHF20L1), transcript variant 2, mRNA [NM_032205]	NM_032205
A_32_P78101	2.91E-06	NM_032880	NM_032880	Homo sapiens immunoglobin superfamily, member 21 (IGSF21), mRNA [NM_032880]	NM_032880
A_23_P404211	2.92E-06	ENST00000267430		Homo sapiens mRNA for KIAA1596 protein, partial cds. [AB046816]	XM_048128
A_23_P40217	2.92E-06	NM_018431	NM_018431	Homo sapiens docking protein 5 (DOK5), transcript variant 1, mRNA [NM_018431]	NM_018431
A_24_P208909	2.92E-06	NM_015271	NM_015271	Homo sapiens tripartite motif-containing 2 (TRIM2), mRNA [NM_015271]	NM_015271
A_32_P65473	2.93E-06	ENST00000330640		Homo sapiens, clone IMAGE:2899977, mRNA, partial cds. [BC022980]	
A_23_P23303	2.93E-06	NM_003686	NM_003686	Homo sapiens exonuclease 1 (EXO1), transcript variant 3, mRNA [NM_003686]	NM_003686
A_23_P388798	2.94E-06	AF467442	AF467442	Homo sapiens Smith-Magenis syndrome chromosome region candidate 5 protein (SMCR5) mRNA, complete cds. [AF467442]	
A_23_P107903	2.95E-06	AK126263	AK126263	Homo sapiens cDNA FLJ44275 fis, clone TOVAR2002800. [AK126263]	XM_290867
A_23_P403398	2.97E-06	NR_002186	NR_002186	Homo sapiens hypothetical protein DKFZp586I1420 (DKFZp586I1420) on chromosome 7 [NR_002186]	NR_002186
A_23_P217528	2.97E-06	NM_007250	NM_007250	Homo sapiens Kruppel-like factor 8 (KLF8), mRNA [NM_007250]	NM_007250

A_23_P52410	2.98E-06	NM_145307	NM_145307	Homo sapiens pleckstrin homology domain containing, family K member 1 (PLEKHK1), mRNA [NM_145307]	NM_145307
A_23_P30020	3.01E-06	NM_030821	NM_030821	Homo sapiens phospholipase A2, group XIIA (PLA2G12A), mRNA [NM_030821]	NM_030821
A_24_P345290	3.03E-06	A_24_P345290			
A_32_P89679	3.03E-06	THC2311626		ALU1_HUMAN (P39188) Alu subfamily J sequence contamination warning entry, partial (10%) [THC2311626]	
A_23_P359647	3.03E-06	NM_138714	NM_138714	Homo sapiens nuclear factor of activated T-cells 5, tonicity-responsive (NFAT5), transcript variant 1, mRNA [NM_138714]	NM_138714
A_23_P4353	3.03E-06	NM_134264	NM_134264	Homo sapiens WD repeat and SOCS box-containing 1 (WSB1), transcript variant 3, mRNA [NM_134264]	NM_134264
A_23_P344568	3.04E-06	NM_145019	NM_145019	Homo sapiens hypothetical protein FLJ30707 (FLJ30707), mRNA [NM_145019]	NM_145019
A_32_P34495	3.04E-06	AK026418	AK026418	Homo sapiens cDNA: FLJ22765 fis, clone KAIA1180. [AK026418]	
A_23_P69738	3.04E-06	NM_023940	NM_023940	Homo sapiens RAS-like, family 11, member B (RASL11B), mRNA [NM_023940]	NM_023940
A_32_P60223	3.04E-06	NM_032329	NM_032329	Homo sapiens inhibitor of growth family, member 5 (ING5), mRNA [NM_032329]	NM_032329
A_24_P119201	3.10E-06	NM_015832	NM_015832	Homo sapiens methyl-CpG binding domain protein 2 (MBD2), transcript variant testis-specific, mRNA [NM_015832]	NM_015832
A_23_P88099	3.11E-06	AK092739	AK092739	Homo sapiens cDNA FLJ35420 fis, clone SMINT2001183. [AK092739]	
A_32_P229818	3.12E-06	AK022044	AK022044	Homo sapiens cDNA FLJ11982 fis, clone HEMBB1001335. [AK022044]	
A_32_P207428	3.12E-06	BC007307	BC007307	Homo sapiens, Similar to zinc finger protein 268, clone IMAGE:3352268, mRNA, partial cds. [BC007307]	XM_039908
A_23_P148919	3.12E-06	NM_000098	NM_000098	Homo sapiens carnitine palmitoyltransferase II (CPT2), nuclear gene encoding mitochondrial protein, mRNA [NM_000098]	NM_000098
A_23_P416468	3.12E-06	ENST00000268043		Homo sapiens DNA helicase homolog (PIF1) mRNA, partial cds. [AF108138]	
A_23_P59138	3.12E-06	NM_002701	NM_002701	Homo sapiens POU domain, class 5, transcription factor 1 (POU5F1), transcript variant 1, mRNA [NM_002701]	NM_002701
A_32_P148745	3.13E-06	AK027618	AK027618	Homo sapiens cDNA FLJ14712 fis, clone NT2RP3000825, weakly similar to NEUROGENIC LOCUS NOTCH 3 PROTEIN. [AK027618]	XM_371878
A_23_P78053	3.17E-06	NM_030802	NM_030802	Homo sapiens C/EBP-induced protein (LOC81558), mRNA [NM_030802]	NM_030802
A_23_P6914	3.17E-06	NM_022776	NM_022776	Homo sapiens oxysterol binding protein-like 11 (OSBPL11), mRNA [NM_022776]	NM_022776
A_23_P131664	3.17E-06	NM_006267	NM_006267	Homo sapiens RAN binding protein 2 (RANBP2), mRNA [NM_006267]	NM_006267
A_23_P19369	3.19E-06	NM_017640	NM_017640	Homo sapiens leucine rich repeat containing 16 (LRRC16), mRNA [NM_017640]	NM_017640
A_23_P163711	3.21E-06	NM_031478	NM_031478	Homo sapiens family with sequence similarity 57, member B (FAM57B), mRNA [NM_031478]	NM_031478
A_23_P153745	3.21E-06	NM_006332	NM_006332	Homo sapiens interferon, gamma-inducible protein 30 (IFI30), mRNA [NM_006332]	NM_006332
A_23_P105251	3.24E-06	NM_005269	NM_005269	Homo sapiens glioma-associated oncogene homolog 1 (zinc finger protein) (GLI1), mRNA [NM_005269]	NM_005269
A_23_P352291	3.24E-06	NM_054016	NM_054016	Homo sapiens FUS interacting protein (serine/arginine-rich) 1 (FUSIP1), transcript variant 2, mRNA [NM_054016]	NM_054016
A_23_P143348	3.29E-06	NM_021220	NM_021220	Homo sapiens ovo-like 2 (Drosophila) (OVOL2), mRNA [NM_021220]	NM_021220
A_23_P82402	3.29E-06	NM_138426	NM_138426	Homo sapiens glucocorticoid induced transcript 1 (GLCCI1), mRNA [NM_138426]	NM_138426
A_23_P146990	3.30E-06	NM_007013	NM_007013	Homo sapiens WW domain containing E3 ubiquitin protein ligase 1 (WWP1), mRNA [NM_007013]	NM_007013
A_23_P2831	3.30E-06	NM_003991	NM_003991	Homo sapiens endothelin receptor type B (EDNRB), transcript variant 2, mRNA [NM_003991]	NM_003991
A_23_P97584	3.30E-06	NM_014597	NM_014597	Homo sapiens estrogen receptor binding protein (ERBP), mRNA [NM_014597]	NM_014597
A_23_P53646	3.30E-06	NM_139207	NM_139207	Homo sapiens nucleosome assembly protein 1-like 1 (NAP1L1), transcript variant 1, mRNA [NM_139207]	NM_139207
A_23_P70297	3.31E-06	AB023174	AB023174	Homo sapiens mRNA for KIAA0957 protein, partial cds. [AB023174]	
A_23_P167256	3.31E-06	AK127326	AK127326	Homo sapiens cDNA FLJ45397 fis, clone BRHIP3027651, weakly similar to Bromodomain-containing protein 1. [AK127326]	
A_23_P304489	3.33E-06	NM_015381	NM_015381	Homo sapiens family with sequence similarity 19 (chemokine (C-C motif)-like), member A5 (FAM19A5), mRNA [NM_015381]	NM_015381
A_32_P140475	3.34E-06	NM_020802	NM_020802	Homo sapiens KIAA1377 protein (KIAA1377), mRNA [NM_020802]	NM_020802
A_24_P214841	3.34E-06	NM_002701	NM_002701	Homo sapiens POU domain, class 5, transcription factor 1 (POU5F1), transcript variant 1, mRNA [NM_002701]	NM_002701
A_23_P366376	3.35E-06	NM_003212	NM_003212	Homo sapiens teratocarcinoma-derived growth factor 1 (TDGF1), mRNA [NM_003212]	NM_003212
A_23_P354894	3.37E-06	NM_152603	NM_152603	Homo sapiens zinc finger protein 567 (ZNF567), mRNA [NM_152603]	NM_152603
A_24_P193295	3.39E-06	NM_198686	NM_198686	Homo sapiens RAB15, member RAS onocogene family (RAB15), mRNA [NM_198686]	NM_198686
A_23_P322756	3.40E-06	AB051463	AB051463	Homo sapiens mRNA for KIAA1676 protein, partial cds. [AB051463]	
A_23_P250800	3.41E-06	NM_006100	NM_006100	Homo sapiens ST3 beta-galactoside alpha-2,3-sialyltransferase 6 (ST3GAL6), mRNA [NM_006100]	NM_006100
A_23_P45875	3.41E-06	NM_003292	NM_003292	Homo sapiens translocated promoter region (to activated MET oncogene) (TPR), mRNA [NM_003292]	NM_003292
A_23_P75071	3.44E-06	NM_016195	NM_016195	Homo sapiens M-phase phosphoprotein 1 (MPHOSPH1), mRNA [NM_016195]	NM_016195

A_23_P157726	3.45E-06	NM_017925	NM_017925	Homo sapiens chromosome 9 open reading frame 55 (C9orf55), mRNA [NM_017925]	NM_017925
A_23_P74895	3.46E-06	NM_024674	NM_024674	Homo sapiens lin-28 homolog (C. elegans) (LIN28), mRNA [NM_024674]	NM_024674
A_24_P844995	3.47E-06	AK054826	AK054826	Homo sapiens cDNA FLJ30264 fis, clone BRACE2002613, moderately similar to Rattus norvegicus rsly1p mRNA. [AK054826]	
A_32_P164246	3.47E-06	NM_033260	NM_033260	Homo sapiens forkhead box Q1 (FOXQ1), mRNA [NM_033260]	NM_033260
A_23_P25587	3.47E-06	NM_007015	NM_007015	Homo sapiens leukocyte cell derived chemotaxin 1 (LECT1), transcript variant 1, mRNA [NM_007015]	NM_007015
A_23_P54517	3.47E-06	NM_006293	NM_006293	Homo sapiens TYRO3 protein tyrosine kinase (TYRO3), mRNA [NM_006293]	NM_006293
A_32_P172141	3.48E-06	NM_016952	NM_016952	Homo sapiens cell adhesion molecule-related/down-regulated by oncogenes (CDON), mRNA [NM_016952]	NM_016952
A_24_P69379	3.48E-06	NM_022900	NM_022900	Homo sapiens O-acetyltransferase (CAS1), mRNA [NM_022900]	NM_022900
A_23_P94911	3.49E-06	AK098175	AK098175	Homo sapiens cDNA FLJ40856 fis, clone TRACH2016498, moderately similar to ZINC FINGER PROTEIN 184. [AK098175]	XM_371174
A_23_P355536	3.51E-06	AK127213	AK127213	Homo sapiens cDNA FLJ45280 fis, clone BRHIP3001360. [AK127213]	
A_23_P110661	3.51E-06	NM_015360	NM_015360	Homo sapiens superkiller viralicidic activity 2-like 2 (S. cerevisiae) (SKIV2L2), mRNA [NM_015360]	NM_015360
A_23_P135239	3.52E-06	NM_005077	NM_005077	Homo sapiens transducin-like enhancer of split 1 (E(sp1) homolog, Drosophila) (TLE1), mRNA [NM_005077]	NM_005077
A_24_P29277	3.53E-06	AK125387	AK125387	Homo sapiens cDNA FLJ43397 fis, clone OCBBF2009788. [AK125387]	
A_23_P393051	3.54E-06	NM_152365	NM_152365	Homo sapiens chromosome 1 open reading frame 172 (C1orf172), mRNA [NM_152365]	NM_152365
A_32_P42197	3.56E-06	NM_031157	NM_031157	Homo sapiens heterogeneous nuclear ribonucleoprotein A1 (HNRPA1), transcript variant 2, mRNA [NM_031157]	NM_031157
A_23_P152420	3.57E-06	NM_014615	NM_014615	Homo sapiens KIAA0182 protein (KIAA0182), mRNA [NM_014615]	NM_014615
A_24_P100996	3.58E-06	ENST00000324559		Homo sapiens mRNA; cDNA DKFZp451A148 (from clone DKFZp451A148). [AL833271]	
A_23_P108028	3.58E-06	NM_007145	NM_007145	Homo sapiens zinc finger protein 146 (ZNF146), mRNA [NM_007145]	NM_007145
A_32_P53558	3.59E-06	ENST00000341569		PREDICTED: Homo sapiens similar to Nedd-4-like E3 ubiquitin-protein ligase WWP1 (WW domain-containing protein 1) (LOC339843), mRNA [XM_290351]	XM_290351
A_32_P167459	3.59E-06	AK021744	AK021744	Homo sapiens cDNA FLJ11682 fis, clone HEMBA1004880. [AK021744]	
A_24_P383609	3.59E-06	NM_199461	NM_199461	Homo sapiens nanos homolog 1 (Drosophila) (NANOS1), transcript variant 1, mRNA [NM_199461]	NM_199461
A_24_P177353	3.59E-06	BC054050	BC054050	Homo sapiens THO complex 2, mRNA (cDNA clone IMAGE:5556338), partial cds. [BC054050]	
A_24_P149023	3.61E-06	NM_004866	NM_004866	Homo sapiens secretory carrier membrane protein 1 (SCAMP1), transcript variant 1, mRNA [NM_004866]	NM_004866
A_23_P148015	3.61E-06	NM_004655	NM_004655	Homo sapiens axin 2 (conductin, axil) (AXIN2), mRNA [NM_004655]	NM_004655
A_32_P208424	3.62E-06	NM_002107	NM_002107	Homo sapiens H3 histone, family 3A (H3F3A), mRNA [NM_002107]	NM_002107
A_24_P943957	3.63E-06	NM_015040	NM_015040	Homo sapiens phosphatidylinositol-3-phosphate/phosphatidylinositol 5-kinase, type III (PIP5K3), transcript variant 2, mRNA [NM_015040]	NM_015040
A_23_P86100	3.76E-06	NM_001007255	NM_001007255	Homo sapiens kelch/ankyrin repeat containing cyclin A1 interacting protein (KARCA1), transcript variant 2, mRNA [NM_001007255]	NM_001007255
A_23_P45345	3.79E-06	NM_014500	NM_014500	Homo sapiens HIV TAT specific factor 1 (HTATSF1), mRNA [NM_014500]	NM_014500
A_23_P125265	3.80E-06	NM_002266	NM_002266	Homo sapiens karyopherin alpha 2 (RAG cohort 1, importin alpha 1) (KPNA2), mRNA [NM_002266]	NM_002266
A_24_P454313	3.82E-06	NM_031157	NM_031157	Homo sapiens heterogeneous nuclear ribonucleoprotein A1 (HNRPA1), transcript variant 2, mRNA [NM_031157]	NM_031157
A_23_P250571	3.84E-06	NM_005509	NM_005509	Homo sapiens Dmx-like 1 (DMXL1), mRNA [NM_005509]	NM_005509
A_23_P208737	3.84E-06	NM_004497	NM_004497	Homo sapiens forkhead box A3 (FOXA3), mRNA [NM_004497]	NM_004497
A_24_P791829	3.84E-06	THC2305590			
A_32_P162443	3.84E-06	A_32_P162443			
A_23_P141484	3.84E-06	NM_018182	NM_018182	Homo sapiens hypothetical protein FLJ10700 (FLJ10700), mRNA [NM_018182]	NM_018182
A_32_P167856	3.85E-06	AF338232	AF338232	Homo sapiens CTAGE-4 protein mRNA, complete cds. [AF338232]	XM_496933
A_23_P158925	3.88E-06	NM_145290	NM_145290	Homo sapiens G protein-coupled receptor 125 (GPR125), mRNA [NM_145290]	NM_145290
A_23_P377267	3.89E-06	AB007940	AB007940	Homo sapiens mRNA for KIAA0471 protein, partial cds. [AB007940]	
A_32_P177955	3.92E-06	BC030123	BC030123	Homo sapiens, clone IMAGE:4815474, mRNA. [BC030123]	XM_499157
A_23_P406025	3.92E-06	NM_015225	NM_015225	Homo sapiens KIAA0367 (KIAA0367), mRNA [NM_015225]	NM_015225
A_23_P385034	3.92E-06	NM_001949	NM_001949	Homo sapiens E2F transcription factor 3 (E2F3), mRNA [NM_001949]	NM_001949
A_23_P393099	3.93E-06	NM_003226	NM_003226	Homo sapiens trefoil factor 3 (intestinal) (TFF3), mRNA [NM_003226]	NM_003226
A_24_P268856	3.94E-06	BC036046	BC036046	Homo sapiens cDNA clone IMAGE:5273244, containing frame-shift errors. [BC036046]	
A_23_P10815	3.94E-06	NM_014676	NM_014676	Homo sapiens pumilio homolog 1 (Drosophila) (PUM1), transcript variant 2, mRNA [NM_014676]	NM_014676
A_23_P103601	3.96E-06	NM_020379	NM_020379	Homo sapiens mannosidase, alpha, class 1C, member 1 (MAN1C1), mRNA [NM_020379]	NM_020379

A_23_P7582	3.97E-06	NM_003202	NM_003202	Homo sapiens transcription factor 7 (T-cell specific, HMG-box) (TCF7), transcript variant 1, mRNA [NM_003202]	NM_003202
A_24_P134653	3.99E-06	NM_003611	NM_003611	Homo sapiens oral-facial-digital syndrome 1 (OFD1), mRNA [NM_003611]	NM_003611
A_23_P136817	4.00E-06	NM_014708	NM_014708	Homo sapiens kinetochore associated 1 (KNTC1), mRNA [NM_014708]	NM_014708
A_23_P7882	4.03E-06	BC022217	BC022217	Homo sapiens chromosome 6 open reading frame 85, mRNA (cDNA clone IMAGE:3846727), complete cds. [BC022217]	
A_23_P256384	4.04E-06	NM_021144	NM_021144	Homo sapiens PC4 and SFRS1 interacting protein 1 (PSIP1), transcript variant 1, mRNA [NM_021144]	NM_021144
A_23_P159053	4.04E-06	NM_002873	NM_002873	Homo sapiens RAD17 homolog (S. pombe) (RAD17), transcript variant 8, mRNA [NM_002873]	NM_002873
A_23_P209320	4.06E-06	NM_022817	NM_022817	Homo sapiens period homolog 2 (Drosophila) (PER2), transcript variant 1, mRNA [NM_022817]	NM_022817
A_24_P372012	4.07E-06	NM_004968	NM_004968	Homo sapiens islet cell autoantigen 1, 69kDa (ICA1), transcript variant 2, mRNA [NM_004968]	NM_004968
A_23_P69537	4.08E-06	NM_006681	NM_006681	Homo sapiens neuromedin U (NMU), mRNA [NM_006681]	NM_006681
A_24_P287756	4.11E-06	NM_007006	NM_007006	Homo sapiens nudix (nucleoside diphosphate linked moiety X)-type motif 21 (NUDT21), mRNA [NM_007006]	NM_007006
A_32_P156851	4.14E-06	NM_005822	NM_005822	Homo sapiens Down syndrome critical region gene 1-like 1 (DSCR1L1), mRNA [NM_005822]	NM_005822
A_23_P57658	4.16E-06	NM_020386	NM_020386	Homo sapiens HRAS-like suppressor (HRASLS), mRNA [NM_020386]	NM_020386
A_24_P177604	4.16E-06	NM_033215	NM_033215	Homo sapiens protein phosphatase 1, regulatory (inhibitor) subunit 3F (PPP1R3F), mRNA [NM_033215]	NM_033215
A_23_P136504	4.17E-06	NM_030631	NM_030631	Homo sapiens solute carrier family 25 (mitochondrial oxodicarboxylate carrier), member 21 (SLC25A21), mRNA [NM_030631]	NM_030631
A_23_P128060	4.19E-06	NM_019591	NM_019591	Homo sapiens zinc finger protein 26 (KOX 20) (ZNF26), mRNA [NM_019591]	NM_019591
A_23_P48713	4.20E-06	NM_152444	NM_152444	Homo sapiens zinc binding alcohol dehydrogenase, domain containing 1 (ZADH1), mRNA [NM_152444]	NM_152444
A_32_P94	4.22E-06	CR602878	CR602878	full-length cDNA clone CS0DF035YB23 of Fetal brain of Homo sapiens (human). [CR602878]	
A_24_P178444	4.22E-06	A_24_P178444			
A_32_P67747	4.23E-06	THC2436814			
A_23_P40817	4.26E-06	NM_032383	NM_032383	Homo sapiens Hermansky-Pudlak syndrome 3 (HPS3), mRNA [NM_032383]	NM_032383
A_23_P5415	4.28E-06	NM_021824	NM_021824	Homo sapiens NIF3 NGG1 interacting factor 3-like 1 (S. pombe) (NIF3L1), mRNA [NM_021824]	NM_021824
A_24_P142503	4.29E-06	NM_018242	NM_018242	Homo sapiens hypothetical protein FLJ10847 (FLJ10847), mRNA [NM_018242]	NM_018242
A_23_P145006	4.29E-06	NM_054023	NM_054023	Homo sapiens secretoglobin, family 3A, member 2 (SCGB3A2), mRNA [NM_054023]	NM_054023
A_24_P166794	4.32E-06	BC047111	BC047111	Homo sapiens, clone IMAGE:5314178, mRNA. [BC047111]	
A_23_P82651	4.33E-06	NM_002523	NM_002523	Homo sapiens neuronal pentraxin II (NPTX2), mRNA [NM_002523]	NM_002523
A_23_P20502	4.33E-06	NM_130849	NM_130849	Homo sapiens solute carrier family 39 (zinc transporter), member 4 (SLC39A4), transcript variant 2, mRNA [NM_130849]	NM_130849
A_23_P119964	4.33E-06	NM_005760	NM_005760	Homo sapiens CCAAT/enhancer binding protein zeta (CEBPZ), mRNA [NM_005760]	NM_005760
A_32_P55462	4.38E-06	CR593500	CR593500	full-length cDNA clone CS0DF014YD20 of Fetal brain of Homo sapiens (human). [CR593500]	XM_373788
A_23_P436484	4.43E-06	ENST00000255136		Q6DEY7 (Q6DEY7) MGC89376 protein, partial (38%) [THC2400949]	
A_32_P84342	4.44E-06	THC2315140		predicted protein {Methanosarcina acetivorans C2A;} , partial (11%) [THC2315140]	
A_23_P88331	4.44E-06	NM_014750	NM_014750	Homo sapiens discs, large homolog 7 (Drosophila) (DLG7), mRNA [NM_014750]	NM_014750
A_32_P123629	4.47E-06	THC2373876		BM069797 ie89f02.y1 Melton Normalized Human Islet 4 N4-HIS 1 Homo sapiens cDNA clone IMAGE:5674130 5', mRNA sequence [BM069797]	
A_23_P208551	4.47E-06	NM_001008701	NM_001008701	Homo sapiens latrophilin 1 (LPHN1), transcript variant 1, mRNA [NM_001008701]	NM_001008701
A_23_P34744	4.49E-06	NM_000396	NM_000396	Homo sapiens cathepsin K (pycnodysostosis) (CTSK), mRNA [NM_000396]	NM_000396
A_23_P65278	4.52E-06	NM_015678	NM_015678	Homo sapiens neurobeachin (NBEA), mRNA [NM_015678]	NM_015678
A_23_P127426	4.52E-06	NM_003273	NM_003273	Homo sapiens transmembrane 7 superfamily member 2 (TM7SF2), mRNA [NM_003273]	NM_003273
A_23_P217304	4.55E-06	NM_021140	NM_021140	Homo sapiens ubiquitously transcribed tetratricopeptide repeat, X chromosome (UTX), mRNA [NM_021140]	NM_021140
A_23_P312840	4.57E-06	NM_020796	NM_020796	Homo sapiens sema domain, transmembrane domain (TM), and cytoplasmic domain, (semaphorin) 6A (SEMA6A), mRNA [NM_020796]	NM_020796
A_23_P217098	4.57E-06	NM_033305	NM_033305	Homo sapiens vacuolar protein sorting 13A (yeast) (VPS13A), transcript variant A, mRNA [NM_033305]	NM_033305
A_23_P50735	4.58E-06	BC043228	BC043228	Homo sapiens, Similar to zinc finger protein 302, clone IMAGE:5295602, mRNA. [BC043228]	XM_290835
A_32_P38623	4.62E-06	BC037849	BC037849	Homo sapiens cDNA clone IMAGE:4815736, partial cds. [BC037849]	
A_23_P24260	4.65E-06	THC2435513		ENP1_HUMAN (P49961) Ectonucleoside triphosphate diphosphohydrolase 1 (NTPDase1) (Ecto-ATP diphosphohydrolase) (ATPDase) (Lymphoid cell activation antigen) (Ecto-apyrase) (CD39 antigen) , partial (92%) [THC2435513]	
A_23_P42045	4.69E-06	NM_181837	NM_181837	Homo sapiens origin recognition complex, subunit 3-like (yeast) (ORC3L), transcript variant 1, mRNA [NM_181837]	NM_181837

A_24_P696507	4.75E-06	AK092810	AK092810	Homo sapiens cDNA FLJ35491 fis, clone SMINT2008625, moderately similar to GLYCINE CLEAVAGE SYSTEM H PROTEIN PRECURSOR. [AK092810]	
A_23_P259741	4.78E-06	NM_002971	NM_002971	Homo sapiens special AT-rich sequence binding protein 1 (binds to nuclear matrix/scaffold-associating DNA's) (SATB1), mRNA [NM_002971]	NM_002971
A_24_P178631	4.81E-06	AK027667	AK027667	Homo sapiens cDNA FLJ14761 fis, clone NT2RP3003302. [AK027667]	
A_23_P94380	4.82E-06	NM_001002260	NM_001002260	Homo sapiens chromosome 9 open reading frame 58 (C9orf58), transcript variant 2, mRNA [NM_001002260]	NM_001002260
A_23_P212639	4.82E-06	NM_004593	NM_004593	Homo sapiens splicing factor, arginine/serine-rich 10 (transformer 2 homolog, Drosophila) (SFRS10), mRNA [NM_004593]	NM_004593
A_24_P497244	4.83E-06	AL050210	AL050210	Homo sapiens mRNA; cDNA DKFZp586G1023 (from clone DKFZp586G1023). [AL050210]	
A_23_P129466	4.84E-06	NM_024997	NM_024997	Homo sapiens activating transcription factor 7 interacting protein 2 (ATF7IP2), mRNA [NM_024997]	NM_024997
A_23_P379864	4.86E-06	BC006267	BC006267	Homo sapiens asparaginase like 1, mRNA (cDNA clone IMAGE:3952485), complete cds. [BC006267]	
A_24_P363408	4.88E-06	NM_012259	NM_012259	Homo sapiens hairy/enhancer-of-split related with YRPW motif 2 (HEY2), mRNA [NM_012259]	NM_012259
A_32_P221822	4.94E-06	AB007976	AB007976	Homo sapiens mRNA, chromosome 1 specific transcript KIAA0507. [AB007976]	
A_23_P149775	4.94E-06	NM_018287	NM_018287	Homo sapiens Rho GTPase activating protein 12 (ARHGAP12), mRNA [NM_018287]	NM_018287
A_32_P182439	4.97E-06	NM_006591	NM_006591	Homo sapiens polymerase (DNA-directed), delta 3, accessory subunit (POLD3), mRNA [NM_006591]	NM_006591
A_23_P95029	5.03E-06	NM_021021	NM_021021	Homo sapiens syntrophin, beta 1 (dystrophin-associated protein A1, 59kDa, basic component 1) (SNTB1), mRNA [NM_021021]	NM_021021
A_24_P348806	5.04E-06	NM_175058	NM_175058	Homo sapiens pleckstrin homology domain containing, family A member 2 (PLEKHA7), mRNA [NM_175058]	NM_175058
A_24_P184803	5.04E-06	NM_004086	NM_004086	Homo sapiens coagulation factor C homolog, cochlin (Limulus polyphemus) (COCH), mRNA [NM_004086]	NM_004086
A_24_P275828	5.04E-06	NM_033419	NM_033419	Homo sapiens per1-like domain containing 1 (PERLD1), mRNA [NM_033419]	NM_033419
A_24_P50666	5.04E-06	A_24_P50666			
A_23_P153026	5.04E-06	NM_000152	NM_000152	Homo sapiens glucosidase, alpha; acid (Pompe disease, glycogen storage disease type II) (GAA), mRNA [NM_000152]	NM_000152
A_32_P214565	5.05E-06	A_32_P214565			
A_23_P501080	5.05E-06	NM_007139	NM_007139	Homo sapiens zinc finger protein 92 (HTF12) (ZNF92), mRNA [NM_007139]	NM_007139
A_23_P163306	5.09E-06	NM_032866	NM_032866	Homo sapiens cingulin-like 1 (CGNL1), mRNA [NM_032866]	NM_032866
A_32_P220523	5.13E-06	THC2439328			
A_24_P664891	5.13E-06	AF020589	AF020589	Homo sapiens cytochrome C oxidase subunit VIa homolog mRNA, complete cds. [AF020589]	
A_23_P115022	5.13E-06	NM_144626	NM_144626	Homo sapiens hypothetical protein MGC17299 (MGC17299), mRNA [NM_144626]	NM_144626
A_23_P166306	5.13E-06	NM_000071	NM_000071	Homo sapiens cystathionine-beta-synthase (CBS), mRNA [NM_000071]	NM_000071
A_23_P46396	5.14E-06	NM_021190	NM_021190	Homo sapiens polypyrimidine tract binding protein 2 (PTBP2), mRNA [NM_021190]	NM_021190
A_24_P365327	5.14E-06	NM_015578	NM_015578	Homo sapiens family with sequence similarity 61, member A (FAM61A), mRNA [NM_015578]	NM_015578
A_32_P31633	5.14E-06	NM_012433	NM_012433	Homo sapiens splicing factor 3b, subunit 1, 155kDa (SF3B1), transcript variant 1, mRNA [NM_012433]	NM_012433
A_23_P100420	5.17E-06	NM_015144	NM_015144	Homo sapiens zinc finger, CCHC domain containing 14 (ZCCHC14), mRNA [NM_015144]	NM_015144
A_23_P98304	5.22E-06	NM_018043	NM_018043	Homo sapiens transmembrane protein 16A (TMEM16A), mRNA [NM_018043]	NM_018043
A_23_P134714	5.22E-06	NM_005836	NM_005836	Homo sapiens heat-responsive protein 12 (HRSP12), mRNA [NM_005836]	NM_005836
A_23_P5875	5.24E-06	NM_000939	NM_000939	Homo sapiens proopiomelanocortin (adrenocorticotropin/ beta-lipotropin/ alpha-melanocyte stimulating hormone/ beta-melanocyte stimulating hormone/ beta-endorphin) (POMC), mRNA [NM_000939]	NM_000939
A_23_P44295	5.26E-06	NM_015097	NM_015097	Homo sapiens cytoplasmic linker associated protein 2 (CLASP2), mRNA [NM_015097]	NM_015097
A_32_P97496	5.30E-06	NM_181722	NM_181722	Homo sapiens hypothetical protein LOC285908 (LOC285908), mRNA [NM_181722]	NM_181722
A_24_P925565	5.34E-06	THC2438118			
A_23_P139388	5.38E-06	NM_016578	NM_016578	Homo sapiens hepatitis B virus x associated protein (HBXAP), mRNA [NM_016578]	NM_016578
A_23_P18123	5.40E-06	NM_014932	NM_014932	Homo sapiens neuroligin 1 (NLGN1), mRNA [NM_014932]	NM_014932
A_23_P163697	5.40E-06	NM_016524	NM_016524	Homo sapiens synaptotagmin XVII (SYT17), mRNA [NM_016524]	NM_016524
A_32_P220696	5.40E-06	NM_017489	NM_017489	Homo sapiens telomeric repeat binding factor (NIMA-interacting) 1 (TERF1), transcript variant 1, mRNA [NM_017489]	NM_017489
A_23_P94216	5.42E-06	NM_152271	NM_152271	Homo sapiens hypothetical protein FLJ23749 (FLJ23749), mRNA [NM_152271]	NM_152271
A_23_P117933	5.42E-06	NM_004483	NM_004483	Homo sapiens glycine cleavage system protein H (aminomethyl carrier) (GCSH), mRNA [NM_004483]	NM_004483
A_23_P254573	5.44E-06	CR598046	CR598046	full-length cDNA clone CS0DI043YJ07 of Placenta Cot 25-normalized of Homo sapiens (human). [CR598046]	XM_168590
A_24_P192434	5.46E-06	NM_017489	NM_017489	Homo sapiens telomeric repeat binding factor (NIMA-interacting) 1 (TERF1), transcript variant 1, mRNA [NM_017489]	NM_017489
A_23_P124742	5.46E-06	NM_001277	NM_001277	Homo sapiens choline kinase alpha (CHKA), transcript variant 1, mRNA [NM_001277]	NM_001277

A_23_P5742	5.47E-06	NM_024584	NM_024584	Homo sapiens hypothetical protein FLJ13646 (FLJ13646), mRNA [NM_024584]	NM_024584
A_32_P104841	5.50E-06	AF087980	AF087980	Homo sapiens full length insert cDNA clone YW27H10. [AF087980]	
A_23_P17814	5.50E-06	NM_015715	NM_015715	Homo sapiens phospholipase A2, group III (PLA2G3), mRNA [NM_015715]	NM_015715
A_24_P240187	5.50E-06	NM_020873	NM_020873	Homo sapiens leucine rich repeat neuronal 1 (LRRN1), mRNA [NM_020873]	NM_020873
A_23_P53057	5.51E-06	NM_013250	NM_013250	Homo sapiens zinc finger protein 215 (ZNF215), mRNA [NM_013250]	NM_013250
A_24_P659113	5.51E-06	NM_152523	NM_152523	Homo sapiens hypothetical protein FLJ40432 (FLJ40432), mRNA [NM_152523]	NM_152523
A_23_P107154	5.51E-06	NM_032582	NM_032582	Homo sapiens ubiquitin specific protease 32 (USP32), mRNA [NM_032582]	NM_032582
A_24_P145122	5.53E-06	THC2301029		Q62VJ0 (Q62VJ0) Small peptidoglycan-associated lipoprotein, partial (13%) [THC2301029]	
A_23_P259692	5.53E-06	NM_058179	NM_058179	Homo sapiens phosphoserine aminotransferase 1 (PSAT1), transcript variant 1, mRNA [NM_058179]	NM_058179
A_23_P73540	5.54E-06	NM_033626	NM_033626	Homo sapiens JM11 protein (JM11), mRNA [NM_033626]	NM_033626
A_23_P211459	5.56E-06	NM_014323	NM_014323	Homo sapiens zinc finger protein 278 (ZNF278), transcript variant 1, mRNA [NM_014323]	NM_014323
A_23_P120863	5.56E-06	NM_004861	NM_004861	Homo sapiens galactose-3-O-sulfotransferase 1 (GAL3ST1), mRNA [NM_004861]	NM_004861
A_23_P57760	5.58E-06	NM_152282	NM_152282	Homo sapiens acid phosphatase-like 2 (ACPL2), mRNA [NM_152282]	NM_152282
A_32_P218671	5.60E-06	BG575983	BG575983	602597328F1 NIH_MGC_87 Homo sapiens cDNA clone IMAGE:4705974 5', mRNA sequence [BG575983]	
A_23_P138507	5.60E-06	NM_001786	NM_001786	Homo sapiens cell division cycle 2, G1 to S and G2 to M (CDC2), transcript variant 1, mRNA [NM_001786]	NM_001786
A_23_P3532	5.61E-06	NM_004862	NM_004862	Homo sapiens lipopolysaccharide-induced TNF factor (LITAF), mRNA [NM_004862]	NM_004862
A_24_P943106	5.62E-06	AB002330	AB002330	Human mRNA for KIAA0332 gene, partial cds. [AB002330]	XM_031553
A_23_P82412	5.62E-06	NM_145645	NM_145645	Homo sapiens Williams-Beuren Syndrome critical region protein 20 copy B (WBSCR20B), mRNA [NM_145645]	NM_145645
A_23_P100883	5.64E-06	NM_015355	NM_015355	Homo sapiens suppressor of zeste 12 homolog (Drosophila) (SUZ12), mRNA [NM_015355]	NM_015355
A_23_P14493	5.71E-06	NM_018139	NM_018139	Homo sapiens chromosome 14 open reading frame 104 (C14orf104), mRNA [NM_018139]	NM_018139
A_23_P253484	5.72E-06	NM_016228	NM_016228	Homo sapiens aminoadipate aminotransferase (AADAT), transcript variant 1, mRNA [NM_016228]	NM_016228
A_24_P639505	5.72E-06	NM_005819	NM_005819	Homo sapiens syntaxin 6 (STX6), mRNA [NM_005819]	NM_005819
A_23_P431360	5.73E-06	NM_016423	NM_016423	Homo sapiens zinc finger protein 219 (ZNF219), mRNA [NM_016423]	NM_016423
A_32_P228124	5.75E-06	AL832747	AL832747	Homo sapiens mRNA; cDNA DKFZp686D0521 (from clone DKFZp686D0521). [AL832747]	
A_23_P165788	5.76E-06	NM_001008489	NM_001008489	Homo sapiens phosphatase, orphan 2 (PHOSPHO2), mRNA [NM_001008489]	NM_001008489
A_23_P19829	5.76E-06	NM_013440	NM_013440	Homo sapiens paired immunoglobin-like type 2 receptor beta (PILRB), transcript variant 1, mRNA [NM_013440]	NM_013440
A_32_P163036	5.77E-06	ENST00000344142		Homo sapiens cDNA PSEC0178 fis, clone OVARC1000636, moderately similar to Sterile alpha motif domain containing protein 4. [AK075484]	XM_291016
A_32_P226801	5.79E-06	THC2453346		AA890297 aj94e10.s1 Soares_parathyroid_tumor_NbHPA Homo sapiens cDNA clone IMAGE:1404138 3', mRNA sequence [AA890297]	
A_23_P76761	5.80E-06	NM_003384	NM_003384	Homo sapiens vaccinia related kinase 1 (VRK1), mRNA [NM_003384]	NM_003384
A_23_P30634	5.81E-06	NM_021813	NM_021813	Homo sapiens BTB and CNC homology 1, basic leucine zipper transcription factor 2 (BACH2), mRNA [NM_021813]	NM_021813
A_32_P41574	5.81E-06	CR626729	CR626729	full-length cDNA clone CS0DF009YE11 of Fetal brain of Homo sapiens (human). [CR626729]	
A_23_P209298	5.83E-06	BC032822	BC032822	Homo sapiens erythrocyte membrane protein band 4.1 like 5, mRNA (cDNA clone MGC:26029 IMAGE:4827274), complete cds. [BC032822]	
A_23_P77079	5.83E-06	NM_016132	NM_016132	Homo sapiens myelin expression factor 2 (MYEF2), mRNA [NM_016132]	NM_016132
A_23_P72157	5.85E-06	NM_032219	NM_032219	Homo sapiens hypothetical protein FLJ22269 (FLJ22269), mRNA [NM_032219]	NM_032219
A_32_P113736	5.87E-06	THC2282618			
A_23_P206290	5.87E-06	NM_153837	NM_153837	Homo sapiens G protein-coupled receptor 114 (GPR114), mRNA [NM_153837]	NM_153837
A_24_P416257	5.88E-06	NM_015044	NM_015044	Homo sapiens golgi associated, gamma adaptin ear containing, ARF binding protein 2 (GGA2), transcript variant 1, mRNA [NM_015044]	NM_015044
A_23_P1286	5.91E-06	NM_144661	NM_144661	Homo sapiens chromosome 10 open reading frame 82 (C10orf82), mRNA [NM_144661]	NM_144661
A_23_P152353	5.91E-06	NM_133451	NM_133451	Homo sapiens KIAA1970 protein (KIAA1970), mRNA [NM_133451]	NM_133451
A_23_P21376	5.94E-06	NM_012301	NM_012301	Homo sapiens membrane associated guanylate kinase, WW and PDZ domain containing 2 (MAGI2), mRNA [NM_012301]	NM_012301
A_32_P24068	5.95E-06	THC2285742			
A_23_P211326	5.96E-06	AK091754	AK091754	Homo sapiens cDNA FLJ34435 fis, clone HLUNG2000955. [AK091754]	
A_24_P898915	5.97E-06	NM_001004321	NM_001004321	Homo sapiens FLJ45445 protein (FLJ45445), mRNA [NM_001004321]	NM_001004321
A_23_P209032	5.98E-06	NM_018443	NM_018443	Homo sapiens zinc finger protein 302 (ZNF302), transcript variant 1, mRNA [NM_018443]	NM_018443

A_32_P95067	5.99E-06	A_32_P95067			
A_32_P33083	5.99E-06	NM_016378	NM_016378	Homo sapiens variable charge, X-linked 2 (VCX2), mRNA [NM_016378]	NM_016378
A_24_P935986	5.99E-06	NM_005504	NM_005504	Homo sapiens branched chain aminotransferase 1, cytosolic (BCAT1), mRNA [NM_005504]	NM_005504
A_24_P317907	6.00E-06	NM_015385	NM_015385	Homo sapiens sorbin and SH3 domain containing 1 (SORBS1), transcript variant 2, mRNA [NM_015385]	NM_015385
A_23_P104617	6.00E-06	NM_152312	NM_152312	Homo sapiens glycosyltransferase-like 1B (GYLTL1B), mRNA [NM_152312]	NM_152312
A_24_P324814	6.06E-06	NM_138363	NM_138363	Homo sapiens hypothetical protein BC009518 (LOC90799), mRNA [NM_138363]	NM_138363
A_23_P20463	6.06E-06	NM_006265	NM_006265	Homo sapiens RAD21 homolog (S. pombe) (RAD21), mRNA [NM_006265]	NM_006265
A_32_P70724	6.08E-06	NM_006618	NM_006618	Homo sapiens Jumonji, AT rich interactive domain 1B (RBP2-like) (JARID1B), mRNA [NM_006618]	NM_006618
A_23_P345830	6.10E-06	NM_014929	NM_014929	Homo sapiens KIAA0971 (KIAA0971), mRNA [NM_014929]	NM_014929
A_24_P114334	6.11E-06	AL050139	AL050139	Homo sapiens mRNA; cDNA DKFZp586M141 (from clone DKFZp586M141). [AL050139]	
A_23_P408675	6.14E-06	NM_003972	NM_003972	Homo sapiens BTAF1 RNA polymerase II, B-TFIID transcription factor-associated, 170kDa (Mot1 homolog, S. cerevisiae) (BTAF1), mRNA [NM_003972]	NM_003972
A_23_P356494	6.21E-06	NM_006846	NM_006846	Homo sapiens serine protease inhibitor, Kazal type 5 (SPINK5), mRNA [NM_006846]	NM_006846
A_23_P146347	6.23E-06	NM_017645	NM_017645	Homo sapiens family with sequence similarity 29, member A (FAM29A), mRNA [NM_017645]	NM_017645
A_32_P148824	6.23E-06	NM_017847	NM_017847	Homo sapiens chromosome 1 open reading frame 27 (C1orf27), mRNA [NM_017847]	NM_017847
A_23_P14986	6.26E-06	NM_000196	NM_000196	Homo sapiens hydroxysteroid (11-beta) dehydrogenase 2 (HSD11B2), mRNA [NM_000196]	NM_000196
A_32_P179746	6.28E-06	THC2405620		Q6DN13 (Q6DN13) MCTP1S, partial (5%) [THC2405620]	
A_23_P160881	6.28E-06	NM_001009568	NM_001009568	Homo sapiens sphingomyelin phosphodiesterase, acid-like 3B (SMPDL3B), transcript variant 2, mRNA [NM_001009568]	NM_001009568
A_32_P148047	6.29E-06	A_32_P148047			
A_32_P8551	6.34E-06	NM_198443	NM_198443	Homo sapiens MRCC2446 (UNQ2446), mRNA [NM_198443]	NM_198443
A_23_P80902	6.34E-06	NM_020242	NM_020242	Homo sapiens kinesin family member 15 (KIF15), mRNA [NM_020242]	NM_020242
A_23_P108501	6.34E-06	NM_004438	NM_004438	Homo sapiens EPH receptor A4 (EPHA4), mRNA [NM_004438]	NM_004438
A_24_P941148	6.40E-06	NM_017645	NM_017645	Homo sapiens family with sequence similarity 29, member A (FAM29A), mRNA [NM_017645]	NM_017645
A_32_P5480	6.41E-06	NM_203463	NM_203463	Homo sapiens LAG1 longevity assurance homolog 6 (S. cerevisiae) (LASS6), mRNA [NM_203463]	NM_203463
A_23_P12784	6.41E-06	NM_012083	NM_012083	Homo sapiens frequently rearranged in advanced T-cell lymphomas 2 (FRAT2), mRNA [NM_012083]	NM_012083
A_23_P72770	6.45E-06	NM_032147	NM_032147	Homo sapiens ubiquitin specific protease 44 (USP44), mRNA [NM_032147]	NM_032147
A_23_P395555	6.45E-06	NM_016444	NM_016444	Homo sapiens zinc finger protein 226 (ZNF226), mRNA [NM_016444]	NM_016444
A_24_P886096	6.48E-06	AK094413	AK094413	Homo sapiens cDNA FLJ37094 fis, clone BRACE2018337. [AK094413]	
A_23_P104471	6.52E-06	NM_001007271	NM_001007271	Homo sapiens dual specificity phosphatase 13 (DUSP13), transcript variant 1, mRNA [NM_001007271]	NM_001007271
A_24_P268210	6.52E-06	CR614186	CR614186	full-length cDNA clone CS0DC007YG11 of Neuroblastoma Cot 25-normalized of Homo sapiens (human). [CR614186]	
A_23_P148879	6.52E-06	NM_000702	NM_000702	Homo sapiens ATPase, Na+/K+ transporting, alpha 2 (+) polypeptide (ATP1A2), mRNA [NM_000702]	NM_000702
A_23_P360754	6.52E-06	NM_005099	NM_005099	Homo sapiens a disintegrin-like and metalloprotease (reprolysin type) with thrombospondin type 1 motif, 4 (ADAMTS4), mRNA [NM_005099]	NM_005099
A_23_P258612	6.53E-06	NM_016529	NM_016529	Homo sapiens ATPase, aminophospholipid transporter-like, Class I, type 8A, member 2 (ATP8A2), mRNA [NM_016529]	NM_016529
A_23_P212397	6.54E-06	NM_015340	NM_015340	Homo sapiens leucyl-tRNA synthetase 2, mitochondrial (LARS2), nuclear gene encoding mitochondrial protein, mRNA [NM_015340]	NM_015340
A_23_P159390	6.56E-06	NM_007027	NM_007027	Homo sapiens topoisomerase (DNA) II binding protein 1 (TOPBP1), mRNA [NM_007027]	NM_007027
A_23_P215060	6.56E-06	NM_005397	NM_005397	Homo sapiens podocalyxin-like (PODXL), transcript variant 2, mRNA [NM_005397]	NM_005397
A_32_P81173	6.58E-06	AL050376	AL050376	Homo sapiens mRNA; cDNA DKFZp586J101 (from clone DKFZp586J101). [AL050376]	
A_32_P113584	6.59E-06	AB011102	AB011102	Homo sapiens mRNA for KIAA0530 protein, partial cds. [AB011102]	
A_23_P257003	6.59E-06	NM_006200	NM_006200	Homo sapiens proprotein convertase subtilisin/kexin type 5 (PCSK5), mRNA [NM_006200]	NM_006200
A_24_P126628	6.59E-06	NM_015257	NM_015257	Homo sapiens KIAA0286 protein (KIAA0286), mRNA [NM_015257]	NM_015257
A_23_P380951	6.63E-06	NM_144689	NM_144689	Homo sapiens zinc finger protein 420 (ZNF420), mRNA [NM_144689]	NM_144689
A_32_P6832	6.63E-06	NM_175921	NM_175921	Homo sapiens hypothetical protein LOC285636 (LOC285636), mRNA [NM_175921]	NM_175921
A_24_P620456	6.66E-06	AK024921	AK024921	Homo sapiens cDNA: FLJ21268 fis, clone COL01718. [AK024921]	
A_23_P350074	6.66E-06	NM_153233	NM_153233	Homo sapiens hypothetical protein FLJ36445 (FLJ36445), mRNA [NM_153233]	NM_153233
A_23_P420417	6.66E-06	NM_138463	NM_138463	Homo sapiens hypothetical protein BC014072 (LOC116238), mRNA [NM_138463]	NM_138463

A_23_P31921	6.69E-06	NM_054012	NM_054012	Homo sapiens argininosuccinate synthetase (ASS), transcript variant 2, mRNA [NM_054012]	NM_054012
A_23_P377888	6.71E-06	NM_014967	NM_014967	Homo sapiens KIAA1018 protein (KIAA1018), mRNA [NM_014967]	NM_014967
A_23_P211099	6.71E-06	NM_003103	NM_003103	Homo sapiens SON DNA binding protein (SON), transcript variant g, mRNA [NM_003103]	NM_003103
A_23_P401675	6.72E-06	NM_144724	NM_144724	Homo sapiens MARVEL domain containing 2 (MARVELD2), mRNA [NM_144724]	NM_144724
A_23_P904	6.74E-06	NM_024603	NM_024603	Homo sapiens chromosome 1 open reading frame 165 (C1orf165), mRNA [NM_024603]	NM_024603
A_23_P212002	6.79E-06	NM_001012651	NM_001012651	Homo sapiens natural killer-tumor recognition sequence (NKTR), transcript variant 2, mRNA [NM_001012651]	NM_001012651
A_23_P115091	6.92E-06	NM_020387	NM_020387	Homo sapiens RAB25, member RAS oncogene family (RAB25), mRNA [NM_020387]	NM_020387
A_23_P311144	6.95E-06	NM_144978	NM_144978	Homo sapiens hypothetical protein FLJ32745 (FLJ32745), mRNA [NM_144978]	NM_144978
A_23_P209269	6.96E-06	NM_002706	NM_002706	Homo sapiens protein phosphatase 1B (formerly 2C), magnesium-dependent, beta isoform (PPM1B), transcript variant 1, mRNA [NM_002706]	NM_002706
A_23_P218817	6.98E-06	NM_152246	NM_152246	Homo sapiens carnitine palmitoyltransferase 1B (muscle) (CPT1B), nuclear gene encoding mitochondrial protein, transcript variant 3, mRNA [NM_152246]	NM_152246
A_23_P83388	6.98E-06	NM_031308	NM_031308	Homo sapiens epiplakin 1 (EPPK1), mRNA [NM_031308]	NM_031308
A_32_P472968	7.00E-06	BC047110	BC047110	Homo sapiens, clone IMAGE:5312754, mRNA. [BC047110]	
A_23_P9472	7.01E-06	NM_033305	NM_033305	Homo sapiens vacuolar protein sorting 13A (yeast) (VPS13A), transcript variant A, mRNA [NM_033305]	NM_033305
A_23_P424002	7.02E-06	NM_002697	NM_002697	Homo sapiens POU domain, class 2, transcription factor 1 (POU2F1), mRNA [NM_002697]	NM_002697
A_23_P148916	7.02E-06	NM_148909	NM_148909	Homo sapiens oxysterol binding protein-like 9 (OSBPL9), transcript variant 7, mRNA [NM_148909]	NM_148909
A_32_P207436	7.03E-06	BC007307	BC007307	Homo sapiens, Similar to zinc finger protein 268, clone IMAGE:3352268, mRNA, partial cds. [BC007307]	XM_039908
A_23_P91697	7.03E-06	NM_004737	NM_004737	Homo sapiens like-glycosyltransferase (LARGE), transcript variant 1, mRNA [NM_004737]	NM_004737
A_23_P127676	7.05E-06	NM_014633	NM_014633	Homo sapiens SH2 domain binding protein 1 (tetratricopeptide repeat containing) (SH2BP1), mRNA [NM_014633]	NM_014633
A_32_P20240	7.07E-06	BX648857	BX648857	Homo sapiens mRNA; cDNA DKFZp686N17231 (from clone DKFZp686N17231). [BX648857]	
A_32_P3742	7.12E-06	AK127804	AK127804	Homo sapiens cDNA FLJ45905 fis, clone OCBBF3026576. [AK127804]	
A_23_P70201	7.14E-06	NM_001270	NM_001270	Homo sapiens chromodomain helicase DNA binding protein 1 (CHD1), mRNA [NM_001270]	NM_001270
A_23_P433132	7.15E-06	NM_173853	NM_173853	Homo sapiens keratinocyte associated protein 3 (KRTCAP3), mRNA [NM_173853]	NM_173853
A_23_P216149	7.16E-06	NM_017489	NM_017489	Homo sapiens telomeric repeat binding factor (NIMA-interacting) 1 (TERF1), transcript variant 1, mRNA [NM_017489]	NM_017489
A_23_P36364	7.16E-06	NM_033209	NM_033209	Homo sapiens Thy-1 co-transcribed (LOC94105), mRNA [NM_033209]	NM_033209
A_23_P20683	7.16E-06	NM_014878	NM_014878	Homo sapiens KIAA0020 (KIAA0020), mRNA [NM_014878]	NM_014878
A_23_P34827	7.19E-06	NM_020897	NM_020897	Homo sapiens hyperpolarization activated cyclic nucleotide-gated potassium channel 3 (HCN3), mRNA [NM_020897]	NM_020897
A_23_P208961	7.19E-06	NM_032853	NM_032853	Homo sapiens melanoma associated antigen (mutated) 1 (MUM1), mRNA [NM_032853]	NM_032853
A_32_P182186	7.22E-06	BC041955	BC041955	Homo sapiens, clone IMAGE:5301910, mRNA. [BC041955]	
A_32_P77665	7.24E-06	THC2384975		ALU7_HUMAN (P39194) Alu subfamily SQ sequence contamination warning entry, partial (6%) [THC2384975]	
A_23_P146134	7.27E-06	NM_024025	NM_024025	Homo sapiens dual specificity phosphatase 26 (putative) (DUSP26), mRNA [NM_024025]	NM_024025
A_23_P89509	7.27E-06	NM_006461	NM_006461	Homo sapiens sperm associated antigen 5 (SPAG5), mRNA [NM_006461]	NM_006461
A_23_P376557	7.31E-06	NM_022718	NM_022718	Homo sapiens matrix metalloproteinase 25 (MMP25), transcript variant 2, mRNA [NM_022718]	NM_022718
A_24_P936319	7.34E-06	BC030115	BC030115	Homo sapiens, clone IMAGE:4801326, mRNA. [BC030115]	
A_24_P47681	7.39E-06	NM_018448	NM_018448	Homo sapiens cullin-associated and neddylation-dissociated 1 (CAND1), mRNA [NM_018448]	NM_018448
A_24_P184799	7.40E-06	NM_004086	NM_004086	Homo sapiens coagulation factor C homolog, cochlin (Limulus polyphemus) (COCH), mRNA [NM_004086]	NM_004086
A_23_P161424	7.42E-06	NM_032812	NM_032812	Homo sapiens plexin domain containing 2 (PLXDC2), mRNA [NM_032812]	NM_032812
A_24_P54847	7.43E-06	CR603201	CR603201	full-length cDNA clone CS0DF008YB18 of Fetal brain of Homo sapiens (human). [CR603201]	
A_24_P916364	7.44E-06	AK025613	AK025613	Homo sapiens cDNA: FLJ21960 fis, clone HEP05517. [AK025613]	
A_23_P53267	7.46E-06	NM_198261	NM_198261	Homo sapiens similar to splicing factor, arginine/serine-rich 4 (FLJ11021), transcript variant 2, mRNA [NM_198261]	NM_198261
A_23_P142075	7.49E-06	NM_001611	NM_001611	Homo sapiens acid phosphatase 5, tartrate resistant (ACP5), mRNA [NM_001611]	NM_001611
A_32_P34404	7.49E-06	THC2279933			
A_24_P329487	7.50E-06	NM_174911	NM_174911	Homo sapiens family with sequence similarity 84, member B (FAM84B), mRNA [NM_174911]	NM_174911
A_23_P115492	7.52E-06	NM_024749	NM_024749	Homo sapiens hypothetical protein FLJ12505 (FLJ12505), mRNA [NM_024749]	NM_024749
A_32_P74120	7.52E-06	BC070363	BC070363	Homo sapiens cDNA clone IMAGE:3960708, partial cds. [BC070363]	XM_378841

A_23_P41280	7.52E-06	NM_006452	NM_006452	Homo sapiens phosphoribosylaminoimidazole carboxylase, phosphoribosylaminoimidazole succinocarboxamide synthetase (PAICS), mRNA [NM_006452]	NM_006452
A_23_P7543	7.53E-06	NM_014733	NM_014733	Homo sapiens zinc finger, FYVE domain containing 16 (ZFYVE16), mRNA [NM_014733]	NM_014733
A_23_P3663	7.59E-06	NM_138418	NM_138418	Homo sapiens hypothetical protein MGC15416 (MGC15416), transcript variant 2, mRNA [NM_138418]	NM_138418
A_24_P254101	7.60E-06	AK125846	AK125846	Homo sapiens cDNA FLJ43858 fis, clone TESTI4007373. [AK125846]	
A_32_P146898	7.62E-06	AA353695	AA353695	AA353695 EST62114 Jurkat T-cells V Homo sapiens cDNA 5' end, mRNA sequence [AA353695]	
A_23_P255591	7.62E-06	X78926	X78926	H.sapiens HZF3 mRNA for zinc finger protein. [X78926]	
A_23_P254702	7.62E-06	NM_003472	NM_003472	Homo sapiens DEK oncogene (DNA binding) (DEK), mRNA [NM_003472]	NM_003472
A_23_P204579	7.62E-06	NM_003211	NM_003211	Homo sapiens thymine-DNA glycosylase (TDG), transcript variant 1, mRNA [NM_003211]	NM_003211
A_32_P109296	7.63E-06	NM_152259	NM_152259	Homo sapiens leucine-rich repeat kinase 1 (MGC45866), mRNA [NM_152259]	NM_152259
A_23_P325661	7.65E-06	NM_003435	NM_003435	Homo sapiens zinc finger protein 134 (clone pHZ-15) (ZNF134), mRNA [NM_003435]	NM_003435
A_23_P99980	7.66E-06	NM_002128	NM_002128	Homo sapiens high-mobility group box 1 (HMGB1), mRNA [NM_002128]	NM_002128
A_24_P127928	7.73E-06	NM_012414	NM_012414	Homo sapiens rab3 GTPase-activating protein, non-catalytic subunit (150kD) (RAB3-GAP150), mRNA [NM_012414]	NM_012414
A_23_P201996	7.76E-06	NM_100264	NM_100264	Homo sapiens WW domain containing adaptor with coiled-coil (WAC), transcript variant 2, mRNA [NM_100264]	NM_100264
A_32_P191746	7.78E-06	THC2455550			
A_24_P89512	7.78E-06	NM_014739	NM_014739	Homo sapiens BCL2-associated transcription factor 1 (BCLAF1), mRNA [NM_014739]	NM_014739
A_23_P427148	7.79E-06	NM_152465	NM_152465	Homo sapiens proline-rich cyclin A1-interacting protein (PROCA1), mRNA [NM_152465]	NM_152465
A_23_P340148	7.83E-06	NM_021998	NM_021998	Homo sapiens zinc finger protein 6 (CMPX1) (ZNF6), mRNA [NM_021998]	NM_021998
A_23_P301051	7.83E-06	ENST00000244221		Homo sapiens mRNA for KIAA1155 protein, partial cds. [AB032981]	XM_376062
A_23_P371966	7.85E-06	AB075826	AB075826	Homo sapiens mRNA for KIAA1946 protein. [AB075826]	
A_24_P113572	7.90E-06	NM_138415	NM_138415	Homo sapiens PHD finger protein 21B (PHF21B), mRNA [NM_138415]	NM_138415
A_24_P941487	7.91E-06	NM_001008401	NM_001008401	Homo sapiens FLJ16231 protein (FLJ16231), mRNA [NM_001008401]	NM_001008401
A_24_P122524	7.92E-06	NM_006784	NM_006784	Homo sapiens WD repeat domain 3 (WDR3), mRNA [NM_006784]	NM_006784
A_24_P235266	7.92E-06	NM_001001555	NM_001001555	Homo sapiens growth factor receptor-bound protein 10 (GRB10), transcript variant 4, mRNA [NM_001001555]	NM_001001555
A_23_P156907	7.93E-06	NM_018013	NM_018013	Homo sapiens hypothetical protein FLJ10159 (FLJ10159), mRNA [NM_018013]	NM_018013
A_24_P372553	7.94E-06	NM_015608	NM_015608	Homo sapiens chromosome 10 open reading frame 137 (C10orf137), mRNA [NM_015608]	NM_015608
A_23_P213255	8.02E-06	NM_020159	NM_020159	Homo sapiens SWI/SNF-related, matrix-associated actin-dependent regulator of chromatin, subfamily a, containing DEAD/H box 1 (SMARCAD1), mRNA [NM_020159]	NM_020159
A_23_P59798	8.02E-06	NM_013446	NM_013446	Homo sapiens makorin, ring finger protein, 1 (MKRN1), mRNA [NM_013446]	NM_013446
A_24_P120934	8.05E-06	NM_006705	NM_006705	Homo sapiens growth arrest and DNA-damage-inducible, gamma (GADD45G), mRNA [NM_006705]	NM_006705
A_23_P5370	8.08E-06	NM_019845	NM_019845	Homo sapiens reprimo, TP53 dependant G2 arrest mediator candidate (RPRM), mRNA [NM_019845]	NM_019845
A_32_P211253	8.08E-06	ENST00000331096			
A_32_P163472	8.14E-06	A_32_P163472			
A_23_P88439	8.14E-06	NM_152332	NM_152332	Homo sapiens membrane targeting (tandem) C2 domain containing 1 (MTAC2D1), mRNA [NM_152332]	NM_152332
A_24_P166613	8.14E-06	NM_017549	NM_017549	Homo sapiens ependymin related protein 1 (zebrafish) (EPDR1), mRNA [NM_017549]	NM_017549
A_24_P225468	8.14E-06	NM_030920	NM_030920	Homo sapiens acidic (leucine-rich) nuclear phosphoprotein 32 family, member E (ANP32E), mRNA [NM_030920]	NM_030920
A_23_P121869	8.17E-06	NM_021036	NM_021036	Homo sapiens SMA5 (SMA5), mRNA [NM_021036]	NM_021036
A_23_P134347	8.17E-06	NM_031311	NM_031311	Homo sapiens carboxypeptidase, vitellogenic-like (CPVL), transcript variant 1, mRNA [NM_031311]	NM_031311
A_23_P325690	8.17E-06	NM_144698	NM_144698	Homo sapiens ankyrin repeat domain 35 (ANKRD35), mRNA [NM_144698]	NM_144698
A_23_P43255	8.17E-06	NM_016623	NM_016623	Homo sapiens family with sequence similarity 49, member B (FAM49B), mRNA [NM_016623]	NM_016623
A_23_P71537	8.19E-06	NM_024790	NM_024790	Homo sapiens centrosome spindle pole associated protein (CSPP), mRNA [NM_024790]	NM_024790
A_24_P364025	8.19E-06	NM_003338	NM_003338	Homo sapiens ubiquitin-conjugating enzyme E2D 1 (UBC4/5 homolog, yeast) (UBE2D1), mRNA [NM_003338]	NM_003338
A_24_P854964	8.19E-06	BC037740	BC037740	Homo sapiens cDNA clone IMAGE:5263531, partial cds. [BC037740]	
A_23_P352266	8.24E-06	NM_000633	NM_000633	Homo sapiens B-cell CLL/lymphoma 2 (BCL2), nuclear gene encoding mitochondrial protein, transcript variant alpha, mRNA [NM_000633]	NM_000633
A_24_P389612	8.29E-06	CR625971	CR625971	full-length cDNA clone CS0DL002YL19 of B cells (Ramos cell line) Cot 25-normalized of Homo sapiens (human). [CR625971]	

A_23_P107552	8.32E-06	NM_014939	NM_014939	Homo sapiens KIAA1012 (KIAA1012), mRNA [NM_014939]	NM_014939
A_32_P108722	8.33E-06	AK127572	AK127572	Homo sapiens cDNA FLJ45665 fis, clone CTONG2027959. [AK127572]	
A_23_P102109	8.33E-06	NM_025019	NM_025019	Homo sapiens tubulin, alpha 4 (TUBA4), mRNA [NM_025019]	NM_025019
A_24_P237389	8.33E-06	NM_001412	NM_001412	Homo sapiens eukaryotic translation initiation factor 1A, X-linked (EIF1AX), mRNA [NM_001412]	NM_001412
A_32_P103837	8.33E-06	NM_021238	NM_021238	Homo sapiens family with sequence similarity 60, member A (FAM60A), mRNA [NM_021238]	NM_021238
A_32_P159289	8.35E-06	A_32_P159289			
A_23_P20622	8.36E-06	NM_003671	NM_003671	Homo sapiens CDC14 cell division cycle 14 homolog B (S. cerevisiae) (CDC14B), transcript variant 1, mRNA [NM_003671]	NM_003671
A_23_P140328	8.37E-06	NM_004713	NM_004713	Homo sapiens serologically defined colon cancer antigen 1 (SDCCAG1), mRNA [NM_004713]	NM_004713
A_23_P252740	8.39E-06	NM_024094	NM_024094	Homo sapiens defective in sister chromatid cohesion homolog 1 (S. cerevisiae) (DCC1), mRNA [NM_024094]	NM_024094
A_24_P941787	8.47E-06	NM_003913	NM_003913	Homo sapiens PRP4 pre-mRNA processing factor 4 homolog B (yeast) (PRPF4B), transcript variant 1, mRNA [NM_003913]	NM_003913
A_23_P395460	8.52E-06	NM_004321	NM_004321	Homo sapiens kinesin family member 1A (KIF1A), mRNA [NM_004321]	NM_004321
A_24_P188941	8.52E-06	NM_002520	NM_002520	Homo sapiens nucleophosmin (nucleolar phosphoprotein B23, numatrin) (NPM1), mRNA [NM_002520]	NM_002520
A_24_P150874	8.53E-06	CR611712	CR611712	full-length cDNA clone CS0DB005YH06 of Neuroblastoma Cot 10-normalized of Homo sapiens (human). [CR611712]	
A_32_P155811	8.56E-06	THC2276639			
A_23_P88880	8.62E-06	NM_015069	NM_015069	Homo sapiens zinc finger protein 423 (ZNF423), mRNA [NM_015069]	NM_015069
A_23_P87049	8.62E-06	NM_003105	NM_003105	Homo sapiens sortilin-related receptor, L(DLR class) A repeats-containing (SORL1), mRNA [NM_003105]	NM_003105
A_23_P74467	8.63E-06	NM_014949	NM_014949	Homo sapiens KIAA0907 (KIAA0907), mRNA [NM_014949]	NM_014949
A_24_P630039	8.69E-06	CR600369	CR600369	full-length cDNA clone CS0DF025YM09 of Fetal brain of Homo sapiens (human). [CR600369]	
A_23_P55376	8.71E-06	NM_015443	NM_015443	Homo sapiens LOC284058 protein (LOC284058), mRNA [NM_015443]	NM_015443
A_23_P45831	8.76E-06	NM_004284	NM_004284	Homo sapiens chromodomain helicase DNA binding protein 1-like (CHD1L), mRNA [NM_004284]	NM_004284
A_32_P206541	8.77E-06	AK128714	AK128714	Homo sapiens cDNA FLJ46881 fis, clone UTERU3015647, moderately similar to Embigin precursor. [AK128714]	
A_23_P92320	8.77E-06	NM_017426	NM_017426	Homo sapiens nucleoporin 54kDa (NUP54), mRNA [NM_017426]	NM_017426
A_32_P154361	8.78E-06	THC2415390			
A_23_P163227	8.84E-06	NM_020990	NM_020990	Homo sapiens creatine kinase, mitochondrial 1B (CKMT1B), nuclear gene encoding mitochondrial protein, mRNA [NM_020990]	NM_020990
A_32_P206293	8.84E-06	BC070371	BC070371	Homo sapiens HLA complex group 12, mRNA (cDNA clone IMAGE:4657554). [BC070371]	
A_23_P309261	8.86E-06	NM_147171	NM_147171	Homo sapiens A kinase (PRKA) anchor protein (yotiao) 9 (AKAP9), transcript variant 1, mRNA [NM_147171]	NM_147171
A_24_P162911	8.87E-06	NM_005813	NM_005813	Homo sapiens protein kinase D3 (PRKD3), mRNA [NM_005813]	NM_005813
A_23_P113245	8.89E-06	D14041	D14041	Homo sapiens mRNA for H-2K binding factor-2, complete cds. [D14041]	
A_23_P325017	8.92E-06	NM_147128	NM_147128	Homo sapiens zinc and ring finger 2 (ZNRF2), mRNA [NM_147128]	NM_147128
A_24_P345781	8.92E-06	THC2428103		Q9W056 (Q9W056) CG1139-PA (LP06969p), partial (4%) [THC2428103]	
A_32_P123514	8.92E-06	BX648831	BX648831	Homo sapiens mRNA; cDNA DKFZp686J06116 (from clone DKFZp686J06116). [BX648831]	XR_000195
A_23_P93311	8.93E-06	NM_013994	NM_013994	Homo sapiens discoidin domain receptor family, member 1 (DDR1), transcript variant 3, mRNA [NM_013994]	NM_013994
A_23_P142389	8.93E-06	NM_205834	NM_205834	Homo sapiens liver-specific bHLH-Zip transcription factor (LISCH7), transcript variant 2, mRNA [NM_205834]	NM_205834
A_23_P388670	8.93E-06	NM_000895	NM_000895	Homo sapiens leukotriene A4 hydrolase (LTA4H), mRNA [NM_000895]	NM_000895
A_24_P228228	8.94E-06	NM_004775	NM_004775	Homo sapiens UDP-Gal:betaGlcNAc beta 1,4- galactosyltransferase, polypeptide 6 (B4GALT6), mRNA [NM_004775]	NM_004775
A_23_P112341	8.94E-06	NM_024945	NM_024945	Homo sapiens chromosome 9 open reading frame 76 (C9orf76), mRNA [NM_024945]	NM_024945
A_24_P23979	9.01E-06	NM_016379	NM_016379	Homo sapiens variable charge, X-linked 3A (VCX3A), mRNA [NM_016379]	NM_016379
A_23_P502832	9.02E-06	NM_006047	NM_006047	Homo sapiens RNA binding motif protein 12 (RBM12), transcript variant 1, mRNA [NM_006047]	NM_006047
A_23_P23705	9.09E-06	BC040655	BC040655	Homo sapiens cDNA clone IMAGE:4798227, partial cds. [BC040655]	
A_23_P218827	9.09E-06	NM_006596	NM_006596	Homo sapiens polymerase (DNA directed), theta (POLQ), transcript variant 1, mRNA [NM_006596]	NM_006596
A_23_P357936	9.14E-06	NM_014010	NM_014010	Homo sapiens astrotactin 2 (ASTN2), transcript variant 1, mRNA [NM_014010]	NM_014010
A_24_P645914	9.18E-06	AK025909	AK025909	Homo sapiens cDNA: FLJ22256 fis, clone HRC02860. [AK025909]	
A_32_P51894	9.18E-06	CR624054	CR624054	full-length cDNA clone CS0DC029YL12 of Neuroblastoma Cot 25-normalized of Homo sapiens (human). [CR624054]	
A_24_P360078	9.18E-06	NM_006726	NM_006726	Homo sapiens LPS-responsive vesicle trafficking, beach and anchor containing (LRBA), mRNA [NM_006726]	NM_006726

A_23_P9574	9.19E-06	NM_018098	NM_018098	Homo sapiens epithelial cell transforming sequence 2 oncogene (ECT2), mRNA [NM_018098]	NM_018098
A_24_P307785	9.20E-06	AK098491	AK098491	Homo sapiens cDNA FLJ25625 fis, clone STM02974. [AK098491]	
A_23_P89145	9.23E-06	NM_018300	NM_018300	Homo sapiens zinc finger protein 83 (HPF1) (ZNF83), mRNA [NM_018300]	NM_018300
A_23_P258088	9.23E-06	NM_020804	NM_020804	Homo sapiens protein kinase C and casein kinase substrate in neurons 1 (PACSIN1), mRNA [NM_020804]	NM_020804
A_24_P240137	9.29E-06	NM_004487	NM_004487	Homo sapiens golgi autoantigen, golgin subfamily b, macrogolgin (with transmembrane signal), 1 (GOLGB1), mRNA [NM_004487]	NM_004487
A_24_P225308	9.30E-06	NM_016374	NM_016374	Homo sapiens AT rich interactive domain 4B (RBP1- like) (ARID4B), transcript variant 1, mRNA [NM_016374]	NM_016374
A_23_P369994	9.33E-06	NM_004734	NM_004734	Homo sapiens doublecortin and CaM kinase-like 1 (DCAMKL1), mRNA [NM_004734]	NM_004734
A_23_P32684	9.48E-06	AF116619	AF116619	Homo sapiens PRO1051 mRNA, complete cds. [AF116619]	
A_23_P411922	9.48E-06	NM_012272	NM_012272	Homo sapiens Huntingtin interacting protein C (HYPC), mRNA [NM_012272]	NM_012272
A_24_P219971	9.50E-06	NM_017896	NM_017896	Homo sapiens chromosome 20 open reading frame 11 (C20orf11), mRNA [NM_017896]	NM_017896
A_24_P929818	9.52E-06	S73202	S73202	argininosuccinate synthetase [human, Japanese classical citrullinemia patient A82, mRNA Partial Mutant, 91 nt]. [S73202]	
A_23_P137876	9.56E-06	NM_001412	NM_001412	Homo sapiens eukaryotic translation initiation factor 1A, X-linked (EIF1AX), mRNA [NM_001412]	NM_001412
A_32_P115277	9.57E-06	THC2279466			
A_24_P174503	9.57E-06	NM_000481	NM_000481	Homo sapiens aminomethyltransferase (glycine cleavage system protein T) (AMT), mRNA [NM_000481]	NM_000481
A_23_P384085	9.59E-06	NM_181453	NM_181453	Homo sapiens GRIP and coiled-coil domain containing 2 (GCC2), transcript variant 1, mRNA [NM_181453]	NM_181453
A_23_P161507	9.60E-06	NM_004923	NM_004923	Homo sapiens metallothionein-like 5, testis-specific (tesmin) (MTL5), mRNA [NM_004923]	NM_004923
A_23_P406957	9.64E-06	NM_138778	NM_138778	Homo sapiens chromosome 9 open reading frame 112 (C9orf112), mRNA [NM_138778]	NM_138778
A_23_P117882	9.65E-06	NM_004378	NM_004378	Homo sapiens cellular retinoic acid binding protein 1 (CRABP1), mRNA [NM_004378]	NM_004378
A_23_P254179	9.68E-06	NM_015339	NM_015339	Homo sapiens activity-dependent neuroprotector (ADNP), transcript variant 1, mRNA [NM_015339]	NM_015339
A_24_P42446	9.69E-06	NM_001015508	NM_001015508	Homo sapiens purine-rich element binding protein G (PURG), transcript variant B, mRNA [NM_001015508]	NM_001015508
A_32_P29632	9.69E-06	NM_001338	NM_001338	Homo sapiens coxsackie virus and adenovirus receptor (CXADR), mRNA [NM_001338]	NM_001338
A_23_P352950	9.72E-06	NM_052926	NM_052926	Homo sapiens paraneoplastic antigen like 5 (PNMA5), mRNA [NM_052926]	NM_052926
A_23_P340263	9.73E-06	NM_173662	NM_173662	Homo sapiens ring finger protein 175 (RNF175), mRNA [NM_173662]	NM_173662
A_23_P424269	9.74E-06	NM_020207	NM_020207	Homo sapiens chromosome 9 open reading frame 102 (C9orf102), mRNA [NM_020207]	NM_020207
A_32_P59673	9.74E-06	NM_199136	NM_199136	Homo sapiens hypothetical protein MGC72075 (MGC72075), mRNA [NM_199136]	NM_199136
A_23_P77612	9.74E-06	NM_145348	NM_145348	Homo sapiens kringle containing transmembrane protein 2 (KREMEN2), transcript variant 3, mRNA [NM_145348]	NM_145348
A_24_P179611	9.74E-06	NM_003292	NM_003292	Homo sapiens translocated promoter region (to activated MET oncogene) (TPR), mRNA [NM_003292]	NM_003292
A_24_P942694	9.75E-06	NM_018017	NM_018017	Homo sapiens chromosome 10 open reading frame 118 (C10orf118), mRNA [NM_018017]	NM_018017
A_23_P38427	9.78E-06	NM_032932	NM_032932	Homo sapiens RAB11 family interacting protein 4 (class II) (RAB11FIP4), mRNA [NM_032932]	NM_032932
A_24_P33989	9.79E-06	NM_022351	NM_022351	Homo sapiens EF-hand calcium binding protein 1 (EFCBP1), mRNA [NM_022351]	NM_022351
A_23_P48350	9.79E-06	NM_182848	NM_182848	Homo sapiens claudin 10 (CLDN10), transcript variant 1, mRNA [NM_182848]	NM_182848
A_23_P96325	9.79E-06	NM_001009954	NM_001009954	Homo sapiens FLJ20105 protein (FLJ20105), transcript variant 2, mRNA [NM_001009954]	NM_001009954
A_23_P306890	9.79E-06	NM_007195	NM_007195	Homo sapiens polymerase (DNA directed) iota (POLI), mRNA [NM_007195]	NM_007195
A_23_P74115	9.81E-06	NM_003579	NM_003579	Homo sapiens RAD54-like (S. cerevisiae) (RAD54L), mRNA [NM_003579]	NM_003579
A_32_P87631	9.83E-06	BC017507	BC017507	Homo sapiens, clone IMAGE:4850148, mRNA. [BC017507]	
A_23_P143285	9.83E-06	AK000809	AK000809	Homo sapiens cDNA FLJ20802 fis, clone ADSU01223. [AK000809]	
A_24_P331882	9.87E-06	AL133028	AL133028	Homo sapiens mRNA; cDNA DKFZp434F117 (from clone DKFZp434F117). [AL133028]	XM_044178
A_24_P921144	9.87E-06	BC056662	BC056662	Homo sapiens cDNA clone IMAGE:4045027, partial cds. [BC056662]	
A_23_P202939	9.87E-06	NM_001642	NM_001642	Homo sapiens amyloid beta (A4) precursor-like protein 2 (APLP2), mRNA [NM_001642]	NM_001642
A_23_P28953	9.87E-06	NM_175850	NM_175850	Homo sapiens DNA (cytosine-5-)-methyltransferase 3 beta (DNMT3B), transcript variant 6, mRNA [NM_175850]	NM_175850
A_23_P47777	9.88E-06	NM_138396	NM_138396	Homo sapiens membrane-associated ring finger (C3HC4) 9 (MARCH9), mRNA [NM_138396]	NM_138396
A_32_P207789	9.88E-06	BQ017638	BQ017638	BQ017638 UI-H-DI0-auv-p-03-0-UI.s1 NCI_CGAP_DI0 Homo sapiens cDNA clone IMAGE:5875058 3', mRNA sequence [BQ017638]	
A_24_P917015	9.90E-06	AF288405	AF288405	Homo sapiens G protein interaction factor 1-like mRNA sequence. [AF288405]	XM_370652
A_23_P133284	9.90E-06	NM_018343	NM_018343	Homo sapiens RIO kinase 2 (yeast) (RIOK2), mRNA [NM_018343]	NM_018343

A_24_P306814	9.92E-06	A_24_P306814			
A_23_P21230	9.93E-06	NM_015294	NM_015294	Homo sapiens tripartite motif-containing 37 (TRIM37), transcript variant 1, mRNA [NM_015294]	NM_015294
A_24_P285179	9.95E-06	THC2337957		Q6IHS4 (Q6IHS4) HDC01261, partial (9%) [THC2337957]	
A_23_P73747	9.95E-06	NM_014782	NM_014782	Homo sapiens armadillo repeat containing, X-linked 2 (ARMCX2), mRNA [NM_014782]	NM_014782
A_32_P148914	9.96E-06	BC037255	BC037255	Homo sapiens hypothetical LOC389634, mRNA (cDNA clone IMAGE:4157715). [BC037255]	
A_23_P2683	9.96E-06	NM_024604	NM_024604	Homo sapiens hypothetical protein FLJ21908 (FLJ21908), mRNA [NM_024604]	NM_024604
A_32_P126311	9.96E-06	NM_173514	NM_173514	Homo sapiens hypothetical protein FLJ90709 (FLJ90709), mRNA [NM_173514]	NM_173514
A_32_P104478	9.97E-06	AK026881	AK026881	Homo sapiens cDNA: FLJ23228 fis, clone CAE06654. [AK026881]	
A_23_P250982	9.97E-06	NM_016048	NM_016048	Homo sapiens isochorismatase domain containing 1 (ISOC1), mRNA [NM_016048]	NM_016048
A_23_P256158	9.98E-06	NM_000683	NM_000683	Homo sapiens adrenergic, alpha-2C-, receptor (ADRA2C), mRNA [NM_000683]	NM_000683
A_23_P349966	1.00E-05	NM_152913	NM_152913	Homo sapiens hypothetical protein DKFZp761L1417 (DKFZp761L1417), mRNA [NM_152913]	NM_152913
A_24_P240338	1.00E-05	NM_001812	NM_001812	Homo sapiens centromere protein C 1 (CENPC1), mRNA [NM_001812]	NM_001812
A_23_P10385	1.00E-05	NM_016448	NM_016448	Homo sapiens denticleless homolog (Drosophila) (DTL), mRNA [NM_016448]	NM_016448
A_32_P113887	1.01E-05	THC2446900			
A_23_P82941	1.01E-05	NM_006421	NM_006421	Homo sapiens ADP-ribosylation factor guanine nucleotide-exchange factor 1(brefeldin A-inhibited) (ARFGEF1), mRNA [NM_006421]	NM_006421
A_32_P74847	1.01E-05	CD048206	CD048206	AGENCOURT_13966160 NIH_MGC_172 Homo sapiens cDNA 5', mRNA sequence [CD048206]	
A_32_P141418	1.02E-05	NM_018076	NM_018076	Homo sapiens armadillo repeat containing 4 (ARMC4), mRNA [NM_018076]	NM_018076
A_23_P344281	1.02E-05	NM_001010879	NM_001010879	Homo sapiens zinc finger protein interacting with K protein 1 (ZIK1), mRNA [NM_001010879]	NM_001010879
A_32_P60343	1.02E-05	AK090778	AK090778	Homo sapiens cDNA FLJ33459 fis, clone BRAMY2000585. [AK090778]	XM_294765
A_23_P170491	1.02E-05	NM_005879	NM_005879	Homo sapiens TRAF interacting protein (TRIP), mRNA [NM_005879]	NM_005879
A_23_P327910	1.02E-05	NM_003413	NM_003413	Homo sapiens Zic family member 3 heterotaxy 1 (odd-paired homolog, Drosophila) (ZIC3), mRNA [NM_003413]	NM_003413
A_23_P397999	1.02E-05	NM_003468	NM_003468	Homo sapiens frizzled homolog 5 (Drosophila) (FZD5), mRNA [NM_003468]	NM_003468
A_32_P71796	1.03E-05	THC2369196		ALU1_HUMAN (P39188) Alu subfamily J sequence contamination warning entry, partial (5%) [THC2369196]	
A_23_P218068	1.03E-05	NM_019012	NM_019012	Homo sapiens pleckstrin homology domain containing, family A member 5 (PLEKHA5), mRNA [NM_019012]	NM_019012
A_24_P937095	1.03E-05	U68494	U68494	Human hbc647 mRNA sequence. [U68494]	
A_23_P56922	1.03E-05	NM_002157	NM_002157	Homo sapiens heat shock 10kDa protein 1 (chaperonin 10) (HSPE1), mRNA [NM_002157]	NM_002157
A_23_P60271	1.04E-05	NM_006444	NM_006444	Homo sapiens SMC2 structural maintenance of chromosomes 2-like 1 (yeast) (SMC2L1), mRNA [NM_006444]	NM_006444
A_24_P938614	1.04E-05	AK026697	AK026697	Homo sapiens cDNA: FLJ23044 fis, clone LNG02454. [AK026697]	
A_23_P148204	1.04E-05	NM_017944	NM_017944	Homo sapiens ubiquitin specific protease 47 (USP47), mRNA [NM_017944]	NM_017944
A_23_P323094	1.04E-05	NM_004426	NM_004426	Homo sapiens polyhomeotic-like 1 (Drosophila) (PHC1), mRNA [NM_004426]	NM_004426
A_23_P147397	1.04E-05	AB082528	AB082528	Homo sapiens mRNA for KIAA1997 protein. [AB082528]	XM_370652
A_23_P157268	1.04E-05	NM_012129	NM_012129	Homo sapiens claudin 12 (CLDN12), mRNA [NM_012129]	NM_012129
A_23_P32955	1.04E-05	U08023	U08023	Human cellular proto-oncogene (c-mer) mRNA, complete cds. [U08023]	
A_23_P133365	1.04E-05	NM_006706	NM_006706	Homo sapiens transcription elongation regulator 1 (TCERG1), mRNA [NM_006706]	NM_006706
A_23_P210176	1.04E-05	NM_000210	NM_000210	Homo sapiens integrin, alpha 6 (ITGA6), mRNA [NM_000210]	NM_000210
A_32_P68533	1.05E-05	ENST00000307507		Homo sapiens mRNA; cDNA DKFZp686O21143 (from clone DKFZp686O21143). [BX648834]	XM_291019
A_23_P143143	1.05E-05	NM_002166	NM_002166	Homo sapiens inhibitor of DNA binding 2, dominant negative helix-loop-helix protein (ID2), mRNA [NM_002166]	NM_002166
A_23_P3204	1.05E-05	NM_002748	NM_002748	Homo sapiens mitogen-activated protein kinase 6 (MAPK6), mRNA [NM_002748]	NM_002748
A_23_P400580	1.06E-05	AB040883	AB040883	Homo sapiens mRNA for KIAA1450 protein, partial cds. [AB040883]	
A_24_P761130	1.06E-05	AK097080	AK097080	Homo sapiens cDNA FLJ39761 fis, clone SPLEN1000083. [AK097080]	XM_496251
A_23_P154500	1.06E-05	NM_175629	NM_175629	Homo sapiens DNA (cytosine-5-)-methyltransferase 3 alpha (DNMT3A), transcript variant 1, mRNA [NM_175629]	NM_175629
A_23_P54006	1.06E-05	NM_015382	NM_015382	Homo sapiens HECT domain containing 1 (HECTD1), mRNA [NM_015382]	NM_015382
A_24_P921823	1.06E-05	THC2399438		HSA270770 T-cell transcription factor-4 long C-terminal isoform 1 {Homo sapiens;} , complete [THC2399438]	
A_32_P231226	1.07E-05	AK095046	AK095046	Homo sapiens cDNA FLJ37727 fis, clone BRHIP2019972. [AK095046]	
A_23_P371266	1.07E-05	NM_015569	NM_015569	Homo sapiens dynamin 3 (DNM3), mRNA [NM_015569]	NM_015569

A_24_P150791	1.07E-05	NM_020655	NM_020655	Homo sapiens junctophilin 3 (JPH3), mRNA [NM_020655]	NM_020655
A_23_P69877	1.07E-05	AK091550	AK091550	Homo sapiens cDNA FLJ34231 fis, clone FCBBF3025905, highly similar to Mus musculus (clone pMLZ-1) zinc finger protein (Zfp) mRNA. [AK091550]	
A_23_P259641	1.07E-05	NM_004456	NM_004456	Homo sapiens enhancer of zeste homolog 2 (Drosophila) (EZH2), transcript variant 1, mRNA [NM_004456]	NM_004456
A_32_P205303	1.08E-05	AF070595	AF070595	Homo sapiens clone 24583 mRNA sequence. [AF070595]	
A_23_P145096	1.08E-05	NM_005084	NM_005084	Homo sapiens phospholipase A2, group VII (platelet-activating factor acetylhydrolase, plasma) (PLA2G7), mRNA [NM_005084]	NM_005084
A_24_P75158	1.08E-05	AL832683	AL832683	Homo sapiens mRNA; cDNA DKFZp313P0917 (from clone DKFZp313P0917). [AL832683]	
A_23_P256021	1.08E-05	NM_031206	NM_031206	Homo sapiens LAS1-like (S. cerevisiae) (LAS1L), mRNA [NM_031206]	NM_031206
A_23_P64051	1.09E-05	A_23_P64051			
A_32_P205792	1.09E-05	A_32_P205792			
A_23_P167920	1.09E-05	NM_005618	NM_005618	Homo sapiens delta-like 1 (Drosophila) (DLL1), mRNA [NM_005618]	NM_005618
A_24_P241792	1.09E-05	NM_198893	NM_198893	Homo sapiens zinc finger protein 160 (ZNF160), transcript variant 2, mRNA [NM_198893]	NM_198893
A_24_P317874	1.09E-05	NM_012311	NM_012311	Homo sapiens KIN, antigenic determinant of recA protein homolog (mouse) (KIN), mRNA [NM_012311]	NM_012311
A_23_P122304	1.09E-05	NM_001527	NM_001527	Homo sapiens histone deacetylase 2 (HDAC2), mRNA [NM_001527]	NM_001527
A_23_P367043	1.10E-05	ENST00000324993		Homo sapiens CDC14 cell division cycle 14 homolog C (S. cerevisiae), mRNA (cDNA clone IMAGE:4826219), partial cds. [BC028690]	
A_24_P652700	1.10E-05	BX648822	BX648822	Homo sapiens mRNA; cDNA DKFZp686C15165 (from clone DKFZp686C15165). [BX648822]	
A_32_P3914	1.10E-05	THC2437430		ALU7_HUMAN (P39194) Alu subfamily SQ sequence contamination warning entry, partial (17%) [THC2437430]	
A_24_P159227	1.10E-05	NM_020168	NM_020168	Homo sapiens p21(CDKN1A)-activated kinase 6 (PAK6), mRNA [NM_020168]	NM_020168
A_23_P51317	1.10E-05	NM_019083	NM_019083	Homo sapiens hypothetical protein FLJ10287 (FLJ10287), mRNA [NM_019083]	NM_019083
A_24_P325176	1.10E-05	AB029032	AB029032	Homo sapiens mRNA for KIAA1109 protein, partial cds. [AB029032]	XM_371706
A_23_P16648	1.10E-05	NM_017573	NM_017573	Homo sapiens proprotein convertase subtilisin/kexin type 4 (PCSK4), mRNA [NM_017573]	NM_017573
A_23_P14105	1.10E-05	NM_001268	NM_001268	Homo sapiens regulator of chromosome condensation (RCC1) and BTB (POZ) domain containing protein 2 (RCBTB2), mRNA [NM_001268]	NM_001268
A_23_P408285	1.10E-05	NM_153026	NM_153026	Homo sapiens prickle-like 1 (Drosophila) (PRICKLE1), mRNA [NM_153026]	NM_153026
A_32_P75425	1.10E-05	NM_001013665	NM_001013665	Homo sapiens hypothetical LOC399744 (LOC399744), mRNA [NM_001013665]	NM_001013665
A_23_P415443	1.10E-05	NM_015341	NM_015341	Homo sapiens barren homolog (Drosophila) (BRRN1), mRNA [NM_015341]	NM_015341
A_24_P66125	1.10E-05	NM_006603	NM_006603	Homo sapiens stromal antigen 2 (STAG2), mRNA [NM_006603]	NM_006603
A_23_P142634	1.10E-05	NM_014168	NM_014168	Homo sapiens methyltransferase like 5 (METTL5), mRNA [NM_014168]	NM_014168
A_23_P349416	1.11E-05	NM_001982	NM_001982	Homo sapiens v-erb-b2 erythroblastic leukemia viral oncogene homolog 3 (avian) (ERBB3), transcript variant 1, mRNA [NM_001982]	NM_001982
A_24_P365901	1.11E-05	NM_178562	NM_178562	Homo sapiens hypothetical protein MGC50844 (MGC50844), mRNA [NM_178562]	NM_178562
A_23_P16022	1.11E-05	NM_005773	NM_005773	Homo sapiens zinc finger protein 256 (ZNF256), mRNA [NM_005773]	NM_005773
A_23_P213661	1.11E-05	NM_015216	NM_015216	Homo sapiens KIAA0433 protein (KIAA0433), mRNA [NM_015216]	NM_015216
A_23_P130359	1.12E-05	NM_030672	NM_030672	Homo sapiens Rho GTPase activating protein 28 (ARHGAP28), transcript variant 2, mRNA [NM_030672]	NM_030672
A_23_P96590	1.12E-05	NM_014710	NM_014710	Homo sapiens G protein-coupled receptor associated sorting protein 1 (GPRASP1), mRNA [NM_014710]	NM_014710
A_24_P87036	1.12E-05	NM_018043	NM_018043	Homo sapiens transmembrane protein 16A (TMEM16A), mRNA [NM_018043]	NM_018043
A_23_P40548	1.12E-05	NM_013313	NM_013313	Homo sapiens yippee-like 1 (Drosophila) (YPEL1), mRNA [NM_013313]	NM_013313
A_23_P345065	1.12E-05	NM_016510	NM_016510	Homo sapiens selenocysteine lyase (SCLY), mRNA [NM_016510]	NM_016510
A_24_P242357	1.12E-05	NM_012262	NM_012262	Homo sapiens heparan sulfate 2-O-sulfotransferase 1 (HS2ST1), mRNA [NM_012262]	NM_012262
A_23_P392384	1.12E-05	NM_001002260	NM_001002260	Homo sapiens chromosome 9 open reading frame 58 (C9orf58), transcript variant 2, mRNA [NM_001002260]	NM_001002260
A_23_P116414	1.12E-05	NM_007069	NM_007069	Homo sapiens HRAS-like suppressor 3 (HRASLS3), mRNA [NM_007069]	NM_007069
A_24_P153880	1.13E-05	NM_198181	NM_198181	Homo sapiens hypothetical protein LOC440295 (LOC440295), mRNA [NM_198181]	NM_198181
A_23_P435002	1.13E-05	NM_152546	NM_152546	Homo sapiens serum response factor binding protein 1 (SRFBP1), mRNA [NM_152546]	NM_152546
A_23_P110851	1.13E-05	NM_003219	NM_003219	Homo sapiens telomerase reverse transcriptase (TERT), transcript variant 1, mRNA [NM_003219]	NM_003219
A_23_P202004	1.13E-05	NM_020200	NM_020200	Homo sapiens phosphoribosyl transferase domain containing 1 (PRTFDC1), mRNA [NM_020200]	NM_020200
A_23_P5339	1.13E-05	NM_030577	NM_030577	Homo sapiens hypothetical protein MGC10993 (MGC10993), mRNA [NM_030577]	NM_030577
A_23_P73192	1.14E-05	NM_024726	NM_024726	Homo sapiens IQ motif containing with AAA domain (IQCA), mRNA [NM_024726]	NM_024726
A_32_P94801	1.14E-05	THC2308876			

A_23_P408167	1.14E-05	NM_001004051	NM_001004051	Homo sapiens G protein-coupled receptor associated sorting protein 2 (GPRASP2), transcript variant 1, mRNA [NM_001004051]	NM_001004051
A_32_P135985	1.14E-05	NM_003212	NM_003212	Homo sapiens teratocarcinoma-derived growth factor 1 (TDGF1), mRNA [NM_003212]	NM_003212
A_32_P205944	1.14E-05	NM_005054	NM_005054	Homo sapiens RAN binding protein 2-like 1 (RANBP2L1), transcript variant 1, mRNA [NM_005054]	NM_005054
A_32_P82179	1.15E-05	THC2318057			
A_32_P19294	1.15E-05	NM_144669	NM_144669	Homo sapiens hypothetical protein FLJ31978 (FLJ31978), mRNA [NM_144669]	NM_144669
A_32_P137382	1.16E-05	BX089701	BX089701	BX089701 BX089701 NCI_CGAP_Lu24 Homo sapiens cDNA clone IMAGp998I115809 ; IMAGE:2341330, mRNA sequence [BX089701]	
A_24_P412486	1.16E-05	CR601315	CR601315	full-length cDNA clone CS0DC001YJ02 of Neuroblastoma Cot 25-normalized of Homo sapiens (human). [CR601315]	
A_24_P22079	1.16E-05	NM_002015	NM_002015	Homo sapiens forkhead box O1A (rhabdomyosarcoma) (FOXO1A), mRNA [NM_002015]	NM_002015
A_32_P199049	1.16E-05	THC2314201		Q7QYY0 (Q7QYY0) GLP_164_21502_20957, partial (9%) [THC2314201]	
A_23_P305060	1.16E-05	NM_005746	NM_005746	Homo sapiens pre-B-cell colony enhancing factor 1 (PBEF1), transcript variant 1, mRNA [NM_005746]	NM_005746
A_24_P190168	1.16E-05	NM_014573	NM_014573	Homo sapiens hypothetical protein MAC30 (MAC30), mRNA [NM_014573]	NM_014573
A_23_P132216	1.16E-05	NM_004175	NM_004175	Homo sapiens small nuclear ribonucleoprotein D3 polypeptide 18kDa (SNRPD3), mRNA [NM_004175]	NM_004175
A_23_P313734	1.17E-05	AB051518	AB051518	Homo sapiens mRNA for KIAA1731 protein, partial cds. [AB051518]	XM_374922
A_23_P139143	1.17E-05	NM_004177	NM_004177	Homo sapiens syntaxin 3A (STX3A), mRNA [NM_004177]	NM_004177
A_23_P353717	1.17E-05	NM_152308	NM_152308	Homo sapiens hypothetical protein MGC24665 (MGC24665), mRNA [NM_152308]	NM_152308
A_23_P74950	1.17E-05	NM_018715	NM_018715	Homo sapiens regulator of chromosome condensation 2 (RCC2), mRNA [NM_018715]	NM_018715
A_32_P167471	1.18E-05	AK125038	AK125038	Homo sapiens cDNA FLJ43048 fis, clone BRTHA3004502. [AK125038]	
A_23_P161719	1.18E-05	NM_152434	NM_152434	Homo sapiens CWF19-like 2, cell cycle control (S. pombe) (CWF19L2), mRNA [NM_152434]	NM_152434
A_23_P82738	1.18E-05	NM_012415	NM_012415	Homo sapiens RAD54 homolog B (S. cerevisiae) (RAD54B), transcript variant 1, mRNA [NM_012415]	NM_012415
A_32_P142440	1.18E-05	NM_174936	NM_174936	Homo sapiens proprotein convertase subtilisin/kexin type 9 (PCSK9), mRNA [NM_174936]	NM_174936
A_23_P76538	1.18E-05	NM_017899	NM_017899	Homo sapiens hypothetical protein FLJ20607 (TSC), mRNA [NM_017899]	NM_017899
A_23_P129064	1.18E-05	NM_001482	NM_001482	Homo sapiens glycine amidinotransferase (L-arginine:glycine amidinotransferase) (GATM), mRNA [NM_001482]	NM_001482
A_23_P101185	1.18E-05	NM_004539	NM_004539	Homo sapiens asparaginyl-tRNA synthetase (NARS), mRNA [NM_004539]	NM_004539
A_23_P212159	1.19E-05	NM_024923	NM_024923	Homo sapiens nucleoporin 210kDa (NUP210), mRNA [NM_024923]	NM_024923
A_23_P66948	1.19E-05	NM_022751	NM_022751	Homo sapiens family with sequence similarity 59, member A (FAM59A), mRNA [NM_022751]	NM_022751
A_23_P422831	1.19E-05	NM_004816	NM_004816	Homo sapiens chromosome 9 open reading frame 61 (C9orf61), mRNA [NM_004816]	NM_004816
A_23_P216257	1.19E-05	NM_005079	NM_005079	Homo sapiens tumor protein D52 (TPD52), transcript variant 3, mRNA [NM_005079]	NM_005079
A_23_P120345	1.20E-05	NM_020651	NM_020651	Homo sapiens pellino homolog 1 (Drosophila) (PELI1), mRNA [NM_020651]	NM_020651
A_23_P253524	1.20E-05	NM_001813	NM_001813	Homo sapiens centromere protein E, 312kDa (CENPE), mRNA [NM_001813]	NM_001813
A_23_P200710	1.20E-05	NM_002646	NM_002646	Homo sapiens phosphoinositide-3-kinase, class 2, beta polypeptide (PIK3C2B), mRNA [NM_002646]	NM_002646
A_23_P65157	1.20E-05	NM_005694	NM_005694	Homo sapiens COX17 homolog, cytochrome c oxidase assembly protein (yeast) (COX17), nuclear gene encoding mitochondrial protein, mRNA [NM_005694]	NM_005694
A_24_P333306	1.21E-05	AK023737	AK023737	Homo sapiens cDNA FLJ13675 fis, clone PLACE1011875, highly similar to Homo sapiens mRNA for KIAA0580 protein. [AK023737]	
A_23_P373799	1.21E-05	NM_020943	NM_020943	Homo sapiens KIAA1604 protein (KIAA1604), mRNA [NM_020943]	NM_020943
A_24_P943997	1.22E-05	NM_178815	NM_178815	Homo sapiens ADP-ribosylation factor-like 8 (ARL8), mRNA [NM_178815]	NM_178815
A_24_P141736	1.22E-05	THC2336852		AMP2_HUMAN (P50579) Methionine aminopeptidase 2 (MetAP 2) (Peptidase M 2) (Initiation factor 2 associated 67 kDa glycoprotein) (p67) (p67eIF2) , partial (89%) [THC2336852]	
A_32_P223140	1.23E-05	NM_145313	NM_145313	Homo sapiens RasGEF domain family, member 1A (RASGEF1A), mRNA [NM_145313]	NM_145313
A_24_P99984	1.23E-05	THC2338942			
A_32_P231086	1.23E-05	NM_198181	NM_198181	Homo sapiens hypothetical protein LOC440295 (LOC440295), mRNA [NM_198181]	NM_198181
A_23_P150950	1.23E-05	NM_144982	NM_144982	Homo sapiens hypothetical protein MGC23401 (MGC23401), mRNA [NM_144982]	NM_144982
A_23_P131227	1.23E-05	NM_017735	NM_017735	Homo sapiens hypothetical protein FLJ20272 (FLJ20272), mRNA [NM_017735]	NM_017735
A_24_P68008	1.23E-05	NM_198545	NM_198545	Homo sapiens hypothetical gene supported by AK075558; BC021286 (LOC374946), mRNA [NM_198545]	NM_198545
A_32_P34003	1.25E-05	THC2313453			
A_23_P150325	1.26E-05	NM_032021	NM_032021	Homo sapiens AD031 protein (AD031), mRNA [NM_032021]	NM_032021
A_24_P56240	1.26E-05	NM_153634	NM_153634	Homo sapiens copine VIII (CPNE8), mRNA [NM_153634]	NM_153634

A_23_P27239	1.26E-05	NM_002548	NM_002548	Homo sapiens olfactory receptor, family 1, subfamily D, member 2 (OR1D2), mRNA [NM_002548]	NM_002548
A_23_P84610	1.26E-05	NM_015450	NM_015450	Homo sapiens POT1 protection of telomeres 1 homolog (S. pombe) (POT1), mRNA [NM_015450]	NM_015450
A_24_P413941	1.26E-05	NM_153689	NM_153689	Homo sapiens hypothetical protein FLJ38973 (FLJ38973), mRNA [NM_153689]	NM_153689
A_23_P10121	1.26E-05	NM_003012	NM_003012	Homo sapiens secreted frizzled-related protein 1 (SFRP1), mRNA [NM_003012]	NM_003012
A_23_P252201	1.27E-05	NM_018456	NM_018456	Homo sapiens ELL associated factor 2 (EAF2), mRNA [NM_018456]	NM_018456
A_23_P163992	1.27E-05	NM_005310	NM_005310	Homo sapiens growth factor receptor-bound protein 7 (GRB7), mRNA [NM_005310]	NM_005310
A_32_P113114	1.27E-05	NM_152289	NM_152289	Homo sapiens zinc finger protein 561 (ZNF561), mRNA [NM_152289]	NM_152289
A_23_P22672	1.27E-05	NM_024810	NM_024810	Homo sapiens chromosome X open reading frame 45 (CXorf45), mRNA [NM_024810]	NM_024810
A_23_P104651	1.27E-05	NM_080668	NM_080668	Homo sapiens cell division cycle associated 5 (CDCA5), mRNA [NM_080668]	NM_080668
A_23_P122937	1.28E-05	NM_014800	NM_014800	Homo sapiens engulfment and cell motility 1 (ced-12 homolog, C. elegans) (ELMO1), transcript variant 1, mRNA [NM_014800]	NM_014800
A_23_P256432	1.28E-05	NM_006243	NM_006243	Homo sapiens protein phosphatase 2, regulatory subunit B (B56), alpha isoform (PPP2R5A), mRNA [NM_006243]	NM_006243
A_23_P67453	1.28E-05	NM_000363	NM_000363	Homo sapiens troponin I, cardiac (TNNI3), mRNA [NM_000363]	NM_000363
A_24_P665504	1.29E-05	BC092421	BC092421	Homo sapiens cDNA clone IMAGE:30378758. [BC092421]	XM_373704
A_24_P797455	1.29E-05	AK056910	AK056910	Homo sapiens cDNA FLJ32348 fis, clone PROST2007200. [AK056910]	
A_24_P246351	1.29E-05	CR615589	CR615589	full-length cDNA clone CS0DC026YJ18 of Neuroblastoma Cot 25-normalized of Homo sapiens (human). [CR615589]	
A_23_P11286	1.29E-05	NM_019597	NM_019597	Homo sapiens heterogeneous nuclear ribonucleoprotein H2 (H') (HNRPH2), mRNA [NM_019597]	NM_019597
A_23_P98483	1.29E-05	NM_021211	NM_021211	Homo sapiens transposon-derived Buster1 transposase-like protein gene (LOC58486), mRNA [NM_021211]	NM_021211
A_23_P13632	1.29E-05	NM_032345	NM_032345	Homo sapiens within bgcn homolog (Drosophila) (WIBG), mRNA [NM_032345]	NM_032345
A_24_P668974	1.29E-05	CD048206	CD048206	AGENCOURT_13966160 NIH_MGC_172 Homo sapiens cDNA 5', mRNA sequence [CD048206]	
A_23_P390148	1.30E-05	NM_014636	NM_014636	Homo sapiens Ral GEF with PH domain and SH3 binding motif 1 (RALGPS1), mRNA [NM_014636]	NM_014636
A_32_P155645	1.30E-05	NM_152549	NM_152549	Homo sapiens hypothetical protein MGC39633 (MGC39633), mRNA [NM_152549]	NM_152549
A_23_P215744	1.30E-05	NM_033427	NM_033427	Homo sapiens cortactin binding protein 2 (CTTNBP2), mRNA [NM_033427]	NM_033427
A_23_P110031	1.30E-05	NM_016089	NM_016089	Homo sapiens zinc finger protein 589 (ZNF589), mRNA [NM_016089]	NM_016089
A_23_P78782	1.31E-05	NM_001217	NM_001217	Homo sapiens carbonic anhydrase XI (CA11), mRNA [NM_001217]	NM_001217
A_23_P46333	1.31E-05	NM_007358	NM_007358	Homo sapiens metal response element binding transcription factor 2 (MTF2), mRNA [NM_007358]	NM_007358
A_24_P940803	1.31E-05	NM_033505	NM_033505	Homo sapiens selenoprotein I (SELI), mRNA [NM_033505]	NM_033505
A_23_P58747	1.31E-05	NM_015111	NM_015111	Homo sapiens Nedd4 binding protein 3 (N4BP3), mRNA [NM_015111]	NM_015111
A_24_P504050	1.31E-05	BX648484	BX648484	Homo sapiens mRNA; cDNA DKFZp686O1555 (from clone DKFZp686O1555). [BX648484]	
A_23_P390734	1.32E-05	NM_015633	NM_015633	Homo sapiens FGFR1 oncogene partner 2 (FGFR1OP2), mRNA [NM_015633]	NM_015633
A_24_P101742	1.32E-05	A_24_P101742			
A_23_P351204	1.32E-05	NM_172109	NM_172109	Homo sapiens potassium voltage-gated channel, KQT-like subfamily, member 2 (KCNQ2), transcript variant 5, mRNA [NM_172109]	NM_172109
A_23_P210608	1.32E-05	NM_006526	NM_006526	Homo sapiens zinc finger protein 217 (ZNF217), mRNA [NM_006526]	NM_006526
A_24_P23546	1.33E-05	NM_198956	NM_198956	Homo sapiens Sp8 transcription factor (SP8), transcript variant 2, mRNA [NM_198956]	NM_198956
A_24_P21447	1.33E-05	NM_006753	NM_006753	Homo sapiens surfeit 6 (SURF6), mRNA [NM_006753]	NM_006753
A_23_P23155	1.33E-05	NM_018836	NM_018836	Homo sapiens adherens junction associated protein 1 (AJAP1), mRNA [NM_018836]	NM_018836
A_24_P9090	1.33E-05	NM_005463	NM_005463	Homo sapiens heterogeneous nuclear ribonucleoprotein D-like (HNRPDL), transcript variant 1, mRNA [NM_005463]	NM_005463
A_23_P90612	1.33E-05	NM_005915	NM_005915	Homo sapiens MCM6 minichromosome maintenance deficient 6 (MIS5 homolog, S. pombe) (S. cerevisiae) (MCM6), mRNA [NM_005915]	NM_005915
A_23_P201376	1.34E-05	NM_014021	NM_014021	Homo sapiens synovial sarcoma, X breakpoint 2 interacting protein (SSX2IP), mRNA [NM_014021]	NM_014021
A_23_P254688	1.34E-05	NM_023943	NM_023943	Homo sapiens hypothetical protein MGC3040 (MGC3040), mRNA [NM_023943]	NM_023943
A_23_P216468	1.34E-05	NM_004170	NM_004170	Homo sapiens solute carrier family 1 (neuronal/epithelial high affinity glutamate transporter, system Xag), member 1 (SLC1A1), mRNA [NM_004170]	NM_004170
A_23_P136058	1.34E-05	NM_001184	NM_001184	Homo sapiens ataxia telangiectasia and Rad3 related (ATR), mRNA [NM_001184]	NM_001184
A_23_P250404	1.34E-05	NM_005732	NM_005732	Homo sapiens RAD50 homolog (S. cerevisiae) (RAD50), transcript variant 1, mRNA [NM_005732]	NM_005732
A_24_P325035	1.34E-05	AK092090	AK092090	Homo sapiens cDNA FLJ34771 fis, clone NT2NE2003150. [AK092090]	
A_23_P436353	1.34E-05	ENST00000351017		Homo sapiens mRNA; cDNA DKFZp761K2213 (from clone DKFZp761K2213); partial cds. [AL161973]	
A_23_P11652	1.34E-05	NM_003368	NM_003368	Homo sapiens ubiquitin specific protease 1 (USP1), transcript variant 1, mRNA [NM_003368]	NM_003368

A_23_P422911	1.35E-05	NM_153456	NM_153456	Homo sapiens heparan sulfate 6-O-sulfotransferase 3 (HS6ST3), mRNA [NM_153456]	NM_153456
A_32_P94160	1.35E-05	BC043195	BC043195	Homo sapiens cDNA clone IMAGE:5288757, partial cds. [BC043195]	
A_24_P818010	1.35E-05	AK097080	AK097080	Homo sapiens cDNA FLJ39761 fis, clone SPLEN1000083. [AK097080]	XM_496672
A_32_P207147	1.35E-05	A_32_P207147			
A_23_P60002	1.35E-05	NM_014673	NM_014673	Homo sapiens KIAA0103 (KIAA0103), mRNA [NM_014673]	NM_014673
A_23_P102320	1.35E-05	NM_138285	NM_138285	Homo sapiens nucleoporin 35kDa (NUP35), transcript variant 1, mRNA [NM_138285]	NM_138285
A_23_P204640	1.35E-05	NM_024865	NM_024865	Homo sapiens Nanog homeobox (NANOG), mRNA [NM_024865]	NM_024865
A_23_P306500	1.35E-05	NM_033360	NM_033360	Homo sapiens v-Ki-ras2 Kirsten rat sarcoma viral oncogene homolog (KRAS), transcript variant a, mRNA [NM_033360]	NM_033360
A_23_P15226	1.36E-05	A_23_P15226			
A_23_P49279	1.36E-05	NM_001001436	NM_001001436	Homo sapiens similar to RIKEN cDNA 4921524J17 (LOC388272), mRNA [NM_001001436]	NM_001001436
A_23_P63897	1.36E-05	NM_022802	NM_022802	Homo sapiens C-terminal binding protein 2 (CTBP2), transcript variant 2, mRNA [NM_022802]	NM_022802
A_23_P253412	1.37E-05	NM_019051	NM_019051	Homo sapiens mitochondrial ribosomal protein L50 (MRPL50), nuclear gene encoding mitochondrial protein, mRNA [NM_019051]	NM_019051
A_24_P937855	1.38E-05	CR936771	CR936771	Homo sapiens mRNA; cDNA DKFZp686A0668 (from clone DKFZp686A0668). [CR936771]	
A_23_P423926	1.38E-05	NM_198935	NM_198935	Homo sapiens synovial sarcoma translocation gene on chromosome 18-like 1 (SS18L1), transcript variant 1, mRNA [NM_198935]	NM_198935
A_23_P206830	1.38E-05	NM_016069	NM_016069	Homo sapiens mitochondria-associated protein involved in granulocyte-macrophage colony-stimulating factor signal transduction (Magmas), nuclear gene encoding mitochondrial protein, mRNA [NM_016069]	NM_016069
A_23_P391506	1.38E-05	NM_016389	NM_016389	Homo sapiens influenza virus NS1A binding protein (IVNS1ABP), transcript variant 2, mRNA [NM_016389]	NM_016389
A_32_P83049	1.38E-05	AF131834	AF131834	Homo sapiens clone 24841 mRNA sequence. [AF131834]	
A_32_P17145	1.39E-05	THC2440409			
A_23_P59999	1.39E-05	THC2437580		Q300_MOUSE (Q02722) Protein Q300, partial (14%) [THC2437580]	
A_24_P14464	1.39E-05	NM_080736	NM_080736	Homo sapiens WAP four-disulfide core domain 2 (WFDC2), transcript variant 2, mRNA [NM_080736]	NM_080736
A_23_P349083	1.39E-05	NM_138782	NM_138782	Homo sapiens FCH domain only 2 (FCHO2), mRNA [NM_138782]	NM_138782
A_24_P406714	1.39E-05	NM_004641	NM_004641	Homo sapiens myeloid/lymphoid or mixed-lineage leukemia (trithorax homolog, Drosophila); translocated to, 10 (MLLT10), transcript variant 1, mRNA [NM_004641]	NM_004641
A_23_P141856	1.39E-05	NM_014480	NM_014480	Homo sapiens zinc finger protein 544 (ZNF544), mRNA [NM_014480]	NM_014480
A_32_P233304	1.39E-05	NM_173083	NM_173083	Homo sapiens lin-9 homolog (C. elegans) (LIN9), mRNA [NM_173083]	NM_173083
A_23_P418413	1.39E-05	NM_005109	NM_005109	Homo sapiens oxidative-stress responsive 1 (OXSR1), mRNA [NM_005109]	NM_005109
A_23_P72737	1.39E-05	NM_003641	NM_003641	Homo sapiens interferon induced transmembrane protein 1 (9-27) (IFITM1), mRNA [NM_003641]	NM_003641
A_32_P19752	1.40E-05	NM_144664	NM_144664	Homo sapiens hypothetical protein MGC33371 (MGC33371), mRNA [NM_144664]	NM_144664
A_23_P386561	1.40E-05	NM_001002926	NM_001002926	Homo sapiens TWIST neighbor (TWISTNB), mRNA [NM_001002926]	NM_001002926
A_23_P59069	1.40E-05	NM_003527	NM_003527	Homo sapiens histone 1, H2bo (HIST1H2BO), mRNA [NM_003527]	NM_003527
A_23_P259344	1.41E-05	NM_031890	NM_031890	Homo sapiens cat eye syndrome chromosome region, candidate 6 (CECR6), mRNA [NM_031890]	NM_031890
A_24_P171058	1.41E-05	AL834364	AL834364	Homo sapiens mRNA; cDNA DKFZp762C1112 (from clone DKFZp762C1112). [AL834364]	
A_24_P63522	1.42E-05	NM_002130	NM_002130	Homo sapiens 3-hydroxy-3-methylglutaryl-Coenzyme A synthase 1 (soluble) (HMGCS1), mRNA [NM_002130]	NM_002130
A_24_P476086	1.43E-05	THC2339518		Q9P2R9 (Q9P2R9) SRp25 nuclear protein (ARL6IP4 protein), partial (6%) [THC2339518]	
A_23_P379034	1.43E-05	NM_025045	NM_025045	Homo sapiens BAI1-associated protein 2-like 2 (BAIAP2L2), mRNA [NM_025045]	NM_025045
A_23_P211007	1.43E-05	NM_003489	NM_003489	Homo sapiens nuclear receptor interacting protein 1 (NRIP1), mRNA [NM_003489]	NM_003489
A_24_P576219	1.44E-05	BC035091	BC035091	Homo sapiens, Similar to hypothetical protein FLJ20489, clone IMAGE:5261717, mRNA. [BC035091]	
A_24_P941166	1.44E-05	NM_001001661	NM_001001661	Homo sapiens zinc finger protein 425 (ZNF425), mRNA [NM_001001661]	NM_001001661
A_24_P277155	1.44E-05	NM_003071	NM_003071	Homo sapiens SWI/SNF related, matrix associated, actin dependent regulator of chromatin, subfamily a, member 3 (SMARCA3), transcript variant 1, mRNA [NM_003071]	NM_003071
A_24_P181998	1.44E-05	ENST00000255896		F lambda 8=Ig lambda-like gene/beta-glucuronidase exon 11 homolog {5' region} [human, fetal liver, mRNA Partial, 452 nt]. [S82637]	
A_24_P166094	1.44E-05	NM_001025595	NM_001025595	Homo sapiens ADP-ribosylation factor interacting protein 1 (arfaptin 1) (ARFIP1), transcript variant 1, mRNA [NM_001025595]	NM_001025595
A_24_P7202	1.45E-05	NM_020738	NM_020738	Homo sapiens kinase D-interacting substance of 220 kDa (KIDINS220), mRNA [NM_020738]	NM_020738
A_23_P317347	1.45E-05	NM_052911	NM_052911	Homo sapiens establishment of cohesion 1 homolog 1 (S. cerevisiae) (ESCO1), mRNA [NM_052911]	NM_052911
A_24_P90878	1.45E-05	NM_017736	NM_017736	Homo sapiens THUMP domain containing 1 (THUMPD1), mRNA [NM_017736]	NM_017736

A_24_P56270	1.45E-05	CR612226	CR612226	full-length cDNA clone CS0DF019YP13 of Fetal brain of Homo sapiens (human). [CR612226]	
A_23_P70328	1.45E-05	NM_018132	NM_018132	Homo sapiens chromosome 6 open reading frame 139 (C6orf139), mRNA [NM_018132]	NM_018132
A_23_P164284	1.45E-05	NM_001307	NM_001307	Homo sapiens claudin 7 (CLDN7), mRNA [NM_001307]	NM_001307
A_32_P35433	1.46E-05	CR600638	CR600638	full-length cDNA clone CS0DI053YD12 of Placenta Cot 25-normalized of Homo sapiens (human). [CR600638]	
A_23_P408996	1.46E-05	AK131269	AK131269	Homo sapiens cDNA FLJ16207 fis, clone CTONG2019822. [AK131269]	XM_371801
A_23_P81392	1.46E-05	NM_015238	NM_015238	Homo sapiens KIBRA protein (KIBRA), mRNA [NM_015238]	NM_015238
A_32_P170444	1.46E-05	CR625990	CR625990	full-length cDNA clone CS0DI074YB14 of Placenta Cot 25-normalized of Homo sapiens (human). [CR625990]	
A_23_P154349	1.46E-05	NM_014362	NM_014362	Homo sapiens 3-hydroxyisobutyryl-Coenzyme A hydrolase (HIBCH), transcript variant 1, mRNA [NM_014362]	NM_014362
A_23_P77459	1.46E-05	NM_003905	NM_003905	Homo sapiens amyloid beta precursor protein binding protein 1 (APPBP1), transcript variant 1, mRNA [NM_003905]	NM_003905
A_32_P41553	1.47E-05	NM_144627	NM_144627	Homo sapiens SSTK-interacting protein (SSTK-IP), mRNA [NM_144627]	NM_144627
A_32_P38404	1.47E-05	BC015133	BC015133	Homo sapiens cDNA clone IMAGE:3934193, partial cds. [BC015133]	
A_32_P170664	1.47E-05	AK024898	AK024898	Homo sapiens cDNA: FLJ21245 fis, clone COL01184. [AK024898]	
A_23_P136909	1.47E-05	NM_030763	NM_030763	Homo sapiens nucleosomal binding protein 1 (NSBP1), mRNA [NM_030763]	NM_030763
A_23_P2233	1.47E-05	NM_006928	NM_006928	Homo sapiens silver homolog (mouse) (SILV), mRNA [NM_006928]	NM_006928
A_23_P7353	1.49E-05	NM_178043	NM_178043	Homo sapiens La ribonucleoprotein domain family, member 2 (LARP2), transcript variant 2, mRNA [NM_178043]	NM_178043
A_32_P169353	1.49E-05	A_32_P169353			
A_23_P254612	1.49E-05	NM_006716	NM_006716	Homo sapiens activator of S phase kinase (ASK), mRNA [NM_006716]	NM_006716
A_24_P775659	1.49E-05	CR593500	CR593500	full-length cDNA clone CS0DF014YD20 of Fetal brain of Homo sapiens (human). [CR593500]	XM_373788
A_23_P207927	1.49E-05	AY163812	AY163812	Homo sapiens HLC-8 mRNA, complete cds. [AY163812]	
A_23_P75509	1.49E-05	NM_003626	NM_003626	Homo sapiens protein tyrosine phosphatase, receptor type, f polypeptide (PTPRF), interacting protein (liprin), alpha 1 (PPFIA1), transcript variant 2, mRNA [NM_003626]	NM_003626
A_23_P136964	1.50E-05	NM_000328	NM_000328	Homo sapiens retinitis pigmentosa GTPase regulator (RPGR), transcript variant A, mRNA [NM_000328]	NM_000328
A_32_P226786	1.50E-05	BC045174	BC045174	Homo sapiens, clone IMAGE:5273245, mRNA. [BC045174]	
A_24_P392475	1.50E-05	ENST00000321892		Homo sapiens cDNA: FLJ23531 fis, clone LNG06065. [AK027184]	
A_23_P83110	1.50E-05	NM_018249	NM_018249	Homo sapiens CDK5 regulatory subunit associated protein 2 (CDK5RAP2), transcript variant 1, mRNA [NM_018249]	NM_018249
A_24_P552987	1.50E-05	A_24_P552987			
A_24_P283225	1.50E-05	NM_004999	NM_004999	Homo sapiens myosin VI (MYO6), mRNA [NM_004999]	NM_004999
A_23_P207940	1.52E-05	NM_080422	NM_080422	Homo sapiens protein tyrosine phosphatase, non-receptor type 2 (PTPN2), transcript variant 2, mRNA [NM_080422]	NM_080422
A_23_P321160	1.53E-05	AB058774	AB058774	Homo sapiens mRNA for KIAA1871 protein, partial cds. [AB058774]	XM_290737
A_24_P904484	1.53E-05	NM_001001873	NM_001001873	Homo sapiens hypothetical protein LOC283174 (LOC283174), mRNA [NM_001001873]	NM_001001873
A_32_P213002	1.53E-05	THC2364440		U84B_HUMAN (Q9UH99) Sad1/unc-84-like protein 2 (Rab5 interacting protein) (Rab5IP), partial (12%) [THC2364440]	
A_32_P18723	1.53E-05	AK095472	AK095472	Homo sapiens cDNA FLJ38153 fis, clone DFNES1000083. [AK095472]	
A_24_P929570	1.54E-05	NM_213589	NM_213589	Homo sapiens Ras association (RalGDS/AF-6) and pleckstrin homology domains 1 (RAPH1), transcript variant 1, mRNA [NM_213589]	NM_213589
A_23_P209200	1.54E-05	NM_001238	NM_001238	Homo sapiens cyclin E1 (CCNE1), transcript variant 1, mRNA [NM_001238]	NM_001238
A_32_P72181	1.55E-05	BC035184	BC035184	Homo sapiens cDNA clone IMAGE:5266408, partial cds. [BC035184]	
A_32_P92171	1.55E-05	BC052596	BC052596	Homo sapiens hypothetical gene supported by BC052596, mRNA (cDNA clone IMAGE:6728287). [BC052596]	XM_371196
A_24_P230009	1.55E-05	A_24_P230009			
A_23_P251785	1.55E-05	NM_024561	NM_024561	Homo sapiens NMDA receptor regulated 1-like (NARG1L), transcript variant 1, mRNA [NM_024561]	NM_024561
A_23_P200551	1.55E-05	NM_032236	NM_032236	Homo sapiens ubiquitin specific protease 48 (USP48), mRNA [NM_032236]	NM_032236
A_24_P380132	1.55E-05	NM_203505	NM_203505	Homo sapiens Ras-GTPase activating protein SH3 domain-binding protein 2 (G3BP2), transcript variant 1, mRNA [NM_203505]	NM_203505
A_23_P92672	1.56E-05	NM_002538	NM_002538	Homo sapiens occludin (OCLN), mRNA [NM_002538]	NM_002538
A_23_P151565	1.56E-05	NM_014990	NM_014990	Homo sapiens GTPase activating Rap/RanGAP domain-like 1 (GARNL1), transcript variant 1, mRNA [NM_014990]	NM_014990
A_32_P6682	1.57E-05	THC2436690		ALU1_HUMAN (P39188) Alu subfamily J sequence contamination warning entry, partial (6%) [THC2436690]	
A_24_P3140	1.57E-05	NM_014497	NM_014497	Homo sapiens zinc finger protein 638 (ZNF638), transcript variant 1, mRNA [NM_014497]	NM_014497
A_24_P123347	1.57E-05	NM_002703	NM_002703	Homo sapiens phosphoribosyl pyrophosphate amidotransferase (PPAT), mRNA [NM_002703]	NM_002703

A_24_P419039	1.58E-05	NM_020766	NM_020766	Homo sapiens protocadherin 19 (PCDH19), mRNA [NM_020766]	NM_020766
A_23_P121106	1.58E-05	NM_003865	NM_003865	Homo sapiens homeo box (expressed in ES cells) 1 (HESX1), mRNA [NM_003865]	NM_003865
A_24_P889070	1.59E-05	AK023647	AK023647	Homo sapiens cDNA FLJ13585 fis, clone PLACE1009150. [AK023647]	
A_24_P931955	1.59E-05	THC2278676			
A_23_P303181	1.59E-05	NM_020126	NM_020126	Homo sapiens sphingosine kinase 2 (SPHK2), mRNA [NM_020126]	NM_020126
A_32_P16989	1.60E-05	A_32_P16989			
A_23_P434919	1.60E-05	NM_152304	NM_152304	Homo sapiens hypothetical protein MGC45806 (MGC45806), mRNA [NM_152304]	NM_152304
A_23_P144684	1.60E-05	NM_032290	NM_032290	Homo sapiens ankyrin repeat domain 32 (ANKRD32), mRNA [NM_032290]	NM_032290
A_23_P215051	1.60E-05	NM_018479	NM_018479	Homo sapiens enoyl Coenzyme A hydratase domain containing 1 (ECHDC1), transcript variant 2, mRNA [NM_018479]	NM_018479
A_23_P26865	1.61E-05	NM_002470	NM_002470	Homo sapiens myosin, heavy polypeptide 3, skeletal muscle, embryonic (MYH3), mRNA [NM_002470]	NM_002470
A_23_P89921	1.61E-05	NM_013256	NM_013256	Homo sapiens zinc finger protein 180 (HHZ168) (ZNF180), mRNA [NM_013256]	NM_013256
A_23_P357995	1.61E-05	NM_178547	NM_178547	Homo sapiens zinc finger and BTB domain containing 8 opposite strand (ZBTB8OS), mRNA [NM_178547]	NM_178547
A_32_P46214	1.62E-05	NM_173653	NM_173653	Homo sapiens solute carrier family 9 (sodium/hydrogen exchanger), isoform 9 (SLC9A9), mRNA [NM_173653]	NM_173653
A_32_P217773	1.62E-05	NM_032872	NM_032872	Homo sapiens synaptotagmin-like 1 (SYTL1), mRNA [NM_032872]	NM_032872
A_23_P398836	1.62E-05	NM_020784	NM_020784	Homo sapiens KIAA1344 (KIAA1344), mRNA [NM_020784]	NM_020784
A_24_P671842	1.62E-05	A_24_P671842			
A_24_P665185	1.63E-05	AL832142	AL832142	Homo sapiens mRNA; cDNA DKFZp686A22111 (from clone DKFZp686A22111). [AL832142]	
A_23_P363647	1.63E-05	NM_182540	NM_182540	Homo sapiens DEAD/H (Asp-Glu-Ala-Asp/His) box polypeptide 26B (DDX26B), mRNA [NM_182540]	NM_182540
A_23_P138137	1.63E-05	NM_145243	NM_145243	Homo sapiens OMA1 homolog, zinc metallopeptidase (S. cerevisiae) (OMA1), mRNA [NM_145243]	NM_145243
A_23_P96775	1.63E-05	NM_020710	NM_020710	Homo sapiens leucine rich repeat containing 47 (LRRC47), mRNA [NM_020710]	NM_020710
A_23_P162795	1.63E-05	NM_006704	NM_006704	Homo sapiens SGT1, suppressor of G2 allele of SKP1 (S. cerevisiae) (SUGT1), mRNA [NM_006704]	NM_006704
A_23_P57697	1.63E-05	NM_020865	NM_020865	Homo sapiens DEAH (Asp-Glu-Ala-His) box polypeptide 36 (DHX36), mRNA [NM_020865]	NM_020865
A_24_P297539	1.63E-05	NM_181803	NM_181803	Homo sapiens ubiquitin-conjugating enzyme E2C (UBE2C), transcript variant 6, mRNA [NM_181803]	NM_181803
A_24_P182539	1.64E-05	ENST00000317868		Homo sapiens cDNA FLJ14867 fis, clone PLACE1002319. [AK027773]	
A_32_P202703	1.64E-05	AL713796	AL713796	Homo sapiens mRNA; cDNA DKFZp667B1610 (from clone DKFZp667B1610). [AL713796]	XM_374317
A_24_P286054	1.64E-05	AB002303	AB002303	Homo sapiens mRNA for KIAA0305 gene, partial cds. [AB002303]	
A_24_P366315	1.64E-05	NM_017641	NM_017641	Homo sapiens kinesin family member 21A (KIF21A), mRNA [NM_017641]	NM_017641
A_24_P541576	1.64E-05	AK026938	AK026938	Homo sapiens cDNA: FLJ23285 fis, clone HEP09071. [AK026938]	XM_374317
A_23_P58898	1.65E-05	NM_012115	NM_012115	Homo sapiens CASP8 associated protein 2 (CASP8AP2), mRNA [NM_012115]	NM_012115
A_23_P53668	1.65E-05	NM_006166	NM_006166	Homo sapiens nuclear transcription factor Y, beta (NFYB), mRNA [NM_006166]	NM_006166
A_23_P366216	1.65E-05	NM_003524	NM_003524	Homo sapiens histone 1, H2bh (HIST1H2BH), mRNA [NM_003524]	NM_003524
A_23_P359738	1.65E-05	NM_015630	NM_015630	Homo sapiens enhancer of polycomb homolog 2 (Drosophila) (EPC2), mRNA [NM_015630]	NM_015630
A_23_P204541	1.66E-05	BX648591	BX648591	Homo sapiens mRNA; cDNA DKFZp686G14198 (from clone DKFZp686G14198). [BX648591]	
A_23_P412059	1.67E-05	BC064616	BC064616	Homo sapiens zinc finger, RAN-binding domain containing 3, mRNA (cDNA clone IMAGE:5575956), complete cds. [BC064616]	
A_23_P332439	1.67E-05	AB007870	AB007870	Homo sapiens KIAA0410 mRNA, partial cds. [AB007870]	
A_23_P7313	1.67E-05	NM_000582	NM_000582	Homo sapiens secreted phosphoprotein 1 (osteopontin, bone sialoprotein I, early T-lymphocyte activation 1) (SPP1), mRNA [NM_000582]	NM_000582
A_23_P252155	1.67E-05	NM_018387	NM_018387	Homo sapiens spermatid perinuclear RNA binding protein (STRBP), mRNA [NM_018387]	NM_018387
A_23_P156117	1.67E-05	NM_014376	NM_014376	Homo sapiens cytoplasmic FMR1 interacting protein 2 (CYFIP2), mRNA [NM_014376]	NM_014376
A_23_P341418	1.67E-05	AK098818	AK098818	Homo sapiens cDNA FLJ25952 fis, clone SYN00911. [AK098818]	
A_23_P25069	1.68E-05	BC039117	BC039117	Homo sapiens ovostatin 2, mRNA (cDNA clone IMAGE:4827636), with apparent retained intron. [BC039117]	XM_495907
A_23_P436281	1.68E-05	NM_003548	NM_003548	Homo sapiens histone 2, H4 (HIST2H4), mRNA [NM_003548]	NM_003548
A_23_P311201	1.68E-05	ENST00000341154		Homo sapiens TLS-associated protein TASR-1 mRNA, complete cds, alternative transcript. [AF419331]	
A_24_P113144	1.69E-05	NM_024857	NM_024857	Homo sapiens chromosome 17 open reading frame 41 (C17orf41), mRNA [NM_024857]	NM_024857
A_23_P343261	1.69E-05	NM_153709	NM_153709	Homo sapiens hypothetical protein MGC40168 (MGC40168), mRNA [NM_153709]	NM_153709

A_23_P106906	1.69E-05	NM_002705	NM_002705	Homo sapiens periplakin (PPL), mRNA [NM_002705]	NM_002705
A_23_P364107	1.69E-05	NM_018353	NM_018353	Homo sapiens chromosome 14 open reading frame 106 (C14orf106), mRNA [NM_018353]	NM_018353
A_23_P372308	1.69E-05	NM_020211	NM_020211	Homo sapiens RGM domain family, member A (RGMA), mRNA [NM_020211]	NM_020211
A_24_P247512	1.70E-05	AK098175	AK098175	Homo sapiens cDNA FLJ40856 fis, clone TRACH2016498, moderately similar to ZINC FINGER PROTEIN 184. [AK098175]	XM_371174
A_23_P87810	1.70E-05	THC2373936		O19057 (O19057) Fertilin alpha protein, partial (18%) [THC2373936]	
A_23_P166698	1.70E-05	NM_016305	NM_016305	Homo sapiens synovial sarcoma translocation gene on chromosome 18-like 2 (SS18L2), mRNA [NM_016305]	NM_016305
A_23_P127033	1.71E-05	NM_024693	NM_024693	Homo sapiens enoyl Coenzyme A hydratase domain containing 3 (ECHDC3), mRNA [NM_024693]	NM_024693
A_24_P158065	1.71E-05	NM_152789	NM_152789	Homo sapiens hypothetical protein MGC40405 (MGC40405), mRNA [NM_152789]	NM_152789
A_23_P129031	1.72E-05	NM_004993	NM_004993	Homo sapiens ataxin 3 (ATXN3), transcript variant 1, mRNA [NM_004993]	NM_004993
A_23_P55073	1.72E-05	NM_015462	NM_015462	Homo sapiens DKFZP586L0724 protein (DKFZP586L0724), mRNA [NM_015462]	NM_015462
A_32_P71113	1.73E-05	A_32_P71113			
A_23_P121480	1.73E-05	NM_001004196	NM_001004196	Homo sapiens CD200 antigen (CD200), transcript variant 2, mRNA [NM_001004196]	NM_001004196
A_32_P38228	1.73E-05	A_32_P38228			
A_24_P148503	1.73E-05	AK024850	AK024850	Homo sapiens cDNA: FLJ21197 fis, clone COL00201. [AK024850]	
A_24_P325520	1.73E-05	NM_002959	NM_002959	Homo sapiens sortilin 1 (SORT1), mRNA [NM_002959]	NM_002959
A_23_P122805	1.73E-05	NM_032842	NM_032842	Homo sapiens hypothetical protein FLJ14803 (FLJ14803), mRNA [NM_032842]	NM_032842
A_23_P383132	1.73E-05	NM_015094	NM_015094	Homo sapiens hypermethylated in cancer 2 (HIC2), mRNA [NM_015094]	NM_015094
A_24_P2995	1.74E-05	NM_015317	NM_015317	Homo sapiens pumilio homolog 2 (Drosophila) (PUM2), mRNA [NM_015317]	NM_015317
A_23_P254472	1.74E-05	NM_024573	NM_024573	Homo sapiens chromosome 6 open reading frame 211 (C6orf211), mRNA [NM_024573]	NM_024573
A_23_P404821	1.75E-05	BC012493	BC012493	Homo sapiens LCHN protein, mRNA (cDNA clone MGC:21082 IMAGE:4470021), complete cds. [BC012493]	
A_32_P63086	1.75E-05	BC041913	BC041913	Homo sapiens, clone IMAGE:5299642, mRNA. [BC041913]	
A_23_P144145	1.75E-05	AF292100	AF292100	Homo sapiens RP42 protein mRNA, complete cds. [AF292100]	
A_23_P211797	1.75E-05	NM_130837	NM_130837	Homo sapiens optic atrophy 1 (autosomal dominant) (OPA1), nuclear gene encoding mitochondrial protein, transcript variant 8, mRNA [NM_130837]	NM_130837
A_24_P208794	1.75E-05	NM_001640	NM_001640	Homo sapiens N-acylaminoacyl-peptide hydrolase (APEH), mRNA [NM_001640]	NM_001640
A_23_P24444	1.75E-05	NM_001360	NM_001360	Homo sapiens 7-dehydrocholesterol reductase (DHCR7), mRNA [NM_001360]	NM_001360
A_24_P345679	1.76E-05	ENST00000355893		Homo sapiens mRNA; cDNA DKFZp686A04129 (from clone DKFZp686A04129) [BX641078]	
A_23_P420442	1.76E-05	NM_153618	NM_153618	Homo sapiens sema domain, transmembrane domain (TM), and cytoplasmic domain, (semaphorin) 6D (SEMA6D), transcript variant 4, mRNA [NM_153618]	NM_153618
A_23_P395595	1.76E-05	NM_015308	NM_015308	Homo sapiens formin binding protein 4 (FNBP4), mRNA [NM_015308]	NM_015308
A_23_P171143	1.76E-05	NM_003270	NM_003270	Homo sapiens tetraspanin 6 (TSPAN6), mRNA [NM_003270]	NM_003270
A_23_P348636	1.77E-05	NM_001454	NM_001454	Homo sapiens forkhead box J1 (FOXJ1), mRNA [NM_001454]	NM_001454
A_24_P376229	1.77E-05	NM_005499	NM_005499	Homo sapiens SUMO-1 activating enzyme subunit 2 (UBA2), mRNA [NM_005499]	NM_005499
A_23_P339705	1.78E-05	NM_173636	NM_173636	Homo sapiens WD repeat domain 62 (WDR62), mRNA [NM_173636]	NM_173636
A_23_P205959	1.78E-05	NM_000693	NM_000693	Homo sapiens aldehyde dehydrogenase 1 family, member A3 (ALDH1A3), mRNA [NM_000693]	NM_000693
A_23_P411723	1.78E-05	NM_002655	NM_002655	Homo sapiens pleiomorphic adenoma gene 1 (PLAG1), mRNA [NM_002655]	NM_002655
A_23_P202594	1.78E-05	NM_024834	NM_024834	Homo sapiens chromosome 10 open reading frame 119 (C10orf119), mRNA [NM_024834]	NM_024834
A_23_P85703	1.79E-05	NM_005686	NM_005686	Homo sapiens SRY (sex determining region Y)-box 13 (SOX13), mRNA [NM_005686]	NM_005686
A_23_P29784	1.79E-05	AL096748	AL096748	Homo sapiens mRNA; cDNA DKFZp434A043 (from clone DKFZp434A043); partial cds. [AL096748]	
A_23_P89755	1.80E-05	NM_016271	NM_016271	Homo sapiens ring finger protein 138 (RNF138), transcript variant 1, mRNA [NM_016271]	NM_016271
A_32_P169500	1.81E-05	THC2433217		ALU1_HUMAN (P39188) Alu subfamily J sequence contamination warning entry, partial (14%) [THC2433217]	
A_23_P1722	1.81E-05	NM_080659	NM_080659	Homo sapiens similar to RIKEN cDNA 2310030G06 gene (MGC14839), mRNA [NM_080659]	NM_080659
A_23_P112004	1.81E-05	NM_012472	NM_012472	Homo sapiens leucine rich repeat containing 6 (LRRC6), mRNA [NM_012472]	NM_012472
A_24_P119036	1.81E-05	ENST00000219746		Homo sapiens cDNA: FLJ23353 fis, clone HEP14321, highly similar to HSU80736 Homo sapiens CAGF9 mRNA. [AK027006]	XM_049037
A_23_P160537	1.81E-05	NM_024037	NM_024037	Homo sapiens chromosome 1 open reading frame 135 (C1orf135), mRNA [NM_024037]	NM_024037
A_23_P49865	1.81E-05	NM_015982	NM_015982	Homo sapiens germ cell specific Y-box binding protein (YBX2), mRNA [NM_015982]	NM_015982
A_32_P211248	1.81E-05	AJ276555	AJ276555	Homo sapiens mRNA for hypothetical protein (ORF1), clone 00275. [AJ276555]	

A_23_P25873	1.82E-05	NM_007086	NM_007086	Homo sapiens WD repeat and HMG-box DNA binding protein 1 (WDHD1), transcript variant 1, mRNA [NM_007086]	NM_007086
A_23_P350070	1.82E-05	NM_015436	NM_015436	Homo sapiens ring finger and CHY zinc finger domain containing 1 (RCHY1), transcript variant 1, mRNA [NM_015436]	NM_015436
A_32_P93736	1.83E-05	THC2379106		Q93NK8 (Q93NK8) YsaW, partial (7%) [THC2379106]	
A_23_P76658	1.83E-05	NM_052818	NM_052818	Homo sapiens hypothetical gene CG018 (CG018), mRNA [NM_052818]	NM_052818
A_24_P175435	1.83E-05	NM_014580	NM_014580	Homo sapiens solute carrier family 2, (facilitated glucose transporter) member 8 (SLC2A8), mRNA [NM_014580]	NM_014580
A_23_P115842	1.83E-05	NM_018237	NM_018237	Homo sapiens cell division cycle and apoptosis regulator 1 (CCAR1), mRNA [NM_018237]	NM_018237
A_23_P95130	1.83E-05	NM_207113	NM_207113	Homo sapiens solute carrier family 37 (glycerol-3-phosphate transporter), member 3 (SLC37A3), transcript variant 1, mRNA [NM_207113]	NM_207113
A_24_P257579	1.84E-05	NM_022140	NM_022140	Homo sapiens erythrocyte membrane protein band 4.1 like 4A (EPB41L4A), mRNA [NM_022140]	NM_022140
A_23_P88303	1.84E-05	NM_021979	NM_021979	Homo sapiens heat shock 70kDa protein 2 (HSPA2), mRNA [NM_021979]	NM_021979
A_23_P347059	1.84E-05	NM_173468	NM_173468	Homo sapiens MOB1, Mps One Binder kinase activator-like 1A (yeast) (MOBKL1A), mRNA [NM_173468]	NM_173468
A_23_P337422	1.84E-05	NM_001018067	NM_001018067	Homo sapiens PAI-1 mRNA binding protein (PAI-RBP1), transcript variant 1, mRNA [NM_001018067]	NM_001018067
A_24_P149266	1.85E-05	NM_020804	NM_020804	Homo sapiens protein kinase C and casein kinase substrate in neurons 1 (PACSIN1), mRNA [NM_020804]	NM_020804
A_24_P295709	1.85E-05	THC2310930			
A_24_P348660	1.85E-05	NM_138271	NM_138271	Homo sapiens alpha thalassemia/mental retardation syndrome X-linked (RAD54 homolog, S. cerevisiae) (ATRX), transcript variant 3, mRNA [NM_138271]	NM_138271
A_23_P131935	1.85E-05	NM_017671	NM_017671	Homo sapiens chromosome 20 open reading frame 42 (C20orf42), mRNA [NM_017671]	NM_017671
A_32_P167631	1.86E-05	THC2320257			
A_24_P91916	1.87E-05	NM_018698	NM_018698	Homo sapiens nuclear transport factor 2-like export factor 2 (NXT2), mRNA [NM_018698]	NM_018698
A_32_P50005	1.87E-05	THC2376384		C40201 artifact-warning sequence (translated ALU class C) - human {Homo sapiens;} , partial (7%) [THC2376384]	
A_32_P217510	1.87E-05	NM_032168	NM_032168	Homo sapiens WD repeat domain 75 (WDR75), mRNA [NM_032168]	NM_032168
A_23_P102000	1.87E-05	NM_001008540	NM_001008540	Homo sapiens chemokine (C-X-C motif) receptor 4 (CXCR4), transcript variant 1, mRNA [NM_001008540]	NM_001008540
A_23_P17163	1.87E-05	S63912	S63912	D10S102=FBRNP [human, fetal brain, mRNA, 3043 nt]. [S63912]	XM_370728
A_24_P940426	1.88E-05	NM_206855	NM_206855	Homo sapiens quaking homolog, KH domain RNA binding (mouse) (QKI), transcript variant 4, mRNA [NM_206855]	NM_206855
A_23_P209146	1.88E-05	NM_003430	NM_003430	Homo sapiens zinc finger protein 91 (HPF7, HTF10) (ZNF91), mRNA [NM_003430]	NM_003430
A_32_P106732	1.88E-05	ENST00000267430		Homo sapiens mRNA for KIAA1596 protein, partial cds. [AB046816]	XM_048128
A_23_P207400	1.88E-05	NM_007295	NM_007295	Homo sapiens breast cancer 1, early onset (BRCA1), transcript variant BRCA1b, mRNA [NM_007295]	NM_007295
A_23_P62081	1.88E-05	NM_003020	NM_003020	Homo sapiens secretory granule, neuroendocrine protein 1 (7B2 protein) (SGNE1), mRNA [NM_003020]	NM_003020
A_23_P108294	1.88E-05	NM_177543	NM_177543	Homo sapiens phosphatidic acid phosphatase type 2C (PPAP2C), transcript variant 3, mRNA [NM_177543]	NM_177543
A_23_P76598	1.88E-05	NM_152726	NM_152726	Homo sapiens EF-hand domain family, member A1 (EFHA1), mRNA [NM_152726]	NM_152726
A_23_P104351	1.88E-05	NM_018472	NM_018472	Homo sapiens family with sequence similarity 45, member B (FAM45B), mRNA [NM_018472]	NM_018472
A_32_P224566	1.89E-05	NM_001004317	NM_001004317	Homo sapiens FLJ16517 protein (FLJ16517), mRNA [NM_001004317]	NM_001004317
A_23_P379327	1.89E-05	AB032990	AB032990	Homo sapiens mRNA for KIAA1164 protein, partial cds. [AB032990]	
A_32_P51518	1.89E-05	AK098220	AK098220	Homo sapiens cDNA FLJ40901 fis, clone UTERU2003704. [AK098220]	
A_24_P368575	1.89E-05	NM_003615	NM_003615	Homo sapiens solute carrier family 4, sodium bicarbonate cotransporter, member 7 (SLC4A7), mRNA [NM_003615]	NM_003615
A_23_P303087	1.89E-05	NM_002825	NM_002825	Homo sapiens pleiotrophin (heparin binding growth factor 8, neurite growth-promoting factor 1) (PTN), mRNA [NM_002825]	NM_002825
A_24_P737553	1.90E-05	AK023774	AK023774	Homo sapiens cDNA FLJ13712 fis, clone PLACE2000394. [AK023774]	
A_23_P18422	1.90E-05	NM_007208	NM_007208	Homo sapiens mitochondrial ribosomal protein L3 (MRPL3), nuclear gene encoding mitochondrial protein, mRNA [NM_007208]	NM_007208
A_23_P29855	1.90E-05	NM_003715	NM_003715	Homo sapiens vesicle docking protein p115 (VDP), mRNA [NM_003715]	NM_003715
A_24_P350890	1.91E-05	NM_006267	NM_006267	Homo sapiens RAN binding protein 2 (RANBP2), mRNA [NM_006267]	NM_006267
A_23_P74981	1.93E-05	NM_033213	NM_033213	Homo sapiens zinc finger protein 670 (ZNF670), mRNA [NM_033213]	NM_033213
A_23_P429491	1.93E-05	NM_145018	NM_145018	Homo sapiens hypothetical protein FLJ25416 (FLJ25416), mRNA [NM_145018]	NM_145018
A_23_P58967	1.93E-05	NM_014827	NM_014827	Homo sapiens zinc finger CCCH-type containing 11A (ZC3H11A), mRNA [NM_014827]	NM_014827
A_23_P353744	1.94E-05	NM_032239	NM_032239	Homo sapiens La ribonucleoprotein domain family, member 2 (LARP2), transcript variant 3, mRNA [NM_032239]	NM_032239
A_23_P259127	1.96E-05	NM_017697	NM_017697	Homo sapiens hypothetical protein FLJ20171 (FLJ20171), mRNA [NM_017697]	NM_017697
A_23_P17593	1.96E-05	NM_001794	NM_001794	Homo sapiens cadherin 4, type 1, R-cadherin (retinal) (CDH4), mRNA [NM_001794]	NM_001794

A_32_P83547	1.97E-05	THC2399645			
A_23_P17620	1.97E-05	NM_052954	NM_052954	Homo sapiens cysteine/tyrosine-rich 1 (CYYR1), mRNA [NM_052954]	NM_052954
A_32_P118657	1.97E-05	AK022044	AK022044	Homo sapiens cDNA FLJ11982 fis, clone HEMBB1001335. [AK022044]	
A_24_P194313	1.97E-05	BC062992	BC062992	Homo sapiens cDNA clone IMAGE:5497083, containing frame-shift errors. [BC062992]	
A_32_P179148	1.97E-05	CN431194	CN431194	CN431194 328775669 GRN_ES Homo sapiens cDNA 5', mRNA sequence [CN431194]	
A_24_P326739	1.98E-05	NM_013267	NM_013267	Homo sapiens glutaminase 2 (liver, mitochondrial) (GLS2), nuclear gene encoding mitochondrial protein, transcript variant 1, mRNA [NM_013267]	NM_013267
A_24_P538708	1.98E-05	AK124263	AK124263	Homo sapiens cDNA FLJ42269 fis, clone TKIDN2015285. [AK124263]	
A_24_P195831	1.98E-05	NM_030771	NM_030771	Homo sapiens NY-REN-41 antigen (NY-REN-41), transcript variant 1, mRNA [NM_030771]	NM_030771
A_32_P459533	1.99E-05	NM_015122	NM_015122	Homo sapiens FCH domain only 1 (FCHO1), mRNA [NM_015122]	NM_015122
A_23_P389919	1.99E-05	NM_014919	NM_014919	Homo sapiens Wolf-Hirschhorn syndrome candidate 1 (WHSC1), transcript variant 4, mRNA [NM_014919]	NM_014919
A_23_P5441	1.99E-05	NM_005689	NM_005689	Homo sapiens ATP-binding cassette, sub-family B (MDR/TAP), member 6 (ABCB6), nuclear gene encoding mitochondrial protein, mRNA [NM_005689]	NM_005689
A_23_P50137	2.00E-05	NM_016626	NM_016626	Homo sapiens ring finger and KH domain containing 2 (RKHD2), mRNA [NM_016626]	NM_016626
A_23_P12199	2.01E-05	NM_052943	NM_052943	Homo sapiens family with sequence similarity 46, member B (FAM46B), mRNA [NM_052943]	NM_052943
A_23_P26557	2.01E-05	NM_025108	NM_025108	Homo sapiens hypothetical protein FLJ13909 (FLJ13909), mRNA [NM_025108]	NM_025108
A_24_P505790	2.02E-05	AK131274	AK131274	Homo sapiens cDNA FLJ16218 fis, clone CTONG3001501, highly similar to Mus musculus glucocorticoid-induced gene 1 mRNA. [AK131274]	
A_23_P405885	2.02E-05	NM_138815	NM_138815	Homo sapiens developmental pluripotency associated 2 (DPPA2), mRNA [NM_138815]	NM_138815
A_23_P386942	2.02E-05	NM_145173	NM_145173	Homo sapiens DIRAS family, GTP-binding RAS-like 1 (DIRAS1), mRNA [NM_145173]	NM_145173
A_23_P169479	2.03E-05	CR595826	CR595826	full-length cDNA clone CS0DC021YA11 of Neuroblastoma Cot 25-normalized of Homo sapiens (human). [CR595826]	
A_24_P917934	2.03E-05	AL832665	AL832665	Homo sapiens mRNA; cDNA DKFZp313J1712 (from clone DKFZp313J1712). [AL832665]	
A_24_P289260	2.03E-05	NM_214462	NM_214462	Homo sapiens dapper, antagonist of beta-catenin, homolog 2 (Xenopus laevis) (DACT2), mRNA [NM_214462]	NM_214462
A_23_P209337	2.03E-05	NM_145280	NM_145280	Homo sapiens similar to hepatocellular carcinoma-associated antigen HCA557b (LOC151194), mRNA [NM_145280]	NM_145280
A_24_P179816	2.05E-05	NM_024330	NM_024330	Homo sapiens solute carrier family 27 (fatty acid transporter), member 3 (SLC27A3), mRNA [NM_024330]	NM_024330
A_23_P205007	2.05E-05	NM_002271	NM_002271	Homo sapiens RAN binding protein 5 (RANBP5), mRNA [NM_002271]	NM_002271
A_23_P70748	2.05E-05	NM_031922	NM_031922	Homo sapiens RALBP1 associated Eps domain containing 1 (REPS1), mRNA [NM_031922]	NM_031922
A_32_P127105	2.06E-05	BC037740	BC037740	Homo sapiens cDNA clone IMAGE:5263531, partial cds. [BC037740]	
A_24_P261734	2.06E-05	CR594705	CR594705	full-length cDNA clone CS0DC003YJ07 of Neuroblastoma Cot 25-normalized of Homo sapiens (human). [CR594705]	
A_23_P308954	2.07E-05	NM_030639	NM_030639	Homo sapiens basic helix-loop-helix domain containing, class B, 9 (BHLHB9), mRNA [NM_030639]	NM_030639
A_32_P232237	2.07E-05	THC2409354			
A_23_P35871	2.07E-05	NM_024680	NM_024680	Homo sapiens E2F transcription factor 8 (E2F8), mRNA [NM_024680]	NM_024680
A_23_P20045	2.07E-05	NM_000466	NM_000466	Homo sapiens peroxisome biogenesis factor 1 (PEX1), mRNA [NM_000466]	NM_000466
A_23_P157022	2.07E-05	NM_152411	NM_152411	Homo sapiens hypothetical protein DKFZp762I137 (DKFZp762I137), mRNA [NM_152411]	NM_152411
A_23_P403955	2.07E-05	NM_007375	NM_007375	Homo sapiens TAR DNA binding protein (TARDBP), mRNA [NM_007375]	NM_007375
A_23_P312851	2.07E-05	NM_006928	NM_006928	Homo sapiens silver homolog (mouse) (SILV), mRNA [NM_006928]	NM_006928
A_23_P135977	2.07E-05	NM_001008938	NM_001008938	Homo sapiens cytoskeleton associated protein 5 (CKAP5), transcript variant 1, mRNA [NM_001008938]	NM_001008938
A_32_P20982	2.07E-05	ENST00000342168		Homo sapiens cDNA FLJ45371 fis, clone BRHIP3017855, highly similar to Homo sapiens nuclear pore complex interacting protein (NPIP). [AK128772]	XM_290670
A_24_P938403	2.08E-05	BC008690	BC008690	Homo sapiens junctophilin 3, mRNA (cDNA clone IMAGE:3867947), complete cds. [BC008690]	
A_24_P133991	2.08E-05	NM_015208	NM_015208	Homo sapiens ankyrin repeat domain 12 (ANKRD12), mRNA [NM_015208]	NM_015208
A_23_P211355	2.08E-05	NM_022720	NM_022720	Homo sapiens DiGeorge syndrome critical region gene 8 (DGCR8), mRNA [NM_022720]	NM_022720
A_23_P62387	2.08E-05	NM_018159	NM_018159	Homo sapiens nudix (nucleoside diphosphate linked moiety X)-type motif 11 (NUDT11), mRNA [NM_018159]	NM_018159
A_32_P128399	2.08E-05	A_32_P128399			
A_23_P51397	2.08E-05	NM_001008493	NM_001008493	Homo sapiens enabled homolog (Drosophila) (ENAH), transcript variant 1, mRNA [NM_001008493]	NM_001008493
A_23_P33759	2.09E-05	NM_004753	NM_004753	Homo sapiens dehydrogenase/reductase (SDR family) member 3 (DHRS3), mRNA [NM_004753]	NM_004753
A_23_P86822	2.11E-05	NM_014679	NM_014679	Homo sapiens translokin (PIG8), mRNA [NM_014679]	NM_014679
A_23_P164047	2.11E-05	NM_012329	NM_012329	Homo sapiens monocyte to macrophage differentiation-associated (MMD), mRNA [NM_012329]	NM_012329

A_24_P242299	2.11E-05	NM_005455	NM_005455	Homo sapiens zinc finger protein 265 (ZNF265), transcript variant 2, mRNA [NM_005455]	NM_005455
A_32_P184746	2.12E-05	THC2378401			
A_32_P96807	2.12E-05	AY726570	AY726570	Homo sapiens clone TESTIS-724 mRNA sequence. [AY726570]	
A_23_P155989	2.14E-05	NM_022145	NM_022145	Homo sapiens leucine zipper protein FKSG14 (FKSG14), mRNA [NM_022145]	NM_022145
A_23_P215751	2.14E-05	NM_005000	NM_005000	Homo sapiens NADH dehydrogenase (ubiquinone) 1 alpha subcomplex, 5, 13kDa (NDUFA5), nuclear gene encoding mitochondrial protein, mRNA [NM_005000]	NM_005000
A_32_P125338	2.15E-05	NM_207334	NM_207334	Homo sapiens family with sequence similarity 43, member B (FAM43B), mRNA [NM_207334]	NM_207334
A_23_P205894	2.15E-05	NM_005159	NM_005159	Homo sapiens actin, alpha, cardiac muscle (ACTC), mRNA [NM_005159]	NM_005159
A_24_P943802	2.16E-05	AK128047	AK128047	Homo sapiens cDNA FLJ46167 fis, clone TESTI4003179. [AK128047]	
A_32_P190404	2.16E-05	AY030238	AY030238	Homo sapiens LQK1 hypothetical protein long isoform (LQK1) mRNA, complete cds, alternatively spliced. [AY030238]	
A_23_P1833	2.17E-05	NM_018644	NM_018644	Homo sapiens beta-1,3-glucuronyltransferase 1 (glucuronosyltransferase P) (B3GAT1), transcript variant 1, mRNA [NM_018644]	NM_018644
A_23_P44684	2.17E-05	NM_018098	NM_018098	Homo sapiens epithelial cell transforming sequence 2 oncogene (ECT2), mRNA [NM_018098]	NM_018098
A_23_P121374	2.17E-05	NM_018987	NM_018987	Homo sapiens sema domain, seven thrombospondin repeats (type 1 and type 1-like), transmembrane domain (TM) and short cytoplasmic domain, (semaphorin) 5B (SEMA5B), mRNA [NM_018987]	NM_018987
A_23_P54556	2.17E-05	NM_014048	NM_014048	Homo sapiens MKL/myocardin-like 2 (MKL2), mRNA [NM_014048]	NM_014048
A_23_P125253	2.19E-05	NM_001290	NM_001290	Homo sapiens LIM domain binding 2 (LDB2), mRNA [NM_001290]	NM_001290
A_23_P71598	2.19E-05	NM_003829	NM_003829	Homo sapiens multiple PDZ domain protein (MPDZ), mRNA [NM_003829]	NM_003829
A_32_P67544	2.19E-05	BX647764	BX647764	Homo sapiens mRNA; cDNA DKFZp686E0352 (from clone DKFZp686E0352). [BX647764]	
A_24_P153713	2.20E-05	BC005052	BC005052	Homo sapiens MARVEL domain containing 3, mRNA (cDNA clone IMAGE:2820819), partial cds. [BC005052]	
A_23_P373100	2.20E-05	BC020879	BC020879	Homo sapiens hypothetical protein MGC24103, mRNA (cDNA clone MGC:24103 IMAGE:4613905), complete cds. [BC020879]	
A_24_P602168	2.21E-05	THC2314177		ALU1_HUMAN (P39188) Alu subfamily J sequence contamination warning entry, partial (9%) [THC2314177]	
A_32_P9127	2.21E-05	THC2406727			
A_23_P252748	2.21E-05	NM_007152	NM_007152	Homo sapiens zinc finger protein 195 (ZNF195), mRNA [NM_007152]	NM_007152
A_32_P212343	2.22E-05	AK057710	AK057710	Homo sapiens cDNA FLJ33148 fis, clone UTERU2000238. [AK057710]	
A_23_P34433	2.22E-05	NM_001009881	NM_001009881	Homo sapiens zinc finger, CCHC domain containing 11 (ZCCHC11), transcript variant 1, mRNA [NM_001009881]	NM_001009881
A_23_P9056	2.22E-05	NM_014781	NM_014781	Homo sapiens RB1-inducible coiled-coil 1 (RB1CC1), mRNA [NM_014781]	NM_014781
A_23_P127995	2.22E-05	NM_001293	NM_001293	Homo sapiens chloride channel, nucleotide-sensitive, 1A (CLNS1A), mRNA [NM_001293]	NM_001293
A_23_P51085	2.23E-05	NM_020675	NM_020675	Homo sapiens spindle pole body component 25 homolog (S. cerevisiae) (SPBC25), mRNA [NM_020675]	NM_020675
A_23_P328323	2.24E-05	NM_018211	NM_018211	Homo sapiens hypothetical protein FLJ10770 (KIAA1579), mRNA [NM_018211]	NM_018211
A_32_P229299	2.24E-05	NM_032458	NM_032458	Homo sapiens PHD finger protein 6 (PHF6), transcript variant 2, mRNA [NM_032458]	NM_032458
A_24_P125311	2.24E-05	A_24_P125311			
A_23_P88209	2.25E-05	NM_016106	NM_016106	Homo sapiens sec1 family domain containing 1 (SCFD1), transcript variant 1, mRNA [NM_016106]	NM_016106
A_24_P649327	2.26E-05	AK124953	AK124953	Homo sapiens cDNA FLJ42963 fis, clone BRSTN2012380. [AK124953]	XM_372556
A_32_P145385	2.26E-05	AK001118	AK001118	Homo sapiens cDNA FLJ10256 fis, clone HEMBB1000870. [AK001118]	
A_23_P201567	2.26E-05	NM_018061	NM_018061	Homo sapiens PRP38 pre-mRNA processing factor 38 (yeast) domain containing B (PRPF38B), mRNA [NM_018061]	NM_018061
A_24_P30923	2.26E-05	NM_003498	NM_003498	Homo sapiens stannin (SNN), mRNA [NM_003498]	NM_003498
A_23_P143906	2.26E-05	NM_022443	NM_022443	Homo sapiens myeloid leukemia factor 1 (MLF1), mRNA [NM_022443]	NM_022443
A_23_P141415	2.27E-05	NM_032133	NM_032133	Homo sapiens MYCBP associated protein (MYCBPAP), mRNA [NM_032133]	NM_032133
A_32_P174398	2.27E-05	AF085351	AF085351	Homo sapiens ELISC-1 mRNA, partial cds. [AF085351]	
A_24_P179183	2.27E-05	NM_015208	NM_015208	Homo sapiens ankyrin repeat domain 12 (ANKRD12), mRNA [NM_015208]	NM_015208
A_24_P102362	2.27E-05	ENST00000320159		Homo sapiens zinc finger protein 553, mRNA (cDNA clone IMAGE:3833591), partial cds. [BC007393]	
A_23_P255104	2.27E-05	NM_005779	NM_005779	Homo sapiens lipoma HMGIC fusion partner-like 2 (LHFPL2), mRNA [NM_005779]	NM_005779
A_23_P431630	2.28E-05	NM_032582	NM_032582	Homo sapiens ubiquitin specific protease 32 (USP32), mRNA [NM_032582]	NM_032582
A_23_P61180	2.28E-05	NM_018390	NM_018390	Homo sapiens phosphatidylinositol-specific phospholipase C, X domain containing 1 (PLCXD1), mRNA [NM_018390]	NM_018390
A_32_P179646	2.29E-05	A_32_P179646			
A_24_P150803	2.29E-05	NM_002773	NM_002773	Homo sapiens protease, serine, 8 (prostasin) (PRSS8), mRNA [NM_002773]	NM_002773

A_24_P664995	2.29E-05	AK055641	AK055641	Homo sapiens cDNA FLJ31079 fis, clone HSYRA2001595. [AK055641]	
A_32_P179807	2.30E-05	A_32_P179807			
A_24_P944714	2.30E-05	AL390129	AL390129	Homo sapiens mRNA; cDNA DKFZp761K0912 (from clone DKFZp761K0912). [AL390129]	
A_24_P91991	2.30E-05	NM_178557	NM_178557	Homo sapiens hypothetical protein FLJ37478 (FLJ37478), mRNA [NM_178557]	NM_178557
A_32_P142077	2.30E-05	THC2300892		A46461 T-cell receptor eta chain - human (fragment) {Homo sapiens;} , complete [THC2300892]	
A_24_P367100	2.30E-05	A_24_P367100			
A_23_P434768	2.30E-05	BC039468	BC039468	Homo sapiens cDNA clone IMAGE:3889720. [BC039468]	
A_24_P105298	2.30E-05	NM_002107	NM_002107	Homo sapiens H3 histone, family 3A (H3F3A), mRNA [NM_002107]	NM_002107
A_23_P339954	2.31E-05	NM_001018059	NM_001018059	Homo sapiens similar to nuclear pore complex interacting protein (LOC440348), mRNA [NM_001018059]	NM_001018059
A_24_P867201	2.32E-05	CR613944	CR613944	full-length cDNA clone CS0CAP007YK21 of Thymus of Homo sapiens (human). [CR613944]	
A_23_P151570	2.32E-05	NM_014990	NM_014990	Homo sapiens GTPase activating Rap/RanGAP domain-like 1 (GARNL1), transcript variant 1, mRNA [NM_014990]	NM_014990
A_24_P380919	2.32E-05	NM_002140	NM_002140	Homo sapiens heterogeneous nuclear ribonucleoprotein K (HNRPK), transcript variant 1, mRNA [NM_002140]	NM_002140
A_24_P509948	2.33E-05	AK091348	AK091348	Homo sapiens cDNA FLJ34029 fis, clone FCBBF2003636. [AK091348]	
A_23_P96008	2.33E-05	NM_006785	NM_006785	Homo sapiens mucosa associated lymphoid tissue lymphoma translocation gene 1 (MALT1), transcript variant 1, mRNA [NM_006785]	NM_006785
A_24_P213478	2.34E-05	NM_020796	NM_020796	Homo sapiens sema domain, transmembrane domain (TM), and cytoplasmic domain, (semaphorin) 6A (SEMA6A), mRNA [NM_020796]	NM_020796
A_23_P91900	2.34E-05	NM_005496	NM_005496	Homo sapiens SMC4 structural maintenance of chromosomes 4-like 1 (yeast) (SMC4L1), transcript variant 1, mRNA [NM_005496]	NM_005496
A_24_P193435	2.34E-05	NM_003257	NM_003257	Homo sapiens tight junction protein 1 (zona occludens 1) (TJP1), transcript variant 1, mRNA [NM_003257]	NM_003257
A_23_P28625	2.34E-05	NM_018256	NM_018256	Homo sapiens WD repeat domain 12 (WDR12), mRNA [NM_018256]	NM_018256
A_23_P145424	2.35E-05	NM_014895	NM_014895	Homo sapiens chromosome 6 open reading frame 84 (C6orf84), mRNA [NM_014895]	NM_014895
A_23_P101121	2.37E-05	THC2404912		Q7XXR9 (Q7XXR9) Katanin, partial (44%) [THC2404912]	
A_23_P34045	2.37E-05	NM_001399	NM_001399	Homo sapiens ectodysplasin A (EDA), transcript variant 1, mRNA [NM_001399]	NM_001399
A_23_P320242	2.37E-05	NM_152748	NM_152748	Homo sapiens KIAA1324-like (KIAA1324L), mRNA [NM_152748]	NM_152748
A_24_P290709	2.38E-05	CR593166	CR593166	full-length cDNA clone CS0DD005YN08 of Neuroblastoma Cot 50-normalized of Homo sapiens (human). [CR593166]	
A_23_P380834	2.39E-05	NM_020193	NM_020193	Homo sapiens chromosome 11 open reading frame 30 (C11orf30), mRNA [NM_020193]	NM_020193
A_24_P103264	2.40E-05	NM_003360	NM_003360	Homo sapiens UDP glycosyltransferase 8 (UDP-galactose ceramide galactosyltransferase) (UGT8), mRNA [NM_003360]	NM_003360
A_23_P87839	2.40E-05	NM_004719	NM_004719	Homo sapiens splicing factor, arginine/serine-rich 2, interacting protein (SFRS2IP), mRNA [NM_004719]	NM_004719
A_23_P50217	2.40E-05	NM_024833	NM_024833	Homo sapiens zinc finger protein 671 (ZNF671), mRNA [NM_024833]	NM_024833
A_24_P28657	2.40E-05	NM_015446	NM_015446	Homo sapiens AT hook containing transcription factor 1 (AHCTF1), mRNA [NM_015446]	NM_015446
A_32_P194182	2.41E-05	A_32_P194182			
A_23_P200524	2.41E-05	NM_001002921	NM_001002921	Homo sapiens adenylate kinase 3-like 2 (AK3L2), mRNA [NM_001002921]	NM_001002921
A_23_P4462	2.41E-05	A_23_P4462			
A_24_P247616	2.41E-05	A_24_P247616			
A_24_P344087	2.42E-05	NM_005132	NM_005132	Homo sapiens REC8-like 1 (yeast) (REC8L1), mRNA [NM_005132]	NM_005132
A_23_P359854	2.42E-05	AB046773	AB046773	Homo sapiens mRNA for KIAA1553 protein, partial cds. [AB046773]	XM_166320
A_32_P108666	2.43E-05	AK091731	AK091731	Homo sapiens cDNA FLJ34412 fis, clone HEART2002432. [AK091731]	
A_23_P395582	2.44E-05	NM_174900	NM_174900	Homo sapiens zinc finger protein 42 (ZFP42), mRNA [NM_174900]	NM_174900
A_23_P115645	2.45E-05	NM_006561	NM_006561	Homo sapiens CUG triplet repeat, RNA binding protein 2 (CUGBP2), transcript variant 2, mRNA [NM_006561]	NM_006561
A_32_P194423	2.45E-05	THC2408398			
A_23_P43079	2.46E-05	NM_017864	NM_017864	Homo sapiens hypothetical protein FLJ20530 (FLJ20530), mRNA [NM_017864]	NM_017864
A_24_P919460	2.46E-05	AB065089	AB065089	Homo sapiens OK/KNS-cl.7 mRNA for ribosomal protein S2, complete cds. [AB065089]	
A_24_P381625	2.46E-05	NM_002806	NM_002806	Homo sapiens proteasome (prosome, macropain) 26S subunit, ATPase, 6 (PSMC6), mRNA [NM_002806]	NM_002806
A_23_P55688	2.47E-05	NM_017879	NM_017879	Homo sapiens zinc finger protein 416 (ZNF416), mRNA [NM_017879]	NM_017879
A_23_P253446	2.48E-05	NM_002045	NM_002045	Homo sapiens growth associated protein 43 (GAP43), mRNA [NM_002045]	NM_002045
A_23_P216068	2.48E-05	NM_014109	NM_014109	Homo sapiens ATPase family, AAA domain containing 2 (ATAD2), mRNA [NM_014109]	NM_014109

A_24_P165595	2.48E-05	THC2310678		Q82VR6 (Q82VR6) Phosphate transport system permease protein, partial (6%) [THC2310678]	
A_24_P379104	2.48E-05	NM_006875	NM_006875	Homo sapiens pim-2 oncogene (PIM2), mRNA [NM_006875]	NM_006875
A_24_P33055	2.49E-05	A_24_P33055			
A_23_P161091	2.49E-05	NM_024772	NM_024772	Homo sapiens zinc finger, MYM domain containing 1 (ZMYM1), mRNA [NM_024772]	NM_024772
A_32_P191004	2.49E-05	ENST00000238789		Homo sapiens mRNA for KIAA1240 protein, partial cds. [AB033066]	XM_039676
A_32_P90740	2.50E-05	AF086427	AF086427	Homo sapiens full length insert cDNA clone ZD79D11. [AF086427]	
A_24_P560519	2.50E-05	AK024346	AK024346	Homo sapiens cDNA FLJ14284 fis, clone PLACE1005898. [AK024346]	
A_23_P349147	2.50E-05	ENST00000305423		Homo sapiens MUC3B mRNA for intestinal mucin, partial cds. [AB038783]	XM_168578
A_24_P319364	2.50E-05	NM_144503	NM_144503	Homo sapiens F11 receptor (F11R), transcript variant 4, mRNA [NM_144503]	NM_144503
A_23_P359111	2.50E-05	NM_015640	NM_015640	Homo sapiens PAI-1 mRNA binding protein (PAI-RBP1), transcript variant 4, mRNA [NM_015640]	NM_015640
A_32_P66934	2.51E-05	BX641014	BX641014	Homo sapiens mRNA; cDNA DKFZp686I19109 (from clone DKFZp686I19109) [BX641014]	
A_23_P154188	2.51E-05	NM_024545	NM_024545	Homo sapiens mSin3A-associated protein 130 (SAP130), mRNA [NM_024545]	NM_024545
A_23_P156562	2.52E-05	A_23_P156562			
A_23_P209987	2.52E-05	NM_019014	NM_019014	Homo sapiens polymerase (RNA) I polypeptide B, 128kDa (POLR1B), mRNA [NM_019014]	NM_019014
A_24_P161973	2.52E-05	NM_015205	NM_015205	Homo sapiens ATPase, Class VI, type 11A (ATP11A), transcript variant 1, mRNA [NM_015205]	NM_015205
A_23_P116743	2.52E-05	AK095700	AK095700	Homo sapiens cDNA FLJ38381 fis, clone FEBRA2003436. [AK095700]	
A_23_P132378	2.53E-05	NM_014246	NM_014246	Homo sapiens cadherin, EGF LAG seven-pass G-type receptor 1 (flamingo homolog, Drosophila) (CELSR1), mRNA [NM_014246]	NM_014246
A_23_P334218	2.53E-05	NM_145647	NM_145647	Homo sapiens WD repeat domain 67 (WDR67), mRNA [NM_145647]	NM_145647
A_23_P36865	2.53E-05	NM_025114	NM_025114	Homo sapiens centrosome protein cep290 (Cep290), mRNA [NM_025114]	NM_025114
A_23_P137470	2.53E-05	NM_020808	NM_020808	Homo sapiens signal-induced proliferation-associated 1 like 2 (SIPA1L2), mRNA [NM_020808]	NM_020808
A_32_P155035	2.55E-05	AK096500	AK096500	Homo sapiens cDNA FLJ39181 fis, clone OCBBF2004235. [AK096500]	
A_23_P48951	2.55E-05	NM_005928	NM_005928	Homo sapiens milk fat globule-EGF factor 8 protein (MFGE8), mRNA [NM_005928]	NM_005928
A_23_P14295	2.56E-05	NM_020181	NM_020181	Homo sapiens chromosome 14 open reading frame 162 (C14orf162), mRNA [NM_020181]	NM_020181
A_24_P56252	2.56E-05	AF086032	AF086032	Homo sapiens full length insert cDNA clone YW25G09. [AF086032]	
A_23_P50455	2.56E-05	NM_002691	NM_002691	Homo sapiens polymerase (DNA directed), delta 1, catalytic subunit 125kDa (POLD1), mRNA [NM_002691]	NM_002691
A_24_P340813	2.58E-05	NM_032870	NM_032870	Homo sapiens chromosome 6 open reading frame 111 (C6orf111), mRNA [NM_032870]	NM_032870
A_23_P112412	2.58E-05	NM_017746	NM_017746	Homo sapiens testis expressed sequence 10 (TEX10), mRNA [NM_017746]	NM_017746
A_23_P106412	2.59E-05	AK130644	AK130644	Homo sapiens cDNA FLJ27134 fis, clone SPL08315. [AK130644]	
A_32_P226186	2.59E-05	THC2266474			
A_24_P332623	2.59E-05	AL133018	AL133018	Homo sapiens mRNA; cDNA DKFZp434F0327 (from clone DKFZp434F0327). [AL133018]	
A_32_P69296	2.59E-05	AK130071	AK130071	Homo sapiens cDNA FLJ26561 fis, clone LNF03981. [AK130071]	
A_32_P161455	2.61E-05	AL831999	AL831999	Homo sapiens mRNA; cDNA DKFZp451K063 (from clone DKFZp451K063). [AL831999]	
A_32_P36582	2.61E-05	AK123649	AK123649	Homo sapiens cDNA FLJ41655 fis, clone FEBRA2025477. [AK123649]	
A_23_P30377	2.61E-05	NM_014829	NM_014829	Homo sapiens DEAD (Asp-Glu-Ala-Asp) box polypeptide 46 (DDX46), mRNA [NM_014829]	NM_014829
A_23_P258463	2.62E-05	NM_006017	NM_006017	Homo sapiens prominin 1 (PROM1), mRNA [NM_006017]	NM_006017
A_23_P110624	2.63E-05	NM_001332	NM_001332	Homo sapiens catenin (cadherin-associated protein), delta 2 (neural plakophilin-related arm-repeat protein) (CTNND2), mRNA [NM_001332]	NM_001332
A_23_P154345	2.63E-05	NM_014362	NM_014362	Homo sapiens 3-hydroxyisobutyryl-Coenzyme A hydrolase (HIBCH), transcript variant 1, mRNA [NM_014362]	NM_014362
A_23_P14508	2.64E-05	ENST00000256367		Homo sapiens tetratricopeptide repeat domain 9, mRNA (cDNA clone IMAGE:5763935), partial cds. [BC047950]	XM_027236
A_24_P379969	2.64E-05	NM_003106	NM_003106	Homo sapiens SRY (sex determining region Y)-box 2 (SOX2), mRNA [NM_003106]	NM_003106
A_23_P359540	2.65E-05	NM_003540	NM_003540	Homo sapiens histone 1, H4f (HIST1H4F), mRNA [NM_003540]	NM_003540
A_23_P202587	2.65E-05	NM_018330	NM_018330	Homo sapiens KIAA1598 (KIAA1598), mRNA [NM_018330]	NM_018330
A_23_P132454	2.65E-05	NM_020307	NM_020307	Homo sapiens cyclin L1 (CCNL1), mRNA [NM_020307]	NM_020307
A_23_P171074	2.66E-05	NM_004867	NM_004867	Homo sapiens integral membrane protein 2A (ITM2A), mRNA [NM_004867]	NM_004867
A_23_P111835	2.66E-05	NM_000108	NM_000108	Homo sapiens dihydrolipoamide dehydrogenase (E3 component of pyruvate dehydrogenase complex, 2-oxo-glutarate complex, branched chain keto acid dehydrogenase complex) (DLD), mRNA [NM_000108]	NM_000108

A_23_P126291	2.66E-05	NM_003094	NM_003094	Homo sapiens small nuclear ribonucleoprotein polypeptide E (SNRPE), mRNA [NM_003094]	NM_003094
A_23_P379945	2.67E-05	AB040942	AB040942	Homo sapiens mRNA for KIAA1509 protein, partial cds. [AB040942]	XM_029353
A_24_P376139	2.68E-05	AK057798	AK057798	Homo sapiens cDNA FLJ25069 fis, clone CBL05145. [AK057798]	
A_23_P110802	2.68E-05	NM_022909	NM_022909	Homo sapiens centromere protein H (CENPH), mRNA [NM_022909]	NM_022909
A_32_P143000	2.69E-05	ENST00000261275		Homo sapiens mRNA for KIAA0574 protein, partial cds. [AB011146]	
A_23_P85969	2.69E-05	NM_182976	NM_182976	Homo sapiens zinc finger protein 326 (ZNF326), transcript variant 1, mRNA [NM_182976]	NM_182976
A_23_P19712	2.69E-05	NM_015895	NM_015895	Homo sapiens geminin, DNA replication inhibitor (GMNN), mRNA [NM_015895]	NM_015895
A_23_P7697	2.69E-05	NM_003100	NM_003100	Homo sapiens sorting nexin 2 (SNX2), mRNA [NM_003100]	NM_003100
A_24_P738859	2.70E-05	AK075186	AK075186	Homo sapiens cDNA FLJ90705 fis, clone PLACE1007591. [AK075186]	
A_24_P943922	2.70E-05	NM_020925	NM_020925	Homo sapiens von Willebrand factor type A and cache domain containing 1 (VWCD1), mRNA [NM_020925]	NM_020925
A_24_P358606	2.70E-05	A_24_P358606			
A_23_P74449	2.70E-05	NM_032756	NM_032756	Homo sapiens glyoxalase domain containing 1 (GLOXD1), mRNA [NM_032756]	NM_032756
A_23_P83931	2.70E-05	NM_005863	NM_005863	Homo sapiens neuroepithelial cell transforming gene 1 (NET1), mRNA [NM_005863]	NM_005863
A_23_P348146	2.72E-05	NM_144595	NM_144595	Homo sapiens hypothetical protein FLJ30046 (FLJ30046), mRNA [NM_144595]	NM_144595
A_24_P261203	2.72E-05	BX537622	BX537622	Homo sapiens mRNA; cDNA DKFZp686J12188 (from clone DKFZp686J12188). [BX537622]	
A_23_P87827	2.72E-05	BC004815	BC004815	Homo sapiens hypothetical protein MGC5139, mRNA (cDNA clone MGC:5139 IMAGE:3448346), complete cds. [BC004815]	
A_23_P159974	2.73E-05	NM_033495	NM_033495	Homo sapiens kelch-like 13 (Drosophila) (KLHL13), mRNA [NM_033495]	NM_033495
A_24_P341504	2.73E-05	NM_017619	NM_017619	Homo sapiens RNA-binding region (RNP1, RRM) containing 3 (RNPC3), mRNA [NM_017619]	NM_017619
A_32_P193646	2.73E-05	NM_002139	NM_002139	Homo sapiens RNA binding motif protein, X-linked (RBMX), mRNA [NM_002139]	NM_002139
A_24_P206427	2.73E-05	NM_001568	NM_001568	Homo sapiens eukaryotic translation initiation factor 3, subunit 6 48kDa (EIF3S6), mRNA [NM_001568]	NM_001568
A_23_P14845	2.74E-05	ENST00000344541		Homo sapiens mRNA; cDNA DKFZp313B2137 (from clone DKFZp313B2137). [BX647599]	
A_23_P141447	2.74E-05	NM_145654	NM_145654	Homo sapiens RAD52 homolog B (S. cerevisiae) (RAD52B), mRNA [NM_145654]	NM_145654
A_24_P109417	2.74E-05	NM_198545	NM_198545	Homo sapiens hypothetical gene supported by AK075558; BC021286 (LOC374946), mRNA [NM_198545]	NM_198545
A_23_P200260	2.74E-05	NM_014801	NM_014801	Homo sapiens pecanex-like 2 (Drosophila) (PCNXL2), transcript variant 1, mRNA [NM_014801]	NM_014801
A_23_P428548	2.74E-05	NM_182641	NM_182641	Homo sapiens fetal Alzheimer antigen (FALZ), transcript variant 1, mRNA [NM_182641]	NM_182641
A_23_P140362	2.74E-05	NM_018228	NM_018228	Homo sapiens chromosome 14 open reading frame 115 (C14orf115), mRNA [NM_018228]	NM_018228
A_24_P791669	2.75E-05	ENST00000356555		PREDICTED: Homo sapiens similar to hypothetical protein 9630041N07 (LOC345462), mRNA [XM_293828]	XM_293828
A_32_P161262	2.76E-05	THC2377294			
A_32_P133090	2.76E-05	XM_379210	XM_379210	PREDICTED: Homo sapiens hypothetical LOC401085 (LOC401085), mRNA [XM_379210]	XM_379210
A_24_P45728	2.76E-05	NM_020770	NM_020770	Homo sapiens cingulin (CGN), mRNA [NM_020770]	NM_020770
A_32_P35969	2.76E-05	CR605947	CR605947	full-length cDNA clone CS0DD004YC02 of Neuroblastoma Cot 50-normalized of Homo sapiens (human). [CR605947]	
A_23_P155969	2.78E-05	NM_014264	NM_014264	Homo sapiens polo-like kinase 4 (Drosophila) (PLK4), mRNA [NM_014264]	NM_014264
A_24_P404458	2.78E-05	NM_014446	NM_014446	Homo sapiens integrin beta 1 binding protein 3 (ITGB1BP3), transcript variant 1, mRNA [NM_014446]	NM_014446
A_23_P331700	2.79E-05	NM_153043	NM_153043	Homo sapiens hypothetical protein FLJ37078 (FLJ37078), mRNA [NM_153043]	NM_153043
A_24_P310630	2.79E-05	NM_080632	NM_080632	Homo sapiens UPF3 regulator of nonsense transcripts homolog B (yeast) (UPF3B), transcript variant 1, mRNA [NM_080632]	NM_080632
A_23_P8311	2.79E-05	NM_016614	NM_016614	Homo sapiens TRAF and TNF receptor associated protein (TTRAP), mRNA [NM_016614]	NM_016614
A_24_P124662	2.80E-05	NM_139078	NM_139078	Homo sapiens mitogen-activated protein kinase-activated protein kinase 5 (MAPKAPK5), transcript variant 2, mRNA [NM_139078]	NM_139078
A_23_P57268	2.82E-05	NM_001338	NM_001338	Homo sapiens coxsackie virus and adenovirus receptor (CXADR), mRNA [NM_001338]	NM_001338
A_23_P137689	2.83E-05	NM_015441	NM_015441	Homo sapiens olfactomedin-like 2B (OLFML2B), mRNA [NM_015441]	NM_015441
A_24_P153511	2.83E-05	NM_020841	NM_020841	Homo sapiens oxysterol binding protein-like 8 (OSBPL8), transcript variant 1, mRNA [NM_020841]	NM_020841
A_23_P131476	2.83E-05	NM_020122	NM_020122	Homo sapiens potassium channel modulatory factor 1 (KCMF1), mRNA [NM_020122]	NM_020122
A_23_P45087	2.84E-05	NM_016220	NM_016220	Homo sapiens zinc finger protein 588 (ZNF588), transcript variant 1, mRNA [NM_016220]	NM_016220
A_32_P475642	2.84E-05	AK025166	AK025166	Homo sapiens cDNA: FLJ21513 fis, clone COL05778. [AK025166]	
A_23_P147199	2.85E-05	NM_006629	NM_006629	Homo sapiens zinc finger protein 271 (ZNF271), mRNA [NM_006629]	NM_006629

A_24_P181149	2.87E-05	NM_024685	NM_024685	Homo sapiens hypothetical protein FLJ23560 (FLJ23560), mRNA [NM_024685]	NM_024685
A_24_P100613	2.87E-05	NM_005559	NM_005559	Homo sapiens laminin, alpha 1 (LAMA1), mRNA [NM_005559]	NM_005559
A_23_P23947	2.88E-05	NM_005204	NM_005204	Homo sapiens mitogen-activated protein kinase kinase kinase 8 (MAP3K8), mRNA [NM_005204]	NM_005204
A_23_P153897	2.88E-05	NM_052847	NM_052847	Homo sapiens guanine nucleotide binding protein (G protein), gamma 7 (GNG7), mRNA [NM_052847]	NM_052847
A_23_P256391	2.88E-05	NM_002078	NM_002078	Homo sapiens golgi autoantigen, golgin subfamily a, 4 (GOLGA4), mRNA [NM_002078]	NM_002078
A_23_P110196	2.89E-05	NM_016323	NM_016323	Homo sapiens hect domain and RLD 5 (HERC5), mRNA [NM_016323]	NM_016323
A_24_P289366	2.89E-05	NM_203463	NM_203463	Homo sapiens LAG1 longevity assurance homolog 6 (S. cerevisiae) (LASS6), mRNA [NM_203463]	NM_203463
A_23_P385246	2.90E-05	NM_153331	NM_153331	Homo sapiens potassium channel tetramerisation domain containing 6 (KCTD6), mRNA [NM_153331]	NM_153331
A_24_P595223	2.90E-05	AK098753	AK098753	Homo sapiens cDNA FLJ25887 fis, clone CBR02996. [AK098753]	
A_24_P916614	2.90E-05	CR749471	CR749471	Homo sapiens mRNA; cDNA DKFZp781I1117 (from clone DKFZp781I1117). [CR749471]	
A_23_P168567	2.90E-05	NM_032999	NM_032999	Homo sapiens general transcription factor II, i (GTF2I), transcript variant 1, mRNA [NM_032999]	NM_032999
A_23_P137514	2.91E-05	NM_016389	NM_016389	Homo sapiens influenza virus NS1A binding protein (IVNS1ABP), transcript variant 2, mRNA [NM_016389]	NM_016389
A_24_P143171	2.92E-05	NM_031442	NM_031442	Homo sapiens transmembrane protein 47 (TMEM47), mRNA [NM_031442]	NM_031442
A_23_P27744	2.92E-05	Y10936	Y10936	H.sapiens mRNA for hypothetical protein downstream of DMPK and DMAHP. [Y10936]	
A_23_P166716	2.92E-05	NM_017819	NM_017819	Homo sapiens RNA (guanine-9-) methyltransferase domain containing 1 (RG9MTD1), mRNA [NM_017819]	NM_017819
A_24_P803885	2.93E-05	NM_207326	NM_207326	Homo sapiens hypothetical protein LOC149134 (LOC149134), mRNA [NM_207326]	NM_207326
A_23_P112201	2.94E-05	NM_015061	NM_015061	Homo sapiens jumonji domain containing 2C (JMJD2C), mRNA [NM_015061]	NM_015061
A_24_P223604	2.94E-05	NM_015496	NM_015496	Homo sapiens DKFZP434I116 protein (DKFZP434I116), transcript variant 1, mRNA [NM_015496]	NM_015496
A_24_P787914	2.96E-05	U25029	U25029	Human glucocorticoid receptor alpha mRNA, variant 3' UTR. [U25029]	
A_23_P9779	2.96E-05	NM_005853	NM_005853	Homo sapiens iroquois homeobox protein 5 (IRX5), mRNA [NM_005853]	NM_005853
A_24_P303420	2.98E-05	NM_001010871	NM_001010871	Homo sapiens hypothetical protein LOC221442 (LOC221442), mRNA [NM_001010871]	NM_001010871
A_23_P94141	2.98E-05	NM_006550	NM_006550	Homo sapiens fibrinogen silencer binding protein (FSBP), mRNA [NM_006550]	NM_006550
A_23_P366559	2.98E-05	NM_144973	NM_144973	Homo sapiens hypothetical protein MGC24039 (MGC24039), mRNA [NM_144973]	NM_144973
A_23_P90273	2.98E-05	NM_022467	NM_022467	Homo sapiens carbohydrate (N-acetylgalactosamine 4-0) sulfotransferase 8 (CHST8), mRNA [NM_022467]	NM_022467
A_32_P24165	2.98E-05	NM_001018115	NM_001018115	Homo sapiens Fanconi anemia, complementation group D2 (FANCD2), transcript variant 2, mRNA [NM_001018115]	NM_001018115
A_24_P337546	2.99E-05	NM_014895	NM_014895	Homo sapiens chromosome 6 open reading frame 84 (C6orf84), mRNA [NM_014895]	NM_014895
A_23_P102471	2.99E-05	NM_000251	NM_000251	Homo sapiens mutS homolog 2, colon cancer, nonpolyposis type 1 (E. coli) (MSH2), mRNA [NM_000251]	NM_000251
A_23_P435407	2.99E-05	NM_001448	NM_001448	Homo sapiens glypican 4 (GPC4), mRNA [NM_001448]	NM_001448
A_32_P164522	3.00E-05	NM_182620	NM_182620	Homo sapiens family with sequence similarity 33, member A (FAM33A), mRNA [NM_182620]	NM_182620
A_24_P37441	3.00E-05	NM_002610	NM_002610	Homo sapiens pyruvate dehydrogenase kinase, isoenzyme 1 (PDK1), nuclear gene encoding mitochondrial protein, mRNA [NM_002610]	NM_002610
A_32_P211080	3.01E-05	NM_001024681	NM_001024681	Homo sapiens D15F37 gene (D15F37), mRNA [NM_001024681]	NM_001024681
A_23_P44139	3.01E-05	NM_000947	NM_000947	Homo sapiens primase, polypeptide 2A, 58kDa (PRIM2A), mRNA [NM_000947]	NM_000947
A_23_P25163	3.02E-05	NM_172178	NM_172178	Homo sapiens mitochondrial ribosomal protein L42 (MRPL42), nuclear gene encoding mitochondrial protein, transcript variant 3, mRNA [NM_172178]	NM_172178
A_23_P73763	3.02E-05	NM_006014	NM_006014	Homo sapiens DNA segment on chromosome X (unique) 9879 expressed sequence (DXS9879E), mRNA [NM_006014]	NM_006014
A_32_P174025	3.03E-05	NM_182704	NM_182704	Homo sapiens selenoprotein V (SELV), mRNA [NM_182704]	NM_182704
A_32_P201773	3.03E-05	NM_015365	NM_015365	Homo sapiens Alport syndrome, mental retardation, midface hypoplasia and elliptocytosis chromosomal region, gene 1 (AMMECR1), transcript variant 1, mRNA [NM_015365]	NM_015365
A_24_P367397	3.03E-05	A_24_P367397			
A_23_P254091	3.03E-05	NM_004520	NM_004520	Homo sapiens kinesin heavy chain member 2 (KIF2), mRNA [NM_004520]	NM_004520
A_24_P267664	3.04E-05	NM_153754	NM_153754	Homo sapiens chromosome 21 open reading frame 88 (C21orf88), mRNA [NM_153754]	NM_153754
A_23_P156355	3.04E-05	NM_153354	NM_153354	Homo sapiens hypothetical protein MGC33214 (MGC33214), mRNA [NM_153354]	NM_153354
A_23_P14062	3.04E-05	NM_020401	NM_020401	Homo sapiens nucleoporin 107kDa (NUP107), mRNA [NM_020401]	NM_020401
A_24_P93948	3.05E-05	BC035285	BC035285	Homo sapiens cDNA clone IMAGE:5197468, with apparent retained intron. [BC035285]	
A_23_P104146	3.05E-05	NM_005095	NM_005095	Homo sapiens zinc finger protein 262 (ZNF262), mRNA [NM_005095]	NM_005095
A_23_P41327	3.07E-05	NM_017816	NM_017816	Homo sapiens hypothetical protein FLJ20425 (LYAR), mRNA [NM_017816]	NM_017816

A_32_P104334	3.07E-05	AW972815	AW972815	EST384910 MAGE resequences, MAGL Homo sapiens cDNA, mRNA sequence [AW972815]	
A_24_P314477	3.07E-05	NM_178012	NM_178012	Homo sapiens tubulin, beta polypeptide paralog (RP11-506K6.1), mRNA [NM_178012]	NM_178012
A_23_P126037	3.08E-05	NM_012421	NM_012421	Homo sapiens rearranged L-myc fusion sequence (RLF), mRNA [NM_012421]	NM_012421
A_23_P152136	3.08E-05	NM_022770	NM_022770	Homo sapiens hypothetical protein FLJ13912 (FLJ13912), mRNA [NM_022770]	NM_022770
A_23_P161706	3.09E-05	THC2317900		Q9H2Q1 (Q9H2Q1) AD031, partial (69%) [THC2317900]	
A_24_P372625	3.09E-05	NM_016422	NM_016422	Homo sapiens ring finger protein 141 (RNF141), mRNA [NM_016422]	NM_016422
A_23_P367628	3.09E-05	NM_014802	NM_014802	Homo sapiens KIAA0528 gene product (KIAA0528), mRNA [NM_014802]	NM_014802
A_23_P124905	3.10E-05	NM_002522	NM_002522	Homo sapiens neuronal pentraxin I (NPTX1), mRNA [NM_002522]	NM_002522
A_23_P8241	3.10E-05	NM_012177	NM_012177	Homo sapiens F-box protein 5 (FBXO5), mRNA [NM_012177]	NM_012177
A_24_P376556	3.10E-05	NM_018947	NM_018947	Homo sapiens cytochrome c, somatic (CYCS), nuclear gene encoding mitochondrial protein, mRNA [NM_018947]	NM_018947
A_24_P166807	3.11E-05	NM_005079	NM_005079	Homo sapiens tumor protein D52 (TPD52), transcript variant 3, mRNA [NM_005079]	NM_005079
A_23_P500734	3.11E-05	NM_015100	NM_015100	Homo sapiens pogo transposable element with ZNF domain (POGZ), transcript variant 1, mRNA [NM_015100]	NM_015100
A_23_P62659	3.11E-05	NM_000310	NM_000310	Homo sapiens palmitoyl-protein thioesterase 1 (ceroid-lipofuscinosis, neuronal 1, infantile) (PPT1), mRNA [NM_000310]	NM_000310
A_23_P83298	3.12E-05	NM_016307	NM_016307	Homo sapiens paired related homeobox 2 (PRRX2), mRNA [NM_016307]	NM_016307
A_24_P352445	3.13E-05	NM_172178	NM_172178	Homo sapiens mitochondrial ribosomal protein L42 (MRPL42), nuclear gene encoding mitochondrial protein, transcript variant 3, mRNA [NM_172178]	NM_172178
A_23_P113462	3.14E-05	NM_017641	NM_017641	Homo sapiens kinesin family member 21A (KIF21A), mRNA [NM_017641]	NM_017641
A_23_P301530	3.14E-05	NM_020987	NM_020987	Homo sapiens ankyrin 3, node of Ranvier (ankyrin G) (ANK3), transcript variant 1, mRNA [NM_020987]	NM_020987
A_23_P145824	3.15E-05	AK000075	AK000075	Homo sapiens cDNA FLJ20068 fis, clone COL01755. [AK000075]	XM_371933
A_23_P65699	3.15E-05	NM_025137	NM_025137	Homo sapiens hypothetical protein FLJ21439 (FLJ21439), mRNA [NM_025137]	NM_025137
A_24_P141995	3.15E-05	NM_032632	NM_032632	Homo sapiens poly(A) polymerase alpha (PAPOLA), mRNA [NM_032632]	NM_032632
A_23_P356598	3.16E-05	NM_018057	NM_018057	Homo sapiens solute carrier family 6, member 15 (SLC6A15), transcript variant 2, mRNA [NM_018057]	NM_018057
A_23_P147786	3.16E-05	NM_014677	NM_014677	Homo sapiens regulating synaptic membrane exocytosis 2 (RIMS2), mRNA [NM_014677]	NM_014677
A_23_P133315	3.16E-05	BC035875	BC035875	Homo sapiens zinc finger protein 131 (clone pHZ-10), mRNA (cDNA clone MGC:46628 IMAGE:5519811), complete cds. [BC035875]	
A_23_P401	3.16E-05	NM_016343	NM_016343	Homo sapiens centromere protein F, 350/400ka (mitosin) (CENPF), mRNA [NM_016343]	NM_016343
A_23_P203283	3.17E-05	AK125902	AK125902	Homo sapiens cDNA FLJ43914 fis, clone TESTI4011161. [AK125902]	
A_23_P162702	3.17E-05	AB014514	AB014514	Homo sapiens mRNA for KIAA0614 protein, partial cds. [AB014514]	
A_23_P253464	3.17E-05	NM_139076	NM_139076	Homo sapiens hypothetical protein FLJ13614 (FLJ13614), mRNA [NM_139076]	NM_139076
A_24_P468810	3.17E-05	AK056550	AK056550	Homo sapiens cDNA FLJ31988 fis, clone NT2RP7008863. [AK056550]	
A_23_P115824	3.17E-05	NM_012207	NM_012207	Homo sapiens heterogeneous nuclear ribonucleoprotein H3 (2H9) (HNRPH3), transcript variant 2H9, mRNA [NM_012207]	NM_012207
A_23_P120316	3.17E-05	NM_006636	NM_006636	Homo sapiens methylenetetrahydrofolate dehydrogenase (NADP+ dependent) 2, methenyltetrahydrofolate cyclohydrolase (MTHFD2), nuclear gene encoding mitochondrial protein, mRNA [NM_006636]	NM_006636
A_23_P88095	3.18E-05	NM_014832	NM_014832	Homo sapiens TBC1 domain family, member 4 (TBC1D4), mRNA [NM_014832]	NM_014832
A_24_P19993	3.18E-05	NM_014827	NM_014827	Homo sapiens zinc finger CCCH-type containing 11A (ZC3H11A), mRNA [NM_014827]	NM_014827
A_23_P434268	3.19E-05	AB029040	AB029040	Homo sapiens mRNA for KIAA1117 protein, partial cds. [AB029040]	
A_24_P46808	3.19E-05	CR590302	CR590302	full-length cDNA clone CS0DF019YL13 of Fetal brain of Homo sapiens (human). [CR590302]	
A_23_P70047	3.20E-05	NM_199189	NM_199189	Homo sapiens matrin 3 (MATR3), transcript variant 1, mRNA [NM_199189]	NM_199189
A_24_P252705	3.21E-05	NM_004412	NM_004412	Homo sapiens DNA (cytosine-5-)-methyltransferase 2 (DNMT2), transcript variant a, mRNA [NM_004412]	NM_004412
A_23_P138139	3.21E-05	NM_145243	NM_145243	Homo sapiens OMA1 homolog, zinc metallopeptidase (S. cerevisiae) (OMA1), mRNA [NM_145243]	NM_145243
A_24_P722216	3.22E-05	NM_018706	NM_018706	Homo sapiens dehydrogenase E1 and transketolase domain containing 1 (DHTKD1), mRNA [NM_018706]	NM_018706
A_24_P302584	3.22E-05	NM_003108	NM_003108	Homo sapiens SRY (sex determining region Y)-box 11 (SOX11), mRNA [NM_003108]	NM_003108
A_32_P61757	3.22E-05	NM_002834	NM_002834	Homo sapiens protein tyrosine phosphatase, non-receptor type 11 (Noonan syndrome 1) (PTPN11), mRNA [NM_002834]	NM_002834
A_32_P10396	3.23E-05	NM_014991	NM_014991	Homo sapiens WD repeat and FYVE domain containing 3 (WDFY3), transcript variant 1, mRNA [NM_014991]	NM_014991
A_23_P103361	3.23E-05	NM_005356	NM_005356	Homo sapiens lymphocyte-specific protein tyrosine kinase (LCK), mRNA [NM_005356]	NM_005356
A_23_P75786	3.24E-05	NM_016582	NM_016582	Homo sapiens solute carrier family 15, member 3 (SLC15A3), mRNA [NM_016582]	NM_016582
A_32_P126375	3.25E-05	NM_198270	NM_198270	Homo sapiens Nance-Horan syndrome (congenital cataracts and dental anomalies) (NHS), mRNA [NM_198270]	NM_198270

A_23_P89710	3.25E-05	NM_018069	NM_018069	Homo sapiens centrosomal protein 192 kDa (Cep192), transcript variant 2, mRNA [NM_018069]	NM_018069
A_24_P110564	3.25E-05	NM_006985	NM_006985	Homo sapiens nuclear pore complex interacting protein (NPIP), mRNA [NM_006985]	NM_006985
A_24_P934546	3.26E-05	NM_003013	NM_003013	Homo sapiens secreted frizzled-related protein 2 (SFRP2), mRNA [NM_003013]	NM_003013
A_23_P106633	3.26E-05	NM_018380	NM_018380	Homo sapiens DEAD (Asp-Glu-Ala-Asp) box polypeptide 28 (DDX28), nuclear gene encoding mitochondrial protein, mRNA [NM_018380]	NM_018380
A_23_P39517	3.27E-05	NM_018079	NM_018079	Homo sapiens hypothetical protein FLJ10379 (FLJ10379), mRNA [NM_018079]	NM_018079
A_23_P124855	3.27E-05	NM_032226	NM_032226	Homo sapiens zinc finger, CCHC domain containing 7 (ZCCHC7), mRNA [NM_032226]	NM_032226
A_24_P271149	3.29E-05	CR592222	CR592222	full-length cDNA clone CS0DB003YO14 of Neuroblastoma Cot 10-normalized of Homo sapiens (human). [CR592222]	
A_23_P314191	3.29E-05	NM_015336	NM_015336	Homo sapiens zinc finger, DHHC-type containing 17 (ZDHHC17), mRNA [NM_015336]	NM_015336
A_32_P222857	3.29E-05	NM_002139	NM_002139	Homo sapiens RNA binding motif protein, X-linked (RBMX), mRNA [NM_002139]	NM_002139
A_23_P104676	3.29E-05	NM_005877	NM_005877	Homo sapiens splicing factor 3a, subunit 1, 120kDa (SF3A1), transcript variant 1, mRNA [NM_005877]	NM_005877
A_32_P505730	3.30E-05	BC017967	BC017967	Homo sapiens major histocompatibility complex, class II, DP beta 2 (pseudogene), mRNA (cDNA clone MGC:24119 IMAGE:4663904), complete cds. [BC017967]	
A_24_P209171	3.31E-05	NM_031469	NM_031469	Homo sapiens SH3 domain binding glutamic acid-rich protein like 2 (SH3BGRL2), mRNA [NM_031469]	NM_031469
A_23_P4551	3.31E-05	NM_015559	NM_015559	Homo sapiens SET binding protein 1 (SETBP1), mRNA [NM_015559]	NM_015559
A_32_P177685	3.32E-05	AA665072	AA665072	AA665072 nu76b01.s1 NCI_CGAP_Alv1 Homo sapiens cDNA clone IMAGE:1216585, mRNA sequence [AA665072]	
A_23_P153197	3.32E-05	NM_170695	NM_170695	Homo sapiens TGFB-induced factor (TALE family homeobox) (TGIF), transcript variant 1, mRNA [NM_170695]	NM_170695
A_23_P89327	3.33E-05	NM_020652	NM_020652	Homo sapiens zinc finger protein 286 (ZNF286), mRNA [NM_020652]	NM_020652
A_23_P121441	3.33E-05	NM_014893	NM_014893	Homo sapiens neuroligin 4, Y-linked (NLGN4Y), mRNA [NM_014893]	NM_014893
A_32_P116840	3.33E-05	NM_203356	NM_203356	Homo sapiens CTAGE family, member 5 (CTAGE5), transcript variant 4, mRNA [NM_203356]	NM_203356
A_23_P250735	3.33E-05	NM_175709	NM_175709	Homo sapiens chromobox homolog 7 (CBX7), mRNA [NM_175709]	NM_175709
A_24_P54253	3.33E-05	NM_152268	NM_152268	Homo sapiens prolyl-tRNA synthetase (mitochondrial)(putative) (PARS2), mRNA [NM_152268]	NM_152268
A_23_P167276	3.33E-05	NM_177453	NM_177453	Homo sapiens progestin and adipoQ receptor family member III (PAQR3), mRNA [NM_177453]	NM_177453
A_23_P88194	3.33E-05	NM_003616	NM_003616	Homo sapiens survival of motor neuron protein interacting protein 1 (SIP1), transcript variant alpha, mRNA [NM_003616]	NM_003616
A_32_P524014	3.36E-05	AK023675	AK023675	Homo sapiens cDNA FLJ13613 fis, clone PLACE1010856. [AK023675]	
A_23_P309865	3.38E-05	NM_152695	NM_152695	Homo sapiens zinc finger protein 449 (ZNF449), mRNA [NM_152695]	NM_152695
A_32_P55241	3.38E-05	NM_001007538	NM_001007538	Homo sapiens transmembrane protein 46 (TMEM46), mRNA [NM_001007538]	NM_001007538
A_23_P161197	3.38E-05	NM_018144	NM_018144	Homo sapiens Sec61 alpha 2 subunit (S. cerevisiae) (SEC61A2), mRNA [NM_018144]	NM_018144
A_24_P261005	3.38E-05	NM_138436	NM_138436	Homo sapiens chromosome 8 open reading frame 40 (C8orf40), mRNA [NM_138436]	NM_138436
A_23_P93690	3.38E-05	NM_182776	NM_182776	Homo sapiens MCM7 minichromosome maintenance deficient 7 (S. cerevisiae) (MCM7), transcript variant 2, mRNA [NM_182776]	NM_182776
A_23_P391228	3.39E-05	NM_152496	NM_152496	Homo sapiens mannosidase, endo-alpha-like (MANEAL), mRNA [NM_152496]	NM_152496
A_23_P119943	3.39E-05	NM_000597	NM_000597	Homo sapiens insulin-like growth factor binding protein 2, 36kDa (IGFBP2), mRNA [NM_000597]	NM_000597
A_23_P141035	3.40E-05	NM_005769	NM_005769	Homo sapiens carbohydrate (N-acetylglucosamine 6-O) sulfotransferase 4 (CHST4), mRNA [NM_005769]	NM_005769
A_23_P33984	3.40E-05	NM_020665	NM_020665	Homo sapiens transmembrane protein 27 (TMEM27), mRNA [NM_020665]	NM_020665
A_23_P396981	3.41E-05	NM_001012506	NM_001012506	Homo sapiens hypothetical protein LOC285331 (LOC285331), mRNA [NM_001012506]	NM_001012506
A_23_P397347	3.43E-05	NM_153255	NM_153255	Homo sapiens minichromosome maintenance deficient domain containing 1 (MCMDC1), mRNA [NM_153255]	NM_153255
A_23_P254442	3.43E-05	NM_001004302	NM_001004302	Homo sapiens hypothetical protein LOC155060 (LOC155060), mRNA [NM_001004302]	NM_001004302
A_23_P56380	3.43E-05	NM_018471	NM_018471	Homo sapiens likely ortholog of mouse immediate early response, erythropoietin 4 (LEREPO4), mRNA [NM_018471]	NM_018471
A_32_P128586	3.44E-05	BI086245	BI086245	602849648F1 NIH_MGC_10 Homo sapiens cDNA clone IMAGE:4991459 5', mRNA sequence [BI086245]	
A_24_P42330	3.44E-05	AK090827	AK090827	Homo sapiens cDNA FLJ33508 fis, clone BRAMY2005094. [AK090827]	
A_23_P120048	3.44E-05	NM_013450	NM_013450	Homo sapiens bromodomain adjacent to zinc finger domain, 2B (BAZ2B), mRNA [NM_013450]	NM_013450
A_23_P99285	3.44E-05	NM_006143	NM_006143	Homo sapiens G protein-coupled receptor 19 (GPR19), mRNA [NM_006143]	NM_006143
A_23_P5611	3.44E-05	NM_018151	NM_018151	Homo sapiens RAP1 interacting factor homolog (yeast) (RIF1), mRNA [NM_018151]	NM_018151
A_23_P347198	3.44E-05	NM_003111	NM_003111	Homo sapiens Sp3 transcription factor (SP3), transcript variant 1, mRNA [NM_003111]	NM_003111
A_32_P174151	3.44E-05	BC036263	BC036263	Homo sapiens KIAA0220-like protein, mRNA (cDNA clone MGC:9515 IMAGE:3903371), complete cds. [BC036263]	XM_496136
A_32_P144999	3.45E-05	XM_374169	XM_374169	PREDICTED: Homo sapiens hypothetical LOC389393 (LOC389393), mRNA [XM_374169]	XM_374169

A_24_P34476	3.45E-05	NM_018151	NM_018151	Homo sapiens RAP1 interacting factor homolog (yeast) (RIF1), mRNA [NM_018151]	NM_018151
A_23_P366468	3.45E-05	ENST00000246228			
A_32_P118372	3.46E-05	NM_015693	NM_015693	Homo sapiens PDZ domain containing 6 (PDZK6), mRNA [NM_015693]	NM_015693
A_24_P374634	3.46E-05	AK002152	AK002152	Homo sapiens cDNA FLJ11290 fis, clone PLACE1009622, weakly similar to MATERNAL EFFECT PROTEIN STAUFEN. [AK002152]	
A_23_P256231	3.48E-05	NM_032145	NM_032145	Homo sapiens F-box protein 30 (FBXO30), mRNA [NM_032145]	NM_032145
A_24_P396720	3.48E-05	NM_002709	NM_002709	Homo sapiens protein phosphatase 1, catalytic subunit, beta isoform (PPP1CB), transcript variant 1, mRNA [NM_002709]	NM_002709
A_23_P109636	3.48E-05	NM_015541	NM_015541	Homo sapiens leucine-rich repeats and immunoglobulin-like domains 1 (LRIG1), mRNA [NM_015541]	NM_015541
A_23_P410965	3.50E-05	NM_020888	NM_020888	Homo sapiens KIAA1522 (KIAA1522), mRNA [NM_020888]	NM_020888
A_23_P114783	3.50E-05	NM_001618	NM_001618	Homo sapiens poly (ADP-ribose) polymerase family, member 1 (PARP1), mRNA [NM_001618]	NM_001618
A_24_P215475	3.51E-05	NM_015394	NM_015394	Homo sapiens zinc finger protein 10 (ZNF10), mRNA [NM_015394]	NM_015394
A_23_P63829	3.51E-05	NM_016299	NM_016299	Homo sapiens heat shock 70kDa protein 14 (HSPA14), mRNA [NM_016299]	NM_016299
A_23_P144531	3.51E-05	AK026323	AK026323	Homo sapiens cDNA: FLJ22670 fis, clone HSI08684. [AK026323]	
A_32_P5276	3.52E-05	AB073386	AB073386	Homo sapiens infant liver cDNA, clone:HMFN1864, full insert sequence. [AB073386]	
A_32_P187817	3.54E-05	AW967501	AW967501	EST379576 MAGE resequences, MAGJ Homo sapiens cDNA, mRNA sequence [AW967501]	
A_24_P78540	3.54E-05	NM_003297	NM_003297	Homo sapiens nuclear receptor subfamily 2, group C, member 1 (NR2C1), mRNA [NM_003297]	NM_003297
A_32_P32406	3.54E-05	X15675	X15675	Human pTR7 mRNA for repetitive sequence. [X15675]	
A_23_P53856	3.54E-05	NM_014887	NM_014887	Homo sapiens phosphonoformate immuno-associated protein 5 (PFAAP5), mRNA [NM_014887]	NM_014887
A_23_P47282	3.55E-05	NM_021978	NM_021978	Homo sapiens suppression of tumorigenicity 14 (colon carcinoma, matriptase, epithin) (ST14), mRNA [NM_021978]	NM_021978
A_32_P89049	3.55E-05	THC2376431		Q7RZ79 (Q7RZ79) Predicted protein, partial (3%) [THC2376431]	
A_24_P683013	3.56E-05	BM696546	BM696546	UI-E-DW0-agk-i-01-0-UI.r1 UI-E-DW0 Homo sapiens cDNA clone UI-E-DW0-agk-i-01-0-UI 5', mRNA sequence [BM696546]	
A_23_P99762	3.56E-05	NM_021239	NM_021239	Homo sapiens RNA binding motif protein 25 (RBM25), mRNA [NM_021239]	NM_021239
A_23_P212284	3.56E-05	NM_015426	NM_015426	Homo sapiens WD repeat domain 51A (WDR51A), mRNA [NM_015426]	NM_015426
A_24_P32920	3.56E-05	CR597240	CR597240	full-length cDNA clone CS0DI085YF14 of Placenta Cot 25-normalized of Homo sapiens (human). [CR597240]	
A_24_P16124	3.56E-05	NR_001590	NR_001590	Homo sapiens interferon induced transmembrane protein 4 pseudogene (IFITM4P) on chromosome 6 [NR_001590]	NR_001590
A_32_P47754	3.57E-05	BC060766	BC060766	Homo sapiens solute carrier family 2 (facilitated glucose transporter), member 14, mRNA (cDNA clone MGC:71510 IMAGE:5297510), complete cds. [BC060766]	
A_32_P147969	3.58E-05	AL080232	AL080232	Homo sapiens mRNA; cDNA DKFZp586A061 (from clone DKFZp586A061) [AL080232]	
A_23_P11507	3.58E-05	NM_015534	NM_015534	Homo sapiens zinc finger, ZZ-type containing 3 (ZZZ3), mRNA [NM_015534]	NM_015534
A_23_P27279	3.58E-05	NM_015277	NM_015277	Homo sapiens neural precursor cell expressed, developmentally down-regulated 4-like (NEDD4L), mRNA [NM_015277]	NM_015277
A_23_P42848	3.59E-05	NM_194455	NM_194455	Homo sapiens KRIT1, ankyrin repeat containing (KRIT1), transcript variant 4, mRNA [NM_194455]	NM_194455
A_24_P20292	3.59E-05	CR626252	CR626252	full-length cDNA clone CS0DD001YO10 of Neuroblastoma Cot 50-normalized of Homo sapiens (human). [CR626252]	
A_23_P164468	3.59E-05	NM_014913	NM_014913	Homo sapiens KIAA0863 protein (KIAA0863), mRNA [NM_014913]	NM_014913
A_23_P300150	3.59E-05	NM_172387	NM_172387	Homo sapiens nuclear factor of activated T-cells, cytoplasmic, calcineurin-dependent 1 (NFATC1), transcript variant 3, mRNA [NM_172387]	NM_172387
A_23_P336670	3.59E-05	NM_015412	NM_015412	Homo sapiens chromosome 3 open reading frame 17 (C3orf17), transcript variant 1, mRNA [NM_015412]	NM_015412
A_23_P356216	3.60E-05	BC026965	BC026965	Homo sapiens cDNA clone MGC:34901 IMAGE:5103002, complete cds. [BC026965]	
A_32_P93852	3.60E-05	NM_138369	NM_138369	Homo sapiens family with sequence similarity 44, member B (FAM44B), mRNA [NM_138369]	NM_138369
A_23_P26094	3.60E-05	NM_024755	NM_024755	Homo sapiens modulator of estrogen induced transcription (FLJ13213), transcript variant 1, mRNA [NM_024755]	NM_024755
A_23_P115902	3.61E-05	A_23_P115902			
A_23_P112652	3.61E-05	NM_015442	NM_015442	Homo sapiens CCR4-NOT transcription complex, subunit 10 (CNOT10), mRNA [NM_015442]	NM_015442
A_23_P71591	3.61E-05	NM_017948	NM_017948	Homo sapiens nucleolar protein 8 (NOL8), mRNA [NM_017948]	NM_017948
A_32_P48043	3.62E-05	AK026980	AK026980	Homo sapiens cDNA: FLJ23327 fis, clone HEP12630, highly similar to HSZNF37 Homo sapiens ZNF37A mRNA for zinc finger protein. [AK026980]	
A_23_P349615	3.62E-05	ENST00000361262		Homo sapiens mRNA for KIAA0594 protein, partial cds. [AB011166]	
A_32_P77502	3.62E-05	S63912	S63912	D10S102=FBRNP [human, fetal brain, mRNA, 3043 nt]. [S63912]	XM_370728
A_23_P48416	3.63E-05	NM_014953	NM_014953	Homo sapiens KIAA1008 (KIAA1008), mRNA [NM_014953]	NM_014953
A_23_P127220	3.64E-05	NM_021800	NM_021800	Homo sapiens DnaJ (Hsp40) homolog, subfamily C, member 12 (DNAJC12), transcript variant 1, mRNA [NM_021800]	NM_021800

A_32_P30874	3.64E-05	AJ420543	AJ420543	Homo sapiens mRNA full length insert cDNA clone EUROIMAGE 1090207. [AJ420543]	
A_23_P78458	3.65E-05	NM_021632	NM_021632	Homo sapiens zinc finger protein 350 (ZNF350), mRNA [NM_021632]	NM_021632
A_23_P160618	3.66E-05	NM_003975	NM_003975	Homo sapiens SH2 domain protein 2A (SH2D2A), mRNA [NM_003975]	NM_003975
A_23_P430181	3.67E-05	NM_024784	NM_024784	Homo sapiens zinc finger and BTB domain containing 3 (ZBTB3), mRNA [NM_024784]	NM_024784
A_32_P174908	3.67E-05	NM_015045	NM_015045	Homo sapiens KIAA0261 (KIAA0261), mRNA [NM_015045]	NM_015045
A_24_P102053	3.68E-05	NM_002538	NM_002538	Homo sapiens occludin (OCLN), mRNA [NM_002538]	NM_002538
A_23_P259090	3.69E-05	NM_031438	NM_031438	Homo sapiens nudix (nucleoside diphosphate linked moiety X)-type motif 12 (NUDT12), mRNA [NM_031438]	NM_031438
A_23_P70168	3.69E-05	NM_152295	NM_152295	Homo sapiens threonyl-tRNA synthetase (TARS), mRNA [NM_152295]	NM_152295
A_23_P44505	3.71E-05	NM_003597	NM_003597	Homo sapiens Kruppel-like factor 11 (KLF11), mRNA [NM_003597]	NM_003597
A_32_P115947	3.72E-05	BC040651	BC040651	Homo sapiens isopentenyl-diphosphate delta isomerase 2, mRNA (cDNA clone IMAGE:4795541), partial cds. [BC040651]	
A_23_P78158	3.72E-05	NM_004703	NM_004703	Homo sapiens rabaptin, RAB GTPase binding effector protein 1 (RABEP1), mRNA [NM_004703]	NM_004703
A_23_P50456	3.72E-05	NM_002691	NM_002691	Homo sapiens polymerase (DNA directed), delta 1, catalytic subunit 125kDa (POLD1), mRNA [NM_002691]	NM_002691
A_23_P255952	3.73E-05	NM_004999	NM_004999	Homo sapiens myosin VI (MYO6), mRNA [NM_004999]	NM_004999
A_32_P83326	3.74E-05	AK130705	AK130705	Homo sapiens cDNA FLJ27195 fis, clone SYN02786. [AK130705]	
A_23_P325501	3.74E-05	NM_015358	NM_015358	Homo sapiens MORC family CW-type zinc finger 3 (MORC3), mRNA [NM_015358]	NM_015358
A_23_P203023	3.74E-05	NM_002906	NM_002906	Homo sapiens radixin (RDX), mRNA [NM_002906]	NM_002906
A_23_P160518	3.78E-05	NM_025188	NM_025188	Homo sapiens tripartite motif-containing 45 (TRIM45), mRNA [NM_025188]	NM_025188
A_23_P120667	3.79E-05	NM_021219	NM_021219	Homo sapiens junctional adhesion molecule 2 (JAM2), mRNA [NM_021219]	NM_021219
A_32_P190294	3.79E-05	ENST00000327721		PREDICTED: Homo sapiens hypothetical protein MGC22265 (MGC22265), mRNA [XM_380175]	XM_380175
A_23_P108394	3.80E-05	THC2352042		Q8IUM9 (Q8IUM9) Acyl-CoA synthetase long-chain family member 3, complete [THC2234200]	
A_23_P25605	3.81E-05	NM_032138	NM_032138	Homo sapiens kelch repeat and BTB (POZ) domain containing 7 (KBTBD7), mRNA [NM_032138]	NM_032138
A_24_P310256	3.82E-05	NM_139284	NM_139284	Homo sapiens leucine-rich repeat LGI family, member 4 (LGI4), mRNA [NM_139284]	NM_139284
A_23_P104138	3.82E-05	BC007286	BC007286	Homo sapiens hypothetical protein MGC15634, mRNA (cDNA clone MGC:15634 IMAGE:3344302), complete cds. [BC007286]	
A_24_P246573	3.82E-05	NM_001018000	NM_001018000	Homo sapiens kazrin (KIAA1026), transcript variant B, mRNA [NM_001018000]	NM_001018000
A_24_P50890	3.82E-05	AK056455	AK056455	Homo sapiens cDNA FLJ31893 fis, clone NT2RP7003632, weakly similar to Microfilarial sheath protein. [AK056455]	
A_32_P128701	3.83E-05	AF085848	AF085848	Homo sapiens full length insert cDNA clone YI48C03. [AF085848]	
A_24_P222997	3.85E-05	NM_032143	NM_032143	Homo sapiens zinc finger, RAN-binding domain containing 3 (ZRANB3), mRNA [NM_032143]	NM_032143
A_32_P875758	3.85E-05	AB046850	AB046850	Homo sapiens mRNA for KIAA1630 protein, partial cds. [AB046850]	
A_23_P3237	3.86E-05	NM_025165	NM_025165	Homo sapiens elongation factor RNA polymerase II-like 3 (ELL3), mRNA [NM_025165]	NM_025165
A_23_P166400	3.86E-05	NM_001007279	NM_001007279	Homo sapiens RAS-related on chromosome 22 (RRP22), transcript variant 2, mRNA [NM_001007279]	NM_001007279
A_23_P372888	3.86E-05	NM_006918	NM_006918	Homo sapiens sterol-C5-desaturase (ERG3 delta-5-desaturase homolog, fungal)-like (SC5DL), transcript variant 1, mRNA [NM_006918]	NM_006918
A_23_P68087	3.86E-05	NM_004044	NM_004044	Homo sapiens 5-aminoimidazole-4-carboxamide ribonucleotide formyltransferase/IMP cyclohydrolase (ATIC), mRNA [NM_004044]	NM_004044
A_32_P44210	3.87E-05	BC037328	BC037328	Homo sapiens cDNA clone IMAGE:5263455, partial cds. [BC037328]	
A_23_P254165	3.88E-05	NM_021785	NM_021785	Homo sapiens retinoic acid induced 2 (RAI2), mRNA [NM_021785]	NM_021785
A_23_P35546	3.88E-05	THC2344956			
A_23_P99260	3.88E-05	NM_006838	NM_006838	Homo sapiens methionyl aminopeptidase 2 (METAP2), mRNA [NM_006838]	NM_006838
A_23_P40295	3.89E-05	NM_012261	NM_012261	Homo sapiens chromosome 20 open reading frame 103 (C20orf103), mRNA [NM_012261]	NM_012261
A_23_P129258	3.89E-05	A_23_P129258			
A_24_P733308	3.91E-05	THC2309960		Q7ZX66 (Q7ZX66) RNPC7 protein (Fragment), partial (9%) [THC2309960]	
A_24_P373286	3.92E-05	NM_014711	NM_014711	Homo sapiens CP110 protein (CP110), mRNA [NM_014711]	NM_014711
A_23_P61854	3.92E-05	ENST00000358746		Homo sapiens mRNA for KIAA0372 gene, partial cds. [AB002370]	
A_23_P80626	3.92E-05	NM_207351	NM_207351	Homo sapiens hypothetical protein FLJ33674 (FLJ33674), mRNA [NM_207351]	NM_207351
A_32_P227027	3.94E-05	THC2288599			
A_32_P23872	3.95E-05	A_32_P23872			

A_24_P585004	3.95E-05	A_24_P585004			
A_23_P72014	3.95E-05	A_23_P72014			
A_23_P73297	3.96E-05	NM_004742	NM_004742	Homo sapiens membrane associated guanylate kinase, WW and PDZ domain containing 1 (MAGI1), mRNA [NM_004742]	NM_004742
A_24_P46953	3.96E-05	NM_013257	NM_013257	Homo sapiens serum/glucocorticoid regulated kinase-like (SGKL), transcript variant 1, mRNA [NM_013257]	NM_013257
A_23_P401472	3.97E-05	NM_000740	NM_000740	Homo sapiens cholinergic receptor, muscarinic 3 (CHRM3), mRNA [NM_000740]	NM_000740
A_23_P78543	3.97E-05	NM_005498	NM_005498	Homo sapiens adaptor-related protein complex 1, mu 2 subunit (AP1M2), mRNA [NM_005498]	NM_005498
A_23_P385861	3.97E-05	NM_152562	NM_152562	Homo sapiens cell division cycle associated 2 (CDCA2), mRNA [NM_152562]	NM_152562
A_23_P160214	3.98E-05	BC028374	BC028374	Homo sapiens chromosome 1 open reading frame 34, mRNA (cDNA clone IMAGE:4827153), partial cds. [BC028374]	XM_375729
A_24_P131580	3.98E-05	NM_031313	NM_031313	Homo sapiens alkaline phosphatase, placental-like 2 (ALPPL2), mRNA [NM_031313]	NM_031313
A_23_P82334	3.98E-05	NM_014251	NM_014251	Homo sapiens solute carrier family 25, member 13 (citrin) (SLC25A13), mRNA [NM_014251]	NM_014251
A_23_P156471	3.99E-05	NM_001253	NM_001253	Homo sapiens CDC5 cell division cycle 5-like (S. pombe) (CDC5L), mRNA [NM_001253]	NM_001253
A_23_P16683	3.99E-05	NM_017722	NM_017722	Homo sapiens hypothetical protein FLJ20244 (FLJ20244), mRNA [NM_017722]	NM_017722
A_23_P100341	3.99E-05	NM_014321	NM_014321	Homo sapiens origin recognition complex, subunit 6 homolog-like (yeast) (ORC6L), mRNA [NM_014321]	NM_014321
A_24_P568190	4.00E-05	NM_207331	NM_207331	Homo sapiens hypothetical protein LOC153561 (LOC153561), mRNA [NM_207331]	NM_207331
A_23_P48495	4.00E-05	NM_199206	NM_199206	Homo sapiens T-cell leukemia/lymphoma 1B (TCL1B), transcript variant 2, mRNA [NM_199206]	NM_199206
A_23_P64019	4.00E-05	NM_201278	NM_201278	Homo sapiens myotubularin related protein 2 (MTMR2), transcript variant 2, mRNA [NM_201278]	NM_201278
A_23_P33511	4.00E-05	AX721087	AX721087	Sequence 47 from Patent WO0220754. [AX721087]	
A_23_P141965	4.00E-05	NM_033417	NM_033417	Homo sapiens sarcoma antigen NY-SAR-48 (NY-SAR-48), transcript variant 1, mRNA [NM_033417]	NM_033417
A_23_P52986	4.00E-05	NM_152718	NM_152718	Homo sapiens hypothetical protein FLJ32009 (FLJ32009), mRNA [NM_152718]	NM_152718
A_23_P60506	4.00E-05	NM_017867	NM_017867	Homo sapiens hypothetical protein FLJ20534 (FLJ20534), mRNA [NM_017867]	NM_017867
A_32_P19431	4.00E-05	NM_001011724	NM_001011724	Homo sapiens heterogeneous nuclear ribonucleoprotein A1-like (LOC144983), transcript variant 1, mRNA [NM_001011724]	NM_001011724
A_23_P163546	4.01E-05	NM_005679	NM_005679	Homo sapiens TATA box binding protein (TBP)-associated factor, RNA polymerase I, C, 110kDa (TAF1C), transcript variant 1, mRNA [NM_005679]	NM_005679
A_23_P206092	4.01E-05	NM_032808	NM_032808	Homo sapiens leucine rich repeat neuronal 6A (LRRN6A), mRNA [NM_032808]	NM_032808
A_24_P20327	4.03E-05	NM_014079	NM_014079	Homo sapiens Kruppel-like factor 15 (KLF15), mRNA [NM_014079]	NM_014079
A_24_P399174	4.03E-05	NM_004703	NM_004703	Homo sapiens rabaptin, RAB GTPase binding effector protein 1 (RABEP1), mRNA [NM_004703]	NM_004703
A_23_P40821	4.03E-05	NM_032383	NM_032383	Homo sapiens Hermansky-Pudlak syndrome 3 (HPS3), mRNA [NM_032383]	NM_032383
A_23_P19182	4.03E-05	NM_016606	NM_016606	Homo sapiens chromosome 5 open reading frame 19 (C5orf19), mRNA [NM_016606]	NM_016606
A_23_P19543	4.03E-05	NM_003137	NM_003137	Homo sapiens SFRS protein kinase 1 (SRPK1), mRNA [NM_003137]	NM_003137
A_23_P317683	4.04E-05	NM_003274	NM_003274	Homo sapiens transmembrane protein 1 (TMEM1), transcript variant 1, mRNA [NM_003274]	NM_003274
A_23_P49060	4.05E-05	NM_003710	NM_003710	Homo sapiens serine protease inhibitor, Kunitz type 1 (SPINT1), transcript variant 2, mRNA [NM_003710]	NM_003710
A_23_P37942	4.05E-05	NM_021195	NM_021195	Homo sapiens claudin 6 (CLDN6), mRNA [NM_021195]	NM_021195
A_32_P3290	4.05E-05	NM_017645	NM_017645	Homo sapiens family with sequence similarity 29, member A (FAM29A), mRNA [NM_017645]	NM_017645
A_32_P118532	4.06E-05	CR625518	CR625518	full-length cDNA clone CS0DL012YA17 of B cells (Ramos cell line) Cot 25-normalized of Homo sapiens (human). [CR625518]	
A_24_P232763	4.06E-05	ENST00000242848		Homo sapiens mRNA for KIAA0853 protein, partial cds. [AB020660]	
A_23_P131074	4.08E-05	NM_016585	NM_016585	Homo sapiens Theg homolog (mouse) (THEG), transcript variant 1, mRNA [NM_016585]	NM_016585
A_24_P370042	4.08E-05	NM_022751	NM_022751	Homo sapiens family with sequence similarity 59, member A (FAM59A), mRNA [NM_022751]	NM_022751
A_23_P17345	4.08E-05	NM_005461	NM_005461	Homo sapiens v-maf musculoaponeurotic fibrosarcoma oncogene homolog B (avian) (MAFB), mRNA [NM_005461]	NM_005461
A_23_P37399	4.09E-05	NM_014216	NM_014216	Homo sapiens inositol 1,3,4-triphosphate 5/6 kinase (ITPK1), mRNA [NM_014216]	NM_014216
A_24_P408297	4.10E-05	NM_018152	NM_018152	Homo sapiens chromosome 20 open reading frame 12 (C20orf12), mRNA [NM_018152]	NM_018152
A_32_P132276	4.10E-05	BE091362	BE091362	BE091362 PM1-BT0728-220300-001-f09 BT0728 Homo sapiens cDNA, mRNA sequence [BE091362]	
A_32_P4985	4.12E-05	NM_015215	NM_015215	Homo sapiens calmodulin binding transcription activator 1 (CAMTA1), mRNA [NM_015215]	NM_015215
A_24_P902728	4.12E-05	NM_203364	NM_203364	Homo sapiens membrane component, chromosome 11, surface marker 1 (M11S1), transcript variant 2, mRNA [NM_203364]	NM_203364
A_32_P109794	4.12E-05	ENST00000360633		Homo sapiens cDNA FLJ42934 fis, clone BRSSN2014112. [AK124924]	
A_23_P44768	4.14E-05	NM_013254	NM_013254	Homo sapiens TANK-binding kinase 1 (TBK1), mRNA [NM_013254]	NM_013254

A_24_P544661	4.15E-05	BC068044	BC068044	Homo sapiens cDNA clone IMAGE:6380649, containing frame-shift errors. [BC068044]	
A_23_P367610	4.16E-05	NM_178123	NM_178123	Homo sapiens SEC14 and spectrin domains 1 (SESTD1), mRNA [NM_178123]	NM_178123
A_23_P152055	4.16E-05	NM_024580	NM_024580	Homo sapiens elongation factor Tu GTP binding domain containing 1 (EFTUD1), mRNA [NM_024580]	NM_024580
A_23_P203013	4.17E-05	NM_002519	NM_002519	Homo sapiens nuclear protein, ataxia-telangiectasia locus (NPAT), mRNA [NM_002519]	NM_002519
A_24_P7157	4.17E-05	NM_020734	NM_020734	Homo sapiens family with sequence similarity 80, member B (FAM80B), mRNA [NM_020734]	NM_020734
A_23_P157527	4.18E-05	NM_033402	NM_033402	Homo sapiens KIAA1764 protein (KIAA1764), mRNA [NM_033402]	NM_033402
A_23_P127279	4.18E-05	NM_019054	NM_019054	Homo sapiens family with sequence similarity 35, member A (FAM35A), mRNA [NM_019054]	NM_019054
A_23_P46539	4.18E-05	NM_032636	NM_032636	Homo sapiens differential display and activated by p53 (DDA3), transcript variant 1, mRNA [NM_032636]	NM_032636
A_24_P192586	4.18E-05	ENST00000337863		full-length cDNA clone CS0DF005YF01 of Fetal brain of Homo sapiens (human). [CR596783]	
A_24_P472055	4.19E-05	AK027134	AK027134	Homo sapiens cDNA: FLJ23481 fis, clone KAIA03003. [AK027134]	
A_23_P412980	4.19E-05	NM_015132	NM_015132	Homo sapiens sorting nexin 13 (SNX13), mRNA [NM_015132]	NM_015132
A_32_P201958	4.19E-05	AK090739	AK090739	Homo sapiens cDNA FLJ33420 fis, clone BRACE2020028. [AK090739]	
A_23_P64669	4.19E-05	NM_017612	NM_017612	Homo sapiens zinc finger, CCHC domain containing 8 (ZCCHC8), mRNA [NM_017612]	NM_017612
A_24_P914649	4.20E-05	BX108121	BX108121	BX108121 Soares_testis_NHT Homo sapiens cDNA clone IMAGp998B051795, mRNA sequence [BX108121]	
A_32_P61439	4.20E-05	THC2416008		U74612 forkhead box M1A {Homo sapiens;} , partial (3%) [THC2416008]	
A_32_P167278	4.20E-05	BC070154	BC070154	Homo sapiens high-mobility group nucleosome binding domain 1, mRNA (cDNA clone MGC:88134 IMAGE:6495327), complete cds. [BC070154]	XM_371117
A_23_P434289	4.21E-05	NM_080865	NM_080865	Homo sapiens G protein-coupled receptor 62 (GPR62), mRNA [NM_080865]	NM_080865
A_23_P29985	4.21E-05	AK022953	AK022953	Homo sapiens cDNA FLJ12891 fis, clone NT2RP2004142. [AK022953]	
A_24_P489480	4.21E-05	BC062438	BC062438	Homo sapiens cDNA clone IMAGE:6494968, containing frame-shift errors. [BC062438]	
A_23_P76951	4.22E-05	NM_030755	NM_030755	Homo sapiens thioredoxin domain containing (TXNDC), mRNA [NM_030755]	NM_030755
A_24_P68019	4.23E-05	NM_138347	NM_138347	Homo sapiens zinc finger protein 551 (ZNF551), mRNA [NM_138347]	NM_138347
A_24_P366107	4.24E-05	ENST00000260916		Homo sapiens, clone IMAGE:4385301, mRNA, partial cds. [BC028188]	XM_166103
A_23_P413184	4.24E-05	NM_173829	NM_173829	Homo sapiens hypothetical protein FLJ36754 (FLJ36754), mRNA [NM_173829]	NM_173829
A_23_P45726	4.24E-05	NM_005826	NM_005826	Homo sapiens heterogeneous nuclear ribonucleoprotein R (HNRPR), mRNA [NM_005826]	NM_005826
A_24_P178602	4.27E-05	NM_198457	NM_198457	Homo sapiens zinc finger protein 600 (ZNF600), mRNA [NM_198457]	NM_198457
A_23_P45560	4.27E-05	NM_000273	NM_000273	Homo sapiens G protein-coupled receptor 143 (GPR143), mRNA [NM_000273]	NM_000273
A_24_P157720	4.28E-05	BQ188033	BQ188033	BQ188033 UI-E-EJ1-aju-o-13-0-UI.r1 UI-E-EJ1 Homo sapiens cDNA clone UI-E-EJ1-aju-o-13-0-UI 5', mRNA sequence [BQ188033]	
A_23_P148969	4.33E-05	NM_017768	NM_017768	Homo sapiens leucine rich repeat containing 40 (LRRC40), mRNA [NM_017768]	NM_017768
A_23_P5551	4.33E-05	NM_005381	NM_005381	Homo sapiens nucleolin (NCL), mRNA [NM_005381]	NM_005381
A_23_P317919	4.34E-05	NM_021148	NM_021148	Homo sapiens zinc finger protein 273 (ZNF273), mRNA [NM_021148]	NM_021148
A_23_P22647	4.34E-05	NM_015686	NM_015686	Homo sapiens transmembrane protein 28 (TMEM28), mRNA [NM_015686]	NM_015686
A_23_P25638	4.35E-05	NM_024546	NM_024546	Homo sapiens chromosome 13 open reading frame 7 (C13orf7), mRNA [NM_024546]	NM_024546
A_23_P500130	4.35E-05	NM_153186	NM_153186	Homo sapiens ankyrin repeat domain 15 (ANKRD15), transcript variant 2, mRNA [NM_153186]	NM_153186
A_24_P174341	4.36E-05	NM_058241	NM_058241	Homo sapiens cyclin T2 (CCNT2), transcript variant b, mRNA [NM_058241]	NM_058241
A_23_P162378	4.37E-05	NM_016122	NM_016122	Homo sapiens NY-REN-58 antigen (NY-REN-58), mRNA [NM_016122]	NM_016122
A_24_P201064	4.37E-05	AK091994	AK091994	Homo sapiens cDNA FLJ34675 fis, clone LIVER2001608. [AK091994]	
A_23_P351215	4.38E-05	NM_005414	NM_005414	Homo sapiens SKI-like (SKIL), mRNA [NM_005414]	NM_005414
A_24_P237757	4.40E-05	AL136621	AL136621	Homo sapiens mRNA; cDNA DKFZp564B162 (from clone DKFZp564B162). [AL136621]	
A_24_P282343	4.40E-05	NM_003159	NM_003159	Homo sapiens cyclin-dependent kinase-like 5 (CDKL5), mRNA [NM_003159]	NM_003159
A_23_P67569	4.40E-05	NM_024888	NM_024888	Homo sapiens plasticity-related gene 2 (PRG2), mRNA [NM_024888]	NM_024888
A_23_P317800	4.40E-05	NM_013367	NM_013367	Homo sapiens anaphase promoting complex subunit 4 (ANAPC4), mRNA [NM_013367]	NM_013367
A_23_P212545	4.40E-05	NM_004162	NM_004162	Homo sapiens RAB5A, member RAS oncogene family (RAB5A), mRNA [NM_004162]	NM_004162
A_23_P502425	4.40E-05	NM_020409	NM_020409	Homo sapiens mitochondrial ribosomal protein L47 (MRPL47), nuclear gene encoding mitochondrial protein, transcript variant 1, mRNA [NM_020409]	NM_020409
A_24_P345131	4.41E-05	D86984	D86984	Human mRNA for KIAA0231 gene, partial cds. [D86984]	
A_24_P260443	4.41E-05	NM_003248	NM_003248	Homo sapiens thrombospondin 4 (THBS4), mRNA [NM_003248]	NM_003248

A_24_P415959	4.41E-05	NM_001969	NM_001969	Homo sapiens eukaryotic translation initiation factor 5 (EIF5), transcript variant 1, mRNA [NM_001969]	NM_001969
A_32_P191290	4.43E-05	BM045853	BM045853	603624848F1 NIH_MGC_40 Homo sapiens cDNA clone IMAGE:5451514 5', mRNA sequence [BM045853]	
A_24_P316305	4.43E-05	ENST00000156471		Homo sapiens cDNA FLJ10311 fis, clone NT2RM2000359, highly similar to Homo sapiens mRNA for KIAA0560 protein. [AK001173]	
A_32_P54186	4.44E-05	THC2343551			
A_23_P143374	4.44E-05	NM_025176	NM_025176	Homo sapiens KIAA0980 protein (KIAA0980), mRNA [NM_025176]	NM_025176
A_23_P379614	4.44E-05	NM_007280	NM_007280	Homo sapiens Opa interacting protein 5 (OIP5), mRNA [NM_007280]	NM_007280
A_24_P172768	4.46E-05	NM_004124	NM_004124	Homo sapiens glia maturation factor, beta (GMFB), mRNA [NM_004124]	NM_004124
A_32_P213637	4.49E-05	AK092942	AK092942	Homo sapiens cDNA FLJ35623 fis, clone SPLEN2010986. [AK092942]	
A_23_P400373	4.50E-05	NM_152519	NM_152519	Homo sapiens hypothetical protein FLJ23861 (FLJ23861), mRNA [NM_152519]	NM_152519
A_32_P86533	4.50E-05	AF038185	AF038185	Homo sapiens clone 23700 mRNA sequence. [AF038185]	
A_23_P302672	4.51E-05	NM_145244	NM_145244	Homo sapiens DNA-damage-inducible transcript 4-like (DDIT4L), mRNA [NM_145244]	NM_145244
A_32_P81806	4.52E-05	THC2406779			
A_23_P170399	4.53E-05	NM_021942	NM_021942	Homo sapiens FLJ12716 protein (FLJ12716), transcript variant 1, mRNA [NM_021942]	NM_021942
A_32_P183983	4.54E-05	BC037838	BC037838	Homo sapiens, clone IMAGE:4813920, mRNA. [BC037838]	
A_23_P142174	4.54E-05	NM_004497	NM_004497	Homo sapiens forkhead box A3 (FOXA3), mRNA [NM_004497]	NM_004497
A_24_P278460	4.55E-05	NM_032228	NM_032228	Homo sapiens male sterility domain containing 2 (MLSTD2), mRNA [NM_032228]	NM_032228
A_24_P351283	4.56E-05	NM_018000	NM_018000	Homo sapiens dilute suppressor (DSU), mRNA [NM_018000]	NM_018000
A_32_P310335	4.56E-05	AK056079	AK056079	Homo sapiens cDNA FLJ31517 fis, clone NT2RI2000007. [AK056079]	
A_23_P155815	4.56E-05	NM_022346	NM_022346	Homo sapiens chromosome condensation protein G (HCAP-G), mRNA [NM_022346]	NM_022346
A_23_P145777	4.57E-05	NM_002489	NM_002489	Homo sapiens NADH dehydrogenase (ubiquinone) 1 alpha subcomplex, 4, 9kDa (NDUFA4), nuclear gene encoding mitochondrial protein, mRNA [NM_002489]	NM_002489
A_32_P79763	4.58E-05	AK023134	AK023134	Homo sapiens cDNA FLJ13072 fis, clone NT2RP3001844. [AK023134]	XM_117117
A_23_P128773	4.58E-05	NM_203356	NM_203356	Homo sapiens CTAGE family, member 5 (CTAGE5), transcript variant 4, mRNA [NM_203356]	NM_203356
A_23_P101374	4.60E-05	NM_030622	NM_030622	Homo sapiens cytochrome P450, family 2, subfamily S, polypeptide 1 (CYP2S1), mRNA [NM_030622]	NM_030622
A_24_P176131	4.60E-05	NM_052950	NM_052950	Homo sapiens WD repeat and FYVE domain containing 2 (WDFY2), mRNA [NM_052950]	NM_052950
A_24_P132703	4.61E-05	ENST00000259676		full-length cDNA clone CS0DB009YI22 of Neuroblastoma Cot 10-normalized of Homo sapiens (human). [CR607939]	
A_23_P408955	4.61E-05	NM_004091	NM_004091	Homo sapiens E2F transcription factor 2 (E2F2), mRNA [NM_004091]	NM_004091
A_23_P123193	4.61E-05	NM_020445	NM_020445	Homo sapiens ARP3 actin-related protein 3 homolog B (yeast) (ACTR3B), mRNA [NM_020445]	NM_020445
A_23_P152047	4.62E-05	NM_138967	NM_138967	Homo sapiens secretory carrier membrane protein 5 (SCAMP5), mRNA [NM_138967]	NM_138967
A_24_P940921	4.63E-05	AK125448	AK125448	Homo sapiens cDNA FLJ43459 fis, clone OCBBF2035564. [AK125448]	
A_32_P121085	4.67E-05	AK097258	AK097258	Homo sapiens cDNA FLJ39939 fis, clone SPLEN2022227, moderately similar to Mus musculus adaptor protein (Dokl) mRNA. [AK097258]	
A_23_P216071	4.67E-05	A_23_P216071			
A_23_P61623	4.68E-05	AL109695	AL109695	Homo sapiens mRNA full length insert cDNA clone EUROIMAGE 39820. [AL109695]	
A_23_P259103	4.68E-05	NM_022090	NM_022090	Homo sapiens transposon-derived Buster3 transposase-like (LOC63920), mRNA [NM_022090]	NM_022090
A_23_P24244	4.69E-05	ENST00000263123		Homo sapiens cDNA FLJ20360 fis, clone HEP16677. [AK000367]	XM_374765
A_23_P70359	4.71E-05	NM_024929	NM_024929	Homo sapiens chromosome 6 open reading frame 59 (C6orf59), mRNA [NM_024929]	NM_024929
A_24_P31275	4.72E-05	NM_001678	NM_001678	Homo sapiens ATPase, Na+/K+ transporting, beta 2 polypeptide (ATP1B2), mRNA [NM_001678]	NM_001678
A_23_P59268	4.75E-05	NM_005493	NM_005493	Homo sapiens RAN binding protein 9 (RANBP9), mRNA [NM_005493]	NM_005493
A_23_P316150	4.76E-05	NM_020800	NM_020800	Homo sapiens WD repeat domain 56 (WDR56), mRNA [NM_020800]	NM_020800
A_32_P204624	4.76E-05	CR591264	CR591264	full-length cDNA clone CS0DC023YK19 of Neuroblastoma Cot 25-normalized of Homo sapiens (human). [CR591264]	XM_498662
A_32_P193792	4.78E-05	AF161353	AF161353	Homo sapiens HSPC090 mRNA, partial cds. [AF161353]	
A_23_P121637	4.78E-05	NM_003619	NM_003619	Homo sapiens protease, serine, 12 (neurotrypsin, motopsin) (PRSS12), mRNA [NM_003619]	NM_003619
A_23_P21473	4.78E-05	NM_024491	NM_024491	Homo sapiens p10-binding protein (Cep70), mRNA [NM_024491]	NM_024491
A_23_P18384	4.79E-05	NM_213654	NM_213654	Homo sapiens armadillo repeat containing 8 (ARMC8), mRNA [NM_213654]	NM_213654
A_24_P226700	4.79E-05	BX648950	BX648950	Homo sapiens mRNA; cDNA DKFZp686E1648 (from clone DKFZp686E1648). [BX648950]	
A_23_P346900	4.81E-05	NM_001005505	NM_001005505	Homo sapiens calcium channel, voltage-dependent, alpha 2/delta subunit 2 (CACNA2D2), transcript variant 1, mRNA [NM_001005505]	NM_001005505

A_23_P111267	4.82E-05	NM_031469	NM_031469	Homo sapiens SH3 domain binding glutamic acid-rich protein like 2 (SH3BGRL2), mRNA [NM_031469]	NM_031469
A_23_P97632	4.82E-05	NM_004446	NM_004446	Homo sapiens glutamyl-prolyl-tRNA synthetase (EPRS), mRNA [NM_004446]	NM_004446
A_24_P320880	4.83E-05	NM_022133	NM_022133	Homo sapiens sorting nexin 16 (SNX16), transcript variant 1, mRNA [NM_022133]	NM_022133
A_24_P273647	4.83E-05	AL833749	AL833749	Homo sapiens mRNA; cDNA DKFZp666L166 (from clone DKFZp666L166). [AL833749]	XM_085463
A_32_P151782	4.83E-05	BG033002	BG033002	602300107F1 NIH_MGC_87 Homo sapiens cDNA clone IMAGE:4401960 5', mRNA sequence [BG033002]	
A_23_P209619	4.83E-05	CR617774	CR617774	full-length cDNA clone CS0DC002YP09 of Neuroblastoma Cot 25-normalized of Homo sapiens (human). [CR617774]	
A_24_P53519	4.84E-05	NM_005483	NM_005483	Homo sapiens chromatin assembly factor 1, subunit A (p150) (CHAF1A), mRNA [NM_005483]	NM_005483
A_23_P48358	4.85E-05	NM_000282	NM_000282	Homo sapiens propionyl Coenzyme A carboxylase, alpha polypeptide (PCCA), mRNA [NM_000282]	NM_000282
A_23_P11390	4.86E-05	NM_004679	NM_004679	Homo sapiens variable charge, Y-linked (VCY), mRNA [NM_004679]	NM_004679
A_32_P185530	4.86E-05	THC2316492			
A_32_P198295	4.86E-05	THC2446045			
A_24_P942604	4.86E-05	NM_006306	NM_006306	Homo sapiens SMC1 structural maintenance of chromosomes 1-like 1 (yeast) (SMC1L1), mRNA [NM_006306]	NM_006306
A_23_P404667	4.87E-05	NM_001197	NM_001197	Homo sapiens BCL2-interacting killer (apoptosis-inducing) (BIK), mRNA [NM_001197]	NM_001197
A_23_P320897	4.91E-05	NM_019049	NM_019049	Homo sapiens hypothetical protein FLJ20054 (FLJ20054), mRNA [NM_019049]	NM_019049
A_32_P75399	4.92E-05	THC2308675		HUMSEF21A SEF2-1A protein {Homo sapiens;} , partial (5%) [THC2308675]	
A_23_P208198	4.94E-05	AK093036	AK093036	Homo sapiens cDNA FLJ35717 fis, clone TESTI2000598. [AK093036]	
A_23_P133543	4.94E-05	NM_017415	NM_017415	Homo sapiens kelch-like 3 (Drosophila) (KLHL3), mRNA [NM_017415]	NM_017415
A_24_P659122	4.94E-05	NM_001013685	NM_001013685	Homo sapiens hypothetical LOC401357 (LOC401357), mRNA [NM_001013685]	NM_001013685
A_24_P814444	4.95E-05	THC2407545			
A_24_P914817	4.95E-05	NM_013255	NM_013255	Homo sapiens muskelin 1, intracellular mediator containing kelch motifs (MKLN1), mRNA [NM_013255]	NM_013255
A_23_P143068	4.96E-05	NM_024726	NM_024726	Homo sapiens IQ motif containing with AAA domain (IQCA), mRNA [NM_024726]	NM_024726
A_24_P371303	4.97E-05	NM_015224	NM_015224	Homo sapiens retinoblastoma-associated protein 140 (RAP140), mRNA [NM_015224]	NM_015224
A_23_P391926	4.99E-05	NM_001008701	NM_001008701	Homo sapiens latrophilin 1 (LPHN1), transcript variant 1, mRNA [NM_001008701]	NM_001008701
A_23_P98022	4.99E-05	NM_012238	NM_012238	Homo sapiens sirtuin (silent mating type information regulation 2 homolog) 1 (S. cerevisiae) (SIRT1), mRNA [NM_012238]	NM_012238
A_23_P71752	4.99E-05	NM_006007	NM_006007	Homo sapiens zinc finger, A20 domain containing 2 (ZA20D2), mRNA [NM_006007]	NM_006007
A_23_P433690	5.00E-05	NM_018555	NM_018555	Homo sapiens zinc finger protein 331 (ZNF331), mRNA [NM_018555]	NM_018555
A_23_P7361	5.00E-05	NM_024090	NM_024090	Homo sapiens ELOVL family member 6, elongation of long chain fatty acids (FEN1/Elo2, SUR4/Elo3-like, yeast) (ELOVL6), mRNA [NM_024090]	NM_024090
A_23_P70069	5.01E-05	AK000420	AK000420	Homo sapiens cDNA FLJ20413 fis, clone KAT02170. [AK000420]	
A_24_P391230	5.02E-05	NM_052954	NM_052954	Homo sapiens cysteine/tyrosine-rich 1 (CYYR1), mRNA [NM_052954]	NM_052954
A_23_P132277	5.02E-05	NM_006739	NM_006739	Homo sapiens MCM5 minichromosome maintenance deficient 5, cell division cycle 46 (S. cerevisiae) (MCM5), mRNA [NM_006739]	NM_006739
A_23_P149818	5.03E-05	NM_080599	NM_080599	Homo sapiens UPF2 regulator of nonsense transcripts homolog (yeast) (UPF2), transcript variant 1, mRNA [NM_080599]	NM_080599
A_24_P938303	5.04E-05	U57365	U57365	Human GRP/bombesin receptor mRNA, partial cds. [U57365]	
A_23_P32577	5.04E-05	NM_080759	NM_080759	Homo sapiens dachshund homolog 1 (Drosophila) (DACH1), transcript variant 1, mRNA [NM_080759]	NM_080759
A_23_P37005	5.06E-05	NM_004294	NM_004294	Homo sapiens mitochondrial translational release factor 1 (MTRF1), nuclear gene encoding mitochondrial protein, mRNA [NM_004294]	NM_004294
A_23_P145114	5.07E-05	NM_001498	NM_001498	Homo sapiens glutamate-cysteine ligase, catalytic subunit (GCLC), mRNA [NM_001498]	NM_001498
A_23_P154315	5.07E-05	NM_182640	NM_182640	Homo sapiens mitochondrial ribosomal protein S9 (MRPS9), nuclear gene encoding mitochondrial protein, mRNA [NM_182640]	NM_182640
A_23_P357185	5.09E-05	NM_018397	NM_018397	Homo sapiens choline dehydrogenase (CHDH), mRNA [NM_018397]	NM_018397
A_23_P431981	5.09E-05	NM_005487	NM_005487	Homo sapiens high-mobility group protein 2-like 1 (HMG2L1), transcript variant 1, mRNA [NM_005487]	NM_005487
A_23_P217872	5.09E-05	NM_006015	NM_006015	Homo sapiens AT rich interactive domain 1A (SWI- like) (ARID1A), transcript variant 1, mRNA [NM_006015]	NM_006015
A_23_P254733	5.09E-05	NM_024629	NM_024629	Homo sapiens MLF1 interacting protein (MLF1IP), mRNA [NM_024629]	NM_024629
A_32_P1772	5.11E-05	NM_020207	NM_020207	Homo sapiens chromosome 9 open reading frame 102 (C9orf102), mRNA [NM_020207]	NM_020207
A_32_P157504	5.12E-05	AK094629	AK094629	Homo sapiens cDNA FLJ37310 fis, clone BRAMY2016706. [AK094629]	
A_32_P482979	5.12E-05	NM_203390	NM_203390	Homo sapiens similar to RIKEN cDNA 3000004N20 (LOC389677), mRNA [NM_203390]	NM_203390
A_23_P416711	5.12E-05	NM_152996	NM_152996	Homo sapiens ST6 (alpha-N-acetyl-neuraminyl-2,3-beta-galactosyl-1,3)-N-acetylgalactosaminide alpha-2,6-sialyltransferase 3 (ST6GALNAC3), mRNA [NM_152996]	NM_152996

A_23_P349267	5.14E-05	NM_012282	NM_012282	Homo sapiens KCNE1-like (KCNE1L), mRNA [NM_012282]	NM_012282
A_23_P10442	5.14E-05	NM_080597	NM_080597	Homo sapiens oxysterol binding protein-like 1A (OSBPL1A), transcript variant OSBPL1B, mRNA [NM_080597]	NM_080597
A_23_P12062	5.14E-05	NM_017646	NM_017646	Homo sapiens tRNA isopentenyltransferase 1 (TRIT1), mRNA [NM_017646]	NM_017646
A_23_P163113	5.15E-05	NM_017922	NM_017922	Homo sapiens PRP39 pre-mRNA processing factor 39 homolog (yeast) (PRPF39), mRNA [NM_017922]	NM_017922
A_32_P164758	5.16E-05	NM_001013685	NM_001013685	Homo sapiens hypothetical LOC401357 (LOC401357), mRNA [NM_001013685]	NM_001013685
A_24_P179585	5.17E-05	NM_018650	NM_018650	Homo sapiens MAP/microtubule affinity-regulating kinase 1 (MARK1), mRNA [NM_018650]	NM_018650
A_23_P159851	5.17E-05	NM_032803	NM_032803	Homo sapiens solute carrier family 7 (cationic amino acid transporter, y+ system), member 3 (SLC7A3), mRNA [NM_032803]	NM_032803
A_23_P428382	5.17E-05	NM_203437	NM_203437	Homo sapiens aftiphilin protein (AFTIPHILIN), transcript variant 1, mRNA [NM_203437]	NM_203437
A_23_P59921	5.17E-05	NM_006713	NM_006713	Homo sapiens activated RNA polymerase II transcription cofactor 4 (PC4), mRNA [NM_006713]	NM_006713
A_24_P913716	5.19E-05	M74509	M74509	Human endogenous retrovirus type C oncovirus sequence. [M74509]	XM_379483
A_32_P54242	5.20E-05	NM_153686	NM_153686	Homo sapiens transcription factor MLR1 (MLR1), mRNA [NM_153686]	NM_153686
A_24_P450596	5.20E-05	CR627133	CR627133	Homo sapiens mRNA; cDNA DKFZp686J154 (from clone DKFZp686J154). [CR627133]	
A_23_P59787	5.20E-05	NM_016019	NM_016019	Homo sapiens LUC7-like 2 (S. cerevisiae) (LUC7L2), mRNA [NM_016019]	NM_016019
A_32_P55414	5.21E-05	A_32_P55414			
A_23_P109452	5.21E-05	NM_001005735	NM_001005735	Homo sapiens CHK2 checkpoint homolog (S. pombe) (CHEK2), transcript variant 3, mRNA [NM_001005735]	NM_001005735
A_23_P362183	5.21E-05	NM_173551	NM_173551	Homo sapiens sterile alpha motif domain containing 6 (SAMD6), mRNA [NM_173551]	NM_173551
A_32_P509964	5.22E-05	AF136408	AF136408	Homo sapiens unknown mRNA. [AF136408]	
A_23_P211748	5.23E-05	NM_005513	NM_005513	Homo sapiens general transcription factor IIE, polypeptide 1 (alpha subunit, 56kD) (GTF2E1), mRNA [NM_005513]	NM_005513
A_23_P254212	5.24E-05	NM_013347	NM_013347	Homo sapiens replication protein A4, 34kDa (RPA4), mRNA [NM_013347]	NM_013347
A_23_P83736	5.24E-05	NM_001564	NM_001564	Homo sapiens inhibitor of growth family, member 2 (ING2), mRNA [NM_001564]	NM_001564
A_32_P80610	5.25E-05	AK124080	AK124080	Homo sapiens cDNA FLJ42086 fis, clone TESOP1000127. [AK124080]	
A_23_P76882	5.25E-05	NM_021178	NM_021178	Homo sapiens cyclin B1 interacting protein 1 (CCNB1IP1), transcript variant 1, mRNA [NM_021178]	NM_021178
A_24_P307759	5.27E-05	NM_015180	NM_015180	Homo sapiens spectrin repeat containing, nuclear envelope 2 (SYNE2), transcript variant 1, mRNA [NM_015180]	NM_015180
A_23_P143331	5.27E-05	NM_001200	NM_001200	Homo sapiens bone morphogenetic protein 2 (BMP2), mRNA [NM_001200]	NM_001200
A_24_P230074	5.29E-05	AK131274	AK131274	Homo sapiens cDNA FLJ16218 fis, clone CTONG3001501, highly similar to Mus musculus glucocorticoid-induced gene 1 mRNA. [AK131274]	
A_24_P903680	5.29E-05	XM_496957	XM_496957	PREDICTED: Homo sapiens similar to FLJ10408 protein (LOC441328), mRNA [XM_496957]	XM_496957
A_23_P97853	5.30E-05	NM_025125	NM_025125	Homo sapiens chromosome 10 open reading frame 57 (C10orf57), mRNA [NM_025125]	NM_025125
A_32_P224888	5.31E-05	AK054895	AK054895	Homo sapiens cDNA FLJ30333 fis, clone BRACE2007262. [AK054895]	
A_24_P926580	5.32E-05	AK001808	AK001808	Homo sapiens cDNA FLJ10946 fis, clone PLACE1000005. [AK001808]	
A_23_P29384	5.32E-05	NM_033210	NM_033210	Homo sapiens zinc finger protein 502 (ZNF502), mRNA [NM_033210]	NM_033210
A_23_P165343	5.32E-05	NM_002830	NM_002830	Homo sapiens protein tyrosine phosphatase, non-receptor type 4 (megakaryocyte) (PTPN4), mRNA [NM_002830]	NM_002830
A_24_P405430	5.32E-05	CR607569	CR607569	full-length cDNA clone CS0DF027YA11 of Fetal brain of Homo sapiens (human). [CR607569]	
A_23_P394166	5.32E-05	NM_013354	NM_013354	Homo sapiens CCR4-NOT transcription complex, subunit 7 (CNOT7), transcript variant 1, mRNA [NM_013354]	NM_013354
A_23_P309619	5.33E-05	AK123704	AK123704	Homo sapiens cDNA FLJ41710 fis, clone HLUNG2011041, weakly similar to Basic proline-rich peptide IB-8a. [AK123704]	XM_371461
A_24_P699896	5.35E-05	BC036637	BC036637	Homo sapiens cDNA clone IMAGE:5296862. [BC036637]	
A_24_P942328	5.36E-05	NM_000791	NM_000791	Homo sapiens dihydrofolate reductase (DHFR), mRNA [NM_000791]	NM_000791
A_24_P524462	5.37E-05	A_24_P524462			
A_24_P680947	5.38E-05	ENST00000335534		Homo sapiens hypothetical protein LOC146909, mRNA (cDNA clone IMAGE:4587138), partial cds. [BC067365]	
A_23_P423074	5.39E-05	CR936791	CR936791	Homo sapiens mRNA; cDNA DKFZp781C2356 (from clone DKFZp781C2356). [CR936791]	XM_032571
A_24_P80571	5.41E-05	CN284574	CN284574	CN284574 17000531534200 GRN_EB Homo sapiens cDNA 5', mRNA sequence [CN284574]	
A_32_P49764	5.41E-05	THC2394165		DBP_HUMAN (Q10586) D-site-binding protein (Albumin D box-binding protein) (TAXREB302), partial (6%) [THC2394165]	
A_23_P92954	5.42E-05	NM_000414	NM_000414	Homo sapiens hydroxysteroid (17-beta) dehydrogenase 4 (HSD17B4), mRNA [NM_000414]	NM_000414
A_23_P385911	5.43E-05	NM_030633	NM_030633	Homo sapiens KIAA1712 (KIAA1712), mRNA [NM_030633]	NM_030633
A_23_P116168	5.43E-05	NM_032424	NM_032424	Homo sapiens KIAA1826 protein (KIAA1826), mRNA [NM_032424]	NM_032424

A_23_P403081	5.44E-05	NM_198566	NM_198566	Homo sapiens FLJ32363 protein (FLJ32363), mRNA [NM_198566]	NM_198566
A_24_P137522	5.45E-05	BC017382	BC017382	Homo sapiens ubiquitin specific protease 53, mRNA (cDNA clone IMAGE:4082351), complete cds. [BC017382]	
A_23_P44631	5.45E-05	NM_014925	NM_014925	Homo sapiens KIAA1002 protein (KIAA1002), mRNA [NM_014925]	NM_014925
A_23_P204427	5.45E-05	NM_002710	NM_002710	Homo sapiens protein phosphatase 1, catalytic subunit, gamma isoform (PPP1CC), mRNA [NM_002710]	NM_002710
A_32_P211363	5.46E-05	NM_133446	NM_133446	Homo sapiens centaurin, gamma-like family, member 1 (CTGLF1), mRNA [NM_133446]	NM_133446
A_23_P210747	5.46E-05	NM_015939	NM_015939	Homo sapiens CGI-09 protein (CGI-09), mRNA [NM_015939]	NM_015939
A_24_P804951	5.47E-05	XM_498560	XM_498560	PREDICTED: Homo sapiens LOC440132 (LOC440132), mRNA [XM_498560]	XM_498560
A_23_P144244	5.47E-05	THC2286151		T09533 COX17 protein - human {Homo sapiens;} , partial (98%) [THC2286151]	
A_24_P277456	5.47E-05	CR605719	CR605719	full-length cDNA clone CS0DK002YG10 of HeLa cells Cot 25-normalized of Homo sapiens (human). [CR605719]	
A_23_P53276	5.47E-05	NM_003920	NM_003920	Homo sapiens timeless homolog (Drosophila) (TIMELESS), mRNA [NM_003920]	NM_003920
A_32_P203300	5.49E-05	THC2306884			
A_23_P59375	5.49E-05	NM_001546	NM_001546	Homo sapiens inhibitor of DNA binding 4, dominant negative helix-loop-helix protein (ID4), mRNA [NM_001546]	NM_001546
A_23_P79032	5.51E-05	BC004943	BC004943	Homo sapiens hypothetical protein MGC10814, mRNA (cDNA clone MGC:10814 IMAGE:3613095), complete cds. [BC004943]	
A_23_P46118	5.52E-05	NM_001821	NM_001821	Homo sapiens choroideremia-like (Rab escort protein 2) (CHML), mRNA [NM_001821]	NM_001821
A_23_P200386	5.52E-05	NM_006559	NM_006559	Homo sapiens KH domain containing, RNA binding, signal transduction associated 1 (KHDRBS1), mRNA [NM_006559]	NM_006559
A_23_P434807	5.54E-05	NM_005440	NM_005440	Homo sapiens Rho family GTPase 2 (RND2), mRNA [NM_005440]	NM_005440
A_24_P551028	5.54E-05	NM_001001664	NM_001001664	Homo sapiens hypothetical protein LOC339745 (LOC339745), mRNA [NM_001001664]	NM_001001664
A_23_P212792	5.54E-05	NM_025009	NM_025009	Homo sapiens centrosomal protein 4 (CEP4), mRNA [NM_025009]	NM_025009
A_24_P870620	5.54E-05	NM_002825	NM_002825	Homo sapiens pleiotrophin (heparin binding growth factor 8, neurite growth-promoting factor 1) (PTN), mRNA [NM_002825]	NM_002825
A_23_P43726	5.54E-05	NM_015231	NM_015231	Homo sapiens nucleoporin 160kDa (NUP160), mRNA [NM_015231]	NM_015231
A_23_P88731	5.54E-05	NM_002875	NM_002875	Homo sapiens RAD51 homolog (RecA homolog, E. coli) (S. cerevisiae) (RAD51), transcript variant 1, mRNA [NM_002875]	NM_002875
A_23_P208210	5.55E-05	NM_014650	NM_014650	Homo sapiens zinc finger protein 432 (ZNF432), mRNA [NM_014650]	NM_014650
A_24_P930926	5.55E-05	ENST00000287322		Homo sapiens BCL2-associated athanogene 4, mRNA (cDNA clone MGC:33671 IMAGE:5259771), complete cds. [BC038505]	
A_23_P328836	5.55E-05	NM_032440	NM_032440	Homo sapiens ligand-dependent corepressor (MLR2), mRNA [NM_032440]	NM_032440
A_24_P357169	5.56E-05	NM_031308	NM_031308	Homo sapiens epiplakin 1 (EPPK1), mRNA [NM_031308]	NM_031308
A_23_P398770	5.56E-05	AK055921	AK055921	Homo sapiens cDNA FLJ31359 fis, clone MESAN2000501, weakly similar to Homo sapiens DNA cytosine methyltransferase 3 alpha (DNMT3A) mRNA. [AK055921]	
A_23_P107644	5.56E-05	NM_006938	NM_006938	Homo sapiens small nuclear ribonucleoprotein D1 polypeptide 16kDa (SNRPD1), mRNA [NM_006938]	NM_006938
A_23_P91001	5.58E-05	NM_019048	NM_019048	Homo sapiens HCV NS3-transactivated protein 1 (NS3TP1), mRNA [NM_019048]	NM_019048
A_23_P131215	5.62E-05	NM_006891	NM_006891	Homo sapiens crystallin, gamma D (CRYGD), mRNA [NM_006891]	NM_006891
A_24_P12660	5.62E-05	THC2303284		Q6GLA9 (Q6GLA9) MGC69246 protein, partial (21%) [THC2303284]	
A_24_P860797	5.65E-05	ENST00000244221		Homo sapiens mRNA for KIAA1155 protein, partial cds. [AB032981]	XM_376062
A_23_P429670	5.65E-05	NM_032440	NM_032440	Homo sapiens ligand-dependent corepressor (MLR2), mRNA [NM_032440]	NM_032440
A_24_P288754	5.65E-05	NM_002641	NM_002641	Homo sapiens phosphatidylinositol glycan, class A (paroxysmal nocturnal hemoglobinuria) (PIGA), transcript variant 1, mRNA [NM_002641]	NM_002641
A_23_P58466	5.66E-05	NM_000344	NM_000344	Homo sapiens survival of motor neuron 1, telomeric (SMN1), transcript variant d, mRNA [NM_000344]	NM_000344
A_23_P363344	5.66E-05	NM_000366	NM_000366	Homo sapiens tropomyosin 1 (alpha) (TPM1), transcript variant 5, mRNA [NM_000366]	NM_000366
A_23_P20558	5.69E-05	NM_017913	NM_017913	Homo sapiens cell division cycle 37 homolog (S. cerevisiae)-like 1 (CDC37L1), mRNA [NM_017913]	NM_017913
A_23_P127840	5.72E-05	NM_013249	NM_013249	Homo sapiens zinc finger protein 214 (ZNF214), mRNA [NM_013249]	NM_013249
A_32_P166356	5.73E-05	THC2350463			
A_32_P98511	5.74E-05	AA807805	AA807805	AA807805 nu88h02.s1 NCI_CGAP_Alv1 Homo sapiens cDNA clone IMAGE:1217811, mRNA sequence [AA807805]	
A_23_P109774	5.76E-05	NM_014415	NM_014415	Homo sapiens zinc finger and BTB domain containing 11 (ZBTB11), mRNA [NM_014415]	NM_014415
A_24_P342316	5.79E-05	AB020633	AB020633	Homo sapiens mRNA for KIAA0826 protein, partial cds. [AB020633]	XM_093839
A_24_P354257	5.80E-05	CR620532	CR620532	full-length cDNA clone CS0DE006YD17 of Placenta of Homo sapiens (human). [CR620532]	
A_23_P127652	5.82E-05	NM_003455	NM_003455	Homo sapiens zinc finger protein 202 (ZNF202), mRNA [NM_003455]	NM_003455
A_23_P158148	5.84E-05	NM_001762	NM_001762	Homo sapiens chaperonin containing TCP1, subunit 6A (zeta 1) (CCT6A), transcript variant 1, mRNA [NM_001762]	NM_001762

A_32_P112078	5.85E-05	THC2440782			
A_23_P5945	5.85E-05	NM_021931	NM_021931	Homo sapiens DEAH (Asp-Glu-Ala-His) box polypeptide 35 (DHX35), mRNA [NM_021931]	NM_021931
A_23_P117928	5.85E-05	NM_014952	NM_014952	Homo sapiens bromo adjacent homology domain containing 1 (BAHD1), mRNA [NM_014952]	NM_014952
A_32_P195291	5.86E-05	CR603272	CR603272	full-length cDNA clone CS0DC013YI10 of Neuroblastoma Cot 25-normalized of Homo sapiens (human). [CR603272]	
A_23_P43846	5.87E-05	NM_001445	NM_001445	Homo sapiens fatty acid binding protein 6, ileal (gastrotropin) (FABP6), mRNA [NM_001445]	NM_001445
A_23_P84399	5.87E-05	NM_014141	NM_014141	Homo sapiens contactin associated protein-like 2 (CNTNAP2), mRNA [NM_014141]	NM_014141
A_24_P83758	5.88E-05	ENST00000292728		Homo sapiens mRNA for KIAA1653 protein, partial cds. [AB051440]	XM_496493
A_23_P159927	5.88E-05	NM_001666	NM_001666	Homo sapiens Rho GTPase activating protein 4 (ARHGAP4), mRNA [NM_001666]	NM_001666
A_23_P314151	5.88E-05	NM_004741	NM_004741	Homo sapiens nucleolar and coiled-body phosphoprotein 1 (NOLC1), mRNA [NM_004741]	NM_004741
A_23_P54389	5.90E-05	NM_024611	NM_024611	Homo sapiens NMDA receptor regulated 2 (NARG2), transcript variant 1, mRNA [NM_024611]	NM_024611
A_23_P156310	5.91E-05	NM_032637	NM_032637	Homo sapiens S-phase kinase-associated protein 2 (p45) (SKP2), transcript variant 2, mRNA [NM_032637]	NM_032637
A_23_P19619	5.92E-05	NM_002114	NM_002114	Homo sapiens human immunodeficiency virus type I enhancer binding protein 1 (HIVEP1), mRNA [NM_002114]	NM_002114
A_23_P109072	5.92E-05	NM_020436	NM_020436	Homo sapiens sal-like 4 (Drosophila) (SALL4), mRNA [NM_020436]	NM_020436
A_24_P84668	5.93E-05	NM_015687	NM_015687	Homo sapiens filamin A interacting protein 1 (FILIP1), mRNA [NM_015687]	NM_015687
A_23_P259251	5.94E-05	NM_012281	NM_012281	Homo sapiens potassium voltage-gated channel, Shal-related subfamily, member 2 (KCND2), mRNA [NM_012281]	NM_012281
A_23_P377141	5.94E-05	NM_015186	NM_015186	Homo sapiens vacuolar protein sorting 13A (yeast) (VPS13A), transcript variant B, mRNA [NM_015186]	NM_015186
A_24_P630916	5.94E-05	THC2366591		ALU7_HUMAN (P39194) Alu subfamily SQ sequence contamination warning entry, partial (12%) [THC2366591]	
A_23_P136693	5.94E-05	AL832747	AL832747	Homo sapiens mRNA; cDNA DKFZp686D0521 (from clone DKFZp686D0521). [AL832747]	
A_23_P23526	5.96E-05	NM_033020	NM_033020	Homo sapiens tripartite motif-containing 33 (TRIM33), transcript variant beta, mRNA [NM_033020]	NM_033020
A_23_P204550	5.99E-05	NM_017988	NM_017988	Homo sapiens SCY1-like 2 (S. cerevisiae) (SCYL2), mRNA [NM_017988]	NM_017988
A_23_P256641	6.00E-05	NM_012282	NM_012282	Homo sapiens KCNE1-like (KCNE1L), mRNA [NM_012282]	NM_012282
A_23_P99473	6.00E-05	NM_023011	NM_023011	Homo sapiens UPF3 regulator of nonsense transcripts homolog A (yeast) (UPF3A), transcript variant 1, mRNA [NM_023011]	NM_023011
A_23_P167227	6.01E-05	NM_005327	NM_005327	Homo sapiens L-3-hydroxyacyl-Coenzyme A dehydrogenase, short chain (HADHSC), mRNA [NM_005327]	NM_005327
A_24_P284584	6.02E-05	NM_032497	NM_032497	Homo sapiens zinc finger protein 559 (ZNF559), mRNA [NM_032497]	NM_032497
A_24_P166311	6.02E-05	NM_022090	NM_022090	Homo sapiens transposon-derived Buster3 transposase-like (LOC63920), mRNA [NM_022090]	NM_022090
A_24_P111096	6.02E-05	NM_004566	NM_004566	Homo sapiens 6-phosphofructo-2-kinase/fructose-2,6-biphosphatase 3 (PFKFB3), mRNA [NM_004566]	NM_004566
A_23_P393401	6.04E-05	BC008178	BC008178	Homo sapiens hypothetical protein LOC339047, mRNA (cDNA clone IMAGE:4184431), complete cds. [BC008178]	
A_24_P256674	6.06E-05	BC040474	BC040474	Homo sapiens Rho guanine nucleotide exchange factor (GEF) 10, mRNA (cDNA clone IMAGE:4250879), complete cds. [BC040474]	
A_24_P355246	6.06E-05	AK023096	AK023096	Homo sapiens cDNA FLJ13034 fis, clone NT2RP3001232. [AK023096]	
A_23_P368794	6.07E-05	NM_174937	NM_174937	Homo sapiens transcription elongation regulator 1-like (TCERG1L), mRNA [NM_174937]	NM_174937
A_23_P121182	6.07E-05	NM_012260	NM_012260	Homo sapiens 2-hydroxyphytanoyl-CoA lyase (HPCL2), mRNA [NM_012260]	NM_012260
A_24_P196400	6.08E-05	NM_017934	NM_017934	Homo sapiens pleckstrin homology domain interacting protein (PHIP), mRNA [NM_017934]	NM_017934
A_23_P345707	6.08E-05	NM_152259	NM_152259	Homo sapiens leucine-rich repeat kinase 1 (MGC45866), mRNA [NM_152259]	NM_152259
A_24_P927537	6.09E-05	THC2269654			
A_23_P204333	6.10E-05	NM_032834	NM_032834	Homo sapiens asparagine-linked glycosylation 10 homolog (yeast, alpha-1,2-glucosyltransferase) (ALG10), mRNA [NM_032834]	NM_032834
A_24_P323598	6.10E-05	NM_001017420	NM_001017420	Homo sapiens establishment of cohesion 1 homolog 2 (S. cerevisiae) (ESCO2), mRNA [NM_001017420]	NM_001017420
A_23_P78888	6.11E-05	NM_001436	NM_001436	Homo sapiens fibrillarin (FBL), mRNA [NM_001436]	NM_001436
A_32_P106523	6.12E-05	THC2441641		B40201 artifact-warning sequence (translated ALU class B) - human {Homo sapiens;} , partial (13%) [THC2441641]	
A_32_P43855	6.12E-05	BX476711	BX476711	DKFZp686M05188_r1 686 (synonym: hlcc3) Homo sapiens cDNA clone DKFZp686M05188 5', mRNA sequence [BX476711]	
A_23_P30805	6.12E-05	NM_021968	NM_021968	Homo sapiens histone 1, H4j (HIST1H4J), mRNA [NM_021968]	NM_021968
A_23_P313828	6.12E-05	NM_181716	NM_181716	Homo sapiens proline rich 6 (PRR6), mRNA [NM_181716]	NM_181716
A_23_P318581	6.12E-05	AB037851	AB037851	Homo sapiens mRNA for KIAA1430 protein, partial cds. [AB037851]	
A_23_P145541	6.14E-05	NM_014845	NM_014845	Homo sapiens KIAA0274 (KIAA0274), mRNA [NM_014845]	NM_014845
A_23_P101193	6.14E-05	AK025336	AK025336	Homo sapiens cDNA: FLJ21683 fis, clone COL09335. [AK025336]	XM_371116

A_23_P129358	6.15E-05	NM_024860	NM_024860	Homo sapiens hypothetical protein FLJ21148 (FLJ21148), mRNA [NM_024860]	NM_024860
A_23_P71017	6.15E-05	NM_001306	NM_001306	Homo sapiens claudin 3 (CLDN3), mRNA [NM_001306]	NM_001306
A_24_P90022	6.15E-05	BC064610	BC064610	Homo sapiens cDNA clone IMAGE:5548247, partial cds. [BC064610]	
A_23_P85598	6.17E-05	NM_020247	NM_020247	Homo sapiens chaperone, ABC1 activity of bc1 complex like (S. pombe) (CABC1), mRNA [NM_020247]	NM_020247
A_24_P913005	6.18E-05	THC2372128		MMFXR1H9 fragile-X-related protein 1 isoform f {Mus musculus;} , partial (12%) [THC2372128]	
A_23_P208143	6.18E-05	AF533250	AF533250	Homo sapiens zinc finger protein (ZNF397) mRNA, complete cds. [AF533250]	
A_24_P135344	6.20E-05	NM_014715	NM_014715	Homo sapiens Rho GTPase-activating protein (RICS), mRNA [NM_014715]	NM_014715
A_23_P37265	6.20E-05	NM_018229	NM_018229	Homo sapiens chromosome 14 open reading frame 108 (C14orf108), mRNA [NM_018229]	NM_018229
A_32_P34881	6.23E-05	THC2305677		HMG1_HUMAN (P09429) High mobility group protein 1 (HMG-1), partial (7%) [THC2305677]	
A_24_P339514	6.23E-05	NM_000767	NM_000767	Homo sapiens cytochrome P450, family 2, subfamily B, polypeptide 6 (CYP2B6), mRNA [NM_000767]	NM_000767
A_23_P209389	6.23E-05	NM_033356	NM_033356	Homo sapiens caspase 8, apoptosis-related cysteine protease (CASP8), transcript variant C, mRNA [NM_033356]	NM_033356
A_32_P25972	6.25E-05	BE826587	BE826587	BE826587 QV1-EN0042-300500-224-e04 EN0042 Homo sapiens cDNA, mRNA sequence [BE826587]	
A_24_P410256	6.25E-05	A_24_P410256			
A_23_P256855	6.27E-05	NM_020750	NM_020750	Homo sapiens exportin 5 (XPO5), mRNA [NM_020750]	NM_020750
A_32_P38645	6.30E-05	NM_182970	NM_182970	Homo sapiens regulating synaptic membrane exocytosis 4 (RIMS4), mRNA [NM_182970]	NM_182970
A_24_P316414	6.31E-05	BC014346	BC014346	Homo sapiens, clone IMAGE:4042988, mRNA, partial cds. [BC014346]	
A_32_P64096	6.31E-05	NM_001012981	NM_001012981	Homo sapiens zinc finger protein 694 (ZNF694), mRNA [NM_001012981]	NM_001012981
A_23_P123905	6.31E-05	NM_016042	NM_016042	Homo sapiens exosome component 3 (EXOSC3), transcript variant 1, mRNA [NM_016042]	NM_016042
A_23_P75430	6.31E-05	NM_020179	NM_020179	Homo sapiens FN5 protein (FN5), mRNA [NM_020179]	NM_020179
A_32_P220472	6.31E-05	NM_019006	NM_019006	Homo sapiens zinc finger, A20 domain containing 3 (ZA20D3), mRNA [NM_019006]	NM_019006
A_24_P173234	6.33E-05	NM_024840	NM_024840	Homo sapiens zinc finger protein 613 (ZNF613), mRNA [NM_024840]	NM_024840
A_23_P202206	6.34E-05	NM_183239	NM_183239	Homo sapiens glutathione S-transferase omega 2 (GSTO2), mRNA [NM_183239]	NM_183239
A_24_P702813	6.34E-05	AK023131	AK023131	Homo sapiens cDNA FLJ13069 fis, clone NT2RP3001752. [AK023131]	
A_32_P84772	6.36E-05	NM_001008401	NM_001008401	Homo sapiens FLJ16231 protein (FLJ16231), mRNA [NM_001008401]	NM_001008401
A_23_P415643	6.36E-05	NM_152652	NM_152652	Homo sapiens zinc finger protein 553 (ZNF553), mRNA [NM_152652]	NM_152652
A_32_P175183	6.37E-05	BC071729	BC071729	Homo sapiens BTB (POZ) domain containing 15, mRNA (cDNA clone MGC:88058 IMAGE:5163748), complete cds. [BC071729]	
A_32_P199263	6.38E-05	BC073929	BC073929	Homo sapiens cDNA clone IMAGE:5196961, partial cds. [BC073929]	
A_32_P219148	6.38E-05	BC030211	BC030211	Homo sapiens, clone IMAGE:5199989, mRNA. [BC030211]	
A_23_P375165	6.40E-05	NM_207459	NM_207459	Homo sapiens FLJ35767 protein (FLJ35767), mRNA [NM_207459]	NM_207459
A_24_P258073	6.40E-05	NM_021830	NM_021830	Homo sapiens progressive external ophthalmoplegia 1 (PEO1), mRNA [NM_021830]	NM_021830
A_23_P132948	6.41E-05	NM_201999	NM_201999	Homo sapiens E74-like factor 2 (ets domain transcription factor) (ELF2), transcript variant 1, mRNA [NM_201999]	NM_201999
A_32_P18824	6.41E-05	NM_013263	NM_013263	Homo sapiens bromodomain containing 7 (BRD7), mRNA [NM_013263]	NM_013263
A_23_P259357	6.41E-05	ENST00000343605		full-length cDNA clone CS0DD009YN24 of Neuroblastoma Cot 50-normalized of Homo sapiens (human). [CR614052]	
A_23_P411335	6.42E-05	NM_152524	NM_152524	Homo sapiens shugoshin-like 2 (S. pombe) (SGOL2), mRNA [NM_152524]	NM_152524
A_23_P153098	6.42E-05	NM_032124	NM_032124	Homo sapiens haloacid dehalogenase-like hydrolase domain containing 2 (HDHD2), mRNA [NM_032124]	NM_032124
A_24_P161725	6.46E-05	BC089388	BC089388	Homo sapiens cDNA clone IMAGE:30390722, containing frame-shift errors. [BC089388]	XM_496028
A_23_P388146	6.48E-05	NM_032828	NM_032828	Homo sapiens zinc finger protein 587 (ZNF587), mRNA [NM_032828]	NM_032828
A_32_P168605	6.49E-05	BC039411	BC039411	Homo sapiens, clone IMAGE:5301690, mRNA. [BC039411]	
A_32_P77933	6.49E-05	AF316855	AF316855	Homo sapiens colon cancer-associated antigen AgSK1-2HT-ECS mRNA, complete cds. [AF316855]	
A_24_P208961	6.50E-05	NM_133370	NM_133370	Homo sapiens splicing factor YT521-B (YT521), mRNA [NM_133370]	NM_133370
A_23_P99967	6.51E-05	NM_033028	NM_033028	Homo sapiens Bardet-Biedl syndrome 4 (BBS4), mRNA [NM_033028]	NM_033028
A_32_P199725	6.51E-05	BU783246	BU783246	BU783246 in01c10.y1 Human insulinoma Homo sapiens cDNA clone IMAGE:6123258 5' similar to SW:ADHX_HUMAN P11766 ALCOHOL DEHYDROGENASE CLASS III CHI CHAIN ;, mRNA sequence [BU783246]	
A_23_P91414	6.52E-05	NM_080625	NM_080625	Homo sapiens chromosome 20 open reading frame 160 (C20orf160), mRNA [NM_080625]	NM_080625
A_23_P252132	6.54E-05	NM_002839	NM_002839	Homo sapiens protein tyrosine phosphatase, receptor type, D (PTPRD), transcript variant 1, mRNA [NM_002839]	NM_002839

A_23_P339601	6.54E-05	NM_152472	NM_152472	Homo sapiens zinc finger protein 578 (ZNF578), mRNA [NM_152472]	NM_152472
A_24_P225970	6.54E-05	NM_001012409	NM_001012409	Homo sapiens shugoshin-like 1 (S. pombe) (SGOL1), transcript variant A1, mRNA [NM_001012409]	NM_001012409
A_24_P3704	6.54E-05	THC2272132		Q64150 (Q64150) Nuclear localization signal binding protein, partial (8%) [THC2272132]	
A_23_P310317	6.54E-05	NM_001008656	NM_001008656	Homo sapiens Treacher Collins-Franceschetti syndrome 1 (TCOF1), transcript variant 1, mRNA [NM_001008656]	NM_001008656
A_23_P81721	6.56E-05	NM_004277	NM_004277	Homo sapiens solute carrier family 25, member 27 (SLC25A27), nuclear gene encoding mitochondrial protein, mRNA [NM_004277]	NM_004277
A_24_P58899	6.56E-05	A_24_P58899			
A_24_P35891	6.56E-05	NM_016423	NM_016423	Homo sapiens zinc finger protein 219 (ZNF219), mRNA [NM_016423]	NM_016423
A_32_P106315	6.57E-05	BX641009	BX641009	Homo sapiens mRNA; cDNA DKFZp686D13227 (from clone DKFZp686D13227) [BX641009]	
A_24_P282043	6.57E-05	NM_006969	NM_006969	Homo sapiens zinc finger protein 28 (KOX 24) (ZNF28), mRNA [NM_006969]	NM_006969
A_32_P162709	6.57E-05	CR624517	CR624517	full-length cDNA clone CS0DC002YA18 of Neuroblastoma Cot 25-normalized of Homo sapiens (human). [CR624517]	
A_32_P132589	6.58E-05	NM_001004339	NM_001004339	Homo sapiens zyg-11 homolog A (C. elegans) (ZYG11A), mRNA [NM_001004339]	NM_001004339
A_23_P12503	6.60E-05	NM_018230	NM_018230	Homo sapiens nucleoporin 133kDa (NUP133), mRNA [NM_018230]	NM_018230
A_32_P34116	6.65E-05	ENST00000284122		Homo sapiens cDNA FLJ43129 fis, clone CTONG3005648. [AK125119]	XM_372205
A_24_P328333	6.66E-05	NM_004850	NM_004850	Homo sapiens Rho-associated, coiled-coil containing protein kinase 2 (ROCK2), mRNA [NM_004850]	NM_004850
A_24_P311845	6.69E-05	CR612518	CR612518	full-length cDNA clone CS0DF004YF08 of Fetal brain of Homo sapiens (human). [CR612518]	
A_23_P207999	6.69E-05	NM_021127	NM_021127	Homo sapiens phorbol-12-myristate-13-acetate-induced protein 1 (PMAIP1), mRNA [NM_021127]	NM_021127
A_24_P260300	6.70E-05	CR622342	CR622342	full-length cDNA clone CS0DJ006YC05 of T cells (Jurkat cell line) Cot 10-normalized of Homo sapiens (human). [CR622342]	
A_23_P203009	6.71E-05	NM_003478	NM_003478	Homo sapiens cullin 5 (CUL5), mRNA [NM_003478]	NM_003478
A_23_P203645	6.71E-05	NM_021212	NM_021212	Homo sapiens HCF-binding transcription factor Zhangfei (ZF), mRNA [NM_021212]	NM_021212
A_32_P222961	6.71E-05	NM_001012968	NM_001012968	Homo sapiens hypothetical protein LOC139886 (LOC139886), mRNA [NM_001012968]	NM_001012968
A_23_P69326	6.72E-05	NM_183393	NM_183393	Homo sapiens Ca2+-dependent secretion activator (CADPS), transcript variant 3, mRNA [NM_183393]	NM_183393
A_24_P308229	6.74E-05	AK095339	AK095339	Homo sapiens cDNA FLJ38020 fis, clone CTONG2012843, weakly similar to Human non-lens beta gamma-crystallin like protein (AIM1) mRNA. [AK095339]	
A_23_P91991	6.74E-05	NM_138381	NM_138381	Homo sapiens hypothetical protein BC008322 (MGC15763), mRNA [NM_138381]	NM_138381
A_32_P121303	6.74E-05	BC039468	BC039468	Homo sapiens cDNA clone IMAGE:3889720. [BC039468]	
A_23_P33539	6.75E-05	NM_020546	NM_020546	Homo sapiens adenylate cyclase 2 (brain) (ADCY2), mRNA [NM_020546]	NM_020546
A_32_P209735	6.77E-05	BC048193	BC048193	Homo sapiens, clone IMAGE:4590099, mRNA. [BC048193]	XM_496741
A_23_P314591	6.77E-05	NM_006166	NM_006166	Homo sapiens nuclear transcription factor Y, beta (NFYB), mRNA [NM_006166]	NM_006166
A_32_P59262	6.79E-05	BM986990	BM986990	BM986990 UI-H-CO0-aqe-h-08-0-UI.s1 NCI_CGAP_Sub9 Homo sapiens cDNA clone IMAGE:3104077 3', mRNA sequence [BM986990]	
A_23_P121825	6.79E-05	NM_024941	NM_024941	Homo sapiens hypothetical protein FLJ13611 (FLJ13611), mRNA [NM_024941]	NM_024941
A_23_P397635	6.79E-05	NM_007131	NM_007131	Homo sapiens zinc finger protein 75 (D8C6) (ZNF75), mRNA [NM_007131]	NM_007131
A_23_P371155	6.81E-05	NM_004906	NM_004906	Homo sapiens Wilms tumor 1 associated protein (WTAP), transcript variant 1, mRNA [NM_004906]	NM_004906
A_23_P252145	6.84E-05	NM_020156	NM_020156	Homo sapiens core 1 synthase, glycoprotein-N-acetylgalactosamine 3-beta-galactosyltransferase, 1 (C1GALT1), mRNA [NM_020156]	NM_020156
A_23_P131111	6.84E-05	NM_198580	NM_198580	Homo sapiens solute carrier family 27 (fatty acid transporter), member 1 (SLC27A1), mRNA [NM_198580]	NM_198580
A_23_P383601	6.86E-05	ENST00000316535		Homo sapiens hypothetical protein FLJ31306, mRNA (cDNA clone IMAGE:4838556), partial cds. [BC034618]	
A_23_P389102	6.86E-05	NM_015194	NM_015194	Homo sapiens myosin ID (MYO1D), mRNA [NM_015194]	NM_015194
A_23_P152066	6.86E-05	AK054969	AK054969	Homo sapiens cDNA FLJ30407 fis, clone BRACE2008553. [AK054969]	
A_32_P210516	6.88E-05	AK024566	AK024566	Homo sapiens cDNA: FLJ20913 fis, clone ADSE00630. [AK024566]	
A_23_P350754	6.89E-05	AF238487	AF238487	Homo sapiens olfactory-like receptor PJCG2 (PJCG2) mRNA, partial cds. [AF238487]	
A_24_P72750	6.89E-05	AL136719	AL136719	Homo sapiens mRNA; cDNA DKFZp566G0346 (from clone DKFZp566G0346). [AL136719]	
A_23_P414420	6.90E-05	NM_018383	NM_018383	Homo sapiens WD repeat domain 33 (WDR33), transcript variant 1, mRNA [NM_018383]	NM_018383
A_23_P161481	6.93E-05	NM_014431	NM_014431	Homo sapiens KIAA1274 (KIAA1274), mRNA [NM_014431]	NM_014431
A_32_P109522	6.94E-05	NM_145062	NM_145062	Homo sapiens chromosome 6 open reading frame 113 (C6orf113), mRNA [NM_145062]	NM_145062
A_24_P57977	6.95E-05	NM_025248	NM_025248	Homo sapiens SNAP25-interacting protein (SNIP), mRNA [NM_025248]	NM_025248
A_24_P195998	6.95E-05	NM_032217	NM_032217	Homo sapiens ankyrin repeat domain 17 (ANKRD17), transcript variant 1, mRNA [NM_032217]	NM_032217

A_23_P118246	6.95E-05	NM_016095	NM_016095	Homo sapiens DNA replication complex GINS protein PSF2 (Pfs2), mRNA [NM_016095]	NM_016095
A_24_P683011	6.97E-05	BM696546	BM696546	UI-E-DW0-agk-i-01-0-UI.r1 UI-E-DW0 Homo sapiens cDNA clone UI-E-DW0-agk-i-01-0-UI 5', mRNA sequence [BM696546]	
A_23_P52597	6.98E-05	NM_031909	NM_031909	Homo sapiens C1q and tumor necrosis factor related protein 4 (C1QTNF4), mRNA [NM_031909]	NM_031909
A_32_P86739	6.99E-05	NM_001010911	NM_001010911	Homo sapiens chromosome 10 open reading frame 114 (C10orf114), mRNA [NM_001010911]	NM_001010911
A_32_P112970	7.01E-05	THC2441612			
A_23_P71053	7.02E-05	NM_016447	NM_016447	Homo sapiens membrane protein, palmitoylated 6 (MAGUK p55 subfamily member 6) (MPP6), mRNA [NM_016447]	NM_016447
A_24_P307869	7.03E-05	NM_001015002	NM_001015002	Homo sapiens lethal giant larvae homolog 2 (Drosophila) (LLGL2), transcript variant 2, mRNA [NM_001015002]	NM_001015002
A_32_P188860	7.05E-05	AK125591	AK125591	Homo sapiens cDNA FLJ43603 fis, clone SPLEN2005767. [AK125591]	
A_23_P129174	7.05E-05	NM_017691	NM_017691	Homo sapiens leucine rich repeat containing 49 (LRRC49), mRNA [NM_017691]	NM_017691
A_32_P88791	7.05E-05	NM_032999	NM_032999	Homo sapiens general transcription factor II, i (GTF2I), transcript variant 1, mRNA [NM_032999]	NM_032999
A_32_P148199	7.06E-05	BM667833	BM667833	BM667833 UI-E-DW0-agj-g-19-0-UI.s1 UI-E-DW0 Homo sapiens cDNA clone UI-E-DW0-agj-g-19-0-UI 3', mRNA sequence [BM667833]	
A_23_P313640	7.06E-05	NM_006609	NM_006609	Homo sapiens mitogen-activated protein kinase kinase kinase 2 (MAP3K2), mRNA [NM_006609]	NM_006609
A_23_P64217	7.07E-05	NM_024891	NM_024891	Homo sapiens hypothetical protein FLJ11783 (FLJ11783), mRNA [NM_024891]	NM_024891
A_24_P942648	7.09E-05	AL133642	AL133642	Homo sapiens mRNA; cDNA DKFZp586G1721 (from clone DKFZp586G1721). [AL133642]	
A_24_P4678	7.09E-05	NM_014384	NM_014384	Homo sapiens acyl-Coenzyme A dehydrogenase family, member 8 (ACAD8), mRNA [NM_014384]	NM_014384
A_32_P171386	7.10E-05	THC2455681			
A_23_P111303	7.11E-05	NM_014892	NM_014892	Homo sapiens RNA binding motif protein 16 (RBM16), mRNA [NM_014892]	NM_014892
A_24_P912871	7.12E-05	A_24_P912871			
A_32_P89415	7.13E-05	A_32_P89415			
A_23_P59657	7.14E-05	NM_003592	NM_003592	Homo sapiens cullin 1 (CUL1), mRNA [NM_003592]	NM_003592
A_23_P218918	7.15E-05	NM_002006	NM_002006	Homo sapiens fibroblast growth factor 2 (basic) (FGF2), mRNA [NM_002006]	NM_002006
A_24_P691775	7.16E-05	ENST00000340510		PREDICTED: Homo sapiens hypothetical gene supported by AK097404; NM_198284 (LOC440726), mRNA [XM_498835]	XM_498835
A_23_P368484	7.18E-05	NM_207387	NM_207387	Homo sapiens FLJ35696 protein (FLJ35696), mRNA [NM_207387]	NM_207387
A_23_P38723	7.18E-05	AK126324	AK126324	Homo sapiens cDNA FLJ44350 fis, clone TRACH3006228. [AK126324]	
A_23_P252664	7.18E-05	NM_000150	NM_000150	Homo sapiens fucosyltransferase 6 (alpha (1,3) fucosyltransferase) (FUT6), mRNA [NM_000150]	NM_000150
A_24_P501698	7.19E-05	THC2302062		ALU2_HUMAN (P39189) Alu subfamily SB sequence contamination warning entry, partial (5%) [THC2302062]	
A_23_P121722	7.19E-05	AK001336	AK001336	Homo sapiens cDNA FLJ10474 fis, clone NT2RP2000067. [AK001336]	XM_371717
A_23_P26021	7.20E-05	NM_004236	NM_004236	Homo sapiens COP9 constitutive photomorphogenic homolog subunit 2 (Arabidopsis) (COPS2), mRNA [NM_004236]	NM_004236
A_23_P346048	7.21E-05	NM_001024681	NM_001024681	Homo sapiens D15F37 gene (D15F37), mRNA [NM_001024681]	NM_001024681
A_24_P169544	7.22E-05	NM_006959	NM_006959	Homo sapiens zinc finger protein 17 (HPF3, KOX 10) (ZNF17), mRNA [NM_006959]	NM_006959
A_32_P14243	7.24E-05	THC2439490		Q835K0 (Q835K0) ATP-dependent RNA helicase, DEAD/DEAH box family, partial (4%) [THC2439490]	
A_32_P232559	7.26E-05	AY007155	AY007155	Homo sapiens clone CDABP0095 mRNA sequence. [AY007155]	XM_498474
A_23_P87769	7.26E-05	NM_017915	NM_017915	Homo sapiens hypothetical protein FLJ20641 (FLJ20641), mRNA [NM_017915]	NM_017915
A_23_P69293	7.30E-05	AJ272267	AJ272267	Homo sapiens partial mRNA for choline dehydrogenase (chdh gene). [AJ272267]	
A_23_P24922	7.32E-05	THC2313644		Q9VN27 (Q9VN27) CG9804-PA (GH04831p), partial (19%) [THC2313644]	
A_23_P90275	7.33E-05	NM_022467	NM_022467	Homo sapiens carbohydrate (N-acetylgalactosamine 4-0) sulfotransferase 8 (CHST8), mRNA [NM_022467]	NM_022467
A_23_P133095	7.33E-05	AB002311	AB002311	Homo sapiens mRNA for KIAA0313 gene, partial cds. [AB002311]	XM_376350
A_23_P98446	7.35E-05	NM_001024956	NM_001024956	Homo sapiens sterol-C5-desaturase (ERG3 delta-5-desaturase homolog, fungal)-like (SC5DL), transcript variant 2, mRNA [NM_001024956]	NM_001024956
A_23_P359052	7.39E-05	NM_148894	NM_148894	Homo sapiens family with sequence similarity 44, member A (FAM44A), mRNA [NM_148894]	NM_148894
A_23_P169838	7.39E-05	NM_025154	NM_025154	Homo sapiens unc-84 homolog A (C. elegans) (UNC84A), mRNA [NM_025154]	NM_025154
A_32_P134657	7.41E-05	NM_015349	NM_015349	Homo sapiens KIAA0240 (KIAA0240), mRNA [NM_015349]	NM_015349
A_32_P213678	7.42E-05	AK123439	AK123439	Homo sapiens cDNA FLJ41445 fis, clone BRSTN2002105. [AK123439]	
A_23_P212749	7.42E-05	NM_002111	NM_002111	Homo sapiens huntingtin (Huntington disease) (HD), mRNA [NM_002111]	NM_002111
A_23_P85218	7.44E-05	NM_005634	NM_005634	Homo sapiens SRY (sex determining region Y)-box 3 (SOX3), mRNA [NM_005634]	NM_005634
A_24_P165423	7.46E-05	NM_052960	NM_052960	Homo sapiens retinol binding protein 7, cellular (RBP7), mRNA [NM_052960]	NM_052960

A_32_P194779	7.46E-05	AB082524	AB082524	Homo sapiens mRNA for KIAA1993 protein. [AB082524]	
A_23_P357365	7.49E-05	NM_005862	NM_005862	Homo sapiens stromal antigen 1 (STAG1), mRNA [NM_005862]	NM_005862
A_23_P144384	7.50E-05	NM_017423	NM_017423	Homo sapiens UDP-N-acetyl-alpha-D-galactosamine:polypeptide N-acetylgalactosaminyltransferase 7 (GalNAc-T7) (GALNT7), mRNA [NM_017423]	NM_017423
A_32_P162524	7.51E-05	THC2339079		Q89FH7 (Q89FH7) Bll6723 protein, partial (3%) [THC2339079]	
A_32_P193322	7.51E-05	NM_152756	NM_152756	Homo sapiens TORC2-specific protein AVO3 (AVO3), mRNA [NM_152756]	NM_152756
A_24_P222184	7.51E-05	AK091439	AK091439	Homo sapiens cDNA FLJ34120 fis, clone FCBBF3009541. [AK091439]	
A_23_P108415	7.53E-05	NM_001349	NM_001349	Homo sapiens aspartyl-tRNA synthetase (DARS), mRNA [NM_001349]	NM_001349
A_23_P155765	7.53E-05	NM_002129	NM_002129	Homo sapiens high-mobility group box 2 (HMGB2), mRNA [NM_002129]	NM_002129
A_23_P77048	7.54E-05	NM_152333	NM_152333	Homo sapiens solute carrier family 25, member 29 (SLC25A29), mRNA [NM_152333]	NM_152333
A_23_P201951	7.55E-05	NM_016374	NM_016374	Homo sapiens AT rich interactive domain 4B (RBP1- like) (ARID4B), transcript variant 1, mRNA [NM_016374]	NM_016374
A_23_P118815	7.55E-05	NM_001012271	NM_001012271	Homo sapiens baculoviral IAP repeat-containing 5 (survivin) (BIRC5), transcript variant 3, mRNA [NM_001012271]	NM_001012271
A_24_P251950	7.57E-05	NM_174914	NM_174914	Homo sapiens UDP glycosyltransferase 3 family, polypeptide A2 (UGT3A2), mRNA [NM_174914]	NM_174914
A_32_P191503	7.58E-05	NM_198284	NM_198284	Homo sapiens hypothetical protein LOC349114 (LOC349114), mRNA [NM_198284]	NM_198284
A_23_P138541	7.59E-05	NM_003739	NM_003739	Homo sapiens aldo-keto reductase family 1, member C3 (3-alpha hydroxysteroid dehydrogenase, type II) (AKR1C3), mRNA [NM_003739]	NM_003739
A_23_P378690	7.59E-05	CR610885	CR610885	full-length cDNA clone CS0DC019YC18 of Neuroblastoma Cot 25-normalized of Homo sapiens (human). [CR610885]	
A_24_P277367	7.59E-05	NM_002994	NM_002994	Homo sapiens chemokine (C-X-C motif) ligand 5 (CXCL5), mRNA [NM_002994]	NM_002994
A_32_P43914	7.60E-05	THC2443137			
A_32_P11451	7.60E-05	NM_015938	NM_015938	Homo sapiens NMD3 homolog (S. cerevisiae) (NMD3), mRNA [NM_015938]	NM_015938
A_23_P128304	7.61E-05	NM_001003398	NM_001003398	Homo sapiens bicaudal D homolog 1 (Drosophila) (BICD1), transcript variant 2, mRNA [NM_001003398]	NM_001003398
A_23_P111961	7.61E-05	NM_032509	NM_032509	Homo sapiens RNA binding motif protein 13 (RBM13), mRNA [NM_032509]	NM_032509
A_24_P261169	7.63E-05	NM_006378	NM_006378	Homo sapiens sema domain, immunoglobulin domain (Ig), transmembrane domain (TM) and short cytoplasmic domain, (semaphorin) 4D (SEMA4D), mRNA [NM_006378]	NM_006378
A_23_P16262	7.63E-05	NM_004533	NM_004533	Homo sapiens myosin binding protein C, fast type (MYBPC2), mRNA [NM_004533]	NM_004533
A_24_P71781	7.64E-05	AL136578	AL136578	Homo sapiens mRNA; cDNA DKFZp761C0824 (from clone DKFZp761C0824). [AL136578]	
A_23_P41054	7.64E-05	NM_002841	NM_002841	Homo sapiens protein tyrosine phosphatase, receptor type, G (PTPRG), mRNA [NM_002841]	NM_002841
A_23_P398275	7.67E-05	AF316855	AF316855	Homo sapiens colon cancer-associated antigen AgSK1-2HT-ECS mRNA, complete cds. [AF316855]	
A_32_P186027	7.68E-05	CR625412	CR625412	full-length cDNA clone CS0DA011YH23 of Neuroblastoma of Homo sapiens (human). [CR625412]	
A_24_P366390	7.70E-05	NM_198079	NM_198079	Homo sapiens golgi autoantigen, golgin subfamily a-like (FLJ40113), mRNA [NM_198079]	NM_198079
A_23_P361448	7.71E-05	NM_144665	NM_144665	Homo sapiens sestrin 3 (SESN3), mRNA [NM_144665]	NM_144665
A_23_P101253	7.71E-05	NM_025040	NM_025040	Homo sapiens zinc finger protein 614 (ZNF614), mRNA [NM_025040]	NM_025040
A_24_P377225	7.75E-05	NM_022832	NM_022832	Homo sapiens ubiquitin specific protease 46 (USP46), mRNA [NM_022832]	NM_022832
A_23_P86550	7.75E-05	NM_003750	NM_003750	Homo sapiens eukaryotic translation initiation factor 3, subunit 10 theta, 150/170kDa (EIF3S10), mRNA [NM_003750]	NM_003750
A_23_P357207	7.80E-05	NM_138409	NM_138409	Homo sapiens chromosome 6 open reading frame 117 (C6orf117), mRNA [NM_138409]	NM_138409
A_23_P257863	7.82E-05	NM_012073	NM_012073	Homo sapiens chaperonin containing TCP1, subunit 5 (epsilon) (CCT5), mRNA [NM_012073]	NM_012073
A_24_P226949	7.84E-05	NM_017645	NM_017645	Homo sapiens family with sequence similarity 29, member A (FAM29A), mRNA [NM_017645]	NM_017645
A_23_P398073	7.85E-05	NM_177968	NM_177968	Homo sapiens protein phosphatase 1B (formerly 2C), magnesium-dependent, beta isoform (PPM1B), transcript variant 2, mRNA [NM_177968]	NM_177968
A_23_P25019	7.86E-05	NM_000946	NM_000946	Homo sapiens primase, polypeptide 1, 49kDa (PRIM1), mRNA [NM_000946]	NM_000946
A_23_P7636	7.87E-05	NM_004219	NM_004219	Homo sapiens pituitary tumor-transforming 1 (PTTG1), mRNA [NM_004219]	NM_004219
A_23_P43197	7.88E-05	NM_004929	NM_004929	Homo sapiens calbindin 1, 28kDa (CALB1), mRNA [NM_004929]	NM_004929
A_23_P17204	7.89E-05	NM_022662	NM_022662	Homo sapiens anaphase promoting complex subunit 1 (ANAPC1), mRNA [NM_022662]	NM_022662
A_24_P191357	7.90E-05	THC2311196			
A_23_P213326	7.90E-05	NM_017747	NM_017747	Homo sapiens ankyrin repeat and KH domain containing 1 (ANKHD1), transcript variant 1, mRNA [NM_017747]	NM_017747
A_23_P30813	7.92E-05	NM_003541	NM_003541	Homo sapiens histone 1, H4k (HIST1H4K), mRNA [NM_003541]	NM_003541
A_32_P50834	7.92E-05	NM_006729	NM_006729	Homo sapiens diaphanous homolog 2 (Drosophila) (DIAPH2), transcript variant 156, mRNA [NM_006729]	NM_006729
A_32_P183022	7.94E-05	NM_001008493	NM_001008493	Homo sapiens enabled homolog (Drosophila) (ENAH), transcript variant 1, mRNA [NM_001008493]	NM_001008493

A_23_P301079	7.95E-05	NM_182620	NM_182620	Homo sapiens family with sequence similarity 33, member A (FAM33A), mRNA [NM_182620]	NM_182620
A_32_P43812	7.95E-05	NM_015115	NM_015115	Homo sapiens KIAA0276 protein (KIAA0276), mRNA [NM_015115]	NM_015115
A_24_P333716	7.95E-05	NM_022459	NM_022459	Homo sapiens exportin 4 (XPO4), mRNA [NM_022459]	NM_022459
A_23_P109907	8.02E-05	NM_175924	NM_175924	Homo sapiens immunoglobulin-like domain containing receptor 1 (ILDR1), mRNA [NM_175924]	NM_175924
A_24_P338757	8.03E-05	NM_005800	NM_005800	Homo sapiens chromosome 13 open reading frame 22 (C13orf22), mRNA [NM_005800]	NM_005800
A_24_P935203	8.03E-05	THC2273930		AF285161 polyubiquitin C {Mus musculus;} , partial (41%) [THC2273930]	
A_23_P85371	8.03E-05	NM_004582	NM_004582	Homo sapiens Rab geranylgeranyltransferase, beta subunit (RABGGTB), mRNA [NM_004582]	NM_004582
A_24_P306063	8.04E-05	NM_020819	NM_020819	Homo sapiens KIAA1411 (KIAA1411), mRNA [NM_020819]	NM_020819
A_24_P247044	8.04E-05	ENST00000355095		Homo sapiens mRNA for KIAA1473 protein, partial cds. [AB040906]	XM_047554
A_23_P13657	8.04E-05	NM_022782	NM_022782	Homo sapiens M-phase phosphoprotein 9 (MPHOSPH9), mRNA [NM_022782]	NM_022782
A_23_P59528	8.04E-05	NM_020186	NM_020186	Homo sapiens ACN9 homolog (S. cerevisiae) (ACN9), mRNA [NM_020186]	NM_020186
A_24_P942850	8.05E-05	NM_024989	NM_024989	Homo sapiens GPI deacylase (PGAP1), mRNA [NM_024989]	NM_024989
A_23_P4653	8.05E-05	NM_145275	NM_145275	Homo sapiens kinesin light chain 2-like (KLC2L), transcript variant 2, mRNA [NM_145275]	NM_145275
A_24_P627984	8.09E-05	THC2437914		ALU1_HUMAN (P39188) Alu subfamily J sequence contamination warning entry, partial (15%) [THC2437914]	
A_24_P860842	8.09E-05	BC027988	BC027988	Homo sapiens hypothetical protein LOC253842, mRNA (cDNA clone IMAGE:4993690), partial cds. [BC027988]	
A_23_P200493	8.11E-05	NM_002296	NM_002296	Homo sapiens lamin B receptor (LBR), transcript variant 1, mRNA [NM_002296]	NM_002296
A_32_P46495	8.12E-05	BX090412	BX090412	BX090412 Soares_testis_NHT Homo sapiens cDNA clone IMAGp998J221862 ; IMAGE:757365, mRNA sequence [BX090412]	
A_32_P147678	8.12E-05	AK123430	AK123430	Homo sapiens cDNA FLJ41436 fis, clone BRHIP2007741. [AK123430]	
A_23_P35916	8.15E-05	NM_000051	NM_000051	Homo sapiens ataxia telangiectasia mutated (includes complementation groups A, C and D) (ATM), transcript variant 1, mRNA [NM_000051]	NM_000051
A_23_P75038	8.15E-05	NM_014881	NM_014881	Homo sapiens DNA cross-link repair 1A (PSO2 homolog, S. cerevisiae) (DCLRE1A), mRNA [NM_014881]	NM_014881
A_24_P163237	8.16E-05	NM_020225	NM_020225	Homo sapiens storkhead box 2 (STOX2), mRNA [NM_020225]	NM_020225
A_23_P162596	8.16E-05	NM_022496	NM_022496	Homo sapiens ARP6 actin-related protein 6 homolog (yeast) (ACTR6), mRNA [NM_022496]	NM_022496
A_24_P346277	8.16E-05	NM_003131	NM_003131	Homo sapiens serum response factor (c-fos serum response element-binding transcription factor) (SRF), mRNA [NM_003131]	NM_003131
A_23_P428468	8.18E-05	BC063594	BC063594	Homo sapiens zinc finger protein 292, mRNA (cDNA clone IMAGE:4526801), partial cds. [BC063594]	XM_048070
A_23_P165408	8.18E-05	NM_144711	NM_144711	Homo sapiens kelch-like 23 (Drosophila) (KLHL23), mRNA [NM_144711]	NM_144711
A_24_P726336	8.19E-05	THC2304714			
A_24_P415260	8.20E-05	ENST00000354185		Homo sapiens cDNA FLJ36123 fis, clone TESTI2022874, weakly similar to ZINC FINGER PROTEIN 135. [AK093442]	
A_23_P134827	8.22E-05	NM_004674	NM_004674	Homo sapiens ash2 (absent, small, or homeotic)-like (Drosophila) (ASH2L), mRNA [NM_004674]	NM_004674
A_24_P928038	8.23E-05	ENST00000265986		Homo sapiens mRNA; cDNA DKFZp781M0420 (from clone DKFZp781M0420). [BX648462]	
A_32_P184039	8.29E-05	A_32_P184039			
A_23_P436336	8.29E-05	NM_198284	NM_198284	Homo sapiens hypothetical protein LOC349114 (LOC349114), mRNA [NM_198284]	NM_198284
A_23_P142616	8.36E-05	NM_016252	NM_016252	Homo sapiens baculoviral IAP repeat-containing 6 (apollon) (BIRC6), mRNA [NM_016252]	NM_016252
A_23_P213126	8.37E-05	AB015331	AB015331	Homo sapiens HRIHFB2017 mRNA, partial cds. [AB015331]	
A_24_P53985	8.37E-05	NM_024772	NM_024772	Homo sapiens zinc finger, MYM domain containing 1 (ZMYM1), mRNA [NM_024772]	NM_024772
A_23_P49646	8.37E-05	NM_002767	NM_002767	Homo sapiens phosphoribosyl pyrophosphate synthetase-associated protein 2 (PRPSAP2), mRNA [NM_002767]	NM_002767
A_24_P930647	8.37E-05	S75896	S75896	clone 39-1 [human, brain, mRNA Mitochondrial Partial Mutant, 201 nt]. [S75896]	
A_23_P151634	8.38E-05	NM_007192	NM_007192	Homo sapiens suppressor of Ty 16 homolog (S. cerevisiae) (SUPT16H), mRNA [NM_007192]	NM_007192
A_32_P45009	8.39E-05	NM_005896	NM_005896	Homo sapiens isocitrate dehydrogenase 1 (NADP+), soluble (IDH1), mRNA [NM_005896]	NM_005896
A_32_P118942	8.40E-05	THC2277556		Q9B8U2 (Q9B8U2) NADH dehydrogenase subunit 6, partial (10%) [THC2277556]	
A_23_P408108	8.43E-05	NM_006980	NM_006980	Homo sapiens mitochondrial transcription termination factor (MTERF), nuclear gene encoding mitochondrial protein, mRNA [NM_006980]	NM_006980
A_23_P212715	8.43E-05	NM_170662	NM_170662	Homo sapiens Cas-Br-M (murine) ecotropic retroviral transforming sequence b (CBLB), mRNA [NM_170662]	NM_170662
A_32_P170888	8.43E-05	NM_013354	NM_013354	Homo sapiens CCR4-NOT transcription complex, subunit 7 (CNOT7), transcript variant 1, mRNA [NM_013354]	NM_013354
A_24_P153643	8.45E-05	NM_004947	NM_004947	Homo sapiens dedicator of cytokinesis 3 (DOCK3), mRNA [NM_004947]	NM_004947
A_32_P40673	8.46E-05	THC2281732			

A_24_P182620	8.46E-05	NM_001408	NM_001408	Homo sapiens cadherin, EGF LAG seven-pass G-type receptor 2 (flamingo homolog, Drosophila) (CELSR2), mRNA [NM_001408]	NM_001408
A_24_P151834	8.50E-05	NM_020740	NM_020740	Homo sapiens ankyrin repeat and FYVE domain containing 1 (ANKFY1), transcript variant 2, mRNA [NM_020740]	NM_020740
A_23_P308150	8.50E-05	NM_152424	NM_152424	Homo sapiens hypothetical protein FLJ39827 (FLJ39827), mRNA [NM_152424]	NM_152424
A_24_P856722	8.51E-05	AI791206	AI791206	nu30f08.y5 NCI_CGAP_Ov5 Homo sapiens cDNA clone IMAGE:1212231 similar to contains Alu repetitive element;contains element MER37 repetitive element ;, mRNA sequence [AI791206]	
A_24_P497843	8.51E-05	THC2290002			
A_24_P297098	8.52E-05	NM_016436	NM_016436	Homo sapiens PHD finger protein 20 (PHF20), mRNA [NM_016436]	NM_016436
A_23_P130642	8.53E-05	NM_178514	NM_178514	Homo sapiens hypothetical protein LOC283487 (LOC283487), mRNA [NM_178514]	NM_178514
A_23_P136013	8.54E-05	A_23_P136013			
A_23_P138805	8.55E-05	NM_012124	NM_012124	Homo sapiens cysteine and histidine-rich domain (CHORD)-containing, zinc binding protein 1 (CHORDC1), mRNA [NM_012124]	NM_012124
A_23_P309850	8.57E-05	NM_152260	NM_152260	Homo sapiens RNA pseudouridylate synthase domain containing 2 (RPUSD2), mRNA [NM_152260]	NM_152260
A_23_P141863	8.59E-05	NM_014480	NM_014480	Homo sapiens zinc finger protein 544 (ZNF544), mRNA [NM_014480]	NM_014480
A_23_P345887	8.59E-05	AF445027	AF445027	Homo sapiens clone 114 tumor rejection antigen mRNA, complete cds. [AF445027]	
A_24_P927650	8.61E-05	AK056744	AK056744	Homo sapiens cDNA FLJ32182 fis, clone PLACE6001823. [AK056744]	
A_24_P291231	8.61E-05	NM_016831	NM_016831	Homo sapiens period homolog 3 (Drosophila) (PER3), mRNA [NM_016831]	NM_016831
A_24_P20120	8.61E-05	NM_018084	NM_018084	Homo sapiens KIAA1212 (KIAA1212), mRNA [NM_018084]	NM_018084
A_32_P180315	8.62E-05	NM_020893	NM_020893	Homo sapiens KIAA1529 (KIAA1529), mRNA [NM_020893]	NM_020893
A_24_P332230	8.62E-05	NM_016648	NM_016648	Homo sapiens HDCMA18P protein (HDCMA18P), mRNA [NM_016648]	NM_016648
A_23_P308731	8.62E-05	NM_138328	NM_138328	Homo sapiens rhomboid, veinlet-like 4 (Drosophila) (RHBDL4), mRNA [NM_138328]	NM_138328
A_24_P287473	8.62E-05	NM_021818	NM_021818	Homo sapiens salvador homolog 1 (Drosophila) (SAV1), mRNA [NM_021818]	NM_021818
A_23_P146284	8.62E-05	NM_003129	NM_003129	Homo sapiens squalene epoxidase (SQLE), mRNA [NM_003129]	NM_003129
A_23_P218517	8.65E-05	NM_013360	NM_013360	Homo sapiens zinc finger protein 222 (ZNF222), mRNA [NM_013360]	NM_013360
A_23_P47788	8.65E-05	NM_005371	NM_005371	Homo sapiens methyltransferase like 1 (METTL1), transcript variant 1, mRNA [NM_005371]	NM_005371
A_23_P80062	8.66E-05	NM_003185	NM_003185	Homo sapiens TAF4 RNA polymerase II, TATA box binding protein (TBP)-associated factor, 135kDa (TAF4), mRNA [NM_003185]	NM_003185
A_23_P72059	8.66E-05	ENST00000359236		Homo sapiens cDNA FLJ20674 fis, clone KAIA4450. [AK000681]	
A_23_P66473	8.66E-05	NM_181671	NM_181671	Homo sapiens phosphatidylinositol transfer protein, cytoplasmic 1 (PITPNC1), transcript variant 2, mRNA [NM_181671]	NM_181671
A_24_P204675	8.66E-05	BC094882	BC094882	Homo sapiens cDNA clone MGC:105000 IMAGE:3093162, complete cds. [BC094882]	XM_290670
A_24_P206047	8.67E-05	NM_001151	NM_001151	Homo sapiens solute carrier family 25 (mitochondrial carrier; adenine nucleotide translocator), member 4 (SLC25A4), nuclear gene encoding mitochondrial protein, mRNA [NM_001151]	NM_001151
A_23_P215625	8.70E-05	NM_021723	NM_021723	Homo sapiens a disintegrin and metalloproteinase domain 22 (ADAM22), transcript variant 1, mRNA [NM_021723]	NM_021723
A_32_P223551	8.70E-05	THC2374771		LPXK_RHIME (Q92RP7) Tetraacyldisaccharide 4'-kinase (Lipid A 4'-kinase) , partial (5%) [THC2374771]	
A_24_P350656	8.70E-05	NM_001005389	NM_001005389	Homo sapiens neurofascin (NFASC), transcript variant 3, mRNA [NM_001005389]	NM_001005389
A_32_P1701	8.70E-05	NM_016937	NM_016937	Homo sapiens polymerase (DNA directed), alpha (POLA), mRNA [NM_016937]	NM_016937
A_23_P44794	8.71E-05	NM_138453	NM_138453	Homo sapiens RAB3C, member RAS oncogene family (RAB3C), mRNA [NM_138453]	NM_138453
A_23_P28590	8.71E-05	NM_024622	NM_024622	Homo sapiens hypothetical protein FLJ21901 (FLJ21901), mRNA [NM_024622]	NM_024622
A_24_P349151	8.72E-05	NM_194292	NM_194292	Homo sapiens spindle assembly abnormal protein 6 (SAS-6), mRNA [NM_194292]	NM_194292
A_24_P298077	8.72E-05	NM_017664	NM_017664	Homo sapiens ankyrin repeat domain 10 (ANKRD10), mRNA [NM_017664]	NM_017664
A_23_P122254	8.73E-05	NM_005124	NM_005124	Homo sapiens nucleoporin 153kDa (NUP153), mRNA [NM_005124]	NM_005124
A_32_P201150	8.75E-05	THC2399007		Q96FS0 (Q96FS0) KRAS2 protein, partial (24%) [THC2399007]	
A_23_P312179	8.76E-05	NM_015120	NM_015120	Homo sapiens Alstrom syndrome 1 (ALMS1), mRNA [NM_015120]	NM_015120
A_23_P213908	8.76E-05	NM_032177	NM_032177	Homo sapiens RNA U, small nuclear RNA export adaptor (phosphorylation regulated) (RNUXA), mRNA [NM_032177]	NM_032177
A_23_P212914	8.80E-05	CR598922	CR598922	full-length cDNA clone CS0DD007YM02 of Neuroblastoma Cot 50-normalized of Homo sapiens (human). [CR598922]	
A_23_P209694	8.81E-05	NM_022894	NM_022894	Homo sapiens poly(A) polymerase gamma (PAPOLG), mRNA [NM_022894]	NM_022894
A_23_P25626	8.82E-05	NM_024808	NM_024808	Homo sapiens FLJ22624 protein (FLJ22624), mRNA [NM_024808]	NM_024808
A_24_P58381	8.84E-05	NM_017780	NM_017780	Homo sapiens chromodomain helicase DNA binding protein 7 (CHD7), mRNA [NM_017780]	NM_017780
A_23_P131967	8.86E-05	NM_016652	NM_016652	Homo sapiens Crn, crooked neck-like 1 (Drosophila) (CRNKL1), mRNA [NM_016652]	NM_016652

A_24_P197196	8.87E-05	NM_005689	NM_005689	Homo sapiens ATP-binding cassette, sub-family B (MDR/TAP), member 6 (ABCB6), nuclear gene encoding mitochondrial protein, mRNA [NM_005689]	NM_005689
A_23_P128991	8.87E-05	NM_031210	NM_031210	Homo sapiens chromosome 14 open reading frame 156 (C14orf156), mRNA [NM_031210]	NM_031210
A_24_P716394	8.88E-05	THC2360841		ALU7_HUMAN (P39194) Alu subfamily SQ sequence contamination warning entry, partial (10%) [THC2360844]	
A_24_P945194	8.88E-05	NM_013374	NM_013374	Homo sapiens programmed cell death 6 interacting protein (PDCD6IP), mRNA [NM_013374]	NM_013374
A_24_P381803	8.92E-05	NM_130839	NM_130839	Homo sapiens ubiquitin protein ligase E3A (human papilloma virus E6-associated protein, Angelman syndrome) (UBE3A), transcript variant 3, mRNA [NM_130839]	NM_130839
A_23_P35645	8.92E-05	NM_032905	NM_032905	Homo sapiens RNA binding motif protein 17 (RBM17), mRNA [NM_032905]	NM_032905
A_23_P153867	8.93E-05	NM_024552	NM_024552	Homo sapiens LAG1 longevity assurance homolog 4 (S. cerevisiae) (LASS4), mRNA [NM_024552]	NM_024552
A_23_P320530	8.94E-05	AK091274	AK091274	Homo sapiens cDNA FLJ33955 fis, clone CTONG2018652, moderately similar to ZINC FINGER PROTEIN MFG-3. [AK091274]	
A_32_P45493	8.96E-05	NM_006625	NM_006625	Homo sapiens FUS interacting protein (serine/arginine-rich) 1 (FUSIP1), transcript variant 1, mRNA [NM_006625]	NM_006625
A_23_P150667	8.97E-05	NM_031217	NM_031217	Homo sapiens kinesin family member 18A (KIF18A), mRNA [NM_031217]	NM_031217
A_24_P179646	9.00E-05	M96956	M96956	Human (clone CR-3) teratocarcinoma-derived growth factor 3 (TDGF3) mRNA, complete cds. [M96956]	
A_32_P73071	9.01E-05	THC2453866			
A_23_P387856	9.01E-05	NM_015277	NM_015277	Homo sapiens neural precursor cell expressed, developmentally down-regulated 4-like (NEDD4L), mRNA [NM_015277]	NM_015277
A_24_P99071	9.02E-05	NM_002271	NM_002271	Homo sapiens RAN binding protein 5 (RANBP5), mRNA [NM_002271]	NM_002271
A_23_P86461	9.03E-05	THC2435505			
A_23_P203030	9.04E-05	NM_001931	NM_001931	Homo sapiens dihydrolipoamide S-acetyltransferase (E2 component of pyruvate dehydrogenase complex) (DLAT), mRNA [NM_001931]	NM_001931
A_32_P138617	9.07E-05	CR590163	CR590163	full-length cDNA clone CS0CAP004YE05 of Thymus of Homo sapiens (human). [CR590163]	
A_32_P748131	9.10E-05	AB075828	AB075828	Homo sapiens mRNA for KIAA1948 protein. [AB075828]	
A_23_P34788	9.12E-05	NM_006845	NM_006845	Homo sapiens kinesin family member 2C (KIF2C), mRNA [NM_006845]	NM_006845
A_23_P99604	9.13E-05	NM_017769	NM_017769	Homo sapiens KIAA1333 (KIAA1333), mRNA [NM_017769]	NM_017769
A_32_P79707	9.14E-05	AI022288	AI022288	AI022288 ow63d08.x1 Soares_senescent_fibroblasts_NbHSF Homo sapiens cDNA clone IMAGE:1651503 3', mRNA sequence [AI022288]	
A_23_P341700	9.14E-05	NM_001007101	NM_001007101	Homo sapiens zinc finger protein 484 (ZNF484), transcript variant 2, mRNA [NM_001007101]	NM_001007101
A_23_P101258	9.14E-05	NM_025040	NM_025040	Homo sapiens zinc finger protein 614 (ZNF614), mRNA [NM_025040]	NM_025040
A_23_P66158	9.15E-05	CR625565	CR625565	full-length cDNA clone CS0DI060YI16 of Placenta Cot 25-normalized of Homo sapiens (human). [CR625565]	
A_23_P92765	9.18E-05	CR604116	CR604116	full-length cDNA clone CS0DF027YM18 of Fetal brain of Homo sapiens (human). [CR604116]	
A_32_P31744	9.20E-05	AK021664	AK021664	Homo sapiens cDNA FLJ11602 fis, clone HEMBA1003908. [AK021664]	
A_23_P1387	9.20E-05	NM_032900	NM_032900	Homo sapiens Rho GTPase activating protein 19 (ARHGAP19), mRNA [NM_032900]	NM_032900
A_23_P100092	9.22E-05	NM_152455	NM_152455	Homo sapiens zinc finger protein 690 (ZNF690), mRNA [NM_152455]	NM_152455
A_24_P854492	9.22E-05	CR620293	CR620293	full-length cDNA clone CS0DF028YD24 of Fetal brain of Homo sapiens (human). [CR620293]	XM_498878
A_24_P913760	9.24E-05	THC2278264			
A_24_P75220	9.25E-05	NM_004742	NM_004742	Homo sapiens membrane associated guanylate kinase, WW and PDZ domain containing 1 (MAGI1), mRNA [NM_004742]	NM_004742
A_32_P196918	9.25E-05	AK057956	AK057956	Homo sapiens cDNA FLJ25227 fis, clone STM01429. [AK057956]	
A_24_P73730	9.25E-05	NM_014157	NM_014157	Homo sapiens HSPC065 protein (HSPC065), mRNA [NM_014157]	NM_014157
A_24_P407704	9.29E-05	THC2434479		DD19_HUMAN (Q9UMR2) ATP-dependent RNA helicase DDX19 (DEAD-box protein 19) (DEAD-box RNA helicase DEAD5), partial (90%) [THC2434479]	
A_23_P169050	9.30E-05	NM_014018	NM_014018	Homo sapiens mitochondrial ribosomal protein S28 (MRPS28), nuclear gene encoding mitochondrial protein, mRNA [NM_014018]	NM_014018
A_23_P16110	9.31E-05	NR_002146	NR_002146	Homo sapiens olfactory receptor, family 7, subfamily E, member 24 (OR7E24) on chromosome 19 [NR_002146]	NR_002146
A_23_P88740	9.32E-05	NM_018455	NM_018455	Homo sapiens uncharacterized bone marrow protein BM039 (BM039), mRNA [NM_018455]	NM_018455
A_23_P68007	9.32E-05	NM_001679	NM_001679	Homo sapiens ATPase, Na+/K+ transporting, beta 3 polypeptide (ATP1B3), mRNA [NM_001679]	NM_001679
A_23_P93464	9.34E-05	NM_000056	NM_000056	Homo sapiens branched chain keto acid dehydrogenase E1, beta polypeptide (maple syrup urine disease) (BCKDHB), nuclear gene encoding mitochondrial protein, transcript variant 2, mRNA [NM_000056]	NM_000056
A_24_P270235	9.34E-05	NM_001759	NM_001759	Homo sapiens cyclin D2 (CCND2), mRNA [NM_001759]	NM_001759
A_23_P106241	9.36E-05	NM_004239	NM_004239	Homo sapiens thyroid hormone receptor interactor 11 (TRIP11), mRNA [NM_004239]	NM_004239
A_23_P163496	9.37E-05	NM_023936	NM_023936	Homo sapiens mitochondrial ribosomal protein S34 (MRPS34), nuclear gene encoding mitochondrial protein, mRNA [NM_023936]	NM_023936
A_24_P309105	9.38E-05	NM_015450	NM_015450	Homo sapiens POT1 protection of telomeres 1 homolog (S. pombe) (POT1), mRNA [NM_015450]	NM_015450

A_23_P130466	9.40E-05	NM_021089	NM_021089	Homo sapiens zinc finger protein 8 (clone HF.18) (ZNF8), mRNA [NM_021089]	NM_021089
A_23_P130187	9.43E-05	A_23_P130187			
A_23_P132874	9.48E-05	NM_032359	NM_032359	Homo sapiens hypothetical protein MGC4308 (MGC4308), mRNA [NM_032359]	NM_032359
A_32_P42976	9.50E-05	THC2381319		BC022074 C6orf102 protein {Homo sapiens;} , partial (6%) [THC2381319]	
A_23_P150129	9.50E-05	NM_018312	NM_018312	Homo sapiens chromosome 11 open reading frame 23 (C11orf23), mRNA [NM_018312]	NM_018312
A_32_P74964	9.53E-05	AK055101	AK055101	Homo sapiens cDNA FLJ30539 fis, clone BRAWH2001255. [AK055101]	
A_23_P68487	9.53E-05	NM_001719	NM_001719	Homo sapiens bone morphogenetic protein 7 (osteogenic protein 1) (BMP7), mRNA [NM_001719]	NM_001719
A_32_P326819	9.55E-05	NM_007043	NM_007043	Homo sapiens HIV-1 rev binding protein 2 (HRB2), mRNA [NM_007043]	NM_007043
A_23_P259393	9.55E-05	NM_001005159	NM_001005159	Homo sapiens Scm-like with four mbt domains 1 (SFMBT1), transcript variant 1, mRNA [NM_001005159]	NM_001005159
A_32_P180920	9.61E-05	AY685922	AY685922	Homo sapiens glioma amplified sequence 64 mRNA, complete cds. [AY685922]	
A_23_P132611	9.63E-05	AF088066	AF088066	Homo sapiens full length insert cDNA clone ZD86C03. [AF088066]	
A_23_P304511	9.64E-05	NM_032347	NM_032347	Homo sapiens zinc finger protein 397 (ZNF397), mRNA [NM_032347]	NM_032347
A_24_P285522	9.65E-05	NM_003618	NM_003618	Homo sapiens mitogen-activated protein kinase kinase kinase kinase 3 (MAP4K3), mRNA [NM_003618]	NM_003618
A_32_P185701	9.67E-05	BC041959	BC041959	Homo sapiens, clone IMAGE:5302136, mRNA. [BC041959]	
A_24_P218509	9.67E-05	THC2434888			
A_23_P375147	9.67E-05	AK125196	AK125196	Homo sapiens cDNA FLJ43206 fis, clone FEBRA2009419. [AK125196]	
A_23_P77066	9.68E-05	NM_022807	NM_022807	Homo sapiens small nuclear ribonucleoprotein polypeptide N (SNRPN), transcript variant 4, mRNA [NM_022807]	NM_022807
A_23_P167194	9.69E-05	NM_001812	NM_001812	Homo sapiens centromere protein C 1 (CENPC1), mRNA [NM_001812]	NM_001812
A_23_P128174	9.69E-05	NM_175623	NM_175623	Homo sapiens RAB3A interacting protein (rabin3) (RAB3IP), transcript variant alpha 2, mRNA [NM_175623]	NM_175623
A_32_P140823	9.72E-05	NM_006985	NM_006985	Homo sapiens nuclear pore complex interacting protein (NPIP), mRNA [NM_006985]	NM_006985
A_23_P431252	9.73E-05	NM_032505	NM_032505	Homo sapiens T-cell activation kelch repeat protein (TA-KRP), mRNA [NM_032505]	NM_032505
A_24_P341731	9.73E-05	A_24_P341731			
A_24_P678620	9.74E-05	THC2405710			
A_23_P346421	9.74E-05	NM_018181	NM_018181	Homo sapiens zinc finger protein 532 (ZNF532), mRNA [NM_018181]	NM_018181
A_23_P48663	9.75E-05	NM_021728	NM_021728	Homo sapiens orthodenticle homolog 2 (Drosophila) (OTX2), transcript variant 1, mRNA [NM_021728]	NM_021728
A_24_P940776	9.76E-05	CR749289	CR749289	Homo sapiens mRNA; cDNA DKFZp686L02246 (from clone DKFZp686L02246). [CR749289]	
A_24_P201879	9.78E-05	NM_021645	NM_021645	Homo sapiens UTP14, U3 small nucleolar ribonucleoprotein, homolog C (yeast) (UTP14C), mRNA [NM_021645]	NM_021645
A_23_P155907	9.78E-05	NM_015143	NM_015143	Homo sapiens methionyl aminopeptidase 1 (METAP1), mRNA [NM_015143]	NM_015143
A_24_P116766	9.79E-05	AL834501	AL834501	Homo sapiens mRNA; cDNA DKFZp761N202 (from clone DKFZp761N202). [AL834501]	
A_23_P150080	9.80E-05	NM_183005	NM_183005	Homo sapiens ribonuclease P/MRP 38kDa subunit (RPP38), transcript variant 1, mRNA [NM_183005]	NM_183005
A_32_P160972	9.82E-05	BC014953	BC014953	Homo sapiens chromosome 6 open reading frame 115, mRNA (cDNA clone MGC:22978 IMAGE:4849571), complete cds. [BC014953]	XM_371848
A_24_P114249	9.83E-05	NM_004482	NM_004482	Homo sapiens UDP-N-acetyl-alpha-D-galactosamine:polypeptide N-acetylgalactosaminyltransferase 3 (GalNAc-T3) (GALNT3), mRNA [NM_004482]	NM_004482
A_24_P56557	9.83E-05	NM_080650	NM_080650	Homo sapiens similar to RIKEN cDNA 5730421E18 gene (MGC14798), mRNA [NM_080650]	NM_080650
A_23_P148602	9.83E-05	NM_003669	NM_003669	Homo sapiens inactivation escape 1 (INE1), mRNA [NM_003669]	NM_003669
A_23_P18465	9.83E-05	NM_002913	NM_002913	Homo sapiens replication factor C (activator 1) 1, 145kDa (RFC1), mRNA [NM_002913]	NM_002913
A_24_P171549	9.87E-05	NM_031942	NM_031942	Homo sapiens cell division cycle associated 7 (CDCA7), transcript variant 1, mRNA [NM_031942]	NM_031942
A_24_P273823	9.91E-05	NM_002519	NM_002519	Homo sapiens nuclear protein, ataxia-telangiectasia locus (NPAT), mRNA [NM_002519]	NM_002519
A_24_P11131	9.93E-05	NM_005839	NM_005839	Homo sapiens serine/arginine repetitive matrix 1 (SRRM1), mRNA [NM_005839]	NM_005839
A_23_P132784	9.94E-05	NM_001013439	NM_001013439	Homo sapiens fragile X mental retardation, autosomal homolog 1 (FXR1), transcript variant 3, mRNA [NM_001013439]	NM_001013439
A_23_P128641	9.95E-05	NM_005800	NM_005800	Homo sapiens chromosome 13 open reading frame 22 (C13orf22), mRNA [NM_005800]	NM_005800
A_23_P62188	9.96E-05	NM_018684	NM_018684	Homo sapiens KIAA1166 (KIAA1166), mRNA [NM_018684]	NM_018684
A_23_P108042	9.97E-05	NM_004829	NM_004829	Homo sapiens natural cytotoxicity triggering receptor 1 (NCR1), mRNA [NM_004829]	NM_004829
A_23_P98431	9.98E-05	NM_000190	NM_000190	Homo sapiens hydroxymethylbilane synthase (HMBS), transcript variant 1, mRNA [NM_000190]	NM_000190
A_24_P586660	9.99E-05	BG292169	BG292169	BG292169 602386485F1 NIH_MGC_93 Homo sapiens cDNA clone IMAGE:4515548 5', mRNA sequence [BG292169]	
A_32_P203592	0.0001	NM_015092	NM_015092	Homo sapiens PI-3-kinase-related kinase SMG-1 (SMG1), mRNA [NM_015092]	NM_015092

A_23_P355447	0.0001	NM_174976	NM_174976	Homo sapiens zinc finger, DHHC-type containing 22 (ZDHHC22), mRNA [NM_174976]	NM_174976
A_23_P33154	0.0001	NM_014393	NM_014393	Homo sapiens staufen, RNA binding protein, homolog 2 (Drosophila) (STAU2), mRNA [NM_014393]	NM_014393
A_23_P137578	0.0001	NM_015176	NM_015176	Homo sapiens F-box protein 28 (FBXO28), mRNA [NM_015176]	NM_015176
A_23_P202143	0.0001	BC006769	BC006769	Homo sapiens nucleolar and coiled-body phosphoprotein 1, mRNA (cDNA clone MGC:5049 IMAGE:2900024), complete cds. [BC006769]	
A_23_P398044	0.0001	NM_052848	NM_052848	Homo sapiens hypothetical protein MGC20255 (MGC20255), mRNA [NM_052848]	NM_052848
A_23_P27873	0.0001	NM_018049	NM_018049	Homo sapiens pleckstrin homology domain containing, family J member 1 (PLEKHJ1), mRNA [NM_018049]	NM_018049
A_24_P161355	0.0001	THC2456735		RS10_HUMAN (P46783) 40S ribosomal protein S10, partial (98%) [THC2456735]	
A_24_P20873	0.000101	NM_003495	NM_003495	Homo sapiens histone 1, H4i (HIST1H4I), mRNA [NM_003495]	NM_003495
A_23_P130158	0.000101	NM_030753	NM_030753	Homo sapiens wingless-type MMTV integration site family, member 3 (WNT3), mRNA [NM_030753]	NM_030753
A_24_P97001	0.000101	NM_032864	NM_032864	Homo sapiens PRP38 pre-mRNA processing factor 38 (yeast) domain containing A (PRPF38A), transcript variant 2, mRNA [NM_032864]	NM_032864
A_32_P63858	0.000102	THC2279910		ALU6_HUMAN (P39193) Alu subfamily SP sequence contamination warning entry, partial (5%) [THC2279910]	
A_23_P5234	0.000102	NM_003423	NM_003423	Homo sapiens zinc finger protein 43 (HTF6) (ZNF43), mRNA [NM_003423]	NM_003423
A_32_P33213	0.000102	THC2358845			
A_24_P668351	0.000102	THC2405936			
A_24_P494454	0.000102	AK127485	AK127485	Homo sapiens cDNA FLJ45577 fis, clone BRTHA3011265, highly similar to Spindlin. [AK127485]	
A_23_P134008	0.000102	BC005991	BC005991	Homo sapiens ubiquitin specific protease 45, mRNA (cDNA clone MGC:14793 IMAGE:4047601), complete cds. [BC005991]	
A_23_P50646	0.000102	BC071811	BC071811	Homo sapiens cDNA clone IMAGE:6452513, partial cds. [BC071811]	
A_23_P59022	0.000102	NM_033502	NM_033502	Homo sapiens transcriptional regulating factor 1 (TRERF1), transcript variant 1, mRNA [NM_033502]	NM_033502
A_23_P410587	0.000102	NM_024900	NM_024900	Homo sapiens PHD finger protein 17 (PHF17), transcript variant S, mRNA [NM_024900]	NM_024900
A_23_P317200	0.000102	NM_153340	NM_153340	Homo sapiens ataxin 7-like 2 (ATXN7L2), mRNA [NM_153340]	NM_153340
A_32_P172864	0.000102	NM_001010883	NM_001010883	Homo sapiens hypothetical protein LOC284611 (LOC284611), mRNA [NM_001010883]	NM_001010883
A_23_P161257	0.000102	THC2306200		Q5W093 (Q5W093) OTTHUMP00000059187 (Programmed cell death 11), complete [THC2306200]	
A_23_P345591	0.000102	NM_002787	NM_002787	Homo sapiens proteasome (prosome, macropain) subunit, alpha type, 2 (PSMA2), mRNA [NM_002787]	NM_002787
A_23_P118544	0.000102	NM_004859	NM_004859	Homo sapiens clathrin, heavy polypeptide (Hc) (CLTC), mRNA [NM_004859]	NM_004859
A_23_P88470	0.000103	NM_017672	NM_017672	Homo sapiens transient receptor potential cation channel, subfamily M, member 7 (TRPM7), mRNA [NM_017672]	NM_017672
A_24_P248606	0.000103	NM_004457	NM_004457	Homo sapiens acyl-CoA synthetase long-chain family member 3 (ACSL3), transcript variant 1, mRNA [NM_004457]	NM_004457
A_23_P7577	0.000103	NM_017665	NM_017665	Homo sapiens zinc finger, CCHC domain containing 10 (ZCCHC10), mRNA [NM_017665]	NM_017665
A_23_P7596	0.000103	NM_012334	NM_012334	Homo sapiens myosin X (MYO10), mRNA [NM_012334]	NM_012334
A_24_P941459	0.000104	AK022156	AK022156	Homo sapiens cDNA FLJ12094 fis, clone HEMBB1002607, highly similar to Homo sapiens vitamin D3 receptor interacting protein (DRIP80) mRNA. [AK022156]	
A_32_P76602	0.000104	THC2281350			
A_32_P85593	0.000104	NM_194324	NM_194324	Homo sapiens hypothetical protein MGC39900 (MGC39900), mRNA [NM_194324]	NM_194324
A_23_P63847	0.000104	NM_003171	NM_003171	Homo sapiens suppressor of var1, 3-like 1 (S. cerevisiae) (SUPV3L1), mRNA [NM_003171]	NM_003171
A_23_P347528	0.000104	NM_005653	NM_005653	Homo sapiens transcription factor CP2 (TFCP2), mRNA [NM_005653]	NM_005653
A_32_P178945	0.000104	NM_018566	NM_018566	Homo sapiens YOD1 OTU deubiquinating enzyme 1 homolog (yeast) (YOD1), mRNA [NM_018566]	NM_018566
A_32_P67577	0.000105	THC2401493			
A_24_P3804	0.000105	NM_002441	NM_002441	Homo sapiens mutS homolog 5 (E. coli) (MSH5), transcript variant 3, mRNA [NM_002441]	NM_002441
A_23_P257296	0.000105	NM_003226	NM_003226	Homo sapiens trefoil factor 3 (intestinal) (TFF3), mRNA [NM_003226]	NM_003226
A_23_P386764	0.000105	NM_020438	NM_020438	Homo sapiens dolichyl pyrophosphate phosphatase 1 (DOLPP1), mRNA [NM_020438]	NM_020438
A_23_P1374	0.000106	NM_006257	NM_006257	Homo sapiens protein kinase C, theta (PRKCQ), mRNA [NM_006257]	NM_006257
A_24_P226116	0.000106	NM_057175	NM_057175	Homo sapiens NMDA receptor regulated 1 (NARG1), mRNA [NM_057175]	NM_057175
A_23_P157333	0.000106	NM_005232	NM_005232	Homo sapiens EPH receptor A1 (EPHA1), mRNA [NM_005232]	NM_005232
A_23_P88909	0.000106	NM_004209	NM_004209	Homo sapiens synaptogyrin 3 (SYNGR3), mRNA [NM_004209]	NM_004209
A_23_P210319	0.000106	NM_183360	NM_183360	Homo sapiens dystrobrevin, beta (DTNB), transcript variant 4, mRNA [NM_183360]	NM_183360
A_32_P103678	0.000106	THC2374684			

A_23_P162807	0.000106	NM_005830	NM_005830	Homo sapiens mitochondrial ribosomal protein S31 (MRPS31), nuclear gene encoding mitochondrial protein, mRNA [NM_005830]	NM_005830
A_32_P214471	0.000107	THC2439581		Q73HM5 (Q73HM5) Phosphatidate cytidylyltransferase , partial (6%) [THC2439581]	
A_23_P161686	0.000107	NM_014715	NM_014715	Homo sapiens Rho GTPase-activating protein (RICS), mRNA [NM_014715]	NM_014715
A_24_P83678	0.000107	NM_198468	NM_198468	Homo sapiens chromosome 6 open reading frame 167 (C6orf167), mRNA [NM_198468]	NM_198468
A_23_P427502	0.000107	AB051436	AB051436	Homo sapiens mRNA for KIAA1133 protein, partial cds. [AB051436]	
A_24_P244952	0.000107	NM_015092	NM_015092	Homo sapiens PI-3-kinase-related kinase SMG-1 (SMG1), mRNA [NM_015092]	NM_015092
A_23_P256716	0.000107	NM_003601	NM_003601	Homo sapiens SWI/SNF related, matrix associated, actin dependent regulator of chromatin, subfamily a, member 5 (SMARCA5), mRNA [NM_003601]	NM_003601
A_23_P332326	0.000107	NM_153213	NM_153213	Homo sapiens Rho guanine nucleotide exchange factor (GEF) 19 (ARHGEF19), mRNA [NM_153213]	NM_153213
A_23_P217596	0.000107	AF022789	AF022789	Homo sapiens ubiquitin hydrolyzing enzyme I (UBH1) mRNA, partial cds. [AF022789]	
A_32_P37733	0.000107	AK098081	AK098081	Homo sapiens cDNA FLJ40762 fis, clone TRACH2002847. [AK098081]	
A_24_P920319	0.000108	NM_021211	NM_021211	Homo sapiens transposon-derived Buster1 transposase-like protein gene (LOC58486), mRNA [NM_021211]	NM_021211
A_32_P216715	0.000108	BM666601	BM666601	UI-E-CQ1-aew-l-18-0-UI.s1 UI-E-CQ1 Homo sapiens cDNA clone UI-E-CQ1-aew-l-18-0-UI 3', mRNA sequence [BM666601]	
A_23_P165247	0.000108	NM_170711	NM_170711	Homo sapiens DAZ associated protein 1 (DAZAP1), transcript variant 1, mRNA [NM_170711]	NM_170711
A_32_P7118	0.000108	NM_002806	NM_002806	Homo sapiens proteasome (prosome, macropain) 26S subunit, ATPase, 6 (PSMC6), mRNA [NM_002806]	NM_002806
A_23_P340382	0.000109	ENST00000295031		Homo sapiens mRNA for KIAA1841 protein, partial cds. [AB058744]	
A_23_P254025	0.000109	NM_003408	NM_003408	Homo sapiens zinc finger protein 37 homolog (mouse) (ZFP37), mRNA [NM_003408]	NM_003408
A_23_P386398	0.000109	NM_153344	NM_153344	Homo sapiens chromosome 6 open reading frame 141 (C6orf141), mRNA [NM_153344]	NM_153344
A_23_P49517	0.000109	NM_006924	NM_006924	Homo sapiens splicing factor, arginine/serine-rich 1 (splicing factor 2, alternate splicing factor) (SFRS1), mRNA [NM_006924]	NM_006924
A_24_P399942	0.00011	NM_173694	NM_173694	Homo sapiens ATPase, Class VI, type 11C (ATP11C), transcript variant 1, mRNA [NM_173694]	NM_173694
A_24_P346126	0.00011	BC021174	BC021174	Homo sapiens small EDRK-rich factor 1A (telomeric), mRNA (cDNA clone MGC:32975 IMAGE:4824358), complete cds. [BC021174]	
A_32_P35512	0.00011	NM_003142	NM_003142	Homo sapiens Sjogren syndrome antigen B (autoantigen La) (SSB), mRNA [NM_003142]	NM_003142
A_23_P324453	0.000111	NM_004876	NM_004876	Homo sapiens zinc finger protein 254 (ZNF254), mRNA [NM_004876]	NM_004876
A_23_P328766	0.000111	NM_145287	NM_145287	Homo sapiens zinc finger protein 519 (ZNF519), mRNA [NM_145287]	NM_145287
A_24_P921933	0.000111	CR611166	CR611166	full-length cDNA clone CS0CAP007YF02 of Thymus of Homo sapiens (human). [CR611166]	
A_24_P311694	0.000111	NM_014757	NM_014757	Homo sapiens mastermind-like 1 (Drosophila) (MAML1), mRNA [NM_014757]	NM_014757
A_23_P54297	0.000111	NM_003758	NM_003758	Homo sapiens eukaryotic translation initiation factor 3, subunit 1 alpha, 35kDa (EIF3S1), mRNA [NM_003758]	NM_003758
A_23_P14482	0.000111	NM_016039	NM_016039	Homo sapiens chromosome 14 open reading frame 166 (C14orf166), mRNA [NM_016039]	NM_016039
A_23_P167698	0.000111	NM_004607	NM_004607	Homo sapiens tubulin-specific chaperone a (TBCA), mRNA [NM_004607]	NM_004607
A_32_P176902	0.000112	A_32_P176902			
A_32_P176550	0.000112	NM_152405	NM_152405	Homo sapiens junction-mediating and regulatory protein (JMY), mRNA [NM_152405]	NM_152405
A_32_P150030	0.000112	NM_003620	NM_003620	Homo sapiens protein phosphatase 1D magnesium-dependent, delta isoform (PPM1D), mRNA [NM_003620]	NM_003620
A_23_P119992	0.000112	NM_006296	NM_006296	Homo sapiens vaccinia related kinase 2 (VRK2), mRNA [NM_006296]	NM_006296
A_23_P339480	0.000112	NM_003642	NM_003642	Homo sapiens histone acetyltransferase 1 (HAT1), mRNA [NM_003642]	NM_003642
A_32_P149536	0.000112	NM_006937	NM_006937	Homo sapiens SMT3 suppressor of mif two 3 homolog 2 (yeast) (SUMO2), transcript variant 1, mRNA [NM_006937]	NM_006937
A_23_P64770	0.000112	NM_004818	NM_004818	Homo sapiens DEAD (Asp-Glu-Ala-Asp) box polypeptide 23 (DDX23), mRNA [NM_004818]	NM_004818
A_23_P30163	0.000113	NM_024614	NM_024614	Homo sapiens hypothetical protein FLJ13197 (FLJ13197), mRNA [NM_024614]	NM_024614
A_24_P307695	0.000113	NM_033402	NM_033402	Homo sapiens KIAA1764 protein (KIAA1764), mRNA [NM_033402]	NM_033402
A_23_P253046	0.000113	NM_006759	NM_006759	Homo sapiens UDP-glucose pyrophosphorylase 2 (UGP2), transcript variant 1, mRNA [NM_006759]	NM_006759
A_23_P9513	0.000113	NM_004689	NM_004689	Homo sapiens metastasis associated 1 (MTA1), mRNA [NM_004689]	NM_004689
A_24_P28165	0.000114	NM_015071	NM_015071	Homo sapiens Rho GTPase activating protein 26 (ARHGAP26), mRNA [NM_015071]	NM_015071
A_24_P15114	0.000114	NM_020935	NM_020935	Homo sapiens ubiquitin specific protease 37 (USP37), mRNA [NM_020935]	NM_020935
A_23_P70827	0.000114	AL136736	AL136736	Homo sapiens mRNA; cDNA DKFZp434O0710 (from clone DKFZp434O0710). [AL136736]	XM_371956
A_23_P66017	0.000114	NM_145239	NM_145239	Homo sapiens similar to lymphocyte antigen 6 complex, locus G5B; G5b protein; open reading frame 31 (LOC112476), mRNA [NM_145239]	NM_145239
A_24_P382253	0.000114	NM_018170	NM_018170	Homo sapiens hypothetical protein FLJ10656 (P15RS), mRNA [NM_018170]	NM_018170

A_23_P60753	0.000114	THC2337941			
A_23_P309701	0.000115	NM_002828	NM_002828	Homo sapiens protein tyrosine phosphatase, non-receptor type 2 (PTPN2), transcript variant 1, mRNA [NM_002828]	NM_002828
A_32_P161554	0.000115	THC2280139			
A_23_P115597	0.000115	NM_014915	NM_014915	Homo sapiens ankyrin repeat domain 26 (ANKRD26), mRNA [NM_014915]	NM_014915
A_23_P38630	0.000115	NM_001050	NM_001050	Homo sapiens somatostatin receptor 2 (SSTR2), mRNA [NM_001050]	NM_001050
A_23_P35444	0.000115	NM_032727	NM_032727	Homo sapiens internexin neuronal intermediate filament protein, alpha (INA), mRNA [NM_032727]	NM_032727
A_32_P104619	0.000116	AK094156	AK094156	Homo sapiens cDNA FLJ36837 fis, clone ASTRO2011422. [AK094156]	
A_32_P220739	0.000116	NM_033160	NM_033160	Homo sapiens zinc finger protein 658 (ZNF658), mRNA [NM_033160]	NM_033160
A_24_P254904	0.000116	NM_032236	NM_032236	Homo sapiens ubiquitin specific protease 48 (USP48), mRNA [NM_032236]	NM_032236
A_23_P145016	0.000116	NM_006696	NM_006696	Homo sapiens bromodomain containing 8 (BRD8), transcript variant 1, mRNA [NM_006696]	NM_006696
A_24_P278747	0.000116	NM_001759	NM_001759	Homo sapiens cyclin D2 (CCND2), mRNA [NM_001759]	NM_001759
A_24_P252497	0.000116	NM_025195	NM_025195	Homo sapiens tribbles homolog 1 (Drosophila) (TRIB1), mRNA [NM_025195]	NM_025195
A_23_P257956	0.000116	NM_004515	NM_004515	Homo sapiens interleukin enhancer binding factor 2, 45kDa (ILF2), mRNA [NM_004515]	NM_004515
A_24_P456452	0.000117	AK074562	AK074562	Homo sapiens cDNA FLJ90081 fis, clone HEMBA1004952. [AK074562]	
A_23_P211727	0.000117	NM_004113	NM_004113	Homo sapiens fibroblast growth factor 12 (FGF12), transcript variant 2, mRNA [NM_004113]	NM_004113
A_23_P17914	0.000117	NM_025225	NM_025225	Homo sapiens adiponutrin (ADPN), mRNA [NM_025225]	NM_025225
A_23_P256342	0.000117	BC045667	BC045667	Homo sapiens sorting nexin 13, mRNA (cDNA clone MGC:44494 IMAGE:5298570), complete cds. [BC045667]	
A_23_P206856	0.000117	NM_030581	NM_030581	Homo sapiens WD repeat domain 59 (WDR59), mRNA [NM_030581]	NM_030581
A_24_P269814	0.000117	NM_001001974	NM_001001974	Homo sapiens pleckstrin homology domain containing, family A (phosphoinositide binding specific) member 1 (PLEKHA1), transcript variant 2, mRNA [NM_001001974]	NM_001001974
A_23_P151405	0.000117	NM_018204	NM_018204	Homo sapiens cytoskeleton associated protein 2 (CKAP2), mRNA [NM_018204]	NM_018204
A_23_P7752	0.000117	NM_020796	NM_020796	Homo sapiens sema domain, transmembrane domain (TM), and cytoplasmic domain, (semaphorin) 6A (SEMA6A), mRNA [NM_020796]	NM_020796
A_23_P56938	0.000118	NM_002908	NM_002908	Homo sapiens v-rel reticuloendotheliosis viral oncogene homolog (avian) (REL), mRNA [NM_002908]	NM_002908
A_24_P208737	0.000118	THC2306718			
A_23_P253622	0.000118	NM_020970	NM_020970	Homo sapiens KIAA1641 (KIAA1641), mRNA [NM_020970]	NM_020970
A_23_P2537	0.000118	NM_052845	NM_052845	Homo sapiens methylmalonic aciduria (cobalamin deficiency) cblB type (MMAB), mRNA [NM_052845]	NM_052845
A_23_P167005	0.000118	NM_014373	NM_014373	Homo sapiens G protein-coupled receptor 160 (GPR160), mRNA [NM_014373]	NM_014373
A_32_P78488	0.000118	A_32_P78488			
A_23_P85441	0.000118	NM_020789	NM_020789	Homo sapiens immunoglobulin superfamily, member 9 (IGSF9), mRNA [NM_020789]	NM_020789
A_23_P19115	0.000118	NM_018094	NM_018094	Homo sapiens G1 to S phase transition 2 (GSPT2), mRNA [NM_018094]	NM_018094
A_23_P146050	0.000118	NM_024699	NM_024699	Homo sapiens zinc finger, AN1-type domain 1 (ZFAND1), mRNA [NM_024699]	NM_024699
A_23_P167269	0.000118	NM_018352	NM_018352	Homo sapiens hypothetical protein FLJ11184 (FLJ11184), mRNA [NM_018352]	NM_018352
A_24_P918266	0.000118	CR749309	CR749309	Homo sapiens mRNA; cDNA DKFZp686O22167 (from clone DKFZp686O22167). [CR749309]	
A_32_P151594	0.000118	NM_003133	NM_003133	Homo sapiens signal recognition particle 9kDa (SRP9), mRNA [NM_003133]	NM_003133
A_23_P140316	0.000119	ENST00000321662		Homo sapiens mRNA; cDNA DKFZp762F0713 (from clone DKFZp762F0713). [AL834372]	XM_290615
A_23_P51797	0.000119	THC2349786		Q5T9S5 (Q5T9S5) Sarcoma antigen NY-SAR-41 (NY-SAR-41), partial (9%) [THC2349786]	
A_32_P13337	0.000119	THC2408277			
A_23_P315386	0.000119	NM_052924	NM_052924	Homo sapiens rhophilin, Rho GTPase binding protein 1 (RHPN1), mRNA [NM_052924]	NM_052924
A_32_P7974	0.000119	A_32_P7974			
A_24_P104538	0.000119	BC000924	BC000924	Homo sapiens neighbor of BRCA1 gene 2, mRNA (cDNA clone MGC:5031 IMAGE:3446931), complete cds. [BC000924]	XM_496240
A_23_P108883	0.000119	NM_022055	NM_022055	Homo sapiens potassium channel, subfamily K, member 12 (KCNK12), mRNA [NM_022055]	NM_022055
A_23_P101811	0.00012	NM_021030	NM_021030	Homo sapiens zinc finger protein 14 (KOX 6) (ZNF14), mRNA [NM_021030]	NM_021030
A_23_P394043	0.00012	BC035808	BC035808	Homo sapiens, clone IMAGE:5745021, mRNA. [BC035808]	XM_048362
A_24_P928765	0.00012	THC2341283		A40201 artifact-warning sequence (translated ALU class A) - human {Homo sapiens;} , partial (13%) [THC2341283]	
A_24_P147263	0.00012	AB033029	AB033029	Homo sapiens mRNA for KIAA1203 protein, partial cds. [AB033029]	
A_23_P115862	0.00012	NM_144589	NM_144589	Homo sapiens catechol-O-methyltransferase domain containing 1 (COMTD1), mRNA [NM_144589]	NM_144589

A_23_P97046	0.000121	NM_000478	NM_000478	Homo sapiens alkaline phosphatase, liver/bone/kidney (ALPL), mRNA [NM_000478]	NM_000478
A_32_P132766	0.000121	THC2406017		Q6C1M3 (Q6C1M3) Similarity, partial (6%) [THC2406017]	
A_23_P45409	0.000121	NM_080632	NM_080632	Homo sapiens UPF3 regulator of nonsense transcripts homolog B (yeast) (UPF3B), transcript variant 1, mRNA [NM_080632]	NM_080632
A_24_P6808	0.000121	NM_004713	NM_004713	Homo sapiens serologically defined colon cancer antigen 1 (SDCCAG1), mRNA [NM_004713]	NM_004713
A_23_P363399	0.000121	NM_030674	NM_030674	Homo sapiens solute carrier family 38, member 1 (SLC38A1), mRNA [NM_030674]	NM_030674
A_23_P20022	0.000121	NM_013332	NM_013332	Homo sapiens hypoxia-inducible protein 2 (HIG2), mRNA [NM_013332]	NM_013332
A_23_P208469	0.000121	NM_004793	NM_004793	Homo sapiens protease, serine, 15 (PRSS15), nuclear gene encoding mitochondrial protein, mRNA [NM_004793]	NM_004793
A_23_P69100	0.000122	NM_015595	NM_015595	Homo sapiens Src homology 3 domain-containing guanine nucleotide exchange factor (SGEF), mRNA [NM_015595]	NM_015595
A_23_P252711	0.000122	NM_016027	NM_016027	Homo sapiens lactamase, beta 2 (LACTB2), mRNA [NM_016027]	NM_016027
A_23_P160377	0.000122	NM_003462	NM_003462	Homo sapiens dynein, axonemal, light intermediate polypeptide 1 (DNALI1), mRNA [NM_003462]	NM_003462
A_24_P332926	0.000122	NM_014884	NM_014884	Homo sapiens splicing factor, arginine/serine-rich 14 (SFRS14), transcript variant 2, mRNA [NM_014884]	NM_014884
A_23_P36658	0.000122	NM_145791	NM_145791	Homo sapiens microsomal glutathione S-transferase 1 (MGST1), transcript variant 1c, mRNA [NM_145791]	NM_145791
A_32_P28365	0.000122	NM_172164	NM_172164	Homo sapiens nuclear autoantigenic sperm protein (histone-binding) (NASP), transcript variant 1, mRNA [NM_172164]	NM_172164
A_23_P143474	0.000122	NM_001697	NM_001697	Homo sapiens ATP synthase, H+ transporting, mitochondrial F1 complex, O subunit (oligomycin sensitivity conferring protein) (ATP5O), nuclear gene encoding mitochondrial protein, mRNA [NM_001697]	NM_001697
A_24_P225719	0.000123	NM_015387	NM_015387	Homo sapiens preimplantation protein 3 (PREI3), transcript variant 1, mRNA [NM_015387]	NM_015387
A_32_P192615	0.000124	AB209316	AB209316	Homo sapiens mRNA for TBP-associated factor 1 isoform 2 variant protein. [AB209316]	
A_23_P92410	0.000124	NM_004346	NM_004346	Homo sapiens caspase 3, apoptosis-related cysteine protease (CASP3), transcript variant alpha, mRNA [NM_004346]	NM_004346
A_23_P380848	0.000124	NM_032731	NM_032731	Homo sapiens thioredoxin-like 5 (TXNL5), mRNA [NM_032731]	NM_032731
A_24_P587938	0.000124	A_24_P587938			
A_32_P168375	0.000125	THC2404028			
A_32_P221958	0.000125	NM_133446	NM_133446	Homo sapiens centaurin, gamma-like family, member 1 (CTGLF1), mRNA [NM_133446]	NM_133446
A_23_P395426	0.000125	NM_022105	NM_022105	Homo sapiens death associated transcription factor 1 (DATF1), transcript variant 1, mRNA [NM_022105]	NM_022105
A_23_P340218	0.000125	NM_152599	NM_152599	Homo sapiens hypothetical protein FLJ35773 (FLJ35773), mRNA [NM_152599]	NM_152599
A_24_P526177	0.000125	NM_003211	NM_003211	Homo sapiens thymine-DNA glycosylase (TDG), transcript variant 1, mRNA [NM_003211]	NM_003211
A_24_P134955	0.000126	NM_006243	NM_006243	Homo sapiens protein phosphatase 2, regulatory subunit B (B56), alpha isoform (PPP2R5A), mRNA [NM_006243]	NM_006243
A_23_P47220	0.000126	A_23_P47220			
A_23_P3242	0.000126	BC064969	BC064969	Homo sapiens cDNA clone IMAGE:6149643, partial cds. [BC064969]	
A_24_P95273	0.000126	NM_015046	NM_015046	Homo sapiens amyotrophic lateral sclerosis 4 (ALS4), mRNA [NM_015046]	NM_015046
A_23_P91930	0.000126	NM_024638	NM_024638	Homo sapiens queuine tRNA-ribosyltransferase domain containing 1 (QTRTD1), mRNA [NM_024638]	NM_024638
A_23_P72568	0.000126	NM_003794	NM_003794	Homo sapiens sorting nexin 4 (SNX4), mRNA [NM_003794]	NM_003794
A_23_P205336	0.000126	NM_016472	NM_016472	Homo sapiens chromosome 14 open reading frame 129 (C14orf129), mRNA [NM_016472]	NM_016472
A_24_P116909	0.000127	NM_006785	NM_006785	Homo sapiens mucosa associated lymphoid tissue lymphoma translocation gene 1 (MALT1), transcript variant 1, mRNA [NM_006785]	NM_006785
A_24_P13285	0.000127	NM_006741	NM_006741	Homo sapiens protein phosphatase 1, regulatory (inhibitor) subunit 1A (PPP1R1A), mRNA [NM_006741]	NM_006741
A_24_P255645	0.000127	NM_020800	NM_020800	Homo sapiens WD repeat domain 56 (WDR56), mRNA [NM_020800]	NM_020800
A_23_P96936	0.000127	NM_017673	NM_017673	Homo sapiens chromosome 1 open reading frame 26 (C1orf26), mRNA [NM_017673]	NM_017673
A_23_P54918	0.000127	NM_153486	NM_153486	Homo sapiens lactate dehydrogenase D (LDHD), nuclear gene encoding mitochondrial protein, transcript variant 1, mRNA [NM_153486]	NM_153486
A_24_P931636	0.000127	BC032312	BC032312	Homo sapiens transforming growth factor beta regulator 1, mRNA (cDNA clone IMAGE:5212572), complete cds. [BC032312]	
A_23_P216307	0.000127	NM_004349	NM_004349	Homo sapiens runt-related transcription factor 1; translocated to, 1 (cyclin D-related) (RUNX1T1), transcript variant 1, mRNA [NM_004349]	NM_004349
A_32_P7783	0.000128	AW377662	AW377662	AW377662 PM0-CT0237-141099-001-e02 CT0237 Homo sapiens cDNA, mRNA sequence [AW377662]	
A_24_P346855	0.000128	NM_002417	NM_002417	Homo sapiens antigen identified by monoclonal antibody Ki-67 (MKI67), mRNA [NM_002417]	NM_002417
A_24_P688133	0.000128	AK124299	AK124299	Homo sapiens cDNA FLJ42306 fis, clone TRACH2001646. [AK124299]	
A_23_P356122	0.000128	NM_015555	NM_015555	Homo sapiens zinc finger protein 451 (ZNF451), mRNA [NM_015555]	NM_015555
A_23_P213638	0.000128	NM_024594	NM_024594	Homo sapiens pantothenate kinase 3 (PANK3), mRNA [NM_024594]	NM_024594

A_23_P87513	0.000128	NM_012463	NM_012463	Homo sapiens ATPase, H+ transporting, lysosomal V0 subunit a isoform 2 (ATP6V0A2), mRNA [NM_012463]	NM_012463
A_24_P686965	0.000128	AK124869	AK124869	Homo sapiens cDNA FLJ42879 fis, clone BRHIP3001283. [AK124869]	XM_375698
A_24_P398898	0.000128	NM_206839	NM_206839	Homo sapiens mortality factor 4 like 1 (MORF4L1), transcript variant 2, mRNA [NM_206839]	NM_206839
A_24_P876772	0.000129	BC032332	BC032332	Homo sapiens cDNA clone MGC:40288 IMAGE:5169056, complete cds. [BC032332]	
A_24_P929807	0.000129	AB052759	AB052759	Homo sapiens hAWMS1 mRNA, complete cds. [AB052759]	
A_23_P404778	0.000129	NM_012465	NM_012465	Homo sapiens tolloid-like 2 (TLL2), mRNA [NM_012465]	NM_012465
A_23_P15621	0.000129	NM_032391	NM_032391	Homo sapiens small nuclear protein PRAC (PRAC), mRNA [NM_032391]	NM_032391
A_24_P20753	0.000129	AK054960	AK054960	Homo sapiens cDNA FLJ30398 fis, clone BRACE2008402, highly similar to Homo sapiens steroid receptor RNA activator isoform 3 mRNA. [AK054960]	
A_23_P333951	0.000129	BC017415	BC017415	Homo sapiens chromosome 1 open reading frame 67, mRNA (cDNA clone IMAGE:4655328). [BC017415]	
A_24_P941441	0.000129	NM_006572	NM_006572	Homo sapiens guanine nucleotide binding protein (G protein), alpha 13 (GNA13), mRNA [NM_006572]	NM_006572
A_23_P204609	0.000129	NM_017599	NM_017599	Homo sapiens transmembrane protein vezatin (VEZATIN), mRNA [NM_017599]	NM_017599
A_23_P6708	0.000129	BC062368	BC062368	Homo sapiens hypothetical protein BC007882, mRNA (cDNA clone MGC:71256 IMAGE:6730791), complete cds. [BC062368]	
A_23_P402952	0.00013	NM_001008747	NM_001008747	Homo sapiens similar to CTAGE6 (LOC441294), mRNA [NM_001008747]	NM_001008747
A_24_P69274	0.00013	NM_024641	NM_024641	Homo sapiens mannosidase, endo-alpha (MANEA), mRNA [NM_024641]	NM_024641
A_24_P68631	0.00013	NM_175065	NM_175065	Homo sapiens histone 2, H2ab (HIST2H2AB), mRNA [NM_175065]	NM_175065
A_24_P241276	0.00013	NM_181503	NM_181503	Homo sapiens exosome component 8 (EXOSC8), mRNA [NM_181503]	NM_181503
A_23_P108342	0.000131	NM_016536	NM_016536	Homo sapiens zinc finger protein 571 (ZNF571), mRNA [NM_016536]	NM_016536
A_23_P126888	0.000131	AB007918	AB007918	Homo sapiens mRNA for KIAA0449 protein, partial cds. [AB007918]	
A_23_P254978	0.000131	NM_032026	NM_032026	Homo sapiens TatD DNase domain containing 1 (TATDN1), mRNA [NM_032026]	NM_032026
A_24_P337058	0.000131	NM_020307	NM_020307	Homo sapiens cyclin L1 (CCNL1), mRNA [NM_020307]	NM_020307
A_23_P404091	0.000132	NM_152407	NM_152407	Homo sapiens GrpE-like 2, mitochondrial (E. coli) (GRPEL2), mRNA [NM_152407]	NM_152407
A_23_P37654	0.000132	ENST00000310492		Homo sapiens mRNA for KIAA0518 protein, partial cds. [AB011090]	XM_031689
A_23_P417994	0.000132	NM_152429	NM_152429	Homo sapiens chromosome 10 open reading frame 13 (C10orf13), mRNA [NM_152429]	NM_152429
A_24_P220618	0.000132	ENST00000253048		Homo sapiens mRNA for KIAA1064 protein, partial cds. [AB028987]	
A_23_P20932	0.000132	NM_000925	NM_000925	Homo sapiens pyruvate dehydrogenase (lipoamide) beta (PDHB), mRNA [NM_000925]	NM_000925
A_24_P146188	0.000133	NM_014942	NM_014942	Homo sapiens ankyrin repeat domain 6 (ANKRD6), mRNA [NM_014942]	NM_014942
A_23_P138524	0.000133	NM_198148	NM_198148	Homo sapiens carboxypeptidase X (M14 family), member 2 (CPXM2), mRNA [NM_198148]	NM_198148
A_23_P211126	0.000133	NM_130436	NM_130436	Homo sapiens dual-specificity tyrosine-(Y)-phosphorylation regulated kinase 1A (DYRK1A), transcript variant 2, mRNA [NM_130436]	NM_130436
A_23_P134517	0.000133	NM_033224	NM_033224	Homo sapiens purine-rich element binding protein B (PURB), mRNA [NM_033224]	NM_033224
A_23_P108871	0.000133	NM_032494	NM_032494	Homo sapiens zinc finger CCCH-type containing 8 (ZC3H8), mRNA [NM_032494]	NM_032494
A_23_P19210	0.000133	NM_032194	NM_032194	Homo sapiens brix domain containing 1 (BXDC1), mRNA [NM_032194]	NM_032194
A_32_P155364	0.000133	NM_000971	NM_000971	Homo sapiens ribosomal protein L7 (RPL7), mRNA [NM_000971]	NM_000971
A_23_P33914	0.000134	NM_017681	NM_017681	Homo sapiens hypothetical protein FLJ20130 (FLJ20130), mRNA [NM_017681]	NM_017681
A_23_P257155	0.000134	NM_000333	NM_000333	Homo sapiens ataxin 7 (ATXN7), mRNA [NM_000333]	NM_000333
A_23_P54781	0.000134	BC051317	BC051317	Homo sapiens retinoblastoma binding protein 6, mRNA (cDNA clone IMAGE:6214974), complete cds. [BC051317]	
A_24_P320221	0.000134	NM_021942	NM_021942	Homo sapiens FLJ12716 protein (FLJ12716), transcript variant 1, mRNA [NM_021942]	NM_021942
A_32_P211141	0.000134	BC022429	BC022429	Homo sapiens cDNA clone MGC:24679 IMAGE:4270959, complete cds. [BC022429]	
A_23_P40072	0.000134	NM_001005369	NM_001005369	Homo sapiens mitochondrial translational initiation factor 2 (MTIF2), nuclear gene encoding mitochondrial protein, transcript variant 1, mRNA [NM_001005369]	NM_001005369
A_23_P141866	0.000135	NM_003435	NM_003435	Homo sapiens zinc finger protein 134 (clone pHZ-15) (ZNF134), mRNA [NM_003435]	NM_003435
A_24_P61467	0.000135	NM_001008895	NM_001008895	Homo sapiens cullin 4A (CUL4A), transcript variant 1, mRNA [NM_001008895]	NM_001008895
A_23_P24926	0.000135	NM_002027	NM_002027	Homo sapiens farnesyltransferase, CAAX box, alpha (FNTA), transcript variant 1, mRNA [NM_002027]	NM_002027
A_23_P26281	0.000135	NR_001565	NR_001565	Homo sapiens pseudogene MGC10997 (MGC10997) on chromosome 15 [NR_001565]	NR_001565
A_24_P183094	0.000135	AJ306929	AJ306929	Homo sapiens ORF for hypothetical protein. [AJ306929]	
A_23_P150092	0.000135	NM_012247	NM_012247	Homo sapiens selenophosphate synthetase 1 (SEPHS1), mRNA [NM_012247]	NM_012247
A_23_P71319	0.000135	NM_004462	NM_004462	Homo sapiens farnesyl-diphosphate farnesyltransferase 1 (FDFT1), mRNA [NM_004462]	NM_004462

A_24_P167877	0.000135	BC036263	BC036263	Homo sapiens KIAA0220-like protein, mRNA (cDNA clone MGC:9515 IMAGE:3903371), complete cds. [BC036263]	XM_370939
A_32_P100109	0.000136	AK097484	AK097484	Homo sapiens cDNA FLJ40165 fis, clone TESTI2015962. [AK097484]	
A_23_P217845	0.000136	NM_002928	NM_002928	Homo sapiens regulator of G-protein signalling 16 (RGS16), mRNA [NM_002928]	NM_002928
A_23_P251987	0.000136	NM_004577	NM_004577	Homo sapiens phosphoserine phosphatase (PSPH), mRNA [NM_004577]	NM_004577
A_23_P159671	0.000136	NM_000292	NM_000292	Homo sapiens phosphorylase kinase, alpha 2 (liver) (PHKA2), mRNA [NM_000292]	NM_000292
A_23_P372516	0.000136	AK094743	AK094743	Homo sapiens cDNA FLJ37424 fis, clone BRAWH2001492. [AK094743]	
A_32_P131940	0.000136	NM_172020	NM_172020	Homo sapiens POM121 membrane glycoprotein (rat) (POM121), mRNA [NM_172020]	NM_172020
A_32_P57013	0.000137	BU540282	BU540282	BU540282 AGENCOURT_10326456 NIH_MGC_141 Homo sapiens cDNA clone IMAGE:6571686 5', mRNA sequence [BU540282]	
A_23_P156811	0.000137	NM_001018022	NM_001018022	Homo sapiens similar to FKSG62 (LOC389286), mRNA [NM_001018022]	NM_001018022
A_32_P70135	0.000137	NM_025138	NM_025138	Homo sapiens chromosome 13 open reading frame 23 (C13orf23), transcript variant 1, mRNA [NM_025138]	NM_025138
A_23_P160336	0.000137	NM_020997	NM_020997	Homo sapiens left-right determination factor 1 (LEFTY1), mRNA [NM_020997]	NM_020997
A_23_P149270	0.000138	THC2249196		CICL_HUMAN (P51801) Chloride channel protein ClC-Kb (Chloride channel Kb) (ClC-K2), complete [THC2249196]	
A_23_P17354	0.000138	NM_024034	NM_024034	Homo sapiens ganglioside-induced differentiation-associated protein 1-like 1 (GDAP1L1), mRNA [NM_024034]	NM_024034
A_23_P125056	0.000138	NM_001702	NM_001702	Homo sapiens brain-specific angiogenesis inhibitor 1 (BAI1), mRNA [NM_001702]	NM_001702
A_24_P355493	0.000138	NM_022126	NM_022126	Homo sapiens phospholysine phosphohistidine inorganic pyrophosphate phosphatase (LHPP), mRNA [NM_022126]	NM_022126
A_24_P402898	0.000138	NM_199324	NM_199324	Homo sapiens HIV-1 induced protein HIN-1 (HSHIN1), transcript variant 1, mRNA [NM_199324]	NM_199324
A_32_P75141	0.000138	A_32_P75141			
A_23_P566	0.000138	NM_014947	NM_014947	Homo sapiens forkhead box J3 (FOXJ3), mRNA [NM_014947]	NM_014947
A_23_P256455	0.000138	NM_002947	NM_002947	Homo sapiens replication protein A3, 14kDa (RPA3), mRNA [NM_002947]	NM_002947
A_23_P35943	0.000138	NM_178510	NM_178510	Homo sapiens ankyrin repeat and kinase domain containing 1 (ANKK1), mRNA [NM_178510]	NM_178510
A_23_P326893	0.000139	NM_145045	NM_145045	Homo sapiens hypothetical protein MGC20983 (MGC20983), mRNA [NM_145045]	NM_145045
A_23_P149938	0.000139	THC2378635			
A_24_P175059	0.000139	NM_004849	NM_004849	Homo sapiens APG5 autophagy 5-like (S. cerevisiae) (APG5L), mRNA [NM_004849]	NM_004849
A_24_P180383	0.000139	NM_015384	NM_015384	Homo sapiens Nipped-B homolog (Drosophila) (NIPBL), transcript variant B, mRNA [NM_015384]	NM_015384
A_32_P76060	0.000139	CR627421	CR627421	Homo sapiens mRNA; cDNA DKFZp781F2227 (from clone DKFZp781F2227). [CR627421]	
A_23_P308800	0.000139	AF158555	AF158555	Homo sapiens glutaminase C mRNA, complete cds. [AF158555]	
A_24_P82142	0.000139	NM_207038	NM_207038	Homo sapiens transcription factor 12 (HTF4, helix-loop-helix transcription factors 4) (TCF12), transcript variant 4, mRNA [NM_207038]	NM_207038
A_23_P124417	0.000139	NM_004336	NM_004336	Homo sapiens BUB1 budding uninhibited by benzimidazoles 1 homolog (yeast) (BUB1), mRNA [NM_004336]	NM_004336
A_32_P137966	0.00014	NM_152317	NM_152317	Homo sapiens DEP domain containing 4 (DEPDC4), mRNA [NM_152317]	NM_152317
A_23_P209578	0.00014	NM_013445	NM_013445	Homo sapiens glutamate decarboxylase 1 (brain, 67kDa) (GAD1), transcript variant GAD25, mRNA [NM_013445]	NM_013445
A_23_P359897	0.000141	AB051505	AB051505	Homo sapiens mRNA for KIAA1718 protein, partial cds. [AB051505]	XM_376680
A_23_P201731	0.000141	NM_004619	NM_004619	Homo sapiens TNF receptor-associated factor 5 (TRAF5), transcript variant 1, mRNA [NM_004619]	NM_004619
A_24_P29876	0.000141	NM_018361	NM_018361	Homo sapiens 1-acylglycerol-3-phosphate O-acyltransferase 5 (lysophosphatidic acid acyltransferase, epsilon) (AGPAT5), mRNA [NM_018361]	NM_018361
A_23_P27947	0.000141	NM_032346	NM_032346	Homo sapiens hypothetical protein MGC13096 (MGC13096), mRNA [NM_032346]	NM_032346
A_23_P98310	0.000141	NM_001326	NM_001326	Homo sapiens cleavage stimulation factor, 3' pre-RNA, subunit 3, 77kDa (CSTF3), mRNA [NM_001326]	NM_001326
A_23_P13969	0.000141	BC052611	BC052611	Homo sapiens cDNA clone MGC:59724 IMAGE:6252827, complete cds. [BC052611]	
A_24_P871940	0.000142	BX537788	BX537788	Homo sapiens mRNA; cDNA DKFZp686P1449 (from clone DKFZp686P1449) [BX537788]	
A_24_P201404	0.000142	NM_014039	NM_014039	Homo sapiens PTD012 protein (PTD012), mRNA [NM_014039]	NM_014039
A_23_P311020	0.000142	NM_138798	NM_138798	Homo sapiens hypothetical protein BC018453 (LOC129531), mRNA [NM_138798]	NM_138798
A_23_P117494	0.000142	NM_005956	NM_005956	Homo sapiens methylenetetrahydrofolate dehydrogenase (NADP+ dependent) 1, methenyltetrahydrofolate cyclohydrolase, formyltetrahydrofolate synthetase (MTHFD1), mRNA [NM_005956]	NM_005956
A_23_P89062	0.000143	NM_024048	NM_024048	Homo sapiens hypothetical protein MGC3020 (MGC3020), mRNA [NM_024048]	NM_024048
A_23_P165130	0.000143	NM_032683	NM_032683	Homo sapiens hypothetical protein MGC12972 (FKSG24), mRNA [NM_032683]	NM_032683
A_23_P201672	0.000143	NM_015935	NM_015935	Homo sapiens KIAA0859 (KIAA0859), transcript variant 1, mRNA [NM_015935]	NM_015935
A_24_P187706	0.000144	NM_002078	NM_002078	Homo sapiens golgi autoantigen, golgin subfamily a, 4 (GOLGA4), mRNA [NM_002078]	NM_002078

A_24_P854913	0.000144	THC2275676			
A_23_P113811	0.000144	A_23_P113811			
A_23_P380998	0.000144	NM_015361	NM_015361	Homo sapiens R3H domain (binds single-stranded nucleic acids) containing (R3HDM), mRNA [NM_015361]	NM_015361
A_23_P308305	0.000145	ENST00000317571		Homo sapiens cDNA FLJ90394 fis, clone NT2RP2005632. [AK074875]	
A_32_P102383	0.000146	THC2323059			
A_24_P378928	0.000146	NM_005160	NM_005160	Homo sapiens adrenergic, beta, receptor kinase 2 (ADRBK2), mRNA [NM_005160]	NM_005160
A_32_P98059	0.000146	AK126329	AK126329	Homo sapiens cDNA FLJ44355 fis, clone TRACH3006699. [AK126329]	
A_23_P103503	0.000146	NM_002393	NM_002393	Homo sapiens Mdm4, transformed 3T3 cell double minute 4, p53 binding protein (mouse) (MDM4), mRNA [NM_002393]	NM_002393
A_23_P327069	0.000147	NM_014743	NM_014743	Homo sapiens KIAA0232 gene product (KIAA0232), mRNA [NM_014743]	NM_014743
A_24_P11791	0.000147	NM_002268	NM_002268	Homo sapiens karyopherin alpha 4 (importin alpha 3) (KPNA4), mRNA [NM_002268]	NM_002268
A_24_P79750	0.000147	NM_006066	NM_006066	Homo sapiens aldo-keto reductase family 1, member A1 (aldehyde reductase) (AKR1A1), transcript variant 1, mRNA [NM_006066]	NM_006066
A_23_P501790	0.000147	NM_006752	NM_006752	Homo sapiens surfeit 5 (SURF5), transcript variant a, mRNA [NM_006752]	NM_006752
A_24_P203964	0.000148	NM_207331	NM_207331	Homo sapiens hypothetical protein LOC153561 (LOC153561), mRNA [NM_207331]	NM_207331
A_23_P343963	0.000148	NM_138435	NM_138435	Homo sapiens family with sequence similarity 83, member F (FAM83F), mRNA [NM_138435]	NM_138435
A_23_P3922	0.000148	AK021772	AK021772	Homo sapiens cDNA FLJ11710 fis, clone HEMBA1005149. [AK021772]	
A_24_P409904	0.000148	AK130705	AK130705	Homo sapiens cDNA FLJ27195 fis, clone SYN02786. [AK130705]	
A_23_P134078	0.000148	NM_170752	NM_170752	Homo sapiens chromodomain protein, Y-like (CDYL), transcript variant 3, mRNA [NM_170752]	NM_170752
A_24_P924752	0.000148	M14087	M14087	Human HL14 gene encoding beta-galactoside-binding lectin, 3' end, clone 2. [M14087]	
A_23_P394259	0.000149	NM_005374	NM_005374	Homo sapiens membrane protein, palmitoylated 2 (MAGUK p55 subfamily member 2) (MPP2), mRNA [NM_005374]	NM_005374
A_23_P123727	0.000149	NM_024617	NM_024617	Homo sapiens zinc finger, CCHC domain containing 6 (ZCCHC6), mRNA [NM_024617]	NM_024617
A_23_P94546	0.000149	NM_025211	NM_025211	Homo sapiens G kinase anchoring protein 1 (GKAP1), mRNA [NM_025211]	NM_025211
A_23_P86838	0.000149	NM_152313	NM_152313	Homo sapiens solute carrier family 36 (proton/amino acid symporter), member 4 (SLC36A4), mRNA [NM_152313]	NM_152313
A_23_P155332	0.000149	NM_020357	NM_020357	Homo sapiens PEST-containing nuclear protein (PCNP), mRNA [NM_020357]	NM_020357
A_32_P186450	0.00015	ENST00000327086		Homo sapiens cDNA clone IMAGE:30334866. [BC092503]	XM_496867
A_24_P620521	0.00015	A_24_P620521			
A_23_P88435	0.00015	NM_005197	NM_005197	Homo sapiens checkpoint suppressor 1 (CHES1), mRNA [NM_005197]	NM_005197
A_24_P400355	0.00015	NM_015949	NM_015949	Homo sapiens chromosome 7 open reading frame 20 (C7orf20), mRNA [NM_015949]	NM_015949
A_23_P65797	0.00015	NM_022480	NM_022480	Homo sapiens BTB/POZ KELCH domain protein (ENC2), mRNA [NM_022480]	NM_022480
A_23_P56069	0.00015	NM_015578	NM_015578	Homo sapiens family with sequence similarity 61, member A (FAM61A), mRNA [NM_015578]	NM_015578
A_24_P7934	0.00015	ENST00000332101		PREDICTED: Homo sapiens similar to histone (15.4 kD) (his-72) (LOC391769), mRNA [XM_373079]	XM_373079
A_24_P588897	0.000151	THC2277195			
A_32_P34516	0.000151	AK056119	AK056119	Homo sapiens cDNA FLJ31557 fis, clone NT2RI2001307. [AK056119]	
A_23_P73220	0.000151	NM_018351	NM_018351	Homo sapiens FYVE, RhoGEF and PH domain containing 6 (FGD6), mRNA [NM_018351]	NM_018351
A_24_P227091	0.000151	NM_004523	NM_004523	Homo sapiens kinesin family member 11 (KIF11), mRNA [NM_004523]	NM_004523
A_23_P56865	0.000151	NM_006773	NM_006773	Homo sapiens DEAD (Asp-Glu-Ala-Asp) box polypeptide 18 (DDX18), mRNA [NM_006773]	NM_006773
A_24_P347447	0.000151	NM_014992	NM_014992	Homo sapiens dishevelled associated activator of morphogenesis 1 (DAAM1), mRNA [NM_014992]	NM_014992
A_24_P385313	0.000151	NM_002840	NM_002840	Homo sapiens protein tyrosine phosphatase, receptor type, F (PTPRF), transcript variant 1, mRNA [NM_002840]	NM_002840
A_23_P201521	0.000151	A_23_P201521			
A_23_P4604	0.000152	NM_198457	NM_198457	Homo sapiens zinc finger protein 600 (ZNF600), mRNA [NM_198457]	NM_198457
A_24_P28622	0.000152	NM_144567	NM_144567	Homo sapiens similar to RIKEN cDNA 2610307I21 (LOC90806), mRNA [NM_144567]	NM_144567
A_23_P252642	0.000152	NM_152384	NM_152384	Homo sapiens Bardet-Biedl syndrome 5 (BBS5), mRNA [NM_152384]	NM_152384
A_23_P97749	0.000152	NM_030588	NM_030588	Homo sapiens DEAH (Asp-Glu-Ala-His) box polypeptide 9 (DHX9), transcript variant 2, mRNA [NM_030588]	NM_030588
A_24_P330309	0.000153	NM_002892	NM_002892	Homo sapiens AT rich interactive domain 4A (RBP1-like) (ARID4A), transcript variant 1, mRNA [NM_002892]	NM_002892
A_23_P39067	0.000153	NM_003121	NM_003121	Homo sapiens Spi-B transcription factor (Spi-1/PU.1 related) (SPIB), mRNA [NM_003121]	NM_003121
A_24_P481375	0.000153	AK021668	AK021668	Homo sapiens cDNA FLJ11606 fis, clone HEMBA1003942. [AK021668]	

A_23_P208812	0.000153	NM_014910	NM_014910	Homo sapiens zinc finger protein 507 (ZNF507), mRNA [NM_014910]	NM_014910
A_32_P183904	0.000153	ENST00000361989		Homo sapiens Src homology 2 domain containing F, mRNA (cDNA clone IMAGE:3162799), partial cds. [BC007586]	
A_23_P14132	0.000153	NM_004128	NM_004128	Homo sapiens general transcription factor IIF, polypeptide 2 (30kD subunit) (GTF2F2), mRNA [NM_004128]	NM_004128
A_24_P270452	0.000154	THC2275804		CB133932 K-EST0185164 L9SNU354 Homo sapiens cDNA clone L9SNU354-10-C01 5', mRNA sequence [CB133932]	
A_23_P169351	0.000154	NM_003026	NM_003026	Homo sapiens SH3-domain GRB2-like 2 (SH3GL2), mRNA [NM_003026]	NM_003026
A_32_P61857	0.000154	ENST00000299783		Homo sapiens mRNA for KIAA1468 protein, partial cds. [AB040901]	
A_23_P17393	0.000154	NM_001316	NM_001316	Homo sapiens CSE1 chromosome segregation 1-like (yeast) (CSE1L), transcript variant 1, mRNA [NM_001316]	NM_001316
A_23_P89841	0.000155	A_23_P89841			
A_23_P22548	0.000155	NM_152579	NM_152579	Homo sapiens hypothetical protein FLJ38564 (FLJ38564), mRNA [NM_152579]	NM_152579
A_23_P78975	0.000155	A_23_P78975			
A_23_P108641	0.000155	NM_032822	NM_032822	Homo sapiens hypothetical protein FLJ14668 (FLJ14668), mRNA [NM_032822]	NM_032822
A_23_P361841	0.000156	ENST00000357529		Homo sapiens mRNA for KIAA1238 protein, partial cds. [AB033064]	
A_23_P211363	0.000156	NM_002073	NM_002073	Homo sapiens guanine nucleotide binding protein (G protein), alpha z polypeptide (GNAZ), mRNA [NM_002073]	NM_002073
A_23_P117163	0.000156	NM_018191	NM_018191	Homo sapiens regulator of chromosome condensation (RCC1) and BTB (POZ) domain containing protein 1 (RCBTB1), mRNA [NM_018191]	NM_018191
A_24_P636332	0.000156	NM_198489	NM_198489	Homo sapiens similar to DLNB14 (DLNB14), mRNA [NM_198489]	NM_198489
A_32_P103633	0.000156	NM_004526	NM_004526	Homo sapiens MCM2 minichromosome maintenance deficient 2, mitotin (S. cerevisiae) (MCM2), mRNA [NM_004526]	NM_004526
A_32_P218249	0.000156	NM_003211	NM_003211	Homo sapiens thymine-DNA glycosylase (TDG), transcript variant 1, mRNA [NM_003211]	NM_003211
A_23_P333998	0.000157	AF090919	AF090919	Homo sapiens clone HQ0327 PRO0327 mRNA, complete cds. [AF090919]	
A_23_P219161	0.000157	NM_006334	NM_006334	Homo sapiens olfactomedin 1 (OLFM1), transcript variant 2, mRNA [NM_006334]	NM_006334
A_32_P197524	0.000158	THC2367807			
A_24_P114339	0.000158	CR604908	CR604908	full-length cDNA clone CS0DF020YB09 of Fetal brain of Homo sapiens (human). [CR604908]	
A_24_P398500	0.000158	NM_022782	NM_022782	Homo sapiens M-phase phosphoprotein 9 (MPHOSPH9), mRNA [NM_022782]	NM_022782
A_23_P45799	0.000158	NM_004153	NM_004153	Homo sapiens origin recognition complex, subunit 1-like (yeast) (ORC1L), mRNA [NM_004153]	NM_004153
A_24_P115774	0.000158	NM_001166	NM_001166	Homo sapiens baculoviral IAP repeat-containing 2 (BIRC2), mRNA [NM_001166]	NM_001166
A_32_P76399	0.000158	NM_016091	NM_016091	Homo sapiens eukaryotic translation initiation factor 3, subunit 6 interacting protein (EIF3S6IP), mRNA [NM_016091]	NM_016091
A_23_P11922	0.000159	AK024925	AK024925	Homo sapiens cDNA: FLJ21272 fis, clone COL01753. [AK024925]	
A_24_P29641	0.000159	NM_148936	NM_148936	Homo sapiens Williams Beuren syndrome chromosome region 20C (WBSCR20C), transcript variant 2, mRNA [NM_148936]	NM_148936
A_23_P208325	0.00016	NM_004234	NM_004234	Homo sapiens zinc finger protein 235 (ZNF235), mRNA [NM_004234]	NM_004234
A_24_P186274	0.00016	NM_001007563	NM_001007563	Homo sapiens insulin-like growth factor binding protein-like 1 (IGFBPL1), mRNA [NM_001007563]	NM_001007563
A_23_P64962	0.00016	A_23_P64962			
A_23_P43476	0.00016	NM_003383	NM_003383	Homo sapiens very low density lipoprotein receptor (VLDLR), transcript variant 1, mRNA [NM_003383]	NM_003383
A_23_P161474	0.00016	NM_182751	NM_182751	Homo sapiens MCM10 minichromosome maintenance deficient 10 (S. cerevisiae) (MCM10), transcript variant 1, mRNA [NM_182751]	NM_182751
A_23_P37327	0.00016	NM_020326	NM_020326	Homo sapiens ATP-binding cassette, sub-family D (ALD), member 4 (ABCD4), transcript variant 5, mRNA [NM_020326]	NM_020326
A_23_P49972	0.00016	NM_001254	NM_001254	Homo sapiens CDC6 cell division cycle 6 homolog (S. cerevisiae) (CDC6), mRNA [NM_001254]	NM_001254
A_23_P100141	0.000161	NM_023076	NM_023076	Homo sapiens chromosome 16 open reading frame 28 (C16orf28), mRNA [NM_023076]	NM_023076
A_23_P171258	0.000161	NM_004299	NM_004299	Homo sapiens ATP-binding cassette, sub-family B (MDR/TAP), member 7 (ABCB7), nuclear gene encoding mitochondrial protein, mRNA [NM_004299]	NM_004299
A_23_P48835	0.000161	NM_138555	NM_138555	Homo sapiens kinesin family member 23 (KIF23), transcript variant 1, mRNA [NM_138555]	NM_138555
A_32_P117697	0.000161	CR623139	CR623139	full-length cDNA clone CS0DI054YB24 of Placenta Cot 25-normalized of Homo sapiens (human). [CR623139]	
A_23_P50368	0.000162	NM_206818	NM_206818	Homo sapiens osteoclast-associated receptor (OSCAR), transcript variant 1, mRNA [NM_206818]	NM_206818
A_23_P36464	0.000162	NM_018164	NM_018164	Homo sapiens chromosome 12 open reading frame 11 (C12orf11), mRNA [NM_018164]	NM_018164
A_23_P99891	0.000162	NM_022566	NM_022566	Homo sapiens mesoderm development candidate 1 (MESDC1), mRNA [NM_022566]	NM_022566
A_23_P163047	0.000163	NM_001008726	NM_001008726	Homo sapiens chromosome 14 open reading frame 150 (C14orf150), transcript variant 1, mRNA [NM_001008726]	NM_001008726
A_23_P72583	0.000163	A_23_P72583			
A_23_P60499	0.000163	NM_021224	NM_021224	Homo sapiens zinc finger protein 462 (ZNF462), mRNA [NM_021224]	NM_021224

A_23_P34800	0.000163	NM_172164	NM_172164	Homo sapiens nuclear autoantigenic sperm protein (histone-binding) (NASP), transcript variant 1, mRNA [NM_172164]	NM_172164
A_24_P230691	0.000164	AK025303	AK025303	Homo sapiens cDNA: FLJ21650 fis, clone COL08516. [AK025303]	
A_23_P59855	0.000164	NM_006524	NM_006524	Homo sapiens zinc finger protein 138 (ZNF138), mRNA [NM_006524]	NM_006524
A_32_P105195	0.000165	NM_014829	NM_014829	Homo sapiens DEAD (Asp-Glu-Ala-Asp) box polypeptide 46 (DDX46), mRNA [NM_014829]	NM_014829
A_32_P36942	0.000166	A_32_P36942			
A_32_P116323	0.000166	NM_015092	NM_015092	Homo sapiens PI-3-kinase-related kinase SMG-1 (SMG1), mRNA [NM_015092]	NM_015092
A_24_P360601	0.000166	NM_017948	NM_017948	Homo sapiens nucleolar protein 8 (NOL8), mRNA [NM_017948]	NM_017948
A_32_P54544	0.000166	NM_001762	NM_001762	Homo sapiens chaperonin containing TCP1, subunit 6A (zeta 1) (CCT6A), transcript variant 1, mRNA [NM_001762]	NM_001762
A_24_P258051	0.000167	NM_032844	NM_032844	Homo sapiens microtubule associated serine/threonine kinase-like (MASTL), mRNA [NM_032844]	NM_032844
A_24_P192338	0.000167	CR608275	CR608275	full-length cDNA clone CS0CAP007YE04 of Thymus of Homo sapiens (human). [CR608275]	
A_23_P336023	0.000167	NM_015928	NM_015928	Homo sapiens androgen-induced proliferation inhibitor (APRIN), transcript variant 2, mRNA [NM_015928]	NM_015928
A_23_P305677	0.000168	NM_181838	NM_181838	Homo sapiens ubiquitin-conjugating enzyme E2D 2 (UBC4/5 homolog, yeast) (UBE2D2), transcript variant 2, mRNA [NM_181838]	NM_181838
A_24_P81900	0.000168	NM_006931	NM_006931	Homo sapiens solute carrier family 2 (facilitated glucose transporter), member 3 (SLC2A3), mRNA [NM_006931]	NM_006931
A_23_P259451	0.000169	NM_007080	NM_007080	Homo sapiens LSM6 homolog, U6 small nuclear RNA associated (S. cerevisiae) (LSM6), mRNA [NM_007080]	NM_007080
A_23_P163481	0.00017	NM_001211	NM_001211	Homo sapiens BUB1 budding uninhibited by benzimidazoles 1 homolog beta (yeast) (BUB1B), mRNA [NM_001211]	NM_001211
A_32_P149298	0.000171	ENST00000295031		Homo sapiens mRNA for KIAA1841 protein, partial cds. [AB058744]	
A_24_P398016	0.000171	NM_018387	NM_018387	Homo sapiens spermatid perinuclear RNA binding protein (STRBP), mRNA [NM_018387]	NM_018387
A_32_P81092	0.000171	THC2373083			
A_23_P336708	0.000171	NM_178502	NM_178502	Homo sapiens deltex 3 homolog (Drosophila) (DTX3), mRNA [NM_178502]	NM_178502
A_24_P206736	0.000171	NM_003442	NM_003442	Homo sapiens zinc finger protein 143 (clone pHZ-1) (ZNF143), mRNA [NM_003442]	NM_003442
A_32_P122703	0.000171	NM_173582	NM_173582	Homo sapiens phosphoglucomutase 2-like 1 (PGM2L1), mRNA [NM_173582]	NM_173582
A_24_P364970	0.000171	NM_020162	NM_020162	Homo sapiens DEAH (Asp-Glu-Ala-His) box polypeptide 33 (DHX33), mRNA [NM_020162]	NM_020162
A_23_P302654	0.000171	NM_018140	NM_018140	Homo sapiens centrosomal protein 72 kDa (Cep72), mRNA [NM_018140]	NM_018140
A_23_P139558	0.000171	NM_022771	NM_022771	Homo sapiens TBC1 domain family, member 15 (TBC1D15), mRNA [NM_022771]	NM_022771
A_23_P41818	0.000171	NM_016107	NM_016107	Homo sapiens zinc finger RNA binding protein (ZFR), mRNA [NM_016107]	NM_016107
A_23_P10463	0.000171	NM_004552	NM_004552	Homo sapiens NADH dehydrogenase (ubiquinone) Fe-S protein 5, 15kDa (NADH-coenzyme Q reductase) (NDUFS5), mRNA [NM_004552]	NM_004552
A_23_P163347	0.000172	NM_016166	NM_016166	Homo sapiens protein inhibitor of activated STAT, 1 (PIAS1), mRNA [NM_016166]	NM_016166
A_24_P784846	0.000173	AK021906	AK021906	Homo sapiens cDNA FLJ11844 fis, clone HEMBA1006665. [AK021906]	
A_24_P940509	0.000173	AK022793	AK022793	Homo sapiens cDNA FLJ12731 fis, clone NT2RP2000108. [AK022793]	
A_24_P914513	0.000173	NM_183050	NM_183050	Homo sapiens branched chain keto acid dehydrogenase E1, beta polypeptide (maple syrup urine disease) (BCKDHB), nuclear gene encoding mitochondrial protein, transcript variant 1, mRNA [NM_183050]	NM_183050
A_23_P160200	0.000173	NM_023070	NM_023070	Homo sapiens zinc finger protein 643 (ZNF643), mRNA [NM_023070]	NM_023070
A_23_P91783	0.000173	NM_181335	NM_181335	Homo sapiens Rho GTPase activating protein 8 (ARHGAP8), transcript variant 2, mRNA [NM_181335]	NM_181335
A_23_P27180	0.000173	NM_032141	NM_032141	Homo sapiens hypothetical protein DKFZp434K1421 (DKFZP434K1421), mRNA [NM_032141]	NM_032141
A_24_P323932	0.000173	ENST00000319822		Homo sapiens, clone IMAGE:2820942, mRNA, partial cds. [BC006474]	XM_031553
A_23_P154522	0.000173	NM_020744	NM_020744	Homo sapiens metastasis associated 1 family, member 3 (MTA3), mRNA [NM_020744]	NM_020744
A_23_P162787	0.000173	NM_006704	NM_006704	Homo sapiens SGT1, suppressor of G2 allele of SKP1 (S. cerevisiae) (SUGT1), mRNA [NM_006704]	NM_006704
A_23_P41025	0.000174	NM_014366	NM_014366	Homo sapiens guanine nucleotide binding protein-like 3 (nucleolar) (GNL3), transcript variant 1, mRNA [NM_014366]	NM_014366
A_24_P881608	0.000174	A_24_P881608			
A_24_P20814	0.000175	NM_030568	NM_030568	Homo sapiens chromosome 6 open reading frame 148 (C6orf148), mRNA [NM_030568]	NM_030568
A_23_P85543	0.000175	NM_007212	NM_007212	Homo sapiens ring finger protein 2 (RNF2), mRNA [NM_007212]	NM_007212
A_23_P18325	0.000175	NM_007217	NM_007217	Homo sapiens programmed cell death 10 (PDCD10), transcript variant 1, mRNA [NM_007217]	NM_007217
A_23_P384748	0.000176	NM_172069	NM_172069	Homo sapiens pleckstrin homology domain containing, family H (with MyTH4 domain) member 2 (PLEKHH2), mRNA [NM_172069]	NM_172069
A_24_P931428	0.000176	AK021980	AK021980	Homo sapiens cDNA FLJ11918 fis, clone HEMBB1000272. [AK021980]	

A_32_P14843	0.000176	ENST00000355290		Homo sapiens mRNA; cDNA DKFZp686B0962 (from clone DKFZp686B0962). [BX647358]	
A_23_P203391	0.000176	BC006267	BC006267	Homo sapiens asparaginase like 1, mRNA (cDNA clone IMAGE:3952485), complete cds. [BC006267]	
A_23_P154488	0.000176	NM_033109	NM_033109	Homo sapiens polyribonucleotide nucleotidyltransferase 1 (PNPT1), mRNA [NM_033109]	NM_033109
A_32_P98313	0.000176	NM_002489	NM_002489	Homo sapiens NADH dehydrogenase (ubiquinone) 1 alpha subcomplex, 4, 9kDa (NDUFA4), nuclear gene encoding mitochondrial protein, mRNA [NM_002489]	NM_002489
A_24_P920880	0.000177	AY279352	AY279352	Homo sapiens truncated zinc finger protein 447 isoform (ZNF447) mRNA, complete cds. [AY279352]	
A_32_P208120	0.000177	NM_153498	NM_153498	Homo sapiens calcium/calmodulin-dependent protein kinase ID (CAMK1D), transcript variant 2, mRNA [NM_153498]	NM_153498
A_23_P69362	0.000177	NM_024661	NM_024661	Homo sapiens hypothetical protein FLJ12436 (FLJ12436), mRNA [NM_024661]	NM_024661
A_23_P66872	0.000177	NM_015721	NM_015721	Homo sapiens gem (nuclear organelle) associated protein 4 (GEMIN4), mRNA [NM_015721]	NM_015721
A_23_P45106	0.000177	NM_017730	NM_017730	Homo sapiens FLJ20259 protein (FLJ20259), transcript variant 1, mRNA [NM_017730]	NM_017730
A_32_P55860	0.000178	NM_182620	NM_182620	Homo sapiens family with sequence similarity 33, member A (FAM33A), mRNA [NM_182620]	NM_182620
A_23_P28664	0.000178	NM_018084	NM_018084	Homo sapiens KIAA1212 (KIAA1212), mRNA [NM_018084]	NM_018084
A_24_P450870	0.000178	THC2334619		RL39_HUMAN (P62891) 60S ribosomal protein L39, partial (60%) [THC2334619]	
A_23_P136721	0.000178	U88896	U88896	Human endogenous retrovirus H protease/integrase-derived ORF1, ORF2, and putative envelope protein mRNA, complete cds. [U88896]	
A_32_P124580	0.000178	AA490192	AA490192	AA490192 aa43f10.r1 Soares_NhHMPu_S1 Homo sapiens cDNA clone IMAGE:823723 5', mRNA sequence [AA490192]	
A_24_P863124	0.000179	THC2276723		O17436 (O17436) Minor ampullate silk protein MiSp2 (Fragment), partial (24%) [THC2276723]	
A_23_P133995	0.000179	NM_016059	NM_016059	Homo sapiens peptidylprolyl isomerase (cyclophilin)-like 1 (PPIL1), mRNA [NM_016059]	NM_016059
A_24_P221668	0.00018	AA359500	AA359500	AA359500 EST68526 Fetal lung II Homo sapiens cDNA 5' end, mRNA sequence [AA359500]	
A_23_P501805	0.00018	NM_145197	NM_145197	Homo sapiens lipoyltransferase 1 (LIPT1), transcript variant 3, mRNA [NM_145197]	NM_145197
A_23_P60899	0.00018	NM_024831	NM_024831	Homo sapiens nuclear receptor coactivator 6 interacting protein (NCOA6IP), mRNA [NM_024831]	NM_024831
A_23_P106505	0.00018	NM_014793	NM_014793	Homo sapiens leucine carboxyl methyltransferase 2 (LCMT2), mRNA [NM_014793]	NM_014793
A_24_P325015	0.00018	BC065520	BC065520	Homo sapiens cDNA clone IMAGE:5787400, partial cds. [BC065520]	
A_32_P183367	0.000181	A_32_P183367			
A_23_P74663	0.000181	NM_005681	NM_005681	Homo sapiens TATA box binding protein (TBP)-associated factor, RNA polymerase I, A, 48kDa (TAF1A), transcript variant 1, mRNA [NM_005681]	NM_005681
A_24_P666795	0.000181	A_24_P666795			
A_23_P12874	0.000181	NM_012341	NM_012341	Homo sapiens GTP binding protein 4 (GTPBP4), mRNA [NM_012341]	NM_012341
A_32_P179205	0.000181	XM_379885	XM_379885	PREDICTED: Homo sapiens similar to Heterogeneous nuclear ribonucleoprotein A1 (Helix-destabilizing protein) (Single-strand binding protein) (hnRNP core protein A1) (HDP-1) (Topoisomerase-inhibitor suppressed) (LOC402562), mRNA [XM_379885]	XM_379885
A_24_P382113	0.000182	NM_006107	NM_006107	Homo sapiens cisplatin resistance-associated overexpressed protein (CROP), transcript variant 2, mRNA [NM_006107]	NM_006107
A_32_P51084	0.000182	NM_015135	NM_015135	Homo sapiens nucleoporin 205kDa (NUP205), mRNA [NM_015135]	NM_015135
A_24_P233995	0.000183	NM_022746	NM_022746	Homo sapiens MOCO sulphurase C-terminal domain containing 1 (MOSC1), mRNA [NM_022746]	NM_022746
A_23_P67952	0.000183	S49953	S49953	N-cym=DNA-binding transcriptional activator homolog {oncogene} [human, Kelly neuroblastoma cell line, mRNA, 778 nt]. [S49953]	
A_23_P432573	0.000183	NM_145015	NM_145015	Homo sapiens MAS-related GPR, member F (MRGPRF), mRNA [NM_145015]	NM_145015
A_32_P138178	0.000184	BE835321	BE835321	BE835321 RC5-FN0022-300600-022-G12 FN0022 Homo sapiens cDNA, mRNA sequence [BE835321]	
A_23_P46390	0.000184	NM_032872	NM_032872	Homo sapiens synaptotagmin-like 1 (SYTL1), mRNA [NM_032872]	NM_032872
A_23_P413051	0.000185	NM_144603	NM_144603	Homo sapiens NADPH oxidase organizer 1 (NOXO1), transcript variant a, mRNA [NM_144603]	NM_144603
A_23_P26124	0.000186	NM_134260	NM_134260	Homo sapiens RAR-related orphan receptor A (RORA), transcript variant 2, mRNA [NM_134260]	NM_134260
A_24_P288116	0.000186	BC040982	BC040982	Homo sapiens, clone IMAGE:4798675, mRNA. [BC040982]	
A_24_P22436	0.000186	NM_024516	NM_024516	Homo sapiens chromosome 16 open reading frame 53 (C16orf53), mRNA [NM_024516]	NM_024516
A_24_P61537	0.000186	NM_001823	NM_001823	Homo sapiens creatine kinase, brain (CKB), mRNA [NM_001823]	NM_001823
A_24_P165082	0.000187	NM_013360	NM_013360	Homo sapiens zinc finger protein 222 (ZNF222), mRNA [NM_013360]	NM_013360
A_24_P350649	0.000187	NM_017990	NM_017990	Homo sapiens pyruvate dehydrogenase phosphatase regulatory subunit (PDPR), mRNA [NM_017990]	NM_017990
A_23_P131526	0.000187	NM_024520	NM_024520	Homo sapiens hypothetical protein FLJ22555 (FLJ22555), mRNA [NM_024520]	NM_024520
A_23_P160406	0.000187	NM_016121	NM_016121	Homo sapiens potassium channel tetramerisation domain containing 3 (KCTD3), mRNA [NM_016121]	NM_016121
A_24_P170357	0.000188	A_24_P170357			

A_24_P330397	0.000188	NM_014574	NM_014574	Homo sapiens striatin, calmodulin binding protein 3 (STRN3), mRNA [NM_014574]	NM_014574
A_24_P107257	0.000189	NM_018362	NM_018362	Homo sapiens lin-7 homolog C (C. elegans) (LIN7C), mRNA [NM_018362]	NM_018362
A_32_P166392	0.000189	ENST00000240349		PREDICTED: Homo sapiens similar to alpha NAC/1.9.2. protein (LOC389240), mRNA [XM_371715]	XM_371715
A_23_P319512	0.00019	NM_003597	NM_003597	Homo sapiens Kruppel-like factor 11 (KLF11), mRNA [NM_003597]	NM_003597
A_23_P75989	0.00019	NM_002576	NM_002576	Homo sapiens p21/Cdc42/Rac1-activated kinase 1 (STE20 homolog, yeast) (PAK1), mRNA [NM_002576]	NM_002576
A_24_P358244	0.000191	NM_014940	NM_014940	Homo sapiens MON1 homolog B (yeast) (MON1B), mRNA [NM_014940]	NM_014940
A_23_P25293	0.000192	NM_014865	NM_014865	Homo sapiens chromosome condensation-related SMC-associated protein 1 (CNAP1), mRNA [NM_014865]	NM_014865
A_24_P41339	0.000192	A_24_P41339			
A_23_P123018	0.000193	NM_025042	NM_025042	Homo sapiens Williams-Beuren syndrome chromosome region 23 (WBSCR23), mRNA [NM_025042]	NM_025042
A_32_P12282	0.000193	A_32_P12282			
A_23_P337424	0.000193	NM_001018067	NM_001018067	Homo sapiens PAI-1 mRNA binding protein (PAI-RBP1), transcript variant 1, mRNA [NM_001018067]	NM_001018067
A_23_P115726	0.000193	NM_194298	NM_194298	Homo sapiens solute carrier family 16 (monocarboxylic acid transporters), member 9 (SLC16A9), mRNA [NM_194298]	NM_194298
A_23_P320250	0.000193	NM_025109	NM_025109	Homo sapiens myosin head domain containing 1 (MYOHD1), mRNA [NM_025109]	NM_025109
A_23_P23206	0.000193	NM_006341	NM_006341	Homo sapiens MAD2 mitotic arrest deficient-like 2 (yeast) (MAD2L2), mRNA [NM_006341]	NM_006341
A_24_P938169	0.000194	AF136171	AF136171	Homo sapiens heparin-binding protein HBp15 mRNA, complete cds. [AF136171]	
A_24_P252310	0.000195	NM_014690	NM_014690	Homo sapiens KIAA0773 gene product (KIAA0773), mRNA [NM_014690]	NM_014690
A_24_P254705	0.000195	NM_020394	NM_020394	Homo sapiens zinc finger protein 695 (ZNF695), mRNA [NM_020394]	NM_020394
A_24_P721898	0.000195	NM_001002860	NM_001002860	Homo sapiens BTB (POZ) domain containing 7 (BTBD7), transcript variant 1, mRNA [NM_001002860]	NM_001002860
A_32_P79504	0.000196	ENST00000267430		Homo sapiens mRNA; cDNA DKFZp667P1220 (from clone DKFZp667P1220). [AL833656]	XM_048128
A_32_P204205	0.000196	NM_017420	NM_017420	Homo sapiens sine oculis homeobox homolog 4 (Drosophila) (SIX4), mRNA [NM_017420]	NM_017420
A_23_P72187	0.000196	NM_004229	NM_004229	Homo sapiens cofactor required for Sp1 transcriptional activation, subunit 2, 150kDa (CRSP2), mRNA [NM_004229]	NM_004229
A_23_P374288	0.000196	NM_007359	NM_007359	Homo sapiens cancer susceptibility candidate 3 (CASC3), mRNA [NM_007359]	NM_007359
A_32_P540407	0.000197	NM_207333	NM_207333	Homo sapiens zinc finger protein like (ZFPL), mRNA [NM_207333]	NM_207333
A_24_P108291	0.000197	NM_018439	NM_018439	Homo sapiens hypothetical protein IMPACT (IMPACT), mRNA [NM_018439]	NM_018439
A_32_P105281	0.000197	AW935085	AW935085	AW935085 RC2-DT0002-290200-017-e02_1 DT0002 Homo sapiens cDNA, mRNA sequence [AW935085]	
A_24_P497226	0.000197	NM_003161	NM_003161	Homo sapiens ribosomal protein S6 kinase, 70kDa, polypeptide 1 (RPS6KB1), mRNA [NM_003161]	NM_003161
A_23_P344853	0.000197	ENST00000296126		Homo sapiens KIAA0007 mRNA, partial cds. [D26488]	XM_087089
A_32_P186921	0.000197	BC032805	BC032805	Homo sapiens cDNA clone MGC:45556 IMAGE:4186857, complete cds. [BC032805]	
A_24_P538403	0.000198	BC041849	BC041849	Homo sapiens, Similar to Rho-associated, coiled-coil containing protein kinase 1, clone IMAGE:5269982, mRNA. [BC041849]	
A_23_P74114	0.000198	NM_182633	NM_182633	Homo sapiens hypothetical protein FLJ39963 (FLJ39963), mRNA [NM_182633]	NM_182633
A_24_P232696	0.000198	NM_139071	NM_139071	Homo sapiens SWI/SNF related, matrix associated, actin dependent regulator of chromatin, subfamily d, member 1 (SMARCD1), transcript variant 2, mRNA [NM_139071]	NM_139071
A_23_P52147	0.000198	NM_003193	NM_003193	Homo sapiens tubulin-specific chaperone e (TBCE), mRNA [NM_003193]	NM_003193
A_23_P415558	0.000199	NM_012256	NM_012256	Homo sapiens zinc finger protein 212 (ZNF212), mRNA [NM_012256]	NM_012256
A_23_P158829	0.000199	NM_004313	NM_004313	Homo sapiens arrestin, beta 2 (ARRB2), transcript variant 1, mRNA [NM_004313]	NM_004313
A_23_P8900	0.000199	NM_004374	NM_004374	Homo sapiens cytochrome c oxidase subunit VIc (COX6C), mRNA [NM_004374]	NM_004374
A_23_P49725	0.0002	ENST00000355067		Homo sapiens cDNA FLJ36169 fis, clone TESTI2026168. [AK093488]	
A_23_P254594	0.0002	NM_000825	NM_000825	Homo sapiens gonadotropin-releasing hormone 1 (luteinizing-releasing hormone) (GNRH1), mRNA [NM_000825]	NM_000825
A_24_P410605	0.0002	BC080541	BC080541	Homo sapiens receptor tyrosine kinase-like orphan receptor 1, mRNA (cDNA clone IMAGE:5477978), complete cds. [BC080541]	
A_23_P385114	0.0002	NM_023929	NM_023929	Homo sapiens zinc finger and BTB domain containing 10 (ZBTB10), mRNA [NM_023929]	NM_023929
A_23_P119418	0.0002	NM_003796	NM_003796	Homo sapiens chromosome 19 open reading frame 2 (C19orf2), transcript variant 1, mRNA [NM_003796]	NM_003796
A_24_P34344	0.0002	NM_033020	NM_033020	Homo sapiens tripartite motif-containing 33 (TRIM33), transcript variant beta, mRNA [NM_033020]	NM_033020
A_32_P16975	0.0002	NM_133450	NM_133450	Homo sapiens KIAA1977 protein (KIAA1977), mRNA [NM_133450]	NM_133450
A_23_P120472	0.000201	NM_003222	NM_003222	Homo sapiens transcription factor AP-2 gamma (activating enhancer binding protein 2 gamma) (TFAP2C), mRNA [NM_003222]	NM_003222
A_32_P187160	0.000201	A_32_P187160			

A_24_P15965	0.000201	NM_203282	NM_203282	Homo sapiens zinc finger protein 539 (ZNF539), mRNA [NM_203282]	NM_203282
A_24_P125690	0.000201	NM_023937	NM_023937	Homo sapiens mitochondrial ribosomal protein L34 (MRPL34), nuclear gene encoding mitochondrial protein, mRNA [NM_023937]	NM_023937
A_24_P136725	0.000202	NM_001010862	NM_001010862	Homo sapiens spindlin family, member 3 (SPIN3), mRNA [NM_001010862]	NM_001010862
A_24_P248345	0.000202	NM_015441	NM_015441	Homo sapiens olfactomedin-like 2B (OLFML2B), mRNA [NM_015441]	NM_015441
A_23_P27734	0.000202	NM_002517	NM_002517	Homo sapiens neuronal PAS domain protein 1 (NPAS1), mRNA [NM_002517]	NM_002517
A_23_P53217	0.000202	NM_152991	NM_152991	Homo sapiens embryonic ectoderm development (EED), transcript variant 2, mRNA [NM_152991]	NM_152991
A_23_P43141	0.000202	NM_001568	NM_001568	Homo sapiens eukaryotic translation initiation factor 3, subunit 6 48kDa (EIF3S6), mRNA [NM_001568]	NM_001568
A_24_P889980	0.000203	BC047753	BC047753	Homo sapiens, Similar to PMS2 postmeiotic segregation increased 2 (S. cerevisiae), clone IMAGE:6193449, mRNA. [BC047753]	XM_496904
A_32_P47200	0.000203	AU121805	AU121805	AU121805 MAMMA1 Homo sapiens cDNA clone MAMMA1001009 5', mRNA sequence [AU121805]	
A_23_P413888	0.000203	NM_001029858	NM_001029858	Homo sapiens solute carrier family 35, member F1 (SLC35F1), mRNA [NM_001029858]	NM_001029858
A_23_P47058	0.000203	NM_022034	NM_022034	Homo sapiens CUB and zona pellucida-like domains 1 (CUZD1), mRNA [NM_022034]	NM_022034
A_32_P2333	0.000203	NM_006713	NM_006713	Homo sapiens activated RNA polymerase II transcription cofactor 4 (PC4), mRNA [NM_006713]	NM_006713
A_23_P107073	0.000203	NM_002945	NM_002945	Homo sapiens replication protein A1, 70kDa (RPA1), mRNA [NM_002945]	NM_002945
A_24_P111511	0.000204	NM_024753	NM_024753	Homo sapiens tetratricopeptide repeat domain 21B (TTC21B), mRNA [NM_024753]	NM_024753
A_32_P49748	0.000204	AK131277	AK131277	Homo sapiens cDNA FLJ16224 fis, clone CTONG3009287. [AK131277]	
A_23_P104109	0.000204	NM_012424	NM_012424	Homo sapiens ribosomal protein S6 kinase, 52kDa, polypeptide 1 (RPS6KC1), mRNA [NM_012424]	NM_012424
A_23_P103201	0.000204	NM_017761	NM_017761	Homo sapiens proline-rich nuclear receptor coactivator 2 (PNRC2), mRNA [NM_017761]	NM_017761
A_23_P47497	0.000204	NM_017840	NM_017840	Homo sapiens mitochondrial ribosomal protein L16 (MRPL16), nuclear gene encoding mitochondrial protein, mRNA [NM_017840]	NM_017840
A_23_P144497	0.000204	NM_001006	NM_001006	Homo sapiens ribosomal protein S3A (RPS3A), mRNA [NM_001006]	NM_001006
A_23_P39441	0.000205	NM_133473	NM_133473	Homo sapiens zinc finger protein 431 (ZNF431), mRNA [NM_133473]	NM_133473
A_23_P430839	0.000205	NM_023002	NM_023002	Homo sapiens hyaluronan and proteoglycan link protein 4 (HAPLN4), mRNA [NM_023002]	NM_023002
A_23_P205738	0.000205	NM_138576	NM_138576	Homo sapiens B-cell CLL/lymphoma 11B (zinc finger protein) (BCL11B), transcript variant 1, mRNA [NM_138576]	NM_138576
A_23_P250353	0.000205	NM_001013002	NM_001013002	Homo sapiens hect domain and RLD 6 (HERC6), transcript variant 2, mRNA [NM_001013002]	NM_001013002
A_23_P350689	0.000205	NM_173570	NM_173570	Homo sapiens zinc finger, DHHC-type containing 23 (ZDHHC23), mRNA [NM_173570]	NM_173570
A_23_P36882	0.000205	NM_006183	NM_006183	Homo sapiens neurotensin (NTS), mRNA [NM_006183]	NM_006183
A_23_P116123	0.000205	NM_001274	NM_001274	Homo sapiens CHK1 checkpoint homolog (S. pombe) (CHEK1), mRNA [NM_001274]	NM_001274
A_23_P50477	0.000205	NM_138639	NM_138639	Homo sapiens BCL2-like 12 (proline rich) (BCL2L12), transcript variant 1, mRNA [NM_138639]	NM_138639
A_24_P15563	0.000206	AB032978	AB032978	Homo sapiens mRNA for KIAA1152 protein, partial cds. [AB032978]	
A_24_P286898	0.000206	AK125150	AK125150	Homo sapiens cDNA FLJ43160 fis, clone FCBBF2000199. [AK125150]	
A_23_P201279	0.000206	NM_006048	NM_006048	Homo sapiens ubiquitination factor E4B (UFD2 homolog, yeast) (UBE4B), mRNA [NM_006048]	NM_006048
A_32_P58606	0.000207	THC2343246			
A_32_P148796	0.000207	ENST00000331398		Homo sapiens mRNA; cDNA DKFZp451I123 (from clone DKFZp451I123). [AL831990]	XM_059929
A_23_P99424	0.000207	NM_003291	NM_003291	Homo sapiens tripeptidyl peptidase II (TPP2), mRNA [NM_003291]	NM_003291
A_23_P63999	0.000207	CR614032	CR614032	full-length cDNA clone CS0DL003YC18 of B cells (Ramos cell line) Cot 25-normalized of Homo sapiens (human). [CR614032]	
A_32_P121908	0.000208	BE973568	BE973568	601680932F1 NIH_MGC_83 Homo sapiens cDNA clone IMAGE:3951222 5', mRNA sequence [BE973568]	
A_24_P190345	0.000208	NM_198480	NM_198480	Homo sapiens zinc finger protein 615 (ZNF615), mRNA [NM_198480]	NM_198480
A_23_P151059	0.000208	NM_018088	NM_018088	Homo sapiens hypothetical protein FLJ10408 (FLJ10408), mRNA [NM_018088]	NM_018088
A_23_P431330	0.000208	NM_175918	NM_175918	Homo sapiens hypothetical protein FLJ34443 (FLJ34443), mRNA [NM_175918]	NM_175918
A_23_P202392	0.000208	NM_024670	NM_024670	Homo sapiens suppressor of variegation 3-9 homolog 2 (Drosophila) (SUV39H2), mRNA [NM_024670]	NM_024670
A_23_P251421	0.000208	NM_031942	NM_031942	Homo sapiens cell division cycle associated 7 (CDCA7), transcript variant 1, mRNA [NM_031942]	NM_031942
A_23_P42695	0.000208	NM_024051	NM_024051	Homo sapiens chromosome 7 open reading frame 24 (C7orf24), mRNA [NM_024051]	NM_024051
A_23_P201287	0.000209	NM_015074	NM_015074	Homo sapiens kinesin family member 1B (KIF1B), transcript variant 1, mRNA [NM_015074]	NM_015074
A_23_P152181	0.000209	NM_018119	NM_018119	Homo sapiens polymerase (RNA) III (DNA directed) polypeptide E (80kD) (POLR3E), mRNA [NM_018119]	NM_018119
A_23_P23318	0.000209	NM_006784	NM_006784	Homo sapiens WD repeat domain 3 (WDR3), mRNA [NM_006784]	NM_006784

A_32_P8120	0.000209	NM_014366	NM_014366	Homo sapiens guanine nucleotide binding protein-like 3 (nucleolar) (GNL3), transcript variant 1, mRNA [NM_014366]	NM_014366
A_32_P8015	0.000209	NM_001011724	NM_001011724	Homo sapiens heterogeneous nuclear ribonucleoprotein A1-like (LOC144983), transcript variant 1, mRNA [NM_001011724]	NM_001011724
A_24_P217804	0.00021	THC2368225			
A_24_P224526	0.00021	NM_022344	NM_022344	Homo sapiens protein kinase Njmu-R1 (NJMU-R1), mRNA [NM_022344]	NM_022344
A_23_P41948	0.00021	NM_017785	NM_017785	Homo sapiens hypothetical protein FLJ20364 (FLJ20364), mRNA [NM_017785]	NM_017785
A_23_P63459	0.00021	NM_001012985	NM_001012985	Homo sapiens chromosome 1 open reading frame 31 (C1orf31), mRNA [NM_001012985]	NM_001012985
A_23_P215111	0.000211	NM_020632	NM_020632	Homo sapiens ATPase, H+ transporting, lysosomal V0 subunit a isoform 4 (ATP6V0A4), transcript variant 1, mRNA [NM_020632]	NM_020632
A_24_P945147	0.000211	NM_004703	NM_004703	Homo sapiens rabaptin, RAB GTPase binding effector protein 1 (RABEP1), mRNA [NM_004703]	NM_004703
A_23_P206441	0.000211	NM_000135	NM_000135	Homo sapiens Fanconi anemia, complementation group A (FANCA), transcript variant 1, mRNA [NM_000135]	NM_000135
A_23_P146431	0.000211	BC021127	BC021127	Homo sapiens KIAA0368, mRNA (cDNA clone MGC:32012 IMAGE:4335177), complete cds. [BC021127]	XM_036708
A_32_P157471	0.000212	CF143262	CF143262	CF143262 UI-HF-BR0p-aqt-a-11-0-UI.r1 NIH_MGC_52 Homo sapiens cDNA clone IMAGE:3101469 5', mRNA sequence [CF143262]	
A_24_P139208	0.000212	NM_013396	NM_013396	Homo sapiens ubiquitin specific protease 25 (USP25), mRNA [NM_013396]	NM_013396
A_23_P502464	0.000212	NM_000625	NM_000625	Homo sapiens nitric oxide synthase 2A (inducible, hepatocytes) (NOS2A), transcript variant 1, mRNA [NM_000625]	NM_000625
A_23_P14216	0.000212	ENST00000239860		Q9H1T5 (Q9H1T5) OTTHUMP00000042268, complete [THC2436901]	
A_23_P317056	0.000212	ENST00000361681			
A_24_P336417	0.000212	NM_004736	NM_004736	Homo sapiens xenotropic and polytropic retrovirus receptor (XPR1), mRNA [NM_004736]	NM_004736
A_23_P129896	0.000213	NM_000382	NM_000382	Homo sapiens aldehyde dehydrogenase 3 family, member A2 (ALDH3A2), mRNA [NM_000382]	NM_000382
A_23_P97309	0.000213	NM_001229	NM_001229	Homo sapiens caspase 9, apoptosis-related cysteine protease (CASP9), transcript variant alpha, mRNA [NM_001229]	NM_001229
A_23_P67708	0.000213	NM_003200	NM_003200	Homo sapiens transcription factor 3 (E2A immunoglobulin enhancer binding factors E12/E47) (TCF3), mRNA [NM_003200]	NM_003200
A_32_P61657	0.000214	NM_006955	NM_006955	Homo sapiens zinc finger protein 11B (ZNF11B), mRNA [NM_006955]	NM_006955
A_24_P189739	0.000214	NM_030640	NM_030640	Homo sapiens dual specificity phosphatase 16 (DUSP16), mRNA [NM_030640]	NM_030640
A_23_P140434	0.000214	NM_018728	NM_018728	Homo sapiens myosin VC (MYO5C), mRNA [NM_018728]	NM_018728
A_32_P144421	0.000214	NM_053042	NM_053042	Homo sapiens KIAA1729 protein (KIAA1729), mRNA [NM_053042]	NM_053042
A_23_P152666	0.000214	NM_004375	NM_004375	Homo sapiens COX11 homolog, cytochrome c oxidase assembly protein (yeast) (COX11), nuclear gene encoding mitochondrial protein, mRNA [NM_004375]	NM_004375
A_23_P80098	0.000214	NM_000819	NM_000819	Homo sapiens phosphoribosylglycinamide formyltransferase, phosphoribosylglycinamide synthetase, phosphoribosylaminoimidazole synthetase (GART), transcript variant 1, mRNA [NM_000819]	NM_000819
A_24_P50458	0.000214	NM_017489	NM_017489	Homo sapiens telomeric repeat binding factor (NIMA-interacting) 1 (TERF1), transcript variant 1, mRNA [NM_017489]	NM_017489
A_23_P349406	0.000215	NM_173642	NM_173642	Homo sapiens family with sequence similarity 80, member A (FAM80A), mRNA [NM_173642]	NM_173642
A_23_P314642	0.000215	NM_017666	NM_017666	Homo sapiens suppressor of hairy wing homolog 3 (Drosophila) (SUHW3), mRNA [NM_017666]	NM_017666
A_23_P12113	0.000215	NM_014053	NM_014053	Homo sapiens feline leukemia virus subgroup C cellular receptor (FLVCR), mRNA [NM_014053]	NM_014053
A_23_P255750	0.000216	NM_024857	NM_024857	Homo sapiens chromosome 17 open reading frame 41 (C17orf41), mRNA [NM_024857]	NM_024857
A_23_P111306	0.000216	NM_014892	NM_014892	Homo sapiens RNA binding motif protein 16 (RBM16), mRNA [NM_014892]	NM_014892
A_23_P134684	0.000216	NM_024567	NM_024567	Homo sapiens hypothetical protein FLJ21616 (FLJ21616), mRNA [NM_024567]	NM_024567
A_23_P14649	0.000216	AK021784	AK021784	Homo sapiens cDNA FLJ11722 fis, clone HEMBA1005311. [AK021784]	
A_32_P110156	0.000216	BC052238	BC052238	Homo sapiens chaperonin containing TCP1, subunit 6A-like, mRNA (cDNA clone IMAGE:5094556). [BC052238]	
A_23_P122876	0.000216	NM_005641	NM_005641	Homo sapiens TAF6 RNA polymerase II, TATA box binding protein (TBP)-associated factor, 80kDa (TAF6), transcript variant 1, mRNA [NM_005641]	NM_005641
A_23_P18818	0.000217	NM_015455	NM_015455	Homo sapiens CCR4-NOT transcription complex, subunit 6 (CNOT6), mRNA [NM_015455]	NM_015455
A_32_P200934	0.000217	AK021443	AK021443	Homo sapiens cDNA FLJ11381 fis, clone HEMBA1000501. [AK021443]	
A_32_P146844	0.000218	THC2406576		ALU2_HUMAN (P39189) Alu subfamily SB sequence contamination warning entry, partial (4%) [THC2406576]	
A_32_P41065	0.000218	NM_001017395	NM_001017395	Homo sapiens transmembrane and coiled-coil domain family 1 (TMCC1), transcript variant 1, mRNA [NM_001017395]	NM_001017395
A_23_P383435	0.000218	NM_004773	NM_004773	Homo sapiens thyroid hormone receptor interactor 3 (TRIP3), mRNA [NM_004773]	NM_004773
A_23_P37704	0.000218	NM_030928	NM_030928	Homo sapiens DNA replication factor (CDT1), mRNA [NM_030928]	NM_030928
A_32_P139079	0.000218	NM_015640	NM_015640	Homo sapiens PAI-1 mRNA binding protein (PAI-RBP1), transcript variant 4, mRNA [NM_015640]	NM_015640
A_24_P510047	0.000219	AK022086	AK022086	Homo sapiens cDNA FLJ12024 fis, clone HEMBB1001797. [AK022086]	

A_23_P34597	0.000219	NM_001785	NM_001785	Homo sapiens cytidine deaminase (CDA), mRNA [NM_001785]	NM_001785
A_23_P130316	0.000219	NM_004671	NM_004671	Homo sapiens protein inhibitor of activated STAT, 2 (PIAS2), transcript variant beta, mRNA [NM_004671]	NM_004671
A_32_P33821	0.000219	BX538015	BX538015	Homo sapiens mRNA; cDNA DKFZp686E2158 (from clone DKFZp686E2158); complete cds. [BX538015]	
A_24_P642771	0.00022	AK024956	AK024956	Homo sapiens cDNA: FLJ21303 fis, clone COL02107. [AK024956]	
A_32_P115505	0.00022	NM_015565	NM_015565	Homo sapiens zinc finger protein 294 (ZNF294), mRNA [NM_015565]	NM_015565
A_32_P197698	0.00022	AK055939	AK055939	Homo sapiens cDNA FLJ31377 fis, clone NESOP1000087. [AK055939]	
A_32_P59678	0.00022	BC042034	BC042034	Homo sapiens hypothetical protein MGC72075, mRNA (cDNA clone IMAGE:5263383), partial cds. [BC042034]	
A_23_P102925	0.00022	NM_005049	NM_005049	Homo sapiens PWP2 periodic tryptophan protein homolog (yeast) (PWP2H), mRNA [NM_005049]	NM_005049
A_23_P59426	0.00022	NM_007349	NM_007349	Homo sapiens PAX interacting (with transcription-activation domain) protein 1 (PAXIP1), mRNA [NM_007349]	NM_007349
A_23_P128663	0.00022	NM_014363	NM_014363	Homo sapiens spastic ataxia of Charlevoix-Saguenay (sacsin) (SACS), mRNA [NM_014363]	NM_014363
A_24_P165259	0.00022	NM_013328	NM_013328	Homo sapiens pyrroline-5-carboxylate reductase family, member 2 (PYCR2), mRNA [NM_013328]	NM_013328
A_23_P28434	0.00022	NM_003761	NM_003761	Homo sapiens vesicle-associated membrane protein 8 (endobrevin) (VAMP8), mRNA [NM_003761]	NM_003761
A_24_P666482	0.000221	NM_001006607	NM_001006607	Homo sapiens c114 SLIT-like testicular protein (LOC474170), mRNA [NM_001006607]	NM_001006607
A_23_P39154	0.000221	NM_003419	NM_003419	Homo sapiens zinc finger protein 345 (ZNF345), mRNA [NM_003419]	NM_003419
A_32_P76035	0.000221	CR749867	CR749867	Homo sapiens mRNA; cDNA DKFZp762K1914 (from clone DKFZp762K1914). [CR749867]	
A_23_P84872	0.000221	NM_024077	NM_024077	Homo sapiens SECIS binding protein 2 (SECISBP2), mRNA [NM_024077]	NM_024077
A_32_P181131	0.000221	AW467174	AW467174	AW467174 ha35g06.x1 NCI_CGAP_Kid12 Homo sapiens cDNA clone IMAGE:2875738 3' similar to gb:X60673_rna1 GTP:AMP PHOSPHOTRANSFERASE MITOCHONDRIAL (HUMAN);, mRNA sequence [AW467174]	
A_23_P164089	0.000221	NM_057178	NM_057178	Homo sapiens rififylin (RFFL), transcript variant 1, mRNA [NM_057178]	NM_057178
A_23_P26916	0.000221	NM_001015053	NM_001015053	Homo sapiens histone deacetylase 5 (HDAC5), transcript variant 3, mRNA [NM_001015053]	NM_001015053
A_23_P52189	0.000221	NM_004837	NM_004837	Homo sapiens geranylgeranyl diphosphate synthase 1 (GGPS1), mRNA [NM_004837]	NM_004837
A_23_P399146	0.000222	NM_153263	NM_153263	Homo sapiens zinc finger protein 549 (ZNF549), mRNA [NM_153263]	NM_153263
A_24_P30670	0.000222	NM_007187	NM_007187	Homo sapiens WW domain binding protein 4 (formin binding protein 21) (WBP4), mRNA [NM_007187]	NM_007187
A_24_P401473	0.000222	AK057719	AK057719	Homo sapiens cDNA FLJ33157 fis, clone UTERU2000393. [AK057719]	
A_24_P59471	0.000222	AK094159	AK094159	Homo sapiens cDNA FLJ36840 fis, clone ASTRO2011461. [AK094159]	
A_23_P323685	0.000222	NM_003543	NM_003543	Homo sapiens histone 1, H4h (HIST1H4H), mRNA [NM_003543]	NM_003543
A_23_P430044	0.000222	NM_032701	NM_032701	Homo sapiens suppressor of variegation 4-20 homolog 2 (Drosophila) (SUV420H2), mRNA [NM_032701]	NM_032701
A_24_P52004	0.000222	NM_015200	NM_015200	Homo sapiens SCC-112 protein (SCC-112), mRNA [NM_015200]	NM_015200
A_23_P133345	0.000222	NM_014666	NM_014666	Homo sapiens enthoprotin (ENTH), mRNA [NM_014666]	NM_014666
A_23_P310350	0.000222	NM_013276	NM_013276	Homo sapiens carbohydrate kinase-like (CARKL), mRNA [NM_013276]	NM_013276
A_24_P414786	0.000222	NM_015891	NM_015891	Homo sapiens cell division cycle 40 homolog (yeast) (CDC40), mRNA [NM_015891]	NM_015891
A_23_P354908	0.000223	NM_015039	NM_015039	Homo sapiens nicotinamide nucleotide adenylyltransferase 2 (NMNAT2), transcript variant 1, mRNA [NM_015039]	NM_015039
A_23_P203564	0.000223	THC2338825		AF071771 SPH-binding factor {Homo sapiens;} , partial (31%) [THC2338825]	
A_24_P568645	0.000223	A_24_P568645			
A_23_P257372	0.000223	AK094065	AK094065	Homo sapiens cDNA FLJ36746 fis, clone UTERU2016757. [AK094065]	
A_23_P416751	0.000224	NM_173530	NM_173530	Homo sapiens zinc finger protein 610 (ZNF610), mRNA [NM_173530]	NM_173530
A_32_P109495	0.000224	A_32_P109495			
A_24_P944049	0.000224	AB011154	AB011154	Homo sapiens mRNA for KIAA0582 protein, partial cds. [AB011154]	
A_23_P5131	0.000224	NM_016368	NM_016368	Homo sapiens myo-inositol 1-phosphate synthase A1 (ISYNA1), mRNA [NM_016368]	NM_016368
A_24_P341292	0.000225	NM_024733	NM_024733	Homo sapiens zinc finger protein 665 (ZNF665), mRNA [NM_024733]	NM_024733
A_24_P914102	0.000225	A_24_P914102			
A_23_P81463	0.000225	NM_019030	NM_019030	Homo sapiens DEAH (Asp-Glu-Ala-His) box polypeptide 29 (DHX29), mRNA [NM_019030]	NM_019030
A_23_P218717	0.000225	NM_014948	NM_014948	Homo sapiens U-box domain containing 5 (UBOX5), transcript variant 1, mRNA [NM_014948]	NM_014948
A_23_P64661	0.000226	NM_032496	NM_032496	Homo sapiens Rho GTPase activating protein 9 (ARHGAP9), mRNA [NM_032496]	NM_032496
A_32_P174832	0.000227	BI523298	BI523298	BI523298 603175578T1 NIH_MGC_121 Homo sapiens cDNA clone IMAGE:5239842 3', mRNA sequence [BI523298]	

A_23_P154972	0.000228	NM_080740	NM_080740	Homo sapiens suppressor of hairy wing homolog 1 (Drosophila) (SUHW1), mRNA [NM_080740]	NM_080740
A_24_P649507	0.000228	THC2438003		Q9BVX4 (Q9BVX4) MGC5566 protein, partial (23%) [THC2438003]	
A_23_P92629	0.000228	NM_005869	NM_005869	Homo sapiens serologically defined colon cancer antigen 10 (SDCCAG10), mRNA [NM_005869]	NM_005869
A_24_P878388	0.000228	XM_373338	XM_373338	PREDICTED: Homo sapiens similar to bA92K2.2 (similar to ubiquitin) (LOC392425), mRNA [XM_373338]	XM_373338
A_23_P212204	0.000229	BX648380	BX648380	Homo sapiens mRNA; cDNA DKFZp686A20205 (from clone DKFZp686A20205). [BX648380]	
A_23_P301336	0.000229	NM_014472	NM_014472	Homo sapiens chromosome 10 open reading frame 28 (C10orf28), mRNA [NM_014472]	NM_014472
A_24_P106112	0.000229	NM_000297	NM_000297	Homo sapiens polycystic kidney disease 2 (autosomal dominant) (PKD2), mRNA [NM_000297]	NM_000297
A_23_P24176	0.000229	NM_019084	NM_019084	Homo sapiens cyclin J (CCNJ), mRNA [NM_019084]	NM_019084
A_23_P216517	0.00023	NM_032818	NM_032818	Homo sapiens chromosome 9 open reading frame 100 (C9orf100), mRNA [NM_032818]	NM_032818
A_23_P81180	0.00023	NM_022832	NM_022832	Homo sapiens ubiquitin specific protease 46 (USP46), mRNA [NM_022832]	NM_022832
A_24_P96897	0.000231	CR594520	CR594520	full-length cDNA clone CS0DF016YO07 of Fetal brain of Homo sapiens (human). [CR594520]	
A_23_P6464	0.000231	NM_003560	NM_003560	Homo sapiens phospholipase A2, group VI (cytosolic, calcium-independent) (PLA2G6), transcript variant 1, mRNA [NM_003560]	NM_003560
A_24_P307665	0.000231	AK021848	AK021848	Homo sapiens cDNA FLJ11786 fis, clone HEMBA1006036. [AK021848]	
A_24_P920188	0.000231	AF230200	AF230200	Homo sapiens OVN6-2 mRNA, partial cds. [AF230200]	
A_23_P323774	0.000232	NM_001001484	NM_001001484	Homo sapiens phosphotriesterase related (PTER), transcript variant 1, mRNA [NM_001001484]	NM_001001484
A_23_P5481	0.000232	NM_025000	NM_025000	Homo sapiens hypothetical protein FLJ13096 (FLJ13096), mRNA [NM_025000]	NM_025000
A_23_P131825	0.000232	NM_003279	NM_003279	Homo sapiens troponin C2, fast (TNNC2), mRNA [NM_003279]	NM_003279
A_23_P115482	0.000232	NM_014176	NM_014176	Homo sapiens ubiquitin-conjugating enzyme E2T (putative) (UBE2T), mRNA [NM_014176]	NM_014176
A_24_P304458	0.000233	AK021751	AK021751	Homo sapiens cDNA FLJ11689 fis, clone HEMBA1004977. [AK021751]	
A_23_P27424	0.000233	NM_133460	NM_133460	Homo sapiens zinc finger protein 418 (ZNF418), mRNA [NM_133460]	NM_133460
A_24_P529168	0.000233	NM_213724	NM_213724	Homo sapiens chromosome 13 open reading frame 25 (C13orf25), transcript variant 1, mRNA [NM_213724]	NM_213724
A_24_P67681	0.000233	ENST00000332148		PREDICTED: Homo sapiens similar to High mobility group protein 4 (HMG-4) (High mobility group protein 2a) (HMG-2a) (LOC441795), mRNA [XM_497547]	XM_497547
A_24_P216421	0.000234	NM_033148	NM_033148	Homo sapiens dystrobrevin, beta (DTNB), transcript variant 3, mRNA [NM_033148]	NM_033148
A_23_P253762	0.000234	BC004565	BC004565	Homo sapiens hypothetical protein MGC12935, mRNA (cDNA clone IMAGE:4309284), partial cds. [BC004565]	
A_24_P247978	0.000234	NM_016089	NM_016089	Homo sapiens zinc finger protein 589 (ZNF589), mRNA [NM_016089]	NM_016089
A_23_P146058	0.000234	NM_001695	NM_001695	Homo sapiens ATPase, H+ transporting, lysosomal 42kDa, V1 subunit C, isoform 1 (ATP6V1C1), transcript variant 1, mRNA [NM_001695]	NM_001695
A_32_P203878	0.000235	THC2373072		CB243285 UI-CF-FN0-agc-l-12-0-UI.s1 UI-CF-FN0 Homo sapiens cDNA clone UI-CF-FN0-agc-l-12-0-UI 3', mRNA sequence [CB243285]	
A_24_P941188	0.000235	AB007928	AB007928	Homo sapiens mRNA for KIAA0459 protein, partial cds. [AB007928]	XM_375697
A_24_P940125	0.000235	NM_015455	NM_015455	Homo sapiens CCR4-NOT transcription complex, subunit 6 (CNOT6), mRNA [NM_015455]	NM_015455
A_24_P73738	0.000235	ENST00000311528		Homo sapiens cDNA FLJ45674 fis, clone D9OST2004417, highly similar to 60S ribosomal protein L13. [AK127579]	
A_23_P154411	0.000235	NM_004792	NM_004792	Homo sapiens peptidyl-prolyl isomerase G (cyclophilin G) (PPIG), mRNA [NM_004792]	NM_004792
A_23_P202458	0.000235	NM_006963	NM_006963	Homo sapiens zinc finger protein 22 (KOX 15) (ZNF22), mRNA [NM_006963]	NM_006963
A_24_P4426	0.000236	NM_014937	NM_014937	Homo sapiens inositol polyphosphate-5-phosphatase F (INPP5F), transcript variant 1, mRNA [NM_014937]	NM_014937
A_23_P41380	0.000236	NM_002940	NM_002940	Homo sapiens ATP-binding cassette, sub-family E (OABP), member 1 (ABCE1), mRNA [NM_002940]	NM_002940
A_23_P104201	0.000236	NM_139313	NM_139313	Homo sapiens YME1-like 1 (S. cerevisiae) (YME1L1), nuclear gene encoding mitochondrial protein, transcript variant 2, mRNA [NM_139313]	NM_139313
A_23_P19020	0.000237	NM_005460	NM_005460	Homo sapiens synuclein, alpha interacting protein (synphilin) (SNCAIP), mRNA [NM_005460]	NM_005460
A_32_P36136	0.000237	XM_378367	XM_378367	PREDICTED: Homo sapiens hypothetical LOC400053 (LOC400053), mRNA [XM_378367]	XM_378367
A_23_P412409	0.000237	NM_015172	NM_015172	Homo sapiens BAT2 domain containing 1 (BAT2D1), mRNA [NM_015172]	NM_015172
A_23_P66774	0.000237	NM_153338	NM_153338	Homo sapiens hypothetical protein FLJ90165 (FLJ90165), mRNA [NM_153338]	NM_153338
A_23_P332584	0.000238	ENST00000176186		Homo sapiens mRNA for KIAA1107 protein, partial cds. [AB029030]	XM_034086
A_23_P134147	0.000238	NM_014797	NM_014797	Homo sapiens zinc finger and BTB domain containing 24 (ZBTB24), mRNA [NM_014797]	NM_014797
A_24_P307175	0.000238	NM_052928	NM_052928	Homo sapiens SET and MYND domain containing 4 (SMYD4), mRNA [NM_052928]	NM_052928
A_23_P85188	0.000239	NM_022838	NM_022838	Homo sapiens armadillo repeat containing, X-linked 5 (ARMCX5), mRNA [NM_022838]	NM_022838
A_24_P76898	0.00024	ENST00000328724		Homo sapiens mRNA; cDNA DKFZp761H0317 (from clone DKFZp761H0317). [AL834350]	

A_24_P519638	0.00024	AK096250	AK096250	Homo sapiens cDNA FLJ38931 fis, clone NT2NE2013189. [AK096250]	
A_23_P23765	0.00024	NM_014288	NM_014288	Homo sapiens integrin beta 3 binding protein (beta3-endonexin) (ITGB3BP), mRNA [NM_014288]	NM_014288
A_32_P25273	0.00024	NM_002156	NM_002156	Homo sapiens heat shock 60kDa protein 1 (chaperonin) (HSPD1), nuclear gene encoding mitochondrial protein, transcript variant 1, mRNA [NM_002156]	NM_002156
A_23_P136805	0.000241	NM_014783	NM_014783	Homo sapiens Rho GTPase activating protein 11A (ARHGAP11A), mRNA [NM_014783]	NM_014783
A_23_P115703	0.000241	NM_001011663	NM_001011663	Homo sapiens polycomb group ring finger 6 (PCGF6), transcript variant 1, mRNA [NM_001011663]	NM_001011663
A_24_P186342	0.000242	NM_020917	NM_020917	Homo sapiens mouse zinc finger protein 14-like (KIAA1559), mRNA [NM_020917]	NM_020917
A_24_P334402	0.000242	NM_024920	NM_024920	Homo sapiens DnaJ (Hsp40) homolog, subfamily B, member 14 (DNAJB14), mRNA [NM_024920]	NM_024920
A_23_P406438	0.000242	NM_182691	NM_182691	Homo sapiens SFRS protein kinase 2 (SRPK2), transcript variant 2, mRNA [NM_182691]	NM_182691
A_23_P93709	0.000243	AK098134	AK098134	Homo sapiens cDNA FLJ40815 fis, clone TRACH2010600. [AK098134]	
A_23_P46769	0.000243	AK126751	AK126751	Homo sapiens cDNA FLJ44798 fis, clone BRACE3040863. [AK126751]	
A_23_P6151	0.000243	NM_002657	NM_002657	Homo sapiens pleiomorphic adenoma gene-like 2 (PLAGL2), mRNA [NM_002657]	NM_002657
A_23_P169887	0.000243	NM_001521	NM_001521	Homo sapiens general transcription factor IIIC, polypeptide 2, beta 110kDa (GTF3C2), mRNA [NM_001521]	NM_001521
A_24_P916288	0.000243	AK026497	AK026497	Homo sapiens cDNA: FLJ22844 fis, clone KAIA5181. [AK026497]	
A_23_P209689	0.000244	NM_004850	NM_004850	Homo sapiens Rho-associated, coiled-coil containing protein kinase 2 (ROCK2), mRNA [NM_004850]	NM_004850
A_23_P156880	0.000245	NM_006208	NM_006208	Homo sapiens ectonucleotide pyrophosphatase/phosphodiesterase 1 (ENPP1), mRNA [NM_006208]	NM_006208
A_32_P166372	0.000246	THC2294276		ALU2_HUMAN (P39189) Alu subfamily SB sequence contamination warning entry, partial (17%) [THC2294276]	
A_23_P166336	0.000246	NM_032262	NM_032262	Homo sapiens hypothetical protein DKFZp434N035 (DKFZp434N035), mRNA [NM_032262]	NM_032262
A_23_P128372	0.000246	NM_002014	NM_002014	Homo sapiens FK506 binding protein 4, 59kDa (FKBP4), mRNA [NM_002014]	NM_002014
A_23_P58353	0.000246	NM_031370	NM_031370	Homo sapiens heterogeneous nuclear ribonucleoprotein D (AU-rich element RNA binding protein 1, 37kDa) (HNRPD), transcript variant 1, mRNA [NM_031370]	NM_031370
A_23_P398947	0.000247	NM_205848	NM_205848	Homo sapiens synaptotagmin VI (SYT6), mRNA [NM_205848]	NM_205848
A_23_P117157	0.000247	NM_003850	NM_003850	Homo sapiens succinate-CoA ligase, ADP-forming, beta subunit (SUCLA2), mRNA [NM_003850]	NM_003850
A_24_P12573	0.000248	NM_006072	NM_006072	Homo sapiens chemokine (C-C motif) ligand 26 (CCL26), mRNA [NM_006072]	NM_006072
A_23_P51117	0.000248	NM_019002	NM_019002	Homo sapiens ETAA16 protein (ETAA16), mRNA [NM_019002]	NM_019002
A_24_P149036	0.000248	NM_001387	NM_001387	Homo sapiens dihydropyrimidinase-like 3 (DPYSL3), mRNA [NM_001387]	NM_001387
A_23_P18684	0.000249	NM_004362	NM_004362	Homo sapiens calmegin (CLGN), mRNA [NM_004362]	NM_004362
A_32_P168388	0.000251	AK123765	AK123765	Homo sapiens cDNA FLJ41771 fis, clone IMR322009807. [AK123765]	
A_23_P419202	0.000251	NM_033160	NM_033160	Homo sapiens zinc finger protein 658 (ZNF658), mRNA [NM_033160]	NM_033160
A_23_P118834	0.000251	NM_001067	NM_001067	Homo sapiens topoisomerase (DNA) II alpha 170kDa (TOP2A), mRNA [NM_001067]	NM_001067
A_23_P420551	0.000251	NM_007174	NM_007174	Homo sapiens citron (rho-interacting, serine/threonine kinase 21) (CIT), mRNA [NM_007174]	NM_007174
A_24_P179336	0.000252	AY029066	AY029066	Homo sapiens Humanin (HN1) mRNA, complete cds. [AY029066]	
A_23_P82959	0.000253	NM_003923	NM_003923	Homo sapiens forkhead box H1 (FOXH1), mRNA [NM_003923]	NM_003923
A_32_P525524	0.000254	NM_178495	NM_178495	Homo sapiens KIAA1754-like (KIAA1754L), transcript variant 1, mRNA [NM_178495]	NM_178495
A_23_P122775	0.000254	NM_032730	NM_032730	Homo sapiens reticulon 4 interacting protein 1 (RTN4IP1), nuclear gene encoding mitochondrial protein, mRNA [NM_032730]	NM_032730
A_23_P98382	0.000254	NM_012459	NM_012459	Homo sapiens translocase of inner mitochondrial membrane 8 homolog B (yeast) (TIMM8B), mRNA [NM_012459]	NM_012459
A_32_P141612	0.000255	NM_002552	NM_002552	Homo sapiens origin recognition complex, subunit 4-like (yeast) (ORC4L), transcript variant 2, mRNA [NM_002552]	NM_002552
A_23_P382654	0.000255	NM_022091	NM_022091	Homo sapiens dJ467N11.1 protein (DJ467N11.1), mRNA [NM_022091]	NM_022091
A_23_P203949	0.000255	NM_004399	NM_004399	Homo sapiens DEAD/H (Asp-Glu-Ala-Asp/His) box polypeptide 11 (CHL1-like helicase homolog, S. cerevisiae) (DDX11), transcript variant 2, mRNA [NM_004399]	NM_004399
A_23_P100196	0.000255	NM_005153	NM_005153	Homo sapiens ubiquitin specific protease 10 (USP10), mRNA [NM_005153]	NM_005153
A_23_P65890	0.000256	NM_006383	NM_006383	Homo sapiens calcium and integrin binding family member 2 (CIB2), mRNA [NM_006383]	NM_006383
A_24_P542375	0.000256	NM_002823	NM_002823	Homo sapiens prothymosin, alpha (gene sequence 28) (PTMA), mRNA [NM_002823]	NM_002823
A_23_P254031	0.000257	NM_007344	NM_007344	Homo sapiens transcription termination factor, RNA polymerase I (TTF1), mRNA [NM_007344]	NM_007344
A_24_P750636	0.000257	THC2267012			
A_23_P164022	0.000257	NM_033375	NM_033375	Homo sapiens myosin IC (MYO1C), mRNA [NM_033375]	NM_033375
A_23_P21804	0.000258	A_23_P21804			

A_23_P210224	0.000258	AL713706	AL713706	Homo sapiens mRNA; cDNA DKFZp761O1810 (from clone DKFZp761O1810). [AL713706]	
A_23_P305938	0.000259	NM_007131	NM_007131	Homo sapiens zinc finger protein 75 (D8C6) (ZNF75), mRNA [NM_007131]	NM_007131
A_24_P902100	0.000259	AL359559	AL359559	Homo sapiens mRNA; cDNA DKFZp762O2215 (from clone DKFZp762O2215). [AL359559]	
A_23_P153441	0.000259	NM_013312	NM_013312	Homo sapiens hook homolog 2 (Drosophila) (HOOK2), mRNA [NM_013312]	NM_013312
A_23_P127079	0.000259	NM_015062	NM_015062	Homo sapiens peroxisome proliferative activated receptor, gamma, coactivator-related 1 (PPRC1), mRNA [NM_015062]	NM_015062
A_32_P145241	0.000259	A_32_P145241			
A_24_P515319	0.00026	AL832996	AL832996	Homo sapiens mRNA; cDNA DKFZp666M073 (from clone DKFZp666M073). [AL832996]	XM_496956
A_24_P48408	0.00026	NM_003799	NM_003799	Homo sapiens RNA (guanine-7-) methyltransferase (RNMT), mRNA [NM_003799]	NM_003799
A_24_P852099	0.00026	THC2301481			
A_32_P107797	0.000261	A_32_P107797			
A_32_P47543	0.000261	BG112935	BG112935	602283549F1 NIH_MGC_86 Homo sapiens cDNA clone IMAGE:4370964 5', mRNA sequence [BG112935]	
A_23_P317591	0.000261	NM_006080	NM_006080	Homo sapiens sema domain, immunoglobulin domain (Ig), short basic domain, secreted, (semaphorin) 3A (SEMA3A), mRNA [NM_006080]	NM_006080
A_23_P84140	0.000261	NM_014642	NM_014642	Homo sapiens IQ motif containing B1 (IQCB1), transcript variant 2, mRNA [NM_014642]	NM_014642
A_24_P375435	0.000261	A_24_P375435			
A_23_P436369	0.000263	NM_015687	NM_015687	Homo sapiens filamin A interacting protein 1 (FILIP1), mRNA [NM_015687]	NM_015687
A_23_P211603	0.000263	A_23_P211603			
A_23_P34578	0.000263	NM_013285	NM_013285	Homo sapiens guanine nucleotide binding protein-like 2 (nucleolar) (GNL2), mRNA [NM_013285]	NM_013285
A_23_P218965	0.000264	NM_020117	NM_020117	Homo sapiens leucyl-tRNA synthetase (LARS), mRNA [NM_020117]	NM_020117
A_23_P213754	0.000264	NM_016480	NM_016480	Homo sapiens poly(A) binding protein interacting protein 2 (PAIP2), mRNA [NM_016480]	NM_016480
A_24_P766865	0.000265	AK096498	AK096498	Homo sapiens cDNA FLJ39179 fis, clone OCBBF2004147. [AK096498]	
A_23_P206228	0.000265	NM_017684	NM_017684	Homo sapiens vacuolar protein sorting 13C (yeast) (VPS13C), transcript variant 1A, mRNA [NM_017684]	NM_017684
A_24_P936393	0.000267	A_24_P936393			
A_23_P131394	0.000267	NM_152527	NM_152527	Homo sapiens solute carrier family 16 (monocarboxylic acid transporters), member 14 (SLC16A14), mRNA [NM_152527]	NM_152527
A_23_P163117	0.000267	NM_024644	NM_024644	Homo sapiens chromosome 14 open reading frame 169 (C14orf169), mRNA [NM_024644]	NM_024644
A_23_P73972	0.000267	NM_015696	NM_015696	Homo sapiens glutathione peroxidase 7 (GPX7), mRNA [NM_015696]	NM_015696
A_32_P83520	0.000268	A_32_P83520			
A_23_P113161	0.000268	NM_030806	NM_030806	Homo sapiens chromosome 1 open reading frame 21 (C1orf21), mRNA [NM_030806]	NM_030806
A_23_P25463	0.000269	NM_025114	NM_025114	Homo sapiens centrosome protein cep290 (Cep290), mRNA [NM_025114]	NM_025114
A_24_P175176	0.000269	NM_020432	NM_020432	Homo sapiens putative homeodomain transcription factor 2 (PHTF2), mRNA [NM_020432]	NM_020432
A_23_P160466	0.000269	NM_006996	NM_006996	Homo sapiens solute carrier family 19 (thiamine transporter), member 2 (SLC19A2), mRNA [NM_006996]	NM_006996
A_23_P310911	0.000269	NM_000386	NM_000386	Homo sapiens bleomycin hydrolase (BLMH), mRNA [NM_000386]	NM_000386
A_23_P97250	0.000269	AB046826	AB046826	Homo sapiens mRNA for KIAA1606 protein, partial cds. [AB046826]	
A_24_P248053	0.000269	NM_052963	NM_052963	Homo sapiens topoisomerase (DNA) I, mitochondrial (TOP1MT), nuclear gene encoding mitochondrial protein, mRNA [NM_052963]	NM_052963
A_23_P5616	0.00027	NM_018151	NM_018151	Homo sapiens RAP1 interacting factor homolog (yeast) (RIF1), mRNA [NM_018151]	NM_018151
A_24_P12281	0.00027	NM_007077	NM_007077	Homo sapiens adaptor-related protein complex 4, sigma 1 subunit (AP4S1), mRNA [NM_007077]	NM_007077
A_24_P205263	0.00027	NM_016649	NM_016649	Homo sapiens chromosome 20 open reading frame 6 (C20orf6), mRNA [NM_016649]	NM_016649
A_24_P205045	0.00027	NM_015576	NM_015576	Homo sapiens CAZ-associated structural protein (CAST), mRNA [NM_015576]	NM_015576
A_24_P18621	0.00027	NM_153207	NM_153207	Homo sapiens AE binding protein 2 (AEBP2), mRNA [NM_153207]	NM_153207
A_23_P90463	0.00027	NM_016199	NM_016199	Homo sapiens LSM7 homolog, U6 small nuclear RNA associated (S. cerevisiae) (LSM7), mRNA [NM_016199]	NM_016199
A_23_P356021	0.000271	NM_152633	NM_152633	Homo sapiens Fanconi anemia, complementation group B (FANCB), transcript variant 2, mRNA [NM_152633]	NM_152633
A_24_P122862	0.000271	NM_017657	NM_017657	Homo sapiens aftiphilin protein (AFTIPHILIN), transcript variant 2, mRNA [NM_017657]	NM_017657
A_23_P213441	0.000271	NM_032175	NM_032175	Homo sapiens Src-associated protein SAW (FLJ12787), mRNA [NM_032175]	NM_032175
A_24_P287785	0.000271	AL833099	AL833099	Homo sapiens mRNA; cDNA DKFZp313E2215 (from clone DKFZp313E2215). [AL833099]	
A_23_P413761	0.000271	NM_003017	NM_003017	Homo sapiens splicing factor, arginine/serine-rich 3 (SFRS3), mRNA [NM_003017]	NM_003017

A_23_P124327	0.000272	NM_022455	NM_022455	Homo sapiens nuclear receptor binding SET domain protein 1 (NSD1), transcript variant 2, mRNA [NM_022455]	NM_022455
A_23_P404730	0.000273	NM_052948	NM_052948	Homo sapiens sorting nexin 26 (SNX26), mRNA [NM_052948]	NM_052948
A_23_P402604	0.000273	NM_012393	NM_012393	Homo sapiens phosphoribosylformylglycinamidine synthase (FGAR amidotransferase) (PFAS), mRNA [NM_012393]	NM_012393
A_23_P69826	0.000273	NM_001358	NM_001358	Homo sapiens DEAH (Asp-Glu-Ala-His) box polypeptide 15 (DHX15), mRNA [NM_001358]	NM_001358
A_32_P47510	0.000274	THC2446452			
A_23_P153266	0.000275	NM_006114	NM_006114	Homo sapiens translocase of outer mitochondrial membrane 40 homolog (yeast) (TOMM40), mRNA [NM_006114]	NM_006114
A_23_P209098	0.000276	NM_001492	NM_001492	Homo sapiens growth differentiation factor 1 (GDF1), mRNA [NM_001492]	NM_001492
A_32_P57140	0.000277	ENST00000279875		Homo sapiens cDNA FLJ14374 fis, clone HEMBA1001635, weakly similar to TESTIS SPECIFIC PROTEIN A. [AK027280]	
A_23_P131149	0.000277	NM_002552	NM_002552	Homo sapiens origin recognition complex, subunit 4-like (yeast) (ORC4L), transcript variant 2, mRNA [NM_002552]	NM_002552
A_23_P16132	0.000278	NM_006387	NM_006387	Homo sapiens calcium homeostasis endoplasmic reticulum protein (CHERP), mRNA [NM_006387]	NM_006387
A_24_P320970	0.000279	BX538061	BX538061	Homo sapiens mRNA; cDNA DKFZp686J16172 (from clone DKFZp686J16172). [BX538061]	
A_24_P55148	0.000279	NM_021058	NM_021058	Homo sapiens histone 1, H2bj (HIST1H2BJ), mRNA [NM_021058]	NM_021058
A_23_P75516	0.00028	NM_003626	NM_003626	Homo sapiens protein tyrosine phosphatase, receptor type, f polypeptide (PTPRF), interacting protein (liprin), alpha 1 (PPFIA1), transcript variant 2, mRNA [NM_003626]	NM_003626
A_32_P211045	0.00028	NM_000791	NM_000791	Homo sapiens dihydrofolate reductase (DHFR), mRNA [NM_000791]	NM_000791
A_32_P9931	0.000281	A_32_P9931			
A_23_P90762	0.000281	NM_013233	NM_013233	Homo sapiens serine threonine kinase 39 (STE20/SPS1 homolog, yeast) (STK39), mRNA [NM_013233]	NM_013233
A_23_P71558	0.000281	NM_004260	NM_004260	Homo sapiens RecQ protein-like 4 (RECQL4), mRNA [NM_004260]	NM_004260
A_23_P147277	0.000281	NM_207346	NM_207346	Homo sapiens tRNA splicing endonuclease 54 homolog (SEN54, S. cerevisiae) (TSEN54), mRNA [NM_207346]	NM_207346
A_24_P125839	0.000282	NM_017447	NM_017447	Homo sapiens chromosome 21 open reading frame 91 (C21orf91), mRNA [NM_017447]	NM_017447
A_32_P159176	0.000282	THC2450500			
A_23_P75097	0.000282	NM_024895	NM_024895	Homo sapiens PDZ domain containing 7 (PDZK7), mRNA [NM_024895]	NM_024895
A_32_P133038	0.000283	THC2358845			
A_32_P12580	0.000283	AY134745	AY134745	Homo sapiens medulloblastoma antigen MU-MB-50.13 mRNA, complete cds. [AY134745]	
A_23_P32217	0.000283	NM_005802	NM_005802	Homo sapiens topoisomerase I binding, arginine/serine-rich (TOPORS), mRNA [NM_005802]	NM_005802
A_32_P190682	0.000284	THC2347318			
A_32_P122590	0.000284	BC035844	BC035844	Homo sapiens, clone IMAGE:5745916, mRNA. [BC035844]	
A_32_P50815	0.000284	AK054852	AK054852	Homo sapiens cDNA FLJ30290 fis, clone BRACE2002884. [AK054852]	
A_23_P160188	0.000284	NM_002857	NM_002857	Homo sapiens peroxisomal biogenesis factor 19 (PEX19), mRNA [NM_002857]	NM_002857
A_23_P311087	0.000284	NM_012482	NM_012482	Homo sapiens zinc finger protein 281 (ZNF281), mRNA [NM_012482]	NM_012482
A_23_P141785	0.000285	A_23_P141785			
A_23_P80409	0.000285	NM_020839	NM_020839	Homo sapiens WD repeat domain 48 (WDR48), mRNA [NM_020839]	NM_020839
A_32_P168853	0.000286	AK123481	AK123481	Homo sapiens cDNA FLJ41487 fis, clone BRTHA2004350. [AK123481]	
A_23_P109733	0.000286	NM_144718	NM_144718	Homo sapiens hypothetical protein AY099107 (LOC152185), mRNA [NM_144718]	NM_144718
A_23_P370989	0.000286	NM_005914	NM_005914	Homo sapiens MCM4 minichromosome maintenance deficient 4 (S. cerevisiae) (MCM4), transcript variant 1, mRNA [NM_005914]	NM_005914
A_23_P101332	0.000286	NM_023008	NM_023008	Homo sapiens hypothetical protein FLJ12949 (FLJ12949), transcript variant 1, mRNA [NM_023008]	NM_023008
A_32_P171328	0.000286	NM_014501	NM_014501	Homo sapiens ubiquitin-conjugating enzyme E2S (UBE2S), mRNA [NM_014501]	NM_014501
A_23_P362759	0.000287	NM_018699	NM_018699	Homo sapiens PR domain containing 5 (PRDM5), mRNA [NM_018699]	NM_018699
A_23_P108842	0.000287	NM_004418	NM_004418	Homo sapiens dual specificity phosphatase 2 (DUSP2), mRNA [NM_004418]	NM_004418
A_24_P686956	0.000287	THC2370457		ALU1_HUMAN (P39188) Alu subfamily J sequence contamination warning entry, partial (3%) [THC2370439]	
A_24_P781846	0.000288	AK024092	AK024092	Homo sapiens cDNA FLJ14030 fis, clone HEMBA1004086. [AK024092]	
A_23_P82181	0.000288	NM_018292	NM_018292	Homo sapiens glutaminyl-tRNA synthase (glutamine-hydrolyzing)-like 1 (QRSL1), mRNA [NM_018292]	NM_018292
A_23_P502488	0.000289	NM_032852	NM_032852	Homo sapiens APG4 autophagy 4 homolog C (S. cerevisiae) (APG4C), transcript variant 1, mRNA [NM_032852]	NM_032852
A_23_P101671	0.000289	AB058775	AB058775	Homo sapiens mRNA for KIAA1872 protein, partial cds. [AB058775]	
A_23_P121122	0.00029	NM_025146	NM_025146	Homo sapiens Mak3 homolog (S. cerevisiae) (MAK3), mRNA [NM_025146]	NM_025146
A_23_P75204	0.00029	NM_017615	NM_017615	Homo sapiens chromosome 10 open reading frame 86 (C10orf86), mRNA [NM_017615]	NM_017615

A_32_P110485	0.000291	THC2315024			
A_23_P107724	0.000291	NM_013380	NM_013380	Homo sapiens zinc finger protein 228 (ZNF228), mRNA [NM_013380]	NM_013380
A_23_P1102	0.000291	NM_001100	NM_001100	Homo sapiens actin, alpha 1, skeletal muscle (ACTA1), mRNA [NM_001100]	NM_001100
A_23_P55601	0.000293	NM_007345	NM_007345	Homo sapiens zinc finger protein 236 (ZNF236), mRNA [NM_007345]	NM_007345
A_24_P135319	0.000293	NM_032866	NM_032866	Homo sapiens cingulin-like 1 (CGNL1), mRNA [NM_032866]	NM_032866
A_24_P35935	0.000293	AK055438	AK055438	Homo sapiens cDNA FLJ30876 fis, clone FEBRA2004412. [AK055438]	
A_23_P156809	0.000293	NM_001018022	NM_001018022	Homo sapiens similar to FKSG62 (LOC389286), mRNA [NM_001018022]	NM_001018022
A_23_P301304	0.000293	NM_023109	NM_023109	Homo sapiens fibroblast growth factor receptor 1 (fms-related tyrosine kinase 2, Pfeiffer syndrome) (FGFR1), transcript variant 7, mRNA [NM_023109]	NM_023109
A_23_P34930	0.000293	NM_005872	NM_005872	Homo sapiens breast carcinoma amplified sequence 2 (BCAS2), mRNA [NM_005872]	NM_005872
A_23_P149798	0.000294	AK027209	AK027209	Homo sapiens cDNA: FLJ23556 fis, clone LNG09443. [AK027209]	
A_23_P35656	0.000294	BC003519	BC003519	Homo sapiens weakly similar to zinc finger protein 195, mRNA (cDNA clone IMAGE:3606289), partial cds. [BC003519]	
A_32_P96641	0.000294	NM_005000	NM_005000	Homo sapiens NADH dehydrogenase (ubiquinone) 1 alpha subcomplex, 5, 13kDa (NDUFA5), nuclear gene encoding mitochondrial protein, mRNA [NM_005000]	NM_005000
A_23_P119857	0.000295	NM_001008237	NM_001008237	Homo sapiens similar to CG14894-PA (LOC130502), mRNA [NM_001008237]	NM_001008237
A_24_P320328	0.000295	NM_006713	NM_006713	Homo sapiens activated RNA polymerase II transcription cofactor 4 (PC4), mRNA [NM_006713]	NM_006713
A_24_P841622	0.000296	AK096589	AK096589	Homo sapiens cDNA FLJ39270 fis, clone OCBBF2010557. [AK096589]	
A_24_P190877	0.000296	NM_030934	NM_030934	Homo sapiens chromosome 1 open reading frame 25 (C1orf25), mRNA [NM_030934]	NM_030934
A_24_P934800	0.000296	AB040937	AB040937	Homo sapiens mRNA for KIAA1504 protein, partial cds. [AB040937]	
A_24_P233960	0.000296	NM_030918	NM_030918	Homo sapiens sorting nexin family member 27 (SNX27), mRNA [NM_030918]	NM_030918
A_23_P45917	0.000296	NM_001826	NM_001826	Homo sapiens CDC28 protein kinase regulatory subunit 1B (CKS1B), mRNA [NM_001826]	NM_001826
A_23_P202750	0.000297	NM_014039	NM_014039	Homo sapiens PTD012 protein (PTD012), mRNA [NM_014039]	NM_014039
A_32_P77225	0.000298	THC2386194			
A_23_P19142	0.000298	NM_004137	NM_004137	Homo sapiens potassium large conductance calcium-activated channel, subfamily M, beta member 1 (KCNMB1), mRNA [NM_004137]	NM_004137
A_23_P128230	0.000298	NM_002135	NM_002135	Homo sapiens nuclear receptor subfamily 4, group A, member 1 (NR4A1), transcript variant 1, mRNA [NM_002135]	NM_002135
A_24_P407930	0.000298	NM_031216	NM_031216	Homo sapiens SEH1-like (S. cerevisiae) (SEH1L), transcript variant 2, mRNA [NM_031216]	NM_031216
A_24_P807883	0.000299	THC2279305			
A_23_P257111	0.000299	NM_000507	NM_000507	Homo sapiens fructose-1,6-bisphosphatase 1 (FBP1), mRNA [NM_000507]	NM_000507
A_23_P101615	0.000299	NM_152477	NM_152477	Homo sapiens zinc finger protein 565 (ZNF565), mRNA [NM_152477]	NM_152477
A_24_P919840	0.000299	AK000954	AK000954	Homo sapiens cDNA FLJ10092 fis, clone HEMBA1002349. [AK000954]	
A_32_P13371	0.000299	THC2437122		Q6DB98 (Q6DB98) EAAT1 protein, partial (3%) [THC2437122]	
A_23_P393686	0.0003	NM_175075	NM_175075	Homo sapiens chromosome 8 open reading frame 42 (C8orf42), mRNA [NM_175075]	NM_175075
A_24_P814872	0.0003	A_24_P814872			
A_24_P873263	0.0003	NM_015355	NM_015355	Homo sapiens suppressor of zeste 12 homolog (Drosophila) (SUZ12), mRNA [NM_015355]	NM_015355
A_24_P56221	0.000301	NM_052845	NM_052845	Homo sapiens methylmalonic aciduria (cobalamin deficiency) cblB type (MMAB), mRNA [NM_052845]	NM_052845
A_23_P502797	0.000301	NM_020830	NM_020830	Homo sapiens WD repeat and FYVE domain containing 1 (WDFY1), mRNA [NM_020830]	NM_020830
A_23_P218096	0.000301	NM_022118	NM_022118	Homo sapiens chromosome 13 open reading frame 10 (C13orf10), mRNA [NM_022118]	NM_022118
A_32_P42895	0.000301	AW138903	AW138903	AW138903 UI-H-BI1-aeq-e-09-0-UI.s1 NCI_CGAP_Sub3 Homo sapiens cDNA clone IMAGE:2720344 3', mRNA sequence [AW138903]	
A_23_P82509	0.000302	NM_021167	NM_021167	Homo sapiens GATA zinc finger domain containing 1 (GATAD1), mRNA [NM_021167]	NM_021167
A_23_P35970	0.000302	NM_001467	NM_001467	Homo sapiens solute carrier family 37 (glycerol-6-phosphate transporter), member 4 (SLC37A4), mRNA [NM_001467]	NM_001467
A_32_P101002	0.000303	THC2448203		ALU1_HUMAN (P39188) Alu subfamily J sequence contamination warning entry, partial (5%) [THC2448203]	
A_23_P435051	0.000303	AB014575	AB014575	Homo sapiens mRNA for KIAA0675 protein, partial cds. [AB014575]	
A_32_P37584	0.000304	AW340352	AW340352	AW340352 hd03g08.x1 Soares_NFL_T_GBC_S1 Homo sapiens cDNA clone IMAGE:2908478 3' similar to TR:P70413 P70413 CYCLIN D-INTERACTING MYB-LIKE PROTEIN. ;, mRNA sequence [AW340352]	
A_24_P269006	0.000304	AK021800	AK021800	Homo sapiens cDNA FLJ11738 fis, clone HEMBA1005474. [AK021800]	
A_23_P35684	0.000304	NM_014937	NM_014937	Homo sapiens inositol polyphosphate-5-phosphatase F (INPP5F), transcript variant 1, mRNA [NM_014937]	NM_014937
A_32_P133780	0.000305	BQ189538	BQ189538	UI-E-EJ1-aka-o-17-0-UI.r1 UI-E-EJ1 Homo sapiens cDNA clone UI-E-EJ1-aka-o-17-0-UI 5', mRNA sequence [BQ189538]	

A_32_P155841	0.000305	AL079294	AL079294	Homo sapiens mRNA full length insert cDNA clone EUROIMAGE 362780. [AL079294]	
A_24_P383834	0.000305	A_24_P383834			
A_24_P67898	0.000305	AF307332	AF307332	Homo sapiens meningioma-expressed antigen 5s splice variant mRNA, complete cds. [AF307332]	
A_24_P7040	0.000305	A_24_P7040			
A_24_P450172	0.000306	AK095151	AK095151	Homo sapiens cDNA FLJ37832 fis, clone BRSSN2009630. [AK095151]	
A_32_P103508	0.000306	AK096022	AK096022	Homo sapiens cDNA FLJ38703 fis, clone KIDNE2002265. [AK096022]	
A_32_P141374	0.000306	NM_178831	NM_178831	Homo sapiens opposite strand transcription unit to STAG3 (GATS), mRNA [NM_178831]	NM_178831
A_23_P37111	0.000306	NM_177438	NM_177438	Homo sapiens Dicer1, Dcr-1 homolog (Drosophila) (DICER1), transcript variant 1, mRNA [NM_177438]	NM_177438
A_24_P165656	0.000306	NM_005813	NM_005813	Homo sapiens protein kinase D3 (PRKD3), mRNA [NM_005813]	NM_005813
A_23_P168306	0.000306	NM_003931	NM_003931	Homo sapiens WAS protein family, member 1 (WASF1), transcript variant 1, mRNA [NM_003931]	NM_003931
A_23_P132226	0.000307	NM_001008566	NM_001008566	Homo sapiens tyrosylprotein sulfotransferase 2 (TPST2), transcript variant 1, mRNA [NM_001008566]	NM_001008566
A_24_P586390	0.000308	AK123446	AK123446	Homo sapiens cDNA FLJ41452 fis, clone BRSTN2010363. [AK123446]	XM_379275
A_23_P130780	0.000309	NM_015711	NM_015711	Homo sapiens glioma tumor suppressor candidate region gene 1 (GLTSCR1), mRNA [NM_015711]	NM_015711
A_23_P151649	0.000309	NM_080649	NM_080649	Homo sapiens APEX nuclease (multifunctional DNA repair enzyme) 1 (APEX1), transcript variant 3, mRNA [NM_080649]	NM_080649
A_23_P250313	0.00031	AB040957	AB040957	Homo sapiens mRNA for KIAA1524 protein, partial cds. [AB040957]	
A_23_P12816	0.00031	NM_018063	NM_018063	Homo sapiens helicase, lymphoid-specific (HELLS), mRNA [NM_018063]	NM_018063
A_24_P314351	0.000311	NM_006777	NM_006777	Homo sapiens zinc finger and BTB domain containing 33 (ZBTB33), mRNA [NM_006777]	NM_006777
A_23_P166964	0.000312	NM_006286	NM_006286	Homo sapiens transcription factor Dp-2 (E2F dimerization partner 2) (TFDP2), mRNA [NM_006286]	NM_006286
A_24_P4171	0.000312	NM_023111	NM_023111	Homo sapiens fibroblast growth factor receptor 1 (fms-related tyrosine kinase 2, Pfeiffer syndrome) (FGFR1), transcript variant 9, mRNA [NM_023111]	NM_023111
A_23_P67312	0.000314	NM_003437	NM_003437	Homo sapiens zinc finger protein 136 (clone pHZ-20) (ZNF136), mRNA [NM_003437]	NM_003437
A_24_P329152	0.000316	NM_012120	NM_012120	Homo sapiens CD2-associated protein (CD2AP), mRNA [NM_012120]	NM_012120
A_24_P111342	0.000316	NM_001229	NM_001229	Homo sapiens caspase 9, apoptosis-related cysteine protease (CASP9), transcript variant alpha, mRNA [NM_001229]	NM_001229
A_23_P99172	0.000317	NM_032358	NM_032358	Homo sapiens hypothetical protein MGC13183 (MGC13183), mRNA [NM_032358]	NM_032358
A_24_P724984	0.000318	AK001846	AK001846	Homo sapiens cDNA FLJ10984 fis, clone PLACE1001810. [AK001846]	
A_32_P20703	0.000319	AK026418	AK026418	Homo sapiens cDNA: FLJ22765 fis, clone KAIA1180. [AK026418]	
A_32_P53338	0.000319	CA312250	CA312250	CA312250 UI-CF-FN0-afm-d-23-0-UI.s1 UI-CF-FN0 Homo sapiens cDNA clone UI-CF-FN0-afm-d-23-0-UI 3', mRNA sequence [CA312250]	
A_23_P55256	0.00032	NM_014897	NM_014897	Homo sapiens zinc finger protein 652 (ZNF652), mRNA [NM_014897]	NM_014897
A_24_P19544	0.00032	NM_013233	NM_013233	Homo sapiens serine threonine kinase 39 (STE20/SPS1 homolog, yeast) (STK39), mRNA [NM_013233]	NM_013233
A_23_P208293	0.00032	CR611629	CR611629	full-length cDNA clone CS0DI066YH20 of Placenta Cot 25-normalized of Homo sapiens (human). [CR611629]	
A_24_P721828	0.000321	AK022015	AK022015	Homo sapiens cDNA FLJ11953 fis, clone HEMBB1000883. [AK022015]	
A_24_P222911	0.000321	NM_006276	NM_006276	Homo sapiens splicing factor, arginine/serine-rich 7, 35kDa (SFRS7), mRNA [NM_006276]	NM_006276
A_23_P62159	0.000321	BC016138	BC016138	Homo sapiens chromosome X open reading frame 17, mRNA (cDNA clone IMAGE:3921622), complete cds. [BC016138]	
A_23_P94053	0.000321	NM_003496	NM_003496	Homo sapiens transformation/transcription domain-associated protein (TRRAP), mRNA [NM_003496]	NM_003496
A_23_P431853	0.000321	ENST00000361453		Homo sapiens cDNA fis, A-COL04217, highly similar to Homo sapiens mitochondrion, NADH dehydrogenase subunit 2. [AK026903]	
A_24_P76521	0.000321	AK056691	AK056691	Homo sapiens cDNA FLJ32129 fis, clone PEBLM2000213, weakly similar to Mus musculus genes for integrin aM290, hapsin. [AK056691]	
A_24_P183864	0.000321	NM_005536	NM_005536	Homo sapiens inositol(myo)-1(or 4)-monophosphatase 1 (IMPA1), mRNA [NM_005536]	NM_005536
A_23_P70231	0.000322	NM_001182	NM_001182	Homo sapiens aldehyde dehydrogenase 7 family, member A1 (ALDH7A1), mRNA [NM_001182]	NM_001182
A_23_P205768	0.000323	NM_006628	NM_006628	Homo sapiens cyclic AMP phosphoprotein, 19 kD (ARPP-19), mRNA [NM_006628]	NM_006628
A_24_P917836	0.000325	AF034187	AF034187	Homo sapiens clone 2.2H12 Ndr Ser/Thr kinase-like protein mRNA, partial cds. [AF034187]	
A_23_P252236	0.000325	NM_000892	NM_000892	Homo sapiens kallikrein B, plasma (Fletcher factor) 1 (KLKB1), mRNA [NM_000892]	NM_000892
A_23_P136683	0.000325	M20432	M20432	Human MHC class II HLA-DQ-beta (DR2-DQw1/DR4 DQw3) mRNA, complete cds, clone ROF2D. [M20432]	
A_24_P35478	0.000325	NM_019619	NM_019619	Homo sapiens par-3 partitioning defective 3 homolog (C. elegans) (PARD3), mRNA [NM_019619]	NM_019619
A_32_P207802	0.000326	THC2405783			
A_24_P96780	0.000326	NM_016343	NM_016343	Homo sapiens centromere protein F, 350/400ka (mitosin) (CENPF), mRNA [NM_016343]	NM_016343

A_23_P106158	0.000326	NM_024884	NM_024884	Homo sapiens L-2-hydroxyglutarate dehydrogenase (L2HGDH), mRNA [NM_024884]	NM_024884
A_23_P77371	0.000326	NM_016284	NM_016284	Homo sapiens CCR4-NOT transcription complex, subunit 1 (CNOT1), transcript variant 1, mRNA [NM_016284]	NM_016284
A_23_P79231	0.000327	NM_134442	NM_134442	Homo sapiens cAMP responsive element binding protein 1 (CREB1), transcript variant B, mRNA [NM_134442]	NM_134442
A_23_P162734	0.000327	NM_005977	NM_005977	Homo sapiens ring finger protein (C3H2C3 type) 6 (RNF6), transcript variant 1, mRNA [NM_005977]	NM_005977
A_23_P163353	0.000328	NM_016166	NM_016166	Homo sapiens protein inhibitor of activated STAT, 1 (PIAS1), mRNA [NM_016166]	NM_016166
A_24_P106794	0.000328	NM_032869	NM_032869	Homo sapiens NudC domain containing 1 (NUDCD1), mRNA [NM_032869]	NM_032869
A_24_P915361	0.000329	AF086536	AF086536	Homo sapiens full length insert cDNA clone ZE08A03. [AF086536]	
A_32_P162374	0.00033	NM_173529	NM_173529	Homo sapiens chromosome 18 open reading frame 54 (C18orf54), mRNA [NM_173529]	NM_173529
A_24_P238499	0.00033	NM_001012716	NM_001012716	Homo sapiens chromosome 18 open reading frame 56 (C18orf56), mRNA [NM_001012716]	NM_001012716
A_23_P157072	0.000331	NM_003751	NM_003751	Homo sapiens eukaryotic translation initiation factor 3, subunit 9 eta, 116kDa (EIF3S9), transcript variant 1, mRNA [NM_003751]	NM_003751
A_23_P93613	0.000332	NM_013255	NM_013255	Homo sapiens muskelin 1, intracellular mediator containing kelch motifs (MKLN1), mRNA [NM_013255]	NM_013255
A_24_P260440	0.000332	AK124768	AK124768	Homo sapiens cDNA FLJ42778 fis, clone BRAWH3005146. [AK124768]	
A_23_P92441	0.000332	NM_002358	NM_002358	Homo sapiens MAD2 mitotic arrest deficient-like 1 (yeast) (MAD2L1), mRNA [NM_002358]	NM_002358
A_23_P134274	0.000333	NM_005837	NM_005837	Homo sapiens processing of precursor 7, ribonuclease P subunit (S. cerevisiae) (POP7), mRNA [NM_005837]	NM_005837
A_32_P58201	0.000334	AK021570	AK021570	Homo sapiens cDNA FLJ11508 fis, clone HEMBA1002162. [AK021570]	
A_23_P326691	0.000334	NM_005610	NM_005610	Homo sapiens retinoblastoma binding protein 4 (RBBP4), mRNA [NM_005610]	NM_005610
A_23_P50096	0.000335	NM_001071	NM_001071	Homo sapiens thymidylate synthetase (TYMS), mRNA [NM_001071]	NM_001071
A_32_P184727	0.000336	NM_002265	NM_002265	Homo sapiens karyopherin (importin) beta 1 (KPNB1), mRNA [NM_002265]	NM_002265
A_32_P81676	0.000337	AL832535	AL832535	Homo sapiens mRNA; cDNA DKFZp547J1816 (from clone DKFZp547J1816). [AL832535]	
A_24_P256337	0.000337	NM_201269	NM_201269	Homo sapiens zinc finger protein 644 (ZNF644), transcript variant 1, mRNA [NM_201269]	NM_201269
A_32_P199217	0.000338	THC2458577			
A_23_P9875	0.000338	NM_007170	NM_007170	Homo sapiens testis-specific kinase 2 (TESK2), mRNA [NM_007170]	NM_007170
A_24_P674924	0.000338	THC2303869		B40201 artifact-warning sequence (translated ALU class B) - human {Homo sapiens;} , partial (15%) [THC2303869]	
A_32_P91156	0.000338	A_32_P91156			
A_32_P22702	0.000339	NM_033222	NM_033222	Homo sapiens PC4 and SFRS1 interacting protein 1 (PSIP1), transcript variant 2, mRNA [NM_033222]	NM_033222
A_23_P100704	0.000339	NM_139033	NM_139033	Homo sapiens mitogen-activated protein kinase 7 (MAPK7), transcript variant 1, mRNA [NM_139033]	NM_139033
A_23_P214798	0.000339	NM_006372	NM_006372	Homo sapiens synaptotagmin binding, cytoplasmic RNA interacting protein (SYNCRIP), mRNA [NM_006372]	NM_006372
A_24_P101402	0.000339	NM_006392	NM_006392	Homo sapiens nucleolar protein 5A (56kDa with KKE/D repeat) (NOL5A), mRNA [NM_006392]	NM_006392
A_23_P101950	0.000339	NM_005917	NM_005917	Homo sapiens malate dehydrogenase 1, NAD (soluble) (MDH1), mRNA [NM_005917]	NM_005917
A_32_P59737	0.000341	THC2438324		ALU1_HUMAN (P39188) Alu subfamily J sequence contamination warning entry, partial (12%) [THC2438324]	
A_23_P354805	0.000341	NM_007249	NM_007249	Homo sapiens Kruppel-like factor 12 (KLF12), transcript variant 1, mRNA [NM_007249]	NM_007249
A_24_P556328	0.000343	A_24_P556328			
A_23_P137573	0.000343	NM_003240	NM_003240	Homo sapiens left-right determination factor 2 (LEFTY2), mRNA [NM_003240]	NM_003240
A_24_P124325	0.000344	CR749800	CR749800	Homo sapiens mRNA; cDNA DKFZp781B0249 (from clone DKFZp781B0249). [CR749800]	
A_23_P5550	0.000344	NM_015317	NM_015317	Homo sapiens pumilio homolog 2 (Drosophila) (PUM2), mRNA [NM_015317]	NM_015317
A_23_P16992	0.000345	NM_003628	NM_003628	Homo sapiens plakophilin 4 (PKP4), transcript variant 1, mRNA [NM_003628]	NM_003628
A_24_P935782	0.000346	NM_001008727	NM_001008727	Homo sapiens zinc finger protein 121 (clone ZHC32) (ZNF121), mRNA [NM_001008727]	NM_001008727
A_23_P306655	0.000347	AB058754	AB058754	Homo sapiens mRNA for KIAA1851 protein, partial cds. [AB058754]	
A_23_P133596	0.000347	NM_013235	NM_013235	Homo sapiens ribonuclease III, nuclear (RNASEN), mRNA [NM_013235]	NM_013235
A_23_P23815	0.000348	NM_021194	NM_021194	Homo sapiens solute carrier family 30 (zinc transporter), member 1 (SLC30A1), mRNA [NM_021194]	NM_021194
A_23_P44586	0.000348	NM_004255	NM_004255	Homo sapiens cytochrome c oxidase subunit Va (COX5A), nuclear gene encoding mitochondrial protein, mRNA [NM_004255]	NM_004255
A_24_P418590	0.000349	A_24_P418590			
A_23_P210496	0.000349	NM_022104	NM_022104	Homo sapiens chromosome 20 open reading frame 67 (C20orf67), mRNA [NM_022104]	NM_022104
A_23_P80129	0.000349	NM_003683	NM_003683	Homo sapiens DNA segment on chromosome 21 (unique) 2056 expressed sequence (D21S2056E), mRNA [NM_003683]	NM_003683

A_32_P87531	0.00035	BC042869	BC042869	Homo sapiens chromosome 1 open reading frame 67, mRNA (cDNA clone MGC:51214 IMAGE:5270407), complete cds. [BC042869]	XM_290922
A_23_P102242	0.00035	NM_022826	NM_022826	Homo sapiens membrane-associated ring finger (C3HC4) 7 (MARCH7), mRNA [NM_022826]	NM_022826
A_23_P201790	0.00035	NM_032105	NM_032105	Homo sapiens protein phosphatase 1, regulatory (inhibitor) subunit 12B (PPP1R12B), transcript variant 2, mRNA [NM_032105]	NM_032105
A_24_P929745	0.000352	AK021842	AK021842	Homo sapiens cDNA FLJ11780 fis, clone HEMBA1005931, weakly similar to ZINC FINGER PROTEIN 83. [AK021842]	
A_23_P42997	0.000353	NM_006693	NM_006693	Homo sapiens cleavage and polyadenylation specific factor 4, 30kDa (CPSF4), mRNA [NM_006693]	NM_006693
A_23_P434900	0.000353	NM_144570	NM_144570	Homo sapiens chromosome 16 open reading frame 34 (C16orf34), mRNA [NM_144570]	NM_144570
A_24_P118541	0.000353	A_24_P118541			
A_23_P69329	0.000354	NM_007312	NM_007312	Homo sapiens hyaluronoglucosaminidase 1 (HYAL1), transcript variant 1, mRNA [NM_007312]	NM_007312
A_24_P191417	0.000354	NM_005966	NM_005966	Homo sapiens NGFI-A binding protein 1 (EGR1 binding protein 1) (NAB1), mRNA [NM_005966]	NM_005966
A_23_P56150	0.000354	NM_032753	NM_032753	Homo sapiens hypothetical protein MGC15631 (MGC15631), mRNA [NM_032753]	NM_032753
A_23_P74954	0.000355	NM_018715	NM_018715	Homo sapiens regulator of chromosome condensation 2 (RCC2), mRNA [NM_018715]	NM_018715
A_32_P87703	0.000355	NM_005911	NM_005911	Homo sapiens methionine adenosyltransferase II, alpha (MAT2A), mRNA [NM_005911]	NM_005911
A_23_P121806	0.000356	NM_021204	NM_021204	Homo sapiens E-1 enzyme (MASA), mRNA [NM_021204]	NM_021204
A_24_P296568	0.000356	NM_006807	NM_006807	Homo sapiens chromobox homolog 1 (HP1 beta homolog Drosophila) (CBX1), mRNA [NM_006807]	NM_006807
A_23_P19291	0.000356	NM_001069	NM_001069	Homo sapiens tubulin, beta 2 (TUBB2), mRNA [NM_001069]	NM_001069
A_32_P111609	0.000358	NM_019059	NM_019059	Homo sapiens translocase of outer mitochondrial membrane 7 homolog (yeast) (TOMM7), mRNA [NM_019059]	NM_019059
A_23_P108948	0.000359	NM_018000	NM_018000	Homo sapiens dilute suppressor (DSU), mRNA [NM_018000]	NM_018000
A_24_P28619	0.000359	NM_144567	NM_144567	Homo sapiens similar to RIKEN cDNA 2610307I21 (LOC90806), mRNA [NM_144567]	NM_144567
A_23_P96990	0.00036	NM_002533	NM_002533	Homo sapiens nuclear VCP-like (NVL), transcript variant 1, mRNA [NM_002533]	NM_002533
A_24_P270376	0.000362	NM_012345	NM_012345	Homo sapiens nuclear fragile X mental retardation protein interacting protein 1 (NUFIP1), mRNA [NM_012345]	NM_012345
A_23_P3212	0.000362	NM_020234	NM_020234	Homo sapiens DTW domain containing 1 (DTWD1), mRNA [NM_020234]	NM_020234
A_23_P85004	0.000363	NM_007309	NM_007309	Homo sapiens diaphanous homolog 2 (Drosophila) (DIAPH2), transcript variant 12C, mRNA [NM_007309]	NM_007309
A_23_P17269	0.000363	NM_018084	NM_018084	Homo sapiens KIAA1212 (KIAA1212), mRNA [NM_018084]	NM_018084
A_23_P111753	0.000363	NM_031903	NM_031903	Homo sapiens mitochondrial ribosomal protein L32 (MRPL32), nuclear gene encoding mitochondrial protein, mRNA [NM_031903]	NM_031903
A_32_P78491	0.000364	NM_004956	NM_004956	Homo sapiens ets variant gene 1 (ETV1), mRNA [NM_004956]	NM_004956
A_23_P78438	0.000364	NM_018255	NM_018255	Homo sapiens signal transducer and activator of transcription 3 interacting protein 1 (STATIP1), mRNA [NM_018255]	NM_018255
A_23_P11224	0.000365	NM_173470	NM_173470	Homo sapiens transmembrane protein 32 (TMEM32), mRNA [NM_173470]	NM_173470
A_32_P111266	0.000367	THC2453189		PEBB_HUMAN (Q13951) Core-binding factor, beta subunit (CBF-beta) (Polyomavirus enhancer binding protein 2 beta subunit) (PEBP2-beta) (PEA2-beta) (SL3-3 enhancer factor 1 beta subunit) (SL3/AKV core-binding factor beta subunit), partial (43%) [THC2453189]	
A_32_P184279	0.000367	NM_005436	NM_005436	Homo sapiens coiled-coil domain containing 6 (CCDC6), mRNA [NM_005436]	NM_005436
A_24_P80915	0.000368	THC2428671			
A_24_P254933	0.000368	A_24_P254933			
A_24_P655268	0.000369	CR609905	CR609905	full-length cDNA clone CS0DD006YF13 of Neuroblastoma Cot 50-normalized of Homo sapiens (human). [CR609905]	
A_32_P64570	0.000369	NM_153186	NM_153186	Homo sapiens ankyrin repeat domain 15 (ANKRD15), transcript variant 2, mRNA [NM_153186]	NM_153186
A_23_P150255	0.000369	NM_006328	NM_006328	Homo sapiens RNA binding motif protein 14 (RBM14), mRNA [NM_006328]	NM_006328
A_32_P28828	0.00037	THC2315330			
A_23_P386450	0.00037	NM_016218	NM_016218	Homo sapiens polymerase (DNA directed) kappa (POLK), mRNA [NM_016218]	NM_016218
A_23_P126825	0.00037	NM_003051	NM_003051	Homo sapiens solute carrier family 16 (monocarboxylic acid transporters), member 1 (SLC16A1), mRNA [NM_003051]	NM_003051
A_23_P99985	0.00037	NM_002128	NM_002128	Homo sapiens high-mobility group box 1 (HMGB1), mRNA [NM_002128]	NM_002128
A_23_P140277	0.000371	NM_015180	NM_015180	Homo sapiens spectrin repeat containing, nuclear envelope 2 (SYNE2), transcript variant 1, mRNA [NM_015180]	NM_015180
A_24_P374943	0.000371	NM_001338	NM_001338	Homo sapiens coxsackie virus and adenovirus receptor (CXADR), mRNA [NM_001338]	NM_001338
A_24_P382130	0.000371	NM_032141	NM_032141	Homo sapiens hypothetical protein DKFZp434K1421 (DKFZP434K1421), mRNA [NM_032141]	NM_032141
A_24_P590537	0.000371	NM_002107	NM_002107	Homo sapiens H3 histone, family 3A (H3F3A), mRNA [NM_002107]	NM_002107
A_32_P24651	0.000372	AK095707	AK095707	Homo sapiens cDNA FLJ38388 fis, clone FEBRA2004485. [AK095707]	

A_24_P128255	0.000373	THC2275950			
A_32_P136982	0.000373	XM_088677	XM_088677	PREDICTED: Homo sapiens similar to UPF3 regulator of nonsense transcripts homolog B isoform 2 (LOC158796), mRNA [XM_088677]	XM_088677
A_24_P234792	0.000373	THC2277727		KC13_RAT (Q62763) Casein kinase I, gamma 3 isoform (CKI-gamma 3) , partial (38%) [THC2277727]	
A_23_P72817	0.000373	NM_020634	NM_020634	Homo sapiens growth differentiation factor 3 (GDF3), mRNA [NM_020634]	NM_020634
A_32_P120484	0.000374	THC2404072		A45981 peptidylprolyl isomerase CyP-40 - human {Homo sapiens;} , partial (12%) [THC2404072]	
A_23_P372925	0.000374	NM_033426	NM_033426	Homo sapiens KIAA1737 (KIAA1737), mRNA [NM_033426]	NM_033426
A_23_P383835	0.000374	NM_005891	NM_005891	Homo sapiens acetyl-Coenzyme A acetyltransferase 2 (acetoacetyl Coenzyme A thiolase) (ACAT2), mRNA [NM_005891]	NM_005891
A_23_P5586	0.000375	NM_005791	NM_005791	Homo sapiens M-phase phosphoprotein 10 (U3 small nucleolar ribonucleoprotein) (MPHOSPH10), mRNA [NM_005791]	NM_005791
A_23_P202501	0.000376	NM_018363	NM_018363	Homo sapiens chromosome 10 open reading frame 59 (C10orf59), mRNA [NM_018363]	NM_018363
A_24_P677734	0.000376	BX648343	BX648343	Homo sapiens mRNA; cDNA DKFZp686I16170 (from clone DKFZp686I16170). [BX648343]	
A_23_P325075	0.000376	NM_003800	NM_003800	Homo sapiens RNA guanylyltransferase and 5'-phosphatase (RNGTT), mRNA [NM_003800]	NM_003800
A_24_P347624	0.000376	NM_022804	NM_022804	Homo sapiens SNRPN upstream reading frame (SNURF), transcript variant 2, mRNA [NM_022804]	NM_022804
A_24_P350126	0.000376	A_24_P350126			
A_24_P41079	0.000377	NM_198181	NM_198181	Homo sapiens hypothetical protein LOC440295 (LOC440295), mRNA [NM_198181]	NM_198181
A_24_P922684	0.000378	BC020539	BC020539	Homo sapiens, clone IMAGE:3048919, mRNA, partial cds. [BC020539]	
A_23_P141893	0.000379	NM_178494	NM_178494	Homo sapiens hypothetical protein FLJ40125 (FLJ40125), mRNA [NM_178494]	NM_178494
A_23_P324633	0.00038	NM_018325	NM_018325	Homo sapiens chromosome 9 open reading frame 72 (C9orf72), transcript variant 1, mRNA [NM_018325]	NM_018325
A_32_P22989	0.000381	A_32_P22989			
A_23_P115608	0.000384	NM_020824	NM_020824	Homo sapiens Rho GTPase activating protein 21 (ARHGAP21), mRNA [NM_020824]	NM_020824
A_32_P224911	0.000384	NM_032026	NM_032026	Homo sapiens TatD DNase domain containing 1 (TATDN1), mRNA [NM_032026]	NM_032026
A_24_P54808	0.000385	NM_020803	NM_020803	Homo sapiens kelch-like 8 (Drosophila) (KLHL8), mRNA [NM_020803]	NM_020803
A_32_P8806	0.000385	A_32_P8806			
A_24_P605190	0.000386	THC2278663		BX491310 DKFZp686K2197_r1 686 (synonym: hlcc3) Homo sapiens cDNA clone DKFZp686K2197 5', mRNA sequence [BX491310]	
A_23_P167263	0.000386	AK127326	AK127326	Homo sapiens cDNA FLJ45397 fis, clone BRHIP3027651, weakly similar to Bromodomain-containing protein 1. [AK127326]	
A_23_P144096	0.000388	NM_145071	NM_145071	Homo sapiens cytokine inducible SH2-containing protein (CISH), mRNA [NM_145071]	NM_145071
A_23_P207445	0.000389	NM_002758	NM_002758	Homo sapiens mitogen-activated protein kinase kinase 6 (MAP2K6), transcript variant 1, mRNA [NM_002758]	NM_002758
A_24_P281988	0.00039	NM_015184	NM_015184	Homo sapiens phospholipase C-like 2 (PLCL2), mRNA [NM_015184]	NM_015184
A_23_P80940	0.00039	NM_002703	NM_002703	Homo sapiens phosphoribosyl pyrophosphate amidotransferase (PPAT), mRNA [NM_002703]	NM_002703
A_23_P170037	0.00039	NM_033290	NM_033290	Homo sapiens midline 1 (Opitz/BBB syndrome) (MID1), transcript variant 3, mRNA [NM_033290]	NM_033290
A_23_P74349	0.00039	NM_145697	NM_145697	Homo sapiens cell division cycle associated 1 (CDCA1), transcript variant 1, mRNA [NM_145697]	NM_145697
A_23_P36226	0.00039	NM_032315	NM_032315	Homo sapiens mitochondrial carrier protein (MGC4399), mRNA [NM_032315]	NM_032315
A_23_P396328	0.000391	AK098775	AK098775	Homo sapiens cDNA FLJ25909 fis, clone CBR04667, highly similar to Homo sapiens multi PDZ domain protein MUPP1 (MUPP1) mRNA. [AK098775]	
A_23_P36458	0.000394	AK001563	AK001563	Homo sapiens cDNA FLJ10701 fis, clone NT2RP3000690. [AK001563]	
A_24_P128741	0.000394	THC2432288		Q15694 (Q15694) Protein immuno-reactive with anti-PTH polyclonal antibodies (Fragment), partial (59%) [THC2432288]	
A_24_P724807	0.000395	A_24_P724807			
A_32_P130968	0.000395	BC035377	BC035377	Homo sapiens, clone IMAGE:4826240, mRNA. [BC035377]	
A_24_P281853	0.000395	A_24_P281853			
A_24_P332739	0.000395	NM_206908	NM_206908	Homo sapiens chromosome 6 open reading frame 216 (C6orf216), transcript variant 2, mRNA [NM_206908]	NM_206908
A_24_P67063	0.000395	A_24_P67063			
A_23_P219105	0.000396	NM_023111	NM_023111	Homo sapiens fibroblast growth factor receptor 1 (fms-related tyrosine kinase 2, Pfeiffer syndrome) (FGFR1), transcript variant 9, mRNA [NM_023111]	NM_023111
A_24_P599496	0.000396	THC2311599		US02_BHV1S (Q08099) Protein US2 homolog, partial (12%) [THC2311599]	
A_23_P52058	0.000396	NM_005646	NM_005646	Homo sapiens TAR (HIV) RNA binding protein 1 (TARBP1), mRNA [NM_005646]	NM_005646
A_23_P375517	0.000397	NM_138286	NM_138286	Homo sapiens zinc finger protein 681 (ZNF681), mRNA [NM_138286]	NM_138286
A_23_P368145	0.000398	NM_152281	NM_152281	Homo sapiens SCY1-like 1 binding protein 1 (SCYL1BP1), mRNA [NM_152281]	NM_152281

A_23_P1956	0.000398	ENST00000307366		Homo sapiens, Similar to RIKEN cDNA 0610006I08 gene, clone IMAGE:3855124, mRNA, partial cds. [BC008643]	XM_495878
A_23_P128930	0.000398	NM_002806	NM_002806	Homo sapiens proteasome (prosome, macropain) 26S subunit, ATPase, 6 (PSMC6), mRNA [NM_002806]	NM_002806
A_23_P67127	0.000401	NM_173633	NM_173633	Homo sapiens hypothetical protein FLJ90805 (FLJ90805), mRNA [NM_173633]	NM_173633
A_23_P10305	0.000401	NM_175859	NM_175859	Homo sapiens CTP synthase II (CTPS2), transcript variant 2, mRNA [NM_175859]	NM_175859
A_23_P411953	0.000401	NM_138300	NM_138300	Homo sapiens pygopus homolog 2 (Drosophila) (PYGO2), mRNA [NM_138300]	NM_138300
A_24_P857404	0.000401	AK125482	AK125482	Homo sapiens cDNA FLJ43493 fis, clone OCBBF3009279. [AK125482]	
A_32_P48086	0.000402	BC015434	BC015434	Homo sapiens, clone IMAGE:4414697, mRNA. [BC015434]	
A_23_P109895	0.000402	NM_022911	NM_022911	Homo sapiens solute carrier family 26, member 6 (SLC26A6), transcript variant 1, mRNA [NM_022911]	NM_022911
A_23_P15906	0.000402	NM_017512	NM_017512	Homo sapiens enolase superfamily member 1 (ENOSF1), transcript variant 2, mRNA [NM_017512]	NM_017512
A_23_P139104	0.000402	THC2373516		Q86UN3 (Q86UN3) Nogo-66 receptor homolog-1 (Nogo-66 receptor-related protein 2), complete [THC2373516]	
A_24_P332862	0.000403	NM_175910	NM_175910	Homo sapiens zinc finger protein 493 (ZNF493), mRNA [NM_175910]	NM_175910
A_23_P119084	0.000403	NM_138347	NM_138347	Homo sapiens zinc finger protein 551 (ZNF551), mRNA [NM_138347]	NM_138347
A_24_P761727	0.000403	A_24_P761727			
A_24_P785293	0.000403	CR603342	CR603342	full-length cDNA clone CS0DL007YF01 of B cells (Ramos cell line) Cot 25-normalized of Homo sapiens (human). [CR603342]	
A_32_P98348	0.000403	AB075859	AB075859	Homo sapiens mRNA for KIAA1979 protein. [AB075859]	
A_24_P418619	0.000403	NM_001014	NM_001014	Homo sapiens ribosomal protein S10 (RPS10), mRNA [NM_001014]	NM_001014
A_23_P48535	0.000404	NM_001173	NM_001173	Homo sapiens Rho GTPase activating protein 5 (ARHGAP5), mRNA [NM_001173]	NM_001173
A_23_P77818	0.000405	NM_006356	NM_006356	Homo sapiens ATP synthase, H+ transporting, mitochondrial F0 complex, subunit d (ATP5H), nuclear gene encoding mitochondrial protein, transcript variant 1, mRNA [NM_006356]	NM_006356
A_32_P15211	0.000406	THC2373821		Q14966 (Q14966) Nuclear protein, NP220, partial (5%) [THC2373821]	
A_23_P88184	0.000406	NM_018167	NM_018167	Homo sapiens BTB (POZ) domain containing 7 (BTBD7), transcript variant 2, mRNA [NM_018167]	NM_018167
A_23_P314584	0.000406	NM_004635	NM_004635	Homo sapiens mitogen-activated protein kinase-activated protein kinase 3 (MAPKAPK3), mRNA [NM_004635]	NM_004635
A_32_P53311	0.000411	AK126245	AK126245	Homo sapiens cDNA FLJ44257 fis, clone TKIDN2015263. [AK126245]	
A_23_P500034	0.000411	NM_030379	NM_030379	Homo sapiens GLI-Kruppel family member GLI2 (GLI2), transcript variant 1, mRNA [NM_030379]	NM_030379
A_23_P354798	0.000411	NM_144576	NM_144576	Homo sapiens hypothetical protein FLJ32452 (FLJ32452), mRNA [NM_144576]	NM_144576
A_23_P127233	0.000411	NM_005871	NM_005871	Homo sapiens survival motor neuron domain containing 1 (SMNDC1), mRNA [NM_005871]	NM_005871
A_24_P8075	0.000412	AY251274	AY251274	Homo sapiens unknown mRNA. [AY251274]	
A_24_P409042	0.000413	ENST00000360515		full-length cDNA clone CS0DC008YI24 of Neuroblastoma Cot 25-normalized of Homo sapiens (human). [CR599493]	
A_32_P135043	0.000414	THC2376245			
A_23_P91590	0.000414	NM_002882	NM_002882	Homo sapiens RAN binding protein 1 (RANBP1), mRNA [NM_002882]	NM_002882
A_23_P208070	0.000415	NM_018439	NM_018439	Homo sapiens hypothetical protein IMPACT (IMPACT), mRNA [NM_018439]	NM_018439
A_23_P212383	0.000415	NM_014016	NM_014016	Homo sapiens SAC1 suppressor of actin mutations 1-like (yeast) (SACM1L), mRNA [NM_014016]	NM_014016
A_24_P74731	0.000416	NM_021120	NM_021120	Homo sapiens discs, large homolog 3 (neuroendocrine-dlg, Drosophila) (DLG3), mRNA [NM_021120]	NM_021120
A_23_P30799	0.000416	NM_021018	NM_021018	Homo sapiens histone 1, H3f (HIST1H3F), mRNA [NM_021018]	NM_021018
A_24_P165736	0.000416	NM_006773	NM_006773	Homo sapiens DEAD (Asp-Glu-Ala-Asp) box polypeptide 18 (DDX18), mRNA [NM_006773]	NM_006773
A_23_P429092	0.000417	AJ459827	AJ459827	Homo sapiens mRNA for HBS1-like protein (HSB1 gene), splice variant 2. [AJ459827]	
A_32_P154021	0.000417	AK054939	AK054939	Homo sapiens cDNA FLJ30377 fis, clone BRACE2007952. [AK054939]	
A_23_P320190	0.000418	NM_173646	NM_173646	Homo sapiens hypothetical protein FLJ39660 (FLJ39660), mRNA [NM_173646]	NM_173646
A_23_P20328	0.000418	CX165016	CX165016	HESC2_23_G02.g1_A035 NIH_MGC_258 Homo sapiens cDNA clone IMAGE:7468613 5', mRNA sequence [CX165016]	
A_24_P935276	0.000418	NM_001010851	NM_001010851	Homo sapiens hypothetical protein LOC90321 (LOC90321), mRNA [NM_001010851]	NM_001010851
A_32_P103771	0.000418	AF527552	AF527552	Homo sapiens B melanoma antigen variant d (BAGE1) mRNA, complete cds, alternatively spliced. [AF527552]	
A_23_P21966	0.00042	NM_022455	NM_022455	Homo sapiens nuclear receptor binding SET domain protein 1 (NSD1), transcript variant 2, mRNA [NM_022455]	NM_022455
A_23_P119583	0.00042	NM_000923	NM_000923	Homo sapiens phosphodiesterase 4C, cAMP-specific (phosphodiesterase E1 dunce homolog, Drosophila) (PDE4C), mRNA [NM_000923]	NM_000923
A_23_P251795	0.000422	NM_152742	NM_152742	Homo sapiens glypican 2 (cerebroglycan) (GPC2), mRNA [NM_152742]	NM_152742
A_24_P268357	0.000422	NM_017951	NM_017951	Homo sapiens hypothetical protein FLJ20297 (FLJ20297), transcript variant 2, mRNA [NM_017951]	NM_017951

A_23_P144999	0.000423	NM_016340	NM_016340	Homo sapiens Rap guanine nucleotide exchange factor (GEF) 6 (RAPGEF6), mRNA [NM_016340]	NM_016340
A_23_P110837	0.000423	NM_016358	NM_016358	Homo sapiens iroquois homeobox protein 4 (IRX4), mRNA [NM_016358]	NM_016358
A_24_P409670	0.000424	AK098175	AK098175	Homo sapiens cDNA FLJ40856 fis, clone TRACH2016498, moderately similar to ZINC FINGER PROTEIN 184. [AK098175]	XM_371174
A_24_P862207	0.000424	BC057844	BC057844	Homo sapiens cDNA clone MGC:71846 IMAGE:5313492, complete cds. [BC057844]	
A_23_P11664	0.000424	NM_004768	NM_004768	Homo sapiens splicing factor, arginine/serine-rich 11 (SFRS11), mRNA [NM_004768]	NM_004768
A_23_P37237	0.000424	NM_145165	NM_145165	Homo sapiens churchill domain containing 1 (CHURC1), mRNA [NM_145165]	NM_145165
A_24_P397489	0.000425	NM_145649	NM_145649	Homo sapiens glucosaminyl (N-acetyl) transferase 2, I-branching enzyme (GCNT2), transcript variant 1, mRNA [NM_145649]	NM_145649
A_24_P941051	0.000425	NM_015235	NM_015235	Homo sapiens cleavage stimulation factor, 3' pre-RNA, subunit 2, 64kDa, tau variant (CSTF2T), mRNA [NM_015235]	NM_015235
A_23_P74034	0.000425	NM_014412	NM_014412	Homo sapiens calcyclin binding protein (CACYBP), transcript variant 1, mRNA [NM_014412]	NM_014412
A_23_P219004	0.000427	NM_152729	NM_152729	Homo sapiens 5'-nucleotidase, cytosolic II-like 1 (NT5C2L1), mRNA [NM_152729]	NM_152729
A_24_P167984	0.000427	NM_015251	NM_015251	Homo sapiens ATM/ATR-Substrate Chk2-Interacting Zn2+-finger protein (ASCIZ), mRNA [NM_015251]	NM_015251
A_23_P211673	0.000427	AL512711	AL512711	Homo sapiens mRNA; cDNA DKFZp761B039 (from clone DKFZp761B039). [AL512711]	
A_23_P8763	0.000429	NM_002835	NM_002835	Homo sapiens protein tyrosine phosphatase, non-receptor type 12 (PTPN12), mRNA [NM_002835]	NM_002835
A_24_P889103	0.00043	CR597846	CR597846	full-length cDNA clone CS0DC012YL18 of Neuroblastoma Cot 25-normalized of Homo sapiens (human). [CR597846]	
A_23_P143994	0.00043	NM_001018115	NM_001018115	Homo sapiens Fanconi anemia, complementation group D2 (FANCD2), transcript variant 2, mRNA [NM_001018115]	NM_001018115
A_32_P165297	0.000432	CR627122	CR627122	Homo sapiens mRNA; cDNA DKFZp779M2422 (from clone DKFZp779M2422). [CR627122]	
A_23_P115861	0.000432	NM_145312	NM_145312	Homo sapiens zinc finger protein 485 (ZNF485), mRNA [NM_145312]	NM_145312
A_24_P312692	0.000432	NM_006595	NM_006595	Homo sapiens apoptosis inhibitor 5 (API5), mRNA [NM_006595]	NM_006595
A_23_P405707	0.000433	NM_020926	NM_020926	Homo sapiens BCL6 co-repressor (BCOR), transcript variant 2, mRNA [NM_020926]	NM_020926
A_24_P491923	0.000433	THC2273623			
A_23_P13176	0.000434	NM_015517	NM_015517	Homo sapiens MBD2 (methyl-CpG-binding protein)-interacting zinc finger protein (MIZF), transcript variant 1, mRNA [NM_015517]	NM_015517
A_24_P225635	0.000435	THC2403280		Q8IX05 (Q8IX05) C-type lectin BIMLEC precursor (Type I transmembrane C-type lectin receptor DCL-1), partial (6%) [THC2403280]	
A_23_P419947	0.000435	NM_022443	NM_022443	Homo sapiens myeloid leukemia factor 1 (MLF1), mRNA [NM_022443]	NM_022443
A_23_P38181	0.000435	NM_138619	NM_138619	Homo sapiens golgi associated, gamma adaptin ear containing, ARF binding protein 3 (GGA3), transcript variant long, mRNA [NM_138619]	NM_138619
A_23_P14636	0.000436	NM_005926	NM_005926	Homo sapiens microfibrillar-associated protein 1 (MFAP1), mRNA [NM_005926]	NM_005926
A_23_P252825	0.000436	NM_012235	NM_012235	Homo sapiens SREBP cleavage-activating protein (SCAP), mRNA [NM_012235]	NM_012235
A_23_P16615	0.000438	NM_005968	NM_005968	Homo sapiens heterogeneous nuclear ribonucleoprotein M (HNRPM), transcript variant 1, mRNA [NM_005968]	NM_005968
A_24_P409182	0.000439	ENST00000303979			
A_23_P13554	0.00044	NM_001007027	NM_001007027	Homo sapiens asparagine-linked glycosylation 8 homolog (yeast, alpha-1,3-glucosyltransferase) (ALG8), transcript variant 2, mRNA [NM_001007027]	NM_001007027
A_32_P53524	0.000441	BC092429	BC092429	Homo sapiens cDNA clone IMAGE:30530513. [BC092429]	
A_23_P73058	0.000441	NM_017774	NM_017774	Homo sapiens CDK5 regulatory subunit associated protein 1-like 1 (CDKAL1), mRNA [NM_017774]	NM_017774
A_23_P145397	0.000441	NM_005190	NM_005190	Homo sapiens cyclin C (CCNC), transcript variant 1, mRNA [NM_005190]	NM_005190
A_23_P215198	0.000442	NM_018264	NM_018264	Homo sapiens radical S-adenosyl methionine and flavodoxin domains 1 (RSAFD1), mRNA [NM_018264]	NM_018264
A_24_P312671	0.000443	NM_005590	NM_005590	Homo sapiens MRE11 meiotic recombination 11 homolog A (S. cerevisiae) (MRE11A), transcript variant 2, mRNA [NM_005590]	NM_005590
A_24_P928574	0.000444	AK023400	AK023400	Homo sapiens cDNA FLJ13338 fis, clone OVARC1001883. [AK023400]	
A_32_P41026	0.000444	THC2270160			
A_23_P112825	0.000445	NM_016015	NM_016015	Homo sapiens leucine carboxyl methyltransferase 1 (LCMT1), mRNA [NM_016015]	NM_016015
A_32_P15829	0.000447	AW389914	AW389914	AW389914 RC4-ST0173-191099-032-f06 ST0173 Homo sapiens cDNA, mRNA sequence [AW389914]	
A_23_P54736	0.000449	NM_016541	NM_016541	Homo sapiens guanine nucleotide binding protein (G protein), gamma 13 (GNG13), mRNA [NM_016541]	NM_016541
A_23_P217411	0.000449	NM_006306	NM_006306	Homo sapiens SMC1 structural maintenance of chromosomes 1-like 1 (yeast) (SMC1L1), mRNA [NM_006306]	NM_006306
A_23_P131626	0.000449	NM_014014	NM_014014	Homo sapiens activating signal cointegrator 1 complex subunit 3-like 1 (ASCC3L1), mRNA [NM_014014]	NM_014014
A_32_P415151	0.00045	NM_182552	NM_182552	Homo sapiens WD repeat domain 27 (WDR27), mRNA [NM_182552]	NM_182552
A_23_P111712	0.00045	NM_006980	NM_006980	Homo sapiens mitochondrial transcription termination factor (MTERF), nuclear gene encoding mitochondrial protein, mRNA [NM_006980]	NM_006980
A_32_P42075	0.00045	NM_198097	NM_198097	Homo sapiens chromosome 7 open reading frame 28B (C7orf28B), mRNA [NM_198097]	NM_198097

A_23_P60339	0.000451	NM_032307	NM_032307	Homo sapiens chromosome 9 open reading frame 64 (C9orf64), mRNA [NM_032307]	NM_032307
A_24_P97221	0.000451	NM_016622	NM_016622	Homo sapiens mitochondrial ribosomal protein L35 (MRPL35), nuclear gene encoding mitochondrial protein, transcript variant 1, mRNA [NM_016622]	NM_016622
A_32_P224040	0.000452	THC2274685		AF326941 5H1 {Caenorhabditis elegans;} , partial (16%) [THC2274685]	
A_23_P109254	0.000452	NM_184244	NM_184244	Homo sapiens RNA-binding region (RNP1, RRM) containing 2 (RNPC2), transcript variant 5, mRNA [NM_184244]	NM_184244
A_23_P336796	0.000454	CR590862	CR590862	full-length cDNA clone CS0DI036YC04 of Placenta Cot 25-normalized of Homo sapiens (human). [CR590862]	XM_290597
A_23_P166280	0.000454	BQ189193	BQ189193	UI-E-EJ1-ajv-o-09-0-UI.r1 UI-E-EJ1 Homo sapiens cDNA clone UI-E-EJ1-ajv-o-09-0-UI 5', mRNA sequence [BQ189193]	
A_23_P200425	0.000454	NM_022753	NM_022753	Homo sapiens S100P binding protein Riken (S100PBPR), transcript variant 1, mRNA [NM_022753]	NM_022753
A_23_P119677	0.000454	NM_001533	NM_001533	Homo sapiens heterogeneous nuclear ribonucleoprotein L (HNRPL), transcript variant 1, mRNA [NM_001533]	NM_001533
A_32_P50924	0.000454	NM_001011724	NM_001011724	Homo sapiens heterogeneous nuclear ribonucleoprotein A1-like (LOC144983), transcript variant 1, mRNA [NM_001011724]	NM_001011724
A_24_P108301	0.000456	NM_173630	NM_173630	Homo sapiens rotatin (RTTN), mRNA [NM_173630]	NM_173630
A_23_P1145	0.000456	NM_018109	NM_018109	Homo sapiens PAP associated domain containing 1 (PAPD1), mRNA [NM_018109]	NM_018109
A_23_P151459	0.000456	NM_015057	NM_015057	Homo sapiens MYC binding protein 2 (MYCBP2), mRNA [NM_015057]	NM_015057
A_32_P49392	0.000456	XM_376150	XM_376150	PREDICTED: Homo sapiens similar to ribosomal protein S3a; 40S ribosomal protein S3a; v-fos transformation effector protein 1 (LOC401016), mRNA [XM_376150]	XM_376150
A_23_P90223	0.000457	NM_152279	NM_152279	Homo sapiens zinc finger protein 585B (ZNF585B), mRNA [NM_152279]	NM_152279
A_24_P287826	0.000457	NM_018124	NM_018124	Homo sapiens ring finger and WD repeat domain 3 (RFWD3), mRNA [NM_018124]	NM_018124
A_23_P69249	0.000457	NM_178042	NM_178042	Homo sapiens actin-like 6A (ACTL6A), transcript variant 3, mRNA [NM_178042]	NM_178042
A_23_P59388	0.000459	NM_001723	NM_001723	Homo sapiens dystonin (DST), transcript variant 1e, mRNA [NM_001723]	NM_001723
A_23_P159877	0.000461	NM_171998	NM_171998	Homo sapiens RAB39B, member RAS oncogene family (RAB39B), mRNA [NM_171998]	NM_171998
A_24_P336705	0.000462	NM_004582	NM_004582	Homo sapiens Rab geranylgeranyltransferase, beta subunit (RABGGTB), mRNA [NM_004582]	NM_004582
A_24_P535219	0.000463	AJ420510	AJ420510	Homo sapiens mRNA full length insert cDNA clone EUROIMAGE 2120537. [AJ420510]	
A_23_P153640	0.000463	NM_032139	NM_032139	Homo sapiens ankyrin repeat domain 27 (VPS9 domain) (ANKRD27), mRNA [NM_032139]	NM_032139
A_23_P65768	0.000463	NM_016304	NM_016304	Homo sapiens chromosome 15 open reading frame 15 (C15orf15), mRNA [NM_016304]	NM_016304
A_24_P94651	0.000463	NM_006321	NM_006321	Homo sapiens ariadne homolog 2 (Drosophila) (ARIH2), mRNA [NM_006321]	NM_006321
A_23_P401098	0.000465	NM_153211	NM_153211	Homo sapiens chromosome 18 open reading frame 17 (C18orf17), mRNA [NM_153211]	NM_153211
A_23_P87532	0.000466	NM_001002259	NM_001002259	Homo sapiens C1q domain containing 1 (C1QDC1), transcript variant 1, mRNA [NM_001002259]	NM_001002259
A_24_P136155	0.000467	A_24_P136155			
A_23_P80648	0.000467	NM_033540	NM_033540	Homo sapiens mitofusin 1 (MFN1), nuclear gene encoding mitochondrial protein, transcript variant 1, mRNA [NM_033540]	NM_033540
A_24_P401670	0.000467	ENST00000333615			
A_23_P125748	0.000468	NM_032441	NM_032441	Homo sapiens zinc finger, matrin type 1 (ZMAT1), transcript variant 3, mRNA [NM_032441]	NM_032441
A_23_P68547	0.000468	NM_182802	NM_182802	Homo sapiens MCM8 minichromosome maintenance deficient 8 (S. cerevisiae) (MCM8), transcript variant 2, mRNA [NM_182802]	NM_182802
A_24_P176374	0.000469	NM_030928	NM_030928	Homo sapiens DNA replication factor (CDT1), mRNA [NM_030928]	NM_030928
A_32_P216872	0.000472	BX647358	BX647358	Homo sapiens mRNA; cDNA DKFZp686B0962 (from clone DKFZp686B0962). [BX647358]	
A_23_P166122	0.000472	NM_006466	NM_006466	Homo sapiens polymerase (RNA) III (DNA directed) polypeptide F, 39 kDa (POLR3F), mRNA [NM_006466]	NM_006466
A_23_P159741	0.000472	NM_017745	NM_017745	Homo sapiens BCL6 co-repressor (BCOR), transcript variant 1, mRNA [NM_017745]	NM_017745
A_23_P428819	0.000472	NM_018064	NM_018064	Homo sapiens chromosome 6 open reading frame 166 (C6orf166), mRNA [NM_018064]	NM_018064
A_32_P211188	0.000475	AK024346	AK024346	Homo sapiens cDNA FLJ14284 fis, clone PLACE1005898. [AK024346]	
A_23_P124427	0.000475	NM_012224	NM_012224	Homo sapiens NIMA (never in mitosis gene a)-related kinase 1 (NEK1), mRNA [NM_012224]	NM_012224
A_23_P142407	0.000475	NM_033204	NM_033204	Homo sapiens zinc finger protein 101 (ZNF101), mRNA [NM_033204]	NM_033204
A_24_P55391	0.000476	BC082970	BC082970	Homo sapiens cDNA clone IMAGE:6598034. [BC082970]	
A_24_P614579	0.000476	BC000206	BC000206	Homo sapiens, clone IMAGE:3350658, mRNA. [BC000206]	
A_24_P320254	0.000477	NM_005520	NM_005520	Homo sapiens heterogeneous nuclear ribonucleoprotein H1 (H) (HNRPH1), mRNA [NM_005520]	NM_005520
A_23_P50108	0.00048	NM_006101	NM_006101	Homo sapiens kinetochore associated 2 (KNTC2), mRNA [NM_006101]	NM_006101
A_32_P155052	0.000481	BC032684	BC032684	Homo sapiens chromosome 18 open reading frame 17, mRNA (cDNA clone IMAGE:5582870), partial cds. [BC032684]	
A_23_P99837	0.000481	NM_017437	NM_017437	Homo sapiens cleavage and polyadenylation specific factor 2, 100kDa (CPSF2), mRNA [NM_017437]	NM_017437

A_24_P739355	0.000481	THC2442931			
A_23_P94063	0.000481	AY260738	AY260738	Homo sapiens truncated zinc finger protein 36 mRNA, complete cds. [AY260738]	
A_23_P380917	0.000481	NM_014300	NM_014300	Homo sapiens SEC11-like 1 (S. cerevisiae) (SEC11L1), mRNA [NM_014300]	NM_014300
A_23_P168810	0.000482	NM_145111	NM_145111	Homo sapiens hypothetical protein DKFZp727G131 (DKFZp727G131), mRNA [NM_145111]	NM_145111
A_23_P99930	0.000484	NM_017858	NM_017858	Homo sapiens timeless-interacting protein (FLJ20516), mRNA [NM_017858]	NM_017858
A_32_P24741	0.000485	AK093729	AK093729	Homo sapiens cDNA FLJ36410 fis, clone THYMU2010637. [AK093729]	XM_496251
A_23_P51966	0.000486	A_23_P51966			
A_24_P277511	0.000487	AK057015	AK057015	Homo sapiens cDNA FLJ32453 fis, clone SKMUS2001703. [AK057015]	
A_32_P142881	0.00049	NM_015092	NM_015092	Homo sapiens PI-3-kinase-related kinase SMG-1 (SMG1), mRNA [NM_015092]	NM_015092
A_32_P61605	0.00049	AF290475	AF290475	Homo sapiens clone 124-1V1, mRNA sequence. [AF290475]	
A_23_P28318	0.00049	NM_018607	NM_018607	Homo sapiens hypothetical protein PRO1853 (PRO1853), transcript variant 2, mRNA [NM_018607]	NM_018607
A_23_P156319	0.00049	NM_033551	NM_033551	Homo sapiens La ribonucleoprotein domain family, member 1 (LARP1), mRNA [NM_033551]	NM_033551
A_24_P411863	0.000493	NM_018682	NM_018682	Homo sapiens myeloid/lymphoid or mixed-lineage leukemia 5 (trithorax homolog, Drosophila) (MLL5), mRNA [NM_018682]	NM_018682
A_24_P61520	0.000494	NM_014381	NM_014381	Homo sapiens mutL homolog 3 (E. coli) (MLH3), mRNA [NM_014381]	NM_014381
A_23_P41734	0.000494	NM_018434	NM_018434	Homo sapiens ring finger protein 130 (RNF130), mRNA [NM_018434]	NM_018434
A_23_P147822	0.000496	NM_022772	NM_022772	Homo sapiens EPS8-like 2 (EPS8L2), mRNA [NM_022772]	NM_022772
A_23_P320658	0.000496	NM_004725	NM_004725	Homo sapiens BUB3 budding uninhibited by benzimidazoles 3 homolog (yeast) (BUB3), transcript variant 1, mRNA [NM_004725]	NM_004725
A_24_P282377	0.000498	AL713660	AL713660	Homo sapiens mRNA; cDNA DKFZp667E0512 (from clone DKFZp667E0512). [AL713660]	
A_24_P298996	0.000499	NM_018451	NM_018451	Homo sapiens centromere protein J (CENPJ), mRNA [NM_018451]	NM_018451
A_24_P242361	0.000499	NM_012262	NM_012262	Homo sapiens heparan sulfate 2-O-sulfotransferase 1 (HS2ST1), mRNA [NM_012262]	NM_012262
A_32_P57702	0.000501	THC2304000		GCSH_HUMAN (P23434) Glycine cleavage system H protein, mitochondrial precursor, partial (80%) [THC2304000]	
A_23_P31372	0.000501	NM_005918	NM_005918	Homo sapiens malate dehydrogenase 2, NAD (mitochondrial) (MDH2), mRNA [NM_005918]	NM_005918
A_24_P649357	0.000502	NM_021652	NM_021652	Homo sapiens SMA4 (SMA4), mRNA [NM_021652]	NM_021652
A_24_P119567	0.000502	NM_144718	NM_144718	Homo sapiens hypothetical protein AY099107 (LOC152185), mRNA [NM_144718]	NM_144718
A_32_P172114	0.000502	BE044472	BE044472	BE044472 ho46b03.x1 Soares_NFL_T_GBC_S1 Homo sapiens cDNA clone IMAGE:3040397 3', mRNA sequence [BE044472]	
A_23_P13847	0.000503	AB032983	AB032983	Homo sapiens mRNA for KIAA1157 protein, partial cds. [AB032983]	XM_350880
A_24_P350196	0.000504	CR600908	CR600908	full-length cDNA clone CS0DL005YJ22 of B cells (Ramos cell line) Cot 25-normalized of Homo sapiens (human). [CR600908]	XM_496332
A_32_P141938	0.000505	THC2453775			
A_32_P131050	0.000505	THC2276742		Q8HBG1 (Q8HBG1) NADH dehydrogenase subunit 4L, partial (13%) [THC2276742]	
A_24_P662972	0.000506	THC2355570			
A_23_P330788	0.000506	NM_015075	NM_015075	Homo sapiens IQ motif and Sec7 domain 2 (IQSEC2), mRNA [NM_015075]	NM_015075
A_23_P155677	0.000506	NM_005339	NM_005339	Homo sapiens huntingtin interacting protein 2 (HIP2), mRNA [NM_005339]	NM_005339
A_23_P301925	0.000506	ENST00000361624		full-length cDNA clone CS0DE002YM12 of Placenta of Homo sapiens (human). [CR606568]	
A_23_P94860	0.000507	NM_024047	NM_024047	Homo sapiens nudix (nucleoside diphosphate linked moiety X)-type motif 9 (NUDT9), transcript variant 1, mRNA [NM_024047]	NM_024047
A_23_P69521	0.000507	NM_006835	NM_006835	Homo sapiens cyclin I (CCNI), mRNA [NM_006835]	NM_006835
A_24_P412088	0.000508	NM_182751	NM_182751	Homo sapiens MCM10 minichromosome maintenance deficient 10 (S. cerevisiae) (MCM10), transcript variant 1, mRNA [NM_182751]	NM_182751
A_23_P258964	0.000509	NM_013417	NM_013417	Homo sapiens isoleucine-tRNA synthetase (IARS), transcript variant long, mRNA [NM_013417]	NM_013417
A_24_P46659	0.000511	THC2372616			
A_23_P318646	0.000511	NM_001014	NM_001014	Homo sapiens ribosomal protein S10 (RPS10), mRNA [NM_001014]	NM_001014
A_23_P100344	0.000512	NM_014321	NM_014321	Homo sapiens origin recognition complex, subunit 6 homolog-like (yeast) (ORC6L), mRNA [NM_014321]	NM_014321
A_23_P156807	0.000513	AF338192	AF338192	Homo sapiens FKSG63 (FKSG63) mRNA, complete cds. [AF338192]	XM_209180
A_24_P25326	0.000513	AB037774	AB037774	Homo sapiens mRNA for KIAA1353 protein, partial cds. [AB037774]	
A_23_P208373	0.000514	NM_000767	NM_000767	Homo sapiens cytochrome P450, family 2, subfamily B, polypeptide 6 (CYP2B6), mRNA [NM_000767]	NM_000767
A_32_P54137	0.000514	NM_006004	NM_006004	Homo sapiens ubiquinol-cytochrome c reductase hinge protein (UQCRH), mRNA [NM_006004]	NM_006004
A_23_P16652	0.000515	NM_152791	NM_152791	Homo sapiens zinc finger protein 555 (ZNF555), mRNA [NM_152791]	NM_152791

A_32_P139229	0.000516	NM_213598	NM_213598	Homo sapiens zinc finger protein 543 (ZNF543), mRNA [NM_213598]	NM_213598
A_23_P164528	0.000516	NM_015285	NM_015285	Homo sapiens WD repeat domain 7 (WDR7), transcript variant 1, mRNA [NM_015285]	NM_015285
A_32_P181891	0.000517	AK097571	AK097571	Homo sapiens cDNA FLJ40252 fis, clone TESTI2024299. [AK097571]	
A_24_P359771	0.000517	ENST00000336112		Homo sapiens mRNA; cDNA DKFZp686L2369 (from clone DKFZp686L2369). [BX648218]	
A_24_P347704	0.000517	NM_006565	NM_006565	Homo sapiens CCCTC-binding factor (zinc finger protein) (CTCF), mRNA [NM_006565]	NM_006565
A_24_P396650	0.000517	NM_002953	NM_002953	Homo sapiens ribosomal protein S6 kinase, 90kDa, polypeptide 1 (RPS6KA1), transcript variant 1, mRNA [NM_002953]	NM_002953
A_23_P71821	0.000517	NM_006195	NM_006195	Homo sapiens pre-B-cell leukemia transcription factor 3 (PBX3), mRNA [NM_006195]	NM_006195
A_23_P57306	0.000517	NM_005441	NM_005441	Homo sapiens chromatin assembly factor 1, subunit B (p60) (CHAF1B), mRNA [NM_005441]	NM_005441
A_24_P122940	0.000519	THC2440296			
A_24_P193482	0.000519	NM_001012969	NM_001012969	Homo sapiens similar to CG11994-PA (FLJ44620), mRNA [NM_001012969]	NM_001012969
A_23_P13604	0.000519	NM_002567	NM_002567	Homo sapiens prostatic binding protein (PBP), mRNA [NM_002567]	NM_002567
A_23_P7732	0.000521	NM_004365	NM_004365	Homo sapiens centrin, EF-hand protein, 3 (CDC31 homolog, yeast) (CETN3), mRNA [NM_004365]	NM_004365
A_24_P710730	0.000521	NM_207331	NM_207331	Homo sapiens hypothetical protein LOC153561 (LOC153561), mRNA [NM_207331]	NM_207331
A_23_P346265	0.000522	NM_024312	NM_024312	Homo sapiens MGC4170 protein (MGC4170), mRNA [NM_024312]	NM_024312
A_23_P63789	0.000523	NM_001005414	NM_001005414	Homo sapiens ZW10 interactor (ZWINT), transcript variant 4, mRNA [NM_001005414]	NM_001005414
A_23_P156620	0.000524	NM_007149	NM_007149	Homo sapiens zinc finger protein 184 (Kruppel-like) (ZNF184), mRNA [NM_007149]	NM_007149
A_23_P208009	0.000524	NM_033280	NM_033280	Homo sapiens SEC11-like 3 (S. cerevisiae) (SEC11L3), mRNA [NM_033280]	NM_033280
A_32_P229104	0.000527	BM983822	BM983822	BM983822 UI-CF-DU1-aay-e-18-0-UI.s1 UI-CF-DU1 Homo sapiens cDNA clone UI-CF-DU1-aay-e-18-0-UI 3', mRNA sequence [BM983822]	
A_23_P47704	0.000528	NM_003355	NM_003355	Homo sapiens uncoupling protein 2 (mitochondrial, proton carrier) (UCP2), nuclear gene encoding mitochondrial protein, mRNA [NM_003355]	NM_003355
A_24_P403244	0.000528	NM_013440	NM_013440	Homo sapiens paired immunoglobin-like type 2 receptor beta (PILRB), transcript variant 1, mRNA [NM_013440]	NM_013440
A_23_P321485	0.000528	NM_173827	NM_173827	Homo sapiens hypothetical protein FLJ38991 (FLJ38991), mRNA [NM_173827]	NM_173827
A_24_P190379	0.000529	NM_018443	NM_018443	Homo sapiens zinc finger protein 302 (ZNF302), transcript variant 1, mRNA [NM_018443]	NM_018443
A_23_P314222	0.00053	NM_138792	NM_138792	Homo sapiens Leo1, Paf1/RNA polymerase II complex component, homolog (S. cerevisiae) (LEO1), mRNA [NM_138792]	NM_138792
A_24_P231132	0.00053	NM_001106	NM_001106	Homo sapiens activin A receptor, type IIB (ACVR2B), mRNA [NM_001106]	NM_001106
A_23_P24044	0.000532	NM_017649	NM_017649	Homo sapiens cyclin M2 (CNNM2), transcript variant 1, mRNA [NM_017649]	NM_017649
A_24_P674479	0.000532	AK090611	AK090611	Homo sapiens cDNA FLJ33292 fis, clone BNGH42000130, weakly similar to Homo sapiens beta glucuronidase isoform. [AK090611]	
A_23_P387000	0.000533	NM_173683	NM_173683	Homo sapiens X Kell blood group precursor-related family, member 6 (XKR6), transcript variant 2, mRNA [NM_173683]	NM_173683
A_24_P772103	0.000534	AK094724	AK094724	Homo sapiens cDNA FLJ37405 fis, clone BRAMY2028269. [AK094724]	
A_24_P367576	0.000534	AK125170	AK125170	Homo sapiens cDNA FLJ43180 fis, clone FCBBF3013846. [AK125170]	
A_24_P106382	0.000535	AK001406	AK001406	Homo sapiens cDNA FLJ10544 fis, clone NT2RP2001601, highly similar to Homo sapiens mRNA for KIAA0797 protein. [AK001406]	
A_24_P838448	0.000535	AK022865	AK022865	Homo sapiens cDNA FLJ12803 fis, clone NT2RP2002172. [AK022865]	
A_23_P318800	0.000536	NM_001050	NM_001050	Homo sapiens somatostatin receptor 2 (SSTR2), mRNA [NM_001050]	NM_001050
A_24_P174613	0.000536	NM_033632	NM_033632	Homo sapiens F-box and WD-40 domain protein 7 (archipelago homolog, Drosophila) (FBXW7), transcript variant 1, mRNA [NM_033632]	NM_033632
A_23_P350886	0.000539	NM_015349	NM_015349	Homo sapiens KIAA0240 (KIAA0240), mRNA [NM_015349]	NM_015349
A_23_P358195	0.00054	NM_198079	NM_198079	Homo sapiens golgi autoantigen, golgin subfamily a-like (FLJ40113), mRNA [NM_198079]	NM_198079
A_23_P339191	0.00054	NM_005188	NM_005188	Homo sapiens Cas-Br-M (murine) ecotropic retroviral transforming sequence (CBL), mRNA [NM_005188]	NM_005188
A_23_P12896	0.000541	NM_022725	NM_022725	Homo sapiens Fanconi anemia, complementation group F (FANCF), mRNA [NM_022725]	NM_022725
A_23_P24796	0.000547	NM_032127	NM_032127	Homo sapiens hypothetical protein DKFZp566M1046 (DKFZP566M1046), mRNA [NM_032127]	NM_032127
A_23_P92490	0.000547	NM_020139	NM_020139	Homo sapiens dehydrogenase/reductase (SDR family) member 6 (DHRS6), mRNA [NM_020139]	NM_020139
A_23_P135381	0.00055	NM_001003845	NM_001003845	Homo sapiens Sp5 transcription factor (SP5), mRNA [NM_001003845]	NM_001003845
A_24_P169493	0.00055	A_24_P169493			
A_24_P592544	0.000551	THC2399998		Q5XI42 (Q5XI42) Fatty aldehyde dehydrogenase-like, partial (5%) [THC2399998]	
A_23_P103628	0.000551	BC011983	BC011983	Homo sapiens protein BAP28, mRNA (cDNA clone IMAGE:3863792), complete cds. [BC011983]	XM_375853
A_23_P25615	0.000552	NM_017826	NM_017826	Homo sapiens hypothetical protein FLJ20449 (FLJ20449), mRNA [NM_017826]	NM_017826

A_32_P200025	0.000552	A_32_P200025			
A_23_P250554	0.000552	NM_018046	NM_018046	Homo sapiens angiogenic factor with G patch and FHA domains 1 (AGGF1), mRNA [NM_018046]	NM_018046
A_24_P305678	0.000552	NM_012399	NM_012399	Homo sapiens phosphatidylinositol transfer protein, beta (PITPNB), mRNA [NM_012399]	NM_012399
A_23_P167324	0.000553	NM_000297	NM_000297	Homo sapiens polycystic kidney disease 2 (autosomal dominant) (PKD2), mRNA [NM_000297]	NM_000297
A_32_P29551	0.000553	THC2450799			
A_23_P208172	0.000554	AK023652	AK023652	Homo sapiens cDNA FLJ13590 fis, clone PLACE1009398, moderately similar to ZINC FINGER PROTEIN 135. [AK023652]	
A_24_P81298	0.000554	THC2336427		PPP6_HUMAN (O00743) Serine/threonine protein phosphatase 6 (PP6) , partial (9%) [THC2336427]	
A_23_P125656	0.000555	NM_001356	NM_001356	Homo sapiens DEAD (Asp-Glu-Ala-Asp) box polypeptide 3, X-linked (DDX3X), transcript variant 2, mRNA [NM_001356]	NM_001356
A_23_P252855	0.000556	NM_018321	NM_018321	Homo sapiens brix domain containing 2 (BXDC2), mRNA [NM_018321]	NM_018321
A_23_P28898	0.000559	NM_000933	NM_000933	Homo sapiens phospholipase C, beta 4 (PLCB4), transcript variant 1, mRNA [NM_000933]	NM_000933
A_24_P206776	0.000559	NM_001885	NM_001885	Homo sapiens crystallin, alpha B (CRYAB), mRNA [NM_001885]	NM_001885
A_23_P11774	0.000559	NM_016037	NM_016037	Homo sapiens UTP11-like, U3 small nucleolar ribonucleoprotein, (yeast) (UTP11L), mRNA [NM_016037]	NM_016037
A_23_P143916	0.00056	NM_014382	NM_014382	Homo sapiens ATPase, Ca++ transporting, type 2C, member 1 (ATP2C1), transcript variant 1, mRNA [NM_014382]	NM_014382
A_23_P117971	0.000562	M15530	M15530	Human B-cell growth factor (BCGF1) mRNA, complete cds. [M15530]	
A_23_P252981	0.000563	NM_021804	NM_021804	Homo sapiens angiotensin I converting enzyme (peptidyl-dipeptidase A) 2 (ACE2), mRNA [NM_021804]	NM_021804
A_24_P332683	0.000565	NM_001008401	NM_001008401	Homo sapiens FLJ16231 protein (FLJ16231), mRNA [NM_001008401]	NM_001008401
A_23_P26905	0.000565	NM_007215	NM_007215	Homo sapiens polymerase (DNA directed), gamma 2, accessory subunit (POLG2), mRNA [NM_007215]	NM_007215
A_32_P9368	0.000565	THC2339078			
A_23_P91328	0.000565	NM_006392	NM_006392	Homo sapiens nucleolar protein 5A (56kDa with KKE/D repeat) (NOL5A), mRNA [NM_006392]	NM_006392
A_24_P782337	0.000566	AK026477	AK026477	Homo sapiens cDNA: FLJ22824 fis, clone KAIA3991. [AK026477]	
A_23_P54447	0.000566	NM_030944	NM_030944	Homo sapiens chromosome 15 open reading frame 5 (C15orf5), mRNA [NM_030944]	NM_030944
A_24_P744818	0.000567	AK021546	AK021546	Homo sapiens cDNA FLJ11484 fis, clone HEMBA1001835. [AK021546]	
A_24_P273716	0.000567	NM_014797	NM_014797	Homo sapiens zinc finger and BTB domain containing 24 (ZBTB24), mRNA [NM_014797]	NM_014797
A_24_P409881	0.000567	ENST00000217341		PREDICTED: Homo sapiens similar to hypothetical protein (LOC338756), mRNA [XM_291989]	XM_291989
A_24_P942773	0.000568	NM_007159	NM_007159	Homo sapiens sarcolemma associated protein (SLMAP), mRNA [NM_007159]	NM_007159
A_32_P61145	0.000569	AK096154	AK096154	Homo sapiens cDNA FLJ38835 fis, clone MESAN2002424. [AK096154]	
A_23_P334990	0.000569	NM_022491	NM_022491	Homo sapiens SDS3 protein (SDS3), mRNA [NM_022491]	NM_022491
A_24_P537188	0.00057	BC032332	BC032332	Homo sapiens cDNA clone MGC:40288 IMAGE:5169056, complete cds. [BC032332]	XM_496438
A_23_P68786	0.00057	NM_016335	NM_016335	Homo sapiens proline dehydrogenase (oxidase) 1 (PRODH), nuclear gene encoding mitochondrial protein, mRNA [NM_016335]	NM_016335
A_24_P284523	0.000571	NM_002446	NM_002446	Homo sapiens mitogen-activated protein kinase kinase kinase 10 (MAP3K10), mRNA [NM_002446]	NM_002446
A_32_P174258	0.000572	BQ719988	BQ719988	AGENCOURT_8103790 Lupski_sympathetic_trunk Homo sapiens cDNA clone IMAGE:6190912 5', mRNA sequence [BQ719988]	
A_23_P209459	0.000573	NM_004482	NM_004482	Homo sapiens UDP-N-acetyl-alpha-D-galactosamine:polypeptide N-acetylgalactosaminyltransferase 3 (GalNAc-T3) (GALNT3), mRNA [NM_004482]	NM_004482
A_23_P204033	0.000574	NM_007007	NM_007007	Homo sapiens cleavage and polyadenylation specific factor 6, 68kDa (CPSF6), mRNA [NM_007007]	NM_007007
A_24_P394361	0.000575	NM_025132	NM_025132	Homo sapiens WD repeat domain 19 (WDR19), mRNA [NM_025132]	NM_025132
A_23_P93737	0.000576	NM_004411	NM_004411	Homo sapiens dynein, cytoplasmic, intermediate polypeptide 1 (DNCI1), mRNA [NM_004411]	NM_004411
A_32_P8732	0.000578	THC2375588			
A_24_P271285	0.000578	NM_052876	NM_052876	Homo sapiens BTB (POZ) domain containing 14B (BTBD14B), mRNA [NM_052876]	NM_052876
A_23_P112859	0.000578	NM_001898	NM_001898	Homo sapiens cystatin SN (CST1), mRNA [NM_001898]	NM_001898
A_23_P373119	0.000578	ENST00000324884		GB|NM_178467.1|NP_848562.1 high-mobility group (nonhistone chromosomal) protein 4-like [Homo sapiens] [NP798948]	
A_24_P884376	0.000579	XM_499519	XM_499519	PREDICTED: Homo sapiens hypothetical gene supported by AK092942; NM_198284 (LOC442539), mRNA [XM_499519]	XM_499519
A_24_P322025	0.000579	NM_004380	NM_004380	Homo sapiens CREB binding protein (Rubinstein-Taybi syndrome) (CREBBP), mRNA [NM_004380]	NM_004380
A_23_P411431	0.000579	ENST00000282366		Homo sapiens mRNA for KIAA1266 protein, partial cds. [AB033092]	
A_24_P181295	0.00058	NM_001001872	NM_001001872	Homo sapiens chromosome 14 open reading frame 37 (C14orf37), mRNA [NM_001001872]	NM_001001872
A_32_P208178	0.000581	NM_001006	NM_001006	Homo sapiens ribosomal protein S3A (RPS3A), mRNA [NM_001006]	NM_001006

A_23_P216396	0.000582	NM_014285	NM_014285	Homo sapiens exosome component 2 (EXOSC2), mRNA [NM_014285]	NM_014285
A_24_P114617	0.000582	NM_014043	NM_014043	Homo sapiens chromatin modifying protein 2B (CHMP2B), mRNA [NM_014043]	NM_014043
A_32_P80255	0.000582	AK021715	AK021715	Homo sapiens cDNA FLJ11653 fis, clone HEMBA1004538. [AK021715]	
A_23_P63908	0.000583	NM_139169	NM_139169	Homo sapiens TruB pseudouridine (psi) synthase homolog 1 (E. coli) (TRUB1), mRNA [NM_139169]	NM_139169
A_23_P82478	0.000583	NM_019042	NM_019042	Homo sapiens hypothetical protein FLJ20485 (FLJ20485), mRNA [NM_019042]	NM_019042
A_32_P109572	0.000583	NM_001533	NM_001533	Homo sapiens heterogeneous nuclear ribonucleoprotein L (HNRPL), transcript variant 1, mRNA [NM_001533]	NM_001533
A_23_P216355	0.000584	NM_013432	NM_013432	Homo sapiens nuclear factor of kappa light polypeptide gene enhancer in B-cells inhibitor-like 2 (NFKBIL2), mRNA [NM_013432]	NM_013432
A_23_P50376	0.000584	NM_018555	NM_018555	Homo sapiens zinc finger protein 331 (ZNF331), mRNA [NM_018555]	NM_018555
A_32_P5571	0.000585	BC088374	BC088374	Homo sapiens cDNA clone IMAGE:6171379, containing frame-shift errors. [BC088374]	
A_24_P165816	0.000586	AK023645	AK023645	Homo sapiens cDNA FLJ13583 fis, clone PLACE1009050. [AK023645]	
A_24_P274987	0.000586	NM_003692	NM_003692	Homo sapiens transmembrane protein with EGF-like and two follistatin-like domains 1 (TMEFF1), mRNA [NM_003692]	NM_003692
A_24_P303160	0.000587	NM_016218	NM_016218	Homo sapiens polymerase (DNA directed) kappa (POLK), mRNA [NM_016218]	NM_016218
A_23_P203947	0.000587	NM_030655	NM_030655	Homo sapiens DEAD/H (Asp-Glu-Ala-Asp/His) box polypeptide 11 (CHL1-like helicase homolog, S. cerevisiae) (DDX11), transcript variant 3, mRNA [NM_030655]	NM_030655
A_23_P254379	0.000587	NM_006620	NM_006620	Homo sapiens HBS1-like (S. cerevisiae) (HBS1L), mRNA [NM_006620]	NM_006620
A_23_P100022	0.000588	NM_014848	NM_014848	Homo sapiens synaptic vesicle glycoprotein 2B (SV2B), mRNA [NM_014848]	NM_014848
A_32_P78649	0.000589	AK123481	AK123481	Homo sapiens cDNA FLJ41487 fis, clone BRTHA2004350. [AK123481]	
A_23_P431890	0.000589	NM_145261	NM_145261	Homo sapiens DnaJ (Hsp40) homolog, subfamily C, member 19 (DNAJC19), transcript variant 1, mRNA [NM_145261]	NM_145261
A_32_P24581	0.000589	NM_002954	NM_002954	Homo sapiens ribosomal protein S27a (RPS27A), mRNA [NM_002954]	NM_002954
A_24_P29733	0.00059	NM_012395	NM_012395	Homo sapiens PFTAIRE protein kinase 1 (PFTK1), mRNA [NM_012395]	NM_012395
A_24_P49371	0.00059	NM_017867	NM_017867	Homo sapiens hypothetical protein FLJ20534 (FLJ20534), mRNA [NM_017867]	NM_017867
A_32_P182156	0.000591	NM_145806	NM_145806	Homo sapiens zinc finger protein 511 (ZNF511), mRNA [NM_145806]	NM_145806
A_23_P36166	0.000592	NM_002645	NM_002645	Homo sapiens phosphoinositide-3-kinase, class 2, alpha polypeptide (PIK3C2A), mRNA [NM_002645]	NM_002645
A_23_P50815	0.000593	NM_020659	NM_020659	Homo sapiens tweety homolog 1 (Drosophila) (TTYH1), transcript variant 1, mRNA [NM_020659]	NM_020659
A_23_P430140	0.000595	AF090930	AF090930	Homo sapiens clone HQ0478 PRO0478 mRNA, complete cds. [AF090930]	
A_32_P111852	0.000596	THC2309459			
A_32_P49116	0.000597	CR617865	CR617865	full-length cDNA clone CS0DF021YD16 of Fetal brain of Homo sapiens (human). [CR617865]	
A_32_P63913	0.000597	NM_006802	NM_006802	Homo sapiens splicing factor 3a, subunit 3, 60kDa (SF3A3), mRNA [NM_006802]	NM_006802
A_24_P219024	0.000598	NM_014953	NM_014953	Homo sapiens KIAA1008 (KIAA1008), mRNA [NM_014953]	NM_014953
A_32_P181077	0.000599	NM_203447	NM_203447	Homo sapiens dedicator of cytokinesis 8 (DOCK8), mRNA [NM_203447]	NM_203447
A_23_P39223	0.0006	NM_021088	NM_021088	Homo sapiens zinc finger protein 2 (ZNF2), transcript variant 1, mRNA [NM_021088]	NM_021088
A_23_P251574	0.000601	NM_017644	NM_017644	Homo sapiens DRE1 protein (DRE1), mRNA [NM_017644]	NM_017644
A_32_P173922	0.000602	A_32_P173922			
A_23_P51082	0.000603	THC2242915		ALU1_HUMAN (P39188) Alu subfamily J sequence contamination warning entry, partial (4%) [THC2242915]	
A_23_P593	0.000603	NM_021639	NM_021639	Homo sapiens GC-rich promoter binding protein 1-like 1 (GPBP1L1), mRNA [NM_021639]	NM_021639
A_24_P479645	0.000604	AK093640	AK093640	Homo sapiens cDNA FLJ36321 fis, clone THYMU2005482. [AK093640]	
A_23_P748	0.000607	NM_006147	NM_006147	Homo sapiens interferon regulatory factor 6 (IRF6), mRNA [NM_006147]	NM_006147
A_23_P163428	0.000608	NM_021823	NM_021823	Homo sapiens phosphopantothenoylcysteine decarboxylase (PPCDC), mRNA [NM_021823]	NM_021823
A_32_P50603	0.000609	ENST00000360206		Homo sapiens cDNA FLJ34164 fis, clone FCBBF3014567. [AK091483]	XM_496547
A_24_P301954	0.000609	BC009492	BC009492	Homo sapiens hypothetical protein MGC16384, mRNA (cDNA clone MGC:16384 IMAGE:3937896), complete cds. [BC009492]	XM_499564
A_23_P164081	0.000609	NM_005964	NM_005964	Homo sapiens myosin, heavy polypeptide 10, non-muscle (MYH10), mRNA [NM_005964]	NM_005964
A_32_P118556	0.000615	A_32_P118556			
A_23_P82748	0.000618	NM_020189	NM_020189	Homo sapiens enhancer of yellow 2 homolog (Drosophila) (ENY2), mRNA [NM_020189]	NM_020189
A_23_P58604	0.000619	NM_016175	NM_016175	Homo sapiens hypothetical LOC51149 (LOC51149), transcript variant 1, mRNA [NM_016175]	NM_016175
A_32_P39093	0.000619	ENST00000258884		Homo sapiens hypothetical protein from EUROIMAGE 588495, mRNA (cDNA clone IMAGE:30346383), partial cds. [BC059401]	XM_051862
A_23_P390116	0.00062	NM_153023	NM_153023	Homo sapiens spermatogenesis associated 13 (SPATA13), mRNA [NM_153023]	NM_153023

A_24_P100387	0.000621	NM_203391	NM_203391	Homo sapiens glycerol kinase (GK), transcript variant 1, mRNA [NM_203391]	NM_203391
A_24_P135092	0.000623	ENST00000359901		Homo sapiens cDNA FLJ90089 fis, clone HEMBA1005267, weakly similar to ANKYRIN, BRAIN VARIANT 1. [AK074570]	
A_24_P363477	0.000623	NM_013393	NM_013393	Homo sapiens FtsJ homolog 2 (E. coli) (FTSJ2), transcript variant 1, mRNA [NM_013393]	NM_013393
A_32_P119204	0.000623	AK025353	AK025353	Homo sapiens cDNA: FLJ21700 fis, clone COL09849, highly similar to HSU14972 Human ribosomal protein S10 mRNA. [AK025353]	XM_016113
A_23_P41166	0.000624	NM_003781	NM_003781	Homo sapiens UDP-Gal:betaGlcNAc beta 1,3-galactosyltransferase, polypeptide 3 (B3GALT3), transcript variant 1, mRNA [NM_003781]	NM_003781
A_24_P16337	0.000625	NM_001013726	NM_001013726	Homo sapiens hypothetical gene supported by AK091508 (LOC441421), mRNA [NM_001013726]	NM_001013726
A_24_P740022	0.000626	A_24_P740022			
A_23_P28688	0.000627	NM_016207	NM_016207	Homo sapiens cleavage and polyadenylation specific factor 3, 73kDa (CPSF3), mRNA [NM_016207]	NM_016207
A_32_P224586	0.000628	THC2342537		ALU1_HUMAN (P39188) Alu subfamily J sequence contamination warning entry, partial (5%) [THC2342537]	
A_24_P8049	0.000628	THC2233934		BC043133 Cnot2 protein {Mus musculus;} , partial (90%) [THC2403846]	
A_32_P109777	0.000629	THC2307981		PHB_HUMAN (P35232) Prohibitin, partial (95%) [THC2307981]	
A_23_P144151	0.000629	AF292100	AF292100	Homo sapiens RP42 protein mRNA, complete cds. [AF292100]	
A_23_P104892	0.000629	NM_001418	NM_001418	Homo sapiens eukaryotic translation initiation factor 4 gamma, 2 (EIF4G2), mRNA [NM_001418]	NM_001418
A_23_P110412	0.000631	AK023055	AK023055	Homo sapiens cDNA FLJ12993 fis, clone NT2RP3000197. [AK023055]	
A_23_P16242	0.000632	NM_021143	NM_021143	Homo sapiens zinc finger protein 20 (KOX 13) (ZNF20), mRNA [NM_021143]	NM_021143
A_23_P101392	0.000633	NM_024074	NM_024074	Homo sapiens transmembrane protein 38A (TMEM38A), mRNA [NM_024074]	NM_024074
A_24_P99046	0.000634	NM_015000	NM_015000	Homo sapiens serine/threonine kinase 38 like (STK38L), mRNA [NM_015000]	NM_015000
A_24_P306585	0.000634	CD359021	CD359021	AGENCOURT_14276711 NIH_MGC_180 Homo sapiens cDNA clone IMAGE:30390221 5', mRNA sequence [CD359021]	
A_23_P78372	0.000634	NM_005131	NM_005131	Homo sapiens THO complex 1 (THOC1), mRNA [NM_005131]	NM_005131
A_23_P134113	0.000634	NM_052831	NM_052831	Homo sapiens chromosome 6 open reading frame 192 (C6orf192), mRNA [NM_052831]	NM_052831
A_24_P353619	0.000635	NM_000478	NM_000478	Homo sapiens alkaline phosphatase, liver/bone/kidney (ALPL), mRNA [NM_000478]	NM_000478
A_23_P215208	0.000635	NM_022116	NM_022116	Homo sapiens fidgetin-like 1 (FIGNL1), mRNA [NM_022116]	NM_022116
A_23_P375104	0.000636	NM_018193	NM_018193	Homo sapiens hypothetical protein FLJ10719 (FLJ10719), mRNA [NM_018193]	NM_018193
A_23_P38860	0.000636	ENST00000252482		Q96GV2 (Q96GV2) XTP7, complete [THC2262919]	
A_23_P68669	0.000637	NM_024944	NM_024944	Homo sapiens chondrolectin (CHODL), mRNA [NM_024944]	NM_024944
A_32_P107752	0.000637	THC2278266		O03734 (O03734) Cytochrome oxidase I (Fragment), partial (6%) [THC2278266]	
A_23_P382688	0.000637	NM_145284	NM_145284	Homo sapiens similar to hypothetical protein MGC17347 (LOC159090), mRNA [NM_145284]	NM_145284
A_23_P111481	0.000637	NM_182800	NM_182800	Homo sapiens arsenate resistance protein ARS2 (ARS2), transcript variant 2, mRNA [NM_182800]	NM_182800
A_24_P367282	0.000638	ENST00000323012			
A_23_P113623	0.000643	NM_032351	NM_032351	Homo sapiens mitochondrial ribosomal protein L45 (MRPL45), nuclear gene encoding mitochondrial protein, mRNA [NM_032351]	NM_032351
A_32_P72477	0.000644	A_32_P72477			
A_24_P204290	0.000645	ENST00000342830			XM_496049
A_23_P392501	0.000645	ENST00000327162		Homo sapiens cDNA FLJ31859 fis, clone NT2RP7001231. [AK056421]	
A_23_P250212	0.000645	ENST00000330777		Homo sapiens mRNA for FLJ00269 protein. [AK122582]	XM_291277
A_32_P141923	0.000645	NM_199290	NM_199290	Homo sapiens alpha-NAC protein (ANAC), mRNA [NM_199290]	NM_199290
A_23_P77468	0.000648	AK024389	AK024389	Homo sapiens cDNA FLJ14327 fis, clone PLACE4000250. [AK024389]	
A_23_P324994	0.000648	NM_018846	NM_018846	Homo sapiens kelch-like 7 (Drosophila) (KLHL7), mRNA [NM_018846]	NM_018846
A_32_P172453	0.000649	THC2442819			
A_23_P342091	0.000652	NM_145063	NM_145063	Homo sapiens chromosome 6 open reading frame 130 (C6orf130), mRNA [NM_145063]	NM_145063
A_24_P270496	0.000653	NM_006491	NM_006491	Homo sapiens neuro-oncological ventral antigen 1 (NOVA1), transcript variant 3, mRNA [NM_006491]	NM_006491
A_32_P179239	0.000654	AK123297	AK123297	Homo sapiens cDNA FLJ41303 fis, clone BRAMY2042131. [AK123297]	
A_23_P98042	0.000654	A_23_P98042			
A_23_P215669	0.000656	NM_032959	NM_032959	Homo sapiens DNA directed RNA polymerase II polypeptide J-related gene (POLR2J2), transcript variant 3, mRNA [NM_032959]	NM_032959
A_23_P205929	0.000656	NM_197960	NM_197960	Homo sapiens dipeptidylpeptidase 8 (DPP8), transcript variant 3, mRNA [NM_197960]	NM_197960

A_24_P362317	0.000656	NM_001111	NM_001111	Homo sapiens adenosine deaminase, RNA-specific (ADAR), transcript variant 1, mRNA [NM_001111]	NM_001111
A_23_P59877	0.000656	NM_001444	NM_001444	Homo sapiens fatty acid binding protein 5 (psoriasis-associated) (FABP5), mRNA [NM_001444]	NM_001444
A_32_P92654	0.000657	NM_021036	NM_021036	Homo sapiens SMA5 (SMA5), mRNA [NM_021036]	NM_021036
A_23_P39704	0.000657	NM_006276	NM_006276	Homo sapiens splicing factor, arginine/serine-rich 7, 35kDa (SFRS7), mRNA [NM_006276]	NM_006276
A_24_P394408	0.000658	NM_014423	NM_014423	Homo sapiens AF4/FMR2 family, member 4 (AFF4), mRNA [NM_014423]	NM_014423
A_23_P208208	0.000658	NM_023074	NM_023074	Homo sapiens zinc finger protein 649 (ZNF649), mRNA [NM_023074]	NM_023074
A_23_P82169	0.000658	NM_003107	NM_003107	Homo sapiens SRY (sex determining region Y)-box 4 (SOX4), mRNA [NM_003107]	NM_003107
A_23_P360769	0.00066	NM_002372	NM_002372	Homo sapiens mannosidase, alpha, class 2A, member 1 (MAN2A1), mRNA [NM_002372]	NM_002372
A_23_P416314	0.00066	BC034222	BC034222	Homo sapiens H-rev107-like protein 5, mRNA (cDNA clone MGC:39540 IMAGE:5268703), complete cds. [BC034222]	
A_32_P146815	0.000663	BC062473	BC062473	Homo sapiens cDNA clone IMAGE:30374677, partial cds. [BC062473]	
A_24_P312164	0.000664	AL110179	AL110179	Homo sapiens mRNA; cDNA DKFZp564D0472 (from clone DKFZp564D0472) [AL110179]	
A_23_P33856	0.000664	NM_006603	NM_006603	Homo sapiens stromal antigen 2 (STAG2), mRNA [NM_006603]	NM_006603
A_32_P128656	0.000666	AF041210	AF041210	Homo sapiens midline 1 fetal kidney isoform 3 (MID1) mRNA, partial cds. [AF041210]	
A_23_P162228	0.000666	NM_005594	NM_005594	Homo sapiens nascent-polypeptide-associated complex alpha polypeptide (NACA), mRNA [NM_005594]	NM_005594
A_24_P171983	0.000667	NM_014165	NM_014165	Homo sapiens chromosome 6 open reading frame 66 (C6orf66), mRNA [NM_014165]	NM_014165
A_23_P132444	0.000671	NM_006756	NM_006756	Homo sapiens transcription elongation factor A (SII), 1 (TCEA1), transcript variant 1, mRNA [NM_006756]	NM_006756
A_23_P259586	0.000672	NM_003318	NM_003318	Homo sapiens TTK protein kinase (TTK), mRNA [NM_003318]	NM_003318
A_23_P210323	0.000673	AB011154	AB011154	Homo sapiens mRNA for KIAA0582 protein, partial cds. [AB011154]	
A_23_P309779	0.000675	NM_018177	NM_018177	Homo sapiens Nedd4 binding protein 2 (N4BP2), mRNA [NM_018177]	NM_018177
A_24_P410627	0.000675	AF117233	AF117233	Homo sapiens znf-xp protein mRNA, complete cds. [AF117233]	
A_23_P133629	0.000675	NM_004661	NM_004661	Homo sapiens CDC23 (cell division cycle 23, yeast, homolog) (CDC23), mRNA [NM_004661]	NM_004661
A_23_P138706	0.000676	NM_000681	NM_000681	Homo sapiens adrenergic, alpha-2A-, receptor (ADRA2A), mRNA [NM_000681]	NM_000681
A_24_P236008	0.000677	AB037781	AB037781	Homo sapiens mRNA for KIAA1360 protein, partial cds. [AB037781]	
A_24_P199500	0.000679	NM_007212	NM_007212	Homo sapiens ring finger protein 2 (RNF2), mRNA [NM_007212]	NM_007212
A_23_P81612	0.000679	NM_003135	NM_003135	Homo sapiens signal recognition particle 19kDa (SRP19), mRNA [NM_003135]	NM_003135
A_24_P487736	0.000684	NM_198279	NM_198279	Homo sapiens chromosome X open reading frame 23 (CXorf23), mRNA [NM_198279]	NM_198279
A_32_P95729	0.000684	NM_018193	NM_018193	Homo sapiens hypothetical protein FLJ10719 (FLJ10719), mRNA [NM_018193]	NM_018193
A_23_P3627	0.000684	NM_003119	NM_003119	Homo sapiens spastic paraplegia 7, paraplegin (pure and complicated autosomal recessive) (SPG7), nuclear gene encoding mitochondrial protein, transcript variant 1, mRNA [NM_003119]	NM_003119
A_32_P165363	0.000685	THC2283809			
A_24_P249187	0.000685	A_24_P249187			
A_23_P104522	0.000685	NM_006393	NM_006393	Homo sapiens nebulette (NEBL), transcript variant 1, mRNA [NM_006393]	NM_006393
A_23_P325924	0.000685	ENST00000288676		Homo sapiens mRNA for FLJ00375 protein. [AK090454]	XM_097977
A_23_P140309	0.000685	NM_144578	NM_144578	Homo sapiens chromosome 14 open reading frame 32 (C14orf32), mRNA [NM_144578]	NM_144578
A_32_P229221	0.000686	AB209318	AB209318	Homo sapiens mRNA for glutamate receptor, ionotropic, delta 2 variant protein. [AB209318]	
A_24_P22657	0.000686	AK096415	AK096415	Homo sapiens cDNA FLJ39096 fis, clone NTONG1000247. [AK096415]	
A_23_P419038	0.000687	NM_004898	NM_004898	Homo sapiens clock homolog (mouse) (CLOCK), mRNA [NM_004898]	NM_004898
A_23_P211015	0.000687	NM_017447	NM_017447	Homo sapiens chromosome 21 open reading frame 91 (C21orf91), mRNA [NM_017447]	NM_017447
A_32_P84640	0.000688	THC2286661		ALU6_HUMAN (P39193) Alu subfamily SP sequence contamination warning entry, partial (14%) [THC2286661]	
A_23_P208477	0.000689	NM_001419	NM_001419	Homo sapiens ELAV (embryonic lethal, abnormal vision, Drosophila)-like 1 (Hu antigen R) (ELAVL1), mRNA [NM_001419]	NM_001419
A_23_P167417	0.00069	NM_015455	NM_015455	Homo sapiens CCR4-NOT transcription complex, subunit 6 (CNOT6), mRNA [NM_015455]	NM_015455
A_23_P170978	0.000694	NM_017865	NM_017865	Homo sapiens zinc finger protein 692 (ZNF692), mRNA [NM_017865]	NM_017865
A_32_P192430	0.000694	NM_001826	NM_001826	Homo sapiens CDC28 protein kinase regulatory subunit 1B (CKS1B), mRNA [NM_001826]	NM_001826
A_23_P165414	0.000697	NM_144711	NM_144711	Homo sapiens kelch-like 23 (Drosophila) (KLHL23), mRNA [NM_144711]	NM_144711
A_24_P146603	0.000697	NM_004728	NM_004728	Homo sapiens DEAD (Asp-Glu-Ala-Asp) box polypeptide 21 (DDX21), mRNA [NM_004728]	NM_004728

A_23_P18931	0.000698	NM_002372	NM_002372	Homo sapiens mannosidase, alpha, class 2A, member 1 (MAN2A1), mRNA [NM_002372]	NM_002372
A_24_P182858	0.000698	NM_030915	NM_030915	Homo sapiens likely ortholog of mouse limb-bud and heart gene (LBH), mRNA [NM_030915]	NM_030915
A_32_P8813	0.0007	AK090515	AK090515	Homo sapiens cDNA FLJ33196 fis, clone ADRGL2006034. [AK090515]	XM_378514
A_23_P63584	0.000702	NM_015446	NM_015446	Homo sapiens AT hook containing transcription factor 1 (AHCTF1), mRNA [NM_015446]	NM_015446
A_32_P175539	0.000702	NM_002902	NM_002902	Homo sapiens reticulocalbin 2, EF-hand calcium binding domain (RCN2), mRNA [NM_002902]	NM_002902
A_24_P194931	0.000703	NM_020463	NM_020463	Homo sapiens KIAA1387 protein (KIAA1387), mRNA [NM_020463]	NM_020463
A_23_P12526	0.000704	NM_005426	NM_005426	Homo sapiens tumor protein p53 binding protein, 2 (TP53BP2), mRNA [NM_005426]	NM_005426
A_23_P428326	0.000705	AK000755	AK000755	Homo sapiens cDNA FLJ20748 fis, clone HEP05772. [AK000755]	
A_23_P141606	0.000706	NM_000386	NM_000386	Homo sapiens bleomycin hydrolase (BLMH), mRNA [NM_000386]	NM_000386
A_32_P55979	0.000707	THC2439252		BC018029 6-pyruvoyltetrahydropterin synthase {Homo sapiens;} , partial (25%) [THC2439252]	
A_23_P31654	0.000707	NM_033301	NM_033301	Homo sapiens ribosomal protein L8 (RPL8), transcript variant 2, mRNA [NM_033301]	NM_033301
A_23_P140427	0.000708	NM_016337	NM_016337	Homo sapiens Enah/Vasp-like (EVL), mRNA [NM_016337]	NM_016337
A_23_P6263	0.000709	NM_002463	NM_002463	Homo sapiens myxovirus (influenza virus) resistance 2 (mouse) (MX2), mRNA [NM_002463]	NM_002463
A_23_P89762	0.000709	NM_194449	NM_194449	Homo sapiens PH domain and leucine rich repeat protein phosphatase (PHLPP), mRNA [NM_194449]	NM_194449
A_24_P926367	0.00071	AK025735	AK025735	Homo sapiens cDNA: FLJ22082 fis, clone HEP14032, highly similar to AF117756 Homo sapiens thyroid hormone receptor-associated protein complex component TRAP150 mRNA. [AK025735]	
A_23_P163006	0.00071	NM_031314	NM_031314	Homo sapiens heterogeneous nuclear ribonucleoprotein C (C1/C2) (HNRPC), transcript variant 1, mRNA [NM_031314]	NM_031314
A_23_P4953	0.000711	NM_018215	NM_018215	Homo sapiens hypothetical protein FLJ10781 (FLJ10781), mRNA [NM_018215]	NM_018215
A_23_P43326	0.000711	NM_006415	NM_006415	Homo sapiens serine palmitoyltransferase, long chain base subunit 1 (SPTLC1), transcript variant 1, mRNA [NM_006415]	NM_006415
A_23_P213153	0.000711	NM_031372	NM_031372	Homo sapiens heterogeneous nuclear ribonucleoprotein D-like (HNRPDL), transcript variant 2, mRNA [NM_031372]	NM_031372
A_23_P41999	0.000712	NM_021940	NM_021940	Homo sapiens stromal membrane-associated protein 1 (SMAP1), mRNA [NM_021940]	NM_021940
A_23_P70007	0.000712	NM_012484	NM_012484	Homo sapiens hyaluronan-mediated motility receptor (RHAMM) (HMMR), transcript variant 1, mRNA [NM_012484]	NM_012484
A_24_P324141	0.000715	ENST00000349637		H.sapiens MUC5B mRNA (clone JER28) for mucin (partial). [X74954]	
A_23_P108404	0.000715	NM_014914	NM_014914	Homo sapiens centaurin, gamma 2 (CENTG2), mRNA [NM_014914]	NM_014914
A_24_P926972	0.000716	AK000923	AK000923	Homo sapiens cDNA FLJ10061 fis, clone HEMBA1001413. [AK000923]	
A_23_P39263	0.000718	NM_173480	NM_173480	Homo sapiens hypothetical protein LOC126295 (LOC126295), mRNA [NM_173480]	NM_173480
A_23_P393034	0.000718	NM_005329	NM_005329	Homo sapiens hyaluronan synthase 3 (HAS3), transcript variant 1, mRNA [NM_005329]	NM_005329
A_32_P10067	0.000718	THC2339836		B40201 artifact-warning sequence (translated ALU class B) - human {Homo sapiens;} , partial (19%) [THC2339836]	
A_24_P239664	0.000719	NM_183050	NM_183050	Homo sapiens branched chain keto acid dehydrogenase E1, beta polypeptide (maple syrup urine disease) (BCKDHB), nuclear gene encoding mitochondrial protein, transcript variant 1, mRNA [NM_183050]	NM_183050
A_23_P46871	0.000719	NM_018344	NM_018344	Homo sapiens solute carrier family 29 (nucleoside transporters), member 3 (SLC29A3), mRNA [NM_018344]	NM_018344
A_23_P208288	0.000719	NM_020657	NM_020657	Homo sapiens zinc finger protein 304 (ZNF304), mRNA [NM_020657]	NM_020657
A_24_P99838	0.00072	NM_013361	NM_013361	Homo sapiens zinc finger protein 223 (ZNF223), mRNA [NM_013361]	NM_013361
A_24_P385119	0.00072	NM_003184	NM_003184	Homo sapiens TAF2 RNA polymerase II, TATA box binding protein (TBP)-associated factor, 150kDa (TAF2), mRNA [NM_003184]	NM_003184
A_32_P72341	0.00072	BX537987	BX537987	Homo sapiens mRNA; cDNA DKFZp686N1030 (from clone DKFZp686N1030). [BX537987]	
A_23_P363206	0.00072	NM_006910	NM_006910	Homo sapiens retinoblastoma binding protein 6 (RBBP6), transcript variant 1, mRNA [NM_006910]	NM_006910
A_24_P736638	0.00072	A_24_P736638			
A_32_P96036	0.00072	THC2430293		Q69Z36 (Q69Z36) MKIAA2009 protein (Fragment), partial (8%) [THC2430293]	
A_24_P410639	0.000721	BC014384	BC014384	Homo sapiens, clone IMAGE:4052238, mRNA, partial cds. [BC014384]	
A_23_P203686	0.000721	ENST00000260045		Homo sapiens cDNA FLJ10660 fis, clone NT2RP2006100, highly similar to Homo sapiens mRNA; cDNA DKFZp564B102. [AK001522]	
A_32_P150012	0.000723	NM_017780	NM_017780	Homo sapiens chromodomain helicase DNA binding protein 7 (CHD7), mRNA [NM_017780]	NM_017780
A_23_P96249	0.000728	NM_020180	NM_020180	Homo sapiens bruno-like 4, RNA binding protein (Drosophila) (BRUNOL4), mRNA [NM_020180]	NM_020180
A_32_P92814	0.000729	BQ929021	BQ929021	AGENCOURT_10034103 NIH_MGC_40 Homo sapiens cDNA clone IMAGE:6482800 5', mRNA sequence [BQ929021]	
A_24_P397107	0.000729	NM_001789	NM_001789	Homo sapiens cell division cycle 25A (CDC25A), transcript variant 1, mRNA [NM_001789]	NM_001789
A_23_P383060	0.000736	NM_021095	NM_021095	Homo sapiens solute carrier family 5 (sodium-dependent vitamin transporter), member 6 (SLC5A6), mRNA [NM_021095]	NM_021095

A_32_P199002	0.000738	BX478538	BX478538	DKFZp686P07204_r1 686 (synonym: hlcc3) Homo sapiens cDNA clone DKFZp686P07204 5', mRNA sequence [BX478538]	
A_32_P58074	0.000738	NM_001006	NM_001006	Homo sapiens ribosomal protein S3A (RPS3A), mRNA [NM_001006]	NM_001006
A_23_P334864	0.000739	NM_173822	NM_173822	Homo sapiens hypothetical protein MGC39518 (MGC39518), mRNA [NM_173822]	NM_173822
A_32_P189845	0.000739	NM_178558	NM_178558	Homo sapiens zinc finger protein 680 (ZNF680), mRNA [NM_178558]	NM_178558
A_23_P354942	0.000741	NM_175910	NM_175910	Homo sapiens zinc finger protein 493 (ZNF493), mRNA [NM_175910]	NM_175910
A_23_P106522	0.000744	NM_032196	NM_032196	Homo sapiens homolog of yeast INO80 (INO80), transcript variant 2, mRNA [NM_032196]	NM_032196
A_24_P145911	0.000745	U87836	U87836	Homo sapiens htra2-beta-2 mRNA, complete cds. [U87836]	
A_23_P390097	0.000752	NM_152574	NM_152574	Homo sapiens chromosome 9 open reading frame 52 (C9orf52), mRNA [NM_152574]	NM_152574
A_24_P405002	0.000752	NM_152835	NM_152835	Homo sapiens PDLIM1 interacting kinase 1 like (PDIK1L), mRNA [NM_152835]	NM_152835
A_32_P14894	0.000752	NM_001014	NM_001014	Homo sapiens ribosomal protein S10 (RPS10), mRNA [NM_001014]	NM_001014
A_32_P231446	0.000753	NM_198268	NM_198268	Homo sapiens homeodomain interacting protein kinase 1 (HIPK1), transcript variant 1, mRNA [NM_198268]	NM_198268
A_24_P214231	0.000754	NM_003035	NM_003035	Homo sapiens TAL1 (SCL) interrupting locus (SIL), mRNA [NM_003035]	NM_003035
A_32_P115749	0.000754	CD104030	CD104030	AGENCOURT_14006900 NIH_MGC_186 Homo sapiens cDNA clone IMAGE:30372058 5', mRNA sequence [CD104030]	
A_32_P220567	0.000758	A_32_P220567			
A_24_P175460	0.000758	AL834528	AL834528	Homo sapiens mRNA; cDNA DKFZp434G2311 (from clone DKFZp434G2311). [AL834528]	
A_23_P300301	0.00076	NM_004241	NM_004241	Homo sapiens jumonji domain containing 1C (JMJD1C), mRNA [NM_004241]	NM_004241
A_23_P151027	0.00076	NM_014802	NM_014802	Homo sapiens KIAA0528 gene product (KIAA0528), mRNA [NM_014802]	NM_014802
A_23_P388855	0.000761	NM_012330	NM_012330	Homo sapiens MYST histone acetyltransferase (monocytic leukemia) 4 (MYST4), mRNA [NM_012330]	NM_012330
A_24_P236091	0.000761	NM_001975	NM_001975	Homo sapiens enolase 2 (gamma, neuronal) (ENO2), mRNA [NM_001975]	NM_001975
A_23_P145054	0.000762	BC038997	BC038997	Homo sapiens chromosome 6 open reading frame 189, mRNA (cDNA clone IMAGE:6059932), partial cds. [BC038997]	
A_23_P258164	0.000763	NM_001302	NM_001302	Homo sapiens cortistatin (CORT), mRNA [NM_001302]	NM_001302
A_23_P144476	0.000764	NM_199327	NM_199327	Homo sapiens sprouty homolog 1, antagonist of FGF signaling (Drosophila) (SPRY1), transcript variant 2, mRNA [NM_199327]	NM_199327
A_23_P409516	0.000765	NM_138333	NM_138333	Homo sapiens chromosome 9 open reading frame 42 (C9orf42), mRNA [NM_138333]	NM_138333
A_23_P70318	0.000767	NM_014936	NM_014936	Homo sapiens ectonucleotide pyrophosphatase/phosphodiesterase 4 (putative function) (ENPP4), mRNA [NM_014936]	NM_014936
A_24_P273561	0.000768	NM_018169	NM_018169	Homo sapiens hypothetical protein FLJ10652 (FLJ10652), mRNA [NM_018169]	NM_018169
A_23_P111126	0.000769	L06175	L06175	Homo Sapiens P5-1 mRNA, complete cds. [L06175]	
A_32_P153773	0.000769	AB209467	AB209467	Homo sapiens mRNA for calcium channel, voltage-dependent, L type, alpha 1B subunit variant protein. [AB209467]	
A_23_P206424	0.00077	NM_025134	NM_025134	Homo sapiens chromodomain helicase DNA binding protein 9 (CHD9), mRNA [NM_025134]	NM_025134
A_24_P636834	0.00077	A_24_P636834			
A_32_P46191	0.000771	ENST00000356194		PREDICTED: Homo sapiens similar to zinc finger protein 91 (HPF7, HTF10) (LOC442695), mRNA [XM_499440]	XM_498210
A_23_P143748	0.000771	NM_015140	NM_015140	Homo sapiens KIAA0153 protein (KIAA0153), mRNA [NM_015140]	NM_015140
A_23_P29994	0.000772	NM_203284	NM_203284	Homo sapiens recombining binding protein suppressor of hairless (Drosophila) (RBPSUH), transcript variant 4, mRNA [NM_203284]	NM_203284
A_32_P163533	0.000773	NM_199005	NM_199005	Homo sapiens zinc finger protein 322B (ZNF322B), mRNA [NM_199005]	NM_199005
A_23_P380724	0.000773	NM_021826	NM_021826	Homo sapiens hypothetical protein FLJ13149 (FLJ13149), mRNA [NM_021826]	NM_021826
A_23_P52278	0.000774	NM_004523	NM_004523	Homo sapiens kinesin family member 11 (KIF11), mRNA [NM_004523]	NM_004523
A_23_P321361	0.000774	NM_138614	NM_138614	Homo sapiens DEAH (Asp-Glu-Ala-His) box polypeptide 30 (DHX30), transcript variant 3, mRNA [NM_138614]	NM_138614
A_23_P150053	0.000775	NM_001613	NM_001613	Homo sapiens actin, alpha 2, smooth muscle, aorta (ACTA2), mRNA [NM_001613]	NM_001613
A_24_P913629	0.000776	A_24_P913629			
A_23_P307430	0.000776	NM_152344	NM_152344	Homo sapiens hypothetical protein FLJ30656 (FLJ30656), mRNA [NM_152344]	NM_152344
A_23_P2293	0.00078	NM_033647	NM_033647	Homo sapiens helicase (DNA) B (HELB), mRNA [NM_033647]	NM_033647
A_23_P216476	0.000784	NM_014872	NM_014872	Homo sapiens zinc finger and BTB domain containing 5 (ZBTB5), mRNA [NM_014872]	NM_014872
A_23_P151018	0.000785	NM_014319	NM_014319	Homo sapiens LEM domain containing 3 (LEMD3), mRNA [NM_014319]	NM_014319
A_24_P193257	0.000787	THC2437177			
A_24_P405190	0.000787	NM_201626	NM_201626	Homo sapiens ubiquitin specific protease 33 (USP33), transcript variant 3, mRNA [NM_201626]	NM_201626
A_24_P270999	0.000788	NM_000334	NM_000334	Homo sapiens sodium channel, voltage-gated, type IV, alpha (SCN4A), mRNA [NM_000334]	NM_000334

A_23_P110492	0.000788	NM_005885	NM_005885	Homo sapiens membrane-associated ring finger (C3HC4) 6 (MARCH6), mRNA [NM_005885]	NM_005885
A_32_P138004	0.000791	NM_207009	NM_207009	Homo sapiens family with sequence similarity 45, member A (FAM45A), mRNA [NM_207009]	NM_207009
A_23_P257335	0.000792	NM_006558	NM_006558	Homo sapiens KH domain containing, RNA binding, signal transduction associated 3 (KHDRBS3), mRNA [NM_006558]	NM_006558
A_24_P557019	0.000795	THC2406147		BF476310 naa21a07.x1 NCI_CGAP_Pr28 Homo sapiens cDNA clone IMAGE:3255444 3' similar to contains Alu repetitive element;contains element MIR MIR repetitive element ;, mRNA sequence [BF476310]	
A_24_P941586	0.000796	AB002381	AB002381	Human mRNA for KIAA0383 gene, partial cds. [AB002381]	
A_32_P80597	0.000796	AK026192	AK026192	Homo sapiens cDNA: FLJ22539 fis, clone HRC13227. [AK026192]	
A_23_P502654	0.000796	NM_004169	NM_004169	Homo sapiens serine hydroxymethyltransferase 1 (soluble) (SHMT1), transcript variant 1, mRNA [NM_004169]	NM_004169
A_24_P42316	0.000797	NM_018429	NM_018429	Homo sapiens B double prime 1, subunit of RNA polymerase III transcription initiation factor IIIB (BDP1), mRNA [NM_018429]	NM_018429
A_24_P339858	0.000797	NM_153204	NM_153204	Homo sapiens chromosome 21 open reading frame 90 (C21orf90), mRNA [NM_153204]	NM_153204
A_23_P67771	0.000798	NM_000465	NM_000465	Homo sapiens BRCA1 associated RING domain 1 (BARD1), mRNA [NM_000465]	NM_000465
A_24_P303589	0.000799	NM_003592	NM_003592	Homo sapiens cullin 1 (CUL1), mRNA [NM_003592]	NM_003592
A_23_P256933	0.000799	NM_000986	NM_000986	Homo sapiens ribosomal protein L24 (RPL24), mRNA [NM_000986]	NM_000986
A_32_P167883	0.0008	THC2344420		ALU7_HUMAN (P39194) Alu subfamily SQ sequence contamination warning entry, partial (21%) [THC2344420]	
A_24_P85123	0.0008	AK024440	AK024440	Homo sapiens mRNA for FLJ00030 protein, partial cds. [AK024440]	
A_23_P67151	0.000801	NM_058164	NM_058164	Homo sapiens olfactomedin 2 (OLFM2), mRNA [NM_058164]	NM_058164
A_32_P44775	0.000801	NM_182505	NM_182505	Homo sapiens chromosome 9 open reading frame 85 (C9orf85), transcript variant 1, mRNA [NM_182505]	NM_182505
A_23_P201400	0.000801	NM_000309	NM_000309	Homo sapiens protoporphyrinogen oxidase (PPOX), nuclear gene encoding mitochondrial protein, mRNA [NM_000309]	NM_000309
A_23_P217236	0.000802	NM_005342	NM_005342	Homo sapiens high-mobility group box 3 (HMGB3), mRNA [NM_005342]	NM_005342
A_24_P323815	0.000803	NM_015057	NM_015057	Homo sapiens MYC binding protein 2 (MYCBP2), mRNA [NM_015057]	NM_015057
A_23_P59950	0.000804	NM_015359	NM_015359	Homo sapiens solute carrier family 39 (zinc transporter), member 14 (SLC39A14), mRNA [NM_015359]	NM_015359
A_23_P93750	0.000806	NM_012322	NM_012322	Homo sapiens LSM5 homolog, U6 small nuclear RNA associated (S. cerevisiae) (LSM5), mRNA [NM_012322]	NM_012322
A_32_P31182	0.000808	NM_000971	NM_000971	Homo sapiens ribosomal protein L7 (RPL7), mRNA [NM_000971]	NM_000971
A_23_P133107	0.000809	NM_032863	NM_032863	Homo sapiens Fraser syndrome 1 (FRAS1), transcript variant 4, mRNA [NM_032863]	NM_032863
A_24_P826348	0.000809	AB111887	AB111887	Homo sapiens mRNA for KIAA2035 protein. [AB111887]	
A_24_P261052	0.000809	NM_015458	NM_015458	Homo sapiens myotubularin related protein 9 (MTMR9), mRNA [NM_015458]	NM_015458
A_32_P204691	0.000812	BC038747	BC038747	Homo sapiens, clone IMAGE:5268658, mRNA. [BC038747]	
A_32_P46510	0.000812	AK131416	AK131416	Homo sapiens cDNA FLJ16526 fis, clone OCBBF2006987. [AK131416]	
A_23_P60565	0.000814	NM_005649	NM_005649	Homo sapiens zinc finger protein 354A (ZNF354A), mRNA [NM_005649]	NM_005649
A_32_P111019	0.000815	THC2443723		BC002736 kelch repeat and BTB (POZ) domain containing 4 {Homo sapiens;} , partial (14%) [THC2443723]	
A_24_P679409	0.000815	NM_005839	NM_005839	Homo sapiens serine/arginine repetitive matrix 1 (SRRM1), mRNA [NM_005839]	NM_005839
A_24_P126417	0.000815	NM_014709	NM_014709	Homo sapiens ubiquitin specific protease 34 (USP34), mRNA [NM_014709]	NM_014709
A_32_P173058	0.000816	NM_015012	NM_015012	Homo sapiens transmembrane protein 41B (TMEM41B), mRNA [NM_015012]	NM_015012
A_32_P183718	0.000817	NM_001003845	NM_001003845	Homo sapiens Sp5 transcription factor (SP5), mRNA [NM_001003845]	NM_001003845
A_32_P28685	0.000817	NM_003090	NM_003090	Homo sapiens small nuclear ribonucleoprotein polypeptide A' (SNRPA1), mRNA [NM_003090]	NM_003090
A_23_P255286	0.000819	NM_017951	NM_017951	Homo sapiens hypothetical protein FLJ20297 (FLJ20297), transcript variant 2, mRNA [NM_017951]	NM_017951
A_24_P158946	0.00082	NM_139241	NM_139241	Homo sapiens FYVE, RhoGEF and PH domain containing 4 (FGD4), mRNA [NM_139241]	NM_139241
A_23_P206347	0.00082	NM_016025	NM_016025	Homo sapiens DORA reverse strand protein 1 (DREV1), mRNA [NM_016025]	NM_016025
A_24_P650011	0.000821	A_24_P650011			
A_24_P77676	0.000821	NM_004134	NM_004134	Homo sapiens heat shock 70kDa protein 9B (mortalin-2) (HSPA9B), nuclear gene encoding mitochondrial protein, mRNA [NM_004134]	NM_004134
A_23_P360209	0.000822	ENST00000361227		Homo sapiens migration-inducing gene 18 protein mRNA, complete cds. [AY423734]	
A_23_P140928	0.000823	NM_024847	NM_024847	Homo sapiens transmembrane channel-like 7 (TMC7), mRNA [NM_024847]	NM_024847
A_23_P406702	0.000823	NM_203459	NM_203459	Homo sapiens calmodulin regulated spectrin-associated protein 1-like 1 (CAMSAP1L1), mRNA [NM_203459]	NM_203459
A_23_P142950	0.000825	NM_015575	NM_015575	Homo sapiens trinucleotide repeat containing 15 (TNRC15), mRNA [NM_015575]	NM_015575
A_23_P162127	0.000825	NM_021825	NM_021825	Homo sapiens hypothetical protein MDS025 (MDS025), mRNA [NM_021825]	NM_021825

A_23_P111517	0.000828	NM_022479	NM_022479	Homo sapiens Williams-Beuren syndrome chromosome region 17 (WBSCR17), mRNA [NM_022479]	NM_022479
A_23_P112026	0.000829	NM_002164	NM_002164	Homo sapiens indoleamine-pyrrole 2,3 dioxygenase (INDO), mRNA [NM_002164]	NM_002164
A_23_P135104	0.00083	NM_016034	NM_016034	Homo sapiens mitochondrial ribosomal protein S2 (MRPS2), nuclear gene encoding mitochondrial protein, mRNA [NM_016034]	NM_016034
A_24_P393449	0.000831	NM_004938	NM_004938	Homo sapiens death-associated protein kinase 1 (DAPK1), mRNA [NM_004938]	NM_004938
A_23_P26895	0.000831	NM_016261	NM_016261	Homo sapiens tubulin, delta 1 (TUBD1), mRNA [NM_016261]	NM_016261
A_24_P380330	0.000832	NM_024594	NM_024594	Homo sapiens pantothenate kinase 3 (PANK3), mRNA [NM_024594]	NM_024594
A_23_P131202	0.000832	NM_018645	NM_018645	Homo sapiens hairy and enhancer of split 6 (Drosophila) (HES6), mRNA [NM_018645]	NM_018645
A_24_P942253	0.000836	NM_015107	NM_015107	Homo sapiens PHD finger protein 8 (PHF8), mRNA [NM_015107]	NM_015107
A_24_P149395	0.000837	BX537624	BX537624	Homo sapiens mRNA; cDNA DKFZp686P02188 (from clone DKFZp686P02188). [BX537624]	
A_23_P120170	0.000838	NM_145702	NM_145702	Homo sapiens tigger transposable element derived 1 (TIGD1), mRNA [NM_145702]	NM_145702
A_23_P135787	0.000841	BX537520	BX537520	Homo sapiens mRNA; cDNA DKFZp686F09142 (from clone DKFZp686F09142). [BX537520]	
A_23_P258002	0.000841	NM_017632	NM_017632	Homo sapiens collaborates/cooperates with ARF (alternate reading frame) protein (CARF), mRNA [NM_017632]	NM_017632
A_23_P169978	0.000844	NM_020747	NM_020747	Homo sapiens zinc finger protein 608 (ZNF608), mRNA [NM_020747]	NM_020747
A_24_P222844	0.000845	NM_018061	NM_018061	Homo sapiens PRP38 pre-mRNA processing factor 38 (yeast) domain containing B (PRPF38B), mRNA [NM_018061]	NM_018061
A_23_P105732	0.000849	A_23_P105732			
A_24_P63969	0.00085	NM_016200	NM_016200	Homo sapiens LSM8 homolog, U6 small nuclear RNA associated (S. cerevisiae) (LSM8), mRNA [NM_016200]	NM_016200
A_23_P202602	0.000852	NM_007190	NM_007190	Homo sapiens SEC23 interacting protein (SEC23IP), mRNA [NM_007190]	NM_007190
A_24_P323545	0.000853	NM_024729	NM_024729	Homo sapiens myosin, heavy polypeptide 14 (MYH14), mRNA [NM_024729]	NM_024729
A_23_P217120	0.000853	NM_024757	NM_024757	Homo sapiens euchromatic histone-lysine N-methyltransferase 1 (EHMT1), mRNA [NM_024757]	NM_024757
A_24_P118862	0.000854	NM_178549	NM_178549	Homo sapiens zinc finger protein 678 (ZNF678), mRNA [NM_178549]	NM_178549
A_24_P114255	0.000854	NM_138799	NM_138799	Homo sapiens O-acyltransferase (membrane bound) domain containing 2 (OACT2), mRNA [NM_138799]	NM_138799
A_24_P107859	0.000856	NM_152594	NM_152594	Homo sapiens sprouty-related, EVH1 domain containing 1 (SPRED1), mRNA [NM_152594]	NM_152594
A_23_P91346	0.000856	BC008667	BC008667	Homo sapiens cDNA clone MGC:17708 IMAGE:3868595, complete cds. [BC008667]	
A_23_P21673	0.000856	NM_017794	NM_017794	Homo sapiens KIAA1797 (KIAA1797), mRNA [NM_017794]	NM_017794
A_24_P675731	0.000857	THC2316878			
A_23_P302568	0.000857	NM_003459	NM_003459	Homo sapiens solute carrier family 30 (zinc transporter), member 3 (SLC30A3), mRNA [NM_003459]	NM_003459
A_24_P418294	0.000861	NM_024548	NM_024548	Homo sapiens leucine-rich repeats and IQ motif containing 2 (LRRIQ2), mRNA [NM_024548]	NM_024548
A_32_P152673	0.000861	NM_002568	NM_002568	Homo sapiens poly(A) binding protein, cytoplasmic 1 (PABPC1), mRNA [NM_002568]	NM_002568
A_32_P132438	0.000864	NM_139275	NM_139275	Homo sapiens A kinase (PRKA) anchor protein 1 (AKAP1), nuclear gene encoding mitochondrial protein, transcript variant 2, mRNA [NM_139275]	NM_139275
A_23_P359655	0.000865	NM_152437	NM_152437	Homo sapiens zinc finger protein 664 (ZNF664), mRNA [NM_152437]	NM_152437
A_23_P413923	0.000866	NM_022160	NM_022160	Homo sapiens DMRT-like family A1 (DMRTA1), mRNA [NM_022160]	NM_022160
A_23_P126197	0.000867	NM_005626	NM_005626	Homo sapiens splicing factor, arginine/serine-rich 4 (SFRS4), mRNA [NM_005626]	NM_005626
A_23_P59507	0.000868	NM_020979	NM_020979	Homo sapiens adaptor protein with pleckstrin homology and src homology 2 domains (APS), mRNA [NM_020979]	NM_020979
A_24_P185484	0.000869	NM_014910	NM_014910	Homo sapiens zinc finger protein 507 (ZNF507), mRNA [NM_014910]	NM_014910
A_24_P601972	0.000869	ENST00000344142		Homo sapiens cDNA clone IMAGE:3950925, partial cds. [BC014578]	XM_291016
A_24_P288149	0.00087	THC2339776			
A_23_P83498	0.000874	NM_006546	NM_006546	Homo sapiens IGF-II mRNA-binding protein 1 (IMP-1), mRNA [NM_006546]	NM_006546
A_23_P57521	0.000874	NM_016091	NM_016091	Homo sapiens eukaryotic translation initiation factor 3, subunit 6 interacting protein (EIF3S6IP), mRNA [NM_016091]	NM_016091
A_24_P311856	0.000875	NM_001851	NM_001851	Homo sapiens collagen, type IX, alpha 1 (COL9A1), transcript variant 1, mRNA [NM_001851]	NM_001851
A_32_P117127	0.000877	ENST00000330336		Homo sapiens ODZ3 (ODZ3) mRNA, partial cds. [AF195420]	XM_497983
A_24_P71938	0.000879	NM_005900	NM_005900	Homo sapiens SMAD, mothers against DPP homolog 1 (Drosophila) (SMAD1), transcript variant 1, mRNA [NM_005900]	NM_005900
A_24_P278299	0.00088	NM_024701	NM_024701	Homo sapiens ankyrin repeat and SOCS box-containing 13 (ASB13), mRNA [NM_024701]	NM_024701
A_23_P351232	0.000885	NM_173680	NM_173680	Homo sapiens hypothetical protein MGC33584 (MGC33584), mRNA [NM_173680]	NM_173680
A_24_P102769	0.000885	NM_006004	NM_006004	Homo sapiens ubiquinol-cytochrome c reductase hinge protein (UQCRH), mRNA [NM_006004]	NM_006004

A_24_P250333	0.000886	NM_004596	NM_004596	Homo sapiens small nuclear ribonucleoprotein polypeptide A (SNRPA), mRNA [NM_004596]	NM_004596
A_24_P102920	0.000888	AK002156	AK002156	Homo sapiens cDNA FLJ11294 fis, clone PLACE1009708. [AK002156]	
A_32_P125558	0.000889	THC2397609		AA411302 zv24g06.r1 Soares_NhHMPu_S1 Homo sapiens cDNA clone IMAGE:754618 5', mRNA sequence [AA411302]	
A_23_P68040	0.000889	NM_006196	NM_006196	Homo sapiens poly(rC) binding protein 1 (PCBP1), mRNA [NM_006196]	NM_006196
A_23_P45699	0.00089	NM_003902	NM_003902	Homo sapiens far upstream element (FUSE) binding protein 1 (FUBP1), mRNA [NM_003902]	NM_003902
A_24_P312417	0.000892	AB046792	AB046792	Homo sapiens mRNA for KIAA1572 protein, partial cds. [AB046792]	
A_23_P201445	0.000892	NM_016076	NM_016076	Homo sapiens chromosome 1 open reading frame 121 (C1orf121), mRNA [NM_016076]	NM_016076
A_23_P163099	0.000893	NM_002692	NM_002692	Homo sapiens polymerase (DNA directed), epsilon 2 (p59 subunit) (POLE2), mRNA [NM_002692]	NM_002692
A_24_P351451	0.000893	NM_130837	NM_130837	Homo sapiens optic atrophy 1 (autosomal dominant) (OPA1), nuclear gene encoding mitochondrial protein, transcript variant 8, mRNA [NM_130837]	NM_130837
A_23_P88703	0.000896	NM_005657	NM_005657	Homo sapiens tumor protein p53 binding protein, 1 (TP53BP1), mRNA [NM_005657]	NM_005657
A_23_P150919	0.000899	ENST00000330079		Homo sapiens cDNA FLJ14466 fis, clone MAMMA1000416. [AK027372]	
A_23_P97810	0.000901	NM_003631	NM_003631	Homo sapiens poly (ADP-ribose) glycohydrolase (PARG), mRNA [NM_003631]	NM_003631
A_23_P109950	0.000902	NM_001106	NM_001106	Homo sapiens activin A receptor, type IIB (ACVR2B), mRNA [NM_001106]	NM_001106
A_23_P142918	0.000905	NM_153689	NM_153689	Homo sapiens hypothetical protein FLJ38973 (FLJ38973), mRNA [NM_153689]	NM_153689
A_23_P37275	0.000906	NM_006568	NM_006568	Homo sapiens cell growth regulator with ring finger domain 1 (CGRRF1), mRNA [NM_006568]	NM_006568
A_23_P161595	0.000906	NM_017490	NM_017490	Homo sapiens MAP/microtubule affinity-regulating kinase 2 (MARK2), transcript variant 1, mRNA [NM_017490]	NM_017490
A_23_P201816	0.000909	NM_014810	NM_014810	Homo sapiens centrosome-associated protein 350 (CAP350), mRNA [NM_014810]	NM_014810
A_32_P40456	0.000911	BU739640	BU739640	UI-E-EJ0-aho-p-08-0-UI.s1 UI-E-EJ0 Homo sapiens cDNA clone UI-E-EJ0-aho-p-08-0-UI 3', mRNA sequence [BU739640]	
A_23_P130470	0.000911	NM_021089	NM_021089	Homo sapiens zinc finger protein 8 (clone HF.18) (ZNF8), mRNA [NM_021089]	NM_021089
A_23_P252075	0.000911	NM_015328	NM_015328	Homo sapiens KIAA0828 protein (KIAA0828), mRNA [NM_015328]	NM_015328
A_32_P179686	0.000911	BX641027	BX641027	Homo sapiens mRNA; cDNA DKFZp686O10247 (from clone DKFZp686O10247). [BX641027]	
A_32_P144908	0.000911	NM_203282	NM_203282	Homo sapiens zinc finger protein 539 (ZNF539), mRNA [NM_203282]	NM_203282
A_23_P128587	0.000914	NM_005870	NM_005870	Homo sapiens sin3-associated polypeptide, 18kDa (SAP18), mRNA [NM_005870]	NM_005870
A_24_P529786	0.000917	AK091744	AK091744	Homo sapiens cDNA FLJ34425 fis, clone HHDPC2008297. [AK091744]	
A_24_P936282	0.000918	AA436686	AA436686	AA436686 zv59a12.s1 Soares_testis_NHT Homo sapiens cDNA clone IMAGE:757918 3' similar to contains Alu repetitive element;, mRNA sequence [AA436686]	
A_23_P259707	0.000922	NM_207299	NM_207299	Homo sapiens plasticity related gene 3 (PRG-3), transcript variant 1, mRNA [NM_207299]	NM_207299
A_23_P26336	0.000924	NM_014153	NM_014153	Homo sapiens zinc finger CCCH-type containing 7A (ZC3H7A), mRNA [NM_014153]	NM_014153
A_23_P111919	0.000926	NM_013357	NM_013357	Homo sapiens purine-rich element binding protein G (PURG), transcript variant A, mRNA [NM_013357]	NM_013357
A_32_P187599	0.000926	NM_015640	NM_015640	Homo sapiens PAI-1 mRNA binding protein (PAI-RBP1), transcript variant 4, mRNA [NM_015640]	NM_015640
A_24_P298320	0.000926	A_24_P298320			
A_23_P349463	0.000927	NM_022097	NM_022097	Homo sapiens hepatocellular carcinoma antigen gene 520 (LOC63928), mRNA [NM_022097]	NM_022097
A_23_P127926	0.000927	NM_003390	NM_003390	Homo sapiens WEE1 homolog (S. pombe) (WEE1), mRNA [NM_003390]	NM_003390
A_24_P86868	0.000927	NM_212554	NM_212554	Homo sapiens similar to CG9643-PA (LOC399818), mRNA [NM_212554]	NM_212554
A_24_P261383	0.000927	NM_024116	NM_024116	Homo sapiens hypothetical protein MGC5306 (MGC5306), mRNA [NM_024116]	NM_024116
A_24_P260325	0.000929	NM_004744	NM_004744	Homo sapiens lecithin retinol acyltransferase (phosphatidylcholine--retinol O-acyltransferase) (LRAT), mRNA [NM_004744]	NM_004744
A_24_P645765	0.00093	NM_020782	NM_020782	Homo sapiens kelch domain containing 5 (KLHDC5), mRNA [NM_020782]	NM_020782
A_23_P86956	0.000932	NM_007037	NM_007037	Homo sapiens a disintegrin-like and metalloprotease (reprolysin type) with thrombospondin type 1 motif, 8 (ADAMTS8), mRNA [NM_007037]	NM_007037
A_32_P159347	0.000932	S63912	S63912	D10S102=FBRNP [human, fetal brain, mRNA, 3043 nt]. [S63912]	XM_370728
A_32_P90210	0.000934	NM_001015892	NM_001015892	Homo sapiens TAF9 RNA polymerase II, TATA box binding protein (TBP)-associated factor, 32kDa (TAF9), transcript variant 4, mRNA [NM_001015892]	NM_001015892
A_23_P16006	0.000936	NM_198457	NM_198457	Homo sapiens zinc finger protein 600 (ZNF600), mRNA [NM_198457]	NM_198457
A_24_P296254	0.000937	NM_014783	NM_014783	Homo sapiens Rho GTPase activating protein 11A (ARHGAP11A), mRNA [NM_014783]	NM_014783
A_32_P104617	0.000937	AK055960	AK055960	Homo sapiens cDNA FLJ31398 fis, clone NT2NE1000175. [AK055960]	
A_24_P686992	0.000938	NM_198555	NM_198555	Homo sapiens ankyrin repeat domain 36 (ANKRD36), mRNA [NM_198555]	NM_198555
A_23_P151856	0.000939	NM_016396	NM_016396	Homo sapiens hypothetical protein HSPC129 (HSPC129), mRNA [NM_016396]	NM_016396

A_23_P15466	0.00094	NM_004589	NM_004589	Homo sapiens SCO cytochrome oxidase deficient homolog 1 (yeast) (SCO1), nuclear gene encoding mitochondrial protein, mRNA [NM_004589]	NM_004589
A_23_P157416	0.000941	NM_032164	NM_032164	Homo sapiens zinc finger protein 394 (ZNF394), mRNA [NM_032164]	NM_032164
A_24_P36285	0.000944	NM_013398	NM_013398	Homo sapiens zinc finger protein 224 (ZNF224), mRNA [NM_013398]	NM_013398
A_23_P253932	0.000944	NM_018147	NM_018147	Homo sapiens Fas apoptotic inhibitory molecule (FAIM), mRNA [NM_018147]	NM_018147
A_23_P23141	0.000944	NM_002107	NM_002107	Homo sapiens H3 histone, family 3A (H3F3A), mRNA [NM_002107]	NM_002107
A_24_P58122	0.000945	BC029660	BC029660	Homo sapiens ubiquitin specific protease 24, mRNA (cDNA clone MGC:29848 IMAGE:4995223), complete cds. [BC029660]	XM_371254
A_24_P341089	0.000946	ENST00000321577		Homo sapiens, clone IMAGE:5167446, mRNA. [BC031698]	
A_23_P62684	0.000947	AF217973	AF217973	Homo sapiens clone PP1345 unknown mRNA. [AF217973]	
A_24_P636390	0.000951	AF009267	AF009267	Homo sapiens clone FBA1 Cri-du-chat region mRNA. [AF009267]	
A_23_P70746	0.000955	NM_017651	NM_017651	Homo sapiens Abelson helper integration site (AHI1), mRNA [NM_017651]	NM_017651
A_23_P424561	0.000955	NM_133639	NM_133639	Homo sapiens ras homolog gene family, member V (RHOV), mRNA [NM_133639]	NM_133639
A_23_P315131	0.000955	AK024388	AK024388	Homo sapiens cDNA FLJ14326 fis, clone PLACE4000247. [AK024388]	
A_24_P201381	0.000955	NM_152429	NM_152429	Homo sapiens chromosome 10 open reading frame 13 (C10orf13), mRNA [NM_152429]	NM_152429
A_23_P154643	0.000958	AK094784	AK094784	Homo sapiens cDNA FLJ37465 fis, clone BRAWH2011823, highly similar to BONE MORPHOGENETIC PROTEIN 7 PRECURSOR. [AK094784]	
A_24_P32790	0.000959	NM_018566	NM_018566	Homo sapiens YOD1 OTU deubiquinating enzyme 1 homolog (yeast) (YOD1), mRNA [NM_018566]	NM_018566
A_23_P121222	0.000961	NM_020165	NM_020165	Homo sapiens RAD18 homolog (S. cerevisiae) (RAD18), mRNA [NM_020165]	NM_020165
A_23_P87591	0.000961	NM_006530	NM_006530	Homo sapiens YEATS domain containing 4 (YEATS4), mRNA [NM_006530]	NM_006530
A_23_P157435	0.000965	NM_015878	NM_015878	Homo sapiens antizyme inhibitor 1 (AZIN1), transcript variant 1, mRNA [NM_015878]	NM_015878
A_24_P924185	0.000966	S81524	S81524	RC1=NADH dehydrogenase subunit 3 homolog/ND3 homolog [human, renal cell carcinoma, mRNA Mitochondrial Partial, 65 nt]. [S81524]	
A_24_P399606	0.000967	NM_000435	NM_000435	Homo sapiens Notch homolog 3 (Drosophila) (NOTCH3), mRNA [NM_000435]	NM_000435
A_32_P221256	0.000967	NM_203481	NM_203481	Homo sapiens hypothetical LOC403340 (MGC70870), mRNA [NM_203481]	NM_203481
A_23_P103334	0.000968	AB095945	AB095945	Homo sapiens mRNA for KIAA2025 protein. [AB095945]	XM_086409
A_32_P4943	0.000968	NM_021927	NM_021927	Homo sapiens hypothetical protein FLJ13220 (FLJ13220), mRNA [NM_021927]	NM_021927
A_24_P210577	0.000972	NM_024755	NM_024755	Homo sapiens modulator of estrogen induced transcription (FLJ13213), transcript variant 1, mRNA [NM_024755]	NM_024755
A_32_P26969	0.000975	NM_207332	NM_207332	Homo sapiens hypothetical protein LOC157697 (LOC157697), mRNA [NM_207332]	NM_207332
A_32_P35800	0.000977	CR749441	CR749441	Homo sapiens mRNA; cDNA DKFZp781J2344 (from clone DKFZp781J2344). [CR749441]	XM_029101
A_23_P8653	0.000981	NM_024788	NM_024788	Homo sapiens hypothetical protein FLJ21062 (FLJ21062), mRNA [NM_024788]	NM_024788
A_24_P532212	0.000981	AK026896	AK026896	Homo sapiens cDNA: FLJ23243 fis, clone COL01757. [AK026896]	
A_24_P913828	0.000986	M55405	M55405	Homo sapiens mucin (MUC-3) mRNA, partial cds. [M55405]	
A_32_P74712	0.000988	NM_021627	NM_021627	Homo sapiens SUMO1/sentrin/SMT3 specific protease 2 (SENP2), mRNA [NM_021627]	NM_021627
A_23_P103414	0.000989	NM_016258	NM_016258	Homo sapiens YTH domain family, member 2 (YTHDF2), mRNA [NM_016258]	NM_016258
A_23_P218841	0.000993	NM_018297	NM_018297	Homo sapiens N-glycanase 1 (NGLY1), mRNA [NM_018297]	NM_018297
A_23_P50320	0.000994	A_23_P50320			
A_24_P55719	0.000998	NM_133640	NM_133640	Homo sapiens surfeit 5 (SURF5), transcript variant b, mRNA [NM_133640]	NM_133640

Supplemental Table S3C
Kmeans Cluster 3 - 2,965 Genes

Gene Name	*P* -value	Common	Genbank	Description	RefSeq
A_23_P52336	9.89E-08	NM_170744	NM_170744	Homo sapiens unc-5 homolog B (C. elegans) (UNC5B), mRNA [NM_170744]	NM_170744
A_24_P128001	1.44E-07	NM_018660	NM_018660	Homo sapiens zinc finger protein 395 (ZNF395), mRNA [NM_018660]	NM_018660
A_23_P300033	1.52E-07	NM_006206	NM_006206	Homo sapiens platelet-derived growth factor receptor, alpha polypeptide (PDGFRA), mRNA [NM_006206]	NM_006206
A_23_P151710	1.60E-07	NM_000956	NM_000956	Homo sapiens prostaglandin E receptor 2 (subtype EP2), 53kDa (PTGER2), mRNA [NM_000956]	NM_000956
A_23_P212968	1.60E-07	NM_001073	NM_001073	Homo sapiens UDP glucuronosyltransferase 2 family, polypeptide B11 (UGT2B11), mRNA [NM_001073]	NM_001073
A_24_P227230	1.60E-07	NM_014333	NM_014333	Homo sapiens immunoglobulin superfamily, member 4 (IGSF4), mRNA [NM_014333]	NM_014333
A_32_P122373	1.60E-07	ENST00000334429		Homo sapiens cDNA FLJ43241 fis, clone HEART1000010, weakly similar to Hepatocyte growth factor-like protein precursor. [AK125231]	
A_23_P347070	1.60E-07	NM_018440	NM_018440	Homo sapiens phosphoprotein associated with glycosphingolipid microdomains 1 (PAG1), mRNA [NM_018440]	NM_018440
A_32_P121549	1.60E-07	BC034319	BC034319	Homo sapiens, clone IMAGE:4837650, mRNA, partial cds. [BC034319]	
A_23_P131614	1.60E-07	NM_004369	NM_004369	Homo sapiens collagen, type VI, alpha 3 (COL6A3), transcript variant 1, mRNA [NM_004369]	NM_004369
A_23_P38167	1.60E-07	NM_022036	NM_022036	Homo sapiens G protein-coupled receptor, family C, group 5, member C (GPRC5C), transcript variant 1, mRNA [NM_022036]	NM_022036
A_23_P44724	1.60E-07	NM_001321	NM_001321	Homo sapiens cysteine and glycine-rich protein 2 (CSRP2), mRNA [NM_001321]	NM_001321
A_24_P802145	1.65E-07	THC2339772			
A_23_P154919	1.65E-07	BC005107	BC005107	Homo sapiens chromosome 21 open reading frame 105, mRNA (cDNA clone IMAGE:3840937), partial cds. [BC005107]	
A_23_P140207	1.65E-07	NM_004563	NM_004563	Homo sapiens phosphoenolpyruvate carboxykinase 2 (mitochondrial) (PCK2), nuclear gene encoding mitochondrial protein, transcript variant 1, mRNA [NM_004563]	NM_004563
A_23_P571	1.65E-07	NM_006516	NM_006516	Homo sapiens solute carrier family 2 (facilitated glucose transporter), member 1 (SLC2A1), mRNA [NM_006516]	NM_006516
A_24_P403561	1.71E-07	NM_002334	NM_002334	Homo sapiens low density lipoprotein receptor-related protein 4 (LRP4), mRNA [NM_002334]	NM_002334
A_23_P67847	1.71E-07	NM_024572	NM_024572	Homo sapiens UDP-N-acetyl-alpha-D-galactosamine:polypeptide N-acetylgalactosaminyltransferase 14 (GalNAc-T14) (GALNT14), mRNA [NM_024572]	NM_024572
A_32_P157945	1.97E-07	NM_004415	NM_004415	Homo sapiens desmoplakin (DSP), transcript variant 1, mRNA [NM_004415]	NM_004415
A_23_P146077	2.04E-07	NM_018660	NM_018660	Homo sapiens zinc finger protein 395 (ZNF395), mRNA [NM_018660]	NM_018660
A_23_P209360	2.04E-07	ENST00000288548		Homo sapiens mRNA for KIAA1921 protein, partial cds. [AB067508]	
A_23_P339119	2.11E-07	NM_024560	NM_024560	Homo sapiens FLJ21963 protein (FLJ21963), mRNA [NM_024560]	NM_024560
A_23_P411296	2.11E-07	NM_005194	NM_005194	Homo sapiens CCAAT/enhancer binding protein (C/EBP), beta (CEBPB), mRNA [NM_005194]	NM_005194
A_23_P145529	2.18E-07	NM_181795	NM_181795	Homo sapiens protein kinase (cAMP-dependent, catalytic) inhibitor beta (PKIB), transcript variant 1, mRNA [NM_181795]	NM_181795
A_23_P154806	2.18E-07	NM_012156	NM_012156	Homo sapiens erythrocyte membrane protein band 4.1-like 1 (EPB41L1), transcript variant 1, mRNA [NM_012156]	NM_012156
A_23_P68851	2.19E-07	AK172861	AK172861	Homo sapiens cDNA FLJ24091 fis, clone COL10314. [AK172861]	
A_32_P113784	2.19E-07	BM680083	BM680083	BM680083 UI-E-EO1-aix-j-14-0-UI.s1 UI-E-EO1 Homo sapiens cDNA clone UI-E-EO1-aix-j-14-0-UI 3', mRNA sequence [BM680083]	
A_23_P99661	2.19E-07	NM_018071	NM_018071	Homo sapiens hypothetical protein FLJ10357 (FLJ10357), mRNA [NM_018071]	NM_018071
A_32_P24832	2.20E-07	NM_020190	NM_020190	Homo sapiens olfactomedin-like 3 (OLFML3), mRNA [NM_020190]	NM_020190
A_24_P129632	2.20E-07	NM_004747	NM_004747	Homo sapiens discs, large homolog 5 (Drosophila) (DLG5), mRNA [NM_004747]	NM_004747
A_32_P196263	2.25E-07	NM_182920	NM_182920	Homo sapiens a disintegrin-like and metalloprotease (reprolysin type) with thrombospondin type 1 motif, 9 (ADAMTS9), mRNA [NM_182920]	NM_182920
A_23_P202327	2.29E-07	NM_003474	NM_003474	Homo sapiens a disintegrin and metalloproteinase domain 12 (meltrin alpha) (ADAM12), transcript variant 1, mRNA [NM_003474]	NM_003474
A_23_P14774	2.29E-07	NM_148979	NM_148979	Homo sapiens cathepsin H (CTSH), transcript variant 2, mRNA [NM_148979]	NM_148979
A_23_P211631	2.32E-07	NM_006486	NM_006486	Homo sapiens fibulin 1 (FBLN1), transcript variant D, mRNA [NM_006486]	NM_006486
A_24_P236251	2.48E-07	NM_003836	NM_003836	Homo sapiens delta-like 1 homolog (Drosophila) (DLK1), mRNA [NM_003836]	NM_003836
A_23_P111860	2.70E-07	NM_018059	NM_018059	Homo sapiens hypothetical protein FLJ10324 (FLJ10324), mRNA [NM_018059]	NM_018059
A_23_P169039	2.70E-07	NM_003068	NM_003068	Homo sapiens snail homolog 2 (Drosophila) (SNAI2), mRNA [NM_003068]	NM_003068
A_23_P114883	2.70E-07	NM_002023	NM_002023	Homo sapiens fibromodulin (FMOD), mRNA [NM_002023]	NM_002023
A_23_P152876	2.70E-07	NM_031934	NM_031934	Homo sapiens RAB34, member RAS oncogene family (RAB34), mRNA [NM_031934]	NM_031934
A_32_P40288	2.77E-07	NM_052913	NM_052913	Homo sapiens KIAA1913 (KIAA1913), mRNA [NM_052913]	NM_052913
A_23_P20443	2.84E-07	NM_021020	NM_021020	Homo sapiens leucine zipper, putative tumor suppressor 1 (LZTS1), mRNA [NM_021020]	NM_021020

A_23_P75299	2.84E-07	NM_022126	NM_022126	Homo sapiens phospholysine phosphohistidine inorganic pyrophosphate phosphatase (LHPP), mRNA [NM_022126]	NM_022126
A_23_P52207	2.84E-07	NM_012342	NM_012342	Homo sapiens BMP and activin membrane-bound inhibitor homolog (Xenopus laevis) (BAMBI), mRNA [NM_012342]	NM_012342
A_24_P277934	3.02E-07	NM_000089	NM_000089	Homo sapiens collagen, type I, alpha 2 (COL1A2), mRNA [NM_000089]	NM_000089
A_23_P37205	3.10E-07	NM_201535	NM_201535	Homo sapiens NDRG family member 2 (NDRG2), transcript variant 1, mRNA [NM_201535]	NM_201535
A_23_P99996	3.16E-07	NM_019066	NM_019066	Homo sapiens MAGE-like 2 (MAGEL2), mRNA [NM_019066]	NM_019066
A_23_P98455	3.24E-07	NM_014622	NM_014622	Homo sapiens loss of heterozygosity, 11, chromosomal region 2, gene A (LOH11CR2A), transcript variant 1, mRNA [NM_014622]	NM_014622
A_32_P108544	3.24E-07	NM_001024457	NM_001024457	Homo sapiens Ran binding protein 2-like 2 (RANBP2L2), mRNA [NM_001024457]	NM_001024457
A_32_P123743	3.24E-07	THC2376725			
A_32_P77742	3.29E-07	THC2338229			
A_23_P86089	3.32E-07	NM_022834	NM_022834	Homo sapiens von Willebrand factor A domain containing 1 (VWA1), transcript variant 1, mRNA [NM_022834]	NM_022834
A_23_P152995	3.46E-07	NM_001045	NM_001045	Homo sapiens solute carrier family 6 (neurotransmitter transporter, serotonin), member 4 (SLC6A4), mRNA [NM_001045]	NM_001045
A_23_P22526	3.46E-07	NM_014799	NM_014799	Homo sapiens hephaestin (HEPH), transcript variant 2, mRNA [NM_014799]	NM_014799
A_23_P214080	3.48E-07	NM_001964	NM_001964	Homo sapiens early growth response 1 (EGR1), mRNA [NM_001964]	NM_001964
A_23_P79562	3.48E-07	NM_001443	NM_001443	Homo sapiens fatty acid binding protein 1, liver (FABP1), mRNA [NM_001443]	NM_001443
A_23_P102391	3.53E-07	NM_014585	NM_014585	Homo sapiens solute carrier family 40 (iron-regulated transporter), member 1 (SLC40A1), mRNA [NM_014585]	NM_014585
A_24_P59667	3.58E-07	BC028068	BC028068	Homo sapiens Janus kinase 3 (a protein tyrosine kinase, leukocyte), mRNA (cDNA clone MGC:39993 IMAGE:5212575), complete cds. [BC028068]	
A_32_P42224	3.72E-07	BX097190	BX097190	BX097190 Soares placenta Nb2HP Homo sapiens cDNA clone IMAGp998G19212, mRNA sequence [BX097190]	
A_23_P217228	3.73E-07	NM_016157	NM_016157	Homo sapiens trophinin (TRO), transcript variant 3, mRNA [NM_016157]	NM_016157
A_23_P150343	3.74E-07	NM_003063	NM_003063	Homo sapiens sarcolipin (SLN), mRNA [NM_003063]	NM_003063
A_23_P63541	3.74E-07	AK001019	AK001019	Homo sapiens cDNA FLJ10157 fis, clone HEMBA1003461. [AK001019]	XM_371354
A_24_P131522	3.74E-07	NM_032208	NM_032208	Homo sapiens anthrax toxin receptor 1 (ANTXR1), transcript variant 1, mRNA [NM_032208]	NM_032208
A_23_P202334	3.74E-07	NM_023028	NM_023028	Homo sapiens fibroblast growth factor receptor 2 (bacteria-expressed kinase, keratinocyte growth factor receptor, craniofacial dysostosis 1, Crouzon syndrome, Pfeiffer syndrome, Jackson-Weiss syndrome) (FGFR2), transcript variant 10, mRNA [NM_023028]	NM_023028
A_32_P105549	3.74E-07	NM_001630	NM_001630	Homo sapiens annexin A8 (ANXA8), mRNA [NM_001630]	NM_001630
A_23_P363778	3.74E-07	NM_001463	NM_001463	Homo sapiens frizzled-related protein (FRZB), mRNA [NM_001463]	NM_001463
A_24_P369232	3.77E-07	NM_031455	NM_031455	Homo sapiens coiled-coil domain containing 3 (CCDC3), mRNA [NM_031455]	NM_031455
A_23_P65518	3.84E-07	NM_016651	NM_016651	Homo sapiens dapper, antagonist of beta-catenin, homolog 1 (Xenopus laevis) (DACT1), mRNA [NM_016651]	NM_016651
A_32_P93045	3.85E-07	AL080082	AL080082	Homo sapiens mRNA; cDNA DKFZp564G1162 (from clone DKFZp564G1162). [AL080082]	
A_32_P34920	3.93E-07	NM_004472	NM_004472	Homo sapiens forkhead box D1 (FOXD1), mRNA [NM_004472]	NM_004472
A_24_P738130	3.94E-07	THC2432280		Q66KD2 (Q66KD2) Lasp1-prov protein, partial (10%) [THC2432280]	
A_24_P610945	3.98E-07	ENST00000311197			
A_23_P125717	4.00E-07	NM_004538	NM_004538	Homo sapiens nucleosome assembly protein 1-like 3 (NAP1L3), mRNA [NM_004538]	NM_004538
A_23_P401718	4.00E-07	NM_207310	NM_207310	Homo sapiens hypothetical protein DKFZp434E2321 (DKFZp434E2321), mRNA [NM_207310]	NM_207310
A_23_P126103	4.00E-07	NM_001902	NM_001902	Homo sapiens cystathionase (cystathionine gamma-lyase) (CTH), transcript variant 1, mRNA [NM_001902]	NM_001902
A_23_P145694	4.02E-07	NM_001673	NM_001673	Homo sapiens asparagine synthetase (ASNS), transcript variant 2, mRNA [NM_001673]	NM_001673
A_23_P86731	4.05E-07	NM_005674	NM_005674	Homo sapiens zinc finger protein 239 (ZNF239), mRNA [NM_005674]	NM_005674
A_24_P23625	4.27E-07	ENST00000245519		Homo sapiens heparan sulfate (glucosamine) 3-O-sulfotransferase 3B1, mRNA (cDNA clone MGC:71688 IMAGE:30343352), complete cds. [BC063301]	
A_24_P334208	4.29E-07	A_24_P334208			
A_23_P22143	4.38E-07	NM_000283	NM_000283	Homo sapiens phosphodiesterase 6B, cGMP-specific, rod, beta (congenital stationary night blindness 3, autosomal dominant) (PDE6B), mRNA [NM_000283]	NM_000283
A_23_P306215	4.39E-07	NM_145175	NM_145175	Homo sapiens family with sequence similarity 84, member A (FAM84A), mRNA [NM_145175]	NM_145175
A_23_P144348	4.45E-07	NM_004787	NM_004787	Homo sapiens slit homolog 2 (Drosophila) (SLIT2), mRNA [NM_004787]	NM_004787
A_24_P62530	4.50E-07	NM_021205	NM_021205	Homo sapiens ras homolog gene family, member U (RHOU), mRNA [NM_021205]	NM_021205
A_23_P395054	4.55E-07	BC008813	BC008813	Homo sapiens annexin A8, mRNA (cDNA clone MGC:10405 IMAGE:3958020), complete cds. [BC008813]	
A_23_P58588	4.58E-07	NM_003062	NM_003062	Homo sapiens slit homolog 3 (Drosophila) (SLIT3), mRNA [NM_003062]	NM_003062

A_23_P142533	4.58E-07	NM_000090	NM_000090	Homo sapiens collagen, type III, alpha 1 (Ehlers-Danlos syndrome type IV, autosomal dominant) (COL3A1), mRNA [NM_000090]	NM_000090
A_23_P82000	4.65E-07	NM_003214	NM_003214	Homo sapiens TEA domain family member 3 (TEAD3), mRNA [NM_003214]	NM_003214
A_23_P391906	4.66E-07	BC044246	BC044246	Homo sapiens KIAA1913, mRNA (cDNA clone MGC:50847 IMAGE:5760073), complete cds. [BC044246]	
A_24_P88763	4.76E-07	NM_032603	NM_032603	Homo sapiens lysyl oxidase-like 3 (LOXL3), mRNA [NM_032603]	NM_032603
A_23_P137097	4.80E-07	NM_006517	NM_006517	Homo sapiens solute carrier family 16 (monocarboxylic acid transporters), member 2 (SLC16A2), mRNA [NM_006517]	NM_006517
A_23_P139260	4.80E-07	NM_183233	NM_183233	Homo sapiens solute carrier family 22 (organic cation transporter), member 18 (SLC22A18), transcript variant 2, mRNA [NM_183233]	NM_183233
A_23_P393645	4.90E-07	NM_139025	NM_139025	Homo sapiens a disintegrin-like and metalloprotease (reprolysin type) with thrombospondin type 1 motif, 13 (ADAMTS13), transcript variant 1, mRNA [NM_139025]	NM_139025
A_23_P372234	4.95E-07	NM_001218	NM_001218	Homo sapiens carbonic anhydrase XII (CA12), transcript variant 1, mRNA [NM_001218]	NM_001218
A_23_P107351	4.95E-07	BC051787	BC051787	Homo sapiens NACHT, leucine rich repeat and PYD (pyrin domain) containing 1, mRNA (cDNA clone MGC:57544 IMAGE:5756099), complete cds. [BC051787]	
A_32_P141768	5.00E-07	NM_001012733	NM_001012733	Homo sapiens 1-acylglycerol-3-phosphate O-acyltransferase 4 (lysophosphatidic acid acyltransferase, delta) (AGPAT4), transcript variant 2, mRNA [NM_001012733]	NM_001012733
A_23_P257834	5.00E-07	NM_000477	NM_000477	Homo sapiens albumin (ALB), mRNA [NM_000477]	NM_000477
A_23_P124084	5.02E-07	NM_005576	NM_005576	Homo sapiens lysyl oxidase-like 1 (LOXL1), mRNA [NM_005576]	NM_005576
A_23_P52697	5.06E-07	NM_020404	NM_020404	Homo sapiens CD248 antigen, endosialin (CD248), mRNA [NM_020404]	NM_020404
A_24_P795230	5.07E-07	AK027541	AK027541	Homo sapiens cDNA FLJ14635 fis, clone NT2RP2001196. [AK027541]	
A_32_P491904	5.07E-07	AK091057	AK091057	Homo sapiens cDNA FLJ33738 fis, clone BRAWH2018527. [AK091057]	
A_23_P158318	5.07E-07	NM_004560	NM_004560	Homo sapiens receptor tyrosine kinase-like orphan receptor 2 (ROR2), mRNA [NM_004560]	NM_004560
A_23_P211522	5.07E-07	NM_145738	NM_145738	Homo sapiens synaptogyrin 1 (SYNGR1), transcript variant 1c, mRNA [NM_145738]	NM_145738
A_23_P253221	5.07E-07	NM_032995	NM_032995	Homo sapiens Rho guanine nucleotide exchange factor (GEF) 4 (ARHGEF4), transcript variant 2, mRNA [NM_032995]	NM_032995
A_23_P163087	5.07E-07	NM_007361	NM_007361	Homo sapiens nidogen 2 (osteonidogen) (NID2), mRNA [NM_007361]	NM_007361
A_24_P109661	5.07E-07	A_24_P109661			
A_23_P107116	5.12E-07	NM_007148	NM_007148	Homo sapiens zinc finger protein 179 (ZNF179), mRNA [NM_007148]	NM_007148
A_23_P138885	5.21E-07	NM_203371	NM_203371	Homo sapiens similar to RIKEN cDNA 1110018M03 (LOC387758), mRNA [NM_203371]	NM_203371
A_23_P201808	5.40E-07	NM_003713	NM_003713	Homo sapiens phosphatidic acid phosphatase type 2B (PPAP2B), transcript variant 1, mRNA [NM_003713]	NM_003713
A_32_P156171	5.66E-07	THC2341051			
A_23_P259071	5.68E-07	NM_001657	NM_001657	Homo sapiens amphiregulin (schwannoma-derived growth factor) (AREG), mRNA [NM_001657]	NM_001657
A_24_P89426	5.70E-07	NM_019101	NM_019101	Homo sapiens apolipoprotein M (APOM), mRNA [NM_019101]	NM_019101
A_23_P320578	5.70E-07	NM_002928	NM_002928	Homo sapiens regulator of G-protein signalling 16 (RGS16), mRNA [NM_002928]	NM_002928
A_23_P216742	5.84E-07	NM_002581	NM_002581	Homo sapiens pregnancy-associated plasma protein A, pappalysin 1 (PAPPA), mRNA [NM_002581]	NM_002581
A_23_P48570	6.01E-07	NM_182908	NM_182908	Homo sapiens dehydrogenase/reductase (SDR family) member 2 (DHRS2), transcript variant 1, mRNA [NM_182908]	NM_182908
A_23_P151805	6.04E-07	NM_006329	NM_006329	Homo sapiens fibulin 5 (FBLN5), mRNA [NM_006329]	NM_006329
A_23_P202448	6.04E-07	NM_199168	NM_199168	Homo sapiens chemokine (C-X-C motif) ligand 12 (stromal cell-derived factor 1) (CXCL12), transcript variant 2, mRNA [NM_199168]	NM_199168
A_32_P183765	6.17E-07	AK126298	AK126298	Homo sapiens cDNA FLJ44318 fis, clone TRACH3000780. [AK126298]	
A_23_P2492	6.18E-07	NM_001734	NM_001734	Homo sapiens complement component 1, s subcomponent (C1S), transcript variant 1, mRNA [NM_001734]	NM_001734
A_24_P108311	6.18E-07	NM_015277	NM_015277	Homo sapiens neural precursor cell expressed, developmentally down-regulated 4-like (NEDD4L), mRNA [NM_015277]	NM_015277
A_23_P91230	6.18E-07	NM_003064	NM_003064	Homo sapiens secretory leukocyte protease inhibitor (antileukoproteinase) (SLPI), mRNA [NM_003064]	NM_003064
A_24_P935491	6.18E-07	NM_000090	NM_000090	Homo sapiens collagen, type III, alpha 1 (Ehlers-Danlos syndrome type IV, autosomal dominant) (COL3A1), mRNA [NM_000090]	NM_000090
A_32_P121079	6.29E-07	AK090467	AK090467	Homo sapiens mRNA for FLJ00388 protein. [AK090467]	
A_23_P83277	6.29E-07	NM_004512	NM_004512	Homo sapiens interleukin 11 receptor, alpha (IL11RA), transcript variant 1, mRNA [NM_004512]	NM_004512
A_23_P15876	6.34E-07	NM_052947	NM_052947	Homo sapiens heart alpha-kinase (HAK), mRNA [NM_052947]	NM_052947
A_23_P35617	6.39E-07	NM_016341	NM_016341	Homo sapiens phospholipase C, epsilon 1 (PLCE1), mRNA [NM_016341]	NM_016341
A_23_P115261	6.39E-07	NM_000029	NM_000029	Homo sapiens angiotensinogen (serine (or cysteine) proteinase inhibitor, clade A (alpha-1 antiproteinase, antitrypsin), member 8) (AGT), mRNA [NM_000029]	NM_000029
A_23_P14798	6.39E-07	NM_002499	NM_002499	Homo sapiens neogenin homolog 1 (chicken) (NEO1), mRNA [NM_002499]	NM_002499
A_32_P84009	6.39E-07	NM_178818	NM_178818	Homo sapiens chemokine-like factor super family 4 (CKLFSF4), transcript variant 1, mRNA [NM_178818]	NM_178818
A_23_P165778	6.39E-07	NM_024101	NM_024101	Homo sapiens melanophilin (MLPH), mRNA [NM_024101]	NM_024101

A_23_P6771	6.39E-07	NM_014583	NM_014583	Homo sapiens LIM and cysteine-rich domains 1 (LMCD1), mRNA [NM_014583]	NM_014583
A_23_P156970	6.39E-07	NM_002402	NM_002402	Homo sapiens mesoderm specific transcript homolog (mouse) (MEST), transcript variant 1, mRNA [NM_002402]	NM_002402
A_23_P57784	6.45E-07	NM_021101	NM_021101	Homo sapiens claudin 1 (CLDN1), mRNA [NM_021101]	NM_021101
A_23_P121533	6.48E-07	NM_012445	NM_012445	Homo sapiens spondin 2, extracellular matrix protein (SPON2), mRNA [NM_012445]	NM_012445
A_23_P42265	6.65E-07	NM_019101	NM_019101	Homo sapiens apolipoprotein M (APOM), mRNA [NM_019101]	NM_019101
A_24_P154037	6.88E-07	NM_003749	NM_003749	Homo sapiens insulin receptor substrate 2 (IRS2), mRNA [NM_003749]	NM_003749
A_23_P135061	6.88E-07	NM_003389	NM_003389	Homo sapiens coronin, actin binding protein, 2A (CORO2A), transcript variant 1, mRNA [NM_003389]	NM_003389
A_23_P328074	6.93E-07	NM_002968	NM_002968	Homo sapiens sal-like 1 (Drosophila) (SALL1), mRNA [NM_002968]	NM_002968
A_23_P500936	6.93E-07	NM_021784	NM_021784	Homo sapiens forkhead box A2 (FOXA2), transcript variant 1, mRNA [NM_021784]	NM_021784
A_23_P154962	6.94E-07	BC035246	BC035246	Homo sapiens KIAA1666 protein, mRNA (cDNA clone MGC:42740 IMAGE:4827837), complete cds. [BC035246]	XM_036936
A_23_P207811	6.98E-07	NM_006451	NM_006451	Homo sapiens poly(A) binding protein interacting protein 1 (PAIP1), transcript variant 1, mRNA [NM_006451]	NM_006451
A_23_P204751	7.01E-07	NM_020039	NM_020039	Homo sapiens amiloride-sensitive cation channel 2, neuronal (ACCN2), transcript variant 1, mRNA [NM_020039]	NM_020039
A_23_P97700	7.01E-07	NM_006472	NM_006472	Homo sapiens thioredoxin interacting protein (TXNIP), mRNA [NM_006472]	NM_006472
A_23_P362228	7.16E-07	NM_138479	NM_138479	Homo sapiens hypothetical protein BC007899 (LOC148898), transcript variant 1, mRNA [NM_138479]	NM_138479
A_23_P63128	7.18E-07	AK024186	AK024186	Homo sapiens cDNA FLJ14124 fis, clone MAMMA1002498. [AK024186]	
A_23_P414654	7.21E-07	NM_175738	NM_175738	Homo sapiens RAB37, member RAS oncogene family (RAB37), mRNA [NM_175738]	NM_175738
A_24_P944588	7.22E-07	AK074843	AK074843	Homo sapiens cDNA FLJ90362 fis, clone NT2RP2003940, weakly similar to ZINC FINGER PROTEIN 43. [AK074843]	
A_23_P151529	7.22E-07	NM_020215	NM_020215	Homo sapiens chromosome 14 open reading frame 132 (C14orf132), mRNA [NM_020215]	NM_020215
A_24_P245838	7.22E-07	AK125361	AK125361	Homo sapiens cDNA FLJ43371 fis, clone NTONG2005969. [AK125361]	
A_23_P106405	7.22E-07	NM_002487	NM_002487	Homo sapiens necdin homolog (mouse) (NDN), mRNA [NM_002487]	NM_002487
A_23_P399078	7.22E-07	NM_000362	NM_000362	Homo sapiens tissue inhibitor of metalloproteinase 3 (Sorsby fundus dystrophy, pseudoinflammatory) (TIMP3), mRNA [NM_000362]	NM_000362
A_23_P414273	7.22E-07	NM_032947	NM_032947	Homo sapiens putative small membrane protein NID67 (NID67), mRNA [NM_032947]	NM_032947
A_24_P418408	7.22E-07	NM_198552	NM_198552	Homo sapiens chromosome 1 open reading frame 153 (C1orf153), mRNA [NM_198552]	NM_198552
A_23_P32444	7.22E-07	NM_032348	NM_032348	Homo sapiens matrix-remodelling associated 8 (MXRA8), mRNA [NM_032348]	NM_032348
A_24_P53778	7.28E-07	NM_080878	NM_080878	Homo sapiens intelectin 2 (ITLN2), mRNA [NM_080878]	NM_080878
A_23_P163666	7.40E-07	NM_053284	NM_053284	Homo sapiens WAP, follistatin/kazal, immunoglobulin, kunitz and netrin domain containing 1 (WFIKKN1), mRNA [NM_053284]	NM_053284
A_23_P165360	7.41E-07	NM_016114	NM_016114	Homo sapiens ankyrin repeat and SOCS box-containing 1 (ASB1), mRNA [NM_016114]	NM_016114
A_24_P346431	7.45E-07	AK092864	AK092864	Homo sapiens cDNA FLJ35545 fis, clone SPLEN2003918, moderately similar to TENSIN. [AK092864]	
A_23_P114814	7.62E-07	NM_021205	NM_021205	Homo sapiens ras homolog gene family, member U (RHOU), mRNA [NM_021205]	NM_021205
A_24_P347566	7.63E-07	NM_015059	NM_015059	Homo sapiens talin 2 (TLN2), mRNA [NM_015059]	NM_015059
A_23_P217704	7.63E-07	NM_003918	NM_003918	Homo sapiens glycogenin 2 (GYG2), mRNA [NM_003918]	NM_003918
A_23_P156327	7.65E-07	NM_000358	NM_000358	Homo sapiens transforming growth factor, beta-induced, 68kDa (TGFBI), mRNA [NM_000358]	NM_000358
A_23_P334955	7.81E-07	NM_053279	NM_053279	Homo sapiens chromosome 8 open reading frame 13 (C8orf13), mRNA [NM_053279]	NM_053279
A_24_P912799	7.92E-07	AF056434	AF056434	Homo sapiens clone FBD8 Cri-du-chat critical region mRNA. [AF056434]	
A_24_P148796	7.98E-07	NM_020998	NM_020998	Homo sapiens macrophage stimulating 1 (hepatocyte growth factor-like) (MST1), mRNA [NM_020998]	NM_020998
A_23_P165927	8.08E-07	NM_015894	NM_015894	Homo sapiens stathmin-like 3 (STMN3), mRNA [NM_015894]	NM_015894
A_23_P400449	8.10E-07	NM_020927	NM_020927	Homo sapiens KIAA1576 protein (KIAA1576), mRNA [NM_020927]	NM_020927
A_24_P40626	8.14E-07	NM_022469	NM_022469	Homo sapiens gremlin 2, cysteine knot superfamily, homolog (Xenopus laevis) (GREM2), mRNA [NM_022469]	NM_022469
A_24_P18137	8.45E-07	BC066952	BC066952	Homo sapiens neurofilament, light polypeptide 68kDa, mRNA (cDNA clone MGC:87388 IMAGE:5264373), complete cds. [BC066952]	
A_23_P160318	8.45E-07	NM_001856	NM_001856	Homo sapiens collagen, type XVI, alpha 1 (COL16A1), mRNA [NM_001856]	NM_001856
A_23_P8834	8.47E-07	NM_001979	NM_001979	Homo sapiens epoxide hydrolase 2, cytoplasmic (EPHX2), mRNA [NM_001979]	NM_001979
A_24_P244706	8.55E-07	NM_001049	NM_001049	Homo sapiens somatostatin receptor 1 (SSTR1), mRNA [NM_001049]	NM_001049
A_23_P53891	8.55E-07	NM_001730	NM_001730	Homo sapiens Kruppel-like factor 5 (intestinal) (KLF5), mRNA [NM_001730]	NM_001730
A_23_P10206	8.55E-07	NM_005328	NM_005328	Homo sapiens hyaluronan synthase 2 (HAS2), mRNA [NM_005328]	NM_005328
A_23_P73787	8.55E-07	NM_153183	NM_153183	Homo sapiens nudix (nucleoside diphosphate linked moiety X)-type motif 10 (NUDT10), mRNA [NM_153183]	NM_153183

A_23_P94338	8.60E-07	NM_006209	NM_006209	Homo sapiens ectonucleotide pyrophosphatase/phosphodiesterase 2 (autotaxin) (ENPP2), mRNA [NM_006209]	NM_006209
A_32_P49284	8.65E-07	XM_372040	XM_372040	PREDICTED: Homo sapiens similar to asparagine synthetase; glutamine-dependent asparagine synthetase; TS11 cell cycle control protein (LOC389652), mRNA [XM_372040]	XM_372040
A_23_P156708	8.72E-07	NM_019105	NM_019105	Homo sapiens tenascin XB (TNXB), transcript variant XB, mRNA [NM_019105]	NM_019105
A_23_P167367	8.78E-07	NM_153426	NM_153426	Homo sapiens paired-like homeodomain transcription factor 2 (PITX2), transcript variant 2, mRNA [NM_153426]	NM_153426
A_23_P209246	8.82E-07	AB209354	AB209354	Homo sapiens mRNA for GLI-Kruppel family member GLI2 isoform beta variant protein. [AB209354]	
A_24_P920447	8.95E-07	NM_020215	NM_020215	Homo sapiens chromosome 14 open reading frame 132 (C14orf132), mRNA [NM_020215]	NM_020215
A_23_P373521	9.05E-07	NM_021973	NM_021973	Homo sapiens heart and neural crest derivatives expressed 2 (HAND2), mRNA [NM_021973]	NM_021973
A_23_P129695	9.05E-07	NM_138440	NM_138440	Homo sapiens slit-like 2 (Drosophila) (SLITL2), mRNA [NM_138440]	NM_138440
A_23_P94800	9.39E-07	NM_002961	NM_002961	Homo sapiens S100 calcium binding protein A4 (calcium protein, calvasculin, metastasin, murine placental homolog) (S100A4), transcript variant 1, mRNA [NM_002961]	NM_002961
A_32_P123255	9.53E-07	CR593492	CR593492	full-length cDNA clone CS0DI054YC18 of Placenta Cot 25-normalized of Homo sapiens (human). [CR593492]	XM_293380
A_24_P310864	9.56E-07	NM_152499	NM_152499	Homo sapiens coiled-coil domain containing 24 (CCDC24), mRNA [NM_152499]	NM_152499
A_23_P120504	9.65E-07	NM_018354	NM_018354	Homo sapiens chromosome 20 open reading frame 46 (C20orf46), mRNA [NM_018354]	NM_018354
A_23_P31124	9.73E-07	NM_030820	NM_030820	Homo sapiens collagen, type XXI, alpha 1 (COL21A1), mRNA [NM_030820]	NM_030820
A_23_P31873	9.73E-07	NM_001002233	NM_001002233	Homo sapiens RAB11 family interacting protein 1 (class I) (RAB11FIP1), transcript variant 2, mRNA [NM_001002233]	NM_001002233
A_24_P639671	9.73E-07	AK095831	AK095831	Homo sapiens cDNA FLJ38512 fis, clone HCHON2000503. [AK095831]	
A_23_P301138	9.73E-07	NM_001005912	NM_001005912	Homo sapiens inositol hexaphosphate kinase 2 (IHPK2), transcript variant 5, mRNA [NM_001005912]	NM_001005912
A_23_P144959	9.73E-07	NM_004385	NM_004385	Homo sapiens chondroitin sulfate proteoglycan 2 (versican) (CSPG2), mRNA [NM_004385]	NM_004385
A_23_P25150	9.78E-07	NM_006897	NM_006897	Homo sapiens homeo box C9 (HOXC9), mRNA [NM_006897]	NM_006897
A_23_P212779	9.99E-07	NM_015393	NM_015393	Homo sapiens DKFZP564O0823 protein (DKFZP564O0823), mRNA [NM_015393]	NM_015393
A_32_P214503	9.99E-07	BC038512	BC038512	Homo sapiens cDNA clone IMAGE:5262734, partial cds. [BC038512]	
A_23_P57667	9.99E-07	NM_032242	NM_032242	Homo sapiens plexin A1 (PLXNA1), mRNA [NM_032242]	NM_032242
A_32_P215866	1.01E-06	THC2312367			
A_23_P253921	1.01E-06	NM_018651	NM_018651	Homo sapiens zinc finger protein 167 (ZNF167), transcript variant 1, mRNA [NM_018651]	NM_018651
A_23_P19030	1.01E-06	ENST00000328668		full-length cDNA clone CS0DI041YK16 of Placenta Cot 25-normalized of Homo sapiens (human). [CR621346]	
A_23_P11843	1.01E-06	NM_201630	NM_201630	Homo sapiens leucine rich repeat neuronal 5 (LRRN5), transcript variant 2, mRNA [NM_201630]	NM_201630
A_23_P75790	1.01E-06	NM_013279	NM_013279	Homo sapiens chromosome 11 open reading frame 9 (C11orf9), mRNA [NM_013279]	NM_013279
A_23_P304897	1.02E-06	NM_000623	NM_000623	Homo sapiens bradykinin receptor B2 (BDKRB2), mRNA [NM_000623]	NM_000623
A_24_P206624	1.02E-06	NM_022974	NM_022974	Homo sapiens fibroblast growth factor receptor 2 (bacteria-expressed kinase, keratinocyte growth factor receptor, craniofacial dysostosis 1, Crouzon syndrome, Pfeiffer syndrome, Jackson-Weiss syndrome) (FGFR2), transcript variant 7, mRNA [NM_022974]	NM_022974
A_24_P128361	1.02E-06	AF289562	AF289562	Homo sapiens clone pp6337 unknown mRNA. [AF289562]	
A_23_P81660	1.02E-06	NM_018368	NM_018368	Homo sapiens LMBR1 domain containing 1 (LMBRD1), mRNA [NM_018368]	NM_018368
A_23_P382	1.02E-06	NM_018125	NM_018125	Homo sapiens Rho guanine nucleotide exchange factor (GEF) 10-like (ARHGEF10L), transcript variant 1, mRNA [NM_018125]	NM_018125
A_24_P109214	1.02E-06	NM_001645	NM_001645	Homo sapiens apolipoprotein C-I (APOC1), mRNA [NM_001645]	NM_001645
A_23_P64873	1.02E-06	NM_001920	NM_001920	Homo sapiens decorin (DCN), transcript variant A1, mRNA [NM_001920]	NM_001920
A_23_P79518	1.03E-06	NM_000576	NM_000576	Homo sapiens interleukin 1, beta (IL1B), mRNA [NM_000576]	NM_000576
A_23_P134085	1.03E-06	NM_173515	NM_173515	Homo sapiens CNKSR family member 3 (CNKSR3), mRNA [NM_173515]	NM_173515
A_23_P2283	1.03E-06	NM_001006667	NM_001006667	Homo sapiens tachykinin 3 (neuromedin K, neurokinin beta) (TAC3), mRNA [NM_001006667]	NM_001006667
A_23_P152305	1.03E-06	NM_001797	NM_001797	Homo sapiens cadherin 11, type 2, OB-cadherin (osteoblast) (CDH11), mRNA [NM_001797]	NM_001797
A_32_P67303	1.04E-06	THC2328806			
A_23_P409093	1.04E-06	NM_178826	NM_178826	Homo sapiens transmembrane protein 16D (TMEM16D), mRNA [NM_178826]	NM_178826
A_23_P170667	1.04E-06	NM_181718	NM_181718	Homo sapiens hypothetical protein LOC253982 (LOC253982), mRNA [NM_181718]	NM_181718
A_23_P146274	1.05E-06	NM_007029	NM_007029	Homo sapiens stathmin-like 2 (STMN2), mRNA [NM_007029]	NM_007029
A_24_P97104	1.06E-06	NM_001935	NM_001935	Homo sapiens dipeptidylpeptidase 4 (CD26, adenosine deaminase complexing protein 2) (DPP4), mRNA [NM_001935]	NM_001935

A_24_P115007	1.06E-06	NM_170740	NM_170740	Homo sapiens aldehyde dehydrogenase 5 family, member A1 (succinate-semialdehyde dehydrogenase) (ALDH5A1), nuclear gene encoding mitochondrial protein, transcript variant 1, mRNA [NM_170740]	NM_170740
A_23_P144656	1.06E-06	NM_006727	NM_006727	Homo sapiens cadherin 10, type 2 (T2-cadherin) (CDH10), mRNA [NM_006727]	NM_006727
A_24_P760368	1.06E-06	THC2434152		Q9HBV3 (Q9HBV3) PP3111, partial (17%) [THC2434152]	
A_24_P940006	1.06E-06	NM_001406	NM_001406	Homo sapiens ephrin-B3 (EFNB3), mRNA [NM_001406]	NM_001406
A_23_P421175	1.06E-06	BC033256	BC033256	Homo sapiens FLJ46072 protein, mRNA (cDNA clone IMAGE:5455669), partial cds. [BC033256]	
A_24_P648880	1.06E-06	NR_002211	NR_002211	Homo sapiens Meis1, myeloid ecotropic viral integration site 1 homolog 4 (mouse) (MEIS4) on chromosome 17 [NR_002211]	NR_002211
A_23_P43337	1.06E-06	NM_144966	NM_144966	Homo sapiens FRAS1 related extracellular matrix 1 (FREM1), mRNA [NM_144966]	NM_144966
A_23_P500353	1.06E-06	NM_021614	NM_021614	Homo sapiens potassium intermediate/small conductance calcium-activated channel, subfamily N, member 2 (KCNN2), transcript variant 1, mRNA [NM_021614]	NM_021614
A_32_P104063	1.06E-06	AF275804	AF275804	Homo sapiens PNAS-108 mRNA, partial sequence. [AF275804]	
A_23_P157147	1.06E-06	NM_018106	NM_018106	Homo sapiens zinc finger, DHHC-type containing 4 (ZDHHC4), mRNA [NM_018106]	NM_018106
A_23_P95930	1.06E-06	NM_001015886	NM_001015886	Homo sapiens high mobility group AT-hook 2 (HMGA2), transcript variant 3, mRNA [NM_001015886]	NM_001015886
A_23_P125233	1.06E-06	NM_001299	NM_001299	Homo sapiens calponin 1, basic, smooth muscle (CNN1), mRNA [NM_001299]	NM_001299
A_23_P304450	1.07E-06	NM_005257	NM_005257	Homo sapiens GATA binding protein 6 (GATA6), mRNA [NM_005257]	NM_005257
A_24_P48069	1.09E-06	NM_018110	NM_018110	Homo sapiens docking protein 4 (DOK4), mRNA [NM_018110]	NM_018110
A_23_P71067	1.11E-06	NM_000474	NM_000474	Homo sapiens twist homolog 1 (acrocephalosyndactyly 3; Saethre-Chotzen syndrome) (Drosophila) (TWIST1), mRNA [NM_000474]	NM_000474
A_32_P2161	1.12E-06	AK023040	AK023040	Homo sapiens cDNA FLJ12978 fis, clone NT2RP2006321. [AK023040]	
A_23_P14673	1.12E-06	NM_020962	NM_020962	Homo sapiens likely ortholog of mouse neighbor of Punc E11 (NOPE), mRNA [NM_020962]	NM_020962
A_23_P42397	1.12E-06	NM_153362	NM_153362	Homo sapiens protease, serine, 35 (PRSS35), mRNA [NM_153362]	NM_153362
A_24_P399980	1.12E-06	NM_014799	NM_014799	Homo sapiens hephaestin (HEPH), transcript variant 2, mRNA [NM_014799]	NM_014799
A_23_P132910	1.12E-06	NM_019027	NM_019027	Homo sapiens RNA-binding protein (FLJ20273), mRNA [NM_019027]	NM_019027
A_23_P332399	1.12E-06	NM_016315	NM_016315	Homo sapiens GULP, engulfment adaptor PTB domain containing 1 (GULP1), mRNA [NM_016315]	NM_016315
A_24_P20630	1.12E-06	NM_016269	NM_016269	Homo sapiens lymphoid enhancer-binding factor 1 (LEF1), mRNA [NM_016269]	NM_016269
A_23_P12928	1.13E-06	ENST00000340797		Homo sapiens solute carrier family 5 (sodium/glucose cotransporter), member 12, mRNA (cDNA clone IMAGE:5187504), partial cds. [BC041454]	
A_23_P70307	1.14E-06	NM_022138	NM_022138	Homo sapiens SPARC related modular calcium binding 2 (SMOC2), mRNA [NM_022138]	NM_022138
A_23_P397376	1.15E-06	NM_005360	NM_005360	Homo sapiens v-maf musculoaponeurotic fibrosarcoma oncogene homolog (avian) (MAF), mRNA [NM_005360]	NM_005360
A_32_P104432	1.15E-06	NM_203306	NM_203306	Homo sapiens hypothetical protein MGC39606 (MGC39606), mRNA [NM_203306]	NM_203306
A_23_P35456	1.15E-06	NM_014631	NM_014631	Homo sapiens SH3 multiple domains 1 (SH3MD1), mRNA [NM_014631]	NM_014631
A_23_P218225	1.16E-06	NM_014298	NM_014298	Homo sapiens quinolinate phosphoribosyltransferase (nicotinate-nucleotide pyrophosphorylase (carboxylating)) (QPRT), mRNA [NM_014298]	NM_014298
A_24_P561165	1.18E-06	A_24_P561165			
A_32_P51237	1.20E-06	NM_181712	NM_181712	Homo sapiens ankyrin repeat domain 38 (ANKRD38), mRNA [NM_181712]	NM_181712
A_23_P406341	1.20E-06	NM_001001936	NM_001001936	Homo sapiens KIAA1914 (KIAA1914), transcript variant 1, mRNA [NM_001001936]	NM_001001936
A_32_P198731	1.21E-06	ENST00000315447		Homo sapiens cDNA FLJ39342 fis, clone OCBBF2018873. [AK096661]	
A_32_P125832	1.22E-06	AK123079	AK123079	Homo sapiens cDNA FLJ41084 fis, clone ADRGL2010974. [AK123079]	
A_23_P37623	1.22E-06	NM_181076	NM_181076	Homo sapiens 88-kDa golgi protein (GM88), transcript variant 2, mRNA [NM_181076]	NM_181076
A_32_P107617	1.23E-06	NM_003019	NM_003019	Homo sapiens surfactant, pulmonary-associated protein D (SFTPD), mRNA [NM_003019]	NM_003019
A_23_P87036	1.23E-06	NM_000482	NM_000482	Homo sapiens apolipoprotein A-IV (APOA4), mRNA [NM_000482]	NM_000482
A_32_P114284	1.24E-06	THC2338091		NQRB_CHLMU (Q9PKB6) Probable Na(+)-translocating NADH-quinone reductase subunit B (Na(+)-translocating NQR subunit B) (Na(+)-NQR subunit B) (NQR complex subunit B) (NQR-1 subunit B) , partial (4%) [THC2338091]	
A_32_P103945	1.24E-06	NM_212543	NM_212543	Homo sapiens UDP-Gal:betaGlcNAc beta 1,4- galactosyltransferase, polypeptide 4 (B4GALT4), transcript variant 1, mRNA [NM_212543]	NM_212543
A_23_P253896	1.24E-06	NM_198278	NM_198278	Homo sapiens likely ortholog of mouse nephronectin (NPNT), mRNA [NM_198278]	NM_198278
A_23_P301133	1.25E-06	NM_001005912	NM_001005912	Homo sapiens inositol hexaphosphate kinase 2 (IHPK2), transcript variant 5, mRNA [NM_001005912]	NM_001005912
A_23_P132718	1.25E-06	NM_004636	NM_004636	Homo sapiens sema domain, immunoglobulin domain (Ig), short basic domain, secreted, (semaphorin) 3B (SEMA3B), transcript variant 1, mRNA [NM_004636]	NM_004636
A_23_P143981	1.25E-06	NM_001004019	NM_001004019	Homo sapiens fibulin 2 (FBLN2), transcript variant 1, mRNA [NM_001004019]	NM_001004019
A_23_P134925	1.25E-06	NM_004331	NM_004331	Homo sapiens BCL2/adenovirus E1B 19kDa interacting protein 3-like (BNIP3L), mRNA [NM_004331]	NM_004331

A_23_P359630	1.26E-06	NM_002665	NM_002665	Homo sapiens plasminogen-like B1 (PLGLB1), mRNA [NM_002665]	NM_002665
A_32_P4626	1.26E-06	AF143325	AF143325	Homo sapiens clone IMAGE:110436 mRNA sequence. [AF143325]	
A_24_P930062	1.27E-06	NM_016463	NM_016463	Homo sapiens CXXC finger 5 (CXXC5), mRNA [NM_016463]	NM_016463
A_23_P37727	1.28E-06	NM_002996	NM_002996	Homo sapiens chemokine (C-X3-C motif) ligand 1 (CX3CL1), mRNA [NM_002996]	NM_002996
A_23_P72411	1.29E-06	NM_178033	NM_178033	Homo sapiens cytochrome P450, family 4, subfamily X, polypeptide 1 (CYP4X1), mRNA [NM_178033]	NM_178033
A_23_P203957	1.29E-06	NM_031920	NM_031920	Homo sapiens ARG99 protein (ARG99), transcript variant 1, mRNA [NM_031920]	NM_031920
A_23_P117582	1.31E-06	NM_130469	NM_130469	Homo sapiens jun dimerization protein 2 (JDP2), mRNA [NM_130469]	NM_130469
A_23_P159237	1.32E-06	AB209066	AB209066	Homo sapiens mRNA for G protein-coupled receptor 20 variant protein. [AB209066]	
A_24_P166443	1.32E-06	NM_002121	NM_002121	Homo sapiens major histocompatibility complex, class II, DP beta 1 (HLA-DPB1), mRNA [NM_002121]	NM_002121
A_23_P371824	1.32E-06	NM_020127	NM_020127	Homo sapiens tuftelin 1 (TUFT1), mRNA [NM_020127]	NM_020127
A_24_P76300	1.33E-06	NM_003444	NM_003444	Homo sapiens zinc finger protein 154 (pHZ-92) (ZNF154), mRNA [NM_003444]	NM_003444
A_23_P371107	1.33E-06	NM_014392	NM_014392	Homo sapiens DNA segment on chromosome 4 (unique) 234 expressed sequence (D4S234E), mRNA [NM_014392]	NM_014392
A_23_P37484	1.34E-06	NM_014918	NM_014918	Homo sapiens carbohydrate (chondroitin) synthase 1 (CHSY1), mRNA [NM_014918]	NM_014918
A_23_P120103	1.35E-06	NM_002252	NM_002252	Homo sapiens potassium voltage-gated channel, delayed-rectifier, subfamily S, member 3 (KCNS3), mRNA [NM_002252]	NM_002252
A_23_P329271	1.35E-06	NM_002386	NM_002386	Homo sapiens melanocortin 1 receptor (alpha melanocyte stimulating hormone receptor) (MC1R), mRNA [NM_002386]	NM_002386
A_23_P372467	1.35E-06	NM_152392	NM_152392	Homo sapiens AHA1, activator of heat shock 90kDa protein ATPase homolog 2 (yeast) (AHSA2), mRNA [NM_152392]	NM_152392
A_23_P93027	1.36E-06	NM_003862	NM_003862	Homo sapiens fibroblast growth factor 18 (FGF18), transcript variant 1, mRNA [NM_003862]	NM_003862
A_23_P1431	1.36E-06	NM_004329	NM_004329	Homo sapiens bone morphogenetic protein receptor, type IA (BMPR1A), mRNA [NM_004329]	NM_004329
A_23_P167401	1.36E-06	NM_018931	NM_018931	Homo sapiens protocadherin beta 11 (PCDHB11), mRNA [NM_018931]	NM_018931
A_24_P318897	1.36E-06	NM_033421	NM_033421	Homo sapiens chromosome 20 open reading frame 161 (C20orf161), transcript variant 1, mRNA [NM_033421]	NM_033421
A_23_P45099	1.36E-06	NM_002125	NM_002125	Homo sapiens major histocompatibility complex, class II, DR beta 5 (HLA-DRB5), mRNA [NM_002125]	NM_002125
A_23_P68759	1.36E-06	NM_003225	NM_003225	Homo sapiens trefoil factor 1 (breast cancer, estrogen-inducible sequence expressed in) (TFF1), mRNA [NM_003225]	NM_003225
A_23_P4611	1.36E-06	NM_012254	NM_012254	Homo sapiens solute carrier family 27 (fatty acid transporter), member 5 (SLC27A5), mRNA [NM_012254]	NM_012254
A_32_P32254	1.38E-06	NM_001848	NM_001848	Homo sapiens collagen, type VI, alpha 1 (COL6A1), mRNA [NM_001848]	NM_001848
A_23_P253451	1.39E-06	NM_020949	NM_020949	Homo sapiens solute carrier family 7 (cationic amino acid transporter, y+ system), member 14 (SLC7A14), mRNA [NM_020949]	NM_020949
A_23_P214079	1.39E-06	NM_003122	NM_003122	Homo sapiens serine protease inhibitor, Kazal type 1 (SPINK1), mRNA [NM_003122]	NM_003122
A_23_P164057	1.39E-06	NM_002404	NM_002404	Homo sapiens microfibrillar-associated protein 4 (MFAP4), mRNA [NM_002404]	NM_002404
A_23_P207520	1.39E-06	NM_000088	NM_000088	Homo sapiens collagen, type I, alpha 1 (COL1A1), mRNA [NM_000088]	NM_000088
A_24_P780319	1.39E-06	A_24_P780319			
A_32_P161855	1.40E-06	NM_018689	NM_018689	Homo sapiens KIAA1199 (KIAA1199), mRNA [NM_018689]	NM_018689
A_23_P170186	1.40E-06	NM_017570	NM_017570	Homo sapiens 5-oxoprolinase (ATP-hydrolysing) (OPLAH), mRNA [NM_017570]	NM_017570
A_23_P421401	1.40E-06	NM_002609	NM_002609	Homo sapiens platelet-derived growth factor receptor, beta polypeptide (PDGFRB), mRNA [NM_002609]	NM_002609
A_24_P91035	1.44E-06	NM_005324	NM_005324	Homo sapiens H3 histone, family 3B (H3.3B) (H3F3B), mRNA [NM_005324]	NM_005324
A_23_P145336	1.45E-06	V00522	V00522	Human mRNA encoding major histocompatibility complex gene HLA-DR beta-I. [V00522]	
A_24_P379413	1.46E-06	NM_000565	NM_000565	Homo sapiens interleukin 6 receptor (IL6R), transcript variant 1, mRNA [NM_000565]	NM_000565
A_23_P9523	1.47E-06	NM_022128	NM_022128	Homo sapiens ribokinase (RBKS), mRNA [NM_022128]	NM_022128
A_23_P70818	1.47E-06	NM_005631	NM_005631	Homo sapiens smoothened homolog (Drosophila) (SMO), mRNA [NM_005631]	NM_005631
A_23_P213375	1.51E-06	NM_018936	NM_018936	Homo sapiens protocadherin beta 2 (PCDHB2), mRNA [NM_018936]	NM_018936
A_23_P427122	1.51E-06	NM_053017	NM_053017	Homo sapiens ADP-ribosyltransferase 5 (ART5), mRNA [NM_053017]	NM_053017
A_23_P359214	1.52E-06	AK096715	AK096715	Homo sapiens cDNA FLJ39396 fis, clone PLACE6008640. [AK096715]	
A_23_P216023	1.52E-06	NM_001146	NM_001146	Homo sapiens angiopoietin 1 (ANGPT1), transcript variant 1, mRNA [NM_001146]	NM_001146
A_24_P253003	1.52E-06	NM_004626	NM_004626	Homo sapiens wingless-type MMTV integration site family, member 11 (WNT11), mRNA [NM_004626]	NM_004626
A_32_P2452	1.52E-06	AK125140	AK125140	Homo sapiens cDNA FLJ43150 fis, clone CTONG3009239. [AK125140]	
A_23_P501183	1.52E-06	NM_148980	NM_148980	Homo sapiens Williams Beuren syndrome chromosome region 20C (WBSCR20C), transcript variant 3, mRNA [NM_148980]	NM_148980
A_23_P20752	1.54E-06	NM_178432	NM_178432	Homo sapiens cell cycle related kinase (CCRK), transcript variant 1, mRNA [NM_178432]	NM_178432

A_23_P7727	1.54E-06	NM_001884	NM_001884	Homo sapiens hyaluronan and proteoglycan link protein 1 (HAPLN1), mRNA [NM_001884]	NM_001884
A_23_P76901	1.56E-06	NM_015549	NM_015549	Homo sapiens pleckstrin homology domain containing, family G (with RhoGef domain) member 3 (PLEKHG3), mRNA [NM_015549]	NM_015549
A_24_P923676	1.58E-06	X15674	X15674	Human pTR5 mRNA for repetitive sequence. [X15674]	
A_23_P79289	1.58E-06	NM_014900	NM_014900	Homo sapiens COBL-like 1 (COBLL1), mRNA [NM_014900]	NM_014900
A_24_P248185	1.58E-06	NM_153685	NM_153685	Homo sapiens hypothetical protein DKFZp547D2210 (DKFZp547D2210), mRNA [NM_153685]	NM_153685
A_23_P99063	1.60E-06	NM_002345	NM_002345	Homo sapiens lumican (LUM), mRNA [NM_002345]	NM_002345
A_23_P35564	1.61E-06	NM_015490	NM_015490	Homo sapiens SEC31-like 2 (S. cerevisiae) (SEC31L2), transcript variant 1, mRNA [NM_015490]	NM_015490
A_32_P122494	1.62E-06	AI652920	AI652920	AI652920 wb40g09.x1 NCI_CGAP_GC6 Homo sapiens cDNA clone IMAGE:2308192 3' similar to SW:NMA_HUMAN Q13145 PUTATIVE TRANSMEMBRANE PROTEIN NMA PRECURSOR. ;, mRNA sequence [AI652920]	
A_23_P120902	1.62E-06	NM_006498	NM_006498	Homo sapiens lectin, galactoside-binding, soluble, 2 (galectin 2) (LGALS2), mRNA [NM_006498]	NM_006498
A_23_P316612	1.64E-06	NM_147193	NM_147193	Homo sapiens GLIS family zinc finger 1 (GLIS1), mRNA [NM_147193]	NM_147193
A_32_P83845	1.64E-06	NM_012258	NM_012258	Homo sapiens hairy/enhancer-of-split related with YRPW motif 1 (HEY1), mRNA [NM_012258]	NM_012258
A_24_P13390	1.64E-06	NM_032814	NM_032814	Homo sapiens hypothetical protein FLJ14627 (FLJ14627), mRNA [NM_032814]	NM_032814
A_23_P399265	1.64E-06	S82024	S82024	SCG10=neuron-specific growth-associated protein/stathmin homolog [human, embryo, mRNA, 696 nt]. [S82024]	
A_23_P217379	1.64E-06	NM_033641	NM_033641	Homo sapiens collagen, type IV, alpha 6 (COL4A6), transcript variant B, mRNA [NM_033641]	NM_033641
A_23_P399001	1.64E-06	NM_016463	NM_016463	Homo sapiens CXXC finger 5 (CXXC5), mRNA [NM_016463]	NM_016463
A_32_P201329	1.69E-06	BC062758	BC062758	Homo sapiens cDNA clone IMAGE:4081583, partial cds. [BC062758]	
A_23_P206022	1.69E-06	NM_001004439	NM_001004439	Homo sapiens integrin, alpha 11 (ITGA11), transcript variant 1, mRNA [NM_001004439]	NM_001004439
A_23_P127565	1.69E-06	NM_178834	NM_178834	Homo sapiens layilin (LOC143903), mRNA [NM_178834]	NM_178834
A_23_P2922	1.69E-06	NM_016586	NM_016586	Homo sapiens MAP3K12 binding inhibitory protein 1 (MBIP), mRNA [NM_016586]	NM_016586
A_23_P338919	1.69E-06	NM_005876	NM_005876	Homo sapiens aortic preferentially expressed gene 1 (APEG1), mRNA [NM_005876]	NM_005876
A_32_P10133	1.71E-06	AK022045	AK022045	Homo sapiens cDNA FLJ11983 fis, clone HEMBB1001337. [AK022045]	
A_23_P11806	1.71E-06	NM_080629	NM_080629	Homo sapiens collagen, type XI, alpha 1 (COL11A1), transcript variant B, mRNA [NM_080629]	NM_080629
A_23_P144911	1.71E-06	NM_152403	NM_152403	Homo sapiens hypothetical protein FLJ39155 (FLJ39155), transcript variant 1, mRNA [NM_152403]	NM_152403
A_23_P110531	1.71E-06	NM_013409	NM_013409	Homo sapiens follistatin (FST), transcript variant FST344, mRNA [NM_013409]	NM_013409
A_23_P7342	1.73E-06	NM_001075	NM_001075	Homo sapiens UDP glucuronosyltransferase 2 family, polypeptide B10 (UGT2B10), mRNA [NM_001075]	NM_001075
A_23_P256542	1.75E-06	NM_014367	NM_014367	Homo sapiens growth and transformation-dependent protein (E2IG5), mRNA [NM_014367]	NM_014367
A_23_P92222	1.76E-06	NM_152673	NM_152673	Homo sapiens mucin 20 (MUC20), mRNA [NM_152673]	NM_152673
A_23_P82503	1.76E-06	ENST00000362013		Homo sapiens MEF3L1 mRNA for MEF3 like 1, complete cds. [AB049150]	XM_499343
A_23_P383986	1.77E-06	NM_015892	NM_015892	Homo sapiens B cell RAG associated protein (GALNAC4S-6ST), mRNA [NM_015892]	NM_015892
A_24_P767725	1.78E-06	XM_496156	XM_496156	PREDICTED: Homo sapiens similar to rhophilin-like protein; RhoB effector; rhophilin-2; rhophilin 2 (LOC440368), mRNA [XM_496156]	XM_496156
A_23_P354827	1.78E-06	NM_153231	NM_153231	Homo sapiens zinc finger protein 550 (ZNF550), mRNA [NM_153231]	NM_153231
A_32_P154445	1.79E-06	CR590573	CR590573	full-length cDNA clone CS0DI042YD07 of Placenta Cot 25-normalized of Homo sapiens (human). [CR590573]	
A_23_P116435	1.79E-06	NM_016412	NM_016412	Homo sapiens insulin-like growth factor 2 antisense (IGF2AS), mRNA [NM_016412]	NM_016412
A_23_P80759	1.79E-06	BC017572	BC017572	Homo sapiens poliovirus receptor-related 3, mRNA (cDNA clone IMAGE:4066485), complete cds. [BC017572]	
A_23_P201319	1.80E-06	NM_032890	NM_032890	Homo sapiens dispatched homolog 1 (Drosophila) (DISP1), mRNA [NM_032890]	NM_032890
A_24_P105564	1.80E-06	NM_005399	NM_005399	Homo sapiens protein kinase, AMP-activated, beta 2 non-catalytic subunit (PRKAB2), mRNA [NM_005399]	NM_005399
A_24_P11315	1.82E-06	NM_020190	NM_020190	Homo sapiens olfactomedin-like 3 (OLFML3), mRNA [NM_020190]	NM_020190
A_24_P787897	1.85E-06	BC045778	BC045778	Homo sapiens, clone IMAGE:4791553, mRNA. [BC045778]	
A_23_P314145	1.85E-06	NM_005715	NM_005715	Homo sapiens uronyl-2-sulfotransferase (UST), mRNA [NM_005715]	NM_005715
A_23_P433820	1.85E-06	NM_152658	NM_152658	Homo sapiens THAP domain containing 8 (THAP8), mRNA [NM_152658]	NM_152658
A_23_P211468	1.87E-06	AA837799	AA837799	AA837799 oe06h09.s1 NCI_CGAP_Ov2 Homo sapiens cDNA clone IMAGE:1385153, mRNA sequence [AA837799]	
A_23_P336198	1.88E-06	AK093655	AK093655	Homo sapiens cDNA FLJ36336 fis, clone THYMU2006303. [AK093655]	
A_23_P58117	1.88E-06	NM_020859	NM_020859	Homo sapiens shroom (SHRM), mRNA [NM_020859]	NM_020859
A_23_P417282	1.89E-06	BC010607	BC010607	Homo sapiens hypothetical protein MGC18216, mRNA (cDNA clone MGC:18216 IMAGE:4156235), complete cds. [BC010607]	

A_23_P50946	1.90E-06	NM_005855	NM_005855	Homo sapiens receptor (calcitonin) activity modifying protein 1 (RAMP1), mRNA [NM_005855]	NM_005855
A_23_P52161	1.92E-06	NM_182901	NM_182901	Homo sapiens chromosome 11 open reading frame 17 (C11orf17), transcript variant 1, mRNA [NM_182901]	NM_182901
A_23_P87013	1.92E-06	NM_001001522	NM_001001522	Homo sapiens transgelin (TAGLN), transcript variant 1, mRNA [NM_001001522]	NM_001001522
A_23_P53247	1.92E-06	NM_178502	NM_178502	Homo sapiens deltex 3 homolog (Drosophila) (DTX3), mRNA [NM_178502]	NM_178502
A_24_P845223	1.93E-06	M27126	M27126	Human lymphocyte antigen (DRw8) mRNA. [M27126]	
A_24_P936758	1.94E-06	AK074614	AK074614	Homo sapiens cDNA FLJ90133 fis, clone HEMBB1000567. [AK074614]	
A_23_P345081	1.94E-06	NM_001009956	NM_001009956	Homo sapiens zinc finger protein 655 (ZNF655), transcript variant 6, mRNA [NM_001009956]	NM_001009956
A_23_P7033	1.94E-06	NM_022149	NM_022149	Homo sapiens melanoma antigen family F, 1 (MAGEF1), mRNA [NM_022149]	NM_022149
A_23_P34233	1.94E-06	NM_014298	NM_014298	Homo sapiens quinolinate phosphoribosyltransferase (nicotinate-nucleotide pyrophosphorylase (carboxylating)) (QPRT), mRNA [NM_014298]	NM_014298
A_32_P52227	1.96E-06	THC2314457		Q6DIG3 (Q6DIG3) Hedgehog interacting protein, partial (3%) [THC2314457]	
A_32_P16323	1.96E-06	ENST00000355077		Homo sapiens cDNA clone IMAGE:30389199, with apparent retained intron. [BC063888]	
A_23_P141362	1.96E-06	NM_001466	NM_001466	Homo sapiens frizzled homolog 2 (Drosophila) (FZD2), mRNA [NM_001466]	NM_001466
A_24_P816844	2.04E-06	THC2433066			
A_24_P406754	2.06E-06	NM_032211	NM_032211	Homo sapiens lysyl oxidase-like 4 (LOXL4), mRNA [NM_032211]	NM_032211
A_23_P130113	2.06E-06	NM_080912	NM_080912	Homo sapiens asialoglycoprotein receptor 2 (ASGR2), transcript variant H2', mRNA [NM_080912]	NM_080912
A_24_P398370	2.06E-06	A_24_P398370			
A_23_P120227	2.06E-06	NM_030915	NM_030915	Homo sapiens likely ortholog of mouse limb-bud and heart gene (LBH), mRNA [NM_030915]	NM_030915
A_23_P57227	2.06E-06	NM_003098	NM_003098	Homo sapiens syntrophin, alpha 1 (dystrophin-associated protein A1, 59kDa, acidic component) (SNTA1), mRNA [NM_003098]	NM_003098
A_24_P190472	2.09E-06	NM_003064	NM_003064	Homo sapiens secretory leukocyte protease inhibitor (antileukoproteinase) (SLPI), mRNA [NM_003064]	NM_003064
A_23_P349676	2.09E-06	AK055121	AK055121	Homo sapiens cDNA FLJ30559 fis, clone BRAWH2004095. [AK055121]	
A_23_P134854	2.09E-06	NM_194284	NM_194284	Homo sapiens claudin 23 (CLDN23), mRNA [NM_194284]	NM_194284
A_23_P216325	2.09E-06	NM_004315	NM_004315	Homo sapiens N-acylsphingosine amidohydrolase (acid ceramidase) 1 (ASAH1), transcript variant 2, mRNA [NM_004315]	NM_004315
A_24_P358474	2.09E-06	A_24_P358474			
A_24_P139901	2.09E-06	NM_002101	NM_002101	Homo sapiens glycophorin C (Gerbich blood group) (GYPC), transcript variant 1, mRNA [NM_002101]	NM_002101
A_24_P343233	2.09E-06	NM_002124	NM_002124	Homo sapiens major histocompatibility complex, class II, DR beta 1 (HLA-DRB1), mRNA [NM_002124]	NM_002124
A_23_P360964	2.09E-06	NM_145056	NM_145056	Homo sapiens thymus expressed gene 3-like (MGC15476), mRNA [NM_145056]	NM_145056
A_23_P10194	2.09E-06	NM_201575	NM_201575	Homo sapiens seizure related 6 homolog (mouse)-like 2 (SEZ6L2), transcript variant 2, mRNA [NM_201575]	NM_201575
A_23_P10902	2.09E-06	NM_001463	NM_001463	Homo sapiens frizzled-related protein (FRZB), mRNA [NM_001463]	NM_001463
A_23_P205031	2.10E-06	NM_001846	NM_001846	Homo sapiens collagen, type IV, alpha 2 (COL4A2), mRNA [NM_001846]	NM_001846
A_23_P67799	2.12E-06	BC046362	BC046362	Homo sapiens transmembrane protein 37, mRNA (cDNA clone MGC:50757 IMAGE:5221396), complete cds. [BC046362]	
A_23_P212500	2.14E-06	NM_001063	NM_001063	Homo sapiens transferrin (TF), mRNA [NM_001063]	NM_001063
A_23_P64785	2.16E-06	NM_152320	NM_152320	Homo sapiens zinc finger protein 641 (ZNF641), mRNA [NM_152320]	NM_152320
A_24_P157926	2.16E-06	NM_006290	NM_006290	Homo sapiens tumor necrosis factor, alpha-induced protein 3 (TNFAIP3), mRNA [NM_006290]	NM_006290
A_23_P4212	2.17E-06	NM_006361	NM_006361	Homo sapiens homeo box B13 (HOXB13), mRNA [NM_006361]	NM_006361
A_23_P376591	2.17E-06	NM_206808	NM_206808	Homo sapiens citrate lyase beta like (CLYBL), transcript variant 2, mRNA [NM_206808]	NM_206808
A_23_P27306	2.17E-06	NM_030781	NM_030781	Homo sapiens collectin sub-family member 12 (COLEC12), transcript variant II, mRNA [NM_030781]	NM_030781
A_23_P19590	2.17E-06	NM_003379	NM_003379	Homo sapiens villin 2 (ezrin) (VIL2), mRNA [NM_003379]	NM_003379
A_23_P76823	2.18E-06	NM_199165	NM_199165	Homo sapiens adenylosuccinate synthase like 1 (ADSSL1), transcript variant 1, mRNA [NM_199165]	NM_199165
A_23_P205057	2.18E-06	NM_014459	NM_014459	Homo sapiens protocadherin 17 (PCDH17), mRNA [NM_014459]	NM_014459
A_23_P128855	2.19E-06	NM_014579	NM_014579	Homo sapiens solute carrier family 39 (zinc transporter), member 2 (SLC39A2), mRNA [NM_014579]	NM_014579
A_32_P109242	2.20E-06	AK055302	AK055302	Homo sapiens cDNA FLJ30740 fis, clone FEBRA2000319. [AK055302]	
A_23_P379054	2.21E-06	NM_148960	NM_148960	Homo sapiens claudin 19 (CLDN19), mRNA [NM_148960]	NM_148960
A_32_P161762	2.22E-06	NM_004348	NM_004348	Homo sapiens runt-related transcription factor 2 (RUNX2), transcript variant 3, mRNA [NM_004348]	NM_004348
A_23_P86021	2.22E-06	NM_003944	NM_003944	Homo sapiens selenium binding protein 1 (SELENBP1), mRNA [NM_003944]	NM_003944

A_23_P76969	2.25E-06	NM_015556	NM_015556	Homo sapiens signal-induced proliferation-associated 1 like 1 (SIPA1L1), mRNA [NM_015556]	NM_015556
A_23_P79302	2.26E-06	NM_177964	NM_177964	Homo sapiens hypothetical protein LOC130576 (LOC130576), mRNA [NM_177964]	NM_177964
A_23_P57199	2.28E-06	NM_080920	NM_080920	Homo sapiens gamma-glutamyltransferase-like activity 4 (GGTLA4), transcript variant C, mRNA [NM_080920]	NM_080920
A_24_P360206	2.30E-06	NM_018902	NM_018902	Homo sapiens protocadherin alpha 11 (PCDHA11), transcript variant 1, mRNA [NM_018902]	NM_018902
A_23_P150807	2.30E-06	NM_003621	NM_003621	Homo sapiens PTPRF interacting protein, binding protein 2 (liprin beta 2) (PPFIBP2), mRNA [NM_003621]	NM_003621
A_24_P213370	2.32E-06	AK129982	AK129982	Homo sapiens cDNA FLJ26472 fis, clone KDN04506. [AK129982]	
A_23_P114947	2.34E-06	NM_002923	NM_002923	Homo sapiens regulator of G-protein signalling 2, 24kDa (RGS2), mRNA [NM_002923]	NM_002923
A_32_P222684	2.35E-06	ENST00000261364		Homo sapiens PR-domain zinc finger protein 6 isoform A (PRDM6) mRNA, partial cds; alternatively spliced. [AF272898]	
A_24_P150466	2.37E-06	ENST00000361956		Homo sapiens mRNA for secreted modular calcium-binding protein (smoc1 gene). [AJ249900]	
A_24_P56310	2.37E-06	NM_148957	NM_148957	Homo sapiens tumor necrosis factor receptor superfamily, member 19 (TNFRSF19), transcript variant 2, mRNA [NM_148957]	NM_148957
A_23_P253661	2.39E-06	NM_024902	NM_024902	Homo sapiens hypothetical protein FLJ13236 (FLJ13236), mRNA [NM_024902]	NM_024902
A_24_P471242	2.39E-06	A_24_P471242			
A_23_P62999	2.42E-06	NM_004455	NM_004455	Homo sapiens exostoses (multiple)-like 1 (EXTL1), mRNA [NM_004455]	NM_004455
A_32_P20523	2.42E-06	NM_145715	NM_145715	Homo sapiens tigger transposable element derived 2 (TIGD2), mRNA [NM_145715]	NM_145715
A_24_P211565	2.45E-06	NM_031910	NM_031910	Homo sapiens C1q and tumor necrosis factor related protein 6 (C1QTNF6), transcript variant 1, mRNA [NM_031910]	NM_031910
A_23_P65779	2.48E-06	NM_022369	NM_022369	Homo sapiens stimulated by retinoic acid gene 6 homolog (mouse) (STRA6), mRNA [NM_022369]	NM_022369
A_32_P117185	2.51E-06	THC2406981		Q9LCV5 (Q9LCV5) Atp operon (Fragment), partial (3%) [THC2406981]	
A_23_P373031	2.53E-06	NM_000719	NM_000719	Homo sapiens calcium channel, voltage-dependent, L type, alpha 1C subunit (CACNA1C), mRNA [NM_000719]	NM_000719
A_23_P96041	2.54E-06	NM_032227	NM_032227	Homo sapiens hypothetical protein FLJ22679 (RP13-360B22.2), mRNA [NM_032227]	NM_032227
A_23_P34915	2.54E-06	NM_004024	NM_004024	Homo sapiens activating transcription factor 3 (ATF3), mRNA [NM_004024]	NM_004024
A_24_P300777	2.55E-06	NM_001109	NM_001109	Homo sapiens a disintegrin and metalloproteinase domain 8 (ADAM8), mRNA [NM_001109]	NM_001109
A_23_P83939	2.55E-06	NM_032796	NM_032796	Homo sapiens synapse associated protein 1, SAP47 homolog (Drosophila) (SYAP1), mRNA [NM_032796]	NM_032796
A_24_P208595	2.56E-06	NM_053034	NM_053034	Homo sapiens anthrax toxin receptor 1 (ANTXR1), transcript variant 2, mRNA [NM_053034]	NM_053034
A_23_P59960	2.57E-06	NM_031461	NM_031461	Homo sapiens cysteine-rich secretory protein LCCL domain containing 1 (CRISPLD1), mRNA [NM_031461]	NM_031461
A_23_P67169	2.57E-06	NM_000641	NM_000641	Homo sapiens interleukin 11 (IL11), mRNA [NM_000641]	NM_000641
A_23_P167030	2.58E-06	NM_000316	NM_000316	Homo sapiens parathyroid hormone receptor 1 (PTHR1), mRNA [NM_000316]	NM_000316
A_23_P22013	2.59E-06	NM_173640	NM_173640	Homo sapiens roof plate-specific spondin (RSPONDIN), mRNA [NM_173640]	NM_173640
A_23_P212937	2.59E-06	NM_002092	NM_002092	Homo sapiens G-rich RNA sequence binding factor 1 (GRSF1), mRNA [NM_002092]	NM_002092
A_32_P232035	2.60E-06	THC2312585		AY175482 antibody variable domain {Oryctolagus cuniculus;} , partial (15%) [THC2312585]	
A_23_P369343	2.63E-06	NM_144505	NM_144505	Homo sapiens kallikrein 8 (neuropsin/ovasin) (KLK8), transcript variant 2, mRNA [NM_144505]	NM_144505
A_23_P205713	2.63E-06	NM_014178	NM_014178	Homo sapiens syntaxin binding protein 6 (amisyn) (STXBP6), mRNA [NM_014178]	NM_014178
A_23_P44244	2.63E-06	NM_003069	NM_003069	Homo sapiens SWI/SNF related, matrix associated, actin dependent regulator of chromatin, subfamily a, member 1 (SMARCA1), transcript variant 1, mRNA [NM_003069]	NM_003069
A_23_P84576	2.68E-06	NM_053034	NM_053034	Homo sapiens anthrax toxin receptor 1 (ANTXR1), transcript variant 2, mRNA [NM_053034]	NM_053034
A_23_P100660	2.71E-06	NM_002615	NM_002615	Homo sapiens serine (or cysteine) proteinase inhibitor, clade F (alpha-2 antiplasmin, pigment epithelium derived factor), member 1 (SERPINF1), mRNA [NM_002615]	NM_002615
A_23_P118493	2.73E-06	NM_005486	NM_005486	Homo sapiens target of myb1-like 1 (chicken) (TOM1L1), mRNA [NM_005486]	NM_005486
A_24_P412156	2.75E-06	NM_000609	NM_000609	Homo sapiens chemokine (C-X-C motif) ligand 12 (stromal cell-derived factor 1) (CXCL12), transcript variant 1, mRNA [NM_000609]	NM_000609
A_23_P56734	2.75E-06	NM_006895	NM_006895	Homo sapiens histamine N-methyltransferase (HNMT), transcript variant 1, mRNA [NM_006895]	NM_006895
A_23_P258698	2.76E-06	NM_005908	NM_005908	Homo sapiens mannosidase, beta A, lysosomal (MANBA), mRNA [NM_005908]	NM_005908
A_23_P162640	2.77E-06	NM_031412	NM_031412	Homo sapiens GABA(A) receptor-associated protein like 1 (GABARAPL1), mRNA [NM_031412]	NM_031412
A_23_P420196	2.80E-06	NM_003745	NM_003745	Homo sapiens suppressor of cytokine signaling 1 (SOCS1), mRNA [NM_003745]	NM_003745
A_24_P320545	2.81E-06	NM_002821	NM_002821	Homo sapiens PTK7 protein tyrosine kinase 7 (PTK7), transcript variant PTK7-1, mRNA [NM_002821]	NM_002821
A_23_P138975	2.81E-06	NM_016090	NM_016090	Homo sapiens RNA binding motif protein 7 (RBM7), mRNA [NM_016090]	NM_016090
A_23_P335695	2.81E-06	AK023854	AK023854	Homo sapiens cDNA FLJ13792 fis, clone THYRO1000072, weakly similar to MYOSIN LIGHT CHAIN KINASE, SMOOTH MUSCLE AND NON-MUSCLE ISOZYMES (EC 2.7.1.117). [AK023854]	

A_23_P91350	2.82E-06	ENST00000265183		Homo sapiens mRNA for KIAA1434 protein, partial cds. [AB037855]	
A_23_P384044	2.83E-06	NM_152495	NM_152495	Homo sapiens cornichon homolog 3 (Drosophila) (CNIH3), mRNA [NM_152495]	NM_152495
A_23_P91870	2.83E-06	NM_032343	NM_032343	Homo sapiens coiled-coil-helix-coiled-coil-helix domain containing 6 (CHCHD6), mRNA [NM_032343]	NM_032343
A_24_P188218	2.83E-06	NM_002476	NM_002476	Homo sapiens myosin, light polypeptide 4, alkali; atrial, embryonic (MYL4), transcript variant 2, mRNA [NM_002476]	NM_002476
A_23_P476	2.85E-06	NM_003953	NM_003953	Homo sapiens myelin protein zero-like 1 (MPZL1), transcript variant 1, mRNA [NM_003953]	NM_003953
A_23_P164387	2.85E-06	NM_003079	NM_003079	Homo sapiens SWI/SNF related, matrix associated, actin dependent regulator of chromatin, subfamily e, member 1 (SMARCE1), mRNA [NM_003079]	NM_003079
A_32_P134427	2.87E-06	BC048201	BC048201	Homo sapiens, clone IMAGE:3660074, mRNA. [BC048201]	
A_32_P61684	2.88E-06	NM_018440	NM_018440	Homo sapiens phosphoprotein associated with glycosphingolipid microdomains 1 (PAG1), mRNA [NM_018440]	NM_018440
A_32_P45168	2.91E-06	NR_002211	NR_002211	Homo sapiens Meis1, myeloid ecotropic viral integration site 1 homolog 4 (mouse) (MEIS4) on chromosome 17 [NR_002211]	NR_002211
A_23_P66137	2.92E-06	NM_014587	NM_014587	Homo sapiens SRY (sex determining region Y)-box 8 (SOX8), mRNA [NM_014587]	NM_014587
A_32_P9963	2.92E-06	NM_004506	NM_004506	Homo sapiens heat shock transcription factor 2 (HSF2), mRNA [NM_004506]	NM_004506
A_23_P11742	2.94E-06	NM_199163	NM_199163	Homo sapiens CXYorf1-related protein (FLJ25222), mRNA [NM_199163]	NM_199163
A_23_P8571	2.95E-06	NM_080744	NM_080744	Homo sapiens scavenger receptor cysteine rich domain containing, group B (4 domains) (SRCRB4D), mRNA [NM_080744]	NM_080744
A_32_P136295	3.00E-06	NM_052847	NM_052847	Homo sapiens guanine nucleotide binding protein (G protein), gamma 7 (GNG7), mRNA [NM_052847]	NM_052847
A_23_P136355	3.00E-06	NM_018194	NM_018194	Homo sapiens hedgehog acyltransferase (HHAT), mRNA [NM_018194]	NM_018194
A_23_P127394	3.02E-06	NM_021117	NM_021117	Homo sapiens cryptochrome 2 (photolyase-like) (CRY2), mRNA [NM_021117]	NM_021117
A_23_P25475	3.03E-06	NM_003578	NM_003578	Homo sapiens sterol O-acyltransferase 2 (SOAT2), mRNA [NM_003578]	NM_003578
A_24_P110914	3.04E-06	AK127569	AK127569	Homo sapiens cDNA FLJ45662 fis, clone CTONG2027150. [AK127569]	
A_24_P320699	3.06E-06	NM_001013398	NM_001013398	Homo sapiens insulin-like growth factor binding protein 3 (IGFBP3), transcript variant 1, mRNA [NM_001013398]	NM_001013398
A_24_P403501	3.06E-06	S80864	S80864	cytochrome c-like polypeptide [human, lung adenocarcinoma A549, mRNA, 1041 nt]. [S80864]	
A_23_P130333	3.06E-06	NM_000371	NM_000371	Homo sapiens transthyretin (prealbumin, amyloidosis type I) (TTR), mRNA [NM_000371]	NM_000371
A_32_P162537	3.08E-06	THC2288797		Q753E4 (Q753E4) AFR372Wp, partial (3%) [THC2288797]	
A_23_P381102	3.08E-06	NM_207310	NM_207310	Homo sapiens hypothetical protein DKFZp434E2321 (DKFZp434E2321), mRNA [NM_207310]	NM_207310
A_23_P157299	3.08E-06	NM_001129	NM_001129	Homo sapiens AE binding protein 1 (AEBP1), mRNA [NM_001129]	NM_001129
A_23_P256473	3.09E-06	NM_006379	NM_006379	Homo sapiens sema domain, immunoglobulin domain (Ig), short basic domain, secreted, (semaphorin) 3C (SEMA3C), mRNA [NM_006379]	NM_006379
A_23_P72001	3.10E-06	NM_174933	NM_174933	Homo sapiens phytanoyl-CoA dioxygenase domain containing 1 (PHYHD1), mRNA [NM_174933]	NM_174933
A_23_P126782	3.12E-06	NM_001993	NM_001993	Homo sapiens coagulation factor III (thromboplastin, tissue factor) (F3), mRNA [NM_001993]	NM_001993
A_23_P171117	3.12E-06	NM_024657	NM_024657	Homo sapiens MORC family CW-type zinc finger 4 (MORC4), mRNA [NM_024657]	NM_024657
A_32_P319200	3.13E-06	NM_199127	NM_199127	Homo sapiens gamma-glutamyltransferase-like 4 (GGTL4), transcript variant 1, mRNA [NM_199127]	NM_199127
A_32_P187009	3.14E-06	AK026295	AK026295	Homo sapiens cDNA: FLJ22642 fis, clone HSI06970. [AK026295]	
A_24_P350838	3.17E-06	X62009	X62009	Homo sapiens partial mRNA for fibrillin 5. [X62009]	
A_24_P478940	3.19E-06	A_24_P478940			
A_23_P55682	3.19E-06	NM_023926	NM_023926	Homo sapiens zinc finger protein 447 (ZNF447), mRNA [NM_023926]	NM_023926
A_23_P122852	3.21E-06	NM_003078	NM_003078	Homo sapiens SWI/SNF related, matrix associated, actin dependent regulator of chromatin, subfamily d, member 3 (SMARCD3), transcript variant 2, mRNA [NM_003078]	NM_003078
A_23_P14072	3.21E-06	NM_002273	NM_002273	Homo sapiens keratin 8 (KRT8), mRNA [NM_002273]	NM_002273
A_32_P23125	3.24E-06	THC2435906		Q8BU53 (Q8BU53) Mus musculus 2 days pregnant adult female oviduct cDNA, RIKEN full-length enriched library, clone:E230038K10 product:procollagen-proline, 2-oxoglutarate 4-dioxygenase (proline 4-hydroxylase), alpha II polypeptide, full insert sequence,...	
A_23_P258944	3.24E-06	NM_012328	NM_012328	Homo sapiens DnaJ (Hsp40) homolog, subfamily B, member 9 (DNAJB9), mRNA [NM_012328]	NM_012328
A_23_P213745	3.24E-06	NM_004887	NM_004887	Homo sapiens chemokine (C-X-C motif) ligand 14 (CXCL14), mRNA [NM_004887]	NM_004887
A_24_P247233	3.24E-06	XM_498018	XM_498018	PREDICTED: Homo sapiens similar to Keratin, type I cytoskeletal 18 (Cytokeratin 18) (K18) (CK 18) (LOC391827), mRNA [XM_498018]	XM_498018
A_23_P76743	3.25E-06	AX781433	AX781433	Sequence 16 from Patent EP1321519. [AX781433]	
A_24_P623814	3.30E-06	AK023526	AK023526	Homo sapiens cDNA FLJ13464 fis, clone PLACE1003478. [AK023526]	
A_23_P326160	3.30E-06	NM_025079	NM_025079	Homo sapiens zinc finger CCCH-type containing 12A (ZC3H12A), mRNA [NM_025079]	NM_025079
A_32_P218355	3.31E-06	AK074567	AK074567	Homo sapiens cDNA FLJ90086 fis, clone HEMBA1005145. [AK074567]	XM_371820

A_23_P82979	3.31E-06	NM_006059	NM_006059	Homo sapiens laminin, gamma 3 (LAMC3), mRNA [NM_006059]	NM_006059
A_23_P157926	3.32E-06	NM_152570	NM_152570	Homo sapiens leucine rich repeat neuronal 6C (LRRN6C), mRNA [NM_152570]	NM_152570
A_23_P83579	3.32E-06	NM_014862	NM_014862	Homo sapiens aryl-hydrocarbon receptor nuclear translocator 2 (ARNT2), mRNA [NM_014862]	NM_014862
A_23_P164451	3.32E-06	NM_005994	NM_005994	Homo sapiens T-box 2 (TBX2), mRNA [NM_005994]	NM_005994
A_24_P219094	3.34E-06	CR936651	CR936651	Homo sapiens mRNA; cDNA DKFZp686G1344 (from clone DKFZp686G1344). [CR936651]	
A_23_P310921	3.34E-06	NM_002589	NM_002589	Homo sapiens BH-protocadherin (brain-heart) (PCDH7), transcript variant a, mRNA [NM_002589]	NM_002589
A_24_P703642	3.34E-06	THC2406981		Q9LCV5 (Q9LCV5) Atp operon (Fragment), partial (3%) [THC2406981]	
A_23_P38816	3.39E-06	NM_130786	NM_130786	Homo sapiens alpha-1-B glycoprotein (A1BG), mRNA [NM_130786]	NM_130786
A_23_P46315	3.40E-06	NM_198459	NM_198459	Homo sapiens FLJ37099 protein (FLJ37099), mRNA [NM_198459]	NM_198459
A_32_P169406	3.40E-06	CR615016	CR615016	full-length cDNA clone CS0DB006YM19 of Neuroblastoma Cot 10-normalized of Homo sapiens (human). [CR615016]	XM_378360
A_23_P112554	3.42E-06	NM_001855	NM_001855	Homo sapiens collagen, type XV, alpha 1 (COL15A1), mRNA [NM_001855]	NM_001855
A_32_P80809	3.42E-06	AA516273	AA516273	AA516273 nf68f09.s1 NCI_CGAP_Co3 Homo sapiens cDNA clone IMAGE:925097 3', mRNA sequence [AA516273]	
A_24_P200023	3.43E-06	NM_000877	NM_000877	Homo sapiens interleukin 1 receptor, type I (IL1R1), mRNA [NM_000877]	NM_000877
A_24_P74070	3.44E-06	NM_032510	NM_032510	Homo sapiens par-6 partitioning defective 6 homolog gamma (C. elegans) (PARD6G), mRNA [NM_032510]	NM_032510
A_23_P128215	3.44E-06	NM_003877	NM_003877	Homo sapiens suppressor of cytokine signaling 2 (SOCS2), mRNA [NM_003877]	NM_003877
A_24_P235429	3.45E-06	NM_005502	NM_005502	Homo sapiens ATP-binding cassette, sub-family A (ABC1), member 1 (ABCA1), mRNA [NM_005502]	NM_005502
A_23_P107432	3.47E-06	NM_207453	NM_207453	Homo sapiens FLJ35934 protein (FLJ35934), mRNA [NM_207453]	NM_207453
A_23_P95221	3.47E-06	NM_005577	NM_005577	Homo sapiens lipoprotein, Lp(a) (LPA), mRNA [NM_005577]	NM_005577
A_23_P211926	3.47E-06	NM_003392	NM_003392	Homo sapiens wingless-type MMTV integration site family, member 5A (WNT5A), mRNA [NM_003392]	NM_003392
A_23_P28927	3.47E-06	A_23_P28927			
A_32_P34522	3.47E-06	BC010544	BC010544	Homo sapiens cDNA clone IMAGE:3462401, partial cds. [BC010544]	
A_32_P206899	3.47E-06	THC2340845			
A_23_P62932	3.47E-06	NM_001677	NM_001677	Homo sapiens ATPase, Na+/K+ transporting, beta 1 polypeptide (ATP1B1), transcript variant 1, mRNA [NM_001677]	NM_001677
A_24_P944640	3.48E-06	NM_020909	NM_020909	Homo sapiens erythrocyte membrane protein band 4.1 like 5 (EPB41L5), mRNA [NM_020909]	NM_020909
A_23_P204048	3.48E-06	NM_006482	NM_006482	Homo sapiens dual-specificity tyrosine-(Y)-phosphorylation regulated kinase 2 (DYRK2), transcript variant 2, mRNA [NM_006482]	NM_006482
A_32_P194891	3.51E-06	THC2282954		Q6CQJ2 (Q6CQJ2) Similarity, partial (13%) [THC2323726]	
A_23_P164814	3.53E-06	NM_024323	NM_024323	Homo sapiens hypothetical protein MGC11271 (MGC11271), mRNA [NM_024323]	NM_024323
A_32_P232214	3.53E-06	THC2316753		Q91TG6 (Q91TG6) T130, partial (7%) [THC2316753]	
A_24_P302249	3.53E-06	NM_001643	NM_001643	Homo sapiens apolipoprotein A-II (APOA2), mRNA [NM_001643]	NM_001643
A_23_P11331	3.53E-06	NM_153333	NM_153333	Homo sapiens transcription elongation factor A (SII)-like 8 (TCEAL8), transcript variant 1, mRNA [NM_153333]	NM_153333
A_23_P169494	3.59E-06	NM_000607	NM_000607	Homo sapiens orosomucoid 1 (ORM1), mRNA [NM_000607]	NM_000607
A_24_P401124	3.62E-06	A_24_P401124			
A_23_P50146	3.64E-06	NM_213602	NM_213602	Homo sapiens CD33 antigen-like 3 (CD33L3), mRNA [NM_213602]	NM_213602
A_23_P31143	3.65E-06	NM_001003395	NM_001003395	Homo sapiens tumor protein D52-like 1 (TPD52L1), transcript variant 2, mRNA [NM_001003395]	NM_001003395
A_32_P129010	3.66E-06	NM_001013615	NM_001013615	Homo sapiens hypothetical LOC541468 protein (LOC541468), mRNA [NM_001013615]	NM_001013615
A_23_P424582	3.70E-06	NM_138934	NM_138934	Homo sapiens palmitoyl-protein thioesterase 2 (PPT2), transcript variant 3, mRNA [NM_138934]	NM_138934
A_23_P152559	3.73E-06	NM_004758	NM_004758	Homo sapiens benzodiazapine receptor (peripheral) associated protein 1 (BZRAP1), mRNA [NM_004758]	NM_004758
A_32_P827528	3.76E-06	BI089613	BI089613	602854489F1 NIH_MGC_10 Homo sapiens cDNA clone IMAGE:4995743 5', mRNA sequence [BI089613]	
A_23_P70660	3.80E-06	NM_017633	NM_017633	Homo sapiens family with sequence similarity 46, member A (FAM46A), mRNA [NM_017633]	NM_017633
A_23_P35414	3.83E-06	NM_005398	NM_005398	Homo sapiens protein phosphatase 1, regulatory (inhibitor) subunit 3C (PPP1R3C), mRNA [NM_005398]	NM_005398
A_24_P16214	3.84E-06	AK090827	AK090827	Homo sapiens cDNA FLJ33508 fis, clone BRAMY2005094. [AK090827]	
A_23_P257583	3.84E-06	NM_015689	NM_015689	Homo sapiens KIAA1277 (KIAA1277), mRNA [NM_015689]	NM_015689
A_23_P83368	3.85E-06	NM_016373	NM_016373	Homo sapiens WW domain containing oxidoreductase (WWOX), transcript variant 1, mRNA [NM_016373]	NM_016373
A_23_P200239	3.85E-06	NM_018982	NM_018982	Homo sapiens Yip1 domain family, member 1 (YIPF1), mRNA [NM_018982]	NM_018982

A_24_P370472	3.88E-06	NM_021983	NM_021983	Homo sapiens major histocompatibility complex, class II, DR beta 4 (HLA-DRB4), mRNA [NM_021983]	NM_021983
A_23_P378364	3.90E-06	NM_032457	NM_032457	Homo sapiens BH-protocadherin (brain-heart) (PCDH7), transcript variant c, mRNA [NM_032457]	NM_032457
A_24_P330518	3.91E-06	NM_001218	NM_001218	Homo sapiens carbonic anhydrase XII (CA12), transcript variant 1, mRNA [NM_001218]	NM_001218
A_23_P8913	3.92E-06	NM_000067	NM_000067	Homo sapiens carbonic anhydrase II (CA2), mRNA [NM_000067]	NM_000067
A_32_P429876	3.93E-06	AK092378	AK092378	Homo sapiens cDNA FLJ35059 fis, clone OCBBF2018827. [AK092378]	
A_23_P75288	3.93E-06	NM_032810	NM_032810	Homo sapiens ATPase family, AAA domain containing 1 (ATAD1), mRNA [NM_032810]	NM_032810
A_24_P383660	3.94E-06	ENST00000312751			
A_24_P358406	3.94E-06	A_24_P358406			
A_23_P12733	3.95E-06	NM_018649	NM_018649	Homo sapiens H2A histone family, member Y2 (H2AFY2), mRNA [NM_018649]	NM_018649
A_23_P122650	3.95E-06	A_23_P122650			
A_24_P417036	3.98E-06	NM_001001930	NM_001001930	Homo sapiens peroxisome proliferative activated receptor, alpha (PPARA), transcript variant 6, mRNA [NM_001001930]	NM_001001930
A_23_P73311	4.00E-06	NM_052958	NM_052958	Homo sapiens chromosome 8 open reading frame 34 (C8orf34), mRNA [NM_052958]	NM_052958
A_23_P85742	4.05E-06	NM_003851	NM_003851	Homo sapiens cellular repressor of E1A-stimulated genes 1 (CREG1), mRNA [NM_003851]	NM_003851
A_23_P105923	4.07E-06	NM_001362	NM_001362	Homo sapiens deiodinase, iodothyronine, type III (DIO3), mRNA [NM_001362]	NM_001362
A_23_P31765	4.12E-06	NM_006823	NM_006823	Homo sapiens protein kinase (cAMP-dependent, catalytic) inhibitor alpha (PKIA), transcript variant 1, mRNA [NM_006823]	NM_006823
A_23_P155514	4.12E-06	NM_001622	NM_001622	Homo sapiens alpha-2-HS-glycoprotein (AHSG), mRNA [NM_001622]	NM_001622
A_24_P62469	4.13E-06	AK122589	AK122589	Homo sapiens mRNA for FLJ00414 protein. [AK122589]	XM_371214
A_24_P247074	4.13E-06	A_24_P247074			
A_24_P317827	4.14E-06	BC043384	BC043384	Homo sapiens chromosome 9 open reading frame 127, mRNA (cDNA clone IMAGE:5246152), partial cds. [BC043384]	
A_23_P132889	4.14E-06	NM_001014809	NM_001014809	Homo sapiens collapsin response mediator protein 1 (CRMP1), transcript variant 1, mRNA [NM_001014809]	NM_001014809
A_23_P79155	4.15E-06	ENST00000329321		Homo sapiens cDNA FLJ16071 fis, clone HSYRA2001003, highly similar to PUTATIVE G PROTEIN-COUPLED RECEPTOR GPR39. [AK122643]	
A_24_P260346	4.16E-06	NM_000671	NM_000671	Homo sapiens alcohol dehydrogenase 5 (class III), chi polypeptide (ADH5), mRNA [NM_000671]	NM_000671
A_32_P16315	4.17E-06	ENST00000308911		full-length cDNA clone CS0DE002YA12 of Placenta of Homo sapiens (human). [CR603249]	XM_371684
A_24_P57528	4.17E-06	NM_139177	NM_139177	Homo sapiens solute carrier family 39 (metal ion transporter), member 11 (SLC39A11), mRNA [NM_139177]	NM_139177
A_23_P132619	4.19E-06	NM_000916	NM_000916	Homo sapiens oxytocin receptor (OXTR), mRNA [NM_000916]	NM_000916
A_23_P325562	4.22E-06	NM_006671	NM_006671	Homo sapiens solute carrier family 1 (glutamate transporter), member 7 (SLC1A7), mRNA [NM_006671]	NM_006671
A_23_P357504	4.26E-06	ENST00000328520		Q765F0 (Q765F0) PreC/core protein, partial (13%) [THC2251776]	
A_23_P217938	4.26E-06	NM_006542	NM_006542	Homo sapiens S-phase response (cyclin-related) (SPHAR), mRNA [NM_006542]	NM_006542
A_23_P340131	4.28E-06	NM_005865	NM_005865	Homo sapiens protease, serine, 16 (thymus) (PRSS16), mRNA [NM_005865]	NM_005865
A_24_P12401	4.28E-06	NM_001025366	NM_001025366	Homo sapiens vascular endothelial growth factor (VEGF), transcript variant 1, mRNA [NM_001025366]	NM_001025366
A_23_P391586	4.29E-06	NM_001018004	NM_001018004	Homo sapiens tropomyosin 1 (alpha) (TPM1), transcript variant 3, mRNA [NM_001018004]	NM_001018004
A_23_P132121	4.32E-06	NM_173354	NM_173354	Homo sapiens SNF1-like kinase (SNF1LK), mRNA [NM_173354]	NM_173354
A_23_P55518	4.32E-06	NM_005904	NM_005904	Homo sapiens SMAD, mothers against DPP homolog 7 (Drosophila) (SMAD7), mRNA [NM_005904]	NM_005904
A_24_P384369	4.32E-06	ENST00000330875			
A_32_P117354	4.34E-06	NM_014988	NM_014988	Homo sapiens KIAA1102 protein (KIAA1102), mRNA [NM_014988]	NM_014988
A_23_P5300	4.35E-06	NM_001875	NM_001875	Homo sapiens carbamoyl-phosphate synthetase 1, mitochondrial (CPS1), mRNA [NM_001875]	NM_001875
A_23_P420326	4.35E-06	NM_153756	NM_153756	Homo sapiens fibronectin type III domain containing 5 (FNDC5), mRNA [NM_153756]	NM_153756
A_24_P227069	4.36E-06	NM_020918	NM_020918	Homo sapiens glycerol-3-phosphate acyltransferase, mitochondrial (GPAM), mRNA [NM_020918]	NM_020918
A_23_P501435	4.37E-06	NM_020536	NM_020536	Homo sapiens CSRP2 binding protein (CSRP2BP), transcript variant 1, mRNA [NM_020536]	NM_020536
A_24_P746314	4.38E-06	THC2403712		aspartate aminotranferase {Xylella fastidiosa Temecula1;} , partial (4%) [THC2403712]	
A_23_P202484	4.41E-06	NM_032772	NM_032772	Homo sapiens zinc finger protein 503 (ZNF503), mRNA [NM_032772]	NM_032772
A_23_P218111	4.44E-06	NM_001002236	NM_001002236	Homo sapiens serine (or cysteine) proteinase inhibitor, clade A (alpha-1 antiproteinase, antitrypsin), member 1 (SERPINA1), transcript variant 2, mRNA [NM_001002236]	NM_001002236
A_23_P103981	4.44E-06	NM_003516	NM_003516	Homo sapiens histone 2, H2aa (HIST2H2AA), mRNA [NM_003516]	NM_003516
A_32_P112546	4.44E-06	A_32_P112546			

A_32_P3385	4.47E-06	CR611098	CR611098	full-length cDNA clone CS0DI060YD22 of Placenta Cot 25-normalized of Homo sapiens (human). [CR611098]	XM_379417
A_32_P115606	4.47E-06	AK131385	AK131385	Homo sapiens cDNA FLJ16460 fis, clone BRCAN2018240. [AK131385]	
A_23_P21976	4.53E-06	NM_001897	NM_001897	Homo sapiens chondroitin sulfate proteoglycan 4 (melanoma-associated) (CSPG4), mRNA [NM_001897]	NM_001897
A_23_P339818	4.53E-06	NM_183376	NM_183376	Homo sapiens arrestin domain containing 4 (ARRDC4), mRNA [NM_183376]	NM_183376
A_23_P211598	4.53E-06	NM_002676	NM_002676	Homo sapiens phosphomannomutase 1 (PMM1), mRNA [NM_002676]	NM_002676
A_24_P334726	4.55E-06	NM_015689	NM_015689	Homo sapiens KIAA1277 (KIAA1277), mRNA [NM_015689]	NM_015689
A_23_P88849	4.55E-06	NM_004165	NM_004165	Homo sapiens Ras-related associated with diabetes (RRAD), mRNA [NM_004165]	NM_004165
A_23_P11071	4.55E-06	NM_203473	NM_203473	Homo sapiens porcupine homolog (Drosophila) (PORCN), transcript variant B, mRNA [NM_203473]	NM_203473
A_23_P133517	4.56E-06	NM_002310	NM_002310	Homo sapiens leukemia inhibitory factor receptor (LIFR), mRNA [NM_002310]	NM_002310
A_23_P10182	4.56E-06	NM_003500	NM_003500	Homo sapiens acyl-Coenzyme A oxidase 2, branched chain (ACOX2), mRNA [NM_003500]	NM_003500
A_23_P374339	4.57E-06	NM_152794	NM_152794	Homo sapiens hypoxia inducible factor 3, alpha subunit (HIF3A), transcript variant 1, mRNA [NM_152794]	NM_152794
A_24_P365571	4.57E-06	NM_032476	NM_032476	Homo sapiens mitochondrial ribosomal protein S6 (MRPS6), nuclear gene encoding mitochondrial protein, mRNA [NM_032476]	NM_032476
A_23_P96285	4.59E-06	NM_022912	NM_022912	Homo sapiens chromosome 2 open reading frame 23 (C2orf23), mRNA [NM_022912]	NM_022912
A_23_P213171	4.62E-06	NM_000253	NM_000253	Homo sapiens microsomal triglyceride transfer protein (large polypeptide, 88kDa) (MTP), mRNA [NM_000253]	NM_000253
A_23_P119562	4.62E-06	NM_001928	NM_001928	Homo sapiens D component of complement (adipsin) (DF), mRNA [NM_001928]	NM_001928
A_23_P66798	4.62E-06	NM_002276	NM_002276	Homo sapiens keratin 19 (KRT19), mRNA [NM_002276]	NM_002276
A_23_P418199	4.65E-06	ENST00000325151		Homo sapiens, clone IMAGE:5248278, mRNA, partial cds. [BC037171]	
A_23_P431569	4.68E-06	ENST00000318291		Homo sapiens, clone IMAGE:3621928, mRNA, partial cds. [BC033133]	
A_24_P42495	4.70E-06	BC010544	BC010544	Homo sapiens cDNA clone IMAGE:3462401, partial cds. [BC010544]	
A_23_P118203	4.74E-06	NM_145252	NM_145252	Homo sapiens similar to common salivary protein 1 (LOC124220), mRNA [NM_145252]	NM_145252
A_23_P155477	4.74E-06	NM_016210	NM_016210	Homo sapiens chromosome 3 open reading frame 18 (C3orf18), mRNA [NM_016210]	NM_016210
A_32_P205431	4.80E-06	AF085962	AF085962	Homo sapiens full length insert cDNA clone YS02G11. [AF085962]	
A_23_P428129	4.82E-06	NM_000076	NM_000076	Homo sapiens cyclin-dependent kinase inhibitor 1C (p57, Kip2) (CDKN1C), mRNA [NM_000076]	NM_000076
A_32_P228804	4.84E-06	BX649112	BX649112	Homo sapiens mRNA; cDNA DKFZp686E02109 (from clone DKFZp686E02109). [BX649112]	
A_24_P333901	4.84E-06	NM_015284	NM_015284	Homo sapiens KIAA0467 (KIAA0467), mRNA [NM_015284]	NM_015284
A_23_P250102	4.84E-06	ENST00000295989		Homo sapiens mRNA for KIAA0667 protein, partial cds. [AB014567]	XM_371617
A_23_P83134	4.84E-06	NM_002048	NM_002048	Homo sapiens growth arrest-specific 1 (GAS1), mRNA [NM_002048]	NM_002048
A_23_P6066	4.87E-06	NM_019609	NM_019609	Homo sapiens carboxypeptidase X (M14 family) (CPXM), mRNA [NM_019609]	NM_019609
A_23_P110791	4.88E-06	NM_005211	NM_005211	Homo sapiens colony stimulating factor 1 receptor, formerly McDonough feline sarcoma viral (v-fms) oncogene homolog (CSF1R), mRNA [NM_005211]	NM_005211
A_24_P306704	4.88E-06	A_24_P306704			
A_23_P35205	4.97E-06	ENST00000236261		Homo sapiens Down syndrome critical region gene 1-like 2, mRNA (cDNA clone MGC:46109 IMAGE:5770066), complete cds. [BC035854]	
A_23_P110122	5.00E-06	NM_004354	NM_004354	Homo sapiens cyclin G2 (CCNG2), mRNA [NM_004354]	NM_004354
A_23_P257043	5.04E-06	NM_005261	NM_005261	Homo sapiens GTP binding protein overexpressed in skeletal muscle (GEM), transcript variant 1, mRNA [NM_005261]	NM_005261
A_23_P93722	5.04E-06	AK074077	AK074077	Homo sapiens mRNA for FLJ00148 protein. [AK074077]	
A_23_P408376	5.05E-06	AB007877	AB007877	Homo sapiens KIAA0417 mRNA, complete cds. [AB007877]	XM_048898
A_24_P890995	5.06E-06	A_24_P890995			
A_32_P13612	5.13E-06	THC2347074			
A_24_P792988	5.13E-06	A_24_P792988			
A_24_P217572	5.14E-06	NM_001957	NM_001957	Homo sapiens endothelin receptor type A (EDNRA), mRNA [NM_001957]	NM_001957
A_23_P21485	5.14E-06	NM_017933	NM_017933	Homo sapiens hypothetical protein FLJ20701 (FLJ20701), mRNA [NM_017933]	NM_017933
A_23_P164958	5.14E-06	NM_032040	NM_032040	Homo sapiens coiled-coil domain containing 8 (CCDC8), mRNA [NM_032040]	NM_032040
A_23_P252541	5.19E-06	NM_177403	NM_177403	Homo sapiens RAB7B, member RAS oncogene family (RAB7B), mRNA [NM_177403]	NM_177403
A_32_P92445	5.21E-06	AK092715	AK092715	Homo sapiens cDNA FLJ35396 fis, clone SKNSH2003483. [AK092715]	
A_23_P37375	5.21E-06	NM_004755	NM_004755	Homo sapiens ribosomal protein S6 kinase, 90kDa, polypeptide 5 (RPS6KA5), transcript variant 1, mRNA [NM_004755]	NM_004755

A_23_P257649	5.22E-06	NM_002899	NM_002899	Homo sapiens retinol binding protein 1, cellular (RBP1), mRNA [NM_002899]	NM_002899
A_23_P100539	5.24E-06	CR609146	CR609146	full-length cDNA clone CS0DM005YF09 of Fetal liver of Homo sapiens (human). [CR609146]	
A_23_P18692	5.25E-06	NM_000671	NM_000671	Homo sapiens alcohol dehydrogenase 5 (class III), chi polypeptide (ADH5), mRNA [NM_000671]	NM_000671
A_23_P335452	5.30E-06	NM_153367	NM_153367	Homo sapiens chromosome 10 open reading frame 56 (C10orf56), mRNA [NM_153367]	NM_153367
A_23_P120435	5.31E-06	NM_080614	NM_080614	Homo sapiens WAP four-disulfide core domain 3 (WFDC3), transcript variant 1, mRNA [NM_080614]	NM_080614
A_24_P523061	5.36E-06	BC031320	BC031320	Homo sapiens, clone IMAGE:5278682, mRNA. [BC031320]	
A_23_P8108	5.37E-06	NM_002123	NM_002123	Homo sapiens major histocompatibility complex, class II, DQ beta 1 (HLA-DQB1), mRNA [NM_002123]	NM_002123
A_23_P256084	5.37E-06	NM_000047	NM_000047	Homo sapiens arylsulfatase E (chondrodysplasia punctata 1) (ARSE), mRNA [NM_000047]	NM_000047
A_23_P162466	5.38E-06	NM_004572	NM_004572	Homo sapiens plakophilin 2 (PKP2), transcript variant 2b, mRNA [NM_004572]	NM_004572
A_23_P201636	5.38E-06	NM_005562	NM_005562	Homo sapiens laminin, gamma 2 (LAMC2), transcript variant 1, mRNA [NM_005562]	NM_005562
A_23_P152262	5.39E-06	NM_004413	NM_004413	Homo sapiens dipeptidase 1 (renal) (DPEP1), mRNA [NM_004413]	NM_004413
A_23_P9485	5.39E-06	NM_000608	NM_000608	Homo sapiens orosomucoid 2 (ORM2), mRNA [NM_000608]	NM_000608
A_24_P109244	5.40E-06	NM_015026	NM_015026	Homo sapiens KIAA1040 protein (KIAA1040), mRNA [NM_015026]	NM_015026
A_23_P101806	5.40E-06	NM_182983	NM_182983	Homo sapiens hepsin (transmembrane protease, serine 1) (HPN), transcript variant 1, mRNA [NM_182983]	NM_182983
A_23_P146444	5.40E-06	BX648086	BX648086	Homo sapiens mRNA; cDNA DKFZp686G19226 (from clone DKFZp686G19226). [BX648086]	
A_23_P430658	5.44E-06	NM_014571	NM_014571	Homo sapiens hairy/enhancer-of-split related with YRPW motif-like (HEYL), mRNA [NM_014571]	NM_014571
A_32_P109516	5.44E-06	XM_374029	XM_374029	PREDICTED: Homo sapiens hypothetical LOC389089 (LOC389089), mRNA [XM_374029]	XM_374029
A_23_P16866	5.46E-06	NM_007127	NM_007127	Homo sapiens villin 1 (VIL1), mRNA [NM_007127]	NM_007127
A_32_P131640	5.47E-06	NM_173078	NM_173078	Homo sapiens SLIT and NTRK-like family, member 4 (SLITRK4), mRNA [NM_173078]	NM_173078
A_24_P923483	5.50E-06	BC020867	BC020867	Homo sapiens solute carrier family 6 (neurotransmitter transporter, GABA), member 13, mRNA (cDNA clone IMAGE:4594185), complete cds. [BC020867]	
A_23_P216596	5.51E-06	AK075235	AK075235	Homo sapiens cDNA FLJ90754 fis, clone PLACE4000354, weakly similar to COMPLEMENT RECEPTOR TYPE 1 PRECURSOR. [AK075235]	
A_23_P306730	5.56E-06	L02932	L02932	Human peroxisome proliferator activated receptor mRNA, complete cds. [L02932]	
A_23_P351270	5.57E-06	NM_002581	NM_002581	Homo sapiens pregnancy-associated plasma protein A, pappalysin 1 (PAPPA), mRNA [NM_002581]	NM_002581
A_23_P90333	5.59E-06	XM_292765	XM_292765	PREDICTED: Homo sapiens zinc finger protein 404 (ZNF404), mRNA [XM_292765]	XM_292765
A_23_P85783	5.59E-06	NM_006623	NM_006623	Homo sapiens phosphoglycerate dehydrogenase (PHGDH), mRNA [NM_006623]	NM_006623
A_23_P41455	5.60E-06	NM_003305	NM_003305	Homo sapiens transient receptor potential cation channel, subfamily C, member 3 (TRPC3), mRNA [NM_003305]	NM_003305
A_24_P934455	5.60E-06	AY358103	AY358103	Homo sapiens clone DNA108759 PIKR2786 (UNQ2786) mRNA, complete cds. [AY358103]	
A_23_P127911	5.60E-06	NM_015430	NM_015430	Homo sapiens regeneration associated muscle protease (DKFZP586H2123), transcript variant 1, mRNA [NM_015430]	NM_015430
A_32_P50066	5.61E-06	CR749274	CR749274	Homo sapiens mRNA; cDNA DKFZp781M044 (from clone DKFZp781M044). [CR749274]	
A_24_P392201	5.65E-06	NM_198517	NM_198517	Homo sapiens TBC1 domain family, member 10C (TBC1D10C), mRNA [NM_198517]	NM_198517
A_24_P410797	5.70E-06	AK125979	AK125979	Homo sapiens cDNA FLJ43991 fis, clone TESTI4019843, highly similar to Rattus norvegicus huntingtin-associated protein interacting protein (duo) (Hapip). [AK125979]	
A_23_P32253	5.70E-06	NM_005384	NM_005384	Homo sapiens nuclear factor, interleukin 3 regulated (NFIL3), mRNA [NM_005384]	NM_005384
A_32_P10936	5.73E-06	NM_004061	NM_004061	Homo sapiens cadherin 12, type 2 (N-cadherin 2) (CDH12), mRNA [NM_004061]	NM_004061
A_32_P71858	5.75E-06	AK124776	AK124776	Homo sapiens cDNA FLJ42786 fis, clone BRAWH3006761. [AK124776]	
A_23_P97402	5.75E-06	NM_020439	NM_020439	Homo sapiens calcium/calmodulin-dependent protein kinase IG (CAMK1G), mRNA [NM_020439]	NM_020439
A_23_P164210	5.77E-06	NM_032258	NM_032258	Homo sapiens TBC1 domain family, member 3 (TBC1D3), mRNA [NM_032258]	NM_032258
A_23_P114232	5.79E-06	NM_006406	NM_006406	Homo sapiens peroxiredoxin 4 (PRDX4), mRNA [NM_006406]	NM_006406
A_23_P56197	5.80E-06	NM_004750	NM_004750	Homo sapiens cytokine receptor-like factor 1 (CRLF1), mRNA [NM_004750]	NM_004750
A_23_P102364	5.83E-06	NM_019850	NM_019850	Homo sapiens neuronal guanine nucleotide exchange factor (NGEF), mRNA [NM_019850]	NM_019850
A_23_P95060	5.83E-06	NM_004443	NM_004443	Homo sapiens EPH receptor B3 (EPHB3), mRNA [NM_004443]	NM_004443
A_23_P16817	5.83E-06	NM_004071	NM_004071	Homo sapiens CDC-like kinase 1 (CLK1), transcript variant 1, mRNA [NM_004071]	NM_004071
A_23_P34142	5.83E-06	NM_016303	NM_016303	Homo sapiens WW domain binding protein 5 (WBP5), transcript variant 1, mRNA [NM_016303]	NM_016303
A_32_P58977	5.85E-06	AW068592	AW068592	AW068592 cn21d01.x1 Normal Human Trabecular Bone Cells Homo sapiens cDNA clone NHTBC_cn21d01 random, mRNA sequence [AW068592]	
A_23_P133438	5.85E-06	NM_019018	NM_019018	Homo sapiens hypothetical protein FLJ11127 (FLJ11127), mRNA [NM_019018]	NM_019018

A_23_P500381	5.85E-06	NM_019859	NM_019859	Homo sapiens 5-hydroxytryptamine (serotonin) receptor 7 (adenylate cyclase-coupled) (HTR7), transcript variant d, mRNA [NM_019859]	NM_019859
A_32_P127248	5.86E-06	CR936742	CR936742	Homo sapiens mRNA; cDNA DKFZp686G1442 (from clone DKFZp686G1442). [CR936742]	
A_23_P308519	5.88E-06	NM_004252	NM_004252	Homo sapiens solute carrier family 9 (sodium/hydrogen exchanger), isoform 3 regulator 1 (SLC9A3R1), mRNA [NM_004252]	NM_004252
A_23_P40194	5.91E-06	NM_017895	NM_017895	Homo sapiens DEAD (Asp-Glu-Ala-Asp) box polypeptide 27 (DDX27), mRNA [NM_017895]	NM_017895
A_23_P135273	5.93E-06	NM_001005336	NM_001005336	Homo sapiens dynamin 1 (DNM1), transcript variant 2, mRNA [NM_001005336]	NM_001005336
A_23_P48988	5.97E-06	NM_003027	NM_003027	Homo sapiens SH3-domain GRB2-like 3 (SH3GL3), mRNA [NM_003027]	NM_003027
A_24_P256063	5.97E-06	ENST00000332292			
A_23_P106682	5.99E-06	NM_001424	NM_001424	Homo sapiens epithelial membrane protein 2 (EMP2), mRNA [NM_001424]	NM_001424
A_24_P895528	6.00E-06	AK022035	AK022035	Homo sapiens cDNA FLJ11973 fis, clone HEMBB1001221. [AK022035]	
A_23_P250164	6.06E-06	NM_000187	NM_000187	Homo sapiens homogentisate 1,2-dioxygenase (homogentisate oxidase) (HGD), mRNA [NM_000187]	NM_000187
A_32_P79483	6.06E-06	BC033993	BC033993	Homo sapiens hypothetical protein LOC283481, mRNA (cDNA clone IMAGE:5296747), partial cds. [BC033993]	
A_23_P204158	6.06E-06	NM_032814	NM_032814	Homo sapiens hypothetical protein FLJ14627 (FLJ14627), mRNA [NM_032814]	NM_032814
A_23_P101905	6.06E-06	NM_005883	NM_005883	Homo sapiens adenomatosis polyposis coli 2 (APC2), mRNA [NM_005883]	NM_005883
A_23_P92929	6.08E-06	NM_016644	NM_016644	Homo sapiens mesenchymal stem cell protein DSC54 (LOC51334), mRNA [NM_016644]	NM_016644
A_23_P74229	6.12E-06	NM_032017	NM_032017	Homo sapiens serine/threonine kinase 40 (STK40), mRNA [NM_032017]	NM_032017
A_24_P233786	6.19E-06	NM_052966	NM_052966	Homo sapiens chromosome 1 open reading frame 24 (C1orf24), transcript variant 2, mRNA [NM_052966]	NM_052966
A_23_P76718	6.20E-06	NM_002498	NM_002498	Homo sapiens NIMA (never in mitosis gene a)-related kinase 3 (NEK3), transcript variant 1, mRNA [NM_002498]	NM_002498
A_24_P268993	6.21E-06	NM_052971	NM_052971	Homo sapiens liver-expressed antimicrobial peptide 2 (LEAP-2), mRNA [NM_052971]	NM_052971
A_23_P72127	6.23E-06	NM_020836	NM_020836	Homo sapiens likely ortholog of rat brain-enriched guanylate kinase-associated protein (KIAA1446), mRNA [NM_020836]	NM_020836
A_24_P267293	6.26E-06	NM_015077	NM_015077	Homo sapiens sterile alpha and TIR motif containing 1 (SARM1), mRNA [NM_015077]	NM_015077
A_23_P214281	6.29E-06	NM_133367	NM_133367	Homo sapiens progestin and adipoQ receptor family member VIII (PAQR8), mRNA [NM_133367]	NM_133367
A_23_P416581	6.31E-06	NM_002073	NM_002073	Homo sapiens guanine nucleotide binding protein (G protein), alpha z polypeptide (GNAZ), mRNA [NM_002073]	NM_002073
A_23_P252783	6.31E-06	NM_014580	NM_014580	Homo sapiens solute carrier family 2, (facilitated glucose transporter) member 8 (SLC2A8), mRNA [NM_014580]	NM_014580
A_23_P17053	6.33E-06	NM_019618	NM_019618	Homo sapiens interleukin 1 family, member 9 (IL1F9), mRNA [NM_019618]	NM_019618
A_23_P214977	6.35E-06	NM_007214	NM_007214	Homo sapiens SEC63-like (S. cerevisiae) (SEC63), mRNA [NM_007214]	NM_007214
A_23_P4649	6.37E-06	NM_001645	NM_001645	Homo sapiens apolipoprotein C-I (APOC1), mRNA [NM_001645]	NM_001645
A_24_P183664	6.40E-06	AB014544	AB014544	Homo sapiens mRNA for KIAA0644 protein, partial cds. [AB014544]	XM_376588
A_23_P93543	6.45E-06	NM_000288	NM_000288	Homo sapiens peroxisomal biogenesis factor 7 (PEX7), mRNA [NM_000288]	NM_000288
A_23_P139500	6.49E-06	NM_030762	NM_030762	Homo sapiens basic helix-loop-helix domain containing, class B, 3 (BHLHB3), mRNA [NM_030762]	NM_030762
A_23_P70719	6.49E-06	NM_000426	NM_000426	Homo sapiens laminin, alpha 2 (merosin, congenital muscular dystrophy) (LAMA2), mRNA [NM_000426]	NM_000426
A_32_P3214	6.49E-06	THC2433340		Q93KJ6 (Q93KJ6) Dissimilatory (Bi-)sulfite reductase beta subunit (Fragment), partial (5%) [THC2433340]	
A_24_P247454	6.53E-06	A_24_P247454			
A_24_P260101	6.60E-06	NM_007289	NM_007289	Homo sapiens membrane metallo-endopeptidase (neutral endopeptidase, enkephalinase, CALLA, CD10) (MME), transcript variant 2b, mRNA [NM_007289]	NM_007289
A_32_P71744	6.63E-06	THC2370609			
A_23_P79482	6.63E-06	NM_001822	NM_001822	Homo sapiens chimerin (chimaerin) 1 (CHN1), transcript variant 1, mRNA [NM_001822]	NM_001822
A_23_P119464	6.72E-06	NM_033103	NM_033103	Homo sapiens rhophilin, Rho GTPase binding protein 2 (RHPN2), mRNA [NM_033103]	NM_033103
A_23_P77328	6.72E-06	NM_005258	NM_005258	Homo sapiens GTP cyclohydrolase I feedback regulator (GCHFR), mRNA [NM_005258]	NM_005258
A_23_P23279	6.74E-06	NM_052862	NM_052862	Homo sapiens RCSD domain containing 1 (RCSD1), mRNA [NM_052862]	NM_052862
A_23_P154585	6.74E-06	NM_033421	NM_033421	Homo sapiens chromosome 20 open reading frame 161 (C20orf161), transcript variant 1, mRNA [NM_033421]	NM_033421
A_23_P95810	6.74E-06	NM_007183	NM_007183	Homo sapiens plakophilin 3 (PKP3), mRNA [NM_007183]	NM_007183
A_23_P52266	6.77E-06	NM_001548	NM_001548	Homo sapiens interferon-induced protein with tetratricopeptide repeats 1 (IFIT1), transcript variant 2, mRNA [NM_001548]	NM_001548
A_23_P383009	6.78E-06	NM_000599	NM_000599	Homo sapiens insulin-like growth factor binding protein 5 (IGFBP5), mRNA [NM_000599]	NM_000599
A_24_P402690	6.78E-06	NM_030926	NM_030926	Homo sapiens integral membrane protein 2C (ITM2C), transcript variant 1, mRNA [NM_030926]	NM_030926
A_24_P57631	6.79E-06	NM_004484	NM_004484	Homo sapiens glypican 3 (GPC3), mRNA [NM_004484]	NM_004484

A_23_P310022	6.85E-06	NM_019590	NM_019590	Homo sapiens KIAA1217 (KIAA1217), mRNA [NM_019590]	NM_019590
A_23_P416395	6.87E-06	NM_003714	NM_003714	Homo sapiens stanniocalcin 2 (STC2), mRNA [NM_003714]	NM_003714
A_24_P344961	6.88E-06	NM_133265	NM_133265	Homo sapiens angiomotin (AMOT), mRNA [NM_133265]	NM_133265
A_24_P924484	6.91E-06	K03200	K03200	Human melanoma-associated antigen p97 (melanotransferrin) mRNA, 3' flank. [K03200]	
A_24_P295010	6.91E-06	NM_004155	NM_004155	Homo sapiens serine (or cysteine) proteinase inhibitor, clade B (ovalbumin), member 9 (SERPINB9), mRNA [NM_004155]	NM_004155
A_23_P65918	6.93E-06	NM_002220	NM_002220	Homo sapiens inositol 1,4,5-trisphosphate 3-kinase A (ITPKA), mRNA [NM_002220]	NM_002220
A_23_P71480	6.94E-06	NM_005218	NM_005218	Homo sapiens defensin, beta 1 (DEFB1), mRNA [NM_005218]	NM_005218
A_23_P304991	6.98E-06	NM_000411	NM_000411	Homo sapiens holocarboxylase synthetase (biotin-(proprionyl-Coenzyme A-carboxylase (ATP-hydrolysing)) ligase) (HLCS), mRNA [NM_000411]	NM_000411
A_23_P69573	7.03E-06	NM_000856	NM_000856	Homo sapiens guanylate cyclase 1, soluble, alpha 3 (GUCY1A3), mRNA [NM_000856]	NM_000856
A_23_P203120	7.04E-06	NM_014333	NM_014333	Homo sapiens immunoglobulin superfamily, member 4 (IGSF4), mRNA [NM_014333]	NM_014333
A_32_P101799	7.11E-06	THC2282944			
A_24_P212539	7.12E-06	NM_138801	NM_138801	Homo sapiens galactose mutarotase (aldose 1-epimerase) (GALM), mRNA [NM_138801]	NM_138801
A_24_P145009	7.12E-06	NM_001013723	NM_001013723	Homo sapiens hypothetical gene supported by AK094370 (LOC441208), mRNA [NM_001013723]	NM_001013723
A_24_P920388	7.12E-06	XM_378398	XM_378398	PREDICTED: Homo sapiens hypothetical LOC400092 (LOC400092), mRNA [XM_378398]	XM_378398
A_32_P32391	7.13E-06	NR_002171	NR_002171	Homo sapiens olfactory receptor, family 7, subfamily E, member 156 pseudogene (OR7E156P) on chromosome 13 [NR_002171]	NR_002171
A_24_P481783	7.16E-06	AK023802	AK023802	Homo sapiens cDNA FLJ13740 fis, clone PLACE3000199. [AK023802]	
A_23_P364504	7.16E-06	ENST00000344233		Homo sapiens cDNA FLJ37034 fis, clone BRACE2011478. [AK094353]	
A_23_P161647	7.16E-06	NM_000920	NM_000920	Homo sapiens pyruvate carboxylase (PC), nuclear gene encoding mitochondrial protein, transcript variant A, mRNA [NM_000920]	NM_000920
A_23_P62021	7.16E-06	L12350	L12350	Human thrombospondin 2 (THBS2) mRNA, complete cds. [L12350]	
A_24_P392060	7.18E-06	NM_198567	NM_198567	Homo sapiens FLJ44216 protein (FLJ44216), mRNA [NM_198567]	NM_198567
A_23_P353704	7.21E-06	NM_019593	NM_019593	Homo sapiens hypothetical protein KIAA1434 (KIAA1434), mRNA [NM_019593]	NM_019593
A_23_P215070	7.22E-06	NM_018718	NM_018718	Homo sapiens testis specific, 14 (TSGA14), mRNA [NM_018718]	NM_018718
A_24_P286114	7.22E-06	NM_004172	NM_004172	Homo sapiens solute carrier family 1 (glial high affinity glutamate transporter), member 3 (SLC1A3), mRNA [NM_004172]	NM_004172
A_23_P29096	7.24E-06	NM_002606	NM_002606	Homo sapiens phosphodiesterase 9A (PDE9A), transcript variant 1, mRNA [NM_002606]	NM_002606
A_23_P114221	7.25E-06	NM_002893	NM_002893	Homo sapiens retinoblastoma binding protein 7 (RBBP7), mRNA [NM_002893]	NM_002893
A_32_P208097	7.27E-06	THC2310998		Q6GN35 (Q6GN35) LOC443687 protein (Fragment), partial (62%) [THC2310998]	
A_23_P200780	7.27E-06	NM_003243	NM_003243	Homo sapiens transforming growth factor, beta receptor III (betaglycan, 300kDa) (TGFBR3), mRNA [NM_003243]	NM_003243
A_23_P117782	7.27E-06	NM_018357	NM_018357	Homo sapiens La ribonucleoprotein domain family, member 6 (LARP6), transcript variant 1, mRNA [NM_018357]	NM_018357
A_23_P431410	7.27E-06	BC065192	BC065192	Homo sapiens chromosome 2 open reading frame 12, mRNA (cDNA clone IMAGE:5205269), partial cds. [BC065192]	
A_23_P97283	7.32E-06	NM_024897	NM_024897	Homo sapiens progestin and adipoQ receptor family member VI (PAQR6), transcript variant 1, mRNA [NM_024897]	NM_024897
A_24_P943575	7.34E-06	NM_032221	NM_032221	Homo sapiens chromodomain helicase DNA binding protein 6 (CHD6), mRNA [NM_032221]	NM_032221
A_23_P107653	7.34E-06	NM_001014763	NM_001014763	Homo sapiens electron-transfer-flavoprotein, beta polypeptide (ETFB), transcript variant 2, mRNA [NM_001014763]	NM_001014763
A_23_P53288	7.42E-06	NM_014255	NM_014255	Homo sapiens transmembrane protein 4 (TMEM4), mRNA [NM_014255]	NM_014255
A_24_P364591	7.45E-06	NM_006329	NM_006329	Homo sapiens fibulin 5 (FBLN5), mRNA [NM_006329]	NM_006329
A_32_P6408	7.50E-06	ENST00000360887		Homo sapiens mRNA; cDNA DKFZp434J1015 (from clone DKFZp434J1015). [AL133055]	XM_499257
A_23_P94879	7.51E-06	NM_000506	NM_000506	Homo sapiens coagulation factor II (thrombin) (F2), mRNA [NM_000506]	NM_000506
A_23_P67198	7.52E-06	NM_015692	NM_015692	Homo sapiens C3 and PZP-like, alpha-2-macroglobulin domain containing 8 (CPAMD8), mRNA [NM_015692]	NM_015692
A_32_P201979	7.53E-06	NM_182765	NM_182765	Homo sapiens HECT domain containing 2 (HECTD2), transcript variant 1, mRNA [NM_182765]	NM_182765
A_23_P164650	7.53E-06	NM_000041	NM_000041	Homo sapiens apolipoprotein E (APOE), mRNA [NM_000041]	NM_000041
A_23_P143127	7.55E-06	NM_019063	NM_019063	Homo sapiens echinoderm microtubule associated protein like 4 (EML4), mRNA [NM_019063]	NM_019063
A_32_P6415	7.60E-06	NM_015088	NM_015088	Homo sapiens trinucleotide repeat containing 6B (TNRC6B), transcript variant 1, mRNA [NM_015088]	NM_015088
A_23_P147025	7.61E-06	NM_004794	NM_004794	Homo sapiens RAB33A, member RAS oncogene family (RAB33A), mRNA [NM_004794]	NM_004794
A_23_P105794	7.63E-06	NM_033255	NM_033255	Homo sapiens epithelial stromal interaction 1 (breast) (EPSTI1), mRNA [NM_033255]	NM_033255
A_23_P51856	7.65E-06	NM_007207	NM_007207	Homo sapiens dual specificity phosphatase 10 (DUSP10), transcript variant 1, mRNA [NM_007207]	NM_007207

A_24_P251962	7.65E-06	NM_018936	NM_018936	Homo sapiens protocadherin beta 2 (PCDHB2), mRNA [NM_018936]	NM_018936
A_32_P103220	7.70E-06	NM_030955	NM_030955	Homo sapiens a disintegrin-like and metalloprotease (reprolysin type) with thrombospondin type 1 motif, 12 (ADAMTS12), mRNA [NM_030955]	NM_030955
A_24_P341546	7.71E-06	A_24_P341546			
A_23_P500244	7.78E-06	NM_080706	NM_080706	Homo sapiens transient receptor potential cation channel, subfamily V, member 1 (TRPV1), transcript variant 3, mRNA [NM_080706]	NM_080706
A_24_P406693	7.78E-06	NM_000917	NM_000917	Homo sapiens procollagen-proline, 2-oxoglutarate 4-dioxygenase (proline 4-hydroxylase), alpha polypeptide I (P4HA1), transcript variant 1, mRNA [NM_000917]	NM_000917
A_24_P180243	7.79E-06	NM_053039	NM_053039	Homo sapiens UDP glucuronosyltransferase 2 family, polypeptide B28 (UGT2B28), mRNA [NM_053039]	NM_053039
A_23_P314101	7.81E-06	NM_019601	NM_019601	Homo sapiens sushi domain containing 2 (SUSD2), mRNA [NM_019601]	NM_019601
A_23_P370666	7.83E-06	NM_080661	NM_080661	Homo sapiens similar to RIKEN cDNA 0610008P16 gene (MGC15937), mRNA [NM_080661]	NM_080661
A_32_P6253	7.86E-06	THC2279919			
A_23_P214739	7.90E-06	NM_012160	NM_012160	Homo sapiens F-box and leucine-rich repeat protein 4 (FBXL4), mRNA [NM_012160]	NM_012160
A_24_P753592	7.90E-06	BC018589	BC018589	Homo sapiens cDNA clone IMAGE:3863301. [BC018589]	
A_23_P92710	7.92E-06	NM_014899	NM_014899	Homo sapiens Rho-related BTB domain containing 3 (RHOBTB3), mRNA [NM_014899]	NM_014899
A_24_P402242	7.93E-06	NM_000090	NM_000090	Homo sapiens collagen, type III, alpha 1 (Ehlers-Danlos syndrome type IV, autosomal dominant) (COL3A1), mRNA [NM_000090]	NM_000090
A_23_P157460	7.96E-06	NM_018660	NM_018660	Homo sapiens zinc finger protein 395 (ZNF395), mRNA [NM_018660]	NM_018660
A_24_P785894	7.96E-06	XM_373881	XM_373881	PREDICTED: Homo sapiens hypothetical LOC388727 (LOC388727), mRNA [XM_373881]	XM_373881
A_23_P341532	7.97E-06	NM_031915	NM_031915	Homo sapiens SET domain, bifurcated 2 (SETDB2), mRNA [NM_031915]	NM_031915
A_32_P41405	8.02E-06	NM_032361	NM_032361	Homo sapiens THO complex 3 (THOC3), mRNA [NM_032361]	NM_032361
A_24_P358131	8.06E-06	ENST00000351766			
A_23_P48198	8.07E-06	NM_031302	NM_031302	Homo sapiens glycosyltransferase 8 domain containing 2 (GLT8D2), mRNA [NM_031302]	NM_031302
A_23_P148584	8.07E-06	NM_144658	NM_144658	Homo sapiens dedicator of cytokinesis 11 (DOCK11), mRNA [NM_144658]	NM_144658
A_24_P10214	8.07E-06	NM_014178	NM_014178	Homo sapiens syntaxin binding protein 6 (amisyn) (STXBP6), mRNA [NM_014178]	NM_014178
A_24_P230057	8.08E-06	ENST00000327707			
A_23_P97181	8.10E-06	NM_022469	NM_022469	Homo sapiens gremlin 2, cysteine knot superfamily, homolog (Xenopus laevis) (GREM2), mRNA [NM_022469]	NM_022469
A_24_P281443	8.10E-06	A_24_P281443			
A_23_P31064	8.13E-06	NM_015529	NM_015529	Homo sapiens monooxygenase, DBH-like 1 (MOXD1), mRNA [NM_015529]	NM_015529
A_23_P325093	8.14E-06	NM_178026	NM_178026	Homo sapiens gamma-glutamyltransferase-like 3 (GGTL3), transcript variant 3, mRNA [NM_178026]	NM_178026
A_23_P214139	8.14E-06	NM_002912	NM_002912	Homo sapiens REV3-like, catalytic subunit of DNA polymerase zeta (yeast) (REV3L), mRNA [NM_002912]	NM_002912
A_24_P159837	8.14E-06	ENST00000328088		Homo sapiens mRNA for PREDICTED: similar to regulator of sex-limitation candidate 11 variant protein. [AB209226]	
A_24_P24645	8.31E-06	XM_497978	XM_497978	PREDICTED: Homo sapiens similar to Keratin, type I cytoskeletal 18 (Cytokeratin 18) (K18) (CK 18) (LOC132391), mRNA [XM_497978]	XM_497978
A_24_P365975	8.32E-06	NM_005202	NM_005202	Homo sapiens collagen, type VIII, alpha 2 (COL8A2), mRNA [NM_005202]	NM_005202
A_23_P56898	8.33E-06	NM_003937	NM_003937	Homo sapiens kynureninase (L-kynurenine hydrolase) (KYNU), mRNA [NM_003937]	NM_003937
A_23_P71867	8.33E-06	NM_147162	NM_147162	Homo sapiens interleukin 11 receptor, alpha (IL11RA), transcript variant 2, mRNA [NM_147162]	NM_147162
A_24_P149124	8.33E-06	NM_004772	NM_004772	Homo sapiens chromosome 5 open reading frame 13 (C5orf13), mRNA [NM_004772]	NM_004772
A_24_P418908	8.38E-06	AK021798	AK021798	Homo sapiens cDNA FLJ11736 fis, clone HEMBA1005468. [AK021798]	
A_32_P220700	8.38E-06	THC2440162			
A_24_P456723	8.44E-06	BC039374	BC039374	Homo sapiens, clone IMAGE:5271446, mRNA. [BC039374]	
A_23_P37892	8.44E-06	NM_133443	NM_133443	Homo sapiens glutamic pyruvate transaminase (alanine aminotransferase) 2 (GPT2), mRNA [NM_133443]	NM_133443
A_32_P4814	8.45E-06	NM_032508	NM_032508	Homo sapiens family with sequence similarity 11, member A (FAM11A), mRNA [NM_032508]	NM_032508
A_24_P123601	8.51E-06	NM_013994	NM_013994	Homo sapiens discoidin domain receptor family, member 1 (DDR1), transcript variant 3, mRNA [NM_013994]	NM_013994
A_23_P211047	8.54E-06	NM_206866	NM_206866	Homo sapiens BTB and CNC homology 1, basic leucine zipper transcription factor 1 (BACH1), transcript variant 1, mRNA [NM_206866]	NM_206866
A_23_P344884	8.57E-06	BC036557	BC036557	Homo sapiens KIAA1394 protein, mRNA (cDNA clone MGC:39385 IMAGE:4310128), complete cds. [BC036557]	XM_208522
A_23_P205228	8.57E-06	NM_000053	NM_000053	Homo sapiens ATPase, Cu++ transporting, beta polypeptide (Wilson disease) (ATP7B), transcript variant 1, mRNA [NM_000053]	NM_000053
A_24_P11462	8.60E-06	NM_052998	NM_052998	Homo sapiens arginine decarboxylase (ADC), mRNA [NM_052998]	NM_052998
A_32_P20221	8.60E-06	AK095738	AK095738	Homo sapiens cDNA FLJ38419 fis, clone FEBRA2009846. [AK095738]	

A_23_P11543	8.64E-06	NM_000147	NM_000147	Homo sapiens fucosidase, alpha-L- 1, tissue (FUCA1), mRNA [NM_000147]	NM_000147
A_23_P113613	8.66E-06	NM_022842	NM_022842	Homo sapiens CUB domain containing protein 1 (CDCP1), transcript variant 1, mRNA [NM_022842]	NM_022842
A_23_P58205	8.75E-06	NM_001134	NM_001134	Homo sapiens alpha-fetoprotein (AFP), mRNA [NM_001134]	NM_001134
A_23_P69179	8.75E-06	NM_018192	NM_018192	Homo sapiens leprecan-like 1 (LEPREL1), mRNA [NM_018192]	NM_018192
A_24_P375360	8.76E-06	A_24_P375360			
A_23_P102950	8.78E-06	NM_080860	NM_080860	Homo sapiens testis specific A2 homolog (mouse) (TSGA2), mRNA [NM_080860]	NM_080860
A_24_P143492	8.78E-06	NM_001010974	NM_001010974	Homo sapiens breast carcinoma amplified sequence 4 (BCAS4), transcript variant 3, mRNA [NM_001010974]	NM_001010974
A_24_P418687	8.78E-06	A_24_P418687			
A_23_P107775	8.88E-06	NM_139172	NM_139172	Homo sapiens MDAC1 (MDAC1), mRNA [NM_139172]	NM_139172
A_24_P321525	8.89E-06	NM_032918	NM_032918	Homo sapiens RAS-like, estrogen-regulated, growth inhibitor (RERG), mRNA [NM_032918]	NM_032918
A_23_P212061	8.92E-06	NM_007289	NM_007289	Homo sapiens membrane metallo-endopeptidase (neutral endopeptidase, enkephalinase, CALLA, CD10) (MME), transcript variant 2b, mRNA [NM_007289]	NM_007289
A_23_P4628	8.93E-06	NM_025027	NM_025027	Homo sapiens zinc finger protein 606 (ZNF606), mRNA [NM_025027]	NM_025027
A_24_P100535	8.93E-06	NM_181519	NM_181519	Homo sapiens synaptotagmin XV (SYT15), transcript variant b, mRNA [NM_181519]	NM_181519
A_23_P147514	8.93E-06	ENST00000305099		Homo sapiens hypothetical protein DKFZp547E052, mRNA (cDNA clone IMAGE:5493862), partial cds. [BC062636]	
A_24_P388433	8.93E-06	NM_002718	NM_002718	Homo sapiens protein phosphatase 2 (formerly 2A), regulatory subunit B'', alpha (PPP2R3A), transcript variant 1, mRNA [NM_002718]	NM_002718
A_23_P205236	8.93E-06	NM_002719	NM_002719	Homo sapiens protein phosphatase 2, regulatory subunit B (B56), gamma isoform (PPP2R5C), transcript variant 1, mRNA [NM_002719]	NM_002719
A_24_P402222	8.94E-06	NM_022555	NM_022555	Homo sapiens major histocompatibility complex, class II, DR beta 3 (HLA-DRB3), mRNA [NM_022555]	NM_022555
A_23_P69670	8.96E-06	NM_018366	NM_018366	Homo sapiens cappuccino homolog (mouse) (CNO), mRNA [NM_018366]	NM_018366
A_23_P73150	8.98E-06	NM_031421	NM_031421	Homo sapiens hypothetical protein DKFZp434H0115 (DKFZP434H0115), mRNA [NM_031421]	NM_031421
A_23_P300220	9.00E-06	NM_145008	NM_145008	Homo sapiens yippee-like 4 (Drosophila) (YPEL4), mRNA [NM_145008]	NM_145008
A_23_P162861	9.09E-06	ENST00000332273		Sequence 1 from Patent WO03046006. [AX772926]	
A_23_P81805	9.09E-06	NM_001025366	NM_001025366	Homo sapiens vascular endothelial growth factor (VEGF), transcript variant 1, mRNA [NM_001025366]	NM_001025366
A_32_P228341	9.11E-06	AK055783	AK055783	Homo sapiens cDNA FLJ31221 fis, clone KIDNE2004279. [AK055783]	
A_32_P62145	9.11E-06	BC013423	BC013423	Homo sapiens, clone IMAGE:3892140, mRNA. [BC013423]	
A_23_P22350	9.13E-06	NM_023927	NM_023927	Homo sapiens HCV NS3-transactivated protein 2 (NS3TP2), mRNA [NM_023927]	NM_023927
A_23_P53176	9.15E-06	NM_016725	NM_016725	Homo sapiens folate receptor 1 (adult) (FOLR1), transcript variant 1, mRNA [NM_016725]	NM_016725
A_23_P149496	9.17E-06	NM_018053	NM_018053	Homo sapiens X Kell blood group precursor-related family, member 8 (XKR8), mRNA [NM_018053]	NM_018053
A_23_P59261	9.18E-06	NM_006670	NM_006670	Homo sapiens trophoblast glycoprotein (TPBG), mRNA [NM_006670]	NM_006670
A_23_P80763	9.20E-06	BC017572	BC017572	Homo sapiens poliovirus receptor-related 3, mRNA (cDNA clone IMAGE:4066485), complete cds. [BC017572]	
A_32_P151544	9.21E-06	NM_000224	NM_000224	Homo sapiens keratin 18 (KRT18), transcript variant 1, mRNA [NM_000224]	NM_000224
A_23_P395438	9.23E-06	NM_053044	NM_053044	Homo sapiens HtrA serine peptidase 3 (HTRA3), mRNA [NM_053044]	NM_053044
A_24_P323072	9.30E-06	NM_178332	NM_178332	Homo sapiens gonadotropin-releasing hormone 2 (GNRH2), transcript variant 2, mRNA [NM_178332]	NM_178332
A_24_P230466	9.35E-06	A_24_P230466			
A_32_P66364	9.38E-06	BC017943	BC017943	Homo sapiens protein phosphatase 1, regulatory (inhibitor) subunit 1C, mRNA (cDNA clone MGC:24041 IMAGE:4288919), complete cds. [BC017943]	
A_24_P89509	9.39E-06	NM_021820	NM_021820	Homo sapiens chromosome 6 open reading frame 75 (C6orf75), mRNA [NM_021820]	NM_021820
A_23_P422115	9.39E-06	NM_144654	NM_144654	Homo sapiens chromosome 9 open reading frame 116 (C9orf116), mRNA [NM_144654]	NM_144654
A_23_P53588	9.41E-06	NM_030775	NM_030775	Homo sapiens wingless-type MMTV integration site family, member 5B (WNT5B), transcript variant 2, mRNA [NM_030775]	NM_030775
A_24_P360269	9.42E-06	NM_003730	NM_003730	Homo sapiens ribonuclease T2 (RNASET2), mRNA [NM_003730]	NM_003730
A_23_P204296	9.46E-06	NM_032918	NM_032918	Homo sapiens RAS-like, estrogen-regulated, growth inhibitor (RERG), mRNA [NM_032918]	NM_032918
A_23_P25964	9.51E-06	NM_000153	NM_000153	Homo sapiens galactosylceramidase (Krabbe disease) (GALC), mRNA [NM_000153]	NM_000153
A_24_P264293	9.51E-06	A_24_P264293			
A_23_P68505	9.52E-06	AL137442	AL137442	Homo sapiens mRNA; cDNA DKFZp566C114 (from clone DKFZp566C114). [AL137442]	
A_24_P79403	9.53E-06	NM_002619	NM_002619	Homo sapiens platelet factor 4 (chemokine (C-X-C motif) ligand 4) (PF4), mRNA [NM_002619]	NM_002619
A_23_P125164	9.53E-06	NM_002012	NM_002012	Homo sapiens fragile histidine triad gene (FHIT), mRNA [NM_002012]	NM_002012

A_23_P106602	9.63E-06	NM_031476	NM_031476	Homo sapiens cysteine-rich secretory protein LCCL domain containing 2 (CRISPLD2), mRNA [NM_031476]	NM_031476
A_23_P128624	9.65E-06	NM_024537	NM_024537	Homo sapiens hypothetical protein FLJ12118 (FLJ12118), mRNA [NM_024537]	NM_024537
A_32_P169550	9.67E-06	BX099371	BX099371	BX099371 BX099371 NCI_CGAP_Lu24 Homo sapiens cDNA clone IMAGp998O205798 ; IMAGE:2337259, mRNA sequence [BX099371]	
A_32_P214011	9.67E-06	THC2277187		Q6PKF4 (Q6PKF4) C20orf77 protein (Fragment), partial (5%) [THC2277187]	
A_23_P390445	9.73E-06	THC2427890		Q8TD54 (Q8TD54) Protein phosphatase 1 regulatory subunit 1A, partial (53%) [THC2427890]	
A_23_P321501	9.74E-06	NM_182908	NM_182908	Homo sapiens dehydrogenase/reductase (SDR family) member 2 (DHRS2), transcript variant 1, mRNA [NM_182908]	NM_182908
A_24_P402510	9.74E-06	NM_152486	NM_152486	Homo sapiens sterile alpha motif domain containing 11 (SAMD11), mRNA [NM_152486]	NM_152486
A_23_P117862	9.78E-06	NM_138704	NM_138704	Homo sapiens necdin-like 2 (NDNL2), mRNA [NM_138704]	NM_138704
A_23_P166929	9.79E-06	NM_005025	NM_005025	Homo sapiens serine (or cysteine) proteinase inhibitor, clade I (neuroserpin), member 1 (SERPINI1), mRNA [NM_005025]	NM_005025
A_23_P67367	9.79E-06	NM_014475	NM_014475	Homo sapiens dihydrodiol dehydrogenase (dimeric) (DHDH), mRNA [NM_014475]	NM_014475
A_23_P377214	9.79E-06	NM_144608	NM_144608	Homo sapiens hexamthylene bis-acetamide inducible 2 (HEXIM2), mRNA [NM_144608]	NM_144608
A_32_P171530	9.87E-06	BC038371	BC038371	Homo sapiens, clone IMAGE:4829282, mRNA. [BC038371]	
A_23_P256504	9.87E-06	NM_001633	NM_001633	Homo sapiens alpha-1-microglobulin/bikunin precursor (AMBP), mRNA [NM_001633]	NM_001633
A_23_P106194	9.87E-06	NM_005252	NM_005252	Homo sapiens v-fos FBJ murine osteosarcoma viral oncogene homolog (FOS), mRNA [NM_005252]	NM_005252
A_24_P374319	9.90E-06	NM_021183	NM_021183	Homo sapiens RAP2C, member of RAS oncogene family (RAP2C), mRNA [NM_021183]	NM_021183
A_23_P90510	9.93E-06	NM_138393	NM_138393	Homo sapiens chromosome 19 open reading frame 32 (C19orf32), mRNA [NM_138393]	NM_138393
A_24_P88850	9.96E-06	NM_012219	NM_012219	Homo sapiens muscle RAS oncogene homolog (MRAS), mRNA [NM_012219]	NM_012219
A_23_P41634	1.00E-05	NM_023039	NM_023039	Homo sapiens ankyrin repeat, family A (RFXANK-like), 2 (ANKRA2), mRNA [NM_023039]	NM_023039
A_23_P133000	1.00E-05	AK021601	AK021601	Homo sapiens cDNA FLJ11539 fis, clone HEMBA1002748. [AK021601]	XM_496724
A_23_P385126	1.01E-05	NM_139160	NM_139160	Homo sapiens novel 58.3 KDA protein (LOC91614), mRNA [NM_139160]	NM_139160
A_24_P412976	1.01E-05	NM_018273	NM_018273	Homo sapiens hypothetical protein FLJ10922 (FLJ10922), mRNA [NM_018273]	NM_018273
A_23_P50775	1.01E-05	NM_024509	NM_024509	Homo sapiens leucine rich repeat and fibronectin type III domain containing 3 (LRFN3), mRNA [NM_024509]	NM_024509
A_23_P28213	1.01E-05	NM_131916	NM_131916	Homo sapiens peptidylprolyl isomerase (cyclophilin)-like 3 (PPIL3), transcript variant PPIL3c, mRNA [NM_131916]	NM_131916
A_23_P373708	1.01E-05	NM_173624	NM_173624	Homo sapiens hypothetical protein FLJ40504 (FLJ40504), mRNA [NM_173624]	NM_173624
A_23_P46639	1.02E-05	NM_000562	NM_000562	Homo sapiens complement component 8, alpha polypeptide (C8A), mRNA [NM_000562]	NM_000562
A_23_P252817	1.02E-05	NM_001048	NM_001048	Homo sapiens somatostatin (SST), mRNA [NM_001048]	NM_001048
A_23_P139418	1.02E-05	NM_198516	NM_198516	Homo sapiens UDP-N-acetyl-alpha-D-galactosamine:polypeptide N-acetylgalactosaminyltransferase-like 4 (GALNTL4), mRNA [NM_198516]	NM_198516
A_23_P254741	1.02E-05	NM_003102	NM_003102	Homo sapiens superoxide dismutase 3, extracellular (SOD3), mRNA [NM_003102]	NM_003102
A_23_P97871	1.03E-05	BX641020	BX641020	Homo sapiens mRNA; cDNA DKFZp686G23148 (from clone DKFZp686G23148). [BX641020]	
A_32_P106615	1.03E-05	AW268902	AW268902	AW268902 xv48h10.x1 Soares_NFL_T_GBC_S1 Homo sapiens cDNA clone IMAGE:2816419 3', mRNA sequence [AW268902]	
A_32_P109029	1.04E-05	NM_022036	NM_022036	Homo sapiens G protein-coupled receptor, family C, group 5, member C (GPRC5C), transcript variant 1, mRNA [NM_022036]	NM_022036
A_23_P118571	1.04E-05	NM_025237	NM_025237	Homo sapiens sclerosteosis (SOST), mRNA [NM_025237]	NM_025237
A_32_P11230	1.04E-05	NM_001013665	NM_001013665	Homo sapiens hypothetical LOC399744 (LOC399744), mRNA [NM_001013665]	NM_001013665
A_23_P218086	1.04E-05	AB032995	AB032995	Homo sapiens mRNA for KIAA1169 protein, partial cds. [AB032995]	
A_23_P94921	1.04E-05	NM_006749	NM_006749	Homo sapiens solute carrier family 20 (phosphate transporter), member 2 (SLC20A2), mRNA [NM_006749]	NM_006749
A_23_P19894	1.04E-05	NM_198098	NM_198098	Homo sapiens aquaporin 1 (channel-forming integral protein, 28kDa) (AQP1), transcript variant 1, mRNA [NM_198098]	NM_198098
A_23_P19592	1.04E-05	NM_015599	NM_015599	Homo sapiens phosphoglucomutase 3 (PGM3), mRNA [NM_015599]	NM_015599
A_32_P31945	1.05E-05	NM_001609	NM_001609	Homo sapiens acyl-Coenzyme A dehydrogenase, short/branched chain (ACADSB), nuclear gene encoding mitochondrial protein, mRNA [NM_001609]	NM_001609
A_23_P302005	1.05E-05	NM_006873	NM_006873	Homo sapiens stoned B-like factor (SBLF), mRNA [NM_006873]	NM_006873
A_23_P142974	1.06E-05	NM_001007231	NM_001007231	Homo sapiens Rho GTPase activating protein 25 (ARHGAP25), transcript variant 1, mRNA [NM_001007231]	NM_001007231
A_23_P124837	1.06E-05	NM_002332	NM_002332	Homo sapiens low density lipoprotein-related protein 1 (alpha-2-macroglobulin receptor) (LRP1), mRNA [NM_002332]	NM_002332
A_24_P125283	1.06E-05	NM_001015053	NM_001015053	Homo sapiens histone deacetylase 5 (HDAC5), transcript variant 3, mRNA [NM_001015053]	NM_001015053
A_23_P41804	1.06E-05	NM_033120	NM_033120	Homo sapiens naked cuticle homolog 2 (Drosophila) (NKD2), mRNA [NM_033120]	NM_033120
A_23_P123555	1.06E-05	NM_032687	NM_032687	Homo sapiens cysteine/histidine-rich 1 (CYHR1), mRNA [NM_032687]	NM_032687

A_32_P175739	1.06E-05	NM_000189	NM_000189	Homo sapiens hexokinase 2 (HK2), mRNA [NM_000189]	NM_000189
A_24_P636441	1.07E-05	AK022223	AK022223	Homo sapiens cDNA FLJ12161 fis, clone MAMMA1000576. [AK022223]	
A_23_P101246	1.07E-05	BC016993	BC016993	Homo sapiens, clone IMAGE:4401841, mRNA. [BC016993]	XM_085831
A_32_P135091	1.07E-05	NM_018301	NM_018301	Homo sapiens hypothetical protein FLJ11016 (FLJ11016), mRNA [NM_018301]	NM_018301
A_23_P165848	1.07E-05	NM_007046	NM_007046	Homo sapiens elastin microfibril interfacer 1 (EMILIN1), mRNA [NM_007046]	NM_007046
A_24_P390928	1.07E-05	NM_024108	NM_024108	Homo sapiens hypothetical protein MGC2650 (MGC2650), mRNA [NM_024108]	NM_024108
A_23_P376627	1.07E-05	NM_152783	NM_152783	Homo sapiens hypothetical protein MGC25181 (MGC25181), mRNA [NM_152783]	NM_152783
A_23_P45999	1.07E-05	NM_012168	NM_012168	Homo sapiens F-box protein 2 (FBXO2), mRNA [NM_012168]	NM_012168
A_23_P214678	1.07E-05	NM_015921	NM_015921	Homo sapiens chromosome 6 open reading frame 82 (C6orf82), transcript variant 2, mRNA [NM_015921]	NM_015921
A_24_P35169	1.08E-05	NM_021167	NM_021167	Homo sapiens GATA zinc finger domain containing 1 (GATAD1), mRNA [NM_021167]	NM_021167
A_23_P396135	1.08E-05	NM_004387	NM_004387	Homo sapiens NK2 transcription factor related, locus 5 (Drosophila) (NKX2-5), mRNA [NM_004387]	NM_004387
A_23_P215634	1.08E-05	NM_001013398	NM_001013398	Homo sapiens insulin-like growth factor binding protein 3 (IGFBP3), transcript variant 1, mRNA [NM_001013398]	NM_001013398
A_32_P204330	1.09E-05	AK093982	AK093982	Homo sapiens cDNA FLJ36663 fis, clone UTERU2002826. [AK093982]	
A_23_P31006	1.09E-05	NM_002125	NM_002125	Homo sapiens major histocompatibility complex, class II, DR beta 5 (HLA-DRB5), mRNA [NM_002125]	NM_002125
A_24_P186746	1.09E-05	A_24_P186746			
A_32_P215700	1.10E-05	NM_181643	NM_181643	Homo sapiens chromosome 1 open reading frame 88 (C1orf88), mRNA [NM_181643]	NM_181643
A_23_P47991	1.10E-05	NM_015335	NM_015335	Homo sapiens thyroid hormone receptor associated protein 2 (THRAP2), mRNA [NM_015335]	NM_015335
A_24_P355944	1.10E-05	NM_004093	NM_004093	Homo sapiens ephrin-B2 (EFNB2), mRNA [NM_004093]	NM_004093
A_23_P3274	1.10E-05	NM_004884	NM_004884	Homo sapiens putative neuronal cell adhesion molecule (PUNC), mRNA [NM_004884]	NM_004884
A_23_P27040	1.10E-05	NM_015544	NM_015544	Homo sapiens DKFZP564K1964 protein (DKFZP564K1964), mRNA [NM_015544]	NM_015544
A_23_P64689	1.11E-05	NM_014871	NM_014871	Homo sapiens ubiquitin specific protease 52 (USP52), mRNA [NM_014871]	NM_014871
A_24_P521994	1.11E-05	NM_017644	NM_017644	Homo sapiens DRE1 protein (DRE1), mRNA [NM_017644]	NM_017644
A_32_P2354	1.11E-05	BX508036	BX508036	BX508036 DKFZp686E08263_r1 686 (synonym: hlcc3) Homo sapiens cDNA clone DKFZp686E08263 5', mRNA sequence [BX508036]	
A_23_P30776	1.11E-05	NM_003523	NM_003523	Homo sapiens histone 1, H2be (HIST1H2BE), mRNA [NM_003523]	NM_003523
A_23_P207742	1.11E-05	NM_003250	NM_003250	Homo sapiens thyroid hormone receptor, alpha (erythroblastic leukemia viral (v-erb-a) oncogene homolog, avian) (THRA), transcript variant 2, mRNA [NM_003250]	NM_003250
A_23_P166826	1.11E-05	NM_018403	NM_018403	Homo sapiens decapping enzyme (DCP1A), mRNA [NM_018403]	NM_018403
A_23_P97952	1.11E-05	NM_198472	NM_198472	Homo sapiens chromosome 10 open reading frame 125 (C10orf125), mRNA [NM_198472]	NM_198472
A_24_P16230	1.11E-05	ENST00000331037			
A_23_P93988	1.12E-05	NM_005435	NM_005435	Homo sapiens Rho guanine nucleotide exchange factor (GEF) 5 (ARHGEF5), transcript variant 1, mRNA [NM_005435]	NM_005435
A_23_P128967	1.12E-05	NM_005589	NM_005589	Homo sapiens aldehyde dehydrogenase 6 family, member A1 (ALDH6A1), nuclear gene encoding mitochondrial protein, mRNA [NM_005589]	NM_005589
A_23_P19389	1.12E-05	NM_006353	NM_006353	Homo sapiens high mobility group nucleosomal binding domain 4 (HMGN4), mRNA [NM_006353]	NM_006353
A_23_P78099	1.13E-05	NM_000638	NM_000638	Homo sapiens vitronectin (serum spreading factor, somatomedin B, complement S-protein) (VTN), mRNA [NM_000638]	NM_000638
A_32_P42989	1.14E-05	A_32_P42989			
A_23_P74993	1.15E-05	AK096104	AK096104	Homo sapiens cDNA FLJ38785 fis, clone LIVER2001329. [AK096104]	
A_24_P133288	1.15E-05	X97675	X97675	H.sapiens mRNA for plakophilin 2a and b. [X97675]	
A_23_P134755	1.15E-05	NM_014112	NM_014112	Homo sapiens trichorhinophalangeal syndrome I (TRPS1), mRNA [NM_014112]	NM_014112
A_24_P844100	1.15E-05	A_24_P844100			
A_24_P48723	1.15E-05	NM_000961	NM_000961	Homo sapiens prostaglandin I2 (prostacyclin) synthase (PTGIS), mRNA [NM_000961]	NM_000961
A_24_P218151	1.16E-05	NM_016352	NM_016352	Homo sapiens carboxypeptidase A4 (CPA4), mRNA [NM_016352]	NM_016352
A_23_P70670	1.16E-05	NM_004233	NM_004233	Homo sapiens CD83 antigen (activated B lymphocytes, immunoglobulin superfamily) (CD83), mRNA [NM_004233]	NM_004233
A_23_P110430	1.16E-05	NM_002448	NM_002448	Homo sapiens msh homeo box homolog 1 (Drosophila) (MSX1), mRNA [NM_002448]	NM_002448
A_23_P58770	1.16E-05	NM_004821	NM_004821	Homo sapiens heart and neural crest derivatives expressed 1 (HAND1), mRNA [NM_004821]	NM_004821
A_23_P253561	1.16E-05	AK056178	AK056178	Homo sapiens cDNA FLJ31616 fis, clone NT2RI2003019. [AK056178]	
A_32_P158786	1.17E-05	ENST00000333722		Homo sapiens, clone IMAGE:5271073, mRNA, partial cds. [BC039369]	XM_056680

A_23_P209527	1.17E-05	THC2310298		A31642 villin [validated] - human {Homo sapiens;} , partial (23%) [THC2310298]	
A_24_P408047	1.17E-05	NM_020904	NM_020904	Homo sapiens pleckstrin homology domain containing, family A (phosphoinositide binding specific) member 4 (PLEKHA4), mRNA [NM_020904]	NM_020904
A_23_P388150	1.18E-05	NM_032562	NM_032562	Homo sapiens phospholipase A2, group XIIB (PLA2G12B), mRNA [NM_032562]	NM_032562
A_23_P152505	1.18E-05	NM_020686	NM_020686	Homo sapiens 4-aminobutyrate aminotransferase (ABAT), nuclear gene encoding mitochondrial protein, transcript variant 1, mRNA [NM_020686]	NM_020686
A_23_P337262	1.18E-05	NM_153000	NM_153000	Homo sapiens adenomatosis polyposis coli down-regulated 1 (APCDD1), mRNA [NM_153000]	NM_153000
A_23_P207058	1.18E-05	NM_003955	NM_003955	Homo sapiens suppressor of cytokine signaling 3 (SOCS3), mRNA [NM_003955]	NM_003955
A_23_P156425	1.19E-05	NM_005907	NM_005907	Homo sapiens mannosidase, alpha, class 1A, member 1 (MAN1A1), mRNA [NM_005907]	NM_005907
A_23_P26426	1.19E-05	NM_014427	NM_014427	Homo sapiens copine VII (CPNE7), transcript variant 2, mRNA [NM_014427]	NM_014427
A_23_P7512	1.19E-05	AK026811	AK026811	Homo sapiens cDNA: FLJ23158 fis, clone LNG09623. [AK026811]	
A_24_P220485	1.19E-05	NM_182487	NM_182487	Homo sapiens olfactomedin-like 2A (OLFML2A), mRNA [NM_182487]	NM_182487
A_24_P161827	1.19E-05	A_24_P161827			
A_24_P570049	1.20E-05	NM_001001930	NM_001001930	Homo sapiens peroxisome proliferative activated receptor, alpha (PPARA), transcript variant 6, mRNA [NM_001001930]	NM_001001930
A_32_P136967	1.20E-05	ENST00000328046		Homo sapiens mRNA for KIAA1677 protein, partial cds. [AB051464]	
A_23_P138435	1.20E-05	NM_020338	NM_020338	Homo sapiens retinoic acid induced 17 (RAI17), mRNA [NM_020338]	NM_020338
A_23_P39955	1.20E-05	NM_001615	NM_001615	Homo sapiens actin, gamma 2, smooth muscle, enteric (ACTG2), mRNA [NM_001615]	NM_001615
A_24_P80500	1.21E-05	NM_020139	NM_020139	Homo sapiens dehydrogenase/reductase (SDR family) member 6 (DHRS6), mRNA [NM_020139]	NM_020139
A_24_P943062	1.21E-05	NM_014615	NM_014615	Homo sapiens KIAA0182 protein (KIAA0182), mRNA [NM_014615]	NM_014615
A_23_P24529	1.21E-05	NM_018195	NM_018195	Homo sapiens hypothetical protein FLJ10726 (FLJ10726), mRNA [NM_018195]	NM_018195
A_23_P72117	1.21E-05	NM_006714	NM_006714	Homo sapiens sphingomyelin phosphodiesterase, acid-like 3A (SMPDL3A), mRNA [NM_006714]	NM_006714
A_32_P353798	1.21E-05	NM_030783	NM_030783	Homo sapiens phosphatidylserine synthase 2 (PTDSS2), mRNA [NM_030783]	NM_030783
A_32_P9468	1.22E-05	AK130822	AK130822	Homo sapiens cDNA FLJ27312 fis, clone TMS06371. [AK130822]	
A_32_P68504	1.22E-05	ENST00000356022		Homo sapiens mRNA for KIAA1571 protein, partial cds. [AB046791]	XM_371590
A_23_P218675	1.22E-05	NM_080733	NM_080733	Homo sapiens WAP four-disulfide core domain 2 (WFDC2), transcript variant 3, mRNA [NM_080733]	NM_080733
A_23_P403424	1.22E-05	NM_005090	NM_005090	Homo sapiens phospholipase A2, group IVB (cytosolic) (PLA2G4B), mRNA [NM_005090]	NM_005090
A_23_P58506	1.22E-05	BX538289	BX538289	Homo sapiens mRNA; cDNA DKFZp779C185 (from clone DKFZp779C185). [BX538289]	
A_24_P255954	1.22E-05	A_24_P255954			
A_23_P123172	1.23E-05	NR_002157	NR_002157	Homo sapiens olfactory receptor, family 2, subfamily A, member 9 pseudogene (OR2A9P) on chromosome 7 [NR_002157]	NR_002157
A_23_P71328	1.23E-05	NM_030583	NM_030583	Homo sapiens matrilin 2 (MATN2), transcript variant 2, mRNA [NM_030583]	NM_030583
A_24_P195974	1.23E-05	A_24_P195974			
A_32_P10495	1.24E-05	THC2274697		ALU7_HUMAN (P39194) Alu subfamily SQ sequence contamination warning entry, partial (31%) [THC2274697]	
A_23_P163567	1.24E-05	NM_018667	NM_018667	Homo sapiens sphingomyelin phosphodiesterase 3, neutral membrane (neutral sphingomyelinase II) (SMPD3), mRNA [NM_018667]	NM_018667
A_24_P116587	1.24E-05	NM_201575	NM_201575	Homo sapiens seizure related 6 homolog (mouse)-like 2 (SEZ6L2), transcript variant 2, mRNA [NM_201575]	NM_201575
A_24_P171041	1.24E-05	AL136922	AL136922	Homo sapiens mRNA; cDNA DKFZp586J151 (from clone DKFZp586J151). [AL136922]	
A_23_P211039	1.25E-05	NM_006988	NM_006988	Homo sapiens a disintegrin-like and metalloprotease (reprolysin type) with thrombospondin type 1 motif, 1 (ADAMTS1), mRNA [NM_006988]	NM_006988
A_23_P111402	1.25E-05	NM_032784	NM_032784	Homo sapiens thrombospondin, type I, domain containing 2 (THSD2), mRNA [NM_032784]	NM_032784
A_32_P209702	1.26E-05	THC2283850			
A_24_P896205	1.26E-05	XM_498676	XM_498676	PREDICTED: Homo sapiens LOC440450 (LOC440450), mRNA [XM_498676]	XM_498676
A_24_P37253	1.28E-05	NM_194317	NM_194317	Homo sapiens hypothetical protein MGC52057 (MGC52057), mRNA [NM_194317]	NM_194317
A_24_P79241	1.28E-05	NM_014948	NM_014948	Homo sapiens U-box domain containing 5 (UBOX5), transcript variant 1, mRNA [NM_014948]	NM_014948
A_23_P401076	1.28E-05	NM_145006	NM_145006	Homo sapiens sushi domain containing 3 (SUSD3), mRNA [NM_145006]	NM_145006
A_23_P58009	1.29E-05	NM_024616	NM_024616	Homo sapiens TPA-induced transmembrane protein (TTMP), mRNA [NM_024616]	NM_024616
A_24_P142095	1.29E-05	NM_006738	NM_006738	Homo sapiens A kinase (PRKA) anchor protein 13 (AKAP13), transcript variant 1, mRNA [NM_006738]	NM_006738
A_23_P115064	1.29E-05	NM_001878	NM_001878	Homo sapiens cellular retinoic acid binding protein 2 (CRABP2), mRNA [NM_001878]	NM_001878
A_23_P149613	1.30E-05	NM_002021	NM_002021	Homo sapiens flavin containing monooxygenase 1 (FMO1), mRNA [NM_002021]	NM_002021

A_24_P59062	1.30E-05	NM_080839	NM_080839	Homo sapiens gamma-glutamyltransferase-like 4 (GGTL4), transcript variant 2, mRNA [NM_080839]	NM_080839
A_23_P167983	1.30E-05	ENST00000314088		Homo sapiens histone 1, H2ac, mRNA (cDNA clone MGC:1730 IMAGE:2988620), complete cds. [BC017379]	
A_23_P337168	1.30E-05	NM_153013	NM_153013	Homo sapiens hypothetical protein FLJ30596 (FLJ30596), mRNA [NM_153013]	NM_153013
A_24_P273799	1.31E-05	AL713659	AL713659	Homo sapiens mRNA; cDNA DKFZp667D1012 (from clone DKFZp667D1012). [AL713659]	
A_24_P284959	1.31E-05	NM_019035	NM_019035	Homo sapiens protocadherin 18 (PCDH18), mRNA [NM_019035]	NM_019035
A_32_P117016	1.32E-05	CR749561	CR749561	Homo sapiens mRNA; cDNA DKFZp686A16126 (from clone DKFZp686A16126). [CR749561]	
A_23_P388900	1.32E-05	NM_018420	NM_018420	Homo sapiens solute carrier family 22 (organic cation transporter), member 15 (SLC22A15), mRNA [NM_018420]	NM_018420
A_23_P324340	1.32E-05	NM_033510	NM_033510	Homo sapiens dispatched homolog 2 (Drosophila) (DISP2), mRNA [NM_033510]	NM_033510
A_23_P142255	1.32E-05	NM_020209	NM_020209	Homo sapiens Src homology 2 domain containing transforming protein D (SHD), mRNA [NM_020209]	NM_020209
A_24_P213336	1.32E-05	A_24_P213336			
A_23_P45294	1.32E-05	NM_207318	NM_207318	Homo sapiens chromosome X open reading frame 39 (CXorf39), mRNA [NM_207318]	NM_207318
A_23_P16806	1.33E-05	BC004487	BC004487	Homo sapiens hypothetical protein MGC10701, mRNA (cDNA clone MGC:10701 IMAGE:3832541), complete cds. [BC004487]	
A_23_P117104	1.33E-05	ENST00000293599		Homo sapiens aquaporin 5, mRNA (cDNA clone MGC:33163 IMAGE:5269384), complete cds. [BC032946]	
A_23_P29067	1.33E-05	NM_005656	NM_005656	Homo sapiens transmembrane protease, serine 2 (TMPRSS2), mRNA [NM_005656]	NM_005656
A_23_P204847	1.33E-05	NM_002298	NM_002298	Homo sapiens lymphocyte cytosolic protein 1 (L-plastin) (LCP1), mRNA [NM_002298]	NM_002298
A_24_P147765	1.34E-05	NM_024955	NM_024955	Homo sapiens hypothetical protein FLJ23322 (FLJ23322), mRNA [NM_024955]	NM_024955
A_32_P18547	1.34E-05	NM_058181	NM_058181	Homo sapiens chromosome 21 open reading frame 57 (C21orf57), transcript variant 1, mRNA [NM_058181]	NM_058181
A_23_P304682	1.34E-05	ENST00000320378		Homo sapiens cDNA FLJ39084 fis, clone NT2RP7018871. [AK096403]	
A_24_P364066	1.34E-05	BC030112	BC030112	Homo sapiens, clone IMAGE:4799578, mRNA. [BC030112]	
A_24_P90774	1.35E-05	NM_001008224	NM_001008224	Homo sapiens uveal autoantigen with coiled-coil domains and ankyrin repeats (UACA), transcript variant 2, mRNA [NM_001008224]	NM_001008224
A_23_P120933	1.36E-05	NM_001675	NM_001675	Homo sapiens activating transcription factor 4 (tax-responsive enhancer element B67) (ATF4), transcript variant 1, mRNA [NM_001675]	NM_001675
A_23_P502047	1.37E-05	NM_177978	NM_177978	Homo sapiens chordin (CHRD), transcript variant 2, mRNA [NM_177978]	NM_177978
A_23_P500892	1.37E-05	NM_003320	NM_003320	Homo sapiens tubby homolog (mouse) (TUB), transcript variant 1, mRNA [NM_003320]	NM_003320
A_23_P409623	1.38E-05	NM_003621	NM_003621	Homo sapiens PTPRF interacting protein, binding protein 2 (liprin beta 2) (PPFIBP2), mRNA [NM_003621]	NM_003621
A_32_P169179	1.38E-05	NR_002307	NR_002307	Homo sapiens msh homeobox homolog 2 (Drosophila) pseudogene (MSX2P) on chromosome 17 [NR_002307]	NR_002307
A_32_P164203	1.38E-05	THC2436337			
A_23_P382584	1.39E-05	NM_001819	NM_001819	Homo sapiens chromogranin B (secretogranin 1) (CHGB), mRNA [NM_001819]	NM_001819
A_23_P342709	1.39E-05	NM_152676	NM_152676	Homo sapiens F-box protein 15 (FBXO15), mRNA [NM_152676]	NM_152676
A_32_P104053	1.39E-05	THC2372182		O13102 (O13102) Activin type IIB receptor precursor, partial (5%) [THC2372182]	
A_23_P168847	1.40E-05	NM_172366	NM_172366	Homo sapiens F-box protein 16 (FBXO16), mRNA [NM_172366]	NM_172366
A_23_P70794	1.40E-05	NM_016277	NM_016277	Homo sapiens RAB23, member RAS oncogene family (RAB23), transcript variant 1, mRNA [NM_016277]	NM_016277
A_23_P144627	1.40E-05	NM_018933	NM_018933	Homo sapiens protocadherin beta 13 (PCDHB13), mRNA [NM_018933]	NM_018933
A_24_P931579	1.41E-05	AK095167	AK095167	Homo sapiens cDNA FLJ37848 fis, clone BRSSN2013544. [AK095167]	
A_24_P148450	1.41E-05	NM_006357	NM_006357	Homo sapiens ubiquitin-conjugating enzyme E2E 3 (UBC4/5 homolog, yeast) (UBE2E3), transcript variant 1, mRNA [NM_006357]	NM_006357
A_23_P250444	1.42E-05	NM_000166	NM_000166	Homo sapiens gap junction protein, beta 1, 32kDa (connexin 32, Charcot-Marie-Tooth neuropathy, X-linked) (GJB1), mRNA [NM_000166]	NM_000166
A_23_P106773	1.42E-05	NM_177528	NM_177528	Homo sapiens sulfotransferase family, cytosolic, 1A, phenol-preferring, member 2 (SULT1A2), transcript variant 2, mRNA [NM_177528]	NM_177528
A_24_P195164	1.42E-05	A_24_P195164			
A_23_P387031	1.43E-05	NM_173465	NM_173465	Homo sapiens collagen, type XXIII, alpha 1 (COL23A1), mRNA [NM_173465]	NM_173465
A_32_P112493	1.43E-05	NM_138370	NM_138370	Homo sapiens hypothetical protein BC007901 (LOC91461), mRNA [NM_138370]	NM_138370
A_32_P211353	1.44E-05	THC2272851		Q7V9T5 (Q7V9T5) Uncharacterized membrane protein, partial (10%) [THC2272851]	
A_24_P272225	1.44E-05	AK126559	AK126559	Homo sapiens cDNA FLJ44595 fis, clone BLADE2004849. [AK126559]	
A_24_P220454	1.44E-05	NM_181552	NM_181552	Homo sapiens cut-like 1, CCAAT displacement protein (Drosophila) (CUTL1), transcript variant 1, mRNA [NM_181552]	NM_181552
A_23_P120794	1.45E-05	NM_004173	NM_004173	Homo sapiens solute carrier family 7 (cationic amino acid transporter, y+ system), member 4 (SLC7A4), mRNA [NM_004173]	NM_004173
A_23_P346670	1.45E-05	AK000249	AK000249	Homo sapiens cDNA FLJ20242 fis, clone COLF6369. [AK000249]	

A_23_P93141	1.45E-05	NM_153699	NM_153699	Homo sapiens glutathione S-transferase A5 (GSTA5), mRNA [NM_153699]	NM_153699
A_32_P56661	1.46E-05	AK074614	AK074614	Homo sapiens cDNA FLJ90133 fis, clone HEMBB1000567. [AK074614]	
A_23_P103011	1.46E-05	NM_004914	NM_004914	Homo sapiens RAB36, member RAS oncogene family (RAB36), mRNA [NM_004914]	NM_004914
A_23_P417415	1.46E-05	NM_147161	NM_147161	Homo sapiens thioesterase, adipose associated (THEA), transcript variant 2, mRNA [NM_147161]	NM_147161
A_23_P48637	1.46E-05	BG108194	BG108194	BG108194 602280063F1 NIH_MGC_86 Homo sapiens cDNA clone IMAGE:4367799 5', mRNA sequence [BG108194]	
A_23_P106280	1.46E-05	AK000900	AK000900	Homo sapiens cDNA FLJ10038 fis, clone HEMBA1000971. [AK000900]	
A_23_P83094	1.46E-05	NM_007005	NM_007005	Homo sapiens transducin-like enhancer of split 4 (E(sp1) homolog, Drosophila) (TLE4), mRNA [NM_007005]	NM_007005
A_23_P369919	1.46E-05	NM_004578	NM_004578	Homo sapiens RAB4A, member RAS oncogene family (RAB4A), mRNA [NM_004578]	NM_004578
A_23_P61127	1.46E-05	NM_024122	NM_024122	Homo sapiens hypothetical protein MGC4825 (MGC4825), mRNA [NM_024122]	NM_024122
A_32_P4581	1.47E-05	AK130118	AK130118	Homo sapiens cDNA FLJ26608 fis, clone LVR00914. [AK130118]	
A_23_P91850	1.47E-05	NM_144717	NM_144717	Homo sapiens fibronectin type III domain containing 6 (FNDC6), mRNA [NM_144717]	NM_144717
A_24_P156911	1.47E-05	NM_003528	NM_003528	Homo sapiens histone 2, H2be (HIST2H2BE), mRNA [NM_003528]	NM_003528
A_23_P42178	1.47E-05	NM_003522	NM_003522	Homo sapiens histone 1, H2bf (HIST1H2BF), mRNA [NM_003522]	NM_003522
A_23_P69586	1.47E-05	NM_005245	NM_005245	Homo sapiens FAT tumor suppressor homolog 1 (Drosophila) (FAT), mRNA [NM_005245]	NM_005245
A_23_P206760	1.47E-05	NM_005143	NM_005143	Homo sapiens haptoglobin (HP), mRNA [NM_005143]	NM_005143
A_23_P87011	1.47E-05	NM_001001522	NM_001001522	Homo sapiens transgelin (TAGLN), transcript variant 1, mRNA [NM_001001522]	NM_001001522
A_32_P123966	1.49E-05	NM_015272	NM_015272	Homo sapiens KIAA1005 protein (KIAA1005), mRNA [NM_015272]	NM_015272
A_23_P39386	1.49E-05	NM_014266	NM_014266	Homo sapiens hematopoietic cell signal transducer (HCST), transcript variant 1, mRNA [NM_014266]	NM_014266
A_24_P299474	1.49E-05	ENST00000314238		Homo sapiens mRNA; cDNA DKFZp761F171 (from clone DKFZp761F171). [AL137500]	XM_047995
A_23_P155509	1.49E-05	NM_001622	NM_001622	Homo sapiens alpha-2-HS-glycoprotein (AHSG), mRNA [NM_001622]	NM_001622
A_24_P584463	1.49E-05	A_24_P584463			
A_23_P65262	1.50E-05	NM_033111	NM_033111	Homo sapiens CG016 (LOC88523), mRNA [NM_033111]	NM_033111
A_23_P392126	1.50E-05	AK055808	AK055808	Homo sapiens cDNA FLJ31246 fis, clone KIDNE2005266. [AK055808]	XM_375430
A_32_P106246	1.50E-05	A_32_P106246			
A_23_P33791	1.50E-05	NM_012446	NM_012446	Homo sapiens single-stranded DNA binding protein 2 (SSBP2), mRNA [NM_012446]	NM_012446
A_23_P3312	1.50E-05	NM_005545	NM_005545	Homo sapiens immunoglobulin superfamily containing leucine-rich repeat (ISLR), transcript variant 1, mRNA [NM_005545]	NM_005545
A_24_P56388	1.50E-05	NM_181054	NM_181054	Homo sapiens hypoxia-inducible factor 1, alpha subunit (basic helix-loop-helix transcription factor) (HIF1A), transcript variant 2, mRNA [NM_181054]	NM_181054
A_23_P46470	1.50E-05	NM_018948	NM_018948	Homo sapiens mitogen-inducible gene 6 (MIG-6), mRNA [NM_018948]	NM_018948
A_32_P175098	1.50E-05	CR613736	CR613736	full-length cDNA clone CS0DI072YA21 of Placenta Cot 25-normalized of Homo sapiens (human). [CR613736]	
A_23_P258221	1.51E-05	NM_005688	NM_005688	Homo sapiens ATP-binding cassette, sub-family C (CFTR/MRP), member 5 (ABCC5), transcript variant 1, mRNA [NM_005688]	NM_005688
A_23_P212508	1.52E-05	NM_001063	NM_001063	Homo sapiens transferrin (TF), mRNA [NM_001063]	NM_001063
A_23_P205177	1.52E-05	NM_000504	NM_000504	Homo sapiens coagulation factor X (F10), mRNA [NM_000504]	NM_000504
A_23_P79591	1.52E-05	NM_000384	NM_000384	Homo sapiens apolipoprotein B (including Ag(x) antigen) (APOB), mRNA [NM_000384]	NM_000384
A_32_P41487	1.52E-05	NM_005517	NM_005517	Homo sapiens high-mobility group nucleosomal binding domain 2 (HMGN2), mRNA [NM_005517]	NM_005517
A_24_P942092	1.53E-05	AK024362	AK024362	Homo sapiens cDNA FLJ14300 fis, clone PLACE1011891. [AK024362]	
A_23_P144916	1.53E-05	NM_005110	NM_005110	Homo sapiens glutamine-fructose-6-phosphate transaminase 2 (GFPT2), mRNA [NM_005110]	NM_005110
A_24_P119685	1.54E-05	NM_052843	NM_052843	Homo sapiens obscurin, cytoskeletal calmodulin and titin-interacting RhoGEF (OBSCN), mRNA [NM_052843]	NM_052843
A_23_P386320	1.55E-05	NM_033316	NM_033316	Homo sapiens antigen p97 (melanoma associated) identified by monoclonal antibodies 133.2 and 96.5 (MFI2), transcript variant 2, mRNA [NM_033316]	NM_033316
A_24_P391591	1.55E-05	AK057596	AK057596	Homo sapiens cDNA FLJ33034 fis, clone THYMU2000236. [AK057596]	XM_499585
A_24_P940517	1.55E-05	NM_016297	NM_016297	Homo sapiens prenylcysteine oxidase 1 (PCYOX1), mRNA [NM_016297]	NM_016297
A_23_P78108	1.55E-05	NM_005165	NM_005165	Homo sapiens aldolase C, fructose-bisphosphate (ALDOC), mRNA [NM_005165]	NM_005165
A_23_P135826	1.55E-05	AK096342	AK096342	Homo sapiens cDNA FLJ39023 fis, clone NT2RP7004348, highly similar to ZINC FINGER PROTEIN 93. [AK096342]	
A_24_P380734	1.55E-05	NM_002998	NM_002998	Homo sapiens syndecan 2 (heparan sulfate proteoglycan 1, cell surface-associated, fibroglycan) (SDC2), mRNA [NM_002998]	NM_002998
A_24_P240487	1.56E-05	NM_000301	NM_000301	Homo sapiens plasminogen (PLG), mRNA [NM_000301]	NM_000301

A_23_P24535	1.56E-05	AK023921	AK023921	Homo sapiens cDNA FLJ13859 fis, clone THYRO1001033, weakly similar to TRANSFORMATION-SENSITIVE PROTEIN IEF SSP 3521. [AK023921]	
A_23_P141005	1.56E-05	NM_001144	NM_001144	Homo sapiens autocrine motility factor receptor (AMFR), transcript variant 1, mRNA [NM_001144]	NM_001144
A_32_P141969	1.57E-05	AK094929	AK094929	Homo sapiens cDNA FLJ37610 fis, clone BRCOC2011398. [AK094929]	
A_24_P62505	1.57E-05	NM_015101	NM_015101	Homo sapiens glycosyltransferase 25 domain containing 2 (GLT25D2), mRNA [NM_015101]	NM_015101
A_23_P148990	1.57E-05	NM_031935	NM_031935	Homo sapiens hemicentin 1 (HMCN1), mRNA [NM_031935]	NM_031935
A_23_P154115	1.57E-05	NM_000599	NM_000599	Homo sapiens insulin-like growth factor binding protein 5 (IGFBP5), mRNA [NM_000599]	NM_000599
A_32_P214665	1.58E-05	ENST00000309878		Homo sapiens cDNA clone IMAGE:4792407, partial cds. [BC067884]	
A_23_P250283	1.58E-05	NM_004161	NM_004161	Homo sapiens RAB1A, member RAS oncogene family (RAB1A), mRNA [NM_004161]	NM_004161
A_24_P275073	1.59E-05	THC2404711		Q7QFH7 (Q7QFH7) ENSANGP00000019298 (Fragment), partial (7%) [THC2404711]	
A_24_P296772	1.59E-05	NM_033256	NM_033256	Homo sapiens protein phosphatase 1, regulatory (inhibitor) subunit 14A (PPP1R14A), mRNA [NM_033256]	NM_033256
A_23_P356004	1.60E-05	NM_013434	NM_013434	Homo sapiens calsenilin, presenilin binding protein, EF-hand transcription factor (CSEN), mRNA [NM_013434]	NM_013434
A_23_P33356	1.60E-05	AF261918	AF261918	Homo sapiens disintegrin metalloproteinase with thrombospondin repeats (ADAMTS9) mRNA, complete cds. [AF261918]	
A_24_P782308	1.60E-05	CR596491	CR596491	full-length cDNA clone CS0DF038YD07 of Fetal brain of Homo sapiens (human). [CR596491]	
A_24_P229726	1.60E-05	A_24_P229726			
A_24_P229728	1.60E-05	A_24_P229728			
A_23_P50276	1.61E-05	NM_031917	NM_031917	Homo sapiens angiopoietin-like 6 (ANGPTL6), mRNA [NM_031917]	NM_031917
A_32_P93144	1.61E-05	XM_372108	XM_372108	PREDICTED: Homo sapiens similar to Ribosome biogenesis protein BMS1 homolog (LOC389748), mRNA [XM_372108]	XM_372108
A_32_P89352	1.62E-05	AK125899	AK125899	Homo sapiens cDNA FLJ43911 fis, clone TESTI4010928. [AK125899]	
A_24_P205427	1.63E-05	AY129023	AY129023	Homo sapiens clone FP18821 unknown mRNA. [AY129023]	
A_23_P91520	1.63E-05	NM_007341	NM_007341	Homo sapiens SH3 domain binding glutamic acid-rich protein (SH3BGR), transcript variant 1, mRNA [NM_007341]	NM_007341
A_23_P213050	1.64E-05	NM_000860	NM_000860	Homo sapiens hydroxyprostaglandin dehydrogenase 15-(NAD) (HPGD), mRNA [NM_000860]	NM_000860
A_23_P214273	1.64E-05	THC2370119		Q5SYZ3 (Q5SYZ3) OTTHUMP00000039926 (Methylmalonyl Coenzyme A mutase), partial (93%) [THC2370119]	
A_32_P6344	1.64E-05	NM_003010	NM_003010	Homo sapiens mitogen-activated protein kinase kinase 4 (MAP2K4), mRNA [NM_003010]	NM_003010
A_23_P205900	1.65E-05	NM_001012338	NM_001012338	Homo sapiens neurotrophic tyrosine kinase, receptor, type 3 (NTRK3), transcript variant 1, mRNA [NM_001012338]	NM_001012338
A_23_P3483	1.65E-05	NM_021098	NM_021098	Homo sapiens calcium channel, voltage-dependent, alpha 1H subunit (CACNA1H), transcript variant 1, mRNA [NM_021098]	NM_021098
A_23_P150394	1.65E-05	NM_022003	NM_022003	Homo sapiens FXYD domain containing ion transport regulator 6 (FXYD6), mRNA [NM_022003]	NM_022003
A_32_P192594	1.65E-05	CR624390	CR624390	full-length cDNA clone CS0DC018YB19 of Neuroblastoma Cot 25-normalized of Homo sapiens (human). [CR624390]	XM_378399
A_24_P409420	1.65E-05	A_24_P409420			
A_32_P127818	1.65E-05	NM_014761	NM_014761	Homo sapiens KIAA0174 (KIAA0174), mRNA [NM_014761]	NM_014761
A_23_P307536	1.66E-05	NM_024676	NM_024676	Homo sapiens chromosome 1 open reading frame 113 (C1orf113), mRNA [NM_024676]	NM_024676
A_24_P98524	1.66E-05	NM_021132	NM_021132	Homo sapiens protein phosphatase 3 (formerly 2B), catalytic subunit, beta isoform (calcineurin A beta) (PPP3CB), mRNA [NM_021132]	NM_021132
A_23_P120860	1.66E-05	NM_003634	NM_003634	Homo sapiens nipsnap homolog 1 (C. elegans) (NIPSNAP1), mRNA [NM_003634]	NM_003634
A_23_P51278	1.66E-05	NM_052940	NM_052940	Homo sapiens leucine rich repeat containing 42 (LRRC42), mRNA [NM_052940]	NM_052940
A_24_P315120	1.67E-05	NM_001855	NM_001855	Homo sapiens collagen, type XV, alpha 1 (COL15A1), mRNA [NM_001855]	NM_001855
A_23_P169197	1.67E-05	NM_032303	NM_032303	Homo sapiens hydroxysteroid dehydrogenase like 2 (HSDL2), mRNA [NM_032303]	NM_032303
A_32_P127153	1.67E-05	NM_003104	NM_003104	Homo sapiens sorbitol dehydrogenase (SORD), mRNA [NM_003104]	NM_003104
A_24_P114739	1.68E-05	NM_172127	NM_172127	Homo sapiens calcium/calmodulin-dependent protein kinase (CaM kinase) II delta (CAMK2D), transcript variant 1, mRNA [NM_172127]	NM_172127
A_23_P369899	1.69E-05	NM_015444	NM_015444	Homo sapiens Ras-induced senescence 1 (RIS1), mRNA [NM_015444]	NM_015444
A_24_P241318	1.69E-05	NM_181340	NM_181340	Homo sapiens WD repeat domain 21A (WDR21A), transcript variant 2, mRNA [NM_181340]	NM_181340
A_23_P65240	1.69E-05	NM_001845	NM_001845	Homo sapiens collagen, type IV, alpha 1 (COL4A1), mRNA [NM_001845]	NM_001845
A_24_P745352	1.70E-05	A_24_P745352			
A_23_P433676	1.70E-05	NM_020951	NM_020951	Homo sapiens zinc finger protein 529 (ZNF529), mRNA [NM_020951]	NM_020951
A_23_P259442	1.70E-05	NM_001873	NM_001873	Homo sapiens carboxypeptidase E (CPE), mRNA [NM_001873]	NM_001873
A_24_P169843	1.70E-05	A_24_P169843			

A_32_P115707	1.71E-05	AI090937	AI090937	AI090937 qa52c01.s1 Soares_NhHMPu_S1 Homo sapiens cDNA clone IMAGE:1690368 3', mRNA sequence [AI090937]	
A_23_P26847	1.71E-05	NM_000346	NM_000346	Homo sapiens SRY (sex determining region Y)-box 9 (campomelic dysplasia, autosomal sex-reversal) (SOX9), mRNA [NM_000346]	NM_000346
A_23_P213877	1.72E-05	NM_023073	NM_023073	Homo sapiens hypothetical protein FLJ13231 (FLJ13231), mRNA [NM_023073]	NM_023073
A_23_P259166	1.72E-05	NM_024863	NM_024863	Homo sapiens transcription elongation factor A (SII)-like 4 (TCEAL4), transcript variant 1, mRNA [NM_024863]	NM_024863
A_32_P79345	1.73E-05	THC2410202		ALU6_HUMAN (P39193) Alu subfamily SP sequence contamination warning entry, partial (11%) [THC2410202]	
A_23_P9402	1.73E-05	NM_147164	NM_147164	Homo sapiens ciliary neurotrophic factor receptor (CNTFR), transcript variant 1, mRNA [NM_147164]	NM_147164
A_23_P145238	1.73E-05	NM_080593	NM_080593	Homo sapiens histone 1, H2bk (HIST1H2BK), mRNA [NM_080593]	NM_080593
A_24_P2584	1.73E-05	BC001892	BC001892	Homo sapiens chromosome 1 open reading frame 63, transcript variant 2, mRNA (cDNA clone MGC:1827 IMAGE:3534629), complete cds. [BC001892]	
A_23_P251499	1.73E-05	NM_002593	NM_002593	Homo sapiens procollagen C-endopeptidase enhancer (PCOLCE), mRNA [NM_002593]	NM_002593
A_23_P157865	1.73E-05	NM_002160	NM_002160	Homo sapiens tenascin C (hexabrachion) (TNC), mRNA [NM_002160]	NM_002160
A_23_P104451	1.73E-05	NM_024045	NM_024045	Homo sapiens DEAD (Asp-Glu-Ala-Asp) box polypeptide 50 (DDX50), mRNA [NM_024045]	NM_024045
A_23_P148088	1.73E-05	NM_000509	NM_000509	Homo sapiens fibrinogen gamma chain (FGG), transcript variant gamma-A, mRNA [NM_000509]	NM_000509
A_23_P165840	1.74E-05	NM_002539	NM_002539	Homo sapiens ornithine decarboxylase 1 (ODC1), mRNA [NM_002539]	NM_002539
A_23_P134191	1.75E-05	NM_021820	NM_021820	Homo sapiens chromosome 6 open reading frame 75 (C6orf75), mRNA [NM_021820]	NM_021820
A_23_P98876	1.77E-05	NM_173596	NM_173596	Homo sapiens solute carrier family 39 (metal ion transporter), member 5 (SLC39A5), mRNA [NM_173596]	NM_173596
A_23_P200792	1.77E-05	NM_024408	NM_024408	Homo sapiens Notch homolog 2 (Drosophila) (NOTCH2), mRNA [NM_024408]	NM_024408
A_24_P637982	1.77E-05	NM_198446	NM_198446	Homo sapiens chromosome 1 open reading frame 122 (C1orf122), mRNA [NM_198446]	NM_198446
A_23_P118392	1.78E-05	NM_016084	NM_016084	Homo sapiens RAS, dexamethasone-induced 1 (RASD1), mRNA [NM_016084]	NM_016084
A_23_P94095	1.79E-05	NM_198401	NM_198401	Homo sapiens ankyrin repeat domain 46 (ANKRD46), mRNA [NM_198401]	NM_198401
A_23_P40315	1.80E-05	NM_018152	NM_018152	Homo sapiens chromosome 20 open reading frame 12 (C20orf12), mRNA [NM_018152]	NM_018152
A_24_P756494	1.81E-05	AK057923	AK057923	Homo sapiens cDNA FLJ25194 fis, clone REC04095. [AK057923]	
A_23_P157247	1.81E-05	NM_152689	NM_152689	Homo sapiens hypothetical protein MGC9712 (MGC9712), mRNA [NM_152689]	NM_152689
A_23_P361014	1.81E-05	NM_020856	NM_020856	Homo sapiens zinc finger protein 537 (ZNF537), mRNA [NM_020856]	NM_020856
A_23_P119794	1.81E-05	CR608907	CR608907	full-length cDNA clone CS0DM002YA18 of Fetal liver of Homo sapiens (human). [CR608907]	
A_23_P355525	1.82E-05	NM_173497	NM_173497	Homo sapiens HECT domain containing 2 (HECTD2), transcript variant 2, mRNA [NM_173497]	NM_173497
A_23_P26928	1.82E-05	ENST00000357776		Homo sapiens cDNA FLJ13094 fis, clone NT2RP3002163. [AK023156]	
A_23_P52657	1.84E-05	AK056607	AK056607	Homo sapiens cDNA FLJ32045 fis, clone NTONG2001014, highly similar to GLYCOGEN PHOSPHORYLASE, MUSCLE FORM (EC 2.4.1.1). [AK056607]	
A_23_P82002	1.84E-05	NM_003214	NM_003214	Homo sapiens TEA domain family member 3 (TEAD3), mRNA [NM_003214]	NM_003214
A_24_P77432	1.84E-05	NM_133631	NM_133631	Homo sapiens roundabout, axon guidance receptor, homolog 1 (Drosophila) (ROBO1), transcript variant 2, mRNA [NM_133631]	NM_133631
A_24_P281374	1.84E-05	ENST00000327852		PREDICTED: Homo sapiens similar to Keratin, type I cytoskeletal 18 (Cytokeratin 18) (K18) (CK 18) (LOC442406), mRNA [XM_498307]	XM_498307
A_32_P76853	1.85E-05	XM_370839	XM_370839	PREDICTED: Homo sapiens similar to hypothetical protein (LOC440234), mRNA [XM_370839]	XM_370839
A_23_P42144	1.85E-05	NM_000287	NM_000287	Homo sapiens peroxisomal biogenesis factor 6 (PEX6), mRNA [NM_000287]	NM_000287
A_23_P99320	1.85E-05	NM_000224	NM_000224	Homo sapiens keratin 18 (KRT18), transcript variant 1, mRNA [NM_000224]	NM_000224
A_23_P379736	1.86E-05	NM_033309	NM_033309	Homo sapiens hypothetical protein MGC4655 (MGC4655), mRNA [NM_033309]	NM_033309
A_24_P80338	1.87E-05	NM_003157	NM_003157	Homo sapiens NIMA (never in mitosis gene a)-related kinase 4 (NEK4), mRNA [NM_003157]	NM_003157
A_23_P121926	1.87E-05	NM_005410	NM_005410	Homo sapiens selenoprotein P, plasma, 1 (SEPP1), mRNA [NM_005410]	NM_005410
A_23_P500410	1.88E-05	NM_130463	NM_130463	Homo sapiens ATPase, H+ transporting, lysosomal 13kDa, V1 subunit G isoform 2 (ATP6V1G2), transcript variant 1, mRNA [NM_130463]	NM_130463
A_32_P107833	1.88E-05	THC2404842		Q8CE45 (Q8CE45) Mus musculus 10 days neonate skin cDNA, RIKEN full-length enriched library, clone:4732483D01 product:long chain fatty acyl elongase, full insert sequence, partial (11%) [THC2404842]	
A_23_P70998	1.88E-05	ENST00000297227		full-length cDNA clone CS0DF028YG12 of Fetal brain of Homo sapiens (human). [CR590623]	
A_23_P46378	1.88E-05	NM_017837	NM_017837	Homo sapiens phosphatidylinositol glycan, class V (PIGV), mRNA [NM_017837]	NM_017837
A_23_P111054	1.88E-05	NM_021062	NM_021062	Homo sapiens histone 1, H2bb (HIST1H2BB), mRNA [NM_021062]	NM_021062
A_23_P203972	1.88E-05	NM_007197	NM_007197	Homo sapiens frizzled homolog 10 (Drosophila) (FZD10), mRNA [NM_007197]	NM_007197
A_23_P99642	1.88E-05	NM_003982	NM_003982	Homo sapiens solute carrier family 7 (cationic amino acid transporter, y+ system), member 7 (SLC7A7), mRNA [NM_003982]	NM_003982
A_24_P103803	1.88E-05	NM_001497	NM_001497	Homo sapiens UDP-Gal:betaGlcNAc beta 1,4- galactosyltransferase, polypeptide 1 (B4GALT1), mRNA [NM_001497]	NM_001497

A_23_P30687	1.89E-05	NM_004155	NM_004155	Homo sapiens serine (or cysteine) proteinase inhibitor, clade B (ovalbumin), member 9 (SERPINB9), mRNA [NM_004155]	NM_004155
A_32_P41594	1.89E-05	A_32_P41594			
A_32_P163858	1.89E-05	NM_005063	NM_005063	Homo sapiens stearoyl-CoA desaturase (delta-9-desaturase) (SCD), mRNA [NM_005063]	NM_005063
A_24_P196658	1.90E-05	NM_005486	NM_005486	Homo sapiens target of myb1-like 1 (chicken) (TOM1L1), mRNA [NM_005486]	NM_005486
A_23_P145711	1.90E-05	NM_024728	NM_024728	Homo sapiens chromosome 7 open reading frame 10 (C7orf10), mRNA [NM_024728]	NM_024728
A_23_P39265	1.90E-05	NM_014400	NM_014400	Homo sapiens GPI-anchored metastasis-associated protein homolog (C4.4A), mRNA [NM_014400]	NM_014400
A_24_P414999	1.90E-05	NM_018407	NM_018407	Homo sapiens lysosomal associated protein transmembrane 4 beta (LAPTM4B), mRNA [NM_018407]	NM_018407
A_32_P22750	1.91E-05	CA421238	CA421238	CA421238 UI-H-FG0-bct-h-02-0-UI.s1 NCI_CGAP_EN1_2 Homo sapiens cDNA clone UI-H-FG0-bct-h-02-0-UI 3', mRNA sequence [CA421238]	
A_23_P421379	1.91E-05	NM_000612	NM_000612	Homo sapiens insulin-like growth factor 2 (somatomedin A) (IGF2), mRNA [NM_000612]	NM_000612
A_24_P303080	1.92E-05	THC2370211			
A_24_P146138	1.92E-05	NM_031410	NM_031410	Homo sapiens protocadherin alpha 1 (PCDHA1), transcript variant 2, mRNA [NM_031410]	NM_031410
A_23_P135634	1.92E-05	AF217963	AF217963	Homo sapiens NRAGE mRNA, complete cds. [AF217963]	
A_23_P118065	1.93E-05	NM_002153	NM_002153	Homo sapiens hydroxysteroid (17-beta) dehydrogenase 2 (HSD17B2), mRNA [NM_002153]	NM_002153
A_23_P208991	1.93E-05	NM_002579	NM_002579	Homo sapiens paralemmin (PALM), mRNA [NM_002579]	NM_002579
A_24_P93111	1.94E-05	A_24_P93111			
A_23_P254917	1.96E-05	NM_006149	NM_006149	Homo sapiens lectin, galactoside-binding, soluble, 4 (galectin 4) (LGALS4), mRNA [NM_006149]	NM_006149
A_24_P182494	1.96E-05	NM_007207	NM_007207	Homo sapiens dual specificity phosphatase 10 (DUSP10), transcript variant 1, mRNA [NM_007207]	NM_007207
A_24_P418717	1.97E-05	NM_004329	NM_004329	Homo sapiens bone morphogenetic protein receptor, type IA (BMPR1A), mRNA [NM_004329]	NM_004329
A_23_P125423	1.97E-05	NM_001733	NM_001733	Homo sapiens complement component 1, r subcomponent (C1R), mRNA [NM_001733]	NM_001733
A_23_P29924	1.97E-05	NM_032927	NM_032927	Homo sapiens hypothetical protein MGC13159 (MGC13159), mRNA [NM_032927]	NM_032927
A_23_P210581	1.97E-05	NM_002237	NM_002237	Homo sapiens potassium voltage-gated channel, subfamily G, member 1 (KCNG1), transcript variant 1, mRNA [NM_002237]	NM_002237
A_32_P38323	1.98E-05	NM_004155	NM_004155	Homo sapiens serine (or cysteine) proteinase inhibitor, clade B (ovalbumin), member 9 (SERPINB9), mRNA [NM_004155]	NM_004155
A_23_P58251	1.98E-05	NM_001014448	NM_001014448	Homo sapiens carboxypeptidase Z (CPZ), transcript variant 3, mRNA [NM_001014448]	NM_001014448
A_23_P314024	1.98E-05	NM_018950	NM_018950	Homo sapiens major histocompatibility complex, class I, F (HLA-F), mRNA [NM_018950]	NM_018950
A_32_P45844	1.99E-05	THC2312104		NAC2_HUMAN (Q9UPR5) Sodium/calcium exchanger 2 precursor (Na(+)/Ca(2+)-exchange protein 2), partial (82%) [THC2312104]	
A_32_P61298	1.99E-05	AK054921	AK054921	Homo sapiens cDNA FLJ30359 fis, clone BRACE2007760, highly similar to 40S RIBOSOMAL PROTEIN S15A. [AK054921]	
A_23_P251984	1.99E-05	NM_004577	NM_004577	Homo sapiens phosphoserine phosphatase (PSPH), mRNA [NM_004577]	NM_004577
A_32_P49350	1.99E-05	ENST00000243927		full-length cDNA clone CS0DH003YG13 of T cells (Jurkat cell line) of Homo sapiens (human). [CR609307]	
A_24_P304636	2.01E-05	NM_004755	NM_004755	Homo sapiens ribosomal protein S6 kinase, 90kDa, polypeptide 5 (RPS6KA5), transcript variant 1, mRNA [NM_004755]	NM_004755
A_23_P16409	2.01E-05	NM_144691	NM_144691	Homo sapiens calpain 12 (CAPN12), mRNA [NM_144691]	NM_144691
A_23_P356616	2.01E-05	NM_145804	NM_145804	Homo sapiens ankyrin repeat and BTB (POZ) domain containing 2 (ABTB2), mRNA [NM_145804]	NM_145804
A_24_P944723	2.01E-05	AK021676	AK021676	Homo sapiens cDNA FLJ11614 fis, clone HEMBA1004015. [AK021676]	
A_23_P163816	2.02E-05	AB002324	AB002324	Human mRNA for KIAA0326 gene, partial cds. [AB002324]	
A_23_P109034	2.03E-05	NM_002999	NM_002999	Homo sapiens syndecan 4 (amphiglycan, ryudocan) (SDC4), mRNA [NM_002999]	NM_002999
A_24_P807031	2.04E-05	NM_001017971	NM_001017971	Homo sapiens hypothetical protein LOC92270 (LOC92270), mRNA [NM_001017971]	NM_001017971
A_24_P121631	2.04E-05	NM_033410	NM_033410	Homo sapiens hypothetical protein MGC13138 (MGC13138), mRNA [NM_033410]	NM_033410
A_24_P215628	2.05E-05	NM_018185	NM_018185	Homo sapiens chromosome 13 open reading frame 17 (C13orf17), transcript variant 1, mRNA [NM_018185]	NM_018185
A_24_P192727	2.05E-05	AF333487	AF333487	Homo sapiens FKSG40 (FKSG40) mRNA, complete cds. [AF333487]	XM_498494
A_24_P302574	2.05E-05	AK055441	AK055441	Homo sapiens cDNA FLJ30879 fis, clone FEBRA2004592, highly similar to Homo sapiens mRNA for putative sialoglycoprotease type 2. [AK055441]	
A_23_P9932	2.05E-05	NM_145341	NM_145341	Homo sapiens programmed cell death 4 (neoplastic transformation inhibitor) (PDCD4), transcript variant 2, mRNA [NM_145341]	NM_145341
A_24_P62783	2.05E-05	NM_004102	NM_004102	Homo sapiens fatty acid binding protein 3, muscle and heart (mammary-derived growth inhibitor) (FABP3), mRNA [NM_004102]	NM_004102
A_23_P102890	2.06E-05	NM_032476	NM_032476	Homo sapiens mitochondrial ribosomal protein S6 (MRPS6), nuclear gene encoding mitochondrial protein, mRNA [NM_032476]	NM_032476
A_23_P390032	2.07E-05	NM_153226	NM_153226	Homo sapiens transmembrane protein 20 (TMEM20), mRNA [NM_153226]	NM_153226
A_24_P59569	2.07E-05	BC000922	BC000922	Homo sapiens hypothetical gene supported by BC000922, mRNA (cDNA clone IMAGE:3447073), partial cds. [BC000922]	XM_378793

A_23_P23996	2.07E-05	NM_000429	NM_000429	Homo sapiens methionine adenosyltransferase I, alpha (MAT1A), mRNA [NM_000429]	NM_000429
A_32_P126259	2.08E-05	THC2443836			
A_23_P373126	2.08E-05	BC035647	BC035647	Homo sapiens, clone IMAGE:5575764, mRNA. [BC035647]	
A_23_P32463	2.08E-05	NM_019037	NM_019037	Homo sapiens exosome component 4 (EXOSC4), mRNA [NM_019037]	NM_019037
A_24_P265856	2.09E-05	NM_020654	NM_020654	Homo sapiens SUMO1/sentrin specific protease 7 (SENP7), mRNA [NM_020654]	NM_020654
A_24_P339157	2.09E-05	NM_014761	NM_014761	Homo sapiens KIAA0174 (KIAA0174), mRNA [NM_014761]	NM_014761
A_23_P15272	2.11E-05	BC050733	BC050733	Homo sapiens ATP-binding cassette, sub-family C (CFTR/MRP), member 6, mRNA (cDNA clone IMAGE:6141205), complete cds. [BC050733]	
A_32_P15799	2.11E-05	NM_005517	NM_005517	Homo sapiens high-mobility group nucleosomal binding domain 2 (HMGN2), mRNA [NM_005517]	NM_005517
A_32_P142652	2.11E-05	AK091931	AK091931	Homo sapiens cDNA FLJ34612 fis, clone KIDNE2014170, highly similar to 40S RIBOSOMAL PROTEIN S4, X ISOFORM. [AK091931]	
A_32_P46817	2.12E-05	THC2280638		RL2A_HUMAN (P46776) 60S ribosomal protein L27a, partial (24%) [THC2280638]	
A_23_P358995	2.12E-05	NM_015525	NM_015525	Homo sapiens inhibitor of Bruton agammaglobulinemia tyrosine kinase (IBTK), mRNA [NM_015525]	NM_015525
A_23_P3111	2.13E-05	NM_006821	NM_006821	Homo sapiens peroxisomal long-chain acyl-coA thioesterase (ZAP128), mRNA [NM_006821]	NM_006821
A_23_P410408	2.14E-05	NM_175883	NM_175883	Homo sapiens olfactory receptor, family 7, subfamily D, member 2 (OR7D2), mRNA [NM_175883]	NM_175883
A_32_P27535	2.14E-05	ENST00000290997		Homo sapiens hypothetical protein FLJ11724, mRNA (cDNA clone IMAGE:5299340), partial cds. [BC045676]	
A_23_P65532	2.14E-05	NM_021255	NM_021255	Homo sapiens pellino homolog 2 (Drosophila) (PELI2), mRNA [NM_021255]	NM_021255
A_24_P415012	2.16E-05	NM_013437	NM_013437	Homo sapiens low density lipoprotein-related protein 12 (LRP12), mRNA [NM_013437]	NM_013437
A_24_P370682	2.16E-05	NM_145214	NM_145214	Homo sapiens tripartite motif-containing 11 (TRIM11), mRNA [NM_145214]	NM_145214
A_24_P264644	2.16E-05	XM_498024	XM_498024	PREDICTED: Homo sapiens similar to Keratin, type I cytoskeletal 18 (Cytokeratin 18) (K18) (CK 18) (LOC345430), mRNA [XM_498024]	XM_498024
A_24_P46377	2.17E-05	NM_017872	NM_017872	Homo sapiens interphase cyctoplasmic foci protein 45 (ICF45), mRNA [NM_017872]	NM_017872
A_23_P135730	2.18E-05	NM_145295	NM_145295	Homo sapiens zinc finger protein 627 (ZNF627), mRNA [NM_145295]	NM_145295
A_32_P219520	2.18E-05	NM_014350	NM_014350	Homo sapiens tumor necrosis factor, alpha-induced protein 8 (TNFAIP8), mRNA [NM_014350]	NM_014350
A_24_P6850	2.18E-05	A_24_P6850			
A_23_P120458	2.19E-05	NM_018683	NM_018683	Homo sapiens zinc finger protein 313 (ZNF313), mRNA [NM_018683]	NM_018683
A_24_P300394	2.19E-05	NM_000846	NM_000846	Homo sapiens glutathione S-transferase A2 (GSTA2), mRNA [NM_000846]	NM_000846
A_23_P45365	2.19E-05	NM_033380	NM_033380	Homo sapiens collagen, type IV, alpha 5 (Alport syndrome) (COL4A5), transcript variant 2, mRNA [NM_033380]	NM_033380
A_24_P847326	2.20E-05	ENST00000335282			
A_23_P116898	2.20E-05	NM_000014	NM_000014	Homo sapiens alpha-2-macroglobulin (A2M), mRNA [NM_000014]	NM_000014
A_23_P313512	2.21E-05	NM_152640	NM_152640	Homo sapiens DCP1 decapping enzyme homolog B (S. cerevisiae) (DCP1B), mRNA [NM_152640]	NM_152640
A_23_P164912	2.22E-05	NM_022165	NM_022165	Homo sapiens lin-7 homolog B (C. elegans) (LIN7B), mRNA [NM_022165]	NM_022165
A_23_P100730	2.23E-05	NM_003726	NM_003726	Homo sapiens src family associated phosphoprotein 1 (SCAP1), mRNA [NM_003726]	NM_003726
A_24_P230675	2.23E-05	NM_003877	NM_003877	Homo sapiens suppressor of cytokine signaling 2 (SOCS2), mRNA [NM_003877]	NM_003877
A_23_P58538	2.23E-05	NM_053000	NM_053000	Homo sapiens TIGA1 (TIGA1), mRNA [NM_053000]	NM_053000
A_32_P174365	2.24E-05	NM_015265	NM_015265	Homo sapiens SATB family member 2 (SATB2), mRNA [NM_015265]	NM_015265
A_23_P119916	2.24E-05	NM_006522	NM_006522	Homo sapiens wingless-type MMTV integration site family, member 6 (WNT6), mRNA [NM_006522]	NM_006522
A_23_P369634	2.25E-05	AF289596	AF289596	Homo sapiens clone pp7882 unknown mRNA. [AF289596]	
A_24_P365515	2.26E-05	NM_021784	NM_021784	Homo sapiens forkhead box A2 (FOXA2), transcript variant 1, mRNA [NM_021784]	NM_021784
A_23_P42306	2.27E-05	NM_006120	NM_006120	Homo sapiens major histocompatibility complex, class II, DM alpha (HLA-DMA), mRNA [NM_006120]	NM_006120
A_24_P106297	2.27E-05	NM_014324	NM_014324	Homo sapiens alpha-methylacyl-CoA racemase (AMACR), transcript variant 1, mRNA [NM_014324]	NM_014324
A_32_P132317	2.28E-05	A_32_P132317			
A_24_P810084	2.28E-05	BC033983	BC033983	Homo sapiens, clone IMAGE:5295564, mRNA. [BC033983]	
A_24_P303193	2.28E-05	CR596214	CR596214	full-length cDNA clone CS0DC006YB07 of Neuroblastoma Cot 25-normalized of Homo sapiens (human). [CR596214]	
A_24_P161733	2.28E-05	A_24_P161733			
A_23_P391396	2.29E-05	NM_001005463	NM_001005463	Homo sapiens early B-cell factor 3 (EBF3), mRNA [NM_001005463]	NM_001005463
A_23_P423331	2.30E-05	NM_032536	NM_032536	Homo sapiens netrin G2 (NTNG2), mRNA [NM_032536]	NM_032536

A_23_P145644	2.30E-05	NM_000790	NM_000790	Homo sapiens dopa decarboxylase (aromatic L-amino acid decarboxylase) (DDC), mRNA [NM_000790]	NM_000790
A_32_P206123	2.30E-05	NM_000301	NM_000301	Homo sapiens plasminogen (PLG), mRNA [NM_000301]	NM_000301
A_23_P57868	2.30E-05	NM_000666	NM_000666	Homo sapiens aminoacylase 1 (ACY1), mRNA [NM_000666]	NM_000666
A_24_P266734	2.30E-05	NM_018276	NM_018276	Homo sapiens slingshot homolog 3 (Drosophila) (SSH3), transcript variant 2, mRNA [NM_018276]	NM_018276
A_23_P33673	2.31E-05	NM_001003818	NM_001003818	Homo sapiens tripartite motif-containing 6 (TRIM6), transcript variant 1, mRNA [NM_001003818]	NM_001003818
A_23_P203920	2.33E-05	NM_005086	NM_005086	Homo sapiens sarcospan (Kras oncogene-associated gene) (SSPN), mRNA [NM_005086]	NM_005086
A_23_P20864	2.33E-05	NM_012098	NM_012098	Homo sapiens angiopoietin-like 2 (ANGPTL2), mRNA [NM_012098]	NM_012098
A_32_P159535	2.35E-05	THC2303047		RS24_HUMAN (P62847) 40S ribosomal protein S24, partial (77%) [THC2303047]	
A_24_P372913	2.36E-05	NM_000545	NM_000545	Homo sapiens transcription factor 1, hepatic; LF-B1, hepatic nuclear factor (HNF1), albumin proximal factor (TCF1), mRNA [NM_000545]	NM_000545
A_23_P381431	2.38E-05	NM_030769	NM_030769	Homo sapiens N-acetylneuraminate pyruvate lyase (dihydrodipicolinate synthase) (NPL), mRNA [NM_030769]	NM_030769
A_24_P542608	2.39E-05	THC2279230		AL563955 AL563955 Homo sapiens FETAL LIVER Homo sapiens cDNA clone CS0DM001YO03 3-PRIME, mRNA sequence [AL563955]	
A_23_P50167	2.41E-05	NM_012319	NM_012319	Homo sapiens solute carrier family 39 (zinc transporter), member 6 (SLC39A6), mRNA [NM_012319]	NM_012319
A_23_P115021	2.41E-05	NM_001103	NM_001103	Homo sapiens actinin, alpha 2 (ACTN2), mRNA [NM_001103]	NM_001103
A_24_P79040	2.43E-05	NM_144691	NM_144691	Homo sapiens calpain 12 (CAPN12), mRNA [NM_144691]	NM_144691
A_24_P650482	2.43E-05	AK056822	AK056822	Homo sapiens cDNA FLJ32260 fis, clone PROST1000334. [AK056822]	
A_23_P4041	2.43E-05	NM_021213	NM_021213	Homo sapiens phosphatidylcholine transfer protein (PCTP), mRNA [NM_021213]	NM_021213
A_32_P41035	2.44E-05	AK092942	AK092942	Homo sapiens cDNA FLJ35623 fis, clone SPLEN2010986. [AK092942]	XM_496251
A_32_P117170	2.45E-05	ENST00000341533		Homo sapiens hypothetical metallo-hydrolase/oxidoreductase structure-containing protein (C7orf18) mRNA, complete cds. [AY357337]	
A_24_P298877	2.45E-05	NM_207356	NM_207356	Homo sapiens hypothetical protein LOC339448 (LOC339448), mRNA [NM_207356]	NM_207356
A_24_P362904	2.46E-05	NM_004567	NM_004567	Homo sapiens 6-phosphofructo-2-kinase/fructose-2,6-biphosphatase 4 (PFKFB4), mRNA [NM_004567]	NM_004567
A_24_P943843	2.46E-05	AK124515	AK124515	Homo sapiens cDNA FLJ42524 fis, clone BRACE3001384. [AK124515]	
A_23_P77859	2.46E-05	NM_203411	NM_203411	Homo sapiens similar to RIKEN cDNA 2600017H02 (LOC92162), mRNA [NM_203411]	NM_203411
A_23_P93780	2.47E-05	NM_001010931	NM_001010931	Homo sapiens hepatocyte growth factor (hepapoietin A; scatter factor) (HGF), transcript variant 2, mRNA [NM_001010931]	NM_001010931
A_32_P220463	2.47E-05	AX721195	AX721195	Sequence 155 from Patent WO0220754. [AX721195]	XM_293903
A_23_P31810	2.47E-05	NM_005195	NM_005195	Homo sapiens CCAAT/enhancer binding protein (C/EBP), delta (CEBPD), mRNA [NM_005195]	NM_005195
A_23_P151394	2.47E-05	NM_004800	NM_004800	Homo sapiens transmembrane 9 superfamily member 2 (TM9SF2), mRNA [NM_004800]	NM_004800
A_23_P214300	2.48E-05	NM_000846	NM_000846	Homo sapiens glutathione S-transferase A2 (GSTA2), mRNA [NM_000846]	NM_000846
A_23_P333498	2.49E-05	NM_030636	NM_030636	Homo sapiens KIAA1706 protein (KIAA1706), mRNA [NM_030636]	NM_030636
A_23_P375494	2.50E-05	NM_004364	NM_004364	Homo sapiens CCAAT/enhancer binding protein (C/EBP), alpha (CEBPA), mRNA [NM_004364]	NM_004364
A_24_P55250	2.50E-05	NM_016063	NM_016063	Homo sapiens chromosome 6 open reading frame 74 (C6orf74), mRNA [NM_016063]	NM_016063
A_23_P431268	2.51E-05	NM_014935	NM_014935	Homo sapiens pleckstrin homology domain containing, family A member 6 (PLEKHA6), mRNA [NM_014935]	NM_014935
A_24_P477048	2.52E-05	A_24_P477048			
A_23_P171366	2.52E-05	NM_004651	NM_004651	Homo sapiens ubiquitin specific protease 11 (USP11), mRNA [NM_004651]	NM_004651
A_23_P122068	2.54E-05	NM_181435	NM_181435	Homo sapiens C1q and tumor necrosis factor related protein 3 (C1QTNF3), transcript variant 2, mRNA [NM_181435]	NM_181435
A_32_P31771	2.54E-05	NM_030650	NM_030650	Homo sapiens KIAA1715 (KIAA1715), mRNA [NM_030650]	NM_030650
A_23_P211493	2.56E-05	NM_153609	NM_153609	Homo sapiens transmembrane protease, serine 6 (TMPRSS6), mRNA [NM_153609]	NM_153609
A_23_P205746	2.56E-05	NM_001008707	NM_001008707	Homo sapiens echinoderm microtubule associated protein like 1 (EML1), transcript variant 1, mRNA [NM_001008707]	NM_001008707
A_23_P44291	2.58E-05	NM_006371	NM_006371	Homo sapiens cartilage associated protein (CRTAP), mRNA [NM_006371]	NM_006371
A_23_P157715	2.58E-05	NM_032902	NM_032902	Homo sapiens protein phosphatase 1, regulatory (inhibitor) subunit 16A (PPP1R16A), mRNA [NM_032902]	NM_032902
A_23_P125519	2.58E-05	NM_001007	NM_001007	Homo sapiens ribosomal protein S4, X-linked (RPS4X), mRNA [NM_001007]	NM_001007
A_23_P330461	2.59E-05	NM_144686	NM_144686	Homo sapiens transmembrane channel-like 4 (TMC4), mRNA [NM_144686]	NM_144686
A_32_P171856	2.59E-05	THC2341414			
A_24_P28977	2.60E-05	NM_003304	NM_003304	Homo sapiens transient receptor potential cation channel, subfamily C, member 1 (TRPC1), mRNA [NM_003304]	NM_003304
A_23_P19723	2.62E-05	NM_021073	NM_021073	Homo sapiens bone morphogenetic protein 5 (BMP5), mRNA [NM_021073]	NM_021073

A_23_P214969	2.63E-05	NM_006079	NM_006079	Homo sapiens Cbp/p300-interacting transactivator, with Glu/Asp-rich carboxy-terminal domain, 2 (CITED2), mRNA [NM_006079]	NM_006079
A_32_P88262	2.64E-05	AK094547	AK094547	Homo sapiens cDNA FLJ37228 fis, clone BRAMY2000411. [AK094547]	
A_23_P214211	2.64E-05	NM_020320	NM_020320	Homo sapiens arginyl-tRNA synthetase-like (RARSL), mRNA [NM_020320]	NM_020320
A_24_P788772	2.64E-05	THC2373524		APE_HUMAN (P02649) Apolipoprotein E precursor (Apo-E), partial (50%) [THC2373524]	
A_23_P94591	2.64E-05	NM_032928	NM_032928	Homo sapiens hypothetical protein MGC14141 (MGC14141), mRNA [NM_032928]	NM_032928
A_24_P106910	2.65E-05	ENST00000331920		Homo sapiens cDNA FLJ42602 fis, clone BRACE3011271, moderately similar to Patched protein. [AK124593]	
A_32_P45297	2.68E-05	THC2441058		Q81D10 (Q81D10) Phage protein, partial (21%) [THC2441058]	
A_23_P360797	2.68E-05	NM_002527	NM_002527	Homo sapiens neurotrophin 3 (NTF3), mRNA [NM_002527]	NM_002527
A_24_P271323	2.70E-05	NM_030777	NM_030777	Homo sapiens solute carrier family 2 (facilitated glucose transporter), member 10 (SLC2A10), mRNA [NM_030777]	NM_030777
A_24_P350136	2.70E-05	A_24_P350136			
A_23_P37914	2.73E-05	NM_052944	NM_052944	Homo sapiens solute carrier family 5 (sodium/glucose cotransporter), member 11 (SLC5A11), mRNA [NM_052944]	NM_052944
A_24_P131392	2.73E-05	NM_144713	NM_144713	Homo sapiens family with sequence similarity 82, member A (FAM82A), mRNA [NM_144713]	NM_144713
A_23_P114903	2.74E-05	NM_002155	NM_002155	Homo sapiens heat shock 70kDa protein 6 (HSP70B') (HSPA6), mRNA [NM_002155]	NM_002155
A_32_P208403	2.74E-05	NM_053064	NM_053064	Homo sapiens guanine nucleotide binding protein (G protein), gamma 2 (GNG2), mRNA [NM_053064]	NM_053064
A_23_P116286	2.74E-05	NM_000480	NM_000480	Homo sapiens adenosine monophosphate deaminase (isoform E) (AMPD3), transcript variant 1, mRNA [NM_000480]	NM_000480
A_23_P375372	2.76E-05	NM_021871	NM_021871	Homo sapiens fibrinogen alpha chain (FGA), transcript variant alpha, mRNA [NM_021871]	NM_021871
A_23_P53736	2.76E-05	NM_033624	NM_033624	Homo sapiens F-box protein 21 (FBXO21), transcript variant 1, mRNA [NM_033624]	NM_033624
A_23_P46238	2.78E-05	NM_033440	NM_033440	Homo sapiens elastase 2A (ELA2A), mRNA [NM_033440]	NM_033440
A_32_P69849	2.79E-05	NM_001012978	NM_001012978	Homo sapiens NGFRAP1-like 1 (NGFRAP1L1), mRNA [NM_001012978]	NM_001012978
A_24_P160263	2.80E-05	NM_201222	NM_201222	Homo sapiens melanoma antigen family D, 2 (MAGED2), transcript variant 3, mRNA [NM_201222]	NM_201222
A_24_P288424	2.82E-05	NM_016045	NM_016045	Homo sapiens chromosome 20 open reading frame 45 (C20orf45), mRNA [NM_016045]	NM_016045
A_24_P79631	2.83E-05	CR598503	CR598503	full-length cDNA clone CS0DF014YO17 of Fetal brain of Homo sapiens (human). [CR598503]	
A_24_P845631	2.84E-05	A_24_P845631			
A_32_P116488	2.84E-05	THC2283842			
A_23_P101908	2.84E-05	NM_005883	NM_005883	Homo sapiens adenomatosis polyposis coli 2 (APC2), mRNA [NM_005883]	NM_005883
A_24_P942211	2.84E-05	NM_014854	NM_014854	Homo sapiens solute carrier family 35, member E2 (SLC35E2), mRNA [NM_014854]	NM_014854
A_23_P82929	2.85E-05	NM_002514	NM_002514	Homo sapiens nephroblastoma overexpressed gene (NOV), mRNA [NM_002514]	NM_002514
A_24_P3783	2.86E-05	NM_003521	NM_003521	Homo sapiens histone 1, H2bm (HIST1H2BM), mRNA [NM_003521]	NM_003521
A_23_P170649	2.86E-05	NM_153225	NM_153225	Homo sapiens RPE-spondin (RPESP), mRNA [NM_153225]	NM_153225
A_24_P48057	2.87E-05	NM_005853	NM_005853	Homo sapiens iroquois homeobox protein 5 (IRX5), mRNA [NM_005853]	NM_005853
A_24_P330234	2.88E-05	NM_007106	NM_007106	Homo sapiens ubiquitin-like 3 (UBL3), mRNA [NM_007106]	NM_007106
A_24_P264832	2.88E-05	NM_005382	NM_005382	Homo sapiens neurofilament 3 (150kDa medium) (NEF3), mRNA [NM_005382]	NM_005382
A_23_P66739	2.90E-05	NM_177550	NM_177550	Homo sapiens solute carrier family 13 (sodium-dependent citrate transporter), member 5 (SLC13A5), mRNA [NM_177550]	NM_177550
A_23_P70648	2.90E-05	NM_000865	NM_000865	Homo sapiens 5-hydroxytryptamine (serotonin) receptor 1E (HTR1E), mRNA [NM_000865]	NM_000865
A_24_P541919	2.90E-05	AF086301	AF086301	Homo sapiens full length insert cDNA clone ZD50F09. [AF086301]	
A_23_P207221	2.90E-05	NM_018242	NM_018242	Homo sapiens hypothetical protein FLJ10847 (FLJ10847), mRNA [NM_018242]	NM_018242
A_23_P408271	2.90E-05	NM_016245	NM_016245	Homo sapiens dehydrogenase/reductase (SDR family) member 8 (DHRS8), mRNA [NM_016245]	NM_016245
A_32_P205624	2.93E-05	ENST00000264554		Homo sapiens mRNA for Sck, partial cds. [AB001451]	
A_23_P421493	2.94E-05	NM_020995	NM_020995	Homo sapiens haptoglobin-related protein (HPR), mRNA [NM_020995]	NM_020995
A_23_P210425	2.95E-05	NM_181526	NM_181526	Homo sapiens myosin, light polypeptide 9, regulatory (MYL9), transcript variant 2, mRNA [NM_181526]	NM_181526
A_23_P156431	2.97E-05	NM_005907	NM_005907	Homo sapiens mannosidase, alpha, class 1A, member 1 (MAN1A1), mRNA [NM_005907]	NM_005907
A_23_P163506	2.98E-05	NM_000101	NM_000101	Homo sapiens cytochrome b-245, alpha polypeptide (CYBA), mRNA [NM_000101]	NM_000101
A_23_P301855	2.99E-05	NM_002338	NM_002338	Homo sapiens limbic system-associated membrane protein (LSAMP), mRNA [NM_002338]	NM_002338
A_23_P256334	2.99E-05	NM_181501	NM_181501	Homo sapiens integrin, alpha 1 (ITGA1), mRNA [NM_181501]	NM_181501

A_23_P163647	2.99E-05	NM_001919	NM_001919	Homo sapiens dodecenoyl-Coenzyme A delta isomerase (3,2 trans-enoyl-Coenzyme A isomerase) (DCI), nuclear gene encoding mitochondrial protein, mRNA [NM_001919]	NM_001919
A_23_P114423	3.00E-05	NM_004683	NM_004683	Homo sapiens regucalcin (senescence marker protein-30) (RGN), transcript variant 1, mRNA [NM_004683]	NM_004683
A_23_P104692	3.00E-05	NM_145065	NM_145065	Homo sapiens pellino 3 alpha (PELI3), mRNA [NM_145065]	NM_145065
A_23_P370035	3.01E-05	NM_181340	NM_181340	Homo sapiens WD repeat domain 21A (WDR21A), transcript variant 2, mRNA [NM_181340]	NM_181340
A_23_P7402	3.02E-05	NM_178140	NM_178140	Homo sapiens PDZ domain containing 3 (PDZK3), transcript variant 1, mRNA [NM_178140]	NM_178140
A_32_P73507	3.03E-05	THC2339389			
A_23_P154986	3.03E-05	NM_013430	NM_013430	Homo sapiens gamma-glutamyltransferase 1 (GGT1), transcript variant 3, mRNA [NM_013430]	NM_013430
A_23_P352684	3.03E-05	NM_003861	NM_003861	Homo sapiens WD repeat domain 22 (WDR22), mRNA [NM_003861]	NM_003861
A_23_P122976	3.03E-05	NM_002069	NM_002069	Homo sapiens guanine nucleotide binding protein (G protein), alpha inhibiting activity polypeptide 1 (GNAI1), mRNA [NM_002069]	NM_002069
A_23_P258048	3.03E-05	AK056630	AK056630	Homo sapiens cDNA FLJ32068 fis, clone OCBBF1000114. [AK056630]	
A_23_P120953	3.03E-05	NM_014509	NM_014509	Homo sapiens serine hydrolase-like 2 (SERHL2), mRNA [NM_014509]	NM_014509
A_24_P510502	3.03E-05	N53226	N53226	N53226 yv57e10.s1 Soares fetal liver spleen 1NFLS Homo sapiens cDNA clone IMAGE:246858 3' similar to gb:X07868_rna1 PUTATIVE INSULIN-LIKE GROWTH FACTOR II ASSOCIATED (HUMAN);, mRNA sequence [N53226]	
A_23_P11244	3.03E-05	NM_145119	NM_145119	Homo sapiens praja 1 (PJA1), mRNA [NM_145119]	NM_145119
A_23_P18276	3.03E-05	NM_005778	NM_005778	Homo sapiens RNA binding motif protein 5 (RBM5), mRNA [NM_005778]	NM_005778
A_23_P20002	3.04E-05	NM_020319	NM_020319	Homo sapiens ankyrin repeat and MYND domain containing 2 (ANKMY2), mRNA [NM_020319]	NM_020319
A_24_P849245	3.04E-05	THC2277104		O13760 (O13760) SPAC17A2.10c protein, partial (12%) [THC2277104]	
A_24_P95154	3.04E-05	NM_178234	NM_178234	Homo sapiens tumor suppressor candidate 3 (TUSC3), transcript variant 2, mRNA [NM_178234]	NM_178234
A_23_P74088	3.05E-05	NM_006983	NM_006983	Homo sapiens matrix metalloproteinase 23B (MMP23B), mRNA [NM_006983]	NM_006983
A_23_P217009	3.07E-05	NM_032596	NM_032596	Homo sapiens chromosome 9 open reading frame 24 (C9orf24), transcript variant 1, mRNA [NM_032596]	NM_032596
A_23_P328511	3.07E-05	NM_001537	NM_001537	Homo sapiens heat shock factor binding protein 1 (HSBP1), mRNA [NM_001537]	NM_001537
A_24_P338648	3.08E-05	NM_001177	NM_001177	Homo sapiens ADP-ribosylation factor-like 1 (ARL1), mRNA [NM_001177]	NM_001177
A_24_P245108	3.09E-05	THC2310236			
A_23_P80382	3.10E-05	NM_015366	NM_015366	Homo sapiens proline rich protein 5 (PRR5), transcript variant 2, mRNA [NM_015366]	NM_015366
A_24_P598406	3.10E-05	BC068459	BC068459	Homo sapiens similar to hypothetical protein FLJ10307, mRNA (cDNA clone MGC:87053 IMAGE:5297972), complete cds. [BC068459]	
A_24_P281605	3.11E-05	A_24_P281605			
A_23_P309599	3.12E-05	AK026760	AK026760	Homo sapiens cDNA: FLJ23107 fis, clone LNG07738. [AK026760]	
A_32_P178635	3.12E-05	BC033590	BC033590	Homo sapiens, clone IMAGE:4344826, mRNA. [BC033590]	
A_24_P102981	3.12E-05	NM_006736	NM_006736	Homo sapiens DnaJ (Hsp40) homolog, subfamily B, member 2 (DNAJB2), mRNA [NM_006736]	NM_006736
A_32_P23854	3.16E-05	NM_020946	NM_020946	Homo sapiens KIAA1608 (KIAA1608), transcript variant 1, mRNA [NM_020946]	NM_020946
A_23_P433016	3.16E-05	NM_001996	NM_001996	Homo sapiens fibulin 1 (FBLN1), transcript variant C, mRNA [NM_001996]	NM_001996
A_23_P80048	3.17E-05	NR_001442	NR_001442	Homo sapiens fer-1-like 4 (C. elegans) (FER1L4) on chromosome 20 [NR_001442]	NR_001442
A_23_P65712	3.18E-05	NM_031452	NM_031452	Homo sapiens hypothetical LOC83640 (MGC2560), mRNA [NM_031452]	NM_031452
A_23_P381979	3.18E-05	NM_003605	NM_003605	Homo sapiens O-linked N-acetylglucosamine (GlcNAc) transferase (UDP-N-acetylglucosamine:polypeptide-N-acetylglucosaminyl transferase) (OGT), transcript variant 3, mRNA [NM_003605]	NM_003605
A_23_P350551	3.18E-05	NM_138425	NM_138425	Homo sapiens likely ortholog of mouse gene rich cluster, C10 gene (GRCC10), mRNA [NM_138425]	NM_138425
A_32_P195924	3.19E-05	THC2378147		Q755F9 (Q755F9) AFL136Wp, partial (4%) [THC2378147]	
A_24_P393838	3.19E-05	NM_014765	NM_014765	Homo sapiens translocase of outer mitochondrial membrane 20 homolog (yeast) (TOMM20), mRNA [NM_014765]	NM_014765
A_32_P147696	3.20E-05	AK090602	AK090602	Homo sapiens cDNA FLJ33283 fis, clone ASTRO2009177. [AK090602]	XM_378312
A_32_P230547	3.20E-05	NM_033407	NM_033407	Homo sapiens dedicator of cytokinesis 7 (DOCK7), mRNA [NM_033407]	NM_033407
A_24_P330668	3.20E-05	ENST00000219638		Homo sapiens cDNA FLJ42145 fis, clone TESTI4000228, highly similar to Homo sapiens ubiquitin binding protein (UBPH). [AK124139]	
A_23_P110108	3.21E-05	NM_024628	NM_024628	Homo sapiens solute carrier family 12 (potassium/chloride transporters), member 8 (SLC12A8), mRNA [NM_024628]	NM_024628
A_32_P217051	3.21E-05	THC2330484			
A_23_P79360	3.21E-05	NM_052946	NM_052946	Homo sapiens nitric oxide synthase trafficker (NOSTRIN), mRNA [NM_052946]	NM_052946
A_32_P170003	3.21E-05	BC059174	BC059174	Homo sapiens TNF receptor-associated factor 3 interacting protein 1, mRNA (cDNA clone MGC:54069 IMAGE:6191726), complete cds. [BC059174]	

A_23_P5983	3.21E-05	NM_006227	NM_006227	Homo sapiens phospholipid transfer protein (PLTP), transcript variant 1, mRNA [NM_006227]	NM_006227
A_32_P16339	3.23E-05	NM_173545	NM_173545	Homo sapiens chromosome 2 open reading frame 13 (C2orf13), mRNA [NM_173545]	NM_173545
A_23_P342275	3.23E-05	NM_006988	NM_006988	Homo sapiens a disintegrin-like and metalloprotease (reprolysin type) with thrombospondin type 1 motif, 1 (ADAMTS1), mRNA [NM_006988]	NM_006988
A_24_P139191	3.27E-05	NM_031483	NM_031483	Homo sapiens itchy homolog E3 ubiquitin protein ligase (mouse) (ITCH), mRNA [NM_031483]	NM_031483
A_24_P129341	3.27E-05	NM_020299	NM_020299	Homo sapiens aldo-keto reductase family 1, member B10 (aldose reductase) (AKR1B10), mRNA [NM_020299]	NM_020299
A_24_P335202	3.28E-05	NM_015424	NM_015424	Homo sapiens chordin-like 2 (CHRDL2), mRNA [NM_015424]	NM_015424
A_23_P200829	3.28E-05	NM_015326	NM_015326	Homo sapiens SLIT-ROBO Rho GTPase activating protein 2 (SRGAP2), mRNA [NM_015326]	NM_015326
A_23_P32454	3.29E-05	NM_003235	NM_003235	Homo sapiens thyroglobulin (TG), mRNA [NM_003235]	NM_003235
A_32_P126222	3.29E-05	AW302758	AW302758	AW302758 xr55g08.x1 NCI_CGAP_Ov26 Homo sapiens cDNA clone IMAGE:2764094 3', mRNA sequence [AW302758]	
A_23_P92623	3.29E-05	NM_000944	NM_000944	Homo sapiens protein phosphatase 3 (formerly 2B), catalytic subunit, alpha isoform (calcineurin A alpha) (PPP3CA), mRNA [NM_000944]	NM_000944
A_32_P38637	3.29E-05	NM_032534	NM_032534	Homo sapiens KIAA1862 protein (KIAA1862), mRNA [NM_032534]	NM_032534
A_23_P87216	3.29E-05	NM_002394	NM_002394	Homo sapiens solute carrier family 3 (activators of dibasic and neutral amino acid transport), member 2 (SLC3A2), transcript variant 3, mRNA [NM_002394]	NM_002394
A_32_P117026	3.30E-05	AK094796	AK094796	Homo sapiens cDNA FLJ37477 fis, clone BRAWH2013047. [AK094796]	
A_32_P192480	3.31E-05	CR627122	CR627122	Homo sapiens mRNA; cDNA DKFZp779M2422 (from clone DKFZp779M2422). [CR627122]	
A_24_P178834	3.31E-05	AK091178	AK091178	Homo sapiens cDNA FLJ33859 fis, clone CTONG2006223, moderately similar to KERATIN, TYPE II CYTOSKELETAL 8. [AK091178]	XM_496418
A_23_P16516	3.31E-05	NM_005224	NM_005224	Homo sapiens AT rich interactive domain 3A (BRIGHT- like) (ARID3A), mRNA [NM_005224]	NM_005224
A_23_P139965	3.33E-05	NM_001010897	NM_001010897	Homo sapiens chromosome 13 open reading frame 21 (C13orf21), mRNA [NM_001010897]	NM_001010897
A_23_P163059	3.33E-05	NM_022495	NM_022495	Homo sapiens chromosome 14 open reading frame 135 (C14orf135), mRNA [NM_022495]	NM_022495
A_24_P134356	3.33E-05	NM_014962	NM_014962	Homo sapiens BTB (POZ) domain containing 3 (BTBD3), transcript variant 1, mRNA [NM_014962]	NM_014962
A_23_P78554	3.33E-05	NM_017703	NM_017703	Homo sapiens F-box and leucine-rich repeat protein 12 (FBXL12), mRNA [NM_017703]	NM_017703
A_32_P93036	3.33E-05	CA307890	CA307890	CA307890 UI-H-FT1-bib-a-05-0-UI.s1 NCI_CGAP_FT1 Homo sapiens cDNA clone UI-H-FT1-bib-a-05-0-UI 3', mRNA sequence [CA307890]	
A_23_P423419	3.33E-05	NM_001005266	NM_001005266	Homo sapiens chromosome 15 open reading frame 21 (C15orf21), transcript variant 1, mRNA [NM_001005266]	NM_001005266
A_23_P200843	3.36E-05	THC2375456		Q7PKG0 (Q7PKG0) ENSANGP00000024462 (Fragment), partial (36%) [THC2375456]	
A_23_P58642	3.36E-05	NM_002653	NM_002653	Homo sapiens paired-like homeodomain transcription factor 1 (PITX1), mRNA [NM_002653]	NM_002653
A_24_P662177	3.37E-05	THC2448843		O57150 (O57150) H88, partial (32%) [THC2448843]	
A_24_P353905	3.37E-05	NM_032348	NM_032348	Homo sapiens matrix-remodelling associated 8 (MXRA8), mRNA [NM_032348]	NM_032348
A_24_P8721	3.37E-05	NM_003517	NM_003517	Homo sapiens histone 2, H2ac (HIST2H2AC), mRNA [NM_003517]	NM_003517
A_32_P225854	3.38E-05	NM_181784	NM_181784	Homo sapiens sprouty-related, EVH1 domain containing 2 (SPRED2), mRNA [NM_181784]	NM_181784
A_23_P156890	3.39E-05	NM_003206	NM_003206	Homo sapiens transcription factor 21 (TCF21), transcript variant 2, mRNA [NM_003206]	NM_003206
A_23_P85008	3.39E-05	NM_000898	NM_000898	Homo sapiens monoamine oxidase B (MAOB), nuclear gene encoding mitochondrial protein, mRNA [NM_000898]	NM_000898
A_24_P40907	3.39E-05	NM_203453	NM_203453	Homo sapiens phosphatidic acid phosphatase type 2 domain containing 2 (PPAPDC2), mRNA [NM_203453]	NM_203453
A_23_P342869	3.40E-05	NM_020066	NM_020066	Homo sapiens formin 2 (FMN2), mRNA [NM_020066]	NM_020066
A_23_P336342	3.40E-05	ENST00000316708		Homo sapiens cDNA FLJ42585 fis, clone BRACE3009237. [AK124576]	
A_24_P166663	3.40E-05	NM_001259	NM_001259	Homo sapiens cyclin-dependent kinase 6 (CDK6), mRNA [NM_001259]	NM_001259
A_32_P197489	3.40E-05	NM_015995	NM_015995	Homo sapiens Kruppel-like factor 13 (KLF13), mRNA [NM_015995]	NM_015995
A_24_P42136	3.40E-05	NM_000224	NM_000224	Homo sapiens keratin 18 (KRT18), transcript variant 1, mRNA [NM_000224]	NM_000224
A_23_P204689	3.41E-05	NM_001004419	NM_001004419	Homo sapiens C-type lectin superfamily 2, member D (CLEC2D), transcript variant 2, mRNA [NM_001004419]	NM_001004419
A_23_P255601	3.41E-05	NM_032508	NM_032508	Homo sapiens family with sequence similarity 11, member A (FAM11A), mRNA [NM_032508]	NM_032508
A_23_P50919	3.42E-05	NM_006216	NM_006216	Homo sapiens serine (or cysteine) proteinase inhibitor, clade E (nexin, plasminogen activator inhibitor type 1), member 2 (SERPINE2), mRNA [NM_006216]	NM_006216
A_23_P168130	3.43E-05	NM_054111	NM_054111	Homo sapiens inositol hexaphosphate kinase 3 (IHPK3), mRNA [NM_054111]	NM_054111
A_23_P4096	3.43E-05	NM_000717	NM_000717	Homo sapiens carbonic anhydrase IV (CA4), mRNA [NM_000717]	NM_000717
A_24_P165346	3.43E-05	NM_014223	NM_014223	Homo sapiens nuclear transcription factor Y, gamma (NFYC), mRNA [NM_014223]	NM_014223
A_23_P119923	3.43E-05	NM_020184	NM_020184	Homo sapiens cyclin M4 (CNNM4), mRNA [NM_020184]	NM_020184
A_24_P562369	3.44E-05	A_24_P562369			

A_23_P422212	3.45E-05	NM_173508	NM_173508	Homo sapiens solute carrier family 35, member F3 (SLC35F3), mRNA [NM_173508]	NM_173508
A_32_P23731	3.45E-05	THC2309960		Q7ZX66 (Q7ZX66) RNPC7 protein (Fragment), partial (9%) [THC2309960]	
A_23_P257417	3.47E-05	NM_152385	NM_152385	Homo sapiens hypothetical protein FLJ31438 (FLJ31438), mRNA [NM_152385]	NM_152385
A_23_P417383	3.48E-05	NM_152792	NM_152792	Homo sapiens hypothetical protein FLJ25084 (FLJ25084), mRNA [NM_152792]	NM_152792
A_23_P90359	3.48E-05	NM_004558	NM_004558	Homo sapiens neurturin (NRTN), mRNA [NM_004558]	NM_004558
A_32_P86400	3.48E-05	CR933624	CR933624	Homo sapiens mRNA; cDNA DKFZp686F0735 (from clone DKFZp686F0735). [CR933624]	XM_371760
A_23_P203183	3.48E-05	NM_000040	NM_000040	Homo sapiens apolipoprotein C-III (APOC3), mRNA [NM_000040]	NM_000040
A_23_P204980	3.48E-05	NM_020121	NM_020121	Homo sapiens UDP-glucose ceramide glucosyltransferase-like 2 (UGCGL2), mRNA [NM_020121]	NM_020121
A_23_P61580	3.50E-05	NM_182543	NM_182543	Homo sapiens NOL1/NOP2/Sun domain family, member 6 (NSUN6), mRNA [NM_182543]	NM_182543
A_23_P206526	3.52E-05	THC2436415			
A_23_P158885	3.54E-05	AK096342	AK096342	Homo sapiens cDNA FLJ39023 fis, clone NT2RP7004348, highly similar to ZINC FINGER PROTEIN 93. [AK096342]	
A_23_P159125	3.54E-05	NM_004695	NM_004695	Homo sapiens solute carrier family 16 (monocarboxylic acid transporters), member 5 (SLC16A5), mRNA [NM_004695]	NM_004695
A_24_P158314	3.55E-05	NM_032293	NM_032293	Homo sapiens GTPase activating Rap/RanGAP domain-like 3 (GARNL3), mRNA [NM_032293]	NM_032293
A_24_P737451	3.55E-05	NM_182501	NM_182501	Homo sapiens MTERF domain containing 2 (MTERFD2), mRNA [NM_182501]	NM_182501
A_23_P393425	3.55E-05	NM_173797	NM_173797	Homo sapiens PAP associated domain containing 4 (PAPD4), mRNA [NM_173797]	NM_173797
A_23_P252536	3.55E-05	NM_030811	NM_030811	Homo sapiens mitochondrial ribosomal protein S26 (MRPS26), nuclear gene encoding mitochondrial protein, mRNA [NM_030811]	NM_030811
A_23_P89799	3.56E-05	NM_006111	NM_006111	Homo sapiens acetyl-Coenzyme A acyltransferase 2 (mitochondrial 3-oxoacyl-Coenzyme A thiolase) (ACAA2), nuclear gene encoding mitochondrial protein, mRNA [NM_006111]	NM_006111
A_24_P95029	3.57E-05	NM_006024	NM_006024	Homo sapiens Tax1 (human T-cell leukemia virus type I) binding protein 1 (TAX1BP1), mRNA [NM_006024]	NM_006024
A_32_P29200	3.58E-05	AK093416	AK093416	Homo sapiens cDNA FLJ36097 fis, clone TESTI2020956. [AK093416]	
A_23_P39910	3.58E-05	NM_001008215	NM_001008215	Homo sapiens hypothetical protein MGC52110 (MGC52110), mRNA [NM_001008215]	NM_001008215
A_23_P258136	3.59E-05	NM_015419	NM_015419	Homo sapiens matrix-remodelling associated 5 (MXRA5), mRNA [NM_015419]	NM_015419
A_23_P34396	3.59E-05	NM_020317	NM_020317	Homo sapiens chromosome 1 open reading frame 63 (C1orf63), transcript variant 2, mRNA [NM_020317]	NM_020317
A_24_P347431	3.60E-05	NM_004496	NM_004496	Homo sapiens forkhead box A1 (FOXA1), mRNA [NM_004496]	NM_004496
A_23_P154338	3.61E-05	NM_025202	NM_025202	Homo sapiens EF-hand domain family, member D1 (EFHD1), mRNA [NM_025202]	NM_025202
A_24_P778844	3.62E-05	AK124841	AK124841	Homo sapiens cDNA FLJ42851 fis, clone BRHIP2005719. [AK124841]	
A_23_P211233	3.62E-05	NM_001849	NM_001849	Homo sapiens collagen, type VI, alpha 2 (COL6A2), transcript variant 2C2, mRNA [NM_001849]	NM_001849
A_32_P80523	3.63E-05	A_32_P80523			
A_23_P113212	3.63E-05	NM_018004	NM_018004	Homo sapiens transmembrane protein 45A (TMEM45A), mRNA [NM_018004]	NM_018004
A_32_P227921	3.64E-05	THC2283605			
A_23_P129738	3.64E-05	NM_004524	NM_004524	Homo sapiens lethal giant larvae homolog 2 (Drosophila) (LLGL2), transcript variant 1, mRNA [NM_004524]	NM_004524
A_23_P163380	3.64E-05	NM_006441	NM_006441	Homo sapiens 5,10-methenyltetrahydrofolate synthetase (5-formyltetrahydrofolate cyclo-ligase) (MTHFS), mRNA [NM_006441]	NM_006441
A_32_P192922	3.65E-05	AL050061	AL050061	Homo sapiens mRNA; cDNA DKFZp566J123 (from clone DKFZp566J123). [AL050061]	
A_32_P190049	3.65E-05	AK056809	AK056809	Homo sapiens cDNA FLJ32247 fis, clone PROST1000120. [AK056809]	
A_23_P384761	3.65E-05	NM_002052	NM_002052	Homo sapiens GATA binding protein 4 (GATA4), mRNA [NM_002052]	NM_002052
A_23_P115919	3.66E-05	NM_006214	NM_006214	Homo sapiens phytanoyl-CoA hydroxylase (Refsum disease) (PHYH), mRNA [NM_006214]	NM_006214
A_24_P257022	3.66E-05	NM_000364	NM_000364	Homo sapiens troponin T2, cardiac (TNNT2), transcript variant 1, mRNA [NM_000364]	NM_000364
A_23_P335069	3.67E-05	AL832613	AL832613	Homo sapiens mRNA; cDNA DKFZp451K1917 (from clone DKFZp451K1917). [AL832613]	
A_24_P589028	3.68E-05	AK026750	AK026750	Homo sapiens cDNA: FLJ23097 fis, clone LNG07418. [AK026750]	
A_32_P147622	3.69E-05	BC064982	BC064982	Homo sapiens cDNA clone IMAGE:2959727, partial cds. [BC064982]	XM_498568
A_32_P153781	3.70E-05	AK123066	AK123066	Homo sapiens cDNA FLJ41071 fis, clone 3NB692003538. [AK123066]	
A_23_P67971	3.70E-05	NM_138801	NM_138801	Homo sapiens galactose mutarotase (aldose 1-epimerase) (GALM), mRNA [NM_138801]	NM_138801
A_23_P51690	3.71E-05	NM_020407	NM_020407	Homo sapiens Rhesus blood group, B glycoprotein (RHBG), mRNA [NM_020407]	NM_020407
A_24_P303647	3.71E-05	ENST00000297145		Homo sapiens mRNA; cDNA DKFZp762M126 (from clone DKFZp762M126). [AL834437]	
A_23_P135616	3.71E-05	NM_016930	NM_016930	Homo sapiens syntaxin 18 (STX18), mRNA [NM_016930]	NM_016930

A_23_P133814	3.72E-05	NM_003531	NM_003531	Homo sapiens histone 1, H3c (HIST1H3C), mRNA [NM_003531]	NM_003531
A_23_P81880	3.72E-05	NM_005730	NM_005730	Homo sapiens CTD (carboxy-terminal domain, RNA polymerase II, polypeptide A) small phosphatase 2 (CTDSP2), mRNA [NM_005730]	NM_005730
A_23_P93641	3.74E-05	NM_020299	NM_020299	Homo sapiens aldo-keto reductase family 1, member B10 (aldose reductase) (AKR1B10), mRNA [NM_020299]	NM_020299
A_23_P103877	3.75E-05	CR622769	CR622769	full-length cDNA clone CS0DI025YD24 of Placenta Cot 25-normalized of Homo sapiens (human). [CR622769]	
A_32_P78816	3.75E-05	NM_004577	NM_004577	Homo sapiens phosphoserine phosphatase (PSPH), mRNA [NM_004577]	NM_004577
A_23_P212756	3.76E-05	NM_001004057	NM_001004057	Homo sapiens G protein-coupled receptor kinase 4 (GRK4), transcript variant 3, mRNA [NM_001004057]	NM_001004057
A_23_P139687	3.76E-05	NM_152321	NM_152321	Homo sapiens hypothetical protein FLJ32115 (FLJ32115), mRNA [NM_152321]	NM_152321
A_23_P341537	3.77E-05	NM_031915	NM_031915	Homo sapiens SET domain, bifurcated 2 (SETDB2), mRNA [NM_031915]	NM_031915
A_23_P105619	3.77E-05	NM_138341	NM_138341	Homo sapiens hypothetical protein BC000282 (LOC89894), mRNA [NM_138341]	NM_138341
A_23_P88810	3.78E-05	NM_012213	NM_012213	Homo sapiens malonyl-CoA decarboxylase (MLYCD), mRNA [NM_012213]	NM_012213
A_32_P42236	3.78E-05	XM_370839	XM_370839	PREDICTED: Homo sapiens similar to hypothetical protein (LOC440234), mRNA [XM_370839]	XM_370839
A_23_P53039	3.80E-05	NM_002301	NM_002301	Homo sapiens lactate dehydrogenase C (LDHC), transcript variant 1, mRNA [NM_002301]	NM_002301
A_32_P210168	3.81E-05	XM_370873	XM_370873	PREDICTED: Homo sapiens similar to RIKEN cDNA 6030419C18 gene (LOC388135), mRNA [XM_370873]	XM_370873
A_23_P212728	3.84E-05	BC020955	BC020955	Homo sapiens hypothetical protein FLJ11046, mRNA (cDNA clone MGC:8800 IMAGE:3847561), complete cds. [BC020955]	
A_24_P405850	3.86E-05	NM_006320	NM_006320	Homo sapiens progesterone receptor membrane component 2 (PGRMC2), mRNA [NM_006320]	NM_006320
A_23_P429977	3.87E-05	NM_000218	NM_000218	Homo sapiens potassium voltage-gated channel, KQT-like subfamily, member 1 (KCNQ1), transcript variant 1, mRNA [NM_000218]	NM_000218
A_24_P567408	3.87E-05	THC2306240		Q8WNH2 (Q8WNH2) NPIP-like protein (Fragment), complete [THC2306240]	
A_24_P144134	3.87E-05	A_24_P144134			
A_23_P130974	3.88E-05	NM_025249	NM_025249	Homo sapiens KIAA1683 (KIAA1683), mRNA [NM_025249]	NM_025249
A_32_P514790	3.88E-05	BC053362	BC053362	Homo sapiens zinc finger CCCH-type containing 5, mRNA (cDNA clone IMAGE:6012927), partial cds. [BC053362]	XM_036115
A_23_P152858	3.88E-05	NM_018405	NM_018405	Homo sapiens hypothetical protein, clone 2746033 (HSA272196), mRNA [NM_018405]	NM_018405
A_23_P130376	3.89E-05	NM_022068	NM_022068	Homo sapiens family with sequence similarity 38, member B (FAM38B), mRNA [NM_022068]	NM_022068
A_23_P428184	3.90E-05	NM_021065	NM_021065	Homo sapiens histone 1, H2ad (HIST1H2AD), mRNA [NM_021065]	NM_021065
A_23_P122443	3.91E-05	NM_005319	NM_005319	Homo sapiens histone 1, H1c (HIST1H1C), mRNA [NM_005319]	NM_005319
A_32_P204048	3.92E-05	CR601260	CR601260	full-length cDNA clone CS0DM001YA20 of Fetal liver of Homo sapiens (human). [CR601260]	
A_32_P146826	3.92E-05	THC2441367			
A_23_P206733	3.93E-05	NM_001266	NM_001266	Homo sapiens carboxylesterase 1 (monocyte/macrophage serine esterase 1) (CES1), transcript variant 3, mRNA [NM_001266]	NM_001266
A_24_P141520	3.93E-05	AK022297	AK022297	Homo sapiens cDNA FLJ12235 fis, clone MAMMA1001243. [AK022297]	
A_23_P66481	3.94E-05	NM_178568	NM_178568	Homo sapiens reticulon 4 receptor-like 1 (RTN4RL1), mRNA [NM_178568]	NM_178568
A_23_P104464	3.97E-05	NM_000698	NM_000698	Homo sapiens arachidonate 5-lipoxygenase (ALOX5), mRNA [NM_000698]	NM_000698
A_23_P102160	3.97E-05	AF435956	AF435956	Homo sapiens unknown mRNA. [AF435956]	
A_23_P424597	3.97E-05	NM_152482	NM_152482	Homo sapiens chromosome 19 open reading frame 25 (C19orf25), mRNA [NM_152482]	NM_152482
A_24_P132787	3.97E-05	NM_021252	NM_021252	Homo sapiens RAB18, member RAS oncogene family (RAB18), mRNA [NM_021252]	NM_021252
A_32_P67610	3.98E-05	CR605444	CR605444	full-length cDNA clone CS0DK008YC22 of HeLa cells Cot 25-normalized of Homo sapiens (human). [CR605444]	
A_24_P64895	3.99E-05	THC2338707			
A_23_P84334	4.00E-05	NM_018266	NM_018266	Homo sapiens transmembrane protein 39A (TMEM39A), mRNA [NM_018266]	NM_018266
A_23_P31407	4.01E-05	NM_006408	NM_006408	Homo sapiens anterior gradient 2 homolog (Xenopus laevis) (AGR2), mRNA [NM_006408]	NM_006408
A_32_P108226	4.01E-05	THC2309737			
A_23_P55606	4.01E-05	ENST00000217537		Homo sapiens mRNA for KIAA0222 gene, partial cds. [D86975]	
A_32_P161661	4.01E-05	BC065226	BC065226	Homo sapiens cDNA clone IMAGE:5768818, partial cds. [BC065226]	
A_23_P215024	4.01E-05	NM_018214	NM_018214	Homo sapiens leucine rich repeat containing 1 (LRRC1), mRNA [NM_018214]	NM_018214
A_24_P630490	4.02E-05	BC020859	BC020859	Homo sapiens, Similar to deafness, autosomal dominant 5 homolog (human), clone IMAGE:4551670, mRNA. [BC020859]	
A_23_P27994	4.02E-05	NM_003332	NM_003332	Homo sapiens TYRO protein tyrosine kinase binding protein (TYROBP), transcript variant 1, mRNA [NM_003332]	NM_003332
A_24_P76267	4.02E-05	NM_002058	NM_002058	Homo sapiens gamma-glutamyltransferase 2 (GGT2), mRNA [NM_002058]	NM_002058

A_32_P144381	4.04E-05	A_32_P144381			
A_23_P161280	4.04E-05	NM_014767	NM_014767	Homo sapiens sparc/osteonectin, cwcv and kazal-like domains proteoglycan (testican) 2 (SPOCK2), mRNA [NM_014767]	NM_014767
A_23_P120809	4.04E-05	NM_080839	NM_080839	Homo sapiens gamma-glutamyltransferase-like 4 (GGTL4), transcript variant 2, mRNA [NM_080839]	NM_080839
A_23_P90419	4.05E-05	NM_025245	NM_025245	Homo sapiens pre-B-cell leukemia transcription factor 4 (PBX4), mRNA [NM_025245]	NM_025245
A_32_P55438	4.07E-05	A_32_P55438			
A_32_P90709	4.07E-05	BC045657	BC045657	Homo sapiens, clone IMAGE:5270591, mRNA. [BC045657]	
A_23_P148768	4.08E-05	NM_000130	NM_000130	Homo sapiens coagulation factor V (proaccelerin, labile factor) (F5), mRNA [NM_000130]	NM_000130
A_32_P149735	4.08E-05	A_32_P149735			
A_24_P267523	4.09E-05	NM_144613	NM_144613	Homo sapiens cytochrome c oxidase subunit VIb polypeptide 2 (testis) (COX6B2), mRNA [NM_144613]	NM_144613
A_32_P44808	4.12E-05	AK124941	AK124941	Homo sapiens cDNA FLJ42951 fis, clone BRSTN2007765. [AK124941]	
A_23_P354217	4.13E-05	NM_153266	NM_153266	Homo sapiens hypothetical protein MGC33486 (MGC33486), mRNA [NM_153266]	NM_153266
A_23_P10743	4.15E-05	NM_000283	NM_000283	Homo sapiens phosphodiesterase 6B, cGMP-specific, rod, beta (congenital stationary night blindness 3, autosomal dominant) (PDE6B), mRNA [NM_000283]	NM_000283
A_23_P129128	4.16E-05	NM_152334	NM_152334	Homo sapiens threonyl-tRNA synthetase-like 2 (TARSL2), mRNA [NM_152334]	NM_152334
A_23_P168610	4.16E-05	NM_014399	NM_014399	Homo sapiens tetraspanin 13 (TSPAN13), mRNA [NM_014399]	NM_014399
A_23_P313603	4.16E-05	AK056855	AK056855	Homo sapiens cDNA FLJ32293 fis, clone PROST2001739. [AK056855]	XM_374902
A_23_P395609	4.17E-05	NM_147189	NM_147189	Homo sapiens hypothetical protein MGC39325 (MGC39325), mRNA [NM_147189]	NM_147189
A_24_P265274	4.19E-05	NM_000089	NM_000089	Homo sapiens collagen, type I, alpha 2 (COL1A2), mRNA [NM_000089]	NM_000089
A_23_P28906	4.19E-05	AB046810	AB046810	Homo sapiens mRNA for KIAA1590 protein, partial cds. [AB046810]	
A_24_P469746	4.19E-05	ENST00000334973		Homo sapiens chromosome 2 unknown mRNA. [AF451986]	XM_371605
A_23_P375281	4.20E-05	NM_080706	NM_080706	Homo sapiens transient receptor potential cation channel, subfamily V, member 1 (TRPV1), transcript variant 3, mRNA [NM_080706]	NM_080706
A_23_P101407	4.20E-05	NM_000064	NM_000064	Homo sapiens complement component 3 (C3), mRNA [NM_000064]	NM_000064
A_24_P307395	4.21E-05	ENST00000332097			
A_24_P284093	4.21E-05	NM_016651	NM_016651	Homo sapiens dapper, antagonist of beta-catenin, homolog 1 (Xenopus laevis) (DACT1), mRNA [NM_016651]	NM_016651
A_23_P8013	4.21E-05	NM_003519	NM_003519	Homo sapiens histone 1, H2bl (HIST1H2BL), mRNA [NM_003519]	NM_003519
A_23_P81581	4.22E-05	NM_001713	NM_001713	Homo sapiens betaine-homocysteine methyltransferase (BHMT), mRNA [NM_001713]	NM_001713
A_23_P13713	4.22E-05	NM_006262	NM_006262	Homo sapiens peripherin (PRPH), mRNA [NM_006262]	NM_006262
A_24_P227121	4.22E-05	CB049198	CB049198	NISC_gj09c06.y1 NCI_CGAP_Pr28 Homo sapiens cDNA clone IMAGE:3271282 5', mRNA sequence [CB049198]	
A_23_P423457	4.23E-05	NM_178276	NM_178276	Homo sapiens chromosome 5 open reading frame 12 (C5orf12), mRNA [NM_178276]	NM_178276
A_24_P390403	4.23E-05	NM_015138	NM_015138	Homo sapiens KIAA0252 (KIAA0252), mRNA [NM_015138]	NM_015138
A_23_P372834	4.23E-05	NM_000385	NM_000385	Homo sapiens aquaporin 1 (channel-forming integral protein, 28kDa) (AQP1), transcript variant 2, mRNA [NM_000385]	NM_000385
A_23_P77381	4.24E-05	NM_018110	NM_018110	Homo sapiens docking protein 4 (DOK4), mRNA [NM_018110]	NM_018110
A_23_P44569	4.25E-05	NM_000392	NM_000392	Homo sapiens ATP-binding cassette, sub-family C (CFTR/MRP), member 2 (ABCC2), mRNA [NM_000392]	NM_000392
A_23_P40847	4.25E-05	NM_004267	NM_004267	Homo sapiens carbohydrate (N-acetylglucosamine-6-O) sulfotransferase 2 (CHST2), mRNA [NM_004267]	NM_004267
A_23_P68628	4.28E-05	NM_031232	NM_031232	Homo sapiens amyloid beta (A4) precursor protein-binding, family A, member 2 binding protein (APBA2BP), transcript variant 2, mRNA [NM_031232]	NM_031232
A_23_P426663	4.30E-05	NM_198159	NM_198159	Homo sapiens microphthalmia-associated transcription factor (MITF), transcript variant 1, mRNA [NM_198159]	NM_198159
A_24_P118196	4.30E-05	ENST00000308603		PREDICTED: Homo sapiens similar to CG9996-PA (LOC389129), mRNA [XM_371647]	XM_371647
A_23_P146554	4.30E-05	NM_000954	NM_000954	Homo sapiens prostaglandin D2 synthase 21kDa (brain) (PTGDS), mRNA [NM_000954]	NM_000954
A_23_P134464	4.30E-05	NM_004279	NM_004279	Homo sapiens peptidase (mitochondrial processing) beta (PMPCB), mRNA [NM_004279]	NM_004279
A_23_P10647	4.32E-05	NM_018659	NM_018659	Homo sapiens cytokine-like 1 (CYTL1), mRNA [NM_018659]	NM_018659
A_32_P122136	4.33E-05	AK057596	AK057596	Homo sapiens cDNA FLJ33034 fis, clone THYMU2000236. [AK057596]	XM_498456
A_23_P202435	4.33E-05	NM_016824	NM_016824	Homo sapiens adducin 3 (gamma) (ADD3), transcript variant 1, mRNA [NM_016824]	NM_016824
A_23_P11295	4.34E-05	NM_014221	NM_014221	Homo sapiens mature T-cell proliferation 1 (MTCP1), nuclear gene encoding mitochondrial protein, transcript variant B1, mRNA [NM_014221]	NM_014221
A_32_P2392	4.40E-05	NM_181076	NM_181076	Homo sapiens 88-kDa golgi protein (GM88), transcript variant 2, mRNA [NM_181076]	NM_181076
A_24_P326511	4.41E-05	NM_015385	NM_015385	Homo sapiens sorbin and SH3 domain containing 1 (SORBS1), transcript variant 2, mRNA [NM_015385]	NM_015385

A_24_P130041	4.41E-05	NM_000786	NM_000786	Homo sapiens cytochrome P450, family 51, subfamily A, polypeptide 1 (CYP51A1), mRNA [NM_000786]	NM_000786
A_24_P941268	4.42E-05	AK057568	AK057568	Homo sapiens cDNA FLJ33006 fis, clone THYMU1000316. [AK057568]	
A_24_P930963	4.42E-05	CR616309	CR616309	full-length cDNA clone CS0DF015YK23 of Fetal brain of Homo sapiens (human). [CR616309]	
A_32_P59475	4.42E-05	BQ019626	BQ019626	BQ019626 UI-H-ED0-axd-f-18-0-UI.s1 NCI_CGAP_ED0 Homo sapiens cDNA clone IMAGE:5827217 3', mRNA sequence [BQ019626]	
A_32_P209791	4.43E-05	BC040051	BC040051	Homo sapiens, clone IMAGE:6018828, mRNA. [BC040051]	
A_23_P126393	4.43E-05	NM_012432	NM_012432	Homo sapiens SET domain, bifurcated 1 (SETDB1), mRNA [NM_012432]	NM_012432
A_32_P119348	4.44E-05	AK130049	AK130049	Homo sapiens cDNA FLJ26539 fis, clone KDN09310. [AK130049]	
A_24_P678418	4.44E-05	NM_207443	NM_207443	Homo sapiens FLJ45244 protein (FLJ45244), mRNA [NM_207443]	NM_207443
A_24_P161036	4.44E-05	NM_006821	NM_006821	Homo sapiens peroxisomal long-chain acyl-coA thioesterase (ZAP128), mRNA [NM_006821]	NM_006821
A_23_P110957	4.53E-05	NM_001452	NM_001452	Homo sapiens forkhead box F2 (FOXF2), mRNA [NM_001452]	NM_001452
A_23_P90679	4.53E-05	NM_018571	NM_018571	Homo sapiens amyotrophic lateral sclerosis 2 (juvenile) chromosome region, candidate 2 (ALS2CR2), mRNA [NM_018571]	NM_018571
A_32_P130265	4.53E-05	NM_014827	NM_014827	Homo sapiens zinc finger CCCH-type containing 11A (ZC3H11A), mRNA [NM_014827]	NM_014827
A_23_P134650	4.53E-05	NM_015545	NM_015545	Homo sapiens pentatricopeptide repeat domain 1 (PTCD1), mRNA [NM_015545]	NM_015545
A_24_P811014	4.54E-05	A_24_P811014			
A_24_P4373	4.54E-05	NM_153045	NM_153045	Homo sapiens chromosome 9 open reading frame 91 (C9orf91), mRNA [NM_153045]	NM_153045
A_23_P251095	4.54E-05	NM_000284	NM_000284	Homo sapiens pyruvate dehydrogenase (lipoamide) alpha 1 (PDHA1), mRNA [NM_000284]	NM_000284
A_23_P27035	4.54E-05	NM_015544	NM_015544	Homo sapiens DKFZP564K1964 protein (DKFZP564K1964), mRNA [NM_015544]	NM_015544
A_32_P183609	4.58E-05	NM_016114	NM_016114	Homo sapiens ankyrin repeat and SOCS box-containing 1 (ASB1), mRNA [NM_016114]	NM_016114
A_24_P335620	4.60E-05	NM_003486	NM_003486	Homo sapiens solute carrier family 7 (cationic amino acid transporter, y+ system), member 5 (SLC7A5), mRNA [NM_003486]	NM_003486
A_32_P224094	4.63E-05	U09850	U09850	Human zinc finger protein (ZNF143) mRNA, complete cds. [U09850]	
A_23_P154379	4.65E-05	NM_003960	NM_003960	Homo sapiens N-acetyltransferase 8 (camello like) (NAT8), mRNA [NM_003960]	NM_003960
A_23_P421811	4.66E-05	NM_152503	NM_152503	Homo sapiens chromosome 20 open reading frame 132 (C20orf132), transcript variant 1, mRNA [NM_152503]	NM_152503
A_32_P159023	4.67E-05	THC2285720		BX114329 BX114329 Soares_NhHMPu_S1 Homo sapiens cDNA clone IMAGp998G064741 ; IMAGE:1932317, mRNA sequence [BX114329]	
A_32_P179005	4.70E-05	THC2363557			
A_24_P7750	4.70E-05	A_24_P7750			
A_23_P60166	4.72E-05	NM_022783	NM_022783	Homo sapiens DEP domain containing 6 (DEPDC6), mRNA [NM_022783]	NM_022783
A_23_P111621	4.73E-05	NM_005685	NM_005685	Homo sapiens GTF2I repeat domain containing 1 (GTF2IRD1), transcript variant 2, mRNA [NM_005685]	NM_005685
A_23_P398637	4.75E-05	NM_152292	NM_152292	Homo sapiens RNA (guanine-9-) methyltransferase domain containing 2 (RG9MTD2), mRNA [NM_152292]	NM_152292
A_24_P384588	4.76E-05	NM_001012452	NM_001012452	Homo sapiens hypothetical protein FLJ32679 (FLJ32679), mRNA [NM_001012452]	NM_001012452
A_23_P92994	4.76E-05	NM_020194	NM_020194	Homo sapiens chromosome 2 open reading frame 33 (C2orf33), mRNA [NM_020194]	NM_020194
A_24_P398940	4.76E-05	NM_138423	NM_138423	Homo sapiens cancer susceptibility candidate 4 (CASC4), transcript variant 1, mRNA [NM_138423]	NM_138423
A_24_P62668	4.78E-05	AK021425	AK021425	Homo sapiens cDNA FLJ11363 fis, clone HEMBA1000251. [AK021425]	
A_23_P95612	4.79E-05	NM_024685	NM_024685	Homo sapiens hypothetical protein FLJ23560 (FLJ23560), mRNA [NM_024685]	NM_024685
A_23_P153146	4.80E-05	NM_004361	NM_004361	Homo sapiens cadherin 7, type 2 (CDH7), transcript variant b, mRNA [NM_004361]	NM_004361
A_32_P48842	4.80E-05	CR595668	CR595668	full-length cDNA clone CS0DB003YC18 of Neuroblastoma Cot 10-normalized of Homo sapiens (human). [CR595668]	
A_23_P131737	4.80E-05	NM_016516	NM_016516	Homo sapiens vacuolar protein sorting 54 (yeast) (VPS54), transcript variant 1, mRNA [NM_016516]	NM_016516
A_23_P58337	4.80E-05	NM_030917	NM_030917	Homo sapiens FIP1 like 1 (S. cerevisiae) (FIP1L1), mRNA [NM_030917]	NM_030917
A_23_P19510	4.81E-05	NM_182549	NM_182549	Homo sapiens major histocompatibility complex, class II, DQ beta 2 (HLA-DQB2), mRNA [NM_182549]	NM_182549
A_23_P209006	4.82E-05	NM_005543	NM_005543	Homo sapiens insulin-like 3 (Leydig cell) (INSL3), mRNA [NM_005543]	NM_005543
A_23_P338981	4.82E-05	NM_134268	NM_134268	Homo sapiens cytoglobin (CYGB), mRNA [NM_134268]	NM_134268
A_23_P1492	4.82E-05	NM_021732	NM_021732	Homo sapiens arginine vasopressin-induced 1 (AVPI1), mRNA [NM_021732]	NM_021732
A_23_P68198	4.86E-05	NM_015677	NM_015677	Homo sapiens SH3 domain containing, Ysc84-like 1 (S. cerevisiae) (SH3YL1), mRNA [NM_015677]	NM_015677
A_23_P52121	4.87E-05	NM_002614	NM_002614	Homo sapiens PDZ domain containing 1 (PDZK1), mRNA [NM_002614]	NM_002614
A_23_P92590	4.87E-05	NM_133370	NM_133370	Homo sapiens splicing factor YT521-B (YT521), mRNA [NM_133370]	NM_133370
A_24_P700052	4.89E-05	AK098638	AK098638	Homo sapiens cDNA FLJ25772 fis, clone TST06461. [AK098638]	

A_24_P148321	4.90E-05	NM_003528	NM_003528	Homo sapiens histone 2, H2be (HIST2H2BE), mRNA [NM_003528]	NM_003528
A_23_P11744	4.92E-05	NM_199163	NM_199163	Homo sapiens CXYorf1-related protein (FLJ25222), mRNA [NM_199163]	NM_199163
A_23_P72680	4.94E-05	NM_014055	NM_014055	Homo sapiens carnitine deficiency-associated, expressed in ventricle 1 (CDV1), transcript variant 1, mRNA [NM_014055]	NM_014055
A_24_P920715	4.96E-05	A_24_P920715			
A_23_P326170	4.96E-05	NM_001743	NM_001743	Homo sapiens calmodulin 2 (phosphorylase kinase, delta) (CALM2), mRNA [NM_001743]	NM_001743
A_23_P24157	4.97E-05	NM_032709	NM_032709	Homo sapiens chromosome 10 open reading frame 33 (C10orf33), mRNA [NM_032709]	NM_032709
A_23_P200930	4.97E-05	NM_000254	NM_000254	Homo sapiens 5-methyltetrahydrofolate-homocysteine methyltransferase (MTR), mRNA [NM_000254]	NM_000254
A_23_P38567	4.99E-05	NM_152904	NM_152904	Homo sapiens spectrin domain with coiled-coils 1 (SPECC1), mRNA [NM_152904]	NM_152904
A_32_P50587	5.01E-05	THC2448178			
A_32_P67623	5.01E-05	AK125664	AK125664	Homo sapiens cDNA FLJ43676 fis, clone SYNOV4009129. [AK125664]	
A_23_P151746	5.02E-05	NM_194279	NM_194279	Homo sapiens HESB like domain containing 1 (HBLD1), mRNA [NM_194279]	NM_194279
A_23_P101642	5.04E-05	NM_002842	NM_002842	Homo sapiens protein tyrosine phosphatase, receptor type, H (PTPRH), mRNA [NM_002842]	NM_002842
A_24_P107674	5.04E-05	NM_144578	NM_144578	Homo sapiens chromosome 14 open reading frame 32 (C14orf32), mRNA [NM_144578]	NM_144578
A_23_P204998	5.05E-05	NM_005766	NM_005766	Homo sapiens FERM, RhoGEF (ARHGEF) and pleckstrin domain protein 1 (chondrocyte-derived) (FARP1), transcript variant 1, mRNA [NM_005766]	NM_005766
A_23_P209735	5.06E-05	NM_025139	NM_025139	Homo sapiens melanoma/melanocyte specific protein KU-MEL-1 (FLJ12584), mRNA [NM_025139]	NM_025139
A_23_P258769	5.06E-05	NM_002121	NM_002121	Homo sapiens major histocompatibility complex, class II, DP beta 1 (HLA-DPB1), mRNA [NM_002121]	NM_002121
A_23_P167017	5.06E-05	NM_022135	NM_022135	Homo sapiens popeye domain containing 2 (POPDC2), mRNA [NM_022135]	NM_022135
A_23_P127054	5.09E-05	NM_148977	NM_148977	Homo sapiens pantothenate kinase 1 (PANK1), transcript variant alpha, mRNA [NM_148977]	NM_148977
A_23_P103371	5.10E-05	NM_052998	NM_052998	Homo sapiens arginine decarboxylase (ADC), mRNA [NM_052998]	NM_052998
A_23_P86195	5.11E-05	NM_152369	NM_152369	Homo sapiens hypothetical protein MGC45474 (MGC45474), mRNA [NM_152369]	NM_152369
A_24_P713729	5.11E-05	THC2373625		ALU8_HUMAN (P39195) Alu subfamily SX sequence contamination warning entry, partial (6%) [THC2373624]	
A_24_P330385	5.12E-05	NM_016609	NM_016609	Homo sapiens solute carrier family 22 (organic cation transporter), member 17 (SLC22A17), transcript variant 2, mRNA [NM_016609]	NM_016609
A_23_P141738	5.12E-05	NM_001007559	NM_001007559	Homo sapiens synovial sarcoma translocation, chromosome 18 (SS18), transcript variant 1, mRNA [NM_001007559]	NM_001007559
A_32_P193378	5.13E-05	AK055370	AK055370	Homo sapiens cDNA FLJ30808 fis, clone FEBRA2001383. [AK055370]	
A_23_P39465	5.13E-05	NM_004335	NM_004335	Homo sapiens bone marrow stromal cell antigen 2 (BST2), mRNA [NM_004335]	NM_004335
A_23_P201342	5.13E-05	NM_181870	NM_181870	Homo sapiens dishevelled, dsh homolog 1 (Drosophila) (DVL1), transcript variant 2, mRNA [NM_181870]	NM_181870
A_23_P59791	5.15E-05	NM_016019	NM_016019	Homo sapiens LUC7-like 2 (S. cerevisiae) (LUC7L2), mRNA [NM_016019]	NM_016019
A_24_P915196	5.18E-05	AF038174	AF038174	Homo sapiens clone 23579 mRNA sequence. [AF038174]	
A_23_P363163	5.18E-05	NM_001008390	NM_001008390	Homo sapiens CGG triplet repeat binding protein 1 (CGGBP1), transcript variant 1, mRNA [NM_001008390]	NM_001008390
A_24_P192627	5.19E-05	NM_004529	NM_004529	Homo sapiens myeloid/lymphoid or mixed-lineage leukemia (trithorax homolog, Drosophila); translocated to, 3 (MLLT3), mRNA [NM_004529]	NM_004529
A_24_P715719	5.21E-05	AL049443	AL049443	Homo sapiens mRNA; cDNA DKFZp586N2020 (from clone DKFZp586N2020). [AL049443]	
A_23_P253495	5.21E-05	NM_000847	NM_000847	Homo sapiens glutathione S-transferase A3 (GSTA3), mRNA [NM_000847]	NM_000847
A_23_P32785	5.21E-05	ENST00000318887		Homo sapiens mRNA; cDNA DKFZp667M2212 (from clone DKFZp667M2212). [AL832398]	
A_23_P430818	5.21E-05	NM_014181	NM_014181	Homo sapiens HSPC159 protein (HSPC159), mRNA [NM_014181]	NM_014181
A_32_P119096	5.21E-05	AF052107	AF052107	Homo sapiens clone 23620 mRNA sequence. [AF052107]	
A_23_P206454	5.21E-05	NM_145039	NM_145039	Homo sapiens hypothetical protein MGC16385 (MGC16385), mRNA [NM_145039]	NM_145039
A_23_P41629	5.23E-05	AB095949	AB095949	Homo sapiens mRNA for KIAA2029 protein. [AB095949]	
A_24_P350644	5.26E-05	AB011087	AB011087	Homo sapiens mRNA for KIAA0515 protein, partial cds. [AB011087]	XM_497080
A_23_P343250	5.30E-05	NM_152303	NM_152303	Homo sapiens zinc finger protein 554 (ZNF554), mRNA [NM_152303]	NM_152303
A_23_P3651	5.31E-05	NM_005332	NM_005332	Homo sapiens hemoglobin, zeta (HBZ), mRNA [NM_005332]	NM_005332
A_32_P387905	5.31E-05	AK024224	AK024224	Homo sapiens cDNA FLJ14162 fis, clone NT2RM4002504. [AK024224]	
A_23_P27107	5.34E-05	NM_003963	NM_003963	Homo sapiens transmembrane 4 L six family member 5 (TM4SF5), mRNA [NM_003963]	NM_003963
A_23_P81650	5.35E-05	NM_020199	NM_020199	Homo sapiens chromosome 5 open reading frame 15 (C5orf15), mRNA [NM_020199]	NM_020199
A_24_P350060	5.35E-05	ENST00000330461		PREDICTED: Homo sapiens similar to Keratin, type I cytoskeletal 18 (Cytokeratin 18) (K18) (CK 18) (LOC391819), mRNA [XM_498013]	XM_498013

A_23_P70897	5.36E-05	NM_017984	NM_017984	Homo sapiens zinc finger, CW type with PWWP domain 1 (ZCWPW1), mRNA [NM_017984]	NM_017984
A_32_P167122	5.39E-05	NM_018254	NM_018254	Homo sapiens REST corepressor 3 (RCOR3), mRNA [NM_018254]	NM_018254
A_24_P129232	5.39E-05	NM_020755	NM_020755	Homo sapiens tumor differentially expressed 2 (TDE2), mRNA [NM_020755]	NM_020755
A_23_P75283	5.39E-05	NM_006744	NM_006744	Homo sapiens retinol binding protein 4, plasma (RBP4), mRNA [NM_006744]	NM_006744
A_32_P75094	5.42E-05	NM_032797	NM_032797	Homo sapiens apoptosis-inducing factor (AIF)-like mitochondrion-associated inducer of death (AMID), mRNA [NM_032797]	NM_032797
A_24_P295791	5.43E-05	NM_032564	NM_032564	Homo sapiens diacylglycerol O-acyltransferase homolog 2 (mouse) (DGAT2), mRNA [NM_032564]	NM_032564
A_23_P114057	5.43E-05	NM_017789	NM_017789	Homo sapiens sema domain, immunoglobulin domain (Ig), transmembrane domain (TM) and short cytoplasmic domain, (semaphorin) 4C (SEMA4C), mRNA [NM_017789]	NM_017789
A_32_P196837	5.44E-05	AA449494	AA449494	AA449494 zx08h11.s1 Soares_total_fetus_Nb2HF8_9w Homo sapiens cDNA clone IMAGE:785925 3' similar to gb:U15981 ADRENAL SPECIFIC 30 KD PROTEIN (HUMAN);, mRNA sequence [AA449494]	
A_23_P202860	5.45E-05	NM_024514	NM_024514	Homo sapiens cytochrome P450, family 2, subfamily R, polypeptide 1 (CYP2R1), mRNA [NM_024514]	NM_024514
A_32_P131143	5.46E-05	AF307448	AF307448	Homo sapiens Cat eye syndrome critical region candidate gene number 4 (CECR4) mRNA, partial cds. [AF307448]	
A_23_P68240	5.46E-05	AK131197	AK131197	Homo sapiens cDNA FLJ16034 fis, clone TESTI2022814, weakly similar to GLYCEROL-3-PHOSPHATE ACYLTRANSFERASE, MITOCHONDRIAL PRECURSOR (EC 2.3.1.15). [AK131197]	
A_24_P224727	5.47E-05	NM_004364	NM_004364	Homo sapiens CCAAT/enhancer binding protein (C/EBP), alpha (CEBPA), mRNA [NM_004364]	NM_004364
A_23_P77201	5.48E-05	NM_032499	NM_032499	Homo sapiens hypothetical protein HH114 (HH114), mRNA [NM_032499]	NM_032499
A_24_P937139	5.49E-05	U97060	U97060	Homo sapiens Slug mRNA, partial cds. [U97060]	
A_23_P8281	5.50E-05	NM_000416	NM_000416	Homo sapiens interferon gamma receptor 1 (IFNGR1), mRNA [NM_000416]	NM_000416
A_23_P27215	5.50E-05	NM_018955	NM_018955	Homo sapiens ubiquitin B (UBB), mRNA [NM_018955]	NM_018955
A_24_P224488	5.51E-05	AK055986	AK055986	Homo sapiens cDNA FLJ31424 fis, clone NT2NE2000392. [AK055986]	
A_23_P259594	5.51E-05	NM_016377	NM_016377	Homo sapiens A kinase (PRKA) anchor protein 7 (AKAP7), transcript variant gamma, mRNA [NM_016377]	NM_016377
A_24_P261470	5.53E-05	NM_138732	NM_138732	Homo sapiens neurexin 2 (NRXN2), transcript variant alpha-2, mRNA [NM_138732]	NM_138732
A_24_P289471	5.55E-05	NM_003730	NM_003730	Homo sapiens ribonuclease T2 (RNASET2), mRNA [NM_003730]	NM_003730
A_24_P936779	5.58E-05	BC009009	BC009009	Homo sapiens phospholipase C, beta 2, mRNA (cDNA clone IMAGE:3446252), complete cds. [BC009009]	
A_23_P390384	5.58E-05	BC037316	BC037316	Homo sapiens, clone IMAGE:5259432, mRNA. [BC037316]	XM_033853
A_23_P114405	5.59E-05	NM_012286	NM_012286	Homo sapiens mortality factor 4 like 2 (MORF4L2), mRNA [NM_012286]	NM_012286
A_23_P149390	5.61E-05	NM_020770	NM_020770	Homo sapiens cingulin (CGN), mRNA [NM_020770]	NM_020770
A_32_P53603	5.61E-05	THC2380864		O61351 (O61351) CG12794-PA (La costa), partial (10%) [THC2380864]	
A_23_P402081	5.63E-05	NM_003520	NM_003520	Homo sapiens histone 1, H2bn (HIST1H2BN), mRNA [NM_003520]	NM_003520
A_24_P37946	5.64E-05	BX537394	BX537394	Homo sapiens mRNA; cDNA DKFZp686F23142 (from clone DKFZp686F23142); complete cds. [BX537394]	
A_24_P19752	5.65E-05	NM_002857	NM_002857	Homo sapiens peroxisomal biogenesis factor 19 (PEX19), mRNA [NM_002857]	NM_002857
A_24_P395610	5.66E-05	NM_201433	NM_201433	Homo sapiens growth arrest-specific 7 (GAS7), transcript variant c, mRNA [NM_201433]	NM_201433
A_32_P48536	5.67E-05	THC2374944			
A_23_P26534	5.67E-05	NM_017885	NM_017885	Homo sapiens host cell factor C1 regulator 1 (XPO1 dependant) (HCFC1R1), transcript variant 1, mRNA [NM_017885]	NM_017885
A_23_P139123	5.70E-05	NM_000062	NM_000062	Homo sapiens serine (or cysteine) proteinase inhibitor, clade G (C1 inhibitor), member 1, (angioedema, hereditary) (SERPING1), mRNA [NM_000062]	NM_000062
A_23_P310956	5.71E-05	NM_058175	NM_058175	Homo sapiens collagen, type VI, alpha 2 (COL6A2), transcript variant 2C2a', mRNA [NM_058175]	NM_058175
A_32_P4595	5.72E-05	AF010236	AF010236	Homo sapiens mRNA from chromosome 5q31-33 region. [AF010236]	
A_23_P11192	5.72E-05	NM_006357	NM_006357	Homo sapiens ubiquitin-conjugating enzyme E2E 3 (UBC4/5 homolog, yeast) (UBE2E3), transcript variant 1, mRNA [NM_006357]	NM_006357
A_23_P112774	5.73E-05	NM_032611	NM_032611	Homo sapiens protein tyrosine phosphatase type IVA, member 3 (PTP4A3), transcript variant 1, mRNA [NM_032611]	NM_032611
A_24_P8892	5.73E-05	NM_006037	NM_006037	Homo sapiens histone deacetylase 4 (HDAC4), mRNA [NM_006037]	NM_006037
A_24_P14634	5.74E-05	NM_133455	NM_133455	Homo sapiens EMI domain containing 1 (EMID1), mRNA [NM_133455]	NM_133455
A_24_P402495	5.74E-05	NM_015017	NM_015017	Homo sapiens ubiquitin specific protease 33 (USP33), transcript variant 1, mRNA [NM_015017]	NM_015017
A_24_P910050	5.75E-05	THC2273889			
A_32_P198325	5.76E-05	NM_207644	NM_207644	Homo sapiens similar to hypothetical protein LOC192734 (LOC388886), mRNA [NM_207644]	NM_207644
A_32_P137075	5.78E-05	BX281073	BX281073	BX281073 BX281073 NCI_CGAP_Lu24 Homo sapiens cDNA clone IMAGp998O175628 ; IMAGE:2273128, mRNA sequence [BX281073]	
A_24_P315256	5.78E-05	A_24_P315256			

A_23_P19102	5.79E-05	AK021777	AK021777	Homo sapiens cDNA FLJ11715 fis, clone HEMBA1005223. [AK021777]	
A_23_P258037	5.81E-05	NM_018433	NM_018433	Homo sapiens jumonji domain containing 1A (JMJD1A), mRNA [NM_018433]	NM_018433
A_32_P102428	5.81E-05	AK129982	AK129982	Homo sapiens cDNA FLJ26472 fis, clone KDN04506. [AK129982]	XM_498522
A_23_P78518	5.81E-05	AK128234	AK128234	Homo sapiens cDNA FLJ46368 fis, clone TESTI4051504. [AK128234]	
A_24_P306925	5.81E-05	BC062739	BC062739	Homo sapiens cDNA clone IMAGE:4270911, partial cds. [BC062739]	
A_23_P327156	5.83E-05	AK094893	AK094893	Homo sapiens cDNA FLJ37574 fis, clone BRCOC2003100. [AK094893]	
A_24_P520767	5.85E-05	BC036441	BC036441	Homo sapiens hypothetical protein LOC149351, mRNA (cDNA clone IMAGE:5273076), with apparent retained intron. [BC036441]	XM_378876
A_23_P58266	5.85E-05	NM_005980	NM_005980	Homo sapiens S100 calcium binding protein P (S100P), mRNA [NM_005980]	NM_005980
A_23_P63178	5.87E-05	NM_005644	NM_005644	Homo sapiens TAF12 RNA polymerase II, TATA box binding protein (TBP)-associated factor, 20kDa (TAF12), mRNA [NM_005644]	NM_005644
A_23_P45025	5.89E-05	NM_138980	NM_138980	Homo sapiens mitogen-activated protein kinase 10 (MAPK10), transcript variant 3, mRNA [NM_138980]	NM_138980
A_23_P429998	5.89E-05	NM_006732	NM_006732	Homo sapiens FBJ murine osteosarcoma viral oncogene homolog B (FOSB), mRNA [NM_006732]	NM_006732
A_23_P139881	5.89E-05	NM_001759	NM_001759	Homo sapiens cyclin D2 (CCND2), mRNA [NM_001759]	NM_001759
A_23_P8482	5.92E-05	NM_001011553	NM_001011553	Homo sapiens septin 7 (SEPT7), transcript variant 2, mRNA [NM_001011553]	NM_001011553
A_23_P70060	5.93E-05	NM_176895	NM_176895	Homo sapiens phosphatidic acid phosphatase type 2A (PPAP2A), transcript variant 2, mRNA [NM_176895]	NM_176895
A_23_P209731	5.94E-05	NM_025139	NM_025139	Homo sapiens melanoma/melanocyte specific protein KU-MEL-1 (FLJ12584), mRNA [NM_025139]	NM_025139
A_23_P320159	5.97E-05	ENST00000314720		Homo sapiens hypothetical protein MGC17403, mRNA (cDNA clone IMAGE:3925366), partial cds. [BC020095]	
A_32_P106864	5.99E-05	AA303143	AA303143	AA303143 EST13031 Uterus tumor I Homo sapiens cDNA 5' end similar to hypothetical protein KIAA0222, mRNA sequence [AA303143]	
A_23_P79599	5.99E-05	NM_152834	NM_152834	Homo sapiens transmembrane protein 18 (TMEM18), mRNA [NM_152834]	NM_152834
A_23_P137948	6.00E-05	NM_013349	NM_013349	Homo sapiens neuron derived neurotrophic factor (NENF), mRNA [NM_013349]	NM_013349
A_23_P333705	6.01E-05	NM_002498	NM_002498	Homo sapiens NIMA (never in mitosis gene a)-related kinase 3 (NEK3), transcript variant 1, mRNA [NM_002498]	NM_002498
A_23_P392541	6.01E-05	NM_015087	NM_015087	Homo sapiens spastic paraplegia 20, spartin (Troyer syndrome) (SPG20), mRNA [NM_015087]	NM_015087
A_32_P154321	6.02E-05	AW804939	AW804939	AW804939 QV4-UM0094-060400-159-g07 UM0094 Homo sapiens cDNA, mRNA sequence [AW804939]	
A_23_P81248	6.02E-05	NM_005642	NM_005642	Homo sapiens TAF7 RNA polymerase II, TATA box binding protein (TBP)-associated factor, 55kDa (TAF7), mRNA [NM_005642]	NM_005642
A_23_P334045	6.05E-05	NM_031501	NM_031501	Homo sapiens protocadherin alpha 5 (PCDHA5), transcript variant 2, mRNA [NM_031501]	NM_031501
A_32_P226700	6.06E-05	THC2438975		Q6STG2 (Q6STG2) DNA polymerase-transactivated protein 3, partial (13%) [THC2438975]	
A_23_P4052	6.07E-05	AK026312	AK026312	Homo sapiens cDNA: FLJ22659 fis, clone HSI07953. [AK026312]	
A_32_P1360	6.08E-05	THC2314369		ALU6_HUMAN (P39193) Alu subfamily SP sequence contamination warning entry, partial (19%) [THC2314369]	
A_23_P215931	6.09E-05	NM_015344	NM_015344	Homo sapiens leptin receptor overlapping transcript-like 1 (LEPROTL1), mRNA [NM_015344]	NM_015344
A_32_P137391	6.11E-05	NM_175885	NM_175885	Homo sapiens hypothetical protein MGC33846 (MGC33846), mRNA [NM_175885]	NM_175885
A_24_P399694	6.11E-05	NM_033089	NM_033089	Homo sapiens zinc finger, CCHC domain containing 3 (ZCCHC3), mRNA [NM_033089]	NM_033089
A_23_P209625	6.12E-05	NM_000104	NM_000104	Homo sapiens cytochrome P450, family 1, subfamily B, polypeptide 1 (CYP1B1), mRNA [NM_000104]	NM_000104
A_23_P321703	6.13E-05	NM_004049	NM_004049	Homo sapiens BCL2-related protein A1 (BCL2A1), mRNA [NM_004049]	NM_004049
A_23_P342910	6.14E-05	ENST00000328046		Homo sapiens mRNA for KIAA1677 protein, partial cds. [AB051464]	
A_24_P14531	6.16E-05	NM_014742	NM_014742	Homo sapiens transmembrane 9 superfamily protein member 4 (TM9SF4), mRNA [NM_014742]	NM_014742
A_23_P29495	6.20E-05	NM_001904	NM_001904	Homo sapiens catenin (cadherin-associated protein), beta 1, 88kDa (CTNNB1), mRNA [NM_001904]	NM_001904
A_24_P408321	6.23E-05	NM_144498	NM_144498	Homo sapiens oxysterol binding protein-like 2 (OSBPL2), transcript variant 2, mRNA [NM_144498]	NM_144498
A_23_P145935	6.26E-05	NM_004445	NM_004445	Homo sapiens EPH receptor B6 (EPHB6), mRNA [NM_004445]	NM_004445
A_23_P3453	6.29E-05	NM_015540	NM_015540	Homo sapiens RNA polymerase II associated protein 1 (RPAP1), mRNA [NM_015540]	NM_015540
A_23_P158976	6.31E-05	NM_000392	NM_000392	Homo sapiens ATP-binding cassette, sub-family C (CFTR/MRP), member 2 (ABCC2), mRNA [NM_000392]	NM_000392
A_23_P65370	6.32E-05	NM_016417	NM_016417	Homo sapiens chromosome 14 open reading frame 87 (C14orf87), mRNA [NM_016417]	NM_016417
A_23_P103720	6.33E-05	NM_024758	NM_024758	Homo sapiens agmatine ureohydrolase (agmatinase) (AGMAT), mRNA [NM_024758]	NM_024758
A_23_P39799	6.34E-05	NM_032603	NM_032603	Homo sapiens lysyl oxidase-like 3 (LOXL3), mRNA [NM_032603]	NM_032603
A_24_P943193	6.34E-05	AB007896	AB007896	Homo sapiens KIAA0436 mRNA, partial cds. [AB007896]	
A_23_P78608	6.37E-05	NM_024898	NM_024898	Homo sapiens family with sequence similarity 31, member C (FAM31C), mRNA [NM_024898]	NM_024898

A_23_P318904	6.37E-05	NM_019605	NM_019605	Homo sapiens SERTA domain containing 4 (SERTAD4), mRNA [NM_019605]	NM_019605
A_23_P66637	6.38E-05	NM_000023	NM_000023	Homo sapiens sarcoglycan, alpha (50kDa dystrophin-associated glycoprotein) (SGCA), mRNA [NM_000023]	NM_000023
A_24_P418216	6.39E-05	A_24_P418216			
A_24_P368023	6.39E-05	BC039457	BC039457	Homo sapiens cDNA clone IMAGE:5312122, partial cds. [BC039457]	
A_23_P120354	6.40E-05	NM_023016	NM_023016	Homo sapiens chromosome 2 open reading frame 26 (C2orf26), mRNA [NM_023016]	NM_023016
A_24_P322229	6.40E-05	NM_033315	NM_033315	Homo sapiens RAS-like, family 10, member B (RASL10B), mRNA [NM_033315]	NM_033315
A_24_P814096	6.40E-05	XM_498623	XM_498623	PREDICTED: Homo sapiens LOC440309 (LOC440309), mRNA [XM_498623]	XM_498623
A_23_P158096	6.40E-05	AK021957	AK021957	Homo sapiens cDNA FLJ11895 fis, clone HEMBA1007301, weakly similar to COLLAGEN ALPHA 1(III) CHAIN. [AK021957]	
A_23_P1014	6.42E-05	NM_032705	NM_032705	Homo sapiens chromosome 1 open reading frame 97 (C1orf97), mRNA [NM_032705]	NM_032705
A_32_P24122	6.47E-05	NM_015894	NM_015894	Homo sapiens stathmin-like 3 (STMN3), mRNA [NM_015894]	NM_015894
A_23_P341938	6.49E-05	NM_005450	NM_005450	Homo sapiens noggin (NOG), mRNA [NM_005450]	NM_005450
A_24_P649747	6.49E-05	NM_014753	NM_014753	Homo sapiens BMS1-like, ribosome assembly protein (yeast) (BMS1L), mRNA [NM_014753]	NM_014753
A_23_P43095	6.56E-05	NM_024721	NM_024721	Homo sapiens zinc finger homeodomain 4 (ZFHX4), mRNA [NM_024721]	NM_024721
A_23_P149301	6.56E-05	NM_033445	NM_033445	Homo sapiens histone 3, H2a (HIST3H2A), mRNA [NM_033445]	NM_033445
A_24_P369898	6.57E-05	ENST00000293201		Homo sapiens mRNA for KIAA1783 protein, partial cds. [AB058686]	XM_496245
A_24_P286935	6.57E-05	NM_004311	NM_004311	Homo sapiens ADP-ribosylation factor-like 3 (ARL3), mRNA [NM_004311]	NM_004311
A_23_P93180	6.58E-05	NM_003526	NM_003526	Homo sapiens histone 1, H2bc (HIST1H2BC), mRNA [NM_003526]	NM_003526
A_23_P66454	6.59E-05	NM_018530	NM_018530	Homo sapiens gasdermin-like (GSDML), mRNA [NM_018530]	NM_018530
A_23_P40470	6.65E-05	NM_017445	NM_017445	Homo sapiens H2B histone family, member S (H2BFS), mRNA [NM_017445]	NM_017445
A_24_P922948	6.68E-05	ENST00000216214		Homo sapiens cDNA FLJ43037 fis, clone BRTHA3002933, highly similar to Homo sapiens uroplakin 3 (UPK3). [AK125027]	
A_24_P194688	6.70E-05	NM_181723	NM_181723	Homo sapiens EF-hand domain family, member A2 (EFHA2), mRNA [NM_181723]	NM_181723
A_23_P50357	6.71E-05	NM_015318	NM_015318	Homo sapiens rho/rac guanine nucleotide exchange factor (GEF) 18 (ARHGEF18), mRNA [NM_015318]	NM_015318
A_23_P84018	6.71E-05	NM_000943	NM_000943	Homo sapiens peptidylprolyl isomerase C (cyclophilin C) (PPIC), mRNA [NM_000943]	NM_000943
A_32_P12214	6.71E-05	NM_001013699	NM_001013699	Homo sapiens similar to H3 histone, family 3B (LOC440093), mRNA [NM_001013699]	NM_001013699
A_23_P25746	6.72E-05	BC007251	BC007251	Homo sapiens chromosome 14 open reading frame 128, mRNA (cDNA clone MGC:15504 IMAGE:2990071), complete cds. [BC007251]	
A_23_P79251	6.72E-05	NM_014600	NM_014600	Homo sapiens EH-domain containing 3 (EHD3), mRNA [NM_014600]	NM_014600
A_23_P206568	6.73E-05	A_23_P206568			
A_23_P96872	6.73E-05	NM_016126	NM_016126	Homo sapiens chromosome 1 open reading frame 41 (C1orf41), mRNA [NM_016126]	NM_016126
A_24_P337746	6.74E-05	NM_014504	NM_014504	Homo sapiens RAB guanine nucleotide exchange factor (GEF) 1 (RABGEF1), mRNA [NM_014504]	NM_014504
A_24_P185615	6.77E-05	THC2307937			
A_23_P42087	6.77E-05	NM_004332	NM_004332	Homo sapiens biphenyl hydrolase-like (serine hydrolase; breast epithelial mucin-associated antigen) (BPHL), mRNA [NM_004332]	NM_004332
A_24_P928522	6.79E-05	AK025142	AK025142	Homo sapiens cDNA: FLJ21489 fis, clone COL05450. [AK025142]	
A_23_P389118	6.79E-05	NM_001025356	NM_001025356	Homo sapiens transmembrane protein 16F (TMEM16F), mRNA [NM_001025356]	NM_001025356
A_23_P387071	6.80E-05	NM_052857	NM_052857	Homo sapiens coiled-coil domain containing 16 (CCDC16), mRNA [NM_052857]	NM_052857
A_23_P26184	6.81E-05	NM_017996	NM_017996	Homo sapiens de-etiolated homolog 1 (Arabidopsis) (DET1), mRNA [NM_017996]	NM_017996
A_23_P354170	6.83E-05	NM_017861	NM_017861	Homo sapiens phosphatidylinositol glycan, class X (PIGX), mRNA [NM_017861]	NM_017861
A_32_P169785	6.85E-05	A_32_P169785			
A_23_P3602	6.86E-05	THC2303868		Q99P30 (Q99P30) Coenzyme A diphosphatase, partial (64%) [THC2303868]	
A_23_P258912	6.86E-05	NM_003970	NM_003970	Homo sapiens myomesin (M-protein) 2, 165kDa (MYOM2), mRNA [NM_003970]	NM_003970
A_23_P43425	6.86E-05	NM_017998	NM_017998	Homo sapiens chromosome 9 open reading frame 40 (C9orf40), mRNA [NM_017998]	NM_017998
A_23_P106204	6.86E-05	NM_145870	NM_145870	Homo sapiens glutathione transferase zeta 1 (maleylacetoacetate isomerase) (GSTZ1), transcript variant 1, mRNA [NM_145870]	NM_145870
A_23_P325411	6.89E-05	THC2310302		Q9Y474 (Q9Y474) DNA-binding protein, complete [THC2310302]	
A_32_P110016	6.91E-05	A_32_P110016			
A_23_P64611	6.93E-05	NM_176798	NM_176798	Homo sapiens pyrimidinergic receptor P2Y, G-protein coupled, 6 (P2RY6), transcript variant 2, mRNA [NM_176798]	NM_176798

A_24_P389714	6.95E-05	AW997516	AW997516	AW997516 RC1-BN0051-280200-011-c05 BN0051 Homo sapiens cDNA, mRNA sequence [AW997516]	
A_24_P97145	6.95E-05	NM_020738	NM_020738	Homo sapiens kinase D-interacting substance of 220 kDa (KIDINS220), mRNA [NM_020738]	NM_020738
A_23_P382081	6.96E-05	NM_144722	NM_144722	Homo sapiens KPL2 protein (FLJ23577), transcript variant 2, mRNA [NM_144722]	NM_144722
A_32_P88310	6.96E-05	BM560112	BM560112	AGENCOURT_6564076 NIH_MGC_119 Homo sapiens cDNA clone IMAGE:5744607 5', mRNA sequence [BM560112]	
A_23_P100802	6.96E-05	BC002698	BC002698	Homo sapiens HSPC009 protein, mRNA (cDNA clone MGC:3372 IMAGE:3609536), complete cds. [BC002698]	
A_32_P72541	6.98E-05	BF803942	BF803942	BF803942 CM2-CI0135-021100-477-g08 CI0135 Homo sapiens cDNA, mRNA sequence [BF803942]	
A_24_P196534	6.98E-05	NM_004571	NM_004571	Homo sapiens PBX/knotted 1 homeobox 1 (PKNOX1), transcript variant 1, mRNA [NM_004571]	NM_004571
A_23_P151915	6.99E-05	ENST00000313774		Homo sapiens glucosaminyl (N-acetyl) transferase 3, mucin type, mRNA (cDNA clone MGC:9086 IMAGE:3851937), complete cds. [BC017032]	
A_32_P209208	7.02E-05	AK091839	AK091839	Homo sapiens cDNA FLJ34520 fis, clone HLUNG2006935, weakly similar to CALCYPHOSINE. [AK091839]	
A_24_P120537	7.08E-05	NM_152550	NM_152550	Homo sapiens SH3 domain containing ring finger 2 (SH3RF2), mRNA [NM_152550]	NM_152550
A_23_P216630	7.10E-05	NM_080546	NM_080546	Homo sapiens CDW92 antigen (CDW92), transcript variant 2, mRNA [NM_080546]	NM_080546
A_24_P90097	7.11E-05	NM_016824	NM_016824	Homo sapiens adducin 3 (gamma) (ADD3), transcript variant 1, mRNA [NM_016824]	NM_016824
A_23_P52031	7.11E-05	NM_002633	NM_002633	Homo sapiens phosphoglucomutase 1 (PGM1), mRNA [NM_002633]	NM_002633
A_23_P2920	7.16E-05	NM_001085	NM_001085	Homo sapiens serine (or cysteine) proteinase inhibitor, clade A (alpha-1 antiproteinase, antitrypsin), member 3 (SERPINA3), mRNA [NM_001085]	NM_001085
A_23_P429959	7.19E-05	NM_020956	NM_020956	Homo sapiens periaxin (PRX), transcript variant 1, mRNA [NM_020956]	NM_020956
A_23_P347048	7.26E-05	NM_030791	NM_030791	Homo sapiens sphingosine-1-phosphate phosphatase 1 (SGPP1), mRNA [NM_030791]	NM_030791
A_23_P170626	7.26E-05	NM_001011667	NM_001011667	Homo sapiens coiled-coil-helix-coiled-coil-helix domain containing 7 (CHCHD7), transcript variant 1, mRNA [NM_001011667]	NM_001011667
A_23_P17316	7.29E-05	NM_152864	NM_152864	Homo sapiens chromosome 20 open reading frame 58 (C20orf58), mRNA [NM_152864]	NM_152864
A_23_P202988	7.29E-05	NM_015423	NM_015423	Homo sapiens aminoadipate-semialdehyde dehydrogenase-phosphopantetheinyl transferase (AASDHPPT), mRNA [NM_015423]	NM_015423
A_23_P130194	7.29E-05	NM_006907	NM_006907	Homo sapiens pyrroline-5-carboxylate reductase 1 (PYCR1), transcript variant 1, mRNA [NM_006907]	NM_006907
A_23_P67785	7.30E-05	NM_024532	NM_024532	Homo sapiens sperm associated antigen 16 (SPAG16), transcript variant 1, mRNA [NM_024532]	NM_024532
A_23_P145786	7.34E-05	NM_032951	NM_032951	Homo sapiens Williams Beuren syndrome chromosome region 14 (WBSCR14), transcript variant 1, mRNA [NM_032951]	NM_032951
A_23_P139575	7.35E-05	NM_001005354	NM_001005354	Homo sapiens DKFZp564J157 protein (DKFZp564J157), transcript variant 3, mRNA [NM_001005354]	NM_001005354
A_24_P397386	7.36E-05	BX648635	BX648635	Homo sapiens mRNA; cDNA DKFZp686C03120 (from clone DKFZp686C03120). [BX648635]	
A_32_P42367	7.37E-05	THC2405114			
A_23_P500464	7.37E-05	NM_001844	NM_001844	Homo sapiens collagen, type II, alpha 1 (primary osteoarthritis, spondyloepiphyseal dysplasia, congenital) (COL2A1), transcript variant 1, mRNA [NM_001844]	NM_001844
A_24_P82948	7.38E-05	NM_018683	NM_018683	Homo sapiens zinc finger protein 313 (ZNF313), mRNA [NM_018683]	NM_018683
A_23_P18082	7.39E-05	NM_032806	NM_032806	Homo sapiens glycosyltransferase (AGO61), mRNA [NM_032806]	NM_032806
A_32_P80901	7.42E-05	BC068045	BC068045	Homo sapiens cDNA clone IMAGE:4140029, partial cds. [BC068045]	
A_23_P78944	7.44E-05	NM_000479	NM_000479	Homo sapiens anti-Mullerian hormone (AMH), mRNA [NM_000479]	NM_000479
A_23_P35995	7.48E-05	NM_024769	NM_024769	Homo sapiens adipocyte-specific adhesion molecule (ASAM), mRNA [NM_024769]	NM_024769
A_23_P377882	7.50E-05	NM_172056	NM_172056	Homo sapiens potassium voltage-gated channel, subfamily H (eag-related), member 2 (KCNH2), transcript variant 2, mRNA [NM_172056]	NM_172056
A_23_P24884	7.50E-05	NM_005418	NM_005418	Homo sapiens suppression of tumorigenicity 5 (ST5), transcript variant 1, mRNA [NM_005418]	NM_005418
A_32_P385587	7.51E-05	NM_000032	NM_000032	Homo sapiens aminolevulinate, delta-, synthase 2 (sideroblastic/hypochromic anemia) (ALAS2), nuclear gene encoding mitochondrial protein, mRNA [NM_000032]	NM_000032
A_23_P332992	7.51E-05	NM_175055	NM_175055	Homo sapiens histone 3, H2bb (HIST3H2BB), mRNA [NM_175055]	NM_175055
A_23_P104224	7.53E-05	NM_138933	NM_138933	Homo sapiens apobec-1 complementation factor (ACF), transcript variant 3, mRNA [NM_138933]	NM_138933
A_23_P131255	7.53E-05	NM_015535	NM_015535	Homo sapiens DNA polymerase-transactivated protein 6 (DNAPTP6), mRNA [NM_015535]	NM_015535
A_23_P307310	7.54E-05	NM_013227	NM_013227	Homo sapiens aggrecan 1 (chondroitin sulfate proteoglycan 1, large aggregating proteoglycan, antigen identified by monoclonal antibody A0122) (AGC1), transcript variant 2, mRNA [NM_013227]	NM_013227
A_24_P117138	7.55E-05	NM_145297	NM_145297	Homo sapiens zinc finger protein 626 (ZNF626), mRNA [NM_145297]	NM_145297
A_23_P150609	7.56E-05	NM_001007139	NM_001007139	Homo sapiens putative insulin-like growth factor II associated protein (LOC492304), mRNA [NM_001007139]	NM_001007139
A_23_P39682	7.59E-05	NM_001079	NM_001079	Homo sapiens zeta-chain (TCR) associated protein kinase 70kDa (ZAP70), transcript variant 1, mRNA [NM_001079]	NM_001079
A_24_P484894	7.61E-05	BC045718	BC045718	Homo sapiens, clone IMAGE:4797078, mRNA. [BC045718]	
A_24_P203000	7.63E-05	NM_000878	NM_000878	Homo sapiens interleukin 2 receptor, beta (IL2RB), mRNA [NM_000878]	NM_000878

A_32_P134968	7.63E-05	NM_001024858	NM_001024858	Homo sapiens spectrin, beta, erythrocytic (includes spherocytosis, clinical type I) (SPTB), transcript variant 1, mRNA [NM_001024858]	NM_001024858
A_32_P13795	7.67E-05	NM_016544	NM_016544	Homo sapiens Ras-associated protein Rap1 (RBJ), mRNA [NM_016544]	NM_016544
A_23_P125303	7.70E-05	NM_001555	NM_001555	Homo sapiens immunoglobulin superfamily, member 1 (IGSF1), transcript variant 1, mRNA [NM_001555]	NM_001555
A_23_P121386	7.71E-05	NM_018010	NM_018010	Homo sapiens estrogen-related receptor beta like 1 (ESRRBL1), mRNA [NM_018010]	NM_018010
A_23_P72550	7.71E-05	NM_003565	NM_003565	Homo sapiens unc-51-like kinase 1 (C. elegans) (ULK1), mRNA [NM_003565]	NM_003565
A_24_P296070	7.76E-05	NM_031431	NM_031431	Homo sapiens component of oligomeric golgi complex 3 (COG3), mRNA [NM_031431]	NM_031431
A_24_P45767	7.80E-05	NM_021831	NM_021831	Homo sapiens hypothetical protein FLJ21839 (FLJ21839), mRNA [NM_021831]	NM_021831
A_23_P144071	7.81E-05	NM_000094	NM_000094	Homo sapiens collagen, type VII, alpha 1 (epidermolysis bullosa, dystrophic, dominant and recessive) (COL7A1), mRNA [NM_000094]	NM_000094
A_24_P20506	7.82E-05	NM_001014809	NM_001014809	Homo sapiens collapsin response mediator protein 1 (CRMP1), transcript variant 1, mRNA [NM_001014809]	NM_001014809
A_24_P220947	7.83E-05	NM_001353	NM_001353	Homo sapiens aldo-keto reductase family 1, member C1 (dihydrodiol dehydrogenase 1; 20-alpha (3-alpha)-hydroxysteroid dehydrogenase) (AKR1C1), mRNA [NM_001353]	NM_001353
A_23_P93524	7.84E-05	NM_001017373	NM_001017373	Homo sapiens sterile alpha motif domain containing 3 (SAMD3), transcript variant 1, mRNA [NM_001017373]	NM_001017373
A_32_P213661	7.87E-05	NM_144974	NM_144974	Homo sapiens hypothetical protein FLJ31846 (FLJ31846), mRNA [NM_144974]	NM_144974
A_23_P15357	7.90E-05	NM_005567	NM_005567	Homo sapiens lectin, galactoside-binding, soluble, 3 binding protein (LGALS3BP), mRNA [NM_005567]	NM_005567
A_32_P147078	7.92E-05	BX648299	BX648299	Homo sapiens mRNA; cDNA DKFZp686J04125 (from clone DKFZp686J04125). [BX648299]	
A_23_P144005	7.93E-05	NM_015931	NM_015931	Homo sapiens fls485 (LOC51066), mRNA [NM_015931]	NM_015931
A_32_P111587	7.95E-05	AF086098	AF086098	Homo sapiens full length insert cDNA clone YZ88E12. [AF086098]	
A_23_P115785	7.97E-05	NM_145235	NM_145235	Homo sapiens fibronectin type III and ankyrin repeat domains 1 (FANK1), mRNA [NM_145235]	NM_145235
A_23_P83838	7.98E-05	NM_004056	NM_004056	Homo sapiens carbonic anhydrase VIII (CA8), mRNA [NM_004056]	NM_004056
A_23_P61674	7.98E-05	NM_020666	NM_020666	Homo sapiens CDC-like kinase 4 (CLK4), mRNA [NM_020666]	NM_020666
A_32_P32207	7.98E-05	NM_173473	NM_173473	Homo sapiens chromosome 10 open reading frame 104 (C10orf104), mRNA [NM_173473]	NM_173473
A_23_P92261	8.05E-05	NM_032331	NM_032331	Homo sapiens hypothetical protein MGC2408 (MGC2408), mRNA [NM_032331]	NM_032331
A_23_P26890	8.11E-05	NM_032950	NM_032950	Homo sapiens matrix metalloproteinase 28 (MMP28), transcript variant 2, mRNA [NM_032950]	NM_032950
A_23_P120973	8.11E-05	NM_017911	NM_017911	Homo sapiens chromosome 22 open reading frame 8 (C22orf8), mRNA [NM_017911]	NM_017911
A_23_P307761	8.17E-05	NM_031215	NM_031215	Homo sapiens Cdk5 and Abl enzyme substrate 2 (CABLES2), mRNA [NM_031215]	NM_031215
A_24_P414376	8.18E-05	THC2371729			
A_24_P304154	8.20E-05	NM_000480	NM_000480	Homo sapiens adenosine monophosphate deaminase (isoform E) (AMPD3), transcript variant 1, mRNA [NM_000480]	NM_000480
A_32_P79584	8.23E-05	NM_001011553	NM_001011553	Homo sapiens septin 7 (SEPT7), transcript variant 2, mRNA [NM_001011553]	NM_001011553
A_23_P13772	8.27E-05	NM_016569	NM_016569	Homo sapiens T-box 3 (ulnar mammary syndrome) (TBX3), transcript variant 2, mRNA [NM_016569]	NM_016569
A_23_P501849	8.29E-05	NM_033251	NM_033251	Homo sapiens ribosomal protein L13 (RPL13), transcript variant 2, mRNA [NM_033251]	NM_033251
A_24_P515815	8.31E-05	AI263220	AI263220	AI263220 qz36f10.x1 NCI_CGAP_Kid11 Homo sapiens cDNA clone IMAGE:2029003 3' similar to TR:Q14041 Q14041 COLLAGEN VI ALPHA-1 N-TERMINAL GLOBULAR DOMAIN PRECURSOR ;, mRNA sequence [AI263220]	
A_24_P268893	8.31E-05	NM_144721	NM_144721	Homo sapiens THAP domain containing 6 (THAP6), mRNA [NM_144721]	NM_144721
A_23_P210829	8.32E-05	NM_018257	NM_018257	Homo sapiens chromosome 20 open reading frame 36 (C20orf36), mRNA [NM_018257]	NM_018257
A_23_P25503	8.32E-05	NM_014923	NM_014923	Homo sapiens fibronectin type III domain containing 3A (FNDC3A), mRNA [NM_014923]	NM_014923
A_23_P22169	8.33E-05	NM_018291	NM_018291	Homo sapiens hypothetical protein FLJ10986 (FLJ10986), mRNA [NM_018291]	NM_018291
A_32_P106944	8.34E-05	NM_001001415	NM_001001415	Homo sapiens zinc finger protein 429 (ZNF429), mRNA [NM_001001415]	NM_001001415
A_23_P109881	8.34E-05	NM_002218	NM_002218	Homo sapiens inter-alpha (globulin) inhibitor H4 (plasma Kallikrein-sensitive glycoprotein) (ITIH4), mRNA [NM_002218]	NM_002218
A_32_P51005	8.34E-05	AL834342	AL834342	Homo sapiens mRNA; cDNA DKFZp761P2314 (from clone DKFZp761P2314). [AL834342]	
A_32_P10083	8.35E-05	BC040293	BC040293	Homo sapiens, clone IMAGE:4820330, mRNA. [BC040293]	
A_32_P164573	8.36E-05	THC2314215		RR12_SPIMX (P42344) Chloroplast 30S ribosomal protein S12, partial (11%) [THC2314215]	
A_23_P112251	8.37E-05	NM_004125	NM_004125	Homo sapiens hypothetical protein LOC552891 (LOC552891), mRNA [NM_004125]	NM_004125
A_24_P940680	8.38E-05	AK074179	AK074179	Homo sapiens mRNA for FLJ00252 protein. [AK074179]	
A_24_P921402	8.42E-05	THC2263973			
A_23_P17575	8.43E-05	NM_000687	NM_000687	Homo sapiens S-adenosylhomocysteine hydrolase (AHCY), mRNA [NM_000687]	NM_000687
A_23_P80778	8.45E-05	NM_032839	NM_032839	Homo sapiens disrupted in renal carcinoma 2 (DIRC2), mRNA [NM_032839]	NM_032839

A_32_P13823	8.50E-05	THC2411757			
A_23_P71855	8.51E-05	NM_001735	NM_001735	Homo sapiens complement component 5 (C5), mRNA [NM_001735]	NM_001735
A_23_P78061	8.51E-05	NM_005568	NM_005568	Homo sapiens LIM homeobox 1 (LHX1), mRNA [NM_005568]	NM_005568
A_24_P707543	8.54E-05	THC2430656		CFA388518 non-histone chromosomal protein HMG-17 {Canis familiaris;} , partial (21%) [THC2430656]	
A_24_P176733	8.55E-05	NM_020198	NM_020198	Homo sapiens GK001 protein (GK001), mRNA [NM_020198]	NM_020198
A_23_P45524	8.57E-05	NM_014380	NM_014380	Homo sapiens nerve growth factor receptor (TNFRSF16) associated protein 1 (NGFRAP1), transcript variant 3, mRNA [NM_014380]	NM_014380
A_23_P205355	8.58E-05	NM_000624	NM_000624	Homo sapiens serine (or cysteine) proteinase inhibitor, clade A (alpha-1 antiproteinase, antitrypsin), member 5 (SERPINA5), mRNA [NM_000624]	NM_000624
A_24_P409857	8.58E-05	NM_004965	NM_004965	Homo sapiens high-mobility group nucleosome binding domain 1 (HMGN1), mRNA [NM_004965]	NM_004965
A_23_P3911	8.60E-05	NM_020405	NM_020405	Homo sapiens plexin domain containing 1 (PLXDC1), mRNA [NM_020405]	NM_020405
A_24_P181101	8.61E-05	NM_022918	NM_022918	Homo sapiens hypothetical protein FLJ22104 (FLJ22104), mRNA [NM_022918]	NM_022918
A_23_P258093	8.62E-05	NM_006411	NM_006411	Homo sapiens 1-acylglycerol-3-phosphate O-acyltransferase 1 (lysophosphatidic acid acyltransferase, alpha) (AGPAT1), transcript variant 1, mRNA [NM_006411]	NM_006411
A_23_P257971	8.63E-05	NM_001353	NM_001353	Homo sapiens aldo-keto reductase family 1, member C1 (dihydrodiol dehydrogenase 1; 20-alpha (3-alpha)-hydroxysteroid dehydrogenase) (AKR1C1), mRNA [NM_001353]	NM_001353
A_23_P258862	8.66E-05	NM_145341	NM_145341	Homo sapiens programmed cell death 4 (neoplastic transformation inhibitor) (PDCD4), transcript variant 2, mRNA [NM_145341]	NM_145341
A_32_P41924	8.67E-05	AF086011	AF086011	Homo sapiens full length insert cDNA clone YW18A11. [AF086011]	
A_23_P75800	8.68E-05	NM_013401	NM_013401	Homo sapiens RAB3A interacting protein (rabin3)-like 1 (RAB3IL1), mRNA [NM_013401]	NM_013401
A_32_P180958	8.68E-05	THC2401540		ALU8_HUMAN (P39195) Alu subfamily SX sequence contamination warning entry, partial (27%) [THC2401540]	
A_23_P120056	8.72E-05	NM_033046	NM_033046	Homo sapiens rhotekin (RTKN), transcript variant 2, mRNA [NM_033046]	NM_033046
A_24_P795594	8.77E-05	AB096249	AB096249	Homo sapiens LOH11CR1J gene, loss of heterozygosity, 11, chromosomal region 1 gene J product. [AB096249]	
A_23_P39814	8.77E-05	NM_004882	NM_004882	Homo sapiens CBF1 interacting corepressor (CIR), transcript variant 1, mRNA [NM_004882]	NM_004882
A_24_P394533	8.80E-05	NM_000434	NM_000434	Homo sapiens sialidase 1 (lysosomal sialidase) (NEU1), mRNA [NM_000434]	NM_000434
A_23_P373017	8.81E-05	NM_002983	NM_002983	Homo sapiens chemokine (C-C motif) ligand 3 (CCL3), mRNA [NM_002983]	NM_002983
A_23_P127140	8.81E-05	NM_014904	NM_014904	Homo sapiens RAB11 family interacting protein 2 (class I) (RAB11FIP2), mRNA [NM_014904]	NM_014904
A_24_P886966	8.82E-05	AK123483	AK123483	Homo sapiens cDNA FLJ41489 fis, clone BRTHA2004582. [AK123483]	
A_24_P40721	8.87E-05	ENST00000217204		Q5TD12 (Q5TD12) OTTHUMP00000030291 (Fragment), partial (46%) [THC2403720]	
A_23_P18413	8.87E-05	NM_016589	NM_016589	Homo sapiens chromosome 3 open reading frame 1 (C3orf1), mRNA [NM_016589]	NM_016589
A_23_P166823	8.89E-05	NM_003280	NM_003280	Homo sapiens troponin C, slow (TNNC1), mRNA [NM_003280]	NM_003280
A_23_P138635	8.89E-05	NM_004052	NM_004052	Homo sapiens BCL2/adenovirus E1B 19kDa interacting protein 3 (BNIP3), nuclear gene encoding mitochondrial protein, mRNA [NM_004052]	NM_004052
A_24_P822704	8.94E-05	NM_001005209	NM_001005209	Homo sapiens similar to RIKEN cDNA A230078I05 gene (MGC99813), mRNA [NM_001005209]	NM_001005209
A_23_P18223	9.01E-05	NM_002215	NM_002215	Homo sapiens inter-alpha (globulin) inhibitor H1 (ITIH1), mRNA [NM_002215]	NM_002215
A_23_P7761	9.02E-05	NM_018356	NM_018356	Homo sapiens hypothetical protein FLJ11193 (FLJ11193), mRNA [NM_018356]	NM_018356
A_24_P67395	9.02E-05	NM_002273	NM_002273	Homo sapiens keratin 8 (KRT8), mRNA [NM_002273]	NM_002273
A_23_P259462	9.03E-05	NM_206841	NM_206841	Homo sapiens Fraser syndrome 1 (FRAS1), transcript variant 2, mRNA [NM_206841]	NM_206841
A_23_P110445	9.03E-05	NM_133175	NM_133175	Homo sapiens amyloid beta (A4) precursor protein-binding, family B, member 3 (APBB3), transcript variant 5, mRNA [NM_133175]	NM_133175
A_24_P329924	9.03E-05	NM_025164	NM_025164	Homo sapiens KIAA0999 protein (KIAA0999), mRNA [NM_025164]	NM_025164
A_24_P285880	9.04E-05	NM_003262	NM_003262	Homo sapiens translocation protein 1 (TLOC1), mRNA [NM_003262]	NM_003262
A_23_P138492	9.07E-05	NM_004210	NM_004210	Homo sapiens neuralized-like (Drosophila) (NEURL), mRNA [NM_004210]	NM_004210
A_23_P415021	9.09E-05	NM_014033	NM_014033	Homo sapiens DKFZP586A0522 protein (DKFZP586A0522), mRNA [NM_014033]	NM_014033
A_23_P371835	9.10E-05	NM_080473	NM_080473	Homo sapiens GATA binding protein 5 (GATA5), mRNA [NM_080473]	NM_080473
A_23_P46936	9.11E-05	NM_000399	NM_000399	Homo sapiens early growth response 2 (Krox-20 homolog, Drosophila) (EGR2), mRNA [NM_000399]	NM_000399
A_23_P398566	9.13E-05	NM_173198	NM_173198	Homo sapiens nuclear receptor subfamily 4, group A, member 3 (NR4A3), transcript variant 2, mRNA [NM_173198]	NM_173198
A_23_P205646	9.14E-05	NM_198794	NM_198794	Homo sapiens mitogen-activated protein kinase kinase kinase kinase 5 (MAP4K5), transcript variant 2, mRNA [NM_198794]	NM_198794
A_24_P409013	9.16E-05	NM_024763	NM_024763	Homo sapiens WD repeat domain 78 (WDR78), transcript variant 1, mRNA [NM_024763]	NM_024763
A_24_P144163	9.16E-05	A_24_P144163			
A_32_P222362	9.20E-05	AK127132	AK127132	Homo sapiens cDNA FLJ45189 fis, clone BRAWH3049068. [AK127132]	

A_24_P225862	9.21E-05	THC2373173			
A_32_P158376	9.25E-05	BF210146	BF210146	601874052F1 NIH_MGC_54 Homo sapiens cDNA clone IMAGE:4098852 5', mRNA sequence [BF210146]	
A_23_P34637	9.25E-05	NM_000537	NM_000537	Homo sapiens renin (REN), mRNA [NM_000537]	NM_000537
A_24_P39211	9.26E-05	NM_198219	NM_198219	Homo sapiens inhibitor of growth family, member 1 (ING1), transcript variant 1, mRNA [NM_198219]	NM_198219
A_23_P149064	9.30E-05	NM_133177	NM_133177	Homo sapiens protein tyrosine phosphatase, receptor type, U (PTPRU), transcript variant 2, mRNA [NM_133177]	NM_133177
A_24_P322474	9.33E-05	NM_006202	NM_006202	Homo sapiens phosphodiesterase 4A, cAMP-specific (phosphodiesterase E2 dunce homolog, Drosophila) (PDE4A), mRNA [NM_006202]	NM_006202
A_23_P379649	9.35E-05	NM_001003940	NM_001003940	Homo sapiens Bcl2 modifying factor (BMF), transcript variant 1, mRNA [NM_001003940]	NM_001003940
A_24_P270728	9.35E-05	NM_012385	NM_012385	Homo sapiens p8 protein (candidate of metastasis 1) (P8), mRNA [NM_012385]	NM_012385
A_23_P53976	9.38E-05	NM_206949	NM_206949	Homo sapiens family with sequence similarity 14, member B (FAM14B), mRNA [NM_206949]	NM_206949
A_23_P396804	9.39E-05	NM_198893	NM_198893	Homo sapiens zinc finger protein 160 (ZNF160), transcript variant 2, mRNA [NM_198893]	NM_198893
A_24_P108517	9.39E-05	NM_174881	NM_174881	Homo sapiens crumbs homolog 3 (Drosophila) (CRB3), transcript variant 3, mRNA [NM_174881]	NM_174881
A_23_P61280	9.40E-05	NM_012133	NM_012133	Homo sapiens coatomer protein complex, subunit gamma 2 (COPG2), mRNA [NM_012133]	NM_012133
A_23_P115375	9.40E-05	NM_001005464	NM_001005464	Homo sapiens histone H3/o (H3/o), mRNA [NM_001005464]	NM_001005464
A_32_P100365	9.46E-05	AI570240	AI570240	AI570240 to76g03.x1 NCI_CGAP_Gas4 Homo sapiens cDNA clone IMAGE:2184244 3', mRNA sequence [AI570240]	
A_23_P99540	9.47E-05	NM_004926	NM_004926	Homo sapiens zinc finger protein 36, C3H type-like 1 (ZFP36L1), mRNA [NM_004926]	NM_004926
A_23_P370707	9.52E-05	A_23_P370707			
A_23_P30693	9.52E-05	NM_000301	NM_000301	Homo sapiens plasminogen (PLG), mRNA [NM_000301]	NM_000301
A_24_P262127	9.53E-05	NM_004165	NM_004165	Homo sapiens Ras-related associated with diabetes (RRAD), mRNA [NM_004165]	NM_004165
A_23_P256470	9.53E-05	NM_000905	NM_000905	Homo sapiens neuropeptide Y (NPY), mRNA [NM_000905]	NM_000905
A_23_P76151	9.53E-05	NM_005967	NM_005967	Homo sapiens NGFI-A binding protein 2 (EGR1 binding protein 2) (NAB2), mRNA [NM_005967]	NM_005967
A_23_P147845	9.53E-05	NM_015027	NM_015027	Homo sapiens KIAA0251 protein (KIAA0251), mRNA [NM_015027]	NM_015027
A_24_P489649	9.54E-05	XM_067448	XM_067448	PREDICTED: Homo sapiens similar to MEST (LOC131572), mRNA [XM_067448]	XM_067448
A_23_P47135	9.55E-05	NM_005693	NM_005693	Homo sapiens nuclear receptor subfamily 1, group H, member 3 (NR1H3), mRNA [NM_005693]	NM_005693
A_32_P22501	9.57E-05	ENST00000313807		Homo sapiens, clone IMAGE:4592052, mRNA, partial cds. [BC026043]	
A_23_P154627	9.58E-05	AF230201	AF230201	Homo sapiens OVC10-2 mRNA, complete cds. [AF230201]	
A_32_P117313	9.59E-05	AF130048	AF130048	Homo sapiens clone FLB3344 PRO0845 mRNA, complete cds. [AF130048]	
A_23_P70398	9.64E-05	NM_003376	NM_003376	Homo sapiens vascular endothelial growth factor (VEGF), transcript variant 2, mRNA [NM_003376]	NM_003376
A_23_P323924	9.65E-05	NM_001002261	NM_001002261	Homo sapiens zinc finger, FYVE domain containing 27 (ZFYVE27), transcript variant 1, mRNA [NM_001002261]	NM_001002261
A_24_P217904	9.66E-05	AF111801	AF111801	Homo sapiens MSTP020 (MST020) mRNA, complete cds. [AF111801]	
A_24_P6381	9.67E-05	NM_178276	NM_178276	Homo sapiens chromosome 5 open reading frame 12 (C5orf12), mRNA [NM_178276]	NM_178276
A_24_P307014	9.67E-05	NM_005104	NM_005104	Homo sapiens bromodomain containing 2 (BRD2), mRNA [NM_005104]	NM_005104
A_24_P15083	9.72E-05	A_24_P15083			
A_23_P120710	9.74E-05	NM_003316	NM_003316	Homo sapiens tetratricopeptide repeat domain 3 (TTC3), transcript variant 1, mRNA [NM_003316]	NM_003316
A_24_P404807	9.75E-05	AK023732	AK023732	Homo sapiens cDNA FLJ13670 fis, clone PLACE1011725. [AK023732]	
A_23_P210253	9.76E-05	NM_152879	NM_152879	Homo sapiens diacylglycerol kinase, delta 130kDa (DGKD), transcript variant 2, mRNA [NM_152879]	NM_152879
A_32_P218806	9.77E-05	AK024516	AK024516	Homo sapiens cDNA: FLJ20863 fis, clone ADKA01804. [AK024516]	
A_23_P68730	9.78E-05	NM_003681	NM_003681	Homo sapiens pyridoxal (pyridoxine, vitamin B6) kinase (PDXK), mRNA [NM_003681]	NM_003681
A_23_P308763	9.78E-05	NM_005766	NM_005766	Homo sapiens FERM, RhoGEF (ARHGEF) and pleckstrin domain protein 1 (chondrocyte-derived) (FARP1), transcript variant 1, mRNA [NM_005766]	NM_005766
A_23_P312652	9.79E-05	AK128423	AK128423	Homo sapiens cDNA FLJ46566 fis, clone THYMU3040829, moderately similar to Cold-inducible RNA-binding protein. [AK128423]	
A_23_P13137	9.82E-05	AY358815	AY358815	Homo sapiens clone DNA108923 SFVP2550 (UNQ2550) mRNA, complete cds. [AY358815]	
A_24_P609323	9.84E-05	AF098968	AF098968	Homo sapiens familial Mediterranean fever locus region, mRNA sequence. [AF098968]	XM_498631
A_24_P200848	9.87E-05	THC2337268			
A_24_P396881	9.89E-05	THC2403165		Q6TXI7 (Q6TXI7) LRRGT00012, partial (4%) [THC2403165]	
A_24_P148151	9.89E-05	NM_005999	NM_005999	Homo sapiens translin-associated factor X (TSNAX), mRNA [NM_005999]	NM_005999

A_24_P278156	9.93E-05	NM_018112	NM_018112	Homo sapiens transmembrane protein 38B (TMEM38B), mRNA [NM_018112]	NM_018112
A_23_P20648	9.93E-05	NM_003172	NM_003172	Homo sapiens surfeit 1 (SURF1), nuclear gene encoding mitochondrial protein, mRNA [NM_003172]	NM_003172
A_23_P27346	9.96E-05	NM_005359	NM_005359	Homo sapiens SMAD, mothers against DPP homolog 4 (Drosophila) (SMAD4), mRNA [NM_005359]	NM_005359
A_23_P109333	9.96E-05	NM_004649	NM_004649	Homo sapiens chromosome 21 open reading frame 33 (C21orf33), nuclear gene encoding mitochondrial protein, transcript variant 1, mRNA [NM_004649]	NM_004649
A_23_P133359	9.98E-05	NM_030613	NM_030613	Homo sapiens zinc finger protein 2 homolog (mouse) (ZFP2), mRNA [NM_030613]	NM_030613
A_32_P73991	9.98E-05	A_32_P73991			
A_23_P170839	9.99E-05	NM_001008491	NM_001008491	Homo sapiens septin 2 (SEPT2), transcript variant 1, mRNA [NM_001008491]	NM_001008491
A_32_P128974	0.0001	AW971123	AW971123	EST383210 MAGE resequences, MAGL Homo sapiens cDNA, mRNA sequence [AW971123]	
A_23_P109320	0.0001	NM_018962	NM_018962	Homo sapiens Down syndrome critical region gene 6 (DSCR6), mRNA [NM_018962]	NM_018962
A_24_P341222	0.0001	ENST00000326140		Homo sapiens hypothetical gene supported by BC032431, mRNA (cDNA clone IMAGE:4521185), partial cds. [BC032431]	XM_371691
A_24_P8220	0.0001	BC001196	BC001196	Homo sapiens, Similar to heparan sulfate 6-O-sulfotransferase, clone IMAGE:3355592, mRNA, partial cds. [BC001196]	
A_32_P154121	0.000101	THC2340670			
A_24_P337397	0.000101	NM_023039	NM_023039	Homo sapiens ankyrin repeat, family A (RFXANK-like), 2 (ANKRA2), mRNA [NM_023039]	NM_023039
A_32_P130522	0.000102	BM457392	BM457392	AGENCOURT_6407446 NIH_MGC_92 Homo sapiens cDNA clone IMAGE:5584854 5', mRNA sequence [BM457392]	
A_23_P131449	0.000102	ENST00000290390			
A_24_P64039	0.000102	NM_006803	NM_006803	Homo sapiens adaptor-related protein complex 3, mu 2 subunit (AP3M2), mRNA [NM_006803]	NM_006803
A_23_P343104	0.000102	AK055463	AK055463	Homo sapiens cDNA FLJ30901 fis, clone FEBRA2005778, weakly similar to INTEGUMENTARY MUCIN A.1 PRECURSOR. [AK055463]	
A_23_P415622	0.000102	NM_014805	NM_014805	Homo sapiens EPM2A (laforin) interacting protein 1 (EPM2AIP1), mRNA [NM_014805]	NM_014805
A_32_P53713	0.000102	THC2306240		Q8WNH2 (Q8WNH2) NPIP-like protein (Fragment), complete [THC2306240]	
A_24_P500422	0.000103	AK095359	AK095359	Homo sapiens cDNA FLJ38040 fis, clone CTONG2013985. [AK095359]	
A_24_P396660	0.000103	NM_147148	NM_147148	Homo sapiens glutathione S-transferase M4 (GSTM4), transcript variant 2, mRNA [NM_147148]	NM_147148
A_24_P510377	0.000103	BC031250	BC031250	Homo sapiens, clone IMAGE:5268928, mRNA. [BC031250]	XM_378859
A_23_P381954	0.000103	NM_000121	NM_000121	Homo sapiens erythropoietin receptor (EPOR), mRNA [NM_000121]	NM_000121
A_23_P57856	0.000103	NM_138931	NM_138931	Homo sapiens B-cell CLL/lymphoma 6 (zinc finger protein 51) (BCL6), transcript variant 2, mRNA [NM_138931]	NM_138931
A_23_P155556	0.000103	NM_019895	NM_019895	Homo sapiens chromosome 3 open reading frame 4 (C3orf4), mRNA [NM_019895]	NM_019895
A_24_P336931	0.000104	NM_020970	NM_020970	Homo sapiens KIAA1641 (KIAA1641), mRNA [NM_020970]	NM_020970
A_23_P216522	0.000104	NM_016446	NM_016446	Homo sapiens chromosome 9 open reading frame 127 (C9orf127), mRNA [NM_016446]	NM_016446
A_23_P310331	0.000104	NM_003624	NM_003624	Homo sapiens RAN binding protein 3 (RANBP3), transcript variant RANBP3-a, mRNA [NM_003624]	NM_003624
A_23_P421032	0.000105	NM_174977	NM_174977	Homo sapiens SEC14-like 4 (S. cerevisiae) (SEC14L4), mRNA [NM_174977]	NM_174977
A_24_P64653	0.000105	NM_152637	NM_152637	Homo sapiens hypothetical protein MGC17301 (MGC17301), mRNA [NM_152637]	NM_152637
A_24_P12521	0.000105	NM_138811	NM_138811	Homo sapiens chromosome 7 open reading frame 31 (C7orf31), mRNA [NM_138811]	NM_138811
A_23_P69617	0.000105	NM_003728	NM_003728	Homo sapiens unc-5 homolog C (C. elegans) (UNC5C), mRNA [NM_003728]	NM_003728
A_32_P225667	0.000105	THC2386962			
A_23_P212511	0.000105	NM_133462	NM_133462	Homo sapiens tetratricopeptide repeat domain 14 (TTC14), mRNA [NM_133462]	NM_133462
A_23_P205910	0.000105	NM_004727	NM_004727	Homo sapiens solute carrier family 24 (sodium/potassium/calcium exchanger), member 1 (SLC24A1), mRNA [NM_004727]	NM_004727
A_24_P226241	0.000105	NM_153234	NM_153234	Homo sapiens Lix1 homolog (mouse) (LIX1), mRNA [NM_153234]	NM_153234
A_23_P98282	0.000105	NM_006946	NM_006946	Homo sapiens spectrin, beta, non-erythrocytic 2 (SPTBN2), mRNA [NM_006946]	NM_006946
A_23_P253949	0.000105	AL137321	AL137321	Homo sapiens mRNA; cDNA DKFZp434P231 (from clone DKFZp434P231). [AL137321]	
A_23_P125078	0.000105	NM_173626	NM_173626	Homo sapiens solute carrier family 26, member 11 (SLC26A11), mRNA [NM_173626]	NM_173626
A_24_P324465	0.000105	NM_001025303	NM_001025303	Homo sapiens similar to histone H2B histone family (LOC440686), mRNA [NM_001025303]	NM_001025303
A_24_P361457	0.000106	NM_173627	NM_173627	Homo sapiens hypothetical protein FLJ35220 (FLJ35220), mRNA [NM_173627]	NM_173627
A_24_P382579	0.000106	NM_000915	NM_000915	Homo sapiens oxytocin, prepro- (neurophysin I) (OXT), mRNA [NM_000915]	NM_000915
A_23_P8906	0.000106	NM_013437	NM_013437	Homo sapiens low density lipoprotein-related protein 12 (LRP12), mRNA [NM_013437]	NM_013437
A_23_P15542	0.000106	NM_000413	NM_000413	Homo sapiens hydroxysteroid (17-beta) dehydrogenase 1 (HSD17B1), mRNA [NM_000413]	NM_000413

A_23_P379142	0.000106	NM_145297	NM_145297	Homo sapiens zinc finger protein 626 (ZNF626), mRNA [NM_145297]	NM_145297
A_23_P205046	0.000106	NM_017664	NM_017664	Homo sapiens ankyrin repeat domain 10 (ANKRD10), mRNA [NM_017664]	NM_017664
A_32_P9924	0.000106	A_32_P9924			
A_32_P216888	0.000107	AK026368	AK026368	Homo sapiens cDNA: FLJ22715 fis, clone HSI13726. [AK026368]	
A_24_P396662	0.000107	NM_147148	NM_147148	Homo sapiens glutathione S-transferase M4 (GSTM4), transcript variant 2, mRNA [NM_147148]	NM_147148
A_24_P154868	0.000107	NM_005588	NM_005588	Homo sapiens meprin A, alpha (PABA peptide hydrolase) (MEP1A), mRNA [NM_005588]	NM_005588
A_23_P23611	0.000107	NM_001008219	NM_001008219	Homo sapiens amylase, alpha 1C; salivary (AMY1C), mRNA [NM_001008219]	NM_001008219
A_24_P365015	0.000107	NM_006361	NM_006361	Homo sapiens homeo box B13 (HOXB13), mRNA [NM_006361]	NM_006361
A_23_P426305	0.000107	NM_003734	NM_003734	Homo sapiens amine oxidase, copper containing 3 (vascular adhesion protein 1) (AOC3), mRNA [NM_003734]	NM_003734
A_24_P207503	0.000107	NM_001009813	NM_001009813	Homo sapiens Meis1, myeloid ecotropic viral integration site 1 homolog 3 (mouse) (MEIS3), transcript variant 2, mRNA [NM_001009813]	NM_001009813
A_23_P117363	0.000107	NM_001756	NM_001756	Homo sapiens serine (or cysteine) proteinase inhibitor, clade A (alpha-1 antiproteinase, antitrypsin), member 6 (SERPINA6), mRNA [NM_001756]	NM_001756
A_32_P114235	0.000107	BX112278	BX112278	BX112278 BX112278 Soares fetal liver spleen 1NFLS Homo sapiens cDNA clone IMAGp998K20129 ; IMAGE:127243, mRNA sequence [BX112278]	
A_23_P8380	0.000107	NM_024033	NM_024033	Homo sapiens hypothetical protein MGC5242 (MGC5242), mRNA [NM_024033]	NM_024033
A_23_P385690	0.000107	NM_033131	NM_033131	Homo sapiens wingless-type MMTV integration site family, member 3A (WNT3A), mRNA [NM_033131]	NM_033131
A_23_P134953	0.000107	NM_001122	NM_001122	Homo sapiens adipose differentiation-related protein (ADFP), mRNA [NM_001122]	NM_001122
A_32_P87872	0.000108	NM_032549	NM_032549	Homo sapiens IMP2 inner mitochondrial membrane protease-like (S. cerevisiae) (IMMP2L), mRNA [NM_032549]	NM_032549
A_23_P10374	0.000108	NM_014925	NM_014925	Homo sapiens KIAA1002 protein (KIAA1002), mRNA [NM_014925]	NM_014925
A_23_P27315	0.000108	NM_032048	NM_032048	Homo sapiens elastin microfibril interfacer 2 (EMILIN2), mRNA [NM_032048]	NM_032048
A_24_P389218	0.000109	NM_005435	NM_005435	Homo sapiens Rho guanine nucleotide exchange factor (GEF) 5 (ARHGEF5), transcript variant 1, mRNA [NM_005435]	NM_005435
A_23_P105747	0.000109	NM_018171	NM_018171	Homo sapiens DIP13 beta (DIP13B), mRNA [NM_018171]	NM_018171
A_32_P91107	0.000109	BC089156	BC089156	Homo sapiens cDNA clone IMAGE:6576427. [BC089156]	
A_32_P168464	0.000109	AK056476	AK056476	Homo sapiens cDNA FLJ31914 fis, clone NT2RP7004884. [AK056476]	
A_23_P64539	0.000109	NM_000559	NM_000559	Homo sapiens hemoglobin, gamma A (HBG1), mRNA [NM_000559]	NM_000559
A_23_P151098	0.00011	NM_032641	NM_032641	Homo sapiens likely ortholog of mouse gene rich cluster, C9 gene (GRCC9), mRNA [NM_032641]	NM_032641
A_23_P80739	0.00011	NM_006225	NM_006225	Homo sapiens phospholipase C, delta 1 (PLCD1), mRNA [NM_006225]	NM_006225
A_24_P83262	0.000111	NM_003270	NM_003270	Homo sapiens tetraspanin 6 (TSPAN6), mRNA [NM_003270]	NM_003270
A_24_P238525	0.000112	ENST00000324709			
A_23_P501831	0.000112	NM_032385	NM_032385	Homo sapiens chromosome 5 open reading frame 4 (C5orf4), transcript variant 2, mRNA [NM_032385]	NM_032385
A_23_P104022	0.000112	NM_052965	NM_052965	Homo sapiens chromosome 1 open reading frame 19 (C1orf19), mRNA [NM_052965]	NM_052965
A_24_P141688	0.000113	AK130583	AK130583	Homo sapiens cDNA FLJ27073 fis, clone SPL02032, highly similar to POLY(RC) BINDING PROTEIN 2. [AK130583]	
A_23_P63711	0.000113	NM_004311	NM_004311	Homo sapiens ADP-ribosylation factor-like 3 (ARL3), mRNA [NM_004311]	NM_004311
A_24_P236935	0.000114	NM_001012964	NM_001012964	Homo sapiens kallikrein 6 (neurosin, zyme) (KLK6), transcript variant B, mRNA [NM_001012964]	NM_001012964
A_32_P42797	0.000114	AK128756	AK128756	Homo sapiens cDNA FLJ44869 fis, clone BRAMY2015516. [AK128756]	XM_498593
A_23_P258887	0.000114	NM_012190	NM_012190	Homo sapiens aldehyde dehydrogenase 1 family, member L1 (ALDH1L1), mRNA [NM_012190]	NM_012190
A_24_P326398	0.000114	NM_173689	NM_173689	Homo sapiens crumbs homolog 2 (Drosophila) (CRB2), mRNA [NM_173689]	NM_173689
A_23_P98963	0.000114	AK002085	AK002085	Homo sapiens cDNA FLJ11223 fis, clone PLACE1008209. [AK002085]	
A_32_P129950	0.000114	NM_001012754	NM_001012754	Homo sapiens similar to RIKEN cDNA 8030451K01 (LOC387921), transcript variant 1, mRNA [NM_001012754]	NM_001012754
A_23_P53137	0.000114	NM_000559	NM_000559	Homo sapiens hemoglobin, gamma A (HBG1), mRNA [NM_000559]	NM_000559
A_23_P109821	0.000114	NM_006354	NM_006354	Homo sapiens transcriptional adaptor 3 (NGG1 homolog, yeast)-like (TADA3L), transcript variant 1, mRNA [NM_006354]	NM_006354
A_23_P207493	0.000115	NM_016424	NM_016424	Homo sapiens cisplatin resistance-associated overexpressed protein (CROP), transcript variant 1, mRNA [NM_016424]	NM_016424
A_23_P404606	0.000116	NM_153607	NM_153607	Homo sapiens adult retina protein (LOC153222), mRNA [NM_153607]	NM_153607
A_23_P31798	0.000117	NM_000015	NM_000015	Homo sapiens N-acetyltransferase 2 (arylamine N-acetyltransferase) (NAT2), mRNA [NM_000015]	NM_000015
A_32_P171143	0.000117	NM_203393	NM_203393	Homo sapiens hypothetical gene supported by BC031661 (LOC389458), mRNA [NM_203393]	NM_203393
A_23_P420692	0.000117	NM_015053	NM_015053	Homo sapiens protein tyrosine phosphatase, receptor type, f polypeptide (PTPRF), interacting protein (liprin), alpha 4 (PPFIA4), mRNA [NM_015053]	NM_015053

A_24_P171182	0.000117	NM_022735	NM_022735	Homo sapiens acyl-Coenzyme A binding domain containing 3 (ACBD3), mRNA [NM_022735]	NM_022735
A_24_P896373	0.000117	THC2300907			
A_32_P201897	0.000118	NM_020066	NM_020066	Homo sapiens formin 2 (FMN2), mRNA [NM_020066]	NM_020066
A_23_P343382	0.000118	AB011086	AB011086	Homo sapiens mRNA for KIAA0514 protein, partial cds. [AB011086]	
A_23_P211299	0.000118	NM_004571	NM_004571	Homo sapiens PBX/knotted 1 homeobox 1 (PKNOX1), transcript variant 1, mRNA [NM_004571]	NM_004571
A_24_P282060	0.000119	AK093006	AK093006	Homo sapiens cDNA FLJ35687 fis, clone SPLEN2019349. [AK093006]	
A_32_P34	0.000119	AK092544	AK092544	Homo sapiens cDNA FLJ35225 fis, clone PROST2001116. [AK092544]	
A_24_P42066	0.000119	NM_002058	NM_002058	Homo sapiens gamma-glutamyltransferase 2 (GGT2), mRNA [NM_002058]	NM_002058
A_23_P933	0.000119	NM_015485	NM_015485	Homo sapiens RWD domain containing 3 (RWDD3), mRNA [NM_015485]	NM_015485
A_24_P116242	0.00012	NM_014315	NM_014315	Homo sapiens kelch domain containing 2 (KLHDC2), mRNA [NM_014315]	NM_014315
A_23_P165819	0.00012	A_23_P165819			
A_24_P170983	0.000121	NM_194312	NM_194312	Homo sapiens hypothetical protein LOC339768 (LOC339768), mRNA [NM_194312]	NM_194312
A_23_P134426	0.000121	NM_001005340	NM_001005340	Homo sapiens glycoprotein (transmembrane) nmb (GPNMB), transcript variant 1, mRNA [NM_001005340]	NM_001005340
A_32_P122268	0.000122	AK131036	AK131036	Homo sapiens cDNA FLJ29001 fis, clone CBL08678. [AK131036]	
A_23_P252193	0.000122	NM_002207	NM_002207	Homo sapiens integrin, alpha 9 (ITGA9), mRNA [NM_002207]	NM_002207
A_23_P18713	0.000123	NM_004827	NM_004827	Homo sapiens ATP-binding cassette, sub-family G (WHITE), member 2 (ABCG2), mRNA [NM_004827]	NM_004827
A_23_P55107	0.000123	NM_014683	NM_014683	Homo sapiens unc-51-like kinase 2 (C. elegans) (ULK2), mRNA [NM_014683]	NM_014683
A_24_P10953	0.000123	AK096342	AK096342	Homo sapiens cDNA FLJ39023 fis, clone NT2RP7004348, highly similar to ZINC FINGER PROTEIN 93. [AK096342]	
A_23_P128783	0.000123	NM_018453	NM_018453	Homo sapiens chromosome 14 open reading frame 11 (C14orf11), mRNA [NM_018453]	NM_018453
A_24_P333733	0.000123	NM_005177	NM_005177	Homo sapiens ATPase, H+ transporting, lysosomal V0 subunit a isoform 1 (ATP6V0A1), mRNA [NM_005177]	NM_005177
A_23_P78265	0.000125	NM_004138	NM_004138	Homo sapiens keratin, hair, acidic, 3A (KRTHA3A), mRNA [NM_004138]	NM_004138
A_23_P161399	0.000125	NM_130439	NM_130439	Homo sapiens MAX interactor 1 (MXI1), transcript variant 2, mRNA [NM_130439]	NM_130439
A_23_P107247	0.000126	NM_018896	NM_018896	Homo sapiens calcium channel, voltage-dependent, alpha 1G subunit (CACNA1G), transcript variant 1, mRNA [NM_018896]	NM_018896
A_23_P387184	0.000126	ENST00000343505		Homo sapiens mRNA for KIAA1357 protein, partial cds. [AB037778]	XM_496826
A_23_P164536	0.000126	NM_002647	NM_002647	Homo sapiens phosphoinositide-3-kinase, class 3 (PIK3C3), mRNA [NM_002647]	NM_002647
A_23_P323943	0.000127	NM_178498	NM_178498	Homo sapiens solute carrier family 5 (sodium/glucose cotransporter), member 12 (SLC5A12), mRNA [NM_178498]	NM_178498
A_23_P109322	0.000127	NM_006198	NM_006198	Homo sapiens Purkinje cell protein 4 (PCP4), mRNA [NM_006198]	NM_006198
A_24_P236437	0.000127	NM_006141	NM_006141	Homo sapiens dynein, cytoplasmic, light intermediate polypeptide 2 (DNCLI2), mRNA [NM_006141]	NM_006141
A_32_P12820	0.000127	THC2400533			
A_23_P332713	0.000129	ENST00000262961		Homo sapiens mRNA for KIAA1086 protein, partial cds. [AB029009]	XM_047610
A_24_P827	0.000129	NM_003599	NM_003599	Homo sapiens suppressor of Ty 3 homolog (S. cerevisiae) (SUPT3H), transcript variant 1, mRNA [NM_003599]	NM_003599
A_23_P116187	0.000129	NM_031938	NM_031938	Homo sapiens beta-carotene dioxygenase 2 (BCDO2), mRNA [NM_031938]	NM_031938
A_32_P211414	0.000129	THC2280849			
A_23_P93431	0.000129	NM_016108	NM_016108	Homo sapiens androgen-induced 1 (AIG1), mRNA [NM_016108]	NM_016108
A_23_P251825	0.000129	NM_001007245	NM_001007245	Homo sapiens interferon-related developmental regulator 1 (IFRD1), transcript variant 2, mRNA [NM_001007245]	NM_001007245
A_23_P20484	0.00013	NM_201553	NM_201553	Homo sapiens fibrinogen-like 1 (FGL1), transcript variant 4, mRNA [NM_201553]	NM_201553
A_23_P40272	0.00013	NM_001501	NM_001501	Homo sapiens gonadotropin-releasing hormone 2 (GNRH2), transcript variant 1, mRNA [NM_001501]	NM_001501
A_23_P78557	0.00013	NM_017703	NM_017703	Homo sapiens F-box and leucine-rich repeat protein 12 (FBXL12), mRNA [NM_017703]	NM_017703
A_32_P52153	0.00013	BE378852	BE378852	601237381F1 NIH_MGC_44 Homo sapiens cDNA clone IMAGE:3609140 5', mRNA sequence [BE378852]	
A_23_P110882	0.00013	NM_021648	NM_021648	Homo sapiens TSPY-like 4 (TSPYL4), mRNA [NM_021648]	NM_021648
A_23_P77568	0.00013	NM_003752	NM_003752	Homo sapiens eukaryotic translation initiation factor 3, subunit 8, 110kDa (EIF3S8), mRNA [NM_003752]	NM_003752
A_23_P135417	0.000131	NM_145740	NM_145740	Homo sapiens glutathione S-transferase A1 (GSTA1), mRNA [NM_145740]	NM_145740
A_23_P23839	0.000131	NM_001017403	NM_001017403	Homo sapiens leucine-rich repeat-containing G protein-coupled receptor 6 (LGR6), transcript variant 1, mRNA [NM_001017403]	NM_001017403
A_23_P114656	0.000131	NM_004964	NM_004964	Homo sapiens histone deacetylase 1 (HDAC1), mRNA [NM_004964]	NM_004964
A_32_P377880	0.000132	BC008580	BC008580	Homo sapiens, clone IMAGE:4179986, mRNA, partial cds. [BC008580]	

A_23_P158570	0.000132	NM_001609	NM_001609	Homo sapiens acyl-Coenzyme A dehydrogenase, short/branched chain (ACADSB), nuclear gene encoding mitochondrial protein, mRNA [NM_001609]	NM_001609
A_24_P96527	0.000132	NM_006476	NM_006476	Homo sapiens ATP synthase, H+ transporting, mitochondrial F0 complex, subunit g (ATP5L), nuclear gene encoding mitochondrial protein, mRNA [NM_006476]	NM_006476
A_24_P414719	0.000132	AK002098	AK002098	Homo sapiens cDNA FLJ11236 fis, clone PLACE1008524. [AK002098]	
A_23_P166109	0.000132	NM_198391	NM_198391	Homo sapiens fibronectin leucine rich transmembrane protein 3 (FLRT3), transcript variant 2, mRNA [NM_198391]	NM_198391
A_23_P51711	0.000133	NM_015849	NM_015849	Homo sapiens elastase 2B (ELA2B), mRNA [NM_015849]	NM_015849
A_23_P168470	0.000133	NM_018842	NM_018842	Homo sapiens BAI1-associated protein 2-like 1 (BAIAP2L1), mRNA [NM_018842]	NM_018842
A_24_P722068	0.000133	THC2268343			
A_23_P147495	0.000133	NM_021946	NM_021946	Homo sapiens BCL6 co-repressor-like 1 (BCORL1), mRNA [NM_021946]	NM_021946
A_24_P126325	0.000134	NM_207035	NM_207035	Homo sapiens chromosome 1 open reading frame 63 (C1orf63), transcript variant 1, mRNA [NM_207035]	NM_207035
A_24_P174367	0.000134	NM_006241	NM_006241	Homo sapiens protein phosphatase 1, regulatory (inhibitor) subunit 2 (PPP1R2), mRNA [NM_006241]	NM_006241
A_23_P60742	0.000134	NM_013292	NM_013292	Homo sapiens fast skeletal myosin light chain 2 (MYLPF), mRNA [NM_013292]	NM_013292
A_32_P117098	0.000135	NM_015532	NM_015532	Homo sapiens glutamate receptor, ionotropic, N-methyl D-aspartate-like 1A (GRINL1A), transcript variant 1, mRNA [NM_015532]	NM_015532
A_23_P137909	0.000135	NM_003493	NM_003493	Homo sapiens histone 3, H3 (HIST3H3), mRNA [NM_003493]	NM_003493
A_23_P142187	0.000136	NM_022462	NM_022462	Homo sapiens hypoxia inducible factor 3, alpha subunit (HIF3A), transcript variant 2, mRNA [NM_022462]	NM_022462
A_23_P207280	0.000136	NM_006380	NM_006380	Homo sapiens amyloid beta precursor protein (cytoplasmic tail) binding protein 2 (APPBP2), mRNA [NM_006380]	NM_006380
A_32_P213692	0.000137	ENST00000216468		Homo sapiens cDNA: FLJ22042 fis, clone HEP09065. [AK025695]	
A_23_P33894	0.000137	NM_201222	NM_201222	Homo sapiens melanoma antigen family D, 2 (MAGED2), transcript variant 3, mRNA [NM_201222]	NM_201222
A_32_P2883	0.000138	THC2269190		Q7PWY5 (Q7PWY5) ENSANGP00000012061, partial (5%) [THC2269190]	
A_23_P135990	0.000138	NM_005630	NM_005630	Homo sapiens solute carrier organic anion transporter family, member 2A1 (SLCO2A1), mRNA [NM_005630]	NM_005630
A_24_P405298	0.000138	NM_002709	NM_002709	Homo sapiens protein phosphatase 1, catalytic subunit, beta isoform (PPP1CB), transcript variant 1, mRNA [NM_002709]	NM_002709
A_23_P134395	0.000138	NM_032988	NM_032988	Homo sapiens transducin (beta)-like 2 (TBL2), transcript variant 2, mRNA [NM_032988]	NM_032988
A_23_P305759	0.000139	NM_138340	NM_138340	Homo sapiens abhydrolase domain containing 3 (ABHD3), mRNA [NM_138340]	NM_138340
A_23_P2967	0.000139	NM_003917	NM_003917	Homo sapiens adaptor-related protein complex 1, gamma 2 subunit (AP1G2), transcript variant 1, mRNA [NM_003917]	NM_003917
A_24_P381199	0.000139	NM_001003818	NM_001003818	Homo sapiens tripartite motif-containing 6 (TRIM6), transcript variant 1, mRNA [NM_001003818]	NM_001003818
A_23_P91552	0.00014	NM_206965	NM_206965	Homo sapiens formiminotransferase cyclodeaminase (FTCD), transcript variant A, mRNA [NM_206965]	NM_206965
A_32_P156237	0.00014	A_32_P156237			
A_23_P502343	0.00014	NM_025220	NM_025220	Homo sapiens a disintegrin and metalloproteinase domain 33 (ADAM33), transcript variant 1, mRNA [NM_025220]	NM_025220
A_23_P124522	0.00014	NM_024819	NM_024819	Homo sapiens hypothetical protein FLJ22955 (FLJ22955), mRNA [NM_024819]	NM_024819
A_32_P85978	0.00014	AK074191	AK074191	Homo sapiens cDNA FLJ23611 fis, clone ADKA02380. [AK074191]	
A_23_P104323	0.000141	NM_002412	NM_002412	Homo sapiens O-6-methylguanine-DNA methyltransferase (MGMT), mRNA [NM_002412]	NM_002412
A_32_P101689	0.000141	NM_014888	NM_014888	Homo sapiens family with sequence similarity 3, member C (FAM3C), mRNA [NM_014888]	NM_014888
A_24_P943205	0.000142	ENST00000313624		Homo sapiens mRNA; cDNA DKFZp667P0410 (from clone DKFZp667P0410). [AL831953]	
A_23_P122796	0.000142	NM_016063	NM_016063	Homo sapiens chromosome 6 open reading frame 74 (C6orf74), mRNA [NM_016063]	NM_016063
A_23_P3193	0.000142	NM_005113	NM_005113	Homo sapiens golgi autoantigen, golgin subfamily a, 5 (GOLGA5), mRNA [NM_005113]	NM_005113
A_23_P421011	0.000143	NM_030929	NM_030929	Homo sapiens Kazal-type serine protease inhibitor domain 1 (KAZALD1), mRNA [NM_030929]	NM_030929
A_23_P66525	0.000143	NM_006042	NM_006042	Homo sapiens heparan sulfate (glucosamine) 3-O-sulfotransferase 3A1 (HS3ST3A1), mRNA [NM_006042]	NM_006042
A_32_P228886	0.000144	BX115350	BX115350	BX115350 Soares fetal liver spleen 1NFLS Homo sapiens cDNA clone IMAGp998L11127, mRNA sequence [BX115350]	
A_23_P254079	0.000144	NM_003943	NM_003943	Homo sapiens genethonin 1 (GENX-3414), mRNA [NM_003943]	NM_003943
A_23_P111041	0.000144	NM_003525	NM_003525	Homo sapiens histone 1, H2bi (HIST1H2BI), mRNA [NM_003525]	NM_003525
A_32_P181297	0.000144	NR_002330	NR_002330	Homo sapiens ST7 overlapping transcript 1 (antisense non-coding RNA) (ST7OT1) on chromosome 7 [NR_002330]	NR_002330
A_24_P89843	0.000144	NM_032687	NM_032687	Homo sapiens cysteine/histidine-rich 1 (CYHR1), mRNA [NM_032687]	NM_032687
A_23_P83953	0.000144	S80864	S80864	cytochrome c-like polypeptide [human, lung adenocarcinoma A549, mRNA, 1041 nt]. [S80864]	
A_23_P34700	0.000144	NM_000364	NM_000364	Homo sapiens troponin T2, cardiac (TNNT2), transcript variant 1, mRNA [NM_000364]	NM_000364
A_24_P69439	0.000145	NM_030780	NM_030780	Homo sapiens mitochondrial folate transporter/carrier (MFTC), mRNA [NM_030780]	NM_030780

A_23_P255714	0.000146	NM_025103	NM_025103	Homo sapiens coiled-coil domain containing 2 (CCDC2), mRNA [NM_025103]	NM_025103
A_24_P243749	0.000146	NM_002612	NM_002612	Homo sapiens pyruvate dehydrogenase kinase, isoenzyme 4 (PDK4), mRNA [NM_002612]	NM_002612
A_24_P83848	0.000147	ENST00000300892		Human kidney gamma-glutamyl transpeptidase type II mRNA, 3' end. [M30474]	
A_23_P89123	0.000147	NM_006324	NM_006324	Homo sapiens craniofacial development protein 1 (CFDP1), mRNA [NM_006324]	NM_006324
A_23_P397208	0.000148	NM_000848	NM_000848	Homo sapiens glutathione S-transferase M2 (muscle) (GSTM2), mRNA [NM_000848]	NM_000848
A_23_P359015	0.000148	NM_016621	NM_016621	Homo sapiens PHD finger protein 21A (PHF21A), mRNA [NM_016621]	NM_016621
A_24_P322847	0.000148	NM_001018051	NM_001018051	Homo sapiens polymerase (RNA) III (DNA directed) polypeptide H (22.9kD) (POLR3H), transcript variant 4, mRNA [NM_001018051]	NM_001018051
A_32_P73139	0.000149	BC047708	BC047708	Homo sapiens, clone IMAGE:5750141, mRNA. [BC047708]	
A_23_P103588	0.000149	NM_005518	NM_005518	Homo sapiens 3-hydroxy-3-methylglutaryl-Coenzyme A synthase 2 (mitochondrial) (HMGCS2), mRNA [NM_005518]	NM_005518
A_32_P136033	0.000149	AK090477	AK090477	Homo sapiens mRNA for FLJ00399 protein. [AK090477]	
A_23_P121783	0.000149	NM_024592	NM_024592	Homo sapiens steroid 5 alpha-reductase 2-like (SRD5A2L), mRNA [NM_024592]	NM_024592
A_23_P146417	0.000149	NM_032012	NM_032012	Homo sapiens chromosome 9 open reading frame 5 (C9orf5), mRNA [NM_032012]	NM_032012
A_32_P55427	0.00015	THC2319816		Q9F8M7 (Q9F8M7) DTDP-glucose 4,6-dehydratase (Fragment), partial (11%) [THC2319816]	
A_24_P259192	0.00015	NM_018957	NM_018957	Homo sapiens SH3-domain binding protein 1 (SH3BP1), mRNA [NM_018957]	NM_018957
A_32_P80850	0.00015	NM_021110	NM_021110	Homo sapiens collagen, type XIV, alpha 1 (undulin) (COL14A1), mRNA [NM_021110]	NM_021110
A_23_P7083	0.00015	NM_052861	NM_052861	Homo sapiens hypothetical protein MGC21675 (MGC21675), mRNA [NM_052861]	NM_052861
A_23_P159907	0.00015	NM_030801	NM_030801	Homo sapiens melanoma antigen family D, 4 (MAGED4), transcript variant 1, mRNA [NM_030801]	NM_030801
A_23_P56680	0.00015	NM_001002256	NM_001002256	Homo sapiens ATP synthase, H+ transporting, mitochondrial F0 complex, subunit c (subunit 9) isoform 3 (ATP5G3), nuclear gene encoding mitochondrial protein, transcript variant 1, mRNA [NM_001002256]	NM_001002256
A_23_P356731	0.000152	NM_152509	NM_152509	Homo sapiens hypothetical protein FLJ31568 (FLJ31568), mRNA [NM_152509]	NM_152509
A_24_P134392	0.000153	NM_006948	NM_006948	Homo sapiens stress 70 protein chaperone, microsome-associated, 60kDa (STCH), mRNA [NM_006948]	NM_006948
A_23_P142310	0.000153	NM_017572	NM_017572	Homo sapiens MAP kinase interacting serine/threonine kinase 2 (MKNK2), mRNA [NM_017572]	NM_017572
A_23_P257763	0.000154	NM_033254	NM_033254	Homo sapiens brother of CDO (BOC), mRNA [NM_033254]	NM_033254
A_23_P117298	0.000156	NM_000131	NM_000131	Homo sapiens coagulation factor VII (serum prothrombin conversion accelerator) (F7), transcript variant 1, mRNA [NM_000131]	NM_000131
A_23_P85664	0.000156	NM_152376	NM_152376	Homo sapiens UBX domain containing 3 (UBXD3), mRNA [NM_152376]	NM_152376
A_24_P459880	0.000156	BC032042	BC032042	Homo sapiens, Similar to cleavage and polyadenylation specific factor 6, 68kD subunit, clone IMAGE:4819488, mRNA. [BC032042]	
A_24_P406480	0.000157	NM_152271	NM_152271	Homo sapiens hypothetical protein FLJ23749 (FLJ23749), mRNA [NM_152271]	NM_152271
A_32_P12430	0.000157	NM_015087	NM_015087	Homo sapiens spastic paraplegia 20, spartin (Troyer syndrome) (SPG20), mRNA [NM_015087]	NM_015087
A_23_P114983	0.000157	NM_032588	NM_032588	Homo sapiens tripartite motif-containing 63 (TRIM63), mRNA [NM_032588]	NM_032588
A_23_P111288	0.000157	NM_016462	NM_016462	Homo sapiens transmembrane protein 14C (TMEM14C), mRNA [NM_016462]	NM_016462
A_23_P124619	0.000158	NM_020672	NM_020672	Homo sapiens S100 calcium binding protein A14 (S100A14), mRNA [NM_020672]	NM_020672
A_23_P202104	0.000159	NM_005729	NM_005729	Homo sapiens peptidylprolyl isomerase F (cyclophilin F) (PPIF), nuclear gene encoding mitochondrial protein, mRNA [NM_005729]	NM_005729
A_32_P87769	0.00016	NM_203301	NM_203301	Homo sapiens F-box protein 33 (FBXO33), mRNA [NM_203301]	NM_203301
A_32_P173744	0.00016	CR603215	CR603215	full-length cDNA clone CS0DI023YC17 of Placenta Cot 25-normalized of Homo sapiens (human). [CR603215]	
A_24_P409500	0.000161	BC000986	BC000986	Homo sapiens cDNA clone IMAGE:3446313, complete cds. [BC000986]	
A_24_P817066	0.000161	NM_182501	NM_182501	Homo sapiens MTERF domain containing 2 (MTERFD2), mRNA [NM_182501]	NM_182501
A_24_P46171	0.000161	NM_017861	NM_017861	Homo sapiens phosphatidylinositol glycan, class X (PIGX), mRNA [NM_017861]	NM_017861
A_32_P202502	0.000161	NM_181723	NM_181723	Homo sapiens EF-hand domain family, member A2 (EFHA2), mRNA [NM_181723]	NM_181723
A_24_P849801	0.000161	NM_000983	NM_000983	Homo sapiens ribosomal protein L22 (RPL22), mRNA [NM_000983]	NM_000983
A_32_P16263	0.000163	AI638367	AI638367	AI638367 tt05f07.x1 NCI_CGAP_GC6 Homo sapiens cDNA clone IMAGE:2239909 3' similar to SW:UBPC_HUMAN P51784 UBIQUITIN CARBOXYL-TERMINAL HYDROLASE 12 ;contains PTR5.t3 PTR5 repetitive element ;, mRNA sequence [AI638367]	
A_24_P321766	0.000163	NM_000624	NM_000624	Homo sapiens serine (or cysteine) proteinase inhibitor, clade A (alpha-1 antiproteinase, antitrypsin), member 5 (SERPINA5), mRNA [NM_000624]	NM_000624
A_32_P52785	0.000163	NM_015345	NM_015345	Homo sapiens dishevelled associated activator of morphogenesis 2 (DAAM2), mRNA [NM_015345]	NM_015345
A_23_P212554	0.000163	NM_024665	NM_024665	Homo sapiens transducin (beta)-like 1X-linked receptor 1 (TBL1XR1), mRNA [NM_024665]	NM_024665
A_24_P295379	0.000163	AB046844	AB046844	Homo sapiens mRNA for KIAA1624 protein, partial cds. [AB046844]	

A_23_P87545	0.000163	NM_021034	NM_021034	Homo sapiens interferon induced transmembrane protein 3 (1-8U) (IFITM3), mRNA [NM_021034]	NM_021034
A_24_P912228	0.000164	AK095564	AK095564	Homo sapiens cDNA FLJ38245 fis, clone FCBBF2007186. [AK095564]	
A_23_P66698	0.000165	AK074161	AK074161	Homo sapiens mRNA for FLJ00234 protein. [AK074161]	
A_23_P31376	0.000165	NM_018334	NM_018334	Homo sapiens leucine rich repeat neuronal 3 (LRRN3), mRNA [NM_018334]	NM_018334
A_23_P39294	0.000165	ENST00000312785		Homo sapiens mRNA full length insert cDNA clone EUROIMAGE 1759349. [AL365412]	
A_24_P346644	0.000165	AL050206	AL050206	Homo sapiens mRNA; cDNA DKFZp586F1523 (from clone DKFZp586F1523). [AL050206]	
A_24_P321511	0.000165	NM_016072	NM_016072	Homo sapiens golgi transport 1 homolog B (S. cerevisiae) (GOLT1B), mRNA [NM_016072]	NM_016072
A_23_P213093	0.000165	NM_017830	NM_017830	Homo sapiens OCIA domain containing 1 (OCIAD1), mRNA [NM_017830]	NM_017830
A_32_P23838	0.000166	NM_207352	NM_207352	Homo sapiens cytochrome P450, family 4, subfamily V, polypeptide 2 (CYP4V2), mRNA [NM_207352]	NM_207352
A_32_P47107	0.000166	CR610759	CR610759	full-length cDNA clone CS0DI026YO05 of Placenta Cot 25-normalized of Homo sapiens (human). [CR610759]	
A_23_P51627	0.000166	NM_024664	NM_024664	Homo sapiens phosphopantothenoylcysteine synthetase (PPCS), mRNA [NM_024664]	NM_024664
A_23_P37514	0.000167	BC011905	BC011905	Homo sapiens DKFZP434H132 protein, mRNA (cDNA clone IMAGE:4121832), complete cds. [BC011905]	
A_24_P576174	0.000167	AK025344	AK025344	Homo sapiens cDNA: FLJ21691 fis, clone COL09555. [AK025344]	
A_23_P153930	0.000169	NM_001616	NM_001616	Homo sapiens activin A receptor, type IIA (ACVR2A), mRNA [NM_001616]	NM_001616
A_32_P192842	0.00017	AI476245	AI476245	AI476245 ti72d11.x1 NCI_CGAP_Kid11 Homo sapiens cDNA clone IMAGE:2137557 3', mRNA sequence [AI476245]	
A_23_P413180	0.000171	NM_017440	NM_017440	Homo sapiens Mdm4, transformed 3T3 cell double minute 1, p53 binding protein (mouse) (MDM1), transcript variant 1, mRNA [NM_017440]	NM_017440
A_24_P943095	0.000171	AY816329	AY816329	Homo sapiens structure protein NSP5b3b mRNA, complete cds. [AY816329]	
A_23_P149019	0.000171	NM_001703	NM_001703	Homo sapiens brain-specific angiogenesis inhibitor 2 (BAI2), mRNA [NM_001703]	NM_001703
A_23_P363896	0.000171	NM_152350	NM_152350	Homo sapiens hypothetical protein MGC40157 (MGC40157), mRNA [NM_152350]	NM_152350
A_24_P230907	0.000172	BC015394	BC015394	Homo sapiens KIAA0974, mRNA (cDNA clone MGC:21703 IMAGE:4431919), complete cds. [BC015394]	
A_32_P218766	0.000173	BC027471	BC027471	Homo sapiens LOC440173, mRNA (cDNA clone MGC:34689 IMAGE:4995424), complete cds. [BC027471]	
A_23_P105803	0.000173	NM_002010	NM_002010	Homo sapiens fibroblast growth factor 9 (glia-activating factor) (FGF9), mRNA [NM_002010]	NM_002010
A_24_P639679	0.000173	AK095831	AK095831	Homo sapiens cDNA FLJ38512 fis, clone HCHON2000503. [AK095831]	
A_23_P76749	0.000173	NM_020692	NM_020692	Homo sapiens UDP-N-acetyl-alpha-D-galactosamine:polypeptide N-acetylgalactosaminyltransferase-like 1 (GALNTL1), mRNA [NM_020692]	NM_020692
A_32_P71675	0.000174	BC035379	BC035379	Homo sapiens, clone IMAGE:5162922, mRNA. [BC035379]	XM_373823
A_23_P151166	0.000174	NM_032369	NM_032369	Homo sapiens hypothetical protein MGC15619 (MGC15619), mRNA [NM_032369]	NM_032369
A_24_P270044	0.000174	NM_024769	NM_024769	Homo sapiens adipocyte-specific adhesion molecule (ASAM), mRNA [NM_024769]	NM_024769
A_23_P370651	0.000174	NM_014883	NM_014883	Homo sapiens family with sequence similarity 13, member A1 (FAM13A1), transcript variant 1, mRNA [NM_014883]	NM_014883
A_23_P407628	0.000174	NM_006766	NM_006766	Homo sapiens MYST histone acetyltransferase (monocytic leukemia) 3 (MYST3), mRNA [NM_006766]	NM_006766
A_24_P53976	0.000174	NM_002065	NM_002065	Homo sapiens glutamate-ammonia ligase (glutamine synthase) (GLUL), mRNA [NM_002065]	NM_002065
A_23_P200477	0.000174	AB043587	AB043587	Homo sapiens gcp60 mRNA for golgi resident protein GCP60, complete cds. [AB043587]	
A_24_P328492	0.000175	NM_144949	NM_144949	Homo sapiens suppressor of cytokine signaling 5 (SOCS5), transcript variant 2, mRNA [NM_144949]	NM_144949
A_24_P183292	0.000175	NM_001025105	NM_001025105	Homo sapiens casein kinase 1, alpha 1 (CSNK1A1), transcript variant 1, mRNA [NM_001025105]	NM_001025105
A_23_P254756	0.000175	NM_006016	NM_006016	Homo sapiens CD164 antigen, sialomucin (CD164), mRNA [NM_006016]	NM_006016
A_32_P34138	0.000176	ENST00000343959		Homo sapiens cDNA clone MGC:72187 IMAGE:4617569, complete cds. [BC062776]	
A_23_P418934	0.000176	NM_001017370	NM_001017370	Homo sapiens similar to RIKEN cDNA 8030451K01 (LOC387921), transcript variant 2, mRNA [NM_001017370]	NM_001017370
A_23_P10401	0.000176	NM_002718	NM_002718	Homo sapiens protein phosphatase 2 (formerly 2A), regulatory subunit B'', alpha (PPP2R3A), transcript variant 1, mRNA [NM_002718]	NM_002718
A_23_P205584	0.000176	THC2372489			
A_24_P24831	0.000176	BC063682	BC063682	Homo sapiens CXYorf1-related protein, mRNA (cDNA clone MGC:75272 IMAGE:4396768), complete cds. [BC063682]	XM_377073
A_23_P69988	0.000176	NM_002887	NM_002887	Homo sapiens arginyl-tRNA synthetase (RARS), mRNA [NM_002887]	NM_002887
A_23_P378288	0.000177	BX647761	BX647761	Homo sapiens mRNA; cDNA DKFZp686K2231 (from clone DKFZp686K2231). [BX647761]	
A_23_P401547	0.000177	NM_015480	NM_015480	Homo sapiens poliovirus receptor-related 3 (PVRL3), mRNA [NM_015480]	NM_015480
A_23_P101208	0.000177	NM_001914	NM_001914	Homo sapiens cytochrome b-5 (CYB5), transcript variant 2, mRNA [NM_001914]	NM_001914
A_24_P294419	0.000179	NM_004636	NM_004636	Homo sapiens sema domain, immunoglobulin domain (Ig), short basic domain, secreted, (semaphorin) 3B (SEMA3B), transcript variant 1, mRNA [NM_004636]	NM_004636

A_23_P254842	0.000179	NM_012080	NM_012080	Homo sapiens haloacid dehalogenase-like hydrolase domain containing 1A (HDHD1A), mRNA [NM_012080]	NM_012080
A_23_P58763	0.000179	NM_015946	NM_015946	Homo sapiens pelota homolog (Drosophila) (PELO), mRNA [NM_015946]	NM_015946
A_32_P62137	0.000181	A_32_P62137			
A_32_P77098	0.000182	NM_001003682	NM_001003682	Homo sapiens cDNA DKFZp434C184 gene (TTMB), mRNA [NM_001003682]	NM_001003682
A_23_P109682	0.000182	NM_022461	NM_022461	Homo sapiens 5-azacytidine induced 2 (AZI2), mRNA [NM_022461]	NM_022461
A_24_P15973	0.000183	A_24_P15973			
A_23_P403588	0.000183	BC000226	BC000226	Homo sapiens ubiquitin specific protease 47, mRNA (cDNA clone IMAGE:3350895), complete cds. [BC000226]	
A_24_P374834	0.000184	AB188491	AB188491	Homo sapiens mRNA, clone: TH020D07. [AB188491]	XM_166659
A_24_P355568	0.000184	NM_005330	NM_005330	Homo sapiens hemoglobin, epsilon 1 (HBE1), mRNA [NM_005330]	NM_005330
A_23_P72537	0.000184	NM_004208	NM_004208	Homo sapiens programmed cell death 8 (apoptosis-inducing factor) (PDCD8), nuclear gene encoding mitochondrial protein, transcript variant 1, mRNA [NM_004208]	NM_004208
A_23_P143692	0.000185	NM_018957	NM_018957	Homo sapiens SH3-domain binding protein 1 (SH3BP1), mRNA [NM_018957]	NM_018957
A_32_P141013	0.000185	ENST00000326261		Homo sapiens mRNA for KIAA1110 protein, partial cds. [AB029033]	
A_23_P77103	0.000185	NM_003104	NM_003104	Homo sapiens sorbitol dehydrogenase (SORD), mRNA [NM_003104]	NM_003104
A_23_P126486	0.000186	BC006312	BC006312	Homo sapiens hypothetical protein MGC12760, mRNA (cDNA clone MGC:12760 IMAGE:4111573), complete cds. [BC006312]	
A_23_P131954	0.000186	NM_014426	NM_014426	Homo sapiens sorting nexin 5 (SNX5), transcript variant 2, mRNA [NM_014426]	NM_014426
A_23_P100203	0.000187	NM_001537	NM_001537	Homo sapiens heat shock factor binding protein 1 (HSBP1), mRNA [NM_001537]	NM_001537
A_23_P255884	0.000187	NM_198252	NM_198252	Homo sapiens gelsolin (amyloidosis, Finnish type) (GSN), transcript variant 2, mRNA [NM_198252]	NM_198252
A_32_P179317	0.000188	BF761348	BF761348	BF761348 RC2-CS0018-041000-015-g01 CS0018 Homo sapiens cDNA, mRNA sequence [BF761348]	
A_23_P66881	0.000188	NM_003835	NM_003835	Homo sapiens regulator of G-protein signalling 9 (RGS9), mRNA [NM_003835]	NM_003835
A_23_P122959	0.000188	BC036337	BC036337	Homo sapiens KIAA0644 gene product, mRNA (cDNA clone MGC:34343 IMAGE:5179761), complete cds. [BC036337]	XM_379800
A_32_P353072	0.000188	NM_018374	NM_018374	Homo sapiens hypothetical protein FLJ11273 (FLJ11273), mRNA [NM_018374]	NM_018374
A_24_P76879	0.000188	NM_003309	NM_003309	Homo sapiens TSPY-like 1 (TSPYL1), mRNA [NM_003309]	NM_003309
A_24_P765795	0.000189	AK055981	AK055981	Homo sapiens cDNA FLJ31419 fis, clone NT2NE2000356. [AK055981]	
A_23_P428738	0.000189	NM_001145	NM_001145	Homo sapiens angiogenin, ribonuclease, RNase A family, 5 (ANG), mRNA [NM_001145]	NM_001145
A_24_P943815	0.00019	NM_004650	NM_004650	Homo sapiens patatin-like phospholipase domain containing 4 (PNPLA4), mRNA [NM_004650]	NM_004650
A_23_P4082	0.00019	NM_006584	NM_006584	Homo sapiens chaperonin containing TCP1, subunit 6B (zeta 2) (CCT6B), mRNA [NM_006584]	NM_006584
A_32_P20912	0.00019	AK025669	AK025669	Homo sapiens cDNA: FLJ22016 fis, clone HEP07422. [AK025669]	
A_23_P38388	0.00019	NM_153229	NM_153229	Homo sapiens hypothetical protein FLJ33318 (FLJ33318), mRNA [NM_153229]	NM_153229
A_24_P287683	0.00019	NM_005829	NM_005829	Homo sapiens adaptor-related protein complex 3, sigma 2 subunit (AP3S2), mRNA [NM_005829]	NM_005829
A_23_P65217	0.000191	NM_014305	NM_014305	Homo sapiens TDP-glucose 4,6-dehydratase (TGDS), mRNA [NM_014305]	NM_014305
A_23_P501193	0.000192	NM_170741	NM_170741	Homo sapiens potassium inwardly-rectifying channel, subfamily J, member 16 (KCNJ16), transcript variant 2, mRNA [NM_170741]	NM_170741
A_24_P246591	0.000192	ENST00000305820			
A_24_P921366	0.000192	NM_033138	NM_033138	Homo sapiens caldesmon 1 (CALD1), transcript variant 1, mRNA [NM_033138]	NM_033138
A_32_P919718	0.000193	NM_178520	NM_178520	Homo sapiens hypothetical protein FLJ38792 (FLJ38792), mRNA [NM_178520]	NM_178520
A_23_P170857	0.000193	NM_002182	NM_002182	Homo sapiens interleukin 1 receptor accessory protein (IL1RAP), transcript variant 1, mRNA [NM_002182]	NM_002182
A_24_P89872	0.000193	NM_197977	NM_197977	Homo sapiens zinc finger protein 189 (ZNF189), mRNA [NM_197977]	NM_197977
A_23_P384355	0.000194	BG547557	BG547557	BG547557 602575410F1 NIH_MGC_77 Homo sapiens cDNA clone IMAGE:4703546 5', mRNA sequence [BG547557]	
A_23_P363472	0.000194	AB032991	AB032991	Homo sapiens mRNA for KIAA1165 protein, partial cds. [AB032991]	
A_23_P93348	0.000194	NM_002341	NM_002341	Homo sapiens lymphotoxin beta (TNF superfamily, member 3) (LTB), transcript variant 1, mRNA [NM_002341]	NM_002341
A_23_P168014	0.000195	NM_021066	NM_021066	Homo sapiens histone 1, H2aj (HIST1H2AJ), mRNA [NM_021066]	NM_021066
A_24_P11061	0.000196	NM_153479	NM_153479	Homo sapiens chondrosarcoma associated gene 1 (CSAG1), transcript variant b, mRNA [NM_153479]	NM_153479
A_23_P218988	0.000196	NM_022902	NM_022902	Homo sapiens solute carrier family 30 (zinc transporter), member 5 (SLC30A5), transcript variant 1, mRNA [NM_022902]	NM_022902
A_23_P167812	0.000197	NM_153020	NM_153020	Homo sapiens RNA binding motif protein 24 (RBM24), mRNA [NM_153020]	NM_153020
A_23_P84836	0.000197	NM_006310	NM_006310	Homo sapiens aminopeptidase puromycin sensitive (NPEPPS), mRNA [NM_006310]	NM_006310

A_24_P127082	0.000197	A_24_P127082			
A_32_P65391	0.000198	XM_370866	XM_370866	PREDICTED: Homo sapiens hypothetical protein FLJ25756 (FLJ25756), mRNA [XM_370866]	XM_370866
A_23_P208302	0.000199	NM_000483	NM_000483	Homo sapiens apolipoprotein C-II (APOC2), mRNA [NM_000483]	NM_000483
A_24_P55356	0.000199	D87682	D87682	Human mRNA for KIAA0241 gene, partial cds. [D87682]	
A_23_P159012	0.000199	NM_023039	NM_023039	Homo sapiens ankyrin repeat, family A (RFXANK-like), 2 (ANKRA2), mRNA [NM_023039]	NM_023039
A_24_P125881	0.000199	NM_005137	NM_005137	Homo sapiens DiGeorge syndrome critical region gene 2 (DGCR2), mRNA [NM_005137]	NM_005137
A_24_P919283	0.000201	A_24_P919283			
A_24_P938006	0.000201	A_24_P938006			
A_24_P123521	0.000202	NM_020666	NM_020666	Homo sapiens CDC-like kinase 4 (CLK4), mRNA [NM_020666]	NM_020666
A_23_P156156	0.000202	NM_032280	NM_032280	Homo sapiens zinc finger, CCHC domain containing 9 (ZCCHC9), mRNA [NM_032280]	NM_032280
A_23_P123228	0.000203	NM_000111	NM_000111	Homo sapiens solute carrier family 26, member 3 (SLC26A3), mRNA [NM_000111]	NM_000111
A_24_P354523	0.000203	THC2338090			
A_23_P30567	0.000203	NM_001882	NM_001882	Homo sapiens corticotropin releasing hormone binding protein (CRHBP), mRNA [NM_001882]	NM_001882
A_24_P83102	0.000204	NM_020070	NM_020070	Homo sapiens immunoglobulin lambda-like polypeptide 1 (IGLL1), transcript variant 1, mRNA [NM_020070]	NM_020070
A_23_P407695	0.000204	NM_176782	NM_176782	Homo sapiens chromosome 1 open reading frame 179 (C1orf179), mRNA [NM_176782]	NM_176782
A_23_P15450	0.000204	NM_018286	NM_018286	Homo sapiens hypothetical protein FLJ10970 (FLJ10970), mRNA [NM_018286]	NM_018286
A_32_P42149	0.000204	AA521057	AA521057	aa71e06.s1 NCI_CGAP_GCB1 Homo sapiens cDNA clone IMAGE:826402 3' similar to contains Alu repetitive element;contains element LTR5 repetitive element ;, mRNA sequence [AA521057]	
A_23_P81859	0.000204	NM_080596	NM_080596	Homo sapiens histone 1, H2ah (HIST1H2AH), mRNA [NM_080596]	NM_080596
A_24_P22050	0.000205	NM_017817	NM_017817	Homo sapiens RAB20, member RAS oncogene family (RAB20), mRNA [NM_017817]	NM_017817
A_23_P79416	0.000205	NM_025133	NM_025133	Homo sapiens F-box protein 11 (FBXO11), transcript variant 1, mRNA [NM_025133]	NM_025133
A_23_P40415	0.000206	NM_007038	NM_007038	Homo sapiens a disintegrin-like and metalloprotease (reprolysin type) with thrombospondin type 1 motif, 5 (aggrecanase-2) (ADAMTS5), mRNA [NM_007038]	NM_007038
A_23_P85140	0.000206	NM_080390	NM_080390	Homo sapiens transcription elongation factor A (SII)-like 2 (TCEAL2), mRNA [NM_080390]	NM_080390
A_24_P19054	0.000206	NM_012121	NM_012121	Homo sapiens CDC42 effector protein (Rho GTPase binding) 4 (CDC42EP4), mRNA [NM_012121]	NM_012121
A_23_P8541	0.000207	AB014518	AB014518	Homo sapiens mRNA for KIAA0618 protein, partial cds. [AB014518]	
A_23_P143484	0.000207	NM_153682	NM_153682	Homo sapiens Down syndrome critical region gene 5 (DSCR5), transcript variant 2, mRNA [NM_153682]	NM_153682
A_23_P5405	0.000208	NM_172070	NM_172070	Homo sapiens zinc finger protein 650 (ZNF650), mRNA [NM_172070]	NM_172070
A_24_P252718	0.000208	BC036529	BC036529	Homo sapiens enhancer of polycomb homolog 1 (Drosophila), mRNA (cDNA clone MGC:33656 IMAGE:4828057), complete cds. [BC036529]	
A_32_P89691	0.000208	NM_003104	NM_003104	Homo sapiens sorbitol dehydrogenase (SORD), mRNA [NM_003104]	NM_003104
A_23_P56356	0.000209	BC042674	BC042674	Homo sapiens phospholipase B1, mRNA (cDNA clone MGC:35447 IMAGE:5191712), complete cds. [BC042674]	
A_23_P5912	0.000209	NM_017798	NM_017798	Homo sapiens YTH domain family, member 1 (YTHDF1), mRNA [NM_017798]	NM_017798
A_23_P217570	0.00021	NM_014289	NM_014289	Homo sapiens calpain 6 (CAPN6), mRNA [NM_014289]	NM_014289
A_24_P274615	0.00021	NM_020801	NM_020801	Homo sapiens arrestin domain containing 3 (ARRDC3), mRNA [NM_020801]	NM_020801
A_23_P345361	0.00021	NM_145055	NM_145055	Homo sapiens chromosome 18 open reading frame 25 (C18orf25), transcript variant 1, mRNA [NM_145055]	NM_145055
A_23_P13852	0.00021	NM_007178	NM_007178	Homo sapiens serine/threonine kinase receptor associated protein (STRAP), mRNA [NM_007178]	NM_007178
A_32_P229760	0.000212	NM_001568	NM_001568	Homo sapiens eukaryotic translation initiation factor 3, subunit 6 48kDa (EIF3S6), mRNA [NM_001568]	NM_001568
A_23_P131289	0.000213	NM_024536	NM_024536	Homo sapiens chondroitin polymerizing factor (CHPF), mRNA [NM_024536]	NM_024536
A_23_P336859	0.000213	AK090467	AK090467	Homo sapiens mRNA for FLJ00388 protein. [AK090467]	
A_32_P192389	0.000214	AW844496	AW844496	AW844496 RC2-CN0051-210200-015-f06 CN0051 Homo sapiens cDNA, mRNA sequence [AW844496]	
A_32_P184417	0.000215	BE181102	BE181102	CM3-HT0629-260400-161-b05 HT0629 Homo sapiens cDNA, mRNA sequence [BE181102]	
A_32_P220078	0.000215	AK092751	AK092751	Homo sapiens cDNA FLJ35432 fis, clone SMINT2002311. [AK092751]	
A_32_P223695	0.000215	AI816119	AI816119	AI816119 au45a07.x1 Schneider fetal brain 00004 Homo sapiens cDNA clone IMAGE:2517684 3' similar to gb:M13934_cds1 40S RIBOSOMAL PROTEIN S14 (HUMAN);, mRNA sequence [AI816119]	
A_23_P128698	0.000215	NM_005842	NM_005842	Homo sapiens sprouty homolog 2 (Drosophila) (SPRY2), mRNA [NM_005842]	NM_005842
A_23_P21644	0.000215	NM_016245	NM_016245	Homo sapiens dehydrogenase/reductase (SDR family) member 8 (DHRS8), mRNA [NM_016245]	NM_016245

A_23_P133923	0.000215	NM_033177	NM_033177	Homo sapiens HLA-B associated transcript 4 (BAT4), mRNA [NM_033177]	NM_033177
A_32_P191285	0.000216	THC2319894			
A_23_P434212	0.000216	NM_177529	NM_177529	Homo sapiens sulfotransferase family, cytosolic, 1A, phenol-preferring, member 1 (SULT1A1), transcript variant 2, mRNA [NM_177529]	NM_177529
A_24_P876522	0.000216	AK022110	AK022110	Homo sapiens cDNA FLJ12048 fis, clone HEMBB1001990. [AK022110]	
A_23_P146855	0.000217	NM_001585	NM_001585	Homo sapiens chromosome 22 open reading frame 1 (C22orf1), mRNA [NM_001585]	NM_001585
A_24_P195785	0.000217	NM_033380	NM_033380	Homo sapiens collagen, type IV, alpha 5 (Alport syndrome) (COL4A5), transcript variant 2, mRNA [NM_033380]	NM_033380
A_24_P500891	0.000217	NM_013411	NM_013411	Homo sapiens adenylate kinase 2 (AK2), transcript variant AK2B, mRNA [NM_013411]	NM_013411
A_32_P105539	0.000218	THC2280145			
A_23_P29153	0.000218	NM_014433	NM_014433	Homo sapiens rhabdoid tumor deletion region gene 1 (RTDR1), mRNA [NM_014433]	NM_014433
A_32_P25050	0.000218	NM_172037	NM_172037	Homo sapiens retinol dehydrogenase 10 (all-trans) (RDH10), mRNA [NM_172037]	NM_172037
A_24_P174313	0.000219	NM_017991	NM_017991	Homo sapiens hypothetical protein FLJ10081 (FLJ10081), mRNA [NM_017991]	NM_017991
A_24_P105761	0.00022	NM_018433	NM_018433	Homo sapiens jumonji domain containing 1A (JMJD1A), mRNA [NM_018433]	NM_018433
A_32_P181103	0.00022	NM_000821	NM_000821	Homo sapiens gamma-glutamyl carboxylase (GGCX), mRNA [NM_000821]	NM_000821
A_23_P168771	0.000222	NM_020879	NM_020879	Homo sapiens KIAA1505 protein (KIAA1505), mRNA [NM_020879]	NM_020879
A_24_P30567	0.000222	NM_139015	NM_139015	Homo sapiens signal peptide peptidase 3 (SPPL3), mRNA [NM_139015]	NM_139015
A_23_P205841	0.000222	NM_006901	NM_006901	Homo sapiens myosin IXA (MYO9A), mRNA [NM_006901]	NM_006901
A_23_P81898	0.000222	NM_006398	NM_006398	Homo sapiens ubiquitin D (UBD), mRNA [NM_006398]	NM_006398
A_24_P365349	0.000222	A_24_P365349			
A_23_P28420	0.000222	NM_013341	NM_013341	Homo sapiens GTP-binding protein PTD004 (PTD004), transcript variant 1, mRNA [NM_013341]	NM_013341
A_23_P99360	0.000223	NM_213590	NM_213590	Homo sapiens ret finger protein 2 (RFP2), transcript variant 3, mRNA [NM_213590]	NM_213590
A_32_P226768	0.000224	BG028463	BG028463	602294372F1 NIH_MGC_86 Homo sapiens cDNA clone IMAGE:4389352 5', mRNA sequence [BG028463]	
A_23_P58228	0.000224	NM_017855	NM_017855	Homo sapiens APin protein (APIN), mRNA [NM_017855]	NM_017855
A_23_P501961	0.000224	NM_032107	NM_032107	Homo sapiens l(3)mbt-like (Drosophila) (L3MBTL), transcript variant II, mRNA [NM_032107]	NM_032107
A_24_P233917	0.000225	NM_014774	NM_014774	Homo sapiens KIAA0494 gene product (KIAA0494), mRNA [NM_014774]	NM_014774
A_24_P358667	0.000226	AF075112	AF075112	Homo sapiens full length insert cDNA YU76E12. [AF075112]	
A_23_P25030	0.000226	NM_003725	NM_003725	Homo sapiens hydroxysteroid (17-beta) dehydrogenase 6 (HSD17B6), mRNA [NM_003725]	NM_003725
A_23_P128050	0.000227	NM_030766	NM_030766	Homo sapiens BCL2-like 14 (apoptosis facilitator) (BCL2L14), transcript variant 2, mRNA [NM_030766]	NM_030766
A_23_P8981	0.000227	NM_000349	NM_000349	Homo sapiens steroidogenic acute regulator (STAR), nuclear gene encoding mitochondrial protein, transcript variant 1, mRNA [NM_000349]	NM_000349
A_23_P15402	0.000227	NM_133491	NM_133491	Homo sapiens spermidine/spermine N1-acetyltransferase 2 (SAT2), mRNA [NM_133491]	NM_133491
A_32_P10311	0.000228	AF305816	AF305816	Homo sapiens PRO0633 mRNA, complete cds. [AF305816]	
A_32_P60632	0.000228	BE175081	BE175081	BE175081 QV2-HT0577-250400-166-d10 HT0577 Homo sapiens cDNA, mRNA sequence [BE175081]	
A_23_P37144	0.000228	AK055367	AK055367	Homo sapiens cDNA FLJ30805 fis, clone FEBRA2001294. [AK055367]	
A_23_P55179	0.000228	NM_001661	NM_001661	Homo sapiens ADP-ribosylation factor 4-like (ARF4L), mRNA [NM_001661]	NM_001661
A_23_P251412	0.00023	NM_006998	NM_006998	Homo sapiens secretagogin, EF-hand calcium binding protein (SCGN), mRNA [NM_006998]	NM_006998
A_23_P106973	0.000232	NM_006640	NM_006640	Homo sapiens septin 9 (SEPT9), mRNA [NM_006640]	NM_006640
A_23_P250002	0.000234	NM_020771	NM_020771	Homo sapiens HECT domain and ankyrin repeat containing, E3 ubiquitin protein ligase 1 (HACE1), mRNA [NM_020771]	NM_020771
A_23_P166536	0.000234	NM_014577	NM_014577	Homo sapiens bromodomain containing 1 (BRD1), mRNA [NM_014577]	NM_014577
A_24_P637257	0.000235	BX537532	BX537532	Homo sapiens mRNA; cDNA DKFZp686L07201 (from clone DKFZp686L07201). [BX537532]	
A_24_P107941	0.000235	NM_024598	NM_024598	Homo sapiens hypothetical protein FLJ13154 (FLJ13154), mRNA [NM_024598]	NM_024598
A_23_P95790	0.000236	NM_017625	NM_017625	Homo sapiens intelectin 1 (galactofuranose binding) (ITLN1), mRNA [NM_017625]	NM_017625
A_24_P365523	0.000237	NM_021784	NM_021784	Homo sapiens forkhead box A2 (FOXA2), transcript variant 1, mRNA [NM_021784]	NM_021784
A_23_P200030	0.000238	NM_003838	NM_003838	Homo sapiens fucose-1-phosphate guanylyltransferase (FPGT), mRNA [NM_003838]	NM_003838
A_24_P57047	0.000238	NM_203486	NM_203486	Homo sapiens delta-like 3 (Drosophila) (DLL3), transcript variant 2, mRNA [NM_203486]	NM_203486
A_23_P170713	0.000238	A_23_P170713			
A_23_P5601	0.000238	NM_001381	NM_001381	Homo sapiens docking protein 1, 62kDa (downstream of tyrosine kinase 1) (DOK1), mRNA [NM_001381]	NM_001381

A_23_P321630	0.000239	NM_020810	NM_020810	Homo sapiens KIAA1393 (KIAA1393), mRNA [NM_020810]	NM_020810
A_23_P317756	0.00024	NM_202000	NM_202000	Homo sapiens SA hypertension-associated homolog (rat) (SAH), transcript variant 2, mRNA [NM_202000]	NM_202000
A_23_P203191	0.00024	NM_000039	NM_000039	Homo sapiens apolipoprotein A-I (APOA1), mRNA [NM_000039]	NM_000039
A_23_P58877	0.000241	NM_020399	NM_020399	Homo sapiens golgi associated PDZ and coiled-coil motif containing (GOPC), transcript variant 1, mRNA [NM_020399]	NM_020399
A_32_P69386	0.000242	ENST00000355251			
A_32_P159612	0.000242	NM_183377	NM_183377	Homo sapiens amiloride-sensitive cation channel 1, neuronal (degenerin) (ACCN1), transcript variant 1, mRNA [NM_183377]	NM_183377
A_23_P88351	0.000242	NM_015915	NM_015915	Homo sapiens spastic paraplegia 3A (autosomal dominant) (SPG3A), mRNA [NM_015915]	NM_015915
A_23_P99437	0.000242	NM_198219	NM_198219	Homo sapiens inhibitor of growth family, member 1 (ING1), transcript variant 1, mRNA [NM_198219]	NM_198219
A_23_P2990	0.000242	NM_001805	NM_001805	Homo sapiens CCAAT/enhancer binding protein (C/EBP), epsilon (CEBPE), mRNA [NM_001805]	NM_001805
A_23_P259328	0.000242	NM_003630	NM_003630	Homo sapiens peroxisomal biogenesis factor 3 (PEX3), mRNA [NM_003630]	NM_003630
A_23_P60579	0.000242	NM_002435	NM_002435	Homo sapiens mannose phosphate isomerase (MPI), mRNA [NM_002435]	NM_002435
A_23_P32903	0.000242	NM_000276	NM_000276	Homo sapiens oculocerebrorenal syndrome of Lowe (OCRL), transcript variant a, mRNA [NM_000276]	NM_000276
A_23_P217151	0.000244	NM_017776	NM_017776	Homo sapiens zinc finger protein 673 (ZNF673), mRNA [NM_017776]	NM_017776
A_24_P226210	0.000244	BX648687	BX648687	Homo sapiens mRNA; cDNA DKFZp686H2338 (from clone DKFZp686H2338). [BX648687]	
A_32_P168247	0.000244	NM_004373	NM_004373	Homo sapiens cytochrome c oxidase subunit VIa polypeptide 1 (COX6A1), nuclear gene encoding mitochondrial protein, mRNA [NM_004373]	NM_004373
A_32_P8604	0.000245	AK093691	AK093691	Homo sapiens cDNA FLJ36372 fis, clone THYMU2008072. [AK093691]	
A_32_P10123	0.000245	BX538256	BX538256	Homo sapiens mRNA; cDNA DKFZp686C1384 (from clone DKFZp686C1384). [BX538256]	
A_24_P174755	0.000247	NM_003060	NM_003060	Homo sapiens solute carrier family 22 (organic cation transporter), member 5 (SLC22A5), mRNA [NM_003060]	NM_003060
A_23_P344988	0.000247	NM_016513	NM_016513	Homo sapiens intestinal cell (MAK-like) kinase (ICK), transcript variant 2, mRNA [NM_016513]	NM_016513
A_24_P924957	0.000248	L32537	L32537	Homo sapiens (clone XP6G6B) mRNA, partial EST. [L32537]	
A_23_P321913	0.000249	NM_022047	NM_022047	Homo sapiens differentially expressed in FDCP 6 homolog (mouse) (DEF6), mRNA [NM_022047]	NM_022047
A_23_P74629	0.000249	NM_001030	NM_001030	Homo sapiens ribosomal protein S27 (metallopanstimulin 1) (RPS27), mRNA [NM_001030]	NM_001030
A_24_P419300	0.00025	AF370427	AF370427	Homo sapiens PP7080 mRNA, complete cds. [AF370427]	
A_23_P385322	0.000251	NM_020799	NM_020799	Homo sapiens associated molecule with the SH3 domain of STAM (AMSH) like protein (AMSH-LP), mRNA [NM_020799]	NM_020799
A_23_P79398	0.000251	NM_004633	NM_004633	Homo sapiens interleukin 1 receptor, type II (IL1R2), transcript variant 1, mRNA [NM_004633]	NM_004633
A_23_P73809	0.000251	NM_020871	NM_020871	Homo sapiens leucine-rich repeats and calponin homology (CH) domain containing 2 (LRCH2), mRNA [NM_020871]	NM_020871
A_23_P394395	0.000251	NM_020433	NM_020433	Homo sapiens junctophilin 2 (JPH2), transcript variant 1, mRNA [NM_020433]	NM_020433
A_23_P91019	0.000251	NM_003690	NM_003690	Homo sapiens protein kinase, interferon-inducible double stranded RNA dependent activator (PRKRA), mRNA [NM_003690]	NM_003690
A_23_P148519	0.000251	NM_014138	NM_014138	Homo sapiens transmembrane protein 29 (TMEM29), mRNA [NM_014138]	NM_014138
A_23_P69437	0.000251	NM_018023	NM_018023	Homo sapiens YEATS domain containing 2 (YEATS2), mRNA [NM_018023]	NM_018023
A_24_P98251	0.000252	NM_017890	NM_017890	Homo sapiens vacuolar protein sorting 13B (yeast) (VPS13B), transcript variant 5, mRNA [NM_017890]	NM_017890
A_23_P112135	0.000252	NM_014294	NM_014294	Homo sapiens translocation associated membrane protein 1 (TRAM1), mRNA [NM_014294]	NM_014294
A_32_P115663	0.000253	THC2405842			
A_32_P158892	0.000253	THC2340773			
A_23_P321959	0.000253	NM_145169	NM_145169	Homo sapiens SFT2 domain containing 1 (SFT2D1), mRNA [NM_145169]	NM_145169
A_23_P32536	0.000254	NM_053064	NM_053064	Homo sapiens guanine nucleotide binding protein (G protein), gamma 2 (GNG2), mRNA [NM_053064]	NM_053064
A_32_P149251	0.000254	NM_152686	NM_152686	Homo sapiens DnaJ (Hsp40) homolog, subfamily C, member 18 (DNAJC18), mRNA [NM_152686]	NM_152686
A_23_P51499	0.000255	NM_002725	NM_002725	Homo sapiens proline/arginine-rich end leucine-rich repeat protein (PRELP), transcript variant 1, mRNA [NM_002725]	NM_002725
A_23_P200252	0.000255	NM_032324	NM_032324	Homo sapiens chromosome 1 open reading frame 57 (C1orf57), mRNA [NM_032324]	NM_032324
A_32_P197720	0.000256	CR738137	CR738137	CR738137 Soares_testis_NHT Homo sapiens cDNA clone IMAGp971D1846 ; IMAGE:731424 5', mRNA sequence [CR738137]	
A_24_P416411	0.000256	NM_000286	NM_000286	Homo sapiens peroxisomal biogenesis factor 12 (PEX12), mRNA [NM_000286]	NM_000286
A_24_P196774	0.000256	BC014395	BC014395	Homo sapiens, clone IMAGE:3029191, mRNA. [BC014395]	
A_24_P315921	0.000257	AK093659	AK093659	Homo sapiens cDNA FLJ36340 fis, clone THYMU2006468. [AK093659]	
A_23_P162199	0.000257	NM_024779	NM_024779	Homo sapiens phosphatidylinositol-4-phosphate 5-kinase, type II, gamma (PIP5K2C), mRNA [NM_024779]	NM_024779

A_23_P127385	0.000257	NM_000256	NM_000256	Homo sapiens myosin binding protein C, cardiac (MYBPC3), mRNA [NM_000256]	NM_000256
A_24_P115621	0.000257	CR627373	CR627373	Homo sapiens mRNA; cDNA DKFZp762K067 (from clone DKFZp762K067). [CR627373]	
A_32_P11381	0.000258	THC2314403		Q7Z2R6 (Q7Z2R6) MSTP132, partial (34%) [THC2314403]	
A_23_P12292	0.000259	NM_023938	NM_023938	Homo sapiens chromosome 1 open reading frame 116 (C1orf116), mRNA [NM_023938]	NM_023938
A_23_P56659	0.000261	NM_133637	NM_133637	Homo sapiens DEAQ box polypeptide 1 (RNA-dependent ATPase) (DQX1), mRNA [NM_133637]	NM_133637
A_24_P117410	0.000261	NM_138433	NM_138433	Homo sapiens hypothetical protein BC009980 (MGC16635), mRNA [NM_138433]	NM_138433
A_23_P356554	0.000261	NM_004282	NM_004282	Homo sapiens BCL2-associated athanogene 2 (BAG2), mRNA [NM_004282]	NM_004282
A_24_P268160	0.000261	NM_178454	NM_178454	Homo sapiens hypothetical protein MGC54289 (MGC54289), mRNA [NM_178454]	NM_178454
A_24_P64918	0.000262	NM_014950	NM_014950	Homo sapiens zinc finger and BTB domain containing 1 (ZBTB1), mRNA [NM_014950]	NM_014950
A_23_P1083	0.000262	NM_002060	NM_002060	Homo sapiens gap junction protein, alpha 4, 37kDa (connexin 37) (GJA4), mRNA [NM_002060]	NM_002060
A_23_P77714	0.000263	NM_024793	NM_024793	Homo sapiens clusterin associated protein 1 (CLUAP1), transcript variant 2, mRNA [NM_024793]	NM_024793
A_32_P44932	0.000264	A_32_P44932			
A_23_P52797	0.000264	NM_030770	NM_030770	Homo sapiens transmembrane protease, serine 5 (spinesin) (TMPRSS5), mRNA [NM_030770]	NM_030770
A_23_P133058	0.000264	NM_152301	NM_152301	Homo sapiens Morf4 family associated protein 1-like 1 (MRFAP1L1), transcript variant 1, mRNA [NM_152301]	NM_152301
A_24_P333663	0.000265	NM_002748	NM_002748	Homo sapiens mitogen-activated protein kinase 6 (MAPK6), mRNA [NM_002748]	NM_002748
A_23_P387552	0.000265	NM_000966	NM_000966	Homo sapiens retinoic acid receptor, gamma (RARG), mRNA [NM_000966]	NM_000966
A_24_P418189	0.000266	XM_495875	XM_495875	PREDICTED: Homo sapiens similar to ribosomal protein S4, X-linked (LOC390183), mRNA [XM_495875]	XM_495875
A_23_P71170	0.000269	NM_018646	NM_018646	Homo sapiens transient receptor potential cation channel, subfamily V, member 6 (TRPV6), mRNA [NM_018646]	NM_018646
A_23_P358662	0.000269	ENST00000296519		Homo sapiens mRNA for KIAA1712 protein, partial cds. [AB051499]	
A_23_P156017	0.000269	NM_022130	NM_022130	Homo sapiens golgi phosphoprotein 3 (coat-protein) (GOLPH3), mRNA [NM_022130]	NM_022130
A_32_P109835	0.00027	THC2316236			
A_23_P42514	0.00027	ENST00000265423		Homo sapiens chromosome 6 open reading frame 62, mRNA (cDNA clone MGC:57512 IMAGE:6499979), complete cds. [BC047866]	
A_23_P120845	0.00027	NM_005080	NM_005080	Homo sapiens X-box binding protein 1 (XBP1), mRNA [NM_005080]	NM_005080
A_23_P164706	0.000271	NM_003451	NM_003451	Homo sapiens zinc finger protein 177 (ZNF177), mRNA [NM_003451]	NM_003451
A_24_P322771	0.000271	NM_003225	NM_003225	Homo sapiens trefoil factor 1 (breast cancer, estrogen-inducible sequence expressed in) (TFF1), mRNA [NM_003225]	NM_003225
A_32_P60459	0.000272	AB188491	AB188491	Homo sapiens mRNA, clone: TH020D07. [AB188491]	
A_23_P129829	0.000272	NM_139280	NM_139280	Homo sapiens ORM1-like 3 (S. cerevisiae) (ORMDL3), mRNA [NM_139280]	NM_139280
A_24_P6030	0.000274	NM_058182	NM_058182	Homo sapiens chromosome 21 open reading frame 51 (C21orf51), mRNA [NM_058182]	NM_058182
A_23_P383819	0.000274	NM_016569	NM_016569	Homo sapiens T-box 3 (ulnar mammary syndrome) (TBX3), transcript variant 2, mRNA [NM_016569]	NM_016569
A_23_P3994	0.000274	NM_152766	NM_152766	Homo sapiens hypothetical protein MGC40107 (MGC40107), mRNA [NM_152766]	NM_152766
A_24_P337774	0.000277	CR618686	CR618686	full-length cDNA clone CS0DC012YO16 of Neuroblastoma Cot 25-normalized of Homo sapiens (human). [CR618686]	
A_32_P156746	0.000277	AI888033	AI888033	wm27e09.x1 NCI_CGAP_Ut4 Homo sapiens cDNA clone IMAGE:2437192 3' similar to contains element PTR7 repetitive element ;, mRNA sequence [AI888033]	
A_23_P94533	0.000277	NM_001912	NM_001912	Homo sapiens cathepsin L (CTSL), transcript variant 1, mRNA [NM_001912]	NM_001912
A_32_P27041	0.000279	AI819757	AI819757	AI819757 wj11h10.x1 NCI_CGAP_Kid12 Homo sapiens cDNA clone IMAGE:2402563 3' similar to gb:A18658 INSULIN RECEPTOR PRECURSOR (HUMAN);, mRNA sequence [AI819757]	
A_23_P125579	0.000279	NM_021242	NM_021242	Homo sapiens MID1 interacting protein 1 (gastrulation specific G12-like (zebrafish)) (MID1IP1), mRNA [NM_021242]	NM_021242
A_24_P355876	0.000279	NM_003217	NM_003217	Homo sapiens testis enhanced gene transcript (BAX inhibitor 1) (TEGT), mRNA [NM_003217]	NM_003217
A_23_P315933	0.00028	NM_031295	NM_031295	Homo sapiens abhydrolase domain containing 11 (ABHD11), transcript variant 4, mRNA [NM_031295]	NM_031295
A_32_P28939	0.000281	NM_001001655	NM_001001655	Homo sapiens similar to hypothetical protein 9530023G02 (MGC90512), mRNA [NM_001001655]	NM_001001655
A_24_P175909	0.000281	NM_004990	NM_004990	Homo sapiens methionine-tRNA synthetase (MARS), mRNA [NM_004990]	NM_004990
A_23_P983	0.000281	NM_004905	NM_004905	Homo sapiens peroxiredoxin 6 (PRDX6), mRNA [NM_004905]	NM_004905
A_32_P156024	0.000282	AK127081	AK127081	Homo sapiens cDNA FLJ45138 fis, clone BRAWH3039258. [AK127081]	
A_32_P131401	0.000284	AI276257	AI276257	AI276257 ql65f01.x1 Soares_NhHMPu_S1 Homo sapiens cDNA clone IMAGE:1877209 3', mRNA sequence [AI276257]	
A_24_P237175	0.000284	NM_001322	NM_001322	Homo sapiens cystatin SA (CST2), mRNA [NM_001322]	NM_001322

A_23_P85053	0.000284	NM_005089	NM_005089	Homo sapiens U2(RNU2) small nuclear RNA auxiliary factor 1-like 2 (U2AF1L2), mRNA [NM_005089]	NM_005089
A_23_P37391	0.000284	AK093969	AK093969	Homo sapiens cDNA FLJ36650 fis, clone UTERU2000023. [AK093969]	
A_32_P71437	0.000284	THC2270648			
A_24_P46334	0.000285	NM_032547	NM_032547	Homo sapiens short coiled-coil protein (SCOC), mRNA [NM_032547]	NM_032547
A_32_P187875	0.000285	THC2306791		AA442092 zw63e06.s1 Soares_total_fetus_Nb2HF8_9w Homo sapiens cDNA clone IMAGE:774754 3', mRNA sequence [AA442092]	
A_32_P114246	0.000286	BC000226	BC000226	Homo sapiens ubiquitin specific protease 47, mRNA (cDNA clone IMAGE:3350895), complete cds. [BC000226]	
A_23_P98910	0.000287	NM_006152	NM_006152	Homo sapiens lymphoid-restricted membrane protein (LRMP), mRNA [NM_006152]	NM_006152
A_32_P62963	0.000289	ENST00000323509			
A_23_P38408	0.000289	NM_024835	NM_024835	Homo sapiens zinc finger protein 403 (ZNF403), mRNA [NM_024835]	NM_024835
A_24_P269598	0.000289	AB002390	AB002390	Human mRNA for KIAA0392 gene, partial cds. [AB002390]	
A_23_P371495	0.000291	AK125140	AK125140	Homo sapiens cDNA FLJ43150 fis, clone CTONG3009239. [AK125140]	
A_23_P166421	0.000293	NM_031937	NM_031937	Homo sapiens TBC1 domain family, member 10A (TBC1D10A), mRNA [NM_031937]	NM_031937
A_32_P43878	0.000295	THC2379275			
A_32_P12344	0.000295	BU618279	BU618279	BU618279 UI-H-FH1-bfh-m-09-0-UI.s1 NCI_CGAP_FH1 Homo sapiens cDNA clone UI-H-FH1-bfh-m-09-0-UI 3', mRNA sequence [BU618279]	
A_24_P941930	0.000296	NM_033083	NM_033083	Homo sapiens ELL associated factor 1 (EAF1), mRNA [NM_033083]	NM_033083
A_23_P401606	0.000297	NM_005711	NM_005711	Homo sapiens EGF-like repeats and discoidin I-like domains 3 (EDIL3), mRNA [NM_005711]	NM_005711
A_24_P372217	0.000297	NM_007175	NM_007175	Homo sapiens SPFH domain family, member 2 (SPFH2), transcript variant 1, mRNA [NM_007175]	NM_007175
A_32_P11372	0.000298	NM_174950	NM_174950	Homo sapiens hypothetical protein FLJ30435 (FLJ30435), mRNA [NM_174950]	NM_174950
A_24_P745883	0.000298	A_24_P745883			
A_23_P7087	0.000298	NM_052861	NM_052861	Homo sapiens hypothetical protein MGC21675 (MGC21675), mRNA [NM_052861]	NM_052861
A_24_P143758	0.000299	AK127569	AK127569	Homo sapiens cDNA FLJ45662 fis, clone CTONG2027150. [AK127569]	
A_24_P218814	0.0003	NM_002905	NM_002905	Homo sapiens retinol dehydrogenase 5 (11-cis and 9-cis) (RDH5), mRNA [NM_002905]	NM_002905
A_23_P148255	0.0003	NM_153488	NM_153488	Homo sapiens melanoma antigen family A, 2B (MAGEA2B), mRNA [NM_153488]	NM_153488
A_23_P133470	0.0003	NM_014819	NM_014819	Homo sapiens praja 2, RING-H2 motif containing (PJA2), mRNA [NM_014819]	NM_014819
A_24_P146211	0.000301	NM_021063	NM_021063	Homo sapiens histone 1, H2bd (HIST1H2BD), transcript variant 1, mRNA [NM_021063]	NM_021063
A_23_P114582	0.000301	THC2335955		Q9BWR1 (Q9BWR1) Mitochondrial ribosomal protein L37, partial (94%) [THC2335955]	
A_24_P936122	0.000301	NM_001001433	NM_001001433	Homo sapiens syntaxin 16 (STX16), transcript variant 1, mRNA [NM_001001433]	NM_001001433
A_23_P108303	0.000302	NM_005001	NM_005001	Homo sapiens NADH dehydrogenase (ubiquinone) 1 alpha subcomplex, 7, 14.5kDa (NDUFA7), mRNA [NM_005001]	NM_005001
A_24_P392991	0.000303	NM_005557	NM_005557	Homo sapiens keratin 16 (focal non-epidermolytic palmoplantar keratoderma) (KRT16), mRNA [NM_005557]	NM_005557
A_23_P79108	0.000303	NM_138813	NM_138813	Homo sapiens ATPase, Class I, type 8B, member 3 (ATP8B3), mRNA [NM_138813]	NM_138813
A_23_P113966	0.000303	NM_018261	NM_018261	Homo sapiens SEC3-like 1 (S. cerevisiae) (SEC3L1), transcript variant 1, mRNA [NM_018261]	NM_018261
A_24_P255543	0.000305	THC2261399			
A_24_P222599	0.000305	NM_002613	NM_002613	Homo sapiens 3-phosphoinositide dependent protein kinase-1 (PDPK1), mRNA [NM_002613]	NM_002613
A_23_P319572	0.000306	NM_005122	NM_005122	Homo sapiens nuclear receptor subfamily 1, group I, member 3 (NR1I3), mRNA [NM_005122]	NM_005122
A_23_P50638	0.000306	NM_052972	NM_052972	Homo sapiens leucine-rich alpha-2-glycoprotein 1 (LRG1), mRNA [NM_052972]	NM_052972
A_24_P152188	0.000306	NM_198859	NM_198859	Homo sapiens prickle-like 2 (Drosophila) (PRICKLE2), mRNA [NM_198859]	NM_198859
A_23_P217917	0.000308	NM_147148	NM_147148	Homo sapiens glutathione S-transferase M4 (GSTM4), transcript variant 2, mRNA [NM_147148]	NM_147148
A_24_P239076	0.000308	NM_001013618	NM_001013618	Homo sapiens similar to omega protein (LOC91353), mRNA [NM_001013618]	NM_001013618
A_24_P103434	0.000309	NM_018974	NM_018974	Homo sapiens unc-93 homolog A (C. elegans) (UNC93A), mRNA [NM_018974]	NM_018974
A_23_P126716	0.000309	NM_178191	NM_178191	Homo sapiens ATPase inhibitory factor 1 (ATPIF1), nuclear gene encoding mitochondrial protein, transcript variant 3, mRNA [NM_178191]	NM_178191
A_24_P643776	0.00031	THC2437143			
A_24_P188105	0.00031	D87470	D87470	Human mRNA for KIAA0280 gene, partial cds. [D87470]	
A_23_P30175	0.00031	NM_018695	NM_018695	Homo sapiens erbb2 interacting protein (ERBB2IP), transcript variant 2, mRNA [NM_018695]	NM_018695
A_24_P391526	0.00031	NM_001005333	NM_001005333	Homo sapiens melanoma antigen family D, 1 (MAGED1), transcript variant 1, mRNA [NM_001005333]	NM_001005333

A_23_P17134	0.000311	NM_002371	NM_002371	Homo sapiens mal, T-cell differentiation protein (MAL), transcript variant a, mRNA [NM_002371]	NM_002371
A_23_P36266	0.000311	NM_003477	NM_003477	Homo sapiens pyruvate dehydrogenase complex, component X (PDHX), mRNA [NM_003477]	NM_003477
A_23_P405148	0.000311	NM_020194	NM_020194	Homo sapiens chromosome 2 open reading frame 33 (C2orf33), mRNA [NM_020194]	NM_020194
A_23_P37127	0.000312	NM_004496	NM_004496	Homo sapiens forkhead box A1 (FOXA1), mRNA [NM_004496]	NM_004496
A_23_P212522	0.000312	NM_014616	NM_014616	Homo sapiens ATPase, Class VI, type 11B (ATP11B), mRNA [NM_014616]	NM_014616
A_23_P56798	0.000312	NM_177554	NM_177554	Homo sapiens acid phosphatase 1, soluble (ACP1), transcript variant 1, mRNA [NM_177554]	NM_177554
A_23_P114929	0.000312	NM_015415	NM_015415	Homo sapiens brain protein 44 (BRP44), mRNA [NM_015415]	NM_015415
A_23_P156402	0.000314	NM_003551	NM_003551	Homo sapiens non-metastatic cells 5, protein expressed in (nucleoside-diphosphate kinase) (NME5), mRNA [NM_003551]	NM_003551
A_23_P61945	0.000314	NM_198159	NM_198159	Homo sapiens microphthalmia-associated transcription factor (MITF), transcript variant 1, mRNA [NM_198159]	NM_198159
A_23_P394605	0.000316	ENST00000265341		Homo sapiens mRNA; cDNA DKFZp564I062 (from clone DKFZp564I062). [AL080064]	
A_32_P75661	0.000317	THC2309312			
A_23_P141946	0.000317	NM_003573	NM_003573	Homo sapiens latent transforming growth factor beta binding protein 4 (LTBP4), mRNA [NM_003573]	NM_003573
A_23_P395075	0.000318	NM_018433	NM_018433	Homo sapiens jumonji domain containing 1A (JMJD1A), mRNA [NM_018433]	NM_018433
A_23_P201963	0.000318	AF196185	AF196185	Homo sapiens atypical PKC isotype-specific interacting protein long variant mRNA, complete cds. [AF196185]	
A_23_P255273	0.000318	NM_002874	NM_002874	Homo sapiens RAD23 homolog B (S. cerevisiae) (RAD23B), mRNA [NM_002874]	NM_002874
A_32_P776626	0.000319	NM_001024457	NM_001024457	Homo sapiens Ran binding protein 2-like 2 (RANBP2L2), mRNA [NM_001024457]	NM_001024457
A_32_P112531	0.000319	A_32_P112531			
A_23_P205913	0.000319	NM_004727	NM_004727	Homo sapiens solute carrier family 24 (sodium/potassium/calcium exchanger), member 1 (SLC24A1), mRNA [NM_004727]	NM_004727
A_24_P335263	0.00032	NM_199040	NM_199040	Homo sapiens nudix (nucleoside diphosphate linked moiety X)-type motif 4 (NUDT4), transcript variant 2, mRNA [NM_199040]	NM_199040
A_23_P56703	0.000321	ENST00000335459		Homo sapiens hypothetical protein LOC129293, mRNA (cDNA clone IMAGE:5762496), partial cds. [BC051789]	
A_23_P61529	0.000322	NM_000404	NM_000404	Homo sapiens galactosidase, beta 1 (GLB1), transcript variant 179423, mRNA [NM_000404]	NM_000404
A_24_P245246	0.000323	CR590530	CR590530	full-length cDNA clone CS0DF027YM02 of Fetal brain of Homo sapiens (human). [CR590530]	
A_23_P169766	0.000324	THC2370681			
A_23_P166686	0.000324	NM_016201	NM_016201	Homo sapiens angiomotin like 2 (AMOTL2), mRNA [NM_016201]	NM_016201
A_24_P246406	0.000325	CR600872	CR600872	full-length cDNA clone CS0DF015YL14 of Fetal brain of Homo sapiens (human). [CR600872]	
A_23_P321320	0.000325	NM_052902	NM_052902	Homo sapiens serine/threonine kinase 11 interacting protein (STK11IP), mRNA [NM_052902]	NM_052902
A_32_P18493	0.000325	A_32_P18493			
A_23_P64980	0.000326	NM_016615	NM_016615	Homo sapiens solute carrier family 6 (neurotransmitter transporter, GABA), member 13 (SLC6A13), mRNA [NM_016615]	NM_016615
A_23_P377965	0.000326	BC010538	BC010538	Homo sapiens chromosome 18 open reading frame 18, mRNA (cDNA clone MGC:17515 IMAGE:3457488), complete cds. [BC010538]	
A_24_P40551	0.000326	BC015794	BC015794	Homo sapiens brain expressed X-linked-like 1, mRNA (cDNA clone IMAGE:3909303), partial cds. [BC015794]	XM_043653
A_24_P335901	0.000327	NM_006631	NM_006631	Homo sapiens zinc finger protein 266 (ZNF266), mRNA [NM_006631]	NM_006631
A_24_P82419	0.000328	NM_005324	NM_005324	Homo sapiens H3 histone, family 3B (H3.3B) (H3F3B), mRNA [NM_005324]	NM_005324
A_23_P412389	0.00033	NM_033649	NM_033649	Homo sapiens fibroblast growth factor 18 (FGF18), transcript variant 2, mRNA [NM_033649]	NM_033649
A_23_P44466	0.000331	NM_024781	NM_024781	Homo sapiens chromosome 18 open reading frame 14 (C18orf14), mRNA [NM_024781]	NM_024781
A_23_P166779	0.000331	NM_013343	NM_013343	Homo sapiens loss of heterozygosity, 3, chromosomal region 2, gene A (LOH3CR2A), mRNA [NM_013343]	NM_013343
A_23_P418485	0.000331	NM_152587	NM_152587	Homo sapiens hypothetical protein MGC33948 (MGC33948), mRNA [NM_152587]	NM_152587
A_24_P527404	0.000331	NM_004329	NM_004329	Homo sapiens bone morphogenetic protein receptor, type IA (BMPR1A), mRNA [NM_004329]	NM_004329
A_23_P215132	0.000331	NM_014149	NM_014149	Homo sapiens HSPC049 protein (HSPC049), mRNA [NM_014149]	NM_014149
A_23_P56654	0.000331	NM_032601	NM_032601	Homo sapiens methylmalonyl CoA epimerase (MCEE), mRNA [NM_032601]	NM_032601
A_23_P207125	0.000331	NM_020795	NM_020795	Homo sapiens neuroligin 2 (NLGN2), mRNA [NM_020795]	NM_020795
A_24_P919899	0.000332	AL137294	AL137294	Homo sapiens mRNA; cDNA DKFZp434L0816 (from clone DKFZp434L0816); partial cds. [AL137294]	
A_32_P137266	0.000332	NM_032437	NM_032437	Homo sapiens KIAA1799 protein (KIAA1799), mRNA [NM_032437]	NM_032437
A_23_P122531	0.000332	NM_016947	NM_016947	Homo sapiens chromosome 6 open reading frame 48 (C6orf48), mRNA [NM_016947]	NM_016947
A_24_P35400	0.000333	NM_007101	NM_007101	Homo sapiens sarcosine dehydrogenase (SARDH), mRNA [NM_007101]	NM_007101

A_23_P258381	0.000333	NM_080862	NM_080862	Homo sapiens SPRY domain-containing SOCS box protein SSB-4 (SSB4), mRNA [NM_080862]	NM_080862
A_23_P136460	0.000335	NM_016603	NM_016603	Homo sapiens chromosome 5 open reading frame 5 (C5orf5), mRNA [NM_016603]	NM_016603
A_24_P367109	0.000335	A_24_P367109			
A_23_P121564	0.000337	NM_000857	NM_000857	Homo sapiens guanylate cyclase 1, soluble, beta 3 (GUCY1B3), mRNA [NM_000857]	NM_000857
A_23_P99811	0.000337	NM_138476	NM_138476	Homo sapiens hypothetical protein MGC5987 (MGC5987), mRNA [NM_138476]	NM_138476
A_23_P140088	0.000338	NM_024570	NM_024570	Homo sapiens hypothetical protein FLJ11712 (FLJ11712), mRNA [NM_024570]	NM_024570
A_23_P99917	0.000338	NM_032856	NM_032856	Homo sapiens WD repeat domain 73 (WDR73), mRNA [NM_032856]	NM_032856
A_24_P737939	0.00034	NM_001012974	NM_001012974	Homo sapiens chromosome 6 open reading frame 154 (C6orf154), mRNA [NM_001012974]	NM_001012974
A_24_P325992	0.000343	NM_002310	NM_002310	Homo sapiens leukemia inhibitory factor receptor (LIFR), mRNA [NM_002310]	NM_002310
A_23_P92824	0.000344	NM_031482	NM_031482	Homo sapiens APG10 autophagy 10-like (S. cerevisiae) (APG10L), mRNA [NM_031482]	NM_031482
A_24_P494230	0.000344	AK123750	AK123750	Homo sapiens cDNA FLJ41756 fis, clone IMR322002035. [AK123750]	XM_499027
A_23_P74852	0.000345	AL035301	AL035301	H.sapiens gene from PAC 106H8. [AL035301]	
A_23_P400378	0.000345	NM_170699	NM_170699	Homo sapiens G protein-coupled bile acid receptor 1 (GPBAR1), mRNA [NM_170699]	NM_170699
A_23_P214425	0.000345	AK025201	AK025201	Homo sapiens cDNA: FLJ21548 fis, clone COL06252. [AK025201]	
A_24_P14010	0.000345	NM_021079	NM_021079	Homo sapiens N-myristoyltransferase 1 (NMT1), mRNA [NM_021079]	NM_021079
A_24_P274640	0.000346	NM_001515	NM_001515	Homo sapiens general transcription factor IIH, polypeptide 2, 44kDa (GTF2H2), mRNA [NM_001515]	NM_001515
A_23_P114616	0.00035	ENST00000262346		Homo sapiens mRNA; cDNA DKFZp566D1346 (from clone DKFZp566D1346). [AL136717]	
A_24_P113341	0.00035	NM_031448	NM_031448	Homo sapiens chromosome 19 open reading frame 12 (C19orf12), mRNA [NM_031448]	NM_031448
A_23_P39237	0.000351	NM_003407	NM_003407	Homo sapiens zinc finger protein 36, C3H type, homolog (mouse) (ZFP36), mRNA [NM_003407]	NM_003407
A_23_P31097	0.000351	NM_014028	NM_014028	Homo sapiens osteopetrosis associated transmembrane protein 1 (OSTM1), mRNA [NM_014028]	NM_014028
A_23_P60259	0.000352	NM_018112	NM_018112	Homo sapiens transmembrane protein 38B (TMEM38B), mRNA [NM_018112]	NM_018112
A_23_P397120	0.000353	ENST00000211092		Homo sapiens HSPC323 mRNA, partial cds. [AF161441]	XM_209196
A_23_P53530	0.000353	NM_025198	NM_025198	Homo sapiens MTERF domain containing 3 (MTERFD3), mRNA [NM_025198]	NM_025198
A_23_P32861	0.000354	NM_015938	NM_015938	Homo sapiens NMD3 homolog (S. cerevisiae) (NMD3), mRNA [NM_015938]	NM_015938
A_24_P879563	0.000354	BC045818	BC045818	Homo sapiens hypothetical protein LOC220906, mRNA (cDNA clone IMAGE:4837455), partial cds. [BC045818]	XM_374781
A_23_P114952	0.000355	NM_016456	NM_016456	Homo sapiens transmembrane protein 9 (TMEM9), mRNA [NM_016456]	NM_016456
A_24_P379820	0.000356	NM_030926	NM_030926	Homo sapiens integral membrane protein 2C (ITM2C), transcript variant 1, mRNA [NM_030926]	NM_030926
A_23_P212258	0.000357	NM_000893	NM_000893	Homo sapiens kininogen 1 (KNG1), mRNA [NM_000893]	NM_000893
A_24_P391568	0.000357	NM_001668	NM_001668	Homo sapiens aryl hydrocarbon receptor nuclear translocator (ARNT), transcript variant 1, mRNA [NM_001668]	NM_001668
A_24_P385012	0.000357	BC030084	BC030084	Homo sapiens cDNA clone IMAGE:4791887, partial cds. [BC030084]	
A_24_P506726	0.000357	AK022936	AK022936	Homo sapiens cDNA FLJ12874 fis, clone NT2RP2003769. [AK022936]	
A_23_P21409	0.000358	NM_025180	NM_025180	Homo sapiens centrosome protein Cep63 (Cep63), mRNA [NM_025180]	NM_025180
A_24_P387869	0.00036	BC029749	BC029749	Homo sapiens, clone IMAGE:4513287, mRNA, partial cds. [BC029749]	
A_32_P92505	0.00036	NM_182551	NM_182551	Homo sapiens lysocardiolipin acyltransferase (LYCAT), transcript variant 1, mRNA [NM_182551]	NM_182551
A_24_P359267	0.000361	ENST00000358335		Homo sapiens, clone IMAGE:3902512, mRNA, partial cds. [BC022779]	
A_23_P133236	0.000362	NM_018934	NM_018934	Homo sapiens protocadherin beta 14 (PCDHB14), mRNA [NM_018934]	NM_018934
A_32_P222383	0.000363	NM_005517	NM_005517	Homo sapiens high-mobility group nucleosomal binding domain 2 (HMGN2), mRNA [NM_005517]	NM_005517
A_23_P365119	0.000365	NM_152675	NM_152675	Homo sapiens hypothetical protein FLJ23754 (FLJ23754), mRNA [NM_152675]	NM_152675
A_23_P82886	0.000366	NM_001926	NM_001926	Homo sapiens defensin, alpha 6, Paneth cell-specific (DEFA6), mRNA [NM_001926]	NM_001926
A_24_P394569	0.000367	AK056855	AK056855	Homo sapiens cDNA FLJ32293 fis, clone PROST2001739. [AK056855]	XM_374902
A_23_P32021	0.000368	NM_000136	NM_000136	Homo sapiens Fanconi anemia, complementation group C (FANCC), mRNA [NM_000136]	NM_000136
A_24_P123720	0.000369	A_24_P123720			
A_32_P201107	0.000369	AI650739	AI650739	AI650739 wa19b02.x1 NCI_CGAP_Kid11 Homo sapiens cDNA clone IMAGE:2298507 3', mRNA sequence [AI650739]	
A_24_P337592	0.00037	NM_181531	NM_181531	Homo sapiens butyrophilin, subfamily 2, member A2 (BTN2A2), transcript variant 2, mRNA [NM_181531]	NM_181531

A_23_P424305	0.00037	AK097877	AK097877	Homo sapiens cDNA FLJ40558 fis, clone THYMU2002756. [AK097877]	
A_32_P69333	0.000371	BX956036	BX956036	DKFZp781J1775_r1 781 (synonym: hlcc4) Homo sapiens cDNA clone DKFZp781J1775 5', mRNA sequence [BX956036]	XM_373489
A_23_P314202	0.000372	NM_173797	NM_173797	Homo sapiens PAP associated domain containing 4 (PAPD4), mRNA [NM_173797]	NM_173797
A_24_P132008	0.000373	NM_002449	NM_002449	Homo sapiens msh homeo box homolog 2 (Drosophila) (MSX2), mRNA [NM_002449]	NM_002449
A_32_P223292	0.000373	A_32_P223292			
A_24_P264909	0.000373	NM_019080	NM_019080	Homo sapiens Nedd4 family interacting protein 2 (NDFIP2), mRNA [NM_019080]	NM_019080
A_32_P69653	0.000374	A_32_P69653			
A_24_P29445	0.000374	NM_030969	NM_030969	Homo sapiens transmembrane protein 14B (TMEM14B), mRNA [NM_030969]	NM_030969
A_24_P50753	0.000375	NM_199040	NM_199040	Homo sapiens nudix (nucleoside diphosphate linked moiety X)-type motif 4 (NUDT4), transcript variant 2, mRNA [NM_199040]	NM_199040
A_32_P147603	0.000376	A_32_P147603			
A_23_P114275	0.000376	NM_006667	NM_006667	Homo sapiens progesterone receptor membrane component 1 (PGRMC1), mRNA [NM_006667]	NM_006667
A_23_P128817	0.000376	NM_004563	NM_004563	Homo sapiens phosphoenolpyruvate carboxykinase 2 (mitochondrial) (PCK2), nuclear gene encoding mitochondrial protein, transcript variant 1, mRNA [NM_004563]	NM_004563
A_32_P163306	0.000377	AK095603	AK095603	Homo sapiens cDNA FLJ38284 fis, clone FCBBF3007152. [AK095603]	
A_24_P225679	0.000377	NM_005544	NM_005544	Homo sapiens insulin receptor substrate 1 (IRS1), mRNA [NM_005544]	NM_005544
A_32_P170406	0.000378	NM_015059	NM_015059	Homo sapiens talin 2 (TLN2), mRNA [NM_015059]	NM_015059
A_24_P382319	0.000378	NM_001712	NM_001712	Homo sapiens carcinoembryonic antigen-related cell adhesion molecule 1 (biliary glycoprotein) (CEACAM1), transcript variant 1, mRNA [NM_001712]	NM_001712
A_32_P96043	0.000379	THC2278239			
A_23_P342688	0.000381	NM_032874	NM_032874	Homo sapiens KIAA1984 (KIAA1984), mRNA [NM_032874]	NM_032874
A_32_P951	0.000382	NM_172006	NM_172006	Homo sapiens WAP four-disulfide core domain 10B (WFDC10B), transcript variant 1, mRNA [NM_172006]	NM_172006
A_23_P86706	0.000382	BC048281	BC048281	Homo sapiens zinc finger, RAN-binding domain containing 1, mRNA (cDNA clone IMAGE:5188569), complete cds. [BC048281]	
A_32_P196072	0.000383	NM_019080	NM_019080	Homo sapiens Nedd4 family interacting protein 2 (NDFIP2), mRNA [NM_019080]	NM_019080
A_32_P126846	0.000384	AI435603	AI435603	AI435603 th79c11.x1 Soares_NhHMPu_S1 Homo sapiens cDNA clone IMAGE:2124884 3', mRNA sequence [AI435603]	
A_23_P39647	0.000384	NM_005070	NM_005070	Homo sapiens solute carrier family 4, anion exchanger, member 3 (SLC4A3), transcript variant 1, mRNA [NM_005070]	NM_005070
A_23_P328545	0.000385	NM_014211	NM_014211	Homo sapiens gamma-aminobutyric acid (GABA) A receptor, pi (GABRP), mRNA [NM_014211]	NM_014211
A_23_P30956	0.000385	NM_015323	NM_015323	Homo sapiens KIAA0776 (KIAA0776), mRNA [NM_015323]	NM_015323
A_24_P262201	0.000386	NM_001017389	NM_001017389	Homo sapiens sulfotransferase family, cytosolic, 1A, phenol-preferring, member 4 (SULT1A4), transcript variant 1, mRNA [NM_001017389]	NM_001017389
A_32_P200537	0.000387	XM_371411	XM_371411	PREDICTED: Homo sapiens similar to GNGT1 protein (LOC388819), mRNA [XM_371411]	XM_371411
A_24_P341538	0.00039	NM_201286	NM_201286	Homo sapiens ubiquitin specific protease 51 (USP51), mRNA [NM_201286]	NM_201286
A_23_P424513	0.00039	NM_005493	NM_005493	Homo sapiens RAN binding protein 9 (RANBP9), mRNA [NM_005493]	NM_005493
A_23_P422732	0.000391	NM_145172	NM_145172	Homo sapiens WD repeat domain 63 (WDR63), mRNA [NM_145172]	NM_145172
A_32_P57775	0.000395	CA313037	CA313037	CA313037 UI-CF-FN0-aex-g-14-0-UI.s1 UI-CF-FN0 Homo sapiens cDNA clone UI-CF-FN0-aex-g-14-0-UI 3', mRNA sequence [CA313037]	
A_23_P29185	0.000397	AB028966	AB028966	Homo sapiens mRNA for KIAA1043 protein, partial cds. [AB028966]	
A_23_P42507	0.000399	NM_139126	NM_139126	Homo sapiens peptidylprolyl isomerase (cyclophilin)-like 4 (PPIL4), mRNA [NM_139126]	NM_139126
A_32_P213103	0.000399	CA414006	CA414006	CA414006 UI-H-EZ0-ban-f-19-0-UI.s1 NCI_CGAP_Ch1 Homo sapiens cDNA clone UI-H-EZ0-ban-f-19-0-UI 3', mRNA sequence [CA414006]	
A_24_P133253	0.000399	NM_000899	NM_000899	Homo sapiens KIT ligand (KITLG), transcript variant b, mRNA [NM_000899]	NM_000899
A_32_P119154	0.0004	AI345640	AI345640	AI345640 tb83h08.x1 NCI_CGAP_Lu26 Homo sapiens cDNA clone IMAGE:2060991 3', mRNA sequence [AI345640]	
A_32_P101334	0.000402	AK130366	AK130366	Homo sapiens cDNA FLJ26856 fis, clone PRS08123. [AK130366]	
A_24_P278234	0.000403	AB075864	AB075864	Homo sapiens mRNA for KIAA1984 protein. [AB075864]	
A_24_P151032	0.000403	NM_002476	NM_002476	Homo sapiens myosin, light polypeptide 4, alkali; atrial, embryonic (MYL4), transcript variant 2, mRNA [NM_002476]	NM_002476
A_23_P213431	0.000403	NM_032380	NM_032380	Homo sapiens G elongation factor, mitochondrial 2 (GFM2), nuclear gene encoding mitochondrial protein, transcript variant 1, mRNA [NM_032380]	NM_032380
A_24_P52697	0.000403	NR_002196	NR_002196	Homo sapiens H19, imprinted maternally expressed untranslated mRNA (H19) on chromosome 11 [NR_002196]	NR_002196
A_24_P41391	0.000404	ENST00000344111		Homo sapiens cDNA FLJ13123 fis, clone NT2RP3002763. [AK023185]	
A_23_P51729	0.000404	NM_020435	NM_020435	Homo sapiens gap junction protein, alpha 12, 47kDa (GJA12), mRNA [NM_020435]	NM_020435
A_23_P8522	0.000404	NM_018374	NM_018374	Homo sapiens hypothetical protein FLJ11273 (FLJ11273), mRNA [NM_018374]	NM_018374

A_23_P26024	0.000406	NM_032413	NM_032413	Homo sapiens normal mucosa of esophagus specific 1 (NMES1), transcript variant 2, mRNA [NM_032413]	NM_032413
A_24_P941643	0.000406	NM_182734	NM_182734	Homo sapiens phospholipase C, beta 1 (phosphoinositide-specific) (PLCB1), transcript variant 2, mRNA [NM_182734]	NM_182734
A_24_P917123	0.000407	NM_013262	NM_013262	Homo sapiens myosin regulatory light chain interacting protein (MYLIP), mRNA [NM_013262]	NM_013262
A_23_P411881	0.000408	NM_006346	NM_006346	Homo sapiens chromosome 13 open reading frame 24 (C13orf24), mRNA [NM_006346]	NM_006346
A_24_P861009	0.000411	NM_001007246	NM_001007246	Homo sapiens bromodomain and WD repeat domain containing 1 (BRWD1), transcript variant 3, mRNA [NM_001007246]	NM_001007246
A_23_P48029	0.000413	NM_016184	NM_016184	Homo sapiens C-type lectin domain family 4, member A (CLEC4A), transcript variant 1, mRNA [NM_016184]	NM_016184
A_23_P385938	0.000413	NM_175854	NM_175854	Homo sapiens PABP1-dependent poly A-specific ribonuclease subunit PAN3 (PAN3), mRNA [NM_175854]	NM_175854
A_32_P36046	0.000414	THC2363557			
A_23_P165214	0.000416	NM_021047	NM_021047	Homo sapiens zinc finger protein 253 (ZNF253), mRNA [NM_021047]	NM_021047
A_24_P276791	0.000416	NM_052940	NM_052940	Homo sapiens leucine rich repeat containing 42 (LRRC42), mRNA [NM_052940]	NM_052940
A_24_P290153	0.000417	NM_000495	NM_000495	Homo sapiens collagen, type IV, alpha 5 (Alport syndrome) (COL4A5), transcript variant 1, mRNA [NM_000495]	NM_000495
A_23_P213137	0.000418	NM_032622	NM_032622	Homo sapiens ligand of numb-protein X (LNX), mRNA [NM_032622]	NM_032622
A_23_P62857	0.000422	A_23_P62857			
A_23_P202275	0.000424	NM_145202	NM_145202	Homo sapiens proline-rich acidic protein 1 (PRAP1), mRNA [NM_145202]	NM_145202
A_24_P354849	0.000424	THC2403680			
A_32_P26721	0.000424	A_32_P26721			
A_23_P502882	0.000424	NM_175864	NM_175864	Homo sapiens core-binding factor, runt domain, alpha subunit 2; translocated to, 2 (CBFA2T2), transcript variant 1, mRNA [NM_175864]	NM_175864
A_24_P839239	0.000424	AK002036	AK002036	Homo sapiens cDNA FLJ11174 fis, clone PLACE1007367. [AK002036]	
A_32_P76992	0.000424	BC036424	BC036424	Homo sapiens, clone IMAGE:4798730, mRNA. [BC036424]	
A_23_P112531	0.000425	NM_203305	NM_203305	Homo sapiens chromosome 9 open reading frame 132 (C9orf132), mRNA [NM_203305]	NM_203305
A_32_P15288	0.000427	THC2285720		BX114329 BX114329 Soares_NhHMPu_S1 Homo sapiens cDNA clone IMAGp998G064741 ; IMAGE:1932317, mRNA sequence [BX114329]	
A_24_P892402	0.000427	AK057652	AK057652	Homo sapiens cDNA FLJ33090 fis, clone TRACH2000559. [AK057652]	
A_24_P205025	0.000428	AK096925	AK096925	Homo sapiens cDNA FLJ39606 fis, clone SKNSH2006234. [AK096925]	
A_23_P122174	0.000428	NM_022550	NM_022550	Homo sapiens X-ray repair complementing defective repair in Chinese hamster cells 4 (XRCC4), transcript variant 3, mRNA [NM_022550]	NM_022550
A_24_P84279	0.000428	NM_032565	NM_032565	Homo sapiens emopamil binding protein-like (EBPL), mRNA [NM_032565]	NM_032565
A_23_P84118	0.000429	NM_004934	NM_004934	Homo sapiens cadherin 18, type 2 (CDH18), mRNA [NM_004934]	NM_004934
A_23_P2601	0.000429	NM_003299	NM_003299	Homo sapiens tumor rejection antigen (gp96) 1 (TRA1), mRNA [NM_003299]	NM_003299
A_23_P141992	0.00043	NM_198706	NM_198706	Homo sapiens short-chain dehydrogenase/reductase 10 (SCDR10), transcript variant b, mRNA [NM_198706]	NM_198706
A_23_P433188	0.000432	NM_022482	NM_022482	Homo sapiens zinc finger protein 336 (ZNF336), mRNA [NM_022482]	NM_022482
A_23_P73835	0.000432	NM_019556	NM_019556	Homo sapiens motile sperm domain containing 1 (MOSPD1), mRNA [NM_019556]	NM_019556
A_32_P53107	0.000433	CR609342	CR609342	full-length cDNA clone CS0DA002YO22 of Neuroblastoma of Homo sapiens (human). [CR609342]	
A_24_P926432	0.000433	AF233453	AF233453	Homo sapiens RACK-like protein PRKCBP1 (PRKCBP1) mRNA, complete cds. [AF233453]	
A_32_P11450	0.000434	NM_014753	NM_014753	Homo sapiens BMS1-like, ribosome assembly protein (yeast) (BMS1L), mRNA [NM_014753]	NM_014753
A_23_P138665	0.000435	NM_005271	NM_005271	Homo sapiens glutamate dehydrogenase 1 (GLUD1), mRNA [NM_005271]	NM_005271
A_24_P128880	0.000436	BC048298	BC048298	Homo sapiens transducin (beta)-like 1X-linked receptor 1, mRNA (cDNA clone IMAGE:5527421), complete cds. [BC048298]	
A_23_P22625	0.00044	NM_006359	NM_006359	Homo sapiens solute carrier family 9 (sodium/hydrogen exchanger), isoform 6 (SLC9A6), mRNA [NM_006359]	NM_006359
A_23_P112481	0.000442	NM_004925	NM_004925	Homo sapiens aquaporin 3 (AQP3), mRNA [NM_004925]	NM_004925
A_32_P190737	0.000444	AK057981	AK057981	Homo sapiens cDNA FLJ25252 fis, clone STM03814. [AK057981]	
A_23_P82206	0.000445	NM_018479	NM_018479	Homo sapiens enoyl Coenzyme A hydratase domain containing 1 (ECHDC1), transcript variant 2, mRNA [NM_018479]	NM_018479
A_23_P90710	0.000445	NM_017569	NM_017569	Homo sapiens family with sequence similarity 48, member A (FAM48A), transcript variant 2, mRNA [NM_017569]	NM_017569
A_23_P68866	0.000448	NM_001003684	NM_001003684	Homo sapiens ubiquinol-cytochrome c reductase complex (7.2 kD) (UCRC), transcript variant 2, mRNA [NM_001003684]	NM_001003684
A_23_P305120	0.000449	NM_144994	NM_144994	Homo sapiens ankyrin repeat domain 23 (ANKRD23), mRNA [NM_144994]	NM_144994
A_23_P418083	0.000449	NM_181714	NM_181714	Homo sapiens chromosome 6 open reading frame 152 (C6orf152), mRNA [NM_181714]	NM_181714
A_24_P345837	0.00045	NM_002448	NM_002448	Homo sapiens msh homeo box homolog 1 (Drosophila) (MSX1), mRNA [NM_002448]	NM_002448

A_24_P158421	0.00045	NM_020150	NM_020150	Homo sapiens SAR1a gene homolog 1 (S. cerevisiae) (SARA1), mRNA [NM_020150]	NM_020150
A_32_P177750	0.000451	NM_018571	NM_018571	Homo sapiens amyotrophic lateral sclerosis 2 (juvenile) chromosome region, candidate 2 (ALS2CR2), mRNA [NM_018571]	NM_018571
A_24_P940815	0.000451	NM_003901	NM_003901	Homo sapiens sphingosine-1-phosphate lyase 1 (SGPL1), mRNA [NM_003901]	NM_003901
A_24_P917026	0.000452	AF055023	AF055023	Homo sapiens clone 24723 mRNA sequence. [AF055023]	
A_32_P150876	0.000452	BC041875	BC041875	Homo sapiens hypothetical gene supported by BC041875; BX648984, mRNA (cDNA clone IMAGE:5271951). [BC041875]	XM_378735
A_23_P417261	0.000454	NM_144715	NM_144715	Homo sapiens EF-hand domain family, member B (EFHB), mRNA [NM_144715]	NM_144715
A_32_P29507	0.000455	NM_005517	NM_005517	Homo sapiens high-mobility group nucleosomal binding domain 2 (HMGN2), mRNA [NM_005517]	NM_005517
A_24_P147910	0.000456	NM_006640	NM_006640	Homo sapiens septin 9 (SEPT9), mRNA [NM_006640]	NM_006640
A_23_P116235	0.000456	NM_001012334	NM_001012334	Homo sapiens midkine (neurite growth-promoting factor 2) (MDK), transcript variant 1, mRNA [NM_001012334]	NM_001012334
A_23_P140725	0.000457	NM_014714	NM_014714	Homo sapiens WD and tetratricopeptide repeats 2 (WDTC2), mRNA [NM_014714]	NM_014714
A_23_P393607	0.000457	NM_053052	NM_053052	Homo sapiens chromosome 1 open reading frame 142 (C1orf142), mRNA [NM_053052]	NM_053052
A_24_P156049	0.000458	NM_012319	NM_012319	Homo sapiens solute carrier family 39 (zinc transporter), member 6 (SLC39A6), mRNA [NM_012319]	NM_012319
A_32_P129968	0.000459	CR936662	CR936662	Homo sapiens mRNA; cDNA DKFZp781F1775 (from clone DKFZp781F1775). [CR936662]	
A_23_P81973	0.000459	NM_014234	NM_014234	Homo sapiens hydroxysteroid (17-beta) dehydrogenase 8 (HSD17B8), mRNA [NM_014234]	NM_014234
A_23_P49816	0.00046	NM_018404	NM_018404	Homo sapiens centaurin, alpha 2 (CENTA2), mRNA [NM_018404]	NM_018404
A_23_P126613	0.000461	NM_080429	NM_080429	Homo sapiens aquaporin 10 (AQP10), mRNA [NM_080429]	NM_080429
A_23_P20882	0.000461	NM_004888	NM_004888	Homo sapiens ATPase, H+ transporting, lysosomal 13kDa, V1 subunit G isoform 1 (ATP6V1G1), mRNA [NM_004888]	NM_004888
A_23_P124177	0.000464	A_23_P124177			
A_24_P923011	0.000465	NM_000301	NM_000301	Homo sapiens plasminogen (PLG), mRNA [NM_000301]	NM_000301
A_23_P374250	0.000466	NM_173812	NM_173812	Homo sapiens dpy-19-like 2 (C. elegans) (DPY19L2), mRNA [NM_173812]	NM_173812
A_24_P79529	0.000467	NM_203311	NM_203311	Homo sapiens CSAG family, member 3A (CSAG3A), mRNA [NM_203311]	NM_203311
A_23_P157365	0.000467	NM_014888	NM_014888	Homo sapiens family with sequence similarity 3, member C (FAM3C), mRNA [NM_014888]	NM_014888
A_32_P206208	0.000468	A_32_P206208			
A_23_P33326	0.000468	THC2290198		O91332 (O91332) EBNA-1, partial (4%) [THC2290198]	
A_23_P205611	0.000473	NM_004124	NM_004124	Homo sapiens glia maturation factor, beta (GMFB), mRNA [NM_004124]	NM_004124
A_32_P49508	0.000478	NM_019036	NM_019036	Homo sapiens 3-hydroxymethyl-3-methylglutaryl-Coenzyme A lyase-like 1 (HMGCLL1), mRNA [NM_019036]	NM_019036
A_23_P157669	0.000479	NM_005005	NM_005005	Homo sapiens NADH dehydrogenase (ubiquinone) 1 beta subcomplex, 9, 22kDa (NDUFB9), mRNA [NM_005005]	NM_005005
A_32_P50670	0.00048	BQ188770	BQ188770	UI-E-EJ1-ajx-c-22-0-UI.r1 UI-E-EJ1 Homo sapiens cDNA clone UI-E-EJ1-ajx-c-22-0-UI 5', mRNA sequence [BQ188770]	
A_32_P3317	0.000481	AK054572	AK054572	Homo sapiens cDNA FLJ30010 fis, clone 3NB692000154. [AK054572]	
A_24_P207150	0.000482	NM_130839	NM_130839	Homo sapiens ubiquitin protein ligase E3A (human papilloma virus E6-associated protein, Angelman syndrome) (UBE3A), transcript variant 3, mRNA [NM_130839]	NM_130839
A_24_P917833	0.000482	NM_006827	NM_006827	Homo sapiens transmembrane trafficking protein (TMP21), mRNA [NM_006827]	NM_006827
A_24_P282363	0.000483	NM_032529	NM_032529	Homo sapiens hypothetical protein KIAA1875 (KIAA1875), mRNA [NM_032529]	NM_032529
A_23_P59613	0.000483	NM_003508	NM_003508	Homo sapiens frizzled homolog 9 (Drosophila) (FZD9), mRNA [NM_003508]	NM_003508
A_23_P161998	0.000484	NM_000613	NM_000613	Homo sapiens hemopexin (HPX), mRNA [NM_000613]	NM_000613
A_23_P257198	0.000484	NM_002495	NM_002495	Homo sapiens NADH dehydrogenase (ubiquinone) Fe-S protein 4, 18kDa (NADH-coenzyme Q reductase) (NDUFS4), mRNA [NM_002495]	NM_002495
A_32_P155043	0.000487	A_32_P155043			
A_24_P333484	0.000488	NM_019008	NM_019008	Homo sapiens hypothetical protein FLJ20232 (FLJ20232), mRNA [NM_019008]	NM_019008
A_23_P25003	0.000488	NM_002956	NM_002956	Homo sapiens restin (Reed-Steinberg cell-expressed intermediate filament-associated protein) (RSN), transcript variant 1, mRNA [NM_002956]	NM_002956
A_23_P161386	0.000489	NM_003061	NM_003061	Homo sapiens slit homolog 1 (Drosophila) (SLIT1), mRNA [NM_003061]	NM_003061
A_23_P45821	0.00049	THC2281747		Q96KY5 (Q96KY5) MGC27169 protein, complete [THC2281747]	
A_32_P41099	0.000492	THC2445411		Q5W952 (Q5W952) NADH dehydrogenase subunit 1 (Fragment), partial (6%) [THC2445411]	
A_23_P101972	0.000492	NM_144575	NM_144575	Homo sapiens calpain 13 (CAPN13), mRNA [NM_144575]	NM_144575
A_23_P207564	0.000494	NM_002984	NM_002984	Homo sapiens chemokine (C-C motif) ligand 4 (CCL4), mRNA [NM_002984]	NM_002984
A_32_P217853	0.000495	THC2281898		Q6VE89 (Q6VE89) VirB1, partial (7%) [THC2281898]	
A_32_P22637	0.000497	THC2280227			

A_24_P130363	0.000499	NM_181482	NM_181482	Homo sapiens chromosome 18 open reading frame 1 (C18orf1), transcript variant a2, mRNA [NM_181482]	NM_181482
A_23_P347194	0.0005	ENST00000295907		Homo sapiens cDNA clone IMAGE:4554992, partial cds. [BC012363]	
A_23_P75811	0.000502	NM_002394	NM_002394	Homo sapiens solute carrier family 3 (activators of dibasic and neutral amino acid transport), member 2 (SLC3A2), transcript variant 3, mRNA [NM_002394]	NM_002394
A_23_P362893	0.000505	NM_021961	NM_021961	Homo sapiens TEA domain family member 1 (SV40 transcriptional enhancer factor) (TEAD1), mRNA [NM_021961]	NM_021961
A_23_P501372	0.000506	NM_139162	NM_139162	Homo sapiens Smith-Magenis syndrome chromosome region, candidate 7 (SMCR7), transcript variant 1, mRNA [NM_139162]	NM_139162
A_24_P244916	0.000507	NM_001018108	NM_001018108	Homo sapiens small EDRK-rich factor 2 (SERF2), mRNA [NM_001018108]	NM_001018108
A_23_P216869	0.000508	NM_197977	NM_197977	Homo sapiens zinc finger protein 189 (ZNF189), mRNA [NM_197977]	NM_197977
A_24_P151622	0.000513	NM_032549	NM_032549	Homo sapiens IMP2 inner mitochondrial membrane protease-like (S. cerevisiae) (IMMP2L), mRNA [NM_032549]	NM_032549
A_24_P315594	0.000514	A_24_P315594			
A_32_P89755	0.000516	NM_003144	NM_003144	Homo sapiens signal sequence receptor, alpha (translocon-associated protein alpha) (SSR1), mRNA [NM_003144]	NM_003144
A_32_P74243	0.000519	BF366211	BF366211	BF366211 IL2-NT0099-300500-096-H08 NT0099 Homo sapiens cDNA, mRNA sequence [BF366211]	
A_23_P142125	0.000519	NM_002152	NM_002152	Homo sapiens histidine rich calcium binding protein (HRC), mRNA [NM_002152]	NM_002152
A_23_P319792	0.000521	NM_019001	NM_019001	Homo sapiens 5'-3' exoribonuclease 1 (XRN1), mRNA [NM_019001]	NM_019001
A_24_P263937	0.000521	NM_199342	NM_199342	Homo sapiens coiled-coil domain containing 23 (CCDC23), mRNA [NM_199342]	NM_199342
A_23_P250607	0.000521	NM_005032	NM_005032	Homo sapiens plastin 3 (T isoform) (PLS3), mRNA [NM_005032]	NM_005032
A_24_P194260	0.000522	CR595063	CR595063	full-length cDNA clone CS0DN001YL07 of Adult brain of Homo sapiens (human). [CR595063]	
A_23_P306964	0.000523	NM_018243	NM_018243	Homo sapiens septin 11 (SEPT11), mRNA [NM_018243]	NM_018243
A_24_P893239	0.000524	BM928197	BM928197	AGENCOURT_6699902 NIH_MGC_121 Homo sapiens cDNA clone IMAGE:5770051 5', mRNA sequence [BM928197]	
A_24_P38276	0.000524	NM_003505	NM_003505	Homo sapiens frizzled homolog 1 (Drosophila) (FZD1), mRNA [NM_003505]	NM_003505
A_23_P49499	0.000525	NM_006456	NM_006456	Homo sapiens ST6 (alpha-N-acetyl-neuraminyl-2,3-beta-galactosyl-1,3)-N-acetylgalactosaminide alpha-2,6-sialyltransferase 2 (ST6GALNAC2), mRNA [NM_006456]	NM_006456
A_32_P87568	0.000525	NM_001008493	NM_001008493	Homo sapiens enabled homolog (Drosophila) (ENAH), transcript variant 1, mRNA [NM_001008493]	NM_001008493
A_32_P2342	0.000526	BX641010	BX641010	Homo sapiens mRNA; cDNA DKFZp686G01227 (from clone DKFZp686G01227). [BX641010]	
A_23_P159797	0.000526	NM_004650	NM_004650	Homo sapiens patatin-like phospholipase domain containing 4 (PNPLA4), mRNA [NM_004650]	NM_004650
A_23_P216340	0.000527	NM_006748	NM_006748	Homo sapiens Src-like-adaptor (SLA), mRNA [NM_006748]	NM_006748
A_23_P161727	0.000527	NM_001541	NM_001541	Homo sapiens heat shock 27kDa protein 2 (HSPB2), mRNA [NM_001541]	NM_001541
A_32_P207360	0.000528	NM_013411	NM_013411	Homo sapiens adenylate kinase 2 (AK2), transcript variant AK2B, mRNA [NM_013411]	NM_013411
A_32_P90812	0.000529	BX647543	BX647543	Homo sapiens mRNA; cDNA DKFZp779F2345 (from clone DKFZp779F2345). [BX647543]	
A_23_P422083	0.000531	NM_018710	NM_018710	Homo sapiens transmembrane protein 55A (TMEM55A), mRNA [NM_018710]	NM_018710
A_24_P227993	0.000531	BC064492	BC064492	Homo sapiens cDNA clone IMAGE:5090094, partial cds. [BC064492]	
A_24_P414269	0.000534	NM_001006940	NM_001006940	Homo sapiens asparagine-linked glycosylation 3 homolog (yeast, alpha-1,3-mannosyltransferase) (ALG3), transcript variant 3, mRNA [NM_001006940]	NM_001006940
A_24_P766716	0.000536	AK126405	AK126405	Homo sapiens cDNA FLJ44441 fis, clone UTERU2020242. [AK126405]	
A_23_P20075	0.000536	NM_013389	NM_013389	Homo sapiens NPC1 (Niemann-Pick disease, type C1, gene)-like 1 (NPC1L1), mRNA [NM_013389]	NM_013389
A_23_P42738	0.000536	NM_032706	NM_032706	Homo sapiens hypothetical protein MGC12966 (MGC12966), mRNA [NM_032706]	NM_032706
A_32_P133916	0.000536	THC2405066		predicted protein {Methanosarcina acetivorans C2A;} , partial (13%) [THC2405066]	
A_32_P108554	0.000539	THC2342491		ALU1_HUMAN (P39188) Alu subfamily J sequence contamination warning entry, partial (13%) [THC2342491]	
A_24_P91472	0.00054	NM_032306	NM_032306	Homo sapiens spermatogenesis associated 11 (SPATA11), mRNA [NM_032306]	NM_032306
A_32_P59355	0.000541	THC2343897			
A_32_P174214	0.000541	BU595528	BU595528	AGENCOURT_8965097 NIH_MGC_142 Homo sapiens cDNA clone IMAGE:6451374 5', mRNA sequence [BU595528]	
A_24_P176714	0.000542	NM_015681	NM_015681	Homo sapiens B9 protein (EPPB9), mRNA [NM_015681]	NM_015681
A_23_P57577	0.000545	NM_014351	NM_014351	Homo sapiens sulfotransferase family 4A, member 1 (SULT4A1), transcript variant 1, mRNA [NM_014351]	NM_014351
A_23_P162142	0.000545	NM_015516	NM_015516	Homo sapiens tsukushi (TSK), mRNA [NM_015516]	NM_015516
A_32_P407245	0.000549	NM_024902	NM_024902	Homo sapiens hypothetical protein FLJ13236 (FLJ13236), mRNA [NM_024902]	NM_024902
A_23_P126225	0.00055	NM_013945	NM_013945	Homo sapiens paired box gene 7 (PAX7), transcript variant 2, mRNA [NM_013945]	NM_013945
A_23_P400515	0.00055	NM_020816	NM_020816	Homo sapiens kinesin family member 17 (KIF17), mRNA [NM_020816]	NM_020816

A_24_P118608	0.00055	NM_032437	NM_032437	Homo sapiens KIAA1799 protein (KIAA1799), mRNA [NM_032437]	NM_032437
A_24_P370471	0.000551	NM_031482	NM_031482	Homo sapiens APG10 autophagy 10-like (S. cerevisiae) (APG10L), mRNA [NM_031482]	NM_031482
A_24_P185854	0.000553	NM_004010	NM_004010	Homo sapiens dystrophin (muscular dystrophy, Duchenne and Becker types) (DMD), transcript variant Dp427p2, mRNA [NM_004010]	NM_004010
A_32_P163125	0.000553	NM_147156	NM_147156	Homo sapiens transmembrane protein 23 (TMEM23), mRNA [NM_147156]	NM_147156
A_32_P84728	0.000554	THC2409283			
A_23_P402000	0.000557	AK091585	AK091585	Homo sapiens cDNA FLJ34266 fis, clone FEBRA2002682, moderately similar to ZINC FINGER PROTEIN 91. [AK091585]	XM_030378
A_23_P217428	0.000558	NM_001174	NM_001174	Homo sapiens Rho GTPase activating protein 6 (ARHGAP6), transcript variant 2, mRNA [NM_001174]	NM_001174
A_24_P321411	0.00056	NM_004705	NM_004705	Homo sapiens protein-kinase, interferon-inducible double stranded RNA dependent inhibitor, repressor of (P58 repressor) (PRKRIR), mRNA [NM_004705]	NM_004705
A_24_P334640	0.000561	NM_133367	NM_133367	Homo sapiens progestin and adipoQ receptor family member VIII (PAQR8), mRNA [NM_133367]	NM_133367
A_23_P99226	0.000561	NM_012240	NM_012240	Homo sapiens sirtuin (silent mating type information regulation 2 homolog) 4 (S. cerevisiae) (SIRT4), mRNA [NM_012240]	NM_012240
A_24_P309360	0.000562	NM_020123	NM_020123	Homo sapiens SM-11044 binding protein (SMBP), mRNA [NM_020123]	NM_020123
A_23_P162559	0.000563	NM_139015	NM_139015	Homo sapiens signal peptide peptidase 3 (SPPL3), mRNA [NM_139015]	NM_139015
A_23_P169319	0.000565	THC2442665		Q5VZ03 (Q5VZ03) OTTHUMP00000021609, partial (8%) [THC2442665]	
A_24_P573514	0.000565	BC033528	BC033528	Homo sapiens, clone IMAGE:4822684, mRNA. [BC033528]	
A_24_P944616	0.000565	AK023129	AK023129	Homo sapiens cDNA FLJ13067 fis, clone NT2RP3001712, highly similar to Homo sapiens HP1-BP74 protein mRNA. [AK023129]	
A_32_P162494	0.000566	THC2375981		Q5SSW7 (Q5SSW7) CAMP responsive elment binding protein-like 1, partial (5%) [THC2375981]	
A_32_P69783	0.000567	THC2445784		Q9F8M7 (Q9F8M7) DTDP-glucose 4,6-dehydratase (Fragment), partial (11%) [THC2445784]	
A_23_P116642	0.000567	NM_133489	NM_133489	Homo sapiens solute carrier family 26, member 10 (SLC26A10), mRNA [NM_133489]	NM_133489
A_24_P189516	0.000571	NM_001609	NM_001609	Homo sapiens acyl-Coenzyme A dehydrogenase, short/branched chain (ACADSB), nuclear gene encoding mitochondrial protein, mRNA [NM_001609]	NM_001609
A_23_P56759	0.000572	NM_016618	NM_016618	Homo sapiens hypothetical protein LOC51315 (LOC51315), mRNA [NM_016618]	NM_016618
A_24_P295620	0.000572	NM_015423	NM_015423	Homo sapiens aminoadipate-semialdehyde dehydrogenase-phosphopantetheinyl transferase (AASDHPPT), mRNA [NM_015423]	NM_015423
A_24_P230734	0.000573	AK091468	AK091468	Homo sapiens cDNA FLJ34149 fis, clone FCBBF3012588, highly similar to Homo sapiens single-strand selective monofunctional uracil DNA glycosylase mRNA. [AK091468]	
A_23_P169756	0.000573	NM_022740	NM_022740	Homo sapiens homeodomain interacting protein kinase 2 (HIPK2), mRNA [NM_022740]	NM_022740
A_23_P255194	0.000578	NM_014827	NM_014827	Homo sapiens zinc finger CCCH-type containing 11A (ZC3H11A), mRNA [NM_014827]	NM_014827
A_24_P23258	0.00058	NM_015124	NM_015124	Homo sapiens death-inducing-protein (DIP), mRNA [NM_015124]	NM_015124
A_23_P57236	0.000581	NM_178026	NM_178026	Homo sapiens gamma-glutamyltransferase-like 3 (GGTL3), transcript variant 3, mRNA [NM_178026]	NM_178026
A_23_P129188	0.000582	NM_033429	NM_033429	Homo sapiens calmodulin-like 4 (CALML4), mRNA [NM_033429]	NM_033429
A_24_P122746	0.000585	AL137722	AL137722	Homo sapiens mRNA; cDNA DKFZp761O051 (from clone DKFZp761O051). [AL137722]	
A_23_P49627	0.000585	NM_006039	NM_006039	Homo sapiens mannose receptor, C type 2 (MRC2), mRNA [NM_006039]	NM_006039
A_23_P368126	0.000589	NM_006621	NM_006621	Homo sapiens S-adenosylhomocysteine hydrolase-like 1 (AHCYL1), mRNA [NM_006621]	NM_006621
A_23_P384635	0.000593	NM_173543	NM_173543	Homo sapiens DAZ interacting protein 1-like (DZIP1L), mRNA [NM_173543]	NM_173543
A_23_P69339	0.000593	NM_001607	NM_001607	Homo sapiens acetyl-Coenzyme A acyltransferase 1 (peroxisomal 3-oxoacyl-Coenzyme A thiolase) (ACAA1), nuclear gene encoding mitochondrial protein, mRNA [NM_001607]	NM_001607
A_24_P126902	0.000593	A_24_P126902			
A_23_P206369	0.000593	NM_014187	NM_014187	Homo sapiens HSPC171 protein (HSPC171), mRNA [NM_014187]	NM_014187
A_24_P330684	0.000596	NM_020686	NM_020686	Homo sapiens 4-aminobutyrate aminotransferase (ABAT), nuclear gene encoding mitochondrial protein, transcript variant 1, mRNA [NM_020686]	NM_020686
A_23_P142708	0.000596	NM_030805	NM_030805	Homo sapiens lectin, mannose-binding 2-like (LMAN2L), mRNA [NM_030805]	NM_030805
A_24_P44462	0.000596	NM_000366	NM_000366	Homo sapiens tropomyosin 1 (alpha) (TPM1), transcript variant 5, mRNA [NM_000366]	NM_000366
A_32_P37592	0.000598	THC2301370		Q7Z5X4 (Q7Z5X4) Intermediate filament-like protein MGC:2625, isoform 1, partial (12%) [THC2301370]	
A_23_P53546	0.000599	M10036	M10036	Human triosephosphate isomerase mRNA, complete cds. [M10036]	
A_24_P25484	0.0006	BC030118	BC030118	Homo sapiens, clone IMAGE:4812643, mRNA, partial cds. [BC030118]	
A_24_P886515	0.000606	THC2314861			
A_24_P35643	0.000608	NM_004626	NM_004626	Homo sapiens wingless-type MMTV integration site family, member 11 (WNT11), mRNA [NM_004626]	NM_004626
A_23_P45786	0.000608	NM_001852	NM_001852	Homo sapiens collagen, type IX, alpha 2 (COL9A2), mRNA [NM_001852]	NM_001852

A_23_P117037	0.000608	NM_015416	NM_015416	Homo sapiens LETM1 domain containing 1 (LETMD1), transcript variant 1, mRNA [NM_015416]	NM_015416
A_23_P140515	0.000608	NM_078474	NM_078474	Homo sapiens BBP-like protein 2 (BLP2), transcript variant 1, mRNA [NM_078474]	NM_078474
A_32_P151557	0.000611	AA779434	AA779434	AA779434 af22b10.s1 Soares_total_fetus_Nb2HF8_9w Homo sapiens cDNA clone IMAGE:1032379 3', mRNA sequence [AA779434]	
A_32_P103695	0.000613	NM_145269	NM_145269	Homo sapiens similar to CG6405 gene product (LOC137392), mRNA [NM_145269]	NM_145269
A_24_P391991	0.000614	AK124132	AK124132	Homo sapiens cDNA FLJ42138 fis, clone TESTI2036684. [AK124132]	XM_295200
A_23_P254353	0.000618	NM_006647	NM_006647	Homo sapiens NADPH oxidase activator 1 (NOXA1), mRNA [NM_006647]	NM_006647
A_23_P43164	0.000622	NM_015170	NM_015170	Homo sapiens sulfatase 1 (SULF1), mRNA [NM_015170]	NM_015170
A_23_P21092	0.000623	NM_001740	NM_001740	Homo sapiens calbindin 2, 29kDa (calretinin) (CALB2), transcript variant CALB2, mRNA [NM_001740]	NM_001740
A_32_P146394	0.000626	AI537201	AI537201	AI537201 tp06e06.x1 NCI_CGAP_Gas4 Homo sapiens cDNA clone IMAGE:2187010 3', mRNA sequence [AI537201]	
A_23_P87310	0.000629	NM_002315	NM_002315	Homo sapiens LIM domain only 1 (rhombotin 1) (LMO1), mRNA [NM_002315]	NM_002315
A_23_P37785	0.00063	BC070103	BC070103	Homo sapiens potassium channel tetramerisation domain containing 19, mRNA (cDNA clone IMAGE:5268205). [BC070103]	XM_085367
A_24_P166789	0.000631	THC2361388			
A_23_P33723	0.000632	NM_004244	NM_004244	Homo sapiens CD163 antigen (CD163), transcript variant 1, mRNA [NM_004244]	NM_004244
A_24_P764595	0.000632	A_24_P764595			
A_23_P5778	0.000633	NM_022449	NM_022449	Homo sapiens RAB17, member RAS oncogene family (RAB17), mRNA [NM_022449]	NM_022449
A_23_P218190	0.000634	NM_000070	NM_000070	Homo sapiens calpain 3, (p94) (CAPN3), transcript variant 1, mRNA [NM_000070]	NM_000070
A_23_P74550	0.000636	NM_178454	NM_178454	Homo sapiens hypothetical protein MGC54289 (MGC54289), mRNA [NM_178454]	NM_178454
A_23_P312181	0.000638	NM_207013	NM_207013	Homo sapiens transcription elongation factor B (SIII), polypeptide 2 (18kDa, elongin B) (TCEB2), transcript variant 2, mRNA [NM_207013]	NM_207013
A_23_P129577	0.000642	NM_033208	NM_033208	Homo sapiens tigger transposable element derived 7 (TIGD7), mRNA [NM_033208]	NM_033208
A_23_P121250	0.000642	NM_001967	NM_001967	Homo sapiens eukaryotic translation initiation factor 4A, isoform 2 (EIF4A2), mRNA [NM_001967]	NM_001967
A_32_P122285	0.000645	A_32_P122285			
A_23_P433111	0.000646	NM_153706	NM_153706	Homo sapiens hypothetical protein MGC33648 (MGC33648), mRNA [NM_153706]	NM_153706
A_32_P185029	0.000648	THC2314901		BC004536 carnitine deficiency-associated gene expressed in ventricle 1 {Homo sapiens;} , partial (7%) [THC2314901]	
A_23_P216679	0.000649	NM_033331	NM_033331	Homo sapiens CDC14 cell division cycle 14 homolog B (S. cerevisiae) (CDC14B), transcript variant 2, mRNA [NM_033331]	NM_033331
A_23_P139958	0.000649	NM_001260	NM_001260	Homo sapiens cyclin-dependent kinase 8 (CDK8), mRNA [NM_001260]	NM_001260
A_23_P29836	0.000649	NM_144638	NM_144638	Homo sapiens transmembrane protein 42 (TMEM42), mRNA [NM_144638]	NM_144638
A_32_P104448	0.000651	THC2397633			
A_23_P30666	0.000651	NM_014452	NM_014452	Homo sapiens tumor necrosis factor receptor superfamily, member 21 (TNFRSF21), mRNA [NM_014452]	NM_014452
A_32_P134679	0.000652	A_32_P134679			
A_23_P157007	0.000652	NM_014020	NM_014020	Homo sapiens LR8 protein (LR8), mRNA [NM_014020]	NM_014020
A_32_P930685	0.000654	BC028359	BC028359	Homo sapiens, clone IMAGE:4828836, mRNA. [BC028359]	
A_23_P138480	0.000654	NM_024886	NM_024886	Homo sapiens chromosome 10 open reading frame 95 (C10orf95), mRNA [NM_024886]	NM_024886
A_23_P5568	0.000654	NM_032740	NM_032740	Homo sapiens SFT2 domain containing 3 (SFT2D3), mRNA [NM_032740]	NM_032740
A_24_P54131	0.000656	NM_022836	NM_022836	Homo sapiens DNA cross-link repair 1B (PSO2 homolog, S. cerevisiae) (DCLRE1B), mRNA [NM_022836]	NM_022836
A_24_P592012	0.000656	AK126720	AK126720	Homo sapiens cDNA FLJ44766 fis, clone BRACE3032537. [AK126720]	
A_23_P252082	0.000656	NM_018487	NM_018487	Homo sapiens hepatocellular carcinoma-associated antigen 112 (HCA112), mRNA [NM_018487]	NM_018487
A_24_P176493	0.000656	NM_015251	NM_015251	Homo sapiens ATM/ATR-Substrate Chk2-Interacting Zn2+-finger protein (ASCIZ), mRNA [NM_015251]	NM_015251
A_23_P338603	0.000656	NM_001011667	NM_001011667	Homo sapiens coiled-coil-helix-coiled-coil-helix domain containing 7 (CHCHD7), transcript variant 1, mRNA [NM_001011667]	NM_001011667
A_23_P47527	0.000659	NM_014206	NM_014206	Homo sapiens chromosome 11 open reading frame 10 (C11orf10), mRNA [NM_014206]	NM_014206
A_32_P199126	0.00066	NM_005559	NM_005559	Homo sapiens laminin, alpha 1 (LAMA1), mRNA [NM_005559]	NM_005559
A_23_P124585	0.000662	AK096342	AK096342	Homo sapiens cDNA FLJ39023 fis, clone NT2RP7004348, highly similar to ZINC FINGER PROTEIN 93. [AK096342]	
A_23_P43175	0.000662	NM_144710	NM_144710	Homo sapiens septin 10 (SEPT10), transcript variant 1, mRNA [NM_144710]	NM_144710
A_24_P7021	0.000663	A_24_P7021			
A_32_P114447	0.000668	BC013799	BC013799	Homo sapiens, clone IMAGE:4386363, mRNA. [BC013799]	
A_23_P127824	0.000669	NM_006757	NM_006757	Homo sapiens troponin T3, skeletal, fast (TNNT3), mRNA [NM_006757]	NM_006757

A_23_P8452	0.000674	NM_002304	NM_002304	Homo sapiens lunatic fringe homolog (Drosophila) (LFNG), mRNA [NM_002304]	NM_002304
A_23_P12755	0.000681	NM_032211	NM_032211	Homo sapiens lysyl oxidase-like 4 (LOXL4), mRNA [NM_032211]	NM_032211
A_23_P157465	0.000681	NM_005671	NM_005671	Homo sapiens reproduction 8 (D8S2298E), mRNA [NM_005671]	NM_005671
A_23_P61688	0.000682	NM_006598	NM_006598	Homo sapiens solute carrier family 12 (potassium/chloride transporters), member 7 (SLC12A7), mRNA [NM_006598]	NM_006598
A_24_P943613	0.000684	NM_015173	NM_015173	Homo sapiens TBC1 (tre-2/USP6, BUB2, cdc16) domain family, member 1 (TBC1D1), mRNA [NM_015173]	NM_015173
A_23_P128554	0.000684	NM_032565	NM_032565	Homo sapiens emopamil binding protein-like (EBPL), mRNA [NM_032565]	NM_032565
A_23_P46017	0.000685	NM_001008661	NM_001008661	Homo sapiens kynurenine aminotransferase III (KAT3), transcript variant 1, mRNA [NM_001008661]	NM_001008661
A_23_P27147	0.000686	NM_001002244	NM_001002244	Homo sapiens APC11 anaphase promoting complex subunit 11 homolog (yeast) (ANAPC11), transcript variant 1, mRNA [NM_001002244]	NM_001002244
A_23_P98844	0.000687	NM_182947	NM_182947	Homo sapiens RAC/CDC42 exchange factor (GEFT), transcript variant 1, mRNA [NM_182947]	NM_182947
A_23_P51565	0.00069	NM_003281	NM_003281	Homo sapiens troponin I, skeletal, slow (TNNI1), mRNA [NM_003281]	NM_003281
A_23_P48339	0.00069	NM_175605	NM_175605	Homo sapiens tetratricopeptide repeat domain 10 (TTC10), transcript variant 1, mRNA [NM_175605]	NM_175605
A_23_P124300	0.000691	NM_017429	NM_017429	Homo sapiens beta-carotene 15,15'-monooxygenase 1 (BCMO1), mRNA [NM_017429]	NM_017429
A_32_P123168	0.000692	BC039397	BC039397	Homo sapiens, clone IMAGE:5299888, mRNA. [BC039397]	
A_24_P245330	0.000695	AK098497	AK098497	Homo sapiens cDNA FLJ25631 fis, clone STM03742. [AK098497]	
A_32_P68076	0.000698	AK123310	AK123310	Homo sapiens cDNA FLJ41316 fis, clone BRAMY2043314. [AK123310]	
A_32_P23010	0.0007	CR600403	CR600403	full-length cDNA clone CS0DN002YE07 of Adult brain of Homo sapiens (human). [CR600403]	
A_23_P57576	0.000704	NM_014351	NM_014351	Homo sapiens sulfotransferase family 4A, member 1 (SULT4A1), transcript variant 1, mRNA [NM_014351]	NM_014351
A_23_P163408	0.000705	NM_020843	NM_020843	Homo sapiens zinc finger protein 291 (ZNF291), mRNA [NM_020843]	NM_020843
A_23_P334608	0.000706	NM_000181	NM_000181	Homo sapiens glucuronidase, beta (GUSB), mRNA [NM_000181]	NM_000181
A_23_P128375	0.000709	NM_032829	NM_032829	Homo sapiens hypothetical protein FLJ14721 (FLJ14721), mRNA [NM_032829]	NM_032829
A_23_P115246	0.000713	NM_003665	NM_003665	Homo sapiens ficolin (collagen/fibrinogen domain containing) 3 (Hakata antigen) (FCN3), transcript variant 1, mRNA [NM_003665]	NM_003665
A_32_P200165	0.000713	NM_001419	NM_001419	Homo sapiens ELAV (embryonic lethal, abnormal vision, Drosophila)-like 1 (Hu antigen R) (ELAVL1), mRNA [NM_001419]	NM_001419
A_24_P396231	0.000714	BC040653	BC040653	Homo sapiens cDNA clone IMAGE:4797120, partial cds. [BC040653]	
A_23_P333138	0.000715	ENST00000261188		O99981 (O99981) NADH dehydrogenase subunit 4 , partial (5%) [THC2339600]	
A_32_P119869	0.000715	THC2314566		Q8LQA6 (Q8LQA6) OJ1125_C04.6 protein, partial (21%) [THC2314566]	
A_24_P122137	0.000715	NM_002309	NM_002309	Homo sapiens leukemia inhibitory factor (cholinergic differentiation factor) (LIF), mRNA [NM_002309]	NM_002309
A_24_P67784	0.000718	BC035246	BC035246	Homo sapiens KIAA1666 protein, mRNA (cDNA clone MGC:42740 IMAGE:4827837), complete cds. [BC035246]	XM_036936
A_23_P153853	0.000718	NM_001398	NM_001398	Homo sapiens enoyl Coenzyme A hydratase 1, peroxisomal (ECH1), mRNA [NM_001398]	NM_001398
A_32_P52251	0.00072	CR612065	CR612065	full-length cDNA clone CS0DC012YI02 of Neuroblastoma Cot 25-normalized of Homo sapiens (human). [CR612065]	
A_23_P6651	0.000721	NM_015224	NM_015224	Homo sapiens retinoblastoma-associated protein 140 (RAP140), mRNA [NM_015224]	NM_015224
A_23_P206310	0.000724	NM_014732	NM_014732	Homo sapiens KIAA0513 (KIAA0513), mRNA [NM_014732]	NM_014732
A_23_P114445	0.000725	NM_020932	NM_020932	Homo sapiens melanoma antigen family E, 1 (MAGEE1), mRNA [NM_020932]	NM_020932
A_24_P732106	0.000726	THC2443960			
A_32_P127978	0.000727	THC2386010			
A_32_P124245	0.000729	NM_175747	NM_175747	Homo sapiens oligodendrocyte transcription factor 3 (OLIG3), mRNA [NM_175747]	NM_175747
A_32_P89310	0.00073	BX538248	BX538248	Homo sapiens mRNA; cDNA DKFZp686O04200 (from clone DKFZp686O04200). [BX538248]	
A_23_P207957	0.000736	NM_003574	NM_003574	Homo sapiens VAMP (vesicle-associated membrane protein)-associated protein A, 33kDa (VAPA), transcript variant 1, mRNA [NM_003574]	NM_003574
A_24_P942469	0.000737	AK129652	AK129652	Homo sapiens cDNA FLJ26141 fis, clone TST03911. [AK129652]	
A_24_P330366	0.000742	D31888	D31888	Homo sapiens KIAA0071 mRNA, partial cds. [D31888]	
A_23_P134314	0.000742	NM_138701	NM_138701	Homo sapiens chromosome 7 open reading frame 11 (C7orf11), mRNA [NM_138701]	NM_138701
A_32_P41471	0.000744	NM_015901	NM_015901	Homo sapiens nudix (nucleoside diphosphate linked moiety X)-type motif 13 (NUDT13), mRNA [NM_015901]	NM_015901
A_23_P373054	0.000745	NM_173826	NM_173826	Homo sapiens chromosome 3 open reading frame 23 (C3orf23), transcript variant 1, mRNA [NM_173826]	NM_173826
A_32_P72622	0.000747	BI771091	BI771091	BI771091 603059664F1 NIH_MGC_122 Homo sapiens cDNA clone IMAGE:5209191 5', mRNA sequence [BI771091]	
A_23_P142560	0.000748	NM_014795	NM_014795	Homo sapiens zinc finger homeobox 1b (ZFHX1B), mRNA [NM_014795]	NM_014795

A_24_P382489	0.000749	NM_198580	NM_198580	Homo sapiens solute carrier family 27 (fatty acid transporter), member 1 (SLC27A1), mRNA [NM_198580]	NM_198580
A_24_P148235	0.000754	NM_001030	NM_001030	Homo sapiens ribosomal protein S27 (metallopanstimulin 1) (RPS27), mRNA [NM_001030]	NM_001030
A_24_P278126	0.000757	NM_002485	NM_002485	Homo sapiens nibrin (NBN), transcript variant 1, mRNA [NM_002485]	NM_002485
A_32_P151133	0.000762	CR601496	CR601496	full-length cDNA clone CS0DI062YF05 of Placenta Cot 25-normalized of Homo sapiens (human). [CR601496]	
A_23_P434929	0.000763	NM_153223	NM_153223	Homo sapiens hypothetical protein FLJ36090 (FLJ36090), mRNA [NM_153223]	NM_153223
A_23_P117546	0.000763	AK129920	AK129920	Homo sapiens cDNA FLJ26410 fis, clone HRT09622. [AK129920]	
A_32_P231415	0.000766	AF132203	AF132203	Homo sapiens PRO1933 mRNA, complete cds. [AF132203]	
A_32_P221631	0.000769	THC2439773			
A_23_P121686	0.000769	NM_004757	NM_004757	Homo sapiens small inducible cytokine subfamily E, member 1 (endothelial monocyte-activating) (SCYE1), mRNA [NM_004757]	NM_004757
A_32_P100379	0.000775	AA599881	AA599881	AA599881 ag32e07.s1 Human bone marrow stromal cells Homo sapiens cDNA clone IMAGE:1091268 3' similar to gb:M21574 ALPHA PLATELET-DERIVED GROWTH FACTOR RECEPTOR PRECURSOR (HUMAN);, mRNA sequence [AA599881]	
A_24_P174824	0.000777	THC2399982		Q9F8M7 (Q9F8M7) DTDP-glucose 4,6-dehydratase (Fragment), partial (11%) [THC2400207]	
A_24_P808100	0.000782	THC2334551			
A_32_P367241	0.000782	NM_080876	NM_080876	Homo sapiens dual specificity phosphatase 19 (DUSP19), mRNA [NM_080876]	NM_080876
A_32_P196115	0.000789	NM_145115	NM_145115	Homo sapiens zinc finger protein 498 (ZNF498), mRNA [NM_145115]	NM_145115
A_32_P191735	0.000796	THC2406457		ALU5_HUMAN (P39192) Alu subfamily SC sequence contamination warning entry, partial (4%) [THC2406457]	
A_32_P180336	0.000796	NM_207429	NM_207429	Homo sapiens FLJ45803 protein (FLJ45803), mRNA [NM_207429]	NM_207429
A_23_P94840	0.000797	NM_130897	NM_130897	Homo sapiens dynein, cytoplasmic, light polypeptide 2B (DNCL2B), mRNA [NM_130897]	NM_130897
A_23_P252808	0.000797	NM_012477	NM_012477	Homo sapiens WW domain binding protein 1 (WBP1), mRNA [NM_012477]	NM_012477
A_32_P31165	0.000798	BC000986	BC000986	Homo sapiens cDNA clone IMAGE:3446313, complete cds. [BC000986]	
A_23_P10291	0.000799	NM_001910	NM_001910	Homo sapiens cathepsin E (CTSE), transcript variant 1, mRNA [NM_001910]	NM_001910
A_32_P184488	0.000803	ENST00000292140		Homo sapiens cDNA clone IMAGE:4299555, partial cds. [BC007947]	
A_23_P211835	0.000807	D13814	D13814	Homo sapiens mRNA for angiotensin II type 1b receptor, complete cds. [D13814]	
A_23_P321473	0.000809	NM_152660	NM_152660	Homo sapiens hypothetical protein MGC34648 (MGC34648), mRNA [NM_152660]	NM_152660
A_24_P246926	0.000809	NM_001302	NM_001302	Homo sapiens cortistatin (CORT), mRNA [NM_001302]	NM_001302
A_32_P180078	0.000812	THC2407930			
A_23_P63254	0.000817	NM_006142	NM_006142	Homo sapiens stratifin (SFN), mRNA [NM_006142]	NM_006142
A_23_P210091	0.000817	NM_014860	NM_014860	Homo sapiens suppressor of Ty 7 (S. cerevisiae)-like (SUPT7L), mRNA [NM_014860]	NM_014860
A_24_P718973	0.00082	NM_198446	NM_198446	Homo sapiens chromosome 1 open reading frame 122 (C1orf122), mRNA [NM_198446]	NM_198446
A_23_P109122	0.000821	AB037863	AB037863	Homo sapiens mRNA for KIAA1442 protein, partial cds. [AB037863]	XM_044921
A_23_P96271	0.000822	NM_003803	NM_003803	Homo sapiens myomesin 1 (skelemin) 185kDa (MYOM1), mRNA [NM_003803]	NM_003803
A_32_P112034	0.000825	AV645774	AV645774	AV645774 AV645774 GLC Homo sapiens cDNA clone GLCAEF02 3', mRNA sequence [AV645774]	
A_23_P210048	0.000825	NM_006037	NM_006037	Homo sapiens histone deacetylase 4 (HDAC4), mRNA [NM_006037]	NM_006037
A_23_P88489	0.000826	NM_172095	NM_172095	Homo sapiens cation channel, sperm associated 2 (CATSPER2), transcript variant 2, mRNA [NM_172095]	NM_172095
A_24_P118874	0.00083	XM_498268	XM_498268	PREDICTED: Homo sapiens similar to 60S ribosomal protein L23a (LOC392181), mRNA [XM_498268]	XM_498268
A_24_P367069	0.000832	A_24_P367069			
A_24_P12149	0.000835	NM_014666	NM_014666	Homo sapiens enthoprotin (ENTH), mRNA [NM_014666]	NM_014666
A_32_P89827	0.000837	AL137411	AL137411	Homo sapiens mRNA; cDNA DKFZp434M082 (from clone DKFZp434M082). [AL137411]	XR_000221
A_23_P121851	0.000839	NM_018935	NM_018935	Homo sapiens protocadherin beta 15 (PCDHB15), mRNA [NM_018935]	NM_018935
A_23_P130753	0.00084	NM_001352	NM_001352	Homo sapiens D site of albumin promoter (albumin D-box) binding protein (DBP), mRNA [NM_001352]	NM_001352
A_32_P42780	0.000841	THC2338292			
A_32_P209582	0.000842	THC2318614		ALU1_HUMAN (P39188) Alu subfamily J sequence contamination warning entry, partial (5%) [THC2318614]	
A_23_P144264	0.000842	NM_014820	NM_014820	Homo sapiens translocase of outer mitochondrial membrane 70 homolog A (yeast) (TOMM70A), nuclear gene encoding mitochondrial protein, mRNA [NM_014820]	NM_014820
A_23_P141029	0.000846	NM_001861	NM_001861	Homo sapiens cytochrome c oxidase subunit IV isoform 1 (COX4I1), mRNA [NM_001861]	NM_001861
A_24_P255253	0.000848	NM_203477	NM_203477	Homo sapiens similar to RPL23AP7 protein (MGC70863), transcript variant 1, mRNA [NM_203477]	NM_203477

A_23_P167066	0.000852	NM_003359	NM_003359	Homo sapiens UDP-glucose dehydrogenase (UGDH), mRNA [NM_003359]	NM_003359
A_32_P67837	0.000857	CR590573	CR590573	full-length cDNA clone CS0DI042YD07 of Placenta Cot 25-normalized of Homo sapiens (human). [CR590573]	
A_24_P405981	0.000859	CR598481	CR598481	full-length cDNA clone CS0DD001YH15 of Neuroblastoma Cot 50-normalized of Homo sapiens (human). [CR598481]	
A_24_P915591	0.000859	BC005305	BC005305	Homo sapiens collagen, type IV, alpha 6, mRNA (cDNA clone IMAGE:3951220), complete cds. [BC005305]	
A_23_P74794	0.00086	NM_002858	NM_002858	Homo sapiens ATP-binding cassette, sub-family D (ALD), member 3 (ABCD3), mRNA [NM_002858]	NM_002858
A_24_P380679	0.000861	NM_182597	NM_182597	Homo sapiens hypothetical protein FLJ39575 (FLJ39575), mRNA [NM_182597]	NM_182597
A_32_P195850	0.000861	NM_173812	NM_173812	Homo sapiens dpy-19-like 2 (C. elegans) (DPY19L2), mRNA [NM_173812]	NM_173812
A_32_P131797	0.000861	BE970058	BE970058	BE970058 601680172F1 NIH_MGC_78 Homo sapiens cDNA clone IMAGE:3950464 5', mRNA sequence [BE970058]	
A_23_P118185	0.000861	NM_031490	NM_031490	Homo sapiens peroxisomal lon protease (LONP), mRNA [NM_031490]	NM_031490
A_32_P52119	0.000866	THC2250585			
A_23_P418434	0.000867	BC032824	BC032824	Homo sapiens chromosome 6 open reading frame 164, mRNA (cDNA clone IMAGE:4824064), partial cds. [BC032824]	
A_24_P414556	0.000867	NM_012382	NM_012382	Homo sapiens osmosis responsive factor (OSRF), mRNA [NM_012382]	NM_012382
A_32_P201434	0.00087	AA719641	AA719641	AA719641 zg52f01.s1 Soares_pineal_gland_N3HPG Homo sapiens cDNA clone IMAGE:396985 3', mRNA sequence [AA719641]	
A_24_P116378	0.000871	NM_015532	NM_015532	Homo sapiens glutamate receptor, ionotropic, N-methyl D-aspartate-like 1A (GRINL1A), transcript variant 1, mRNA [NM_015532]	NM_015532
A_24_P731549	0.000871	AB188495	AB188495	Homo sapiens mRNA, clone: TH072F08. [AB188495]	
A_23_P53198	0.000874	NM_032564	NM_032564	Homo sapiens diacylglycerol O-acyltransferase homolog 2 (mouse) (DGAT2), mRNA [NM_032564]	NM_032564
A_23_P52727	0.000878	NM_182964	NM_182964	Homo sapiens neuron navigator 2 (NAV2), transcript variant 1, mRNA [NM_182964]	NM_182964
A_24_P902653	0.00088	CR616041	CR616041	full-length cDNA clone CS0DC015YF20 of Neuroblastoma Cot 25-normalized of Homo sapiens (human). [CR616041]	
A_24_P16036	0.000881	NM_001013663	NM_001013663	Homo sapiens similar to CG14903-PA (LOC391356), mRNA [NM_001013663]	NM_001013663
A_23_P46819	0.000885	NM_033637	NM_033637	Homo sapiens beta-transducin repeat containing (BTRC), transcript variant 1, mRNA [NM_033637]	NM_033637
A_24_P928830	0.000887	A_24_P928830			
A_23_P90659	0.000893	NM_014713	NM_014713	Homo sapiens lysosomal-associated protein transmembrane 4 alpha (LAPTM4A), mRNA [NM_014713]	NM_014713
A_32_P209094	0.000901	NM_018291	NM_018291	Homo sapiens hypothetical protein FLJ10986 (FLJ10986), mRNA [NM_018291]	NM_018291
A_23_P111112	0.000902	NM_020442	NM_020442	Homo sapiens valyl-tRNA synthetase 2-like (VARS2L), mRNA [NM_020442]	NM_020442
A_23_P88503	0.000902	D42039	D42039	Homo sapiens KIAA0081 mRNA, partial cds. [D42039]	
A_23_P83659	0.000906	NM_003399	NM_003399	Homo sapiens X-prolyl aminopeptidase (aminopeptidase P) 2, membrane-bound (XPNPEP2), mRNA [NM_003399]	NM_003399
A_32_P3021	0.000908	BM989848	BM989848	UI-H-DI0-ato-k-13-0-UI.s1 NCI_CGAP_DI0 Homo sapiens cDNA clone IMAGE:5862276 3', mRNA sequence [BM989848]	
A_23_P18824	0.00091	NM_006999	NM_006999	Homo sapiens polymerase (DNA directed) sigma (POLS), mRNA [NM_006999]	NM_006999
A_23_P311232	0.000912	NM_015282	NM_015282	Homo sapiens cytoplasmic linker associated protein 1 (CLASP1), mRNA [NM_015282]	NM_015282
A_32_P179526	0.000917	BC010934	BC010934	Homo sapiens zinc finger and BTB domain containing 20, mRNA (cDNA clone IMAGE:4291354), partial cds. [BC010934]	
A_23_P70387	0.000918	NM_006789	NM_006789	Homo sapiens apolipoprotein B mRNA editing enzyme, catalytic polypeptide-like 2 (APOBEC2), mRNA [NM_006789]	NM_006789
A_23_P3514	0.000918	NM_013242	NM_013242	Homo sapiens gene trap locus 3 (mouse) (GTL3), mRNA [NM_013242]	NM_013242
A_32_P208039	0.000926	AL049390	AL049390	Homo sapiens mRNA; cDNA DKFZp586O1318 (from clone DKFZp586O1318) [AL049390]	
A_23_P301896	0.000926	NM_015866	NM_015866	Homo sapiens PR domain containing 2, with ZNF domain (PRDM2), transcript variant 2, mRNA [NM_015866]	NM_015866
A_32_P225536	0.000928	BC045098	BC045098	Homo sapiens, clone IMAGE:5171383, mRNA. [BC045098]	
A_24_P944144	0.000933	NM_080797	NM_080797	Homo sapiens death associated transcription factor 1 (DATF1), transcript variant 3, mRNA [NM_080797]	NM_080797
A_23_P24469	0.000933	NM_003476	NM_003476	Homo sapiens cysteine and glycine-rich protein 3 (cardiac LIM protein) (CSRP3), mRNA [NM_003476]	NM_003476
A_23_P211212	0.000935	NM_030582	NM_030582	Homo sapiens collagen, type XVIII, alpha 1 (COL18A1), transcript variant 1, mRNA [NM_030582]	NM_030582
A_24_P364072	0.000936	THC2364724			
A_23_P124384	0.000936	NM_006884	NM_006884	Homo sapiens short stature homeobox 2 (SHOX2), transcript variant SHOX2a, mRNA [NM_006884]	NM_006884
A_24_P252575	0.000936	NM_012197	NM_012197	Homo sapiens RAB GTPase activating protein 1 (RABGAP1), mRNA [NM_012197]	NM_012197
A_24_P943894	0.000937	NM_152753	NM_152753	Homo sapiens signal peptide, CUB domain, EGF-like 3 (SCUBE3), mRNA [NM_152753]	NM_152753
A_32_P80587	0.00094	A_32_P80587			
A_24_P867868	0.000941	BX537532	BX537532	Homo sapiens mRNA; cDNA DKFZp686L07201 (from clone DKFZp686L07201). [BX537532]	

A_23_P308603	0.000944	NM_005417	NM_005417	Homo sapiens v-src sarcoma (Schmidt-Ruppin A-2) viral oncogene homolog (avian) (SRC), transcript variant 1, mRNA [NM_005417]	NM_005417
A_32_P82111	0.000945	NM_020737	NM_020737	Homo sapiens leucine rich repeat and fibronectin type III domain containing 2 (LRFN2), mRNA [NM_020737]	NM_020737
A_23_P145916	0.000951	NM_001129	NM_001129	Homo sapiens AE binding protein 1 (AEBP1), mRNA [NM_001129]	NM_001129
A_23_P141730	0.000953	NM_001943	NM_001943	Homo sapiens desmoglein 2 (DSG2), mRNA [NM_001943]	NM_001943
A_24_P940135	0.000955	BC024007	BC024007	Homo sapiens chitobiase, di-N-acetyl-, mRNA (cDNA clone IMAGE:4823479), complete cds. [BC024007]	
A_24_P123245	0.000955	NM_031370	NM_031370	Homo sapiens heterogeneous nuclear ribonucleoprotein D (AU-rich element RNA binding protein 1, 37kDa) (HNRPD), transcript variant 1, mRNA [NM_031370]	NM_031370
A_23_P304000	0.000959	NM_015026	NM_015026	Homo sapiens KIAA1040 protein (KIAA1040), mRNA [NM_015026]	NM_015026
A_23_P386168	0.000961	BC022076	BC022076	Homo sapiens hypothetical protein LOC255783, mRNA (cDNA clone IMAGE:4817351). [BC022076]	
A_23_P152516	0.000962	NM_005324	NM_005324	Homo sapiens H3 histone, family 3B (H3.3B) (H3F3B), mRNA [NM_005324]	NM_005324
A_24_P37264	0.000963	NM_014746	NM_014746	Homo sapiens ring finger protein 144 (RNF144), mRNA [NM_014746]	NM_014746
A_24_P125273	0.000965	NM_001661	NM_001661	Homo sapiens ADP-ribosylation factor 4-like (ARF4L), mRNA [NM_001661]	NM_001661
A_24_P606239	0.000968	AK056073	AK056073	Homo sapiens cDNA FLJ31511 fis, clone NT2RI1000035. [AK056073]	
A_24_P128563	0.000971	NM_012316	NM_012316	Homo sapiens karyopherin alpha 6 (importin alpha 7) (KPNA6), mRNA [NM_012316]	NM_012316
A_32_P150418	0.000972	CR624880	CR624880	full-length cDNA clone CS0DB005YI23 of Neuroblastoma Cot 10-normalized of Homo sapiens (human). [CR624880]	
A_23_P215642	0.000979	NM_022748	NM_022748	Homo sapiens tensin 3 (TNS3), mRNA [NM_022748]	NM_022748
A_32_P73413	0.000982	XM_379036	XM_379036	PREDICTED: Homo sapiens hypothetical LOC400890 (LOC400890), mRNA [XM_379036]	XM_379036
A_23_P98085	0.000983	NM_000314	NM_000314	Homo sapiens phosphatase and tensin homolog (mutated in multiple advanced cancers 1) (PTEN), mRNA [NM_000314]	NM_000314
A_23_P68072	0.000983	NM_032118	NM_032118	Homo sapiens WD repeat domain 54 (WDR54), mRNA [NM_032118]	NM_032118
A_23_P90743	0.000984	NM_002909	NM_002909	Homo sapiens regenerating islet-derived 1 alpha (pancreatic stone protein, pancreatic thread protein) (REG1A), mRNA [NM_002909]	NM_002909
A_32_P26956	0.000984	AW090036	AW090036	AW090036 xd01b06.x1 NCI_CGAP_Brn35 Homo sapiens cDNA clone IMAGE:2592467 3', mRNA sequence [AW090036]	
A_24_P101694	0.000984	A_24_P101694			
A_23_P361049	0.000985	NM_012223	NM_012223	Homo sapiens myosin IB (MYO1B), mRNA [NM_012223]	NM_012223
A_32_P76627	0.000991	ENST00000322533		full-length cDNA clone CS0DI013YN06 of Placenta Cot 25-normalized of Homo sapiens (human). [CR597597]	
A_24_P883629	0.000995	ENST00000258457		Homo sapiens cDNA FLJ45759 fis, clone MESAN2017133. [AK127661]	
A_23_P204929	0.000996	NM_016248	NM_016248	Homo sapiens A kinase (PRKA) anchor protein 11 (AKAP11), transcript variant 1, mRNA [NM_016248]	NM_016248
A_23_P101960	0.000999	NM_006887	NM_006887	Homo sapiens zinc finger protein 36, C3H type-like 2 (ZFP36L2), mRNA [NM_006887]	NM_006887

Supplemental Table S3D
Kmeans Cluster 4 - 2,952 Genes

Gene Name	*P* -value	Common	Genbank	Description	RefSeq
A_23_P204286	1.60E-07	NM_000900	NM_000900	Homo sapiens matrix Gla protein (MGP), mRNA [NM_000900]	NM_000900
A_24_P54879	1.65E-07	NM_005506	NM_005506	Homo sapiens scavenger receptor class B, member 2 (SCARB2), mRNA [NM_005506]	NM_005506
A_23_P143885	1.66E-07	NM_019555	NM_019555	Homo sapiens Rho guanine nucleotide exchange factor (GEF) 3 (ARHGEF3), mRNA [NM_019555]	NM_019555
A_24_P139993	1.71E-07	NM_032977	NM_032977	Homo sapiens caspase 10, apoptosis-related cysteine protease (CASP10), transcript variant D, mRNA [NM_032977]	NM_032977
A_23_P38106	1.71E-07	NM_021972	NM_021972	Homo sapiens sphingosine kinase 1 (SPHK1), transcript variant 1, mRNA [NM_021972]	NM_021972
A_23_P41854	2.04E-07	NM_032587	NM_032587	Homo sapiens caspase recruitment domain family, member 6 (CARD6), mRNA [NM_032587]	NM_032587
A_23_P1691	2.09E-07	NM_002421	NM_002421	Homo sapiens matrix metalloproteinase 1 (interstitial collagenase) (MMP1), mRNA [NM_002421]	NM_002421
A_24_P246825	2.11E-07	THC2295607			
A_23_P427023	2.32E-07	NM_130759	NM_130759	Homo sapiens GTPase, IMAP family member 1 (GIMAP1), mRNA [NM_130759]	NM_130759
A_23_P314250	2.39E-07	NM_033387	NM_033387	Homo sapiens family with sequence similarity 78, member A (FAM78A), mRNA [NM_033387]	NM_033387
A_23_P331928	2.39E-07	AK095888	AK095888	Homo sapiens cDNA FLJ38569 fis, clone HCHON2006459. [AK095888]	
A_23_P55544	2.77E-07	NM_133459	NM_133459	Homo sapiens collagen and calcium binding EGF domains 1 (CCBE1), mRNA [NM_133459]	NM_133459
A_23_P53390	2.77E-07	NM_002837	NM_002837	Homo sapiens protein tyrosine phosphatase, receptor type, B (PTPRB), mRNA [NM_002837]	NM_002837
A_23_P207911	2.84E-07	NM_016113	NM_016113	Homo sapiens transient receptor potential cation channel, subfamily V, member 2 (TRPV2), mRNA [NM_016113]	NM_016113
A_23_P152655	3.00E-07	NM_000873	NM_000873	Homo sapiens intercellular adhesion molecule 2 (ICAM2), mRNA [NM_000873]	NM_000873
A_23_P162425	3.18E-07	NM_016072	NM_016072	Homo sapiens golgi transport 1 homolog B (S. cerevisiae) (GOLT1B), mRNA [NM_016072]	NM_016072
A_23_P421423	3.48E-07	NM_006291	NM_006291	Homo sapiens tumor necrosis factor, alpha-induced protein 2 (TNFAIP2), mRNA [NM_006291]	NM_006291
A_23_P119353	3.65E-07	NM_017805	NM_017805	Homo sapiens Ras interacting protein 1 (RASIP1), mRNA [NM_017805]	NM_017805
A_32_P77252	3.73E-07	THC2375310			
A_24_P323114	3.74E-07	NR_001446	NR_001446	Homo sapiens annexin A2 pseudogene 3 (ANXA2P3) on chromosome 10 [NR_001446]	NR_001446
A_32_P56001	3.83E-07	NM_012072	NM_012072	Homo sapiens complement component 1, q subcomponent, receptor 1 (C1QR1), mRNA [NM_012072]	NM_012072
A_24_P406334	3.85E-07	NM_012449	NM_012449	Homo sapiens six transmembrane epithelial antigen of the prostate 1 (STEAP1), mRNA [NM_012449]	NM_012449
A_23_P127964	4.09E-07	NM_199418	NM_199418	Homo sapiens prolylcarboxypeptidase (angiotensinase C) (PRCP), transcript variant 2, mRNA [NM_199418]	NM_199418
A_23_P200815	4.12E-07	BF678528	BF678528	602085985F1 NIH_MGC_83 Homo sapiens cDNA clone IMAGE:4250205 5', mRNA sequence [BF678528]	
A_23_P74609	4.31E-07	NM_015714	NM_015714	Homo sapiens putative lymphocyte G0/G1 switch gene (G0S2), mRNA [NM_015714]	NM_015714
A_23_P253982	4.38E-07	NM_002141	NM_002141	Homo sapiens homeo box A4 (HOXA4), mRNA [NM_002141]	NM_002141
A_23_P502520	4.39E-07	NM_172374	NM_172374	Homo sapiens interleukin 4 induced 1 (IL4I1), transcript variant 2, mRNA [NM_172374]	NM_172374
A_24_P924697	4.39E-07	AK055915	AK055915	Homo sapiens cDNA FLJ31353 fis, clone MESAN2000264. [AK055915]	
A_23_P87150	4.41E-07	NM_004811	NM_004811	Homo sapiens leupaxin (LPXN), mRNA [NM_004811]	NM_004811
A_23_P160025	4.41E-07	NM_005531	NM_005531	Homo sapiens interferon, gamma-inducible protein 16 (IFI16), mRNA [NM_005531]	NM_005531
A_23_P75369	4.58E-07	NM_006019	NM_006019	Homo sapiens T-cell, immune regulator 1, ATPase, H+ transporting, lysosomal V0 protein a isoform 3 (TCIRG1), transcript variant 1, mRNA [NM_006019]	NM_006019
A_23_P91910	4.80E-07	NM_020353	NM_020353	Homo sapiens phospholipid scramblase 4 (PLSCR4), mRNA [NM_020353]	NM_020353
A_24_P156490	4.82E-07	NM_002247	NM_002247	Homo sapiens potassium large conductance calcium-activated channel, subfamily M, alpha member 1 (KCNMA1), transcript variant 2, mRNA [NM_002247]	NM_002247
A_23_P43810	4.85E-07	NM_206943	NM_206943	Homo sapiens latent transforming growth factor beta binding protein 1 (LTBP1), transcript variant 1, mRNA [NM_206943]	NM_206943
A_32_P168326	5.05E-07	NM_001012421	NM_001012421	Homo sapiens OTTHUMP00000064580 (LOC441430), mRNA [NM_001012421]	NM_001012421
A_24_P385611	5.07E-07	NM_003113	NM_003113	Homo sapiens nuclear antigen Sp100 (SP100), mRNA [NM_003113]	NM_003113
A_24_P274831	5.07E-07	NM_153236	NM_153236	Homo sapiens GTPase, IMAP family member 7 (GIMAP7), mRNA [NM_153236]	NM_153236
A_23_P6321	5.07E-07	NM_003277	NM_003277	Homo sapiens claudin 5 (transmembrane protein deleted in velocardiofacial syndrome) (CLDN5), mRNA [NM_003277]	NM_003277
A_23_P128574	5.11E-07	NM_017993	NM_017993	Homo sapiens hypothetical protein FLJ10094 (FLJ10094), mRNA [NM_017993]	NM_017993
A_23_P206280	5.11E-07	NM_201525	NM_201525	Homo sapiens G protein-coupled receptor 56 (GPR56), transcript variant 3, mRNA [NM_201525]	NM_201525
A_23_P70968	6.39E-07	NM_006896	NM_006896	Homo sapiens homeo box A7 (HOXA7), mRNA [NM_006896]	NM_006896

A_23_P120594	6.76E-07	NM_032501	NM_032501	Homo sapiens acetyl-Coenzyme A synthetase 2 (AMP forming)-like (ACAS2L), nuclear gene encoding mitochondrial protein, mRNA [NM_032501]	NM_032501
A_32_P47874	6.85E-07	THC2440787			
A_23_P131089	7.01E-07	NM_198471	NM_198471	Homo sapiens FLJ46061 protein (FLJ46061), mRNA [NM_198471]	NM_198471
A_23_P86283	7.12E-07	NM_006762	NM_006762	Homo sapiens lysosomal associated multispanning membrane protein 5 (LAPTM5), mRNA [NM_006762]	NM_006762
A_23_P92517	7.16E-07	NM_031956	NM_031956	Homo sapiens NYD-SP14 protein (NYD-SP14), mRNA [NM_031956]	NM_031956
A_23_P91334	7.22E-07	NM_052970	NM_052970	Homo sapiens heat shock 70kD protein 12B (HSPA12B), mRNA [NM_052970]	NM_052970
A_23_P78405	7.22E-07	NM_006033	NM_006033	Homo sapiens lipase, endothelial (LIPG), mRNA [NM_006033]	NM_006033
A_23_P80040	7.41E-07	NM_006404	NM_006404	Homo sapiens protein C receptor, endothelial (EPCR) (PROCR), mRNA [NM_006404]	NM_006404
A_32_P11262	7.65E-07	AK124396	AK124396	Homo sapiens cDNA FLJ42405 fis, clone ASTRO3000474. [AK124396]	
A_24_P273726	8.08E-07	NM_198080	NM_198080	Homo sapiens methionine sulfoxide reductase B3 (MSRB3), mRNA [NM_198080]	NM_198080
A_23_P145555	8.39E-07	NM_138569	NM_138569	Homo sapiens chromosome 6 open reading frame 142 (C6orf142), mRNA [NM_138569]	NM_138569
A_32_P211558	8.45E-07	THC2312818		CJ11_MOUSE (Q9D9B4) Protein C10orf11 homolog, partial (95%) [THC2312818]	
A_24_P303480	8.45E-07	NM_006834	NM_006834	Homo sapiens RAB32, member RAS oncogene family (RAB32), mRNA [NM_006834]	NM_006834
A_23_P205567	8.45E-07	NM_006255	NM_006255	Homo sapiens protein kinase C, eta (PRKCH), mRNA [NM_006255]	NM_006255
A_23_P252062	8.59E-07	NM_138711	NM_138711	Homo sapiens peroxisome proliferative activated receptor, gamma (PPARG), transcript variant 3, mRNA [NM_138711]	NM_138711
A_24_P360674	8.78E-07	NM_078487	NM_078487	Homo sapiens cyclin-dependent kinase inhibitor 2B (p15, inhibits CDK4) (CDKN2B), transcript variant 2, mRNA [NM_078487]	NM_078487
A_24_P734060	9.09E-07	AL832183	AL832183	Homo sapiens mRNA; cDNA DKFZp686D0720 (from clone DKFZp686D0720). [AL832183]	
A_23_P64617	9.73E-07	NM_012193	NM_012193	Homo sapiens frizzled homolog 4 (Drosophila) (FZD4), mRNA [NM_012193]	NM_012193
A_23_P24843	9.73E-07	NM_014632	NM_014632	Homo sapiens microtubule associated monoxygenase, calponin and LIM domain containing 2 (MICAL2), mRNA [NM_014632]	NM_014632
A_23_P210158	9.91E-07	THC2278254		Q9VK63 (Q9VK63) CG5776-PA (LD25466p), partial (3%) [THC2278254]	
A_24_P189533	9.91E-07	ENST00000278505		Homo sapiens mRNA for KIAA0830 protein, partial cds. [AB020637]	XM_290546
A_24_P739344	9.99E-07	NM_016931	NM_016931	Homo sapiens NADPH oxidase 4 (NOX4), mRNA [NM_016931]	NM_016931
A_24_P98613	9.99E-07	NM_030927	NM_030927	Homo sapiens tetraspanin 14 (TSPAN14), mRNA [NM_030927]	NM_030927
A_23_P382065	1.01E-06	NM_016242	NM_016242	Homo sapiens endomucin (EMCN), mRNA [NM_016242]	NM_016242
A_23_P336040	1.02E-06	NM_020399	NM_020399	Homo sapiens golgi associated PDZ and coiled-coil motif containing (GOPC), transcript variant 1, mRNA [NM_020399]	NM_020399
A_23_P306987	1.02E-06	NM_031439	NM_031439	Homo sapiens SRY (sex determining region Y)-box 7 (SOX7), mRNA [NM_031439]	NM_031439
A_23_P90357	1.02E-06	NM_201636	NM_201636	Homo sapiens thromboxane A2 receptor (TBXA2R), transcript variant 1, mRNA [NM_201636]	NM_201636
A_23_P213562	1.02E-06	NM_001992	NM_001992	Homo sapiens coagulation factor II (thrombin) receptor (F2R), mRNA [NM_001992]	NM_001992
A_23_P156826	1.02E-06	NM_032744	NM_032744	Homo sapiens chromosome 6 open reading frame 105 (C6orf105), mRNA [NM_032744]	NM_032744
A_23_P83098	1.02E-06	NM_000689	NM_000689	Homo sapiens aldehyde dehydrogenase 1 family, member A1 (ALDH1A1), mRNA [NM_000689]	NM_000689
A_23_P58390	1.02E-06	NM_152400	NM_152400	Homo sapiens hypothetical protein FLJ39370 (FLJ39370), mRNA [NM_152400]	NM_152400
A_23_P252471	1.02E-06	NM_000442	NM_000442	Homo sapiens platelet/endothelial cell adhesion molecule (CD31 antigen) (PECAM1), mRNA [NM_000442]	NM_000442
A_24_P491643	1.02E-06	THC2365017		HSU02032 ribosomal protein L23a {Homo sapiens;} , partial (89%) [THC2365017]	
A_23_P27381	1.04E-06	NM_005786	NM_005786	Homo sapiens serologically defined colon cancer antigen 33 (SDCCAG33), mRNA [NM_005786]	NM_005786
A_23_P30069	1.06E-06	AK023743	AK023743	Homo sapiens cDNA FLJ13681 fis, clone PLACE2000014, weakly similar to HYPOTHETICAL HELICASE C28H8.3 IN CHROMOSOME III. [AK023743]	
A_24_P236235	1.06E-06	NM_013231	NM_013231	Homo sapiens fibronectin leucine rich transmembrane protein 2 (FLRT2), mRNA [NM_013231]	NM_013231
A_24_P98249	1.06E-06	NM_006283	NM_006283	Homo sapiens transforming, acidic coiled-coil containing protein 1 (TACC1), mRNA [NM_006283]	NM_006283
A_23_P158593	1.06E-06	NM_000093	NM_000093	Homo sapiens collagen, type V, alpha 1 (COL5A1), mRNA [NM_000093]	NM_000093
A_23_P37983	1.06E-06	NM_005947	NM_005947	Homo sapiens metallothionein 1B (functional) (MT1B), mRNA [NM_005947]	NM_005947
A_23_P20035	1.08E-06	NM_138445	NM_138445	Homo sapiens G protein-coupled receptor 146 (GPR146), mRNA [NM_138445]	NM_138445
A_23_P218928	1.09E-06	NM_016613	NM_016613	Homo sapiens hypothetical protein DKFZp434L142 (DKFZp434L142), mRNA [NM_016613]	NM_016613
A_23_P151075	1.10E-06	NM_001175	NM_001175	Homo sapiens Rho GDP dissociation inhibitor (GDI) beta (ARHGDIB), mRNA [NM_001175]	NM_001175
A_24_P795371	1.12E-06	AK000872	AK000872	Homo sapiens cDNA FLJ10010 fis, clone HEMBA1000302. [AK000872]	XM_498625
A_23_P202978	1.12E-06	NM_033292	NM_033292	Homo sapiens caspase 1, apoptosis-related cysteine protease (interleukin 1, beta, convertase) (CASP1), transcript variant alpha, mRNA [NM_033292]	NM_033292

A_23_P216920	1.12E-06	NM_014397	NM_014397	Homo sapiens NIMA (never in mitosis gene a)-related kinase 6 (NEK6), mRNA [NM_014397]	NM_014397
A_23_P390504	1.12E-06	NM_001453	NM_001453	Homo sapiens forkhead box C1 (FOXC1), mRNA [NM_001453]	NM_001453
A_23_P7212	1.13E-06	NM_000204	NM_000204	Homo sapiens I factor (complement) (IF), mRNA [NM_000204]	NM_000204
A_23_P119202	1.15E-06	NM_001252	NM_001252	Homo sapiens tumor necrosis factor (ligand) superfamily, member 7 (TNFSF7), mRNA [NM_001252]	NM_001252
A_23_P211207	1.16E-06	NM_015833	NM_015833	Homo sapiens adenosine deaminase, RNA-specific, B1 (RED1 homolog rat) (ADARB1), transcript variant DRABA2b, mRNA [NM_015833]	NM_015833
A_32_P217655	1.16E-06	XM_496405	XM_496405	PREDICTED: Homo sapiens similar to D(1B) dopamine receptor (D(5) dopamine receptor) (D1beta dopamine receptor) (LOC440684), mRNA [XM_496405]	XM_496405
A_23_P145965	1.20E-06	NM_003596	NM_003596	Homo sapiens tyrosylprotein sulfotransferase 1 (TPST1), mRNA [NM_003596]	NM_003596
A_24_P209455	1.24E-06	NM_018326	NM_018326	Homo sapiens GTPase, IMAP family member 4 (GIMAP4), mRNA [NM_018326]	NM_018326
A_24_P389916	1.24E-06	NM_005512	NM_005512	Homo sapiens leucine rich repeat containing 32 (LRRC32), mRNA [NM_005512]	NM_005512
A_23_P217866	1.24E-06	AK094968	AK094968	Homo sapiens cDNA FLJ37649 fis, clone BRHIP2000534, moderately similar to GAMMA-INTERFERON-INDUCIBLE PROTEIN IFI-16. [AK094968]	
A_32_P111072	1.30E-06	THC2389705			
A_23_P82449	1.32E-06	NM_004403	NM_004403	Homo sapiens deafness, autosomal dominant 5 (DFNA5), mRNA [NM_004403]	NM_004403
A_23_P254512	1.33E-06	NM_004428	NM_004428	Homo sapiens ephrin-A1 (EFNA1), transcript variant 1, mRNA [NM_004428]	NM_004428
A_23_P127584	1.34E-06	NM_006169	NM_006169	Homo sapiens nicotinamide N-methyltransferase (NNMT), mRNA [NM_006169]	NM_006169
A_23_P68155	1.35E-06	NM_022168	NM_022168	Homo sapiens interferon induced with helicase C domain 1 (IFIH1), mRNA [NM_022168]	NM_022168
A_24_P601695	1.36E-06	AK026194	AK026194	Homo sapiens cDNA: FLJ22541 fis, clone HSI00130. [AK026194]	
A_23_P19936	1.36E-06	NM_006854	NM_006854	Homo sapiens KDEL (Lys-Asp-Glu-Leu) endoplasmic reticulum protein retention receptor 2 (KDELR2), mRNA [NM_006854]	NM_006854
A_23_P85164	1.36E-06	NM_006730	NM_006730	Homo sapiens deoxyribonuclease I-like 1 (DNASE1L1), transcript variant 1, mRNA [NM_006730]	NM_006730
A_23_P381261	1.39E-06	NM_139247	NM_139247	Homo sapiens adenylate cyclase 4 (ADCY4), mRNA [NM_139247]	NM_139247
A_23_P321511	1.40E-06	NM_178450	NM_178450	Homo sapiens membrane-associated ring finger (C3HC4) 3 (MARCH3), mRNA [NM_178450]	NM_178450
A_24_P322741	1.45E-06	NM_000628	NM_000628	Homo sapiens interleukin 10 receptor, beta (IL10RB), mRNA [NM_000628]	NM_000628
A_32_P167239	1.45E-06	NM_152406	NM_152406	Homo sapiens hypothetical protein FLJ36748 (FLJ36748), mRNA [NM_152406]	NM_152406
A_24_P134488	1.46E-06	NM_052880	NM_052880	Homo sapiens HGFL gene (MGC17330), mRNA [NM_052880]	NM_052880
A_23_P126836	1.46E-06	NM_003326	NM_003326	Homo sapiens tumor necrosis factor (ligand) superfamily, member 4 (tax-transcriptionally activated glycoprotein 1, 34kDa) (TNFSF4), mRNA [NM_003326]	NM_003326
A_23_P42375	1.47E-06	NM_006834	NM_006834	Homo sapiens RAB32, member RAS oncogene family (RAB32), mRNA [NM_006834]	NM_006834
A_23_P76731	1.48E-06	NM_014226	NM_014226	Homo sapiens renal tumor antigen (RAGE), mRNA [NM_014226]	NM_014226
A_32_P186157	1.52E-06	NM_030626	NM_030626	Homo sapiens leucine rich repeat containing 27 (LRRC27), mRNA [NM_030626]	NM_030626
A_23_P208866	1.52E-06	NM_004877	NM_004877	Homo sapiens glia maturation factor, gamma (GMFG), mRNA [NM_004877]	NM_004877
A_32_P18440	1.52E-06	BX641020	BX641020	Homo sapiens mRNA; cDNA DKFZp686G23148 (from clone DKFZp686G23148). [BX641020]	
A_23_P101380	1.53E-06	NM_198540	NM_198540	Homo sapiens UDP-Gal:betaGal beta 1,3-galactosyltransferase polypeptide 7 (B3GALT7), mRNA [NM_198540]	NM_198540
A_24_P14260	1.56E-06	NM_014959	NM_014959	Homo sapiens caspase recruitment domain family, member 8 (CARD8), mRNA [NM_014959]	NM_014959
A_23_P69242	1.56E-06	NM_182760	NM_182760	Homo sapiens sulfatase modifying factor 1 (SUMF1), mRNA [NM_182760]	NM_182760
A_24_P385148	1.58E-06	NM_006991	NM_006991	Homo sapiens zinc finger protein 197 (ZNF197), transcript variant 1, mRNA [NM_006991]	NM_006991
A_32_P452655	1.59E-06	NM_009587	NM_009587	Homo sapiens lectin, galactoside-binding, soluble, 9 (galectin 9) (LGALS9), transcript variant long, mRNA [NM_009587]	NM_009587
A_24_P912985	1.60E-06	A_24_P912985			
A_23_P414343	1.62E-06	NM_005951	NM_005951	Homo sapiens metallothionein 1H (MT1H), mRNA [NM_005951]	NM_005951
A_24_P408981	1.64E-06	ENST00000276672			
A_24_P810290	1.64E-06	AK098668	AK098668	Homo sapiens cDNA FLJ25802 fis, clone TST07145. [AK098668]	XM_113641
A_24_P272523	1.64E-06	A_24_P272523			
A_23_P218463	1.64E-06	NM_013376	NM_013376	Homo sapiens SERTA domain containing 1 (SERTAD1), mRNA [NM_013376]	NM_013376
A_23_P154605	1.64E-06	NM_018837	NM_018837	Homo sapiens sulfatase 2 (SULF2), transcript variant 1, mRNA [NM_018837]	NM_018837
A_23_P125643	1.66E-06	NM_024087	NM_024087	Homo sapiens ankyrin repeat and SOCS box-containing 9 (ASB9), mRNA [NM_024087]	NM_024087
A_23_P154507	1.71E-06	NM_022334	NM_022334	Homo sapiens integrin beta 1 binding protein 1 (ITGB1BP1), transcript variant 2, mRNA [NM_022334]	NM_022334
A_23_P74359	1.74E-06	NM_004078	NM_004078	Homo sapiens cysteine and glycine-rich protein 1 (CSRP1), mRNA [NM_004078]	NM_004078

A_23_P24870	1.76E-06	NM_000610	NM_000610	Homo sapiens CD44 antigen (homing function and Indian blood group system) (CD44), transcript variant 1, mRNA [NM_000610]	NM_000610
A_23_P396666	1.77E-06	NM_015079	NM_015079	Homo sapiens KIAA1055 protein (KIAA1055), mRNA [NM_015079]	NM_015079
A_24_P298179	1.79E-06	ENST00000354530			
A_24_P194881	1.79E-06	ENST00000262795		Homo sapiens mRNA for KIAA1650 protein, partial cds. [AB051437]	
A_24_P277657	1.82E-06	NM_006877	NM_006877	Homo sapiens guanosine monophosphate reductase (GMPR), mRNA [NM_006877]	NM_006877
A_23_P57323	1.82E-06	NM_004449	NM_004449	Homo sapiens v-ets erythroblastosis virus E26 oncogene like (avian) (ERG), transcript variant 2, mRNA [NM_004449]	NM_004449
A_32_P200697	1.85E-06	NM_181709	NM_181709	Homo sapiens hypothetical protein LOC144347 (LOC144347), mRNA [NM_181709]	NM_181709
A_23_P150379	1.88E-06	NM_144765	NM_144765	Homo sapiens epithelial V-like antigen 1 (EVA1), transcript variant 2, mRNA [NM_144765]	NM_144765
A_23_P79069	1.96E-06	AK024488	AK024488	Homo sapiens mRNA for FLJ00087 protein, partial cds. [AK024488]	XM_029084
A_23_P131816	1.96E-06	NM_052951	NM_052951	Homo sapiens deoxynucleotidyltransferase, terminal, interacting protein 1 (DNTTIP1), mRNA [NM_052951]	NM_052951
A_23_P315571	2.00E-06	NM_015150	NM_015150	Homo sapiens raft-linking protein (RAFTLIN), mRNA [NM_015150]	NM_015150
A_23_P160968	2.02E-06	NM_018891	NM_018891	Homo sapiens laminin, gamma 2 (LAMC2), transcript variant 2, mRNA [NM_018891]	NM_018891
A_32_P129265	2.04E-06	U92981	U92981	Homo sapiens clone DT1P1B6 mRNA, CAG repeat region. [U92981]	
A_32_P512061	2.04E-06	NR_002188	NR_002188	Homo sapiens glucosidase, beta; acid, pseudogene (GBAP) on chromosome 1 [NR_002188]	NR_002188
A_23_P79889	2.06E-06	NM_020673	NM_020673	Homo sapiens RAB22A, member RAS oncogene family (RAB22A), mRNA [NM_020673]	NM_020673
A_23_P23221	2.06E-06	NM_001924	NM_001924	Homo sapiens growth arrest and DNA-damage-inducible, alpha (GADD45A), mRNA [NM_001924]	NM_001924
A_23_P73012	2.07E-06	NM_032823	NM_032823	Homo sapiens chromosome 9 open reading frame 3 (C9orf3), mRNA [NM_032823]	NM_032823
A_23_P317324	2.09E-06	NM_005241	NM_005241	Homo sapiens ecotropic viral integration site 1 (EVI1), mRNA [NM_005241]	NM_005241
A_24_P171873	2.09E-06	NM_012176	NM_012176	Homo sapiens F-box protein 4 (FBXO4), transcript variant 1, mRNA [NM_012176]	NM_012176
A_23_P211561	2.09E-06	AK093341	AK093341	Homo sapiens cDNA FLJ36022 fis, clone TESTI2016599. [AK093341]	
A_23_P146367	2.09E-06	NM_032310	NM_032310	Homo sapiens chromosome 9 open reading frame 89 (C9orf89), mRNA [NM_032310]	NM_032310
A_23_P168388	2.15E-06	NM_175571	NM_175571	Homo sapiens GTPase, IMAP family member 8 (GIMAP8), mRNA [NM_175571]	NM_175571
A_24_P18105	2.15E-06	NM_032466	NM_032466	Homo sapiens aspartate beta-hydroxylase (ASPH), transcript variant 3, mRNA [NM_032466]	NM_032466
A_32_P228348	2.17E-06	NM_207505	NM_207505	Homo sapiens FLJ45248 protein (FLJ45248), mRNA [NM_207505]	NM_207505
A_23_P13822	2.17E-06	NM_018423	NM_018423	Homo sapiens serine/threonine/tyrosine kinase 1 (STYK1), mRNA [NM_018423]	NM_018423
A_24_P596251	2.17E-06	A_24_P596251			
A_23_P102571	2.17E-06	NM_020062	NM_020062	Homo sapiens SLC2A4 regulator (SLC2A4RG), mRNA [NM_020062]	NM_020062
A_24_P127641	2.21E-06	AK128882	AK128882	Homo sapiens cDNA FLJ46914 fis, clone SPLEN2027852. [AK128882]	XM_499014
A_23_P404481	2.21E-06	NM_001400	NM_001400	Homo sapiens endothelial differentiation, sphingolipid G-protein-coupled receptor, 1 (EDG1), mRNA [NM_001400]	NM_001400
A_23_P24507	2.22E-06	AL833655	AL833655	Homo sapiens mRNA; cDNA DKFZp667O0320 (from clone DKFZp667O0320). [AL833655]	
A_24_P327886	2.22E-06	NM_003196	NM_003196	Homo sapiens transcription elongation factor A (SII), 3 (TCEA3), mRNA [NM_003196]	NM_003196
A_23_P66682	2.22E-06	NM_156036	NM_156036	Homo sapiens homeo box B6 (HOXB6), transcript variant 3, mRNA [NM_156036]	NM_156036
A_24_P329065	2.25E-06	NM_007048	NM_007048	Homo sapiens butyrophilin, subfamily 3, member A1 (BTN3A1), transcript variant 1, mRNA [NM_007048]	NM_007048
A_24_P211064	2.25E-06	NM_020648	NM_020648	Homo sapiens twisted gastrulation homolog 1 (Drosophila) (TWSG1), mRNA [NM_020648]	NM_020648
A_23_P200976	2.25E-06	NM_031207	NM_031207	Homo sapiens hydroxypyruvate isomerase homolog (E. coli) (HYI), mRNA [NM_031207]	NM_031207
A_32_P232588	2.26E-06	AA987695	AA987695	AA987695 oq88b08.s1 NCI_CGAP_Kid6 Homo sapiens cDNA clone IMAGE:1593399 3', mRNA sequence [AA987695]	
A_23_P23748	2.26E-06	NM_014969	NM_014969	Homo sapiens WD repeat domain 47 (WDR47), mRNA [NM_014969]	NM_014969
A_23_P354187	2.28E-06	NM_144628	NM_144628	Homo sapiens TBC1 domain family, member 20 (TBC1D20), mRNA [NM_144628]	NM_144628
A_24_P110983	2.32E-06	AK055109	AK055109	Homo sapiens cDNA FLJ30547 fis, clone BRAWH2001439. [AK055109]	
A_24_P178167	2.32E-06	A_24_P178167			
A_23_P206724	2.32E-06	NM_175617	NM_175617	Homo sapiens metallothionein 1E (functional) (MT1E), mRNA [NM_175617]	NM_175617
A_23_P60296	2.37E-06	NM_012383	NM_012383	Homo sapiens osteoclast stimulating factor 1 (OSTF1), mRNA [NM_012383]	NM_012383
A_24_P329795	2.41E-06	NM_007021	NM_007021	Homo sapiens chromosome 10 open reading frame 10 (C10orf10), mRNA [NM_007021]	NM_007021
A_23_P369456	2.44E-06	NM_033542	NM_033542	Homo sapiens chromosome 20 open reading frame 35 (C20orf35), mRNA [NM_033542]	NM_033542

A_24_P914434	2.47E-06	NM_000849	NM_000849	Homo sapiens glutathione S-transferase M3 (brain) (GSTM3), mRNA [NM_000849]	NM_000849
A_23_P86682	2.51E-06	NM_013451	NM_013451	Homo sapiens fer-1-like 3, myoferlin (C. elegans) (FER1L3), transcript variant 1, mRNA [NM_013451]	NM_013451
A_23_P133133	2.52E-06	NM_025144	NM_025144	Homo sapiens alpha-kinase 1 (ALPK1), mRNA [NM_025144]	NM_025144
A_23_P259506	2.52E-06	NM_032412	NM_032412	Homo sapiens putative nuclear protein ORF1-FL49 (ORF1-FL49), mRNA [NM_032412]	NM_032412
A_23_P341567	2.55E-06	NM_178833	NM_178833	Homo sapiens hypothetical protein BC009732 (LOC133308), mRNA [NM_178833]	NM_178833
A_23_P427703	2.56E-06	X97261	X97261	H.sapiens mRNA for metallothionein isoform 1R. [X97261]	
A_23_P119390	2.58E-06	NM_014601	NM_014601	Homo sapiens EH-domain containing 2 (EHD2), mRNA [NM_014601]	NM_014601
A_23_P159255	2.68E-06	NM_002845	NM_002845	Homo sapiens protein tyrosine phosphatase, receptor type, M (PTPRM), mRNA [NM_002845]	NM_002845
A_23_P329353	2.69E-06	NM_015463	NM_015463	Homo sapiens chromosome 2 open reading frame 32 (C2orf32), mRNA [NM_015463]	NM_015463
A_32_P217261	2.69E-06	AK023660	AK023660	Homo sapiens cDNA FLJ13598 fis, clone PLACE1009921. [AK023660]	
A_23_P21324	2.73E-06	NM_057179	NM_057179	Homo sapiens twist homolog 2 (Drosophila) (TWIST2), mRNA [NM_057179]	NM_057179
A_32_P141338	2.75E-06	BE930053	BE930053	BE930053 CM2-GN0054-230800-344-a10 GN0054 Homo sapiens cDNA, mRNA sequence [BE930053]	
A_23_P210619	2.75E-06	NM_016407	NM_016407	Homo sapiens chromosome 20 open reading frame 43 (C20orf43), mRNA [NM_016407]	NM_016407
A_23_P370005	2.80E-06	NM_003804	NM_003804	Homo sapiens receptor (TNFRSF)-interacting serine-threonine kinase 1 (RIPK1), mRNA [NM_003804]	NM_003804
A_24_P364807	2.83E-06	BX641069	BX641069	Homo sapiens mRNA; cDNA DKFZp686H22112 (from clone DKFZp686H22112). [BX641069]	
A_23_P96383	2.83E-06	NM_006307	NM_006307	Homo sapiens sushi-repeat-containing protein, X-linked (SRPX), mRNA [NM_006307]	NM_006307
A_23_P55936	2.83E-06	NM_004107	NM_004107	Homo sapiens Fc fragment of IgG, receptor, transporter, alpha (FCGRT), mRNA [NM_004107]	NM_004107
A_23_P75523	2.83E-06	NM_203330	NM_203330	Homo sapiens CD59 antigen p18-20 (antigen identified by monoclonal antibodies 16.3A5, EJ16, EJ30, EL32 and G344) (CD59), transcript variant 1, mRNA [NM_203330]	NM_203330
A_24_P10137	2.84E-06	NM_014059	NM_014059	Homo sapiens response gene to complement 32 (RGC32), mRNA [NM_014059]	NM_014059
A_23_P421843	2.89E-06	ENST00000285206		Homo sapiens hypothetical protein LOC201191, mRNA (cDNA clone IMAGE:5296646), partial cds. [BC047081]	
A_24_P564396	2.92E-06	THC2335868		ALU5_HUMAN (P39192) Alu subfamily SC sequence contamination warning entry, partial (8%) [THC2335868]	
A_23_P103775	2.97E-06	NM_032270	NM_032270	Homo sapiens leucine rich repeat containing 8 family, member C (LRRC8C), mRNA [NM_032270]	NM_032270
A_24_P411749	2.98E-06	NM_198569	NM_198569	Homo sapiens G protein-coupled receptor 126 (GPR126), mRNA [NM_198569]	NM_198569
A_23_P368681	3.03E-06	NM_015660	NM_015660	Homo sapiens GTPase, IMAP family member 2 (GIMAP2), mRNA [NM_015660]	NM_015660
A_24_P501520	3.04E-06	AK098185	AK098185	Homo sapiens cDNA FLJ40866 fis, clone TRACH2018718. [AK098185]	
A_23_P57137	3.05E-06	NM_018347	NM_018347	Homo sapiens chromosome 20 open reading frame 29 (C20orf29), mRNA [NM_018347]	NM_018347
A_23_P65401	3.06E-06	NM_021914	NM_021914	Homo sapiens cofilin 2 (muscle) (CFL2), transcript variant 1, mRNA [NM_021914]	NM_021914
A_23_P111995	3.12E-06	NM_002318	NM_002318	Homo sapiens lysyl oxidase-like 2 (LOXL2), mRNA [NM_002318]	NM_002318
A_23_P364024	3.13E-06	NM_006851	NM_006851	Homo sapiens GLI pathogenesis-related 1 (glioma) (GLIPR1), mRNA [NM_006851]	NM_006851
A_23_P200067	3.13E-06	NM_004442	NM_004442	Homo sapiens EPH receptor B2 (EPHB2), transcript variant 2, mRNA [NM_004442]	NM_004442
A_23_P105442	3.13E-06	NM_181711	NM_181711	Homo sapiens GRP1 (general receptor for phosphoinositides 1)-associated scaffold protein (GRASP), mRNA [NM_181711]	NM_181711
A_24_P682550	3.16E-06	A_24_P682550			
A_23_P163467	3.20E-06	NM_207380	NM_207380	Homo sapiens FLJ43339 protein (FLJ43339), mRNA [NM_207380]	NM_207380
A_23_P53126	3.22E-06	NM_005574	NM_005574	Homo sapiens LIM domain only 2 (rhombotin-like 1) (LMO2), mRNA [NM_005574]	NM_005574
A_23_P62741	3.24E-06	BC025721	BC025721	Homo sapiens EGF, latrophilin and seven transmembrane domain containing 1, mRNA (cDNA clone MGC:34204 IMAGE:5229055), complete cds. [BC025721]	XM_371262
A_23_P39074	3.24E-06	NM_006270	NM_006270	Homo sapiens related RAS viral (r-ras) oncogene homolog (RRAS), mRNA [NM_006270]	NM_006270
A_23_P89780	3.28E-06	NM_198129	NM_198129	Homo sapiens laminin, alpha 3 (LAMA3), transcript variant 1, mRNA [NM_198129]	NM_198129
A_23_P161156	3.29E-06	NM_182755	NM_182755	Homo sapiens hypothetical protein LOC220929 (LOC220929), mRNA [NM_182755]	NM_182755
A_23_P391264	3.30E-06	NM_007047	NM_007047	Homo sapiens butyrophilin, subfamily 3, member A2 (BTN3A2), mRNA [NM_007047]	NM_007047
A_24_P868583	3.31E-06	NM_001012421	NM_001012421	Homo sapiens OTTHUMP00000064580 (LOC441430), mRNA [NM_001012421]	NM_001012421
A_23_P29023	3.38E-06	NM_032910	NM_032910	Homo sapiens chromosome 21 open reading frame 119 (C21orf119), mRNA [NM_032910]	NM_032910
A_23_P36689	3.40E-06	NM_006992	NM_006992	Homo sapiens B7 gene (B7), transcript variant 2, mRNA [NM_006992]	NM_006992
A_24_P356830	3.41E-06	AK129956	AK129956	Homo sapiens cDNA FLJ26446 fis, clone KDN02743. [AK129956]	
A_23_P128919	3.44E-06	NM_002306	NM_002306	Homo sapiens lectin, galactoside-binding, soluble, 3 (galectin 3) (LGALS3), mRNA [NM_002306]	NM_002306

A_24_P38815	3.45E-06	NM_000391	NM_000391	Homo sapiens tripeptidyl peptidase I (TPP1), mRNA [NM_000391]	NM_000391
A_23_P39840	3.45E-06	NM_006634	NM_006634	Homo sapiens vesicle-associated membrane protein 5 (myobrevin) (VAMP5), mRNA [NM_006634]	NM_006634
A_24_P145134	3.46E-06	BC010943	BC010943	Homo sapiens oncostatin M receptor, mRNA (cDNA clone IMAGE:4043935), complete cds. [BC010943]	
A_23_P85682	3.47E-06	NM_005595	NM_005595	Homo sapiens nuclear factor I/A (NFIA), mRNA [NM_005595]	NM_005595
A_23_P62907	3.47E-06	NM_007348	NM_007348	Homo sapiens activating transcription factor 6 (ATF6), mRNA [NM_007348]	NM_007348
A_32_P44453	3.48E-06	NM_002194	NM_002194	Homo sapiens inositol polyphosphate-1-phosphatase (INPP1), mRNA [NM_002194]	NM_002194
A_23_P342138	3.48E-06	NM_152702	NM_152702	Homo sapiens chromosome 9 open reading frame 94 (C9orf94), mRNA [NM_152702]	NM_152702
A_24_P125469	3.48E-06	NM_006033	NM_006033	Homo sapiens lipase, endothelial (LIPG), mRNA [NM_006033]	NM_006033
A_23_P61886	3.49E-06	AK055659	AK055659	Homo sapiens cDNA FLJ31097 fis, clone IMR321000210. [AK055659]	
A_23_P106425	3.51E-06	NM_182616	NM_182616	Homo sapiens hypothetical protein MGC61550 (MGC61550), mRNA [NM_182616]	NM_182616
A_23_P21560	3.53E-06	NM_030797	NM_030797	Homo sapiens family with sequence similarity 49, member A (FAM49A), mRNA [NM_030797]	NM_030797
A_23_P169030	3.53E-06	NM_003842	NM_003842	Homo sapiens tumor necrosis factor receptor superfamily, member 10b (TNFRSF10B), transcript variant 1, mRNA [NM_003842]	NM_003842
A_32_P32722	3.53E-06	CR603845	CR603845	full-length cDNA clone CS0DI026YH22 of Placenta Cot 25-normalized of Homo sapiens (human). [CR603845]	
A_23_P149975	3.53E-06	NM_031453	NM_031453	Homo sapiens chromosome 10 open reading frame 45 (C10orf45), mRNA [NM_031453]	NM_031453
A_32_P497742	3.57E-06	AK091271	AK091271	Homo sapiens cDNA FLJ33952 fis, clone CTONG2018614. [AK091271]	
A_24_P148261	3.57E-06	AK021874	AK021874	Homo sapiens cDNA FLJ11812 fis, clone HEMBA1006364. [AK021874]	
A_24_P313354	3.58E-06	NM_021005	NM_021005	Homo sapiens nuclear receptor subfamily 2, group F, member 2 (NR2F2), mRNA [NM_021005]	NM_021005
A_23_P12343	3.64E-06	NM_000849	NM_000849	Homo sapiens glutathione S-transferase M3 (brain) (GSTM3), mRNA [NM_000849]	NM_000849
A_23_P83007	3.66E-06	NM_203403	NM_203403	Homo sapiens chromosome 9 open reading frame 150 (C9orf150), mRNA [NM_203403]	NM_203403
A_23_P115407	3.69E-06	NM_146421	NM_146421	Homo sapiens glutathione S-transferase M1 (GSTM1), transcript variant 2, mRNA [NM_146421]	NM_146421
A_24_P119141	3.70E-06	NM_000313	NM_000313	Homo sapiens protein S (alpha) (PROS1), mRNA [NM_000313]	NM_000313
A_23_P142294	3.71E-06	NM_014297	NM_014297	Homo sapiens ethylmalonic encephalopathy 1 (ETHE1), mRNA [NM_014297]	NM_014297
A_23_P417404	3.80E-06	AF151783	AF151783	Homo sapiens MEG3 (MEG3) mRNA, complete cds. [AF151783]	
A_24_P229911	3.82E-06	A_24_P229911			
A_23_P2793	3.84E-06	NM_001629	NM_001629	Homo sapiens arachidonate 5-lipoxygenase-activating protein (ALOX5AP), mRNA [NM_001629]	NM_001629
A_23_P216610	3.84E-06	NM_022486	NM_022486	Homo sapiens sushi domain containing 1 (SUSD1), mRNA [NM_022486]	NM_022486
A_24_P354715	3.85E-06	NM_002526	NM_002526	Homo sapiens 5'-nucleotidase, ecto (CD73) (NT5E), mRNA [NM_002526]	NM_002526
A_23_P137848	3.85E-06	NM_145729	NM_145729	Homo sapiens mitochondrial ribosomal protein L24 (MRPL24), nuclear gene encoding mitochondrial protein, transcript variant 1, mRNA [NM_145729]	NM_145729
A_32_P210642	3.85E-06	NM_201446	NM_201446	Homo sapiens EGF-like-domain, multiple 7 (EGFL7), transcript variant 2, mRNA [NM_201446]	NM_201446
A_32_P140489	3.88E-06	NM_001001557	NM_001001557	Homo sapiens growth differentiation factor 6 (GDF6), mRNA [NM_001001557]	NM_001001557
A_23_P105002	3.89E-06	NM_000327	NM_000327	Homo sapiens retinal outer segment membrane protein 1 (ROM1), mRNA [NM_000327]	NM_000327
A_24_P858698	3.91E-06	NM_002501	NM_002501	Homo sapiens nuclear factor I/X (CCAAT-binding transcription factor) (NFIX), mRNA [NM_002501]	NM_002501
A_23_P360340	3.92E-06	NM_001008224	NM_001008224	Homo sapiens uveal autoantigen with coiled-coil domains and ankyrin repeats (UACA), transcript variant 2, mRNA [NM_001008224]	NM_001008224
A_23_P146644	3.93E-06	NM_001002857	NM_001002857	Homo sapiens annexin A2 (ANXA2), transcript variant 2, mRNA [NM_001002857]	NM_001002857
A_23_P361773	3.95E-06	NM_001760	NM_001760	Homo sapiens cyclin D3 (CCND3), mRNA [NM_001760]	NM_001760
A_23_P61426	3.96E-06	NM_012331	NM_012331	Homo sapiens methionine sulfoxide reductase A (MSRA), mRNA [NM_012331]	NM_012331
A_23_P212688	3.97E-06	NM_005241	NM_005241	Homo sapiens ecotropic viral integration site 1 (EVI1), mRNA [NM_005241]	NM_005241
A_23_P34375	4.04E-06	NM_003196	NM_003196	Homo sapiens transcription elongation factor A (SII), 3 (TCEA3), mRNA [NM_003196]	NM_003196
A_23_P4662	4.06E-06	NM_005178	NM_005178	Homo sapiens B-cell CLL/lymphoma 3 (BCL3), mRNA [NM_005178]	NM_005178
A_23_P31399	4.07E-06	NM_000305	NM_000305	Homo sapiens paraoxonase 2 (PON2), transcript variant 1, mRNA [NM_000305]	NM_000305
A_23_P353035	4.07E-06	NM_001553	NM_001553	Homo sapiens insulin-like growth factor binding protein 7 (IGFBP7), mRNA [NM_001553]	NM_001553
A_23_P59616	4.11E-06	NM_173537	NM_173537	Homo sapiens GTF2I repeat domain containing 2 (GTF2IRD2), mRNA [NM_173537]	NM_173537
A_23_P160460	4.11E-06	NM_003115	NM_003115	Homo sapiens UDP-N-acteylglucosamine pyrophosphorylase 1 (UAP1), mRNA [NM_003115]	NM_003115
A_23_P316012	4.12E-06	NM_020663	NM_020663	Homo sapiens ras homolog gene family, member J (RHOJ), mRNA [NM_020663]	NM_020663

A_23_P62583	4.14E-06	NM_001409	NM_001409	Homo sapiens EGF-like-domain, multiple 3 (EGFL3), mRNA [NM_001409]	NM_001409
A_23_P40975	4.16E-06	NM_014674	NM_014674	Homo sapiens ER degradation enhancer, mannosidase alpha-like 1 (EDEM1), mRNA [NM_014674]	NM_014674
A_24_P86755	4.20E-06	NM_018956	NM_018956	Homo sapiens chromosome 9 open reading frame 9 (C9orf9), mRNA [NM_018956]	NM_018956
A_32_P66881	4.22E-06	THC2280549			
A_23_P1594	4.22E-06	NM_003377	NM_003377	Homo sapiens vascular endothelial growth factor B (VEGFB), mRNA [NM_003377]	NM_003377
A_23_P250619	4.23E-06	NM_153746	NM_153746	Homo sapiens zinc finger, DHHC-type containing 14 (ZDHHC14), transcript variant 2, mRNA [NM_153746]	NM_153746
A_23_P84063	4.31E-06	NM_016522	NM_016522	Homo sapiens neurotrimin (HNT), mRNA [NM_016522]	NM_016522
A_23_P1602	4.32E-06	NM_006779	NM_006779	Homo sapiens CDC42 effector protein (Rho GTPase binding) 2 (CDC42EP2), mRNA [NM_006779]	NM_006779
A_24_P937405	4.33E-06	NM_007173	NM_007173	Homo sapiens protease, serine, 23 (PRSS23), mRNA [NM_007173]	NM_007173
A_23_P46170	4.34E-06	NM_201542	NM_201542	Homo sapiens mediator of RNA polymerase II transcription, subunit 8 homolog (yeast) (MED8), transcript variant 1, mRNA [NM_201542]	NM_201542
A_24_P300952	4.39E-06	NM_001642	NM_001642	Homo sapiens amyloid beta (A4) precursor-like protein 2 (APLP2), mRNA [NM_001642]	NM_001642
A_23_P151870	4.48E-06	NM_015554	NM_015554	Homo sapiens glucuronyl C5-epimerase (GLCE), mRNA [NM_015554]	NM_015554
A_24_P79300	4.50E-06	NM_003277	NM_003277	Homo sapiens claudin 5 (transmembrane protein deleted in velocardiofacial syndrome) (CLDN5), mRNA [NM_003277]	NM_003277
A_32_P92642	4.52E-06	BE710245	BE710245	BE710245 IL3-HT0619-120700-212-G05 HT0619 Homo sapiens cDNA, mRNA sequence [BE710245]	
A_23_P65584	4.52E-06	NM_182476	NM_182476	Homo sapiens coenzyme Q6 homolog (yeast) (COQ6), transcript variant 1, mRNA [NM_182476]	NM_182476
A_23_P121196	4.52E-06	NM_024334	NM_024334	Homo sapiens transmembrane protein 43 (TMEM43), mRNA [NM_024334]	NM_024334
A_23_P30363	4.52E-06	NM_004199	NM_004199	Homo sapiens procollagen-proline, 2-oxoglutarate 4-dioxygenase (proline 4-hydroxylase), alpha polypeptide II (P4HA2), transcript variant 1, mRNA [NM_004199]	NM_004199
A_23_P31903	4.52E-06	NM_183057	NM_183057	Homo sapiens vacuolar protein sorting 28 (yeast) (VPS28), transcript variant 2, mRNA [NM_183057]	NM_183057
A_24_P305933	4.54E-06	NM_020698	NM_020698	Homo sapiens transmembrane and coiled-coil domain family 3 (TMCC3), mRNA [NM_020698]	NM_020698
A_23_P136978	4.55E-06	NM_014467	NM_014467	Homo sapiens sushi-repeat-containing protein, X-linked 2 (SRPX2), mRNA [NM_014467]	NM_014467
A_32_P85813	4.57E-06	BF326020	BF326020	BF326020 QV1-AN0059-040800-295-e05 AN0059 Homo sapiens cDNA, mRNA sequence [BF326020]	
A_24_P40306	4.60E-06	NM_000185	NM_000185	Homo sapiens serine (or cysteine) proteinase inhibitor, clade D (heparin cofactor), member 1 (SERPIND1), mRNA [NM_000185]	NM_000185
A_24_P943301	4.60E-06	ENST00000292357		Homo sapiens cDNA FLJ25943 fis, clone JTH10559. [AK098809]	
A_23_P160582	4.61E-06	NM_031207	NM_031207	Homo sapiens hydroxypyruvate isomerase homolog (E. coli) (HYI), mRNA [NM_031207]	NM_031207
A_24_P217489	4.64E-06	NM_000824	NM_000824	Homo sapiens glycine receptor, beta (GLRB), mRNA [NM_000824]	NM_000824
A_23_P101093	4.68E-06	NM_016429	NM_016429	Homo sapiens coatomer protein complex, subunit zeta 2 (COPZ2), mRNA [NM_016429]	NM_016429
A_24_P413988	4.68E-06	NM_006464	NM_006464	Homo sapiens trans-golgi network protein 2 (TGOLN2), mRNA [NM_006464]	NM_006464
A_23_P46852	4.71E-06	NM_024928	NM_024928	Homo sapiens oligonucleotide/oligosaccharide-binding fold containing 1 (OBFC1), mRNA [NM_024928]	NM_024928
A_32_P74814	4.75E-06	THC2314823			
A_23_P72387	4.75E-06	NM_021638	NM_021638	Homo sapiens actin filament associated protein (AFAP), transcript variant 1, mRNA [NM_021638]	NM_021638
A_23_P203994	4.75E-06	NM_002813	NM_002813	Homo sapiens proteasome (prosome, macropain) 26S subunit, non-ATPase, 9 (PSMD9), mRNA [NM_002813]	NM_002813
A_23_P6674	4.80E-06	NM_020169	NM_020169	Homo sapiens latexin (LXN), mRNA [NM_020169]	NM_020169
A_23_P97296	4.80E-06	NM_018090	NM_018090	Homo sapiens adaptin-ear-binding coat-associated protein 2 (NECAP2), mRNA [NM_018090]	NM_018090
A_32_P107372	4.82E-06	NM_002053	NM_002053	Homo sapiens guanylate binding protein 1, interferon-inducible, 67kDa (GBP1), mRNA [NM_002053]	NM_002053
A_23_P423309	4.84E-06	NM_016580	NM_016580	Homo sapiens protocadherin 12 (PCDH12), mRNA [NM_016580]	NM_016580
A_23_P91390	4.84E-06	NM_000361	NM_000361	Homo sapiens thrombomodulin (THBD), mRNA [NM_000361]	NM_000361
A_23_P54649	4.87E-06	NM_153425	NM_153425	Homo sapiens TNFRSF1A-associated via death domain (TRADD), transcript variant 2, mRNA [NM_153425]	NM_153425
A_32_P120330	4.88E-06	THC2329478			
A_23_P79122	4.98E-06	NM_018467	NM_018467	Homo sapiens uncharacterized hematopoietic stem/progenitor cells protein MDS032 (MDS032), mRNA [NM_018467]	NM_018467
A_23_P218706	5.06E-06	NM_024325	NM_024325	Homo sapiens zinc finger protein 343 (ZNF343), mRNA [NM_024325]	NM_024325
A_23_P166677	5.08E-06	NM_022736	NM_022736	Homo sapiens major facilitator superfamily domain containing 1 (MFSD1), mRNA [NM_022736]	NM_022736
A_24_P104174	5.10E-06	NM_016281	NM_016281	Homo sapiens TAO kinase 3 (TAOK3), mRNA [NM_016281]	NM_016281
A_23_P211244	5.13E-06	NM_206962	NM_206962	Homo sapiens HMT1 hnRNP methyltransferase-like 1 (S. cerevisiae) (HRMT1L1), transcript variant 1, mRNA [NM_206962]	NM_206962

A_23_P166248	5.13E-06	NM_004414	NM_004414	Homo sapiens Down syndrome critical region gene 1 (DSCR1), transcript variant 1, mRNA [NM_004414]	NM_004414
A_23_P404120	5.14E-06	NM_173802	NM_173802	Homo sapiens hypothetical protein MGC50559 (MGC50559), mRNA [NM_173802]	NM_173802
A_23_P423108	5.15E-06	NM_182499	NM_182499	Homo sapiens tudor domain containing 10 (TDRD10), mRNA [NM_182499]	NM_182499
A_24_P686014	5.15E-06	A_24_P686014			
A_32_P88965	5.17E-06	BX537651	BX537651	Homo sapiens mRNA; cDNA DKFZp686C0390 (from clone DKFZp686C0390). [BX537651]	
A_24_P637651	5.22E-06	THC2314600			
A_23_P77174	5.24E-06	NM_004855	NM_004855	Homo sapiens phosphatidylinositol glycan, class B (PIGB), mRNA [NM_004855]	NM_004855
A_23_P114084	5.26E-06	NM_000444	NM_000444	Homo sapiens phosphate regulating endopeptidase homolog, X-linked (hypophosphatemia, vitamin D resistant rickets) (PHEX), transcript variant 1843529, mRNA [NM_000444]	NM_000444
A_23_P41664	5.28E-06	ENST00000334994		Homo sapiens synleurin mRNA, complete cds. [AY280614]	
A_32_P188953	5.30E-06	NM_001005732	NM_001005732	Homo sapiens chromosome 21 open reading frame 34 (C21orf34), transcript variant 1, mRNA [NM_001005732]	NM_001005732
A_24_P288890	5.32E-06	NM_181709	NM_181709	Homo sapiens hypothetical protein LOC144347 (LOC144347), mRNA [NM_181709]	NM_181709
A_24_P84130	5.36E-06	AK024680	AK024680	Homo sapiens cDNA: FLJ21027 fis, clone CAE07110. [AK024680]	
A_24_P209765	5.38E-06	NM_007234	NM_007234	Homo sapiens dynactin 3 (p22) (DCTN3), transcript variant 1, mRNA [NM_007234]	NM_007234
A_23_P33256	5.40E-06	NM_000174	NM_000174	Homo sapiens glycoprotein IX (platelet) (GP9), mRNA [NM_000174]	NM_000174
A_23_P51986	5.40E-06	NM_006697	NM_006697	Homo sapiens myotubularin related protein 11 (MTMR11), transcript variant 1, mRNA [NM_006697]	NM_006697
A_23_P63017	5.40E-06	NM_006499	NM_006499	Homo sapiens lectin, galactoside-binding, soluble, 8 (galectin 8) (LGALS8), transcript variant 1, mRNA [NM_006499]	NM_006499
A_24_P74371	5.42E-06	NM_000308	NM_000308	Homo sapiens protective protein for beta-galactosidase (galactosialidosis) (PPGB), mRNA [NM_000308]	NM_000308
A_23_P53567	5.45E-06	NM_016053	NM_016053	Homo sapiens CGI-116 protein (CGI-116), mRNA [NM_016053]	NM_016053
A_23_P209636	5.46E-06	NM_006449	NM_006449	Homo sapiens CDC42 effector protein (Rho GTPase binding) 3 (CDC42EP3), mRNA [NM_006449]	NM_006449
A_23_P149562	5.51E-06	NM_004815	NM_004815	Homo sapiens Rho GTPase activating protein 29 (ARHGAP29), mRNA [NM_004815]	NM_004815
A_23_P121527	5.54E-06	NM_015990	NM_015990	Homo sapiens kelch-like 5 (Drosophila) (KLHL5), mRNA [NM_015990]	NM_015990
A_23_P50426	5.56E-06	NM_015493	NM_015493	Homo sapiens ankyrin repeat domain 25 (ANKRD25), mRNA [NM_015493]	NM_015493
A_24_P930088	5.60E-06	AK123264	AK123264	Homo sapiens cDNA FLJ41270 fis, clone BRAMY2036387. [AK123264]	
A_23_P203882	5.60E-06	NM_022791	NM_022791	Homo sapiens matrix metalloproteinase 19 (MMP19), transcript variant rasi-6, mRNA [NM_022791]	NM_022791
A_24_P148750	5.60E-06	NM_004844	NM_004844	Homo sapiens SH3-domain binding protein 5 (BTK-associated) (SH3BP5), transcript variant 1, mRNA [NM_004844]	NM_004844
A_32_P166693	5.60E-06	THC2265769		Q9GEJ2 (Q9GEJ2) NADH dehydrogenase subunit F (Fragment), partial (5%) [THC2265769]	
A_23_P131435	5.64E-06	NM_014880	NM_014880	Homo sapiens CD302 antigen (CD302), mRNA [NM_014880]	NM_014880
A_24_P39378	5.68E-06	NM_004748	NM_004748	Homo sapiens cell cycle progression 1 (CCPG1), transcript variant 1, mRNA [NM_004748]	NM_004748
A_23_P100711	5.68E-06	NM_000304	NM_000304	Homo sapiens peripheral myelin protein 22 (PMP22), transcript variant 1, mRNA [NM_000304]	NM_000304
A_23_P394154	5.72E-06	NM_173457	NM_173457	Homo sapiens phosphodiesterase 8A (PDE8A), transcript variant 5, mRNA [NM_173457]	NM_173457
A_24_P345451	5.72E-06	NM_024843	NM_024843	Homo sapiens cytochrome b reductase 1 (CYBRD1), mRNA [NM_024843]	NM_024843
A_23_P43276	5.73E-06	NM_032777	NM_032777	Homo sapiens G protein-coupled receptor 124 (GPR124), mRNA [NM_032777]	NM_032777
A_23_P61371	5.79E-06	NM_198282	NM_198282	Homo sapiens hypothetical protein LOC340061 (LOC340061), mRNA [NM_198282]	NM_198282
A_32_P342064	5.79E-06	NM_002032	NM_002032	Homo sapiens ferritin, heavy polypeptide 1 (FTH1), mRNA [NM_002032]	NM_002032
A_24_P361896	5.81E-06	NM_005953	NM_005953	Homo sapiens metallothionein 2A (MT2A), mRNA [NM_005953]	NM_005953
A_32_P227870	5.85E-06	BC042520	BC042520	Homo sapiens, clone IMAGE:4828750, mRNA. [BC042520]	
A_23_P159956	5.85E-06	NM_012216	NM_012216	Homo sapiens midline 2 (MID2), transcript variant 1, mRNA [NM_012216]	NM_012216
A_24_P942945	5.85E-06	NM_198569	NM_198569	Homo sapiens G protein-coupled receptor 126 (GPR126), mRNA [NM_198569]	NM_198569
A_32_P235159	5.91E-06	CR623684	CR623684	full-length cDNA clone CS0DK004YM05 of HeLa cells Cot 25-normalized of Homo sapiens (human). [CR623684]	XM_087225
A_23_P353056	5.91E-06	NM_014807	NM_014807	Homo sapiens transmembrane protein 24 (TMEM24), mRNA [NM_014807]	NM_014807
A_24_P636882	5.96E-06	THC2337176		ALU1_HUMAN (P39188) Alu subfamily J sequence contamination warning entry, partial (30%) [THC2337176]	
A_23_P6335	5.97E-06	NM_000185	NM_000185	Homo sapiens serine (or cysteine) proteinase inhibitor, clade D (heparin cofactor), member 1 (SERPIND1), mRNA [NM_000185]	NM_000185
A_23_P54636	5.99E-06	NM_004691	NM_004691	Homo sapiens ATPase, H+ transporting, lysosomal 38kDa, V0 subunit d isoform 1 (ATP6V0D1), mRNA [NM_004691]	NM_004691
A_23_P347169	6.00E-06	NM_001001927	NM_001001927	Homo sapiens mitochondrial tumor suppressor 1 (MTUS1), nuclear gene encoding mitochondrial protein, transcript variant 3, mRNA [NM_001001927]	NM_001001927

A_23_P162589	6.06E-06	NM_001017535	NM_001017535	Homo sapiens vitamin D (1,25- dihydroxyvitamin D3) receptor (VDR), transcript variant 2, mRNA [NM_001017535]	NM_001017535
A_23_P10995	6.11E-06	NM_014483	NM_014483	Homo sapiens RNA binding motif, single stranded interacting protein (RBMS3), transcript variant 2, mRNA [NM_014483]	NM_014483
A_23_P121657	6.12E-06	NM_005114	NM_005114	Homo sapiens heparan sulfate (glucosamine) 3-O-sulfotransferase 1 (HS3ST1), mRNA [NM_005114]	NM_005114
A_32_P447001	6.13E-06	AK130862	AK130862	Homo sapiens cDNA FLJ27352 fis, clone TST05165. [AK130862]	XM_085236
A_23_P5221	6.21E-06	NM_032433	NM_032433	Homo sapiens zinc finger protein 333 (ZNF333), mRNA [NM_032433]	NM_032433
A_23_P94230	6.23E-06	NM_015364	NM_015364	Homo sapiens lymphocyte antigen 96 (LY96), mRNA [NM_015364]	NM_015364
A_24_P90005	6.26E-06	NM_005203	NM_005203	Homo sapiens collagen, type XIII, alpha 1 (COL13A1), transcript variant 1, mRNA [NM_005203]	NM_005203
A_24_P129277	6.27E-06	NM_006092	NM_006092	Homo sapiens caspase recruitment domain family, member 4 (CARD4), mRNA [NM_006092]	NM_006092
A_23_P347632	6.28E-06	NM_014751	NM_014751	Homo sapiens metastasis suppressor 1 (MTSS1), mRNA [NM_014751]	NM_014751
A_23_P135164	6.39E-06	NM_207309	NM_207309	Homo sapiens UDP-N-acteylglucosamine pyrophosphorylase 1-like 1 (UAP1L1), mRNA [NM_207309]	NM_207309
A_23_P354387	6.40E-06	NM_013451	NM_013451	Homo sapiens fer-1-like 3, myoferlin (C. elegans) (FER1L3), transcript variant 1, mRNA [NM_013451]	NM_013451
A_23_P62901	6.41E-06	NM_006763	NM_006763	Homo sapiens BTG family, member 2 (BTG2), mRNA [NM_006763]	NM_006763
A_24_P240065	6.41E-06	NM_016079	NM_016079	Homo sapiens vacuolar protein sorting 24 (yeast) (VPS24), transcript variant 1, mRNA [NM_016079]	NM_016079
A_23_P46131	6.42E-06	NM_024869	NM_024869	Homo sapiens glycine/arginine rich protein 1 (GRRP1), mRNA [NM_024869]	NM_024869
A_23_P168587	6.45E-06	NM_138771	NM_138771	Homo sapiens alpha-1,3(6)-mannosylglycoprotein beta-1,6-N-acetyl-glucosaminyltransferase-like (LOC90693), mRNA [NM_138771]	NM_138771
A_23_P152727	6.48E-06	NM_014798	NM_014798	Homo sapiens pleckstrin homology domain containing, family M (with RUN domain) member 1 (PLEKHM1), mRNA [NM_014798]	NM_014798
A_23_P69941	6.49E-06	AK002097	AK002097	Homo sapiens cDNA FLJ11235 fis, clone PLACE1008488. [AK002097]	XM_496773
A_23_P109201	6.49E-06	NM_020531	NM_020531	Homo sapiens chromosome 20 open reading frame 3 (C20orf3), mRNA [NM_020531]	NM_020531
A_23_P365614	6.50E-06	NM_004557	NM_004557	Homo sapiens Notch homolog 4 (Drosophila) (NOTCH4), mRNA [NM_004557]	NM_004557
A_23_P144796	6.52E-06	NM_003687	NM_003687	Homo sapiens PDZ and LIM domain 4 (PDLIM4), mRNA [NM_003687]	NM_003687
A_24_P71973	6.54E-06	NM_002253	NM_002253	Homo sapiens kinase insert domain receptor (a type III receptor tyrosine kinase) (KDR), mRNA [NM_002253]	NM_002253
A_32_P226620	6.57E-06	THC2441546			
A_24_P396702	6.58E-06	NM_014880	NM_014880	Homo sapiens CD302 antigen (CD302), mRNA [NM_014880]	NM_014880
A_23_P207399	6.61E-06	NM_031858	NM_031858	Homo sapiens neighbor of BRCA1 gene 1 (NBR1), transcript variant 2, mRNA [NM_031858]	NM_031858
A_23_P63660	6.63E-06	NM_032333	NM_032333	Homo sapiens chromosome 10 open reading frame 58 (C10orf58), mRNA [NM_032333]	NM_032333
A_32_P69930	6.71E-06	NM_153376	NM_153376	Homo sapiens hypothetical protein FLJ90575 (FLJ90575), mRNA [NM_153376]	NM_153376
A_23_P159027	6.71E-06	NM_015461	NM_015461	Homo sapiens zinc finger protein 521 (ZNF521), mRNA [NM_015461]	NM_015461
A_24_P320796	6.72E-06	NM_182827	NM_182827	Homo sapiens FK506 binding protein 9-like (FKBP9L), mRNA [NM_182827]	NM_182827
A_24_P59607	6.77E-06	BC019938	BC019938	Homo sapiens hypothetical protein LOC339263, mRNA (cDNA clone IMAGE:3924994), partial cds. [BC019938]	XM_378661
A_23_P79703	6.77E-06	NM_000183	NM_000183	Homo sapiens hydroxyacyl-Coenzyme A dehydrogenase/3-ketoacyl-Coenzyme A thiolase/enoyl-Coenzyme A hydratase (trifunctional protein), beta subunit (HADHB), mRNA [NM_000183]	NM_000183
A_23_P58002	6.81E-06	NM_022171	NM_022171	Homo sapiens T-cell leukemia translocation altered gene (TCTA), mRNA [NM_022171]	NM_022171
A_23_P143964	6.87E-06	NM_004844	NM_004844	Homo sapiens SH3-domain binding protein 5 (BTK-associated) (SH3BP5), transcript variant 1, mRNA [NM_004844]	NM_004844
A_23_P73114	6.88E-06	NM_000313	NM_000313	Homo sapiens protein S (alpha) (PROS1), mRNA [NM_000313]	NM_000313
A_23_P109345	6.88E-06	NM_004339	NM_004339	Homo sapiens pituitary tumor-transforming 1 interacting protein (PTTG1IP), mRNA [NM_004339]	NM_004339
A_23_P48717	6.92E-06	NM_006432	NM_006432	Homo sapiens Niemann-Pick disease, type C2 (NPC2), mRNA [NM_006432]	NM_006432
A_23_P368996	6.98E-06	NM_198075	NM_198075	Homo sapiens hypothetical protein DKFZp761L1518 (DKFZp761L1518), mRNA [NM_198075]	NM_198075
A_23_P41390	7.01E-06	NM_018986	NM_018986	Homo sapiens SH3 domain and tetratricopeptide repeats 1 (SH3TC1), mRNA [NM_018986]	NM_018986
A_23_P333330	7.02E-06	BC005015	BC005015	Homo sapiens zinc finger, DHHC-type containing 24, mRNA (cDNA clone IMAGE:3636082), partial cds. [BC005015]	
A_32_P117453	7.03E-06	BE764339	BE764339	BE764339 IL5-NT0071-180700-116-h04 NT0071 Homo sapiens cDNA, mRNA sequence [BE764339]	
A_24_P921086	7.04E-06	THC2376213		Q78EJ8 (Q78EJ8) Calpain , partial (78%) [THC2376213]	
A_23_P168188	7.10E-06	NM_001009991	NM_001009991	Homo sapiens synaptotagmin-like 3 (SYTL3), mRNA [NM_001009991]	NM_001009991
A_24_P209109	7.12E-06	NM_030571	NM_030571	Homo sapiens Nedd4 family interacting protein 1 (NDFIP1), mRNA [NM_030571]	NM_030571
A_23_P307544	7.20E-06	AK055196	AK055196	Homo sapiens cDNA FLJ30634 fis, clone CTONG2002453. [AK055196]	XM_498440
A_23_P14083	7.25E-06	NM_181847	NM_181847	Homo sapiens adhesion molecule with Ig-like domain 2 (AMIGO2), mRNA [NM_181847]	NM_181847

A_23_P114172	7.31E-06	NM_017752	NM_017752	Homo sapiens FLJ20298 protein (FLJ20298), transcript variant 1, mRNA [NM_017752]	NM_017752
A_24_P136453	7.34E-06	NM_022648	NM_022648	Homo sapiens tensin 1 (TNS1), mRNA [NM_022648]	NM_022648
A_32_P170736	7.39E-06	AK098422	AK098422	Homo sapiens cDNA FLJ25556 fis, clone JTH02629. [AK098422]	
A_23_P132915	7.41E-06	NM_138389	NM_138389	Homo sapiens hypothetical protein BC001096 (LOC92689), mRNA [NM_138389]	NM_138389
A_24_P397928	7.41E-06	NM_147780	NM_147780	Homo sapiens cathepsin B (CTSB), transcript variant 2, mRNA [NM_147780]	NM_147780
A_23_P350249	7.42E-06	AB065086	AB065086	Homo sapiens mRNA for OK/KNS-CL.4, complete cds. [AB065086]	
A_32_P71736	7.42E-06	NM_004569	NM_004569	Homo sapiens phosphatidylinositol glycan, class H (PIGH), mRNA [NM_004569]	NM_004569
A_23_P148047	7.45E-06	NM_000958	NM_000958	Homo sapiens prostaglandin E receptor 4 (subtype EP4) (PTGER4), mRNA [NM_000958]	NM_000958
A_24_P376339	7.46E-06	BC016333	BC016333	Homo sapiens cyclin L2, mRNA (cDNA clone MGC:23944 IMAGE:4076039), complete cds. [BC016333]	
A_24_P942321	7.62E-06	NM_018027	NM_018027	Homo sapiens FERM domain containing 4A (FRMD4A), mRNA [NM_018027]	NM_018027
A_23_P257871	7.65E-06	NM_001343	NM_001343	Homo sapiens disabled homolog 2, mitogen-responsive phosphoprotein (Drosophila) (DAB2), mRNA [NM_001343]	NM_001343
A_23_P323180	7.67E-06	NM_006898	NM_006898	Homo sapiens homeo box D3 (HOXD3), mRNA [NM_006898]	NM_006898
A_23_P215900	7.73E-06	NM_016240	NM_016240	Homo sapiens scavenger receptor class A, member 3 (SCARA3), transcript variant 1, mRNA [NM_016240]	NM_016240
A_23_P2884	7.79E-06	NM_004569	NM_004569	Homo sapiens phosphatidylinositol glycan, class H (PIGH), mRNA [NM_004569]	NM_004569
A_24_P347378	7.83E-06	NM_001629	NM_001629	Homo sapiens arachidonate 5-lipoxygenase-activating protein (ALOX5AP), mRNA [NM_001629]	NM_001629
A_23_P53557	7.83E-06	NM_002342	NM_002342	Homo sapiens lymphotoxin beta receptor (TNFR superfamily, member 3) (LTBR), mRNA [NM_002342]	NM_002342
A_23_P55076	7.90E-06	NM_130766	NM_130766	Homo sapiens skeletal muscle and kidney enriched inositol phosphatase (SKIP), transcript variant 2, mRNA [NM_130766]	NM_130766
A_32_P123088	7.97E-06	NM_021649	NM_021649	Homo sapiens toll-like receptor adaptor molecule 2 (TICAM2), mRNA [NM_021649]	NM_021649
A_32_P98975	8.02E-06	NM_052849	NM_052849	Homo sapiens hypothetical protein MGC20481 (MGC20481), mRNA [NM_052849]	NM_052849
A_24_P75920	8.03E-06	ENST00000327804		Homo sapiens hypothetical protein LOC126075, mRNA (cDNA clone IMAGE:5171832). [BC038439]	XM_496289
A_23_P68807	8.03E-06	BC021857	BC021857	Homo sapiens, clone IMAGE:4994346, mRNA. [BC021857]	
A_23_P200160	8.05E-06	NM_001014975	NM_001014975	Homo sapiens complement factor H (CFH), transcript variant 2, mRNA [NM_001014975]	NM_001014975
A_24_P96961	8.06E-06	NM_025106	NM_025106	Homo sapiens SPRY domain-containing SOCS box protein SSB-1 (SSB1), mRNA [NM_025106]	NM_025106
A_23_P63371	8.07E-06	NM_003189	NM_003189	Homo sapiens T-cell acute lymphocytic leukemia 1 (TAL1), mRNA [NM_003189]	NM_003189
A_24_P31929	8.08E-06	NM_000950	NM_000950	Homo sapiens proline rich Gla (G-carboxyglutamic acid) 1 (PRRG1), mRNA [NM_000950]	NM_000950
A_23_P435394	8.12E-06	NM_000958	NM_000958	Homo sapiens prostaglandin E receptor 4 (subtype EP4) (PTGER4), mRNA [NM_000958]	NM_000958
A_23_P330611	8.14E-06	NM_003387	NM_003387	Homo sapiens Wiskott-Aldrich syndrome protein interacting protein (WASPIP), mRNA [NM_003387]	NM_003387
A_23_P70355	8.14E-06	NM_004568	NM_004568	Homo sapiens serine (or cysteine) proteinase inhibitor, clade B (ovalbumin), member 6 (SERPINB6), mRNA [NM_004568]	NM_004568
A_23_P320739	8.17E-06	NM_002397	NM_002397	Homo sapiens MADS box transcription enhancer factor 2, polypeptide C (myocyte enhancer factor 2C) (MEF2C), mRNA [NM_002397]	NM_002397
A_23_P160559	8.17E-06	NM_004425	NM_004425	Homo sapiens extracellular matrix protein 1 (ECM1), transcript variant 1, mRNA [NM_004425]	NM_004425
A_24_P156113	8.20E-06	NM_014601	NM_014601	Homo sapiens EH-domain containing 2 (EHD2), mRNA [NM_014601]	NM_014601
A_24_P251053	8.21E-06	NM_024329	NM_024329	Homo sapiens EF-hand domain family, member D2 (EFHD2), mRNA [NM_024329]	NM_024329
A_23_P334123	8.24E-06	NM_030790	NM_030790	Homo sapiens T-cell immunomodulatory protein (CDA08), mRNA [NM_030790]	NM_030790
A_23_P256051	8.27E-06	NM_203302	NM_203302	Homo sapiens similar to RPL23AP7 protein (MGC70863), transcript variant 2, mRNA [NM_203302]	NM_203302
A_23_P99802	8.32E-06	NM_013231	NM_013231	Homo sapiens fibronectin leucine rich transmembrane protein 2 (FLRT2), mRNA [NM_013231]	NM_013231
A_23_P329261	8.35E-06	NM_000891	NM_000891	Homo sapiens potassium inwardly-rectifying channel, subfamily J, member 2 (KCNJ2), mRNA [NM_000891]	NM_000891
A_24_P639629	8.37E-06	AK056245	AK056245	Homo sapiens cDNA FLJ31683 fis, clone NT2RI2005353. [AK056245]	
A_23_P360804	8.39E-06	NM_020939	NM_020939	Homo sapiens copine V (CPNE5), mRNA [NM_020939]	NM_020939
A_32_P116556	8.41E-06	AB058761	AB058761	Homo sapiens mRNA for KIAA1858 protein, partial cds. [AB058761]	XM_040592
A_23_P210210	8.44E-06	NM_001430	NM_001430	Homo sapiens endothelial PAS domain protein 1 (EPAS1), mRNA [NM_001430]	NM_001430
A_23_P160433	8.47E-06	NM_024709	NM_024709	Homo sapiens chromosome 1 open reading frame 115 (C1orf115), mRNA [NM_024709]	NM_024709
A_24_P247931	8.47E-06	NM_183420	NM_183420	Homo sapiens F-box protein 25 (FBXO25), transcript variant 2, mRNA [NM_183420]	NM_183420
A_23_P76059	8.47E-06	ENST00000354743		full-length cDNA clone CS0DA004YM06 of Neuroblastoma of Homo sapiens (human). [CR594105]	
A_23_P121716	8.63E-06	NM_005139	NM_005139	Homo sapiens annexin A3 (ANXA3), mRNA [NM_005139]	NM_005139

A_24_P753161	8.72E-06	NM_001204	NM_001204	Homo sapiens bone morphogenetic protein receptor, type II (serine/threonine kinase) (BMPR2), transcript variant 1, mRNA [NM_001204]	NM_001204
A_23_P23443	8.72E-06	NM_024329	NM_024329	Homo sapiens EF-hand domain family, member D2 (EFHD2), mRNA [NM_024329]	NM_024329
A_23_P38864	8.72E-06	NM_006423	NM_006423	Homo sapiens Rab acceptor 1 (prenylated) (RABAC1), mRNA [NM_006423]	NM_006423
A_23_P156289	8.77E-06	NM_003999	NM_003999	Homo sapiens oncostatin M receptor (OSMR), mRNA [NM_003999]	NM_003999
A_24_P925611	8.78E-06	NM_006386	NM_006386	Homo sapiens DEAD (Asp-Glu-Ala-Asp) box polypeptide 17 (DDX17), transcript variant 1, mRNA [NM_006386]	NM_006386
A_32_P182511	8.83E-06	THC2281182			
A_23_P91827	8.83E-06	NM_032778	NM_032778	Homo sapiens MYC induced nuclear antigen (MINA), transcript variant 2, mRNA [NM_032778]	NM_032778
A_23_P213518	8.83E-06	NM_173060	NM_173060	Homo sapiens calpastatin (CAST), transcript variant 2, mRNA [NM_173060]	NM_173060
A_23_P501583	8.84E-06	NM_002505	NM_002505	Homo sapiens nuclear transcription factor Y, alpha (NFYA), transcript variant 1, mRNA [NM_002505]	NM_002505
A_23_P133902	8.88E-06	NM_014068	NM_014068	Homo sapiens psoriasis susceptibility 1 candidate 1 (PSORS1C1), mRNA [NM_014068]	NM_014068
A_24_P212565	8.88E-06	A_24_P212565			
A_32_P37474	8.89E-06	ENST00000340836		PREDICTED: Homo sapiens similar to supervillin isoform 2; membrane-associated F-actin binding protein p205; archvillin (LOC387648), mRNA [XM_370542]	XM_370542
A_23_P209183	8.92E-06	NM_024656	NM_024656	Homo sapiens glycosyltransferase 25 domain containing 1 (GLT25D1), mRNA [NM_024656]	NM_024656
A_23_P151506	8.93E-06	NM_016445	NM_016445	Homo sapiens pleckstrin 2 (PLEK2), mRNA [NM_016445]	NM_016445
A_23_P209408	8.93E-06	NM_032977	NM_032977	Homo sapiens caspase 10, apoptosis-related cysteine protease (CASP10), transcript variant D, mRNA [NM_032977]	NM_032977
A_23_P145957	8.93E-06	NM_022445	NM_022445	Homo sapiens thiamin pyrophosphokinase 1 (TPK1), mRNA [NM_022445]	NM_022445
A_24_P100830	8.96E-06	NM_207337	NM_207337	Homo sapiens hypothetical protein LOC196394 (LOC196394), mRNA [NM_207337]	NM_207337
A_24_P945113	9.01E-06	CR596233	CR596233	full-length cDNA clone CS0DF012YN06 of Fetal brain of Homo sapiens (human). [CR596233]	
A_23_P250379	9.07E-06	NM_015274	NM_015274	Homo sapiens mannosidase, alpha, class 2B, member 2 (MAN2B2), mRNA [NM_015274]	NM_015274
A_23_P53081	9.07E-06	NM_020896	NM_020896	Homo sapiens oxysterol binding protein-like 5 (OSBPL5), transcript variant 1, mRNA [NM_020896]	NM_020896
A_23_P350574	9.20E-06	NM_152378	NM_152378	Homo sapiens Fc receptor-like and mucin-like 2 (FCRLM2), mRNA [NM_152378]	NM_152378
A_23_P151690	9.23E-06	NM_018477	NM_018477	Homo sapiens actin-related protein 10 homolog (S. cerevisiae) (ACTR10), mRNA [NM_018477]	NM_018477
A_24_P602871	9.28E-06	THC2278542			
A_24_P929083	9.29E-06	THC2275899		BC073800 BTB (POZ) domain containing 4 {Homo sapiens;} , partial (3%) [THC2275899]	
A_23_P256735	9.30E-06	NM_016134	NM_016134	Homo sapiens plasma glutamate carboxypeptidase (PGCP), mRNA [NM_016134]	NM_016134
A_23_P251075	9.33E-06	NM_005491	NM_005491	Homo sapiens chromosome X open reading frame 6 (CXorf6), mRNA [NM_005491]	NM_005491
A_23_P62890	9.39E-06	NM_002053	NM_002053	Homo sapiens guanylate binding protein 1, interferon-inducible, 67kDa (GBP1), mRNA [NM_002053]	NM_002053
A_23_P210708	9.39E-06	NM_080792	NM_080792	Homo sapiens protein tyrosine phosphatase, non-receptor type substrate 1 (PTPNS1), mRNA [NM_080792]	NM_080792
A_23_P14458	9.48E-06	NM_144581	NM_144581	Homo sapiens chromosome 14 open reading frame 149 (C14orf149), mRNA [NM_144581]	NM_144581
A_23_P94370	9.48E-06	NM_003909	NM_003909	Homo sapiens copine III (CPNE3), mRNA [NM_003909]	NM_003909
A_24_P192805	9.60E-06	NM_001007232	NM_001007232	Homo sapiens inhibitory caspase recruitment domain (CARD) protein (INCA), mRNA [NM_001007232]	NM_001007232
A_24_P209772	9.60E-06	NM_007234	NM_007234	Homo sapiens dynactin 3 (p22) (DCTN3), transcript variant 1, mRNA [NM_007234]	NM_007234
A_23_P250380	9.65E-06	NM_015274	NM_015274	Homo sapiens mannosidase, alpha, class 2B, member 2 (MAN2B2), mRNA [NM_015274]	NM_015274
A_24_P763655	9.67E-06	THC2271508		BOVCALP calpactin I heavy chain (p36) {Bos taurus;} , partial (53%) [THC2271508]	
A_23_P116512	9.68E-06	NM_024841	NM_024841	Homo sapiens hypothetical protein FLJ14213 (FLJ14213), mRNA [NM_024841]	NM_024841
A_23_P110167	9.68E-06	NM_002413	NM_002413	Homo sapiens microsomal glutathione S-transferase 2 (MGST2), mRNA [NM_002413]	NM_002413
A_23_P46045	9.68E-06	NM_003617	NM_003617	Homo sapiens regulator of G-protein signalling 5 (RGS5), mRNA [NM_003617]	NM_003617
A_23_P255111	9.68E-06	A_23_P255111			
A_23_P158725	9.70E-06	NM_004207	NM_004207	Homo sapiens solute carrier family 16 (monocarboxylic acid transporters), member 3 (SLC16A3), mRNA [NM_004207]	NM_004207
A_23_P42435	9.74E-06	NM_183041	NM_183041	Homo sapiens dystrobrevin binding protein 1 (DTNBP1), transcript variant 3, mRNA [NM_183041]	NM_183041
A_23_P8640	9.77E-06	NM_001505	NM_001505	Homo sapiens G protein-coupled receptor 30 (GPR30), mRNA [NM_001505]	NM_001505
A_32_P172920	9.86E-06	THC2344124		BC043584 MGC50273 protein {Homo sapiens;} , partial (73%) [THC2344124]	
A_23_P132405	9.90E-06	NM_014049	NM_014049	Homo sapiens acyl-Coenzyme A dehydrogenase family, member 9 (ACAD9), mRNA [NM_014049]	NM_014049
A_23_P422724	9.93E-06	NM_000943	NM_000943	Homo sapiens peptidylprolyl isomerase C (cyclophilin C) (PPIC), mRNA [NM_000943]	NM_000943

A_23_P358709	9.96E-06	AB033060	AB033060	Homo sapiens mRNA for KIAA1234 protein, partial cds. [AB033060]	
A_23_P353005	9.96E-06	NM_152553	NM_152553	Homo sapiens IBR domain containing 1 (IBRDC1), mRNA [NM_152553]	NM_152553
A_24_P294124	1.00E-05	NM_014755	NM_014755	Homo sapiens SERTA domain containing 2 (SERTAD2), mRNA [NM_014755]	NM_014755
A_24_P12438	1.01E-05	NM_181782	NM_181782	Homo sapiens nuclear receptor coactivator 7 (NCOA7), mRNA [NM_181782]	NM_181782
A_23_P147423	1.02E-05	NM_182920	NM_182920	Homo sapiens a disintegrin-like and metalloprotease (reprolysin type) with thrombospondin type 1 motif, 9 (ADAMTS9), mRNA [NM_182920]	NM_182920
A_24_P237686	1.02E-05	ENST00000236199			
A_24_P120380	1.02E-05	NM_032906	NM_032906	Homo sapiens hypothetical protein MGC14156 (MGC14156), mRNA [NM_032906]	NM_032906
A_23_P53623	1.03E-05	NM_002560	NM_002560	Homo sapiens purinergic receptor P2X, ligand-gated ion channel, 4 (P2RX4), transcript variant 1, mRNA [NM_002560]	NM_002560
A_23_P86002	1.04E-05	NM_016040	NM_016040	Homo sapiens transmembrane emp24 protein transport domain containing 5 (TMED5), mRNA [NM_016040]	NM_016040
A_23_P500998	1.04E-05	NM_152739	NM_152739	Homo sapiens homeo box A9 (HOXA9), transcript variant 1, mRNA [NM_152739]	NM_152739
A_32_P104263	1.04E-05	THC2432735			
A_24_P393958	1.05E-05	NM_007034	NM_007034	Homo sapiens DnaJ (Hsp40) homolog, subfamily B, member 4 (DNAJB4), mRNA [NM_007034]	NM_007034
A_24_P16913	1.06E-05	NM_005845	NM_005845	Homo sapiens ATP-binding cassette, sub-family C (CFTR/MRP), member 4 (ABCC4), mRNA [NM_005845]	NM_005845
A_23_P202219	1.06E-05	NM_015916	NM_015916	Homo sapiens family with sequence similarity 26, member B (FAM26B), mRNA [NM_015916]	NM_015916
A_32_P125402	1.06E-05	THC2314822		NOA1_HUMAN (Q9NY12) Nucleolar protein family A member 1 (snoRNP protein GAR1) (H/ACA ribonucleoprotein GAR1), partial (18%) [THC2314822]	
A_23_P116207	1.06E-05	NM_020153	NM_020153	Homo sapiens hypothetical protein FLJ21827 (FLJ21827), mRNA [NM_020153]	NM_020153
A_24_P294233	1.06E-05	NM_014905	NM_014905	Homo sapiens glutaminase (GLS), mRNA [NM_014905]	NM_014905
A_24_P230486	1.07E-05	A_24_P230486			
A_23_P10391	1.07E-05	NM_000393	NM_000393	Homo sapiens collagen, type V, alpha 2 (COL5A2), mRNA [NM_000393]	NM_000393
A_23_P357929	1.08E-05	NM_015139	NM_015139	Homo sapiens solute carrier family 35 (UDP-glucuronic acid/UDP-N-acetylgalactosamine dual transporter), member D1 (SLC35D1), mRNA [NM_015139]	NM_015139
A_23_P311640	1.09E-05	NM_006076	NM_006076	Homo sapiens HIV-1 Rev binding protein-like (HRBL), mRNA [NM_006076]	NM_006076
A_23_P203488	1.09E-05	NM_000543	NM_000543	Homo sapiens sphingomyelin phosphodiesterase 1, acid lysosomal (acid sphingomyelinase) (SMPD1), transcript variant 1, mRNA [NM_000543]	NM_000543
A_23_P70290	1.09E-05	NM_018247	NM_018247	Homo sapiens transmembrane protein 30A (TMEM30A), mRNA [NM_018247]	NM_018247
A_23_P91379	1.10E-05	NM_018993	NM_018993	Homo sapiens Ras and Rab interactor 2 (RIN2), mRNA [NM_018993]	NM_018993
A_23_P104073	1.10E-05	NM_002960	NM_002960	Homo sapiens S100 calcium binding protein A3 (S100A3), mRNA [NM_002960]	NM_002960
A_23_P376188	1.10E-05	BC031618	BC031618	Homo sapiens chromosome 10 open reading frame 30, mRNA (cDNA clone MGC:35247 IMAGE:5172600), complete cds. [BC031618]	
A_23_P75609	1.10E-05	NM_014956	NM_014956	Homo sapiens KIAA1052 protein (Cep164), mRNA [NM_014956]	NM_014956
A_23_P82420	1.10E-05	NM_004603	NM_004603	Homo sapiens syntaxin 1A (brain) (STX1A), mRNA [NM_004603]	NM_004603
A_23_P102770	1.10E-05	NM_016100	NM_016100	Homo sapiens N-acetyltransferase 5 (ARD1 homolog, S. cerevisiae) (NAT5), transcript variant 1, mRNA [NM_016100]	NM_016100
A_24_P223518	1.11E-05	NM_007270	NM_007270	Homo sapiens FK506 binding protein 9, 63 kDa (FKBP9), mRNA [NM_007270]	NM_007270
A_23_P13094	1.11E-05	NM_002425	NM_002425	Homo sapiens matrix metalloproteinase 10 (stromelysin 2) (MMP10), mRNA [NM_002425]	NM_002425
A_32_P170925	1.11E-05	ENST00000360201		Homo sapiens thioredoxin reductase 3, mRNA (cDNA clone IMAGE:5270485), partial cds. [BC050032]	XM_051264
A_23_P203933	1.12E-05	NM_002223	NM_002223	Homo sapiens inositol 1,4,5-triphosphate receptor, type 2 (ITPR2), mRNA [NM_002223]	NM_002223
A_23_P34546	1.12E-05	NM_006642	NM_006642	Homo sapiens serologically defined colon cancer antigen 8 (SDCCAG8), mRNA [NM_006642]	NM_006642
A_23_P32029	1.12E-05	NM_007001	NM_007001	Homo sapiens solute carrier family 35, member D2 (SLC35D2), mRNA [NM_007001]	NM_007001
A_23_P61222	1.12E-05	AJ002425	AJ002425	Homo sapiens mRNA for p65 protein. [AJ002425]	
A_23_P415401	1.12E-05	NM_001206	NM_001206	Homo sapiens Kruppel-like factor 9 (KLF9), mRNA [NM_001206]	NM_001206
A_23_P27584	1.12E-05	NM_001020818	NM_001020818	Homo sapiens myeloid-associated differentiation marker (MYADM), transcript variant 1, mRNA [NM_001020818]	NM_001020818
A_32_P201723	1.13E-05	BU624603	BU624603	BU624603 UI-H-FG1-bgk-p-15-0-UI.s1 NCI_CGAP_FG1 Homo sapiens cDNA clone UI-H-FG1-bgk-p-15-0-UI 3', mRNA sequence [BU624603]	
A_23_P55477	1.13E-05	NM_000676	NM_000676	Homo sapiens adenosine A2b receptor (ADORA2B), mRNA [NM_000676]	NM_000676
A_23_P208516	1.13E-05	NM_024298	NM_024298	Homo sapiens leukocyte receptor cluster (LRC) member 4 (LENG4), mRNA [NM_024298]	NM_024298
A_24_P49183	1.15E-05	NM_017820	NM_017820	Homo sapiens hypothetical protein FLJ20433 (FLJ20433), mRNA [NM_017820]	NM_017820
A_23_P104624	1.16E-05	ENST00000278505		Homo sapiens mRNA for KIAA0830 protein, partial cds. [AB020637]	XM_290546
A_24_P154080	1.17E-05	NM_001397	NM_001397	Homo sapiens endothelin converting enzyme 1 (ECE1), mRNA [NM_001397]	NM_001397

A_23_P155857	1.17E-05	NM_198041	NM_198041	Homo sapiens nudix (nucleoside diphosphate linked moiety X)-type motif 6 (NUDT6), transcript variant 2, mRNA [NM_198041]	NM_198041
A_24_P916496	1.17E-05	NM_002737	NM_002737	Homo sapiens protein kinase C, alpha (PRKCA), mRNA [NM_002737]	NM_002737
A_23_P147918	1.17E-05	NM_080388	NM_080388	Homo sapiens S100 calcium binding protein A16 (S100A16), mRNA [NM_080388]	NM_080388
A_32_P49035	1.18E-05	A_32_P49035			
A_23_P138717	1.18E-05	NM_001005339	NM_001005339	Homo sapiens regulator of G-protein signalling 10 (RGS10), transcript variant 1, mRNA [NM_001005339]	NM_001005339
A_24_P204244	1.18E-05	NR_001562	NR_001562	Homo sapiens annexin A2 pseudogene 1 (ANXA2P1) on chromosome 4 [NR_001562]	NR_001562
A_32_P139708	1.19E-05	THC2290220			
A_24_P817209	1.19E-05	AK125077	AK125077	Homo sapiens cDNA FLJ43087 fis, clone BRTHA3019105. [AK125077]	
A_24_P252078	1.19E-05	BC067086	BC067086	Homo sapiens cDNA clone MGC:71335 IMAGE:6088873, complete cds. [BC067086]	
A_23_P416142	1.19E-05	NM_004087	NM_004087	Homo sapiens discs, large homolog 1 (Drosophila) (DLG1), mRNA [NM_004087]	NM_004087
A_23_P49597	1.19E-05	NM_020360	NM_020360	Homo sapiens phospholipid scramblase 3 (PLSCR3), mRNA [NM_020360]	NM_020360
A_23_P363034	1.21E-05	ENST00000307201		Homo sapiens hypothetical protein LOC116236, mRNA (cDNA clone MGC:21749 IMAGE:4537124), complete cds. [BC012476]	
A_24_P170667	1.21E-05	ENST00000279873		Homo sapiens AT rich interactive domain 5B (MRF1-like), mRNA (cDNA clone IMAGE:30345306), partial cds. [BC066345]	XM_084482
A_24_P71700	1.21E-05	NM_145166	NM_145166	Homo sapiens zinc finger protein 651 (ZNF651), mRNA [NM_145166]	NM_145166
A_23_P359091	1.21E-05	U68019	U68019	Homo sapiens mad protein homolog (hMAD-3) mRNA, complete cds. [U68019]	
A_23_P37569	1.22E-05	NM_006122	NM_006122	Homo sapiens mannosidase, alpha, class 2A, member 2 (MAN2A2), mRNA [NM_006122]	NM_006122
A_23_P149494	1.22E-05	NM_015600	NM_015600	Homo sapiens chromosome 20 open reading frame 22 (C20orf22), mRNA [NM_015600]	NM_015600
A_23_P88381	1.22E-05	NM_001005743	NM_001005743	Homo sapiens numb homolog (Drosophila) (NUMB), transcript variant 1, mRNA [NM_001005743]	NM_001005743
A_23_P61987	1.22E-05	NM_025268	NM_025268	Homo sapiens hole gene (MGC4659), mRNA [NM_025268]	NM_025268
A_23_P52647	1.22E-05	NM_006795	NM_006795	Homo sapiens EH-domain containing 1 (EHD1), mRNA [NM_006795]	NM_006795
A_23_P48886	1.23E-05	NM_001110	NM_001110	Homo sapiens a disintegrin and metalloproteinase domain 10 (ADAM10), mRNA [NM_001110]	NM_001110
A_24_P707530	1.24E-05	THC2336549		S10030 urease 11K chain - Ureaplasma urealyticum {Ureaplasma urealyticum;} , partial (10%) [THC2336549]	
A_23_P10858	1.24E-05	ENST00000318139		Homo sapiens KIAA0692 protein, mRNA (cDNA clone IMAGE:5473979), partial cds. [BC062373]	
A_23_P22957	1.24E-05	NM_016009	NM_016009	Homo sapiens SH3-domain GRB2-like endophilin B1 (SH3GLB1), mRNA [NM_016009]	NM_016009
A_23_P410613	1.24E-05	NM_152261	NM_152261	Homo sapiens hypothetical protein MGC17943 (MGC17943), mRNA [NM_152261]	NM_152261
A_23_P401084	1.25E-05	NM_174945	NM_174945	Homo sapiens zinc finger protein 575 (ZNF575), mRNA [NM_174945]	NM_174945
A_23_P161074	1.26E-05	NM_015602	NM_015602	Homo sapiens torsin A interacting protein 1 (TOR1AIP1), mRNA [NM_015602]	NM_015602
A_24_P748377	1.26E-05	AL833456	AL833456	Homo sapiens mRNA; cDNA DKFZp686I18116 (from clone DKFZp686I18116). [AL833456]	
A_23_P157970	1.29E-05	NM_014425	NM_014425	Homo sapiens inversin (INVS), transcript variant 1, mRNA [NM_014425]	NM_014425
A_23_P14184	1.29E-05	NM_018676	NM_018676	Homo sapiens thrombospondin, type I, domain containing 1 (THSD1), transcript variant 1, mRNA [NM_018676]	NM_018676
A_24_P402438	1.30E-05	NM_003238	NM_003238	Homo sapiens transforming growth factor, beta 2 (TGFB2), mRNA [NM_003238]	NM_003238
A_24_P102080	1.30E-05	AF070629	AF070629	Homo sapiens clone 24484 RAB2 mRNA, partial cds. [AF070629]	
A_23_P201619	1.30E-05	AL080111	AL080111	Homo sapiens mRNA; cDNA DKFZp586G2222 (from clone DKFZp586G2222). [AL080111]	
A_24_P269619	1.30E-05	NM_001359	NM_001359	Homo sapiens 2,4-dienoyl CoA reductase 1, mitochondrial (DECR1), nuclear gene encoding mitochondrial protein, mRNA [NM_001359]	NM_001359
A_23_P394064	1.30E-05	NM_012232	NM_012232	Homo sapiens polymerase I and transcript release factor (PTRF), mRNA [NM_012232]	NM_012232
A_24_P238131	1.31E-05	ENST00000309558		Homo sapiens mRNA for KIAA0527 protein, partial cds. [AB011099]	XM_171054
A_32_P66222	1.32E-05	NM_001012421	NM_001012421	Homo sapiens OTTHUMP00000064580 (LOC441430), mRNA [NM_001012421]	NM_001012421
A_24_P277295	1.32E-05	NM_198490	NM_198490	Homo sapiens RAB43, member RAS oncogene family (RAB43), mRNA [NM_198490]	NM_198490
A_23_P156061	1.33E-05	NM_005575	NM_005575	Homo sapiens leucyl/cystinyl aminopeptidase (LNPEP), transcript variant 1, mRNA [NM_005575]	NM_005575
A_24_P234094	1.33E-05	NM_003929	NM_003929	Homo sapiens RAB7, member RAS oncogene family-like 1 (RAB7L1), mRNA [NM_003929]	NM_003929
A_24_P164894	1.33E-05	NM_006052	NM_006052	Homo sapiens Down syndrome critical region gene 3 (DSCR3), mRNA [NM_006052]	NM_006052
A_32_P146764	1.34E-05	THC2382264			
A_32_P30600	1.34E-05	THC2284350			
A_23_P87421	1.34E-05	BC063022	BC063022	Homo sapiens cDNA clone IMAGE:5246259, partial cds. [BC063022]	

A_23_P62920	1.34E-05	NM_014970	NM_014970	Homo sapiens kinesin-associated protein 3 (KIFAP3), mRNA [NM_014970]	NM_014970
A_23_P34478	1.35E-05	NM_017655	NM_017655	Homo sapiens GIPC PDZ domain containing family, member 2 (GIPC2), mRNA [NM_017655]	NM_017655
A_24_P56317	1.35E-05	NM_144778	NM_144778	Homo sapiens muscleblind-like 2 (Drosophila) (MBNL2), transcript variant 1, mRNA [NM_144778]	NM_144778
A_23_P303155	1.36E-05	NM_032824	NM_032824	Homo sapiens hypothetical protein FLJ14681 (FLJ14681), mRNA [NM_032824]	NM_032824
A_23_P155335	1.36E-05	NM_002662	NM_002662	Homo sapiens phospholipase D1, phophatidylcholine-specific (PLD1), mRNA [NM_002662]	NM_002662
A_24_P97785	1.37E-05	CR611332	CR611332	full-length cDNA clone CS0DF014YA22 of Fetal brain of Homo sapiens (human). [CR611332]	
A_32_P36313	1.38E-05	THC2310680		AF262221 SNARE Vti1a protein {Rattus norvegicus;} , complete [THC2310680]	
A_32_P171253	1.40E-05	THC2442210		Q9F8M7 (Q9F8M7) DTDP-glucose 4,6-dehydratase (Fragment), partial (11%) [THC2442210]	
A_23_P168556	1.40E-05	NM_004603	NM_004603	Homo sapiens syntaxin 1A (brain) (STX1A), mRNA [NM_004603]	NM_004603
A_23_P341392	1.40E-05	NM_144611	NM_144611	Homo sapiens hypothetical protein MGC32124 (MGC32124), mRNA [NM_144611]	NM_144611
A_32_P131929	1.40E-05	AK000028	AK000028	Homo sapiens cDNA FLJ20021 fis, clone ADSE01233. [AK000028]	XM_028217
A_32_P171043	1.40E-05	BI090438	BI090438	602853723F1 NIH_MGC_10 Homo sapiens cDNA clone IMAGE:4995269 5', mRNA sequence [BI090438]	
A_23_P109143	1.40E-05	NM_000311	NM_000311	Homo sapiens prion protein (p27-30) (Creutzfeld-Jakob disease, Gerstmann-Strausler-Scheinker syndrome, fatal familial insomnia) (PRNP), transcript variant 1, mRNA [NM_000311]	NM_000311
A_24_P860781	1.41E-05	AK092921	AK092921	Homo sapiens cDNA FLJ35602 fis, clone SPLEN2009088, highly similar to HLA CLASS I HISTOCOMPATIBILITY ANTIGEN. [AK092921]	
A_24_P307025	1.41E-05	NR_000029	NR_000029	Homo sapiens ribosomal protein L23a pseudogene 7 (RPL23AP7) on chromosome 2 [NR_000029]	NR_000029
A_23_P48217	1.42E-05	NM_030817	NM_030817	Homo sapiens hypothetical protein DKFZp434F0318 (DKFZP434F0318), mRNA [NM_030817]	NM_030817
A_23_P51339	1.43E-05	NM_007034	NM_007034	Homo sapiens DnaJ (Hsp40) homolog, subfamily B, member 4 (DNAJB4), mRNA [NM_007034]	NM_007034
A_23_P8400	1.43E-05	NM_004935	NM_004935	Homo sapiens cyclin-dependent kinase 5 (CDK5), mRNA [NM_004935]	NM_004935
A_23_P354208	1.43E-05	NM_152348	NM_152348	Homo sapiens hypothetical protein FLJ33817 (FLJ33817), mRNA [NM_152348]	NM_152348
A_23_P106844	1.43E-05	NM_005953	NM_005953	Homo sapiens metallothionein 2A (MT2A), mRNA [NM_005953]	NM_005953
A_32_P128661	1.44E-05	BC023274	BC023274	Homo sapiens cDNA clone IMAGE:4500064, partial cds. [BC023274]	
A_24_P772488	1.44E-05	THC2311602			
A_24_P218265	1.44E-05	NM_003842	NM_003842	Homo sapiens tumor necrosis factor receptor superfamily, member 10b (TNFRSF10B), transcript variant 1, mRNA [NM_003842]	NM_003842
A_24_P200854	1.45E-05	NM_006735	NM_006735	Homo sapiens homeo box A2 (HOXA2), mRNA [NM_006735]	NM_006735
A_24_P49190	1.45E-05	NM_181655	NM_181655	Homo sapiens hypothetical protein LOC284018 (LOC284018), transcript variant 1, mRNA [NM_181655]	NM_181655
A_23_P319565	1.45E-05	NM_170753	NM_170753	Homo sapiens piggyBac transposable element derived 3 (PGBD3), mRNA [NM_170753]	NM_170753
A_23_P79931	1.45E-05	NM_139322	NM_139322	Homo sapiens attractin (ATRN), transcript variant 2, mRNA [NM_139322]	NM_139322
A_23_P65678	1.45E-05	NM_000138	NM_000138	Homo sapiens fibrillin 1 (Marfan syndrome) (FBN1), mRNA [NM_000138]	NM_000138
A_23_P212119	1.46E-05	NM_054110	NM_054110	Homo sapiens UDP-N-acetyl-alpha-D-galactosamine:polypeptide N-acetylgalactosaminyltransferase-like 2 (GALNTL2), mRNA [NM_054110]	NM_054110
A_24_P26496	1.46E-05	NM_004557	NM_004557	Homo sapiens Notch homolog 4 (Drosophila) (NOTCH4), mRNA [NM_004557]	NM_004557
A_24_P1167	1.46E-05	NM_017987	NM_017987	Homo sapiens RUN and FYVE domain containing 2 (RUFY2), mRNA [NM_017987]	NM_017987
A_23_P207837	1.46E-05	NM_001304	NM_001304	Homo sapiens carboxypeptidase D (CPD), mRNA [NM_001304]	NM_001304
A_32_P71032	1.46E-05	THC2407039			
A_23_P120883	1.46E-05	NM_002133	NM_002133	Homo sapiens heme oxygenase (decycling) 1 (HMOX1), mRNA [NM_002133]	NM_002133
A_23_P311732	1.47E-05	NM_018841	NM_018841	Homo sapiens guanine nucleotide binding protein (G protein), gamma 12 (GNG12), mRNA [NM_018841]	NM_018841
A_23_P157600	1.47E-05	NM_015214	NM_015214	Homo sapiens DDHD domain containing 2 (DDHD2), mRNA [NM_015214]	NM_015214
A_24_P411899	1.47E-05	NM_183419	NM_183419	Homo sapiens ring finger protein 19 (RNF19), transcript variant 1, mRNA [NM_183419]	NM_183419
A_23_P212696	1.47E-05	NM_007085	NM_007085	Homo sapiens follistatin-like 1 (FSTL1), mRNA [NM_007085]	NM_007085
A_23_P120243	1.48E-05	NM_024501	NM_024501	Homo sapiens homeo box D1 (HOXD1), mRNA [NM_024501]	NM_024501
A_23_P58082	1.48E-05	NM_199511	NM_199511	Homo sapiens steroid sensitive gene 1 (URB), transcript variant 1, mRNA [NM_199511]	NM_199511
A_23_P170273	1.49E-05	NM_014933	NM_014933	Homo sapiens SEC31-like 1 (S. cerevisiae) (SEC31L1), transcript variant 1, mRNA [NM_014933]	NM_014933
A_24_P17710	1.50E-05	NM_015990	NM_015990	Homo sapiens kelch-like 5 (Drosophila) (KLHL5), mRNA [NM_015990]	NM_015990
A_24_P838743	1.50E-05	NM_004568	NM_004568	Homo sapiens serine (or cysteine) proteinase inhibitor, clade B (ovalbumin), member 6 (SERPINB6), mRNA [NM_004568]	NM_004568
A_23_P56933	1.50E-05	NM_020532	NM_020532	Homo sapiens reticulon 4 (RTN4), transcript variant 1, mRNA [NM_020532]	NM_020532

A_23_P72853	1.51E-05	NM_032889	NM_032889	Homo sapiens hypothetical protein MGC11308 (MGC11308), mRNA [NM_032889]	NM_032889
A_23_P68970	1.51E-05	NM_014570	NM_014570	Homo sapiens ADP-ribosylation factor GTPase activating protein 3 (ARFGAP3), mRNA [NM_014570]	NM_014570
A_24_P213503	1.52E-05	NM_006504	NM_006504	Homo sapiens protein tyrosine phosphatase, receptor type, E (PTPRE), transcript variant 1, mRNA [NM_006504]	NM_006504
A_23_P53439	1.52E-05	NM_138432	NM_138432	Homo sapiens serine dehydratase-like (SDSL), mRNA [NM_138432]	NM_138432
A_32_P123527	1.53E-05	THC2366170		ALU5_HUMAN (P39192) Alu subfamily SC sequence contamination warning entry, partial (3%) [THC2366170]	
A_32_P215856	1.53E-05	A_32_P215856			
A_23_P134734	1.53E-05	NM_017786	NM_017786	Homo sapiens hypothetical protein FLJ20366 (FLJ20366), mRNA [NM_017786]	NM_017786
A_24_P238186	1.53E-05	A_24_P238186			
A_23_P214464	1.53E-05	NM_001760	NM_001760	Homo sapiens cyclin D3 (CCND3), mRNA [NM_001760]	NM_001760
A_24_P135322	1.53E-05	NM_003873	NM_003873	Homo sapiens neuropilin 1 (NRP1), transcript variant 1, mRNA [NM_003873]	NM_003873
A_24_P401601	1.53E-05	A_24_P401601			
A_23_P211957	1.54E-05	NM_001024847	NM_001024847	Homo sapiens transforming growth factor, beta receptor II (70/80kDa) (TGFBR2), transcript variant 1, mRNA [NM_001024847]	NM_001024847
A_23_P351724	1.56E-05	NM_022648	NM_022648	Homo sapiens tensin 1 (TNS1), mRNA [NM_022648]	NM_022648
A_23_P30784	1.56E-05	NM_013375	NM_013375	Homo sapiens activator of basal transcription 1 (ABT1), mRNA [NM_013375]	NM_013375
A_24_P133475	1.56E-05	NM_138452	NM_138452	Homo sapiens dehydrogenase/reductase (SDR family) member 1 (DHRS1), mRNA [NM_138452]	NM_138452
A_23_P159986	1.56E-05	BC007360	BC007360	Homo sapiens hypothetical protein MGC16121, mRNA (cDNA clone MGC:16121 IMAGE:3627113), complete cds. [BC007360]	
A_32_P460973	1.56E-05	NM_005516	NM_005516	Homo sapiens major histocompatibility complex, class I, E (HLA-E), mRNA [NM_005516]	NM_005516
A_23_P397417	1.57E-05	NM_153261	NM_153261	Homo sapiens hypothetical protein FLJ38101 (FLJ38101), mRNA [NM_153261]	NM_153261
A_24_P753760	1.57E-05	A_24_P753760			
A_24_P277955	1.58E-05	NM_016068	NM_016068	Homo sapiens tetratricopeptide repeat domain 11 (TTC11), mRNA [NM_016068]	NM_016068
A_23_P28869	1.58E-05	NM_002836	NM_002836	Homo sapiens protein tyrosine phosphatase, receptor type, A (PTPRA), transcript variant 1, mRNA [NM_002836]	NM_002836
A_23_P157128	1.59E-05	NM_003930	NM_003930	Homo sapiens src family associated phosphoprotein 2 (SCAP2), mRNA [NM_003930]	NM_003930
A_23_P10873	1.61E-05	NM_003263	NM_003263	Homo sapiens toll-like receptor 1 (TLR1), mRNA [NM_003263]	NM_003263
A_23_P148162	1.61E-05	NM_018269	NM_018269	Homo sapiens membrane-type 1 matrix metalloproteinase cytoplasmic tail binding protein-1 (MTCBP-1), mRNA [NM_018269]	NM_018269
A_23_P130856	1.63E-05	NM_032816	NM_032816	Homo sapiens hypothetical protein FLJ14640 (FLJ14640), mRNA [NM_032816]	NM_032816
A_23_P414964	1.63E-05	NM_173548	NM_173548	Homo sapiens zinc finger protein 584 (ZNF584), mRNA [NM_173548]	NM_173548
A_23_P99163	1.63E-05	NM_018370	NM_018370	Homo sapiens hypothetical protein FLJ11259 (FLJ11259), mRNA [NM_018370]	NM_018370
A_24_P273857	1.64E-05	NM_012082	NM_012082	Homo sapiens zinc finger protein, multitype 2 (ZFPM2), mRNA [NM_012082]	NM_012082
A_32_P144920	1.64E-05	NM_015509	NM_015509	Homo sapiens adaptin-ear-binding coat-associated protein 1 (NECAP1), mRNA [NM_015509]	NM_015509
A_24_P944390	1.64E-05	AL050107	AL050107	Homo sapiens mRNA; cDNA DKFZp586I1419 (from clone DKFZp586I1419); partial cds. [AL050107]	
A_24_P255123	1.64E-05	A_24_P255123			
A_23_P144578	1.64E-05	NM_138335	NM_138335	Homo sapiens glucosamine-6-phosphate deaminase 2 (GNPDA2), mRNA [NM_138335]	NM_138335
A_24_P270890	1.64E-05	NM_001007226	NM_001007226	Homo sapiens speckle-type POZ protein (SPOP), transcript variant 1, mRNA [NM_001007226]	NM_001007226
A_23_P131596	1.64E-05	NM_032319	NM_032319	Homo sapiens chromosome 2 open reading frame 7 (C2orf7), mRNA [NM_032319]	NM_032319
A_23_P415006	1.64E-05	NM_015470	NM_015470	Homo sapiens RAB11 family interacting protein 5 (class I) (RAB11FIP5), mRNA [NM_015470]	NM_015470
A_23_P41777	1.64E-05	NM_024715	NM_024715	Homo sapiens chromosome 5 open reading frame 14 (C5orf14), mRNA [NM_024715]	NM_024715
A_23_P73721	1.65E-05	NM_016656	NM_016656	Homo sapiens Ras-related GTP binding B (RRAGB), transcript variant RAGBl, mRNA [NM_016656]	NM_016656
A_24_P85300	1.65E-05	AB033063	AB033063	Homo sapiens mRNA for KIAA1237 protein, partial cds. [AB033063]	XM_087386
A_24_P825942	1.66E-05	AK001796	AK001796	Homo sapiens cDNA FLJ10934 fis, clone OVARC1000640. [AK001796]	
A_32_P29806	1.66E-05	NM_003805	NM_003805	Homo sapiens CASP2 and RIPK1 domain containing adaptor with death domain (CRADD), mRNA [NM_003805]	NM_003805
A_24_P945059	1.67E-05	BC022556	BC022556	Homo sapiens myc target 1, mRNA (cDNA clone MGC:26933 IMAGE:4793564), complete cds. [BC022556]	
A_24_P233560	1.67E-05	A_24_P233560			
A_23_P1782	1.67E-05	NM_002231	NM_002231	Homo sapiens CD82 antigen (CD82), transcript variant 1, mRNA [NM_002231]	NM_002231
A_24_P127051	1.67E-05	A_24_P127051			

A_32_P147063	1.68E-05	AL355688	AL355688	Homo sapiens EST from clone 208499, full insert. [AL355688]	
A_24_P634530	1.68E-05	THC2281463			
A_23_P318284	1.68E-05	NM_015141	NM_015141	Homo sapiens glycerol-3-phosphate dehydrogenase 1-like (GPD1L), mRNA [NM_015141]	NM_015141
A_23_P339098	1.68E-05	NM_017515	NM_017515	Homo sapiens solute carrier family 35, member F2 (SLC35F2), mRNA [NM_017515]	NM_017515
A_24_P280558	1.69E-05	ENST00000309771		Homo sapiens melanoma-associated antigen mRNA, partial cds. [AF543495]	
A_23_P218434	1.69E-05	NM_015919	NM_015919	Homo sapiens zinc finger protein 226 (ZNF226), mRNA [NM_015919]	NM_015919
A_32_P207169	1.69E-05	BC094794	BC094794	Homo sapiens cDNA clone IMAGE:30520069. [BC094794]	
A_24_P42517	1.69E-05	NM_006854	NM_006854	Homo sapiens KDEL (Lys-Asp-Glu-Leu) endoplasmic reticulum protein retention receptor 2 (KDELR2), mRNA [NM_006854]	NM_006854
A_23_P147950	1.70E-05	AK057293	AK057293	Homo sapiens cDNA FLJ32731 fis, clone TESTI2001134. [AK057293]	XM_372038
A_32_P37089	1.70E-05	AK097700	AK097700	Homo sapiens cDNA FLJ40381 fis, clone TESTI2035688. [AK097700]	
A_24_P887092	1.70E-05	THC2339791			
A_32_P175301	1.70E-05	NM_014957	NM_014957	Homo sapiens KIAA0870 protein (KIAA0870), mRNA [NM_014957]	NM_014957
A_23_P166807	1.70E-05	NM_033010	NM_033010	Homo sapiens poly(rC) binding protein 4 (PCBP4), transcript variant 4, mRNA [NM_033010]	NM_033010
A_23_P114740	1.71E-05	NM_002113	NM_002113	Homo sapiens complement factor H-related 1 (CFHL1), mRNA [NM_002113]	NM_002113
A_23_P417363	1.73E-05	AK057267	AK057267	Homo sapiens cDNA FLJ32705 fis, clone TESTI2000600, weakly similar to RESTIN. [AK057267]	
A_23_P29141	1.73E-05	NM_004782	NM_004782	Homo sapiens synaptosomal-associated protein, 29kDa (SNAP29), mRNA [NM_004782]	NM_004782
A_24_P313334	1.73E-05	AK094846	AK094846	Homo sapiens cDNA FLJ37527 fis, clone BRCAN2011946. [AK094846]	
A_23_P68006	1.74E-05	NM_000877	NM_000877	Homo sapiens interleukin 1 receptor, type I (IL1R1), mRNA [NM_000877]	NM_000877
A_23_P111635	1.74E-05	NM_016086	NM_016086	Homo sapiens dual specificity phosphatase 24 (putative) (DUSP24), mRNA [NM_016086]	NM_016086
A_23_P365685	1.75E-05	NM_033514	NM_033514	Homo sapiens LIM and senescent cell antigen-like domains 3 (LIMS3), mRNA [NM_033514]	NM_033514
A_23_P217737	1.76E-05	L06133	L06133	Human putative Cu++-transporting P-type ATPase mRNA, complete cds. [L06133]	
A_23_P32036	1.77E-05	NM_017881	NM_017881	Homo sapiens chromosome 9 open reading frame 95 (C9orf95), mRNA [NM_017881]	NM_017881
A_23_P435604	1.77E-05	NM_015047	NM_015047	Homo sapiens KIAA0090 (KIAA0090), mRNA [NM_015047]	NM_015047
A_23_P307392	1.79E-05	NM_012074	NM_012074	Homo sapiens D4, zinc and double PHD fingers, family 3 (DPF3), mRNA [NM_012074]	NM_012074
A_24_P919304	1.79E-05	BC012204	BC012204	Homo sapiens fibronectin type III domain containing 3B, mRNA (cDNA clone IMAGE:3882800), complete cds. [BC012204]	
A_23_P62768	1.79E-05	NM_033504	NM_033504	Homo sapiens transmembrane protein 54 (TMEM54), mRNA [NM_033504]	NM_033504
A_23_P140648	1.79E-05	NM_014608	NM_014608	Homo sapiens cytoplasmic FMR1 interacting protein 1 (CYFIP1), mRNA [NM_014608]	NM_014608
A_23_P336992	1.80E-05	NM_182491	NM_182491	Homo sapiens hypothetical protein LOC90637 (LOC90637), mRNA [NM_182491]	NM_182491
A_23_P117175	1.80E-05	NM_006437	NM_006437	Homo sapiens poly (ADP-ribose) polymerase family, member 4 (PARP4), mRNA [NM_006437]	NM_006437
A_23_P51518	1.81E-05	NM_003617	NM_003617	Homo sapiens regulator of G-protein signalling 5 (RGS5), mRNA [NM_003617]	NM_003617
A_23_P79331	1.82E-05	NM_022152	NM_022152	Homo sapiens PP1201 protein (PP1201), mRNA [NM_022152]	NM_022152
A_24_P303770	1.82E-05	NM_147780	NM_147780	Homo sapiens cathepsin B (CTSB), transcript variant 2, mRNA [NM_147780]	NM_147780
A_23_P69218	1.82E-05	NM_018447	NM_018447	Homo sapiens 30 kDa protein (LOC55831), mRNA [NM_018447]	NM_018447
A_23_P212655	1.83E-05	NM_130446	NM_130446	Homo sapiens kelch-like 6 (Drosophila) (KLHL6), mRNA [NM_130446]	NM_130446
A_24_P398319	1.83E-05	NM_020644	NM_020644	Homo sapiens chromosome 11 open reading frame 15 (C11orf15), mRNA [NM_020644]	NM_020644
A_24_P141481	1.83E-05	NM_203330	NM_203330	Homo sapiens CD59 antigen p18-20 (antigen identified by monoclonal antibodies 16.3A5, EJ16, EJ30, EL32 and G344) (CD59), transcript variant 1, mRNA [NM_203330]	NM_203330
A_23_P144357	1.84E-05	NM_172250	NM_172250	Homo sapiens methylmalonic aciduria (cobalamin deficiency) cblA type (MMAA), mRNA [NM_172250]	NM_172250
A_24_P890536	1.85E-05	CR627148	CR627148	Homo sapiens mRNA; cDNA DKFZp779F2127 (from clone DKFZp779F2127). [CR627148]	
A_23_P118633	1.85E-05	NM_022827	NM_022827	Homo sapiens spermatogenesis associated 20 (SPATA20), mRNA [NM_022827]	NM_022827
A_23_P103824	1.85E-05	THC2249577		Q5VWB9 (Q5VWB9) Phosphatidylinositol 4-kinase, catalytic, beta polypeptide , complete [THC2249577]	
A_24_P177795	1.85E-05	ENST00000343149			
A_24_P655849	1.86E-05	BF514799	BF514799	UI-H-BW1-anj-a-01-0-UI.s1 NCI_CGAP_Sub7 Homo sapiens cDNA clone IMAGE:3082272 3', mRNA sequence [BF514799]	
A_23_P363316	1.87E-05	NM_002147	NM_002147	Homo sapiens homeo box B5 (HOXB5), mRNA [NM_002147]	NM_002147
A_32_P163089	1.87E-05	NM_207376	NM_207376	Homo sapiens hypothetical protein (LOC387882), mRNA [NM_207376]	NM_207376

A_23_P202905	1.88E-05	NM_148910	NM_148910	Homo sapiens toll-interleukin 1 receptor (TIR) domain containing adaptor protein (TIRAP), transcript variant 2, mRNA [NM_148910]	NM_148910
A_23_P500861	1.88E-05	NM_182961	NM_182961	Homo sapiens spectrin repeat containing, nuclear envelope 1 (SYNE1), transcript variant longest, mRNA [NM_182961]	NM_182961
A_23_P137196	1.88E-05	NM_001560	NM_001560	Homo sapiens interleukin 13 receptor, alpha 1 (IL13RA1), mRNA [NM_001560]	NM_001560
A_23_P167040	1.88E-05	NM_006810	NM_006810	Homo sapiens protein disulfide isomerase family A, member 5 (PDIA5), mRNA [NM_006810]	NM_006810
A_24_P414980	1.88E-05	NM_016139	NM_016139	Homo sapiens coiled-coil-helix-coiled-coil-helix domain containing 2 (CHCHD2), mRNA [NM_016139]	NM_016139
A_23_P202672	1.88E-05	NM_002939	NM_002939	Homo sapiens ribonuclease/angiogenin inhibitor 1 (RNH1), transcript variant 1, mRNA [NM_002939]	NM_002939
A_23_P419213	1.89E-05	NM_020817	NM_020817	Homo sapiens KIAA1407 (KIAA1407), mRNA [NM_020817]	NM_020817
A_24_P13190	1.89E-05	NM_138961	NM_138961	Homo sapiens endothelial cell adhesion molecule (ESAM), mRNA [NM_138961]	NM_138961
A_24_P201353	1.89E-05	NM_144591	NM_144591	Homo sapiens chromosome 10 open reading frame 32 (C10orf32), mRNA [NM_144591]	NM_144591
A_23_P141180	1.89E-05	AK055959	AK055959	Homo sapiens cDNA FLJ31397 fis, clone NT2NE1000163. [AK055959]	
A_23_P70843	1.89E-05	NM_199186	NM_199186	Homo sapiens 2,3-bisphosphoglycerate mutase (BPGM), transcript variant 2, mRNA [NM_199186]	NM_199186
A_24_P926993	1.90E-05	A_24_P926993			
A_24_P944383	1.91E-05	AL050107	AL050107	Homo sapiens mRNA; cDNA DKFZp586I1419 (from clone DKFZp586I1419); partial cds. [AL050107]	
A_24_P98975	1.92E-05	NM_033121	NM_033121	Homo sapiens ankyrin repeat domain 13 (ANKRD13), mRNA [NM_033121]	NM_033121
A_23_P112726	1.94E-05	NM_002977	NM_002977	Homo sapiens sodium channel, voltage-gated, type IX, alpha (SCN9A), mRNA [NM_002977]	NM_002977
A_23_P6909	1.94E-05	NM_178445	NM_178445	Homo sapiens chemokine (C-C motif) receptor-like 1 (CCRL1), transcript variant 1, mRNA [NM_178445]	NM_178445
A_23_P501538	1.96E-05	NM_153631	NM_153631	Homo sapiens homeo box A3 (HOXA3), transcript variant 2, mRNA [NM_153631]	NM_153631
A_23_P72643	1.96E-05	NM_003816	NM_003816	Homo sapiens a disintegrin and metalloproteinase domain 9 (meltrin gamma) (ADAM9), transcript variant 1, mRNA [NM_003816]	NM_003816
A_23_P120488	1.96E-05	NM_024663	NM_024663	Homo sapiens aminopeptidase-like 1 (NPEPL1), mRNA [NM_024663]	NM_024663
A_32_P94798	1.96E-05	NM_001002857	NM_001002857	Homo sapiens annexin A2 (ANXA2), transcript variant 2, mRNA [NM_001002857]	NM_001002857
A_24_P89887	1.97E-05	NM_032823	NM_032823	Homo sapiens chromosome 9 open reading frame 3 (C9orf3), mRNA [NM_032823]	NM_032823
A_23_P252413	1.97E-05	ENST00000245185		Homo sapiens metallothionein 2A, mRNA (cDNA clone MGC:12397 IMAGE:4051220), complete cds. [BC007034]	
A_23_P71526	1.98E-05	NM_018482	NM_018482	Homo sapiens development and differentiation enhancing factor 1 (DDEF1), mRNA [NM_018482]	NM_018482
A_24_P204971	1.99E-05	NM_012257	NM_012257	Homo sapiens HMG-box transcription factor 1 (HBP1), mRNA [NM_012257]	NM_012257
A_23_P206041	1.99E-05	NM_006122	NM_006122	Homo sapiens mannosidase, alpha, class 2A, member 2 (MAN2A2), mRNA [NM_006122]	NM_006122
A_23_P90021	2.01E-05	NM_024341	NM_024341	Homo sapiens zinc finger protein 557 (ZNF557), mRNA [NM_024341]	NM_024341
A_24_P242204	2.02E-05	NM_145799	NM_145799	Homo sapiens septin 6 (SEPT6), transcript variant I, mRNA [NM_145799]	NM_145799
A_23_P369983	2.02E-05	NM_174905	NM_174905	Homo sapiens hypothetical protein LOC147965 (LOC147965), mRNA [NM_174905]	NM_174905
A_23_P86012	2.02E-05	NM_001017402	NM_001017402	Homo sapiens laminin, beta 3 (LAMB3), transcript variant 2, mRNA [NM_001017402]	NM_001017402
A_23_P210482	2.02E-05	NM_000022	NM_000022	Homo sapiens adenosine deaminase (ADA), mRNA [NM_000022]	NM_000022
A_23_P210690	2.02E-05	NM_021158	NM_021158	Homo sapiens tribbles homolog 3 (Drosophila) (TRIB3), mRNA [NM_021158]	NM_021158
A_23_P11685	2.03E-05	NM_024420	NM_024420	Homo sapiens phospholipase A2, group IVA (cytosolic, calcium-dependent) (PLA2G4A), mRNA [NM_024420]	NM_024420
A_23_P201035	2.03E-05	NM_001005749	NM_001005749	Homo sapiens glucosidase, beta; acid (includes glucosylceramidase) (GBA), transcript variant 4, mRNA [NM_001005749]	NM_001005749
A_23_P17095	2.03E-05	NM_006287	NM_006287	Homo sapiens tissue factor pathway inhibitor (lipoprotein-associated coagulation inhibitor) (TFPI), mRNA [NM_006287]	NM_006287
A_24_P388659	2.04E-05	NM_031305	NM_031305	Homo sapiens Rho GTPase activating protein 24 (ARHGAP24), mRNA [NM_031305]	NM_031305
A_23_P47073	2.04E-05	NM_014023	NM_014023	Homo sapiens WD repeat domain 37 (WDR37), mRNA [NM_014023]	NM_014023
A_23_P420361	2.04E-05	NM_018462	NM_018462	Homo sapiens chromosome 3 open reading frame 10 (C3orf10), mRNA [NM_018462]	NM_018462
A_23_P430120	2.05E-05	NM_001430	NM_001430	Homo sapiens endothelial PAS domain protein 1 (EPAS1), mRNA [NM_001430]	NM_001430
A_24_P50543	2.05E-05	BC031266	BC031266	Homo sapiens, clone IMAGE:5277162, mRNA. [BC031266]	XM_378511
A_32_P140501	2.05E-05	A_32_P140501			
A_23_P87902	2.05E-05	NM_003845	NM_003845	Homo sapiens dual-specificity tyrosine-(Y)-phosphorylation regulated kinase 4 (DYRK4), mRNA [NM_003845]	NM_003845
A_24_P143440	2.05E-05	NM_177953	NM_177953	Homo sapiens dynein, cytoplasmic, light polypeptide 2A (DNCL2A), transcript variant 2, mRNA [NM_177953]	NM_177953
A_24_P186414	2.07E-05	NM_021943	NM_021943	Homo sapiens testis expressed sequence 27 (TEX27), mRNA [NM_021943]	NM_021943
A_24_P934563	2.07E-05	NM_014342	NM_014342	Homo sapiens mitochondrial carrier homolog 2 (C. elegans) (MTCH2), nuclear gene encoding mitochondrial protein, mRNA [NM_014342]	NM_014342

A_23_P145	2.08E-05	NM_000191	NM_000191	Homo sapiens 3-hydroxymethyl-3-methylglutaryl-Coenzyme A lyase (hydroxymethylglutaricaciduria) (HMGCL), mRNA [NM_000191]	NM_000191
A_23_P162746	2.08E-05	NM_015974	NM_015974	Homo sapiens crystallin, lambda 1 (CRYL1), mRNA [NM_015974]	NM_015974
A_24_P110558	2.09E-05	NM_001007189	NM_001007189	Homo sapiens similar to bovine IgA regulatory protein (LOC492311), mRNA [NM_001007189]	NM_001007189
A_24_P147461	2.09E-05	NM_198833	NM_198833	Homo sapiens serine (or cysteine) proteinase inhibitor, clade B (ovalbumin), member 8 (SERPINB8), transcript variant 2, mRNA [NM_198833]	NM_198833
A_23_P16451	2.09E-05	NM_025241	NM_025241	Homo sapiens UBX domain containing 1 (UBXD1), mRNA [NM_025241]	NM_025241
A_23_P157580	2.09E-05	NM_005625	NM_005625	Homo sapiens syndecan binding protein (syntenin) (SDCBP), transcript variant 1, mRNA [NM_005625]	NM_005625
A_24_P842962	2.10E-05	THC2379232			
A_23_P367026	2.10E-05	NM_015600	NM_015600	Homo sapiens chromosome 20 open reading frame 22 (C20orf22), mRNA [NM_015600]	NM_015600
A_23_P141248	2.11E-05	NM_021947	NM_021947	Homo sapiens serine racemase (SRR), mRNA [NM_021947]	NM_021947
A_23_P213288	2.11E-05	AF389338	AF389338	Homo sapiens acyl-CoA-desaturase mRNA, complete cds. [AF389338]	
A_23_P205531	2.11E-05	NM_194430	NM_194430	Homo sapiens ribonuclease, RNase A family, 4 (RNASE4), transcript variant 1, mRNA [NM_194430]	NM_194430
A_23_P345692	2.11E-05	NM_138284	NM_138284	Homo sapiens interleukin 17D (IL17D), mRNA [NM_138284]	NM_138284
A_23_P96350	2.11E-05	NM_007213	NM_007213	Homo sapiens PRA1 domain family, member 2 (PRAF2), mRNA [NM_007213]	NM_007213
A_24_P928052	2.11E-05	NM_003873	NM_003873	Homo sapiens neuropilin 1 (NRP1), transcript variant 1, mRNA [NM_003873]	NM_003873
A_23_P389525	2.11E-05	NM_152516	NM_152516	Homo sapiens copper metabolism (Murr1) domain containing 1 (COMMD1), mRNA [NM_152516]	NM_152516
A_23_P25336	2.12E-05	NM_016433	NM_016433	Homo sapiens glycolipid transfer protein (GLTP), mRNA [NM_016433]	NM_016433
A_32_P61693	2.12E-05	AK131287	AK131287	Homo sapiens cDNA FLJ16240 fis, clone HCASM2003357. [AK131287]	
A_23_P113005	2.13E-05	NM_004428	NM_004428	Homo sapiens ephrin-A1 (EFNA1), transcript variant 1, mRNA [NM_004428]	NM_004428
A_24_P941572	2.14E-05	AK126071	AK126071	Homo sapiens cDNA FLJ44083 fis, clone TESTI4040939. [AK126071]	
A_32_P59302	2.15E-05	BC080552	BC080552	Homo sapiens cDNA clone IMAGE:6254031. [BC080552]	
A_23_P102731	2.15E-05	NM_175839	NM_175839	Homo sapiens spermine oxidase (SMOX), transcript variant 1, mRNA [NM_175839]	NM_175839
A_23_P357811	2.15E-05	NM_021038	NM_021038	Homo sapiens muscleblind-like (Drosophila) (MBNL1), transcript variant 1, mRNA [NM_021038]	NM_021038
A_24_P101282	2.16E-05	BC031342	BC031342	Homo sapiens, clone IMAGE:5019307, mRNA. [BC031342]	
A_32_P155026	2.16E-05	THC2305309			
A_24_P622468	2.17E-05	BU076424	BU076424	BU076424 im51d09.x1 HR85 islet Homo sapiens cDNA clone IMAGE:6038465 3' similar to SW:MIC2_HUMAN P14209 T-CELL SURFACE GLYCOPROTEIN E2 PRECURSOR ;, mRNA sequence [BU076424]	
A_23_P253368	2.18E-05	NM_018951	NM_018951	Homo sapiens homeo box A10 (HOXA10), transcript variant 1, mRNA [NM_018951]	NM_018951
A_23_P99853	2.18E-05	NM_019600	NM_019600	Homo sapiens hypothetical protein FLJ10980 (FLJ10980), mRNA [NM_019600]	NM_019600
A_23_P33482	2.19E-05	NM_003369	NM_003369	Homo sapiens UV radiation resistance associated gene (UVRAG), mRNA [NM_003369]	NM_003369
A_23_P96144	2.19E-05	NM_001204	NM_001204	Homo sapiens bone morphogenetic protein receptor, type II (serine/threonine kinase) (BMPR2), transcript variant 1, mRNA [NM_001204]	NM_001204
A_23_P203900	2.19E-05	NM_005505	NM_005505	Homo sapiens scavenger receptor class B, member 1 (SCARB1), mRNA [NM_005505]	NM_005505
A_23_P25706	2.20E-05	NM_024734	NM_024734	Homo sapiens calmin (calponin-like, transmembrane) (CLMN), mRNA [NM_024734]	NM_024734
A_32_P190181	2.21E-05	THC2345075			
A_24_P1545	2.21E-05	NM_015633	NM_015633	Homo sapiens FGFR1 oncogene partner 2 (FGFR1OP2), mRNA [NM_015633]	NM_015633
A_23_P41645	2.21E-05	NM_012081	NM_012081	Homo sapiens elongation factor, RNA polymerase II, 2 (ELL2), mRNA [NM_012081]	NM_012081
A_24_P69538	2.22E-05	NM_138557	NM_138557	Homo sapiens toll-like receptor 4 (TLR4), transcript variant 4, mRNA [NM_138557]	NM_138557
A_23_P29124	2.22E-05	NM_000407	NM_000407	Homo sapiens glycoprotein Ib (platelet), beta polypeptide (GP1BB), mRNA [NM_000407]	NM_000407
A_23_P127128	2.24E-05	NM_022365	NM_022365	Homo sapiens DnaJ (Hsp40) homolog, subfamily C, member 1 (DNAJC1), mRNA [NM_022365]	NM_022365
A_24_P88801	2.25E-05	NM_000272	NM_000272	Homo sapiens nephronophthisis 1 (juvenile) (NPHP1), transcript variant 1, mRNA [NM_000272]	NM_000272
A_23_P141126	2.25E-05	NM_000154	NM_000154	Homo sapiens galactokinase 1 (GALK1), mRNA [NM_000154]	NM_000154
A_23_P88626	2.25E-05	NM_001150	NM_001150	Homo sapiens alanyl (membrane) aminopeptidase (aminopeptidase N, aminopeptidase M, microsomal aminopeptidase, CD13, p150) (ANPEP), mRNA [NM_001150]	NM_001150
A_23_P252903	2.25E-05	NM_022464	NM_022464	Homo sapiens endoplasmic reticulum chaperone SIL1, homolog of yeast (SIL1), mRNA [NM_022464]	NM_022464
A_24_P123190	2.26E-05	NM_002662	NM_002662	Homo sapiens phospholipase D1, phophatidylcholine-specific (PLD1), mRNA [NM_002662]	NM_002662
A_23_P65823	2.26E-05	AF180519	AF180519	Homo sapiens GABA-A receptor-associated protein mRNA, complete cds. [AF180519]	
A_23_P46618	2.26E-05	NM_025179	NM_025179	Homo sapiens plexin A2 (PLXNA2), mRNA [NM_025179]	NM_025179

A_32_P215002	2.27E-05	AI342696	AI342696	AI342696 qo35e02.x1 NCI_CGAP_Lu5 Homo sapiens cDNA clone IMAGE:1910522 3' similar to gb:M59040 CD44 ANTIGEN, EPITHELIAL FORM PRECURSOR (HUMAN);, mRNA sequence [AI342696]	
A_24_P6517	2.28E-05	NM_001029884	NM_001029884	Homo sapiens pleckstrin homology domain containing, family G (with RhoGef domain) member 1 (PLEKHG1), mRNA [NM_001029884]	NM_001029884
A_23_P116902	2.28E-05	NM_021071	NM_021071	Homo sapiens Dombrock blood group (DO), mRNA [NM_021071]	NM_021071
A_24_P385134	2.28E-05	AF389338	AF389338	Homo sapiens acyl-CoA-desaturase mRNA, complete cds. [AF389338]	
A_24_P250964	2.28E-05	NM_003768	NM_003768	Homo sapiens phosphoprotein enriched in astrocytes 15 (PEA15), mRNA [NM_003768]	NM_003768
A_24_P304051	2.28E-05	NM_004832	NM_004832	Homo sapiens glutathione S-transferase omega 1 (GSTO1), mRNA [NM_004832]	NM_004832
A_24_P357465	2.30E-05	NM_021202	NM_021202	Homo sapiens tumor protein p53 inducible nuclear protein 2 (TP53INP2), mRNA [NM_021202]	NM_021202
A_24_P116233	2.30E-05	NM_020456	NM_020456	Homo sapiens chromosome 13 open reading frame 1 (C13orf1), mRNA [NM_020456]	NM_020456
A_23_P71889	2.30E-05	NM_153437	NM_153437	Homo sapiens outer dense fiber of sperm tails 2 (ODF2), transcript variant 2, mRNA [NM_153437]	NM_153437
A_23_P130089	2.30E-05	NM_174887	NM_174887	Homo sapiens intraflagellar transport protein IFT20 (IFT20), mRNA [NM_174887]	NM_174887
A_23_P329198	2.30E-05	NM_022837	NM_022837	Homo sapiens hypothetical protein FLJ22833 (FLJ22833), mRNA [NM_022837]	NM_022837
A_24_P278393	2.31E-05	NM_183013	NM_183013	Homo sapiens cAMP responsive element modulator (CREM), transcript variant 19, mRNA [NM_183013]	NM_183013
A_23_P383532	2.32E-05	AK090988	AK090988	Homo sapiens cDNA FLJ33669 fis, clone BRAMY2028740. [AK090988]	
A_24_P393312	2.34E-05	AK090554	AK090554	Homo sapiens cDNA FLJ33235 fis, clone ASTRO2002202. [AK090554]	
A_24_P46417	2.34E-05	NM_005990	NM_005990	Homo sapiens serine/threonine kinase 10 (STK10), mRNA [NM_005990]	NM_005990
A_23_P99741	2.35E-05	NM_004196	NM_004196	Homo sapiens cyclin-dependent kinase-like 1 (CDC2-related kinase) (CDKL1), mRNA [NM_004196]	NM_004196
A_23_P54376	2.36E-05	NM_004809	NM_004809	Homo sapiens stomatin (EPB72)-like 1 (STOML1), mRNA [NM_004809]	NM_004809
A_23_P6935	2.37E-05	NM_198793	NM_198793	Homo sapiens CD47 antigen (Rh-related antigen, integrin-associated signal transducer) (CD47), transcript variant 2, mRNA [NM_198793]	NM_198793
A_23_P11800	2.38E-05	BC020630	BC020630	Homo sapiens calcium/calmodulin-dependent protein kinase II inhibitor 1, mRNA (cDNA clone MGC:22256 IMAGE:4703846), complete cds. [BC020630]	
A_23_P15864	2.38E-05	NM_003927	NM_003927	Homo sapiens methyl-CpG binding domain protein 2 (MBD2), transcript variant 1, mRNA [NM_003927]	NM_003927
A_24_P409250	2.39E-05	ENST00000308436		GB|AK074061.1|BAB84887.1 FLJ00132 protein [Homo sapiens] [NP434181]	
A_24_P798709	2.42E-05	AK025015	AK025015	Homo sapiens cDNA: FLJ21362 fis, clone COL02886. [AK025015]	
A_23_P101707	2.42E-05	NM_005817	NM_005817	Homo sapiens mannose-6-phosphate receptor binding protein 1 (M6PRBP1), mRNA [NM_005817]	NM_005817
A_23_P387630	2.43E-05	NM_014725	NM_014725	Homo sapiens START domain containing 8 (STARD8), mRNA [NM_014725]	NM_014725
A_23_P27256	2.43E-05	NM_020648	NM_020648	Homo sapiens twisted gastrulation homolog 1 (Drosophila) (TWSG1), mRNA [NM_020648]	NM_020648
A_24_P108005	2.43E-05	NM_022818	NM_022818	Homo sapiens microtubule-associated protein 1 light chain 3 beta (MAP1LC3B), mRNA [NM_022818]	NM_022818
A_24_P96403	2.46E-05	NM_001001890	NM_001001890	Homo sapiens runt-related transcription factor 1 (acute myeloid leukemia 1; aml1 oncogene) (RUNX1), transcript variant 2, mRNA [NM_001001890]	NM_001001890
A_24_P387839	2.46E-05	NM_144780	NM_144780	Homo sapiens degenerative spermatocyte homolog 1, lipid desaturase (Drosophila) (DEGS1), transcript variant 2, mRNA [NM_144780]	NM_144780
A_23_P161194	2.46E-05	NM_003380	NM_003380	Homo sapiens vimentin (VIM), mRNA [NM_003380]	NM_003380
A_32_P198179	2.47E-05	BQ354462	BQ354462	BQ354462 CM2-HT0970-131100-502-h12 HT0970 Homo sapiens cDNA, mRNA sequence [BQ354462]	
A_32_P158181	2.47E-05	THC2437757			
A_23_P24414	2.47E-05	NM_016938	NM_016938	Homo sapiens EGF-containing fibulin-like extracellular matrix protein 2 (EFEMP2), mRNA [NM_016938]	NM_016938
A_24_P184555	2.47E-05	NM_002859	NM_002859	Homo sapiens paxillin (PXN), mRNA [NM_002859]	NM_002859
A_24_P68342	2.48E-05	NM_001845	NM_001845	Homo sapiens collagen, type IV, alpha 1 (COL4A1), mRNA [NM_001845]	NM_001845
A_23_P203344	2.48E-05	NM_053023	NM_053023	Homo sapiens zinc finger protein 91 homolog (mouse) (ZFP91), transcript variant 1, mRNA [NM_053023]	NM_053023
A_32_P215938	2.48E-05	BC048343	BC048343	Homo sapiens G-protein signalling modulator 1 (AGS3-like, C. elegans), mRNA (cDNA clone IMAGE:5242824), complete cds. [BC048343]	
A_32_P222695	2.49E-05	NM_001001669	NM_001001669	Homo sapiens FLJ41603 protein (FLJ41603), mRNA [NM_001001669]	NM_001001669
A_23_P55127	2.49E-05	NM_020233	NM_020233	Homo sapiens x 006 protein (MDS006), mRNA [NM_020233]	NM_020233
A_23_P20107	2.49E-05	NM_015917	NM_015917	Homo sapiens glutathione S-transferase kappa 1 (GSTK1), mRNA [NM_015917]	NM_015917
A_32_P89709	2.49E-05	NM_001018004	NM_001018004	Homo sapiens tropomyosin 1 (alpha) (TPM1), transcript variant 3, mRNA [NM_001018004]	NM_001018004
A_23_P130919	2.50E-05	NM_130807	NM_130807	Homo sapiens MOB1, Mps One Binder kinase activator-like 2A (yeast) (MOBKL2A), mRNA [NM_130807]	NM_130807
A_24_P183128	2.51E-05	NM_016619	NM_016619	Homo sapiens placenta-specific 8 (PLAC8), mRNA [NM_016619]	NM_016619
A_23_P47867	2.51E-05	NM_003622	NM_003622	Homo sapiens PTPRF interacting protein, binding protein 1 (liprin beta 1) (PPFIBP1), transcript variant 1, mRNA [NM_003622]	NM_003622
A_23_P33376	2.52E-05	NM_002595	NM_002595	Homo sapiens PCTAIRE protein kinase 2 (PCTK2), mRNA [NM_002595]	NM_002595

A_23_P204238	2.52E-05	NM_018416	NM_018416	Homo sapiens forkhead box J2 (FOXJ2), mRNA [NM_018416]	NM_018416
A_32_P39394	2.53E-05	AB209404	AB209404	Homo sapiens mRNA for GLIS family zinc finger 3 variant protein. [AB209404]	
A_23_P110345	2.53E-05	NM_012110	NM_012110	Homo sapiens cysteine-rich hydrophobic domain 2 (CHIC2), mRNA [NM_012110]	NM_012110
A_23_P167096	2.53E-05	NM_005429	NM_005429	Homo sapiens vascular endothelial growth factor C (VEGFC), mRNA [NM_005429]	NM_005429
A_23_P20183	2.53E-05	NM_016068	NM_016068	Homo sapiens tetratricopeptide repeat domain 11 (TTC11), mRNA [NM_016068]	NM_016068
A_23_P103454	2.53E-05	NM_032264	NM_032264	Homo sapiens hypothetical protein AE2 (AE2), mRNA [NM_032264]	NM_032264
A_32_P150735	2.55E-05	NM_001008407	NM_001008407	Homo sapiens B-cell receptor-associated protein 29 (BCAP29), transcript variant 4, mRNA [NM_001008407]	NM_001008407
A_23_P48807	2.55E-05	NM_001001556	NM_001001556	Homo sapiens galactokinase 2 (GALK2), transcript variant 2, mRNA [NM_001001556]	NM_001001556
A_23_P111701	2.55E-05	NM_004126	NM_004126	Homo sapiens guanine nucleotide binding protein (G protein), gamma 11 (GNG11), mRNA [NM_004126]	NM_004126
A_24_P762613	2.56E-05	AK021543	AK021543	Homo sapiens cDNA FLJ11481 fis, clone HEMBA1001803. [AK021543]	
A_23_P63038	2.56E-05	NM_022356	NM_022356	Homo sapiens leucine proline-enriched proteoglycan (leprecan) 1 (LEPRE1), mRNA [NM_022356]	NM_022356
A_23_P206661	2.57E-05	NM_000903	NM_000903	Homo sapiens NAD(P)H dehydrogenase, quinone 1 (NQO1), transcript variant 1, mRNA [NM_000903]	NM_000903
A_32_P190036	2.58E-05	XM_497783	XM_497783	PREDICTED: Homo sapiens similar to transcript increased in spermiogenesis 78 (LOC441964), mRNA [XM_497783]	XM_497783
A_23_P168882	2.58E-05	NM_033285	NM_033285	Homo sapiens tumor protein p53 inducible nuclear protein 1 (TP53INP1), mRNA [NM_033285]	NM_033285
A_24_P367602	2.59E-05	AK092260	AK092260	Homo sapiens cDNA FLJ34941 fis, clone NT2RP7007480. [AK092260]	
A_23_P2705	2.59E-05	NM_005767	NM_005767	Homo sapiens purinergic receptor P2Y, G-protein coupled, 5 (P2RY5), mRNA [NM_005767]	NM_005767
A_24_P279760	2.59E-05	A_24_P279760			
A_23_P157371	2.59E-05	NM_014888	NM_014888	Homo sapiens family with sequence similarity 3, member C (FAM3C), mRNA [NM_014888]	NM_014888
A_24_P344516	2.62E-05	NM_024924	NM_024924	Homo sapiens hypothetical protein FLJ12985 (FLJ12985), mRNA [NM_024924]	NM_024924
A_32_P96692	2.62E-05	THC2340838		Q9BX12 (Q9BX12) GTP binding protein 2 (Fragment), partial (26%) [THC2340838]	
A_23_P257423	2.62E-05	NM_144988	NM_144988	Homo sapiens hypothetical protein MGC19780 (MGC19780), mRNA [NM_144988]	NM_144988
A_24_P389959	2.62E-05	NM_016057	NM_016057	Homo sapiens coatomer protein complex, subunit zeta 1 (COPZ1), mRNA [NM_016057]	NM_016057
A_24_P86389	2.62E-05	NM_003514	NM_003514	Homo sapiens histone 1, H2am (HIST1H2AM), mRNA [NM_003514]	NM_003514
A_23_P211345	2.63E-05	NM_080647	NM_080647	Homo sapiens T-box 1 (TBX1), transcript variant C, mRNA [NM_080647]	NM_080647
A_24_P332953	2.63E-05	A_24_P332953			
A_23_P344531	2.63E-05	ENST00000307662		Homo sapiens mRNA for KIAA1029 protein, partial cds. [AB028952]	
A_23_P96965	2.64E-05	NM_030786	NM_030786	Homo sapiens syncoilin, intermediate filament 1 (SYNC1), mRNA [NM_030786]	NM_030786
A_23_P80156	2.64E-05	NM_206962	NM_206962	Homo sapiens HMT1 hnRNP methyltransferase-like 1 (S. cerevisiae) (HRMT1L1), transcript variant 1, mRNA [NM_206962]	NM_206962
A_32_P135348	2.64E-05	NM_033394	NM_033394	Homo sapiens TPR domain, ankyrin-repeat and coiled-coil-containing (TANC), mRNA [NM_033394]	NM_033394
A_24_P414371	2.64E-05	NM_000944	NM_000944	Homo sapiens protein phosphatase 3 (formerly 2B), catalytic subunit, alpha isoform (calcineurin A alpha) (PPP3CA), mRNA [NM_000944]	NM_000944
A_24_P228027	2.65E-05	NM_144611	NM_144611	Homo sapiens hypothetical protein MGC32124 (MGC32124), mRNA [NM_144611]	NM_144611
A_24_P45367	2.65E-05	NM_020448	NM_020448	Homo sapiens NIPA-like domain containing 3 (NPAL3), mRNA [NM_020448]	NM_020448
A_32_P148345	2.67E-05	NM_001002857	NM_001002857	Homo sapiens annexin A2 (ANXA2), transcript variant 2, mRNA [NM_001002857]	NM_001002857
A_23_P4223	2.67E-05	NM_005831	NM_005831	Homo sapiens nuclear domain 10 protein (NDP52), mRNA [NM_005831]	NM_005831
A_23_P412392	2.67E-05	NM_004892	NM_004892	Homo sapiens SEC22 vesicle trafficking protein-like 1 (S. cerevisiae) (SEC22L1), mRNA [NM_004892]	NM_004892
A_24_P133171	2.67E-05	AK026078	AK026078	Homo sapiens cDNA: FLJ22425 fis, clone HRC08686. [AK026078]	
A_24_P942068	2.68E-05	ENST00000313631		Homo sapiens mRNA for KIAA1636 protein, partial cds. [AB046856]	XM_371074
A_32_P166921	2.69E-05	AV692191	AV692191	AV692191 AV692191 GKC Homo sapiens cDNA clone GKCFFD09 5', mRNA sequence [AV692191]	
A_23_P48897	2.69E-05	NM_004748	NM_004748	Homo sapiens cell cycle progression 1 (CCPG1), transcript variant 1, mRNA [NM_004748]	NM_004748
A_23_P99141	2.69E-05	NM_019858	NM_019858	Homo sapiens gene rich cluster, A gene (GRCA), transcript variant A-2, mRNA [NM_019858]	NM_019858
A_23_P60283	2.71E-05	NM_000380	NM_000380	Homo sapiens xeroderma pigmentosum, complementation group A (XPA), mRNA [NM_000380]	NM_000380
A_24_P49383	2.71E-05	NM_024684	NM_024684	Homo sapiens PTD015 protein (PTD015), mRNA [NM_024684]	NM_024684
A_23_P156049	2.71E-05	NM_000521	NM_000521	Homo sapiens hexosaminidase B (beta polypeptide) (HEXB), mRNA [NM_000521]	NM_000521
A_23_P21785	2.72E-05	NM_022072	NM_022072	Homo sapiens NOL1/NOP2/Sun domain family, member 3 (NSUN3), mRNA [NM_022072]	NM_022072

A_23_P85460	2.72E-05	NM_001262	NM_001262	Homo sapiens cyclin-dependent kinase inhibitor 2C (p18, inhibits CDK4) (CDKN2C), transcript variant 1, mRNA [NM_001262]	NM_001262
A_23_P502710	2.72E-05	NM_152237	NM_152237	Homo sapiens growth arrest-specific 2 like 1 (GAS2L1), transcript variant 3, mRNA [NM_152237]	NM_152237
A_24_P169343	2.73E-05	NM_153698	NM_153698	Homo sapiens chromosome 9 open reading frame 21 (C9orf21), mRNA [NM_153698]	NM_153698
A_23_P162087	2.73E-05	NM_020644	NM_020644	Homo sapiens chromosome 11 open reading frame 15 (C11orf15), mRNA [NM_020644]	NM_020644
A_23_P85180	2.74E-05	NM_003492	NM_003492	Homo sapiens chromosome X open reading frame 12 (CXorf12), mRNA [NM_003492]	NM_003492
A_23_P253434	2.74E-05	NM_022763	NM_022763	Homo sapiens fibronectin type III domain containing 3B (FNDC3B), mRNA [NM_022763]	NM_022763
A_23_P30435	2.75E-05	NM_006058	NM_006058	Homo sapiens TNFAIP3 interacting protein 1 (TNIP1), mRNA [NM_006058]	NM_006058
A_23_P125117	2.75E-05	NM_173546	NM_173546	Homo sapiens hypothetical protein MGC35097 (MGC35097), mRNA [NM_173546]	NM_173546
A_24_P75230	2.75E-05	NM_033546	NM_033546	Homo sapiens myosin regulatory light chain MRLC2 (MRLC2), mRNA [NM_033546]	NM_033546
A_32_P177040	2.76E-05	NM_175064	NM_175064	Homo sapiens Williams Beuren syndrome chromosome region 19 (WBSCR19), mRNA [NM_175064]	NM_175064
A_23_P85693	2.76E-05	NM_004120	NM_004120	Homo sapiens guanylate binding protein 2, interferon-inducible (GBP2), mRNA [NM_004120]	NM_004120
A_23_P136347	2.76E-05	NM_004447	NM_004447	Homo sapiens epidermal growth factor receptor pathway substrate 8 (EPS8), mRNA [NM_004447]	NM_004447
A_23_P408095	2.76E-05	NM_001011546	NM_001011546	Homo sapiens destrin (actin depolymerizing factor) (DSTN), transcript variant 2, mRNA [NM_001011546]	NM_001011546
A_23_P34835	2.76E-05	NM_005572	NM_005572	Homo sapiens lamin A/C (LMNA), transcript variant 2, mRNA [NM_005572]	NM_005572
A_23_P61551	2.77E-05	NM_006110	NM_006110	Homo sapiens CD2 antigen (cytoplasmic tail) binding protein 2 (CD2BP2), mRNA [NM_006110]	NM_006110
A_24_P22746	2.78E-05	THC2336861		Q96FJ6 (Q96FJ6) Zinc binding alcohol dehydrogenase, domain containing 2, complete [THC2336861]	
A_23_P69226	2.80E-05	NM_018447	NM_018447	Homo sapiens 30 kDa protein (LOC55831), mRNA [NM_018447]	NM_018447
A_24_P97703	2.80E-05	NM_000919	NM_000919	Homo sapiens peptidylglycine alpha-amidating monooxygenase (PAM), transcript variant 1, mRNA [NM_000919]	NM_000919
A_23_P59349	2.80E-05	NM_016217	NM_016217	Homo sapiens headcase homolog (Drosophila) (HECA), mRNA [NM_016217]	NM_016217
A_23_P88589	2.81E-05	NM_021005	NM_021005	Homo sapiens nuclear receptor subfamily 2, group F, member 2 (NR2F2), mRNA [NM_021005]	NM_021005
A_24_P296689	2.82E-05	AK124426	AK124426	Homo sapiens cDNA FLJ42435 fis, clone BLADE2006849. [AK124426]	
A_23_P17955	2.82E-05	NM_012157	NM_012157	Homo sapiens F-box and leucine-rich repeat protein 2 (FBXL2), mRNA [NM_012157]	NM_012157
A_23_P168909	2.82E-05	NM_012082	NM_012082	Homo sapiens zinc finger protein, multitype 2 (ZFPM2), mRNA [NM_012082]	NM_012082
A_24_P69150	2.82E-05	NM_004290	NM_004290	Homo sapiens ring finger protein 14 (RNF14), transcript variant 1, mRNA [NM_004290]	NM_004290
A_23_P117286	2.82E-05	NM_006493	NM_006493	Homo sapiens ceroid-lipofuscinosis, neuronal 5 (CLN5), mRNA [NM_006493]	NM_006493
A_23_P54055	2.82E-05	NM_032876	NM_032876	Homo sapiens jub, ajuba homolog (Xenopus laevis) (JUB), transcript variant 1, mRNA [NM_032876]	NM_032876
A_23_P71464	2.82E-05	NM_001359	NM_001359	Homo sapiens 2,4-dienoyl CoA reductase 1, mitochondrial (DECR1), nuclear gene encoding mitochondrial protein, mRNA [NM_001359]	NM_001359
A_32_P80198	2.83E-05	A_32_P80198			
A_24_P213494	2.83E-05	NM_006504	NM_006504	Homo sapiens protein tyrosine phosphatase, receptor type, E (PTPRE), transcript variant 1, mRNA [NM_006504]	NM_006504
A_24_P314179	2.83E-05	NM_005239	NM_005239	Homo sapiens v-ets erythroblastosis virus E26 oncogene homolog 2 (avian) (ETS2), mRNA [NM_005239]	NM_005239
A_24_P652537	2.84E-05	BC034006	BC034006	Homo sapiens, clone IMAGE:5295612, mRNA. [BC034006]	
A_23_P83798	2.84E-05	NM_006982	NM_006982	Homo sapiens cartilage paired-class homeoprotein 1 (CART1), mRNA [NM_006982]	NM_006982
A_23_P151368	2.84E-05	NM_174928	NM_174928	Homo sapiens hypothetical protein LOC221143 (LOC221143), mRNA [NM_174928]	NM_174928
A_23_P385206	2.84E-05	NM_177424	NM_177424	Homo sapiens syntaxin 12 (STX12), mRNA [NM_177424]	NM_177424
A_23_P20494	2.84E-05	NM_006096	NM_006096	Homo sapiens N-myc downstream regulated gene 1 (NDRG1), mRNA [NM_006096]	NM_006096
A_23_P257144	2.84E-05	ENST00000252804		Homo sapiens Melanoma associated gene, mRNA (cDNA clone IMAGE:3948774), partial cds. [BC009496]	XM_056455
A_24_P280983	2.85E-05	BC025338	BC025338	Homo sapiens, clone IMAGE:4865533, mRNA. [BC025338]	
A_23_P12044	2.85E-05	NM_018022	NM_018022	Homo sapiens transmembrane protein 51 (TMEM51), mRNA [NM_018022]	NM_018022
A_23_P250671	2.85E-05	NM_201397	NM_201397	Homo sapiens glutathione peroxidase 1 (GPX1), transcript variant 2, mRNA [NM_201397]	NM_201397
A_23_P83857	2.87E-05	NM_000240	NM_000240	Homo sapiens monoamine oxidase A (MAOA), nuclear gene encoding mitochondrial protein, mRNA [NM_000240]	NM_000240
A_23_P216966	2.88E-05	NM_000962	NM_000962	Homo sapiens prostaglandin-endoperoxide synthase 1 (prostaglandin G/H synthase and cyclooxygenase) (PTGS1), transcript variant 1, mRNA [NM_000962]	NM_000962
A_24_P17719	2.88E-05	NM_015990	NM_015990	Homo sapiens kelch-like 5 (Drosophila) (KLHL5), mRNA [NM_015990]	NM_015990
A_24_P333857	2.90E-05	NM_032291	NM_032291	Homo sapiens SH3-domain GRB2-like (endophilin) interacting protein 1 (SGIP1), mRNA [NM_032291]	NM_032291
A_23_P81399	2.90E-05	NM_003900	NM_003900	Homo sapiens sequestosome 1 (SQSTM1), mRNA [NM_003900]	NM_003900

A_24_P39101	2.91E-05	NM_031954	NM_031954	Homo sapiens potassium channel tetramerisation domain containing 10 (KCTD10), mRNA [NM_031954]	NM_031954
A_24_P180424	2.91E-05	NM_018247	NM_018247	Homo sapiens transmembrane protein 30A (TMEM30A), mRNA [NM_018247]	NM_018247
A_23_P358944	2.91E-05	NM_033245	NM_033245	Homo sapiens promyelocytic leukemia (PML), transcript variant 12, mRNA [NM_033245]	NM_033245
A_23_P68211	2.92E-05	NM_003124	NM_003124	Homo sapiens sepiapterin reductase (7,8-dihydrobiopterin:NADP+ oxidoreductase) (SPR), mRNA [NM_003124]	NM_003124
A_32_P29582	2.93E-05	NM_203477	NM_203477	Homo sapiens similar to RPL23AP7 protein (MGC70863), transcript variant 1, mRNA [NM_203477]	NM_203477
A_24_P250922	2.94E-05	NM_000963	NM_000963	Homo sapiens prostaglandin-endoperoxide synthase 2 (prostaglandin G/H synthase and cyclooxygenase) (PTGS2), mRNA [NM_000963]	NM_000963
A_23_P12463	2.95E-05	NM_002826	NM_002826	Homo sapiens quiescin Q6 (QSCN6), transcript variant 1, mRNA [NM_002826]	NM_002826
A_23_P110712	2.96E-05	NM_004417	NM_004417	Homo sapiens dual specificity phosphatase 1 (DUSP1), mRNA [NM_004417]	NM_004417
A_23_P158024	2.96E-05	NM_007234	NM_007234	Homo sapiens dynactin 3 (p22) (DCTN3), transcript variant 1, mRNA [NM_007234]	NM_007234
A_23_P23855	2.96E-05	NM_032305	NM_032305	Homo sapiens polymerase (RNA) III (DNA directed) polypeptide G (32kD) like (POLR3GL), mRNA [NM_032305]	NM_032305
A_23_P118122	2.97E-05	NM_003834	NM_003834	Homo sapiens regulator of G-protein signalling 11 (RGS11), transcript variant 2, mRNA [NM_003834]	NM_003834
A_23_P259632	2.97E-05	NM_031925	NM_031925	Homo sapiens transmembrane protein induced by tumor necrosis factor alpha (TMPIT), mRNA [NM_031925]	NM_031925
A_23_P34710	2.99E-05	ENST00000357180		full-length cDNA clone CS0DF032YO23 of Fetal brain of Homo sapiens (human). [CR604521]	
A_24_P96171	3.00E-05	NM_030578	NM_030578	Homo sapiens hypothetical protein MGC4093 (MGC4093), mRNA [NM_030578]	NM_030578
A_23_P65442	3.01E-05	NM_006084	NM_006084	Homo sapiens interferon-stimulated transcription factor 3, gamma 48kDa (ISGF3G), mRNA [NM_006084]	NM_006084
A_24_P166045	3.02E-05	AF070525	AF070525	Homo sapiens clone 24706 mRNA sequence. [AF070525]	
A_23_P118370	3.02E-05	AK022252	AK022252	Homo sapiens cDNA FLJ12190 fis, clone MAMMA1000842. [AK022252]	
A_23_P166297	3.02E-05	NM_207630	NM_207630	Homo sapiens ATP-binding cassette, sub-family G (WHITE), member 1 (ABCG1), transcript variant 1, mRNA [NM_207630]	NM_207630
A_24_P205316	3.03E-05	AK074041	AK074041	Homo sapiens mRNA for FLJ00095 protein. [AK074041]	
A_23_P417331	3.03E-05	NM_004586	NM_004586	Homo sapiens ribosomal protein S6 kinase, 90kDa, polypeptide 3 (RPS6KA3), mRNA [NM_004586]	NM_004586
A_24_P319736	3.03E-05	NM_002398	NM_002398	Homo sapiens Meis1, myeloid ecotropic viral integration site 1 homolog (mouse) (MEIS1), mRNA [NM_002398]	NM_002398
A_23_P500601	3.03E-05	NM_033017	NM_033017	Homo sapiens tripartite motif-containing 4 (TRIM4), transcript variant alpha, mRNA [NM_033017]	NM_033017
A_32_P10633	3.03E-05	BC022417	BC022417	Homo sapiens cDNA clone IMAGE:4243782, partial cds. [BC022417]	
A_23_P61149	3.03E-05	NM_001017915	NM_001017915	Homo sapiens inositol polyphosphate-5-phosphatase, 145kDa (INPP5D), transcript variant 1, mRNA [NM_001017915]	NM_001017915
A_24_P345993	3.04E-05	NM_001746	NM_001746	Homo sapiens calnexin (CANX), transcript variant 1, mRNA [NM_001746]	NM_001746
A_23_P331479	3.04E-05	BC066644	BC066644	Homo sapiens KIAA1949, mRNA (cDNA clone MGC:71094 IMAGE:5575643), complete cds. [BC066644]	
A_23_P414913	3.04E-05	NM_022343	NM_022343	Homo sapiens chromosome 9 open reading frame 19 (C9orf19), mRNA [NM_022343]	NM_022343
A_24_P832426	3.07E-05	BC032021	BC032021	Homo sapiens beta 3-glycosyltransferase-like, mRNA (cDNA clone IMAGE:4825471), partial cds. [BC032021]	
A_23_P18559	3.09E-05	NM_003866	NM_003866	Homo sapiens inositol polyphosphate-4-phosphatase, type II, 105kDa (INPP4B), mRNA [NM_003866]	NM_003866
A_23_P111888	3.09E-05	NM_138455	NM_138455	Homo sapiens collagen triple helix repeat containing 1 (CTHRC1), mRNA [NM_138455]	NM_138455
A_32_P233019	3.10E-05	NM_012430	NM_012430	Homo sapiens SEC22 vesicle trafficking protein-like 2 (S. cerevisiae) (SEC22L2), mRNA [NM_012430]	NM_012430
A_23_P139632	3.11E-05	NM_002076	NM_002076	Homo sapiens glucosamine (N-acetyl)-6-sulfatase (Sanfilippo disease IIID) (GNS), mRNA [NM_002076]	NM_002076
A_23_P258978	3.11E-05	NM_002077	NM_002077	Homo sapiens golgi autoantigen, golgin subfamily a, 1 (GOLGA1), mRNA [NM_002077]	NM_002077
A_23_P8744	3.11E-05	NM_020728	NM_020728	Homo sapiens family with sequence similarity 62 (C2 domain containing) member B (FAM62B), mRNA [NM_020728]	NM_020728
A_23_P126416	3.12E-05	NM_005424	NM_005424	Homo sapiens tyrosine kinase with immunoglobulin-like and EGF-like domains 1 (TIE1), mRNA [NM_005424]	NM_005424
A_24_P303354	3.12E-05	NM_021064	NM_021064	Homo sapiens histone 1, H2ag (HIST1H2AG), mRNA [NM_021064]	NM_021064
A_23_P125788	3.15E-05	NM_152278	NM_152278	Homo sapiens transcription elongation factor A (SII)-like 7 (TCEAL7), mRNA [NM_152278]	NM_152278
A_23_P214185	3.15E-05	NM_016021	NM_016021	Homo sapiens ubiquitin-conjugating enzyme E2, J1 (UBC6 homolog, yeast) (UBE2J1), mRNA [NM_016021]	NM_016021
A_23_P156180	3.16E-05	NM_003059	NM_003059	Homo sapiens solute carrier family 22 (organic cation transporter), member 4 (SLC22A4), mRNA [NM_003059]	NM_003059
A_24_P106057	3.16E-05	AK129838	AK129838	Homo sapiens cDNA FLJ26328 fis, clone HRT01493. [AK129838]	
A_23_P67466	3.16E-05	NM_002812	NM_002812	Homo sapiens proteasome (prosome, macropain) 26S subunit, non-ATPase, 8 (PSMD8), mRNA [NM_002812]	NM_002812
A_23_P97064	3.17E-05	NM_018438	NM_018438	Homo sapiens F-box protein 6 (FBXO6), mRNA [NM_018438]	NM_018438
A_23_P416289	3.17E-05	NM_033425	NM_033425	Homo sapiens DIX domain containing 1 (DIXDC1), mRNA [NM_033425]	NM_033425
A_32_P33937	3.18E-05	ENST00000355190		Homo sapiens mRNA; cDNA DKFZp686L01145 (from clone DKFZp686L01145). [BX538108]	

A_32_P217750	3.18E-05	NM_002183	NM_002183	Homo sapiens interleukin 3 receptor, alpha (low affinity) (IL3RA), mRNA [NM_002183]	NM_002183
A_24_P257348	3.18E-05	NM_006407	NM_006407	Homo sapiens ADP-ribosylation-like factor 6 interacting protein 5 (ARL6IP5), mRNA [NM_006407]	NM_006407
A_23_P97932	3.18E-05	NM_012228	NM_012228	Homo sapiens methionine sulfoxide reductase B2 (MSRB2), mRNA [NM_012228]	NM_012228
A_23_P126939	3.19E-05	NM_003929	NM_003929	Homo sapiens RAB7, member RAS oncogene family-like 1 (RAB7L1), mRNA [NM_003929]	NM_003929
A_23_P406424	3.19E-05	NM_175744	NM_175744	Homo sapiens ras homolog gene family, member C (RHOC), mRNA [NM_175744]	NM_175744
A_23_P54891	3.20E-05	NM_022744	NM_022744	Homo sapiens hypothetical protein FLJ13868 (FLJ13868), mRNA [NM_022744]	NM_022744
A_24_P207139	3.23E-05	NM_033238	NM_033238	Homo sapiens promyelocytic leukemia (PML), transcript variant 1, mRNA [NM_033238]	NM_033238
A_23_P41066	3.23E-05	NM_170717	NM_170717	Homo sapiens Ras association (RalGDS/AF-6) domain family 1 (RASSF1), transcript variant G, mRNA [NM_170717]	NM_170717
A_24_P271527	3.23E-05	NM_014876	NM_014876	Homo sapiens KIAA0063 gene product (KIAA0063), mRNA [NM_014876]	NM_014876
A_23_P35791	3.24E-05	NM_003942	NM_003942	Homo sapiens ribosomal protein S6 kinase, 90kDa, polypeptide 4 (RPS6KA4), transcript variant 1, mRNA [NM_003942]	NM_003942
A_32_P46840	3.24E-05	CR596712	CR596712	full-length cDNA clone CS0DI028YF10 of Placenta Cot 25-normalized of Homo sapiens (human). [CR596712]	
A_24_P767222	3.25E-05	THC2404909			
A_32_P87074	3.25E-05	AK022150	AK022150	Homo sapiens cDNA FLJ12088 fis, clone HEMBB1002545. [AK022150]	
A_23_P157545	3.25E-05	NM_006167	NM_006167	Homo sapiens NK3 transcription factor related, locus 1 (Drosophila) (NKX3-1), mRNA [NM_006167]	NM_006167
A_23_P218858	3.25E-05	NM_015429	NM_015429	Homo sapiens ABI gene family, member 3 (NESH) binding protein (ABI3BP), mRNA [NM_015429]	NM_015429
A_23_P200396	3.25E-05	NM_019118	NM_019118	Homo sapiens chromosome 1 open reading frame 91 (C1orf91), mRNA [NM_019118]	NM_019118
A_23_P73801	3.26E-05	NM_001006640	NM_001006640	Homo sapiens transcription elongation factor A (SII)-like 1 (TCEAL1), transcript variant 3, mRNA [NM_001006640]	NM_001006640
A_23_P50907	3.26E-05	NM_002210	NM_002210	Homo sapiens integrin, alpha V (vitronectin receptor, alpha polypeptide, antigen CD51) (ITGAV), mRNA [NM_002210]	NM_002210
A_23_P49192	3.28E-05	NM_198061	NM_198061	Homo sapiens carboxylesterase 2 (intestine, liver) (CES2), transcript variant 2, mRNA [NM_198061]	NM_198061
A_24_P50057	3.28E-05	A_24_P50057			
A_23_P201079	3.29E-05	NM_012231	NM_012231	Homo sapiens PR domain containing 2, with ZNF domain (PRDM2), transcript variant 1, mRNA [NM_012231]	NM_012231
A_23_P363968	3.29E-05	NM_016546	NM_016546	Homo sapiens complement component 1, r subcomponent-like (C1RL), mRNA [NM_016546]	NM_016546
A_24_P79054	3.29E-05	NM_000660	NM_000660	Homo sapiens transforming growth factor, beta 1 (Camurati-Engelmann disease) (TGFB1), mRNA [NM_000660]	NM_000660
A_23_P206697	3.29E-05	NM_030579	NM_030579	Homo sapiens outer mitochondrial membrane cytochrome b5 (CYB5-M), mRNA [NM_030579]	NM_030579
A_23_P141394	3.29E-05	NM_017983	NM_017983	Homo sapiens WD40 repeat protein Interacting with phosphoInositides of 49kDa (WIPI49), mRNA [NM_017983]	NM_017983
A_24_P410086	3.30E-05	NM_032627	NM_032627	Homo sapiens single stranded DNA binding protein 4 (SSBP4), transcript variant 1, mRNA [NM_032627]	NM_032627
A_23_P132973	3.31E-05	NM_032906	NM_032906	Homo sapiens hypothetical protein MGC14156 (MGC14156), mRNA [NM_032906]	NM_032906
A_23_P98205	3.32E-05	NM_003654	NM_003654	Homo sapiens carbohydrate (keratan sulfate Gal-6) sulfotransferase 1 (CHST1), mRNA [NM_003654]	NM_003654
A_23_P15564	3.32E-05	NM_016627	NM_016627	Homo sapiens hypothetical protein LOC51321 (LOC51321), mRNA [NM_016627]	NM_016627
A_23_P150931	3.33E-05	NM_018113	NM_018113	Homo sapiens limb region 1 homolog (mouse)-like (LMBR1L), mRNA [NM_018113]	NM_018113
A_23_P26223	3.33E-05	NM_000048	NM_000048	Homo sapiens argininosuccinate lyase (ASL), transcript variant 2, mRNA [NM_000048]	NM_000048
A_23_P167051	3.34E-05	NM_014556	NM_014556	Homo sapiens Ellis van Creveld syndrome (EVC), transcript variant 1, mRNA [NM_014556]	NM_014556
A_32_P140289	3.34E-05	CR601567	CR601567	full-length cDNA clone CS0DN003YI06 of Adult brain of Homo sapiens (human). [CR601567]	
A_23_P210100	3.35E-05	NM_019885	NM_019885	Homo sapiens cytochrome P450, family 26, subfamily B, polypeptide 1 (CYP26B1), mRNA [NM_019885]	NM_019885
A_23_P337753	3.36E-05	NM_138402	NM_138402	Homo sapiens hypothetical protein BC004921 (LOC93349), mRNA [NM_138402]	NM_138402
A_23_P301984	3.36E-05	NM_001007090	NM_001007090	Homo sapiens hypothetical protein FLJ25402 (FLJ25402), mRNA [NM_001007090]	NM_001007090
A_23_P170280	3.37E-05	NM_020453	NM_020453	Homo sapiens ATPase, Class V, type 10D (ATP10D), mRNA [NM_020453]	NM_020453
A_23_P139722	3.37E-05	NM_001065	NM_001065	Homo sapiens tumor necrosis factor receptor superfamily, member 1A (TNFRSF1A), mRNA [NM_001065]	NM_001065
A_23_P138899	3.37E-05	NM_016451	NM_016451	Homo sapiens coatomer protein complex, subunit beta (COPB), mRNA [NM_016451]	NM_016451
A_23_P34144	3.38E-05	NM_014061	NM_014061	Homo sapiens melanoma antigen family H, 1 (MAGEH1), mRNA [NM_014061]	NM_014061
A_23_P88819	3.38E-05	NM_017458	NM_017458	Homo sapiens major vault protein (MVP), transcript variant 1, mRNA [NM_017458]	NM_017458
A_23_P110151	3.39E-05	NM_031305	NM_031305	Homo sapiens Rho GTPase activating protein 24 (ARHGAP24), mRNA [NM_031305]	NM_031305
A_23_P107313	3.39E-05	NM_006923	NM_006923	Homo sapiens stromal cell-derived factor 2 (SDF2), mRNA [NM_006923]	NM_006923
A_23_P4821	3.39E-05	NM_002229	NM_002229	Homo sapiens jun B proto-oncogene (JUNB), mRNA [NM_002229]	NM_002229

A_23_P209215	3.40E-05	NM_005843	NM_005843	Homo sapiens signal transducing adaptor molecule (SH3 domain and ITAM motif) 2 (STAM2), mRNA [NM_005843]	NM_005843
A_32_P116206	3.40E-05	AK025431	AK025431	Homo sapiens cDNA: FLJ21778 fis, clone HEP00201. [AK025431]	
A_32_P21993	3.40E-05	NM_003290	NM_003290	Homo sapiens tropomyosin 4 (TPM4), mRNA [NM_003290]	NM_003290
A_23_P87853	3.41E-05	NM_020698	NM_020698	Homo sapiens transmembrane and coiled-coil domain family 3 (TMCC3), mRNA [NM_020698]	NM_020698
A_23_P217088	3.41E-05	NM_000476	NM_000476	Homo sapiens adenylate kinase 1 (AK1), mRNA [NM_000476]	NM_000476
A_23_P377957	3.44E-05	NM_138444	NM_138444	Homo sapiens potassium channel tetramerisation domain containing 12 (KCTD12), mRNA [NM_138444]	NM_138444
A_23_P98995	3.45E-05	NM_020898	NM_020898	Homo sapiens calcium binding and coiled-coil domain 1 (CALCOCO1), mRNA [NM_020898]	NM_020898
A_23_P452	3.46E-05	NM_001014796	NM_001014796	Homo sapiens discoidin domain receptor family, member 2 (DDR2), transcript variant 1, mRNA [NM_001014796]	NM_001014796
A_23_P388993	3.46E-05	ENST00000278590		Homo sapiens mRNA for KIAA1726 protein, partial cds. [AB051513]	XM_370654
A_23_P373687	3.47E-05	NM_144709	NM_144709	Homo sapiens hypothetical protein FLJ32312 (FLJ32312), mRNA [NM_144709]	NM_144709
A_23_P25433	3.47E-05	NM_020374	NM_020374	Homo sapiens chromosome 12 open reading frame 4 (C12orf4), mRNA [NM_020374]	NM_020374
A_23_P142455	3.47E-05	NM_012237	NM_012237	Homo sapiens sirtuin (silent mating type information regulation 2 homolog) 2 (S. cerevisiae) (SIRT2), transcript variant 1, mRNA [NM_012237]	NM_012237
A_24_P345948	3.49E-05	NM_001012732	NM_001012732	Homo sapiens dCMP deaminase (DCTD), transcript variant 1, mRNA [NM_001012732]	NM_001012732
A_32_P35486	3.50E-05	THC2317058			
A_24_P290856	3.50E-05	NM_130853	NM_130853	Homo sapiens protein tyrosine phosphatase, receptor type, S (PTPRS), transcript variant 3, mRNA [NM_130853]	NM_130853
A_32_P233049	3.52E-05	G36726	G36726	SHGC-52890 Human Homo sapiens STS cDNA, sequence tagged site. [G36726]	
A_24_P416370	3.52E-05	NM_024015	NM_024015	Homo sapiens homeo box B4 (HOXB4), mRNA [NM_024015]	NM_024015
A_24_P194845	3.52E-05	NM_198829	NM_198829	Homo sapiens ras-related C3 botulinum toxin substrate 1 (rho family, small GTP binding protein Rac1) (RAC1), transcript variant Rac1c, mRNA [NM_198829]	NM_198829
A_32_P19608	3.54E-05	THC2439900			
A_23_P31453	3.54E-05	NM_012449	NM_012449	Homo sapiens six transmembrane epithelial antigen of the prostate 1 (STEAP1), mRNA [NM_012449]	NM_012449
A_24_P261567	3.55E-05	NM_030792	NM_030792	Homo sapiens glycerophosphodiester phosphodiesterase domain containing 5 (GDPD5), mRNA [NM_030792]	NM_030792
A_23_P92967	3.55E-05	NM_004531	NM_004531	Homo sapiens molybdenum cofactor synthesis 2 (MOCS2), transcript variant 3, mRNA [NM_004531]	NM_004531
A_23_P205499	3.55E-05	NM_014045	NM_014045	Homo sapiens low density lipoprotein receptor-related protein 10 (LRP10), mRNA [NM_014045]	NM_014045
A_23_P91487	3.56E-05	NM_016940	NM_016940	Homo sapiens chromosome 21 open reading frame 6 (C21orf6), mRNA [NM_016940]	NM_016940
A_32_P46981	3.58E-05	BC017654	BC017654	Homo sapiens, clone IMAGE:3887603, mRNA. [BC017654]	
A_24_P357576	3.58E-05	NM_019600	NM_019600	Homo sapiens hypothetical protein FLJ10980 (FLJ10980), mRNA [NM_019600]	NM_019600
A_23_P21363	3.58E-05	NM_024060	NM_024060	Homo sapiens AHNAK nucleoprotein (desmoyokin) (AHNAK), transcript variant 2, mRNA [NM_024060]	NM_024060
A_23_P110571	3.59E-05	ENST00000261569		Human mRNA for KIAA0303 gene, partial cds. [AB002301]	XM_291141
A_24_P244096	3.59E-05	NM_001002762	NM_001002762	Homo sapiens DnaJ (Hsp40) homolog, subfamily B, member 12 (DNAJB12), transcript variant 1, mRNA [NM_001002762]	NM_001002762
A_23_P12514	3.59E-05	NM_175744	NM_175744	Homo sapiens ras homolog gene family, member C (RHOC), mRNA [NM_175744]	NM_175744
A_24_P925664	3.60E-05	THC2429167			
A_24_P205268	3.60E-05	NM_015299	NM_015299	Homo sapiens KIAA0323 (KIAA0323), mRNA [NM_015299]	NM_015299
A_23_P139912	3.63E-05	NM_002178	NM_002178	Homo sapiens insulin-like growth factor binding protein 6 (IGFBP6), mRNA [NM_002178]	NM_002178
A_32_P143216	3.64E-05	AK026322	AK026322	Homo sapiens cDNA: FLJ22669 fis, clone HSI08594. [AK026322]	
A_24_P77904	3.65E-05	NM_018951	NM_018951	Homo sapiens homeo box A10 (HOXA10), transcript variant 1, mRNA [NM_018951]	NM_018951
A_23_P143356	3.65E-05	AY007156	AY007156	Homo sapiens clone CDABP0105 mRNA sequence. [AY007156]	
A_23_P420428	3.65E-05	NM_053041	NM_053041	Homo sapiens COMM domain containing 7 (COMMD7), mRNA [NM_053041]	NM_053041
A_23_P7642	3.65E-05	NM_003118	NM_003118	Homo sapiens secreted protein, acidic, cysteine-rich (osteonectin) (SPARC), mRNA [NM_003118]	NM_003118
A_24_P244100	3.66E-05	NM_001002762	NM_001002762	Homo sapiens DnaJ (Hsp40) homolog, subfamily B, member 12 (DNAJB12), transcript variant 1, mRNA [NM_001002762]	NM_001002762
A_24_P75072	3.66E-05	NM_014311	NM_014311	Homo sapiens single-strand selective monofunctional uracil DNA glycosylase (SMUG1), mRNA [NM_014311]	NM_014311
A_23_P388400	3.69E-05	NM_033394	NM_033394	Homo sapiens TPR domain, ankyrin-repeat and coiled-coil-containing (TANC), mRNA [NM_033394]	NM_033394
A_23_P26697	3.70E-05	NM_033452	NM_033452	Homo sapiens tripartite motif-containing 47 (TRIM47), mRNA [NM_033452]	NM_033452
A_23_P39076	3.70E-05	NM_006270	NM_006270	Homo sapiens related RAS viral (r-ras) oncogene homolog (RRAS), mRNA [NM_006270]	NM_006270
A_23_P21382	3.70E-05	NM_002292	NM_002292	Homo sapiens laminin, beta 2 (laminin S) (LAMB2), mRNA [NM_002292]	NM_002292

A_23_P143414	3.71E-05	NM_080748	NM_080748	Homo sapiens chromosome 20 open reading frame 52 (C20orf52), mRNA [NM_080748]	NM_080748
A_24_P912684	3.72E-05	NM_001620	NM_001620	Homo sapiens AHNAK nucleoprotein (desmoyokin) (AHNAK), transcript variant 1, mRNA [NM_001620]	NM_001620
A_23_P393727	3.72E-05	NM_201266	NM_201266	Homo sapiens neuropilin 2 (NRP2), transcript variant 1, mRNA [NM_201266]	NM_201266
A_23_P143845	3.72E-05	NM_015508	NM_015508	Homo sapiens TCDD-inducible poly(ADP-ribose) polymerase (TIPARP), mRNA [NM_015508]	NM_015508
A_23_P97339	3.74E-05	NM_004696	NM_004696	Homo sapiens solute carrier family 16 (monocarboxylic acid transporters), member 4 (SLC16A4), mRNA [NM_004696]	NM_004696
A_24_P177763	3.74E-05	NM_030911	NM_030911	Homo sapiens cytidine and dCMP deaminase domain containing 1 (CDADC1), mRNA [NM_030911]	NM_030911
A_23_P255812	3.74E-05	NM_016127	NM_016127	Homo sapiens hypothetical protein MGC8721 (MGC8721), mRNA [NM_016127]	NM_016127
A_32_P131377	3.74E-05	NM_005669	NM_005669	Homo sapiens chromosome 5 open reading frame 18 (C5orf18), mRNA [NM_005669]	NM_005669
A_24_P297078	3.76E-05	NM_020531	NM_020531	Homo sapiens chromosome 20 open reading frame 3 (C20orf3), mRNA [NM_020531]	NM_020531
A_23_P1254	3.76E-05	NM_004832	NM_004832	Homo sapiens glutathione S-transferase omega 1 (GSTO1), mRNA [NM_004832]	NM_004832
A_23_P110961	3.77E-05	NM_016098	NM_016098	Homo sapiens brain protein 44-like (BRP44L), mRNA [NM_016098]	NM_016098
A_23_P314070	3.77E-05	NM_004308	NM_004308	Homo sapiens Rho GTPase activating protein 1 (ARHGAP1), mRNA [NM_004308]	NM_004308
A_23_P219197	3.78E-05	NM_134427	NM_134427	Homo sapiens regulator of G-protein signalling 3 (RGS3), transcript variant 4, mRNA [NM_134427]	NM_134427
A_23_P143514	3.79E-05	BC004343	BC004343	Homo sapiens chromosome 21 open reading frame 122, mRNA (cDNA clone MGC:10960 IMAGE:3633193), complete cds. [BC004343]	
A_23_P5422	3.80E-05	AB082523	AB082523	Homo sapiens mRNA for KIAA1992 protein. [AB082523]	
A_23_P69863	3.82E-05	NM_015669	NM_015669	Homo sapiens protocadherin beta 5 (PCDHB5), mRNA [NM_015669]	NM_015669
A_24_P879740	3.84E-05	CR595522	CR595522	full-length cDNA clone CS0DC001YI12 of Neuroblastoma Cot 25-normalized of Homo sapiens (human). [CR595522]	
A_24_P192840	3.86E-05	THC2437510		ALU4_HUMAN (P39191) Alu subfamily SB2 sequence contamination warning entry, partial (27%) [THC2437510]	
A_23_P431305	3.86E-05	NM_152421	NM_152421	Homo sapiens hypothetical protein MGC20262 (MGC20262), mRNA [NM_152421]	NM_152421
A_23_P138514	3.86E-05	NM_012071	NM_012071	Homo sapiens COMM domain containing 3 (COMMD3), mRNA [NM_012071]	NM_012071
A_32_P131608	3.87E-05	AW627852	AW627852	AW627852 hh96d01.x1 NCI_CGAP_GU1 Homo sapiens cDNA clone IMAGE:2970625 3', mRNA sequence [AW627852]	
A_23_P65022	3.87E-05	NM_000017	NM_000017	Homo sapiens acyl-Coenzyme A dehydrogenase, C-2 to C-3 short chain (ACADS), nuclear gene encoding mitochondrial protein, mRNA [NM_000017]	NM_000017
A_24_P74374	3.88E-05	NM_000308	NM_000308	Homo sapiens protective protein for beta-galactosidase (galactosialidosis) (PPGB), mRNA [NM_000308]	NM_000308
A_24_P937231	3.89E-05	AK092744	AK092744	Homo sapiens cDNA FLJ35425 fis, clone SMINT2001615, highly similar to FERRITIN LIGHT CHAIN. [AK092744]	
A_32_P174385	3.89E-05	A_32_P174385			
A_23_P162766	3.90E-05	NM_015296	NM_015296	Homo sapiens dedicator of cytokinesis 9 (DOCK9), mRNA [NM_015296]	NM_015296
A_23_P205273	3.91E-05	NM_015316	NM_015316	Homo sapiens protein phosphatase 1, regulatory (inhibitor) subunit 13B (PPP1R13B), mRNA [NM_015316]	NM_015316
A_23_P121064	3.92E-05	NM_002852	NM_002852	Homo sapiens pentraxin-related gene, rapidly induced by IL-1 beta (PTX3), mRNA [NM_002852]	NM_002852
A_23_P48936	3.93E-05	NM_005902	NM_005902	Homo sapiens SMAD, mothers against DPP homolog 3 (Drosophila) (SMAD3), mRNA [NM_005902]	NM_005902
A_24_P279489	3.95E-05	AK123107	AK123107	Homo sapiens cDNA FLJ41112 fis, clone BRACE1000239. [AK123107]	
A_23_P91151	3.96E-05	NM_003038	NM_003038	Homo sapiens solute carrier family 1 (glutamate/neutral amino acid transporter), member 4 (SLC1A4), mRNA [NM_003038]	NM_003038
A_23_P168788	3.96E-05	NM_001084	NM_001084	Homo sapiens procollagen-lysine, 2-oxoglutarate 5-dioxygenase 3 (PLOD3), mRNA [NM_001084]	NM_001084
A_23_P16976	3.97E-05	NM_001153	NM_001153	Homo sapiens annexin A4 (ANXA4), mRNA [NM_001153]	NM_001153
A_24_P237270	3.98E-05	NM_000675	NM_000675	Homo sapiens adenosine A2a receptor (ADORA2A), mRNA [NM_000675]	NM_000675
A_24_P926666	3.98E-05	AK000925	AK000925	Homo sapiens cDNA FLJ10063 fis, clone HEMBA1001446. [AK000925]	
A_24_P256583	3.98E-05	NM_194291	NM_194291	Homo sapiens hypothetical protein BC017881 (LOC157378), mRNA [NM_194291]	NM_194291
A_23_P212781	3.98E-05	THC2364375		BC008083 SEPT11 protein {Homo sapiens;} , partial (17%) [THC2364375]	
A_23_P141044	3.98E-05	NM_145271	NM_145271	Homo sapiens zinc finger protein 688 (ZNF688), mRNA [NM_145271]	NM_145271
A_23_P144677	3.99E-05	AF178574	AF178574	Homo sapiens MSTP146 (MST146) mRNA, complete cds. [AF178574]	
A_23_P120915	3.99E-05	NM_138797	NM_138797	Homo sapiens hypothetical protein BC014641 (LOC129138), mRNA [NM_138797]	NM_138797
A_24_P100551	3.99E-05	NM_020870	NM_020870	Homo sapiens SH3 multiple domains 2 (SH3MD2), mRNA [NM_020870]	NM_020870
A_23_P69267	4.01E-05	NM_015407	NM_015407	Homo sapiens abhydrolase domain containing 14A (ABHD14A), mRNA [NM_015407]	NM_015407
A_24_P280833	4.02E-05	ENST00000307896		Homo sapiens cDNA clone IMAGE:3954254, partial cds. [BC014218]	
A_23_P29029	4.03E-05	NM_032910	NM_032910	Homo sapiens chromosome 21 open reading frame 119 (C21orf119), mRNA [NM_032910]	NM_032910

A_32_P162250	4.03E-05	NM_033515	NM_033515	Homo sapiens Rho GTPase activating protein 18 (ARHGAP18), mRNA [NM_033515]	NM_033515
A_23_P135878	4.04E-05	NM_020148	NM_020148	Homo sapiens spire homolog 1 (Drosophila) (SPIRE1), mRNA [NM_020148]	NM_020148
A_24_P66001	4.04E-05	NM_001003684	NM_001003684	Homo sapiens ubiquinol-cytochrome c reductase complex (7.2 kD) (UCRC), transcript variant 2, mRNA [NM_001003684]	NM_001003684
A_23_P431591	4.04E-05	NM_001014279	NM_001014279	Homo sapiens similar to annexin II receptor (LOC389289), mRNA [NM_001014279]	NM_001014279
A_24_P109511	4.06E-05	NM_016139	NM_016139	Homo sapiens coiled-coil-helix-coiled-coil-helix domain containing 2 (CHCHD2), mRNA [NM_016139]	NM_016139
A_24_P148777	4.07E-05	NM_170717	NM_170717	Homo sapiens Ras association (RalGDS/AF-6) domain family 1 (RASSF1), transcript variant G, mRNA [NM_170717]	NM_170717
A_32_P181166	4.12E-05	NM_174896	NM_174896	Homo sapiens chromosome 1 open reading frame 162 (C1orf162), mRNA [NM_174896]	NM_174896
A_24_P37903	4.13E-05	THC2371798			
A_32_P4199	4.14E-05	NM_173557	NM_173557	Homo sapiens ring finger protein 152 (RNF152), mRNA [NM_173557]	NM_173557
A_24_P818005	4.14E-05	NM_024107	NM_024107	Homo sapiens hypothetical protein MGC3123 (MGC3123), transcript variant 1, mRNA [NM_024107]	NM_024107
A_23_P208389	4.15E-05	NM_021913	NM_021913	Homo sapiens AXL receptor tyrosine kinase (AXL), transcript variant 1, mRNA [NM_021913]	NM_021913
A_23_P318300	4.16E-05	NM_133646	NM_133646	Homo sapiens sterile alpha motif and leucine zipper containing kinase AZK (ZAK), transcript variant 2, mRNA [NM_133646]	NM_133646
A_23_P63319	4.17E-05	NM_003101	NM_003101	Homo sapiens sterol O-acyltransferase (acyl-Coenzyme A: cholesterol acyltransferase) 1 (SOAT1), transcript variant 688113, mRNA [NM_003101]	NM_003101
A_23_P48771	4.17E-05	NM_024952	NM_024952	Homo sapiens chromosome 14 open reading frame 159 (C14orf159), mRNA [NM_024952]	NM_024952
A_24_P298360	4.18E-05	NM_021070	NM_021070	Homo sapiens latent transforming growth factor beta binding protein 3 (LTBP3), mRNA [NM_021070]	NM_021070
A_24_P146670	4.20E-05	NM_014720	NM_014720	Homo sapiens STE20-like kinase (yeast) (SLK), mRNA [NM_014720]	NM_014720
A_32_P157846	4.21E-05	AK092260	AK092260	Homo sapiens cDNA FLJ34941 fis, clone NT2RP7007480. [AK092260]	
A_23_P75921	4.21E-05	NM_145803	NM_145803	Homo sapiens TNF receptor-associated factor 6 (TRAF6), transcript variant 1, mRNA [NM_145803]	NM_145803
A_23_P118300	4.23E-05	NM_031208	NM_031208	Homo sapiens fumarylacetoacetate hydrolase domain containing 1 (FAHD1), transcript variant 2, mRNA [NM_031208]	NM_031208
A_24_P396375	4.23E-05	CR595983	CR595983	full-length cDNA clone CS0DI039YA23 of Placenta Cot 25-normalized of Homo sapiens (human). [CR595983]	
A_32_P163937	4.24E-05	BQ064481	BQ064481	AGENCOURT_6853651 NIH_MGC_99 Homo sapiens cDNA clone IMAGE:5926217 5', mRNA sequence [BQ064481]	
A_24_P376309	4.24E-05	BC008564	BC008564	Homo sapiens cDNA clone IMAGE:4178802, partial cds. [BC008564]	
A_23_P213102	4.24E-05	NM_016081	NM_016081	Homo sapiens palladin (KIAA0992), mRNA [NM_016081]	NM_016081
A_24_P271049	4.27E-05	BC093004	BC093004	Homo sapiens cDNA sequence BC022357, mRNA (cDNA clone MGC:110854 IMAGE:5273608), complete cds. [BC093004]	
A_23_P39364	4.27E-05	NM_004838	NM_004838	Homo sapiens homer homolog 3 (Drosophila) (HOMER3), mRNA [NM_004838]	NM_004838
A_24_P82200	4.28E-05	NM_170676	NM_170676	Homo sapiens Meis1, myeloid ecotropic viral integration site 1 homolog 2 (mouse) (MEIS2), transcript variant d, mRNA [NM_170676]	NM_170676
A_24_P122337	4.32E-05	NM_080737	NM_080737	Homo sapiens synaptotagmin-like 4 (granuphilin-a) (SYTL4), mRNA [NM_080737]	NM_080737
A_32_P82650	4.33E-05	BM701175	BM701175	UI-E-EJ0-ahj-e-13-0-UI.r1 UI-E-EJ0 Homo sapiens cDNA clone UI-E-EJ0-ahj-e-13-0-UI 5', mRNA sequence [BM701175]	
A_24_P120827	4.34E-05	BC012452	BC012452	Homo sapiens hypothetical protein FLJ32731, mRNA (cDNA clone MGC:16804 IMAGE:3880903), complete cds. [BC012452]	XM_372038
A_23_P102494	4.34E-05	NM_017877	NM_017877	Homo sapiens chromosome 2 open reading frame 18 (C2orf18), mRNA [NM_017877]	NM_017877
A_23_P166640	4.35E-05	NM_006407	NM_006407	Homo sapiens ADP-ribosylation-like factor 6 interacting protein 5 (ARL6IP5), mRNA [NM_006407]	NM_006407
A_23_P124132	4.39E-05	NM_024648	NM_024648	Homo sapiens hypothetical protein FLJ22222 (FLJ22222), transcript variant 1, mRNA [NM_024648]	NM_024648
A_32_P111565	4.39E-05	NM_002032	NM_002032	Homo sapiens ferritin, heavy polypeptide 1 (FTH1), mRNA [NM_002032]	NM_002032
A_23_P166100	4.40E-05	NM_021156	NM_021156	Homo sapiens hypothetical protein DJ971N18.2 (DJ971N18.2), mRNA [NM_021156]	NM_021156
A_23_P92025	4.41E-05	NM_022094	NM_022094	Homo sapiens cell death-inducing DFFA-like effector c (CIDEC), mRNA [NM_022094]	NM_022094
A_23_P74526	4.41E-05	NM_203456	NM_203456	Homo sapiens peptidylprolyl isomerase E (cyclophilin E) (PPIE), transcript variant 2, mRNA [NM_203456]	NM_203456
A_23_P301521	4.42E-05	AK055602	AK055602	Homo sapiens cDNA FLJ31040 fis, clone HSYRA2000224. [AK055602]	
A_23_P99303	4.43E-05	NM_023071	NM_023071	Homo sapiens spermatogenesis associated, serine-rich 2 (SPATS2), mRNA [NM_023071]	NM_023071
A_32_P195065	4.44E-05	NM_004263	NM_004263	Homo sapiens sema domain, immunoglobulin domain (Ig), transmembrane domain (TM) and short cytoplasmic domain, (semaphorin) 4F (SEMA4F), mRNA [NM_004263]	NM_004263
A_23_P207896	4.45E-05	NM_001893	NM_001893	Homo sapiens casein kinase 1, delta (CSNK1D), transcript variant 1, mRNA [NM_001893]	NM_001893
A_24_P49106	4.47E-05	NM_152278	NM_152278	Homo sapiens transcription elongation factor A (SII)-like 7 (TCEAL7), mRNA [NM_152278]	NM_152278
A_24_P135769	4.48E-05	NM_198315	NM_198315	Homo sapiens loss of heterozygosity, 11, chromosomal region 2, gene A (LOH11CR2A), transcript variant 2, mRNA [NM_198315]	NM_198315
A_23_P423695	4.48E-05	NM_006454	NM_006454	Homo sapiens MAX dimerization protein 4 (MXD4), mRNA [NM_006454]	NM_006454

A_23_P252721	4.48E-05	NM_182643	NM_182643	Homo sapiens deleted in liver cancer 1 (DLC1), transcript variant 1, mRNA [NM_182643]	NM_182643
A_23_P206018	4.48E-05	NM_001018004	NM_001018004	Homo sapiens tropomyosin 1 (alpha) (TPM1), transcript variant 3, mRNA [NM_001018004]	NM_001018004
A_23_P92073	4.50E-05	NM_001003935	NM_001003935	Homo sapiens poly (ADP-ribose) polymerase family, member 3 (PARP3), transcript variant 3, mRNA [NM_001003935]	NM_001003935
A_24_P305623	4.50E-05	NM_006134	NM_006134	Homo sapiens transmembrane protein 50B (TMEM50B), mRNA [NM_006134]	NM_006134
A_24_P272290	4.51E-05	NM_183373	NM_183373	Homo sapiens chromosome 6 open reading frame 145 (C6orf145), mRNA [NM_183373]	NM_183373
A_24_P277349	4.51E-05	NM_014933	NM_014933	Homo sapiens SEC31-like 1 (S. cerevisiae) (SEC31L1), transcript variant 1, mRNA [NM_014933]	NM_014933
A_23_P9340	4.53E-05	NM_020064	NM_020064	Homo sapiens BarH-like 1 (Drosophila) (BARHL1), mRNA [NM_020064]	NM_020064
A_23_P120572	4.53E-05	NM_018993	NM_018993	Homo sapiens Ras and Rab interactor 2 (RIN2), mRNA [NM_018993]	NM_018993
A_23_P123454	4.53E-05	NM_024815	NM_024815	Homo sapiens nudix (nucleoside diphosphate linked moiety X)-type motif 18 (NUDT18), mRNA [NM_024815]	NM_024815
A_23_P48747	4.53E-05	NM_138452	NM_138452	Homo sapiens dehydrogenase/reductase (SDR family) member 1 (DHRS1), mRNA [NM_138452]	NM_138452
A_23_P112596	4.53E-05	NM_001123	NM_001123	Homo sapiens adenosine kinase (ADK), transcript variant ADK-short, mRNA [NM_001123]	NM_001123
A_24_P152649	4.54E-05	AK092522	AK092522	Homo sapiens cDNA FLJ35203 fis, clone PLACE6018441, moderately similar to Mus musculus peroxisomal long chain acyl-CoA thioesterase Ib (Pte1b) gene. [AK092522]	
A_23_P62335	4.54E-05	NM_018196	NM_018196	Homo sapiens trimethyllysine hydroxylase, epsilon (TMLHE), mRNA [NM_018196]	NM_018196
A_23_P103282	4.54E-05	NM_004872	NM_004872	Homo sapiens transmembrane protein 59 (TMEM59), mRNA [NM_004872]	NM_004872
A_23_P94030	4.55E-05	NM_002291	NM_002291	Homo sapiens laminin, beta 1 (LAMB1), mRNA [NM_002291]	NM_002291
A_24_P410453	4.57E-05	NM_033071	NM_033071	Homo sapiens spectrin repeat containing, nuclear envelope 1 (SYNE1), transcript variant longer, mRNA [NM_033071]	NM_033071
A_23_P406122	4.57E-05	ENST00000252032		Homo sapiens mRNA; cDNA DKFZp434N061 (from clone DKFZp434N061); partial cds. [AL110249]	XM_045421
A_23_P304524	4.58E-05	NM_152619	NM_152619	Homo sapiens doublecortin and CaM kinase-like 2 (DCAMKL2), mRNA [NM_152619]	NM_152619
A_23_P410017	4.58E-05	ENST00000284259		Homo sapiens leucine rich repeat containing 35, mRNA (cDNA clone IMAGE:3913004). [BC020501]	
A_23_P127367	4.58E-05	NM_021173	NM_021173	Homo sapiens polymerase (DNA-directed), delta 4 (POLD4), mRNA [NM_021173]	NM_021173
A_23_P316381	4.60E-05	NM_003501	NM_003501	Homo sapiens acyl-Coenzyme A oxidase 3, pristanoyl (ACOX3), mRNA [NM_003501]	NM_003501
A_23_P204879	4.62E-05	NM_030925	NM_030925	Homo sapiens calcium binding protein 39-like (CAB39L), mRNA [NM_030925]	NM_030925
A_23_P136573	4.62E-05	NM_003896	NM_003896	Homo sapiens ST3 beta-galactoside alpha-2,3-sialyltransferase 5 (ST3GAL5), mRNA [NM_003896]	NM_003896
A_23_P126899	4.62E-05	NM_003846	NM_003846	Homo sapiens peroxisomal biogenesis factor 11B (PEX11B), mRNA [NM_003846]	NM_003846
A_23_P79842	4.62E-05	NM_015937	NM_015937	Homo sapiens phosphatidylinositol glycan, class T (PIGT), mRNA [NM_015937]	NM_015937
A_32_P28284	4.62E-05	NM_003290	NM_003290	Homo sapiens tropomyosin 4 (TPM4), mRNA [NM_003290]	NM_003290
A_23_P129221	4.63E-05	NM_000137	NM_000137	Homo sapiens fumarylacetoacetate hydrolase (fumarylacetoacetase) (FAH), mRNA [NM_000137]	NM_000137
A_24_P265051	4.64E-05	AK095606	AK095606	Homo sapiens cDNA FLJ38287 fis, clone FCBBF3008362, moderately similar to PLEXIN 4 PRECURSOR. [AK095606]	XM_039393
A_23_P385105	4.65E-05	NM_032726	NM_032726	Homo sapiens phospholipase C, delta 4 (PLCD4), mRNA [NM_032726]	NM_032726
A_23_P165608	4.65E-05	NM_004263	NM_004263	Homo sapiens sema domain, immunoglobulin domain (Ig), transmembrane domain (TM) and short cytoplasmic domain, (semaphorin) 4F (SEMA4F), mRNA [NM_004263]	NM_004263
A_23_P129157	4.65E-05	NM_024608	NM_024608	Homo sapiens nei endonuclease VIII-like 1 (E. coli) (NEIL1), mRNA [NM_024608]	NM_024608
A_24_P918397	4.66E-05	NM_031212	NM_031212	Homo sapiens solute carrier family 25, member 28 (SLC25A28), mRNA [NM_031212]	NM_031212
A_24_P349039	4.67E-05	NM_020754	NM_020754	Homo sapiens Cdc42 GTPase-activating protein (CDGAP), mRNA [NM_020754]	NM_020754
A_23_P158969	4.67E-05	NM_139177	NM_139177	Homo sapiens solute carrier family 39 (metal ion transporter), member 11 (SLC39A11), mRNA [NM_139177]	NM_139177
A_23_P305140	4.67E-05	NM_144591	NM_144591	Homo sapiens chromosome 10 open reading frame 32 (C10orf32), mRNA [NM_144591]	NM_144591
A_23_P99614	4.67E-05	NM_033271	NM_033271	Homo sapiens BTB (POZ) domain containing 6 (BTBD6), mRNA [NM_033271]	NM_033271
A_23_P344400	4.68E-05	NM_020731	NM_020731	Homo sapiens aryl-hydrocarbon receptor repressor (AHRR), mRNA [NM_020731]	NM_020731
A_23_P214330	4.68E-05	NM_030666	NM_030666	Homo sapiens serine (or cysteine) proteinase inhibitor, clade B (ovalbumin), member 1 (SERPINB1), mRNA [NM_030666]	NM_030666
A_23_P388433	4.69E-05	ENST00000296496		full-length cDNA clone CS0DI016YF21 of Placenta Cot 25-normalized of Homo sapiens (human). [CR597270]	
A_32_P529165	4.70E-05	AB040895	AB040895	Homo sapiens mRNA for KIAA1462 protein, partial cds. [AB040895]	XM_166132
A_24_P484797	4.71E-05	AF279614	AF279614	Homo sapiens cell death-inducing CIDE-like effector (CICE) pseudogene, mRNA sequence. [AF279614]	XR_000206
A_23_P209564	4.71E-05	NM_024843	NM_024843	Homo sapiens cytochrome b reductase 1 (CYBRD1), mRNA [NM_024843]	NM_024843
A_24_P56330	4.72E-05	NM_152324	NM_152324	Homo sapiens hypothetical protein MGC35169 (MGC35169), mRNA [NM_152324]	NM_152324
A_23_P305100	4.72E-05	NM_153369	NM_153369	Homo sapiens KIAA1919 (KIAA1919), mRNA [NM_153369]	NM_153369

A_24_P242391	4.73E-05	NM_003953	NM_003953	Homo sapiens myelin protein zero-like 1 (MPZL1), transcript variant 1, mRNA [NM_003953]	NM_003953
A_23_P209232	4.75E-05	NM_024692	NM_024692	Homo sapiens restin-like 2 (RSNL2), mRNA [NM_024692]	NM_024692
A_23_P3866	4.75E-05	NM_021149	NM_021149	Homo sapiens coactosin-like 1 (Dictyostelium) (COTL1), mRNA [NM_021149]	NM_021149
A_24_P380061	4.76E-05	NM_031305	NM_031305	Homo sapiens Rho GTPase activating protein 24 (ARHGAP24), mRNA [NM_031305]	NM_031305
A_23_P36647	4.76E-05	NM_177441	NM_177441	Homo sapiens hypothetical protein MGC3123 (MGC3123), transcript variant 2, mRNA [NM_177441]	NM_177441
A_24_P329353	4.76E-05	NM_032831	NM_032831	Homo sapiens chromosome 7 open reading frame 19 (C7orf19), mRNA [NM_032831]	NM_032831
A_23_P423389	4.76E-05	NM_006368	NM_006368	Homo sapiens cAMP responsive element binding protein 3 (CREB3), mRNA [NM_006368]	NM_006368
A_24_P200583	4.76E-05	NM_003337	NM_003337	Homo sapiens ubiquitin-conjugating enzyme E2B (RAD6 homolog) (UBE2B), mRNA [NM_003337]	NM_003337
A_24_P105391	4.77E-05	NM_001017922	NM_001017922	Homo sapiens erythroblast membrane-associated protein (ERMAP), transcript variant 1, mRNA [NM_001017922]	NM_001017922
A_23_P153489	4.77E-05	NM_002975	NM_002975	Homo sapiens C-type lectin domain family 11, member A (CLEC11A), mRNA [NM_002975]	NM_002975
A_24_P810697	4.78E-05	NM_001008529	NM_001008529	Homo sapiens matrix-remodelling associated 7 (MXRA7), transcript variant 2, mRNA [NM_001008529]	NM_001008529
A_24_P170496	4.78E-05	ENST00000333546		PREDICTED: Homo sapiens similar to 60S ribosomal protein L23a (LOC391282), mRNA [XM_372878]	XM_372878
A_32_P52609	4.80E-05	NM_145693	NM_145693	Homo sapiens lipin 1 (LPIN1), mRNA [NM_145693]	NM_145693
A_24_P324314	4.81E-05	NM_001008529	NM_001008529	Homo sapiens matrix-remodelling associated 7 (MXRA7), transcript variant 2, mRNA [NM_001008529]	NM_001008529
A_32_P25357	4.83E-05	NM_004933	NM_004933	Homo sapiens cadherin 15, M-cadherin (myotubule) (CDH15), mRNA [NM_004933]	NM_004933
A_23_P207367	4.84E-05	NM_003152	NM_003152	Homo sapiens signal transducer and activator of transcription 5A (STAT5A), mRNA [NM_003152]	NM_003152
A_23_P205997	4.86E-05	NM_031301	NM_031301	Homo sapiens anterior pharynx defective 1 homolog B (C. elegans) (APH1B), mRNA [NM_031301]	NM_031301
A_23_P258124	4.89E-05	NM_012279	NM_012279	Homo sapiens zinc finger protein 346 (ZNF346), mRNA [NM_012279]	NM_012279
A_24_P118938	4.93E-05	ENST00000359676		Homo sapiens mRNA; cDNA DKFZp566G1424 (from clone DKFZp566G1424). [AL122043]	
A_23_P58419	4.96E-05	NM_002253	NM_002253	Homo sapiens kinase insert domain receptor (a type III receptor tyrosine kinase) (KDR), mRNA [NM_002253]	NM_002253
A_23_P125726	4.96E-05	NM_032227	NM_032227	Homo sapiens hypothetical protein FLJ22679 (RP13-360B22.2), mRNA [NM_032227]	NM_032227
A_32_P5800	4.97E-05	CR602075	CR602075	full-length cDNA clone CS0DD009YF23 of Neuroblastoma Cot 50-normalized of Homo sapiens (human). [CR602075]	
A_23_P320304	4.99E-05	NM_201252	NM_201252	Homo sapiens aflatoxin B1 aldehyde reductase 3 (AFAR3), mRNA [NM_201252]	NM_201252
A_23_P158277	5.00E-05	NM_181719	NM_181719	Homo sapiens hypothetical protein LOC255104 (LOC255104), mRNA [NM_181719]	NM_181719
A_23_P250564	5.00E-05	NM_005400	NM_005400	Homo sapiens protein kinase C, epsilon (PRKCE), mRNA [NM_005400]	NM_005400
A_23_P126186	5.03E-05	NM_003676	NM_003676	Homo sapiens degenerative spermatocyte homolog 1, lipid desaturase (Drosophila) (DEGS1), transcript variant 1, mRNA [NM_003676]	NM_003676
A_23_P207650	5.04E-05	NM_000018	NM_000018	Homo sapiens acyl-Coenzyme A dehydrogenase, very long chain (ACADVL), nuclear gene encoding mitochondrial protein, mRNA [NM_000018]	NM_000018
A_24_P328524	5.05E-05	NM_003947	NM_003947	Homo sapiens kalirin, RhoGEF kinase (KALRN), transcript variant 2, mRNA [NM_003947]	NM_003947
A_23_P47642	5.06E-05	NM_018222	NM_018222	Homo sapiens parvin, alpha (PARVA), mRNA [NM_018222]	NM_018222
A_23_P3221	5.06E-05	NM_021199	NM_021199	Homo sapiens sulfide quinone reductase-like (yeast) (SQRDL), mRNA [NM_021199]	NM_021199
A_24_P97526	5.09E-05	NM_017801	NM_017801	Homo sapiens chemokine-like factor super family 6 (CKLFSF6), mRNA [NM_017801]	NM_017801
A_23_P114662	5.11E-05	NM_001889	NM_001889	Homo sapiens crystallin, zeta (quinone reductase) (CRYZ), mRNA [NM_001889]	NM_001889
A_32_P150748	5.12E-05	G31710	G31710	sWSS2257 Eric D. Green Homo sapiens STS cDNA, sequence tagged site. [G31710]	
A_24_P703830	5.12E-05	THC2375654		NNS3_HUMAN (P60323) Nanos homolog 3, partial (31%) [THC2375654]	
A_23_P153676	5.12E-05	NM_003260	NM_003260	Homo sapiens transducin-like enhancer of split 2 (E(sp1) homolog, Drosophila) (TLE2), mRNA [NM_003260]	NM_003260
A_23_P115417	5.13E-05	NM_015149	NM_015149	Homo sapiens ral guanine nucleotide dissociation stimulator-like 1 (RGL1), mRNA [NM_015149]	NM_015149
A_24_P57700	5.14E-05	NM_015035	NM_015035	Homo sapiens zinc fingers and homeoboxes 3 (ZHX3), mRNA [NM_015035]	NM_015035
A_23_P420334	5.15E-05	NM_152595	NM_152595	Homo sapiens piggyBac transposable element derived 4 (PGBD4), mRNA [NM_152595]	NM_152595
A_23_P429461	5.15E-05	NM_145059	NM_145059	Homo sapiens fucokinase (FUK), mRNA [NM_145059]	NM_145059
A_23_P46928	5.15E-05	NM_002627	NM_002627	Homo sapiens phosphofructokinase, platelet (PFKP), mRNA [NM_002627]	NM_002627
A_24_P208567	5.16E-05	NM_003855	NM_003855	Homo sapiens interleukin 18 receptor 1 (IL18R1), mRNA [NM_003855]	NM_003855
A_23_P130598	5.16E-05	NM_024103	NM_024103	Homo sapiens solute carrier family 25 (mitochondrial carrier; phosphate carrier), member 23 (SLC25A23), mRNA [NM_024103]	NM_024103
A_23_P204937	5.16E-05	NM_014059	NM_014059	Homo sapiens response gene to complement 32 (RGC32), mRNA [NM_014059]	NM_014059
A_23_P17811	5.18E-05	NM_012429	NM_012429	Homo sapiens SEC14-like 2 (S. cerevisiae) (SEC14L2), mRNA [NM_012429]	NM_012429

A_23_P95599	5.18E-05	NM_001012731	NM_001012731	Homo sapiens hypothetical protein LOC283874 (LOC283874), mRNA [NM_001012731]	NM_001012731
A_24_P247303	5.18E-05	A_24_P247303			
A_24_P304629	5.19E-05	NM_014909	NM_014909	Homo sapiens KIAA1036 (KIAA1036), mRNA [NM_014909]	NM_014909
A_23_P76480	5.19E-05	BF213738	BF213738	BF213738 601847628F1 NIH_MGC_55 Homo sapiens cDNA clone IMAGE:4078519 5', mRNA sequence [BF213738]	
A_23_P414978	5.19E-05	NM_177533	NM_177533	Homo sapiens nudix (nucleoside diphosphate linked moiety X)-type motif 14 (NUDT14), mRNA [NM_177533]	NM_177533
A_23_P130626	5.19E-05	NM_024075	NM_024075	Homo sapiens tRNA splicing endonuclease 34 homolog (SEN34, S. cerevisiae) (TSEN34), mRNA [NM_024075]	NM_024075
A_32_P7308	5.20E-05	THC2407640			
A_24_P683917	5.20E-05	AK022486	AK022486	Homo sapiens cDNA FLJ12424 fis, clone MAMMA1003099. [AK022486]	
A_23_P170587	5.20E-05	NM_020197	NM_020197	Homo sapiens SET and MYND domain containing 2 (SMYD2), mRNA [NM_020197]	NM_020197
A_24_P228026	5.22E-05	NM_144611	NM_144611	Homo sapiens hypothetical protein MGC32124 (MGC32124), mRNA [NM_144611]	NM_144611
A_23_P436259	5.23E-05	NM_152461	NM_152461	Homo sapiens endoplasmic reticulum to nucleus signalling 1 (ERN1), transcript variant 2, mRNA [NM_152461]	NM_152461
A_32_P27373	5.24E-05	CV569217	CV569217	CV569217 od02h01.y1 Human keratoconus cornea, unamplified, (od/oe) Homo sapiens cDNA clone od02h01 5', mRNA sequence [CV569217]	
A_24_P322191	5.24E-05	NM_005831	NM_005831	Homo sapiens nuclear domain 10 protein (NDP52), mRNA [NM_005831]	NM_005831
A_24_P109652	5.24E-05	AB082533	AB082533	Homo sapiens mRNA for KIAA2002 protein. [AB082533]	XM_370878
A_23_P340848	5.27E-05	NM_000960	NM_000960	Homo sapiens prostaglandin I2 (prostacyclin) receptor (IP) (PTGIR), mRNA [NM_000960]	NM_000960
A_24_P379750	5.31E-05	NM_002357	NM_002357	Homo sapiens MAX dimerization protein 1 (MXD1), mRNA [NM_002357]	NM_002357
A_32_P147149	5.32E-05	AF088062	AF088062	Homo sapiens full length insert cDNA clone ZD74E10. [AF088062]	
A_24_P69691	5.32E-05	NM_145011	NM_145011	Homo sapiens zinc finger protein 25 (KOX 19) (ZNF25), mRNA [NM_145011]	NM_145011
A_24_P364381	5.33E-05	NM_052845	NM_052845	Homo sapiens methylmalonic aciduria (cobalamin deficiency) cblB type (MMAB), mRNA [NM_052845]	NM_052845
A_32_P112881	5.33E-05	CR624966	CR624966	full-length cDNA clone CS0DK008YO11 of HeLa cells Cot 25-normalized of Homo sapiens (human). [CR624966]	
A_23_P382154	5.34E-05	NM_176794	NM_176794	Homo sapiens mitochondrial ribosomal protein L43 (MRPL43), nuclear gene encoding mitochondrial protein, transcript variant 4, mRNA [NM_176794]	NM_176794
A_24_P92771	5.34E-05	NR_002205	NR_002205	Homo sapiens ferritin, heavy polypeptide-like 12 (FTHL12) on chromosome 9 [NR_002205]	NR_002205
A_32_P195387	5.35E-05	AK097956	AK097956	Homo sapiens cDNA FLJ40637 fis, clone THYMU2015984. [AK097956]	
A_23_P135548	5.37E-05	NM_000110	NM_000110	Homo sapiens dihydropyrimidine dehydrogenase (DPYD), mRNA [NM_000110]	NM_000110
A_24_P373174	5.39E-05	NM_004580	NM_004580	Homo sapiens RAB27A, member RAS oncogene family (RAB27A), transcript variant 1, mRNA [NM_004580]	NM_004580
A_24_P288685	5.39E-05	NM_001560	NM_001560	Homo sapiens interleukin 13 receptor, alpha 1 (IL13RA1), mRNA [NM_001560]	NM_001560
A_24_P19810	5.39E-05	NM_024664	NM_024664	Homo sapiens phosphopantothenoylcysteine synthetase (PPCS), mRNA [NM_024664]	NM_024664
A_24_P156566	5.39E-05	NM_003928	NM_003928	Homo sapiens CAAX box 1 (CXX1), mRNA [NM_003928]	NM_003928
A_23_P143713	5.40E-05	NM_021822	NM_021822	Homo sapiens apolipoprotein B mRNA editing enzyme, catalytic polypeptide-like 3G (APOBEC3G), mRNA [NM_021822]	NM_021822
A_23_P27013	5.40E-05	NM_024017	NM_024017	Homo sapiens homeo box B9 (HOXB9), mRNA [NM_024017]	NM_024017
A_24_P84970	5.41E-05	ENST0000034014 1		PREDICTED: Homo sapiens similar to Keratin, type I cytoskeletal 18 (Cytokeratin 18) (K18) (CK 18) (LOC391819), mRNA [XM_498013]	XM_498013
A_23_P201097	5.46E-05	NM_000858	NM_000858	Homo sapiens guanylate kinase 1 (GUK1), mRNA [NM_000858]	NM_000858
A_23_P136916	5.47E-05	NM_017883	NM_017883	Homo sapiens WD repeat domain 13 (WDR13), mRNA [NM_017883]	NM_017883
A_24_P246173	5.48E-05	NM_004145	NM_004145	Homo sapiens myosin IXB (MYO9B), mRNA [NM_004145]	NM_004145
A_24_P278192	5.50E-05	NM_000476	NM_000476	Homo sapiens adenylate kinase 1 (AK1), mRNA [NM_000476]	NM_000476
A_24_P65616	5.51E-05	NM_006505	NM_006505	Homo sapiens poliovirus receptor (PVR), mRNA [NM_006505]	NM_006505
A_23_P162579	5.53E-05	NM_014365	NM_014365	Homo sapiens heat shock 22kDa protein 8 (HSPB8), mRNA [NM_014365]	NM_014365
A_23_P211252	5.55E-05	NM_001001438	NM_001001438	Homo sapiens lanosterol synthase (2,3-oxidosqualene-lanosterol cyclase) (LSS), transcript variant 2, mRNA [NM_001001438]	NM_001001438
A_23_P110941	5.56E-05	NM_001512	NM_001512	Homo sapiens glutathione S-transferase A4 (GSTA4), mRNA [NM_001512]	NM_001512
A_32_P69492	5.59E-05	AK025631	AK025631	Homo sapiens cDNA: FLJ21978 fis, clone HEP06046, highly similar to AF158185 Homo sapiens xeroderma pigmentosum variant RAD30 (RAD30) mRNA. [AK025631]	
A_23_P215790	5.60E-05	NM_005228	NM_005228	Homo sapiens epidermal growth factor receptor (erythroblastic leukemia viral (v-erb-b) oncogene homolog, avian) (EGFR), transcript variant 1, mRNA [NM_005228]	NM_005228
A_23_P21853	5.60E-05	NM_012305	NM_012305	Homo sapiens adaptor-related protein complex 2, alpha 2 subunit (AP2A2), mRNA [NM_012305]	NM_012305
A_24_P58337	5.60E-05	NM_002032	NM_002032	Homo sapiens ferritin, heavy polypeptide 1 (FTH1), mRNA [NM_002032]	NM_002032
A_24_P71661	5.62E-05	NM_006371	NM_006371	Homo sapiens cartilage associated protein (CRTAP), mRNA [NM_006371]	NM_006371

A_24_P511686	5.62E-05	CR616845	CR616845	full-length cDNA clone CS0DF020YJ04 of Fetal brain of Homo sapiens (human). [CR616845]	XM_378309
A_23_P73457	5.62E-05	NM_025158	NM_025158	Homo sapiens RUN and FYVE domain containing 1 (RUFY1), mRNA [NM_025158]	NM_025158
A_23_P1331	5.63E-05	NM_005203	NM_005203	Homo sapiens collagen, type XIII, alpha 1 (COL13A1), transcript variant 1, mRNA [NM_005203]	NM_005203
A_32_P171793	5.64E-05	THC2317110			
A_23_P69908	5.64E-05	NM_002064	NM_002064	Homo sapiens glutaredoxin (thioltransferase) (GLRX), mRNA [NM_002064]	NM_002064
A_23_P72850	5.65E-05	NM_032889	NM_032889	Homo sapiens hypothetical protein MGC11308 (MGC11308), mRNA [NM_032889]	NM_032889
A_23_P166453	5.66E-05	NM_152243	NM_152243	Homo sapiens CDC42 effector protein (Rho GTPase binding) 1 (CDC42EP1), transcript variant 1, mRNA [NM_152243]	NM_152243
A_23_P256375	5.66E-05	NM_004604	NM_004604	Homo sapiens syntaxin 4A (placental) (STX4A), mRNA [NM_004604]	NM_004604
A_24_P355649	5.67E-05	NM_002017	NM_002017	Homo sapiens Friend leukemia virus integration 1 (FLI1), mRNA [NM_002017]	NM_002017
A_23_P74278	5.72E-05	AB209081	AB209081	Homo sapiens mRNA for phosphodiesterase 4B, cAMP-specific (phosphodiesterase E4 dunce homolog, Drosophila) variant protein. [AB209081]	
A_32_P50275	5.72E-05	AK095453	AK095453	Homo sapiens cDNA FLJ38134 fis, clone D9OST2000440. [AK095453]	
A_23_P77000	5.72E-05	NM_014909	NM_014909	Homo sapiens KIAA1036 (KIAA1036), mRNA [NM_014909]	NM_014909
A_23_P83751	5.76E-05	NM_024806	NM_024806	Homo sapiens hypothetical protein FLJ23554 (FLJ23554), transcript variant 1, mRNA [NM_024806]	NM_024806
A_23_P57401	5.76E-05	NM_006767	NM_006767	Homo sapiens leucine-zipper-like transcription regulator 1 (LZTR1), mRNA [NM_006767]	NM_006767
A_23_P19257	5.76E-05	NM_178148	NM_178148	Homo sapiens solute carrier family 35, member B2 (SLC35B2), mRNA [NM_178148]	NM_178148
A_23_P407112	5.78E-05	NM_145263	NM_145263	Homo sapiens spermatogenesis associated 18 homolog (rat) (SPATA18), mRNA [NM_145263]	NM_145263
A_32_P98683	5.78E-05	AL832481	AL832481	Homo sapiens mRNA; cDNA DKFZp686J1719 (from clone DKFZp686J1719). [AL832481]	
A_23_P355244	5.79E-05	NM_017654	NM_017654	Homo sapiens sterile alpha motif domain containing 9 (SAMD9), mRNA [NM_017654]	NM_017654
A_24_P902195	5.79E-05	A_24_P902195			
A_24_P681563	5.83E-05	A_24_P681563			
A_23_P154208	5.84E-05	NM_017567	NM_017567	Homo sapiens N-acetylglucosamine kinase (NAGK), mRNA [NM_017567]	NM_017567
A_24_P141214	5.86E-05	NM_198194	NM_198194	Homo sapiens stomatin (STOM), transcript variant 2, mRNA [NM_198194]	NM_198194
A_23_P107283	5.86E-05	NM_002145	NM_002145	Homo sapiens homeo box B2 (HOXB2), mRNA [NM_002145]	NM_002145
A_23_P129856	5.87E-05	THC2375716		Q9V9M3 (Q9V9M3) CG16937-PA, partial (6%) [THC2375716]	
A_23_P255263	5.87E-05	NM_198194	NM_198194	Homo sapiens stomatin (STOM), transcript variant 2, mRNA [NM_198194]	NM_198194
A_23_P20606	5.87E-05	NM_015469	NM_015469	Homo sapiens nipsnap homolog 3A (C. elegans) (NIPSNAP3A), mRNA [NM_015469]	NM_015469
A_23_P85236	5.87E-05	NM_003928	NM_003928	Homo sapiens CAAX box 1 (CXX1), mRNA [NM_003928]	NM_003928
A_23_P110585	5.89E-05	NM_138492	NM_138492	Homo sapiens hypothetical protein MGC21644 (MGC21644), transcript variant 3, mRNA [NM_138492]	NM_138492
A_32_P820503	5.89E-05	NM_002032	NM_002032	Homo sapiens ferritin, heavy polypeptide 1 (FTH1), mRNA [NM_002032]	NM_002032
A_24_P595460	5.90E-05	AK097398	AK097398	Homo sapiens cDNA FLJ40079 fis, clone TESTI2001498, highly similar to DNA-BINDING PROTEIN NEFA PRECURSOR. [AK097398]	
A_24_P873764	5.92E-05	ENST00000359540		Homo sapiens cDNA FLJ36027 fis, clone TESTI2016888, weakly similar to BREAKPOINT CLUSTER REGION PROTEIN (EC 2.7.1.-). [AK093346]	XM_496519
A_23_P170733	5.92E-05	NM_058172	NM_058172	Homo sapiens anthrax toxin receptor 2 (ANTXR2), mRNA [NM_058172]	NM_058172
A_23_P214026	5.94E-05	NM_001999	NM_001999	Homo sapiens fibrillin 2 (congenital contractural arachnodactyly) (FBN2), mRNA [NM_001999]	NM_001999
A_23_P136012	5.97E-05	NM_012180	NM_012180	Homo sapiens F-box protein 8 (FBXO8), mRNA [NM_012180]	NM_012180
A_24_P70993	5.98E-05	NM_002414	NM_002414	Homo sapiens CD99 antigen (CD99), mRNA [NM_002414]	NM_002414
A_23_P217688	5.99E-05	NM_004089	NM_004089	Homo sapiens TSC22 domain family, member 3 (TSC22D3), transcript variant 2, mRNA [NM_004089]	NM_004089
A_24_P124370	5.99E-05	NM_018222	NM_018222	Homo sapiens parvin, alpha (PARVA), mRNA [NM_018222]	NM_018222
A_24_P940115	6.00E-05	NM_182643	NM_182643	Homo sapiens deleted in liver cancer 1 (DLC1), transcript variant 1, mRNA [NM_182643]	NM_182643
A_23_P255317	6.00E-05	NM_004290	NM_004290	Homo sapiens ring finger protein 14 (RNF14), transcript variant 1, mRNA [NM_004290]	NM_004290
A_23_P52552	6.00E-05	NM_004281	NM_004281	Homo sapiens BCL2-associated athanogene 3 (BAG3), mRNA [NM_004281]	NM_004281
A_23_P9883	6.02E-05	NM_004895	NM_004895	Homo sapiens cold autoinflammatory syndrome 1 (CIAS1), transcript variant 1, mRNA [NM_004895]	NM_004895
A_23_P1292	6.04E-05	NM_000124	NM_000124	Homo sapiens excision repair cross-complementing rodent repair deficiency, complementation group 6 (ERCC6), mRNA [NM_000124]	NM_000124
A_24_P332595	6.05E-05	A_24_P332595			
A_23_P347623	6.06E-05	NM_019891	NM_019891	Homo sapiens ERO1-like beta (S. cerevisiae) (ERO1LB), mRNA [NM_019891]	NM_019891

A_24_P532232	6.06E-05	NM_182898	NM_182898	Homo sapiens cAMP responsive element binding protein 5 (CREB5), transcript variant 1, mRNA [NM_182898]	NM_182898
A_23_P92042	6.06E-05	NM_002222	NM_002222	Homo sapiens inositol 1,4,5-triphosphate receptor, type 1 (ITPR1), mRNA [NM_002222]	NM_002222
A_24_P251411	6.07E-05	NM_032824	NM_032824	Homo sapiens hypothetical protein FLJ14681 (FLJ14681), mRNA [NM_032824]	NM_032824
A_23_P162982	6.07E-05	NM_021004	NM_021004	Homo sapiens dehydrogenase/reductase (SDR family) member 4 (DHRS4), mRNA [NM_021004]	NM_021004
A_23_P374695	6.07E-05	NM_000459	NM_000459	Homo sapiens TEK tyrosine kinase, endothelial (venous malformations, multiple cutaneous and mucosal) (TEK), mRNA [NM_000459]	NM_000459
A_23_P137016	6.09E-05	NM_002970	NM_002970	Homo sapiens spermidine/spermine N1-acetyltransferase (SAT), mRNA [NM_002970]	NM_002970
A_32_P2730	6.11E-05	BC005081	BC005081	Homo sapiens brevican, mRNA (cDNA clone IMAGE:3618761), partial cds. [BC005081]	
A_23_P39202	6.11E-05	NM_033520	NM_033520	Homo sapiens chromosome 19 open reading frame 33 (C19orf33), mRNA [NM_033520]	NM_033520
A_24_P233915	6.13E-05	NM_001001653	NM_001001653	Homo sapiens mediator of RNA polymerase II transcription, subunit 8 homolog (yeast) (MED8), transcript variant 4, mRNA [NM_001001653]	NM_001001653
A_23_P43273	6.13E-05	NM_000127	NM_000127	Homo sapiens exostoses (multiple) 1 (EXT1), mRNA [NM_000127]	NM_000127
A_24_P409265	6.15E-05	NM_005848	NM_005848	Homo sapiens c-myc promoter binding protein (MYCPBP), mRNA [NM_005848]	NM_005848
A_23_P27573	6.15E-05	NM_013342	NM_013342	Homo sapiens TCF3 (E2A) fusion partner (in childhood Leukemia) (TFPT), mRNA [NM_013342]	NM_013342
A_23_P160481	6.17E-05	NM_018442	NM_018442	Homo sapiens IQ motif and WD repeats 1 (IQWD1), transcript variant 1, mRNA [NM_018442]	NM_018442
A_23_P113283	6.18E-05	NM_022470	NM_022470	Homo sapiens p53 target zinc finger protein (WIG1), transcript variant 1, mRNA [NM_022470]	NM_022470
A_24_P118472	6.18E-05	A_24_P118472			
A_24_P919633	6.19E-05	AF281279	AF281279	Homo sapiens unknown mRNA, complete cds. [AF281279]	
A_32_P186725	6.21E-05	THC2433670		G01958 homeobox protein - human (fragment) {Homo sapiens;} , partial (5%) [THC2433670]	
A_23_P160503	6.21E-05	NM_016066	NM_016066	Homo sapiens glutaredoxin 2 (GLRX2), transcript variant 1, mRNA [NM_016066]	NM_016066
A_24_P219324	6.22E-05	NM_004614	NM_004614	Homo sapiens thymidine kinase 2, mitochondrial (TK2), mRNA [NM_004614]	NM_004614
A_23_P63447	6.23E-05	THC2373845			
A_23_P339773	6.27E-05	NM_182752	NM_182752	Homo sapiens family with sequence similarity 79, member A (FAM79A), mRNA [NM_182752]	NM_182752
A_24_P392146	6.29E-05	BC036911	BC036911	Homo sapiens KIAA1632, mRNA (cDNA clone IMAGE:5139756), complete cds. [BC036911]	
A_32_P101420	6.38E-05	A_32_P101420			
A_23_P35412	6.40E-05	NM_001549	NM_001549	Homo sapiens interferon-induced protein with tetratricopeptide repeats 3 (IFIT3), mRNA [NM_001549]	NM_001549
A_23_P19657	6.41E-05	NM_032832	NM_032832	Homo sapiens low density lipoprotein receptor-related protein 11 (LRP11), mRNA [NM_032832]	NM_032832
A_23_P431939	6.42E-05	NM_001531	NM_001531	Homo sapiens major histocompatibility complex, class I-related (MR1), mRNA [NM_001531]	NM_001531
A_23_P33196	6.44E-05	NM_000393	NM_000393	Homo sapiens collagen, type V, alpha 2 (COL5A2), mRNA [NM_000393]	NM_000393
A_23_P120921	6.45E-05	NM_138797	NM_138797	Homo sapiens hypothetical protein BC014641 (LOC129138), mRNA [NM_138797]	NM_138797
A_32_P217709	6.45E-05	NM_198829	NM_198829	Homo sapiens ras-related C3 botulinum toxin substrate 1 (rho family, small GTP binding protein Rac1) (RAC1), transcript variant Rac1c, mRNA [NM_198829]	NM_198829
A_24_P34155	6.51E-05	D43967	D43967	Homo sapiens mRNA for AML1a protein, complete cds. [D43967]	
A_32_P101653	6.51E-05	XM_499110	XM_499110	PREDICTED: Homo sapiens LOC441345 (LOC441345), mRNA [XM_499110]	XM_499110
A_32_P125568	6.51E-05	THC2416690		Q9EQG5 (Q9EQG5) Nedd4-binding brain specific protein BEAN (Fragment), partial (5%) [THC2416690]	
A_23_P311869	6.51E-05	NM_003896	NM_003896	Homo sapiens ST3 beta-galactoside alpha-2,3-sialyltransferase 5 (ST3GAL5), mRNA [NM_003896]	NM_003896
A_23_P64173	6.51E-05	NM_001017534	NM_001017534	Homo sapiens CARD only protein (COPl), transcript variant 1, mRNA [NM_001017534]	NM_001017534
A_23_P115573	6.51E-05	NM_198149	NM_198149	Homo sapiens transmembrane protein 58 (TMEM58), mRNA [NM_198149]	NM_198149
A_32_P197620	6.55E-05	BC009550	BC009550	Homo sapiens FAM51A1 pseudogene, mRNA (cDNA clone MGC:10091 IMAGE:3898110), complete cds. [BC009550]	
A_24_P179504	6.57E-05	NM_144668	NM_144668	Homo sapiens WD repeat domain 66 (WDR66), mRNA [NM_144668]	NM_144668
A_32_P161681	6.58E-05	NM_001008528	NM_001008528	Homo sapiens matrix-remodelling associated 7 (MXRA7), transcript variant 1, mRNA [NM_001008528]	NM_001008528
A_24_P33048	6.62E-05	NM_001017923	NM_001017923	Homo sapiens chromosome 14 open reading frame 28 (C14orf28), mRNA [NM_001017923]	NM_001017923
A_23_P157620	6.62E-05	NM_022749	NM_022749	Homo sapiens retinoic acid induced 16 (RAI16), mRNA [NM_022749]	NM_022749
A_23_P90311	6.63E-05	NM_182919	NM_182919	Homo sapiens toll-like receptor adaptor molecule 1 (TICAM1), transcript variant 2, mRNA [NM_182919]	NM_182919
A_23_P93677	6.67E-05	NM_017994	NM_017994	Homo sapiens hypothetical protein FLJ10099 (FLJ10099), mRNA [NM_017994]	NM_017994
A_24_P201702	6.68E-05	NM_005127	NM_005127	Homo sapiens C-type lectin domain family 2, member B (CLEC2B), mRNA [NM_005127]	NM_005127
A_32_P52911	6.68E-05	NM_018269	NM_018269	Homo sapiens membrane-type 1 matrix metalloproteinase cytoplasmic tail binding protein-1 (MTCBP-1), mRNA [NM_018269]	NM_018269

A_23_P109934	6.71E-05	NM_016173	NM_016173	Homo sapiens HemK methyltransferase family member 1 (HEMK1), mRNA [NM_016173]	NM_016173
A_23_P47879	6.71E-05	NM_003153	NM_003153	Homo sapiens signal transducer and activator of transcription 6, interleukin-4 induced (STAT6), mRNA [NM_003153]	NM_003153
A_23_P24004	6.74E-05	NM_001547	NM_001547	Homo sapiens interferon-induced protein with tetratricopeptide repeats 2 (IFIT2), mRNA [NM_001547]	NM_001547
A_23_P76829	6.75E-05	NM_020834	NM_020834	Homo sapiens KIAA1443 (KIAA1443), mRNA [NM_020834]	NM_020834
A_23_P70148	6.77E-05	NM_182977	NM_182977	Homo sapiens nicotinamide nucleotide transhydrogenase (NNT), mRNA [NM_182977]	NM_182977
A_23_P405216	6.79E-05	NM_173656	NM_173656	Homo sapiens zinc finger protein 619 (ZNF619), mRNA [NM_173656]	NM_173656
A_23_P165186	6.79E-05	NM_019104	NM_019104	Homo sapiens protein F25965 (F25965), mRNA [NM_019104]	NM_019104
A_23_P165668	6.80E-05	AK025657	AK025657	Homo sapiens cDNA: FLJ22004 fis, clone HEP06871. [AK025657]	
A_24_P305570	6.82E-05	NM_018993	NM_018993	Homo sapiens Ras and Rab interactor 2 (RIN2), mRNA [NM_018993]	NM_018993
A_24_P316364	6.83E-05	NM_015523	NM_015523	Homo sapiens small fragment nuclease (DKFZP566E144), mRNA [NM_015523]	NM_015523
A_23_P69810	6.84E-05	NM_032717	NM_032717	Homo sapiens hypothetical protein MGC11324 (MGC11324), mRNA [NM_032717]	NM_032717
A_32_P177300	6.85E-05	BQ186377	BQ186377	UI-E-EJ1-ajr-a-19-0-UI.r1 UI-E-EJ1 Homo sapiens cDNA clone UI-E-EJ1-ajr-a-19-0-UI 5', mRNA sequence [BQ186377]	
A_24_P463989	6.88E-05	THC2376306		Q7QRW0 (Q7QRW0) GLP_69_6195_6536, partial (14%) [THC2376306]	
A_23_P129956	6.92E-05	NM_004090	NM_004090	Homo sapiens dual specificity phosphatase 3 (vaccinia virus phosphatase VH1-related) (DUSP3), mRNA [NM_004090]	NM_004090
A_24_P314688	6.93E-05	NM_018218	NM_018218	Homo sapiens ubiquitin specific protease 40 (USP40), mRNA [NM_018218]	NM_018218
A_23_P343594	6.95E-05	BC067300	BC067300	Homo sapiens cDNA clone IMAGE:30389677, partial cds. [BC067300]	
A_24_P290719	6.95E-05	AY047582	AY047582	Homo sapiens neural precursor cell expressed developmentally down-regulated protein 8 (NEDD8) mRNA, complete cds. [AY047582]	
A_23_P211806	6.96E-05	NM_017724	NM_017724	Homo sapiens leucine rich repeat (in FLII) interacting protein 2 (LRRFIP2), transcript variant 2, mRNA [NM_017724]	NM_017724
A_23_P151586	6.96E-05	NM_006405	NM_006405	Homo sapiens transmembrane 9 superfamily member 1 (TM9SF1), transcript variant 1, mRNA [NM_006405]	NM_006405
A_24_P217848	6.96E-05	NM_003510	NM_003510	Homo sapiens histone 1, H2ak (HIST1H2AK), mRNA [NM_003510]	NM_003510
A_24_P277331	6.96E-05	NM_002337	NM_002337	Homo sapiens low density lipoprotein receptor-related protein associated protein 1 (LRPAP1), mRNA [NM_002337]	NM_002337
A_23_P359588	6.98E-05	NM_032098	NM_032098	Homo sapiens protocadherin gamma subfamily B, 4 (PCDHGB4), transcript variant 2, mRNA [NM_032098]	NM_032098
A_24_P913339	6.98E-05	BC016389	BC016389	Homo sapiens chromosome 2 open reading frame 18, mRNA (cDNA clone IMAGE:3860139), complete cds. [BC016389]	
A_23_P123672	6.98E-05	NM_014290	NM_014290	Homo sapiens tudor domain containing 7 (TDRD7), mRNA [NM_014290]	NM_014290
A_24_P162211	6.99E-05	NM_001001438	NM_001001438	Homo sapiens lanosterol synthase (2,3-oxidosqualene-lanosterol cyclase) (LSS), transcript variant 2, mRNA [NM_001001438]	NM_001001438
A_23_P21134	7.01E-05	NM_004083	NM_004083	Homo sapiens DNA-damage-inducible transcript 3 (DDIT3), mRNA [NM_004083]	NM_004083
A_23_P143551	7.01E-05	NM_001696	NM_001696	Homo sapiens ATPase, H+ transporting, lysosomal 31kDa, V1 subunit E isoform 1 (ATP6V1E1), mRNA [NM_001696]	NM_001696
A_23_P122815	7.03E-05	NM_001219	NM_001219	Homo sapiens calumenin (CALU), mRNA [NM_001219]	NM_001219
A_23_P257538	7.10E-05	NM_006606	NM_006606	Homo sapiens retinoblastoma binding protein 9 (RBBP9), transcript variant 1, mRNA [NM_006606]	NM_006606
A_24_P162718	7.10E-05	NM_005572	NM_005572	Homo sapiens lamin A/C (LMNA), transcript variant 2, mRNA [NM_005572]	NM_005572
A_23_P211504	7.15E-05	NM_016657	NM_016657	Homo sapiens KDEL (Lys-Asp-Glu-Leu) endoplasmic reticulum protein retention receptor 3 (KDELR3), transcript variant 2, mRNA [NM_016657]	NM_016657
A_23_P427075	7.19E-05	NM_004937	NM_004937	Homo sapiens cystinosis, nephropathic (CTNS), mRNA [NM_004937]	NM_004937
A_23_P20285	7.21E-05	NM_021630	NM_021630	Homo sapiens PDZ and LIM domain 2 (mystique) (PDLIM2), transcript variant 2, mRNA [NM_021630]	NM_021630
A_32_P119744	7.24E-05	BC039550	BC039550	Homo sapiens, clone IMAGE:5760770, mRNA. [BC039550]	XM_298151
A_23_P361569	7.24E-05	NM_025181	NM_025181	Homo sapiens solute carrier family 35, member F5 (SLC35F5), mRNA [NM_025181]	NM_025181
A_32_P177953	7.24E-05	CR598364	CR598364	full-length cDNA clone CS0CAP007YJ17 of Thymus of Homo sapiens (human). [CR598364]	
A_23_P144369	7.26E-05	NM_153757	NM_153757	Homo sapiens nucleosome assembly protein 1-like 5 (NAP1L5), mRNA [NM_153757]	NM_153757
A_24_P217758	7.27E-05	AL049404	AL049404	Homo sapiens mRNA; cDNA DKFZp586F0219 (from clone DKFZp586F0219). [AL049404]	
A_24_P324449	7.29E-05	NM_012089	NM_012089	Homo sapiens ATP-binding cassette, sub-family B (MDR/TAP), member 10 (ABCB10), nuclear gene encoding mitochondrial protein, mRNA [NM_012089]	NM_012089
A_24_P81789	7.29E-05	NM_019034	NM_019034	Homo sapiens ras homolog gene family, member F (in filopodia) (RHOF), mRNA [NM_019034]	NM_019034
A_32_P154601	7.34E-05	AK125850	AK125850	Homo sapiens cDNA FLJ43862 fis, clone TESTI4007775. [AK125850]	
A_32_P231617	7.36E-05	NM_014220	NM_014220	Homo sapiens transmembrane 4 L six family member 1 (TM4SF1), mRNA [NM_014220]	NM_014220
A_24_P99216	7.37E-05	NM_014045	NM_014045	Homo sapiens low density lipoprotein receptor-related protein 10 (LRP10), mRNA [NM_014045]	NM_014045
A_24_P32715	7.37E-05	ENST00000313481			

A_23_P327426	7.40E-05	NM_152902	NM_152902	Homo sapiens TIP41, TOR signalling pathway regulator-like (S. cerevisiae) (TIPRL), mRNA [NM_152902]	NM_152902
A_24_P19983	7.46E-05	THC2437888		Q6XZE5 (Q6XZE5) CINCIN1, partial (5%) [THC2437888]	
A_23_P58815	7.50E-05	NM_012176	NM_012176	Homo sapiens F-box protein 4 (FBXO4), transcript variant 1, mRNA [NM_012176]	NM_012176
A_24_P370887	7.51E-05	NM_004781	NM_004781	Homo sapiens vesicle-associated membrane protein 3 (cellubrevin) (VAMP3), mRNA [NM_004781]	NM_004781
A_24_P85200	7.52E-05	NM_194278	NM_194278	Homo sapiens chromosome 14 open reading frame 43 (C14orf43), mRNA [NM_194278]	NM_194278
A_24_P103060	7.53E-05	NM_139279	NM_139279	Homo sapiens multiple coagulation factor deficiency 2 (MCFD2), mRNA [NM_139279]	NM_139279
A_23_P166910	7.53E-05	NM_173823	NM_173823	Homo sapiens DnaJ (Hsp40) homolog, subfamily C, member 13 (DNAJC13), mRNA [NM_173823]	NM_173823
A_32_P151933	7.54E-05	THC2279436			
A_23_P140170	7.55E-05	NM_006364	NM_006364	Homo sapiens Sec23 homolog A (S. cerevisiae) (SEC23A), mRNA [NM_006364]	NM_006364
A_23_P35796	7.60E-05	NM_006244	NM_006244	Homo sapiens protein phosphatase 2, regulatory subunit B (B56), beta isoform (PPP2R5B), mRNA [NM_006244]	NM_006244
A_24_P30194	7.61E-05	NM_012420	NM_012420	Homo sapiens interferon-induced protein with tetratricopeptide repeats 5 (IFIT5), mRNA [NM_012420]	NM_012420
A_24_P458479	7.64E-05	NM_001013404	NM_001013404	Homo sapiens hypothetical LOC504188 (LOC504188), mRNA [NM_001013404]	NM_001013404
A_23_P155301	7.65E-05	NM_145910	NM_145910	Homo sapiens NIMA (never in mitosis gene a)- related kinase 11 (NEK11), transcript variant 2, mRNA [NM_145910]	NM_145910
A_23_P201264	7.67E-05	AF088049	AF088049	Homo sapiens full length insert cDNA clone ZD62D09. [AF088049]	
A_24_P303524	7.67E-05	NM_182924	NM_182924	Homo sapiens MICAL-like 2 (MICAL-L2), transcript variant 1, mRNA [NM_182924]	NM_182924
A_23_P257638	7.69E-05	NM_006384	NM_006384	Homo sapiens calcium and integrin binding 1 (calmyrin) (CIB1), mRNA [NM_006384]	NM_006384
A_24_P392742	7.70E-05	A_24_P392742			
A_23_P311608	7.70E-05	NM_054022	NM_054022	Homo sapiens golgi SNAP receptor complex member 2 (GOSR2), transcript variant B, mRNA [NM_054022]	NM_054022
A_23_P49708	7.71E-05	NM_002087	NM_002087	Homo sapiens granulin (GRN), transcript variant 1, mRNA [NM_002087]	NM_002087
A_23_P136552	7.76E-05	NM_017733	NM_017733	Homo sapiens GPI7 protein (GPI7), mRNA [NM_017733]	NM_017733
A_23_P312246	7.76E-05	NM_024725	NM_024725	Homo sapiens hypothetical protein FLJ23518 (FLJ23518), mRNA [NM_024725]	NM_024725
A_23_P1461	7.79E-05	NM_001008211	NM_001008211	Homo sapiens optineurin (OPTN), transcript variant 1, mRNA [NM_001008211]	NM_001008211
A_23_P2097	7.80E-05	NM_018073	NM_018073	Homo sapiens tripartite motif-containing 68 (TRIM68), mRNA [NM_018073]	NM_018073
A_23_P330999	7.81E-05	NM_015979	NM_015979	Homo sapiens cofactor required for Sp1 transcriptional activation, subunit 3, 130kDa (CRSP3), transcript variant 2, mRNA [NM_015979]	NM_015979
A_24_P72420	7.82E-05	NM_014800	NM_014800	Homo sapiens engulfment and cell motility 1 (ced-12 homolog, C. elegans) (ELMO1), transcript variant 1, mRNA [NM_014800]	NM_014800
A_23_P133075	7.82E-05	NM_033115	NM_033115	Homo sapiens hypothetical protein MGC16169 (MGC16169), mRNA [NM_033115]	NM_033115
A_23_P62115	7.83E-05	NM_003254	NM_003254	Homo sapiens tissue inhibitor of metalloproteinase 1 (erythroid potentiating activity, collagenase inhibitor) (TIMP1), mRNA [NM_003254]	NM_003254
A_23_P316239	7.87E-05	NM_001013845	NM_001013845	Homo sapiens hypothetical LOC541578 protein (LOC541578), mRNA [NM_001013845]	NM_001013845
A_23_P59397	7.90E-05	NM_031924	NM_031924	Homo sapiens radial spokehead-like 2 (RSHL2), mRNA [NM_031924]	NM_031924
A_23_P14769	7.91E-05	NM_002005	NM_002005	Homo sapiens feline sarcoma oncogene (FES), mRNA [NM_002005]	NM_002005
A_23_P309803	7.93E-05	NM_015694	NM_015694	Homo sapiens KIAA1285 protein (KIAA1285), mRNA [NM_015694]	NM_015694
A_23_P87752	7.94E-05	NM_014262	NM_014262	Homo sapiens leprecan-like 2 (LEPREL2), mRNA [NM_014262]	NM_014262
A_23_P93236	7.95E-05	NM_006355	NM_006355	Homo sapiens tripartite motif-containing 38 (TRIM38), mRNA [NM_006355]	NM_006355
A_23_P143274	7.95E-05	NM_024958	NM_024958	Homo sapiens chromosome 20 open reading frame 98 (C20orf98), mRNA [NM_024958]	NM_024958
A_23_P69491	7.98E-05	NM_001664	NM_001664	Homo sapiens ras homolog gene family, member A (RHOA), mRNA [NM_001664]	NM_001664
A_32_P96134	8.02E-05	ENST00000310974		Homo sapiens mRNA for KIAA0877 protein, partial cds. [AB020684]	XM_371891
A_24_P80181	8.02E-05	NM_017849	NM_017849	Homo sapiens hypothetical protein FLJ20507 (FLJ20507), mRNA [NM_017849]	NM_017849
A_23_P103180	8.05E-05	NM_138467	NM_138467	Homo sapiens chromosome 1 open reading frame 171 (C1orf171), mRNA [NM_138467]	NM_138467
A_23_P108835	8.06E-05	NM_016061	NM_016061	Homo sapiens yippee-like 5 (Drosophila) (YPEL5), mRNA [NM_016061]	NM_016061
A_23_P50504	8.07E-05	NM_000146	NM_000146	Homo sapiens ferritin, light polypeptide (FTL), mRNA [NM_000146]	NM_000146
A_23_P390677	8.08E-05	NM_004991	NM_004991	Homo sapiens myelodysplasia syndrome 1 (MDS1), mRNA [NM_004991]	NM_004991
A_24_P75019	8.08E-05	AF274938	AF274938	Homo sapiens PNAS-13 mRNA, complete cds. [AF274938]	XM_496861
A_23_P81993	8.09E-05	NM_178508	NM_178508	Homo sapiens chromosome 6 open reading frame 1 (C6orf1), transcript variant 1, mRNA [NM_178508]	NM_178508
A_24_P374741	8.10E-05	AK057828	AK057828	Homo sapiens cDNA FLJ25099 fis, clone CBR01272. [AK057828]	
A_23_P218237	8.11E-05	NM_000229	NM_000229	Homo sapiens lecithin-cholesterol acyltransferase (LCAT), mRNA [NM_000229]	NM_000229

A_24_P196019	8.11E-05	XM_171154	XM_171154	PREDICTED: Homo sapiens similar to myelin protein zero-like 1 isoform a; protein zero related (LOC253883), mRNA [XM_171154]	XM_171154
A_24_P311917	8.14E-05	NM_006994	NM_006994	Homo sapiens butyrophilin, subfamily 3, member A3 (BTN3A3), transcript variant 1, mRNA [NM_006994]	NM_006994
A_32_P123771	8.14E-05	NM_006886	NM_006886	Homo sapiens ATP synthase, H+ transporting, mitochondrial F1 complex, epsilon subunit (ATP5E), nuclear gene encoding mitochondrial protein, mRNA [NM_006886]	NM_006886
A_23_P396842	8.15E-05	NM_152734	NM_152734	Homo sapiens chromosome 6 open reading frame 89 (C6orf89), mRNA [NM_152734]	NM_152734
A_23_P211428	8.16E-05	NM_134269	NM_134269	Homo sapiens smoothelin (SMTN), transcript variant 2, mRNA [NM_134269]	NM_134269
A_24_P50567	8.16E-05	A_24_P50567			
A_24_P942517	8.19E-05	ENST00000246024		Homo sapiens mRNA for KIAA1162 protein, partial cds. [AB032988]	
A_32_P203749	8.20E-05	AF086547	AF086547	Homo sapiens full length insert cDNA clone ZE12B03. [AF086547]	
A_24_P835500	8.23E-05	AK026647	AK026647	Homo sapiens cDNA: FLJ22994 fis, clone KAT11918. [AK026647]	
A_23_P48166	8.26E-05	NM_198974	NM_198974	Homo sapiens PTK9 protein tyrosine kinase 9 (PTK9), transcript variant 2, mRNA [NM_198974]	NM_198974
A_23_P129935	8.28E-05	NM_030938	NM_030938	Homo sapiens transmembrane protein 49 (TMEM49), mRNA [NM_030938]	NM_030938
A_23_P141960	8.29E-05	NM_203344	NM_203344	Homo sapiens SERTA domain containing 3 (SERTAD3), transcript variant 2, mRNA [NM_203344]	NM_203344
A_23_P56709	8.29E-05	NM_005667	NM_005667	Homo sapiens ring finger protein 103 (RNF103), mRNA [NM_005667]	NM_005667
A_23_P200404	8.29E-05	NM_001625	NM_001625	Homo sapiens adenylate kinase 2 (AK2), transcript variant AK2A, mRNA [NM_001625]	NM_001625
A_24_P110591	8.31E-05	A_24_P110591			
A_32_P108592	8.33E-05	THC2312637		moxR3 {Mycobacterium smegmatis str. MC2 155;} , partial (3%) [THC2312637]	
A_32_P11670	8.34E-05	BC030083	BC030083	Homo sapiens, clone IMAGE:4791783, mRNA. [BC030083]	
A_32_P218883	8.34E-05	NM_018339	NM_018339	Homo sapiens riboflavin kinase (RFK), mRNA [NM_018339]	NM_018339
A_23_P218770	8.35E-05	NM_002872	NM_002872	Homo sapiens ras-related C3 botulinum toxin substrate 2 (rho family, small GTP binding protein Rac2) (RAC2), mRNA [NM_002872]	NM_002872
A_24_P944519	8.36E-05	NM_006203	NM_006203	Homo sapiens phosphodiesterase 4D, cAMP-specific (phosphodiesterase E3 dunce homolog, Drosophila) (PDE4D), mRNA [NM_006203]	NM_006203
A_32_P85835	8.36E-05	AK094979	AK094979	Homo sapiens cDNA FLJ37660 fis, clone BRHIP2010723. [AK094979]	
A_23_P217447	8.37E-05	NM_002294	NM_002294	Homo sapiens lysosomal-associated membrane protein 2 (LAMP2), transcript variant LAMP2A, mRNA [NM_002294]	NM_002294
A_23_P204277	8.41E-05	NM_177925	NM_177925	Homo sapiens H2A histone family, member J (H2AFJ), transcript variant 2, mRNA [NM_177925]	NM_177925
A_24_P414282	8.43E-05	THC2277620		Q9NQR4 (Q9NQR4) Nit protein 2 (CUA002), partial (92%) [THC2277620]	
A_23_P61646	8.44E-05	NM_004604	NM_004604	Homo sapiens syntaxin 4A (placental) (STX4A), mRNA [NM_004604]	NM_004604
A_24_P376391	8.45E-05	NM_015103	NM_015103	Homo sapiens plexin D1 (PLXND1), mRNA [NM_015103]	NM_015103
A_24_P931476	8.48E-05	BC043603	BC043603	Homo sapiens, Similar to hypothetical protein FLJ33215, clone IMAGE:5503684, mRNA. [BC043603]	
A_23_P122216	8.48E-05	NM_002317	NM_002317	Homo sapiens lysyl oxidase (LOX), mRNA [NM_002317]	NM_002317
A_23_P76136	8.50E-05	AY358804	AY358804	Homo sapiens clone DNA100312 VSSW1971 (UNQ1971) mRNA, complete cds. [AY358804]	
A_23_P13701	8.50E-05	NM_016056	NM_016056	Homo sapiens CGI-119 protein (CGI-119), mRNA [NM_016056]	NM_016056
A_23_P330070	8.50E-05	BC015514	BC015514	Homo sapiens tissue factor pathway inhibitor (lipoprotein-associated coagulation inhibitor), mRNA (cDNA clone MGC:9251 IMAGE:3902987), complete cds. [BC015514]	
A_23_P254626	8.50E-05	NM_003919	NM_003919	Homo sapiens sarcoglycan, epsilon (SGCE), mRNA [NM_003919]	NM_003919
A_23_P167201	8.52E-05	NM_016955	NM_016955	Homo sapiens soluble liver antigen/liver pancreas antigen (SLA/LP), transcript variant 1, mRNA [NM_016955]	NM_016955
A_23_P132115	8.53E-05	NM_173354	NM_173354	Homo sapiens SNF1-like kinase (SNF1LK), mRNA [NM_173354]	NM_173354
A_23_P159775	8.53E-05	NM_021990	NM_021990	Homo sapiens gamma-aminobutyric acid (GABA) A receptor, epsilon (GABRE), transcript variant 4, mRNA [NM_021990]	NM_021990
A_32_P62997	8.53E-05	NM_018492	NM_018492	Homo sapiens PDZ binding kinase (PBK), mRNA [NM_018492]	NM_018492
A_23_P212034	8.55E-05	AL831922	AL831922	Homo sapiens mRNA; cDNA DKFZp761P0818 (from clone DKFZp761P0818). [AL831922]	
A_24_P128312	8.60E-05	NM_007135	NM_007135	Homo sapiens zinc finger protein 79 (pT7) (ZNF79), mRNA [NM_007135]	NM_007135
A_23_P213085	8.61E-05	AK075364	AK075364	Homo sapiens cDNA PSEC0051 fis, clone NT2RP2000168. [AK075364]	
A_24_P762886	8.61E-05	AB007954	AB007954	Homo sapiens mRNA, chromosome 1 specific transcript KIAA0485. [AB007954]	
A_23_P70915	8.61E-05	NM_032831	NM_032831	Homo sapiens chromosome 7 open reading frame 19 (C7orf19), mRNA [NM_032831]	NM_032831
A_32_P155247	8.61E-05	NM_000146	NM_000146	Homo sapiens ferritin, light polypeptide (FTL), mRNA [NM_000146]	NM_000146
A_24_P88870	8.62E-05	NM_033169	NM_033169	Homo sapiens UDP-Gal:betaGlcNAc beta 1,3-galactosyltransferase, polypeptide 3 (B3GALT3), transcript variant 4, mRNA [NM_033169]	NM_033169

A_23_P156209	8.62E-05	NM_033414	NM_033414	Homo sapiens zinc finger protein 622 (ZNF622), mRNA [NM_033414]	NM_033414
A_24_P334130	8.65E-05	NM_054034	NM_054034	Homo sapiens fibronectin 1 (FN1), transcript variant 7, mRNA [NM_054034]	NM_054034
A_23_P79661	8.68E-05	BC005078	BC005078	Homo sapiens cDNA clone IMAGE:3609481, complete cds. [BC005078]	
A_23_P122915	8.70E-05	NM_015379	NM_015379	Homo sapiens brain protein I3 (BRI3), mRNA [NM_015379]	NM_015379
A_23_P88404	8.72E-05	NM_003239	NM_003239	Homo sapiens transforming growth factor, beta 3 (TGFB3), mRNA [NM_003239]	NM_003239
A_24_P205230	8.75E-05	NM_001004333	NM_001004333	Homo sapiens similar to DNA segment, Chr 11, Brigham & Womens Genetics 0434 expressed (MGC71993), mRNA [NM_001004333]	NM_001004333
A_23_P29248	8.79E-05	NM_003312	NM_003312	Homo sapiens thiosulfate sulfurtransferase (rhodanese) (TST), nuclear gene encoding mitochondrial protein, mRNA [NM_003312]	NM_003312
A_23_P21207	8.81E-05	NM_003335	NM_003335	Homo sapiens ubiquitin-activating enzyme E1-like (UBE1L), mRNA [NM_003335]	NM_003335
A_23_P13183	8.83E-05	NM_000401	NM_000401	Homo sapiens exostoses (multiple) 2 (EXT2), transcript variant 1, mRNA [NM_000401]	NM_000401
A_23_P379630	8.85E-05	NM_138570	NM_138570	Homo sapiens hypothetical protein MGC15523 (MGC15523), mRNA [NM_138570]	NM_138570
A_23_P368886	8.87E-05	NM_175856	NM_175856	Homo sapiens chondroitin sulfate synthase 3 (CSS3), mRNA [NM_175856]	NM_175856
A_23_P113825	8.87E-05	NM_144653	NM_144653	Homo sapiens BTB (POZ) domain containing 14A (BTBD14A), mRNA [NM_144653]	NM_144653
A_23_P365719	8.87E-05	NM_003190	NM_003190	Homo sapiens TAP binding protein (tapasin) (TAPBP), transcript variant 1, mRNA [NM_003190]	NM_003190
A_23_P59700	8.88E-05	NM_005868	NM_005868	Homo sapiens BET1 homolog (S. cerevisiae) (BET1), mRNA [NM_005868]	NM_005868
A_23_P153311	8.88E-05	NM_003331	NM_003331	Homo sapiens tyrosine kinase 2 (TYK2), mRNA [NM_003331]	NM_003331
A_23_P39718	8.88E-05	NM_005102	NM_005102	Homo sapiens fasciculation and elongation protein zeta 2 (zygin II) (FEZ2), mRNA [NM_005102]	NM_005102
A_32_P128023	8.89E-05	AK125351	AK125351	Homo sapiens cDNA FLJ43361 fis, clone NT2RP7015512. [AK125351]	
A_23_P256172	8.92E-05	NM_199417	NM_199417	Homo sapiens nuclear protein E3-3 (DKFZP564J0123), transcript variant 5, mRNA [NM_199417]	NM_199417
A_23_P376211	8.94E-05	NM_007270	NM_007270	Homo sapiens FK506 binding protein 9, 63 kDa (FKBP9), mRNA [NM_007270]	NM_007270
A_24_P167473	8.94E-05	NM_005719	NM_005719	Homo sapiens actin related protein 2/3 complex, subunit 3, 21kDa (ARPC3), mRNA [NM_005719]	NM_005719
A_23_P395172	8.98E-05	AK095685	AK095685	Homo sapiens cDNA FLJ38366 fis, clone FEBRA2001134. [AK095685]	
A_23_P67432	8.99E-05	NM_152478	NM_152478	Homo sapiens zinc finger protein 583 (ZNF583), mRNA [NM_152478]	NM_152478
A_23_P168974	8.99E-05	AK128645	AK128645	Homo sapiens cDNA FLJ46804 fis, clone TRACH3032570, highly similar to Homo sapiens syndecan binding protein (syntenin) (SDCBP). [AK128645]	
A_24_P88554	8.99E-05	NM_003846	NM_003846	Homo sapiens peroxisomal biogenesis factor 11B (PEX11B), mRNA [NM_003846]	NM_003846
A_23_P250462	9.00E-05	NM_001183	NM_001183	Homo sapiens ATPase, H+ transporting, lysosomal accessory protein 1 (ATP6AP1), mRNA [NM_001183]	NM_001183
A_23_P108932	9.01E-05	NR_002229	NR_002229	Homo sapiens ribosomal protein L23a pseudogene 13 (RPL23AP13) on chromosome 2 [NR_002229]	NR_002229
A_24_P240732	9.01E-05	NM_017813	NM_017813	Homo sapiens myo-inositol monophosphatase A3 (IMPA3), mRNA [NM_017813]	NM_017813
A_23_P216708	9.01E-05	NM_018339	NM_018339	Homo sapiens riboflavin kinase (RFK), mRNA [NM_018339]	NM_018339
A_23_P358555	9.01E-05	NM_032356	NM_032356	Homo sapiens hypothetical protein MGC14151 (MGC14151), mRNA [NM_032356]	NM_032356
A_23_P62764	9.03E-05	NM_024296	NM_024296	Homo sapiens hypothetical protein MGC1203 (MGC1203), mRNA [NM_024296]	NM_024296
A_23_P146922	9.04E-05	NM_000820	NM_000820	Homo sapiens growth arrest-specific 6 (GAS6), mRNA [NM_000820]	NM_000820
A_23_P28084	9.05E-05	ENST0000034358 7		Homo sapiens syntaxin 10, mRNA (cDNA clone MGC:19625 IMAGE:4125873), complete cds. [BC017237]	
A_23_P139476	9.10E-05	NM_001780	NM_001780	Homo sapiens CD63 antigen (melanoma 1 antigen) (CD63), mRNA [NM_001780]	NM_001780
A_23_P395566	9.11E-05	NM_024735	NM_024735	Homo sapiens F-box protein 31 (FBXO31), mRNA [NM_024735]	NM_024735
A_23_P67299	9.18E-05	NM_020812	NM_020812	Homo sapiens dedicator of cytokinesis 6 (DOCK6), mRNA [NM_020812]	NM_020812
A_23_P154108	9.18E-05	NM_001378	NM_001378	Homo sapiens dynein, cytoplasmic, intermediate polypeptide 2 (DNCI2), mRNA [NM_001378]	NM_001378
A_23_P29504	9.24E-05	THC2337271		BC008589 SENP5 protein {Homo sapiens;} , partial (10%) [THC2337271]	
A_23_P80570	9.26E-05	NM_001086	NM_001086	Homo sapiens arylacetamide deacetylase (esterase) (AADAC), mRNA [NM_001086]	NM_001086
A_23_P37441	9.26E-05	NM_004048	NM_004048	Homo sapiens beta-2-microglobulin (B2M), mRNA [NM_004048]	NM_004048
A_24_P45446	9.29E-05	NM_052941	NM_052941	Homo sapiens guanylate binding protein 4 (GBP4), mRNA [NM_052941]	NM_052941
A_23_P170719	9.33E-05	A_23_P170719			
A_24_P643587	9.34E-05	NM_024095	NM_024095	Homo sapiens ankyrin repeat and SOCS box-containing 8 (ASB8), mRNA [NM_024095]	NM_024095
A_23_P58207	9.34E-05	NM_021159	NM_021159	Homo sapiens RAP1, GTP-GDP dissociation stimulator 1 (RAP1GDS1), mRNA [NM_021159]	NM_021159
A_24_P132518	9.43E-05	NM_001556	NM_001556	Homo sapiens inhibitor of kappa light polypeptide gene enhancer in B-cells, kinase beta (IKBKB), mRNA [NM_001556]	NM_001556
A_23_P157038	9.43E-05	NM_152755	NM_152755	Homo sapiens hypothetical protein MGC40499 (MGC40499), mRNA [NM_152755]	NM_152755

A_24_P413920	9.50E-05	NM_145175	NM_145175	Homo sapiens family with sequence similarity 84, member A (FAM84A), mRNA [NM_145175]	NM_145175
A_23_P41145	9.55E-05	NM_138805	NM_138805	Homo sapiens family with sequence similarity 3, member D (FAM3D), mRNA [NM_138805]	NM_138805
A_32_P140656	9.55E-05	CR621148	CR621148	full-length cDNA clone CS0DC029YJ02 of Neuroblastoma Cot 25-normalized of Homo sapiens (human). [CR621148]	
A_24_P160104	9.56E-05	NM_018943	NM_018943	Homo sapiens tubulin, alpha 8 (TUBA8), mRNA [NM_018943]	NM_018943
A_23_P117416	9.57E-05	NM_145685	NM_145685	Homo sapiens BRF1 homolog, subunit of RNA polymerase III transcription initiation factor IIIB (S. cerevisiae) (BRF1), transcript variant 3, mRNA [NM_145685]	NM_145685
A_23_P252052	9.57E-05	NM_182909	NM_182909	Homo sapiens downregulated in ovarian cancer 1 (DOC1), transcript variant 1, mRNA [NM_182909]	NM_182909
A_23_P209669	9.58E-05	NM_201266	NM_201266	Homo sapiens neuropilin 2 (NRP2), transcript variant 1, mRNA [NM_201266]	NM_201266
A_24_P186354	9.58E-05	A_24_P186354			
A_23_P259521	9.59E-05	NM_018268	NM_018268	Homo sapiens WD repeat domain 41 (WDR41), mRNA [NM_018268]	NM_018268
A_23_P111672	9.60E-05	NM_152829	NM_152829	Homo sapiens testis derived transcript (3 LIM domains) (TES), transcript variant 2, mRNA [NM_152829]	NM_152829
A_32_P3113	9.61E-05	AF070529	AF070529	Homo sapiens clone 24525 mRNA sequence. [AF070529]	
A_24_P326082	9.64E-05	NM_005516	NM_005516	Homo sapiens major histocompatibility complex, class I, E (HLA-E), mRNA [NM_005516]	NM_005516
A_23_P253052	9.65E-05	NM_031462	NM_031462	Homo sapiens CD99 antigen-like 2 (CD99L2), transcript variant 1, mRNA [NM_031462]	NM_031462
A_23_P124570	9.66E-05	NM_005246	NM_005246	Homo sapiens fer (fps/fes related) tyrosine kinase (phosphoprotein NCP94) (FER), mRNA [NM_005246]	NM_005246
A_23_P76914	9.66E-05	NM_005982	NM_005982	Homo sapiens sine oculis homeobox homolog 1 (Drosophila) (SIX1), mRNA [NM_005982]	NM_005982
A_23_P388200	9.67E-05	NM_153705	NM_153705	Homo sapiens KDEL (Lys-Asp-Glu-Leu) containing 2 (KDELC2), mRNA [NM_153705]	NM_153705
A_23_P206177	9.73E-05	NM_003825	NM_003825	Homo sapiens synaptosomal-associated protein, 23kDa (SNAP23), transcript variant 1, mRNA [NM_003825]	NM_003825
A_24_P140391	9.75E-05	NM_032906	NM_032906	Homo sapiens hypothetical protein MGC14156 (MGC14156), mRNA [NM_032906]	NM_032906
A_23_P79836	9.75E-05	NM_006811	NM_006811	Homo sapiens tumor differentially expressed 1 (TDE1), transcript variant 1, mRNA [NM_006811]	NM_006811
A_23_P255827	9.77E-05	NM_021631	NM_021631	Homo sapiens apoptosis inhibitor (FKSG2), mRNA [NM_021631]	NM_021631
A_24_P196175	9.77E-05	A_24_P196175			
A_24_P4816	9.77E-05	NM_031412	NM_031412	Homo sapiens GABA(A) receptor-associated protein like 1 (GABARAPL1), mRNA [NM_031412]	NM_031412
A_24_P47467	9.77E-05	NM_024092	NM_024092	Homo sapiens hypothetical protein MGC5508 (MGC5508), mRNA [NM_024092]	NM_024092
A_23_P393620	9.77E-05	NM_006528	NM_006528	Homo sapiens tissue factor pathway inhibitor 2 (TFPI2), mRNA [NM_006528]	NM_006528
A_23_P7144	9.77E-05	NM_001511	NM_001511	Homo sapiens chemokine (C-X-C motif) ligand 1 (melanoma growth stimulating activity, alpha) (CXCL1), mRNA [NM_001511]	NM_001511
A_24_P8371	9.89E-05	ENST00000329078		Homo sapiens, Similar to spinster-like protein, clone IMAGE:4814561, mRNA, partial cds. [BC041772]	
A_23_P150365	9.89E-05	NM_015523	NM_015523	Homo sapiens small fragment nuclease (DKFZP566E144), mRNA [NM_015523]	NM_015523
A_24_P270424	9.90E-05	AK124946	AK124946	Homo sapiens cDNA FLJ42956 fis, clone BRSTN2009899. [AK124946]	
A_24_P214506	9.90E-05	NM_183352	NM_183352	Homo sapiens SEC13-like 1 (S. cerevisiae) (SEC13L1), transcript variant 2, mRNA [NM_183352]	NM_183352
A_32_P95397	9.92E-05	NM_002211	NM_002211	Homo sapiens integrin, beta 1 (fibronectin receptor, beta polypeptide, antigen CD29 includes MDF2, MSK12) (ITGB1), transcript variant 1A, mRNA [NM_002211]	NM_002211
A_24_P200942	9.95E-05	NM_030935	NM_030935	Homo sapiens TSC22 domain family, member 4 (TSC22D4), mRNA [NM_030935]	NM_030935
A_23_P129704	9.95E-05	NM_018975	NM_018975	Homo sapiens telomeric repeat binding factor 2, interacting protein (TERF2IP), mRNA [NM_018975]	NM_018975
A_23_P35399	9.98E-05	NM_145869	NM_145869	Homo sapiens annexin A11 (ANXA11), transcript variant c, mRNA [NM_145869]	NM_145869
A_23_P392897	0.0001	A_23_P392897			
A_23_P338233	0.0001	NM_181708	NM_181708	Homo sapiens hypothetical protein LOC144233 (LOC144233), mRNA [NM_181708]	NM_181708
A_24_P32766	0.0001	A_24_P32766			
A_23_P360167	0.0001	NM_006400	NM_006400	Homo sapiens dynactin 2 (p50) (DCTN2), mRNA [NM_006400]	NM_006400
A_24_P942636	0.000101	NM_014423	NM_014423	Homo sapiens AF4/FMR2 family, member 4 (AFF4), mRNA [NM_014423]	NM_014423
A_24_P870509	0.000101	AF086261	AF086261	Homo sapiens full length insert cDNA clone ZD42A11. [AF086261]	
A_24_P139742	0.000101	NM_022787	NM_022787	Homo sapiens nicotinamide nucleotide adenylyltransferase 1 (NMNAT1), mRNA [NM_022787]	NM_022787
A_23_P316511	0.000101	NM_002146	NM_002146	Homo sapiens homeo box B3 (HOXB3), mRNA [NM_002146]	NM_002146
A_32_P149492	0.000101	AF419616	AF419616	Homo sapiens clone DKFZp564A057 AG02 mRNA, partial cds. [AF419616]	XM_496399
A_24_P135444	0.000101	NM_001144	NM_001144	Homo sapiens autocrine motility factor receptor (AMFR), transcript variant 1, mRNA [NM_001144]	NM_001144
A_23_P110403	0.000101	NM_014476	NM_014476	Homo sapiens PDZ and LIM domain 3 (PDLIM3), mRNA [NM_014476]	NM_014476

A_23_P89941	0.000101	NM_001800	NM_001800	Homo sapiens cyclin-dependent kinase inhibitor 2D (p19, inhibits CDK4) (CDKN2D), transcript variant 1, mRNA [NM_001800]	NM_001800
A_24_P44514	0.000101	NM_006384	NM_006384	Homo sapiens calcium and integrin binding 1 (calmyrin) (CIB1), mRNA [NM_006384]	NM_006384
A_24_P294842	0.000102	NM_000332	NM_000332	Homo sapiens ataxin 1 (ATXN1), mRNA [NM_000332]	NM_000332
A_23_P129801	0.000102	NM_006822	NM_006822	Homo sapiens RAB40B, member RAS oncogene family (RAB40B), mRNA [NM_006822]	NM_006822
A_23_P144877	0.000102	NM_004045	NM_004045	Homo sapiens ATX1 antioxidant protein 1 homolog (yeast) (ATOX1), mRNA [NM_004045]	NM_004045
A_23_P28652	0.000102	NM_016085	NM_016085	Homo sapiens chromosome 2 open reading frame 28 (C2orf28), transcript variant 1, mRNA [NM_016085]	NM_016085
A_23_P378722	0.000102	NM_002970	NM_002970	Homo sapiens spermidine/spermine N1-acetyltransferase (SAT), mRNA [NM_002970]	NM_002970
A_23_P131463	0.000103	NM_016552	NM_016552	Homo sapiens ankyrin repeat and MYND domain containing 1 (ANKMY1), transcript variant 1, mRNA [NM_016552]	NM_016552
A_23_P165707	0.000103	NM_174898	NM_174898	Homo sapiens hypothetical protein LOC129530 (LOC129530), mRNA [NM_174898]	NM_174898
A_23_P37505	0.000103	NM_130810	NM_130810	Homo sapiens dyslexia susceptibility 1 candidate 1 (DYX1C1), mRNA [NM_130810]	NM_130810
A_23_P201459	0.000103	NM_022873	NM_022873	Homo sapiens interferon, alpha-inducible protein (clone IFI-6-16) (G1P3), transcript variant 3, mRNA [NM_022873]	NM_022873
A_24_P126741	0.000103	ENST00000309178		PREDICTED: Homo sapiens similar to Glutaredoxin (Thioltransferase) (TTase) (LOC441113), mRNA [XM_496782]	XM_496782
A_23_P311468	0.000103	NM_182498	NM_182498	Homo sapiens hypothetical protein MGC51082 (MGC51082), mRNA [NM_182498]	NM_182498
A_23_P52806	0.000103	NM_012104	NM_012104	Homo sapiens beta-site APP-cleaving enzyme 1 (BACE1), transcript variant a, mRNA [NM_012104]	NM_012104
A_24_P854643	0.000104	AL833114	AL833114	Homo sapiens mRNA; cDNA DKFZp313L2229 (from clone DKFZp313L2229). [AL833114]	
A_23_P34548	0.000104	NM_006642	NM_006642	Homo sapiens serologically defined colon cancer antigen 8 (SDCCAG8), mRNA [NM_006642]	NM_006642
A_24_P73075	0.000104	NM_017868	NM_017868	Homo sapiens tetratricopeptide repeat domain 12 (TTC12), mRNA [NM_017868]	NM_017868
A_23_P7706	0.000104	NM_198321	NM_198321	Homo sapiens UDP-N-acetyl-alpha-D-galactosamine:polypeptide N-acetylgalactosaminyltransferase 10 (GalNAc-T10) (GALNT10), transcript variant 1, mRNA [NM_198321]	NM_198321
A_23_P255436	0.000104	NM_000487	NM_000487	Homo sapiens arylsulfatase A (ARSA), mRNA [NM_000487]	NM_000487
A_32_P194563	0.000104	THC2281660			
A_24_P202567	0.000104	NM_025194	NM_025194	Homo sapiens inositol 1,4,5-trisphosphate 3-kinase C (ITPKC), mRNA [NM_025194]	NM_025194
A_23_P85392	0.000104	NM_007033	NM_007033	Homo sapiens RER1 retention in endoplasmic reticulum 1 homolog (S. cerevisiae) (RER1), mRNA [NM_007033]	NM_007033
A_32_P156564	0.000105	THC2312748			
A_23_P201764	0.000105	NM_024887	NM_024887	Homo sapiens dehydrodolichyl diphosphate synthase (DHDDS), transcript variant 1, mRNA [NM_024887]	NM_024887
A_23_P257743	0.000105	NM_003028	NM_003028	Homo sapiens Src homology 2 domain containing adaptor protein B (SHB), mRNA [NM_003028]	NM_003028
A_23_P8196	0.000105	CR591849	CR591849	full-length cDNA clone CS0DC003YO01 of Neuroblastoma Cot 25-normalized of Homo sapiens (human). [CR591849]	
A_23_P46844	0.000105	NM_030912	NM_030912	Homo sapiens tripartite motif-containing 8 (TRIM8), mRNA [NM_030912]	NM_030912
A_23_P52286	0.000105	NM_015448	NM_015448	Homo sapiens deleted in a mouse model of primary ciliary dyskinesia (RP11-529I10.4), mRNA [NM_015448]	NM_015448
A_23_P201338	0.000105	NM_016176	NM_016176	Homo sapiens stromal cell derived factor 4 (SDF4), mRNA [NM_016176]	NM_016176
A_23_P140146	0.000105	NM_032036	NM_032036	Homo sapiens family with sequence similarity 14, member A (FAM14A), mRNA [NM_032036]	NM_032036
A_24_P377144	0.000106	NM_058172	NM_058172	Homo sapiens anthrax toxin receptor 2 (ANTXR2), mRNA [NM_058172]	NM_058172
A_23_P129476	0.000106	NM_004913	NM_004913	Homo sapiens chromosome 16 open reading frame 7 (C16orf7), mRNA [NM_004913]	NM_004913
A_23_P81087	0.000106	NM_020395	NM_020395	Homo sapiens PHD finger protein 22 (PHF22), mRNA [NM_020395]	NM_020395
A_23_P39971	0.000106	NM_015701	NM_015701	Homo sapiens chromosome 2 open reading frame 30 (C2orf30), mRNA [NM_015701]	NM_015701
A_23_P140738	0.000106	NM_014745	NM_014745	Homo sapiens family with sequence similarity 38, member A (FAM38A), mRNA [NM_014745]	NM_014745
A_23_P23924	0.000106	NM_001748	NM_001748	Homo sapiens calpain 2, (m/II) large subunit (CAPN2), mRNA [NM_001748]	NM_001748
A_24_P383480	0.000107	NM_000313	NM_000313	Homo sapiens protein S (alpha) (PROS1), mRNA [NM_000313]	NM_000313
A_23_P90125	0.000107	ENST00000253720		Homo sapiens aspartyl protease 3 mRNA, partial cds. [AF200344]	XR_000169
A_32_P121234	0.000107	A_32_P121234			
A_23_P153529	0.000107	NM_017636	NM_017636	Homo sapiens transient receptor potential cation channel, subfamily M, member 4 (TRPM4), mRNA [NM_017636]	NM_017636
A_23_P120557	0.000107	NM_017714	NM_017714	Homo sapiens chromosome 20 open reading frame 13 (C20orf13), mRNA [NM_017714]	NM_017714
A_24_P416055	0.000107	NM_031452	NM_031452	Homo sapiens hypothetical LOC83640 (MGC2560), mRNA [NM_031452]	NM_031452
A_23_P14948	0.000107	NM_003791	NM_003791	Homo sapiens membrane-bound transcription factor protease, site 1 (MBTPS1), transcript variant 1, mRNA [NM_003791]	NM_003791

A_24_P318967	0.000107	NM_003681	NM_003681	Homo sapiens pyridoxal (pyridoxine, vitamin B6) kinase (PDXK), mRNA [NM_003681]	NM_003681
A_24_P341006	0.000108	A_24_P341006			
A_23_P15394	0.000108	NM_001251	NM_001251	Homo sapiens CD68 antigen (CD68), mRNA [NM_001251]	NM_001251
A_24_P388570	0.000108	NM_032778	NM_032778	Homo sapiens MYC induced nuclear antigen (MINA), transcript variant 2, mRNA [NM_032778]	NM_032778
A_24_P340066	0.000108	NM_001421	NM_001421	Homo sapiens E74-like factor 4 (ets domain transcription factor) (ELF4), mRNA [NM_001421]	NM_001421
A_23_P124022	0.000108	NM_032286	NM_032286	Homo sapiens hypothetical protein MGC5309 (TRG20), mRNA [NM_032286]	NM_032286
A_23_P150403	0.000109	NM_021729	NM_021729	Homo sapiens vacuolar protein sorting 11 (yeast) (VPS11), mRNA [NM_021729]	NM_021729
A_23_P124024	0.000109	NM_032286	NM_032286	Homo sapiens hypothetical protein MGC5309 (TRG20), mRNA [NM_032286]	NM_032286
A_23_P50020	0.000109	NM_018714	NM_018714	Homo sapiens component of oligomeric golgi complex 1 (COG1), mRNA [NM_018714]	NM_018714
A_23_P103968	0.000109	NM_012067	NM_012067	Homo sapiens aldo-keto reductase family 7, member A3 (aflatoxin aldehyde reductase) (AKR7A3), mRNA [NM_012067]	NM_012067
A_23_P55251	0.000109	NM_002204	NM_002204	Homo sapiens integrin, alpha 3 (antigen CD49C, alpha 3 subunit of VLA-3 receptor) (ITGA3), transcript variant a, mRNA [NM_002204]	NM_002204
A_32_P85999	0.000109	NM_001257	NM_001257	Homo sapiens cadherin 13, H-cadherin (heart) (CDH13), mRNA [NM_001257]	NM_001257
A_23_P99186	0.00011	NM_183415	NM_183415	Homo sapiens ubiquitin protein ligase E3B (UBE3B), transcript variant 3, mRNA [NM_183415]	NM_183415
A_24_P354451	0.00011	THC2311186		CAO3_HUMAN (O15254) Acyl-coenzyme A oxidase 3, peroxisomal (Pristanoyl-CoA oxidase) , partial (13%) [THC2311186]	
A_23_P116809	0.00011	NM_016057	NM_016057	Homo sapiens coatomer protein complex, subunit zeta 1 (COPZ1), mRNA [NM_016057]	NM_016057
A_24_P101859	0.00011	XM_373252	XM_373252	PREDICTED: Homo sapiens similar to coiled-coil-helix-coiled-coil-helix domain containing 2; 16.7kD protein; chromosome 7 open reading frame 17 (LOC392221), mRNA [XM_373252]	XM_373252
A_23_P17030	0.00011	NM_018226	NM_018226	Homo sapiens arginyl aminopeptidase (aminopeptidase B)-like 1 (RNPEPL1), mRNA [NM_018226]	NM_018226
A_23_P404259	0.000111	NM_001008397	NM_001008397	Homo sapiens similar to RIKEN cDNA 2310016C16 (LOC493869), mRNA [NM_001008397]	NM_001008397
A_23_P218774	0.000111	NM_002872	NM_002872	Homo sapiens ras-related C3 botulinum toxin substrate 2 (rho family, small GTP binding protein Rac2) (RAC2), mRNA [NM_002872]	NM_002872
A_23_P76992	0.000112	NM_002632	NM_002632	Homo sapiens placental growth factor, vascular endothelial growth factor-related protein (PGF), mRNA [NM_002632]	NM_002632
A_23_P90752	0.000112	NM_015909	NM_015909	Homo sapiens neuroblastoma-amplified protein (NAG), mRNA [NM_015909]	NM_015909
A_23_P13364	0.000112	NM_005013	NM_005013	Homo sapiens nucleobindin 2 (NUCB2), mRNA [NM_005013]	NM_005013
A_32_P179258	0.000113	NM_020673	NM_020673	Homo sapiens RAB22A, member RAS oncogene family (RAB22A), mRNA [NM_020673]	NM_020673
A_32_P181222	0.000113	NM_002247	NM_002247	Homo sapiens potassium large conductance calcium-activated channel, subfamily M, alpha member 1 (KCNMA1), transcript variant 2, mRNA [NM_002247]	NM_002247
A_23_P129144	0.000113	NM_152451	NM_152451	Homo sapiens GRINL1A combined protein (Gcom1), transcript variant 8, mRNA [NM_152451]	NM_152451
A_24_P108863	0.000113	NM_006746	NM_006746	Homo sapiens sex comb on midleg-like 1 (Drosophila) (SCML1), mRNA [NM_006746]	NM_006746
A_32_P122940	0.000113	AL390181	AL390181	Homo sapiens mRNA; cDNA DKFZp547J125 (from clone DKFZp547J125). [AL390181]	
A_23_P340890	0.000113	CR601567	CR601567	full-length cDNA clone CS0DN003YI06 of Adult brain of Homo sapiens (human). [CR601567]	
A_32_P89837	0.000113	AF110908	AF110908	Homo sapiens TNF-receptor associated factor-3 (TRAF-3) mRNA, partial cds; and 3'UTR. [AF110908]	
A_23_P307940	0.000113	NM_006136	NM_006136	Homo sapiens capping protein (actin filament) muscle Z-line, alpha 2 (CAPZA2), mRNA [NM_006136]	NM_006136
A_23_P251695	0.000113	NM_013248	NM_013248	Homo sapiens NTF2-like export factor 1 (NXT1), mRNA [NM_013248]	NM_013248
A_24_P325333	0.000114	NM_002600	NM_002600	Homo sapiens phosphodiesterase 4B, cAMP-specific (phosphodiesterase E4 dunce homolog, Drosophila) (PDE4B), mRNA [NM_002600]	NM_002600
A_23_P82047	0.000114	THC2273080			
A_23_P22200	0.000114	NM_024095	NM_024095	Homo sapiens ankyrin repeat and SOCS box-containing 8 (ASB8), mRNA [NM_024095]	NM_024095
A_23_P75903	0.000114	NM_019009	NM_019009	Homo sapiens toll interacting protein (TOLLIP), mRNA [NM_019009]	NM_019009
A_24_P923142	0.000114	BC020784	BC020784	Homo sapiens similar to RIKEN cDNA 1200014N16 gene, mRNA (cDNA clone IMAGE:4773508), complete cds. [BC020784]	
A_24_P164731	0.000114	NM_006858	NM_006858	Homo sapiens transmembrane emp24 domain containing 1 (TMED1), mRNA [NM_006858]	NM_006858
A_23_P146512	0.000114	NM_016548	NM_016548	Homo sapiens golgi phosphoprotein 2 (GOLPH2), transcript variant 1, mRNA [NM_016548]	NM_016548
A_23_P143676	0.000114	A_23_P143676			
A_23_P353097	0.000114	NM_178121	NM_178121	Homo sapiens HBV pre-s2 binding protein 1 (SBP1), mRNA [NM_178121]	NM_178121
A_23_P12680	0.000114	NM_002778	NM_002778	Homo sapiens prosaposin (variant Gaucher disease and variant metachromatic leukodystrophy) (PSAP), mRNA [NM_002778]	NM_002778
A_32_P93792	0.000115	A_32_P93792			
A_23_P170453	0.000115	NM_001900	NM_001900	Homo sapiens cystatin D (CST5), mRNA [NM_001900]	NM_001900

A_24_P322635	0.000115	NM_182764	NM_182764	Homo sapiens engulfment and cell motility 2 (ced-12 homolog, C. elegans) (ELMO2), transcript variant 3, mRNA [NM_182764]	NM_182764
A_23_P105592	0.000115	BC016140	BC016140	Homo sapiens mevalonate kinase (mevalonic aciduria), mRNA (cDNA clone MGC:9001 IMAGE:3921543), complete cds. [BC016140]	
A_32_P221590	0.000116	A_32_P221590			
A_23_P213319	0.000116	NM_197941	NM_197941	Homo sapiens a disintegrin-like and metalloprotease (reprolysin type) with thrombospondin type 1 motif, 6 (ADAMTS6), mRNA [NM_197941]	NM_197941
A_24_P282578	0.000116	NM_003145	NM_003145	Homo sapiens signal sequence receptor, beta (translocon-associated protein beta) (SSR2), mRNA [NM_003145]	NM_003145
A_23_P356965	0.000117	ENST00000327026		Homo sapiens mRNA for FLJ00130 protein. [AK074059]	
A_32_P184888	0.000117	THC2343253			
A_24_P19337	0.000117	NM_015338	NM_015338	Homo sapiens additional sex combs like 1 (Drosophila) (ASXL1), mRNA [NM_015338]	NM_015338
A_23_P54929	0.000117	NM_020424	NM_020424	Homo sapiens hypothetical protein A-211C6.1 (LOC57149), mRNA [NM_020424]	NM_020424
A_23_P217367	0.000117	NM_178271	NM_178271	Homo sapiens APG4 autophagy 4 homolog A (S. cerevisiae) (APG4A), transcript variant 3, mRNA [NM_178271]	NM_178271
A_23_P96165	0.000118	NM_024650	NM_024650	Homo sapiens hypothetical protein FLJ22531 (FLJ22531), mRNA [NM_024650]	NM_024650
A_24_P933704	0.000118	NM_138822	NM_138822	Homo sapiens peptidylglycine alpha-amidating monooxygenase (PAM), transcript variant 4, mRNA [NM_138822]	NM_138822
A_24_P379649	0.000118	NR_002229	NR_002229	Homo sapiens ribosomal protein L23a pseudogene 13 (RPL23AP13) on chromosome 2 [NR_002229]	NR_002229
A_24_P251841	0.000118	ENST00000274031		Homo sapiens mRNA for KIAA1717 protein, partial cds. [AB051504]	
A_24_P417996	0.000118	A_24_P417996			
A_32_P194372	0.000119	AK129547	AK129547	Homo sapiens cDNA FLJ26036 fis, clone PRS00145. [AK129547]	
A_24_P404033	0.000119	NM_182616	NM_182616	Homo sapiens hypothetical protein MGC61550 (MGC61550), mRNA [NM_182616]	NM_182616
A_23_P209944	0.000119	NM_017750	NM_017750	Homo sapiens all-trans-13,14-dihydroretinol saturase (RetSat), mRNA [NM_017750]	NM_017750
A_23_P79622	0.000119	NM_016105	NM_016105	Homo sapiens FK506 binding protein 7 (FKBP7), transcript variant 1, mRNA [NM_016105]	NM_016105
A_24_P612441	0.000119	CR591566	CR591566	full-length cDNA clone CS0DL004YB03 of B cells (Ramos cell line) Cot 25-normalized of Homo sapiens (human). [CR591566]	
A_24_P627415	0.00012	THC2268216		NCB2_HUMAN (P80303) Nucleobindin 2 precursor (DNA-binding protein NEFA), partial (49%) [THC2268216]	
A_23_P151820	0.00012	NM_024832	NM_024832	Homo sapiens Ras and Rab interactor 3 (RIN3), mRNA [NM_024832]	NM_024832
A_24_P551067	0.00012	CR603951	CR603951	full-length cDNA clone CS0DM011YC22 of Fetal liver of Homo sapiens (human). [CR603951]	XM_499585
A_24_P148521	0.00012	NM_022152	NM_022152	Homo sapiens PP1201 protein (PP1201), mRNA [NM_022152]	NM_022152
A_24_P111912	0.00012	NM_032042	NM_032042	Homo sapiens hypothetical protein DKFZp564D172 (DKFZP564D172), mRNA [NM_032042]	NM_032042
A_24_P274842	0.00012	NM_007233	NM_007233	Homo sapiens TP53 activated protein 1 (TP53AP1), mRNA [NM_007233]	NM_007233
A_23_P358628	0.00012	ENST00000310974		Homo sapiens mRNA for KIAA0877 protein, partial cds. [AB020684]	XM_371891
A_23_P216549	0.00012	NM_014806	NM_014806	Homo sapiens RUN and SH3 domain containing 2 (RUSC2), mRNA [NM_014806]	NM_014806
A_23_P332509	0.00012	NM_015909	NM_015909	Homo sapiens neuroblastoma-amplified protein (NAG), mRNA [NM_015909]	NM_015909
A_23_P97394	0.00012	NM_003567	NM_003567	Homo sapiens breast cancer anti-estrogen resistance 3 (BCAR3), mRNA [NM_003567]	NM_003567
A_23_P54846	0.00012	NM_014685	NM_014685	Homo sapiens homocysteine-inducible, endoplasmic reticulum stress-inducible, ubiquitin-like domain member 1 (HERPUD1), transcript variant 1, mRNA [NM_014685]	NM_014685
A_24_P918044	0.000121	AK023572	AK023572	Homo sapiens cDNA FLJ13510 fis, clone PLACE1005146. [AK023572]	
A_23_P135190	0.000121	NM_006266	NM_006266	Homo sapiens ral guanine nucleotide dissociation stimulator (RALGDS), mRNA [NM_006266]	NM_006266
A_23_P14302	0.000121	NM_024633	NM_024633	Homo sapiens chromosome 14 open reading frame 139 (C14orf139), mRNA [NM_024633]	NM_024633
A_24_P410587	0.000121	NM_153032	NM_153032	Homo sapiens hypothetical protein FLJ32065 (FLJ32065), mRNA [NM_153032]	NM_153032
A_23_P426501	0.000122	BC007329	BC007329	Homo sapiens transducin-like enhancer of split 6 (E(sp1) homolog, Drosophila), mRNA (cDNA clone IMAGE:3687767). [BC007329]	
A_23_P335661	0.000122	AB028976	AB028976	Homo sapiens mRNA for KIAA1053 protein, partial cds. [AB028976]	
A_23_P141764	0.000122	NM_003826	NM_003826	Homo sapiens N-ethylmaleimide-sensitive factor attachment protein, gamma (NAPG), mRNA [NM_003826]	NM_003826
A_23_P18966	0.000122	NM_001017973	NM_001017973	Homo sapiens procollagen-proline, 2-oxoglutarate 4-dioxygenase (proline 4-hydroxylase), alpha polypeptide II (P4HA2), transcript variant 2, mRNA [NM_001017973]	NM_001017973
A_23_P157784	0.000122	NM_032593	NM_032593	Homo sapiens histidine triad nucleotide binding protein 2 (HINT2), mRNA [NM_032593]	NM_032593
A_32_P223777	0.000123	THC2315176		IL6B_HUMAN (P40189) Interleukin-6 receptor beta chain precursor (IL-6R-beta) (Interleukin 6 signal transducer) (Membrane glycoprotein 130) (gp130) (Oncostatin M receptor) (CDw130) (CD130 antigen), partial (27%) [THC2315176]	
A_24_P80532	0.000123	NM_004354	NM_004354	Homo sapiens cyclin G2 (CCNG2), mRNA [NM_004354]	NM_004354

A_24_P234732	0.000123	NM_006454	NM_006454	Homo sapiens MAX dimerization protein 4 (MXD4), mRNA [NM_006454]	NM_006454
A_23_P347100	0.000123	A_23_P347100			
A_23_P102258	0.000123	NM_053050	NM_053050	Homo sapiens mitochondrial ribosomal protein L53 (MRPL53), nuclear gene encoding mitochondrial protein, mRNA [NM_053050]	NM_053050
A_32_P117908	0.000124	A_32_P117908			
A_24_P308506	0.000124	NM_016347	NM_016347	Homo sapiens putative N-acetyltransferase Camello 2 (CML2), mRNA [NM_016347]	NM_016347
A_23_P216043	0.000124	NM_024295	NM_024295	Homo sapiens Der1-like domain family, member 1 (DERL1), mRNA [NM_024295]	NM_024295
A_32_P58999	0.000124	BE671816	BE671816	BE671816 7a47d01.x1 NCI_CGAP_GC6 Homo sapiens cDNA clone IMAGE:3221857 3' similar to gb:X12433 PROTEIN PHPS1-2 (HUMAN);, mRNA sequence [BE671816]	
A_23_P2661	0.000124	NM_015646	NM_015646	Homo sapiens RAP1B, member of RAS oncogene family (RAP1B), transcript variant 1, mRNA [NM_015646]	NM_015646
A_23_P4754	0.000124	NM_006012	NM_006012	Homo sapiens ClpP caseinolytic protease, ATP-dependent, proteolytic subunit homolog (E. coli) (CLPP), nuclear gene encoding mitochondrial protein, mRNA [NM_006012]	NM_006012
A_32_P35906	0.000125	BQ010172	BQ010172	BQ010172 UI-H-ED0-axz-j-15-0-UI.s1 NCI_CGAP_ED0 Homo sapiens cDNA clone IMAGE:5835758 3', mRNA sequence [BQ010172]	
A_23_P21072	0.000125	NM_130781	NM_130781	Homo sapiens RAB24, member RAS oncogene family (RAB24), mRNA [NM_130781]	NM_130781
A_24_P944458	0.000125	NM_016133	NM_016133	Homo sapiens insulin induced gene 2 (INSIG2), mRNA [NM_016133]	NM_016133
A_23_P26254	0.000125	NM_016013	NM_016013	Homo sapiens NADH dehydrogenase (ubiquinone) 1 alpha subcomplex, assembly factor 1 (NDUFAF1), mRNA [NM_016013]	NM_016013
A_23_P63798	0.000125	NM_001300	NM_001300	Homo sapiens Kruppel-like factor 6 (KLF6), transcript variant 2, mRNA [NM_001300]	NM_001300
A_32_P192804	0.000126	BF195626	BF195626	BF195626 7n86a01.x1 NCI_CGAP_Ov18 Homo sapiens cDNA clone IMAGE:3571464 3' similar to SW:ER72_HUMAN P13667 PROTEIN DISULFIDE ISOMERASE-RELATED PROTEIN ERP-72 PRECURSOR ;, mRNA sequence [BF195626]	
A_24_P917384	0.000126	BC009467	BC009467	Homo sapiens, clone IMAGE:3613029, mRNA, partial cds. [BC009467]	
A_23_P200928	0.000126	NM_002508	NM_002508	Homo sapiens nidogen 1 (NID1), mRNA [NM_002508]	NM_002508
A_23_P87401	0.000126	NM_030792	NM_030792	Homo sapiens glycerophosphodiester phosphodiesterase domain containing 5 (GDPD5), mRNA [NM_030792]	NM_030792
A_32_P16854	0.000126	NM_015391	NM_015391	Homo sapiens anaphase promoting complex subunit 13 (ANAPC13), mRNA [NM_015391]	NM_015391
A_32_P103291	0.000126	NM_022743	NM_022743	Homo sapiens SET and MYND domain containing 3 (SMYD3), mRNA [NM_022743]	NM_022743
A_23_P252322	0.000126	NM_006886	NM_006886	Homo sapiens ATP synthase, H+ transporting, mitochondrial F1 complex, epsilon subunit (ATP5E), nuclear gene encoding mitochondrial protein, mRNA [NM_006886]	NM_006886
A_32_P121537	0.000127	A_32_P121537			
A_23_P127027	0.000127	NM_001081	NM_001081	Homo sapiens cubilin (intrinsic factor-cobalamin receptor) (CUBN), mRNA [NM_001081]	NM_001081
A_23_P427039	0.000127	NM_152667	NM_152667	Homo sapiens haloacid dehalogenase-like hydrolase domain containing 4 (HDHD4), mRNA [NM_152667]	NM_152667
A_23_P114144	0.000127	NM_014008	NM_014008	Homo sapiens coiled-coil domain containing 22 (CCDC22), mRNA [NM_014008]	NM_014008
A_23_P98350	0.000128	NM_001165	NM_001165	Homo sapiens baculoviral IAP repeat-containing 3 (BIRC3), transcript variant 1, mRNA [NM_001165]	NM_001165
A_23_P9209	0.000128	NM_018376	NM_018376	Homo sapiens nipsnap homolog 3B (C. elegans) (NIPSNAP3B), mRNA [NM_018376]	NM_018376
A_23_P131899	0.000128	NM_080489	NM_080489	Homo sapiens syndecan binding protein (syntenin) 2 (SDCBP2), transcript variant 1, mRNA [NM_080489]	NM_080489
A_24_P261929	0.000128	NM_206949	NM_206949	Homo sapiens family with sequence similarity 14, member B (FAM14B), mRNA [NM_206949]	NM_206949
A_24_P409219	0.000129	NM_017667	NM_017667	Homo sapiens hypothetical protein FLJ20097 (FLJ20097), transcript variant 1, mRNA [NM_017667]	NM_017667
A_32_P223859	0.000129	NM_006714	NM_006714	Homo sapiens sphingomyelin phosphodiesterase, acid-like 3A (SMPDL3A), mRNA [NM_006714]	NM_006714
A_23_P342727	0.000129	NM_178006	NM_178006	Homo sapiens START domain containing 13 (STARD13), transcript variant alpha, mRNA [NM_178006]	NM_178006
A_24_P944570	0.000129	AF200348	AF200348	Homo sapiens melanoma-associated antigen MG50 mRNA, partial cds. [AF200348]	
A_32_P121755	0.000129	THC2406579		Q7Z6F8 (Q7Z6F8) MGC18079, partial (7%) [THC2406579]	
A_24_P281683	0.000129	A_24_P281683			
A_32_P165611	0.000129	NM_015254	NM_015254	Homo sapiens kinesin family member 13B (KIF13B), mRNA [NM_015254]	NM_015254
A_23_P122052	0.000129	NM_001008397	NM_001008397	Homo sapiens similar to RIKEN cDNA 2310016C16 (LOC493869), mRNA [NM_001008397]	NM_001008397
A_23_P31893	0.000129	NM_003033	NM_003033	Homo sapiens ST3 beta-galactoside alpha-2,3-sialyltransferase 1 (ST3GAL1), transcript variant 1, mRNA [NM_003033]	NM_003033
A_24_P270144	0.00013	NM_001780	NM_001780	Homo sapiens CD63 antigen (melanoma 1 antigen) (CD63), mRNA [NM_001780]	NM_001780
A_24_P257151	0.000131	NM_004071	NM_004071	Homo sapiens CDC-like kinase 1 (CLK1), transcript variant 1, mRNA [NM_004071]	NM_004071
A_23_P35609	0.000131	NM_000375	NM_000375	Homo sapiens uroporphyrinogen III synthase (congenital erythropoietic porphyria) (UROS), mRNA [NM_000375]	NM_000375

A_24_P263767	0.000131	ENST00000355804		GB|M80470.1|AAA66955.1 MHC class I HLA-J antigen [NP111779]	
A_23_P91802	0.000131	NM_001953	NM_001953	Homo sapiens endothelial cell growth factor 1 (platelet-derived) (ECGF1), mRNA [NM_001953]	NM_001953
A_32_P112623	0.000132	NM_175923	NM_175923	Homo sapiens hypothetical protein MGC42630 (MGC42630), mRNA [NM_175923]	NM_175923
A_23_P502350	0.000132	NM_000635	NM_000635	Homo sapiens regulatory factor X, 2 (influences HLA class II expression) (RFX2), transcript variant 1, mRNA [NM_000635]	NM_000635
A_23_P410998	0.000132	NM_002868	NM_002868	Homo sapiens RAB5B, member RAS oncogene family (RAB5B), mRNA [NM_002868]	NM_002868
A_23_P91221	0.000132	NM_181805	NM_181805	Homo sapiens protein kinase (cAMP-dependent, catalytic) inhibitor gamma (PKIG), transcript variant 1, mRNA [NM_181805]	NM_181805
A_32_P186865	0.000133	THC2406944		Q9F8M7 (Q9F8M7) DTDP-glucose 4,6-dehydratase (Fragment), partial (11%) [THC2406944]	
A_23_P310590	0.000133	NM_178507	NM_178507	Homo sapiens NS5ATP13TP2 protein (NS5ATP13TP2), mRNA [NM_178507]	NM_178507
A_23_P123732	0.000133	NM_001001551	NM_001001551	Homo sapiens chromosome 9 open reading frame 103 (C9orf103), mRNA [NM_001001551]	NM_001001551
A_23_P201368	0.000133	NM_004388	NM_004388	Homo sapiens chitobiase, di-N-acetyl- (CTBS), mRNA [NM_004388]	NM_004388
A_32_P191141	0.000133	A_32_P191141			
A_24_P337334	0.000133	NM_001012732	NM_001012732	Homo sapiens dCMP deaminase (DCTD), transcript variant 1, mRNA [NM_001012732]	NM_001012732
A_23_P137532	0.000133	NM_000302	NM_000302	Homo sapiens procollagen-lysine 1, 2-oxoglutarate 5-dioxygenase 1 (PLOD1), mRNA [NM_000302]	NM_000302
A_23_P254254	0.000134	NM_000199	NM_000199	Homo sapiens N-sulfoglucosamine sulfohydrolase (sulfamidase) (SGSH), mRNA [NM_000199]	NM_000199
A_23_P25515	0.000135	NM_004685	NM_004685	Homo sapiens myotubularin related protein 6 (MTMR6), mRNA [NM_004685]	NM_004685
A_24_P400324	0.000135	ENST00000262042		Homo sapiens mRNA for KIAA0960 protein, partial cds. [AB023177]	
A_32_P49728	0.000135	ENST00000308092		Homo sapiens cDNA FLJ13794 fis, clone THYRO1000092. [AK023856]	
A_23_P34107	0.000135	NM_004699	NM_004699	Homo sapiens family with sequence similarity 50, member A (FAM50A), mRNA [NM_004699]	NM_004699
A_23_P351069	0.000136	NM_003955	NM_003955	Homo sapiens suppressor of cytokine signaling 3 (SOCS3), mRNA [NM_003955]	NM_003955
A_23_P251717	0.000136	NM_007075	NM_007075	Homo sapiens WD repeat domain 45 (WDR45), transcript variant 1, mRNA [NM_007075]	NM_007075
A_23_P101272	0.000136	NM_017908	NM_017908	Homo sapiens zinc finger protein 446 (ZNF446), mRNA [NM_017908]	NM_017908
A_32_P69166	0.000137	NM_182603	NM_182603	Homo sapiens ankyrin repeat domain 42 (ANKRD42), mRNA [NM_182603]	NM_182603
A_23_P120970	0.000137	NM_001008572	NM_001008572	Homo sapiens tubulin tyrosine ligase-like family, member 1 (TTLL1), transcript variant 2, mRNA [NM_001008572]	NM_001008572
A_23_P170467	0.000137	NM_006537	NM_006537	Homo sapiens ubiquitin specific protease 3 (USP3), mRNA [NM_006537]	NM_006537
A_23_P145264	0.000137	NM_018950	NM_018950	Homo sapiens major histocompatibility complex, class I, F (HLA-F), mRNA [NM_018950]	NM_018950
A_23_P6413	0.000137	NM_080430	NM_080430	Homo sapiens selenoprotein M (SELM), mRNA [NM_080430]	NM_080430
A_24_P205213	0.000138	NM_198709	NM_198709	Homo sapiens arylsulfatase B (ARSB), transcript variant 2, mRNA [NM_198709]	NM_198709
A_23_P2203	0.000138	NM_016281	NM_016281	Homo sapiens TAO kinase 3 (TAOK3), mRNA [NM_016281]	NM_016281
A_23_P148785	0.000138	NM_199344	NM_199344	Homo sapiens SFT2 domain containing 2 (SFT2D2), mRNA [NM_199344]	NM_199344
A_23_P125668	0.000138	NM_145305	NM_145305	Homo sapiens similar to solute carrier family 25 , member 16 (LOC203427), mRNA [NM_145305]	NM_145305
A_23_P211973	0.000139	NM_024800	NM_024800	Homo sapiens NIMA (never in mitosis gene a)- related kinase 11 (NEK11), transcript variant 1, mRNA [NM_024800]	NM_024800
A_24_P714618	0.000139	BC011779	BC011779	Homo sapiens cDNA clone IMAGE:3941306, partial cds. [BC011779]	
A_24_P141019	0.000139	NM_018412	NM_018412	Homo sapiens suppression of tumorigenicity 7 (ST7), transcript variant a, mRNA [NM_018412]	NM_018412
A_24_P221327	0.000139	BC020847	BC020847	Homo sapiens, clone IMAGE:4730995, mRNA, partial cds. [BC020847]	
A_24_P171075	0.000139	NM_183013	NM_183013	Homo sapiens cAMP responsive element modulator (CREM), transcript variant 19, mRNA [NM_183013]	NM_183013
A_24_P920207	0.000139	THC2363099		Q69ZH9 (Q69ZH9) MKIAA1501 protein (Fragment), partial (98%) [THC2363099]	
A_24_P155016	0.00014	NM_018718	NM_018718	Homo sapiens testis specific, 14 (TSGA14), mRNA [NM_018718]	NM_018718
A_24_P322353	0.00014	NM_024430	NM_024430	Homo sapiens proline-serine-threonine phosphatase interacting protein 2 (PSTPIP2), mRNA [NM_024430]	NM_024430
A_24_P570583	0.00014	NM_194319	NM_194319	Homo sapiens zinc finger protein 542 (ZNF542), mRNA [NM_194319]	NM_194319
A_23_P75056	0.00014	NM_001002295	NM_001002295	Homo sapiens GATA binding protein 3 (GATA3), transcript variant 1, mRNA [NM_001002295]	NM_001002295
A_24_P213924	0.00014	NM_020947	NM_020947	Homo sapiens KIAA1609 protein (KIAA1609), mRNA [NM_020947]	NM_020947
A_24_P111737	0.000141	NM_014616	NM_014616	Homo sapiens ATPase, Class VI, type 11B (ATP11B), mRNA [NM_014616]	NM_014616
A_23_P132595	0.000141	NM_014667	NM_014667	Homo sapiens vestigial like 4 (Drosophila) (VGLL4), mRNA [NM_014667]	NM_014667
A_24_P80135	0.000142	NM_014369	NM_014369	Homo sapiens protein tyrosine phosphatase, non-receptor type 18 (brain-derived) (PTPN18), mRNA [NM_014369]	NM_014369

A_24_P398781	0.000142	NM_052873	NM_052873	Homo sapiens MGC16028 similar to RIKEN cDNA 1700019E19 gene (MGC16028), mRNA [NM_052873]	NM_052873
A_23_P106056	0.000142	NM_001344	NM_001344	Homo sapiens defender against cell death 1 (DAD1), mRNA [NM_001344]	NM_001344
A_24_P48204	0.000143	NM_003004	NM_003004	Homo sapiens secreted and transmembrane 1 (SECTM1), mRNA [NM_003004]	NM_003004
A_23_P60079	0.000143	NM_001147	NM_001147	Homo sapiens angiopoietin 2 (ANGPT2), mRNA [NM_001147]	NM_001147
A_24_P399680	0.000143	NM_080821	NM_080821	Homo sapiens chromosome 20 open reading frame 108 (C20orf108), mRNA [NM_080821]	NM_080821
A_23_P11874	0.000143	NM_003953	NM_003953	Homo sapiens myelin protein zero-like 1 (MPZL1), transcript variant 1, mRNA [NM_003953]	NM_003953
A_23_P24716	0.000143	NM_017870	NM_017870	Homo sapiens heat shock 70kDa protein 5 (glucose-regulated protein, 78kDa) binding protein 1 (HSPA5BP1), transcript variant 1, mRNA [NM_017870]	NM_017870
A_23_P97990	0.000143	NM_002775	NM_002775	Homo sapiens protease, serine, 11 (IGF binding) (PRSS11), mRNA [NM_002775]	NM_002775
A_24_P162319	0.000144	NM_006746	NM_006746	Homo sapiens sex comb on midleg-like 1 (Drosophila) (SCML1), mRNA [NM_006746]	NM_006746
A_23_P428139	0.000144	NM_004093	NM_004093	Homo sapiens ephrin-B2 (EFNB2), mRNA [NM_004093]	NM_004093
A_24_P7330	0.000144	A_24_P7330			
A_23_P27285	0.000144	NM_138608	NM_138608	Homo sapiens metallophosphoesterase 1 (MPPE1), transcript variant 2, mRNA [NM_138608]	NM_138608
A_24_P159916	0.000144	NM_032627	NM_032627	Homo sapiens single stranded DNA binding protein 4 (SSBP4), transcript variant 1, mRNA [NM_032627]	NM_032627
A_32_P95034	0.000145	BC091525	BC091525	Homo sapiens cDNA clone IMAGE:30512240. [BC091525]	
A_23_P419239	0.000145	NM_018638	NM_018638	Homo sapiens ethanolamine kinase 1 (ETNK1), mRNA [NM_018638]	NM_018638
A_23_P203658	0.000145	NM_007166	NM_007166	Homo sapiens phosphatidylinositol binding clathrin assembly protein (PICALM), transcript variant 1, mRNA [NM_007166]	NM_007166
A_23_P111005	0.000145	NM_015388	NM_015388	Homo sapiens Yip1 domain family, member 3 (YIPF3), mRNA [NM_015388]	NM_015388
A_23_P406330	0.000146	NM_022733	NM_022733	Homo sapiens stromal membrane-associated protein 1-like (SMAP1L), mRNA [NM_022733]	NM_022733
A_32_P135450	0.000146	A_32_P135450			
A_24_P566891	0.000146	AK023803	AK023803	Homo sapiens cDNA FLJ13741 fis, clone PLACE3000208. [AK023803]	
A_24_P287503	0.000146	NM_021260	NM_021260	Homo sapiens zinc finger, FYVE domain containing 1 (ZFYVE1), transcript variant 1, mRNA [NM_021260]	NM_021260
A_23_P119196	0.000146	NM_016270	NM_016270	Homo sapiens Kruppel-like factor 2 (lung) (KLF2), mRNA [NM_016270]	NM_016270
A_32_P226356	0.000147	THC2317058			
A_32_P222751	0.000147	AA253216	AA253216	AA253216 zr53g08.s1 Soares_NhHMPu_S1 Homo sapiens cDNA clone IMAGE:667166 3', mRNA sequence [AA253216]	
A_23_P134910	0.000147	NM_003878	NM_003878	Homo sapiens gamma-glutamyl hydrolase (conjugase, folylpolygammaglutamyl hydrolase) (GGH), mRNA [NM_003878]	NM_003878
A_23_P81219	0.000148	NM_016619	NM_016619	Homo sapiens placenta-specific 8 (PLAC8), mRNA [NM_016619]	NM_016619
A_23_P374082	0.000148	NM_033274	NM_033274	Homo sapiens a disintegrin and metalloproteinase domain 19 (meltrin beta) (ADAM19), transcript variant 2, mRNA [NM_033274]	NM_033274
A_23_P123608	0.000149	NM_004972	NM_004972	Homo sapiens Janus kinase 2 (a protein tyrosine kinase) (JAK2), mRNA [NM_004972]	NM_004972
A_24_P659980	0.000149	THC2281837			
A_23_P82051	0.000149	NM_058182	NM_058182	Homo sapiens chromosome 21 open reading frame 51 (C21orf51), mRNA [NM_058182]	NM_058182
A_32_P507580	0.000149	BC011481	BC011481	Homo sapiens cDNA clone IMAGE:4151212, partial cds. [BC011481]	
A_24_P93321	0.000149	A_24_P93321			
A_24_P372613	0.000149	NM_001164	NM_001164	Homo sapiens amyloid beta (A4) precursor protein-binding, family B, member 1 (Fe65) (APBB1), transcript variant 1, mRNA [NM_001164]	NM_001164
A_24_P117620	0.000149	NM_018584	NM_018584	Homo sapiens calcium/calmodulin-dependent protein kinase II inhibitor 1 (CAMK2N1), mRNA [NM_018584]	NM_018584
A_23_P8175	0.000149	NM_006718	NM_006718	Homo sapiens pleiomorphic adenoma gene-like 1 (PLAGL1), transcript variant 2, mRNA [NM_006718]	NM_006718
A_23_P2446	0.000149	NM_014254	NM_014254	Homo sapiens transmembrane protein 5 (TMEM5), mRNA [NM_014254]	NM_014254
A_23_P355067	0.00015	NM_019026	NM_019026	Homo sapiens transmembrane and coiled-coil domains 1 (TMCO1), mRNA [NM_019026]	NM_019026
A_23_P110005	0.000151	NM_032316	NM_032316	Homo sapiens nicolin 1 (NICN1), mRNA [NM_032316]	NM_032316
A_24_P58054	0.000152	NM_015266	NM_015266	Homo sapiens solute carrier family 9 (sodium/hydrogen exchanger), isoform 8 (SLC9A8), mRNA [NM_015266]	NM_015266
A_24_P53600	0.000152	NM_139321	NM_139321	Homo sapiens attractin (ATRN), transcript variant 1, mRNA [NM_139321]	NM_139321
A_23_P259874	0.000152	NM_012430	NM_012430	Homo sapiens SEC22 vesicle trafficking protein-like 2 (S. cerevisiae) (SEC22L2), mRNA [NM_012430]	NM_012430
A_23_P141974	0.000152	NM_003290	NM_003290	Homo sapiens tropomyosin 4 (TPM4), mRNA [NM_003290]	NM_003290
A_23_P502470	0.000153	NM_002184	NM_002184	Homo sapiens interleukin 6 signal transducer (gp130, oncostatin M receptor) (IL6ST), transcript variant 1, mRNA [NM_002184]	NM_002184
A_32_P101860	0.000153	A_32_P101860			

A_23_P253012	0.000153	NM_017577	NM_017577	Homo sapiens hypothetical protein DKFZp434C0328 (DKFZp434C0328), mRNA [NM_017577]	NM_017577
A_23_P819	0.000153	NM_005101	NM_005101	Homo sapiens interferon, alpha-inducible protein (clone IFI-15K) (G1P2), mRNA [NM_005101]	NM_005101
A_32_P100338	0.000153	A_32_P100338			
A_24_P123166	0.000154	NM_022171	NM_022171	Homo sapiens T-cell leukemia translocation altered gene (TCTA), mRNA [NM_022171]	NM_022171
A_24_P410363	0.000154	NM_022051	NM_022051	Homo sapiens egl nine homolog 1 (C. elegans) (EGLN1), mRNA [NM_022051]	NM_022051
A_24_P416177	0.000154	NM_001114	NM_001114	Homo sapiens adenylate cyclase 7 (ADCY7), mRNA [NM_001114]	NM_001114
A_23_P93032	0.000154	NM_032367	NM_032367	Homo sapiens zinc finger, BED-type containing 3 (ZBED3), mRNA [NM_032367]	NM_032367
A_32_P27020	0.000154	NM_173638	NM_173638	Homo sapiens hypothetical protein MGC8902 (MGC8902), mRNA [NM_173638]	NM_173638
A_24_P610375	0.000156	CA306742	CA306742	CA306742 UI-H-FT1-bht-f-03-0-UI.s1 NCI_CGAP_FT1 Homo sapiens cDNA clone UI-H-FT1-bht-f-03-0-UI 3', mRNA sequence [CA306742]	
A_23_P94683	0.000156	NM_002504	NM_002504	Homo sapiens nuclear transcription factor, X-box binding 1 (NFX1), transcript variant 1, mRNA [NM_002504]	NM_002504
A_23_P57836	0.000156	A_23_P57836			
A_23_P115356	0.000156	NM_003689	NM_003689	Homo sapiens aldo-keto reductase family 7, member A2 (aflatoxin aldehyde reductase) (AKR7A2), mRNA [NM_003689]	NM_003689
A_23_P121326	0.000157	NM_007022	NM_007022	Homo sapiens cytochrome b-561 domain containing 2 (CYB561D2), mRNA [NM_007022]	NM_007022
A_24_P221445	0.000158	NM_181354	NM_181354	Homo sapiens oxidation resistance 1 (OXR1), mRNA [NM_181354]	NM_181354
A_23_P303763	0.000158	NM_007135	NM_007135	Homo sapiens zinc finger protein 79 (pT7) (ZNF79), mRNA [NM_007135]	NM_007135
A_32_P137408	0.000158	THC2317808		Q8CFL1 (Q8CFL1) Stxbp4 protein (Syntaxin binding protein 4), partial (3%) [THC2317808]	
A_23_P392222	0.000158	ENST00000320188		Homo sapiens chromosome 9 open reading frame 119, mRNA (cDNA clone IMAGE:5168627), partial cds. [BC029911]	XM_372143
A_24_P228667	0.000158	NM_003776	NM_003776	Homo sapiens mitochondrial ribosomal protein L40 (MRPL40), nuclear gene encoding mitochondrial protein, mRNA [NM_003776]	NM_003776
A_23_P356466	0.000159	BC004219	BC004219	Homo sapiens cDNA clone IMAGE:3354271, complete cds. [BC004219]	
A_23_P401774	0.00016	NM_018712	NM_018712	Homo sapiens ELMO domain containing 1 (ELMOD1), mRNA [NM_018712]	NM_018712
A_24_P826046	0.00016	AK024177	AK024177	Homo sapiens cDNA FLJ14115 fis, clone MAMMA1001760. [AK024177]	XM_499165
A_23_P160546	0.00016	NM_018379	NM_018379	Homo sapiens family with sequence similarity 63, member A (FAM63A), mRNA [NM_018379]	NM_018379
A_23_P9591	0.00016	NM_006816	NM_006816	Homo sapiens lectin, mannose-binding 2 (LMAN2), mRNA [NM_006816]	NM_006816
A_23_P26173	0.00016	NM_007364	NM_007364	Homo sapiens transmembrane emp24 domain containing 3 (TMED3), mRNA [NM_007364]	NM_007364
A_24_P134568	0.000161	NM_006915	NM_006915	Homo sapiens retinitis pigmentosa 2 (X-linked recessive) (RP2), mRNA [NM_006915]	NM_006915
A_24_P943792	0.000161	NM_001777	NM_001777	Homo sapiens CD47 antigen (Rh-related antigen, integrin-associated signal transducer) (CD47), transcript variant 1, mRNA [NM_001777]	NM_001777
A_24_P180654	0.000161	BC063666	BC063666	Homo sapiens cAMP responsive element binding protein 3-like 2, mRNA (cDNA clone IMAGE:4185677), complete cds. [BC063666]	
A_23_P115743	0.000162	NM_022063	NM_022063	Homo sapiens chromosome 10 open reading frame 84 (C10orf84), mRNA [NM_022063]	NM_022063
A_24_P285768	0.000162	NM_014674	NM_014674	Homo sapiens ER degradation enhancer, mannosidase alpha-like 1 (EDEM1), mRNA [NM_014674]	NM_014674
A_23_P256059	0.000162	NM_203477	NM_203477	Homo sapiens similar to RPL23AP7 protein (MGC70863), transcript variant 1, mRNA [NM_203477]	NM_203477
A_23_P215944	0.000162	NM_147780	NM_147780	Homo sapiens cathepsin B (CTSB), transcript variant 2, mRNA [NM_147780]	NM_147780
A_24_P135841	0.000163	ENST00000328043		Homo sapiens low density lipoprotein receptor-related protein 11, mRNA (cDNA clone IMAGE:4072521), partial cds. [BC016788]	
A_23_P154256	0.000163	NM_032213	NM_032213	Homo sapiens RNA binding motif and ELMO domain 1 (RBED1), mRNA [NM_032213]	NM_032213
A_23_P62351	0.000163	NM_019007	NM_019007	Homo sapiens armadillo repeat containing, X-linked 6 (ARMCX6), transcript variant 1, mRNA [NM_019007]	NM_019007
A_23_P502930	0.000164	NM_006712	NM_006712	Homo sapiens Fas-activated serine/threonine kinase (FASTK), transcript variant 1, mRNA [NM_006712]	NM_006712
A_24_P503710	0.000165	AK000038	AK000038	Homo sapiens cDNA FLJ20031 fis, clone ADSU02180. [AK000038]	
A_24_P612446	0.000165	CR591566	CR591566	full-length cDNA clone CS0DL004YB03 of B cells (Ramos cell line) Cot 25-normalized of Homo sapiens (human). [CR591566]	
A_23_P88163	0.000165	NM_018036	NM_018036	Homo sapiens chromosome 14 open reading frame 103 (C14orf103), mRNA [NM_018036]	NM_018036
A_24_P53282	0.000165	NM_001304	NM_001304	Homo sapiens carboxypeptidase D (CPD), mRNA [NM_001304]	NM_001304
A_24_P118531	0.000165	BP290435	BP290435	BP290435 BP290435 Sugano cDNA library, lung fibroblast Homo sapiens cDNA clone LNF05072, mRNA sequence [BP290435]	
A_24_P298894	0.000165	ENST00000332888			
A_23_P431789	0.000165	NM_001660	NM_001660	Homo sapiens ADP-ribosylation factor 4 (ARF4), mRNA [NM_001660]	NM_001660
A_24_P55225	0.000166	NM_152732	NM_152732	Homo sapiens chromosome 6 open reading frame 206 (C6orf206), mRNA [NM_152732]	NM_152732
A_23_P101319	0.000166	NM_152354	NM_152354	Homo sapiens zinc finger protein 285 (ZNF285), mRNA [NM_152354]	NM_152354

A_23_P169117	0.000166	NM_006570	NM_006570	Homo sapiens Ras-related GTP binding A (RRAGA), mRNA [NM_006570]	NM_006570
A_24_P222237	0.000167	NM_001024681	NM_001024681	Homo sapiens D15F37 gene (D15F37), mRNA [NM_001024681]	NM_001024681
A_32_P228618	0.000168	NM_001003793	NM_001003793	Homo sapiens RNA binding motif, single stranded interacting protein (RBMS3), transcript variant 1, mRNA [NM_001003793]	NM_001003793
A_32_P4608	0.000169	A_32_P4608			
A_32_P46526	0.000169	NM_173491	NM_173491	Homo sapiens LSM11, U7 small nuclear RNA associated (LSM11), mRNA [NM_173491]	NM_173491
A_23_P150207	0.000169	NM_004322	NM_004322	Homo sapiens BCL2-antagonist of cell death (BAD), transcript variant 1, mRNA [NM_004322]	NM_004322
A_23_P213678	0.000169	NM_000919	NM_000919	Homo sapiens peptidylglycine alpha-amidating monooxygenase (PAM), transcript variant 1, mRNA [NM_000919]	NM_000919
A_23_P434352	0.00017	NM_001750	NM_001750	Homo sapiens calpastatin (CAST), transcript variant 1, mRNA [NM_001750]	NM_001750
A_23_P120153	0.00017	NM_173647	NM_173647	Homo sapiens ring finger protein 149 (RNF149), mRNA [NM_173647]	NM_173647
A_23_P57304	0.000171	NM_006134	NM_006134	Homo sapiens transmembrane protein 50B (TMEM50B), mRNA [NM_006134]	NM_006134
A_23_P88602	0.000171	NM_170677	NM_170677	Homo sapiens Meis1, myeloid ecotropic viral integration site 1 homolog 2 (mouse) (MEIS2), transcript variant a, mRNA [NM_170677]	NM_170677
A_23_P300714	0.000171	NM_005775	NM_005775	Homo sapiens vinexin beta (SH3-containing adaptor molecule-1) (SCAM-1), transcript variant 1, mRNA [NM_005775]	NM_005775
A_23_P203790	0.000171	NM_006812	NM_006812	Homo sapiens amplified in osteosarcoma (OS9), transcript variant 1, mRNA [NM_006812]	NM_006812
A_24_P116017	0.000172	NM_002813	NM_002813	Homo sapiens proteasome (prosome, macropain) 26S subunit, non-ATPase, 9 (PSMD9), mRNA [NM_002813]	NM_002813
A_23_P303455	0.000172	NM_153832	NM_153832	Homo sapiens G protein-coupled receptor 161 (GPR161), transcript variant 2, mRNA [NM_153832]	NM_153832
A_32_P149174	0.000172	NM_001008739	NM_001008739	Homo sapiens similar to RIKEN cDNA 2310039H08 (LOC441150), mRNA [NM_001008739]	NM_001008739
A_24_P83944	0.000173	NM_017652	NM_017652	Homo sapiens zinc finger protein 586 (ZNF586), mRNA [NM_017652]	NM_017652
A_23_P83403	0.000173	NM_014988	NM_014988	Homo sapiens KIAA1102 protein (KIAA1102), mRNA [NM_014988]	NM_014988
A_23_P26878	0.000173	NM_030808	NM_030808	Homo sapiens nudE nuclear distribution gene E homolog like 1 (A. nidulans) (NDEL1), transcript variant 2, mRNA [NM_030808]	NM_030808
A_23_P149153	0.000173	NM_022359	NM_022359	Homo sapiens phosphodiesterase 4D interacting protein (myomegalin) (PDE4DIP), transcript variant 3, mRNA [NM_022359]	NM_022359
A_23_P204511	0.000173	NM_017842	NM_017842	Homo sapiens hypothetical protein FLJ20489 (FLJ20489), mRNA [NM_017842]	NM_017842
A_32_P20454	0.000173	THC2369831		Q99JH4 (Q99JH4) TAFII140 protein (Fragment), partial (4%) [THC2369831]	
A_23_P346006	0.000174	NM_020739	NM_020739	Homo sapiens cell cycle progression 1 (CCPG1), transcript variant 2, mRNA [NM_020739]	NM_020739
A_24_P236799	0.000174	NM_006868	NM_006868	Homo sapiens RAB31, member RAS oncogene family (RAB31), mRNA [NM_006868]	NM_006868
A_23_P110212	0.000175	NM_001995	NM_001995	Homo sapiens acyl-CoA synthetase long-chain family member 1 (ACSL1), mRNA [NM_001995]	NM_001995
A_24_P160969	0.000176	NM_006034	NM_006034	Homo sapiens tumor protein p53 inducible protein 11 (TP53I11), mRNA [NM_006034]	NM_006034
A_32_P213624	0.000176	BU537444	BU537444	AGENCOURT_10294432 NIH_MGC_126 Homo sapiens cDNA clone IMAGE:6567406 5', mRNA sequence [BU537444]	
A_23_P62429	0.000176	BC000393	BC000393	Homo sapiens DKFZP564B147 protein, mRNA (cDNA clone MGC:8471 IMAGE:2821721), complete cds. [BC000393]	
A_23_P94159	0.000177	NM_183421	NM_183421	Homo sapiens F-box protein 25 (FBXO25), transcript variant 1, mRNA [NM_183421]	NM_183421
A_23_P41487	0.000177	NM_015130	NM_015130	Homo sapiens KIAA0882 protein (KIAA0882), mRNA [NM_015130]	NM_015130
A_23_P351679	0.000178	NM_004614	NM_004614	Homo sapiens thymidine kinase 2, mitochondrial (TK2), mRNA [NM_004614]	NM_004614
A_23_P209712	0.000179	CR749288	CR749288	Homo sapiens mRNA; cDNA DKFZp686E07254 (from clone DKFZp686E07254). [CR749288]	
A_23_P120182	0.000179	NM_004544	NM_004544	Homo sapiens NADH dehydrogenase (ubiquinone) 1 alpha subcomplex, 10, 42kDa (NDUFA10), nuclear gene encoding mitochondrial protein, mRNA [NM_004544]	NM_004544
A_23_P62881	0.00018	NM_032291	NM_032291	Homo sapiens SH3-domain GRB2-like (endophilin) interacting protein 1 (SGIP1), mRNA [NM_032291]	NM_032291
A_23_P76983	0.00018	NM_025057	NM_025057	Homo sapiens chromosome 14 open reading frame 45 (C14orf45), mRNA [NM_025057]	NM_025057
A_32_P153937	0.000181	THC2339839		T4S9_HUMAN (P62079) Transmembrane 4 superfamily member 9 (Tetraspanin 5) (Tspan-5) (Tetraspan NET-4), partial (35%) [THC2339839]	
A_24_P618928	0.000181	CR602022	CR602022	full-length cDNA clone CS0DJ004YB09 of T cells (Jurkat cell line) Cot 10-normalized of Homo sapiens (human). [CR602022]	
A_24_P526623	0.000181	CR606637	CR606637	full-length cDNA clone CS0DI007YA21 of Placenta Cot 25-normalized of Homo sapiens (human). [CR606637]	XM_375527
A_24_P283395	0.000181	NM_052853	NM_052853	Homo sapiens aarF domain containing kinase 2 (ADCK2), mRNA [NM_052853]	NM_052853
A_23_P86424	0.000181	NM_005437	NM_005437	Homo sapiens nuclear receptor coactivator 4 (NCOA4), mRNA [NM_005437]	NM_005437
A_23_P62967	0.000182	NM_018662	NM_018662	Homo sapiens disrupted in schizophrenia 1 (DISC1), transcript variant L, mRNA [NM_018662]	NM_018662
A_32_P90551	0.000182	NM_014230	NM_014230	Homo sapiens signal recognition particle 68kDa (SRP68), mRNA [NM_014230]	NM_014230
A_23_P255785	0.000183	NM_006153	NM_006153	Homo sapiens NCK adaptor protein 1 (NCK1), mRNA [NM_006153]	NM_006153
A_32_P48256	0.000184	BF448317	BF448317	BF448317 nad18a05.x1 NCI_CGAP_Lu24 Homo sapiens cDNA clone IMAGE:3365672 3', mRNA sequence [BF448317]	

A_24_P26897	0.000184	NM_005539	NM_005539	Homo sapiens inositol polyphosphate-5-phosphatase, 40kDa (INPP5A), mRNA [NM_005539]	NM_005539
A_32_P97798	0.000185	AU184995	AU184995	AU184995 AU184995 Homo sapiens T-cell library (Sugita Y) Homo sapiens cDNA clone B00751-019, mRNA sequence [AU184995]	
A_23_P63896	0.000185	NM_000043	NM_000043	Homo sapiens Fas (TNF receptor superfamily, member 6) (FAS), transcript variant 1, mRNA [NM_000043]	NM_000043
A_23_P88309	0.000185	NM_153811	NM_153811	Homo sapiens solute carrier family 38, member 6 (SLC38A6), mRNA [NM_153811]	NM_153811
A_23_P502312	0.000186	NM_078481	NM_078481	Homo sapiens CD97 antigen (CD97), transcript variant 1, mRNA [NM_078481]	NM_078481
A_23_P14975	0.000186	NM_032140	NM_032140	Homo sapiens chromosome 16 open reading frame 48 (C16orf48), mRNA [NM_032140]	NM_032140
A_24_P328646	0.000186	NM_005778	NM_005778	Homo sapiens RNA binding motif protein 5 (RBM5), mRNA [NM_005778]	NM_005778
A_24_P503669	0.000189	AK093628	AK093628	Homo sapiens cDNA FLJ36309 fis, clone THYMU2004986. [AK093628]	
A_23_P92614	0.00019	NM_133636	NM_133636	Homo sapiens DNA helicase HEL308 (HEL308), mRNA [NM_133636]	NM_133636
A_24_P599453	0.000191	CR610404	CR610404	full-length cDNA clone CS0DA008YA11 of Neuroblastoma of Homo sapiens (human). [CR610404]	
A_24_P146575	0.000191	NM_003028	NM_003028	Homo sapiens Src homology 2 domain containing adaptor protein B (SHB), mRNA [NM_003028]	NM_003028
A_23_P15944	0.000191	NM_018696	NM_018696	Homo sapiens elaC homolog 1 (E. coli) (ELAC1), mRNA [NM_018696]	NM_018696
A_23_P138849	0.000191	NM_006388	NM_006388	Homo sapiens HIV-1 Tat interacting protein, 60kDa (HTATIP), transcript variant 2, mRNA [NM_006388]	NM_006388
A_23_P111171	0.000192	NM_003782	NM_003782	Homo sapiens UDP-Gal:betaGlcNAc beta 1,3-galactosyltransferase, polypeptide 4 (B3GALT4), mRNA [NM_003782]	NM_003782
A_23_P257131	0.000192	NM_002618	NM_002618	Homo sapiens peroxisome biogenesis factor 13 (PEX13), mRNA [NM_002618]	NM_002618
A_23_P108708	0.000192	NM_016494	NM_016494	Homo sapiens hypothetical protein LOC51255 (LOC51255), mRNA [NM_016494]	NM_016494
A_23_P309515	0.000193	NM_152387	NM_152387	Homo sapiens potassium channel tetramerisation domain containing 18 (KCTD18), mRNA [NM_152387]	NM_152387
A_24_P191971	0.000193	AK027248	AK027248	Homo sapiens cDNA: FLJ23595 fis, clone LNG15262. [AK027248]	
A_24_P89257	0.000193	BC012766	BC012766	Homo sapiens endoplasmic reticulum-golgi intermediate compartment 32 kDa protein, mRNA (cDNA clone MGC:16233 IMAGE:3677787), complete cds. [BC012766]	
A_23_P59210	0.000193	NM_000389	NM_000389	Homo sapiens cyclin-dependent kinase inhibitor 1A (p21, Cip1) (CDKN1A), transcript variant 1, mRNA [NM_000389]	NM_000389
A_32_P69987	0.000194	A_32_P69987			
A_23_P70867	0.000194	NM_016038	NM_016038	Homo sapiens Shwachman-Bodian-Diamond syndrome (SBDS), mRNA [NM_016038]	NM_016038
A_23_P435501	0.000195	NM_198941	NM_198941	Homo sapiens tumor differentially expressed 1 (TDE1), transcript variant 2, mRNA [NM_198941]	NM_198941
A_24_P114671	0.000196	AL117599	AL117599	Homo sapiens mRNA; cDNA DKFZp564I0463 (from clone DKFZp564I0463). [AL117599]	
A_24_P6949	0.000196	NM_025029	NM_025029	Homo sapiens hypothetical protein FLJ14346 (FLJ14346), mRNA [NM_025029]	NM_025029
A_24_P191750	0.000197	NM_153365	NM_153365	Homo sapiens hypothetical protein FLJ90013 (FLJ90013), mRNA [NM_153365]	NM_153365
A_23_P83976	0.000197	NM_145036	NM_145036	Homo sapiens hypothetical protein MGC33887 (MGC33887), mRNA [NM_145036]	NM_145036
A_32_P233834	0.000197	THC2392085			
A_23_P51754	0.000197	NM_001010935	NM_001010935	Homo sapiens RAP1A, member of RAS oncogene family (RAP1A), transcript variant 1, mRNA [NM_001010935]	NM_001010935
A_23_P100501	0.000197	NM_002134	NM_002134	Homo sapiens heme oxygenase (decycling) 2 (HMOX2), mRNA [NM_002134]	NM_002134
A_23_P30035	0.000199	NM_018409	NM_018409	Homo sapiens LRP2 binding protein (LRP2BP), mRNA [NM_018409]	NM_018409
A_24_P303145	0.000199	AK054966	AK054966	Homo sapiens cDNA FLJ30404 fis, clone BRACE2008481. [AK054966]	
A_32_P225328	0.000199	A_32_P225328			
A_23_P25403	0.0002	NM_013320	NM_013320	Homo sapiens host cell factor C2 (HCFC2), mRNA [NM_013320]	NM_013320
A_23_P47077	0.0002	NM_004281	NM_004281	Homo sapiens BCL2-associated athanogene 3 (BAG3), mRNA [NM_004281]	NM_004281
A_23_P368278	0.0002	NM_014824	NM_014824	Homo sapiens FCH and double SH3 domains 2 (FCHSD2), mRNA [NM_014824]	NM_014824
A_23_P306919	0.0002	NM_001013845	NM_001013845	Homo sapiens hypothetical LOC541578 protein (LOC541578), mRNA [NM_001013845]	NM_001013845
A_24_P118721	0.000201	ENST00000308448			
A_23_P107894	0.000201	NM_024029	NM_024029	Homo sapiens Yip1 domain family, member 2 (YIPF2), mRNA [NM_024029]	NM_024029
A_24_P88800	0.000201	NM_004718	NM_004718	Homo sapiens cytochrome c oxidase subunit VIIa polypeptide 2 like (COX7A2L), nuclear gene encoding mitochondrial protein, mRNA [NM_004718]	NM_004718
A_23_P22119	0.000201	NM_201380	NM_201380	Homo sapiens plectin 1, intermediate filament binding protein 500kDa (PLEC1), transcript variant 6, mRNA [NM_201380]	NM_201380
A_23_P133770	0.000201	NM_138493	NM_138493	Homo sapiens chromosome 6 open reading frame 129 (C6orf129), mRNA [NM_138493]	NM_138493
A_24_P489695	0.000202	ENST00000340455		Homo sapiens cDNA FLJ46111 fis, clone TESTI2034913, moderately similar to Keratin, type II cytoskeletal 8. [AK127993]	
A_24_P151498	0.000202	NM_206962	NM_206962	Homo sapiens HMT1 hnRNP methyltransferase-like 1 (S. cerevisiae) (HRMT1L1), transcript variant 1, mRNA [NM_206962]	NM_206962

A_23_P116016	0.000203	CR623195	CR623195	full-length cDNA clone CS0DF011YN02 of Fetal brain of Homo sapiens (human). [CR623195]	
A_24_P19228	0.000204	NM_138924	NM_138924	Homo sapiens guanidinoacetate N-methyltransferase (GAMT), transcript variant 2, mRNA [NM_138924]	NM_138924
A_23_P98645	0.000205	NM_003737	NM_003737	Homo sapiens dachsous 1 (Drosophila) (DCHS1), mRNA [NM_003737]	NM_003737
A_32_P62342	0.000205	BC039145	BC039145	Homo sapiens hypothetical protein LOC283464, mRNA (cDNA clone MGC:21651 IMAGE:4508300), complete cds. [BC039145]	XM_290597
A_24_P169634	0.000206	AF019382	AF019382	Homo sapiens mannose-binding protein-A pseudogene (MBL1P1) mRNA sequence. [AF019382]	XR_000276
A_23_P124962	0.000206	NM_152832	NM_152832	Homo sapiens Mouse Mammary Turmor Virus Receptor homolog 1 (MTVR1), mRNA [NM_152832]	NM_152832
A_23_P348979	0.000207	NM_175923	NM_175923	Homo sapiens hypothetical protein MGC42630 (MGC42630), mRNA [NM_175923]	NM_175923
A_23_P72025	0.000207	NM_000387	NM_000387	Homo sapiens solute carrier family 25 (carnitine/acylcarnitine translocase), member 20 (SLC25A20), nuclear gene encoding mitochondrial protein, mRNA [NM_000387]	NM_000387
A_23_P23941	0.000208	NM_001007094	NM_001007094	Homo sapiens zinc finger protein 37a (KOX 21) (ZNF37A), transcript variant 1, mRNA [NM_001007094]	NM_001007094
A_32_P66148	0.000208	A_32_P66148			
A_24_P408457	0.000208	NM_000262	NM_000262	Homo sapiens N-acetylgalactosaminidase, alpha- (NAGA), mRNA [NM_000262]	NM_000262
A_24_P594721	0.000208	BC062753	BC062753	Homo sapiens cDNA clone IMAGE:3933366, partial cds. [BC062753]	
A_23_P48295	0.000209	NM_030911	NM_030911	Homo sapiens cytidine and dCMP deaminase domain containing 1 (CDADC1), mRNA [NM_030911]	NM_030911
A_24_P235520	0.000209	NM_175923	NM_175923	Homo sapiens hypothetical protein MGC42630 (MGC42630), mRNA [NM_175923]	NM_175923
A_23_P344555	0.000209	NM_006403	NM_006403	Homo sapiens neural precursor cell expressed, developmentally down-regulated 9 (NEDD9), transcript variant 1, mRNA [NM_006403]	NM_006403
A_23_P200126	0.000209	NM_003684	NM_003684	Homo sapiens MAP kinase interacting serine/threonine kinase 1 (MKNK1), transcript variant 1, mRNA [NM_003684]	NM_003684
A_23_P12572	0.000209	NM_033339	NM_033339	Homo sapiens caspase 7, apoptosis-related cysteine protease (CASP7), transcript variant gamma, mRNA [NM_033339]	NM_033339
A_23_P124122	0.000209	NM_018663	NM_018663	Homo sapiens peroxisomal membrane protein 2, 22kDa (PXMP2), mRNA [NM_018663]	NM_018663
A_23_P404871	0.000209	BC012766	BC012766	Homo sapiens endoplasmic reticulum-golgi intermediate compartment 32 kDa protein, mRNA (cDNA clone MGC:16233 IMAGE:3677787), complete cds. [BC012766]	
A_24_P407311	0.00021	NM_014584	NM_014584	Homo sapiens ERO1-like (S. cerevisiae) (ERO1L), mRNA [NM_014584]	NM_014584
A_23_P146576	0.00021	NM_021996	NM_021996	Homo sapiens globoside alpha-1,3-N-acetylgalactosaminyltransferase 1 (GBGT1), mRNA [NM_021996]	NM_021996
A_23_P120002	0.00021	NM_004510	NM_004510	Homo sapiens SP110 nuclear body protein (SP110), transcript variant b, mRNA [NM_004510]	NM_004510
A_23_P12282	0.00021	NM_001004023	NM_001004023	Homo sapiens dual-specificity tyrosine-(Y)-phosphorylation regulated kinase 3 (DYRK3), transcript variant 2, mRNA [NM_001004023]	NM_001004023
A_23_P91076	0.00021	CR621710	CR621710	full-length cDNA clone CS0DL008YP09 of B cells (Ramos cell line) Cot 25-normalized of Homo sapiens (human). [CR621710]	
A_23_P315364	0.000211	NM_002089	NM_002089	Homo sapiens chemokine (C-X-C motif) ligand 2 (CXCL2), mRNA [NM_002089]	NM_002089
A_32_P39944	0.000211	AK095791	AK095791	Homo sapiens cDNA FLJ38472 fis, clone FEBRA2022148. [AK095791]	
A_24_P276583	0.000211	NM_019026	NM_019026	Homo sapiens transmembrane and coiled-coil domains 1 (TMCO1), mRNA [NM_019026]	NM_019026
A_24_P290348	0.000212	A_24_P290348			
A_23_P207967	0.000213	NM_014772	NM_014772	Homo sapiens KIAA0427 (KIAA0427), mRNA [NM_014772]	NM_014772
A_23_P329890	0.000213	NM_174926	NM_174926	Homo sapiens hypothetical protein MGC17839 (MGC17839), mRNA [NM_174926]	NM_174926
A_23_P119362	0.000214	NM_001425	NM_001425	Homo sapiens epithelial membrane protein 3 (EMP3), mRNA [NM_001425]	NM_001425
A_24_P66592	0.000215	NM_173515	NM_173515	Homo sapiens CNKSR family member 3 (CNKSR3), mRNA [NM_173515]	NM_173515
A_23_P19938	0.000215	NM_006854	NM_006854	Homo sapiens KDEL (Lys-Asp-Glu-Leu) endoplasmic reticulum protein retention receptor 2 (KDELR2), mRNA [NM_006854]	NM_006854
A_32_P107777	0.000216	THC2339241		Q822A8 (Q822A8) Ribonucleoside-diphosphate reductase, beta subunit, partial (5%) [THC2339241]	
A_23_P29621	0.000216	NM_145262	NM_145262	Homo sapiens CG9886-like (GLYCTK), mRNA [NM_145262]	NM_145262
A_23_P217778	0.000216	NM_078629	NM_078629	Homo sapiens male-specific lethal 3-like 1 (Drosophila) (MSL3L1), transcript variant 1, mRNA [NM_078629]	NM_078629
A_32_P93584	0.000217	BG423475	BG423475	602449331F1 NIH_MGC_14 Homo sapiens cDNA clone IMAGE:4587846 5', mRNA sequence [BG423475]	
A_24_P306214	0.000217	NM_020947	NM_020947	Homo sapiens KIAA1609 protein (KIAA1609), mRNA [NM_020947]	NM_020947
A_24_P249072	0.000217	NM_181531	NM_181531	Homo sapiens butyrophilin, subfamily 2, member A2 (BTN2A2), transcript variant 2, mRNA [NM_181531]	NM_181531
A_32_P197511	0.000217	NM_138439	NM_138439	Homo sapiens hypothetical protein BC014089 (LOC114984), mRNA [NM_138439]	NM_138439
A_24_P48898	0.000217	NM_145637	NM_145637	Homo sapiens apolipoprotein L, 2 (APOL2), transcript variant beta, mRNA [NM_145637]	NM_145637
A_23_P303803	0.000219	NM_152474	NM_152474	Homo sapiens chromosome 19 open reading frame 18 (C19orf18), mRNA [NM_152474]	NM_152474
A_23_P166376	0.000219	NM_004121	NM_004121	Homo sapiens gamma-glutamyltransferase-like activity 1 (GGTLA1), mRNA [NM_004121]	NM_004121

A_23_P103864	0.000219	NM_024525	NM_024525	Homo sapiens tetratricopeptide repeat domain 13 (TTC13), mRNA [NM_024525]	NM_024525
A_24_P185036	0.000219	NM_018348	NM_018348	Homo sapiens hypothetical protein FLJ11171 (FLJ11171), mRNA [NM_018348]	NM_018348
A_24_P252130	0.000219	NM_006238	NM_006238	Homo sapiens peroxisome proliferative activated receptor, delta (PPARD), transcript variant 1, mRNA [NM_006238]	NM_006238
A_23_P29555	0.000219	NM_183352	NM_183352	Homo sapiens SEC13-like 1 (S. cerevisiae) (SEC13L1), transcript variant 2, mRNA [NM_183352]	NM_183352
A_32_P205110	0.00022	AK021858	AK021858	Homo sapiens cDNA FLJ11796 fis, clone HEMBA1006158, highly similar to Homo sapiens transcription factor forkhead-like 7 (FKHL7) gene. [AK021858]	
A_23_P433829	0.000221	NM_152409	NM_152409	Homo sapiens hypothetical protein FLJ37562 (FLJ37562), mRNA [NM_152409]	NM_152409
A_24_P282031	0.000221	NM_015914	NM_015914	Homo sapiens thioredoxin domain containing 11 (TXNDC11), mRNA [NM_015914]	NM_015914
A_32_P185682	0.000221	CR613361	CR613361	full-length cDNA clone CS0DJ002YF02 of T cells (Jurkat cell line) Cot 10-normalized of Homo sapiens (human). [CR613361]	
A_23_P2814	0.000221	NM_005905	NM_005905	Homo sapiens SMAD, mothers against DPP homolog 9 (Drosophila) (SMAD9), mRNA [NM_005905]	NM_005905
A_23_P374104	0.000221	NM_012098	NM_012098	Homo sapiens angiopoietin-like 2 (ANGPTL2), mRNA [NM_012098]	NM_012098
A_32_P18470	0.000221	NM_001012979	NM_001012979	Homo sapiens transcription elongation factor A (SII)-like 5 (TCEAL5), mRNA [NM_001012979]	NM_001012979
A_23_P14946	0.000221	NM_003791	NM_003791	Homo sapiens membrane-bound transcription factor protease, site 1 (MBTPS1), transcript variant 1, mRNA [NM_003791]	NM_003791
A_23_P92687	0.000221	NM_004394	NM_004394	Homo sapiens death-associated protein (DAP), mRNA [NM_004394]	NM_004394
A_24_P277807	0.000221	NM_152828	NM_152828	Homo sapiens sorting nexin 3 (SNX3), transcript variant 3, mRNA [NM_152828]	NM_152828
A_23_P24345	0.000221	NM_152264	NM_152264	Homo sapiens solute carrier family 39 (zinc transporter), member 13 (SLC39A13), mRNA [NM_152264]	NM_152264
A_23_P99747	0.000222	NM_004196	NM_004196	Homo sapiens cyclin-dependent kinase-like 1 (CDC2-related kinase) (CDKL1), mRNA [NM_004196]	NM_004196
A_23_P149695	0.000222	BC069003	BC069003	Homo sapiens cDNA clone IMAGE:5441406, partial cds. [BC069003]	
A_24_P414205	0.000222	NM_017541	NM_017541	Homo sapiens crystallin, gamma S (CRYGS), mRNA [NM_017541]	NM_017541
A_24_P566916	0.000222	CR618687	CR618687	full-length cDNA clone CS0CAP008YE23 of Thymus of Homo sapiens (human). [CR618687]	
A_23_P502224	0.000222	NM_000398	NM_000398	Homo sapiens cytochrome b5 reductase 3 (CYB5R3), transcript variant M, mRNA [NM_000398]	NM_000398
A_23_P201086	0.000223	NM_001658	NM_001658	Homo sapiens ADP-ribosylation factor 1 (ARF1), transcript variant 4, mRNA [NM_001658]	NM_001658
A_23_P31584	0.000223	NM_022777	NM_022777	Homo sapiens RAB, member RAS oncogene family-like 5 (RABL5), mRNA [NM_022777]	NM_022777
A_24_P919279	0.000224	NM_206894	NM_206894	Homo sapiens hypothetical protein LOC388536 (MGC62100), mRNA [NM_206894]	NM_206894
A_32_P120638	0.000225	NM_033547	NM_033547	Homo sapiens hypothetical gene MGC16733 similar to CG12113 (MGC16733), mRNA [NM_033547]	NM_033547
A_23_P147711	0.000225	NM_000906	NM_000906	Homo sapiens natriuretic peptide receptor A/guanylate cyclase A (atrionatriuretic peptide receptor A) (NPR1), mRNA [NM_000906]	NM_000906
A_24_P719948	0.000225	AK023629	AK023629	Homo sapiens cDNA FLJ13567 fis, clone PLACE1008331. [AK023629]	
A_23_P200801	0.000225	NM_001002811	NM_001002811	Homo sapiens phosphodiesterase 4D interacting protein (myomegalin) (PDE4DIP), transcript variant 5, mRNA [NM_001002811]	NM_001002811
A_23_P259892	0.000226	X66610	X66610	H.sapiens mRNA for enolase. [X66610]	
A_32_P35751	0.000227	THC2288230			
A_23_P404134	0.000227	NM_014828	NM_014828	Homo sapiens chromosome 14 open reading frame 92 (C14orf92), mRNA [NM_014828]	NM_014828
A_23_P250506	0.000227	NM_018973	NM_018973	Homo sapiens dolichyl-phosphate mannosyltransferase polypeptide 3 (DPM3), transcript variant 1, mRNA [NM_018973]	NM_018973
A_24_P253318	0.000227	NM_018477	NM_018477	Homo sapiens actin-related protein 10 homolog (S. cerevisiae) (ACTR10), mRNA [NM_018477]	NM_018477
A_24_P378368	0.000228	NM_003791	NM_003791	Homo sapiens membrane-bound transcription factor protease, site 1 (MBTPS1), transcript variant 1, mRNA [NM_003791]	NM_003791
A_23_P18649	0.000229	AK026709	AK026709	Homo sapiens cDNA: FLJ23056 fis, clone LNG03287. [AK026709]	
A_32_P100683	0.000229	AK092338	AK092338	Homo sapiens cDNA FLJ35019 fis, clone OCBBF2014541. [AK092338]	
A_24_P922877	0.000229	L04733	L04733	Homo sapiens kinesin light chain mRNA, complete cds. [L04733]	
A_24_P268676	0.00023	NM_003670	NM_003670	Homo sapiens basic helix-loop-helix domain containing, class B, 2 (BHLHB2), mRNA [NM_003670]	NM_003670
A_23_P83438	0.00023	NM_023079	NM_023079	Homo sapiens hypothetical protein FLJ13855 (FLJ13855), mRNA [NM_023079]	NM_023079
A_24_P101503	0.000231	A_24_P101503			
A_23_P51548	0.000231	NM_004528	NM_004528	Homo sapiens microsomal glutathione S-transferase 3 (MGST3), mRNA [NM_004528]	NM_004528
A_23_P408328	0.000232	NM_138632	NM_138632	Homo sapiens Tara-like protein (HRIHFB2122), transcript variant 2, mRNA [NM_138632]	NM_138632
A_24_P287974	0.000233	NM_017949	NM_017949	Homo sapiens CUE domain containing 1 (CUEDC1), mRNA [NM_017949]	NM_017949
A_23_P102122	0.000233	NM_152862	NM_152862	Homo sapiens actin related protein 2/3 complex, subunit 2, 34kDa (ARPC2), transcript variant 1, mRNA [NM_152862]	NM_152862

A_23_P393856	0.000234	AK021929	AK021929	Homo sapiens cDNA FLJ11867 fis, clone HEMBA1006976, weakly similar to H.sapiens mRNA for Gal-beta(1-3/1-4)GlcNAc alpha-2.3-sialyltransferase. [AK021929]	
A_23_P316472	0.000235	NM_144666	NM_144666	Homo sapiens hypothetical protein FLJ32752 (FLJ32752), mRNA [NM_144666]	NM_144666
A_23_P128956	0.000235	NM_021260	NM_021260	Homo sapiens zinc finger, FYVE domain containing 1 (ZFYVE1), transcript variant 1, mRNA [NM_021260]	NM_021260
A_23_P202964	0.000235	NM_052932	NM_052932	Homo sapiens pro-oncosis receptor inducing membrane injury gene (PORIMIN), mRNA [NM_052932]	NM_052932
A_23_P29851	0.000235	NM_002337	NM_002337	Homo sapiens low density lipoprotein receptor-related protein associated protein 1 (LRPAP1), mRNA [NM_002337]	NM_002337
A_23_P89621	0.000236	NM_003655	NM_003655	Homo sapiens chromobox homolog 4 (Pc class homolog, Drosophila) (CBX4), mRNA [NM_003655]	NM_003655
A_24_P910297	0.000238	NM_004486	NM_004486	Homo sapiens golgi autoantigen, golgin subfamily a, 2 (GOLGA2), mRNA [NM_004486]	NM_004486
A_23_P165333	0.000238	NM_139346	NM_139346	Homo sapiens bridging integrator 1 (BIN1), transcript variant 4, mRNA [NM_139346]	NM_139346
A_24_P52714	0.000238	NM_032193	NM_032193	Homo sapiens AYP1 protein (AYP1), mRNA [NM_032193]	NM_032193
A_32_P41021	0.000239	NM_015338	NM_015338	Homo sapiens additional sex combs like 1 (Drosophila) (ASXL1), mRNA [NM_015338]	NM_015338
A_23_P37988	0.000239	NM_152727	NM_152727	Homo sapiens copine II (CPNE2), mRNA [NM_152727]	NM_152727
A_24_P286845	0.00024	CR936686	CR936686	Homo sapiens mRNA; cDNA DKFZp686O06231 (from clone DKFZp686O06231). [CR936686]	
A_24_P273074	0.00024	A_24_P273074			
A_23_P143867	0.00024	NM_007114	NM_007114	Homo sapiens TATA element modulatory factor 1 (TMF1), mRNA [NM_007114]	NM_007114
A_23_P219060	0.00024	NM_022107	NM_022107	Homo sapiens G-protein signalling modulator 3 (AGS3-like, C. elegans) (GPSM3), mRNA [NM_022107]	NM_022107
A_23_P78244	0.000241	BC053363	BC053363	Homo sapiens cDNA clone IMAGE:6141118, partial cds. [BC053363]	
A_23_P51639	0.000242	NM_015284	NM_015284	Homo sapiens KIAA0467 (KIAA0467), mRNA [NM_015284]	NM_015284
A_23_P431638	0.000242	BC029662	BC029662	Homo sapiens chromosome 20 open reading frame 142, mRNA (cDNA clone IMAGE:4933017), with apparent retained intron. [BC029662]	
A_24_P45005	0.000242	NM_024663	NM_024663	Homo sapiens aminopeptidase-like 1 (NPEPL1), mRNA [NM_024663]	NM_024663
A_23_P64102	0.000243	NM_004292	NM_004292	Homo sapiens Ras and Rab interactor 1 (RIN1), mRNA [NM_004292]	NM_004292
[truncated: 123,172 more chars]
