# Supplementary material for: Functional and Transcriptional Characterization of Human Embryonic Stem Cell-Derived Endothelial Cells for Treatment of Myocardial Infarction
Source: PLoS One. 2009 Dec 31;4(12):e8443. doi: 10.1371/journal.pone.0008443 (PMC2795856; doi:10.1371/journal.pone.0008443)
Supplement: Table S4 — Over-represented GO terms in the K-means clustering significant gene lists. (1.25 MB PDF) [file pone.0008443.s010.pdf]

Supplemental Table S4A
GO Biological process Categories Overrepresented in Kmeans Cluster Set 1

GO Category	Genes in Category	% of Genes in Category	Genes in List in Category	% of Genes in List in Category	*P*-Value
GO:19538: protein metabolism	5170	25.15	929	29.99	2.04E-11
GO:43170: macromolecule metabolism	6957	33.85	1210	39.06	2.55E-11
GO:44267: cellular protein metabolism	5126	24.94	920	29.7	3.63E-11
GO:44260: cellular macromolecule metabolism	5196	25.28	928	29.95	9.03E-11
GO:6412: protein biosynthesis	1104	5.371	231	7.456	5.35E-08
GO:74: regulation of progression through cell cycle	732	3.562	164	5.294	5.51E-08
GO:7049: cell cycle	1151	5.6	239	7.715	6.06E-08
GO:43412: biopolymer modification	2787	13.56	512	16.53	1.78E-07
GO:6915: apoptosis	790	3.844	170	5.487	5.23E-07
GO:9059: macromolecule biosynthesis	1197	5.824	241	7.779	7.10E-07
GO:50875: cellular physiological process	15952	77.61	2506	80.89	8.16E-07
GO:12501: programmed cell death	796	3.873	170	5.487	8.63E-07
GO:43037: translation	289	1.406	75	2.421	9.58E-07
GO:6399: tRNA metabolism	132	0.642	42	1.356	9.65E-07
GO:50790: regulation of enzyme activity	377	1.834	92	2.97	1.15E-06
GO:6464: protein modification	2713	13.2	492	15.88	1.60E-06
GO:16265: death	844	4.106	175	5.649	4.09E-06
GO:8219: cell death	840	4.087	174	5.617	4.64E-06
GO:51338: regulation of transferase activity	197	0.958	54	1.743	5.50E-06
GO:7582: physiological process	17631	85.78	2735	88.28	5.82E-06
GO:43085: positive regulation of enzyme activity	170	0.827	48	1.549	7.49E-06
GO:43283: biopolymer metabolism	4478	21.79	767	24.76	9.72E-06
GO:8104: protein localization	1076	5.235	213	6.875	1.04E-05
GO:9058: biosynthesis	1974	9.604	363	11.72	1.34E-05
GO:45184: establishment of protein localization	1045	5.084	206	6.649	1.90E-05
GO:45859: regulation of protein kinase activity	191	0.929	51	1.646	2.16E-05
GO:15031: protein transport	1009	4.909	199	6.423	2.56E-05
GO:7243: protein kinase cascade	482	2.345	105	3.389	4.54E-05
GO:7010: cytoskeleton organization and biogenesis	602	2.929	126	4.067	5.78E-05
GO:6364: rRNA processing	71	0.345	24	0.775	6.65E-05
GO:16072: rRNA metabolism	76	0.37	25	0.807	7.86E-05
GO:43038: amino acid activation	59	0.287	21	0.678	8.00E-05
GO:43039: tRNA aminoacylation	59	0.287	21	0.678	8.00E-05
GO:6418: tRNA aminoacylation for protein translation	59	0.287	21	0.678	8.00E-05
GO:16192: vesicle-mediated transport	580	2.822	121	3.906	9.29E-05
GO:8632: apoptotic program	95	0.462	29	0.936	0.000102
GO:6457: protein folding	387	1.883	85	2.744	0.000173
GO:8033: tRNA processing	71	0.345	23	0.742	0.000194
GO:6914: autophagy	34	0.165	14	0.452	0.000212
GO:6917: induction of apoptosis	213	1.036	52	1.679	0.000224
GO:12502: induction of programmed cell death	214	1.041	52	1.679	0.000253

GO:44249: cellular biosynthesis	1765	8.588	316	10.2	0.00037
GO:42981: regulation of apoptosis	469	2.282	98	3.163	0.000389
GO:7046: ribosome biogenesis	102	0.496	29	0.936	0.000393
GO:9062: fatty acid catabolism	6	0.0292	5	0.161	0.000407
GO:51641: cellular localization	1047	5.094	197	6.359	0.000418
GO:16043: cell organization and biogenesis	2519	12.26	437	14.11	0.00044
GO:43065: positive regulation of apoptosis	235	1.143	55	1.775	0.000467
GO:6512: ubiquitin cycle	997	4.851	188	6.068	0.000504
GO:6432: phenylalanyl-tRNA aminoacylation	4	0.0195	4	0.129	0.000515
GO:42985: negative regulation of amyloid precursor protein biosynthesis	4	0.0195	4	0.129	0.000515
GO:45210: FasL biosynthesis	4	0.0195	4	0.129	0.000515
GO:6858: extracellular transport	4	0.0195	4	0.129	0.000515
GO:6092: main pathways of carbohydrate metabolism	153	0.744	39	1.259	0.000526
GO:51258: protein polymerization	62	0.302	20	0.646	0.000527
GO:51649: establishment of cellular localization	1034	5.031	194	6.262	0.000535
GO:48518: positive regulation of biological process	975	4.744	184	5.939	0.000553
GO:43068: positive regulation of programmed cell death	238	1.158	55	1.775	0.000646
GO:43067: regulation of programmed cell death	476	2.316	98	3.163	0.000658
GO:46907: intracellular transport	1025	4.987	191	6.165	0.000831
GO:30029: actin filament-based process	246	1.197	56	1.808	0.000851
GO:278: mitotic cell cycle	339	1.649	73	2.356	0.000858
GO:8637: apoptotic mitochondrial changes	19	0.0924	9	0.291	0.000868
GO:45069: regulation of viral genome replication	16	0.0778	8	0.258	0.00109
GO:51347: positive regulation of transferase activity	84	0.409	24	0.775	0.00111
GO:42157: lipoprotein metabolism	94	0.457	26	0.839	0.0012
GO:154: rRNA modification	7	0.0341	5	0.161	0.00125
GO:9186: deoxyribonucleoside diphosphate metabolism	7	0.0341	5	0.161	0.00125
GO:9451: RNA modification	28	0.136	11	0.355	0.00159
GO:42982: amyloid precursor protein metabolism	17	0.0827	8	0.258	0.00179
GO:51345: positive regulation of hydrolase activity	54	0.263	17	0.549	0.00182
GO:7028: cytoplasm organization and biogenesis	148	0.72	36	1.162	0.00205
GO:165: MAPKKK cascade	164	0.798	39	1.259	0.00214
GO:7021: tubulin folding	14	0.0681	7	0.226	0.00226
GO:42984: regulation of amyloid precursor protein biosynthesis	5	0.0243	4	0.129	0.00227
GO:42983: amyloid precursor protein biosynthesis	5	0.0243	4	0.129	0.00227
GO:30036: actin cytoskeleton organization and biogenesis	229	1.114	51	1.646	0.00231
GO:6996: organelle organization and biogenesis	1378	6.705	245	7.908	0.00243
GO:51329: interphase of mitotic cell cycle	79	0.384	22	0.71	0.00251
GO:9103: lipopolysaccharide biosynthesis	11	0.0535	6	0.194	0.00272
GO:8653: lipopolysaccharide metabolism	11	0.0535	6	0.194	0.00272
GO:50435: beta-amyloid metabolism	18	0.0876	8	0.258	0.00279
GO:6533: aspartate catabolism	8	0.0389	5	0.161	0.00291
GO:6531: aspartate metabolism	8	0.0389	5	0.161	0.00291
GO:6919: caspase activation	52	0.253	16	0.516	0.00317
GO:43280: positive regulation of caspase activity	52	0.253	16	0.516	0.00317
GO:43281: regulation of caspase activity	52	0.253	16	0.516	0.00317
GO:42254: ribosome biogenesis and assembly	131	0.637	32	1.033	0.00328

GO:44248: cellular catabolism	730	3.552	137	4.422	0.00328
GO:45786: negative regulation of progression through cell cycle	255	1.241	55	1.775	0.00334
GO:15980: energy derivation by oxidation of organic compounds	222	1.08	49	1.582	0.00335
GO:9826: unidimensional cell growth	3	0.0146	3	0.0968	0.00342
GO:18345: protein palmitoylation	3	0.0146	3	0.0968	0.00342
GO:9226: nucleotide-sugar biosynthesis	3	0.0146	3	0.0968	0.00342
GO:917: barrier septum formation	3	0.0146	3	0.0968	0.00342
GO:51325: interphase	81	0.394	22	0.71	0.00352
GO:6413: translational initiation	101	0.491	26	0.839	0.00359
GO:6561: proline biosynthesis	12	0.0584	6	0.194	0.00475
GO:45860: positive regulation of protein kinase activity	78	0.38	21	0.678	0.0048
GO:43123: positive regulation of I-kappaB kinase/NF-kappaB cascade	150	0.73	35	1.13	0.00482
GO:8624: induction of apoptosis by extracellular signals	45	0.219	14	0.452	0.00502
GO:6777: Mo-molybdopterin cofactor biosynthesis	9	0.0438	5	0.161	0.00574
GO:19720: Mo-molybdopterin cofactor metabolism	9	0.0438	5	0.161	0.00574
GO:79: regulation of cyclin dependent protein kinase activity	55	0.268	16	0.516	0.00584
GO:9249: protein-lipoylation	6	0.0292	4	0.129	0.00598
GO:6099: tricarboxylic acid cycle	37	0.18	12	0.387	0.00631
GO:6904: vesicle docking during exocytosis	37	0.18	12	0.387	0.00631
GO:9132: nucleoside diphosphate metabolism	13	0.0633	6	0.194	0.0077
GO:8625: induction of apoptosis via death domain receptors	13	0.0633	6	0.194	0.0077
GO:48522: positive regulation of cellular process	815	3.965	148	4.777	0.0079
GO:46356: acetyl-CoA catabolism	38	0.185	12	0.387	0.00798
GO:7242: intracellular signaling cascade	1818	8.845	310	10.01	0.00809
GO:6098: pentose-phosphate shunt	17	0.0827	7	0.226	0.00848
GO:6740: NADPH regeneration	17	0.0827	7	0.226	0.00848
GO:7249: I-kappaB kinase/NF-kappaB cascade	200	0.973	43	1.388	0.00918
GO:9068: aspartate family amino acid catabolism	10	0.0487	5	0.161	0.0101
GO:43405: regulation of MAPK activity	83	0.404	21	0.678	0.0102
GO:7017: microtubule-based process	241	1.173	50	1.614	0.0106
GO:43122: regulation of I-kappaB kinase/NF-kappaB cascade	158	0.769	35	1.13	0.0111
GO:7033: vacuole organization and biogenesis	22	0.107	8	0.258	0.0117
GO:50792: regulation of viral life cycle	22	0.107	8	0.258	0.0117
GO:9396: folic acid and derivative biosynthesis	14	0.0681	6	0.194	0.0118
GO:7006: mitochondrial membrane organization and biogenesis	14	0.0681	6	0.194	0.0118
GO:18377: protein myristoylation	4	0.0195	3	0.0968	0.0121
GO:18319: protein amino acid myristoylation	4	0.0195	3	0.0968	0.0121
GO:6499: N-terminal protein myristoylation	4	0.0195	3	0.0968	0.0121
GO:9133: nucleoside diphosphate biosynthesis	4	0.0195	3	0.0968	0.0121
GO:9189: deoxyribonucleoside diphosphate biosynthesis	4	0.0195	3	0.0968	0.0121
GO:9138: pyrimidine nucleoside diphosphate metabolism	4	0.0195	3	0.0968	0.0121
GO:50709: negative regulation of protein secretion	4	0.0195	3	0.0968	0.0121
GO:46685: response to arsenic	4	0.0195	3	0.0968	0.0121
GO:185: activation of MAPKKK activity	7	0.0341	4	0.129	0.0123
GO:16601: Rac protein signal transduction	7	0.0341	4	0.129	0.0123
GO:9100: glycoprotein metabolism	226	1.1	47	1.517	0.0124
GO:6887: exocytosis	116	0.564	27	0.872	0.0126

GO:51187: cofactor catabolism	45	0.219	13	0.42	0.0131
GO:9060: aerobic respiration	45	0.219	13	0.42	0.0131
GO:51348: negative regulation of transferase activity	60	0.292	16	0.516	0.0141
GO:6469: negative regulation of protein kinase activity	60	0.292	16	0.516	0.0141
GO:6100: tricarboxylic acid cycle intermediate metabolism	36	0.175	11	0.355	0.0141
GO:9967: positive regulation of signal transduction	172	0.837	37	1.194	0.0147
GO:6091: generation of precursor metabolites and energy	916	4.457	162	5.229	0.0147
GO:9109: coenzyme catabolism	41	0.199	12	0.387	0.0151
GO:48278: vesicle docking	41	0.199	12	0.387	0.0151
GO:51179: localization	4468	21.74	720	23.24	0.0152
GO:44265: cellular macromolecule catabolism	480	2.335	90	2.905	0.0152
GO:19058: viral infectious cycle	56	0.272	15	0.484	0.0164
GO:19438: aromatic compound biosynthesis	15	0.073	6	0.194	0.0172
GO:42559: pteridine and derivative biosynthesis	15	0.073	6	0.194	0.0172
GO:42558: pteridine and derivative metabolism	15	0.073	6	0.194	0.0172
GO:19941: modification-dependent protein catabolism	219	1.066	45	1.453	0.0173
GO:6511: ubiquitin-dependent protein catabolism	219	1.066	45	1.453	0.0173
GO:6118: electron transport	567	2.759	104	3.357	0.0177
GO:9615: response to virus	82	0.399	20	0.646	0.0178
GO:6897: endocytosis	248	1.207	50	1.614	0.0179
GO:6084: acetyl-CoA metabolism	47	0.229	13	0.42	0.0189
GO:9101: glycoprotein biosynthesis	192	0.934	40	1.291	0.0192
GO:51336: regulation of hydrolase activity	120	0.584	27	0.872	0.0194
GO:6886: intracellular protein transport	654	3.182	118	3.809	0.0195
GO:9056: catabolism	845	4.111	149	4.81	0.0205
GO:9303: rRNA transcription	8	0.0389	4	0.129	0.0217
GO:44257: cellular protein catabolism	274	1.333	54	1.743	0.0218
GO:51603: proteolysis during cellular protein catabolism	274	1.333	54	1.743	0.0218
GO:30705: cytoskeleton-dependent intracellular transport	149	0.725	32	1.033	0.0225
GO:7018: microtubule-based movement	149	0.725	32	1.033	0.0225
GO:16070: RNA metabolism	786	3.824	139	4.487	0.0225
GO:30033: microvillus biogenesis	2	0.00973	2	0.0646	0.0227
GO:40016: embryonic cleavage	2	0.00973	2	0.0646	0.0227
GO:16139: glycoside catabolism	2	0.00973	2	0.0646	0.0227
GO:16142: O-glycoside catabolism	2	0.00973	2	0.0646	0.0227
GO:42353: fucose biosynthesis	2	0.00973	2	0.0646	0.0227
GO:6005: L-fucose biosynthesis	2	0.00973	2	0.0646	0.0227
GO:42350: GDP-L-fucose biosynthesis	2	0.00973	2	0.0646	0.0227
GO:42351: 'de novo' GDP-L-fucose biosynthesis	2	0.00973	2	0.0646	0.0227
GO:9139: pyrimidine nucleoside diphosphate biosynthesis	2	0.00973	2	0.0646	0.0227
GO:9197: pyrimidine deoxyribonucleoside diphosphate biosynthesis	2	0.00973	2	0.0646	0.0227
GO:6233: dTDP biosynthesis	2	0.00973	2	0.0646	0.0227
GO:9148: pyrimidine nucleoside triphosphate biosynthesis	2	0.00973	2	0.0646	0.0227
GO:9212: pyrimidine deoxyribonucleoside triphosphate biosynthesis	2	0.00973	2	0.0646	0.0227
GO:6235: dTTP biosynthesis	2	0.00973	2	0.0646	0.0227
GO:9202: deoxyribonucleoside triphosphate biosynthesis	2	0.00973	2	0.0646	0.0227
GO:46368: GDP-L-fucose metabolism	2	0.00973	2	0.0646	0.0227

GO:16137: glycoside metabolism	2	0.00973	2	0.0646	0.0227
GO:16140: O-glycoside metabolism	2	0.00973	2	0.0646	0.0227
GO:351: assembly of spliceosomal tri-snRNP U4/U6.U5	2	0.00973	2	0.0646	0.0227
GO:6374: nuclear mRNA splicing via U2-type spliceosome	2	0.00973	2	0.0646	0.0227
GO:1522: pseudouridine synthesis	2	0.00973	2	0.0646	0.0227
GO:46075: dTTP metabolism	2	0.00973	2	0.0646	0.0227
GO:9196: pyrimidine deoxyribonucleoside diphosphate metabolism	2	0.00973	2	0.0646	0.0227
GO:46072: dTDP metabolism	2	0.00973	2	0.0646	0.0227
GO:42448: progesterone metabolism	2	0.00973	2	0.0646	0.0227
GO:19915: sequestering of lipid	2	0.00973	2	0.0646	0.0227
GO:45908: negative regulation of vasodilation	2	0.00973	2	0.0646	0.0227
GO:45907: positive regulation of vasoconstriction	2	0.00973	2	0.0646	0.0227
GO:48302: regulation of isotype switching to IgG isotypes	2	0.00973	2	0.0646	0.0227
GO:48291: isotype switching to IgG isotypes	2	0.00973	2	0.0646	0.0227
GO:15853: adenine transport	2	0.00973	2	0.0646	0.0227
GO:30997: regulation of centriole-centriole cohesion	2	0.00973	2	0.0646	0.0227
GO:46586: regulation of calcium-dependent cell-cell adhesion	2	0.00973	2	0.0646	0.0227
GO:46588: negative regulation of calcium-dependent cell-cell adhesion	2	0.00973	2	0.0646	0.0227
GO:51354: negative regulation of oxidoreductase activity	2	0.00973	2	0.0646	0.0227
GO:51001: negative regulation of nitric-oxide synthase activity	2	0.00973	2	0.0646	0.0227
GO:51234: establishment of localization	4439	21.6	712	22.98	0.0228
GO:271: polysaccharide biosynthesis	29	0.141	9	0.291	0.0229
GO:43284: biopolymer biosynthesis	29	0.141	9	0.291	0.0229
GO:59: protein import into nucleus, docking	29	0.141	9	0.291	0.0229
GO:6560: proline metabolism	16	0.0778	6	0.194	0.024
GO:87: M phase of mitotic cell cycle	264	1.284	52	1.679	0.0242
GO:6108: malate metabolism	12	0.0584	5	0.161	0.0244
GO:9262: deoxyribonucleotide metabolism	12	0.0584	5	0.161	0.0244
GO:43086: negative regulation of enzyme activity	90	0.438	21	0.678	0.0249
GO:51186: cofactor metabolism	288	1.401	56	1.808	0.0253
GO:7067: mitosis	259	1.26	51	1.646	0.0255
GO:8152: metabolism	12150	59.12	1881	60.72	0.0256
GO:19079: viral genome replication	39	0.19	11	0.355	0.0257
GO:6414: translational elongation	54	0.263	14	0.452	0.0264
GO:9263: deoxyribonucleotide biosynthesis	5	0.0243	3	0.0968	0.027
GO:19217: regulation of fatty acid metabolism	5	0.0243	3	0.0968	0.027
GO:209: protein polyubiquitination	5	0.0243	3	0.0968	0.027
GO:15074: DNA integration	5	0.0243	3	0.0968	0.027
GO:9649: entrainment of circadian clock	5	0.0243	3	0.0968	0.027
GO:31571: G1 DNA damage checkpoint	5	0.0243	3	0.0968	0.027
GO:6977: DNA damage response, signal transduction by p53 class mediator resulting in cell cycle arrest	5	0.0243	3	0.0968	0.027
GO:19752: carboxylic acid metabolism	639	3.109	114	3.68	0.0288
GO:6739: NADP metabolism	21	0.102	7	0.226	0.0294
GO:7040: lysosome organization and biogenesis	21	0.102	7	0.226	0.0294
GO:48519: negative regulation of biological process	1223	5.95	208	6.714	0.0296
GO:6468: protein amino acid phosphorylation	905	4.403	157	5.068	0.0298

GO:8360: regulation of cell shape	55	0.268	14	0.452	0.0306
GO:187: activation of MAPK activity	45	0.219	12	0.387	0.0311
GO:6082: organic acid metabolism	641	3.119	114	3.68	0.0311
GO:6509: membrane protein ectodomain proteolysis	17	0.0827	6	0.194	0.0325
GO:6605: protein targeting	293	1.426	56	1.808	0.0341
GO:7025: beta-tubulin folding	9	0.0438	4	0.129	0.0345
GO:51085: chaperone cofactor dependent protein folding	9	0.0438	4	0.129	0.0345
GO:18065: protein-cofactor linkage	9	0.0438	4	0.129	0.0345
GO:51048: negative regulation of secretion	9	0.0438	4	0.129	0.0345
GO:6622: protein targeting to lysosome	9	0.0438	4	0.129	0.0345
GO:6626: protein targeting to mitochondrion	31	0.151	9	0.291	0.0349
GO:19320: hexose catabolism	104	0.506	23	0.742	0.0351
GO:16311: dephosphorylation	235	1.143	46	1.485	0.0356
GO:45333: cellular respiration	51	0.248	13	0.42	0.036
GO:48511: rhythmic process	46	0.224	12	0.387	0.0364
GO:7015: actin filament organization	46	0.224	12	0.387	0.0364
GO:43406: positive regulation of MAPK activity	46	0.224	12	0.387	0.0364
GO:7623: circadian rhythm	22	0.107	7	0.226	0.0376
GO:80: G1 phase of mitotic cell cycle	22	0.107	7	0.226	0.0376
GO:46365: monosaccharide catabolism	105	0.511	23	0.742	0.0388
GO:16032: viral life cycle	78	0.38	18	0.581	0.0397
GO:6221: pyrimidine nucleotide biosynthesis	27	0.131	8	0.258	0.0407
GO:46903: secretion	374	1.82	69	2.227	0.0411
GO:42158: lipoprotein biosynthesis	52	0.253	13	0.42	0.0416
GO:6497: protein lipidation	52	0.253	13	0.42	0.0416
GO:6470: protein amino acid dephosphorylation	220	1.07	43	1.388	0.0419
GO:6760: folic acid and derivative metabolism	18	0.0876	6	0.194	0.0427
GO:45045: secretory pathway	297	1.445	56	1.808	0.0428
GO:6007: glucose catabolism	90	0.438	20	0.646	0.045
GO:6810: transport	4061	19.76	647	20.88	0.0468
GO:42168: heme metabolism	23	0.112	7	0.226	0.0473
GO:7162: negative regulation of cell adhesion	23	0.112	7	0.226	0.0473
GO:16054: organic acid catabolism	14	0.0681	5	0.161	0.0475
GO:46395: carboxylic acid catabolism	14	0.0681	5	0.161	0.0475
GO:6999: nuclear pore organization and biogenesis	6	0.0292	3	0.0968	0.0479
GO:30330: DNA damage response, signal transduction by p53 class mediator	6	0.0292	3	0.0968	0.0479
GO:44238: primary metabolism	11033	53.68	1706	55.07	0.0483

Supplemental Table S4B
GO Biological process Categories Overrepresented in Kmeans Cluster Set 2

GO Category	Genes in Category	% of Genes in Category	Genes in List in Category	% of Genes in List in Category	*P* -Value
GO:6139: nucleobase, nucleoside, nucleotide and nucleic acid metabolism	5227	25.43	911	38.67	1.58E-51
GO:44238: primary metabolism	11033	53.68	1514	64.26	1.68E-28
GO:43283: biopolymer metabolism	4478	21.79	713	30.26	8.93E-25
GO:16070: RNA metabolism	786	3.824	189	8.022	4.10E-24
GO:8152: metabolism	12150	59.12	1616	68.59	4.39E-24
GO:44237: cellular metabolism	11473	55.82	1540	65.37	8.87E-24
GO:6396: RNA processing	656	3.192	162	6.876	4.88E-22
GO:19219: regulation of nucleobase, nucleoside, nucleotide and nucleic acid metabolism	3417	16.63	558	23.68	4.17E-21
GO:6350: transcription	3517	17.11	569	24.15	1.27E-20
GO:31323: regulation of cellular metabolism	3642	17.72	584	24.79	2.56E-20
GO:6351: transcription, DNA-dependent	3244	15.78	531	22.54	3.38E-20
GO:6355: regulation of transcription, DNA-dependent	3150	15.33	512	21.73	9.17E-19
GO:45449: regulation of transcription	3356	16.33	539	22.88	1.01E-18
GO:19222: regulation of metabolism	3747	18.23	590	25.04	1.04E-18
GO:16071: mRNA metabolism	427	2.078	113	4.796	4.47E-18
GO:6259: DNA metabolism	971	4.724	200	8.489	3.05E-17
GO:51244: regulation of cellular physiological process	5009	24.37	741	31.45	6.35E-17
GO:50875: cellular physiological process	15952	77.61	1980	84.04	1.22E-16
GO:6260: DNA replication	257	1.25	78	3.311	2.02E-16
GO:50791: regulation of physiological process	5180	25.2	758	32.17	3.55E-16
GO:50794: regulation of cellular process	5371	26.13	777	32.98	2.14E-15
GO:50789: regulation of biological process	5781	28.13	821	34.85	2.23E-14
GO:6397: mRNA processing	379	1.844	96	4.075	2.91E-14
GO:279: M phase	321	1.562	83	3.523	4.94E-13
GO:51301: cell division	301	1.465	79	3.353	7.80E-13
GO:8380: RNA splicing	300	1.46	77	3.268	5.11E-12
GO:278: mitotic cell cycle	339	1.649	83	3.523	1.10E-11
GO:6996: organelle organization and biogenesis	1378	6.705	239	10.14	1.35E-11
GO:7049: cell cycle	1151	5.6	206	8.744	2.30E-11
GO:43170: macromolecule metabolism	6957	33.85	941	39.94	2.96E-11
GO:87: M phase of mitotic cell cycle	264	1.284	67	2.844	2.06E-10
GO:6261: DNA-dependent DNA replication	129	0.628	41	1.74	5.59E-10
GO:7067: mitosis	259	1.26	65	2.759	6.30E-10
GO:6974: response to DNA damage stimulus	355	1.727	81	3.438	7.73E-10
GO:51276: chromosome organization and biogenesis	506	2.462	102	4.329	7.44E-09
GO:6281: DNA repair	312	1.518	71	3.014	9.56E-09
GO:9719: response to endogenous stimulus	375	1.825	81	3.438	1.12E-08
GO:7059: chromosome segregation	56	0.272	23	0.976	1.50E-08
GO:398: nuclear mRNA splicing, via spliceosome	204	0.993	52	2.207	1.81E-08
GO:375: RNA splicing, via transesterification reactions	204	0.993	52	2.207	1.81E-08
GO:377: RNA splicing, via transesterification reactions with bulged adenosine as nucleophile	204	0.993	52	2.207	1.81E-08

GO:16043: cell organization and biogenesis	2519	12.26	372	15.79	4.26E-08
GO:7001: chromosome organization and biogenesis (sensu Eukaryota)	485	2.36	95	4.032	1.02E-07
GO:6270: DNA replication initiation	29	0.141	15	0.637	1.19E-07
GO:7582: physiological process	17631	85.78	2095	88.92	1.09E-06
GO:6403: RNA localization	98	0.477	28	1.188	3.17E-06
GO:7051: spindle organization and biogenesis	25	0.122	12	0.509	6.18E-06
GO:7046: ribosome biogenesis	102	0.496	28	1.188	7.40E-06
GO:51327: M phase of meiotic cell cycle	66	0.321	21	0.891	8.47E-06
GO:7126: meiosis	66	0.321	21	0.891	8.47E-06
GO:51321: meiotic cell cycle	66	0.321	21	0.891	8.47E-06
GO:67: DNA replication and chromosome cycle	19	0.0924	10	0.424	1.33E-05
GO:50657: nucleic acid transport	95	0.462	26	1.104	1.64E-05
GO:50658: RNA transport	95	0.462	26	1.104	1.64E-05
GO:51236: establishment of RNA localization	95	0.462	26	1.104	1.64E-05
GO:6323: DNA packaging	427	2.078	78	3.311	2.00E-05
GO:75: cell cycle checkpoint	55	0.268	18	0.764	2.39E-05
GO:51169: nuclear transport	189	0.92	41	1.74	3.97E-05
GO:70: mitotic sister chromatid segregation	29	0.141	12	0.509	3.97E-05
GO:6325: establishment and/or maintenance of chromatin architecture	415	2.019	75	3.183	4.14E-05
GO:51168: nuclear export	89	0.433	24	1.019	4.45E-05
GO:6913: nucleocytoplasmic transport	203	0.988	43	1.825	4.73E-05
GO:7028: cytoplasm organization and biogenesis	148	0.72	34	1.443	5.26E-05
GO:819: sister chromatid segregation	30	0.146	12	0.509	5.93E-05
GO:42254: ribosome biogenesis and assembly	131	0.637	31	1.316	6.10E-05
GO:9987: cellular process	18591	90.45	2181	92.57	7.54E-05
GO:51325: interphase	81	0.394	22	0.934	8.11E-05
GO:7017: microtubule-based process	241	1.173	48	2.037	9.09E-05
GO:723: telomere maintenance	41	0.199	14	0.594	0.000113
GO:9069: serine family amino acid metabolism	52	0.253	16	0.679	0.000153
GO:6405: RNA export from nucleus	79	0.384	21	0.891	0.000163
GO:51329: interphase of mitotic cell cycle	79	0.384	21	0.891	0.000163
GO:15931: nucleobase, nucleoside, nucleotide and nucleic acid transport	108	0.525	26	1.104	0.000172
GO:51028: mRNA transport	75	0.365	20	0.849	0.00022
GO:6364: rRNA processing	71	0.345	19	0.806	0.000299
GO:6402: mRNA catabolism	36	0.175	12	0.509	0.000445
GO:51252: regulation of RNA metabolism	27	0.131	10	0.424	0.000512
GO:9071: serine family amino acid catabolism	15	0.073	7	0.297	0.000719
GO:7093: mitotic checkpoint	15	0.073	7	0.297	0.000719
GO:7088: regulation of mitosis	59	0.287	16	0.679	0.000745
GO:16072: rRNA metabolism	76	0.37	19	0.806	0.000752
GO:6520: amino acid metabolism	348	1.693	60	2.547	0.000807
GO:9083: branched chain family amino acid catabolism	8	0.0389	5	0.212	0.000818
GO:6406: mRNA export from nucleus	61	0.297	16	0.679	0.0011
GO:6546: glycine catabolism	12	0.0584	6	0.255	0.00113
GO:6544: glycine metabolism	25	0.122	9	0.382	0.00123
GO:30705: cytoskeleton-dependent intracellular transport	149	0.725	30	1.273	0.00146
GO:7018: microtubule-based movement	149	0.725	30	1.273	0.00146

GO:6310: DNA recombination	112	0.545	24	1.019	0.00175
GO:184: mRNA catabolism, nonsense-mediated decay	31	0.151	10	0.424	0.00175
GO:7131: meiotic recombination	31	0.151	10	0.424	0.00175
GO:7052: mitotic spindle organization and biogenesis	17	0.0827	7	0.297	0.00176
GO:43487: regulation of RNA stability	13	0.0633	6	0.255	0.00189
GO:43488: regulation of mRNA stability	13	0.0633	6	0.255	0.00189
GO:46834: lipid phosphorylation	6	0.0292	4	0.17	0.00213
GO:46854: phosphoinositide phosphorylation	6	0.0292	4	0.17	0.00213
GO:51272: positive regulation of cell motility	6	0.0292	4	0.17	0.00213
GO:46928: regulation of neurotransmitter secretion	6	0.0292	4	0.17	0.00213
GO:7094: mitotic spindle checkpoint	6	0.0292	4	0.17	0.00213
GO:31577: spindle checkpoint	6	0.0292	4	0.17	0.00213
GO:40017: positive regulation of locomotion	6	0.0292	4	0.17	0.00213
GO:48520: positive regulation of behavior	6	0.0292	4	0.17	0.00213
GO:226: microtubule cytoskeleton organization and biogenesis	89	0.433	20	0.849	0.00226
GO:82: G1/S transition of mitotic cell cycle	33	0.161	10	0.424	0.00295
GO:51052: regulation of DNA metabolism	50	0.243	13	0.552	0.00341
GO:74: regulation of progression through cell cycle	732	3.562	108	4.584	0.00344
GO:6399: tRNA metabolism	132	0.642	26	1.104	0.00398
GO:6333: chromatin assembly or disassembly	221	1.075	39	1.655	0.00405
GO:9063: amino acid catabolism	81	0.394	18	0.764	0.00416
GO:9081: branched chain family amino acid metabolism	15	0.073	6	0.255	0.0045
GO:30902: hindbrain development	7	0.0341	4	0.17	0.00453
GO:6400: tRNA modification	7	0.0341	4	0.17	0.00453
GO:245: spliceosome assembly	35	0.17	10	0.424	0.00473
GO:7004: telomerase-dependent telomere maintenance	20	0.0973	7	0.297	0.00516
GO:6284: base-excision repair	25	0.122	8	0.34	0.00528
GO:6352: transcription initiation	91	0.443	19	0.806	0.00672
GO:6519: amino acid and derivative metabolism	411	2	64	2.716	0.00674
GO:8543: fibroblast growth factor receptor signaling pathway	26	0.127	8	0.34	0.00688
GO:9310: amine catabolism	85	0.414	18	0.764	0.00705
GO:7127: meiosis I	37	0.18	10	0.424	0.00726
GO:6268: DNA unwinding during replication	12	0.0584	5	0.212	0.0078
GO:6359: regulation of transcription from RNA polymerase III promoter	8	0.0389	4	0.17	0.00823
GO:6312: mitotic recombination	8	0.0389	4	0.17	0.00823
GO:44270: nitrogen compound catabolism	87	0.423	18	0.764	0.00902
GO:16568: chromatin modification	226	1.1	38	1.613	0.00999
GO:45934: negative regulation of nucleobase, nucleoside, nucleotide and nucleic acid metabolism	292	1.421	47	1.995	0.0103
GO:45893: positive regulation of transcription, DNA-dependent	143	0.696	26	1.104	0.0115
GO:6461: protein complex assembly	422	2.053	64	2.716	0.0119
GO:6275: regulation of DNA replication	23	0.112	7	0.297	0.012
GO:6607: NLS-bearing substrate import into nucleus	18	0.0876	6	0.255	0.0124
GO:30032: lamellipodium biogenesis	5	0.0243	3	0.127	0.0126
GO:7164: establishment of tissue polarity	5	0.0243	3	0.127	0.0126
GO:51103: DNA ligation during DNA repair	5	0.0243	3	0.127	0.0126
GO:6288: base-excision repair, DNA ligation	5	0.0243	3	0.127	0.0126

GO:1946: lymphangiogenesis	2	0.00973	2	0.0849	0.0131
GO:1945: lymph vessel development	2	0.00973	2	0.0849	0.0131
GO:46716: muscle maintenance	2	0.00973	2	0.0849	0.0131
GO:6335: DNA replication-dependent nucleosome assembly	2	0.00973	2	0.0849	0.0131
GO:46785: microtubule polymerization	2	0.00973	2	0.0849	0.0131
GO:19896: axon transport of mitochondrion	2	0.00973	2	0.0849	0.0131
GO:266: mitochondrial fission	2	0.00973	2	0.0849	0.0131
GO:19067: viral assembly, maturation, egress, and release	2	0.00973	2	0.0849	0.0131
GO:19076: viral release	2	0.00973	2	0.0849	0.0131
GO:30324: lung development	9	0.0438	4	0.17	0.0135
GO:16441: posttranscriptional gene silencing	9	0.0438	4	0.17	0.0135
GO:35194: RNA-mediated posttranscriptional gene silencing	9	0.0438	4	0.17	0.0135
GO:9113: purine base biosynthesis	9	0.0438	4	0.17	0.0135
GO:6266: DNA ligation	9	0.0438	4	0.17	0.0135
GO:31047: RNA-mediated gene silencing	9	0.0438	4	0.17	0.0135
GO:7062: sister chromatid cohesion	9	0.0438	4	0.17	0.0135
GO:35295: tube development	29	0.141	8	0.34	0.0138
GO:6473: protein amino acid acetylation	29	0.141	8	0.34	0.0138
GO:46907: intracellular transport	1025	4.987	140	5.942	0.0149
GO:48015: phosphoinositide-mediated signaling	105	0.511	20	0.849	0.0152
GO:16126: sterol biosynthesis	41	0.199	10	0.424	0.0153
GO:6383: transcription from RNA polymerase III promoter	41	0.199	10	0.424	0.0153
GO:51649: establishment of cellular localization	1034	5.031	141	5.985	0.0154
GO:7076: mitotic chromosome condensation	14	0.0681	5	0.212	0.0162
GO:31570: DNA integrity checkpoint	19	0.0924	6	0.255	0.0163
GO:51641: cellular localization	1047	5.094	142	6.027	0.0178
GO:16569: covalent chromatin modification	48	0.234	11	0.467	0.0179
GO:7010: cytoskeleton organization and biogenesis	602	2.929	86	3.65	0.0183
GO:45935: positive regulation of nucleobase, nucleoside, nucleotide and nucleic acid metabolism	193	0.939	32	1.358	0.0204
GO:30323: respiratory tube development	10	0.0487	4	0.17	0.0205
GO:6144: purine base metabolism	10	0.0487	4	0.17	0.0205
GO:8652: amino acid biosynthesis	75	0.365	15	0.637	0.0218
GO:86: G2/M transition of mitotic cell cycle	15	0.073	5	0.212	0.0221
GO:30879: mammary gland development	6	0.0292	3	0.127	0.023
GO:42471: ear morphogenesis	6	0.0292	3	0.127	0.023
GO:42472: inner ear morphogenesis	6	0.0292	3	0.127	0.023
GO:76: DNA replication checkpoint	6	0.0292	3	0.127	0.023
GO:69: centromere and kinetochore complex maturation	6	0.0292	3	0.127	0.023
GO:8053: mitochondrial fusion	6	0.0292	3	0.127	0.023
GO:48284: organelle fusion	6	0.0292	3	0.127	0.023
GO:31324: negative regulation of cellular metabolism	324	1.576	49	2.08	0.0262
GO:6401: RNA catabolism	64	0.311	13	0.552	0.0278
GO:9070: serine family amino acid biosynthesis	27	0.131	7	0.297	0.029
GO:45941: positive regulation of transcription	191	0.929	31	1.316	0.029
GO:16481: negative regulation of transcription	273	1.328	42	1.783	0.0291
GO:1508: regulation of action potential	16	0.0778	5	0.212	0.0291

GO:51123: transcriptional preinitiation complex formation	11	0.0535	4	0.17	0.0293
GO:45898: regulation of transcriptional preinitiation complex formation	11	0.0535	4	0.17	0.0293
GO:18: regulation of DNA recombination	11	0.0535	4	0.17	0.0293
GO:6367: transcription initiation from RNA polymerase II promoter	58	0.282	12	0.509	0.0296
GO:30163: protein catabolism	320	1.557	48	2.037	0.0313
GO:7276: gametogenesis	260	1.265	40	1.698	0.0325
GO:45005: maintenance of fidelity during DNA-dependent DNA replication	46	0.224	10	0.424	0.033
GO:6298: mismatch repair	46	0.224	10	0.424	0.033
GO:6512: ubiquitin cycle	997	4.851	133	5.645	0.0337
GO:16458: gene silencing	28	0.136	7	0.297	0.035
GO:30916: otic vesicle formation	3	0.0146	2	0.0849	0.0364
GO:48645: organ formation	3	0.0146	2	0.0849	0.0364
GO:6424: glutamyl-tRNA aminoacylation	3	0.0146	2	0.0849	0.0364
GO:6433: prolyl-tRNA aminoacylation	3	0.0146	2	0.0849	0.0364
GO:9236: cobalamin biosynthesis	3	0.0146	2	0.0849	0.0364
GO:35104: positive regulation of sterol regulatory element binding-protein target gene transcription	3	0.0146	2	0.0849	0.0364
GO:6370: mRNA capping	3	0.0146	2	0.0849	0.0364
GO:9452: RNA capping	3	0.0146	2	0.0849	0.0364
GO:6393: RNA transcription termination from mitochondrial promoter	3	0.0146	2	0.0849	0.0364
GO:9235: cobalamin metabolism	3	0.0146	2	0.0849	0.0364
GO:51493: regulation of cytoskeleton organization and biogenesis	3	0.0146	2	0.0849	0.0364
GO:7064: mitotic sister chromatid cohesion	3	0.0146	2	0.0849	0.0364
GO:7080: mitotic metaphase plate congression	3	0.0146	2	0.0849	0.0364
GO:51310: metaphase plate congression	3	0.0146	2	0.0849	0.0364
GO:45010: actin nucleation	3	0.0146	2	0.0849	0.0364
GO:50000: chromosome localization	3	0.0146	2	0.0849	0.0364
GO:51303: establishment of chromosome localization	3	0.0146	2	0.0849	0.0364
GO:8298: intracellular mRNA localization	3	0.0146	2	0.0849	0.0364
GO:43331: response to dsRNA	3	0.0146	2	0.0849	0.0364
GO:42553: cellular nerve ensheathment	7	0.0341	3	0.127	0.0369
GO:7272: ionic insulation of neurons by glial cells	7	0.0341	3	0.127	0.0369
GO:42552: myelination	7	0.0341	3	0.127	0.0369
GO:16246: RNA interference	7	0.0341	3	0.127	0.0369
GO:6534: cysteine metabolism	7	0.0341	3	0.127	0.0369
GO:51253: negative regulation of RNA metabolism	7	0.0341	3	0.127	0.0369
GO:50686: negative regulation of mRNA processing	7	0.0341	3	0.127	0.0369
GO:6474: N-terminal protein amino acid acetylation	7	0.0341	3	0.127	0.0369
GO:6301: postreplication repair	7	0.0341	3	0.127	0.0369
GO:7089: traversing start control point of mitotic cell cycle	7	0.0341	3	0.127	0.0369
GO:6357: regulation of transcription from RNA polymerase II promoter	440	2.141	63	2.674	0.037
GO:6605: protein targeting	293	1.426	44	1.868	0.0372
GO:7030: Golgi organization and biogenesis	17	0.0827	5	0.212	0.0375
GO:9064: glutamine family amino acid metabolism	60	0.292	12	0.509	0.0376
GO:6730: one-carbon compound metabolism	60	0.292	12	0.509	0.0376
GO:6606: protein import into nucleus	108	0.525	19	0.806	0.0376
GO:51170: nuclear import	108	0.525	19	0.806	0.0376

GO:16570: histone modification	47	0.229	10	0.424	0.0377
GO:31325: positive regulation of cellular metabolism	226	1.1	35	1.486	0.0397
GO:6895: Golgi to endosome transport	12	0.0584	4	0.17	0.04
GO:7185: transmembrane receptor protein tyrosine phosphatase signaling pathway	12	0.0584	4	0.17	0.04
GO:59: protein import into nucleus, docking	29	0.141	7	0.297	0.0417
GO:17038: protein import	125	0.608	21	0.891	0.0465
GO:31124: mRNA 3'-end processing	18	0.0876	5	0.212	0.0471
GO:31123: RNA 3'-end processing	18	0.0876	5	0.212	0.0471
GO:42325: regulation of phosphorylation	30	0.146	7	0.297	0.0492
GO:1932: regulation of protein amino acid phosphorylation	24	0.117	6	0.255	0.0494
GO:51318: G1 phase	24	0.117	6	0.255	0.0494
GO:31023: microtubule organizing center organization and biogenesis	24	0.117	6	0.255	0.0494
GO:51297: centrosome organization and biogenesis	24	0.117	6	0.255	0.0494

Supplemental Table S4C

GO Biological process Categories Overrepresented in Kmeans Cluster Set 3

GO Category	Genes in Category	% of Genes in Category	Genes in List in Category	% of Genes in List in Category	*P* -Value
GO:48513: organ development	907	4.413	145	8.146	1.97E-13
GO:7275: development	2960	14.4	362	20.34	5.79E-13
GO:6817: phosphate transport	146	0.71	38	2.135	4.50E-10
GO:9653: morphogenesis	940	4.574	129	7.247	8.55E-08
GO:1501: skeletal development	204	0.993	41	2.303	2.79E-07
GO:6334: nucleosome assembly	134	0.652	31	1.742	3.21E-07
GO:7155: cell adhesion	1002	4.875	133	7.472	3.67E-07
GO:31497: chromatin assembly	149	0.725	33	1.854	3.98E-07
GO:43062: extracellular structure organization and biogenesis	78	0.38	21	1.18	1.90E-06
GO:30198: extracellular matrix organization and biogenesis	78	0.38	21	1.18	1.90E-06
GO:8015: circulation	171	0.832	33	1.854	9.70E-06
GO:6750: glutathione biosynthesis	19	0.0924	9	0.506	1.11E-05
GO:7167: enzyme linked receptor protein signaling pathway	310	1.508	50	2.809	1.33E-05
GO:15698: inorganic anion transport	229	1.114	40	2.247	1.46E-05
GO:19886: antigen processing, exogenous antigen via MHC class II	34	0.165	12	0.674	1.54E-05
GO:6629: lipid metabolism	870	4.233	110	6.18	3.31E-05
GO:7517: muscle development	255	1.241	42	2.36	3.84E-05
GO:19884: antigen presentation, exogenous antigen	32	0.156	11	0.618	4.64E-05
GO:7169: transmembrane receptor protein tyrosine kinase signaling pathway	228	1.109	38	2.135	6.79E-05
GO:6749: glutathione metabolism	23	0.112	9	0.506	7.14E-05
GO:7399: nervous system development	735	3.576	93	5.225	0.000129
GO:48731: system development	739	3.596	93	5.225	0.000156
GO:6820: anion transport	271	1.319	42	2.36	0.000157
GO:6869: lipid transport	119	0.579	23	1.292	0.000201
GO:44255: cellular lipid metabolism	687	3.343	87	4.888	0.000204
GO:44272: sulfur compound biosynthesis	56	0.272	14	0.787	0.000227
GO:51259: protein oligomerization	32	0.156	10	0.562	0.000252
GO:51241: negative regulation of organismal physiological process	32	0.156	10	0.562	0.000252
GO:42060: wound healing	145	0.705	26	1.461	0.000283
GO:6325: establishment and/or maintenance of chromatin architecture	415	2.019	57	3.202	0.00034
GO:6333: chromatin assembly or disassembly	221	1.075	35	1.966	0.000353
GO:7596: blood coagulation	132	0.642	24	1.348	0.000385
GO:7156: homophilic cell adhesion	181	0.881	30	1.685	0.000415
GO:9887: organ morphogenesis	310	1.508	45	2.528	0.00042
GO:50878: regulation of body fluids	157	0.764	27	1.517	0.000436
GO:6461: protein complex assembly	422	2.053	57	3.202	0.000519
GO:50793: regulation of development	127	0.618	23	1.292	0.000534
GO:51239: regulation of organismal physiological process	193	0.939	31	1.742	0.000584
GO:19400: alditol metabolism	3	0.0146	3	0.169	0.000649
GO:6059: hexitol metabolism	3	0.0146	3	0.169	0.000649
GO:6060: sorbitol metabolism	3	0.0146	3	0.169	0.000649

GO:6323: DNA packaging	427	2.078	57	3.202	0.000693
GO:7154: cell communication	5496	26.74	534	30	0.000708
GO:30195: negative regulation of blood coagulation	19	0.0924	7	0.393	0.000715
GO:30193: regulation of blood coagulation	19	0.0924	7	0.393	0.000715
GO:50819: negative regulation of coagulation	30	0.146	9	0.506	0.000717
GO:50818: regulation of coagulation	30	0.146	9	0.506	0.000717
GO:19752: carboxylic acid metabolism	639	3.109	79	4.438	0.000805
GO:50817: coagulation	139	0.676	24	1.348	0.000833
GO:6082: organic acid metabolism	641	3.119	79	4.438	0.000882
GO:40007: growth	303	1.474	43	2.416	0.000891
GO:7599: hemostasis	140	0.681	24	1.348	0.000924
GO:8286: insulin receptor signaling pathway	31	0.151	9	0.506	0.000934
GO:9308: amine metabolism	500	2.433	64	3.596	0.00102
GO:45445: myoblast differentiation	21	0.102	7	0.393	0.00141
GO:30154: cell differentiation	820	3.99	96	5.393	0.00143
GO:16337: cell-cell adhesion	302	1.469	42	2.36	0.00151
GO:42730: fibrinolysis	16	0.0778	6	0.337	0.00156
GO:9966: regulation of signal transduction	442	2.151	57	3.202	0.00157
GO:902: cellular morphogenesis	453	2.204	58	3.258	0.0017
GO:16477: cell migration	147	0.715	24	1.348	0.00184
GO:51260: protein homooligomerization	22	0.107	7	0.393	0.00192
GO:6519: amino acid and derivative metabolism	411	2	53	2.978	0.00225
GO:8361: regulation of cell size	245	1.192	35	1.966	0.00228
GO:16049: cell growth	245	1.192	35	1.966	0.00228
GO:45661: regulation of myoblast differentiation	4	0.0195	3	0.169	0.00243
GO:45663: positive regulation of myoblast differentiation	4	0.0195	3	0.169	0.00243
GO:6542: glutamine biosynthesis	4	0.0195	3	0.169	0.00243
GO:51291: protein heterooligomerization	4	0.0195	3	0.169	0.00243
GO:48010: vascular endothelial growth factor receptor signaling pathway	4	0.0195	3	0.169	0.00243
GO:30947: regulation of vascular endothelial growth factor receptor signaling pathway	4	0.0195	3	0.169	0.00243
GO:30949: positive regulation of vascular endothelial growth factor receptor signaling pathway	4	0.0195	3	0.169	0.00243
GO:48637: skeletal muscle development	29	0.141	8	0.449	0.00256
GO:48741: skeletal muscle fiber development	29	0.141	8	0.449	0.00256
GO:48747: muscle fiber development	29	0.141	8	0.449	0.00256
GO:7416: synaptogenesis	42	0.204	10	0.562	0.00259
GO:51056: regulation of small GTPase mediated signal transduction	94	0.457	17	0.955	0.00274
GO:6790: sulfur metabolism	102	0.496	18	1.011	0.00276
GO:6953: acute-phase response	36	0.175	9	0.506	0.00294
GO:6807: nitrogen compound metabolism	532	2.588	65	3.652	0.00296
GO:16055: Wnt receptor signaling pathway	162	0.788	25	1.404	0.00329
GO:7179: transforming growth factor beta receptor signaling pathway	44	0.214	10	0.562	0.00373
GO:6575: amino acid derivative metabolism	81	0.394	15	0.843	0.00375
GO:9968: negative regulation of signal transduction	98	0.477	17	0.955	0.00428
GO:50777: negative regulation of immune response	25	0.122	7	0.393	0.0043
GO:7001: chromosome organization and biogenesis (sensu Eukaryota)	485	2.36	59	3.315	0.00492
GO:6349: imprinting	9	0.0438	4	0.225	0.00495

GO:8105: asymmetric protein localization	9	0.0438	4	0.225	0.00495
GO:50920: regulation of chemotaxis	9	0.0438	4	0.225	0.00495
GO:50921: positive regulation of chemotaxis	9	0.0438	4	0.225	0.00495
GO:6829: zinc ion transport	14	0.0681	5	0.281	0.00499
GO:6631: fatty acid metabolism	203	0.988	29	1.629	0.00515
GO:50808: synapse organization and biogenesis	46	0.224	10	0.562	0.00523
GO:51094: positive regulation of development	39	0.19	9	0.506	0.00523
GO:50874: organismal physiological process	2704	13.16	270	15.17	0.00536
GO:42692: muscle cell differentiation	26	0.127	7	0.393	0.00545
GO:30279: negative regulation of ossification	5	0.0243	3	0.169	0.00567
GO:9448: gamma-aminobutyric acid metabolism	5	0.0243	3	0.169	0.00567
GO:46851: negative regulation of bone remodeling	5	0.0243	3	0.169	0.00567
GO:45176: apical protein localization	5	0.0243	3	0.169	0.00567
GO:9309: amine biosynthesis	109	0.53	18	1.011	0.00571
GO:44271: nitrogen compound biosynthesis	109	0.53	18	1.011	0.00571
GO:6936: muscle contraction	232	1.129	32	1.798	0.00583
GO:40008: regulation of growth	207	1.007	29	1.629	0.00676
GO:9888: tissue development	263	1.28	35	1.966	0.00714
GO:6541: glutamine metabolism	21	0.102	6	0.337	0.00728
GO:7499: ectoderm and mesoderm interaction	2	0.00973	2	0.112	0.0075
GO:30219: megakaryocyte differentiation	2	0.00973	2	0.112	0.0075
GO:30325: adrenal gland development	2	0.00973	2	0.112	0.0075
GO:35270: endocrine system development	2	0.00973	2	0.112	0.0075
GO:9128: purine nucleoside monophosphate catabolism	2	0.00973	2	0.112	0.0075
GO:9169: purine ribonucleoside monophosphate catabolism	2	0.00973	2	0.112	0.0075
GO:6196: AMP catabolism	2	0.00973	2	0.112	0.0075
GO:9125: nucleoside monophosphate catabolism	2	0.00973	2	0.112	0.0075
GO:9158: ribonucleoside monophosphate catabolism	2	0.00973	2	0.112	0.0075
GO:42271: susceptibility to natural killer cell mediated cytotoxicity	2	0.00973	2	0.112	0.0075
GO:6907: pinocytosis	2	0.00973	2	0.112	0.0075
GO:46627: negative regulation of insulin receptor signaling pathway	2	0.00973	2	0.112	0.0075
GO:1570: vasculogenesis	10	0.0487	4	0.225	0.00769
GO:6658: phosphatidylserine metabolism	10	0.0487	4	0.225	0.00769
GO:51276: chromosome organization and biogenesis	506	2.462	60	3.371	0.00789
GO:6937: regulation of muscle contraction	49	0.238	10	0.562	0.00832
GO:45595: regulation of cell differentiation	80	0.389	14	0.787	0.00835
GO:15669: gas transport	35	0.17	8	0.449	0.0088
GO:15671: oxygen transport	35	0.17	8	0.449	0.0088
GO:30182: neuron differentiation	166	0.808	24	1.348	0.00888
GO:9605: response to external stimulus	1052	5.118	113	6.348	0.00946
GO:1558: regulation of cell growth	185	0.9	26	1.461	0.00955
GO:48519: negative regulation of biological process	1223	5.95	129	7.247	0.0103
GO:30333: antigen processing	74	0.36	13	0.73	0.0105
GO:43118: negative regulation of physiological process	1056	5.138	113	6.348	0.0105
GO:19605: butyrate metabolism	6	0.0292	3	0.169	0.0106
GO:50766: positive regulation of phagocytosis	6	0.0292	3	0.169	0.0106
GO:50764: regulation of phagocytosis	6	0.0292	3	0.169	0.0106

GO:50868: negative regulation of T cell activation	11	0.0535	4	0.225	0.0113
GO:48523: negative regulation of cellular process	1122	5.459	119	6.685	0.0115
GO:45597: positive regulation of cell differentiation	23	0.112	6	0.337	0.0117
GO:6576: biogenic amine metabolism	67	0.326	12	0.674	0.0117
GO:6730: one-carbon compound metabolism	60	0.292	11	0.618	0.013
GO:7519: striated muscle development	68	0.331	12	0.674	0.0131
GO:6508: proteolysis	950	4.622	102	5.73	0.0134
GO:15674: di-, tri-valent inorganic cation transport	209	1.017	28	1.573	0.0137
GO:7223: frizzled-2 signaling pathway	24	0.117	6	0.337	0.0144
GO:42157: lipoprotein metabolism	94	0.457	15	0.843	0.0149
GO:50776: regulation of immune response	94	0.457	15	0.843	0.0149
GO:9611: response to wounding	554	2.695	63	3.539	0.0156
GO:35023: regulation of Rho protein signal transduction	46	0.224	9	0.506	0.0157
GO:48589: developmental growth	12	0.0584	4	0.225	0.0158
GO:6596: polyamine biosynthesis	12	0.0584	4	0.225	0.0158
GO:8210: estrogen metabolism	12	0.0584	4	0.225	0.0158
GO:51181: cofactor transport	12	0.0584	4	0.225	0.0158
GO:7411: axon guidance	54	0.263	10	0.562	0.0164
GO:6732: coenzyme metabolism	250	1.216	32	1.798	0.0166
GO:9628: response to abiotic stimulus	617	3.002	69	3.876	0.0167
GO:48514: blood vessel morphogenesis	113	0.55	17	0.955	0.0174
GO:1568: blood vessel development	113	0.55	17	0.955	0.0174
GO:9261: ribonucleotide catabolism	7	0.0341	3	0.169	0.0174
GO:45017: glycerolipid biosynthesis	7	0.0341	3	0.169	0.0174
GO:46463: acylglycerol biosynthesis	7	0.0341	3	0.169	0.0174
GO:46460: neutral lipid biosynthesis	7	0.0341	3	0.169	0.0174
GO:46504: glycerol ether biosynthesis	7	0.0341	3	0.169	0.0174
GO:6644: phospholipid metabolism	149	0.725	21	1.18	0.0179
GO:6643: membrane lipid metabolism	215	1.046	28	1.573	0.0194
GO:7409: axonogenesis	97	0.472	15	0.843	0.0195
GO:1944: vasculature development	115	0.56	17	0.955	0.0204
GO:7178: transmembrane receptor protein serine/threonine kinase signaling pathway	64	0.311	11	0.618	0.0206
GO:50767: regulation of neurogenesis	33	0.161	7	0.393	0.0208
GO:8283: cell proliferation	810	3.941	87	4.888	0.0209
GO:8354: germ cell migration	3	0.0146	2	0.112	0.0212
GO:45666: positive regulation of neuron differentiation	3	0.0146	2	0.112	0.0212
GO:42063: gliogenesis	3	0.0146	2	0.112	0.0212
GO:6420: arginyl-tRNA aminoacylation	3	0.0146	2	0.112	0.0212
GO:1912: positive regulation of immune cell mediated cytotoxicity	3	0.0146	2	0.112	0.0212
GO:45954: positive regulation of natural killer cell mediated cytotoxicity	3	0.0146	2	0.112	0.0212
GO:1913: T cell mediated cytotoxicity	3	0.0146	2	0.112	0.0212
GO:31343: positive regulation of cell killing	3	0.0146	2	0.112	0.0212
GO:46626: regulation of insulin receptor signaling pathway	3	0.0146	2	0.112	0.0212
GO:50770: regulation of axonogenesis	13	0.0633	4	0.225	0.0212
GO:8016: regulation of heart contraction rate	41	0.199	8	0.449	0.0225
GO:9108: coenzyme biosynthesis	162	0.788	22	1.236	0.0232

GO:48667: neuron morphogenesis during differentiation	108	0.525	16	0.899	0.0235
GO:31175: neurite morphogenesis	108	0.525	16	0.899	0.0235
GO:1525: angiogenesis	108	0.525	16	0.899	0.0235
GO:42493: response to drug	34	0.165	7	0.393	0.0243
GO:7565: pregnancy	74	0.36	12	0.674	0.0246
GO:6584: catecholamine metabolism	20	0.0973	5	0.281	0.025
GO:7498: mesoderm development	42	0.204	8	0.449	0.0258
GO:17015: regulation of transforming growth factor beta receptor signaling pathway	8	0.0389	3	0.169	0.0261
GO:50918: positive chemotaxis	8	0.0389	3	0.169	0.0261
GO:50926: regulation of positive chemotaxis	8	0.0389	3	0.169	0.0261
GO:50927: positive regulation of positive chemotaxis	8	0.0389	3	0.169	0.0261
GO:50930: induction of positive chemotaxis	8	0.0389	3	0.169	0.0261
GO:31214: biomineral formation	58	0.282	10	0.562	0.0261
GO:1503: ossification	58	0.282	10	0.562	0.0261
GO:8652: amino acid biosynthesis	75	0.365	12	0.674	0.027
GO:45807: positive regulation of endocytosis	14	0.0681	4	0.225	0.0277
GO:19439: aromatic compound catabolism	21	0.102	5	0.281	0.0305
GO:7031: peroxisome organization and biogenesis	21	0.102	5	0.281	0.0305
GO:6650: glycerophospholipid metabolism	85	0.414	13	0.73	0.0307
GO:48666: neuron development	139	0.676	19	1.067	0.0312
GO:50876: reproductive physiological process	94	0.457	14	0.787	0.0314
GO:48609: reproductive organismal physiological process	94	0.457	14	0.787	0.0314
GO:46849: bone remodeling	60	0.292	10	0.562	0.0323
GO:42221: response to chemical stimulus	527	2.564	58	3.258	0.0347
GO:46364: monosaccharide biosynthesis	29	0.141	6	0.337	0.0352
GO:19319: hexose biosynthesis	29	0.141	6	0.337	0.0352
GO:46165: alcohol biosynthesis	29	0.141	6	0.337	0.0352
GO:6957: complement activation, alternative pathway	15	0.073	4	0.225	0.0353
GO:9636: response to toxin	15	0.073	4	0.225	0.0353
GO:48627: myoblast development	9	0.0438	3	0.169	0.0366
GO:48628: myoblast maturation	9	0.0438	3	0.169	0.0366
GO:6703: estrogen biosynthesis	9	0.0438	3	0.169	0.0366
GO:42036: negative regulation of cytokine biosynthesis	9	0.0438	3	0.169	0.0366
GO:9435: NAD biosynthesis	9	0.0438	3	0.169	0.0366
GO:19674: NAD metabolism	9	0.0438	3	0.169	0.0366
GO:1818: negative regulation of cytokine production	9	0.0438	3	0.169	0.0366
GO:45103: intermediate filament-based process	9	0.0438	3	0.169	0.0366
GO:45104: intermediate filament cytoskeleton organization and biogenesis	9	0.0438	3	0.169	0.0366
GO:46466: membrane lipid catabolism	22	0.107	5	0.281	0.0367
GO:17148: negative regulation of protein biosynthesis	22	0.107	5	0.281	0.0367
GO:18958: phenol metabolism	22	0.107	5	0.281	0.0367
GO:9596: detection of pest, pathogen or parasite	22	0.107	5	0.281	0.0367
GO:42445: hormone metabolism	70	0.341	11	0.618	0.0375
GO:16051: carbohydrate biosynthesis	115	0.56	16	0.899	0.0394
GO:51186: cofactor metabolism	288	1.401	34	1.91	0.0399
GO:30516: regulation of axon extension	4	0.0195	2	0.112	0.04

GO:30502: negative regulation of bone mineralization	4	0.0195	2	0.112	0.04
GO:7520: myoblast fusion	4	0.0195	2	0.112	0.04
GO:6195: purine nucleotide catabolism	4	0.0195	2	0.112	0.04
GO:9154: purine ribonucleotide catabolism	4	0.0195	2	0.112	0.04
GO:16572: histone phosphorylation	4	0.0195	2	0.112	0.04
GO:46033: AMP metabolism	4	0.0195	2	0.112	0.04
GO:1910: regulation of immune cell mediated cytotoxicity	4	0.0195	2	0.112	0.04
GO:42269: regulation of natural killer cell mediated cytotoxicity	4	0.0195	2	0.112	0.04
GO:31341: regulation of cell killing	4	0.0195	2	0.112	0.04
GO:51014: actin filament severing	4	0.0195	2	0.112	0.04
GO:9642: response to light intensity	4	0.0195	2	0.112	0.04
GO:10043: response to zinc ion	4	0.0195	2	0.112	0.04
GO:6520: amino acid metabolism	348	1.693	40	2.247	0.04
GO:8285: negative regulation of cell proliferation	240	1.168	29	1.629	0.0423
GO:31327: negative regulation of cellular biosynthesis	23	0.112	5	0.281	0.0437
GO:6595: polyamine metabolism	16	0.0778	4	0.225	0.0439
GO:51250: negative regulation of lymphocyte activation	16	0.0778	4	0.225	0.0439
GO:16338: calcium-independent cell-cell adhesion	31	0.151	6	0.337	0.0471
GO:7588: excretion	64	0.311	10	0.562	0.0475
GO:16042: lipid catabolism	109	0.53	15	0.843	0.0488
GO:41: transition metal ion transport	82	0.399	12	0.674	0.0494

Supplemental Table S4D

GO Biological process Categories Overrepresented in Kmeans Cluster Set 4

GO Category	Genes in Category	% of Genes in Category	Genes in List in Category	% of Genes in List in Category	*P* -Value
GO:15031: protein transport	1009	4.909	146	8.216	2.77E-10
GO:45184: establishment of protein localization	1045	5.084	148	8.329	9.50E-10
GO:8104: protein localization	1076	5.235	149	8.385	3.93E-09
GO:16192: vesicle-mediated transport	580	2.822	92	5.177	7.93E-09
GO:6886: intracellular protein transport	654	3.182	100	5.627	1.24E-08
GO:46907: intracellular transport	1025	4.987	141	7.935	1.61E-08
GO:51649: establishment of cellular localization	1034	5.031	141	7.935	2.80E-08
GO:51641: cellular localization	1047	5.094	142	7.991	3.50E-08
GO:43122: regulation of I-kappaB kinase/NF-kappaB cascade	158	0.769	36	2.026	5.38E-08
GO:43123: positive regulation of I-kappaB kinase/NF-kappaB cascade	150	0.73	34	1.913	1.43E-07
GO:7249: I-kappaB kinase/NF-kappaB cascade	200	0.973	41	2.307	1.52E-07
GO:9967: positive regulation of signal transduction	172	0.837	37	2.082	1.70E-07
GO:48514: blood vessel morphogenesis	113	0.55	28	1.576	2.52E-07
GO:1568: blood vessel development	113	0.55	28	1.576	2.52E-07
GO:1525: angiogenesis	108	0.525	27	1.519	3.39E-07
GO:1944: vasculature development	115	0.56	28	1.576	3.73E-07
GO:48193: Golgi vesicle transport	137	0.667	30	1.688	1.64E-06
GO:7050: cell cycle arrest	100	0.487	24	1.351	3.27E-06
GO:6888: ER to Golgi transport	55	0.268	16	0.9	1.04E-05
GO:48522: positive regulation of cellular process	815	3.965	106	5.965	1.31E-05
GO:30029: actin filament-based process	246	1.197	42	2.364	1.54E-05
GO:50817: coagulation	139	0.676	28	1.576	1.91E-05
GO:7596: blood coagulation	132	0.642	27	1.519	2.00E-05
GO:7599: hemostasis	140	0.681	28	1.576	2.19E-05
GO:50878: regulation of body fluids	157	0.764	30	1.688	2.85E-05
GO:6506: GPI anchor biosynthesis	36	0.175	12	0.675	2.94E-05
GO:6505: GPI anchor metabolism	36	0.175	12	0.675	2.94E-05
GO:30036: actin cytoskeleton organization and biogenesis	229	1.114	39	2.195	3.23E-05
GO:45786: negative regulation of progression through cell cycle	255	1.241	42	2.364	3.70E-05
GO:42060: wound healing	145	0.705	28	1.576	4.26E-05
GO:45601: regulation of endothelial cell differentiation	4	0.0195	4	0.225	5.57E-05
GO:48518: positive regulation of biological process	975	4.744	118	6.64	0.000107
GO:30203: glycosaminoglycan metabolism	53	0.258	14	0.788	0.000118
GO:6022: aminoglycan metabolism	54	0.263	14	0.788	0.000147
GO:45045: secretory pathway	297	1.445	45	2.532	0.000153
GO:15781: pyrimidine nucleotide-sugar transport	8	0.0389	5	0.281	0.000215
GO:7243: protein kinase cascade	482	2.345	65	3.658	0.000219
GO:51234: establishment of localization	4439	21.6	443	24.93	0.000241
GO:6980: redox signal response	5	0.0243	4	0.225	0.000259
GO:48523: negative regulation of cellular process	1122	5.459	130	7.316	0.00032
GO:51179: localization	4468	21.74	444	24.99	0.000344

GO:46489: phosphoinositide biosynthesis	46	0.224	12	0.675	0.000405
GO:46903: secretion	374	1.82	52	2.926	0.000436
GO:46916: transition metal ion homeostasis	48	0.234	12	0.675	0.000618
GO:31179: peptide modification	3	0.0146	3	0.169	0.000645
GO:1519: peptide amidation	3	0.0146	3	0.169	0.000645
GO:45454: cell redox homeostasis	19	0.0924	7	0.394	0.000708
GO:45446: endothelial cell differentiation	6	0.0292	4	0.225	0.000724
GO:30503: regulation of cell redox homeostasis	6	0.0292	4	0.225	0.000724
GO:6897: endocytosis	248	1.207	37	2.082	0.00076
GO:46474: glycerophospholipid biosynthesis	56	0.272	13	0.732	0.000797
GO:6012: galactose metabolism	10	0.0487	5	0.281	0.000836
GO:48519: negative regulation of biological process	1223	5.95	137	7.71	0.000901
GO:9966: regulation of signal transduction	442	2.151	58	3.264	0.000915
GO:7242: intracellular signaling cascade	1818	8.845	194	10.92	0.000991
GO:7264: small GTPase mediated signal transduction	520	2.53	66	3.714	0.00103
GO:42158: lipoprotein biosynthesis	52	0.253	12	0.675	0.00132
GO:6497: protein lipidation	52	0.253	12	0.675	0.00132
GO:44262: cellular carbohydrate metabolism	460	2.238	59	3.32	0.00144
GO:6810: transport	4061	19.76	400	22.51	0.00146
GO:6878: copper ion homeostasis	7	0.0341	4	0.225	0.00157
GO:6880: intracellular sequestering of iron ion	7	0.0341	4	0.225	0.00157
GO:50796: regulation of insulin secretion	7	0.0341	4	0.225	0.00157
GO:5975: carbohydrate metabolism	688	3.347	82	4.615	0.00179
GO:43118: negative regulation of physiological process	1056	5.138	118	6.64	0.00218
GO:6013: mannose metabolism	17	0.0827	6	0.338	0.00222
GO:6635: fatty acid beta-oxidation	17	0.0827	6	0.338	0.00222
GO:51243: negative regulation of cellular physiological process	1016	4.943	114	6.415	0.00224
GO:15780: nucleotide-sugar transport	12	0.0584	5	0.281	0.00227
GO:19885: antigen processing, endogenous antigen via MHC class I	35	0.17	9	0.506	0.00236
GO:16559: peroxisome fission	4	0.0195	3	0.169	0.00241
GO:19883: antigen presentation, endogenous antigen	29	0.141	8	0.45	0.00253
GO:43161: proteasomal ubiquitin-dependent protein catabolism	23	0.112	7	0.394	0.00253
GO:51238: sequestering of metal ion	8	0.0389	4	0.225	0.00293
GO:16265: death	844	4.106	96	5.402	0.00325
GO:1502: cartilage condensation	13	0.0633	5	0.281	0.00342
GO:30384: phosphoinositide metabolism	58	0.282	12	0.675	0.00353
GO:8219: cell death	840	4.087	95	5.346	0.00401
GO:6979: response to oxidative stress	74	0.36	14	0.788	0.00404
GO:6515: misfolded or incompletely synthesized protein catabolism	19	0.0924	6	0.338	0.00419
GO:30433: ER-associated protein catabolism	19	0.0924	6	0.338	0.00419
GO:6826: iron ion transport	52	0.253	11	0.619	0.00429
GO:8654: phospholipid biosynthesis	91	0.443	16	0.9	0.00471
GO:16043: cell organization and biogenesis	2519	12.26	253	14.24	0.00489
GO:45930: negative regulation of progression through mitotic cell cycle	9	0.0438	4	0.225	0.00492
GO:46467: membrane lipid biosynthesis	108	0.525	18	1.013	0.00509
GO:43119: positive regulation of physiological process	681	3.313	78	4.389	0.00632
GO:42345: regulation of NF-kappaB import into nucleus	15	0.073	5	0.281	0.0069

GO:42348: NF-kappaB import into nucleus	15	0.073	5	0.281	0.0069
GO:30968: unfolded protein response	15	0.073	5	0.281	0.0069
GO:12501: programmed cell death	796	3.873	89	5.008	0.00708
GO:7040: lysosome organization and biogenesis	21	0.102	6	0.338	0.00722
GO:1947: heart looping	2	0.00973	2	0.113	0.00747
GO:48699: neurogenesis	2	0.00973	2	0.113	0.00747
GO:45602: negative regulation of endothelial cell differentiation	2	0.00973	2	0.113	0.00747
GO:19471: 4-hydroxyproline metabolism	2	0.00973	2	0.113	0.00747
GO:18401: peptidyl-proline hydroxylation to 4-hydroxy-L-proline	2	0.00973	2	0.113	0.00747
GO:1574: ganglioside biosynthesis	2	0.00973	2	0.113	0.00747
GO:50760: negative regulation of thymidylate synthase biosynthesis	2	0.00973	2	0.113	0.00747
GO:50758: regulation of thymidylate synthase biosynthesis	2	0.00973	2	0.113	0.00747
GO:50757: thymidylate synthase biosynthesis	2	0.00973	2	0.113	0.00747
GO:9231: riboflavin biosynthesis	2	0.00973	2	0.113	0.00747
GO:18208: peptidyl-proline modification	2	0.00973	2	0.113	0.00747
GO:19511: peptidyl-proline hydroxylation	2	0.00973	2	0.113	0.00747
GO:42726: riboflavin and derivative metabolism	2	0.00973	2	0.113	0.00747
GO:6771: riboflavin metabolism	2	0.00973	2	0.113	0.00747
GO:42727: riboflavin and derivative biosynthesis	2	0.00973	2	0.113	0.00747
GO:15782: CMP-sialic acid transport	2	0.00973	2	0.113	0.00747
GO:15789: UDP-N-acetylgalactosamine transport	2	0.00973	2	0.113	0.00747
GO:45444: fat cell differentiation	10	0.0487	4	0.225	0.00764
GO:6915: apoptosis	790	3.844	88	4.952	0.00812
GO:7033: vacuole organization and biogenesis	22	0.107	6	0.338	0.00921
GO:19370: leukotriene biosynthesis	16	0.0778	5	0.281	0.00933
GO:30865: cortical cytoskeleton organization and biogenesis	16	0.0778	5	0.281	0.00933
GO:51242: positive regulation of cellular physiological process	653	3.177	74	4.164	0.00978
GO:41: transition metal ion transport	82	0.399	14	0.788	0.0102
GO:6729: tetrahydrobiopterin biosynthesis	6	0.0292	3	0.169	0.0106
GO:46146: tetrahydrobiopterin metabolism	6	0.0292	3	0.169	0.0106
GO:6893: Golgi to plasma membrane transport	6	0.0292	3	0.169	0.0106
GO:48285: organelle fission	6	0.0292	3	0.169	0.0106
GO:6643: membrane lipid metabolism	215	1.046	29	1.632	0.011
GO:9893: positive regulation of metabolism	243	1.182	32	1.801	0.0111
GO:30833: regulation of actin filament polymerization	11	0.0535	4	0.225	0.0112
GO:30073: insulin secretion	11	0.0535	4	0.225	0.0112
GO:8064: regulation of actin polymerization and/or depolymerization	53	0.258	10	0.563	0.0143
GO:6879: iron ion homeostasis	38	0.185	8	0.45	0.0144
GO:9611: response to wounding	554	2.695	63	3.545	0.0151
GO:16477: cell migration	147	0.715	21	1.182	0.0153
GO:44255: cellular lipid metabolism	687	3.343	76	4.277	0.0153
GO:8285: negative regulation of cell proliferation	240	1.168	31	1.745	0.0158
GO:6984: ER-nuclear signaling pathway	18	0.0876	5	0.281	0.0158
GO:51235: maintenance of localization	32	0.156	7	0.394	0.0175
GO:9887: organ morphogenesis	310	1.508	38	2.138	0.0182
GO:8154: actin polymerization and/or depolymerization	72	0.35	12	0.675	0.0199
GO:9266: response to temperature stimulus	19	0.0924	5	0.281	0.02

GO:30832: regulation of actin filament length	56	0.272	10	0.563	0.0206
GO:46883: regulation of hormone secretion	13	0.0633	4	0.225	0.0211
GO:9401: phosphoenolpyruvate-dependent sugar phosphotransferase system	13	0.0633	4	0.225	0.0211
GO:30866: cortical actin cytoskeleton organization and biogenesis	13	0.0633	4	0.225	0.0211
GO:30072: peptide hormone secretion	13	0.0633	4	0.225	0.0211
GO:31529: ruffle organization and biogenesis	3	0.0146	2	0.113	0.0211
GO:42733: embryonic digit morphogenesis	3	0.0146	2	0.113	0.0211
GO:8065: establishment of blood-nerve barrier	3	0.0146	2	0.113	0.0211
GO:48638: regulation of developmental growth	3	0.0146	2	0.113	0.0211
GO:48639: positive regulation of developmental growth	3	0.0146	2	0.113	0.0211
GO:19509: methionine salvage	3	0.0146	2	0.113	0.0211
GO:43102: amino acid salvage	3	0.0146	2	0.113	0.0211
GO:6600: creatine metabolism	3	0.0146	2	0.113	0.0211
GO:6601: creatine biosynthesis	3	0.0146	2	0.113	0.0211
GO:6517: protein deglycosylation	3	0.0146	2	0.113	0.0211
GO:6788: heme oxidation	3	0.0146	2	0.113	0.0211
GO:45861: negative regulation of proteolysis	3	0.0146	2	0.113	0.0211
GO:45862: positive regulation of proteolysis	3	0.0146	2	0.113	0.0211
GO:30047: actin modification	3	0.0146	2	0.113	0.0211
GO:7042: lysosomal lumen acidification	3	0.0146	2	0.113	0.0211
GO:31575: G1/S transition checkpoint	3	0.0146	2	0.113	0.0211
GO:43001: Golgi to plasma membrane protein transport	3	0.0146	2	0.113	0.0211
GO:40018: positive regulation of body size	3	0.0146	2	0.113	0.0211
GO:6891: intra-Golgi transport	41	0.199	8	0.45	0.0223
GO:6118: electron transport	567	2.759	63	3.545	0.0234
GO:46456: icosanoid biosynthesis	34	0.165	7	0.394	0.0241
GO:6928: cell motility	356	1.732	42	2.364	0.0243
GO:51674: localization of cell	356	1.732	42	2.364	0.0243
GO:40011: locomotion	356	1.732	42	2.364	0.0243
GO:51093: negative regulation of development	20	0.0973	5	0.281	0.0248
GO:43450: alkene biosynthesis	20	0.0973	5	0.281	0.0248
GO:6518: peptide metabolism	20	0.0973	5	0.281	0.0248
GO:8643: carbohydrate transport	66	0.321	11	0.619	0.0252
GO:51223: regulation of protein transport	27	0.131	6	0.338	0.0252
GO:6066: alcohol metabolism	377	1.834	44	2.476	0.0254
GO:8277: regulation of G-protein coupled receptor protein signaling pathway	42	0.204	8	0.45	0.0256
GO:43094: metabolic compound salvage	8	0.0389	3	0.169	0.0259
GO:51453: regulation of cellular pH	8	0.0389	3	0.169	0.0259
GO:51452: cellular pH reduction	8	0.0389	3	0.169	0.0259
GO:45851: pH reduction	8	0.0389	3	0.169	0.0259
GO:30336: negative regulation of cell migration	8	0.0389	3	0.169	0.0259
GO:46824: positive regulation of nucleocytoplasmic transport	8	0.0389	3	0.169	0.0259
GO:42307: positive regulation of protein import into nucleus	8	0.0389	3	0.169	0.0259
GO:42993: positive regulation of transcription factor import into nucleus	8	0.0389	3	0.169	0.0259
GO:42346: positive regulation of NF-kappaB import into nucleus	8	0.0389	3	0.169	0.0259
GO:51222: positive regulation of protein transport	8	0.0389	3	0.169	0.0259
GO:6929: substrate-bound cell migration	8	0.0389	3	0.169	0.0259

GO:30162: regulation of proteolysis	14	0.0681	4	0.225	0.0276
GO:6081: aldehyde metabolism	14	0.0681	4	0.225	0.0276
GO:6917: induction of apoptosis	213	1.036	27	1.519	0.0286
GO:6790: sulfur metabolism	102	0.496	15	0.844	0.029
GO:9144: purine nucleoside triphosphate metabolism	111	0.54	16	0.9	0.0292
GO:9205: purine ribonucleoside triphosphate metabolism	111	0.54	16	0.9	0.0292
GO:9199: ribonucleoside triphosphate metabolism	111	0.54	16	0.9	0.0292
GO:42364: water-soluble vitamin biosynthesis	28	0.136	6	0.338	0.0298
GO:6687: glycosphingolipid metabolism	28	0.136	6	0.338	0.0298
GO:12502: induction of programmed cell death	214	1.041	27	1.519	0.0301
GO:6650: glycerophospholipid metabolism	85	0.414	13	0.732	0.0303
GO:42157: lipoprotein metabolism	94	0.457	14	0.788	0.031
GO:6800: oxygen and reactive oxygen species metabolism	103	0.501	15	0.844	0.0313
GO:6644: phospholipid metabolism	149	0.725	20	1.125	0.0322
GO:6690: icosanoid metabolism	52	0.253	9	0.506	0.0328
GO:51247: positive regulation of protein metabolism	44	0.214	8	0.45	0.033
GO:43067: regulation of programmed cell death	476	2.316	53	2.983	0.034
GO:19395: fatty acid oxidation	29	0.141	6	0.338	0.0349
GO:19438: aromatic compound biosynthesis	15	0.073	4	0.225	0.0351
GO:42559: pteridine and derivative biosynthesis	15	0.073	4	0.225	0.0351
GO:42558: pteridine and derivative metabolism	15	0.073	4	0.225	0.0351
GO:6903: vesicle targeting	15	0.073	4	0.225	0.0351
GO:7034: vacuolar transport	15	0.073	4	0.225	0.0351
GO:19318: hexose metabolism	198	0.963	25	1.407	0.0356
GO:8203: cholesterol metabolism	96	0.467	14	0.788	0.0363
GO:30970: retrograde protein transport, ER to cytosol	9	0.0438	3	0.169	0.0365
GO:6691: leukotriene metabolism	22	0.107	5	0.281	0.0365
GO:42306: regulation of protein import into nucleus	22	0.107	5	0.281	0.0365
GO:42990: regulation of transcription factor import into nucleus	22	0.107	5	0.281	0.0365
GO:42991: transcription factor import into nucleus	22	0.107	5	0.281	0.0365
GO:48513: organ development	907	4.413	94	5.29	0.0367
GO:6508: proteolysis	950	4.622	98	5.515	0.0371
GO:6944: membrane fusion	45	0.219	8	0.45	0.0372
GO:42981: regulation of apoptosis	469	2.282	52	2.926	0.0379
GO:7165: signal transduction	4419	21.5	412	23.19	0.0385
GO:42127: regulation of cell proliferation	439	2.136	49	2.757	0.0388
GO:6986: response to unfolded protein	88	0.428	13	0.732	0.0389
GO:6950: response to stress	1501	7.303	149	8.385	0.0389
GO:6787: porphyrin catabolism	4	0.0195	2	0.113	0.0398
GO:42396: phosphagen biosynthesis	4	0.0195	2	0.113	0.0398
GO:46835: carbohydrate phosphorylation	4	0.0195	2	0.113	0.0398
GO:6599: phosphagen metabolism	4	0.0195	2	0.113	0.0398
GO:17121: phospholipid scrambling	4	0.0195	2	0.113	0.0398
GO:46329: negative regulation of JNK cascade	4	0.0195	2	0.113	0.0398
GO:10165: response to X-ray	4	0.0195	2	0.113	0.0398
GO:9746: response to hexose stimulus	4	0.0195	2	0.113	0.0398
GO:9749: response to glucose stimulus	4	0.0195	2	0.113	0.0398

GO:7626: locomotory behavior	369	1.795	42	2.364	0.0404
GO:9110: vitamin biosynthesis	30	0.146	6	0.338	0.0406
GO:7032: endosome organization and biogenesis	54	0.263	9	0.506	0.0406
GO:8610: lipid biosynthesis	319	1.552	37	2.082	0.0409
GO:74: regulation of progression through cell cycle	732	3.562	77	4.333	0.0412
GO:5996: monosaccharide metabolism	201	0.978	25	1.407	0.0415
GO:6029: proteoglycan metabolism	38	0.185	7	0.394	0.0419
GO:6024: glycosaminoglycan biosynthesis	23	0.112	5	0.281	0.0434
GO:6004: fucose metabolism	23	0.112	5	0.281	0.0434
GO:45596: negative regulation of cell differentiation	16	0.0778	4	0.225	0.0437
GO:7250: activation of NF-kappaB-inducing kinase	16	0.0778	4	0.225	0.0437
GO:9141: nucleoside triphosphate metabolism	117	0.569	16	0.9	0.0445
GO:79: regulation of cyclin dependent protein kinase activity	55	0.268	9	0.506	0.045
GO:6892: post-Golgi transport	31	0.151	6	0.338	0.0468
GO:1501: skeletal development	204	0.993	25	1.407	0.0481
GO:46034: ATP metabolism	91	0.443	13	0.732	0.049
GO:9117: nucleotide metabolism	304	1.479	35	1.97	0.0499
