# Supplementary material for: Functional and Transcriptional Characterization of Human Embryonic Stem Cell-Derived Endothelial Cells for Treatment of Myocardial Infarction
Source: PLoS One. 2009 Dec 31;4(12):e8443. doi: 10.1371/journal.pone.0008443 (PMC2795856; doi:10.1371/journal.pone.0008443)
Supplement: Supplemental Microarray Analysis S1 — (0.15 MB DOC) [file pone.0008443.s012.doc]

**SUPPLEMENTAL MICROARRAY ANALYSIS**

**K-means clustering analysis shows broad transcriptional trends during differentiation**

Given the enormous amount of data in microarrays, it is often useful to first obtain an overview of the broader trends in the data before proceeding to specific genes. Using K-means clustering analysis, we can easily identify four major trends in the microarray data (**Figure 3D,** see also **Tables S3** and **S4**). Cluster 1 is composed of 5,089 genes whose expression is increased at the hESC-EC and HUVEC stages compared to hESC and EB (**Figure 3D**). To better understand which cellular processes are important in this cluster, we performed statistical Gene Ontology (GO) biological process overrepresentation analysis. GO analysis of cluster 1 shows that the processes to which these genes contribute include many basic differentiated cell functions such as protein and macromolecule metabolism, cell cycle, vesicle-mediated transport, cytoskeletal biogenesis, and control of apoptosis (**Table S4A**). The reverse expression pattern is seen in the 3,998 genes composing cluster 2, which are downregulated in the hESC-EC and HUVEC stages compared to hESC and EB (**Table S3A**). The processes overrepresented in this cluster primarily involve nucleic acid synthesis, DNA replication and chromatin maintenance, cell cycle and mitosis, and transcription in general (**Table S4B**). This theme is consistent with patterns seen in normal embryonic development in both Drosophila and mouse [1], and reflects the fact that earlier undifferentiated cells are undergoing rapid replication and production of broad ranges of transcripts; later in development, cell cycling slows dramatically as cells begin to express a more limited number of genes that are appropriate for the differentiated state. Cluster 3 is comprised of 2,965 genes whose expression increases when hESCs transition to the EB stage, then progressively decrease in the hESC-EC and HUVEC stages (**Table S3C**). The overrepresented processes in this cluster correspond to development and differentiation pathways – the critical steps for EBs, including many aspects of development: organ, skeletal, nervous system, and morphogenesis (**Table S4C**). The final interesting cluster of genes is cluster 4 (**Table S3D**), which represents 2,952 genes whose expression progressively increases from the hESC to EB to hESC-EC to HUVEC stages. Similar to cluster 1, many of the overrepresented biological processes are related to basic differentiated cell pathways such as protein transport and cellular localization; however, the endothelial cells processes (blood vessel morphogenesis and development, angiogenesis and vasculature development) are also highly represented here (**Table S4D**). Overall, hESC-ECs express endothelial cell genes at levels similar to those found in HUVEC, making this population a good source of potential replacement cells *in vivo*.

**Transcriptional changes at each stage of differentiation**

**hESCs exhibit unique biologic processes and molecular signature.** GO analysis reveals that the most highly upregulated processes in hESCs are almost exclusively cell cycling and mitosis, as well as nucleic acid synthesis and metabolism (**Table S2A**). This was not surprising given that hESCs’ primary mission is to self renew. hESCs are also characterized by a network of genes important for pluripotency, including the unique homeobox transcription factor NANOG, which is the main downstream effector of this network. When we compared expression patterns in hESCs to EB cells, we found that there were 2,170 genes expressed much more highly in hESCs. The most dramatically elevated transcript in hESCs was NANOG, which is expressed at a level 60 times higher in hESCs than in EB [2,3] (**Table S1A**). POU5F1 (also known as Oct4), upstream of NANOG, is one of the critical regulators of pluripotency in the mammalian embryo, and our results show that it is expressed 37-50 fold more highly in the undifferentiated hESCs when compared to EB. SOX2, another key pluripotency gene, is expressed at 14.5 times the level in hESCs as in EB [4]. Other known markers of ES cell status are also clearly present at high levels: TDGF1 and 3 (Crypto1 and 3) [5], expressed >10 fold higher levels in hESCs; LIN28 (7 fold higher); the ES cell markers such as developmental pluripotency-associated 4 (Dppa4) (8.5 fold) [6] and homeobox expressed in ES cells 1 (Hesx1, 8 fold) [7], and left-right determination factor 1 (LEFTY1, up 47 fold), galanin (GAL, up 23 fold), DNA (cytosine-5-)-methyltransferase 3 beta (DNMT3B, up 19 fold), fibroblast growth factor 2 (basic) (FGF2, up 7 fold).

**EB cells express many mesodermal and endothelial specific gene programs.** Differentiation to the EB stage is a critical step as development occurs spontaneously down many paths. Analysis of the biological processes upregulated in EBs reflects this heterogeneity **(Table S2B**), with overrepresentation of vasculature and blood vessel development, skeletal development, lung development, and others in the microarray data. We noted many angiogenic and endothelial-specific genes that were upregulated in EBs, including the endothelial transcription factor endothelial PAS domain protein 1 (EPAS1, also called hypoxia-inducible factor-2, up 2.1 fold) [8], endothelial TEK tyrosine kinase (TEK, also known as TIE2, up 12 fold) [9], kinase insert domain receptor (KDR, up 5-8 fold), the ephrin receptor protein-tyrosine kinases EFNA1 (up 5-14 fold) EFNB2 (up 3 fold) [10,11,12], endothelial protein C receptor (EPCR , up 2-3 fold) [13], and the claudin family member CLDN5 (up 4.2 fold) that is a component of endothelial and epithelial tight junctions [14]. VEGF variant 1 (VEGF, up 2.4 fold) and VEGFC (up 11.8 fold) were also upregulated in EBs. Interestingly we also observed upregulation in EBs of the estrogen receptor GPR30 (up 20 fold), as well as a number of blood coagulation genes indicating hematopoiesis, including plasminogen (PLG, up >5 fold), tissue plasminogen activator (PLAT, up 30 fold), and coagulation factors II (F2, also known as thrombin, up 56 fold), III (F3), VII (F7, up 8.5 fold), and X (F10, up 5 fold), among others. The coagulation cascade has been shown to modulate endothelial cell function in developing blood vessels, and thrombin's actions on endothelial cells contributes to vascular development and hemostasis [15]. Lastly, confirming previous reports that the Wnt signaling pathway is important for stem cells and endothelial commitement [16,17], we observed upregulation of WNT5A (up 9.2 fold) and WNT5B (up 5 fold).

We do still see at this stage expression of genes characteristic of all three cell layers, endoderm, mesoderm and ectoderm. These include endodermal genes such as FOXA1 (HNF3A, up 11 fold) [18,19], the mesodermal genes such as the musculoskeletal transcription factors runt-related transcription factor 2 (RUNX2, up 8 fold)[20] and SOX9 (up 5.1 fold), cardiogenic/mesodermal and endodermal zinc finger transcription factors GATA4 (up 2.8 fold), GATA5 (up 17.4 fold) and GATA6 (up 12.6 fold) [21,22,23], and heart and neural crest derivatives expressed 1 (HAND1, up 9.6 fold), as well as the neuro-developmental gene CLN5 (up 3.3 fold) [24], demonstrating that EBs still contain cells from each lineage. Several hemoglobin genes (HBG1, HBE1, HBZ) are also upregulated in EBs, indicating development of erythropoietic cells. We also noted upregulation of the multi-functional factors bone morphogenetic proteins 4 (BMP4, up 6.3 fold) and 5 (BMP5, up 14.8 fold), LIM domain only 2 (rhombotin-like 1) (LMO2, up 7.2 fold), the transcription factor forkhead box P1, transcript variant 1 (FOXP1, up 2.3 fold). Many developmental regulatory genes such as the homeobox genes (HOXA3, A4, A9, A10, B2, B3, B4, B5, B8, B9, B13, and D1) [25] and limb bud and cardiac T-box family of transcription factors TBX2 and TBX3 [26,27] were also upregulated, further illustrating the multiplicity of developmental lineages in EBs.

**hESC-ECs downregulate early developmental genes and upregulate endothelial genes**. Differentiation of EBs to endothelial cells is marked by a downregulation of processes such as transcription, non-endothelial developmental pathways (including lung, skeletal and nervous system), and blood coagulation (**Table S2C**). In looking at the GO pathways that are overrepresented in hESC-ECs, apoptotic pathways are significantly upregulated, as well as angiogenesis, blood vessel and vasculature development. The downregulation of developmental programs is evidenced by the decreased expression of a number of homeobox genes (HOXB2, 3, 4, HOXA2, HOXD1, IRX5, HESX1), as well as the limb bud developmental regulators PITX1 and PITX2[28]. Also noteworthy are the mesodermal transcription factors GATA5, GATA6, MEF2C and FOXC1, whose expression decreases between the EB and EC stages (**Table S1C**) indicating that hESC-ECs have progressed beyond the mesodermal stage. Nevertheless, there is still transcriptional evidence for the presence of other mesodermal derivatives such as hematopoietic derivatives with the increased expression of skeletogenic genes such as RUNX2 (6.6 fold).

Interestingly, **Table S1C** also reveals downregulation of many transforming growth factor-beta (TGF-beta) superfamily signaling proteins, including Noggin (NOG, down 3 fold), TGFB3, and BMP2, BMP4, and BMP5, suggesting that this pathway is less important for endothelial development as these cells mature. Interestingly, the endothelial-specific receptor NOTCH4 (down 2.3 fold) and its ligands delta-like 1 (DLL1) and jagged 2 (JAG2) were all downregulated, indicating a reduction in NOTCH signaling that is required for early cell fate determination [29,30]. However there is still evidence of continued developmental programs that are active in hESC-ECs compared to EBs, as demonstrated by the upregulation of BMP1, BMP6, and the homeobox genes HOXA7, A9, A10, and C9 (**Table S1D**). Interestingly, the chemokine (C-X-C motif) receptor 4 (CXCR4, down 21 fold), which is important for chemotaxis of endothelial cells, is down in hESC-ECs compared to EBs.

Concurrent with the downregulation of early developmental gene programs, we observe substantial increases in expression of many vasculature-related genes in hESC-ECs compared to EBs (**Table S1D**). These genes include the endothelial-specific transcription factors Kruppel-like factor 2 (KLF2, up 3.5-5 fold)[31] and EPAS1 (up 2.3 fold), PECAM1 (up 3.1 fold), endothelial lipase (LIPG, up 2.9-3.8 fold), and endothelin 1 (EDN1, up 16 fold). A number of genes demonstrated continued upregulation from the hESC to EB to EC stages, including endothelial protein C receptor (EPCR, up 2.3 fold) peroxisome proliferative activated receptor, gamma (PPARG, up 12.2 fold), and endothelial cell adhesion molecule (ESAM, up 2.5-3 fold). In the endothelium, caveolin-1 (CAV1, up 23-31 fold) regulates nitric oxide signaling by binding to and inhibiting endothelial nitric oxide synthase (eNOS) [32]. GATA2 expression was increased 4.6 fold and is known to be involved with regulation of PECAM1, endothelin-1, and ICAM2 [33]. The endothelial-specific gene Robo4 (up 2.6-8.1 fold) maintains vascular integrity and inhibits endothelial cell migration and angiogenesis [34]. Other genes upregulated in hESC-ECs include the tyrosine kinase receptor for both VEGF and semaphorin, neuropilin 1 (NRP1, up 3-5 fold), that is expressed in both endothelial and neuronal cells [35], apelin (APLN, up 2-20 fold), ecto-5'-nucleotidase (CD73, also known as NT5E, up 20-26 fold) [36], ITGB3 (up 7-12 fold), and CD109 antigen (up 3-4 fold). Lastly, compared to EBs, hESC-ECs further upregulate of WNT5A and WNT5B (up 2.4 and 3.6 fold, respectively), but downregulate of WNT2B, 3, 6, and 11. Of note, the WNT signaling pathway inhibitor dickkopf homolog 1 (DKK1, up 26 fold) is upregulated in hESC-ECs, which has recently been shown to be important for *in vitro* differentiation of hESCs to cardiovascular progenitors [37].

**Significant changes between hESC-ECs and HUVECs.** One of our goals in this study is to compare our hESC-EC population to a cell population that would likely be optimal for cell transplantation into ischemic tissue. An optimal cell type would be committed to the endothelial lineage but would still retain the capacity to undergo mitosis and thereby regenerate damaged vasculature. Assessing the similarities, as well as differences, in gene expression between hESC-ECs and HUVECs will thus give insight into the overall maturation of our differentiated cells. Many of the increased processes in HUVECs include angiogenesis, blood vessel morphogenesis and development, iron ion sequestering and transport, and phosphorylation, suggesting that HUVECs may be a somewhat more differentiated or “matured” endothelial cell (**Table S2F**). We were curious about the increased iron metabolism, and found that recent evidence has uncovered a mechanism for feedback regulation of EPAS1 translation in response to iron, suggesting a relationship between iron levels and EPAS1 regulation [38]. Downregulated processes in HUVECs reflect the continued differentiation of HUVECs when compared to hESC-ECs, including skeletal and nervous system development, as well as a reduction in VEGF and Wnt receptor signaling (**Table S2E**). Specific genes that are downregulated in HUVECs include RUNX2 (down 320 fold) and SOX9 (down 49 fold), as well as the hemoglobin genes (HBG1, HBE1, HBZ), indicating that there is further differentiation towards endothelial rather than musculoskeletal of erythropoietic lineages. Compared to hESC-ECs, HUVECs markedly downregulate WNT5A (down 256 fold) and WNT5B (down 91 fold), and to a lesser degree WNT3A (down 2.9 fold), WNT3 (down 2.1 fold), WNT6 (down 3 fold), and WNT11 (down 3.2 fold). As in EBs, the estrogen receptor (GPR30, up 7.5 fold) is again upregulated in HUVECs, and may contribute to mobilization of endothelial cells [39].

A number of early developmental genes are further downregulated in HUVECs. These include the mesodermal transcription factors NKX2.5 (down 5.6 fold) and HAND2 (down 6.7 fold). Bone morphogenetic protein regulation is alternately regulated depending on the BMP. For example, HUVECs downregulate BMPR1A 15-20 fold and BMP5 3 fold, yet upregulate BMP4 (up 7.8 fold) and BMP6 (up 14.4 fold). Likewise, homeobox genes do not exhibit a clear pattern of regulation in HUVECs vs. hESC-ECs, where HOXA2, A3, A4, A5, A7, B4, D1, and D3 are upregulated while HOXB6, B9, B13, and C9 are downregulated. The early developmental T-box transcription factors, TBX1, TBX2, TBX3, are also all downregulated in HUVECs. A number of forkhead box transcription factors were downregulated (Fox A1, A2, A3, D1, F1, F2), with FOXD1 (down 38 fold) and FOXA1 (down 19-34 fold) the most dramatically downregulated. Interestingly, FOXC1 was the only forkhead transcription factor that was upregulated (6.6 fold) in HUVECs.

In contrast, HUVECs further upregulate a number of important genes related to angiogenesis and vasculature development. These genes include the intercellular adhesion molecule KDR (up 6.2-7.5 fold), fms-related tyrosine kinase 4 (FLT4, up 5.2 fold), ICAM2 (up 51 fold), CLDN5 (up 8.7-10.3 fold), ROBO4 (up 6-7 fold), von Willebrand factor (VWF, up 97 fold), EPCR (up 4 fold), PECAM1 (up 6.9 fold), nitric oxide synthase 3 (NOS3, up 6.7 fold), EFNA1 (up 5-10 fold), ITGB3 (up 4-5 fold), APL (up 3-8 fold), EPHA4 (up 5-14 fold)[40], cadherin 5, type 2, VE-cadherin (CDH5, up 6.5 fold), the tight junction protein 2 (TJP2, up 4.5 fold), endothelial cell-specific molecule 1 (ESM1, up 7.5 fold), angiopoietin 2 (ANGPT2, up 2.9 fold – this is in contrast to angiopoietin 1, ANGPT1, which is downregulated 13.5 fold), endothelin 1 (EDN1, up 6.4 fold), TEK (or TIE2, up 4.5 fold), NOTCH1 (up 4 fold), CD109 antigen (Gov platelet alloantigens) (CD109, up 5.4 to 6.4 fold). The chemokine (C-X-C motif) receptor 4 (CXCR4, up 24 fold) is important for chemotaxis of endothelial cells. Taken together, HUVECs likely represent a slightly more differentiated endothelial cell type compared to hESC-ECs.

**Supplemental Microarray References**

1. Wagner RA, Tabibiazar R, Liao A, Quertermous T (2005) Genome-wide expression dynamics during mouse embryonic development reveal similarities to Drosophila development. Dev Biol 288: 595-611.

2. Chambers I, Colby D, Robertson M, Nichols J, Lee S, et al. (2003) Functional expression cloning of Nanog, a pluripotency sustaining factor in embryonic stem cells. Cell 113: 643-655.

3. Pan G, Thomson JA (2007) Nanog and transcriptional networks in embryonic stem cell pluripotency. Cell Res 17: 42-49.

4. Schulz WA, Hoffmann MJ (2007) Transcription factor networks in embryonic stem cells and testicular cancer and the definition of epigenetics. Epigenetics 2: 37-42.

5. Hentschke M, Kurth I, Borgmeyer U, Hubner CA (2006) Germ cell nuclear factor is a repressor of CRIPTO-1 and CRIPTO-3. J Biol Chem 281: 33497-33504.

6. Masaki H, Nishida T, Kitajima S, Asahina K, Teraoka H (2007) Developmental pluripotency-associated 4 (DPPA4) localized in active chromatin inhibits mouse embryonic stem cell differentiation into a primitive ectoderm lineage. J Biol Chem 282: 33034-33042.

7. Webb GC, Thomas PQ, Ford JH, Rathjen PD (1993) Hesx1, a homeobox gene expressed by murine embryonic stem cells, maps to mouse chromosome 14, bands A3-B. Genomics 18: 464-466.

8. Tian H, McKnight SL, Russell DW (1997) Endothelial PAS domain protein 1 (EPAS1), a transcription factor selectively expressed in endothelial cells. Genes & Development 11: 72-82.

9. Sato TN, Qin Y, Kozak CA, Audus KL (1993) Tie-1 and tie-2 define another class of putative receptor tyrosine kinase genes expressed in early embryonic vascular system. Proceedings of the National Academy of Sciences of the United States of America 90: 9355-9358.

10. Korff T, Braun J, Pfaff D, Augustin HG, Hecker M (2008) Role of ephrinB2 expression in endothelial cells during arteriogenesis: impact on smooth muscle cell migration and monocyte recruitment. Blood 112: 73-81.

11. Aasheim H-C, Delabie J, Finne EF (2005) Ephrin-A1 binding to CD4+ T lymphocytes stimulates migration and induces tyrosine phosphorylation of PYK2. Blood 105: 2869-2876.

12. Gale NW, Yancopoulos GD (1999) Growth factors acting via endothelial cell-specific receptor tyrosine kinases: VEGFs, angiopoietins, and ephrins in vascular development. Genes & Development 13: 1055-1066.

13. Bae J-S, Yang L, Manithody C, Rezaie AR (2007) The ligand occupancy of endothelial protein C receptor switches the protease-activated receptor 1-dependent signaling specificity of thrombin from a permeability-enhancing to a barrier-protective response in endothelial cells. Blood 110: 3909-3916.

14. Taddei A, Giampietro C, Conti A, Orsenigo F, Breviario F, et al. (2008) Endothelial adherens junctions control tight junctions by VE-cadherin-mediated upregulation of claudin-5. Nature Cell Biology 10: 923-934.

15. Griffin CT, Srinivasan Y, Zheng YW, Huang W, Coughlin SR (2001) A role for thrombin receptor signaling in endothelial cells during embryonic development. Science 293: 1666-1670.

16. Wang H, Charles PC, Wu Y, Ren R, Pi X, et al. (2006) Gene expression profile signatures indicate a role for Wnt signaling in endothelial commitment from embryonic stem cells. Circulation Research 98: 1331-1339.

17. Matushansky I, Maki RG, Cordon-Cardo C (2008) A context dependent role for Wnt signaling in tumorigenesis and stem cells. Cell Cycle 7: 720-724.

18. Kaestner KH (2005) The making of the liver: developmental competence in foregut endoderm and induction of the hepatogenic program. Cell Cycle 4: 1146-1148.

19. Lee CS, Friedman JR, Fulmer JT, Kaestner KH (2005) The initiation of liver development is dependent on Foxa transcription factors. Nature 435: 944-947.

20. Schroeder TM, Jensen ED, Westendorf JJ (2005) Runx2: a master organizer of gene transcription in developing and maturing osteoblasts. Birth Defects Research Part C, Embryo Today 75: 213-225.

21. Molkentin JD, Lin Q, Duncan SA, Olson EN (1997) Requirement of the transcription factor GATA4 for heart tube formation and ventral morphogenesis. Genes & Development 11: 1061-1072.

22. Xin M, Davis CA, Molkentin JD, Lien C-L, Duncan SA, et al. (2006) A threshold of GATA4 and GATA6 expression is required for cardiovascular development. Proceedings of the National Academy of Sciences of the United States of America 103: 11189-11194.

23. Reiter JF, Alexander J, Rodaway A, Yelon D, Patient R, et al. (1999) Gata5 is required for the development of the heart and endoderm in zebrafish. Genes & Development 13: 2983-2995.

24. Heinonen O, Salonen T, Jalanko A, Peltonen L, Copp A (2000) CLN-1 and CLN-5, genes for infantile and variant late infantile neuronal ceroid lipofuscinoses, are expressed in the embryonic human brain. Journal of Comparative Neurology 426: 406-412.

25. Pearson JC, Lemons D, McGinnis W (2005) Modulating Hox gene functions during animal body patterning. Nat Rev Genet 6: 893-904.

26. King M, Arnold JS, Shanske A, Morrow BE (2006) T-genes and limb bud development. American Journal of Medical Genetics Part A 140: 1407-1413.

27. Plageman TF, Yutzey KE (2005) T-box genes and heart development: putting the "T" in heart. Developmental Dynamics 232: 11-20.

28. Marcil A, Dumontier E, Chamberland M, Camper SA, Drouin J (2003) Pitx1 and Pitx2 are required for development of hindlimb buds. Development 130: 45-55.

29. Uyttendaele H, Marazzi G, Wu G, Yan Q, Sassoon D, et al. (1996) Notch4/int-3, a mammary proto-oncogene, is an endothelial cell-specific mammalian Notch gene. Development 122: 2251-2259.

30. Uyttendaele H, Ho J, Rossant J, Kitajewski J (2001) Vascular patterning defects associated with expression of activated Notch4 in embryonic endothelium. Proceedings of the National Academy of Sciences of the United States of America 98: 5643-5648.

31. Atkins GB, Jain MK (2007) Role of KrÃ¼ppel-like transcription factors in endothelial biology. Circulation Research 100: 1686-1695.

32. Minshall RD, Sessa WC, Stan RV, Anderson RGW, Malik AB (2003) Caveolin regulation of endothelial function. American Journal of Physiology Lung Cellular and Molecular Physiology 285: L1179-1183.

33. Cowan PJ, Tsang D, Pedic CM, Abbott LR, Shinkel TA, et al. (1998) The human ICAM-2 promoter is endothelial cell-specific in vitro and in vivo and contains critical Sp1 and GATA binding sites. The Journal of Biological Chemistry 273: 11737-11744.

34. Jones CA, London NR, Chen H, Park KW, Sauvaget D, et al. (2008) Robo4 stabilizes the vascular network by inhibiting pathologic angiogenesis and endothelial hyperpermeability. Nature Medicine 14: 448-453.

35. Kawasaki T, Kitsukawa T, Bekku Y, Matsuda Y, Sanbo M, et al. (1999) A requirement for neuropilin-1 in embryonic vessel formation. Development 126: 4895-4902.

36. Airas L, Niemela J, Salmi M, Puurunen T, Smith DJ, et al. (1997) Differential regulation and function of CD73, a glycosyl-phosphatidylinositol-linked 70-kD adhesion molecule, on lymphocytes and endothelial cells. The Journal of Cell Biology 136: 421-431.

37. Yang L, Soonpaa MH, Adler ED, Roepke TK, Kattman SJ, et al. (2008) Human cardiovascular progenitor cells develop from a KDR+ embryonic-stem-cell-derived population. Nature 453: 524-528.

38. Sanchez M, Galy B, Muckenthaler MU, Hentze MW (2007) Iron-regulatory proteins limit hypoxia-inducible factor-2alpha expression in iron deficiency. Nature Structural & Molecular Biology 14: 420-426.

39. Urbich C, Dimmeler S (2004) Endothelial progenitor cells: characterization and role in vascular biology. Circulation Research 95: 343-353.

40. Brantley-Sieders DM, Caughron J, Hicks D, Pozzi A, Ruiz JC, et al. (2004) EphA2 receptor tyrosine kinase regulates endothelial cell migration and vascular assembly through phosphoinositide 3-kinase-mediated Rac1 GTPase activation. Journal of Cell Science 117: 2037-2049.
